# Supplementary material for: Eurasian aspen (Populus tremula L.): Central Europe’s keystone species ‘hiding in plain sight’
Source: PLoS One. 2024 Mar 27;19(3):e0301109. doi: 10.1371/journal.pone.0301109 (PMC10971661; doi:10.1371/journal.pone.0301109)
Supplement: S1 File — (DOCX) [file pone.0301109.s001.docx]

**S1**

**Geomorphometric indices for terrain topography and soil moisture related characteristics**

*Internet-based information*

**Topographic Wetness Index (SAGA)**

Topographic wetness index (TWI), also called Topographic Index or Compound Topographic Index is a parameter describing the tendency of a site to accumulate water.

The 'SAGA Wetness Index' is, as the name says, similar to the 'Topographic Wetness Index' (TWI), but it is based on a modified catchment area calculation ('Modified Catchment Area'), which does not think of the flow as very thin film. As result it predicts for cells situated in valley floors with a small vertical distance to a channel a more realistic, higher potential soil moisture compared to the standard TWI calculation.

References:

Boehner, J., Koethe, R. Conrad, O., Gross, J., Ringeler, A., Selige, T. (2002): Soil Regionalisation by Means of Terrain Analysis and Process Parameterisation. In: Micheli, E., Nachtergaele, F., Montanarella, L. [Ed.]: Soil Classification 2001. European Soil Bureau, Research Report No. 7, EUR 20398 EN, Luxembourg. pp.213-222.

Boehner, J., Selige, T. (2006): Spatial prediction of soil attributes using terrain analysis and climate regionalisation. In: Boehner, J., McCloy, K.R., Strobl, J. [Ed.]: SAGA - Analysis and Modelling Applications, Goettinger Geographische Abhandlungen, Goettingen: 13-28.

<http://www.saga-gis.org/saga_tool_doc/2.2.2/ta_hydrology_15.html>

**Module Topographic Openness**

Topographic openness expresses the dominance (positive) or enclosure (negative) of a landscape location. See Yokoyama et al. (2002) for a precise definition. Openness has been related to how wide a landscape can be viewed from any position. It has been proven to be a meaningful input for computer aided geomorphological mapping.

References:

Yokoyama, R. Shirasawa, M. Pike, R.J. (2002): Visualizing topography by openness: A new application of image processing to digital elevation models. Photogrammetric Engineering and Remote Sensing, Vol.68, pp.251-266. online at ASPRS.

<http://www.saga-gis.org/saga_tool_doc/2.2.5/ta_lighting_5.html>

A new parameter, here termed openness, expressing the degree of dominance or enclosure of a location on an irregular surface, is developed to visualize topographic character. Openness is an angular measure of the relation between surface relief and horizontal distance. For angles less than 90", it is equivalent to the internal angle of a cone, its apex at a DEM location, constrained by neighboring elevations within a specified radial distance. Openness incorporates the terrain line-of-sight, or viewshed, concept and is calculated from multiple zenith and nadir angles-here along eight azimuths. Openness has two viewer perspectives. Positive values, expressing openness above the surface, are high for convex forms, whereas negative values describe this attribute below the surface and are high for concave forms.

<https://www.semanticscholar.org/paper/Visualizing-topography-by-openness:-A-new-of-image-Shirasawa-Yokoyama/c3d9a561fdb9e8c34a2b79152aea72b46090bb2e>

# Topographic Position Index

The Topographic Position Index (TPI) compares the elevation of each cell in a DEM to the mean elevation of a specified neighborhood around that cell. Since the only input required is a digital elevation model, TPI can be readily generated almost anywhere.

Positive TPI values represent locations that are higher than the average of their surroundings, as defined by the neighborhood (ridges). Negative TPI values represent locations that are lower than their surroundings (valleys). TPI values near zero are either flat areas (where the slope is near zero) or areas of constant slope (where the slope of the point is significantly greater than zero).

http://www.jennessent.com/downloads/tpi-poster-tnc_18x22.pdf

**Module Multiresolution Index of Valley Bottom Flatness (MRVBF)**

Calculation of the 'multiresolution index of valley bottom flatness' (MRVBF) and the complementary 'multiresolution index of the ridge top flatness' (MRRTF).

References:

Gallant, J.C., Dowling, T.I. (2003): 'A multiresolution index of valley bottom flatness for mapping depositional areas', Water Resources Research, 39/12:1347-1359

<http://www.saga-gis.org/saga_tool_doc/2.1.4/ta_morphometry_8.html>

Valley bottoms function as hydrological buffers that significantly affect runoff behavior. Distinguishing valley bottoms from hillslopes is an important first step in identifying and characterizing sediment deposits for hydrologic and geomorphic purposes. Valley bottoms occur at a range of scales from a few meters to hundreds of kilometers in extent. This paper describes an algorithm for using digital elevation models to identify valley bottoms based on their topographic signature as flat low-lying areas. The algorithm operates at a range of scales and combines the results at different scales into a single multiresolution index. This index classifies degrees of valley bottom flatness, which may be related to depth of deposit. The index can also be used to identify groundwater constrictions and to delineate hydrologic and geomorphic units.

<https://www.researchgate.net/publication/248808482_A_Multi-Resolution_Index_of_Valley_Bottom_Flatness_for_Mapping_Depositional_Areas>

The MRVBF index utilizes the flatness and lowness characteristics of valley bottoms. Flatness is measured by the inverse of slope, and lowness is measured by a ranking of elevation with respect to a circular surrounding area. The two measures, both scaled to the range 0 to 1, are combined by multiplication and could be interpreted as membership functions of fuzzy sets. The method draws on ideas in the Fuzzy Landscape Analysis GIS (FLAG) method for fuzzy landscape indices (Roberts et al., 1997; Dowling et al., 2003).

While MRVBF is a continuous measure, it naturally divides into classes corresponding to the different resolutions and slope thresholds. Values less than 0.5 are not valley bottom areas. Values from 0.5 to 1.5 are considered to be the steepest and smallest resolvable valley bottoms.

<https://agupubs.onlinelibrary.wiley.com/doi/full/10.1029/2002WR001426>
