# Supplementary figures and images for: Eurasian aspen (Populus tremula L.): Central Europe’s keystone species ‘hiding in plain sight’
Source: PLoS One. 2024 Mar 27;19(3):e0301109. doi: 10.1371/journal.pone.0301109 (PMC10971661; doi:10.1371/journal.pone.0301109)

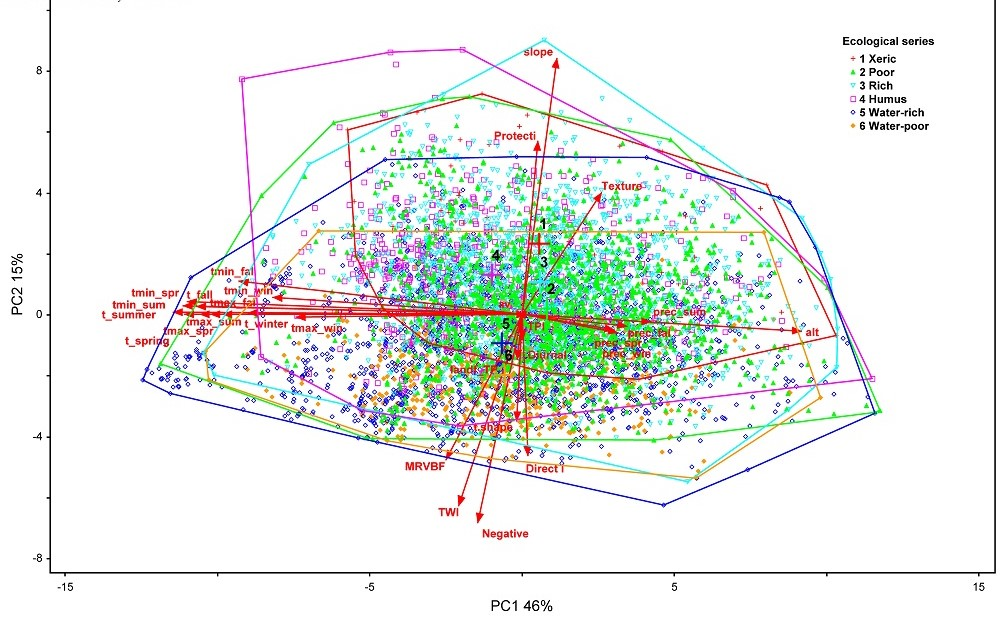

Supplement: S1 Fig — (TIF) [file pone.0301109.s004.tif]
