# Supplementary material for: Eurasian aspen (Populus tremula L.): Central Europe’s keystone species ‘hiding in plain sight’
Source: PLoS One. 2024 Mar 27;19(3):e0301109. doi: 10.1371/journal.pone.0301109 (PMC10971661; doi:10.1371/journal.pone.0301109)
Supplement: S1 Data — (PDF) [file pone.0301109.s005.pdf]

plot	ecoseries	alt	av	slope	t.shape	landf_TPI	Direct Ins	Diurnal An	MRVBF	Negative O	Protection	Texture	TPI	TWI	t_spring	t_summer	t_fall	t_winter	tmax_spring	tmax_summer	tmax_fall	tmax_winter
os00011	5	568	0.4	4	3	6	6.84	-0.02	1.5	1.54	0	97.11	-1.8	7.84	6.41	15.02	6.92	-1.31	10.93	20.28	10.57	1.15
os00248	5	616	0.08	3	3	6	6.94	0.05	1.78	1.55	0.05	81.39	-0.64	8.71	6.01	14.62	6.47	-1.64	10.4	19.73	9.95	0.81
os00259	7	537	0.65	8	2	7	6.52	-0.12	0.75	1.54	0	96.45	-4.37	6.88	6.38	14.98	6.9	-1.32	10.87	20.22	10.49	1.13
os00373	7	640	0.21	2	3	6	6.89	0.02	2.46	1.55	0	73.1	-0.29	9.24	5.93	14.56	6.45	-1.73	10.37	19.7	10.03	0.74
os00383	5	614	0.47	2	2	0	6.89	0	2.93	1.56	0.06	93.23	-0.4	8.82	6.02	14.63	6.51	-1.65	10.49	19.82	10.1	0.8
os00388	5	644	0.4	4	3	6	6.98	0.04	0.63	1.53	0	98.91	0.37	7.43	5.93	14.56	6.44	-1.71	10.29	19.62	9.88	0.74
os00528	3	636	0.49	11	1	4	6.95	0.04	0.01	1.45	0.06	95.52	4.01	5.78	6.27	15	6.78	-1.6	10.78	20.11	10.38	0.87
os00713	5	609	0.4	1	3	6	6.88	0	2.83	1.53	0.02	28.86	-0.57	9.13	6.38	15.09	6.78	-1.59	11.06	20.35	10.49	0.99
os00734	5	503	0.87	3	3	6	6.77	-0.04	0.83	1.52	0.02	93.68	0.05	7.58	7.26	16.03	7.45	-1.08	12.23	21.58	11.29	1.58
os00833	7	521	1	15	2	7	6.24	-0.23	0.06	1.48	0.15	94.36	-5.55	6.51	6.78	15.45	7.08	-1.34	11.52	20.81	10.8	1.21
os00942	5	579	0.78	10	2	3	6.48	-0.11	0.27	1.49	0.09	92.51	-2.77	7.59	6.38	15.07	6.8	-1.56	10.99	20.29	10.44	0.97
os00950	7	695	0.37	17	3	5	6.45	0.02	0	1.45	0.1	100	0.63	6.6	5.55	14.24	6.27	-1.95	9.84	19.14	9.68	0.43
os00978	6	872	0.18	5	3	6	7.1	0.05	0.8	1.5	0.04	98.92	-0.42	7.15	4.48	13.24	5.53	-2.78	8.69	17.87	8.78	-0.43
os01022	7	582	0.03	16	2	7	6.78	0.22	0	1.49	0.23	99.97	-4.76	7.76	5.66	14.36	6.39	-2.18	10.14	19.33	9.87	0.26
os01028	7	791	0.2	13	2	7	7.14	0.15	0.01	1.49	0.14	100	-2.72	7.06	4.86	13.6	5.74	-2.55	9.14	18.32	9.05	-0.15
os01083	7	525	0	9	2	7	6.89	0.15	0.62	1.54	0.2	99.64	-6.33	7.99	6.38	15.09	6.97	-1.71	10.85	20.07	10.49	0.72
os01087	3	512	0.46	15	1	4	6.35	-0.01	0	1.37	0.11	97.26	5.5	5.13	6.99	15.66	7.45	-1.3	11.52	20.72	11	1.2
os01092	7	511	0.09	12	2	7	6.95	0.13	0.44	1.51	0.17	100	-7.28	7.59	6.74	15.44	7.25	-1.46	11.23	20.43	10.75	1.03
os01129	7	657	0	11	2	0	7.04	0.19	0.1	1.46	0.14	99.81	-2.21	7.29	5.63	14.39	6.39	-2.21	10.03	19.27	9.82	0.18
os01446	3	341	0.05	20	1	4	6.88	0.32	0	1.42	0.11	97.59	3.33	5.07	8.19	17.02	8.43	-0.35	12.58	21.87	11.84	2.12
os01514	3	460	0.3	9	1	1	7	0.12	0	1.35	0.06	90.98	8.02	5.23	7.56	16.25	7.9	-0.9	12.2	21.42	11.53	1.64
os01543	6	741	0.08	3	2	0	7.02	0.05	1.97	1.55	0.07	84.79	-1.3	8.36	5.14	13.91	6.09	-2.46	9.41	18.64	9.41	-0.09
os01547	4	302	0.98	4	3	6	6.68	-0.04	0.58	1.51	0.03	93.32	0.76	6.81	8.82	17.55	8.86	0.07	13.57	22.89	12.63	2.71
os01584	4	377	0.53	6	2	0	6.63	-0.01	0.46	1.52	0.07	78.01	-1.63	7.5	8.35	17.27	8.3	-0.55	13	22.7	12.06	2.19
os01614	5	408	0.45	3	3	6	6.69	-0.05	0.55	1.54	0.02	88.38	0.45	8	8.12	17.04	8.28	-0.56	12.73	22.48	11.98	2.13
os01636	7	387	0.01	8	2	3	6.93	0.14	0.62	1.48	0.08	99.24	-3.21	7.66	8.35	17.19	8.39	-0.36	12.94	22.54	12.05	2.35
os01637	7	387	0.02	8	2	3	6.87	0.11	0.72	1.49	0.08	99.23	-3.37	7.86	8.35	17.19	8.39	-0.36	12.94	22.54	12.05	2.35
os01646	4	375	0.26	6	2	3	6.9	0.07	0.9	1.47	0.12	98.95	-4.05	7.01	8.38	17.19	8.38	-0.34	12.93	22.48	11.98	2.35
os01685	4	391	0.98	12	3	5	6.45	-0.16	0.09	1.45	0.1	97.15	-1.25	7.21	8.35	17.24	8.32	-0.54	13.01	22.65	12	2.15
os01690	4	335	0.4	17	2	7	6.8	0.1	0	1.47	0.19	82.59	-5.02	6.06	8.49	17.39	8.43	-0.46	13.19	22.83	12.15	2.22
os01701	4	387	0.43	6	2	3	6.66	0	0.89	1.52	0.08	98.56	-2.72	8.06	8.42	17.35	8.36	-0.54	13.12	22.84	12.12	2.2
os01712	7	520	0.53	3	3	6	6.75	-0.02	2.21	1.52	0.04	77.17	-1.54	8.41	7.74	16.72	7.79	-0.99	12.33	22.19	11.45	1.74
os01713	7	522	0.23	3	3	6	6.81	0.01	2.64	1.52	0.03	74.07	-0.38	8.19	7.74	16.72	7.79	-0.99	12.32	22.19	11.45	1.74
os01718	7	433	0.71	6	3	5	6.78	-0.03	1.14	1.49	0.06	86.89	-1.4	8.25	8.23	17.2	8.22	-0.61	12.84	22.69	11.96	2.13
os01739	4	372	0.95	3	3	6	6.67	-0.05	1.91	1.54	0.04	74.57	-1.59	8.66	8.29	17.17	8.42	-0.45	12.98	22.7	12.2	2.31
os01751	3	383	0.95	4	3	6	6.67	-0.06	0.57	1.5	0.03	89.97	1.83	7.57	8.31	17.21	8.43	-0.45	12.99	22.75	12.28	2.23

os01752	3	381	0.97	7	1	4	6.5	-0.13	0.22	1.48	0.04	90.87	2.07	7.22	8.31	17.21	8.43	-0.46	12.99	22.75	12.27	2.23
os01753	3	381	0.97	7	1	4	6.51	-0.12	0.13	1.47	0.04	90.56	2.59	7.07	8.32	17.22	8.43	-0.45	12.99	22.75	12.28	2.24
os01775	5	474	0.95	4	3	6	6.74	-0.05	1.86	1.54	0.06	99.46	-1.15	7.62	7.94	16.89	7.98	-0.83	12.6	22.3	11.66	1.94
os01802	5	433	0.81	7	3	5	6.51	-0.11	0.3	1.51	0.05	92.15	-0.31	7.32	8.29	17.29	8.23	-0.65	12.96	22.77	11.97	2.07
os01807	4	433	0.01	5	3	6	6.92	0.08	1.04	1.51	0.07	97.45	-1.9	7.73	8.1	17.04	8.09	-0.72	12.78	22.5	11.85	2.01
os01866	7	396	0.77	3	2	3	6.7	-0.03	2.6	1.56	0.07	86.71	-3.57	8.78	8.29	17.23	8.25	-0.58	12.98	22.69	12.01	2.15
os01927	5	414	0.3	7	2	7	6.91	0.07	1.08	1.53	0.09	93.96	-2.77	8.44	8.23	17.18	8.17	-0.68	12.83	22.58	11.87	2
os01972	5	471	0.99	1	3	6	6.77	-0.02	1.71	1.54	0.01	39.82	-0.31	8.26	8.09	17.14	8.04	-0.8	12.79	22.61	11.79	1.91
os02020	7	284	0.92	0	2	0	6.71	0	2.99	1.57	0.12	40.71	-0.79	8.92	8.65	17.42	8.66	-0.08	13.15	22.65	12.25	2.53
os02037	4	451	0.8	7	2	3	6.74	-0.05	0.85	1.54	0.09	97.66	-3.14	7.42	8	16.9	8.02	-0.81	12.67	22.36	11.79	1.95
os02042	4	422	0.38	5	2	3	6.92	0.07	0.89	1.5	0.07	99.24	-2.4	7.26	8.2	17.09	8.16	-0.66	12.88	22.56	11.94	2.08
os02043	4	430	0.3	7	2	3	6.92	0.08	0.85	1.5	0.09	99.51	-3.75	7.16	8.18	17.07	8.15	-0.67	12.86	22.55	11.93	2.07
os02044	4	463	0.89	10	2	3	6.64	-0.09	0.72	1.52	0.11	97.73	-5.26	8.28	7.99	16.92	8	-0.79	12.68	22.37	11.77	1.95
os02081	5	503	0.79	5	3	6	6.78	-0.04	1.13	1.52	0.06	99.82	-1.9	7.82	7.78	16.75	7.84	-0.93	12.42	22.14	11.52	1.84
os02093	5	477	0.6	5	2	0	6.68	-0.02	0.82	1.54	0.08	99.92	-1.09	7.71	7.78	16.71	7.84	-0.95	12.52	22.15	11.61	1.89
os02112	4	440	0.39	13	2	0	6.48	0.02	0	1.43	0.11	97.19	1.55	6.77	7.92	16.9	7.95	-0.9	12.7	22.36	11.77	1.93
os02126	5	403	0.08	1	3	6	6.82	0.02	2.9	1.56	0.05	71.21	-0.9	9.65	8.18	17.16	8.17	-0.76	13.03	22.71	12.08	2.13
os02128	5	407	0.77	1	2	0	6.78	-0.01	2.93	1.56	0.05	76.35	-0.81	8.67	8.16	17.14	8.15	-0.78	13.01	22.68	12.06	2.12
os02209	5	456	0.4	3	3	6	6.87	0.02	1.66	1.54	0.06	83.19	-0.17	9.01	7.68	16.65	7.77	-1.08	12.5	22.11	11.69	1.76
os02303	4	471	0.42	8	3	9	6.92	0.05	0.51	1.48	0.07	99.87	-0.97	6.66	8	16.98	7.99	-0.88	12.82	22.47	11.84	1.99
os02306	5	461	0.92	7	2	7	6.53	-0.1	1.02	1.55	0.09	91.55	-4.07	8.42	7.87	16.87	7.87	-1.12	12.77	22.39	11.77	1.79
os02385	5	475	0	3	3	6	6.86	0.04	1.88	1.52	0.05	97.42	-1.38	8.03	7.71	16.72	7.83	-0.96	12.53	22.19	11.7	1.92
os02399	7	500	0.84	6	2	3	6.77	-0.04	1.57	1.54	0.07	77.43	-1.5	8.39	7.47	16.47	7.57	-1.43	12.49	22.14	11.75	1.59
os02400	7	499	0.77	3	2	3	6.79	-0.02	2.53	1.56	0.08	77.52	-2.82	8.61	7.46	16.46	7.56	-1.44	12.49	22.14	11.75	1.58
os02401	7	502	0.65	7	3	5	6.8	-0.03	0.31	1.52	0.06	77.49	-0.25	8.24	7.47	16.47	7.57	-1.43	12.5	22.15	11.75	1.59
os02402	7	502	0.67	6	3	5	6.84	-0.01	0.41	1.53	0.06	77.62	-0.57	8.31	7.47	16.47	7.57	-1.43	12.5	22.15	11.76	1.59
os02419	5	488	0	1	3	6	6.85	0.02	4.94	1.53	0.01	61.66	-0.16	8.03	7.69	16.67	7.72	-1.27	12.74	22.37	11.89	1.74
os02433	6	528	0.77	5	2	3	6.77	-0.05	1.06	1.54	0.07	94.87	-2.21	6.89	7.23	16.23	7.36	-1.55	12.13	21.79	11.48	1.41
os02439	5	555	0.6	2	3	6	6.87	0	1.96	1.53	0.02	72.98	-0.77	8.23	7.2	16.19	7.33	-1.56	12.08	21.74	11.43	1.39
os02455	4	377	0.6	8	2	0	6.47	-0.1	0.44	1.52	0.08	99.66	-1.08	7.25	8.26	17.21	8.25	-0.59	13.09	22.69	12.12	2.28
os02506	5	404	0.08	3	3	6	6.86	0.04	3.3	1.54	0.03	38.62	-0.16	8.93	8.28	17.25	8.24	-0.69	13.16	22.84	12.19	2.19
os02527	4	305	0.05	4	2	7	6.77	0.04	1.76	1.55	0.12	95.27	-5.33	8.41	8.59	17.5	8.53	-0.32	13.43	22.99	12.43	2.47
os02569	4	353	0.98	8	3	5	6.43	-0.14	0.45	1.5	0.09	97.32	-1.7	7.62	8.53	17.46	8.47	-0.47	13.39	23.03	12.47	2.35
os02573	7	409	0.89	5	2	3	6.69	-0.06	1.41	1.53	0.08	97.19	-2.55	8.59	8.17	17.06	8.16	-0.72	12.9	22.48	12.02	2.1
os02599	7	447	0.94	3	3	6	6.76	-0.03	2.66	1.53	0.02	72.5	-0.72	8.48	8.04	16.97	8.05	-0.82	12.8	22.44	11.89	2
os02632	7	386	0.17	5	2	3	6.78	0.04	1.43	1.55	0.07	87.69	-2.71	8.02	8.33	17.23	8.31	-0.6	13.15	22.73	12.19	2.26
os02661	4	431	0.84	11	3	5	6.33	-0.16	0.03	1.48	0.08	92.89	-0.6	6.62	8.18	17.07	8.16	-0.74	12.97	22.55	11.97	2.13

os02677	4	507	0.77	5	3	5	6.72	-0.07	0.89	1.52	0.07	99.84	-2.16	8.29	7.7	16.62	7.75	-1.07	12.46	22.07	11.52	1.79
os02694	5	554	0.6	6	2	3	6.67	-0.04	0.93	1.51	0.08	99.57	-2.82	7.52	7.55	16.49	7.62	-1.18	12.27	21.91	11.36	1.69
os02748	5	617	0.33	4	3	6	6.82	0.02	1.28	1.52	0.06	99.61	-1.06	7.73	7.22	16.19	7.33	-1.45	11.82	21.56	11	1.38
os02793	5	542	0.4	2	3	6	6.88	0.01	2.75	1.55	0.04	72.73	-0.63	9.14	7.55	16.56	7.54	-1.34	12.25	22	11.35	1.51
os02800	5	548	0.17	1	3	6	6.84	0.01	0.29	1.53	0.02	87.6	0.47	7.59	7.59	16.61	7.58	-1.31	12.31	22.07	11.42	1.55
os02801	5	547	0.13	2	3	6	6.86	0.02	0.28	1.53	0.02	87.21	0.41	7.59	7.59	16.61	7.58	-1.3	12.31	22.07	11.42	1.55
os02802	5	546	0.03	1	3	6	6.88	0.03	0.2	1.53	0.02	86.7	0.58	7.55	7.6	16.62	7.59	-1.3	12.32	22.07	11.43	1.56
os02809	4	332	0.13	4	2	0	6.79	0.01	1.27	1.53	0.09	87.01	-0.97	8.23	8.57	17.38	8.52	-0.27	13.31	22.76	12.32	2.5
os02819	7	427	0.47	9	2	3	6.48	-0.06	0.51	1.53	0.1	95.17	-3.79	7.76	8.09	16.98	8.1	-0.7	12.78	22.36	11.83	2.06
os02969	5	331	0.3	5	2	3	6.83	0.04	1.31	1.53	0.08	96.34	-3.19	7.41	8.61	17.41	8.56	-0.2	13.36	22.81	12.41	2.56
os02975	4	341	0.23	5	2	3	6.75	0.05	1.38	1.54	0.06	98.68	-2.48	7.86	8.61	17.4	8.57	-0.25	13.36	22.8	12.39	2.51
os03100	7	308	0.01	9	2	7	6.76	0.14	1.05	1.54	0.18	93.43	-5.91	6.99	8.48	17.22	8.46	-0.26	13.08	22.4	12.18	2.4
os03164	4	339	0.05	4	2	3	6.68	0.01	1.18	1.51	0.08	97.35	-2.71	8.14	8.66	17.49	8.64	-0.15	13.32	22.81	12.39	2.53
os03276	4	390	0.96	6	3	5	6.64	-0.08	0.84	1.49	0.07	99.47	-1.98	7.29	8.2	17.03	8.33	-0.36	12.73	22.22	12	2.38
os03369	7	367	0.7	5	2	3	6.72	-0.03	1.55	1.53	0.09	98.75	-3.8	7.56	8.28	17.08	8.28	-0.55	13.01	22.48	12.13	2.23
os03389	4	343	0.79	6	3	5	6.52	-0.08	0.43	1.51	0.05	95.87	-0.32	6.41	8.67	17.43	8.6	-0.13	13.33	22.74	12.38	2.62
os03436	4	352	0.92	11	3	5	6.27	-0.16	0.01	1.47	0.08	97.08	0.44	7.57	8.52	17.35	8.51	-0.35	13.19	22.73	12.25	2.38
os03449	4	361	0.81	4	2	3	6.64	-0.06	2.51	1.55	0.07	94.07	-2.71	9.68	8.41	17.27	8.4	-0.47	13.09	22.64	12.15	2.26
os03492	4	333	0.27	6	2	0	6.79	0.01	0.42	1.52	0.07	87.48	0.41	8.29	8.57	17.38	8.52	-0.27	13.31	22.76	12.32	2.5
os03497	4	411	0.83	9	3	5	6.66	-0.08	0.15	1.5	0.08	97.21	-1.21	6.17	8.21	17.02	8.22	-0.6	12.91	22.45	12.04	2.19
os03501	4	354	0.71	7	2	0	6.55	-0.1	0.29	1.51	0.1	98.3	-1.17	8.03	8.33	17.13	8.33	-0.51	13.07	22.52	12.18	2.26
os03502	5	353	0.77	6	2	7	6.54	-0.06	0.77	1.53	0.09	98.7	-2.45	7.95	8.32	17.11	8.32	-0.52	13.05	22.51	12.16	2.25
os03508	5	438	0.77	7	3	5	6.75	-0.04	0.22	1.51	0.06	98.96	-0.23	7.44	7.94	16.81	8.01	-0.82	12.64	22.18	11.83	1.98
os03585	5	458	0.62	2	3	9	6.74	-0.02	1.57	1.51	0.04	97.39	-0.32	6.79	7.99	16.85	8.04	-0.8	12.69	22.24	11.87	2.02
os03588	5	436	0.7	5	3	6	6.77	-0.03	0.24	1.52	0.05	96.26	0.15	6.71	8.04	16.91	8.08	-0.75	12.75	22.3	11.91	2.05
os03589	5	405	0.11	5	2	0	6.61	-0.08	0.92	1.54	0.06	85.13	-1.09	8.38	8.06	16.91	8.09	-0.73	12.76	22.28	11.94	2.06
os03594	5	461	0.6	6	3	5	6.64	-0.04	0.56	1.52	0.05	94.69	-0.22	7.3	7.89	16.77	7.95	-0.86	12.59	22.17	11.76	1.93
os03678	7	390	0.76	5	3	6	6.62	-0.06	1.89	1.54	0.07	86.43	-1.13	8.96	8.26	17.12	8.26	-0.53	12.91	22.44	11.95	2.2
os03780	4	334	0.75	10	2	3	6.45	-0.07	0.58	1.5	0.11	94.78	-4.55	7.42	8.52	17.27	8.53	-0.25	13.13	22.56	12.22	2.38
os03785	5	461	0.8	2	3	6	6.77	-0.03	2.27	1.54	0.05	80.78	-0.5	8.39	7.77	16.56	7.95	-0.74	12.2	21.68	11.44	1.82
os03789	7	432	0.81	15	2	3	6.21	-0.17	0.1	1.48	0.14	94.6	-4.68	7.71	7.97	16.77	8.09	-0.63	12.47	21.99	11.67	2.01
os03791	5	519	0.02	6	3	9	6.88	0.08	0.44	1.49	0.04	95.83	-0.34	6.29	7.63	16.49	7.82	-0.86	12.1	21.67	11.39	1.81
os03794	4	470	0.97	5	3	6	6.73	-0.06	1.5	1.52	0.07	99.58	-1.74	7.95	7.76	16.61	7.93	-0.76	12.25	21.83	11.52	1.9
os03795	4	468	0.96	5	2	3	6.7	-0.07	1.33	1.52	0.07	99.66	-1.88	7.98	7.77	16.61	7.93	-0.75	12.26	21.84	11.53	1.9
os03801	4	374	0.43	5	2	3	6.82	0.03	1.65	1.53	0.09	97.68	-4.08	8.06	8.35	17.16	8.39	-0.37	12.94	22.47	12.08	2.3
os03802	4	374	0.8	5	2	3	6.75	-0.02	1.4	1.52	0.09	97.76	-3.33	8.05	8.35	17.16	8.4	-0.36	12.94	22.47	12.08	2.3
os03803	4	373	0	6	2	3	6.83	0.06	1.39	1.53	0.1	97.57	-3.74	8.16	8.35	17.16	8.4	-0.36	12.94	22.47	12.08	2.3

os03804	4	372	0.24	6	2	3	6.8	0.04	1.22	1.53	0.1	97.58	-3.59	8.15	8.36	17.16	8.4	-0.36	12.95	22.48	12.09	2.31
os03836	5	491	0.92	0	3	6	6.83	0	2.45	1.54	0.01	17.31	-0.1	10.84	7.67	16.46	7.88	-0.8	12.05	21.56	11.33	1.74
os03872	5	434	0.55	2	3	6	6.8	-0.01	2.51	1.54	0.05	83.81	-0.07	7.88	7.98	16.79	8.08	-0.71	12.62	22.19	11.85	1.99
os03934	7	421	0.15	6	3	5	6.92	0.07	1.06	1.52	0.05	92.22	-2.12	7.96	8.02	16.77	8.09	-0.62	12.67	22.08	11.84	2.07
os03937	7	349	0.12	6	2	7	6.73	0.08	1.21	1.54	0.16	93.31	-7.46	6.89	8.18	16.92	8.22	-0.49	12.78	22.19	11.95	2.23
os04010	4	311	0.05	6	2	3	6.53	-0.09	1.33	1.52	0.1	95.96	-3.06	7.27	8.49	17.17	8.52	-0.28	13.27	22.57	12.39	2.46
os04075	5	389	0.76	7	3	5	6.53	-0.08	0.79	1.51	0.07	99.95	-1.99	8.15	8.08	16.82	8.15	-0.56	12.81	22.19	12	2.23
os04150	5	399	0.92	3	3	6	6.68	-0.04	1.49	1.54	0.02	62.3	-0.35	8.19	8.15	16.93	8.33	-0.36	12.68	22.13	11.93	2.18
os04155	5	405	0.65	2	3	6	6.7	-0.03	1.6	1.54	0.01	51.97	0.15	8.62	8.14	16.92	8.32	-0.37	12.67	22.12	11.92	2.17
os04291	5	563	0.87	5	3	6	6.81	-0.04	1	1.52	0.03	71.93	-0.03	8.06	7.14	16.17	7.31	-1.56	12.05	21.72	11.28	1.34
os04362	4	335	0.41	3	2	3	6.73	-0.01	2.53	1.52	0.1	92.21	-2.55	7.35	8.5	17.35	8.6	-0.2	13.08	22.56	12.28	2.52
os04418	5	488	0.92	1	3	6	6.78	-0.02	0.72	1.52	0.01	68.08	1.26	8.28	7.71	16.69	7.72	-1.25	12.73	22.39	11.91	1.74
os04446	6	545	1	10	3	5	6.5	-0.16	0.23	1.5	0.07	89.58	-1.57	6.39	7.22	16.22	7.34	-1.55	12.11	21.77	11.46	1.41
os04682	5	373	0.92	2	3	6	6.68	-0.04	2.06	1.54	0.04	67.77	-0.8	8.15	8.19	17.02	8.25	-0.55	12.89	22.41	12.1	2.24
os04787	6	537	0.08	2	3	6	6.89	0.02	2.49	1.55	0.03	53.37	-1	8.37	7.31	16.31	7.41	-1.33	12.22	21.79	11.52	1.73
os04804	5	530	0.08	4	3	6	6.93	0.05	1.12	1.55	0.03	51.83	-0.51	8.24	7.25	16.22	7.37	-1.56	12.21	21.87	11.54	1.42
os04837	6	430	0.92	0	3	6	6.79	0	3.98	1.57	0.02	5.07	0.02	11.51	7.95	16.96	7.89	-1.02	12.97	22.6	12.07	2.05
os04852	5	508	0.31	2	3	6	6.89	0.03	1.9	1.54	0.03	87.65	-0.56	7.46	7.45	16.41	7.53	-1.35	12.35	21.92	11.63	1.65
os04876	6	537	0.92	1	3	6	6.84	0	1.98	1.54	0.03	76.88	-1.04	8.29	7.36	16.35	7.42	-1.32	12.24	21.82	11.51	1.73
os04884	6	499	0.23	3	3	6	6.8	0.01	3.98	1.54	0.02	9.63	0.33	9.59	7.59	16.6	7.62	-1.21	12.52	22.16	11.74	1.86
os04887	5	432	0.92	0	3	6	6.79	0	4.99	1.57	0.02	10.36	-0.03	11.98	7.93	16.93	7.89	-1.01	12.98	22.61	12.08	2.07
os04918	5	514	0.77	1	3	6	6.84	0	3.01	1.56	0.03	46.03	-0.1	8.59	7.34	16.3	7.46	-1.4	12.31	21.88	11.61	1.62
os04943	5	509	0.65	3	3	6	6.74	-0.04	1.88	1.55	0.03	55.63	-0.99	8.37	7.38	16.35	7.5	-1.4	12.35	21.94	11.65	1.63
os04944	5	511	0.88	2	3	6	6.76	-0.03	1.9	1.55	0.03	57.9	-0.65	8.54	7.38	16.35	7.5	-1.4	12.35	21.94	11.65	1.63
os04946	5	514	0.21	2	3	6	6.88	0.03	2.43	1.56	0.04	65.52	-0.95	8.16	7.35	16.31	7.44	-1.42	12.21	21.8	11.51	1.54
os04964	7	474	0.6	4	3	6	6.68	-0.03	1.34	1.52	0.07	82.45	-1.94	7.74	7.71	16.71	7.7	-1.17	12.72	22.34	11.86	1.89
os04981	5	437	0.92	0	3	6	6.79	0	3.97	1.57	0.02	21.35	-0.19	10.95	7.83	16.79	7.79	-1.06	12.86	22.45	11.98	2.02
os04984	5	435	0.77	2	3	6	6.77	-0.02	4.95	1.57	0.02	8.81	-0.32	9.29	7.87	16.85	7.84	-1.05	12.92	22.5	12.03	2.04
os05023	7	427	0.77	4	2	7	6.74	0	1.08	1.56	0.13	68.95	-5.46	6.91	7.87	16.84	7.86	-1.07	12.83	22.46	12.01	1.92
os05024	4	430	0.22	5	1	4	6.84	0.07	0.18	1.5	0.05	80.44	2.66	7.41	7.99	16.99	7.95	-0.97	12.94	22.58	12.07	2
os05064	5	454	0.92	1	3	6	6.83	0.02	3.1	1.54	0.03	36.6	-0.33	9.36	7.66	16.56	7.77	-0.98	12.37	21.9	11.52	1.85
os05081	7	330	1	6	2	3	6.56	-0.1	0.95	1.53	0.08	95.16	-2.75	8.11	8.5	17.33	8.61	-0.2	13.11	22.59	12.29	2.52
os05082	4	329	0.92	6	2	3	6.52	-0.09	1.04	1.52	0.08	95.23	-2.65	7.9	8.5	17.34	8.61	-0.2	13.11	22.58	12.29	2.52
os05103	4	379	0.18	8	3	5	6.92	0.08	0.61	1.51	0.07	98.46	-1.55	7.13	8.26	17.02	8.36	-0.37	12.7	22.18	11.91	2.22
os05157	5	411	0.9	2	3	6	6.71	-0.04	1.94	1.54	0.04	86.01	-1.87	8.23	8.18	16.99	8.29	-0.45	12.8	22.29	11.99	2.2
os05166	5	429	0.47	3	3	6	6.82	0	0.87	1.53	0.02	72.41	0.19	7.87	8.09	16.89	8.2	-0.52	12.65	22.16	11.88	2.12
os05228	7	395	0	4	2	3	6.64	-0.06	1.87	1.53	0.08	97.39	-3.74	7.82	8.24	17.04	8.35	-0.4	12.82	22.31	12.02	2.21

os05261	4	344	0.58	8	3	5	6.57	-0.02	0.73	1.49	0.06	94.16	-1.46	8.38	8.51	17.31	8.57	-0.23	13.19	22.74	12.35	2.44
os05286	4	317	0.2	11	2	3	6.87	0.1	0.05	1.5	0.13	89.1	-3.95	8.58	8.62	17.35	8.69	-0.07	13.31	22.67	12.41	2.54
os05425	7	469	0.55	9	3	5	6.56	-0.04	0.15	1.49	0.05	98.17	1.06	6.58	8.22	17.26	8.13	-0.67	12.99	22.71	12.04	2.36
os05437	7	435	0.92	2	2	0	6.78	-0.02	2.95	1.56	0.08	91.36	-2.38	8.85	8.26	17.39	8.14	-0.73	13.15	22.9	12.1	2.37
os05494	7	461	0.02	9	3	5	6.94	0.12	0.64	1.5	0.09	95.63	-2.22	8.39	8.12	17.11	8.08	-0.82	13.42	23.07	12.51	2.58
os05495	7	460	0	6	2	7	6.82	0.04	1.8	1.55	0.12	95.77	-5.41	8.33	8.11	17.1	8.08	-0.82	13.41	23.07	12.51	2.58
os05523	7	427	0.73	9	2	3	6.42	-0.12	0.43	1.51	0.13	96.96	-5.07	7.82	8.39	17.38	8.26	-0.53	13.14	22.79	12.12	2.48
os05590	5	400	0.6	2	2	0	6.72	-0.02	3.93	1.56	0.06	77.18	-1.27	8.71	8.41	17.4	8.32	-0.58	13.44	23.12	12.51	2.59
os05593	7	470	0.04	8	2	3	6.57	-0.11	0.88	1.5	0.07	99.11	-2.84	7.22	8.27	17.32	8.21	-0.65	13.33	23.04	12.43	2.55
os05614	7	430	0.82	8	2	0	6.5	-0.1	0.72	1.52	0.09	94.67	-2.58	8.67	8.18	17.18	8.13	-0.73	13.1	22.74	12.22	2.39
os05619	7	420	0.99	6	2	3	6.59	-0.1	1.05	1.53	0.08	97.87	-1.79	8.04	8.36	17.4	8.24	-0.61	13.16	22.89	12.18	2.42
os05663	7	443	0.01	3	3	6	6.86	0.05	1.94	1.56	0.04	46.95	-1.32	8.74	8.14	17.23	8.03	-0.89	12.91	22.71	11.89	2.08
os05711	5	487	0.98	5	3	6	6.76	-0.05	1.34	1.54	0.04	86.53	-1.48	8.49	7.99	17.11	7.91	-0.99	12.84	22.71	11.88	1.98
os05721	5	482	0.98	4	3	6	6.74	-0.05	1.67	1.54	0.05	91.79	-1.7	8.53	8	17.12	7.92	-0.98	12.85	22.72	11.89	1.99
os05726	5	482	0.95	5	3	5	6.71	-0.06	1.02	1.53	0.04	92.15	-1.05	8.26	8	17.12	7.92	-0.98	12.85	22.73	11.89	1.99
os05735	5	494	0.23	2	3	6	6.84	0.02	1.39	1.54	0.01	75.64	-0.04	7.77	7.99	17.12	7.92	-0.99	12.83	22.7	11.88	1.98
os05737	5	493	0.07	2	3	6	6.84	0.02	1.54	1.54	0.01	76.41	-0.03	7.8	7.99	17.12	7.92	-0.99	12.83	22.7	11.88	1.99
os05739	5	492	0.11	2	3	6	6.84	0.02	1.52	1.54	0.01	78.81	0.01	7.86	7.99	17.12	7.92	-0.98	12.83	22.69	11.88	1.99
os05740	5	492	0.04	2	3	6	6.84	0.02	1.62	1.54	0.01	79.76	-0.04	7.89	7.99	17.12	7.92	-0.98	12.83	22.69	11.88	1.99
os05744	5	465	0.23	2	3	6	6.81	0.02	2.41	1.55	0.02	53.54	-0.25	8.96	8.09	17.2	8.01	-0.92	12.92	22.78	11.97	2.05
os05745	5	495	0.01	2	3	6	6.84	0.02	1.48	1.54	0.01	73.83	-0.06	7.77	7.99	17.12	7.92	-0.99	12.83	22.7	11.88	1.98
os05799	5	472	0.05	2	3	6	6.83	0.03	1.89	1.55	0.02	63.21	-0.82	8.65	8.06	17.15	7.97	-0.93	12.85	22.69	11.9	2.02
os05937	5	451	0.99	2	3	6	6.76	-0.03	1.93	1.55	0.03	77.96	-0.82	8.81	8.17	17.24	8.04	-0.95	12.99	22.81	11.98	1.97
os05992	5	491	0.74	3	3	6	6.85	0.01	3.97	1.55	0.02	23.82	0.13	8.97	7.92	17.02	7.82	-1.12	12.75	22.54	11.78	1.8
os06008	7	406	0.44	8	2	3	6.61	-0.01	0.83	1.47	0.07	91.85	-2.78	6.99	8.36	17.46	8.21	-0.69	13.27	23.04	12.19	2.3
os06056	7	483	0.35	2	3	6	6.84	0.02	4.06	1.55	0.02	25.28	-0.22	8.61	8.01	17.12	7.9	-1.05	12.86	22.67	11.89	1.88
os06057	5	486	0.01	2	3	6	6.88	0.04	3.7	1.55	0.02	22.87	-0.38	8.81	7.99	17.1	7.89	-1.07	12.85	22.65	11.87	1.86
os06117	5	458	0.92	0	3	6	6.79	-0.01	3.81	1.55	0.02	24.06	-0.28	9.11	7.89	16.92	7.98	-0.86	12.59	22.26	11.83	2.01
os06118	5	459	0.01	1	3	6	6.83	0.02	1.98	1.55	0.03	26.42	-0.89	8.95	7.9	16.95	8	-0.83	12.6	22.25	11.82	2.02
os06145	5	432	0.53	2	3	6	6.77	0	2.55	1.55	0.01	18	0.45	8.57	8.1	17.14	8.07	-0.86	13	22.72	12.15	2.07
os06180	5	404	0.95	3	2	3	6.81	0.01	5.62	1.55	0.05	48.86	-1.9	8.53	8.2	17.2	8.11	-0.8	13.15	22.81	12.21	2.17
os06253	5	497	0.12	3	3	6	6.83	0.03	1.67	1.55	0.05	82.23	-0.78	7.95	7.84	16.94	7.78	-1.13	12.67	22.42	11.69	1.78
os06395	7	502	0.65	7	2	3	6.86	0.01	0.88	1.53	0.12	97.33	-5.59	6.68	7.74	16.75	7.74	-1.23	12.62	22.26	11.62	1.65
os06478	4	388	0.24	4	2	3	6.65	-0.05	1.94	1.53	0.11	90.83	-5.08	8.28	8.27	17.3	8.34	-0.55	12.95	22.62	12.18	2.35
os06578	5	458	0.08	1	3	6	6.83	0.02	2.9	1.55	0.02	70.86	-0.15	8.99	8.01	17.07	7.97	-0.93	12.8	22.52	11.87	2
os06597	7	403	0.09	10	2	3	6.96	0.14	0.56	1.5	0.11	88.96	-4.75	8.24	8.33	17.38	8.26	-0.67	13.16	22.85	12.16	2.25
os06599	7	406	0.21	4	3	6	6.83	0.06	1.31	1.54	0.04	80.25	-0.73	8.44	8.32	17.37	8.24	-0.7	13.15	22.85	12.17	2.23

os06605	5	474	0.99	5	3	5	6.66	-0.08	0.59	1.52	0.04	93.38	-0.09	6.93	7.94	17.02	7.89	-1.02	12.77	22.47	11.81	1.9
os06649	5	543	0.2	3	3	6	6.93	0.05	0.54	1.51	0.02	90.37	1.2	7.91	7.71	16.78	7.68	-1.3	12.55	22.29	11.62	1.66
os06650	5	498	0.01	4	3	6	6.9	0.06	1.19	1.54	0.04	94.38	-0.6	8.34	7.85	16.91	7.8	-1.2	12.71	22.45	11.75	1.75
os06651	5	500	0.01	4	3	6	6.9	0.07	1.05	1.54	0.04	94.52	-0.64	8.29	7.84	16.91	7.79	-1.21	12.7	22.44	11.74	1.74
os06652	5	500	0.01	4	3	6	6.9	0.07	0.98	1.54	0.04	94.24	-0.55	8.2	7.84	16.91	7.79	-1.21	12.7	22.44	11.74	1.74
os06683	5	492	0.79	6	3	5	6.59	-0.09	0.48	1.52	0.05	95.61	-0.69	6.88	7.91	16.92	7.83	-1.19	12.78	22.45	11.76	1.76
os06691	5	485	0	5	3	6	6.93	0.09	0.97	1.53	0.05	95.61	-1.26	6.97	7.99	17	7.89	-1.13	12.86	22.53	11.84	1.8
os06735	5	444	0.99	5	3	6	6.65	-0.08	0.9	1.54	0.04	92.08	-0.58	7.57	8.23	17.3	8.1	-0.91	13.09	22.88	12.08	2.01
os06812	5	519	0.92	1	3	6	6.81	-0.01	2.99	1.55	0.03	37.27	-0.14	8.13	7.73	16.84	7.71	-1.18	12.51	22.26	11.57	1.76
os06823	5	509	0.04	2	3	6	6.9	0.04	1.57	1.54	0.03	65.92	-0.25	8.27	7.86	16.95	7.8	-1.13	12.68	22.45	11.7	1.77
os06869	5	477	0.92	1	3	6	6.78	-0.02	0.32	1.55	0.01	29.02	0.08	9.26	8.1	17.23	7.99	-0.98	12.94	22.81	12	1.91
os06877	5	474	0.77	2	3	6	6.82	0	1.38	1.55	0.01	51.16	0.22	8.41	8.12	17.24	8	-0.97	12.95	22.82	12	1.93
os06879	5	472	0.91	3	3	6	6.76	-0.04	1.02	1.55	0.02	65.32	0.17	8.32	8.12	17.24	8	-0.97	12.95	22.81	12.01	1.92
os06884	5	468	0.99	2	3	6	6.76	-0.03	1.51	1.55	0.02	73.18	-0.05	8.62	8.12	17.24	8.01	-0.97	12.95	22.82	12.01	1.93
os06891	5	470	0.4	2	3	6	6.85	0.01	2.03	1.55	0.02	76.75	-0.1	8.43	8.12	17.23	8	-0.98	12.94	22.8	12	1.92
os06931	5	449	0.53	3	3	6	6.84	0.01	2.05	1.55	0.02	49.82	-0.07	9.06	8.28	17.45	8.14	-0.85	13.16	23.05	12.14	2.12
os06932	5	451	0.99	2	3	6	6.76	-0.03	0.47	1.55	0.01	36.78	0.33	8.93	8.28	17.44	8.14	-0.86	13.16	23.05	12.14	2.12
os06945	5	454	0.99	2	3	6	6.75	-0.03	1.48	1.55	0.02	53.99	-0.25	8.78	8.24	17.42	8.11	-0.88	13.14	23.02	12.11	2.09
os07085	4	479	0.77	6	2	3	6.82	-0.02	0.58	1.54	0.08	93.29	-2.75	7.45	7.67	16.69	7.74	-1.23	12.65	22.37	11.9	1.75
os07112	4	375	0.77	5	2	7	6.74	-0.03	1.06	1.56	0.1	73.07	-3.86	7.86	8.29	17.3	8.18	-0.68	13.1	22.74	12.13	2.23
os07165	7	458	1	5	3	5	6.65	-0.08	0.77	1.53	0.04	98.39	-1.16	7.08	8.11	17.22	8.05	-0.81	12.9	22.66	11.98	2.13
os07167	7	466	0.96	5	3	5	6.69	-0.07	0.58	1.51	0.04	98.18	-0.03	6.83	8.1	17.21	8.04	-0.82	12.89	22.65	11.97	2.12
os07243	7	388	0.1	12	2	3	6.46	-0.13	0.95	1.54	0.16	93.42	-7.26	8.82	8.46	17.58	8.35	-0.61	13.23	23.03	12.2	2.31
os07422	5	431	0.23	1	3	6	6.79	0.01	0.55	1.54	0.02	56.54	0.26	9.42	8.28	17.34	8.15	-0.77	13.27	23.01	12.28	2.27
os07480	7	415	0.86	10	3	5	6.48	-0.1	0.31	1.52	0.07	86.63	-0.83	8.65	8.41	17.54	8.23	-0.66	13.26	23.08	12.16	2.31
os07490	7	456	0.92	5	3	5	6.62	-0.08	0.84	1.53	0.05	94.45	-1.3	8.13	8.19	17.33	8.05	-0.81	13.07	22.84	12.01	2.16
os07515	5	464	0.08	2	3	6	6.85	0.02	1.2	1.55	0.04	68.29	-0.21	8.42	8.28	17.44	8.13	-0.78	13.17	22.94	12.05	2.28
os07549	5	455	0.99	2	3	6	6.75	-0.03	3.25	1.55	0.02	48.24	-0.33	8.25	7.93	16.89	7.89	-1.02	12.88	22.48	12.05	2.05
os07593	5	387	0.17	5	3	6	6.84	0.07	1.46	1.53	0.05	86.68	-1.23	7.4	8.53	17.62	8.34	-0.6	13.59	23.38	12.48	2.49
os07601	3	453	0.06	13	1	1	6.87	0.18	0.02	1.38	0.08	96.64	4.94	6.53	8.35	17.46	8.18	-0.67	13.22	22.96	12.06	2.28
os07604	5	409	0.81	3	3	6	6.67	-0.04	1.17	1.55	0.03	79.27	0.19	8.81	8.38	17.42	8.22	-0.7	13.39	23.04	12.35	2.42
os07609	7	425	0.85	5	2	3	6.71	0	1.48	1.54	0.1	83.83	-4.59	7.03	8.4	17.45	8.22	-0.63	13.19	22.82	12.05	2.34
os07628	7	516	0.86	9	2	3	6.68	-0.08	0.36	1.5	0.11	99.85	-2.89	6.8	7.79	16.99	7.78	-1.05	12.67	22.48	11.71	1.97
os07654	5	468	0.22	5	2	0	6.6	-0.09	0.48	1.51	0.08	97.99	0.74	6.32	7.73	16.67	7.71	-1.14	12.83	22.31	11.95	2.01
os07718	7	381	0.78	5	2	3	6.59	-0.05	0.92	1.53	0.12	94.84	-5.35	7.8	8.64	17.79	8.41	-0.6	13.63	23.45	12.44	2.4
os07759	5	517	0.92	2	3	6	6.77	-0.02	2.96	1.54	0.04	79.67	-0.11	8.68	8.09	17.31	7.94	-0.94	12.96	22.79	11.86	2.14
os07763	5	467	0.35	4	3	6	6.9	0.04	0.49	1.54	0.06	82.42	-0.04	8.03	8.24	17.39	8.1	-0.8	13.11	22.89	12.01	2.27

os07835	5	426	0.99	4	3	6	6.72	-0.04	2.86	1.55	0.03	59.12	-0.35	9.36	8.27	17.35	8.13	-0.84	13.07	22.86	12.06	2.04
os07944	5	416	0.4	3	3	6	6.84	0.01	0.64	1.55	0.02	86.05	0.13	7.97	8.39	17.44	8.22	-0.73	13.17	22.94	12.12	2.15
os07949	5	460	0.04	3	3	6	6.87	0.05	2.05	1.55	0.03	58.29	-1.01	8.83	8.1	17.12	8	-1.01	12.87	22.67	11.86	1.86
os07951	5	459	0	3	3	6	6.86	0.04	2.23	1.55	0.03	59.67	-1.19	8.93	8.1	17.12	8	-1.01	12.87	22.67	11.86	1.86
os07978	7	487	0.98	5	2	3	6.69	-0.07	2.32	1.55	0.06	78	-2.54	9.63	7.97	17.05	7.88	-1.02	12.63	22.46	11.69	1.85
os08054	5	410	0.9	8	3	5	6.56	-0.12	0.17	1.51	0.07	97.56	-0.91	6.32	8.45	17.61	8.26	-0.7	13.37	23.23	12.28	2.28
os08062	5	400	1	5	3	6	6.62	-0.09	0.7	1.53	0.07	96.83	-1.84	7.97	8.52	17.67	8.33	-0.65	13.46	23.28	12.32	2.34
os08083	7	394	0.6	2	3	6	6.78	-0.01	5.53	1.56	0.06	78.74	-1.17	7.63	8.46	17.53	8.25	-0.66	13.34	23.13	12.19	2.33
os08086	5	454	0.2	5	3	6	6.91	0.05	2.01	1.55	0.04	63.72	-0.85	8.3	8.1	17.19	7.99	-0.93	12.87	22.69	11.88	2.02
os08105	5	522	0.01	5	3	5	6.93	0.09	0.3	1.52	0.06	97.93	0.2	6.28	7.72	16.75	7.65	-1.31	12.6	22.27	11.59	1.66
os08132	7	360	0.02	16	3	5	6.03	-0.24	0	1.48	0.14	71.49	-2.37	5.94	8.7	17.82	8.46	-0.56	13.7	23.52	12.5	2.44
os08152	6	412	0.4	2	3	6	6.84	0.02	4.56	1.56	0.03	68.08	-0.49	9.7	8.36	17.39	8.16	-0.8	13.31	23.1	12.24	2.17
os08175	5	460	0.6	4	3	6	6.65	-0.05	1.16	1.55	0.05	87.64	-1.58	8.6	8.14	17.22	8.02	-0.95	13.03	22.86	12.01	2
os08212	5	457	0	5	3	6	6.64	-0.07	1.46	1.55	0.05	86.01	-1.95	8.61	8.14	17.23	8.02	-0.95	13.04	22.86	12.02	2.01
os08213	5	451	0.92	3	2	3	6.7	-0.04	2.05	1.55	0.05	79.4	-2.81	8.73	8.16	17.24	8.04	-0.94	13.06	22.89	12.04	2.02
os08214	5	453	0.75	5	2	3	6.64	-0.07	1.6	1.55	0.05	81.42	-2.03	8.62	8.15	17.24	8.03	-0.94	13.05	22.88	12.03	2.01
os08215	5	456	0.65	5	3	5	6.61	-0.06	1.05	1.54	0.05	83.55	-1.45	8.55	8.15	17.23	8.03	-0.95	13.05	22.87	12.03	2.01
os08219	5	471	0.6	2	3	6	6.77	0	2.59	1.55	0.03	66.62	-0.73	8.66	8.08	17.15	7.96	-1	12.87	22.71	11.91	1.94
os08285	5	469	0.97	2	3	6	6.78	-0.02	2.84	1.55	0.03	80.27	-1.09	9.13	8.04	17.1	7.93	-1.02	12.81	22.63	11.85	1.9
os08308	5	485	0.69	4	3	6	6.74	-0.01	2.34	1.55	0.04	76.95	-1.24	8.61	8	17.13	7.94	-0.95	12.71	22.58	11.77	1.94
os08309	5	485	0.84	3	3	6	6.74	-0.02	2.31	1.55	0.04	76.57	-1.13	8.65	8	17.13	7.94	-0.96	12.71	22.57	11.77	1.94
os08310	5	486	0.61	4	3	6	6.73	-0.02	2.22	1.54	0.04	76.09	-0.9	8.73	8	17.13	7.94	-0.96	12.71	22.57	11.77	1.94
os08317	7	467	0	4	2	3	6.71	-0.06	1.98	1.55	0.08	93.41	-3.48	9.4	8.01	17.07	7.91	-1	12.67	22.49	11.72	1.88
os08319	7	470	0.3	3	2	3	6.74	-0.02	2.14	1.55	0.09	93.43	-3.93	9.46	8	17.07	7.9	-1	12.66	22.49	11.71	1.87
os08321	7	471	0.02	3	2	3	6.73	-0.04	1.98	1.55	0.09	93.47	-3.57	9.44	8	17.07	7.9	-1	12.66	22.48	11.71	1.87
os08323	7	474	0.8	3	2	3	6.76	-0.03	1.92	1.54	0.09	92.9	-4.01	9.45	7.99	17.07	7.9	-1	12.65	22.48	11.71	1.87
os08326	5	492	0.72	2	3	6	6.74	-0.03	2.87	1.55	0.03	80.54	-0.43	9.35	7.91	16.99	7.83	-1.05	12.56	22.39	11.64	1.83
os08336	5	501	0.19	2	3	6	6.74	-0.04	2.43	1.55	0.03	79.19	-0.55	8.76	7.88	16.98	7.82	-1.06	12.54	22.38	11.63	1.82
os08390	5	420	0.4	3	3	6	6.85	0.02	1.86	1.53	0.02	63.49	-0.53	8.62	8.46	17.53	8.27	-0.72	13.33	23.14	12.26	2.23
os08391	5	415	0.4	3	3	6	6.84	0.01	0.59	1.55	0.02	85.72	0.19	8	8.41	17.45	8.24	-0.72	13.2	22.95	12.14	2.17
os08422	5	465	0.99	2	3	6	6.75	-0.03	0.97	1.54	0.02	90.48	0.13	7.65	8.2	17.33	8.06	-0.84	13	22.84	11.95	2.1
os08453	5	394	0.31	3	3	6	6.74	0	2.09	1.51	0.04	84.04	-1.77	8.61	8.58	17.69	8.36	-0.61	13.43	23.27	12.32	2.39
os08454	5	395	0.41	2	3	6	6.75	-0.01	2.07	1.51	0.04	83.63	-1.44	8.6	8.58	17.69	8.36	-0.6	13.43	23.27	12.32	2.39
os08455	5	395	0.43	2	3	6	6.75	0	2.07	1.51	0.04	83.44	-1.34	8.61	8.58	17.69	8.36	-0.6	13.43	23.27	12.32	2.39
os08456	5	395	0.32	1	3	6	6.76	0	2.07	1.51	0.04	83.3	-1.31	8.62	8.58	17.69	8.36	-0.6	13.43	23.27	12.32	2.39
os08457	7	381	0.32	8	3	5	6.78	0.09	0.42	1.48	0.08	89.81	-1.03	8.63	8.58	17.69	8.36	-0.6	13.44	23.27	12.32	2.39
os08458	7	383	0.53	7	3	5	6.74	0.04	0.74	1.49	0.08	89.7	-0.73	8.45	8.58	17.69	8.36	-0.6	13.44	23.27	12.32	2.39

os08461	7	356	0.08	6	2	7	6.89	0.12	0.83	1.55	0.15	77.5	-6.35	7.19	8.6	17.71	8.37	-0.58	13.46	23.29	12.35	2.4
os08462	7	356	0.07	6	2	7	6.88	0.12	0.86	1.55	0.15	77.47	-6.56	7.18	8.6	17.71	8.37	-0.58	13.46	23.29	12.35	2.4
os08463	7	356	0.08	6	2	7	6.88	0.12	0.86	1.55	0.16	77.43	-6.77	7.16	8.6	17.71	8.37	-0.58	13.46	23.29	12.35	2.4
os08472	5	410	0.01	4	3	6	6.87	0.07	0.49	1.52	0.02	69.02	0.18	8.99	8.49	17.55	8.29	-0.7	13.31	23.13	12.24	2.25
os08539	7	478	0.87	7	3	5	6.71	-0.07	0.35	1.51	0.04	92.49	-0.26	6.73	8.03	17.06	7.92	-1.08	12.93	22.61	11.91	1.84
os08582	5	501	0.03	3	3	6	6.87	0.04	1.88	1.55	0.05	96.27	-1.52	8.96	7.84	16.88	7.79	-1.2	12.6	22.42	11.66	1.68
os08587	4	505	0.78	3	3	6	6.73	-0.05	1.46	1.54	0.03	74.79	-0.03	8.32	7.87	16.91	7.8	-1.15	12.65	22.44	11.71	1.74
os08593	5	470	0.01	2	3	6	6.86	0.03	1.55	1.56	0.02	59.39	-0.16	8.35	8.04	17.1	7.92	-1.03	12.87	22.64	11.86	1.87
os08619	5	451	0.03	4	3	6	6.65	-0.07	0.73	1.53	0.03	82.64	-0.41	7.64	8.26	17.33	8.08	-0.88	13.13	22.94	12.05	2.06
os08654	5	467	0.32	4	3	6	6.66	-0.07	0.44	1.54	0.04	96.44	-0.14	7.58	8.29	17.45	8.14	-0.79	13.25	23.05	12.18	2.32
os08656	5	380	0.91	19	1	4	6.12	-0.24	0.01	1.41	0.1	85.23	3.36	6.41	8.58	17.65	8.43	-0.48	13.43	23.14	12.34	2.52
os08659	5	417	0.98	7	2	3	6.6	-0.1	0.81	1.53	0.08	95.95	-2.7	6.97	8.4	17.51	8.25	-0.63	13.24	23.02	12.15	2.38
os08664	5	373	0.99	2	3	6	6.72	-0.03	1.47	1.56	0.04	46.94	0.01	9.27	8.58	17.62	8.41	-0.48	13.42	23.12	12.33	2.53
os08666	5	412	0.97	8	2	7	6.59	-0.09	1.33	1.54	0.13	93.77	-6.39	7.24	8.4	17.48	8.25	-0.62	13.21	22.98	12.16	2.41
os08669	5	409	0.97	9	2	7	6.54	-0.11	1.02	1.53	0.11	93.89	-3.56	7.53	8.41	17.49	8.26	-0.61	13.22	22.98	12.17	2.42
os08670	5	403	0.92	8	2	0	6.56	-0.11	0.52	1.52	0.1	92.32	-2.07	7.98	8.42	17.48	8.26	-0.6	13.22	22.96	12.18	2.43
os08671	5	402	0.98	9	2	0	6.57	-0.11	0.33	1.52	0.1	92.21	-1.31	8.08	8.42	17.48	8.26	-0.6	13.22	22.96	12.18	2.43
os08763	5	435	0.72	5	3	5	6.63	-0.04	0.56	1.54	0.05	86.22	-0.83	8.1	8.23	17.29	8.15	-0.7	13.03	22.77	12.07	2.33
os08774	6	523	0.01	2	3	6	6.86	0.02	2.79	1.55	0.02	15.95	-0.09	8.94	7.8	16.86	7.79	-0.94	12.47	22.23	11.58	2.07
os08984	7	389	0.02	10	2	7	6.49	0.06	0.61	1.54	0.26	88.79	-12.95	6.44	8.24	17.25	8.08	-0.92	13.18	22.86	12.1	2.01
os08999	7	495	0.68	1	3	6	6.82	0	2.93	1.56	0.07	38.34	0.19	10.36	7.04	15.85	7.25	-1.47	12.17	21.46	11.58	1.84
os09000	6	494	0.01	2	2	3	6.84	0.03	1.41	1.56	0.09	46.25	-2.48	7.9	7.05	15.85	7.25	-1.47	12.17	21.46	11.58	1.84
os09001	6	492	0.92	0	3	6	6.82	0	3.38	1.56	0.05	23.98	0.04	11.49	7.07	15.89	7.28	-1.46	12.2	21.5	11.6	1.85
os09003	7	494	0.92	0	2	0	6.83	0	3.33	1.57	0.07	35.35	-0.39	10.51	7.03	15.84	7.23	-1.48	12.16	21.44	11.57	1.83
os09004	6	493	0.08	1	3	6	6.83	0.01	3.85	1.56	0.06	33.35	-0.03	10.92	7.05	15.86	7.25	-1.48	12.17	21.46	11.58	1.84
os09025	5	497	0.23	6	3	5	6.81	0.05	0.69	1.53	0.05	70.65	-0.89	7.29	7.36	16.25	7.46	-1.33	12.35	21.86	11.66	1.76
os09032	7	414	0.92	0	3	6	6.8	0.01	3.97	1.56	0.03	24.69	0.11	11.06	7.87	16.83	7.86	-1.05	13.15	22.7	12.28	2.16
os09045	6	571	0.64	6	1	4	6.65	-0.06	0.31	1.49	0.06	95.11	2.26	6.75	6.7	15.54	7	-1.62	11.73	21.04	11.24	1.68
os09051	7	491	0.07	1	3	6	6.84	0.01	2.95	1.57	0.04	24.65	-0.32	8.94	7.08	15.9	7.29	-1.45	12.21	21.51	11.6	1.85
os09052	6	491	0.92	0	3	6	6.82	0	3.69	1.57	0.04	21.13	0.03	10.11	7.09	15.9	7.29	-1.44	12.21	21.52	11.6	1.85
os09097	6	523	0.21	6	3	6	6.93	0.04	0.18	1.53	0.05	80.15	1.31	8.2	6.95	15.82	7.18	-1.56	12.1	21.45	11.5	1.72
os09116	5	415	0.47	1	3	6	6.79	0	5.88	1.57	0.03	22.81	-0.56	9.05	7.87	16.85	7.84	-1.02	13.1	22.63	12.23	2.13
os09120	5	469	0.04	4	3	6	6.9	0.06	1.58	1.54	0.05	79.92	-0.46	7.85	7.59	16.53	7.65	-1.16	12.8	22.25	12.02	2.09
os09144	5	582	0.9	6	3	5	6.62	-0.09	0.39	1.5	0.04	94.49	0.64	6.54	6.8	15.7	7.09	-1.63	11.86	21.26	11.32	1.62
os09152	5	516	0.19	2	3	6	6.87	0.01	3.02	1.56	0.05	34.34	0.21	9.12	7.02	15.9	7.24	-1.53	12.14	21.55	11.55	1.74
os09168	5	654	0.23	2	3	6	6.9	0.02	1.27	1.55	0.01	54.51	0.07	8.03	6.48	15.38	6.83	-1.83	11.17	20.64	10.7	1.12
os09224	7	379	0.92	0	3	6	6.76	0	2.68	1.55	0.03	61.32	-1.26	8.87	7.99	16.82	8.21	-0.84	12.7	22.31	11.9	1.86

os09225	7	378	0.85	2	3	6	6.71	-0.02	1.94	1.54	0.02	55.4	-0.4	8.6	8	16.83	8.22	-0.83	12.71	22.33	11.91	1.87
os09226	7	372	0.07	2	3	6	6.7	-0.03	2.2	1.54	0.02	55.7	-0.28	8.2	8.03	16.86	8.25	-0.81	12.73	22.36	11.93	1.89
os09227	7	365	0.92	5	3	6	6.6	-0.07	1.77	1.54	0.04	75.63	-1.63	8.81	8.04	16.87	8.26	-0.8	12.75	22.38	11.95	1.91
os09229	7	364	0	2	3	5	6.72	0	2	1.54	0.06	88.03	-2.59	9.08	8	16.82	8.22	-0.85	12.68	22.3	11.89	1.85
os09231	7	371	0.1	4	2	3	6.77	0.03	1.76	1.54	0.05	84.3	-2.06	8.91	7.98	16.8	8.2	-0.87	12.66	22.28	11.87	1.83
os09232	7	378	0.97	5	2	3	6.63	-0.08	1.22	1.54	0.05	75.24	-1.25	8.7	7.95	16.77	8.18	-0.9	12.64	22.25	11.85	1.81
os09235	7	319	0.08	6	3	5	6.78	0.07	0.42	1.54	0.07	72.41	-2	7.75	8.33	17.16	8.47	-0.59	13.13	22.73	12.25	2.16
os09236	7	321	0.16	7	3	5	6.75	0.08	0.31	1.52	0.06	73.33	-1.42	7.52	8.33	17.16	8.47	-0.59	13.13	22.72	12.25	2.16
os09237	4	305	1	5	2	7	6.52	-0.08	1.44	1.56	0.11	88.2	-4.87	7.83	8.36	17.2	8.5	-0.58	13.17	22.77	12.29	2.21
os09238	4	306	1	7	2	7	6.53	-0.08	1.22	1.55	0.12	88.69	-5.27	7.63	8.35	17.19	8.5	-0.58	13.17	22.77	12.29	2.21
os09244	6	369	0.92	1	3	6	6.74	-0.02	3.63	1.56	0.04	72.82	-1.51	9.7	8.03	16.84	8.19	-0.83	12.73	22.37	11.9	1.86
os09247	7	349	0.85	6	2	3	6.61	-0.04	1.49	1.55	0.1	94.33	-4.82	8.3	8.1	16.91	8.25	-0.77	12.82	22.45	11.96	1.93
os09255	4	374	0.6	6	2	7	6.69	-0.03	2.81	1.57	0.14	86.12	-7.28	8.02	7.8	16.63	8.03	-1.06	12.47	22.1	11.64	1.6
os09261	5	475	0.83	4	3	6	6.66	-0.06	0.14	1.52	0.03	96.7	1.26	7.75	7.4	16.25	7.7	-1.37	12.03	21.72	11.29	1.31
os09268	5	493	0.85	4	3	6	6.66	-0.06	0.79	1.53	0.03	91.45	-0.23	7.68	7.36	16.21	7.66	-1.41	11.99	21.68	11.24	1.27
os09324	4	340	0.08	4	3	6	6.72	0.02	1.42	1.55	0.06	99.78	-0.95	7.1	8.19	17.03	8.43	-0.66	12.91	22.52	12.16	2.06
os09344	7	311	0.83	2	3	6	6.71	-0.03	2.71	1.55	0.02	34.84	-0.09	9.39	8.4	17.2	8.57	-0.5	13.19	22.77	12.36	2.24
os09392	5	312	0.93	1	3	6	6.71	-0.02	1.74	1.55	0.02	51.08	-0.19	8.51	8.3	17.05	8.53	-0.59	12.96	22.44	12.2	2.13
os09445	7	309	0.66	3	3	6	6.66	-0.04	2.57	1.55	0.02	42.96	-0.86	9.43	8.4	17.2	8.57	-0.5	13.19	22.77	12.36	2.24
os09481	5	403	0.01	4	3	6	6.87	0.07	0.52	1.51	0.03	78.17	0.68	8.87	7.94	16.82	8.14	-0.78	12.59	22.29	11.82	1.93
os09489	6	380	0.23	2	3	6	6.74	0	3.13	1.55	0.02	35	-0.18	9.79	8.18	17.09	8.34	-0.54	12.84	22.57	12.07	2.2
os09490	6	382	0.92	2	3	6	6.72	-0.02	2.94	1.55	0.02	30.74	-0.16	9.41	8.17	17.08	8.34	-0.54	12.83	22.56	12.06	2.19
os09514	5	384	0.03	1	3	6	6.82	0.03	2.97	1.55	0.02	21.96	-0.26	9.12	8.18	17.08	8.35	-0.52	12.76	22.54	12.05	2.21
os09577	7	371	0.12	6	2	3	6.79	0.09	1.12	1.54	0.14	87.22	-6.99	7.22	8.23	17.14	8.39	-0.49	12.95	22.69	12.19	2.27
os09586	5	284	0.92	1	3	6	6.73	0	3.79	1.56	0.02	25.2	-0.35	9.7	8.48	17.25	8.63	-0.48	13.24	22.7	12.39	2.24
os09589	3	265	1	13	3	5	6.23	-0.21	0.01	1.49	0.07	88.85	-0.29	6.04	8.65	17.42	8.77	-0.41	13.53	22.94	12.61	2.38
os09601	3	276	0.15	9	3	5	6.7	0.08	0.12	1.51	0.1	79.57	-1.55	6.89	8.61	17.42	8.69	-0.36	13.51	23.09	12.59	2.46
os09604	5	349	0.92	1	3	6	6.75	-0.01	2.74	1.57	0.02	38.77	-0.3	8.99	8.26	17.1	8.38	-0.59	13.1	22.69	12.19	2.19
os09614	6	331	0.79	2	3	6	6.68	-0.02	2.78	1.56	0.03	60.26	-1.06	8.14	8.38	17.21	8.49	-0.48	13.25	22.84	12.35	2.29
os09683	5	421	0.55	2	3	6	6.76	0	2.87	1.55	0.03	48.25	-0.95	8.16	8.28	17.29	8.24	-0.59	12.82	22.71	11.9	2.15
os09731	4	410	0.4	8	2	3	6.87	0.07	0.74	1.51	0.12	99.53	-5.87	7.4	8.11	17.04	8.13	-0.82	12.68	22.46	11.74	1.88
os09752	7	454	0.42	3	3	6	6.75	-0.01	1.88	1.54	0.03	84.98	-0.86	8.93	7.98	16.95	8.05	-0.85	12.49	22.32	11.6	1.81
os09926	7	462	0.86	2	2	0	6.73	-0.03	2.1	1.56	0.07	62	-0.01	8.44	7	15.73	7.3	-1.11	11.68	20.94	11.01	1.62
os09933	5	592	0.99	6	3	5	6.68	-0.09	0.47	1.5	0.07	99.57	-0.73	6.16	6.51	15.27	6.9	-1.39	11.1	20.36	10.58	1.38
os09952	5	501	1	10	2	3	6.43	-0.16	0.39	1.52	0.1	99.81	-3.81	7.34	6.92	15.68	7.23	-1.17	11.69	20.94	11.02	1.62
os09953	5	492	0.03	8	3	5	6.5	-0.13	0.22	1.52	0.07	99.56	-0.28	6.46	6.95	15.68	7.25	-1.16	11.67	20.92	11.01	1.63
os09965	5	501	0.4	3	3	6	6.89	0.02	1.44	1.54	0.06	76.55	-0.37	7.6	6.92	15.71	7.23	-1.19	11.74	21.01	11.07	1.65

os09985	5	567	0.6	9	3	5	6.86	-0.01	0.14	1.5	0.08	99.75	-1.04	7.36	6.51	15.3	6.9	-1.44	11.22	20.47	10.67	1.38
os10054	4	544	0.13	6	3	5	6.86	0.06	0.47	1.52	0.05	97.9	-0.51	6.91	6.83	15.52	7.12	-1.29	11.45	20.67	10.78	1.41
os10097	5	482	0.88	11	3	5	6.57	-0.12	0.04	1.51	0.06	86.24	-0.73	6.14	7.04	15.86	7.31	-1.12	11.98	21.28	11.23	1.75
os10135	5	514	0.99	2	3	6	6.77	-0.03	1.77	1.56	0	64.1	-0.11	7.95	6.91	15.61	7.15	-1.25	11.58	20.79	10.87	1.45
os10139	7	508	0.23	5	3	5	6.76	0.02	0.19	1.5	0	99.19	1.79	5.99	6.79	15.67	7.11	-1.48	11.93	21.24	11.3	1.67
os10186	5	516	0.77	9	3	5	6.71	-0.07	0.14	1.5	0.07	99.87	-1.15	6.65	6.76	15.65	7.1	-1.41	11.93	21.27	11.38	1.84
os10236	5	429	0.23	2	3	6	6.79	0.02	1.38	1.55	0	58.22	0.11	8.62	7.06	15.95	7.32	-1.33	12.28	21.63	11.51	1.76
os10301	5	614	0.92	1	2	7	6.79	-0.02	2.98	1.56	0	86.12	-2.68	7.47	6.25	14.9	6.63	-1.73	10.76	19.86	10.18	0.92
os10302	6	675	0.84	8	3	5	6.59	-0.11	0.25	1.51	0.05	94.65	0.28	6.51	5.88	14.57	6.37	-1.82	10.15	19.35	9.77	0.79
os10328	7	569	0.9	7	2	7	6.73	-0.07	0.47	1.54	0.09	85.27	-2.52	7.46	6.47	15.2	6.84	-1.54	11.02	20.29	10.46	1.11
os10340	5	543	0.5	5	3	6	6.85	-0.01	0.54	1.53	0.04	99.47	-0.11	7.05	6.72	15.51	7.05	-1.37	11.45	20.77	10.82	1.35
os10365	7	528	0.88	7	3	5	6.53	-0.12	0.32	1.51	0.05	73.64	0.01	6.64	6.76	15.53	7.05	-1.39	11.49	20.77	10.84	1.35
os10369	5	536	0.67	4	2	0	6.7	-0.05	1.33	1.54	0.06	74.11	-0.88	8.16	6.67	15.41	6.99	-1.43	11.31	20.57	10.68	1.24
os10371	5	561	0.92	5	2	0	6.67	-0.08	0.56	1.53	0.07	95.95	-0.38	6.99	6.5	15.25	6.86	-1.52	11.08	20.34	10.51	1.14
os10372	5	584	0.79	4	3	6	6.69	-0.07	0.59	1.53	0.05	95.39	-0.39	7.34	6.43	15.19	6.82	-1.56	11.02	20.29	10.48	1.13
os10382	4	671	0.34	11	3	5	6.39	-0.19	0.01	1.47	0.08	99.57	0.86	5.88	6.17	14.95	6.61	-1.71	10.73	20.02	10.28	1.03
os10387	5	554	0.25	4	3	6	6.93	0.03	0.99	1.54	0.03	93.31	-0.47	7.66	6.71	15.51	7.04	-1.4	11.59	20.92	10.96	1.45
os10389	5	593	0.99	8	2	0	6.63	-0.12	0.3	1.51	0.07	99.98	-1.04	6.87	6.41	15.2	6.81	-1.56	11.08	20.38	10.55	1.19
os10390	5	616	0.74	7	3	5	6.83	-0.04	0.36	1.5	0.06	99.98	0.43	6.74	6.35	15.15	6.77	-1.6	10.98	20.29	10.48	1.17
os10394	6	577	0.77	6	3	5	6.8	-0.05	0.27	1.52	0.04	97.01	0.61	7.48	6.51	15.31	6.9	-1.48	11.21	20.52	10.67	1.3
os10403	5	562	0.77	10	3	5	6.72	-0.08	0.09	1.5	0.08	99.05	-0.58	6.01	6.61	15.37	6.96	-1.4	11.21	20.53	10.69	1.38
os10419	5	581	0.04	8	3	5	6.93	0.12	0.1	1.5	0.05	97.93	-0.13	5.78	6.66	15.4	6.99	-1.39	11.21	20.5	10.66	1.32
os10424	5	600	0.68	3	3	6	6.77	-0.03	1.22	1.53	0.06	99.07	0.25	7.57	6.41	15.17	6.81	-1.51	10.88	20.19	10.4	1.19
os10469	5	662	0.95	5	3	6	6.83	-0.05	0.57	1.52	0.06	94.34	-0.3	7.22	6.05	14.83	6.53	-1.73	10.48	19.79	10.1	0.98
os10493	5	573	0.08	5	3	5	7	0.09	0.5	1.51	0.05	93.12	-0.07	7.04	6.52	15.22	6.85	-1.53	11.21	20.41	10.61	1.22
os10542	5	519	0.7	2	3	6	6.84	0	4.92	1.55	0.02	45.53	0.21	8.7	6.91	15.61	7.14	-1.29	11.65	20.88	10.94	1.42
os10593	5	567	0.6	5	2	0	6.71	-0.03	0.55	1.53	0.07	96.03	-0.31	8.3	6.5	15.23	6.83	-1.56	11.22	20.47	10.62	1.18
os10616	5	515	0.4	1	3	6	6.84	0	4.8	1.55	0.02	40.76	0.35	8.74	6.87	15.57	7.09	-1.33	11.64	20.88	10.94	1.4
os10636	8	560	0.01	8	3	5	6.98	0.13	0.18	1.5	0.07	88.36	-0.89	5.94	6.61	15.31	6.92	-1.5	11.34	20.53	10.7	1.26
os10713	5	595	0.97	6	3	5	6.64	-0.1	0.47	1.52	0.05	98.11	0.37	6.51	6.37	15.13	6.76	-1.59	10.92	20.2	10.4	1.09
os10716	4	661	0.92	8	3	5	6.54	-0.13	0.08	1.49	0.07	99.94	0.46	5.89	6.18	14.96	6.61	-1.7	10.73	20.02	10.28	1.03
os10717	3	683	0.6	8	1	4	6.65	-0.04	0.03	1.46	0.06	97.61	2.76	6.03	6.14	14.92	6.58	-1.72	10.68	19.97	10.24	1.01
os10720	5	537	0.76	5	2	0	6.68	-0.05	0.58	1.53	0.05	75.03	-0.24	8.02	6.67	15.41	6.99	-1.43	11.31	20.57	10.68	1.24
os10721	3	583	0.39	9	3	5	6.68	0.01	0.13	1.5	0.06	98.32	-0.48	6.41	6.56	15.34	6.91	-1.49	11.23	20.51	10.65	1.23
os10722	3	592	0.36	3	3	9	6.93	0.03	0.29	1.5	0.02	97.37	1.49	6.71	6.55	15.33	6.9	-1.5	11.23	20.51	10.64	1.23
os10774	5	616	0.77	7	3	5	6.77	-0.07	0.29	1.51	0.06	99.94	-0.41	6.83	6.37	15.17	6.79	-1.58	11.01	20.31	10.51	1.19
os10801	5	514	0.77	2	3	6	6.81	-0.02	3.14	1.56	0.04	65.68	-0.24	8.43	6.89	15.59	7.11	-1.3	11.64	20.87	10.93	1.41

os10808	5	517	0.92	1	3	6	6.83	-0.01	4.93	1.55	0.01	42.73	0.11	8.8	6.91	15.61	7.14	-1.29	11.65	20.88	10.94	1.42
os10826	5	503	0.92	1	3	6	6.85	0.02	0.7	1.56	0.01	31.96	0.31	8.45	6.93	15.61	7.14	-1.25	11.72	20.92	10.99	1.5
os10859	5	601	0.08	7	2	0	7.03	0.12	0.36	1.52	0.07	99.76	-0.7	6.85	6.28	15.02	6.67	-1.68	10.91	20.16	10.38	1.04
os10860	5	601	0.45	6	3	5	6.75	-0.01	0.64	1.53	0.07	99.94	-0.93	7.13	6.23	14.98	6.63	-1.7	10.83	20.09	10.33	1.02
os10889	5	661	0.01	5	3	5	6.98	0.08	0.23	1.51	0.04	99.62	0.14	6.53	6.15	14.92	6.58	-1.73	10.68	19.96	10.2	0.96
os10919	7	589	0.12	5	3	5	7	0.08	0.61	1.54	0.05	93.82	-0.69	8.67	6.31	14.98	6.72	-1.56	10.71	19.97	10.26	1.05
os10923	6	559	0	6	2	0	6.98	0.1	0.53	1.54	0.08	85.55	-0.97	8.22	6.34	15.01	6.74	-1.53	10.77	20	10.28	1.06
os10932	7	604	0.95	7	2	0	6.68	-0.1	0.27	1.52	0.07	90.39	0.03	6.67	6.09	14.75	6.55	-1.7	10.41	19.63	9.98	0.88
os10975	7	522	0.4	3	2	7	6.87	0.03	2.42	1.56	0.09	87.65	-3.24	8.53	6.47	15.16	6.83	-1.5	11.01	20.32	10.56	1.18
os10977	7	593	1	5	3	6	6.71	-0.07	1.88	1.53	0.03	51.9	-0.35	8.72	6.42	15.14	6.79	-1.54	11.03	20.34	10.58	1.2
os11025	4	546	0.99	9	3	9	6.58	-0.13	0.12	1.44	0.06	95.21	1.18	6.04	6.74	15.46	7.04	-1.35	11.5	20.75	10.84	1.37
os11049	8	678	0.27	4	3	6	6.86	0.02	1.07	1.52	0.05	99.72	-0.47	7.45	5.93	14.68	6.4	-1.82	10.44	19.67	10.01	0.87
os11086	5	606	0.04	4	3	6	6.92	0.06	1.03	1.53	0.03	77.71	-0.22	7.66	6.32	14.99	6.74	-1.6	10.86	20.13	10.34	1
os11090	6	605	0.04	3	3	6	6.88	0.03	1.92	1.53	0.05	90.56	-1.27	8.13	6.17	14.88	6.6	-1.76	10.83	20.16	10.33	0.93
os11121	5	662	0.19	8	3	5	7.05	0.09	0.29	1.5	0.05	99.96	-0.5	6.2	6.05	14.78	6.51	-1.71	10.51	19.8	10.17	0.98
os11172	5	598	0.15	5	3	6	7	0.08	0.74	1.53	0.06	95.65	-1.3	7.69	6.29	14.96	6.7	-1.57	10.69	19.94	10.24	1.04
os11195	5	622	0.58	5	3	5	6.76	-0.02	0.49	1.52	0.04	95.05	-0.07	6.56	6.31	15.09	6.73	-1.54	10.91	20.15	10.43	1.25
os11196	5	616	0.99	2	3	6	6.84	-0.03	0.24	1.52	0.03	95.55	0.32	7.81	6.39	15.17	6.8	-1.53	11.06	20.28	10.52	1.28
os11230	5	533	0.51	6	3	5	6.9	0.02	0.62	1.52	0.07	99.99	-1.6	8.11	6.75	15.53	7.1	-1.31	11.42	20.69	10.79	1.46
os11247	4	605	0.35	6	2	0	6.76	0.01	0.48	1.51	0.09	95.06	0.06	7.21	6.28	15.06	6.74	-1.54	10.69	19.98	10.26	1.19
os11260	5	582	0.45	6	3	5	6.73	0	0.55	1.5	0.07	99.74	-0.4	7.62	6.52	15.33	6.91	-1.42	11.18	20.45	10.59	1.37
os11319	5	583	0.13	5	2	0	6.89	0.06	0.55	1.53	0	98.26	-0.32	6.69	6.52	15.28	6.89	-1.48	11.1	20.35	10.55	1.31
os11345	7	506	0.08	2	2	0	6.85	0.01	3.52	1.56	0	42.2	-0.2	7.82	6.88	15.62	7.17	-1.29	11.64	20.85	10.96	1.52
os11353	6	528	0.23	3	3	6	6.82	0.02	1.47	1.55	0.06	74.98	-0.34	7.87	6.71	15.46	7.03	-1.35	11.34	20.57	10.73	1.42
os11387	7	614	0.39	7	2	0	6.75	0.01	0.36	1.51	0	96.25	-0.16	7.17	6.3	15.07	6.71	-1.58	10.89	20.14	10.42	1.24
os11395	7	639	0.92	0	2	0	6.85	-0.01	2.75	1.56	0	91.49	-1.63	8.6	6.02	14.8	6.54	-1.68	10.42	19.69	10.1	1.08
os11473	5	443	0.37	13	3	5	6.56	0.03	0.01	1.49	0	65.93	-1.69	6.87	7.05	15.91	7.34	-1.28	12.16	21.54	11.46	1.77
os11500	5	590	0.76	7	3	5	6.82	-0.04	0.68	1.52	0.06	97.79	-1.6	7.47	6.51	15.27	6.86	-1.47	11.18	20.44	10.61	1.35
os11543	5	475	0.83	4	2	0	6.64	-0.07	0.56	1.54	0.07	86.1	0.15	7.34	6.94	15.73	7.23	-1.21	11.77	21.05	11.06	1.62
os11549	5	569	0.31	5	3	5	6.96	0.05	0.67	1.51	0.05	98.29	-0.89	6.78	6.64	15.45	6.99	-1.34	11.4	20.66	10.74	1.49
os11550	4	485	0.59	8	3	5	6.82	-0.02	0.25	1.52	0.06	99.87	-0.49	6.61	7.04	15.87	7.31	-1.13	11.98	21.26	11.19	1.77
os11551	5	495	0.01	6	3	5	6.94	0.1	0.48	1.52	0.08	97.05	-0.37	7.55	6.85	15.68	7.16	-1.22	11.69	20.94	10.96	1.61
os11552	5	463	0.71	2	3	6	6.76	-0.01	3.09	1.55	0.03	53.86	0.5	8.74	6.95	15.83	7.28	-1.1	11.94	21.33	11.28	1.85
os11560	5	473	0.04	4	3	6	6.86	0.06	0.58	1.53	0	99.41	-0.18	7.58	6.88	15.77	7.17	-1.44	12.06	21.41	11.38	1.68
os11610	7	550	0.4	8	2	3	6.95	0.05	0.5	1.52	0.09	99.99	-2.27	6.68	6.67	15.47	7.02	-1.37	11.42	20.73	10.84	1.47
os11613	7	504	0	4	2	0	6.67	-0.08	0.49	1.53	0.07	93.4	0.8	8.54	6.83	15.59	7.12	-1.3	11.6	20.85	10.92	1.52
os11637	4	575	0.6	9	1	1	6.53	-0.08	0.08	1.46	0.05	99.02	2.48	6.07	6.57	15.49	7	-1.39	11.51	20.91	10.94	1.63

os11682	5	536	0.98	2	3	6	6.81	-0.02	1.56	1.54	0.03	83.28	-0.09	7.56	6.81	15.58	7.11	-1.3	11.63	20.89	10.97	1.54
os11683	5	536	0.95	2	3	6	6.81	-0.02	1.54	1.54	0.03	83.92	-0.11	7.54	6.81	15.58	7.11	-1.3	11.63	20.89	10.97	1.54
os11684	5	535	0.77	2	3	6	6.82	-0.02	1.99	1.54	0.03	81.79	0	7.74	6.82	15.59	7.12	-1.29	11.64	20.9	10.98	1.55
os11692	5	506	0.72	9	3	5	6.74	-0.06	0.15	1.5	0.07	99.21	-0.37	7.79	6.83	15.61	7.16	-1.26	11.54	20.81	10.89	1.51
os11694	5	528	0.99	6	2	0	6.63	-0.1	0.53	1.52	0.07	97.39	-0.66	8.18	6.66	15.46	7.02	-1.34	11.34	20.61	10.71	1.44
os11696	5	534	0.61	9	2	0	6.55	-0.07	0.19	1.5	0.07	97.04	0.57	8.14	6.65	15.45	7.02	-1.35	11.3	20.57	10.68	1.43
os11719	7	648	0.35	6	1	2	6.78	0	0.04	1.47	0	89.29	4.26	7.27	6.05	14.83	6.56	-1.66	10.44	19.72	10.12	1.1
os11721	7	640	0.05	8	2	0	6.9	0.09	0.3	1.52	0	90.55	-1.7	8.21	6.03	14.81	6.55	-1.67	10.42	19.7	10.1	1.09
os11727	7	548	0.88	10	2	0	6.65	-0.11	0.06	1.51	0.09	99.98	-0.68	6.78	6.46	15.27	6.85	-1.43	11.05	20.35	10.52	1.39
os11728	7	548	0.89	9	2	0	6.65	-0.11	0.07	1.51	0.09	99.98	-0.71	7.05	6.46	15.27	6.85	-1.43	11.05	20.35	10.52	1.39
os11731	7	529	0.87	8	2	0	6.7	-0.08	0.39	1.53	0.09	99.85	-1.16	7.53	6.56	15.36	6.92	-1.39	11.19	20.49	10.61	1.44
os11732	7	522	0.93	7	2	0	6.68	-0.09	0.76	1.54	0.09	99.76	-1.86	7.87	6.59	15.4	6.96	-1.37	11.24	20.55	10.66	1.46
os11740	5	449	0.8	5	3	6	6.65	-0.04	0.54	1.53	0.03	93.23	-0.29	6.67	7.18	16.03	7.43	-1.02	12.25	21.6	11.45	1.93
os11741	7	663	0.63	7	3	5	6.9	-0.02	0.32	1.48	0.07	100	-0.16	6.29	6.06	14.92	6.58	-1.62	10.51	19.83	10.17	1.2
os11751	7	556	0.92	9	3	5	6.49	-0.14	0.24	1.49	0.08	94.86	-0.69	6.59	6.64	15.46	6.99	-1.35	11.39	20.66	10.75	1.49
os11757	5	578	0.23	5	2	0	6.8	0.03	0.79	1.53	0.09	99.77	-1.45	8.71	6.36	15.13	6.77	-1.53	10.92	20.18	10.45	1.27
os11865	7	514	0.93	8	3	5	6.64	-0.1	0.16	1.52	0.08	78.2	-0.77	8.07	6.83	15.52	7.12	-1.35	11.67	20.95	10.86	1.34
os11932	4	578	0.14	15	1	4	6.77	0.15	0	1.44	0.07	99.89	1.88	5.43	6.94	15.87	7.14	-1.61	11.87	21.37	11.2	1.43
os11933	5	554	0.19	5	3	6	6.86	0.05	0.54	1.53	0.07	99.83	-1.29	6.71	6.97	15.9	7.16	-1.58	11.91	21.39	11.22	1.45
os11947	5	466	1	6	2	0	6.62	-0.09	0.46	1.54	0.08	79.93	-0.76	7.94	7.33	16.22	7.45	-1.32	12.3	21.75	11.54	1.71
os11987	5	609	0.77	5	2	0	6.71	-0.08	0.53	1.53	0.09	99.95	-0.23	7.29	7.07	16	7.35	-1.31	11.92	21.39	11.46	1.92
os11992	5	521	0.92	0	3	6	6.82	0	2.66	1.54	0.04	67.78	-0.36	8.05	7.74	16.66	7.86	-0.96	12.74	22.28	12.14	2.31
os11996	5	692	0.99	5	3	5	6.76	-0.08	0.31	1.49	0.05	99.81	0.15	6.32	6.76	15.75	7.13	-1.49	11.56	21.12	11.24	1.75
os12004	6	662	0.38	6	2	3	6.97	0.04	0.87	1.52	0.1	100	-2.41	7.99	6.76	15.65	7.11	-1.43	11.51	20.91	11.13	1.74
os12031	5	630	0	7	2	7	6.99	0.12	0.66	1.52	0.13	99.44	-2.08	7.17	6.8	15.66	7.17	-1.33	11.45	20.87	11.16	1.87
os12049	7	497	0.33	9	3	9	6.69	0.03	0.14	1.47	0.06	91.37	0.35	7.18	7.92	16.78	8.01	-0.85	13.02	22.49	12.39	2.44
os12102	7	612	0.33	11	2	3	6.73	0.05	0.25	1.51	0.12	99.8	-4.02	7.47	7.04	15.89	7.31	-1.49	12.09	21.43	11.6	1.88
os12111	4	628	0.43	16	1	1	6.73	0	0	1.29	0.06	97.86	13.26	5.61	7.36	16.21	7.51	-1.29	12.54	21.85	11.88	2.11
os12394	5	589	0.82	2	3	6	6.79	-0.03	2.85	1.55	0.02	62.32	-0.3	8.74	7.05	15.89	7.32	-1.55	12.32	21.69	11.91	2.02
os12430	7	612	0.53	14	2	7	6.36	-0.07	0	1.48	0.13	99.59	-3.59	8.23	6.99	15.9	7.28	-1.37	11.75	21.24	11.34	1.8
os12502	5	604	0	7	3	5	7.01	0.12	0.45	1.52	0.07	99.94	-0.87	6.67	7.06	15.89	7.34	-1.25	11.82	21.2	11.42	1.95
os12506	5	614	0.47	5	2	0	6.88	-0.01	0.75	1.54	0.1	99.8	-1.39	7.56	6.94	15.87	7.24	-1.36	11.73	21.19	11.3	1.84
os12507	5	615	0.77	6	2	0	6.81	-0.05	0.59	1.53	0.1	99.75	-0.86	7.17	6.95	15.88	7.25	-1.36	11.74	21.2	11.31	1.84
os12508	5	612	0.77	5	2	0	6.83	-0.04	0.56	1.53	0.09	99.49	0.2	6.88	6.97	15.9	7.27	-1.34	11.77	21.23	11.34	1.85
os12509	5	608	0.77	7	2	0	6.82	-0.04	0.37	1.52	0.09	99.12	-0.14	6.9	6.99	15.92	7.28	-1.33	11.79	21.26	11.35	1.86
os12537	7	521	1	6	3	5	6.6	-0.11	0.58	1.52	0.05	99.2	-0.81	7.4	7.61	16.52	7.78	-1.01	12.58	22.09	12.04	2.23
os12558	4	442	0.89	11	2	7	6.44	-0.12	0.34	1.53	0.2	85.59	-5.83	6.82	7.87	16.74	7.97	-0.86	12.91	22.39	12.32	2.4

os12559	4	443	0.87	12	2	7	6.38	-0.14	0.34	1.52	0.19	85.41	-5.97	6.84	7.88	16.74	7.98	-0.86	12.91	22.39	12.32	2.4
os12591	5	537	0.7	3	3	6	6.76	-0.03	1.29	1.53	0.03	95.28	0.14	7.22	7.61	16.5	7.77	-1.06	12.71	22.21	12.18	2.28
os12593	5	544	0.6	3	3	6	6.75	-0.02	0.97	1.53	0.04	99.51	-0.01	7.16	7.58	16.47	7.74	-1.08	12.69	22.19	12.15	2.27
os12630	3	518	0.73	7	2	3	6.61	-0.06	0.86	1.5	0.07	88.61	-2.42	7.92	7.69	16.54	7.84	-1	12.79	22.27	12.23	2.33
os12631	3	516	0.78	8	2	3	6.59	-0.05	0.8	1.49	0.08	88.62	-2.7	7.94	7.69	16.54	7.85	-1	12.79	22.27	12.23	2.33
os12632	3	514	0.69	9	2	3	6.55	-0.06	0.64	1.49	0.09	88.42	-3	7.96	7.7	16.55	7.85	-1	12.8	22.28	12.24	2.34
os12634	7	579	0.87	12	3	9	6.64	-0.11	0.01	1.47	0.07	99.95	0.36	5.65	7.49	16.41	7.68	-1.15	12.62	22.13	12.09	2.21
os12635	7	563	0.92	11	3	5	6.62	-0.12	0.03	1.48	0.07	99.87	-1.3	6.27	7.51	16.43	7.69	-1.14	12.65	22.16	12.11	2.22
os12642	5	558	0.03	7	3	5	6.9	0.09	0.54	1.51	0.06	99.74	-0.72	6.86	7.5	16.39	7.68	-1.13	12.59	22.11	12.08	2.23
os12664	6	561	0.92	0	3	6	6.85	0	2.39	1.55	0.01	5.35	0.07	10.47	7.47	16.33	7.64	-1.26	12.67	22.14	12.15	2.13
os12668	7	510	0.26	4	2	3	6.83	0.07	1.21	1.52	0.12	94.92	-5.5	7.73	7.67	16.53	7.82	-1.04	12.8	22.27	12.25	2.27
os12669	7	506	0.29	19	2	3	6.84	0.18	0.11	1.48	0.18	95.74	-8.18	6.55	7.68	16.53	7.83	-1.03	12.81	22.28	12.25	2.28
os12682	7	606	0.01	5	2	0	6.95	0.09	0.69	1.53	0.12	99.96	-1.25	8.45	6.91	15.77	7.26	-1.27	11.59	21.01	11.25	1.93
os12683	5	605	0	5	2	0	6.96	0.09	0.67	1.53	0.12	99.95	-0.79	8.11	6.93	15.78	7.27	-1.27	11.61	21.03	11.26	1.94
os12686	5	605	0.01	5	2	0	6.93	0.09	0.77	1.53	0.12	99.94	-0.85	7.67	6.94	15.79	7.28	-1.26	11.63	21.04	11.27	1.94
os12707	5	531	1	6	3	5	6.63	-0.1	0.22	1.52	0.06	85.39	0.74	7.71	7.45	16.29	7.62	-1.27	12.65	22.05	12.11	2.09
os12711	7	552	0.4	2	3	6	6.81	0	2.05	1.54	0.03	80.95	-0.28	8.86	7.47	16.31	7.64	-1.19	12.62	22.04	12.07	2.14
os12791	7	625	0.79	16	2	0	6.54	-0.14	0	1.46	0.13	99.97	-1.45	6.39	6.91	15.77	7.2	-1.55	11.95	21.25	11.47	1.82
os12795	7	634	0.43	6	3	5	6.76	0	0.52	1.51	0.07	100	-0.59	6.96	7.02	15.9	7.27	-1.46	12.1	21.47	11.61	1.93
os12798	5	642	0.45	13	3	5	6.54	-0.02	0	1.46	0.07	99.77	0.76	5.83	7.02	15.89	7.28	-1.47	12.1	21.45	11.59	1.92
os12809	5	653	0.08	5	3	6	7.02	0.09	0.66	1.52	0.09	99.88	-0.8	6.54	6.88	15.76	7.18	-1.41	11.66	21.11	11.26	1.79
os12828	5	706	0.82	7	3	5	6.63	-0.12	0.33	1.5	0.06	100	-0.3	6.76	6.64	15.57	7.02	-1.5	11.38	20.84	11.08	1.7
os12836	5	607	0.81	5	2	0	6.66	-0.1	0.5	1.53	0.1	97.06	-0.28	6.87	6.93	15.82	7.24	-1.38	11.72	21.17	11.32	1.83
os12909	5	531	0.92	1	3	6	6.79	-0.02	2.89	1.55	0.03	58.42	-0.97	9.1	7.65	16.51	7.79	-1.12	12.85	22.37	12.26	2.28
os12912	5	461	0.77	3	3	6	6.8	-0.02	2.7	1.56	0.05	83.75	-1.52	9.71	8.01	16.98	8.03	-0.82	13	22.54	12.23	2.34
os12913	5	460	0.59	3	3	6	6.81	-0.01	2.9	1.57	0.05	82.37	-1.78	9.33	8.02	16.99	8.03	-0.81	13.01	22.55	12.24	2.34
os12920	5	516	0.63	4	3	6	6.7	-0.05	1.22	1.54	0.05	89.72	-1.1	6.97	7.74	16.72	7.82	-0.93	12.63	22.24	12.03	2.25
os12923	5	561	0.99	2	3	6	6.8	-0.03	1.48	1.55	0.01	6.04	0.12	8.78	7.45	16.3	7.62	-1.29	12.66	22.12	12.13	2.11
os12951	7	697	0.35	10	2	7	6.77	0.05	0.4	1.52	0.11	94.55	-2.21	6.88	6.23	15.06	6.72	-2.14	11.26	20.69	11.19	1.4
os12962	5	702	0.23	2	3	6	6.92	0.02	2.37	1.55	0.03	52.44	-0.32	8.22	6.37	15.2	6.83	-2.07	11.41	20.85	11.34	1.45
os13005	5	666	0.77	2	3	6	6.88	-0.02	1.94	1.54	0.03	68.2	-0.64	8.09	6.62	15.42	6.95	-1.96	11.88	21.22	11.57	1.68
os13056	7	497	0.54	11	2	3	6.89	0.04	0.32	1.49	0.1	76.02	-2.63	7.73	7.73	16.6	7.86	-1.04	12.9	22.38	12.3	2.32
os13057	7	509	0.8	8	3	5	6.76	-0.05	0.72	1.49	0.08	83.12	-2.18	8.35	7.69	16.56	7.83	-1.07	12.86	22.34	12.28	2.3
os13112	5	633	0.89	3	3	6	6.79	-0.03	0.98	1.54	0.03	94.39	0.18	7.13	6.89	15.76	7.23	-1.57	11.9	21.34	11.55	1.82
os13259	5	559	0.92	3	3	6	6.75	-0.04	1.06	1.55	0.02	50.49	0.07	8.85	7.07	15.93	7.25	-1.48	12.09	21.6	11.54	1.9
os13272	5	533	0.92	1	3	6	6.79	-0.02	1.57	1.55	0.02	43.35	-0.14	7.65	7.37	16.27	7.48	-1.35	12.49	22.04	11.89	2.08
os13278	7	541	0.76	2	3	6	6.8	-0.02	2.92	1.56	0.03	39.2	-1.05	9.03	7.22	16.09	7.37	-1.42	12.29	21.81	11.72	1.99

os13296	7	527	0.98	12	1	4	6.43	-0.18	0	1.44	0.06	92.89	1.98	6.06	7.52	16.36	7.63	-1.2	12.64	22.11	12.08	2.19
os13320	5	614	0.92	1	3	6	6.9	0.02	3.86	1.56	0.04	33.11	-0.69	8.39	6.86	15.63	7.16	-1.88	12.13	21.45	11.78	1.69
os13406	7	446	0.48	4	3	6	6.76	0.01	0.59	1.53	0.04	78.99	0.82	8.38	7.87	16.7	7.9	-0.99	12.95	22.44	12.27	2.36
os13428	5	479	0.63	5	2	0	6.62	-0.07	0.76	1.53	0.08	96.74	-0.82	7.38	7.65	16.48	7.75	-1.09	12.75	22.25	12.13	2.29
os13433	5	449	0.6	5	3	6	6.8	-0.02	1.63	1.55	0.07	74.3	-0.95	8.16	7.78	16.61	7.81	-1.04	12.89	22.38	12.23	2.35
os13463	7	641	0.6	6	2	3	6.87	-0.02	0.99	1.54	0.08	90.9	-3.74	8.11	6.64	15.44	6.96	-1.93	11.86	21.23	11.57	1.69
os13492	7	617	0.15	6	2	7	6.96	0.08	1.25	1.55	0.12	96.9	-6.19	8.14	6.84	15.68	7.18	-1.57	11.78	21.19	11.46	1.8
os13498	5	593	0.6	7	2	0	6.66	-0.05	0.34	1.52	0.08	92.58	-0.96	8.21	6.85	15.71	7.12	-1.7	12.08	21.47	11.67	1.87
os13566	5	613	0.92	0	3	6	6.88	0.01	2.51	1.54	0.02	73.13	-0.5	9.17	7.08	15.97	7.35	-1.47	12.17	21.62	11.79	1.96
os13577	7	557	1	6	2	0	6.62	-0.1	0.62	1.53	0.08	95.26	-1.61	8.26	7.25	16.1	7.48	-1.39	12.37	21.83	11.93	2.05
os13604	5	624	0	2	3	6	6.91	0.03	1.63	1.54	0.02	38.73	-0.16	8.95	7	15.88	7.3	-1.51	12.05	21.5	11.71	1.91
os13609	7	641	0.98	6	2	0	6.73	-0.08	0.37	1.52	0.08	99.63	-0.59	7.19	6.7	15.54	7.06	-1.64	11.59	21	11.32	1.73
os13612	5	644	0.49	5	3	5	6.93	0.01	0.95	1.53	0.06	97.61	-1.78	7.01	6.77	15.6	7.12	-1.61	11.74	21.12	11.43	1.79
os13618	5	513	0.81	4	3	6	6.71	-0.06	0.31	1.54	0.06	84.56	0.53	7.95	7.36	16.2	7.53	-1.3	12.46	21.96	11.97	2.1
os13622	5	590	0.6	4	3	6	6.72	-0.04	0.7	1.54	0.04	83.28	-0.65	7.81	7.06	15.94	7.29	-1.46	12.13	21.62	11.71	1.95
os13630	5	598	0.88	7	3	5	6.6	-0.1	0.36	1.52	0.05	95.66	-0.83	7.33	7.08	15.94	7.32	-1.45	12.18	21.64	11.76	2
os13655	5	575	0.82	6	3	5	6.61	-0.08	0.36	1.51	0.05	99.57	0.16	7.05	7.23	16.11	7.47	-1.4	12.38	21.83	11.93	2.06
os13656	5	572	0.92	5	3	5	6.66	-0.07	0.49	1.52	0.04	99.44	-0.08	7.34	7.24	16.11	7.48	-1.39	12.38	21.83	11.93	2.06
os13662	5	599	0.23	4	3	6	6.86	0.02	1.23	1.52	0.03	76.29	-0.47	7.97	7.13	16.01	7.4	-1.46	12.28	21.73	11.86	2.01
os13666	5	584	0.28	6	3	5	6.79	0.03	0.38	1.49	0.04	91.3	1.38	6.74	7.18	16.03	7.44	-1.45	12.33	21.78	11.92	2.03
os13681	5	644	0.65	5	3	5	6.9	-0.01	0.5	1.53	0.06	99.58	-0.27	6.89	6.79	15.64	7.15	-1.59	11.72	21.14	11.41	1.77
os13684	7	581	0.01	4	2	3	6.95	0.06	1.72	1.55	0.06	91.5	-2.94	8.15	7.1	15.93	7.38	-1.47	12.16	21.59	11.77	1.97
os13685	5	582	0.01	7	3	5	6.98	0.1	1.16	1.54	0.06	91.15	-2.15	8.05	7.1	15.93	7.38	-1.47	12.16	21.6	11.78	1.97
os13693	5	577	0.63	5	3	6	6.68	-0.07	0.55	1.52	0.04	97.55	-0.24	7.36	7.19	16.03	7.41	-1.43	12.31	21.79	11.89	2.06
os13720	7	650	0.08	6	3	5	7.03	0.1	0.13	1.51	0.06	95.01	-0.38	7.91	6.78	15.65	7.08	-1.65	11.76	21.24	11.41	1.8
os13721	7	572	0.35	12	2	7	6.6	0.02	0.44	1.53	0.15	96.73	-5.91	6.92	7.01	15.86	7.26	-1.55	12.09	21.5	11.66	1.92
os13732	5	483	0.75	5	2	0	6.75	-0.05	0.76	1.52	0.08	93.88	-0.26	7.72	7.64	16.52	7.82	-1.12	12.82	22.29	12.27	2.27
os13785	5	632	0.81	3	3	6	6.75	-0.06	0.97	1.54	0.03	92.69	-0.2	8.07	6.79	15.55	7.12	-1.7	11.92	21.2	11.6	1.82
os13794	5	645	0.77	4	3	6	6.86	-0.03	1.08	1.53	0.03	96.75	-0.24	7.15	6.79	15.55	7.1	-1.82	12.04	21.33	11.71	1.74
os13799	5	592	1	6	3	5	6.64	-0.1	0.97	1.53	0.05	88.11	-1.54	8.93	7.03	15.85	7.33	-1.52	12.15	21.55	11.79	1.95
os13801	7	592	0.7	2	3	6	6.8	-0.03	2.62	1.55	0.05	84.22	-1.57	8.95	7.03	15.84	7.31	-1.55	12.2	21.57	11.82	1.98
os13819	5	637	0.95	9	3	5	6.63	-0.12	0.35	1.51	0.06	85.64	-0.46	7.7	6.76	15.52	7.06	-1.88	12.04	21.3	11.67	1.69
os13831	7	702	0.9	9	3	5	6.69	-0.12	0.17	1.47	0.07	99.98	0.07	6.34	6.46	15.32	6.84	-1.73	11.23	20.74	10.97	1.64
os13854	7	607	0.53	7	2	3	6.75	0	0.95	1.53	0.08	98.03	-3.3	7.86	6.95	15.8	7.18	-1.69	12.26	21.61	11.8	1.93
os13856	7	641	0.49	8	3	5	6.96	0.03	0.62	1.52	0.07	95.9	-2.04	7.06	6.7	15.5	7.01	-1.89	11.95	21.31	11.63	1.74
os13857	5	621	0.92	2	2	3	6.78	-0.03	1.85	1.56	0.06	82.95	-2.37	8.49	6.77	15.56	7.05	-1.84	12.02	21.39	11.67	1.78
os13860	5	644	0.6	2	3	6	6.82	-0.01	1.96	1.55	0.05	94.42	-1.16	8.34	6.61	15.39	6.97	-1.9	11.71	21.07	11.45	1.66

os13862	5	646	0.7	3	3	6	6.79	-0.02	2	1.55	0.05	93.59	-0.98	8.4	6.6	15.38	6.96	-1.9	11.7	21.06	11.45	1.65
os13865	7	667	1	6	3	5	6.67	-0.11	0.42	1.51	0.05	97.71	0.28	6.91	6.63	15.45	6.95	-1.93	11.92	21.27	11.62	1.68
os13906	7	584	0.73	3	3	6	6.78	-0.05	1.94	1.55	0.04	93.72	-1.34	8.25	7.16	16	7.42	-1.43	12.26	21.67	11.84	1.95
os13923	6	505	0.05	9	2	3	6.86	0.12	0.54	1.54	0.1	68.79	-4.27	6.88	7.43	16.32	7.53	-1.29	12.46	22.03	11.85	2.11
os13966	7	635	0.27	2	2	7	6.88	0.02	2.23	1.56	0.08	75.24	-2.22	9.26	6.53	15.29	6.89	-1.9	11.61	20.93	11.32	1.64
os13970	5	544	0	3	3	6	6.74	-0.05	1.6	1.56	0	79.57	-0.53	7.25	7.04	15.94	7.22	-1.47	11.95	21.54	11.43	1.89
os14009	7	492	0.05	8	3	5	6.92	0.11	0.58	1.52	0.06	94.04	-0.35	7.65	7.66	16.56	7.76	-1.12	12.71	22.27	12.08	2.21
os14013	5	476	0.3	4	3	6	6.76	0	2.22	1.54	0.05	86.89	-0.6	9.23	7.63	16.55	7.71	-1.17	12.69	22.29	12.06	2.18
os14023	7	458	0.95	10	3	5	6.48	-0.15	0.11	1.5	0.06	90.13	0.42	6.14	7.91	16.77	7.98	-0.9	13.05	22.51	12.36	2.43
os14028	4	460	0.14	12	2	3	7	0.16	0.42	1.48	0.1	97.34	-3.94	8.07	7.92	16.81	7.97	-0.87	13.09	22.6	12.39	2.48
os14040	6	522	0.3	2	3	6	6.89	0.03	1.91	1.54	0.01	60.18	0.3	8.78	7.63	16.5	7.7	-1.12	12.63	22.2	12.02	2.22
os14068	4	414	0.42	2	3	6	6.76	0	4.08	1.57	0.05	71.6	-2.06	8.39	8.12	16.97	8.15	-0.77	13.31	22.78	12.57	2.56
os14069	4	415	0.38	3	3	6	6.72	-0.01	3.98	1.56	0.05	65.43	-1.46	8.51	8.12	16.97	8.15	-0.77	13.3	22.78	12.57	2.55
os14070	4	416	0.5	4	3	6	6.72	-0.01	4.29	1.56	0.04	50.9	-1.09	8.35	8.11	16.97	8.15	-0.77	13.3	22.77	12.57	2.55
os14074	6	429	0.99	6	3	6	6.63	-0.08	0.56	1.54	0.05	81.47	-0.72	8.93	8.09	16.94	8.13	-0.78	13.26	22.74	12.55	2.53
os14089	7	473	0.74	4	2	7	6.7	-0.06	1.7	1.55	0.09	92.36	-3.26	9.05	7.74	16.6	7.88	-0.99	12.84	22.32	12.27	2.35
os14094	7	492	0.1	7	3	5	6.8	0.06	0.33	1.5	0.06	79.39	0.16	6.38	7.76	16.63	7.88	-0.99	12.88	22.38	12.3	2.33
os14102	5	518	0.9	3	3	6	6.72	-0.03	0.59	1.53	0.02	93.68	0.09	7.44	7.6	16.48	7.76	-1.09	12.67	22.18	12.16	2.27
os14106	5	462	0.26	2	3	6	6.74	-0.04	1.69	1.55	0.03	73.58	-0.25	8.6	7.81	16.65	7.84	-1.03	12.88	22.39	12.21	2.34
os14118	6	444	0.62	11	3	5	6.46	-0.06	0.11	1.52	0.07	79.49	-0.98	7.57	7.8	16.67	7.85	-1.03	12.89	22.42	12.23	2.31
os14119	5	465	0.92	7	3	5	6.54	-0.1	0.29	1.52	0.04	97.08	0.09	7.14	7.75	16.63	7.81	-1.06	12.84	22.38	12.2	2.29
os14123	5	482	0.99	4	3	6	6.71	-0.06	0.55	1.54	0.03	79.34	0.08	8.05	7.64	16.54	7.72	-1.14	12.78	22.33	12.15	2.27
os14124	5	481	0.99	3	3	6	6.72	-0.06	0.44	1.54	0.03	78.58	0	8.1	7.64	16.54	7.72	-1.14	12.78	22.33	12.15	2.27
os14130	7	475	0.11	4	2	3	6.88	0.03	1.7	1.54	0.05	93.02	-2.52	8.08	7.72	16.63	7.75	-1.14	12.86	22.44	12.19	2.28
os14181	5	435	0.49	5	2	0	6.62	-0.09	0.97	1.55	0.06	54.67	-1.35	7.91	7.81	16.66	7.88	-0.98	12.93	22.41	12.25	2.36
os14182	5	472	0.97	5	2	0	6.77	-0.04	1.56	1.54	0.08	97.5	-1.06	7.99	7.67	16.49	7.76	-1.07	12.76	22.26	12.13	2.29
os14183	5	483	0.85	4	2	7	6.72	-0.06	1.46	1.55	0.08	98.42	-2.83	7.92	7.63	16.46	7.73	-1.1	12.72	22.22	12.11	2.28
os14184	5	497	0.42	6	2	3	6.89	0.02	1.36	1.54	0.09	99.5	-3.53	7.92	7.6	16.43	7.71	-1.11	12.68	22.19	12.08	2.26
os14207	5	545	0.23	5	3	5	6.79	0.02	0.88	1.54	0.05	89.88	-0.87	8.38	7.35	16.21	7.54	-1.28	12.46	21.94	11.98	2.16
os14225	5	495	0.96	1	3	6	6.84	0.01	2.75	1.54	0.03	66.57	0.72	9.53	7.53	16.41	7.6	-1.26	12.68	22.24	12.03	2.18
os14230	7	500	0.35	4	3	6	6.73	-0.01	0.42	1.53	0.04	79.58	0.82	7.41	7.46	16.36	7.55	-1.3	12.62	22.18	12	2.15
os14243	6	535	0.4	2	3	6	6.86	0.01	1.73	1.53	0.02	73.32	-0.1	8.48	7.37	16.29	7.47	-1.3	12.42	22	11.81	2.12
os14276	7	597	0.41	10	3	5	6.65	0	0.14	1.5	0.07	96.74	-0.86	6.6	6.89	15.76	7.15	-1.53	11.84	21.34	11.43	1.88
os14298	7	441	0.07	2	2	3	6.83	0.02	2.08	1.56	0.08	61.45	-2.23	7.42	7.81	16.68	7.85	-1.04	12.88	22.44	12.22	2.3
os14355	5	571	0.9	10	1	4	6.4	-0.16	0.02	1.46	0.06	98.99	2.88	5.75	7.31	16.24	7.44	-1.31	12.32	21.77	11.65	1.95
os14502	4	476	0.4	3	2	0	6.87	0.02	2.7	1.55	0.07	78.9	0.07	8.66	7.87	16.77	7.83	-0.94	12.84	22.27	12	2.29
os14520	5	495	0.92	7	2	3	6.54	-0.1	0.56	1.52	0.1	99.91	-2.32	6.31	7.69	16.66	7.74	-1.07	12.84	22.38	12.06	2.17

os14530	7	518	0.88	8	2	3	6.5	-0.12	0.61	1.52	0.1	99.5	-2.43	7.09	7.43	16.4	7.54	-1.22	12.58	22.1	11.87	2.04
os14590	5	505	0.53	6	3	5	6.89	0.02	0.53	1.52	0.07	100	-0.73	6.71	7.61	16.55	7.66	-1.09	12.69	22.22	11.91	2.15
os14599	7	549	0.78	2	2	7	6.81	-0.02	2.43	1.55	0.11	93.65	-3.79	8.68	7.2	16.12	7.36	-1.34	12.19	21.62	11.56	1.89
os14613	5	574	0.11	5	3	5	6.87	0.06	0.88	1.52	0.08	99.93	-2.28	7.48	7.35	16.35	7.47	-1.3	12.36	21.93	11.75	1.95
os14638	7	526	0.84	6	3	5	6.61	-0.07	0.35	1.52	0.08	98.62	-0.16	7.43	7.49	16.46	7.55	-1.21	12.54	22.06	11.8	2.04
os14658	5	459	0.07	5	2	7	6.64	-0.08	1.37	1.55	0.1	96.32	-3.7	8.16	7.63	16.54	7.69	-1.11	12.65	22.16	11.91	2.09
os14836	7	450	1	8	3	5	6.54	-0.13	0.43	1.52	0.07	97.59	-1.87	7	8.2	17.23	8.07	-0.81	13.17	22.77	12.17	2.35
os14853	5	450	0.89	2	3	6	6.76	-0.03	1.94	1.55	0.02	38.39	-0.13	8.55	8.22	17.27	8.1	-0.8	13.29	22.91	12.28	2.38
os14854	5	451	0.77	2	3	6	6.77	-0.02	1.78	1.56	0.02	36.66	-0.07	8.57	8.22	17.26	8.1	-0.8	13.29	22.91	12.28	2.38
os14920	7	792	0.63	9	3	5	6.9	-0.04	0.19	1.5	0.07	100	-1.67	8.06	5.84	14.66	6.43	-1.91	10.56	19.79	10.49	1.34
os14939	7	741	0.85	10	3	5	6.74	-0.1	0.05	1.5	0.08	99.51	-0.58	6.82	6.14	14.96	6.68	-1.79	10.74	20.02	10.61	1.37
os14967	7	741	0.77	10	3	5	6.83	-0.06	0.05	1.49	0.08	99.95	-1.02	6.96	6.44	15.27	6.85	-1.63	11.15	20.46	10.9	1.6
os14996	7	631	0.73	5	2	7	6.69	-0.08	1.12	1.55	0.11	99.11	-2.48	7.34	6.78	15.61	7.11	-1.46	11.53	20.87	11.14	1.72
os15003	7	696	0.4	10	2	0	6.66	0	0.06	1.49	0.11	100	-0.11	6.94	6.57	15.43	6.97	-1.55	11.3	20.64	10.97	1.64
os15033	7	732	0.26	7	3	5	6.8	0.02	0.35	1.51	0.11	99.93	-0.76	6.8	6.29	15.14	6.78	-1.64	10.78	20.14	10.58	1.45
os15065	5	566	1	6	3	5	6.62	-0.1	0.45	1.51	0.06	99.9	0.07	6.28	7.5	16.4	7.58	-1.13	12.41	21.84	11.7	2.04
os15089	4	827	0	8	3	9	7.09	0.13	0.03	1.42	0.04	99.92	1.46	5.62	6.31	15.2	6.77	-1.68	10.99	20.36	10.76	1.52
os15118	5	712	0.55	7	2	0	6.95	0.01	0.34	1.52	0.11	99.67	0.08	6.11	6.25	15.09	6.75	-1.7	10.84	20.12	10.65	1.44
os15207	7	550	0.6	6	3	5	6.69	-0.02	0.78	1.51	0.08	99.57	-1.81	6.74	7.7	16.65	7.73	-1.04	12.65	22.12	11.83	2.12
os15220	7	581	0.02	5	2	0	6.83	0.03	0.96	1.53	0.07	99.28	-1.4	8.67	7.29	16.19	7.46	-1.17	12.06	21.48	11.47	1.95
os15277	6	597	0.05	5	2	3	6.94	0.08	1.31	1.54	0.09	72.48	-2.11	8.89	7.18	16.07	7.4	-1.24	11.97	21.38	11.41	1.89
os15297	7	656	0.92	6	2	0	6.66	-0.1	0.76	1.54	0.1	99.95	-1.67	7.57	6.63	15.47	7.01	-1.54	11.36	20.69	11.02	1.65
os15321	7	644	0.85	8	2	0	6.55	-0.11	0.2	1.5	0.1	100	-0.07	7.38	6.9	15.77	7.19	-1.4	11.67	21.04	11.21	1.78
os15326	7	637	0.83	11	2	0	6.44	-0.13	0.03	1.49	0.11	100	-1.34	7.55	6.92	15.79	7.2	-1.39	11.69	21.07	11.23	1.79
os15346	7	588	0.62	11	2	0	6.87	0	0.02	1.5	0.1	88.86	-0.29	6.57	7.15	16.02	7.38	-1.27	11.99	21.33	11.45	1.88
os15455	5	543	0.92	6	2	0	6.6	-0.1	0.6	1.52	0.08	85.46	-0.55	8.08	7.5	16.4	7.59	-1.11	12.41	21.82	11.71	2.08
os15462	5	526	0.2	11	2	7	6.99	0.14	0.26	1.52	0.11	99.98	-4.54	7.36	7.54	16.54	7.65	-1.15	12.56	22.13	11.9	2.07
os15527	5	584	0.55	8	3	5	6.66	-0.02	0.33	1.5	0.09	99.64	-0.56	6.75	7.33	16.22	7.47	-1.18	12.18	21.6	11.57	2
os15587	5	555	0.31	1	2	0	6.81	0.01	1.99	1.56	0.16	75.5	-1.89	8.22	7.01	15.86	7.25	-1.41	12.01	21.32	11.47	1.87
os15631	5	470	0.75	2	3	6	6.82	0	1.82	1.55	0.04	82.21	-0.61	8.34	8.03	16.98	7.98	-0.84	13.07	22.58	12.22	2.37
os15655	7	570	0.76	8	2	0	6.55	-0.09	0.22	1.51	0.08	99.8	-0.63	6.75	7.41	16.38	7.55	-1.1	12.26	21.74	11.6	2.06
os15660	7	584	0.6	7	2	0	6.61	-0.06	0.27	1.51	0.08	99.54	-0.21	6.24	7.26	16.22	7.44	-1.17	12.05	21.53	11.44	1.97
os15661	7	585	0.59	7	2	0	6.62	-0.04	0.24	1.5	0.08	99.46	-0.08	6.16	7.28	16.23	7.45	-1.16	12.07	21.55	11.45	1.98
os15670	7	623	0.58	5	3	5	6.75	-0.01	0.18	1.49	0.07	100	1.19	6.1	7.14	16.06	7.35	-1.23	11.86	21.32	11.34	1.89
os15672	5	630	0.71	7	3	5	6.63	-0.07	0.22	1.5	0.07	100	0.12	6.14	7.07	16	7.3	-1.27	11.78	21.25	11.28	1.86
os15691	7	441	0.23	2	2	3	6.73	-0.01	2.76	1.56	0.1	73.86	-3.08	7.56	7.8	16.67	7.84	-1.04	12.87	22.43	12.21	2.3
os15734	7	668	0.98	9	2	0	6.56	-0.14	0.17	1.51	0.1	99.92	-0.75	7	6.49	15.36	6.88	-1.73	11.32	20.66	10.99	1.52

os15756	5	726	0.97	6	3	5	6.76	-0.09	0.86	1.48	0.07	99.43	-1.89	7.81	6.43	15.34	6.82	-1.73	11.37	20.7	11.05	1.61
os15854	5	588	0.4	7	3	5	6.7	0	0.23	1.49	0.06	98.02	-0.32	6.74	7.14	16.02	7.32	-1.42	12.17	21.49	11.56	1.83
os15882	7	673	0.9	9	2	0	6.52	-0.13	0.11	1.51	0.07	100	-0.68	6.35	6.5	15.36	6.9	-1.74	11.37	20.68	11.01	1.53
os15933	4	696	0.01	14	3	9	7.09	0.23	0	1.43	0.12	100	-1.31	5.57	6.55	15.41	6.93	-1.65	11.49	20.77	11.14	1.66
os15940	5	694	0.41	4	3	6	6.94	0	1.28	1.53	0.09	100	-0.72	8.05	6.31	15.14	6.73	-1.74	11.14	20.39	10.89	1.54
os15952	4	741	0.99	14	3	9	6.3	-0.24	0	1.45	0.09	99.95	0.12	5.76	6.36	15.18	6.77	-1.72	11.2	20.47	10.94	1.59
os15953	4	735	1	13	3	9	6.36	-0.22	0	1.46	0.09	99.97	-0.59	5.82	6.36	15.19	6.78	-1.71	11.22	20.48	10.95	1.59
os15975	6	618	0.6	6	2	0	6.66	-0.05	0.61	1.53	0.11	97.48	-0.94	7.68	6.61	15.46	6.97	-1.61	11.5	20.83	11.13	1.65
os16344	7	604	0.27	22	2	3	6.21	0.04	0	1.43	0	89.03	-1.5	6.72	6.86	15.62	7.19	-1.88	12	21.29	11.73	1.63
os16429	5	644	0.08	2	3	6	6.93	0.03	0.88	1.54	0.04	86.39	0.27	8.68	6.74	15.48	7.1	-1.71	11.71	20.9	11.4	1.67
os16441	5	631	0.02	8	3	5	6.93	0.11	0.2	1.49	0.07	84.98	0.99	6.77	6.73	15.49	7.11	-1.77	11.69	20.95	11.44	1.68
os16447	3	650	0.02	16	3	5	7	0.24	0.01	1.44	0.11	94.3	0.01	7.18	6.58	15.36	7.02	-1.89	11.54	20.83	11.36	1.55
os16462	5	779	0.74	10	3	5	6.52	-0.13	0.04	1.47	0.09	98.54	-0.35	6.29	6.11	14.92	6.66	-2	10.96	20.2	10.88	1.36
os16464	7	731	0.83	10	2	0	6.47	-0.15	0.03	1.48	0.1	99.02	0.1	6.96	6.22	15.03	6.73	-1.97	11.1	20.36	10.97	1.41
os16479	7	613	0.65	5	2	0	6.88	-0.01	1.86	1.53	0.08	86.34	-0.82	8.02	6.78	15.51	7.16	-1.65	11.69	20.86	11.39	1.65
os16510	7	529	0.88	21	2	0	6.19	-0.22	0	1.41	0.15	81.66	0.48	5.83	7.29	16.02	7.49	-1.41	12.38	21.61	11.85	1.93
os16600	5	540	0.45	6	2	0	6.68	-0.02	0.61	1.53	0.09	98.27	-1.6	7.07	7.03	15.88	7.24	-1.43	12.06	21.41	11.46	1.83
os16602	5	542	0.91	3	2	0	6.73	-0.04	3.08	1.56	0.07	35.13	-0.27	9.87	6.89	15.66	7.18	-1.43	11.72	21	11.25	1.72
os16634	5	563	0.37	3	3	6	6.75	-0.05	0.98	1.55	0.05	57.04	-0.63	8.33	6.72	15.55	7	-1.67	11.78	21.12	11.29	1.6
os16672	7	499	0.8	5	2	0	6.62	-0.06	0.62	1.55	0.11	48.37	-1.11	7.78	6.86	15.62	7.13	-1.53	11.88	21.09	11.42	1.78
os16697	7	545	0.22	2	2	0	6.89	0.02	2.85	1.55	0.07	62.71	0.07	7.98	6.72	15.51	7.03	-1.65	11.75	20.99	11.31	1.68
os16817	4	605	0.26	14	2	7	7.04	0.17	0	1.47	0.13	99.99	-2.35	5.99	6.4	15.21	6.75	-1.8	11.45	20.7	11.09	1.55
os16843	6	547	0.92	6	2	0	6.62	-0.09	0.49	1.53	0.11	98.65	-0.15	7.2	6.55	15.38	6.93	-1.64	11.58	20.89	11.24	1.68
os16860	6	493	1	4	2	7	6.72	-0.05	2.09	1.55	0.1	89.96	-2.66	7.87	7.19	16.09	7.32	-1.37	12.42	21.81	11.76	1.99
os16861	6	489	0.92	3	2	7	6.71	-0.05	2.54	1.56	0.11	91.11	-3.38	7.64	7.22	16.12	7.34	-1.35	12.46	21.85	11.78	2
os16899	4	560	1	5	3	5	6.66	-0.09	0.33	1.5	0.05	88	0.63	7.34	7.2	16.16	7.35	-1.36	12.34	21.83	11.68	1.91
os16922	5	608	0.17	10	2	0	7.07	0.14	0.06	1.48	0.12	100	-1.35	6.38	6.51	15.37	6.87	-1.61	11.4	20.67	11	1.66
os16927	5	658	0.47	7	3	5	6.95	0.02	0.43	1.51	0.08	99.98	-0.6	6.65	6.22	15.04	6.64	-1.86	11.17	20.4	10.87	1.47
os16943	7	632	0.81	9	2	3	6.5	-0.12	0.28	1.49	0.12	99.41	-2.96	7.39	6.52	15.36	6.88	-1.63	11.36	20.64	10.99	1.65
os16984	5	542	0.35	13	2	0	6.54	0	0	1.48	0.09	98.55	0.45	5.8	7.05	15.9	7.25	-1.43	12.08	21.43	11.47	1.83
os16993	5	533	0.99	6	2	0	6.64	-0.1	0.35	1.53	0.08	98	0.13	7.02	7.05	15.91	7.24	-1.43	12.08	21.44	11.49	1.84
os17003	5	538	0.92	1	2	0	6.8	-0.02	3.97	1.56	0.07	34.46	0.25	10.29	6.91	15.69	7.19	-1.43	11.75	21.04	11.27	1.73
os17037	7	567	0.6	9	2	7	6.54	-0.06	0.56	1.52	0.16	99.45	-6.27	8.13	6.58	15.35	6.93	-1.59	11.38	20.65	11.02	1.64
os17093	6	661	0.01	6	3	5	6.65	-0.11	0.42	1.52	0.06	98.29	-0.48	6.8	6.3	15.13	6.76	-1.68	10.95	20.24	10.72	1.51
os17113	7	433	0.22	3	2	7	6.8	0.03	1.33	1.56	0.08	50.47	-2.2	7.85	7.63	16.56	7.68	-1.13	12.89	22.37	12.1	2.12
os17114	7	434	0.92	1	2	0	6.78	0	2.45	1.56	0.07	49.91	-1.01	7.99	7.61	16.54	7.67	-1.14	12.87	22.34	12.08	2.11
os17133	5	598	0.23	6	2	0	6.81	0.04	0.54	1.51	0.08	100	-0.18	7	6.23	15.12	6.71	-1.69	11.23	20.56	10.99	1.64

os17147	5	520	0.13	8	3	5	6.79	0.07	0.17	1.51	0.09	98.27	-1.6	6.13	6.95	15.86	7.24	-1.45	12.03	21.45	11.53	1.74
os17215	5	614	0.98	3	3	6	6.78	-0.04	1.96	1.52	0.06	95.71	-1.08	7.06	6.58	15.4	6.9	-1.73	11.62	20.95	11.19	1.55
os17217	5	616	0.92	1	3	6	6.83	-0.02	2.17	1.52	0.06	95.49	-0.85	6.93	6.58	15.4	6.89	-1.73	11.61	20.94	11.19	1.55
os17218	6	580	0.99	4	3	6	6.74	-0.07	0.21	1.53	0.05	90.45	0.64	6.98	6.65	15.47	6.96	-1.7	11.7	21.04	11.24	1.58
os17219	6	577	0.04	4	3	6	6.72	-0.07	0.29	1.54	0.05	87.65	0.25	7.22	6.66	15.48	6.96	-1.69	11.71	21.04	11.25	1.58
os17220	6	579	0.98	5	3	6	6.76	-0.06	0.4	1.53	0.06	89.86	0.2	7.16	6.66	15.48	6.96	-1.69	11.7	21.04	11.25	1.58
os17225	7	542	0.91	10	2	0	6.58	-0.13	0.05	1.5	0.1	99.9	-1.12	6.39	6.53	15.44	6.92	-1.55	11.71	21.05	11.32	1.82
os17226	5	559	0.96	11	3	5	6.54	-0.15	0.02	1.48	0.1	99.96	0.29	6.45	6.5	15.41	6.9	-1.56	11.68	21.02	11.29	1.81
os17227	5	522	0.99	8	2	0	6.56	-0.13	0.58	1.52	0.11	99.72	-2.13	7.15	6.58	15.48	6.95	-1.53	11.77	21.11	11.36	1.85
os17233	5	592	0.08	8	3	5	7.05	0.13	0.34	1.52	0.09	97.83	-1.88	5.98	6.54	15.38	6.91	-1.7	11.53	20.86	11.17	1.59
os17237	5	579	0.77	4	3	6	6.81	-0.04	0.98	1.53	0.04	92.45	0.02	7.68	6.65	15.49	6.98	-1.67	11.67	21.02	11.26	1.62
os17243	7	486	0.97	5	2	7	6.63	-0.08	1.27	1.54	0.1	98.91	-2.11	8.02	6.86	15.78	7.15	-1.47	12.06	21.47	11.56	1.87
os17255	5	578	0.21	3	3	6	6.93	0.03	2.66	1.53	0.06	79.92	-0.58	8.31	6.68	15.59	7.02	-1.58	11.64	21.05	11.21	1.58
os17264	5	647	0.34	8	3	9	6.78	0.04	0.24	1.48	0.07	100	-0.5	6.27	6.23	15.14	6.71	-1.68	11.32	20.66	11.04	1.69
os17267	5	622	0.92	5	3	6	6.67	-0.08	0.9	1.52	0.06	98.65	-1.27	7.23	6.41	15.25	6.81	-1.71	11.38	20.7	11.1	1.64
os17348	7	501	0.77	4	2	7	6.81	-0.02	1.95	1.55	0.09	89.63	-3.34	8.71	7.23	16.12	7.38	-1.4	12.18	21.65	11.47	1.66
os17368	5	514	0.6	9	2	3	6.86	0.01	0.13	1.5	0.09	98.73	-2.22	6.71	7.02	15.95	7.29	-1.44	12.05	21.53	11.54	1.68
os17376	5	511	0.6	5	3	6	6.7	-0.03	0.91	1.54	0.05	94.89	-1.34	7.71	7.07	15.96	7.32	-1.43	12.11	21.57	11.56	1.7
os17433	4	520	0.98	16	2	7	6.25	-0.22	0.01	1.49	0.18	75.04	-4.71	5.54	6.73	15.48	7.04	-1.58	11.64	20.88	11.27	1.71
os17442	7	475	0.92	0	2	0	6.82	0	3.99	1.57	0.07	18.69	-0.46	10.15	7.15	15.99	7.33	-1.39	12.32	21.62	11.7	1.92
os17496	5	599	0.92	4	3	6	6.71	-0.06	1.63	1.5	0.07	96.11	-0.34	7.48	6.53	15.43	6.94	-1.61	11.61	20.99	11.29	1.78
os17526	5	634	0.98	10	3	5	6.56	-0.14	0.17	1.5	0.13	100	-1.85	6.55	6.22	15.11	6.7	-1.78	11.2	20.52	10.99	1.59
os17528	5	641	1	10	3	5	6.46	-0.18	0.05	1.5	0.12	100	-1.34	6.69	6.21	15.1	6.69	-1.79	11.18	20.5	10.98	1.58
os17572	3	465	0.4	8	2	7	6.43	0.04	0.61	1.56	0.32	84.31	-15.51	6.2	7.64	16.48	7.8	-1.02	12.68	22.13	12.1	2.27
os17628	5	733	0.83	7	2	7	6.62	-0.11	0.73	1.52	0.11	99.91	-2.49	8.14	6.22	15.04	6.71	-2.03	11.23	20.4	10.99	1.34
os17629	5	733	0.84	9	2	7	6.81	-0.06	0.64	1.53	0.12	99.9	-3.94	8.28	6.21	15.03	6.71	-2.03	11.22	20.39	10.98	1.34
os17656	5	735	0.79	7	2	7	6.8	-0.07	0.83	1.53	0.11	99.87	-3.26	8.48	6.2	15.02	6.7	-2.04	11.21	20.38	10.98	1.33
os17731	5	775	0.01	4	2	0	7.01	0.07	0.79	1.55	0.08	66.29	-0.56	7.01	5.66	14.38	6.32	-2.19	10.21	19.31	10.31	0.96
os17744	7	752	0.08	5	2	3	7.06	0.08	1.33	1.57	0.06	40.95	-2.59	7.86	5.89	14.6	6.44	-2.17	10.56	19.63	10.49	1
os17760	4	898	0.4	8	2	0	7.1	0.05	0.48	1.5	0.09	100	-0.32	6.71	5.01	13.74	5.86	-2.55	9.4	18.5	9.76	0.55
os17814	3	866	0.62	11	2	0	7.01	0.01	0.02	1.48	0.1	99.94	1.5	6.32	5.18	13.96	6.05	-2.11	9.39	18.64	9.72	1
os18008	7	780	0.55	7	3	5	6.98	0.02	0.34	1.51	0.08	99.71	0.26	6	5.95	14.76	6.53	-1.89	10.5	19.79	10.44	1.28
os18097	5	811	0.93	11	3	5	6.61	-0.16	0.01	1.48	0.1	100	-0.19	6.19	5.64	14.45	6.32	-1.99	10.16	19.39	10.22	1.2
os18308	5	826	0.7	4	2	0	6.97	-0.01	0.56	1.52	0.05	95.94	-0.05	7.33	5.51	14.3	6.2	-2.24	10.06	19.24	10.14	0.94
os18437	5	850	0.57	7	3	5	7.02	0.01	0.21	1.52	0.07	98.66	-0.12	6.77	5.26	13.99	6.1	-2.15	9.66	18.8	9.93	1.03
os18453	5	794	0.12	7	3	5	7.12	0.1	0.36	1.5	0.07	99.52	-0.19	7.71	5.84	14.72	6.47	-1.96	10.46	19.79	10.42	1.27
os18465	5	816	0.6	10	3	5	6.63	-0.07	0.17	1.49	0.09	99.99	-1.35	7.56	5.62	14.41	6.31	-1.98	10.19	19.39	10.27	1.27

os18471	7	720	0.77	5	2	3	6.86	-0.04	1.37	1.52	0.09	99.88	-2.34	8.35	6.13	14.94	6.63	-1.78	10.88	20.12	10.72	1.49
os18474	5	773	0.55	9	3	5	6.94	-0.01	0.05	1.48	0.07	99.99	0.13	6.97	5.92	14.72	6.49	-1.86	10.58	19.8	10.53	1.39
os18479	3	810	0	22	3	9	6.97	0.35	0	1.4	0.14	99.98	-0.87	5.14	5.83	14.63	6.47	-1.91	10.52	19.72	10.5	1.33
os18500	7	836	0.77	8	3	5	6.91	-0.04	0.44	1.49	0.09	99.81	-0.95	7.45	5.56	14.37	6.26	-2.04	10.08	19.29	10.17	1.16
os18631	7	783	0.88	9	3	5	6.81	-0.08	0.09	1.51	0	99.23	-0.43	6.17	5.92	14.73	6.63	-2.12	10.6	19.95	10.76	1.27
os18681	7	641	0.17	15	2	7	7.1	0.2	0	1.45	0.16	96.27	-2.64	6.2	6.46	15.22	6.94	-1.77	11.21	20.46	11.06	1.54
os18720	6	726	0.92	1	3	6	6.92	0.01	5.52	1.55	0.01	19.48	0.93	12.06	6.22	15.16	6.9	-2.03	10.69	20.05	10.75	1.13
os18721	3	701	0.26	11	2	0	7.09	0.12	0.03	1.48	0.12	97.99	0.16	6.22	6.15	14.9	6.74	-1.85	10.69	19.93	10.68	1.37
os18724	5	688	0.9	7	2	0	6.59	-0.11	0.26	1.52	0.11	95.6	0.17	6.94	6.19	14.95	6.77	-1.83	10.73	19.96	10.7	1.38
os18732	5	770	0.92	2	3	6	6.89	-0.03	3.66	1.55	0.02	39.51	0.08	8.58	6.16	14.97	6.68	-2.14	11.14	20.35	10.95	1.22
os18733	8	766	0.81	1	3	6	6.91	-0.02	3.82	1.55	0.02	38.94	-0.14	8.96	6.16	14.97	6.68	-2.14	11.14	20.35	10.95	1.22
os18734	6	769	0.66	1	3	6	6.9	-0.02	3.76	1.55	0.02	38.36	0.01	8.95	6.16	14.97	6.68	-2.14	11.14	20.35	10.95	1.22
os18735	5	773	0.77	2	3	6	6.94	-0.01	1.85	1.55	0.02	39.05	-0.59	8.15	6.16	14.97	6.68	-2.15	11.14	20.35	10.95	1.21
os18822	5	771	0.98	3	3	6	6.9	-0.03	1.55	1.54	0.04	97.4	-0.03	7.26	6.1	14.89	6.64	-2.08	10.98	20.13	10.81	1.2
os18846	7	865	0.99	7	3	5	6.79	-0.1	0.39	1.51	0.08	99.98	0.2	7.05	5.35	14.07	6.14	-2.23	9.73	18.91	9.93	0.91
os18857	7	865	0.35	6	2	0	7.02	0.01	0.89	1.53	0.12	98.45	-2.28	9.1	5.09	13.87	5.99	-2.35	9.43	18.64	9.72	0.79
os18860	7	863	0	6	2	0	7.1	0.1	0.44	1.52	0.09	99.9	-0.23	7.29	5.28	14.02	6.09	-2.29	9.7	18.89	9.92	0.85
os18919	5	755	0.38	5	3	5	7.04	0.05	0.76	1.54	0.06	86.12	-1.1	7.86	6.11	14.9	6.66	-2.1	10.9	20.07	10.8	1.22
os19014	6	827	0.05	11	3	5	7.02	0.15	0.02	1.48	0.08	100	-0.77	6.13	5.47	14.21	6.24	-1.97	9.84	19.01	9.96	1.16
os19066	7	580	0.38	8	2	7	6.54	-0.03	0.49	1.54	0.22	88.75	-2.42	8.6	6.04	14.74	6.57	-1.84	10.67	19.81	10.57	1.4
os19068	7	749	0.35	5	2	7	6.87	0.04	1.06	1.54	0.15	97.55	-2.3	7.73	5.45	14.11	6.23	-1.99	9.75	18.88	9.92	1.12
os19079	7	675	0.36	16	2	0	6.41	0	0	1.45	0.15	96.09	-0.76	6.56	5.79	14.5	6.38	-2.01	10.43	19.58	10.43	1.25
os19202	7	644	0.49	8	1	1	6.71	-0.01	0.06	1.45	0.05	99.83	2.72	5.7	6.05	14.89	6.64	-1.75	10.81	20.16	10.68	1.47
os19207	6	631	0.12	9	2	0	6.87	0.1	0.1	1.5	0.1	100	-0.66	6.34	6.01	14.89	6.57	-1.78	10.84	20.19	10.68	1.48
os19224	7	463	0.01	3	3	6	6.87	0.05	1.64	1.54	0.05	72.92	-0.94	8.19	6.96	15.83	7.26	-1.29	12.24	21.57	11.61	1.99
os19289	5	812	0.06	5	3	5	7.01	0.07	1.32	1.5	0.07	97.09	-2.39	8.33	5.36	14.22	6.1	-2.12	9.94	19.21	10.05	1.08
os19300	5	688	0.54	12	3	5	6.51	-0.06	0.01	1.47	0.08	100	0.89	6.12	5.93	14.8	6.48	-1.85	10.78	20.07	10.64	1.46
os19339	6	811	0.07	8	3	5	7.02	0.11	0.21	1.49	0.06	100	-0.1	6.32	5.54	14.27	6.29	-1.94	9.93	19.11	10.03	1.19
os19379	5	621	0.3	10	2	0	6.72	0.04	0.05	1.5	0.11	99.51	-0.81	6.39	6.14	15.02	6.65	-1.74	11.03	20.35	10.82	1.56
os19382	5	644	1	8	2	7	6.55	-0.12	0.4	1.53	0.15	96.88	-1.55	8.8	5.71	14.57	6.37	-1.95	10.39	19.67	10.38	1.31
os19416	7	724	0.92	1	2	7	6.87	-0.01	2.05	1.55	0.08	89.99	-2.06	8.54	5.65	14.47	6.3	-2.08	10.39	19.61	10.4	1.2
os19417	6	740	0.15	7	2	0	6.92	0.06	0.44	1.51	0.08	97.27	-1.17	7.39	5.62	14.45	6.27	-2.1	10.35	19.56	10.36	1.18
os19609	7	749	0.92	2	2	7	6.86	-0.01	1.98	1.56	0.13	81.99	-5.33	7.8	5.76	14.53	6.37	-2.22	10.45	19.86	10.55	1.2
os19623	5	448	0.77	4	3	6	6.74	-0.04	1.64	1.55	0.04	96.92	-1.3	7.38	8.25	17.22	8.18	-0.72	13.31	22.88	12.46	2.5
os19631	5	413	0.99	2	3	6	6.72	-0.04	1.39	1.56	0.03	62.87	-0.15	7.62	8.38	17.37	8.31	-0.62	13.49	23.07	12.61	2.58
os19641	5	471	0.44	2	3	6	6.82	-0.01	2.76	1.55	0.06	88.66	-1.34	8.48	7.94	16.87	8	-0.83	12.94	22.5	12.27	2.38
os19666	3	456	1	14	2	3	6.31	-0.2	0	1.43	0.1	96.99	1.51	6.58	8.08	16.96	8.13	-0.76	13.31	22.78	12.57	2.57

os19673	5	452	0.31	4	3	6	6.88	0.03	1.5	1.54	0.04	91.6	-0.67	7.91	8.23	17.2	8.16	-0.73	13.27	22.84	12.43	2.48
os19675	5	419	0.96	4	3	6	6.67	-0.07	0.54	1.55	0.04	80.92	-0.58	6.87	8.39	17.37	8.31	-0.62	13.49	23.07	12.61	2.58
os19681	5	419	0.99	4	3	6	6.67	-0.06	1.01	1.55	0.04	79.16	-0.74	7.1	8.39	17.37	8.31	-0.62	13.49	23.07	12.61	2.58
os19689	7	431	0.15	11	2	3	6.7	0.1	0.51	1.52	0.12	76.59	-3.57	7.07	8.3	17.27	8.22	-0.67	13.34	22.95	12.5	2.52
os19740	5	498	0.92	2	3	6	6.74	-0.04	1.93	1.54	0.02	57.18	-0.43	9.17	7.74	16.66	7.77	-1.14	12.83	22.4	12.12	2.15
os19760	5	429	0.92	0	3	6	6.79	0	5.79	1.57	0.01	1.87	-0.21	11.41	8.21	17.25	8.13	-0.81	13.08	22.88	12.22	2.33
os19761	5	431	0.92	0	3	6	6.79	0	5.71	1.57	0.01	8.19	-0.23	10.42	8.21	17.24	8.14	-0.8	13.09	22.92	12.24	2.33
os19785	5	393	0.92	0	3	6	6.76	-0.01	4.34	1.56	0.01	8.03	-0.16	10.17	8.66	17.7	8.48	-0.51	13.67	23.35	12.66	2.68
os19787	5	396	0.92	0	3	6	6.78	0	4.33	1.55	0.01	32.8	0.38	10.15	8.67	17.71	8.49	-0.5	13.7	23.4	12.7	2.72
os19836	6	465	0.92	0	3	6	6.81	0	3.97	1.56	0.03	33.42	-0.07	10.78	7.78	16.71	7.81	-1.09	12.82	22.41	12.11	2.15
os19866	5	393	0.99	1	3	6	6.74	-0.02	2.86	1.56	0.01	2.94	-0.24	8.99	8.6	17.58	8.48	-0.44	13.79	23.38	12.86	2.83
os19875	5	404	0.92	1	3	6	6.76	-0.01	2.97	1.56	0.02	9.23	-0.01	9.2	8.51	17.47	8.42	-0.48	13.68	23.26	12.78	2.79
os19882	5	523	0.95	3	3	6	6.79	-0.04	0.58	1.54	0.02	80.43	0.39	7.67	7.66	16.57	7.71	-1.18	12.74	22.33	12.05	2.11
os19924	5	393	0.77	1	3	6	6.76	-0.01	4.17	1.56	0.02	11.25	0	9.65	8.66	17.7	8.49	-0.51	13.66	23.34	12.65	2.68
os19941	5	397	0.92	1	3	6	6.77	0	3.94	1.56	0.04	34.72	-0.88	9.12	8.56	17.59	8.45	-0.5	13.46	23.1	12.48	2.62
os19945	5	383	0.92	0	3	6	6.77	0	5.06	1.56	0.02	35.51	-0.61	9.03	8.67	17.67	8.54	-0.46	13.65	23.31	12.7	2.74
os19946	5	383	0.92	0	3	6	6.76	-0.01	5.51	1.56	0.02	32.43	-0.41	9.6	8.67	17.67	8.54	-0.46	13.66	23.31	12.7	2.74
os19947	5	383	0.93	1	3	6	6.76	-0.01	5.49	1.56	0.02	32.52	-0.34	9.32	8.67	17.67	8.54	-0.46	13.66	23.31	12.7	2.74
os19948	5	384	0.92	1	3	6	6.75	-0.01	5.56	1.54	0.02	30.54	0.4	9.43	8.68	17.67	8.54	-0.46	13.66	23.31	12.7	2.74
os19949	5	384	0.77	1	3	6	6.76	0	5.62	1.55	0.02	26.51	0.95	10.03	8.68	17.67	8.54	-0.46	13.66	23.31	12.7	2.74
os19950	5	384	0.77	1	3	6	6.75	-0.01	5.16	1.54	0.02	26.13	1.04	9.17	8.67	17.67	8.54	-0.46	13.66	23.31	12.7	2.74
os19951	5	385	0.82	1	3	6	6.74	-0.01	4.1	1.53	0.02	26.12	1.18	8.85	8.67	17.67	8.54	-0.46	13.66	23.31	12.71	2.74
os19952	5	385	0.85	1	3	6	6.74	-0.01	3.72	1.53	0.02	26.27	1.23	8.77	8.67	17.67	8.54	-0.46	13.66	23.31	12.7	2.74
os19953	5	385	0.91	1	3	6	6.74	-0.01	3.52	1.53	0.02	26.48	1.25	8.73	8.68	17.67	8.55	-0.46	13.67	23.32	12.71	2.75
os19954	5	384	0.92	0	3	6	6.75	-0.01	5.04	1.56	0.02	33.77	-0.5	8.86	8.67	17.67	8.54	-0.46	13.66	23.31	12.7	2.74
os19958	5	404	0.79	3	3	6	6.71	-0.03	3.82	1.56	0.02	2.43	-0.26	8.83	8.53	17.6	8.41	-0.55	13.65	23.31	12.73	2.69
os19966	5	421	0.92	1	3	6	6.81	0.01	2	1.56	0.01	2.35	0.08	9.02	8.45	17.49	8.33	-0.6	13.55	23.21	12.66	2.64
os19968	5	419	0.24	3	3	6	6.88	0.04	1.52	1.55	0.03	81.83	-0.56	7.46	8.42	17.45	8.34	-0.6	13.55	23.15	12.65	2.64
os20010	5	397	0.92	0	3	6	6.78	0	2.3	1.56	0.01	22.34	-0.37	8.79	8.58	17.61	8.43	-0.51	13.73	23.39	12.75	2.72
os20026	5	433	0.95	4	3	6	6.7	-0.06	1.07	1.54	0.04	69.09	-1.31	6.89	8.32	17.34	8.27	-0.64	13.42	23.04	12.59	2.58
os20030	5	435	0.98	4	3	6	6.68	-0.07	0.71	1.54	0.03	74.28	-0.54	7.55	8.32	17.34	8.26	-0.64	13.42	23.04	12.59	2.58
os20035	5	433	0.99	3	3	6	6.71	-0.05	1.23	1.54	0.03	67.93	-0.44	7.35	8.32	17.34	8.26	-0.64	13.42	23.04	12.58	2.59
os20066	5	435	0.99	2	3	6	6.73	-0.04	1.51	1.55	0.02	78.85	-0.25	7.5	8.32	17.33	8.26	-0.64	13.43	23.04	12.58	2.59
os20104	5	433	0.92	1	3	6	6.78	-0.02	2.58	1.56	0.02	43.37	-0.04	8.26	8.31	17.31	8.27	-0.64	13.45	23.04	12.62	2.62
os20106	5	415	0.69	4	3	6	6.65	-0.04	0.73	1.54	0.03	74.81	-0.1	7.76	8.43	17.39	8.36	-0.53	13.58	23.13	12.74	2.74
os20121	5	453	0.33	3	3	6	6.84	0.01	1.96	1.55	0.08	94.24	-2.8	8.23	8.1	17.03	8.13	-0.74	13.2	22.72	12.47	2.51
os20134	5	431	0.62	4	3	6	6.67	-0.04	1.04	1.55	0.03	62.14	-0.45	8.85	8.28	17.19	8.25	-0.63	13.41	22.93	12.63	2.63

os20149	5	456	0.34	6	3	5	6.68	0	0.83	1.52	0.06	89.85	-1.56	8.08	8.08	16.94	8.12	-0.78	13.31	22.76	12.59	2.56
os20150	5	474	0.92	4	3	6	6.66	-0.06	0.87	1.51	0.03	82.07	-0.07	7.67	8.06	16.91	8.09	-0.8	13.28	22.73	12.56	2.54
os20158	5	440	0.05	7	3	5	6.88	0.11	0.31	1.52	0.05	91.11	-0.08	6.89	8.18	17.07	8.17	-0.72	13.47	22.95	12.68	2.63
os20170	5	461	0.01	3	3	6	6.88	0.06	0.74	1.52	0.03	94.43	0.27	7.38	8.1	16.99	8.11	-0.76	13.39	22.87	12.6	2.58
os20172	5	472	0.08	4	3	6	6.91	0.06	1.58	1.52	0.03	83.99	-0.96	7.21	8.11	17	8.12	-0.76	13.39	22.89	12.61	2.59
os20176	5	459	0.06	4	3	6	6.91	0.06	1.32	1.53	0.03	87.82	-0.44	7.63	8.06	16.95	8.09	-0.78	13.31	22.82	12.56	2.57
os20177	5	459	0	4	3	6	6.91	0.06	1.29	1.53	0.03	88.15	-0.65	7.64	8.06	16.95	8.09	-0.78	13.31	22.82	12.55	2.57
os20200	5	466	0.09	6	3	5	6.95	0.08	0.62	1.52	0.04	77.56	-0.05	7.53	8.01	16.87	8.07	-0.82	13.2	22.68	12.5	2.5
os20201	5	465	0.02	7	3	5	6.97	0.1	0.5	1.51	0.04	78.01	-0.19	7.44	8.01	16.87	8.07	-0.82	13.2	22.68	12.5	2.5
os20222	5	464	0.91	3	3	6	6.72	-0.03	0.71	1.54	0.02	68.19	0.32	7.62	8.05	16.92	8.04	-0.84	13.24	22.76	12.44	2.52
os20232	6	512	0.77	2	3	6	6.8	-0.02	0.39	1.54	0.02	57.1	0.73	8.16	7.72	16.66	7.77	-1.13	12.84	22.42	12.11	2.17
os20236	6	506	0.49	5	3	6	6.68	-0.04	1.08	1.54	0.04	76.26	-0.9	7.94	7.71	16.65	7.76	-1.14	12.83	22.4	12.12	2.16
os20252	5	530	0.99	4	3	6	6.72	-0.06	0.8	1.53	0.02	95.04	-0.16	7.35	7.68	16.63	7.74	-1.14	12.81	22.41	12.06	2.19
os20254	5	533	0.99	4	3	6	6.72	-0.06	0.67	1.53	0.02	96.78	0.03	7.22	7.67	16.62	7.73	-1.14	12.8	22.41	12.06	2.18
os20268	5	502	0.92	1	3	6	6.85	0.01	4.01	1.56	0.02	22.89	-0.32	10.16	7.77	16.7	7.8	-1.11	12.9	22.48	12.11	2.22
os20327	5	521	1	5	3	5	6.66	-0.09	0.47	1.52	0.04	97.37	-0.18	6.57	7.8	16.77	7.82	-1.06	12.77	22.44	11.99	2.13
os20355	5	508	0.9	5	3	5	6.72	-0.07	0.6	1.52	0.04	97.16	0.15	7.47	7.79	16.78	7.81	-1.05	12.74	22.41	11.96	2.12
os20377	5	510	0.77	1	3	6	6.82	-0.01	2.81	1.55	0.01	10.49	-0.22	8.98	7.83	16.83	7.84	-1	12.77	22.46	11.96	2.15
os20394	7	418	0.92	0	3	6	6.79	0	5.97	1.57	0.01	3.18	-0.07	11.8	8.1	17.07	8	-0.84	12.9	22.56	11.98	2.18
os20433	5	425	0.01	7	2	3	6.54	-0.11	0.34	1.54	0.06	47.77	-1.51	7.22	8.22	17.27	8.09	-0.9	13.06	22.91	12.07	2.11
os20468	5	471	0.51	2	3	6	6.73	-0.04	1.95	1.55	0.03	35.63	-1.28	8.09	7.74	16.75	7.73	-1.21	12.64	22.38	11.8	1.93
os20487	6	418	0.92	0	3	6	6.79	0	6.64	1.57	0.01	0.29	-0.34	9.98	8.33	17.44	8.21	-0.7	13.23	23.1	12.26	2.27
os20495	5	432	0.74	3	3	6	6.69	-0.03	1.56	1.55	0.03	58.56	-0.74	7.57	8.25	17.32	8.12	-0.82	13.16	22.99	12.17	2.23
os20563	7	484	0.08	3	3	6	6.83	0.03	2.41	1.55	0.03	75.25	-0.96	9.24	7.89	16.96	7.85	-1.06	12.71	22.52	11.73	1.88
os20584	7	406	0.92	0	3	6	6.78	0	6.95	1.57	0.02	3.73	0.01	12.24	8.43	17.52	8.26	-0.73	13.51	23.28	12.36	2.39
os20591	5	437	0.92	4	3	6	6.66	-0.06	0.96	1.54	0.03	50.23	-0.38	7.68	8.22	17.27	8.08	-0.83	13.08	22.9	12.12	2.23
os20593	5	427	0.92	2	3	6	6.82	0.02	3.98	1.56	0.03	32.12	-0.8	9.15	8.24	17.3	8.11	-0.83	13.13	22.95	12.15	2.23
os20597	5	437	0.92	2	3	6	6.72	-0.04	0.92	1.55	0.02	33.55	0.22	8.35	8.25	17.32	8.1	-0.82	13.17	22.99	12.16	2.25
os20616	5	449	0.92	1	3	6	6.74	-0.03	3.78	1.56	0.03	52.17	-0.9	8.69	8.09	17.15	7.99	-0.96	13	22.82	12.03	2.1
os20639	5	441	0.76	2	3	6	6.83	0.01	2.81	1.55	0.02	47.5	0.25	9.09	8.21	17.26	8.04	-0.91	13.02	22.9	12.03	2.09
os20653	7	405	0.92	1	3	6	6.76	-0.01	6.92	1.57	0.02	2.63	-0.39	9.74	8.44	17.51	8.26	-0.73	13.48	23.29	12.37	2.35
os20654	7	405	0.92	0	3	6	6.78	0	6.9	1.57	0.01	7.49	-0.43	10.03	8.43	17.51	8.26	-0.73	13.47	23.29	12.36	2.34
os20677	5	430	0.92	1	3	6	6.81	0.01	4.77	1.56	0.02	28.47	-0.26	9.18	8.24	17.31	8.09	-0.86	13.13	22.99	12.15	2.18
os20679	5	440	0.98	4	2	3	6.74	-0.04	1.89	1.54	0.05	76.94	-2.02	8.24	8.18	17.25	8.08	-0.92	13.04	22.88	12.04	2.1
os20692	5	415	0.92	0	3	6	6.78	0	6.23	1.57	0.01	1.58	-0.32	9.53	8.29	17.41	8.19	-0.78	13.27	23.21	12.37	2.27
os20708	5	431	0.7	2	3	6	6.75	-0.03	6.77	1.55	0.01	24.57	0.32	8.85	8.28	17.42	8.16	-0.79	13.32	23.2	12.32	2.3
os20718	7	406	0.92	0	3	6	6.78	0	6.97	1.57	0.02	3.4	0	12.18	8.43	17.52	8.26	-0.73	13.49	23.27	12.37	2.39

os20721	5	415	0.92	0	3	6	6.8	0	6.66	1.57	0.01	0.63	-0.3	9.69	8.3	17.42	8.2	-0.78	13.28	23.22	12.37	2.28
os20722	6	415	0.92	0	3	6	6.8	0.01	6.47	1.57	0.01	1.38	-0.49	9.56	8.29	17.42	8.19	-0.78	13.27	23.2	12.37	2.28
os20757	5	458	0.23	1	3	6	6.79	0	2.98	1.56	0.02	12.59	-0.55	9.12	8	17.02	7.91	-0.93	12.8	22.54	11.87	2.11
os20770	6	471	0.92	1	3	6	6.82	0	2.96	1.56	0.02	23.13	0.03	9.05	7.73	16.73	7.73	-1.23	12.61	22.33	11.78	1.93
os20776	5	476	0.99	2	3	6	6.77	-0.03	1.58	1.55	0.01	35.2	-0.05	8.01	7.74	16.74	7.73	-1.22	12.64	22.37	11.8	1.93
os20834	5	485	0.04	2	3	6	6.83	0.02	0.23	1.55	0.02	68.24	0.37	9.05	7.63	16.62	7.63	-1.33	12.49	22.18	11.6	1.74
os20841	6	521	0.4	1	3	6	6.85	0.01	2.94	1.55	0.03	37.47	-0.95	9.02	7.43	16.43	7.47	-1.46	12.23	21.93	11.4	1.61
os20843	5	520	0.08	2	3	6	6.89	0.03	0.79	1.54	0.02	49.91	0.07	8.69	7.43	16.42	7.47	-1.46	12.21	21.91	11.38	1.6
os20877	5	444	0.15	1	3	6	6.8	0.01	3.94	1.56	0.01	2.22	0.26	9.31	7.88	16.86	7.82	-1.06	12.69	22.36	11.86	2.1
os20892	5	496	0.07	5	3	6	6.65	-0.08	0.52	1.54	0	69.69	-0.8	6.79	7.07	15.97	7.25	-1.41	12.02	21.6	11.47	1.91
os20911	6	495	0.16	2	3	6	6.83	0.02	3.43	1.55	0	37.6	0.07	8.89	7.16	16.05	7.29	-1.35	12.02	21.62	11.48	1.95
os20916	6	463	0.92	0	3	6	6.8	0	3.96	1.57	0.02	36.41	-0.69	9.67	7.49	16.43	7.53	-1.23	12.53	22.13	11.77	2.07
os20940	5	464	0.92	1	3	6	6.77	-0.02	6.02	1.56	0.01	0.18	0.03	9.21	7.48	16.4	7.5	-1.26	12.66	22.2	11.87	2.1
os20949	5	470	0.23	1	3	6	6.8	0	5.9	1.56	0.01	19.81	0.32	9.17	7.46	16.34	7.52	-1.2	12.36	21.92	11.68	2.11
os21009	5	493	0.18	7	3	5	6.99	0.1	0.46	1.52	0.05	73.42	-0.75	7.98	7.7	16.61	7.74	-1.14	12.75	22.35	12.05	2.17
os21010	5	476	0.98	4	3	6	6.76	-0.04	0.87	1.54	0.03	81.32	-0.48	7.8	7.77	16.66	7.79	-1.11	12.84	22.42	12.13	2.23
os21015	6	449	0.92	1	3	6	6.84	0.02	4.35	1.57	0.03	29.49	-0.57	9.05	7.87	16.76	7.86	-1.07	13.01	22.58	12.27	2.32
os21017	6	477	0.13	1	3	6	6.78	-0.02	3.34	1.56	0.04	73.15	-1.69	8.4	7.62	16.56	7.69	-1.16	12.7	22.31	11.99	2.2
os21018	5	484	0.05	3	3	6	6.86	0.04	2.68	1.55	0.03	54.78	-1.29	9.29	7.63	16.59	7.67	-1.14	12.67	22.29	11.94	2.21
os21021	5	507	0.23	1	3	6	6.82	0.01	0.65	1.55	0.01	10.19	0.11	9.17	7.53	16.48	7.6	-1.24	12.63	22.22	11.91	2.17
os21032	5	490	0.4	2	3	6	6.85	0.01	0.9	1.54	0.01	57.64	0.8	8.9	7.56	16.5	7.61	-1.23	12.65	22.27	11.91	2.15
os21033	5	489	0.4	3	3	6	6.87	0.02	0.68	1.54	0.02	58.89	0.73	8.75	7.56	16.5	7.61	-1.23	12.65	22.27	11.91	2.15
os21056	6	457	0.92	1	3	6	6.82	0.02	2.57	1.56	0.02	10.06	0.14	8.37	7.72	16.64	7.76	-1.15	12.87	22.45	12.12	2.29
os21058	6	478	0.08	3	3	6	6.9	0.05	1.21	1.55	0.02	67.97	0.07	8.42	7.65	16.57	7.66	-1.19	12.76	22.33	12	2.23
os21085	6	463	0.88	1	3	6	6.79	-0.01	2.82	1.56	0.02	24.77	-0.09	8.29	7.69	16.59	7.72	-1.16	12.79	22.35	12.09	2.29
os21086	6	461	0.92	1	3	6	6.8	-0.01	3.86	1.57	0.02	1.44	-0.3	9.62	7.72	16.62	7.75	-1.14	12.83	22.38	12.12	2.31
os21094	5	483	0.92	0	3	6	6.8	-0.01	3.74	1.57	0.02	23.87	-0.57	9.42	7.51	16.4	7.59	-1.2	12.51	22.06	11.84	2.17
os21096	5	483	0.24	1	3	6	6.78	-0.02	3.67	1.57	0.02	25.57	-0.64	8.94	7.51	16.4	7.59	-1.2	12.51	22.06	11.84	2.17
os21127	5	490	0.01	3	3	6	6.88	0.04	1.69	1.55	0.04	78.3	-0.4	7.78	7.7	16.6	7.74	-1.13	12.75	22.34	12.05	2.17
os21133	5	490	0.51	4	3	6	6.89	0.02	1.62	1.53	0.03	63.19	0.09	8.69	7.67	16.6	7.71	-1.13	12.7	22.32	12.01	2.18
os21151	5	467	0.23	1	3	6	6.8	0.01	5.55	1.56	0.01	4.96	0.02	9.06	7.49	16.4	7.51	-1.23	12.53	22.11	11.79	2.1
os21162	5	463	0.92	0	3	6	6.8	0	5.98	1.56	0.01	1.12	-0.09	9.85	7.47	16.38	7.5	-1.25	12.63	22.17	11.85	2.1
os21164	5	465	0.92	0	3	6	6.8	0	5.98	1.56	0	0	0.16	11.14	7.46	16.36	7.49	-1.27	12.63	22.17	11.85	2.09
os21165	6	464	0.92	1	3	6	6.79	-0.01	6.19	1.56	0.01	0.67	-0.68	9.35	7.47	16.39	7.49	-1.29	12.67	22.21	11.87	2.09
os21169	6	463	0.99	1	3	6	6.78	-0.01	5.91	1.56	0.01	3.02	-0.05	9.47	7.46	16.37	7.48	-1.29	12.66	22.19	11.86	2.08
os21185	6	473	0.92	0	3	6	6.81	0	5.82	1.57	0.01	7.74	0.09	11.37	7.27	16.16	7.37	-1.31	12.25	21.79	11.6	2.01
os21187	5	490	0.92	1	3	6	6.86	0.02	3.36	1.56	0	42.45	-0.27	9.05	7.09	16.02	7.21	-1.45	12.08	21.68	11.49	1.88

os21190	5	493	0.05	3	3	6	6.89	0.04	0.45	1.54	0	37.33	0.29	8.65	7.12	16.04	7.23	-1.43	12.12	21.72	11.5	1.89
os21191	6	491	0.92	0	3	6	6.82	0	2.98	1.55	0	22.16	0.42	11.23	7.11	16.04	7.23	-1.44	12.11	21.72	11.5	1.89
os21197	5	496	0.91	2	3	6	6.77	-0.03	2.61	1.55	0.01	1.67	0.05	8.84	7.41	16.35	7.49	-1.27	12.36	21.99	11.65	2.02
os21199	5	479	0.77	2	3	6	6.81	-0.02	1.71	1.55	0.02	54.91	-0.29	8.45	7.44	16.38	7.49	-1.25	12.42	22.04	11.7	2.03
os21203	5	469	0.95	3	3	6	6.74	-0.04	0.93	1.55	0.02	56.41	-0.01	9.34	7.49	16.44	7.53	-1.23	12.53	22.13	11.77	2.06
os21211	5	467	0.6	2	3	6	6.78	0	2.93	1.55	0.02	29.5	-0.09	9.09	7.49	16.43	7.53	-1.23	12.54	22.13	11.78	2.08
os21213	5	468	0.23	2	3	6	6.78	0	1.47	1.55	0.02	34.1	0.22	8.88	7.49	16.43	7.53	-1.23	12.54	22.13	11.78	2.08
os21221	5	446	0.08	2	3	6	6.86	0.04	1.07	1.55	0.04	77.96	-0.15	7.67	8.36	17.5	8.19	-0.73	13.33	23.08	12.22	2.38
os21255	5	408	0.92	0	3	6	6.78	0	5.47	1.57	0.01	0.06	0	11.73	8.5	17.6	8.33	-0.64	13.6	23.31	12.6	2.55
os21367	7	398	0.92	0	3	6	6.79	0.01	5.88	1.56	0.01	0.01	-0.12	10.26	8.63	17.74	8.42	-0.57	13.72	23.46	12.64	2.66
os21371	7	396	0.08	1	3	6	6.8	0.01	6.7	1.57	0.02	17.8	-0.34	8.96	8.6	17.7	8.4	-0.62	13.69	23.4	12.6	2.61
os21372	7	397	0.06	1	3	6	6.81	0.02	6.67	1.56	0.02	10.23	0.04	9.85	8.6	17.7	8.4	-0.61	13.69	23.4	12.6	2.61
os21397	5	437	0.99	3	3	6	6.72	-0.04	1.32	1.55	0.02	69.07	0.04	7.69	8.02	16.98	7.97	-0.88	13.19	22.73	12.29	2.34
os21425	5	426	0.08	4	3	6	6.89	0.07	1.72	1.53	0.05	91.37	-1.03	7.36	8.11	17.09	8.04	-0.91	13.12	22.76	12.21	2.15
os21470	5	418	0.77	3	3	6	6.79	-0.01	1.78	1.54	0.05	86.01	-1.69	7.52	8.49	17.56	8.31	-0.65	13.57	23.25	12.44	2.51
os21507	6	418	0.92	1	3	6	6.82	0.02	3.73	1.56	0.02	11.72	-0.32	9.85	8.44	17.5	8.32	-0.65	13.62	23.27	12.68	2.62
os21508	5	418	0.13	2	3	6	6.84	0.03	3.66	1.56	0.02	11.47	-0.34	9.6	8.44	17.5	8.32	-0.64	13.62	23.27	12.68	2.62
os21509	5	416	0.92	1	3	6	6.81	0.01	3.71	1.56	0.02	10.31	-0.63	9.57	8.45	17.51	8.32	-0.64	13.62	23.27	12.69	2.62
os21527	5	404	0.92	0	3	6	6.78	0	5.9	1.56	0.02	2.33	-0.12	11.78	8.14	17.06	8.07	-0.86	13.36	22.86	12.45	2.34
os21528	5	419	0.92	1	3	6	6.76	-0.02	2.59	1.56	0.04	60.35	-1.81	8.43	7.99	16.94	7.95	-0.93	13.21	22.72	12.32	2.28
os21530	5	421	0.76	7	2	3	6.51	-0.12	0.32	1.55	0.06	66.68	-2.14	7.55	7.99	16.93	7.95	-0.93	13.2	22.71	12.32	2.28
os21546	5	452	0.92	1	3	6	6.83	0.02	1.31	1.55	0.01	35.59	0.12	8.76	8.42	17.6	8.25	-0.74	13.48	23.3	12.36	2.38
os21547	5	449	0.08	2	3	6	6.84	0.03	1.59	1.55	0.01	18.8	0.02	8.99	8.42	17.6	8.25	-0.73	13.49	23.31	12.37	2.39
os21548	5	451	0.01	2	3	6	6.83	0.03	1.25	1.55	0.01	30.12	0.08	8.78	8.42	17.61	8.25	-0.73	13.48	23.3	12.36	2.39
os21549	5	449	0.01	2	3	6	6.84	0.03	1.48	1.55	0.01	20.69	0.05	8.94	8.43	17.61	8.25	-0.73	13.49	23.31	12.37	2.39
os21558	5	430	0.93	2	3	6	6.76	-0.02	3.87	1.55	0.02	9.1	0.06	8.96	8.47	17.6	8.29	-0.69	13.48	23.21	12.36	2.45
os21569	5	429	0.23	4	3	6	6.78	0.02	0.72	1.54	0.02	59.53	0.14	7.72	8.56	17.71	8.37	-0.65	13.65	23.41	12.54	2.54
os21572	5	413	0.92	3	3	6	6.7	-0.04	3.06	1.55	0.02	43.07	-0.38	8.43	8.58	17.73	8.39	-0.63	13.67	23.43	12.57	2.57
os21573	5	413	0.02	3	3	6	6.69	-0.04	2.87	1.55	0.03	45.49	-0.65	8.43	8.58	17.73	8.39	-0.64	13.67	23.43	12.57	2.57
os21592	7	398	0.92	0	3	6	6.78	0	5.55	1.57	0.01	0	0	11.73	8.64	17.77	8.44	-0.55	13.78	23.49	12.67	2.69
os21608	6	386	0.01	2	3	6	6.8	0.02	2.79	1.56	0.03	17.8	0.14	9.2	8.68	17.78	8.49	-0.51	13.83	23.5	12.76	2.73
os21631	5	390	0.92	0	3	6	6.77	0	6.01	1.57	0.02	20.73	-0.12	9.7	8.62	17.69	8.41	-0.6	13.72	23.4	12.64	2.6
os21636	5	393	0.06	1	3	6	6.81	0.02	3.2	1.57	0.02	40.25	-0.6	8.47	8.57	17.66	8.37	-0.61	13.67	23.36	12.62	2.59
os21702	5	434	0.23	2	3	6	6.79	0.01	2.86	1.56	0.03	42.7	-0.65	8.94	8.48	17.64	8.29	-0.72	13.52	23.32	12.39	2.42
os21705	5	437	0.83	2	3	6	6.74	-0.01	2.01	1.55	0.03	57.56	-0.54	8.8	8.47	17.63	8.28	-0.72	13.51	23.32	12.38	2.41
os21706	5	438	0.65	3	3	6	6.71	-0.03	1.53	1.55	0.03	61.37	-0.51	8.71	8.47	17.63	8.28	-0.72	13.51	23.31	12.38	2.41
os21711	5	437	0.18	2	3	6	6.81	0.02	1.43	1.55	0.02	69.75	-0.12	8.33	8.47	17.64	8.29	-0.72	13.53	23.33	12.4	2.42

os21732	5	489	0.6	8	2	3	6.55	-0.05	0.06	1.52	0.08	89.8	-2.31	8.41	7.91	16.86	7.89	-0.87	12.89	22.39	12.09	2.3
os21786	6	420	0.92	0	3	6	6.79	0	5.91	1.57	0.01	2.1	0	11.73	8.31	17.34	8.1	-0.77	13.11	22.89	11.99	2.18
os21793	5	415	0.82	3	3	6	6.68	-0.04	4.68	1.55	0.03	19.57	-0.2	8.95	8.26	17.3	8.11	-0.78	12.98	22.78	11.91	2.15
os21797	5	433	0.92	0	3	6	6.79	0	6.3	1.57	0.01	0	-0.03	12.27	8.27	17.35	8.13	-0.76	13.02	22.86	11.99	2.2
os21838	6	430	0.92	0	3	6	6.79	0	6.3	1.57	0.01	0.83	-0.27	10.16	8.26	17.34	8.13	-0.74	13.03	22.85	11.98	2.22
os21839	5	430	0.92	0	3	6	6.79	0	6.28	1.57	0.01	1.46	-0.18	11.41	8.27	17.34	8.13	-0.74	13.04	22.85	11.98	2.22
os21840	6	430	0.92	0	3	6	6.79	0	6.62	1.57	0.01	0.07	-0.38	10.08	8.26	17.35	8.13	-0.74	13.03	22.86	11.99	2.22
os21841	5	430	0.92	0	3	6	6.79	0	6.02	1.57	0.01	2.62	-0.16	11.38	8.26	17.34	8.13	-0.74	13.03	22.85	11.98	2.22
os21855	5	485	0.12	2	3	6	6.85	0.02	3.62	1.55	0.01	8.76	0.11	9.05	8.06	17.14	7.94	-0.99	12.8	22.63	11.82	1.92
os21859	5	404	0.92	2	3	6	6.7	-0.03	1.41	1.55	0.02	50.27	0.09	8.16	8.42	17.46	8.22	-0.68	13.25	23.05	12.11	2.3
os21875	5	513	0.52	6	3	5	6.57	-0.11	0.49	1.52	0.04	99	-0.49	6.47	7.91	16.95	7.84	-1.11	12.74	22.49	11.66	1.83
os21900	5	448	0.01	2	3	6	6.83	0.03	3.95	1.56	0.02	7.1	-0.65	9.36	8.25	17.41	8.14	-0.83	13.17	23.06	12.13	2.2
os21903	5	421	0.13	1	3	6	6.8	0.02	5.98	1.56	0.02	17.26	-0.6	8.44	8.35	17.4	8.15	-0.77	13.19	22.98	12.06	2.18
os21909	5	470	0.92	0	3	6	6.83	0.01	3.95	1.56	0.02	5.72	0	9.69	8.07	17.15	7.96	-0.95	12.78	22.62	11.81	1.97
os21910	5	470	0.92	1	3	6	6.83	0.01	3.99	1.55	0.02	6.4	0.16	9.7	8.08	17.15	7.96	-0.95	12.78	22.62	11.81	1.97
os21928	5	492	0.91	1	3	6	6.8	0	2.56	1.55	0.01	38.77	0.02	8.76	8.01	17.14	7.97	-0.94	12.9	22.76	11.97	2.1
os21962	5	474	0.02	1	3	6	6.84	0.02	3.95	1.56	0.02	12.5	-0.55	9.46	8.05	17.13	7.94	-0.98	12.78	22.6	11.8	1.94
os22009	5	455	0.92	0	3	6	6.81	0.01	3.58	1.56	0.01	9.55	-0.56	8.92	8.22	17.37	8.1	-0.85	13.13	23.03	12.1	2.17
os22038	5	435	0.6	2	3	6	6.76	-0.01	4.89	1.55	0.01	6.08	-0.12	8.58	8.26	17.27	8.09	-0.83	13.01	22.8	11.92	2.07
os22041	5	438	0.23	1	3	6	6.79	0	1.88	1.55	0.02	49.41	-0.21	8.23	8.23	17.26	8.09	-0.87	13.01	22.78	11.9	2.05
os22042	5	437	0.23	1	3	6	6.8	0.01	1.91	1.55	0.02	43.55	-0.14	8.37	8.23	17.26	8.09	-0.87	13.01	22.78	11.9	2.05
os22043	5	436	0.92	1	3	6	6.82	0.02	2.21	1.55	0.02	36.59	-0.34	8.58	8.23	17.26	8.09	-0.87	13.01	22.78	11.9	2.05
os22050	5	481	0.6	4	2	3	6.74	0	1.92	1.54	0.06	94.83	-2.52	8.61	7.99	17.01	7.91	-1	12.7	22.46	11.65	1.87
os22051	5	482	0.53	5	2	3	6.74	0	1.97	1.55	0.06	94.54	-2.64	8.56	7.98	17.01	7.91	-1.01	12.69	22.45	11.65	1.86
os22052	5	483	0.6	5	2	3	6.75	0.01	1.79	1.55	0.05	94.28	-2.5	8.52	7.98	17	7.9	-1.01	12.69	22.45	11.65	1.86
os22065	5	451	0.01	3	3	6	6.86	0.04	1.51	1.55	0.02	97.16	-0.05	7.71	8.15	17.19	8.03	-0.91	12.91	22.66	11.83	1.98
os22066	5	452	0.01	2	3	6	6.86	0.04	1.37	1.55	0.02	97.63	0.07	7.54	8.14	17.19	8.03	-0.92	12.91	22.66	11.82	1.98
os22082	5	448	0.83	4	3	5	6.65	-0.05	1.18	1.55	0.06	75.78	-1.92	8.33	8.13	17.16	8	-0.97	12.96	22.72	11.82	1.97
os22084	5	448	0.79	6	3	6	6.61	-0.07	1.02	1.54	0.06	77.71	-1.53	8.19	8.14	17.16	8	-0.96	12.97	22.73	11.83	1.98
os22122	5	416	0.23	2	3	6	6.78	0.02	1.68	1.55	0.03	71.96	-0.55	7.74	8.29	17.33	8.13	-0.76	13.01	22.81	11.94	2.17
os22124	5	424	0.01	3	3	6	6.84	0.04	1.27	1.55	0.03	76.52	-0.08	8.67	8.27	17.31	8.11	-0.78	12.98	22.78	11.92	2.15
os22131	6	401	0.92	1	3	6	6.76	-0.01	5.48	1.56	0.02	20.67	-0.22	9.29	8.42	17.48	8.22	-0.67	13.28	23.08	12.13	2.32
os22135	7	415	0.31	2	3	6	6.71	-0.03	1.95	1.56	0.04	84.21	-2.02	7.78	8.37	17.41	8.18	-0.72	13.18	22.98	12.08	2.26
os22138	6	402	0.01	2	3	6	6.84	0.04	2.33	1.56	0.03	61.32	-0.5	8.13	8.43	17.5	8.2	-0.68	13.29	23.09	12.16	2.32
os22143	6	419	0.73	13	3	5	6.3	-0.15	0.04	1.45	0.07	83.44	0.8	5.96	8.38	17.44	8.18	-0.73	13.21	23.03	12.11	2.27
os22145	5	435	0.7	4	3	6	6.68	-0.03	1.86	1.54	0.03	81.16	-1.03	8.84	8.26	17.35	8.12	-0.79	13.09	22.93	12.03	2.18
os22171	5	497	0.92	0	3	6	6.82	0	1.76	1.55	0	8.67	0.12	11.33	8.02	17.17	7.96	-0.96	12.94	22.83	11.99	2.07

os22172	5	497	0.92	0	3	6	6.82	0	1.91	1.55	0	9.36	0.06	11.35	8.02	17.17	7.96	-0.96	12.93	22.83	12	2.07
os22176	5	493	0.92	0	3	6	6.83	0.01	2.89	1.55	0.01	11.7	0.26	10.84	8.03	17.2	7.98	-0.94	12.95	22.86	12.01	2.08
os22224	5	453	0.92	0	3	6	6.8	0	4.72	1.56	0.01	7.93	-0.07	9.53	8.14	17.2	8.02	-0.89	12.85	22.68	11.87	2.07
os22255	6	421	0.92	1	3	6	6.75	-0.02	5.96	1.56	0.01	0.1	-0.11	9.09	8.3	17.43	8.21	-0.77	13.32	23.29	12.46	2.36
os22281	6	431	0.6	3	3	6	6.7	-0.03	6.92	1.54	0.02	11.35	0.73	8	8.01	16.97	7.92	-0.92	12.77	22.43	11.92	2.2
os22282	6	432	0.99	2	3	6	6.73	-0.04	6.96	1.56	0.02	8.82	-0.3	8.24	8.01	16.97	7.92	-0.92	12.77	22.43	11.92	2.2
os22314	5	429	0.08	2	3	6	6.83	0.03	6.9	1.55	0.01	17.03	0.89	8.45	8.04	17.02	7.97	-0.91	12.79	22.49	11.95	2.19
os22339	5	441	0.92	1	3	6	6.81	0.01	3.71	1.57	0.02	15.32	-0.51	8.99	7.82	16.77	7.77	-1.1	12.53	22.2	11.76	2.1
os22352	6	434	0.92	0	3	6	6.79	0	6.76	1.57	0	0.1	0	11.68	7.87	16.78	7.8	-1.01	12.56	22.19	11.77	2.17
os22362	6	441	0.23	1	3	6	6.8	0.01	3.82	1.57	0.02	15.22	-0.28	8.44	7.78	16.73	7.71	-1.1	12.49	22.12	11.67	2.09
os22368	7	436	0.73	1	3	6	6.79	0	6.86	1.56	0.01	3.36	-0.03	10.09	7.76	16.68	7.73	-1.03	12.45	22.09	11.68	2.14
os22373	6	469	0.92	0	3	6	6.79	-0.01	4.04	1.56	0.01	4.99	-0.36	9.08	7.68	16.64	7.65	-1.2	12.51	22.19	11.69	1.94
os22391	5	473	0.92	1	3	6	6.79	-0.01	5.27	1.54	0	25.36	0.72	10.17	7.32	16.21	7.39	-1.32	12.36	21.9	11.65	2.01
os22395	6	452	0	1	3	6	6.83	0.02	4.71	1.56	0.01	3.47	-0.05	9.6	7.75	16.71	7.76	-1.06	12.73	22.34	11.91	2.2
os22422	6	442	0.92	0	3	6	6.79	0	6.32	1.56	0.01	0.96	0.1	10.48	7.88	16.84	7.86	-0.98	12.81	22.42	11.97	2.22
os22423	5	443	0.92	0	3	6	6.77	-0.01	6.22	1.56	0.01	4.29	-0.17	8.75	7.88	16.83	7.85	-0.98	12.8	22.41	11.96	2.21
os22450	5	486	0.08	3	3	6	6.91	0.05	1.88	1.56	0.05	75.77	-1.77	8.26	7.7	16.61	7.74	-1.14	12.75	22.35	12.05	2.17
os22578	5	462	0.9	4	3	6	6.67	-0.06	3.91	1.55	0.03	29.12	-0.54	8.15	7.71	16.6	7.73	-1.14	12.78	22.34	12.02	2.27
os22580	5	482	0.92	0	3	6	6.8	-0.01	0.59	1.55	0	8.78	0.4	9.19	7.72	16.66	7.76	-1.06	12.69	22.32	11.98	2.2
os22590	5	453	0.92	0	3	6	6.8	0	4.88	1.56	0.01	16.77	0	12.17	7.71	16.64	7.74	-1.07	12.71	22.32	11.92	2.19
os22598	5	458	0.08	1	3	6	6.84	0.02	3.91	1.56	0.01	5.11	-0.15	9.76	7.75	16.7	7.77	-1.07	12.73	22.37	11.96	2.2
os22645	6	422	0.23	1	3	6	6.8	0.01	6.91	1.56	0.01	13.23	-0.01	8.61	8.15	17.15	8.06	-0.85	12.91	22.63	12.04	2.25
os22647	5	430	0.21	6	3	5	6.78	0.05	0.26	1.53	0.03	29.27	0.06	7.02	8.05	17.04	7.98	-0.87	12.78	22.49	11.95	2.18
os22649	6	432	0.92	0	3	6	6.79	0	3.87	1.57	0.01	16.28	-0.16	10.47	8.02	16.99	7.95	-0.89	12.74	22.44	11.92	2.16
os22664	5	424	0.92	0	3	6	6.79	0	6.98	1.56	0.01	1.19	0.23	10.81	8.26	17.31	8.17	-0.83	13.11	22.87	12.24	2.31
os22666	5	424	0.92	0	3	6	6.77	-0.01	6.98	1.56	0.01	1.17	0.16	10.76	8.26	17.31	8.17	-0.83	13.11	22.87	12.25	2.32
os22707	8	431	0	1	3	6	6.74	-0.02	5.62	1.56	0.02	7.56	-0.78	8.86	8.15	17.16	8.08	-0.81	12.93	22.69	12.11	2.3
os22711	5	433	0.42	2	3	6	6.82	0	6.77	1.56	0.01	4.6	-0.04	8.77	7.97	16.94	7.9	-0.95	12.67	22.37	11.86	2.15
os22718	5	431	0.92	1	3	6	6.76	-0.02	2.57	1.56	0.01	18.36	-0.35	9.11	8.15	17.17	8.1	-0.9	13.03	22.75	12.25	2.31
os22747	5	437	0.92	0	3	6	6.79	0	3.93	1.57	0.01	1.48	-0.3	9.81	7.85	16.81	7.79	-1.07	12.55	22.2	11.78	2.12
os22762	6	469	0.92	0	3	6	6.79	-0.01	2.25	1.55	0.03	59.08	-0.31	8.65	7.7	16.67	7.68	-1.17	12.47	22.13	11.69	2.01
os22763	6	469	0.92	0	3	6	6.8	0	2.73	1.56	0.04	57.17	-0.15	8.17	7.7	16.66	7.68	-1.17	12.47	22.13	11.69	2.01
os22770	6	441	0.08	1	3	6	6.8	0.01	3.69	1.57	0.01	3.99	-0.16	9.08	7.81	16.75	7.75	-1.1	12.52	22.13	11.7	2.1
os22774	6	441	0.92	0	3	6	6.8	0	3.84	1.57	0.01	14.17	-0.05	11.38	7.78	16.73	7.72	-1.1	12.5	22.12	11.67	2.09
os22779	6	434	0.99	2	3	6	6.77	-0.02	6.85	1.56	0.01	8.1	0.31	8.94	7.95	16.9	7.9	-0.97	12.83	22.44	12	2.23
os22780	5	437	0.92	1	3	6	6.82	0.02	5.94	1.56	0.01	7.62	-0.11	9.51	7.94	16.91	7.91	-0.97	12.87	22.48	12.02	2.23
os22781	6	436	0.92	0	3	6	6.79	0	6.79	1.57	0.01	4.34	-0.28	9.4	7.96	16.9	7.91	-0.97	12.84	22.45	12	2.24

os22784	6	442	0.92	0	3	6	6.8	0	5.95	1.57	0.01	11	-0.21	11.39	7.85	16.79	7.82	-1.01	12.79	22.4	11.96	2.21
os22785	6	442	0.92	0	3	6	6.79	0	5.95	1.57	0.01	8.96	-0.21	10.51	7.85	16.79	7.82	-1.01	12.79	22.4	11.96	2.21
os22787	6	445	0.92	0	3	6	6.8	0	5.93	1.56	0.01	0.58	-0.06	9.27	7.79	16.72	7.78	-1.04	12.74	22.35	11.9	2.2
os22788	6	445	0.92	0	3	6	6.8	0	5.93	1.57	0.01	0.6	-0.11	9.57	7.79	16.72	7.78	-1.04	12.74	22.35	11.9	2.2
os22789	6	445	0.92	0	3	6	6.81	0	5.93	1.57	0.01	0.75	-0.26	9.49	7.79	16.72	7.78	-1.04	12.74	22.35	11.91	2.2
os22790	6	445	0.92	0	3	6	6.81	0.01	5.92	1.57	0.01	0.98	-0.32	9.47	7.79	16.72	7.78	-1.04	12.74	22.35	11.91	2.2
os22791	6	445	0.92	0	3	6	6.81	0.01	5.92	1.57	0.01	1.07	-0.37	9.44	7.79	16.72	7.78	-1.04	12.74	22.35	11.91	2.2
os22792	6	445	0.92	0	3	6	6.81	0.01	5.92	1.57	0.01	1.14	-0.44	9.41	7.79	16.72	7.78	-1.04	12.74	22.35	11.91	2.2
os22793	6	445	0.92	0	3	6	6.81	0	5.92	1.57	0.01	1.22	-0.47	9.24	7.79	16.72	7.78	-1.05	12.74	22.35	11.91	2.2
os22794	6	445	0.77	1	3	6	6.81	0	5.91	1.57	0.01	1.34	-0.6	8.96	7.79	16.73	7.78	-1.05	12.74	22.35	11.91	2.2
os22802	6	459	0.01	2	3	6	6.72	-0.04	2.07	1.56	0.02	18.18	-0.46	8.55	7.71	16.69	7.73	-1.11	12.73	22.35	11.93	2.2
os22828	7	440	0.92	0	3	6	6.8	0	6.82	1.57	0.01	5.89	-0.33	9.49	7.66	16.63	7.68	-1.08	12.4	22.03	11.62	2.12
os22835	6	465	0.16	1	3	6	6.81	0.01	4.16	1.56	0.02	40.74	-0.17	8.58	7.57	16.56	7.6	-1.2	12.4	22.07	11.6	1.93
os22866	5	479	0.92	0	3	6	6.8	-0.01	2.67	1.56	0.01	8.06	-0.1	9.02	7.49	16.42	7.55	-1.2	12.42	22.05	11.69	2.04
os22882	7	415	0.65	3	3	6	6.81	0	6.98	1.55	0.02	12.59	0.01	8.42	8.24	17.25	8.15	-0.8	13.04	22.77	12.15	2.29
os22887	6	453	0.92	0	3	6	6.8	0	6.91	1.56	0.01	13.39	-0.23	10.89	7.58	16.54	7.59	-1.23	12.73	22.31	11.89	2.1
os22902	5	430	0.6	1	3	6	6.76	-0.01	3.77	1.56	0.01	9.99	0.14	9.27	8.15	17.16	8.1	-0.87	13	22.72	12.2	2.31
os22940	5	447	0.92	2	3	6	6.73	-0.04	4.92	1.56	0.03	18.89	-0.26	9.25	7.91	16.85	7.91	-0.99	12.83	22.46	12.05	2.25
os22991	7	442	0.77	2	3	6	6.8	-0.01	4.26	1.56	0.04	57.73	-0.98	9.13	8.01	17.02	7.98	-0.96	13.09	22.83	12.26	2.26
os22992	5	560	0.01	2	3	6	6.89	0.03	1.82	1.54	0.02	71.75	-0.3	7.64	7.6	16.59	7.64	-1.17	12.63	22.27	11.85	2.06
os23003	5	510	0.66	2	3	6	6.77	-0.03	0.84	1.54	0.01	49.66	0.31	8.26	7.76	16.69	7.77	-1.13	12.83	22.41	12.04	2.13
os23010	5	455	0.92	3	3	6	6.7	-0.05	4.76	1.56	0.04	61.84	-0.62	8.55	7.96	16.93	7.96	-1.01	13.06	22.72	12.24	2.2
os23012	5	485	0.08	1	3	6	6.84	0.01	2.83	1.55	0.01	1.35	0.25	8.89	7.85	16.8	7.86	-1.07	12.92	22.52	12.12	2.14
os23025	6	437	0.2	2	3	6	6.86	0.03	1.63	1.55	0.02	35.03	-0.35	8.11	8.25	17.13	8.23	-0.65	13.42	22.92	12.64	2.63
os23026	5	439	0.92	2	3	6	6.85	0.03	1.53	1.55	0.02	46.3	-0.3	8.03	8.25	17.14	8.25	-0.64	13.45	22.95	12.67	2.65
os23027	5	434	0.99	2	3	6	6.76	-0.02	1.14	1.55	0.01	10.28	0.2	9.65	8.27	17.17	8.27	-0.62	13.49	22.99	12.7	2.67
os23041	7	414	0.6	2	3	6	6.75	-0.01	6.98	1.57	0.02	13.21	-0.67	8.58	8.24	17.26	8.16	-0.79	13.04	22.77	12.15	2.29
os23044	7	413	0.92	0	3	6	6.79	0	6.99	1.57	0.02	10.41	-0.55	8.85	8.26	17.28	8.16	-0.77	13.07	22.82	12.19	2.3
os23067	5	392	0.92	0	3	6	6.77	0	5.91	1.56	0.01	0.51	0.16	11.68	8.61	17.67	8.41	-0.6	13.71	23.39	12.66	2.59
os23075	5	468	0.23	1	3	6	6.8	0	4.29	1.56	0.01	0.25	0.04	9.08	7.42	16.36	7.45	-1.31	12.48	22.07	11.68	1.97
os23100	5	446	0.26	2	3	6	6.84	0.02	1.51	1.55	0.01	34.44	-0.05	8.18	8.31	17.46	8.14	-0.8	13.16	23.09	12.14	2.19
os23118	7	414	0.7	1	3	6	6.79	0	6.27	1.56	0.03	28.29	-1.05	8.16	8.25	17.27	8.16	-0.78	13.06	22.81	12.18	2.3
os23129	5	477	0.92	0	3	6	6.81	0	2.95	1.55	0.02	27.51	-0.53	9.07	8.04	17.06	8	-0.84	12.67	22.54	11.68	1.92
os23131	7	470	0.97	8	3	5	6.62	-0.1	0.4	1.53	0.06	80.33	-1.06	8.08	7.92	16.89	7.92	-0.99	12.72	22.41	11.79	1.83
os23153	4	484	0.24	6	2	0	6.74	0.02	0.48	1.54	0.07	99.55	-0.43	6.32	7.87	16.87	7.85	-1.04	12.54	22.28	11.5	1.76
os23193	7	427	0.92	1	2	3	6.78	-0.01	2.95	1.56	0.05	86.42	-2.2	9.17	8.05	16.98	8.17	-0.68	12.67	22.44	11.88	2.01
os23194	5	451	0.92	2	3	6	6.71	-0.04	1.63	1.54	0.02	83.06	-0.18	7.89	7.99	16.91	8.14	-0.67	12.58	22.36	11.82	2.02

os23209	7	496	0.11	2	3	6	6.85	0.03	2.59	1.54	0.04	77.46	-0.36	9.73	7.5	16.38	7.69	-1.16	11.89	21.65	11.18	1.45
os23212	7	512	0.87	2	3	6	6.74	-0.04	1.89	1.54	0.03	78.66	-0.64	8.57	7.6	16.54	7.81	-0.97	12.13	21.95	11.45	1.68
os23259	5	480	0.92	3	3	6	6.71	-0.04	2.75	1.55	0.04	75.12	-0.77	8.87	7.89	16.89	7.86	-0.94	12.43	22.25	11.48	1.78
os23272	3	442	0.33	24	2	3	5.97	0.05	0	1.44	0.21	88.84	-5.52	5.7	8.11	17.08	8.07	-0.82	12.89	22.6	11.88	2
os23296	5	456	0.4	3	2	3	6.85	0.02	2.97	1.55	0.06	85.88	-2.47	9.19	8.1	17.11	8.03	-0.8	12.76	22.62	11.75	1.97
os23341	5	379	0.82	4	2	0	6.72	-0.04	0.75	1.53	0.07	89.76	-0.55	7.13	8.48	17.41	8.39	-0.5	13.12	22.86	12.11	2.21
os23350	5	492	0.81	4	2	0	6.68	-0.07	1.62	1.55	0.05	87.47	-1.23	8.33	7.83	16.84	7.82	-0.98	12.37	22.2	11.43	1.74
os23373	5	486	0.8	4	3	6	6.71	-0.03	0.76	1.54	0.06	73.46	-0.68	7.59	7.92	16.95	7.9	-0.94	12.59	22.4	11.63	1.82
os23392	5	568	0.03	4	3	6	6.88	0.04	1.84	1.54	0.04	84.6	-0.46	9.22	7.49	16.5	7.53	-1.24	12.05	21.83	11.08	1.52
os23393	5	569	0.23	4	3	6	6.86	0.03	1.98	1.54	0.04	84.02	-0.61	9.24	7.49	16.5	7.53	-1.25	12.04	21.83	11.08	1.52
os23400	5	629	0.11	4	3	6	6.98	0.06	1.3	1.53	0.03	94.17	0	7.7	7.15	16.18	7.25	-1.49	11.75	21.46	10.85	1.29
os23440	5	436	0.15	4	3	6	6.67	-0.06	2.03	1.54	0.05	90.92	-1.76	9.18	8.16	17.17	8.1	-0.81	12.95	22.71	11.92	1.99
os23488	6	515	0.08	1	3	6	6.86	0.01	2.95	1.55	0.03	67.87	-0.96	8.65	7.84	16.91	7.82	-1.14	12.6	22.44	11.62	1.7
os23589	5	557	0.76	2	2	0	6.85	-0.01	1.94	1.55	0.07	97.43	-1.49	8.06	7.38	16.36	7.46	-1.38	12.01	21.75	11.18	1.46
os23591	7	556	0.76	3	2	0	6.88	0.01	1.92	1.55	0.07	97.14	-1.37	8.06	7.39	16.37	7.47	-1.37	12.02	21.76	11.19	1.47
os23714	5	500	0.05	6	2	7	6.88	0.08	0.76	1.53	0.13	97.16	-1.82	7.55	7.67	16.65	7.66	-1.21	12.42	22.05	11.46	1.67
os23771	4	605	0.65	12	1	1	6.32	-0.2	0	1.42	0.09	100	5.01	5.9	7.35	16.36	7.39	-1.37	12.04	21.71	11.16	1.47
os23781	5	582	0.85	7	3	5	6.58	-0.09	0.63	1.51	0.09	99.3	-1.98	6.65	7.34	16.34	7.4	-1.43	12.05	21.73	11.16	1.44
os23829	6	658	0.77	2	3	6	6.89	-0.01	2.68	1.55	0.03	59.99	-0.75	7.8	6.45	15.44	6.72	-2.08	10.97	20.69	10.37	0.83
os23964	5	536	0.97	5	3	5	6.75	-0.06	0.71	1.55	0.05	90.89	-1.39	7.15	7.03	16.02	7.17	-1.78	11.6	21.34	10.85	1.03
os23994	5	622	0.83	2	3	6	6.86	-0.02	2.91	1.55	0.04	59.48	-0.51	8.83	6.51	15.51	6.78	-2	10.89	20.66	10.34	0.8
os24018	5	663	0.23	2	3	6	6.86	0	2.68	1.55	0.04	45.73	-1.21	9.33	6.35	15.32	6.65	-2.13	10.85	20.54	10.26	0.74
os24048	6	500	0.07	5	2	7	6.92	0.08	0.99	1.55	0.08	77.73	-2.47	8.17	7.14	16.15	7.27	-1.72	11.75	21.54	11	1.07
os24080	5	519	0.99	2	2	0	6.8	-0.03	3.97	1.55	0.07	63.64	-0.94	9.05	7.21	16.19	7.33	-1.59	11.88	21.59	11.09	1.4
os24108	4	488	0.92	0	3	6	6.83	0.01	4.73	1.56	0.01	22.77	0.48	9.69	7.75	16.79	7.7	-1.3	12.7	22.48	11.79	1.73
os24109	5	501	0.92	1	3	6	6.85	0.01	2.62	1.56	0.02	35.13	0.04	8.51	7.67	16.72	7.67	-1.31	12.56	22.33	11.65	1.7
os24115	5	517	0	7	3	5	6.6	-0.11	0.82	1.54	0.06	92.83	-1.93	7.15	7.6	16.66	7.61	-1.39	12.48	22.27	11.61	1.64
os24116	5	495	0.77	1	3	6	6.8	-0.01	2.56	1.56	0.03	44.6	-0.03	8.2	7.7	16.74	7.68	-1.33	12.59	22.38	11.7	1.71
os24119	5	480	0.92	2	3	6	6.76	-0.02	3.7	1.56	0.03	36.37	-0.1	8.52	7.76	16.79	7.7	-1.31	12.74	22.53	11.82	1.77
os24122	4	482	0.92	2	3	6	6.74	-0.03	3.61	1.55	0.02	34.05	0.5	8.66	7.77	16.81	7.71	-1.3	12.76	22.54	11.83	1.78
os24123	7	485	0.73	1	3	6	6.82	-0.01	3.12	1.56	0.02	26.4	0.01	9.41	7.78	16.81	7.73	-1.3	12.77	22.55	11.84	1.78
os24143	5	530	0.99	5	3	5	6.65	-0.09	0.47	1.52	0.04	96.81	0.22	7.4	7.58	16.64	7.57	-1.4	12.41	22.18	11.54	1.65
os24144	7	559	0.92	2	2	3	6.81	0.01	1.99	1.56	0.08	71.02	-4	7.48	7.32	16.39	7.39	-1.51	12.06	21.85	11.2	1.44
os24170	5	522	0.6	2	3	6	6.79	-0.01	2.98	1.56	0.02	14.73	-0.35	9.1	7.44	16.44	7.45	-1.52	12.2	21.92	11.33	1.46
os24192	5	576	0.1	4	3	6	6.89	0.05	0.86	1.54	0.05	87.21	-0.21	7.15	7.24	16.32	7.33	-1.56	11.96	21.77	11.13	1.39
os24197	5	565	0.23	2	3	6	6.88	0.03	2.42	1.54	0.03	55.81	-0.65	8.14	7.37	16.42	7.43	-1.47	12.13	21.9	11.32	1.54
os24213	5	529	0.23	1	3	6	6.83	0.01	2.45	1.55	0.02	41.35	-0.14	9.09	7.38	16.38	7.41	-1.55	12.11	21.84	11.27	1.42

os24221	5	539	0.92	1	3	6	6.81	-0.02	2.97	1.56	0.03	62.6	-0.56	8.75	7.54	16.59	7.55	-1.35	12.28	22.11	11.4	1.61
os24252	5	611	0.01	5	3	6	6.89	0.05	0.59	1.53	0.05	99.89	-0.17	7.49	7.02	16.1	7.17	-1.67	11.68	21.51	10.91	1.24
os24264	5	574	0.76	6	3	5	6.65	-0.1	0.63	1.53	0.06	98.25	-1.28	6.86	7.27	16.32	7.34	-1.54	11.96	21.76	11.06	1.36
os24267	5	592	0.01	3	3	6	6.77	-0.05	1.91	1.54	0.05	93.19	-1.66	8.25	7.08	16.13	7.19	-1.65	11.65	21.45	10.84	1.2
os24282	5	559	0.47	2	3	6	6.86	0	2.95	1.56	0.03	64.21	-0.96	8.61	7.22	16.26	7.32	-1.56	11.83	21.64	10.98	1.29
os24292	7	488	0.39	4	3	6	6.76	0	1.63	1.55	0.02	49.99	0.17	8.51	7.81	16.88	7.79	-1.16	12.63	22.41	11.75	1.83
os24297	5	479	0.6	1	3	6	6.79	-0.01	3.57	1.56	0.02	38.01	-0.22	8.57	7.93	16.99	7.89	-1.1	12.81	22.61	11.82	1.9
os24325	5	499	0.99	3	3	6	6.76	-0.04	1.76	1.55	0.03	72.5	-0.4	7.78	7.68	16.72	7.67	-1.34	12.58	22.37	11.7	1.69
os24331	7	507	0.81	5	3	5	6.63	-0.09	0.61	1.54	0.05	89.48	-1.01	7.25	7.68	16.71	7.67	-1.35	12.59	22.37	11.7	1.69
os24332	5	498	0.99	2	3	6	6.76	-0.03	2.3	1.56	0.03	68.89	-0.56	7.81	7.69	16.72	7.67	-1.34	12.59	22.37	11.7	1.7
os24343	5	510	0.01	2	3	6	6.85	0.02	3.67	1.56	0.03	54.62	-0.7	8.93	7.62	16.66	7.63	-1.38	12.54	22.32	11.68	1.67
os24367	5	493	0.08	3	3	6	6.86	0.05	1.68	1.55	0.03	53.78	-0.71	8.18	7.71	16.76	7.7	-1.3	12.62	22.39	11.7	1.72
os24387	5	484	0.31	4	3	6	6.78	0.02	0.84	1.54	0.03	63.35	0.05	7.74	7.77	16.85	7.7	-1.33	12.72	22.51	11.74	1.73
os24418	5	537	0.43	5	3	6	6.74	-0.01	0.63	1.54	0.04	96.29	-0.31	7.28	7.33	16.32	7.37	-1.57	12.06	21.77	11.22	1.43
os24419	5	538	0.51	5	3	6	6.74	-0.01	0.61	1.54	0.04	96.39	-0.28	7.25	7.33	16.32	7.37	-1.58	12.06	21.77	11.22	1.42
os24430	5	491	0.92	0	3	6	6.82	0	4.91	1.55	0.01	2.4	0.15	9.63	7.74	16.79	7.67	-1.36	12.68	22.43	11.65	1.68
os24453	5	488	0.66	3	3	6	6.77	-0.01	2.88	1.55	0.03	72.24	-0.56	8.45	7.71	16.73	7.63	-1.38	12.56	22.35	11.56	1.62
os24463	5	496	0.08	2	3	6	6.87	0.02	5.02	1.56	0.02	15.91	-0.37	8.4	7.63	16.68	7.6	-1.43	12.45	22.3	11.53	1.58
os24478	5	528	0.92	1	3	6	6.86	0.02	1.74	1.56	0.01	18.06	0.11	8.78	7.46	16.48	7.45	-1.55	12.23	21.99	11.27	1.37
os24533	5	576	0.36	3	3	6	6.94	0.04	0.93	1.54	0.03	82.75	-0.12	8.07	7.1	16.11	7.2	-1.77	11.73	21.49	10.9	1.1
os24538	7	569	0.27	5	3	5	6.97	0.05	0.6	1.53	0.05	72.04	-0.69	7.21	7.1	16.11	7.2	-1.76	11.71	21.48	10.89	1.11
os24569	5	550	0.92	0	3	6	6.83	0	3.91	1.55	0.02	32.96	-0.02	9.24	7.22	16.24	7.3	-1.68	11.86	21.56	11.02	1.18
os24632	5	643	0.21	4	3	5	6.99	0.05	0.94	1.53	0.05	97.17	-1.11	6.84	6.82	15.83	6.99	-2.01	11.51	21.26	10.72	0.87
os24677	5	543	0.18	1	3	6	6.83	0	2.63	1.56	0.03	51.14	-0.5	8.28	7.19	16.17	7.28	-1.66	11.86	21.57	11.01	1.23
os24701	7	612	0.17	6	2	3	6.67	-0.09	1.39	1.54	0.11	96.11	-5.29	8.19	6.55	15.54	6.81	-1.97	10.96	20.7	10.32	0.83
os24704	7	635	0.61	3	2	3	6.77	-0.04	1.98	1.55	0.07	93.97	-3.59	7.98	6.49	15.48	6.77	-2.02	10.91	20.64	10.25	0.78
os24706	6	674	0.01	4	3	6	6.97	0.06	0.79	1.54	0.02	74.74	-0.19	7.66	6.38	15.36	6.67	-2.12	10.9	20.59	10.29	0.78
os24712	5	646	0.23	4	3	6	6.89	0.03	0.77	1.54	0.02	82.05	-0.12	7.46	6.58	15.57	6.82	-1.99	11.22	20.94	10.56	0.98
os24718	7	632	0.31	8	3	5	6.74	0.01	0.3	1.52	0	89.04	-1.78	7.42	6.52	15.56	6.81	-2.04	11.19	20.94	10.59	0.96
os24819	5	592	0.35	3	3	6	6.79	-0.03	2.95	1.55	0.06	83.77	-1.27	8.16	7	15.98	7.15	-1.76	11.61	21.34	10.78	1.07
os24863	7	539	0.7	9	2	3	6.51	-0.14	0.61	1.5	0.1	92.98	-3.74	7.68	7.66	16.67	7.63	-1.18	12.31	22.06	11.33	1.62
os24883	5	668	0.4	5	3	6	6.98	0.03	0.8	1.52	0.03	99.87	-0.45	7.33	6.77	15.74	6.93	-1.93	11.35	21.04	10.54	0.92
os24925	5	580	0.36	2	3	6	6.9	0.02	2.98	1.56	0.04	78.81	-0.89	9.42	7.25	16.28	7.3	-1.55	11.93	21.69	11.01	1.32
os24947	5	503	0.76	2	3	6	6.75	-0.02	2.05	1.55	0.03	82.54	-0.91	8.65	7.69	16.74	7.68	-1.22	12.41	22.23	11.49	1.73
os24974	5	658	0.08	2	3	6	6.96	0.04	1.76	1.54	0.02	78.29	-0.1	8.38	6.81	15.81	6.96	-1.82	11.39	21.14	10.56	1.01
os24977	5	602	0.46	4	3	6	6.93	0.02	3.85	1.56	0.04	57.31	-0.53	8.99	7.02	16	7.11	-1.72	11.59	21.33	10.73	1.13
os25026	5	607	0.97	4	3	6	6.82	-0.04	1.45	1.54	0.05	97.53	-0.69	7.19	7.03	16.02	7.12	-1.72	11.62	21.34	10.74	1.11

os25029	7	595	0.44	7	3	5	6.71	-0.02	0.1	1.52	0.05	71.5	-0.38	7.92	7.07	16.06	7.15	-1.72	11.66	21.35	10.75	1.12
os25079	5	627	0.92	2	3	6	6.91	0.03	0.66	1.53	0.02	91.23	-0.28	6.68	7.09	16.08	7.16	-1.74	11.76	21.44	10.82	1.13
os25082	5	623	0	4	3	6	6.98	0.07	0.68	1.52	0.03	93.54	-0.22	6.72	7.08	16.07	7.15	-1.75	11.75	21.43	10.81	1.12
os25087	5	671	0.4	5	3	6	6.99	0.04	0.67	1.52	0.03	99.79	-0.16	7.3	6.76	15.73	6.92	-1.94	11.34	21.03	10.53	0.91
os25098	5	623	0.36	3	3	6	6.94	0.02	2.11	1.55	0.05	86.82	-0.81	7.83	6.9	15.87	7.04	-1.86	11.46	21.17	10.64	0.99
os25124	7	640	0.89	5	3	6	6.68	-0.07	0.55	1.52	0.05	92.74	-0.19	7.5	6.68	15.64	6.88	-2.09	11.3	20.99	10.53	0.74
os25147	5	648	0.12	6	1	4	6.89	0.05	0.31	1.52	0.02	53.66	1.53	7.45	6.74	15.74	6.93	-2.08	11.44	21.16	10.63	0.76
os25152	6	647	0.01	2	3	6	6.91	0.02	2.89	1.55	0.02	48.38	-0.86	8.58	6.68	15.68	6.9	-2.1	11.37	21.07	10.58	0.73
os25168	7	490	0.13	5	2	7	6.85	0.08	1.31	1.55	0.13	86.36	-5.04	8.79	7.69	16.72	7.67	-1.24	12.4	22.2	11.37	1.55
os25171	5	484	1	7	2	3	6.57	-0.11	0.88	1.54	0.06	98.54	-2.49	8.16	7.81	16.83	7.78	-1.16	12.54	22.36	11.52	1.67
os25218	7	510	0.65	3	3	6	6.85	0	1.98	1.54	0.04	88.95	-0.63	8.81	7.74	16.79	7.69	-1.2	12.49	22.36	11.48	1.6
os25223	4	526	0.71	18	3	5	6.61	-0.08	0	1.44	0.12	89.92	-1.65	5.9	7.68	16.73	7.64	-1.24	12.42	22.26	11.41	1.56
os25248	5	600	0.11	2	3	6	6.91	0.02	2.65	1.54	0.02	44.37	-0.3	8.15	7.29	16.38	7.37	-1.49	12.01	21.81	11.08	1.34
os25257	5	539	0.37	9	3	5	6.72	0.03	0.43	1.5	0.07	86.48	-0.47	8.47	7.54	16.56	7.54	-1.38	12.28	22.07	11.27	1.45
os25319	5	542	0.23	3	3	6	6.83	0.02	1.65	1.53	0.02	76.81	0.48	8.54	7.77	16.87	7.72	-1.08	12.41	22.37	11.43	1.71
os25348	7	484	0.98	3	3	6	6.73	-0.05	2.24	1.55	0.04	85.21	-1.46	8.71	7.95	17	7.89	-0.95	12.56	22.52	11.58	1.82
os25354	5	505	0.99	4	3	6	6.71	-0.06	0.6	1.53	0.03	93.71	-0.09	6.95	7.95	16.99	7.88	-0.96	12.56	22.52	11.57	1.81
os25355	5	514	0.99	4	3	6	6.7	-0.07	0.52	1.52	0.02	87.86	0.91	7.03	7.94	16.99	7.87	-0.97	12.56	22.51	11.57	1.81
os25357	5	496	0.8	3	3	6	6.77	-0.04	0.77	1.54	0.03	93.54	-0.22	7.21	7.95	16.99	7.88	-0.97	12.57	22.52	11.58	1.81
os25384	7	608	0.78	3	3	6	6.84	-0.03	1.96	1.52	0.05	97.79	-1.93	7.81	7.29	16.27	7.32	-1.48	11.93	21.66	11.04	1.36
os25447	7	526	0.92	5	3	6	6.65	-0.06	0.86	1.54	0.05	96.47	-1.52	8.2	7.49	16.46	7.54	-1.42	12.15	21.97	11.25	1.4
os25454	7	515	0.23	2	3	6	6.81	0.01	2.97	1.56	0.04	50.86	-0.35	8.99	7.61	16.63	7.63	-1.33	12.36	22.17	11.4	1.5
os25506	5	599	0.35	3	3	6	6.82	0.01	1.86	1.54	0.05	94.62	-1.53	7.96	6.96	15.9	7.16	-1.81	11.62	21.32	10.77	0.99
os25511	5	654	0.77	3	3	6	6.87	-0.02	2.78	1.55	0.03	61.45	-0.25	8.87	6.6	15.55	6.83	-2.06	11.19	20.89	10.43	0.7
os25516	5	573	0.61	2	3	6	6.8	-0.02	3.88	1.56	0.04	63.86	-0.4	9.29	7.21	16.19	7.33	-1.61	11.85	21.63	10.98	1.2
os25517	5	586	0.68	3	3	6	6.87	-0.01	0.63	1.53	0.03	77.94	0.31	8.11	7.21	16.2	7.33	-1.61	11.88	21.66	10.99	1.21
os25534	5	578	0.77	4	3	6	6.85	-0.02	0.64	1.53	0.05	81.22	-0.31	7.99	7.23	16.21	7.35	-1.6	11.89	21.68	11.01	1.22
os25538	7	547	0.69	6	3	5	6.63	-0.06	0.83	1.52	0.06	87.66	-1.19	8.12	7.5	16.52	7.51	-1.4	12.23	22	11.23	1.44
os25546	5	596	0.01	3	3	6	6.95	0.05	1.44	1.52	0.04	89.82	-0.37	6.78	7.32	16.34	7.37	-1.52	12.09	21.86	11.12	1.35
os25549	5	588	0.4	2	3	6	6.88	0	1.33	1.52	0.02	70.74	1.55	8.46	7.34	16.34	7.37	-1.51	12.1	21.86	11.13	1.36
os25567	5	576	0.46	3	3	6	6.82	0.01	1.86	1.55	0.03	64.88	-0.21	8.59	7.29	16.28	7.35	-1.58	12.02	21.76	11.09	1.31
os25580	7	540	0.07	3	2	3	6.75	-0.04	2.13	1.55	0.07	93.51	-2.17	8.5	7.42	16.42	7.47	-1.49	12.15	21.92	11.22	1.39
os25582	7	541	0.85	3	2	3	6.74	-0.05	2.04	1.55	0.07	93.44	-2.39	8.42	7.42	16.42	7.47	-1.49	12.15	21.92	11.22	1.39
os25589	5	563	0.6	4	3	6	6.72	-0.03	0.87	1.54	0.05	94.74	-0.79	7.68	7.3	16.35	7.39	-1.57	12.07	21.86	11.12	1.3
os25717	8	580	0.06	7	3	5	6.93	0.1	0.2	1.52	0.06	91.86	-0.16	8.05	7.01	15.95	7.15	-1.8	11.6	21.37	10.78	0.98
os25718	8	583	0.12	8	3	5	6.87	0.09	0.15	1.51	0.07	93.5	-0.06	7.96	7	15.95	7.14	-1.8	11.6	21.37	10.77	0.98
os25720	8	582	0.01	8	3	5	6.96	0.13	0.06	1.51	0.07	91.59	0.38	7.67	7.01	15.96	7.15	-1.79	11.6	21.38	10.78	0.98

os25757	5	648	0.56	3	3	6	6.89	0.03	1.78	1.54	0.03	83.97	-0.45	8.54	6.69	15.63	6.89	-1.95	11.24	20.94	10.48	0.86
os25831	5	623	0.77	5	2	3	6.86	-0.03	1.52	1.55	0.07	95.25	-2.47	7.81	6.84	15.8	7	-1.9	11.37	21.13	10.63	0.94
os25843	5	608	0.33	7	3	5	6.73	-0.01	0.31	1.53	0.06	93.58	-0.46	8.72	6.91	15.86	7.06	-1.84	11.48	21.21	10.68	0.99
os25854	5	679	0.97	2	3	6	6.85	-0.03	2.54	1.54	0.02	41.57	-0.31	8.42	6.56	15.55	6.82	-2.07	11.12	20.83	10.41	0.76
os25872	5	598	0.35	3	3	6	6.94	0.03	1.51	1.55	0.03	80.5	-0.3	8.07	6.98	15.93	7.11	-1.85	11.67	21.34	10.8	1.01
os25910	5	628	0.03	2	3	6	6.9	0.03	2.82	1.55	0.03	62.95	-0.96	8.93	6.8	15.78	6.98	-1.95	11.37	21.09	10.59	0.85
os25933	5	584	0.4	4	3	6	6.93	0.03	1.08	1.54	0.04	93.85	-0.66	8.01	7.38	16.46	7.43	-1.32	11.99	21.84	11.1	1.47
os25960	7	570	0.77	3	3	6	6.82	-0.03	2.55	1.55	0.04	85.32	-0.58	8.76	7.4	16.44	7.41	-1.43	12.13	21.88	11.15	1.45
os25965	7	600	0.89	3	3	6	6.83	-0.03	2.07	1.55	0.04	88.54	-1.97	8.63	7.33	16.35	7.35	-1.49	12.03	21.8	11.09	1.39
os26004	4	543	0.98	3	3	6	6.75	-0.05	0.15	1.52	0.04	93.57	1.74	8.15	7.54	16.59	7.52	-1.33	12.29	22.06	11.28	1.52
os26066	7	622	0.84	9	3	5	6.74	-0.08	0.06	1.47	0.07	99.75	1.74	6.02	7.09	16.09	7.19	-1.63	11.82	21.46	10.94	1.24
os26105	5	599	0.21	2	3	6	6.91	0.02	2.89	1.55	0.03	59.05	-0.67	8.61	7.25	16.25	7.29	-1.6	12.03	21.68	11.12	1.32
os26106	5	598	0.73	2	3	6	6.89	0.01	2.94	1.55	0.03	56.23	-0.69	8.78	7.26	16.25	7.3	-1.59	12.03	21.69	11.13	1.32
os26119	5	573	0.85	9	3	5	6.67	-0.1	0.13	1.5	0.08	95.95	-1.37	6.05	7.34	16.32	7.36	-1.53	12.15	21.77	11.24	1.38
os26121	5	574	0.88	11	2	3	6.63	-0.11	0.08	1.5	0.09	96.42	-1.77	5.92	7.35	16.33	7.37	-1.53	12.16	21.77	11.25	1.38
os26127	7	569	0.37	11	3	5	6.63	0.03	0.02	1.49	0.11	97.32	-1.24	6.9	7.4	16.38	7.43	-1.49	12.23	21.85	11.31	1.42
os26128	7	546	0.08	13	2	0	6.78	0.13	0.01	1.48	0.13	94.08	-2.12	7.04	7.45	16.43	7.47	-1.47	12.28	21.9	11.34	1.45
os26249	4	647	0.99	10	3	9	6.58	-0.15	0.09	1.46	0.07	99.79	0.21	5.76	7.28	16.31	7.36	-1.54	12.09	21.73	11.19	1.38
os26265	5	581	0.47	4	2	7	6.87	0.05	1.39	1.53	0.1	99.38	-3.23	8.36	7.23	16.23	7.3	-1.49	11.96	21.6	11.03	1.34
os26268	7	478	0.96	5	2	7	6.7	-0.06	2.29	1.56	0.09	83.16	-3.75	8.23	7.89	16.89	7.82	-1.16	12.62	22.37	11.67	1.69
os26290	5	543	0.09	2	3	6	6.9	0.03	2.8	1.54	0.02	52.69	0.43	8.7	7.53	16.58	7.55	-1.35	12.27	22.11	11.4	1.6
os26306	7	640	0.06	2	3	9	6.92	0.03	1.9	1.51	0.04	66.38	-0.34	7.66	7.14	16.15	7.24	-1.57	11.84	21.52	10.97	1.29
os26346	7	504	0.17	10	2	3	6.56	-0.04	1.05	1.55	0.15	96.81	-7.03	8.57	7.76	16.76	7.71	-1.21	12.48	22.23	11.49	1.66
os26355	7	497	0.92	1	2	3	6.81	-0.01	2.9	1.56	0.08	85.57	-3.16	8.81	7.78	16.78	7.75	-1.19	12.5	22.24	11.49	1.68
os26358	7	548	0.36	5	2	3	6.76	0	1.5	1.53	0.08	97.91	-3.09	8.29	7.55	16.6	7.55	-1.36	12.3	22.04	11.32	1.56
os26365	6	458	0.83	5	3	6	6.83	0	3.19	1.55	0.06	64.55	-0.25	8.48	8.04	17.09	7.94	-1	12.79	22.64	11.78	1.97
os26378	7	593	0.23	1	3	6	6.88	0.01	3.99	1.56	0.04	57.95	-1.18	8.8	7.3	16.31	7.33	-1.56	12.08	21.74	11.14	1.34
os26396	5	547	0.27	4	3	6	6.8	0.02	0.66	1.53	0.04	90.07	0.29	8.66	7.62	16.67	7.58	-1.38	12.52	22.2	11.51	1.55
os26450	4	693	0.92	15	1	1	6.51	-0.16	0	1.38	0.09	93.31	5.76	6.12	6.99	16.02	7.09	-1.64	11.66	21.33	10.85	1.23
os26456	5	638	0.4	2	3	6	6.93	0.02	0.56	1.53	0.02	55.71	0.24	8.58	7.08	16.07	7.19	-1.64	11.8	21.44	10.94	1.23
os26457	5	638	0.32	1	3	6	6.86	0	0.5	1.53	0.03	63.18	0.4	8.86	7.08	16.08	7.18	-1.64	11.79	21.44	10.93	1.23
os26505	7	639	0	2	3	9	6.93	0.03	2.14	1.51	0.04	61.43	-0.32	7.92	7.14	16.15	7.24	-1.57	11.84	21.52	10.97	1.29
os26508	5	648	0.47	4	3	9	6.81	-0.01	1.75	1.5	0.07	90.27	-1.99	6.47	7.13	16.13	7.23	-1.58	11.82	21.5	10.95	1.26
os26513	5	628	0.4	2	3	6	6.9	0	1.93	1.54	0.03	69.2	-0.67	7.92	7.13	16.13	7.22	-1.61	11.84	21.48	10.97	1.24
os26516	5	631	0.53	2	3	6	6.9	0	1.53	1.54	0.03	61.74	-0.1	8.02	7.1	16.11	7.21	-1.61	11.79	21.46	10.92	1.23
os26517	6	621	0.77	1	3	6	6.88	0	2.97	1.54	0.03	61.59	-0.19	8.7	7.1	16.11	7.2	-1.61	11.78	21.45	10.92	1.22
os26569	7	598	0	5	2	0	6.99	0.09	0.62	1.53	0.08	99.87	-1.04	7.4	7.16	16.24	7.29	-1.53	11.74	21.55	10.91	1.3

os26583	7	527	0.91	9	2	3	6.53	-0.12	1.01	1.53	0.09	95.77	-3.34	8.57	7.71	16.71	7.69	-1.24	12.42	22.18	11.44	1.63
os26585	5	491	0.15	14	2	3	6.69	0.12	0.2	1.52	0.13	90.35	-4.87	7.6	7.88	16.9	7.81	-1.12	12.65	22.39	11.58	1.78
os26591	7	503	0.99	8	3	5	6.56	-0.13	0.52	1.52	0.08	90.07	-1.85	7.57	7.75	16.78	7.72	-1.22	12.5	22.25	11.49	1.67
os26603	5	560	0.13	3	2	3	6.93	0.05	2.23	1.55	0.06	95.32	-2.99	9.18	7.39	16.43	7.42	-1.44	12.07	21.85	11.17	1.46
os26610	7	485	0.71	7	2	0	6.81	-0.02	0.36	1.54	0.1	66.29	-0.83	8.1	7.74	16.77	7.7	-1.21	12.45	22.23	11.49	1.71
os26625	7	509	0.01	5	3	5	6.95	0.09	0.56	1.53	0.05	88.53	-0.63	9.18	7.59	16.66	7.6	-1.28	12.32	22.12	11.41	1.64
os26628	5	582	0.6	4	3	6	6.74	-0.03	0.85	1.53	0.03	95.88	-0.14	7.54	7.39	16.47	7.46	-1.43	12.13	21.95	11.21	1.52
os26629	5	479	0.92	1	3	6	6.81	0	3.59	1.56	0.03	61.58	-0.65	9.42	7.94	17.01	7.88	-1.05	12.68	22.54	11.69	1.92
os26630	5	479	0.6	2	3	6	6.81	0.01	3.46	1.56	0.03	60.34	-1.46	9.19	7.94	17	7.87	-1.06	12.67	22.53	11.68	1.91
os26646	5	485	0	3	3	6	6.71	-0.05	1.55	1.53	0.05	94.2	-0.22	7.87	7.43	16.34	7.6	-1.19	12.17	21.73	11.5	1.66
os26737	4	524	0.98	7	2	7	6.58	-0.11	0.62	1.54	0.12	76.65	-1.62	7.6	6.47	15.28	6.91	-2.11	11.07	20.7	10.46	0.55
os26738	7	543	0.68	11	2	7	6.42	-0.12	0.08	1.51	0.11	91.92	-2.25	7.7	6.45	15.26	6.89	-2.12	11.04	20.67	10.43	0.54
os26763	7	588	0.92	5	3	6	6.68	-0.07	1.49	1.51	0.06	98.37	-1.35	7.43	6.51	15.35	6.9	-2.12	11.16	20.79	10.58	0.54
os26775	5	498	0.77	2	3	6	6.8	-0.02	2.57	1.56	0.02	52.09	-0.06	9.03	6.95	15.77	7.4	-1.67	11.64	21.31	11.05	0.98
os26782	5	513	0.6	2	3	6	6.76	-0.03	0.87	1.54	0.01	63.09	0.34	8.3	6.94	15.74	7.39	-1.63	11.63	21.28	11.03	1.03
os26786	7	634	0.1	5	2	3	6.7	-0.08	1.6	1.54	0.07	98.07	-2.94	9.42	6.05	14.85	6.68	-2.23	10.46	20.11	10.07	0.34
os26792	5	587	0.92	4	3	6	6.71	-0.07	0.85	1.54	0.04	92.35	-0.4	8.8	6.32	15.11	6.88	-2.04	10.74	20.4	10.29	0.54
os26802	5	501	0.85	3	3	6	6.77	-0.04	1.05	1.55	0.02	55.53	0.02	8.68	6.94	15.76	7.39	-1.68	11.63	21.3	11.04	0.97
os26803	5	500	0.9	2	3	6	6.78	-0.03	1.26	1.55	0.02	55.41	-0.17	8.77	6.94	15.76	7.4	-1.68	11.63	21.3	11.05	0.97
os26827	4	586	0.6	6	3	5	6.71	-0.03	1.1	1.54	0.06	92.8	-2.04	8.52	6.38	15.17	6.94	-2.01	10.91	20.57	10.41	0.54
os26871	5	606	0.04	2	3	6	6.94	0.04	2.86	1.54	0.04	86.87	-0.6	8.57	6.26	15.07	6.77	-2.23	10.88	20.5	10.37	0.43
os26874	5	614	0.6	2	3	6	6.84	0	1.61	1.52	0.03	87.02	-0.12	7.83	6.3	15.11	6.8	-2.2	10.94	20.57	10.41	0.45
os26900	7	567	0.16	4	2	0	6.86	0.04	0.98	1.54	0.09	76.05	-1.12	7.4	6.41	15.23	6.89	-2.14	11.06	20.71	10.5	0.52
os26909	4	603	0.13	12	3	5	6.76	0.09	0.01	1.45	0.08	93.97	0.77	5.94	6.4	15.24	6.9	-2.13	11.08	20.71	10.51	0.52
os26946	6	586	0.23	1	3	6	6.85	0	2.97	1.54	0.04	46.43	-0.15	8.55	6.42	15.27	6.92	-2.12	11.15	20.76	10.57	0.53
os26947	5	587	0.88	1	3	6	6.82	-0.02	2.9	1.54	0.03	51.16	-0.33	8.18	6.42	15.26	6.92	-2.12	11.15	20.76	10.57	0.53
os26987	5	656	0.9	4	3	6	6.75	-0.06	1.02	1.52	0.03	99.44	-0.22	7.3	6.14	14.99	6.64	-2.37	10.74	20.38	10.24	0.29
os26999	7	531	0.92	5	2	7	6.63	-0.09	1	1.55	0.11	85.85	-3.31	7.46	6.6	15.41	6.98	-2.08	11.28	20.9	10.66	0.6
os27016	6	666	0.58	3	3	6	6.82	-0.02	1.81	1.54	0.04	83.26	-0.53	7.8	6.06	14.92	6.56	-2.44	10.63	20.25	10.16	0.2
os27045	6	555	0.92	3	3	6	6.73	-0.05	2.33	1.54	0.05	90.03	-0.42	8.51	6.53	15.34	7.02	-2.07	11.15	20.81	10.63	0.66
os27052	5	567	0.92	2	3	6	6.78	-0.03	0.48	1.52	0.02	69.92	0.37	8.54	6.72	15.54	7.23	-1.71	11.22	20.9	10.74	0.97
os27100	5	557	0.92	2	3	6	6.79	-0.02	0.99	1.54	0.01	32.56	0.02	8.76	6.81	15.65	7.23	-1.81	11.42	21.15	10.84	0.9
os27119	5	483	0.79	3	3	6	6.74	-0.02	0.95	1.54	0.01	47.86	0.55	8.36	7.48	16.38	7.61	-1.41	12.2	21.97	11.38	1.34
os27165	7	439	0.98	3	3	6	6.69	-0.06	0.16	1.54	0.02	80.47	1.08	8.01	7.45	16.3	7.75	-1.4	12.13	21.89	11.42	1.32
os27192	5	617	0.27	2	3	6	6.81	-0.03	1.61	1.53	0.02	81.13	-0.08	7.88	6.38	15.24	6.91	-2.05	10.91	20.64	10.45	0.68
os27193	5	616	0.92	2	3	6	6.81	-0.03	1.59	1.53	0.02	76.73	-0.06	8	6.38	15.24	6.91	-2.05	10.91	20.64	10.45	0.69
os27208	5	602	0.92	0	3	6	6.86	0	2.05	1.54	0	26.57	0.07	11.09	6.51	15.34	6.99	-2.06	11.14	20.88	10.64	0.74

os27217	7	584	0.77	5	3	5	6.67	-0.07	0.87	1.53	0.04	95.82	-1.12	7.75	6.47	15.3	6.94	-2.09	11.09	20.8	10.54	0.64
os27225	7	584	0.76	10	2	7	6.41	-0.08	0.44	1.54	0.2	93.72	-10.77	7.94	6.26	15.11	6.79	-2.16	10.63	20.4	10.17	0.46
os27246	7	597	0	3	3	6	6.95	0.06	1.85	1.54	0.04	81.58	-0.79	8.53	6.46	15.36	6.95	-2.06	11.12	20.94	10.61	0.64
os27260	5	518	0.92	1	3	6	6.85	0	2.72	1.55	0.01	27.58	0.16	8.82	7.08	15.92	7.44	-1.62	11.66	21.38	10.99	1.07
os27263	7	524	0.47	4	2	3	6.8	0.01	1.77	1.55	0.04	89.97	-1.83	8.85	7.08	15.95	7.36	-1.66	11.69	21.43	10.99	1.05
os27266	5	491	0.92	1	3	6	6.79	-0.01	3.97	1.56	0.03	30.93	-0.43	9.16	7.18	16.04	7.47	-1.61	11.82	21.54	11.08	1.08
os27279	5	461	0.81	4	3	6	6.68	-0.04	0.56	1.55	0.04	97.18	-0.15	7.43	7.21	16.01	7.57	-1.45	11.91	21.52	11.26	1.26
os27280	5	465	0.68	5	3	5	6.64	-0.05	0.51	1.54	0.04	98.43	-0.13	6.98	7.19	15.99	7.56	-1.46	11.89	21.51	11.25	1.25
os27310	7	519	0	9	2	3	6.5	-0.14	0.69	1.52	0.11	99.15	-4.67	8.7	6.92	15.71	7.4	-1.53	11.41	21.05	10.9	1.12
os27387	5	451	0.99	3	3	6	6.69	-0.06	1.18	1.56	0.05	88.69	-0.83	8.13	7.23	16.02	7.61	-1.43	11.93	21.55	11.29	1.29
os27395	5	546	0.9	3	3	6	6.73	-0.05	1.7	1.55	0.04	92.61	-0.42	8.69	6.59	15.41	7.1	-1.97	11.24	20.89	10.69	0.67
os27401	5	558	0.92	3	3	6	6.73	-0.05	1.3	1.55	0.03	91.78	0.04	7.91	6.55	15.37	7.06	-2	11.19	20.84	10.65	0.65
os27405	5	606	0.23	2	3	6	6.87	0.02	1.43	1.54	0.02	57.92	-0.15	8.41	6.39	15.2	6.9	-2.13	11	20.67	10.48	0.55
os27417	7	581	0.99	9	2	3	6.53	-0.15	0.28	1.51	0.09	95.97	-2.37	7.5	6.41	15.21	6.94	-2.11	11.01	20.66	10.52	0.61
os27423	5	537	0.83	1	3	6	6.81	-0.02	2.92	1.56	0.04	59.95	-0.32	8.99	6.65	15.47	7.13	-1.96	11.25	20.94	10.73	0.77
os27424	4	536	0.76	6	3	5	6.83	-0.02	0.37	1.53	0.07	67.95	-1.35	7.29	6.63	15.43	7.08	-2.02	11.27	20.96	10.73	0.73
os27453	5	562	0.04	3	3	6	6.86	0.03	0.98	1.55	0.03	51.81	-0.3	7.81	6.47	15.27	6.96	-2.11	11.03	20.72	10.51	0.58
os27498	5	610	0.9	4	3	6	6.69	-0.07	0.55	1.52	0.03	98.83	0.43	6.86	6.33	15.16	6.9	-2.12	10.93	20.61	10.49	0.53
os27547	5	620	0.03	4	3	6	6.98	0.06	1.78	1.53	0.04	96.99	-1.37	8.62	6.16	14.98	6.72	-2.28	10.64	20.31	10.18	0.38
os27549	5	611	0.98	3	3	6	6.81	-0.04	0.58	1.53	0.03	76	0.19	8.11	6.24	15.06	6.79	-2.21	10.71	20.39	10.21	0.43
os27556	5	601	0.99	6	3	6	6.67	-0.09	0.71	1.54	0.06	81.1	-1.78	8.75	6.25	15.07	6.79	-2.22	10.72	20.4	10.22	0.43
os27586	5	441	0.73	2	3	6	6.76	-0.01	2.94	1.56	0.02	10.44	-0.31	9.72	7.71	16.56	7.91	-0.99	12.3	22	11.52	1.68
os27589	5	618	0.82	10	3	5	6.44	-0.13	0.39	1.51	0.09	79.85	-2.22	6.76	6.3	15.16	6.81	-2.29	10.86	20.6	10.34	0.43
os27619	7	490	0.01	9	2	3	6.92	0.13	0.56	1.53	0.1	91.01	-3.41	8.82	6.93	15.81	7.24	-1.89	11.68	21.48	11.04	0.85
os27632	5	447	0.09	3	3	6	6.68	-0.06	0.55	1.53	0.02	77.06	0.56	8.03	7.7	16.6	7.8	-1.26	12.43	22.19	11.59	1.51
os27645	5	552	0.92	2	3	6	6.8	-0.03	3.87	1.56	0.05	42.07	-0.19	9.03	6.49	15.33	6.98	-1.99	10.92	20.68	10.4	0.64
os27668	5	560	0.42	5	3	6	6.8	0.02	1.28	1.55	0.04	36.77	-0.76	8.04	6.67	15.54	7.05	-2.04	11.2	21.03	10.64	0.66
os27672	7	624	0	5	2	3	6.77	-0.01	1.37	1.54	0.12	97.72	-5.5	9.36	6.1	14.93	6.65	-2.26	10.42	20.16	9.98	0.33
os27746	5	512	0.08	2	3	6	6.87	0.02	2.95	1.55	0.04	80.53	-1.02	9.09	6.91	15.76	7.24	-1.86	11.69	21.51	11.06	0.9
os27747	5	511	0.08	2	3	6	6.89	0.03	2.89	1.55	0.04	79.97	-1.27	9.14	6.91	15.77	7.24	-1.85	11.69	21.52	11.06	0.9
os27766	4	501	0.08	2	3	6	6.87	0.03	3.24	1.55	0.04	71.13	-1.33	9.77	7	15.86	7.31	-1.78	11.77	21.58	11.11	0.97
os27867	5	488	0.01	3	3	6	6.88	0.05	1.9	1.54	0.02	71.78	-0.64	8.33	7.23	16.11	7.46	-1.57	11.97	21.71	11.2	1.18
os27919	5	498	0.6	2	3	6	6.85	0.01	2.23	1.55	0.02	27.01	0.03	8.65	7.26	16.15	7.47	-1.58	12	21.76	11.2	1.17
os27921	5	474	0.08	4	3	6	6.91	0.05	1.06	1.54	0.03	88.88	-0.14	8.15	7.36	16.22	7.54	-1.51	12.09	21.83	11.27	1.23
os28040	7	554	0.92	3	3	6	6.78	-0.04	1.68	1.55	0.03	81.63	-0.32	8.56	7.06	15.95	7.23	-1.7	11.71	21.42	10.87	1.03
os28083	5	575	0.6	4	3	6	6.74	-0.03	0.98	1.52	0.04	99.38	-0.42	6.95	6.91	15.81	7.16	-1.83	11.59	21.35	10.83	0.95
os28093	4	509	0.19	9	3	5	6.75	0.06	0.02	1.48	0.06	94.78	2.64	6.91	7.23	16.11	7.43	-1.63	11.96	21.71	11.14	1.12

os28107	7	552	0.24	3	3	6	6.73	-0.06	1.7	1.54	0.04	88.46	-0.53	8.18	7.02	15.91	7.25	-1.74	11.7	21.44	10.9	1
os28150	4	487	0.08	3	2	7	6.9	0.04	2.41	1.55	0.07	83.44	-2.34	7.66	7.22	16.1	7.4	-1.58	11.81	21.57	11.04	1.16
os28152	5	513	0.56	3	3	6	6.86	0.01	2.9	1.54	0.03	80.87	0.79	8.3	7.19	16.07	7.38	-1.59	11.76	21.53	10.98	1.16
os28155	5	517	0.96	5	3	6	6.68	-0.07	1.25	1.54	0.04	78.4	-0.34	8.61	7.18	16.06	7.37	-1.6	11.74	21.51	10.96	1.15
os28176	5	481	0.4	2	3	6	6.85	0.01	2.92	1.55	0.02	71.04	-0.39	8.86	7.44	16.31	7.69	-1.32	12.06	21.78	11.31	1.35
os28187	5	497	0.92	1	3	6	6.78	-0.02	2.81	1.55	0.01	19.02	-0.02	9.05	7.37	16.24	7.62	-1.42	12.02	21.76	11.28	1.26
os28200	5	499	0.92	0	3	6	6.82	0	3.9	1.55	0.02	6.41	-0.19	10.63	7.23	16.11	7.53	-1.53	11.9	21.64	11.15	1.17
os28203	5	542	0.92	2	3	6	6.75	-0.04	0.62	1.54	0.02	61.52	0.37	7.9	7.14	16.05	7.41	-1.59	11.74	21.49	11.04	1.11
os28204	5	541	0.92	1	3	6	6.85	0.01	2.78	1.54	0.02	38.7	-0.39	8.76	7.13	16.04	7.43	-1.61	11.79	21.55	11.06	1.09
os28213	5	492	0.92	2	3	6	6.75	-0.03	2.36	1.56	0.03	56.18	-0.39	8.8	7.23	16.11	7.5	-1.55	11.91	21.62	11.16	1.13
os28214	7	511	0.97	5	3	6	6.7	-0.07	1.21	1.54	0.04	93.06	-1.08	8.63	7.14	16.02	7.42	-1.62	11.79	21.52	11.06	1.08
os28294	7	410	0	3	3	6	6.82	0.04	1.79	1.54	0.06	80.58	-1.2	8.57	7.75	16.64	7.84	-1.22	12.51	22.27	11.66	1.54
os28375	7	477	0.08	6	2	3	6.96	0.09	1.03	1.53	0.08	79.67	-3.38	7.9	7.32	16.19	7.52	-1.53	12.06	21.83	11.3	1.24
os28413	7	469	0.02	3	3	6	6.69	-0.05	2.27	1.55	0.04	87.24	-1.74	8.99	7.51	16.4	7.73	-1.36	12.18	21.9	11.36	1.34
os28417	5	499	0	3	3	6	6.89	0.05	2.87	1.54	0.03	60.4	-1.18	9.47	7.39	16.29	7.57	-1.41	11.98	21.73	11.2	1.3
os28418	7	499	0.23	4	3	6	6.79	0.02	1.91	1.54	0.04	67.36	-1.75	9.1	7.39	16.29	7.57	-1.41	11.98	21.73	11.2	1.3
os28451	5	459	0.92	1	3	6	6.84	0.02	3.94	1.56	0.03	30.94	-0.1	8.32	7.71	16.59	7.78	-1.22	12.38	22.09	11.47	1.48
os28452	5	457	0.92	1	3	6	6.83	0.02	4.95	1.56	0.03	24.61	-0.42	8.51	7.72	16.6	7.78	-1.21	12.38	22.1	11.48	1.49
os28584	5	572	0.92	0	3	6	6.86	0	2	1.55	0.01	18.07	0.22	8.94	6.64	15.56	7.03	-2.05	11.22	21.04	10.66	0.67
os28642	7	628	0.08	3	3	6	6.95	0.04	2.64	1.54	0.03	68.34	-0.07	8.31	6.71	15.64	6.97	-1.99	11.28	20.98	10.53	0.79
os28770	7	401	0.13	18	2	7	6.94	0.2	0.07	1.49	0.17	96.84	-6.08	7.97	8.13	17.13	8.09	-0.92	12.84	22.65	11.86	1.87
os28784	7	490	0.67	5	2	3	6.66	-0.06	2.09	1.54	0.07	91.3	-2.52	8.6	7.94	16.97	7.88	-0.91	12.55	22.49	11.58	1.85
os28839	7	499	0.09	4	2	3	6.84	0.04	1.66	1.55	0.08	88.77	-3.77	7.94	7.29	16.19	7.5	-1.49	11.86	21.63	11.12	1.23
os28845	4	446	0.08	4	3	6	6.91	0.07	0.54	1.54	0.04	63.91	-0.57	9.09	7.61	16.48	7.79	-1.25	12.26	21.95	11.44	1.44
os28847	4	448	0	4	3	6	6.9	0.07	0.44	1.54	0.04	66.95	0.32	8.98	7.62	16.49	7.8	-1.24	12.27	21.96	11.45	1.45
os28850	7	535	0.97	2	3	6	6.79	-0.03	1.93	1.54	0.02	89.31	-0.43	8.11	7.33	16.23	7.52	-1.45	11.91	21.63	11.1	1.27
os28854	7	467	0.18	8	2	3	6.91	0.05	0.77	1.51	0.1	98.7	-3.59	8.13	7.7	16.64	7.81	-1.15	12.3	22.06	11.43	1.56
os28928	4	446	0.69	4	2	0	6.83	0.01	1.99	1.55	0.07	81.96	-2.89	7.76	7.71	16.59	7.78	-1.19	12.34	22.06	11.46	1.55
os28970	7	436	0.92	2	3	6	6.7	-0.04	1.78	1.55	0.03	89.6	-0.88	8.56	7.81	16.71	7.87	-1.18	12.5	22.26	11.61	1.56
os28986	6	461	0.77	1	3	6	6.8	-0.01	2.98	1.55	0.03	38	-0.64	8.73	7.64	16.54	7.74	-1.28	12.31	22.08	11.44	1.47
os29033	6	588	0.6	5	3	5	6.7	-0.04	0.74	1.53	0.05	95.64	-0.54	7.32	6.99	15.88	7.15	-1.83	11.61	21.3	10.76	0.95
os29039	6	594	0.47	5	3	5	6.87	-0.01	0.51	1.53	0.06	98.29	-1.4	7.12	6.95	15.87	7.14	-1.84	11.57	21.26	10.73	0.96
os29050	7	601	0.17	2	3	6	6.88	0.02	2.66	1.55	0.04	62.62	-0.56	9.42	6.87	15.77	7.09	-1.91	11.47	21.19	10.66	0.9
os29055	7	600	0.01	2	3	6	6.9	0.03	2.73	1.55	0.04	59.21	-0.38	9.46	6.87	15.77	7.09	-1.91	11.47	21.18	10.66	0.9
os29068	7	436	0.58	11	3	5	6.48	-0.06	0.36	1.46	0.09	96.24	-0.78	7.05	8.03	17.05	8.08	-0.81	12.58	22.45	11.7	1.91
os29128	7	596	0.9	3	3	6	6.74	-0.05	1.73	1.52	0.04	82	-0.4	8.02	7.13	16.09	7.28	-1.6	11.7	21.5	10.86	1.15
os29276	7	575	0.18	4	2	0	6.88	0.04	2.01	1.55	0.06	92.69	-1.69	9.02	6.91	15.82	7.12	-1.82	11.51	21.23	10.7	0.95

os29277	7	590	0.09	4	3	6	6.97	0.06	0.59	1.53	0.06	99.04	-0.07	7.23	6.92	15.82	7.12	-1.81	11.51	21.24	10.7	0.96
os29278	7	575	0.92	2	2	7	6.87	0.02	2.61	1.56	0.06	94.25	-2.46	9.12	6.91	15.81	7.11	-1.82	11.5	21.23	10.7	0.95
os29356	5	487	1	5	3	5	6.63	-0.09	0.35	1.53	0.04	71.86	0.49	6.97	7.39	16.45	7.55	-1.51	12.02	21.95	11.23	1.24
os29376	5	556	0.23	4	2	3	6.84	0.03	1.96	1.54	0.07	94.24	-2.34	8.54	6.89	15.91	7.15	-1.89	11.53	21.41	10.85	0.83
os29412	7	498	0.78	5	2	3	6.62	-0.04	1.22	1.54	0.12	93.07	-5.03	8.72	7.28	16.3	7.46	-1.67	11.93	21.87	11.18	1.1
os29468	6	574	0.73	7	3	5	6.61	-0.07	0.25	1.51	0.05	92.44	0.12	7.32	6.99	16.01	7.17	-1.87	11.73	21.66	10.91	0.96
os29491	7	579	0.92	1	3	6	6.87	0.01	3.3	1.56	0.07	78.32	-1.42	8.07	6.66	15.61	6.94	-1.98	11.09	20.86	10.45	0.75
os29492	5	605	0.08	2	3	6	6.93	0.03	2.35	1.55	0.05	82.44	-1.42	8.74	6.61	15.58	6.9	-1.98	10.94	20.75	10.33	0.7
os29507	5	627	0.08	3	3	6	6.94	0.04	2.93	1.54	0.04	82.25	-0.99	9.15	6.31	15.21	6.81	-2.22	10.94	20.79	10.49	0.47
os29512	7	559	0.92	2	3	6	6.79	-0.02	3.55	1.55	0.02	32.06	0.08	10.36	6.69	15.56	7.1	-1.98	11.44	21.26	10.88	0.76
os29522	6	482	1	6	3	5	6.6	-0.1	0.76	1.53	0.04	84.88	-1.02	7.97	7.36	16.23	7.55	-1.5	12.1	21.86	11.33	1.26
os29530	7	496	0.06	4	2	3	6.85	0.04	1.95	1.54	0.04	91.72	-2.14	8.74	7.17	16.03	7.42	-1.66	11.87	21.63	11.16	1.09
os29531	7	459	0.19	3	2	3	6.84	0.01	1.92	1.55	0.05	82.85	-2.44	8.97	7.36	16.23	7.55	-1.5	12.1	21.84	11.29	1.24
os29539	6	603	0.85	7	3	5	6.59	-0.1	0.49	1.52	0.06	80.2	-1.6	7.94	6.75	15.64	7.02	-1.89	11.23	20.98	10.51	0.83
os29554	5	502	0.23	1	3	6	6.82	0.01	1.53	1.55	0.01	35.83	-0.07	8.95	7.3	16.23	7.44	-1.6	11.94	21.79	11.16	1.18
os29555	5	551	0.92	1	3	6	6.82	-0.01	4.22	1.54	0.01	18.33	0.17	9.23	7.06	16.04	7.24	-1.82	11.75	21.67	11	1.01
os29622	7	639	0.01	2	3	6	6.93	0.04	2.3	1.55	0.02	77.12	-0.71	8.87	6.57	15.6	6.9	-2.1	11.09	20.91	10.5	0.64
os29742	7	467	0.34	4	2	3	6.71	-0.02	1.86	1.54	0.08	82.85	-3.28	9.29	7.32	16.17	7.53	-1.53	12.04	21.76	11.26	1.2
os29747	5	470	0	2	3	6	6.86	0.03	0.93	1.54	0.02	64.27	0.34	8.48	7.34	16.21	7.53	-1.52	12.07	21.82	11.26	1.22
os29749	5	495	0.79	1	3	6	6.81	-0.01	0.18	1.54	0.01	35.56	0.33	8.62	7.26	16.12	7.47	-1.57	11.96	21.74	11.22	1.18
os29761	5	533	0.23	2	3	6	6.85	0.02	0.36	1.53	0.02	85.47	0.92	7.85	7.13	16.01	7.34	-1.65	11.74	21.5	10.98	1.1
os29776	4	602	0.74	11	3	5	6.37	-0.14	0.01	1.46	0.07	89.69	1.47	6.31	6.84	15.82	7.05	-1.96	11.51	21.28	10.72	0.86
os29790	5	495	0.08	2	3	6	6.89	0.04	2.71	1.55	0.03	44.86	-0.23	8.44	7.28	16.22	7.45	-1.56	11.91	21.71	11.1	1.23
os29812	5	488	0.92	2	3	6	6.75	-0.04	2.18	1.55	0.02	58.43	-0.38	8.63	7.31	16.22	7.47	-1.6	11.98	21.81	11.21	1.18
os29826	5	467	0	3	2	7	6.73	-0.03	3.19	1.56	0.08	92.26	-4.3	8.94	7.25	16.14	7.43	-1.62	11.98	21.78	11.24	1.15
os29902	5	508	0.92	2	3	6	6.84	0	0.65	1.53	0.02	66.98	1.15	8.77	7.21	16.16	7.37	-1.71	11.86	21.77	11.12	1.1
os29903	5	520	0.92	0	3	6	6.83	0	5.7	1.56	0.02	43.01	-0.17	9.99	7.1	16.07	7.29	-1.81	11.75	21.67	11.03	0.98
os29914	5	552	0.77	1	3	6	6.85	0	0.23	1.53	0.02	71.82	1.11	8.85	7.06	15.96	7.24	-1.69	11.59	21.31	10.79	1.03
os29926	5	579	0.08	1	3	6	6.88	0.01	4.64	1.56	0.04	62.77	-0.55	9.3	6.86	15.75	7.06	-1.89	11.45	21.12	10.66	0.87
os29966	6	610	0.23	4	2	7	6.85	0.03	1.86	1.54	0.08	91.15	-2.85	7.3	6.62	15.59	6.85	-2.02	11.12	20.88	10.41	0.75
os29967	6	612	0.01	4	2	7	6.9	0.04	1.92	1.54	0.08	91.36	-2.53	7.38	6.61	15.59	6.85	-2.03	11.12	20.88	10.4	0.75
os29968	5	581	0.77	4	3	6	6.84	-0.03	0.94	1.54	0.04	62.62	-0.63	8.15	6.9	15.83	7.1	-1.87	11.5	21.2	10.71	0.89
os29992	4	525	0.89	4	2	0	6.75	-0.05	0.51	1.51	0.07	68.95	1.49	8.52	7.15	16.13	7.32	-1.74	11.74	21.54	10.95	1.02
os30003	7	589	0.99	7	3	5	6.65	-0.11	0.33	1.52	0.05	87.81	-0.37	7.17	6.82	15.82	7.03	-1.94	11.42	21.24	10.68	0.84
os30019	7	552	0.82	6	2	0	6.77	-0.05	1.34	1.54	0.07	93.63	-2.2	8.51	6.9	15.86	7.11	-1.92	11.55	21.33	10.81	0.86
os30069	7	601	0.59	3	3	6	6.78	-0.02	1.59	1.53	0.03	89	-1.1	7.12	6.86	15.8	7.07	-1.9	11.3	21.07	10.6	0.81
os30165	5	549	0.92	5	3	5	6.65	-0.09	0.81	1.53	0.06	98.26	-1.91	7.47	6.88	15.81	7.15	-1.94	11.61	21.44	10.89	0.81

os30169	5	559	0.08	3	3	6	6.92	0.04	2.61	1.55	0.04	71.22	-0.47	8.42	6.72	15.65	7.04	-2.05	11.3	21.16	10.65	0.64
os30241	7	595	0.03	2	3	6	6.93	0.04	2.36	1.54	0.04	70.15	-0.73	8.81	6.72	15.7	7.01	-2.04	11.35	21.22	10.67	0.67
os30295	7	533	0.63	4	2	7	6.73	-0.02	1.91	1.55	0.1	83.22	-3.26	7.85	7.09	16.09	7.28	-1.67	11.58	21.42	10.85	1.04
os30296	7	533	0.53	3	2	7	6.74	-0.01	2.18	1.55	0.09	82.72	-2.94	7.96	7.08	16.09	7.28	-1.67	11.58	21.42	10.85	1.04
os30408	7	523	0.95	4	3	6	6.72	-0.06	0.74	1.56	0.06	58.18	-1	8.44	6.9	15.8	7.21	-1.94	11.56	21.38	10.96	0.79
os30467	4	608	0.97	12	1	1	6.38	-0.19	0	1.39	0.05	99.94	5.08	5.34	6.77	15.67	7.11	-1.87	11.26	20.91	10.63	0.82
os30504	6	518	0.6	3	2	0	6.74	-0.02	1.48	1.55	0.06	77.54	-0.91	8.87	7.08	16.05	7.29	-1.86	11.78	21.54	11.04	0.85
os30507	5	629	0.24	4	3	5	6.75	-0.07	1.52	1.52	0.05	92.06	-1.99	7.61	6.7	15.7	7	-2.06	11.31	21.1	10.7	0.64
os30509	5	640	0.99	4	3	9	6.82	-0.05	0.81	1.49	0.03	95.87	0.23	6.71	6.72	15.7	7.01	-2.05	11.31	21.1	10.7	0.65
os30603	7	619	0.01	4	3	6	6.74	-0.07	0.62	1.52	0.04	98.38	-0.43	6.89	6.3	15.21	6.78	-2.25	10.84	20.63	10.32	0.49
os30621	5	634	0.92	6	3	5	6.67	-0.09	0.35	1.53	0.06	95.1	-0.36	9	6.16	15.02	6.64	-2.4	10.65	20.4	10.13	0.26
os30630	5	619	0.03	2	3	6	6.79	-0.03	2.74	1.55	0.04	87.82	-1.21	8.86	6.31	15.18	6.75	-2.31	10.88	20.66	10.32	0.36
os30693	5	640	0.73	4	2	7	6.72	-0.07	1.71	1.55	0.09	98.85	-2.03	8.71	5.95	14.78	6.51	-2.49	10.43	20.1	9.99	0.16
os30701	5	682	0.01	6	2	0	7.03	0.09	0.99	1.54	0.07	99.51	-1.81	8.38	5.88	14.78	6.39	-2.63	10.37	20.12	9.91	0.05
os30725	5	630	1	6	2	7	6.72	-0.08	1.14	1.54	0.12	97.72	-3.37	8.22	6.04	14.89	6.51	-2.45	10.47	20.18	10.02	0.19
os30729	5	605	0.92	1	2	0	6.86	-0.01	3.9	1.56	0.05	72.36	-0.95	8.67	6.39	15.3	6.77	-2.35	11.07	20.85	10.47	0.36
os30758	5	509	0.34	6	2	3	6.79	0.03	1.5	1.54	0.07	89.09	-1.84	8.32	7.05	15.97	7.3	-1.91	11.82	21.57	11.1	0.82
os30763	5	547	0.08	2	3	6	6.89	0.02	2.77	1.56	0.05	80.91	-1.15	8.35	6.72	15.62	7.06	-2.15	11.44	21.21	10.84	0.59
os30785	7	648	0.07	2	3	6	6.93	0.03	2.97	1.55	0.05	78.63	-0.67	8.57	6.13	15.02	6.59	-2.45	10.65	20.42	10.15	0.24
os30835	5	685	1	5	3	5	6.75	-0.08	0.81	1.52	0.07	97.53	-1.34	7.47	5.98	14.83	6.49	-2.47	10.5	20.12	10.05	0.19
os30850	4	505	0.3	13	2	7	6.55	0.04	0.24	1.5	0.16	94.84	-4.04	7.6	6.75	15.63	7.1	-1.97	11.41	21.01	10.76	0.72
os30862	5	584	0.51	7	2	0	6.96	0.05	0.6	1.48	0.11	99.98	0.04	7.8	6.49	15.37	6.96	-2.02	10.95	20.59	10.45	0.62
os30865	5	627	0.96	12	3	5	6.53	-0.16	0.01	1.46	0.11	100	-0.88	6.06	6.3	15.2	6.74	-2.22	10.82	20.47	10.31	0.41
os31080	5	594	0.23	5	3	6	6.79	0.02	0.41	1.51	0.05	99.19	0.67	6.87	6.54	15.44	6.93	-2.09	11.07	20.72	10.48	0.55
os31109	7	646	0.17	7	2	7	6.95	0.05	0.8	1.54	0.11	92.4	-3.63	7.71	6.57	15.59	6.79	-2.18	11.21	20.93	10.47	0.67
os31137	5	523	0.96	3	3	6	6.78	-0.04	0.55	1.53	0.02	87.73	0.53	8.19	7.14	16.12	7.35	-1.75	11.83	21.72	11.09	1.03
os31171	5	525	0.08	1	3	6	6.84	0	2.95	1.56	0.03	76.7	-1.59	8.97	7.2	16.29	7.4	-1.69	11.81	21.7	11.11	1.07
os31172	5	524	0.77	2	3	6	6.81	-0.02	2.97	1.55	0.03	67.34	-0.7	9.38	7.23	16.32	7.42	-1.65	11.82	21.73	11.12	1.09
os31259	5	622	0.39	5	3	5	6.8	0	0.92	1.54	0.05	72.2	-1.62	7.51	6.76	15.71	6.94	-2.02	11.48	21.18	10.66	0.82
os31261	5	621	0.6	3	3	6	6.81	-0.01	2.79	1.55	0.04	58.97	-0.71	8.02	6.75	15.71	6.93	-2.02	11.47	21.16	10.65	0.81
os31273	5	637	0.77	4	3	6	6.81	-0.05	1.7	1.54	0.06	99.67	-1.8	8.29	6.62	15.62	6.85	-2.05	11.22	20.98	10.5	0.73
os31308	5	631	0.24	7	3	5	6.81	0.03	0.53	1.52	0.06	93.41	-0.66	6.84	6.65	15.63	6.9	-2.05	11.17	20.95	10.5	0.73
os31314	5	581	0.77	4	2	7	6.82	-0.03	2.48	1.55	0.08	92.37	-2.45	7.76	6.84	15.84	7.05	-1.99	11.44	21.24	10.73	0.78
os31549	6	648	0.83	2	3	6	6.86	-0.02	2.89	1.55	0.03	68.52	-0.94	9.05	6.51	15.53	6.86	-2.16	11.03	20.88	10.46	0.62
os31571	5	547	0.4	1	3	6	6.84	-0.01	3	1.56	0.05	83.21	-1.26	9.35	7.1	16.16	7.3	-1.79	11.76	21.62	11.06	1
os31599	5	594	0.75	7	3	5	6.6	-0.08	0.51	1.5	0.06	92.22	-0.93	7.85	6.93	15.96	7.09	-1.95	11.57	21.35	10.77	0.87
os31613	5	619	0.92	1	3	6	6.88	0	2.92	1.55	0.04	70.74	-0.89	8.38	6.76	15.79	6.97	-1.99	11.29	21.07	10.58	0.81

os31624	5	603	0.09	6	3	5	7.01	0.08	0.17	1.5	0.07	95.57	1.03	6.18	6.76	15.79	7	-1.93	11.25	21.02	10.54	0.86
os31667	5	521	0.99	3	3	6	6.79	-0.03	1.58	1.53	0.03	92.65	0.53	8.12	7.27	16.34	7.42	-1.64	11.91	21.76	11.14	1.13
os31668	5	520	0.93	4	3	6	6.79	-0.03	2.23	1.53	0.03	92.56	0.41	8.1	7.27	16.34	7.42	-1.63	11.91	21.77	11.14	1.13
os31678	5	622	0.79	2	3	6	6.84	-0.02	2.8	1.55	0.04	60.46	-0.5	8.78	6.52	15.52	6.78	-2	10.89	20.67	10.35	0.8
os31690	5	539	0.77	3	3	6	6.83	-0.02	2.75	1.56	0.04	63.75	-0.55	9.23	7.02	16.06	7.14	-1.83	11.67	21.48	10.98	1
os31691	6	538	0.53	3	3	6	6.85	-0.01	2.5	1.56	0.04	63.31	-1.06	9.27	7.03	16.06	7.14	-1.83	11.67	21.49	10.99	1
os31692	5	538	0.77	4	3	6	6.81	-0.03	1.83	1.55	0.04	65.84	-1.34	9.17	7.02	16.06	7.14	-1.83	11.67	21.49	10.99	1
os31693	5	539	0.92	5	3	5	6.77	-0.05	1.2	1.54	0.04	67.53	-1.23	9.08	7.02	16.06	7.14	-1.83	11.67	21.48	10.99	1
os31694	5	540	0.91	5	3	5	6.74	-0.06	1	1.54	0.04	70.54	-0.63	8.91	7.02	16.06	7.14	-1.83	11.67	21.48	10.98	0.99
os31746	4	527	0.99	12	3	5	6.5	-0.15	0.45	1.51	0.1	85.94	-2.66	6.42	7.14	16.19	7.28	-1.75	11.74	21.59	10.98	1.05
os31747	4	532	0.08	13	3	5	7.05	0.2	0.05	1.48	0.09	87.14	-0.68	6.41	7.14	16.18	7.27	-1.76	11.73	21.57	10.97	1.05
os31811	5	462	0.57	3	3	6	6.71	-0.03	1.84	1.55	0.03	60.07	-0.61	9.14	8.05	17.08	8	-0.82	12.71	22.53	11.7	1.93
os31820	7	642	0.86	3	3	6	6.84	-0.03	1.92	1.52	0.06	95.76	-2.03	8.29	6.53	15.54	6.79	-2	10.96	20.72	10.35	0.82
os31829	5	535	0.79	4	3	6	6.78	-0.05	0.8	1.55	0.05	89.82	-1.39	7.22	7.03	16.02	7.17	-1.77	11.6	21.35	10.86	1.03
os31830	5	622	0.77	2	3	6	6.85	-0.02	2.89	1.55	0.04	60.02	-0.5	8.82	6.52	15.52	6.78	-2	10.89	20.67	10.34	0.8
os31886	3	465	0.42	13	2	3	6.92	0.1	0.01	1.49	0.13	88.03	-3.41	6.73	7.15	16	7.42	-1.73	11.91	21.71	11.23	1.02
os31897	5	513	0.71	3	3	6	6.85	0	2.87	1.54	0.03	80.85	0.92	8.29	7.19	16.07	7.38	-1.59	11.76	21.53	10.98	1.16
os31909	7	575	0.23	3	2	7	6.86	0.02	2.42	1.56	0.06	94.15	-2.31	9.1	6.91	15.81	7.11	-1.82	11.5	21.23	10.7	0.95
os31915	7	502	0.37	3	3	5	6.89	0.03	2.15	1.55	0.1	93.46	-2.76	9.12	7.26	16.28	7.45	-1.69	11.91	21.84	11.16	1.09
os31919	7	555	0.21	6	2	7	6.98	0.08	0.93	1.5	0.11	99.66	-3.11	7.93	6.73	15.61	7.1	-1.92	11.25	20.88	10.68	0.76
os31974	5	179	0.09	1	3	6	6.72	0.01	6.71	1.56	0.01	6.31	-0.51	9	9.48	18.18	9.48	0.76	14.11	23.32	13.07	3.32
os31976	5	187	0.92	0	3	6	6.69	0	4.13	1.55	0	32.52	0.44	10.1	9.47	18.18	9.43	0.75	14.06	23.28	13.04	3.31
os32028	7	184	0.92	0	3	6	6.69	0	2.93	1.57	0.05	34.95	-0.46	8.19	9.27	18.02	9.27	0.48	14.03	23.39	13	3.05
os32122	7	160	0.77	1	3	6	6.67	-0.01	4.96	1.56	0.02	12.29	-0.15	9.72	9.8	18.6	9.74	1.02	14.62	23.81	13.49	3.69
os32123	7	160	0.92	0	3	6	6.67	0	4.94	1.57	0.01	7.38	0.02	11.82	9.79	18.61	9.74	1.03	14.61	23.8	13.48	3.68
os32155	4	188	0.31	30	2	7	5.78	0.09	0	1.41	0.23	68.13	-7.24	6.28	9.54	18.37	9.56	0.91	14.08	23.34	13.16	3.5
os32182	5	252	0.87	3	3	6	6.61	-0.04	0.54	1.54	0.02	73.18	0.47	7.49	8.71	17.41	8.92	-0.03	13.29	22.62	12.53	2.55
os32197	5	258	0.02	3	3	6	6.76	0.04	1.68	1.55	0.02	41.9	-0.3	8.13	8.6	17.32	8.83	-0.1	13.11	22.45	12.38	2.47
os32201	5	250	0.82	3	3	6	6.68	-0.03	1.31	1.55	0.03	73.77	-0.51	7.65	8.54	17.27	8.8	-0.21	13.12	22.5	12.44	2.39
os32236	4	228	0.67	6	2	3	6.64	0.07	1.24	1.53	0.15	80.29	-7.38	8	8.58	17.3	8.8	-0.05	13.29	22.65	12.49	2.51
os32238	4	223	0.01	2	3	6	6.76	0.04	5.91	1.56	0.02	19.38	-0.38	8.8	8.76	17.45	8.93	-0.07	13.6	22.98	12.76	2.58
os32244	4	225	0.99	4	3	6	6.61	-0.06	1.6	1.55	0.05	65.94	-2.06	7.93	8.63	17.33	8.83	-0.09	13.43	22.78	12.6	2.52
os32245	4	294	0.23	3	2	3	6.77	0.04	1.36	1.5	0.07	98.18	-1.6	6.46	8.47	17.25	8.68	-0.19	13.32	22.72	12.46	2.46
os32247	7	259	0.99	2	3	6	6.66	-0.04	1.44	1.55	0.05	79.14	-1.24	7.6	8.48	17.21	8.78	-0.27	13.17	22.57	12.5	2.32
os32281	7	229	0.92	0	3	6	6.7	-0.01	6.06	1.57	0.03	38.08	-0.78	9.03	8.72	17.41	8.91	-0.06	13.62	22.9	12.72	2.56
os32308	4	268	0.6	8	2	3	6.41	-0.1	0.57	1.52	0.09	99.37	-3.44	7.5	8.44	17.17	8.67	-0.23	13.27	22.65	12.44	2.41
os32312	4	265	0.94	6	3	5	6.56	-0.09	0.23	1.51	0.05	98.55	1.06	7.05	8.45	17.18	8.68	-0.22	13.28	22.66	12.45	2.41

os32314	7	270	0.81	15	2	3	6.03	-0.19	0.03	1.48	0.13	98.88	-4.76	5.98	8.46	17.19	8.68	-0.21	13.28	22.64	12.47	2.43
os32334	7	252	0.85	8	3	5	6.38	-0.12	0.33	1.51	0.07	93.98	-0.96	6.77	8.54	17.25	8.74	-0.16	13.34	22.68	12.53	2.47
os32341	7	234	0.38	3	3	6	6.65	-0.01	1.93	1.55	0.06	85.19	-1.58	7.89	8.53	17.24	8.74	-0.16	13.33	22.68	12.52	2.46
os32349	4	277	0.97	10	2	3	6.43	-0.14	0.44	1.49	0.11	98.84	-4.49	6.59	8.46	17.19	8.68	-0.21	13.28	22.64	12.47	2.43
os32360	4	308	0.05	11	3	9	6.77	0.15	0.01	1.47	0.07	97.78	-0.62	5.8	8.45	17.22	8.66	-0.21	13.31	22.7	12.45	2.45
os32398	4	251	0.37	5	2	7	6.51	-0.07	1.48	1.54	0.14	95.8	-5.31	7.41	8.74	17.54	8.88	0.06	13.68	23.09	12.75	2.74
os32538	7	227	0.92	0	3	6	6.68	-0.01	5.99	1.56	0.03	24.86	-0.71	8.75	8.7	17.43	8.91	-0.06	13.51	22.86	12.67	2.53
os32608	4	242	0.9	8	2	7	6.41	-0.12	0.67	1.53	0.09	98.35	-2.99	7.76	8.77	17.56	8.91	0.06	13.79	23.19	12.82	2.75
os32612	5	242	0.99	2	3	6	6.65	-0.04	2.2	1.55	0.04	79.1	-0.79	7.75	8.54	17.27	8.84	-0.26	13.25	22.65	12.55	2.33
os32616	7	282	0.95	5	3	6	6.61	-0.07	1.23	1.54	0.07	87.53	-1.5	8.54	8.38	17.1	8.66	-0.29	13	22.38	12.3	2.31
os32638	4	237	0.65	3	2	3	6.6	-0.04	1.95	1.55	0.09	84.27	-4.8	8.06	8.58	17.31	8.79	-0.06	13.31	22.68	12.49	2.52
os32640	4	236	0.47	2	3	6	6.68	0	1.95	1.55	0.05	82.14	-0.97	7.53	8.59	17.31	8.79	-0.11	13.39	22.72	12.58	2.51
os32692	4	265	0.17	7	2	3	6.69	0.05	0.85	1.53	0.09	96.88	-3.04	7.28	8.38	17.11	8.69	-0.36	12.97	22.38	12.31	2.25
os32694	4	269	0.37	9	3	5	6.54	0.01	0.38	1.51	0.1	97.61	-2.02	6.91	8.37	17.1	8.68	-0.37	12.95	22.37	12.3	2.24
os32705	7	217	0.23	2	3	6	6.68	0.01	2.99	1.55	0.06	22.32	-0.05	8.95	8.56	17.27	8.84	-0.11	13.2	22.61	12.51	2.43
os32707	4	223	0	2	3	6	6.76	0.04	5.91	1.56	0.02	18.04	-0.33	8.81	8.76	17.45	8.93	-0.07	13.6	22.97	12.76	2.57
os32715	7	227	0.92	2	3	6	6.64	-0.03	4.71	1.56	0.03	47.46	-1.06	8.32	8.68	17.42	8.92	-0.1	13.54	22.88	12.7	2.51
os32721	4	270	0.19	3	2	3	6.76	0.02	2.63	1.55	0.1	78.03	-5.23	7.95	8.11	16.81	8.52	-0.45	12.73	22.09	12.16	2.06
os32722	4	257	0.98	12	2	3	6.32	-0.17	0.28	1.51	0.13	88.3	-5.37	7.69	8.21	16.89	8.58	-0.41	12.84	22.2	12.26	2.12
os32870	7	178	0.92	0	3	6	6.68	0	6.99	1.56	0	2.92	-0.04	11.68	9.46	18.2	9.41	0.59	14.31	23.6	13.2	3.23
os32871	7	179	0.92	0	3	6	6.67	-0.01	6.99	1.55	0	11.31	0.56	10.18	9.43	18.15	9.4	0.55	14.25	23.57	13.22	3.23
os32916	6	185	0.92	0	3	6	6.69	0	7.89	1.57	0.01	4.49	-0.28	10.71	9.38	18.19	9.37	0.36	14.4	23.83	13.32	3.13
os32948	7	187	0.92	0	3	6	6.69	0	7.97	1.57	0.01	17.47	-0.04	11.51	9.3	18.07	9.31	0.24	14.37	23.77	13.31	3.07
os32965	5	184	0.92	0	3	6	6.69	0	6.23	1.57	0	0	0	11.63	9.37	18.04	9.36	0.53	14.09	23.32	13.07	3.17
os32966	5	184	0.92	0	3	6	6.69	0	6.05	1.57	0	0	0	11.63	9.37	18.04	9.35	0.53	14.08	23.3	13.06	3.17
os32967	5	184	0.92	0	3	6	6.69	0	6.15	1.57	0	0	0	11.63	9.37	18.04	9.35	0.53	14.09	23.32	13.07	3.17
os32978	4	275	0	12	3	5	6.86	0.19	0.01	1.49	0.08	86.08	-0.95	6.16	8.39	17.1	8.68	-0.5	13.13	22.47	12.41	2.18
os32980	5	209	0.92	0	3	6	6.7	0	5.61	1.57	0.01	0.58	-0.08	11.65	8.81	17.52	9.02	-0.17	13.59	22.92	12.83	2.54
os32990	4	268	0.94	10	3	5	6.46	-0.13	0.74	1.53	0.1	80.92	-2.62	7.75	8.96	17.71	8.92	-0.02	13.92	23.3	12.87	2.74
os32991	4	269	0.95	13	2	3	6.38	-0.16	0.13	1.54	0.12	77.87	-3.51	7.7	8.96	17.71	8.92	-0.02	13.92	23.3	12.87	2.74
os33003	5	227	0.92	0	3	6	6.7	0	6.98	1.57	0.01	0	0.01	11.42	8.97	17.68	9.01	-0.12	13.84	23.17	12.83	2.63
os33023	4	235	0.23	3	3	6	6.68	0.01	0.5	1.54	0.02	68.66	0.55	7.55	8.88	17.6	8.95	-0.23	13.77	23.08	12.82	2.5
os33034	5	242	0.08	3	3	6	6.8	0.05	2.65	1.54	0.02	47.15	0.21	8.84	8.88	17.62	8.95	-0.16	13.81	23.16	12.84	2.59
os33041	5	245	0.6	8	3	5	6.51	-0.03	0.36	1.53	0.06	80.02	-1.33	7.58	8.65	17.38	8.87	-0.44	13.5	22.85	12.76	2.29
os33088	5	250	0.08	2	3	6	6.76	0.03	3.33	1.56	0.02	22.98	-0.28	8.67	9.15	17.93	9.13	0.38	13.91	23.28	12.96	3.08
os33089	5	250	0.92	2	3	6	6.66	-0.02	5.06	1.57	0.02	4.68	-0.66	9.2	9.14	17.92	9.14	0.39	13.91	23.27	12.95	3.06
os33172	7	251	0.92	1	3	6	6.74	0.02	3.56	1.56	0.02	20.33	-0.3	9.03	9.13	17.91	9.12	0.38	13.9	23.26	12.94	3.07

os33187	7	230	0.13	4	3	6	6.81	0.06	1.18	1.55	0.06	63.69	-1.72	7.26	9.38	18.27	9.35	0.59	14.2	23.68	13.2	3.31
os33192	4	310	1	19	1	4	5.8	-0.31	0	1.4	0.13	92.5	1.94	6.15	8.45	17.31	8.52	-0.25	13.27	22.74	12.37	2.54
os33319	5	220	0.92	0	3	6	6.69	0	3.42	1.56	0.01	3.42	0.34	9.51	8.98	17.77	9.14	0.19	13.65	23.15	12.86	2.81
os33321	4	227	0.92	0	3	6	6.7	0	2.99	1.55	0.03	58.76	-0.74	11.22	9.08	17.85	9.1	0.3	13.97	23.25	12.9	3
os33371	7	249	0.26	9	2	3	6.42	-0.13	0.76	1.51	0.09	95.16	-3.48	8.1	8.72	17.45	8.88	0.05	13.3	22.56	12.51	2.61
os33473	4	248	1	8	3	5	6.5	-0.11	0.27	1.53	0.08	63.17	-1.82	6.53	8.24	16.88	8.54	-1.14	13.17	22.38	12.61	1.62
os33475	4	275	0.92	1	3	6	6.68	-0.02	1.85	1.56	0.02	42.55	-0.45	8.03	8.26	16.92	8.53	-1.06	13.1	22.31	12.41	1.69
os33476	4	275	0.99	3	3	6	6.65	-0.04	1.68	1.55	0.03	53.85	-0.75	7.79	8.26	16.92	8.53	-1.06	13.1	22.31	12.41	1.69
os33522	5	275	0.23	2	3	6	6.74	0.02	1.47	1.55	0.03	43.66	-0.5	7.44	8.16	16.9	8.5	-1.04	12.88	22.28	12.28	1.66
os33540	5	261	0.92	0	3	6	6.73	0.01	5.27	1.56	0.01	10.2	0.18	10.53	8.33	17.05	8.62	-0.9	13.18	22.5	12.5	1.83
os33545	5	280	0.02	2	3	6	6.8	0.04	1.44	1.55	0.01	43.59	-0.11	7.56	8.29	17.06	8.59	-0.89	13.1	22.59	12.42	1.83
os33567	5	264	0.16	2	3	6	6.65	-0.04	0.83	1.55	0.02	39.39	0.28	7.89	8.37	17.07	8.64	-0.87	13.25	22.54	12.54	1.87
os33571	5	249	0	6	3	5	6.85	0.1	0.81	1.54	0.05	62.99	-1.86	8.32	8.45	17.16	8.73	-0.75	13.32	22.61	12.65	2.02
os33585	5	236	0.92	0	3	6	6.71	0	4.94	1.56	0.01	1.47	0.11	9.41	8.68	17.4	8.93	-0.47	13.56	22.91	12.84	2.27
os33616	5	256	0.01	1	3	6	6.75	0.02	2.26	1.56	0.02	16.47	0.02	9.2	8.28	17.09	8.62	-0.86	12.99	22.56	12.55	1.87
os33637	5	279	0.92	2	3	6	6.67	-0.03	1.71	1.55	0.02	52.96	-0.41	7.82	8.26	16.93	8.55	-1.04	13.12	22.33	12.42	1.7
os33648	5	276	0.13	2	3	6	6.73	0.02	2.79	1.55	0.03	78.09	-0.53	8.56	8.28	16.96	8.58	-1.02	13.15	22.35	12.45	1.73
os33732	4	272	0.92	1	3	6	6.71	-0.01	3.9	1.56	0.02	14.82	-0.02	8.28	8.27	16.93	8.54	-1.05	13.11	22.32	12.42	1.69
os33742	7	254	0.6	1	3	6	6.7	0	4.93	1.56	0.01	1.32	0.21	10.21	8.43	17.1	8.7	-0.93	13.38	22.64	12.67	1.83
os33774	5	278	0.92	1	3	6	6.74	0	0.36	1.55	0	25.99	0.45	9.44	8.25	16.98	8.56	-0.96	13.06	22.39	12.4	1.75
os33775	5	278	0.92	0	3	6	6.74	0	1.44	1.56	0.01	30.28	-0.07	9.06	8.25	16.97	8.55	-0.97	13.05	22.38	12.39	1.74
os33776	5	278	0.77	1	3	6	6.74	0	0.71	1.55	0	25.2	0.3	9.32	8.25	16.97	8.55	-0.96	13.06	22.39	12.4	1.75
os33836	5	205	0.92	0	3	6	6.69	0	6.98	1.57	0	0.04	0	12.54	9.02	17.75	9.07	-0.07	13.95	23.33	12.97	2.7
os33845	6	285	0.99	2	3	9	6.69	-0.03	1.78	1.51	0.02	82.08	-0.32	8.12	8.56	17.31	8.81	-0.48	13.45	22.8	12.68	2.24
os33849	6	286	0.92	1	3	6	6.75	0.01	0.66	1.54	0.01	68.87	0.14	9.07	8.43	17.18	8.72	-0.62	13.25	22.65	12.58	2.1
os33880	5	267	0	3	3	6	6.79	0.05	1.57	1.55	0.02	79.44	-0.41	7.67	8.28	16.99	8.61	-0.96	13.03	22.39	12.49	1.8
os33881	5	252	0.92	1	3	6	6.71	-0.01	1.99	1.56	0.02	25.16	0	8.42	8.42	17.1	8.7	-0.76	13.16	22.42	12.5	1.97
os33892	5	276	0.93	3	3	6	6.62	-0.06	0.95	1.54	0.03	73.58	-0.19	7.89	8.29	17.01	8.6	-0.89	13.04	22.36	12.45	1.86
os33905	5	258	0.33	10	3	9	6.52	0.02	0.04	1.48	0.06	90.49	0.97	5.91	8.97	17.72	9.05	-0.08	13.81	23.16	12.85	2.67
os33942	5	225	0.92	0	3	6	6.7	0	2.11	1.56	0.01	0.16	0.17	11.37	8.78	17.56	8.94	-0.31	13.56	23.05	12.81	2.41
os33953	5	243	0.88	1	3	6	6.68	-0.02	1.48	1.56	0.01	11.88	-0.12	8.66	8.7	17.42	8.92	-0.38	13.58	22.91	12.81	2.31
os33966	6	244	0.92	1	3	6	6.72	0.01	6.81	1.55	0.02	33.48	-0.07	8.69	8.73	17.47	8.94	-0.38	13.61	22.96	12.84	2.34
os34011	5	228	0.01	2	3	6	6.76	0.03	2.46	1.56	0.02	28.97	-0.59	8.58	8.78	17.53	8.96	-0.31	13.65	23.07	12.87	2.43
os34016	7	229	0.35	2	3	6	6.79	0.05	4.16	1.56	0.04	12.05	-0.57	7.74	8.73	17.51	8.94	-0.33	13.57	23.01	12.83	2.4
os34046	7	249	0.92	1	3	6	6.66	-0.02	1.98	1.56	0.01	6.31	-0.06	8.41	8.5	17.3	8.77	-0.58	13.26	22.74	12.63	2.14
os34047	5	245	0.92	1	3	6	6.67	-0.02	2.57	1.56	0.01	2.86	-0.31	8.59	8.52	17.31	8.78	-0.57	13.28	22.76	12.65	2.15
os34049	5	248	0.35	1	3	6	6.68	-0.02	2.21	1.56	0.01	5.97	0.06	8.95	8.5	17.3	8.77	-0.59	13.27	22.75	12.64	2.14

os34059	5	249	0.63	3	3	6	6.6	-0.06	1.69	1.54	0.03	85.08	-0.73	7.36	8.68	17.47	8.88	-0.43	13.46	22.98	12.77	2.32
os34082	7	247	0.99	2	3	6	6.67	-0.03	4.2	1.56	0.04	69.03	-0.62	8.21	8.54	17.3	8.77	-0.56	13.28	22.77	12.62	2.17
os34120	7	238	0.08	1	3	6	6.74	0.01	4.96	1.56	0.01	6.75	0.01	9.77	8.41	17.11	8.69	-0.98	13.39	22.63	12.77	1.8
os34121	7	238	0.03	1	3	6	6.73	0.02	4.96	1.56	0.01	6.72	0	9.97	8.41	17.11	8.69	-0.98	13.39	22.63	12.77	1.81
os34132	5	264	0.77	2	3	6	6.71	-0.02	2.81	1.55	0.02	71.12	-0.03	9.03	8.3	17.02	8.62	-0.88	13.04	22.39	12.47	1.86
os34135	5	266	0.76	2	3	6	6.73	-0.01	2.59	1.55	0.02	72.26	-0.08	8.91	8.3	17.02	8.62	-0.88	13.04	22.39	12.47	1.86
os34169	5	229	0.92	0	3	6	6.7	0	7.66	1.57	0.01	8.07	-0.26	11.57	8.57	17.37	8.87	-0.6	13.36	22.93	12.78	2.17
os34186	5	237	0.92	1	3	6	6.66	-0.03	1.57	1.56	0.01	15.16	-0.07	8.26	8.75	17.56	8.96	-0.43	13.59	23.17	12.93	2.3
os34189	5	247	0.92	2	3	6	6.68	-0.01	2.73	1.56	0.02	12.74	-0.42	9.16	8.57	17.37	8.84	-0.55	13.33	22.85	12.72	2.17
os34197	5	239	0.77	1	3	6	6.7	-0.01	2.89	1.56	0.01	27.19	-0.13	9.49	8.69	17.44	8.93	-0.4	13.52	22.91	12.8	2.31
os34202	5	236	0.77	1	3	6	6.69	-0.02	1.88	1.56	0.01	13.07	-0.01	8.64	8.66	17.44	8.91	-0.41	13.46	22.9	12.79	2.31
os34230	5	244	0.53	6	3	5	6.58	-0.02	0.66	1.54	0.04	53.43	-0.68	8.02	8.53	17.34	8.84	-0.63	13.29	22.89	12.76	2.12
os34231	5	257	0.56	4	1	4	6.64	-0.01	0.18	1.52	0.02	69.83	2.05	7.7	8.55	17.33	8.82	-0.61	13.4	22.9	12.76	2.15
os34232	6	235	0.7	2	3	6	6.73	0.02	1.97	1.55	0.04	64.67	-1.42	8.17	8.71	17.55	8.95	-0.42	13.54	23.14	12.93	2.34
os34233	7	237	0.4	1	3	6	6.72	0.01	0.03	1.53	0	72.05	1.11	8.57	8.72	17.55	8.95	-0.41	13.54	23.15	12.93	2.34
os34235	7	236	0.11	2	3	6	6.73	0.02	0.53	1.54	0.01	70.72	0.36	8.36	8.72	17.55	8.95	-0.41	13.54	23.15	12.93	2.34
os34245	5	270	0.92	1	3	6	6.71	-0.01	2.64	1.55	0.02	65.09	-0.71	8.58	8.48	17.24	8.78	-0.58	13.3	22.69	12.61	2.13
os34258	5	266	0.92	0	3	6	6.72	0	6.66	1.56	0	0.87	0	11.76	8.16	16.89	8.49	-1.17	13.25	22.51	12.57	1.69
os34260	5	264	0.23	1	3	6	6.72	0.01	6.55	1.56	0.01	8.83	-0.25	9.33	8.19	16.89	8.5	-1.16	13.25	22.51	12.59	1.7
os34271	5	269	0.92	0	3	6	6.72	0	6.07	1.56	0	0.09	0.04	12.02	8.11	16.86	8.46	-1.19	13.14	22.45	12.51	1.67
os34333	4	249	0.92	1	3	6	6.68	-0.01	5.85	1.56	0.01	8.53	0.36	9.11	8.22	16.91	8.48	-0.87	13.03	22.4	12.38	1.92
os34406	4	258	0.33	1	3	6	6.7	0	4.97	1.56	0.02	6.91	-0.17	8.89	8.28	16.91	8.5	-1.08	13.5	22.61	12.59	1.85
os34421	5	298	0.92	0	3	6	6.74	0	5.73	1.54	0	38.99	0.89	9.59	8.11	16.78	8.4	-1.24	13.3	22.42	12.44	1.66
os34425	5	266	0.92	0	3	6	6.73	0.01	6.69	1.56	0.01	1.58	-0.36	10.36	8.17	16.88	8.48	-1.15	13.28	22.5	12.58	1.71
os34476	4	258	0.39	4	2	3	6.73	0.02	2.28	1.56	0.09	81.3	-4.83	7.84	8.19	16.89	8.47	-1.01	13.13	22.43	12.43	1.85
os34487	7	246	0.55	2	3	6	6.65	-0.03	5.88	1.56	0.02	6.92	-0.37	9.03	8.25	16.92	8.49	-0.83	13.02	22.39	12.4	1.94
os34516	7	247	0.23	1	3	6	6.73	0.01	5.97	1.56	0.01	7.13	0.23	10.02	8.24	16.92	8.49	-0.86	13.06	22.42	12.41	1.94
os34525	5	260	0.92	0	3	6	6.72	0	5.97	1.57	0.01	4	-0.06	11.65	8.2	16.9	8.42	-0.84	13.03	22.5	12.3	1.96
os34581	5	289	0	6	3	5	6.87	0.1	0.44	1.53	0.05	39.92	-0.38	7.9	7.99	16.67	8.24	-0.95	12.78	22.25	12.05	1.81
os34584	5	288	0.02	8	3	5	6.9	0.13	0.25	1.52	0.07	42.78	-1.18	7.67	7.98	16.67	8.24	-0.96	12.78	22.25	12.04	1.8
os34594	5	260	0.92	0	3	6	6.72	0	5.95	1.57	0.01	12.67	-0.02	11.26	8.21	16.9	8.42	-0.83	13.04	22.5	12.3	1.96
os34596	6	283	0.23	1	3	6	6.72	0	2.97	1.56	0.03	49.21	-0.31	8.61	8.09	16.78	8.32	-0.86	12.92	22.4	12.17	1.92
os34597	6	283	0.92	1	3	6	6.72	0	2.97	1.56	0.03	50.29	-0.42	8.62	8.08	16.78	8.32	-0.86	12.92	22.4	12.17	1.92
os34613	4	260	0.92	0	3	6	6.72	0	5.97	1.57	0.01	4.03	-0.06	11.63	8.2	16.9	8.42	-0.84	13.03	22.5	12.3	1.96
os34671	7	260	0.99	6	2	3	6.52	-0.1	0.68	1.54	0.06	57.15	-2.29	6.99	8.13	16.83	8.4	-0.89	12.89	22.29	12.27	1.87
os34711	5	257	0.92	1	3	6	6.69	-0.01	1.71	1.53	0.02	35.56	1.15	9.2	8.35	17.08	8.6	-0.71	13.09	22.54	12.48	2.06
os34722	5	276	0.56	3	3	6	6.75	0	0.29	1.52	0.01	57.95	1.44	7.61	8.11	16.82	8.36	-0.86	12.84	22.32	12.18	1.87

os34760	7	218	0.92	0	3	6	6.72	0.01	6.99	1.57	0.02	8.28	-0.34	9.68	8.79	17.55	8.95	-0.34	13.67	23.06	12.88	2.49
os34763	6	218	0.48	1	3	6	6.73	0.01	6.99	1.56	0.01	0.78	-0.05	9.37	8.79	17.55	8.96	-0.34	13.66	23.05	12.86	2.49
os34764	7	218	0.08	1	3	6	6.72	0.01	6.99	1.57	0.01	1.66	-0.51	9.65	8.79	17.55	8.96	-0.34	13.66	23.05	12.87	2.49
os34771	7	218	0.6	1	3	6	6.68	0	6.99	1.56	0.01	1.35	-0.02	9.29	8.79	17.55	8.96	-0.34	13.66	23.04	12.86	2.49
os34779	6	219	0.92	0	3	6	6.7	0	6.99	1.57	0.01	2.25	0	11.66	8.77	17.52	8.94	-0.35	13.64	23.02	12.86	2.46
os34822	7	232	0.92	0	3	6	6.71	0	6.91	1.56	0	12.11	0.19	10.64	8.72	17.54	8.86	-0.39	13.74	23.31	12.92	2.45
os34845	6	251	0.92	2	3	6	6.65	-0.03	1.69	1.55	0.04	61.74	-0.07	7.5	8.26	17.03	8.57	-0.78	12.95	22.36	12.45	1.97
os34846	5	252	0.66	2	3	6	6.65	-0.02	1.82	1.55	0.04	64.46	-0.38	7.84	8.27	17.05	8.57	-0.78	12.97	22.38	12.46	1.97
os34849	5	227	0.92	0	3	6	6.7	0	6.91	1.57	0	0.34	0	11.42	8.49	17.34	8.77	-0.72	13.34	22.94	12.86	2.07
os34860	5	236	0.62	1	3	6	6.67	-0.01	6.83	1.56	0.01	0	0.04	8.96	8.27	16.93	8.54	-0.8	12.89	22.19	12.41	1.91
os34963	6	270	0.04	1	3	6	6.68	-0.03	2.37	1.56	0.03	32.06	0.02	7.53	8.05	16.71	8.34	-0.89	12.63	22.05	12.08	1.82
os35030	5	262	0.92	0	3	6	6.73	0	3.54	1.56	0.01	15.43	-0.46	9.24	8.33	17.08	8.55	-0.66	13.2	22.68	12.41	2.07
os35036	5	264	0.51	2	3	6	6.75	0.01	2.73	1.55	0.04	40.24	-0.79	8.67	8.12	16.82	8.46	-0.85	12.63	22.01	12.22	1.82
os35056	5	257	0.23	2	3	6	6.69	0	2.48	1.56	0.03	62.23	-1.4	8.16	8.31	17.04	8.54	-0.75	13.1	22.58	12.44	2.02
os35063	5	306	0.99	2	3	6	6.69	-0.03	2.3	1.55	0.02	32.29	-0.71	8.32	7.96	16.69	8.27	-0.96	12.63	22.11	12.03	1.78
os35088	5	221	0.92	0	3	6	6.7	0	6.98	1.56	0	0.01	0.12	11.84	8.69	17.41	8.87	-0.42	13.53	22.88	12.84	2.36
os35089	5	221	0.92	0	3	6	6.7	0	6.98	1.57	0	0.02	0.06	11.85	8.69	17.41	8.87	-0.42	13.53	22.88	12.84	2.36
os35096	5	228	0.08	2	3	6	6.76	0.03	6.98	1.55	0.02	36.56	0.08	8.3	8.76	17.53	8.91	-0.37	13.69	23.07	12.9	2.48
os35097	5	228	0.92	1	3	6	6.68	0	6.99	1.55	0.01	49.7	-0.2	8.65	8.78	17.54	8.94	-0.35	13.69	23.08	12.9	2.48
os35104	5	216	0.6	2	3	6	6.68	-0.01	6.99	1.56	0.01	2.87	-0.05	9.4	8.82	17.6	8.97	-0.32	13.76	23.17	12.96	2.53
os35137	5	237	0.4	3	3	6	6.77	0.02	1.99	1.55	0.03	48.68	-0.99	8.61	8.46	17.23	8.72	-0.64	13.3	22.71	12.7	2.12
os35167	7	225	0.08	1	3	6	6.72	0.01	5.01	1.57	0.02	7.84	-0.5	9.06	8.65	17.42	8.78	-0.51	13.67	23.18	12.94	2.3
os35168	7	224	0.92	1	3	6	6.67	-0.02	6.98	1.56	0.01	3.32	0.38	9.43	8.7	17.49	8.86	-0.44	13.69	23.2	12.96	2.39
os35179	7	234	0.99	6	3	5	6.55	-0.08	1.2	1.52	0.02	20.74	1.28	7.39	8.63	17.44	8.77	-0.47	13.71	23.23	12.95	2.4
os35180	7	233	1	6	3	5	6.56	-0.08	1.19	1.53	0.03	18.74	0.88	7.43	8.63	17.44	8.77	-0.47	13.71	23.23	12.95	2.4
os35185	5	227	0.82	1	3	6	6.67	-0.01	6.93	1.57	0.01	9.67	-0.31	9.2	8.7	17.5	8.84	-0.42	13.71	23.27	12.94	2.41
os35186	5	228	0.92	0	3	6	6.69	-0.01	6.92	1.56	0.01	12.09	0.03	9.37	8.7	17.5	8.84	-0.42	13.71	23.27	12.94	2.41
os35196	5	235	0.92	2	3	6	6.63	-0.04	1.8	1.55	0.03	40.38	-0.6	8.12	8.5	17.35	8.74	-0.68	13.39	22.9	12.78	2.14
os35203	6	230	0.92	1	3	6	6.71	0.01	2.76	1.56	0.02	32.25	-0.13	9.29	8.54	17.31	8.79	-0.6	13.4	22.83	12.79	2.19
os35211	7	238	0.92	0	3	6	6.71	0	6.92	1.57	0.01	0.31	-0.03	11.4	8.25	16.9	8.54	-0.82	12.93	22.22	12.4	1.9
os35221	5	247	0.92	0	3	6	6.71	0	4.11	1.56	0.01	0.03	0.27	10.82	8.19	16.86	8.48	-0.86	12.83	22.17	12.31	1.86
os35264	5	222	0.92	0	3	6	6.69	-0.01	0.8	1.55	0	19.4	0.46	10.88	8.9	17.65	9	-0.17	13.76	23.16	12.87	2.58
os35267	5	213	0.92	0	3	6	6.7	0	6.98	1.57	0	0	0	11.83	8.86	17.6	8.98	-0.23	13.63	23.09	12.82	2.49
os35277	7	217	0.92	0	3	6	6.7	0	6.95	1.57	0.01	10.05	0.01	11.76	8.78	17.53	8.93	-0.3	13.53	23	12.79	2.45
os35286	5	212	0.92	0	3	6	6.7	0	6.94	1.57	0.01	20.46	-0.32	8.89	8.82	17.56	8.94	-0.25	13.54	22.98	12.76	2.5
os35291	7	207	0.92	0	3	6	6.68	-0.01	4.7	1.56	0.01	7	0.39	10.36	8.98	17.76	9	-0.1	13.8	23.24	12.82	2.63
os35341	5	239	0.92	1	3	6	6.7	-0.01	3.94	1.57	0.03	37.3	-0.89	8.8	8.65	17.39	8.9	-0.45	13.54	22.87	12.76	2.26

os35379	7	207	0.92	0	3	6	6.71	0.01	6.43	1.55	0.01	34.52	0.77	9.76	8.91	17.64	8.99	-0.19	13.7	23.09	12.8	2.58
os35395	7	218	0.92	3	3	6	6.61	-0.04	5.18	1.56	0.03	6.68	-0.83	8.05	8.81	17.59	8.94	-0.33	13.76	23.19	12.91	2.56
os35400	5	240	0.92	0	3	6	6.71	0	2.97	1.56	0.01	1.21	-0.01	11.61	8.68	17.44	8.82	-0.39	13.55	22.99	12.69	2.44
os35554	7	302	0.83	13	3	5	6.56	-0.09	0.18	1.49	0.12	76.18	-2.69	7.5	8.36	17.04	8.61	-0.13	12.8	22.11	12.16	2.34
os35571	5	384	0.79	5	2	3	6.66	-0.02	1.42	1.5	0.07	94.97	-2.53	6.75	7.93	16.63	8.27	-0.38	12.2	21.5	11.58	2.04
os35609	3	287	0.02	13	1	4	6.17	-0.21	0	1.38	0.08	90.57	5.82	5.96	8.36	17.03	8.63	-0.12	12.78	22.02	12.1	2.35
os35740	4	257	1	3	2	0	6.69	-0.02	2.72	1.55	0.1	85.82	-2.6	7.8	8.5	17.23	8.73	-0.07	12.97	22.26	12.27	2.41
os35816	7	298	0.46	15	2	7	5.89	-0.25	0.34	1.51	0.21	89.4	-8.73	7.26	8.07	16.74	8.37	-0.25	12.33	21.61	11.72	2.17
os35845	4	218	0.93	18	2	7	6.03	-0.25	0	1.44	0.18	90.43	-2.89	5.77	8.79	17.49	9	0.3	13.19	22.41	12.46	2.75
os35959	4	235	0.74	6	3	5	6.65	-0.04	1.06	1.52	0.08	75.99	-3.17	8.91	9.02	17.81	9.13	0.38	13.72	22.99	12.77	2.97
os35960	7	296	0.77	9	2	7	6.24	-0.14	0.65	1.54	0.2	89.78	-9.84	7.45	8.07	16.74	8.37	-0.25	12.33	21.61	11.71	2.17
os35965	7	300	0.99	6	2	7	6.6	-0.05	2.84	1.55	0.13	94.77	-6.97	7.01	8.22	16.89	8.51	-0.19	12.56	21.82	11.93	2.26
os35968	4	336	0.17	12	2	7	6.64	0.1	0.64	1.51	0.2	95.5	-7.18	6.96	8.03	16.71	8.32	-0.31	12.3	21.59	11.71	2.12
os35997	4	254	0.9	20	2	7	5.77	-0.25	0	1.48	0.24	95.24	-6.7	5.51	8.43	17.11	8.69	0	12.73	21.96	12.07	2.43
os36080	7	300	0.96	12	3	5	6.36	-0.16	0.64	1.51	0.13	75.48	-4.46	7.22	8.36	17.04	8.61	-0.13	12.81	22.12	12.17	2.34
os36144	4	251	0.77	8	2	7	6.63	-0.06	0.72	1.52	0.13	86.86	-2.98	6.61	8.6	17.32	8.82	0.06	13.12	22.42	12.4	2.59
os36167	3	257	0.88	20	2	7	5.79	-0.26	0	1.45	0.15	86.73	-4.79	6.1	8.7	17.4	8.9	0.16	13.24	22.47	12.43	2.67
os36168	4	270	0.99	12	2	0	6.24	-0.2	0.02	1.49	0.1	84.33	-1.18	6.87	8.53	17.24	8.76	0.02	13.01	22.3	12.32	2.52
os36228	3	327	0.43	23	3	5	6.72	0.11	0.01	1.35	0.12	96.56	3.68	5.7	8.15	16.83	8.44	-0.21	12.37	21.64	11.78	2.22
os36251	3	322	0.96	19	2	3	5.8	-0.3	0	1.41	0.16	95.87	-4.43	5.61	8.22	16.88	8.5	-0.17	12.44	21.69	11.82	2.27
os36275	3	354	0.92	10	2	3	6.44	-0.12	0.66	1.47	0.09	94.94	-2.96	6.96	8.07	16.76	8.36	-0.3	12.37	21.64	11.73	2.16
os36291	3	364	0.11	10	1	4	6.75	0.1	0.04	1.38	0.04	95.9	7.22	6.06	8.01	16.69	8.32	-0.32	12.25	21.55	11.65	2.1
os36293	3	359	0.61	12	3	5	6.53	-0.12	0.06	1.41	0.08	96.35	2.89	5.98	8.01	16.69	8.32	-0.32	12.25	21.55	11.65	2.1
os36315	4	271	0.33	9	2	7	6.58	0.04	0.61	1.54	0.17	93.51	-6.82	6.89	8.22	16.87	8.5	-0.15	12.48	21.73	11.86	2.27
os36408	4	263	0.98	29	1	4	5.08	-0.44	0	1.29	0.1	69.34	7.74	5.8	8.5	17.2	8.74	-0.07	13.09	22.44	12.35	2.44
os36458	4	350	0.07	10	1	1	6.8	0.13	0.02	1.39	0.04	99.32	5.12	5.68	8.22	16.94	8.53	-0.44	12.91	22.27	12.18	2.12
os36510	7	268	0.77	5	2	7	6.48	-0.03	1.36	1.55	0.24	82.35	-10.55	6.73	8.11	16.78	8.48	-0.56	12.63	21.97	12.02	1.98
os36669	7	361	0.6	5	2	3	6.74	-0.02	1.6	1.53	0.08	94.42	-4.24	8.44	7.77	16.52	8.21	-0.96	12.36	21.77	11.78	1.68
os36670	4	362	0.99	10	3	5	6.35	-0.17	0.14	1.48	0.07	96.87	-1.14	7.6	7.78	16.53	8.21	-0.95	12.37	21.78	11.79	1.68
os36680	5	340	0.92	5	3	5	6.64	-0.07	0.88	1.53	0.08	94.22	-1.93	7.15	7.82	16.58	8.24	-0.94	12.44	21.86	11.84	1.72
os36690	7	348	0.87	9	2	3	6.57	-0.1	0.73	1.52	0.11	95.51	-4.44	7.71	7.79	16.57	8.22	-0.98	12.39	21.85	11.82	1.65
os36707	4	325	0.88	12	2	3	6.25	-0.16	0.22	1.46	0.11	96.01	-4.17	6.94	7.99	16.68	8.4	-0.67	12.44	21.79	11.85	1.84
os36730	4	322	1	11	2	3	6.38	-0.15	0.5	1.5	0.1	97.14	-3.77	7.12	7.97	16.66	8.39	-0.68	12.44	21.78	11.84	1.85
os36783	4	295	1	19	1	4	6.02	-0.27	0.01	1.39	0.11	89.16	3.14	6.12	8.14	16.81	8.51	-0.38	12.69	22.04	12.12	2.11
os36788	4	238	0.97	6	2	7	6.48	-0.1	0.33	1.53	0.17	84.46	-5.13	6.11	8.21	16.88	8.57	-0.35	12.8	22.12	12.19	2.16
os36789	4	240	0.84	11	2	7	6.29	-0.17	0.11	1.52	0.18	85.41	-6.28	6.05	8.21	16.87	8.57	-0.35	12.8	22.12	12.19	2.16
os36810	4	302	0.47	5	3	6	6.67	-0.01	2.01	1.51	0.04	47.64	-1.18	8.72	8.11	16.81	8.51	-0.38	12.66	22.04	12.08	2.12

os36822	6	278	0.51	5	2	3	6.57	-0.03	1.32	1.54	0.15	99.38	-5.94	6.04	8.24	16.94	8.58	-0.43	12.96	22.33	12.3	2.15
os36841	4	310	0.13	16	3	5	6.96	0.22	0	1.45	0.12	97.34	-1.56	5.51	8.07	16.79	8.42	-0.53	12.71	22.04	12.01	2.02
os36883	3	340	1	15	3	9	6.16	-0.24	0	1.4	0.11	97.82	-0.45	6.37	7.84	16.52	8.29	-0.57	12.33	21.67	11.77	1.89
os36902	4	316	0.09	4	3	6	6.83	0.07	0.76	1.51	0.04	94.97	0.66	7.93	7.9	16.59	8.35	-0.6	12.47	21.82	11.94	1.92
os36912	4	319	0.73	6	2	3	6.72	-0.03	1.32	1.53	0.09	91.49	-4.41	7.73	7.84	16.56	8.32	-0.67	12.43	21.82	11.93	1.87
os36913	4	326	0.29	4	3	6	6.87	0.06	0.46	1.51	0.02	95.3	0.82	6.98	7.88	16.59	8.35	-0.64	12.47	21.85	11.96	1.89
os37018	5	335	0.11	4	2	3	6.85	0.06	1.8	1.54	0.09	92.95	-4.03	7.15	8.13	16.9	8.4	-0.39	12.47	21.86	11.84	2.07
os37216	4	233	0.76	8	2	7	6.35	-0.12	0.53	1.54	0.15	80.64	-6.4	6.96	8.58	17.31	8.8	-0.07	13.13	22.38	12.39	2.47
os37249	3	344	0.27	9	2	3	6.68	0.06	0.53	1.43	0.08	98.03	-1.63	6.81	8.13	16.9	8.42	-0.42	12.57	21.93	11.91	2.08
os37254	4	369	0.06	9	2	3	6.87	0.13	0.61	1.49	0.08	92.35	-2.5	7.56	7.98	16.75	8.29	-0.54	12.4	21.75	11.76	1.92
os37273	4	310	0.92	7	2	3	6.6	-0.07	1.49	1.53	0.09	94.22	-4.18	8.32	8.26	17.06	8.55	-0.28	12.68	22.06	12.03	2.17
os37309	5	315	0	8	2	3	6.88	0.12	0.74	1.52	0.12	86.06	-5.51	7.57	8.31	17.04	8.56	-0.21	12.76	22.06	12.07	2.26
os37474	3	283	0.35	20	3	5	6.29	0.04	0	1.42	0.1	84.26	1.36	5.63	8.18	16.86	8.49	-0.25	12.83	22.13	12.2	2.32
os37485	5	308	0.97	6	3	5	6.61	-0.08	0.51	1.53	0.09	93.83	-1.08	7.57	7.98	16.7	8.34	-0.39	12.63	21.91	12.01	2.18
os37549	4	312	0.67	16	2	3	6.13	-0.23	0.02	1.46	0.14	93.14	-3.56	6.74	8.11	16.8	8.41	-0.35	12.6	21.83	11.94	2.18
os37554	4	366	0.91	10	2	7	6.26	-0.15	0.27	1.5	0.19	90.91	-3.73	7.32	7.7	16.47	8.01	-0.72	12.03	21.4	11.42	1.72
os37556	4	365	0.99	14	2	7	6.19	-0.18	0.32	1.5	0.19	91.81	-4.67	7.14	7.71	16.48	8.02	-0.72	12.03	21.41	11.43	1.73
os37558	4	337	0.69	14	3	5	6.17	-0.16	0	1.46	0.09	99.6	-1.08	5.85	7.99	16.73	8.29	-0.47	12.36	21.69	11.74	1.98
os37644	4	313	0.92	6	1	4	6.51	-0.08	1.03	1.43	0.05	86.74	3.76	7.51	8.11	16.82	8.41	-0.35	12.57	21.81	11.94	2.14
os37718	7	304	0.16	6	2	3	6.73	0.06	1.11	1.54	0.08	78.82	-3.25	7.2	7.89	16.6	8.34	-0.49	12.6	21.88	12.02	2.05
os37722	5	363	0.63	6	2	3	6.65	-0.02	1.13	1.52	0.1	90.16	-4.05	7.63	7.61	16.3	8.06	-0.7	12.17	21.48	11.66	1.79
os37829	3	369	0.15	11	2	3	6.93	0.12	0.43	1.5	0.14	95.26	-4.45	6.37	7.84	16.61	8.14	-0.57	12.17	21.52	11.57	1.89
os37842	4	362	0.05	14	2	3	6.93	0.2	0	1.47	0.12	90.39	-1.24	6.59	7.86	16.62	8.21	-0.52	12.16	21.5	11.57	1.92
os37870	4	347	0.63	4	2	3	6.86	0.05	1.26	1.52	0.09	97.1	-1.88	6.24	8.04	16.76	8.35	-0.41	12.37	21.67	11.74	2.07
os37950	5	303	0.52	6	2	7	6.5	-0.04	1.15	1.55	0.16	87.85	-7.34	6.79	8.11	16.81	8.41	-0.35	12.56	21.8	11.92	2.14
os37993	5	320	0.6	7	3	5	6.45	-0.1	0.56	1.52	0.07	81.09	-1.41	7.07	7.88	16.59	8.26	-0.48	12.42	21.73	11.86	2
os38115	7	285	0.87	18	3	5	5.97	-0.24	0.27	1.49	0.14	93.76	-3.47	6.26	8.05	16.75	8.39	-0.34	12.69	22.01	12.1	2.23
os38174	4	315	0.4	11	3	5	6.49	-0.02	0.23	1.47	0.08	93.4	-1.33	6.92	8.14	16.84	8.47	-0.27	12.51	21.84	11.93	2.2
os38205	5	323	0.69	5	2	7	6.62	-0.03	2.36	1.56	0.19	92.1	-9.03	6.9	7.97	16.65	8.31	-0.4	12.31	21.61	11.72	2.08
os38212	5	334	0.27	7	2	3	6.64	0.03	0.98	1.52	0.11	99.2	-4.54	7.13	8.03	16.72	8.33	-0.39	12.53	21.77	11.9	2.12
os38233	4	262	0.1	4	3	6	6.62	-0.06	0.47	1.53	0.03	53.38	1.13	8.11	8.34	17.02	8.6	-0.12	13.01	22.28	12.34	2.47
os38235	7	324	0	15	3	5	6.92	0.25	0	1.46	0.08	81.31	0.04	5.51	7.9	16.59	8.32	-0.5	12.53	21.82	11.98	2.02
os38249	5	341	0	17	2	3	6.54	0.28	0.39	1.51	0.3	86.54	-14.55	5.61	7.92	16.68	8.21	-0.56	12.31	21.66	11.67	1.89
os38255	5	379	0.16	9	2	3	6.75	0.09	0.74	1.5	0.1	90.38	-3.99	7.62	7.84	16.6	8.14	-0.62	12.19	21.54	11.55	1.82
os38405	5	310	0.98	14	3	5	6.05	-0.24	0.05	1.43	0.07	68.89	1.73	7.13	8.13	16.81	8.42	-0.24	12.56	21.93	11.9	2.22
os38502	4	269	0.01	8	2	3	6.56	0.03	2.15	1.55	0.16	77.71	-8.48	8.32	8.38	17.09	8.63	-0.16	12.98	22.37	12.25	2.36
os38523	5	257	1	13	2	7	6.18	-0.21	0.24	1.52	0.26	91.14	-11.07	5.45	8.33	17.02	8.56	-0.16	12.89	22.23	12.19	2.33

os38563	5	349	0.23	4	3	6	6.75	0.03	0.72	1.52	0.03	96.49	0.13	7.83	7.8	16.52	8.21	-0.53	12.38	21.7	11.84	1.99
os38598	5	280	0.95	3	2	7	6.69	-0.01	2.92	1.56	0.09	80.65	-3.74	7.24	8.21	16.93	8.49	-0.24	12.74	21.96	12.07	2.3
os38608	4	313	0.08	6	2	3	6.7	-0.03	1.16	1.5	0.09	95.98	-2.64	6.23	8.16	16.87	8.46	-0.28	12.66	21.9	12.02	2.24
os38614	4	382	0.84	13	2	3	6.37	-0.17	0.29	1.45	0.12	94.36	-1.94	7.35	7.88	16.64	8.18	-0.58	12.23	21.58	11.59	1.86
os38615	3	396	0.37	18	1	4	5.87	-0.28	0.06	1.41	0.13	92.07	1.33	5.88	7.74	16.51	8.05	-0.69	12.07	21.44	11.46	1.75
os38632	5	384	0.01	14	2	3	6.83	0.2	0.31	1.5	0.13	98.59	-4.59	5.79	7.85	16.63	8.16	-0.61	12.2	21.56	11.57	1.82
os38686	3	315	0.01	7	3	6	6.48	-0.1	0.63	1.46	0.08	85.93	0.09	7.57	8.27	16.95	8.53	-0.23	12.62	21.88	11.96	2.24
os38722	4	306	0.6	13	3	5	6.22	-0.13	0.01	1.46	0.11	94.26	0.01	6.37	8.19	16.87	8.49	-0.19	12.49	21.75	11.86	2.25
os38774	4	334	0.87	7	2	3	6.51	-0.1	1.52	1.53	0.14	87.3	-7.47	7.82	8.03	16.72	8.36	-0.35	12.37	21.65	11.78	2.12
os38797	4	315	0.51	11	3	5	6.41	-0.08	0.32	1.51	0.08	89.02	-1.92	6.73	8.19	16.91	8.5	-0.24	12.61	21.89	12	2.22
os38858	4	297	0	6	2	7	6.79	0.08	1.65	1.55	0.19	97.03	-8.87	7.24	8.09	16.86	8.39	-0.45	12.55	21.88	11.89	2.04
os38960	5	358	0	5	2	3	6.61	-0.05	1.87	1.53	0.11	83.43	-5.74	7.35	7.57	16.23	8.1	-0.69	12.14	21.42	11.62	1.81
os38963	5	331	0.76	9	2	7	6.4	-0.1	0.66	1.54	0.15	87.17	-6.54	7.41	7.6	16.27	8.13	-0.67	12.2	21.5	11.68	1.85
os39130	5	440	0.25	10	2	3	6.68	0.06	0.52	1.52	0.11	94.46	-3.74	7.35	7.13	15.86	7.71	-1.46	11.68	21.13	11.23	1.09
os39169	3	354	0.96	19	1	1	6.05	-0.26	0	1.24	0.12	85.11	9.22	4.59	7.92	16.63	8.35	-0.9	12.56	21.93	11.94	1.65
os39398	3	430	0.08	18	3	5	6.96	0.31	0	1.42	0.15	99.89	-0.6	6.03	7.09	15.8	7.68	-1.42	11.68	21.05	11.19	1.08
os39543	3	388	0.01	5	2	3	6.84	0.08	1.31	1.52	0.13	97.98	-3.52	6.57	7.45	16.11	8.01	-0.73	11.92	21.22	11.46	1.7
os39556	7	326	0.23	6	2	7	6.74	0.06	1.18	1.55	0.12	87.65	-4.99	7.22	7.7	16.38	8.17	-0.64	12.34	21.67	11.82	1.9
os39557	5	332	0.13	9	3	5	6.71	0.08	0.24	1.51	0.11	87.95	-1.98	6.91	7.7	16.38	8.17	-0.65	12.34	21.66	11.81	1.9
os39558	5	329	0.1	9	2	7	6.73	0.09	0.14	1.52	0.12	87.78	-3.26	7.02	7.7	16.38	8.17	-0.65	12.34	21.66	11.81	1.9
os39606	5	346	0.08	2	3	6	6.79	0.01	3.95	1.55	0.06	62.26	-0.77	8.9	7.52	16.19	8.06	-0.7	11.98	21.32	11.52	1.76
os39800	3	407	0.93	7	1	4	6.62	-0.1	0.12	1.47	0.03	96.35	2.31	6.28	7.44	16.13	7.97	-0.88	11.97	21.29	11.43	1.61
os39947	4	315	0.61	6	2	0	6.46	-0.1	0.55	1.51	0.12	84.72	-0.3	7.13	7.6	16.33	8.09	-0.97	12.21	21.65	11.72	1.56
os40017	7	403	0	25	2	7	6.78	0.39	0	1.39	0.24	99.9	-3.84	5.32	7.13	15.84	7.72	-1.48	11.66	21.04	11.2	1.02
os40125	5	322	0	9	2	3	6.9	0.13	0.63	1.53	0.08	92.43	-3.35	8.63	7.83	16.54	8.29	-0.72	12.42	21.83	11.93	1.8
os40206	5	308	1	5	2	3	6.54	-0.1	0.99	1.53	0.1	95.92	-3.49	7.23	7.96	16.68	8.35	-0.91	12.56	21.92	11.96	1.72
os40299	4	325	0.98	12	3	5	6.26	-0.19	0.04	1.43	0.09	94.26	-1.14	5.98	7.91	16.57	8.3	-0.87	12.39	21.72	11.81	1.69
os40317	3	272	0.06	21	2	0	6.67	0.27	0	1.4	0.17	83.27	-0.74	5.98	8.02	16.71	8.42	-0.71	12.5	21.85	11.91	1.84
os40388	7	360	0.59	9	3	5	6.51	-0.04	0.08	1.49	0.05	90	1.13	8.52	7.58	16.31	8.05	-1.33	12.18	21.55	11.66	1.27
os40410	3	336	0.19	16	3	5	6.96	0.19	0	1.4	0.11	99.33	0.14	6.02	7.98	16.68	8.37	-0.69	12.52	21.89	11.9	1.84
os40485	5	421	0	5	3	9	6.89	0.08	0.55	1.44	0.02	99.46	0.64	6.82	7.51	16.22	8.01	-1.19	12.1	21.44	11.51	1.37
os40557	5	377	0.92	9	3	9	6.39	-0.14	0.06	1.48	0.05	99.81	-0.08	6.02	7.74	16.44	8.16	-1.09	12.29	21.66	11.71	1.53
os40571	4	321	0.41	13	3	5	6.42	-0.02	0.07	1.49	0.09	81.23	-1.28	7.46	7.95	16.7	8.33	-0.89	12.53	21.99	11.96	1.72
os40572	4	321	0.45	11	3	5	6.51	-0.01	0.33	1.51	0.08	78.7	-1.53	7.8	7.94	16.69	8.32	-0.9	12.52	21.98	11.95	1.71
os40602	5	333	0.01	13	2	3	6.94	0.21	0.09	1.49	0.1	96.24	-3.35	6.04	7.85	16.56	8.24	-1.09	12.44	21.83	11.85	1.54
os40661	5	277	0.92	0	3	6	6.73	0	4.1	1.57	0.02	10.02	-0.24	11.9	8.21	16.9	8.56	-0.14	12.71	22.02	12.05	2.3
os40695	5	316	0.92	1	3	6	6.74	0	4.64	1.55	0	18.24	0.35	10.03	8.01	16.66	8.36	-0.22	12.35	21.61	11.75	2.21

os40696	5	312	0.23	3	3	6	6.74	0.02	1.7	1.55	0.02	37.39	-0.16	8.5	8.01	16.66	8.36	-0.22	12.34	21.6	11.75	2.21
os40741	5	302	0.09	2	3	6	6.69	-0.02	2.96	1.56	0.05	47.05	-0.67	9.45	7.91	16.55	8.35	-0.36	12.37	21.6	11.78	2.05
os40828	4	405	0.53	19	3	9	6.71	0.02	0	1.38	0.12	99.85	0.8	5.27	7.78	16.5	8.23	-0.54	12.35	21.63	11.77	1.96
os40959	7	309	0.77	6	2	7	6.73	-0.03	0.87	1.53	0.12	92.86	-2.52	7.73	7.98	16.71	8.29	-0.45	12.48	21.74	11.84	2.08
os41052	5	337	0.02	5	3	6	6.87	0.08	0.43	1.53	0.07	97.3	0.34	6.87	7.94	16.65	8.24	-0.63	12.41	21.58	11.68	1.79
os41073	7	213	0.18	9	2	7	6.59	0.05	1.16	1.5	0.19	91.57	-6.81	5.45	8.32	17.02	8.72	0.03	12.61	21.92	12.15	2.41
os41153	7	315	0.01	4	2	3	6.77	0.04	2.21	1.53	0.09	84.01	-4.52	9.04	7.98	16.76	8.42	-0.29	12.23	21.58	11.78	2.05
os41154	7	315	0.21	7	2	7	6.52	-0.05	1.01	1.53	0.14	95.14	-7.44	8.74	7.96	16.75	8.4	-0.31	12.17	21.54	11.74	2.03
os41166	7	295	0.86	7	3	5	6.5	-0.09	0.4	1.49	0.03	86.84	1.05	7.62	8.12	16.86	8.54	-0.18	12.36	21.71	11.92	2.17
os41253	5	536	0.77	6	2	0	6.83	-0.01	0.82	1.52	0	99.86	-2.1	7.28	6.39	15.19	7.12	-1.53	10.48	19.92	10.19	0.73
os41284	7	483	0	5	2	0	6.95	0.1	0.6	1.53	0.09	99.8	-0.33	6.86	6.91	15.72	7.45	-1.27	11.17	20.54	10.68	1.12
os41303	6	445	0.34	11	2	3	6.6	0.03	0.06	1.49	0.11	99.45	-2.94	6	7.2	15.99	7.67	-1.08	11.52	20.83	10.95	1.27
os41412	5	467	0.77	3	3	6	6.75	-0.04	1.75	1.54	0.04	84.05	-0.8	7.76	7.23	15.89	7.33	-0.97	12.08	21.44	11.2	1.69
os41424	5	456	0.77	6	3	5	6.77	-0.04	0.77	1.53	0.05	87.6	-0.71	7	7.27	15.99	7.43	-1.01	12.18	21.57	11.31	1.67
os41433	5	464	0.53	5	3	6	6.7	-0.01	0.71	1.54	0.07	84.1	-1.59	7.16	7.18	15.93	7.41	-1.09	12.06	21.46	11.29	1.6
os41451	5	509	0.14	2	3	6	6.86	0.02	0.34	1.54	0.01	18.48	0.16	8.49	6.96	15.59	7.23	-1.21	11.81	21.15	11.12	1.38
os41466	5	494	0.92	0	3	6	6.84	0.01	0.63	1.53	0.01	68.43	0.42	8.69	6.99	15.64	7.26	-1.16	11.85	21.21	11.14	1.41
os41504	5	436	0.92	0	3	6	6.79	0	5.95	1.57	0.01	1.99	-0.04	11.52	7.43	16.07	7.52	-0.96	12.62	21.99	11.66	1.77
os41505	8	435	0	1	3	6	6.76	-0.02	5.93	1.56	0.01	1.25	-0.36	9.74	7.42	16.07	7.52	-0.96	12.61	21.98	11.65	1.77
os41549	5	442	0.01	4	3	6	6.9	0.06	0.61	1.56	0.07	35.17	-2.05	7.63	7.32	16.04	7.49	-0.98	12.24	21.63	11.38	1.7
os41554	6	455	0.77	2	2	3	6.8	0	1.97	1.56	0.09	77.03	-3.76	7.63	7.16	15.86	7.35	-1.07	12.04	21.41	11.18	1.59
os41592	7	435	0.23	5	3	5	6.81	0.06	0.92	1.55	0.06	53.03	-2.07	8.31	7.39	16.06	7.54	-1.03	12.37	21.73	11.5	1.67
os41606	5	443	0.43	11	3	5	6.89	0.05	0.1	1.5	0.08	87.13	-0.32	6.1	7.48	16.21	7.78	-0.99	12.31	21.64	11.66	1.66
os41622	7	494	0.92	3	3	6	6.73	-0.05	1.78	1.51	0.02	84.03	0.29	7.69	7.24	15.99	7.53	-1.13	12.16	21.48	11.45	1.55
os41648	5	417	0.01	7	3	5	6.47	-0.13	0.25	1.51	0.08	82.88	-0.96	6.22	7.62	16.37	7.82	-0.94	12.46	21.83	11.69	1.72
os41712	5	410	0.92	1	3	6	6.81	0.01	2.39	1.55	0.02	47.69	0.7	8.95	7.7	16.51	7.83	-0.88	12.9	22.28	11.88	1.88
os41728	5	436	0.68	5	3	6	6.62	-0.08	0.54	1.53	0.06	82.3	-1.34	8.03	7.54	16.34	7.7	-0.94	12.64	22.02	11.63	1.78
os41917	5	462	0.25	10	1	1	6.93	0.08	0.37	1.47	0.06	96.62	1.75	6.37	7.57	16.33	7.72	-0.85	12.5	21.91	11.53	1.76
os42048	5	437	0.77	1	3	6	6.81	0	1.1	1.52	0.03	72.7	0.1	7.77	7.58	16.33	7.74	-0.84	12.51	21.87	11.55	1.77
os42057	5	485	0.4	2	3	9	6.87	0.02	1.47	1.47	0.01	72.33	0.15	7.61	7.5	16.26	7.64	-0.89	12.42	21.84	11.46	1.71
os42058	5	487	0.08	2	3	9	6.87	0.03	1.38	1.49	0.01	45.11	0.16	7.78	7.46	16.24	7.61	-0.92	12.4	21.81	11.44	1.69
os42059	5	486	0.08	2	3	9	6.86	0.02	1.38	1.48	0.01	69.89	0.2	7.57	7.49	16.26	7.64	-0.9	12.42	21.84	11.46	1.71
os42128	5	463	0.92	1	2	0	6.76	-0.02	1.93	1.55	0.06	84.74	0.07	7.72	7.2	15.92	7.4	-1.13	12.04	21.37	11.15	1.43
os42213	3	362	0.08	4	3	6	6.89	0.07	0.28	1.53	0.03	62.35	-0.3	7.56	8.19	16.9	8.4	-0.41	12.94	22.17	12.13	2.21
os42218	4	321	0.22	3	2	3	6.8	0.05	1.52	1.56	0.06	60.15	-2.17	8.36	8.32	17.09	8.52	-0.3	13.2	22.48	12.32	2.39
os42272	6	255	0.47	4	3	6	6.77	0.01	1.17	1.55	0.05	62.61	-0.73	7.78	8.92	17.73	9.03	0.11	13.76	23.04	12.84	2.85
os42275	6	286	0.01	2	2	3	6.74	0.02	2.04	1.54	0.05	73.76	-2.82	8.4	8.81	17.61	8.94	0.05	13.61	22.88	12.72	2.75

os42387	7	233	0.16	9	1	4	6.7	0.1	0.05	1.49	0.08	83.31	2.14	6.25	8.98	17.9	9.05	0.12	14.2	23.65	13.29	3.05
os42401	7	262	0.92	0	3	6	6.72	0	4.94	1.56	0.02	39.85	0.06	10.43	8.81	17.71	8.95	-0.06	13.96	23.4	13.12	2.74
os42453	6	437	0.26	5	3	5	6.91	0.05	0.86	1.52	0	95	-0.85	7.48	7.11	15.87	7.74	-0.9	11.21	20.57	10.93	1.43
os42497	5	472	0.23	5	3	6	6.78	0.03	1.74	1.53	0.05	83.41	-1.27	9.22	6.88	15.66	7.54	-1.15	11.28	20.64	10.86	1.25
os42511	7	401	0.93	4	2	0	6.74	-0.04	0.83	1.54	0	99.94	-0.73	8.06	7.22	15.95	7.84	-0.89	11.68	21	11.22	1.52
os42596	5	420	0.01	2	3	6	6.83	0.04	1.59	1.54	0.02	57.62	0.51	8.46	7.25	16.02	7.88	-0.78	11.66	21.05	11.29	1.65
os42634	5	414	0.99	6	2	7	6.62	-0.09	0.87	1.54	0.09	97.27	-2.72	8.59	7.05	15.85	7.72	-0.92	11.4	20.77	11.1	1.48
os42820	5	406	0.77	3	3	6	6.75	-0.03	1.35	1.55	0.03	90.02	-0.2	8.16	7.29	16.09	7.93	-0.74	11.74	21.17	11.38	1.7
os42825	5	426	0.92	1	3	6	6.81	0.01	0.2	1.55	0	31.03	0.68	9.25	7.24	16.06	7.86	-0.83	11.8	21.25	11.36	1.66
os42857	4	405	0.92	4	3	6	6.65	-0.06	1.5	1.54	0	96.75	-0.41	7.56	7.27	15.99	7.88	-0.85	11.72	21.03	11.26	1.56
os42859	7	447	0.77	6	3	5	6.76	-0.04	0.73	1.51	0.06	99.98	0.01	7.01	7.03	15.79	7.68	-0.99	11.3	20.66	10.88	1.31
os42878	5	285	0.39	3	2	0	6.78	0.01	2.33	1.57	0	80.09	-1.74	8.91	7.86	16.55	8.42	-0.29	12.52	21.88	11.99	2.24
os42888	5	293	0.53	8	2	3	6.58	0	0.49	1.53	0.07	95.83	-1.89	7.12	7.83	16.48	8.42	-0.26	12.3	21.56	11.79	2.09
os42898	5	290	0.92	1	3	6	6.7	-0.02	2.42	1.54	0.07	86.77	0.22	8.87	7.84	16.47	8.42	-0.27	12.28	21.53	11.77	2.08
os42972	5	414	0.36	15	2	3	6.42	0.02	0.17	1.49	0.18	93.41	-7.41	6.83	7.24	16.03	7.85	-1.1	11.94	21.41	11.39	1.43
os43043	5	279	0.92	0	3	6	6.73	0	2.96	1.56	0	74.81	-0.64	9.18	7.99	16.7	8.54	-0.14	12.65	22.01	12.1	2.38
os43131	4	352	0.99	6	3	9	6.6	-0.09	0.28	1.49	0.03	90.33	0.8	6.32	7.69	16.36	8.28	-0.39	12.1	21.41	11.61	1.96
os43178	7	336	0.6	8	3	5	6.47	-0.07	0.34	1.5	0.06	99.08	-0.42	7.67	7.53	16.19	8.19	-0.66	12.14	21.38	11.65	1.8
os43179	5	336	0.27	4	3	6	6.62	-0.06	1.72	1.54	0	83.65	-0.79	7.9	7.6	16.23	8.23	-0.53	12.26	21.49	11.75	1.96
os43229	5	400	0.92	0	3	6	6.78	0	0.99	1.55	0	25.96	0.5	10.48	7.25	15.87	7.97	-1.08	11.85	21.02	11.52	1.51
os43239	6	265	0.3	13	3	5	6.52	0.04	0.09	1.5	0	86.83	-1.49	7.52	7.89	16.54	8.47	-0.42	12.58	21.85	12.19	2.2
os43241	5	278	0.35	7	3	5	6.62	0.01	0.62	1.51	0	88.61	-1.36	7.51	7.89	16.54	8.47	-0.43	12.58	21.85	12.18	2.19
os43244	4	277	0.78	11	1	4	6.33	-0.12	0.06	1.48	0	97.49	0.88	7.01	7.92	16.6	8.5	-0.2	12.6	21.87	12.19	2.36
os43266	5	351	0.81	9	3	5	6.4	-0.12	0.12	1.51	0.06	99.03	-1.12	7.21	7.48	16.11	8.15	-0.67	12.09	21.32	11.63	1.81
os43267	4	408	0.99	6	3	9	6.57	-0.1	0.49	1.51	0.03	97.66	0.03	6.5	7.35	16	8.03	-0.8	11.91	21.17	11.48	1.68
os43274	5	278	0.59	7	2	8	6.57	-0.02	0.91	1.5	0	96.27	-2.51	7.62	7.98	16.63	8.55	-0.21	12.65	21.88	12.14	2.34
os43314	7	330	0.6	10	3	5	6.39	-0.08	0.11	1.51	0.07	98.87	-1.21	7.59	7.54	16.2	8.2	-0.66	12.15	21.39	11.67	1.81
os43437	7	390	0.77	5	2	0	6.76	-0.02	1.14	1.54	0.09	88.64	-1.15	7.9	7.23	15.93	7.82	-1.17	11.9	21.28	11.33	1.33
os43479	3	655	0.53	20	3	5	6.11	-0.04	0	1.41	0.18	98.6	-0.66	5.09	5.49	14.23	6.43	-2.81	9.59	19.04	9.66	-0.41
os43487	6	714	0.07	4	2	0	7.02	0.07	0.59	1.52	0.09	78.03	-1.1	7.22	5.32	14.11	6.26	-2.86	9.49	18.94	9.47	-0.46
os43490	4	607	0.96	19	2	0	6.12	-0.27	0	1.44	0.16	95.33	-0.45	6.04	5.68	14.41	6.57	-2.67	9.85	19.24	9.83	-0.27
os43577	3	633	0.1	13	2	3	6.75	0.09	0.03	1.49	0.15	94.71	-3.82	7.4	5.83	14.68	6.69	-2.55	10.14	19.65	9.98	-0.11
os43617	7	447	0.01	10	2	3	6.96	0.15	0.75	1.52	0.12	97.58	-5.68	6.86	7.09	15.9	7.74	-1.13	11.76	21.23	11.25	1.37
os43665	6	596	0.23	6	3	5	6.82	0.04	0.35	1.5	0.06	100	0.27	6.47	6.21	14.93	6.98	-1.75	10.39	19.78	10.19	0.63
os43727	3	635	0.88	12	3	5	6.3	-0.19	0	1.45	0.08	96.61	1.19	5.75	5.96	14.78	6.77	-2.51	10.31	19.8	10.09	-0.08
os43771	5	772	0.77	8	3	5	6.63	-0.1	0.29	1.47	0.07	99.71	-0.96	7.02	5.02	13.81	6.02	-3.26	9.12	18.53	9.28	-0.86
os43805	5	497	0.54	10	3	5	6.48	-0.07	0.2	1.49	0.1	99.97	-2.33	7.18	6.66	15.3	7.42	-1.34	11.18	20.4	10.79	1.09

os43812	5	452	0	6	3	5	6.95	0.11	0.56	1.52	0.06	98.48	-0.45	7.33	6.81	15.47	7.55	-1.2	11.36	20.58	10.95	1.21
os43813	5	458	0	7	3	5	6.93	0.11	0.39	1.51	0.06	98.05	-0.5	6.92	6.79	15.45	7.53	-1.22	11.33	20.55	10.93	1.19
os43827	5	421	0.87	9	2	3	6.4	-0.14	0.29	1.49	0.1	98.58	-3.17	6.58	7.06	15.66	7.74	-1.06	11.61	20.81	11.17	1.36
os43851	7	465	0.23	3	2	3	6.77	0	1.97	1.56	0.06	94.27	-2.04	8.75	6.74	15.38	7.59	-1.5	11.14	20.33	10.9	0.98
os43852	7	472	0.23	4	2	7	6.72	-0.03	1.96	1.56	0.09	96.5	-3.11	8.66	6.61	15.26	7.49	-1.59	10.99	20.19	10.77	0.86
os43862	5	346	0.77	1	3	6	6.75	-0.01	2.94	1.56	0.04	29.83	-0.1	8.66	7.29	15.89	8.02	-0.98	11.6	20.8	11.3	1.4
os43872	5	428	1	10	2	3	6.37	-0.13	0.38	1.47	0.11	94.35	-3.14	6.78	7.01	15.6	7.7	-1.11	11.51	20.72	11.07	1.3
os43933	7	740	0.88	22	3	5	5.77	-0.29	0	1.39	0.14	100	-1.4	6.28	5.33	14.1	6.26	-3.3	9.52	18.87	9.59	-0.84
os43935	7	698	0.79	20	2	3	5.89	-0.24	0	1.42	0.16	99.94	-2.72	6.71	5.4	14.16	6.32	-3.26	9.59	18.94	9.65	-0.81
os43979	4	675	0	14	2	7	6.52	0.2	0.41	1.51	0.29	98.35	-8.87	7.16	4.9	13.66	5.97	-3.53	8.93	18.34	9.21	-1.06
os44024	4	571	0.18	14	2	7	7.03	0.24	0	1.47	0.19	96.65	-3.18	5.97	5.52	14.26	6.49	-3.26	9.58	18.99	9.78	-0.79
os44029	7	638	1	11	2	7	6.44	-0.18	0.3	1.49	0.18	97.86	-6.56	7.75	5.31	14.06	6.3	-3.35	9.36	18.78	9.57	-0.89
os44176	5	445	0.88	2	3	6	6.72	-0.04	2.24	1.54	0.06	85.53	-1.87	8.61	6.9	15.6	7.52	-1.64	11.45	20.79	11.01	0.86
os44181	7	425	0.99	12	2	7	6.35	-0.17	0.32	1.51	0.11	92.14	-3.34	7.95	6.98	15.72	7.52	-2.33	11.56	20.88	11.18	0.22
os44276	4	450	0.99	12	3	5	6.4	-0.18	0.01	1.42	0.1	99.98	-0.11	6.3	6.92	15.65	7.52	-2.05	11.45	20.8	11.08	0.43
os44323	4	552	0	8	3	9	6.97	0.12	0.11	1.47	0.03	95.71	1.34	6.11	6.69	15.45	7.28	-2.37	11.22	20.59	10.86	0.18
os44437	7	517	0.77	10	2	3	6.74	-0.05	0.38	1.49	0.12	99.65	-3.66	6.9	6.43	15.17	7.18	-2.47	10.85	20.17	10.67	0.02
os44460	7	477	0.34	10	2	7	6.91	0.09	0.61	1.52	0.13	99.98	-5.86	7.95	6.53	15.24	7.21	-2.29	10.95	20.29	10.7	0.18
os44533	6	515	0.08	3	3	6	6.92	0.05	1.56	1.53	0.05	71.65	-0.01	8.8	6.47	15.22	7.17	-2.31	10.93	20.3	10.68	0.18
os44559	7	423	0.77	7	2	3	6.69	-0.07	0.62	1.49	0.08	99.47	-2.38	7.74	6.97	15.68	7.58	-2.01	11.47	20.8	11.15	0.48
os44619	7	466	0.01	4	2	7	6.84	0.05	1.68	1.54	0.14	97.3	-7.46	8.2	6.62	15.35	7.27	-2.03	11.07	20.43	10.73	0.44
os44636	6	550	0.02	9	2	0	7.04	0.16	0.1	1.49	0.09	100	0.84	6.89	6.3	15.07	7.01	-2.24	10.75	20.13	10.4	0.21
os44683	7	413	0.8	12	2	7	6.34	-0.06	0.26	1.46	0.17	99.29	-7.26	8.09	7.04	15.75	7.6	-1.78	11.56	20.87	11.12	0.74
os44685	7	453	0.54	10	2	3	6.55	-0.03	0.65	1.48	0.13	99.25	-6.24	7.8	6.93	15.67	7.52	-1.86	11.44	20.76	11.03	0.66
os44693	7	397	0.3	12	2	0	6.94	0.14	0.11	1.47	0.15	98.7	-1.88	7.5	7.06	15.78	7.61	-1.79	11.56	20.88	11.14	0.73
os44731	7	325	0.57	18	2	7	5.72	-0.14	0.05	1.48	0.23	97.52	-8.11	6.05	7.45	16.13	7.91	-1.4	12	21.28	11.43	1.13
os44744	7	339	0.98	17	2	7	6.08	-0.25	0	1.43	0.21	95.26	-5.41	7.27	7.39	16.08	7.88	-1.54	11.91	21.22	11.41	0.98
os44953	4	634	0.25	12	2	0	7.05	0.11	0.01	1.48	0.11	99.96	-1.42	6.65	5.61	14.37	6.51	-3	9.81	19.21	9.89	-0.57
os44976	7	486	0.95	5	3	5	6.69	-0.08	0.52	1.53	0.06	92.56	-0.26	6.92	6.63	15.39	7.27	-2.75	11.17	20.48	10.9	-0.19
os45023	7	657	0.05	7	2	3	7.06	0.11	0.76	1.49	0.1	99.62	-3.05	7.79	5.76	14.55	6.59	-3.15	10.07	19.43	10.03	-0.63
os45053	4	468	0.78	16	2	0	6.05	-0.19	0	1.43	0.15	97.44	-0.59	6.52	6.58	15.31	7.23	-2.67	11.09	20.39	10.82	-0.1
os45124	7	514	0.32	19	2	7	6.34	0.07	0	1.43	0.18	97.65	-2.06	6.08	6.26	14.98	6.97	-2.85	10.67	19.99	10.49	-0.31
os45213	7	591	0.78	17	2	3	6.08	-0.19	0	1.44	0.15	99.9	-4.47	6.27	6.02	14.77	6.81	-2.87	10.39	19.74	10.28	-0.4
os45314	7	391	0.32	8	2	3	6.87	0.05	0.68	1.48	0.13	100	-5.06	7.25	7.22	15.94	7.69	-1.79	11.79	21.14	11.31	0.73
os45454	7	474	0.7	20	2	0	5.87	-0.19	0	1.43	0.17	96.24	-0.99	5.55	6.56	15.28	7.2	-2.53	11.05	20.36	10.78	-0.01
os45648	7	631	0.6	9	3	5	6.48	-0.16	0.11	1.47	0.07	99.87	-0.51	6.98	5.9	14.67	6.68	-2.96	10.29	19.66	10.17	-0.48
os45717	7	458	0.21	5	2	3	6.9	0.06	1.84	1.52	0.08	98.95	-3.66	8.7	6.85	15.58	7.42	-2.39	11.38	20.71	11.06	0.17

os45727	4	505	0.23	13	2	0	6.65	0.09	0	1.45	0.1	95.14	-0.12	6.31	6.55	15.3	7.18	-2.5	11.08	20.4	10.78	0.04
os45738	4	379	0.44	25	2	7	6.69	0.12	0	1.43	0.21	94.29	-4.65	6.12	7.06	15.81	7.58	-2.16	11.65	20.97	11.23	0.39
os45756	7	477	0.08	9	2	3	6.96	0.12	0.72	1.5	0.11	99.52	-4.14	7.71	6.81	15.56	7.38	-2.25	11.38	20.74	11	0.3
os45765	4	388	0.02	9	3	5	6.96	0.14	0.21	1.49	0.1	97.11	-1.07	7.74	7.23	15.96	7.73	-1.95	11.87	21.2	11.37	0.6
os45806	7	415	0.39	8	2	3	6.95	0.1	0.64	1.47	0.1	98.01	-2.47	7.97	7.24	15.97	7.7	-1.95	11.85	21.2	11.35	0.59
os45871	7	546	0.6	4	3	5	6.67	-0.06	0.57	1.5	0.05	99.63	-0.62	6.81	6.61	15.37	7.23	-1.99	11.09	20.45	10.69	0.58
os45952	4	407	0.83	16	1	2	5.87	-0.24	0	1.39	0.13	90.02	4.79	6.09	7.01	15.72	7.57	-2.34	11.58	20.88	11.22	0.24
os45979	7	428	0.8	9	2	7	6.44	-0.13	0.72	1.52	0.11	94.54	-3.94	8.17	6.96	15.69	7.5	-2.34	11.53	20.85	11.15	0.21
os45980	7	428	0.98	9	2	7	6.44	-0.14	0.49	1.52	0.1	93.64	-3.16	8.03	6.97	15.7	7.51	-2.34	11.54	20.86	11.17	0.21
os45982	7	425	0.88	12	2	7	6.32	-0.18	0.26	1.5	0.11	91.67	-3.01	7.9	6.99	15.72	7.53	-2.33	11.57	20.88	11.19	0.22
os45993	4	477	0.85	9	2	3	6.46	-0.13	0.57	1.51	0.1	97.96	-2.27	7.23	6.87	15.61	7.43	-2.38	11.45	20.81	11.08	0.18
os45997	4	435	0.33	6	2	7	6.75	0.02	1.47	1.55	0.08	90.26	-3.34	7.69	6.91	15.65	7.47	-2.39	11.49	20.85	11.13	0.18
os45998	7	476	0.01	3	2	3	6.85	0.02	2.84	1.54	0.06	87.95	-2.58	8.88	6.77	15.5	7.35	-2.53	11.29	20.67	10.99	0.05
os46177	7	503	0.81	14	2	3	6.31	-0.17	0.2	1.49	0.1	98.2	-1.51	6.81	6.54	15.26	7.17	-2.67	11.02	20.31	10.81	-0.13
os46255	4	436	0.28	13	1	4	6.22	-0.19	0.01	1.43	0.08	93.4	3.45	5.98	6.99	15.69	7.56	-2.62	11.62	20.88	11.24	-0.01
os46286	4	407	0.65	20	3	5	5.93	-0.15	0	1.41	0.13	94.16	1.57	5.6	7.11	15.81	7.64	-2.49	11.76	21	11.35	0.12
os46330	7	403	0.15	5	2	3	6.76	0.03	1.77	1.53	0.07	99.16	-3.1	8.04	7.14	15.85	7.66	-2.37	11.72	20.97	11.31	0.2
os46441	4	451	0.32	15	3	5	6.51	0.05	0	1.46	0.08	91.19	0.3	6.57	6.86	15.56	7.43	-2.77	11.51	20.75	11.13	-0.19
os46450	7	440	0.4	5	3	6	6.84	0.01	0.89	1.53	0.06	87.33	-0.72	7.79	6.9	15.59	7.46	-2.75	11.56	20.79	11.17	-0.17
os46452	7	460	0.68	7	2	3	6.83	0	0.72	1.53	0.11	77.61	-3.49	7.39	6.75	15.46	7.36	-2.82	11.36	20.61	11.01	-0.23
os46488	4	390	0.99	15	2	7	6.17	-0.24	0	1.48	0.14	70.59	-3.75	6.05	7.09	15.84	7.62	-2.5	11.79	21.08	11.39	0.12
os46598	7	456	0.77	11	2	3	6.35	-0.14	0.62	1.51	0.15	98.24	-5.51	7.11	6.81	15.53	7.39	-2.46	11.31	20.62	11	0.09
os46723	4	526	0.72	10	2	8	6.82	-0.02	0.16	1.48	0.11	99.24	-3.28	6.79	6.67	15.4	7.25	-2.57	11.22	20.54	10.87	0.03
os46739	7	450	0.86	6	2	0	6.74	-0.05	0.89	1.52	0.1	98.75	-1.5	6.98	6.89	15.6	7.43	-2.4	11.44	20.73	11.02	0.21
os46775	4	520	0.15	9	2	7	6.89	0.11	0.22	1.55	0.19	88.6	-7.95	7.09	6.15	14.9	6.93	-3	10.53	19.84	10.4	-0.44
os46788	7	409	0.17	5	2	3	6.87	0.06	1.57	1.53	0.13	98.17	-5.17	8.06	7.06	15.79	7.58	-2.55	11.75	21.04	11.34	0.09
os46809	7	458	1	9	3	5	6.52	-0.14	0.32	1.5	0.05	94.83	-0.36	7.42	7	15.74	7.54	-2.5	11.68	20.98	11.25	0.1
os46831	7	456	0.7	6	2	3	6.68	0.01	1.15	1.51	0.11	97.68	-4.22	7.11	6.95	15.72	7.5	-2.63	11.65	20.98	11.24	-0.01
os46855	7	482	0.82	6	2	3	6.65	-0.04	1.23	1.52	0.07	98.7	-3.09	8.77	6.7	15.43	7.33	-2.81	11.29	20.58	10.97	-0.21
os46879	7	462	1	11	2	3	6.54	-0.13	0.57	1.49	0.1	93.49	-4.45	7.6	6.85	15.56	7.41	-2.62	11.44	20.7	11.09	-0.04
os46900	7	410	0.03	11	2	3	6.94	0.17	0.27	1.48	0.1	99.28	-3.23	6.99	7.1	15.8	7.63	-2.47	11.75	20.99	11.33	0.13
os46909	4	378	0.05	18	1	4	6.76	0.24	0	1.41	0.1	74.94	2.02	6.49	7.19	15.92	7.71	-2.41	11.85	21.1	11.44	0.18
os46952	5	446	0.77	2	3	6	6.79	-0.02	1.37	1.53	0.01	40.78	0.28	8.93	6.95	15.63	7.57	-2.18	11.34	20.63	11.12	0.37
os46963	4	309	0.47	3	2	3	6.8	0.05	1.53	1.53	0.1	79.48	-3.65	8.67	7.74	16.46	8.18	-1.63	12.44	21.81	12.11	1.12
os47123	4	479	0.14	13	3	5	7.02	0.16	0	1.42	0.1	82.58	3.44	5.97	6.59	15.31	7.25	-2.72	10.99	20.3	10.81	-0.18
os47124	4	491	0.03	8	1	4	7	0.14	0.04	1.42	0.07	92.05	1.51	7.28	6.57	15.3	7.24	-2.73	10.97	20.28	10.8	-0.2
os47215	7	395	1	9	2	3	6.52	-0.12	0.62	1.51	0.11	98.67	-4.91	7.14	7.24	15.91	7.73	-2.31	11.84	21.09	11.43	0.28

os47256	4	387	0.6	18	3	5	6.25	-0.05	0.17	1.47	0.15	99.22	-4.36	6.27	7.2	15.89	7.69	-2.35	11.76	21.04	11.38	0.24
os47338	7	280	0.88	7	2	7	6.54	-0.09	0.73	1.55	0.16	72.68	-6.93	7.83	7.72	16.41	8.2	-1.69	12.51	21.86	12.13	1.07
os47361	7	332	0.27	3	2	7	6.73	0.02	2.19	1.56	0.19	89.58	-5.87	7.33	7.22	15.95	7.81	-2.06	11.75	21.14	11.54	0.59
os47486	7	302	0.23	3	2	7	6.67	0	1.9	1.57	0.09	64.58	-3.58	7.63	7.84	16.54	8.26	-1.49	12.56	21.87	12.06	1.2
os47487	7	364	0.92	6	3	5	6.55	-0.09	0.76	1.51	0.06	99.95	-0.72	7.13	7.75	16.45	8.19	-1.54	12.46	21.78	11.99	1.15
os47521	4	407	0.04	8	2	7	6.91	0.12	1.48	1.53	0.15	94.58	-6.39	7.31	7.09	15.83	7.6	-2.13	11.65	21	11.21	0.48
os47578	5	311	0.77	2	3	6	6.74	-0.01	1.92	1.55	0.03	65.36	-0.5	8	7.89	16.64	8.3	-1.31	12.48	21.88	12	1.33
os47654	3	407	0.95	11	2	8	6.35	-0.18	0.11	1.5	0.09	87.57	-2.75	6.67	7.5	16.22	7.97	-1.78	12.17	21.54	11.85	0.98
os47682	4	514	1	10	1	1	6.47	-0.16	0.01	1.37	0.04	98.08	5.27	5.62	6.95	15.67	7.49	-2.3	11.52	20.82	11.1	0.28
os47744	7	426	0.73	5	2	7	6.65	-0.03	1.54	1.53	0.08	94.21	-2.47	7.77	7.1	15.81	7.59	-2.15	11.67	20.97	11.21	0.45
os47793	7	354	0.59	7	2	3	6.77	0.01	1.04	1.55	0.12	93.35	-4.95	8.15	7.52	16.25	7.99	-1.45	12.11	21.46	11.58	1.13
os47800	7	503	0.03	11	2	3	6.41	-0.11	0.37	1.48	0.13	99.12	-3.08	7.28	6.75	15.52	7.32	-2.07	11.23	20.59	10.78	0.48
os47813	7	388	0.01	6	2	7	6.55	-0.1	1.03	1.51	0.14	96.69	-3.21	7.83	7.19	15.95	7.64	-1.88	11.82	21.19	11.29	0.71
os47843	7	454	0.31	5	3	6	6.76	0.02	0.97	1.51	0.06	99.31	-1.77	7.7	7.02	15.76	7.53	-2.12	11.61	20.97	11.17	0.43
os47898	4	558	0.87	7	3	5	6.75	-0.07	0.32	1.49	0.07	99.93	-0.92	7.21	6.64	15.41	7.21	-2.25	11.16	20.54	10.74	0.29
os47899	4	563	0.9	6	2	3	6.82	-0.03	0.78	1.49	0.09	99.98	-2.8	7.41	6.63	15.4	7.19	-2.26	11.13	20.52	10.72	0.28
os47946	7	446	0.46	12	2	7	6.33	-0.18	0.14	1.52	0.12	90.29	-4.19	8.3	6.94	15.69	7.44	-2.1	11.5	20.86	11.02	0.45
os47951	5	489	0.04	3	3	6	6.9	0.05	0.76	1.52	0.02	73.52	0.07	8.14	6.85	15.6	7.4	-2.3	11.43	20.77	11.03	0.27
os47953	7	415	0.87	11	2	3	6.35	-0.13	0.43	1.5	0.14	97.75	-7.02	8.23	7.14	15.87	7.65	-2.06	11.77	21.1	11.3	0.5
os47954	7	411	0.8	12	3	5	6.27	-0.16	0.01	1.46	0.1	98.28	-1.1	6.06	7.23	15.96	7.72	-1.98	11.87	21.19	11.38	0.57
os47996	4	317	0.1	11	1	4	6.74	0.13	0.02	1.44	0.05	85.7	3.92	6.54	7.85	16.57	8.26	-1.49	12.66	22	12.31	1.27
os47997	4	315	0.13	14	3	5	6.65	0.13	0.02	1.43	0.07	85.13	2.33	6.61	7.85	16.56	8.26	-1.49	12.66	22	12.31	1.27
os48018	7	367	0.92	3	3	6	6.73	0.01	1.47	1.53	0.07	91.6	-1.52	8.54	7.46	16.17	7.91	-1.53	12.04	21.37	11.5	1.01
os48025	7	365	0.21	5	2	3	6.81	0.06	1.98	1.54	0.08	93.58	-3.27	8.25	7.49	16.21	7.96	-1.48	12.07	21.43	11.54	1.1
os48027	7	324	0.92	5	2	7	6.48	-0.1	1.11	1.55	0.16	83.73	-6.05	7.16	7.61	16.33	8.07	-1.35	12.19	21.55	11.66	1.24
os48042	7	434	0.07	15	3	5	6.77	0.17	0	1.43	0.11	94.3	2.37	6.01	7.06	15.79	7.57	-2.21	11.71	21.04	11.24	0.39
os48062	7	386	0.77	4	2	3	6.78	-0.01	1.87	1.52	0.09	93.98	-2.26	8.02	7.43	16.13	7.9	-1.89	11.98	21.3	11.57	0.77
os48067	7	440	0.82	9	2	3	6.45	-0.14	0.45	1.48	0.13	97.32	-3.96	7.6	7	15.72	7.58	-2.21	11.5	20.85	11.16	0.4
os48116	4	283	0.98	12	1	4	6.33	-0.17	0.19	1.45	0.05	62.77	2.12	7.04	7.85	16.55	8.28	-1.56	12.55	21.84	12.18	1.17
os48146	7	268	0.27	13	2	7	6.09	-0.21	0.52	1.52	0.19	90.84	-8.65	6.98	7.81	16.5	8.26	-1.56	12.52	21.82	12.17	1.17
os48184	4	301	0.92	1	3	6	6.74	0.01	2	1.55	0.05	75.33	-1.51	7.52	7.93	16.61	8.33	-1.44	12.72	21.95	12.25	1.34
os48218	7	322	0.97	8	2	7	6.6	-0.06	1.64	1.54	0.13	88.32	-6.54	7.21	7.47	16.13	8	-1.85	11.86	21.17	11.59	0.7
os48276	7	323	0.99	5	3	6	6.62	-0.07	1.67	1.55	0.07	84.15	-2.27	8.76	7.74	16.46	8.13	-1.58	12.34	21.68	11.83	1.07
os48294	7	395	0.71	3	2	7	6.82	0.05	2.18	1.56	0.14	88.05	-5.57	7.21	7.18	15.89	7.65	-2.02	11.78	21.12	11.3	0.54
os48302	7	418	0.23	8	2	7	6.71	0.06	0.85	1.53	0.14	97.24	-5.97	7.89	7.04	15.77	7.54	-2.11	11.61	20.93	11.14	0.45
os48377	7	399	0.47	2	2	7	6.75	-0.01	1.22	1.55	0.14	95.1	-6.42	7.59	7.12	15.85	7.62	-2.12	11.69	21.02	11.24	0.49
os48393	7	442	1	6	2	3	6.57	-0.1	0.94	1.52	0.11	95.01	-3.06	6.74	6.94	15.64	7.47	-2.33	11.51	20.8	11.08	0.26

os48444	5	503	0	4	3	6	6.93	0.06	1.77	1.53	0.03	82.85	-0.63	8.3	6.58	15.28	7.21	-2.75	11.25	20.48	10.96	-0.15
os48445	5	501	0.08	4	3	6	6.93	0.06	2.06	1.54	0.04	79.7	-1.26	8.4	6.58	15.28	7.21	-2.76	11.25	20.48	10.96	-0.15
os48468	5	465	0.99	4	3	9	6.69	-0.07	0.4	1.49	0.02	86	0.96	6.73	6.94	15.65	7.5	-2.39	11.6	20.9	11.26	0.27
os48509	4	464	0.98	12	2	3	6.32	-0.18	0.1	1.47	0.16	98.99	-6.96	6.39	6.85	15.6	7.42	-2.6	11.51	20.83	11.14	0.02
os48607	7	553	0.83	7	3	5	6.56	-0.1	0.4	1.48	0.08	99.52	-0.84	6.41	6.23	14.91	6.96	-2.89	10.72	19.95	10.52	-0.28
os48700	4	330	0.94	12	2	7	6.43	-0.15	0.06	1.5	0.1	57.94	-0.63	7.47	7.35	16.06	7.87	-2.03	12.02	21.37	11.65	0.65
os48764	5	422	0.06	3	3	6	6.87	0.05	2.39	1.55	0.04	71.47	-1.42	7.76	7.03	15.77	7.59	-2.2	11.74	21.14	11.37	0.5
os48765	5	422	0.01	4	3	6	6.86	0.06	1.65	1.55	0.05	71.8	-1.31	7.68	7.03	15.77	7.59	-2.2	11.74	21.14	11.37	0.49
os48808	4	343	0.8	13	1	4	6.52	-0.11	0.04	1.44	0.06	83.11	2.46	6.9	7.47	16.17	8	-1.92	12.13	21.53	11.85	0.8
os48814	4	328	0.13	19	2	7	6.55	0.19	0	1.45	0.16	96.99	-2.8	6.66	7.38	16.09	7.88	-1.93	12.02	21.39	11.67	0.77
os48818	7	342	0.54	11	2	3	6.56	0	0.86	1.52	0.1	97.28	-3.81	8.39	7.44	16.18	7.94	-1.84	12.11	21.53	11.72	0.85
os48894	4	387	0.99	11	2	3	6.51	-0.1	0.8	1.53	0	95.57	-8.57	7.31	7.13	15.85	7.63	-1.94	11.9	21.27	11.47	0.79
os48920	6	473	0.47	2	3	9	6.76	-0.03	1.98	1.49	0.04	89.2	-1.85	7.91	6.91	15.64	7.5	-2.3	11.49	20.86	11.21	0.34
os48931	7	400	0.48	8	2	3	6.58	-0.01	1.47	1.54	0.15	89.16	-6.62	6.95	7.08	15.82	7.63	-2.16	11.76	21.16	11.4	0.55
os49005	7	495	0.12	8	2	3	6.73	0.05	0.81	1.53	0.14	93.27	-5.61	7.37	6.31	14.99	7.04	-3.04	10.93	20.16	10.67	-0.44
os49009	4	541	0.08	6	2	0	6.97	0.1	0.49	1.54	0	91.2	-1.82	7.82	6.06	14.74	6.82	-3.14	10.67	19.89	10.47	-0.56
os49050	4	450	0.9	13	2	7	6.22	-0.21	0.01	1.49	0.14	83.37	-3.53	7.07	6.45	15.12	7.16	-2.91	10.99	20.26	10.78	-0.32
os49052	7	486	0.83	13	2	3	6.51	-0.14	0.03	1.47	0.15	98.09	-4.35	6.55	6.42	15.09	7.13	-2.93	10.97	20.23	10.74	-0.33
os49065	4	531	0.77	9	2	0	6.51	-0.1	0.35	1.5	0.12	97.21	-1.65	7.56	6.11	14.8	6.87	-3.11	10.73	19.95	10.52	-0.53
os49110	4	503	0.88	6	3	5	6.59	-0.1	0.65	1.5	0.07	94.05	0.69	7.5	6.24	14.93	6.96	-2.63	10.87	20.19	10.66	0.05
os49122	4	366	0.79	10	2	7	6.38	-0.11	0.42	1.53	0.11	60.27	-4.72	7.09	7.01	15.73	7.58	-1.8	11.91	21.27	11.5	0.98
os49173	4	567	0.99	19	2	7	6	-0.3	0	1.45	0.17	94.55	-2.59	7.33	5.82	14.51	6.62	-3.11	10.28	19.57	10.16	-0.52
os49177	5	543	0.23	10	2	0	6.43	-0.15	0.18	1.5	0.11	97.96	-0.13	7.18	5.96	14.62	6.74	-3.06	10.41	19.64	10.23	-0.48
os49182	7	557	0.47	12	2	7	6.47	-0.12	1.19	1.51	0.17	97.71	-8.74	6.93	5.88	14.57	6.67	-3	10.26	19.56	10.13	-0.42
os49265	7	550	0.31	7	2	7	6.76	0.04	0.78	1.52	0.12	98.08	-3.98	7.36	6.01	14.68	6.77	-2.97	10.4	19.67	10.24	-0.38
os49355	7	512	0.39	4	2	7	6.88	0.02	1.46	1.52	0	95.35	-1.85	8.33	6.16	14.86	6.86	-2.5	10.71	20.05	10.48	0.09
os49362	7	472	0.44	11	2	3	6.46	-0.05	0.71	1.52	0.19	99.37	-8.69	7.69	6.46	15.14	7.12	-2.53	10.98	20.29	10.73	0.1
os49379	7	460	0.12	9	2	7	6.72	0.07	0.19	1.51	0.16	73.52	-2.69	7.42	6.38	15.05	7.08	-2.55	10.93	20.25	10.7	0.07
os49438	4	546	0.93	11	2	7	6.56	-0.12	0.63	1.5	0.16	97.58	-5.45	7.73	5.91	14.59	6.69	-2.9	10.3	19.59	10.17	-0.31
os49480	4	415	0.36	7	2	7	6.91	0.07	0.57	1.52	0.13	81.06	-1.81	8.01	6.59	15.29	7.26	-2.19	11.25	20.55	10.96	0.56
os49494	4	375	0.32	7	2	3	6.55	-0.05	1.28	1.56	0	85.99	-6.03	7.8	7	15.68	7.58	-1.8	11.89	21.2	11.44	1.01
os49501	7	418	0.02	2	3	6	6.82	0.03	2.92	1.55	0.03	48.94	-0.36	9.17	6.81	15.51	7.43	-2.08	11.52	20.87	11.18	0.59
os49507	7	392	0.69	6	2	7	6.63	-0.03	1.52	1.57	0	92.31	-4.91	8.45	6.85	15.52	7.47	-1.81	11.62	20.96	11.27	0.95
os49521	5	551	0.23	9	2	0	6.75	0.06	0.11	1.49	0.07	98.71	1.07	6.35	6.04	14.7	6.81	-3.01	10.49	19.71	10.29	-0.44
os49527	7	502	0.98	2	2	7	6.78	-0.02	1.94	1.54	0.13	85.46	-2.94	7.43	6.08	14.76	6.83	-2.87	10.46	19.75	10.3	-0.27
os49578	4	537	0.23	5	2	7	6.8	0.03	1.29	1.52	0.11	97.75	-2.35	7.3	6.15	14.88	6.86	-2.56	10.69	20.05	10.48	0.05
os49584	4	509	1	5	2	0	6.68	-0.08	1.37	1.54	0	97.76	-1.01	7.81	6.2	14.91	6.91	-2.45	10.74	20.07	10.49	0.14

os49597	4	481	1	13	3	5	6.31	-0.21	0.08	1.42	0.07	95.32	2.05	7.07	6.56	15.23	7.18	-2.4	11.23	20.53	10.9	0.28
os49617	7	475	0.15	10	2	3	6.43	-0.14	0.52	1.51	0.17	99.42	-6.76	7.68	6.46	15.14	7.12	-2.53	10.98	20.29	10.73	0.1
os49672	7	390	0.05	6	2	7	6.89	0.1	1.55	1.55	0.09	88.88	-3.39	7.72	6.94	15.62	7.48	-2.06	11.69	21	11.29	0.63
os49688	4	492	0.82	22	3	5	6.28	-0.18	0	1.37	0.15	96.12	-0.34	5.35	6.43	15.13	7.08	-2.38	11.1	20.41	10.78	0.28
os49708	7	368	0.52	7	3	5	6.63	-0.01	0.09	1.5	0	71.52	1.44	7.27	7.23	15.94	7.73	-1.85	12.06	21.36	11.58	0.89
os49764	7	664	0.23	7	2	0	6.84	0.04	0.18	1.52	0.09	96.41	0.76	7.78	5.45	14.16	6.27	-2.85	9.84	19.24	9.7	-0.23
os49765	5	699	0.81	7	2	0	6.77	-0.07	0.35	1.51	0.08	97.16	-0.31	7.52	5.31	14.03	6.14	-2.95	9.73	19.1	9.6	-0.34
os49802	4	714	0.21	11	3	5	6.84	0.1	0.01	1.48	0.09	100	-1.04	6.47	5.3	14.03	6.14	-2.89	9.66	19.06	9.51	-0.31
os49889	4	570	0.64	12	3	5	6.89	0.03	0.18	1.49	0	98.85	-2.35	7.24	6.13	14.86	6.67	-2.36	10.89	20.29	10.35	0.28
os49981	4	294	0.92	0	3	6	6.74	0.01	1.19	1.53	0.01	36.33	0.89	10.58	7.97	16.64	8.31	-1.32	12.96	22.2	12.31	1.53
os50066	4	300	0	3	3	6	6.8	0.05	0.97	1.55	0.02	47.26	-0.03	8.81	7.79	16.49	8.21	-1.5	12.53	21.87	12.07	1.28
os50102	7	411	0.02	7	2	7	6.5	-0.12	0.9	1.55	0.13	93.32	-6.29	9.29	7.09	15.83	7.64	-1.8	11.88	21.28	11.42	0.99
os50116	4	289	0.87	7	3	5	6.49	-0.08	0.78	1.52	0.05	35.42	0.46	8.19	7.91	16.58	8.27	-1.32	12.86	22.16	12.26	1.52
os50181	4	324	0.6	4	2	3	6.72	-0.02	1.64	1.54	0.08	76.63	-3.95	7.65	7.72	16.45	8.1	-1.3	12.58	22.02	12	1.47
os50183	7	324	0.87	4	2	3	6.6	-0.05	1.46	1.55	0.09	75.04	-4.12	7.67	7.72	16.45	8.11	-1.3	12.59	22.02	12	1.47
os50192	4	415	0.12	6	3	5	6.59	-0.09	0.89	1.52	0.05	84.18	-1.37	7.72	7.29	15.98	7.76	-1.66	12.13	21.52	11.6	1.16
os50206	7	477	0.16	6	2	7	6.95	0.09	0.91	1.51	0.11	93.56	-2.99	7.28	6.72	15.43	7.3	-2.04	11.48	20.86	11.05	0.74
os50279	4	392	1	12	3	5	6.33	-0.2	0.01	1.48	0.08	84.83	-0.71	7.99	7.32	16.02	7.79	-1.66	12.16	21.54	11.64	1.18
os50280	4	390	0.99	13	2	3	6.33	-0.19	0.06	1.5	0.09	84.97	-1.68	8.1	7.32	16.02	7.79	-1.66	12.16	21.54	11.64	1.18
os50296	4	452	0.55	4	2	3	6.73	-0.01	1.67	1.53	0.05	96.78	-2.03	8.31	7	15.72	7.53	-1.87	11.81	21.2	11.32	0.94
os50306	7	457	0.38	6	2	3	6.73	0.03	1.11	1.55	0.15	95.99	-7.28	7.22	6.91	15.64	7.48	-1.94	11.7	21.1	11.26	0.87
os50335	4	628	0.23	9	3	5	6.77	0.05	0.04	1.49	0.06	99.43	1.67	5.92	5.99	14.7	6.69	-2.54	10.62	19.97	10.33	0.19
os50411	7	505	0.08	5	2	3	6.81	0.02	1.79	1.54	0.08	91.2	-4.05	9.04	6.52	15.24	7.02	-2.08	11.22	20.76	10.71	0.58
os50475	4	380	0.57	4	3	6	6.71	-0.01	1.98	1.55	0.05	82.81	-1.42	9.23	7.48	16.26	7.84	-1.53	12.47	21.97	11.73	1.3
os50499	5	314	0.47	2	3	6	6.7	0	1.57	1.56	0.02	39.95	0.01	8.48	7.88	16.58	8.18	-1.27	12.94	22.36	12.2	1.6
os50541	5	480	0.23	4	3	6	6.79	0.03	0.88	1.5	0.03	99.86	-0.47	7.76	7.08	15.86	7.52	-1.57	11.75	21.38	11.2	1.11
os50549	4	356	0.83	16	2	3	6.48	-0.12	0.08	1.47	0.13	88.02	-3.47	6.36	7.58	16.28	7.95	-1.47	12.58	21.97	11.9	1.38
os50570	7	306	0.01	2	3	6	6.77	0.03	2.9	1.56	0.07	77.42	-1.78	8.21	7.87	16.62	8.15	-1.22	12.9	22.4	12.13	1.59
os50571	7	305	0.08	1	2	3	6.78	0.03	2.36	1.57	0.07	75.7	-2.23	8.23	7.87	16.63	8.16	-1.22	12.91	22.41	12.13	1.6
os50572	7	305	0.08	2	3	6	6.78	0.03	2.33	1.56	0.07	75.32	-2.19	8.21	7.88	16.63	8.16	-1.22	12.91	22.41	12.13	1.6
os50573	7	304	0.06	2	2	3	6.78	0.03	2.18	1.56	0.07	75.05	-2.2	8.26	7.88	16.63	8.16	-1.22	12.91	22.41	12.14	1.6
os50574	7	304	0.08	2	2	3	6.78	0.03	2.12	1.56	0.08	74.78	-2.34	8.24	7.88	16.64	8.16	-1.22	12.91	22.42	12.14	1.6
os50575	7	304	0.09	2	2	3	6.79	0.03	2.12	1.56	0.08	74.55	-2.49	8.17	7.88	16.64	8.17	-1.21	12.91	22.42	12.14	1.61
os50576	7	304	0.38	2	2	3	6.78	0.03	1.95	1.57	0.08	74.27	-2.55	8.19	7.88	16.64	8.17	-1.21	12.92	22.42	12.14	1.61
os50577	7	304	0.23	2	2	3	6.78	0.02	2.02	1.56	0.08	74	-2.4	8.1	7.89	16.64	8.17	-1.21	12.92	22.42	12.14	1.61
os50578	7	303	0.13	2	3	6	6.78	0.02	2.14	1.56	0.08	73.49	-2.16	8.12	7.89	16.64	8.17	-1.21	12.92	22.42	12.14	1.61
os50579	7	303	0.4	2	3	6	6.78	0.02	2.05	1.56	0.08	72.98	-1.98	8.06	7.89	16.65	8.17	-1.21	12.92	22.43	12.15	1.61

os50591	7	409	0.68	7	2	7	6.58	-0.04	0.69	1.54	0.12	83.9	-3.6	7.65	7.15	15.87	7.56	-1.74	12.04	21.51	11.34	1.02
os50604	7	408	0.4	3	2	7	6.78	0	1.62	1.55	0.16	85.71	-5.04	7.08	7.12	15.85	7.55	-1.76	12.01	21.48	11.31	1.01
os50605	7	408	0.58	10	2	7	6.72	-0.01	0.43	1.52	0.16	85.21	-5.27	6.81	7.14	15.87	7.56	-1.75	12.04	21.5	11.34	1.02
os50635	5	525	0.02	5	3	5	6.92	0.08	0.7	1.52	0.03	99.32	-0.18	7.66	6.6	15.35	7.1	-2.06	11.4	20.87	10.83	0.67
os50637	4	450	0.01	5	3	6	6.88	0.08	0.55	1.54	0.05	98.73	0.31	8.18	6.92	15.65	7.36	-1.84	11.75	21.22	11.12	0.88
os50638	4	493	0.53	13	2	3	6.36	-0.05	0.05	1.49	0.1	96.18	-3.51	8.76	6.67	15.42	7.16	-2.02	11.48	20.96	10.9	0.72
os50654	7	326	0.7	4	3	6	6.63	-0.03	1.71	1.55	0.06	85.93	-2.04	7.28	7.8	16.55	8.09	-1.26	12.82	22.35	12.01	1.54
os50655	4	387	0.02	16	2	3	6.88	0.26	0	1.47	0.14	94.38	-2.87	7.56	7.42	16.19	7.77	-1.46	12.31	21.89	11.57	1.28
os50666	4	465	0.02	10	2	3	6.88	0.15	0.17	1.48	0.1	99.88	-2.96	7.86	6.83	15.55	7.24	-1.94	11.63	21.16	11.05	0.76
os50679	4	497	0.53	8	2	7	6.63	0	0.57	1.51	0.13	93.68	-2.58	7.2	6.51	15.23	6.99	-2.15	11.3	20.78	10.77	0.55
os50684	7	520	0.94	5	3	6	6.71	-0.07	2.17	1.53	0.05	87.91	-1.36	8.92	6.44	15.17	6.95	-2.15	11.2	20.69	10.67	0.52
os50685	7	507	0.63	6	2	7	6.73	-0.04	2.2	1.55	0.1	93.77	-4.47	8.51	6.48	15.19	6.98	-2.12	11.22	20.73	10.69	0.52
os50689	4	498	0.17	5	2	0	6.83	0.04	1.82	1.55	0.08	91.25	-1.56	8.84	6.51	15.23	7.01	-2.1	11.25	20.77	10.72	0.54
os50695	7	428	0.71	4	2	3	6.74	0	1.63	1.55	0.07	86.94	-2.66	8.89	7.09	15.82	7.43	-1.76	11.97	21.52	11.31	0.97
os50711	5	460	0.13	3	3	6	6.86	0.02	1.9	1.54	0.04	61.06	-0.07	8.27	6.96	15.71	7.33	-1.85	11.88	21.44	11.24	0.92
os50716	4	457	0.2	6	2	3	6.79	0.06	1.09	1.52	0.11	91.75	-5.28	7.72	6.91	15.66	7.31	-1.87	11.8	21.33	11.15	0.87
os50718	7	428	0.48	7	2	3	6.63	-0.01	0.85	1.54	0.09	85.83	-4.2	8.7	7.04	15.79	7.42	-1.79	11.97	21.53	11.3	0.96
os50719	5	455	0.6	2	3	6	6.77	-0.01	1.54	1.52	0.02	51.88	0.14	8.87	7.03	15.77	7.4	-1.8	11.94	21.49	11.28	0.95
os50720	7	434	0.91	7	2	3	6.6	0	0.82	1.54	0.17	85.71	-8.42	8.58	7.03	15.77	7.38	-1.81	11.94	21.49	11.27	0.96
os50752	4	398	0.27	5	1	4	6.62	-0.05	0.02	1.44	0.04	49.59	5.24	7.33	7.29	16.06	7.6	-1.65	12.27	21.83	11.52	1.1
os50753	4	398	0.98	5	1	4	6.62	-0.08	2.56	1.46	0.04	50.15	4.12	7.92	7.29	16.06	7.6	-1.65	12.27	21.83	11.52	1.11
os50773	7	327	1	6	3	5	6.54	-0.09	0.65	1.53	0.06	96.25	-1.17	7.32	7.79	16.55	8.08	-1.17	12.68	22.25	11.9	1.56
os50781	4	363	0.92	3	2	3	6.83	0.04	2.31	1.54	0.07	96.33	-2.9	9.1	7.6	16.38	7.98	-1.23	12.43	22.01	11.76	1.54
os50782	4	362	0.59	3	3	6	6.81	0.02	2.77	1.54	0.06	96	-2.05	9.23	7.62	16.39	7.99	-1.22	12.44	22.02	11.77	1.54
os50783	4	307	0.13	1	3	6	6.74	0.01	4.43	1.56	0.02	15.15	0.12	9.75	7.96	16.68	8.25	-0.96	12.79	22.33	12.08	1.81
os50784	4	307	0.92	0	3	6	6.74	0	4.16	1.56	0.02	17.4	0.17	11.79	7.96	16.68	8.25	-0.97	12.79	22.33	12.07	1.81
os50785	4	306	0.09	1	3	6	6.76	0.02	4.1	1.56	0.02	14.78	-0.12	9.67	7.96	16.68	8.25	-0.96	12.79	22.33	12.08	1.81
os50786	4	307	0.92	1	3	6	6.74	0	3.97	1.57	0.03	20.31	-0.26	9.37	7.96	16.67	8.25	-0.97	12.79	22.32	12.07	1.81
os50787	4	306	0.06	1	3	6	6.75	0.01	3.99	1.56	0.03	14.96	-0.34	9.62	7.96	16.68	8.25	-0.97	12.79	22.33	12.07	1.81
os50789	7	305	0.19	13	3	5	6.63	0.06	0.49	1.48	0.09	88.19	-2.21	6.33	7.97	16.69	8.25	-1.11	12.89	22.35	12.19	1.72
os50802	7	377	0.92	6	2	7	6.52	-0.09	1.34	1.54	0.1	89.85	-3.62	8.35	7.44	16.23	7.8	-1.33	12.22	21.82	11.55	1.36
os50835	4	431	0.2	5	3	6	6.85	0.07	0.81	1.54	0.08	96.19	-1.78	7.43	7.11	15.94	7.46	-1.74	11.97	21.6	11.28	1
os50870	7	649	0.81	10	2	3	6.48	-0.12	0.29	1.5	0.13	99.99	-4.27	7.05	5.68	14.53	6.33	-2.67	10.22	19.73	9.86	-0.06
os50872	7	661	0.77	12	3	5	6.37	-0.14	0.01	1.46	0.11	100	-1.2	6.58	5.66	14.51	6.32	-2.68	10.2	19.71	9.84	-0.08
os50873	7	667	0.79	11	3	5	6.4	-0.14	0.02	1.47	0.11	100	-1.25	6.53	5.66	14.51	6.31	-2.68	10.19	19.71	9.84	-0.08
os50874	7	678	0.75	12	3	5	6.37	-0.15	0.01	1.46	0.1	100	-1.41	6.41	5.65	14.49	6.3	-2.69	10.18	19.69	9.82	-0.09
os50884	7	555	0.41	6	2	7	6.75	0.01	1.03	1.53	0.11	95.95	-3.92	7.91	6.23	15.06	6.75	-2.32	10.9	20.47	10.39	0.35

os50915	5	399	0.51	5	2	3	6.67	-0.01	1.11	1.55	0.1	86.65	-3.71	6.67	7.32	16.1	7.6	-1.57	12.17	21.78	11.42	1.16
os50926	7	484	0.7	13	2	3	6.24	-0.18	0.05	1.49	0.11	99.87	-2.23	6.35	6.93	15.71	7.31	-1.82	11.76	21.38	11.11	0.88
os50933	7	453	0.3	7	2	3	6.88	0.04	1.06	1.52	0.11	96.49	-5.45	7.59	6.96	15.74	7.33	-1.85	11.83	21.44	11.16	0.87
os50934	7	453	0.9	4	2	3	6.66	-0.05	1.73	1.53	0.1	96.58	-3.91	7.41	6.97	15.74	7.33	-1.85	11.84	21.44	11.17	0.87
os50935	7	465	1	6	2	3	6.62	-0.09	1.2	1.54	0.09	94.71	-4.11	7.17	6.95	15.73	7.32	-1.85	11.81	21.42	11.15	0.86
os50936	7	457	1	7	2	3	6.59	-0.11	0.73	1.52	0.1	96.29	-4.04	7.41	6.96	15.73	7.32	-1.85	11.83	21.43	11.16	0.86
os50941	4	402	0.98	1	3	6	6.74	-0.02	2.87	1.56	0.06	28.76	-0.52	7.66	7.23	16	7.54	-1.69	12.19	21.77	11.45	1.06
os50951	7	429	0.65	3	2	7	6.76	0	1.91	1.55	0.15	95.2	-6.77	7.26	7	15.81	7.35	-1.82	11.92	21.52	11.18	0.9
os50959	4	467	0.1	4	2	3	6.74	-0.01	1.49	1.53	0.08	94.86	-3.85	7.5	6.94	15.75	7.3	-1.81	11.76	21.38	11.11	0.89
os50971	4	417	0.83	15	2	7	6.15	-0.19	0.03	1.49	0.14	95.75	-4.39	6.35	7.14	15.93	7.47	-1.72	12.05	21.67	11.32	1.02
os50983	4	395	0.01	2	3	6	6.77	0.01	2.95	1.54	0.05	70.77	-1.46	8.81	7.38	16.14	7.64	-1.56	12.46	21.99	11.58	1.23
os51045	7	434	0.14	10	2	7	6.35	-0.16	0.75	1.52	0.17	94.09	-8.53	6.9	6.91	15.68	7.28	-1.83	11.69	21.25	11.03	0.88
os51049	4	383	0.07	4	3	6	6.9	0.08	0.55	1.54	0.08	35.36	-1.03	8.22	7.32	16.1	7.61	-1.55	12.21	21.78	11.45	1.21
os51084	7	612	0	7	2	0	7	0.12	0.66	1.5	0.1	98.26	-2	8.27	5.84	14.66	6.47	-2.55	10.4	19.9	9.98	0.07
os51085	7	657	0.24	3	2	3	6.91	0.04	1.85	1.51	0.1	99.34	-3.69	8.11	5.67	14.51	6.34	-2.65	10.17	19.66	9.81	-0.06
os51104	4	455	0.95	14	2	3	6.5	-0.14	0.19	1.46	0.12	98.54	-3.58	6.32	7.08	15.84	7.44	-1.64	11.77	21.38	11.14	1.05
os51183	7	583	0.4	8	3	5	6.97	0.05	0.48	1.51	0.08	100	-0.23	6.71	5.8	14.54	6.42	-2.6	10.42	19.77	10.07	0.08
os51248	5	712	0.89	11	2	7	6.67	-0.12	0.02	1.52	0	100	-2.12	6.57	4.59	13.38	5.56	-3.1	8.51	18.02	8.77	-0.62
os51343	3	647	0.76	20	1	1	6.63	-0.05	0	1.35	0.16	98.66	4.28	4.82	5.47	14.22	6.3	-2.26	9.48	18.89	9.46	0.18
os51584	3	527	0.92	15	3	5	6.09	-0.24	0	1.46	0.12	98.84	-1.18	5.56	6.12	14.94	6.83	-1.99	10.45	19.8	10.26	0.69
os51657	7	572	0.46	2	3	6	6.88	0.01	3.96	1.56	0.05	57.81	-0.67	8.88	5.87	14.68	6.53	-2.47	10.49	19.82	10.23	0.22
os51665	7	422	0.77	3	2	0	6.84	0.03	1.89	1.55	0.11	80.77	-1.91	7.35	6.57	15.36	7.18	-1.77	11.06	20.45	10.76	0.93
os51725	4	563	0.98	14	1	4	6.38	-0.15	0	1.33	0.09	98.78	8.37	6.39	6.08	14.84	6.64	-2.28	10.53	19.88	10.15	0.36
os51771	7	555	0.65	6	2	3	6.59	-0.08	1.38	1.52	0.14	98.54	-7.38	7.55	6.04	14.78	6.63	-2.37	10.49	19.79	10.16	0.24
os51791	7	441	0.55	9	2	7	6.43	-0.02	0.16	1.53	0.19	85.3	-2.76	6.49	6.37	15.1	6.85	-2.15	10.94	20.28	10.52	0.48
os51810	7	555	0.02	14	3	5	6.94	0.2	0	1.43	0.08	99.62	-0.39	5.84	6.19	14.92	6.75	-2.29	10.67	20	10.33	0.33
os51834	4	473	0.22	16	1	2	6.65	0.13	0	1.39	0.13	66.39	5.33	4.84	6.35	15.05	6.86	-2.2	10.89	20.24	10.5	0.43
os51842	4	592	0.18	9	1	4	6.93	0.03	0.03	1.41	0.06	99.31	6.16	5.69	5.84	14.58	6.42	-2.57	10.44	19.76	10.12	0.09
os51847	4	491	0.68	19	2	7	6.56	-0.06	0	1.46	0.25	94.29	-6.7	6.28	6.1	14.86	6.65	-2.37	10.68	20.01	10.31	0.26
os51900	4	419	0.92	5	2	7	6.84	0.12	0.83	1.56	0.22	63.98	-7.33	7.39	6.34	15.08	6.86	-2.16	10.92	20.29	10.49	0.49
os51918	7	566	0.3	9	2	3	6.7	0.02	0.64	1.49	0.11	97.04	-3.67	7.96	6	14.74	6.57	-2.43	10.67	19.98	10.29	0.25
os51977	4	678	0.23	8	3	5	7.06	0.09	0.18	1.49	0.08	100	-0.48	6.26	5.28	14.01	6.01	-2.85	9.72	19	9.52	-0.22
os52084	4	561	1	19	1	4	6.09	-0.27	0	1.38	0.12	98.56	3.36	5.34	6.07	14.83	6.63	-2.29	10.52	19.88	10.15	0.35
os52109	7	622	0.93	11	2	7	6.39	-0.14	0.76	1.54	0.22	98.6	-9.4	8.17	5.19	13.97	6	-2.79	9.38	18.79	9.36	-0.21
os52112	4	640	0.11	8	1	1	7.08	0.13	0.15	1.43	0.04	99.64	2.35	5.86	5.83	14.52	6.43	-2.53	10.42	19.66	10.07	0.17
os52128	7	757	0.76	6	3	5	6.92	-0.02	0.46	1.5	0.05	94.62	0.1	6.81	4.72	13.51	5.66	-2.97	8.78	18.12	8.9	-0.42
os52163	4	351	0.45	8	1	4	6.53	-0.03	0.04	1.47	0.06	97.84	2.46	6.13	7.5	16.31	7.82	-1.41	12.09	21.63	11.57	1.34

os52197	5	736	0.93	6	3	5	6.79	-0.08	0.57	1.51	0.05	97.88	-0.47	7.2	4.79	13.63	5.66	-3.07	8.81	18.21	8.9	-0.5
os52374	7	487	0.89	4	2	7	6.7	-0.06	1.79	1.52	0.11	99.08	-2.52	8.4	6.21	14.91	7.01	-1.52	10.47	19.8	10.38	1.11
os52460	7	527	1	10	2	7	6.36	-0.15	1.03	1.55	0.24	98.16	-11.98	7.42	5.53	14.23	6.45	-2.01	9.59	18.93	9.65	0.55
os52527	7	492	0.92	6	2	7	6.61	-0.09	0.99	1.53	0.12	98.99	-2.6	7.4	6.13	14.89	6.9	-1.66	10.32	19.65	10.23	0.91
os52529	7	606	0.23	14	2	0	6.65	0.08	0	1.44	0.12	100	0.04	6.28	5.59	14.4	6.39	-2.16	9.95	19.24	9.82	0.48
os52589	3	494	0.06	16	2	0	6.79	0.21	0	1.45	0.12	99.67	-1.52	5.75	6.26	14.98	7.01	-1.5	10.48	19.77	10.29	1.03
os52672	7	507	0.08	1	2	0	6.81	-0.01	2.96	1.57	0.11	45.44	-1.3	7.79	5.88	14.63	6.63	-2.06	9.98	19.31	9.91	0.53
os52766	7	570	0.99	12	2	7	6.34	-0.2	0.03	1.49	0.13	99.71	-2.93	7.73	5.76	14.56	6.42	-2.46	9.95	19.35	9.75	0.08
os52813	7	445	0.08	3	2	7	6.86	0.04	1.77	1.57	0.12	50.53	-1.9	7.9	6.55	15.29	7.06	-1.94	11.01	20.33	10.61	0.7
os53161	7	490	0.35	13	2	7	6.63	0.1	0.01	1.48	0.18	92.64	-5.63	6.73	6.41	15.24	6.89	-2.18	11.07	20.57	10.55	0.49
os53253	4	438	0.64	11	2	3	6.48	-0.04	0.16	1.5	0.09	99.58	-1.96	7.26	6.93	15.74	7.38	-1.76	11.38	20.92	10.95	0.88
os53309	7	323	0.08	2	3	6	6.78	0.03	1.82	1.54	0.04	90.57	-0.49	8.33	7.73	16.6	7.99	-1.26	12.55	22.14	11.88	1.49
os53400	5	380	0	2	2	0	6.84	0.04	2.37	1.54	0.06	80.11	-0.05	7.78	7.26	16.12	7.62	-1.5	11.73	21.37	11.24	1.22
os53409	7	339	0.66	4	2	0	6.75	-0.02	1.94	1.55	0.09	96.83	-2.06	8.16	7.36	16.2	7.71	-1.49	11.94	21.45	11.42	1.26
os53555	4	646	0	11	3	9	7.06	0.19	0.02	1.45	0.07	100	-0.52	5.85	5.89	14.71	6.53	-2.36	10.08	19.54	9.9	0.22
os53624	7	289	0.62	4	3	6	6.62	-0.05	2.16	1.55	0.06	92.16	-1.61	8.79	7.91	16.78	8.17	-1.16	12.68	22.29	12.09	1.62
os53660	4	240	0.12	6	2	3	6.76	0.07	1.33	1.53	0.08	97.1	-3.71	8.53	8.57	17.5	8.66	-0.74	13.52	23.23	12.76	2.15
os53667	4	292	0.23	7	3	5	6.68	0.05	0.61	1.5	0.05	95.64	0.31	6.95	8.3	17.26	8.46	-0.92	13.18	22.96	12.52	1.95
os53668	4	292	0.23	8	3	5	6.67	0.05	0.55	1.5	0.05	95.85	0.36	6.86	8.3	17.26	8.46	-0.92	13.18	22.96	12.53	1.95
os53669	4	291	0.26	9	3	5	6.61	0.04	0.16	1.5	0.06	97.09	-1.98	7	8.31	17.26	8.47	-0.92	13.19	22.96	12.53	1.95
os53670	4	298	0.3	8	3	5	6.61	0.03	0.17	1.49	0.05	98.03	1.06	6.26	8.32	17.27	8.48	-0.91	13.2	22.97	12.53	1.96
os53671	4	296	0.4	8	3	5	6.61	0.03	0.14	1.49	0.05	97.93	0.94	6.3	8.32	17.27	8.48	-0.91	13.2	22.97	12.54	1.96
os53672	4	290	0.38	11	3	5	6.54	0.03	0.03	1.48	0.06	97.26	0.91	6.37	8.32	17.28	8.48	-0.91	13.21	22.98	12.54	1.97
os53675	4	271	0.11	9	2	3	6.7	0.09	0.36	1.51	0.09	94.51	-3.14	6.9	8.34	17.31	8.5	-0.9	13.24	23	12.56	1.98
os53676	7	276	0.84	10	2	3	6.37	-0.11	0.53	1.5	0.09	95.78	-2.75	6.68	8.35	17.31	8.51	-0.9	13.25	23.01	12.56	1.99
os53680	5	324	0.08	2	3	6	6.81	0.03	1	1.53	0.02	74.59	0.36	7.95	7.93	16.9	8.18	-1.18	12.66	22.45	12.11	1.67
os53815	7	385	0.77	1	2	7	6.76	-0.01	2.6	1.55	0.1	93.43	-3.64	8.08	7.09	15.96	7.63	-1.21	11.7	21.16	11.37	1.49
os53840	7	464	0.09	5	2	3	6.94	0.08	1.15	1.53	0.07	99.59	-2.61	8.46	6.74	15.62	7.34	-1.52	11.27	20.76	11.02	1.23
os53856	5	537	0.08	3	3	6	6.93	0.05	1.63	1.53	0.03	85.31	-0.34	8.95	6.35	15.25	7.01	-1.81	10.81	20.33	10.61	0.88
os53858	4	486	0.91	7	3	9	6.54	-0.12	0.33	1.49	0.05	99.19	-0.08	6.02	6.82	15.72	7.42	-1.49	11.39	20.92	11.12	1.29
os53890	5	562	0.4	2	3	6	6.89	0.02	2.75	1.54	0.05	94.18	-1.58	9.11	6.08	14.94	6.74	-2.11	10.56	19.97	10.26	0.6
os53956	5	598	0.12	6	2	3	7.01	0.08	0.97	1.52	0.08	98.94	-2.32	7.74	5.82	14.68	6.43	-2.58	10.36	19.78	10.05	0.07
os54056	5	549	0.36	3	3	6	6.91	0.03	1.08	1.53	0.03	66.02	0.31	8.25	6.22	15.12	6.9	-2.08	10.65	20.16	10.4	0.59
os54140	5	541	0.35	4	3	6	6.94	0.05	1.25	1.54	0.04	95.65	-0.82	7.42	6.28	15.14	6.91	-2.01	10.76	20.26	10.49	0.72
os54175	5	625	0.01	2	3	6	6.93	0.04	1.68	1.55	0.03	76.36	-0.16	7.92	5.68	14.56	6.29	-2.76	10.1	19.61	9.81	-0.17
os54195	7	448	0.26	10	2	7	6.59	0	0.5	1.53	0.12	96.6	-3.08	8.72	6.46	15.28	7.05	-1.84	10.9	20.31	10.59	0.82
os54202	7	520	0.36	4	2	7	6.64	-0.08	1.54	1.54	0.12	98.92	-3	9.02	6.15	15.01	6.76	-2.13	10.6	20.04	10.29	0.52

os54215	4	555	0.79	5	3	5	6.78	-0.05	0.6	1.52	0.04	99.93	-0.24	6.7	6.15	15.01	6.79	-2.23	10.56	20.01	10.28	0.4
os54229	5	549	0.11	3	3	6	6.91	0.03	1.15	1.53	0.03	65.54	0.36	8.27	6.21	15.12	6.89	-2.08	10.65	20.15	10.4	0.59
os54243	5	518	0.25	3	3	6	6.78	0.01	1.63	1.53	0.04	88.86	-0.9	7.89	6.58	15.51	7.07	-2	11.12	20.71	10.74	0.71
os54328	7	300	0.01	7	3	6	6.83	0.09	0.2	1.5	0.04	80.77	1.22	7.26	8.08	17.09	8.28	-1.05	12.97	22.79	12.33	1.85
os54354	7	447	0.97	5	2	7	6.73	-0.03	2.54	1.55	0.1	75.74	-5.14	7.94	6.89	15.83	7.46	-1.52	11.51	21.15	11.24	1.27
os54403	7	459	0.63	8	2	7	6.77	-0.03	0.49	1.53	0.09	93.67	-2.35	8.68	6.72	15.63	7.28	-1.66	11.26	20.88	10.99	1.07
os54685	7	321	0.41	4	2	0	6.81	0.02	2.37	1.54	0.08	91.54	-1.55	8.98	7.61	16.51	8.07	-0.96	12.25	21.86	11.84	1.88
os54788	5	289	0.51	4	2	0	6.79	0.02	0.13	1.51	0.09	92.23	1.62	7.86	7.86	16.75	8.26	-0.77	12.6	22.24	12.16	2.08
os54885	5	369	0.23	3	3	6	6.76	0.02	3.62	1.54	0.02	53.62	-0.07	9.2	7.38	16.29	7.89	-1.09	12.06	21.74	11.79	1.71
os54924	5	365	0.41	3	3	6	6.78	-0.01	1.69	1.55	0.02	46.38	-0.2	8.45	7.46	16.36	7.97	-1.03	12.15	21.82	11.89	1.79
os54926	5	367	0.92	0	3	6	6.73	-0.01	2.69	1.55	0.02	36.34	-0.62	9.56	7.43	16.34	7.95	-1.05	12.11	21.79	11.85	1.77
os55085	5	343	0.83	4	3	6	6.61	-0.05	0.58	1.54	0.06	95	-0.15	7.96	7.49	16.41	7.95	-1.05	12.24	21.85	11.83	1.77
os55193	7	295	0.21	10	2	7	6.72	0.08	0.72	1.53	0.18	86.25	-8.91	6.54	7.72	16.6	8.11	-0.89	12.44	22.02	11.97	1.95
os55273	7	279	0.99	5	2	3	6.57	-0.09	1.14	1.52	0.06	93.16	-1.95	8.58	8.06	16.97	8.41	-0.75	12.88	22.57	12.42	2.21
os55334	5	348	0.83	2	3	6	6.7	-0.03	3.44	1.54	0.05	96.12	-0.31	7.87	7.57	16.47	8.02	-1	12.2	21.81	11.79	1.85
os55352	5	300	0.4	2	3	6	6.79	0.02	2.38	1.54	0.06	92.64	0.15	8.04	7.87	16.78	8.3	-0.79	12.55	22.19	12.13	2.11
os55353	5	297	0.38	3	3	6	6.8	0.02	2.93	1.54	0.06	90.83	-0.8	8.16	7.87	16.78	8.3	-0.78	12.55	22.19	12.14	2.11
os55373	7	287	0.71	9	2	7	6.72	-0.02	0.7	1.53	0.11	89.09	-2.76	7.8	7.91	16.8	8.3	-0.77	12.57	22.21	12.16	2.12
os55488	7	283	0.92	4	3	6	6.64	-0.05	1.7	1.55	0.05	85.54	-0.84	8.82	7.99	16.95	8.35	-0.82	12.86	22.58	12.41	2.12
os55489	7	282	0.99	4	3	6	6.65	-0.05	1.78	1.55	0.05	84.8	-0.81	8.84	7.99	16.95	8.35	-0.82	12.86	22.58	12.41	2.12
os55565	7	355	0.08	5	2	7	6.58	0.04	0.91	1.55	0.24	90.45	-4.02	7.54	6.83	15.61	7.25	-1.92	11.33	20.77	10.85	0.73
os55727	5	602	0.15	2	3	6	6.92	0.02	0.79	1.54	0.02	75.1	0.21	8.57	5.98	14.88	6.55	-2.56	10.56	20.04	10.24	0.14
os55776	5	607	0.4	3	3	6	6.91	0.01	2.42	1.55	0.05	85.48	-1.27	9.05	5.86	14.76	6.45	-2.58	10.37	19.86	10.04	0.07
os55834	5	608	0.35	4	3	6	6.83	0.02	0.62	1.53	0.05	99.59	-0.07	7.53	5.97	14.84	6.56	-2.57	10.46	19.9	10.13	0.12
os55874	5	585	0.99	5	3	5	6.72	-0.08	0.27	1.51	0.03	94.8	0.57	6.87	6.21	15.15	6.76	-2.36	10.75	20.3	10.41	0.35
os55880	5	584	0	5	3	5	6.97	0.08	0.54	1.51	0.05	99.32	-0.3	7.5	6.15	15.1	6.69	-2.42	10.67	20.25	10.36	0.31
os55916	5	588	0.92	0	3	6	6.85	-0.01	1.07	1.52	0.01	85.49	0.21	8.68	6.15	15.09	6.72	-2.32	10.63	20.18	10.32	0.38
os55966	7	514	0.52	8	2	0	6.92	0.04	0.37	1.53	0.1	72.08	-1.03	8.12	6.3	15.21	6.82	-2.37	10.91	20.44	10.53	0.35
os55969	7	305	0.01	5	2	3	6.73	0.03	1.03	1.54	0.07	87.22	-1.71	8.13	7.95	16.92	8.2	-1.18	12.69	22.5	12.14	1.68
os56067	5	639	0.77	4	3	6	6.84	-0.04	1.43	1.51	0.03	86.79	-0.68	7.64	5.85	14.76	6.46	-2.66	10.38	19.91	10.1	0.06
os56088	7	452	0.49	7	2	3	6.65	-0.02	0.92	1.48	0.08	88.85	-4.13	8.63	7.23	16.28	7.66	-1.44	11.92	21.72	11.47	1.41
os56201	4	390	0.73	27	2	7	6.34	-0.07	0	1.43	0.23	85.95	-7.13	6.04	7.03	15.87	7.51	-1.55	11.61	21.16	11.22	1.21
os56211	7	370	0.92	3	2	7	6.61	-0.04	1.5	1.57	0.27	83.04	-8.65	6.44	6.93	15.82	7.44	-1.65	11.57	21.16	11.19	1.12
os56337	5	496	0.83	2	3	6	6.77	-0.03	0.56	1.53	0.02	67.35	0.75	8.62	6.65	15.57	7.19	-1.9	11.32	20.92	10.95	0.88
os56395	7	270	0.71	3	2	3	6.67	0	1.86	1.55	0.1	91.17	-3.88	8.73	8.08	17.1	8.38	-0.83	12.97	22.83	12.45	2.11
os56400	7	274	0.92	2	2	3	6.74	0.03	3.17	1.56	0.06	72.11	-2.04	8.5	8.09	17.07	8.38	-0.83	12.98	22.77	12.45	2.1
os56414	5	409	0.6	1	3	6	6.81	0.01	2.97	1.53	0.05	75.2	-1.71	8.95	7.3	16.27	7.74	-1.35	12.03	21.74	11.66	1.53

os56424	7	412	0.53	19	2	7	6.02	-0.1	0.15	1.46	0.15	97.76	-4.34	7.5	7.27	16.3	7.68	-1.45	11.96	21.78	11.53	1.42
os56441	4	351	0.4	7	2	0	6.89	0.06	0.38	1.53	0.08	99.6	-0.63	6.47	7.61	16.58	7.93	-1.34	12.39	22.15	11.86	1.54
os56475	7	387	0.37	5	2	7	6.57	0	0.97	1.55	0.19	77.46	-7.24	6.86	6.99	15.86	7.5	-1.55	11.59	21.14	11.21	1.2
os56562	5	640	0.08	2	3	6	6.94	0.04	0.96	1.53	0.02	73.14	0.53	8.62	5.8	14.69	6.39	-2.64	10.37	19.79	9.99	0.12
os56568	5	595	0.79	1	3	6	6.83	-0.01	0.56	1.53	0.01	31.24	0	8.61	6.12	14.97	6.66	-2.37	10.52	19.98	10.16	0.37
os56671	5	596	0.2	4	3	6	6.95	0.03	0.86	1.52	0.03	98.89	-0.75	7.44	6.04	14.89	6.57	-2.45	10.64	20.04	10.21	0.31
os56688	7	532	0.19	7	2	0	6.85	0.07	0.37	1.54	0.09	78.83	-1.6	8.62	6.23	15.09	6.74	-2.36	10.83	20.28	10.4	0.4
os56734	5	610	0.62	4	3	6	6.9	0	0.52	1.52	0.02	98.08	0.39	7.22	6	14.87	6.54	-2.49	10.45	19.91	10.11	0.22
os56745	5	628	0.92	3	3	6	6.76	-0.05	0.73	1.51	0.02	84.86	0.46	7.38	5.95	14.84	6.51	-2.54	10.39	19.86	10.05	0.19
os56747	5	623	0.44	2	3	6	6.86	0.01	1.73	1.51	0.02	72.36	0.2	7.82	5.91	14.79	6.48	-2.54	10.3	19.76	9.99	0.17
os56820	4	512	0.92	8	2	7	6.47	-0.13	0.22	1.52	0.13	86.17	-3.3	7.01	6.29	15.12	6.8	-2.25	10.75	20.19	10.33	0.45
os57014	7	610	0.19	8	2	7	6.85	0.11	1.09	1.54	0.14	92.49	-6.7	7.9	6.1	14.91	6.66	-1.91	10.78	20.16	10.45	0.82
os57044	7	569	0.77	3	2	7	6.81	-0.02	1.99	1.55	0.09	91.53	-3.03	8.51	6.34	15.06	6.78	-1.81	11.14	20.41	10.67	0.97
os57077	7	574	0.77	1	2	0	6.86	-0.01	3.2	1.55	0.06	82.76	0.19	9.51	6.34	15.06	6.79	-1.77	11.02	20.33	10.63	0.96
os57124	5	515	0.4	8	2	7	6.93	0.05	0.51	1.51	0.11	93.39	-3.2	7.41	6.75	15.52	7.12	-1.52	11.55	20.91	11.07	1.28
os57156	7	588	0.93	4	3	6	6.74	-0.06	2.15	1.54	0.06	90.4	-1.61	8.8	6.36	15.01	6.77	-1.57	10.93	20.2	10.38	1.04
os57251	5	673	0	9	2	0	7.05	0.14	0.18	1.52	0.07	97.53	-0.13	7.79	5.71	14.4	6.23	-2.16	10.33	19.53	9.96	0.57
os57263	7	593	0.06	6	2	7	6.74	0.06	0.95	1.55	0.19	95.1	-9.16	7.41	6.17	14.87	6.61	-1.87	10.92	20.14	10.43	0.9
os57403	4	744	0.07	21	2	3	6.96	0.33	0	1.41	0.18	97.83	-2.89	5.97	5.57	14.36	6.19	-2.21	10.19	19.53	9.94	0.48
os57457	5	556	0	6	2	0	6.96	0.1	1.06	1.55	0.11	92.67	-1.96	7.88	6.37	15.13	6.85	-1.76	11.09	20.38	10.66	0.96
os57497	3	559	0.65	28	1	4	6.47	-0.05	0	1.32	0.17	95.66	4.52	5.24	6.49	15.16	6.93	-1.58	11.24	20.41	10.68	1.05
os57541	4	696	0	15	1	4	7.08	0.24	0	1.43	0.09	99.18	2.97	5.53	5.84	14.54	6.34	-2.1	10.59	19.77	10.15	0.69
os57559	5	664	0.19	2	3	6	6.95	0.03	2.76	1.55	0.03	55.91	-0.34	8.7	5.98	14.77	6.54	-1.95	10.57	19.92	10.26	0.73
os57574	4	706	0.43	10	3	5	7.03	0.06	0.04	1.48	0.07	99.8	-0.22	5.95	5.85	14.63	6.43	-2.03	10.48	19.82	10.16	0.66
os57582	6	658	0.6	3	3	6	6.81	-0.03	1.54	1.55	0.03	83.67	-0.23	7.45	6	14.73	6.53	-2.04	10.76	20.07	10.38	0.68
os57583	6	659	0.75	3	3	6	6.79	-0.03	1.11	1.55	0.03	86.45	-0.23	7.3	5.99	14.72	6.52	-2.04	10.74	20.06	10.37	0.68
os57586	5	649	0.92	0	3	6	6.87	-0.01	3.95	1.56	0.03	33.87	-1.18	9.07	6.01	14.73	6.53	-2.06	10.8	20.07	10.43	0.67
os57640	7	546	1	14	2	7	6.26	-0.23	0.01	1.49	0.14	88.82	-2.65	6.3	6.5	15.22	6.94	-1.69	11.26	20.58	10.85	1.06
os57663	5	646	0.85	4	3	6	6.79	-0.06	0.81	1.53	0.03	89.47	0.18	7.06	6.13	14.93	6.68	-1.89	10.86	20.24	10.52	0.8
os57687	3	592	0.23	13	2	3	6.64	0.05	0.37	1.49	0.14	98.41	-4.18	6.96	6.29	14.97	6.77	-1.71	10.95	20.14	10.48	0.91
os57691	5	683	0.72	5	3	6	6.89	-0.02	0.42	1.53	0.03	96.27	-0.01	7.1	5.89	14.61	6.38	-2.16	10.74	19.94	10.28	0.63
os57713	5	708	0.92	0	3	6	6.91	0	2.51	1.55	0.01	14.98	0.14	11.1	5.7	14.39	6.22	-2.26	10.46	19.68	10.1	0.54
os57717	5	693	0.92	1	3	6	6.9	-0.01	2.13	1.54	0.02	58.27	0.44	9.21	5.76	14.47	6.27	-2.24	10.55	19.76	10.17	0.58
os57718	5	691	0.23	1	3	6	6.92	0.01	3.97	1.55	0.02	17.92	-0.55	9.76	5.73	14.44	6.27	-2.22	10.5	19.69	10.12	0.58
os57736	5	680	0.01	3	3	6	6.97	0.05	0.83	1.53	0.03	63.08	-0.25	7.74	5.91	14.65	6.42	-2.14	10.72	19.98	10.33	0.67
os57765	5	663	0.01	4	2	7	6.96	0.06	2.02	1.54	0.11	97.7	-5.6	8.21	5.83	14.62	6.43	-2.06	10.4	19.8	10.14	0.65
os57813	5	661	0	3	3	6	6.95	0.05	3.36	1.55	0.04	50.72	-1.16	8.82	5.98	14.77	6.54	-1.94	10.56	19.91	10.25	0.73

os57820	5	622	0.6	2	3	6	6.84	0	1.9	1.54	0.03	41.96	-0.38	8.86	6.26	15.03	6.72	-1.9	11.13	20.47	10.69	0.83
os57824	5	609	0.92	3	2	3	6.71	-0.05	1.76	1.56	0.09	63.28	-2.85	7.22	6.23	15.02	6.74	-1.9	11.08	20.43	10.67	0.82
os57923	5	672	0.92	5	3	5	6.69	-0.07	0.5	1.51	0.04	94.54	-0.04	7.82	5.85	14.57	6.43	-1.9	10.43	19.66	10.06	0.7
os58064	5	634	0.6	3	3	6	6.82	-0.01	1.83	1.53	0.03	74.97	-0.43	7.8	6.23	15.01	6.76	-1.87	10.99	20.31	10.66	0.84
os58065	4	664	0.47	9	3	9	6.97	0.03	0.12	1.47	0.09	100	-1.41	5.73	6.17	14.92	6.7	-1.87	10.87	20.17	10.54	0.87
os58066	4	673	0.41	11	3	9	6.99	0.05	0.01	1.46	0.08	100	-0.09	5.63	6.13	14.88	6.66	-1.9	10.84	20.14	10.5	0.84
os58071	5	641	0.77	3	3	6	6.89	-0.01	1.87	1.54	0.04	93.21	-0.52	7.66	6.2	14.92	6.71	-1.93	11.01	20.27	10.64	0.79
os58175	7	524	0.76	4	2	7	6.72	-0.05	1.59	1.55	0.11	95.42	-5.41	8.69	6.68	15.4	7.09	-1.47	11.35	20.63	10.83	1.15
os58313	5	704	0.03	2	3	6	6.95	0.03	0.45	1.53	0.02	59.24	0.5	7.48	5.76	14.52	6.36	-2.02	10.35	19.63	10.09	0.61
os58318	4	727	0.76	14	3	9	6.26	-0.17	0	1.45	0.09	99.4	-0.84	5.68	5.81	14.55	6.41	-1.99	10.31	19.61	10.06	0.62
os58320	4	721	0.97	15	1	1	6.44	-0.2	0	1.43	0.07	98.53	3.09	5.36	5.73	14.49	6.36	-2.03	10.26	19.58	10.03	0.6
os58457	5	686	0.23	2	3	6	6.9	0.01	1.63	1.54	0.04	71.21	0.06	7.38	5.76	14.51	6.37	-2.01	10.25	19.56	10.01	0.6
os58472	5	622	1	5	2	7	6.72	-0.08	1.46	1.54	0.08	94.56	-2.5	8.8	6.04	14.77	6.57	-1.99	10.8	20.04	10.42	0.74
os58481	4	665	0.63	7	3	9	6.9	-0.02	0.17	1.48	0.05	100	0.6	6.01	6.16	14.91	6.69	-1.87	10.87	20.17	10.53	0.87
os58483	4	674	0.37	11	3	9	6.71	0.05	0.01	1.46	0.08	100	-1.39	5.51	6.13	14.88	6.67	-1.9	10.86	20.16	10.52	0.84
os58504	5	642	0.45	3	3	6	6.93	0.01	0.23	1.54	0.02	55.62	0.69	7.9	6.15	14.87	6.66	-1.92	10.9	20.18	10.54	0.8
os58544	7	648	0.19	9	2	0	7.03	0.08	0.38	1.5	0.1	98.89	-1.51	7.52	5.93	14.76	6.52	-1.96	10.5	19.92	10.28	0.74
os58564	5	652	0.96	4	3	6	6.79	-0.06	1.69	1.52	0.04	95.32	-1.09	8.36	6.14	14.98	6.69	-1.86	10.79	20.23	10.53	0.88
os58571	7	552	0	5	2	0	6.95	0.08	0.93	1.54	0.11	93.14	-1.08	7.65	6.31	15.06	6.81	-1.8	11	20.31	10.61	0.94
os58606	3	580	0.27	22	2	0	6.47	0.14	0	1.43	0.18	94.49	-3.15	6.61	6.23	14.96	6.71	-1.77	10.85	20.17	10.43	0.85
os58614	7	577	0.93	6	3	5	6.75	-0.07	0.97	1.52	0.07	98.09	-1.74	7.96	6.46	15.25	6.97	-1.66	11.19	20.56	10.84	1.07
os58619	7	639	0.6	5	2	3	6.96	0.04	1.06	1.52	0.1	99.91	-4.61	7.61	6.17	15.01	6.73	-1.79	10.88	20.29	10.6	0.93
os58620	5	591	0.08	4	3	6	6.97	0.06	1.29	1.54	0.06	97.2	-0.32	8.29	6.41	15.23	6.91	-1.68	11.15	20.56	10.84	1.07
os58662	7	538	0.27	9	2	3	6.75	0.05	0.87	1.51	0.14	92.29	-6.09	8.6	6.74	15.49	7.16	-1.4	11.35	20.69	10.87	1.2
os58776	5	795	0.66	5	3	6	6.78	-0.08	0.56	1.52	0.03	76.55	-0.1	7.22	5.06	13.76	5.85	-2.35	9.4	18.63	9.3	0.13
os58777	6	794	0.45	6	3	5	6.72	-0.09	0.48	1.52	0.03	76.95	-0.07	6.83	5.06	13.75	5.85	-2.35	9.39	18.61	9.3	0.12
os58792	5	713	0.53	20	3	5	6.12	-0.08	0	1.39	0.14	94.88	0.45	5.73	5.51	14.18	6.19	-2.12	9.93	19.16	9.72	0.4
os58802	7	635	0.03	20	3	5	7.03	0.32	0	1.42	0.15	99.44	-1.98	6.58	5.97	14.63	6.52	-1.87	10.6	19.77	10.19	0.75
os58817	7	716	0.87	20	3	5	6.32	-0.22	0	1.42	0.11	94.33	1.55	6.64	5.51	14.18	6.19	-2.14	10	19.18	9.75	0.43
os58826	7	713	0.66	6	3	9	6.95	0	0.82	1.48	0.05	97.72	-1.53	7.98	5.7	14.37	6.34	-2.03	10.22	19.42	9.94	0.55
os59010	5	621	0.03	2	3	6	6.93	0.03	0.64	1.55	0.03	78.36	0.29	7.41	6.14	14.81	6.62	-1.67	10.6	19.86	10.14	0.89
os59011	5	622	0.08	3	3	6	6.97	0.05	1.13	1.55	0.04	86.5	-0.05	7.05	6.11	14.78	6.6	-1.69	10.56	19.82	10.11	0.87
os59022	6	655	0.92	1	3	6	6.85	-0.02	0.72	1.54	0.03	67.77	0.08	7.68	5.94	14.62	6.51	-1.8	10.41	19.69	10.09	0.71
os59029	6	775	0.92	0	3	6	6.94	0	3.62	1.53	0.01	28.77	0.36	9.61	5.2	13.9	5.96	-2.25	9.55	18.77	9.4	0.2
os59033	5	674	0.92	5	3	6	6.71	-0.08	0.39	1.5	0.03	94.01	0.07	7.37	5.85	14.57	6.43	-1.9	10.44	19.67	10.06	0.7
os59060	3	770	0.92	7	1	1	6.84	-0.07	0.22	1.43	0.03	99.2	1.88	5.97	5.6	14.31	6.28	-2.05	10.06	19.29	9.82	0.49
os59064	5	800	0.53	5	3	6	6.76	-0.09	0.53	1.51	0.02	71.53	0.65	7.19	5.06	13.76	5.85	-2.35	9.4	18.62	9.3	0.12

os59065	5	621	0.08	2	3	6	6.95	0.04	1.16	1.55	0.04	83.74	0.04	7.12	6.12	14.79	6.61	-1.68	10.58	19.83	10.12	0.88
os59074	7	681	0	11	3	5	7.07	0.19	0.02	1.5	0.1	100	-0.95	6.17	5.65	14.34	6.27	-1.92	9.93	19.22	9.66	0.61
os59113	5	596	0.92	0	3	6	6.87	0.01	2.31	1.52	0.01	15.41	-0.16	9.81	6.47	15.29	6.94	-1.57	11.07	20.47	10.66	1.07
os59131	7	595	0.05	5	3	9	6.89	0.06	0.98	1.48	0.06	91.8	-0.51	7.56	6.63	15.43	7.04	-1.45	11.23	20.64	10.76	1.16
os59214	5	425	0.92	7	3	5	6.49	-0.11	0.22	1.51	0.07	99.41	-0.08	6.78	7.35	16.23	7.6	-1.54	11.96	21.54	11.23	1.14
os59410	4	369	0.08	2	2	0	6.82	0.04	1.96	1.54	0.1	92.8	-1.69	7.75	8.12	17.13	8.15	-1.06	12.69	22.38	11.73	1.62
os59459	7	532	0.83	6	2	3	6.81	-0.03	0.82	1.54	0.07	98.98	-1.93	9.05	6.65	15.59	7.07	-2.04	11.21	20.87	10.7	0.7
os59499	4	397	0.37	6	2	7	6.62	-0.01	1.13	1.55	0.13	97.48	-7.06	8.87	7.34	16.27	7.63	-1.5	12.03	21.71	11.39	1.27
os59642	5	632	0.77	1	3	6	6.87	-0.01	2.22	1.55	0.01	28.72	0.32	9.34	6.1	15.04	6.62	-2.34	10.35	20	10.01	0.29
os59644	6	612	0.21	2	3	6	6.92	0.02	2.98	1.55	0.04	82.61	-1.42	9.45	6.13	15.08	6.65	-2.33	10.44	20.1	10.08	0.3
os59645	7	632	0.44	2	3	6	6.89	-0.01	1.79	1.55	0.03	81.65	-0.15	8.95	6.08	15.03	6.59	-2.37	10.39	20.04	10.02	0.24
os59677	4	476	0.21	9	2	3	6.98	0.1	0.55	1.5	0.08	99.28	-2.69	8.05	6.93	15.86	7.29	-1.75	11.48	21.14	10.95	0.96
os59709	4	463	0.98	18	1	1	5.97	-0.3	0	1.39	0.09	97.47	2.2	5.54	7.24	16.08	7.51	-1.54	11.77	21.38	11.17	1.18
os59744	4	310	0.29	7	3	5	6.92	0.1	0.25	1.49	0.07	91.49	0.62	7.01	7.98	16.84	8.14	-1.05	12.81	22.39	12.07	1.73
os59746	4	302	0.09	12	2	0	6.97	0.19	0.01	1.46	0.08	89.7	0.66	6.99	7.98	16.85	8.14	-1.05	12.81	22.39	12.07	1.74
os59753	5	501	0.92	2	3	6	6.76	-0.03	2.78	1.52	0.02	44.14	-0.33	8.69	6.93	15.86	7.28	-1.72	11.46	21.15	10.97	0.97
os59754	5	501	0.92	2	3	6	6.76	-0.03	2.69	1.52	0.02	45.6	-0.36	8.73	6.93	15.86	7.28	-1.72	11.46	21.15	10.97	0.97
os59815	5	600	0.12	2	3	6	6.89	0.03	1.81	1.54	0.02	77.02	0.18	9.18	6.32	15.25	6.8	-2.24	10.84	20.45	10.37	0.48
os59817	5	619	0.6	3	3	6	6.81	-0.02	0.23	1.54	0.02	76.7	0.67	8.05	6.25	15.19	6.74	-2.29	10.75	20.37	10.31	0.42
os59835	5	610	0.63	3	3	6	6.76	-0.04	0.84	1.53	0.03	98.74	0.15	8.1	6.28	15.18	6.75	-2.25	10.69	20.31	10.24	0.45
os59853	4	566	0.61	12	1	4	6.41	-0.09	0	1.45	0.07	94.44	3.61	5.52	6.5	15.42	6.94	-2.09	10.99	20.6	10.48	0.61
os59887	4	553	0.23	11	3	5	6.72	0.06	0.16	1.48	0.07	95.99	-1.01	6.85	6.7	15.61	7.11	-1.95	11.24	20.84	10.7	0.8
os59914	5	542	0.92	2	3	6	6.78	-0.03	2.98	1.54	0.05	62.11	-1	8.57	6.6	15.49	7.03	-2.01	11.08	20.7	10.57	0.73
os59934	4	580	0.21	8	3	9	6.82	0.07	0.03	1.45	0.04	97.48	2.25	5.74	6.65	15.56	7.07	-2	11.21	20.82	10.68	0.78
os60014	4	378	0.23	10	2	7	6.82	0.03	0.55	1.52	0.14	94.22	-5.03	8.09	7.48	16.42	7.74	-1.36	12.12	21.82	11.47	1.41
os60015	4	382	0.55	12	2	7	6.78	0.01	0.26	1.51	0.14	95.5	-5.02	8.13	7.48	16.41	7.74	-1.36	12.11	21.82	11.46	1.41
os60225	4	352	0.72	11	2	3	6.61	-0.06	0.33	1.5	0.14	99.6	-6.39	6.94	8.17	17.12	8.19	-1.09	13.04	22.65	12.02	1.67
os60249	4	374	0.81	7	3	5	6.5	-0.1	0.35	1.51	0.09	94.05	-0.42	6.27	7.95	16.91	8	-1.22	12.7	22.33	11.73	1.5
os60250	4	375	0.65	7	3	5	6.49	-0.1	0.33	1.51	0.09	94.09	-0.6	6.22	7.95	16.91	8	-1.22	12.7	22.32	11.73	1.5
os60279	4	369	0.09	15	3	9	6.85	0.2	0.03	1.42	0.09	99.28	-1.43	6.33	8.2	17.15	8.21	-1.08	13.06	22.67	12.03	1.68
os60280	4	365	0.03	18	2	8	6.79	0.25	0	1.41	0.13	99.22	-2.66	6.04	8.2	17.15	8.22	-1.08	13.06	22.67	12.04	1.69
os60282	4	301	0.68	14	2	7	6.15	-0.23	0.01	1.52	0.15	70.07	-4.22	7.39	8.31	17.22	8.27	-0.99	13.12	22.71	12.06	1.74
os60300	5	538	0.08	8	2	3	6.89	0.12	0.87	1.53	0.13	94.46	-5.3	6.92	6.9	15.87	7.22	-1.88	11.31	21	10.7	0.76
os60311	7	480	0.37	5	2	3	6.91	0.05	2.16	1.51	0.1	95.31	-4.54	8.38	7.27	16.26	7.49	-1.64	11.72	21.4	10.99	1.02
os60411	5	658	0.74	2	3	6	6.82	-0.02	1.68	1.54	0.03	81.97	-0.39	8.4	6.07	15.02	6.57	-2.35	10.32	19.98	9.93	0.24
os60467	4	535	0.74	5	2	3	6.65	-0.06	1.27	1.5	0.07	97.6	-2.73	8.09	7.04	16.05	7.3	-1.72	11.57	21.31	10.82	0.93
os60805	4	369	0.08	2	2	0	6.81	0.03	1.96	1.54	0.1	92.8	-1.9	7.74	8.11	17.13	8.15	-1.06	12.69	22.38	11.73	1.61

os60807	7	315	0.41	6	2	7	6.68	-0.03	2	1.55	0.14	93.91	-6.77	7.48	7.85	16.73	8.02	-1.11	12.61	22.21	11.93	1.66
os60827	4	303	0.99	5	3	5	6.58	-0.09	0.58	1.51	0.05	97.43	-0.71	7.56	8.39	17.32	8.43	-0.82	13.42	23.03	12.5	2.1
os60843	7	399	0.25	3	2	3	6.84	0.03	1.87	1.54	0.05	90.93	-2.34	7.89	7.41	16.25	7.7	-1.55	12.37	22.03	11.62	1.19
os60859	5	406	0.92	2	3	6	6.71	-0.04	1.93	1.54	0.04	82.44	-0.36	7.99	7.64	16.48	7.98	-1.02	12.44	22.05	11.74	1.75
os60911	5	287	0.92	1	3	6	6.75	0.02	3.07	1.56	0.02	29.88	-0.58	9.02	8.23	17.02	8.41	-0.75	13.27	22.83	12.38	2.13
os60924	5	290	0.92	1	3	6	6.72	0	2.89	1.55	0.02	45.71	1.06	9.98	8.21	16.97	8.39	-0.77	13.22	22.75	12.35	2.1
os60951	7	278	0.92	1	3	6	6.71	-0.01	3.39	1.56	0.04	17.41	-0.57	9.73	8.31	17.08	8.44	-0.69	13.4	22.98	12.48	2.18
os60960	4	344	0.98	2	3	6	6.72	-0.02	0.35	1.54	0.01	92.43	0.33	8.65	7.94	16.74	8.16	-0.96	12.89	22.54	12.11	1.83
os60996	4	383	0.7	6	2	3	6.79	0	1	1.53	0.1	96.85	-4.3	8.29	7.61	16.44	7.92	-1.17	12.48	22.15	11.8	1.58
os61066	7	416	0.1	5	2	3	6.76	0.03	1.51	1.55	0.11	95.72	-6.13	7.61	7.22	16.01	7.53	-1.62	12.06	21.66	11.3	1.07
os61088	5	432	0.66	5	2	3	6.67	-0.01	1.32	1.53	0.1	93.54	-4.08	6.82	7.26	16.05	7.56	-1.59	12.11	21.7	11.34	1.09
os61099	6	397	0	2	3	6	6.72	-0.04	1.82	1.55	0.03	72.25	-0.33	8.66	7.35	16.14	7.63	-1.56	12.22	21.85	11.47	1.16
os61103	5	397	0.67	3	3	6	6.83	0.02	1.95	1.54	0.03	81.87	-0.85	9.05	7.37	16.18	7.66	-1.55	12.26	21.9	11.52	1.17
os61114	4	402	0.24	5	2	3	6.81	0.06	1.38	1.53	0.08	97.35	-3.5	6.76	7.44	16.26	7.71	-1.53	12.36	22.01	11.61	1.22
os61116	5	397	0.89	3	3	6	6.74	-0.03	1.92	1.55	0.04	86.46	-1.28	8.06	7.42	16.26	7.71	-1.55	12.38	22.05	11.64	1.2
os61136	4	424	0.05	6	2	0	6.93	0.1	0.43	1.51	0.1	98.82	-0.17	7.84	7.1	15.92	7.44	-1.66	11.83	21.47	11.13	1.03
os61182	4	422	0.76	7	2	7	6.77	-0.03	1.04	1.54	0.09	93.7	-3.93	6.96	7.15	15.94	7.49	-1.66	11.95	21.57	11.24	1.02
os61183	7	418	0.82	6	2	0	6.64	-0.06	1.45	1.54	0.09	85.86	-1.14	7.53	7.2	15.99	7.53	-1.63	12.01	21.63	11.29	1.05
os61212	5	411	0.98	2	3	6	6.74	-0.03	2.94	1.56	0.03	59.53	-0.83	9.04	7.38	16.26	7.69	-1.44	12.22	21.9	11.4	1.29
os61215	5	417	0.92	1	3	6	6.8	0	3.56	1.56	0.03	26.34	-0.11	8.91	7.35	16.18	7.63	-1.49	12.16	21.84	11.37	1.26
os61217	5	424	0.08	1	3	6	6.84	0.03	0.65	1.54	0.02	56.44	0.65	8.77	7.37	16.23	7.66	-1.47	12.21	21.89	11.4	1.28
os61313	5	388	0.75	6	3	5	6.76	-0.03	0.71	1.52	0.07	97.49	-1.4	7.2	7.41	16.2	7.72	-1.38	12.19	21.86	11.47	1.33
os61387	7	454	0.32	7	2	3	6.86	0.06	1.03	1.53	0.1	97.89	-4.82	7.53	7.03	15.85	7.4	-1.73	11.86	21.45	11.16	0.99
os61393	7	431	0.3	5	3	5	6.91	0.07	1.18	1.54	0.05	92.52	-1.52	8.03	7.19	15.99	7.49	-1.66	12.04	21.64	11.31	1.06
os61398	5	417	0.6	2	3	6	6.71	-0.03	3.04	1.56	0.03	56.55	-1.09	8.26	7.33	16.19	7.63	-1.5	12.13	21.8	11.35	1.23
os61399	5	418	0.38	3	3	6	6.74	0	2.04	1.54	0.02	50.8	0.33	8.27	7.36	16.22	7.65	-1.48	12.16	21.84	11.37	1.26
os61413	5	407	0.08	3	3	6	6.85	0.04	4.94	1.55	0.03	51.96	-0.15	8.81	7.39	16.26	7.7	-1.44	12.21	21.89	11.39	1.3
os61435	4	381	0.22	16	2	3	6.56	0.15	0.37	1.52	0.18	95.49	-8.31	6.27	7.39	16.19	7.66	-1.5	12.31	21.93	11.49	1.22
os61505	7	480	0.38	9	2	3	6.61	-0.01	0.58	1.48	0.08	99.28	-2.94	6.84	7	15.79	7.34	-1.75	11.82	21.41	11.1	0.97
os61514	5	410	0.99	6	3	5	6.62	-0.09	0.24	1.53	0.05	71.16	0.27	7.85	7.22	16.04	7.54	-1.63	12.09	21.68	11.34	1.11
os61515	5	418	0.85	2	3	6	6.77	-0.02	0.16	1.53	0.03	79.2	1.38	8.8	7.2	16.01	7.52	-1.67	12.05	21.64	11.32	1.08
os61523	5	447	0.08	6	3	5	6.87	0.09	0.48	1.53	0.05	94.67	-0.49	7.33	7.01	15.84	7.38	-1.83	11.85	21.43	11.17	0.92
os61524	5	454	0.01	5	3	5	6.89	0.08	0.42	1.52	0.04	94.93	0.59	6.72	7	15.83	7.37	-1.84	11.84	21.42	11.15	0.91
os61525	5	456	0	7	3	5	6.92	0.11	0.67	1.52	0.05	95.76	-0.7	6.67	6.99	15.82	7.36	-1.84	11.83	21.41	11.15	0.91
os61530	5	447	0	7	2	3	6.88	0.1	0.98	1.54	0.09	96.45	-4.37	7.18	6.93	15.75	7.3	-1.89	11.75	21.32	11.07	0.84
os61540	5	439	0.01	7	3	5	6.94	0.13	0.45	1.52	0.08	99.16	-2.07	6.95	7.12	15.94	7.46	-1.72	11.98	21.59	11.27	1.02
os61544	5	405	0.79	2	3	6	6.74	-0.03	0.41	1.54	0.02	75.79	0.56	8.58	7.38	16.19	7.67	-1.55	12.27	21.91	11.53	1.18

os61561	7	413	0.76	3	2	0	6.78	-0.01	0.61	1.54	0.08	90.46	0.34	7.29	7.22	16.07	7.55	-1.58	11.94	21.61	11.24	1.12
os61566	7	425	0.57	10	2	0	6.82	0.01	0.03	1.5	0.09	96.71	-0.43	5.93	7.13	15.96	7.48	-1.63	11.85	21.51	11.17	1.05
os61586	7	406	0.95	5	2	3	6.72	-0.04	1.66	1.54	0.08	91.98	-2.87	7.65	7.31	16.08	7.58	-1.6	12.28	21.88	11.49	1.15
os61587	7	409	0.12	5	2	3	6.58	-0.09	1.2	1.54	0.1	95.53	-4.35	6.91	7.3	16.08	7.58	-1.6	12.27	21.87	11.48	1.14
os61605	4	413	0.76	5	2	7	6.8	0.02	1.48	1.54	0.11	91.4	-3.96	7.12	7.22	16.03	7.53	-1.64	12.15	21.78	11.38	1.1
os61682	4	378	0.99	17	3	5	5.99	-0.28	0	1.42	0.11	87.64	0.02	5.48	7.55	16.33	7.8	-1.39	12.48	22.12	11.67	1.35
os61683	4	381	1	15	1	4	6.17	-0.24	0.01	1.42	0.08	89.37	1.25	5.59	7.54	16.32	7.79	-1.4	12.47	22.11	11.66	1.35
os61752	4	336	0.23	12	2	7	6.42	0.07	0.77	1.53	0.25	85.74	-5.45	6.79	7.58	16.4	7.88	-1.24	12.4	22.07	11.66	1.47
os61767	5	418	0.75	3	3	6	6.67	-0.05	1.63	1.55	0.03	47.54	0.24	8.23	7.36	16.22	7.65	-1.47	12.16	21.84	11.37	1.26
os61768	4	436	0.6	16	3	5	6.22	-0.07	0	1.45	0.14	89.99	-2.97	6.99	7.17	16	7.52	-1.57	11.82	21.49	11.09	1.12
os61784	7	424	0.01	4	2	3	6.87	0.07	1.36	1.54	0.11	90.7	-3.83	7.72	7.21	16.01	7.56	-1.52	11.85	21.53	11.15	1.11
os61813	5	418	0.92	1	3	6	6.79	0	2.19	1.55	0.02	50.45	0.22	8.88	7.38	16.25	7.68	-1.45	12.23	21.92	11.41	1.29
os61814	5	411	0.78	2	3	6	6.78	-0.01	3.71	1.56	0.03	59.25	-0.69	9.18	7.38	16.26	7.69	-1.44	12.22	21.9	11.4	1.29
os61818	5	409	0	2	3	6	6.84	0.04	3.96	1.54	0.03	53.83	-0.27	8.56	7.39	16.26	7.7	-1.43	12.21	21.9	11.4	1.3
os61824	5	445	0.54	2	3	6	6.77	0	2.01	1.55	0.02	36.96	-0.09	8.81	7.32	16.17	7.58	-1.51	12.24	21.93	11.54	1.26
os61839	5	457	0.86	2	3	6	6.78	-0.02	3.38	1.55	0.02	41.19	0.48	9.09	7.19	16.07	7.51	-1.59	12.04	21.8	11.38	1.15
os61887	4	453	0.7	4	2	7	6.72	-0.02	2.72	1.56	0.13	85.13	-6.64	8	7.13	15.96	7.46	-1.66	11.83	21.51	11.21	1
os62022	4	378	0.91	11	2	0	6.49	-0.11	0.03	1.47	0.15	94.19	-0.88	6.82	7.34	16.22	7.65	-1.4	11.9	21.61	11.29	1.24
os62039	4	414	0.01	2	3	6	6.75	-0.03	0.38	1.51	0.03	96.18	1.17	7.18	7.55	16.42	7.76	-1.36	12.28	21.92	11.59	1.38
os62071	7	442	0.77	4	2	3	6.79	-0.02	1.65	1.54	0.08	90.2	-3.16	7.79	7.28	16.11	7.55	-1.54	11.9	21.53	11.21	1.14
os62103	5	466	0.92	0	3	6	6.81	0	3.76	1.54	0.01	54.73	0.26	11.71	7.15	16.02	7.46	-1.61	11.96	21.72	11.27	1.12
os62125	7	393	0.89	5	2	7	6.71	-0.04	1.51	1.55	0.12	73.8	-4	8.28	7.45	16.29	7.75	-1.3	12.35	22.05	11.58	1.43
os62202	7	383	0.77	4	2	7	6.69	-0.05	1.9	1.55	0.11	93.03	-4.81	7.61	7.47	16.32	7.74	-1.35	12.12	21.8	11.46	1.33
os62246	4	353	0.92	10	2	3	6.49	-0.06	0.54	1.52	0.18	89.23	-7.97	7.97	7.67	16.51	7.89	-1.27	12.47	22.11	11.76	1.48
os62273	4	517	0.15	11	2	3	6.78	0.08	0.4	1.5	0.09	92.17	-2.38	8.75	6.86	15.67	7.23	-1.79	11.57	21.23	10.92	0.85
os62310	5	452	0.92	0	3	6	6.8	0	3.44	1.56	0.02	24.62	0.02	10.89	7.23	16.12	7.52	-1.57	12.09	21.84	11.43	1.17
os62313	5	453	0.08	1	3	6	6.83	0.01	2.94	1.57	0.03	30.6	-0.49	9	7.22	16.11	7.51	-1.58	12.07	21.82	11.41	1.16
os62329	4	455	0.44	5	2	7	6.63	-0.04	1.74	1.56	0.15	82.79	-8.39	7.29	7.12	15.94	7.44	-1.67	11.81	21.49	11.19	0.99
os62389	4	450	0.62	8	2	3	6.46	-0.07	0.6	1.49	0.16	99.46	-7.11	7.55	7.26	16.1	7.51	-1.55	11.91	21.52	11.17	1.14
os62435	7	429	0.99	7	2	3	6.45	-0.09	1.22	1.53	0.11	99.07	-3.71	7.04	7.21	16.09	7.53	-1.51	11.9	21.64	11.28	1.15
os62442	7	383	0.08	2	3	6	6.82	0.03	3.83	1.56	0.03	74.03	-1.08	9.19	7.5	16.38	7.75	-1.33	12.23	21.96	11.55	1.37
os62444	7	383	0.08	1	3	6	6.81	0.02	3.87	1.56	0.03	75.69	-0.44	9.23	7.51	16.38	7.75	-1.33	12.25	21.98	11.56	1.38
os62448	7	391	0.73	4	2	3	6.65	-0.06	1.69	1.56	0.08	82.11	-3.06	7.78	7.46	16.35	7.75	-1.33	12.2	21.93	11.54	1.36
os62451	7	402	0.72	5	3	5	6.6	-0.09	1.42	1.52	0.05	73.15	0.73	8.96	7.43	16.31	7.7	-1.44	12.27	22	11.59	1.27
os62453	5	401	0.04	4	3	6	6.64	-0.07	0.57	1.54	0.03	70.67	0.15	7.48	7.45	16.34	7.73	-1.42	12.31	22.03	11.62	1.3
os62459	4	410	0.99	4	2	3	6.71	-0.05	1.67	1.55	0.06	86.89	-2.3	7.77	7.37	16.23	7.65	-1.45	12.12	21.84	11.47	1.23
os62460	5	412	0.19	3	3	6	6.86	0.03	1.82	1.55	0.06	86.45	-1.33	7.92	7.37	16.23	7.64	-1.46	12.12	21.84	11.47	1.23

os62464	4	444	0.13	6	2	7	6.57	-0.09	1.08	1.54	0.13	99.93	-4.47	6.51	7.1	16	7.44	-1.61	11.85	21.59	11.24	1.06
os62491	4	372	0.78	9	3	5	6.44	-0.12	0.56	1.51	0.09	98.92	-2.56	6.96	7.55	16.4	7.8	-1.44	12.5	22.14	11.74	1.33
os62507	4	464	0.13	5	2	3	6.78	0.02	1.68	1.52	0.06	99.11	-2.94	8.22	7.14	15.97	7.45	-1.69	11.86	21.46	11.22	1.05
os62508	4	453	0.09	6	2	3	6.81	0.06	1.07	1.52	0.06	99.84	-2.57	8.32	7.15	15.98	7.47	-1.68	11.88	21.48	11.24	1.06
os62519	7	422	0	4	3	6	6.88	0.06	0.63	1.54	0.03	94.69	0.3	7.83	7.4	16.24	7.67	-1.53	12.28	21.88	11.57	1.26
os62539	7	372	0.92	12	2	3	6.18	-0.2	0.14	1.51	0.14	98.83	-4.9	5.63	7.56	16.4	7.79	-1.33	12.27	21.94	11.52	1.39
os62551	4	437	0.99	8	3	5	6.57	-0.11	0.66	1.49	0.06	98.64	-0.1	7.52	7.32	16.17	7.62	-1.47	11.95	21.64	11.28	1.22
os62556	4	377	0.99	8	3	5	6.49	-0.12	0.3	1.53	0.06	75.49	-1.24	7.44	7.57	16.44	7.81	-1.29	12.32	22.04	11.62	1.39
os62600	4	357	0.71	14	3	5	6.17	-0.21	0	1.47	0.11	96.12	-0.61	6.52	7.69	16.52	7.91	-1.23	12.38	22.05	11.65	1.5
os62656	4	309	0.73	15	2	7	6.07	-0.2	0	1.46	0.16	75.24	-2.88	5.44	7.79	16.64	8.01	-1.13	12.39	22.01	11.62	1.55
os62678	7	391	0.91	7	3	5	6.65	-0.08	0.29	1.52	0.07	100	-0.92	7.71	7.52	16.4	7.79	-1.31	12.2	21.86	11.53	1.4
os62683	4	418	0.4	4	2	7	6.81	0.03	1.14	1.55	0.13	97.13	-5.62	7.49	7.31	16.17	7.59	-1.47	11.89	21.59	11.28	1.18
os62698	7	450	0.99	3	2	3	6.77	-0.02	1.89	1.55	0.07	86.33	-3.72	8.56	7.25	16.09	7.53	-1.55	11.87	21.5	11.18	1.12
os62713	4	444	0.93	6	2	3	6.75	-0.04	0.97	1.51	0.08	96.27	-2.42	7.52	7.3	16.13	7.56	-1.52	11.92	21.55	11.23	1.14
os62746	4	397	1	16	2	0	6.13	-0.25	0	1.47	0.14	74.13	-1.57	6.9	7.45	16.31	7.65	-1.44	12.1	21.76	11.41	1.23
os62800	7	394	0.2	3	3	6	6.71	-0.04	2.41	1.55	0.05	76.27	-0.64	8.49	7.46	16.34	7.73	-1.48	12.39	22.1	11.66	1.24
os62846	7	596	0.89	5	3	6	6.78	-0.06	0.56	1.54	0.05	80.38	-0.24	7.55	6.35	15.15	6.78	-2.28	11.03	20.57	10.42	0.34
os62895	5	604	0.08	3	3	6	6.75	-0.06	0.74	1.53	0.05	92.84	0.01	6.69	6.32	15.12	6.76	-2.3	10.99	20.54	10.39	0.32
os62898	7	598	0.85	5	3	6	6.77	-0.06	0.63	1.53	0.05	84.84	0.13	7.57	6.34	15.14	6.78	-2.29	11.02	20.56	10.41	0.33
os62911	4	546	0.92	2	2	3	6.79	-0.02	2.95	1.56	0.06	81.96	-2.17	8.95	6.64	15.47	7.05	-1.95	11.29	20.94	10.69	0.64
os62912	4	553	0.92	2	2	3	6.78	-0.01	2.3	1.56	0.08	79.58	-4.06	8.96	6.6	15.44	7.02	-1.98	11.25	20.9	10.65	0.61
os62935	4	519	0.64	9	3	5	6.49	-0.09	0.15	1.49	0.06	97.78	-0.45	7.65	6.8	15.58	7.27	-1.74	11.46	21.12	10.88	0.91
os62954	7	406	0.77	2	3	6	6.75	-0.02	2.36	1.54	0.04	79.54	-1.3	8.41	7.65	16.48	7.98	-1.01	12.45	22.05	11.74	1.75
os62960	5	405	0.23	2	3	6	6.81	0.03	2.48	1.54	0.05	78.07	-0.13	7.41	7.56	16.36	7.89	-1.13	12.32	21.96	11.67	1.64
os62963	7	425	0.01	5	3	5	6.61	-0.08	1.29	1.54	0.07	92.83	-1.54	8.05	7.44	16.23	7.8	-1.2	12.15	21.79	11.52	1.51
os62982	4	474	0.77	4	2	3	6.77	-0.03	1.75	1.55	0.06	88.13	-2.25	8.79	7.09	15.87	7.51	-1.49	11.76	21.42	11.14	1.17
os62983	7	488	0.23	9	2	3	6.75	0.05	0.5	1.53	0.07	75.48	-1.88	7.9	6.97	15.77	7.4	-1.61	11.61	21.27	11.03	1.05
os63015	5	390	0.91	4	2	3	6.66	-0.04	2.26	1.56	0.05	56.69	-2.45	9.43	7.61	16.43	7.92	-1.14	12.51	22.24	11.78	1.58
os63082	5	600	0.92	3	3	6	6.76	-0.05	1.41	1.54	0.03	81.5	-0.47	7.26	6.4	15.22	6.84	-2.21	11.1	20.71	10.52	0.45
os63115	4	598	0.04	16	3	5	7.08	0.26	0	1.42	0.1	95.6	0.31	5.95	6.39	15.18	6.8	-2.22	11.03	20.61	10.46	0.4
os63147	7	574	0.67	12	2	7	6.38	-0.1	0.05	1.48	0.12	93.31	-2.36	6.67	6.47	15.28	6.85	-2.18	11.09	20.69	10.48	0.44
os63189	7	508	0.1	8	2	3	6.76	0.06	0.86	1.51	0.13	99.56	-6.09	7.64	6.96	15.81	7.26	-1.78	11.62	21.25	10.95	0.9
os63254	5	519	0.65	3	3	6	6.86	0	0.77	1.52	0.02	85.22	0.08	7.28	7.05	15.87	7.55	-1.35	11.47	21.09	10.96	1.24
os63358	6	507	0.45	2	3	6	6.74	-0.04	1.77	1.52	0.02	88.83	-0.45	8.02	7.14	15.97	7.64	-1.31	11.64	21.3	11.1	1.3
os63375	5	372	0.92	2	3	6	6.73	-0.01	2.92	1.55	0.03	65.63	-0.83	8.62	7.76	16.57	8.13	-0.88	12.32	21.95	11.75	1.76
os63408	7	298	0.6	12	2	7	6.16	-0.08	0.41	1.55	0.18	79.37	-6.14	6.76	8.13	16.93	8.41	-0.63	12.97	22.53	12.26	2.15
os63439	4	419	0.9	4	3	6	6.64	-0.06	1.44	1.55	0.04	86.46	-1.17	8.98	7.46	16.27	7.86	-1.11	12.06	21.67	11.49	1.58

os63448	7	499	0.4	5	3	6	6.75	0	1.64	1.52	0.07	99.81	-1.92	7.86	7.05	15.88	7.54	-1.42	11.57	21.23	11.06	1.24
os63540	4	408	0.18	11	3	5	7	0.16	0.02	1.48	0.08	98.37	0.23	5.81	7.73	16.63	7.84	-1.27	12.54	22.16	11.68	1.47
os63895	5	527	0.6	2	3	6	6.79	-0.01	2.69	1.53	0.03	88.34	0.2	8.25	6.95	15.88	7.26	-1.79	11.59	21.25	10.96	0.95
os63923	7	492	0.08	5	3	6	6.94	0.07	1.58	1.53	0.05	95.86	-1.32	8.5	7.07	15.94	7.36	-1.72	11.7	21.34	11.04	1.01
os63925	7	455	0.24	9	2	0	6.72	0.05	0.07	1.51	0.08	86.95	-0.38	7.65	7.19	16.06	7.45	-1.65	11.85	21.47	11.14	1.09
os63950	4	410	0.11	4	2	7	6.84	0.06	1.88	1.56	0.12	87.86	-5.87	8.59	7.37	16.27	7.62	-1.5	12.07	21.72	11.41	1.21
os64043	4	394	0.16	9	3	5	6.97	0.11	0.35	1.51	0.08	94.28	-1.45	7.52	7.7	16.61	7.82	-1.29	12.52	22.13	11.68	1.44
os64085	4	405	0.03	8	3	5	6.97	0.11	0.24	1.49	0.06	98.55	0.44	6.66	7.76	16.66	7.86	-1.25	12.58	22.19	11.71	1.49
os64124	5	477	0.77	2	3	6	6.8	-0.02	0.53	1.53	0.03	86.43	0.02	7.06	7.33	16.26	7.56	-1.46	11.95	21.62	11.21	1.18
os64184	7	366	0.85	11	3	5	6.3	-0.15	0.02	1.48	0.07	97.18	-0.03	6.85	7.59	16.48	7.85	-1.23	12.15	21.87	11.5	1.47
os64200	4	394	0.87	10	2	3	6.63	-0.09	0.6	1.51	0.09	99.46	-3.59	7.48	7.51	16.39	7.78	-1.29	12.07	21.77	11.42	1.41
os64247	4	322	0	7	2	3	6.9	0.12	0.88	1.52	0.08	92.07	-3.04	6.64	7.86	16.73	8.07	-1.07	12.53	22.18	11.81	1.67
os64383	7	366	0.68	3	3	6	6.82	0.02	1.52	1.51	0.05	97.73	-1.22	7.5	7.51	16.41	7.77	-1.37	12.2	21.87	11.49	1.36
os64399	4	355	0.49	14	2	3	6.39	-0.01	0.25	1.49	0.12	95.84	-4.5	7.79	7.64	16.44	7.88	-1.33	12.4	22	11.66	1.38
os64456	4	364	0.91	7	3	5	6.45	-0.11	0.37	1.48	0.08	94.5	-1.21	7.05	7.62	16.42	7.88	-1.33	12.37	21.96	11.62	1.38
os64577	4	431	0.19	9	2	3	6.93	0.11	0.74	1.5	0.14	99.86	-6.49	7.01	7.02	15.87	7.39	-1.7	11.73	21.27	11.13	1
os64593	7	439	0.35	13	2	7	6.46	0.02	0.01	1.47	0.17	81.9	-3.51	6.27	6.79	15.59	7.18	-1.99	11.55	21.07	10.95	0.75
os64596	7	444	0.07	19	2	7	6.78	0.29	0	1.48	0.21	92.24	-6.88	7.56	6.69	15.51	7.09	-2.04	11.43	20.97	10.87	0.69
os64649	7	484	0.76	11	2	7	6.34	-0.13	0.31	1.51	0.15	94.19	-5.55	7.77	6.6	15.45	7.04	-2.08	11.3	20.82	10.74	0.62
os64659	7	483	0.39	6	2	3	6.88	0.03	1.19	1.52	0.11	99.76	-5.19	7.43	6.73	15.59	7.14	-1.93	11.4	20.96	10.84	0.76
os64758	5	466	0.92	0	3	6	6.81	0	4.09	1.54	0.01	56.68	0.36	11.22	7.15	16.02	7.46	-1.61	11.95	21.71	11.26	1.12
os64760	4	498	0.27	11	2	3	6.63	0.05	0.03	1.49	0.1	99.51	-2.6	5.94	7.01	15.87	7.34	-1.69	11.64	21.28	11.04	0.98
os64761	5	404	0.27	6	2	3	6.59	-0.09	1.19	1.54	0.05	71.73	-1.78	7.89	7.39	16.28	7.69	-1.35	12.07	21.82	11.44	1.31
os64776	7	344	0.19	2	3	6	6.69	-0.03	3.82	1.56	0.04	85.14	-1.43	8.8	7.82	16.67	7.99	-1.2	12.68	22.31	11.92	1.55
os64801	4	450	0	8	3	5	6.93	0.13	0.48	1.53	0.09	97.73	-2.5	7.21	7.3	16.21	7.53	-1.56	11.98	21.64	11.28	1.17
os64820	7	336	0.23	6	2	7	6.77	0.05	0.92	1.54	0.2	99.05	-8.99	6.56	7.45	16.28	7.73	-1.38	12.12	21.7	11.43	1.35
os64910	5	305	0.61	2	3	6	6.76	0	0.77	1.53	0.01	82.1	-0.04	7.91	7.91	16.8	8.31	-0.74	12.72	22.39	12.32	2.11
os64961	5	259	0.02	3	2	3	6.76	0.04	2.1	1.55	0.05	83.76	-2.41	8.57	8.09	16.93	8.46	-0.72	12.81	22.45	12.43	2.22
os65069	5	249	0.62	2	3	6	6.73	0	1.76	1.54	0.01	49.57	0.02	8.46	8.36	17.18	8.65	-0.58	13.21	22.77	12.65	2.4
os65082	5	255	0.92	3	2	3	6.62	-0.03	1.9	1.54	0.06	92.23	-3.24	8.11	8.21	17.06	8.54	-0.69	13.01	22.65	12.55	2.28
os65095	5	255	0.93	6	2	3	6.58	-0.06	1.77	1.55	0.1	92.78	-5.47	8.07	8.13	17	8.48	-0.68	12.93	22.58	12.48	2.24
os65096	5	251	0.99	6	2	3	6.58	-0.07	2.07	1.53	0.08	91.99	-3.67	7.64	8.14	17.01	8.49	-0.68	12.94	22.59	12.49	2.25
os65118	7	246	0.39	3	2	3	6.63	-0.04	2.77	1.55	0.09	92.2	-4.87	8.36	8.29	17.17	8.6	-0.62	13.14	22.76	12.63	2.35
os65119	7	246	0.86	7	2	3	6.47	-0.1	0.87	1.54	0.08	92.33	-3.97	8.12	8.3	17.17	8.6	-0.62	13.15	22.76	12.63	2.35
os65149	7	325	0.63	10	2	3	6.36	-0.09	0.97	1.54	0.12	59.13	-4.59	7.09	7.4	16.18	7.98	-0.74	12.16	21.59	11.82	2.11
os65164	5	372	0	4	3	6	6.64	-0.06	0.37	1.53	0	76.18	1.05	7.5	7.15	15.92	7.8	-0.96	11.62	20.95	11.36	1.79
os65198	5	363	0.75	6	3	5	6.57	-0.06	0.63	1.54	0	76.67	-0.41	8.22	7.1	15.87	7.75	-0.99	11.55	20.89	11.31	1.75

os65206	5	230	0.02	12	3	5	6.92	0.19	0.01	1.47	0.05	63.15	1.07	7.49	8.28	17.09	8.59	-0.6	13.07	22.66	12.59	2.34
os65251	4	485	0.96	7	3	5	6.61	-0.11	0.24	1.51	0	95.13	1.16	6.55	7.5	16.58	7.57	-1.51	12.23	22.05	11.37	1.34
os65252	4	478	1	9	3	5	6.5	-0.14	0.13	1.51	0	95.37	0.11	6.5	7.5	16.58	7.57	-1.5	12.23	22.04	11.37	1.34
os65350	4	317	0.96	26	2	0	5.45	-0.37	0	1.41	0.22	82.59	-3	5.07	8.56	17.72	8.53	-0.65	13.22	23.14	12.27	2.1
os65394	5	394	0.36	2	3	6	6.82	0.01	2.88	1.55	0.02	54.63	-0.79	8.89	8.4	17.59	8.39	-0.86	13.17	23.15	12.2	1.95
os65663	7	459	0.56	4	3	6	6.74	0.01	0.28	1.54	0.07	51.23	-1.78	7.93	7.7	16.84	7.8	-1.32	12.46	22.39	11.61	1.48
os65664	7	459	0.17	5	2	3	6.76	0.03	0.46	1.54	0.07	51.15	-2.03	7.76	7.71	16.85	7.81	-1.32	12.46	22.39	11.61	1.48
os65829	5	534	0.96	6	3	5	6.66	-0.1	0.31	1.51	0.05	84.87	0.17	7.51	7.25	16.34	7.46	-1.74	11.91	21.84	11.21	1.02
os65871	4	518	0.42	7	3	5	6.68	-0.01	0.68	1.51	0.06	90.03	-1.17	7.22	7.19	16.24	7.39	-1.71	11.95	21.87	11.17	1.02
os65977	7	494	0.95	8	2	7	6.64	-0.1	0.63	1.53	0.11	97.64	-3.89	7.13	7.22	16.27	7.45	-1.72	11.89	21.82	11.23	1.01
os65986	5	536	0.92	0	3	6	6.83	0	2.56	1.55	0.01	15.25	0.08	9.37	7.05	16.03	7.29	-1.83	11.68	21.59	11.08	0.89
os66077	7	487	0.3	4	2	3	6.76	0.01	1.9	1.55	0.08	79.52	-2.45	7.8	7.38	16.43	7.54	-1.6	12.12	22.05	11.3	1.17
os66082	7	518	0.32	7	3	5	6.73	0.01	0.34	1.52	0.06	90.09	-0.04	7.91	7.21	16.27	7.38	-1.7	11.89	21.82	11.1	1.05
os66150	7	465	0.92	1	2	7	6.75	0	2.55	1.56	0.14	93.95	-6.02	7.19	7.31	16.34	7.47	-1.66	12.08	21.99	11.3	1.09
os66275	4	327	0.34	13	2	0	6.41	0.04	0	1.47	0.23	94.3	-0.56	5.74	7.87	16.81	7.96	-1.19	12.46	22.17	11.59	1.51
os66320	7	309	0.91	2	2	0	6.7	-0.02	2.38	1.56	0.16	46.22	-0.67	8.16	8.1	17.01	8.12	-1.11	12.71	22.41	11.79	1.57
os66420	4	430	0.99	7	3	9	6.56	-0.11	0.73	1.45	0.07	96.78	-0.91	7.31	7.97	16.93	7.99	-1.24	12.67	22.43	11.79	1.48
os66427	7	423	0.39	4	3	6	6.84	0.01	1.51	1.49	0.05	95.31	-0.49	7.66	7.78	16.78	7.87	-1.35	12.5	22.31	11.67	1.37
os66445	4	359	0.88	30	1	4	5.8	-0.27	0	1.29	0.19	96	3.29	4.61	8.16	17.11	8.16	-1.03	12.83	22.55	11.9	1.66
os66493	3	450	0.47	29	1	4	6.57	0.11	0	1.27	0.14	97.48	11.68	4.5	7.75	16.77	7.84	-1.38	12.43	22.27	11.63	1.32
os66563	4	468	0.49	21	3	9	6.01	-0.06	0	1.35	0.14	99.18	2.54	5.7	7.61	16.55	7.76	-1.36	12.15	21.87	11.34	1.35
os66564	4	310	0.09	8	2	0	6.76	0.1	0.25	1.53	0.13	81.84	-1.93	7.54	8.21	17.15	8.23	-0.97	12.84	22.54	11.88	1.74
os66631	4	374	0.21	12	2	3	6.83	0.13	0.2	1.49	0.15	99.95	-7.46	7.43	8.08	17.08	8.14	-1.12	12.83	22.51	11.89	1.62
os66644	4	398	0.28	18	2	0	6.92	0.19	0	1.45	0.14	98.74	-1.74	6.79	7.95	16.97	8.02	-1.2	12.51	22.28	11.65	1.5
os66646	3	475	0.03	12	1	1	6.92	0.17	0.01	1.45	0.05	95.91	3	5.5	7.82	16.86	7.92	-1.26	12.36	22.15	11.56	1.42
os66716	7	452	0.55	15	2	3	6.18	-0.15	0.08	1.47	0.14	95.12	-3.9	7.45	7.62	16.66	7.73	-1.46	12.28	22.09	11.47	1.26
os66739	4	455	0.99	11	2	3	6.43	-0.16	0.53	1.49	0.11	86.07	-4.23	7.7	7.5	16.48	7.69	-1.42	12.14	21.89	11.34	1.3
os66744	4	439	1	10	3	5	6.52	-0.13	0.51	1.48	0.08	98.9	-2.85	7.86	7.6	16.58	7.75	-1.38	12.31	22.01	11.46	1.35
os66745	4	441	0.99	9	3	5	6.55	-0.12	0.6	1.49	0.08	98.84	-2.63	7.89	7.6	16.58	7.75	-1.38	12.3	22.01	11.46	1.35
os66746	4	441	0.99	8	3	5	6.61	-0.1	0.74	1.49	0.08	98.76	-2.75	8	7.59	16.58	7.75	-1.39	12.3	22.01	11.46	1.35
os66811	4	492	0.27	17	2	7	6.49	0.09	0	1.45	0.15	98.41	-3.62	7.31	6.95	15.84	7.26	-1.81	11.53	21.17	10.87	0.85
os66866	5	517	0.92	1	3	6	6.83	0	2.98	1.55	0.04	90.59	-0.84	9.2	7.35	16.46	7.54	-1.68	11.99	21.93	11.25	1.07
os66982	7	423	0.77	4	2	7	6.76	-0.02	1.34	1.54	0.1	76.39	-2.51	7.93	7.91	17.01	7.99	-1.21	12.57	22.4	11.69	1.53
os67175	4	326	0.02	11	2	7	6.94	0.18	0.26	1.51	0.13	95.14	-4.75	6.66	8.61	17.7	8.52	-0.77	13.4	23.06	12.28	2.05
os67231	4	415	0.03	16	3	5	6.95	0.16	0	1.43	0.14	90.53	-1.13	7.62	7.96	17.06	7.99	-1.32	12.74	22.69	11.84	1.48
os67374	5	438	0.02	13	2	7	6.97	0.21	0.23	1.51	0.14	93.64	-3.65	7.85	7.68	16.78	7.77	-1.49	12.39	22.35	11.56	1.28
os67393	5	429	0.37	2	3	6	6.84	0.03	3.92	1.55	0.03	37.55	0.02	8.82	7.93	17.05	8.01	-1.23	12.67	22.67	11.83	1.57

os67478	4	418	0.07	3	3	6	6.87	0.05	2.8	1.54	0.07	93.09	-1.85	8.7	8.18	17.26	8.2	-0.96	12.81	22.6	11.86	1.79
os67479	4	418	0.92	1	3	6	6.8	0.01	2.92	1.54	0.06	92.7	-1.62	8.98	8.18	17.26	8.2	-0.97	12.8	22.6	11.86	1.79
os67480	4	417	0.02	3	2	3	6.87	0.05	2.79	1.54	0.07	93.19	-2.08	8.66	8.18	17.26	8.2	-0.97	12.81	22.6	11.86	1.79
os67481	4	418	0.76	1	3	6	6.83	0.02	2.76	1.54	0.06	92.8	-1.89	8.87	8.18	17.26	8.2	-0.97	12.8	22.6	11.85	1.79
os67482	4	418	0.53	2	3	6	6.85	0.03	2.48	1.54	0.06	92.84	-1.98	8.79	8.18	17.26	8.2	-0.97	12.8	22.6	11.85	1.79
os67483	4	419	0.3	3	3	6	6.87	0.04	2.19	1.54	0.06	92.93	-1.8	8.69	8.18	17.26	8.19	-0.97	12.8	22.6	11.85	1.79
os67504	4	390	0.06	8	2	3	6.92	0.1	0.83	1.53	0.09	97.76	-3.57	8.18	8.32	17.42	8.32	-0.9	12.96	22.79	12.01	1.87
os67505	4	389	0.05	7	2	3	6.91	0.09	0.89	1.53	0.09	97.73	-3.45	8.12	8.32	17.42	8.32	-0.9	12.96	22.79	12.01	1.87
os67506	4	388	0.03	6	2	3	6.9	0.09	1.01	1.53	0.09	97.71	-3.4	8.1	8.32	17.43	8.32	-0.9	12.96	22.8	12.02	1.87
os67507	4	388	0.03	7	2	3	6.9	0.08	1.26	1.53	0.09	97.69	-3.59	8.16	8.32	17.43	8.32	-0.9	12.96	22.8	12.02	1.87
os67508	4	378	0.89	7	2	7	6.62	-0.08	1.37	1.54	0.11	94.92	-3.69	7.1	8.34	17.45	8.34	-0.89	12.99	22.82	12.03	1.88
os67509	4	378	0.97	8	2	7	6.61	-0.09	1.21	1.53	0.1	95	-3.15	7.13	8.34	17.45	8.34	-0.89	12.99	22.82	12.04	1.88
os67513	4	384	0.98	17	1	4	6.02	-0.27	0	1.43	0.13	94.12	-0.43	6.62	8.25	17.37	8.27	-0.94	12.9	22.76	11.96	1.79
os67578	4	265	0.99	6	3	5	6.53	-0.1	0.28	1.53	0.08	91.07	-1.78	7.52	9.16	18.28	8.97	-0.4	14.06	23.82	12.87	2.44
os67624	4	308	0.58	7	3	5	6.81	0.02	0.52	1.51	0.06	89.04	-0.39	7.03	8.79	17.84	8.65	-0.67	13.6	23.24	12.43	2.13
os67635	4	393	0	8	2	3	6.95	0.14	0.64	1.5	0.08	95.4	-3.34	8.38	8.33	17.41	8.3	-0.89	13	22.66	11.95	1.87
os67643	7	247	0.73	3	2	7	6.59	-0.03	1.81	1.55	0.15	83.76	-4.13	7.45	9.06	18.15	8.87	-0.56	14.01	23.71	12.81	2.28
os67646	7	280	1	8	2	3	6.45	-0.13	0.62	1.52	0.09	95.29	-3.27	7.03	9.06	18.2	8.86	-0.57	14.07	23.81	12.83	2.3
os67693	5	491	0.08	3	3	6	6.91	0.04	1.38	1.54	0.02	83.04	-0.06	8.63	7.44	16.47	7.59	-1.61	12.14	22.01	11.39	1.11
os67822	7	493	0.89	6	2	3	6.73	-0.04	1.44	1.54	0.11	89.44	-5.65	7.28	7.16	16.07	7.38	-1.86	11.96	21.7	11.2	0.88
os67874	7	585	0.01	6	3	5	6.93	0.09	0.55	1.49	0.06	95.32	-1.13	8.2	6.67	15.62	7	-2.13	11.31	21.08	10.72	0.57
os67908	4	543	0.92	3	3	9	6.71	-0.06	0.24	1.46	0.03	97.52	1.57	6.66	7.15	16.12	7.34	-1.87	11.89	21.65	11.17	0.84
os67929	5	525	0.39	3	3	6	6.9	0.02	3.41	1.55	0.04	73.2	-0.57	8.71	7.09	16.02	7.3	-1.85	11.78	21.48	11.06	0.87
os68097	7	460	0.92	0	3	6	6.78	-0.01	4.96	1.57	0.04	51.18	-1.12	8.91	7.57	16.66	7.65	-1.48	12.32	22.19	11.5	1.36
os68128	5	490	0.31	5	3	6	6.72	-0.01	0.54	1.52	0.03	72.69	0.39	8.28	7.44	16.48	7.49	-1.6	12.18	22.04	11.36	1.16
os68145	4	502	0.97	7	2	7	6.69	-0.08	0.87	1.54	0.11	89.53	-3.51	8.09	7.35	16.42	7.5	-1.58	11.93	21.81	11.18	1.16
os68153	4	486	0.81	9	3	5	6.52	-0.08	0.2	1.48	0.06	94.77	0.48	6	7.54	16.63	7.66	-1.5	12.3	22.18	11.46	1.31
os68243	4	431	1	5	2	3	6.68	-0.07	1.27	1.52	0.05	94.79	-1.9	8.33	7.91	17.01	7.94	-1.17	12.61	22.43	11.77	1.74
os68298	7	358	0.48	6	2	7	6.83	0.08	0.59	1.54	0.16	81.73	-4.26	7.55	8.05	17.11	8.04	-1.09	12.8	22.53	11.87	1.82
os68369	4	426	0.98	10	3	5	6.51	-0.14	0.48	1.51	0.08	98.29	-2.02	7.84	8.08	17.23	8.12	-1.12	12.84	22.7	11.94	1.78
os68402	4	426	0.39	3	2	3	6.7	-0.04	2.46	1.54	0.07	81.62	-3.75	8.35	7.94	17.02	7.92	-1.28	12.78	22.57	11.82	1.65
os68463	4	318	0.92	4	3	6	6.61	-0.05	2.49	1.54	0.05	77.04	-1.31	8.88	9.14	18.34	8.91	-0.43	14.07	23.92	12.82	2.48
os68642	4	336	0.3	15	3	5	6.44	0.06	0	1.45	0.09	94.23	0.39	6.05	8.97	18.22	8.81	-0.57	13.93	23.91	12.8	2.36
os68645	4	372	0.23	4	2	0	6.71	0.01	1.45	1.53	0.09	79.95	-1.7	7.33	8.45	17.67	8.41	-0.84	13.25	23.25	12.27	2
os68725	4	313	0.92	13	2	7	6.42	-0.15	0.43	1.5	0.15	99.4	-5.34	7.52	8.74	17.84	8.62	-0.66	13.57	23.28	12.41	2.14
os68782	4	438	0.35	3	2	3	6.74	-0.01	2.51	1.51	0.07	84.18	-3.39	8.67	7.97	17.05	8	-1.14	12.66	22.43	11.79	1.78
os68783	4	439	0.41	4	2	3	6.71	-0.03	2.89	1.51	0.06	83.52	-3.31	8.74	7.96	17.04	8	-1.14	12.65	22.43	11.79	1.78

os68784	4	450	0.08	2	3	9	6.84	0.03	0.12	1.48	0.01	78.26	0.75	8.49	7.97	17.06	8.01	-1.14	12.67	22.44	11.8	1.78
os68817	7	192	0.92	0	3	6	6.69	0	7.54	1.57	0	2.05	0.03	12.24	8.98	17.84	8.94	-0.4	14.04	23.57	13.09	2.67
os68831	7	191	0.92	1	3	6	6.68	-0.01	5.96	1.56	0	0.6	0.27	10.06	9.07	17.94	8.99	-0.4	14.22	23.82	13.2	2.68
os68838	7	190	0.92	0	3	6	6.69	0	6.5	1.57	0	0	0	12.06	9.08	17.98	9.01	-0.39	14.25	23.88	13.21	2.68
os68898	7	199	0.92	0	3	6	6.69	0	6.96	1.57	0	0.52	0	11.59	8.94	17.82	8.93	-0.47	14.02	23.59	13.06	2.57
os68902	7	199	0.92	0	3	6	6.69	0	6.97	1.57	0	0.15	0	11.59	8.93	17.81	8.92	-0.47	14	23.57	13.06	2.57
os68903	7	199	0.92	0	3	6	6.69	0	6.96	1.57	0	0.05	-0.01	11.59	8.93	17.8	8.92	-0.47	13.99	23.55	13.04	2.57
os68919	7	194	0.92	0	3	6	6.69	0	7.31	1.57	0	0	0	12.02	8.98	17.83	8.97	-0.37	13.89	23.4	12.96	2.62
os68920	7	210	0.92	1	3	6	6.66	-0.02	4.8	1.57	0.03	32.96	-0.64	8.1	8.83	17.75	8.86	-0.57	13.9	23.61	13.02	2.42
os68922	7	209	0.77	6	3	5	6.67	-0.03	3.51	1.53	0.04	30.4	-0.53	7.33	8.82	17.74	8.86	-0.57	13.88	23.6	13.01	2.42
os68924	7	211	0.23	1	3	6	6.69	0	4.78	1.56	0.02	32.49	-0.43	8.53	8.82	17.74	8.86	-0.57	13.89	23.6	13.01	2.41
os68928	7	206	0.92	0	3	6	6.69	0	5.76	1.57	0.01	8.34	-0.01	11.59	8.92	17.82	8.93	-0.53	14.04	23.7	13.13	2.46
os68930	7	203	0.92	1	3	6	6.66	-0.02	5.51	1.57	0.02	11.08	-0.79	9.01	8.91	17.8	8.93	-0.53	14.03	23.7	13.13	2.46
os68932	7	195	0.92	0	3	6	6.69	0	7.68	1.57	0	0.11	0	11.95	8.89	17.74	8.91	-0.41	13.83	23.35	12.92	2.58
os68954	4	227	0.77	10	3	5	6.61	-0.06	0.08	1.48	0.05	73.85	1.59	6.74	8.81	17.73	8.78	-0.58	13.98	23.6	13	2.39
os68955	4	209	0.6	11	2	3	6.71	0	0.36	1.53	0.1	58.48	-4.65	7.17	8.82	17.75	8.8	-0.57	14	23.62	13.02	2.4
os68956	4	227	0.65	11	3	5	6.7	-0.01	0.03	1.48	0.05	70.3	0.71	6.99	8.81	17.73	8.79	-0.58	13.99	23.57	13	2.39
os68967	7	243	0.28	3	3	6	6.63	-0.05	1.25	1.54	0.02	69.1	0.13	8.03	8.87	17.71	8.81	-0.6	14.15	23.64	13.05	2.38
os68969	7	198	0.92	0	3	6	6.69	0	6.99	1.57	0.01	0.55	-0.2	9.52	8.97	17.83	8.96	-0.47	13.99	23.6	13.09	2.5
os68971	7	196	0.69	1	3	6	6.73	0.02	6.99	1.56	0.03	31.53	-0.78	8.58	8.97	17.83	8.97	-0.44	13.93	23.56	13.06	2.54
os68972	7	198	0.68	3	3	6	6.71	0	6.99	1.55	0.02	29.98	-0.47	8.54	8.97	17.83	8.97	-0.44	13.93	23.56	13.06	2.54
os68973	7	196	0.92	1	3	6	6.65	-0.02	7	1.55	0	21.25	0.32	8.89	8.93	17.82	8.96	-0.39	13.86	23.43	12.97	2.57
os68974	7	195	0.92	1	3	6	6.65	-0.02	7	1.56	0.01	20.64	-0.19	8.86	8.93	17.82	8.96	-0.39	13.86	23.43	12.97	2.57
os68987	7	194	0.6	1	3	6	6.69	0	6.1	1.56	0	0.14	0.42	9.55	9.05	17.9	9	-0.36	14.13	23.66	13.11	2.68
os68988	4	304	0.99	8	2	3	6.5	-0.11	1.03	1.54	0.1	95.82	-3.82	8.16	8.01	16.99	8.26	-1.11	12.96	22.69	12.34	1.81
os68989	4	307	0	4	2	0	6.83	0.07	1.46	1.54	0.07	88.85	-1.4	8.02	7.96	16.93	8.21	-1.15	12.89	22.63	12.29	1.77
os68991	7	269	0.48	1	3	6	6.74	0.01	5.87	1.57	0.01	2.92	-0.27	9.77	8.28	17.23	8.49	-0.95	13.2	22.94	12.56	2.06
os68992	7	270	0.4	1	3	6	6.72	0.01	5.86	1.56	0.01	2.44	0.12	9.51	8.28	17.23	8.48	-0.95	13.2	22.94	12.56	2.06
os68993	7	271	1	3	3	6	6.7	-0.01	5.48	1.53	0.02	11.34	0.98	9.14	8.28	17.23	8.48	-0.94	13.2	22.94	12.57	2.05
os69000	7	257	0.92	1	3	6	6.68	-0.02	5.65	1.56	0.02	1.18	0.11	9.78	8.35	17.24	8.52	-0.93	13.3	22.99	12.62	2.05
os69027	5	251	0.04	1	3	6	6.69	-0.01	2.63	1.56	0.01	8.78	-0.12	8.95	8.32	17.06	8.42	-0.77	13.2	22.59	12.32	2.07
os69102	7	214	0.92	0	3	6	6.7	0	5.79	1.57	0	0.73	0.08	11.23	8.85	17.92	8.85	-0.52	13.95	23.86	13.04	2.43
os69119	7	203	0.92	0	3	6	6.69	0	5.98	1.57	0	0.51	0	11.73	8.78	17.7	8.81	-0.6	13.86	23.44	12.98	2.37
os69120	7	203	0.92	0	3	6	6.69	0	5.98	1.57	0	0.32	0	11.73	8.78	17.7	8.81	-0.6	13.86	23.44	12.97	2.37
os69127	7	204	0.92	0	3	6	6.69	0	5.93	1.57	0.01	0	-0.02	11.82	8.79	17.69	8.81	-0.58	13.82	23.42	12.93	2.38
os69168	7	287	0.73	2	3	6	6.69	-0.01	4.72	1.56	0.02	1.58	0.11	10.09	7.98	16.85	8.21	-1.13	12.92	22.56	12.18	1.67
os69170	7	287	0.92	0	3	6	6.72	0	4.67	1.56	0.02	1.98	0.29	11.12	7.98	16.85	8.21	-1.13	12.92	22.56	12.18	1.67

os69172	7	287	0.92	0	3	6	6.74	0	4.49	1.56	0.02	2.79	0.28	10.91	7.97	16.85	8.2	-1.13	12.91	22.56	12.18	1.67
os69192	6	239	0.92	0	3	6	6.71	0	5.94	1.57	0.01	2.85	-0.15	11.08	8.46	17.25	8.55	-0.76	13.5	22.93	12.61	2.12
os69198	7	254	0.92	0	3	6	6.72	0	5.96	1.56	0.01	3.19	0	10.51	8.34	17.22	8.44	-0.84	13.35	22.97	12.47	1.97
os69199	7	254	0.92	0	3	6	6.73	0.01	5.96	1.56	0	0.22	0.29	11.6	8.36	17.23	8.46	-0.84	13.41	22.98	12.49	2
os69203	7	254	0.92	0	3	6	6.73	0.01	4.99	1.57	0.01	0	-0.4	10.5	8.36	17.22	8.46	-0.85	13.41	22.97	12.49	1.99
os69213	7	222	0.92	0	3	6	6.7	0	6.9	1.57	0	0.3	0.1	11.59	8.58	17.4	8.65	-0.67	13.58	23.07	12.71	2.24
os69227	7	217	0.01	2	3	6	6.72	0.02	5.99	1.56	0.01	8.23	0.23	9.01	8.73	17.62	8.8	-0.58	13.74	23.36	12.92	2.38
os69228	7	217	0.03	1	3	6	6.73	0.02	5.99	1.56	0.01	8.06	0.1	9.01	8.73	17.62	8.8	-0.58	13.74	23.37	12.92	2.38
os69240	7	275	0.01	2	3	6	6.75	0.02	4.08	1.56	0.03	0.04	0	10.2	8.12	17.04	8.27	-1.04	13.12	22.84	12.32	1.76
os69241	6	274	0.92	1	3	6	6.75	0.01	4.99	1.56	0.01	3.97	0.51	10.04	8.14	17.07	8.29	-1.01	13.14	22.9	12.38	1.77
os69250	7	255	0.92	0	3	6	6.72	0	4.99	1.57	0.01	0	0	12.62	8.35	17.21	8.45	-0.86	13.39	22.95	12.47	1.98
os69252	7	256	0.92	0	3	6	6.72	0	3.95	1.57	0.02	2.92	-0.04	12.71	8.3	17.14	8.39	-0.88	13.27	22.85	12.4	1.92
os69256	7	255	0.92	0	3	6	6.72	0	4.75	1.57	0.01	0	-0.08	12.62	8.33	17.17	8.43	-0.87	13.32	22.9	12.44	1.95
os69258	7	255	0.92	0	3	6	6.73	0.01	3.96	1.57	0.01	0.32	-0.25	10.62	8.33	17.16	8.43	-0.87	13.32	22.89	12.43	1.95
os69262	7	264	0.92	0	3	6	6.72	0	4.99	1.57	0.01	0.07	0.09	12.31	8.23	17.13	8.34	-0.97	13.13	22.85	12.31	1.83
os69268	7	286	0.23	1	3	6	6.72	0	5.52	1.56	0.01	5.21	0.11	9.57	8	16.88	8.21	-1.1	12.97	22.59	12.22	1.69
os69269	7	285	0.43	1	3	6	6.72	0	5.59	1.57	0.02	4.08	-0.38	9.94	8.02	16.89	8.23	-1.1	12.98	22.62	12.23	1.7
os69270	7	285	0.92	1	3	6	6.73	0	4.99	1.57	0.01	0	-0.03	9.99	8.02	16.89	8.23	-1.1	12.98	22.62	12.23	1.7
os69274	7	204	0.08	1	3	6	6.73	0.02	5.92	1.56	0.01	2.82	0.08	9.35	8.97	17.89	8.93	-0.52	14.11	23.75	13.17	2.5
os69281	7	248	0.92	0	3	6	6.71	0	3.48	1.57	0.01	7.59	-0.12	9.8	8.4	17.23	8.52	-0.87	13.4	22.94	12.64	1.98
os69324	7	169	0.92	0	3	6	6.68	0	6.96	1.57	0	7.92	-0.04	10.66	9.57	18.47	9.35	-0.17	14.69	24.3	13.52	2.82
os69328	7	169	0.92	0	3	6	6.68	0	6.91	1.57	0	0	0.01	11.92	9.59	18.46	9.36	-0.17	14.7	24.31	13.52	2.83
os69338	5	177	0.92	1	3	6	6.7	0.01	3.95	1.57	0.01	3.53	-0.33	9.19	9.62	18.53	9.33	-0.07	14.42	23.91	13.19	2.78
os69412	7	174	0.92	1	3	6	6.68	0.01	5.67	1.57	0.01	0.1	-0.34	9.54	9.43	18.38	9.31	-0.24	14.56	24.22	13.51	2.77
os69440	5	180	0.23	1	3	6	6.68	0.01	3.93	1.56	0.01	7.36	-0.28	9.05	9.69	18.67	9.4	-0.05	14.56	24.15	13.31	2.82
os69454	7	188	0.92	2	2	3	6.59	-0.04	1.32	1.56	0.06	42.76	-2.36	7.73	10.16	19.28	9.63	0.05	15.45	25.02	13.78	3.05
os69456	7	188	0.92	0	3	6	6.69	0	6.9	1.57	0.01	4.3	0.05	10.78	10.31	19.49	9.75	0.1	15.51	25.19	13.88	3.03
os69484	7	193	0.92	0	3	6	6.69	0	5.86	1.57	0.01	18.79	-0.07	11.58	10.15	19.3	9.66	0.13	15.46	25.11	13.84	3.16
os69510	7	183	0.92	1	3	6	6.65	-0.02	4.81	1.56	0.02	10.4	-0.08	9.28	9.89	19.06	9.5	-0.14	15.02	24.87	13.63	2.73
os69511	7	183	0.92	1	3	6	6.66	-0.01	4.76	1.56	0.02	10.2	-0.06	9.41	9.89	19.06	9.5	-0.14	15.02	24.87	13.63	2.73
os69513	7	182	0.95	3	3	6	6.62	-0.04	4.79	1.55	0.03	23.01	-0.06	8.14	9.88	19.05	9.49	-0.14	15.02	24.87	13.62	2.73
os69515	7	180	0.4	1	3	6	6.71	0.01	5.98	1.57	0.02	3.33	-0.69	9.34	9.96	19.14	9.56	-0.1	15.14	24.91	13.65	2.8
os69516	7	182	0.92	0	3	6	6.68	0	5.97	1.57	0	0.16	0.02	12.13	9.95	19.13	9.54	-0.1	15.12	24.91	13.66	2.78
os69525	7	177	0.07	1	3	6	6.72	0.02	6.24	1.56	0.02	15.89	-0.39	9.1	10.11	19.29	9.65	0.03	15.17	25	13.65	2.88
os69530	7	175	0.92	0	3	6	6.68	0	6.99	1.57	0.01	5.64	0	11.39	10.07	19.2	9.63	0.02	14.99	24.69	13.47	2.87
os69531	7	175	0.92	0	3	6	6.69	0.01	6.99	1.57	0.01	7.37	-0.31	9.04	10.07	19.2	9.63	0.01	14.99	24.69	13.47	2.87
os69534	7	176	0.92	1	3	6	6.7	0.01	6.98	1.56	0.01	7.36	0.06	9.23	10.07	19.19	9.63	0	15.02	24.74	13.51	2.87

os69539	7	172	0.92	0	3	6	6.68	0	6.99	1.57	0.01	6.51	-0.15	11.32	10.06	19.18	9.63	0.05	14.87	24.58	13.42	2.91
os69553	7	169	0.92	0	3	6	6.68	0	6.88	1.57	0.01	0.03	0	12.2	10.08	19.22	9.69	0.1	14.74	24.45	13.35	2.9
os69565	7	169	0.92	0	3	6	6.68	0	6.2	1.57	0.01	5.4	-0.02	12.4	10.05	19.14	9.63	0.06	14.72	24.4	13.31	2.84
os69566	7	169	0.92	0	3	6	6.68	0	5.9	1.57	0.01	3.93	-0.01	11.5	10.05	19.13	9.63	0.05	14.74	24.41	13.32	2.84
os69579	7	170	0.92	0	3	6	6.68	0	6.81	1.57	0.01	3.89	0	12.18	10.3	19.46	9.81	0.16	15.38	25.06	13.76	3.03
os69580	7	170	0.92	0	3	6	6.68	0	3.97	1.57	0.02	7.45	0	12.18	10.31	19.47	9.82	0.17	15.4	25.07	13.76	3.03
os69597	4	233	0.58	1	2	7	6.69	0	2.98	1.56	0.12	85.41	-2.21	8.56	9.62	18.8	9.3	-0.25	14.38	24.09	12.99	2.54
os69621	7	164	0.92	0	3	6	6.67	0	4.47	1.55	0.01	12.78	0.48	10.55	10.2	19.28	9.73	0.1	15.27	24.85	13.74	3
os69625	7	161	0.92	0	3	6	6.68	0	3.98	1.57	0.01	1.4	-0.01	11.93	10.18	19.25	9.72	0.1	15.24	24.83	13.78	2.99
os69670	7	157	0.03	1	3	6	6.68	0.01	6.69	1.56	0.01	8.77	-0.23	9.57	10.25	19.35	9.78	0.23	15.25	24.93	13.83	3.1
os69672	7	159	0.92	0	3	6	6.68	0	7.69	1.56	0	2.45	0.42	11.23	10.23	19.28	9.73	0.16	15.29	24.85	13.79	3.07
os69674	7	158	0.92	0	3	6	6.67	0	7.68	1.57	0	0.86	0	11.75	10.23	19.28	9.73	0.18	15.29	24.85	13.79	3.08
os69682	7	150	0.7	2	3	6	6.69	0	7.86	1.56	0	14.51	-0.85	8.32	10.23	19.46	9.84	0.17	15.24	25.31	14.12	3.09
os69721	7	207	0.42	5	2	7	6.57	-0.01	1.32	1.55	0.11	83.11	-4.44	7.24	9.59	18.73	9.27	-0.31	14.66	24.33	13.23	2.59
os69722	7	207	0.36	5	2	7	6.58	-0.01	1.37	1.55	0.11	83.27	-4.26	7.31	9.59	18.72	9.27	-0.31	14.65	24.32	13.23	2.59
os69723	7	207	0.43	5	2	7	6.59	-0.01	1.58	1.55	0.11	83.4	-4.14	7.41	9.59	18.72	9.27	-0.31	14.65	24.32	13.23	2.58
os69729	6	211	0.92	0	2	0	6.71	0.01	2.92	1.56	0.1	77.42	-1.66	7.98	9.52	18.66	9.22	-0.36	14.57	24.24	13.17	2.54
os69730	6	211	0.08	1	2	0	6.72	0.02	2.9	1.56	0.11	77.07	-1.89	7.98	9.52	18.66	9.21	-0.36	14.57	24.24	13.17	2.54
os69731	6	211	0	1	2	0	6.72	0.02	2.84	1.56	0.11	76.83	-2.01	7.98	9.52	18.65	9.21	-0.36	14.57	24.23	13.16	2.54
os69732	6	212	0.06	2	2	0	6.72	0.02	2.82	1.56	0.11	76.63	-2.06	7.98	9.52	18.65	9.21	-0.37	14.56	24.23	13.16	2.53
os69733	6	212	0.23	2	2	0	6.72	0.02	2.63	1.56	0.11	76.37	-2.13	7.98	9.51	18.65	9.21	-0.37	14.56	24.23	13.16	2.53
os69734	6	212	0.13	2	2	0	6.71	0.02	2.57	1.56	0.11	76.11	-2.08	8.01	9.51	18.64	9.2	-0.37	14.55	24.22	13.15	2.53
os69737	6	215	0.92	0	2	0	6.7	0	3.05	1.56	0.11	77.29	-1.46	10.62	9.49	18.62	9.18	-0.39	14.52	24.2	13.13	2.51
os69738	6	215	0.92	0	2	0	6.7	0	2.85	1.56	0.11	77.86	-1.77	10.23	9.49	18.62	9.18	-0.39	14.52	24.19	13.13	2.51
os69739	6	215	0.92	0	2	0	6.7	0	2.86	1.56	0.11	78.23	-1.91	11.39	9.48	18.62	9.18	-0.39	14.52	24.19	13.13	2.51
os69740	6	215	0.92	0	2	7	6.7	0	2.93	1.56	0.11	78.56	-2.15	9.88	9.48	18.61	9.18	-0.39	14.51	24.19	13.12	2.51
os69741	6	215	0.92	1	2	7	6.7	0.01	3.04	1.56	0.11	78.91	-2.3	8.45	9.48	18.61	9.17	-0.39	14.51	24.18	13.12	2.51
os69742	6	215	0.23	1	2	0	6.7	0.01	3.11	1.56	0.11	79.21	-2.14	8.44	9.48	18.61	9.17	-0.4	14.51	24.18	13.12	2.5
os69743	7	216	0.22	2	2	0	6.71	0.02	3.35	1.56	0.11	79.5	-1.98	8.42	9.47	18.61	9.17	-0.4	14.5	24.18	13.11	2.5
os69744	7	216	0.23	2	2	0	6.71	0.02	3.5	1.56	0.11	79.77	-1.61	8.43	9.47	18.6	9.17	-0.4	14.5	24.17	13.11	2.5
os69745	7	217	0.23	1	2	0	6.71	0.01	3.02	1.56	0.11	80	-1.36	8.42	9.47	18.6	9.16	-0.4	14.49	24.17	13.11	2.5
os69746	7	217	0.92	1	2	0	6.7	0	2.99	1.56	0.1	80.06	-1.2	8.43	9.46	18.6	9.16	-0.41	14.49	24.16	13.1	2.49
os69747	6	217	0.92	1	2	0	6.7	0.01	2.98	1.56	0.1	80.18	-1.37	8.43	9.46	18.59	9.16	-0.41	14.49	24.16	13.1	2.49
os69748	6	218	0.23	1	2	0	6.71	0.02	2.97	1.56	0.1	80.26	-1.33	8.44	9.46	18.59	9.16	-0.41	14.48	24.16	13.1	2.49
os69750	7	219	0.08	2	2	0	6.75	0.04	1.85	1.56	0.11	81.29	-1.97	8.27	9.45	18.58	9.15	-0.42	14.47	24.15	13.09	2.48
os69752	7	221	0.08	5	2	7	6.82	0.07	1.49	1.54	0.11	82.75	-2.37	8.13	9.46	18.59	9.15	-0.41	14.48	24.16	13.1	2.49
os69753	7	224	0.45	4	2	7	6.77	0.03	1.58	1.54	0.11	85.21	-2.52	7.97	9.46	18.59	9.16	-0.41	14.49	24.16	13.1	2.49

os69754	7	225	0.6	4	2	7	6.74	0.01	1.81	1.54	0.12	86.81	-2.41	7.96	9.46	18.6	9.16	-0.41	14.5	24.17	13.1	2.5
os69755	7	226	0	7	2	7	6.82	0.1	0.88	1.53	0.11	86.65	-2.12	7.73	9.46	18.59	9.15	-0.41	14.49	24.16	13.1	2.49
os69756	7	227	0.01	4	2	7	6.79	0.08	1.21	1.54	0.12	87.98	-2.59	7.9	9.46	18.59	9.16	-0.41	14.49	24.16	13.1	2.49
os69757	7	228	0.01	5	2	7	6.78	0.08	1.24	1.54	0.12	89.27	-3.04	7.85	9.46	18.59	9.16	-0.41	14.49	24.16	13.1	2.49
os69758	6	228	0.19	3	2	0	6.75	0.04	2.16	1.55	0.1	89.61	-1.73	8.16	9.41	18.54	9.11	-0.45	14.41	24.1	13.04	2.45
os69759	7	227	0.6	1	2	7	6.67	-0.01	2.18	1.56	0.1	89.43	-2.17	8.21	9.41	18.55	9.11	-0.45	14.41	24.1	13.04	2.45
os69779	4	215	0.01	4	2	0	6.77	0.06	1.57	1.55	0.12	86.1	-2.28	7.77	9.56	18.71	9.25	-0.34	14.58	24.29	13.19	2.54
os69780	4	215	0.01	4	2	7	6.79	0.07	1.2	1.55	0.12	84.95	-2.63	7.64	9.56	18.71	9.25	-0.34	14.58	24.29	13.19	2.54
os69787	4	234	0.65	6	2	7	6.52	-0.04	0.66	1.53	0.11	83.78	-2.08	8.02	9.63	18.81	9.31	-0.24	14.38	24.09	12.99	2.54
os69789	7	180	0.92	0	3	6	6.68	0	5.98	1.57	0.01	10.64	-0.27	11.65	9.97	19.15	9.56	-0.1	15.17	24.93	13.66	2.81
os69791	7	181	0.4	2	3	6	6.72	0.01	5.98	1.56	0.01	3	-0.15	9.21	9.96	19.14	9.56	-0.1	15.14	24.91	13.66	2.8
os69792	7	181	0.19	2	3	6	6.72	0.01	5.98	1.56	0.01	2.85	-0.23	9.21	9.96	19.14	9.56	-0.1	15.14	24.91	13.65	2.8
os69793	7	181	0.1	2	3	6	6.72	0.01	5.97	1.56	0.01	2.72	-0.19	9.18	9.96	19.14	9.56	-0.1	15.13	24.91	13.65	2.8
os69795	7	180	0.6	2	3	6	6.66	-0.01	5.98	1.56	0.01	10.38	-0.13	9.75	9.97	19.15	9.56	-0.1	15.16	24.93	13.66	2.81
os69796	7	183	0.92	0	3	6	6.68	0	5.98	1.57	0	0.04	0.08	12.03	9.96	19.13	9.55	-0.1	15.16	24.94	13.67	2.8
os69797	7	183	0.92	0	3	6	6.68	0	5.98	1.57	0	0	0.15	12.03	9.97	19.13	9.55	-0.1	15.17	24.95	13.67	2.8
os69801	7	184	0.92	0	3	6	6.69	0	5.67	1.57	0.01	3.5	-0.08	11.98	9.89	19	9.46	-0.13	15.06	24.82	13.63	2.73
os69802	7	184	0.92	0	3	6	6.69	0	5.68	1.57	0.01	3.81	-0.06	11.99	9.89	19	9.46	-0.13	15.06	24.82	13.63	2.73
os69803	7	183	0.03	2	3	6	6.73	0.03	5.71	1.56	0.02	23.83	-0.17	9.56	9.89	19.01	9.47	-0.13	15.05	24.83	13.63	2.73
os69804	7	183	0.23	2	3	6	6.71	0.02	5.71	1.56	0.02	26.05	-0.46	9.37	9.89	19.01	9.47	-0.13	15.05	24.83	13.63	2.73
os69805	7	188	0.05	8	3	5	6.86	0.11	0.15	1.49	0.02	37.04	1.86	7.45	9.9	19.02	9.48	-0.13	15.05	24.85	13.63	2.73
os69808	7	171	0.92	0	3	6	6.68	0	6.98	1.57	0.01	14.39	-0.12	12.02	10.09	19.22	9.66	0.08	14.87	24.59	13.42	2.92
os69823	7	183	0.92	0	3	6	6.68	0	5.51	1.57	0.01	4.07	0.02	11.56	9.9	19.07	9.51	-0.12	15.06	24.89	13.64	2.73
os69824	7	183	0.92	0	3	6	6.68	0	5.54	1.56	0.01	4.16	0.11	11.75	9.9	19.07	9.51	-0.12	15.06	24.89	13.64	2.73
os69829	6	181	0.25	1	3	6	6.72	0.01	3.94	1.56	0.02	16.72	-0.26	9.76	9.9	19.09	9.47	-0.15	15.03	24.88	13.53	2.75
os69830	6	181	0.08	1	3	6	6.71	0.01	4.22	1.57	0.02	16.24	-0.56	9.71	9.9	19.09	9.47	-0.15	15.03	24.88	13.53	2.75
os69835	6	182	0.92	2	3	6	6.63	-0.02	4.28	1.56	0.02	16.15	-0.14	8.97	9.9	19.09	9.47	-0.15	15.04	24.88	13.53	2.75
os69845	7	185	0.92	1	3	6	6.69	0	5.98	1.56	0.01	0.05	0.37	10.05	9.69	18.79	9.33	-0.25	14.85	24.55	13.41	2.66
os69846	7	185	0.77	1	3	6	6.69	0	5.98	1.56	0.01	0.05	0.27	9.9	9.69	18.79	9.33	-0.25	14.85	24.55	13.41	2.66
os69849	7	201	0.92	0	3	6	6.69	0	4.18	1.57	0.03	44.05	-0.73	9.3	9.44	18.53	9.15	-0.44	14.65	24.33	13.29	2.48
os69852	7	187	0.92	0	3	6	6.69	0	4.93	1.57	0.01	4.32	0.03	12.02	9.66	18.75	9.32	-0.27	14.8	24.5	13.4	2.65
os69857	7	218	0.05	1	3	6	6.72	0.01	3.07	1.56	0.02	34.08	-0.54	8.95	9.45	18.55	9.17	-0.45	14.65	24.39	13.33	2.46
os69858	7	218	0.08	1	3	6	6.72	0.01	3.07	1.56	0.02	33.86	-0.5	8.99	9.45	18.55	9.17	-0.45	14.65	24.39	13.33	2.46
os69859	7	218	0.08	2	3	6	6.75	0.02	3.07	1.56	0.02	33.14	-0.33	9.06	9.45	18.55	9.16	-0.45	14.65	24.39	13.33	2.46
os69861	7	218	0.11	2	3	6	6.75	0.02	3.05	1.56	0.03	32.6	-0.49	9.09	9.45	18.55	9.16	-0.45	14.65	24.39	13.33	2.45
os69862	7	218	0.19	2	3	6	6.75	0.02	3.06	1.56	0.03	32.36	-0.69	9.14	9.44	18.55	9.16	-0.45	14.65	24.39	13.33	2.45
os69863	7	218	0.08	2	3	6	6.75	0.02	3.05	1.56	0.03	32.13	-0.61	9.11	9.44	18.55	9.16	-0.45	14.65	24.39	13.33	2.45

os69864	7	218	0.35	2	3	6	6.74	0.02	3.05	1.56	0.02	31.71	-0.46	9.11	9.44	18.55	9.16	-0.45	14.65	24.39	13.33	2.45
os69865	7	163	0.92	1	3	6	6.71	0.02	2.82	1.57	0	11.19	-0.9	8.87	9.79	18.76	9.44	0	14.79	24.42	13.49	2.91
os69867	7	188	0.92	0	3	6	6.69	0	3.17	1.57	0.02	9.69	-0.28	10.12	9.76	18.92	9.39	-0.18	14.98	24.74	13.54	2.73
os69961	4	373	0.37	6	2	3	6.59	-0.05	0.92	1.52	0.07	95.53	-2.6	8.16	8.13	17.09	8.18	-1.13	13.1	22.65	12.16	1.73
os70178	4	246	0.29	14	2	3	6.06	-0.21	0.01	1.48	0.13	85.24	-3.14	6.37	8.78	17.7	8.73	-0.67	13.61	23.3	12.73	2.25
os70183	4	339	0	7	2	7	6.9	0.13	0.73	1.51	0.16	100	-4.98	7.26	8.27	17.22	8.34	-0.99	12.99	22.68	12.21	1.89
os70236	4	278	0.87	15	2	3	6.07	-0.21	0	1.48	0.12	95.49	-3.07	6.04	8.43	17.27	8.52	-0.76	12.98	22.6	12.22	2.02
os70237	4	280	0.85	5	2	3	6.58	-0.04	1.38	1.54	0.08	94.67	-4.08	8.19	8.41	17.26	8.51	-0.8	13.05	22.66	12.26	2.04
os70240	4	347	0.96	9	3	5	6.5	-0.13	0.35	1.48	0.11	98.97	-1.54	6.78	8.29	17.23	8.36	-0.97	12.84	22.52	12	1.78
os70241	4	371	0.95	17	3	5	6.19	-0.23	0.01	1.45	0.14	99.47	-2.11	6.36	8.21	17.15	8.3	-1.02	12.74	22.43	11.93	1.73
os70253	4	245	0.92	3	2	7	6.67	-0.02	2.53	1.56	0.15	88.8	-4.39	7.46	8.36	17.2	8.49	-0.82	12.98	22.61	12.28	2.03
os70309	4	224	0.79	11	2	3	6.25	-0.15	0.33	1.51	0.15	98.1	-6.36	7.72	8.84	17.77	8.86	-0.65	13.88	23.64	13.08	2.36
os70386	4	315	0.87	7	2	3	6.65	-0.06	1.08	1.52	0.09	98.26	-4.48	7.47	8.39	17.29	8.47	-0.84	13.28	22.84	12.39	2.05
os70508	7	256	0.04	2	2	7	6.74	0	3.4	1.56	0.09	92	-4.45	8.53	8.4	17.29	8.55	-0.84	13.31	22.98	12.54	2.01
os70512	7	282	0.22	3	3	6	6.73	0.02	2.13	1.56	0.05	61.84	-1.69	8.43	8.24	17.17	8.46	-0.98	13.15	22.88	12.46	1.93
os70535	7	329	0.8	13	2	7	6.2	-0.14	0.22	1.51	0.14	93.48	-4.9	6.97	7.82	16.72	8.09	-1.2	12.59	22.26	11.99	1.63
os70542	4	278	0.02	9	3	5	6.38	-0.15	0.26	1.5	0.08	97.13	-0.94	6.77	8.36	17.29	8.51	-0.9	13.39	23.06	12.63	2.01
os70555	4	283	0.6	8	3	5	6.51	-0.03	0.18	1.51	0.06	95.7	-0.06	7.08	8.3	17.2	8.46	-0.91	13.2	22.86	12.45	1.95
os70596	7	364	0.03	7	3	5	6.89	0.11	0.76	1.51	0.06	96.09	-1.19	7.54	7.72	16.65	8.01	-1.24	12.41	22.1	11.85	1.59
os70597	4	293	0.63	6	1	4	6.5	-0.06	0.03	1.43	0.04	86.85	5.57	6.12	8.08	17.01	8.31	-1.05	12.97	22.66	12.34	1.86
os70616	7	311	0.92	6	3	5	6.52	-0.08	0.81	1.53	0.08	95.95	-1.56	7.86	7.93	16.91	8.2	-1.18	12.88	22.64	12.31	1.78
os70624	4	363	0.85	10	2	3	6.36	-0.12	0.51	1.49	0.12	99.65	-5.78	6.66	7.84	16.84	8.12	-1.21	12.76	22.53	12.18	1.77
os70687	4	489	0.01	7	3	5	6.9	0.11	0.23	1.48	0.06	99.2	1.1	6.3	7.23	16.13	7.57	-1.8	11.94	21.59	11.39	1.15
os70914	7	420	0.81	5	2	7	6.74	-0.03	1.45	1.55	0.12	91.98	-5.3	7.43	7.42	16.33	7.65	-1.86	12.41	22.06	11.71	1.19
os70966	7	504	0.04	7	2	7	6.49	-0.13	0.41	1.51	0.2	99.52	-3.12	7.17	6.69	15.64	7.1	-2.34	11.54	21.18	11.08	0.71
os70968	7	569	0.1	16	2	3	6.97	0.22	0	1.45	0.14	99.82	-4.73	6.71	6.71	15.65	7.1	-2.33	11.57	21.21	11.1	0.74
os71022	4	265	0.21	12	2	0	6.17	-0.2	0.01	1.49	0.09	93.06	-1.4	6.67	8.4	17.25	8.56	-0.93	13.24	22.92	12.51	2.02
os71023	7	283	0.34	4	2	7	6.72	0.03	1.7	1.54	0.11	98.05	-3.89	8.29	8.46	17.29	8.55	-0.93	13.43	23	12.61	2.11
os71024	7	293	0.02	3	2	7	6.62	-0.05	2.14	1.55	0.1	97.94	-2.91	8.26	8.41	17.24	8.51	-0.96	13.36	22.94	12.56	2.07
os71174	4	366	0.24	4	3	6	6.62	-0.06	1.38	1.55	0.06	97.98	-0.98	7.68	7.96	16.8	8.15	-1.32	12.85	22.38	12.04	1.64
os71217	7	210	0.98	2	2	7	6.64	-0.03	2.78	1.56	0.12	87.23	-3.08	8.08	8.92	17.8	8.87	-0.66	14.09	23.69	13.09	2.37
os71277	4	494	1	11	2	7	6.42	-0.15	0.45	1.51	0.24	99.3	-11.38	7.47	6.95	15.89	7.35	-1.87	11.57	21.22	11.11	1.04
os71416	4	416	0.42	9	3	5	6.6	0	0.06	1.51	0.07	99.25	1.22	7.42	7.67	16.52	7.91	-1.5	12.47	22.01	11.71	1.4
os71417	4	416	0.4	8	3	5	6.59	-0.02	0.04	1.51	0.07	99.21	1.58	7.44	7.67	16.52	7.91	-1.5	12.48	22.01	11.71	1.4
os71545	7	222	0.4	6	2	7	6.82	0.06	1.23	1.55	0.14	92.85	-4.2	7.7	8.74	17.61	8.76	-0.72	13.82	23.45	12.93	2.29
os71546	7	221	0.21	4	2	7	6.78	0.04	1.69	1.56	0.13	92.72	-4.04	7.8	8.75	17.61	8.76	-0.72	13.82	23.45	12.94	2.29
os71571	4	256	0.45	11	2	0	6.44	0.01	0.02	1.48	0.11	97.39	-0.07	6.82	8.72	17.62	8.71	-0.81	13.75	23.39	12.82	2.19

os71572	4	256	0.45	11	2	0	6.43	0	0.02	1.48	0.11	97.43	-0.2	6.81	8.72	17.62	8.72	-0.81	13.75	23.39	12.82	2.19
os71575	7	225	0.99	2	2	0	6.64	-0.03	2.83	1.55	0.09	86.61	-1.73	8.34	8.89	17.82	8.84	-0.7	14.08	23.75	13.1	2.36
os71576	7	233	0.53	5	2	7	6.59	-0.02	1.7	1.54	0.11	95.28	-3	7.08	8.85	17.77	8.82	-0.71	14.04	23.69	13.06	2.33
os71577	7	232	0.47	5	2	7	6.61	-0.02	1.71	1.54	0.11	95.21	-3.38	7.06	8.86	17.78	8.82	-0.71	14.04	23.7	13.06	2.33
os71578	7	231	0.52	5	2	7	6.62	-0.01	1.75	1.55	0.12	95.13	-3.74	7.05	8.86	17.78	8.82	-0.71	14.04	23.7	13.06	2.33
os71614	7	404	0.17	11	2	3	6.71	0.09	0.29	1.51	0.1	98.61	-3.83	7.23	7.72	16.56	7.95	-1.46	12.61	22.19	11.94	1.52
os71672	7	378	0.69	4	3	6	6.63	-0.05	1.64	1.54	0.08	91.63	-1.33	8.36	7.64	16.53	7.92	-1.53	12.35	22	11.72	1.4
os71715	4	242	1	9	2	3	6.37	-0.15	0.41	1.52	0.1	89.63	-3.2	7.62	8.73	17.61	8.77	-0.68	13.67	23.38	12.82	2.29
os71769	4	324	0.79	16	2	0	6.03	-0.19	0.09	1.46	0.15	98.42	-2.23	6.99	7.99	16.86	8.2	-1.17	12.73	22.38	12.05	1.76
os71792	4	272	0.99	10	2	0	6.38	-0.16	0.04	1.5	0.08	92.69	0.33	6.86	8.78	17.63	8.71	-0.84	14.07	23.55	12.99	2.24
os71857	4	501	0.84	9	2	7	6.45	-0.12	0.63	1.53	0.14	99.35	-4.48	7.89	7.17	15.99	7.43	-1.83	11.71	21.16	11.02	0.94
os71970	4	305	0.8	11	3	5	6.36	-0.08	0.11	1.49	0.08	97.38	-1.62	6.83	8.1	17.12	8.28	-1.05	13.03	22.85	12.36	1.86
os71983	7	310	0.49	3	3	6	6.66	-0.04	1.77	1.55	0.03	73.39	-0.71	9.26	8.06	17.01	8.37	-0.95	13.01	22.65	12.45	2.17
os72014	5	319	0.59	7	3	5	6.75	-0.01	0.52	1.5	0.05	92.63	-0.42	7.12	8.03	17	8.29	-1.07	12.92	22.73	12.34	1.85
os72038	7	319	0.23	2	3	6	6.73	0	1.97	1.55	0.06	99.35	-1.85	7.66	7.93	16.88	8.3	-1.01	12.75	22.42	12.21	1.99
os72156	7	461	0.97	12	2	0	6.31	-0.19	0.07	1.49	0.14	99.8	-0.85	6.35	6.99	15.94	7.54	-1.69	11.8	21.39	11.48	1.44
os72200	7	450	0.62	6	3	5	6.59	-0.07	0.66	1.53	0.09	97.46	-1.09	6.85	7.03	15.99	7.53	-1.65	11.62	21.3	11.22	1.23
os72217	7	250	0.92	1	3	6	6.7	-0.01	5.94	1.56	0.01	1.92	0.2	10.49	8.3	17.29	8.53	-0.8	13.32	23.07	12.63	2.18
os72218	7	250	0.92	0	3	6	6.71	0	5.93	1.57	0.01	1.08	0.05	12	8.3	17.29	8.52	-0.8	13.31	23.07	12.63	2.17
os72221	7	241	0.92	0	3	6	6.71	0	3.96	1.57	0.02	6.8	0	11.87	8.34	17.3	8.57	-0.71	13.36	23.1	12.69	2.29
os72222	7	241	0.92	0	3	6	6.71	0	3.96	1.57	0.02	7.65	0	11.87	8.34	17.3	8.57	-0.71	13.36	23.1	12.69	2.3
os72223	5	247	0.89	7	2	3	6.58	-0.07	1.34	1.54	0.06	74.71	-2.06	8.02	8.32	17.29	8.55	-0.71	13.3	23.07	12.69	2.28
os72266	5	379	0.92	1	3	6	6.71	-0.03	1.79	1.55	0.05	78.58	-0.44	7.54	7.58	16.54	7.97	-1.32	12.34	22.05	11.8	1.6
os72271	7	251	0.92	0	3	6	6.71	0	3.03	1.56	0.01	16.13	0.02	9.81	8.3	17.26	8.53	-0.77	13.33	23.06	12.63	2.2
os72274	7	246	0.92	0	3	6	6.71	0	5.91	1.57	0.01	3.89	-0.12	11.14	8.3	17.29	8.53	-0.76	13.35	23.07	12.64	2.22
os72305	7	268	0.13	4	3	6	6.58	-0.07	0.92	1.53	0.03	85.85	-0.04	7.61	8.34	17.3	8.58	-0.73	13.37	23.08	12.73	2.36
os72307	5	284	0.39	3	3	6	6.81	0.03	1.23	1.53	0.02	95.05	0.09	7.44	8.29	17.26	8.56	-0.77	13.3	23	12.65	2.32
os72308	5	285	0.1	3	3	6	6.81	0.03	1.28	1.53	0.02	95.32	-0.14	7.37	8.29	17.26	8.56	-0.77	13.29	23	12.65	2.32
os72338	7	311	0.01	4	2	3	6.63	-0.04	1.59	1.55	0.09	90.22	-3.19	7.87	8.04	17.01	8.33	-0.92	12.94	22.66	12.3	2.11
os72339	7	314	0.03	5	2	3	6.6	-0.05	1.62	1.54	0.07	90.49	-2.25	7.92	8.03	17	8.33	-0.92	12.94	22.65	12.29	2.11
os72345	4	297	0.65	14	2	3	6.72	0	0.02	1.5	0.11	49.46	-1.62	6.43	8.1	17.08	8.39	-0.87	13.01	22.75	12.37	2.14
os72375	7	216	0.08	0	3	6	6.71	0	2.51	1.56	0.02	33.14	0.01	9.25	8.6	17.59	8.81	-0.44	13.59	23.29	12.9	2.59
os72430	5	268	0.08	1	3	6	6.76	0.02	1.01	1.54	0.01	63.9	-0.05	8.38	8.44	17.37	8.66	-0.64	13.5	23.07	12.83	2.53
os72460	5	238	1	5	2	3	6.64	-0.05	1.94	1.55	0.09	85.57	-3.51	7.81	8.6	17.46	8.75	-0.64	13.67	23.12	13.02	2.6
os72497	5	266	0.53	5	2	3	6.63	-0.03	2.44	1.55	0.07	70.23	-2.79	8.15	8.39	17.21	8.66	-0.87	13.41	22.81	12.88	2.4
os72509	7	304	0.97	7	3	5	6.68	-0.05	0.41	1.51	0.05	87.69	-1.07	7.43	8.26	17.06	8.53	-0.99	13.31	22.66	12.78	2.3
os72520	5	286	0.88	10	3	5	6.35	-0.15	0.26	1.51	0.08	96.4	-2.31	7.31	8.22	17.09	8.51	-1.01	13.26	22.69	12.8	2.29

os72539	5	267	0.06	8	3	5	6.89	0.12	0.35	1.52	0.05	67.92	-0.64	7.46	8.48	17.39	8.68	-0.72	13.57	23.08	12.9	2.54
os72546	5	262	0.07	6	2	3	6.7	0.04	2.13	1.55	0.07	58.81	-2.94	8.1	8.49	17.39	8.7	-0.73	13.57	23.05	12.93	2.55
os72570	5	269	0.21	3	3	6	6.79	0.03	2.96	1.55	0.04	67.36	-1.04	8.25	8.47	17.39	8.68	-0.73	13.56	23.09	12.87	2.53
os72649	5	250	0.79	9	3	5	6.49	-0.12	0.37	1.53	0.08	66.56	-2.03	7.04	8.53	17.43	8.72	-0.71	13.6	23.09	12.98	2.56
os72679	3	239	0.6	3	2	3	6.65	-0.04	1.59	1.55	0.06	82.59	-2.51	7.49	8.61	17.5	8.78	-0.55	13.67	23.21	12.98	2.64
os72716	5	274	0.13	4	2	3	6.62	-0.05	1.89	1.55	0.05	37.24	-1.99	8.04	8.26	17.24	8.54	-0.78	13.22	22.91	12.62	2.31
os72721	7	260	0.77	1	2	3	6.68	-0.02	3.11	1.56	0.06	74.21	-2.89	8.8	8.28	17.27	8.57	-0.76	13.24	22.95	12.64	2.33
os72748	7	349	0.6	8	3	5	6.53	-0.05	0.36	1.51	0.08	97.55	-1.2	7.78	7.83	16.7	8.19	-1.39	12.97	22.4	12.54	1.99
os72765	5	279	0.92	1	3	6	6.71	-0.01	2.39	1.55	0.01	16.87	0.01	9.32	8.27	17.25	8.54	-0.77	13.25	22.94	12.64	2.31
os72766	5	279	0.92	1	3	6	6.71	-0.01	2.47	1.55	0.01	18.56	-0.11	9.05	8.27	17.25	8.54	-0.77	13.25	22.94	12.64	2.31
os72775	5	259	0.09	1	3	6	6.68	-0.02	3.97	1.56	0.02	6.04	-0.2	10.21	8.45	17.46	8.7	-0.67	13.58	23.28	12.91	2.52
os72777	5	273	0.92	1	3	6	6.68	-0.02	1.3	1.56	0.01	10.72	0.08	9.48	8.34	17.34	8.6	-0.74	13.4	23.11	12.78	2.4
os72778	5	273	0.92	0	3	6	6.71	-0.01	1.36	1.56	0.01	7.16	0.09	9.77	8.34	17.34	8.59	-0.74	13.4	23.11	12.78	2.4
os72785	5	306	0.29	6	3	5	6.65	0.02	0.4	1.51	0.03	79.82	1.07	7.87	8.15	17.05	8.44	-0.89	13.13	22.74	12.57	2.29
os72797	7	287	0.85	6	2	3	6.53	-0.08	0.84	1.54	0.07	88.05	-1.56	7.87	8.29	17.3	8.55	-0.8	13.43	23.14	12.77	2.41
os72815	5	301	0.88	9	3	5	6.39	-0.13	0.16	1.49	0.05	88.81	0.36	7.66	8.15	17	8.42	-0.96	13.18	22.68	12.6	2.26
os72871	5	334	0.73	4	3	6	6.61	-0.05	0.59	1.52	0.02	95.3	0.31	6.9	8.04	16.9	8.34	-1.1	13.12	22.6	12.6	2.19
os72872	5	334	0.89	4	3	6	6.62	-0.05	0.54	1.52	0.02	95.53	0.31	6.9	8.04	16.9	8.34	-1.1	13.12	22.6	12.6	2.19
os72888	7	207	0.92	0	3	6	6.7	0	5.88	1.57	0	7.65	-0.13	11.57	8.73	17.5	8.9	-0.53	13.83	23.16	13.16	2.67
os72895	7	208	0.92	0	3	6	6.69	0	5.89	1.56	0	11.04	0.18	9.64	8.73	17.51	8.9	-0.54	13.83	23.19	13.17	2.67
os72906	7	218	0.92	1	3	6	6.68	-0.01	5.89	1.56	0.01	15.74	0.16	9.98	8.73	17.57	8.9	-0.59	13.77	23.23	13.18	2.68
os72907	7	219	0.92	3	3	6	6.65	-0.03	5.88	1.53	0.02	21.99	1.29	8.29	8.73	17.57	8.9	-0.59	13.77	23.23	13.18	2.68
os72915	7	228	0.92	0	3	6	6.7	0	4.99	1.56	0.01	15.32	0.15	10.16	8.79	17.72	8.94	-0.57	13.9	23.43	13.26	2.72
os72951	7	232	1	5	3	5	6.59	-0.07	1.42	1.54	0.05	66.4	-2	8.28	8.52	17.48	8.75	-0.52	13.47	23.16	12.83	2.51
os72958	5	229	0.45	6	2	3	6.55	-0.02	0.66	1.54	0.06	42.41	-2.84	7.5	8.49	17.42	8.7	-0.63	13.48	23.14	12.83	2.43
os73064	5	388	0.92	1	3	6	6.73	-0.02	1.13	1.54	0.01	24.62	0.49	9.27	7.69	16.47	8.14	-1.3	12.54	21.95	12.19	1.92
os73092	5	391	0.92	1	3	6	6.73	-0.02	1.41	1.54	0.01	13.94	0.12	8.88	7.65	16.43	8.1	-1.33	12.49	21.91	12.15	1.89
os73093	5	391	0.92	1	3	6	6.74	-0.02	1.29	1.54	0.01	13.57	0.09	8.86	7.64	16.43	8.1	-1.34	12.49	21.91	12.15	1.89
os73108	7	416	0.81	8	2	7	6.48	-0.12	0.96	1.56	0.13	95.96	-5.86	8.57	7.23	16.04	7.76	-1.63	12.04	21.48	11.77	1.62
os73135	7	362	0.21	4	2	3	6.79	0.04	1.74	1.55	0.08	93.79	-2.14	7.45	7.79	16.58	8.22	-1.3	12.72	22.14	12.33	1.97
os73150	5	356	0.92	2	3	6	6.71	-0.02	3.84	1.56	0.02	14.29	-0.19	9.54	7.78	16.65	8.19	-1.31	12.75	22.18	12.33	1.91
os73190	4	331	0.07	14	3	5	6.13	-0.22	0	1.44	0.08	87.63	0.62	5.44	8.06	17.02	8.37	-0.94	13.03	22.68	12.46	2.18
os73196	5	321	0.92	1	3	6	6.73	-0.01	0.84	1.55	0.01	29.7	0.12	9.38	8.07	17.02	8.39	-1.01	13.1	22.75	12.54	2.19
os73220	7	284	0.23	6	2	3	6.67	0.04	0.98	1.55	0.1	64.48	-3.89	7.68	8.18	17.06	8.46	-1.05	13.27	22.78	12.74	2.22
os73253	7	323	0.92	0	3	6	6.75	0	3.95	1.56	0.02	14.16	-0.18	10.93	8.01	16.92	8.33	-1.13	13.11	22.66	12.57	2.13
os73254	5	348	0.92	1	3	6	6.72	-0.02	1.11	1.56	0.01	13.9	0.14	9.21	7.95	16.86	8.29	-1.21	13.04	22.59	12.54	2.06
os73255	5	348	0.92	1	3	6	6.72	-0.02	0.64	1.56	0.01	15.72	0.25	9.35	7.95	16.85	8.29	-1.21	13.04	22.58	12.54	2.06

os73256	5	347	0.92	0	3	6	6.75	0	1.49	1.56	0.01	14.5	0.09	10.25	7.95	16.86	8.29	-1.21	13.05	22.59	12.54	2.07
os73258	7	306	0.91	4	3	5	6.65	-0.06	1.12	1.55	0.06	83.25	-1.42	8.43	8.06	16.94	8.37	-1.18	13.14	22.66	12.65	2.12
os73264	5	382	0.92	2	3	6	6.72	-0.02	1.87	1.56	0.03	76.57	-0.5	8.15	7.69	16.59	8.1	-1.37	12.67	22.23	12.24	1.87
os73280	4	296	0.78	8	1	4	6.45	-0.09	0.1	1.5	0.04	93.38	1.61	7.21	8.26	17.23	8.54	-0.8	13.32	22.99	12.72	2.31
os73301	5	329	0.93	6	3	5	6.59	-0.09	0.3	1.52	0.05	91.08	0.79	7.59	8.06	17.03	8.36	-1.02	13.19	22.82	12.6	2.24
os73330	7	371	0.4	11	2	3	6.5	-0.03	0.72	1.53	0.18	98.93	-8.76	7.43	7.72	16.64	8.1	-1.31	12.78	22.31	12.28	1.92
os73343	7	326	0.6	1	3	6	6.72	-0.01	2.84	1.56	0.04	25.42	-0.18	8.32	7.98	16.87	8.32	-1.11	13.02	22.59	12.49	2.1
os73345	5	376	0.66	3	3	6	6.66	-0.05	0.88	1.53	0.03	72.49	0.59	8.05	7.74	16.63	8.13	-1.34	12.74	22.29	12.25	1.89
os73346	5	378	0.74	3	3	6	6.68	-0.04	1.45	1.53	0.03	72.47	-0.02	8.06	7.74	16.62	8.12	-1.35	12.73	22.29	12.25	1.89
os73367	7	386	0.01	5	3	5	6.83	0.07	0.92	1.52	0.04	34.99	0.41	8.42	7.54	16.44	7.97	-1.44	12.45	21.97	12.02	1.73
os73373	5	361	0.45	9	3	5	6.41	-0.15	0.07	1.5	0.07	87.27	0	7.52	7.71	16.64	8.09	-1.35	12.71	22.26	12.24	1.88
os73416	7	249	0.92	1	3	6	6.7	-0.01	5.97	1.56	0.01	3.88	0.11	9.95	8.3	17.31	8.53	-0.78	13.34	23.07	12.65	2.2
os73433	4	285	0.53	2	3	6	6.75	0.01	3.56	1.56	0.06	79.42	-2.22	8.6	8.08	17.06	8.33	-1.05	12.98	22.76	12.38	1.87
os73437	5	304	0.12	3	3	6	6.81	0.04	0.84	1.53	0.02	93.17	0.16	7.57	8.15	17.19	8.41	-0.84	13.16	23	12.48	2.16
os73483	5	470	0.92	9	3	5	6.6	-0.11	0.08	1.49	0.07	98.57	-1.03	6.14	7.43	16.17	7.72	-1.05	12.33	21.66	11.64	1.63
os73497	4	453	0.99	15	1	1	6.14	-0.25	0	1.43	0.08	99.58	4.07	5.91	7.55	16.33	7.84	-0.99	12.48	21.83	11.78	1.67
os73534	5	406	0.04	7	2	7	6.77	0.07	0.76	1.51	0.12	98.15	-3.81	8.18	7.5	16.2	7.8	-0.94	12.24	21.51	11.59	1.64
os73576	3	371	0.36	27	3	5	5.87	0.07	0	1.36	0.22	97.06	-0.34	4.59	7.76	16.36	8.01	-0.78	12.3	21.45	11.6	1.7
os73581	7	332	0.61	3	3	6	6.78	0.01	2.08	1.56	0.06	77.5	-1.92	7.85	8.12	16.78	8.33	-0.51	12.84	21.97	12.03	2.08
os73582	7	311	0.92	1	3	6	6.77	0.02	4.5	1.56	0.07	70.77	-2.09	8.78	8.22	16.89	8.43	-0.44	12.97	22.14	12.17	2.19
os73583	7	315	0.92	0	3	6	6.75	0.01	3.94	1.56	0.05	58.96	-0.49	10.68	8.21	16.87	8.41	-0.45	12.95	22.11	12.15	2.17
os73584	7	312	0.01	1	3	6	6.78	0.02	4.8	1.56	0.05	68.97	-0.43	8.8	8.21	16.88	8.42	-0.44	12.96	22.12	12.16	2.18
os73586	7	332	0.45	6	3	6	6.82	0.02	1.45	1.54	0.06	77.39	-0.78	7.82	8.12	16.77	8.32	-0.52	12.83	21.97	12.03	2.08
os73587	7	320	0.92	0	3	6	6.74	0	3.57	1.56	0.05	51.02	-0.32	10.67	8.16	16.82	8.37	-0.48	12.89	22.03	12.09	2.12
os73589	7	313	0.13	1	3	6	6.77	0.01	4.51	1.56	0.05	65.92	-0.51	8.71	8.2	16.87	8.41	-0.45	12.95	22.1	12.14	2.17
os73590	7	313	0.08	1	3	6	6.77	0.02	4.71	1.56	0.05	68.75	-0.34	8.78	8.21	16.88	8.42	-0.44	12.96	22.13	12.16	2.18
os73598	7	312	0.01	1	3	6	6.77	0.02	4.81	1.56	0.05	69.1	-0.53	8.87	8.22	16.88	8.42	-0.44	12.97	22.13	12.17	2.19
os73605	4	368	0	25	2	0	6.78	0.41	0	1.39	0.18	96.58	-0.18	5.32	7.65	16.28	7.94	-0.86	12.24	21.42	11.56	1.66
os73610	4	341	0.29	6	2	3	6.75	0.07	0.74	1.52	0.15	91.07	-4.93	6.86	8.05	16.67	8.29	-0.59	12.76	21.89	11.99	1.99
os73611	7	312	0.92	1	3	6	6.77	0.02	4.85	1.56	0.06	69.5	-0.86	8.87	8.22	16.88	8.42	-0.44	12.97	22.13	12.17	2.19
os73648	3	445	0.77	4	1	4	6.83	0	0.07	1.49	0.03	95.94	3.01	7.55	7.76	16.6	8	-0.81	12.7	22.1	12.06	2.08
os73754	4	605	0	6	2	7	6.94	0.08	1.37	1.53	0.1	98.16	-3.08	8.03	6.16	14.8	6.69	-1.93	10.66	19.79	10.19	0.55
os73755	4	596	1	15	3	5	6.23	-0.25	0	1.43	0.1	99.58	2.27	5.75	6.28	14.93	6.78	-1.86	10.78	19.91	10.28	0.62
os73883	4	661	0.19	13	3	5	6.79	0.11	0	1.46	0.11	99.92	-0.04	5.94	5.98	14.65	6.56	-1.97	10.52	19.61	10.09	0.53
os73891	4	512	0.17	8	2	7	6.91	0.12	0.53	1.51	0.15	98.9	-3.69	7.14	6.7	15.35	7.13	-1.55	11.31	20.45	10.77	1.03
os73961	5	655	0	2	3	6	6.94	0.03	2.38	1.55	0.05	81.47	-0.63	8.17	6.03	14.72	6.56	-2.06	10.84	20.03	10.43	0.65
os73986	5	769	0.77	3	3	6	6.9	-0.03	0.64	1.53	0.04	85.1	-0.19	8.2	5.3	14.03	6.01	-2.43	9.98	19.07	9.68	0.22

os73994	4	554	0.01	9	1	4	6.92	0.1	0.09	1.49	0.06	86.49	2.29	8.59	6.78	15.51	7.2	-1.46	11.43	20.64	10.92	1.2
os74001	4	486	0.04	4	3	6	6.67	-0.07	0.95	1.51	0.05	96.84	-0.91	7.07	7.37	16.15	7.7	-1.02	12.24	21.48	11.6	1.71
os74005	4	484	0.38	3	3	6	6.87	0.02	1.98	1.51	0.06	95.45	-1.57	7.89	7.38	16.15	7.71	-1.02	12.24	21.48	11.61	1.71
os74072	3	601	0.01	5	3	6	6.95	0.08	0.95	1.54	0.06	90.16	-1.75	7.31	6.48	15.32	6.96	-1.74	11.36	20.76	10.92	0.99
os74073	4	661	0.41	14	3	5	6.68	0.09	0	1.45	0.13	93.13	-1.33	5.89	6.17	14.97	6.7	-1.91	10.96	20.28	10.54	0.78
os74091	4	486	1	17	2	3	6.16	-0.25	0	1.48	0.11	89.98	-3.14	7.74	7.24	16.05	7.6	-1.16	12.06	21.38	11.49	1.61
os74173	7	439	0.92	1	3	6	6.78	-0.01	2.78	1.54	0.06	78.15	-0.09	9.4	7.15	15.99	7.65	-1.81	12.11	21.6	11.82	1.54
os74183	7	417	0.17	6	2	7	6.82	0.06	0.98	1.54	0.07	98.24	-2.4	6.54	7.37	16.21	7.87	-1.63	12.36	21.79	12.02	1.69
os74186	7	383	0.76	11	2	7	6.48	-0.09	0.76	1.53	0.13	97.09	-4.18	8.35	7.55	16.35	8.02	-1.47	12.48	21.91	12.13	1.8
os74242	7	457	0.02	5	2	3	6.85	0.07	1.47	1.55	0	97.86	-2.48	8	7.09	15.93	7.58	-2.05	12.15	21.64	11.91	1.39
os74272	7	420	0.08	7	2	3	6.86	0.1	0.65	1.53	0.07	98.43	-2.34	6.38	7.36	16.2	7.86	-1.64	12.34	21.78	12	1.68
os74287	7	405	0.4	4	3	6	6.71	0.01	0.94	1.54	0.06	94.05	-0.76	7.54	7.49	16.33	7.96	-1.56	12.49	21.93	12.13	1.77
os74288	7	409	0.54	3	3	5	6.77	0.03	1.42	1.55	0.07	96.48	-1.7	7	7.44	16.28	7.93	-1.59	12.44	21.87	12.08	1.74
os74304	7	738	0.74	8	2	7	6.44	-0.12	0.71	1.55	0	99.28	-11.15	7.35	4.84	13.77	5.7	-3.41	9.22	18.85	9.42	-0.28
os74424	7	344	0.85	4	3	6	6.61	-0.06	2.39	1.54	0.05	72.12	-1.74	8.45	7.95	16.78	8.33	-1.19	12.95	22.37	12.5	2.07
os74427	5	659	0.9	10	2	0	6.24	-0.17	0.2	1.46	0.25	99.91	-2.33	5.69	5.34	14.31	6.19	-2.79	9.66	19.24	9.7	0.13
os74444	5	533	0.4	4	3	6	6.92	0.03	0.84	1.51	0.08	95.79	-0.12	6.85	6.43	15.32	7.05	-2.24	10.94	20.53	10.85	0.86
os74528	7	526	0.47	6	2	7	6.63	-0.05	0.69	1.54	0.2	89.84	-1.58	6.83	5.85	14.71	6.61	-2.45	10.25	19.77	10.31	0.6
os74612	5	454	0.01	5	2	0	6.9	0.08	0.55	1.53	0.08	99.2	-0.37	6.44	7.05	16.03	7.5	-1.77	11.86	21.55	11.47	1.31
os74633	5	444	0.26	7	3	5	6.93	0.07	0.37	1.51	0.08	99.07	-0.89	6.08	7.12	16.07	7.59	-1.69	11.81	21.45	11.42	1.36
os74688	7	662	0.54	14	2	7	6.34	-0.09	0	1.46	0.17	99.06	-3.17	6.65	5.52	14.46	6.27	-2.9	9.86	19.49	9.88	0.03
os74701	5	426	0.23	3	3	6	6.78	0.02	2	1.53	0.06	97.59	-0.03	7.29	7.45	16.43	7.82	-1.52	12.31	22.03	11.83	1.55
os74821	4	357	0.19	25	2	0	6.57	0.28	0	1.43	0.21	96.47	-3.42	5.93	7.54	16.46	7.87	-1.42	12.11	21.8	11.55	1.34
os74848	4	512	0.45	8	2	3	6.63	-0.01	0.58	1.49	0.15	99.92	-3.55	6.58	6.95	15.92	7.4	-1.86	11.61	21.33	11.17	0.96
os74851	7	267	0.69	3	2	3	6.6	-0.06	1.79	1.54	0.07	91.62	-2.78	7.97	8.36	17.26	8.56	-0.88	13.08	22.82	12.43	1.99
os74918	7	400	0.98	7	2	7	6.65	-0.06	1.07	1.53	0.17	97.14	-6.76	7.25	7.34	16.25	7.7	-1.65	11.93	21.61	11.47	1.27
os74949	4	266	0.15	10	3	5	6.69	0.1	0.04	1.48	0.09	89.81	0.21	6.39	8.43	17.33	8.57	-0.85	13.15	22.9	12.47	2.02
os74951	4	269	0.21	10	3	5	6.69	0.1	0.03	1.47	0.08	90.54	0.36	6.37	8.43	17.33	8.57	-0.85	13.15	22.9	12.48	2.02
os74959	4	267	0.38	2	2	3	6.78	0.03	2.34	1.56	0.04	86.66	-1.91	8.19	8.49	17.38	8.64	-0.79	13.21	22.94	12.51	2.06
os74960	4	267	0.29	3	3	6	6.8	0.04	2.1	1.55	0.04	87.33	-1.21	8.3	8.5	17.38	8.64	-0.78	13.22	22.95	12.52	2.06
os74970	4	210	0.4	3	3	6	6.75	0.02	2.72	1.56	0.06	61.21	-0.95	8.37	8.86	17.77	8.89	-0.61	13.84	23.61	12.98	2.34
os74975	4	238	0.74	6	2	3	6.5	-0.08	1.21	1.53	0.11	98.38	-4	7.78	8.63	17.55	8.72	-0.72	13.49	23.29	12.75	2.18
os74986	7	441	0.08	17	2	7	6.64	0.19	0.22	1.48	0.19	96.52	-6.63	5.66	7.06	15.95	7.46	-1.71	11.48	21.08	11.06	1.09
os74991	4	335	0.79	11	3	5	6.31	-0.13	0.04	1.48	0.08	92.34	1.61	7.2	7.97	16.86	8.21	-1.18	12.66	22.35	12.07	1.69
os74992	4	338	0.67	9	3	5	6.41	-0.1	0.1	1.48	0.07	91.89	1.69	7.24	7.97	16.86	8.21	-1.18	12.66	22.35	12.07	1.69
os74995	4	332	0.21	12	2	7	6.68	0.11	0.74	1.53	0.19	94.76	-9.13	6.11	7.79	16.64	8.08	-1.2	12.3	21.92	11.76	1.6
os75006	4	272	0.02	7	2	3	6.47	-0.05	0.78	1.51	0.13	98.07	-4.41	7.49	8.37	17.28	8.53	-0.98	13.23	22.99	12.55	1.98

os75010	7	284	0.41	6	2	7	6.79	0.03	1.8	1.53	0.13	95.8	-5.94	7.84	8.24	17.2	8.42	-1.1	13.17	22.96	12.49	1.88
os75013	7	291	0.41	7	2	7	6.74	0.01	0.86	1.52	0.13	97.19	-6.04	7.76	8.23	17.19	8.41	-1.11	13.16	22.95	12.48	1.87
os75014	7	289	0.96	7	2	7	6.6	-0.07	1.24	1.52	0.13	96.55	-5.7	7.76	8.23	17.19	8.42	-1.1	13.17	22.96	12.49	1.88
os75019	4	280	0.99	2	2	7	6.65	-0.05	1.85	1.56	0.1	78.17	-3.96	7.74	8.22	17.15	8.41	-1.11	13.13	22.88	12.46	1.9
os75022	4	300	1	13	3	5	6.2	-0.22	0	1.47	0.09	87.04	0.12	6.34	8.25	17.17	8.44	-1.09	13.15	22.91	12.48	1.91
os75069	4	362	0.92	7	3	5	6.51	-0.08	0.61	1.48	0.06	94.79	-0.48	7.71	7.97	16.84	8.19	-1.26	12.71	22.37	12.02	1.67
os75159	7	523	0.49	6	3	5	6.71	-0.01	0.84	1.52	0.1	99.69	-1.34	6.93	6.58	15.49	7.1	-2.28	11.32	20.92	11.04	0.78
os75176	7	546	0.88	11	2	0	6.61	-0.12	0.02	1.47	0.16	96.91	-0.44	5.87	6.22	15.14	6.79	-2.48	10.84	20.42	10.59	0.53
os75250	7	446	0.42	12	2	3	6.92	0.08	0.08	1.46	0.15	88.95	-4.05	6.56	7.14	16.06	7.58	-1.73	11.83	21.42	11.41	1.31
os75296	4	522	0.42	3	1	1	6.71	-0.05	0.01	1.42	0	100	5.5	6.34	7.25	16.2	7.64	-1.72	12.09	21.77	11.62	1.37
os75452	7	459	0.7	7	2	7	6.51	-0.09	0.73	1.53	0.13	98.75	-4.42	7.34	6.92	15.83	7.37	-2.03	11.57	21.21	11.17	0.98
os75459	4	551	1	20	2	3	5.88	-0.32	0	1.38	0.17	96.57	-1.16	5.11	6.54	15.45	7.06	-2.31	11.13	20.79	10.83	0.68
os75534	7	456	0.99	9	2	7	6.5	-0.1	1.07	1.54	0.19	95.08	-7.91	7.37	6.75	15.69	7.25	-1.96	11.22	20.87	10.82	0.83
os75538	4	514	0.95	16	2	3	6.33	-0.2	0	1.43	0.12	99.71	-1.85	6.22	6.85	15.77	7.31	-1.92	11.34	21	10.92	0.89
os75589	4	580	0.98	21	2	7	5.86	-0.31	0	1.41	0.2	99.57	-1.61	5.72	6.15	15.1	6.69	-2.64	10.8	20.41	10.46	0.36
os75862	4	410	0.64	14	2	3	6.78	0.02	0.01	1.48	0.12	99.85	-2.63	6.71	7.37	16.29	7.72	-1.57	12.02	21.72	11.48	1.29
os75893	4	268	1	13	2	3	6.15	-0.22	0.02	1.47	0.14	96.52	-4.48	6.06	8.77	17.63	8.86	0.01	13.52	22.86	12.61	2.68
os75905	4	387	0.91	14	1	4	6.37	-0.18	0	1.43	0.08	87.99	2.1	6.08	7.96	16.75	8.2	-0.63	12.46	21.66	11.69	1.88
os75928	5	425	0.99	4	3	6	6.66	-0.07	0.59	1.52	0.05	90.9	-0.59	7.76	7.66	16.5	7.93	-0.98	12.19	21.47	11.42	1.54
os76031	4	238	0.87	18	2	0	6.31	-0.16	0	1.46	0.17	87.96	-1.4	6.13	8.28	16.99	8.55	-0.33	12.79	22	12	2.11
os76040	5	548	0.89	3	3	6	6.75	-0.05	1.55	1.53	0.04	95.8	-0.23	6.88	6.91	15.72	7.32	-1.59	11.35	20.69	10.71	0.82
os76042	7	516	0.01	3	3	6	6.9	0.05	1.52	1.53	0.04	77.21	-0.37	9.29	6.98	15.79	7.38	-1.56	11.44	20.76	10.78	0.89
os76057	4	296	0.76	20	3	9	5.76	-0.24	0	1.36	0.14	99.98	1.2	5.03	8.63	17.43	8.87	0.06	13.09	22.44	12.36	2.53
os76182	4	304	0.62	12	2	3	6.46	-0.1	0.35	1.42	0.14	99.48	-4.54	7.75	8.55	17.38	8.72	-0.19	13.15	22.49	12.27	2.32
os76217	4	394	0.46	4	3	6	6.73	0.02	1.72	1.49	0.09	99.81	-1.93	8.08	7.84	16.64	8.2	-0.63	12.22	21.52	11.57	1.78
os76219	4	308	0.99	24	2	7	5.42	-0.38	0	1.42	0.22	99.33	-5.79	5.92	8.19	17.02	8.49	-0.29	12.59	21.91	11.88	2.17
os76276	5	555	0.55	5	3	6	6.76	0	0.57	1.51	0.04	93.8	0.14	6.53	6.92	15.73	7.33	-1.57	11.36	20.7	10.71	0.83
os76296	7	322	0.47	3	2	0	6.62	-0.06	1.92	1.54	0.09	96.4	-2.07	7.5	7.93	16.68	8.3	-0.46	12.41	21.73	11.78	1.96
os76328	5	358	1	14	3	9	6.15	-0.24	0	1.44	0.11	99.18	-0.45	5.62	7.99	16.74	8.33	-0.48	12.53	21.82	11.84	2
os76341	4	268	0.03	3	1	4	6.8	0.06	0.03	1.49	0.07	97	3.45	7.79	8.37	17.17	8.69	-0.07	12.77	22.1	12.15	2.36
os76346	4	228	0.35	27	2	7	6.62	0.19	0	1.42	0.29	99.59	-8.29	5.73	8.32	17.09	8.63	-0.18	12.77	22.05	12.09	2.24
os76358	5	341	1	5	3	6	6.56	-0.08	0.51	1.51	0.08	100	-0.32	7.34	7.92	16.71	8.28	-0.5	12.48	21.77	11.83	1.99
os76360	4	385	0.96	14	2	3	6.08	-0.23	0.07	1.47	0.15	99.63	-6.14	7.41	7.71	16.5	8.08	-0.7	12.24	21.54	11.6	1.78
os76374	4	421	0.96	18	3	5	6.12	-0.26	0	1.44	0.14	98.72	-1.72	5.14	7.58	16.33	7.93	-0.91	12.03	21.29	11.32	1.47
os76384	4	485	0.35	15	2	3	6.89	0.13	0	1.46	0.15	99.8	-4.32	5.1	7.2	15.99	7.58	-1.27	11.64	20.92	10.93	1.09
os76385	5	549	0.02	3	3	6	6.73	-0.05	1.17	1.53	0.05	96.62	-0.35	6.63	6.9	15.71	7.31	-1.59	11.34	20.68	10.7	0.82
os76386	7	517	0	2	3	6	6.88	0.03	1.47	1.53	0.04	80.89	-0.13	9.42	6.98	15.79	7.39	-1.56	11.45	20.77	10.78	0.89

os76393	7	424	0.03	8	2	3	6.96	0.14	0.55	1.46	0.11	99.94	-3.15	6.93	7.61	16.45	7.98	-0.83	12	21.36	11.36	1.6
os76409	4	405	1	20	2	3	5.88	-0.28	0.01	1.4	0.18	99.76	-6.65	7.32	7.72	16.49	8.07	-0.78	12.06	21.32	11.39	1.58
os76455	4	524	0.66	11	3	5	6.94	0.07	0	1.43	0.09	99.96	3.56	5.42	7.29	16.12	7.53	-1.35	11.69	20.96	10.85	1.11
os76500	4	522	0.01	10	3	5	7.04	0.17	0.04	1.48	0.1	100	-0.01	6.04	7.22	16.04	7.46	-1.4	11.55	20.8	10.71	1.01
os76554	4	363	0.58	8	2	3	6.57	-0.03	0.89	1.47	0.1	99.54	-4.96	7.82	8.39	17.36	8.52	-0.29	13.02	22.5	12.11	2.26
os76846	4	190	0.6	8	3	5	6.41	-0.08	0.38	1.51	0.11	98.81	-2.54	7.67	9.12	17.87	9.2	0.35	13.73	22.95	12.82	2.96
os76876	7	411	0.98	8	2	0	6.55	-0.12	0.21	1.49	0.09	99.44	0.81	7.01	7.48	16.27	7.82	-1.09	12	21.29	11.27	1.4
os76885	5	447	0.92	2	2	7	6.73	-0.03	1.6	1.49	0.15	93.88	-3.3	7.22	7.17	15.97	7.57	-1.25	11.56	20.86	10.94	1.18
os76887	4	399	0.04	12	2	7	6.98	0.21	0.01	1.49	0.13	98.44	-2.18	5.94	7.52	16.27	7.87	-0.97	11.92	21.21	11.27	1.48
os76897	7	392	0.9	6	2	0	6.68	-0.07	0.4	1.51	0.09	99.34	0.24	7.14	7.6	16.35	7.95	-0.9	12.01	21.31	11.37	1.57
os76920	7	266	0.09	1	2	3	6.71	0	2.61	1.56	0.05	80.1	-2.61	8.08	8.55	17.26	8.76	-0.13	13.13	22.36	12.38	2.45
os77011	7	479	0.01	5	3	5	6.9	0.08	0.8	1.51	0.06	99.59	-0.25	7.56	7.48	16.35	7.68	-1.24	12.02	21.31	11.15	1.31
os77026	4	555	0.77	6	3	5	6.81	-0.04	0.48	1.49	0.05	100	0.31	6.56	7.18	16.03	7.43	-1.45	11.54	20.82	10.71	0.99
os77065	4	437	0.01	4	1	1	6.87	0.07	0.14	1.39	0.05	99.55	2.69	6.4	7.92	16.8	8.14	-0.71	12.52	21.85	11.73	1.85
os77094	3	401	0.34	11	3	9	6.92	0.08	0.03	1.46	0.09	98.49	-1.31	5.65	7.95	16.72	8.26	-0.58	12.44	21.74	11.81	1.93
os77102	7	357	0.4	2	3	6	6.78	0	0.21	1.53	0.04	97.09	0.6	7.59	8.02	16.75	8.29	-0.6	12.55	21.78	11.8	1.92
os77107	7	304	0	11	2	7	6.87	0.16	0.47	1.52	0.12	100	-4.71	7.26	8.27	17	8.51	-0.38	12.81	22.01	12.03	2.18
os77134	4	411	0.01	9	2	0	6.91	0.14	0.36	1.51	0.13	99.47	-2.66	7.28	7.49	16.27	7.82	-1.07	11.94	21.21	11.22	1.42
os77151	7	507	0.19	8	3	5	6.98	0.09	0.37	1.51	0.08	99.34	-1.92	7.87	7.02	15.84	7.41	-1.5	11.5	20.85	10.82	0.98
os77187	7	382	0.15	12	2	7	6.67	0.11	0.06	1.49	0.15	99.99	-3.18	7.01	7.81	16.65	8.04	-0.78	12.29	21.62	11.54	1.78
os77241	4	539	0.78	3	3	6	6.74	-0.03	0.94	1.52	0.04	92.69	-0.66	6.84	7.34	16.26	7.59	-1.38	11.91	21.28	11.06	1.16
os77275	4	228	0.4	23	3	5	6.6	0.08	0	1.38	0.15	96.86	0.38	5.98	9.08	17.91	9.14	0.22	13.97	23.23	12.99	2.91
os77301	4	535	0.23	1	3	6	6.83	0.01	0.99	1.52	0.03	87.86	0.47	8.43	7.39	16.3	7.64	-1.34	11.97	21.33	11.12	1.2
os77302	4	536	0.92	2	3	6	6.78	-0.02	0.62	1.52	0.03	90.19	-0.05	7.45	7.38	16.29	7.62	-1.35	11.95	21.31	11.1	1.19
os77314	4	372	1	12	2	7	6.29	-0.2	0.05	1.47	0.12	98.5	-2.47	7.79	8.04	16.88	8.24	-0.52	12.49	21.82	11.71	2.05
os77347	4	311	0.98	8	1	4	6.52	-0.12	0.17	1.49	0.05	82.44	3.05	6.25	8.33	17.06	8.58	-0.3	12.88	22.11	12.13	2.23
os77357	7	348	0.94	4	3	6	6.66	-0.06	0.42	1.53	0.05	94.03	0.18	8.1	8.05	16.78	8.32	-0.57	12.58	21.81	11.83	1.95
os77395	7	257	0.58	6	2	7	6.78	0.02	1.67	1.55	0.09	91.36	-4.34	8.47	8.58	17.31	8.76	-0.14	13.15	22.34	12.3	2.39
os77416	4	548	0.02	10	3	5	7.05	0.17	0.03	1.45	0.07	100	1.9	5.74	7.18	16.03	7.44	-1.45	11.56	20.84	10.73	1
os77418	4	540	0.21	10	1	4	7.05	0.15	0.01	1.45	0.07	99.99	2.28	5.66	7.26	16.1	7.5	-1.38	11.65	20.92	10.81	1.08
os77464	4	318	0.93	15	2	7	6.27	-0.2	0	1.45	0.13	98.6	-2.9	7.18	8.17	16.94	8.41	-0.44	12.49	21.76	11.75	1.99
os77487	4	525	0.51	22	3	5	6.76	0.07	0	1.4	0.15	100	-2.24	5.88	7.23	16.08	7.56	-1.26	11.49	20.84	10.8	1.12
os77499	4	522	1	9	2	3	6.5	-0.14	0.56	1.46	0.12	100	-3.92	7.26	7.28	16.14	7.58	-1.2	11.52	20.9	10.85	1.25
os77536	4	353	0.39	13	3	9	6.84	0.1	0	1.46	0.11	99.42	-1.95	5.39	8.06	16.79	8.36	-0.49	12.55	21.77	11.83	1.95
os77579	5	401	0.97	5	3	5	6.66	-0.07	0.45	1.52	0.07	98.6	0.2	7.23	7.6	16.39	8	-0.79	12.06	21.39	11.48	1.7
os77646	5	303	0.2	14	2	3	6.87	0.16	0.13	1.49	0.13	100	-4.37	5.58	8.19	16.93	8.49	-0.27	12.79	22.02	12.1	2.29
os77676	7	308	0.4	5	2	7	6.79	0.05	1.34	1.54	0.12	99.44	-5.01	7.64	7.93	16.66	8.26	-0.52	12.45	21.67	11.79	1.98

os77725	4	323	0.43	8	3	5	6.83	0.03	0.11	1.51	0.07	89.54	0.58	6.95	8.02	16.76	8.33	-0.53	12.49	21.76	11.83	1.96
os77755	4	278	0.04	14	2	0	6.93	0.23	0	1.44	0.19	98.65	0.08	5.45	7.97	16.77	8.33	-0.45	12.38	21.68	11.77	1.97
os77764	4	486	0.01	8	2	3	6.98	0.14	0.39	1.52	0.08	97.18	-2.63	6.19	7.27	16.06	7.61	-1.3	11.76	21.03	11.04	1.19
os77767	4	272	0.76	19	2	7	5.75	-0.18	0.23	1.45	0.27	100	-11.06	6.98	8.42	17.25	8.64	-0.13	12.79	22.12	11.99	2.31
os77815	5	520	0.77	2	3	6	6.83	-0.01	2.66	1.54	0.04	74.11	0.02	8.58	7	15.92	7.34	-1.4	11.9	21.43	11.41	1.61
os77868	5	483	0.99	2	3	6	6.75	-0.04	1.17	1.54	0.04	74.29	0.01	8.29	7.24	16.15	7.53	-1.19	12.23	21.72	11.68	1.87
os77998	5	430	0.65	4	3	6	6.66	-0.07	0.57	1.53	0.04	82.83	0.34	8.58	7.34	16.19	7.64	-1.04	12.31	21.8	11.71	2.01
os78033	5	457	0.02	4	2	0	6.87	0.07	0.78	1.53	0.08	97.02	-0.78	7.87	7.1	16.01	7.36	-1.21	12.29	21.7	11.68	2.06
os78046	7	568	0.17	9	3	5	7.05	0.14	0.24	1.5	0.11	99.98	-1.83	6.6	6.48	15.42	6.95	-1.51	11.41	20.86	11.06	1.62
os78055	5	368	0.92	1	3	6	6.71	-0.03	1.12	1.55	0.02	17.83	0	8.35	7.89	16.84	8.11	-0.8	13.13	22.78	12.5	2.26
os78207	4	396	0.92	6	3	6	6.56	-0.1	0.84	1.51	0.05	92.49	-0.4	8.88	7.67	16.65	7.95	-0.95	12.6	22.35	12.1	1.97
os78215	5	419	0.88	5	3	5	6.73	-0.04	0.71	1.51	0.03	92.4	0.9	7.45	7.74	16.76	8	-0.86	12.71	22.45	12.14	2.09
os78216	5	438	0.39	3	3	6	6.71	-0.04	0.5	1.52	0.02	93.93	0.19	7.3	7.76	16.79	8.01	-0.85	12.74	22.48	12.15	2.12
os78228	5	400	0.08	2	3	6	6.82	0.02	2.72	1.55	0.01	21.34	-0.16	8.8	7.82	16.81	8.04	-0.81	13.02	22.64	12.35	2.23
os78256	4	380	0.59	13	1	4	6.25	-0.19	0	1.45	0.05	87	2.14	6.96	7.73	16.72	8	-0.82	12.78	22.46	12.16	2.13
os78262	5	395	0.35	7	2	3	6.56	-0.02	1.19	1.53	0.13	97.33	-7.11	8.21	7.58	16.54	7.86	-0.94	12.55	22.18	11.92	2
os78339	5	355	0.03	2	3	6	6.68	-0.04	1.44	1.55	0.03	71.74	-0.04	7.36	7.94	16.88	8.13	-0.72	13.19	22.82	12.5	2.34
os78352	5	373	0.12	2	3	6	6.83	0.03	0.55	1.54	0.01	67.12	0.52	7.75	7.93	16.9	8.14	-0.76	13.21	22.85	12.53	2.35
os78353	5	448	0.31	4	3	6	6.67	-0.07	1.62	1.54	0.04	90.84	-0.78	8.16	7.22	16.13	7.52	-1.15	12.23	21.77	11.65	1.96
os78413	5	465	0.92	6	2	3	6.57	-0.1	0.69	1.53	0.07	98.51	-2.14	6.17	7.19	16.09	7.48	-1.19	12.27	21.77	11.69	1.96
os78431	5	472	0.99	3	3	6	6.73	-0.04	1.97	1.54	0.04	92.05	-1.59	9.16	7.37	16.32	7.67	-1.18	12.38	21.97	11.85	1.86
os78469	3	456	0.39	4	3	6	6.8	-0.01	0.86	1.52	0.08	85.97	-0.84	7.27	7.28	16.21	7.58	-1.13	12.39	21.91	11.8	2.01
os78516	5	494	0.2	4	3	6	6.84	0.04	0.58	1.53	0.04	98.82	-0.06	8.97	7.19	16.13	7.51	-1.29	12.19	21.72	11.65	1.76
os78573	5	463	0.81	6	3	5	6.58	-0.08	0.46	1.52	0.04	94.48	0.23	6.24	7.21	16.16	7.54	-1.16	12.39	21.95	11.83	1.98
os78582	5	461	0.99	4	3	6	6.7	-0.06	1	1.54	0.06	96.94	-0.54	7.58	7.13	16.06	7.46	-1.2	12.2	21.74	11.66	1.94
os78601	5	510	0.99	4	3	5	6.65	-0.08	0.55	1.52	0.04	99.1	-0.4	6.62	7.02	15.98	7.38	-1.24	12.09	21.65	11.62	1.89
os78625	7	538	0.92	3	3	6	6.71	-0.05	0.33	1.52	0.04	98.95	0.47	6.53	6.75	15.68	7.19	-1.4	11.73	21.21	11.29	1.72
os78627	7	543	0.63	5	3	6	6.66	-0.08	0.56	1.52	0.04	99.57	0.07	6.63	6.71	15.64	7.16	-1.42	11.69	21.17	11.26	1.7
os78665	5	488	0.39	4	3	6	6.79	0.02	1.57	1.54	0.08	99.37	-1.04	7.83	6.99	15.91	7.32	-1.3	12.04	21.52	11.51	1.88
os78667	5	516	0.9	5	3	6	6.65	-0.09	0.54	1.52	0.05	99.12	-0.54	7.1	6.96	15.88	7.29	-1.32	11.99	21.49	11.48	1.86
os78682	5	477	0.52	6	2	3	6.62	-0.03	0.81	1.51	0.12	98.26	-2.22	7.51	7.1	16.01	7.38	-1.22	12.2	21.6	11.6	1.99
os78786	5	469	0.76	9	3	9	6.45	-0.1	0.04	1.47	0.05	97.33	1.38	5.95	7.35	16.24	7.58	-1.12	12.52	21.99	11.89	2.13
os78822	5	409	0.92	2	3	6	6.72	-0.03	3.32	1.56	0.04	34.4	0.13	8.44	7.52	16.36	7.69	-1.05	12.64	22.1	11.97	2.12
os78824	7	397	0.88	2	3	6	6.71	-0.04	1.78	1.55	0.06	95.93	-0.69	8.82	7.58	16.46	7.77	-0.99	12.76	22.21	12.07	2.2
os78830	4	592	0.17	11	1	1	7.03	0.13	0	1.36	0.06	98.25	8.59	5.26	6.84	15.77	7.19	-1.38	11.84	21.34	11.33	1.78
os78854	5	434	0.47	3	2	0	6.73	0	1.78	1.55	0.05	89.87	-1.05	8.5	7.51	16.45	7.77	-1.11	12.54	22.11	11.96	1.96
os78860	5	498	0.92	2	3	6	6.79	-0.02	2.89	1.55	0.03	41.52	-0.25	8.89	7.1	16	7.41	-1.36	12.06	21.55	11.48	1.7

os78866	5	416	0.67	3	3	6	6.66	-0.06	0.61	1.54	0.04	99.6	0.01	6.93	7.61	16.53	7.8	-1.01	12.73	22.22	12.05	2.1
os78868	5	454	0.6	5	3	6	6.67	-0.03	1.5	1.54	0.05	99.78	-1.42	8.17	7.38	16.28	7.62	-1.12	12.43	21.93	11.84	1.97
os78880	5	418	0	3	2	0	6.85	0.04	2.45	1.55	0.06	96.2	-1.46	9.41	7.44	16.34	7.69	-1.06	12.52	22	11.88	2.03
os78885	5	464	0.23	2	3	6	6.82	0.03	1.34	1.55	0.04	96.48	0.08	7.8	7.28	16.17	7.56	-1.22	12.23	21.71	11.67	1.8
os78952	5	381	0.92	1	3	6	6.79	0.02	2.15	1.55	0.01	32.73	-0.19	8.35	7.86	16.79	8.07	-0.85	13.09	22.69	12.44	2.28
os78974	4	365	0.92	1	3	6	6.75	0	2.89	1.56	0.04	56.77	-0.47	9.46	7.99	16.94	8.16	-0.77	13.05	22.67	12.38	2.25
os78995	5	430	0.48	3	3	6	6.73	-0.01	2.42	1.54	0.04	89.74	-1.3	8.21	7.35	16.28	7.61	-1.05	12.54	22.07	11.91	2.07
os79029	5	486	0.08	2	3	6	6.87	0.03	3.54	1.55	0.03	11.98	-0.47	9.18	7.01	15.84	7.28	-1.18	12.01	21.36	11.32	1.73
os79141	5	453	0.01	3	3	6	6.86	0.04	2.62	1.56	0.06	53	-0.82	7.87	7.14	16	7.42	-1.11	12.11	21.51	11.46	1.81
os79170	5	417	0.92	0	3	6	6.79	0	4.99	1.57	0.02	1.36	0.04	12.29	7.35	16.2	7.55	-0.94	12.46	21.78	11.59	2.03
os79247	5	383	0.6	2	3	6	6.71	-0.02	2.67	1.56	0.05	78.4	-1.31	8.06	7.55	16.44	7.78	-0.89	12.52	22.09	11.9	2.08
os79322	4	405	1	6	3	5	6.58	-0.1	0.32	1.52	0.06	95.56	-0.7	6.79	7.48	16.37	7.66	-0.94	12.71	22.12	11.9	2.18
os79356	5	430	0.99	7	2	3	6.6	-0.11	0.6	1.53	0.09	98.16	-3.26	7.47	7.31	16.24	7.6	-1.08	12.51	22.03	11.88	2.05
os79408	5	428	0.99	6	3	5	6.64	-0.08	0.91	1.54	0.04	73.47	-1.24	8.49	7.42	16.37	7.7	-0.97	12.53	22.15	11.96	2.04
os79412	4	411	0	2	3	6	6.73	-0.03	2.6	1.54	0.02	56.5	-0.04	8.72	7.55	16.47	7.8	-0.9	12.65	22.24	12.01	2.1
os79451	5	463	0.07	5	3	5	6.86	0.07	0.94	1.54	0.06	74.46	-0.85	7.61	7.09	15.88	7.36	-1.11	11.96	21.24	11.26	1.74
os79557	6	441	0.1	2	3	6	6.85	0.03	1.98	1.56	0.04	71.98	-1.67	8.22	7.22	16.08	7.48	-1.09	12.3	21.69	11.57	1.84
os79579	5	424	0.93	3	3	6	6.73	-0.04	1.85	1.54	0.03	86.58	-0.88	8.11	7.49	16.4	7.72	-0.97	12.61	22.15	11.91	2.1
os79602	5	469	0.13	3	3	6	6.88	0.04	3.03	1.55	0.04	78.83	-0.23	9.51	7.13	16.01	7.39	-1.14	12.14	21.53	11.49	1.85
os79614	5	480	0.4	2	3	6	6.84	0	3.58	1.55	0.03	55.37	-0.67	8.76	7.08	15.98	7.37	-1.17	12.09	21.55	11.46	1.82
os79671	5	422	0.96	2	3	6	6.75	-0.03	1.8	1.55	0.03	85.22	-0.09	8.73	7.35	16.22	7.56	-0.95	12.57	21.94	11.76	2.14
os79672	5	434	0.99	3	3	6	6.73	-0.04	1.06	1.54	0.03	80.97	0.11	7.73	7.29	16.16	7.51	-0.98	12.47	21.86	11.7	2.1
os79696	5	401	0.36	5	3	6	6.67	-0.01	0.86	1.53	0.06	98.3	-1.76	7.57	7.49	16.39	7.74	-0.9	12.68	22.18	11.96	2.18
os79730	5	481	0.92	1	3	6	6.79	-0.01	4.33	1.56	0.02	22.68	-0.35	9.44	7.09	15.88	7.34	-1.17	12.11	21.44	11.37	1.75
os79813	5	403	0.24	3	3	6	6.84	0.03	2.07	1.55	0.05	85.22	-1.88	9.44	7.47	16.37	7.72	-0.92	12.62	22.14	11.94	2.18
os79815	5	399	0	2	2	3	6.82	0.03	2.81	1.56	0.06	85.02	-3.73	9.58	7.48	16.38	7.73	-0.91	12.64	22.16	11.95	2.19
os79829	5	409	0.37	6	2	3	6.65	0	1.01	1.54	0.1	81.12	-4.08	7.85	7.39	16.24	7.6	-0.92	12.59	21.95	11.77	2.17
os79950	5	487	0	6	3	5	6.96	0.1	0.79	1.52	0.06	99.56	-1.6	7.94	6.96	15.87	7.29	-1.13	12.03	21.46	11.38	1.91
os79957	5	483	0.7	4	3	6	6.7	-0.03	1.03	1.53	0.03	86.27	-0.39	7.26	7.04	15.95	7.33	-1.1	12.16	21.58	11.47	1.95
os80040	7	411	0.08	1	3	6	6.81	0.01	4.98	1.57	0.02	4.93	-0.41	9.91	7.36	16.24	7.56	-1.14	12.78	22.16	12.1	2.2
os80044	5	433	0.01	4	3	6	6.91	0.07	0.72	1.52	0.03	81.59	0.15	8.2	7.36	16.3	7.62	-1.04	12.56	22.09	11.92	2.07
os80109	5	413	0.15	5	2	3	6.91	0.07	0.8	1.53	0.06	90.22	-2.24	6.6	7.53	16.43	7.73	-1.04	12.78	22.25	12.08	2.19
os80115	5	392	0.92	1	3	6	6.73	-0.02	2.72	1.56	0.03	43.13	-0.2	9	7.65	16.57	7.82	-1.01	12.97	22.44	12.2	2.25
os80133	4	429	0.47	10	3	5	6.91	0.06	0.11	1.5	0.07	93.08	-1.13	7.49	7.5	16.41	7.69	-1.06	12.7	22.18	12.02	2.18
os80161	3	461	0.98	8	1	1	6.48	-0.13	0.08	1.46	0.04	99.91	2.37	6.17	7.42	16.32	7.63	-1.09	12.61	22.11	11.96	2.17
os80170	5	662	0.56	7	3	5	6.93	0	0.29	1.51	0.05	92.97	-0.57	7.04	6.06	14.87	6.6	-1.91	10.69	20.11	10.41	0.81
os80181	5	555	0.76	3	3	6	6.85	-0.01	1.69	1.55	0.04	96.61	-0.5	8.79	6.65	15.54	7.12	-1.53	11.41	20.93	11.07	1.21

os80182	5	552	0.77	3	3	6	6.84	-0.02	1.71	1.55	0.04	95.1	-0.43	8.78	6.66	15.56	7.13	-1.53	11.43	20.95	11.08	1.22
os80240	5	529	0.97	3	3	6	6.78	-0.04	1.73	1.53	0.03	79.42	-0.53	7.92	6.89	15.76	7.26	-1.43	11.67	21.23	11.26	1.4
os80271	4	488	0.43	3	2	3	6.76	0	1.77	1.53	0.07	94.42	-2.6	9.05	7.1	16.02	7.46	-1.28	11.95	21.52	11.48	1.57
os80320	5	452	0.61	2	3	6	6.83	0.01	1.5	1.55	0.03	63.56	0.13	8.78	7.36	16.34	7.68	-1.08	12.04	21.75	11.59	1.75
os80325	5	438	0.48	2	3	6	6.84	0.01	1.68	1.54	0.02	84.88	-0.19	8.12	7.46	16.44	7.79	-1	12.19	21.89	11.69	1.85
os80328	5	437	0.4	2	3	6	6.84	0.02	1.78	1.54	0.02	88.19	-0.24	8.12	7.47	16.45	7.8	-0.99	12.2	21.9	11.7	1.86
os80329	5	446	0.08	2	3	6	6.85	0.02	1.22	1.54	0.03	61.77	0.13	8.95	7.37	16.34	7.68	-1.08	12.03	21.74	11.58	1.75
os80340	5	431	0.78	4	3	6	6.66	-0.05	1.9	1.55	0.05	86.47	-2.17	9.34	7.45	16.42	7.75	-1.03	12.18	21.87	11.66	1.83
os80382	5	432	0.27	8	3	5	6.69	0.04	0.38	1.51	0.05	100	-1.18	6.91	7.51	16.45	7.81	-1.05	12.23	21.99	11.82	1.76
os80497	5	450	0.1	3	3	6	6.84	0.05	1.51	1.54	0.04	98.25	-0.76	8.42	7.39	16.35	7.71	-1.11	12.09	21.87	11.69	1.69
os80660	7	416	0.42	8	2	3	6.6	-0.02	0.8	1.53	0.12	96.5	-4.68	8.41	7.54	16.51	7.81	-0.96	12.32	22.02	11.84	1.88
os80668	4	371	0.24	9	2	0	6.93	0.08	0.09	1.5	0.07	97.43	0.99	6.6	7.81	16.74	8.05	-0.81	12.61	22.3	12.08	2.06
os80669	4	404	0.32	9	2	3	6.7	0.08	0.53	1.53	0.18	99.26	-8.45	7.53	7.7	16.64	7.94	-0.88	12.48	22.17	11.97	1.97
os80680	4	421	0.79	11	2	3	6.44	-0.08	0.67	1.5	0.15	95.9	-6.84	8.11	7.53	16.5	7.85	-0.97	12.34	22.05	11.88	1.86
os80718	4	469	0	10	2	0	6.9	0.12	0.46	1.52	0.13	92.3	-3.56	6.28	7.01	15.89	7.42	-1.28	11.79	21.33	11.35	1.45
os80841	4	451	0.44	7	3	5	6.92	0.06	0.72	1.52	0.08	90.96	-0.29	7.54	7.26	16.13	7.6	-1.15	12.04	21.59	11.58	1.6
os80851	5	557	0.08	6	3	5	7	0.09	0.5	1.53	0.05	98.11	-1.14	7.25	6.63	15.52	7.13	-1.52	11.39	20.92	11.07	1.19
os80908	3	528	0.12	13	1	4	7.07	0.18	0.02	1.39	0.06	97.83	3.94	5.7	7.02	15.99	7.43	-1.29	11.7	21.4	11.37	1.51
os80909	3	527	0.1	14	1	4	7.08	0.2	0	1.39	0.07	97.86	3.65	5.64	7.02	15.99	7.43	-1.29	11.7	21.4	11.37	1.51
os80983	4	456	0.86	13	2	7	6.24	-0.19	0.1	1.51	0.16	97.61	-5.39	8.54	7.09	16.07	7.49	-1.29	11.76	21.48	11.4	1.53
os80984	4	459	0.17	13	2	7	6.25	-0.2	0.09	1.5	0.15	97.65	-4.51	8.33	7.09	16.06	7.48	-1.29	11.76	21.48	11.4	1.53
os81006	5	443	0.92	2	3	6	6.73	-0.03	2.66	1.55	0.02	69.76	-0.15	8.59	7.41	16.39	7.73	-1.04	12.11	21.82	11.64	1.8
os81007	5	440	0.92	3	3	6	6.7	-0.04	2.41	1.54	0.03	69.9	-0.24	8.94	7.42	16.4	7.74	-1.04	12.13	21.83	11.65	1.81
os81051	5	431	0.39	3	3	6	6.86	0.03	1.13	1.54	0.02	93.06	0.26	7.77	7.47	16.44	7.79	-0.99	12.19	21.89	11.69	1.86
os81053	5	430	0.23	3	3	6	6.86	0.03	0.95	1.54	0.03	93.69	0.18	7.74	7.47	16.45	7.79	-0.99	12.19	21.89	11.69	1.86
os81069	5	423	0.39	2	3	6	6.84	0.02	2.95	1.55	0.04	81.76	-0.34	8.94	7.4	16.38	7.74	-1.03	12.1	21.81	11.62	1.8
os81094	4	436	0.77	7	2	0	6.77	-0.03	0.79	1.51	0.08	99.15	-2.15	8.26	7.41	16.39	7.69	-1.07	12.19	21.91	11.7	1.84
os81156	5	395	0.08	3	3	6	6.78	0.03	1.9	1.54	0.05	98.22	-1.65	8.24	7.81	16.88	8.11	-0.82	12.99	22.78	12.43	2.23
os81253	4	356	0.6	6	2	3	6.6	-0.04	0.9	1.55	0.06	79.49	-2.25	7.94	8.22	17.28	8.31	-0.62	13.5	23.26	12.62	2.49
os81325	5	361	0.92	0	3	6	6.76	0	7.59	1.55	0	2.43	0.22	11.29	8.06	17.1	8.24	-0.66	13.16	22.9	12.49	2.38
os81416	5	356	0.96	2	3	6	6.73	-0.03	2.57	1.54	0.03	64.42	-0.65	8.87	7.98	16.94	8.19	-0.74	13.26	22.91	12.58	2.37
os81444	5	527	0.4	2	2	3	6.86	0	2.51	1.55	0.05	84.62	-2.23	8.67	6.81	15.75	7.21	-1.51	11.64	21.23	11.23	1.3
os81713	5	434	0.23	3	3	6	6.79	0.03	0.72	1.53	0.05	64.93	0	8.28	7.41	16.4	7.73	-0.98	12.39	22.06	11.82	1.98
os81733	4	418	0.03	8	2	3	6.47	-0.14	0.49	1.54	0.09	57.64	-3.07	8.13	7.44	16.4	7.74	-1.11	12.44	22.07	11.92	1.79
os81849	4	344	0.21	6	2	7	6.89	0.08	1.19	1.54	0.13	94.24	-4.05	8.05	7.9	16.83	8.11	-0.76	12.66	22.32	12.05	2.09
os81954	7	448	0.92	0	3	6	6.79	0	2.99	1.54	0.03	34.21	-1.11	9.1	7.35	16.31	7.67	-1.03	12.28	21.9	11.71	1.89
os82006	5	358	0.92	1	3	6	6.78	0	3.49	1.54	0.01	27.95	-0.03	8.94	8.05	17.07	8.27	-0.65	13.19	22.92	12.52	2.38

os82049	5	376	0.06	4	3	6	6.87	0.07	0.75	1.54	0.03	77.55	-0.14	7.48	7.85	16.84	8.08	-0.78	13.13	22.78	12.45	2.31
os82059	5	362	0.92	0	3	6	6.75	-0.01	7.56	1.55	0	3.15	0.73	10.13	8.05	17.09	8.24	-0.66	13.15	22.89	12.49	2.38
os82060	5	361	0.92	0	3	6	6.76	0	7.6	1.55	0	2.79	-0.02	10.86	8.05	17.09	8.24	-0.66	13.16	22.89	12.49	2.38
os82070	5	370	0.92	4	3	6	6.64	-0.06	1.8	1.54	0.05	91.61	-1.3	7.81	8.11	17.2	8.27	-0.61	13.24	23.04	12.5	2.41
os82082	4	296	0.99	12	2	7	6.34	-0.18	0.05	1.51	0.11	69.61	-2.82	7.75	8.3	17.26	8.42	-0.57	13.32	22.96	12.54	2.38
os82092	5	442	0.14	2	3	6	6.79	0	1.51	1.54	0.03	87.5	-0.06	8.84	7.69	16.7	7.89	-0.95	12.71	22.41	12.02	2.08
os82095	5	430	0.01	3	3	6	6.84	0.04	2.74	1.54	0.03	66.42	-0.31	8.7	7.7	16.71	7.9	-0.94	12.72	22.4	12.02	2.09
os82107	5	425	0.6	3	3	6	6.72	-0.01	0.94	1.53	0.03	63.21	-0.36	7.33	7.78	16.77	7.99	-0.91	12.88	22.52	12.19	2.15
os82110	5	467	0.56	3	3	6	6.81	-0.01	1.5	1.53	0.02	82.01	0.17	8.19	7.48	16.44	7.75	-1.13	12.52	22.09	11.92	1.93
os82153	5	432	0.92	5	3	6	6.62	-0.07	0.95	1.53	0.04	99.99	-0.88	8.47	7.68	16.64	7.87	-1.07	12.64	22.28	11.98	1.92
os82178	5	347	0.99	2	3	6	6.72	-0.03	3.5	1.56	0.02	23.11	-0.22	9.13	7.94	16.92	8.17	-0.69	13.17	22.83	12.5	2.32
os82248	5	452	0.92	1	3	9	6.76	-0.02	0.42	1.52	0	91.04	1.11	7.97	7.61	16.61	7.9	-0.97	12.62	22.38	12.14	2.01
os82340	5	359	0.92	1	3	6	6.74	-0.02	2.98	1.55	0.02	37.59	0.21	9.44	8.06	17.06	8.23	-0.65	12.98	22.67	12.25	2.27
os82357	5	385	0.27	6	3	6	6.86	0.03	0.93	1.53	0.04	86.48	-0.97	8.21	7.95	16.92	8.15	-0.72	12.8	22.51	12.12	2.2
os82413	4	379	0.99	3	3	9	6.72	-0.04	1.04	1.5	0.01	87.29	0.45	7.56	8.08	17.06	8.27	-0.68	13.06	22.72	12.35	2.25
os82433	7	480	0.76	7	3	5	6.78	-0.04	0.52	1.53	0.06	81.57	-1.35	8.59	7.08	16	7.46	-1.24	11.77	21.4	11.4	1.56
os82458	5	465	0.92	1	3	6	6.76	-0.02	2.78	1.55	0.02	57.24	-0.5	9.41	7.26	16.23	7.6	-1.2	12.16	21.86	11.72	1.72
os82459	5	465	0.47	2	3	6	6.76	-0.03	2.73	1.55	0.02	59.4	0.09	9.32	7.26	16.24	7.6	-1.2	12.17	21.87	11.72	1.72
os82460	5	465	0.92	2	3	6	6.73	-0.04	1.57	1.54	0.02	86.48	0.05	8.36	7.29	16.25	7.61	-1.19	12.19	21.89	11.74	1.73
os82470	4	491	0.23	4	2	3	6.73	0.04	0.79	1.56	0.12	76.11	-4.79	6.98	6.97	15.76	7.22	-1.26	11.91	21.25	11.19	1.56
os82472	7	491	0.23	4	2	3	6.81	0.05	1.45	1.56	0.09	72.2	-3.53	7.17	6.96	15.75	7.22	-1.26	11.9	21.24	11.18	1.56
os82486	5	493	0.08	1	3	6	6.85	0.02	5.9	1.55	0	3.46	0.48	9.09	7.09	15.88	7.3	-1.2	12.14	21.48	11.39	1.71
os82488	5	493	0.23	1	3	6	6.82	0.01	5.74	1.55	0.01	26.06	0.01	8.18	7.06	15.85	7.3	-1.2	12.14	21.45	11.4	1.69
os82492	6	473	0	6	3	6	6.92	0.09	2.86	1.54	0.04	26.15	-0.48	7.72	7.18	16	7.39	-1.15	12.26	21.66	11.52	1.8
os82493	5	472	0.85	2	3	6	6.77	-0.03	4.98	1.56	0.02	25.92	-0.39	9.17	7.18	16	7.39	-1.15	12.26	21.66	11.52	1.8
os82495	5	468	0.89	2	3	6	6.77	-0.02	4.98	1.56	0.03	31.87	-0.23	8.97	7.21	16	7.4	-1.14	12.28	21.69	11.54	1.82
os82521	4	424	1	18	2	7	5.91	-0.3	0	1.44	0.2	89.82	-2.97	5.8	7.1	15.91	7.34	-1.23	12.06	21.44	11.32	1.66
os82543	5	472	0.5	4	3	6	6.84	0.01	4.74	1.56	0.05	56.21	-1.44	7.92	7.14	15.96	7.36	-1.16	12.18	21.6	11.47	1.76
os82558	5	480	0.01	3	3	6	6.72	-0.05	1.02	1.55	0.02	67.2	-0.09	7.63	7.13	15.9	7.33	-1.19	12.21	21.58	11.48	1.7
os82564	5	508	0.71	8	3	5	6.61	-0.06	0.31	1.51	0.05	74.47	0.82	6.71	7.01	15.85	7.25	-1.23	12.02	21.45	11.33	1.71
os82637	6	467	0.92	1	3	6	6.83	0	1.14	1.55	0.02	46.36	0.23	8.58	7.17	16.01	7.42	-1.13	12.17	21.56	11.48	1.74
os82655	5	462	0.77	5	3	6	6.76	-0.04	0.55	1.54	0.06	47.19	-0.58	8.85	7.13	15.96	7.38	-1.12	12.07	21.47	11.38	1.73
os82667	5	488	0.92	1	3	6	6.84	0.01	1.61	1.56	0.02	66.06	-0.34	8.61	7.07	15.86	7.31	-1.19	12.08	21.41	11.36	1.73
os82798	5	485	0.13	2	3	6	6.79	-0.01	3.97	1.54	0.02	34.47	0.65	9.98	7.12	15.96	7.34	-1.16	12.15	21.6	11.45	1.77
os82799	5	484	0.92	0	3	6	6.82	0	3.88	1.56	0.03	56.38	-0.36	9.86	7.09	15.93	7.32	-1.19	12.11	21.56	11.42	1.76
os82811	6	493	0.92	1	3	6	6.82	0	5.89	1.56	0.01	6.05	0.07	9.31	7.09	15.88	7.3	-1.2	12.14	21.48	11.39	1.7
os82812	5	493	0.92	0	3	6	6.82	0	5.9	1.56	0	4.79	0.37	11.24	7.09	15.88	7.3	-1.2	12.14	21.49	11.39	1.71

os82830	5	489	0.56	2	3	6	6.84	0.01	4.67	1.56	0.02	23.56	-0.11	9.3	7.04	15.81	7.28	-1.2	12.08	21.39	11.33	1.66
os82842	5	470	0.92	0	3	6	6.81	0	4.99	1.56	0.02	25.91	-0.07	9.49	7.21	16	7.4	-1.13	12.28	21.7	11.55	1.82
os82843	6	473	0.99	2	3	6	6.77	-0.03	4.97	1.56	0.02	24.05	-0.11	9.13	7.18	16	7.39	-1.14	12.26	21.66	11.52	1.8
os82935	3	505	0.08	6	3	5	6.98	0.1	0.28	1.51	0.04	81.22	1.51	7.45	6.99	15.78	7.24	-1.25	11.99	21.33	11.31	1.65
os82936	6	494	0.77	4	2	3	6.78	-0.03	1.57	1.56	0.09	69.59	-3.75	8.79	6.99	15.78	7.24	-1.25	11.98	21.32	11.3	1.65
os82937	5	494	0.83	4	3	5	6.76	-0.04	1.5	1.55	0.07	72.3	-2.14	8.77	7	15.79	7.25	-1.24	11.99	21.33	11.31	1.65
os82979	6	478	0.92	0	3	6	6.81	0	5.97	1.57	0.01	1.65	0	11.64	7.13	15.9	7.3	-1.19	12.17	21.53	11.44	1.76
os82980	6	474	0.92	0	3	6	6.81	0	5.74	1.57	0.03	44.94	-0.79	9.5	7.15	15.97	7.35	-1.14	12.22	21.61	11.49	1.77
os82986	5	495	0.95	4	3	6	6.72	-0.06	1.73	1.54	0.04	78.06	-1.26	7.79	7.02	15.78	7.24	-1.24	12.01	21.35	11.28	1.59
os83004	5	459	0.02	1	3	6	6.81	0.01	2.95	1.56	0.05	53.94	-0.82	9.02	7.11	15.93	7.36	-1.13	12.04	21.43	11.36	1.71
os83018	5	537	0.92	2	3	6	6.87	0.02	4.91	1.54	0.02	52.14	0.71	8.59	6.74	15.47	7.03	-1.45	11.6	20.92	10.97	1.37
os83022	5	540	0.92	0	3	6	6.84	0	4.98	1.55	0.01	31.68	0.07	11.19	6.75	15.48	7.04	-1.44	11.61	20.94	10.99	1.38
os83030	5	540	0.65	2	3	6	6.8	-0.01	4.97	1.54	0.01	35.95	0.61	8.98	6.74	15.48	7.04	-1.45	11.61	20.94	10.98	1.37
os83042	5	536	0.59	1	3	6	6.8	-0.02	4.03	1.56	0.01	23.28	0.12	8.93	6.74	15.5	7.05	-1.41	11.62	20.99	11.02	1.42
os83108	5	623	0.77	5	3	6	6.86	-0.02	0.39	1.49	0.04	92.88	0.73	7.41	6.4	15.16	6.77	-1.73	11.19	20.54	10.71	1.08
os83155	4	541	0.79	6	2	3	6.76	-0.05	1.15	1.5	0.1	99.12	-4.69	7.99	6.74	15.49	7.05	-1.52	11.58	20.91	10.99	1.32
os83181	5	518	0.73	6	2	3	6.59	-0.11	0.93	1.52	0.09	99.71	-3.52	8.56	6.81	15.6	7.12	-1.43	11.72	21.1	11.11	1.44
os83188	3	468	0.01	31	2	0	6.58	0.48	0	1.33	0.25	91.93	0.91	4.72	6.9	15.72	7.18	-1.39	11.86	21.24	11.21	1.52
os83238	5	531	0.92	0	3	6	6.83	0	2.77	1.56	0.01	9.14	0.01	12.34	6.83	15.58	7.07	-1.39	11.83	21.13	11.17	1.53
os83239	5	532	0.23	1	3	6	6.84	0.01	0.21	1.55	0.01	6.89	0.42	10.25	6.83	15.59	7.07	-1.39	11.83	21.14	11.18	1.53
os83240	5	531	0.92	0	3	6	6.83	0	2.37	1.56	0.01	10.57	-0.1	12.08	6.83	15.58	7.07	-1.39	11.83	21.13	11.17	1.53
os83249	5	491	0.92	0	3	6	6.83	0.01	2.2	1.55	0.01	25.01	-0.01	8.93	7.07	15.84	7.28	-1.25	12.15	21.48	11.45	1.68
os83254	5	564	0.92	0	3	6	6.84	0	3.18	1.55	0.02	42.64	-0.45	9.4	6.62	15.36	6.94	-1.46	11.45	20.74	10.84	1.34
os83385	7	448	0.63	4	2	7	6.65	-0.04	1.09	1.55	0.14	84.1	-6.24	7.58	7.11	15.9	7.31	-1.24	12.12	21.5	11.41	1.69
os83403	7	474	0.06	8	2	7	6.83	0.1	0.29	1.54	0.11	74.62	-3.64	6.63	7.02	15.78	7.26	-1.34	11.99	21.34	11.34	1.58
os83432	5	528	0.4	1	3	6	6.86	0.01	2.95	1.55	0.01	21.74	0.2	9.39	6.86	15.61	7.1	-1.36	11.88	21.18	11.22	1.55
os83468	5	498	0.36	2	3	6	6.8	0	2.8	1.55	0.02	20.03	-0.3	9.21	7.03	15.8	7.25	-1.28	12.09	21.43	11.41	1.66
os83552	5	468	0.23	2	3	6	6.8	0.01	1.79	1.55	0.03	63.42	-0.23	8.34	7.45	16.35	7.71	-1.28	12.36	21.91	11.82	1.75
os83712	5	441	0.07	5	3	6	6.91	0.06	2.16	1.55	0.05	72.52	-1.34	8.63	7.48	16.39	7.64	-1.24	12.63	22.09	11.9	1.87
os83716	5	577	0.04	5	3	6	6.98	0.08	0.55	1.53	0.04	100	0.28	7.28	6.87	15.76	7.16	-1.63	11.72	21.2	11.16	1.41
os83718	5	586	0.08	5	3	6	6.98	0.07	0.57	1.53	0.04	100	-0.28	6.88	6.83	15.72	7.12	-1.64	11.66	21.14	11.12	1.39
os83785	5	400	1	7	3	5	6.54	-0.11	0.54	1.52	0.06	90.9	-0.61	7.52	7.64	16.56	7.88	-1.05	12.7	22.25	12.08	2.03
os83809	5	490	0.92	0	3	6	6.83	0.01	4.63	1.56	0.06	60.4	-0.52	8.77	7.13	16.04	7.36	-1.48	12.24	21.74	11.63	1.68
os83876	5	405	0.65	3	3	6	6.67	-0.03	1.6	1.55	0.05	81.4	-0.83	7.73	7.63	16.54	7.83	-1.06	12.65	22.21	12.02	2
os83880	5	435	0.01	2	3	6	6.84	0.03	2.55	1.55	0.04	81.84	-0.77	8.83	7.54	16.46	7.78	-1.11	12.58	22.12	11.98	2.01
os83882	5	436	0.23	2	3	6	6.81	0.02	2.84	1.55	0.04	77.37	-0.72	8.85	7.55	16.46	7.78	-1.11	12.58	22.12	11.98	2.01
os83908	5	554	0.6	3	3	6	6.76	-0.02	1.48	1.54	0.04	100	-0.13	7.26	6.9	15.83	7.21	-1.54	11.72	21.24	11.2	1.48

os83911	5	618	0.19	1	3	6	6.89	0.02	1.92	1.54	0.01	21.3	0.04	9.73	6.72	15.62	7.04	-1.7	11.58	21.05	11.02	1.3
os83912	5	589	0.08	4	3	6	6.99	0.08	0.54	1.53	0.04	100	-0.57	6.79	6.82	15.71	7.11	-1.65	11.64	21.13	11.11	1.39
os83944	5	442	0.29	4	3	6	6.67	-0.07	0.59	1.53	0.04	98.05	0.18	6.85	7.53	16.44	7.76	-1.13	12.36	21.92	11.76	1.85
os83945	5	449	0.99	7	3	5	6.6	-0.1	0.3	1.52	0.05	97.43	0.45	6.42	7.5	16.41	7.73	-1.15	12.32	21.88	11.73	1.83
os83960	5	419	0.76	6	3	5	6.55	-0.09	0.44	1.52	0.04	98.01	0.1	6.31	7.78	16.72	7.98	-0.92	12.66	22.26	11.98	2.05
os83961	5	434	0.4	3	3	6	6.85	0.02	0.81	1.54	0.04	87.6	-0.18	7.72	7.65	16.53	7.85	-1.13	12.55	22.05	11.85	1.81
os84033	5	582	0.02	2	3	6	6.89	0.03	2.87	1.55	0.03	83.17	-0.67	9.42	6.5	15.31	6.94	-1.67	11.23	20.67	10.81	1.1
os84111	4	599	0.12	10	3	5	7.07	0.13	0.08	1.46	0.07	99.56	-0.22	5.96	6.46	15.18	6.85	-1.68	11.27	20.54	10.78	1.14
os84190	5	591	0.92	1	3	6	6.85	0.01	2.58	1.54	0.02	55.88	-0.35	8.74	6.52	15.43	6.99	-1.68	11.26	20.83	10.91	1.12
os84246	4	457	0.07	18	1	4	7	0.3	0.01	1.37	0.07	92.04	7.63	5.98	7.22	16.04	7.48	-1.16	12.15	21.61	11.56	1.72
os84285	5	539	0.53	5	3	5	6.7	-0.03	0.77	1.5	0.05	98.84	-0.48	6.83	6.8	15.6	7.14	-1.45	11.68	21.05	11.15	1.44
os84464	5	467	0.61	5	3	6	6.64	-0.08	0.63	1.53	0.05	99.35	-0.89	7.15	7.12	16.01	7.4	-1.18	12.1	21.61	11.47	1.79
os84485	5	501	0.77	1	3	6	6.81	-0.01	2.66	1.54	0.02	71.69	-0.62	8.43	6.98	15.85	7.29	-1.23	11.81	21.29	11.2	1.64
os84609	7	506	0.15	13	2	7	6.69	0.1	0.02	1.47	0.12	99.72	-1.17	6.84	6.85	15.65	7.14	-1.37	11.7	21.07	11.04	1.52
os84936	5	557	0.08	3	3	6	6.92	0.04	1.5	1.54	0.02	79.22	0.14	8.02	6.7	15.6	7.11	-1.57	11.5	21.06	11.08	1.24
os85036	4	505	0.77	7	3	5	6.68	-0.09	0.3	1.49	0.05	91.75	1.49	6.91	6.93	15.7	7.24	-1.38	11.87	21.21	11.27	1.52
os85039	5	539	0.08	1	3	6	6.88	0.02	2.94	1.55	0.02	20.17	-0.35	9.3	6.73	15.52	7.1	-1.48	11.6	20.97	11.08	1.39
os85040	5	539	0.34	1	3	6	6.87	0.02	2.95	1.55	0.02	22.07	-0.44	9.36	6.74	15.53	7.11	-1.48	11.61	20.97	11.09	1.39
os85263	5	537	0.72	2	3	6	6.85	0	2.61	1.54	0.05	89.65	-1.39	8.81	6.74	15.68	7.16	-1.55	11.55	21.15	11.16	1.26
os85283	4	466	0.4	3	3	6	6.86	0.02	1.69	1.54	0.02	68.82	-0.01	8.01	7.27	16.24	7.61	-1.05	12.25	21.91	11.72	1.9
os85390	5	485	0.55	6	3	5	6.83	-0.01	0.58	1.52	0.08	98.23	-1.28	6.86	7	15.92	7.3	-1.25	12.14	21.61	11.46	1.86
os85399	5	439	0.6	2	3	6	6.73	-0.02	1.77	1.53	0.03	82.07	-0.13	7.52	7.32	16.16	7.53	-0.93	12.43	21.75	11.58	2.02
os85409	5	489	0.72	5	3	5	6.65	-0.05	0.48	1.53	0.06	99.97	-0.19	6.76	6.89	15.84	7.26	-1.29	11.93	21.42	11.38	1.77
os85419	5	520	0.36	4	2	3	6.89	0.02	1.94	1.54	0.07	96.49	-2.95	8.55	6.73	15.65	7.12	-1.34	11.78	21.2	11.16	1.7
os85425	5	461	0.77	2	3	6	6.79	-0.02	1.99	1.55	0.07	98.69	-2.02	8.17	7.04	15.97	7.34	-1.22	12.19	21.65	11.51	1.87
os85426	5	475	0.03	6	3	5	6.59	-0.1	0.47	1.52	0.07	99.61	-0.2	6.97	7.01	15.93	7.31	-1.24	12.15	21.61	11.48	1.86
os85427	5	483	0.55	6	3	5	6.84	0	0.6	1.52	0.08	98.36	-1.08	6.85	7	15.92	7.3	-1.24	12.15	21.61	11.47	1.86
os85430	5	553	0.75	7	3	5	6.58	-0.08	0.21	1.51	0.08	100	-0.02	6.38	6.61	15.55	7.04	-1.41	11.51	20.98	11.03	1.62
os85433	5	431	0.75	6	3	5	6.58	-0.07	0.47	1.53	0.06	96.4	0.25	7.38	7.16	16.09	7.47	-1.09	12.3	21.78	11.68	1.98
os85434	5	433	0.65	5	3	5	6.59	-0.07	0.49	1.53	0.06	96.73	0.28	7.16	7.16	16.09	7.47	-1.1	12.3	21.77	11.68	1.98
os85441	5	435	0.02	6	3	6	6.94	0.08	0.87	1.53	0.06	93.89	-1.65	7.25	7.33	16.27	7.6	-1.08	12.52	22.04	11.88	2.05
os85442	5	436	0.01	5	3	5	6.94	0.09	0.75	1.53	0.05	94.18	-1.47	7.44	7.32	16.25	7.59	-1.08	12.5	22.02	11.87	2.05
os85443	5	433	0.21	5	2	3	6.61	-0.09	1.38	1.53	0.08	95.36	-3.08	7.69	7.3	16.25	7.62	-1.08	12.5	22.05	11.9	2.04
os85450	5	558	0.33	7	3	5	6.97	0.06	0.36	1.51	0.07	100	-0.92	6.68	6.61	15.54	7.04	-1.46	11.53	20.99	11.1	1.62
os85451	5	554	0.4	7	3	5	6.96	0.05	0.64	1.5	0.07	100	-0.65	6.7	6.63	15.55	7.05	-1.45	11.55	21.01	11.12	1.63
os85453	5	531	0.43	5	3	5	6.86	-0.01	0.79	1.53	0.07	99.99	-1.65	7.85	6.7	15.62	7.11	-1.42	11.64	21.1	11.18	1.67
os85481	5	451	0.6	6	3	5	6.63	-0.04	0.49	1.53	0.04	94.58	-0.36	6.51	7.17	16.02	7.42	-1.03	12.25	21.59	11.45	1.93

os85484	5	435	0.69	4	3	6	6.66	-0.05	0.69	1.55	0.03	75.01	-0.36	7.49	7.21	16.06	7.46	-1	12.3	21.64	11.49	1.94
os85521	5	545	0.65	2	3	6	6.85	-0.01	4.97	1.56	0.03	32.82	-0.26	9.24	6.65	15.37	6.99	-1.43	11.36	20.68	10.77	1.28
os85529	5	575	0.47	6	2	3	6.88	0.01	1.64	1.55	0.14	95.78	-6.51	8.48	6.49	15.35	7.01	-1.63	11.2	20.69	10.9	1.1
os85530	5	577	0.66	7	2	3	6.53	-0.09	0.91	1.53	0.15	96.18	-7.22	8.12	6.48	15.34	7	-1.64	11.2	20.68	10.9	1.09
os85546	5	545	1	6	3	5	6.66	-0.1	0.32	1.53	0.04	99.99	0.03	7.11	6.75	15.63	7.22	-1.5	11.51	21.04	11.16	1.25
os85632	5	420	0.92	3	3	6	6.68	-0.05	1.43	1.55	0.04	93.64	-0.2	8.38	7.62	16.53	7.83	-1.06	12.45	22.02	11.84	1.91
os85788	4	446	0.48	14	3	5	6.83	0.02	0.03	1.47	0.09	98.36	0.14	7.5	7.27	16.14	7.63	-1.11	12.11	21.63	11.62	1.66
os85789	5	442	0.48	2	3	6	6.8	0.01	1.47	1.53	0.03	87.6	0.02	8.77	7.69	16.7	7.89	-0.95	12.71	22.41	12.02	2.09
os85792	5	349	0.77	1	3	6	6.74	-0.01	1.97	1.56	0.02	57.67	-0.88	8.59	7.93	16.91	8.16	-0.7	13.15	22.83	12.5	2.31
os85793	5	343	0.99	2	3	6	6.72	-0.03	3.24	1.54	0.01	71.23	0.7	7.61	8.15	17.11	8.3	-0.63	13.43	23.05	12.64	2.48
os85795	5	485	0.34	3	3	6	6.87	0.02	3.18	1.55	0.04	60.23	-0.09	8.92	7.09	15.93	7.31	-1.19	12.11	21.56	11.42	1.75
os85797	5	535	0.92	2	3	6	6.85	0	4.68	1.56	0.02	49.01	0.16	8.98	6.7	15.43	7.01	-1.45	11.46	20.81	10.85	1.31
os85816	7	484	1	5	2	7	6.65	-0.08	1.54	1.57	0.07	74.86	-2.31	7.83	7.3	16.21	7.5	-1.32	11.98	21.54	11.33	1.5
os85824	5	597	0.21	3	3	6	6.95	0.04	1.59	1.55	0.03	97.61	-0.45	7.77	6.71	15.68	7	-1.79	11.44	21.03	10.92	1.13
os85825	5	599	0.4	3	3	6	6.95	0.04	1.55	1.55	0.03	98.25	-0.42	7.64	6.69	15.66	6.99	-1.8	11.42	21.01	10.91	1.12
os85829	5	554	0.98	3	3	6	6.79	-0.04	1.27	1.55	0.02	72.39	0.03	7.94	7.04	16	7.24	-1.62	11.82	21.44	11.24	1.33
os85830	5	610	0.44	4	3	6	6.93	0.02	1.24	1.55	0.04	99.47	-0.54	8.03	6.56	15.49	6.87	-1.85	11.14	20.74	10.71	1.05
os85831	5	612	0.53	4	3	6	6.93	0.02	0.82	1.54	0.04	99.47	-0.21	7.97	6.55	15.49	6.87	-1.85	11.14	20.73	10.71	1.05
os85844	5	566	0.4	2	3	6	6.87	0	2.27	1.56	0.02	19.48	-0.19	9.56	6.82	15.71	7.06	-1.69	11.45	20.99	10.97	1.22
os85924	5	612	0.4	2	3	6	6.91	0.02	1.43	1.54	0.01	24.19	-0.17	8.41	6.77	15.77	7.05	-1.8	11.58	21.21	11.05	1.14
os85925	5	611	0.92	1	3	6	6.87	0	2.08	1.54	0.01	22.32	0.01	8.81	6.78	15.77	7.05	-1.8	11.59	21.22	11.06	1.15
os86049	5	518	0.77	2	3	6	6.83	-0.01	2.81	1.55	0.03	53.4	-0.08	9.28	7.09	15.99	7.39	-1.43	11.76	21.27	11.2	1.37
os86133	4	434	0.68	3	3	6	6.72	-0.02	1.92	1.53	0.05	98.86	-2.02	8.56	7.76	16.7	7.88	-0.99	12.74	22.26	11.92	1.92
os86134	4	440	0.78	4	3	6	6.65	-0.05	1.54	1.53	0.06	98.93	-2.02	8.42	7.72	16.67	7.85	-1.01	12.68	22.22	11.88	1.89
os86138	7	420	0.71	4	3	6	6.66	-0.06	2.33	1.54	0.05	93.21	-1.01	9.22	7.85	16.8	7.94	-0.94	12.86	22.4	12	2
os86140	7	418	0.81	8	3	5	6.51	-0.09	0.26	1.53	0.06	87.87	-0.68	9.37	7.69	16.61	7.85	-1	12.51	22.02	11.71	1.82
os86141	7	426	0.92	3	2	0	6.68	-0.04	1.96	1.55	0.05	88.43	-0.4	9.51	7.62	16.53	7.79	-1.05	12.4	21.9	11.62	1.76
os86148	5	449	0.67	5	3	5	6.63	-0.05	0.37	1.53	0.04	99.95	0.28	7.58	7.69	16.54	7.83	-0.97	12.6	22.01	11.78	1.94
os86149	5	453	0.74	5	3	6	6.63	-0.05	0.42	1.52	0.04	99.96	0.35	7.5	7.67	16.52	7.81	-0.99	12.57	21.98	11.76	1.92
os86150	5	455	0.84	5	3	6	6.64	-0.06	0.57	1.53	0.04	99.97	0.31	7.5	7.66	16.51	7.8	-0.99	12.55	21.97	11.74	1.91
os86151	5	456	0.83	5	3	6	6.64	-0.06	0.56	1.53	0.04	99.97	0.39	7.47	7.65	16.5	7.8	-1	12.55	21.96	11.74	1.91
os86152	5	457	0.88	4	3	6	6.65	-0.06	0.57	1.53	0.04	99.96	0.36	7.42	7.65	16.5	7.79	-1	12.54	21.96	11.73	1.9
os86153	5	457	0.92	4	3	6	6.66	-0.05	0.59	1.53	0.04	99.95	0.23	7.35	7.64	16.49	7.79	-1	12.53	21.95	11.73	1.9
os86163	5	531	0.08	3	3	6	6.92	0.05	1.6	1.55	0.02	57.02	-0.12	8.17	7.17	16.11	7.36	-1.47	12.05	21.59	11.41	1.52
os86164	5	619	0.73	4	3	6	6.91	0	0.58	1.54	0.04	99.38	0.01	7.84	6.51	15.42	6.82	-1.86	11.08	20.64	10.68	1.03
os86194	4	451	0.19	7	3	5	6.96	0.09	0.24	1.5	0.08	99.82	-0.71	6.52	7.61	16.44	7.76	-0.92	12.1	21.63	11.3	1.81
os86228	6	642	0.23	2	3	6	6.88	0.02	1.66	1.55	0.02	42.22	-0.16	8.02	6.55	15.43	6.88	-1.81	11.28	20.74	10.78	1.16

os86233	5	660	0.01	2	3	6	6.93	0.03	0.27	1.54	0.01	79.3	0.28	7.35	6.47	15.38	6.82	-1.83	11.17	20.64	10.68	1.12
os86368	5	609	0.4	3	3	6	6.92	0.02	1.45	1.55	0.03	94.34	0.13	8.55	6.51	15.41	6.84	-1.85	11.04	20.59	10.64	1.01
os86383	5	574	0.77	4	3	6	6.88	0	0.37	1.53	0.04	90.85	0.92	9.39	6.84	15.77	7.08	-1.66	11.46	21.04	10.93	1.26
os86389	5	500	0.9	7	3	5	6.7	-0.08	0.34	1.51	0.05	99.78	0.16	7.3	7.25	16.14	7.53	-1.37	12.17	21.64	11.55	1.65
os86398	5	646	0.01	2	3	6	6.94	0.04	0.97	1.54	0.01	88.24	0.11	7.64	6.55	15.45	6.89	-1.81	11.3	20.75	10.79	1.17
os86444	5	548	0.84	5	3	6	6.74	-0.06	0.53	1.53	0.04	100	0.26	7.26	7.01	15.93	7.33	-1.51	11.87	21.37	11.32	1.52
os86461	5	530	0.92	3	3	6	6.71	-0.06	0.97	1.54	0.04	99.42	0.09	8.3	7.06	15.95	7.34	-1.51	11.87	21.38	11.32	1.49
os86465	5	582	0.23	3	3	6	6.86	0.02	1.77	1.55	0.03	81.57	-0.17	10.06	6.77	15.63	7.1	-1.66	11.47	20.97	10.95	1.27
os86468	5	550	0.95	4	3	6	6.78	-0.05	0.58	1.53	0.04	100	0.32	7.27	7	15.92	7.32	-1.51	11.86	21.36	11.32	1.51
os86469	5	517	0.1	7	3	5	6.54	-0.12	0.31	1.52	0.05	99.03	0.09	7.1	7.2	16.12	7.49	-1.38	12.15	21.65	11.57	1.67
os86477	5	534	0.6	2	3	6	6.77	-0.02	1.26	1.55	0.02	99.69	0.07	8.22	7.15	16.05	7.45	-1.52	12.05	21.55	11.49	1.48
os86505	5	400	0.02	14	2	7	6.9	0.18	0.04	1.48	0.13	66.18	-2.01	6.43	7.77	16.66	7.91	-1.03	12.66	22.2	11.94	1.91
os86507	5	408	0.04	11	3	5	6.99	0.19	0.07	1.49	0.1	73.67	-1.5	6.56	7.76	16.64	7.9	-1.04	12.64	22.19	11.92	1.9
os86508	5	401	0.15	12	2	0	7	0.17	0.01	1.49	0.1	68.4	-1.48	6.7	7.75	16.64	7.9	-1.04	12.64	22.18	11.92	1.9
os86520	5	454	0.92	0	3	6	6.8	0	4.58	1.56	0.02	29.12	0.04	11.14	7.51	16.39	7.7	-1.21	12.23	21.73	11.57	1.62
os86537	7	450	0.92	1	3	6	6.79	-0.01	5.51	1.56	0.02	22.18	-0.3	9.44	7.57	16.47	7.73	-1.15	12.35	21.85	11.65	1.68
os86538	7	450	0.92	0	3	6	6.81	0	5.36	1.56	0.02	21.12	0.19	10.06	7.57	16.47	7.73	-1.15	12.35	21.85	11.65	1.68
os86550	5	489	0.4	5	3	6	6.91	0.04	0.78	1.53	0.03	92.45	-0.35	7.17	7.34	16.25	7.55	-1.29	12.05	21.59	11.42	1.54
os86601	5	448	0.99	9	1	4	6.42	-0.16	0.06	1.47	0.07	76.96	2.18	7.17	7.46	16.38	7.68	-1.18	12.24	21.8	11.64	1.73
os86654	7	443	0.34	3	3	6	6.73	0	1.8	1.54	0.05	91.4	-1.01	8.68	7.55	16.52	7.74	-1.19	12.41	22.04	11.72	1.69
os86666	5	641	0.23	2	3	6	6.88	0.01	1.66	1.55	0.01	44.79	0.03	7.95	6.55	15.44	6.89	-1.81	11.29	20.75	10.79	1.16
os86668	5	641	0.14	2	3	6	6.92	0.03	0.94	1.55	0.01	81.49	0.12	7.77	6.56	15.46	6.89	-1.8	11.31	20.77	10.79	1.17
os86691	4	403	0.88	4	3	6	6.75	-0.03	0.82	1.52	0.04	94.92	-0.36	8.36	7.91	16.85	8.03	-0.96	12.76	22.36	11.99	1.82
os86700	5	433	0.92	7	2	0	6.53	-0.11	0.71	1.53	0.08	95.37	-1.98	7.11	7.61	16.59	7.8	-1.22	12.49	22.13	11.8	1.57
os86703	7	487	0.92	2	2	3	6.73	-0.05	2.25	1.55	0.09	88.99	-4.66	8.51	7.25	16.24	7.52	-1.35	12	21.71	11.48	1.45
os86713	4	409	0.92	1	3	6	6.77	-0.01	2.98	1.54	0.04	89.1	-1.76	9.14	7.82	16.78	7.96	-1.03	12.68	22.29	11.92	1.76
os86735	4	452	0.08	6	3	5	6.59	-0.1	0.49	1.53	0.06	99.59	-0.87	6.94	7.51	16.48	7.73	-1.18	12.3	21.96	11.7	1.62
os86783	4	376	0.68	14	1	4	6.21	-0.09	0	1.38	0.07	97.77	5.87	5.75	8.04	16.91	8.15	-0.8	12.78	22.29	12.03	1.93
os86992	7	397	0.49	5	2	7	6.7	0	1.82	1.54	0.16	95.76	-7.44	7.94	7.66	16.62	7.83	-1.17	12.53	22.22	11.92	1.69
os87001	7	419	0.09	6	2	7	6.92	0.09	1.43	1.53	0.1	95.11	-4.21	7.63	7.61	16.59	7.81	-1.21	12.5	22.19	11.89	1.66
os87046	5	349	0.97	9	2	7	6.5	-0.11	0.18	1.52	0.13	86.65	-1.57	8.04	7.93	16.85	8.03	-0.98	12.76	22.39	12.07	1.82
os87047	5	360	0.82	5	2	0	6.74	-0.03	0.97	1.53	0.1	92.1	-1.27	7.71	7.92	16.84	8.02	-0.99	12.75	22.38	12.07	1.81
os87049	4	346	0.97	19	2	0	5.82	-0.31	0	1.43	0.14	96.69	-0.63	6.64	7.97	16.86	8.09	-0.88	12.71	22.27	11.99	1.87
os87054	4	366	0.64	13	3	5	6.69	-0.04	0	1.46	0.1	99.57	0.7	7.12	7.98	16.9	8.08	-0.89	12.76	22.35	12	1.86
os87055	4	428	0.99	11	2	3	6.29	-0.19	0.03	1.48	0.08	95.82	-1.76	7.54	7.75	16.68	7.88	-1.09	12.56	22.18	11.82	1.68
os87102	4	390	0.49	6	2	3	6.61	-0.08	1.32	1.52	0.1	94.95	-4.53	8.76	7.92	16.87	8.01	-0.88	12.77	22.38	12.02	1.96
os87104	4	388	0.83	3	2	3	6.7	-0.04	2.7	1.53	0.07	96.11	-2.37	8.67	7.93	16.88	8.02	-0.87	12.78	22.38	12.03	1.96

os87107	4	387	0.17	3	2	3	6.71	-0.04	2.77	1.53	0.07	96.36	-2.49	8.54	7.93	16.88	8.02	-0.87	12.78	22.38	12.03	1.97
os87108	5	393	0.23	5	3	6	6.74	0.02	1.44	1.49	0.06	94.16	-1.76	7.3	7.98	16.9	8.07	-0.82	12.78	22.36	12.04	1.99
os87164	4	365	0.25	11	2	7	6.94	0.12	0.1	1.52	0.11	59.34	-2.74	6.99	8.11	17.04	8.17	-0.75	13.22	22.73	12.3	2.18
os87169	5	385	0.58	3	3	6	6.82	0.01	0.76	1.54	0.04	92.38	0.12	7.27	8.1	17.05	8.14	-0.75	13.2	22.74	12.28	2.17
os87187	4	349	0.99	3	3	6	6.65	-0.05	1.62	1.53	0.03	85.44	-0.5	8.09	8.29	17.21	8.34	-0.56	13.42	22.93	12.5	2.35
os87338	5	455	0.92	0	3	6	6.81	0	2.83	1.54	0.01	6.81	-0.26	9.14	7.46	16.3	7.63	-1.16	12.03	21.54	11.37	1.58
os87355	3	405	0.03	10	3	9	7	0.16	0.04	1.44	0.05	94.59	1.19	6.17	7.99	16.81	8.06	-0.76	12.82	22.21	11.94	2.13
os87439	5	345	0.75	6	2	0	6.79	0.06	0.6	1.5	0.14	98.7	-3.62	6.94	7.95	16.78	8.03	-0.77	12.7	22.12	11.88	2.03
os87459	4	355	0.54	5	2	7	6.63	-0.06	1.37	1.52	0.15	99.14	-5.49	7.83	7.89	16.7	8.02	-0.77	12.62	22.02	11.87	2.06
os87468	7	374	0.01	4	3	6	6.77	0.04	1	1.52	0.07	98.11	-0.82	7.81	8	16.83	8.1	-0.73	12.78	22.22	11.94	2.11
os87474	4	408	0.51	6	3	9	6.57	-0.1	0.77	1.47	0.05	99.82	-1.06	6.71	8.06	16.82	8.09	-0.68	12.79	22.15	11.96	2.15
os87560	4	279	0.83	20	2	7	6.03	-0.21	0	1.47	0.22	66.74	-7.81	6.22	8.34	17.1	8.42	-0.41	13.12	22.44	12.28	2.39
os87567	7	408	0.64	4	3	6	6.67	-0.03	1.74	1.53	0.05	89.87	-1.62	8.28	7.62	16.44	7.81	-1.02	12.18	21.64	11.48	1.68
os87679	4	442	0.4	3	3	6	6.85	0.02	1.24	1.52	0.02	82.67	0.14	7.58	7.71	16.65	7.88	-1.07	12.6	22.3	12.03	1.81
os87701	4	360	0.33	7	1	4	6.89	0.06	0.26	1.47	0.06	94.11	2.6	6.81	7.98	16.92	8.17	-0.74	12.83	22.52	12.24	2.12
os87720	3	422	0.19	16	2	7	6.63	0.14	0.13	1.49	0.15	94.36	-3.8	7.23	7.58	16.54	7.77	-1.18	12.45	22.13	11.88	1.66
os87721	4	431	0.6	9	2	7	6.58	0	0.76	1.5	0.12	94.31	-2.88	8.42	7.57	16.53	7.77	-1.18	12.44	22.12	11.87	1.66
os87792	4	366	0.18	7	3	5	6.92	0.09	0.52	1.52	0.04	97.34	-0.51	7.95	7.93	16.87	8.13	-0.73	12.75	22.43	12.14	2.11
os87892	4	447	0.08	3	3	6	6.88	0.04	1.65	1.53	0.02	70.87	0.1	7.83	7.67	16.63	7.86	-1.09	12.56	22.26	12	1.79
os87893	4	446	0.08	3	3	6	6.88	0.05	1.62	1.53	0.02	73.61	0.11	7.85	7.67	16.63	7.86	-1.09	12.56	22.26	12	1.79
os87894	4	446	0.08	3	3	6	6.89	0.05	1.61	1.53	0.02	76.79	0.12	7.87	7.67	16.63	7.86	-1.09	12.56	22.26	12	1.79
os87895	4	445	0.08	3	3	6	6.89	0.05	1.49	1.53	0.02	79.47	0.14	7.88	7.67	16.63	7.86	-1.09	12.56	22.26	12	1.79
os87897	4	444	0.11	3	3	6	6.88	0.04	1.61	1.53	0.02	74.62	0.04	8.05	7.68	16.64	7.87	-1.08	12.57	22.28	12.01	1.8
os87932	4	431	0.45	12	2	3	6.83	0.02	0.43	1.5	0.1	89.92	-3.31	8.36	7.63	16.59	7.86	-1.06	12.55	22.28	12.02	1.81
os87934	4	432	0.74	9	2	3	6.73	-0.04	0.78	1.51	0.09	89.79	-3.43	8.43	7.63	16.59	7.86	-1.06	12.55	22.28	12.02	1.81
os87935	4	431	0.39	15	3	5	6.91	0.08	0.1	1.45	0.09	89.95	0.4	7.17	7.63	16.59	7.86	-1.06	12.55	22.28	12.02	1.81
os87938	4	363	0.04	7	2	3	6.73	0.05	1.17	1.52	0.11	92.26	-5.52	7.78	7.93	16.87	8.12	-0.77	12.77	22.45	12.17	2.09
os87958	4	459	0.4	4	3	6	6.86	0.01	0.23	1.51	0.02	94.68	1.43	7.52	7.56	16.52	7.75	-1.18	12.43	22.12	11.87	1.66
os87987	4	425	0.01	4	2	3	6.78	-0.01	1.91	1.55	0.08	96.22	-3.97	8.35	7.61	16.57	7.82	-1.16	12.44	22.1	11.84	1.68
os87996	4	287	0.87	8	2	0	6.42	-0.13	0.24	1.52	0.11	82.19	-1.98	7.65	8.26	17.17	8.39	-0.6	13.13	22.83	12.47	2.26
os87998	4	342	0.33	11	2	3	6.59	0.03	0.45	1.51	0.1	94.28	-2.1	8.11	8.03	16.97	8.21	-0.67	12.87	22.56	12.26	2.17
os88011	4	355	0.99	5	3	6	6.6	-0.09	0.78	1.51	0.05	98.03	-0.63	7.11	8.08	17.01	8.2	-0.8	12.99	22.65	12.34	2.08
os88065	5	430	0.01	7	3	5	6.88	0.1	0.72	1.49	0.08	98.61	-0.87	7.18	7.71	16.58	7.85	-0.95	12.38	21.88	11.66	1.83
os88160	7	353	0.97	6	2	0	6.63	-0.07	0.38	1.53	0.1	82.12	-1.39	6.78	7.98	16.92	8.11	-0.88	12.93	22.55	12.19	2.01
os88162	7	348	0.68	3	2	0	6.77	-0.01	2.19	1.54	0.08	79.08	-0.01	8.22	8	16.94	8.12	-0.87	12.94	22.56	12.2	2.02
os88172	7	403	0.23	1	3	6	6.8	0.02	2.97	1.54	0.05	87.05	-1.83	8.83	7.78	16.73	7.96	-0.95	12.67	22.33	12.03	1.93
os88200	4	367	0.88	10	2	7	6.55	-0.11	0.46	1.49	0.13	97.11	-2.73	8.05	7.98	16.91	8.06	-0.83	12.8	22.4	12.06	1.99

os88224	3	530	0.77	4	3	6	6.82	-0.02	1.57	1.53	0.03	85.27	-0.64	8.56	7.12	16.12	7.42	-1.42	11.84	21.55	11.34	1.38
os88240	5	443	0.99	2	3	6	6.74	-0.04	1.73	1.55	0.03	89.5	-0.32	8	7.54	16.39	7.73	-1.18	12.12	21.57	11.44	1.5
os88267	7	362	0.02	2	2	3	6.68	-0.03	1.54	1.56	0.09	66.55	-3	7.29	8.03	16.9	8.16	-0.86	12.83	22.31	12.11	1.87
os88328	7	373	0.27	3	2	3	6.76	0.02	1.83	1.55	0.08	94.79	-3.5	7.23	8.09	16.98	8.22	-0.86	13.02	22.48	12.27	1.95
os88344	4	383	0.01	3	3	6	6.82	0.04	1.68	1.54	0.04	97.11	-0.81	8.35	7.97	16.88	8.13	-0.81	12.93	22.47	12.24	2.06
os88376	7	346	0.6	3	2	7	6.64	-0.04	2.01	1.55	0.12	87.26	-3.76	7.01	7.94	16.79	8.09	-0.81	12.57	22.06	11.86	1.89
os88390	5	366	0.4	2	3	6	6.8	0.01	1.56	1.55	0.01	54.25	-0.17	9.01	8.12	17.05	8.25	-0.75	13	22.55	12.28	2.04
os88437	4	423	0.82	3	3	6	6.68	-0.04	1.8	1.54	0.03	96.4	-0.54	8.62	7.71	16.67	7.92	-1.05	12.56	22.2	11.91	1.76
os88440	4	470	0.74	15	1	4	6.16	-0.2	0	1.4	0.1	99.62	3.51	7.06	7.39	16.34	7.65	-1.2	12.14	21.8	11.6	1.58
os88481	5	441	0.92	0	3	6	6.78	-0.01	3.45	1.55	0.03	20.21	-0.25	9.14	7.56	16.4	7.73	-1.18	12.16	21.61	11.44	1.5
os88492	5	457	0.3	2	3	6	6.74	-0.01	1.7	1.54	0.03	71.26	-0.38	8.38	7.47	16.32	7.66	-1.22	12	21.46	11.32	1.43
os88537	4	331	0.6	12	2	3	6.36	-0.06	0.08	1.53	0.09	54.65	-3.25	6.89	8.22	17.12	8.32	-0.69	13.07	22.63	12.32	2.12
os88538	7	338	0.7	9	2	3	6.45	-0.06	0.33	1.5	0.09	62.42	-2.09	6.03	8.21	17.12	8.32	-0.69	13.06	22.63	12.32	2.12
os88539	5	336	0.78	9	2	3	6.47	-0.06	0.73	1.53	0.1	71.84	-3.38	7.62	8.23	17.14	8.33	-0.68	13.09	22.66	12.34	2.13
os88567	4	388	0.15	7	2	3	6.71	0.03	1.18	1.54	0.08	94.98	-3.77	7.67	8.02	16.9	8.16	-0.94	12.95	22.4	12.19	1.88
os88569	4	389	0	4	2	3	6.87	0.06	1.69	1.55	0.05	85.3	-2.51	8.75	8	16.86	8.13	-0.94	12.89	22.35	12.14	1.85
os88571	4	387	0.26	4	2	3	6.84	0.04	1.93	1.55	0.06	83.38	-3.2	9.05	8.01	16.86	8.13	-0.94	12.89	22.35	12.15	1.85
os88572	4	386	0.01	5	2	3	6.84	0.06	1.55	1.55	0.06	81.9	-3.26	8.87	8.01	16.86	8.13	-0.93	12.89	22.35	12.15	1.85
os88573	4	391	0.17	7	3	5	6.87	0.11	0.72	1.52	0.06	87.25	-1.56	7.82	8	16.86	8.13	-0.94	12.88	22.34	12.14	1.84
os88583	4	353	0.77	1	3	6	6.76	-0.01	2.57	1.56	0.02	39.75	-0.82	8.48	8.19	17.05	8.32	-0.72	13.07	22.56	12.36	2.05
os88592	4	353	0.77	2	3	6	6.75	-0.01	1.96	1.56	0.06	86.48	-1.81	8.97	8.12	17.01	8.24	-0.75	13.12	22.66	12.41	2.1
os88598	4	343	0.4	7	3	5	6.69	0.04	0.67	1.52	0.09	74.43	-1.91	7.48	8.18	17.1	8.3	-0.72	13.15	22.72	12.43	2.14
os88601	7	365	0.08	4	1	4	6.81	0.05	0.14	1.49	0.03	94.35	2.88	7.36	8.16	17.08	8.28	-0.73	13.14	22.7	12.41	2.13
os88609	7	389	0.37	4	3	6	6.86	0.04	1.81	1.55	0.05	96.95	-1.75	8.58	7.93	16.84	8.1	-0.85	12.89	22.43	12.22	2.03
os88620	4	387	0.01	4	3	6	6.86	0.07	1.27	1.54	0.05	98.82	-1.75	8.25	7.96	16.87	8.12	-0.82	12.92	22.46	12.23	2.05
os88621	4	384	0.23	3	3	6	6.78	0.03	1.64	1.54	0.04	98.09	-0.89	8.33	7.97	16.88	8.13	-0.81	12.93	22.47	12.24	2.06
os88637	4	360	0.96	4	3	6	6.6	-0.07	1.38	1.54	0.06	92.89	-1.84	7.97	8.11	17.07	8.21	-0.82	13.04	22.69	12.27	2
os88651	7	289	0.89	8	2	3	6.45	-0.11	0.31	1.53	0.07	79.92	-1.19	7.91	8.47	17.33	8.56	-0.33	13.33	22.75	12.45	2.42
os88659	4	314	0.93	7	3	5	6.55	-0.1	0.78	1.52	0.09	96.31	-2.56	7.4	8.3	17.14	8.4	-0.48	12.95	22.4	12.16	2.22
os88675	7	357	0.9	10	2	3	6.41	-0.14	0.37	1.52	0.13	92.61	-3.21	6.86	7.94	16.8	8.11	-0.8	12.6	22.11	11.91	1.93
os88686	7	332	0.84	8	2	3	6.46	-0.1	0.52	1.52	0.08	87.45	-2.76	7.44	8.23	17.08	8.37	-0.51	12.88	22.3	12.09	2.17
os88791	5	432	0.92	0	3	9	6.79	0	0.86	1.49	0	44.22	0.78	9.23	7.81	16.65	7.91	-0.97	12.6	22.06	11.78	1.77
os88819	5	402	0.92	0	3	6	6.76	-0.01	1.44	1.54	0.01	50.02	0.66	9.46	7.78	16.62	7.89	-0.93	12.5	21.95	11.71	1.79
os88867	5	431	0.35	9	3	5	6.93	0.07	0.08	1.51	0.05	96.65	-0.23	6.57	7.68	16.53	7.8	-1.05	12.59	22.02	11.75	1.81
os88870	5	396	0	7	3	5	6.89	0.1	0.38	1.53	0.05	88.11	-0.32	7.64	7.81	16.67	7.92	-0.92	12.57	22.01	11.75	1.79
os88884	5	439	0.62	2	3	6	6.74	-0.01	2.5	1.54	0.02	57.12	-0.85	8.7	7.51	16.34	7.68	-1.11	12.04	21.45	11.31	1.58
os89013	5	480	0.01	8	3	5	6.98	0.13	0.25	1.52	0.08	100	-0.97	6.67	7.04	15.94	7.45	-1.25	11.88	21.41	11.44	1.52

os89019	4	495	0.96	5	3	5	6.7	-0.07	0.72	1.51	0.05	99.68	-1.02	7.77	7.14	16.04	7.5	-1.24	12	21.57	11.54	1.57
os89025	4	398	0.48	8	1	2	6.82	0.02	0.02	1.45	0.1	93.34	4	6.11	7.52	16.43	7.84	-1	12.27	21.92	11.82	1.78
os89057	4	482	0	11	2	3	6.97	0.17	0.26	1.5	0.09	93.71	-3.05	7.96	7.21	16.18	7.6	-1.17	11.89	21.6	11.53	1.59
os89121	4	470	0.08	6	3	5	6.96	0.08	0.49	1.52	0.05	97.04	0.29	7.11	7.29	16.19	7.62	-1.16	12.17	21.75	11.69	1.65
os89164	4	433	0.59	10	2	3	6.81	0.01	0.6	1.5	0.16	98.75	-5.23	7.22	7.32	16.21	7.66	-1.12	12.14	21.73	11.68	1.68
os89165	4	437	0.4	9	2	3	6.57	-0.08	0.64	1.51	0.18	99.09	-6.42	7.1	7.32	16.21	7.66	-1.12	12.14	21.73	11.68	1.68
os89166	4	442	0.01	6	2	3	6.63	-0.06	0.99	1.52	0.15	98.92	-6.59	6.98	7.32	16.21	7.65	-1.12	12.13	21.72	11.67	1.68
os89167	4	450	0.64	9	2	3	6.74	-0.05	0.92	1.51	0.14	98.27	-5.09	6.83	7.31	16.21	7.65	-1.12	12.13	21.71	11.67	1.68
os89222	4	423	0.71	6	3	5	6.8	-0.01	0.99	1.53	0.06	91.51	-0.74	8.32	7.46	16.39	7.81	-1.07	12.18	21.89	11.79	1.73
os89321	5	500	0.81	3	3	6	6.73	-0.05	2.21	1.55	0.03	54.13	-0.46	8.35	7.17	16.14	7.49	-1.38	11.88	21.54	11.42	1.4
os89400	4	522	0.94	14	2	0	6.41	-0.18	0	1.48	0.11	93.18	-0.98	5.95	6.77	15.61	7.23	-1.51	11.63	21.06	11.23	1.22
os89410	7	513	0.17	10	2	7	6.99	0.15	0.59	1.54	0.15	77.08	-6.26	7.31	6.75	15.56	7.21	-1.52	11.53	20.92	11.15	1.21
os89772	5	501	0	7	3	5	6.95	0.11	0.37	1.51	0.08	99.94	-0.31	6.39	7	15.92	7.42	-1.27	11.85	21.39	11.42	1.51
os89775	5	517	0.01	8	3	5	6.97	0.13	0.3	1.5	0.1	99.43	-0.39	6.06	6.95	15.87	7.37	-1.31	11.79	21.34	11.37	1.48
os89794	4	592	0.03	18	3	9	6.84	0.27	0	1.42	0.13	96.07	-1.39	5.38	6.76	15.69	7.23	-1.45	11.55	21.09	11.18	1.33
os89819	7	546	0.45	7	3	5	6.67	-0.02	0.35	1.5	0.07	99.81	-1.3	6.43	6.86	15.77	7.31	-1.41	11.66	21.21	11.25	1.37
os89873	7	533	0	8	2	3	6.89	0.12	1.06	1.52	0.15	96.95	-7.45	7.21	6.79	15.61	7.21	-1.5	11.66	21.09	11.25	1.21
os89939	4	535	0.98	5	3	6	6.68	-0.09	0.3	1.5	0.07	95.24	0.8	6.25	6.76	15.6	7.23	-1.53	11.63	21.05	11.22	1.21
os90038	5	554	0.21	2	3	6	6.88	0.01	3.87	1.56	0.02	0.04	-0.06	9.87	6.64	15.47	7.11	-1.56	11.39	20.84	11.05	1.18
os90039	5	555	0.4	1	3	6	6.86	0	3.85	1.56	0.02	0.01	0.07	9.39	6.68	15.52	7.14	-1.53	11.46	20.91	11.12	1.21
os90059	6	515	0.86	9	2	7	6.44	-0.15	0.52	1.55	0.17	82.45	-6.62	6.73	6.79	15.64	7.25	-1.46	11.66	21.11	11.28	1.29
os90066	5	547	0.48	2	3	6	6.86	0	0.4	1.54	0.01	55.59	0.35	8.3	6.81	15.67	7.27	-1.44	11.69	21.14	11.31	1.31
os90110	7	517	0.98	5	2	7	6.7	-0.07	1.14	1.55	0.1	89.67	-3.1	8.21	6.77	15.61	7.23	-1.52	11.64	21.06	11.24	1.22
os90220	7	405	0.68	8	2	0	6.48	-0.13	0.26	1.52	0.07	92.91	-1.22	6.43	7.58	16.45	7.9	-0.9	12.33	21.84	11.79	1.81
os90228	6	400	0.48	3	2	0	6.69	-0.05	1.74	1.55	0.08	87.38	-1.17	8.05	7.64	16.49	7.93	-0.88	12.44	21.95	11.87	1.86
os90347	6	504	0.99	2	3	6	6.77	-0.03	2.98	1.55	0.04	69.12	-0.24	9.33	7.1	16.03	7.46	-1.31	11.73	21.32	11.34	1.45
os90465	4	347	0.23	7	2	3	6.66	0.03	0.71	1.54	0.11	93.69	-3.61	8.32	8.11	16.96	8.23	-0.74	12.94	22.35	12.17	2.03
os90521	7	398	0.57	8	2	3	6.86	0.04	0.8	1.51	0.12	100	-4.69	6.79	7.87	16.75	8.08	-0.85	12.43	21.87	11.74	1.79
os90534	7	376	0.85	10	2	3	6.53	-0.11	0.72	1.52	0.15	96.58	-6.45	7.67	7.89	16.76	8.11	-0.85	12.47	21.91	11.81	1.79
os90665	7	428	0.98	7	2	7	6.5	-0.13	0.4	1.53	0.16	94.7	-2.85	7.2	7.37	16.3	7.68	-1.17	12.11	21.68	11.61	1.61
os90711	4	482	1	22	3	5	5.64	-0.37	0	1.42	0.13	94.22	-0.32	6.07	7.46	16.43	7.7	-1.19	12.24	21.9	11.66	1.61
os90750	7	550	0	5	3	5	6.97	0.08	1.02	1.53	0.04	88.66	-0.65	9.42	6.87	15.85	7.25	-1.55	11.53	21.22	11.14	1.22
os90834	7	431	0.99	4	3	6	6.65	-0.07	1.62	1.54	0.06	91.47	-0.77	8.61	7.43	16.33	7.77	-1.01	12.08	21.63	11.61	1.69
os90937	7	429	0.87	5	2	7	6.56	-0.03	1.6	1.55	0.22	94.6	-9.6	7.03	7.36	16.29	7.66	-1.18	12.1	21.65	11.6	1.61
os91001	7	456	0.23	1	3	6	6.82	0.02	2.99	1.56	0.05	70.93	-0.35	9.01	7.23	16.17	7.57	-1.25	11.94	21.57	11.5	1.52
os91004	7	511	0.88	6	3	5	6.6	-0.09	0.64	1.52	0.06	89.11	-1.08	8.15	7.08	16.01	7.44	-1.33	11.7	21.32	11.31	1.4
os91007	6	499	0.6	3	2	3	6.73	-0.03	2.16	1.55	0.06	94.05	-2.67	8.27	7.1	16.03	7.46	-1.33	11.73	21.35	11.34	1.41

os91017	5	518	0.99	2	3	6	6.79	-0.02	1.29	1.54	0.02	51.29	0.17	9.16	7.08	16.02	7.45	-1.31	11.71	21.32	11.32	1.43
os91214	7	468	0.81	5	2	7	6.74	-0.04	1.57	1.55	0.1	97.06	-2.82	8.01	7.09	15.96	7.49	-1.22	11.58	21.15	11.18	1.46
os91225	3	440	0.08	20	2	7	6.79	0.26	0.06	1.49	0.2	96.55	-7.6	5.91	7.31	16.25	7.66	-1.18	12.01	21.68	11.61	1.59
os91284	7	315	0.23	11	2	3	6.66	0.1	0.24	1.52	0.14	94.32	-5.28	6.98	8.34	17.16	8.42	-0.57	13.24	22.6	12.38	2.23
os91332	7	426	0.03	6	2	0	6.57	-0.11	0.43	1.52	0.16	93.65	-0.95	7.12	7.37	16.3	7.68	-1.17	12.11	21.69	11.62	1.62
os91367	5	512	0.63	2	3	6	6.78	-0.02	1.09	1.54	0.02	64.71	0.32	9.24	7.11	16.05	7.47	-1.3	11.75	21.35	11.36	1.45
os91370	5	502	0.23	3	3	6	6.83	0.03	1.85	1.54	0.03	90.48	-0.48	8.2	7.12	16.06	7.48	-1.28	11.77	21.38	11.38	1.46
os91373	7	473	0.01	1	3	6	6.82	0.01	2.09	1.56	0.05	89.5	-1.34	8	7.18	16.09	7.51	-1.28	11.83	21.46	11.42	1.47
os91374	7	473	0.92	1	2	3	6.83	0.01	1.99	1.56	0.05	90.14	-2.36	7.89	7.18	16.09	7.51	-1.28	11.83	21.46	11.42	1.47
os91379	6	502	0.74	4	3	5	6.67	-0.05	1.45	1.54	0.06	94.32	-2.16	7.85	7.1	16.02	7.45	-1.33	11.72	21.35	11.33	1.41
os91380	5	506	0.71	3	3	6	6.72	-0.04	1.65	1.53	0.04	96.81	-1.11	6.94	7.11	16.04	7.46	-1.33	11.74	21.37	11.35	1.41
os91385	7	511	0.98	8	2	3	6.56	-0.13	0.42	1.52	0.06	77.62	-1.27	8.44	7.07	16.05	7.43	-1.42	11.74	21.44	11.32	1.35
os91398	6	534	0.16	2	3	6	6.86	0.01	2.49	1.54	0.05	86.96	-1.5	8.92	7	15.99	7.35	-1.45	11.68	21.34	11.21	1.34
os91400	6	536	0.65	2	3	6	6.85	-0.01	2.46	1.53	0.03	69.27	-0.82	8.58	7.07	16.05	7.37	-1.43	11.77	21.45	11.29	1.36
os91421	7	402	0.92	8	2	3	6.46	-0.12	0.9	1.54	0.12	92.98	-4.41	7.59	7.8	16.69	8.01	-0.98	12.54	22	11.88	1.75
os91455	3	476	0.41	8	3	5	6.61	0	0.05	1.48	0.11	95.55	-0.77	5.48	7.61	16.52	7.81	-1.2	12.39	21.85	11.68	1.56
os91587	7	281	0.33	5	2	7	6.57	-0.02	1.31	1.56	0.13	91.13	-5.06	8.06	8.41	17.25	8.52	-0.45	13.23	22.54	12.33	2.3
os91590	4	261	0.76	16	2	7	6.07	-0.16	0.01	1.47	0.14	78.55	-0.81	6.72	8.62	17.44	8.68	-0.28	13.47	22.86	12.54	2.46
os91600	7	500	0.65	2	3	6	6.75	-0.04	2.57	1.55	0.03	54.5	-0.68	8.47	7.17	16.14	7.49	-1.38	11.88	21.53	11.42	1.4
os91609	7	436	0.95	7	2	7	6.61	-0.09	0.72	1.53	0.12	99.66	-4.79	8.43	7.47	16.41	7.72	-1.15	12.23	21.88	11.68	1.64
os00002	1	620	0.95	5	3	6	6.81	-0.05	0.54	1.52	0	91.43	0.83	7.21	6.11	14.74	6.71	-1.52	10.63	19.98	10.35	1
os00114	1	586	0.45	14	1	4	6.51	0.01	0	1.43	0	92.37	3.78	5.42	6.22	14.82	6.67	-1.5	10.75	20.06	10.23	0.94
os00320	1	641	0.08	4	3	6	6.99	0.06	0.15	1.52	0	85.51	1.07	8.48	6.04	14.7	6.5	-1.7	10.66	20.03	10.17	0.83
os00379	1	657	0.4	3	3	6	6.93	0.01	2.51	1.55	0	87.17	-0.28	8.36	5.81	14.46	6.38	-1.79	10.24	19.57	9.94	0.68
os00550	1	553	0.16	14	3	5	7.07	0.2	0	1.48	0.09	95.78	-0.37	6.08	6.83	15.71	7.15	-1.4	11.79	21.29	10.97	1.24
os00643	1	661	0.25	7	3	9	7.05	0.08	0.28	1.48	0.05	100	-0.16	6.66	6.01	14.68	6.6	-1.83	10.4	19.7	10.15	0.6
os00880	1	465	0.77	9	2	0	6.71	-0.05	0.15	1.51	0.1	61.29	-1.69	6.8	7.16	15.86	7.39	-1.14	12.11	21.45	11.32	1.52
os00971	1	777	0.99	3	3	6	6.87	-0.04	1.21	1.53	0	96.4	-0.11	7.29	5.11	13.83	6.07	-2.37	9.28	18.45	9.32	-0.01
os01128	1	667	0.01	17	2	0	7.01	0.26	0.06	1.44	0.14	99.86	-1.44	6.78	5.65	14.4	6.4	-2.2	10.04	19.28	9.83	0.19
os01130	1	656	0.53	11	1	2	6.96	0.06	0	1.37	0.11	99.76	8.07	5.14	5.69	14.44	6.43	-2.17	10.09	19.33	9.87	0.23
os01324	1	401	0.03	13	3	5	7	0.22	0	1.5	0.1	99.86	-1.58	6.29	7.5	16.16	7.86	-0.98	12.13	21.3	11.48	1.55
os01519	1	370	0.42	5	3	6	6.86	0.04	0.37	1.52	0.05	83.83	-0.38	7.57	8.06	16.82	8.32	-0.59	12.86	22.11	12.05	2.02
os01520	1	407	0.55	4	3	6	6.83	0.01	0.99	1.55	0.05	84.41	-0.87	7.67	7.63	16.36	7.98	-0.87	12.16	21.41	11.58	1.67
os01549	1	235	0.01	4	3	6	6.78	0.06	0.7	1.54	0.04	94.78	0.09	7.04	8.94	17.79	8.99	-0.11	13.81	23.35	12.82	2.64
os01645	1	375	0.76	8	2	3	6.77	-0.02	0.63	1.47	0.12	99.33	-3.33	7.08	8.37	17.18	8.37	-0.35	12.92	22.47	11.97	2.34
os01665	1	406	0.89	9	1	4	6.6	-0.1	0.03	1.45	0.08	96.01	2.5	5.91	8.24	17.14	8.23	-0.61	12.83	22.51	11.86	2.08
os01676	1	429	0.92	11	3	5	6.31	-0.18	0.03	1.46	0.07	96.4	-0.18	6.37	8.25	17.18	8.24	-0.63	12.93	22.6	11.94	2.11

os01714	1	480	0	6	2	3	6.93	0.1	0.99	1.53	0.06	95.51	-1.47	8.03	7.84	16.82	7.88	-0.91	12.42	22.28	11.54	1.83
os01716	1	473	0.27	10	3	5	6.62	0.02	0.07	1.48	0.08	99.81	-0.69	6.69	7.85	16.83	7.88	-0.91	12.45	22.3	11.57	1.83
os01717	1	392	0.04	14	3	5	7	0.22	0	1.45	0.08	97.08	0.88	5.57	8.4	17.4	8.42	-0.43	12.99	22.86	12.12	2.31
os01756	1	363	0.65	5	3	5	6.81	0.01	2.15	1.55	0.07	82.62	-2.36	8.84	8.26	17.17	8.39	-0.48	12.9	22.67	12.14	2.23
os01904	1	362	0.5	16	3	5	6.8	0.06	0	1.44	0.11	96.28	1.87	6.13	8.56	17.42	8.46	-0.41	13.2	22.84	12.21	2.26
os02026	1	422	0.09	10	3	5	6.39	-0.16	0.01	1.48	0.07	95.9	0.05	7.23	8.17	17.06	8.19	-0.64	12.81	22.44	11.9	2.08
os02027	1	429	0.33	12	3	5	6.61	0.04	0.05	1.47	0.08	94.85	-0.4	7.64	8.14	17.05	8.16	-0.66	12.79	22.43	11.88	2.06
os02054	1	368	0	10	2	7	6.52	0.07	1.2	1.53	0.16	82.25	-5.79	6.71	8.3	17.14	8.27	-0.49	12.85	22.44	11.92	2.19
os02061	1	466	1	13	3	9	6.33	-0.2	0	1.45	0.07	99.97	0.63	5.39	8.14	17.07	8.16	-0.68	12.81	22.51	11.91	2.07
os02064	1	449	0.83	9	3	5	6.7	-0.07	0.08	1.48	0.05	99.77	0.68	6.06	8.16	17.1	8.19	-0.66	12.82	22.51	11.91	2.08
os02114	1	524	0.99	7	1	1	6.6	-0.11	0.2	1.44	0.04	99.75	2.63	6.09	7.76	16.77	7.83	-1.01	12.51	22.22	11.68	1.84
os02309	1	367	0.65	20	2	7	6.55	-0.04	0	1.41	0.19	90.73	-1.19	5.24	8.22	17.2	8.35	-0.37	12.83	22.45	12.06	2.44
os02368	1	348	0.76	7	3	5	6.49	-0.11	0.3	1.5	0.07	85.79	0.39	7.5	8.45	17.38	8.41	-0.39	13.37	22.96	12.39	2.47
os02414	1	567	0.04	2	3	9	6.9	0.03	0.11	1.48	0.01	98.58	1.89	7.18	7.56	16.59	7.69	-1.23	12.37	22.09	11.65	1.69
os02472	1	476	1	9	1	1	6.5	-0.13	0.01	1.4	0.04	99.81	6.52	5.58	8.09	17.06	8.06	-0.83	12.9	22.54	11.94	2.03
os02657	1	510	0.8	4	1	1	6.75	-0.05	0.19	1.48	0.01	94.63	2.99	6.98	7.86	16.77	7.89	-0.96	12.61	22.25	11.71	1.9
os02668	1	540	0.58	11	3	5	6.47	-0.07	0.05	1.47	0.07	99.95	0.14	6.14	7.65	16.58	7.7	-1.11	12.39	22	11.49	1.76
os02701	1	503	0.41	13	1	4	6.47	0	0	1.42	0.07	98.71	5.11	5.57	7.8	16.73	7.83	-1.02	12.57	22.21	11.66	1.86
os02974	1	318	0.71	5	2	3	6.64	-0.01	1.32	1.55	0.07	90.21	-3.76	8.19	8.7	17.48	8.64	-0.2	13.44	22.87	12.46	2.57
os03173	1	421	0.4	8	3	5	6.91	0.06	0.18	1.48	0.07	88.88	0.71	6.59	8.08	16.93	8.15	-0.51	12.57	22.16	11.73	2.12
os03231	1	332	0.93	13	2	3	6.39	-0.16	0.56	1.5	0.13	97.54	-5.44	6.78	8.47	17.23	8.51	-0.2	13.09	22.49	12.23	2.57
os03500	1	383	0.55	10	1	4	6.77	-0.01	0.03	1.38	0.06	83	4.26	5.47	8.4	17.18	8.39	-0.45	13.14	22.58	12.25	2.31
os03602	1	424	0.76	5	3	6	6.63	-0.06	0.64	1.53	0.04	79.37	0.34	7.47	8	16.86	8.06	-0.78	12.7	22.27	11.85	2.02
os03627	1	435	0.99	8	3	5	6.51	-0.12	0.29	1.51	0.05	90.97	-0.28	6.66	8	16.89	8.05	-0.78	12.71	22.28	11.87	2.05
os03715	1	431	0.41	10	3	5	6.61	0.02	0.06	1.48	0.05	99.19	0.43	5.96	8.28	17.2	8.26	-0.61	13.05	22.69	12.13	2.25
os03739	1	395	0.96	19	3	5	6.13	-0.24	0	1.43	0.12	97.35	-0.24	5.17	8.18	16.96	8.23	-0.52	12.81	22.26	11.96	2.16
os03744	1	333	0.44	5	2	7	6.77	0	1.4	1.54	0.12	92.78	-3.35	7.18	8.37	17.13	8.38	-0.36	12.98	22.37	12.1	2.32
os03839	1	437	0.73	11	2	3	6.34	-0.14	0.04	1.5	0.11	95.5	-2.94	7.5	7.93	16.68	8.08	-0.62	12.39	21.86	11.62	1.94
os03852	1	415	0.91	10	2	7	6.38	-0.15	0.05	1.51	0.11	84.63	-3.22	6.93	7.99	16.8	8.11	-0.69	12.65	22.21	11.88	2
os03855	1	443	1	10	1	4	6.44	-0.16	0.01	1.46	0.05	89.85	2.75	5.65	7.99	16.8	8.1	-0.69	12.64	22.21	11.87	2
os03864	1	415	0.98	13	3	5	6.31	-0.2	0	1.47	0.1	90.56	-0.05	5.46	8.03	16.82	8.13	-0.63	12.67	22.17	11.87	2.03
os03884	1	443	0.92	12	2	3	6.48	-0.15	0.08	1.49	0.11	93.63	-4	6.09	7.95	16.77	8.05	-0.72	12.58	22.14	11.81	1.96
os03975	1	389	0.14	3	3	6	6.78	0.02	0.31	1.53	0.02	70.53	0.73	8.2	8.22	17	8.29	-0.46	13	22.46	12.14	2.25
os04722	1	390	0.77	4	2	3	6.72	-0.04	1.53	1.52	0.07	96.21	-2.11	7.56	8.22	17.13	8.33	-0.45	12.83	22.42	12.08	2.3
os04764	1	346	1	9	3	5	6.41	-0.16	0.09	1.45	0.07	99.16	0.65	5.7	8.6	17.5	8.62	-0.21	13.38	22.94	12.47	2.63
os04765	1	346	1	8	3	5	6.42	-0.15	0.13	1.45	0.07	99.18	-0.13	5.78	8.59	17.49	8.61	-0.21	13.37	22.93	12.47	2.63
os04774	1	438	0.99	9	3	9	6.52	-0.14	0.26	1.45	0.08	100	-0.6	6.1	8.19	17.15	8.23	-0.57	13.06	22.68	12.16	2.32

os05095	1	298	0.28	6	1	4	6.69	0.03	0.06	1.48	0.04	90.92	2.77	7.8	8.82	17.59	8.85	0.04	13.61	23.03	12.69	2.73
os05232	1	372	0.2	8	3	5	6.96	0.12	0.18	1.48	0.08	92.21	0.41	6.67	8.36	17.15	8.45	-0.31	12.92	22.4	12.1	2.27
os05272	1	364	0.4	5	3	6	6.79	0	1.6	1.54	0.06	87.45	-2.01	8.66	8.37	17.24	8.44	-0.35	12.98	22.66	12.18	2.28
os05325	1	449	0.98	5	3	5	6.67	-0.08	0.44	1.49	0.04	97.1	0.7	6.44	8.35	17.34	8.22	-0.55	13.09	22.74	12.07	2.46
os05382	1	475	0.01	4	3	6	6.88	0.07	0.57	1.5	0.03	97.35	-0.19	7.08	7.94	16.83	7.99	-0.86	13.12	22.62	12.41	2.49
os05417	1	474	0.09	5	3	6	6.82	0.05	0.55	1.51	0.04	97.16	0.05	6.94	8.1	17.1	8.07	-0.77	13.07	22.74	12.2	2.35
os05452	1	415	0.8	4	3	6	6.74	-0.04	0.58	1.52	0.04	96.75	-0.22	6.85	8.49	17.49	8.33	-0.51	13.29	22.94	12.25	2.52
os05513	1	500	0.02	12	2	0	7.05	0.19	0.03	1.47	0.09	94.65	0.38	6.88	7.83	16.83	7.85	-1.02	13.05	22.7	12.23	2.35
os05518	1	500	0	4	2	7	6.88	0.06	2.7	1.55	0.08	90.84	-4.35	7.92	7.79	16.78	7.82	-1.06	12.98	22.61	12.18	2.31
os05519	1	410	0.35	13	3	5	6.94	0.13	0	1.48	0.13	97.47	-2.49	7.24	8.34	17.37	8.25	-0.54	13.12	22.76	12.12	2.47
os05592	1	434	0.48	13	2	3	6.41	-0.05	0.03	1.49	0.12	97.83	-2.91	6.37	8.3	17.32	8.23	-0.65	13.35	23.06	12.45	2.55
os05709	1	443	0.99	8	2	3	6.59	-0.1	1.01	1.54	0.09	91.84	-4.18	7.63	8.21	17.32	8.09	-0.85	13.14	23.03	12.12	2.18
os05903	1	436	0.03	3	3	6	6.72	-0.03	2.35	1.55	0.04	69.75	-1.03	8.36	8.08	17.06	7.97	-0.83	12.9	22.51	11.91	2.13
os06133	1	433	0.05	3	3	6	6.87	0.05	2.21	1.55	0.03	56.25	-0.83	7.83	8.03	17.07	8.01	-0.91	12.97	22.68	12.12	2.03
os06317	1	573	0.97	14	3	9	6.37	-0.21	0	1.44	0.09	99.91	1	5.51	7.61	16.61	7.57	-1.4	12.49	22.13	11.5	1.57
os06320	1	533	0.04	9	1	4	6.87	0.11	0.03	1.47	0.05	98.95	3.19	5.65	7.73	16.72	7.67	-1.32	12.61	22.24	11.61	1.64
os06326	1	555	0	7	3	9	6.99	0.13	0.15	1.49	0.05	96.53	0.76	6.04	7.67	16.7	7.63	-1.34	12.56	22.23	11.57	1.63
os06351	1	559	0.24	10	3	5	6.99	0.09	0.05	1.46	0.07	99.01	0.8	5.79	7.62	16.63	7.59	-1.38	12.5	22.17	11.51	1.6
os06355	1	527	0.31	13	3	5	7.01	0.13	0.01	1.48	0.1	99.06	-0.43	7.24	7.64	16.66	7.61	-1.36	12.53	22.19	11.53	1.62
os06460	1	625	0.77	8	3	9	6.79	-0.06	0.24	1.47	0.05	94.92	0.87	6.31	7.29	16.29	7.38	-1.56	12.11	21.76	11.22	1.38
os06463	1	575	0.74	7	3	5	6.62	-0.07	0.36	1.51	0.05	87.41	0.49	7.01	7.38	16.37	7.44	-1.53	12.23	21.84	11.31	1.4
os06468	1	642	0.05	2	3	9	6.91	0.02	0.02	1.5	0.01	97.85	1.92	7.42	7.17	16.17	7.25	-1.64	11.99	21.59	11.1	1.3
os06471	1	627	0.99	13	3	5	6.39	-0.2	0.03	1.48	0.09	95.81	-1.16	6.75	7.18	16.18	7.26	-1.63	11.99	21.6	11.09	1.3
os06559	1	447	0.69	9	3	5	6.48	-0.08	0.12	1.51	0.06	72.14	-0.61	7.13	8.03	17.05	8	-0.93	12.84	22.51	11.9	2.02
os06562	1	460	0.92	6	3	5	6.6	-0.08	0.2	1.52	0.03	79.8	0.56	7.15	8.02	17.05	7.99	-0.92	12.8	22.49	11.87	2.01
os06574	1	391	0.17	10	2	0	6.7	0.09	0.45	1.51	0.14	92.73	-4.04	6.69	8.27	17.31	8.2	-0.7	13.08	22.76	12.08	2.21
os06646	1	583	0.6	3	3	9	6.79	-0.02	0.24	1.5	0.02	99.46	0.48	7.01	7.55	16.6	7.56	-1.45	12.4	22.09	11.49	1.51
os06660	1	606	0.08	2	1	1	6.91	0.03	0.05	1.47	0	96.94	2.14	7.29	7.46	16.48	7.48	-1.49	12.29	21.96	11.38	1.45
os06662	1	578	0.03	9	1	4	6.94	0.12	0.04	1.47	0.04	97.08	2.9	5.81	7.48	16.51	7.49	-1.47	12.32	21.99	11.4	1.46
os06687	1	467	0.67	13	3	5	6.75	-0.03	0	1.47	0.08	91.79	0.18	6.13	8.02	17.02	7.91	-1.12	12.87	22.53	11.84	1.82
os06827	1	397	0.25	17	2	3	6.97	0.18	0	1.47	0.13	94.9	-1.94	6.28	8.31	17.35	8.23	-0.71	13.08	22.78	12.08	2.18
os06836	1	396	0.6	10	1	4	6.43	-0.07	0.06	1.44	0.05	92.77	2.82	6.46	8.43	17.5	8.34	-0.58	13.13	22.85	12.11	2.29
os07078	1	476	0.98	6	3	5	6.7	-0.07	0.45	1.52	0.05	97.52	-0.36	6.62	7.8	16.84	7.83	-1.12	12.76	22.47	11.96	1.86
os07117	1	410	0.99	3	3	6	6.69	-0.05	1.18	1.53	0.02	83.36	0.43	7.46	8.22	17.25	8.13	-0.78	13.12	22.8	12.21	2.16
os07129	1	427	0.77	2	3	6	6.72	-0.03	1.82	1.56	0.03	40.75	-0.9	7.81	8.07	17.08	7.99	-0.9	13.08	22.73	12.15	2.12
os07149	1	444	0.03	3	3	6	6.73	-0.04	2.29	1.55	0.03	63.2	-0.42	7.63	7.98	17	7.9	-0.9	12.82	22.46	11.87	2.09
os07182	1	456	0.92	4	3	6	6.64	-0.07	0.84	1.53	0.04	86.9	0.03	7.62	8.05	17.2	8	-0.91	13.01	22.81	11.99	2.09

os07183	1	448	0.92	3	2	3	6.69	-0.05	1.89	1.52	0.06	97.11	-2.34	7.04	8.14	17.25	8.05	-0.83	13.01	22.79	12.01	2.15
os07317	1	519	0.59	5	3	6	6.64	-0.08	0.37	1.52	0.03	94.51	0.23	6.75	7.91	17	7.82	-1.1	12.7	22.55	11.77	1.86
os07623	1	477	0.89	7	3	5	6.66	-0.09	0.31	1.51	0.05	91.04	0.5	6.75	7.85	16.86	7.82	-1.05	12.84	22.46	11.96	1.99
os07702	1	547	0.24	3	3	6	6.77	-0.01	1.3	1.51	0.04	90.23	-0.6	8.15	7.37	16.35	7.45	-1.35	12.42	21.96	11.67	1.78
os07774	1	478	0.16	5	3	6	6.94	0.06	0.55	1.53	0.04	68.22	-0.17	8.16	7.99	17.1	7.93	-1	12.78	22.61	11.81	1.96
os07950	1	466	0.01	2	3	6	6.84	0.03	1.74	1.55	0.01	46.73	0.07	8.78	8.1	17.12	7.99	-1.01	12.87	22.67	11.86	1.85
os08240	1	437	0.87	8	1	4	6.5	-0.09	0.13	1.48	0.04	71.33	1.99	7.57	8.27	17.34	8.12	-0.88	13.18	22.97	12.15	2.06
os08361	1	434	0	4	3	6	6.91	0.08	0.7	1.51	0.05	91.95	0.47	7.31	8.28	17.32	8.13	-0.86	13.02	22.79	11.98	2.04
os08372	1	466	0.92	4	3	6	6.65	-0.06	0.57	1.52	0.03	97.5	0.05	8.2	8.13	17.18	7.99	-0.95	12.82	22.61	11.83	1.95
os08373	1	462	0.92	3	3	6	6.69	-0.05	0.43	1.53	0.04	97.39	0.15	8.72	8.13	17.18	8	-0.95	12.83	22.62	11.83	1.95
os08533	1	484	0.16	3	3	6	6.9	0.05	0.68	1.55	0.02	72.58	0.23	8.08	7.98	17	7.88	-1.11	12.91	22.57	11.86	1.8
os08534	1	485	0.08	4	3	6	6.93	0.07	0.7	1.54	0.03	74	-0.25	7.89	7.97	16.99	7.86	-1.11	12.9	22.55	11.84	1.8
os08620	1	399	0.56	11	2	3	6.75	0.04	0.41	1.53	0.15	79.19	-5.76	7.74	8.42	17.49	8.22	-0.73	13.32	23.12	12.22	2.19
os08868	1	364	0.1	22	2	3	5.55	-0.33	0.06	1.47	0.21	86.24	-6.46	6.71	8.62	17.75	8.41	-0.59	13.54	23.35	12.39	2.4
os08896	1	430	0.4	9	2	3	6.9	0.07	0.78	1.53	0.13	96.56	-5.17	6.73	8.27	17.35	8.12	-0.81	13.15	22.99	12.1	2.17
os08978	1	443	0.32	12	1	4	6.97	0.11	0.02	1.44	0.06	88.02	3.41	5.67	8.22	17.24	8.06	-0.94	13.17	22.85	12.1	1.99
os08981	1	444	0.96	11	1	4	6.54	-0.13	0.01	1.43	0.06	87.58	2.54	6.14	8.22	17.24	8.05	-0.95	13.17	22.85	12.1	1.99
os09039	1	553	0.91	13	3	9	6.55	-0.14	0	1.43	0.08	99.31	2.06	5.99	7.02	15.86	7.22	-1.45	12.11	21.43	11.54	1.82
os09059	1	572	0.76	4	3	5	6.8	-0.04	0.07	1.5	0.02	84.62	1.67	6.62	6.97	15.85	7.17	-1.49	12.13	21.5	11.54	1.84
os09220	1	389	0.08	4	1	4	6.61	-0.07	0.04	1.48	0.03	89.03	3.42	6.99	7.96	16.76	8.2	-0.9	12.6	22.23	11.86	1.81
os09233	1	368	0.98	4	3	6	6.68	-0.06	0.39	1.53	0.03	98.48	0.29	7.53	8	16.81	8.23	-0.87	12.68	22.29	11.9	1.84
os09320	1	526	0.23	5	1	4	6.83	0.04	0.13	1.46	0.04	96.05	2.99	6.55	7.14	15.97	7.58	-1.32	11.54	21.19	10.99	1.29
os09345	1	311	0.91	2	3	6	6.71	-0.03	2.68	1.55	0.02	34.09	-0.06	9.37	8.4	17.2	8.57	-0.5	13.19	22.77	12.36	2.24
os09347	1	310	0.92	3	3	6	6.64	-0.04	2.02	1.54	0.02	49.22	-0.1	9.31	8.4	17.2	8.57	-0.5	13.18	22.77	12.36	2.24
os09434	1	329	0.08	2	3	6	6.82	0.04	0.51	1.53	0.01	81.19	0.31	7.27	8.3	17.04	8.52	-0.61	13.06	22.51	12.27	2.14
os09600	1	275	0.35	7	2	3	6.68	0.04	0.24	1.53	0.09	80.25	-2.25	7.35	8.62	17.43	8.69	-0.35	13.52	23.09	12.6	2.46
os09607	1	365	0.76	2	3	6	6.7	-0.03	2.95	1.56	0.04	80.17	-0.61	8.69	8.12	16.98	8.27	-0.67	12.88	22.51	12.02	2.06
os09610	1	396	0.23	2	3	6	6.75	0	0.19	1.52	0.01	71.99	0.57	8.62	8.03	16.86	8.17	-0.73	12.7	22.39	11.91	1.98
os09624	1	446	1	9	3	5	6.52	-0.14	0.61	1.49	0.06	87.85	-1.57	8.15	7.64	16.48	7.87	-1.18	12.25	21.96	11.5	1.5
os09748	1	414	0.55	4	3	6	6.74	0.01	1.68	1.53	0.05	98.27	-1.75	8.63	8.19	17.18	8.21	-0.67	12.69	22.55	11.81	2.06
os09758	1	368	0.84	11	3	5	6.3	-0.15	0.02	1.48	0.06	66.71	1.01	7.19	8.51	17.47	8.45	-0.45	13.18	22.96	12.19	2.28
os09762	1	428	0.99	4	3	6	6.71	-0.05	0.7	1.54	0.04	34.58	0.1	7.67	8.27	17.28	8.24	-0.6	12.82	22.7	11.89	2.15
os09778	1	411	0.08	10	2	3	6.9	0.15	0.58	1.5	0.12	96.67	-4.66	7.91	8.18	17.2	8.19	-0.67	12.7	22.57	11.76	2.03
os09954	1	521	0.53	8	1	1	6.61	-0.04	0.01	1.45	0.03	97.99	5.12	5.7	7.07	15.83	7.34	-1.12	11.93	21.18	11.21	1.73
os10029	1	476	0.65	8	2	3	6.77	-0.04	0.49	1.52	0.08	85.03	-2.2	6.89	7.03	15.76	7.3	-1.11	11.75	21.01	11.08	1.66
os10084	1	502	0.83	9	3	9	6.45	-0.12	0.05	1.48	0.05	98.39	1.72	5.67	7.09	15.85	7.36	-1.1	11.96	21.21	11.23	1.74
os10087	1	554	0.6	9	3	5	6.84	-0.02	0.12	1.5	0.08	98.24	-0.63	6.32	6.6	15.37	6.98	-1.34	11.26	20.51	10.7	1.44

os10093	1	483	0.99	8	3	5	6.51	-0.13	0.16	1.51	0.08	93.71	-0.6	8.35	6.95	15.73	7.24	-1.16	11.71	20.97	11.06	1.63
os10238	1	565	0.15	12	1	1	6.79	0.12	0	1.44	0.07	99.91	2.5	5.62	6.74	15.63	7.07	-1.48	11.79	21.16	11.18	1.66
os10243	1	556	1	17	3	9	6.06	-0.28	0	1.44	0.12	98.33	-0.18	5.61	6.72	15.61	7.06	-1.45	11.74	21.12	11.15	1.66
os10245	1	570	1	15	1	1	6.17	-0.25	0	1.42	0.09	97.53	3.32	5.34	6.71	15.61	7.06	-1.45	11.74	21.12	11.15	1.65
os10263	1	552	0.77	10	3	9	6.75	-0.06	0.01	1.46	0.07	99.96	1.76	5.69	6.69	15.56	7.05	-1.44	11.69	21.07	11.14	1.67
os10268	1	535	0.28	14	3	5	7.01	0.15	0	1.46	0.1	99.55	-0.01	5.66	6.68	15.55	7.05	-1.44	11.69	21.07	11.14	1.67
os10272	1	535	0.27	14	3	5	7.02	0.15	0	1.46	0.1	99.58	-0.14	5.7	6.68	15.55	7.05	-1.44	11.69	21.07	11.14	1.67
os10354	1	630	0.93	11	3	5	6.39	-0.18	0.07	1.48	0.08	92.92	-0.41	6.5	6.22	14.96	6.63	-1.69	10.82	20.09	10.31	1
os10976	1	591	0.77	3	3	6	6.85	-0.02	0.92	1.51	0.02	71.98	0.7	7.82	6.41	15.13	6.79	-1.54	11.01	20.32	10.56	1.18
os11008	1	488	0.6	4	3	6	6.71	-0.03	0.98	1.54	0.04	61.97	-0.21	8.72	6.99	15.71	7.21	-1.31	12	21.32	11.36	1.62
os11016	1	535	0.09	8	2	3	6.98	0.13	0.29	1.55	0.12	60.83	-5.16	7.4	6.6	15.27	6.91	-1.44	11.21	20.43	10.62	1.25
os11239	1	574	0.6	6	3	5	6.86	-0.02	0.56	1.52	0.08	98.44	-0.92	8.48	6.46	15.25	6.87	-1.45	11.01	20.28	10.47	1.31
os11262	1	564	0.42	8	3	5	6.65	-0.02	0.1	1.49	0.05	99.95	1.2	6.03	6.63	15.43	7	-1.36	11.33	20.6	10.71	1.43
os11265	1	555	0.3	7	3	5	6.75	0.02	0.35	1.5	0.07	99.86	-0.19	6.59	6.6	15.4	6.97	-1.38	11.28	20.55	10.67	1.41
os11347	1	547	0.62	12	3	5	6.8	-0.02	0	1.46	0.09	97.85	-0.17	5.58	6.76	15.49	7.08	-1.32	11.33	20.56	10.72	1.43
os11434	1	467	0.05	7	2	0	6.88	0.09	0.61	1.54	0	88.25	-2.21	7.7	6.8	15.67	7.16	-1.24	11.63	20.99	10.99	1.71
os11518	1	526	0.99	8	3	5	6.59	-0.12	0.62	1.51	0.1	98.18	-2.31	7.03	6.68	15.59	7.04	-1.46	11.71	21.09	11.14	1.65
os11642	1	499	0.03	16	3	5	6.89	0.23	0	1.45	0.09	96.45	1.05	5.22	6.87	15.8	7.23	-1.32	12.01	21.42	11.35	1.77
os11651	1	539	0.01	7	2	3	6.96	0.12	0.92	1.51	0.09	94.82	-3.07	8.13	6.57	15.5	7	-1.41	11.55	20.96	10.98	1.63
os11653	1	540	0.01	8	2	3	6.96	0.12	0.77	1.51	0.09	94.63	-2.59	8.1	6.57	15.5	7	-1.41	11.55	20.96	10.98	1.63
os11655	1	542	0.04	10	2	3	6.93	0.14	0.14	1.49	0.09	93.95	-1.01	7.64	6.58	15.5	7.01	-1.41	11.56	20.97	10.99	1.63
os11675	1	548	0.46	13	1	4	6.46	-0.04	0	1.45	0.1	99.36	2.65	5.63	6.57	15.49	7	-1.46	11.54	20.96	11.06	1.64
os11691	1	578	0.34	5	3	9	6.98	0.06	0.55	1.49	0.05	98.29	0.4	6.52	6.63	15.45	6.99	-1.36	11.42	20.7	10.8	1.49
os11753	1	527	0.84	9	3	5	6.69	-0.09	0.11	1.5	0.06	99.9	-0.59	6.24	6.84	15.66	7.15	-1.23	11.69	20.96	10.98	1.62
os11754	1	529	0.84	9	3	5	6.68	-0.09	0.09	1.5	0.06	99.87	-0.36	6.05	6.83	15.65	7.14	-1.24	11.68	20.95	10.97	1.62
os11938	1	514	0.23	4	3	6	6.8	0.02	0.87	1.54	0.06	99.81	-0.47	6.99	7.18	16.06	7.35	-1.42	12.13	21.57	11.39	1.6
os11946	1	465	0.76	7	2	0	6.58	-0.08	0.44	1.52	0.08	80.39	0.28	7.69	7.34	16.23	7.46	-1.32	12.32	21.76	11.56	1.71
os11980	1	691	0	9	2	3	7.07	0.15	0.28	1.49	0.11	100	-2.11	7.68	6.74	15.7	7.07	-1.44	11.44	20.94	11.1	1.74
os12127	1	704	0.88	10	3	5	6.48	-0.14	0.04	1.45	0.08	95.29	0.58	5.75	6.67	15.49	7.05	-1.7	11.59	20.86	11.24	1.6
os12157	1	741	0.01	9	2	3	7.07	0.15	0.19	1.49	0.1	98.77	-2.59	5.82	6.43	15.28	6.88	-1.81	11.33	20.63	11.04	1.53
os12192	1	704	0.87	11	3	9	6.49	-0.14	0.01	1.45	0.05	99.7	1.83	5.45	6.75	15.56	7.07	-1.7	11.77	21.01	11.37	1.64
os12243	1	604	0.12	6	2	0	7.01	0.08	0.52	1.52	0.13	99.8	-0.78	7.04	6.96	15.81	7.29	-1.25	11.66	21.07	11.3	1.95
os12436	1	543	0.99	6	3	5	6.7	-0.08	0.46	1.53	0.05	99.41	0.4	6.69	7.5	16.44	7.68	-1.12	12.47	21.99	11.93	2.09
os12713	1	522	0.65	14	1	4	6.15	-0.23	0	1.38	0.07	90.4	5.37	5.42	7.62	16.46	7.77	-1.1	12.77	22.22	12.22	2.22
os12742	1	622	0.95	14	3	9	6.47	-0.18	0	1.45	0.08	99.45	1.51	5.32	7.22	16.15	7.46	-1.25	12.13	21.61	11.64	2
os12823	1	588	0.99	4	3	6	6.73	-0.07	0.16	1.49	0.03	98.82	2.07	6.54	7.26	16.04	7.46	-1.4	12.32	21.61	11.76	1.94
os13020	1	620	0.92	4	3	6	6.7	-0.07	0.41	1.54	0.05	82.68	-0.04	8.67	6.78	15.57	7.05	-1.78	11.97	21.31	11.57	1.8

os13417	1	474	0.96	9	3	5	6.54	-0.13	0.16	1.52	0.05	93.7	0.13	6.88	7.69	16.58	7.74	-1.13	12.85	22.39	12.2	2.28
os13613	1	653	0.21	7	3	5	7.03	0.08	0.38	1.51	0.06	97.43	0.87	6.84	6.72	15.54	7.08	-1.65	11.66	21.04	11.37	1.76
os13770	1	780	0.23	6	3	9	6.9	0.04	0.54	1.48	0.04	100	1.23	5.99	6.22	15.03	6.73	-1.92	11.06	20.36	10.94	1.49
os14027	1	461	0.05	11	3	5	7.02	0.18	0.1	1.45	0.06	96.9	0.81	6.83	7.95	16.83	8	-0.85	13.13	22.63	12.42	2.5
os14186	1	507	0.31	7	3	5	6.93	0.05	0.79	1.52	0.07	99.63	-1.78	7.7	7.58	16.41	7.7	-1.12	12.66	22.17	12.07	2.25
os14369	1	454	0.99	3	3	6	6.73	-0.05	1.02	1.55	0.02	63.33	-0.04	8.46	8.2	17.24	8.07	-0.81	13.27	22.87	12.24	2.36
os14455	1	520	0.81	6	3	5	6.72	-0.07	0.17	1.5	0.05	97.78	1.51	6.66	7.83	16.83	7.8	-0.99	12.83	22.38	11.9	2.22
os14478	1	477	0.01	8	3	5	6.91	0.13	0.15	1.51	0.07	80.69	-0.97	7.21	7.95	16.9	7.9	-0.91	12.92	22.42	12	2.26
os14533	1	598	0.89	12	3	5	6.57	-0.14	0.01	1.46	0.08	99.96	-0.15	5.77	7.03	15.89	7.23	-1.45	12	21.39	11.46	1.82
os14565	1	534	0.98	11	2	0	6.37	-0.19	0.01	1.48	0.09	99.4	0.96	5.75	7.21	16.12	7.37	-1.36	12.26	21.69	11.65	1.91
os14659	1	527	0.91	11	3	5	6.34	-0.16	0.01	1.46	0.07	99.03	1.12	6.19	7.55	16.47	7.62	-1.16	12.56	22.08	11.84	2.06
os14704	1	532	0.24	8	1	4	6.99	0.09	0.04	1.47	0.05	87.21	3.11	5.65	7.67	16.64	7.71	-1.13	12.65	22.24	11.86	2.01
os14710	1	512	0.44	9	2	0	6.58	-0.02	0.1	1.51	0.09	96.25	-1.68	7.31	7.61	16.61	7.67	-1.17	12.63	22.22	11.89	2
os14725	1	519	1	8	1	1	6.61	-0.11	0	1.41	0.03	96.53	5.84	5.42	7.88	16.87	7.85	-1.01	12.87	22.46	11.98	2.11
os14816	1	499	0.19	11	3	9	7.03	0.15	0.02	1.47	0.06	99.53	0.73	5.73	8.05	17.05	7.98	-0.9	13.13	22.72	12.26	2.29
os14855	1	450	0.08	1	3	6	6.83	0.01	2.04	1.55	0.02	47.52	0.45	9.55	8.25	17.3	8.12	-0.78	13.33	22.93	12.3	2.39
os14954	1	735	1	5	1	1	6.75	-0.08	0.03	1.45	0.02	99.66	4.1	6.11	6.46	15.27	6.9	-1.63	11.14	20.43	10.88	1.56
os14998	1	665	0.81	13	3	5	6.33	-0.16	0.01	1.48	0.11	100	-0.82	5.36	6.67	15.5	7.03	-1.52	11.4	20.74	11.05	1.67
os15000	1	681	0.95	15	3	5	6.44	-0.19	0	1.46	0.12	100	0.99	5.56	6.77	15.6	7.1	-1.46	11.52	20.86	11.13	1.71
os15035	1	747	0.23	11	3	5	6.78	0.06	0.04	1.48	0.12	99.87	-2.08	6.19	6.23	15.09	6.73	-1.67	10.73	20.08	10.54	1.43
os15288	1	747	0.91	8	3	5	6.73	-0.11	0.27	1.49	0.09	100	-0.39	6.26	6.39	15.3	6.82	-1.65	11.1	20.48	10.83	1.56
os15296	1	679	0.38	10	3	5	7.04	0.08	0.09	1.49	0.09	100	-0.36	7.03	6.62	15.47	6.99	-1.54	11.35	20.69	11.01	1.65
os15301	1	690	0.6	9	2	3	6.59	-0.06	0.22	1.49	0.1	99.98	-2.08	6.21	6.63	15.46	7.01	-1.54	11.36	20.69	11.02	1.65
os15332	1	670	0.48	11	3	5	6.57	-0.04	0.02	1.48	0.11	99.98	0.25	6.3	6.74	15.6	7.08	-1.47	11.48	20.84	11.1	1.71
os15629	1	481	0.99	5	3	6	6.67	-0.08	0.69	1.53	0.03	85.27	0.18	6.64	8.06	17.02	8	-0.83	13.14	22.65	12.26	2.39
os15784	1	733	0.93	11	3	9	6.59	-0.16	0.02	1.46	0.08	99.89	-1.15	5.94	6.43	15.29	6.83	-1.7	11.34	20.65	11.03	1.62
os15900	1	705	0.44	12	3	9	6.57	-0.02	0.01	1.42	0.08	99.61	1.46	5.87	6.59	15.44	6.93	-1.65	11.55	20.79	11.14	1.63
os16067	1	768	0.77	5	3	5	6.87	-0.05	0.16	1.49	0.05	98.46	1.99	7.45	6.15	14.96	6.67	-2.06	11.12	20.41	11.02	1.44
os16505	1	689	0.65	17	3	5	6.79	-0.03	0	1.44	0.1	99.88	0.41	5.24	6.6	15.37	6.97	-1.81	11.6	20.85	11.34	1.6
os16529	1	594	0.81	8	3	5	6.56	-0.1	0.26	1.51	0.1	96.78	-1.38	6.1	7.23	16.04	7.42	-1.36	12.27	21.56	11.72	1.99
os16583	1	483	0.48	9	3	5	6.88	0.02	0.08	1.5	0.06	83.68	0.44	6.77	7.64	16.53	7.71	-1.13	12.76	22.31	12.13	2.26
os16590	1	598	0.02	8	3	5	6.96	0.13	0.37	1.51	0.09	99.31	-1.82	7.39	7.02	15.92	7.22	-1.45	12.05	21.46	11.5	1.84
os16601	1	636	0.99	9	3	9	6.52	-0.15	0.11	1.47	0.07	100	0.1	5.91	6.67	15.51	6.99	-1.6	11.56	20.87	11.12	1.68
os16622	1	637	0.75	3	3	6	6.87	-0.02	1.94	1.51	0.06	98.78	-1.57	7.85	6.47	15.3	6.82	-1.75	11.5	20.73	11.1	1.58
os16694	1	650	0.06	16	3	5	6.88	0.21	0	1.46	0.12	99.67	-1.84	5.72	6.18	14.97	6.63	-1.86	11.04	20.26	10.83	1.43
os16836	1	674	0.7	13	1	1	6.39	-0.11	0	1.39	0.07	97.36	5.73	5.2	6.26	15.1	6.72	-1.78	11.23	20.49	10.97	1.53
os16850	1	620	0.82	9	3	9	6.75	-0.08	0.04	1.45	0.05	99.6	2.01	5.52	6.82	15.72	7.07	-1.54	11.93	21.31	11.42	1.8

os16859	1	475	0.23	6	2	0	6.78	0.04	0.2	1.52	0.07	57.84	0.68	7.35	7.24	16.09	7.39	-1.34	12.41	21.71	11.74	1.97
os16878	1	570	0.02	21	3	5	6.98	0.34	0	1.41	0.15	99.99	0.37	5.17	6.91	15.8	7.14	-1.49	12.05	21.44	11.5	1.85
os16916	1	452	0.87	2	2	0	6.76	-0.02	1.55	1.55	0.14	48.56	-0.3	8.78	7.37	16.24	7.46	-1.22	12.59	21.93	11.85	2.1
os17020	1	611	1	17	3	5	6.13	-0.28	0	1.42	0.13	100	-1.03	5.64	6.8	15.61	7.07	-1.55	11.69	21	11.22	1.72
os17039	1	633	0.88	14	3	5	6.22	-0.22	0	1.43	0.1	99.63	1.43	5.47	6.29	15.07	6.73	-1.82	11.2	20.37	10.95	1.49
os17211	1	560	0.61	15	3	5	6.81	0.01	0	1.44	0.11	98.42	-1.02	5.85	6.8	15.71	7.14	-1.48	11.9	21.31	11.48	1.84
os17222	1	660	0.47	10	3	5	6.65	-0.02	0.04	1.46	0.06	100	1.19	6.09	6.15	15.05	6.65	-1.72	11.19	20.53	10.95	1.65
os17223	1	603	0.07	15	3	5	6.84	0.19	0	1.45	0.09	100	-0.85	5.47	6.44	15.32	6.84	-1.6	11.52	20.86	11.17	1.74
os17224	1	608	0.04	14	3	9	6.93	0.2	0	1.45	0.1	100	-1.82	5.94	6.45	15.33	6.85	-1.6	11.55	20.88	11.19	1.74
os17282	1	634	0.21	8	1	4	7.05	0.12	0.17	1.45	0.05	89.93	3.46	5.79	6.45	15.33	6.86	-1.7	11.41	20.77	11.1	1.61
os17306	1	662	0.52	2	3	9	6.84	-0.01	1.35	1.49	0.02	85.76	0.08	7.18	6.51	15.42	6.87	-1.73	11.33	20.79	10.91	1.43
os17353	1	512	0.08	6	3	5	6.97	0.09	0.44	1.52	0.05	89.2	-0.94	6.4	7.12	16.01	7.36	-1.4	12.2	21.65	11.61	1.72
os17400	1	624	0.08	10	3	9	7.09	0.15	0.09	1.45	0.07	98.21	0.89	6.5	6.51	15.23	6.88	-1.68	11.29	20.54	11.01	1.6
os17409	1	634	0.3	5	1	1	6.96	0.04	0.01	1.45	0.03	98.76	4.9	6.15	6.45	15.24	6.85	-1.74	11.37	20.6	11.08	1.55
os17446	1	597	0.9	5	3	5	6.68	-0.07	0.72	1.5	0.04	90.12	0.57	6.79	6.61	15.45	6.98	-1.66	11.68	21.01	11.3	1.72
os17525	1	587	0.57	17	3	9	6.78	0	0	1.44	0.14	100	-1.71	6	6.56	15.44	6.96	-1.61	11.64	20.97	11.3	1.75
os17658	1	779	0.23	10	1	4	7.11	0.11	0	1.43	0.06	96.45	3.97	5.2	6.19	14.97	6.68	-2.1	11.06	20.26	10.88	1.16
os17775	1	873	0.26	9	1	1	7.16	0.11	0.03	1.4	0.08	98.4	2.62	5.57	5.41	14.15	6.17	-2.29	9.89	18.99	10.09	0.84
os18007	1	791	0.62	9	2	3	6.96	0.01	0.05	1.5	0.1	99.75	-2.57	5.74	5.93	14.74	6.52	-1.9	10.47	19.77	10.42	1.26
os18208	1	813	0.31	5	1	4	6.88	0.01	0	1.47	0.03	99.16	3.8	6.4	5.74	14.47	6.34	-2.22	10.37	19.48	10.35	1.01
os18340	1	663	1	13	3	5	6.38	-0.21	0	1.47	0.09	99.61	-0.74	6.15	6.73	15.62	7.07	-1.49	11.53	20.88	11.12	1.69
os18346	1	782	0.75	11	3	5	6.51	-0.11	0.02	1.47	0.07	94.98	0.93	6.4	6.02	14.85	6.56	-1.88	10.7	19.92	10.54	1.3
os18682	1	646	0.6	14	2	0	6.87	0	0	1.46	0.15	98.02	0.2	5.96	6.43	15.19	6.91	-1.79	11.16	20.42	11.03	1.53
os18912	1	860	0.09	12	1	4	7.21	0.18	0.01	1.46	0.06	99.62	3.15	5.62	5.58	14.36	6.33	-2.14	10.09	19.25	10.18	1.03
os18932	1	742	0.99	3	3	6	6.84	-0.05	1.22	1.54	0.04	86.92	-0.35	7.49	6.24	15.04	6.75	-2.05	11.11	20.29	10.94	1.26
os18935	1	860	0.91	7	1	1	6.83	-0.08	0.01	1.43	0.05	99.51	5.77	5.87	5.74	14.48	6.38	-2.18	10.4	19.49	10.37	1.03
os18939	1	735	0.92	1	3	6	6.91	-0.01	3.66	1.56	0.04	24.29	-0.35	9.82	6.21	14.93	6.69	-2	10.97	20.04	10.73	1.21
os19082	1	678	1	12	3	5	6.41	-0.2	0	1.46	0.09	98.97	1.16	5.45	6.21	14.98	6.73	-1.67	10.76	20.01	10.6	1.48
os19083	1	708	0.75	7	3	5	6.64	-0.12	0.35	1.51	0.08	97.83	-0.53	6.15	6	14.77	6.59	-1.76	10.51	19.76	10.4	1.38
os19100	1	573	0.13	12	2	8	7.04	0.18	0.01	1.45	0.15	100	-3.59	5.6	6.5	15.4	6.92	-1.51	11.69	21	11.26	1.84
os19260	1	634	1	13	2	0	6.36	-0.21	0	1.46	0.1	100	1.42	5.96	6.02	14.91	6.56	-1.77	10.93	20.23	10.74	1.51
os19365	1	702	0.07	17	3	9	6.88	0.21	0	1.44	0.12	100	-1.52	5.89	5.98	14.85	6.5	-1.83	10.86	20.15	10.7	1.5
os19377	1	634	0.28	9	2	0	6.73	0.03	0.09	1.51	0.09	100	-1.18	7.18	6.11	14.97	6.61	-1.77	11.02	20.32	10.82	1.55
os20127	1	435	0.23	6	3	5	6.7	0.01	0.48	1.53	0.06	84.93	-1.37	7.61	8.43	17.52	8.3	-0.63	13.42	23.16	12.39	2.51
os20163	1	475	0.02	5	3	9	6.87	0.08	0.34	1.48	0.03	95.04	1.9	6.65	8.15	17.05	8.15	-0.74	13.44	22.93	12.65	2.61
os20619	1	432	0.87	3	3	6	6.84	0.01	1.9	1.55	0.03	63.38	-0.62	8.76	8.2	17.25	8.07	-0.91	13.01	22.88	12	2.08
os20837	1	527	0.58	6	3	5	6.84	-0.02	0.4	1.52	0.05	74.73	-0.17	6.73	7.32	16.28	7.4	-1.46	12.02	21.73	11.22	1.59

os21291	1	410	0.4	3	3	6	6.83	0.01	1.77	1.55	0.04	79.5	-0.59	8.04	8.38	17.45	8.21	-0.7	13.52	23.18	12.42	2.47
os21380	1	464	0.46	2	3	6	6.78	0	2.95	1.55	0.03	61.47	-0.18	9.62	8.33	17.45	8.2	-0.73	13.32	23.07	12.25	2.41
os21390	1	475	0.04	3	3	6	6.82	0.03	0.38	1.53	0.02	86	0.97	8	8.29	17.43	8.17	-0.77	13.29	23.1	12.25	2.4
os21417	1	450	0.02	4	1	4	6.88	0.07	0.05	1.51	0.02	66.54	2.3	7.71	8.06	17.04	7.98	-0.96	13.05	22.7	12.18	2.11
os21423	1	436	0.02	9	3	5	6.97	0.14	0.29	1.52	0.07	90.07	-1.52	7.68	8.04	17.04	7.97	-0.97	13.04	22.73	12.17	2.11
os21517	1	428	0.98	4	3	6	6.73	-0.05	0.36	1.52	0.05	94.16	1.12	7.5	8.11	17.07	8	-0.84	13.27	22.8	12.3	2.38
os21521	1	449	0.6	4	3	6	6.71	-0.02	0.13	1.52	0.03	80.36	2.01	7.64	7.88	16.81	7.85	-1.01	13.14	22.64	12.26	2.22
os21523	1	443	0.47	3	3	6	6.74	-0.01	1.31	1.54	0.04	88.11	-0.03	7.66	7.89	16.82	7.86	-1.01	13.15	22.65	12.27	2.23
os21524	1	443	0.47	3	3	6	6.74	-0.01	1.26	1.54	0.04	87.64	-0.05	7.62	7.89	16.82	7.86	-1.01	13.15	22.65	12.27	2.23
os21655	1	416	0.3	2	3	6	6.76	0	2.83	1.56	0.02	38.89	0.01	8.79	8.51	17.6	8.34	-0.65	13.6	23.28	12.53	2.55
os21908	1	476	0.92	3	3	6	6.71	-0.05	0.8	1.54	0.02	70.95	0.3	7.79	8.04	17.11	7.96	-0.97	12.75	22.58	11.71	1.97
os21925	1	426	0.4	4	3	6	6.85	0.02	1.65	1.54	0.04	90	-0.96	8.07	8.33	17.4	8.16	-0.8	13.24	23.09	12.16	2.2
os22083	1	450	0.85	6	3	5	6.57	-0.08	0.45	1.53	0.06	76.81	-1.4	8.04	8.13	17.16	8	-0.97	12.96	22.72	11.82	1.97
os23237	1	497	0.66	10	3	9	6.8	-0.02	0.04	1.46	0.06	96.63	0.14	6.18	8.07	17.07	8.02	-0.89	12.78	22.52	11.74	1.9
os23317	1	511	0.12	13	3	9	6.78	0.14	0	1.44	0.08	99.97	1.56	5.53	8.02	17.02	7.99	-0.82	12.59	22.38	11.66	1.9
os23526	1	650	0.09	9	1	4	6.91	0.11	0.01	1.47	0.07	94.25	2.6	6.2	6.97	16.03	7.14	-1.59	11.48	21.31	10.69	1.21
os23569	1	511	0.08	11	2	0	6.85	0.15	0.02	1.47	0.12	100	-1.69	6.32	7.68	16.66	7.72	-1.06	12.39	22.06	11.41	1.79
os23575	1	601	0.81	8	3	5	6.55	-0.11	0.2	1.5	0.06	97.43	-0.29	7.15	7.32	16.32	7.45	-1.32	11.95	21.65	11.06	1.51
os23595	1	509	0.99	2	3	6	6.83	-0.01	2.86	1.55	0.03	77.57	-1.5	8.86	7.77	16.74	7.78	-1.11	12.53	22.26	11.63	1.74
os23692	1	532	0.4	12	2	7	6.57	0.01	0.03	1.5	0.12	81.29	-3.1	6.15	7.58	16.58	7.6	-1.25	12.27	22	11.35	1.57
os23740	1	528	0.53	8	1	4	6.61	-0.04	0.02	1.38	0.04	98.58	7.06	5.87	7.74	16.73	7.74	-1.13	12.47	22.08	11.45	1.67
os23779	1	597	0.07	13	3	5	6.89	0.17	0	1.44	0.09	99.74	0.78	5.66	7.38	16.39	7.44	-1.41	12.1	21.77	11.2	1.46
os23820	1	540	0.6	9	3	5	6.58	-0.05	0.55	1.51	0.09	99.62	-2.46	7.88	7.52	16.52	7.55	-1.25	12.23	21.91	11.31	1.59
os23951	1	486	1	11	3	5	6.54	-0.13	0.16	1.51	0.07	65.47	0.23	7.55	7.4	16.45	7.49	-1.64	12.11	21.96	11.3	1.14
os23982	1	608	0.04	5	3	6	6.94	0.07	0.7	1.51	0.03	86.06	0.34	7.01	6.79	15.82	6.98	-1.95	11.38	21.19	10.75	0.89
os24026	1	553	0.34	5	3	6	6.72	-0.07	0.24	1.54	0.03	74.44	-0.06	7.35	7.07	16.12	7.21	-1.8	11.76	21.57	11.04	1.01
os24058	1	570	0.17	10	3	5	7.05	0.14	0.12	1.5	0.07	92.42	-1.37	5.93	7.1	16.12	7.21	-1.86	11.81	21.54	10.98	0.99
os24062	1	546	0	6	3	5	7.01	0.11	0.42	1.52	0.05	72.91	-0.21	7.16	7.24	16.25	7.32	-1.66	11.91	21.6	11.04	1.18
os24340	1	522	0.47	2	3	6	6.85	0	1.24	1.55	0.02	61.88	0.17	7.96	7.59	16.65	7.6	-1.4	12.5	22.29	11.65	1.64
os24438	1	527	0.92	4	3	6	6.69	-0.07	1.6	1.53	0.03	75.67	-0.4	7.53	7.47	16.5	7.49	-1.49	12.3	22.01	11.39	1.5
os24452	1	470	0.77	1	3	6	6.78	-0.02	4.14	1.55	0.04	70.42	-0.15	8.74	7.74	16.78	7.66	-1.35	12.62	22.41	11.62	1.65
os24686	1	582	0.72	12	3	5	6.41	-0.09	0.08	1.49	0.08	91.62	-1.79	7.66	6.92	15.93	7.07	-1.85	11.52	21.25	10.71	0.95
os24765	1	562	0.47	8	3	5	6.67	0	0.4	1.49	0.07	97.86	-1.48	7.69	7.58	16.63	7.55	-1.31	12.35	22.14	11.34	1.55
os24799	1	595	0.2	3	3	6	6.95	0.04	1.62	1.55	0.03	79.31	-0.65	8.47	7.07	16.06	7.16	-1.8	11.73	21.43	10.85	1.07
os24910	1	611	0.01	7	3	5	6.97	0.1	0.16	1.51	0.04	89.97	0.77	6.37	6.89	15.84	7.06	-1.87	11.53	21.22	10.69	0.95
os25028	1	637	0.75	7	3	5	6.87	-0.02	0.26	1.51	0.05	94.01	0.54	6.58	6.9	15.88	7.02	-1.79	11.46	21.2	10.61	1.02
os25076	1	573	0.65	9	3	5	6.9	0.01	0.58	1.53	0.08	70.41	-1.91	8.38	7.2	16.17	7.23	-1.65	11.85	21.54	10.91	1.2

os25092	1	668	0.28	5	3	5	7.01	0.06	0.53	1.52	0.03	99.99	-0.38	6.95	6.77	15.74	6.93	-1.93	11.34	21.03	10.54	0.91
os25104	1	660	0.76	6	3	5	6.89	-0.02	0.43	1.51	0.04	98.27	0.27	6.71	6.79	15.77	6.96	-1.91	11.34	21.05	10.54	0.93
os25170	1	474	0.81	16	2	3	6.18	-0.17	0.01	1.48	0.1	91.46	-1.85	7.43	7.97	17	7.88	-1.04	12.68	22.5	11.65	1.75
os25191	1	524	0.15	11	1	4	6.75	0.09	0.01	1.45	0.06	79.89	4.1	5.66	7.76	16.79	7.71	-1.18	12.44	22.3	11.43	1.61
os25208	1	528	0.23	6	3	5	6.77	0.04	0.56	1.52	0.05	99.98	-0.37	6.7	7.73	16.79	7.68	-1.2	12.48	22.36	11.47	1.59
os25214	1	475	0.04	15	2	3	7	0.22	0.05	1.47	0.11	91.72	-3.01	7.49	8	17.04	7.89	-1.04	12.79	22.63	11.73	1.75
os25226	1	529	0.23	2	2	3	6.83	0.02	2.69	1.54	0.06	97.11	-2.77	8.84	7.7	16.76	7.66	-1.23	12.42	22.3	11.43	1.56
os25263	1	492	0.76	8	2	7	6.55	-0.06	0.71	1.54	0.09	85.71	-3.06	8.36	7.71	16.73	7.68	-1.24	12.42	22.21	11.39	1.55
os25373	1	465	0.4	7	2	3	6.53	-0.11	0.8	1.51	0.07	99.22	-2.78	7.83	8.06	17.09	7.95	-0.95	12.76	22.63	11.72	1.85
os25382	1	602	0.79	4	3	6	6.84	-0.03	1.57	1.54	0.06	96.53	-1.3	8.44	7.2	16.22	7.27	-1.55	11.88	21.61	11.02	1.3
os25467	1	502	0.33	7	3	5	6.69	-0.01	0.75	1.53	0.06	96.94	-1.13	7.75	7.71	16.74	7.69	-1.28	12.5	22.32	11.48	1.59
os25579	1	537	0.92	2	2	7	6.79	-0.02	2.96	1.56	0.07	93.19	-2.62	8.74	7.43	16.43	7.48	-1.48	12.16	21.94	11.23	1.39
os25583	1	552	0.76	6	3	5	6.61	-0.08	0.45	1.52	0.05	92.16	-0.42	7.05	7.41	16.41	7.47	-1.5	12.15	21.92	11.22	1.38
os25595	1	573	0.23	12	3	5	6.67	0.06	0.03	1.49	0.1	92.96	-1.9	5.73	7.19	16.18	7.31	-1.6	11.83	21.6	10.93	1.18
os25612	1	570	0.77	4	3	6	6.83	-0.03	0.72	1.53	0.05	98.08	0.18	7.95	7.23	16.24	7.33	-1.61	11.97	21.71	11.07	1.24
os25627	1	594	1	5	3	5	6.69	-0.09	0.42	1.5	0.05	98.1	0.55	6.54	7.22	16.21	7.33	-1.59	11.92	21.7	11.01	1.21
os25678	1	616	0.03	7	3	5	6.96	0.1	0.15	1.5	0.04	91.71	1.6	6.17	6.89	15.83	7.06	-1.87	11.52	21.22	10.69	0.95
os25707	1	588	0.76	1	3	6	6.86	-0.01	3.91	1.55	0.03	30.87	0.04	9.43	6.98	15.93	7.11	-1.82	11.64	21.34	10.78	1.01
os25733	1	670	0.79	6	3	5	6.65	-0.11	0.45	1.52	0.05	89.56	-1.03	6.72	6.63	15.59	6.86	-1.97	11.16	20.86	10.42	0.8
os25734	1	675	1	7	3	5	6.65	-0.11	0.16	1.5	0.03	94.5	1.63	5.92	6.64	15.59	6.87	-1.97	11.18	20.88	10.43	0.81
os25745	1	678	0	10	3	5	7.04	0.16	0.03	1.49	0.06	85.29	1.28	6.2	6.61	15.56	6.84	-1.99	11.15	20.83	10.4	0.83
os25767	1	613	0.81	4	3	6	6.71	-0.06	1.22	1.53	0.04	98.76	-0.85	7.5	7	16.01	7.15	-1.73	11.66	21.4	10.8	1.08
os25776	1	592	0.53	10	3	5	6.92	0.02	0.06	1.48	0.06	91.89	0.3	5.9	7.16	16.17	7.27	-1.65	11.85	21.58	10.98	1.19
os25787	1	571	1	6	3	5	6.65	-0.1	0.45	1.51	0.05	94.29	0.41	6.46	7.3	16.27	7.38	-1.58	12.03	21.77	11.11	1.27
os25926	1	529	0.03	5	3	6	6.95	0.07	0.75	1.54	0.04	79.01	-0.4	8.11	7.72	16.74	7.69	-1.1	12.3	22.09	11.31	1.66
os25964	1	600	0.47	4	3	6	6.75	-0.06	1.53	1.54	0.03	89.97	-0.58	8.47	7.33	16.36	7.35	-1.48	12.03	21.8	11.09	1.39
os26008	1	593	0.41	3	3	6	6.92	0.02	0.3	1.52	0.02	88.74	0.5	7.14	7.44	16.47	7.42	-1.42	12.14	21.95	11.19	1.46
os26053	1	538	0.97	13	3	5	6.41	-0.19	0.01	1.47	0.09	99.59	-0.28	6.25	7.62	16.62	7.64	-1.3	12.44	22.05	11.43	1.6
os26120	1	579	0.9	13	3	5	6.55	-0.14	0	1.48	0.09	96.84	-0.85	5.81	7.34	16.32	7.36	-1.53	12.16	21.77	11.24	1.38
os26132	1	564	0.77	5	3	6	6.83	-0.03	0.93	1.52	0.07	99.7	-0.99	7.64	7.48	16.46	7.46	-1.46	12.31	21.92	11.36	1.48
os26152	1	611	0.97	5	3	6	6.77	-0.06	0.28	1.51	0.03	93.98	2.13	6.79	7.3	16.31	7.37	-1.6	12.14	21.74	11.22	1.35
os26155	1	618	0.81	3	1	4	6.84	-0.03	0.02	1.5	0.01	86.39	2.63	6.64	7.28	16.29	7.36	-1.61	12.13	21.73	11.21	1.34
os26164	1	630	0.09	6	1	4	6.87	0.05	0.03	1.46	0.03	99.03	4.2	6.24	7.18	16.17	7.26	-1.66	12.03	21.61	11.12	1.28
os26166	1	625	0.02	9	1	4	6.94	0.11	0.07	1.46	0.04	99.14	2.55	5.77	7.2	16.18	7.27	-1.65	12.04	21.63	11.13	1.29
os26173	1	615	0.03	5	1	1	6.91	0.06	0.02	1.48	0.02	98.51	3.86	6.56	7.32	16.3	7.36	-1.58	12.16	21.76	11.24	1.36
os26174	1	616	0.07	4	3	9	6.98	0.07	0.28	1.5	0.02	97.04	1.94	7.03	7.31	16.29	7.35	-1.58	12.15	21.74	11.23	1.35
os26201	1	528	0.23	14	1	4	7.04	0.16	0	1.44	0.09	98.14	4.09	5.43	7.65	16.62	7.6	-1.37	12.49	22.11	11.51	1.56

os26222	1	581	0.95	15	3	5	6.35	-0.21	0	1.45	0.1	97.16	0.59	5.6	7.47	16.48	7.49	-1.47	12.33	21.94	11.37	1.45
os26230	1	580	0.03	13	3	9	7.09	0.2	0	1.42	0.06	99.75	2.65	5.67	7.64	16.62	7.59	-1.38	12.49	22.11	11.5	1.55
os26255	1	540	0.57	9	3	5	6.45	-0.16	0.09	1.51	0.08	99.48	-0.93	5.97	7.54	16.55	7.57	-1.35	12.33	21.95	11.35	1.54
os26292	1	642	0.88	12	3	9	6.36	-0.17	0.01	1.44	0.07	97.8	2.25	5.49	7.28	16.29	7.37	-1.55	12.1	21.74	11.2	1.37
os26294	1	682	0.04	5	3	6	6.99	0.08	0.29	1.5	0.05	92.29	-0.68	6.84	6.88	15.9	7.03	-1.72	11.55	21.22	10.75	1.15
os26305	1	542	0.4	8	3	5	6.97	0.06	0.32	1.48	0.07	99.21	-0.52	6.3	7.63	16.61	7.65	-1.13	12.31	21.94	11.34	1.64
os26328	1	483	0.81	6	2	3	6.75	-0.05	1.96	1.52	0.1	88.86	-1.56	7.22	7.9	16.92	7.83	-1.09	12.63	22.37	11.59	1.8
os26354	1	529	0.4	2	3	6	6.88	0.02	1.75	1.54	0.02	80.39	-0.07	8.1	7.8	16.8	7.75	-1.18	12.54	22.29	11.52	1.74
os26399	1	538	0	6	3	5	6.96	0.1	0.49	1.53	0.03	88.38	-0.07	6.6	7.7	16.75	7.64	-1.34	12.59	22.29	11.58	1.61
os26507	1	653	0.89	8	3	9	6.57	-0.12	0.1	1.47	0.06	90.57	0.99	5.95	7.12	16.13	7.23	-1.58	11.81	21.5	10.95	1.26
os26520	1	561	0.08	2	2	0	6.92	0.04	1.5	1.55	0.05	80.99	-0.06	8.37	7.41	16.44	7.42	-1.47	12.23	21.87	11.26	1.4
os26577	1	484	0.47	11	2	3	6.48	-0.05	0.21	1.51	0.1	83.38	-3.2	6.86	7.95	16.97	7.87	-1.06	12.64	22.4	11.63	1.84
os26592	1	504	0.98	11	1	4	6.35	-0.19	0.03	1.47	0.07	90.45	1.61	6.43	7.78	16.8	7.74	-1.2	12.53	22.28	11.51	1.68
os26594	1	537	1	14	2	7	6.28	-0.23	0	1.48	0.1	97.7	-1.14	6.98	7.53	16.58	7.54	-1.36	12.29	22.03	11.31	1.56
os26765	1	596	0.84	10	1	4	6.43	-0.14	0.03	1.45	0.05	95.42	4.2	5.94	6.52	15.36	6.9	-2.11	11.17	20.8	10.58	0.54
os26873	1	622	0.03	5	3	5	6.99	0.09	0.44	1.5	0.03	93.35	0.39	6.65	6.28	15.1	6.8	-2.21	10.92	20.54	10.4	0.45
os26973	1	618	0.56	10	3	5	6.9	0.01	0.12	1.49	0.11	100	-1.66	6.26	6.26	15.08	6.74	-2.24	10.81	20.46	10.31	0.42
os27039	1	518	0.77	4	3	6	6.79	-0.04	1.7	1.54	0.05	90.18	-0.75	9.08	6.83	15.66	7.34	-1.61	11.38	21.04	10.89	1.08
os27125	1	462	0.99	1	3	6	6.77	-0.02	4.85	1.57	0.04	68.11	-0.95	8.96	7.52	16.42	7.65	-1.38	12.25	22.02	11.4	1.38
os27244	1	541	0.01	5	3	5	6.97	0.09	0.83	1.54	0.06	94.22	-1.66	8.12	6.7	15.57	7.13	-1.92	11.42	21.18	10.89	0.83
os27329	1	583	0.4	4	3	6	6.96	0.04	0.4	1.51	0.03	97.5	0.64	7.12	6.63	15.44	7.17	-1.78	11.09	20.79	10.63	0.86
os27370	1	579	0.1	8	3	5	7.04	0.11	0.21	1.48	0.04	98.06	2.06	6.23	6.53	15.35	7.03	-1.99	11.06	20.73	10.6	0.72
os27374	1	604	0.62	8	3	5	6.85	-0.02	0.17	1.49	0.06	99.38	0.1	6.04	6.43	15.25	6.97	-2.07	10.9	20.63	10.45	0.64
os27388	1	453	1	4	3	6	6.66	-0.07	0.74	1.55	0.04	89.49	-0.58	8.09	7.23	16.02	7.61	-1.43	11.93	21.55	11.29	1.29
os27422	1	542	0.99	3	3	6	6.78	-0.04	0.67	1.54	0.03	68.87	0.52	8.76	6.63	15.45	7.11	-1.98	11.21	20.92	10.72	0.76
os27429	1	552	0.92	0	3	6	6.83	0	4.89	1.53	0.02	53.22	1.45	10.55	6.63	15.45	7.09	-2	11.26	20.98	10.73	0.73
os27582	1	524	0.02	3	3	6	6.93	0.06	1.68	1.53	0.03	63.73	-0.5	8.33	7.31	16.19	7.52	-1.47	11.89	21.65	11.14	1.21
os27585	1	443	0.92	1	3	6	6.76	-0.02	2.94	1.56	0.01	8.49	-0.08	9.59	7.7	16.55	7.91	-1	12.29	21.99	11.52	1.67
os27627	1	485	0.51	17	3	5	6.27	-0.05	0.04	1.46	0.1	94.85	-0.83	7.41	7.19	16.06	7.41	-1.64	11.94	21.72	11.19	1.08
os27655	1	553	0.67	4	3	6	6.76	-0.04	0.32	1.51	0.03	79.11	1.14	7.63	6.8	15.73	7.14	-1.98	11.37	21.23	10.81	0.68
os27729	1	488	0.63	17	1	4	6.09	-0.13	0	1.43	0.09	89.32	3.3	5.96	7.03	15.88	7.36	-1.75	11.81	21.59	11.16	1
os27733	1	494	0.9	7	1	4	6.55	-0.1	0.05	1.47	0.06	94.04	3.63	6.78	7	15.85	7.34	-1.77	11.78	21.56	11.13	0.97
os27799	1	498	0.24	3	3	6	6.9	0.04	2.94	1.55	0.04	84	-1.25	9.28	7.05	15.89	7.35	-1.78	11.81	21.62	11.15	0.97
os27801	1	498	0.11	14	1	4	6.76	0.13	0	1.45	0.08	91.51	0.76	7.13	7.05	15.91	7.36	-1.78	11.83	21.64	11.17	0.98
os27891	1	470	0.89	6	3	5	6.57	-0.09	0.57	1.5	0.04	93.18	1.05	7.32	7.38	16.23	7.57	-1.49	12.14	21.85	11.31	1.24
os27917	1	475	0.57	4	3	6	6.78	-0.03	0.71	1.53	0.05	86.33	0.4	8.55	7.24	16.11	7.45	-1.61	12	21.74	11.19	1.15
os27941	1	572	0.23	2	3	6	6.85	0.01	1.07	1.53	0.02	76.29	0.05	7.78	6.95	15.85	7.21	-1.79	11.67	21.43	10.91	0.98

os27989	1	490	0.87	7	3	5	6.58	-0.1	0.5	1.52	0.05	76.83	-0.21	8.87	7.43	16.34	7.57	-1.44	12.14	21.89	11.29	1.31
os28067	1	534	0.94	7	3	5	6.68	-0.09	0.4	1.51	0.04	98.36	0.46	6.61	7.22	16.13	7.4	-1.6	11.94	21.69	11.14	1.15
os28076	1	537	0.23	7	3	5	6.77	0.04	0.55	1.5	0.04	99.23	-0.43	6.67	7.2	16.11	7.4	-1.63	11.96	21.72	11.16	1.13
os28113	1	522	0.69	11	3	5	6.41	-0.1	0.03	1.49	0.08	92.38	-0.46	7.13	7.15	16.03	7.34	-1.68	11.82	21.57	11.01	1.07
os28115	1	592	0.06	2	3	6	6.91	0.03	1.42	1.52	0.03	85.5	-0.65	7.55	6.88	15.8	7.14	-1.8	11.47	21.23	10.72	0.93
os28120	1	545	0.74	7	2	0	6.61	-0.07	0.26	1.52	0.06	94.35	-0.57	7.78	6.97	15.88	7.21	-1.77	11.58	21.33	10.8	0.98
os28142	1	550	0.24	10	3	5	7.03	0.11	0.29	1.5	0.06	82.98	0.56	6.38	7.05	15.93	7.28	-1.72	11.67	21.43	10.91	1.04
os28151	1	490	1	7	3	5	6.58	-0.11	0.26	1.52	0.06	76.99	0.03	7.75	7.21	16.09	7.4	-1.58	11.81	21.58	11.05	1.16
os28163	1	519	0.77	2	3	6	6.85	0	0.32	1.54	0.03	66.72	0.86	9.27	7.1	16.01	7.3	-1.63	11.6	21.36	10.85	1.11
os28166	1	593	0.92	6	3	5	6.63	-0.09	0.55	1.51	0.03	90.59	0.01	7	6.85	15.77	7.12	-1.82	11.41	21.17	10.68	0.92
os28218	1	545	0.76	3	3	6	6.85	-0.01	1.66	1.53	0.02	77.44	-0.35	7.89	7.11	16.01	7.4	-1.61	11.76	21.5	11.03	1.07
os28267	1	524	0.95	4	3	5	6.78	-0.04	1.53	1.54	0.06	92.32	-1.79	8.69	7.05	15.98	7.34	-1.66	11.68	21.47	10.99	1.09
os28307	1	532	0.02	5	1	1	6.96	0.08	0.01	1.46	0.01	92.46	5.05	6.05	7.24	16.14	7.47	-1.55	11.93	21.71	11.18	1.2
os28363	1	482	0.92	6	3	5	6.76	-0.04	0.96	1.52	0.05	98.45	-1.61	7.69	7.38	16.26	7.57	-1.48	12.16	21.94	11.34	1.28
os28367	1	445	0	9	2	7	6.77	0.13	1.13	1.53	0.17	96.47	-8.72	8.46	7.46	16.4	7.62	-1.42	12.29	22.12	11.44	1.37
os28438	1	489	0.92	2	2	0	6.82	-0.01	2.1	1.55	0.06	80.47	-1.26	7.86	7.24	16.14	7.5	-1.54	11.9	21.63	11.14	1.17
os28446	1	486	0.91	7	2	3	6.51	-0.1	0.39	1.51	0.07	97.68	-1.98	6.89	7.61	16.49	7.68	-1.3	12.28	21.98	11.38	1.43
os28463	1	474	0.92	1	3	6	6.76	-0.02	0.43	1.54	0.03	72.56	0.62	8.97	7.57	16.45	7.67	-1.34	12.19	21.92	11.34	1.39
os28541	1	478	0.76	4	2	0	6.68	-0.05	3.1	1.55	0.06	73.61	-0.86	8.34	6.99	15.86	7.31	-1.84	11.74	21.56	11.09	0.89
os28570	1	508	0.77	14	3	5	6.59	-0.11	0	1.46	0.08	88	1.58	6.63	6.99	15.85	7.3	-1.83	11.72	21.53	11.08	0.91
os28644	1	599	0.92	5	3	5	6.67	-0.07	0.66	1.53	0.07	96.07	-1.26	7.05	6.79	15.7	7.02	-1.9	11.41	21.11	10.61	0.86
os28669	1	434	0.31	27	2	3	6.07	0.09	0.02	1.45	0.19	90.48	-5.01	6.06	7.64	16.57	7.75	-1.29	12.48	22.28	11.61	1.48
os28672	1	430	0.92	6	2	3	6.63	-0.04	1.28	1.52	0.06	96.76	-1.77	8.48	7.85	16.76	7.9	-1.13	12.58	22.31	11.65	1.63
os28785	1	495	0.92	5	3	6	6.63	-0.07	0.4	1.53	0.05	91.36	-0.28	8.24	7.93	16.97	7.87	-0.92	12.55	22.49	11.57	1.85
os28817	1	545	0.04	3	3	6	6.73	-0.05	1.38	1.52	0.03	66.76	-0.78	7.79	7.61	16.64	7.61	-1.29	12.28	22.14	11.34	1.51
os28820	1	486	1	10	3	5	6.45	-0.17	0.03	1.45	0.05	93.67	1.82	6.04	7.94	16.98	7.87	-1.03	12.64	22.52	11.62	1.76
os28826	1	482	0.17	8	3	5	6.99	0.1	0.13	1.49	0.06	89.61	0.33	5.87	7.82	16.78	7.83	-1.18	12.44	22.25	11.49	1.62
os28836	1	483	0.92	2	3	6	6.76	-0.03	0.85	1.55	0.01	40.05	0.16	9.3	7.57	16.43	7.75	-1.14	12.05	21.73	11.27	1.52
os28896	1	439	0.99	9	2	0	6.5	-0.14	0.06	1.51	0.08	95.54	-0.59	8.12	7.66	16.54	7.78	-1.22	12.24	21.97	11.38	1.48
os28925	1	402	0.03	5	2	7	6.88	0.09	1.51	1.55	0.12	96.83	-6.03	8.39	7.91	16.76	7.95	-1.03	12.54	22.25	11.58	1.67
os28933	1	421	0.3	9	1	2	6.58	-0.01	0.06	1.47	0.07	86.34	2.1	7.57	7.74	16.62	7.84	-1.19	12.34	22.08	11.47	1.53
os28936	1	426	0.14	14	3	5	6.68	0.11	0	1.48	0.08	92.05	-0.09	6.65	7.82	16.71	7.9	-1.13	12.46	22.21	11.58	1.57
os28996	1	440	1	8	1	4	6.5	-0.14	0.07	1.46	0.05	97.61	2.59	6.17	7.91	16.8	7.92	-1.01	12.52	22.23	11.57	1.7
os29191	1	519	0.6	6	3	5	6.66	-0.03	0.14	1.51	0.05	98.7	0.42	5.92	7.35	16.31	7.51	-1.42	11.88	21.67	11.07	1.27
os29217	1	424	0.92	12	1	4	6.25	-0.18	0	1.44	0.08	93.38	2.83	6.14	8.17	17.19	8.05	-0.88	12.91	22.74	11.86	1.92
os29224	1	434	0.12	5	2	3	6.86	0.03	1.45	1.54	0.06	97.99	-2.15	8.4	8.11	17.13	8.02	-0.94	12.87	22.7	11.83	1.87
os29292	1	505	0.92	3	3	6	6.74	-0.02	0.48	1.52	0.06	87.92	0.39	6.97	7.54	16.51	7.59	-1.37	12.15	21.95	11.21	1.42

os29332	1	539	0.23	4	2	0	6.8	0.02	0.52	1.54	0.05	80.84	0.05	7.89	7.24	16.13	7.35	-1.65	11.87	21.56	10.98	1.14
os29364	1	582	0.94	8	3	5	6.64	-0.11	0.47	1.5	0.06	98	-0.61	7.35	6.92	15.96	7.19	-1.87	11.56	21.49	10.87	0.84
os29368	1	618	0.51	7	1	1	6.91	0	0.03	1.47	0.02	99.08	3.01	5.95	6.86	15.91	7.15	-1.91	11.49	21.42	10.82	0.82
os29394	1	561	0.89	9	1	4	6.63	-0.12	0.09	1.47	0.05	92.81	2.68	6.27	6.97	16.03	7.23	-1.81	11.59	21.49	10.89	0.91
os29400	1	649	0.01	6	1	1	7.01	0.09	0.03	1.48	0.02	98.83	3.56	6.59	6.7	15.73	7	-2.01	11.26	21.17	10.62	0.68
os29436	1	614	0.92	1	3	6	6.83	-0.02	0.04	1.49	0.02	94.64	2.07	7.52	6.84	15.88	7.12	-1.9	11.45	21.34	10.77	0.8
os29457	1	485	0.96	5	3	6	6.69	-0.07	0.39	1.54	0.07	84.07	-1.64	7.73	7.46	16.55	7.61	-1.47	12.19	22.1	11.37	1.33
os29463	1	552	0.13	4	2	3	6.95	0.05	1.61	1.56	0.1	70.04	-2.52	7.98	7.06	16.14	7.28	-1.82	11.73	21.59	11.05	0.94
os29484	1	572	0.99	3	3	6	6.78	-0.04	1.55	1.54	0.03	89.69	-0.35	7.72	6.91	15.86	7.13	-1.88	11.51	21.27	10.77	0.89
os29620	1	555	1	10	2	7	6.47	-0.16	0.74	1.53	0.12	92.68	-4.76	6.83	6.82	15.79	7.08	-1.92	11.33	21.13	10.65	0.82
os29748	1	443	0.2	4	2	3	6.72	0	1.56	1.54	0.09	87	-3.45	7.78	7.43	16.3	7.6	-1.47	12.16	21.9	11.35	1.28
os29760	1	540	0.01	5	3	6	6.97	0.09	0.54	1.52	0.04	96.37	-0.2	6.91	7.09	15.97	7.32	-1.68	11.7	21.47	10.95	1.09
os29818	1	473	0.99	5	3	6	6.66	-0.08	1.15	1.54	0.05	69.81	-1.37	8.13	7.28	16.17	7.46	-1.57	11.94	21.71	11.17	1.21
os29911	1	527	0.01	7	3	5	6.94	0.09	1.67	1.54	0.07	86.43	-2.64	8.7	7.09	16.09	7.29	-1.82	11.8	21.71	11.04	1.01
os29939	1	521	0.12	3	2	3	6.91	0.05	1.87	1.54	0.05	85.76	-1.96	8.61	7.19	16.23	7.34	-1.7	11.92	21.85	11.11	1.13
os29989	1	536	0.05	15	2	3	7.05	0.22	0	1.46	0.11	89.89	-0.76	7.32	7.07	16.05	7.26	-1.77	11.64	21.44	10.86	0.96
os30072	1	617	0.69	7	1	4	6.63	-0.06	0.04	1.47	0.05	98	2.81	5.67	6.81	15.79	7.03	-1.94	11.25	21.07	10.54	0.77
os30074	1	537	0.23	8	3	5	6.78	0.06	0.22	1.5	0.07	95.84	-0.66	6.76	7.16	16.25	7.28	-1.74	12	22	11.07	1.12
os30078	1	525	0.05	9	3	5	7.02	0.14	0.47	1.51	0.07	99.71	-1.45	8.01	7.07	16	7.3	-1.83	11.77	21.6	11.03	0.96
os30111	1	512	0.09	8	3	5	6.83	0.08	0.13	1.48	0.07	97.83	0.72	5.85	7.18	16.12	7.35	-1.78	11.89	21.74	11.12	0.98
os30115	1	582	0.36	9	3	5	6.69	0.02	0.06	1.5	0.07	88.28	-0.24	7.14	6.77	15.73	7.04	-2.03	11.39	21.24	10.7	0.69
os30121	1	615	0.11	8	3	5	6.89	0.09	0.14	1.49	0.04	96.59	1.06	6.12	6.69	15.67	6.98	-2.05	11.29	21.12	10.64	0.65
os30172	1	541	0.13	10	1	4	6.77	0.07	0.07	1.48	0.05	82.03	1.12	6.85	6.93	15.84	7.21	-1.88	11.48	21.31	10.83	0.8
os30174	1	610	0.76	6	2	3	6.89	0	1.36	1.54	0.07	94.21	-2.53	8.48	6.68	15.63	6.98	-2.09	11.3	21.15	10.66	0.63
os30192	1	460	0.66	17	3	5	6.05	-0.15	0	1.45	0.14	95.94	-2.3	6.01	7.59	16.61	7.68	-1.44	12.24	22.15	11.39	1.31
os30214	1	579	0.89	6	2	7	6.7	-0.04	1.87	1.53	0.1	99.61	-4.62	8.36	6.79	15.75	7.05	-2.04	11.44	21.3	10.77	0.74
os30264	1	579	0.99	5	3	5	6.71	-0.08	0.66	1.52	0.04	97.99	-0.22	6.79	6.91	15.92	7.16	-1.87	11.55	21.44	10.85	0.85
os30265	1	585	0.84	4	3	6	6.77	-0.06	0.41	1.52	0.03	97.76	0.41	6.49	6.91	15.92	7.16	-1.88	11.54	21.43	10.84	0.84
os30268	1	560	0.8	6	1	4	6.63	-0.07	0.13	1.49	0.04	91.5	3.08	6.59	7.03	16.04	7.26	-1.8	11.7	21.58	10.97	0.91
os30364	1	532	0.78	3	2	3	6.83	-0.01	1.87	1.55	0.07	93.47	-3.01	8.31	6.99	15.96	7.23	-1.86	11.72	21.55	10.98	0.85
os30395	1	584	0.01	10	3	5	7.05	0.16	0.07	1.49	0.06	97.46	1.22	6.33	6.85	15.87	7.12	-1.93	11.56	21.44	10.87	0.79
os30458	1	573	0.72	10	3	5	6.82	-0.03	0.06	1.47	0.09	99.99	-1.27	7.08	6.83	15.77	7.15	-1.85	11.28	20.98	10.62	0.83
os30477	1	505	0.15	17	3	9	6.62	0.15	0	1.36	0.13	98.9	1.55	5.74	7.45	16.34	7.65	-1.49	12.03	21.69	11.25	1.15
os30495	1	558	0.2	12	2	3	6.72	0.09	0.05	1.46	0.12	100	-3.23	5.77	6.99	15.9	7.27	-1.73	11.48	21.16	10.8	0.93
os30608	1	629	0.13	5	3	6	7	0.06	1.04	1.53	0.05	90.74	-0.63	9.63	6.24	15.12	6.74	-2.33	10.76	20.52	10.25	0.33
os30668	1	528	0.92	6	3	5	6.62	-0.08	0.51	1.53	0.06	74.62	-0.73	7.9	6.88	15.79	7.21	-1.96	11.53	21.35	10.94	0.74
os30726	1	639	0.77	4	2	0	6.85	-0.03	1.78	1.54	0.06	84.42	-0.65	8.3	6.14	15.04	6.61	-2.44	10.71	20.45	10.18	0.23

os30738	1	547	0.43	7	3	5	6.67	0	0.21	1.47	0.05	94.86	1.37	6.1	7.01	15.92	7.27	-1.94	11.77	21.52	11.06	0.8
os30741	1	514	0.23	4	1	4	6.77	0	0.03	1.44	0.02	89.72	4.44	6.78	7.18	16.12	7.39	-1.83	12.07	21.78	11.29	0.89
os30773	1	614	0	10	3	5	7.01	0.17	0.01	1.5	0.06	89.82	0.67	6.03	6.47	15.39	6.86	-2.31	11.16	20.96	10.59	0.45
os30778	1	590	0.62	10	3	5	6.85	-0.02	0.06	1.5	0.07	94.09	-0.26	7.84	6.49	15.4	6.88	-2.25	11.14	20.94	10.58	0.45
os30806	1	555	0.48	13	3	5	6.49	-0.02	0.01	1.45	0.08	99.81	0.38	6.11	6.99	15.98	7.27	-1.83	11.54	21.42	10.92	0.86
os30817	1	580	0.6	6	2	3	6.67	-0.05	0.82	1.5	0.11	99.97	-1.8	6.85	6.52	15.37	6.89	-2.16	11.13	20.8	10.58	0.53
os30838	1	581	0.01	16	2	0	7.01	0.25	0	1.47	0.14	99.89	-1.36	6.87	6.33	15.23	6.77	-2.2	10.87	20.51	10.34	0.43
os30869	1	588	0.29	14	1	1	7.04	0.14	0	1.36	0.09	96.15	2.69	5.26	6.83	15.7	7.16	-1.89	11.37	21.03	10.76	0.76
os30880	1	675	0.12	4	3	9	6.77	-0.06	1.87	1.41	0.04	97.09	-1.16	7.59	6.53	15.44	6.94	-2.05	11.04	20.73	10.51	0.6
os30896	1	536	0.12	9	2	3	6.79	0.07	0.48	1.49	0.1	96.13	-3.85	7.77	6.86	15.81	7.16	-2.02	11.57	21.33	10.93	0.68
os30899	1	485	0.17	14	2	7	6.65	0.13	0.04	1.49	0.14	85.18	-4.04	6.04	7.12	16.09	7.35	-1.89	11.94	21.7	11.2	0.87
os30964	1	601	0.67	8	1	1	6.86	-0.02	0.02	1.44	0.05	100	3.21	5.7	6.7	15.62	7.07	-1.93	11.3	20.94	10.71	0.7
os30975	1	515	0.62	16	1	4	6.79	0.01	0	1.39	0.09	99.74	4.71	5.28	7.14	16.04	7.42	-1.62	11.7	21.36	11.01	1.03
os30979	1	459	0.94	25	2	0	5.77	-0.31	0	1.41	0.19	95.43	-2.76	5.48	7.2	16.11	7.47	-1.57	11.78	21.44	11.07	1.08
os31022	1	575	0.85	23	2	8	6.21	-0.21	0	1.39	0.18	99.99	-4.98	5.3	6.89	15.76	7.22	-1.84	11.46	21.1	10.84	0.84
os31036	1	620	0	7	1	1	6.97	0.06	0	1.39	0.01	97.8	9.04	6.21	6.66	15.55	7.01	-2.04	11.26	20.91	10.65	0.6
os31044	1	570	0.45	12	3	5	6.54	0.01	0.01	1.48	0.1	92.16	-1.79	6.9	6.69	15.56	7.04	-2.02	11.28	20.92	10.66	0.62
os31047	1	561	0.1	13	3	5	6.81	0.14	0.01	1.48	0.09	89.88	-2.19	6.81	6.73	15.61	7.07	-1.99	11.34	20.97	10.69	0.65
os31078	1	587	0.07	13	3	9	6.25	-0.21	0	1.43	0.07	96.57	1.35	5.51	6.77	15.64	7.11	-1.98	11.36	20.99	10.7	0.68
os31081	1	544	0.76	17	1	2	6.16	-0.16	0	1.44	0.1	94.98	2.68	6.78	6.65	15.54	7.01	-2.04	11.19	20.82	10.55	0.6
os31086	1	618	0.61	9	1	4	6.58	-0.05	0.01	1.44	0.04	99.89	4.55	5.5	6.53	15.43	6.91	-2.09	11.05	20.68	10.44	0.55
os31088	1	620	0.34	12	3	5	6.69	0.05	0.01	1.45	0.07	99.94	0.87	5.81	6.51	15.41	6.9	-2.1	11.03	20.66	10.42	0.54
os31135	1	652	0.08	8	3	5	7.08	0.14	0.08	1.48	0.04	99.79	1.81	6.03	6.67	15.71	6.92	-2.01	11.18	20.97	10.5	0.81
os31136	1	455	0.32	22	2	0	5.72	-0.32	0	1.42	0.14	89.23	0.33	5.68	7.32	16.26	7.47	-1.67	12.07	21.89	11.27	1.11
os31322	1	603	0.5	6	2	7	6.69	-0.04	1.19	1.55	0.08	92.86	-3.93	8.18	6.66	15.65	6.93	-2.04	11.12	20.92	10.46	0.68
os31378	1	599	0.01	4	3	6	6.96	0.07	0.11	1.52	0.02	96.77	1.85	7.43	6.94	15.98	7.09	-1.92	11.63	21.41	10.84	0.94
os31468	1	566	0.47	7	1	1	6.67	-0.02	0.28	1.48	0.03	90.96	2.5	6.08	7.24	16.29	7.37	-1.71	11.96	21.83	11.17	1.09
os31526	1	574	0.4	10	3	5	6.45	-0.15	0.1	1.49	0.08	99.85	-0.92	5.99	7.01	16.05	7.21	-1.85	11.65	21.46	10.86	0.91
os31532	1	666	0.99	2	1	1	6.84	-0.03	0.06	1.48	0.01	98.1	2.26	6.74	6.73	15.75	6.98	-2.01	11.28	21.08	10.58	0.77
os31612	1	613	0.9	1	3	6	6.85	-0.02	2.99	1.56	0.05	71.77	-1.3	8.58	6.74	15.77	6.96	-2	11.27	21.04	10.56	0.8
os31640	1	484	0.4	12	2	0	6.94	0.08	0.1	1.51	0.12	84.46	-1.26	7.62	7.29	16.39	7.45	-1.64	11.9	21.77	11.11	1.09
os31641	1	482	0.41	8	2	7	6.89	0.05	0.95	1.54	0.13	85.07	-3.29	7.73	7.29	16.38	7.45	-1.64	11.89	21.77	11.11	1.09
os31646	1	570	0.65	11	3	5	6.8	-0.04	0.02	1.42	0.09	99.52	1.78	5.64	7.1	16.16	7.29	-1.73	11.63	21.47	10.89	0.99
os31647	1	570	0.65	11	3	5	6.79	-0.04	0.01	1.42	0.08	99.5	2.14	5.6	7.1	16.17	7.29	-1.73	11.63	21.47	10.89	1
os31652	1	493	0.32	25	1	4	6.35	0.08	0	1.41	0.11	83.89	1.29	6.08	7.49	16.56	7.62	-1.53	12.16	22.03	11.37	1.22
os31708	1	552	0	6	3	5	6.98	0.1	0.59	1.52	0.05	94.61	0.13	7.83	6.96	15.94	7.11	-1.81	11.5	21.25	10.78	1.02
os31768	1	603	0.08	7	3	9	6.93	0.1	0.11	1.48	0.04	97.42	1.24	5.99	7	16.04	7.17	-1.86	11.6	21.43	10.87	0.93

os31773	1	542	0.82	13	1	4	6.2	-0.18	0.01	1.42	0.07	83.3	2.95	5.75	7.27	16.34	7.44	-1.69	11.91	21.79	11.19	1.08
os31807	1	481	0.48	8	2	3	6.63	-0.02	0.62	1.53	0.08	81.69	-3.51	8.26	7.97	16.97	7.94	-0.99	12.74	22.57	11.8	1.85
os31809	1	595	0.59	11	2	3	6.52	-0.05	0.13	1.49	0.08	99.78	-1.83	6.82	7.38	16.42	7.42	-1.36	12.03	21.77	11.07	1.46
os31854	1	594	1	5	3	5	6.69	-0.09	0.37	1.5	0.04	98.3	0.73	6.54	7.22	16.21	7.33	-1.59	11.92	21.7	11.01	1.21
os31861	1	540	1	5	2	0	6.68	-0.08	0.61	1.53	0.07	81.83	-0.51	8.98	7.35	16.32	7.42	-1.55	12.09	21.83	11.16	1.31
os31891	1	476	0.35	12	2	3	6.89	0.06	0.14	1.51	0.14	99.19	-5.21	8.03	7.51	16.41	7.63	-1.37	12.19	21.96	11.36	1.38
os31906	1	546	0.6	7	3	5	6.59	-0.07	0.4	1.52	0.06	94.43	-0.87	7.55	6.97	15.88	7.21	-1.77	11.57	21.32	10.79	0.98
os31928	1	545	0.06	9	3	5	6.91	0.13	0.04	1.49	0.04	80.46	0.94	7.25	7.24	16.3	7.35	-1.79	12.02	21.91	11.16	1.08
os31955	1	176	0.92	1	3	6	6.7	0.01	7.42	1.56	0.01	13.84	0.05	9.08	9.47	18.19	9.44	0.73	14.08	23.35	13.05	3.27
os32124	1	212	0.89	7	2	0	6.41	-0.1	0.39	1.52	0.09	69.35	-1.98	7.6	9.03	17.73	9.12	0.38	13.7	22.89	12.75	2.93
os32130	1	267	0.85	3	3	9	6.67	-0.04	1.73	1.51	0.01	55.5	0.48	8.37	9.07	17.88	9.1	0.34	13.92	23.17	12.93	3.03
os32223	1	253	0.92	5	3	5	6.51	-0.09	0.31	1.52	0.06	80.19	0.37	8.11	8.76	17.61	8.82	-0.1	13.8	23.1	12.78	2.71
os32428	1	277	1	8	3	5	6.42	-0.14	0.19	1.49	0.06	98.39	0.16	7.11	8.72	17.46	8.85	-0.01	13.73	23.06	12.77	2.67
os32572	1	334	0.28	4	3	9	6.87	0.06	0.49	1.48	0.03	99.36	0.76	6.4	8.4	17.16	8.63	-0.25	13.26	22.64	12.41	2.41
os32642	1	244	0.77	14	3	5	6.6	-0.07	0	1.47	0.07	79.61	0.31	6.53	8.74	17.51	8.88	-0.01	13.62	23.04	12.67	2.64
os32983	1	213	0.88	4	3	6	6.61	-0.06	0.68	1.54	0.02	78.53	-0.02	7.71	9.3	18.03	9.29	0.46	14.16	23.42	13.08	3.13
os32989	1	231	0.4	8	2	3	6.8	0.04	1.05	1.54	0.08	63.5	-2.83	8.42	9.2	18.01	9.17	0.24	14.19	23.67	13.22	3.04
os33012	1	222	0.07	8	3	5	6.77	0.11	0.2	1.5	0.05	80.3	1.24	7.18	9.05	17.82	9.09	-0.03	14	23.45	13	2.75
os33047	1	189	0.92	0	3	6	6.69	0	6.64	1.57	0.01	4.38	-0.19	10.51	9.41	18.16	9.33	0.43	14.48	23.8	13.32	3.2
os33082	1	270	0.65	6	1	4	6.53	-0.04	0.32	1.43	0.03	65.05	5.96	7.01	8.96	17.78	8.96	-0.01	13.87	23.39	12.9	2.78
os33420	1	311	0.92	4	3	6	6.6	-0.07	0.59	1.51	0.03	98.29	0.44	6.99	8.6	17.38	8.76	-0.15	13.31	22.56	12.51	2.52
os33423	1	329	0.86	6	3	9	6.56	-0.09	0.34	1.49	0.03	99.68	1.23	6.32	8.58	17.37	8.75	-0.15	13.31	22.56	12.5	2.52
os33437	1	275	0.9	12	3	5	6.18	-0.19	0.01	1.49	0.07	98.9	-0.77	6.42	8.72	17.59	8.8	-0.2	13.77	23.12	12.77	2.59
os34194	1	283	0	5	1	4	6.55	-0.08	0.09	1.48	0.02	74.99	3.88	7.66	8.15	16.81	8.48	-1.21	12.99	22.24	12.44	1.55
os34454	1	253	0.23	4	3	6	6.67	0.01	1.14	1.54	0.06	80.64	-1.55	6.6	8.28	16.97	8.54	-0.93	13.16	22.44	12.55	1.83
os34609	1	257	0.92	5	3	6	6.66	-0.04	2.23	1.54	0.02	18.31	0.47	8.5	8.2	16.89	8.45	-0.83	12.95	22.38	12.29	1.91
os35304	1	236	0.92	2	3	6	6.64	-0.03	1.48	1.55	0.01	26.1	0.02	8.53	8.75	17.5	8.86	-0.34	13.69	23.06	12.76	2.49
os35963	1	314	0.98	12	2	3	6.36	-0.17	0.54	1.51	0.09	90.77	-3.13	7.1	8.2	16.86	8.5	-0.18	12.5	21.76	11.87	2.24
os36141	1	267	0.09	11	2	7	6.89	0.16	0.28	1.51	0.16	93.59	-6.45	6.65	8.56	17.27	8.78	0.01	13.08	22.39	12.38	2.54
os36181	1	234	0.97	14	3	5	6.05	-0.23	0	1.44	0.1	90.35	0.49	6	9.03	17.79	9.16	0.4	13.73	22.98	12.81	2.96
os36183	1	268	0.34	7	2	7	6.75	0.05	1.14	1.54	0.16	93.27	-5.89	7.15	8.51	17.23	8.76	0.04	12.9	22.14	12.21	2.5
os36195	1	284	0.15	23	2	7	6.47	0.3	0	1.47	0.25	96.8	-11.09	5.53	8.26	16.93	8.55	-0.1	12.46	21.72	11.82	2.29
os36205	1	209	0.92	4	2	7	6.55	-0.06	2.48	1.54	0.08	90.77	-3.32	7.93	9.08	17.86	9.21	0.51	13.64	22.93	12.78	3.03
os36212	1	351	0.96	20	3	5	6	-0.27	0	1.36	0.12	96.21	0.99	5.79	8	16.71	8.33	-0.32	12.25	21.52	11.64	2.11
os36237	1	334	0.37	13	1	4	6.8	0.03	0.02	1.34	0.07	96.65	6.07	6.06	8.19	16.87	8.49	-0.19	12.44	21.68	11.82	2.25
os36268	1	304	0	7	1	4	6.49	-0.12	0.17	1.43	0.05	95.95	3.03	6.48	8.47	17.16	8.7	0.03	12.75	22.01	12.08	2.44
os36282	1	380	0.77	12	3	5	6.72	-0.02	0.16	1.43	0.06	95.62	1.01	6.38	7.94	16.65	8.28	-0.37	12.22	21.52	11.6	2.06

os36306	1	310	0.98	17	3	5	6.02	-0.27	0	1.41	0.11	98.47	1.27	5.68	8.24	16.89	8.53	-0.1	12.49	21.73	11.86	2.31
os36316	1	364	0.01	20	1	4	6.87	0.31	0	1.37	0.12	98.73	2.4	5.42	7.9	16.58	8.24	-0.41	12.2	21.5	11.62	2.03
os36657	1	339	0.02	2	3	9	6.8	0.04	1.35	1.51	0.01	63.14	0.52	7.91	8.07	16.76	8.42	-0.54	12.6	21.95	11.96	2
os36658	1	338	0.01	2	3	9	6.81	0.04	1.24	1.51	0.01	64.69	0.51	7.75	8.07	16.75	8.42	-0.54	12.6	21.94	11.96	2
os36700	1	257	0.99	10	2	0	6.35	-0.16	0.07	1.51	0.07	88.28	-0.9	7.42	8.32	17.06	8.67	-0.51	12.99	22.39	12.34	2.13
os36701	1	357	0.92	1	3	6	6.81	0.03	2.74	1.55	0.04	94.27	-1.27	8.93	7.8	16.59	8.25	-0.93	12.38	21.82	11.8	1.67
os36812	1	242	0.13	16	2	7	6.57	0.15	0	1.51	0.2	72.65	-7.03	6.13	8.2	16.9	8.57	-0.33	12.75	22.13	12.17	2.19
os36814	1	360	0.93	14	2	3	6.36	-0.18	0	1.46	0.12	94.71	-2	6.92	7.62	16.27	8.11	-0.7	12.06	21.39	11.53	1.74
os36836	1	341	0.17	3	1	1	6.82	0.06	0.08	1.44	0.03	99.14	3.93	6.23	8.18	16.9	8.5	-0.47	12.87	22.24	12.15	2.09
os36853	1	269	0.97	16	2	3	6.09	-0.23	0.2	1.5	0.17	92.48	-6.83	5.63	8.28	16.98	8.59	-0.38	12.89	22.18	12.16	2.16
os36869	1	269	0.81	4	3	6	6.55	-0.07	0.4	1.53	0.05	93.61	0.7	6.31	8.24	16.94	8.58	-0.43	12.85	22.18	12.16	2.11
os36919	1	261	0.99	24	2	7	5.61	-0.34	0	1.44	0.2	87.05	-4.28	5.5	8.13	16.82	8.53	-0.34	12.65	22	12.07	2.12
os36968	1	271	0.75	11	2	3	6.29	-0.1	0.49	1.5	0.2	99.73	-7.68	6.49	8.58	17.31	8.8	-0.07	13.13	22.38	12.38	2.48
os37026	1	243	0.98	7	2	7	6.42	-0.13	0.23	1.54	0.11	79.48	-3.31	7.1	8.57	17.29	8.78	-0.04	13.04	22.33	12.32	2.43
os37036	1	282	0.02	7	3	5	6.88	0.12	0.72	1.47	0.07	95.67	0.63	6.26	8.55	17.29	8.73	-0.09	13.14	22.4	12.36	2.46
os37058	1	375	0.64	12	1	1	6.28	-0.11	0	1.4	0.06	91.98	3.67	5.9	8.06	16.84	8.37	-0.47	12.52	21.87	11.87	2.03
os37155	1	238	0.58	5	2	3	6.58	-0.02	1.11	1.54	0.06	62.93	-1.94	8	8.95	17.74	9.12	0.3	13.59	22.89	12.8	2.88
os37283	1	306	0	4	2	3	6.6	-0.06	1.57	1.52	0.11	96.18	-3.89	7.4	8.33	17.08	8.57	-0.24	12.84	22.15	12.14	2.27
os37292	1	237	0.73	8	2	0	6.37	-0.11	0.14	1.51	0.11	69.87	-2.07	7.25	8.64	17.38	8.86	0	13.18	22.44	12.44	2.51
os37367	1	319	0.94	20	2	3	6.03	-0.26	0	1.45	0.16	89.26	-4.2	5.69	8.07	16.78	8.39	-0.35	12.48	21.81	11.89	2.16
os37515	1	327	0.08	2	3	9	6.8	0.03	0.47	1.49	0.02	95.65	0.67	7.77	8.14	16.85	8.47	-0.23	12.73	21.99	12.07	2.28
os37582	1	350	0.9	5	3	6	6.56	-0.08	1.37	1.49	0.04	81.02	-0.14	8.44	8	16.73	8.34	-0.43	12.37	21.67	11.76	2.03
os37593	1	340	0.68	16	3	5	6.1	-0.23	0	1.44	0.11	94.54	-1.42	6.86	7.95	16.71	8.26	-0.47	12.28	21.61	11.67	1.98
os37652	1	291	0	16	2	7	6.88	0.25	0	1.48	0.14	95.23	-3.02	7.17	8.17	16.87	8.47	-0.3	12.65	21.9	11.99	2.19
os37694	1	299	0.54	9	2	3	6.78	0.01	0.53	1.48	0.11	98.2	-3.9	6.2	8.43	17.15	8.66	-0.08	12.86	22.12	12.17	2.36
os37815	1	325	0.02	7	2	7	6.7	0.08	0.92	1.54	0.14	91.53	-7.45	6.07	8.03	16.8	8.33	-0.44	12.37	21.76	11.74	1.99
os37878	1	245	0.17	11	1	2	6.91	0.18	0	1.46	0.1	66.47	3.51	6.02	8.55	17.25	8.77	0	12.95	22.19	12.24	2.45
os37922	1	317	0.04	15	2	3	6.81	0.22	0	1.47	0.13	82.27	-2.14	6.64	8.1	16.81	8.41	-0.31	12.56	21.82	11.93	2.15
os37949	1	322	0.52	7	2	3	6.64	0.01	0.91	1.52	0.07	78.94	-2.54	7.78	8.1	16.81	8.4	-0.37	12.55	21.8	11.9	2.13
os37963	1	365	0.46	5	2	3	6.84	0.05	1.43	1.52	0.11	96.19	-3.02	6.66	7.84	16.54	8.19	-0.51	12.3	21.58	11.71	2
os38163	1	308	1	10	2	3	6.36	-0.17	0.12	1.51	0.08	90.09	-1.83	7.83	8.14	16.84	8.47	-0.27	12.51	21.83	11.92	2.2
os38209	1	351	0	11	3	5	6.91	0.18	0.02	1.47	0.07	99.9	-0.08	6.41	7.99	16.67	8.29	-0.42	12.48	21.72	11.85	2.09
os38248	1	343	0	13	2	7	6.86	0.19	0.16	1.5	0.17	83.92	-5.85	7.28	7.96	16.71	8.24	-0.54	12.35	21.69	11.7	1.92
os38264	1	339	0.99	15	2	0	6.07	-0.25	0	1.45	0.13	96.48	0.39	5.93	7.99	16.76	8.29	-0.53	12.43	21.78	11.78	1.95
os38403	1	313	0.92	8	1	4	6.35	-0.15	0.28	1.46	0.04	63.1	2.64	7.42	8.13	16.81	8.42	-0.24	12.57	21.93	11.9	2.22
os38447	1	301	0.08	3	1	4	6.82	0.05	0.32	1.52	0.02	77.71	2.05	8.61	8.24	16.94	8.49	-0.2	12.74	22.12	12.07	2.27
os38516	1	312	1	12	2	3	6.24	-0.21	0.06	1.46	0.09	83.29	-1.69	6.94	8.18	16.86	8.45	-0.23	12.65	21.97	11.99	2.21

os38540	1	284	0.98	5	3	6	6.61	-0.07	0.44	1.51	0.03	69.55	1.53	7.52	8.51	17.2	8.69	-0.07	13.19	22.5	12.4	2.47
os38557	1	335	0.82	8	2	3	6.67	-0.06	0.69	1.53	0.07	92.87	-2.73	7.94	7.79	16.5	8.22	-0.55	12.37	21.68	11.82	1.94
os38558	1	329	0.03	9	2	3	6.93	0.13	0.69	1.52	0.11	96.24	-3.93	7.78	7.83	16.56	8.26	-0.5	12.44	21.75	11.9	2
os38603	1	365	0.08	8	3	5	6.95	0.13	0.46	1.49	0.06	97.91	-1.25	6.33	7.93	16.68	8.23	-0.57	12.35	21.66	11.71	1.91
os38612	1	351	0.99	22	3	5	5.79	-0.33	0	1.41	0.11	83.09	1.74	6.01	8.05	16.8	8.34	-0.48	12.48	21.8	11.82	2
os38613	1	330	0.77	12	2	3	6.48	-0.07	0.71	1.52	0.19	81.34	-6.53	5.92	8.04	16.79	8.33	-0.47	12.47	21.79	11.8	1.98
os38813	1	305	0	7	3	5	6.87	0.1	1.02	1.53	0.08	92.27	-2.18	8.23	8.14	16.83	8.43	-0.27	12.55	21.86	11.93	2.21
os38816	1	303	0.95	14	2	7	6.34	-0.18	0.08	1.5	0.12	93.5	-3.97	7.39	8.14	16.83	8.43	-0.27	12.54	21.86	11.93	2.21
os38817	1	314	0.99	10	3	5	6.39	-0.17	0.01	1.46	0.07	95.03	2.02	6.22	8.14	16.83	8.43	-0.27	12.53	21.85	11.92	2.2
os38864	1	338	0.98	15	2	0	6.07	-0.25	0	1.45	0.13	96.52	0	5.98	7.99	16.76	8.29	-0.53	12.43	21.78	11.78	1.95
os38885	1	352	0	20	1	1	6.81	0.32	0	1.36	0.1	92.66	4.78	4.65	7.98	16.71	8.35	-0.41	12.49	21.81	11.86	2.09
os38891	1	388	0.13	12	3	9	6.73	0.12	0.01	1.42	0.09	99.99	0.75	5.46	7.86	16.6	8.22	-0.54	12.41	21.71	11.81	2
os39159	1	360	0.94	15	3	9	6.31	-0.19	0	1.45	0.1	98.26	0.22	5.84	7.74	16.49	8.22	-1	12.41	21.85	11.84	1.58
os39240	1	305	0.77	12	2	0	6.66	-0.05	0.14	1.5	0.09	75.92	-0.43	6.52	7.74	16.4	8.2	-0.72	12.33	21.62	11.74	1.77
os39299	1	323	0.85	19	2	7	6.11	-0.21	0	1.48	0.2	86.32	-6.28	5.46	7.61	16.26	8.11	-0.94	12.2	21.56	11.67	1.6
os39360	1	392	1	6	3	9	6.55	-0.11	0.37	1.49	0.06	95.53	-0.85	5.68	7.58	16.23	8.11	-0.68	12.14	21.42	11.62	1.81
os39363	1	428	0.07	10	3	9	6.84	0.13	0.03	1.47	0.07	97.68	1.21	5.72	7.34	16.01	7.9	-0.85	11.89	21.22	11.45	1.65
os39627	1	433	0.74	3	3	6	6.83	0	2.56	1.53	0.04	69.73	0.11	8.08	7.1	15.75	7.7	-1.12	11.56	20.9	11.13	1.37
os39643	1	413	0.52	15	2	3	6.8	0.04	0	1.46	0.11	97.82	-2.81	5.27	7.33	15.98	7.87	-0.99	11.85	21.16	11.37	1.52
os39677	1	376	0.99	13	3	5	6.31	-0.2	0	1.48	0.12	70.79	-1.59	6.08	7.38	16.03	7.93	-1.05	11.97	21.29	11.47	1.5
os39846	1	405	0.87	8	1	1	6.47	-0.1	0.26	1.45	0.04	95.11	2.42	6.47	7.42	16.1	7.96	-0.99	11.99	21.34	11.48	1.53
os39902	1	340	0.02	23	1	4	6.75	0.35	0	1.35	0.15	95.2	3.37	5.61	7.67	16.35	8.14	-0.88	12.24	21.62	11.71	1.65
os39985	1	314	0.7	17	2	7	6.55	-0.04	0	1.45	0.19	89.57	-3.09	5.33	7.77	16.47	8.23	-1.03	12.37	21.69	11.78	1.51
os40001	1	431	0.01	13	1	4	6.85	0.19	0	1.41	0.08	96.99	5.17	5.88	7.24	15.96	7.77	-1.38	11.76	21.09	11.26	1.15
os40058	1	396	0.12	8	2	3	6.63	0.05	0.75	1.53	0.16	96.69	-8.09	6.96	7.3	16.05	7.86	-1.32	11.88	21.32	11.41	1.22
os40312	1	312	0.88	8	1	1	6.43	-0.12	0.03	1.44	0.03	97.12	5.07	6.15	8.07	16.73	8.44	-0.76	12.62	21.92	12	1.79
os40316	1	305	0	24	1	4	6.75	0.39	0	1.36	0.16	86.07	2.49	5.71	8.02	16.7	8.41	-0.71	12.49	21.85	11.91	1.83
os40324	1	302	0.47	11	2	3	6.84	0.09	0.39	1.47	0.15	88.61	-6.5	6.43	7.98	16.66	8.36	-0.83	12.5	21.8	11.9	1.74
os40523	1	345	0.01	14	2	3	6.88	0.23	0	1.49	0.17	99.8	-6.46	6.37	7.68	16.37	8.11	-1.07	12.26	21.6	11.67	1.49
os40527	1	347	0.6	3	3	6	6.67	-0.02	0.6	1.52	0.03	88.65	0.93	7.08	7.75	16.44	8.19	-1.19	12.3	21.62	11.71	1.42
os40575	1	364	0.01	8	3	5	6.92	0.13	0.41	1.51	0.07	89.27	-1.72	7.9	7.79	16.54	8.2	-1.02	12.36	21.81	11.8	1.6
os40676	1	312	0.08	12	1	4	6.97	0.19	0.01	1.43	0.06	81.56	2	6.71	8.12	16.8	8.41	-0.24	12.56	21.92	11.89	2.21
os40678	1	312	0.49	5	3	6	6.78	0.01	0.85	1.52	0.05	69.36	-0.17	7.63	8.11	16.8	8.4	-0.25	12.55	21.91	11.89	2.2
os40735	1	327	0.09	10	2	3	6.73	0.1	0.65	1.52	0.12	95.29	-5.25	8.18	7.71	16.35	8.14	-0.49	12.11	21.36	11.52	1.89
os40848	1	371	0.29	10	2	7	6.94	0.11	0.06	1.5	0.16	93.89	-3.67	6.67	7.41	16.05	7.92	-0.87	11.85	21.21	11.38	1.6
os41082	1	299	0.01	2	3	6	6.79	0.04	1.25	1.51	0.05	84.99	-1.37	7.06	8.15	16.9	8.58	-0.14	12.4	21.75	11.96	2.2
os41207	1	393	0.6	4	3	9	6.65	-0.03	0.23	1.41	0.04	99.97	2.04	5.94	7.95	16.7	8.38	-0.35	12.35	21.7	11.85	2.1

os41222	1	466	0.8	11	2	3	6.4	-0.13	0.28	1.46	0.12	99.41	-0.97	6.72	7.06	15.84	7.56	-1.17	11.36	20.66	10.81	1.17
os41226	1	314	0.66	22	1	4	5.59	-0.23	0	1.37	0.12	91.99	5.88	5.68	8.1	16.86	8.48	-0.26	12.56	21.88	11.97	2.15
os41234	1	368	0.53	11	2	3	6.37	-0.07	0.03	1.49	0.13	99.7	-2.14	6.22	7.64	16.41	8.11	-0.64	12.02	21.35	11.48	1.76
os41236	1	471	0.66	9	2	7	6.75	-0.04	0.57	1.5	0.14	99.86	-4.15	7.52	6.93	15.72	7.44	-1.3	11.23	20.54	10.68	1.05
os41249	1	457	0.01	13	1	1	6.87	0.17	0	1.41	0.06	99.92	3.86	5.49	7.22	15.95	7.8	-0.88	11.44	20.78	11.03	1.46
os41593	1	449	0.01	9	3	5	7	0.15	0.13	1.5	0.06	64.63	0.48	7.12	7.32	16	7.49	-1.07	12.25	21.6	11.4	1.61
os41605	1	460	0.92	3	1	1	6.79	-0.01	0.01	1.47	0.01	90.12	5.12	7.71	7.46	16.2	7.77	-1	12.29	21.62	11.64	1.65
os41729	1	429	0.64	2	3	6	6.71	-0.03	1.76	1.55	0.03	56.94	-0.12	8.55	7.58	16.37	7.73	-0.93	12.69	22.07	11.69	1.8
os41767	1	489	0.08	3	3	6	6.91	0.05	0.48	1.52	0.02	70.82	0.97	8.37	7.36	16.17	7.57	-0.99	12.37	21.79	11.43	1.64
os42217	1	321	0.92	0	3	6	6.76	0.01	2.42	1.56	0.05	57.35	-0.81	8.66	8.32	17.09	8.52	-0.3	13.2	22.48	12.32	2.39
os42271	1	265	0.25	7	3	5	6.84	0.06	0.32	1.53	0.06	78.54	-0.6	6.72	8.95	17.77	9.06	0.13	13.8	23.08	12.88	2.87
os42410	1	284	0.73	12	2	3	6.25	-0.1	0.01	1.49	0.11	81.15	-2.21	6.45	8.74	17.55	8.79	-0.23	14	23.24	13.01	2.71
os42483	1	460	0.87	7	3	9	6.66	-0.09	0.5	1.51	0.05	97.34	-0.71	7.04	7.1	15.87	7.73	-0.99	11.6	20.94	11.15	1.46
os42553	1	365	0.47	6	3	5	6.65	0	0.22	1.51	0	76.24	1.47	6.87	7.66	16.47	8.3	-0.37	12.04	21.49	11.75	2.05
os42559	1	376	0.47	14	3	5	6.74	0	0	1.47	0	96.75	0	5.66	7.56	16.34	8.17	-0.59	11.9	21.26	11.58	1.82
os42563	1	380	0.4	5	3	6	6.78	-0.01	1.08	1.53	0	90.99	-0.8	7.86	7.42	16.2	8.05	-0.56	11.77	21.21	11.47	1.86
os42600	1	418	0.92	2	3	6	6.72	-0.03	2.12	1.55	0.02	65.84	0.1	8.15	7.22	16	7.84	-0.82	11.64	21.03	11.27	1.62
os42649	1	455	0.25	6	2	3	6.92	0.06	0.74	1.51	0.08	99.95	-2.47	7.65	6.95	15.72	7.62	-0.98	11.05	20.44	10.79	1.37
os42708	1	410	0.9	7	3	9	6.69	-0.07	0.64	1.47	0	96.5	-0.14	6.44	7.47	16.25	8.07	-0.69	11.76	21.14	11.44	1.74
os42715	1	433	0.99	13	2	0	6.2	-0.23	0	1.48	0	99.77	-0.57	5.75	7.08	15.9	7.72	-0.97	11.3	20.7	11.03	1.4
os42717	1	451	0.92	10	3	5	6.35	-0.16	0.06	1.49	0	95.45	-1.41	5.7	7.14	15.97	7.78	-0.93	11.35	20.74	11.06	1.44
os42759	1	440	0.53	3	3	6	6.74	0	0.4	1.53	0.03	85.81	0.39	8.16	7.13	15.89	7.76	-0.96	11.53	20.87	11.12	1.47
os42766	1	416	0.21	5	3	6	6.77	0.03	0.59	1.51	0.03	95.39	0.29	6.95	7.37	16.17	8.02	-0.63	11.76	21.19	11.44	1.81
os42786	1	368	0.5	6	3	5	6.62	-0.02	0.37	1.52	0.06	87.01	0.38	8.7	7.44	16.21	8.06	-0.6	11.88	21.25	11.5	1.83
os42831	1	425	0.08	6	3	5	6.96	0.11	0.77	1.52	0.06	92.79	-1.39	7.33	7.2	15.97	7.83	-0.86	11.66	21.01	11.26	1.58
os42856	1	482	0.6	7	3	5	6.63	-0.03	0.41	1.5	0	99	0.19	6.66	7.05	15.84	7.67	-0.99	11.16	20.56	10.89	1.35
os42886	1	301	0.81	9	2	0	6.35	-0.13	0.05	1.49	0	91.8	1.07	6.1	7.78	16.47	8.36	-0.34	12.43	21.78	11.92	2.18
os42892	1	322	0.51	9	2	3	6.42	-0.11	0.74	1.53	0.1	95.71	-4.32	7.56	7.7	16.34	8.32	-0.38	12.24	21.55	11.78	2.08
os42908	1	334	0.92	7	3	5	6.47	-0.11	0.58	1.52	0.07	95.96	-1.39	6.84	7.53	16.14	8.13	-0.53	11.86	21.15	11.44	1.82
os42917	1	327	0.99	8	3	5	6.53	-0.1	0.56	1.52	0.06	94.17	-0.92	6.54	7.57	16.19	8.16	-0.55	12.08	21.34	11.65	1.93
os42918	1	333	0.87	7	3	5	6.5	-0.11	0.4	1.51	0.06	95.26	-0.11	6.34	7.54	16.16	8.13	-0.57	12.05	21.31	11.62	1.9
os42919	1	312	0.92	5	3	5	6.55	-0.08	0.76	1.53	0.06	88.85	-0.45	7.07	7.65	16.27	8.24	-0.48	12.18	21.43	11.72	1.99
os43060	1	304	0.23	6	3	5	6.7	0.04	0.21	1.52	0.05	82.6	0.77	6.84	7.75	16.39	8.36	-0.34	12.27	21.58	11.81	2.1
os43074	1	409	0.09	9	2	3	6.95	0.11	0.28	1.51	0.11	90.1	-4.12	6.65	7.08	15.71	7.75	-1.02	11.76	20.98	11.3	1.49
os43257	1	353	0.36	6	2	0	6.58	-0.08	1.42	1.54	0.09	91.17	-3.11	7.71	7.3	15.94	8	-0.99	11.98	21.16	11.63	1.64
os43320	1	411	0.84	11	2	0	6.3	-0.17	0.05	1.5	0.12	99.66	-2.03	5.65	7.01	15.65	7.68	-0.96	11.27	20.58	10.92	1.37
os43327	1	365	0.98	15	1	4	6.21	-0.23	0	1.45	0.1	99.99	2.31	5.96	7.29	15.93	7.97	-0.68	11.62	20.91	11.26	1.68

os43488	1	718	0.06	4	3	6	7.03	0.07	1.68	1.51	0.08	98.51	-0.85	7.63	5.36	14.16	6.31	-2.85	9.54	18.99	9.54	-0.45
os43595	1	519	1	5	3	9	6.69	-0.07	0.29	1.42	0.03	96.07	2.19	6.64	6.75	15.5	7.42	-1.69	11.25	20.65	10.8	0.8
os43681	1	437	0.7	10	2	7	6.7	-0.01	0.59	1.49	0.16	99.06	-6.84	7.04	7.01	15.79	7.69	-1.27	11.61	21.04	11.15	1.24
os43753	1	590	0.71	11	1	2	6.41	-0.11	0	1.43	0.1	98.56	4.54	5.71	5.77	14.51	6.63	-2.68	10	19.41	9.93	-0.23
os43769	1	714	0.01	15	3	5	7.01	0.23	0	1.45	0.11	99.94	-0.02	6.23	5.29	14.06	6.26	-3.1	9.45	18.84	9.53	-0.69
os43908	1	604	0.02	16	2	7	6.85	0.24	0.06	1.5	0.17	99.89	-5.52	7.1	5.62	14.4	6.54	-3.08	9.85	19.25	9.91	-0.65
os44331	1	488	0.99	17	1	1	5.98	-0.28	0	1.35	0.09	99.88	4.21	4.88	7.12	15.83	7.64	-1.58	11.6	20.94	11.12	0.95
os44382	1	420	1	18	1	4	5.98	-0.29	0	1.35	0.12	99.51	2.93	5.64	7.12	15.83	7.67	-1.81	11.62	20.96	11.22	0.69
os44416	1	747	0.48	13	3	9	6.56	-0.02	0	1.42	0.07	99.57	1.8	5.87	5.5	14.27	6.41	-3.02	9.71	19.11	9.77	-0.6
os44429	1	471	0.53	23	1	2	5.91	-0.06	0	1.37	0.15	89.85	4.31	4.92	6.47	15.21	7.21	-2.5	10.88	20.21	10.7	-0.02
os44444	1	664	0.09	8	2	8	6.86	0.08	0.25	1.46	0.07	100	-1.85	5.8	5.91	14.64	6.75	-2.77	10.17	19.52	10.14	-0.34
os44470	1	442	0.92	14	1	4	6.52	-0.12	0	1.39	0.11	94.49	7.18	5.05	6.78	15.49	7.44	-2.18	11.27	20.6	10.96	0.33
os44479	1	453	0.8	5	3	6	6.62	-0.08	0.42	1.4	0.07	98.07	1.18	5.98	6.83	15.56	7.49	-2.16	11.32	20.63	11.02	0.31
os44515	1	649	0	9	3	5	7.05	0.16	0.15	1.47	0.08	99.44	-1.29	6.32	5.94	14.71	6.71	-2.62	10.29	19.69	10.1	-0.16
os44529	1	515	0.12	14	2	7	7.05	0.19	0	1.47	0.14	97.09	-2.58	6.42	6.43	15.17	7.14	-2.35	10.87	20.25	10.63	0.13
os44764	1	457	1	19	1	1	5.88	-0.32	0	1.36	0.12	99.36	3.35	5.04	7.17	15.89	7.71	-1.52	11.69	21	11.18	1.01
os44836	1	569	0.86	13	1	4	6.26	-0.19	0	1.35	0.1	97.85	5.21	5.26	6.29	15.05	7.02	-2.86	10.74	20.05	10.55	-0.31
os44908	1	576	0.3	14	2	7	6.53	0.05	0.01	1.47	0.15	98.89	-4.39	7.01	6.03	14.8	6.81	-2.84	10.44	19.82	10.3	-0.37
os44932	1	402	0.97	17	2	7	6.12	-0.25	0	1.46	0.17	84.2	-2.85	6.11	6.87	15.58	7.53	-2.12	11.34	20.66	11.05	0.35
os45006	1	606	0.81	19	3	9	5.94	-0.22	0	1.41	0.14	99.48	-0.74	5.59	6.14	14.91	6.9	-2.93	10.56	19.87	10.39	-0.41
os45010	1	630	0.99	16	1	1	6.14	-0.26	0	1.38	0.11	99.96	2.84	5.27	6.07	14.84	6.85	-2.96	10.47	19.8	10.31	-0.45
os45016	1	638	0.81	18	3	9	6.01	-0.21	0	1.39	0.12	99.9	-0.11	5.38	6.12	14.89	6.89	-2.94	10.53	19.86	10.37	-0.42
os45019	1	655	0.79	17	1	1	6.14	-0.17	0	1.36	0.08	99.98	5.22	5.11	6.15	14.91	6.9	-2.93	10.56	19.88	10.39	-0.4
os45072	1	471	0.04	20	3	5	6.91	0.32	0	1.4	0.13	97.21	-0.31	5.39	6.86	15.6	7.45	-2.47	11.42	20.69	11.08	0.09
os45122	1	557	0.54	14	3	5	6.35	-0.08	0	1.44	0.12	99.87	-0.51	5.53	6.4	15.13	7.06	-2.77	10.84	20.17	10.64	-0.21
os45136	1	543	0.62	17	1	2	6.65	-0.04	0	1.4	0.19	95.02	2.06	5.62	6.04	14.77	6.8	-2.98	10.35	19.69	10.26	-0.47
os45150	1	613	0.43	21	2	0	6.13	-0.03	0	1.42	0.15	99.51	-1.36	6.45	5.86	14.6	6.66	-3.07	10.14	19.49	10.09	-0.57
os45152	1	566	0.83	21	1	4	5.7	-0.28	0	1.38	0.16	97.91	2.44	5.59	6.13	14.9	6.9	-2.94	10.55	19.86	10.38	-0.41
os45154	1	634	0.89	13	3	5	6.29	-0.18	0.01	1.44	0.12	99.66	0.95	6.87	5.71	14.44	6.57	-3.14	9.94	19.27	9.93	-0.67
os45164	1	701	0.06	6	3	9	6.98	0.09	0.39	1.44	0.04	99.91	0.71	6.61	5.68	14.45	6.51	-3.13	10.02	19.35	9.96	-0.62
os45182	1	588	0.16	22	1	4	7.03	0.29	0	1.35	0.14	98.83	4.37	5.43	5.99	14.71	6.77	-2.94	10.3	19.59	10.18	-0.51
os45241	1	595	0.77	2	1	1	6.85	-0.01	0.49	1.4	0.06	99.98	2.04	6.53	6.17	14.89	6.93	-2.77	10.52	19.85	10.41	-0.33
os45247	1	503	0.4	23	2	7	6.78	0.13	0	1.42	0.2	95.17	-1.44	5.33	6.16	14.9	6.93	-2.82	10.51	19.84	10.39	-0.38
os45301	1	458	0.63	9	3	9	6.79	-0.02	0.25	1.47	0.07	100	-0.83	7.56	7.18	15.91	7.65	-1.84	11.78	21.16	11.26	0.73
os45547	1	423	0.29	18	1	4	6.45	0.11	0	1.43	0.11	96.17	2.07	5.53	6.98	15.69	7.58	-2.05	11.5	20.84	11.14	0.45
os45571	1	562	0.4	14	1	4	6.98	0.1	0	1.41	0.09	99.6	3.67	5.19	6.22	14.98	6.97	-2.7	10.66	20.01	10.5	-0.21
os45627	1	621	0.6	8	3	5	6.61	-0.05	0.39	1.48	0.07	99.86	-0.26	6.51	5.93	14.69	6.69	-2.91	10.33	19.71	10.19	-0.43

os45628	1	617	0.65	9	3	5	6.56	-0.07	0.26	1.48	0.07	99.79	-0.47	6.55	5.93	14.7	6.69	-2.91	10.33	19.71	10.19	-0.43
os45679	1	553	0.34	13	3	5	6.59	0.03	0	1.45	0.09	95.89	0.64	6.9	6.3	15.05	6.99	-2.7	10.79	20.11	10.56	-0.17
os45714	1	395	0.45	13	2	0	6.87	0.09	0	1.47	0.13	92.72	0.34	5.89	7.09	15.78	7.58	-2.25	11.63	20.94	11.25	0.32
os45715	1	443	0.92	15	3	5	6.49	-0.13	0.21	1.46	0.13	98.49	-1.61	7.64	6.96	15.69	7.51	-2.31	11.51	20.83	11.15	0.24
os45720	1	485	0.77	6	1	1	6.79	-0.03	0.06	1.44	0.04	99.1	3.88	5.99	6.89	15.62	7.45	-2.35	11.43	20.76	11.09	0.2
os45732	1	510	0.78	12	1	4	6.33	-0.14	0	1.38	0.08	98.48	4.06	5.5	6.68	15.43	7.29	-2.4	11.22	20.55	10.89	0.14
os45734	1	443	0.34	17	1	2	6.37	0.05	0	1.41	0.15	97.03	1.99	5.55	6.82	15.56	7.4	-2.29	11.37	20.71	11.01	0.25
os45755	1	475	0.04	10	1	4	6.89	0.15	0.05	1.41	0.06	99.26	2.97	6.14	6.91	15.65	7.46	-2.21	11.48	20.83	11.09	0.35
os45829	1	524	0.08	3	3	5	6.95	0.07	0.92	1.49	0.05	99.16	-1.03	6.68	6.7	15.45	7.27	-2.38	11.23	20.59	10.86	0.18
os45833	1	490	0.11	2	3	6	6.87	0.02	0.07	1.52	0.02	97.37	1.91	8.15	6.79	15.54	7.37	-2.4	11.33	20.69	11	0.15
os45947	1	498	0.53	11	1	4	6.87	0.02	0	1.38	0.07	97.69	5.8	5.24	6.7	15.4	7.29	-2.55	11.23	20.51	10.91	0
os45977	1	455	0.83	11	3	5	6.56	-0.12	0.01	1.48	0.09	99.61	-1.25	5.93	6.98	15.7	7.52	-2.34	11.56	20.86	11.17	0.23
os45991	1	472	0.01	2	3	6	6.86	0.03	0.48	1.53	0.03	77.98	0.58	8.36	6.84	15.59	7.41	-2.38	11.38	20.76	11.04	0.19
os45992	1	510	0.23	3	3	9	6.82	0.02	0.66	1.51	0.02	93.44	0.27	7.17	6.78	15.54	7.36	-2.43	11.34	20.73	11	0.15
os46038	1	467	0.09	12	2	3	6.41	-0.12	0.61	1.51	0.12	97.76	-5.43	7.91	6.93	15.66	7.47	-2.35	11.57	20.87	11.12	0.27
os46045	1	502	0.34	7	3	5	6.73	0.03	0.53	1.52	0.07	99.49	-1.45	7.77	6.68	15.45	7.29	-2.57	11.26	20.61	10.91	0.03
os46150	1	404	0.24	13	2	3	6.96	0.17	0.01	1.48	0.16	95.41	-5.05	6.48	7.11	15.83	7.63	-2.48	11.8	21.07	11.39	0.12
os46278	1	456	0.99	10	3	5	6.48	-0.15	0.02	1.45	0.07	99.33	2.13	5.95	6.87	15.59	7.44	-2.61	11.46	20.73	11.11	-0.03
os46285	1	420	0.73	18	1	4	5.99	-0.18	0	1.4	0.12	99.37	1.16	6.23	7.1	15.8	7.63	-2.51	11.75	20.99	11.34	0.11
os46304	1	376	0.97	11	2	0	6.38	-0.18	0.01	1.5	0.09	77.27	-1.26	6.2	7.23	15.95	7.73	-2.39	11.88	21.13	11.49	0.21
os46352	1	427	0.89	12	3	5	6.6	-0.1	0.11	1.48	0.08	98.37	-0.28	5.93	7.07	15.78	7.6	-2.4	11.66	20.94	11.27	0.21
os46384	1	379	1	11	2	7	6.33	-0.18	0.35	1.52	0.16	93.83	-5.85	7.56	7.15	15.85	7.71	-2.13	11.58	20.87	11.3	0.41
os46386	1	387	0.9	12	2	3	6.42	-0.16	0.01	1.48	0.13	95.19	-2.45	6.94	7.13	15.84	7.69	-2.15	11.56	20.86	11.28	0.39
os46414	1	452	0.31	11	1	4	6.97	0.1	0	1.44	0.06	99.65	3.46	5.46	6.99	15.67	7.54	-2.66	11.62	20.85	11.23	-0.07
os46430	1	429	0.19	11	3	5	6.99	0.13	0.03	1.47	0.07	98.16	0.31	5.9	6.98	15.68	7.54	-2.64	11.63	20.85	11.23	-0.05
os46473	1	418	0.98	15	3	5	6.14	-0.24	0	1.42	0.11	99.5	-0.7	5.82	7.09	15.82	7.64	-2.51	11.76	20.99	11.34	0.1
os46641	1	442	0.02	9	1	4	6.41	-0.14	0.01	1.4	0.06	98.11	5.54	5.45	7	15.7	7.56	-2.57	11.64	20.9	11.24	0.04
os46647	1	689	0.24	6	1	1	6.86	0.03	0.05	1.43	0.06	98.05	3.76	6.06	5.56	14.3	6.48	-3.31	9.77	19.13	9.81	-0.8
os46659	1	440	0.55	13	2	0	6.8	0.01	0	1.47	0.11	93.99	0.53	5.8	6.86	15.57	7.46	-2.76	11.47	20.69	11.11	-0.19
os46686	1	374	0.27	10	2	0	6.59	0.03	0.04	1.5	0.11	84.47	-1.87	7.11	7.13	15.83	7.65	-2.44	11.75	21.01	11.36	0.16
os46731	1	491	0.97	7	1	4	6.63	-0.1	0.02	1.46	0.05	91.19	4.58	6.61	6.73	15.48	7.32	-2.53	11.29	20.61	10.93	0.08
os46734	1	521	0.08	7	1	1	6.89	0.1	0.25	1.46	0.04	95.76	2.9	6.23	6.8	15.5	7.33	-2.47	11.36	20.66	10.94	0.14
os46830	1	462	0.08	11	3	9	7.03	0.17	0.01	1.47	0.08	97.91	-0.58	6.34	6.96	15.73	7.51	-2.63	11.66	20.98	11.25	-0.01
os46839	1	461	0.94	9	3	5	6.55	-0.13	0.13	1.51	0.07	78	-0.79	7.76	6.86	15.63	7.44	-2.65	11.51	20.85	11.17	-0.07
os46861	1	493	0.87	9	3	9	6.72	-0.07	0.13	1.46	0.06	99.72	-0.57	5.99	6.87	15.59	7.43	-2.74	11.54	20.81	11.14	-0.13
os46885	1	416	0.5	14	2	3	6.42	0	0	1.45	0.1	97.81	0.93	6.46	7.03	15.74	7.58	-2.53	11.65	20.9	11.27	0.07
os46896	1	396	0.13	17	2	0	6.64	0.19	0	1.44	0.13	84.21	-0.92	5.93	7.14	15.85	7.67	-2.46	11.8	21.04	11.38	0.14

os46988	1	475	0	11	2	0	6.95	0.19	0.01	1.49	0.12	75.1	-1.08	5.82	6.46	15.16	7.15	-2.8	10.79	20.11	10.65	-0.28
os47157	1	462	0.76	3	3	6	6.82	0	0.41	1.54	0.08	88.69	0.41	9.22	6.57	15.3	7.22	-2.73	11.09	20.4	10.84	-0.18
os47160	1	464	0.79	12	3	5	6.3	-0.15	0.02	1.46	0.09	81.93	-0.17	5.98	6.73	15.45	7.33	-2.65	11.3	20.61	11	-0.07
os47163	1	474	0.02	12	3	5	7.02	0.19	0.13	1.48	0.09	99.1	-1.62	7.44	6.55	15.25	7.22	-2.75	11.05	20.32	10.82	-0.18
os47172	1	401	0.21	18	2	7	6.93	0.2	0.03	1.43	0.17	76.85	-3.2	7.47	6.88	15.57	7.5	-2.48	11.47	20.69	11.13	0.15
os47182	1	383	0.64	9	2	3	6.69	-0.04	0.82	1.52	0.15	94.48	-6.2	6.39	7.15	15.84	7.65	-2.48	11.81	21.07	11.42	0.12
os47185	1	386	0.24	22	3	5	6.8	0.23	0	1.43	0.17	94.26	-2.68	5.67	7.18	15.87	7.68	-2.45	11.85	21.1	11.45	0.15
os47195	1	387	0.23	10	2	3	6.96	0.13	0.5	1.51	0.1	98.52	-2.41	7.29	7.12	15.84	7.67	-2.41	11.75	21	11.36	0.17
os47204	1	372	0	4	3	6	6.87	0.07	0.52	1.51	0.04	87.71	0.8	6.97	7.29	15.97	7.78	-2.27	11.87	21.13	11.5	0.31
os47205	1	357	0.17	10	3	5	6.97	0.16	0.03	1.49	0.07	70.53	1.45	6.83	7.28	15.97	7.77	-2.27	11.86	21.13	11.5	0.31
os47225	1	471	0.2	2	1	1	6.72	-0.04	0.05	1.47	0.02	96.1	3.61	7.86	7.02	15.75	7.56	-2.44	11.65	20.92	11.25	0.16
os47238	1	383	0.92	4	2	7	6.66	-0.02	1.79	1.55	0.14	94.66	-5.53	7.13	7.05	15.77	7.6	-2.36	11.61	20.89	11.27	0.19
os47255	1	398	0.57	13	1	4	6.36	-0.05	0.02	1.42	0.09	99.16	1.99	5.97	7.21	15.91	7.71	-2.33	11.77	21.05	11.39	0.27
os47278	1	364	0.53	14	2	0	6.34	-0.06	0.01	1.46	0.09	91.7	1.21	5.75	7.24	15.95	7.77	-2.19	11.78	21.08	11.43	0.38
os47288	1	382	0.5	11	3	5	6.58	0.03	0.04	1.49	0.1	96.68	-1.45	6.51	7.17	15.86	7.69	-2.36	11.79	21.02	11.38	0.23
os47337	1	282	0.83	11	2	7	6.56	-0.07	0.29	1.52	0.16	75.27	-5.43	7.55	7.71	16.4	8.19	-1.69	12.5	21.84	12.12	1.07
os47423	1	438	0.79	13	1	2	6.21	-0.17	0	1.44	0.14	85.07	3.58	5.57	6.95	15.66	7.47	-2.29	11.54	20.87	11.1	0.32
os47651	1	420	0.87	4	3	9	6.7	-0.05	1.24	1.5	0.03	78.44	-0.59	7.53	7.46	16.22	7.95	-1.78	12.13	21.54	11.81	0.96
os47746	1	451	1	6	3	5	6.58	-0.1	0.42	1.51	0.06	97.09	-0.25	8.76	6.81	15.52	7.4	-2.39	11.25	20.58	10.98	0.15
os47866	1	441	1	14	1	4	6.22	-0.23	0	1.4	0.09	97.73	2.37	6.36	7.17	15.91	7.64	-1.98	11.78	21.13	11.27	0.58
os47925	1	486	0.02	4	2	3	6.92	0.07	1.57	1.52	0.06	95.06	-2.3	8.16	6.95	15.69	7.44	-2.13	11.49	20.85	11.03	0.44
os47974	1	404	0.36	10	1	2	6.58	0	0.01	1.41	0.09	88.76	7.19	6.28	7.1	15.82	7.59	-2.13	11.73	21.07	11.26	0.46
os47989	1	409	0.01	14	1	4	6.95	0.24	0	1.41	0.09	92.09	5.53	5.63	7.2	15.92	7.67	-2.02	11.82	21.15	11.33	0.54
os47995	1	323	0.19	4	1	4	6.7	0.01	0.11	1.47	0.02	68.12	4.05	7.91	7.82	16.55	8.23	-1.51	12.63	21.98	12.27	1.26
os48072	1	398	0.7	13	2	0	6.21	-0.12	0.01	1.47	0.15	93.25	-1.76	5.52	7.19	15.91	7.72	-2.07	11.72	21.06	11.34	0.56
os48137	1	485	0.99	11	3	5	6.44	-0.17	0.01	1.49	0.09	100	-0.59	6.04	6.66	15.39	7.3	-2.42	11.04	20.42	10.82	0.1
os48143	1	450	0.85	10	2	0	6.66	-0.09	0.09	1.5	0.08	99.94	0.33	6.04	6.87	15.6	7.5	-2.26	11.28	20.63	11.05	0.26
os48165	1	372	0	3	3	6	6.86	0.06	0.8	1.54	0.02	84.59	-0.03	7.37	7.52	16.25	8.01	-1.71	12.18	21.51	11.81	1
os48169	1	351	0.29	7	3	5	6.88	0.06	0.62	1.52	0.04	93.23	-0.42	8.97	7.62	16.31	8.09	-1.66	12.25	21.56	11.89	1.06
os48269	1	344	0.01	7	3	5	6.87	0.11	0.28	1.52	0.1	97.28	-0.74	6.19	7.59	16.34	8.02	-1.57	12.13	21.52	11.65	1.04
os48281	1	355	0.02	8	3	5	6.82	0.11	0.24	1.51	0.07	99.75	-0.2	6.58	7.51	16.22	7.97	-1.79	12.09	21.4	11.62	0.84
os48284	1	443	0.83	4	1	1	6.68	-0.04	0.01	1.38	0.01	98.42	6.39	6.55	7.39	16.11	7.88	-1.89	11.93	21.26	11.5	0.71
os48290	1	406	0.42	10	1	4	6.37	-0.17	0.03	1.46	0.09	88.12	3.72	7.25	7.25	15.95	7.7	-2	11.85	21.18	11.37	0.57
os48300	1	451	0.99	10	3	5	6.57	-0.12	0.14	1.48	0.1	99.38	-1.12	6.02	7.06	15.79	7.55	-2.1	11.63	20.95	11.16	0.46
os48313	1	467	0.8	6	3	9	6.57	-0.08	0.6	1.49	0.04	99.72	0.54	6.76	7.07	15.78	7.57	-2.13	11.63	20.94	11.17	0.45
os48342	1	468	0	13	2	3	6.87	0.23	0	1.48	0.12	99.57	-3.04	5.69	7.06	15.78	7.55	-2.03	11.57	20.92	11.13	0.56
os48352	1	400	0.48	3	3	9	6.75	0.01	0.45	1.49	0.02	94.58	1.12	7.24	7.51	16.25	7.97	-1.63	12.03	21.41	11.55	0.99

os48368	1	507	0.07	11	3	5	6.86	0.14	0.01	1.47	0.06	94.98	1.88	5.78	6.83	15.57	7.39	-2.35	11.42	20.73	10.98	0.25
os48370	1	530	0.76	15	3	5	6.66	-0.07	0	1.43	0.09	99.77	0.26	5.22	6.72	15.44	7.27	-2.5	11.33	20.64	10.89	0.11
os48410	1	473	0.88	19	3	5	5.87	-0.27	0	1.42	0.13	95.92	-2.23	5.25	6.77	15.53	7.36	-2.61	11.41	20.72	11.07	0.02
os48433	1	493	0.81	12	3	5	6.31	-0.16	0.14	1.47	0.09	95.54	-1.59	7.44	6.65	15.35	7.27	-2.61	11.21	20.51	10.92	0.01
os48443	1	519	0.98	13	3	5	6.36	-0.2	0.01	1.45	0.07	97.36	1.38	6.22	6.6	15.31	7.23	-2.74	11.27	20.51	10.97	-0.12
os48451	1	449	1	17	2	3	6.06	-0.28	0.02	1.47	0.12	94.17	-3.06	6.82	6.81	15.5	7.41	-2.55	11.5	20.73	11.17	0.09
os48513	1	502	0.84	15	1	4	6.07	-0.22	0	1.41	0.08	97.98	3.64	6.22	6.68	15.41	7.27	-2.66	11.27	20.57	10.95	-0.03
os48545	1	490	0	4	3	9	6.91	0.06	0.48	1.45	0.03	88.43	0.56	8.12	6.79	15.47	7.4	-2.51	11.37	20.62	11.05	0.12
os48565	1	490	0.01	16	2	3	6.91	0.24	0	1.41	0.1	92.13	-1.51	5.88	6.69	15.4	7.32	-2.58	11.24	20.51	10.94	0.04
os48612	1	418	0.02	21	2	0	6.74	0.3	0	1.45	0.14	96.11	-1.06	6.22	6.79	15.44	7.41	-2.51	11.31	20.55	11.02	0.11
os48676	1	393	0.73	15	2	3	6.14	-0.14	0.17	1.49	0.17	98.3	-7.55	6.74	7.07	15.74	7.64	-2.34	11.73	20.98	11.36	0.34
os48757	1	396	1	7	3	5	6.53	-0.11	1.1	1.51	0.06	88.07	-2.02	8.78	7.14	15.86	7.68	-2.13	11.8	21.14	11.43	0.56
os48804	1	374	0.72	14	1	4	6.56	-0.09	0	1.43	0.08	92.71	1.39	6.07	7.24	15.97	7.8	-2.04	11.87	21.27	11.58	0.64
os48856	1	395	0.98	13	2	0	6.3	-0.2	0	1.47	0.11	94.92	0.83	6.2	7.04	15.75	7.59	-2.05	11.7	21.08	11.35	0.62
os48882	1	429	0.61	11	2	3	6.51	-0.03	0.69	1.5	0.16	98.75	-8.25	7.75	6.93	15.64	7.51	-2.11	11.61	20.98	11.26	0.56
os48885	1	442	0.63	15	2	7	6.17	-0.14	0	1.43	0.15	97.4	-2.48	7.65	6.76	15.48	7.36	-2.22	11.43	20.81	11.1	0.46
os48904	1	355	0.23	1	3	6	6.79	0.02	2.75	1.54	0.03	73.9	-0.99	9.26	7.42	16.13	7.95	-1.96	12.05	21.45	11.78	0.75
os48909	1	428	0.44	9	3	5	6.56	-0.03	0.14	1.48	0.06	89.85	1.45	6.75	6.96	15.68	7.58	-2.26	11.5	20.85	11.25	0.36
os48960	1	471	0.87	9	3	5	6.66	-0.09	0.09	1.49	0.07	97.95	0.04	6.8	6.79	15.52	7.37	-2.8	11.45	20.71	11.06	-0.22
os48989	1	500	0.4	4	1	1	6.91	0.05	0.04	1.47	0.02	98.98	3.35	6.17	6.72	15.48	7.31	-2.65	11.4	20.7	11.03	-0.06
os48999	1	497	0.29	4	3	5	6.9	0.05	0.16	1.5	0.08	83.65	1.83	7.52	6.35	15.04	7.06	-3.02	10.94	20.17	10.69	-0.42
os49010	1	541	0.01	12	1	4	6.96	0.18	0	1.44	0.06	96.03	4.71	5.97	6.19	14.87	6.94	-3.08	10.84	20.03	10.6	-0.49
os49066	1	539	0.77	3	3	6	6.84	-0.02	0.47	1.51	0.03	94.4	0.57	7.22	6.18	14.86	6.93	-3.08	10.81	20.02	10.58	-0.5
os49068	1	516	0.35	18	1	4	6.42	0.06	0	1.43	0.11	89.68	1.59	5.24	6.29	14.97	7.02	-3.04	10.92	20.14	10.67	-0.44
os49069	1	525	0.04	14	3	5	7.05	0.22	0.02	1.48	0.08	91.39	-1.62	6.8	6.29	14.97	7.02	-3.04	10.92	20.14	10.66	-0.44
os49084	1	446	0.91	15	1	4	6.38	-0.18	0	1.41	0.11	85.91	4.04	5.99	6.66	15.33	7.31	-2.72	11.19	20.44	10.93	-0.15
os49092	1	380	0.23	8	2	7	6.64	0.05	0.71	1.55	0.15	65.04	-5.42	6.82	6.86	15.53	7.47	-2.49	11.43	20.64	11.11	0.13
os49169	1	554	0.66	6	3	9	6.67	-0.04	0.16	1.51	0	99.65	0.81	6.3	6.27	14.97	6.93	-2.45	10.9	20.21	10.61	0.21
os49264	1	550	0.04	6	2	7	6.98	0.1	0.97	1.54	0.11	98.11	-4.97	7.39	6	14.68	6.77	-2.98	10.39	19.66	10.23	-0.39
os49358	1	451	1	16	2	0	6.07	-0.27	0	1.43	0.15	94.6	2.06	6.51	6.51	15.17	7.18	-2.52	11.03	20.35	10.78	0.13
os49380	1	473	0.7	14	2	7	6.32	-0.1	0.24	1.48	0.17	85.77	-3.93	6.97	6.37	15.04	7.07	-2.56	10.93	20.24	10.69	0.07
os49383	1	502	0.35	13	2	3	6.95	0.12	0.07	1.5	0.13	93.55	-5.97	6.51	6.27	14.95	7.01	-2.61	10.84	20.15	10.65	0.01
os49458	1	513	0.95	13	3	5	6.49	-0.15	0	1.44	0.12	98.84	1.55	5.26	6.24	14.93	6.97	-2.64	10.71	20.05	10.54	-0.03
os49512	1	543	0.42	13	3	5	6.84	0.06	0.02	1.48	0.13	92.73	-2.01	5.92	6.09	14.78	6.85	-2.75	10.69	19.97	10.5	-0.09
os49541	1	453	0.11	16	2	0	6.64	0.16	0	1.46	0.14	99.57	-1.51	5.71	6.61	15.3	7.26	-2.59	11.12	20.39	10.84	0.04
os49593	1	471	0.38	5	1	4	6.6	-0.1	0.35	1.47	0.03	83.17	3.06	7.58	6.58	15.29	7.19	-2.35	11.28	20.61	10.94	0.33
os49599	1	465	0.88	22	3	5	5.76	-0.27	0	1.4	0.13	91.69	2.31	5.4	6.57	15.26	7.21	-2.41	11.21	20.54	10.88	0.27

os49656	1	409	0	20	1	2	6.91	0.31	0	1.43	0.17	91.47	1.6	5.9	6.74	15.38	7.32	-2.3	11.42	20.68	11.07	0.36
os49673	1	400	0.24	12	3	5	6.56	0.02	0.29	1.47	0.09	77.11	-0.82	6.84	6.99	15.67	7.52	-2.02	11.75	21.06	11.34	0.67
os49694	1	497	1	24	1	1	5.66	-0.36	0	1.27	0	98.44	11.8	4.56	6.77	15.45	7.33	-2.13	11.5	20.79	11.14	0.57
os49745	1	660	0.33	3	3	9	6.85	0.01	0.61	1.48	0.03	91.9	1.02	7.09	5.84	14.54	6.56	-2.6	10.36	19.74	10.11	0.04
os49803	1	668	0.63	11	3	5	6.49	-0.09	0.01	1.46	0.1	99.9	0.73	5.98	5.52	14.24	6.31	-2.75	9.95	19.33	9.73	-0.15
os49867	1	523	0.35	14	1	4	6.61	0.09	0	1.4	0.1	94.78	5.96	5.29	6.35	15.1	6.9	-2.16	11.06	20.55	10.55	0.48
os49872	1	607	0.88	5	2	3	6.66	-0.06	1.35	1.52	0.11	96.62	-4.11	8.49	5.93	14.74	6.5	-2.57	10.52	19.98	10.1	0.12
os49886	1	504	0.99	14	2	0	6.17	-0.24	0	1.44	0.12	91.54	0.34	5.8	6.5	15.26	7	-2.05	11.15	20.65	10.6	0.58
os49900	1	569	0.1	16	2	3	6.82	0.19	0	1.47	0.11	89.98	-2.32	7.56	6.22	15.01	6.73	-2.38	10.87	20.37	10.38	0.3
os49907	1	565	1	16	3	5	6.11	-0.24	0	1.41	0.1	95.66	0.68	6.49	6.22	15.02	6.73	-2.39	10.89	20.36	10.39	0.3
os49952	1	530	0.11	13	2	0	7.05	0.18	0	1.48	0	88.14	-1.04	6.34	6.1	14.87	6.67	-2.34	10.77	20.22	10.26	0.28
os49963	1	529	0.92	0	3	6	6.82	0	2.93	1.56	0	43.38	-0.05	9.89	6.3	15.01	6.84	-2.24	11.1	20.5	10.54	0.41
os49969	1	572	0.91	8	2	0	6.71	-0.08	0.26	1.52	0.08	99.98	-0.92	6.65	5.94	14.73	6.52	-2.42	10.39	19.91	10.01	0.2
os49987	1	646	0.82	15	1	4	6.16	-0.2	0	1.42	0.09	92.34	3.45	5.54	5.74	14.47	6.52	-2.7	10.3	19.66	10.09	-0.02
os50095	1	496	0.52	3	3	6	6.8	0.01	0.29	1.53	0.02	97.03	1	7.46	6.78	15.5	7.37	-2	11.48	20.9	11.11	0.78
os50131	1	280	0.78	15	2	3	6.12	-0.17	0	1.49	0.09	34.45	-0.85	6.81	7.91	16.6	8.33	-1.45	12.73	22.02	12.26	1.35
os50140	1	492	0.98	21	2	0	5.7	-0.33	0	1.41	0.16	100	-1.68	5.87	6.6	15.36	7.2	-2.2	11.27	20.71	10.94	0.52
os50159	1	436	0.08	21	2	0	6.7	0.25	0	1.43	0.15	99.6	-0.98	5.39	6.88	15.65	7.49	-1.97	11.54	21	11.2	0.77
os50217	1	496	0.58	16	2	3	6.31	-0.06	0	1.47	0.12	99.81	-3.32	5.99	6.76	15.49	7.34	-1.99	11.56	20.93	11.11	0.79
os50224	1	541	0.01	6	3	5	6.92	0.09	0.76	1.51	0.04	96.44	-1.36	6.86	6.66	15.38	7.21	-2.06	11.43	20.83	10.99	0.7
os50278	1	387	0.97	11	3	5	6.43	-0.16	0.03	1.5	0.07	83.53	-1.09	8.8	7.32	16.02	7.79	-1.66	12.16	21.54	11.64	1.17
os50336	1	593	0.03	11	2	7	6.88	0.13	0.08	1.51	0.11	95.44	-3.74	7.62	6.04	14.75	6.74	-2.51	10.69	20.03	10.38	0.22
os50397	1	448	0.23	5	3	5	6.79	0.04	0.63	1.49	0.04	99.88	-0.52	7.16	7.27	16.05	7.67	-1.46	12.01	21.63	11.39	1.23
os50612	1	409	0.69	15	2	0	6.14	-0.12	0.03	1.42	0.09	89.71	3.24	5.96	7.23	15.97	7.62	-1.68	12.13	21.6	11.42	1.08
os50639	1	551	0.6	3	3	6	6.89	0.01	1.12	1.43	0.03	91.78	0.93	7.51	6.43	15.15	6.95	-2.18	11.17	20.65	10.68	0.54
os50662	1	433	0.03	22	2	0	6.9	0.34	0	1.43	0.17	89.62	-1.04	6.58	6.91	15.63	7.31	-1.84	11.71	21.22	11.11	0.84
os50676	1	501	0.55	11	2	7	6.43	-0.07	0.32	1.53	0.16	95.88	-6.95	6.48	6.46	15.19	6.94	-2.18	11.24	20.7	10.72	0.51
os50722	1	541	0.98	15	3	5	6.12	-0.26	0	1.42	0.08	80.05	1.43	5.83	6.5	15.26	7	-2.09	11.27	20.8	10.75	0.58
os50726	1	464	0.95	10	2	3	6.41	-0.12	0.57	1.51	0.14	97.66	-5.55	7.73	6.79	15.54	7.23	-1.91	11.65	21.15	11	0.77
os50743	1	511	0.89	3	1	4	6.74	-0.03	0.24	1.51	0.02	83.87	2.07	8.71	6.56	15.28	7.05	-2.06	11.27	20.82	10.75	0.61
os50744	1	511	0.6	4	1	4	6.71	-0.04	0.1	1.5	0.03	84.79	2.35	8.29	6.57	15.28	7.05	-2.06	11.27	20.82	10.75	0.61
os50747	1	439	0.89	13	2	3	6.51	-0.14	0.1	1.47	0.1	91	-3.43	6.67	7.1	15.84	7.47	-1.58	11.76	21.33	11.11	1.09
os50871	1	655	0.74	13	2	3	6.35	-0.14	0.01	1.47	0.12	99.99	-2.71	6.62	5.67	14.52	6.32	-2.67	10.21	19.72	9.85	-0.07
os50882	1	574	0.47	9	3	5	6.61	-0.03	0.31	1.48	0.07	95.49	0.74	6.61	6.19	15.02	6.72	-2.35	10.85	20.41	10.36	0.33
os50896	1	523	0.91	8	1	4	6.48	-0.12	0.04	1.41	0.05	97.01	5.4	5.74	6.65	15.46	7.07	-2.07	11.42	21	10.79	0.64
os50924	1	458	0.07	13	3	5	6.17	-0.21	0	1.45	0.09	99.81	0.7	5.33	7.05	15.8	7.42	-1.72	11.81	21.4	11.15	1
os50939	1	396	0.62	5	3	6	6.59	-0.08	0.82	1.54	0.06	64.58	-0.71	8.01	7.27	16.04	7.58	-1.66	12.24	21.81	11.5	1.09

os50940	1	402	0.8	3	3	6	6.78	-0.01	0.39	1.53	0.04	53.99	0.34	8.53	7.26	16.03	7.57	-1.67	12.23	21.79	11.48	1.08
os50960	1	451	1	11	2	7	6.44	-0.15	0.57	1.51	0.15	97.81	-5.15	7.32	6.93	15.72	7.3	-1.83	11.75	21.4	11.09	0.86
os50964	1	511	0.96	26	2	8	5.13	-0.42	0	1.36	0.2	99.53	-3.22	5.05	6.91	15.71	7.28	-1.86	11.75	21.39	11.08	0.83
os50980	1	410	0.16	16	1	4	6.65	0.14	0	1.43	0.09	87.83	2.67	5.58	7.34	16.11	7.6	-1.62	12.39	21.94	11.54	1.17
os50999	1	439	1	21	3	5	5.86	-0.32	0	1.42	0.12	67.72	1.01	5.65	7.06	15.81	7.41	-1.73	11.95	21.49	11.24	0.99
os51002	1	499	0.99	10	3	5	6.44	-0.17	0.09	1.47	0.09	91.74	-0.72	6.48	6.65	15.41	7.11	-2	11.4	20.94	10.81	0.69
os51081	1	665	0.02	9	3	5	7.1	0.16	0.07	1.45	0.08	99.91	0.2	5.86	5.78	14.6	6.42	-2.58	10.31	19.81	9.91	0.02
os51086	1	612	0.51	9	2	0	6.68	0	0.44	1.51	0.09	97.79	-1.99	8.25	5.84	14.66	6.46	-2.56	10.39	19.9	9.98	0.07
os51100	1	509	0.8	14	1	4	6.19	-0.17	0.02	1.43	0.08	99.89	2.48	6.48	6.74	15.56	7.17	-1.99	11.56	21.15	10.91	0.74
os51225	1	637	0.4	8	2	0	7.02	0.07	0.19	1.51	0.1	100	-0.44	6.82	5.4	14.14	6.11	-2.82	9.92	19.24	9.67	-0.18
os51527	1	475	0.89	13	3	5	6.24	-0.19	0	1.44	0.1	100	0.77	5.82	6.76	15.6	7.36	-1.48	11.24	20.65	10.93	1.24
os51589	1	352	0.4	8	2	0	6.86	0.05	0.2	1.52	0.09	81.86	-0.26	6.1	7.27	16.08	7.77	-1.14	11.83	21.28	11.47	1.63
os51597	1	539	0.78	5	3	6	6.73	-0.07	0.54	1.46	0.07	99.78	0.22	6.26	6.24	15.05	6.9	-1.97	10.65	20	10.41	0.72
os51693	1	547	0.86	15	3	5	6.48	-0.16	0	1.44	0.13	99.98	-0.8	5.76	6.13	14.89	6.68	-2.28	10.61	19.97	10.23	0.35
os51794	1	495	0.57	15	3	5	6.14	-0.24	0.01	1.46	0.14	98.11	-2.77	6.67	6.34	15.08	6.83	-2.17	10.87	20.21	10.47	0.46
os51846	1	557	0.91	12	1	4	6.49	-0.17	0.01	1.44	0.1	95.95	1.75	6.35	5.93	14.68	6.5	-2.5	10.56	19.9	10.2	0.16
os51888	1	625	0.51	11	2	3	6.62	0	0.43	1.51	0.14	100	-6.28	7.08	5.48	14.23	6.16	-2.74	10.01	19.31	9.85	-0.09
os51896	1	596	0.3	15	3	5	6.98	0.09	0.02	1.46	0.1	93.3	-0.39	5.94	5.67	14.44	6.33	-2.64	10.21	19.52	9.97	0.01
os51925	1	626	0.33	13	3	9	6.67	0.07	0	1.45	0.07	99.92	-0.02	5.52	5.85	14.6	6.43	-2.57	10.49	19.82	10.16	0.11
os51998	1	587	0.23	12	1	4	6.77	0.11	0	1.4	0.07	98.73	5.13	5.62	5.89	14.6	6.5	-2.47	10.37	19.71	9.98	0.13
os52079	1	589	0.92	12	3	5	6.31	-0.19	0.01	1.47	0.1	99.53	-1.18	6.32	5.87	14.66	6.48	-2.43	10.32	19.68	9.98	0.21
os52111	1	521	0.01	17	2	0	6.03	-0.28	0	1.42	0.15	97.95	0.25	6.77	5.99	14.72	6.57	-2.38	10.56	19.86	10.24	0.29
os52120	1	465	0.64	16	1	2	6.09	-0.11	0	1.4	0.15	97.26	4.57	5.26	6.38	15.11	6.88	-2.16	11.01	20.34	10.54	0.52
os52127	1	760	0.77	6	3	5	6.91	-0.03	0.23	1.5	0.05	95.65	0.51	6.77	4.72	13.51	5.66	-2.97	8.77	18.12	8.9	-0.42
os52188	1	396	0.98	12	3	5	6.37	-0.18	0.01	1.48	0.1	99.77	-0.26	6.29	7.14	16.02	7.54	-1.63	11.75	21.3	11.21	1.04
os52338	1	474	0.76	9	3	5	6.47	-0.09	0.05	1.48	0.08	99.37	1.18	6	6.35	15.07	7.1	-1.4	10.54	19.84	10.36	1.14
os52363	1	433	0.94	9	3	5	6.53	-0.14	0.03	1.48	0.08	97.69	1.38	6.44	6.63	15.32	7.33	-1.22	10.81	20.11	10.59	1.33
os52366	1	351	0.73	12	3	5	6.3	-0.12	0.04	1.5	0.08	80.81	-1.09	6.32	7.15	15.87	7.78	-0.84	11.5	20.83	11.19	1.78
os52528	1	599	0.11	12	2	7	6.84	0.12	0.06	1.47	0.12	100	-3.17	7.22	5.6	14.4	6.39	-2.16	9.95	19.24	9.82	0.48
os52635	1	430	1	6	2	0	6.6	-0.1	0.59	1.54	0.06	100	-1.16	7.88	6.64	15.39	7.33	-1.29	10.95	20.28	10.78	1.37
os52824	1	457	0.33	13	3	5	6.5	0.03	0	1.46	0.09	99.58	-0.66	6.1	6.85	15.62	7.28	-1.83	11.46	20.85	10.98	0.88
os52858	1	579	0.7	18	1	4	6.59	-0.09	0	1.36	0.11	99.48	4.33	5.66	6.07	14.84	6.65	-2.27	10.53	19.87	10.14	0.36
os52874	1	379	1	16	1	2	6.07	-0.26	0	1.43	0.15	96.37	2.77	5.18	7.06	15.86	7.43	-1.7	11.74	21.24	11.16	1.01
os52917	1	419	0.02	10	3	5	6.99	0.18	0.07	1.47	0.09	98.36	-0.56	6.21	6.99	15.76	7.38	-1.7	11.63	21.06	11.1	0.99
os52922	1	417	0.78	11	2	3	6.28	-0.15	0.09	1.5	0.15	99.99	-4.39	6.59	6.94	15.75	7.36	-1.73	11.51	20.96	10.99	0.92
os52929	1	396	0.23	6	2	0	6.77	0.05	0.61	1.53	0.08	99.35	-0.51	7.15	7.05	15.81	7.45	-1.64	11.49	20.95	10.99	0.96
os52930	1	366	0.62	10	2	0	6.37	-0.09	0.03	1.51	0.08	81.71	-1.41	6.24	7.29	16.06	7.64	-1.51	11.93	21.39	11.34	1.15

os52959	1	476	0.86	9	3	5	6.44	-0.13	0.12	1.45	0.08	99.39	0.16	6.07	6.82	15.66	7.26	-1.79	11.44	20.94	10.95	0.92
os52979	1	385	0.47	8	2	3	6.78	0	0.57	1.51	0.14	98.83	-4.86	6.28	7.26	16.05	7.63	-1.48	11.89	21.32	11.31	1.21
os53022	1	442	0.01	15	1	4	6.89	0.23	0	1.42	0.11	98.15	2.93	5.27	6.88	15.7	7.31	-1.76	11.5	21	11	0.95
os53034	1	440	0.55	21	2	7	6.61	0.02	0	1.43	0.21	97.97	-4.9	6.27	6.41	15.14	6.9	-2.13	11.04	20.37	10.57	0.54
os53072	1	372	0.76	25	2	3	5.37	-0.26	0	1.41	0.18	85.78	-3.65	5.59	7.33	16.15	7.67	-1.52	11.92	21.46	11.37	1.14
os53122	1	617	0.7	15	1	1	6.27	-0.12	0	1.38	0.08	99.93	2.27	5.61	6.11	14.87	6.69	-2.22	10.53	19.89	10.16	0.4
os53125	1	358	0.04	21	2	7	6.73	0.3	0	1.43	0.19	88.75	-2.13	5.63	7.14	15.93	7.51	-1.64	11.82	21.32	11.23	1.08
os53138	1	520	0.99	13	3	5	6.39	-0.19	0	1.44	0.11	100	1.62	5.63	6.3	15.09	6.84	-2.06	10.73	20.2	10.36	0.6
os53175	1	424	0.63	17	2	7	6.68	-0.01	0	1.45	0.23	99.95	-4.4	5.24	6.87	15.68	7.28	-1.84	11.52	21.04	10.99	0.86
os53191	1	458	0.09	13	2	0	7.02	0.17	0.01	1.47	0.13	99.35	-0.19	5.74	6.55	15.35	7.05	-1.95	11.11	20.51	10.67	0.71
os53209	1	389	0.28	12	2	0	6.64	0.09	0.01	1.48	0.1	98.8	-1.07	5.68	7.09	15.88	7.49	-1.64	11.69	21.15	11.15	1.03
os53224	1	377	0.67	16	2	3	6.05	-0.17	0	1.47	0.11	85.5	-2.42	6.19	7.32	16.09	7.66	-1.49	11.97	21.44	11.37	1.17
os53233	1	375	0.02	5	2	3	6.84	0.08	0.91	1.52	0.07	98.67	-1.91	6.66	7.43	16.28	7.78	-1.47	12.13	21.73	11.53	1.22
os53284	1	378	0.76	3	3	6	6.78	0	2.02	1.55	0.07	88.8	-1.53	7.73	7.28	16.06	7.63	-1.52	11.96	21.42	11.36	1.2
os53286	1	360	0.96	18	3	5	6.12	-0.24	0	1.44	0.13	79.55	-2.06	5.75	7.52	16.33	7.8	-1.4	12.29	21.8	11.63	1.32
os53307	1	310	0.01	10	2	3	6.85	0.15	0.14	1.51	0.09	68.78	-1.47	6.67	7.69	16.51	7.95	-1.29	12.5	22.06	11.8	1.47
os53329	1	365	0	17	3	5	6.88	0.28	0	1.4	0.1	93.71	0.96	5.31	7.53	16.38	7.83	-1.37	12.33	21.89	11.66	1.34
os53337	1	403	0.48	11	1	2	6.84	0.04	0	1.45	0.1	98.75	3.18	5.29	7.18	16	7.54	-1.58	11.88	21.41	11.3	1.13
os53389	1	409	0.73	15	2	3	6.6	-0.07	0.01	1.47	0.13	99.8	-4.27	5.91	7.11	15.95	7.49	-1.59	11.74	21.25	11.19	1.1
os53408	1	385	0.09	13	2	0	6.76	0.15	0	1.44	0.11	98.96	1.16	5.68	7.09	15.89	7.5	-1.63	11.55	21.02	11.09	1.03
os53429	1	336	0.77	9	2	0	6.65	-0.06	0.16	1.5	0.09	98.22	-1.64	6.78	7.55	16.45	7.88	-1.35	12.18	21.84	11.66	1.38
os53431	1	310	0.07	8	3	5	6.94	0.14	0.16	1.51	0.07	97.39	-0.55	6.34	7.78	16.62	8.04	-1.19	12.41	22.02	11.82	1.57
os53433	1	304	0.08	9	3	5	6.94	0.14	0.15	1.51	0.07	95.85	-0.41	6.42	7.8	16.64	8.06	-1.18	12.44	22.04	11.84	1.58
os53436	1	302	0.03	11	3	5	6.95	0.17	0.03	1.47	0.07	95.18	1.2	6.97	7.94	16.76	8.17	-1.07	12.68	22.26	11.99	1.7
os53440	1	295	0.33	12	3	5	6.54	0.04	0.12	1.48	0.1	89.71	-0.66	6.92	7.89	16.71	8.12	-1.09	12.58	22.15	11.91	1.66
os53493	1	388	0	9	3	5	6.92	0.15	0.09	1.49	0.08	99.74	-0.92	6.69	7.4	16.26	7.7	-1.48	12.01	21.64	11.42	1.23
os53524	1	381	0.01	15	2	3	6.92	0.25	0	1.45	0.12	96.74	-1.15	5.61	7.34	16.24	7.68	-1.55	12.07	21.75	11.52	1.16
os53533	1	413	0.93	17	2	0	6.21	-0.22	0	1.44	0.18	99.6	-1.82	6.32	6.84	15.63	7.31	-1.72	11.19	20.66	10.81	0.94
os53540	1	348	0.99	10	3	5	6.42	-0.16	0.03	1.48	0.09	96.59	-0.91	5.84	7.53	16.38	7.85	-1.39	12.15	21.68	11.61	1.37
os53570	1	544	0.89	6	3	5	6.65	-0.1	0.43	1.48	0.06	99.6	0.37	6.64	6.24	15.05	6.83	-2.05	10.42	19.89	10.22	0.55
os53572	1	317	0.02	11	2	7	6.86	0.18	0.22	1.52	0.13	90.74	-3.12	7.95	7.6	16.45	7.91	-1.38	12.27	21.87	11.71	1.38
os53573	1	347	0.81	16	3	5	6.48	-0.12	0	1.44	0.09	97.53	1.86	5.28	7.64	16.48	7.93	-1.36	12.31	21.91	11.76	1.4
os53618	1	434	1	6	2	0	6.59	-0.1	0.67	1.53	0.06	100	-0.81	7.83	6.57	15.32	7.27	-1.34	10.85	20.18	10.7	1.31
os53625	1	305	0.08	8	3	5	6.94	0.14	0.13	1.51	0.06	96.06	0.04	6.27	7.8	16.64	8.06	-1.18	12.44	22.04	11.84	1.58
os53630	1	385	0.12	7	3	5	6.77	0.07	0.41	1.51	0.08	98.21	-0.84	6.99	7.31	16.19	7.65	-1.55	11.92	21.53	11.35	1.15
os53639	1	305	0.04	8	3	5	6.83	0.11	0.27	1.5	0.04	94.84	1.39	7.27	8.17	17.11	8.35	-1.03	12.98	22.76	12.39	1.84
os53648	1	238	0.47	7	2	3	6.55	0	1.02	1.54	0.12	90.49	-5.35	7.96	8.52	17.47	8.62	-0.8	13.49	23.22	12.76	2.13

os53702	1	297	0.63	18	2	3	6.66	0.01	0	1.46	0.13	73.73	-2.85	6.34	8.08	17.05	8.29	-1.09	12.88	22.71	12.33	1.77
os53730	1	296	0.1	9	3	5	6.73	0.09	0.16	1.51	0.06	90.4	-0.39	6.83	8.17	17.13	8.37	-1.04	12.99	22.79	12.39	1.85
os53783	1	430	1	18	3	9	5.99	-0.29	0	1.43	0.12	99.98	-0.73	4.98	7.27	16.13	7.78	-1.11	11.89	21.38	11.54	1.62
os53789	1	434	0.96	13	3	9	6.44	-0.16	0.03	1.47	0.1	96.47	-1.12	5.24	7.14	16	7.67	-1.19	11.74	21.22	11.42	1.52
os53805	1	476	0.92	7	3	9	6.55	-0.11	0.44	1.48	0.06	99.97	-0.45	6.01	6.92	15.82	7.51	-1.4	11.53	21.04	11.24	1.38
os53816	1	425	0.98	11	2	3	6.29	-0.18	0.08	1.51	0.11	96.01	-3.48	6.39	7.03	15.91	7.59	-1.25	11.6	21.08	11.3	1.46
os53824	1	667	0.23	6	3	5	6.86	0.04	0.46	1.51	0.04	97.73	0.32	6.62	5.52	14.39	6.17	-2.83	9.86	19.35	9.61	-0.26
os53826	1	665	0.23	5	3	6	6.86	0.04	0.62	1.51	0.04	97.93	0.39	6.5	5.54	14.4	6.19	-2.82	9.88	19.37	9.63	-0.25
os53963	1	633	0	7	3	9	7.03	0.13	0.43	1.5	0.05	97.15	-0.37	6.49	5.83	14.69	6.42	-2.68	10.47	19.87	10.09	-0.03
os54179	1	429	0.62	7	2	0	6.55	-0.06	0.3	1.52	0.1	97.93	-0.74	7.02	6.66	15.47	7.22	-1.67	11.12	20.53	10.78	1
os54193	1	515	0.35	4	3	6	6.94	0.06	1.17	1.5	0.03	74.53	0.51	7.98	6.47	15.37	7.11	-1.91	10.96	20.49	10.68	0.82
os54197	1	447	0.51	15	2	0	6.27	-0.06	0	1.47	0.12	92.08	-1.15	6.1	6.51	15.31	7.06	-1.84	10.95	20.36	10.6	0.8
os54214	1	518	0.75	7	2	0	6.58	-0.08	0.35	1.53	0.09	79.73	-0.76	6.84	6.17	15	6.71	-2.34	10.75	20.17	10.36	0.31
os54218	1	427	0.27	3	3	6	6.85	0.02	1.54	1.53	0.03	94.14	-0.13	7.27	7	15.95	7.57	-1.38	11.6	21.31	11.36	1.37
os54253	1	460	0.59	5	3	6	6.78	-0.03	0.63	1.49	0.04	75.73	0.26	8.49	7.09	16.13	7.52	-1.68	11.83	21.55	11.38	1.18
os54338	1	355	0.69	11	3	5	6.65	-0.05	0.02	1.49	0.11	99.94	-1.4	5.47	7.47	16.37	7.92	-1.06	12.08	21.71	11.7	1.72
os54412	1	501	0.27	8	2	0	6.69	0.02	0.39	1.5	0.08	82.99	-0.86	9.35	6.42	15.34	7.05	-1.89	10.89	20.47	10.65	0.83
os54462	1	373	0.08	1	3	6	6.8	0.02	1.5	1.54	0.03	80.96	-0.26	7.92	7.3	16.18	7.83	-1.09	11.9	21.5	11.66	1.68
os54547	1	275	1	10	3	5	6.33	-0.16	0.07	1.51	0.07	94.06	-1.58	6.07	7.98	16.88	8.33	-0.71	12.79	22.48	12.35	2.17
os54628	1	375	0.99	6	3	5	6.58	-0.1	0.67	1.51	0.06	97.53	-0.72	7.16	7.45	16.33	7.92	-1.1	12.07	21.67	11.68	1.71
os54672	1	311	0.96	10	3	5	6.49	-0.13	0.08	1.49	0.07	98.78	-0.8	6.2	7.92	16.83	8.35	-0.75	12.62	22.27	12.22	2.16
os54674	1	323	0.93	10	3	5	6.51	-0.12	0.08	1.47	0.06	98.8	0.7	6.08	7.91	16.83	8.34	-0.75	12.61	22.26	12.21	2.15
os54677	1	279	0.85	8	2	3	6.41	-0.11	0.44	1.52	0.08	98.92	-2.67	6.77	7.99	16.9	8.41	-0.7	12.72	22.36	12.3	2.21
os54849	1	322	0.62	5	3	5	6.56	-0.07	0.53	1.52	0.03	84.36	0.47	7.99	7.7	16.59	8.15	-0.87	12.48	22.12	12.15	1.97
os54989	1	470	0.92	1	3	6	6.79	-0.01	1.19	1.5	0.01	80.46	0.37	8.85	6.94	15.88	7.49	-1.48	11.52	21.13	11.22	1.27
os55002	1	367	0.97	9	2	0	6.53	-0.13	0.03	1.49	0.09	95.97	1.18	6.45	7.41	16.33	7.85	-1.1	12.03	21.72	11.66	1.7
os55005	1	424	0.93	6	3	5	6.68	-0.07	0.61	1.5	0.04	98.55	-0.53	6.81	7.22	16.16	7.71	-1.23	11.8	21.46	11.47	1.56
os55017	1	460	0.4	4	3	6	6.85	0.01	0.46	1.51	0.05	93.6	0.49	8.06	6.87	15.78	7.43	-1.47	11.3	20.9	11.02	1.27
os55019	1	457	0.77	6	3	5	6.81	-0.02	0.32	1.5	0.05	92.87	0.12	8.27	6.87	15.78	7.43	-1.47	11.3	20.9	11.02	1.27
os55093	1	325	0.92	4	3	6	6.59	-0.06	0.59	1.54	0.06	97.46	-0.25	6.68	7.61	16.53	8.06	-0.95	12.38	22	11.97	1.87
os55172	1	377	0.81	14	3	5	6.2	-0.16	0.01	1.44	0.09	97.59	-0.49	7.08	7.58	16.53	8.03	-1.07	12.29	21.99	11.89	1.78
os55173	1	345	0.4	20	2	7	6.74	0.1	0	1.46	0.18	99.61	-4.57	6.5	7.55	16.51	8.01	-1.08	12.27	21.95	11.86	1.76
os55272	1	278	0.03	8	3	5	6.4	-0.14	0.25	1.51	0.06	92.29	-1.4	8.48	8.09	17	8.44	-0.73	12.92	22.61	12.45	2.23
os55274	1	268	0.4	7	3	5	6.85	0.06	0.52	1.52	0.06	96.44	-0.41	8.03	8.1	17.01	8.45	-0.7	12.88	22.57	12.41	2.24
os55413	1	288	0.99	8	3	5	6.46	-0.13	0.11	1.48	0.06	97.12	0.4	7.7	8.02	16.94	8.4	-0.75	12.79	22.48	12.34	2.19
os55446	1	300	0.11	4	2	0	6.8	0.06	1.92	1.55	0.13	88.78	-2	7.29	7.74	16.68	8.15	-0.98	12.51	22.22	12.1	1.94
os55460	1	269	0.3	7	3	5	6.86	0.07	0.08	1.51	0.05	83.36	0.84	7.26	8.2	17.18	8.49	-0.74	13.12	22.9	12.6	2.22

os55462	1	300	0.15	8	2	3	6.9	0.1	1.05	1.51	0.11	95.59	-4.26	8.09	7.81	16.7	8.24	-0.83	12.48	22.09	12.07	2.05
os55486	1	320	0.38	4	2	3	6.68	-0.04	1.52	1.53	0.09	97.25	-3.65	8.18	7.8	16.75	8.19	-0.94	12.6	22.33	12.2	1.99
os55494	1	295	0.6	12	3	5	6.31	-0.08	0	1.47	0.06	85.35	1.39	6.41	8.05	17.01	8.41	-0.78	12.95	22.67	12.48	2.18
os55511	1	304	0.81	4	3	9	6.61	-0.04	0.53	1.47	0.02	91.56	1.14	6.95	8.13	17.09	8.47	-0.72	13.03	22.74	12.53	2.24
os55517	1	571	0.77	4	3	6	6.82	-0.03	1.32	1.55	0.06	83.14	-0.7	8.1	5.92	14.75	6.51	-2.62	10.58	19.97	10.23	0.07
os55519	1	665	0.62	8	3	5	6.93	0	0.33	1.5	0.05	98.42	0.34	6.61	5.45	14.3	6.14	-2.95	9.94	19.34	9.72	-0.27
os55580	1	501	0.01	6	2	7	6.85	0.06	1.59	1.54	0.13	98.68	-5.15	8.55	6.27	15.08	6.78	-2.29	10.69	20.1	10.3	0.36
os55596	1	597	0.01	3	3	6	6.92	0.05	1.36	1.53	0.04	91.6	-0.24	9.14	5.84	14.69	6.43	-2.72	10.56	19.97	10.17	-0.07
os55634	1	692	0.4	11	3	5	6.67	0.02	0.02	1.49	0.09	98.86	-1.68	5.63	5.26	14.08	6.02	-2.9	9.54	18.94	9.45	-0.25
os55673	1	611	0.47	4	1	4	6.91	0	0.07	1.45	0.03	95.67	2.75	6.54	5.78	14.6	6.4	-2.73	10.31	19.72	10.02	-0.08
os55677	1	621	0.37	10	3	5	6.71	0.02	0.06	1.48	0.05	99.82	1.6	6.9	5.84	14.7	6.43	-2.66	10.51	19.92	10.13	-0.04
os55683	1	741	0.31	5	3	5	7.04	0.05	0.65	1.51	0.04	100	-0.43	7.25	5	13.89	5.8	-3.13	9.26	18.67	9.19	-0.51
os55697	1	520	0.93	9	1	1	6.52	-0.15	0.1	1.4	0.06	97.78	3.55	6.01	6.61	15.43	7.06	-2.08	11.06	20.51	10.64	0.6
os55701	1	530	0.24	14	2	8	6.58	0.06	0.01	1.44	0.12	99.35	-3.9	6.63	6.46	15.28	6.96	-2.13	10.89	20.3	10.48	0.54
os55829	1	523	0.99	21	2	0	5.93	-0.3	0.16	1.46	0.15	76.22	-3.98	7.24	6.35	15.22	6.86	-2.26	10.87	20.3	10.48	0.41
os55830	1	528	0.51	15	2	0	6.3	-0.07	0	1.45	0.1	80.66	1.14	6.8	6.37	15.23	6.88	-2.24	10.87	20.31	10.49	0.44
os55871	1	535	0.01	7	3	5	6.95	0.11	0.74	1.51	0.06	94.05	-1.44	8.52	6.34	15.25	6.86	-2.23	10.73	20.3	10.41	0.47
os55978	1	419	0.65	8	3	5	6.77	-0.03	0.36	1.47	0.08	97.8	-0.87	7.21	7.13	15.95	7.53	-1.69	11.69	21.18	11.19	1.01
os55983	1	315	0.39	12	2	0	6.91	0.1	0.06	1.5	0.11	95.52	-1.74	6.77	7.65	16.47	7.95	-1.29	12.23	21.76	11.67	1.42
os56004	1	283	0.01	6	3	5	6.81	0.08	1.13	1.53	0.07	82.59	-0.46	9.37	8.05	17.04	8.26	-1.08	12.82	22.6	12.19	1.76
os56065	1	536	0.03	13	2	3	7.04	0.21	0.01	1.49	0.1	89.22	-1.83	6.91	6.25	15.1	6.75	-2.46	10.92	20.4	10.51	0.29
os56105	1	423	0.4	14	3	5	6.4	0	0	1.44	0.11	96.26	1.44	6.47	7.16	16.11	7.64	-1.42	11.85	21.54	11.51	1.41
os56134	1	353	0.99	12	3	5	6.27	-0.2	0.01	1.46	0.1	98.64	-0.59	6.36	7.65	16.64	8.06	-1.12	12.45	22.23	12.03	1.81
os56159	1	308	0.23	17	2	7	6.46	0.11	0.01	1.49	0.17	87.94	-6.01	6.43	7.78	16.78	8.18	-1	12.59	22.37	12.16	1.94
os56193	1	510	0.8	6	1	1	6.74	-0.05	0	1.41	0.02	95.16	5.49	6.53	6.8	15.67	7.33	-1.67	11.35	20.9	11.04	1.06
os56309	1	330	0.85	6	3	5	6.64	-0.06	0.95	1.53	0.07	99.88	-1.9	8.33	7.75	16.72	8.13	-1.06	12.56	22.32	12.11	1.85
os56369	1	531	0.67	2	3	6	6.77	-0.04	2.46	1.54	0.04	93.31	-1.56	8.69	6.41	15.28	6.91	-2.1	11.11	20.57	10.67	0.66
os56374	1	543	0.4	5	3	5	6.94	0.04	0.92	1.53	0.06	96.67	-1.23	8	6.31	15.17	6.82	-2.17	10.93	20.38	10.5	0.56
os56376	1	605	0	4	3	6	7	0.08	0.9	1.49	0.05	95.95	-0.4	7.78	6.04	14.95	6.66	-2.26	10.52	20.02	10.2	0.44
os56390	1	315	0.86	7	3	5	6.5	-0.1	0.97	1.51	0.06	97.55	-2.06	7.93	7.9	16.91	8.25	-0.92	12.75	22.59	12.31	1.99
os56464	1	526	0.93	1	3	6	6.81	-0.01	1.08	1.54	0.01	35.81	0.1	9.1	6.58	15.54	7.18	-1.9	11.17	20.8	10.9	0.94
os56469	1	484	0.04	4	2	7	6.92	0.07	2.15	1.54	0.08	96	-3.78	9.39	6.69	15.64	7.26	-1.82	11.3	20.94	11	1.04
os56499	1	365	0.16	17	2	7	6.59	0.15	0	1.45	0.14	97.51	-1.96	5.8	7.07	15.88	7.48	-1.65	11.53	21	11.07	1.01
os56670	1	602	0.21	5	3	5	6.98	0.05	0.37	1.52	0.02	99.49	1.17	6.91	6.2	15.04	6.72	-2.31	10.81	20.2	10.34	0.45
os56871	1	670	0.77	5	3	6	6.8	-0.06	0.69	1.53	0.04	97.74	-0.2	7.46	5.43	14.32	6.09	-3.03	9.98	19.42	9.67	-0.42
os56882	1	556	0.83	6	3	5	6.79	-0.05	0.63	1.52	0.04	99.93	-0.17	6.61	6.15	15.01	6.78	-2.24	10.55	20	10.27	0.4
os56922	1	684	0.15	14	3	5	7.14	0.2	0	1.47	0.1	100	-0.46	5.46	5.62	14.3	6.24	-1.93	9.87	19.17	9.62	0.6

os56985	1	514	0.18	10	2	0	7.03	0.13	0.02	1.48	0.14	88.05	1.01	6.47	6.64	15.37	7.06	-1.59	11.38	20.7	10.9	1.15
os57065	1	598	0.76	10	2	3	6.54	-0.14	0.97	1.52	0.12	98.71	-4.87	8.01	6.26	15.02	6.76	-1.83	10.95	20.24	10.56	0.91
os57279	1	562	0.89	17	1	4	6.44	-0.15	0	1.33	0.09	98.5	12.14	4.84	6.58	15.31	6.89	-1.64	11.43	20.69	10.85	1.15
os57498	1	540	0.59	25	2	7	6.62	0.02	0	1.41	0.23	96.27	-3.18	6.3	6.5	15.17	6.94	-1.57	11.25	20.42	10.69	1.06
os57523	1	591	0.42	13	2	3	6.26	-0.19	0.06	1.45	0.12	96.48	-3.03	7.13	6.38	15.11	6.84	-1.68	11.05	20.35	10.59	0.94
os57527	1	648	0.01	9	1	4	6.83	0.07	0.04	1.44	0.06	90.62	3.46	7.06	5.97	14.68	6.52	-2.04	10.67	19.92	10.3	0.63
os57616	1	644	0.77	1	3	9	6.87	-0.01	0.47	1.49	0.01	70.64	0.19	8.15	6.21	14.96	6.72	-1.86	11.02	20.3	10.63	0.87
os57622	1	715	0.01	11	1	1	7.13	0.18	0.02	1.44	0.04	97.06	4.73	5.85	5.8	14.53	6.36	-2.19	10.52	19.77	10.19	0.54
os57752	1	652	0.04	11	3	9	7.09	0.19	0.03	1.44	0.06	99.9	-0.31	5.56	6.34	15.04	6.73	-1.76	11.17	20.4	10.63	1.05
os57764	1	581	1	12	1	4	6.42	-0.19	0	1.45	0.09	99.78	3.27	5.51	6.43	15.18	6.9	-1.73	11.15	20.44	10.71	1.02
os57793	1	584	0.21	9	3	5	6.79	0.07	0.12	1.48	0.08	92.07	0.77	7.29	6.43	15.16	6.89	-1.6	11.09	20.38	10.62	1.01
os57804	1	674	0.22	6	3	5	7.05	0.08	0.45	1.52	0.06	96.13	-0.79	7.23	5.76	14.42	6.3	-2.16	10.54	19.69	10.15	0.57
os57817	1	649	0.29	3	3	6	6.85	0	2.28	1.54	0.02	65.41	0.69	8.52	6.04	14.77	6.53	-2.03	10.86	20.11	10.42	0.74
os57878	1	459	0.99	12	3	5	6.31	-0.2	0.02	1.43	0.09	94.82	-0.97	5.37	7.41	16.12	7.66	-0.9	12.04	21.4	11.32	1.63
os57894	1	556	0.02	22	2	0	6.93	0.34	0	1.4	0.18	97.1	-2.42	5.67	6.41	15.1	6.87	-1.52	11.01	20.27	10.51	1.04
os58027	1	589	0.83	14	2	0	6.58	-0.13	0	1.48	0.11	97.5	-0.14	7.23	6.37	15.17	6.88	-1.7	11.08	20.48	10.78	1.02
os58063	1	636	0.37	2	3	6	6.94	0.03	2.51	1.53	0.03	53.04	0.07	8.26	6.22	15	6.76	-1.87	10.97	20.3	10.65	0.83
os58067	1	593	0.5	18	2	3	6.29	-0.04	0.07	1.44	0.12	99.02	-1.12	6	6.35	15.09	6.85	-1.75	11.08	20.4	10.71	0.99
os58095	1	604	0.01	13	3	5	6.99	0.2	0.01	1.48	0.09	99.7	-0.66	6.18	6.29	15.01	6.78	-1.82	11.05	20.33	10.66	0.93
os58096	1	618	0.08	11	3	5	7.1	0.17	0.01	1.46	0.08	99.17	0.78	6.33	6.28	15	6.78	-1.82	11.06	20.33	10.66	0.92
os58118	1	642	0.33	11	1	4	6.49	-0.05	0.01	1.43	0.1	99.96	1.93	6.47	6.13	14.98	6.7	-1.83	10.78	20.23	10.53	0.87
os58153	1	654	0.14	13	1	4	6.82	0.13	0	1.43	0.09	99.29	3.55	5.39	5.99	14.74	6.55	-1.81	10.55	19.82	10.18	0.79
os58161	1	565	0.23	4	3	6	6.85	0.04	0.41	1.5	0.04	97.57	0.92	7.38	6.6	15.34	7.03	-1.56	11.28	20.6	10.78	1.07
os58198	1	552	0.72	7	2	7	6.79	-0.04	1.06	1.52	0.11	94.96	-4.26	8.03	6.55	15.27	6.98	-1.54	11.21	20.5	10.72	1.08
os58437	1	442	0.47	13	2	0	6.4	-0.03	0	1.47	0.16	68.2	-0.01	5.83	7.1	15.79	7.41	-1.11	11.62	20.98	11.03	1.4
os58480	1	708	0.08	15	1	1	6.92	0.18	0	1.37	0.05	99.99	5.19	5.22	6.16	14.91	6.69	-1.87	10.87	20.17	10.54	0.87
os58574	1	693	0.92	2	3	6	6.87	-0.01	1.88	1.52	0.04	91.12	0.04	8.4	5.89	14.73	6.46	-1.99	10.48	19.9	10.23	0.73
os58799	1	653	0.12	21	3	5	6.97	0.31	0	1.41	0.14	98.35	-0.78	5.28	5.93	14.6	6.5	-1.9	10.49	19.69	10.15	0.68
os59058	1	625	0.99	20	1	4	5.99	-0.3	0	1.43	0.14	99.95	-0.2	7.36	6.25	14.98	6.68	-1.65	10.87	20.13	10.26	0.87
os59067	1	683	0.01	9	3	5	7.08	0.16	0.03	1.48	0.08	100	0.89	5.6	5.64	14.33	6.26	-1.92	9.91	19.21	9.65	0.62
os59070	1	697	0.16	17	3	5	7.08	0.24	0	1.44	0.12	100	0.13	5.27	5.64	14.33	6.26	-1.92	9.91	19.21	9.65	0.62
os59073	1	683	0	14	3	5	7.06	0.23	0	1.49	0.1	100	-1.46	5.76	5.67	14.36	6.28	-1.91	9.96	19.24	9.68	0.62
os59084	1	662	0.73	8	3	5	6.97	0.03	0.26	1.47	0.08	99.12	-0.72	7.07	5.97	14.67	6.45	-1.99	10.67	19.88	10.23	0.75
os59216	1	436	0.41	9	2	3	6.65	0.03	0.24	1.52	0.07	100	-2.26	6.99	7.35	16.22	7.6	-1.54	11.97	21.55	11.23	1.14
os59486	1	398	0.99	1	3	6	6.76	-0.01	0.32	1.48	0.01	67.1	0.87	9.03	7.56	16.46	7.82	-1.3	12.26	21.95	11.67	1.45
os59704	1	460	0.74	11	3	5	6.35	-0.17	0.03	1.47	0.07	97.68	-0.3	5.87	7.24	16.08	7.51	-1.54	11.77	21.37	11.17	1.18
os59795	1	538	0.15	9	2	7	6.8	0.08	0.75	1.51	0.13	97.36	-4.51	8.5	6.58	15.52	7	-2.03	11.08	20.75	10.61	0.67

os59798	1	540	0.04	11	2	7	7.06	0.18	0.04	1.49	0.11	91.36	-2.07	7.8	6.5	15.44	6.93	-2.09	10.99	20.65	10.52	0.61
os59857	1	543	0.41	7	3	5	6.93	0.04	0.79	1.53	0.07	99	-1.69	8.68	6.62	15.56	7.04	-2.06	11.17	20.83	10.67	0.68
os59948	1	486	0.45	7	3	5	6.65	0	0.27	1.51	0.08	96.09	-0.76	6.72	6.9	15.79	7.25	-1.82	11.51	21.14	10.98	0.94
os59968	1	398	0.7	12	3	5	6.7	-0.04	0	1.45	0.06	90.44	2.26	7.74	7.47	16.4	7.76	-1.39	12.16	21.86	11.59	1.36
os60029	1	371	0.73	8	3	5	6.74	-0.03	0.14	1.49	0.06	96.84	0.63	6.04	7.68	16.61	7.91	-1.22	12.45	22.11	11.78	1.58
os60030	1	373	0.66	9	3	5	6.76	-0.02	0.06	1.48	0.06	97.16	1.6	5.86	7.67	16.61	7.91	-1.22	12.45	22.11	11.78	1.58
os60058	1	366	0.88	11	3	5	6.49	-0.14	0.01	1.48	0.07	98.48	0.08	6.83	7.85	16.79	8.05	-1.12	12.73	22.37	12.02	1.75
os60100	1	343	0.01	1	3	6	6.77	0.01	2.79	1.52	0.02	43.85	-0.29	8.35	8.04	16.95	8.19	-0.98	12.98	22.59	12.19	1.92
os60239	1	405	0.68	7	3	9	6.77	-0.02	0.19	1.42	0.06	99.28	1.64	6.38	8.01	16.99	8.04	-1.15	12.86	22.53	11.87	1.6
os60245	1	344	0.85	13	2	7	6.19	-0.17	0	1.5	0.14	86.93	-4.94	6.09	8.05	17	8.08	-1.11	12.64	22.26	11.68	1.52
os60246	1	402	0.66	15	1	1	6.19	-0.09	0	1.38	0.06	94.77	6.1	5.27	8.14	17.08	8.15	-1.09	12.97	22.57	11.95	1.64
os60296	1	320	0.74	6	2	7	6.75	0	1.57	1.54	0.18	97.43	-5.46	7.83	8.19	17.15	8.18	-1.05	12.89	22.55	11.88	1.66
os60310	1	356	0.59	11	3	5	6.41	-0.07	0.02	1.48	0.08	99.58	-0.09	6.75	8.07	17.04	8.11	-1.12	12.73	22.4	11.75	1.59
os60318	1	306	0.98	19	2	3	5.91	-0.28	0	1.46	0.15	96.59	-3.6	5.72	8.39	17.39	8.35	-0.87	13.09	22.77	11.97	1.83
os60336	1	424	0.97	16	3	9	6.26	-0.22	0.01	1.4	0.08	99.28	1.97	6.13	7.97	16.99	8.04	-1.1	12.56	22.27	11.56	1.58
os60695	1	593	0.11	3	3	6	6.95	0.04	1.23	1.54	0.03	95.49	-0.52	7.89	6.39	15.32	6.84	-2.2	10.89	20.53	10.43	0.53
os60813	1	567	0.1	6	3	9	7	0.09	0.46	1.49	0.05	98.79	-0.68	6.35	6.62	15.53	7.03	-2.02	11.13	20.76	10.63	0.75
os60831	1	460	0.32	11	3	5	6.34	-0.18	0.02	1.47	0.07	97.49	-0.18	5.93	7.24	16.08	7.51	-1.54	11.77	21.37	11.17	1.18
os60835	1	485	0.97	5	3	6	6.73	-0.06	0.95	1.52	0.05	99.68	-0.72	8.16	6.92	15.87	7.29	-1.75	11.5	21.17	11	0.95
os60863	1	406	0.96	15	2	0	6.24	-0.22	0	1.45	0.12	91.8	0.17	6.15	7.39	16.21	7.75	-1.32	12.17	21.9	11.55	1.42
os60908	1	277	0.96	8	2	3	6.4	-0.15	0.23	1.52	0.08	93.92	-1.88	6.15	8.24	16.98	8.42	-0.77	13.16	22.66	12.42	2.01
os60945	1	317	0.92	1	3	6	6.73	0	3.84	1.55	0.02	22.44	0.11	8.44	8.1	16.89	8.3	-0.84	13.09	22.73	12.26	2.01
os60954	1	335	0.92	2	3	6	6.68	-0.04	1.35	1.55	0.02	91.4	0.04	8.11	7.98	16.79	8.2	-0.93	12.95	22.59	12.16	1.86
os60959	1	340	1	4	3	6	6.64	-0.06	0.66	1.54	0.02	89.26	-0.1	8.36	7.96	16.76	8.18	-0.94	12.92	22.56	12.13	1.85
os60968	1	358	0.99	2	3	6	6.7	-0.03	1.49	1.55	0.02	95.59	-0.14	8.46	7.86	16.66	8.11	-1.01	12.78	22.43	12.05	1.77
os60988	1	379	0.89	22	1	4	6.03	-0.26	0	1.39	0.14	74.05	2.17	6.21	7.66	16.44	7.97	-1.16	12.52	22.11	11.85	1.59
os60995	1	390	0.4	13	3	5	6.88	0.06	0	1.48	0.09	97.2	-0.74	7.32	7.6	16.43	7.91	-1.18	12.47	22.14	11.79	1.57
os61053	1	468	0.04	18	3	9	6.86	0.25	0	1.42	0.11	96.5	-2.71	5.62	7.06	15.85	7.39	-1.72	11.89	21.5	11.18	1.01
os61065	1	426	0.6	7	3	5	6.52	-0.09	0.49	1.51	0.09	97.14	0.35	6.26	7.17	15.97	7.5	-1.64	12.02	21.62	11.27	1.04
os61113	1	399	0.18	8	2	3	6.72	0.05	0.54	1.5	0.08	96.7	-1.28	7.32	7.43	16.25	7.71	-1.53	12.35	22	11.6	1.21
os61126	1	410	0.4	3	2	0	6.83	0.01	0.58	1.54	0.07	96.08	-0.3	6.5	7.24	16.06	7.57	-1.61	12.07	21.69	11.34	1.1
os61240	1	409	0	5	3	5	6.87	0.08	0.53	1.51	0.03	92.56	0.25	7.5	7.33	16.18	7.66	-1.58	12.25	21.89	11.5	1.15
os61267	1	430	0.18	7	1	4	6.77	0.05	0.02	1.43	0.09	98.49	5.36	6.44	7.17	15.97	7.5	-1.65	12	21.61	11.24	1.04
os61351	1	479	0.57	10	3	5	6.54	-0.03	0.29	1.45	0.14	99.8	0.16	6.68	7.06	15.95	7.41	-1.62	11.83	21.57	11.11	1.1
os61414	1	406	0.66	2	3	6	6.81	0.01	4.96	1.55	0.03	58.01	-0.49	8.9	7.39	16.26	7.7	-1.44	12.2	21.89	11.39	1.3
os61417	1	401	0.77	1	3	6	6.75	-0.02	2.26	1.56	0.03	56.27	-0.26	8.72	7.52	16.35	7.79	-1.35	12.37	22.1	11.53	1.38
os61618	1	351	0.51	13	3	5	6.78	0.04	0.07	1.51	0.11	49.56	-1.71	6.98	7.6	16.37	7.83	-1.38	12.57	22.17	11.72	1.37

os61668	1	344	0.01	20	2	7	6.79	0.31	0	1.47	0.23	61.53	-6.41	6.24	7.43	16.22	7.72	-1.44	12.26	21.97	11.51	1.27
os61687	1	382	0.09	15	2	0	6.99	0.23	0	1.46	0.14	91.45	-0.04	6.16	7.35	16.14	7.64	-1.5	12.16	21.86	11.42	1.2
os61688	1	378	0.12	15	2	0	6.95	0.23	0	1.47	0.14	91.88	-0.35	6.02	7.34	16.13	7.64	-1.51	12.15	21.86	11.41	1.2
os61689	1	355	0.23	15	2	0	6.4	0.07	0	1.44	0.16	81.33	0.86	5.59	7.38	16.17	7.65	-1.48	12.24	21.89	11.48	1.23
os61691	1	381	0.47	5	2	7	6.57	-0.05	1.45	1.54	0.14	95.28	-5.62	7.25	7.36	16.17	7.65	-1.49	12.26	21.9	11.45	1.21
os61695	1	353	0.76	9	1	4	6.38	-0.15	0.03	1.43	0.06	83.01	4.95	7.57	7.67	16.45	7.92	-1.26	12.52	22.23	11.74	1.45
os61703	1	420	1	13	1	4	6.22	-0.22	0	1.42	0.12	98.57	2.75	5.21	7.3	16.13	7.62	-1.52	12.11	21.82	11.38	1.19
os61709	1	404	0.03	9	3	5	6.84	0.11	0.13	1.5	0.05	94.97	0.99	7.73	7.37	16.18	7.67	-1.42	12.13	21.83	11.39	1.24
os61710	1	403	0.09	9	3	5	6.84	0.13	0.07	1.5	0.06	94.79	0.37	7.88	7.37	16.18	7.67	-1.43	12.13	21.82	11.39	1.24
os61748	1	408	0.7	14	2	3	6.65	-0.06	0	1.46	0.1	85.9	0.03	6.73	7.39	16.23	7.72	-1.36	12.15	21.86	11.48	1.36
os61774	1	459	0.96	16	3	5	6.03	-0.26	0	1.45	0.11	94.79	-1.27	5.8	7.15	16	7.5	-1.65	11.85	21.55	11.16	1.04
os61780	1	397	0.08	7	2	7	6.82	0.11	1.24	1.54	0.19	94.47	-8.36	7.12	7.24	16.06	7.59	-1.48	11.9	21.57	11.19	1.15
os61795	1	388	0.87	10	2	7	6.59	-0.1	0.25	1.52	0.11	97.41	-3.5	7.15	7.44	16.28	7.73	-1.36	12.28	22.01	11.5	1.35
os61809	1	455	1	14	2	3	6.31	-0.21	0.03	1.49	0.12	99.96	-4.1	6.98	7.16	16.04	7.49	-1.56	11.95	21.7	11.2	1.15
os61820	1	396	0.11	9	3	5	6.76	0.07	0.1	1.5	0.05	46.86	0.89	7.72	7.52	16.36	7.8	-1.35	12.41	22.1	11.55	1.41
os61825	1	428	0.47	3	3	6	6.8	-0.01	2.79	1.55	0.02	43.73	-0.17	8.3	7.39	16.24	7.65	-1.47	12.29	21.97	11.59	1.3
os61876	1	489	0.84	16	2	7	6.43	-0.13	0	1.47	0.19	100	-2.84	5.17	6.62	15.57	7.07	-1.94	11.35	21.06	10.8	0.76
os61904	1	361	0.93	16	2	3	6.27	-0.2	0	1.47	0.13	75.07	-3.12	6.28	7.71	16.55	7.94	-1.36	12.73	22.36	11.94	1.44
os61906	1	366	0.97	17	3	5	5.98	-0.28	0	1.41	0.09	75.18	3.08	5.65	7.71	16.55	7.94	-1.35	12.73	22.36	11.95	1.44
os61933	1	481	0.85	8	1	1	6.46	-0.12	0.04	1.42	0.03	96.48	5.53	6.03	7.14	16	7.5	-1.65	11.85	21.54	11.15	1.04
os62170	1	483	0.01	16	3	9	7.02	0.27	0	1.41	0.11	98	0.77	6.19	7.29	16.11	7.53	-1.55	11.95	21.54	11.2	1.15
os62173	1	503	0.25	19	1	4	6.4	0.09	0	1.38	0.13	93.35	3.43	5.4	6.9	15.74	7.25	-1.82	11.45	21.09	10.87	0.82
os62197	1	442	0.24	7	1	4	6.95	0.09	0.07	1.46	0.06	99.65	3.14	5.85	7.28	16.13	7.58	-1.5	11.89	21.58	11.24	1.19
os62214	1	483	0.23	4	3	9	6.79	0.03	0.84	1.5	0.02	96.75	0.14	7.31	7.1	15.93	7.42	-1.71	11.81	21.41	11.16	1.01
os62242	1	493	0.21	16	2	3	6.62	0.12	0	1.47	0.12	99.85	-3.23	6.86	6.96	15.83	7.31	-1.72	11.58	21.23	11	0.97
os62268	1	461	0.1	6	3	5	6.95	0.08	0.44	1.49	0.07	97.12	-0.46	6.29	7.17	16.03	7.46	-1.6	11.81	21.47	11.2	1.09
os62271	1	375	1	6	3	5	6.57	-0.1	0.59	1.5	0.08	95.52	-0.41	6.05	7.7	16.56	7.91	-1.27	12.44	22.07	11.68	1.43
os62289	1	452	0	13	3	5	6.96	0.22	0	1.46	0.08	91.35	1.11	6.3	7.23	16.09	7.54	-1.64	11.97	21.67	11.25	1.09
os62296	1	457	0.23	6	3	5	6.77	0.04	1.45	1.55	0.07	69.8	-1.95	7.86	7.11	15.98	7.46	-1.73	11.9	21.66	11.26	0.99
os62325	1	530	0.99	14	1	4	6.27	-0.23	0	1.43	0.08	86.73	3.49	6.46	6.77	15.62	7.18	-1.9	11.31	20.99	10.82	0.73
os62333	1	429	0.35	10	3	5	6.64	0.02	0.18	1.51	0.06	56.22	-0.65	7.22	7.42	16.25	7.66	-1.44	12.3	21.97	11.58	1.3
os62360	1	487	0.92	3	3	6	6.72	-0.03	2.15	1.54	0.04	83.21	-1.52	9.15	7.13	15.98	7.41	-1.65	11.87	21.5	11.17	1.06
os62369	1	466	0.65	16	2	3	6.63	-0.07	0.01	1.46	0.14	99.54	-4.36	6.64	7.25	16.1	7.5	-1.54	12	21.61	11.24	1.15
os62371	1	440	0.08	12	2	0	6.22	-0.2	0	1.45	0.13	97.72	-0.83	5.96	7.28	16.14	7.52	-1.53	12.03	21.64	11.27	1.17
os62388	1	579	0.12	14	2	3	6.73	0.11	0.02	1.48	0.13	94.11	-5.04	7.37	6.61	15.48	7	-1.99	11.15	20.75	10.56	0.69
os62434	1	455	0.54	12	3	5	6.81	0.02	0	1.49	0.09	99.77	0.29	5.2	7.12	16	7.46	-1.57	11.8	21.54	11.2	1.1
os62440	1	376	1	8	3	5	6.49	-0.13	0.21	1.52	0.06	80.52	-0.88	7.49	7.58	16.47	7.82	-1.31	12.42	22.14	11.7	1.42

os62441	1	370	0.99	4	3	6	6.69	-0.05	1.84	1.55	0.05	78.2	-0.81	8.03	7.62	16.53	7.87	-1.28	12.53	22.26	11.79	1.45
os62474	1	395	0.99	2	3	6	6.71	-0.04	1.9	1.56	0.04	77.8	-0.91	8.25	7.44	16.33	7.73	-1.47	12.38	22.09	11.64	1.23
os62481	1	378	0.38	12	1	4	6.46	0	0	1.43	0.04	82.36	1.98	6.44	7.61	16.49	7.87	-1.39	12.61	22.28	11.84	1.36
os62490	1	388	0.99	8	3	5	6.56	-0.12	0.3	1.5	0.07	99.91	-1.14	6.01	7.52	16.37	7.78	-1.46	12.48	22.12	11.73	1.32
os62503	1	397	0.95	8	2	0	6.54	-0.12	0.09	1.5	0.09	92.73	1.21	6.75	7.27	16.09	7.57	-1.58	11.94	21.55	11.24	1.13
os62506	1	409	0.99	7	2	3	6.53	-0.12	0.47	1.52	0.12	96.22	-3.03	6.27	7.26	16.09	7.56	-1.59	11.95	21.55	11.24	1.12
os62513	1	455	0.6	5	3	5	6.69	-0.01	0.31	1.5	0.03	98.53	1.2	6.59	7.27	16.1	7.57	-1.61	12.1	21.71	11.42	1.15
os62518	1	464	0.07	6	3	5	6.86	0.08	0.67	1.53	0.04	99.1	-0.81	7.36	7.09	15.96	7.44	-1.74	11.87	21.47	11.25	1.03
os62531	1	373	0.9	7	2	3	6.66	-0.06	1.07	1.54	0.08	78.58	-2.05	8.07	7.59	16.44	7.84	-1.36	12.46	22.09	11.74	1.41
os62534	1	397	0.37	18	3	5	6	-0.26	0	1.45	0.12	89.63	-0.81	6.29	7.42	16.26	7.7	-1.49	12.25	21.88	11.59	1.29
os62557	1	402	0.62	2	3	6	6.79	0	1.91	1.56	0.03	80.73	0	8.22	7.41	16.28	7.69	-1.38	12.04	21.75	11.38	1.3
os62570	1	478	0.1	10	3	5	6.83	0.12	0.11	1.47	0.09	100	-2	6.23	7.14	16.02	7.48	-1.57	11.81	21.5	11.21	1.1
os62577	1	371	0.82	11	2	3	6.63	-0.08	0.05	1.51	0.09	63.59	-1.43	7.12	7.61	16.46	7.85	-1.28	12.37	22.06	11.66	1.43
os62593	1	473	0.98	12	3	5	6.46	-0.16	0.01	1.47	0.11	99.95	1.02	5.5	7.06	15.94	7.39	-1.63	11.65	21.33	11.06	1.02
os62699	1	428	0.64	13	2	7	6.28	-0.09	0.09	1.51	0.14	81.47	-5.21	6.5	7.16	16.01	7.44	-1.6	11.77	21.39	11.12	1.05
os62706	1	450	0.77	4	3	6	6.75	-0.04	0.34	1.51	0.06	97.25	0.79	6.31	7.25	16.11	7.5	-1.58	11.88	21.47	11.18	1.1
os62707	1	449	0.77	4	3	6	6.8	-0.02	0.49	1.52	0.06	97.39	-0.2	6.49	7.25	16.11	7.5	-1.58	11.89	21.47	11.19	1.1
os62785	1	367	0.8	16	3	5	6	-0.21	0	1.47	0.11	99.04	-1.82	5.38	7.84	16.68	7.99	-1.24	12.76	22.32	11.94	1.52
os62847	1	647	0.9	7	3	5	6.6	-0.09	0.22	1.48	0.04	99.44	1.07	7.47	6.21	15.02	6.68	-2.36	10.86	20.43	10.3	0.27
os62940	1	360	0.04	13	2	3	6.8	0.2	0	1.48	0.18	91.96	-4.36	5.96	7.74	16.54	8.05	-1.03	12.55	22.16	11.88	1.72
os62964	1	455	0.21	8	3	5	6.75	0.06	0.07	1.49	0.08	90.68	-1.39	6.59	7.3	16.12	7.69	-1.3	12	21.65	11.39	1.41
os62975	1	461	0.68	5	3	5	6.62	-0.06	0.33	1.49	0.03	94.45	1.97	6.9	7.25	16.08	7.66	-1.35	11.96	21.63	11.36	1.39
os62989	1	441	0.08	15	2	3	6.95	0.25	0	1.47	0.11	84.69	-2.71	5.73	7.38	16.2	7.77	-1.34	12.16	21.86	11.57	1.41
os63046	1	584	0.92	4	3	6	6.71	-0.06	0.73	1.54	0.06	84.16	-0.98	7.32	6.41	15.21	6.84	-2.2	11.11	20.72	10.55	0.46
os63117	1	589	0.64	19	1	4	5.91	-0.3	0	1.41	0.11	90.16	2.82	5.96	6.43	15.22	6.83	-2.21	11.09	20.67	10.48	0.42
os63132	1	641	0.24	5	3	6	6.76	-0.07	0.66	1.52	0.03	96.41	0.85	6.98	6.33	15.13	6.76	-2.3	10.97	20.52	10.38	0.35
os63143	1	583	0.99	14	2	0	6.34	-0.21	0.11	1.48	0.12	92.42	-0.79	7.54	6.36	15.2	6.78	-2.24	10.96	20.56	10.39	0.38
os63146	1	609	0	15	3	5	7.01	0.24	0	1.41	0.11	95.7	-0.26	5.52	6.4	15.22	6.81	-2.21	11.01	20.61	10.42	0.4
os63232	1	391	0.88	4	3	9	6.67	-0.06	0.55	1.52	0.03	80.22	-0.21	6.52	7.8	16.6	8.14	-0.86	12.5	22.1	11.89	1.91
os63245	1	357	0.92	3	3	6	6.65	-0.05	1.19	1.52	0.02	74.41	0	7.76	7.99	16.85	8.31	-0.75	12.79	22.45	12.17	2.02
os63256	1	335	0.89	5	3	5	6.66	-0.06	0.49	1.51	0.03	99.91	0.31	6.29	8.16	16.99	8.42	-0.66	12.98	22.62	12.32	2.14
os63289	1	347	0.11	4	3	6	6.59	-0.08	0.19	1.5	0.05	93.63	1.03	7.81	7.89	16.7	8.21	-0.8	12.63	22.22	11.98	1.97
os63326	1	333	0.9	10	2	7	6.52	-0.11	0.4	1.53	0.12	89.16	-4.29	7.1	7.94	16.73	8.25	-0.8	12.57	22.15	11.9	1.89
os63343	1	469	0.1	9	2	3	6.91	0.14	0.58	1.5	0.13	99.35	-6.01	7.05	7.2	16	7.66	-1.3	11.71	21.36	11.17	1.3
os63378	1	420	0.96	4	3	6	6.7	-0.05	0.94	1.53	0.03	98.13	-0.11	7.93	7.55	16.36	7.95	-1.03	12.08	21.71	11.51	1.6
os63446	1	471	0.53	16	3	5	6.8	0.01	0	1.43	0.1	98.79	0	7.44	7.2	16.01	7.68	-1.3	11.76	21.4	11.22	1.36
os63535	1	368	0.06	6	3	5	6.76	0.05	0.94	1.51	0.06	86.51	-1.05	8.11	7.9	16.79	7.97	-1.17	12.7	22.32	11.83	1.59

os63550	1	395	0.85	13	3	9	6.19	-0.18	0	1.44	0.07	99.68	1.22	5.69	7.99	16.93	8.03	-1.18	12.86	22.5	11.92	1.6
os63563	1	425	0.9	30	1	2	5.07	-0.41	0	1.32	0.22	96.23	0.27	5.21	7.43	16.31	7.63	-1.43	12.15	21.78	11.37	1.29
os63611	1	476	0.61	6	3	6	6.71	0	1.01	1.48	0.08	98.36	-0.83	7.89	7.3	16.19	7.53	-1.51	11.94	21.6	11.24	1.14
os63637	1	391	0.81	13	2	7	6.31	-0.19	0.02	1.48	0.11	94.27	-1.8	7.05	7.64	16.53	7.83	-1.28	12.31	21.98	11.54	1.41
os63647	1	460	0	6	1	1	6.91	0.1	0.1	1.42	0.02	99.6	3.07	6.02	7.73	16.68	7.82	-1.26	12.53	22.16	11.67	1.48
os63716	1	355	1	15	3	5	6.15	-0.23	0.1	1.46	0.09	87.04	-0.81	6.96	7.9	16.8	7.98	-1.17	12.7	22.33	11.84	1.59
os63733	1	479	0.4	3	3	9	6.89	0.04	0.62	1.51	0.03	96.45	-0.52	6.7	7.36	16.26	7.56	-1.5	12.08	21.73	11.36	1.23
os63767	1	476	0.82	17	3	9	6.48	-0.13	0	1.39	0.13	98.74	0.35	5.61	7.42	16.29	7.63	-1.43	12.14	21.78	11.35	1.31
os63887	1	489	0.43	10	3	5	6.96	0.07	0.06	1.47	0.06	97.87	0.86	6.59	7.23	16.15	7.48	-1.61	11.92	21.57	11.22	1.13
os63926	1	511	0.57	4	2	3	6.75	-0.02	1.66	1.53	0.07	92.32	-3.42	8.22	7.01	15.89	7.31	-1.75	11.64	21.29	11	0.97
os63930	1	521	1	15	1	4	6.15	-0.26	0	1.43	0.08	98.24	1.89	5.91	7	15.89	7.31	-1.75	11.64	21.3	11.01	0.98
os64057	1	373	0	9	2	3	6.93	0.14	0.53	1.52	0.11	97.03	-3.61	7.35	7.91	16.85	7.98	-1.25	12.81	22.43	11.88	1.55
os64080	1	425	0.99	5	3	5	6.61	-0.09	0.71	1.5	0.05	98.51	-1.06	6.47	7.71	16.61	7.82	-1.28	12.51	22.12	11.65	1.45
os64093	1	403	0.07	11	3	5	7.01	0.18	0.01	1.48	0.09	98.21	0.01	5.81	7.73	16.63	7.84	-1.27	12.54	22.16	11.68	1.47
os64123	1	431	0.98	5	3	5	6.71	-0.06	0.32	1.49	0.04	98.23	1.54	6.41	7.6	16.53	7.74	-1.31	12.29	21.95	11.47	1.38
os64137	1	579	0.86	17	2	3	6.44	-0.17	0.02	1.45	0.15	99.06	-3.46	5.89	6.67	15.55	7.01	-2.02	11.32	20.98	10.71	0.67
os64148	1	296	0.59	5	3	6	6.58	-0.04	0.22	1.5	0.03	91.69	1.56	6.67	8.06	16.92	8.24	-0.94	12.78	22.45	12.02	1.79
os64158	1	376	0.43	4	3	6	6.78	-0.01	0.57	1.51	0.03	96.8	0.48	6.77	7.5	16.38	7.77	-1.32	12.07	21.74	11.39	1.35
os64214	1	378	0	11	1	4	6.98	0.18	0.03	1.41	0.08	97.23	5.12	5.95	7.39	16.29	7.66	-1.4	12.01	21.67	11.33	1.29
os64242	1	266	0.09	6	2	7	6.59	0.07	0.53	1.54	0.2	80.94	-2.66	7.16	7.88	16.74	8.09	-1.04	12.52	22.16	11.78	1.66
os64243	1	266	0.07	6	2	7	6.61	0.07	0.86	1.54	0.2	79.37	-3.65	7.2	7.88	16.74	8.09	-1.04	12.52	22.16	11.78	1.66
os64246	1	325	0.27	5	3	6	6.85	0.04	0.51	1.52	0.03	95.2	0.86	7.44	7.83	16.7	8.05	-1.09	12.49	22.14	11.78	1.64
os64248	1	322	0	8	3	5	6.88	0.12	0.37	1.5	0.06	90.6	-0.6	6.95	7.87	16.74	8.08	-1.07	12.54	22.19	11.82	1.67
os64363	1	527	0.95	12	3	5	6.46	-0.17	0	1.43	0.08	98.57	1.24	5.85	6.74	15.59	7.16	-1.85	11.17	20.81	10.65	0.77
os64400	1	355	0.22	13	2	3	6.87	0.12	0.87	1.49	0.13	95.63	-4.72	8.06	7.64	16.44	7.89	-1.33	12.4	22	11.66	1.38
os64405	1	339	0.04	13	3	5	6.76	0.15	0.12	1.48	0.1	91.96	-1.07	8.04	7.65	16.46	7.89	-1.32	12.43	22.02	11.68	1.38
os64447	1	467	0.04	9	3	9	6.88	0.12	0.15	1.47	0.04	99.42	0.85	6.54	7.08	15.87	7.44	-1.67	11.75	21.31	11.1	1.03
os64501	1	356	0.05	9	3	5	6.96	0.14	0.19	1.48	0.07	97.34	0.5	6.64	7.54	16.5	7.8	-1.34	12.32	22.03	11.62	1.41
os64505	1	415	0.54	13	3	9	6.34	-0.09	0.02	1.41	0.08	99.18	0.8	6.37	7.56	16.43	7.8	-1.34	12.28	21.95	11.56	1.4
os64509	1	314	1	9	2	0	6.39	-0.16	0.03	1.47	0.07	95.78	2.21	6.96	7.69	16.61	7.91	-1.28	12.49	22.2	11.78	1.49
os64510	1	382	0.08	1	3	6	6.82	0.02	2.44	1.52	0.03	84.54	0.41	8.87	7.48	16.42	7.74	-1.4	12.24	21.93	11.53	1.36
os64523	1	321	0	6	2	3	6.89	0.1	0.77	1.51	0.07	99.4	-2.06	6.73	7.91	16.79	8.07	-1.14	12.73	22.45	11.95	1.64
os64560	1	310	0.1	17	2	7	6.94	0.24	0	1.47	0.19	79.1	-2.14	6.09	7.38	16.17	7.67	-1.41	11.97	21.52	11.3	1.28
os64576	1	436	0.75	11	2	3	6.73	-0.04	0.22	1.47	0.09	99.63	-2.28	6.28	7.07	15.9	7.43	-1.67	11.77	21.31	11.16	1.03
os64614	1	507	1	13	3	5	6.28	-0.22	0	1.44	0.1	99.38	1.05	5.55	6.61	15.39	7.02	-2.11	11.32	20.8	10.74	0.61
os64648	1	452	0.01	21	1	4	6.83	0.31	0	1.38	0.09	84.99	4.98	6.01	6.98	15.83	7.32	-1.79	11.72	21.26	11.08	0.93
os64650	1	518	0.08	7	1	1	7	0.11	0.11	1.44	0.03	99.77	2.57	5.95	6.78	15.62	7.16	-1.94	11.49	21.03	10.89	0.76

os64658	1	490	0.87	10	3	5	6.7	-0.07	0.12	1.48	0.1	99.65	-0.82	6.82	6.72	15.58	7.13	-1.94	11.39	20.95	10.84	0.75
os64660	1	485	0.81	12	3	5	6.71	-0.06	0.26	1.48	0.1	99.72	-1.86	7.2	6.73	15.59	7.14	-1.93	11.4	20.96	10.84	0.76
os64662	1	362	0.15	9	2	3	6.39	-0.13	0.38	1.5	0.1	99.46	-2.59	7.19	7.45	16.28	7.74	-1.43	12.21	21.77	11.53	1.29
os64664	1	363	0.73	14	1	4	6.17	-0.16	0	1.44	0.1	99.06	2.8	5.82	7.49	16.3	7.77	-1.41	12.25	21.81	11.56	1.31
os64711	1	424	0.26	16	3	9	6.98	0.18	0	1.41	0.09	99.32	1.16	5.66	7.24	16.1	7.55	-1.59	11.97	21.56	11.31	1.15
os64766	1	413	0.99	15	2	3	6.11	-0.24	0.05	1.46	0.13	97.53	-3.28	5.61	7.52	16.4	7.76	-1.41	12.45	22.17	11.71	1.37
os64767	1	464	0.07	6	3	5	6.86	0.08	0.65	1.52	0.04	99.1	-0.76	7.33	7.09	15.96	7.44	-1.74	11.87	21.47	11.25	1.03
os64772	1	477	0.47	13	3	5	6.5	0.01	0.01	1.41	0.1	98.82	0.98	5.8	7.01	15.92	7.41	-1.62	11.58	21.22	11.01	1.06
os64786	1	458	0.05	13	3	5	6.89	0.19	0	1.46	0.1	97.57	-1.05	6	7.24	16.11	7.5	-1.57	11.86	21.5	11.2	1.11
os64826	1	315	0.12	16	2	0	6.98	0.24	0	1.44	0.17	79.74	0.05	5.75	7.38	16.17	7.66	-1.42	11.96	21.52	11.29	1.28
os64839	1	448	0.48	4	3	6	6.88	0.07	0.8	1.51	0.07	98.73	-1.72	7.44	7.01	15.81	7.38	-1.67	11.61	21.14	10.99	1.03
os64891	1	245	0.15	11	1	4	6.23	-0.19	0.02	1.46	0	83.29	1.4	5.95	8.07	16.93	8.54	-0.54	12.82	22.33	12.43	2.27
os64909	1	245	0.01	13	3	5	6.22	-0.19	0	1.47	0	90.54	1.45	6.54	8.14	16.96	8.54	-0.59	12.99	22.56	12.57	2.26
os64934	1	269	0	7	3	5	6.88	0.12	0.23	1.5	0.05	85.06	0.22	6.69	8.02	16.84	8.43	-0.64	12.71	22.31	12.33	2.24
os65093	1	241	0.85	7	3	5	6.49	-0.09	0.28	1.51	0.04	52.78	0.36	8.23	8.21	17.08	8.59	-0.56	13	22.68	12.64	2.39
os65204	1	381	0.92	1	3	6	6.75	-0.01	0.28	1.54	0	82.57	0.75	9.24	7.08	15.84	7.73	-1	11.52	20.86	11.28	1.74
os65224	1	522	0.06	6	3	5	6.9	0.09	0.29	1.5	0	90.12	0.3	6.75	7.09	16.14	7.23	-1.75	11.7	21.53	11	1.04
os65426	1	428	0.01	4	3	6	6.87	0.07	0.3	1.53	0.04	84.6	0.46	7.62	8.14	17.3	8.11	-1.06	12.89	22.87	11.94	1.78
os65585	1	462	0.89	6	3	5	6.68	-0.08	0.34	1.51	0.03	96.45	0.47	7.16	7.76	16.85	7.83	-1.32	12.46	22.45	11.59	1.41
os65589	1	423	0.61	6	3	5	6.65	-0.02	0.57	1.54	0.07	88	-1.18	7.49	7.75	16.8	7.82	-1.32	12.44	22.38	11.56	1.45
os65679	1	432	0.99	10	3	5	6.38	-0.17	0.03	1.48	0.07	81.82	0.69	7.05	8.1	17.28	8.1	-1.11	12.88	22.91	11.94	1.7
os65729	1	508	0.01	7	3	5	6.95	0.11	0.37	1.52	0.03	90.88	0.1	6.49	7.61	16.82	7.71	-1.44	12.34	22.47	11.56	1.36
os65730	1	508	0.01	7	3	5	6.95	0.11	0.37	1.52	0.03	90.68	0.07	6.46	7.61	16.82	7.71	-1.44	12.34	22.47	11.56	1.36
os65760	1	465	0.02	6	3	5	6.88	0.08	1.34	1.53	0.04	68.72	-0.68	8.28	7.68	16.77	7.77	-1.42	12.43	22.36	11.55	1.36
os65862	1	482	0.46	12	2	7	6.52	0.02	0.08	1.53	0.15	91.77	-7.4	7.46	7.21	16.24	7.39	-1.7	11.98	21.88	11.22	1.03
os65870	1	520	0.99	8	3	5	6.56	-0.13	0.16	1.5	0.04	94.12	1.26	6.49	7.22	16.27	7.42	-1.69	11.99	21.91	11.2	1.05
os65881	1	581	0.65	5	1	1	6.71	-0.05	0.01	1.49	0.01	84.99	3.47	6.57	7.03	16.05	7.25	-1.81	11.71	21.64	10.98	0.89
os65947	1	519	0.99	8	3	5	6.54	-0.13	0.27	1.5	0.05	90.93	-0.59	6.39	7.41	16.5	7.55	-1.56	12.08	22.02	11.29	1.19
os65949	1	517	1	9	3	5	6.5	-0.15	0.2	1.5	0.06	90.68	-0.45	6.37	7.41	16.5	7.55	-1.56	12.09	22.02	11.29	1.19
os65957	1	497	0.85	11	3	5	6.36	-0.16	0.05	1.49	0.08	84.46	-1.13	6.67	7.26	16.26	7.48	-1.68	11.91	21.83	11.23	1.04
os66153	1	525	0.01	4	3	6	6.96	0.07	0.36	1.53	0.04	99.46	-0.01	7.88	7.14	16.16	7.34	-1.75	11.88	21.79	11.16	0.99
os66246	1	480	0.67	15	2	0	6.34	-0.04	0.04	1.48	0.15	98.65	-1.35	6.27	7.04	15.92	7.32	-1.7	11.56	21.21	10.89	0.97
os66364	1	501	0.14	8	3	5	6.8	0.07	0.36	1.48	0.07	99.28	-1.67	6.43	7.38	16.36	7.54	-1.59	11.97	21.78	11.24	1.1
os66474	1	374	0.71	16	2	8	6.6	-0.07	0	1.43	0.13	97.73	-3.09	6.14	8.26	17.24	8.23	-1.01	12.99	22.74	12.03	1.71
os66490	1	439	0.99	19	1	4	6.01	-0.29	0	1.4	0.11	94.46	4.17	5.8	7.77	16.77	7.84	-1.39	12.47	22.31	11.64	1.34
os66492	1	386	1	21	1	2	5.74	-0.34	0	1.37	0.15	84.73	3.67	5.43	7.9	16.9	7.95	-1.3	12.6	22.43	11.74	1.42
os66512	1	408	1	19	2	3	5.81	-0.26	0.03	1.43	0.18	96.32	-7.25	6.83	7.88	16.89	7.94	-1.28	12.6	22.4	11.73	1.43

os66553	1	493	0.12	9	2	3	6.48	-0.14	0.45	1.46	0.11	99.61	-2.16	6.65	7.39	16.36	7.57	-1.49	11.92	21.65	11.17	1.24
os66582	1	462	0.93	9	2	3	6.41	-0.16	0.17	1.44	0.1	99.28	-2.41	6.55	7.6	16.6	7.76	-1.33	12.2	21.9	11.38	1.4
os66603	1	379	0.55	5	3	9	6.9	0.08	0.81	1.47	0.03	87.25	1.29	7.16	8.18	17.17	8.22	-1.06	12.92	22.58	11.94	1.63
os66623	1	422	0.14	5	3	9	6.83	0.06	0.9	1.48	0.03	92.3	-0.27	7.63	8.03	17.03	8.08	-1.14	12.76	22.45	11.82	1.59
os66655	1	376	0.5	4	2	3	6.69	-0.01	2.06	1.53	0.07	94.5	-3.17	7.39	8.18	17.14	8.17	-1.07	12.84	22.49	11.85	1.64
os66659	1	317	0.7	10	2	3	6.37	-0.11	0.25	1.52	0.12	97.66	-3.06	6.39	8.5	17.51	8.46	-0.88	13.34	23	12.26	1.88
os66679	1	298	0.98	12	1	4	6.35	-0.18	0	1.44	0.05	99.35	3.83	5.44	8.71	17.7	8.61	-0.75	13.6	23.25	12.48	2.04
os66729	1	459	0.03	5	2	3	6.93	0.09	1.38	1.54	0.07	94.23	-2.23	8.43	7.6	16.63	7.72	-1.42	12.16	21.97	11.38	1.26
os66755	1	449	0.98	10	2	3	6.4	-0.16	0.15	1.49	0.08	98.62	-1.78	7.56	7.57	16.56	7.74	-1.4	12.27	21.98	11.43	1.33
os66765	1	455	0.44	11	1	4	6.93	0.07	0.01	1.44	0.05	98.1	2.4	6.16	7.55	16.54	7.72	-1.41	12.23	21.94	11.41	1.31
os66766	1	455	0.4	11	3	5	6.93	0.07	0.01	1.44	0.06	97.92	1.91	6.21	7.55	16.54	7.72	-1.41	12.23	21.94	11.41	1.31
os66833	1	600	0.59	10	1	1	6.91	0.01	0.01	1.42	0.05	100	3.39	5.41	6.98	15.93	7.26	-1.87	11.56	21.31	10.92	0.78
os66912	1	455	0.08	1	3	6	6.84	0.02	3.09	1.56	0.04	57.31	-1.57	8.28	7.73	16.87	7.84	-1.35	12.45	22.47	11.64	1.44
os67008	1	403	0.9	4	3	6	6.74	-0.04	1.04	1.51	0.03	92.72	-0.09	7.59	8.33	17.41	8.32	-0.86	12.99	22.81	12.09	1.92
os67048	1	384	0.95	10	2	3	6.56	-0.11	0.16	1.49	0.07	93.47	-0.44	6.82	8.45	17.56	8.41	-0.82	13.19	23.01	12.19	1.96
os67342	1	530	0.96	5	3	5	6.7	-0.08	0.53	1.53	0.03	97.69	0.02	7.02	7.27	16.35	7.47	-1.72	11.9	21.84	11.22	1.02
os67352	1	488	0.74	15	1	4	6.22	-0.12	0.01	1.43	0.07	89.27	3.79	5.89	7.55	16.6	7.67	-1.55	12.24	22.15	11.44	1.18
os67415	1	430	0.76	9	1	4	6.44	-0.09	0.01	1.4	0.02	79.44	6.06	6.69	8.13	17.27	8.16	-1.08	12.83	22.87	12	1.69
os67444	1	431	0.35	4	3	6	6.69	-0.05	2.02	1.54	0.05	74.41	-2.2	8.59	8.06	17.21	8.17	-1.01	12.65	22.62	11.89	1.75
os67469	1	329	0	6	3	5	6.89	0.11	0.5	1.52	0.05	96.21	-0.54	7.25	8.74	17.8	8.67	-0.62	13.49	23.26	12.45	2.17
os67475	1	381	0.14	11	3	5	7	0.18	0.01	1.47	0.08	96.82	1.43	6.66	8.41	17.52	8.4	-0.83	13.12	22.91	12.12	1.98
os67486	1	422	0.12	2	2	3	6.79	0	2.7	1.54	0.08	93.12	-3.61	7.99	8.16	17.24	8.18	-0.98	12.78	22.58	11.83	1.77
os67487	1	423	0.08	3	2	3	6.87	0.04	2.73	1.54	0.07	93.24	-3.54	7.88	8.16	17.24	8.18	-0.98	12.78	22.58	11.83	1.77
os67488	1	422	0.02	2	2	3	6.73	-0.03	2.63	1.54	0.08	92.91	-3.74	8.02	8.16	17.24	8.18	-0.98	12.78	22.58	11.83	1.77
os67573	1	323	0.6	4	2	7	6.63	0.01	2.34	1.55	0.16	91.88	-7.62	8.46	8.53	17.58	8.46	-0.75	13.1	22.82	12.05	1.97
os67575	1	324	0.24	3	2	7	6.61	0	2.03	1.55	0.15	92.25	-6.99	8.53	8.52	17.57	8.45	-0.75	13.08	22.8	12.04	1.96
os67595	1	256	0.11	9	2	7	6.76	0.12	0.13	1.51	0.1	76.82	-2.31	7.28	9.21	18.33	8.96	-0.48	14.26	23.95	12.94	2.4
os67619	1	324	1	9	3	5	6.42	-0.15	0.09	1.48	0.07	96.97	1.06	6.59	8.72	17.78	8.59	-0.7	13.51	23.15	12.36	2.09
os67626	1	300	0.01	5	3	5	6.87	0.1	0.25	1.5	0.07	92.85	0.61	7.26	8.77	17.84	8.62	-0.65	13.58	23.21	12.41	2.13
os67685	1	480	0.03	4	3	6	6.93	0.08	0.41	1.52	0.04	84.65	0.95	7.6	7.41	16.45	7.57	-1.63	12.11	21.99	11.39	1.07
os67737	1	521	0.04	18	1	1	7.01	0.27	0	1.41	0.09	96.03	0.35	5.49	7.45	16.5	7.6	-1.58	12.2	22.13	11.39	1.17
os67744	1	494	0.04	12	2	3	6.95	0.19	0.02	1.47	0.1	95.68	-2.18	6.87	7.43	16.48	7.55	-1.59	12.18	22.12	11.37	1.17
os67756	1	457	0.87	8	2	0	6.5	-0.12	0.13	1.5	0.07	86.61	0.37	7.99	7.44	16.45	7.63	-1.59	12.14	22.04	11.39	1.13
os67821	1	501	0.59	4	3	6	6.77	0	0.41	1.47	0.03	89.18	2.14	6.92	7.2	16.13	7.4	-1.82	12.07	21.78	11.31	0.89
os67856	1	503	0.87	16	1	4	6.04	-0.23	0	1.42	0.09	90.12	3.36	5.98	7.2	16.15	7.36	-1.84	11.99	21.76	11.23	0.89
os67910	1	506	0.09	11	3	5	7.03	0.14	0.09	1.47	0.1	97.26	-1.31	6.31	7.21	16.18	7.4	-1.83	11.91	21.68	11.2	0.88
os67943	1	422	0.54	8	2	7	6.81	0.02	0.62	1.53	0.15	95.24	-6.32	7.72	7.51	16.49	7.66	-1.57	12.24	22.01	11.47	1.14

os67974	1	461	0.53	14	1	1	6.85	0.04	0.01	1.4	0.06	99.03	4.7	6	7.75	16.71	7.86	-1.27	12.56	22.2	11.67	1.47
os67990	1	545	0.92	3	3	6	6.74	-0.04	1.15	1.54	0.03	80.57	-0.06	7.11	7.15	16.22	7.34	-1.71	11.8	21.62	11.05	1.11
os68045	1	482	0.95	3	3	6	6.73	-0.05	3.99	1.55	0.05	66.91	-0.44	8.9	7.4	16.45	7.5	-1.64	12.12	21.97	11.31	1.14
os68055	1	478	0.05	2	1	4	6.87	0.03	5.01	1.47	0.01	79.09	3.22	7.31	7.58	16.68	7.63	-1.52	12.39	22.31	11.49	1.28
os68100	1	464	0.29	3	3	6	6.72	-0.05	2.81	1.56	0.04	46.26	-1.45	8.45	7.56	16.65	7.64	-1.49	12.33	22.17	11.5	1.36
os68102	1	463	0.92	3	3	6	6.71	-0.04	3.11	1.56	0.04	47.19	-1.43	8.66	7.56	16.65	7.64	-1.49	12.33	22.17	11.5	1.36
os68103	1	462	0.07	2	3	6	6.74	-0.04	3.56	1.56	0.04	50.98	-1.47	9.24	7.56	16.66	7.64	-1.49	12.33	22.18	11.51	1.36
os68126	1	454	1	5	3	5	6.65	-0.08	0.5	1.54	0.05	63.45	-0.49	7.87	7.57	16.63	7.62	-1.51	12.33	22.19	11.45	1.3
os68129	1	493	0.92	4	3	6	6.65	-0.07	0.64	1.53	0.03	65.16	-0.07	7.29	7.44	16.49	7.5	-1.61	12.2	22.07	11.37	1.17
os68146	1	471	1	10	3	5	6.51	-0.14	0.08	1.52	0.08	79.22	-1.01	8.13	7.54	16.62	7.66	-1.5	12.24	22.11	11.43	1.28
os68157	1	521	0.08	6	3	5	6.99	0.1	0.64	1.52	0.05	95.21	-1.01	7.69	7.37	16.48	7.55	-1.53	11.98	21.9	11.23	1.24
os68175	1	486	0.99	4	3	6	6.7	-0.07	0.56	1.53	0.03	87.63	0.03	6.48	7.6	16.64	7.67	-1.48	12.44	22.23	11.58	1.4
os68176	1	486	0.99	4	3	6	6.71	-0.06	0.54	1.53	0.03	87.18	0.09	6.49	7.6	16.64	7.67	-1.48	12.44	22.23	11.58	1.4
os68210	1	428	0.4	12	1	4	6.53	0.01	0.02	1.4	0.09	83.79	3.21	7.06	8.12	17.26	8.2	-0.94	12.74	22.56	11.87	1.92
os68372	1	429	0.52	9	2	3	6.8	-0.01	0.59	1.52	0.12	89.43	-4.43	8.52	8.02	17.16	8.06	-1.18	12.79	22.65	11.9	1.73
os68378	1	451	0.92	3	3	6	6.68	-0.05	0.82	1.54	0	53.75	-0.35	8.52	7.92	17.08	8	-1.22	12.69	22.56	11.85	1.71
os68450	1	399	0.21	4	3	6	6.76	0.02	1.39	1.54	0.04	77.11	-0.04	7.47	8.29	17.46	8.26	-0.89	13.01	22.9	12.02	1.98
os68470	1	487	0.92	5	3	6	6.64	-0.07	0.44	1.51	0.03	92.76	0.69	7.41	7.62	16.73	7.71	-1.48	12.46	22.34	11.57	1.38
os68483	1	505	0.92	8	3	5	6.48	-0.13	0.47	1.5	0.06	89.3	-1.08	8.35	7.49	16.65	7.63	-1.49	12.22	22.15	11.43	1.36
os68495	1	449	0.08	2	3	6	6.87	0.04	1.62	1.55	0.02	52.75	-0.04	8.78	7.87	17.02	7.94	-1.21	12.57	22.5	11.7	1.64
os68496	1	422	0.17	7	3	5	6.58	-0.09	0.28	1.51	0.03	47.35	0.96	7.98	8.06	17.22	8.07	-1.09	12.84	22.74	11.93	1.81
os68502	1	421	0.84	15	1	4	5.99	-0.24	0	1.35	0.09	87.88	5.95	5.32	7.94	17.06	7.95	-1.27	12.83	22.69	11.87	1.63
os68600	1	244	0.99	7	3	5	6.46	-0.12	0.22	1.49	0.04	83.2	1.45	6.71	9.48	18.61	9.18	-0.29	14.42	24.21	13.11	2.56
os68601	1	245	1	7	3	5	6.47	-0.12	0.21	1.49	0.04	82.83	1.84	6.77	9.48	18.61	9.18	-0.29	14.42	24.21	13.11	2.56
os68665	1	456	0.98	4	2	3	6.72	-0.05	1.44	1.52	0.05	73.68	-2.09	8.91	7.99	17.16	8.09	-1.04	12.63	22.5	11.78	1.84
os68684	1	264	0.23	7	2	0	6.66	0.04	0.36	1.51	0.09	92.68	0.1	7.11	8.9	18.05	8.75	-0.54	13.73	23.43	12.49	2.25
os68726	1	387	0.07	5	3	9	6.91	0.08	0.37	1.48	0.03	91.25	0.35	6.71	8.61	17.76	8.51	-0.72	13.4	23.16	12.24	2.08
os68728	1	306	0.01	4	2	0	6.81	0.07	1.22	1.54	0.11	92.8	-0.78	7.95	8.53	17.57	8.46	-0.83	13.23	22.92	12.14	1.95
os68769	1	450	0.34	3	3	9	6.77	0.01	1.54	1.5	0.02	78.3	-0.66	7.42	8.22	17.29	8.22	-0.96	12.86	22.64	11.92	1.82
os68770	1	441	0.34	5	3	9	6.71	0	0.56	1.47	0.03	94.59	0.59	7.71	8.24	17.31	8.25	-0.94	12.9	22.67	11.95	1.83
os69008	1	264	0.23	3	3	6	6.73	0.03	1.53	1.55	0.03	59.02	-0.23	8.45	8.17	16.92	8.31	-0.87	13.02	22.4	12.18	1.94
os69209	1	245	0.76	2	3	6	6.71	-0.01	3.12	1.57	0.04	26.2	-1.29	8.39	8.35	17.14	8.45	-0.75	13.15	22.66	12.28	2.01
os69279	1	225	0.92	1	2	3	6.68	-0.01	2.96	1.57	0.05	69.86	-2.33	8.12	8.86	17.7	8.79	-0.62	14.1	23.61	13.04	2.36
os69397	1	206	0.1	2	3	6	6.71	0.02	2.23	1.56	0.01	7.62	-0.3	9.06	9.5	18.45	9.26	-0.16	14.34	23.95	13.15	2.68
os69444	1	225	0.06	1	3	6	6.71	0.02	2.8	1.55	0.01	19.76	0.26	8.8	9.36	18.38	9.13	-0.32	14.3	24.01	13.14	2.52
os70056	1	268	0.88	7	2	3	6.64	-0.06	0.38	1.52	0.1	91.15	-2.76	8.22	8.61	17.52	8.67	-0.76	13.46	23.18	12.67	2.16
os70119	1	252	0.92	8	2	3	6.59	-0.08	0.76	1.53	0.09	94.8	-3.42	7.1	8.74	17.67	8.78	-0.7	13.67	23.4	12.85	2.25

os70127	1	262	0.94	9	3	5	6.57	-0.09	0.31	1.5	0.07	95.22	-0.48	6.19	8.74	17.67	8.78	-0.7	13.66	23.39	12.84	2.25
os70149	1	261	0.96	9	3	5	6.56	-0.09	0.29	1.5	0.07	95.2	-0.63	6.23	8.74	17.67	8.78	-0.7	13.66	23.39	12.84	2.25
os70179	1	230	0.77	8	2	7	6.35	-0.11	0.32	1.53	0.15	81.16	-4.79	7.43	8.77	17.69	8.73	-0.67	13.59	23.28	12.71	2.25
os70356	1	329	0.6	8	3	5	6.5	-0.06	0.1	1.49	0.05	97.82	1.91	6.25	8.35	17.27	8.41	-0.91	13.33	22.95	12.42	2.02
os70414	1	290	1	5	3	5	6.55	-0.09	0.31	1.51	0.03	98.65	1.15	6.59	8.59	17.51	8.57	-0.76	13.68	23.27	12.68	2.16
os70516	1	302	0.92	6	3	5	6.48	-0.1	0.19	1.5	0.04	92.51	0.98	6.1	8.26	17.17	8.47	-0.96	13.16	22.87	12.46	1.92
os70551	1	286	0.74	6	3	5	6.54	-0.07	0.85	1.53	0.04	88.12	-1.46	8.17	8.32	17.22	8.51	-0.92	13.25	23	12.55	1.97
os70553	1	266	0.37	9	3	5	6.68	0.06	0.39	1.51	0.07	72.99	-1.11	8.07	8.41	17.31	8.58	-0.86	13.36	23.05	12.6	2.04
os70591	1	325	0.35	10	3	5	6.54	0.01	0.11	1.5	0.06	96.57	-0.86	6.51	8.14	17.04	8.32	-1.04	13.02	22.74	12.36	1.83
os70604	1	351	1	11	1	4	6.3	-0.19	0	1.46	0.05	98.46	3.23	5.64	7.91	16.84	8.16	-1.23	12.87	22.56	12.23	1.71
os70627	1	315	0.09	8	2	0	6.39	-0.15	0.1	1.5	0.09	91.53	-0.34	6.89	7.91	16.9	8.18	-1.18	12.83	22.59	12.26	1.8
os70725	1	459	0.05	7	3	5	6.97	0.11	0.31	1.48	0.05	93.74	1.03	6.44	7.41	16.3	7.69	-1.73	12.23	21.86	11.62	1.25
os70796	1	353	0.89	17	1	4	5.93	-0.25	0	1.42	0.1	88.32	2.95	5.38	8	16.84	8.18	-1.31	12.86	22.5	12.14	1.67
os70856	1	397	0.07	12	1	4	7.01	0.18	0.01	1.44	0.08	83.46	3.65	5.61	7.67	16.54	7.85	-1.69	12.65	22.26	11.95	1.38
os70864	1	385	0	10	2	0	6.94	0.17	0.04	1.5	0.11	89.02	0.02	6.45	7.6	16.48	7.82	-1.72	12.59	22.21	11.91	1.34
os70866	1	400	0.01	9	3	5	6.92	0.16	0.11	1.48	0.1	91.18	-0.13	6.38	7.6	16.48	7.83	-1.72	12.59	22.22	11.91	1.35
os70867	1	403	0.01	10	3	5	6.9	0.15	0.12	1.48	0.09	91.98	-0.31	6.32	7.6	16.48	7.83	-1.72	12.59	22.22	11.91	1.35
os70910	1	462	0.66	9	1	4	6.74	-0.05	0.08	1.46	0.06	99.54	2.06	5.88	7.42	16.33	7.64	-1.86	12.41	22.07	11.71	1.19
os70911	1	489	0.19	4	1	1	6.92	0.05	0.04	1.44	0.02	98.45	3.16	6.08	7.35	16.29	7.59	-1.91	12.33	22	11.65	1.14
os70916	1	570	0.65	11	3	5	6.41	-0.18	0.02	1.45	0.09	100	1.62	5.5	6.65	15.58	7.01	-2.33	11.42	21.05	10.92	0.67
os70965	1	528	0.92	18	2	7	5.88	-0.28	0	1.42	0.21	99.72	-3.45	5.4	6.69	15.63	7.09	-2.34	11.54	21.17	11.08	0.71
os71013	1	367	0.41	6	3	5	6.65	-0.01	0.18	1.51	0.06	86.43	1.64	6.71	7.89	16.73	8.08	-1.42	12.83	22.42	12.08	1.55
os71014	1	369	0.59	5	3	5	6.7	0.02	0.17	1.51	0.06	87.14	1.63	6.61	7.89	16.72	8.08	-1.42	12.83	22.42	12.08	1.55
os71037	1	329	0.92	7	3	5	6.48	-0.1	0.8	1.5	0.05	90.05	-0.58	7.81	8.57	17.49	8.57	-0.92	13.63	23.33	12.75	2.08
os71105	1	409	0.55	12	2	0	6.82	0.02	0.01	1.47	0.14	98.19	-1.15	7.17	7.44	16.33	7.76	-1.57	12.09	21.76	11.54	1.36
os71123	1	695	0.01	8	3	9	7	0.12	0.18	1.44	0.05	99.75	0.05	6.49	6.38	15.29	6.84	-2.38	10.99	20.54	10.52	0.51
os71156	1	689	0.71	5	3	9	6.91	-0.01	0.18	1.44	0.03	99.39	1.25	6.75	6.43	15.33	6.88	-2.36	11.06	20.58	10.59	0.57
os71280	1	309	0.91	7	2	3	6.49	-0.1	0.81	1.5	0.08	99	-2.71	7.31	8.46	17.3	8.54	-0.95	13.59	23.14	12.71	2.1
os71299	1	335	1	6	3	5	6.55	-0.1	0.6	1.52	0.06	93	-0.51	6.86	8.36	17.2	8.48	-1.06	13.49	23.03	12.58	1.98
os71303	1	293	0.45	10	3	5	6.49	-0.01	0.05	1.5	0.08	95.77	-0.65	6.35	8.45	17.28	8.54	-1	13.63	23.2	12.71	2.07
os71321	1	336	0.4	10	2	7	6.56	0.03	0.47	1.52	0.15	99.16	-5.39	7.55	8.02	16.89	8.18	-1.29	13.04	22.62	12.23	1.75
os71323	1	335	0.21	10	3	9	6.71	0.09	0.05	1.45	0.06	94.37	1.31	6.12	8.37	17.17	8.48	-1	13.39	22.93	12.54	2.02
os71387	1	734	0.36	5	1	1	7.03	0.05	0.11	1.42	0.02	99.37	2.53	6.24	6.38	15.28	6.84	-2.38	10.99	20.54	10.52	0.51
os71388	1	731	0.25	6	3	9	7.04	0.06	0.17	1.42	0.03	99.34	1.77	6.18	6.38	15.28	6.83	-2.38	10.99	20.53	10.51	0.51
os71389	1	730	0.33	5	3	9	7.05	0.07	0.23	1.42	0.03	99.3	1.28	6.16	6.37	15.28	6.83	-2.39	10.98	20.53	10.51	0.51
os71420	1	626	0.32	21	2	0	6.91	0.16	0	1.39	0	100	2.03	5.32	6.29	15.19	6.76	-2.38	10.76	20.25	10.27	0.41
os71423	1	395	0.69	5	3	6	6.61	-0.06	0.56	1.52	0.05	99.74	-0.08	6.51	7.94	16.77	8.12	-1.33	12.87	22.37	12	1.58

os71425	1	395	0.91	4	3	6	6.62	-0.06	0.57	1.52	0.05	99.66	-0.01	6.53	7.94	16.78	8.12	-1.32	12.87	22.37	12	1.59
os71429	1	393	0.81	4	3	6	6.63	-0.06	0.5	1.52	0.05	99.44	0.4	6.54	7.95	16.79	8.13	-1.32	12.88	22.39	12.01	1.59
os71434	1	391	0.11	5	3	6	6.6	-0.08	0.5	1.51	0.05	98.84	0.86	6.54	7.95	16.79	8.14	-1.32	12.88	22.39	12.02	1.6
os71435	1	390	0.05	5	3	6	6.59	-0.08	0.51	1.51	0.05	98.75	0.89	6.54	7.95	16.79	8.14	-1.31	12.89	22.39	12.02	1.6
os71436	1	385	0.92	5	3	5	6.59	-0.08	0.54	1.52	0.05	98.72	0.07	6.57	7.98	16.82	8.16	-1.29	12.93	22.43	12.05	1.62
os71574	1	303	0.38	16	3	5	6.33	0.03	0	1.42	0.11	96.77	0.48	6.29	8.6	17.5	8.6	-0.89	13.62	23.27	12.73	2.11
os71587	1	263	0.99	10	2	7	6.4	-0.11	0.7	1.52	0.12	97.54	-5.1	8.14	8.7	17.62	8.71	-0.8	13.82	23.47	12.89	2.23
os71592	1	226	0.23	5	2	3	6.63	0.01	1.28	1.53	0.08	99.8	-3.17	8.06	9.04	18.01	8.94	-0.59	14.25	24.01	13.21	2.48
os71622	1	399	0.79	17	1	4	5.99	-0.19	0	1.42	0.11	93.22	2.94	5.62	7.81	16.66	8.03	-1.38	12.68	22.27	12	1.62
os71753	1	338	0.05	8	3	5	6.81	0.11	0.13	1.47	0.06	96.72	0.74	6.25	8.16	17	8.32	-1.03	12.87	22.51	12.17	1.88
os71764	1	401	0.7	14	1	1	6.21	-0.13	0	1.42	0.06	100	3.78	5.61	8	16.82	8.17	-1.18	12.71	22.35	12.02	1.76
os71844	1	453	0.54	3	3	6	6.76	0.01	0.29	1.5	0.02	98.23	1.49	7.06	7.85	16.68	8.03	-1.39	12.79	22.24	11.94	1.56
os71879	1	631	0.65	19	3	9	6.01	-0.16	0	1.37	0.12	99.85	0.07	5.28	6.64	15.61	7.06	-2.31	11.39	21.05	10.93	0.66
os71929	1	294	0.77	9	3	5	6.4	-0.1	0.16	1.49	0.05	88.36	0.46	7.07	8.16	17.18	8.45	-0.8	13.14	22.92	12.53	2.23
os71939	1	245	0.77	4	3	6	6.71	-0.02	0.51	1.55	0.05	67.29	-0.46	8.19	8.37	17.32	8.61	-0.69	13.39	23.1	12.75	2.38
os71949	1	319	0.27	2	3	6	6.79	0.02	2.85	1.56	0.04	63.08	-0.6	7.86	7.86	16.84	8.14	-1.17	12.69	22.45	12.13	1.72
os72041	1	335	0.91	7	3	5	6.45	-0.11	0.36	1.51	0.07	97.62	-1.2	6.27	7.97	16.96	8.3	-1	12.89	22.6	12.31	2.06
os72043	1	335	0.7	10	3	5	6.35	-0.12	0.03	1.49	0.08	98.46	-0.46	6.15	7.96	16.95	8.28	-1.01	12.87	22.59	12.3	2.05
os72162	1	382	0.81	12	3	5	6.25	-0.15	0	1.47	0.13	98.92	-0.96	5.02	7.62	16.58	8	-1.3	12.58	22.24	12.05	1.82
os72176	1	400	1	19	3	5	5.91	-0.31	0	1.42	0.11	96.79	0.39	5.42	7.65	16.59	8.01	-1.31	12.67	22.31	12.09	1.85
os72197	1	443	0.53	5	3	6	6.65	-0.03	0.37	1.52	0.08	95.46	0.55	7.1	7.11	16.08	7.6	-1.59	11.74	21.4	11.32	1.33
os72303	1	262	0.08	2	3	6	6.79	0.04	0.12	1.53	0.02	79.43	1.37	7.88	8.32	17.28	8.58	-0.72	13.34	23.03	12.69	2.35
os72348	1	368	0.6	10	1	4	6.41	-0.1	0.02	1.47	0.08	99.4	1.79	8.07	7.72	16.67	8.07	-1.18	12.58	22.23	12.01	1.86
os72378	1	237	0	6	2	3	6.5	-0.08	1.39	1.54	0.09	87.66	-2.65	8.19	8.36	17.31	8.61	-0.72	13.35	23.02	12.73	2.36
os72720	1	259	0.92	1	2	3	6.73	0	3.5	1.56	0.06	80.55	-2.35	8.26	8.29	17.27	8.57	-0.75	13.25	22.96	12.64	2.33
os72750	1	252	0.99	7	3	5	6.51	-0.11	0.56	1.52	0.06	89.94	-1.09	7.02	8.38	17.35	8.62	-0.73	13.45	23.16	12.8	2.4
os72792	1	295	0.23	8	2	3	6.76	0.08	0.79	1.51	0.08	92.46	-2.85	8.21	8.27	17.27	8.54	-0.8	13.36	23.01	12.73	2.38
os72793	1	273	0.77	7	3	5	6.47	-0.08	0.47	1.53	0.07	82.95	-0.87	8.08	8.33	17.33	8.58	-0.76	13.46	23.15	12.8	2.43
os72886	1	306	0.92	8	3	5	6.41	-0.12	0.24	1.5	0.05	84.81	0.31	7.37	8.11	16.98	8.43	-1.16	13.26	22.7	12.76	2.17
os72957	1	232	0.04	7	2	3	6.86	0.11	1.06	1.54	0.09	82.36	-3.34	7.47	8.41	17.35	8.65	-0.68	13.42	23.08	12.77	2.39
os72973	1	257	0.43	7	3	5	6.75	0	0.84	1.53	0.07	94.38	-1.87	7.64	8.35	17.32	8.6	-0.73	13.42	23.12	12.76	2.39
os72988	1	273	0.41	3	3	6	6.78	0.01	0.28	1.53	0.02	82.87	1.15	8.49	8.28	17.25	8.54	-0.77	13.28	22.98	12.68	2.32
os72994	1	253	0.08	7	3	5	6.89	0.12	0.08	1.5	0.05	82.4	1.95	7.16	8.33	17.3	8.6	-0.75	13.37	23.06	12.73	2.36
os72999	1	276	0.11	6	3	6	6.54	-0.09	1.08	1.53	0.05	83.23	-1.59	7.8	8.27	17.26	8.54	-0.8	13.32	23.03	12.7	2.33
os73007	1	242	0.11	6	2	3	6.73	0.06	0.95	1.54	0.07	94.33	-3.1	8.02	8.48	17.46	8.71	-0.56	13.45	23.16	12.81	2.49
os73034	1	272	0.93	5	2	3	6.66	-0.04	1.69	1.53	0.07	88.67	-3.42	8.38	8.45	17.44	8.66	-0.66	13.52	23.22	12.88	2.51
os73119	1	394	0.43	15	2	3	6.71	0.03	0.33	1.47	0.16	86.74	-5.09	7.65	7.63	16.42	8.07	-1.43	12.58	22.01	12.21	1.87

os73132	1	390	0.1	2	3	6	6.79	0.02	1.92	1.55	0.06	91.25	-1.4	8.33	7.62	16.46	8.07	-1.46	12.63	22.09	12.25	1.87
os73148	1	344	0.99	4	3	6	6.63	-0.07	0.78	1.53	0.05	97.53	-0.49	7.04	7.95	16.77	8.29	-1.31	13.12	22.54	12.66	2.07
os73209	1	338	0.64	5	3	6	6.58	-0.06	0.54	1.51	0.03	97.83	0.26	7.1	8	16.88	8.3	-1.12	13.07	22.58	12.53	2.16
os73302	1	319	1	5	3	5	6.61	-0.07	1.26	1.54	0.05	86.84	-0.55	8.62	8.06	17.02	8.36	-1.01	13.19	22.81	12.62	2.25
os73314	1	379	0.6	9	2	3	6.58	-0.09	0.65	1.5	0.1	99.4	-2.5	6.5	7.7	16.64	8.08	-1.26	12.7	22.28	12.2	1.95
os73324	1	385	0.89	10	3	5	6.36	-0.15	0.09	1.48	0.07	98.9	-1.54	5.81	7.77	16.7	8.14	-1.27	12.84	22.37	12.33	1.96
os73326	1	387	0.89	10	3	9	6.34	-0.16	0.05	1.47	0.06	99.25	-1.11	5.78	7.76	16.69	8.14	-1.28	12.83	22.37	12.32	1.95
os73327	1	388	0.92	10	3	9	6.32	-0.17	0.04	1.47	0.06	99.56	-0.94	5.8	7.75	16.69	8.13	-1.28	12.82	22.36	12.32	1.94
os73329	1	350	0.94	5	3	5	6.6	-0.09	0.87	1.52	0.06	95.59	-1.21	7	7.81	16.75	8.18	-1.25	12.89	22.42	12.37	1.99
os73334	1	352	0.13	12	3	5	6.74	0.13	0.01	1.47	0.08	93.65	-0.79	6.05	7.89	16.8	8.24	-1.19	12.94	22.5	12.42	2.05
os73408	1	393	0.08	14	3	5	7.01	0.23	0	1.45	0.08	99.55	0.16	5.48	7.74	16.73	8.07	-1.29	12.63	22.33	12.02	1.67
os73431	1	332	0.35	5	2	0	6.85	0.05	0.18	1.53	0.05	91.58	0.65	6.85	7.74	16.71	8.04	-1.26	12.55	22.31	12	1.63
os73518	1	459	0.43	22	2	0	6.75	0.12	0	1.41	0.2	98.52	-0.34	5.97	7.02	15.69	7.44	-1.2	11.56	20.75	11.03	1.33
os73522	1	512	1	27	3	9	5.45	-0.4	0	1.37	0.16	99.3	0.07	6	7.26	15.95	7.59	-1.05	11.93	21.18	11.36	1.52
os73549	1	414	0.88	12	1	1	6.53	-0.13	0.01	1.42	0.08	98.47	2.36	6.48	7.79	16.42	8.01	-0.78	12.54	21.73	11.79	1.77
os73555	1	479	0.33	9	1	1	6.97	0.08	0.03	1.47	0.06	99.43	2.5	6.07	7.32	15.98	7.66	-1.03	11.97	21.17	11.37	1.54
os73560	1	678	0.08	7	3	5	7.07	0.11	0.4	1.51	0.04	99.39	0.78	7.2	5.91	14.65	6.49	-2.11	10.72	19.92	10.36	0.59
os73625	1	454	1	7	3	5	6.57	-0.11	0.35	1.51	0.03	96.42	0.6	6.33	7.61	16.41	7.9	-0.9	12.57	21.91	11.93	1.9
os73636	1	527	0.54	4	3	9	6.7	-0.06	0.41	1.47	0.02	87.05	-0.28	6.8	7.35	16.17	7.71	-1.05	12.18	21.53	11.62	1.73
os73652	1	512	0.6	9	3	5	6.52	-0.06	0.31	1.48	0.07	92.49	-1.21	6.42	7.31	16.13	7.68	-1.09	12.13	21.49	11.58	1.69
os73662	1	529	0.11	4	3	6	6.73	-0.06	0.56	1.51	0.03	91.61	0.24	7.33	7.11	15.91	7.49	-1.23	11.86	21.16	11.33	1.53
os73705	1	540	0.85	7	2	3	6.71	-0.08	0.7	1.48	0.07	98.55	-1.88	7.31	6.8	15.43	7.19	-1.55	11.38	20.52	10.77	0.96
os73723	1	478	0.19	23	3	5	6.29	0.12	0	1.39	0.16	99.94	-1.35	6.25	7.28	15.94	7.6	-1.13	11.96	21.15	11.3	1.41
os73744	1	540	0	13	1	4	7.01	0.22	0	1.37	0.07	97.51	6.52	5.33	6.85	15.49	7.26	-1.48	11.46	20.61	10.86	1.04
os73759	1	591	0.7	11	3	5	6.45	-0.11	0.05	1.48	0.08	99.9	-0.42	6.26	6.44	15.07	6.91	-1.76	10.96	20.08	10.42	0.71
os73786	1	653	0.09	10	2	0	6.93	0.14	0.04	1.48	0.1	99.91	-0.42	7.15	5.91	14.6	6.5	-2.1	10.45	19.6	10.03	0.41
os73960	1	617	0.11	8	3	5	7.05	0.11	0.34	1.5	0.07	91.84	-0.03	6.41	6.3	15.03	6.79	-1.9	11.17	20.44	10.74	0.82
os74097	1	654	0.95	3	3	6	6.83	-0.04	1.87	1.52	0.04	94.05	-0.88	6.98	6.22	14.98	6.74	-1.92	11.07	20.36	10.68	0.79
os74185	1	393	0.76	7	2	0	6.49	-0.1	0.15	1.48	0.11	97.08	1.22	7.71	7.54	16.35	8.01	-1.48	12.47	21.9	12.12	1.8
os74271	1	420	0.08	11	2	0	6.97	0.13	0.1	1.5	0.15	95.64	-3.08	6.97	7.25	16.09	7.75	-1.76	12.23	21.71	11.92	1.56
os74273	1	424	0.01	8	3	5	6.88	0.12	0.4	1.51	0.07	98.61	-1.3	6.17	7.34	16.18	7.85	-1.65	12.32	21.76	11.99	1.67
os74286	1	406	0.23	5	3	5	6.76	0.03	1.09	1.54	0.07	95.15	-1.15	7.16	7.47	16.31	7.95	-1.56	12.48	21.92	12.11	1.77
os74289	1	411	0.35	6	3	5	6.75	0.03	1.02	1.54	0.07	96.94	-1.57	6.95	7.42	16.26	7.91	-1.6	12.42	21.86	12.06	1.73
os74312	1	463	0.87	4	2	0	6.67	-0.07	0.45	1.53	0.11	84.86	1.34	6.96	6.73	15.56	7.3	-2.04	11.47	20.99	11.31	1.23
os74314	1	471	0.77	15	1	2	6.18	-0.2	0.01	1.48	0.15	78.32	-0.76	7.01	6.6	15.42	7.23	-2.07	11.23	20.73	11.12	1.16
os74368	1	497	0.84	12	2	3	6.31	-0.19	0.1	1.49	0.08	99.76	-1.55	6.91	6.87	15.74	7.4	-2.25	11.95	21.44	11.7	1.18
os74392	1	553	0.04	11	2	3	6.82	0.14	0.38	1.49	0	99.33	-5.95	6.74	6.45	15.32	6.98	-2.76	11.66	21.12	11.35	0.79

os74394	1	526	0.03	9	2	7	6.98	0.13	0.45	1.51	0	98.97	-3.56	6.95	6.5	15.36	7.02	-2.73	11.7	21.17	11.39	0.82
os74426	1	658	0.89	8	1	2	6.41	-0.13	0.14	1.44	0.24	99.89	3.02	5.64	5.33	14.3	6.18	-2.79	9.64	19.22	9.69	0.12
os74438	1	536	0.6	7	3	5	6.64	-0.03	0.3	1.5	0.09	98.51	0.35	6.7	6.54	15.42	7.14	-2.05	11.05	20.64	10.81	0.93
os74442	1	530	0.64	13	1	4	6.32	-0.11	0.01	1.43	0.1	84.22	3.51	5.92	6.44	15.33	7.06	-2.23	10.95	20.55	10.86	0.87
os74521	1	639	0.99	14	3	5	6.36	-0.22	0	1.41	0.13	99.19	0.95	5.6	5.84	14.72	6.56	-2.47	10.16	19.73	10.16	0.47
os74604	1	378	0.54	9	2	3	6.62	-0.01	0.86	1.53	0.07	92.12	-2.33	7.8	7.67	16.64	8	-1.39	12.6	22.29	12.08	1.72
os74667	1	538	0.45	13	3	9	6.44	-0.03	0	1.42	0.12	99.83	-1.82	5.49	6.77	15.67	7.27	-2.03	11.45	21.01	11.12	1.02
os74690	1	652	0.6	13	2	3	6.39	-0.09	0.02	1.44	0.13	98.99	-2.98	5.65	5.85	14.78	6.54	-2.72	10.31	19.95	10.23	0.25
os74691	1	710	0	8	1	4	7.02	0.12	0.01	1.35	0.06	97.76	9.02	5.45	5.55	14.48	6.28	-2.91	9.9	19.55	9.9	0.04
os74693	1	673	0.53	8	2	7	6.66	-0.02	0.4	1.5	0.17	98.68	-2.83	6.73	5.46	14.39	6.21	-2.97	9.78	19.44	9.82	-0.01
os74739	1	567	0.95	10	3	5	6.64	-0.11	0.27	1.46	0.12	99.68	-0.63	5.9	6.59	15.55	7	-2.45	11.5	21.15	11.01	0.62
os74746	1	580	0.7	19	3	5	6	-0.19	0	1.43	0.14	99.55	0.68	5.24	6.5	15.47	6.92	-2.51	11.4	21.05	10.94	0.55
os74755	1	624	0.4	10	1	1	6.65	0	0.01	1.42	0.05	100	3.56	5.68	6.56	15.53	6.97	-2.48	11.49	21.13	11.01	0.6
os74819	1	401	0.4	3	3	9	6.85	0.03	0.53	1.49	0.03	95.63	1.01	7.05	7.77	16.67	8.05	-1.36	12.47	22.18	11.94	1.57
os74853	1	315	0.7	8	2	3	6.75	-0.01	0.51	1.51	0.1	90.95	-0.86	7.15	8.01	16.85	8.26	-1.05	12.56	22.2	11.98	1.76
os74957	1	246	0.32	4	3	6	6.8	0.04	2.32	1.55	0.06	89.98	-1.45	8.58	8.55	17.45	8.69	-0.75	13.28	23.04	12.58	2.1
os75028	1	257	0.8	9	2	3	6.42	-0.1	0.29	1.53	0.09	74.57	-3.16	7.74	8.4	17.28	8.57	-0.88	13.18	22.91	12.51	2.01
os75030	1	254	0.59	15	3	5	6.22	-0.08	0	1.47	0.09	63.88	0.13	6.87	8.48	17.38	8.64	-0.84	13.27	23.03	12.61	2.07
os75051	1	431	0.42	9	2	3	6.63	0	0.53	1.51	0.1	97.12	-2.38	7.06	7.35	16.27	7.68	-1.67	12.02	21.69	11.49	1.28
os75079	1	344	0.7	17	2	0	5.89	-0.26	0.05	1.44	0.14	82.28	-0.86	6.04	7.84	16.73	8.09	-1.37	12.65	22.31	12.02	1.61
os75082	1	413	0.87	12	3	5	6.49	-0.14	0.01	1.45	0.11	98.02	-0.67	5.95	7.54	16.44	7.83	-1.61	12.3	21.95	11.73	1.37
os75210	1	744	0.86	19	1	1	6.41	-0.2	0	1.34	0.09	99.94	6.15	5.06	5.68	14.64	6.34	-2.8	10.12	19.73	9.96	0.14
os75237	1	674	1	15	1	1	6.22	-0.25	0	1.33	0.07	99.97	6.35	5.19	6.46	15.37	6.96	-2.33	11.19	20.77	10.84	0.71
os75437	1	358	0.9	7	3	5	6.61	-0.09	0.05	1.5	0.09	95.51	1.23	7.02	7.77	16.82	8.05	-1.29	12.72	22.55	12.08	1.69
os75453	1	545	0.96	17	1	1	6.24	-0.24	0	1.35	0.11	99.9	4.93	5.03	6.82	15.74	7.28	-2.1	11.47	21.11	11.08	0.9
os75482	1	612	0.7	16	2	0	6.22	-0.15	0.12	1.5	0.18	90.72	-2.8	6.39	5.71	14.66	6.38	-2.93	10.22	19.9	10.11	0.03
os75494	1	768	0.7	9	1	1	6.62	-0.07	0.02	1.37	0.07	100	5.16	5.64	5.4	14.37	6.16	-3.05	9.79	19.42	9.76	-0.13
os75496	1	556	0.01	10	3	5	6.95	0.15	0.02	1.45	0.07	98.07	1.97	5.58	6.75	15.7	7.19	-2.16	11.34	21.04	10.93	0.75
os75553	1	450	0.4	17	3	5	6.91	0.13	0	1.42	0.14	97.76	0.39	5.08	7.11	16.06	7.5	-1.85	11.74	21.43	11.24	1.04
os75704	1	535	0.34	14	1	2	6.57	0.05	0	1.43	0.12	99.26	1.67	6.27	6.55	15.51	7.02	-2.37	11.23	20.89	10.78	0.55
os75724	1	539	1	16	3	5	6.08	-0.28	0	1.41	0.11	97.87	2.54	5.37	6.84	15.79	7.26	-2.1	11.44	21.16	11.03	0.83
os75776	1	498	0.83	19	3	5	5.87	-0.24	0	1.41	0.15	99.66	0.47	5.9	6.83	15.8	7.27	-2.04	11.47	21.14	11.02	0.86
os75780	1	567	0.6	8	1	4	6.57	-0.07	0.17	1.42	0.07	99.9	2.81	6.08	6.66	15.63	7.14	-2.15	11.23	20.91	10.84	0.73
os75828	1	586	0.99	20	1	1	5.97	-0.31	0	1.32	0.13	100	3.27	5.06	6.86	15.83	7.27	-2.16	11.58	21.27	11.1	0.8
os75861	1	413	0.75	14	3	5	6.53	-0.11	0	1.46	0.11	99.95	-1.07	6.73	7.37	16.29	7.72	-1.57	12.02	21.72	11.48	1.29
os75913	1	223	1	8	3	5	6.41	-0.14	0.18	1.5	0.06	98.02	0.45	6.23	9.06	17.9	9.17	0.26	13.86	23.13	12.93	2.93
os75960	1	246	0.99	11	1	2	6.25	-0.18	0	1.46	0.2	99.73	2.45	5.33	8.26	17.02	8.56	-0.3	12.7	21.96	12	2.12

os75996	1	321	0.9	16	1	1	6.33	-0.17	0	1.39	0.08	100	5.56	5.19	8.24	16.99	8.56	-0.25	12.72	21.96	12.06	2.18
os75999	1	278	0	12	3	5	6.93	0.2	0.01	1.47	0.1	99.95	-0.29	7.55	8.26	17.02	8.57	-0.23	12.74	22.01	12.09	2.2
os76065	1	441	0.92	11	3	9	6.27	-0.19	0.01	1.42	0.09	99.97	0.41	6.01	7.75	16.58	8.09	-0.71	12.12	21.5	11.49	1.74
os76090	1	434	0.43	21	2	0	6.02	-0.04	0	1.37	0.16	99.89	2.59	5.34	7.34	16.15	7.75	-1.14	11.7	21.02	11.06	1.26
os76104	1	277	0.53	25	2	7	5.58	-0.09	0	1.4	0.25	99.74	-5.18	5.33	8.21	16.93	8.49	-0.4	12.54	21.76	11.82	2.03
os76110	1	370	0.11	10	1	1	6.67	-0.01	0	1.27	0.07	98.61	13.48	5.62	8.19	16.88	8.47	-0.45	12.55	21.73	11.81	1.98
os76115	1	472	0.17	15	2	0	7.01	0.21	0	1.42	0.15	100	1.64	5.52	7.13	15.93	7.52	-1.34	11.49	20.78	10.84	1.07
os76123	1	551	0.18	11	3	5	6.99	0.11	0.21	1.48	0.1	99.41	-1.99	6.37	6.93	15.73	7.33	-1.6	11.4	20.74	10.73	0.87
os76172	1	259	0.62	15	2	3	6.62	-0.03	0	1.45	0.16	99.45	-3.1	5.99	8.65	17.38	8.87	0.05	13.04	22.26	12.26	2.5
os76212	1	286	0.41	8	3	5	6.55	-0.02	0.14	1.46	0.06	99.97	0.8	6.1	8.49	17.26	8.79	-0.08	12.94	22.22	12.22	2.34
os76242	1	390	0.82	9	3	9	6.41	-0.13	0.02	1.41	0.07	99.64	1.99	5.59	8.04	16.78	8.33	-0.5	12.45	21.71	11.77	1.88
os76246	1	488	0.19	7	3	5	6.99	0.1	0.35	1.47	0.05	97.88	0.28	6.77	7.25	16.07	7.67	-1.2	11.61	20.93	10.99	1.18
os76259	1	332	0.01	4	2	8	6.79	0.04	1.73	1.44	0.16	99.37	-6.64	6.09	8.17	16.91	8.46	-0.39	12.6	21.85	11.91	2
os76273	1	544	0.37	7	3	5	6.72	0	0.4	1.47	0.05	99.95	0.53	6.24	6.99	15.8	7.4	-1.47	11.43	20.75	10.78	0.93
os76310	1	526	0.98	10	3	9	6.45	-0.16	0.09	1.47	0.06	99.34	0.32	6.24	7.3	16.18	7.7	-1.15	11.66	21.02	11.05	1.23
os76312	1	417	0.11	9	2	8	6.78	0.1	0.14	1.46	0.09	99.67	-2.21	5.88	7.93	16.76	8.25	-0.5	12.27	21.62	11.66	1.9
os76322	1	357	0.79	19	3	9	5.84	-0.22	0	1.37	0.18	99.91	-1.91	4.93	8.15	16.9	8.47	-0.42	12.49	21.7	11.8	1.98
os76335	1	391	1	10	1	4	6.54	-0.12	0	1.44	0.06	97.54	5.44	5.52	7.67	16.41	8.07	-0.71	12.04	21.31	11.43	1.67
os76363	1	335	0.4	8	3	5	6.52	-0.03	0.14	1.49	0.08	99.23	-0.36	6.51	8.01	16.78	8.33	-0.46	12.56	21.82	11.88	2.02
os76382	1	520	0.41	2	3	6	6.84	0	0.26	1.52	0.04	80.05	0.69	9.08	7	15.81	7.4	-1.48	11.46	20.79	10.78	0.92
os76390	1	424	0.99	14	3	9	6.23	-0.23	0	1.42	0.12	98.75	-0.79	5.29	7.81	16.66	8.15	-0.65	12.19	21.58	11.55	1.8
os76399	1	419	0.64	9	1	4	6.47	-0.07	0.02	1.41	0.07	99.59	4.21	5.8	7.67	16.48	8.03	-0.79	12.06	21.38	11.41	1.61
os76405	1	440	1	12	3	9	6.37	-0.19	0.01	1.44	0.08	96.53	-0.88	5.66	7.69	16.48	8.05	-0.79	12.02	21.34	11.39	1.62
os76426	1	319	0	15	2	7	6.76	0.24	0.21	1.48	0.24	99.81	-8	5.75	8.1	16.95	8.34	-0.55	12.66	22	11.85	1.97
os76437	1	412	0.92	3	1	4	6.67	-0.04	0.01	1.48	0.03	98.98	4.48	6.52	7.53	16.26	7.94	-0.83	11.88	21.17	11.28	1.54
os76461	1	467	0.75	7	3	5	6.55	-0.08	0.16	1.49	0.04	99.41	1.91	6.56	7.73	16.61	7.94	-1.05	12.34	21.69	11.45	1.51
os76683	1	320	0.98	16	1	1	6.09	-0.25	0	1.34	0.09	99.84	6.27	5.84	8.36	17.08	8.63	-0.23	12.7	21.9	11.96	2.17
os76695	1	393	1	24	2	7	5.49	-0.39	0	1.39	0.16	100	-1.04	4.96	7.76	16.54	8.07	-0.76	12.05	21.34	11.38	1.64
os76702	1	394	0.47	16	2	7	6.22	-0.03	0.03	1.45	0.17	99.96	-6.76	5.8	7.74	16.54	8.06	-0.75	12.02	21.35	11.34	1.63
os76710	1	276	0.7	24	3	5	6.44	-0.05	0	1.4	0.19	99.26	0.56	5.48	8.39	17.16	8.59	-0.22	12.77	22.04	11.97	2.18
os76713	1	269	0.64	24	3	5	6.35	-0.09	0	1.38	0.19	99.63	-0.22	5.35	8.53	17.28	8.73	-0.11	12.91	22.14	12.11	2.3
os76735	1	359	0.79	7	1	1	6.48	-0.11	0.02	1.36	0.05	99.99	6.34	5.34	8.64	17.58	8.76	-0.04	13.17	22.66	12.24	2.43
os76836	1	363	0.03	4	2	7	6.83	0.07	1.4	1.53	0.15	99.33	-2.85	6.3	7.95	16.78	8.17	-0.66	12.44	21.74	11.67	1.9
os76848	1	370	0.77	11	2	7	6.27	-0.14	0.14	1.49	0.18	98.56	-6.26	7.74	7.82	16.59	8.1	-0.74	12.13	21.4	11.42	1.7
os76849	1	410	0.5	9	1	4	6.88	0.04	0.03	1.42	0.09	98.36	2.62	5.72	7.76	16.53	8.04	-0.8	12.07	21.34	11.36	1.65
os76878	1	370	0.08	5	3	5	6.9	0.07	0.14	1.51	0.06	99.98	1.11	7	7.8	16.54	8.11	-0.78	12.3	21.55	11.58	1.7
os76882	1	373	0.83	7	1	4	6.66	-0.07	0.07	1.47	0.07	99.54	2.41	7.17	7.77	16.53	8.1	-0.75	12.24	21.5	11.56	1.72

os76914	1	411	0.99	6	3	9	6.61	-0.09	0.2	1.47	0.04	99.48	1.19	6.35	7.92	16.68	8.23	-0.59	12.39	21.7	11.77	1.91
os76925	1	325	0.4	11	3	5	6.88	0.08	0.05	1.5	0.07	99.9	-0.3	6.07	8.11	16.85	8.38	-0.51	12.62	21.84	11.87	2.04
os76932	1	374	0.35	15	3	5	6.91	0.12	0	1.46	0.08	100	0.58	5.68	7.97	16.73	8.26	-0.63	12.47	21.71	11.74	1.92
os76934	1	368	0.34	15	3	5	6.92	0.13	0	1.47	0.09	100	-0.62	5.78	7.98	16.74	8.27	-0.62	12.48	21.72	11.75	1.93
os77036	1	503	0.99	8	3	5	6.57	-0.13	0.24	1.5	0.1	99.9	-0.29	5.82	7.29	16.14	7.5	-1.39	11.65	20.95	10.8	1.04
os77045	1	458	0.47	5	3	5	6.71	0.02	0.32	1.5	0.06	100	0.97	6.17	7.75	16.62	7.9	-1	12.3	21.63	11.36	1.48
os77059	1	531	0.01	4	3	6	6.92	0.06	0.6	1.5	0.03	94.92	1.07	7.11	7.42	16.33	7.66	-1.31	12.02	21.37	11.16	1.25
os77221	1	495	0.53	17	3	5	6.77	0.02	0	1.42	0.12	99.86	-2.03	5.22	7.45	16.28	7.76	-1.06	11.73	21.05	11.04	1.32
os77230	1	479	0.05	13	3	5	7.04	0.21	0.01	1.42	0.1	98.34	-0.06	5.36	7.68	16.53	7.88	-0.95	12.22	21.51	11.36	1.55
os77259	1	573	1	16	3	9	6.22	-0.25	0	1.41	0.09	100	-0.61	5.57	7.18	16.15	7.49	-1.27	11.46	20.94	10.76	1.13
os77283	1	522	0.94	10	3	5	6.61	-0.12	0.1	1.48	0.08	99.97	-0.14	6.56	7.38	16.27	7.61	-1.27	11.81	21.13	10.95	1.17
os77303	1	555	0.35	9	3	5	6.97	0.06	0.3	1.47	0.08	99.53	-1.12	6.83	7.1	16	7.4	-1.38	11.41	20.77	10.71	1.08
os77305	1	408	0.4	7	2	0	6.85	0.02	0.64	1.51	0.12	100	-1.93	6.16	7.65	16.45	7.91	-0.91	12.01	21.28	11.27	1.56
os77342	1	381	0.01	8	3	5	6.94	0.14	0.1	1.49	0.06	98.44	1.08	6.46	7.68	16.44	8.02	-0.82	12.12	21.4	11.45	1.64
os77363	1	330	0.13	9	3	5	6.96	0.14	0.07	1.5	0.07	100	-0.15	7.27	8.11	16.86	8.38	-0.51	12.63	21.85	11.88	2.04
os77379	1	343	0.26	12	3	5	6.96	0.14	0.01	1.49	0.08	99.97	-0.83	5.89	7.97	16.73	8.26	-0.62	12.47	21.71	11.74	1.92
os77407	1	447	0.21	9	1	4	6.77	0.08	0.04	1.48	0.05	99.32	2.35	5.82	7.52	16.27	7.84	-1.09	12.03	21.22	11.26	1.38
os77448	1	376	0.74	8	2	3	6.49	-0.06	0.87	1.5	0.11	93.49	-4.27	7.92	8.08	16.9	8.26	-0.58	12.67	21.9	11.8	1.94
os77470	1	232	0.56	11	2	7	6.67	-0.02	0.16	1.5	0.19	99.63	-5.29	6.57	8.54	17.29	8.72	-0.1	12.93	22.17	12.11	2.31
os77473	1	280	0.89	17	1	4	6.18	-0.22	0	1.37	0.12	99.47	3.81	5.18	8.54	17.29	8.74	-0.11	12.91	22.15	12.12	2.31
os77493	1	409	1	16	3	5	6.1	-0.26	0	1.45	0.11	99.98	-1.08	5.69	7.76	16.57	8.07	-0.78	12.1	21.37	11.4	1.66
os77571	1	389	0.73	10	3	9	6.73	-0.04	0.05	1.47	0.06	99.46	0.13	5.84	7.81	16.53	8.15	-0.63	12.29	21.54	11.65	1.87
os77573	1	376	1	7	3	5	6.54	-0.11	0.39	1.49	0.05	99.8	0.4	6.72	7.78	16.52	8.14	-0.65	12.26	21.52	11.62	1.85
os77586	1	366	0.27	11	3	5	6.94	0.11	0.04	1.48	0.09	100	-0.6	6.19	7.77	16.5	8.11	-0.63	12.23	21.5	11.62	1.88
os77604	1	368	0.37	9	3	5	6.56	0	0.07	1.47	0.07	100	0.68	6.24	7.91	16.66	8.27	-0.58	12.43	21.68	11.74	1.9
os77667	1	463	0.17	5	3	5	6.84	0.06	0.57	1.51	0.06	96.98	-0.62	8.23	7.2	15.99	7.63	-1.09	11.6	20.91	11.05	1.4
os77669	1	412	0.6	9	3	5	6.49	-0.06	0.29	1.46	0.06	97.74	1.48	7.21	7.54	16.3	7.93	-0.83	11.97	21.24	11.37	1.64
os77675	1	307	0.02	6	2	7	6.86	0.09	1.17	1.54	0.11	99.32	-3.36	7.81	7.95	16.68	8.28	-0.5	12.48	21.69	11.81	2
os77723	1	409	0.36	13	2	3	6.9	0.1	0.02	1.48	0.12	99.44	-2.51	5.66	7.65	16.41	8.01	-0.85	12.08	21.39	11.45	1.64
os77747	1	282	0.5	18	2	3	6.65	0.05	0	1.44	0.16	99.85	-5.86	6.38	8.49	17.26	8.76	-0.03	12.88	22.14	12.15	2.42
os77784	1	489	0	4	2	7	6.73	0.01	1.75	1.55	0.14	91.95	-7.07	8.67	6.94	15.84	7.3	-1.44	11.81	21.41	11.36	1.4
os78260	1	407	0.38	8	2	3	6.65	0.01	0.9	1.51	0.09	96.16	-2.89	7.07	7.58	16.54	7.86	-0.94	12.55	22.19	11.92	2
os78282	1	484	1	12	1	4	6.46	-0.15	0	1.45	0.06	95.83	4.75	5.54	7.07	15.94	7.37	-1.26	12.16	21.58	11.58	1.89
os78718	1	532	0.75	9	3	5	6.48	-0.1	0.15	1.5	0.09	100	-0.95	6.21	6.78	15.68	7.15	-1.4	11.85	21.27	11.37	1.83
os78747	1	499	0.6	13	3	5	6.38	-0.06	0	1.48	0.08	99.85	-0.89	5.61	6.97	15.87	7.3	-1.3	12.1	21.51	11.53	1.88
os78777	1	453	0.08	6	2	0	6.96	0.09	0.76	1.53	0.07	100	-1.2	8.17	7.14	16.03	7.4	-1.23	12.22	21.67	11.64	1.96
os78778	1	458	0.02	6	3	5	6.96	0.11	0.43	1.52	0.07	100	-0.35	7.26	7.13	16.02	7.39	-1.23	12.21	21.65	11.63	1.95

os78779	1	481	0.77	6	2	0	6.79	-0.03	0.49	1.52	0.07	100	-0.2	6.41	7.03	15.92	7.31	-1.29	12.07	21.53	11.52	1.9
os78816	1	430	0.6	4	3	6	6.68	-0.03	0.47	1.53	0.04	92.96	0.22	7.17	7.43	16.33	7.67	-1.05	12.55	22.05	11.95	2.12
os78826	1	388	0.98	12	3	5	6.33	-0.19	0.04	1.49	0.07	91.19	-0.61	6.06	7.73	16.59	7.89	-0.9	12.94	22.41	12.21	2.29
os78859	1	499	0.86	3	3	6	6.77	-0.03	1.51	1.55	0.03	54.72	-0.19	8.23	7.12	16.02	7.42	-1.35	12.07	21.56	11.5	1.7
os78916	1	423	0	9	3	5	6.4	-0.15	0.09	1.48	0.05	93.23	1.93	6.06	7.59	16.48	7.82	-0.94	12.68	22.18	12.06	2.17
os78966	1	404	0.59	7	2	3	6.57	-0.03	0.72	1.54	0.09	95.72	-4.06	8.55	7.51	16.42	7.77	-1.02	12.57	22.1	11.97	2.12
os79275	1	483	0	5	3	6	6.91	0.08	0.54	1.5	0.03	96.42	0.64	6.64	7.19	16.04	7.45	-1.1	12.2	21.6	11.54	1.87
os79458	1	441	0.37	9	2	0	6.66	0.03	0.04	1.5	0.08	84.75	0.24	6.68	7.21	16	7.45	-1.08	12.22	21.53	11.44	1.83
os79463	1	401	0.18	5	3	6	6.89	0.05	0.94	1.53	0.03	84.87	-0.25	8.23	7.54	16.48	7.8	-0.86	12.59	22.17	11.92	2.13
os79631	1	438	0.79	3	3	6	6.73	-0.04	0.19	1.54	0.03	83.71	0.71	8.62	7.28	16.2	7.58	-1.02	12.27	21.82	11.67	1.92
os79720	1	508	0.53	4	2	3	6.9	0.03	1.78	1.54	0.07	92.43	-2.74	8.1	6.94	15.88	7.29	-1.23	11.81	21.42	11.32	1.71
os79778	1	384	0.01	12	3	5	6.96	0.19	0.04	1.5	0.08	76.17	-0.69	7.05	7.59	16.51	7.83	-0.85	12.67	22.26	12.02	2.13
os79801	1	450	1	6	3	5	6.59	-0.1	0.5	1.53	0.06	86.13	-1.64	7.05	7.28	16.15	7.53	-1.06	12.39	21.8	11.69	1.97
os79867	1	451	0.47	4	3	6	6.83	0	0.51	1.51	0.03	98.19	0.16	6.99	7.22	16.14	7.51	-1.06	12.35	21.83	11.7	2.03
os79954	1	488	0.99	2	3	6	6.78	-0.02	0.49	1.54	0.01	66.21	0.59	8.76	7.06	15.97	7.34	-1.1	12.19	21.6	11.49	1.95
os79956	1	484	0.6	4	3	6	6.69	-0.03	0.78	1.53	0.03	86.58	-0.3	7.2	7.04	15.95	7.33	-1.1	12.16	21.58	11.47	1.95
os79958	1	487	0.92	2	3	6	6.77	-0.01	1.91	1.54	0.02	70.21	-0.21	8.43	7.06	15.97	7.34	-1.1	12.19	21.6	11.49	1.95
os80091	1	424	0.2	7	3	5	6.92	0.07	0.47	1.53	0.06	92.39	-0.89	8	7.43	16.32	7.64	-1.17	12.63	22.09	11.98	2.09
os80097	1	554	1	12	2	0	6.38	-0.2	0.02	1.48	0.1	99.97	-0.57	5.51	6.64	15.52	7	-1.57	11.69	21.09	11.32	1.77
os80495	1	471	0.29	7	3	5	6.7	0.02	0.29	1.5	0.06	99.72	-0.09	7.24	7.32	16.27	7.64	-1.17	12	21.79	11.62	1.64
os80714	1	534	0.19	2	3	6	6.88	0.01	1.94	1.54	0.03	86.19	-0.94	7.88	6.85	15.74	7.29	-1.41	11.64	21.18	11.24	1.35
os81092	1	451	0.23	5	3	6	6.62	-0.09	0.54	1.51	0.03	98.91	0	7.09	7.52	16.5	7.78	-1	12.32	22.03	11.8	1.91
os81718	1	379	0.92	3	3	6	6.66	-0.05	0.84	1.53	0.03	81.97	0.12	7.66	7.8	16.79	8.06	-0.79	12.85	22.54	12.23	2.18
os82081	1	298	1	6	3	5	6.48	-0.12	0.39	1.52	0.1	70.26	-1.24	7.79	8.3	17.26	8.42	-0.57	13.33	22.97	12.54	2.38
os82116	1	475	0.03	5	3	6	6.89	0.07	0.39	1.52	0.03	98.14	0.41	6.77	7.39	16.35	7.69	-1.18	12.38	21.98	11.82	1.86
os82522	1	424	0.87	11	2	0	6.51	-0.13	0.01	1.48	0.14	85.69	-0.13	6.58	7.2	16.01	7.41	-1.17	12.18	21.54	11.4	1.72
os82654	1	475	0.3	7	2	3	6.73	0.03	0.57	1.53	0.06	87.22	-2.18	7.66	7.05	15.85	7.3	-1.18	11.93	21.31	11.29	1.69
os82682	1	495	0.92	5	3	6	6.65	-0.07	0.53	1.53	0.04	63.51	-0.14	7.99	6.98	15.78	7.24	-1.27	11.98	21.32	11.27	1.63
os82877	1	448	0.73	11	2	7	6.35	-0.14	0.34	1.53	0.13	76.26	-3.87	7.67	7.14	15.92	7.36	-1.2	12.16	21.54	11.46	1.71
os82895	1	522	0.43	7	3	5	6.73	0.02	0.36	1.51	0.04	81.19	0.75	6.14	6.87	15.65	7.16	-1.32	11.76	21.02	11.05	1.45
os82983	1	507	0.82	7	2	3	6.56	-0.09	0.51	1.53	0.07	86.75	-2.26	6.97	6.9	15.7	7.17	-1.3	11.87	21.18	11.17	1.55
os83044	1	659	0.24	2	3	6	6.94	0.02	1.82	1.54	0.01	66.28	-0.29	8.64	6.05	14.77	6.5	-1.84	10.7	20	10.29	0.9
os83048	1	568	0.08	3	3	6	6.93	0.04	2.74	1.55	0.02	37.44	0.37	8.69	6.62	15.35	6.93	-1.5	11.43	20.8	10.87	1.29
os83049	1	571	0.09	3	1	4	6.92	0.03	0.07	1.51	0.01	35.74	2.88	8.69	6.62	15.36	6.93	-1.5	11.43	20.78	10.87	1.29
os83119	1	597	0	7	2	3	7	0.1	0.88	1.5	0.08	99.03	-2.81	7.66	6.43	15.17	6.83	-1.69	11.21	20.49	10.73	1.12
os83178	1	516	1	12	3	5	6.36	-0.2	0.01	1.47	0.08	99.23	-1.28	7.05	6.84	15.63	7.15	-1.42	11.75	21.12	11.14	1.44
os83311	1	492	0.15	5	3	6	6.86	0.06	0.39	1.53	0.04	58.63	-0.15	7.92	6.98	15.69	7.21	-1.31	11.97	21.28	11.33	1.6

os83469	1	549	0.34	13	2	3	6.65	0.08	0.01	1.5	0.1	90.7	-2.84	5.64	6.65	15.4	6.96	-1.58	11.52	20.83	10.96	1.27
os83611	1	380	0.75	7	2	0	6.48	-0.12	0.4	1.52	0.06	90.31	-0.21	8.32	7.72	16.6	7.93	-0.99	12.69	22.22	12.05	2.01
os83701	1	496	0.35	7	2	3	6.96	0.07	0.3	1.53	0.07	92.57	-2.2	6.34	7.12	16.05	7.38	-1.45	12.23	21.76	11.67	1.69
os83870	1	447	0.3	4	3	6	6.9	0.04	0.57	1.52	0.03	100	0.14	7.76	7.57	16.52	7.82	-1.09	12.62	22.23	12.04	1.99
os83877	1	445	0.5	5	3	5	6.68	-0.01	0.51	1.53	0.04	97.1	-0.19	6.47	7.54	16.43	7.77	-1.12	12.54	22.09	11.91	1.94
os83883	1	468	0.16	4	3	6	6.83	0.04	0.68	1.54	0.02	97.25	-0.03	7.67	7.48	16.4	7.7	-1.16	12.51	22.07	11.89	1.91
os83893	1	495	0.55	6	3	5	6.88	0.01	0.47	1.5	0.04	97.65	0.51	6.57	7.28	16.18	7.56	-1.25	12.24	21.75	11.66	1.81
os83931	1	419	0.77	6	3	5	6.76	-0.04	0.57	1.54	0.07	76.93	-1.17	7.23	7.67	16.57	7.77	-1.14	12.84	22.3	12.02	1.98
os84194	1	600	0.06	4	3	6	6.97	0.07	0.6	1.53	0.02	73.72	-0.16	7.14	6.51	15.41	6.98	-1.68	11.24	20.8	10.89	1.11
os84284	1	544	0.6	7	3	5	6.65	-0.03	0.23	1.48	0.04	98.03	1.02	6.37	6.82	15.62	7.16	-1.44	11.7	21.08	11.17	1.45
os84300	1	541	0.02	8	3	5	6.95	0.12	0.33	1.49	0.07	99.38	0.25	6.12	6.78	15.59	7.13	-1.44	11.64	21.06	11.13	1.45
os84463	1	463	0.99	3	3	6	6.7	-0.06	1.12	1.54	0.05	99	-0.55	7.44	7.13	16.02	7.41	-1.17	12.11	21.62	11.48	1.79
os84564	1	529	0.15	10	3	5	6.77	0.1	0.08	1.48	0.1	99.3	-1.54	6.05	6.81	15.62	7.11	-1.39	11.66	21.04	11.01	1.51
os84565	1	547	0.37	9	2	3	6.63	0.01	0.06	1.49	0.13	97.28	-3.08	6.04	6.82	15.63	7.11	-1.38	11.67	21.05	11.01	1.51
os84568	1	588	0.48	10	3	5	6.96	0.05	0.1	1.46	0.08	96.92	-0.58	6.77	6.58	15.41	6.93	-1.47	11.36	20.76	10.8	1.38
os84570	1	597	0.27	9	3	5	7.02	0.1	0.15	1.48	0.08	96.28	-1.58	6.96	6.55	15.38	6.91	-1.48	11.33	20.72	10.78	1.36
os84816	1	566	0.16	9	3	5	7.04	0.12	0.34	1.47	0.06	98.93	0.02	6.92	6.62	15.36	7.03	-1.59	11.36	20.69	10.9	1.17
os84878	1	613	0.08	8	3	5	7.06	0.12	0.2	1.49	0.06	99.2	-0.13	6.56	6.41	15.22	6.87	-1.77	11.14	20.54	10.75	1.03
os85037	1	499	0.93	11	1	4	6.54	-0.14	0.02	1.48	0.08	90.96	1.27	7.7	6.92	15.7	7.24	-1.39	11.87	21.2	11.27	1.51
os85351	1	494	0.6	4	3	6	6.72	-0.02	0.56	1.51	0.06	99.13	-0.42	6.07	7.02	15.94	7.35	-1.19	12.04	21.52	11.47	1.82
os85408	1	538	0.6	12	3	5	6.41	-0.09	0.01	1.49	0.11	100	-1.89	5.51	6.77	15.72	7.17	-1.34	11.75	21.24	11.23	1.71
os85475	1	438	0.99	2	3	6	6.77	-0.02	0.25	1.53	0.02	59.59	0.49	9.05	7.23	16.08	7.48	-0.99	12.31	21.64	11.49	1.94
os85501	1	478	0.67	6	3	5	6.63	-0.05	1.03	1.52	0.06	98.11	-2.36	8.06	6.99	15.88	7.29	-1.11	12.03	21.4	11.29	1.83
os85560	1	567	0.01	2	3	6	6.89	0.03	2.91	1.55	0.02	34.41	-0.14	9.05	6.62	15.35	6.93	-1.5	11.42	20.79	10.86	1.29
os86115	1	480	0.07	9	3	5	7.01	0.14	0.29	1.5	0.1	94.58	-1.66	6.03	7.22	16.14	7.51	-1.38	11.96	21.45	11.33	1.44
os86131	1	416	0.6	6	3	5	6.6	-0.04	0.54	1.53	0.05	99.7	-0.39	7.2	7.83	16.76	7.95	-0.94	12.81	22.33	11.99	1.95
os86132	1	417	0.6	7	3	5	6.59	-0.04	0.37	1.52	0.05	99.75	-0.03	7.18	7.83	16.76	7.94	-0.94	12.8	22.32	11.98	1.95
os86313	1	681	0.85	5	1	1	6.7	-0.07	0.38	1.49	0.03	90.88	2.07	6.66	6.28	15.18	6.73	-1.97	10.78	20.27	10.46	0.88
os86475	1	531	0.99	4	3	6	6.69	-0.07	0.58	1.54	0.03	94.94	0.03	7.19	7.15	16.05	7.45	-1.5	12	21.49	11.44	1.46
os86478	1	546	0.92	6	3	5	6.62	-0.09	0.51	1.53	0.04	94.59	-0.19	6.89	7.13	16.03	7.43	-1.54	12.03	21.53	11.49	1.47
os86479	1	593	0.55	2	3	6	6.89	0.01	0.21	1.54	0.02	60.39	0.6	8.63	6.82	15.73	7.17	-1.7	11.53	21.03	11.08	1.24
os86485	1	661	0.99	4	3	6	6.78	-0.06	0.56	1.51	0.03	99.14	0.87	6.68	6.43	15.33	6.87	-1.88	10.99	20.48	10.63	0.98
os86683	1	518	0.34	5	3	6	6.95	0.06	0.38	1.51	0.03	96.11	0.32	6.73	7.3	16.31	7.53	-1.39	12.09	21.78	11.52	1.4
os86706	1	490	0.08	3	3	6	6.92	0.05	2.24	1.53	0.05	82.78	-1.08	9.08	7.32	16.3	7.57	-1.31	12.09	21.78	11.55	1.5
os86719	1	409	0.83	7	3	5	6.49	-0.1	0.29	1.52	0.07	89.38	-0.69	6.97	7.8	16.78	7.95	-1.08	12.69	22.34	11.98	1.72
os86742	1	482	0.99	11	3	5	6.31	-0.21	0.01	1.49	0.09	94.3	-1.47	6.42	7.44	16.43	7.69	-1.24	12.23	21.92	11.67	1.58
os87093	1	410	0.92	9	3	5	6.42	-0.14	0.33	1.48	0.05	97.65	-0.27	6.75	7.84	16.7	7.95	-0.88	12.51	22.01	11.8	1.87

os87343	1	456	0.92	0	3	6	6.8	0	0.82	1.54	0	35.23	0.43	10.51	7.47	16.32	7.66	-1.15	12.06	21.59	11.4	1.58
os87466	1	433	0.94	6	1	1	6.7	-0.06	0.02	1.4	0.05	99.67	7.46	5.61	7.9	16.73	7.96	-0.78	12.64	22.09	11.79	2.01
os87514	1	362	0.28	3	2	0	6.83	0.02	2.79	1.54	0.05	52.96	-0.7	7.81	8.14	17.06	8.15	-0.7	13.19	22.7	12.21	2.2
os87546	1	366	0.51	9	3	5	6.46	-0.04	0.06	1.46	0.1	97.68	-0.95	7.07	7.97	16.77	8.05	-0.74	12.67	22.07	11.86	2.06
os87913	1	376	0.96	8	3	5	6.55	-0.12	0.19	1.46	0.06	97.04	1.57	6.47	7.87	16.81	8.1	-0.82	12.71	22.42	12.16	2.02
os87933	1	434	1	14	1	4	6.27	-0.21	0.01	1.43	0.06	88.06	4.67	5.95	7.63	16.59	7.85	-1.06	12.54	22.27	12.01	1.81
os88017	1	396	0.13	3	3	6	6.82	0.04	2.24	1.52	0.03	64.1	-0.51	8.2	7.9	16.88	8.06	-0.87	12.82	22.45	12.18	2.02
os88266	1	377	0.92	8	3	5	6.44	-0.14	0.24	1.51	0.07	84.92	0.21	6.14	7.94	16.8	8.09	-0.92	12.73	22.21	12	1.8
os88270	1	406	0.96	7	3	5	6.55	-0.11	0.43	1.49	0.06	98.33	0.05	6.2	7.93	16.79	8.07	-0.92	12.71	22.19	11.98	1.8
os88280	1	421	0.04	3	2	3	6.82	0.04	1.93	1.54	0.08	98.87	-3.79	8.04	7.67	16.54	7.87	-1.09	12.3	21.81	11.65	1.63
os88559	1	412	0.79	5	2	3	6.74	-0.04	1.51	1.54	0.06	64.51	-3.05	8.39	7.94	16.84	8.08	-0.97	12.92	22.36	12.18	1.85
os88584	1	395	0.24	6	3	6	6.74	0.03	0.18	1.51	0.03	65.29	0.97	7.98	8.01	16.92	8.16	-0.86	12.94	22.47	12.21	1.95
os88653	1	338	0.68	5	3	5	6.54	-0.08	0.49	1.5	0.03	91.53	0.93	7.86	8.36	17.23	8.47	-0.42	13.16	22.64	12.35	2.34
os88660	1	341	1	13	3	5	6.2	-0.22	0.06	1.47	0.13	97.89	-1.93	6.67	8.14	16.96	8.25	-0.62	12.74	22.17	11.96	2.04
os88740	1	444	0.31	5	3	6	6.68	-0.02	0.18	1.51	0.02	98.98	2.08	6.64	7.66	16.52	7.78	-1.04	12.52	21.96	11.7	1.8
os89016	1	482	0.96	7	2	3	6.62	-0.09	0.89	1.5	0.13	98.41	-5.31	8.17	7.16	16.12	7.54	-1.21	11.89	21.57	11.53	1.58
os89129	1	490	0.07	12	2	0	6.87	0.16	0.01	1.49	0.11	97.92	-0.76	6.45	6.95	15.85	7.38	-1.35	11.74	21.29	11.35	1.44
os89198	1	553	0.06	4	2	3	6.92	0.07	1.6	1.5	0.06	99.4	-2.31	8.15	6.82	15.72	7.24	-1.42	11.32	20.92	11.02	1.3
os89214	1	482	0.55	12	2	3	6.75	0	0.02	1.48	0.12	97.9	-3.48	5.18	7.3	16.23	7.64	-1.15	11.98	21.69	11.61	1.64
os89230	1	486	0.28	14	3	5	6.62	0.06	0	1.44	0.07	97.4	1	5.65	7.22	16.17	7.59	-1.25	11.91	21.6	11.53	1.51
os89374	1	596	0.47	9	3	5	6.95	0.04	0.11	1.43	0.08	96.56	1.18	5.57	6.48	15.26	6.99	-1.64	11.16	20.51	10.85	1.04
os89377	1	579	0.65	14	2	3	6.78	-0.03	0.01	1.45	0.12	97.84	-2.78	7.38	6.48	15.26	6.98	-1.64	11.15	20.51	10.84	1.04
os89407	1	587	0.53	11	3	5	6.56	-0.03	0.08	1.47	0.12	99.77	-0.61	6.55	6.43	15.26	6.96	-1.7	11.17	20.55	10.85	1.02
os89441	1	572	0.7	12	1	4	6.78	-0.04	0.01	1.43	0.08	96.92	3.31	5.98	6.59	15.34	7.05	-1.6	11.3	20.61	10.92	1.12
os89548	1	557	0.6	13	3	5	6.86	0.01	0	1.43	0.1	94.21	0.77	5.8	6.79	15.6	7.21	-1.51	11.66	21.09	11.24	1.2
os89574	1	392	0.01	12	2	3	6.92	0.19	0.17	1.51	0.1	88.17	-2.63	6.09	7.83	16.68	8.1	-0.77	12.78	22.24	12.17	2.05
os89910	1	601	0.38	7	2	3	6.97	0.06	1.07	1.49	0.09	97.91	-4.06	8	6.48	15.29	6.97	-1.64	11.24	20.65	10.93	1.09
os89933	1	474	0.69	11	2	0	6.76	-0.03	0.03	1.5	0.12	68.44	-2.01	6.28	7.08	15.92	7.48	-1.33	12.02	21.47	11.58	1.45
os89938	1	553	0.92	9	3	5	6.61	-0.12	0.05	1.48	0.06	98.45	1.01	6.12	6.74	15.59	7.22	-1.53	11.61	21.03	11.21	1.21
os90005	1	499	0.03	12	2	0	7.02	0.21	0.01	1.48	0.17	66.48	-1.35	5.54	6.77	15.56	7.21	-1.49	11.59	20.98	11.2	1.24
os90030	1	590	0.26	8	2	3	6.92	0.06	0.86	1.52	0.13	97.19	-6.76	8.73	6.46	15.29	6.97	-1.66	11.23	20.67	10.93	1.09
os90050	1	560	1	11	2	3	6.44	-0.18	0.08	1.48	0.07	95.15	-1.12	7.32	6.79	15.64	7.26	-1.48	11.66	21.1	11.27	1.28
os90134	1	427	0.34	5	2	7	6.88	0.06	1.42	1.54	0.1	91.74	-2.37	7.62	7.43	16.32	7.77	-1.01	12.08	21.64	11.62	1.69
os90158	1	349	1	11	3	5	6.36	-0.17	0.22	1.49	0.08	97.17	-2.16	7.99	8.27	17.15	8.37	-0.59	13.33	22.71	12.47	2.31
os90182	1	314	0.97	11	3	5	6.36	-0.17	0.23	1.49	0.11	93.71	-2.95	6.72	8.42	17.35	8.56	-0.42	13.56	23.11	12.79	2.53
os90184	1	371	0.53	3	3	9	6.71	-0.02	0.68	1.52	0.03	84.92	0.28	8.15	8.22	17.17	8.39	-0.52	13.35	22.89	12.61	2.42
os90185	1	341	0.23	2	1	4	6.72	0.01	0.03	1.44	0.01	75.25	6.13	6.58	8.32	17.2	8.49	-0.45	13.4	22.9	12.68	2.45

os90313	1	433	0.13	7	2	7	6.85	0.09	0.77	1.53	0.12	83.36	-2.49	8	7.24	16.13	7.63	-1.18	11.89	21.49	11.51	1.53
os90372	1	397	0.95	1	3	6	6.74	-0.02	0.27	1.51	0.01	80.19	0.35	8.3	8.03	16.86	8.21	-0.73	12.66	22.08	11.95	1.94
os90384	1	389	0.96	3	3	6	6.67	-0.05	1.91	1.54	0.05	92.37	-1.8	8.73	7.94	16.82	8.16	-0.83	12.69	22.12	11.99	1.88
os90456	1	293	0.34	2	3	6	6.66	-0.04	1.08	1.54	0.03	99.28	0.1	7.88	8.63	17.47	8.73	-0.27	13.43	22.86	12.58	2.47
os90572	1	456	0.13	19	1	1	7.01	0.26	0	1.37	0.09	97.1	4.55	5.28	7.76	16.62	7.93	-1.03	12.43	21.85	11.72	1.63
os90814	1	530	0.1	2	1	1	6.89	0.03	0.03	1.43	0	84.53	3.15	8.12	7.58	16.49	7.78	-1.23	12.36	21.82	11.65	1.53
os90880	1	357	0.76	8	3	5	6.66	-0.06	0.74	1.52	0.07	97.09	-2.23	7.38	8.19	17.08	8.34	-0.59	13.19	22.63	12.41	2.24
os91101	1	321	0.06	17	2	7	6.62	0.2	0	1.49	0.15	94.31	-5.67	6.12	8.28	17.13	8.42	-0.54	13.05	22.44	12.26	2.19
os91166	1	372	0.81	6	3	5	6.54	-0.09	0.39	1.5	0.04	95.79	1.01	6.63	8.13	17.02	8.33	-0.58	13.21	22.69	12.53	2.31
os91193	1	319	0.92	3	2	7	6.63	-0.05	1.96	1.55	0.1	85.91	-3.09	7.46	8.23	17.13	8.42	-0.52	13.31	22.81	12.62	2.38
os91242	1	344	0.72	13	2	3	6.33	-0.1	0.08	1.48	0.11	97.15	-2.51	7.7	8.18	17.09	8.34	-0.61	13.29	22.73	12.46	2.32
os91246	1	321	0.85	13	2	3	6.17	-0.18	0.06	1.5	0.11	96.34	-2.19	6.84	8.36	17.2	8.44	-0.52	13.36	22.73	12.48	2.3
os91249	1	330	0.67	6	3	6	6.58	-0.05	0.69	1.51	0.05	93.6	-0.76	7.07	8.34	17.25	8.51	-0.45	13.45	23.01	12.71	2.49
os91292	1	413	0.01	6	2	8	6.83	0.07	1.31	1.5	0.1	96.1	-5.32	7.38	8.02	16.89	8.15	-0.8	13	22.4	12.18	2.04
os91298	1	388	0.84	17	1	4	6.04	-0.17	0	1.4	0.09	92.81	3.87	5.73	8	16.86	8.14	-0.83	13	22.41	12.19	2.01
os91387	1	570	0.28	3	3	6	6.76	-0.05	1.31	1.53	0.02	84.27	-0.23	7.94	6.9	15.89	7.28	-1.49	11.58	21.24	11.16	1.29
os91389	1	567	0.99	3	3	6	6.75	-0.05	1.49	1.54	0.03	85.29	-0.53	7.82	6.9	15.88	7.28	-1.49	11.58	21.24	11.16	1.29
os91470	1	436	0.4	5	3	6	6.89	0.04	0.45	1.51	0.06	98.71	0.66	7.67	7.68	16.57	7.86	-1.12	12.41	21.86	11.71	1.61
os91511	1	374	0.93	7	2	3	6.51	-0.13	0.32	1.51	0.07	97.47	-2.49	7	8.22	17.08	8.36	-0.58	12.88	22.31	12.12	2.12
os91521	1	377	0.99	8	3	5	6.5	-0.13	0.51	1.5	0.07	97.66	-1.58	7.03	8.21	17.08	8.35	-0.59	12.88	22.31	12.11	2.11
os91523	1	344	0.95	16	3	5	6.07	-0.24	0	1.47	0.11	97.57	-0.56	5.99	8.16	16.99	8.32	-0.62	12.79	22.19	12.04	2.04
os91527	1	372	0.98	10	3	5	6.41	-0.16	0.35	1.49	0.08	98.59	-1.31	7.35	8.1	16.93	8.26	-0.67	12.72	22.14	12	1.99
os91557	1	358	0.86	6	2	3	6.78	-0.01	0.93	1.52	0.1	97.43	-4.06	7.67	8.11	16.95	8.25	-0.72	12.74	22.13	11.92	1.93
os91579	1	405	0.5	22	1	1	5.93	-0.07	0	1.41	0.13	81.94	0.7	6.82	8	16.82	8.16	-0.77	12.58	21.98	11.8	1.86
os91581	1	364	0.32	15	2	3	6.84	0.08	0.03	1.47	0.12	99.66	-3.53	7.07	8.12	16.96	8.27	-0.69	12.83	22.18	11.98	2.02
os91613	1	492	0.61	4	3	6	6.7	-0.04	0.44	1.51	0.03	97.44	0.46	6.7	7.3	16.25	7.62	-1.22	12.03	21.59	11.54	1.58
os00281	2	525	0	4	2	3	6.89	0.04	2.72	1.55	0.06	63.2	-1.9	8.2	6.73	15.37	6.96	-1.33	11.62	20.99	10.85	1.33
os00398	2	516	0.4	3	3	6	6.88	0.01	0.6	1.54	0.03	89.08	0.22	8.36	6.82	15.47	7.02	-1.3	11.81	21.16	10.97	1.4
os00408	2	524	0.76	11	2	3	6.5	-0.08	0.75	1.52	0.12	97.88	-4.89	8.09	6.75	15.39	7.07	-1.32	11.52	20.84	10.89	1.21
os00556	2	599	0.01	4	1	4	6.93	0.06	0.29	1.5	0.05	91.49	1.75	8.62	6.32	15.05	6.82	-1.59	10.81	20.13	10.4	0.92
os00572	2	587	0.42	6	3	5	6.67	-0.04	0.21	1.48	0.07	96.21	0.82	7.56	6.53	15.3	6.99	-1.45	11.16	20.5	10.65	1.11
os00594	2	490	0.47	5	3	6	6.89	0.02	0.91	1.53	0.05	86	-0.3	8.29	7.1	15.85	7.37	-1.2	11.97	21.33	11.16	1.41
os00644	2	728	0.54	7	3	9	6.98	0.02	0.24	1.48	0.05	99.63	-0.08	6.32	5.61	14.28	6.27	-2.07	9.92	19.2	9.78	0.34
os00669	2	637	0.07	14	3	5	7.11	0.23	0	1.47	0.09	99.82	-0.79	7.12	5.95	14.62	6.55	-1.87	10.34	19.63	10.1	0.56
os00683	2	696	0.53	22	3	5	6.75	0.04	0	1.38	0	99.14	1.71	5.34	5.65	14.31	6.34	-1.98	10.01	19.29	9.8	0.38
os00685	2	723	0	17	3	9	7.1	0.28	0	1.42	0.1	99.99	-0.14	5.83	5.55	14.23	6.23	-1.93	9.81	19.15	9.65	0.47
os00725	2	559	0.4	2	3	6	6.89	0.01	0.21	1.53	0.01	74.8	0.56	8.96	6.81	15.52	7.11	-1.32	11.63	20.93	10.87	1.26

os00987	2	877	0.97	3	3	6	6.91	-0.05	0.56	1.51	0.02	98.83	-0.03	7.33	4.47	13.21	5.47	-2.86	8.79	17.94	8.79	-0.45
os01108	2	906	0.63	4	3	9	6.84	-0.05	0.43	1.51	0.03	93.42	0.08	6.7	4.45	13.27	5.51	-2.92	8.66	17.95	8.79	-0.62
os01138	2	416	0.26	12	2	7	6.91	0.12	0.01	1.51	0.22	93.44	-4.8	6.82	7.22	15.92	7.64	-1.14	11.83	21.04	11.23	1.38
os01226	2	787	0.07	4	3	6	6.95	0.04	0.58	1.53	0	98.31	-0.23	6.97	5.08	13.86	6.04	-2.54	9.21	18.48	9.31	-0.22
os01241	2	746	0.99	6	3	5	6.73	-0.1	0.42	1.52	0.04	73.45	0.18	7.74	5.23	14	6.2	-2.35	9.45	18.67	9.45	-0.02
os01286	2	504	0.79	8	3	5	6.5	-0.13	0.18	1.46	0.06	99.91	1.8	6.25	7.24	16.02	7.68	-1.12	11.74	21	11.23	1.4
os01346	2	650	0.53	10	3	5	6.99	0.05	0.18	1.49	0.09	97.89	-0.59	7.56	5.92	14.69	6.64	-2.12	10.25	19.48	9.98	0.23
os01355	2	601	0.88	13	3	5	6.31	-0.17	0.01	1.45	0.1	97.77	0.4	7.85	6.25	15.01	6.9	-1.88	10.62	19.82	10.29	0.5
os01521	2	414	0.01	12	3	5	6.99	0.2	0.03	1.49	0.1	99.38	-1.7	6.07	7.61	16.38	7.97	-0.86	12.17	21.4	11.58	1.7
os01527	2	378	0.36	6	3	5	6.57	-0.1	0.59	1.51	0.06	99.32	-0.04	6.86	8.12	16.88	8.4	-0.49	12.73	22.01	12.04	2.11
os01542	2	564	0.19	3	1	4	6.92	0.04	0.01	1.48	0	79.72	3.21	7.8	6.61	15.25	6.88	-1.37	11.37	20.72	10.67	1.22
os01575	2	458	0.6	11	1	4	6.45	-0.06	0.01	1.46	0.05	99.61	2.67	5.88	8.06	16.96	8.07	-0.7	12.67	22.31	11.75	2
os01677	2	388	0.62	16	1	2	6.72	0	0	1.4	0.12	84.03	4.58	5.38	8.29	17.21	8.26	-0.58	12.97	22.65	11.98	2.15
os02092	2	491	0.42	7	3	5	6.66	0	0.29	1.52	0.07	99.57	-0.28	6.68	7.73	16.67	7.8	-0.98	12.47	22.1	11.56	1.86
os02160	2	342	0.14	17	2	3	6.59	0.18	0	1.44	0.18	99.07	-4.28	5.89	8.44	17.37	8.41	-0.45	13.26	22.83	12.27	2.41
os02229	2	543	0.42	20	1	1	6.3	-0.02	0	1.44	0.11	98.42	-0.47	5.48	7.71	16.7	7.76	-1.08	12.53	22.2	11.61	1.81
os02340	2	574	0.82	2	3	6	6.8	-0.02	2.91	1.54	0.05	80.34	-0.83	8.02	7.09	16.09	7.27	-1.67	12	21.66	11.31	1.26
os02346	2	533	0.23	6	1	4	6.77	0.03	0.12	1.49	0.05	93.68	2.39	6.22	7.39	16.37	7.49	-1.46	12.3	21.92	11.51	1.47
os02393	2	537	0.77	8	3	5	6.75	-0.06	0.19	1.52	0.06	79.74	-0.4	7.32	7.16	16.14	7.3	-1.56	11.96	21.61	11.33	1.38
os02411	2	496	0.77	11	3	9	6.64	-0.09	0.02	1.45	0.1	99.85	-1.24	5.51	7.76	16.78	7.88	-0.88	12.46	22.11	11.63	1.95
os02426	2	533	0.13	3	3	6	6.92	0.04	0.55	1.51	0.02	79.62	0.71	7.13	7.4	16.4	7.51	-1.42	12.29	21.96	11.61	1.53
os02428	2	540	0.92	7	3	5	6.59	-0.1	0.88	1.53	0.08	86.23	-2.71	8.16	7.31	16.3	7.43	-1.49	12.17	21.82	11.49	1.48
os02518	2	419	0.85	7	3	9	6.74	-0.05	0.66	1.47	0.06	99.95	-1.56	7.34	8.3	17.25	8.32	-0.53	13.13	22.75	12.28	2.32
os02530	2	402	0.81	14	1	4	6.17	-0.18	0	1.41	0.08	97.83	4.37	5.58	8.29	17.24	8.25	-0.61	13.12	22.69	12.11	2.25
os02531	2	377	0.24	9	3	5	6.63	0.02	0.06	1.47	0.06	95.04	1.93	6.67	8.47	17.39	8.4	-0.5	13.31	22.91	12.29	2.33
os02542	2	496	0.89	17	3	9	6.01	-0.24	0	1.42	0.1	99.11	0.89	5.63	7.86	16.85	7.9	-0.94	12.64	22.3	11.71	1.89
os02546	2	445	0.45	17	2	3	6.86	0.08	0	1.42	0.13	99.71	-1.48	5.92	8.1	17.07	8.09	-0.8	12.89	22.53	11.93	2.03
os02556	2	363	0.91	11	2	8	6.41	-0.16	0.27	1.47	0.1	99.73	-2.96	6.57	8.54	17.4	8.56	-0.28	13.25	22.8	12.43	2.47
os02622	2	539	0.1	14	3	9	6.48	-0.01	0.01	1.45	0.09	98.35	0.21	5.49	7.71	16.7	7.76	-1.08	12.53	22.2	11.62	1.81
os02664	2	559	0.01	6	3	9	6.94	0.09	0.2	1.46	0.03	99.75	1.67	6.17	7.67	16.6	7.73	-1.11	12.41	22.07	11.52	1.78
os02691	2	534	0.6	11	3	9	6.83	-0.01	0.03	1.48	0.08	99.44	-1.08	6.03	7.7	16.66	7.76	-1.08	12.47	22.08	11.47	1.79
os02714	2	472	0	11	1	4	6.97	0.17	0	1.44	0.06	95.99	4.5	5.82	7.94	16.86	7.94	-0.91	12.7	22.34	11.76	1.94
os02717	2	452	0.25	11	3	5	6.66	0.08	0.01	1.47	0.09	86.21	-0.36	6.01	7.98	16.9	7.97	-0.89	12.78	22.41	11.83	1.99
os02735	2	591	0.45	7	3	5	6.73	0	0.12	1.5	0.04	99.36	1.61	6.08	7.38	16.35	7.47	-1.32	12.03	21.74	11.15	1.5
os02745	2	644	0	5	3	5	7	0.09	0.83	1.51	0.05	96.62	-1.02	7.33	7.14	16.12	7.26	-1.5	11.72	21.45	10.92	1.32
os02757	2	571	0.49	8	1	1	6.68	-0.01	0.12	1.47	0.04	97.68	2.36	6.03	7.57	16.56	7.6	-1.25	12.26	21.98	11.34	1.56
os02760	2	583	0.24	6	3	5	7.02	0.09	0.47	1.51	0.07	98.77	-0.25	6.16	7.4	16.37	7.46	-1.34	12.04	21.77	11.17	1.46

os02770	2	548	0.86	4	3	6	6.73	-0.07	0.75	1.52	0.06	96.74	-0.08	7	7.56	16.56	7.61	-1.23	12.29	21.99	11.35	1.61
os02825	2	351	0.81	7	1	4	6.63	-0.08	0.09	1.44	0.07	100	3.09	6.57	8.4	17.15	8.45	-0.21	13.05	22.41	12.23	2.56
os02827	2	398	0.75	7	3	9	6.56	-0.05	0.06	1.46	0.04	99.64	1.88	5.88	8.31	17.11	8.41	-0.35	12.88	22.36	12.12	2.39
os03047	2	397	0.71	7	3	9	6.58	-0.05	0.06	1.46	0.04	99.62	2.02	5.87	8.32	17.11	8.41	-0.35	12.88	22.36	12.12	2.39
os03186	2	428	0.45	7	2	3	6.6	-0.03	1.29	1.53	0.11	91.2	-5.79	8.49	8.03	16.89	8.09	-0.58	12.56	22.16	11.7	2.04
os03295	2	429	0.02	12	1	4	6.9	0.18	0	1.44	0.06	99.85	2.8	5.43	8.11	16.96	8.14	-0.66	12.78	22.29	11.95	2.09
os03325	2	409	0.99	6	3	5	6.62	-0.09	0.83	1.52	0.08	99.95	-1.76	7.12	8	16.85	8.08	-0.65	12.67	22.19	11.85	2.12
os03326	2	420	0.85	9	3	5	6.41	-0.13	0.11	1.49	0.08	99.75	-0.22	6.31	8.05	16.9	8.12	-0.62	12.72	22.23	11.89	2.15
os03387	2	316	0.92	8	3	5	6.41	-0.13	0.27	1.52	0.09	85.63	-1.14	6.03	8.68	17.44	8.61	-0.12	13.34	22.74	12.39	2.63
os03409	2	312	0.69	3	3	6	6.68	-0.01	1.72	1.54	0.05	89.72	-1.27	8.48	8.69	17.48	8.62	-0.16	13.43	22.85	12.45	2.59
os03411	2	351	0.92	4	3	6	6.63	-0.05	1.47	1.53	0.04	86.17	-1.19	7.72	8.56	17.37	8.53	-0.24	13.3	22.72	12.36	2.53
os03601	2	440	0.66	6	3	5	6.6	-0.06	0.36	1.51	0.05	94.07	0.55	6.34	7.99	16.84	8.05	-0.78	12.68	22.25	11.84	2.01
os03632	2	419	0.99	5	1	4	6.61	-0.09	0.16	1.5	0.03	95.37	2.38	6.81	8.15	17.03	8.17	-0.67	12.88	22.48	12.02	2.16
os03672	2	388	0.19	8	2	3	6.95	0.11	0.3	1.5	0.06	90.84	-1.63	7.5	8.47	17.33	8.43	-0.41	13.21	22.77	12.3	2.39
os03865	2	417	0.83	10	2	3	6.47	-0.11	0.62	1.51	0.08	95.01	-2.11	7.66	8	16.8	8.1	-0.68	12.64	22.19	11.86	2
os03871	2	452	0.64	4	1	4	6.64	-0.07	0.15	1.49	0.02	86.11	2.74	7.25	7.96	16.78	8.06	-0.72	12.6	22.18	11.84	1.98
os03924	2	448	0.08	3	3	9	6.88	0.05	0.55	1.51	0.01	91.13	0.59	7.18	8	16.73	8.07	-0.67	12.71	22.11	11.88	2.05
os04062	2	402	1	8	3	5	6.49	-0.14	0.18	1.48	0.06	98.7	1.55	6.35	8.07	16.84	8.16	-0.58	12.79	22.2	11.93	2.12
os04090	2	318	0.33	9	3	5	6.89	0.08	0.47	1.5	0.09	95.81	-0.31	6.44	8.6	17.35	8.59	-0.12	13.23	22.62	12.35	2.62
os04106	2	358	0.78	3	2	3	6.76	-0.01	2.93	1.52	0.06	88.82	-2.84	8.9	8.26	17.06	8.34	-0.37	13	22.46	12.19	2.41
os04407	2	363	0.43	1	3	6	6.74	-0.01	0.05	1.52	0.01	50.3	1.44	8.68	8.33	17.09	8.37	-0.32	13.03	22.44	12.14	2.46
os04409	2	546	0.62	5	3	6	6.68	-0.05	0.53	1.53	0.05	82.38	-0.05	7.84	7.06	16	7.26	-1.61	11.83	21.41	11.2	1.27
os04439	2	506	0.58	7	3	9	6.65	-0.02	0.71	1.45	0.06	99.68	-0.74	6.62	7.76	16.79	7.91	-0.85	12.44	22.08	11.64	1.99
os04508	2	543	0.53	7	3	5	6.89	0.01	0.52	1.51	0.05	87.56	-0.04	8.53	7.31	16.33	7.43	-1.45	12.29	21.97	11.44	1.5
os04690	2	399	0.25	4	3	6	6.61	-0.08	0.65	1.53	0.04	94.46	-0.64	7.39	8.12	16.98	8.21	-0.57	12.82	22.35	12.03	2.22
os04706	2	419	0.98	9	3	5	6.43	-0.15	0.07	1.49	0.05	99.5	0.45	6.27	8.13	17.02	8.2	-0.64	12.9	22.46	12.11	2.16
os04732	2	415	0.54	7	2	3	6.81	0	0.92	1.51	0.09	99.91	-4.52	6.98	8.12	17.05	8.27	-0.48	12.71	22.31	11.99	2.28
os04785	2	559	0.01	2	3	6	6.89	0.03	1.73	1.53	0.03	67.6	-0.34	8.07	7.28	16.31	7.39	-1.38	12.28	21.88	11.58	1.7
os04809	2	493	0.91	1	3	6	6.79	-0.01	0.82	1.53	0.02	65.99	1.31	9.15	7.59	16.56	7.63	-1.35	12.62	22.28	11.87	1.67
os04819	2	542	0.23	7	2	3	6.96	0.06	0.68	1.51	0.06	91.01	-1.66	7.88	7.31	16.3	7.43	-1.49	12.18	21.83	11.5	1.49
os04822	2	539	0.6	6	3	9	6.66	-0.05	0.16	1.5	0.03	92.53	0.77	6.36	7.53	16.54	7.62	-1.32	12.46	22.14	11.77	1.66
os04935	2	525	0.55	2	1	4	6.81	0	0	1.48	0	80.19	4.49	6.79	7.37	16.34	7.49	-1.41	12.36	21.96	11.66	1.64
os04947	2	515	0.26	7	3	5	6.98	0.08	0.43	1.52	0.06	91.28	-1.52	6.64	7.43	16.4	7.51	-1.38	12.3	21.89	11.56	1.59
os04991	2	560	0.11	4	3	6	6.95	0.05	1.09	1.52	0.04	89.59	-0.72	7.96	7.28	16.29	7.37	-1.39	12.14	21.77	11.48	1.66
os05004	2	497	0.87	3	3	6	6.73	-0.05	0.75	1.52	0.02	64.1	1.75	8.71	7.5	16.48	7.55	-1.3	12.53	22.1	11.75	1.78
os05037	2	548	0.15	4	3	6	6.95	0.05	0.62	1.53	0.03	93.45	-0.45	6.99	7.21	16.15	7.35	-1.49	12.23	21.77	11.52	1.63
os05047	2	247	0.82	12	2	3	6.52	-0.04	0.52	1.46	0.17	98.3	-5.82	7.34	9.04	17.77	9.02	0.32	13.73	22.98	12.78	3.07

os05057	2	349	0.99	4	3	6	6.67	-0.06	1.03	1.51	0.04	99.99	-0.85	8.06	8.35	17.14	8.39	-0.34	13.1	22.56	12.26	2.45
os05077	2	369	0.99	7	3	5	6.56	-0.11	0.69	1.5	0.05	98.59	-0.4	7.44	8.42	17.27	8.52	-0.29	13.05	22.55	12.24	2.44
os05250	2	395	0.97	11	3	5	6.4	-0.17	0.06	1.49	0.07	94.72	0.35	6.59	8.2	16.99	8.31	-0.43	12.67	22.25	11.93	2.18
os05416	2	441	0.07	8	2	0	6.86	0.1	0.39	1.52	0.08	95.82	-0.3	8.08	8.13	17.13	8.09	-0.77	13.04	22.68	12.17	2.36
os05522	2	405	0.95	15	2	3	6.12	-0.23	0.26	1.46	0.13	98.6	-3.69	7.44	8.44	17.42	8.31	-0.5	13.2	22.83	12.17	2.5
os05575	2	547	0.2	8	2	3	6.77	0.06	0.65	1.52	0.1	96.39	-4.37	6.41	7.76	16.78	7.77	-1.06	12.87	22.54	12.04	2.24
os05584	2	495	0.15	6	2	7	6.77	0.06	0.79	1.54	0.14	97.84	-7.11	7.89	7.89	16.92	7.87	-0.98	13.06	22.75	12.19	2.32
os05646	2	488	0.92	4	3	6	6.74	-0.04	1.04	1.52	0.02	63.68	0.91	8.78	8.08	17.08	8.05	-0.8	13.05	22.71	12.2	2.34
os05720	2	489	0.65	3	3	6	6.83	-0.01	0.71	1.53	0.02	89.14	0.36	7.3	8.01	17.15	7.94	-0.97	12.88	22.75	11.91	2
os05724	2	490	0.74	3	3	6	6.84	0	0.7	1.53	0.02	88.35	0.35	7.32	8.01	17.15	7.94	-0.97	12.88	22.75	11.91	2
os05729	2	476	0.99	2	3	6	6.76	-0.04	0.86	1.54	0.03	90.89	-0.04	8.3	8.03	17.16	7.95	-0.96	12.91	22.78	11.93	2.02
os05733	2	491	0.65	3	3	6	6.84	0	0.62	1.54	0.01	87.3	0.31	7.33	8.01	17.15	7.94	-0.97	12.87	22.75	11.91	2
os05752	2	450	0	7	3	5	6.9	0.11	0.14	1.52	0.04	80.79	0.77	8.16	8.14	17.22	8.04	-0.86	12.95	22.75	11.95	2.1
os05756	2	471	0.6	3	3	6	6.73	-0.02	2.61	1.54	0.03	73.68	-0.31	9.4	8.03	17.13	7.94	-0.95	12.82	22.65	11.87	2
os05777	2	490	0.85	4	3	6	6.68	-0.05	0.77	1.53	0.03	89.1	0.06	8.5	7.97	17.06	7.88	-1	12.75	22.61	11.81	1.96
os05810	2	449	0.48	3	3	6	6.77	0.01	2.35	1.55	0.03	73.18	-0.83	8.99	8.15	17.21	8.04	-0.87	12.93	22.72	11.91	2.09
os05817	2	439	0.1	3	3	6	6.82	0.04	0.44	1.54	0.02	78.64	0.4	8.48	8.21	17.3	8.13	-0.82	13.04	22.89	12.07	2.14
os05836	2	501	0.23	6	3	5	6.8	0.04	0.49	1.52	0.05	97.82	-0.29	6.74	7.79	16.77	7.78	-1.17	12.64	22.28	11.64	1.74
os05953	2	560	0.89	12	1	1	6.32	-0.17	0.01	1.44	0.05	96.92	2.71	5.65	7.63	16.66	7.66	-1.28	12.49	22.16	11.56	1.65
os06221	2	406	0.07	5	3	6	6.89	0.07	0.14	1.47	0.04	90.93	0.99	7.36	8.38	17.44	8.27	-0.64	13.1	22.83	12.09	2.25
os06407	2	539	0.4	8	3	5	6.65	-0.03	0.38	1.51	0.05	93.47	-1.01	7.86	7.57	16.64	7.6	-1.34	12.43	22.13	11.5	1.56
os06427	2	572	0.99	7	3	5	6.61	-0.12	0.24	1.49	0.04	79.19	0.49	6.03	7.5	16.58	7.55	-1.39	12.35	22.06	11.45	1.53
os06433	2	529	1	5	3	5	6.68	-0.08	1.2	1.54	0.05	89.75	-2.03	8.33	7.64	16.69	7.66	-1.32	12.48	22.16	11.52	1.57
os06438	2	561	0.01	16	1	4	6.88	0.24	0	1.43	0.1	99.84	2.76	5.04	7.59	16.6	7.6	-1.35	12.43	22.07	11.46	1.55
os06444	2	557	0.56	4	1	4	6.76	-0.01	0.04	1.49	0.02	99.28	3.16	6.83	7.58	16.59	7.61	-1.36	12.44	22.07	11.46	1.55
os06454	2	630	0.38	10	1	1	6.69	0.01	0.01	1.46	0.03	83.88	3.68	5.58	7.34	16.34	7.41	-1.51	12.16	21.82	11.25	1.43
os06509	2	495	1	4	3	6	6.71	-0.06	1.8	1.54	0.05	94.03	-0.73	8.07	7.87	16.89	7.83	-1.18	12.73	22.41	11.75	1.73
os06548	2	503	0.86	4	3	6	6.79	-0.03	0.88	1.5	0.03	88.26	1.31	6.81	7.85	16.9	7.84	-1.16	12.75	22.44	11.81	1.77
os06676	2	557	0.92	9	3	5	6.47	-0.14	0.18	1.48	0.07	93.78	0.48	6.33	7.59	16.66	7.59	-1.38	12.43	22.16	11.51	1.57
os06701	2	458	0.99	5	3	6	6.69	-0.07	0.61	1.53	0.03	88.7	0.5	7.59	8.12	17.18	8.01	-0.99	12.96	22.72	11.96	1.93
os06861	2	476	0.23	2	3	6	6.8	0.01	0.33	1.54	0.01	41.67	0.84	8.57	8.11	17.25	8.01	-0.97	12.96	22.84	12.01	1.92
os06865	2	479	0.92	0	3	6	6.82	0	0.91	1.55	0	23.78	0.23	10.26	8.11	17.23	8	-0.97	12.95	22.82	12	1.92
os06868	2	478	0.92	1	3	6	6.78	-0.02	0.35	1.55	0.01	29.07	0.28	9.05	8.11	17.23	7.99	-0.98	12.94	22.82	12	1.91
os06874	2	477	0.99	2	3	6	6.78	-0.03	1.07	1.55	0.01	40.24	0.25	8.6	8.11	17.23	8	-0.97	12.95	22.82	12	1.92
os06953	2	450	0.92	0	3	6	6.79	0	4.7	1.53	0.01	55.03	0.41	10.01	8.27	17.42	8.14	-0.86	13.15	23.03	12.14	2.08
os06987	2	569	1	7	1	1	6.6	-0.12	0.09	1.47	0.03	97.6	2.58	5.72	7.6	16.63	7.64	-1.31	12.46	22.14	11.55	1.64
os06990	2	540	0.99	12	2	3	6.37	-0.17	0.42	1.51	0.13	94.5	-5.38	6.24	7.6	16.63	7.64	-1.31	12.46	22.14	11.55	1.63

os06997	2	500	0.22	6	1	4	6.78	0.03	0.01	1.47	0.03	88.98	5.4	6.42	7.84	16.83	7.83	-1.13	12.72	22.37	11.76	1.78
os07054	2	537	0.77	10	3	5	6.78	-0.04	0.02	1.48	0.06	99.76	1.31	5.78	7.67	16.69	7.7	-1.26	12.55	22.2	11.59	1.65
os07102	2	440	0.22	2	3	6	6.81	0.02	2.6	1.55	0.01	11.9	-0.34	8.66	8.05	17.11	8.07	-0.75	12.77	22.5	11.93	2.13
os07132	2	467	0.15	7	3	5	6.98	0.11	0.26	1.51	0.08	96.79	-0.79	7.23	7.95	16.98	7.87	-1.01	12.9	22.59	11.97	2.03
os07278	2	472	0.08	5	3	5	6.95	0.08	0.8	1.52	0.03	74.3	0.13	7.01	8.23	17.43	8.1	-0.86	13.12	23	12.08	2.11
os07282	2	464	0.08	3	3	6	6.89	0.05	1.18	1.55	0.02	43.9	-0.42	8.23	8.24	17.44	8.11	-0.85	13.13	23.01	12.08	2.12
os07283	2	468	0.24	2	3	6	6.88	0.03	1.45	1.54	0.02	49.88	0.11	8.03	8.23	17.43	8.1	-0.86	13.13	23	12.08	2.11
os07284	2	471	0.13	2	3	6	6.86	0.02	1.42	1.54	0.01	52.17	0.1	8.05	8.24	17.44	8.11	-0.85	13.13	23.01	12.09	2.12
os07287	2	470	0.4	2	3	6	6.86	0.02	1.68	1.54	0.01	49.52	0.15	8.26	8.24	17.44	8.11	-0.85	13.13	23.01	12.09	2.12
os07516	2	483	0.08	5	3	5	6.95	0.08	1.08	1.53	0.05	88.4	-1.37	8.85	8.23	17.44	8.05	-0.85	13.13	22.94	11.99	2.22
os07548	2	459	0.83	3	3	6	6.7	-0.05	0.86	1.54	0.02	64	0	7.88	7.94	16.9	7.9	-1.01	12.9	22.49	12.06	2.06
os07558	2	522	0.43	2	1	1	6.78	-0.03	0.05	1.48	0.01	99.1	2.4	6.71	7.87	16.88	7.85	-1.07	12.9	22.5	12.06	2.05
os07671	2	425	0	10	3	5	6.98	0.17	0.11	1.5	0.09	98.55	-2.36	7.16	8.19	17.2	8.1	-0.86	13.23	22.91	12.3	2.26
os07685	2	441	0.58	4	3	6	6.75	0.01	0.7	1.51	0.03	74.02	0.69	7.45	8.13	17.13	8.05	-0.91	13.13	22.8	12.24	2.18
os07700	2	496	0.08	5	1	1	6.95	0.08	0.15	1.49	0.03	91.2	2.13	6.4	7.81	16.79	7.78	-1.09	12.78	22.38	11.92	2.02
os07744	2	463	0.08	2	3	6	6.88	0.04	2.07	1.54	0.05	90.42	-0.63	8.87	8.32	17.55	8.15	-0.83	13.31	23.18	12.13	2.24
os07754	2	464	0.92	9	3	5	6.42	-0.14	0.11	1.47	0.03	78.31	1.17	7.4	8.33	17.55	8.15	-0.81	13.31	23.19	12.14	2.26
os07765	2	509	0.92	2	3	6	6.75	-0.04	0.38	1.53	0.02	82.17	0.14	6.85	8.2	17.39	8.09	-0.83	13.2	23.02	12.14	2.3
os07779	2	423	0.5	7	3	6	6.86	0.02	1.39	1.53	0.07	80.33	-0.41	8.8	8.33	17.47	8.2	-0.73	13.18	23.08	12.15	2.27
os07823	2	523	0.72	10	2	3	6.8	-0.03	0.28	1.5	0.11	99.09	-4.3	7.07	7.68	16.64	7.72	-1.1	12.42	22.06	11.47	1.75
os07945	2	430	0.63	3	3	6	6.84	0.01	1.65	1.55	0.03	85.38	-0.53	8.44	8.26	17.36	8.1	-0.82	13.04	22.85	12.02	2.09
os08093	2	458	0.67	8	3	5	6.62	-0.02	0.23	1.51	0.06	69.49	0.99	7.12	8.1	17.19	7.99	-0.93	12.87	22.69	11.88	2.03
os08100	2	499	0.08	3	3	6	6.89	0.05	1.79	1.54	0.02	74.45	-0.19	8.38	7.94	17.04	7.87	-1.02	12.74	22.6	11.79	1.94
os08101	2	457	0.05	2	3	6	6.82	0.02	1.21	1.55	0.01	62.17	0.11	9.07	8.11	17.2	8.01	-0.91	12.88	22.7	11.88	2.05
os08169	2	476	0.92	13	3	9	6.25	-0.2	0	1.45	0.07	99.47	-0.19	5.71	8.1	17.21	8.02	-0.86	12.96	22.76	11.97	2.13
os08220	2	481	0.6	4	3	6	6.71	-0.03	0.75	1.54	0.02	85.7	-0.23	7.72	8.04	17.12	7.94	-1.02	12.83	22.68	11.87	1.92
os08312	2	531	0.92	0	3	6	6.83	0	1.13	1.54	0	24.1	0.3	11.12	7.83	16.94	7.79	-1.11	12.5	22.34	11.59	1.8
os08428	2	452	0.77	3	3	6	6.79	-0.02	1.52	1.54	0.02	98.87	-0.05	7.81	8.26	17.39	8.1	-0.8	13.05	22.88	12	2.14
os08442	2	397	0.4	8	3	5	6.88	0.05	0.48	1.49	0.07	98.23	-1.98	6.75	8.56	17.69	8.35	-0.63	13.47	23.29	12.32	2.37
os08509	2	423	0.07	2	3	6	6.85	0.04	2.39	1.55	0.03	80.67	-0.56	9.43	8.36	17.46	8.2	-0.75	13.13	22.98	12.09	2.19
os08576	2	487	0.92	2	2	3	6.85	0.02	2.92	1.56	0.05	71.67	-2.04	9.05	7.88	16.91	7.83	-1.15	12.65	22.46	11.71	1.7
os08675	2	392	0.83	7	3	5	6.66	-0.07	0.48	1.51	0.05	95.97	-0.5	7.32	8.61	17.72	8.43	-0.49	13.5	23.24	12.37	2.49
os08690	2	460	0.4	3	3	6	6.85	0.01	0.92	1.54	0.02	82.34	0.39	7.58	8.26	17.44	8.13	-0.84	13.15	23.03	12.12	2.13
os08761	2	447	0.98	5	2	3	6.7	-0.06	1.89	1.53	0.1	98.44	-4.93	8.31	8.21	17.3	8.13	-0.73	13.05	22.82	12.1	2.32
os08766	2	504	0.23	5	3	5	6.76	0.02	0.06	1.5	0.04	96.65	2.32	7.41	7.94	17	7.91	-0.86	12.66	22.41	11.73	2.15
os08775	2	468	0.92	5	3	6	6.62	-0.08	0.54	1.53	0.03	81.97	-0.12	8.79	8.18	17.3	8.07	-0.87	13.09	22.97	12.12	2.17
os08788	2	487	0.05	2	3	6	6.82	0.02	2.26	1.55	0.02	30.27	-0.66	8.65	8.07	17.22	8	-0.92	12.99	22.89	12.04	2.11

os08849	2	440	0.79	13	3	9	6.65	-0.07	0	1.42	0.08	96.83	0.45	6.49	8.52	17.66	8.3	-0.69	13.46	23.31	12.3	2.32
os08850	2	438	0.76	12	3	5	6.63	-0.08	0.01	1.41	0.08	96.3	0.71	6.37	8.52	17.66	8.31	-0.68	13.46	23.32	12.3	2.32
os08883	2	414	0.39	7	3	5	6.88	0.04	0.64	1.54	0.06	91.26	-1.61	7.88	8.35	17.42	8.18	-0.77	13.21	23.03	12.15	2.21
os09073	2	548	0.99	7	3	5	6.63	-0.11	0.26	1.51	0.04	95.37	0.98	6.24	7.17	16.09	7.33	-1.44	12.16	21.64	11.52	1.68
os09099	2	510	0.23	10	3	5	6.76	0.08	0.11	1.49	0.07	90.37	-0.26	7.01	7.21	16.09	7.34	-1.4	12.46	21.85	11.74	1.88
os09100	2	508	0.23	9	3	5	6.78	0.08	0.22	1.5	0.07	89.93	-1.02	7.1	7.21	16.09	7.34	-1.4	12.46	21.85	11.75	1.88
os09103	2	440	0.09	3	2	3	6.87	0.05	2.58	1.56	0.06	61.47	-2.96	7.89	7.78	16.73	7.76	-1.12	12.94	22.47	12.07	2
os09105	2	474	0.01	4	3	6	6.91	0.06	1.76	1.54	0.05	75.83	-0.98	8.32	7.39	16.3	7.49	-1.29	12.67	22.09	11.93	2.02
os09125	2	484	0.08	1	3	6	6.86	0.02	0.15	1.51	0.01	82.95	1.85	8.42	7.72	16.69	7.72	-1.16	12.84	22.4	12	1.95
os09174	2	538	0.44	11	1	4	6.6	0.01	0.01	1.45	0.06	95.37	2.88	5.52	7.21	16.12	7.38	-1.4	12.17	21.64	11.51	1.68
os09175	2	588	0.4	9	1	1	6.99	0.06	0.23	1.46	0.04	95.85	1.44	6.52	7.07	16	7.29	-1.47	12	21.47	11.36	1.6
os09176	2	542	0.98	9	3	5	6.49	-0.15	0.11	1.5	0.08	99.34	-0.37	6.42	7.1	16.03	7.31	-1.46	12.02	21.51	11.37	1.6
os09588	2	268	0.89	9	3	5	6.36	-0.13	0.1	1.5	0.05	94.61	0.67	6.06	8.65	17.42	8.77	-0.4	13.53	22.95	12.61	2.38
os09832	2	406	0.83	13	2	3	6.27	-0.16	0.03	1.5	0.1	93.21	-3.06	6.88	8.44	17.61	8.28	-0.71	13.35	23.22	12.29	2.26
os09835	2	545	0.55	5	3	5	6.91	0.02	0.35	1.52	0.04	76.2	-0.8	7.34	7.28	16.3	7.4	-1.38	12.25	21.82	11.55	1.7
os10131	2	510	0.3	5	3	5	6.74	0	0.43	1.52	0.04	96.01	0.99	7.11	6.95	15.72	7.26	-1.16	11.76	21.01	11.07	1.65
os10335	2	548	0.38	5	3	6	6.96	0.06	0.65	1.53	0.05	99.72	-0.5	7.15	6.68	15.47	7.01	-1.39	11.4	20.73	10.8	1.35
os10350	2	608	0.01	6	2	0	7.01	0.1	0.21	1.51	0.06	92.32	1.11	7.92	6.27	15.01	6.67	-1.66	10.88	20.14	10.35	1.03
os10391	2	654	0.37	7	3	5	7.01	0.06	0.25	1.49	0.05	98.64	0.46	6.59	6.25	15.06	6.7	-1.64	10.85	20.15	10.38	1.13
os10429	2	576	0.67	14	3	5	6.75	-0.04	0	1.45	0.1	95.47	0.51	5.85	6.58	15.35	6.95	-1.42	11.17	20.49	10.66	1.35
os10443	2	597	0.35	11	3	5	7.01	0.08	0.09	1.49	0.09	99.18	0.13	6.67	6.39	15.16	6.8	-1.54	10.91	20.24	10.47	1.21
os10451	2	695	0.89	10	3	9	6.73	-0.1	0.04	1.47	0.06	99.83	-0.18	6.53	6.03	14.84	6.52	-1.74	10.53	19.83	10.15	1
os10452	2	663	0.77	3	3	6	6.9	-0.01	0.88	1.52	0.05	97.38	-0.31	7.31	6.1	14.92	6.58	-1.71	10.63	19.94	10.22	1.05
os10507	2	573	0.19	7	3	5	7.02	0.09	0.36	1.51	0.06	97.48	0.29	6.38	6.48	15.21	6.82	-1.58	11.23	20.44	10.61	1.18
os10508	2	612	0.11	8	3	5	6.91	0.1	0.23	1.5	0.06	97.57	-0.38	6.87	6.41	15.14	6.76	-1.61	11.11	20.35	10.53	1.14
os10511	2	617	0.01	8	3	9	7	0.13	0.19	1.49	0.06	99.99	-0.04	5.8	6.48	15.18	6.81	-1.57	11.15	20.39	10.57	1.17
os10543	2	516	0.23	5	1	4	6.8	0.03	0.08	1.51	0.02	58.83	2.33	8.12	6.91	15.61	7.13	-1.3	11.65	20.87	10.93	1.42
os10559	2	533	0.6	2	3	6	6.8	-0.01	2.65	1.56	0	49.42	-0.32	9.6	6.77	15.5	7.01	-1.35	11.49	20.72	10.8	1.37
os10598	2	626	0.08	6	3	5	6.91	0.08	0.27	1.49	0.04	99.84	0.28	6.02	6.37	15.11	6.73	-1.62	11	20.26	10.44	1.1
os10635	2	555	0.01	7	3	5	6.96	0.11	0.35	1.51	0.06	88.15	-0.36	6.19	6.61	15.31	6.92	-1.5	11.35	20.54	10.71	1.26
os10642	2	591	0.71	4	3	6	6.73	-0.04	0.62	1.53	0.04	82.49	-0.36	7.55	6.51	15.21	6.83	-1.56	11.17	20.4	10.57	1.18
os10649	2	601	0.03	5	3	6	6.95	0.08	0.63	1.52	0.04	96.18	-0.61	6.38	6.44	15.17	6.79	-1.59	11.16	20.39	10.56	1.16
os10666	2	556	0.26	5	3	5	6.76	0.01	0.52	1.52	0.09	92.71	-0.8	6.92	6.64	15.4	7.01	-1.39	11.29	20.6	10.76	1.39
os10675	2	602	0.33	2	3	6	6.93	0.03	1.18	1.53	0.04	87.66	-0.02	7.5	6.41	15.21	6.83	-1.53	11.03	20.34	10.55	1.25
os10698	2	556	0.73	5	3	5	6.65	-0.06	0.65	1.52	0.07	90.3	-0.42	8.03	6.6	15.39	6.95	-1.46	11.35	20.66	10.77	1.31
os10735	2	629	0.75	6	3	5	6.67	-0.06	0.48	1.52	0.06	99.46	-0.79	7.09	6.32	15.09	6.72	-1.64	10.92	20.21	10.42	1.11
os10749	2	558	0.15	4	3	6	6.95	0.05	0.39	1.51	0.03	97.62	1.22	6.55	6.73	15.51	7.04	-1.37	11.47	20.8	10.85	1.38

os10750	2	567	0.22	5	3	5	6.98	0.06	0.38	1.5	0.03	99.44	1.26	6.33	6.67	15.46	6.99	-1.39	11.38	20.71	10.79	1.34
os10762	2	563	1	6	3	5	6.62	-0.11	0.41	1.52	0.06	94.38	-0.19	6.33	6.5	15.23	6.86	-1.52	11	20.28	10.45	1.13
os10851	2	649	0.79	5	1	4	6.8	-0.06	0.16	1.48	0.03	96.27	3.68	8.35	6.27	15.09	6.72	-1.62	10.88	20.18	10.41	1.15
os10853	2	556	0.76	7	3	5	6.82	-0.03	0.31	1.52	0.05	96.05	-0.2	6.92	6.64	15.44	7	-1.41	11.37	20.68	10.82	1.41
os10903	2	598	0.7	7	3	5	6.6	-0.07	0.27	1.5	0.05	98.46	0.4	6.23	6.44	15.21	6.82	-1.57	11.09	20.36	10.53	1.17
os10996	2	611	0.53	5	3	5	6.94	0.02	0.43	1.52	0.04	98.46	0.1	6.38	6.3	15	6.73	-1.56	10.71	20	10.3	1.07
os11004	2	591	0.01	4	3	6	6.92	0.06	0.21	1.52	0.03	91.04	1.17	8.1	6.31	15.01	6.72	-1.54	10.74	20.01	10.32	1.08
os11029	2	539	0.53	10	3	5	6.91	0.04	0.04	1.48	0.08	96.02	-0.01	5.62	6.65	15.34	6.95	-1.4	11.29	20.51	10.68	1.28
os11064	2	569	0.23	2	2	3	6.85	0.01	2.66	1.54	0.05	88.56	-2.11	8.97	6.61	15.38	6.93	-1.45	11.38	20.65	10.74	1.3
os11079	2	645	0.69	4	3	6	6.87	-0.02	0.38	1.53	0.03	97.96	-0.47	7.01	6.18	14.88	6.61	-1.71	10.8	20.1	10.27	0.95
os11089	2	565	1	6	3	5	6.67	-0.09	0.72	1.53	0.06	81.46	-0.74	7.22	6.45	15.14	6.83	-1.54	11.01	20.32	10.5	1.09
os11114	2	584	0.47	3	3	6	6.8	0	1.78	1.53	0.04	75.88	-0.45	8.29	6.42	15.12	6.82	-1.49	10.99	20.28	10.5	1.17
os11258	2	570	0.76	7	3	5	6.58	-0.08	0.42	1.51	0.07	99.98	-0.89	6.76	6.47	15.28	6.87	-1.45	11.11	20.38	10.54	1.34
os11259	2	578	0.81	8	3	5	6.56	-0.1	0.26	1.51	0.07	99.95	-0.36	6.63	6.44	15.25	6.84	-1.47	11.06	20.33	10.5	1.32
os11261	2	581	0.81	8	3	5	6.54	-0.11	0.2	1.5	0.07	99.93	-0.15	6.35	6.44	15.25	6.84	-1.47	11.06	20.34	10.51	1.32
os11300	2	547	0.9	4	2	0	6.79	-0.04	1.55	1.55	0.08	83.64	-0.48	8.41	6.53	15.25	6.89	-1.41	10.96	20.23	10.45	1.28
os11400	2	557	1	12	3	9	6.37	-0.2	0.01	1.47	0.08	99.46	0.23	5.69	6.67	15.5	7.04	-1.31	11.34	20.67	10.76	1.55
os11617	2	494	0.01	5	2	0	6.82	0.05	0.57	1.53	0.07	88.5	-0.47	7.49	6.8	15.7	7.15	-1.22	11.7	21.1	11.09	1.77
os11639	2	536	0.83	8	3	5	6.51	-0.12	0.2	1.52	0.08	99.95	-1.34	6.74	6.56	15.47	6.99	-1.39	11.47	20.87	10.91	1.63
os11700	2	625	0.5	8	1	4	6.71	0	0.16	1.47	0.09	97.47	1.91	5.98	6.23	15.04	6.7	-1.55	10.63	19.91	10.22	1.16
os11701	2	533	0.08	4	2	0	6.95	0.07	1.22	1.53	0	89.91	-1.36	7.53	6.66	15.39	6.98	-1.42	11.35	20.58	10.74	1.4
os11703	2	555	0.79	8	2	0	6.54	-0.08	0.16	1.53	0	97.75	-0.16	6.54	6.63	15.36	6.95	-1.44	11.3	20.54	10.71	1.39
os11733	2	610	0.38	16	3	5	6.98	0.1	0	1.45	0.1	100	0.84	5.98	6.28	15.14	6.75	-1.52	10.84	20.16	10.4	1.31
os11734	2	618	0.46	15	3	5	6.97	0.09	0	1.44	0.09	100	1.91	5.96	6.25	15.11	6.73	-1.53	10.8	20.12	10.38	1.3
os11742	2	634	0.97	7	3	5	6.72	-0.09	0.2	1.47	0.05	100	1.87	6.05	6.25	15.11	6.73	-1.53	10.79	20.11	10.36	1.3
os11743	2	631	0.87	6	3	5	6.77	-0.07	0.16	1.47	0.04	100	1.86	6.04	6.27	15.13	6.75	-1.52	10.82	20.14	10.39	1.31
os11750	2	459	0.92	1	3	6	6.77	-0.02	3.83	1.56	0.04	38.96	-0.6	8.53	6.97	15.84	7.28	-1.09	11.94	21.33	11.27	1.85
os11829	2	563	0.92	10	1	4	6.43	-0.15	0.02	1.46	0.06	91.51	3.06	6.31	6.52	15.19	6.88	-1.5	11.16	20.43	10.53	1.14
os11840	2	523	0.15	12	3	5	6.75	0.11	0.01	1.47	0.11	93.12	-1.09	6.22	6.82	15.5	7.11	-1.36	11.6	20.93	10.85	1.32
os11841	2	543	0.92	4	3	6	6.76	-0.05	0.6	1.5	0.02	97.74	0.96	6.78	6.85	15.55	7.14	-1.33	11.68	20.98	10.89	1.35
os11843	2	621	0	6	3	5	7.02	0.1	0.47	1.51	0.05	71.5	1.53	7.55	6.17	14.86	6.58	-1.77	10.81	20.09	10.28	0.91
os11872	2	648	0.77	7	3	9	6.86	-0.03	0.36	1.47	0.04	99.98	1.08	6.14	6.36	15.1	6.76	-1.58	11.04	20.35	10.44	1.05
os11881	2	569	0.23	2	2	3	6.85	0.01	2.64	1.54	0.05	88.57	-2.13	8.97	6.61	15.38	6.93	-1.45	11.38	20.65	10.74	1.3
os12051	2	501	0.32	7	3	9	6.67	0	0.37	1.46	0.05	89.85	1.51	6.55	7.91	16.77	8	-0.86	13.01	22.48	12.39	2.43
os12100	2	669	0.4	7	1	1	7.01	0.05	0.01	1.4	0.03	99.87	6.13	5.85	6.95	15.82	7.24	-1.53	11.99	21.34	11.54	1.84
os12101	2	610	0.45	12	2	3	6.58	0	0.1	1.49	0.12	99.8	-3.15	7.01	7.05	15.9	7.32	-1.49	12.1	21.44	11.61	1.88
os12109	2	682	0.08	8	3	9	6.94	0.1	0.12	1.48	0.05	99.37	-0.02	5.59	6.94	15.82	7.23	-1.51	12	21.36	11.54	1.87

os12139	2	688	0.19	10	3	9	7.1	0.13	0.05	1.48	0.06	97.35	0.69	5.69	6.85	15.64	7.15	-1.67	11.84	21.1	11.44	1.69
os12183	2	630	0.09	13	3	5	6.86	0.16	0	1.46	0.11	95.97	-0.02	5.6	6.97	15.78	7.22	-1.53	12.02	21.27	11.55	1.82
os12205	2	726	0.23	10	3	5	6.81	0.06	0.05	1.49	0.08	98.57	0.03	6.37	6.36	15.2	6.8	-1.96	11.34	20.59	11.07	1.38
os12215	2	647	0.49	10	3	5	6.95	0.03	0.05	1.49	0.1	99.99	-1.16	6.02	6.86	15.67	7.14	-1.6	11.89	21.12	11.46	1.75
os12251	2	660	0.7	11	3	5	6.81	-0.05	0.02	1.46	0.08	99.05	0.12	5.69	6.94	15.84	7.21	-1.51	12	21.4	11.52	1.88
os12261	2	617	0.14	9	2	3	6.84	0.09	0.37	1.51	0.12	99.83	-4.47	7.65	7.02	15.87	7.3	-1.5	12.07	21.41	11.59	1.87
os12396	2	787	0.78	8	3	9	6.88	-0.05	0.35	1.46	0.1	100	-2.05	6.08	6.25	15.06	6.75	-1.95	11.2	20.43	11.05	1.45
os12431	2	620	0.68	14	2	7	6.33	-0.08	0	1.46	0.13	99.68	-1.99	7.65	6.94	15.86	7.24	-1.39	11.69	21.18	11.3	1.78
os12432	2	623	0.54	14	2	0	6.36	-0.07	0	1.46	0.13	99.73	-1.51	7.63	6.92	15.84	7.22	-1.41	11.67	21.15	11.28	1.77
os12444	2	567	1	8	3	5	6.56	-0.14	0.2	1.51	0.06	99.4	-0.17	6.36	7.34	16.29	7.56	-1.21	12.23	21.78	11.76	2
os12496	2	582	0.09	8	3	9	7.04	0.11	0.19	1.45	0.05	95.71	0.77	5.9	7.42	16.22	7.6	-1.17	12.43	21.69	11.83	2.08
os12533	2	555	0.33	12	3	5	6.62	0.03	0.01	1.46	0.09	98.42	0.9	5.6	7.54	16.44	7.71	-1.06	12.49	22.03	11.96	2.17
os12619	2	533	0.69	5	3	5	6.65	-0.06	0.54	1.53	0.05	97.38	0.08	7.74	7.7	16.66	7.8	-1	12.61	22.19	11.98	2.19
os12622	2	606	0.59	4	3	5	6.73	-0.05	0.31	1.49	0.07	99.99	1.47	6.83	7.2	16.09	7.47	-1.15	12	21.49	11.6	2.11
os12637	2	549	0.6	5	3	6	6.87	0	0.57	1.52	0.05	83.46	-1.08	6.68	7.53	16.42	7.71	-1.14	12.66	22.19	12.14	2.22
os12645	2	507	0.42	9	1	4	6.63	0.01	0.03	1.45	0.05	77.46	2.84	7.35	7.74	16.6	7.86	-1.04	12.96	22.44	12.36	2.31
os12647	2	505	0.02	11	3	5	6.9	0.15	0.01	1.44	0.05	78.52	2.22	7.44	7.74	16.6	7.86	-1.05	12.96	22.44	12.36	2.31
os12648	2	504	0	12	2	3	6.97	0.18	0.01	1.48	0.08	77.74	0.33	7.52	7.74	16.6	7.86	-1.05	12.96	22.44	12.36	2.31
os12702	2	532	0.68	12	2	7	6.4	-0.07	0.54	1.52	0.13	87.91	-4.98	7.16	7.45	16.28	7.62	-1.28	12.64	22.04	12.11	2.09
os12751	2	576	0.92	5	3	6	6.68	-0.08	0.41	1.53	0.04	82.68	0.17	7.41	7.27	16.12	7.48	-1.33	12.39	21.79	11.88	2.02
os12802	2	671	0.37	9	1	4	6.79	0.05	0.02	1.45	0.05	99.9	3.87	5.58	6.88	15.76	7.19	-1.56	11.92	21.28	11.49	1.83
os12816	2	721	0.92	10	3	9	6.46	-0.16	0.01	1.44	0.06	99.41	0.75	5.56	6.63	15.49	7	-1.68	11.53	20.94	11.24	1.66
os12825	2	631	0.1	7	3	5	7.03	0.09	0.42	1.51	0.04	93.29	0.12	7.22	7	15.82	7.27	-1.54	12.01	21.3	11.55	1.84
os12838	2	714	0.4	14	3	5	7	0.08	0	1.46	0.09	100	-1.05	5.44	6.57	15.38	6.98	-1.74	11.57	20.74	11.25	1.61
os12841	2	652	0.81	16	2	0	6.62	-0.11	0	1.47	0.13	87.16	-0.55	6.68	6.66	15.43	7.02	-1.74	11.67	20.84	11.31	1.6
os12902	2	511	0.79	4	3	6	6.67	-0.08	0.58	1.53	0.02	75.46	0.39	7.13	7.8	16.65	7.9	-1.01	13.02	22.49	12.4	2.35
os12908	2	512	0.08	1	3	6	6.86	0.02	1.78	1.53	0.02	82.19	0.48	8.08	7.8	16.66	7.87	-1	13	22.49	12.36	2.36
os12918	2	520	0.91	5	3	5	6.72	-0.07	0.34	1.52	0.06	95.26	0.65	7.73	7.69	16.69	7.79	-0.96	12.6	22.2	11.99	2.22
os13036	2	578	0.63	10	3	5	6.51	-0.07	0.22	1.42	0.08	88.55	1.47	6.38	7.19	16.04	7.41	-1.48	12.45	21.87	11.98	2.06
os13045	2	683	0.63	5	3	9	6.72	-0.06	0.75	1.48	0.04	99.46	-0.24	6.56	6.69	15.51	7	-1.82	11.88	21.24	11.5	1.78
os13055	2	503	0.11	12	1	4	7.04	0.19	0.08	1.41	0.06	73.32	4.8	6.72	7.75	16.62	7.88	-1.02	12.91	22.4	12.32	2.33
os13102	2	804	0.25	6	3	5	7.09	0.07	0.61	1.49	0.09	100	-1.17	7.27	5.93	14.83	6.51	-2.01	10.63	20.06	10.58	1.32
os13122	2	522	0.85	5	2	0	6.65	-0.06	1.28	1.54	0.08	91.43	-1.57	7.75	7.36	16.2	7.56	-1.31	12.48	21.95	12.02	2.13
os13160	2	663	0.23	5	3	6	6.86	0.03	0.58	1.51	0.04	97.71	0.5	6.85	6.66	15.49	6.98	-1.88	11.96	21.31	11.65	1.71
os13196	2	692	0.38	3	3	6	7.01	0.06	1	1.52	0.02	88.56	0.58	8.21	6.49	15.34	6.92	-2.03	11.58	21.02	11.45	1.52
os13209	2	660	0.19	8	3	5	7.07	0.1	0.21	1.5	0.05	95.08	1	6.74	6.66	15.43	7	-1.9	11.91	21.16	11.58	1.66
os13237	2	628	0.45	7	2	3	6.71	-0.01	0.9	1.53	0.1	96.88	-4.76	7.74	6.79	15.55	7.1	-1.8	12.02	21.3	11.69	1.75

os13269	2	593	0.28	3	3	6	6.93	0.02	2.85	1.54	0.03	62.36	0.01	8.78	6.93	15.81	7.15	-1.62	12.09	21.57	11.59	1.86
os13419	2	450	0.6	15	3	5	6.25	-0.1	0	1.46	0.1	67.42	0.08	6.33	7.77	16.63	7.82	-1.03	12.91	22.4	12.24	2.36
os13462	2	664	0.67	4	3	6	6.89	-0.02	0.69	1.53	0.03	84.05	0.41	7.77	6.64	15.44	6.96	-1.94	11.89	21.24	11.58	1.69
os13464	2	646	0.26	8	3	5	6.79	0.04	0.36	1.51	0.08	79.63	-0.65	6.83	6.65	15.46	6.97	-1.9	11.86	21.25	11.57	1.7
os13583	2	550	0.92	6	3	5	6.59	-0.1	0.42	1.47	0.04	93.11	1.57	6.9	7.44	16.29	7.63	-1.27	12.58	22.03	12.09	2.15
os13592	2	540	0.89	8	2	3	6.55	-0.13	0.44	1.52	0.08	95.01	-2.05	8.53	7.4	16.25	7.6	-1.3	12.53	21.98	12.05	2.12
os13629	2	579	0.96	8	3	5	6.57	-0.13	0.44	1.52	0.06	85.36	-1.1	7.57	7.11	15.98	7.33	-1.43	12.2	21.68	11.78	2
os13678	2	566	0.93	7	3	5	6.68	-0.1	0.19	1.48	0.04	93.31	1.77	6.51	7.33	16.15	7.53	-1.35	12.5	21.94	12.04	2.11
os13687	2	564	0.02	13	2	3	6.94	0.19	0.01	1.5	0.09	90.96	-1.93	8.09	7.16	15.99	7.42	-1.46	12.29	21.7	11.89	2.02
os13696	2	599	0	11	3	5	7	0.18	0.04	1.5	0.09	97.76	-1.76	5.64	7.02	15.88	7.28	-1.51	12.14	21.59	11.73	1.97
os13735	2	508	0.76	4	3	6	6.83	-0.01	0.31	1.52	0.04	98.94	0.7	6.52	7.63	16.51	7.8	-1.11	12.82	22.28	12.27	2.26
os13754	2	582	0.6	1	3	6	6.83	-0.01	0.58	1.54	0.02	79.5	-0.06	7.86	7.08	15.89	7.28	-1.63	12.43	21.78	11.94	2.01
os13762	2	665	0.83	11	3	5	6.65	-0.12	0.01	1.46	0.09	98.14	0.42	5.63	6.63	15.39	7.03	-1.75	11.6	20.84	11.35	1.69
os13777	2	604	0.76	5	3	6	6.69	-0.06	0.37	1.51	0.03	99.28	0.4	6.77	7.23	16.09	7.46	-1.41	12.41	21.82	11.94	1.99
os13811	2	568	1	6	3	5	6.64	-0.1	0.3	1.52	0.04	99.61	0.45	6.6	7.34	16.18	7.56	-1.34	12.5	21.95	12.03	2.07
os13816	2	612	0.01	4	3	6	6.96	0.06	1.95	1.52	0.03	74.85	0.57	6.91	6.9	15.66	7.16	-1.85	12.24	21.55	11.87	1.71
os13822	2	568	0.7	9	3	5	6.47	-0.14	0.08	1.49	0.08	92.44	0.77	6.36	6.99	15.71	7.22	-1.74	12.2	21.39	11.74	1.8
os13829	2	684	0.9	11	1	4	6.44	-0.16	0.01	1.45	0.07	99.46	3.06	5.45	6.57	15.44	6.92	-1.68	11.39	20.9	11.11	1.71
os13843	2	736	0.01	10	3	5	7.05	0.17	0.09	1.49	0.08	99.8	-0.96	5.83	6.26	15.07	6.69	-2.04	11.35	20.68	11.09	1.52
os13853	2	611	0.37	4	2	3	6.8	0	1.32	1.53	0.06	97.32	-2.25	7.86	6.93	15.78	7.17	-1.7	12.23	21.58	11.78	1.92
os13871	2	699	0.92	5	1	4	6.74	-0.07	0.34	1.5	0.03	77.16	1.93	7.11	6.39	15.25	6.85	-2.06	11.48	20.92	11.36	1.47
os13907	2	584	0.1	3	3	6	6.76	-0.05	1.93	1.55	0.04	93.53	-1.48	8.23	7.16	16	7.42	-1.43	12.27	21.67	11.84	1.95
os13935	2	587	0.77	3	3	6	6.86	-0.01	1.18	1.54	0.03	90.54	-0.17	7.52	7.04	15.93	7.23	-1.49	12.1	21.63	11.64	1.95
os13937	2	631	0.08	2	3	9	6.93	0.03	0.19	1.52	0.02	68.66	0.62	8.29	6.89	15.79	7.13	-1.54	11.9	21.42	11.47	1.89
os13946	2	612	0.4	2	3	6	6.91	0.01	0.52	1.54	0.02	54.18	0.02	8.42	6.85	15.72	7.12	-1.59	11.83	21.33	11.43	1.84
os14099	2	488	0.81	7	1	4	6.55	-0.1	0.07	1.48	0.06	94.25	2.36	7.51	7.71	16.58	7.85	-1.02	12.8	22.29	12.25	2.33
os14159	2	563	0.82	6	3	5	6.61	-0.08	0.46	1.51	0.06	95.15	-0.52	6.59	7.27	16.15	7.5	-1.33	12.34	21.84	11.9	2.1
os14190	2	452	0.23	12	3	5	6.66	0.08	0.01	1.49	0.1	85.39	-1.67	6.12	7.79	16.64	7.85	-1	12.91	22.39	12.25	2.34
os14197	2	480	0.01	7	3	5	6.97	0.13	0.3	1.5	0.05	92.1	0	7.44	7.71	16.58	7.77	-1.09	12.84	22.35	12.2	2.31
os14203	2	526	0.08	3	1	4	6.92	0.05	0.02	1.37	0.03	92.76	6.13	7.82	7.55	16.4	7.74	-1.18	12.7	22.15	12.18	2.22
os14242	2	534	0.87	4	3	6	6.78	-0.04	1.88	1.53	0.03	77.16	-0.68	8.15	7.37	16.29	7.47	-1.3	12.43	22	11.81	2.12
os14255	2	565	0.23	4	3	6	6.84	0.03	0.86	1.54	0.05	87.74	-0.99	8.38	7.16	16.05	7.32	-1.43	12.24	21.76	11.76	2.02
os14256	2	587	0.72	4	3	6	6.72	-0.05	0.58	1.54	0.03	81.79	0.61	7.85	7.05	15.95	7.24	-1.48	12.12	21.65	11.66	1.96
os14262	2	573	0.26	5	2	3	6.81	0.03	1.45	1.53	0.06	90.63	-2.14	8.05	7.1	15.98	7.27	-1.49	12.2	21.72	11.7	1.98
os14263	2	564	0.6	9	2	3	6.62	-0.04	0.43	1.52	0.06	85.94	-1.33	8.83	7.13	16.01	7.29	-1.48	12.23	21.75	11.72	1.99
os14266	2	589	0.12	3	2	3	6.74	-0.05	1.68	1.55	0.07	85.66	-3.21	8.17	7	15.89	7.2	-1.53	12.07	21.58	11.59	1.94
os14346	2	525	0.3	7	3	5	6.95	0.05	0.67	1.51	0.11	99.88	-1.99	7.19	7.37	16.34	7.49	-1.25	12.52	22.04	11.84	2

os14514	2	488	0.89	8	3	5	6.69	-0.08	0.39	1.51	0.06	98.92	-1.76	6.37	7.78	16.74	7.8	-1.02	12.93	22.45	12.12	2.22
os14536	2	611	0.89	6	3	5	6.75	-0.08	0.21	1.49	0.05	100	1.73	6.42	6.9	15.76	7.16	-1.5	11.84	21.22	11.34	1.78
os14601	2	574	0.67	6	3	5	6.65	-0.06	0.66	1.51	0.09	99.21	-0.67	6.85	7.23	16.16	7.39	-1.34	12.24	21.68	11.59	1.92
os14602	2	605	0.65	10	1	1	6.54	-0.05	0	1.41	0.06	99.97	4.76	5.31	7.26	16.19	7.41	-1.33	12.27	21.72	11.61	1.93
os14609	2	541	0.92	5	1	1	6.62	-0.09	0	1.42	0.02	98.6	7.26	5.6	7.64	16.55	7.66	-1.14	12.67	22.14	11.91	2.08
os14611	2	558	0.75	12	3	5	6.36	-0.13	0	1.47	0.08	99.63	1	5.78	7.4	16.37	7.5	-1.27	12.45	21.93	11.76	1.99
os14618	2	564	0.25	5	3	5	6.76	0	0.51	1.52	0.06	99.96	-0.85	6.9	7.45	16.44	7.57	-1.24	12.48	22.04	11.85	2.02
os14631	2	545	0.82	5	3	5	6.63	-0.1	0.55	1.51	0.07	97.57	0.08	6.77	7.44	16.41	7.52	-1.24	12.5	22.01	11.78	2.02
os14641	2	617	0.47	11	3	9	6.96	0.04	0.02	1.43	0.07	99.08	1.66	5.64	7.32	16.32	7.45	-1.32	12.33	21.89	11.65	1.89
os14649	2	462	0.1	14	1	4	6.82	0.2	0	1.42	0.08	82.06	3.17	5.71	7.82	16.75	7.8	-1	12.95	22.44	12.06	2.23
os14679	2	533	0.92	13	1	1	6.23	-0.2	0	1.4	0.06	93.49	4.64	5.34	7.76	16.73	7.75	-1.09	12.8	22.34	11.9	2.07
os14731	2	586	0.08	12	1	1	6.9	0.15	0	1.42	0.07	99.97	3.18	5.36	7.28	16.25	7.42	-1.31	12.39	21.9	11.75	1.95
os14755	2	587	0.08	11	1	1	7.09	0.18	0.01	1.43	0.05	97.75	4.03	5.39	7.57	16.57	7.63	-1.15	12.46	22.07	11.76	2.03
os14756	2	581	0.14	12	3	9	7.09	0.18	0	1.44	0.07	98.02	1.56	5.43	7.57	16.57	7.62	-1.15	12.46	22.07	11.76	2.03
os14778	2	450	0.63	10	3	5	6.8	-0.01	0.05	1.48	0.06	93.34	1.63	5.77	8.15	17.17	8.06	-0.83	13.2	22.82	12.24	2.32
os14781	2	439	0.01	9	3	5	6.97	0.16	0.09	1.5	0.08	83.92	-0.46	6.15	8.09	17.11	8.02	-0.87	13.16	22.79	12.25	2.3
os14869	2	426	0.99	7	3	5	6.61	-0.1	0.39	1.52	0.07	95.25	-0.78	6.87	8.29	17.34	8.16	-0.75	13.33	22.98	12.31	2.4
os14894	2	473	0.1	11	3	5	6.34	-0.18	0.02	1.48	0.07	97.88	0.1	5.42	7.98	16.99	7.95	-0.94	12.93	22.59	12.04	2.13
os15002	2	700	0.55	11	2	0	6.51	-0.07	0.04	1.49	0.11	100	-0.93	5.92	6.55	15.41	6.95	-1.56	11.28	20.61	10.96	1.63
os15189	2	607	0.41	4	3	9	6.9	0	0.22	1.5	0.02	95.63	0.67	6.88	7.26	16.16	7.45	-1.24	12.22	21.6	11.62	2.01
os15218	2	614	0.6	6	1	4	6.87	-0.01	0.03	1.47	0.05	92.49	3.99	6.31	7.26	16.13	7.46	-1.2	12.05	21.45	11.46	1.92
os15239	2	587	0.67	12	3	5	6.4	-0.1	0.01	1.48	0.1	92.53	-0.95	5.96	7.37	16.28	7.54	-1.13	12.2	21.59	11.55	1.96
os15248	2	635	0.84	15	3	5	6.59	-0.13	0	1.45	0.1	99.99	0.54	5.17	7.09	15.99	7.32	-1.3	11.88	21.27	11.34	1.86
os15278	2	615	0.03	12	3	5	7.08	0.2	0.01	1.46	0.1	84.39	-0.83	6.54	7.2	16.1	7.42	-1.22	12	21.41	11.43	1.9
os15327	2	679	0.27	16	3	5	6.53	0.05	0	1.43	0.12	99.94	0.69	5.3	6.8	15.66	7.12	-1.44	11.55	20.92	11.14	1.74
os15329	2	674	0.33	14	3	5	6.54	0.02	0	1.46	0.13	99.97	-1.91	5.62	6.76	15.63	7.1	-1.46	11.51	20.87	11.11	1.72
os15330	2	697	0.83	14	3	5	6.28	-0.17	0	1.43	0.09	99.99	2.51	5.3	6.76	15.63	7.09	-1.46	11.5	20.87	11.11	1.72
os15370	2	719	0.06	13	3	5	7.13	0.21	0	1.47	0.1	96.8	-0.87	5.96	6.35	15.16	6.84	-1.67	11	20.28	10.81	1.5
os15435	2	619	0.49	7	1	1	6.72	-0.02	0.18	1.47	0.04	99.13	2.41	6.35	7.34	16.25	7.47	-1.2	12.22	21.65	11.57	1.96
os15439	2	625	0.98	6	3	9	6.71	-0.09	0.17	1.47	0.03	99.93	2.29	6.01	7.27	16.16	7.42	-1.23	12.12	21.53	11.51	1.95
os15474	2	657	0.36	11	3	9	7.04	0.09	0.02	1.43	0.08	98.67	0.21	5.88	7.16	16.11	7.34	-1.32	12.06	21.53	11.47	1.89
os15520	2	578	0.73	12	1	4	6.35	-0.13	0	1.44	0.08	97.18	3.01	5.32	7.49	16.4	7.59	-1.11	12.41	21.82	11.71	2.09
os15531	2	593	0.96	9	3	5	6.64	-0.12	0.2	1.49	0.09	99.92	-1.03	6.32	7.29	16.17	7.46	-1.2	12.15	21.5	11.53	1.97
os15532	2	587	1	10	3	5	6.5	-0.16	0.01	1.49	0.08	99.85	1.21	5.61	7.37	16.26	7.51	-1.16	12.25	21.61	11.59	2
os15591	2	614	0.18	4	1	4	6.94	0.07	0.16	1.47	0.07	94.33	2.02	6.65	6.95	15.79	7.21	-1.42	11.9	21.18	11.39	1.82
os15613	2	613	0.95	9	2	0	6.64	-0.12	0.05	1.5	0.12	98.48	-0.18	7.61	6.87	15.76	7.16	-1.41	11.68	21.04	11.21	1.75
os15714	2	701	0.03	5	2	0	6.98	0.08	0.54	1.51	0.08	98.44	-0.31	7.34	6.37	15.26	6.78	-1.73	11.25	20.57	10.97	1.58

os15721	2	644	0.45	7	3	9	6.7	-0.03	0.06	1.48	0.04	99.98	1.81	5.95	6.89	15.78	7.16	-1.5	11.87	21.23	11.39	1.76
os15723	2	581	0.23	15	1	4	6.67	0.11	0	1.42	0.1	98.96	2.42	5.49	7.24	16.14	7.38	-1.34	12.24	21.66	11.59	1.89
os15750	2	767	0.6	8	3	9	6.68	-0.06	0.07	1.46	0.04	95.58	2.5	6.17	6.1	14.87	6.57	-1.89	10.99	20.15	10.83	1.47
os15760	2	657	0.02	11	3	5	7.11	0.18	0.02	1.48	0.08	100	-0.45	6.03	6.67	15.57	7	-1.63	11.69	20.99	11.27	1.73
os15790	2	743	0.02	10	2	8	7.13	0.18	0.04	1.46	0.1	99.45	-2.78	5.72	6.35	15.23	6.79	-1.74	11.21	20.54	10.94	1.55
os15791	2	735	0.33	14	1	4	7.09	0.12	0	1.43	0.07	99.37	2.9	5.61	6.34	15.23	6.78	-1.74	11.21	20.53	10.94	1.55
os15817	2	647	0.64	11	3	5	6.48	-0.09	0.02	1.49	0.08	99.76	-0.38	5.91	6.77	15.67	7.07	-1.55	11.64	21.04	11.19	1.68
os15823	2	595	0.4	13	3	5	6.53	-0.01	0	1.46	0.1	98.06	0.98	5.72	7.15	16.04	7.33	-1.38	12.07	21.48	11.49	1.83
os15824	2	615	0.01	12	1	1	6.34	-0.18	0	1.42	0.05	98.67	6.15	5.22	7.14	16.04	7.32	-1.38	12.06	21.48	11.49	1.83
os15828	2	709	0.58	9	1	1	6.59	-0.08	0.01	1.42	0.05	100	4.97	5.5	6.68	15.58	7.01	-1.59	11.51	20.91	11.11	1.63
os15829	2	689	0.71	16	3	9	6.19	-0.16	0	1.44	0.1	100	0.47	5.26	6.75	15.65	7.06	-1.56	11.59	21	11.17	1.66
os15831	2	739	0.51	8	1	1	7	0.03	0.02	1.42	0.04	99.57	4.83	5.52	6.61	15.52	6.96	-1.62	11.42	20.82	11.06	1.6
os15833	2	721	0.58	7	3	9	6.7	-0.04	0.33	1.49	0.08	99.34	-1.31	6.16	6.54	15.44	6.9	-1.66	11.33	20.73	10.98	1.57
os15834	2	679	0.6	6	2	0	6.71	-0.04	0.61	1.51	0.09	98.82	-0.18	6.39	6.6	15.5	6.96	-1.61	11.4	20.8	11.04	1.6
os15865	2	643	0.28	9	1	1	6.75	0.03	0.12	1.46	0.06	95.4	3.49	6.45	6.9	15.73	7.17	-1.57	11.83	21.16	11.34	1.72
os15866	2	645	0.41	4	3	6	6.79	-0.01	0.21	1.48	0.05	94.57	2.13	6.71	6.87	15.71	7.15	-1.58	11.8	21.13	11.32	1.71
os15868	2	650	0.92	2	3	6	6.86	-0.02	0.09	1.47	0.05	93.44	2.48	6.6	6.82	15.65	7.11	-1.61	11.73	21.06	11.27	1.68
os15869	2	636	0.27	12	1	4	6.67	0.04	0.01	1.46	0.09	95.33	1.45	5.88	6.9	15.74	7.17	-1.56	11.84	21.17	11.35	1.72
os15870	2	687	0.98	18	1	4	6.07	-0.28	0	1.41	0.08	99.95	5.33	4.95	6.55	15.4	6.92	-1.72	11.38	20.71	11.04	1.55
os15916	2	724	0.96	13	3	9	6.38	-0.21	0.01	1.44	0.08	99.66	-0.1	5.87	6.43	15.28	6.85	-1.72	11.27	20.53	10.95	1.53
os15921	2	603	0.48	13	2	7	6.46	-0.05	0.26	1.51	0.17	87.76	-3.85	7.39	6.61	15.46	6.95	-1.63	11.56	20.81	11.16	1.66
os15971	2	566	0.85	16	3	5	6.11	-0.21	0	1.43	0.1	97.39	1.45	5.44	7.12	16.01	7.32	-1.38	12.06	21.45	11.5	1.83
os15976	2	685	0.76	9	2	3	6.62	-0.12	0.81	1.5	0.09	97.02	-4.06	8.08	6.55	15.44	6.93	-1.65	11.43	20.78	11.1	1.62
os15982	2	791	0.6	6	3	9	6.76	-0.04	0.19	1.46	0.03	99.99	1.48	6.21	6.26	15.16	6.72	-1.78	11.12	20.45	10.87	1.51
os15989	2	571	0.4	8	3	5	6.68	0	0.38	1.5	0.08	99.86	-1.14	7.03	7.12	16.02	7.31	-1.39	12.03	21.48	11.47	1.83
os16004	2	574	0.86	15	3	9	6.17	-0.21	0	1.44	0.08	99.98	0.62	5.56	7.4	16.35	7.49	-1.27	12.43	21.91	11.74	2
os16015	2	578	0.76	17	3	9	6.07	-0.18	0	1.44	0.1	99.96	0.55	5.47	7.38	16.34	7.47	-1.28	12.41	21.88	11.72	1.99
os16037	2	543	0.79	11	2	3	6.7	-0.07	0.08	1.48	0.11	96.91	-2.44	7.02	7.39	16.32	7.5	-1.26	12.44	21.87	11.74	1.98
os16195	2	718	0.17	10	3	5	7.13	0.14	0.01	1.46	0.06	99.85	1.2	5.51	6.43	15.22	6.86	-1.9	11.46	20.7	11.25	1.54
os16343	2	600	0.5	22	3	5	6.02	-0.06	0	1.43	0	86.57	-1.57	6.43	6.87	15.63	7.2	-1.87	12.01	21.3	11.73	1.64
os16350	2	667	0.92	1	3	9	6.89	0	0.05	1.49	0.01	97.7	1.17	7.91	6.76	15.53	7.1	-1.89	11.9	21.16	11.6	1.63
os16372	2	661	0.08	12	3	9	6.88	0.14	0.01	1.46	0.08	99.97	-0.43	5.85	6.82	15.54	7.17	-1.66	11.75	20.95	11.42	1.7
os16421	2	646	0.74	16	1	4	6.14	-0.17	0	1.42	0.11	97.4	2.63	5.85	6.74	15.5	7.09	-1.74	11.79	21.03	11.46	1.68
os16426	2	732	0.23	12	3	9	7.07	0.15	0.07	1.45	0.08	99.33	0.89	5.08	6.51	15.29	6.92	-1.85	11.51	20.72	11.27	1.57
os16434	2	692	0.6	10	3	5	6.59	-0.06	0.23	1.49	0.07	99.51	-0.82	6.48	6.55	15.32	6.96	-1.82	11.54	20.75	11.28	1.61
os16445	2	710	0.03	10	3	9	7.12	0.17	0.04	1.45	0.08	99.66	0.53	5.65	6.52	15.3	6.93	-1.95	11.63	20.88	11.36	1.56
os16448	2	659	0.4	3	1	4	6.94	0.02	0.1	1.45	0.03	98.14	3.29	6.91	6.71	15.47	7.1	-1.86	11.74	21.01	11.51	1.6

os16481	2	618	0.01	6	3	5	6.98	0.1	0.46	1.5	0.08	96.34	-0.76	5.91	6.89	15.6	7.23	-1.6	11.78	20.96	11.45	1.7
os16491	2	656	0.05	10	3	5	6.94	0.14	0.06	1.49	0.08	93.15	-1.39	6.9	6.68	15.43	7.08	-1.71	11.62	20.8	11.34	1.63
os16777	2	550	0.79	9	3	5	6.72	-0.07	0.14	1.49	0.09	98.88	0.81	6.07	6.88	15.75	7.11	-1.6	11.99	21.38	11.43	1.68
os16784	2	587	0	9	3	5	7	0.15	0.06	1.51	0.07	86.28	0.16	6.06	6.57	15.39	6.89	-1.72	11.62	20.95	11.19	1.56
os17033	2	646	0.65	20	3	5	6.72	-0.02	0	1.4	0.14	99.96	2	5.49	6.39	15.22	6.8	-1.69	11.2	20.48	10.86	1.54
os17091	2	662	0.6	7	3	5	6.94	0.01	0.14	1.49	0.07	92.47	1.02	6.38	6.37	15.19	6.8	-1.67	11.06	20.35	10.76	1.51
os17106	2	569	0.87	9	2	3	6.62	-0.08	0.36	1.51	0.14	99.92	-4.75	6.15	6.81	15.68	7.06	-1.64	11.91	21.29	11.36	1.65
os17109	2	548	0.96	8	1	4	6.62	-0.12	0	1.43	0.03	96.21	6.84	5.94	7.05	15.92	7.22	-1.51	12.23	21.61	11.6	1.76
os17117	2	542	0.54	12	3	5	6.87	0.02	0.01	1.46	0.09	98.38	-0.9	6.28	7.24	16.26	7.39	-1.34	12.42	21.97	11.76	1.93
os17119	2	596	0.03	4	3	9	6.97	0.07	0.45	1.48	0.03	84.24	0.67	7.08	6.87	15.75	7.09	-1.61	12.02	21.38	11.42	1.69
os17248	2	553	0.47	10	2	0	6.55	-0.04	0.04	1.5	0.12	97.36	-0.19	6.52	6.59	15.45	6.98	-1.61	11.63	20.97	11.26	1.71
os17257	2	542	0.05	13	3	5	6.88	0.19	0	1.44	0.11	98.85	-0.08	5.13	6.75	15.68	7.11	-1.48	11.97	21.37	11.5	1.88
os17280	2	629	0.01	6	3	5	7.02	0.1	0.93	1.5	0.06	92.57	-0.81	7.68	6.42	15.29	6.84	-1.71	11.37	20.73	11.07	1.6
os17326	2	551	0.35	11	3	5	6.59	0	0.01	1.48	0.09	99.33	0.5	5.65	7.07	15.98	7.26	-1.5	11.97	21.47	11.32	1.56
os17344	2	486	0.6	7	1	4	6.65	-0.02	0.04	1.47	0.05	85.56	2.42	6.18	7.35	16.24	7.47	-1.31	12.34	21.79	11.59	1.72
os17355	2	553	0.02	8	3	5	7.02	0.14	0.19	1.5	0.08	99.03	-1.48	6.49	6.97	15.9	7.2	-1.55	11.87	21.35	11.26	1.52
os17369	2	512	0.71	9	3	5	6.76	-0.04	0.27	1.51	0.09	98.5	-2.02	6.22	7.03	15.95	7.3	-1.44	12.05	21.52	11.54	1.69
os17494	2	565	0.17	13	3	9	6.75	0.12	0	1.46	0.1	100	-1.47	5.63	6.75	15.65	7.1	-1.49	11.92	21.3	11.49	1.9
os17503	2	534	0.83	9	3	5	6.46	-0.12	0.11	1.51	0.07	99.5	-0.8	5.81	6.75	15.64	7.1	-1.48	11.9	21.24	11.46	1.85
os17507	2	539	0.08	13	3	9	7.06	0.21	0.01	1.45	0.1	100	-0.55	5.39	6.84	15.71	7.13	-1.43	12.08	21.43	11.59	1.96
os17508	2	585	0.41	8	3	9	6.96	0.04	0.07	1.46	0.05	100	2.39	5.82	6.84	15.72	7.13	-1.43	12.08	21.43	11.59	1.96
os17509	2	562	0.25	12	2	8	7.03	0.13	0.02	1.46	0.1	100	-2.46	5.65	6.76	15.63	7.07	-1.47	11.98	21.33	11.52	1.93
os17596	2	599	0.15	12	3	5	6.76	0.09	0.04	1.47	0.09	99.59	-1.45	6.71	7.01	15.95	7.21	-1.45	12.04	21.5	11.5	1.84
os17609	2	692	0.13	11	3	5	6.84	0.09	0.02	1.47	0.09	98.56	-0.17	5.68	6.55	15.32	6.98	-1.78	11.48	20.65	11.24	1.56
os17664	2	780	0.25	12	3	9	7.12	0.13	0.01	1.45	0.1	100	-1.74	5.76	6.33	15.14	6.78	-1.99	11.36	20.55	11.09	1.39
os17677	2	936	0.43	7	3	9	7.09	0.03	0.15	1.47	0.06	100	1.35	6.11	5.02	13.73	5.91	-2.45	9.4	18.47	9.77	0.64
os17794	2	832	0.31	9	2	0	7.12	0.09	0.07	1.48	0.09	97.79	0.99	8.37	5.37	14.16	6.17	-2.01	9.61	18.9	9.88	1.12
os17826	2	691	0.91	17	1	2	6.05	-0.26	0	1.43	0.17	95.89	0.59	6.01	6.1	14.91	6.65	-1.7	10.62	19.88	10.54	1.46
os17880	2	906	0.89	9	3	5	6.59	-0.14	0.05	1.47	0.05	97.5	1.71	6.94	5.22	14.05	6.05	-2.14	9.54	18.82	9.8	1
os17909	2	676	0.34	11	3	5	6.71	0.04	0.02	1.46	0.11	98.81	0.46	6.1	6.56	15.37	6.95	-1.58	11.28	20.56	10.99	1.65
os17918	2	829	0.07	4	1	4	7.01	0.06	0.19	1.5	0.02	93.58	2.56	7.3	5.79	14.65	6.41	-2	10.41	19.71	10.38	1.21
os17955	2	719	0.23	24	2	7	6.89	0.26	0	1.43	0.25	92.86	-7.28	5.84	5.86	14.65	6.45	-1.89	10.5	19.7	10.46	1.3
os17959	2	798	0.62	8	2	0	6.97	0	0.16	1.5	0.07	98.81	0.83	6.43	5.7	14.47	6.35	-1.95	10.28	19.48	10.32	1.27
os18010	2	758	0.01	8	2	0	7.04	0.13	0.35	1.52	0.1	88.75	-2.4	7.21	5.88	14.67	6.47	-1.95	10.4	19.67	10.39	1.21
os18021	2	940	0.21	5	3	9	7	0.04	0.28	1.48	0.03	99.84	1.79	6.36	5.2	14.02	6	-2.37	9.73	18.97	9.92	0.83
os18096	2	820	0.77	10	3	5	6.82	-0.08	0.11	1.49	0.1	99.96	-1.9	7.66	5.6	14.41	6.29	-2.02	10.13	19.34	10.2	1.18
os18226	2	858	0.83	12	3	9	6.79	-0.09	0.01	1.43	0.08	99.98	1.4	5.75	5.63	14.39	6.25	-2.23	10.25	19.4	10.25	0.98

os18332	2	835	0.01	8	3	5	7.14	0.13	0.11	1.49	0.04	95.31	1.13	6.38	5.74	14.6	6.37	-2.03	10.34	19.65	10.35	1.2
os18429	2	785	0.92	1	1	4	6.89	-0.03	0.13	1.49	0.03	96.45	2.74	7.23	5.84	14.59	6.45	-1.97	10.52	19.7	10.52	1.33
os18430	2	765	0.56	12	3	9	6.49	-0.08	0	1.46	0.08	99.96	0.57	5.78	5.97	14.73	6.54	-1.9	10.7	19.87	10.62	1.38
os18447	2	798	0.92	12	3	5	6.4	-0.19	0.01	1.46	0.08	99.98	-0.03	5.7	6.07	14.96	6.6	-1.92	10.87	20.17	10.69	1.33
os18483	2	796	0.04	11	3	5	7.16	0.19	0.01	1.47	0.09	100	-0.96	6.39	5.76	14.57	6.4	-1.93	10.37	19.59	10.4	1.31
os18703	2	705	0.21	6	3	6	7.03	0.06	0.39	1.48	0.05	96.16	0.76	6.85	6.53	15.3	6.98	-1.87	11.42	20.69	11.28	1.54
os19056	2	805	0.73	10	3	9	6.54	-0.11	0.03	1.46	0.06	99.96	1.53	6.09	5.61	14.41	6.24	-2.13	10.3	19.51	10.34	1.16
os19165	2	594	0.31	11	3	5	6.35	-0.19	0.04	1.49	0.11	99.72	-1.45	6.79	6.04	14.9	6.63	-1.72	10.8	20.11	10.68	1.5
os19172	2	638	0.88	9	2	0	6.51	-0.13	0.11	1.5	0.08	99.99	-0.47	6.73	6.01	14.9	6.55	-1.78	10.91	20.2	10.74	1.5
os19266	2	621	0.53	12	3	5	6.49	-0.04	0.03	1.47	0.11	100	-0.79	6.42	6.16	15.04	6.66	-1.72	11.12	20.44	10.87	1.59
os19287	2	753	0.99	9	3	5	6.67	-0.13	0.04	1.47	0.06	92.33	2	6	5.62	14.43	6.28	-2.05	10.29	19.54	10.35	1.19
os19358	2	634	0.35	13	2	0	6.28	-0.22	0.01	1.49	0.09	99.99	-1.57	6.35	6.01	14.9	6.55	-1.78	10.91	20.2	10.74	1.5
os19366	2	655	0.12	11	3	9	6.79	0.09	0.02	1.46	0.09	100	-1.14	6.05	6.29	15.17	6.72	-1.7	11.28	20.6	11	1.66
os19404	2	676	0.12	13	3	5	6.83	0.14	0	1.46	0.11	100	-1.69	5.93	6.05	14.92	6.55	-1.8	10.96	20.25	10.77	1.54
os19405	2	596	0.45	7	3	5	6.68	-0.02	0.48	1.52	0.08	100	-0.12	7.07	6.29	15.15	6.74	-1.7	11.26	20.56	10.99	1.64
os19415	2	724	0.86	2	2	0	6.85	-0.04	1.84	1.55	0.08	88.58	-1.39	8.96	5.64	14.47	6.3	-2.07	10.39	19.6	10.4	1.19
os19433	2	734	0.77	15	3	5	6.26	-0.16	0	1.45	0.1	100	0.39	6.04	5.8	14.65	6.39	-2	10.61	19.86	10.54	1.3
os19439	2	783	0.54	6	2	0	7.03	0.03	0.65	1.51	0.09	97.04	-0.5	7.85	5.33	14.1	6.06	-2.25	9.92	19.11	10.06	1
os19608	2	750	0.68	7	2	7	6.87	0.03	0.97	1.54	0.15	83.04	-5.99	7.33	5.76	14.53	6.38	-2.22	10.45	19.87	10.55	1.2
os19619	2	473	1	7	3	5	6.53	-0.13	0.15	1.5	0.04	87.04	1.16	6.29	8.09	17.07	8.03	-0.79	13.18	22.69	12.28	2.41
os19626	2	467	0.95	3	3	6	6.76	-0.04	1.54	1.54	0.03	84.02	-0.93	7.67	8.19	17.16	8.13	-0.74	13.22	22.78	12.38	2.45
os19651	2	450	0.84	4	2	3	6.69	-0.06	1.58	1.53	0.07	97.02	-3.58	8	8.19	17.07	8.22	-0.68	13.46	22.93	12.67	2.63
os19686	2	464	0.83	3	3	6	6.75	-0.04	1.48	1.55	0.04	93.48	-0.43	8.11	8.08	17.07	8.08	-0.77	13.08	22.62	12.29	2.39
os19845	2	418	0.77	1	3	6	6.78	-0.01	6.73	1.56	0.01	18.66	0	8.69	8.29	17.34	8.18	-0.78	13.15	22.9	12.24	2.33
os19884	2	466	0.6	6	3	5	6.67	-0.01	0.65	1.54	0.06	70.3	-1.56	7.25	7.71	16.62	7.75	-1.13	12.9	22.5	12.26	2.33
os19892	2	420	0.92	0	3	6	6.79	0	5.96	1.57	0.01	0.46	-0.18	11.76	8.28	17.35	8.19	-0.77	13.19	23.01	12.33	2.34
os19894	2	420	0.92	0	3	6	6.78	0	5.96	1.57	0.01	0.26	-0.19	9.8	8.28	17.35	8.19	-0.77	13.18	23.01	12.32	2.34
os20025	2	437	0.99	6	3	5	6.63	-0.09	0.44	1.52	0.04	76.46	-0.76	6.61	8.32	17.34	8.26	-0.64	13.42	23.04	12.59	2.58
os20233	2	518	0.92	1	3	6	6.82	-0.01	0.36	1.54	0.01	79.49	0.5	7.76	7.71	16.64	7.76	-1.13	12.83	22.41	12.1	2.17
os20332	2	427	0.89	4	3	5	6.72	-0.05	1.73	1.54	0.05	73.69	-1.85	8.73	8.37	17.38	8.25	-0.65	13.52	23.2	12.6	2.58
os20333	2	429	0.67	3	3	6	6.77	-0.02	1.93	1.54	0.04	74.07	-0.82	8.61	8.37	17.38	8.25	-0.65	13.52	23.2	12.61	2.58
os20401	2	417	0.85	1	3	6	6.75	-0.01	6.96	1.56	0.02	18.3	0.15	9.35	8.19	17.18	8.07	-0.81	12.99	22.67	12.08	2.23
os20469	2	463	0.58	5	3	5	6.65	-0.04	0.37	1.53	0.04	66.87	-0.68	6.63	7.98	17.02	7.92	-1.04	12.79	22.54	11.84	1.97
os20562	2	481	0.15	4	3	6	6.79	0.02	1.83	1.55	0.04	70.8	-1.36	9.13	7.89	16.96	7.85	-1.06	12.71	22.53	11.74	1.88
os20570	2	409	0.06	1	3	6	6.79	0	6.99	1.56	0.01	5.97	0.22	10.2	8.39	17.43	8.26	-0.71	13.26	23	12.29	2.33
os20571	2	409	0.06	1	3	6	6.77	-0.01	6.99	1.56	0.01	5.51	0.45	9.66	8.38	17.42	8.26	-0.71	13.25	22.99	12.29	2.33
os20581	2	405	0.92	0	3	6	6.78	0	6.97	1.57	0.02	0.86	0	12.33	8.44	17.53	8.26	-0.73	13.52	23.29	12.37	2.4

os20582	2	405	0.92	0	3	6	6.78	0	6.97	1.57	0.02	0.76	0	12.33	8.44	17.53	8.26	-0.73	13.52	23.29	12.38	2.4
os20586	2	407	0.92	1	3	6	6.78	0.01	2.04	1.57	0.03	38.33	-0.87	8.9	8.41	17.5	8.25	-0.73	13.47	23.25	12.34	2.37
os20650	2	400	0.92	0	3	6	6.78	0	3.99	1.55	0.03	45.16	0.62	11.76	8.43	17.5	8.24	-0.72	13.38	23.21	12.28	2.31
os20674	2	441	0.16	2	3	6	6.77	-0.01	2.98	1.56	0.03	49.18	-1.36	9.82	8.15	17.21	8.03	-0.94	13	22.85	12.01	2.1
os20687	2	421	0.92	1	3	6	6.8	0.01	3.71	1.57	0.02	27.76	-0.53	9.8	8.26	17.35	8.17	-0.8	13.15	23.02	12.2	2.2
os20787	2	522	0.23	4	3	6	6.79	0.02	0.57	1.53	0.02	93.84	0.04	6.85	7.61	16.63	7.62	-1.39	12.58	22.31	11.7	1.75
os20810	2	516	0.18	1	3	6	6.84	0.02	1.56	1.54	0.02	42.36	-0.17	8.92	7.71	16.74	7.67	-1.26	12.52	22.33	11.57	1.7
os21194	2	455	0.92	1	3	6	6.81	0	6.02	1.55	0.01	26.16	0.63	9.51	7.6	16.53	7.59	-1.22	12.71	22.3	11.9	2.11
os21357	2	412	0.99	1	3	6	6.77	-0.01	0.61	1.56	0.01	4.56	0.18	8.64	8.57	17.69	8.39	-0.63	13.66	23.37	12.58	2.59
os21561	2	424	0.91	5	1	4	6.6	-0.07	0.13	1.51	0.02	60.96	2.19	7.29	8.57	17.69	8.37	-0.66	13.64	23.37	12.52	2.53
os21575	2	408	0.92	3	3	6	6.69	-0.04	1.02	1.54	0.02	56.33	0.35	8.38	8.53	17.63	8.38	-0.63	13.6	23.31	12.57	2.57
os21680	2	434	0.23	4	3	6	6.65	-0.07	0.43	1.53	0.03	90.05	0.09	6.6	8.43	17.51	8.25	-0.69	13.6	23.27	12.48	2.49
os21681	2	433	1	5	3	6	6.63	-0.08	0.58	1.53	0.03	89.13	-0.24	6.76	8.44	17.51	8.25	-0.69	13.6	23.27	12.48	2.49
os21777	2	426	0.15	2	3	6	6.85	0.03	4.86	1.54	0.02	50.13	0.28	8.53	8.31	17.33	8.1	-0.78	13.07	22.83	11.97	2.1
os22144	2	421	0.01	7	3	5	6.89	0.11	0.57	1.53	0.05	93.25	-1.1	8.45	8.33	17.4	8.16	-0.76	13.16	23.01	12.09	2.23
os22265	2	417	0.59	2	3	6	6.81	0.01	6.98	1.56	0.01	4.4	-0.37	8.98	8.32	17.38	8.22	-0.74	13.18	22.94	12.3	2.31
os22267	2	416	0.92	0	3	6	6.78	0	6.99	1.56	0.01	4.4	-0.23	9.66	8.32	17.38	8.22	-0.74	13.18	22.94	12.3	2.31
os22665	2	431	0.6	2	3	6	6.75	-0.01	6.94	1.55	0.01	26.98	0.08	8.14	8.24	17.29	8.16	-0.83	13.08	22.85	12.22	2.29
os22675	2	416	0.99	5	3	6	6.66	-0.07	0.96	1.55	0.03	18.74	-0.88	7.94	8.27	17.32	8.17	-0.79	13.12	22.88	12.22	2.33
os22681	2	420	0.92	1	3	6	6.75	-0.02	6.94	1.56	0.01	4.18	0.11	9.33	8.28	17.36	8.2	-0.81	13.18	22.98	12.33	2.34
os22682	2	420	0.92	1	3	6	6.74	-0.02	6.94	1.56	0.01	3.92	0.04	9.27	8.28	17.36	8.2	-0.81	13.18	22.98	12.33	2.34
os22684	2	430	0.92	0	3	6	6.79	0	6.36	1.56	0.01	2.79	-0.06	9.51	8.27	17.31	8.18	-0.8	13.12	22.94	12.27	2.33
os22685	2	430	0.92	0	3	6	6.8	0	6.36	1.56	0.01	2.52	-0.08	9.16	8.27	17.31	8.18	-0.8	13.12	22.93	12.27	2.33
os22687	2	430	0.92	0	3	6	6.79	0	6.36	1.56	0.01	3.13	0.05	10.04	8.27	17.31	8.18	-0.8	13.12	22.94	12.27	2.33
os22701	2	422	0.92	0	3	6	6.79	0	6.97	1.56	0.01	2.93	0.03	10.95	8.29	17.36	8.2	-0.8	13.17	22.96	12.31	2.33
os22749	2	458	0.05	2	3	6	6.84	0.02	2.55	1.55	0.02	45.5	-0.65	8.42	7.79	16.77	7.74	-1.13	12.6	22.28	11.8	2.04
os22752	2	474	0.89	2	3	6	6.76	-0.03	3.69	1.55	0.02	17.94	-0.13	8.43	7.62	16.58	7.62	-1.23	12.44	22.13	11.64	1.93
os22838	2	421	0.92	0	3	6	6.78	0	6.98	1.56	0.01	11.85	0.1	9.8	8.2	17.2	8.09	-0.86	13.03	22.75	12.16	2.29
os22839	2	421	0.92	0	3	6	6.79	0	6.98	1.56	0.01	10.9	0.03	9.83	8.2	17.2	8.09	-0.86	13.02	22.75	12.16	2.29
os22870	2	489	0.13	2	3	6	6.84	0.03	1.93	1.55	0.03	45.68	-1.29	8.22	7.45	16.39	7.54	-1.22	12.39	22.02	11.71	2.05
os22955	2	501	0.04	5	3	5	6.9	0.08	0.48	1.53	0.03	71.44	0.52	7.64	7.73	16.64	7.75	-1.15	12.82	22.39	12.1	2.12
os23040	2	414	0.92	0	3	6	6.78	0	6.92	1.57	0.03	24.24	-0.66	9.09	8.24	17.25	8.15	-0.8	13.04	22.77	12.15	2.29
os23048	2	410	0.92	0	3	6	6.78	0	6.99	1.57	0.01	3.31	0	12.04	8.37	17.44	8.27	-0.73	13.24	23.06	12.33	2.35
os23086	2	430	0.08	2	3	6	6.83	0.03	2.81	1.55	0.03	57.19	-1.25	9.48	8.34	17.52	8.18	-0.77	13.27	23.17	12.2	2.24
os23140	2	450	0.3	18	2	3	6.94	0.17	0	1.44	0.14	97.67	-1.49	5.77	8.13	17.13	8.1	-0.73	12.73	22.5	11.77	1.98
os23324	2	524	0.99	5	1	1	6.68	-0.08	0.07	1.44	0.04	100	3.37	6.68	7.96	16.96	7.93	-0.87	12.52	22.33	11.61	1.86
os23326	2	502	0.6	10	3	9	6.49	-0.07	0.06	1.48	0.1	100	-1.57	5.84	7.96	16.96	7.93	-0.87	12.51	22.33	11.6	1.86

os23342	2	461	0.11	7	3	5	6.81	0.08	0.32	1.52	0.09	99.45	-1.61	6.38	8.07	17.08	8.04	-0.82	12.76	22.55	11.79	1.92
os23348	2	502	1	5	3	5	6.64	-0.09	0.66	1.54	0.05	91.87	-0.54	7.63	7.83	16.86	7.83	-0.99	12.38	22.2	11.44	1.7
os23349	2	525	0.43	6	3	5	6.73	0	0.36	1.53	0.06	95.98	-0.01	7.22	7.67	16.7	7.7	-1.08	12.18	22.04	11.26	1.62
os23355	2	537	0.05	9	2	7	6.84	0.09	0.54	1.53	0.09	83.7	-2.41	8.25	7.62	16.67	7.63	-1.12	12.18	22.05	11.25	1.65
os23368	2	568	0.1	10	3	5	6.9	0.13	0.04	1.47	0.06	98.72	1.12	5.91	7.59	16.62	7.63	-1.13	12.09	21.95	11.19	1.57
os23370	2	530	0.03	11	1	4	6.92	0.16	0	1.47	0.08	99.25	2.43	5.65	7.75	16.77	7.76	-1.07	12.29	22.11	11.35	1.66
os23371	2	529	0.02	10	3	5	7	0.17	0.08	1.49	0.09	99.25	0.39	6.25	7.72	16.75	7.74	-1.08	12.27	22.09	11.33	1.64
os23372	2	488	0	13	3	5	6.95	0.22	0	1.47	0.1	79.32	-1.45	5.38	7.95	16.97	7.93	-0.91	12.63	22.43	11.67	1.83
os23375	2	536	0.11	10	3	9	7.06	0.16	0.01	1.45	0.05	100	1.8	5.87	7.94	16.97	7.92	-0.95	12.62	22.43	11.66	1.82
os23493	2	510	0.74	4	3	6	6.7	-0.04	1.34	1.53	0.04	92.12	-0.59	7.53	7.9	16.97	7.86	-1.1	12.67	22.52	11.67	1.74
os23521	2	626	0.6	10	3	5	6.56	-0.06	0.06	1.47	0.06	99.99	0.23	6.02	7.16	16.2	7.26	-1.49	11.75	21.51	10.89	1.32
os23533	2	620	0	10	3	5	7.03	0.17	0.04	1.48	0.07	98.14	0.31	5.91	7.18	16.25	7.29	-1.49	11.78	21.58	10.92	1.3
os23619	2	436	0.01	9	2	0	6.92	0.15	0.09	1.51	0.09	87.61	-0.92	7.48	8.07	17.07	8.06	-0.78	12.82	22.57	11.87	2
os23775	2	555	0.99	7	1	1	6.64	-0.1	0.08	1.48	0.03	99.13	2.66	6	7.66	16.65	7.67	-1.16	12.38	22.06	11.44	1.66
os23895	2	588	0.01	2	3	6	6.91	0.03	0.18	1.53	0.06	98.25	0.86	8.63	6.66	15.66	6.89	-1.93	11.11	20.85	10.48	0.89
os23903	2	610	0.63	5	3	6	6.73	-0.08	0.2	1.52	0.03	81.63	1.23	8.29	6.85	15.83	7.01	-1.85	11.49	21.14	10.7	1.02
os23958	2	566	0.47	6	3	5	6.94	0.04	0.63	1.53	0.05	92.18	-0.33	8.62	6.86	15.86	7.04	-1.86	11.39	21.12	10.69	0.97
os23984	2	599	0.65	4	3	6	6.75	-0.04	0.06	1.51	0.03	91.25	1.97	7.58	6.84	15.88	7.01	-1.92	11.45	21.27	10.81	0.93
os23990	2	597	0.09	4	3	6	6.97	0.06	0.33	1.53	0.02	83.15	0.89	7.5	6.79	15.83	6.98	-1.95	11.4	21.2	10.76	0.88
os24045	2	559	0	8	2	3	6.96	0.11	0.4	1.53	0.07	85.03	-1.68	7.4	6.97	15.99	7.13	-1.87	11.52	21.35	10.8	0.96
os24154	2	529	0.23	2	3	6	6.82	0.01	3.6	1.56	0.05	65.54	-1.39	7.95	7.27	16.31	7.35	-1.61	11.95	21.68	11.13	1.3
os24245	2	549	0.45	11	3	5	6.96	0.08	0.07	1.5	0.08	97.5	-0.77	5.69	7.41	16.46	7.45	-1.5	12.04	21.87	11.21	1.39
os24249	2	543	0.6	5	3	5	6.74	-0.01	0.53	1.52	0.04	84.31	-0.06	6.56	7.45	16.51	7.51	-1.46	12.11	21.92	11.27	1.46
os24250	2	521	0.35	5	3	6	6.92	0.04	1.14	1.54	0.04	62.64	-0.42	8.31	7.5	16.55	7.54	-1.43	12.17	21.97	11.3	1.47
os24293	2	479	0.02	7	3	5	6.85	0.07	0.4	1.53	0.04	85.37	-0.03	7.39	7.9	16.95	7.84	-1.13	12.74	22.54	11.8	1.88
os24307	2	505	0.08	3	3	6	6.9	0.05	1.52	1.55	0.03	80.58	-0.67	8.77	7.72	16.78	7.69	-1.26	12.52	22.34	11.65	1.77
os24328	2	499	0.99	2	3	6	6.75	-0.04	1.65	1.55	0.03	75.46	-0.28	7.67	7.69	16.72	7.67	-1.34	12.59	22.38	11.7	1.7
os24369	2	496	0.92	1	3	6	6.86	0.02	1.19	1.56	0.01	18.29	-0.02	8.21	7.72	16.78	7.71	-1.3	12.66	22.42	11.74	1.73
os24386	2	485	0.49	3	3	6	6.78	0	0.7	1.54	0.02	65.36	0.56	8.39	7.77	16.84	7.7	-1.33	12.71	22.51	11.73	1.73
os24421	2	547	0.45	6	3	5	6.71	-0.01	0.47	1.53	0.04	98.24	-0.17	7.07	7.3	16.3	7.35	-1.59	12.03	21.74	11.2	1.41
os24431	2	468	0.92	2	2	3	6.86	0.02	1.94	1.57	0.06	47.75	-2.36	8.75	7.75	16.82	7.68	-1.35	12.71	22.45	11.67	1.69
os24509	2	501	0.45	10	2	0	6.54	-0.04	0.06	1.49	0.09	86.16	0.58	6.63	7.46	16.47	7.47	-1.52	12.25	21.96	11.35	1.47
os24517	2	539	0.99	4	3	6	6.72	-0.07	0.64	1.54	0.03	84.68	-0.09	7.29	7.26	16.29	7.34	-1.62	11.94	21.67	11.13	1.3
os24521	2	554	0.6	4	3	6	6.71	-0.04	0.31	1.52	0.02	98.42	0.51	6.59	7.33	16.34	7.38	-1.61	12.01	21.75	11.17	1.3
os24616	2	561	0.59	23	3	5	6.66	-0.01	0	1.39	0.11	75.38	3.13	5.05	7.16	16.17	7.25	-1.81	11.87	21.61	11.03	1.03
os24665	2	594	0.23	2	3	6	6.85	0.01	0.17	1.53	0.02	95.64	1.34	7.82	6.95	15.96	7.1	-1.89	11.59	21.31	10.78	0.95
os24705	2	671	0.2	2	3	6	6.91	0.02	0.46	1.54	0.02	50.41	0.29	8.02	6.41	15.4	6.69	-2.08	10.93	20.62	10.32	0.82

os24831	2	584	0.94	6	3	5	6.74	-0.07	0.31	1.51	0.03	99.29	0.99	6.06	7.47	16.48	7.47	-1.43	12.2	21.98	11.22	1.41
os24856	2	675	0.23	5	3	6	7.03	0.07	0.53	1.51	0.04	100	0.35	7	6.88	15.97	7.04	-1.8	11.48	21.32	10.72	1.09
os24877	2	500	0.98	3	3	6	6.76	-0.04	1.87	1.54	0.04	91.07	-1.02	8.13	7.87	16.89	7.8	-1.08	12.55	22.37	11.52	1.68
os24881	2	501	0.88	3	3	6	6.73	-0.02	0.35	1.53	0.04	97.68	0.42	7.59	7.77	16.81	7.75	-1.18	12.49	22.31	11.48	1.64
os24886	2	521	0.19	4	3	6	6.94	0.05	0.77	1.54	0.04	87.19	-0.25	8.35	7.47	16.49	7.47	-1.53	12.23	21.97	11.28	1.41
os24911	2	631	0.06	5	3	5	7.01	0.09	0.31	1.52	0.05	85.79	1.16	8.04	6.72	15.69	6.92	-2.01	11.38	21.05	10.58	0.83
os24987	2	651	0.88	2	1	4	6.81	-0.03	0.02	1.5	0.01	97.02	2.41	6.86	6.96	15.98	7.1	-1.74	11.59	21.34	10.74	1.09
os25052	2	591	0.4	3	3	6	6.92	0.02	0.2	1.53	0.03	90.94	0.81	8.76	7.23	16.28	7.3	-1.56	11.94	21.71	11.06	1.34
os25070	2	579	0.92	4	3	6	6.73	-0.06	1.74	1.55	0.05	84.98	-1.79	9.06	7.11	16.13	7.21	-1.62	11.69	21.44	10.81	1.21
os25108	2	501	0.12	6	3	6	6.84	0.06	0.57	1.55	0.05	59.26	-0.77	8.53	7.56	16.56	7.54	-1.5	12.34	22.09	11.36	1.45
os25125	2	681	0.23	3	3	6	6.88	0.02	0.45	1.52	0.02	96.65	0.84	6.9	6.59	15.57	6.84	-2.16	11.23	20.93	10.48	0.69
os25139	2	566	0.92	3	3	6	6.75	-0.04	1.58	1.55	0.04	90.66	-0.18	7.79	7.15	16.16	7.22	-1.8	11.86	21.59	10.99	1.08
os25165	2	495	0.77	6	3	6	6.79	-0.04	1.24	1.53	0.07	95.43	-1.88	8.58	7.76	16.77	7.72	-1.2	12.46	22.28	11.44	1.58
os25172	2	480	0.66	13	2	0	6.41	-0.08	0.13	1.49	0.08	92.2	-0.48	8.14	7.83	16.87	7.79	-1.14	12.56	22.37	11.53	1.67
os25195	2	556	0.23	3	3	6	6.84	0.02	1.94	1.51	0.04	96.4	-0.7	7.44	7.66	16.72	7.61	-1.24	12.37	22.24	11.39	1.56
os25225	2	560	0.45	6	3	5	6.71	-0.02	1.01	1.54	0.06	92.16	-1.63	8.57	7.44	16.49	7.46	-1.4	12.14	21.97	11.19	1.39
os25234	2	518	0.99	3	3	6	6.75	-0.04	1.81	1.55	0.04	86.89	-0.68	8.33	7.7	16.74	7.68	-1.2	12.37	22.22	11.38	1.57
os25253	2	533	0.04	9	2	7	6.9	0.11	0.61	1.53	0.08	89.36	-2.48	7.88	7.56	16.58	7.55	-1.37	12.31	22.09	11.29	1.46
os25259	2	515	0.4	8	3	5	6.62	-0.03	0.66	1.51	0.07	93.93	-1.26	7.45	7.69	16.71	7.67	-1.25	12.41	22.19	11.39	1.54
os25284	2	603	0.77	2	3	6	6.85	-0.02	0.97	1.55	0.02	48.71	0.35	8.73	7.26	16.33	7.34	-1.52	11.97	21.77	11.05	1.31
os25330	2	535	0.37	6	3	5	6.98	0.08	0.71	1.53	0.06	93.29	-1.2	7.87	7.76	16.84	7.68	-1.15	12.48	22.39	11.46	1.68
os25331	2	556	0.99	3	3	6	6.78	-0.04	1.42	1.53	0.02	80.67	-0.17	7.61	7.73	16.84	7.66	-1.16	12.46	22.38	11.48	1.66
os25363	2	490	0.79	7	3	5	6.54	-0.09	0.47	1.51	0.04	94.09	0.37	7.42	7.96	17	7.88	-0.98	12.6	22.53	11.61	1.82
os25375	2	606	0.6	4	3	6	6.89	-0.01	0.36	1.52	0.04	71.39	0.76	9.15	7.19	16.22	7.26	-1.58	11.87	21.64	11.05	1.3
os25378	2	613	0.08	3	3	6	6.97	0.05	0.63	1.54	0.03	87.64	0.09	8.33	7.15	16.19	7.24	-1.6	11.83	21.58	11.02	1.28
os25389	2	594	0.02	4	3	6	6.94	0.06	0.96	1.54	0.05	66	-1.48	8.27	7.26	16.25	7.3	-1.47	11.91	21.62	10.99	1.37
os25391	2	614	0.07	3	3	6	6.95	0.04	2.24	1.54	0.05	87.76	-1.93	8.44	7.17	16.16	7.23	-1.52	11.77	21.5	10.89	1.31
os25430	2	532	0.17	18	3	5	6.73	0.16	0	1.46	0.11	89.82	-0.66	6.09	7.35	16.28	7.45	-1.51	12.01	21.73	11.11	1.28
os25497	2	656	0.67	6	1	1	6.69	-0.05	0.04	1.47	0.02	97.48	3.61	5.96	6.9	15.83	7.12	-1.86	11.56	21.25	10.72	0.94
os25519	2	615	0.08	4	3	6	6.98	0.06	0.98	1.53	0.04	81.3	-0.37	7.68	7	15.97	7.2	-1.75	11.6	21.34	10.8	1.03
os25554	2	542	1	5	3	5	6.65	-0.08	0.86	1.53	0.05	98.83	-1.05	8.03	7.55	16.56	7.56	-1.37	12.34	22.12	11.31	1.47
os25596	2	624	0	7	3	5	7.03	0.13	0.26	1.5	0.07	91.29	0.39	6.76	6.96	15.95	7.13	-1.73	11.51	21.28	10.69	1.03
os25609	2	538	0.77	7	3	5	6.78	-0.05	0.17	1.51	0.06	84.72	0.94	6.16	7.4	16.39	7.45	-1.52	12.16	21.9	11.22	1.35
os25632	2	653	0	7	1	4	7.02	0.11	0.03	1.48	0.03	95.41	3.87	5.92	6.87	15.82	7.07	-1.78	11.32	21.06	10.54	0.94
os25643	2	672	0.77	7	3	5	6.88	-0.03	0.33	1.5	0.05	95.09	0.23	6.7	6.61	15.55	6.87	-2.01	11.17	20.86	10.42	0.77
os25648	2	653	0.95	8	2	3	6.78	-0.06	0.46	1.51	0.09	97.74	-2.99	6.95	6.68	15.63	6.92	-1.96	11.21	20.91	10.45	0.8
os25650	2	612	0.01	9	3	5	7.01	0.14	0.11	1.5	0.06	92.01	0.53	7.93	6.84	15.78	7.03	-1.9	11.4	21.1	10.57	0.88

os25701	2	614	0.3	7	3	5	6.77	0.03	0.31	1.51	0.06	94.57	-0.3	6.34	6.88	15.84	7.06	-1.88	11.51	21.23	10.68	0.94
os25727	2	686	0.92	4	3	6	6.77	-0.05	0.14	1.5	0.02	95.75	1.89	6.49	6.62	15.57	6.84	-1.97	11.15	20.84	10.41	0.81
os25730	2	676	0.23	2	3	6	6.87	0	0.2	1.5	0.02	84.07	1.76	7.22	6.62	15.58	6.85	-1.97	11.15	20.85	10.41	0.8
os25740	2	644	0	4	3	6	6.99	0.07	0.52	1.51	0.02	76.16	1.09	7.13	6.8	15.73	6.98	-1.92	11.42	21.12	10.6	0.89
os25749	2	646	0.58	6	3	5	6.9	-0.01	0.47	1.52	0.03	94.66	0.31	6.52	6.76	15.71	6.96	-1.94	11.36	21.05	10.56	0.89
os25759	2	623	0.77	1	3	6	6.9	0.01	2.69	1.56	0.05	78.66	-1.38	8.64	6.76	15.72	6.95	-1.92	11.36	21.03	10.55	0.89
os25779	2	604	0.88	8	3	5	6.78	-0.06	0.3	1.51	0.05	99.04	-0.52	7.02	7.08	16.1	7.21	-1.7	11.75	21.5	10.91	1.14
os25781	2	594	0.98	10	3	5	6.59	-0.14	0.08	1.48	0.07	98.83	0.3	6.71	7.11	16.13	7.23	-1.68	11.78	21.53	10.94	1.16
os25794	2	575	0.78	5	3	5	6.79	-0.05	0.51	1.51	0.06	96.12	0.03	6.32	7.12	16.13	7.24	-1.68	11.77	21.51	10.93	1.15
os25797	2	636	0.08	3	3	6	6.96	0.04	0.51	1.5	0.03	96.55	0.23	6.94	6.96	15.95	7.12	-1.79	11.56	21.32	10.76	1.06
os25825	2	644	0.92	0	3	6	6.89	0	3.55	1.54	0.02	66.68	0.75	10.51	6.82	15.79	6.99	-1.93	11.35	21.12	10.62	0.9
os25828	2	637	0.02	7	3	5	7.06	0.12	0.18	1.49	0.06	97.73	1.04	7.37	6.84	15.8	7	-1.89	11.37	21.13	10.63	0.94
os25829	2	636	0.19	9	3	5	7.08	0.12	0.07	1.48	0.06	97.4	2.13	6.89	6.84	15.8	7.01	-1.89	11.38	21.13	10.63	0.94
os25836	2	592	0.53	3	3	6	6.93	0.02	0.23	1.53	0.05	93.32	0.5	6.99	7.01	15.98	7.15	-1.77	11.62	21.35	10.79	1.07
os25862	2	615	0.08	4	3	6	6.97	0.05	0.87	1.52	0.03	91.42	0.44	7.67	6.92	15.87	7.07	-1.9	11.61	21.28	10.77	0.95
os25878	2	593	0.6	11	3	5	6.47	-0.08	0.02	1.5	0.07	70.31	-0.84	6.28	6.99	15.94	7.13	-1.85	11.71	21.39	10.84	0.98
os25879	2	642	0.83	2	3	9	6.84	-0.02	0.01	1.5	0.01	93.83	2.11	7.31	6.86	15.82	7.03	-1.94	11.59	21.27	10.74	0.89
os25887	2	667	0.99	6	3	5	6.7	-0.1	0.45	1.52	0.04	89.79	0.38	6.68	6.54	15.51	6.79	-2.13	11.14	20.86	10.41	0.68
os25895	2	641	0.19	3	3	6	6.96	0.03	1.8	1.54	0.05	89.55	-0.74	8.74	6.62	15.58	6.84	-2.07	11.25	20.92	10.49	0.77
os25934	2	575	0.77	5	3	6	6.84	-0.03	0.51	1.52	0.04	99.79	-0.19	6.79	7.55	16.59	7.54	-1.2	12.11	21.97	11.23	1.59
os25947	2	467	0.66	10	2	0	6.78	-0.01	0.74	1.53	0.11	83.5	-3.22	7.5	7.9	16.94	7.79	-1.1	12.67	22.52	11.64	1.73
os25962	2	576	0.4	5	3	6	6.94	0.03	1.22	1.54	0.06	92.19	-1.58	8.91	7.39	16.41	7.39	-1.44	12.1	21.86	11.13	1.44
os25967	2	575	0.49	3	2	3	6.84	-0.02	1.94	1.55	0.06	92.84	-2.96	9.03	7.39	16.41	7.39	-1.44	12.1	21.86	11.13	1.43
os25980	2	576	0.86	3	3	6	6.74	-0.06	1.52	1.55	0.05	90.22	-1.03	8.2	7.27	16.33	7.33	-1.49	11.96	21.76	11.05	1.36
os26045	2	519	0.49	4	2	3	6.82	0.03	1.9	1.52	0.05	88.68	-2.63	8.86	7.75	16.8	7.75	-1.19	12.45	22.31	11.5	1.61
os26049	2	532	0.65	7	3	5	6.59	-0.06	0.2	1.5	0.04	87.78	1.36	6.43	7.74	16.79	7.74	-1.2	12.45	22.3	11.49	1.61
os26210	2	618	0.6	8	3	9	6.64	-0.04	0.14	1.45	0.05	98.3	1.16	6.14	7.45	16.44	7.46	-1.5	12.28	21.9	11.33	1.44
os26215	2	607	0	13	1	1	7.01	0.2	0	1.44	0.06	99.32	1.59	6.06	7.52	16.52	7.51	-1.46	12.36	21.98	11.4	1.48
os26216	2	608	0.01	11	1	1	6.96	0.16	0	1.44	0.05	99.33	1.87	6.09	7.52	16.51	7.51	-1.46	12.36	21.98	11.4	1.48
os26271	2	548	0.73	16	2	3	6.17	-0.15	0.16	1.49	0.14	97.59	-5.08	7.53	7.56	16.64	7.6	-1.28	12.24	22.06	11.29	1.51
os26405	2	561	0.08	8	3	5	7.03	0.12	0.28	1.51	0.06	99.21	-0.85	6.2	7.58	16.61	7.56	-1.39	12.45	22.09	11.44	1.5
os26448	2	708	0.01	9	1	1	7.11	0.13	0.33	1.4	0.05	92.71	3.06	5.99	7.01	16.04	7.1	-1.62	11.68	21.35	10.86	1.25
os26455	2	665	0	7	1	1	6.99	0.11	0.07	1.44	0.03	90.93	4.13	5.88	7.11	16.12	7.2	-1.61	11.81	21.48	10.96	1.24
os26460	2	488	0.27	7	2	3	6.71	0.03	1.16	1.54	0.1	75.92	-3.84	7.33	7.81	16.83	7.75	-1.18	12.59	22.3	11.63	1.67
os26470	2	595	1	6	3	9	6.63	-0.11	0.33	1.5	0.04	99.54	0.32	6.18	7.49	16.55	7.52	-1.4	12.23	22.03	11.32	1.45
os26563	2	611	0.17	3	3	6	6.94	0.04	1.86	1.54	0.03	82.27	-0.75	7.98	7.22	16.22	7.27	-1.61	11.99	21.64	11.1	1.31
os26598	2	546	0.52	15	3	5	6.37	-0.06	0	1.47	0.08	88.88	0.09	6.97	7.5	16.55	7.52	-1.36	12.21	21.99	11.29	1.54

os26607	2	569	0.08	1	3	6	6.89	0.02	2.96	1.55	0.04	59.52	-1.04	9.92	7.43	16.51	7.48	-1.41	12.17	21.98	11.24	1.54
os26707	2	680	0.06	9	1	1	7.1	0.14	0.05	1.45	0.05	100	2.78	5.61	6.07	14.92	6.58	-2.36	10.59	20.22	10.1	0.29
os26790	2	586	0.13	4	3	6	6.87	0.04	1.39	1.54	0.04	91.69	-0.01	9.36	6.32	15.1	6.87	-2.04	10.74	20.39	10.28	0.54
os26818	2	465	0.03	8	3	5	6.83	0.1	0.09	1.49	0.04	72.64	1.5	7.95	7.24	16.03	7.63	-1.43	11.94	21.57	11.29	1.25
os27251	2	569	0.79	7	3	5	6.74	-0.07	0.33	1.51	0.05	87.47	0.31	7.08	6.64	15.49	7.09	-1.95	11.33	21.09	10.77	0.8
os27253	2	560	0.23	1	3	6	6.84	0.01	1.72	1.54	0.01	61.49	0.88	9.59	6.72	15.55	7.14	-1.87	11.33	21.08	10.77	0.85
os27286	2	493	0.71	9	3	5	6.76	-0.05	0.15	1.51	0.06	92.14	-0.32	6.57	7.06	15.84	7.48	-1.57	11.74	21.36	11.11	1.1
os27428	2	552	1	5	3	6	6.67	-0.08	0.6	1.52	0.04	90.05	0.27	8.33	6.6	15.43	7.08	-2	11.17	20.89	10.68	0.74
os27464	2	642	0.99	5	3	9	6.74	-0.08	0.37	1.47	0.03	94.87	1.02	6.48	6.26	15.05	6.8	-2.22	10.81	20.47	10.35	0.46
os27473	2	614	0.83	9	3	5	6.77	-0.07	0.09	1.49	0.05	96.11	0.95	6.31	6.29	15.09	6.83	-2.18	10.86	20.5	10.38	0.5
os27511	2	607	0.91	9	3	5	6.63	-0.12	0.2	1.5	0.05	95.98	0.75	7.78	6.23	15.06	6.81	-2.19	10.8	20.5	10.37	0.46
os27590	2	590	0.16	6	2	3	6.89	0.06	1.19	1.54	0.06	80.2	-1.65	8.31	6.46	15.37	6.87	-2.21	11.08	20.89	10.46	0.47
os27593	2	601	0.95	10	3	5	6.62	-0.12	0.06	1.49	0.06	91.12	-0.49	5.76	6.94	15.83	7.12	-1.79	11.58	21.29	10.76	0.94
os27785	2	450	0.58	5	2	0	6.66	-0.03	0.12	1.51	0.06	76.41	1.76	7.54	7.28	16.12	7.54	-1.62	12.03	21.78	11.29	1.14
os27887	2	503	0.08	3	3	6	6.9	0.04	1.5	1.53	0.02	94.54	-0.31	7.58	7.22	16.1	7.46	-1.57	11.96	21.71	11.2	1.18
os27943	2	568	0.52	5	1	1	6.74	-0.01	0.2	1.5	0.02	98.05	2.27	6.55	7.08	15.97	7.31	-1.72	11.82	21.58	11.06	1.06
os28005	2	492	0.01	2	3	6	6.85	0.03	2.8	1.54	0.04	67.21	-0.04	9.47	7.45	16.36	7.59	-1.42	12.15	21.92	11.3	1.32
os28056	2	497	0.92	5	3	6	6.64	-0.06	0.11	1.49	0.04	89.69	2.9	6.96	7.33	16.25	7.5	-1.5	12.05	21.81	11.24	1.22
os28097	2	503	0.83	6	3	6	6.64	-0.05	0.16	1.49	0.04	91.72	1.28	6.26	7.31	16.22	7.48	-1.57	12.08	21.81	11.22	1.19
os28117	2	564	0.66	6	3	5	6.59	-0.09	0.44	1.51	0.06	97.79	-0.3	7.86	6.94	15.85	7.18	-1.79	11.53	21.29	10.76	0.96
os28118	2	560	0.87	8	3	5	6.55	-0.1	0.21	1.51	0.06	97.35	0.08	7.58	6.94	15.85	7.18	-1.79	11.53	21.28	10.76	0.96
os28124	2	599	0.02	8	3	5	7.04	0.13	0.3	1.5	0.05	92.19	0.14	6.56	6.87	15.79	7.13	-1.82	11.42	21.18	10.67	0.93
os28125	2	609	0.47	7	1	1	7.01	0.07	0.05	1.46	0.03	94.33	3.42	5.67	6.88	15.8	7.14	-1.82	11.44	21.19	10.68	0.93
os28132	2	572	0.92	4	3	6	6.73	-0.05	2.09	1.54	0.04	80.75	0.14	8.44	6.82	15.72	7.08	-1.84	11.33	21.07	10.59	0.89
os28136	2	595	0.28	3	3	6	6.95	0.04	1.2	1.54	0.05	86.64	-0.84	8.94	6.76	15.65	7.02	-1.87	11.22	20.96	10.49	0.84
os28159	2	501	0.57	4	3	6	6.71	-0.03	0.59	1.52	0.03	70.26	0.89	8.69	7.23	16.11	7.41	-1.57	11.79	21.54	11.02	1.18
os28246	2	513	0.09	4	3	6	6.93	0.05	0.21	1.49	0.03	90.17	2.05	6.32	7.17	16.02	7.43	-1.64	11.85	21.59	11.13	1.11
os28250	2	481	0.76	10	2	3	6.73	-0.04	0.68	1.53	0.12	89.17	-3.45	6.91	7.23	16.07	7.48	-1.6	11.91	21.65	11.18	1.14
os28258	2	559	0.21	4	3	6	6.85	0.04	0.46	1.51	0.03	90.08	0.81	6.97	7.03	15.93	7.31	-1.7	11.65	21.42	10.96	1.03
os28351	2	552	0.77	3	3	6	6.81	-0.03	2.89	1.53	0.03	84.71	-0.27	8.85	6.74	15.62	7.13	-1.89	11.38	21.14	10.82	0.87
os28634	2	610	0.01	4	3	9	6.97	0.07	0.45	1.5	0.02	94.1	0.8	6.43	6.99	15.92	7.23	-1.67	11.55	21.34	10.82	1.08
os28723	2	588	0.23	6	3	5	6.81	0.03	0.42	1.5	0.05	97.62	1.15	6.47	7.19	16.18	7.32	-1.56	11.83	21.62	10.96	1.2
os28726	2	529	0.19	5	3	6	6.96	0.06	1.06	1.53	0.04	86.5	-1.16	7.77	7.44	16.4	7.53	-1.42	12.09	21.93	11.2	1.35
os28909	2	439	0.43	8	3	5	6.48	-0.14	0.18	1.5	0.05	97.36	0.97	6.17	7.96	16.85	7.98	-0.96	12.42	22.15	11.44	1.69
os28921	2	469	0.05	8	2	3	6.96	0.09	0.56	1.5	0.09	97.11	-2.01	7.56	7.67	16.57	7.76	-1.17	12.26	22.01	11.35	1.54
os28940	2	468	0.02	6	3	5	6.87	0.08	0.57	1.53	0.05	83.49	-0.53	8.49	7.54	16.46	7.68	-1.31	12.16	21.94	11.32	1.41
os28947	2	516	0.92	1	3	6	6.86	0.02	2.57	1.55	0.02	44.23	0.28	9.09	7.24	16.14	7.47	-1.51	11.79	21.57	11.06	1.21

os29041	2	596	0.92	5	3	5	6.66	-0.07	0.65	1.52	0.05	87.72	0.03	7.85	6.93	15.85	7.11	-1.86	11.54	21.23	10.72	0.92
os29082	2	476	0.37	5	2	3	6.73	0	1.81	1.54	0.06	72.92	-2.24	8.42	7.95	16.98	7.91	-0.93	12.52	22.44	11.58	1.82
os29097	2	536	0.23	7	3	5	6.73	0.03	0.16	1.48	0.06	99.81	1.22	5.74	7.61	16.58	7.67	-1.24	12.16	21.99	11.29	1.51
os29119	2	603	0.07	4	3	6	6.98	0.07	0.11	1.51	0.02	77.5	1.92	7.35	7.17	16.14	7.32	-1.56	11.73	21.51	10.86	1.18
os29126	2	582	0.08	2	3	6	6.91	0.03	3.83	1.54	0.04	65.18	-1	9.36	7.17	16.1	7.32	-1.57	11.7	21.51	10.87	1.16
os29138	2	598	0.23	7	3	9	6.84	0.07	0.18	1.49	0.05	95.29	-0.24	5.78	7.21	16.16	7.35	-1.55	11.82	21.57	10.97	1.2
os29151	2	541	0.87	6	1	4	6.59	-0.1	0.04	1.48	0.03	93.95	3.93	6.45	7.38	16.31	7.5	-1.44	12.02	21.76	11.13	1.31
os29154	2	508	1	5	3	6	6.68	-0.08	0.23	1.51	0.02	95.72	1	6.36	7.62	16.52	7.69	-1.27	12.29	22	11.35	1.45
os29159	2	458	0.92	0	3	6	6.8	0	3.83	1.56	0.03	37.6	-0.45	9.01	7.74	16.63	7.79	-1.2	12.44	22.13	11.5	1.53
os29160	2	474	0.77	3	2	3	6.79	-0.02	1.85	1.54	0.06	94.59	-2.78	8.24	7.64	16.54	7.7	-1.26	12.31	22.02	11.37	1.46
os29166	2	559	0.98	9	3	5	6.57	-0.14	0.06	1.49	0.07	99.69	-0.05	6.63	7.28	16.22	7.41	-1.51	11.9	21.65	11.03	1.24
os29171	2	585	0.93	14	3	9	6.48	-0.17	0	1.45	0.07	99.95	0.91	5.87	7.26	16.2	7.39	-1.52	11.88	21.64	11.02	1.23
os29174	2	568	0.08	2	3	6	6.89	0.01	0.16	1.52	0.03	99.6	1.03	7.14	7.3	16.24	7.43	-1.5	11.93	21.69	11.06	1.25
os29176	2	572	0.92	1	3	9	6.86	0.01	0.12	1.5	0.01	98.29	1.79	7.59	7.32	16.26	7.45	-1.48	11.95	21.71	11.08	1.26
os29177	2	561	0.01	5	1	4	6.98	0.08	0.3	1.5	0.04	98.65	1.84	7.34	7.33	16.27	7.46	-1.48	11.97	21.73	11.09	1.27
os29178	2	560	0.07	6	3	5	7	0.1	0.4	1.5	0.04	98.57	1.45	7.35	7.32	16.26	7.45	-1.48	11.96	21.72	11.08	1.27
os29239	2	515	0.08	6	3	5	6.98	0.08	0.64	1.52	0.06	94.6	-1.05	7.47	7.54	16.52	7.59	-1.36	12.18	21.99	11.26	1.42
os29271	2	518	0.22	7	3	5	6.84	0.07	0.76	1.53	0.06	90.11	-1.74	8.13	7.49	16.45	7.57	-1.39	12.15	21.97	11.25	1.38
os29327	2	596	0.6	6	3	5	6.67	-0.05	0.45	1.52	0.05	96.91	-0.27	6.37	6.96	15.88	7.16	-1.81	11.57	21.28	10.74	1
os29381	2	592	0.28	3	3	6	6.78	-0.04	0.08	1.51	0.03	87.77	1.82	7.77	6.85	15.87	7.12	-1.91	11.47	21.35	10.79	0.8
os29483	2	580	0.39	10	3	5	6.66	0.02	0.03	1.47	0.07	95.32	-0.09	6.26	6.99	15.96	7.19	-1.82	11.53	21.34	10.77	0.91
os29590	2	664	0.01	8	3	5	7.03	0.14	0.24	1.5	0.05	97.87	-0.26	6.66	6.59	15.48	6.86	-2.08	11.16	20.84	10.4	0.69
os29631	2	585	0.01	3	3	6	6.88	0.03	0.25	1.52	0.02	92.76	0.57	7.19	6.99	15.99	7.16	-1.87	11.7	21.53	10.89	0.95
os29784	2	638	0.88	5	3	6	6.81	-0.05	0.54	1.53	0.03	88.08	-0.82	6.9	6.63	15.53	6.92	-1.94	11.07	20.81	10.37	0.77
os29796	2	502	0.08	2	3	6	6.88	0.03	1.56	1.55	0.02	46.81	0.19	7.98	7.27	16.2	7.43	-1.57	11.88	21.68	11.08	1.23
os29835	2	458	0.49	12	1	4	6.49	-0.02	0.03	1.45	0.06	88.9	2.92	7.09	7.37	16.23	7.55	-1.51	12.1	21.86	11.33	1.23
os29916	2	628	0.26	5	3	5	7	0.06	0.65	1.51	0.03	89.65	0.41	7.06	6.73	15.62	6.96	-1.95	11.27	20.96	10.52	0.79
os29928	2	619	0.47	6	3	5	6.78	0.01	0.39	1.51	0.03	92.39	0.96	6.31	6.97	15.9	7.15	-1.85	11.6	21.3	10.75	0.95
os29948	2	685	0.23	4	3	6	6.86	0.02	0.72	1.52	0.04	99.93	-0.22	7.13	6.48	15.45	6.76	-2.17	11.08	20.78	10.35	0.63
os29950	2	682	0.12	8	3	9	6.93	0.09	0.21	1.49	0.05	96.67	0.09	6.14	6.59	15.55	6.84	-2.09	11.17	20.87	10.42	0.69
os29961	2	683	0.01	9	3	5	6.98	0.13	0.12	1.49	0.06	99.91	0.11	6.23	6.51	15.46	6.78	-2.14	11.05	20.75	10.33	0.66
os29979	2	629	0.99	8	3	5	6.68	-0.11	0.27	1.5	0.05	99.4	-0.33	6.4	6.78	15.75	7.01	-1.92	11.39	21.16	10.64	0.86
os29980	2	633	0.99	8	3	5	6.63	-0.12	0.2	1.5	0.06	99.72	-0.77	6.5	6.75	15.72	6.98	-1.94	11.35	21.12	10.61	0.84
os29981	2	629	0.96	8	3	5	6.67	-0.11	0.19	1.5	0.06	99.25	-0.57	6.36	6.79	15.75	7.01	-1.92	11.39	21.17	10.65	0.86
os29988	2	539	0.85	5	3	5	6.67	-0.08	0.39	1.52	0.05	94.1	1.08	7.39	7.03	16.01	7.21	-1.77	11.62	21.42	10.84	0.99
os30018	2	542	0.44	6	2	0	6.7	-0.02	0.34	1.54	0.08	67.85	-1.18	7.31	6.95	15.89	7.16	-1.89	11.55	21.3	10.8	0.9
os30038	2	564	0.44	5	1	4	6.95	0.05	0.03	1.46	0.03	91.31	5.28	6.1	7.07	16.06	7.24	-1.8	11.72	21.5	10.91	0.96

os30057	2	539	0.87	5	3	5	6.67	-0.06	0.98	1.54	0.06	66.2	-0.28	8.97	7.14	16.1	7.28	-1.66	11.64	21.45	10.84	1.1
os30061	2	569	0.09	11	3	5	6.86	0.12	0.04	1.49	0.06	88.58	0.08	6.61	7.11	16.08	7.25	-1.7	11.7	21.49	10.89	1.05
os30065	2	588	0.72	11	2	3	6.44	-0.09	0.09	1.49	0.08	90.23	-2.05	7.2	6.76	15.73	7.03	-1.97	11.27	21.06	10.6	0.77
os30073	2	625	0.06	11	1	1	6.94	0.14	0.01	1.46	0.05	98.73	2.97	5.53	6.79	15.77	7.02	-1.94	11.24	21.05	10.53	0.76
os30176	2	645	0.92	0	3	6	6.88	0	0.58	1.53	0.01	53.4	0.4	10.46	6.63	15.66	6.95	-2.05	11.13	20.96	10.52	0.66
os30180	2	645	0.07	1	3	6	6.89	0.01	0.36	1.53	0.01	45.91	0.21	9.03	6.63	15.66	6.95	-2.05	11.13	20.96	10.52	0.66
os30235	2	548	0.99	11	1	4	6.4	-0.18	0.04	1.47	0.07	90.95	2.09	5.85	7.04	16.04	7.27	-1.8	11.73	21.61	10.98	0.93
os30248	2	620	0.76	5	1	4	6.89	-0.01	0.19	1.48	0.04	99.88	2.76	6.11	6.81	15.82	7.09	-1.95	11.39	21.29	10.72	0.75
os30385	2	555	0.48	3	3	6	6.88	0.01	0.26	1.52	0.02	97.32	1.13	6.38	7.06	16.07	7.28	-1.78	11.76	21.67	11.04	0.93
os30539	2	608	0.08	15	1	4	6.13	-0.25	0	1.4	0.07	99.35	4.7	5.36	6.6	15.48	6.95	-2.14	11.27	20.9	10.67	0.56
os30547	2	625	0.61	12	3	9	6.44	-0.09	0.01	1.45	0.1	100	-0.26	5.95	6.52	15.4	6.9	-2.14	11.15	20.76	10.56	0.54
os30628	2	594	0.66	12	3	5	6.42	-0.1	0.02	1.49	0.07	67.99	-0.27	7.53	6.44	15.29	6.89	-2.22	11	20.75	10.45	0.47
os30641	2	609	0.08	2	3	6	6.91	0.02	0.7	1.55	0.02	59.81	0.53	9.49	6.44	15.35	6.85	-2.23	11.06	20.88	10.49	0.47
os30651	2	602	0.77	3	3	6	6.86	-0.02	0.33	1.53	0.03	77.95	0.7	8.41	6.5	15.41	6.9	-2.22	11.11	20.92	10.56	0.49
os30749	2	514	0.09	4	3	6	6.87	0.05	3.79	1.53	0.04	70.37	0.99	8.5	7.05	15.96	7.29	-1.87	11.72	21.49	11.03	0.84
os30770	2	563	1	5	3	5	6.67	-0.09	0.68	1.52	0.05	88.38	-0.14	8.75	6.7	15.62	7.04	-2.14	11.43	21.2	10.82	0.58
os30805	2	526	0.38	6	3	6	6.7	-0.01	0.17	1.5	0.04	93.18	1.14	7.63	7.16	16.14	7.38	-1.79	11.85	21.7	11.13	0.91
os30812	2	605	0.14	4	3	6	6.87	0.04	0.32	1.51	0.03	98.65	0.9	6.69	6.83	15.89	7.14	-1.97	11.45	21.33	10.8	0.74
os30827	2	563	0.47	14	2	0	6.97	0.09	0	1.45	0.12	89.91	2.3	5.88	6.56	15.45	6.94	-2.14	11.22	20.89	10.63	0.55
os30881	2	426	0.25	22	2	0	6.2	0.12	0	1.4	0.22	95.62	-1.01	6.33	7.1	15.99	7.41	-1.7	11.64	21.31	10.98	0.94
os30936	2	623	0.9	9	1	1	6.51	-0.13	0.09	1.43	0.04	99.82	2.29	5.87	6.65	15.55	7.02	-1.99	11.3	20.95	10.7	0.66
os31001	2	601	0.47	16	3	5	6.94	0.11	0	1.44	0.13	99.88	-2.01	5.66	6.64	15.51	6.98	-2.11	11.32	20.94	10.7	0.58
os31006	2	668	0.56	9	1	1	6.64	-0.04	0.01	1.36	0.03	100	6.45	5.35	6.64	15.53	7.01	-2.04	11.28	20.88	10.65	0.64
os31007	2	674	0.48	6	1	1	6.79	-0.01	0	1.36	0.01	100	8.07	5.65	6.64	15.52	7	-2.05	11.27	20.88	10.64	0.63
os31106	2	702	0.71	9	3	5	6.86	-0.03	0.09	1.5	0.08	96.22	-1.81	6.18	6.33	15.33	6.64	-2.27	10.88	20.59	10.24	0.54
os31121	2	616	0.14	6	3	5	7.01	0.08	0.31	1.52	0.05	95.21	0.47	7.83	6.67	15.68	6.88	-2.01	11.21	20.96	10.5	0.8
os31194	2	657	0.28	6	1	4	6.79	0.02	0.05	1.49	0.02	93.55	2.93	6.22	6.75	15.7	6.93	-2.03	11.45	21.16	10.65	0.81
os31214	2	513	0.75	13	3	5	6.29	-0.14	0	1.48	0.09	93.75	-0.01	6.07	7.26	16.36	7.43	-1.65	11.87	21.75	11.09	1.08
os31239	2	633	0.37	9	3	5	6.68	-0.01	0.05	1.49	0.05	90.17	1.78	6.52	6.68	15.71	6.92	-2.03	11.2	20.97	10.51	0.77
os31243	2	666	0.4	3	3	6	6.94	0.01	1.86	1.54	0.04	90.57	-0.76	8.47	6.53	15.5	6.79	-2.18	11.12	20.83	10.42	0.66
os31277	2	589	0.78	7	3	5	6.81	-0.04	0.17	1.51	0.05	60.57	0.78	8.35	6.9	15.9	7.05	-1.93	11.62	21.36	10.8	0.9
os31283	2	599	0.5	4	1	4	6.79	0	0.4	1.51	0.03	80.87	2.2	7.98	6.91	15.88	7.06	-1.93	11.65	21.37	10.82	0.93
os31284	2	603	0.23	6	3	5	6.85	0.05	0.56	1.52	0.05	93.43	-0.08	7.28	6.92	15.91	7.08	-1.91	11.67	21.39	10.84	0.94
os31289	2	595	0.65	5	3	6	6.72	-0.04	0.66	1.54	0.04	97.9	-0.45	6.93	6.9	15.87	7.06	-1.95	11.59	21.35	10.77	0.9
os31305	2	627	0.56	3	3	6	6.8	-0.02	0.24	1.53	0.03	79.76	0.71	7.69	6.74	15.72	6.97	-2.01	11.31	21.09	10.59	0.79
os31317	2	618	0.4	11	3	5	6.98	0.06	0.03	1.48	0.06	96.89	-0.44	5.97	6.81	15.8	7.03	-1.99	11.41	21.19	10.67	0.79
os31335	2	596	0.44	16	1	4	6.93	0.07	0	1.47	0.09	92.59	0.92	6.18	6.85	15.86	7.04	-1.96	11.47	21.23	10.71	0.9

os31352	2	577	0.02	10	3	5	6.96	0.15	0.06	1.5	0.07	93.72	-0.26	6.11	6.99	16.03	7.17	-1.89	11.68	21.53	10.9	0.93
os31362	2	558	0.1	6	3	5	6.87	0.06	0.88	1.53	0.05	82.74	-1.1	8.79	7.14	16.2	7.27	-1.83	11.91	21.78	11.07	1.03
os31417	2	508	0.73	6	3	5	6.81	-0.02	0.51	1.54	0.06	63.13	-0.49	8.63	7.32	16.35	7.42	-1.67	12.02	21.83	11.21	1.13
os31428	2	537	0.6	3	3	6	6.76	-0.02	0.05	1.51	0.02	86.98	2.09	7.74	7.27	16.32	7.39	-1.7	11.97	21.82	11.17	1.09
os31447	2	531	0.96	5	3	6	6.7	-0.08	0.37	1.51	0.03	80.27	1.17	7.09	7.36	16.41	7.46	-1.67	12.07	21.94	11.24	1.13
os31479	2	638	0.4	8	3	5	6.69	-0.02	0.27	1.52	0.05	95.39	-0.4	6.66	6.7	15.71	6.9	-2.09	11.39	21.1	10.61	0.75
os31480	2	638	0.4	8	3	5	6.69	-0.02	0.26	1.52	0.05	95.53	-0.27	6.69	6.69	15.71	6.9	-2.09	11.39	21.1	10.61	0.75
os31487	2	646	0.6	7	3	5	6.69	-0.04	0.31	1.52	0.05	97.71	0.24	7.02	6.67	15.69	6.88	-2.11	11.36	21.07	10.59	0.73
os31522	2	627	0.11	2	3	6	6.93	0.02	2.87	1.54	0.03	30.73	-0.09	9.14	6.65	15.65	6.91	-2.05	11.11	20.91	10.45	0.74
os31672	2	549	0.57	3	3	6	6.88	0	0.45	1.52	0.03	99.11	0.68	7.09	7.18	16.26	7.34	-1.7	11.81	21.67	11.05	1.06
os31676	2	544	0.91	6	3	5	6.71	-0.07	0.9	1.54	0.06	96.08	-1.68	6.99	7	16.05	7.16	-1.84	11.69	21.52	10.97	0.99
os31685	2	607	0.01	7	3	5	6.95	0.1	0.27	1.5	0.05	81.27	0.32	6.73	6.76	15.79	6.95	-1.96	11.34	21.15	10.71	0.87
os31688	2	594	0.19	4	3	6	6.96	0.05	0.66	1.53	0.02	83.94	0.06	7.45	6.8	15.83	6.99	-1.95	11.4	21.2	10.76	0.88
os31711	2	595	0.97	4	3	6	6.79	-0.05	1.75	1.53	0.05	95.23	-1.28	7.88	6.77	15.82	6.99	-1.89	11.33	21.11	10.64	0.94
os31739	2	584	0.66	9	3	5	6.48	-0.14	0.04	1.48	0.06	84.95	2.22	6.18	6.89	15.93	7.07	-1.9	11.44	21.27	10.73	0.93
os31812	2	435	0.01	4	2	3	6.91	0.07	1.55	1.55	0.06	87.9	-2.07	8.51	8.16	17.16	8.07	-0.76	12.84	22.68	11.81	2
os31813	2	504	0	5	3	5	6.63	-0.09	0.66	1.53	0.05	92.42	-0.46	7.43	7.83	16.86	7.83	-0.99	12.38	22.2	11.44	1.7
os31845	2	654	0.82	7	1	1	6.61	-0.09	0.07	1.48	0.03	97.08	2.95	5.81	6.9	15.83	7.12	-1.86	11.56	21.25	10.72	0.94
os31846	2	615	0.08	4	3	6	6.98	0.06	0.87	1.53	0.04	81.27	-0.37	7.64	7	15.97	7.2	-1.75	11.6	21.34	10.8	1.03
os31866	2	606	0.65	9	3	5	6.89	0	0.09	1.5	0.05	84.6	0.52	6.14	6.95	15.9	7.09	-1.89	11.64	21.31	10.79	0.96
os31867	2	504	0.75	7	2	3	6.6	-0.05	0.86	1.53	0.08	91.9	-4.07	7.62	7.65	16.66	7.7	-1.25	12.29	22.15	11.38	1.54
os31869	2	441	0.99	2	3	6	6.76	-0.02	2.6	1.56	0.05	66.11	-1.37	8.52	7.29	16.1	7.63	-1.5	11.98	21.66	11.28	1.23
os31877	2	518	0.08	6	3	5	6.9	0.08	0.32	1.53	0.05	89.72	-0.67	7.96	7.1	16.01	7.36	-1.64	11.73	21.51	11.03	1.1
os31887	2	508	0.03	3	3	6	6.92	0.06	0.63	1.52	0.02	96.86	0.05	7.22	7.23	16.1	7.46	-1.57	11.96	21.72	11.2	1.18
os31890	2	470	0.01	6	3	6	6.95	0.11	0.26	1.5	0.05	76.09	2.05	8.05	7.29	16.14	7.48	-1.58	12.03	21.75	11.21	1.18
os31905	2	542	1	10	1	4	6.47	-0.17	0.02	1.47	0.05	96.64	2.98	5.6	7.34	16.24	7.5	-1.48	11.93	21.7	11.15	1.27
os32040	2	247	0.01	1	3	6	6.67	-0.03	2	1.55	0.01	22.89	-0.12	8.69	9.02	17.78	9.06	0.27	13.87	23.16	12.82	2.95
os32111	2	216	0.99	7	3	5	6.45	-0.11	0.5	1.52	0.05	69.71	-0.78	8.23	9.24	17.99	9.29	0.57	14.01	23.23	13.01	3.15
os32112	2	225	0.09	2	3	6	6.65	-0.03	1.02	1.53	0.01	55.12	0.77	8.73	9.25	17.99	9.29	0.58	14.02	23.23	13.01	3.15
os32164	2	162	0.92	0	3	6	6.67	0	7.94	1.57	0.01	2.7	-0.33	10.29	9.78	18.59	9.73	1	14.61	23.83	13.51	3.68
os32598	2	224	0.92	1	3	6	6.66	-0.02	5.81	1.55	0.01	28.42	0.84	9.19	8.81	17.51	8.99	0	13.64	22.9	12.77	2.61
os33007	2	252	0.26	5	3	6	6.66	0.02	0.54	1.52	0.02	91.36	0.59	6.75	8.9	17.67	8.99	-0.07	13.65	23.1	12.72	2.62
os33010	2	239	0.75	1	3	6	6.72	0	2.08	1.55	0.02	42.89	0.07	8.56	8.9	17.69	8.96	-0.09	13.68	23.14	12.75	2.61
os33015	2	210	0.92	0	3	6	6.7	0	4.43	1.56	0.01	0.31	0.29	10.42	9.05	17.78	9.07	-0.05	14	23.38	12.99	2.72
os33065	2	214	0.92	0	3	6	6.68	-0.01	5.98	1.54	0.01	37.06	0.43	9.47	9.23	18.02	9.2	0.15	14.19	23.64	13.09	2.96
os33404	2	210	0.92	1	3	6	6.69	-0.01	1.71	1.53	0.01	44.59	0.5	9.39	9.28	18.1	9.34	0.58	13.97	23.2	12.96	3.17
os33414	2	212	0.99	11	3	5	6.21	-0.2	0.01	1.48	0.08	76.74	-0.42	6.26	9.37	18.19	9.43	0.67	14.18	23.46	13.19	3.29

os33431	2	229	0.87	4	3	6	6.59	-0.06	0.98	1.5	0.02	63.19	0.82	7.64	9.11	17.9	9.24	0.4	13.84	23.11	12.98	3.04
os33446	2	165	0.91	3	3	6	6.58	-0.05	1.63	1.54	0.02	49.31	-0.14	8.94	9.52	18.27	9.48	0.66	14.46	23.57	13.27	3.37
os33978	2	267	0.47	4	1	1	6.76	0.01	0.85	1.5	0.01	88.34	2.59	6.55	8.69	17.45	8.91	-0.4	13.53	22.93	12.8	2.32
os33979	2	263	0.88	2	3	6	6.65	-0.02	0.19	1.52	0.02	90.62	0.44	6.8	8.7	17.46	8.91	-0.4	13.54	22.94	12.8	2.32
os34070	2	209	0.08	2	3	6	6.77	0.04	6.91	1.56	0.03	7.53	-0.53	8.46	8.87	17.59	8.95	-0.18	13.62	23.01	12.75	2.54
os34084	2	245	0.78	3	3	6	6.68	-0.03	1.65	1.55	0.04	66.81	-1.12	8.19	8.54	17.31	8.78	-0.55	13.3	22.79	12.64	2.19
os35039	2	258	0.1	2	3	6	6.79	0.04	1.26	1.55	0.02	60.07	-0.28	8.14	8.28	16.98	8.56	-0.75	12.92	22.31	12.4	1.96
os35136	2	242	0.08	2	3	6	6.75	0.03	0.98	1.54	0	60.71	1.24	8.54	8.52	17.26	8.76	-0.6	13.35	22.79	12.76	2.18
os35139	2	239	0.64	3	3	6	6.7	-0.01	4.27	1.53	0.01	45.44	1.24	7.66	8.55	17.31	8.77	-0.59	13.44	22.88	12.82	2.23
os35433	2	216	0.99	7	3	5	6.45	-0.11	0.5	1.52	0.05	69.71	-0.78	8.23	9.24	17.99	9.29	0.57	14.01	23.23	13.01	3.15
os35548	2	323	0.47	7	3	5	6.52	-0.05	0.19	1.48	0.06	97.89	0.23	7.83	8.17	16.85	8.46	-0.21	12.5	21.77	11.87	2.22
os35600	2	325	1	13	2	7	6.16	-0.21	0.23	1.52	0.18	92.65	-7.78	6.58	7.97	16.65	8.31	-0.4	12.31	21.61	11.72	2.08
os35617	2	293	0.16	6	2	3	6.59	-0.04	1.08	1.46	0.09	95.58	-0.9	7.17	8.3	16.97	8.58	-0.16	12.68	21.93	12.01	2.31
os35698	2	282	0.98	12	2	0	6.19	-0.2	0.01	1.44	0.16	95.25	1.35	6.36	8.3	16.97	8.57	-0.07	12.5	21.76	11.88	2.32
os35720	2	233	0.81	13	2	7	6.03	-0.17	0.1	1.54	0.3	94.79	-12.8	6.06	8.53	17.21	8.78	0.11	12.84	22.03	12.13	2.51
os35733	2	323	0.99	9	1	4	6.45	-0.14	0.05	1.46	0.04	92.23	3.06	6.63	8.33	17.05	8.57	-0.2	12.76	22.05	12.07	2.27
os35783	2	360	0	8	3	5	6.87	0.11	0.48	1.48	0.06	90.52	1.18	6.88	7.99	16.68	8.3	-0.33	12.33	21.61	11.71	2.08
os35838	2	364	0.88	17	3	5	6.3	-0.18	0	1.4	0.11	96.97	0.34	5.92	7.98	16.67	8.31	-0.32	12.28	21.56	11.67	2.1
os35844	2	206	0.8	18	2	7	5.76	-0.24	0	1.47	0.19	87.48	-5.36	5.6	8.8	17.5	9.01	0.3	13.2	22.42	12.47	2.76
os35857	2	308	0	12	2	3	6.86	0.17	0.28	1.41	0.12	88.12	-2.42	7.12	8.32	16.97	8.59	-0.05	12.59	21.82	11.95	2.35
os35882	2	282	0.99	15	2	0	6.07	-0.25	0	1.41	0.14	92.45	0.32	6.06	8.35	17.05	8.59	-0.09	12.66	21.91	11.97	2.35
os35979	2	349	0.46	16	1	4	6.74	0.03	0	1.33	0.07	90.59	9.91	5.73	8.12	16.8	8.41	-0.24	12.39	21.67	11.79	2.19
os36001	2	329	0.81	6	3	5	6.53	-0.06	0.58	1.47	0.04	73.15	0.54	7.45	8.41	17.09	8.65	-0.04	12.73	21.98	12.09	2.39
os36004	2	319	1	14	1	4	6.18	-0.23	0	1.36	0.08	97.26	6.64	5.98	8.38	17.07	8.63	-0.08	12.73	21.98	12.1	2.36
os36007	2	237	0.42	17	2	7	5.92	0.01	0.11	1.5	0.27	84.78	-9.52	6.16	8.49	17.14	8.73	0.01	12.8	22.03	12.15	2.45
os36226	2	312	0.05	29	3	5	6.67	0.43	0	1.34	0.18	95.5	3.84	5.25	8.17	16.84	8.45	-0.2	12.38	21.65	11.79	2.23
os36241	2	261	0.5	10	2	7	6.56	0.02	0.78	1.55	0.28	93.78	-12.44	6.4	8.28	16.95	8.57	-0.11	12.5	21.74	11.89	2.32
os36265	2	331	0.98	17	3	5	6.13	-0.24	0	1.4	0.12	95.46	1.7	5.52	8.17	16.87	8.42	-0.25	12.49	21.79	11.83	2.21
os36298	2	331	0.07	13	1	2	6.9	0.14	0	1.42	0.08	91.55	4.99	5.51	8	16.68	8.32	-0.32	12.3	21.56	11.68	2.08
os36300	2	330	0.87	16	2	3	5.99	-0.23	0.02	1.48	0.17	92.92	-5.18	6.11	8.02	16.7	8.33	-0.31	12.33	21.58	11.7	2.1
os36459	2	318	0.15	19	2	3	6.63	0.22	0	1.45	0.13	99.3	-3.34	5.58	8.24	16.96	8.55	-0.43	12.94	22.3	12.21	2.13
os36590	2	255	0.25	21	2	7	5.82	0.05	0.22	1.51	0.35	86.29	-15.9	7.24	7.93	16.61	8.36	-0.48	12.41	21.78	11.86	1.98
os36749	2	296	1	16	3	5	6.11	-0.24	0	1.46	0.1	95.75	-1.23	6.33	8.01	16.71	8.41	-0.72	12.54	21.87	11.93	1.84
os36770	2	377	0.01	5	3	9	6.82	0.06	0.58	1.48	0.03	95.98	0.13	6.79	7.87	16.58	8.29	-0.84	12.4	21.78	11.82	1.77
os36781	2	303	0	13	1	4	6.88	0.22	0	1.41	0.08	92.19	2.89	6.63	8.05	16.73	8.46	-0.4	12.56	21.93	12	2.06
os36830	2	312	0.77	10	1	4	6.62	-0.07	0.03	1.35	0.06	95.07	7.05	6.18	8.03	16.71	8.44	-0.4	12.52	21.88	11.96	2.05
os36856	2	254	0.99	15	2	7	6.05	-0.24	0.05	1.48	0.14	87.3	-2.98	6.36	8.35	17.03	8.65	-0.33	12.95	22.24	12.22	2.2

os36864	2	347	0.04	14	3	5	7	0.21	0.02	1.43	0.08	92.81	0.67	6.63	7.71	16.37	8.16	-0.62	12.15	21.47	11.61	1.8
os36875	2	366	0.45	13	1	1	6.44	0	0.01	1.39	0.07	88.94	1.79	6.14	7.75	16.42	8.21	-0.61	12.22	21.57	11.67	1.84
os36901	2	295	0.85	18	3	5	5.85	-0.24	0	1.4	0.11	87.9	0.56	6.31	8.24	16.95	8.58	-0.42	12.87	22.24	12.18	2.13
os36970	2	306	0.61	9	3	5	6.59	-0.08	0.24	1.39	0.07	99.56	3.4	5.97	8.51	17.26	8.75	-0.11	13.04	22.32	12.32	2.42
os36988	2	380	0.71	12	3	9	6.34	-0.1	0.09	1.43	0.09	91.59	-1.79	6.59	8.09	16.86	8.4	-0.45	12.55	21.9	11.89	2.05
os36990	2	266	0.48	15	3	5	6.31	-0.01	0	1.43	0.13	96.11	-0.21	5.33	8.64	17.37	8.84	-0.01	13.2	22.46	12.42	2.53
os37111	2	231	0.6	4	2	7	6.58	-0.04	1.48	1.56	0.11	60.82	-3.15	8.07	8.7	17.44	8.9	0.04	13.25	22.51	12.49	2.56
os37112	2	273	0.77	9	1	4	6.38	-0.09	0.03	1.42	0.04	92.02	5.68	5.98	8.61	17.35	8.83	-0.02	13.15	22.41	12.41	2.49
os37198	2	259	0.03	23	2	3	6.68	0.32	0	1.42	0.17	98.85	-3.02	5.61	8.59	17.33	8.82	-0.06	13.15	22.4	12.4	2.49
os37209	2	292	0.99	17	3	5	6.09	-0.25	0	1.39	0.12	99.07	2.01	5.13	8.58	17.31	8.8	-0.07	13.13	22.38	12.38	2.47
os37224	2	360	0.89	14	1	4	6.09	-0.22	0.01	1.41	0.09	92.49	2.49	6.33	8	16.77	8.31	-0.52	12.45	21.8	11.8	1.96
os37227	2	389	0.92	5	3	9	6.59	-0.07	0.78	1.46	0.02	84.5	1.86	7.11	7.99	16.76	8.3	-0.53	12.43	21.79	11.78	1.95
os37232	2	276	0.01	16	2	3	6.76	0.23	0	1.46	0.18	97.81	-8.5	5.98	8.64	17.38	8.84	-0.01	13.21	22.46	12.43	2.53
os37265	2	343	0.99	8	1	4	6.58	-0.1	0.08	1.45	0.05	85.82	2.32	6.79	8.14	16.92	8.44	-0.39	12.54	21.92	11.91	2.06
os37285	2	281	0.88	6	3	5	6.49	-0.09	0.4	1.49	0.04	92.29	1.08	6.61	8.51	17.23	8.71	-0.07	12.94	22.22	12.21	2.4
os37287	2	319	0.59	6	3	5	6.6	-0.02	0.56	1.5	0.04	95.17	0.26	6.72	8.36	17.11	8.6	-0.19	12.83	22.12	12.11	2.28
os37313	2	282	0.91	6	2	3	6.55	-0.08	0.78	1.52	0.06	95.76	-1.05	7.58	8.5	17.24	8.71	-0.09	12.98	22.27	12.29	2.41
os37366	2	346	0.03	15	1	4	6.9	0.25	0	1.36	0.06	91.65	8.89	5.2	8.04	16.77	8.38	-0.37	12.45	21.78	11.87	2.14
os37369	2	311	1	5	3	6	6.6	-0.08	0.59	1.53	0.04	72.89	0.58	7.54	8.07	16.76	8.34	-0.3	12.54	21.88	11.88	2.17
os37552	2	333	1	15	2	3	6.11	-0.25	0	1.42	0.11	96.29	-1.98	6.7	8.13	16.82	8.43	-0.33	12.63	21.86	11.98	2.21
os37587	2	339	1	11	2	0	6.35	-0.17	0.54	1.51	0.13	92	-3.82	6.34	7.88	16.64	8.19	-0.52	12.2	21.54	11.6	1.92
os37588	2	345	1	14	2	0	6.21	-0.22	0.01	1.47	0.12	90.84	-0.12	6.25	7.87	16.63	8.18	-0.53	12.19	21.53	11.59	1.91
os37706	2	370	0.23	4	3	6	6.77	0.05	0.1	1.48	0.05	87.66	2.02	8.06	7.57	16.32	8.02	-0.73	12.11	21.46	11.58	1.74
os37732	2	319	1	13	2	7	6.21	-0.21	0.2	1.51	0.13	59.01	-3.97	6.46	7.83	16.52	8.21	-0.54	12.37	21.7	11.82	1.95
os37776	2	348	0.08	8	3	5	6.94	0.12	0.1	1.5	0.06	95.18	0.75	6.72	7.74	16.49	8.16	-0.57	12.23	21.56	11.66	1.91
os37855	2	353	0.56	6	3	5	6.56	-0.06	0.9	1.51	0.08	95.26	-1.87	6.6	7.99	16.72	8.32	-0.44	12.33	21.63	11.7	2.03
os37913	2	308	1	9	3	5	6.47	-0.13	0.02	1.44	0.08	98.73	0.97	5.48	8.31	16.98	8.57	-0.15	12.68	21.92	12	2.32
os38078	2	375	0.4	2	3	9	6.82	0.02	0.46	1.5	0.01	97.17	0.85	7.08	7.78	16.56	8.17	-0.58	12.27	21.61	11.63	1.88
os38079	2	369	0.08	6	3	5	6.9	0.07	0.41	1.5	0.03	98.01	0.83	6.75	7.79	16.57	8.18	-0.58	12.28	21.62	11.64	1.89
os38203	2	305	0.52	5	2	3	6.72	-0.02	1.67	1.55	0.11	95.47	-5.08	7.71	8.2	16.91	8.51	-0.25	12.64	21.98	12.04	2.25
os38219	2	353	0.81	3	3	6	6.66	-0.05	0.36	1.49	0.03	82.98	1.56	7.04	8.05	16.75	8.36	-0.36	12.54	21.8	11.9	2.11
os38254	2	380	0.4	3	1	1	6.77	-0.01	0.02	1.37	0.02	91.44	6.31	6.83	7.94	16.71	8.24	-0.53	12.31	21.67	11.68	1.92
os38286	2	320	0.99	16	2	3	6.04	-0.26	0	1.45	0.11	97.5	-1.83	5.83	8.2	16.86	8.47	-0.21	12.53	21.8	11.89	2.25
os38381	2	385	0.92	2	3	9	6.75	0	1.84	1.41	0.02	87.92	0.84	7.69	8.2	16.9	8.45	-0.26	12.56	21.85	11.89	2.2
os38404	2	315	0.92	3	1	4	6.62	-0.06	0.7	1.47	0.01	59.23	2.65	7.9	8.15	16.82	8.44	-0.22	12.58	21.94	11.91	2.23
os38411	2	315	0.78	12	1	4	6.18	-0.19	0	1.33	0.08	93.62	9.14	5.48	8.14	16.82	8.42	-0.24	12.54	21.85	11.91	2.22
os38432	2	298	0.95	15	1	4	6.3	-0.18	0.02	1.37	0.09	93.49	3.91	5.69	8.24	16.92	8.5	-0.17	12.74	22.05	12.05	2.26

os38468	2	312	0	17	3	5	6.9	0.27	0	1.43	0.08	81.93	-0.18	6.85	8.19	16.89	8.46	-0.23	12.69	22.01	12.02	2.23
os38520	2	298	0.76	14	1	4	6.13	-0.16	0.02	1.39	0.06	83.24	5.99	6.28	8.33	17.03	8.57	-0.18	12.88	22.21	12.19	2.32
os38550	2	302	0.2	6	3	6	6.77	0.06	1.06	1.54	0.06	80.35	-0.77	7.85	7.96	16.66	8.37	-0.46	12.57	21.88	12.03	2.05
os38554	2	324	0.49	4	3	6	6.68	-0.01	1.14	1.51	0.03	95.93	-0.38	6.99	8.07	16.77	8.38	-0.35	12.75	22.01	12.08	2.25
os38571	2	298	0.53	4	3	6	6.69	0.01	1.66	1.55	0.05	87.55	-1.85	8.28	8.14	16.86	8.51	-0.28	12.76	22.07	12.13	2.22
os38623	2	335	0.03	21	3	5	6.89	0.33	0	1.39	0.13	94.86	1.72	5.97	8.03	16.73	8.34	-0.42	12.4	21.69	11.78	2.06
os38625	2	330	0.23	9	1	4	6.68	0.08	0.3	1.39	0.05	89.98	4.82	6.33	8.08	16.79	8.38	-0.38	12.5	21.76	11.87	2.12
os38653	2	331	0.05	11	3	5	6.82	0.15	0.57	1.51	0.1	93.26	-2.39	7.19	8.1	16.82	8.4	-0.37	12.47	21.76	11.83	2.13
os38665	2	326	0.27	2	2	3	6.75	0	2.98	1.56	0.06	89.48	-3	8.36	8.09	16.79	8.39	-0.38	12.61	21.83	11.93	2.12
os38666	2	315	0	4	3	6	6.83	0.07	0.77	1.49	0.05	95.08	1.32	7.26	8.16	16.86	8.46	-0.31	12.65	21.89	11.99	2.18
os38698	2	283	0.84	18	3	5	6.27	-0.18	0	1.4	0.14	94.94	0.31	6.34	8.32	16.99	8.6	-0.15	12.73	21.96	12.06	2.32
os38768	2	304	0.49	4	1	4	6.79	0.02	0.1	1.52	0.04	83.27	2.11	7.52	8.21	16.93	8.54	-0.23	12.61	21.95	12.02	2.26
os38822	2	316	0.15	7	1	4	6.72	0.05	0.16	1.5	0.04	90.74	1.78	6.5	8.07	16.77	8.44	-0.33	12.67	21.97	12.01	2.16
os38829	2	406	0.15	13	3	9	7	0.17	0	1.41	0.09	97.6	2.32	5.11	7.47	16.11	7.97	-0.82	11.93	21.27	11.44	1.64
os38830	2	381	0.17	19	1	4	6.91	0.25	0	1.35	0.15	94.16	4.07	4.76	7.42	16.06	7.94	-0.87	11.88	21.22	11.39	1.61
os38877	2	347	0.89	3	3	6	6.77	0	2.49	1.52	0.06	81.59	-0.04	8.37	7.6	16.28	8.04	-0.7	12.14	21.46	11.64	1.78
os38899	2	425	0.15	15	2	8	6.96	0.2	0	1.45	0.11	100	-3.03	5.47	7.6	16.35	8.01	-0.72	12.17	21.51	11.56	1.85
os38916	2	373	0.03	9	2	7	6.91	0.14	0.81	1.54	0.12	85.77	-4.09	7.03	7.31	15.96	7.94	-0.78	11.72	21.02	11.28	1.61
os38937	2	425	0.25	14	3	5	6.92	0.13	0	1.46	0.11	93.62	-0.5	5.41	7.13	15.81	7.76	-0.95	11.53	20.81	11.13	1.45
os38968	2	361	0.54	22	3	5	5.92	-0.09	0	1.41	0.12	79.68	1.58	5.97	7.56	16.2	8.09	-0.65	12.04	21.32	11.56	1.78
os38972	2	371	0.09	8	3	5	6.96	0.13	0.1	1.48	0.07	94.47	0.18	6.57	7.49	16.16	8.03	-0.71	12.01	21.3	11.53	1.73
os38988	2	327	0.67	12	3	5	6.31	-0.1	0	1.47	0.07	78.85	1.33	5.46	7.73	16.43	8.21	-0.61	12.39	21.67	11.84	1.93
os39021	2	389	0.23	10	2	3	6.96	0.12	0.05	1.49	0.13	80.49	-2.14	5.39	7.28	15.91	7.88	-0.88	11.74	21.03	11.3	1.59
os39026	2	428	0.04	18	3	5	6.82	0.24	0	1.43	0.1	88.28	2.29	5.47	7.11	15.75	7.74	-1.01	11.48	20.81	11.11	1.39
os39115	2	409	0.4	4	3	6	6.89	0.05	0.32	1.51	0.03	96.53	1.55	6.48	7.33	15.99	7.88	-0.86	11.77	21.13	11.37	1.6
os39182	2	430	0.12	4	3	6	6.91	0.07	1.11	1.54	0.06	77.25	-1.43	7.27	7.12	15.77	7.72	-1.1	11.58	20.92	11.15	1.38
os39222	2	382	0.21	13	3	5	6.78	0.15	0.01	1.45	0.09	98.41	-1.16	5.98	7.53	16.19	8.03	-0.78	12.02	21.35	11.47	1.67
os39295	2	395	0.02	8	3	5	6.86	0.12	0.4	1.51	0.11	96.18	-1.72	5.81	7.23	15.92	7.81	-1.3	11.83	21.19	11.31	1.21
os39296	2	439	0.03	20	2	3	6.78	0.28	0	1.43	0.15	99.88	-1.47	6	7.07	15.78	7.66	-1.43	11.67	21.03	11.17	1.07
os39356	2	380	0.08	5	2	3	6.84	0.08	1	1.54	0.11	91.2	-4.5	6.4	7.41	16.07	8	-0.76	11.92	21.22	11.47	1.72
os39397	2	436	0.03	21	3	5	6.78	0.31	0	1.41	0.14	99.96	0.75	5.83	7.08	15.79	7.67	-1.42	11.67	21.04	11.17	1.07
os39461	2	428	0.36	6	2	3	6.88	0.04	0.93	1.52	0.08	96.74	-3.53	7.22	7.14	15.82	7.73	-1.11	11.65	20.99	11.19	1.4
os39472	2	408	0.07	5	3	6	6.91	0.09	0.5	1.47	0.04	93.01	1.4	7.06	7.39	16.07	7.93	-1.02	11.95	21.31	11.45	1.5
os39488	2	466	0.05	11	2	0	6.82	0.14	0.01	1.48	0.09	99.28	0.32	5.62	6.79	15.46	7.47	-1.21	11.09	20.4	10.76	1.18
os39489	2	469	0.01	9	2	0	6.94	0.15	0.29	1.51	0.1	98.84	-1.85	5.92	6.76	15.43	7.45	-1.24	11.06	20.37	10.72	1.15
os39498	2	320	0.14	10	3	5	6.7	0.08	0.05	1.52	0.09	74.69	-1.94	7.1	7.78	16.45	8.22	-0.59	12.42	21.73	11.9	1.95
os39513	2	469	0.4	5	3	5	6.9	0.03	0.24	1.51	0.04	97.34	0.89	6.65	6.96	15.6	7.57	-1.23	11.33	20.67	10.95	1.23

os39530	2	425	0	5	3	5	6.89	0.08	0.63	1.5	0.07	96.58	1.72	7.17	7.13	15.77	7.76	-0.94	11.49	20.79	11.11	1.44
os39532	2	425	0.52	18	2	3	6.74	0.07	0.01	1.47	0.15	92.55	-5.31	5.78	7.12	15.8	7.75	-0.96	11.52	20.8	11.12	1.45
os39593	2	396	0.28	6	3	5	6.89	0.05	0.44	1.52	0.05	97.11	-0.25	6.86	7.34	16	7.89	-0.85	11.79	21.14	11.38	1.61
os39598	2	428	0.04	6	3	5	6.89	0.1	0.44	1.51	0.05	94.73	-0.1	6.44	7.24	15.92	7.83	-0.94	11.7	21.04	11.3	1.53
os39612	2	388	0.98	8	3	5	6.51	-0.12	0.28	1.5	0.06	80.88	0.27	7.25	7.35	15.99	7.92	-0.9	11.82	21.15	11.35	1.58
os39628	2	449	0.11	4	3	6	6.91	0.06	1.52	1.53	0.04	73.5	-0.59	8.24	7.04	15.69	7.65	-1.17	11.46	20.79	11.05	1.3
os39632	2	406	0.05	9	3	5	6.99	0.15	0.12	1.5	0.07	94.2	-0.53	6.44	7.2	15.86	7.79	-1.07	11.67	21	11.25	1.45
os39634	2	426	0.46	13	3	5	6.86	0.06	0	1.48	0.1	98.79	-1.28	5.53	7.11	15.79	7.71	-1.12	11.59	20.94	11.17	1.38
os39636	2	462	0.67	10	3	5	6.78	-0.02	0.09	1.48	0.06	98.39	-0.18	6.11	7.07	15.76	7.67	-1.15	11.57	20.92	11.12	1.35
os39640	2	391	0.76	16	2	3	6.62	-0.07	0	1.47	0.11	89.32	-0.57	5.83	7.26	15.92	7.82	-1.04	11.74	21.07	11.32	1.48
os39642	2	395	0.55	6	3	5	6.81	0.01	0.18	1.51	0.07	95.88	1.06	6.27	7.37	16.02	7.9	-0.97	11.9	21.2	11.41	1.54
os39653	2	451	0.69	12	2	3	6.36	-0.07	0.03	1.47	0.12	97.39	-2.61	5.83	7.01	15.66	7.63	-1.21	11.42	20.74	11.03	1.27
os39679	2	396	0.92	0	3	6	6.79	0.01	1.97	1.53	0.02	18.71	-0.24	9.02	7.39	16.04	7.93	-1.03	11.96	21.29	11.48	1.51
os39690	2	428	0.23	9	1	4	6.4	-0.15	0.01	1.46	0.08	98.25	2.83	5.59	7.21	15.88	7.79	-1.03	11.68	21.02	11.22	1.45
os39710	2	420	0.23	6	3	5	6.75	0.05	0.55	1.48	0.05	85.11	1.5	7.73	7.3	15.96	7.83	-1	11.81	21.14	11.31	1.5
os39714	2	385	0.18	21	1	4	6.42	0.14	0	1.39	0.11	87.87	3.29	5.73	7.46	16.14	7.98	-0.87	11.99	21.32	11.45	1.61
os39788	2	343	0.77	5	1	1	6.69	-0.05	0.02	1.31	0.03	94.18	8.66	5.94	7.88	16.54	8.33	-0.55	12.41	21.69	11.83	1.93
os39849	2	402	0.76	17	2	3	6.05	-0.16	0	1.44	0.11	89.21	-1.81	6.65	7.39	16.07	7.94	-1.01	11.96	21.31	11.46	1.5
os39970	2	385	0.99	10	1	4	6.44	-0.16	0.11	1.41	0.06	99.12	4.63	6.33	7.58	16.32	8.09	-0.94	12.18	21.61	11.66	1.61
os39997	2	362	0.41	9	1	1	6.61	0.01	0.02	1.38	0.03	96.13	5.28	5.53	7.88	16.59	8.31	-0.94	12.54	21.94	11.94	1.63
os40023	2	404	0.92	1	3	6	6.77	-0.01	0.49	1.51	0.02	89.01	1.32	9.24	7.42	16.15	7.95	-1.25	12.01	21.43	11.52	1.29
os40031	2	446	0.56	16	3	5	6.81	0.05	0	1.47	0.14	95.54	-1.21	6.13	7.13	15.86	7.71	-1.46	11.69	21.13	11.24	1.08
os40035	2	464	0.1	8	1	4	6.99	0.11	0.02	1.44	0.04	95.19	3.65	5.76	7.13	15.86	7.71	-1.46	11.69	21.13	11.23	1.08
os40041	2	465	0.08	6	3	5	6.97	0.1	0.49	1.5	0.04	97.79	0.83	6.34	7.11	15.85	7.7	-1.46	11.67	21.08	11.22	1.05
os40099	2	465	0.26	4	3	6	6.91	0.05	0.44	1.51	0.03	99.89	0.48	7.39	7.09	15.84	7.7	-1.44	11.67	21.12	11.21	1.09
os40292	2	333	0.78	10	1	4	6.67	-0.04	0	1.39	0.04	95.53	7.89	5.33	7.89	16.57	8.3	-0.83	12.38	21.71	11.79	1.73
os40293	2	319	0.35	8	2	3	6.8	0.11	0.8	1.5	0.14	95.68	-5.6	5.9	7.88	16.57	8.3	-0.82	12.37	21.71	11.78	1.73
os40296	2	273	0.88	4	2	0	6.67	-0.03	0.02	1.47	0.1	67.34	1.74	7.02	8.02	16.69	8.39	-0.79	12.52	21.83	11.92	1.76
os40368	2	327	0.08	10	2	3	6.82	0.14	0.43	1.51	0.09	97.33	-3.24	7.37	7.82	16.52	8.23	-1.02	12.42	21.79	11.85	1.58
os40414	2	298	0.12	30	1	2	6.11	0.22	0	1.28	0.17	94.21	2.01	6.88	7.96	16.66	8.35	-0.72	12.46	21.83	11.85	1.82
os40441	2	358	0.83	16	1	4	6.46	-0.11	0	1.37	0.1	93.56	5.97	5.55	7.74	16.43	8.18	-0.88	12.21	21.59	11.64	1.64
os40442	2	358	0.84	16	1	4	6.39	-0.15	0	1.38	0.11	96.88	3.17	6.04	7.72	16.42	8.17	-0.89	12.2	21.58	11.62	1.62
os40448	2	387	0.03	10	3	9	6.88	0.16	0.04	1.44	0.07	90.3	1.32	5.54	7.75	16.46	8.17	-0.89	12.26	21.67	11.68	1.64
os40449	2	386	0.03	12	3	9	6.9	0.19	0.01	1.43	0.08	88.62	0.37	5.85	7.74	16.45	8.17	-0.89	12.25	21.66	11.67	1.64
os40506	2	314	0.76	14	1	4	6.14	-0.15	0	1.41	0.07	82.62	4.57	5.94	8.07	16.74	8.44	-0.78	12.67	22.02	12.02	1.77
os40525	2	401	0.79	6	3	9	6.59	-0.06	0.58	1.47	0.04	98.3	1.56	6.93	7.55	16.25	8.01	-1.17	12.12	21.47	11.55	1.39
os40576	2	380	0.08	7	3	5	6.95	0.11	0.3	1.5	0.05	94.37	0.34	6.98	7.81	16.57	8.22	-0.99	12.38	21.82	11.81	1.63

os40638	2	362	0.1	3	3	6	6.67	-0.05	0.61	1.53	0.01	72.54	0.99	7.86	7.73	16.37	8.13	-0.45	12.06	21.35	11.49	1.95
os40641	2	457	0.31	14	1	1	6.53	0.04	0	1.38	0.05	98.39	4.35	5.47	7.26	15.91	7.84	-0.97	11.72	21.06	11.26	1.49
os40665	2	311	0.82	20	3	5	6.5	-0.06	0.19	1.42	0.16	93.41	-2.4	6.08	8.03	16.74	8.41	-0.21	12.27	21.59	11.74	2.2
os40717	2	336	0.92	1	3	6	6.73	-0.01	0.86	1.5	0.01	75.12	0.77	9.17	7.86	16.52	8.27	-0.41	12.31	21.58	11.67	2.04
os40764	2	366	0.77	2	1	4	6.77	-0.01	0.92	1.48	0.02	85.79	3.3	8.06	7.69	16.3	8.11	-0.58	12.21	21.41	11.53	1.86
os40831	2	343	0.78	5	3	5	6.65	-0.07	0.38	1.51	0.05	95.79	0.11	6.2	7.85	16.55	8.3	-0.48	12.43	21.72	11.85	2.01
os40855	2	389	0.08	7	3	5	6.94	0.1	0.3	1.48	0.06	87.21	1.71	6.51	7.39	16.06	7.95	-0.84	11.86	21.2	11.41	1.61
os40900	2	321	0.02	5	3	6	6.8	0.06	1.32	1.54	0.07	84.32	-0.79	7.78	7.8	16.48	8.26	-0.46	12.32	21.59	11.74	1.99
os40925	2	327	0.11	17	1	4	6.75	0.22	0	1.33	0.08	98.98	7.54	5.48	8.28	16.94	8.57	-0.13	12.52	21.77	11.93	2.31
os40938	2	285	0.01	10	2	3	6.85	0.14	0.59	1.47	0.13	97.95	-6.93	7.65	8.59	17.28	8.83	0.13	12.91	22.14	12.21	2.58
os40947	2	368	0.23	7	3	5	6.63	0.01	0.44	1.44	0.07	94.36	0.12	6.82	7.63	16.29	8.13	-0.68	12.09	21.43	11.55	1.76
os40958	2	331	0.05	11	3	5	6.82	0.15	0.57	1.51	0.1	93.26	-2.39	7.19	8.1	16.82	8.4	-0.37	12.47	21.76	11.83	2.13
os40966	2	305	0.77	3	3	6	6.74	-0.01	0.1	1.52	0.04	83.59	1.62	7.44	8.21	16.93	8.54	-0.23	12.61	21.95	12.02	2.26
os40967	2	304	0.42	4	1	4	6.8	0.02	0.12	1.52	0.04	83.34	2.1	7.51	8.21	16.93	8.54	-0.23	12.61	21.95	12.02	2.26
os40969	2	287	0.5	3	3	6	6.73	-0.01	0.82	1.49	0.02	60.71	2.04	8.03	8.2	16.89	8.51	-0.14	12.56	21.93	11.93	2.28
os40979	2	292	0	14	3	5	6.91	0.23	0	1.42	0	91.87	1.5	6.22	8.24	16.99	8.66	-0.03	12.45	21.81	12	2.33
os41151	2	288	0.47	7	3	5	6.6	-0.01	0.4	1.48	0.05	80.02	0.32	7.87	8.14	16.88	8.56	-0.15	12.38	21.72	11.93	2.18
os41161	2	348	0.6	4	3	6	6.79	0	0.26	1.5	0.03	87.55	1.24	9.14	7.86	16.65	8.3	-0.44	12.07	21.47	11.64	1.92
os41227	2	314	0.73	20	1	4	5.71	-0.23	0	1.36	0.11	91.59	7.2	5.59	8.11	16.86	8.48	-0.26	12.57	21.89	11.97	2.16
os41250	2	401	0.97	7	2	3	6.56	-0.11	0.71	1.5	0.11	95.01	-2.25	6.27	7.35	16.11	7.91	-0.79	11.6	20.92	11.14	1.55
os41270	2	400	0.38	5	3	5	6.88	0.04	1.35	1.55	0.07	72.24	-1.93	7.86	7.26	15.97	7.8	-0.9	11.51	20.89	11.11	1.44
os41274	2	398	0.32	17	3	5	6.32	0.03	0	1.42	0.13	90.63	2.99	6.18	7.25	15.95	7.8	-0.92	11.57	20.91	11.16	1.43
os41278	2	377	0.19	3	3	6	6.82	0.05	0.11	1.52	0.05	84.32	1.6	8.28	7.43	16.11	7.96	-0.77	11.81	21.15	11.39	1.64
os41285	2	442	0.04	10	2	3	6.95	0.17	0.44	1.51	0.11	96.23	-3.78	6.79	7.16	15.95	7.67	-1.03	11.45	20.85	10.99	1.38
os41314	2	400	0.64	6	2	3	6.88	0.08	0.92	1.54	0.12	87.83	-3.48	6.6	7.24	15.96	7.79	-0.92	11.54	20.89	11.13	1.43
os41319	2	470	0.96	15	3	5	6.05	-0.26	0	1.43	0.14	94.24	-0.73	5.04	7.11	15.9	7.64	-1.07	11.39	20.78	10.93	1.33
os41343	2	389	0.08	7	1	1	6.94	0.1	0.18	1.45	0.03	94.64	2.13	5.92	7.51	16.19	8.05	-0.71	11.98	21.29	11.52	1.74
os41359	2	350	0.44	5	2	3	6.78	0.01	1.83	1.55	0.11	96.34	-3.89	7.31	7.47	16.15	8.03	-0.71	11.9	21.2	11.46	1.71
os41416	2	473	0.98	3	1	4	6.73	-0.04	0.06	1.51	0.02	79.29	1.72	7.52	7.25	15.91	7.36	-0.96	12.08	21.45	11.21	1.69
os41574	2	481	0.25	6	3	5	6.95	0.07	0.46	1.52	0.03	85.95	-0.31	6.51	7.13	15.8	7.29	-1.11	12.18	21.52	11.25	1.63
os41575	2	472	0.23	1	3	6	6.8	0	0.22	1.54	0.02	62.79	0.82	8.77	7.21	15.87	7.34	-1.05	12.26	21.59	11.32	1.68
os41579	2	475	0.97	4	3	6	6.8	-0.02	1.71	1.54	0.05	78.89	-2.11	7.81	7.15	15.81	7.3	-1.1	12.18	21.53	11.26	1.65
os41654	2	451	0.47	3	3	6	6.76	0	0.35	1.52	0.02	86.75	0.95	7.41	7.59	16.37	7.79	-0.94	12.42	21.81	11.65	1.71
os41693	2	433	0.01	2	3	6	6.81	0.02	4.68	1.55	0.02	11.75	-0.4	9.04	7.59	16.35	7.74	-0.96	12.64	21.97	11.74	1.78
os41877	2	453	0.6	4	3	6	6.7	-0.02	1.64	1.52	0.05	94.74	-1.19	7.69	7.56	16.33	7.77	-0.96	12.38	21.77	11.62	1.7
os42140	2	490	0.05	3	3	9	6.71	-0.06	0.39	1.48	0.01	68.33	1.81	7.02	7.54	16.32	7.68	-0.88	12.51	21.93	11.52	1.74
os42227	2	397	0.03	2	3	6	6.82	0.01	0.96	1.54	0.04	62.22	0.15	8.25	7.83	16.58	8.14	-0.74	12.43	21.68	11.78	1.83

os42481	2	443	0	13	1	4	6.98	0.22	0	1.42	0.07	84.08	5.94	5.78	7.1	15.87	7.74	-0.95	11.4	20.76	11.02	1.43
os42539	2	379	0.12	9	3	5	6.97	0.13	0.13	1.48	0	99.42	-1.28	6.95	7.67	16.46	8.25	-0.5	11.99	21.38	11.66	1.9
os42572	2	447	0.77	10	3	5	6.7	-0.07	0.13	1.47	0.06	96.58	0.64	6.29	7.2	16.01	7.87	-0.74	11.55	20.99	11.26	1.66
os42585	2	432	0.04	2	3	6	6.81	0.03	1.55	1.53	0	55.04	-0.02	8.89	7.2	16	7.87	-0.72	11.55	21.01	11.26	1.68
os42648	2	506	0.18	11	3	5	7.03	0.13	0.03	1.46	0.06	99.76	1.38	6.33	6.86	15.65	7.55	-1.05	10.96	20.37	10.72	1.3
os42661	2	394	0.18	5	2	0	6.61	-0.08	0.72	1.54	0.05	92.31	-0.65	8.18	7.25	16.05	7.92	-0.74	11.58	20.98	11.29	1.65
os42830	2	464	0.31	5	3	5	6.94	0.07	0.48	1.52	0.03	88.36	0.12	6.8	7.05	15.84	7.71	-0.99	11.5	20.87	11.13	1.47
os42863	2	279	0.92	1	3	6	6.75	0.01	2.95	1.55	0	75.64	-0.34	8.98	7.99	16.7	8.54	-0.14	12.65	22.01	12.1	2.38
os42870	2	297	0.29	10	3	5	6.69	0.09	0.11	1.48	0.08	93.59	-1.27	6.85	7.91	16.61	8.47	-0.21	12.5	21.82	11.96	2.22
os42873	2	299	0.09	7	2	3	6.78	0.09	0.65	1.52	0	82.47	-2.28	6.8	7.82	16.49	8.41	-0.28	12.4	21.71	11.92	2.19
os42964	2	429	0.52	10	3	5	6.83	0.01	0.11	1.44	0.06	95.5	1.12	6.27	7.2	15.86	7.85	-0.96	11.9	21.15	11.41	1.53
os42971	2	370	0.67	22	2	7	5.82	-0.2	0	1.48	0.19	90.94	-4.26	6.18	7.34	16.12	7.93	-0.91	12.08	21.53	11.51	1.58
os43042	2	279	0.72	1	3	6	6.72	0.01	2.98	1.56	0	67.73	-1.35	8.97	7.99	16.7	8.54	-0.14	12.65	22.02	12.1	2.38
os43092	2	334	0.03	11	3	5	6.82	0.13	0.03	1.49	0.07	85.29	1.22	7.3	7.45	16.08	8.07	-0.67	11.98	21.22	11.51	1.77
os43100	2	417	0.25	10	3	5	6.7	0.07	0.02	1.47	0.07	89	1.85	7.01	7.08	15.71	7.74	-1.08	11.74	20.93	11.25	1.36
os43105	2	425	0.12	20	3	5	7	0.25	0	1.43	0.1	98.29	0.26	5.51	7.15	15.79	7.8	-1.01	11.83	21.06	11.35	1.47
os43110	2	420	0.01	12	1	4	6.98	0.21	0.01	1.43	0.05	99.05	4.67	5.66	7.28	16.01	7.88	-0.93	12.01	21.39	11.46	1.54
os43117	2	323	0.72	8	3	5	6.45	-0.09	0.22	1.5	0	99.92	0.92	6.63	7.72	16.4	8.29	-0.38	12.1	21.47	11.65	2.01
os43182	2	431	1	16	3	5	6.1	-0.26	0	1.41	0	96.94	1.24	6.28	7.06	15.72	7.76	-0.94	11.68	20.94	11.26	1.54
os43204	2	364	0.35	2	3	6	6.76	0.01	1.37	1.53	0	55.84	0.55	8.29	7.59	16.31	8.23	-0.45	12.25	21.6	11.84	2.09
os43223	2	372	0.98	8	3	5	6.54	-0.12	0.22	1.51	0	91.67	0.4	6.87	7.28	15.89	8.01	-1.23	11.79	20.96	11.51	1.3
os43226	2	437	0.92	5	3	6	6.61	-0.08	0.54	1.53	0.04	98.23	0.08	7.04	7	15.61	7.77	-1.31	11.47	20.64	11.16	1.24
os43287	2	385	0.1	11	1	4	6.75	0.13	0	1.45	0.09	90.45	2.01	5.52	7.17	15.77	7.83	-0.95	11.8	21	11.34	1.54
os43396	2	468	0.6	12	3	5	6.45	-0.04	0	1.45	0.07	98.79	1.8	5.38	7.03	15.73	7.68	-1.13	11.68	21	11.21	1.33
os43402	2	475	0.35	13	1	4	6.57	0.07	0	1.42	0.1	98.98	4.89	5.92	6.88	15.68	7.58	-1.35	11.46	20.94	11.04	1.16
os43471	2	487	0.17	12	2	7	6.66	0.08	0.24	1.49	0.11	93.7	-2.78	6.86	6.75	15.49	7.4	-1.59	11.33	20.69	10.88	0.91
os43485	2	581	0.02	15	1	2	6.9	0.22	0	1.43	0.15	95.6	3.99	6.22	5.86	14.61	6.74	-2.38	10.08	19.49	9.96	0.03
os43528	2	499	0.99	4	2	7	6.71	-0.05	1.44	1.54	0.16	85.49	-2.94	7.27	6.31	15	7.08	-2.42	10.62	19.96	10.46	0.04
os43532	2	521	0.99	22	1	4	5.81	-0.34	0	1.35	0.16	78.11	5.89	5.04	6.32	15.01	7.1	-2.43	10.63	19.98	10.49	0.03
os43572	2	553	0.4	5	1	1	6.86	0.01	0.01	1.42	0.04	96.53	5.69	5.85	6.51	15.26	7.25	-1.88	10.89	20.3	10.58	0.58
os43589	2	551	0.02	12	3	5	6.33	-0.18	0.01	1.44	0.09	94.02	0.31	5.43	6.53	15.33	7.23	-1.98	10.97	20.48	10.61	0.48
os43596	2	521	0.23	2	1	1	6.78	-0.03	0.09	1.42	0.01	96.4	2.93	7.37	6.75	15.5	7.42	-1.69	11.26	20.66	10.8	0.81
os43675	2	462	0.01	9	3	5	6.95	0.15	0.04	1.45	0.09	99.41	0.99	5.31	6.97	15.6	7.65	-1.18	11.58	20.8	11.13	1.25
os43734	2	685	0.1	8	3	5	7.07	0.13	0.03	1.46	0.08	99.97	2.44	5.56	5.69	14.53	6.56	-2.61	9.98	19.5	9.85	-0.17
os43745	2	636	0.14	17	2	0	6.76	0.17	0	1.43	0.13	99.99	1.87	5.95	5.62	14.34	6.5	-2.77	9.78	19.21	9.77	-0.36
os43746	2	670	0.07	14	3	5	6.91	0.19	0	1.44	0.11	99.99	-1.9	6.2	5.57	14.3	6.45	-2.8	9.73	19.15	9.73	-0.4
os43760	2	575	1	13	1	4	6.26	-0.22	0	1.39	0.1	98.15	4.37	5.47	6.08	14.81	6.9	-2.52	10.37	19.74	10.26	-0.08

os43762	2	549	0.01	4	1	4	6.9	0.06	0.07	1.46	0.08	90.8	4.2	6.69	6.22	14.93	6.99	-2.44	10.54	19.89	10.38	0.01
os43773	2	709	0.22	13	3	9	7.13	0.17	0	1.44	0.08	99.69	0.38	5.92	5.68	14.47	6.53	-2.78	10.01	19.42	9.88	-0.31
os43786	2	633	0.06	7	3	5	6.96	0.1	0.69	1.49	0.08	99.79	-1.27	7.53	5.9	14.67	6.69	-2.62	10.26	19.67	10.06	-0.14
os43816	2	450	0.23	3	1	4	6.74	0.01	0.03	1.43	0.06	98.64	3.63	6.4	6.87	15.47	7.59	-1.22	11.27	20.49	10.91	1.16
os43826	2	433	0.7	11	3	5	6.37	-0.1	0.02	1.47	0.08	99.4	-0.22	6.22	7.03	15.63	7.71	-1.09	11.56	20.76	11.12	1.33
os43856	2	489	0.05	5	1	1	6.88	0.07	0.01	1.45	0.04	99.48	5.52	6.15	6.68	15.32	7.53	-1.63	10.94	20.22	10.72	0.77
os43916	2	628	0.02	10	1	4	7.08	0.16	0.09	1.45	0.08	99.24	3.15	6.38	5.76	14.52	6.64	-3.04	9.99	19.39	10.03	-0.63
os43940	2	823	0.82	9	1	1	6.84	-0.07	0.05	1.44	0.06	100	2.32	6.19	4.95	13.76	5.98	-3.32	9	18.44	9.25	-0.91
os44127	2	492	0.25	6	2	0	6.59	-0.11	0.34	1.46	0.07	97.98	2.25	6.25	6.62	15.35	7.28	-2.03	11.08	20.44	10.73	0.45
os44218	2	591	0.05	17	3	5	7.04	0.27	0	1.41	0.12	98.76	0.23	5.81	6.14	14.91	6.89	-2.77	10.57	19.94	10.41	-0.28
os44281	2	462	0.92	7	3	5	6.5	-0.12	0.3	1.48	0.08	99.57	-0.8	7.55	6.75	15.49	7.37	-2.14	11.23	20.58	10.94	0.33
os44315	2	435	0.05	20	1	4	6.72	0.26	0	1.38	0.13	99.42	2.86	5.01	7.1	15.85	7.62	-1.9	11.71	21.08	11.25	0.62
os44344	2	411	0.64	10	2	0	6.76	-0.02	0.09	1.51	0.11	83.23	-1.49	5.94	6.89	15.59	7.53	-2.08	11.37	20.68	11.06	0.39
os44399	2	516	0.37	6	3	5	6.94	0.05	0.49	1.49	0.05	99.68	0.98	6.7	6.62	15.36	7.31	-1.83	11.15	20.49	10.72	0.67
os44422	2	545	0.37	9	1	4	6.71	0.03	0.04	1.43	0.09	98.12	3.21	5.78	6.16	14.9	6.96	-2.7	10.5	19.88	10.42	-0.24
os44449	2	496	0.92	16	1	1	6.04	-0.24	0	1.38	0.11	98.82	2.65	5.62	6.78	15.47	7.45	-2.17	11.23	20.54	10.96	0.28
os44466	2	499	0.68	13	2	0	6.75	-0.04	0.01	1.48	0.1	99.56	-0.06	6.31	6.53	15.25	7.23	-2.29	10.95	20.26	10.7	0.2
os44485	2	456	0.1	20	1	4	6.98	0.31	0	1.33	0.12	93.18	6.97	5.13	6.75	15.46	7.4	-2.23	11.21	20.54	10.93	0.25
os44487	2	452	0.19	20	1	4	6.99	0.28	0	1.3	0.11	92.06	9.86	4.87	6.74	15.45	7.4	-2.24	11.21	20.54	10.93	0.24
os44517	2	622	0.19	8	3	5	7.05	0.1	0.36	1.5	0.07	99.94	-1.14	7.64	6	14.77	6.76	-2.59	10.36	19.76	10.15	-0.12
os44521	2	548	0.68	8	3	5	6.84	-0.02	0.07	1.46	0.06	99.85	1.61	5.75	6.34	15.1	7.06	-2.39	10.77	20.15	10.54	0.08
os44522	2	545	0.76	8	3	5	6.79	-0.04	0.05	1.46	0.06	99.87	1.26	5.78	6.35	15.11	7.07	-2.39	10.78	20.16	10.55	0.09
os44565	2	583	0.47	3	1	1	6.82	0	0.04	1.47	0.01	98.02	2.4	6.73	6.41	15.17	7.1	-2.28	10.85	20.23	10.6	0.21
os44596	2	458	0.99	9	1	2	6.51	-0.14	0.01	1.47	0.12	94.7	2.09	7.01	6.67	15.43	7.34	-2.04	11.14	20.52	10.83	0.43
os44608	2	480	0	12	3	5	6.94	0.19	0	1.46	0.1	95.31	0.24	6.05	6.73	15.47	7.36	-1.96	11.21	20.56	10.85	0.5
os44616	2	562	0.95	7	3	5	6.64	-0.11	0.34	1.49	0.06	99.7	-0.73	6.15	6.45	15.19	7.13	-2.18	10.88	20.26	10.57	0.3
os44627	2	524	0.92	8	3	5	6.51	-0.13	0.47	1.46	0.07	99.61	-0.34	6.78	6.69	15.44	7.33	-1.97	11.16	20.51	10.78	0.52
os44684	2	458	0.81	9	3	5	6.43	-0.12	0.09	1.42	0.07	96.9	1.94	7.02	7.03	15.76	7.6	-1.78	11.56	20.88	11.13	0.74
os44687	2	471	0.35	4	3	9	6.9	0.04	0.72	1.43	0.03	96.21	1.38	7.01	7.11	15.84	7.67	-1.71	11.65	20.96	11.2	0.8
os44698	2	546	0.43	14	3	5	6.9	0.05	0	1.43	0.09	99.83	0.75	6.17	6.51	15.26	7.18	-2.19	10.96	20.33	10.67	0.31
os44710	2	431	0.15	19	1	4	6.55	0.18	0	1.32	0.1	98.87	8.11	4.97	7.03	15.76	7.6	-1.81	11.52	20.85	11.13	0.69
os44711	2	396	0.73	15	1	4	6.12	-0.16	0	1.36	0.13	96.96	3.65	5.4	7.19	15.9	7.72	-1.7	11.69	21.01	11.26	0.81
os44754	2	423	0.98	8	3	9	6.47	-0.14	0.16	1.46	0.05	98.77	0.16	6.46	7.3	16	7.8	-1.59	11.83	21.15	11.32	0.93
os44760	2	390	0.74	20	1	1	6.47	-0.07	0	1.29	0.12	99.49	10.59	5.01	7.39	16.08	7.87	-1.4	11.93	21.22	11.39	1.12
os44771	2	395	0.53	12	1	1	6.36	-0.06	0	1.39	0.09	99.77	3.62	5.22	7.51	16.19	8	-1.28	12.05	21.34	11.5	1.22
os44792	2	511	0.21	8	3	9	6.99	0.09	0.17	1.46	0.06	100	0.93	6.25	6.88	15.62	7.5	-1.71	11.38	20.75	10.96	0.79
os44797	2	447	0.6	10	2	0	6.81	0.01	0.1	1.48	0.1	97.79	0.95	6.57	6.87	15.59	7.48	-1.78	11.39	20.72	10.92	0.7

os44803	2	419	0.12	13	2	7	6.74	0.15	0.01	1.49	0.12	99.75	-3.76	5.61	7.18	15.91	7.72	-1.52	11.7	21.02	11.2	1.04
os44862	2	687	0.81	12	3	9	6.38	-0.2	0.01	1.43	0.08	98.91	0.42	5.83	5.7	14.47	6.57	-3.09	9.95	19.32	9.95	-0.66
os45084	2	536	1	16	3	5	6.15	-0.26	0	1.42	0.1	99.51	0.83	6.24	6.51	15.26	7.16	-2.64	11.03	20.34	10.74	-0.09
os45148	2	576	0.3	21	2	0	6.97	0.21	0	1.4	0.18	94.88	1.02	4.97	5.89	14.65	6.72	-3.04	10.25	19.58	10.13	-0.55
os45231	2	579	1	16	3	5	6.08	-0.28	0	1.39	0.12	99.85	1.69	5.47	6.11	14.86	6.92	-2.76	10.43	19.77	10.37	-0.32
os45239	2	659	0.87	11	3	9	6.7	-0.1	0.04	1.42	0.07	99.76	-0.63	6.22	5.93	14.69	6.76	-2.87	10.25	19.58	10.19	-0.42
os45517	2	448	0.17	13	2	0	6.7	0.11	0	1.47	0.13	92.23	-0.48	5.81	6.73	15.44	7.35	-2.3	11.22	20.57	10.93	0.2
os45575	2	597	0.8	9	3	5	6.52	-0.1	0.31	1.47	0.09	99.3	-1.45	7.13	6.05	14.82	6.82	-2.83	10.46	19.84	10.31	-0.35
os45580	2	617	0.04	8	3	5	6.98	0.12	0.5	1.49	0.07	98.02	-0.54	6.97	5.94	14.71	6.71	-2.9	10.34	19.72	10.21	-0.43
os45615	2	561	0.04	18	2	3	6.87	0.23	0	1.45	0.15	99.64	-3.91	7.15	6.19	14.95	6.92	-2.75	10.65	20	10.46	-0.25
os45636	2	645	0.2	10	3	5	6.82	0.07	0.22	1.48	0.07	97.19	-1.29	7.01	5.85	14.61	6.62	-2.97	10.24	19.61	10.12	-0.49
os45642	2	622	0.4	12	3	5	6.99	0.08	0.02	1.47	0.1	99.76	-1.06	5.7	5.87	14.62	6.63	-2.97	10.23	19.62	10.12	-0.51
os45644	2	614	0.44	11	2	0	6.99	0.07	0.05	1.47	0.1	99.66	-0.04	5.71	5.88	14.63	6.65	-2.97	10.24	19.63	10.13	-0.5
os45848	2	422	0.36	13	3	5	6.91	0.09	0	1.44	0.12	91.53	-1.71	6.77	7.12	15.84	7.65	-1.7	11.66	20.99	11.15	0.83
os45909	2	532	0.95	16	1	4	6.29	-0.22	0	1.38	0.1	98.82	3.24	5.74	6.45	15.17	7.13	-2.66	10.88	20.18	10.65	-0.19
os45923	2	621	0.65	6	1	1	6.65	-0.1	0.04	1.39	0.03	99.95	4.13	5.73	6.19	14.94	6.93	-2.81	10.62	19.94	10.43	-0.33
os45987	2	517	0.52	7	1	1	6.89	0.02	0.05	1.46	0.03	99.5	3.94	5.7	6.76	15.52	7.34	-2.43	11.29	20.66	10.97	0.12
os46051	2	555	0.7	4	1	1	6.84	-0.02	0.04	1.47	0.01	90.55	3.49	6.52	6.61	15.36	7.22	-2.59	11.22	20.54	10.84	0.01
os46063	2	410	0.62	4	2	3	6.81	0.01	1.94	1.55	0.09	95.11	-4.42	8.06	7.08	15.8	7.58	-2.43	11.72	21.02	11.29	0.18
os46073	2	459	0.29	13	1	4	6.52	0.03	0	1.34	0.06	93.22	9.34	5.09	6.91	15.64	7.44	-2.43	11.52	20.85	11.08	0.18
os46118	2	339	0.01	5	3	5	6.86	0.09	0.33	1.51	0.03	96.9	1.36	7.89	7.48	16.17	7.98	-1.88	12.05	21.32	11.75	0.75
os46387	2	405	0.95	13	3	5	6.38	-0.18	0.02	1.46	0.09	94.92	1.84	6.04	7.1	15.82	7.67	-2.16	11.54	20.84	11.25	0.37
os46600	2	512	0.08	3	1	1	6.93	0.06	0.73	1.41	0.02	98.59	1.97	7.04	6.82	15.56	7.39	-2.41	11.34	20.66	10.97	0.19
os46851	2	469	0.35	8	3	5	6.93	0.06	0.4	1.52	0.06	85.71	-0.67	6.96	6.78	15.55	7.39	-2.72	11.42	20.73	11.08	-0.13
os46928	2	403	0.94	14	3	5	6.33	-0.19	0	1.46	0.12	91.12	-0.26	6.37	7.07	15.78	7.64	-2.19	11.5	20.81	11.22	0.34
os46940	2	439	0	3	3	6	6.87	0.05	1.22	1.54	0.02	99.64	0.01	8.34	6.97	15.65	7.56	-2.28	11.44	20.75	11.23	0.32
os47038	2	454	0.08	2	3	6	6.87	0.03	1.71	1.53	0.01	47.31	-0.02	8.4	6.87	15.58	7.49	-2.31	11.37	20.68	11.16	0.29
os47083	2	528	0.04	13	3	5	7.06	0.2	0.03	1.46	0.07	96.91	1.95	6.54	6.46	15.19	7.11	-2.7	10.93	20.23	10.72	-0.14
os47190	2	358	0.45	19	2	7	6.68	0.08	0	1.49	0.19	80.29	-7.6	5.71	7.21	15.92	7.74	-2.36	11.85	21.08	11.44	0.24
os47200	2	371	0.01	5	3	5	6.87	0.09	0.36	1.53	0.07	94.71	-0.51	7.85	7.24	15.96	7.75	-2.3	11.87	21.13	11.48	0.28
os47365	2	463	0.07	8	3	5	6.83	0.1	0.18	1.49	0.06	95.46	0.8	6.7	6.69	15.39	7.35	-2.48	11.19	20.51	10.98	0.09
os47684	2	475	0.53	13	1	4	6.35	-0.08	0	1.39	0.08	98.44	3.33	5.58	6.99	15.7	7.52	-2.28	11.57	20.86	11.13	0.3
os47872	2	465	0.91	8	1	4	6.55	-0.1	0.01	1.45	0.05	87.9	4.04	8.18	6.92	15.66	7.43	-2.1	11.51	20.88	11.03	0.45
os47912	2	458	0.18	9	2	3	6.98	0.12	0.64	1.51	0.09	98.38	-4.52	6.77	7.06	15.8	7.56	-2.09	11.65	21.01	11.18	0.47
os47914	2	435	0.92	6	1	4	6.68	-0.07	0.13	1.47	0.04	97.68	3.34	6.4	7.16	15.87	7.63	-2.04	11.75	21.11	11.27	0.52
os47915	2	457	0.11	12	2	3	6.81	0.12	0.43	1.49	0.08	94.86	-2.29	7.44	7.04	15.79	7.55	-2.09	11.64	21	11.17	0.47
os47938	2	492	0.6	10	3	5	6.53	-0.04	0.05	1.48	0.08	99.67	-0.05	6.9	6.83	15.59	7.35	-2.16	11.39	20.76	10.94	0.38

os48275	2	367	0.19	8	3	5	6.94	0.1	0.16	1.5	0.04	94.46	0.98	6.59	7.56	16.31	8.01	-1.69	12.14	21.51	11.64	0.97
os48288	2	415	0.53	6	1	4	6.86	0.02	0.17	1.48	0.05	97	2.44	6.36	7.32	16.03	7.81	-1.95	11.87	21.21	11.46	0.71
os48316	2	483	0.01	10	3	5	6.94	0.15	0.16	1.48	0.07	99.27	-0.85	5.99	6.99	15.72	7.5	-2.17	11.54	20.85	11.08	0.42
os48494	2	455	0.74	7	3	9	6.8	-0.02	0.44	1.49	0.04	90.55	-0.08	6.87	6.96	15.68	7.52	-2.36	11.63	20.94	11.28	0.3
os48642	2	400	0	11	2	0	6.96	0.2	0.02	1.48	0.13	96.57	-0.8	7.51	6.9	15.58	7.52	-2.45	11.49	20.74	11.16	0.18
os48715	2	418	0.21	12	3	5	6.99	0.16	0	1.47	0.09	98.77	-0.66	5.95	7.07	15.8	7.62	-2.29	11.77	21.08	11.4	0.38
os48723	2	461	0.01	13	3	9	6.91	0.2	0.01	1.42	0.07	99.69	-0.54	5.8	7	15.68	7.58	-2.39	11.69	20.92	11.33	0.26
os48724	2	410	0.76	11	2	3	6.3	-0.14	0.17	1.49	0.13	95.92	-3.33	7.69	7	15.67	7.57	-2.4	11.7	20.93	11.33	0.24
os48842	2	445	0.34	2	3	6	6.74	-0.02	0.83	1.53	0.02	80.35	0.18	7.74	6.93	15.67	7.5	-2.22	11.6	21.02	11.29	0.45
os48913	2	463	0	6	1	4	6.92	0.11	0.16	1.48	0.04	96.26	2.18	6.51	6.84	15.57	7.46	-2.33	11.4	20.76	11.16	0.3
os48915	2	438	0.03	7	3	5	6.96	0.12	0.26	1.48	0.04	98.83	1.89	6.82	6.94	15.68	7.54	-2.26	11.53	20.89	11.25	0.4
os49153	2	519	0.93	6	3	9	6.7	-0.08	0.25	1.4	0.05	98.09	2.02	6.2	6.48	15.12	7.14	-2.53	10.98	20.28	10.72	0.08
os49233	2	548	0.36	13	1	4	6.62	0.05	0	1.42	0.07	99.78	3.56	5.8	6.25	14.93	6.98	-2.88	10.76	19.97	10.55	-0.29
os49256	2	561	0.15	7	3	5	6.9	0.09	0.37	1.51	0.07	95.3	0.96	6.67	6.06	14.74	6.83	-2.97	10.5	19.73	10.33	-0.39
os49466	2	461	0.99	5	3	6	6.67	-0.07	0.93	1.54	0.04	56.47	0.5	9.06	6.5	15.19	7.17	-2.38	11.21	20.5	10.93	0.32
os49543	2	408	0.92	5	3	5	6.58	-0.09	0.59	1.51	0.06	90.11	-1.15	7.18	6.98	15.67	7.57	-2.4	11.58	20.85	11.24	0.24
os49622	2	483	0.38	20	1	4	6.83	0.09	0	1.37	0.12	95.67	4.19	4.89	6.49	15.15	7.14	-2.5	11.02	20.32	10.77	0.13
os49670	2	465	0.81	13	3	5	6.19	-0.19	0	1.45	0.14	99.05	-1.35	5.52	6.54	15.19	7.17	-2.43	11.13	20.43	10.85	0.2
os49718	2	569	0.97	7	3	9	6.69	-0.09	0.36	1.42	0.05	98.15	0.32	6.03	6.32	14.99	7.03	-2.65	10.79	20.09	10.57	-0.03
os49960	2	606	0.54	7	1	1	6.92	0.01	0.06	1.42	0.04	99.96	2.89	5.76	6.26	15.02	6.81	-2.19	10.93	20.4	10.4	0.4
os50211	2	620	0.98	14	3	5	6.23	-0.22	0.03	1.44	0.11	97.58	-1.3	6.71	6.06	14.79	6.75	-2.48	10.72	20.09	10.4	0.25
os50293	2	481	0.11	16	2	7	6.99	0.24	0	1.47	0.15	91.7	-2.3	6.59	6.7	15.42	7.29	-2.06	11.47	20.84	11.03	0.72
os50319	2	608	0.81	9	3	9	6.52	-0.08	0.08	1.48	0.07	99.49	-0.49	5.81	6.21	14.93	6.89	-2.39	10.86	20.25	10.56	0.36
os50464	2	549	0.99	7	3	9	6.57	-0.13	0.18	1.43	0.06	94.9	0.33	6.05	6.49	15.27	7.01	-2.11	11.26	20.75	10.7	0.55
os50592	2	427	0.62	5	3	6	6.83	0	0.45	1.51	0.04	86.92	0.43	7.04	7.22	15.95	7.62	-1.7	12.14	21.6	11.42	1.08
os50792	2	333	0.08	4	3	6	6.88	0.08	0.74	1.53	0.04	96.73	-0.52	7.18	7.79	16.53	8.13	-1.2	12.65	22.08	12	1.6
os50902	2	457	0.01	9	3	5	6.99	0.14	0.02	1.48	0.07	98.33	0.95	7.49	6.95	15.77	7.32	-1.86	11.77	21.39	11.12	0.88
os50973	2	418	0	12	2	3	6.9	0.2	0.19	1.5	0.13	97.67	-2.64	5.74	7.3	16.07	7.58	-1.64	12.33	21.89	11.51	1.14
os50974	2	426	0.01	5	3	6	6.68	-0.05	0.21	1.48	0.07	98.21	1.95	6.31	7.31	16.08	7.58	-1.64	12.33	21.89	11.51	1.14
os51040	2	510	0.89	8	3	5	6.74	-0.06	0.39	1.51	0.09	98.78	-1.39	7.04	6.52	15.32	7	-2.06	11.26	20.83	10.68	0.62
os51107	2	487	0.17	7	2	3	6.92	0.05	0.9	1.51	0.07	97.03	-3.28	7.07	6.98	15.78	7.37	-1.72	11.76	21.38	11.11	0.98
os51342	2	625	0.7	11	2	3	6.43	-0.11	0.04	1.49	0.14	98.89	-2.39	6.8	5.3	14.06	6.15	-2.4	9.27	18.69	9.27	0.02
os51603	2	508	0.44	9	3	5	6.92	0.04	0.06	1.48	0.09	97.18	1.38	6.65	6.24	15.07	6.91	-1.97	10.73	20.07	10.47	0.73
os51662	2	567	0.33	14	2	3	6.57	0.04	0	1.46	0.09	95.58	-1.02	5.69	6.08	14.9	6.79	-2.05	10.49	19.86	10.27	0.64
os51759	2	685	0.99	18	3	9	6.11	-0.29	0	1.4	0.11	99.99	-0.64	5.48	5.6	14.41	6.28	-2.63	9.98	19.32	9.76	-0.02
os51806	2	564	0.73	5	1	1	6.69	-0.05	0.03	1.36	0.02	98.77	6.67	6.07	6.21	14.95	6.78	-2.29	10.7	20.04	10.36	0.34
os51904	2	620	1	11	3	9	6.4	-0.19	0.01	1.4	0.07	99.94	0.64	5.61	5.97	14.76	6.55	-2.41	10.57	19.9	10.2	0.22

os52088	2	531	0.95	18	3	5	5.94	-0.29	0	1.39	0.13	97.38	1.89	5.25	6.22	14.95	6.79	-2.28	10.71	20.06	10.37	0.34
os52089	2	501	1	13	2	0	6.16	-0.23	0	1.47	0.18	99.41	-2.92	7.17	6.16	14.9	6.74	-2.32	10.64	19.98	10.31	0.3
os52373	2	602	0.97	14	3	5	6.2	-0.23	0	1.47	0.09	100	0	5.87	5.64	14.34	6.52	-2	9.75	19.1	9.76	0.57
os52573	2	418	0.05	11	1	4	6.31	-0.17	0	1.41	0.06	87.3	6.4	5.3	6.93	15.62	7.55	-1.01	11.31	20.62	11.02	1.63
os52634	2	427	0.26	10	3	5	6.59	0.02	0.06	1.51	0.08	99.67	-1.31	7.15	6.65	15.39	7.33	-1.29	10.95	20.28	10.77	1.38
os53477	2	356	0.7	10	3	5	6.36	-0.1	0.03	1.47	0.08	99.98	-0.19	5.86	7.69	16.62	7.95	-1.33	12.59	22.3	11.91	1.46
os53479	2	331	0.3	11	3	5	6.91	0.1	0.03	1.46	0.1	96.01	0.25	5.55	7.72	16.63	7.98	-1.32	12.58	22.27	11.91	1.46
os53480	2	327	0.21	14	3	5	6.93	0.16	0	1.45	0.11	96.21	1.03	5.23	7.73	16.63	7.99	-1.32	12.59	22.28	11.92	1.46
os56832	2	317	0.01	2	3	6	6.78	0.03	0.28	1.54	0.01	76.32	0.24	7.57	7.98	16.96	8.22	-1.11	12.75	22.5	12.13	1.73
os56897	2	309	0.2	4	3	6	6.74	0.04	0.57	1.53	0.03	92.52	0.38	8.06	7.99	16.97	8.22	-1.11	12.75	22.52	12.14	1.73
os56918	2	589	0	10	3	5	7.01	0.16	0.09	1.48	0.06	97	1.64	6.27	6.38	15.17	6.86	-1.76	11.12	20.49	10.73	1
os57013	2	636	0.6	14	1	4	6.37	-0.08	0	1.44	0.08	93	2.34	5.58	6.15	14.96	6.7	-1.88	10.85	20.22	10.51	0.84
os57015	2	656	0.69	18	3	5	6.1	-0.15	0	1.41	0.1	92.81	1.65	5.51	6.11	14.93	6.67	-1.9	10.8	20.18	10.47	0.82
os57054	2	701	0.87	5	1	1	6.88	-0.02	0	1.46	0.02	96.95	4.5	6.7	5.89	14.63	6.45	-2.06	10.54	19.81	10.24	0.66
os57067	2	610	0.12	7	3	9	7.04	0.1	0.18	1.47	0.05	99.11	1.73	6.19	6.39	15.14	6.87	-1.75	11.1	20.39	10.66	0.99
os57120	2	642	1	8	3	5	6.62	-0.13	0.18	1.5	0.05	97.51	0.79	7.08	6.08	14.81	6.56	-1.98	10.83	20.1	10.44	0.8
os57122	2	526	0.67	22	1	2	6.55	-0.06	0	1.4	0.15	95.76	4.42	5.59	6.71	15.42	7.08	-1.55	11.45	20.78	10.98	1.21
os57134	2	642	0.44	4	3	6	6.96	0.02	0.64	1.52	0.03	96	-0.32	7.08	6.15	14.9	6.64	-1.91	10.89	20.18	10.5	0.87
os57157	2	563	0.79	4	1	4	6.71	-0.04	0.03	1.48	0.05	82.57	3.45	7.41	6.47	15.13	6.85	-1.53	11.08	20.34	10.47	1.11
os57160	2	627	0.96	9	3	5	6.48	-0.15	0.09	1.49	0.05	85.37	0.93	6.49	6.33	15.01	6.73	-1.62	10.97	20.26	10.39	1.04
os57161	2	429	0.6	24	1	2	6.64	0.03	0	1.36	0.14	72.2	3.19	5.17	7.24	15.92	7.44	-1.05	12.12	21.4	11.26	1.57
os57162	2	428	0.06	22	2	7	6.78	0.34	0	1.41	0.16	71.54	-2.07	5.36	7.33	16	7.51	-1.01	12.22	21.5	11.34	1.63
os57167	2	568	0.01	17	2	7	6.89	0.28	0.14	1.48	0.16	99.89	-6.06	7.52	6.36	15.05	6.77	-1.57	10.94	20.23	10.35	1.02
os57280	2	557	0.07	19	1	4	7.06	0.26	0	1.33	0.12	98.75	8.76	4.72	6.59	15.32	6.9	-1.64	11.43	20.7	10.86	1.15
os57360	2	675	0.39	4	1	1	6.84	0.01	0.21	1.42	0.03	99.52	2.5	6.21	6.1	14.76	6.6	-1.83	10.72	19.91	10.32	0.79
os57375	2	696	0.6	6	3	5	6.76	-0.02	0.22	1.51	0.04	91.89	1.25	7.52	5.76	14.48	6.29	-2.23	10.61	19.83	10.22	0.58
os57404	2	687	0.02	15	3	5	7.12	0.24	0	1.46	0.14	99.99	-0.51	5.45	5.72	14.5	6.32	-2.12	10.35	19.69	10.07	0.57
os57420	2	646	0.57	7	1	4	6.68	-0.03	0.11	1.47	0.03	86.29	3.16	6.16	6.15	14.86	6.6	-1.96	11.01	20.27	10.55	0.82
os57467	2	686	0.6	1	3	6	6.92	0	0.2	1.53	0.01	59.81	0.53	8.15	6.01	14.84	6.57	-1.94	10.63	20.04	10.36	0.79
os57493	2	565	0.81	19	2	0	5.92	-0.23	0	1.4	0.14	96.85	2.33	5.05	6.44	15.09	6.88	-1.61	11.1	20.32	10.59	1.01
os57505	2	578	0.98	13	2	7	6.24	-0.22	0.01	1.48	0.15	98.75	-2.14	5.58	6.28	14.97	6.75	-1.7	10.92	20.13	10.45	0.92
os57555	2	644	0	20	3	5	6.98	0.31	0	1.41	0.12	97.99	-0.06	5.46	6.1	14.8	6.55	-1.93	10.83	20.04	10.36	0.85
os57566	2	647	0.25	8	2	0	7.03	0.08	0.04	1.49	0.09	95.93	1.66	7.39	5.93	14.69	6.48	-2.06	10.64	19.93	10.29	0.67
os57621	2	709	0.06	16	3	5	7.13	0.24	0	1.44	0.08	95.96	1.81	5.73	5.8	14.53	6.37	-2.19	10.52	19.77	10.19	0.54
os57688	2	579	0.62	6	2	3	6.67	-0.04	1.64	1.52	0.13	98.56	-5.29	7.89	6.33	15.01	6.8	-1.68	11	20.18	10.52	0.94
os57762	2	645	0.73	12	1	4	6.79	-0.05	0	1.45	0.08	99.98	2.31	5.64	6.12	14.88	6.64	-1.9	10.78	20.07	10.44	0.82
os57826	2	681	0.58	2	3	6	6.85	-0.01	0.38	1.54	0.03	59.13	1.08	8.95	5.92	14.66	6.46	-2.09	10.66	19.94	10.29	0.65

os57827	2	665	0.99	8	1	2	6.64	-0.12	0.06	1.48	0.09	95.36	1.99	7.11	5.87	14.64	6.44	-2.1	10.58	19.88	10.24	0.63
os57829	2	642	0.88	5	2	0	6.82	-0.04	0.22	1.51	0.08	95.49	1.5	8.1	5.97	14.73	6.52	-2.04	10.69	19.98	10.33	0.7
os57837	2	611	0	16	3	5	7.05	0.27	0	1.45	0.13	87.88	0.37	5.9	6.09	14.8	6.58	-1.88	10.78	20.02	10.35	0.81
os57941	2	664	0.86	12	3	5	6.33	-0.19	0	1.44	0.08	99.88	-0.23	6.05	6.02	14.73	6.56	-1.82	10.69	19.88	10.25	0.8
os57957	2	632	0.99	4	3	6	6.8	-0.05	0.73	1.51	0.03	93.52	0.2	6.91	6.29	15.05	6.78	-1.8	11.04	20.37	10.68	0.94
os57961	2	617	0.4	2	1	4	6.88	0.02	0.01	1.47	0.02	97.18	4.34	9.06	6.3	15.06	6.78	-1.81	11.09	20.39	10.7	0.93
os57962	2	611	0.06	7	3	5	6.92	0.1	0.17	1.45	0.06	95.84	1.75	6.33	6.33	15.09	6.82	-1.77	11.09	20.41	10.72	0.97
os58012	2	538	1	7	1	4	6.62	-0.11	0.03	1.49	0.09	93.82	2.86	7.27	6.62	15.41	7.08	-1.57	11.33	20.74	11.01	1.15
os58087	2	618	0.6	2	3	6	6.82	-0.01	3.96	1.55	0.04	45.76	-1.67	8.96	6.23	14.97	6.75	-1.86	10.98	20.27	10.62	0.88
os58119	2	663	0.86	14	3	5	6.25	-0.19	0	1.46	0.09	99.44	-0.12	6.53	6.06	14.91	6.63	-1.87	10.69	20.13	10.45	0.83
os58299	2	658	0.07	6	3	9	6.9	0.06	0.73	1.5	0.05	93.96	-1.11	6.57	6.16	14.88	6.68	-1.88	10.94	20.18	10.56	0.87
os58393	2	466	0.77	8	1	4	6.49	-0.07	0	1.32	0.04	96.75	13.06	5.47	7.32	16.06	7.55	-0.98	12.1	21.44	11.25	1.58
os58408	2	510	0.96	3	3	6	6.76	-0.04	0.24	1.49	0.06	99.26	0.9	6.55	6.95	15.67	7.25	-1.2	11.6	20.96	10.85	1.32
os58474	2	586	0.13	24	2	7	6.51	0.23	0	1.42	0.19	92.33	-4.5	5.84	6.28	15.01	6.78	-1.83	11.07	20.35	10.66	0.92
os58486	2	640	0.19	18	1	4	6.64	0.15	0	1.41	0.09	91.89	4.19	5.55	6.09	14.82	6.61	-1.88	10.81	20.06	10.45	0.82
os58487	2	607	0.81	10	3	5	6.69	-0.09	0.03	1.45	0.08	98.62	1.51	6.2	6.31	15.02	6.8	-1.81	11.13	20.39	10.73	0.95
os58517	2	648	0.92	0	3	6	6.88	0	1.69	1.54	0.01	20.1	0.23	9.04	6.08	14.82	6.6	-1.88	10.81	20.08	10.43	0.81
os58575	2	680	0.16	5	3	5	7.04	0.08	0.35	1.52	0.04	93.82	0.64	7.54	5.98	14.81	6.55	-1.95	10.59	20.01	10.33	0.77
os58642	2	544	0.72	16	2	3	6.12	-0.15	0	1.42	0.16	98.46	-4.63	6.49	6.71	15.39	7.1	-1.43	11.46	20.69	10.85	1.18
os58653	2	492	0.83	8	1	4	6.85	0.01	0.01	1.36	0.05	99.5	7.74	5.58	7.17	15.91	7.47	-1.06	11.88	21.23	11.17	1.51
os58661	2	542	0.17	19	2	3	7.01	0.25	0	1.42	0.13	91.99	-2.4	6.28	6.75	15.5	7.17	-1.39	11.36	20.71	10.87	1.22
os58711	2	607	0.09	9	3	9	7.06	0.12	0.21	1.45	0.05	97.6	1	6.18	6.46	15.17	6.91	-1.44	10.94	20.26	10.43	1.06
os58753	2	648	0.6	2	3	6	6.83	-0.02	2.14	1.54	0.03	65.85	0.17	8.42	5.95	14.63	6.53	-1.78	10.41	19.69	10.09	0.73
os58775	2	792	0.79	4	3	6	6.79	-0.07	0.6	1.52	0.03	84.93	0.21	7.49	5.07	13.78	5.86	-2.34	9.42	18.64	9.31	0.14
os58827	2	689	0.13	7	1	4	7.06	0.09	0	1.43	0.03	99.64	5.65	6.27	5.85	14.52	6.45	-1.95	10.41	19.61	10.09	0.64
os58832	2	718	0.13	21	1	4	6.71	0.23	0	1.38	0.13	91.65	2.91	5.16	5.57	14.22	6.21	-2.15	10.15	19.29	9.87	0.48
os58833	2	717	0.09	10	3	5	6.93	0.12	0.07	1.44	0.06	90.66	-0.05	6.69	5.65	14.3	6.25	-2.13	10.24	19.39	9.95	0.51
os58835	2	709	0.04	23	3	5	6.8	0.31	0	1.39	0.15	93.23	0.26	5.48	5.57	14.22	6.21	-2.14	10.15	19.29	9.87	0.47
os58858	2	658	0.91	15	2	3	6.44	-0.19	0	1.47	0.12	100	-2.13	6.97	5.91	14.57	6.46	-1.94	10.51	19.67	10.14	0.66
os58862	2	644	0.67	19	1	4	6.68	-0.06	0	1.4	0.11	99.96	1.74	5.57	6.01	14.67	6.54	-1.89	10.64	19.79	10.24	0.71
os58894	2	506	0.76	9	2	0	6.47	-0.1	0.17	1.52	0.09	99.99	-1.85	6.2	6.85	15.58	7.14	-1.33	11.59	20.91	10.8	1.21
os58895	2	527	0.85	14	3	5	6.15	-0.2	0	1.48	0.11	100	-1.53	5.77	6.81	15.53	7.1	-1.35	11.53	20.86	10.76	1.18
os59012	2	735	0.35	11	1	4	6.69	0.01	0.02	1.47	0.07	91.83	1.91	5.95	5.5	14.13	6.14	-2.24	10.07	19.22	9.82	0.41
os59032	2	703	0	17	1	1	7.08	0.29	0	1.37	0.08	97.84	5.9	5.56	5.86	14.54	6.45	-1.93	10.4	19.61	10.08	0.64
os59063	2	804	0.92	4	3	6	6.82	-0.06	0.65	1.52	0.02	65.35	0.95	7.71	5.05	13.74	5.85	-2.36	9.39	18.6	9.29	0.11
os59187	2	685	0.24	13	3	5	6.7	0.05	0.04	1.47	0.09	98.98	-0.32	7.03	5.81	14.51	6.3	-2.13	10.52	19.7	10.1	0.65
os59770	2	528	0.93	6	3	5	6.67	-0.09	0.59	1.52	0.05	94.22	-0.62	7.28	6.74	15.68	7.14	-1.94	11.29	20.97	10.78	0.78

os59771	2	523	0.95	6	3	5	6.65	-0.1	1.03	1.53	0.06	92.74	-2.25	8.02	6.75	15.69	7.15	-1.93	11.3	20.98	10.78	0.79
os59773	2	557	0.71	4	3	6	6.82	-0.03	1.45	1.53	0.04	98.22	-0.89	7.9	6.66	15.61	7.08	-1.98	11.2	20.88	10.7	0.73
os59860	2	572	0.08	6	3	5	7	0.09	0.33	1.51	0.04	88.87	1.02	7.45	6.47	15.39	6.92	-2.12	10.97	20.6	10.49	0.63
os59879	2	463	0.22	9	1	4	6.78	0.09	0.11	1.44	0.06	93.29	4.11	6.58	7.17	16.07	7.48	-1.66	11.82	21.49	11.22	1.12
os59883	2	475	0.91	5	2	3	6.75	-0.05	1.79	1.55	0.08	99.45	-3.6	8.37	6.97	15.87	7.32	-1.79	11.56	21.22	10.99	0.98
os59982	2	437	0.13	11	3	5	6.8	0.13	0.02	1.48	0.07	83.89	0.38	6.21	7.25	16.21	7.58	-1.56	11.99	21.67	11.41	1.22
os60165	2	655	0.05	3	3	6	6.97	0.05	0.77	1.52	0.03	93.93	0.95	9.08	6.08	15.02	6.58	-2.38	10.4	20.02	9.96	0.25
os60391	2	677	0.6	4	3	6	6.79	-0.03	0.6	1.52	0.03	99.59	0.09	7.12	6.01	14.95	6.52	-2.4	10.33	19.95	9.92	0.22
os60418	2	370	0.66	15	3	5	6.13	-0.12	0	1.48	0.11	66.49	-1.76	5.89	7.87	16.73	7.95	-1.28	12.66	22.2	11.71	1.44
os60481	2	424	0.05	8	3	5	6.87	0.11	0.21	1.5	0.07	96	0.16	6.58	7.59	16.53	7.76	-1.42	12.27	21.89	11.39	1.26
os60814	2	478	0.83	3	2	3	6.75	-0.04	1.7	1.55	0.07	99.34	-3.13	8.36	6.96	15.86	7.31	-1.8	11.54	21.2	10.97	0.96
os60965	2	362	0.92	2	3	6	6.7	-0.03	1.53	1.55	0.02	90.33	0.33	8.95	7.82	16.61	8.07	-1.04	12.71	22.37	11.99	1.72
os60983	2	368	0.99	4	3	6	6.67	-0.06	0.6	1.54	0.03	99.33	-0.4	7.22	7.75	16.57	8.04	-1.08	12.64	22.31	11.93	1.7
os61001	2	427	0.08	2	3	6	6.84	0.03	2.42	1.53	0.06	77.41	0.58	8.71	7.36	16.18	7.75	-1.35	12.13	21.82	11.53	1.37
os61010	2	463	0.01	3	3	6	6.72	-0.05	0.46	1.52	0.01	83.33	0.51	7.34	7.32	16.16	7.7	-1.37	12.11	21.81	11.5	1.37
os61086	2	414	0.25	6	2	3	6.74	0.04	0.7	1.52	0.09	92.29	-2.11	7.1	7.27	16.05	7.57	-1.59	12.11	21.72	11.34	1.09
os61100	2	407	0.99	4	3	6	6.68	-0.06	0.4	1.53	0.03	85.14	0.44	7.72	7.34	16.12	7.62	-1.57	12.21	21.82	11.45	1.16
os61127	2	400	0.89	3	3	6	6.71	-0.04	1.92	1.55	0.06	90.83	-1.18	7.16	7.39	16.2	7.69	-1.51	12.24	21.87	11.5	1.19
os61304	2	440	0.01	5	3	5	6.92	0.09	0.52	1.51	0.05	88	0.55	7.08	7.23	16.1	7.58	-1.48	11.99	21.76	11.33	1.22
os61424	2	409	0.99	8	3	5	6.49	-0.13	0.14	1.49	0.05	98.53	2.02	6.43	7.3	16.11	7.58	-1.55	12.23	21.86	11.48	1.17
os61528	2	426	0.79	7	3	6	6.73	-0.05	0.32	1.51	0.06	83.7	0.38	8.8	7.08	15.89	7.43	-1.78	11.9	21.49	11.22	0.95
os61576	2	379	0.34	8	3	5	6.47	-0.13	0.33	1.52	0.05	90.03	-1.89	6.94	7.61	16.48	7.87	-1.42	12.66	22.34	11.87	1.36
os61581	2	357	0.92	5	2	3	6.58	-0.07	1.64	1.56	0.07	58.76	-2.97	7.56	7.65	16.51	7.88	-1.36	12.7	22.37	11.88	1.41
os61610	2	373	0.39	9	3	5	6.41	-0.14	0.2	1.5	0.1	88.88	-1.14	6.8	7.43	16.21	7.69	-1.5	12.46	22.04	11.61	1.27
os61636	2	390	0.67	4	2	7	6.64	-0.05	1.83	1.55	0.08	88.1	-2.24	8.1	7.28	16.09	7.58	-1.57	12.19	21.81	11.43	1.15
os61644	2	454	0.12	9	3	5	6.46	-0.11	0.3	1.45	0.06	98.88	0.81	6.05	7.17	15.96	7.5	-1.65	12.04	21.64	11.28	1.07
os61697	2	368	0.01	5	3	5	6.88	0.09	0.52	1.53	0.05	90.94	0.2	7.35	7.59	16.39	7.85	-1.28	12.42	22.14	11.64	1.41
os61763	2	449	0.16	7	1	1	6.97	0.09	0.05	1.39	0.04	98.99	4.52	5.95	7.36	16.2	7.65	-1.41	12.21	21.95	11.48	1.31
os61826	2	463	0.02	2	3	6	6.83	0.03	0.42	1.49	0.02	67.18	0.69	9.07	7.3	16.16	7.57	-1.5	12.17	21.81	11.46	1.22
os61840	2	454	0.99	2	3	6	6.76	-0.03	3.79	1.56	0.03	41.78	-0.62	9.22	7.18	16.06	7.5	-1.59	12.03	21.79	11.37	1.15
os62098	2	437	0.99	1	3	6	6.77	-0.02	2.8	1.55	0.01	41.07	0.37	8.88	7.25	16.08	7.55	-1.6	12.03	21.71	11.28	1.14
os62115	2	443	0.08	2	3	6	6.84	0.03	2.82	1.56	0.02	51.03	-0.75	9.09	7.27	16.17	7.55	-1.57	12.2	21.96	11.48	1.21
os62119	2	487	0.01	10	3	5	6.96	0.16	0.11	1.46	0.07	96.74	-1.13	6.28	6.98	15.87	7.37	-1.78	11.69	21.31	11.11	0.97
os62308	2	449	0.77	3	3	6	6.78	-0.02	2.98	1.55	0.02	38.52	0.15	8.86	7.27	16.16	7.55	-1.6	12.17	21.97	11.47	1.17
os62365	2	488	0.37	9	3	5	6.64	0.02	0.11	1.46	0.05	87.59	1.8	6.97	7.19	16.03	7.45	-1.6	11.94	21.57	11.23	1.1
os62394	2	480	0.23	10	3	5	6.76	0.09	0.04	1.45	0.08	99.41	-0.17	6.44	7.21	16.03	7.47	-1.59	11.89	21.45	11.13	1.07
os62482	2	354	0.32	6	2	3	6.71	0.03	0.74	1.53	0.09	72.8	-3.25	7.51	7.67	16.52	7.91	-1.36	12.66	22.34	11.91	1.41

os62515	2	470	0	2	3	6	6.84	0.03	0.33	1.51	0.02	97.17	0.5	7.28	7.19	16.02	7.5	-1.67	12	21.6	11.33	1.1
os62516	2	438	0.27	10	3	5	6.98	0.11	0.24	1.51	0.08	98.03	-0.77	7.97	7.26	16.1	7.56	-1.63	12.09	21.69	11.42	1.15
os62705	2	460	0.32	5	2	0	6.92	0.05	0.5	1.53	0.07	88.63	0.02	8.07	7.1	15.93	7.38	-1.69	11.67	21.26	10.99	1
os62737	2	482	0.92	10	3	5	6.35	-0.17	0.16	1.46	0.06	95.04	1.13	7.18	7.14	16.02	7.43	-1.63	11.76	21.42	11.15	1.06
os62854	2	581	0.7	5	3	5	6.65	-0.08	0.41	1.52	0.05	88.99	0.26	8.07	6.43	15.21	6.83	-2.23	11.11	20.66	10.47	0.4
os62973	2	472	0.8	2	3	9	6.74	-0.03	0.59	1.45	0.01	92.42	0.92	7.22	7.32	16.14	7.69	-1.34	12.02	21.73	11.45	1.37
os62991	2	375	0.6	2	3	6	6.72	-0.02	0.46	1.49	0.03	83.29	0.31	8.18	7.78	16.56	8.08	-1.05	12.62	22.22	11.92	1.71
os63032	2	519	0.6	4	3	6	6.72	-0.02	0.21	1.5	0.03	92.62	1.23	6.77	6.85	15.63	7.34	-1.62	11.37	21.07	10.86	0.99
os63201	2	328	0.79	2	3	6	6.67	-0.04	2.45	1.55	0.02	68.08	-0.01	9.1	8.06	16.86	8.33	-0.7	12.98	22.52	12.23	2.12
os63426	2	445	0.92	5	3	5	6.7	-0.06	0.28	1.52	0.03	94.77	1.29	6.95	7.43	16.24	7.81	-1.23	12.16	21.78	11.49	1.51
os63427	2	455	0.79	4	3	6	6.75	-0.04	0.35	1.52	0.02	93.74	0.76	6.81	7.44	16.24	7.8	-1.22	12.16	21.77	11.49	1.53
os63751	2	401	0.01	3	1	4	6.73	0.01	0.04	1.39	0.02	84.92	7.86	6.39	7.83	16.73	7.93	-1.22	12.61	22.26	11.79	1.55
os63834	2	521	0.01	9	3	5	7.02	0.15	0.1	1.46	0.05	99.99	1.47	6.4	7	15.88	7.29	-1.74	11.64	21.26	10.95	0.98
os63890	2	482	0.35	23	2	3	6.8	0.17	0	1.42	0.16	98.81	-3.64	6.02	7.07	16	7.36	-1.69	11.7	21.37	11.07	1.02
os63893	2	557	0.78	9	1	1	6.75	-0.06	0.01	1.43	0.04	99.53	3.63	5.26	6.94	15.88	7.25	-1.8	11.6	21.23	10.95	0.95
os63969	2	448	0.08	12	2	7	7.02	0.19	0.03	1.5	0.13	95.55	-3.75	7.02	7.15	16.05	7.41	-1.66	11.79	21.46	11.13	1.06
os64041	2	400	0.7	8	2	3	6.7	-0.06	0.68	1.52	0.08	94.55	-1.77	7.17	7.72	16.62	7.83	-1.28	12.53	22.14	11.69	1.45
os64108	2	389	0.85	10	3	9	6.53	-0.12	0.04	1.45	0.06	99.08	1.72	6.65	7.93	16.85	8.02	-1.14	12.75	22.37	11.83	1.59
os64210	2	389	0.92	6	3	5	6.53	-0.09	0.41	1.51	0.07	93.27	-0.02	7.01	7.46	16.34	7.73	-1.32	12.01	21.66	11.35	1.4
os64270	2	448	0.85	16	1	1	6.01	-0.22	0	1.37	0.08	97.8	4.13	5.34	7.24	16.11	7.55	-1.54	11.88	21.53	11.22	1.18
os64672	2	358	0.19	8	3	5	6.94	0.11	0.21	1.48	0.06	98.58	1.44	6.05	7.52	16.36	7.79	-1.38	12.27	21.87	11.58	1.33
os64739	2	435	0.92	3	3	6	6.7	-0.04	2.82	1.55	0.04	56.43	-0.85	8.03	7.35	16.18	7.59	-1.48	12.23	21.89	11.49	1.26
os65138	2	375	0.04	2	3	6	6.7	-0.04	0.54	1.54	0	87.54	0.25	8.73	7.16	15.96	7.79	-0.99	11.76	21.16	11.48	1.81
os65271	2	543	0.01	7	2	3	6.94	0.11	0.79	1.53	0.07	94.94	-2.82	7.67	7.21	16.29	7.4	-1.69	11.87	21.84	11.1	1.05
os65278	2	458	0.25	8	3	5	6.97	0.09	0.35	1.51	0.07	93.15	-0.48	7.07	7.77	16.93	7.85	-1.34	12.54	22.58	11.72	1.46
os65531	2	461	0.64	3	2	3	6.71	-0.03	1.85	1.54	0.05	79.64	-1.97	8.38	7.7	16.81	7.79	-1.4	12.44	22.43	11.6	1.38
os65556	2	462	0.91	2	3	6	6.8	-0.01	0.02	1.53	0.01	73.5	1.77	8.59	7.77	16.89	7.86	-1.37	12.53	22.55	11.69	1.44
os65806	2	495	0.17	5	3	5	6.94	0.06	0.51	1.51	0.04	93.58	0.17	6.73	7.64	16.8	7.78	-1.35	12.36	22.32	11.57	1.45
os65885	2	582	0.39	2	3	6	6.83	-0.02	1.97	1.53	0.02	70.27	1.15	8.3	6.96	15.99	7.2	-1.86	11.62	21.54	10.91	0.86
os65915	2	496	0.89	13	2	3	6.53	-0.14	0.11	1.49	0.11	98.97	-3.84	6.32	7.47	16.53	7.58	-1.56	12.19	22.11	11.32	1.21
os65934	2	458	0.11	8	3	5	6.98	0.11	0.63	1.51	0.06	83.02	-0.7	8.53	7.55	16.66	7.68	-1.47	12.27	22.17	11.43	1.29
os65975	2	539	0.76	9	3	5	6.48	-0.15	0.11	1.47	0.04	94.28	1.11	6.04	7.16	16.21	7.41	-1.75	11.82	21.76	11.18	0.96
os66043	2	504	0.93	11	2	3	6.6	-0.12	0.37	1.48	0.08	99.27	-2.25	7.25	7.38	16.42	7.51	-1.62	12.12	22.05	11.32	1.14
os66064	2	535	0.03	13	2	8	6.94	0.2	0.01	1.45	0.08	99.14	-1.58	5.42	7.46	16.53	7.6	-1.55	12.21	22.15	11.38	1.18
os66073	2	461	0.75	10	2	7	6.33	-0.12	0.84	1.52	0.18	94.71	-6.68	6.74	7.45	16.52	7.6	-1.55	12.19	22.13	11.38	1.19
os66119	2	475	0.08	14	3	5	7.05	0.22	0	1.46	0.09	97.66	1.09	5.68	7.43	16.48	7.56	-1.58	12.2	22.12	11.36	1.18
os66388	2	499	0.27	10	1	4	6.59	0.01	0.04	1.42	0.06	89.86	3.74	6.17	7.39	16.39	7.55	-1.63	12.11	21.94	11.37	1.09

os66426	2	425	0.66	6	3	5	6.76	-0.03	0.75	1.49	0.05	95.68	-0.41	7.16	7.78	16.78	7.87	-1.35	12.5	22.31	11.67	1.36
os66441	2	326	0.71	23	3	5	5.55	-0.24	0	1.36	0.21	89.69	1.27	5.04	8.19	17.13	8.19	-1.02	12.86	22.57	11.92	1.67
os66510	2	422	0.65	13	2	3	6.21	-0.18	0.26	1.44	0.15	98.27	-5.41	6.83	7.83	16.85	7.91	-1.31	12.55	22.35	11.69	1.4
os66595	2	396	0.24	19	3	9	6.89	0.17	0	1.4	0.11	99.74	-0.4	5.43	8.15	17.13	8.16	-1.03	12.8	22.53	11.86	1.67
os66712	2	487	0.92	1	3	6	6.84	0.02	2.97	1.54	0.05	85.88	-1.15	9.11	7.5	16.54	7.65	-1.48	12.11	21.89	11.33	1.21
os66713	2	487	0.09	2	3	6	6.86	0.03	2.92	1.54	0.05	86.37	-1.16	9.06	7.5	16.54	7.65	-1.48	12.11	21.89	11.33	1.22
os66715	2	493	0.92	1	3	6	6.8	0	2.99	1.53	0.03	77.6	-1.08	9.1	7.48	16.51	7.63	-1.49	12.04	21.84	11.28	1.21
os66733	2	536	0.84	5	3	5	6.74	-0.06	0.48	1.48	0.04	99.78	1.23	6.05	7.11	16.12	7.37	-1.65	11.61	21.4	10.94	1.03
os66809	2	567	0.81	10	2	8	6.43	-0.13	0.04	1.47	0.1	99.48	-2.07	5.93	6.87	15.77	7.18	-1.82	11.41	21.05	10.76	0.85
os66820	2	465	0.85	14	1	1	6.52	-0.13	0	1.41	0.1	99.66	2.26	5.42	7.51	16.39	7.7	-1.49	12.12	21.8	11.36	1.15
os66850	2	483	0.44	15	3	5	6.33	-0.04	0	1.41	0.12	98.89	0.8	5.61	7.34	16.28	7.52	-1.66	11.94	21.68	11.19	1.01
os67137	2	311	0.99	7	3	5	6.49	-0.12	0.28	1.51	0.07	88.09	-1.01	7.19	8.91	18.02	8.78	-0.53	13.72	23.5	12.61	2.28
os67341	2	511	0.08	1	3	6	6.87	0.02	1.86	1.55	0.02	46.96	-0.33	8.68	7.38	16.47	7.55	-1.67	12.06	21.99	11.32	1.07
os67364	2	420	0.05	8	3	5	6.86	0.11	0.27	1.52	0.05	85.82	0.09	6.91	8.02	17.09	8.03	-1.23	12.81	22.72	11.87	1.52
os67382	2	415	0.23	4	1	4	6.79	0.03	0.16	1.45	0.02	93.03	3.08	6.43	8.14	17.28	8.16	-1.15	12.92	22.89	12.01	1.63
os67521	2	457	0.08	4	3	6	6.9	0.05	0.72	1.53	0.03	91.66	0.2	8.17	7.88	16.98	7.98	-1.23	12.4	22.33	11.58	1.47
os67544	2	370	0.38	8	2	3	6.87	0.05	0.9	1.53	0.1	96.65	-3.36	8.13	8.21	17.26	8.22	-0.99	12.84	22.58	11.9	1.72
os67556	2	359	0.7	7	2	7	6.78	-0.01	0.92	1.53	0.09	94.92	-3.1	8.41	8.26	17.3	8.25	-0.97	12.89	22.63	11.94	1.75
os67659	2	391	0.6	9	2	3	6.68	-0.06	0.27	1.51	0.1	83.71	-2.66	6.97	8.05	17.13	8.05	-1.18	12.85	22.74	11.88	1.57
os67664	2	443	0.68	2	3	6	6.82	0	2.22	1.52	0.03	60.83	1.71	8.23	7.86	17	7.97	-1.26	12.61	22.59	11.79	1.53
os67666	2	457	0	4	3	6	6.89	0.07	0.62	1.53	0.03	75.7	-0.19	7.15	7.84	17.03	7.96	-1.2	12.54	22.58	11.74	1.57
os67707	2	518	0.18	8	3	9	6.89	0.11	0.26	1.47	0.06	97.18	0.74	6.44	7.36	16.42	7.53	-1.63	12.09	22.04	11.33	1.13
os67759	2	488	0.09	8	2	3	6.8	0.08	0.79	1.53	0.13	99.2	-5.6	8.34	7.35	16.42	7.52	-1.63	12.08	22.01	11.29	1.11
os67761	2	511	0.81	8	3	5	6.52	-0.1	0.2	1.49	0.04	95.59	2.15	7.01	7.35	16.4	7.53	-1.64	12.05	21.99	11.3	1.11
os67790	2	416	0.01	7	1	4	6.95	0.13	0.25	1.48	0.05	79.55	2.99	6.55	7.88	16.92	7.93	-1.32	12.62	22.51	11.72	1.45
os67797	2	568	0.19	9	3	9	6.8	0.07	0.1	1.46	0.09	99.68	-0.14	5.73	6.83	15.72	7.19	-1.84	11.33	20.96	10.75	0.83
os67801	2	591	0.08	2	1	1	6.87	0.01	0.25	1.38	0.02	99.46	3.34	7.11	6.85	15.77	7.17	-1.96	11.46	21.15	10.83	0.73
os67945	2	514	0.67	15	3	5	6.22	-0.13	0	1.43	0.1	99.54	-0.14	5.79	7.27	16.23	7.46	-1.72	11.94	21.7	11.21	1
os70187	2	357	0.52	11	2	3	6.76	0	0.14	1.48	0.14	99.98	-4.15	6.85	8.31	17.26	8.38	-0.95	13.03	22.73	12.26	1.93
os70189	2	348	0.39	16	2	3	6.8	0.09	0.01	1.46	0.16	99.96	-5.52	6.53	8.32	17.27	8.39	-0.95	13.04	22.75	12.27	1.94
os70231	2	371	0.69	7	3	9	6.75	-0.03	0.28	1.45	0.05	98.1	1.17	6.27	8.35	17.31	8.41	-0.91	12.99	22.71	12.17	1.9
os70232	2	370	0.76	7	3	9	6.75	-0.03	0.3	1.45	0.05	97.97	1.04	6.28	8.35	17.31	8.41	-0.91	12.99	22.71	12.17	1.9
os74208	2	505	0.56	15	2	7	6.78	0.02	0.19	1.52	0.17	97.32	-6.15	7.88	6.47	15.32	7.14	-2.11	11.11	20.59	11.05	1.15
os75318	2	509	0.97	11	3	9	6.48	-0.16	0.02	1.43	0.08	99.99	1.05	5.88	7.05	15.99	7.48	-1.87	11.85	21.47	11.38	1.18
os76406	2	443	0.18	7	1	1	6.81	0.08	0.08	1.4	0.05	100	4.13	5.81	7.77	16.55	8.12	-0.73	12.08	21.4	11.45	1.68
os76796	2	273	0.99	8	2	0	6.32	-0.14	0.06	1.48	0.13	99.29	0.99	6.09	8.48	17.29	8.68	-0.08	12.9	22.23	12.11	2.44
os76874	2	347	0.7	11	3	5	6.71	-0.04	0.04	1.49	0.07	97.07	1.33	7.22	7.93	16.68	8.22	-0.68	12.46	21.7	11.72	1.83

os76884	2	579	0.9	4	3	9	6.71	-0.07	0.26	1.49	0.02	95.6	1.51	6.59	6.92	15.73	7.33	-1.6	11.4	20.74	10.72	0.87
os77079	2	415	0.08	9	3	5	6.99	0.14	0.07	1.5	0.07	100	0.68	6.32	7.53	16.31	7.9	-0.94	11.98	21.27	11.33	1.51
os77294	2	506	0.01	2	3	6	6.85	0.02	0.25	1.52	0.02	83.82	0.21	8.02	7.59	16.48	7.82	-1.16	12.24	21.59	11.38	1.43
os77333	2	447	0.06	8	2	7	6.77	0.09	0.66	1.51	0.18	99.4	-5.67	6.82	7.36	16.16	7.7	-1.18	11.82	21.1	11.1	1.3
os77496	2	495	0.3	9	3	5	6.99	0.09	0.06	1.46	0.07	97.74	1.99	6.91	7.36	16.17	7.66	-1.13	11.64	20.97	10.96	1.3
os77497	2	491	0.34	10	3	5	6.98	0.09	0.04	1.45	0.07	98.64	1.61	6.95	7.37	16.18	7.68	-1.12	11.66	20.98	10.97	1.32
os77774	2	369	0.08	3	1	4	6.84	0.03	0.15	1.51	0.02	85.84	2.49	7.85	8.02	16.98	8.2	-0.73	13.28	22.92	12.57	2.4
os77946	2	432	0.04	5	3	5	6.63	-0.03	0.23	1.49	0.06	98.78	1.47	6.29	7.51	16.4	7.77	-1	12.58	22.04	11.94	2.11
os78101	2	352	0	3	3	6	6.65	-0.05	0.91	1.54	0.02	84.38	-0.15	7.41	8.06	17.01	8.24	-0.71	13.33	22.97	12.61	2.43
os78152	2	419	0.92	6	3	9	6.54	-0.1	0.42	1.47	0.04	100	0.01	6.14	7.85	16.8	8.08	-0.81	12.8	22.43	12.2	2.15
os78181	2	503	0.08	11	3	5	7.05	0.18	0.06	1.48	0.07	96.55	0.41	6.66	7.01	15.94	7.37	-1.38	11.92	21.5	11.46	1.48
os78261	2	409	1	6	3	5	6.59	-0.1	0.21	1.5	0.03	98.8	1.85	7.41	7.58	16.53	7.85	-0.95	12.54	22.17	11.91	1.99
os78293	2	412	0.07	4	3	6	6.64	-0.07	0.58	1.54	0.05	98.98	-0.13	6.81	7.53	16.41	7.73	-1.02	12.69	22.15	12.02	2.17
os78335	2	351	0.92	2	3	6	6.68	-0.04	1.53	1.56	0.03	58.23	-0.39	7.51	7.95	16.88	8.14	-0.72	13.2	22.82	12.5	2.34
os78414	2	465	0.92	3	3	6	6.69	-0.05	1.84	1.54	0.06	96.34	-1.18	7.44	7.18	16.09	7.48	-1.19	12.27	21.77	11.69	1.96
os78508	2	363	0.72	5	3	6	6.62	-0.06	0.21	1.53	0.03	55.58	0.83	7.5	7.89	16.89	8.13	-0.74	13.1	22.81	12.46	2.29
os78528	2	437	0.54	8	3	5	6.56	-0.04	0.59	1.52	0.07	91.08	-2.11	7.93	7.21	16.12	7.48	-1.06	12.39	21.82	11.63	2.03
os78539	2	418	0.48	7	3	5	6.63	-0.02	0.52	1.52	0.06	85.38	0	7.03	7.35	16.29	7.65	-1.08	12.55	22.11	11.93	2.06
os78543	2	445	0.95	7	3	5	6.52	-0.12	0.18	1.49	0.05	77.41	1.58	6.69	7.28	16.22	7.59	-1.12	12.45	22.01	11.87	2.03
os78660	2	468	0.99	7	2	3	6.59	-0.11	0.28	1.5	0.07	99.32	-2.31	5.72	7.25	16.15	7.54	-1.15	12.36	21.86	11.78	2.02
os78711	2	498	1	11	3	9	6.39	-0.18	0.07	1.47	0.08	99.99	0.63	5.88	7.15	16.05	7.39	-1.19	12.31	21.72	11.71	2.09
os78726	2	586	0.84	8	3	9	6.53	-0.11	0.2	1.47	0.07	100	0.79	5.78	6.68	15.58	7.06	-1.46	11.75	21.15	11.27	1.77
os78756	2	501	0.67	15	2	3	6.19	-0.15	0	1.48	0.12	100	-3.15	5.49	6.88	15.79	7.24	-1.35	12	21.4	11.46	1.84
os78807	2	494	0.43	5	3	6	6.9	0.03	0.75	1.52	0.06	98.77	-0.4	6.54	7.18	16.1	7.47	-1.19	12.25	21.77	11.72	2.02
os78809	2	452	0.72	3	3	6	6.68	-0.05	0.91	1.53	0.04	94.28	0.23	7.86	7.3	16.25	7.58	-1.12	12.43	21.94	11.88	2.07
os78852	2	419	0.6	2	3	6	6.73	-0.02	2.8	1.54	0.04	86.59	-0.72	8.01	7.65	16.59	7.83	-1	12.91	22.45	12.19	2.16
os78861	2	516	0.77	2	3	6	6.84	-0.01	1.46	1.54	0.03	62.95	-0.25	8.18	7.03	15.94	7.34	-1.39	11.96	21.44	11.45	1.66
os78956	2	385	0.99	1	3	6	6.74	-0.02	5.87	1.55	0.01	15.15	0.16	8.9	7.8	16.74	8.04	-0.87	12.98	22.57	12.35	2.21
os78973	2	389	0.08	4	3	6	6.88	0.07	1.17	1.53	0.03	90.76	-0.4	7.73	7.96	16.94	8.16	-0.79	13.06	22.71	12.39	2.27
os79106	2	452	0	3	3	6	6.89	0.05	1.08	1.54	0.02	76.68	0.27	7.76	7.28	16.18	7.57	-1.02	12.36	21.85	11.69	2.03
os79119	2	518	0.87	5	1	4	6.67	-0.05	0.03	1.49	0.03	82.96	2.92	7.51	6.95	15.82	7.26	-1.22	11.86	21.28	11.26	1.7
os79127	2	565	0.54	3	3	6	6.78	-0.01	0.44	1.51	0.02	93.32	0.86	7.1	6.76	15.63	7.1	-1.34	11.61	21.04	11.07	1.58
os79130	2	443	0.64	3	3	6	6.71	-0.03	1.51	1.55	0.02	53.67	-0.15	8.41	7.25	16.11	7.5	-1.09	12.38	21.76	11.61	1.87
os79218	2	456	0.4	5	3	6	6.89	0.03	0.81	1.52	0.04	97	-0.41	7.45	7.12	16.06	7.45	-1.12	12.28	21.78	11.64	1.95
os79222	2	437	0.29	3	2	3	6.83	0.01	1.89	1.54	0.07	96.86	-2.77	7.53	7.26	16.19	7.55	-1.08	12.43	21.92	11.76	2
os79227	2	473	0.05	3	3	6	6.87	0.05	0.1	1.52	0.02	87.47	1.81	7.93	7.19	16.13	7.54	-1.07	12.15	21.77	11.61	1.88
os79230	2	464	0.29	3	3	6	6.7	-0.05	0.62	1.53	0.02	94.25	0.23	7.65	7.2	16.11	7.5	-1.06	12.15	21.69	11.58	1.9

os79353	2	425	0.99	8	3	5	6.55	-0.12	0.49	1.52	0.05	94.68	-0.78	7.7	7.31	16.23	7.6	-1.02	12.47	21.96	11.79	2.05
os79397	2	484	0.35	7	3	5	6.95	0.07	0.34	1.52	0.05	96.68	-0.46	6.77	7.11	15.95	7.39	-1.13	12.06	21.47	11.42	1.81
os79433	2	390	0.23	3	3	6	6.75	0.02	1.12	1.54	0.03	83.99	-0.36	7.44	7.69	16.66	7.94	-0.86	12.95	22.57	12.27	2.25
os79462	2	424	0.91	6	3	5	6.58	-0.08	0.65	1.53	0.04	89.6	-0.61	7.62	7.41	16.33	7.68	-1.03	12.6	22.11	11.92	2.1
os79540	2	453	0.09	3	3	6	6.9	0.05	1.36	1.53	0.02	80.12	-0.12	7.58	7.28	16.18	7.57	-1.02	12.36	21.84	11.69	2.03
os79542	2	452	0.07	4	3	6	6.91	0.06	1.1	1.53	0.02	80.16	-0.24	7.66	7.29	16.19	7.57	-1.02	12.36	21.84	11.69	2.03
os79648	2	404	0.73	7	3	5	6.53	-0.11	0.37	1.52	0.03	73.2	0.64	7	7.55	16.4	7.71	-0.83	12.84	22.19	11.98	2.27
os79721	2	526	0.92	1	3	6	6.82	0	0.72	1.53	0.01	66.75	0.22	8.65	6.92	15.86	7.28	-1.25	11.8	21.39	11.3	1.69
os79727	2	491	0.61	3	3	6	6.85	0	0.47	1.55	0.02	75.58	0.35	8.22	7.06	15.85	7.31	-1.19	12.07	21.39	11.35	1.72
os79762	2	416	0.56	4	2	3	6.7	-0.02	1.79	1.52	0.07	84.68	-3.48	7.27	7.47	16.34	7.64	-0.9	12.83	22.15	11.95	2.22
os79773	2	401	0.3	4	3	6	6.65	-0.07	0.62	1.53	0.04	91.03	-0.33	7.28	7.46	16.38	7.7	-0.9	12.57	22.1	11.9	2.16
os79776	2	415	0	5	3	6	6.88	0.07	0.48	1.52	0.03	77.2	0.95	7.49	7.48	16.41	7.77	-0.94	12.57	22.17	11.96	2.06
os79777	2	445	0.23	4	3	6	6.85	0.06	0.93	1.53	0.04	93.82	-0.82	7.14	7.33	16.27	7.6	-0.97	12.37	21.91	11.75	2.06
os79785	2	396	0.99	6	3	5	6.62	-0.09	0.62	1.53	0.05	85.33	0.21	7.83	7.47	16.39	7.71	-0.89	12.57	22.11	11.91	2.16
os79790	2	402	1	6	3	5	6.6	-0.09	0.48	1.53	0.04	91.93	-0.24	7.24	7.46	16.37	7.7	-0.9	12.57	22.1	11.91	2.16
os79791	2	419	0.6	3	3	6	6.71	-0.02	1.9	1.52	0.04	88.85	-1.59	8.3	7.46	16.36	7.7	-0.91	12.54	22.09	11.9	2.15
os79803	2	461	0.08	5	2	3	6.91	0.06	2.06	1.54	0.06	83.4	-2.49	8.75	7.2	16.11	7.47	-1.1	12.27	21.71	11.6	1.91
os79807	2	428	0.72	3	2	3	6.81	0	1.91	1.55	0.04	96.78	-1.87	9.07	7.32	16.21	7.56	-1	12.39	21.88	11.72	2.03
os79808	2	421	0.23	3	2	3	6.83	0.05	1.77	1.56	0.09	82.45	-3.89	8.36	7.36	16.24	7.6	-0.99	12.44	21.91	11.74	2.05
os79811	2	468	0.09	4	3	6	6.93	0.07	0.52	1.53	0.03	86.27	0.83	7.69	7.15	16.05	7.44	-1.13	12.17	21.65	11.53	1.86
os79814	2	401	0.01	3	2	3	6.85	0.04	1.92	1.55	0.06	85.42	-2.29	9.42	7.47	16.37	7.72	-0.92	12.62	22.15	11.94	2.18
os79821	2	401	0.08	5	3	6	6.91	0.08	0.73	1.53	0.03	94.2	-0.34	7.05	7.59	16.48	7.78	-0.86	12.75	22.25	12.02	2.24
os79875	2	432	0.32	7	3	5	6.91	0.05	0.36	1.51	0.05	95.7	-0.33	8.1	7.25	16.18	7.55	-1.04	12.4	21.88	11.74	2.04
os79878	2	427	0.77	1	3	6	6.81	0	2.63	1.55	0.05	95.06	-1.56	8.61	7.27	16.19	7.56	-1.04	12.42	21.9	11.75	2.04
os79880	2	441	0.09	5	3	6	6.64	-0.07	0.38	1.51	0.03	98.16	1.13	6.64	7.27	16.19	7.57	-1.04	12.43	21.91	11.76	2.04
os79903	2	435	0.17	5	3	6	6.63	-0.08	0.56	1.54	0.05	96.64	-0.41	7.87	7.19	16.13	7.5	-1.1	12.37	21.87	11.71	1.98
os79910	2	419	0.99	2	3	6	6.71	-0.04	1.7	1.55	0.05	74.09	-0.97	7.91	7.3	16.23	7.57	-1.07	12.49	21.99	11.81	2.02
os79911	2	384	0.5	7	3	5	6.86	0.03	0.39	1.52	0.05	96.22	0.05	6.69	7.65	16.55	7.86	-0.83	12.77	22.31	12.06	2.23
os79912	2	384	0.51	6	3	5	6.87	0.04	0.22	1.52	0.04	95.73	0.57	6.74	7.65	16.55	7.86	-0.83	12.78	22.31	12.06	2.23
os79914	2	401	0.21	4	3	6	6.87	0.04	0.58	1.52	0.03	99.09	0.38	6.83	7.6	16.5	7.82	-0.85	12.73	22.26	12.02	2.21
os79922	2	507	0.77	8	3	5	6.53	-0.09	0.24	1.5	0.07	88.61	-0.67	7.87	6.95	15.81	7.26	-1.19	11.84	21.22	11.2	1.71
os79924	2	496	0.07	4	3	6	6.88	0.06	1.64	1.55	0.06	86.97	-1.79	7.79	6.96	15.83	7.27	-1.21	11.88	21.29	11.27	1.71
os79926	2	515	0.03	4	1	4	6.89	0.05	0.06	1.49	0.02	90.19	3.58	7.44	6.98	15.85	7.29	-1.2	11.91	21.32	11.29	1.72
os79930	2	432	0.99	2	3	6	6.73	-0.04	1.33	1.55	0.04	84.68	-0.08	7.83	7.28	16.13	7.49	-0.94	12.41	21.76	11.59	2.07
os80009	2	440	0.05	6	2	3	6.89	0.1	0.95	1.54	0.09	98.33	-4.67	8.16	7.15	16.08	7.46	-1.1	12.29	21.78	11.67	1.97
os80011	2	467	0.92	7	3	5	6.55	-0.11	0.61	1.51	0.04	96.72	-0.26	6.86	7.16	16.09	7.47	-1.13	12.31	21.81	11.66	1.96
os80026	2	436	0.51	3	2	3	6.85	0.02	1.94	1.55	0.06	96.31	-2.79	7.59	7.27	16.2	7.56	-1.08	12.44	21.93	11.77	2

os80033	2	436	0.89	3	3	6	6.77	-0.03	1.92	1.54	0.05	95.79	-1.34	7.7	7.27	16.2	7.56	-1.08	12.44	21.93	11.77	2
os80134	2	428	0.86	14	3	5	6.17	-0.18	0.01	1.47	0.11	97.46	-1.39	5.41	7.55	16.46	7.73	-1.02	12.78	22.25	12.08	2.22
os80167	2	649	0.25	8	3	5	7.05	0.09	0.48	1.51	0.07	97.66	-1.44	7.22	6.1	14.91	6.64	-1.89	10.74	20.15	10.44	0.83
os80332	2	439	0.37	3	3	6	6.86	0.02	0.92	1.54	0.03	83.67	0.08	8.62	7.36	16.34	7.7	-1.05	12.03	21.75	11.55	1.79
os80756	2	580	0.77	6	3	5	6.83	-0.03	0.45	1.51	0.03	98.85	1.22	6.87	6.58	15.41	7.03	-1.59	11.26	20.74	10.88	1.15
os80826	2	623	0.92	10	3	5	6.43	-0.17	0.02	1.48	0.08	97.83	-0.11	7.63	6.21	15.03	6.72	-1.83	10.85	20.28	10.54	0.9
os80840	2	517	0.5	6	3	5	6.89	0.01	0.51	1.52	0.06	99.99	-0.52	6.43	6.98	15.86	7.39	-1.31	11.73	21.28	11.32	1.43
os80857	2	538	0.15	10	1	4	6.78	0.07	0.01	1.46	0.06	83.69	2.52	8.23	6.72	15.56	7.19	-1.52	11.46	20.96	11.12	1.23
os80859	2	539	0.76	18	1	4	6.05	-0.18	0	1.4	0.11	85.65	2.08	5.33	6.77	15.63	7.23	-1.49	11.52	21.03	11.17	1.26
os80871	2	476	0.92	6	3	5	6.58	-0.1	0.46	1.52	0.04	100	-0.37	7.61	7.15	16.05	7.52	-1.17	11.96	21.5	11.51	1.57
os80873	2	432	0.91	5	3	5	6.64	-0.09	0.86	1.54	0.08	74.16	-0.9	8.57	7.28	16.16	7.65	-1.1	12.13	21.65	11.64	1.64
os80887	2	525	0.92	5	3	6	6.66	-0.07	0.58	1.53	0.03	97	0.14	8.03	6.9	15.84	7.31	-1.34	11.56	21.2	11.21	1.43
os80888	2	534	0.23	2	3	6	6.74	-0.04	0.39	1.53	0.02	95.5	0.77	7.68	6.9	15.84	7.31	-1.35	11.56	21.2	11.21	1.43
os80890	2	529	0.76	11	3	5	6.38	-0.14	0.02	1.49	0.09	97.74	-1.25	6.56	6.91	15.85	7.33	-1.36	11.58	21.21	11.25	1.41
os80898	2	525	0.92	9	2	3	6.43	-0.15	0.22	1.5	0.09	88.63	-2.17	7.08	6.84	15.79	7.28	-1.4	11.48	21.15	11.17	1.4
os80913	2	531	0.93	5	1	1	6.67	-0.08	0.19	1.48	0.02	88.87	2.2	6.17	7.13	16.12	7.52	-1.24	11.82	21.56	11.47	1.58
os80921	2	538	0.01	4	3	6	6.96	0.08	0.56	1.5	0.02	81.05	1.3	7.26	6.96	15.89	7.36	-1.31	11.64	21.31	11.3	1.46
os80928	2	417	0.68	10	2	0	6.77	-0.02	0.04	1.5	0.09	97.94	-0.22	6.76	7.51	16.47	7.82	-0.99	12.25	21.99	11.83	1.83
os81054	2	429	0.4	4	3	6	6.86	0.03	0.86	1.54	0.03	96.24	-0.01	8.13	7.48	16.46	7.81	-0.98	12.21	21.91	11.71	1.87
os81067	2	423	0.4	3	3	6	6.85	0.02	1.79	1.55	0.03	95.24	-0.4	8.32	7.49	16.47	7.81	-0.98	12.21	21.91	11.71	1.87
os81072	2	459	0.99	6	3	5	6.67	-0.08	0.86	1.5	0.05	88.24	-1.5	7.55	7.43	16.42	7.76	-1.05	12.17	21.92	11.76	1.77
os81099	2	367	0.04	16	1	4	7	0.26	0	1.42	0.08	96.7	3.16	6.09	7.87	16.8	8.09	-0.75	12.64	22.27	12.02	2.08
os81101	2	435	0.23	5	3	6	6.79	0.04	1.19	1.53	0.05	98.66	-1.51	8.13	7.45	16.42	7.74	-1.02	12.25	21.93	11.69	1.84
os81102	2	457	0.12	6	3	5	6.83	0.07	0.47	1.51	0.04	99.89	-0.12	6.67	7.39	16.37	7.68	-1.06	12.18	21.87	11.65	1.8
os81112	2	482	0.22	5	3	5	6.84	0.05	0.15	1.51	0.03	98.42	1.65	7.21	7.22	16.14	7.55	-1.24	12.13	21.7	11.63	1.63
os81120	2	391	0.27	5	3	5	6.91	0.07	0.65	1.52	0.03	97.52	0.88	7.19	7.87	16.87	8.11	-0.78	12.75	22.43	12.13	2.11
os81438	2	450	0.73	8	2	3	6.75	-0.04	0.41	1.52	0.09	89.7	-2.53	8.61	7.29	16.2	7.59	-1.21	12.29	21.88	11.76	1.71
os81443	2	536	0.56	6	3	5	6.87	0	0.2	1.51	0.03	89.08	1.07	7.47	6.8	15.73	7.2	-1.52	11.62	21.22	11.22	1.29
os81466	2	479	0.24	3	3	6	6.89	0.03	0.65	1.53	0.03	90.18	0.02	8.08	7.19	16.08	7.47	-1.13	12.11	21.64	11.56	1.79
os81479	2	424	0.92	2	3	6	6.71	-0.04	1.6	1.54	0.02	86.09	-0.07	8.74	7.64	16.65	7.91	-0.97	12.73	22.41	12.16	2.1
os81521	2	367	0.92	1	3	6	6.76	-0.01	1.67	1.53	0.01	11.3	0.11	8.47	8.01	16.99	8.23	-0.74	13.3	22.96	12.62	2.39
os81574	2	359	0.77	3	3	6	6.75	-0.02	1.34	1.55	0.01	26.21	-0.13	7.82	8.01	16.99	8.21	-0.72	13.29	22.93	12.59	2.4
os81600	2	383	0.08	2	3	6	6.82	0.03	2.29	1.55	0.02	74.79	-0.87	9.3	7.81	16.76	8.04	-0.77	12.93	22.57	12.29	2.23
os81688	2	443	0	2	3	9	6.83	0.03	0.14	1.52	0.01	88.6	0.72	7.47	7.72	16.66	7.92	-0.94	12.6	22.21	11.95	2.03
os81735	2	422	0.05	3	3	6	6.87	0.06	1.33	1.54	0.04	74.26	0.24	8.25	7.48	16.42	7.77	-1.09	12.46	22.08	11.92	1.81
os81902	2	408	0.17	3	3	6	6.68	-0.05	1.49	1.54	0.04	76.25	-0.44	8.7	7.59	16.55	7.86	-1.01	12.56	22.22	12.02	1.89
os82057	2	396	0.6	2	3	6	6.73	-0.01	0.73	1.54	0.02	84.56	0.28	8.64	7.77	16.79	8.05	-0.88	12.86	22.56	12.27	2.16

os82141	2	437	0.65	5	3	9	6.64	-0.04	0.52	1.5	0.06	100	-0.75	6.16	7.64	16.6	7.92	-0.93	12.53	22.17	11.99	2.02
os82158	2	437	0.25	7	3	5	6.92	0.06	0.48	1.51	0.05	99.98	-0.45	6.75	7.72	16.67	7.93	-0.99	12.67	22.27	12	2.01
os82159	2	373	0.92	0	3	6	6.77	0	1.98	1.54	0	19.13	-0.03	11.28	7.98	16.95	8.16	-0.75	13.23	22.88	12.53	2.38
os82208	2	407	0.84	5	3	6	6.61	-0.08	1.08	1.53	0.04	73.03	-0.73	7.62	7.69	16.66	7.98	-0.94	12.54	22.29	12.04	1.97
os82232	2	398	0	1	2	3	6.79	0.01	2.98	1.56	0.05	79.78	-2.13	8.63	7.64	16.61	7.93	-0.96	12.49	22.22	12	1.93
os82233	2	389	0.92	1	3	6	6.73	-0.02	3.55	1.56	0.05	81.34	-0.58	9.46	7.68	16.64	7.97	-0.94	12.52	22.26	12.03	1.96
os82234	2	398	0	1	2	3	6.79	0.01	2.97	1.56	0.05	80.2	-2.19	8.59	7.64	16.61	7.93	-0.96	12.49	22.22	12	1.93
os82235	2	397	0.92	1	3	6	6.79	0	2.98	1.56	0.05	82.31	-1.8	8.6	7.65	16.61	7.93	-0.96	12.49	22.23	12	1.93
os82236	2	391	0.12	2	3	6	6.7	-0.04	2.34	1.55	0.04	84.8	-0.81	9.07	7.68	16.65	7.97	-0.93	12.53	22.27	12.03	1.97
os82237	2	390	0.11	3	3	6	6.69	-0.04	2.38	1.55	0.04	84.71	-0.84	9.07	7.68	16.65	7.97	-0.93	12.53	22.27	12.03	1.97
os82316	2	326	0.99	14	2	7	6.16	-0.21	0.21	1.52	0.18	90.06	-7.35	8.49	7.93	16.87	8.13	-0.73	12.7	22.37	12.08	2.16
os82409	2	366	0.67	5	3	6	6.78	-0.01	0.4	1.51	0.04	94.29	-0.4	7.86	8.08	17.06	8.26	-0.68	13.05	22.72	12.35	2.25
os82410	2	364	0.98	5	2	3	6.71	-0.04	1.4	1.52	0.05	94.7	-2.01	8.01	8.08	17.06	8.27	-0.67	13.06	22.72	12.35	2.25
os82412	2	363	0.89	4	3	6	6.71	-0.04	1.48	1.52	0.05	95.33	-1.55	8.04	8.09	17.07	8.27	-0.67	13.06	22.72	12.35	2.25
os82432	2	482	0.71	8	3	5	6.8	-0.02	0.32	1.52	0.05	81.48	-0.41	8.56	7.07	16	7.46	-1.24	11.76	21.39	11.4	1.56
os82454	2	383	0.15	7	2	3	6.7	0.03	0.9	1.54	0.1	89.36	-4.51	7.65	7.68	16.61	7.97	-0.94	12.44	22.18	11.99	1.87
os82465	2	477	0.68	5	3	5	6.87	0.01	0.54	1.53	0.04	98.92	-0.02	7.51	7.19	16.16	7.53	-1.23	12.06	21.73	11.62	1.67
os82466	2	478	0.73	6	3	5	6.88	0.02	0.45	1.52	0.04	99.03	0.14	7.47	7.18	16.16	7.53	-1.23	12.05	21.73	11.62	1.67
os82481	2	504	0.98	2	3	6	6.76	-0.04	1.79	1.54	0.01	60.37	0.04	8.37	7.04	15.8	7.26	-1.23	12.04	21.35	11.31	1.64
os82482	2	487	0.77	1	3	6	6.82	0	4.77	1.56	0.02	24.92	-0.15	9.32	7.05	15.82	7.28	-1.2	12.09	21.4	11.35	1.66
os82491	2	478	0.29	2	3	6	6.88	0.03	2.05	1.55	0.02	42.04	-0.54	8.93	7.14	15.96	7.37	-1.17	12.23	21.62	11.49	1.77
os82527	2	439	0.98	11	2	0	6.44	-0.17	0.01	1.46	0.11	87.83	1.79	6.1	7.18	16	7.4	-1.18	12.16	21.53	11.39	1.71
os82533	2	572	0	3	3	9	6.74	-0.06	0.14	1.5	0.02	67.04	1.28	7.1	6.79	15.62	7.1	-1.4	11.72	21.13	11.07	1.52
os82593	2	464	0.92	2	3	6	6.81	0	4.98	1.56	0.03	38.17	-0.63	8.7	7.23	16.05	7.41	-1.13	12.28	21.67	11.55	1.78
os82594	2	464	0.01	3	3	6	6.84	0.03	4.98	1.55	0.02	34.16	0.07	8.83	7.23	16.05	7.41	-1.13	12.28	21.67	11.55	1.78
os82633	2	461	0.04	2	3	6	6.86	0.03	0.88	1.55	0.02	49.97	0.24	8.47	7.2	16.03	7.44	-1.12	12.19	21.59	11.5	1.76
os82642	2	482	0.77	6	3	5	6.78	-0.04	0.59	1.52	0.04	97.99	0.15	6.93	7.12	15.97	7.37	-1.16	12.1	21.49	11.41	1.74
os82680	2	513	0.77	3	3	6	6.8	-0.03	3.1	1.55	0.03	57.85	-0.43	8.98	6.93	15.72	7.18	-1.3	11.93	21.26	11.25	1.62
os82727	2	490	0.02	3	3	6	6.7	-0.06	0.8	1.55	0.02	41.2	-0.13	7.96	7.05	15.82	7.28	-1.21	12.09	21.39	11.34	1.66
os82762	2	528	0.63	5	3	6	6.81	-0.03	1.27	1.53	0.05	97.6	-1.84	7.84	6.88	15.72	7.14	-1.3	11.74	21.19	11.09	1.56
os82813	2	487	0.74	2	3	6	6.86	0.01	5.59	1.55	0.02	33.75	0.16	8.47	7.12	15.9	7.3	-1.17	12.16	21.53	11.46	1.76
os82824	2	494	0.08	2	3	6	6.87	0.03	3.78	1.55	0.02	26.53	0.29	9.14	7.04	15.82	7.28	-1.2	12.08	21.4	11.35	1.66
os82887	2	494	0.89	8	3	5	6.74	-0.06	0.54	1.53	0.05	73.38	-0.66	8.62	7.03	15.82	7.27	-1.22	12.03	21.37	11.34	1.68
os82888	2	494	1	9	3	5	6.48	-0.15	0.12	1.5	0.06	84.17	-0.04	6.49	7.04	15.83	7.28	-1.21	12.05	21.37	11.36	1.69
os82890	2	495	0.95	7	3	5	6.63	-0.1	0.18	1.51	0.04	83.6	0.56	6.41	7.03	15.82	7.27	-1.22	12.03	21.36	11.34	1.68
os82912	2	560	0.45	5	3	6	6.76	0.01	0.33	1.5	0.03	96.07	1.83	6.63	6.76	15.63	7.1	-1.35	11.61	21.04	11.06	1.58
os82913	2	557	0.51	5	3	5	6.71	-0.03	0.41	1.51	0.03	96.8	1.02	6.89	6.77	15.64	7.11	-1.34	11.63	21.06	11.08	1.59

os82919	2	540	0.31	5	3	5	6.94	0.04	0.58	1.52	0.06	97.97	-0.88	7.56	6.82	15.68	7.16	-1.32	11.7	21.11	11.14	1.59
os82949	2	484	0.01	5	3	5	6.91	0.09	0.79	1.53	0.06	93.96	-1.05	7.1	7.08	15.86	7.3	-1.21	12.07	21.43	11.34	1.7
os82987	2	489	0.98	3	3	6	6.73	-0.04	1.06	1.55	0.02	40.8	-0.21	8.06	7.06	15.83	7.29	-1.2	12.11	21.41	11.35	1.67
os82992	2	488	0.32	2	3	6	6.76	-0.01	2.77	1.55	0.04	65.76	-1.55	8.41	7.07	15.9	7.3	-1.23	12.08	21.49	11.4	1.73
os82993	2	481	0.92	1	3	6	6.83	0.01	5.75	1.56	0.01	23.33	-0.11	8.99	7.11	15.9	7.3	-1.2	12.16	21.51	11.42	1.74
os83001	2	447	0.92	2	3	6	6.73	-0.03	1.26	1.55	0.02	62.37	0.2	8.1	7.25	16.11	7.5	-1.09	12.38	21.75	11.61	1.87
os83088	2	630	0.6	12	3	5	6.42	-0.08	0.01	1.46	0.09	98.98	-0.61	5.78	6.29	15.03	6.72	-1.82	11.08	20.34	10.6	0.98
os83101	2	595	0.99	10	3	5	6.5	-0.16	0.03	1.47	0.06	98.41	1.16	6.27	6.47	15.25	6.83	-1.7	11.3	20.6	10.75	1.09
os83156	2	584	0.23	1	3	9	6.83	0	0.05	1.5	0.01	88.35	1.53	8.32	6.66	15.43	6.98	-1.55	11.51	20.84	10.95	1.28
os83161	2	576	0.5	11	3	5	6.92	0.02	0.05	1.49	0.07	92.39	-0.04	7.61	6.54	15.35	6.91	-1.61	11.42	20.77	10.87	1.22
os83204	2	571	0.99	3	1	4	6.75	-0.06	0.12	1.49	0.01	67.8	2.21	7.43	6.71	15.51	7.03	-1.51	11.66	21.03	11.08	1.41
os83267	2	552	0	11	2	7	6.92	0.17	0.65	1.5	0.14	97.76	-5.51	7.71	6.47	15.2	6.86	-1.71	11.32	20.58	10.77	1.08
os83300	2	479	0.96	8	3	5	6.6	-0.11	0.99	1.54	0.07	62.45	-1.39	7.66	7.07	15.81	7.27	-1.28	12.05	21.37	11.39	1.6
os83335	2	607	0.24	9	3	5	7.02	0.08	0.25	1.47	0.08	98.61	-0.43	7.21	6.4	15.2	6.79	-1.73	11.22	20.53	10.69	1.06
os83391	2	500	1	8	3	5	6.59	-0.12	0.35	1.49	0.06	70.8	0.35	7.02	7.05	15.86	7.27	-1.25	12.07	21.45	11.36	1.68
os83392	2	492	0.94	12	2	3	6.48	-0.16	0.16	1.5	0.1	77.22	-2.91	7.18	7.05	15.86	7.27	-1.26	12.06	21.44	11.35	1.68
os83414	2	543	0.07	5	3	6	6.97	0.08	0.36	1.51	0.02	88.94	1.95	7.15	6.84	15.61	7.08	-1.34	11.85	21.14	11.15	1.5
os83479	2	582	1	15	3	5	6.23	-0.24	0	1.46	0.11	99.87	-1.15	6.3	6.46	15.24	6.83	-1.71	11.28	20.59	10.74	1.08
os83490	2	498	0	2	3	6	6.88	0.04	1.45	1.54	0.02	87.13	-0.11	8.32	7.05	15.83	7.27	-1.26	12.11	21.47	11.43	1.69
os83613	2	448	0.09	7	3	9	6.87	0.1	0.11	1.49	0.03	98.76	1.99	6.3	7.66	16.56	7.88	-1.02	12.66	22.2	12.03	2.02
os83777	2	510	0.08	9	3	9	6.43	-0.15	0.2	1.49	0.06	99.96	-1.04	6.37	7.31	16.2	7.5	-1.34	12.39	21.87	11.75	1.76
os83779	2	514	0.02	8	3	9	6.92	0.12	0.1	1.47	0.06	98.39	0.2	5.99	7.32	16.22	7.5	-1.33	12.38	21.83	11.72	1.76
os83802	2	494	0.77	2	3	6	6.83	0	1.05	1.54	0.02	54.47	0.2	8.5	7.2	16.1	7.43	-1.4	12.32	21.8	11.7	1.74
os83858	2	453	0.87	5	3	6	6.63	-0.07	0.72	1.53	0.04	97.48	-0.35	6.95	7.61	16.53	7.83	-1.13	12.53	22.04	11.83	1.82
os83934	2	510	0.19	9	3	5	7.01	0.11	0.14	1.5	0.08	91.32	-2.07	6.12	7.14	16.05	7.37	-1.48	12.25	21.75	11.64	1.68
os83936	2	513	0.09	6	3	5	6.85	0.07	0.44	1.5	0.04	89.79	1.49	6.44	7.19	16.09	7.41	-1.43	12.28	21.75	11.63	1.72
os83995	2	551	0.95	9	1	4	6.62	-0.12	0.08	1.45	0.06	97.92	2.28	6.23	6.69	15.46	7.08	-1.55	11.47	20.83	11.01	1.24
os84023	2	454	0.28	6	2	7	6.9	0.07	0.82	1.55	0.19	83.19	-6.7	7.65	7.02	15.81	7.35	-1.35	11.89	21.29	11.34	1.49
os84067	2	526	0.77	2	3	6	6.82	-0.01	0.31	1.53	0.02	86.79	0.38	8.48	6.83	15.61	7.17	-1.43	11.65	21.05	11.12	1.4
os84100	2	538	1	13	1	4	6.32	-0.21	0.01	1.43	0.08	96.94	3.6	5.81	6.73	15.45	7.09	-1.51	11.46	20.81	11.01	1.24
os84115	2	627	0.76	3	3	6	6.79	-0.03	1.9	1.51	0.02	82.13	-0.28	7.94	6.31	15.06	6.74	-1.81	11.09	20.38	10.64	0.99
os84116	2	629	0.92	1	3	9	6.84	-0.01	1.53	1.51	0.01	80.44	0.31	8.12	6.31	15.07	6.75	-1.81	11.1	20.38	10.65	0.99
os84125	2	599	0.99	1	3	9	6.84	-0.01	0.29	1.47	0.01	93.78	1.61	8	6.56	15.28	6.95	-1.65	11.41	20.68	10.89	1.13
os84140	2	549	0.79	5	3	9	6.77	-0.05	0.28	1.48	0.03	96.61	1.14	6.13	6.88	15.67	7.21	-1.41	11.79	21.13	11.22	1.42
os84181	2	535	0.08	6	3	5	7	0.11	0.39	1.53	0.05	90.13	-1.02	7.95	6.76	15.6	7.15	-1.55	11.55	21.04	11.1	1.27
os84251	2	510	0	14	3	5	7	0.23	0	1.45	0.08	98	0.35	5.95	6.89	15.69	7.22	-1.39	11.77	21.18	11.23	1.49
os84287	2	531	0.23	6	3	5	6.75	0.01	0.48	1.51	0.05	98.16	-0.23	6.88	6.82	15.62	7.16	-1.44	11.71	21.08	11.18	1.46

os84373	2	486	0.89	3	3	6	6.71	-0.04	1.69	1.54	0.02	83.82	-0.56	7.73	7.22	16.2	7.55	-1.08	12.17	21.8	11.66	1.88
os84397	2	426	1	9	3	5	6.44	-0.16	0.14	1.51	0.05	82.59	0.09	7.2	7.52	16.48	7.79	-0.93	12.64	22.34	12.1	2.09
os84402	2	383	0.66	9	3	5	6.48	-0.08	0.26	1.53	0.06	57.48	-0.97	8.6	7.67	16.64	7.93	-0.84	12.82	22.5	12.23	2.17
os84438	2	406	0.82	13	1	4	6.18	-0.17	0	1.39	0.08	79.47	5.63	5.36	7.49	16.4	7.76	-0.94	12.44	22	11.77	1.97
os84511	2	509	0.77	5	3	6	6.76	-0.05	0.58	1.52	0.03	86.88	-0.01	7.34	7.02	15.9	7.33	-1.18	11.88	21.35	11.29	1.67
os84524	2	367	0.81	9	2	0	6.58	-0.08	1.1	1.51	0.16	82.9	0.35	7.99	7.5	16.42	7.75	-0.92	12.48	22.02	11.79	2
os84607	2	448	0.87	19	2	0	5.88	-0.25	0	1.43	0.16	89.76	-0.75	6.6	7.1	15.84	7.33	-1.25	12	21.35	11.31	1.62
os84645	2	539	0.7	1	3	6	6.8	-0.01	1.96	1.55	0.02	52.89	-0.07	8.97	6.77	15.56	7.1	-1.45	11.56	20.97	11.02	1.36
os84722	2	475	0.23	6	3	5	6.95	0.07	0.38	1.5	0.03	96.07	1	6.44	7.22	16.11	7.52	-1.11	12.21	21.69	11.55	1.82
os84777	2	487	0.97	4	3	6	6.76	-0.04	0.34	1.52	0.03	99.12	0.61	6.89	7.05	15.92	7.35	-1.2	11.92	21.38	11.28	1.68
os84820	2	514	0.83	26	2	7	5.35	-0.32	0	1.39	0.17	91.94	-1.8	5.82	6.73	15.46	7.11	-1.53	11.49	20.82	11	1.23
os84910	2	572	0.43	3	3	6	6.91	0.02	1.49	1.54	0.04	81.83	-1.09	9.36	6.58	15.39	6.99	-1.64	11.3	20.77	10.89	1.16
os84959	2	515	0.78	4	3	6	6.79	-0.04	0.58	1.49	0.04	95.67	0.07	7.89	7	15.82	7.25	-1.23	11.88	21.26	11.19	1.64
os84965	2	469	0.42	24	2	7	5.95	-0.01	0	1.43	0.18	98.91	-4.37	5.88	7.08	15.85	7.32	-1.25	11.97	21.35	11.3	1.62
os84976	2	513	0.09	2	3	6	6.88	0.02	1.79	1.55	0.02	70.85	-0.44	8.77	6.91	15.72	7.23	-1.33	11.75	21.15	11.15	1.47
os85020	2	492	0	10	3	5	6.97	0.16	0.08	1.46	0.07	89.26	1.69	6.77	7.01	15.82	7.32	-1.31	11.9	21.32	11.35	1.53
os85104	2	545	0.27	5	3	5	6.98	0.07	0.64	1.52	0.04	95.17	-0.59	7.89	6.67	15.41	7.02	-1.56	11.53	20.82	10.97	1.26
os85112	2	605	0.22	3	3	6	6.88	0.03	0.86	1.53	0.03	54.28	-0.17	7.96	6.39	15.15	6.81	-1.71	11.17	20.47	10.71	1.09
os85113	2	607	0.56	3	3	6	6.83	0	0.19	1.53	0.02	57.75	0.46	8.12	6.39	15.15	6.82	-1.71	11.17	20.48	10.71	1.09
os85116	2	582	0.04	7	3	5	7.03	0.12	0.44	1.51	0.05	97.05	-1.51	6.44	6.55	15.3	6.93	-1.59	11.33	20.66	10.82	1.21
os85121	2	573	0.08	8	1	4	7.02	0.11	0.02	1.47	0.02	95.28	3.73	6.76	6.61	15.37	6.99	-1.55	11.41	20.75	10.88	1.26
os85146	2	508	0.92	18	2	0	6.3	-0.2	0	1.45	0.16	98.11	-2.36	6.53	6.78	15.49	7.13	-1.47	11.52	20.88	11.06	1.3
os85161	2	488	0.77	3	3	6	6.8	-0.02	1.26	1.54	0.01	55.52	0	8.34	7.1	15.94	7.36	-1.17	12.02	21.45	11.42	1.73
os85182	2	402	0.92	3	2	3	6.67	-0.05	2.05	1.54	0.08	87.25	-3.87	8.11	7.42	16.31	7.67	-0.98	12.44	21.93	11.73	1.95
os85230	2	516	0.37	14	2	3	6.47	0	0	1.49	0.11	82.47	-2.41	6.99	6.88	15.66	7.18	-1.37	11.71	21.08	11.12	1.47
os85245	2	618	0.08	9	2	3	7.07	0.14	0.56	1.51	0.1	99.8	-4.07	7.89	6.27	15.07	6.75	-1.83	10.93	20.36	10.58	0.93
os85246	2	603	0.19	7	2	3	6.79	0.04	0.94	1.53	0.12	99.06	-5.74	8.17	6.29	15.09	6.76	-1.82	10.95	20.38	10.61	0.94
os85256	2	548	0.41	7	3	5	6.68	-0.01	0.34	1.51	0.04	84.42	0.11	7.35	6.77	15.6	7.16	-1.55	11.58	21.06	11.12	1.28
os85282	2	450	0.4	4	3	6	6.89	0.04	1.07	1.54	0.03	97.86	-0.47	7.98	7.32	16.29	7.65	-1.02	12.32	21.98	11.78	1.93
os85302	2	475	0.25	7	3	5	6.94	0.06	0.35	1.51	0.05	99.17	-0.14	7.31	7.13	15.93	7.42	-1.24	12.05	21.47	11.48	1.64
os85308	2	479	0.92	4	3	6	6.67	-0.06	1.17	1.53	0.03	84.14	-0.63	7.71	7.26	16.22	7.58	-1.06	12.22	21.83	11.68	1.89
os85323	2	398	0.82	12	3	5	6.27	-0.14	0.01	1.47	0.09	91.51	0.63	6.44	7.44	16.33	7.69	-0.97	12.4	21.91	11.72	1.93
os85337	2	483	0.92	4	3	6	6.65	-0.07	0.56	1.53	0.02	88.9	0.27	7.64	7.1	15.99	7.42	-1.16	12.02	21.49	11.42	1.72
os85432	2	481	0.06	7	3	5	6.87	0.09	0.18	1.5	0.04	99.55	1.4	5.94	7.07	16	7.39	-1.15	12.17	21.64	11.57	1.91
os85459	2	430	0.92	1	3	6	6.74	-0.02	3.9	1.56	0.02	11.6	0.39	10.17	7.25	16.08	7.48	-1	12.29	21.59	11.44	1.91
os85460	2	430	0.92	1	3	6	6.74	-0.02	3.9	1.56	0.02	11.76	0.41	10.19	7.25	16.09	7.48	-0.99	12.3	21.6	11.45	1.91
os85480	2	431	0.02	8	3	5	6.98	0.14	0.18	1.51	0.08	55.8	-0.58	7.34	7.23	16.08	7.47	-1	12.3	21.63	11.49	1.93

os85494	2	449	0.92	1	3	6	6.75	-0.03	2.86	1.56	0.04	81.54	-1.32	8.77	7.09	15.98	7.38	-1.06	12.23	21.6	11.46	1.98
os85503	2	491	0.47	8	3	5	6.66	0	0.38	1.51	0.08	91.24	-1.11	7.47	6.93	15.82	7.23	-1.13	11.95	21.34	11.25	1.82
os85504	2	486	0.43	8	3	5	6.65	0.01	0.16	1.5	0.07	92.42	-0.2	6.92	6.95	15.83	7.24	-1.12	11.96	21.35	11.26	1.82
os85528	2	623	0.99	12	2	3	6.42	-0.19	0.26	1.5	0.11	99.03	-4.44	7.08	6.4	15.27	6.93	-1.68	11.1	20.58	10.81	1.04
os85543	2	605	0.55	6	3	5	6.9	0	0.23	1.49	0.04	88.06	0.67	6.37	6.46	15.31	6.95	-1.67	11.15	20.65	10.85	1.07
os85573	2	382	0.92	1	3	6	6.75	0	5.79	1.56	0.01	3.43	-0.27	8.9	7.79	16.72	8.03	-0.87	12.96	22.57	12.34	2.21
os85799	2	559	0.66	5	2	3	6.95	0.07	1.02	1.54	0.1	95.87	-3.65	8.38	6.54	15.28	6.93	-1.65	11.37	20.66	10.85	1.16
os85803	2	491	0.32	10	2	7	6.28	-0.16	0.72	1.53	0.21	85.72	-6.9	6.23	6.66	15.38	6.99	-1.59	11.5	20.77	10.94	1.24
os85817	2	484	0.96	5	2	7	6.64	-0.09	1.19	1.56	0.07	76.04	-2.12	7.82	7.31	16.22	7.5	-1.32	11.98	21.55	11.34	1.5
os85934	2	495	0.01	5	3	6	6.66	-0.08	0.52	1.53	0.04	82.46	0.28	7.17	7.36	16.31	7.55	-1.29	12.1	21.68	11.42	1.54
os85940	2	372	0.82	5	3	6	6.59	-0.06	0.53	1.52	0.03	99.97	0.14	6.92	8.18	17.11	8.26	-0.63	13.29	22.82	12.4	2.28
os86015	2	511	0.97	13	3	9	6.23	-0.21	0	1.46	0.09	99.56	-1.01	5.6	7.55	16.57	7.66	-1.22	12.53	22.16	11.68	1.7
os86193	2	523	0.4	6	1	1	6.94	0.04	0.17	1.41	0.04	99.29	2.29	7.14	7.47	16.33	7.64	-1.02	11.95	21.5	11.17	1.71
os86197	2	429	0.39	5	3	6	6.89	0.04	0.57	1.53	0.05	99.85	-0.61	7.06	7.68	16.53	7.82	-0.89	12.22	21.74	11.4	1.84
os86382	2	582	0.4	4	3	6	6.91	0.02	0.68	1.54	0.04	94.22	-0.19	8.96	6.76	15.68	7.02	-1.7	11.34	20.93	10.83	1.22
os86506	2	416	0.08	8	3	5	6.98	0.14	0.35	1.5	0.08	80.2	-0.02	6.59	7.73	16.62	7.88	-1.06	12.61	22.16	11.89	1.89
os86745	2	508	0.99	11	1	1	6.37	-0.19	0.03	1.46	0.05	89.25	3.16	6.12	7.42	16.4	7.67	-1.25	12.21	21.89	11.65	1.58
os87183	2	428	0.8	13	3	9	6.19	-0.21	0.05	1.42	0.07	99.65	1.01	6.39	8.16	17.07	8.21	-0.71	13.27	22.73	12.27	2.23
os87188	2	421	0.65	3	3	6	6.69	-0.03	1.24	1.55	0.04	99.89	-0.15	7.39	7.86	16.7	7.97	-0.86	12.81	22.21	11.97	2.06
os87317	2	403	0.4	5	1	1	6.87	0.03	0.02	1.47	0.01	98.15	3.97	6.28	7.98	16.78	8.09	-0.81	12.9	22.3	12.08	2.06
os87790	2	357	0.7	5	2	7	6.72	-0.03	1.49	1.54	0.1	96.81	-3.32	7.49	7.91	16.85	8.12	-0.76	12.71	22.37	12.1	2.1
os87855	2	364	0.4	4	3	6	6.83	0.02	1.66	1.49	0.04	96.39	-1.22	8.01	8.11	17.05	8.22	-0.78	13.02	22.67	12.38	2.09
os87867	2	416	0.57	4	3	6	6.8	-0.01	0.45	1.52	0.03	90.18	0.89	7.89	7.78	16.74	7.96	-0.99	12.74	22.39	12.14	1.9
os87887	2	419	0.95	7	2	3	6.68	-0.07	0.83	1.53	0.07	92.61	-3.04	8.19	7.74	16.71	7.94	-1.03	12.68	22.37	12.09	1.84
os87888	2	417	0.88	7	2	3	6.7	-0.06	0.94	1.54	0.07	93.46	-3.4	8.26	7.74	16.71	7.94	-1.03	12.69	22.38	12.09	1.84
os87931	2	433	0.98	9	3	5	6.39	-0.17	0.06	1.45	0.05	91.59	2.22	6.51	7.64	16.6	7.87	-1.05	12.57	22.29	12.04	1.82
os87943	2	364	0.9	9	3	5	6.52	-0.13	0.09	1.49	0.08	97.23	0.78	6.35	7.93	16.87	8.14	-0.74	12.74	22.4	12.12	2.11
os87945	2	366	0.9	9	3	5	6.54	-0.12	0.08	1.49	0.07	97.29	1.02	6.31	7.94	16.87	8.14	-0.74	12.74	22.41	12.12	2.11
os87959	2	458	1	6	3	5	6.61	-0.1	0.33	1.49	0.04	94.72	1.69	6.59	7.55	16.49	7.73	-1.19	12.39	22.09	11.84	1.65
os88053	2	457	0.4	9	2	3	6.42	-0.15	0.09	1.5	0.1	99.78	-2.3	6.1	7.55	16.44	7.7	-1.1	12.26	21.77	11.55	1.75
os88181	2	383	0.28	9	2	3	6.9	0.08	0.87	1.54	0.13	92.41	-6.48	7.83	7.88	16.79	8.02	-0.97	12.82	22.44	12.09	1.94
os88341	2	427	0.01	3	3	9	6.85	0.05	0.21	1.52	0.02	90.09	0.96	6.88	7.88	16.79	8.06	-0.89	12.83	22.37	12.17	1.99
os88342	2	423	0.88	5	3	5	6.73	-0.05	0.87	1.53	0.03	81.34	-0.43	7.03	7.89	16.79	8.06	-0.89	12.85	22.37	12.17	1.99
os88369	2	447	0.92	1	3	6	6.81	0	0.47	1.51	0.01	55.18	0.16	9.08	7.71	16.56	7.89	-1.02	12.31	21.79	11.6	1.64
os88456	2	523	0.94	4	3	9	6.77	-0.04	1.16	1.51	0.02	82.69	-0.16	7.44	7.29	16.27	7.57	-1.31	12.03	21.72	11.5	1.51
os88469	2	395	0.92	6	3	5	6.55	-0.09	0.7	1.52	0.13	97.27	-2.03	7.24	7.79	16.61	7.97	-0.94	12.32	21.74	11.58	1.72
os88499	2	471	0	11	2	3	6.95	0.19	0.11	1.5	0.1	95.03	-3.21	5.83	7.53	16.43	7.72	-1.28	12.31	21.78	11.6	1.47

os88519	2	354	0.54	5	2	3	6.64	-0.03	1.27	1.55	0.06	80.33	-2.19	7.42	8.05	16.91	8.17	-0.76	12.7	22.23	12.01	1.95
os88550	2	355	0.02	18	1	4	6.89	0.3	0	1.39	0.11	73.12	2.23	5.01	8.24	17.17	8.32	-0.68	13.1	22.69	12.32	2.14
os88555	2	407	0.17	4	3	6	6.79	0.03	1.14	1.54	0.03	80.37	-0.57	8.29	7.96	16.83	8.08	-0.92	12.95	22.39	12.17	1.93
os88557	2	400	0.88	9	3	5	6.43	-0.12	0.23	1.51	0.07	80.05	0.26	7.34	7.95	16.83	8.12	-0.93	12.8	22.26	12.08	1.86
os88608	2	402	0.01	3	3	6	6.87	0.06	1.67	1.54	0.03	96.47	-0.31	7.9	7.91	16.82	8.08	-0.87	12.88	22.41	12.2	2.01
os88640	2	374	0.19	7	1	4	6.78	0.07	0.2	1.49	0.05	88.64	2.21	7.14	8.04	16.97	8.17	-0.87	12.9	22.52	12.23	1.94
os88644	2	367	0.37	7	3	5	6.68	0.03	0.58	1.52	0.06	87.94	-0.85	7.09	8.08	17.01	8.21	-0.84	12.95	22.57	12.28	1.97
os88655	2	230	0.87	8	3	5	6.39	-0.11	0.23	1.52	0.09	87.01	-1.87	7.22	8.91	17.8	8.93	0.08	13.92	23.36	12.93	2.9
os88742	2	441	0.92	4	3	6	6.65	-0.06	0.4	1.52	0.02	97.42	0.19	6.53	7.68	16.54	7.79	-1.03	12.55	21.99	11.72	1.81
os88799	2	415	0.11	1	3	6	6.75	-0.02	2.46	1.55	0.01	26.69	0.05	8.47	7.76	16.61	7.89	-0.92	12.43	21.88	11.63	1.8
os88824	2	402	0.06	4	3	6	6.79	0.04	0.62	1.54	0.03	67.84	-0.29	8.32	7.74	16.58	7.88	-0.97	12.4	21.85	11.65	1.75
os88855	2	423	0.72	5	3	5	6.61	-0.07	0.65	1.53	0.05	89.76	-0.73	6.39	7.71	16.58	7.85	-1.12	12.54	22.04	11.75	1.64
os88968	2	469	0.22	7	3	9	6.83	0.07	0.48	1.47	0.05	95.28	-0.37	6.72	7.39	16.33	7.74	-1.06	12.08	21.8	11.7	1.69
os88969	2	404	0.3	9	1	1	6.95	0.1	0.08	1.44	0.06	89.26	3.1	6.73	7.75	16.7	8.02	-0.85	12.56	22.25	12.07	1.97
os88975	2	490	0.13	4	1	4	6.83	0.04	0.26	1.46	0.03	95.92	2.5	6.73	7.39	16.35	7.63	-1.26	12.16	21.89	11.69	1.59
os89056	2	488	0.02	16	3	5	7.05	0.24	0	1.44	0.07	94.23	1.01	6.7	7.22	16.18	7.61	-1.16	11.89	21.61	11.54	1.6
os89063	2	421	0.51	6	2	3	6.73	0.01	1.36	1.53	0.08	96.39	-2.82	7.84	7.51	16.43	7.83	-1.05	12.24	21.94	11.84	1.75
os89094	2	527	0.97	11	3	5	6.36	-0.19	0.02	1.46	0.08	90.49	-0.02	6.4	6.93	15.84	7.36	-1.35	11.49	21.13	11.16	1.39
os89108	2	468	0.21	7	3	9	6.8	0.06	0.44	1.47	0.06	95.6	-0.58	6.86	7.4	16.34	7.75	-1.05	12.09	21.8	11.7	1.7
os89160	2	406	0.97	15	2	0	6.13	-0.23	0	1.47	0.13	82.73	-0.45	6.33	7.37	16.25	7.69	-1.09	12.2	21.78	11.73	1.7
os89208	2	443	0.17	9	3	5	6.99	0.12	0.1	1.51	0.07	98.48	-0.77	6.31	7.43	16.35	7.77	-1.09	12.13	21.83	11.75	1.7
os89220	2	488	0.36	12	3	5	6.94	0.07	0.03	1.49	0.09	95.82	-1.47	5.77	7.19	16.14	7.57	-1.22	11.86	21.54	11.51	1.56
os89239	2	568	0.91	8	3	9	6.52	-0.12	0.21	1.47	0.04	93.12	1.45	6.51	6.93	15.91	7.33	-1.47	11.6	21.31	11.27	1.31
os89241	2	522	0.87	8	3	5	6.51	-0.12	0.21	1.51	0.07	99.25	-1.01	6.3	6.98	15.96	7.37	-1.43	11.66	21.36	11.31	1.35
os89274	2	382	0.77	4	2	0	6.73	-0.03	1.6	1.55	0.09	79.38	-1.15	8.19	7.62	16.55	7.9	-0.98	12.37	22.07	11.93	1.84
os89276	2	383	0.67	9	2	0	6.71	-0.05	0.15	1.51	0.1	69.65	-1.02	7.54	7.66	16.59	7.93	-0.95	12.42	22.11	11.97	1.87
os89278	2	380	0.77	10	2	3	6.64	-0.08	0.06	1.52	0.12	69.55	-3.13	7.88	7.7	16.64	7.98	-0.92	12.48	22.17	12.01	1.9
os89279	2	382	0.81	12	3	5	6.58	-0.1	0.01	1.48	0.1	73.12	-0.3	7.47	7.72	16.65	7.99	-0.91	12.5	22.18	12.02	1.91
os89280	2	377	0.77	10	3	5	6.64	-0.08	0.12	1.51	0.1	71.57	-1.57	8.06	7.74	16.67	8.01	-0.9	12.52	22.21	12.04	1.92
os89287	2	393	0.03	13	1	2	6.81	0.2	0.01	1.38	0.1	85.97	6.97	5.56	7.54	16.46	7.86	-0.98	12.26	21.95	11.83	1.8
os89295	2	450	0.99	9	3	5	6.57	-0.12	0.35	1.51	0.08	85	-0.49	8.26	7.39	16.37	7.67	-1.2	12.16	21.87	11.68	1.61
os89345	2	543	0.27	9	1	4	6.99	0.08	0.03	1.46	0.1	95.16	1.6	7.01	6.6	15.4	7.07	-1.58	11.31	20.72	10.99	1.15
os89352	2	530	0.38	13	3	5	6.45	-0.02	0	1.45	0.11	73.56	0.87	6.24	6.72	15.51	7.17	-1.52	11.48	20.86	11.09	1.21
os89580	2	459	0.08	6	2	0	6.95	0.09	0.52	1.54	0.08	80.32	-1.51	7.36	7.21	16.07	7.6	-1.23	12.12	21.63	11.69	1.51
os89584	2	462	0.47	12	2	0	6.86	0.03	0.05	1.48	0.09	62.92	1.28	7.08	7.23	16.1	7.62	-1.24	12.17	21.66	11.71	1.54
os89585	2	463	0.75	8	3	5	6.77	-0.04	0.25	1.49	0.08	63.81	1.4	7.03	7.24	16.11	7.63	-1.23	12.18	21.67	11.72	1.55
os89588	2	475	1	7	3	5	6.56	-0.12	0.3	1.52	0.05	99.96	-0.23	6.43	7.32	16.18	7.69	-1.13	12.1	21.59	11.61	1.61

os89604	2	462	0.91	9	3	5	6.45	-0.13	0.43	1.51	0.07	100	-2.17	7.71	7.42	16.31	7.77	-1.02	12.17	21.7	11.66	1.73
os89822	2	543	0.23	13	3	5	6.68	0.1	0	1.45	0.08	99.79	0.2	5.86	6.9	15.81	7.35	-1.37	11.7	21.26	11.28	1.4
os89845	2	560	0.12	8	2	3	6.87	0.1	0.85	1.51	0.13	94.01	-6.51	7.65	6.68	15.5	7.13	-1.63	11.57	20.97	11.14	1.12
os89850	2	560	0.01	20	1	4	7	0.33	0	1.38	0.1	93.1	6.01	5.43	6.68	15.51	7.14	-1.62	11.58	20.97	11.15	1.13
os89851	2	570	0.67	7	3	5	6.62	-0.07	0.72	1.47	0.07	95.3	0.01	6.91	6.69	15.51	7.14	-1.62	11.58	20.98	11.15	1.13
os89934	2	508	0.23	16	1	4	6.57	0.09	0	1.4	0.1	82.09	3.16	5.36	7.05	15.9	7.46	-1.34	11.99	21.45	11.56	1.44
os89974	2	551	0.6	14	3	5	6.85	0.02	0	1.47	0.11	94.24	0.87	6.53	6.64	15.46	7.11	-1.66	11.53	20.92	11.09	1.09
os90027	2	609	0.4	6	3	5	6.94	0.02	0.65	1.47	0.04	99.5	0.81	6.94	6.5	15.32	7	-1.64	11.29	20.72	10.97	1.11
os90120	2	557	0.1	18	2	0	6.8	0.21	0.01	1.48	0.14	88.39	-2.34	7.58	6.57	15.38	7.05	-1.7	11.47	20.85	11.03	1.04
os90121	2	558	0.23	14	2	7	6.66	0.09	0	1.49	0.13	93.61	-3.18	7.14	6.58	15.4	7.07	-1.69	11.49	20.87	11.05	1.06
os90457	2	296	0.48	2	3	6	6.65	-0.04	1.23	1.54	0.03	98.46	0.05	7.97	8.59	17.43	8.69	-0.3	13.36	22.79	12.53	2.43
os90536	2	457	0.01	5	3	6	6.84	0.05	0.17	1.5	0.02	97.25	1.71	6.64	7.74	16.62	7.94	-1.01	12.39	21.85	11.76	1.69
os90576	2	395	0.99	7	3	9	6.56	-0.11	0.32	1.49	0.05	99.1	0.02	6.19	8.05	16.89	8.2	-0.77	12.65	22.02	11.86	1.86
os90645	2	425	0.37	6	1	4	6.9	0.05	0.06	1.47	0.04	91.7	1.96	7.57	7.76	16.67	7.98	-0.91	12.63	22.11	11.98	1.91
os90660	2	468	0.1	14	2	3	6.75	0.15	0.11	1.49	0.12	91.06	-3.17	6.79	7.4	16.33	7.68	-1.15	12.14	21.72	11.63	1.65
os90789	2	277	0.99	8	3	5	6.41	-0.14	0.17	1.5	0.04	94.43	0.72	7.1	8.8	17.64	8.86	-0.16	13.74	23.12	12.84	2.62
os90813	2	461	0.01	5	1	4	6.86	0.07	0.14	1.49	0.06	95.09	2.82	6.67	7.64	16.54	7.83	-1.18	12.43	21.88	11.71	1.58
os90820	2	456	0.04	12	3	5	7.02	0.2	0.02	1.47	0.1	98.23	-1.44	6.11	7.64	16.55	7.84	-1.19	12.45	21.91	11.74	1.58
os90822	2	459	0.06	6	3	5	6.86	0.08	0.79	1.52	0.07	98.45	-1.66	6.8	7.6	16.51	7.8	-1.23	12.41	21.89	11.69	1.54
os90839	2	460	0.4	7	2	0	6.89	0.05	0.67	1.52	0.09	98.99	-1.21	6.6	7.21	16.11	7.57	-1.15	11.78	21.34	11.34	1.54
os91025	2	578	0.23	3	3	6	6.85	0.02	0.1	1.51	0.02	84.75	1.46	6.88	6.78	15.76	7.19	-1.59	11.4	21.07	11.05	1.18
os91075	2	323	0.27	14	2	3	6.53	0.07	0.01	1.51	0.12	88.04	-2.71	7.95	8.28	17.12	8.39	-0.59	13.17	22.54	12.32	2.19
os91200	2	471	0.52	3	3	6	6.76	0	1.33	1.54	0.03	87.01	-0.13	8.46	7.42	16.33	7.71	-1.12	12.18	21.68	11.64	1.68
os91201	2	491	0.92	6	3	5	6.58	-0.09	0.52	1.53	0.05	98.69	-0.22	6.91	7.21	16.17	7.54	-1.28	11.93	21.51	11.46	1.5
os91208	2	434	0.14	7	2	3	6.82	0.08	0.59	1.53	0.07	84.69	-1.86	7.19	7.55	16.43	7.82	-1.01	12.29	21.88	11.8	1.75
os91314	2	476	0	3	3	6	6.69	-0.06	1.76	1.52	0.04	88.82	-0.86	8.62	7.46	16.39	7.73	-1.09	12.24	21.77	11.68	1.71
os91320	2	424	0.55	8	3	5	6.83	0.01	0.28	1.51	0.08	99.93	-0.7	6.33	7.66	16.61	7.89	-1	12.51	22.1	11.88	1.83
os91333	2	520	0.56	8	1	4	6.57	-0.05	0.04	1.45	0.04	95.65	5.1	5.82	7.19	16.15	7.53	-1.31	11.89	21.52	11.45	1.49
os91352	2	411	0.92	3	3	6	6.65	-0.06	1.35	1.54	0.03	88.35	-0.22	7.61	7.83	16.75	8.06	-0.86	12.66	22.31	12.06	1.95
os91361	2	408	0.01	3	3	6	6.85	0.05	0.6	1.54	0.02	84.53	0.13	8.48	7.88	16.79	8.06	-0.88	12.81	22.35	12.14	1.99
os91399	2	547	0.9	3	3	6	6.8	-0.04	1.67	1.53	0.03	83.6	-0.25	8.25	6.98	15.97	7.33	-1.48	11.64	21.31	11.19	1.32
os91405	2	530	0.6	8	3	5	6.6	-0.05	0.39	1.51	0.05	89.64	-0.89	7.05	7.1	16.09	7.41	-1.43	11.82	21.5	11.33	1.4
os91440	2	465	0.16	14	3	5	7.03	0.19	0	1.44	0.13	88.54	0.17	5.71	7.65	16.52	7.84	-1.12	12.3	21.75	11.62	1.58
os91450	2	469	0.09	11	2	3	6.74	0.12	0.04	1.49	0.1	98.66	-2.71	5.34	7.61	16.51	7.82	-1.18	12.36	21.81	11.66	1.56
os91459	2	457	0.01	7	1	4	6.83	0.07	0.02	1.46	0.05	99	5.19	5.78	7.68	16.58	7.86	-1.17	12.5	21.96	11.78	1.6
os91473	2	437	0.39	3	1	4	6.75	-0.01	0.12	1.51	0.05	98.34	2.68	8.09	7.71	16.61	7.91	-1.11	12.48	21.92	11.77	1.63
os91481	2	438	0.92	1	1	4	6.76	-0.01	0.05	1.51	0.04	98.41	2.41	8.36	7.71	16.61	7.9	-1.12	12.48	21.92	11.76	1.63

os91588	2	313	0.57	10	3	5	6.39	-0.06	0.02	1.47	0.1	91.15	-0.14	6.5	8.5	17.32	8.59	-0.39	13.33	22.65	12.39	2.36
os91612	2	399	0.79	4	3	6	6.72	-0.04	1.51	1.53	0.04	98.36	-0.76	7.58	7.93	16.85	8.12	-0.83	12.9	22.36	12.15	2.02
os91634	2	428	0.77	4	3	6	6.76	-0.03	0.18	1.52	0.05	97.04	1.55	7.5	7.62	16.57	7.85	-1.09	12.4	22.04	11.81	1.75

tmin_spring	tmin_summer	tmin_fall	tmin_winter	prec_spring	prec_summer	prec_fall	prec_winter
1.85	9.76	3.28	-3.81	159.93	242.27	172.2	166.99
1.62	9.49	2.96	-4.1	160.53	245.17	175.59	178.38
1.87	9.76	3.29	-3.8	159.92	242.27	172.48	167.54
1.49	9.36	2.84	-4.23	169.68	253.76	182.99	191.93
1.53	9.44	2.96	-4.14	166.59	250.73	179.31	184.95
1.54	9.48	2.99	-4.13	162.6	248.31	179.45	178.43
1.78	9.9	3.22	-4.11	162.36	264.58	173.14	153.21
1.71	9.84	3.1	-4.15	146.78	242.63	165.02	148.28
2.26	10.45	3.61	-3.71	132.72	229.27	148.09	124.24
2.02	10.12	3.38	-3.91	140.63	237.74	157.79	135.98
1.79	9.86	3.16	-4.1	146.24	243.38	165.36	146.8
1.28	9.33	2.84	-4.36	173.56	274.98	193.18	178.25
0.28	8.59	2.28	-5.13	207.18	300.58	205.06	200.55
1.19	9.42	2.91	-4.62	190.01	285.11	186.65	176.07
0.57	8.85	2.43	-4.97	199.38	296.4	199.34	192.42
1.91	10.12	3.46	-4.17	187.67	280.54	180.12	170.27
2.45	10.64	3.91	-3.78	177.57	272.49	171.67	158.68
2.23	10.45	3.74	-3.91	181.67	276.5	176.1	164.35
1.26	9.48	2.96	-4.58	199.05	290.69	189.73	185.51
3.81	12.18	5.02	-2.82	162.85	249.61	156.98	136.28
2.91	11.1	4.28	-3.46	171.19	265.75	164.21	150.53
0.88	9.19	2.76	-4.81	202.85	293.96	195.86	188.92
4.08	12.19	5.1	-2.55	122.5	207	100.02	69.01
3.69	11.82	4.56	-3.26	131.73	216.32	107.31	78.15
3.54	11.6	4.57	-3.27	125.9	214.23	103.56	72.09
3.76	11.84	4.69	-3.07	127.77	213.61	104.16	72.4
3.76	11.84	4.69	-3.07	127.73	213.61	104.16	72.42
3.82	11.93	4.75	-3.03	130.53	213.3	103.76	73.3
3.72	11.85	4.65	-3.25	130.23	214.3	105.08	74.66
3.82	11.95	4.72	-3.15	129.11	213.77	103.65	72.64
3.7	11.84	4.58	-3.27	131.07	214.81	105.02	73.93
3.16	11.28	4.12	-3.74	134.02	222.73	110.85	79.91
3.16	11.28	4.11	-3.74	134.03	222.73	110.85	79.91
3.59	11.7	4.47	-3.37	129.32	215.57	106.64	74.7
3.59	11.66	4.64	-3.2	123.36	214.04	102.45	70.16
3.6	11.68	4.61	-3.16	121.82	211.91	102.21	70.74

3.6	11.67	4.61	-3.16	121.85	211.93	102.24	70.76
3.6	11.68	4.62	-3.15	121.83	211.88	102.16	70.66
3.28	11.47	4.26	-3.58	137.33	220.85	111.06	83.25
3.63	11.8	4.5	-3.37	131.59	218.95	107.17	77.57
3.42	11.58	4.34	-3.46	132.78	219.13	108.77	78.73
3.6	11.78	4.49	-3.34	133.54	218.65	108.25	79.01
3.61	11.75	4.48	-3.38	133.04	219.22	108.44	77.52
3.44	11.62	4.32	-3.51	134.16	221.1	109.83	80.8
4.15	12.21	5.05	-2.71	126.36	211.16	101.86	70.24
3.3	11.48	4.26	-3.57	134.92	217.87	109.86	80.04
3.49	11.66	4.4	-3.41	131.31	218.17	107.5	77.96
3.48	11.65	4.39	-3.42	131.43	218.34	107.66	78.15
3.31	11.47	4.23	-3.56	132.81	219.72	108.98	80.09
3.14	11.34	4.14	-3.71	138.73	223.77	112.3	85.77
3.05	11.29	4.08	-3.79	139.78	222.73	114.83	87.97
3.14	11.47	4.12	-3.75	145.74	228.13	124.16	103.29
3.33	11.61	4.23	-3.63	139.8	222.66	118.87	95.92
3.31	11.59	4.22	-3.65	140.09	223.15	119.25	96.43
2.87	11.17	3.87	-3.95	146.64	228.28	129.34	111.6
3.18	11.49	4.13	-3.74	146.26	227.65	125.5	106.11
2.99	11.35	3.95	-4.04	147.58	231.53	129.64	111.36
2.94	11.26	3.91	-3.84	150.02	231.39	133.21	116.26
2.46	10.77	3.37	-4.49	154.98	239.35	144.7	133.8
2.45	10.76	3.37	-4.5	155.05	239.4	144.75	133.88
2.46	10.77	3.37	-4.49	154.94	239.3	144.67	133.75
2.46	10.77	3.37	-4.49	154.9	239.28	144.65	133.72
2.63	10.98	3.52	-4.3	153.34	238.76	145.73	134.49
2.34	10.66	3.24	-4.51	157.96	247.91	154.75	148.71
2.32	10.65	3.24	-4.53	159.08	249.11	155.72	149.84
3.44	11.75	4.38	-3.46	143.47	226.45	125.77	105.71
3.41	11.68	4.29	-3.56	139.69	222.28	117.95	94.65
3.75	12.03	4.64	-3.14	139.1	218.55	113.26	90.19
3.64	11.85	4.5	-3.28	136.2	215.78	111.75	86.19
3.4	11.6	4.31	-3.52	138.17	218.8	114.78	87.77
3.27	11.5	4.2	-3.65	139.48	220.05	116.25	91.35
3.53	11.7	4.43	-3.46	133.1	215.69	110.07	82.48
3.39	11.58	4.35	-3.58	136.6	218.83	112.8	85.91

2.95	11.2	3.98	-3.95	142.53	226.7	119.57	94.4
2.82	11.08	3.89	-4.08	144.93	228.55	120.8	96.8
2.62	10.88	3.66	-4.26	150.29	235.28	128.02	103.7
2.82	11.13	3.76	-4.18	148.54	233.8	126.52	102.4
2.88	11.17	3.78	-4.15	148.09	232.46	125.63	102
2.88	11.17	3.78	-4.15	148.07	232.43	125.6	101.99
2.89	11.17	3.79	-4.15	148.05	232.36	125.55	101.95
3.84	12.01	4.7	-3.04	132.23	209.7	104.93	75.91
3.4	11.59	4.37	-3.47	135.34	216.92	109.38	80.47
3.88	12.04	4.73	-2.96	132.22	209.11	103.21	73.9
3.88	12.03	4.73	-3.01	130.86	210.3	103.68	75.55
3.89	11.98	4.77	-2.93	129.78	208.98	102.03	72.83
4.02	12.15	4.88	-2.83	128.54	208.35	102.22	71.72
3.7	11.85	4.66	-3.1	133.27	210.55	107.58	80.21
3.55	11.65	4.43	-3.32	132.4	210.77	106.12	79
4	12.13	4.82	-2.87	129.94	208.54	103.19	73.23
3.84	12	4.71	-3.06	131.97	210.52	104.21	74.86
3.72	11.87	4.65	-3.18	130.59	212.3	105.85	76.1
3.84	12.01	4.7	-3.04	132.22	209.71	104.92	75.9
3.5	11.64	4.42	-3.38	133.73	212.66	107.92	80.8
3.6	11.75	4.51	-3.28	131.97	210.26	106.25	78.1
3.59	11.73	4.5	-3.29	132.05	210.52	106.38	78.22
3.26	11.42	4.17	-3.6	135.54	216.55	112.66	85.67
3.29	11.45	4.2	-3.58	136.2	216.52	112.45	85.7
3.34	11.51	4.25	-3.53	134.93	216.16	111.98	84.86
3.35	11.51	4.27	-3.52	134.15	215.87	110.85	83.74
3.21	11.38	4.16	-3.66	136.83	218.54	113.16	87
3.63	11.81	4.58	-3.26	132.77	215.04	107.45	78.45
3.9	12.02	4.86	-2.87	129.13	210.21	102.47	71.79
3.34	11.44	4.45	-3.32	132.04	214.02	107.31	77.5
3.45	11.56	4.48	-3.26	131.69	214.15	106.32	76.65
3.16	11.29	4.24	-3.48	133.27	217.09	108.61	79.98
3.28	11.4	4.34	-3.39	132.74	215.89	107.32	78.7
3.28	11.4	4.34	-3.38	132.73	215.87	107.27	78.64
3.74	11.85	4.72	-3.04	128.83	212.57	104.3	73.34
3.74	11.86	4.73	-3.04	128.77	212.53	104.26	73.29
3.74	11.86	4.73	-3.04	128.77	212.53	104.27	73.27

3.75	11.86	4.73	-3.04	128.72	212.5	104.24	73.23
3.26	11.36	4.41	-3.34	131.53	213.23	107.85	77.97
3.32	11.41	4.31	-3.36	131.82	211.49	105.13	75.95
3.35	11.45	4.36	-3.34	131.67	211.1	104.08	76.13
3.58	11.68	4.52	-3.18	129.51	210	103.9	75.29
3.7	11.76	4.65	-3.04	129.33	205.55	101.56	71.76
3.36	11.48	4.34	-3.35	131.03	208.73	104.22	76.06
3.67	11.75	4.76	-2.92	126.31	208.72	103.47	73.34
3.67	11.74	4.75	-2.93	126.42	208.9	103.59	73.39
2.26	10.63	3.36	-4.49	155.89	241.41	142.12	127.59
3.93	12.13	4.92	-2.92	132.59	210.28	107.59	79.69
2.66	11.01	3.55	-4.28	152.88	238.98	145.64	133.77
2.33	10.66	3.24	-4.52	158.42	248.4	155.12	149.18
3.45	11.63	4.42	-3.35	133.86	212.27	108.04	82.14
2.43	10.84	3.3	-4.4	171.64	261.66	176.63	178.67
2.27	10.56	3.2	-4.55	158.11	243.58	151.04	142.37
2.91	11.28	3.71	-4.08	160.46	246.23	158.95	153.88
2.55	10.91	3.43	-4.35	163.84	250.59	161.66	158.51
2.45	10.86	3.33	-4.4	172.06	260.98	174.79	177.41
2.65	11.02	3.5	-4.25	166.51	254.46	168.34	167.63
2.92	11.29	3.73	-4.07	160.55	246.12	159.95	155.04
2.36	10.7	3.32	-4.44	166.38	252.17	166.67	164.43
2.43	10.76	3.33	-4.46	164.97	252.45	164.18	162.56
2.43	10.76	3.33	-4.46	164.93	252.52	164.21	162.59
2.5	10.83	3.38	-4.4	163.4	250.53	162.65	158.26
2.71	11.09	3.55	-4.23	162.33	249.05	160.94	156.57
2.82	11.18	3.61	-4.12	161.72	248.87	162.3	159.21
2.84	11.21	3.64	-4.1	160.97	248.85	161.3	156.97
2.9	11.24	3.7	-4.08	159.61	244.61	155	146.51
3	11.35	3.82	-3.96	158.63	241.7	151.29	141.95
2.95	11.17	4.02	-3.81	137.37	215.01	112.37	88.5
3.92	12.12	4.9	-2.92	132.75	209.67	107.6	79.38
3.92	12.12	4.9	-2.91	132.68	209.6	107.52	79.32
3.79	11.86	4.8	-2.97	128.55	210.9	104.27	72.63
3.58	11.7	4.59	-3.1	126.69	212.71	103.98	72.9
3.52	11.63	4.55	-3.14	127.02	213.16	104.22	73.88
3.67	11.77	4.67	-2.99	126.82	212.63	104.48	72.45

3.81	11.89	4.8	-2.91	124.19	211.32	102.11	70.1
3.93	12.02	4.97	-2.69	124.33	208.64	101.19	70.19
3.44	11.83	4.26	-3.72	158.17	246.51	143.62	129.66
3.35	11.82	4.18	-3.8	159.74	249.44	148.73	137.02
2.81	11.17	3.65	-4.2	164.52	257.8	150.12	136.6
2.81	11.16	3.64	-4.2	164.56	257.9	150.2	136.69
3.66	11.98	4.41	-3.56	155.8	245.1	142.85	129.51
3.38	11.71	4.14	-3.73	158.14	247.78	143.75	129.41
3.24	11.6	4	-3.85	160.16	250.62	146.79	134.59
3.27	11.61	4.05	-3.83	161.23	250.46	145.44	131.21
3.51	11.91	4.31	-3.68	154.87	244.64	141.15	125.95
3.39	11.73	4.17	-3.8	151.58	236.13	130.05	109.29
3.14	11.52	3.97	-3.99	152.27	237.94	131.72	110
3.15	11.52	3.97	-3.98	152.01	237.77	131.55	109.75
3.15	11.52	3.97	-3.98	151.95	237.73	131.51	109.7
3.16	11.53	3.98	-3.96	152.89	238.39	132.09	110.68
3.16	11.53	3.98	-3.96	152.92	238.42	132.11	110.71
3.16	11.53	3.98	-3.96	152.98	238.47	132.13	110.75
3.17	11.53	3.98	-3.96	153	238.49	132.15	110.77
3.26	11.61	4.06	-3.89	152.28	237.62	131.3	109.43
3.16	11.53	3.98	-3.96	152.86	238.36	132.08	110.66
3.29	11.61	4.06	-3.9	153.14	236.87	130.89	110.19
3.33	11.7	4.06	-3.85	150.51	233.24	133.04	114.69
3.06	11.48	3.88	-4.04	152.46	240.03	139.46	122.4
3.47	11.89	4.23	-3.66	156.17	243.21	146.46	136.33
3.14	11.57	3.93	-3.98	152.33	238.34	138.67	122.28
3.13	11.56	3.92	-3.99	152.25	238.51	138.77	122.17
3.16	11.59	4.16	-3.72	150.15	236.87	139.37	125.19
3.19	11.63	4.19	-3.68	150.09	236.67	139.52	125.07
3.17	11.54	3.97	-3.8	153.18	237.77	142.71	129.85
3.27	11.63	4.02	-3.74	153.48	236.55	144	131.63
3.01	11.42	3.89	-4.06	155.36	239.56	141.1	124.49
2.9	11.27	3.87	-4.14	149.2	232.45	133.42	115.22
3.58	11.99	4.52	-3.44	144.35	229.26	131.95	114.42
3.21	11.6	4.06	-3.87	152.22	239.64	138.95	123.9
3.51	11.92	4.32	-3.61	150.05	234.62	136.03	120.56
3.5	11.9	4.31	-3.62	150.07	235.03	136.55	121.33

3.1	11.52	3.98	-3.95	152.92	237.43	138.74	122.01
2.86	11.26	3.74	-4.26	152.51	237.56	137.11	118.33
2.98	11.38	3.84	-4.17	151.34	235.98	135.33	116.17
2.97	11.38	3.83	-4.17	151.38	236.03	135.4	116.24
2.97	11.38	3.83	-4.17	151.38	236.04	135.41	116.26
3.05	11.41	3.9	-4.12	149.11	233.51	131.33	111.31
3.13	11.48	3.97	-4.06	148.48	233.06	130.3	110.29
3.35	11.72	4.09	-3.81	149.55	232.34	130.54	112.58
2.96	11.4	3.84	-4.09	156.36	243.3	143.66	129
3.05	11.49	3.9	-4.04	154.09	241.44	141.37	125.29
3.24	11.64	4.01	-3.88	152.09	237.23	137.06	119.99
3.26	11.67	4.02	-3.87	151.65	237.03	136.66	119.53
3.26	11.67	4.02	-3.87	151.63	236.9	136.66	119.48
3.27	11.68	4.03	-3.86	151.46	236.83	136.51	119.27
3.26	11.65	4.01	-3.88	151.61	236.76	136.7	119.25
3.4	11.85	4.13	-3.79	154.04	238.21	138.44	122.29
3.4	11.84	4.13	-3.8	154.22	238.2	138.46	122.36
3.37	11.82	4.1	-3.82	154.52	238.73	138.97	123.33
2.7	11.01	3.55	-4.22	156.65	243.96	149.21	139.87
3.49	11.83	4.25	-3.58	154.01	234.21	142.55	129.57
3.32	11.77	4.15	-3.76	155.61	241.56	146.36	133.68
3.31	11.76	4.14	-3.77	155.89	241.76	146.63	134.04
3.72	12.12	4.48	-3.49	149.16	236.18	137.4	122.19
3.32	11.67	4.02	-3.82	158.54	244.67	153.27	144.82
3.55	12.03	4.34	-3.64	156.76	243.03	146.19	131.5
3.33	11.79	4.11	-3.79	157.02	244.34	148.98	136.67
3.37	11.95	4.2	-3.85	163	249.9	152.64	142.28
2.97	11.32	3.72	-4.08	164.04	252.13	163.18	158.69
3.46	11.88	4.16	-3.7	157.87	243.81	151.88	143.36
3.51	11.96	4.3	-3.64	159.09	247.74	150.88	141.91
3.36	11.75	4.1	-3.85	161.54	247.23	159.28	152.97
3.62	12.08	4.37	-3.61	157.94	245.98	151.63	139.93
2.95	11.46	3.81	-4.03	163.97	251.85	155.33	147.86
2.62	11.02	3.47	-4.32	171.79	259.29	177.59	178.96
3.63	12.12	4.39	-3.61	153.24	238.4	138.97	122.93
3.21	11.81	4.06	-3.98	165.73	252.32	155.11	144.07
3.34	11.93	4.17	-3.87	163.61	251.13	153.64	143.63

3.47	11.84	4.19	-3.73	147.77	233.85	132.5	114.09
3.58	11.97	4.3	-3.63	148.73	231.92	131.73	113.24
3.33	11.6	4.12	-3.88	142.96	227.27	120.42	94.12
3.33	11.6	4.12	-3.87	142.98	227.28	120.42	94.12
3.31	11.65	4.07	-3.89	150.92	236.55	131.78	112.28
3.56	11.95	4.27	-3.67	146.82	234.82	131.59	112.7
3.61	12.06	4.35	-3.65	151.61	238.24	137.79	120.29
3.58	11.93	4.3	-3.64	143.35	226.82	121.01	97.03
3.34	11.68	4.13	-3.85	151.32	236.8	130.37	109.61
2.83	11.22	3.69	-4.28	150.72	235.33	133.13	114.59
3.67	12.15	4.43	-3.56	151.96	236.98	137.91	121.4
3.4	11.72	4.12	-3.76	143.68	229.04	123.59	100.18
3.25	11.58	4.01	-3.91	150.12	232.26	128.48	106.95
3.26	11.58	4.01	-3.91	150.03	232.14	128.37	106.82
3.27	11.6	4.02	-3.9	149.76	231.79	128.07	106.43
3.27	11.59	4.02	-3.9	149.85	231.9	128.16	106.56
3.26	11.59	4.02	-3.9	149.94	232.02	128.27	106.69
3.25	11.57	4	-3.95	151.41	235.34	129.76	109.39
3.29	11.59	4.03	-3.93	150.25	235.02	128.64	108.43
3.34	11.69	4.11	-3.88	152.43	238.82	133.74	114.08
3.33	11.69	4.11	-3.88	152.44	238.84	133.76	114.09
3.33	11.69	4.11	-3.88	152.46	238.86	133.77	114.1
3.34	11.67	4.1	-3.87	149.97	235.73	131.04	111.3
3.34	11.67	4.1	-3.87	150.15	235.85	131.04	111.38
3.33	11.67	4.1	-3.87	150.22	235.91	131.1	111.45
3.33	11.67	4.09	-3.87	150.36	236.02	131.18	111.57
3.26	11.61	4.02	-3.94	151.88	237.94	133.51	114.14
3.24	11.59	4.01	-3.95	152.32	237.9	133.68	114.37
3.57	11.93	4.27	-3.67	146.45	233.12	131.2	110.45
3.6	11.98	4.31	-3.62	148.49	231.66	131.24	112.87
3.39	11.85	4.15	-3.82	153.03	238.78	137.44	120.98
3.7	12.11	4.4	-3.57	148.73	233.3	132.65	115.22
3.7	12.11	4.4	-3.57	148.74	233.28	132.64	115.22
3.7	12.11	4.4	-3.57	148.74	233.28	132.63	115.21
3.7	12.11	4.4	-3.57	148.74	233.27	132.62	115.21
3.71	12.12	4.41	-3.57	148.57	233.42	132.83	115.19
3.71	12.12	4.41	-3.57	148.58	233.42	132.82	115.2

3.72	12.13	4.42	-3.56	148.36	232.87	132.4	114.81
3.72	12.13	4.42	-3.56	148.37	232.86	132.39	114.81
3.72	12.13	4.42	-3.56	148.37	232.85	132.38	114.81
3.63	11.99	4.35	-3.64	146.4	231.9	129.59	109.37
3.13	11.49	3.96	-3.98	145.76	230.11	123.46	99.4
3.06	11.34	3.93	-4.08	143.96	231.76	122.85	97.52
3.09	11.39	3.9	-4.04	146.37	231.64	123.43	99.61
3.21	11.53	3.99	-3.93	144.57	231.14	122.7	98.08
3.35	11.72	4.09	-3.84	144.28	230.19	123.93	100.98
3.32	11.84	4.13	-3.86	160.98	250.42	149.85	138.65
3.74	12.14	4.49	-3.48	150.86	240	137.24	120.9
3.55	12.02	4.32	-3.64	154.61	244.54	142.07	127.57
3.75	12.13	4.48	-3.5	150.47	240.23	137.3	121.36
3.54	12	4.33	-3.64	156.35	246.1	142.96	128.53
3.56	12.02	4.35	-3.62	155.62	245.73	142.47	127.85
3.57	12.02	4.36	-3.61	155.14	245.48	142.12	127.48
3.57	12.02	4.36	-3.61	155.1	245.46	142.08	127.44
3.45	11.81	4.26	-3.71	155.7	245.02	140.09	124.54
3.12	11.48	3.99	-3.94	160.69	250.38	145.68	130.7
3.32	11.64	4.04	-3.86	144.58	229.2	124.72	100.72
1.91	10.26	2.9	-4.76	184.77	279.07	203.47	216.43
1.91	10.26	2.91	-4.76	184.78	278.96	203.46	216.36
1.94	10.28	2.93	-4.74	184.76	278.58	202.65	215.49
1.89	10.25	2.9	-4.77	184.91	279.29	203.79	216.82
1.91	10.26	2.9	-4.76	184.78	279.32	203.37	216.5
2.35	10.68	3.27	-4.44	175.46	262.25	180.86	185.05
2.56	10.93	3.4	-4.23	169.15	257.81	178.05	180.52
1.67	10.04	2.74	-4.92	190.3	286.06	210.01	227.87
1.95	10.29	2.94	-4.73	184.82	278.33	202.46	215.18
1.95	10.29	2.95	-4.73	184.85	278.24	202.34	215.04
1.82	10.17	2.86	-4.8	184.43	276.1	198.21	209.85
2.64	11.02	3.46	-4.19	167.58	255.83	174.51	176.54
2.4	10.78	3.28	-4.4	176.01	264.87	188.77	196.26
1.77	10.11	2.82	-4.87	183.61	275.86	196.97	209.08
1.91	10.24	2.92	-4.79	180.6	272.8	193.48	203.25
1.8	10.09	2.97	-4.78	177.73	269.49	179.73	183.01
3.27	11.29	4.5	-3.59	123.4	213.09	103.2	70.73

3.28	11.3	4.51	-3.58	123.17	213.04	103.12	70.63
3.3	11.32	4.52	-3.57	122.67	212.94	103	70.42
3.32	11.34	4.54	-3.56	122.21	212.81	102.91	70.26
3.32	11.29	4.51	-3.61	123.22	213.37	103.43	70.66
3.3	11.28	4.5	-3.62	123.58	213.62	103.7	70.81
3.28	11.26	4.48	-3.64	123.98	213.91	104.03	71.01
3.52	11.56	4.7	-3.34	120.43	209.7	102.78	68.07
3.52	11.56	4.7	-3.34	120.44	209.7	102.78	68.07
3.53	11.56	4.7	-3.31	120.81	210.65	101.97	68.01
3.53	11.56	4.7	-3.31	120.84	210.67	102	68.03
3.3	11.32	4.49	-3.56	124.36	214.07	104.62	70.37
3.37	11.37	4.54	-3.51	123.14	212.58	104.14	69.87
3.18	11.15	4.43	-3.72	125.98	214.9	105.35	72.06
2.77	10.76	4.07	-4.03	129.33	220.46	109.2	76.27
2.72	10.72	4.04	-4.07	130.04	221.24	109.84	76.68
3.5	11.54	4.7	-3.37	123	214.76	103.89	69.14
3.63	11.66	4.78	-3.25	120.21	209.34	101.02	67.46
3.61	11.68	4.81	-3.3	120.53	209.25	101.11	66.75
3.63	11.66	4.78	-3.25	120.2	209.31	101.01	67.42
3.31	11.33	4.46	-3.47	125.97	216.73	104.48	71.77
3.54	11.59	4.62	-3.27	123.29	214.33	103.08	70.34
3.54	11.58	4.62	-3.27	123.45	214.52	103.14	70.39
3.59	11.63	4.67	-3.24	123.76	214.99	102.77	70.68
3.52	11.62	4.58	-3.26	124.8	214.47	102.9	70.9
3.71	11.79	4.89	-3.22	119.04	210.28	100.3	66.07
3.79	11.9	4.94	-3.16	118.8	208.62	99.41	64.86
3.74	11.76	4.83	-3.15	119.4	207.96	99.4	65.83
3.46	11.5	4.59	-3.34	123.68	212.74	101.55	69.33
3.52	11.56	4.63	-3.26	121.82	212.02	101.07	67.89
3.7	11.87	4.62	-3.31	129.22	216.15	106.85	76.09
3.53	11.59	4.49	-3.52	129.42	217.26	105.84	74.46
3.46	11.59	4.47	-3.54	131.03	220.29	107.74	76.53
2.34	10.54	3.56	-3.88	151.72	246.14	160.44	154.66
1.89	10.16	3.21	-4.16	162.65	258.18	175.03	174.51
2.16	10.41	3.41	-3.99	152.36	248.55	162.46	157.78
2.2	10.43	3.44	-3.96	152.25	247.55	161.6	157.31
2.14	10.39	3.39	-4.02	152.19	249.57	162.32	157.08

1.84	10.14	3.15	-4.25	162.93	260.84	175.12	175.29
2.21	10.4	3.47	-3.96	156.35	248.49	166.98	163.14
2.11	10.44	3.4	-4.02	157.67	257.85	168.5	163.44
2.25	10.42	3.48	-3.95	155.17	245.44	165.76	161.53
1.64	10.07	2.93	-4.62	183.36	283.02	196.58	198.82
1.55	9.99	2.8	-4.65	184.94	283.55	197.82	204.92
1.85	10.26	3.14	-4.43	176.25	275.56	187.2	184.74
1.75	9.94	3.07	-4.39	167.57	251.15	179.69	182.38
1.61	9.78	2.96	-4.43	164.88	253.41	180.02	180.53
1.93	10.11	3.21	-4.16	147.18	242.48	158.96	154.25
2.04	10.25	3.28	-4.08	138.53	239.28	150.44	142.47
2.01	10.24	3.27	-4.13	141.67	241.29	152.45	144.43
2.03	10.25	3.3	-4.13	143.94	245.34	155.95	149.68
1.91	10.16	3.2	-4.22	148.61	249.92	161.93	157.68
1.86	10.11	3.15	-4.26	150.19	251.46	163.66	159.73
1.61	9.92	2.97	-4.42	158.36	258.42	171.98	171.24
1.85	10.09	3.1	-4.25	149.68	248.28	157.21	152.82
1.74	10.01	3.05	-4.33	154.68	253.63	165.65	163.18
1.71	9.98	3.02	-4.36	157.07	255.05	168.01	166.12
1.84	10.1	3.12	-4.28	153.9	251.71	163.69	160.81
1.98	10.22	3.24	-4.18	154.89	251.69	165.95	162.01
2.11	10.3	3.35	-4.1	155.2	249.83	168.02	163.65
1.92	10.17	3.2	-4.21	161.05	255.58	172.81	170.67
1.62	9.9	2.97	-4.43	163.62	262.66	179.44	180.64
1.85	10.03	3.09	-4.27	152.01	245.26	164.25	159.83
2.14	10.33	3.34	-4	147.83	243.52	157.9	151.27
1.78	10.01	3.03	-4.32	153.83	248.64	164.03	160.83
2.08	10.24	3.25	-4.08	147.12	240.97	158.66	151.7
1.89	10.06	3.13	-4.24	150.3	243.67	162.23	157.17
1.82	10.08	3.11	-4.29	151.93	253.08	166.02	162.8
1.62	9.93	2.98	-4.42	157.94	258.17	171.75	170.79
1.59	9.9	2.95	-4.44	159.32	259.26	173.17	172.64
2.03	10.24	3.3	-4.13	143.98	245.38	156	149.75
1.9	10.14	3.2	-4.21	146.02	247.34	158.43	152.99
1.89	10.13	3.19	-4.22	146.42	247.87	159.02	153.46
1.73	10	3.04	-4.34	157.06	254.54	167.75	165.63
2.13	10.29	3.31	-4.01	147.33	241.98	158.58	152.18

2.14	10.33	3.34	-4	147.87	243.57	157.91	151.27
2.16	10.33	3.31	-4.01	148.81	242.38	160	154.91
1.65	9.89	2.93	-4.4	158.5	253.41	169.73	168.55
1.63	9.88	2.91	-4.41	159.22	254.31	170.88	170.01
1.62	9.88	2.93	-4.42	159.45	256.05	172.77	172.14
1.9	10.01	3.17	-4.16	142.74	237.36	153.96	149.08
1.92	10.03	3.22	-4.15	144.26	236.44	155.83	151.19
1.78	9.87	3.1	-4.25	151.83	242.39	165.69	163.06
1.92	10.03	3.1	-4.19	132.59	231.92	142.28	132.44
1.8	9.94	3	-4.28	132.65	234.22	143.4	135.51
2	10.18	3.22	-4.09	134.29	232.52	145.47	136.7
1.42	9.66	2.79	-4.5	159.21	252.91	173.83	174.36
1.77	9.84	3.14	-4.21	132.07	238.07	144.62	132.75
1.51	9.6	2.86	-4.46	137.68	240.16	150.77	141.86
1.6	9.75	2.88	-4.39	144.72	244.87	158.7	154.73
1.88	9.99	3.16	-4.17	143.15	237.73	154.58	149.69
1.71	10	3.03	-4.32	170.14	264.77	181.86	184.37
1.75	10.04	3.07	-4.34	167.23	263.73	179.7	181.46
2.08	10.37	3.36	-4.1	162.65	260.98	173.42	171.59
1.85	10.16	3.19	-4.26	175.23	272.5	187.25	189.58
1.87	10.21	3.21	-4.21	167.9	266.67	179.89	180.36
1.96	10.23	3.22	-4.24	175.01	269.69	185.12	188.15
2.15	10.4	3.41	-4.09	168.14	263.15	175.44	173.66
2.09	10.32	3.34	-4.1	170.8	267.33	178.06	177.55
1.7	10.01	3.03	-4.4	182.36	279.89	193.61	198.43
1.63	9.94	3	-4.44	187.61	285.75	199.81	206.7
1.92	10.32	3.22	-4.34	174.1	274.21	184.85	181.82
1.82	10.12	3.15	-4.28	166.9	263.18	178.95	178.64
2.09	10.39	3.38	-4.04	157.77	257.62	169.42	165.97
1.89	10.25	3.23	-4.21	167.4	267.28	179.67	178.21
2.1	10.46	3.41	-4	161.12	260.15	170.81	165.73
2.05	10.38	3.38	-4.08	163.91	262.98	175.09	171.28
1.97	10.36	3.3	-4.07	165.99	269.37	178.92	174.26
1.69	10.13	2.96	-4.55	178.59	279.96	192.02	192.13
1.91	10.2	3.2	-4.21	159.55	259.16	170.63	168.91
2.02	10.3	3.3	-4.14	157.38	255.41	168.97	165.57
1.64	10.08	3.03	-4.42	177.96	281.54	191.58	192.71

1.99	10.28	3.28	-4.16	159.1	257.12	170.21	167.03
1.98	10.28	3.28	-4.16	159.13	257.13	170.24	167.05
1.99	10.28	3.29	-4.15	158.89	256.98	169.96	166.66
2.12	10.41	3.4	-4.06	160.75	259.28	171.09	168.67
1.98	10.3	3.31	-4.13	164.64	263.19	176.02	174.75
2.01	10.32	3.33	-4.13	165.23	263.44	176.07	174.91
1.65	9.96	3.02	-4.42	186.89	284.99	198.91	205.29
1.64	9.95	3.01	-4.43	187.33	285.47	199.44	206.08
1.83	10.19	3.2	-4.25	175.15	274.97	187.11	188.24
1.83	10.19	3.2	-4.25	175.16	275.02	187.16	188.31
1.89	10.24	3.25	-4.21	173.12	273.07	184.69	184.6
1.91	10.26	3.27	-4.19	172.22	272.26	183.66	183.06
2.09	10.44	3.38	-4	160.76	262.28	171.01	165.08
1.62	9.95	3.01	-4.43	183.3	282.86	197.27	204.03
1.87	10.24	3.22	-4.22	166.76	267.55	178.93	177.96
1.81	10.1	3.1	-4.31	178.07	276.04	188.8	191.13
2.03	10.1	3.36	-4.03	129.51	222.84	139.66	125.18
2.03	10.38	3.1	-4.63	172.54	264.58	178.45	180.9
2.05	10.4	3.13	-4.61	172.25	264.18	177.98	180.07
2.35	10.7	3.4	-4.34	168.65	259.03	173.27	174.1
2.25	10.62	3.21	-4.52	194.02	292.87	191.71	196.29
2.72	11.05	3.56	-4.21	175.88	273.5	167.25	162.5
1.97	10.38	3	-4.72	199.01	301.11	190.77	195
2.02	10.39	3.05	-4.63	202.72	300.77	199.27	208.46
2.17	10.45	3.2	-4.53	204.02	304.45	193.67	197.01
2.83	11.08	3.63	-4.12	173.61	269.99	162	154.93
1.99	10.33	3.01	-4.83	199.08	298.19	186.7	186.84
2.18	10.57	3.14	-4.66	193.98	291.08	184.09	183.83
1.79	10.05	2.72	-5.13	192.42	291.27	172.05	166.34
2.23	10.58	3.2	-4.54	195.67	294.36	189.09	193.19
2.26	10.56	3.28	-4.48	200.14	299.54	190.08	194.07
2.17	10.56	3.18	-4.54	196.54	296.02	194.51	200.77
2.18	10.56	3.18	-4.54	196.39	295.85	194.35	200.53
2.2	10.58	3.2	-4.53	195.9	295.21	193.79	199.7
2.21	10.59	3.21	-4.52	195.52	294.72	193.35	199.06
2.66	10.95	3.51	-4.27	180.3	279.6	171.05	167.59
2.84	11.08	3.63	-4.11	175.43	272.06	164.07	158.23

2.84	11.08	3.64	-4.11	175.34	271.97	164.02	158.17
2.49	10.79	3.36	-4.39	180.8	279.6	168.06	163.7
2.45	10.77	3.33	-4.43	181.33	280.38	168.57	164.47
2.57	10.85	3.43	-4.34	179.56	278.1	167.29	162.84
2.58	10.85	3.44	-4.33	179.46	277.97	167.17	162.68
2.59	10.86	3.44	-4.33	179.35	277.82	167.05	162.52
2.34	10.65	3.22	-4.54	181.77	281.85	170.07	166.07
2.36	10.66	3.24	-4.53	181.27	281.3	169.58	165.4
2.37	10.69	3.27	-4.48	182.77	282.36	170.74	166.87
2.23	10.53	3.13	-4.63	181.46	280.6	169.36	163.95
2.52	10.79	3.39	-4.39	180.17	277.55	168.01	163
2.53	10.79	3.4	-4.38	180.09	277.38	167.83	162.8
2.24	10.54	3.26	-4.47	201.47	301	190.65	193.02
2.25	10.55	3.27	-4.47	201.17	300.57	190.3	192.55
2.26	10.55	3.27	-4.46	200.98	300.3	190.08	192.25
2.23	10.52	3.13	-4.62	183.78	280.03	168.93	164.36
2.33	10.61	3.24	-4.51	186.47	283.21	173.06	167.95
1.88	10.26	2.93	-4.9	203.45	303.89	193.65	197.08
1.91	10.32	2.95	-4.82	199.96	298.14	189.13	189.83
1.95	10.32	2.95	-4.85	199.42	298.44	188.95	190.1
2.08	10.45	3.08	-4.63	198.02	298.4	196.5	204.07
1.91	10.3	2.98	-4.75	203.79	304.69	201.51	209.94
2.14	10.49	3.14	-4.59	197.61	296.95	195.53	202.44
2.41	10.69	3.28	-4.51	178.36	274.66	163.09	156.09
3.03	11.39	3.82	-3.96	173.89	266.78	166.7	164.72
3.04	11.4	3.82	-3.96	173.62	266.56	166.49	164.41
2.83	11.19	3.63	-4.1	176.56	272.81	168.86	166.3
2.22	10.5	3.11	-4.68	181.77	280.4	168.93	163.05
1.2	9.44	2.25	-5.68	208.82	314.36	185.79	177.9
1.31	9.53	2.31	-5.63	206.21	311.24	182.77	173.56
1.37	9.63	2.33	-5.55	200.71	302.75	178.3	172.31
2.55	10.8	3.4	-4.41	175.21	270.85	159.94	150.58
2.51	10.76	3.36	-4.45	176.09	271.87	161.05	151.99
1.89	10.16	2.86	-4.95	192.2	291.39	178.96	174.8
2.03	10.29	2.97	-4.88	173.86	268.46	150.97	131.47
2.24	10.49	3.05	-4.79	172.81	268.61	150.3	134.36
2.11	10.41	2.99	-4.86	173.64	268.58	150.54	132.92

2.4	10.6	3.19	-4.6	171.01	268.48	152.96	140.92
1.59	9.83	2.53	-5.41	199.64	301.24	178.52	173.37
2.77	10.99	3.52	-4.31	164.96	259.86	147.56	132.74
2.55	10.76	3.33	-4.47	170.04	266.79	152.25	139.55
2.64	10.82	3.39	-4.42	165.69	262.44	148.13	134.33
1.39	9.66	2.36	-5.54	199.68	301.98	177.71	171.01
1.9	10.13	2.89	-4.96	196.98	293.16	178.24	174.27
1.65	9.95	2.59	-5.26	192.03	292.04	170.89	163.85
2	10.29	2.94	-4.9	189.27	288.08	174.05	167.58
2.1	10.39	3.05	-4.83	184.54	281.52	168.94	161.17
1.96	10.24	2.91	-4.93	190.78	289.46	175.3	169.69
1.81	10.05	2.82	-5.02	200.34	297.18	181.6	178.68
1.82	10.07	2.8	-5.03	199.19	296.77	179.95	176.74
2.24	10.46	3.08	-4.69	176.46	274.44	158.06	146.54
2	10.25	2.9	-4.9	183.97	284.58	165.93	156.68
1.99	10.25	2.9	-4.92	186.02	284.99	166.8	158.58
2.11	10.37	3.04	-4.84	183	281.39	167.4	159.96
2.11	10.37	3.04	-4.83	182.94	281.26	167.36	159.89
2.01	10.26	2.93	-4.92	186.59	285	170.55	162.85
2.03	10.27	2.96	-4.9	186.26	284.54	169.26	161.89
1.87	10.12	2.88	-4.97	197.33	294.03	179.36	175.79
2.05	10.26	2.97	-4.88	189.86	286.78	171.49	165.74
2.05	10.27	2.97	-4.88	189.84	286.76	171.46	165.71
2.05	10.3	2.94	-4.88	183.98	282.71	164.52	156.98
1.76	10.04	2.72	-5.06	190.91	293.24	171.94	163.76
1.95	10.19	2.84	-4.98	187.75	286.74	166.45	157.69
2.49	10.74	3.36	-4.51	176.5	271.39	159.8	151.84
1.65	9.93	2.63	-5.22	200.47	300.86	180.71	177.81
1.52	9.77	2.48	-5.38	200.75	301.94	180.46	175.97
1.93	10.16	2.86	-5	192.19	291.05	173.97	168.35
1.86	10.1	2.8	-5.04	192.13	291.82	174.26	168.06
1.48	9.73	2.45	-5.43	203.03	303.78	182.51	177.68
1.69	9.93	2.7	-5.12	195.15	295.94	173.67	162.96
1.66	9.96	2.56	-5.3	190.83	289.53	169.33	161.6
1.44	9.72	2.39	-5.5	198.37	300.1	176.11	169.65
1.51	9.78	2.43	-5.45	196.52	297.82	173.82	167.19
1.52	9.73	2.49	-5.45	198.18	299.45	175.52	166.03

1.52	9.72	2.49	-5.45	198.29	299.56	175.61	166.1
1.32	9.62	2.31	-5.54	199.73	303.36	180.48	175
2.05	10.33	2.98	-4.8	189.79	286.57	173.97	170.7
2.38	10.63	3.18	-4.71	171.25	265.78	148.45	130.81
1.46	9.65	2.46	-5.47	197.5	298.51	172.3	162.38
2.11	10.34	2.99	-4.83	172.26	267.89	147.4	126.56
2.62	10.84	3.39	-4.47	167.33	261.5	149.7	134.48
2.58	10.82	3.37	-4.51	166.83	262.18	147.94	132.97
2.79	11.01	3.58	-4.23	166.48	261.62	149.9	136.84
2.76	11	3.57	-4.22	167.2	260.96	152.22	140.01
2.58	10.81	3.4	-4.46	168.88	263.65	153.44	138.6
2.95	11.14	3.72	-4.06	163.71	259.11	150.14	137.22
2.95	11.14	3.72	-4.06	163.65	259.21	150.12	137.28
2.95	11.14	3.72	-4.07	163.5	259.43	150.11	137.49
2.93	11.14	3.71	-4.1	163.46	260.23	150.36	138.12
2.65	10.86	3.47	-4.35	173.19	269.09	156.03	146.31
2.63	10.89	3.47	-4.35	173.93	269.34	157.67	148.36
2.52	10.75	3.37	-4.46	174.82	271.55	158.87	148.3
2.71	10.93	3.46	-4.36	166.07	260.34	148.21	133.2
2.74	10.93	3.49	-4.37	164.59	258.15	147.08	130.1
2.69	10.9	3.44	-4.41	165.41	259.32	147.57	131.29
2.54	10.76	3.3	-4.54	166.57	262.36	148.27	132.09
2.54	10.76	3.3	-4.54	166.56	262.35	148.25	132.07
2.57	10.82	3.33	-4.56	164.06	259.7	145.54	129.03
2.72	10.9	3.49	-4.32	165.67	262.44	149.02	135.7
2.56	10.77	3.34	-4.46	169.66	266.6	152.15	139.33
2.52	10.74	3.32	-4.48	170.56	267.6	152.88	140.37
2.49	10.72	3.29	-4.5	171.24	268.5	153.57	141.36
2.25	10.47	3.11	-4.71	177.92	278.06	161.42	152.44
2.36	10.6	3.13	-4.71	168.98	265.59	148.66	131.19
2.3	10.53	3.08	-4.75	170.46	267.04	149.56	132.77
2.32	10.58	3.14	-4.74	172.14	267.01	149.39	131.81
1.98	10.19	2.88	-4.93	185.03	282.98	163.73	151.58
2.74	10.94	3.49	-4.39	164.73	257.3	146.07	129.75
2.33	10.73	3.24	-4.52	186.41	280.08	196.59	210.65
2.85	11.24	3.64	-4.16	179.18	270.26	176.41	178.47
2.56	10.95	3.42	-4.31	175.74	264.03	185.17	189.62

2.33	10.74	3.22	-4.48	179.73	269.55	191.42	199.59
2.54	10.9	3.38	-4.35	179.06	265.78	186.29	193.44
2.26	10.63	3.19	-4.58	189.4	282.63	200.01	215.74
2.32	10.75	3.2	-4.54	186.02	277.43	190.17	199.49
2.41	10.84	3.32	-4.46	182.34	272.97	188.52	198.52
2.61	10.95	3.47	-4.33	179.54	269.99	187.04	196.19
3.2	11.65	3.96	-3.96	167.64	258.91	165.68	162.25
3.17	11.66	3.94	-3.98	165.22	257.86	163.38	160.02
3.17	11.66	3.93	-3.99	165.27	257.95	163.48	160.16
1.14	9.56	2.38	-5.19	225.41	324.8	244.92	284.5
1.57	9.91	2.75	-4.95	217.47	317.14	218.86	237.37
1.72	10.09	2.83	-4.85	210.91	311.36	209.65	223.76
2.02	10.39	3.08	-4.64	204.13	300.65	201.74	214.86
1.84	10.22	2.93	-4.77	207.32	306.92	206.56	221.23
1.78	10.14	2.98	-4.73	212.33	313.1	215.73	234.53
2.58	10.98	3.46	-4.32	183.84	278.59	186.67	191.49
1.61	10.03	2.76	-4.92	211.11	313.57	212.78	228.48
1.66	10.01	2.88	-4.85	213.77	313.83	216.29	234.05
2.77	11.19	3.61	-4.22	178.14	272.64	179	181.98
2.52	10.87	3.46	-4.26	191.67	288.58	188.25	193.59
2.41	10.75	3.35	-4.34	193.25	292.41	191.32	198.44
1.9	10.27	2.99	-4.72	207.47	304.18	205.6	219.33
2.1	10.49	3.13	-4.58	200.05	298.74	199.38	210.44
2.11	10.5	3.15	-4.56	199.7	298.18	198.9	209.81
2.33	10.7	3.31	-4.42	195.46	291.87	192.91	202.73
2.61	11	3.48	-4.32	185.15	279.74	186.31	191.86
2.53	10.97	3.38	-4.38	182.42	273.12	186.64	192.49
2.44	10.85	3.36	-4.39	191.42	285.18	188.71	196.19
1.99	10.42	3.06	-4.7	199.4	295.45	206.88	224.94
2.96	11.38	3.74	-4.06	173.64	265.16	171.18	168.62
2.6	11.04	3.51	-4.25	185.31	279.1	182.31	185.53
2.49	10.92	3.43	-4.33	189.6	282.95	186.44	190.96
2.5	10.93	3.44	-4.32	189.28	282.57	186.03	190.41
2.43	10.81	3.38	-4.34	194.03	289.28	190.3	196.57
2.37	10.76	3.34	-4.38	195.31	290.77	191.6	198.34
2.74	10.94	3.49	-4.39	164.9	257.6	146.75	130.15
1.66	10.07	2.77	-4.97	207.67	305.6	221.58	248.36

1.51	9.95	2.57	-5.05	208.55	304.28	223.48	252.5
2.12	10.53	3.08	-4.66	192.73	288.01	204.5	223.65
1.63	10.03	2.74	-4.99	207.73	306.02	222.18	249.84
1.64	10.04	2.72	-4.94	207.86	303.52	224.64	253.1
1.46	9.89	2.58	-5.02	215.05	311.42	232.04	264.85
1.48	9.91	2.61	-5.02	212.42	309.4	231.27	262.09
1.48	9.92	2.61	-5.02	212.21	309.13	231	261.71
1.73	10.1	2.76	-4.88	202.96	300.23	220.16	246.25
1.7	9.93	2.66	-5.38	209.56	315.87	190.11	187.74
1.76	10.05	2.77	-5.09	210.13	312.81	195.95	198.55
1.77	10.02	2.79	-5.2	212.39	315.95	194.91	194.39
1.64	9.89	2.67	-5.32	215.49	323.59	198.64	198.01
1.26	9.64	2.44	-5.37	223.76	330.88	213	219.65
1.33	9.71	2.48	-5.34	220.87	326.46	209.29	216.14
1.88	10.15	2.91	-4.96	208.6	311.45	195.35	198.73
2.18	10.41	3.12	-4.76	196.67	294.41	178.66	178.57
1.99	10.36	3.02	-4.7	187.04	281.82	206.41	225.36
2.05	10.35	3.11	-4.62	193.01	287.67	210.71	232
1.65	10	2.73	-4.93	188.71	282.39	204.44	218.9
1.84	10.14	2.85	-4.86	192.99	289.48	212.2	232.13
1.7	10.05	2.74	-4.95	195.07	291.86	213.64	233.79
1.34	9.73	2.45	-5.16	196.14	295.1	219.13	242.37
1.52	9.9	2.62	-5.01	193.95	292.11	211.28	231.8
1.94	10.35	2.9	-4.7	183.15	274.97	199.87	212.92
1.96	10.36	2.92	-4.69	182.87	274.16	199.26	211.94
2.07	10.5	3.03	-4.63	182.78	275.56	198.75	211.83
1.62	10.05	2.75	-4.9	199.26	297.13	222.3	248.57
1.27	9.67	2.43	-5.19	202.84	303.21	226.81	255.11
1.68	10.05	2.79	-4.89	199.64	297.18	223.07	248.46
2	10.37	3.02	-4.7	186.9	281.41	206.06	224.82
2	10.38	3.02	-4.69	186.68	280.24	205.15	223.48
2.05	10.35	3.11	-4.63	192.08	287.21	210.1	231.24
1.77	10.1	2.88	-4.86	199.86	296.12	221.25	246.43
1.63	9.98	2.81	-4.85	203.07	300.95	226.14	255.97
2.36	10.74	3.28	-4.39	173.64	265.78	187.13	194.57
2.36	10.73	3.26	-4.4	174.03	266.29	187.93	195.54
1.23	9.67	2.45	-5.03	198.37	300.57	217.93	236.78

1.89	10.27	2.95	-4.67	179.84	276.15	193.24	202.49
1.51	9.88	2.62	-5.02	191.76	286.33	208.37	226.21
1.51	9.88	2.62	-5.02	191.81	286.45	208.51	226.45
1.59	9.95	2.68	-4.98	190.11	284.35	206.43	222.52
1.6	9.96	2.68	-4.97	190.03	284.2	206.27	222.23
1.59	9.96	2.68	-4.98	190.04	284.29	206.37	222.4
1.34	9.8	2.51	-4.95	189.53	290.12	209.57	222.06
1.32	9.78	2.49	-4.96	190.01	290.65	210.24	223.13
1.37	9.83	2.53	-4.92	188.72	289.25	208.41	220.3
1.53	9.91	2.64	-4.98	190.45	287.51	209.5	228.52
1.59	9.97	2.7	-4.93	188.55	284.33	205.68	223.18
1.68	10.09	2.75	-4.78	181.93	282.44	198.8	209.87
1.71	10.09	2.82	-4.79	185.37	281.32	199.74	213.28
1.14	9.61	2.35	-5.09	195.15	297.86	218.11	235.08
1.4	9.82	2.53	-5.06	196.3	296.81	216.98	238.45
2.26	10.61	3.3	-4.46	173.27	262.52	178.45	181.02
2.01	10.34	3.06	-4.62	176.64	271.65	187.25	194.5
2	10.34	3.07	-4.62	175.85	269.92	185.1	191.67
1.78	10.09	2.84	-4.87	197.36	292.05	217.08	237.62
1.97	10.35	2.96	-4.71	183.4	279.51	201.72	215.47
1.46	9.87	2.6	-4.99	194.15	292.9	211.52	229.73
1.27	9.68	2.43	-5.15	200.2	300.19	219.58	245.18
1.26	9.67	2.42	-5.16	200.51	300.55	219.97	245.87
2.58	10.84	3.46	-4.29	183.93	280.24	172.05	167.34
1.24	9.66	2.43	-5.38	224.58	325.97	217.74	232.79
1.23	9.66	2.43	-5.38	224.87	326.3	218.06	233.16
1.23	9.65	2.42	-5.39	225.04	326.52	218.23	233.39
1.12	9.44	2.36	-5.35	239.61	340.27	255.1	297.79
1.24	9.6	2.41	-5.33	233.33	334.65	250.6	288.51
0.6	8.96	1.96	-5.65	257.88	361.14	277.61	328.74
0.96	9.32	2.36	-5.23	241.01	348.25	253.12	292.6
1.4	9.71	2.62	-5.08	222.08	323.3	223.98	244.08
1.13	9.5	2.45	-5.17	231.6	332.5	247.87	288.15
0.97	9.36	2.24	-5.43	235.84	338.92	246.54	279.67
0.85	9.14	2.26	-5.33	239.88	340.42	262.35	313.7
1.23	9.65	2.48	-5.17	221.59	323.35	237.04	269.15
1.08	9.46	2.38	-5.18	230.65	331.96	250.39	294.46

1.39	9.76	2.55	-5.04	215.84	315.06	237.34	272.78
1.26	9.65	2.48	-5.1	221.44	321	243.94	281.81
1.17	9.57	2.4	-5.16	224.08	323.27	246.26	286.13
1.04	9.43	2.38	-5.22	233.29	335.08	250.24	291.84
1.23	9.53	2.47	-5.48	230.31	345.15	218.02	219.15
1.73	9.98	2.82	-5.09	215.29	322.92	203.41	205.57
1.76	10.27	3.02	-5.21	225.53	330.99	224.94	240.84
1.6	9.87	2.79	-5.03	223.96	335.14	213.65	217.61
1.65	9.91	2.84	-5	223.32	334.25	212.8	216.25
1.18	9.6	2.37	-5.48	223.3	329	217.98	229.07
1.18	9.6	2.37	-5.48	223.31	328.96	217.86	228.97
1.19	9.6	2.37	-5.47	223.3	328.93	217.86	228.95
1.18	9.59	2.37	-5.48	223.34	329.15	218.16	229.25
1.24	9.64	2.46	-5.37	225.53	330.29	224.23	239.63
0.93	9.24	2.35	-5.38	242.95	348.41	240.15	265.3
0.77	9.08	2.23	-5.48	248.62	355.1	246.4	272.54
0.85	9.17	2.28	-5.44	242.77	347.64	240.43	265.43
1.31	9.74	2.52	-5.42	226.38	332.42	228.73	247.9
1.11	9.41	2.53	-5.07	229.97	332.19	254.35	301.31
1.42	9.68	2.56	-5.08	216.82	315.79	238.79	275.44
1.12	9.35	2.52	-5.08	231.29	332.45	256.87	305.94
1.17	9.44	2.34	-5.27	219.65	318.64	239.43	276.23
1.3	9.65	2.58	-4.97	204.3	306.24	219.24	238.21
1.17	9.59	2.44	-5.05	204.9	308.28	222.59	244.85
1.67	10.09	2.89	-4.56	181.46	280.37	194.36	199.39
0.77	9.23	2.15	-5.33	221.25	325.22	242.47	280.96
1.09	9.53	2.34	-5.16	205.84	309.9	226.81	253.69
1.16	9.45	2.55	-5.06	228.6	330.39	252.52	298.66
1.27	9.7	2.48	-5.05	202.93	305.19	220.3	243.46
1.02	9.42	2.36	-5.19	212.6	316.84	231.24	261.93
0.88	9.31	2.18	-5.34	215.12	317.91	236.65	272.75
0.86	9.29	2.16	-5.35	215.71	318.91	237.98	274.66
1.06	9.23	2.24	-5.62	212.09	319.83	187.14	176.29
3.17	11.57	3.9	-3.91	168.48	261.28	163.82	158.76
3.27	11.67	4	-3.84	164.35	256.97	158.83	153.75
2.92	11.27	3.73	-4.06	171.76	267.33	163.01	157.99
2.86	11.14	3.67	-4.06	169.47	263.44	157.79	148.07

3.16	11.56	3.9	-3.91	168.88	261.95	164.07	159.44
3.27	11.68	4	-3.83	164.24	256.82	158.54	153.48
3.27	11.67	4	-3.84	164.29	256.9	158.7	153.63
3.23	11.62	3.98	-3.84	165.83	259.24	159.24	154.26
2.64	10.88	3.45	-4.43	166.01	259.4	147.71	132.85
3.32	11.59	4.03	-3.9	150.49	239.42	133.51	113.51
3.3	11.57	4	-3.9	150.52	239.57	133.82	114.04
3.63	12.04	4.31	-3.7	154.23	246.19	147.49	135.4
3.62	12.04	4.3	-3.7	155.04	246.23	147.66	135.81
2.75	10.99	3.54	-4.35	161.88	255.54	144.09	127.05
3.39	11.76	4.11	-3.73	158.44	249.65	146.26	134.75
3.33	11.7	4.05	-3.76	159.86	251.38	148.28	137.46
2.56	10.8	3.38	-4.5	167.64	261.86	149.76	135.81
3.64	12.04	4.31	-3.69	154.23	246.12	147.4	135.24
3.69	12.05	4.39	-3.65	156.56	245.67	145.52	132.08
3.69	12.05	4.37	-3.64	155.81	243.9	144.98	132.96
3.69	12.05	4.37	-3.64	155.77	243.89	144.98	132.97
3.69	12.05	4.37	-3.64	155.76	243.9	144.98	132.97
3.69	12.05	4.37	-3.64	155.73	243.88	144.99	132.98
3.69	12.05	4.36	-3.64	155.71	243.88	144.99	132.98
3.69	12.05	4.36	-3.65	155.71	243.9	144.99	132.98
3.69	12.05	4.36	-3.65	155.7	243.9	144.99	132.98
3.69	12.05	4.36	-3.65	155.72	243.92	144.99	132.98
3.69	12.05	4.36	-3.65	155.63	243.86	144.99	133
3.69	12.05	4.36	-3.65	155.72	243.92	144.99	132.98
3.43	11.88	4.11	-3.77	159.52	250.65	152.2	144.2
3.33	11.77	4.01	-3.85	162.1	255.52	156.46	148.9
3.33	11.76	4.01	-3.82	163.82	255.32	156.27	149.56
3.43	11.85	4.1	-3.74	157.68	250.12	149.99	141.43
3.23	11.61	3.96	-3.87	165.78	257.6	157.27	150.67
3.23	11.61	3.95	-3.87	165.87	257.74	157.32	150.71
3.23	11.6	3.95	-3.87	165.87	257.77	157.31	150.68
3.22	11.59	3.94	-3.88	165.81	258.12	157.32	150.45
3.19	11.56	3.94	-3.88	164.73	258.05	155.84	148.24
3.28	11.62	3.99	-3.82	161.21	252.91	150.49	139.95
3.01	11.32	3.77	-4.01	167.38	262.84	159.2	151.78
3.13	11.43	3.89	-3.9	163.7	256.95	154.59	144.06

2.85	11.1	3.65	-4.11	169.08	263.58	156.39	147.16
2.82	11.08	3.63	-4.13	169.44	264.25	157.16	147.88
2.91	11.16	3.7	-4.05	167.67	260.09	154.02	143.51
2.83	11.11	3.64	-4.11	168.91	261.33	155.05	144.95
2.83	11.11	3.64	-4.11	168.77	261.05	155.01	144.76
2.8	11.07	3.6	-4.15	169.43	261.8	155.5	145.05
2.8	11.07	3.6	-4.15	169.43	261.8	155.5	145.04
2.82	11.06	3.61	-4.15	167.56	262.01	153.85	142.27
2.82	11.06	3.62	-4.15	167.52	261.97	153.82	142.21
2.83	11.09	3.63	-4.19	163.25	257.17	149.03	135.3
2.62	10.9	3.44	-4.45	165.96	260.21	149.48	134.81
2.61	10.87	3.44	-4.44	166.74	260.23	148.35	134.41
2.56	10.87	3.4	-4.47	167.31	261.27	151.86	138.53
2.56	10.86	3.39	-4.48	167.38	261.43	151.97	138.65
2.64	10.94	3.45	-4.42	165.81	259.01	150.44	135.93
2.8	11.12	3.62	-4.22	162.88	255.01	145.73	130.69
2.79	11.12	3.62	-4.22	163.12	255.38	146.18	131.27
2.89	11.22	3.72	-4.15	164.16	254.77	146.63	131.72
3.33	11.6	4.02	-3.85	148.46	236.37	126.32	102.41
3.35	11.63	4.11	-3.91	144.85	231.63	124.32	99.54
2.86	11.11	3.66	-4.37	151.07	238.66	127.28	101.67
3.43	11.74	4.15	-3.75	149.64	234.95	130.03	108.75
3.34	11.67	4.09	-3.88	145.41	235.05	125.68	101.71
3.15	11.41	4.02	-3.99	147.21	234.39	124.57	100.55
3.39	11.77	4.13	-3.8	146.06	233.32	126.02	102.18
3.31	11.65	4.06	-3.91	146.16	235.62	126.96	102.41
3.33	11.67	4.09	-3.88	145.92	235.92	126.71	102.18
3.32	11.68	4.07	-3.89	145.73	236.17	127.21	103.17
3.16	11.47	3.94	-4.02	147.37	235.2	126.16	101.18
3.32	11.61	4.1	-3.91	146.03	232.64	124.71	100.29
3.37	11.76	4.13	-3.8	144.78	232.25	125.29	101.55
3.37	11.75	4.13	-3.8	144.48	231.76	124.92	101.22
3.33	11.62	4.07	-3.92	144.89	232.41	125.57	100.76
3.34	11.61	4.09	-3.92	145	231.77	124.75	99.73
3.32	11.6	4.04	-3.81	147.54	234.58	128.37	106.02
3.25	11.6	4	-3.88	148.3	235.84	128.81	105.66
3.39	11.78	4.13	-3.8	146.6	233.7	126.08	102.53

3.32	11.6	4.04	-3.8	147.77	234.17	128.52	106.05
3.32	11.61	4.04	-3.8	147.62	234.37	128.44	106.03
3.21	11.51	3.98	-3.95	148.6	235.93	126.91	102.96
2.84	11.1	3.62	-4.4	151.81	240.31	128.22	102.88
2.86	11.11	3.66	-4.38	151.27	238.73	127.36	101.72
2.79	11.08	3.67	-4.4	152.73	240.25	126.13	98.77
2.62	10.91	3.54	-4.5	155.54	243.12	128.6	101.56
2.64	10.92	3.55	-4.48	155.55	242.86	128.44	101.34
3.08	11.33	3.77	-4.25	150.58	239.25	127	102.74
2.16	10.39	3	-4.72	166.74	259.38	140.6	113.82
2.26	10.46	3.12	-4.64	166.16	259.95	140.05	115.77
2.45	10.72	3.29	-4.55	159.24	252.68	135.94	111.87
2.31	10.6	3.14	-4.62	158.47	251.67	134.76	109.96
2.53	10.74	3.33	-4.5	164.64	258.02	140.34	118.32
2.65	10.88	3.41	-4.44	164.71	260.09	145.94	129.48
2.67	10.93	3.44	-4.45	163.52	259.07	144.13	127.82
2.71	10.95	3.45	-4.44	161.55	256	142.46	126.13
2.54	10.81	3.36	-4.54	161.19	257.69	143.58	125.8
2.57	10.83	3.41	-4.5	161.33	257.03	142.7	123.98
2.44	10.71	3.29	-4.66	161.34	258.12	143.85	125.86
2.45	10.73	3.31	-4.63	160.86	256.37	141.62	121.83
2.44	10.73	3.31	-4.63	160.87	256.39	141.62	121.83
2.58	10.83	3.38	-4.57	160.32	255.55	140.6	123.72
2.54	10.78	3.35	-4.61	161.01	257.13	142.21	123.43
2.61	10.84	3.38	-4.56	162.36	257.69	143.63	124.82
2.64	10.87	3.41	-4.54	161.57	256.73	142.73	123.98
2.53	10.74	3.33	-4.57	166.91	260.59	144.58	124.84
2.53	10.74	3.33	-4.57	166.97	260.65	144.61	124.89
2.65	10.89	3.42	-4.43	164.32	259.76	145.15	128.88
2.63	10.89	3.42	-4.46	161.91	257.25	143.87	126.68
2.41	10.67	3.25	-4.57	158.41	253.27	135.65	111.81
2.31	10.59	3.14	-4.62	158.52	251.78	135.11	110.4
2.28	10.56	3.11	-4.64	159.49	251.79	135.27	110.66
2.27	10.56	3.1	-4.65	159.29	250.95	134.66	109.27
2.25	10.55	3.09	-4.66	159.65	250.3	134.81	109.23
2.3	10.55	3.13	-4.6	161.45	253.25	135.99	110.27
2.1	10.35	2.96	-4.75	163.83	258.63	139.1	111.73

2.12	10.38	2.98	-4.74	163.62	257.79	138.74	111.04
2.11	10.36	2.97	-4.76	164	258.07	138.94	111.34
2.44	10.7	3.3	-4.55	160.88	255.77	138.45	115.55
2.42	10.7	3.28	-4.55	160.01	254.07	136.88	114.1
2.46	10.73	3.31	-4.54	159.44	252.78	136	112.06
2.44	10.71	3.29	-4.55	159.13	252.65	135.89	111.73
2.44	10.71	3.29	-4.55	159.15	252.6	135.88	111.69
3.39	11.93	4.19	-3.86	160.29	249.36	152.69	142.83
3.39	11.9	4.1	-3.86	158.82	249.35	154.86	147.06
3.53	12.03	4.2	-3.8	155.87	246.66	149.08	138.49
3.5	12.02	4.2	-3.8	155.85	247.39	151.46	142
3.5	12.02	4.2	-3.8	155.76	247.35	151.34	141.85
2.83	11.24	3.66	-4.1	170.82	257.13	178.26	178.25
3.11	11.46	3.85	-4.01	163.56	248.95	161.01	154.15
3.42	11.94	4.16	-3.82	158.94	246.52	152.86	144.02
3.28	11.74	3.95	-3.89	161.4	255.72	157.58	151.12
3.28	11.73	3.95	-3.88	161.5	255.79	157.64	151.17
3.28	11.74	3.95	-3.88	161.42	255.69	157.54	151.05
2.9	11.26	3.69	-4.07	164.51	254.88	172.79	171.44
2.78	11.15	3.59	-4.15	168.81	256.5	177.3	178.04
2.78	11.15	3.58	-4.15	168.93	256.64	177.48	178.31
3.38	11.92	4.15	-3.87	159.6	249.82	150.08	138.22
3.38	11.92	4.15	-3.86	159.46	249.57	150.06	138.17
3.38	11.92	4.15	-3.86	159.55	249.73	150.07	138.19
3.38	11.92	4.15	-3.86	159.44	249.55	150.05	138.14
3.46	11.98	4.24	-3.82	158.47	247.21	150.84	140.69
3.45	11.96	4.18	-3.83	155.96	246.59	149.31	138
3.48	12.02	4.2	-3.81	155.96	246.83	148.34	138
3.48	12.02	4.2	-3.81	155.96	246.85	148.32	138
3.53	12.03	4.2	-3.8	156.2	247.09	148.37	138.13
3.55	12.03	4.22	-3.78	156.01	247.39	148.21	137.68
3.49	11.96	4.21	-3.79	157.97	247.48	152.5	144.48
3.47	11.95	4.18	-3.81	157.8	247.17	152.52	144.41
3.42	11.94	4.18	-3.85	158.44	247.67	149.38	137.69
3.41	11.94	4.17	-3.85	158.72	248.04	149.46	137.78
3.41	11.94	4.17	-3.85	158.76	248.12	149.49	137.79
3.41	11.93	4.17	-3.85	158.44	248.15	149.59	137.79

2.92	11.33	3.72	-4.07	176.03	267.94	173.64	171.91
3.5	11.78	4.22	-3.72	142.86	226.47	119.26	94.55
3.54	11.83	4.29	-3.71	142.33	226.6	119.4	95.68
3.49	11.83	4.26	-3.7	152.08	235.92	132.39	112.78
3.52	11.83	4.27	-3.68	151.68	235.27	132.19	112.37
3.52	11.83	4.27	-3.68	151.66	235.22	132.18	112.34
3.5	11.83	4.27	-3.69	151.91	235.71	132.26	112.53
3.52	11.83	4.27	-3.68	151.67	235.25	132.18	112.36
3.29	11.63	4.05	-3.92	151.67	236.66	130.82	110.88
3.58	11.9	4.3	-3.65	144.86	227.89	121.26	97.08
3.13	11.43	4.03	-4.04	145.65	231.39	121.78	97.5
3.33	11.73	4.12	-3.84	151.76	239.54	136.11	118.04
3.52	11.83	4.24	-3.71	142.68	226.81	119.03	94.24
3.35	11.68	4.12	-3.86	150.61	236.55	130.85	111.08
3.35	11.68	4.12	-3.86	150.59	236.52	130.81	111.07
3.13	11.5	3.97	-4	155.76	242.59	140.28	122.5
3.31	11.65	4.09	-3.9	151.19	237.05	131.07	111.13
3.3	11.7	4.1	-3.85	152.46	240.04	136.42	118.42
3.5	11.77	4.26	-3.72	141.57	226.8	119.29	94
3.48	11.73	4.27	-3.77	141.93	226.42	118.74	94
3.48	11.73	4.27	-3.77	141.89	226.42	118.73	94
3.48	11.74	4.26	-3.76	141.86	226.41	118.75	94
3.3	11.58	4.14	-3.89	144.1	229.37	122.16	96.78
3.29	11.58	4.14	-3.89	144.18	229.41	122.2	96.85
3.29	11.57	4.13	-3.89	144.26	229.46	122.25	96.92
3.42	11.69	4.24	-3.8	142.36	227.37	120.04	94.53
3.42	11.68	4.24	-3.8	142.45	227.38	120.07	94.54
3.31	11.61	4.18	-3.9	143.81	229.3	119.12	95.19
3.31	11.62	4.18	-3.9	143.72	229.21	119.05	95.1
3.56	11.84	4.3	-3.69	142.94	226.93	119.89	96.52
3.54	11.83	4.29	-3.71	143.33	227.59	120.24	96.82
3.56	11.9	4.29	-3.66	144.49	228	121.59	97.28
3.57	11.86	4.28	-3.68	144.82	228.83	121.3	97.36
3.54	11.87	4.27	-3.68	143.16	228.33	122	97.8
3.53	11.85	4.26	-3.71	143.38	229.62	122.08	98.14
3.45	11.76	4.19	-3.76	144.95	231.1	123.2	99.65
3.1	11.51	3.94	-3.99	154.82	242.44	139.86	122.12

3.09	11.51	3.94	-3.99	154.82	242.47	139.88	122.15
3.1	11.52	3.96	-3.99	154.58	242.58	140.23	122.53
3.4	11.72	4.16	-3.84	151.65	236.35	131.38	110.7
3.3	11.57	4.03	-3.87	149.34	234.81	131.62	109.86
3.24	11.5	3.92	-4.04	151.38	239.66	129.13	106.93
3.23	11.5	3.91	-4.05	151.39	239.93	129.35	107.06
3.31	11.56	4	-4.01	151.45	238.05	129.96	106.97
3.09	11.36	3.8	-4.28	151.3	239.52	127.52	104.99
3.15	11.37	3.85	-4.17	150.6	239.16	127.52	104.77
3.06	11.34	3.77	-4.31	150.88	239.29	127.86	104.21
3.04	11.27	3.78	-4.21	153.57	243.06	129.63	105.58
2.84	11.11	3.63	-4.37	151.84	240.65	128.86	104.02
2.29	10.55	3.12	-4.63	161.91	251.51	134.5	107.99
2.78	11.04	3.58	-4.33	154.57	247.92	135.73	113.31
2.97	11.25	3.76	-4.25	152.95	243.8	132.26	110.3
2.96	11.24	3.75	-4.25	152.93	243.75	132.21	110.2
2.65	10.89	3.41	-4.44	164.53	260.02	145.65	129.17
2.62	10.85	3.42	-4.53	161.82	256.09	141.1	121.14
2.74	11	3.56	-4.35	159.77	254.67	141.26	122.27
2.73	10.97	3.56	-4.35	157.35	250.53	135.29	114.68
2.78	11.06	3.59	-4.33	156.67	250.64	137.74	117.3
3.39	11.69	4.09	-3.92	148.71	236.94	128.01	106.21
3.33	11.6	4.02	-3.97	149.76	237.96	129	106.23
3.31	11.55	3.99	-3.97	150.25	238.78	129.27	105.5
3.4	11.73	4.1	-3.93	148.85	236.19	129.75	107.96
3.4	11.73	4.1	-3.94	148.89	236.35	129.95	108.16
3.36	11.62	4.06	-3.91	150.61	240.77	132.45	114.26
3.27	11.51	3.94	-4.03	151.94	238.78	129.4	106.55
3.26	11.61	3.98	-4.09	150.34	240.84	132.59	112.67
3.13	11.37	3.82	-4.24	150.86	239.19	127.36	104.55
2.91	11.22	3.68	-4.36	151.58	240.65	128.88	105
2.91	11.21	3.67	-4.36	151.65	240.72	128.92	105
3.11	11.38	3.81	-4.28	150.81	239.37	127.1	104.47
3.07	11.34	3.78	-4.3	150.86	239.29	127.78	104.18
3.04	11.34	3.8	-4.17	151.91	242.59	131.89	109.65
3.01	11.31	3.79	-4.24	152.45	243.64	132.18	110.66
3.03	11.34	3.8	-4.17	152.7	242.83	132.09	109.89

2.9	11.18	3.69	-4.27	152.89	246.68	132.47	111.9
2.9	11.18	3.69	-4.27	152.91	246.71	132.44	111.82
2.85	11.11	3.65	-4.27	153.03	247.25	133.29	111.86
2.85	11.11	3.65	-4.27	153.03	247.24	133.35	111.9
2.85	11.11	3.65	-4.27	153.03	247.22	133.44	111.97
2.85	11.11	3.65	-4.27	153.03	247.22	133.51	112.03
2.85	11.11	3.65	-4.27	153.03	247.21	133.54	112.05
2.85	11.11	3.65	-4.27	153.03	247.21	133.56	112.07
2.85	11.11	3.65	-4.27	153.03	247.2	133.6	112.09
2.85	11.11	3.65	-4.27	153.03	247.19	133.66	112.13
2.73	11.01	3.56	-4.38	156.58	250.3	137.36	115.98
2.94	11.21	3.75	-4.27	153.48	243.19	130.01	105.09
2.8	11.05	3.61	-4.38	152.11	242.62	129.45	104.01
2.57	10.8	3.4	-4.46	159.67	252.73	136.24	113.42
3.45	11.76	4.14	-3.86	148.31	235.66	128.43	106.13
2.43	10.74	3.29	-4.57	158.76	247.81	132.9	107.79
3.28	11.62	4.02	-4.04	150.28	240.68	133.08	113.49
2.99	11.26	3.76	-4.23	156.31	249.43	137.5	117.39
2.93	11.23	3.72	-4.14	154.73	246.01	136.42	118.89
2.58	10.91	3.45	-4.41	167.34	261.91	150.93	137.49
2.67	10.97	3.48	-4.38	163	256.38	145.65	129.88
2.87	11.13	3.66	-4.21	158.34	248.91	139.58	121.29
2.77	11.07	3.58	-4.3	160.73	253.18	141.58	124.97
3.06	11.37	3.84	-3.92	164.42	257.94	154.89	145.19
3.05	11.37	3.84	-3.91	164.2	257.49	154.32	144.75
3.07	11.37	3.84	-3.91	163.66	257.02	153.51	143.96
3.46	11.77	4.15	-3.86	148.2	235.63	128.34	106.14
3.44	11.76	4.12	-3.85	148.15	235.77	128.22	105.76
3.48	11.96	4.2	-3.77	158.37	247.83	153.55	145.6
2.35	10.63	3.21	-4.6	157.18	248.25	132.19	105.64
3.43	11.85	4.17	-3.79	149.7	237.75	133.21	116.61
3.44	11.76	4.11	-3.86	148.22	235.74	128.35	106
3.38	11.56	4.3	-3.57	134.92	221.19	110.62	81
3.13	11.39	4.08	-3.85	142.02	225.32	116.07	89.72
3.19	11.44	4.17	-3.8	142.86	228.67	118.09	90.58
3.42	11.51	4.46	-3.35	129.89	217.32	105.83	75.48
3.4	11.46	4.44	-3.34	129.11	217.46	106.63	75.77

3.1	11.14	4.18	-3.77	131.53	224.23	108.84	78.44
3.09	11.15	4.16	-3.64	131.62	220.11	108.92	78.72
3.34	11.53	4.25	-3.64	136.51	225.04	112.75	84.59
3.32	11.58	4.27	-3.61	137.8	223.04	112.85	84.93
3.42	11.63	4.31	-3.56	134.33	221.08	109.66	80.66
3.8	11.99	4.65	-3.22	132.36	217.7	107.61	78.45
3.28	11.48	4.21	-3.69	137.69	226.87	114.1	85.8
3.26	11.49	4.17	-3.7	137.68	226.05	113.85	86.74
2.94	11.17	3.93	-4	143.05	230.75	119.04	91.39
2.94	11.17	3.93	-4	143.08	230.75	119.09	91.45
2.58	10.84	3.64	-4.29	148.89	237.41	125.02	98.61
3.38	11.64	4.29	-3.61	137.54	223.75	113.13	85.73
3.1	11.38	4.02	-3.96	143.59	232.42	120.59	95.75
2.72	10.98	3.72	-4.2	147.17	233.23	124.61	100.02
2.72	10.99	3.73	-4.19	147.13	233.1	124.51	99.88
2.92	11.22	3.88	-4.04	148.01	232.17	126.23	104.55
2.67	10.95	3.66	-4.24	153.27	236.41	131.02	110.49
2.62	10.98	3.66	-4.28	152.59	237.45	130.75	109.74
1.89	10.15	3.06	-5	166.94	256.31	139.7	110.91
2.44	10.7	3.47	-4.61	151.52	240.95	127.09	93.83
2.12	10.37	3.21	-4.79	162.88	253.06	135.24	103.3
1.87	10.12	3.03	-5	165.05	257.04	140.2	110.92
2.53	10.79	3.53	-4.53	149.54	238.88	123.97	90.89
2.53	10.79	3.53	-4.58	154.32	243.71	128.47	100.65
2.81	11.14	3.67	-4.35	148.41	238.22	124.15	97.34
2.81	11.12	3.67	-4.35	150.57	239.46	125.34	100.48
2.75	11.05	3.59	-4.36	151.96	241.57	128.14	102.91
2.81	11.13	3.64	-4.35	149.99	239.67	126.49	100.71
2.75	11.08	3.63	-4.39	147.43	237.07	124.34	98.03
2.76	11.09	3.63	-4.39	147.18	236.68	124.05	97.82
2.77	11.1	3.63	-4.39	147.39	236.59	123.87	97.99
2.76	11.07	3.64	-4.4	150.83	240.99	126.2	101.21
2.59	10.89	3.56	-4.47	153.19	244.87	130.41	104.36
2.68	10.96	3.62	-4.5	150.57	239.36	125.18	97.46
2.55	10.85	3.53	-4.49	154.37	246.33	130.7	105.4
2.61	10.92	3.53	-4.47	153.12	245.11	130.2	106.45
2.64	10.94	3.6	-4.51	151.3	240.15	125.63	98.01

2.79	11.07	3.71	-4.3	152.61	240.41	127.06	103.01
2.38	10.69	3.43	-4.57	156.82	249.65	133.36	109.16
2.58	10.88	3.6	-4.42	154.25	242.28	128.74	103.98
2.51	10.79	3.55	-4.52	154.44	244.24	130.58	103.38
2.63	10.91	3.64	-4.43	153.11	241.16	127.3	100.19
3.01	11.32	3.85	-4.19	149.41	238.95	126.33	101.16
3.09	11.38	3.93	-4.1	147.19	234.9	124.09	99.6
2.78	11.1	3.62	-4.37	149.88	240.01	126.81	100.93
2.76	11.08	3.6	-4.39	149.72	239.64	126.86	100.94
2.79	11.1	3.62	-4.37	149.79	239.86	126.76	100.86
2.71	11.02	3.56	-4.42	151.76	241.62	126.78	102.27
2.83	11.14	3.68	-4.34	149.56	238.38	125.35	99.49
2.82	11.17	3.67	-4.38	147.16	235.98	122.05	96.09
2.57	10.89	3.56	-4.57	153.07	242.24	126.98	99.89
2.57	10.88	3.56	-4.57	153.1	242.26	127	99.91
2.81	11.16	3.68	-4.41	148.71	235.7	122.32	94.11
2.84	11.15	3.69	-4.4	147	236.04	121.47	93.84
2.79	11.1	3.67	-4.41	147.69	237.21	123.14	95.53
2.67	10.96	3.63	-4.46	147.86	235.86	123.76	95.19
2.46	10.77	3.49	-4.65	151.28	242.56	127.76	98.39
2.47	10.79	3.5	-4.64	151.14	242.94	127.94	98.49
2.58	10.9	3.59	-4.54	150.11	241.57	127.13	97.23
2.11	10.4	3.24	-4.89	153.75	246.74	130.48	101.25
2.51	10.81	3.56	-4.56	151.79	241.72	127.1	98.33
2.15	10.39	3.31	-4.79	157.98	250.89	133.08	103.36
2.08	10.32	3.28	-4.82	158.66	251.23	133.69	103.91
1.88	10.12	3.05	-5	166.37	256.23	140.05	111.41
1.93	10.23	3.08	-4.98	164.12	256.15	138.38	110.69
1.86	10.12	2.98	-5.04	165.03	255.69	138.3	110.46
2.4	10.62	3.51	-4.6	148.94	240.15	125.69	95.26
3.02	11.28	3.94	-3.97	144	231.36	118.95	91.54
2.17	10.45	3.32	-4.79	157.46	248.19	131.18	104.32
2.57	10.87	3.59	-4.42	155.84	243.22	127.79	103.05
2.97	11.23	3.85	-4.16	149.27	237.99	124.75	100.64
2.24	10.52	3.35	-4.66	158	246.35	129.83	104.45
2.43	10.72	3.53	-4.56	155.1	242.18	126.56	100.63
2.44	10.69	3.52	-4.56	151.41	242.53	125.48	98.3

2.47	10.71	3.52	-4.56	152	242.57	125.6	99.12
2.41	10.71	3.48	-4.62	153.14	242.41	126.48	100.38
2.4	10.7	3.47	-4.63	153.18	242.74	126.53	100.6
2.16	10.44	3.31	-4.79	157.58	248.32	131.28	104.51
2.33	10.56	3.43	-4.71	154.88	245.86	128.8	101.03
2.04	10.29	3.21	-4.91	155.6	248.07	130.27	100.45
2.06	10.34	3.21	-4.93	154.09	247.75	130.02	100.75
2.03	10.3	3.21	-4.94	154.59	248.52	130.51	101.61
3	11.21	3.99	-4.04	140.04	228.34	116.07	86.32
3.11	11.33	4.05	-3.95	137.97	226.76	113.61	83.97
3.01	11.26	3.91	-4.01	139.94	230.49	116.71	87.9
2.95	11.22	3.86	-4.06	141.27	231.08	117.45	89.07
2.64	10.9	3.64	-4.32	146.45	235.53	121.08	93.81
2.8	11.05	3.78	-4.2	144.86	231.18	119.07	90
3.13	11.36	4.01	-3.86	140.02	228.32	115.85	86.53
3.32	11.51	4.17	-3.71	134.91	224.47	113.07	82.62
3.3	11.49	4.15	-3.73	135.23	224.06	113.04	82.84
3.29	11.48	4.15	-3.73	135.44	224.14	113.16	82.96
3.3	11.49	4.16	-3.72	135.01	223.78	112.74	82.67
2.6	10.89	3.59	-4.3	148.57	237.23	123.43	97.97
2.84	11	3.85	-4.24	140.11	229.35	116.1	86.14
2.9	11.09	3.9	-4.18	140.01	227.94	115.53	84.86
2.32	10.48	3.56	-4.59	146.13	235.5	121.44	92.04
2	10.2	3.3	-4.86	149.55	241.4	126.86	96.13
2.56	10.75	3.69	-4.44	144.86	234.49	120.48	91.02
2.54	10.74	3.66	-4.45	145.12	234.75	120.73	91.52
2.56	10.75	3.68	-4.44	144.32	234.21	120.34	90.88
2.79	10.99	3.8	-4.24	143.16	231.16	118.84	89.1
2.57	10.8	3.64	-4.38	146.34	234.07	121.56	92.2
2.57	10.82	3.66	-4.38	146.18	233.99	121.65	91.67
2.57	10.79	3.61	-4.43	146.52	235.68	122.25	92.76
2.67	10.91	3.71	-4.35	144.09	233.55	119.88	90.11
2.67	10.91	3.71	-4.35	144.09	233.56	119.89	90.12
2.56	10.78	3.66	-4.41	146.4	234.28	120.09	91.11
2.38	10.56	3.5	-4.58	146.53	237.05	124.23	91.28
2.38	10.55	3.49	-4.59	146.62	237.15	124.31	91.37
2.38	10.56	3.5	-4.58	146.47	237	124.22	91.22

2.12	10.35	3.32	-4.76	152.79	244.98	129.33	98.19
2.24	10.49	3.39	-4.71	150.7	244.71	127.64	99.04
2.31	10.55	3.45	-4.67	149.82	243.05	126.46	96.55
2	10.26	3.21	-4.89	156.11	249.37	130.56	102
2.3	10.51	3.42	-4.7	148.22	238.49	124.01	92.58
2.22	10.48	3.36	-4.77	153.31	245.17	128	99.28
2.8	11.06	3.75	-4.14	144.21	233.88	121.48	93.13
2.67	11	3.67	-4.32	149.1	236.98	122.45	96.68
2.6	10.93	3.62	-4.35	149.61	238.31	123.55	98.04
2.83	11.1	3.77	-4.2	147.24	232.9	119.71	94.3
2.33	10.72	3.46	-4.5	158.21	240.9	137.1	120.18
2.45	10.82	3.47	-4.5	157.94	239.57	134.96	116.39
2.46	10.83	3.47	-4.49	157.87	239.45	134.91	116.32
2.52	10.87	3.52	-4.45	157.68	238.82	135.89	117.74
2.53	10.87	3.52	-4.45	157.53	238.69	135.71	117.61
2.58	10.95	3.54	-4.44	156.8	238.3	135.68	117.57
2.62	10.99	3.57	-4.42	156.33	237.54	135.25	117
2.45	10.85	3.52	-4.48	155.27	239.02	136.53	119.39
2.49	10.88	3.61	-4.33	155.15	238.28	134.45	115.95
3.11	11.38	3.97	-4.03	144.18	230.64	122.01	96.96
2.78	11.07	3.71	-4.31	152.73	240.41	127.05	103.05
2.44	10.81	3.51	-4.42	155.7	240.73	133.97	113.2
3.03	11.33	3.94	-4.09	147.63	232.34	121.09	97.06
3.04	11.35	3.97	-4.1	147.14	232.66	121.69	96.83
2.84	11.15	3.8	-4.23	149.5	235.88	124.43	100.77
3.27	11.54	4.11	-3.99	146.59	234.68	124.1	99.66
2.51	10.87	3.49	-4.47	156.91	238.38	134.55	115.13
2.7	11.09	3.62	-4.35	152.82	236.41	133.22	113.99
2.32	10.65	3.37	-4.53	158.81	242.74	136.63	118.27
2.37	10.73	3.42	-4.54	157.58	241.47	136.37	117.01
2.38	10.73	3.42	-4.53	157.27	241.54	136.21	116.87
2.44	10.8	3.51	-4.43	155.73	240.79	134.04	113.29
2.43	10.8	3.51	-4.43	156.03	240.89	134.02	113.5
2.42	10.79	3.45	-4.49	156.48	240.6	135.52	116.31
2.41	10.79	3.48	-4.46	156.45	241.3	134.54	114.33
2.42	10.81	3.48	-4.46	156.33	241.46	134.48	114.41
2.58	10.91	3.64	-4.38	151.65	242.53	130.15	106.25

3	11.3	3.92	-4.12	148.32	233.53	121.52	97.55
3.12	11.43	4.04	-4.04	146.32	231.19	121.16	97.22
3.03	11.32	3.95	-4.09	147.14	232.25	121.83	97.65
2.69	11	3.67	-4.34	153.53	241.08	128.23	103.41
3.02	11.29	3.91	-4.13	149.46	235.55	123.81	98.57
2.9	11.18	3.82	-4.2	148.81	238.55	125.35	101.08
2.69	11	3.69	-4.38	154.08	242.07	129.41	105.96
3.21	11.49	4.07	-4.02	148.66	235.99	124.74	100.73
3.2	11.48	4.07	-4.03	148.72	236.03	124.8	100.77
2.68	10.97	3.71	-4.04	150.51	238.51	141.18	128.06
1.9	9.89	3.34	-4.76	135.01	239.58	121.6	86.53
1.88	9.88	3.32	-4.78	135.36	240.09	121.91	87
1.89	9.91	3.25	-4.8	133.07	238.47	124.16	87.9
2.28	10.21	3.75	-4.32	127.56	231.88	114.7	79.22
2.26	10.2	3.74	-4.31	128.08	232.28	115.63	79.16
1.67	9.6	3.29	-4.77	135.5	245.66	124.68	90.11
1.92	9.8	3.49	-4.61	134.02	241.05	120.08	86.12
2.27	10.2	3.74	-4.32	127.51	231.96	114.79	79.31
2.27	10.21	3.74	-4.32	127.54	231.95	114.79	79.31
1.86	9.79	3.45	-4.59	131.75	240.93	121.01	85.71
1.61	9.62	3.17	-4.88	136.32	243.54	125.64	89.43
1.64	9.65	3.2	-4.86	135.59	242.65	125.21	88.72
1.74	9.76	3.24	-4.81	134.53	241.68	124.03	87.14
1.74	9.75	3.24	-4.8	134.19	241.79	124.31	86.95
1.72	9.75	3.23	-4.81	132.9	240.75	124.84	86.66
1.72	9.75	3.23	-4.81	132.99	240.68	124.92	86.74
1.57	9.64	3.02	-5.01	138.49	246.46	129.47	93.69
1.91	9.91	3.33	-4.75	132.25	238.05	122.13	85.64
1.48	9.59	2.96	-5.05	140.57	248.6	131.5	95.53
1.93	9.86	3.42	-4.78	134.55	235.9	117.53	82.72
2.2	10.18	3.72	-4.39	132.69	231.56	116.78	82.61
2.19	10.16	3.61	-4.55	132.61	228.4	116.26	81.85
2.73	10.78	3.83	-4.18	131.98	221.76	112.69	78.06
2.76	10.7	4.07	-4.14	125.91	218.99	109.11	74.3
1.89	9.85	3.4	-4.78	137.61	238.34	121.19	87.45
1.89	9.85	3.39	-4.78	137.59	238.39	121.23	87.51
1.85	9.84	3.34	-4.83	135.86	235.35	118.91	85.23

1.85	9.78	3.34	-4.84	136.38	233.93	118.13	84.77
1.91	9.84	3.42	-4.8	139.29	239.22	121.73	87.66
1.78	9.76	3.27	-4.8	136.62	235.44	119.29	86.3
2.52	10.45	3.86	-4.33	130.07	223.64	113	79.93
2.46	10.46	3.75	-4.37	132.58	224.46	113.45	81.27
2.55	10.56	3.86	-4.29	130.52	222.67	111.64	78.72
2.51	10.45	3.92	-4.16	126.25	226.61	111.25	75.89
2.5	10.43	3.91	-4.17	126.38	226.84	111.36	76.05
2.41	10.35	3.9	-4.18	131.96	227.77	112.93	80.18
2.54	10.48	3.95	-4.14	125.75	225.91	110.89	75.76
1.95	9.91	3.48	-4.63	131.15	234.85	117.09	82.66
1.92	9.88	3.46	-4.66	131.68	235.61	117.53	83.2
1.79	9.73	3.3	-4.83	136.82	238.62	118.87	85.22
1.83	9.77	3.35	-4.84	136.03	237.22	118.52	84.59
2.06	9.98	3.53	-4.7	133.07	233.1	116.23	81.69
1.99	9.92	3.47	-4.75	132.76	234.17	117.52	82.03
1.91	9.81	3.41	-4.8	135.39	235.49	117.64	84.62
1.76	9.7	3.33	-4.75	131.65	239.17	120.68	86.61
1.69	9.62	3.23	-4.93	137.72	239.2	120.78	88.69
1.77	9.69	3.31	-4.86	137.27	238.26	120.13	87.82
1.77	9.69	3.31	-4.87	137.22	238.08	120.03	87.73
3.13	11.15	4.31	-3.67	127.09	218.94	107.46	74.14
1.74	9.72	3.24	-5.02	140.19	239.23	123.81	88.58
2.19	10.11	3.48	-4.63	133.71	226.04	115.79	81.74
2.98	11	4.03	-4.01	130.2	219.08	110	75.18
2.07	9.98	3.54	-4.64	136.02	234.48	118.1	84.45
2.11	10.06	3.46	-4.8	135.27	231.04	120.22	85.09
1.79	9.71	3.33	-4.87	141.27	241.78	124.18	89.63
2.15	10.05	3.46	-4.57	132.07	228.07	115.57	81.71
2.15	10.06	3.47	-4.57	132.01	227.97	115.54	81.65
2.23	10.14	3.52	-4.53	131.45	225.29	115.56	80.65
2.51	10.49	3.74	-4.35	132.67	224.85	113.19	78.95
2.55	10.54	3.76	-4.34	133.12	223.7	114.33	79.55
2.64	10.62	3.8	-4.26	131.58	222.42	112.52	78
2.39	10.48	3.59	-4.45	139.76	230.61	116.86	86.16
2.24	10.27	3.5	-4.57	138.73	231.72	118.47	86.14
2.5	10.52	3.7	-4.39	136.15	226.29	114.9	81.38

2.35	10.41	3.59	-4.5	137.97	229.97	117.64	85.56
2.61	10.61	3.79	-4.35	133.97	224.57	114.87	81.12
2.61	10.62	3.79	-4.35	135.22	225.29	115.35	81.31
2.6	10.62	3.78	-4.35	135.55	225.68	115.54	81.47
2.82	10.84	4.08	-4	130.51	221.14	109.65	77.09
2.69	10.72	3.98	-4.1	131.28	220.99	109.89	77.41
2.59	10.59	3.89	-4.22	131.87	222.31	111.37	79.26
2.56	10.58	3.82	-4.29	133.08	224.02	112.7	81.02
2.52	10.51	3.78	-4.31	132.07	224.03	112.64	80.66
2.6	10.59	3.87	-4.23	130.91	220.19	111.11	78.85
2.51	10.52	3.8	-4.31	131.86	222.27	112.42	80.07
2.99	11.02	4.02	-3.99	128.62	216.3	109.18	75.06
2.55	10.54	3.77	-4.31	130.2	222.93	112.63	78.35
2.88	10.91	4.07	-4.04	129.64	220.82	108.66	77.14
2.81	10.84	3.99	-4.12	132.53	222.61	110.69	79.42
2.81	10.83	3.98	-4.12	132.54	222.57	110.76	79.42
3.05	11.11	4.1	-3.94	133.63	221.78	110.15	78.15
3.05	11.11	4.11	-3.93	133.62	221.71	110.05	78.11
2.1	10.1	3.42	-4.8	133.57	231.84	121	85.57
2.14	10.28	3.38	-4.74	146.59	238.69	123.52	92.99
3.43	11.6	4.3	-3.7	131.05	218.86	108.01	77.12
3.3	11.48	4.18	-3.69	136.56	225.26	111.88	82.81
2.71	10.73	3.87	-4.21	133.55	224.34	112.75	79.71
3	11.02	4.16	-3.93	129.18	220.02	107.73	76.27
3.01	11.03	4.17	-3.93	129.27	219.98	107.57	76.21
2.74	10.86	3.94	-4.16	133.44	225.03	111.61	81.27
3.11	11.21	4.18	-3.89	132.11	222.06	109.16	77.52
3.09	11.13	4.13	-3.93	131.2	222.21	110.13	76.98
3.13	11.16	4.14	-3.89	130.89	219.3	108.15	75.62
2.97	11.01	4.04	-4.02	132.93	221.13	109.95	77.55
2.35	10.48	3.52	-4.62	144.78	234.66	121.09	90.31
2.34	10.47	3.53	-4.63	143.82	234.82	120.8	89.92
2.27	10.4	3.5	-4.66	144.43	235.69	122.12	90.01
2.27	10.4	3.5	-4.66	144.55	235.83	122.17	90.16
3.52	11.67	4.48	-3.53	131.26	220.51	108.06	77.04
2.54	10.69	3.69	-4.35	141.73	233.11	118.29	88.54
2.29	10.42	3.53	-4.59	143.34	233.92	120.33	89.71

2.3	10.43	3.54	-4.57	143.34	234.05	120.28	89.8
2.29	10.42	3.52	-4.59	143.38	233.97	120.38	89.75
2.78	10.97	3.85	-4.25	133.61	225.39	118.12	82.69
2.25	10.42	3.46	-4.6	138.95	231.58	123.66	88.67
2.59	10.74	3.73	-4.44	135.14	227.35	119.32	83.62
2.24	10.37	3.41	-4.71	138.45	232.75	121.52	87.53
2.25	10.36	3.44	-4.7	145.35	240.65	125.42	92.19
2.27	10.4	3.47	-4.66	147.17	241.5	126.34	93.72
1.66	9.66	3.13	-4.92	139.07	240.41	123.56	89.99
1.95	9.88	3.33	-4.72	135.5	233.26	118.69	84.56
2.58	10.57	3.78	-4.28	131.64	222.42	112.62	77.83
2.46	10.39	3.68	-4.41	131.99	224.51	113.29	79.1
2.65	10.63	3.82	-4.25	131.14	222.12	112.18	77.95
2.26	10.3	3.52	-4.62	141.21	234.93	120.93	88.96
2.61	10.68	3.73	-4.4	134.39	226.68	115.14	81.01
2.37	10.44	3.51	-4.62	135.35	230.89	119	84.62
2.06	10.29	3.32	-4.83	146.12	243.51	130.7	97.13
2.6	10.57	3.8	-4.29	130.52	223.45	111.74	77.81
2.63	10.61	3.79	-4.27	131.57	222.5	112.79	78.01
2.56	10.53	3.71	-4.3	131.98	223.79	113.9	79.41
2.5	10.52	3.7	-4.41	135.44	227.33	116.06	82.11
2.21	10.4	3.36	-4.76	146.38	239.55	125.46	92.66
2.67	10.73	3.79	-4.35	133.84	228.76	114.76	81
2.6	10.61	3.71	-4.38	133.28	223.43	114.94	79.59
2.52	10.49	3.65	-4.38	132.37	224.75	114.17	80.39
2.51	10.54	3.62	-4.5	134.82	226.69	116.14	81.53
2.41	10.48	3.56	-4.6	134.76	228.19	116.85	83.2
2.54	10.61	3.68	-4.44	138.18	231.59	118.65	84.64
2.27	10.4	3.46	-4.66	143.53	234.94	121.92	89.81
2.09	10.27	3.31	-4.78	149.61	241.95	127.33	95.27
2.08	10.27	3.31	-4.78	149.63	242	127.37	95.32
2.27	10.43	3.47	-4.64	144.29	236.76	122.47	89.94
2.55	10.7	3.69	-4.48	141.67	232.56	119.53	85.84
2.22	10.4	3.41	-4.7	142.13	237.22	122.21	90.57
2.27	10.41	3.44	-4.71	140.11	234.59	122.26	88.29
2.4	10.53	3.55	-4.6	144.88	238.54	124.7	91.21
2.19	10.19	3.43	-4.7	135.33	229.41	119.14	84.73

2.12	10.13	3.43	-4.71	136.55	231.47	120.97	86.84
2.11	10.21	3.34	-4.78	139.7	235.25	124.69	90.89
2.58	10.78	3.73	-4.4	138.46	230.94	122.04	87.47
2.58	10.78	3.72	-4.4	138.49	230.96	122.05	87.49
2.24	10.22	3.48	-4.65	133.17	229.61	118.97	84.17
2.3	10.46	3.58	-4.51	133.6	237.09	125.52	90.22
2.39	10.56	3.54	-4.56	132.16	228.89	120.3	86.9
2.1	10.31	3.31	-4.76	136.39	235.58	126.05	93.52
2.11	10.32	3.33	-4.75	136.39	235.67	125.89	93.63
1.79	9.77	3.26	-5.02	139.36	237.14	123.61	89.31
1.64	9.61	3.14	-5.04	140.1	242.63	126.17	92.12
1.74	9.73	3.19	-4.99	138.32	239.74	125.17	90.96
1.47	9.42	3.04	-5.13	140.92	245.96	127.68	93.31
1.37	9.42	2.86	-5.28	143.38	246.57	131.18	97.43
1.58	9.62	3.03	-5.11	139.27	246.36	129.04	94.6
1.68	9.77	3.08	-5.05	139.87	241.71	125.98	91.58
2.28	10.36	3.48	-4.64	130.92	230.27	119.46	83.7
2.02	10.04	3.29	-4.88	134.13	232.8	121.54	85.8
1.62	9.61	3.05	-5.11	139.11	242.31	127.21	93.3
1.45	9.54	2.92	-5.1	142.01	251.15	133.82	98.37
2.12	10.25	3.43	-4.66	133.29	238.06	125.24	88.48
2.03	10.15	3.43	-4.65	134.7	241.07	127.32	91.6
1.79	9.9	3.16	-4.85	135.69	243.94	128.85	93.49
2.02	10.15	3.38	-4.73	134.88	241.12	129.64	93.3
1.92	10.23	3.14	-5.02	157.03	250.02	134.13	103.46
2.48	10.52	3.61	-4.52	135.79	226.71	117.14	82.53
2.6	10.87	3.69	-4.44	142.51	233.44	124.05	88.46
2.65	10.93	3.72	-4.39	141.16	232.97	123.4	87.87
2.05	10.28	3.22	-4.87	150.48	243.42	127.48	96.53
2.05	10.28	3.22	-4.87	150.76	243.54	127.76	96.8
2.01	10.24	3.21	-4.87	150.72	244.89	129.61	98.02
2.11	10.29	3.33	-4.82	151.61	243.76	128.12	95.9
2.24	10.44	3.38	-4.78	147.43	239.74	125.64	93.08
2	10.21	3.25	-4.91	149.76	243.67	130.49	97.44
2.42	10.73	3.57	-4.58	145.23	234.61	124.64	89.97
2.3	10.57	3.41	-4.74	152.93	243.96	128.39	97.95
2.22	10.52	3.36	-4.78	155.18	247.02	131.05	99.7

2.31	10.57	3.43	-4.72	154.76	243.4	129.05	97.8
2.62	10.91	3.69	-4.42	142.24	233.71	122.75	88.67
2.62	10.91	3.7	-4.42	142.2	233.66	122.7	88.62
2.12	10.38	3.21	-4.79	162.84	252.98	135.19	103.2
2.34	10.6	3.36	-4.67	152.33	242.35	126.81	93.9
2.34	10.61	3.36	-4.66	152.33	242.29	126.76	93.85
2.34	10.6	3.35	-4.67	152.4	242.33	126.8	93.9
2.34	10.6	3.35	-4.67	152.44	242.34	126.82	93.93
2.34	10.6	3.35	-4.67	152.51	242.4	126.87	94
2.52	10.78	3.56	-4.56	147.8	238.15	124.26	91.66
2.52	10.77	3.56	-4.57	147.95	238.23	124.37	91.82
3.42	11.61	4.31	-3.55	134.01	221.16	109.83	81.12
2.09	10.35	3.25	-4.83	158.95	251.72	134.14	103.21
2.44	10.7	3.47	-4.61	151.51	240.97	127.02	93.79
2.12	10.38	3.21	-4.79	162.8	252.97	135.17	103.21
2.36	10.28	3.62	-4.49	129.46	225.2	112.9	78.84
2.61	10.62	3.79	-4.35	135.21	225.27	115.34	81.3
2.29	10.42	3.52	-4.59	143.38	233.97	120.38	89.75
2.57	10.72	3.72	-4.45	135.45	227.84	119.57	84.02
2.2	10.33	3.53	-4.6	132.75	236.97	126.01	88.79
4.86	13.07	5.87	-1.77	122.55	209.35	102.98	68.64
4.85	13.03	5.87	-1.81	123.07	209.05	102.07	68.8
4.47	12.65	5.51	-2.11	123.87	215.9	105.85	72.35
5	13.39	6.02	-1.64	123.35	214.1	107.2	73.08
5	13.39	6.03	-1.64	123.12	214.15	107.28	73.09
4.99	13.39	5.97	-1.65	122.09	202.48	99.1	67
4.11	12.17	5.32	-2.59	126.87	218.43	108.81	75.2
4.07	12.14	5.28	-2.66	126.09	218.26	108.73	75.08
3.96	12	5.17	-2.82	126.33	216.96	108.29	75
3.86	11.97	5.11	-2.63	133.03	227.8	116.98	83.81
3.9	11.96	5.13	-2.67	134.26	226.84	115	82.36
3.8	11.9	5.04	-2.7	132.64	227.77	116.47	83.65
3.63	11.76	4.92	-2.78	133.04	226.54	116.54	83.42
3.78	11.83	5.05	-2.87	128.96	221.07	111.49	78.59
3.84	11.94	5.09	-2.7	132.71	224.15	112.66	80.79
3.61	11.72	4.89	-2.82	132.48	227.4	116.38	83.63
3.62	11.73	4.89	-2.81	132.46	227.44	116.36	83.61

3.64	11.75	4.91	-2.8	132.38	227.19	117.14	83.79
3.71	11.83	4.98	-2.75	132.2	227.31	117.33	83.72
3.7	11.81	4.97	-2.76	132.24	227.29	117.24	83.75
3.64	11.75	4.91	-2.8	132.36	227.14	117.31	83.8
3.61	11.74	4.9	-2.79	132.98	226.55	116.51	83.49
3.8	11.99	5.03	-2.65	130.06	223.5	111.54	80.14
3.9	11.98	5.13	-2.67	134.45	229.93	117.81	83.88
3.75	11.95	5.02	-2.67	128.85	225.72	111.36	79.04
3.84	11.88	5.09	-2.84	129.04	220.53	112.26	78.97
3.75	11.83	5.02	-2.88	129.13	220.54	111.15	78.02
3.83	11.95	5.09	-2.65	133	227.54	117.04	83.85
3.78	11.9	5.03	-2.7	131.99	227.49	117.71	83.45
3.81	11.84	5.05	-2.97	128.9	218.67	110.27	77.48
3.8	11.83	5.04	-2.98	128.9	218.75	110.32	77.55
3.91	11.94	5.16	-2.68	137.61	234.61	118.85	86.61
3.9	11.96	5.13	-2.67	134.23	226.84	115	82.33
3.85	11.93	5.12	-2.74	132.65	225.39	114.32	81.02
3.54	11.52	4.88	-2.98	140.8	238.18	124.23	92.08
3.6	11.59	4.91	-2.95	140	237.3	123.74	91.26
4.6	12.8	5.61	-2.07	120.59	206.49	100.2	67.05
4.59	12.75	5.58	-2.1	120.72	207.95	100.53	67.26
4.35	12.56	5.4	-2.42	118.77	206.66	98.98	67
4.23	12.41	5.33	-2.53	117.82	206.58	98.93	66.07
4.65	12.79	5.65	-2.06	119.53	205.6	99.59	66.2
4.65	12.79	5.65	-2.05	119.53	205.56	99.56	66.23
4.65	12.79	5.65	-2.06	119.52	205.6	99.59	66.19
3.67	11.74	4.95	-3.18	127.66	216.39	109.8	77.05
4.03	12.09	5.2	-2.87	124.48	213.49	106.09	72.24
4	12.11	4.98	-2.76	118.85	206.55	99.09	67
4	12.11	4.98	-2.76	118.81	206.5	99.09	67
4.08	12.21	5.21	-2.84	117.41	206.75	98.09	65.02
3.99	12.12	5.13	-2.95	118.17	207.03	98.71	65.81
3.95	12.08	5.08	-2.92	118.6	206.81	98.52	66.2
3.77	11.91	5.01	-3.14	119.4	208.21	102.72	69.24
4.38	12.57	5.33	-2.3	123.09	206.61	101.04	68.01
4.39	12.55	5.32	-2.3	123.06	206.38	100.94	68
4.36	12.55	5.32	-2.31	123.01	206.68	101.04	68.03

4.55	12.86	5.51	-2.12	122.98	207.46	99.1	67.45
3.65	11.92	4.65	-3.03	124.4	202.06	98	67
4.3	12.39	5.42	-2.42	123.72	216.96	107.23	73.16
4.2	12.41	5.29	-2.37	124.81	213.12	104.62	71.56
4.1	12.34	5.27	-2.55	137.24	235.89	132.03	98.47
3.3	11.42	4.49	-3.93	117.73	215.21	105.44	69.96
3.4	11.52	4.67	-3.8	120.24	214.51	107.97	73.35
3.41	11.52	4.68	-3.79	120.08	214.38	107.78	73.32
3.46	11.53	4.73	-3.73	124.54	216.14	109	76
3.48	11.59	4.76	-3.65	124.03	214.5	107.89	74.88
3.46	11.55	4.74	-3.63	126.82	215.94	109.95	76.11
3.48	11.6	4.77	-3.63	124.1	213.5	108.02	74.09
3.58	11.69	4.79	-3.5	119.92	211.71	104.05	71.91
3.81	11.9	4.97	-3.19	117.12	208.83	101.06	69
3.55	11.63	4.7	-3.59	116.2	211.9	102.45	67.97
3.41	11.54	4.68	-3.79	120.03	214.4	108	73.21
3.44	11.55	4.7	-3.76	119.25	213.27	107.65	73.05
3.42	11.53	4.68	-3.79	120.47	214.7	107.95	73.26
3.5	11.62	4.74	-3.68	121.07	213.28	107.24	72.99
3.45	11.54	4.73	-3.7	124.3	215.09	108.88	75.88
3.45	11.53	4.73	-3.7	124.36	215.15	108.92	75.92
3.45	11.54	4.73	-3.7	124.32	215.12	108.89	75.9
4.07	12.18	5.16	-2.8	115.01	205.14	98	64
3.7	11.84	4.96	-3.21	121.47	210.26	102.85	70.14
3.61	11.7	4.86	-3.35	122.22	210.98	104.26	71.94
3.52	11.61	4.74	-3.7	116.96	212.02	104.18	70.37
3.69	11.78	4.88	-3.49	117.92	210.32	103.56	68
3.55	11.64	4.76	-3.64	117.51	211.36	103.99	70.37
4.13	12.28	5.25	-2.81	116.9	207.67	99.79	66.97
3.98	12.07	5.05	-3.03	116.09	206.03	99.9	64.76
3.82	11.96	5.03	-3.12	118.42	209.52	102.01	68.89
3.84	11.97	5.03	-3.1	118.18	209.03	100.86	67.95
3.91	12.03	5.07	-3.05	118.58	207.28	100.98	66.7
3.9	12.01	5.06	-3.08	118.3	207.64	100.96	67.02
3.79	11.83	4.93	-3.31	115.34	209.45	100.99	66.26
3.8	11.85	4.94	-3.3	115.21	209.25	100.93	65.99
3.78	11.83	4.92	-3.32	115.49	209.61	101.03	66.29

3.89	11.97	4.97	-3.17	115.57	208.44	99.59	65.18
3.78	11.85	4.92	-3.26	116.54	209.45	100.46	66.3
3.45	11.6	4.6	-3.76	115.52	213.84	104	68
3.45	11.6	4.6	-3.76	115.54	213.84	103.99	68
3.56	11.65	4.78	-3.64	117.49	211.25	103.95	69.65
3.56	11.65	4.78	-3.64	117.49	211.26	103.95	69.72
3.81	11.83	4.96	-3.34	115.48	209	100.82	65.23
3.9	11.98	4.97	-3.19	116.98	208.33	100.22	65.01
3.83	11.89	4.96	-3.29	115.47	210	100.73	66.49
3.85	11.98	5.04	-3.12	117.35	208.01	100.98	67.57
3.87	11.98	5.02	-3.12	116.79	207.83	101	67.16
3.75	11.78	4.87	-3.37	118.16	210.87	101.07	67.86
3.71	11.76	4.87	-3.34	118.55	211.19	101.4	67.99
3.87	11.94	4.98	-3.18	116.48	207.93	101.47	66.02
3.87	11.94	4.99	-3.18	116.46	207.87	101.4	66.01
3.87	11.94	4.98	-3.18	116.48	207.87	101.41	66.02
3.66	11.79	4.92	-3.29	120.28	211.58	103.04	71.08
3.11	11.28	4.4	-4.01	116.49	219.22	106	68.87
3.12	11.29	4.4	-4	116.29	219.36	105.98	68.73
3.11	11.26	4.4	-4.04	116.22	220.69	106.82	69.49
3.42	11.41	4.58	-3.64	117.18	218.26	104.88	66
3.06	11.2	4.4	-4.03	116.39	221.15	106.05	68.25
2.94	11.12	4.34	-4.13	118.6	222.82	107.76	70.94
3.09	11.26	4.4	-4.04	115.76	219.07	106.02	68.8
3.26	11.35	4.51	-3.84	117.19	218.92	105.21	68.43
3.47	11.46	4.61	-3.6	117.58	218.14	103.66	66
3.43	11.42	4.59	-3.63	117.16	218.3	104.63	66
3.34	11.32	4.5	-3.6	118.45	220.09	105	66
3.17	11.1	4.44	-3.72	120.43	227.07	107.52	66.93
3.17	11.09	4.44	-3.72	120.5	227.14	107.63	66.99
3.35	11.31	4.5	-3.6	118.53	219.83	105	66
3.24	11.17	4.49	-3.64	121.06	221.24	106.19	66.85
3.23	11.17	4.49	-3.64	121.08	221.29	106.24	66.88
3.34	11.32	4.5	-3.6	118.45	220.08	105	66
3.42	11.38	4.55	-3.66	117.57	220.08	104.94	66
3.6	11.62	4.7	-3.47	116.39	214.21	101.89	65.87
3.38	11.34	4.55	-3.59	119.38	221.57	105	66

3.92	12.04	4.97	-3.16	115.23	206.69	98.73	63.91
3.93	12.05	4.98	-3.16	115.17	207.08	98.53	63.93
3.93	12.05	4.98	-3.16	115.18	207.03	98.55	63.93
3.93	12.06	4.99	-3.16	115.22	207.28	98.45	63.94
3.92	12.02	4.97	-3.18	114.68	206.62	98.51	63.59
3.68	11.79	4.77	-3.23	114.22	207.74	98.89	63
3.57	11.68	4.68	-3.53	115.08	214.38	102.66	66.72
3.57	11.69	4.68	-3.53	115.11	214.35	102.56	66.63
3.67	11.73	4.67	-3.49	114.22	211.51	100.75	65
3.61	11.67	4.72	-3.52	114.48	213.19	101.95	65.5
3.46	11.4	4.58	-3.59	118.1	220.04	104.18	66.47
3.49	11.49	4.68	-3.41	115.04	214.42	103.75	65.3
3.58	11.59	4.69	-3.52	114.36	215.7	102.81	66.96
3.51	11.53	4.64	-3.52	116.95	215.72	103.37	65.87
3.29	11.26	4.48	-3.66	118.44	220.24	105.53	68.22
3.87	11.93	4.91	-3.23	113.04	206.36	98.02	64
3.87	11.93	4.91	-3.22	113.02	206.35	98.02	63.99
3.87	12	4.96	-3.18	115.73	207.2	98.93	63.93
3.88	11.99	4.97	-3.17	115.36	207.24	98.83	63.84
3.93	12.06	4.97	-3.14	115.56	207.13	98.25	63.12
3.67	11.73	4.72	-3.43	113.24	211.64	101.02	64.68
3.6	11.64	4.65	-3.36	113.21	208.23	99.06	63.11
3.67	11.74	4.73	-3.28	113.97	206.63	98.17	63
3.57	11.63	4.66	-3.3	113.94	207.86	99.27	63
3.57	11.63	4.66	-3.3	113.88	207.83	99.29	63
3.66	11.73	4.72	-3.28	113.9	207	98.82	63
3.66	11.73	4.72	-3.28	113.91	207.06	98.84	63
3.66	11.81	4.69	-3.47	114	211.95	101	66.05
3.69	11.78	4.78	-3.4	112.35	211.05	101.24	64.92
3.58	11.6	4.67	-3.57	114.18	214.8	101.91	66
3.53	11.56	4.64	-3.58	115.84	215.57	102.06	66
4.04	12.14	5.12	-2.9	117.11	206.02	98	64.04
4.1	12.14	5.13	-2.93	115.26	205.8	98.01	64
4	12.08	5.07	-3.06	113.99	207.32	97.79	63.78
4.08	12.14	5.12	-3	114.37	206.52	97.05	63.61
4.14	12.23	5.2	-2.85	115.9	205.29	98.09	63.1
3.77	11.91	5.02	-3.18	120.37	209.83	102.25	69.6

4.11	12.19	5.16	-2.95	113.59	204.99	97	63
3.86	11.99	4.94	-3.14	114.47	205.79	97.18	63
3.8	11.9	4.92	-3.2	114.57	206.41	98.44	63.88
3.91	11.98	5.07	-2.62	132.74	230.73	117.04	84.13
3.67	11.78	4.91	-2.83	139.53	236.94	125.27	93.91
3.97	12.06	5.16	-2.57	140.61	240.55	129.22	96.8
4.02	12.2	5.19	-2.56	135.52	235.17	127.23	94.15
3.83	11.88	5.03	-2.67	138.38	234.96	123.44	89.67
4.43	12.59	5.57	-2.16	132.04	229.24	119.54	85.9
4.34	12.6	5.44	-2.18	129.51	225.31	116.65	81.82
3.83	11.88	5.03	-2.67	138.38	234.96	123.44	89.68
3.9	11.95	5.08	-2.61	135.66	233.63	121.2	87.9
3.75	11.81	4.96	-2.75	134.73	232.16	119.86	88.37
4.14	12.24	5.3	-2.44	134.55	231.49	119.51	87.19
3.91	11.98	5.07	-2.62	132.64	230.67	117	84.07
4.08	12.2	5.24	-2.46	129.92	228.32	114.92	81.5
4.19	12.35	5.38	-2.33	129.24	227.68	114.52	81.05
4.06	12.17	5.22	-2.48	130.06	228.34	115.28	82.48
3.92	12	5.12	-2.64	137.81	234.77	124.85	91.32
4.01	12.09	5.18	-2.6	136.92	233.47	122.92	90.48
3.77	11.88	5	-2.72	139.42	237.76	126.62	93.99
3.75	11.87	5	-2.76	138.79	236.14	125.09	93.36
3.75	11.87	4.99	-2.76	139.03	236.09	125.13	93.27
3.95	12.01	5.15	-2.56	137.73	235.51	123.77	90.02
3.89	11.95	5.12	-2.61	133.46	230.03	117.39	84.37
3.52	11.6	4.89	-3	136.83	228.96	118	86.62
3.58	11.61	4.95	-3.11	134.14	224.9	115.93	83.54
3.18	11.28	4.63	-3.56	133.5	224.62	116.26	84.68
3.18	11.29	4.63	-3.55	133.51	224.52	116.15	84.55
3.2	11.31	4.63	-3.56	133.76	223.18	115.48	83.55
3.22	11.31	4.61	-3.6	131.25	221.28	113.79	82
3.51	11.54	4.93	-3.19	134.27	223.59	117.07	84.84
3.5	11.53	4.92	-3.21	132.83	224.52	117.04	84.73
3.55	11.59	4.92	-2.89	140.82	239.92	124.19	91.95
3.61	11.63	4.97	-2.89	140.53	238.86	123.83	91.38
3.6	11.62	4.97	-2.89	140.54	238.88	123.84	91.39
3.56	11.59	4.93	-2.88	141.67	239.6	124.05	92.01

3.5	11.57	4.87	-3.02	136.01	230.59	119.31	87.51
3.46	11.52	4.84	-3.07	135.81	227.88	118.34	87
3.36	11.36	4.8	-3.02	143.1	242.17	127.43	95.03
3.36	11.36	4.76	-3.13	141.45	239.76	125.93	93.42
3.28	11.28	4.71	-3.21	142.09	238.24	125.95	94.27
3.31	11.32	4.73	-3.19	141.77	238.41	125.58	93.98
3.77	11.93	4.99	-2.81	143.02	243.15	135.9	103.25
4.04	12.23	5.21	-2.59	143.86	243.14	138.42	106.42
3.69	11.9	4.9	-2.9	142.67	243.23	138.29	106.83
3.56	11.77	4.79	-3	143.02	243.36	138.62	107.69
3.87	12.05	5.07	-2.74	141.09	240.88	134.99	102.5
3.87	12.04	5.05	-2.69	137.64	238.41	129.24	95.98
3.49	11.59	4.81	-2.86	155.65	265.51	146.39	115.92
3.36	11.47	4.69	-2.96	156.59	266.25	148.68	117.69
3.59	11.76	4.86	-2.87	146.9	249.43	137.86	105.61
3.37	11.54	4.67	-3.16	145.72	244.27	139.08	108.96
3.38	11.55	4.67	-3.15	145.74	244.34	139.11	108.98
3.59	11.79	4.84	-2.94	145.93	245.8	140.9	109.71
3.67	11.83	4.91	-2.84	144.29	246.59	136.79	104.31
3.23	11.34	4.65	-3.04	164.2	276.38	151.18	121.72
3.02	11.14	4.45	-3.21	164.66	274.45	152.74	124.97
3.53	11.67	4.79	-3.01	144.93	244.47	139.03	106.98
3.57	11.74	4.83	-2.98	143.36	244.68	135.53	104.71
3.69	11.84	4.93	-2.87	142.77	243.31	135.06	103.08
3.68	11.83	4.93	-2.84	144.18	245.91	136.02	103.79
3.34	11.42	4.68	-3	161.66	274.26	151.99	122.52
3.39	11.49	4.7	-2.93	155.78	265.24	146.23	116.26
3.76	11.84	4.98	-2.75	140.57	241.88	127.09	94.08
3.62	11.71	4.87	-2.87	140.6	240.41	125.99	94
3.55	11.67	4.81	-2.9	146.24	248.11	135.57	104.55
3.65	11.77	4.92	-2.69	152.29	261.07	142.36	111.14
3.26	11.36	4.66	-3.02	162.29	272.63	150.16	120.76
3.53	11.73	4.79	-3.03	142.98	244.85	139.83	109.26
3.48	11.66	4.74	-3.07	144.7	245.1	139.5	109.33
3.69	11.72	4.93	-2.65	140.95	243.88	125.46	91.68
3.79	11.81	4.96	-2.67	133.36	230.42	117.03	84.38
3.78	11.81	4.96	-2.64	134.14	233.14	118.5	85.72

3.23	11.34	4.58	-3.04	157.63	269.05	148.44	119.56
3.71	11.89	4.91	-2.8	148.13	252.16	144.93	114.34
3.65	11.8	4.91	-2.79	147.94	250.01	138.65	106.05
3.53	11.69	4.79	-3.03	145.59	245.57	139.68	109.27
3.41	11.58	4.7	-3.13	145.69	244.57	139.12	108.94
3.47	11.67	4.74	-3.07	143.83	243.89	137.71	107.34
3.88	12.01	5.08	-2.69	141.24	240.32	131.02	98.8
3.93	11.99	5.12	-2.63	138.62	238.2	125.34	92.62
3.69	11.77	4.92	-2.82	141.24	241.18	126.85	94.12
3.78	11.88	5	-2.7	141.98	245.25	131.42	99.36
3.64	11.85	4.86	-2.92	143.6	244.52	141.21	110.77
2.99	11.01	4.55	-3.17	161.86	267.6	146.23	117.03
3	11.05	4.54	-3.17	162.17	268.39	146.41	117.08
2.56	10.58	4.18	-3.97	143.9	233.58	127.77	97.9
3.31	11.34	4.76	-3.47	139.24	229.25	122.15	90.57
2.51	10.54	4.17	-3.94	146.04	234.21	128.62	99.73
2.94	10.98	4.54	-3.17	163.27	268.41	147.76	119.63
3.03	11.09	4.51	-3.17	160.56	268.73	146.58	117.3
3.02	11.09	4.51	-3.17	160.58	268.69	146.56	117.29
3.03	11.09	4.51	-3.17	160.57	268.71	146.57	117.29
3.07	11.06	4.6	-3.16	152.7	257.59	138.44	107.46
2.94	10.97	4.5	-3.37	146.98	244.99	131.07	99.26
3	10.99	4.51	-3.52	141.92	236.64	127.11	96
2.58	10.64	4.22	-3.99	143.45	231.4	126.86	97.48
3.26	11.26	4.68	-3.26	141.94	238.51	126.39	94.38
3.37	11.44	4.73	-3.54	130.98	221.41	114.92	81.94
3.39	11.44	4.8	-3.4	133.69	224.24	116.87	85.61
3.54	11.57	4.92	-3.27	133.26	222.36	116.01	84
2.98	11.08	4.46	-3.94	135.49	225.8	120.14	88.14
3.47	11.46	4.87	-3.21	134.98	225.63	118.22	86.4
2.96	11	4.48	-3.73	137.96	228.82	122.57	92.47
3.18	11.25	4.59	-3.71	130.1	222.97	115.6	83.43
3.34	11.42	4.71	-3.52	130.65	220.97	113.27	82
3.33	11.41	4.7	-3.53	130.67	220.95	113.3	82
3.25	11.3	4.6	-3.71	129.01	221.16	112.61	81.34
3.74	11.81	5.06	-2.58	150.98	256.42	135.62	102.15
3.67	11.71	4.97	-2.64	143.34	247.52	129.42	95.86

3.68	11.72	4.97	-2.63	143.38	247.59	129.39	96.06
3.48	11.49	4.92	-2.77	152	260	136.91	104.82
3.2	11.33	4.7	-3.02	155.26	263.08	144.31	112.26
3.49	11.69	4.75	-2.99	147.58	251.74	145.82	115.3
3.48	11.73	4.82	-3.07	155.09	255.61	164.36	135.85
4.01	12.13	5.3	-2.39	157.65	259.94	169.77	146.12
3.75	11.93	5.07	-2.65	157.53	265.85	177.28	148.99
3.74	11.93	5.06	-2.66	157.59	265.35	177.88	148.95
3.88	12.02	5.14	-2.51	156.43	264.84	176.61	149.94
2.34	10.45	4.02	-3.78	162.78	259.03	156.61	134.56
2.65	10.85	4.19	-3.64	162.46	260.91	158.25	132.85
2.88	11.13	4.41	-3.45	160.26	262.96	161.58	136.36
2.35	10.34	3.48	-3.62	128	204.91	140.03	122.26
2.38	10.39	3.54	-3.68	122.49	200.73	134.63	114.25
2.3	10.38	3.57	-3.78	123.92	207.98	136.33	116.85
2.1	10.03	3.34	-3.78	141.43	227.57	155.43	133.49
2.15	10.09	3.38	-3.72	139.04	223.93	153.05	130.52
2.21	10.17	3.4	-3.68	125.24	207.28	137.76	113.91
2.21	10.17	3.39	-3.69	125.37	207.38	137.88	114.19
2.41	10.43	3.6	-3.66	122.42	201.26	134.05	112.99
2.3	10.3	3.47	-3.71	124.92	202.59	136.41	117.68
2.37	10.41	3.56	-3.76	120.91	211.95	134.01	111.06
2.65	10.77	3.88	-3.65	143.15	248.7	152.02	127.17
2.34	10.51	3.6	-3.79	141.22	249.21	154.11	129.42
2.76	10.94	3.96	-3.59	139.74	245.18	149.05	124.19
2.52	10.73	3.8	-3.67	131.35	237.48	145.07	118.28
2.45	10.66	3.75	-3.64	132.05	237.64	145.98	120.45
2.64	10.76	3.88	-3.47	130	235.83	142.74	117.13
2.67	10.79	3.93	-3.46	130.13	234.88	143.04	116.5
2.55	10.7	3.82	-3.52	131.25	236.64	143.75	118.49
2.52	10.68	3.8	-3.54	131.86	237.03	144.39	119.09
2.55	10.69	3.82	-3.52	131.32	236.68	143.81	118.54
2.33	10.47	3.68	-3.69	130.37	232.88	142.13	120.43
3.44	11.62	4.69	-3.07	161.99	258.08	155.24	137.31
3.47	11.73	4.7	-3	160.17	256.51	153	135.44
4.06	12.44	5.19	-2.62	161.19	250.78	150.92	133.43
4	12.38	5.15	-2.68	163.32	252.6	152.72	136.02

3.74	12.16	4.83	-2.83	148.95	239.47	138.61	119.67
3.66	12.01	4.74	-2.84	153.09	245.93	145.06	127.57
2.99	11.16	4.54	-3.23	162.61	262.02	178.59	163.58
2.5	10.68	4.24	-3.57	164.5	263.73	166.74	150.58
2.75	10.91	4.48	-3.31	165.01	268.71	163.97	148.37
2.82	11.01	4.47	-3.19	165.43	259.05	172.06	161.62
2.73	10.93	4.37	-3.33	163.58	258.83	173.07	162.53
2.83	11	4.48	-3.17	167.22	260.64	169.81	158.09
2.69	10.89	4.37	-3.32	168.2	264.83	166.72	153.12
2.8	10.96	4.52	-3.26	165.63	269.59	163.87	147.9
2.77	10.9	4.47	-3.29	166.93	267.62	162.56	144.92
3.16	11.2	4.86	-2.8	164.99	262.8	145.04	122.23
3.37	11.38	5.03	-2.67	164.6	270.06	147.48	120.7
3.4	11.41	5.05	-2.65	164.18	270.11	147.18	120.24
2.54	10.66	4.33	-3.62	152.01	244.84	134.94	108.01
3.32	11.37	4.94	-2.66	169.76	274.1	149.89	124.45
3.27	11.29	4.93	-2.76	164.45	273.62	148.23	122.19
2.91	10.96	4.74	-3.13	157.56	245.97	139.37	118.68
2.93	10.98	4.73	-3.04	162.07	248.38	142.65	120.54
2.64	10.71	4.4	-3.66	156.06	236.29	136.05	115.97
3.16	11.24	4.73	-3.04	156.74	232.12	139.82	122.12
3.16	11.24	4.72	-3.04	156.72	232.25	139.81	122.2
3.24	11.32	4.82	-2.76	157.29	238.09	144.45	126.43
2.87	10.91	4.7	-3.14	158.81	247	141.5	120.01
2.77	10.8	4.6	-3.25	157.96	247.18	140.68	119.34
3.31	11.39	4.93	-2.77	158.32	239.18	140.65	122.9
2.92	10.97	4.75	-3.13	157.55	245.92	139.34	118.67
2.57	10.63	4.3	-3.67	150.94	243.34	134.24	105.85
1.37	9.42	3.2	-5.21	150.84	241.93	137.91	112.59
1.14	9.29	3.06	-5.26	153.47	241.97	138.43	114.42
1.52	9.56	3.32	-5.06	150.54	241.46	137.17	111.09
1.54	9.66	3.39	-4.96	150.5	240.28	134.87	109.34
2.43	10.57	4.24	-3.63	152.98	247.17	136.85	110.76
2.03	10.1	3.81	-4.13	151.1	242.79	136.82	109.55
1.62	9.74	3.43	-4.94	149.36	239.27	133.2	107.79
0.92	9.07	2.78	-5.68	155.65	247.91	139.51	115.14
2.13	10.19	4.03	-3.79	157.19	247.46	141.06	119.52

2.32	10.34	4.15	-3.65	158.11	249.95	140.6	117.34
2.31	10.32	4.14	-3.67	157.79	249.82	140.48	117.37
2.5	10.52	4.31	-3.49	158.15	250.99	139.56	115.73
2.33	10.46	4.26	-4	156.39	239.34	135.34	116.16
2.25	10.38	4.2	-4.07	156.25	239.52	135.72	116.31
3	10.97	4.72	-3.38	154.15	239.99	133.83	112.36
2.5	10.5	4.3	-3.51	157.16	250.51	139.08	115.84
1.13	9.34	2.91	-5.73	152.97	245.45	135.99	110.21
1.19	9.4	2.95	-5.69	152.53	244.74	135.43	109.28
0.89	9.01	2.75	-5.98	152.91	259.65	140.27	112.82
1.48	9.55	3.21	-5.72	144.86	250	133.75	102.37
1.27	9.34	3.05	-5.81	147.53	253.13	135.59	105.74
2.33	10.4	4.04	-4.1	147.55	236.22	129.83	101.02
2.4	10.55	3.89	-4.89	136.22	229.76	121.72	91.58
2.41	10.5	3.93	-4.55	140.3	229.63	124.28	95.19
2.17	10.31	3.7	-4.92	136.95	231.09	124.33	93.86
2.04	10.18	3.69	-4.92	144.3	235.23	129.77	101.3
2.07	10.2	3.72	-4.78	146.54	236.33	129.35	102.12
2.02	10.12	3.64	-4.77	146.08	236.47	129.03	101.49
2.45	10.56	4.01	-4.49	142.16	230.22	125.09	95.88
2.15	10.28	3.82	-4.51	145.22	235.19	128.55	100.53
1.83	9.98	3.63	-4.68	148.7	234.73	130.98	103.31
2.53	10.64	4.08	-4.26	142.3	231.63	125.22	96.96
2.44	10.55	4.01	-4.34	142.38	231.61	125.6	97.18
2.56	10.68	4.09	-4.26	141.79	230.53	125.04	95.09
2.89	10.98	4.38	-3.92	139.97	229.41	123.24	92.96
2.86	10.95	4.32	-4.06	139.91	228.43	123.14	92.61
1.4	9.53	3.13	-5.42	151.63	242.86	134.7	107.99
2.1	10.27	3.66	-5.3	137.89	234.45	124.88	94.46
1.42	9.61	3.15	-5.65	145.48	241.84	132.82	104.51
2.07	10.23	3.64	-5.2	141.04	233.53	126.16	96.64
1.81	9.98	3.43	-5.36	144.29	235.66	128.57	99.73
1.65	9.81	3.34	-5.32	146.06	237.68	129.31	103.07
2.63	10.74	4.13	-4.34	137.69	227.84	122.11	92.79
2.02	10.18	3.64	-5.02	143.03	230.83	127.12	98.39
1.51	9.69	3.19	-5.44	144.6	236.22	130.78	103.86
2.32	10.44	3.81	-4.92	137.63	228.87	123.15	94.68

2.02	10.18	3.58	-5.02	139.3	231.27	125.71	97.34
2.48	10.62	3.96	-4.68	136.66	227.43	122.08	92.72
2.24	10.39	3.77	-4.8	138.05	229.48	123.89	94.54
2.61	10.74	4.05	-4.51	136.1	227.3	121.61	91.07
2.61	10.73	4.05	-4.5	133.63	226.82	120.71	89.39
2.15	10.31	3.8	-4.54	140.27	231.1	125.86	96.92
2.45	10.57	3.91	-4.88	136.96	229.04	122.83	92.98
2.38	10.53	3.87	-4.9	136.39	229.79	121.91	91.8
2.39	10.54	3.88	-4.9	136.31	229.78	121.82	91.7
2.4	10.55	3.89	-4.89	136.19	229.76	121.69	91.55
2.29	10.44	3.79	-4.97	135.4	229.14	122.45	91.99
2.33	10.48	3.84	-4.97	135.8	229.16	122.14	91.63
2.22	10.36	3.72	-5.06	137.29	231.37	123.39	93.51
2.04	10.19	3.58	-5.24	131.71	238	122.81	89.27
2.38	10.53	3.87	-5.21	134.41	231.69	120.22	87.78
2.47	10.63	3.92	-5.09	130.38	230.15	118.6	85.92
2.52	10.69	3.98	-4.97	129.2	229.98	118.08	84.44
2.21	10.37	3.73	-5.38	136.06	232.45	122.64	90.07
2.23	10.4	3.76	-5.36	135.57	232.03	122.32	89.74
2.13	10.33	3.7	-5.4	137.62	233.93	123.99	92.21
2.44	10.58	3.85	-5.11	131.52	230.01	118.18	86.73
2.3	10.44	3.78	-5.02	132.2	231.77	119.14	87.24
2.12	10.27	3.67	-5.17	133.05	231.32	121.04	90.59
2.34	10.46	3.84	-4.99	132.82	230.4	119.69	87.93
1.8	9.96	3.48	-5.51	143.58	241.38	128.47	98.51
2.37	10.55	3.81	-5.14	132.77	229.7	118.29	88.03
2.34	10.54	3.81	-5.13	132.33	228.4	119.67	89.02
2.25	10.46	3.74	-5.24	133.36	230.15	122.11	90.26
2.11	10.29	3.67	-5.39	138.93	235.38	124.33	92.9
2.24	10.43	3.77	-5.24	131.84	233.4	121.79	88.54
2.47	10.64	3.93	-5.07	130.1	230.43	118.33	85.66
2.55	10.72	3.97	-5.03	130.73	228.95	117.51	85.17
2.59	10.65	4.01	-4.79	130.67	231.27	118.86	83.56
3.01	11.1	4.26	-4.37	122.2	223.41	111.78	74.78
2.2	10.3	3.72	-5.27	136.22	241.13	124.9	91.22
2.18	10.29	3.71	-5.28	136.38	241.27	125.18	91.49
2.61	10.75	4	-4.9	130.99	226.69	116.1	84.61

2.57	10.74	4.02	-4.9	129.23	227.69	117.5	84.9
2.93	10.99	4.24	-4.43	122.63	226.5	111.99	74.03
2.69	10.76	4.07	-4.69	126.21	231.01	115.61	79.09
3.14	11.24	4.44	-4.19	122.54	221.76	110.58	76.03
3.04	11.15	4.37	-4.23	123.12	222.75	111.35	77.05
2.53	10.68	4	-4.76	131.67	228.22	117.72	85.99
3.31	11.4	4.58	-3.98	126.24	217.71	110.94	77.92
2.78	10.91	4.08	-4.53	124.78	225.91	113.78	78.36
2.42	10.53	3.91	-4.92	132.88	229.83	119.01	86.69
2.51	10.64	3.96	-4.74	133.72	226.49	118.47	87.06
2.96	11.05	4.42	-4.02	134.03	225.21	119.17	87.57
2.26	10.4	3.85	-4.62	134.23	229.17	121.46	91.52
2.58	10.68	4.02	-4.44	135.3	226.85	120.53	90.5
2.42	10.56	3.89	-4.67	134.67	228.73	122.05	91.54
2.12	10.27	3.67	-4.82	134.03	229.25	121.08	91.92
2.11	10.25	3.66	-4.83	134.08	229.44	121.21	92.05
2.38	10.48	3.86	-4.67	134.24	228.73	119.75	89.57
2.27	10.4	3.78	-4.87	133.29	230.17	122.65	91.63
2.52	10.65	3.98	-4.62	131.06	227.03	119.31	88.88
2.62	10.73	4.04	-4.55	130.94	226.52	118.81	88.09
3.02	11.11	4.25	-4.32	122.36	222.35	111.41	74.2
3.02	11.11	4.25	-4.32	122.37	222.37	111.4	74.23
2.89	10.96	4.35	-4.12	134.87	226.15	119.66	87.91
2.92	11.01	4.39	-4.05	134.71	225.61	119.6	88.08
3.03	11.13	4.5	-3.94	132.09	224.41	118.34	87.19
2.4	10.54	3.89	-4.82	131.42	226.86	119.7	89.15
2.87	10.95	4.24	-4.54	130.28	225.83	114.78	81.13
2.53	10.61	3.99	-4.81	132.19	228.47	118.06	85.01
3.16	11.23	4.38	-4.31	121.95	221.25	110.56	74.51
3.12	11.18	4.34	-4.32	121.3	222.29	110.85	74.72
3.15	11.25	4.39	-4.21	119	217.24	108.2	73.73
3.07	11.11	4.38	-4.42	125.95	224.99	113.67	78.48
3.12	11.22	4.41	-4.24	126.95	221.07	112.94	78.97
2.6	10.7	4.01	-4.6	133.01	226.6	117.53	86.72
2.49	10.61	3.92	-4.69	133.27	227.11	117.85	86.98
2.55	10.71	4.02	-4.74	131.57	227.95	117.42	85.84
2.39	10.51	3.89	-4.94	132.94	229.93	119.42	87.02

1.89	10.06	3.47	-5.37	133.2	239.87	123.89	89.5
1.9	10.06	3.47	-5.37	133.25	239.94	123.9	89.49
2.25	10.38	3.75	-5.05	130.28	237.71	120.6	84.49
2.19	10.38	3.69	-5.19	133.21	239.4	123.25	87.95
1.75	9.87	3.43	-5.47	138.23	247.3	129.31	93.77
2.68	10.75	4.06	-4.75	125.87	233.43	116.76	77.86
2.33	10.42	3.8	-4.88	129.66	239.6	119.67	81.48
2.33	10.42	3.8	-4.88	129.66	239.58	119.68	81.51
2.81	10.83	4.13	-4.58	124.1	230.28	113.96	76.18
2.73	10.78	4.11	-4.62	125.64	232.81	116	77.65
2.78	10.82	4.16	-4.54	124.16	232.18	115.16	76.74
2.36	10.42	3.79	-4.65	127.95	241.1	119	78.8
2.3	10.4	3.78	-4.98	129.43	237.85	120.46	83.57
2.41	10.49	3.86	-4.84	129.36	238.33	119.11	81.44
1.69	9.82	3.37	-5.64	139.06	243.92	127.74	95
1.44	9.59	3.17	-5.73	140.98	248.02	130.06	98.32
1.9	10	3.51	-5.53	136.38	241.93	125.57	92.29
1.88	9.99	3.49	-5.55	136.4	242.45	125.79	92.84
1.5	9.65	3.23	-5.7	140.19	247.42	129.57	97.89
1.6	9.67	3.29	-5.28	136.48	252	128.45	91.6
2.1	10.16	3.66	-4.6	129.43	247.09	122.6	80.45
1.38	9.45	3.09	-5.66	144.7	252.88	133.14	99.1
1.53	9.6	3.24	-5.63	141.4	252.01	131.81	96.51
1.52	9.6	3.22	-5.59	142.2	253.45	133.06	96.67
1.6	9.68	3.31	-5.56	142.03	251.11	131.2	95.4
1.62	9.69	3.25	-5.11	137.74	257.49	129.77	90.81
1.93	9.98	3.53	-5.15	134.75	248.15	125.72	87.88
1.83	9.86	3.46	-5.19	135.99	252.31	125.17	88.89
1.54	9.59	3.23	-5.48	141.22	257.08	131.02	96.21
1.96	10.02	3.55	-4.91	134.81	250.98	126.63	87.39
2.11	10.13	3.66	-4.6	131.52	251.15	123.53	80.08
2.12	10.17	3.69	-4.79	131.28	247.24	123.95	83.92
2.08	10.1	3.64	-4.57	131.02	248.86	121.99	81.03
1.6	9.66	3.29	-5.6	140.75	251.52	130.81	95.57
1.71	9.79	3.38	-5.48	140.69	250.74	130.74	94.26
1.61	9.67	3.26	-5.19	136.1	257.67	130.39	91.66
1.69	9.74	3.32	-5.05	136.47	256.71	129.14	89.67

1.88	9.95	3.48	-5.08	133.1	248.72	124.42	86.53
1.93	9.98	3.53	-5.16	134.77	248.18	125.77	87.91
2.18	10.25	3.66	-4.78	131.03	244.83	121.65	81.43
1.79	9.85	3.39	-5.01	135.34	253.39	126.72	86.99
2.46	10.5	3.86	-4.59	128.94	240.63	118.17	77.92
1.02	9.05	2.81	-5.43	148.03	271.25	135.13	97.77
0.87	8.96	2.68	-5.57	147.85	273.35	137.02	100.74
0.93	8.99	2.74	-5.46	147.87	275.08	137.38	100.41
1.36	9.42	2.99	-5.02	137.09	262.98	131.18	89.91
3.01	11.09	4.33	-4.17	118.42	226.25	110.27	70.37
3.02	11.09	4.36	-4.27	121.32	226.79	111.29	72.71
2.33	10.38	3.85	-4.6	130.95	244.63	118.34	78.83
2.97	11.03	4.3	-4.16	121.47	229.98	110.76	71.01
2.88	10.87	4.19	-4.08	121.86	231.45	110.42	71.4
2.88	10.87	4.19	-4.08	121.87	231.4	110.38	71.35
2.43	10.45	3.9	-4.46	128.63	242.26	116.37	76.5
1.96	9.98	3.53	-4.81	133.26	251.08	121.51	82.64
2.47	10.49	3.95	-4.47	127.07	239.89	115.36	76.14
2.47	10.49	3.95	-4.47	127.09	239.92	115.39	76.15
2.2	10.24	3.74	-4.66	130.89	245.67	118.63	79.92
2.14	10.19	3.7	-4.72	131.58	246.28	119.66	80.54
1.38	9.43	3.07	-5.26	139.54	260.34	129.84	92.46
1.81	9.76	3.31	-4.74	135.66	256.94	125.71	83.2
2.48	10.51	3.95	-4.35	127.29	243.2	114.75	73.89
2.81	10.83	4.17	-4.15	122.53	234.07	110.91	69.35
2.39	10.35	3.82	-4.25	129.17	241.01	118.44	78.5
2.57	10.6	4.01	-4.34	126.47	239.32	114.25	73.07
2.86	10.84	4.21	-4.08	123.95	233.82	111.58	69.84
2.87	10.85	4.21	-4.07	123.84	233.65	111.49	69.77
2.87	10.85	4.22	-4.07	123.8	233.6	111.46	69.74
2.87	10.85	4.22	-4.07	123.77	233.55	111.44	69.72
2.87	10.85	4.22	-4.07	123.74	233.51	111.41	69.71
2.88	10.85	4.22	-4.07	123.71	233.46	111.39	69.69
2.88	10.86	4.22	-4.07	123.68	233.42	111.37	69.67
2.88	10.86	4.22	-4.06	123.65	233.37	111.34	69.65
2.88	10.86	4.22	-4.06	123.61	233.31	111.31	69.63
2.88	10.86	4.22	-4.06	123.58	233.26	111.28	69.61

2.26	10.25	3.76	-4.51	130.03	246.62	118.08	77.39
2.24	10.23	3.74	-4.52	130.2	246.83	118.32	77.61
2.25	10.25	3.76	-4.51	130.03	246.56	118.08	77.37
1.83	9.84	3.4	-4.78	136.27	254.01	123.62	83.39
2.09	10.08	3.59	-4.57	133.88	250	119.97	79.31
1.9	9.89	3.43	-4.73	135.83	252.91	122.71	82.23
2.79	10.79	4.15	-4.05	125.35	234.6	111.47	70.71
2.54	10.52	3.96	-4.17	126.47	240.3	115.77	74.1
2	9.96	3.42	-4.65	133.35	251.23	120.89	79.94
1.72	9.7	3.22	-4.85	135	255.23	123.81	83.3
1.69	9.67	3.22	-4.84	136.33	256.82	125.36	84.21
1.72	9.69	3.26	-4.8	135.62	256.87	125.19	83.71
1.75	9.72	3.28	-4.78	135.42	256.41	125.04	83.3
2.19	10.12	3.56	-4.51	130.52	247.27	118.92	76.22
2.05	10.01	3.45	-4.61	131.34	248.74	121.15	78.62
2.03	9.98	3.46	-4.61	132.2	249.98	122.14	79.08
2.11	10.06	3.51	-4.56	130.17	248.33	121.19	77.85
2.11	10.05	3.51	-4.55	130.36	248.72	121.57	77.92
2.11	10.07	3.51	-4.54	130.36	249	121.42	77.28
2.33	10.27	3.65	-4.39	128.46	241.38	117.28	74.43
2.33	10.27	3.65	-4.39	128.45	241.4	117.28	74.44
2.89	10.84	4.25	-3.91	121.74	231.25	110.93	70.73
2.83	10.76	4.19	-3.97	122.37	233.42	112.68	71.95
2.84	10.77	4.2	-3.96	122.35	233.2	112.59	71.78
3.14	11.04	4.46	-3.76	121.28	226.48	109	68.61
3.14	11.04	4.46	-3.76	121.26	226.58	109	68.66
3.14	11.04	4.46	-3.76	121.34	226.5	109	68.65
3.13	11.04	4.46	-3.76	121.31	226.63	109	68.71
3.13	11.04	4.46	-3.76	121.38	226.54	109	68.69
3.03	11.04	4.3	-3.95	120.97	227.92	109.06	68.84
2.69	10.61	4.06	-4.04	125.43	235.83	114	74.08
2.26	10.29	3.63	-4.51	129.7	245.8	122.64	78.93
1.16	9.32	2.84	-5.28	143.38	270.74	137.19	99.13
1.14	9.31	2.82	-5.3	143.55	271.03	137.42	99.44
1.14	9.3	2.82	-5.3	143.6	271.13	137.51	99.55
1.13	9.29	2.81	-5.31	143.72	271.34	137.7	99.78
1.57	9.66	3.11	-5	138.82	260.27	132.03	90.57

2.44	10.4	3.78	-4.32	127.21	241.73	118.6	75.86
2.1	10.08	3.5	-4.54	130.47	246.51	120.51	79.34
2.09	10.07	3.48	-4.55	129	245.4	120.08	79.03
2.09	10.07	3.48	-4.55	128.97	245.39	120.01	78.99
2.09	10.06	3.48	-4.54	128.96	245.58	120.31	79.15
2.09	10.06	3.48	-4.55	128.97	245.48	120.15	79.07
2.27	10.23	3.62	-4.42	128.47	241.93	117.85	75.42
2.09	10.1	3.49	-4.55	128.91	246.13	120.17	78.21
2.13	10.1	3.52	-4.51	129.29	245.81	120.45	79.7
2.23	10.21	3.62	-4.43	128.1	244	119.01	76.76
2.31	10.3	3.69	-4.34	126.38	242.75	118.12	74.75
2.11	10.13	3.57	-4.51	131.47	251.4	122.79	80.39
2.46	10.42	3.79	-4.3	125.83	241.49	118.33	75.42
1.29	9.44	2.95	-5.14	141.98	267.89	134.96	96.16
1.19	9.34	2.87	-5.22	142.57	270.65	136.59	98.53
2.35	10.29	3.74	-4.32	128.75	245.79	118.18	77.66
1.17	9.29	2.78	-5.25	143.35	275.55	139.32	97.16
0.67	8.73	2.38	-5.61	154.59	295.68	149.66	112.35
1.46	9.57	3.16	-4.68	149.87	286.98	143.75	104.93
1.81	10.08	3.41	-4.66	154.46	292.49	152.94	111.03
1.22	9.53	2.82	-5.17	154.06	290.15	152.65	111.48
2.1	10.31	3.59	-4.46	149.98	282.23	148.66	102.83
1.62	9.81	3.12	-4.91	142.32	272.53	138.89	96.49
1.57	9.75	3.08	-5	144.78	273.72	140.12	96.97
1.8	9.92	3.22	-4.82	141.6	267.87	135.29	91.49
1.68	9.83	3.15	-4.92	144	270.79	138.14	94.64
1.77	9.91	3.22	-4.84	143.05	268.11	136.06	92.31
1.21	9.4	2.68	-5.22	144.63	275.97	139.93	99.06
1.53	9.7	3.02	-5.02	143.89	271.82	138.26	95.02
1.76	9.91	3.19	-4.82	140.25	267.67	135.26	91.48
1.35	9.54	2.83	-5.1	143.66	273.14	138.11	96.14
0.84	9.02	2.5	-5.47	149.72	284.81	146.06	105.45
1.61	9.8	3.11	-4.92	142.41	272.72	139.01	96.7
1.02	9.13	2.64	-5.35	149.78	285.21	144.61	103.57
1.22	9.38	2.77	-5.23	145.15	275.02	140.09	98.4
0.65	8.94	2.42	-5.55	159.88	304.78	160.18	125.06
2.88	11.01	4.08	-4.15	133.44	249.14	128.8	84.31

0.79	9.04	2.41	-5.59	158.14	296.98	158.14	121.29
1.98	10.04	3.65	-4.15	139.05	273.11	135.09	93.6
1.47	9.59	3.24	-4.58	144.64	283.11	141.61	103.89
1.92	10.1	3.58	-4.24	146.46	282.25	142.4	102.57
1.22	9.55	2.97	-4.82	151.67	292.1	146.55	110.36
2.06	10.19	3.75	-4.06	144.69	276.93	138.94	97.23
1.76	9.92	3.35	-4.6	152.18	290.79	150	108.49
1.59	9.77	3.09	-4.98	145.97	280.29	145.4	104.51
2.08	10.25	3.5	-4.59	141.71	270.69	136.1	92.85
1.77	9.9	3.22	-4.87	136.29	260.78	131.9	89.81
2.43	10.58	3.74	-4.4	136.49	257.12	134.24	89.61
2.9	11	4.09	-4.03	127.56	244.65	123.12	77.32
2.78	10.92	4.01	-4.19	132.79	249.63	130.18	87.52
2.8	10.93	3.98	-4.22	135.12	251.24	130.55	86.26
1.68	9.89	3.19	-4.94	145.14	275.62	144.19	104.44
3.15	11.24	4.24	-3.94	128.85	242.17	126.74	79.98
3.62	11.79	4.58	-3.63	132.8	243.76	132.95	85
3.39	11.56	4.38	-3.84	134.64	248.58	135.45	88.37
3.4	11.56	4.38	-3.84	134.63	248.56	135.45	88.36
3.4	11.56	4.39	-3.84	134.74	248.54	135.55	88.42
3.41	11.57	4.39	-3.84	134.71	248.39	135.55	88.36
3.41	11.57	4.39	-3.83	134.67	248.33	135.53	88.34
3.42	11.57	4.4	-3.83	134.54	248.14	135.48	88.24
3.45	11.6	4.42	-3.81	134.17	247.58	135.3	87.91
3.45	11.6	4.42	-3.8	134.12	247.46	135.28	87.85
3.18	11.33	4.23	-4.04	141.6	258.67	140.35	92.1
2.51	10.76	3.91	-3.95	149	279.63	148.95	102.3
2.19	10.46	3.65	-4.3	151.49	287.88	151.57	106.89
1.88	10.19	3.41	-4.52	154.73	294.36	156.43	112.4
2.28	10.53	3.73	-4.26	151.86	284.88	149.33	103.6
1.6	9.93	3.22	-4.78	156.25	295.53	156.63	114.81
1.29	9.56	2.83	-5.22	152.45	289.08	153.49	113.06
1.76	10.1	3.37	-4.76	156.12	289.21	153.79	111.64
1.77	10.05	3.29	-4.74	156.4	292.1	156.12	112.82
1.26	9.53	2.79	-5.35	154.59	291.18	157.44	117.08
2.03	10.28	3.53	-4.47	152.4	286.95	151.82	106.64
1.68	10	3.25	-4.76	153.67	290.32	153.72	111.5

1.74	10.03	3.27	-4.87	152.16	286.65	152.51	110.35
1.76	10.1	3.36	-4.76	156.11	289.23	153.79	111.65
2.07	10.26	3.38	-4.76	162.35	292.64	159.17	113.71
3.18	11.39	4.26	-3.93	154.74	272.57	149.48	95.52
2.28	10.54	3.69	-4.28	160.26	289.78	156.76	108.18
2.18	10.38	3.62	-4.36	156.07	287.65	153.14	107.74
2.99	11.17	4.29	-3.81	163.68	285.91	155.09	100.42
3.13	11.24	4.35	-3.63	157.79	274.91	149.1	94.31
2.7	10.9	4	-3.89	150.53	276.17	147.62	98.13
2.76	10.94	4.03	-3.86	149.69	276.3	147.39	97.71
2.73	10.93	4.01	-3.87	149.55	275.98	147.69	97.95
2.75	10.95	4.05	-3.86	152.77	276.83	148.28	97.58
2.98	11.18	4.27	-3.73	156.11	277.03	148.56	95.59
3.25	11.4	4.44	-3.68	162.99	279.6	151.63	93.81
2.94	11.11	4.25	-3.85	164.46	287.44	155.89	101.05
3.2	11.36	4.45	-3.69	159.32	279.51	151.78	96.02
3.2	11.36	4.45	-3.69	159.31	279.41	151.78	96.01
3.22	11.38	4.48	-3.68	159.56	278.66	151.8	95.96
3.14	11.31	4.32	-3.76	164.96	284.21	153.74	95.4
3.14	11.31	4.32	-3.76	164.94	284.15	153.71	95.35
2.35	10.47	3.66	-4.58	140.91	261.87	140.57	94.81
1.42	9.71	2.88	-5.32	152.85	285.37	155.05	113.6
1.35	9.64	2.87	-5.3	162.72	297.97	162.83	120.72
1.49	9.79	2.99	-5.24	151.36	282.54	152.68	112.34
1.68	9.97	3.1	-5.09	152.84	281.94	152.49	111.42
1.62	9.92	3.05	-5.17	154.86	282.91	154.32	113.21
1.68	9.99	3.12	-5.05	151.92	283.1	152.69	111.63
1.72	9.98	3.1	-5.08	150.49	281.52	152.59	109.83
3.2	11.33	4.23	-4.04	141.34	257.95	140.24	92.06
1.35	9.65	2.81	-5.36	158.19	291.99	157.44	118.62
2.55	10.82	3.84	-4.31	169.07	296.05	160.12	108.86
2.42	10.59	3.77	-4.28	158.58	283.93	152.3	104.09
2.29	10.49	3.69	-4.41	160.46	285.68	153.59	105.41
1.98	10.21	3.43	-4.7	164.8	291.49	157.3	111.35
3.19	11.4	4.29	-3.74	163.01	285.51	152.17	93.5
3.18	11.34	4.3	-3.78	162.07	280.26	151.56	93.37
2.59	10.8	3.85	-4.21	167.16	295.83	159.74	106.29

2.54	10.8	3.83	-4.34	167.69	296.29	160.44	108.76
2.81	11.01	4	-4.23	161.42	283.61	154.56	102.8
2.41	10.56	3.78	-4.27	159.19	284.77	152.64	104.45
1.25	9.58	2.78	-5.37	162.6	298.83	162.62	122.36
1.72	9.99	3.15	-5.1	158.59	290.58	158.64	116.32
1.44	9.75	2.94	-5.22	162.25	293.88	159.67	118.03
1.64	9.91	3.07	-5.13	158.95	289.63	157.09	113.68
1.53	9.81	2.98	-5.24	159.14	292.28	158.38	117.44
1.51	9.8	2.97	-5.24	159.16	292.41	158.61	118.46
1.51	9.81	2.97	-5.24	159.54	292.8	159.63	118.99
1.8	10.06	3.25	-4.94	159	289.65	155.95	113.85
1.47	9.66	2.85	-4.65	157.23	265.41	161.56	153.36
1.53	9.71	2.85	-4.6	142.29	252.83	148.76	139.07
1.63	9.8	2.96	-4.52	147.21	257.35	153.25	142.86
1.94	10.11	3.2	-4.34	144.17	253.29	147.95	137.54
1.79	9.85	3.16	-4.2	132.65	236.9	145.27	134.03
1.08	9.26	2.53	-4.86	150.07	258.23	159.5	153.68
1.43	9.6	2.78	-4.64	135.39	248.76	146.89	137.19
0.92	9.2	2.46	-4.92	165.96	273.94	170.95	166.2
1.66	9.83	3.04	-4.48	148.72	258.46	152.12	141.85
1.79	9.91	3.19	-4.22	137.52	248.18	145.66	129.4
1.1	9.31	2.51	-4.87	147.23	259.5	158.22	152.27
1.37	9.61	2.83	-4.61	158.37	266.75	163.12	154.6
1.23	9.46	2.7	-4.71	160.92	269.03	165.66	158.33
1.2	9.43	2.65	-4.79	156.96	265.06	161.33	152.23
1.19	9.43	2.65	-4.79	157.22	265.28	161.54	152.54
1.2	9.39	2.65	-4.84	154.18	262.69	159.42	150.04
1.68	9.86	3.02	-4.43	151.59	258.37	155.91	142.26
1.43	9.66	2.84	-4.6	154.57	263.04	159.84	150.16
1.64	9.77	3.06	-4.32	141.57	252.53	149.67	135.03
1.09	9.27	2.51	-4.94	151.89	263.65	159.7	150.45
0.92	9.12	2.37	-5.05	152.01	261.08	160.48	153.33
0.95	9.17	2.39	-5.04	151.17	260.41	159.17	151.68
0.97	9.2	2.44	-5.02	147.19	258.56	158.74	151.82
1.11	9.31	2.49	-4.95	148.82	260.79	158.36	150.5
1.24	9.49	2.71	-4.76	162.27	270.11	166.7	160.41
1.38	9.62	2.84	-4.61	158.41	266.73	162.97	154.64

1.37	9.58	2.77	-4.67	151.28	260.22	156.93	144.16
1.41	9.63	2.8	-4.62	152.62	260.78	157.81	144.92
1.31	9.44	2.82	-4.47	152.22	261.99	161.11	150.25
1.46	9.7	2.87	-4.59	161.81	266.56	163.02	153.5
1.44	9.66	2.84	-4.61	158.66	264.38	162.87	151.56
1.4	9.62	2.8	-4.65	159.55	265.1	163.36	152.55
1.4	9.56	2.79	-4.66	161.77	265.14	164.65	153.07
2.02	10.16	3.38	-4.06	143.32	253.66	151.77	132.76
1.21	9.37	2.67	-4.67	162.73	268.63	167.18	156.84
1.29	9.46	2.74	-4.6	161.16	269.12	165.9	156.41
1.2	9.4	2.68	-4.66	162.62	270.05	167.05	157.6
1.24	9.43	2.71	-4.61	162.21	269.05	166.88	157.29
1.29	9.47	2.73	-4.7	160.51	267.18	164.82	154.06
1.44	9.66	2.84	-4.61	158.83	264.54	162.84	151.69
1.4	9.63	2.8	-4.64	158.87	264.41	163.54	152.19
1.38	9.58	2.78	-4.65	159.37	265.39	163.4	152.42
1.35	9.59	2.77	-4.65	161.91	268.2	165.83	157.72
1.47	9.72	2.85	-4.59	158.94	264.76	161.87	154.43
1.63	9.8	3	-4.53	151.71	261.66	155.28	146.27
1.6	9.77	3.02	-4.38	151.15	261.64	158.72	144.14
1.76	9.94	3.07	-4.39	153.27	259.56	155.58	144
1.51	9.75	2.87	-4.53	158.03	265.41	160.58	151.97
1.68	9.9	3	-4.41	155.01	261.77	157.08	147.26
2.15	10.29	3.45	-3.99	143.19	252.66	151.77	131.95
0.73	8.87	2.41	-4.83	168.27	272.67	178.69	173.89
0.73	8.86	2.41	-4.83	168.59	272.88	179.17	174.09
1.07	9.21	2.65	-4.62	156.15	264.43	167.49	157.95
1.31	9.5	2.84	-4.5	149.44	259	158.2	146.49
1.03	9.17	2.59	-4.68	155.74	264.97	166.54	157.81
1.16	9.35	2.72	-4.58	150.57	261.77	162.05	151.93
1.68	9.75	3.08	-4.26	136.85	242.76	149.9	138.89
1.66	9.73	3.07	-4.27	137.52	243.57	150.61	139.78
1.44	9.58	2.96	-4.31	147.84	259.24	158.29	146.05
0.86	9	2.52	-4.73	164.83	270.58	176.21	170.28
1.31	9.44	2.82	-4.47	152.26	262.03	161.16	150.23
1.16	9.3	2.75	-4.55	154	264.91	166.6	155.91
0.72	8.87	2.41	-4.83	168.38	272.75	178.83	173.98

1.67	9.74	3.08	-4.26	137.26	243.28	150.34	139.44
1.36	9.45	2.86	-4.47	150.06	256.49	163.01	156.78
1.88	10.09	3.24	-4.18	150.92	259.64	159.08	141.78
2.01	10.21	3.35	-4.07	149.68	257.58	157.62	139.6
2.73	10.89	4	-4.19	128	238.54	124.93	85.2
3.55	11.87	4.56	-3.73	121.23	213.67	116.23	81.62
2.09	10.3	3.44	-4.75	133.32	252.61	134.9	96.29
2.65	10.83	3.87	-4.28	127.74	242.04	128.91	87.46
1.85	10.08	3.2	-4.95	139.24	258.39	140.41	105.4
1.86	10.08	3.19	-4.94	139.28	257.36	139.9	105.58
1.81	10.02	3.15	-4.98	140.65	258.94	141	106.32
2.4	10.56	3.64	-4.44	130.9	250.41	131.92	92.14
2.69	10.81	3.87	-4.23	129.52	244.37	128.58	87.51
3.16	11.29	4.23	-3.8	124.34	238.06	126	81.39
3.16	11.29	4.23	-3.8	124.3	238.03	125.94	81.39
2.38	10.55	3.63	-4.42	131.84	251.15	132.7	93.42
2.38	10.56	3.63	-4.42	131.83	251.14	132.7	93.38
1.84	10.05	3.23	-4.97	138.21	257.65	137.08	100.29
1.79	10.02	3.19	-5	138.91	258.63	138.58	101.38
1.85	10.06	3.25	-4.94	139.43	257.78	137.09	101.38
2.03	10.23	3.4	-4.82	136.75	253.96	133.45	97.1
2.16	10.35	3.51	-4.72	133.78	251.53	132.69	94.76
2.09	10.28	3.47	-4.76	134.07	251.75	133.34	95.72
2.09	10.29	3.44	-4.76	133.75	252.44	133.33	95.57
2.87	11.02	4.04	-4.12	126.13	239.02	126	85.62
2.87	11.02	4.03	-4.12	126.15	239.1	126.03	85.66
3.3	11.58	4.34	-3.88	118.52	216.56	114.77	77.48
3.2	11.5	4.29	-3.89	122	216.93	117.3	79.74
3.2	11.5	4.29	-3.89	122	216.95	117.3	79.75
3.34	11.62	4.38	-3.86	118.23	215.93	114.72	77.37
3.35	11.63	4.38	-3.85	118.21	215.87	114.71	77.35
3.48	11.77	4.5	-3.73	117.73	212.23	112.97	76.25
2.5	10.76	3.73	-4.52	134.52	241.03	130.09	96.47
2.81	11.1	4	-4.31	129.13	232.85	126.44	92.19
1.82	10.04	3.2	-4.95	143.08	257.49	140.04	106.67
2.52	10.82	3.77	-4.38	131.13	239.49	128.06	92.76
3.55	11.87	4.56	-3.73	121.26	213.71	116.26	81.66

3.08	11.22	4.16	-3.88	125.05	240.04	126.18	82.82
3.36	11.6	4.37	-3.73	126.72	235.75	126.74	84.68
2.46	10.49	3.77	-4.32	127.69	238.22	119.18	76.75
2.9	10.88	4.22	-3.8	122.13	221.29	108.39	72.17
3.18	11.19	4.46	-3.63	117.91	218.37	106.09	66.26
3.21	11.18	4.47	-3.64	118.88	220.13	106.07	66.29
3.19	11.18	4.42	-3.58	114.38	217	105.29	66
2.99	10.96	4.22	-3.71	116.94	221.44	107.54	69.14
2.78	10.75	4.05	-3.92	119.9	224.16	110.23	72.21
2.38	10.35	3.77	-4.32	127.91	240.07	119.81	77.43
2.41	10.39	3.8	-4.29	127.35	238.96	119.54	76.69
2.47	10.45	3.8	-4.32	126.66	238.81	119.97	76.03
2.49	10.49	3.82	-4.3	126.35	237.95	119.83	76
2.5	10.51	3.8	-4.27	127.73	235.34	118.37	75.98
2.47	10.5	3.77	-4.31	127.71	238.2	119.08	76.58
2.37	10.39	3.71	-4.35	127.81	239.51	120.55	79.82
2.36	10.34	3.73	-4.36	129.44	239.8	120.33	78.15
2.39	10.38	3.76	-4.33	129.01	238.89	119.83	77.53
2.6	10.59	3.97	-4.15	126.21	232.61	117.67	76
2.52	10.52	3.9	-4.23	126.53	232.49	118.07	76.33
2.55	10.54	3.93	-4.2	126.57	232.5	117.98	76.09
2.63	10.53	3.95	-4.08	125.85	234.93	115.35	74.4
2.22	10.21	3.63	-4.45	128.96	243.17	121.82	79.57
2.34	10.33	3.7	-4.41	126.62	240.98	120.62	78.2
2.55	10.55	3.91	-4.23	124.89	232.83	117.55	76.43
2.58	10.58	3.94	-4.21	124.87	232.2	117.52	76.16
2.61	10.6	3.98	-4.14	126.04	232.91	117.52	76.01
2.48	10.44	3.82	-4.22	126.74	238.75	117.54	74.63
2.17	10.18	3.59	-4.47	129.74	245.04	121.29	80.2
2.38	10.36	3.74	-4.39	127.22	241.9	119.87	76.96
2.35	10.34	3.71	-4.42	127.14	242.38	120.25	77.52
2.23	10.24	3.59	-4.55	129.34	247.04	123.19	80.2
2.21	10.23	3.58	-4.56	129.33	247.23	123.24	80.37
2.21	10.22	3.58	-4.57	129.43	247.4	123.29	80.47
2.14	10.18	3.54	-4.61	129.6	248.16	123.83	81.37
2.3	10.3	3.65	-4.49	127.06	242.72	121.96	78.91
2.49	10.5	3.82	-4.29	126.42	237.56	119.85	76

2.5	10.53	3.86	-4.27	127.11	237.75	119.21	77.97
2.43	10.46	3.77	-4.3	127.67	238.91	120.13	79.05
2.34	10.29	3.69	-4.34	127.1	241.13	116.94	75.62
2.33	10.29	3.69	-4.35	127.13	241.22	117.02	75.69
2.31	10.26	3.66	-4.37	127.45	241.62	117.66	76.3
2.61	10.52	3.92	-4.13	124.62	236.5	115.4	72.54
2.6	10.52	3.91	-4.14	124.63	236.58	115.44	72.61
2.8	10.72	4.1	-3.95	122.44	231.55	113.8	72.58
2.59	10.58	3.95	-4.21	124.85	232.24	117.5	76.17
2.55	10.54	3.95	-4.26	127.4	236.74	119.18	78.94
2.53	10.5	3.94	-4.15	126.55	238.59	118.88	77.36
2.57	10.56	3.95	-4.17	126.45	232.73	117.81	76.02
2.6	10.59	3.97	-4.15	126.21	232.62	117.67	76
2.61	10.6	3.98	-4.14	126.02	232.95	117.52	76
2.4	10.43	3.65	-4.27	125.57	230.95	117.87	79.1
2.33	10.35	3.62	-4.35	127.57	234.19	119.29	79.38
2.42	10.43	3.67	-4.35	126.15	235.41	121.49	82.52
2.79	10.82	3.99	-4.05	125.46	236.9	121.74	80.88
2.8	10.89	3.92	-4.09	125.43	233.25	121.46	80.17
2.65	10.72	3.87	-4.21	126.45	235.05	121.36	81.96
2.31	10.33	3.66	-4.38	126.21	235.75	119	80.01
2.57	10.55	3.92	-4.02	123.73	229.33	115.41	75.63
2.8	10.85	4	-4.05	125.31	235.72	120.64	79.46
2.85	10.89	4.03	-4.02	123.27	233.3	119.41	78
2.13	10.11	3.56	-4.42	130.81	237.32	122.15	83.3
2.37	10.35	3.61	-4.34	126.56	233.77	118.86	79.18
2.36	10.35	3.61	-4.34	126.78	233.94	118.98	79.29
2.41	10.42	3.67	-4.36	126.55	235.79	121.7	82.7
2.61	10.67	3.81	-4.23	127.04	233.67	121.78	82.26
2.52	10.55	3.78	-4.2	126.79	238.61	120.55	80.18
2.76	10.77	3.97	-4.03	122.52	233.57	118.49	76.97
2.76	10.77	3.97	-4.03	122.6	233.46	118.48	76.96
2.76	10.76	3.95	-4.03	122.5	234.59	118.07	76.82
2.58	10.62	3.83	-4.16	124.76	234.44	119.44	77.4
2.61	10.65	3.84	-4.14	124.51	234.77	119.35	76.96
2.59	10.62	3.84	-4.16	126.14	236.08	119.57	78.5
2.58	10.62	3.83	-4.17	126.14	236.1	119.65	78.56

2.38	10.43	3.66	-4.33	127.35	239.41	122.59	81.87
2.59	10.65	3.87	-4.22	122.59	235.32	119.03	77.03
2.39	10.48	3.73	-4.41	127.97	244.31	124.88	82.95
2.41	10.49	3.74	-4.4	127.82	244.03	124.76	82.7
2.52	10.58	3.79	-4.34	126.39	239.24	122.67	80.82
2.84	10.87	4.04	-4.04	124.36	235	119.45	77.87
2.68	10.73	3.92	-4.15	127.29	238.35	122.19	80.95
2.81	10.82	4	-3.98	122.69	232.31	117.95	76.93
2.97	11.04	4.15	-3.91	122.03	232.28	118.64	77.32
3.2	11.27	4.38	-3.83	124	230.68	120.72	76.84
2.83	10.91	4.02	-4	122.58	232.59	121.36	79.7
2.71	10.78	3.93	-4.13	126.16	236.94	122.91	81.8
2.64	10.7	3.86	-4.22	126.58	235.41	121.48	82.08
2.68	10.74	3.88	-4.2	126.53	234.3	121.54	81.83
2.79	10.86	3.92	-4.15	125.91	230.57	121.08	80.95
2.51	10.58	3.8	-4.21	124.6	233.89	118.12	76.66
1.68	9.74	3.11	-4.92	135.9	244.91	127.4	91.27
1.66	9.72	3.09	-4.94	136.27	245.34	127.75	91.68
1.67	9.74	3.11	-4.93	135.99	245.04	127.49	91.38
1.97	9.97	3.43	-4.56	132.58	240.53	123.98	86.34
1.95	9.95	3.41	-4.58	132.77	240.99	124.37	86.78
2.12	10.07	3.65	-4.39	129.24	235.36	116.95	80.73
2.91	10.89	4.23	-3.8	122.13	221.24	108.33	72.14
2.77	10.77	4.11	-3.87	121.63	223.69	110.61	73.16
2.73	10.69	4.1	-3.92	123.84	225.4	110.85	73.97
2.4	10.36	3.89	-4.16	126.33	230.76	114.36	77.19
2.3	10.25	3.8	-4.25	127.41	232.95	115.63	78.57
2.71	10.65	4.06	-3.87	122.56	226.05	111.69	72.8
1.71	9.73	3.14	-4.85	135.1	241.22	125.68	88.89
1.73	9.74	3.14	-4.86	134.46	243.98	127.74	91.25
1.82	9.87	3.22	-4.8	133.56	242.79	126.93	90.31
2.3	10.35	3.56	-4.45	129.32	236.52	122.93	85.86
2.66	10.64	4.14	-3.95	128.02	226.87	111.91	78.33
2.68	10.64	4.13	-3.93	127.89	225.76	111.03	77.63
3.2	11.18	4.51	-3.53	120.14	218.85	104.93	70.31
3.31	11.35	4.54	-3.46	117.01	214.27	102.53	66.76
2.86	10.84	4.23	-3.81	125	223.58	107.38	73.31

2.54	10.5	4.02	-4.04	129.31	226.43	111.37	78.31
2.92	11.13	3.98	-4.04	123.43	225.25	118.74	79.69
2.32	10.46	3.57	-4.52	130.17	239.63	126.26	88.41
2.43	10.56	3.67	-4.43	129.55	237.37	124.29	85.7
2.52	10.64	3.74	-4.38	128.84	235.06	122.33	84.28
2.72	10.8	3.84	-4.23	126.74	231.71	121.41	82.23
2.9	11.1	3.97	-4.07	124.68	225.88	119.32	80.22
2.95	11.15	4	-4.04	123.46	224.85	118.49	79.32
2.72	10.91	3.89	-4.13	124.59	230.86	120.51	84.43
3.03	11.11	4.23	-3.91	124.11	236.96	122.52	79.48
2.94	11.03	4.15	-3.98	125.07	238.8	123.57	80.94
3.19	11.28	4.34	-3.77	121.43	232.99	120.37	78.01
2.82	10.94	4.04	-4.1	125.33	238.84	123.26	79.4
2.86	10.88	4.11	-4.08	124.94	235.45	119.08	75.59
2.87	10.89	4.12	-4.07	125	235.83	119.23	75.69
2.33	10.47	3.67	-4.41	129.12	250.91	127.1	84.03
2.04	10.12	3.39	-4.72	130.83	252.64	126.33	83.9
1.96	10.07	3.33	-4.77	131.36	254.39	127.49	85.35
1.91	10.05	3.35	-4.76	134.16	255.53	129.56	87.65
2.06	10.21	3.46	-4.64	131.11	254.81	129.65	87.84
2.31	10.33	3.66	-4.38	126.17	235.74	119	80.07
2.4	10.48	3.64	-4.37	129.53	241.17	125.87	85.88
2.73	10.74	3.94	-4.04	123.98	236.01	118.26	77.29
2.97	11.04	4.06	-3.95	122.48	229.95	118.73	76.75
2.61	10.76	3.77	-4.3	127.46	234.19	123.1	84.35
2.79	10.85	4.03	-4.12	124.73	239.03	122.14	78.5
3.11	11.23	4.33	-3.63	151.82	267.82	146.93	94.37
3.34	11.41	4.48	-3.63	155.45	268.01	148.38	92.98
3.51	11.61	4.66	-3.56	156.75	269.59	149.71	94.44
3.41	11.47	4.53	-3.62	154.94	267.13	149.16	93.2
3.35	11.44	4.51	-3.65	155.17	269.34	149.99	93.91
3.36	11.45	4.52	-3.65	155.07	268.84	149.95	93.74
3.44	11.55	4.57	-3.59	157.95	272.24	150.7	93.62
3.44	11.56	4.57	-3.59	157.92	272.16	150.69	93.57
2.66	10.8	4.15	-3.58	133.53	259.07	127.14	83.43
2.7	10.89	4.21	-3.7	140.1	266.32	134.87	91.5
2.67	10.86	4.19	-3.72	140.77	267.34	135.36	92.13

3.48	11.51	4.6	-3.54	151.83	262.81	146.79	91.29
2.77	11.09	3.78	-4.36	141.95	233.5	120.19	85.19
2.78	11.09	3.79	-4.35	141.94	233.53	120.26	85.17
3.88	12.26	4.82	-3.42	125.77	213.97	114.08	78.95
3.65	12.03	4.59	-3.66	126	215.5	115.71	79.54
2.97	11.27	4.01	-4.12	133.78	224.13	116.78	81.82
2.97	11.27	4.02	-4.12	133.79	224.09	116.77	81.79
2.59	10.82	3.73	-4.48	134.73	226.12	121.77	87.45
2.44	10.6	3.57	-4.44	132.18	226.08	118.67	84.18
2.53	10.68	3.66	-4.43	131.25	224.64	118.72	83.92
2.36	10.46	3.52	-4.55	133.21	226.1	119.5	84.74
2.64	10.85	3.75	-4.36	130.09	224.62	116.41	82.35
2.53	10.73	3.68	-4.44	133.36	227.04	119.51	85.49
2.52	10.65	3.61	-4.38	129.94	223.61	116.07	82.47
3.28	11.47	4.34	-3.87	122.86	215.23	113.24	78.42
3.48	11.62	4.45	-3.76	120.24	210.94	110.56	73.96
3.24	11.43	4.2	-3.95	122.16	214.96	112.69	77.33
3.07	11.27	4.06	-4.07	124.86	218.21	114.02	79.13
3.52	11.69	4.47	-3.73	119.78	210.63	110.8	75.23
3.05	11.24	4.04	-4.09	124.57	220.2	116.11	80.71
3.04	11.24	4.17	-4.05	125.16	221.09	117.84	81.82
3.57	11.81	4.59	-3.68	118.06	209.52	111.81	74.79
3.35	11.67	4.4	-3.87	122.09	213.74	114.42	79.01
3.37	11.66	4.37	-3.86	124.4	216.65	116.76	81.18
3.29	11.58	4.29	-3.94	125.22	219.84	117.75	82.85
2.97	11.22	4.01	-4.14	127.32	221.67	119.02	84.37
2.84	11.09	4.01	-4.12	124.88	225.9	119.33	83.29
2.89	11.16	4.03	-4.09	124.32	223.54	118.56	82.31
2.88	11.16	4.03	-4.09	124.34	223.57	118.58	82.34
2.88	11.16	4.03	-4.09	124.37	223.61	118.59	82.36
2.39	10.52	3.67	-4.46	130.81	233.52	122.81	86.38
2.7	10.97	3.78	-4.42	133.65	228.17	121.33	88.2
3.26	11.61	4.3	-3.97	129.84	220.01	117.13	83.86
3.82	12.33	4.74	-3.58	120.86	208.6	114.44	81.25
3.17	11.44	4.14	-4.09	127.24	217.09	114.4	80.72
2.95	11.23	3.98	-4.26	130.03	221.46	117.34	83.51
3.16	11.45	4.16	-3.99	129.28	218.24	115.1	80.16

3.53	11.93	4.52	-3.75	128.2	216.49	117.33	83.78
3.53	11.92	4.52	-3.75	128.22	216.51	117.34	83.79
3.52	11.92	4.52	-3.75	128.22	216.52	117.35	83.79
3.52	11.92	4.52	-3.75	128.24	216.55	117.37	83.8
3.52	11.92	4.52	-3.75	128.26	216.58	117.4	83.81
3.52	11.92	4.52	-3.75	128.28	216.6	117.42	83.82
3.66	12.05	4.62	-3.65	126.95	213.77	115.36	81.37
3.66	12.05	4.62	-3.64	126.94	213.73	115.34	81.35
3.66	12.05	4.63	-3.64	126.92	213.71	115.32	81.33
3.66	12.05	4.63	-3.64	126.9	213.67	115.3	81.31
3.68	12.07	4.64	-3.63	126.81	213.25	115.13	80.9
3.68	12.07	4.64	-3.63	126.79	213.23	115.11	80.88
3.63	11.97	4.6	-3.68	126.15	214.33	114.96	81.26
4.27	12.72	5.08	-3.21	117.52	201.93	106.34	73.29
3.97	12.45	4.87	-3.46	117.92	205.06	111.22	77.69
3.7	12.13	4.67	-3.65	122.17	211.95	116.12	82.59
4.11	12.59	4.93	-3.41	114.78	200.15	111.2	76.5
4.05	12.57	4.89	-3.44	115.81	201.23	111.87	77.83
2.72	10.94	3.77	-4.34	128.81	226.03	118.79	84.45
2.33	10.44	3.52	-4.6	130.34	227.18	118.38	82.65
2.04	10.17	3.27	-4.82	135.44	237.34	125.54	91.43
2.42	10.57	3.55	-4.58	130.15	227.93	119.74	84.38
2.41	10.56	3.57	-4.57	130.96	227.95	119.62	85.04
2.82	11.11	3.83	-4.28	138.72	229.66	118.31	83.3
2.67	10.91	3.65	-4.43	143.16	232.78	120.76	86.88
2.75	11.02	3.83	-4.35	141.57	233.38	121.44	86.89
2.78	11.08	3.85	-4.31	140.62	229.5	118.93	84.99
3.2	11.58	4.11	-4.07	134.64	227.74	118.28	81.73
3.3	11.69	4.22	-3.97	133.76	222.89	115.56	78.15
3.32	11.76	4.29	-4.03	131.57	223.67	118.11	81.47
3.08	11.46	4.06	-4.16	134.32	225.69	116.31	79.87
4.16	12.76	5.01	-3.36	124.67	209.61	111.72	76.53
3.99	12.51	4.83	-3.47	123.14	209.19	111.16	76.12
3.65	12.09	4.56	-3.7	127.19	217.95	114.76	79.76
3.91	12.42	4.85	-3.48	118	206.3	113.42	79.38
3.3	11.68	4.19	-4.03	133.76	224.95	116.97	80.76
3.3	11.68	4.19	-4.03	133.79	224.99	116.99	80.81

3.3	11.69	4.2	-4.03	133.84	224.98	116.74	80.89
3.93	12.09	4.81	-3.46	132.48	238.89	131.17	85
3.92	12.08	4.82	-3.44	132.61	238.87	132.58	85.25
3.92	12.09	4.81	-3.44	132.88	241.16	133.09	86.67
3.85	12.04	4.8	-3.5	130.49	239.32	131.26	84.25
3.85	12.04	4.8	-3.5	130.6	239.39	131.36	84.48
3.85	12.04	4.8	-3.5	130.55	239.59	131.32	84.4
4.03	12.23	4.99	-3.33	128.56	235.78	129.73	83.46
3.73	11.89	4.67	-3.54	135.79	245.98	135.13	86.65
3.74	11.9	4.67	-3.54	135.62	245.96	135.02	86.41
3.73	11.89	4.67	-3.54	135.97	245.98	135.07	86.52
3.8	11.97	4.73	-3.53	131.46	240.49	131.99	85
3.81	11.97	4.73	-3.53	131.16	239.36	131.92	84.55
3.95	12.14	4.9	-3.4	129.68	238.11	130.41	84.92
3.64	11.87	4.57	-3.56	128.13	236.55	126.61	80.68
3.66	11.89	4.58	-3.55	127.89	236.41	126.36	80.55
3.64	11.86	4.56	-3.55	127.89	236.28	126.87	80.57
3.58	11.83	4.54	-3.6	127.05	237.8	128.11	83.88
3.89	12.08	4.81	-3.43	126.91	235.73	128.67	82.53
3.95	12.16	4.87	-3.43	126.67	236.19	129.61	82.03
3.95	12.15	4.87	-3.43	126.65	236.19	129.58	82.04
4.02	12.19	4.95	-3.38	128.94	235.45	130.02	82.99
4.02	12.19	4.95	-3.38	128.94	235.47	130.03	82.99
3.98	12.17	4.87	-3.38	129.29	235.09	130.02	84.28
3.07	11.29	4.18	-4.06	157.82	274.82	152.29	97.96
3.02	11.26	4.14	-4.09	158.74	275.82	152.92	98.96
3.35	11.52	4.44	-3.98	157.25	270.14	150.47	95.8
3.35	11.52	4.44	-3.98	157.3	270.18	150.48	95.92
3.34	11.52	4.43	-3.98	157.13	269.72	150.74	96.47
3.38	11.53	4.42	-3.87	152.49	265.38	147.1	93.37
3.44	11.54	4.52	-3.6	121.91	231.78	121.84	76
3.77	12	4.69	-3.47	127.08	237.19	127.07	80.18
3.75	11.9	4.67	-3.53	127.86	237.52	129.22	81.42
3.75	11.9	4.67	-3.53	127.9	237.48	129.19	81.4
3.77	11.92	4.68	-3.5	128.78	237.8	128.74	81.51
3.06	11.15	4.21	-3.95	125.23	238.67	120.09	75.14
3.06	11.15	4.21	-3.95	125.25	238.69	120.11	75.16

3.05	11.15	4.21	-3.95	125.32	238.75	120.2	75.2
3.44	11.54	4.49	-3.63	123.81	231.59	121.91	76
3.33	11.44	4.43	-3.64	120.8	230.4	118.71	73.11
3.32	11.45	4.43	-3.64	121.85	230.11	118.78	73.52
3.32	11.46	4.42	-3.65	122.45	230.16	118.96	73.96
3.6	11.73	4.56	-3.57	125.09	234.44	125.01	78
3.7	11.88	4.66	-3.57	124.72	234.37	126	78.02
3.7	11.88	4.66	-3.57	124.73	234.37	126	78.02
3.15	11.24	4.24	-3.81	123.92	234.01	119.02	73.77
3.15	11.25	4.24	-3.8	123.88	233.91	118.87	73.18
3.33	11.46	4.41	-3.66	122.36	230.34	118.96	73.98
3.32	11.43	4.4	-3.7	122.99	232.03	119.24	74.22
3.33	11.44	4.4	-3.69	122.56	231.1	119.03	74.05
3.33	11.44	4.4	-3.69	122.53	231.16	119.05	74.09
3.29	11.39	4.36	-3.76	121.96	231.35	118.97	73.19
3.07	11.16	4.2	-3.9	125.01	238.04	120.15	74.1
3.07	11.17	4.22	-3.9	125.11	237.87	119.93	74.16
3.07	11.18	4.23	-3.92	125.28	237.75	119.67	74.53
3.81	11.98	4.73	-3.53	131.87	240.41	132.11	85
3.41	11.52	4.39	-3.78	129.91	240.8	127.54	80.06
4.43	12.66	5.14	-3.19	138.26	248.64	141.08	96
4.44	12.67	5.16	-3.17	138.72	249.41	141.47	96
4.83	13.14	5.51	-2.94	138.71	249.75	139.92	96
4.35	12.54	5.12	-3.26	136.47	244.74	139.13	93.85
4.84	13.14	5.5	-2.92	142.61	254.25	141.66	97.06
4.86	13.54	5.5	-2.94	120.76	197.35	108.13	75.04
5.07	13.77	5.67	-2.83	121.9	199.21	111.65	78.55
4.86	13.54	5.49	-2.93	120.33	197.68	107.47	74
4.73	13.25	5.37	-3.03	114.8	198.01	108.29	74.02
4.73	13.25	5.37	-3.03	114.82	198.01	108.31	74.02
4.73	13.24	5.36	-3.03	114.37	198.01	108.09	74
4.8	13.35	5.45	-2.97	114.44	197.63	109.74	74
4.77	13.33	5.42	-2.97	114.62	197.35	109.14	74
5.03	13.61	5.65	-2.84	116.37	197.26	110.72	76.41
5.15	13.7	5.75	-2.83	116.73	198.3	111.44	77
5.15	13.7	5.75	-2.83	116.69	198.3	111.4	77
5.09	13.65	5.71	-2.84	117.21	197.46	111.17	76.99

5.25	13.79	5.84	-2.8	117.87	198.73	112.17	77.34
5.43	13.99	6.05	-2.68	120.67	200.27	115.22	80.07
5.34	13.9	5.97	-2.73	118.99	200.54	114.58	79.98
5.31	13.88	5.95	-2.74	118.83	200.62	114.57	79.95
5.24	13.88	5.88	-2.72	120	199.3	113.61	78.46
5.21	13.84	5.86	-2.73	119.68	199.39	113.18	78.65
4.88	13.51	5.6	-3.04	128.32	216.53	126.66	91.73
5.17	13.73	5.67	-2.81	128.98	221.34	126.2	88.31
5.16	13.65	5.64	-2.8	130.97	227.18	127.96	90.73
5.27	13.81	5.76	-2.67	139.53	241.21	134.83	95.36
5.17	13.71	5.67	-2.74	135.28	234.77	131.98	93
5.17	13.72	5.67	-2.72	135.39	235.44	132	93
5.21	13.61	5.6	-2.72	148.13	260.26	144.82	104.49
4.55	13.13	5.29	-3.22	120.75	210.17	118.7	83.22
4.55	13.13	5.29	-3.23	120.77	210.2	118.73	83.25
4.55	13.12	5.29	-3.23	120.79	210.23	118.75	83.28
4.49	13.07	5.25	-3.27	121.3	211.12	119.4	84.02
4.49	13.07	5.25	-3.27	121.33	211.17	119.44	84.05
4.48	13.07	5.25	-3.27	121.35	211.21	119.46	84.08
4.48	13.07	5.25	-3.28	121.37	211.24	119.49	84.11
4.48	13.06	5.24	-3.28	121.4	211.3	119.54	84.15
4.47	13.06	5.24	-3.28	121.43	211.36	119.58	84.19
4.45	13.04	5.22	-3.3	121.56	211.67	119.83	84.39
4.45	13.04	5.22	-3.3	121.57	211.73	119.87	84.42
4.44	13.03	5.22	-3.3	121.58	211.76	119.9	84.43
4.44	13.03	5.22	-3.3	121.6	211.8	119.94	84.46
4.44	13.03	5.22	-3.3	121.61	211.84	119.97	84.48
4.44	13.03	5.21	-3.3	121.62	211.88	120	84.5
4.43	13.03	5.21	-3.31	121.63	211.92	120.04	84.52
4.43	13.02	5.21	-3.31	121.64	211.98	120.09	84.55
4.43	13.02	5.21	-3.31	121.65	212.04	120.14	84.58
4.42	13.02	5.21	-3.31	121.66	212.09	120.19	84.61
4.42	13.01	5.2	-3.31	121.67	212.14	120.23	84.63
4.42	13.01	5.2	-3.31	121.68	212.19	120.27	84.66
4.41	13	5.19	-3.32	121.66	212.35	120.42	84.73
4.41	13	5.19	-3.32	121.6	212.14	120.24	84.59
4.42	13.01	5.19	-3.32	121.56	212	120.11	84.5

4.42	13.01	5.19	-3.32	121.53	211.92	120.05	84.46
4.41	13	5.19	-3.32	121.54	212.05	120.16	84.52
4.41	13	5.19	-3.32	121.52	211.99	120.11	84.48
4.41	13	5.19	-3.32	121.51	211.96	120.09	84.46
4.38	12.97	5.18	-3.33	121.82	213.32	121.29	85.45
4.38	12.97	5.18	-3.33	121.86	213.29	121.26	85.45
4.56	13.13	5.33	-3.23	122.39	214.05	121.99	85.66
4.56	13.13	5.33	-3.22	122.37	214.01	121.95	85.61
4.89	13.52	5.61	-3.03	128.14	216.34	126.54	91.6
4.79	13.35	5.44	-2.97	114.25	197.82	109.91	74
4.8	13.35	5.45	-2.97	114.44	197.61	109.72	74
4.8	13.35	5.45	-2.97	114.46	197.59	109.71	74
4.8	13.35	5.45	-2.97	114.49	197.56	109.7	74
4.8	13.35	5.45	-2.97	114.25	197.82	109.88	74
4.75	13.32	5.4	-2.97	114.48	198.21	109.81	74
4.76	13.33	5.4	-2.97	114.45	198.12	109.89	74
4.71	13.21	5.31	-3.03	112.64	198.17	108.04	73.97
4.71	13.21	5.31	-3.03	112.62	198.18	108.04	73.97
4.73	13.23	5.32	-3.03	112.51	198.17	107.73	74
4.73	13.23	5.32	-3.03	112.51	198.17	107.72	74
4.73	13.23	5.32	-3.03	112.65	198.17	107.54	74
5.31	13.85	5.9	-2.76	117.97	199.32	112.83	77.77
4.73	13.26	5.37	-3.01	114.07	198.11	108.06	74
4.73	13.26	5.37	-3.01	114.05	198.12	108.06	74
4.73	13.29	5.39	-3.04	114.95	197.4	111.05	74.33
4.73	13.29	5.39	-3.04	114.92	197.36	111.04	74.29
4.74	13.3	5.39	-3.04	114.95	197.38	111.07	74.33
4.53	13.02	5.23	-3.14	110.23	194.74	108.31	74
4.53	13.02	5.23	-3.14	110.24	194.73	108.32	74
4.23	12.74	5.02	-3.36	111.56	196.97	110.81	75.13
4.5	13.01	5.23	-3.13	112.39	194.26	107.07	72.02
4.23	12.74	5	-3.37	110.73	194.5	109.6	74.55
4.23	12.74	5	-3.37	110.74	194.5	109.6	74.56
4.23	12.73	5	-3.37	110.75	194.5	109.61	74.57
4.23	12.73	5	-3.37	110.78	194.48	109.62	74.59
4.23	12.73	5	-3.37	110.78	194.47	109.62	74.6
4.23	12.73	5	-3.37	110.79	194.47	109.62	74.61

4.23	12.73	5	-3.37	110.8	194.46	109.62	74.62
4.79	13.1	5.42	-2.9	145.23	257.67	141.87	98.05
4.58	13.09	5.26	-3.09	113.89	198.01	107.82	73.33
3.15	11.49	4.21	-4	135.38	246.64	137.23	96.8
3.94	12.07	4.76	-3.6	140.78	250.33	141.88	98.28
3.55	11.77	4.46	-3.85	145.51	259.8	148.34	107
3.88	11.93	4.83	-3.57	141.6	252.63	141.69	97.37
3.79	11.86	4.75	-3.61	140.69	252.96	142.72	98.25
3.74	11.98	4.69	-3.7	142.83	252.32	141.44	101.19
3.67	11.91	4.64	-3.75	143.61	253.94	142.66	102.63
3.77	11.81	4.7	-3.66	142.09	253.68	143.4	100.07
3.8	11.9	4.63	-3.67	139.68	247.73	140.81	96.26
3.51	11.74	4.54	-3.73	137.99	250.04	140.41	96.55
3.48	11.61	4.54	-3.72	141.99	256.04	142.53	94.22
3.34	11.44	4.45	-3.86	149.45	260.28	145.8	97.08
3.04	11.23	4.18	-4.06	152.01	272.02	151.18	100.92
3.34	11.51	4.36	-3.83	143.27	255.65	141.92	92.13
3.41	11.51	4.49	-3.79	145.33	257.87	143.4	96.35
3.03	11.21	4.19	-4.07	155.9	274.32	151.45	102.29
3.2	11.34	4.31	-3.96	152.63	267.89	149.03	96.61
2.98	11.16	4.11	-4.15	159.13	275.45	155.07	102.32
2.92	11.16	4.05	-4.22	166.01	283.42	159.81	105.91
2.52	10.69	3.74	-4.75	170.38	283.66	168.95	126.03
2.44	10.61	3.6	-4.93	173.18	279.35	171.87	126.86
1.85	10.11	3.12	-5.4	184.33	299.85	181.93	139.06
1.84	10.1	3.1	-5.42	184.18	298.89	181.57	139.28
3.58	11.59	4.57	-3.91	150.09	258.01	147.65	101.18
3.51	11.58	4.48	-3.95	149.11	253.47	150.92	107.5
3.47	11.54	4.44	-3.98	150.11	254.52	151.67	108.39
3.08	11.22	4.28	-4.23	157.69	256.2	159.21	118.91
3.76	11.9	4.65	-3.69	143.72	247.41	145.41	101.47
2.36	10.54	3.61	-4.79	170.05	289.32	170.39	130.87
2.87	11.03	4.11	-4.39	161	262.26	162.68	123.87
2.87	11.04	4.11	-4.39	160.98	262.21	162.64	123.82
3.66	11.76	4.59	-3.75	144.69	249.44	146.22	103.34
3.66	11.76	4.6	-3.75	144.67	249.38	146.17	103.29
3.69	11.83	4.59	-3.8	146.29	252.93	148.85	104.96

3.69	11.84	4.59	-3.8	146.27	252.88	148.82	104.93
3.73	11.89	4.62	-3.75	141.55	249.17	144.93	101
3.71	11.87	4.6	-3.77	141.86	249.87	145.2	101.39
3.71	11.87	4.6	-3.77	141.85	249.86	145.19	101.37
3.71	11.87	4.6	-3.77	141.84	249.85	145.18	101.36
2.84	10.94	3.97	-4.46	162.92	266.34	161.96	120.25
2.95	11.05	4.12	-4.43	164.74	274.76	162.9	116.49
3.78	11.85	4.69	-3.67	141.72	249.36	143.94	98.46
3.28	11.34	4.36	-4.09	153.73	265.59	153.31	107.73
3.5	11.73	4.45	-3.92	147.57	251.33	150.66	108.37
2.64	10.83	3.82	-4.61	167.28	273.57	172.67	134.75
3.16	11.37	4.25	-3.96	153.09	273.75	149.68	96.16
3.11	11.37	4.28	-4.06	175.71	294.46	164.19	106.89
3.12	11.29	4.25	-4	159.27	275.69	153.39	97
3.1	11.33	4.35	-4.01	177.81	296.61	164.74	105.53
2.19	10.49	3.6	-4.83	192.35	322.35	184.73	134.25
2.43	10.71	3.83	-4.59	180.67	308.49	174.7	122.76
3.33	11.5	4.45	-3.79	158.02	274.36	150.01	91.15
3.33	11.49	4.45	-3.79	158.05	274.42	150.01	91.24
3.34	11.52	4.42	-3.71	160.38	277.33	151.85	90.23
3.34	11.52	4.42	-3.71	160.36	277.42	151.84	90.21
3.31	11.51	4.4	-3.73	160.5	279.28	152.42	90.95
2.84	11.06	4.12	-4.2	172.67	292.53	164.48	109.49
3.32	11.48	4.42	-3.74	158.38	274.9	150.05	90.66
3.33	11.49	4.43	-3.72	158.28	274.7	150.02	90.35
3.31	11.53	4.44	-3.82	167.37	283.76	156.36	94.18
3.27	11.54	4.46	-3.83	169.63	285.7	157.04	95.35
3.27	11.54	4.45	-3.83	169.69	285.76	157.04	95.38
3.12	11.35	4.34	-3.96	170.91	289.16	161.71	100.72
3.11	11.34	4.33	-3.96	171	289.29	161.84	100.91
3.19	11.42	4.4	-3.88	168.75	286.94	159.93	98.72
3.66	11.87	4.73	-3.47	160.03	273.35	148.38	90.13
3.4	11.66	4.49	-3.8	166.26	281	156.98	100.28
3.51	11.8	4.54	-3.89	167.27	279.63	159.15	103
3.34	11.63	4.4	-4.13	176.02	289.89	167.15	110.42
3.19	11.47	4.28	-4.26	178.67	293.81	169.61	113.28
3.19	11.45	4.26	-4.3	176.42	291.97	167.77	112.2

3.4	11.72	4.46	-3.95	169.21	283.06	160.15	104.58
3.43	11.71	4.48	-4	168.72	283.32	160.66	105.15
3.37	11.7	4.44	-3.96	168.26	281.95	159.94	104.91
3.44	11.78	4.49	-4	168.61	283.21	160.71	105.14
3.55	11.77	4.59	-3.71	163.23	274.98	154.52	98.74
3.29	11.55	4.43	-3.86	168.16	284.73	156.56	97.68
3.31	11.58	4.46	-3.84	167.54	284.34	155.92	97.32
2.69	11	3.84	-4.74	185.13	305.49	176.73	123.46
3.28	11.54	4.42	-3.86	168.7	284.84	156.89	97.77
3.28	11.54	4.42	-3.86	168.71	284.85	156.91	97.78
3.37	11.64	4.46	-3.85	169.61	283.62	158.08	99.53
3.29	11.57	4.42	-3.89	169.89	284.87	158.08	99.3
3.29	11.57	4.42	-3.89	169.93	284.96	158.1	99.36
3.13	11.39	4.29	-4.08	173.88	290.39	163.87	106.08
3.18	11.49	4.31	-4.01	173.62	290.06	162.47	104.93
3.08	11.32	4.19	-4.22	171.84	288.98	163.4	106.67
2.99	11.24	4.08	-4.38	175.05	293.34	166.49	109.89
2.99	11.24	4.08	-4.38	175.05	293.35	166.5	109.91
3.63	11.83	4.61	-3.77	163.24	269.63	153.12	97.2
3.63	11.83	4.61	-3.77	163.97	270.87	153.59	97.71
3.67	11.91	4.6	-3.8	165.42	273.94	155.96	100
3.66	11.91	4.6	-3.8	165.3	273.98	155.97	100
3.63	11.96	4.61	-3.86	169.1	281.13	158.24	102.98
3.57	11.81	4.65	-3.56	161.82	275.56	149.7	90.54
3.48	11.68	4.56	-3.7	164.07	276.47	152.21	90.7
2.83	11.02	4.13	-4.55	195.53	316.91	185.98	135.31
2.79	10.97	4.09	-4.57	196.34	318.52	186.75	136.88
2.79	10.97	4.09	-4.58	196.4	318.64	186.81	136.98
2.43	10.58	3.74	-4.91	203.17	329.54	195.08	147.62
2.83	11.04	4.1	-4.54	197.52	318.67	186.31	135.04
2.84	11.1	4.05	-4.53	186.03	306.51	177.28	123.67
3.09	11.36	4.28	-4.04	176.03	294.08	163.39	105.54
3.02	11.31	4.21	-4.18	176.82	296.52	166.53	109.45
3.08	11.34	4.17	-4.35	176.75	291.31	166.55	109.12
2.92	11.2	4.11	-4.39	179.97	297.19	170.17	113.27
2.85	11.13	4.03	-4.51	181	299.46	171.39	115.76
2.85	11.13	4.03	-4.51	181.04	299.59	171.51	115.82

2.85	11.13	4.03	-4.5	180.96	299.38	171.29	115.73
2.97	11.22	4.08	-4.48	180.17	295.35	169.2	113.51
2.69	11	3.95	-4.59	186.69	308.6	177.13	123.03
3.2	11.45	4.36	-3.95	171.33	287.46	158.94	98.83
2.93	11.26	4.1	-4.3	177.65	296.29	167.91	111.42
2.69	10.97	3.95	-4.53	184.61	304.31	173.64	119.19
2.91	11.18	4.14	-4.33	180.65	297.8	168.83	113.02
2.74	10.99	3.97	-4.57	185.05	305.97	175.01	120.11
2.74	10.99	3.97	-4.57	185.07	306	175.01	120.13
2.67	10.9	3.93	-4.61	187.75	310.75	179.02	125.72
2.71	10.96	3.96	-4.56	184.93	305.43	174.94	119.91
3.33	11.5	4.46	-3.76	157.82	274.04	150.01	90.59
3.18	11.36	4.27	-3.97	159.8	273.59	151.09	95.9
3.14	11.4	4.33	-3.82	166.37	286.28	156.41	96.55
2.55	10.72	3.82	-3.71	144.43	250.5	153.61	129.06
2.63	10.82	3.86	-3.65	145.25	250.18	152.27	128.27
2.74	10.87	3.98	-3.52	147.86	251.12	154.06	131.45
3.22	11.3	4.43	-3.33	153.43	252.75	153.33	132.5
3.41	11.55	4.66	-3.11	158.01	254.04	151.62	133.56
3.46	11.62	4.7	-3.07	154.8	251.71	150.15	130.77
3.46	11.61	4.7	-3.08	155.36	251.72	149.96	130.83
3.46	11.62	4.7	-3.07	155.14	251.86	150.14	130.91
3.41	11.55	4.66	-3.12	158.08	254.13	151.7	133.67
3.44	11.58	4.68	-3.09	156.5	252.13	150.55	131.79
3.46	11.61	4.7	-3.08	155.51	251.86	150.03	130.97
3.46	11.62	4.7	-3.07	155.12	251.88	150.18	130.93
3.46	11.62	4.7	-3.07	155.02	251.85	150.2	130.91
3.08	11.12	4.28	-3.4	149.82	250.92	153.6	130.42
3.34	11.5	4.6	-3.18	156.77	252.67	152.47	133.76
3.46	11.62	4.7	-3.07	154.94	251.8	150.18	130.85
2.76	11.1	3.96	-3.64	157.64	252.09	149.58	134.26
1.65	9.84	3.19	-4.41	165.21	268.81	169.11	156.87
1.77	9.94	3.31	-4.36	162.5	267.55	166.14	153.82
1.45	9.72	3.01	-4.49	180.2	277.27	179.2	168.86
2.08	10.24	3.49	-4.11	160.26	262.52	162.91	147.99
1.18	9.44	2.7	-4.77	168.88	270.36	168.14	160.14
0.65	8.97	2.33	-5.07	183.13	284.15	180.85	177.63

2.1	10.37	3.47	-4.1	163.88	265.46	159.82	149.35
2.54	10.8	3.8	-3.75	159.15	256.76	154.7	140.6
2.55	10.81	3.81	-3.75	159.23	256.7	154.52	140.39
1.57	9.87	2.98	-4.48	166.65	267.47	163.05	154.77
1.37	9.68	2.83	-4.6	172.25	272.09	167.08	160.09
2.41	10.7	3.69	-3.9	161.57	257.63	154.02	141.42
2.2	10.39	3.48	-5.15	204.99	331.42	197.46	150.94
2.39	10.6	3.7	-4.94	204.66	331.82	196.46	148.26
2.59	10.81	3.89	-4.73	202.28	326.72	191.46	142.57
2.01	10.24	3.29	-5.47	211.35	336.49	204.96	157.65
2.37	10.59	3.69	-4.95	204.91	332.25	196.77	148.66
2.48	10.69	3.78	-4.87	202.7	328.36	194.05	145.18
2.44	10.66	3.75	-4.9	203.53	329.84	195.06	146.48
0.44	8.7	1.99	-6.56	236.29	388.45	231.39	204.85
2.95	11.18	4.18	-4.44	191.63	312.34	180.13	127.11
1.05	9.38	2.67	-5.73	218.01	368.18	211.93	178.71
1.92	10.1	3.27	-5.32	204	328.84	193.32	147.13
1.42	9.67	2.93	-5.51	218.38	358.82	210.01	171.11
2.26	10.55	3.57	-4.84	186.12	309.76	176.57	126.03
2.45	10.68	3.73	-4.7	186.49	308.25	175.5	124.74
1.13	9.39	2.64	-5.87	214.38	349.72	205.68	167.77
2.6	10.84	3.81	-4.59	177.24	297.79	169.17	115.82
3	11.13	4.22	-4.18	156.89	274.5	155.03	109.81
2.28	10.53	3.62	-4.66	170.26	292.91	166.81	120.75
3.65	11.71	4.68	-3.76	146.53	256.69	147.68	99.58
2.78	10.88	3.96	-4.53	170.61	283.89	164.16	116.58
3.7	11.77	4.68	-3.73	145.27	253.81	145.46	98.82
3.7	11.77	4.68	-3.73	145.27	253.8	145.46	98.81
3.76	11.8	4.78	-3.65	147.18	255.13	146.37	97.14
3.77	11.8	4.78	-3.65	147.11	255	146.37	97.04
3.89	11.93	4.8	-3.53	140.96	245.68	140.9	92.8
3.78	11.83	4.69	-3.64	141.34	250.33	143.33	96.04
2.68	10.8	3.88	-4.48	166.46	285.89	162.82	119.95
3.29	11.35	4.34	-4.07	157.38	266.85	153.35	105.31
3.29	11.35	4.34	-4.07	157.38	266.88	153.35	105.33
3.27	11.36	4.41	-4	156.7	270.44	153.74	107.85
3.49	11.55	4.5	-3.94	151.28	257.8	147.8	101.03

3.32	11.4	4.34	-4.09	151.65	260.87	150.65	103.8
3.31	11.39	4.33	-4.09	151.69	261.04	150.77	103.99
3.31	11.4	4.33	-4.09	151.66	260.92	150.68	103.87
3.3	11.39	4.35	-4.11	156.22	263.93	151.68	103.78
3.33	11.41	4.37	-4.09	156.07	263.55	151.44	103.66
3.24	11.29	4.38	-4.19	162.76	267.3	157.97	110.47
1.84	10.06	3.18	-5.35	197.42	322.14	186.93	141.8
1.62	9.87	2.99	-5.48	198.85	329.91	189.37	148.9
2.46	10.65	3.74	-4.76	185.12	307.61	175.63	124.56
2.37	10.63	3.66	-4.82	181.33	302.64	172.68	122.17
2.26	10.44	3.56	-5.02	187.59	303.29	179.64	133.17
1.92	10.12	3.28	-5.31	194.63	314.5	186.92	142.81
2.31	10.52	3.64	-4.77	174.8	296.8	170.54	125.07
2.37	10.56	3.69	-4.77	174.22	295.07	170.6	123.14
1.47	9.8	2.91	-5.6	195.14	321.7	190.91	152.13
2.71	10.85	3.93	-4.44	169.13	287.5	164.54	113.88
3.97	12.45	5.13	-2.65	151.04	244	151.47	123.69
3.46	11.84	4.72	-3.15	155.24	249.01	157.76	131.56
3.11	11.52	4.41	-3.46	154.04	249.87	157.84	131.31
3.76	11.96	5.08	-2.75	149.09	253.98	156.8	126.47
2.42	10.74	3.93	-4.01	149.56	242.52	149.95	121.11
2.48	10.79	3.98	-3.98	149.02	242.35	149.44	120.56
4.14	12.42	5.38	-2.41	152.47	254.97	161.93	132.03
3.96	12.31	5.15	-2.71	149.44	246.08	153.14	123.55
3.47	11.77	4.82	-3.06	150.71	253	160.09	130.8
3.81	12.12	5.09	-2.74	148.68	248.48	157.34	127.17
2.43	10.74	3.94	-3.99	149.6	243.03	149.96	121.29
3.44	11.64	4.83	-2.92	156.49	262.93	165.26	139.64
3.45	11.68	4.82	-2.96	153.4	260.96	161.86	134.67
3.98	12.23	5.25	-2.5	154.67	258.91	168.11	138.03
3.91	12.14	5.18	-2.62	153.77	255.52	162.71	133.51
3.4	11.63	4.75	-2.97	152.25	259.34	162.39	134.78
3.2	11.47	4.58	-3.16	152.2	258	161.49	134.14
3.12	11.38	4.51	-3.32	149.27	251.57	155.42	126.51
2.76	11.05	4.22	-3.64	149.61	246.75	151.21	123.34
2.41	10.73	3.93	-4.01	149.61	242.55	149.95	121.16
2.48	10.79	3.99	-3.97	149.04	242.45	149.47	120.55

3.24	11.55	4.61	-3.2	150.6	254.07	160.69	132.79
3.38	11.67	4.77	-3.17	149.06	249.75	156.11	126.4
2.89	11.29	4.21	-3.85	151.61	234.64	139.12	116.89
2.88	11.3	4.21	-3.86	152.28	235.17	139.4	117.16
3.79	12.23	4.96	-2.84	149.25	242.45	148.12	120.37
4.5	12.81	5.58	-2.23	138.19	231.81	136.44	105.48
2.98	11.25	4.37	-3.58	147.1	243.58	146.21	116.4
2.8	11.04	4.21	-3.68	147.72	245.15	146.51	118.01
3.08	11.33	4.45	-3.4	147.44	247.66	148.02	118.79
3.16	11.4	4.51	-3.35	147.02	247.64	147.48	118
3.96	12.18	5.16	-2.68	142.22	244.36	141.08	109.79
2.94	11.37	4.22	-3.81	151.24	236.09	139.06	118.48
2.81	11.23	4.14	-3.92	151.99	234.36	139.29	116.87
3.33	11.76	4.58	-3.27	154.13	248.41	157.07	129.89
3.47	11.68	4.73	-3.09	143.38	244.29	142.95	112.19
3.48	11.74	4.8	-3.12	145.19	243.41	143.94	113.56
3.71	11.97	4.98	-2.93	144.17	241.59	141.89	111.16
3.02	11.32	4.4	-3.57	145.67	240.42	143.32	114.14
2.51	10.82	3.97	-4	147.76	241.58	145.06	117.08
3.32	11.69	4.54	-3.35	141.87	235.3	138.59	109.35
2.79	11.26	4.11	-3.9	155.81	238.41	141.95	122.94
4.17	12.59	5.27	-2.46	148.24	236.53	143.44	117.51
2.83	11.29	4.14	-3.87	156.04	238.83	142.28	123.16
2.82	11.28	4.13	-3.88	155.91	238.72	142.11	123.08
3.59	11.97	4.79	-3.08	142.47	235.72	139.24	111.03
3.78	12.01	5.02	-2.85	144.02	244.83	143.66	111.9
3.51	11.76	4.83	-3.09	145.13	243.68	144.06	113.61
4.02	12.3	5.22	-2.69	142.01	239	140.07	108.12
2.8	11.22	4.13	-3.94	151.94	233.95	139.02	116.79
2.86	11.27	4.18	-3.87	151.72	234.47	139.11	116.87
3.85	12.09	5.07	-2.84	145.37	242.84	146.11	117.46
2.99	11.33	4.3	-3.66	145.55	237.42	143.27	115.44
3	11.38	4.3	-3.63	144.04	235.19	139.79	112.4
3.58	11.79	4.91	-2.92	148.61	254.82	154.07	124.15
3.17	11.4	4.53	-3.28	148.87	250.81	149	119.09
3.62	11.83	4.88	-2.84	148.58	254.11	147.47	117.52
3.41	11.64	4.75	-3.05	151.72	258.19	154.56	126.02

3.56	11.76	4.84	-3	145.89	248.1	146.61	115.87
3.59	11.85	4.9	-2.88	155.13	257.51	164.77	136.01
2.77	11.07	4.19	-3.8	146.08	241.61	144.4	115.27
4.07	12.37	5.27	-2.58	144.88	238.37	143.96	113.8
2.09	10.44	3.3	-4.42	167.43	265.7	174.02	176.3
2.24	10.57	3.38	-4.29	167.94	266.64	175.07	177.69
2.35	10.64	3.54	-4.12	158.26	260.37	166.18	162.42
1.94	10.32	3.08	-4.51	172.51	273.75	185.99	190.56
1.56	9.98	2.85	-4.64	180.3	284.93	194.01	201.95
2.66	10.92	3.74	-3.92	150.9	250.81	156.77	147.6
2.73	10.96	3.81	-3.89	146.8	243.91	147.82	135.67
2.75	11.06	3.86	-3.84	148.12	245.66	148.18	137.36
2.77	11.08	3.86	-3.82	148.21	246.33	148.2	137.66
2.63	10.96	3.76	-3.84	145.61	246.74	150.15	139.01
2.7	10.98	3.85	-3.8	141.1	245.75	146.33	132.68
2.64	10.9	3.78	-3.86	140.69	243.61	145.67	133.15
2.68	10.95	3.78	-3.8	143.03	245.6	149.55	137.58
2.64	10.94	3.77	-3.87	146.39	247.47	152.35	142.17
2.19	10.5	3.39	-4.21	159.52	264.85	170.01	166.67
2.08	10.42	3.25	-4.38	163.96	267.6	176.52	175.44
2.37	10.71	3.51	-4.26	162.26	259.75	165.99	163.15
2.12	10.48	3.34	-4.28	160.18	266.45	171.45	169.47
2.24	10.56	3.36	-4.33	165.46	263.56	171.11	171.22
2.05	10.41	3.25	-4.32	159.34	266.3	173.83	170.03
2.02	10.4	3.23	-4.36	162.85	268.78	175.49	174.21
1.95	10.32	3.16	-4.37	164.12	272.4	179.21	179.07
1.75	10.14	3.05	-4.5	174.92	279.29	187.1	190.91
1.72	10.12	3.03	-4.52	175.49	280.01	187.79	192.16
1.92	10.27	3.13	-4.49	169.23	273.44	181.81	183.99
1.89	10.25	3.11	-4.51	171.07	275.25	183.51	186.17
2.02	10.4	3.19	-4.45	170.99	272.34	182.33	186.07
2.15	10.5	3.24	-4.36	169.05	268.69	178.53	180.43
2.36	10.65	3.45	-4.24	166.34	265.29	174.01	174.33
2.4	10.69	3.47	-4.17	165.47	262.2	171.68	171.59
1.84	10.22	3.08	-4.54	173.2	277.14	185.64	190.7
2.46	10.78	3.56	-4.2	159.32	258.73	164.32	161.79
2.14	10.45	3.3	-4.43	169.64	265.05	177.99	180.6

2.49	10.8	3.57	-4.12	161.17	257.94	167.74	165.47
2.3	10.63	3.42	-4.25	165.15	264.06	172.33	173.28
2.38	10.68	3.49	-4.19	167.72	263.94	171.77	171.93
2.33	10.64	3.42	-4.27	167.58	263.56	173.77	174.54
2.65	10.92	3.71	-3.97	153.92	251.42	158.69	151.33
2.89	11.17	3.94	-3.83	151.48	247.43	152.12	142.83
2.14	10.48	3.34	-4.17	160.46	263.13	171.39	165.46
2.01	10.3	3.26	-4.12	147.84	246.59	155.84	149.79
2.17	10.51	3.4	-4.05	149.03	254.64	157.34	151.14
2.23	10.59	3.5	-3.88	158.86	257.13	166.63	158.22
2.54	10.8	3.67	-3.86	150.67	254.13	158.72	150.05
2.2	10.59	3.42	-4.05	153.9	257	162.84	155.39
2.11	10.44	3.32	-4.21	159.8	264.49	172.83	167.3
2.33	10.59	3.49	-3.96	145.73	250.91	154.91	145.49
2.43	10.69	3.58	-3.9	146.05	250.44	155.26	144.76
2.24	10.49	3.49	-3.96	147.55	243.71	156.52	147.77
2.18	10.47	3.35	-4.02	139.86	245.94	149.97	138.93
2.35	10.64	3.53	-4.08	154.27	258.29	164.03	157.72
2.11	10.44	3.3	-4.08	151.31	254.06	159.69	153.01
2.07	10.39	3.3	-4.15	150.89	257.97	161.58	154.58
2.12	10.49	3.38	-4.02	156.46	257.66	165.77	158.36
2.09	10.46	3.35	-4.06	156.91	259.12	167.37	160.34
2.32	10.63	3.52	-4	155.88	258.99	165.36	157.16
2.05	10.32	3.28	-4.09	146.59	243.72	154.43	146.75
2.32	10.62	3.49	-4	149.7	255.3	160.4	153.06
2.33	10.63	3.5	-3.99	149.64	255.03	160.3	152.79
2.17	10.53	3.4	-3.97	157.06	256.05	164.63	156.43
1.92	10.29	3.19	-4.19	165.38	268.37	176.2	172.54
1.95	10.35	3.24	-4.14	163.98	267.48	175.34	171.03
1.93	10.31	3.01	-4.47	174.49	271.03	185.44	188.12
2.15	10.49	3.35	-4.16	160.17	262.61	170.67	164.68
2.29	10.6	3.37	-4.32	170.04	265.88	175.17	177.25
2.34	10.67	3.39	-4.27	166.14	263.83	174.01	174.85
2.29	10.59	3.37	-4.28	167.85	266.78	174.03	176.42
2.2	10.54	3.27	-4.34	167.64	268.12	177.68	179.63
1.42	9.64	2.8	-4.67	160.05	265.9	163.74	157.07
1.9	10.1	3.17	-4.3	152.31	257.36	153.69	143.4

1.91	10.11	3.18	-4.29	152.14	257.22	153.55	143.18
2.09	10.32	3.27	-4.25	149.99	256.27	153.91	143.63
2.25	10.5	3.41	-4.14	150.2	254.22	151.59	142.53
2.67	10.9	3.8	-3.91	152.86	251.31	149.3	137.6
2.78	11.01	3.89	-3.82	151.2	248.96	146.71	134.62
2.79	11.01	3.89	-3.82	151.14	248.9	146.64	134.46
2.67	10.91	3.81	-3.91	152.8	251.08	149.41	137.62
2.74	11	3.86	-3.86	152.62	247.65	146.85	136.05
2.75	10.94	3.81	-3.85	148.63	246.48	145.63	132.3
2.67	10.87	3.72	-3.91	150.18	248.38	147.18	134.45
2.75	10.98	3.82	-3.83	152.46	248.42	147.02	133.08
2.99	11.19	4	-3.67	148.53	244.75	143.55	128.52
2.9	11.09	3.93	-3.74	150.24	246.58	145.15	130.5
2.75	10.98	3.83	-3.8	149.17	246.93	146.04	131.47
2.23	10.46	3.44	-4.04	149.7	253.33	149.22	137.47
2.44	10.65	3.62	-3.9	148.08	250.15	147.03	133.14
1.9	10.12	3.19	-4.24	153.31	258.63	154.89	144.32
2.34	10.58	3.5	-4.09	153.92	257.14	153.03	141.9
2.34	10.58	3.5	-4.09	153.93	257.16	153.04	141.92
2.4	10.62	3.53	-4.1	153.3	255.56	152.04	141.51
2.39	10.62	3.53	-4.1	153.34	255.61	152.07	141.56
2.72	10.96	3.84	-3.86	151.65	250.21	147.97	136.23
2.73	10.97	3.85	-3.85	151.47	250.04	147.74	136.01
2.79	11.01	3.89	-3.82	151.33	248.87	146.74	134.53
2.79	11.01	3.9	-3.82	151.31	248.84	146.71	134.45
2.74	10.97	3.86	-3.86	151.99	249.78	147.91	136.23
2.62	10.89	3.73	-3.95	152.49	248.02	148.81	136.64
2.68	10.97	3.74	-3.87	146.75	245.55	147.4	136.03
2.93	11.31	3.99	-3.72	149.01	244.39	149.4	137.28
2.94	11.26	3.98	-3.7	145.36	245.95	149.03	137.47
2.69	10.98	3.8	-3.84	145.86	246.8	151.88	141.28
1.99	10.22	3.19	-4.35	148.83	255.08	153.27	143.95
2.45	10.77	3.62	-3.95	143.32	249.59	150.53	139.68
2.46	10.74	3.58	-4.02	147.7	248.91	148.69	138.15
3.11	11.33	4.19	-3.61	146.34	240.76	143.56	130.21
2.45	10.74	3.58	-3.98	141.66	247.3	149.21	137.92
2.93	11.24	3.98	-3.7	144.82	245.51	148.66	136.46

2.59	10.88	3.7	-3.84	143.27	247.07	150.88	139.05
2.94	11.25	3.97	-3.71	145.18	245.94	149.1	137.48
2.94	11.25	3.98	-3.71	145.29	245.95	149.08	137.51
2.96	11.33	4.02	-3.66	146.05	243.63	146.24	135.53
3.29	11.54	4.3	-3.49	143.47	234.92	140.31	124.82
2.64	11.01	3.77	-3.98	153.92	249.28	154.56	145.54
2.66	11.02	3.78	-3.96	153.33	248.99	154.08	145.35
2.69	11.01	3.77	-3.96	153.13	250.12	155.07	147.02
2.45	10.76	3.57	-4.18	157.93	254.02	160.2	155.19
2.69	11.01	3.75	-4.06	152.14	243.73	147.49	136.03
2.74	10.99	3.82	-3.73	141.89	244.24	148.21	134.7
2.56	10.85	3.66	-3.96	147.97	247.79	148.6	137.74
3.16	11.42	4.2	-3.56	146.05	240.29	143.14	130.17
3.08	11.34	4.17	-3.62	147.45	242.4	145.32	131.6
3.12	11.39	4.16	-3.6	146.41	236.92	142.08	127.35
2.39	10.59	3.54	-4.07	151.68	254.57	151.16	140.51
2.37	10.58	3.47	-4.13	150.17	253.45	150.73	139.52
2.37	10.58	3.47	-4.13	150.09	253.45	150.68	139.52
2.38	10.61	3.49	-4.12	149.89	253.32	150.37	139.64
2.02	10.24	3.23	-4.04	133.29	235.23	142.95	131.95
2.02	10.24	3.23	-4.04	133.32	235.25	142.96	132.04
2.02	10.25	3.17	-4.1	132.39	235.31	142.82	131.15
2	10.25	3.17	-4.11	133.87	236.69	143.91	133.02
2.1	10.33	3.25	-4.08	133.48	237.47	143.86	131.57
2.1	10.34	3.25	-4.08	133.61	237.55	143.85	131.62
2.12	10.35	3.26	-4.08	132.92	236.99	143.43	131.4
2.15	10.34	3.34	-4.11	127.34	239.69	138.25	126.17
2.1	10.31	3.22	-4.11	129.82	236.5	139.34	127.49
2.06	10.23	3.17	-4.1	127.29	231.31	135.84	123.18
2.02	10.26	3.18	-4.16	133.89	240.64	144.84	135.02
2.19	10.46	3.36	-4.02	139.25	244.62	149.39	138.77
2.21	10.44	3.38	-4.01	143.01	244.49	151.13	142.18
2.03	10.3	3.26	-4.11	145.99	243.6	154.35	146.94
2.07	10.29	3.22	-4.12	130.73	237.85	140.93	129.6
2.06	10.28	3.22	-4.14	130.53	238.32	141.33	129.87
2.02	10.25	3.17	-4.1	132.37	235.29	142.81	131.15
2.02	10.25	3.17	-4.1	132.37	235.31	142.73	131.06

2.02	10.27	3.2	-4.07	133.79	237	144.25	134.17
2.12	10.36	3.27	-4.08	133.08	237.07	143.39	131.57
2.1	10.34	3.25	-4.08	133.67	237.58	143.85	131.65
1.97	10.23	3.2	-4.15	144.39	244.16	154.35	147.18
1.98	10.24	3.21	-4.15	144.28	244.02	154.4	147.27
1.98	10.24	3.21	-4.15	144.22	243.96	154.3	147.17
2.06	10.27	3.19	-4.1	131.47	236.2	140.15	128.31
2.09	10.3	3.22	-4.1	130.87	236.18	140.11	128.54
2	10.25	3.22	-4.07	132.59	234.98	142.19	130.71
2.19	10.42	3.37	-4.03	143.33	245.21	151.62	142.99
1.92	10.04	3.1	-4.27	118.18	232.66	129.32	115.85
1.9	10.03	3.09	-4.28	118.02	232.79	128.98	115.75
1.9	10.03	3.09	-4.28	118.08	232.78	129.09	115.88
1.86	10.05	3.05	-4.27	118.72	231.42	130.32	118.43
1.57	9.82	2.88	-4.52	136.3	249.11	146.3	137.36
1.88	10.08	3.09	-4.34	130.12	242.22	138.86	128.49
1.92	10.14	3.13	-4.3	130.38	241.52	139.08	128.88
1.94	10.18	3.15	-4.27	128.63	238.18	138.61	127.27
1.83	9.99	2.97	-4.27	127.18	232.05	138.7	127.51
1.83	9.99	2.97	-4.28	126.54	231.72	138.37	127
1.83	9.99	2.98	-4.27	127.36	232.14	138.78	127.64
1.98	10.19	3.11	-4.18	127.03	231.21	135.41	122.52
1.78	9.98	3.01	-4.25	136.8	236.64	145.89	138.37
2.07	10.29	3.22	-4.17	127.21	234.85	136.73	123.2
2.03	10.24	3.16	-4.23	122.69	233.74	132.22	119.43
1.84	10.02	2.99	-4.27	127.06	231.61	138.26	126.88
1.95	10.17	3.08	-4.2	126.54	231.97	135.71	123.21
2.49	10.78	3.57	-4.29	157.74	248.78	156.92	150.58
2.36	10.68	3.38	-4.37	166.73	259.93	171.76	171.58
2	10.31	3.14	-4.62	173.56	263.5	175.47	177.41
1.97	10.29	3.12	-4.64	174.14	264.42	176.65	179.05
2.6	10.89	3.67	-4.13	157.3	250.19	158.08	151.67
2.02	10.36	3.09	-4.62	171.45	266.76	180.71	184.93
2.59	10.85	3.66	-4.1	158.91	253.75	161.73	157.15
2.5	10.79	3.56	-4.2	159.65	253.15	161.52	156.45
2.5	10.79	3.56	-4.2	159.68	253.06	161.57	156.44
2.11	10.4	3.24	-4.55	170.59	263.53	175.03	176.84

1.88	10.2	3.07	-4.72	175.66	265.89	176.64	179.81
1.96	10.28	3.11	-4.64	174.33	264.63	176.84	179.34
2.7	10.98	3.79	-4.08	153.66	248.86	152.78	144.88
2.67	10.95	3.76	-4.1	154.01	249.31	153.24	145.58
2.89	11.16	3.97	-3.88	151.78	245.39	150.03	139.61
2.79	11.05	3.85	-4.06	152.98	244.99	148.51	139.03
1.77	9.98	3.08	-4.46	151.72	259.7	155.28	146.66
1.62	9.82	2.9	-4.54	141.95	253.72	150.38	141.72
1.78	10.02	3.05	-4.47	152.64	260.04	157.32	150.23
2.28	10.48	3.42	-4.05	137.28	244.01	141.38	128.58
1.91	10.11	3.12	-4.32	139.29	251.07	146.97	136.54
2.11	10.43	3.35	-4.15	145.39	250.26	153.99	144.85
2.15	10.41	3.37	-4.06	136.94	245.62	145.94	136.69
1.96	10.22	3.22	-4.22	135.69	246.05	143.97	134.42
1.91	10.13	3.16	-4.39	149.03	255.57	152.96	144.18
2.01	10.21	3.22	-4.27	136.09	247.53	143.28	131.64
1.89	10.09	3.13	-4.33	140.14	252.71	147	136.37
1.89	10.09	3.13	-4.33	140.07	252.66	146.94	136.29
1.95	10.18	3.16	-4.38	149.72	255.79	154.2	145.44
2.29	10.58	3.46	-4.02	145.38	250.48	152.32	142.79
1.81	10.25	3.13	-4.34	170.99	275.55	186.18	182.25
2.2	10.57	3.47	-3.91	156.49	255.97	164.63	156.95
1.87	10.26	3.15	-4.35	168.82	274.3	183.14	182.58
1.7	10.14	3.07	-4.41	177.27	279.97	189.19	188.45
1.85	10.28	3.15	-4.31	170.4	274.55	185.05	181.01
1.82	10.26	3.13	-4.34	171.02	275.44	185.89	182.28
1.82	10.25	3.13	-4.34	170.96	275.53	186.14	182.21
1.73	10.13	3.05	-4.48	177.65	283.6	192.44	195.45
2.05	10.4	3.31	-4.15	163.45	267.84	175.82	172.35
2.05	10.4	3.31	-4.15	163.49	267.9	175.88	172.44
2.13	10.47	3.32	-4.17	160.78	264.06	172.5	167.08
2.12	10.46	3.31	-4.19	160.95	264.45	173.05	167.72
2.13	10.47	3.33	-4.2	159.65	264.7	172.68	167.36
1.66	10.07	2.98	-4.51	177.12	281.24	191.15	196.08
1.67	10.07	2.99	-4.51	176.9	280.93	190.81	195.54
1.73	10.12	3.04	-4.47	175.97	279.48	189.28	193.03
2.09	10.44	3.38	-4.01	160.82	262.4	171.14	165.28

2.13	10.47	3.41	-3.98	160.37	261.21	170.06	163.63
1.95	10.07	3.21	-4.17	121.43	235.04	133.18	119.71
1.79	9.99	3.08	-4.37	154.46	258.94	155.49	145.87
1.79	9.99	3.08	-4.37	154.54	259.03	155.62	146.02
1.99	10.2	3.23	-4.21	151.95	256.05	151.14	140.83
2.77	11.04	3.84	-4.02	152.98	247.38	151.95	143.07
2.44	10.66	3.63	-3.9	149.53	249.99	146.99	133.72
2.64	11.01	3.77	-3.98	153.91	249.27	154.55	145.52
2.73	10.98	3.81	-3.74	142.17	244.45	148.22	134.91
2.86	11.19	3.95	-3.73	144.15	243.7	147.08	134.9
2.06	10.28	3.21	-4.14	130.51	238.43	141.37	129.93
1.94	10.05	3.14	-4.22	119.47	233.86	131.83	116.93
2.6	10.89	3.66	-4.15	152.29	238.26	137.38	123.91
2	10.3	3.06	-4.71	164.88	252.12	158.21	151.82
1.99	10.29	3.06	-4.72	165.06	252.32	158.43	152.16
2.27	10.56	3.24	-4.54	162.36	251.38	158.36	152.18
1.98	10.23	3.04	-4.73	167.81	256.68	162.62	158.03
1.98	10.22	3.04	-4.73	167.83	256.76	162.7	158.12
2.16	10.41	3.18	-4.6	166.96	253.68	163.27	158.3
1.96	10.28	3.01	-4.74	163.04	249.45	154.79	147.17
1.97	10.28	3.02	-4.74	163.01	249.37	154.79	147.15
2.47	10.74	3.58	-4.26	154.76	241.63	144.4	133.62
2.8	11.13	3.84	-3.9	142.28	231.62	130.7	115.25
2.77	11.1	3.82	-3.92	142.72	231.96	131.12	115.79
2.85	11.19	3.87	-3.84	141.81	230.03	129.61	111.98
2.9	11.19	3.97	-3.83	143.11	229.62	130.38	113.29
2.87	11.14	3.94	-3.86	143.95	230.16	131.28	114.6
2.78	11.07	3.85	-3.87	140.8	222.52	122.5	101.53
2.77	11.06	3.83	-3.88	141.03	222.75	122.74	101.85
2.76	11.05	3.83	-3.89	141.15	222.89	122.88	102.03
2.76	11.05	3.83	-3.89	141.2	222.93	122.92	102.09
2.75	11.05	3.82	-3.89	141.24	222.98	122.97	102.16
2.75	11.04	3.82	-3.9	141.29	223.03	123.02	102.22
2.28	10.59	3.28	-4.48	166.53	255.34	167.99	166.7
1.93	10.18	2.99	-4.77	170.68	258.52	166.24	163.01
3.11	11.28	4.2	-3.64	133.52	214.39	112.08	86.68
1.83	10.13	3	-4.77	176.82	268.41	178.87	182.39

1.8	10.09	2.96	-4.78	177.74	269.22	179.86	183.2
1.98	10.23	3.05	-4.73	169.94	260.04	168.04	165.05
2.18	10.47	3.22	-4.58	170.07	258.27	167.61	166.72
2.38	10.66	3.5	-4.38	164.35	254.31	163.73	160.76
1.83	10.13	3	-4.76	176.48	268.1	178.8	182.41
2.17	10.48	3.32	-4.51	168.5	259.25	169.15	168.5
2.23	10.5	3.38	-4.5	167.34	258.37	167.67	166.1
2.06	10.33	3.22	-4.61	171.7	263.01	171.73	172.48
2.16	10.47	3.31	-4.52	168.63	259.42	169.33	168.75
2.27	10.59	3.4	-4.42	166.31	256.78	165.86	163.4
2.23	10.54	3.39	-4.52	162.89	253.99	160.07	155.02
2.86	11.13	3.91	-3.98	151.09	245.33	147.31	136.21
2.85	11.11	3.9	-3.99	151.19	245.55	147.52	136.53
2.85	11.11	3.9	-3.99	151.24	245.54	147.54	136.55
2.79	11.05	3.83	-4.03	150.2	237.49	140.58	127.01
2.78	11.06	3.82	-4.01	148.62	236.94	138.82	125.17
2.78	11.06	3.82	-4.01	148.66	236.92	138.77	125.16
2.63	10.91	3.7	-4.13	151.33	240.48	143.62	131.23
2.68	10.97	3.71	-4.11	153.11	244.11	148.56	136.45
2.71	11	3.76	-4.09	151	242.93	145.09	133.23
1.84	10.13	3	-4.77	176.69	268.29	178.78	182.33
1.84	10.14	3	-4.76	176.38	268	178.7	182.33
3.09	11.34	4.07	-3.74	144.34	231.76	129.99	110.55
2.78	11.08	3.8	-4.01	148.11	237.16	133.98	117.48
2.5	10.77	3.56	-4.16	154.66	246.39	143.72	129.89
3	11.27	4	-3.8	145.34	232.58	131	111.81
2.72	10.98	3.76	-3.99	151.52	243.55	140.11	124.94
3.31	11.52	4.25	-3.54	141.07	226.22	125.8	104.71
2.77	11.02	3.73	-4	146.93	238.91	135.67	120.08
2.73	10.98	3.7	-4.04	147.55	239.7	135.57	120.82
3.08	11.3	4.02	-3.77	143.11	229.68	130.17	110.33
3.06	11.29	4.01	-3.79	143.2	229.88	130.35	110.56
3.21	11.42	4.16	-3.62	141.96	227.29	127.56	106.85
3.19	11.44	4.16	-3.64	142.53	228.83	128.68	108.11
2.93	11.21	3.94	-3.83	145.78	232.18	131.44	112.31
3.08	11.34	4.02	-3.72	144.17	233.22	132.8	116.73
3.1	11.35	4.03	-3.71	143.98	233.05	132.58	116.38

3.1	11.36	4.03	-3.71	143.94	233.01	132.55	116.32
3.18	11.42	4.1	-3.65	143.06	232.46	131.96	114.69
3.01	11.36	4.03	-3.7	139.01	228.44	127.67	109.48
2.98	11.33	3.99	-3.7	139.4	226.77	125.77	107.98
3.18	11.51	4.16	-3.47	134.87	220.49	120.03	99.38
2.89	11.06	3.89	-3.87	136.91	217.62	116.87	92.34
3.16	11.4	4.17	-3.61	131.76	208.77	107.49	80.87
3.18	11.4	4.2	-3.59	133.97	213.11	111.21	85.37
3.2	11.37	4.18	-3.59	130.64	209.43	107.71	80.75
3.25	11.48	4.23	-3.54	132.68	210.78	111.02	83.75
3.27	11.46	4.27	-3.52	129.56	208.99	106.57	80.12
3.61	11.8	4.56	-3.22	128.4	206.79	103.87	76.72
3.08	11.27	4.08	-3.72	135.62	217	116.8	91.55
2.78	11.04	3.73	-3.93	149.47	240.75	139.97	125.17
3.15	11.35	4.12	-3.6	142.81	237.89	140.08	124.6
2.7	10.94	3.67	-4.03	151.91	244.45	140.6	125.78
2.7	10.93	3.67	-4.03	152.03	244.54	140.7	125.85
3.11	11.31	4.13	-3.58	146.51	238.27	141.53	126.97
2.76	11.02	3.72	-3.95	149.48	240.82	140.26	125.28
2.76	11.02	3.72	-3.95	149.49	240.8	140.25	125.28
2.76	11.02	3.72	-3.95	149.5	240.78	140.23	125.27
2.76	11.02	3.72	-3.95	149.51	240.75	140.21	125.27
2.77	11.03	3.72	-3.94	149.51	240.89	140.18	125.34
2.72	10.94	3.75	-3.94	149.57	247.44	143.66	129.22
2.72	10.94	3.74	-3.94	149.57	247.45	143.67	129.22
2.72	10.94	3.75	-3.94	149.56	247.41	143.63	129.2
3.1	11.3	4.08	-3.64	143.95	238.73	141.31	126.04
2.68	10.92	3.66	-4.04	151.89	244.99	141	126.67
2.79	11.04	3.77	-3.99	150.41	240.14	138.75	123.03
3.34	11.53	4.31	-3.45	141.05	233.5	136.46	120.66
3.18	11.38	4.2	-3.53	144.95	237.24	140.26	125.12
3.14	11.37	4.05	-3.66	145.77	235.79	135.26	118.91
3.02	11.28	4.01	-3.76	146.27	234.73	134.48	119.38
3.04	11.28	4.03	-3.77	144.49	234.88	139.39	123.24
3.06	11.3	4.05	-3.75	143.9	234.9	138.98	122.85
2.88	11.16	3.9	-3.85	146.83	239.87	143	128.14
3.16	11.41	4.08	-3.66	143.18	232.47	132.04	115.18

2.4	10.67	3.47	-4.22	156.67	249.12	146.12	133.19
2.98	11.22	4.05	-3.84	143.39	228.59	127.06	106.55
3.23	11.51	4.23	-3.58	144.3	231.3	128.92	108.83
3.16	11.46	4.16	-3.66	146.63	235.9	131.69	112.8
3.04	11.28	4.03	-3.66	148.81	240.22	135.8	118.85
3.3	11.52	4.3	-3.5	135.49	219.92	120.68	92.96
3.24	11.5	4.21	-3.56	144.29	231.82	130.13	110.14
2.9	11.14	3.9	-3.89	150.4	240.47	137.95	120.66
2.64	10.89	3.7	-4.01	151.06	246.25	141.43	128.71
2.98	11.22	4.06	-3.84	144.48	229.54	127.98	107.44
2.94	11.17	4.01	-3.87	144.75	229.34	128.32	107.93
3.38	11.61	4.31	-3.47	142.43	229.23	128.94	107.94
3.37	11.61	4.31	-3.47	142.47	229.22	128.98	107.99
3.37	11.62	4.31	-3.47	142.65	229.03	128.89	107.9
3.09	11.39	4.12	-3.73	147.73	235.8	132.17	113.15
3.11	11.41	4.12	-3.71	146.81	234.92	131.34	111.99
3.11	11.42	4.13	-3.71	146.76	234.86	131.3	111.91
3.12	11.42	4.13	-3.71	146.73	234.81	131.28	111.85
3.11	11.41	4.12	-3.72	146.87	234.87	131.41	111.97
3.3	11.56	4.28	-3.51	144.55	232.55	130.09	109.82
3.09	11.38	4.08	-3.62	148.03	236.53	133.69	115.39
3.21	11.49	4.17	-3.54	146.16	234.6	131.48	112.99
3.19	11.47	4.16	-3.56	146.52	235.02	131.83	113.35
2.98	11.25	4	-3.7	149.96	241.77	136.35	120.17
3.02	11.27	4.02	-3.67	149.07	240.43	136.01	119.03
3.04	11.28	4.03	-3.66	148.84	240.19	135.83	118.82
3.19	11.47	4.15	-3.64	144.58	233.69	129.87	111.24
3.62	11.91	4.66	-3.09	134.03	219.42	121.1	94.07
3.64	11.87	4.64	-3.16	135.28	219.77	120.49	93.38
3.29	11.49	4.31	-3.51	134.88	216.91	117.08	89.5
3.56	11.84	4.63	-3.2	137.39	221.68	122.31	96.52
3.04	11.24	4.02	-3.69	135.09	215.67	115.3	88.64
3.08	11.28	4.09	-3.64	133.42	214.68	113.27	86.78
2.79	11.05	3.83	-3.88	132.14	211.89	110.01	83.08
3.07	11.3	4.07	-3.66	134.31	215.05	114.48	87.56
3.01	11.2	4.04	-3.8	137.85	219.98	118.81	94.7
2.2	10.49	3.44	-4.04	152.97	254.45	151.61	139.09

2.27	10.52	3.45	-4.02	152.54	254.73	151.28	137.85
2.76	10.95	3.84	-3.78	146.28	247.39	145.29	130.09
2.56	10.75	3.66	-3.95	150.4	252.54	148.85	136.72
2.42	10.65	3.55	-3.95	150.37	252.08	149.11	134.95
2.49	10.71	3.64	-3.91	149.42	251.41	147.92	134.5
2.49	10.71	3.64	-3.91	149.42	251.41	147.92	134.48
2.49	10.71	3.64	-3.91	149.42	251.42	147.97	134.52
2.48	10.71	3.63	-3.91	149.42	251.41	148	134.53
2.74	10.9	3.82	-3.86	149.6	250.09	145.11	132.33
2.48	10.73	3.58	-4.17	157.31	252.13	147.83	134.25
1.92	10.15	3.21	-4.25	155.23	260.11	155.26	143.44
1.95	10.18	3.26	-4.23	152.85	257.63	153.83	141.63
2.17	10.46	3.41	-4.05	153.73	255.64	152.04	139.79
2.11	10.42	3.36	-4.09	154.49	256.73	153.01	141.18
1.98	10.28	3.25	-4.21	155.52	256.96	154.73	145.01
2.05	10.33	3.31	-4.17	154.03	255.77	152.98	142.26
1.9	10.13	3.2	-4.26	156.33	260.92	155.35	143.46
1.91	10.14	3.21	-4.26	154.91	260.15	155.22	143.76
1.88	10.07	3.16	-4.29	151.69	257.21	153.53	141.67
1.92	10.1	3.17	-4.26	151.5	257.04	153.04	141.14
1.93	10.15	3.19	-4.24	152.06	257.28	152.18	141.4
1.95	10.18	3.21	-4.21	152.03	256.91	151.16	141.16
1.91	10.14	3.21	-4.26	154.99	260.16	155.16	143.62
2.85	11.08	4.01	-3.65	151.05	250.48	145.01	130.96
2.82	11.07	3.99	-3.66	150.54	250.1	145.48	131.43
2.46	10.72	3.59	-4.06	158.48	255.54	150.71	138.2
3.3	11.58	4.33	-3.51	151.63	240.77	138.37	120.05
3.29	11.59	4.39	-3.5	151.73	241.69	138.48	121.19
3.35	11.61	4.41	-3.49	150.69	239.76	136.92	119.55
2.62	10.92	3.75	-3.98	155.85	250.62	145.38	131.85
2.7	10.95	3.74	-3.98	151.98	243.97	141.4	126.29
2.22	10.52	3.38	-4.35	161.72	257.72	152.06	140.28
2.82	11.03	3.96	-3.73	153.77	249.57	145.86	133.07
2.62	10.93	3.74	-3.97	155.97	251.01	145.71	132.15
2.53	10.75	3.63	-4	155.31	254.75	149.26	135.96
2.46	10.69	3.57	-4.09	159.31	255.19	150.41	138.55
2.48	10.7	3.58	-4.08	158.91	254.45	149.83	137.97

2.45	10.7	3.57	-4.07	158.73	255.7	151	138.66
2.58	10.79	3.78	-3.91	156.94	255.58	150.83	138.86
2.63	10.82	3.72	-3.96	153.66	252.78	148.78	134.52
3.41	11.72	4.47	-3.36	149.82	240.28	136.51	119.03
2.63	10.92	3.75	-3.97	155.79	250.59	145.33	131.8
2.47	10.72	3.59	-4.05	158.14	255.43	150.68	138.12
2.47	10.72	3.59	-4.05	157.71	255.39	150.75	137.91
2.5	10.73	3.62	-4.03	156.45	256.08	149.93	136.51
2.5	10.73	3.62	-4.03	156.51	256.09	149.96	136.52
2.47	10.7	3.58	-4.08	159	254.44	149.86	138.06
2.48	10.7	3.59	-4.08	158.72	254.09	149.6	137.73
2.43	10.67	3.54	-4.19	157.56	253.57	149.02	136.59
2.35	10.61	3.47	-4.25	158.29	252.31	148.26	136.3
2.38	10.66	3.5	-4.22	155.26	250.29	145.4	134
3.07	11.37	4.14	-3.71	152.29	241.46	139.08	121.82
2.84	11.18	3.94	-3.94	151.16	238.67	136.58	118.32
3.64	11.94	4.73	-3.16	141.93	227.01	128.32	105.98
3.76	12.05	4.84	-3.03	140.12	224.97	127.09	103.21
2.48	10.73	3.58	-4.17	157.31	252.14	147.83	134.25
2.71	10.95	3.75	-3.96	150.56	245.42	140.66	127.39
1.59	9.52	3.05	-4.03	167.57	250	180.45	180.37
1.68	9.59	3.11	-3.96	156.14	241.31	171.06	168.61
1.47	9.4	2.84	-4.22	164.91	249.22	178.28	186.66
1.43	9.32	2.82	-4.26	170.53	256.15	185.86	194.94
1.86	10.1	3.31	-4.02	145.08	250	163.36	141.12
1.6	9.67	3.02	-4.25	168.22	268.94	180.43	159.14
2.21	10.24	3.46	-3.79	130.85	222.55	147.39	122.36
0.94	9.19	2.83	-4.71	201.52	297.24	195.97	190.45
1.28	9.5	2.97	-4.57	198.88	290.48	189.53	185.29
1.32	9.53	3	-4.55	198.26	290.05	189.09	184.53
2.86	11	4.22	-3.52	164.94	262.06	161.33	143.73
3.27	11.51	4.55	-3.16	165.82	260.2	157.06	142.27
3.09	11.33	4.39	-3.38	173.61	268.47	164.49	154.24
4.05	12.2	5.16	-2.87	117.15	206.58	98.21	64.99
3.81	11.92	4.74	-3.04	130.66	213.42	103.86	73.42
3.62	11.75	4.59	-3.29	131.1	215.51	106.39	75.75
3.56	11.74	4.52	-3.34	130.67	216.97	105.92	75.26

3.28	11.4	4.21	-3.65	132.47	221.29	109.81	79.16
3.24	11.37	4.18	-3.65	132.64	221.49	110.14	79.14
3.82	11.96	4.7	-3.16	129.52	214.85	104.45	72.59
3.64	11.66	4.65	-3.19	125.4	212.42	101.92	70.28
3.91	12.01	4.71	-3.08	127.94	213.66	103.23	73.46
3.51	11.65	4.45	-3.36	132.57	218.09	106.61	77.35
3.49	11.63	4.43	-3.38	132.77	218.27	106.97	77.65
3.71	11.85	4.62	-3.15	130.73	216.17	105.94	75.52
3.47	11.63	4.4	-3.41	132.45	218.43	108.14	79.45
3.49	11.65	4.42	-3.4	132.39	218.52	107.63	78.92
3.02	11.33	3.99	-3.88	145.09	227.66	124.26	102.38
3.6	11.96	4.64	-3.19	144.25	225.2	125.55	106.45
3.52	11.81	4.44	-3.29	139.6	221.7	119.62	97.23
2.72	11.1	3.73	-4.15	153.74	241.54	144.95	131.38
3.26	11.56	4.22	-3.69	144.61	226.93	123.63	102.85
3.09	11.32	4.07	-3.84	139.38	223.07	116.35	91.54
2.88	11.14	3.91	-4	144.22	227.48	120.53	95.9
3.01	11.26	4	-3.89	142.9	225.53	119.71	95.21
3.97	12.1	4.8	-2.94	130.18	209.45	102.7	74.57
3.61	11.71	4.58	-3.13	131.69	214.36	106.3	75.92
3.86	11.96	4.74	-2.97	129.4	207.14	103.86	75.1
3.67	11.81	4.56	-3.23	131.63	209.42	105.34	77.59
3.3	11.47	4.23	-3.58	135.2	216.51	111.3	84.98
3.3	11.48	4.23	-3.57	135.62	216.51	110.58	84.37
3.5	11.68	4.41	-3.46	135.09	217.23	109.82	81.48
3.57	11.66	4.49	-3.21	131.53	209.81	104.17	74.61
3.78	11.87	4.67	-3.03	130.37	210.24	102.66	73.45
3.44	11.53	4.5	-3.21	130	212.86	105.73	76.02
3.33	11.43	4.33	-3.36	131.72	211.49	105.32	75.4
3.33	11.43	4.33	-3.35	131.52	211.5	105.13	75.58
3.39	11.5	4.39	-3.3	130.57	211.07	104.41	75.28
3.33	11.38	4.28	-3.4	132.25	210.7	105.27	76.5
3.44	11.55	4.43	-3.16	127.72	209.58	103.74	73.69
3.6	11.83	4.56	-3.21	136.44	214.64	110.89	85.79
3.79	12.07	4.74	-3.07	136.33	214.86	112.32	88.07
3.79	12.06	4.74	-3.07	136.38	214.93	112.37	88.13
3.34	11.64	4.31	-3.44	140.23	222.49	119.35	97.92

4	12.14	5.03	-2.65	121.06	207.63	100.78	69.02
3.81	11.91	4.79	-2.89	126.75	211.17	102.89	71.6
3.75	11.81	4.74	-3.01	127.37	212.3	104.08	72.54
3.62	11.95	4.38	-3.59	156.68	246	143.79	130.86
2.77	11.02	3.58	-4.2	166.87	260.46	151.88	139.78
3.12	11.48	3.92	-3.93	162.94	253.64	147.36	133.26
3.7	12.06	4.45	-3.53	154.13	242.95	140.03	126.37
2.6	10.96	3.46	-4.41	167.46	261.73	151.95	139.8
2.59	10.92	3.44	-4.43	167.76	261.52	151.99	139.69
3.63	11.96	4.39	-3.55	156.38	244.69	142.25	126.82
3.25	11.59	4.02	-3.84	159.98	250.45	146.3	132.61
3.26	11.62	4.04	-3.88	149.25	234.79	128.44	105.52
3.26	11.63	4.03	-3.78	158.49	242.44	149.06	139.25
3.08	11.49	3.91	-3.86	153.44	238.53	142.41	129.96
2.72	11.11	3.64	-4.36	153	234.31	133.04	114.5
2.85	11.23	3.74	-4.26	151.46	233.25	131.68	112.9
2.79	11.19	3.69	-4.3	151.22	234.76	133	114.37
2.72	11.12	3.63	-4.34	151.59	235.22	133.93	115.28
2.74	11.15	3.64	-4.32	151.23	235.25	133.83	115.18
2.44	10.86	3.52	-4.48	155.75	239.38	138.3	121.16
2.51	10.91	3.55	-4.45	154.49	237.75	138.22	119.56
2.32	10.75	3.42	-4.57	158.08	240.75	139.84	122.71
2.32	10.76	3.44	-4.55	158.17	240.51	139.23	121.93
3.23	11.61	4.11	-3.86	150.2	234.06	137.17	121.37
3.23	11.61	4.09	-3.86	150.86	236.92	137.88	122.27
3.47	11.86	4.28	-3.65	149.53	234.29	136.99	121.9
2.7	11.12	3.64	-4.39	155.74	239.52	138.49	120.58
2.62	11.02	3.59	-4.43	156.19	239.4	138.32	120.26
2.64	11.03	3.59	-4.44	156.44	239.62	138.26	120.62
3.16	11.52	3.97	-4.04	148.86	233.31	130.65	111.24
3.54	11.94	4.38	-3.57	150.22	237.28	138.4	121.52
3.73	12.13	4.56	-3.44	148.89	234.25	137.69	121.57
2.84	11.19	3.68	-4.1	156.94	241.2	148.22	137.67
3.3	11.66	4.06	-3.71	152.2	236.67	143.64	130.1
3.08	11.44	3.84	-3.94	156.85	241.13	150.32	140.65
3.15	11.53	3.93	-3.86	159.8	243.92	150.85	142.01
3.16	11.63	3.99	-3.89	160.2	245.91	149.15	140

3.27	11.7	4.06	-3.83	158.96	245.23	148.8	138.92
3.11	11.48	3.92	-4.04	150.72	238.52	131.76	111.58
2.85	11.24	3.7	-4.06	161.14	246.14	156.34	150.26
2.34	10.76	3.24	-4.48	172.66	264.67	181.92	185.47
3.23	11.59	4.03	-3.94	151.28	237.86	131.54	110.75
3.33	11.59	4.12	-3.88	142.87	227.27	120.47	94.11
3.39	11.69	4.1	-3.84	144.73	230.5	124.67	102.51
3.52	11.84	4.25	-3.76	146.7	231.14	127.38	106.68
3.4	11.74	4.17	-3.83	148.07	234.28	129.17	109.1
3.41	11.74	4.17	-3.82	147.95	234.18	129.05	108.99
3.06	11.44	3.9	-4.03	146.34	230.75	124.99	102
3.05	11.43	3.89	-4.04	146.44	230.95	125.21	102.05
3.52	11.87	4.23	-3.69	143.34	228.21	120.98	97.89
3.69	12.11	4.41	-3.58	150.3	235.71	135.15	117.74
3.38	11.71	4.14	-3.81	146.81	232.79	126.12	103.6
3.29	11.62	4.01	-3.88	144.8	229.53	124.83	101.16
3.28	11.61	4	-3.89	144.87	229.64	124.88	101.33
1.93	10.28	2.94	-4.73	185.07	279.7	204.2	217.5
1.8	10.22	2.77	-4.83	185.57	280.24	206.58	221.11
3.33	11.32	4.55	-3.61	123.74	213.08	104.24	70.17
3.34	11.33	4.55	-3.58	123.3	213.24	103.28	70.11
2.69	10.74	4.16	-3.94	129.6	227.46	111.01	78.99
3.63	11.65	4.78	-3.25	120.21	209.35	101.02	67.46
3.63	11.66	4.79	-3.25	120.14	209.26	101	67.32
3.52	11.59	4.77	-3.35	118.9	208.94	101.35	66.85
3.74	11.76	4.83	-3.14	119.39	207.95	99.42	65.79
3.38	11.44	4.52	-3.4	124.74	213.56	103.01	70.19
3.33	11.35	4.46	-3.46	125.25	216.1	103.61	71.18
3.01	11.03	4.27	-3.82	128.84	216.99	107.69	74.16
3.7	11.81	4.63	-3.36	129.33	215.11	106.31	75.08
3.86	12.01	4.72	-3.18	130.24	213.33	104.81	72.9
3.69	11.85	4.61	-3.33	129.41	216.35	106.93	76.34
3.67	11.8	4.6	-3.37	129.5	216.65	106.17	75.7
2.23	10.47	3.47	-3.94	149.72	246.89	158.58	151.65
2.32	10.52	3.54	-3.89	150.01	245.13	159	153.41
2.25	10.49	3.49	-3.93	149.33	246.37	158.03	150.82
1.95	10.21	3.25	-4.12	160.41	256.53	172.1	170.47

2.21	10.46	3.46	-3.95	150.5	246.97	159.72	154.7
1.66	10.1	2.95	-4.61	183	282.99	194.69	197.92
1.7	10.1	2.99	-4.57	182.72	283.94	194.09	198.56
1.69	10.09	2.98	-4.57	182.82	284.07	194.19	198.89
1.69	10.08	2.96	-4.56	183.48	284.89	195.3	200.8
1.67	10.07	2.94	-4.58	183.62	285	195.36	201.15
1.68	10.07	2.94	-4.58	183.6	284.98	195.34	201.11
1.63	9.83	2.94	-4.41	157.33	252.05	170	168.48
1.8	9.94	3.01	-4.28	132.95	234.23	143.65	135.79
1.97	10.11	3.07	-4.23	122.47	228.85	132.7	120.02
1.98	10.12	3.23	-4.11	139.24	233.78	148.9	141.65
1.9	10.22	3.24	-4.21	170.57	268.14	181.68	182.29
1.93	10.26	3.25	-4.17	164.99	264.12	176.92	176.63
1.91	10.25	3.24	-4.18	165.97	264.89	177.84	177.71
2.15	10.4	3.42	-4.05	169.32	264.08	176.02	175.24
1.96	10.33	3.33	-4.19	175.51	277.94	187.63	184.2
1.66	10.06	2.95	-4.59	183.33	284.73	194.86	200.48
1.76	10.17	3.11	-4.42	174.07	277.64	188.01	185.39
1.62	10.07	3.01	-4.45	178.52	282.15	192.74	193.11
1.62	10.07	3.01	-4.45	178.5	282.12	192.7	193.05
1.62	10.08	3.01	-4.45	178.46	282.05	192.62	192.86
1.65	10.03	2.92	-4.58	184.93	286.18	197.36	203.94
1.85	10.21	3.19	-4.22	165.23	265.3	177.47	177.18
2	10.35	3.33	-4.1	162.95	263.47	174.4	171.13
2	10.35	3.33	-4.11	162.96	263.55	174.45	171.25
2.23	10.56	3.28	-4.46	169.48	262.61	175.11	175.56
2.35	10.7	3.4	-4.34	168.62	258.89	173.24	173.96
2.02	10.44	3.05	-4.65	200.14	298.32	198.11	205.47
1.77	10.12	2.84	-5.01	211.86	312.38	200.39	206.87
1.54	9.96	2.68	-5.13	216.82	318.1	206.62	213.72
1.69	10.1	2.76	-5.04	210.15	310.63	201.71	209.46
2.28	10.56	3.28	-4.46	200.69	299.94	189.82	192.03
2.57	10.88	3.43	-4.32	184.32	282.41	175.28	174.69
2.48	10.74	3.35	-4.41	181.98	278.45	168.81	163.68
2.34	10.7	3.27	-4.48	191.72	289.84	187.98	192.25
2.18	10.46	3.13	-4.74	196.66	294.28	182.21	181.81
1.6	9.86	2.5	-5.36	191.79	292.97	170.67	161.34

2.52	10.74	3.24	-4.55	167.37	263.18	146.34	131.41
1.77	10.04	2.77	-5.06	200.99	298.84	181.48	178.59
1.39	9.69	2.51	-5.31	215.23	319.5	197.91	197.97
2.78	11.03	3.59	-4.2	166.72	260.34	151.75	139.55
2.48	10.71	3.28	-4.51	171.61	269.02	153.99	141.99
3.15	11.65	3.93	-3.99	166.13	258.4	163.96	160.84
2.86	11.32	3.67	-4.19	175.37	268.17	174.36	174.15
2.97	11.4	3.78	-4.07	173.68	266.33	172.69	172.07
2	10.37	3	-4.71	189.25	282.33	205.26	221.7
2.13	10.51	3.07	-4.62	184.81	277.25	199.63	212.84
2.55	10.9	3.41	-4.39	181.46	271.36	188.6	198.98
2.67	11.06	3.53	-4.3	174.56	266.42	180.81	186.26
2.61	11.02	3.46	-4.35	177.63	268.69	181.45	187.68
2.89	11.27	3.75	-4.12	170.89	260.52	175.84	176.51
2.95	11.37	3.71	-4.1	170.59	261.67	171.46	172.36
3.19	11.69	3.96	-3.96	164.89	257.19	162.59	158.89
1.78	10.12	2.9	-4.82	211.22	310.19	209.74	224.55
1.93	10.3	3.01	-4.7	206.67	303.4	204.61	218.16
2.01	10.38	3.07	-4.64	204.23	301.16	201.92	215.27
1.71	10.08	2.93	-4.78	213.61	314.59	217.2	236.04
1.71	10.12	2.83	-4.83	208.92	311.56	210.21	223.43
1.9	10.29	2.99	-4.73	207.03	304.49	205.81	219.33
1.9	10.27	2.99	-4.72	207.6	304.49	205.49	219.29
1.96	10.35	3.03	-4.68	203.55	302.92	202.89	216.07
2.97	11.39	3.73	-4.06	172.96	264.49	170.32	167.91
1.55	9.96	2.62	-5	208.83	305.48	226.26	255.86
1.65	10.08	2.72	-4.94	208.43	303.82	222.95	252.06
1.17	9.51	2.31	-5.56	219.66	324.88	201	200.89
1.57	9.91	2.6	-5.23	214.7	316.1	197.9	202.09
2.17	10.5	3.12	-4.68	197.4	295.95	188.77	192.56
2.56	10.77	3.31	-4.53	166.75	262.88	148.65	133.03
1.98	10.34	2.94	-4.76	189.59	283.96	204.77	223.2
1.77	10.15	2.85	-4.84	195.58	292.85	218.18	241.96
1.45	9.85	2.55	-5.09	197.38	295.86	219.81	242.92
1.3	9.69	2.45	-5.16	203.52	305.27	227.74	256.99
1.3	9.71	2.46	-5.14	199.16	300.02	219.83	245.01
1.7	10.13	2.7	-4.88	190.15	284.67	211.23	229.02

2.06	10.45	3.04	-4.63	183.35	278.19	200.46	213.07
1.76	10.2	2.77	-4.83	187.79	282.01	208.54	224.95
2.14	10.53	3.09	-4.57	180.53	271.69	196.76	208.9
1.89	10.24	2.95	-4.78	191.75	290.44	214.05	236.46
1.41	9.75	2.51	-5.14	204.14	304.36	226.06	253.83
1.72	10.1	2.79	-4.79	183.7	283.18	202.29	215.04
1.09	9.57	2.31	-5.12	197.35	300.55	220.57	239.28
1.31	9.76	2.51	-4.96	193.27	294.4	212.05	226.84
1.32	9.77	2.52	-4.96	192.81	294.04	211.61	226.13
1.47	9.85	2.61	-5.01	191.92	292.31	212.85	232.57
1.69	10.03	2.79	-4.87	185.07	278.85	199.89	212.29
2.04	10.37	3.11	-4.6	174.35	269.02	183.15	188.9
1.68	9.97	2.74	-4.95	204.27	300	223.77	249.67
1.54	9.87	2.6	-5.03	197.94	298.24	219.09	243.96
1.56	9.9	2.65	-5.01	189.62	288.29	208.96	226.37
1.52	9.9	2.64	-4.99	193.41	291.66	210.14	229.97
1.3	9.7	2.48	-5.4	225.89	328.57	222.01	238.53
0.97	9.3	2.25	-5.41	245.56	347.79	263.18	311.25
1.39	9.7	2.61	-5.09	222.48	323.67	224.49	244.81
1.11	9.47	2.32	-5.43	235.93	336.73	249.89	285.69
1.91	10.34	3.01	-4.7	204.79	302.98	214.26	232.52
1.37	9.78	2.58	-5.11	224.17	324.06	232.93	260.93
1.7	9.97	2.81	-5.1	215.9	323.94	204.27	206.55
1.08	9.45	2.48	-5.32	240.58	348.27	244.62	269.91
1.39	9.8	2.58	-5.35	224.32	327.34	222.36	240.67
1.07	9.45	2.41	-5.4	237.4	345	244.5	270.65
1.47	9.84	2.68	-5.24	228.24	330.75	232.16	256.57
1.64	9.94	2.86	-4.81	209.35	308.01	230.12	263.54
1.48	9.8	2.76	-4.9	214.07	314.24	236.97	273.83
1.33	9.8	2.57	-4.86	190.77	290.38	208.46	219.28
1.13	9.56	2.38	-5.08	203.83	307.57	223.99	246.39
1.1	9.55	2.35	-5.15	204.33	308.21	225.44	251.32
1.21	9.66	2.42	-5.09	202.32	305.45	221.67	245.35
3.45	11.89	4.24	-3.76	156.6	248.83	147.87	136.65
2.88	11.14	3.68	-4.07	168.01	260.41	154.35	144.07
3.34	11.63	4.13	-3.9	144.7	231.9	123.78	98.84
2.62	10.87	3.56	-4.48	155.85	245.38	129.35	103.08

3.23	11.71	4.01	-3.86	159.85	248.83	158.6	151.76
3.34	11.81	4.12	-3.85	159.8	250.21	151.06	139.13
3.28	11.78	4.08	-3.89	161.01	250.74	152.27	140.92
3.06	11.39	3.78	-4.04	162.4	248.97	159.22	153.57
3.06	11.37	3.77	-4.06	162.81	248.87	159.87	153.54
2.95	11.32	3.74	-4.07	170.5	255.68	174.56	175.59
2.64	11.01	3.45	-4.25	168.34	259.39	176.68	178.74
2.65	11.01	3.45	-4.24	168.25	259.34	176.62	178.58
2.65	11.02	3.45	-4.24	168.23	259.33	176.6	178.54
3.43	11.93	4.15	-3.86	156.98	248.91	152.07	143.24
3.34	11.63	4.2	-3.91	146.31	230.52	121.59	98.12
3.42	11.72	4.16	-3.81	144.46	230.57	123.1	100.16
3.3	11.61	4.18	-3.9	143.82	229.33	119.14	95.24
3.35	11.63	4.3	-3.65	139.91	225.8	113.95	87.48
3.44	11.65	4.34	-3.53	136.37	223.22	111.93	83.94
2.47	10.78	3.57	-4.4	152.71	243.83	130.07	106.69
2.96	11.23	4.04	-3.94	141.26	225.32	118.45	92.9
2.71	10.98	3.81	-4.17	146.93	233.19	124.16	98.96
2.96	11.22	3.93	-3.98	143.61	227.82	118.27	92.9
2.9	11.17	3.86	-4.08	147.94	232.6	125.59	101.16
3.01	11.33	4.02	-3.93	147.39	229.9	126.33	105.35
2.65	11	3.68	-4.26	151.67	237.18	130.04	109.44
2.83	11.11	3.81	-4.1	147.56	232.46	125.08	101.07
2.66	10.91	3.67	-4.44	143.27	232.76	120.77	87.54
2.17	10.46	3.23	-4.77	155.87	247.04	130.41	98.65
2.37	10.65	3.38	-4.64	152.2	241.26	125.76	93.06
2.38	10.68	3.45	-4.7	149.21	241.35	127.12	96.64
2.58	10.91	3.6	-4.52	150.44	240.46	126.34	96.22
2.7	11	3.56	-4.43	151.8	241.99	127.52	102.71
2.67	10.97	3.58	-4.52	150.25	239.49	125.56	97.59
2.85	11.16	3.71	-4.38	147.11	235.04	121.79	93.74
2.32	10.61	3.44	-4.68	153.55	244.05	129.69	99.29
2.85	11.12	3.77	-4.19	145.81	233.28	119.75	92.26
2.42	10.66	3.46	-4.66	151.55	241.6	126.95	99.09
2.28	10.45	3.44	-4.69	148.54	239.14	124.68	93.29
2.33	10.58	3.45	-4.64	152.81	245.94	127.4	100.66
2.54	10.81	3.58	-4.53	152.07	240.15	125.4	97.95

2.18	10.45	3.32	-4.79	157.42	248.28	131.18	104.21
2.24	10.47	3.36	-4.76	156.26	247.29	130.19	102.97
3.25	11.47	4.16	-3.84	136.43	224.81	111.74	82.17
3.07	11.32	4	-3.95	138.98	227.65	115.03	86.21
3.01	11.24	3.9	-4.01	141.08	229.44	115.85	87.53
3.23	11.46	4.05	-3.84	139.07	225.44	112.13	84.02
2.98	11.24	3.89	-4.02	139.71	230.4	115.8	88.01
2.99	11.21	3.98	-4.03	140.29	228.22	115.68	86.32
3.35	11.55	4.17	-3.76	134.19	222.34	110.19	80.09
2.51	10.81	3.54	-4.39	148.71	239.88	126.21	100.91
2.93	11.14	3.91	-4.16	140.22	227.31	114.87	84.59
2.68	10.92	3.72	-4.34	144.06	233.42	119.67	89.93
2.66	10.91	3.7	-4.36	144.07	233.61	119.99	90.21
2.54	10.76	3.67	-4.43	146.23	236.23	122.01	91.07
2.52	10.73	3.58	-4.47	145.95	234.94	122.42	92.4
2.54	10.75	3.64	-4.45	145.8	235.25	121.67	91.3
2.27	10.45	3.44	-4.7	148.55	239.24	124.75	93.36
2.33	10.51	3.46	-4.66	148.25	237.34	123.64	92.36
2.09	10.32	3.33	-4.79	152.72	244.42	129.26	98.4
2.1	10.32	3.33	-4.79	152.6	244.28	129.12	98.12
2.07	10.3	3.3	-4.81	153.95	245.63	130.37	99.48
2.36	10.57	3.48	-4.58	148.5	238.48	124.84	94.79
2.49	10.71	3.57	-4.51	148.44	236.35	124.45	93.14
2.58	10.81	3.64	-4.43	146.21	233.66	121.63	91.12
3.15	11.37	4.06	-3.85	141.81	229.23	117.08	88.12
2.61	10.94	3.62	-4.35	149.53	238.22	123.48	97.98
2.73	10.99	3.7	-4.27	148.71	234.64	121.3	95.78
2.81	11.17	3.84	-4.17	151.15	233.76	130.18	111.11
2.52	10.87	3.52	-4.45	157.55	238.73	135.75	117.66
2.63	10.98	3.58	-4.4	155.2	237.76	133.77	115.69
2.45	10.84	3.49	-4.51	156.22	240.58	139.09	121.57
2.44	10.83	3.49	-4.53	156.11	240.67	139.47	121.84
2.36	10.73	3.39	-4.56	158.75	240.73	138.95	121.53
2.37	10.74	3.4	-4.56	158.61	240.54	138.79	121.34
2.49	10.86	3.49	-4.48	157.23	238.6	137.47	119.7
2.48	10.85	3.48	-4.49	157.31	238.84	137.66	119.89
2.79	11.16	3.71	-4.31	153.9	234.37	133.29	114.89

2.62	11.01	3.6	-4.4	155.72	237.43	136.12	118.19
2.78	11.15	3.72	-4.3	153.77	235.18	133.67	115.74
2.73	11.12	3.79	-4.2	152.11	234.61	131.49	112.4
2.43	10.85	3.52	-4.49	155.06	239.44	137.79	120.89
2.22	10.56	3.29	-4.58	161.3	245.02	138.98	120.92
2.96	11.26	3.96	-3.93	147.67	231.39	126.11	105.09
3.16	11.49	4.09	-4	147.12	230.81	122.56	97.05
3.04	11.36	3.98	-4.09	147.34	232.86	122.17	98.24
2.78	11.18	3.69	-4.3	152.28	235.71	132.21	112.78
2.43	10.79	3.5	-4.43	156.09	240.95	134.07	113.6
2.6	11.01	3.61	-4.35	155.88	237.06	132.9	112.34
3.25	11.55	4.14	-3.94	146.56	230.34	120.96	96.75
3.05	11.34	3.96	-4.07	146.86	231.83	121.48	97.24
2.78	11.13	3.77	-4.27	151	237.47	124.69	101.54
1.9	9.92	3.26	-4.79	133.47	238.66	124.22	87.69
1.64	9.64	3.19	-4.86	135.56	242.83	125.33	88.94
1.69	9.71	3.2	-4.88	136.34	242.67	125.3	89.41
2.3	10.29	3.78	-4.27	131.31	232.29	113.61	79.98
2.79	10.83	3.87	-4.13	131.28	221.19	112.32	78.13
2.02	9.95	3.42	-4.67	134.91	230.99	116.92	83.4
2.17	10.11	3.67	-4.44	134.39	234.17	116.54	83.31
2.03	9.92	3.5	-4.69	134.89	234.27	117.07	83.47
1.94	9.87	3.44	-4.76	137.05	236.33	118.39	84.47
2.54	10.48	3.95	-4.14	125.8	225.99	110.92	75.8
2.04	9.97	3.53	-4.7	133.77	233.44	116.45	82.06
2	9.92	3.45	-4.76	133.67	232.89	117.8	82.24
2.73	10.75	3.94	-4.16	131.91	223.88	110.6	79.93
3.12	11.14	4.3	-3.67	127.21	219.04	107.52	74.22
2.46	10.4	3.61	-4.38	133.4	224.59	114.06	80.03
2.25	10.26	3.48	-4.67	132.05	232.87	120.11	85.31
2.23	10.17	3.56	-4.5	132.17	225.49	114.41	79.88
2.19	10.14	3.54	-4.52	132.47	225.96	114.5	80.26
2.26	10.19	3.57	-4.53	131.08	226	114.56	80.37
2.27	10.19	3.55	-4.54	130.19	225.92	113.76	79.75
2.64	10.62	3.84	-4.26	130.99	223.4	111.91	77.3
2.52	10.5	3.74	-4.35	133.39	223.39	113.8	79.86
2.25	10.3	3.5	-4.55	137.61	230.99	118.8	85.16

2.74	10.8	3.85	-4.19	133.5	222.69	112.16	79.9
2.51	10.57	3.67	-4.34	135.92	224.73	115.74	81.59
2.46	10.49	3.68	-4.4	134.74	226.93	115.54	81.77
2.46	10.51	3.7	-4.43	137.06	228.14	115.74	82.52
2.31	10.36	3.58	-4.54	139.46	231.79	119.28	86.16
2.37	10.42	3.62	-4.49	138.65	231.25	117.79	84.89
2.42	10.43	3.64	-4.46	138.72	228.37	116.71	83.68
2.6	10.6	3.77	-4.35	133.77	224.47	114.98	81.06
2.61	10.62	3.78	-4.36	136.26	228.9	116.43	82.75
2.29	10.34	3.57	-4.55	139.65	232.06	119.86	86.6
2.51	10.52	3.77	-4.31	132.59	223.87	113	80.93
2.46	10.48	3.64	-4.37	134.26	227.43	115.21	81.93
2.54	10.56	3.75	-4.29	132.1	225.1	113.81	79.81
2.6	10.61	3.79	-4.28	129.76	221.98	112.49	77.74
2.64	10.68	3.79	-4.22	129.67	222.57	112.63	77.63
2.63	10.64	3.84	-4.24	132.14	222.33	111.82	79.72
2.94	11.01	4.01	-4.03	134.57	223.76	111.35	79.32
2.93	10.97	4.01	-4.08	132.44	221.86	111.84	77.8
2.24	10.17	3.52	-4.58	131.05	224.9	115.06	80.36
2.25	10.18	3.51	-4.59	130.87	225.06	115.01	81.23
2.18	10.29	3.44	-4.67	143.4	235.85	120.74	90.23
2.79	10.85	3.88	-4.09	130.05	221.57	110.72	75.88
3.15	11.2	4.17	-3.87	130.64	219.82	108.34	75.39
3.3	11.47	4.18	-3.69	136.61	225.31	111.96	82.84
2.91	11.12	3.89	-4.11	140.61	229.34	115.33	86.65
3.25	11.47	4.11	-3.83	139.09	225.19	112.55	83.34
3.19	11.31	4.14	-3.96	136.2	224.22	111.99	80.42
3.09	11.13	4.23	-3.8	130.96	221.45	108.26	76.68
3.06	11.1	4.16	-3.94	130.51	220.66	108.54	76.82
3.28	11.33	4.28	-3.76	130.19	218.72	107.82	75.66
3.14	11.15	4.19	-3.9	128.53	219.07	107.56	75.74
3.19	11.21	4.22	-3.86	128.59	219.56	107.7	74.83
3.3	11.35	4.3	-3.73	129.65	219.81	108.2	75.58
2.84	10.91	3.95	-4.09	135.5	227.57	114.14	82.32
3.44	11.62	4.28	-3.67	132.61	220	108.17	77.55
3.35	11.56	4.2	-3.75	134.15	220.82	109.25	78.67
2.92	11.06	3.97	-4.15	137.87	228.81	114.23	83.83

2.56	10.69	3.73	-4.46	141.32	230.69	118.42	86.94
2.27	10.45	3.48	-4.58	138.8	231.83	122.83	88.83
2.23	10.42	3.45	-4.61	139.45	232.87	123.49	89.88
2.4	10.56	3.58	-4.52	138.09	231.25	122.3	88.29
2.12	10.29	3.38	-4.69	140.94	237.03	125.75	92.46
2.22	10.41	3.46	-4.64	139.83	232.67	124.65	90.34
2.75	11.03	3.83	-4.28	134.92	227.41	118.72	84.42
2.41	10.69	3.54	-4.59	145.04	235.71	125.13	90.61
2.35	10.48	3.48	-4.68	141.06	236.34	122.33	88.5
2.32	10.44	3.49	-4.69	140.44	235.77	123.54	89.71
2.7	10.67	3.85	-4.22	130.14	220.76	111.32	77.2
2.46	10.47	3.67	-4.43	136.49	228.32	116.36	82.52
2.63	10.63	3.75	-4.32	132.84	223.31	113.35	79.56
2.38	10.46	3.51	-4.62	136.15	230.2	117.66	84.19
2.47	10.57	3.58	-4.53	135.27	231.32	117.28	83.71
2.5	10.65	3.63	-4.5	142.36	234.21	120.91	86.74
2.35	10.5	3.5	-4.62	146.81	239.73	125.18	92.45
2.34	10.53	3.49	-4.58	136.98	231.44	120.35	85.55
2.4	10.43	3.55	-4.57	135.53	227.8	118.81	84.02
2.43	10.51	3.58	-4.55	133.99	226.94	117.51	83.07
2.14	10.24	3.38	-4.74	139.07	234.87	123.43	90.04
2.1	10.17	3.36	-4.77	138.49	234.85	123.54	89.62
2.37	10.35	3.57	-4.55	134.69	228.16	119.05	84.58
2.04	10.11	3.29	-4.8	138.26	234.41	123.66	90.19
2.94	11.06	3.98	-4.21	130.81	222.86	115.4	79.39
2.11	10.21	3.34	-4.79	139.39	234.33	123.51	89.67
2.29	10.43	3.49	-4.59	138.75	230.52	122.94	88.31
2.29	10.43	3.49	-4.59	138.88	230.57	123.16	88.43
2.37	10.51	3.54	-4.53	137.16	229.36	121.43	86.2
2.27	10.36	3.47	-4.59	133.13	227.81	120.04	85.65
2.14	10.28	3.36	-4.68	136.55	230.63	122.35	88.3
2.37	10.55	3.65	-4.54	134.75	234.9	125.58	92.11
2.88	11	4.02	-4.15	126.77	223.01	116.14	80.15
2.49	10.65	3.71	-4.4	130.46	232.57	123.28	88.27
1.72	9.68	3.22	-4.99	139.79	240.54	125.07	90
2.25	10.21	3.47	-4.67	132.63	229.86	118.97	83.82
1.57	9.63	3.04	-5.12	141.15	244.4	128.95	94.32

2.25	10.33	3.46	-4.67	131.21	230.93	119.88	84.3
2.35	10.47	3.52	-4.59	128.25	226.49	118.1	82.51
1.78	9.84	3.13	-5.03	137.49	238.3	124.89	89.45
1.85	9.85	3.21	-4.98	135.71	237.7	123.98	88.95
2.43	10.52	3.6	-4.51	134.57	229.29	120.15	86.18
1.9	9.93	3.21	-4.84	134.34	238.16	124.76	88.71
1.81	9.92	3.19	-4.84	135.37	243.49	128.27	92.87
2.27	10.38	3.59	-4.52	132.07	234.85	121.68	86.27
2.01	10.16	3.39	-4.7	135.1	240.53	126.03	91.18
2.14	10.27	3.37	-4.72	133.02	233.67	123.15	88.07
2.35	10.46	3.47	-4.62	131.27	228.57	119.79	83.81
2.13	10.27	3.43	-4.56	131.69	239.46	126.29	90.43
2.59	10.73	3.82	-4.26	128.22	232.48	122.81	86.21
2.64	10.79	3.86	-4.22	127.54	231.18	122.15	85.4
2.3	10.44	3.58	-4.52	131.15	234.62	123.67	86.73
2.03	10.16	3.37	-4.69	133.97	241.06	128.29	91.51
2.08	10.2	3.4	-4.66	133.34	239.65	128.12	91.16
2.13	10.25	3.44	-4.64	132.66	238.24	127.69	90.52
2.19	10.32	3.53	-4.63	133.06	237.9	126.72	89.71
2.15	10.26	3.48	-4.66	134.68	238.96	127.43	91.06
2.04	10.18	3.4	-4.73	136.04	241.06	129.53	92.82
2.03	10.17	3.39	-4.73	136.16	241.29	129.74	93.15
2.16	10.45	3.31	-4.82	154.96	245.93	131.82	100.86
2.57	10.62	3.68	-4.46	133.78	225.09	116.45	81.28
2.2	10.36	3.39	-4.78	149.15	241.48	127.58	94.52
2.26	10.53	3.37	-4.78	150.42	240.35	127.26	94.18
2.53	10.76	3.57	-4.52	145.29	234.9	121.01	88.95
2.39	10.6	3.55	-4.63	147.23	236.99	124.84	91.08
2.19	10.4	3.4	-4.77	150.09	242.36	129.05	95.68
2.22	10.52	3.36	-4.78	155.21	246.91	131.22	99.96
2.69	10.97	3.8	-4.36	142.05	234.71	122.44	88
2.69	10.96	3.79	-4.36	142.09	234.76	122.47	88.04
2.57	10.86	3.71	-4.44	145.07	237.4	125.18	90.67
2.57	10.86	3.71	-4.44	145.04	237.37	125.15	90.64
2.8	11.07	3.88	-4.29	139.05	230.74	119.59	84.63
2.37	10.65	3.42	-4.63	151.74	242.53	127.79	95.46
2.4	10.66	3.47	-4.64	150.62	238.73	126.67	94.18

2.62	10.89	3.69	-4.44	141.89	233.06	122.72	87.7
3.17	11.41	4.07	-3.86	143.94	227.09	117.62	91.51
2.75	11.06	3.82	-4.14	147.85	234.97	123.31	99.01
2.54	10.75	3.64	-4.45	145.8	235.22	121.64	91.28
2.6	10.84	3.67	-4.41	145.56	232.74	121.01	90.35
2.82	10.85	3.91	-4.14	131.93	222.71	112.73	78.03
2.37	10.42	3.62	-4.49	138.65	231.31	117.81	84.92
2.46	10.68	3.52	-4.65	145.27	236.22	122.48	88.78
4.85	13.04	5.85	-1.82	123.5	210.67	102	68.45
4.36	12.56	5.48	-2.18	127.76	223.02	113.08	78.1
4.19	12.58	5.28	-2.37	126.52	216.61	113.83	79.61
3.73	12.12	4.84	-2.9	129.12	217.96	118.35	88.6
3.69	11.86	4.98	-2.72	128.1	224.29	111.3	79.21
3.56	11.68	4.85	-2.83	132.8	226.65	116.36	83.73
3.86	12	5.1	-2.6	133.13	226.58	115.1	81.94
4.46	12.62	5.46	-2.17	119.63	205.13	100.22	66.65
4.21	12.34	5.14	-2.53	119.02	207.99	98.81	66.37
4.1	12.2	5.17	-2.81	116.24	206.52	98.01	64
4.37	12.54	5.31	-2.4	117.23	206.73	98.27	65
4.03	12.17	5.04	-2.83	118.78	207.53	97.57	64.52
3.85	12.18	5.02	-2.8	137.8	236.93	134.99	102.91
3.82	12.17	5	-2.82	137.49	236.47	134.82	102.81
3.67	12.05	4.78	-3	135.65	222.57	125.59	98.25
3.27	11.4	4.5	-3.95	118.1	215.07	105.33	71.3
3.38	11.52	4.56	-3.71	116.39	216.13	103.66	67.49
3.44	11.42	4.59	-3.57	117.73	219.83	104.86	66
3.83	11.96	4.95	-3.16	115.29	206.93	98.65	63.8
3.9	11.95	5.07	-2.59	135.51	233.42	121.3	88.05
4.02	12.15	5.18	-2.5	129.99	228.75	115.06	82.05
4.37	12.64	5.49	-2.16	129.04	222.72	115.51	80.78
4.1	12.26	5.31	-2.43	134.41	228.93	119.67	86
4.04	12.15	5.24	-2.5	136.14	234.54	123.82	91.42
4.52	12.79	5.63	-2.05	130.42	225.78	117.85	83.2
3.76	11.89	5	-2.74	137.49	235.87	124.97	92.92
3.97	12.03	5.16	-2.62	137.72	234.45	124.5	91.26
4.15	12.29	5.32	-2.41	134.68	233.21	124.17	91.67
3.69	11.79	4.92	-2.81	139.74	236.74	125.31	93.79

3.98	12.03	5.18	-2.55	136.08	234.51	122.19	88.36
3.6	11.68	4.84	-2.85	137.92	235.88	123.98	91
3.54	11.57	4.9	-3.07	135.29	226.64	116.72	84.85
3.53	11.56	4.9	-3.08	135.31	226.65	116.74	84.83
3.63	11.71	4.98	-3.17	129.92	220.72	112.13	80
3.25	11.35	4.66	-3.54	131.84	221.48	113.6	81.98
3.66	11.67	4.98	-2.82	141.41	238.9	123.6	91.6
3.15	11.15	4.68	-3.15	146.04	245.32	131.06	98.76
3.49	11.56	4.86	-3.03	136.82	228.99	118	87.01
3.68	11.75	5.03	-2.91	135.29	228.04	117.44	85.41
3.63	11.67	5.01	-2.99	134.83	226.94	118.11	84.98
3.64	11.63	4.98	-2.79	142.71	240.85	125.24	92.54
4.04	12.25	5.21	-2.59	143.71	242.31	137.61	105.52
4.09	12.28	5.25	-2.52	137.37	237.37	130.71	98.14
3.94	12.19	5.13	-2.63	136.22	236.2	129.28	96.37
3.61	11.84	4.83	-2.96	142.94	243.83	139.55	108.05
4.28	12.57	5.42	-2.29	132.25	232.05	123.65	89.95
3.81	12.02	5.02	-2.75	135.99	236.75	129.6	97.68
4.12	12.32	5.27	-2.5	137.01	237.67	132.22	97.76
3.66	11.74	4.89	-2.84	140.77	241.03	126.08	93.31
3.56	11.7	4.85	-2.76	149.46	256.79	139.84	107.72
3.64	11.81	4.89	-2.89	145.03	245.74	137.94	106.34
3.64	11.78	4.88	-2.92	144.32	244.86	138.25	106.2
3.71	11.85	4.93	-2.77	141.24	243.47	132.13	99.84
4.03	12.18	5.2	-2.55	139.72	237.81	128.57	95.57
3.68	11.86	4.9	-2.86	143.86	244.4	135.76	104.61
4.16	12.3	5.31	-2.45	139.74	237.97	128	95.68
3.66	11.79	4.93	-2.79	141.52	244.27	132.19	99.58
3.68	11.82	4.92	-2.85	144	245.38	135.17	102.85
3.37	11.52	4.68	-3.02	144.98	247.36	134.68	103.57
3.78	11.85	4.99	-2.75	140.35	242.08	127.18	94.19
3.51	11.64	4.78	-2.94	146.43	248.09	135.5	104.69
3.55	11.75	4.81	-3	142.98	245.06	140.03	109.33
3.55	11.76	4.78	-3	143.02	243.83	139.82	108.7
3.7	11.72	4.94	-2.65	140.9	243.84	125.42	91.65
3.74	11.75	4.93	-2.68	137.17	235.26	120.47	87.6
3.7	11.75	4.88	-2.7	136.69	233.88	120.59	87.91

3.83	11.89	5	-2.66	132.99	227.79	114.99	82.46
3.21	11.34	4.6	-3.05	159.88	273.03	149.86	120.95
3.23	11.35	4.61	-3.03	158.29	269.59	147.43	118.79
3.49	11.69	4.76	-3.04	145.12	245.95	141.51	111.3
3.61	11.81	4.85	-2.95	144.53	245.86	141.36	110.87
3.61	11.81	4.85	-2.95	144.23	246.05	140.99	110.41
3.71	11.77	4.95	-2.74	139.34	241.07	124.97	91.43
3.71	11.77	4.95	-2.74	139.3	241.02	124.85	91.33
3.71	11.77	4.95	-2.74	139.23	240.93	124.67	91.21
3.55	11.76	4.78	-3	143.02	243.83	139.82	108.7
3.48	11.64	4.83	-2.92	159.78	268.85	155.03	126.86
3.3	11.47	4.64	-3.08	154.97	263.22	154.98	127.3
3.09	11.12	4.57	-3.56	141.42	230.93	124.52	93.05
3.15	11.15	4.64	-3.25	148.39	246.25	132.27	100.07
3	11	4.54	-3.46	145.67	240.21	129.95	98.72
3	11.01	4.56	-3.17	161.52	266.69	145.7	116.76
2.77	10.8	4.36	-3.37	155.16	259.51	142.89	113.13
2.63	10.64	4.27	-3.6	150.48	249.43	135.57	106.27
2.79	10.8	4.38	-3.51	148.72	247.36	133.9	103.16
2.81	10.82	4.39	-3.58	146.99	243.62	132.63	101.18
2.84	10.87	4.4	-3.49	146.81	243.97	131.58	100.22
3.05	11.06	4.55	-3.4	145.22	239.59	128.45	96.99
3.18	11.24	4.68	-3.55	140.49	229.31	123.12	92.59
2.72	10.81	4.29	-3.92	140.8	229.65	123.73	94.48
2.71	10.75	4.31	-3.85	142.79	233.02	126.57	96.57
3.52	11.54	4.88	-3.31	134.02	223.85	116.27	84.77
3.54	11.56	4.92	-3.27	133.46	222.37	116.04	84.02
3.45	11.52	4.86	-3.39	133	223.3	115.92	84.24
3.12	11.16	4.59	-3.62	138.04	228.42	120.61	90.53
3.2	11.29	4.66	-3.79	132.19	224.32	116.55	85.22
3.21	11.28	4.59	-3.61	132.25	220.18	113.3	82
3.68	11.71	4.93	-2.66	141.13	244.14	125.63	91.85
3.67	11.7	4.91	-2.67	141.05	243.88	125.59	91.79
3.31	11.37	4.75	-2.92	148.02	254.14	133.9	101.2
2.92	10.91	4.48	-3.33	150.31	251.86	134.9	103.88
3.9	12.05	5.18	-2.48	157.76	265.53	177.97	150.06
3.56	11.75	4.89	-2.76	156.6	261.82	170.33	144.37

2.79	11.02	4.34	-3.52	160.28	261.28	159.74	135.94
3.63	11.82	4.99	-2.73	157.62	262.45	167.37	141.95
3.27	11.48	4.73	-3.05	159.51	264.01	167.05	143
2.66	10.91	4.21	-3.65	158.99	259.56	158.43	134.73
2.96	11.13	4.58	-3.2	164.23	264.6	167.86	149.18
2.38	10.41	3.59	-3.75	121.53	214.08	134.17	112.4
2.64	10.76	3.87	-3.66	143.4	248.8	152.23	127.47
2.47	10.67	3.77	-3.65	131.65	237.04	145.09	119.25
2.35	10.56	3.69	-3.62	131.66	229.86	147.32	121.65
3.47	11.73	4.7	-3	160.08	256.36	152.93	135.32
4.09	12.47	5.21	-2.6	161.18	250.76	150.54	133.04
3.52	11.87	4.61	-3.17	146.73	238.04	136.58	117.17
2.62	10.79	4.33	-3.42	167.91	263.9	168.73	155.96
3.29	11.42	4.84	-2.79	165.3	249.86	172.85	167.92
3.22	11.43	4.78	-3.02	162.04	262.11	186.59	171.96
3.05	11.2	4.65	-2.96	166.53	255.94	170.6	161.3
2.78	10.97	4.45	-3.23	165.32	259.3	171.4	160.91
2.83	11.01	4.42	-3.34	164.06	261.36	176.08	161.74
3.14	11.37	4.7	-3.08	162.04	261.22	183.11	170.21
2.84	11.1	4.43	-3.36	162.31	261.15	179.89	166.04
2.93	11.17	4.5	-3.31	162.33	262.62	181.39	165.11
2.72	10.91	4.37	-3.36	167.56	263.88	169.68	157.15
2.97	11.15	4.59	-3.02	166.84	257.61	173.68	164.99
3.02	11.21	4.65	-3.04	164.48	257.8	173.37	166.58
2.74	10.92	4.4	-3.28	165.78	260.33	171.57	160.04
2.91	11.12	4.44	-3.33	161.6	262.3	179.02	163.2
3.11	11.14	4.81	-2.84	164.54	262.61	144.94	122.37
3.13	11.18	4.84	-2.83	164.46	264.36	145.35	121.29
3.16	11.15	4.86	-2.86	160.96	263.84	144.7	117.63
3.04	11.05	4.69	-3.02	159.24	257.55	140.85	114.21
3.01	11.03	4.68	-3.04	159.03	257.33	140.79	114.25
3.12	11.13	4.76	-2.94	160.06	258.46	141.08	114.09
3.19	11.23	4.88	-2.79	164.9	265.69	146.12	121.03
2.42	10.45	4.19	-3.54	157.83	250.04	139.33	114.1
2.61	10.71	4.35	-3.63	155.76	238.15	137.91	118.78
2.72	10.74	4.44	-3.3	158.65	259.7	146	120.2
2.98	10.97	4.67	-3.07	157.89	258.65	142.27	116.78

1.19	9.31	3.1	-5.26	153.09	242.08	138.38	113.41
2.25	10.33	4.02	-4.16	148.72	238.24	131.84	104.86
2.42	10.53	4.24	-3.78	151.47	245.18	135.38	108.81
1.54	9.62	3.32	-5.11	151.59	242.34	135.5	109.49
1.12	9.28	2.96	-5.53	154.74	245.67	137.4	112.23
1.4	9.58	3.17	-5.49	152.38	244.24	134.81	108.18
2.59	10.71	4.16	-4.13	141.23	229.52	124.75	95.03
2.63	10.71	4.11	-4.31	139.29	230.1	123.8	93.88
1.28	9.45	3.04	-5.48	151.56	243.81	135.67	109.65
2.06	10.19	3.68	-4.95	143.99	234.78	129.47	100.37
1.6	9.75	3.33	-5.21	149.04	240.74	133.14	106.54
2.31	10.4	3.91	-4.62	144.61	234.94	128.16	99.22
2.36	10.46	3.94	-4.63	143.05	233.45	127.77	97.7
1.56	9.71	3.3	-5.09	146.45	238.39	132.96	106.48
1.97	10.09	3.63	-4.82	145.96	236.98	129.54	102.42
2.65	10.75	4.22	-4.03	141.32	229.87	124.9	95.06
1.87	10.05	3.49	-5.38	140.9	235.35	128.24	98.89
1.61	9.81	3.3	-5.34	144.24	234.92	130.69	102.9
2.38	10.49	3.98	-4.6	143.42	233.9	128.01	97.41
1.74	9.92	3.39	-5.47	142.66	236.46	129.63	100.3
1.68	9.86	3.35	-5.51	143.36	237.25	130.1	100.91
1.73	9.91	3.38	-5.48	142.76	236.56	129.78	100.45
1.74	9.92	3.39	-5.47	142.47	236.28	129.66	100.27
2.33	10.44	3.81	-5.04	138.19	230.87	124.31	93.54
1.9	10.06	3.48	-5.31	142.34	234.77	127.53	98.89
1.69	9.86	3.38	-5.48	145.94	240.2	129.67	101.68
1.53	9.71	3.24	-5.57	146.79	241.9	131.25	103.39
1.74	9.92	3.38	-5.47	142.82	236.7	129.62	100.37
1.46	9.63	3.19	-5.62	147.61	243.35	132.18	104.09
1.35	9.56	3.08	-5.63	148.68	241.57	131.57	105.99
1.67	9.84	3.36	-5.4	148.17	239.01	130.44	102.92
1.83	9.94	3.47	-5.22	147.22	238.01	129.84	102.21
1.83	9.96	3.48	-5.25	146.88	236.63	129.66	102.22
2.54	10.68	4.02	-4.41	135.94	228.06	121.7	91.18
2.47	10.57	3.98	-4.54	141.59	229.4	124.5	95.07
1.77	9.94	3.44	-5.2	143.58	233.3	129.5	101.17
1.52	9.72	3.2	-5.41	145.24	235.41	131.41	103.67

1.52	9.72	3.2	-5.41	145.22	235.42	131.41	103.68
1.82	9.99	3.44	-5.21	143.16	233.6	128.45	100.23
2.53	10.63	3.98	-4.78	136.44	227.97	122.55	92.1
2.42	10.54	3.9	-4.84	137.15	228.07	122.55	93.47
2.35	10.48	3.84	-4.88	137.19	228.48	122.76	94.36
2.14	10.29	3.67	-4.92	138.19	229.82	124.49	96.23
2.26	10.4	3.78	-4.83	137.51	228.67	123.68	94.77
2.32	10.47	3.84	-4.75	137.61	228.7	123.11	93.77
2.18	10.32	3.7	-4.92	137.81	231.69	124.24	93.6
2.26	10.41	3.76	-4.94	136.49	230.48	123.41	93.15
2.16	10.31	3.69	-5.1	139.19	231.41	125.88	95.42
2.41	10.54	3.88	-4.88	135.82	229.37	122.13	91.86
2.3	10.44	3.78	-4.92	136.12	229.94	123.13	92.52
2.23	10.36	3.72	-4.98	135.38	229.47	122.9	92.89
2.3	10.45	3.82	-4.96	131.95	228.73	120.22	89.8
2.1	10.27	3.66	-5.16	137.04	231.01	122.49	92.98
2.43	10.61	3.86	-5.1	130.19	231.25	119.3	84.55
2.27	10.44	3.79	-5.23	132.2	233.24	121.52	88.29
2.45	10.62	3.91	-5.1	130.51	230.27	118.76	86.09
2.59	10.73	3.98	-5	130.4	228.8	117.14	84.8
2.47	10.61	3.88	-4.99	131.84	228.15	117.36	86.43
2.73	10.82	4.13	-4.67	129.68	228.6	116.81	82.64
2.71	10.8	4.11	-4.69	129.96	228.77	117.04	82.81
2.35	10.51	3.85	-5.26	134.98	230.42	120.86	88.32
2.34	10.51	3.84	-5.24	134.14	230.38	120.69	88.1
2.44	10.63	3.9	-5.11	130.84	229.9	119.02	86.75
2.36	10.54	3.86	-5.16	131.46	231.47	119.7	87.05
1.36	9.47	3.11	-5.79	147.6	248.96	133.52	104.02
2.27	10.42	3.8	-5.37	137.05	233.25	123.07	90.13
2.5	10.68	3.97	-5.04	129.66	229.13	118.32	84.76
2.2	10.37	3.73	-5.11	133.17	231.79	120.36	89.67
2.22	10.37	3.75	-5.06	133.05	230.87	120.06	88.89
2.25	10.46	3.74	-5.24	133.38	230.09	122.1	90.23
2.21	10.41	3.75	-5.24	135.73	233.47	123.06	91.8
2.2	10.37	3.7	-5.35	134.05	231.72	121.95	90.11
2.4	10.56	3.89	-5.14	130.29	232.15	119.73	86.22
2.5	10.66	3.95	-5.06	130.16	229.79	118.22	85.41

2.11	10.2	3.65	-5.33	137.99	241.09	126.74	92.91
2.08	10.22	3.64	-5.33	134.8	241.15	124.59	91.01
2.16	10.3	3.68	-5.26	133.94	238.92	123.96	88.83
2.05	10.16	3.62	-5.35	134.95	240.18	125.68	90.73
2.34	10.45	3.85	-5.1	134.71	239.51	121.55	86.39
2.46	10.63	3.88	-5.11	129.13	230.59	119.05	84.11
2.49	10.66	3.91	-5.07	128.99	229.93	118.52	83.7
2.5	10.68	3.97	-5.03	129.52	228.28	118.57	84.05
2.67	10.81	4.05	-4.87	128.39	226.11	116.24	83.37
2.67	10.81	4.05	-4.86	128.34	226.14	116.17	83.21
2.4	10.57	3.83	-5.05	132.15	228.97	117.95	87.25
2.49	10.63	3.92	-4.93	130.45	228.87	116.99	85.03
2.6	10.75	4.04	-4.88	129.12	227.27	117.22	84.67
2.69	10.84	4.14	-4.77	127.77	227.18	115.92	81.96
2.54	10.68	4.01	-4.97	127.41	230.11	118.02	83.5
2.92	10.99	4.23	-4.44	122.7	226.65	112.04	74.09
2.34	10.48	3.85	-4.89	133.49	227.59	120.08	89.09
2.78	10.89	4.1	-4.56	124.57	225.92	113.73	78.65
2.36	10.48	3.84	-4.93	131.94	230.67	119.5	86.48
2.54	10.66	4	-4.53	133.88	227.09	119.84	89.51
2.4	10.51	3.84	-4.67	134.18	228.1	119.39	88.99
2.48	10.6	3.94	-4.72	131.55	225.68	119.23	88.28
2.59	10.7	4.01	-4.59	130.58	224.7	118.43	87.62
2.99	11.09	4.23	-4.34	122.21	223.5	111.84	74.22
2.66	10.76	4.09	-4.69	130.36	227.46	116.9	83.06
2.26	10.36	3.77	-4.94	133.55	233.26	120.99	87.61
2.48	10.57	3.94	-4.8	131.09	230.9	119.29	85.17
2.86	10.97	4.2	-4.45	122.88	224.47	113.06	77.85
2.98	11.07	4.28	-4.38	122.3	223.42	112.52	76.95
3.06	11.17	4.41	-4.19	128.06	221.81	113.24	81.37
2.94	11.03	4.31	-4.43	128.16	223.52	113.69	81.06
2.86	10.95	4.25	-4.53	129.45	224.63	114.66	82.16
2.64	10.75	4.05	-4.58	132.49	225.94	117.4	86.39
2.5	10.62	3.92	-4.68	133.28	227.2	117.93	87.08
2.48	10.62	3.94	-4.73	134	226.55	118.89	87.58
2.53	10.65	3.97	-4.62	133.65	226.78	117.99	86.97
2.98	11.09	4.35	-4.24	129.65	222.82	114.77	81.99

2.25	10.4	3.76	-4.98	134.89	229.49	120.15	88.9
2.11	10.29	3.65	-5.1	134.5	230.27	121.88	90.62
2.15	10.3	3.67	-5.21	133.46	239.24	123.54	88.27
2.06	10.22	3.61	-5.24	135.59	241.36	123.78	89.18
1.91	10.07	3.48	-5.36	133.1	239.3	123.7	89.15
2.1	10.24	3.63	-5.18	131.33	238.05	121.89	86.06
2.08	10.23	3.62	-5.24	134.94	241.08	123.93	89.08
2.22	10.34	3.74	-5.13	133.94	239.93	122.7	86.79
2.14	10.28	3.69	-5.21	136.4	241.28	123.4	88.87
2.24	10.36	3.79	-5.13	133.65	240.05	123.29	87.37
2.41	10.52	3.9	-5.03	131.12	237.15	120.08	83.03
2.51	10.58	3.92	-4.84	127.93	236.6	118.22	80.68
2.62	10.68	4.01	-4.73	126.47	233.67	116.81	78.76
2.36	10.44	3.87	-4.73	127.6	239.56	119.4	80.43
2.24	10.3	3.73	-4.8	129.06	243.2	120.77	81.81
2.07	10.16	3.6	-4.9	130.6	245.97	122.63	83.98
2.77	10.8	4.1	-4.61	124.57	231.02	114.78	77.06
2.43	10.49	3.89	-4.89	129.22	235.68	119.46	82.77
2.12	10.32	3.67	-5.41	137.4	233.37	123.41	91.45
2.03	10.24	3.59	-5.28	133.33	238.92	123.49	88.79
1.75	9.86	3.43	-5.61	139.51	244.33	127.42	93.81
1.56	9.72	3.27	-5.66	139.92	246.26	129.43	97.24
1.56	9.72	3.27	-5.67	139.9	246.33	129	97.15
1.67	9.79	3.35	-5.64	138.96	244.07	128.2	95.39
1.67	9.8	3.35	-5.64	139.12	244.19	128.13	95.4
2.14	10.25	3.7	-5.3	135.76	239.74	123.57	89.27
2.32	10.43	3.83	-5.11	134.55	239.81	121.91	86.5
1.65	9.74	3.25	-5.1	137.16	256.37	129.29	89.4
1.59	9.67	3.31	-5.57	142.09	251.19	131.23	95.52
1.97	10.01	3.56	-5.13	134.62	248.28	125.57	87.19
1.81	9.85	3.45	-5.2	136.16	252.45	125.42	89.06
1.7	9.74	3.35	-5.26	137.25	254.26	126.98	91.48
1.76	9.78	3.4	-5.25	137.46	253.39	126.59	90.74
1.5	9.58	3.21	-5.4	137.54	254.98	129.88	93.79
2.1	10.2	3.69	-5.23	135.77	243.36	126.1	88.39
1.89	9.94	3.45	-5.04	132.56	249.23	124.77	85.51
1.93	10	3.52	-5.06	133.7	250.01	124.63	86.69

2.06	10.11	3.6	-4.98	131.78	245.23	122.8	83.81
2.22	10.29	3.7	-4.75	130.74	244.04	121.13	80.83
2.04	10.12	3.54	-4.83	132.32	248	123.95	82.44
1.3	9.34	3.02	-5.23	143.19	265.75	131.42	92.92
1.11	9.15	2.89	-5.33	146.06	271.99	134.77	97.1
1.68	9.63	3.25	-4.81	135.17	257.98	127.96	85.44
1.32	9.46	2.88	-5.25	140.86	265.75	135.98	96.22
1.82	9.84	3.38	-4.65	134.32	257.05	127.11	84.79
1.56	9.66	3.08	-5.06	139.15	260.94	132.59	91.27
1.56	9.67	3.06	-5.07	137.92	260.21	131.91	91.47
1.43	9.49	3.08	-4.96	136.45	262.27	131.6	90.21
1.51	9.53	3.11	-4.92	135.25	259.56	129.97	87.1
1.48	9.56	3.05	-5.07	139.07	265.51	134.78	93.34
1.18	9.25	2.94	-5.38	141.44	264.58	131.89	95.22
2.08	10.13	3.66	-4.8	132.74	249.44	122.28	82.49
3.06	11.18	4.4	-4.25	123.24	224.34	110.03	72.74
1.91	10.02	3.51	-4.93	133.03	247.92	123.45	86.42
2.22	10.3	3.76	-4.7	129.76	243.27	121.09	82.18
2	10.03	3.57	-4.78	133.51	250.66	121.08	81.69
1.9	9.92	3.45	-4.84	135.6	252.95	122.71	83.86
2.47	10.49	3.95	-4.47	127.06	239.86	115.32	76.14
1.42	9.46	3.11	-5.23	139.3	259.44	129.26	91.85
2.51	10.47	3.95	-4.17	126.25	238.75	116.73	76.33
2.34	10.3	3.8	-4.46	129.53	245.95	117.45	76.44
1.67	9.67	3.21	-4.9	136.22	256.63	124.32	84.83
2.09	10.04	3.53	-4.59	133.06	249.68	119.51	78.98
1.68	9.66	3.2	-4.88	135.53	256.13	124.34	84.06
1.73	9.72	3.27	-4.77	136.49	258	127.79	84.25
1.95	9.9	3.42	-4.62	134.66	253.32	124.01	80.06
1.83	9.78	3.33	-4.72	135.33	256.28	125.22	82.71
1.84	9.78	3.33	-4.72	135.3	256.23	125.17	82.67
2.45	10.36	3.88	-4.23	128.81	245.58	118.46	77.3
1.15	9.31	2.83	-5.29	143.49	270.94	137.34	99.34
1.54	9.65	3.09	-5.02	139.21	261.18	132.71	91.14
1.89	9.93	3.33	-4.8	134.46	252.33	127.19	84.45
2.27	10.22	3.67	-4.38	128.71	246.36	119.38	77.88
2.32	10.26	3.64	-4.4	128.58	241.41	117.38	74.62

2.3	10.25	3.63	-4.4	128.66	241.51	117.53	74.86
2.09	10.08	3.51	-4.53	129.31	246.07	120.76	79.58
2.07	10.05	3.48	-4.56	129.45	246.59	121.18	79.91
2.27	10.27	3.66	-4.37	126.65	242.3	117.62	75.67
2.16	10.11	3.59	-4.45	130.33	248.26	121.42	78.45
1.88	9.9	3.42	-4.66	134.03	255.15	125.94	82.98
1.26	9.4	2.93	-5.15	141.82	268.64	135.64	96.63
1.29	9.44	2.95	-5.14	142.02	267.95	134.98	96.24
1.93	9.98	3.39	-4.69	133.29	251.75	125.18	83.11
0.87	9.04	2.54	-5.45	147.8	282.57	144.99	103.99
2.3	10.55	3.8	-4.19	151.81	283.85	148.98	104.87
2.67	10.9	4.08	-3.87	146.74	275.37	144.73	98.54
1.85	10.07	3.38	-4.65	154.96	289.15	151.65	108.04
1.62	9.8	3.11	-4.91	141.48	271.66	137.95	95.4
1.8	9.94	3.24	-4.82	141.79	268.4	135.9	92.08
1.32	9.51	2.82	-5.16	145.56	273.76	139.34	97.24
0.96	9.14	2.5	-5.38	147.68	284.82	144.37	102.66
1.17	9.35	2.68	-5.26	145.74	280.64	142.3	101.33
1.2	9.41	2.68	-5.22	144.69	275.98	139.87	98.82
1.4	9.5	2.99	-5.06	141.77	272.38	137.53	94.85
1.41	9.63	2.96	-5.07	144.38	276.24	141.41	99.75
1.43	9.53	2.91	-5.05	142.74	273.76	137.23	94.06
1.75	9.89	3.18	-4.81	140.99	267.68	134.33	90.26
0.65	8.94	2.42	-5.55	159.88	304.82	160.2	125.07
2.59	10.74	3.85	-4.32	134.23	252.76	131.54	87.32
2.16	10.29	3.84	-3.94	142.62	276.11	137.38	96.21
2.43	10.52	4.06	-3.73	141.63	271.83	135.32	92.99
2.83	10.89	4.35	-3.48	138.24	262.3	130.86	86.36
1.22	9.55	2.97	-4.82	151.58	292.04	146.54	110.26
2.35	10.49	3.94	-3.92	144.24	273.63	138.7	96.12
2.22	10.38	3.57	-4.52	138.49	263.93	132.41	89.57
1.65	9.82	3.13	-4.9	142.36	272.14	138.82	95.9
2.36	10.46	3.71	-4.4	130.62	251.93	125.33	82.41
2.34	10.47	3.68	-4.41	136.38	260.62	130.79	87.35
2.42	10.53	3.75	-4.35	136.41	260.62	133.35	87.88
2.58	10.68	3.91	-4.2	135.72	259.25	132.23	86.91
2.66	10.74	3.94	-4.17	133.63	254.86	129.72	83.12

2.19	10.37	3.57	-4.45	135.92	259.42	131.58	87.93
2.65	10.74	3.95	-4.18	133.65	251.84	126.65	81.87
2.22	10.39	3.6	-4.42	135.87	258.54	130.66	86.92
1.78	9.91	3.22	-4.78	140.73	266.97	133.8	89.68
2.75	10.84	3.99	-4.16	133.03	251.63	129.6	84.7
1.7	9.84	3.21	-4.85	141.43	271.1	137.95	95.39
2.44	10.52	3.78	-4.34	130.38	251.04	124.54	81.31
1.86	9.99	3.33	-4.72	138.27	265.58	134.8	92.14
2.18	10.31	3.57	-4.54	131.64	254.16	127.71	85.17
2.02	10.17	3.43	-4.66	138.86	267.95	135.56	92.34
2.53	10.62	3.83	-4.27	135.2	258.11	131.59	85.72
2.69	10.77	3.96	-4.15	133.48	254.3	129.38	82.8
2.73	10.86	3.98	-4.15	131.59	250.24	127.41	82.72
2.57	10.69	3.87	-4.22	134.92	254.33	128.77	83.24
2.72	10.83	3.99	-4.1	133.57	250.03	126.85	81.03
2.88	10.96	4.08	-4.02	125.77	242.4	123.18	77.01
2.75	10.87	3.98	-4.07	127.92	246.19	124.17	79.03
2.48	10.62	3.8	-4.28	131.28	252.4	127.89	83.53
2.48	10.62	3.81	-4.28	134.93	254.81	129.28	84.46
2.64	10.76	3.9	-4.33	138.93	255.98	133.98	89.28
2.95	11.05	4.11	-4.11	130.41	246.98	129.64	83.99
3.11	11.23	4.27	-3.93	129.34	242	126.94	81.25
3.12	11.23	4.28	-3.92	129.22	241.76	126.82	81.05
3.19	11.29	4.34	-3.84	125.15	237.74	122.13	77.94
3.19	11.29	4.35	-3.85	126.26	238.24	122.98	78.49
2.75	10.88	3.98	-4.2	129.47	247.03	127.98	84.05
2.57	10.72	3.84	-4.28	130.68	250.16	128.51	84.2
2.49	10.59	3.8	-4.36	140.48	260.83	135.64	91.92
2.93	11.05	4.09	-4.14	134.21	248.47	129.28	84.51
2.05	10.18	3.46	-4.67	146.08	270.68	142.23	99.44
2.92	11.03	4.08	-4.15	131.31	247.55	127.75	82.72
2.94	11.06	4.1	-4.14	131.32	247.11	128.15	82.83
2.29	10.44	3.9	-3.96	144.8	274.87	139.36	97.05
3.12	11.23	4.28	-3.92	129.22	241.76	126.82	81.04
2.69	10.83	3.94	-4.25	130.06	249.03	129.13	85.04
3.36	11.45	4.33	-3.92	139.96	253.92	138.43	90.11
3.54	11.69	4.47	-3.7	134.15	246.09	135.37	86.92

3.3	11.4	4.29	-3.97	139.23	254.2	138.53	90.33
3.35	11.46	4.34	-3.9	139.6	253.16	138.31	89.65
2.63	10.87	4.02	-3.86	147.12	277.88	146.5	99.9
2.54	10.78	3.94	-3.93	148.24	279.32	148.21	101.66
2.35	10.58	3.77	-4.19	150.48	282.76	148.82	103.51
2.46	10.72	3.88	-3.99	150.1	280.87	149.72	102.56
1.18	9.45	2.72	-5.41	157.01	295.47	159.88	120.9
1.2	9.46	2.73	-5.4	156.91	295.09	159.63	120.57
1.19	9.5	2.71	-5.33	151.47	287.62	154.72	113.7
2.21	10.43	3.67	-4.33	151.2	284.98	149.47	103.93
1.99	10.25	3.5	-4.64	155.47	285.46	152.44	108.15
2.05	10.29	3.55	-4.48	149.65	282.88	149.13	105.06
1.6	9.84	3.09	-5	152.46	285.19	151.46	109.2
2.41	10.59	3.78	-4.13	152	282.69	149.18	102.15
2.41	10.65	3.67	-4.5	164.4	292.73	159.24	110.32
2.85	11.03	4.15	-3.86	157.56	280.82	150.42	98.9
1.93	10.19	3.45	-4.61	155.44	288.11	154.6	110.02
2.69	10.89	4.01	-3.88	148.79	276.27	146.73	97.91
3.16	11.3	4.34	-3.6	155.26	271.12	146.45	92.92
2.84	11.02	4.22	-3.9	166.35	288.93	156.91	102.48
3.23	11.38	4.45	-3.66	158.64	277.94	150.67	95
3.23	11.38	4.45	-3.67	158.75	278.18	150.82	95.14
3.29	11.43	4.49	-3.61	157.55	275.42	149.23	93.46
2.93	11.11	4.13	-3.72	149.62	273.11	146.29	95.01
2.34	10.59	3.76	-4.24	161.14	290.22	156.92	108.29
2.76	10.95	4.08	-3.91	162.63	289.11	155.19	102.24
2.61	10.83	3.95	-4.02	164.9	293.17	156.94	106.94
2.41	10.64	3.87	-4.22	168.28	300.46	162.24	112.18
2.41	10.65	3.87	-4.21	168.31	300.42	162.22	112.11
2.86	11.03	4.13	-3.79	151.95	274.08	147.92	95.84
2.87	11.06	4.16	-3.95	168.05	290.01	156.28	101.03
2.86	11.04	4.16	-3.94	168.09	289.86	156.43	101.17
3.27	11.42	4.46	-3.67	162.74	279.1	151.53	93.57
3.29	11.47	4.49	-3.64	162.22	278.1	150.49	94.4
3.23	11.42	4.45	-3.67	163.47	279.93	151.27	95.36
2.97	11.15	4.19	-3.89	167.65	288.49	156.36	98.91
3.26	11.46	4.36	-3.73	162.81	282.38	152.61	92.31

3.15	11.31	4.41	-3.71	159.63	281	152.14	97.14
2.99	11.17	4.21	-3.87	167.22	289.17	156.31	98.79
3.19	11.38	4.36	-3.75	164.79	282.47	152.79	94.13
3.25	11.45	4.4	-3.7	164.2	280.76	152.06	94.16
1.24	9.51	2.79	-5.3	151.78	282.81	151.9	110.66
0.97	9.26	2.56	-5.6	153.08	286.98	154.64	114.49
1.85	10.07	3.27	-4.97	147.62	273.61	147.58	105.33
1.13	9.44	2.67	-5.39	151.95	286.15	152.78	112.72
0.96	9.21	2.56	-5.52	155.62	291.95	155.43	116.12
1.26	9.53	2.8	-5.39	150.59	281.13	151.39	109.65
1.18	9.49	2.71	-5.33	150.51	286.7	154.02	113.07
0.79	9.1	2.42	-5.72	155.96	293.55	158.12	120.98
2.11	10.31	3.46	-4.75	142.84	265.19	142.44	98.37
2.02	10.24	3.43	-4.81	144.03	270.07	145.17	102.33
1.85	10.11	3.27	-4.96	148.55	276.05	148.21	105.87
1.88	10.15	3.29	-4.94	148.17	275.5	147.87	105.7
1.95	10.18	3.3	-4.9	153.13	283.4	153.92	111.52
2.59	10.74	3.86	-4.39	139.48	255.2	135.16	89.96
3.06	11.18	4.23	-4.01	134.55	248.55	131.94	85.11
3.3	11.46	4.34	-3.91	137	255.47	137.99	89.59
1.57	9.82	3	-5.19	152.99	282.61	153.75	110.38
2.48	10.7	3.8	-4.3	168.72	296.65	160.27	108.23
2.84	11.07	4.05	-4.01	166.47	291.81	157.35	101.36
2.96	11.16	4.17	-3.91	165.69	287.6	155.15	98.4
2.24	10.45	3.63	-4.38	157.67	287.14	154.1	107.92
2.94	11.15	4.14	-3.96	164.89	287.43	155.4	98.43
1.72	9.99	3.17	-4.87	161.52	291.53	158.76	113.3
1.7	9.95	3.15	-4.92	161.1	291.78	159.98	115.04
1.55	9.88	3.11	-4.99	160.42	296.51	160.68	118.28
3.06	11.24	4.21	-3.86	164.94	286.46	154.54	96.7
2	10.25	3.43	-4.73	171.45	302.73	163.47	116.85
2.07	10.34	3.47	-4.67	171.2	300.99	163.33	115.48
2.62	10.75	3.89	-4.34	139.1	256.26	134.17	89.52
1.61	9.89	3.06	-5.04	160.81	291.11	158.3	115.18
0.89	9.22	2.5	-5.63	156.22	293.49	159.52	120.52
1.74	10.02	3.27	-4.88	152.17	286.7	152.55	110.42
1.35	9.42	2.84	-4.48	150.8	257.7	163.17	157.74

1.91	10.06	3.18	-4.34	144.31	254.67	147.84	136.96
1.59	9.77	2.95	-4.56	148.76	260.22	154.71	144.74
1.74	9.92	2.98	-4.43	129.26	241.99	138.17	126.78
1.8	9.92	3.2	-4.21	137.44	248.17	145.5	129.28
1.73	9.89	3.12	-4.3	148.32	259.4	156.12	140.19
1.25	9.44	2.74	-4.69	163.35	268.66	166.39	155.82
1.4	9.6	2.81	-4.6	153.27	260.78	157.8	146.22
1.09	9.27	2.54	-4.94	158.6	267.37	163.65	155.94
1.54	9.72	2.84	-4.57	132.82	245.48	143.01	132.48
1.75	9.9	3.08	-4.44	146.71	257.97	151.28	140.94
1.78	9.94	3.17	-4.22	147.1	258.58	155.45	139.01
0.99	9.15	2.45	-4.9	147.16	259.91	157.6	149.27
1.22	9.41	2.61	-4.83	149.98	260.86	157.2	146.74
2.8	10.85	4	-3.42	135.03	237.88	143.51	118.82
1.82	9.91	3.23	-4.07	142.2	252.24	151.3	135.49
1.66	9.87	2.99	-4.41	154.87	262.4	157.65	147.38
1.45	9.7	2.86	-4.59	162.44	266.69	163.17	154.17
1.59	9.8	2.96	-4.49	155.22	261.29	159.47	146.7
1.52	9.72	2.89	-4.55	156.18	262.1	160.81	148.19
1.51	9.71	2.88	-4.55	156.32	262.04	160.72	148.25
1.5	9.71	2.87	-4.56	159.65	265.15	161.94	153.82
1.44	9.64	2.93	-4.41	151.17	264.07	161.39	149.54
1.91	10.07	3.26	-4.19	145.09	254.95	152.93	135.55
1.89	10.05	3.27	-4.14	145.34	256.26	153.66	136.02
2.57	10.63	3.8	-3.63	136.16	243.48	146.73	122.59
1.43	9.66	2.83	-4.62	158.59	264.29	163.09	151.66
1.32	9.54	2.72	-4.71	162.11	269.31	166.29	159.68
1.32	9.48	2.85	-4.49	149.49	259.29	159.12	147.3
1.62	9.81	3.07	-4.19	144.82	249.14	158.96	144.66
1.37	9.44	2.85	-4.47	150.04	256.86	162.5	156.74
1.37	9.44	2.85	-4.47	150.05	256.93	162.42	156.72
1.38	9.46	2.87	-4.46	149.4	255.9	162.21	155.86
1.28	9.46	2.66	-4.74	141.5	253.18	152.02	144.13
2.73	10.89	3.99	-4.19	127.15	237.94	124.55	85.1
2.87	11	4	-4.08	126.56	240.75	126.06	85.05
2.69	10.81	3.87	-4.23	129.43	244.28	128.63	87.39
2.08	10.28	3.41	-4.75	136.1	254.02	135.43	97.83

2.02	10.24	3.35	-4.79	136.72	254.9	136.31	98.7
2.06	10.27	3.42	-4.77	133.66	253.29	135.41	96.72
2.28	10.45	3.56	-4.61	131.92	248.99	131.73	92.55
2.77	10.93	3.91	-4.13	127.31	242.81	127.81	86.3
2.93	11.09	4.05	-4.03	123.68	239.27	125.71	84.68
2.93	11.09	4.04	-4.04	123.72	239.3	125.73	84.72
2.98	11.19	4.09	-3.97	123.49	239.38	126.55	84.46
3.13	11.36	4.16	-3.88	124.15	237.47	125.76	83.48
3.16	11.42	4.21	-3.91	121.55	221.66	115.05	78.71
3.44	11.73	4.5	-3.79	121.37	214.49	115.85	79.79
3.32	11.6	4.36	-3.82	119.66	213.37	114.3	77.15
3.46	11.77	4.52	-3.75	120.05	214.59	115.08	78.19
3.37	11.67	4.44	-3.83	121.25	217.97	117.16	80.75
3.74	12.01	4.75	-3.56	119.33	213.37	112.89	76.87
3.44	11.73	4.49	-3.77	122.52	218.05	117.81	82.61
1.86	10.1	3.25	-4.93	136.95	256.66	138.02	100.77
2.1	10.29	3.46	-4.77	132.8	251.64	133.66	95.38
2.69	10.81	3.87	-4.23	129.44	244.28	128.62	87.39
2.37	10.54	3.61	-4.45	131.64	250.84	131.95	92.47
2.6	10.53	3.97	-4.05	123.13	228.49	111.86	74.56
3.32	11.29	4.46	-3.55	115.13	216.25	104.06	65.93
3.09	11.03	4.33	-3.7	118.56	221.18	108.31	68.7
3.02	11	4.25	-3.69	116.8	220.66	107.23	68.85
3	10.98	4.23	-3.7	116.8	221.08	107.41	69.02
2.94	10.9	4.18	-3.78	117.4	222.19	108.3	70.01
2.8	10.78	4.07	-3.9	119.55	222.93	109.44	72.05
2.77	10.74	4.05	-3.93	119.98	224.3	110.32	72.32
2.22	10.2	3.6	-4.45	128.14	244.78	120.75	79.34
2.34	10.32	3.74	-4.34	128.57	241.03	120.03	78.04
2.49	10.5	3.79	-4.27	127.7	235.52	118.44	76.09
2.45	10.44	3.78	-4.31	127.78	238.06	118.78	77.17
2.46	10.46	3.79	-4.33	126.42	238.98	119.91	76.14
2.36	10.33	3.76	-4.31	127.72	241.21	119.85	77.93
2.28	10.31	3.74	-4.35	129.98	239.73	121.03	81.17
2.62	10.6	3.98	-4.14	126.03	232.94	117.52	76.03
2.64	10.61	4.02	-4.07	126.14	234.15	116.9	75.27
2.62	10.55	3.93	-4.12	124.4	236.22	115.14	72.17

2.56	10.48	3.9	-4.12	123.9	236.77	117.18	74.58
2.49	10.42	3.85	-4.19	124.96	238.15	117.81	75.65
2.48	10.41	3.85	-4.19	125.03	238.19	117.79	75.68
2.52	10.45	3.86	-4.18	125.04	238.06	117.49	74.68
2.46	10.42	3.82	-4.24	126.08	238.91	117.52	75.13
2.78	10.68	4.06	-3.98	122.48	233.08	114.59	71.91
2.49	10.42	3.85	-4.2	125.33	238.04	117.94	76.28
2.62	10.54	3.97	-4.09	126.08	236.04	117.63	75.62
2.62	10.54	3.97	-4.09	126.09	236.03	117.65	75.64
2.65	10.58	3.96	-4.04	124.58	233.83	116.57	74.96
2.41	10.46	3.78	-4.36	126.97	237.76	120.39	79.57
2.58	10.52	3.98	-4.13	125.72	237.11	118.33	76.88
2.61	10.55	3.99	-4.09	124.35	234.83	116.46	75.34
2.35	10.36	3.79	-4.29	129.03	238.84	120.17	79.96
2.65	10.63	4.02	-4.07	125.84	233.94	116.49	75.21
2.49	10.5	3.7	-4.23	124.31	230.03	117.44	78.64
1.95	10.07	3.36	-4.65	131.37	247.41	127.23	87.67
2.7	10.74	3.93	-4.13	122.59	233.19	117.76	75.21
2.7	10.75	3.94	-4.13	122.54	233.09	117.78	75.18
2.4	10.45	3.78	-4.36	126.99	237.78	120.48	79.68
2.62	10.68	3.84	-4.22	126.61	233.46	121.2	81.68
2.33	10.4	3.64	-4.45	130.42	240.11	124.96	85.8
2.64	10.7	3.9	-4.17	128.05	238.8	122.69	81.63
2.37	10.46	3.71	-4.43	128.41	245.04	125.14	83.62
2.35	10.46	3.61	-4.39	129.35	242.4	126.64	86.09
2.51	10.59	3.77	-4.27	126.53	239.11	123.59	83.15
2.95	11.06	4.16	-3.99	124.02	232.1	121.01	79.75
2.48	10.5	3.86	-4.38	126.96	235.77	118.84	79.07
2.31	10.33	3.67	-4.46	129.31	237.88	120.64	81.34
2.21	10.26	3.55	-4.52	129.9	242.36	126.69	87.09
2.53	10.52	3.73	-4.2	122.76	230.78	117	78.44
2.37	10.45	3.65	-4.34	127.4	236.12	122.63	83.36
2.51	10.61	3.76	-4.24	126.21	233.4	120.51	81.99
2.54	10.63	3.78	-4.22	125.87	232.56	120.32	81.52
2.07	10.21	3.43	-4.67	133.65	245.23	128.81	92.24
2.45	10.48	3.72	-4.25	127.13	240.16	121.48	81.29
2.76	10.78	3.94	-4.01	122.69	233.22	118.08	76.35

2.74	10.78	3.93	-4	122.76	232.51	117.5	76.22
2.51	10.57	3.8	-4.2	124.71	233.9	118.24	76.8
2.62	10.68	3.88	-4.16	122.91	232.02	116.93	75.73
2.56	10.62	3.84	-4.24	122.69	235.73	119.18	77.43
2.61	10.64	3.91	-4.29	126.79	242.57	123.18	80.7
2.59	10.63	3.9	-4.3	127	242.77	123.33	80.83
2.45	10.52	3.75	-4.39	127.2	241.05	123.75	82.14
2.31	10.39	3.62	-4.49	128.53	245.38	125.79	85.14
2.74	10.79	3.97	-4.14	123.67	235.28	120.24	79
2.59	10.66	3.85	-4.26	124.81	238.6	122.69	80.3
2.79	10.79	4	-4.05	122.72	235.51	119.22	78.42
2.47	10.55	3.74	-4.26	126.73	241.11	123.24	82.87
2.85	10.87	4.04	-3.99	121.89	233.04	117.81	76.24
2.46	10.53	3.75	-4.29	127.69	241.92	125.15	84.54
2.56	10.62	3.77	-4.29	127.87	237.79	123.79	83.99
2.63	10.69	3.85	-4.26	127.96	233.7	121.8	82.4
2.64	10.7	3.86	-4.26	127.92	233.54	121.77	82.34
2.91	11.06	4.05	-3.98	122.68	229.21	119.66	78.02
1.58	9.65	3.02	-5	137.43	247.05	129.18	93.17
2.92	10.91	4.23	-3.8	119.34	220.5	108.07	70.77
2.62	10.57	4.01	-4.01	125.34	227.52	112.51	75.52
2.56	10.52	3.96	-4.07	125.35	228.04	113.22	76
2.6	10.57	3.97	-4.07	121.96	228.45	113.12	74.73
1.68	9.71	3.15	-4.87	135.63	241.28	125.5	88.55
1.76	9.77	3.16	-4.84	134.58	242.87	127.05	90.57
1.67	9.72	3.11	-4.93	136.85	244.2	128.34	92.21
1.74	9.82	3.17	-4.85	136.17	245.17	128.26	92.05
1.77	9.84	3.19	-4.83	135.04	244.62	127.92	91.31
3.1	11.09	4.4	-3.6	119.71	218.08	105	69.86
3.21	11.25	4.48	-3.54	117.98	214.47	104.05	68.67
3.31	11.35	4.55	-3.45	116.82	212.58	102.69	66.85
3.16	11.17	4.43	-3.55	119.58	218.47	104.5	69.02
3.29	11.3	4.61	-3.51	121.05	214.11	104.13	69.19
2.72	10.68	4.15	-3.93	127.17	223.52	110.41	76.45
3.01	10.98	4.38	-3.66	122.48	220.71	106.87	72.58
2.68	10.65	4.1	-3.93	127.29	226.8	110.09	76.37
3.1	11.27	4.11	-3.9	121.49	222.34	116.58	77.13

3.1	11.34	4.13	-3.96	121.64	222.7	117.79	78.16
2.7	10.84	3.88	-4.18	124.67	229.72	120.39	81.58
2.65	10.76	3.87	-4.18	126.3	229.92	120.43	82.37
2.97	11.09	4.1	-3.95	122.4	224.72	117.2	78.64
2.92	11.15	4	-4.03	121.1	223.55	117.46	80.06
3.11	11.27	4.11	-3.9	121.26	222.2	116.42	77.02
2.63	10.8	3.79	-4.25	125.93	231.69	122.03	83.56
2.71	10.81	3.89	-4.15	124.53	229.19	119.79	81.66
2.54	10.71	3.73	-4.34	128.04	235.45	123.74	85.25
2.38	10.51	3.64	-4.49	130.72	237.98	125.65	86.9
2.38	10.51	3.63	-4.47	130.89	237.89	125.34	87.42
3.01	11.27	4.06	-4.01	123.17	224.6	118.59	79.48
2.9	11.11	3.97	-4.05	123.37	225.6	118.89	79.96
2.92	11.13	3.98	-4.04	123.42	225.27	118.75	79.71
2.9	11.09	4.02	-4.01	122.75	226.45	117.51	81.07
2.03	10.13	3.35	-4.69	131.66	240.85	126.42	90.22
3.34	11.41	4.44	-3.66	120.33	230.25	118.64	75.95
2.96	11.03	4.16	-4	125.27	237.84	123.67	80.18
2.8	10.91	4.04	-4.1	125.43	239.64	124.51	81.26
3.23	11.29	4.37	-3.77	121.36	232.97	120.43	77
3.24	11.29	4.37	-3.77	121.4	232.96	120.43	77.01
3.17	11.26	4.32	-3.79	121.5	233.44	120.73	78.31
3.2	11.29	4.34	-3.77	121.35	232.9	120.31	77.91
2.28	10.4	3.66	-4.51	130.6	250.23	130.11	89.71
2.86	10.88	4.11	-4.08	124.92	235.41	119.06	75.56
2.86	10.89	4.11	-4.08	124.92	235.14	118.86	75.46
2.44	10.47	3.78	-4.39	128.32	243.74	124.62	81.57
2.8	10.93	4	-4.09	124.93	238.38	122.84	78.82
2.83	10.91	4.01	-4.08	124.59	238.98	121.73	78.37
2.89	11.01	4.08	-4.03	124.25	237.57	121.84	78
2.75	10.87	3.97	-4.13	125.43	239	123.58	79.83
3.06	11.18	4.2	-3.93	122.48	234.87	120.09	76.26
2.78	10.81	4.05	-4.11	126.47	239.84	122.01	79.03
2.36	10.5	3.7	-4.39	128.81	250.2	126.76	83.48
1.9	9.99	3.31	-4.81	132.27	254.08	128.18	86.65
2.28	10.41	3.63	-4.48	129.35	249.46	126.75	84.26
2.09	10.22	3.47	-4.63	131.29	252.72	128.61	86.96

2.05	10.21	3.45	-4.65	131.16	254.8	129.68	87.88
2.06	10.22	3.46	-4.64	131.08	254.72	129.61	87.77
2.71	10.79	3.96	-4.14	125.54	243.68	123.87	78.95
2.73	10.82	3.98	-4.12	125.35	243.34	123.64	78.56
2.49	10.62	3.78	-4.31	127.78	245.39	125.14	81.65
2.58	10.66	3.8	-4.17	123.16	235.42	119.43	77.96
2.31	10.39	3.62	-4.49	128.53	245.38	125.79	85.14
2.49	10.61	3.81	-4.31	129.38	244.18	127.69	86.68
2.62	10.68	3.8	-4.26	127.79	234.04	122.94	83.27
2.78	10.81	4.05	-4.11	126.52	239.89	122.06	79.06
2.44	10.49	3.75	-4.35	129.47	244.94	126.01	83.27
3.31	11.55	4.64	-3.36	138.01	254.15	136.95	90.95
3.29	11.36	4.49	-3.45	146.95	259.4	142.73	90.71
3.33	11.38	4.53	-3.54	153.12	266.31	145.85	93.34
3.42	11.46	4.53	-3.5	153.24	264.35	144.2	90.35
2.66	10.85	4.18	-3.73	140.98	267.77	135.58	92.4
2.45	10.75	3.48	-4.57	153.09	242.04	126.48	93.68
3.36	11.71	4.29	-3.91	129.61	220.58	115.02	79.81
3.04	11.27	4.07	-4.07	132.64	220.68	115.27	80.23
3.09	11.24	4.08	-4.07	129.97	221.02	113.8	77.52
3.32	11.65	4.25	-3.91	129.79	220.26	116.25	80.13
2.86	11.16	3.89	-4.23	134.77	227.5	119.02	85.4
2.86	11.16	3.89	-4.22	134.77	227.51	119.04	85.41
2.93	11.21	3.97	-4.19	131.84	220.7	117.05	81.8
2.45	10.59	3.57	-4.43	132.08	224.54	117.48	83.79
2.47	10.63	3.59	-4.43	131.09	225.67	118.19	83.64
2.31	10.5	3.49	-4.55	136.72	230.37	121.76	87.7
2.75	10.96	3.83	-4.35	132.87	226.09	118.63	83.88
2.75	10.96	3.83	-4.34	132.8	225.99	118.66	83.87
2.59	10.71	3.72	-4.39	131.67	223.88	117.5	82.94
2.41	10.52	3.52	-4.48	133.6	225.18	118	84.23
2.52	10.64	3.77	-4.37	130.76	231.33	121.75	86.07
2.76	10.94	3.84	-4.29	128.06	225.64	118.81	83.83
3.56	11.75	4.43	-3.73	119.96	209.72	111.11	74.22
3.05	11.25	4.04	-4.09	124.88	218.84	115.46	79.97
3.17	11.36	4.13	-4.01	122.88	216.28	113.91	78.53
3.19	11.4	4.15	-3.99	122.8	216.96	114.83	78.79

2.82	11.07	3.98	-4.17	128.1	224.4	120.75	86.24
3.04	11.28	4.13	-4.03	125.69	220.22	118.22	83.59
3.45	11.75	4.49	-3.76	119.37	210.64	113.22	77.13
3.32	11.62	4.36	-3.87	123.82	214.96	115.13	79.13
3.47	11.82	4.49	-3.8	120.58	212.43	114.23	80.03
3.69	12.03	4.65	-3.66	117.35	206.82	111.69	75.7
3.83	12.16	4.74	-3.55	116.2	202.47	107.73	71.5
3	11.25	4.04	-4.11	127.74	223.33	120.07	84.95
2.87	11.15	4.02	-4.1	124.69	223.61	119.12	82.71
2.87	11.14	4.01	-4.11	124.79	223.76	119.93	83.11
2.87	11.14	4.01	-4.11	124.8	223.79	119.95	83.13
2.4	10.57	3.6	-4.53	130.54	231.26	121.94	87.21
3	11.29	4.04	-4.12	130.27	221.41	116.95	81.87
3.67	12.01	4.61	-3.65	125.42	213.39	112.85	79.56
3.71	12.14	4.67	-3.61	123.98	212.24	114.7	80.31
2.64	10.85	3.74	-4.46	130.99	227.62	121.46	87.14
2.85	11.07	3.88	-4.28	127.58	223.14	118.59	84.25
3.39	11.68	4.33	-3.85	128.24	217.28	114.21	79.94
3.45	11.77	4.43	-3.77	126.7	218.19	115.35	81.54
4	12.35	4.87	-3.41	121.6	207.98	109.31	75.36
3.73	12.13	4.69	-3.61	125.67	211.8	114.51	80.61
3.5	11.9	4.51	-3.76	128.5	216.94	117.71	83.95
3.5	11.9	4.51	-3.76	128.52	216.98	117.74	83.96
3.5	11.9	4.51	-3.76	128.5	216.94	117.71	83.95
3.95	12.34	4.87	-3.46	121.29	210.24	114.4	80.62
3.94	12.34	4.86	-3.46	121.43	210.39	114.49	80.76
4.16	12.7	4.99	-3.37	117.01	202.31	111.27	77
3.93	12.41	4.84	-3.49	118.32	205.79	111.83	78.23
3.98	12.44	4.89	-3.46	118.23	204.81	111.72	78.23
2.7	10.89	3.74	-4.36	128.23	225.69	118.16	84.58
2.67	10.85	3.81	-4.34	130.1	223.91	116.9	81.78
2.64	10.83	3.73	-4.33	128.88	223.14	116.42	82.03
2.74	10.87	3.85	-4.3	129.72	222.29	115.89	81.52
2.38	10.48	3.54	-4.58	128.72	226	118.13	82.02
2.41	10.55	3.51	-4.59	129.77	227.34	119.75	83.51
2.49	10.66	3.62	-4.52	129.84	227.43	119.66	84.03
2.8	10.94	3.84	-4.28	125.35	221.7	116.01	80.44

2.94	11.22	4.05	-4.04	123.66	222.45	117.9	80.8
2.5	10.81	3.6	-4.53	146.84	240.16	125.42	91.33
2.67	10.92	3.67	-4.44	143.15	232.8	120.71	87.49
2.76	11.04	3.73	-4.34	140.59	229.84	118.41	84.02
2.81	11.1	3.81	-4.29	139.1	230.61	118.4	83.54
2.81	11.1	3.81	-4.29	139.08	230.61	118.44	83.56
2.81	11.1	3.81	-4.29	139.04	230.59	118.54	83.62
2.8	11.08	3.79	-4.32	140.08	230.21	118.37	84.07
2.68	10.92	3.65	-4.42	142.67	232.62	120.71	86.7
2.85	11.13	3.92	-4.27	138.26	230.11	118.69	83.48
2.76	11.1	3.86	-4.3	138.62	232.54	121.94	86.55
2.74	11.07	3.77	-4.36	140.1	231.38	120.22	83.75
2.74	11.07	3.77	-4.36	140.1	231.37	120.22	83.75
3.54	12.01	4.56	-3.8	131.63	220.76	116.72	79.71
3.24	11.68	4.23	-4.09	132.98	225.03	118.89	82.64
3.18	11.62	4.12	-4.15	135.27	227.64	119.99	83.33
3.57	11.99	4.52	-3.77	130.73	219.49	115.67	78.94
2.77	11.13	3.83	-4.34	138.96	230.44	119.16	84.36
2.79	11.15	3.81	-4.32	141.9	232.52	121.42	86.29
3.18	11.55	4.13	-4.07	134.35	224.04	117.6	81.24
3.29	11.66	4.21	-3.99	130.6	221.43	115.1	79.25
3.09	11.45	4.05	-4.16	135.4	225.13	115.22	79.51
4.53	13	5.27	-3.11	115.95	199.37	106.06	72.5
4.53	13	5.27	-3.11	115.95	199.35	106.07	72.51
3.35	11.81	4.39	-3.91	135.41	226.2	121.17	85.42
4.1	12.6	4.99	-3.35	116.82	204.03	110.8	76.06
3.82	12.36	4.76	-3.53	120.83	209.78	115.3	81.08
3.83	12.25	4.76	-3.59	118.42	207.38	113.26	78.23
3.53	11.94	4.53	-3.72	127.3	215.34	116.4	82.54
3.56	11.96	4.56	-3.7	126.59	214.64	115.99	82.02
3.31	11.42	4.45	-3.68	123.79	232.95	121.97	76.64
3.55	11.63	4.6	-3.47	120.83	229.36	119	74.01
3.62	11.82	4.53	-3.61	124.06	235	127.35	83.84
4.67	12.97	5.39	-3.01	142.42	256.18	142.42	99.17
4.43	12.76	5.15	-3.17	140.39	255.47	142.81	99.18
3.78	11.89	4.67	-3.68	142.34	251.36	143.51	98.87
3.85	11.95	4.7	-3.64	141.79	249.75	142.28	97.3

3.85	11.95	4.7	-3.64	141.73	249.81	142.34	97.38
3.85	11.95	4.7	-3.64	141.73	249.81	142.34	97.37
3.94	12.07	4.76	-3.6	140.83	250.28	141.96	98.36
3.36	11.59	4.38	-3.8	137.59	251.64	140.35	96.34
3.49	11.74	4.48	-3.67	133.91	244.77	135.93	91.12
3.36	11.46	4.47	-3.84	149.68	260.33	145.6	97.3
3.38	11.48	4.46	-3.83	147.37	258.78	144.22	94.61
3.47	11.56	4.54	-3.74	144.31	256.91	143.37	93.94
3.22	11.37	4.28	-3.9	147.7	261.15	146.07	96.08
2.94	11.12	4.06	-4.18	155.78	274.42	152.72	101.28
3.01	11.21	4.14	-4.15	161.95	278.79	157.52	103.24
2.61	10.73	3.74	-4.71	167.47	277.43	165.27	123.34
3.14	11.19	4.19	-4.28	160.52	263.17	156.95	112.55
2.65	10.78	3.78	-4.73	166.48	271.54	163.59	119.58
2.6	10.75	3.73	-4.78	167.68	271.61	164.25	120.63
2.61	10.75	3.73	-4.78	167.6	271.51	164.32	120.55
2.61	10.75	3.73	-4.78	167.6	271.52	164.35	120.55
2.44	10.6	3.59	-4.92	173.54	278.64	171.47	127.09
2.38	10.54	3.54	-4.97	174.09	280.36	172.31	128.29
1.88	10.12	3.17	-5.34	184.94	299.56	185.12	144.43
1.84	10.1	3.11	-5.41	184.44	299.82	182.01	139.18
2.98	11.04	4.08	-4.42	161.43	261.92	160.37	117.87
2.98	11.04	4.08	-4.42	161.45	261.95	160.39	117.89
3.48	11.67	4.41	-3.93	147.83	255.45	151.63	108.09
2.78	10.92	3.95	-4.47	163.28	276.42	162.9	120.68
1.79	10.04	3.16	-5.29	177.76	294.15	183.88	149.81
1.84	10.07	3.18	-5.27	177.75	291.9	183.4	148.86
3.32	11.47	4.37	-4.04	150.46	254.03	152.23	108.25
3.22	11.39	4.37	-4.09	154.44	250.69	154.96	112.71
3.3	11.4	4.37	-4.1	154.34	249.21	153.34	109.23
2.98	11.16	4.17	-4.32	156.82	256.04	158.92	117.64
3.36	11.4	4.43	-4.05	155.07	253.08	153.57	109.04
1.79	10.03	3.16	-5.29	178.8	294.41	183.81	149.93
1.79	10.03	3.15	-5.29	178.86	294.49	183.9	150.05
1.78	10.03	3.15	-5.3	178.86	294.55	183.96	150.12
1.85	10.12	3.24	-5.18	177.98	293.3	184.34	150.31
3.03	11.19	4.21	-4.23	156.72	256.99	159.21	119.68

3.03	11.19	4.21	-4.23	156.7	256.92	159.17	119.63
3.03	11.19	4.22	-4.23	156.6	256.68	159.01	119.43
3.03	11.2	4.22	-4.23	156.57	256.56	158.92	119.36
3.04	11.2	4.22	-4.23	156.56	256.53	158.9	119.34
3.06	11.22	4.24	-4.21	156.28	255.86	158.48	118.74
3.54	11.71	4.47	-3.9	147.76	255.21	150.73	107.44
3.6	11.75	4.53	-3.83	144.26	251.81	146.41	103.77
3.8	12.01	4.68	-3.67	141.09	248.62	143.18	99.25
2.95	11.05	4.08	-4.36	162.33	267.66	160.8	118.05
3.43	11.48	4.47	-3.97	150.01	261.03	151.08	106.08
3.24	11.29	4.3	-4.14	154.03	266.29	153.76	110
2.94	11.17	4.1	-4.32	158.7	264.9	162.04	122.24
1.93	10.17	3.23	-5.28	182.5	301.32	181.38	139.3
3.2	11.45	4.37	-3.85	169.43	287.77	157.33	95.28
3.35	11.58	4.49	-3.79	166.96	282.93	155.19	93.11
3.05	11.24	4.16	-4.04	156.23	276.3	151.39	97.89
3.04	11.3	4.28	-4.05	174.65	294.93	163.23	104.4
3.03	11.29	4.28	-4.06	174.72	295.01	163.39	104.66
2.66	10.91	3.96	-4.43	181.06	301.28	170.49	113.1
2.62	10.88	3.93	-4.48	180.89	300.05	170.26	112.66
2.5	10.76	3.88	-4.54	181.98	305.94	173.79	121.28
3.3	11.56	4.47	-3.8	168.31	284.53	156.29	93.51
2.84	11.08	4.12	-4.19	172.8	296.09	164.47	107.23
3.36	11.6	4.5	-3.79	166.54	280.19	153.83	93.94
3.32	11.58	4.47	-3.83	167.36	284.17	155.74	97.17
3.3	11.55	4.44	-3.85	167.84	283.68	156.08	94.66
3.2	11.5	4.35	-3.99	172.01	288.17	161.72	103.62
3.23	11.54	4.36	-3.99	173	288.36	161.36	103.36
2.98	11.25	4.08	-4.51	179.32	295.7	170.55	114.68
3.41	11.65	4.53	-3.74	165.25	278.17	153.04	92.59
3.29	11.55	4.45	-3.83	167.72	284.02	155.56	94.45
3.27	11.53	4.41	-3.89	168.3	284.19	156.92	96.96
3.3	11.54	4.44	-3.86	169.22	283.06	155.9	95.36
3.24	11.49	4.38	-3.92	169.4	285.36	157.44	97.39
3.51	11.77	4.65	-3.62	163.75	277.65	152.09	93.38
3.38	11.64	4.47	-3.82	169.28	284.15	158.35	99.72
2.68	10.85	3.92	-4.69	197.62	320.66	189.22	138.98

2.61	10.8	3.89	-4.77	200.24	323.87	191.02	141.33
2.76	11.04	3.92	-4.66	185.54	304.47	176.25	121.96
2.94	11.19	4.07	-4.38	174.87	294.5	167.59	110.86
2.94	11.26	4.12	-4.31	177.76	295.48	167.82	111.14
2.7	11.01	3.97	-4.43	182.76	304.2	173.22	118.33
2.73	11.01	3.99	-4.5	184.1	302.85	172.72	118.08
2.73	11.01	3.98	-4.5	184.18	303.03	172.84	118.23
2.72	11	3.98	-4.51	184.27	303.24	172.99	118.41
2.77	11.04	4.01	-4.47	183.62	301.71	172.11	117.03
2.84	11.1	4.07	-4.4	182.79	299.91	170.34	115.22
2.84	11.12	4.09	-4.26	167.47	286.99	161.67	107.2
2.91	11.1	4.08	-4.15	160.27	280.99	152.96	100.44
2.52	10.63	3.85	-3.72	159.5	262.09	164.68	144.92
2.58	10.72	3.85	-3.63	153.96	257.26	159.55	138.54
3.03	11.12	4.23	-3.35	144.23	246.15	148.23	125.11
2.7	10.83	3.97	-3.57	154.3	256.83	158.89	137.75
1.13	9.33	2.62	-4.82	165.37	270.53	169.07	159.29
2.63	10.92	3.85	-3.73	158.36	253.58	150.55	136.13
2.52	10.82	3.77	-3.83	159.81	256.22	152.65	140.98
2.48	10.78	3.73	-3.85	160.03	256.64	152.96	141.17
2.36	10.64	3.63	-3.97	160.22	259.03	154.92	143.31
2.22	10.35	3.62	-4.05	155.07	259.04	158.2	142.59
2.6	10.71	3.88	-3.66	149.69	250.51	153.25	133.31
2.26	10.38	3.65	-4.02	154.7	258.04	158.27	142.21
1.91	10.06	3.4	-4.27	160.35	265.3	163.67	150.2
1.39	9.6	2.96	-4.6	167.59	273.58	171.25	160.5
1.41	9.64	2.85	-4.62	163.79	266.67	163.93	153.67
1.37	9.6	2.82	-4.64	163.77	267.83	164.59	154.34
2.58	10.8	3.88	-4.74	202.52	327.09	191.73	142.94
2.26	10.46	3.57	-5.09	207.31	334.85	200.12	151.42
2.36	10.58	3.68	-4.96	205.19	332.73	197.11	149.09
2.47	10.68	3.78	-4.87	202.92	328.75	194.31	145.52
2.43	10.64	3.74	-4.91	203.8	330.31	195.39	146.9
1.98	10.16	3.29	-5.31	211.28	339.48	203.54	159.41
1.97	10.12	3.31	-5.31	213.46	341.9	204.98	161.11
1.81	10.03	3.1	-5.7	212.58	339.82	207.2	163.15
1.26	9.53	2.59	-6.28	219.66	348.58	215.23	177.41

1.3	9.57	2.63	-6.25	219.01	347.2	214.45	176.13
1.04	9.37	2.66	-5.73	218.13	368.45	212.07	178.99
2.02	10.21	3.48	-5.03	199.92	331.95	190.31	143.21
1.94	10.11	3.29	-5.31	204.14	328.81	193.35	146.9
1.46	9.69	2.99	-5.45	213.03	354.71	204.17	165.97
2.75	10.96	3.94	-4.49	177.47	295.74	166.82	111.81
2.12	10.33	3.45	-5.04	191.73	316.36	182.34	133.85
1.35	9.61	2.84	-5.72	210.23	340.51	200.16	159.79
1.17	9.39	2.64	-5.88	212.95	347.99	205.51	168.15
1.13	9.34	2.6	-5.93	213.54	349.97	206.78	170.45
1.7	9.99	2.96	-5.54	187.4	305.36	184.57	143.13
1.63	9.93	2.9	-5.6	188.62	308.13	186.1	145.35
1.66	9.95	2.91	-5.58	187.97	306.9	185.18	143.96
3.04	11.15	4.13	-4.29	160.13	271.32	156.56	109.51
3.46	11.52	4.54	-3.88	153.96	266.23	151.56	104.4
3.81	11.82	4.8	-3.63	145.69	253.65	146.35	96.08
3.63	11.68	4.62	-3.78	149.33	256.81	148.35	98.76
3.68	11.73	4.69	-3.74	146.85	255.18	146.53	98.06
2.68	10.83	3.89	-4.62	169.48	282.18	165.97	120.39
3.04	11.15	4.19	-4.36	164.05	270.77	159.1	112.26
2.79	10.91	3.96	-4.6	168.45	277.89	164.08	118.01
1.23	9.55	2.73	-5.73	203.86	343.12	196.43	160.98
1.72	9.99	3.09	-5.39	195.83	324.57	185.6	143.46
2.84	11.1	4	-4.31	170.06	288.29	162.35	109.21
2.17	10.37	3.48	-5.09	188.3	305.58	180.62	135.33
1.17	9.4	2.64	-5.88	209.99	342.26	204.5	167.04
1.03	9.32	2.52	-5.98	211.75	353.12	205.65	171.25
2.14	10.35	3.44	-5.08	177.68	297.15	176.23	133.9
2.48	10.66	3.76	-4.74	172.45	291.52	168.35	119.42
1.87	10.13	3.25	-5.3	187.69	308.21	184.35	141.05
2.23	10.41	3.5	-5.02	177.41	294.32	174.78	132.13
2.21	10.44	3.54	-4.93	181.6	305.71	174.17	129.6
2.07	10.32	3.44	-5.03	183.43	310.14	176.74	133.45
2.17	10.38	3.42	-5.07	180.68	297.52	177.51	132.88
2.72	10.85	3.93	-4.44	169.1	287.41	164.57	113.9
4.26	12.66	5.4	-2.41	147.88	236.38	145.93	117.63
3.82	12.08	5.13	-2.7	150.12	254.12	160.45	131.3

3.75	12	5.07	-2.69	152.74	254.9	161.68	132.56
3.77	12.01	5.08	-2.68	152.86	255.61	161.96	133.21
3.36	11.67	4.71	-3.09	151.28	254.77	161.79	133.35
2.98	11.3	4.42	-3.5	148.62	248.32	154.28	126.37
3.85	12.11	5.15	-2.82	148.37	245.21	150.58	119.78
3.81	12.06	5.1	-2.86	148.6	245.31	150.01	119.52
2.77	11.11	4.22	-3.76	148.8	242.01	148.76	120.28
2.41	10.72	3.9	-4.04	148.09	242.7	146.37	117.98
4.27	12.51	5.49	-2.41	148.07	245.28	151.07	120.8
4.05	12.31	5.33	-2.52	149.45	251.3	159.17	128.24
3.6	11.85	4.94	-2.92	149.03	254.21	159.31	130.54
2.88	11.21	4.33	-3.59	149.2	249.13	155.47	127.49
3.73	11.97	5.05	-2.81	149.03	254.15	159.37	130.38
2.55	10.88	4.04	-3.88	148.85	245.94	151.53	125.47
2.95	11.33	4.31	-3.52	152.72	249.01	158.33	131.72
3.57	11.9	4.87	-2.91	153.91	255.71	164.9	134.91
3.81	12.07	5.14	-2.8	148.39	249.73	156.41	125.92
3.29	11.48	4.75	-3.09	160.02	265.76	164.48	138.53
3.47	11.72	4.81	-2.92	152.02	258.53	161.45	133.18
2.54	10.82	4.01	-3.87	149.42	244.68	150.71	122.83
3.41	11.73	4.76	-3.05	151.42	255.32	162.56	133.7
3.28	11.6	4.66	-3.17	150.5	253.85	160.32	132.32
3.32	11.62	4.68	-3.21	150.71	251.55	158.9	130.28
3.55	11.93	4.81	-3.05	151.91	248.79	155.8	126.07
3.15	11.36	4.63	-3.22	159.7	264.79	163.74	137.79
3.11	11.55	4.39	-3.6	156.89	240.85	143.64	123.62
4.02	12.26	5.27	-2.67	147.78	245.1	149.14	119.34
3.44	11.73	4.75	-3.2	147.89	243.24	148.65	118.98
3.45	11.76	4.75	-3.17	148.83	244.09	147.99	119.49
4.04	12.29	5.25	-2.65	146.51	244.73	147.7	118.67
4.19	12.4	5.36	-2.54	146.23	243.4	146.96	117.16
4.1	12.47	5.26	-2.51	148.02	242.49	148.03	119.18
3.45	11.81	4.66	-3.24	141.94	235.78	138.82	109.39
3.47	11.76	4.77	-3.17	145.85	243.19	146.63	116.88
3.42	11.71	4.72	-3.22	145.9	242.94	146.81	117.09
3.31	11.54	4.66	-3.27	145.63	244.04	145.75	114.99
3.34	11.56	4.66	-3.23	146.49	245.3	146.52	115.68

3.45	11.67	4.73	-3.11	143.81	244.67	143	112.89
3.59	11.86	4.88	-3.05	144.21	241.57	141.98	110.93
3.45	11.73	4.76	-3.17	144.69	241.44	142.29	111.66
3.46	11.73	4.76	-3.16	144.69	241.45	142.29	111.65
2.9	11.32	4.21	-3.82	153.99	236.86	140.32	118.82
3.19	11.64	4.48	-3.49	154.7	240.86	143.65	122.91
2.85	11.31	4.16	-3.85	155.85	239.24	142.13	123.26
3.17	11.5	4.49	-3.47	145.8	239.85	144.24	116.1
3.16	11.61	4.42	-3.47	150.41	237.34	143.43	118.79
2.91	11.34	4.22	-3.66	146.22	235.65	142.81	115.32
2.93	11.4	4.25	-3.71	153.46	236.33	142.74	120.23
2.79	11.21	4.11	-3.84	142.86	232.15	137.87	110.11
3.29	11.6	4.58	-3.37	144.51	239.17	141.98	112.92
3.25	11.47	4.58	-3.3	145.8	245.41	145.74	116.02
3.58	11.85	4.87	-3.06	144.4	241.52	142.1	111.21
3.46	11.74	4.77	-3.16	144.55	241.44	142.19	111.5
3.02	11.32	4.43	-3.57	146.05	244.22	146.77	116.45
3.52	11.9	4.73	-3.11	147.29	237.23	142.8	115.71
4.18	12.41	5.37	-2.53	146.12	244.16	147.22	117.81
4.19	12.41	5.37	-2.53	146.19	243.4	146.94	117.14
3.45	11.73	4.73	-3.21	145.69	242.39	144.72	115.35
3.3	11.53	4.65	-3.15	151.97	258.98	155.66	127.54
3.3	11.52	4.64	-3.17	152.24	259.6	156.17	127.84
3.27	11.5	4.61	-3.16	152.66	258.73	156.11	128.55
3.41	11.65	4.78	-3.09	151.29	257	156.88	126.77
2.77	11.04	4.21	-3.58	153.54	256.6	156.94	130.08
3.09	11.33	4.48	-3.32	154.28	259.28	158.18	130.72
3.43	11.66	4.76	-3.03	151.73	258.23	154.51	125.93
3.22	11.45	4.57	-3.3	145.95	247.12	146.46	116.76
4.11	12.38	5.38	-2.47	148.3	246.65	155.16	125.02
2.09	10.3	3.29	-4.28	146.12	252.41	150.89	140.42
2.63	10.9	3.78	-3.86	140.77	243.69	145.78	133.29
1.97	10.32	3.18	-4.4	173.86	274.79	182.46	185.57
1.71	10.11	2.91	-4.65	179.04	279.6	191.71	198.72
1.86	10.23	3.05	-4.49	175.99	276.63	187.2	191.68
2.05	10.39	3.19	-4.43	170.53	272.41	180.76	184.16
2.04	10.38	3.18	-4.44	170.57	272.58	180.98	184.51

1.95	10.32	3.14	-4.49	172.25	274.82	183.26	187.69
2.31	10.62	3.42	-4.21	161.15	262.49	171.62	168.2
2.51	10.79	3.58	-4.09	163.29	258.9	168.11	165.88
2.16	10.47	3.31	-4.41	169.31	264.63	177.48	179.84
2.48	10.77	3.6	-4.09	157.5	257.02	165.16	159.26
2.46	10.75	3.55	-4.11	158.94	259.1	166.56	161.85
2.16	10.46	3.35	-4.04	148.48	252.32	158.31	150.95
2.21	10.49	3.47	-3.97	146.87	246.49	156.13	148
2.51	10.79	3.67	-3.84	148.51	252.18	156.11	147.91
2.31	10.58	3.48	-3.94	148.59	253.23	156.56	147.91
2.04	10.34	3.28	-4.17	149.66	256.15	160.37	153.31
2.5	10.75	3.64	-3.85	145.63	249.15	153.87	143.81
2.18	10.52	3.38	-4.08	149.26	253.68	159.3	150.75
2.07	10.45	3.31	-4.13	159.57	265.11	172.89	166.66
1.95	10.36	3.24	-4.13	163.6	267.52	175.41	170.86
1.95	10.35	3.24	-4.14	164.01	267.53	175.37	171.08
1.95	10.36	3.24	-4.13	163.53	267.4	175.36	170.8
2.24	10.56	3.3	-4.36	169.44	267.21	177.61	179.92
1.59	9.97	2.68	-4.91	188.04	287.84	207.16	224
2.6	10.81	3.66	-3.96	151.54	249.48	148.36	135.99
2.07	10.28	3.31	-4.16	150.45	255.16	151.29	139.14
2.7	10.95	3.79	-3.87	150.94	247.7	146.82	134.35
2.75	11.03	3.88	-3.76	141.18	244.77	145.19	131.79
3.29	11.54	4.31	-3.49	143.4	234.83	140.28	124.73
2.41	10.73	3.55	-4.23	160.1	256.08	163.39	159.66
2.24	10.44	3.42	-4.05	127.54	238.28	136.97	124.69
2.13	10.38	3.32	-4.07	145.55	247.54	154.7	147.39
1.96	10.22	3.19	-4.17	144.13	244.4	151.85	144.26
2.1	10.33	3.23	-4.15	127.09	233.71	135.66	122.76
2.04	10.28	3.27	-4.09	137.97	238.03	148.54	139.2
1.96	10.2	3.18	-4.16	144.23	244.18	151.9	144.42
1.4	9.53	2.7	-4.58	140.25	245.64	155.4	147.12
1.81	9.93	2.97	-4.33	123.52	232.61	135.95	124.01
1.81	9.94	2.98	-4.33	124.1	232.3	136.36	124.57
1.65	9.86	2.95	-4.5	136.46	247.72	146.2	135.85
1.95	10.16	3.15	-4.29	129.75	240.93	138.05	127.47
1.98	10.12	3.08	-4.23	122.23	229.22	132.36	120.17

1.79	9.97	2.98	-4.42	126.36	239.98	135.53	124.31
2.75	11.01	3.8	-4.01	155.07	247.61	153.71	145.89
2.03	10.37	3.13	-4.6	173.7	268.51	181.65	186.81
2.53	10.8	3.62	-4.15	158.76	253.41	160.58	156.61
2.52	10.78	3.6	-4.14	161.21	255.29	163.2	160.52
2.45	10.74	3.52	-4.25	163	254.59	163.21	159.68
2.32	10.59	3.45	-4.31	165.69	259.73	169.94	169.87
2.53	10.84	3.51	-4.25	165.69	254.34	166.51	165.05
1.79	10.02	3.06	-4.47	152.99	260.34	157.45	150.3
1.93	10.13	3.14	-4.31	138.94	250.64	146.63	135.97
1.9	10.11	3.12	-4.31	140.64	251.08	147.49	137.77
2.13	10.45	3.36	-4.14	145.16	250	153.75	144.45
1.93	10.19	3.2	-4.23	136.29	247.17	144.86	135.8
1.94	10.19	3.2	-4.22	136.12	247.26	144.74	135.81
1.77	10.04	3.06	-4.33	140.9	252.13	149.85	142.52
1.75	10.02	3.05	-4.35	141.47	252.52	150.4	143.45
1.87	10.02	3.14	-4.36	146.03	255.44	149.55	139.04
1.66	9.88	2.98	-4.55	152.35	260.78	155.84	147.97
2	10.2	3.22	-4.27	136.18	247.7	143.35	131.71
1.98	10.35	3.26	-4.22	169.23	273.02	181.82	179.14
1.79	10.18	3.09	-4.4	172.18	276.81	186	187.09
2.17	10.51	3.45	-3.96	160.07	260.52	169.31	162.68
1.94	10.32	3.28	-4.11	163.31	266.91	175.95	172.14
1.81	9.93	2.97	-4.33	123.6	232.65	136.07	124.17
2.52	10.81	3.65	-4.2	150.45	237.16	141.29	127.94
2.86	11.19	3.91	-3.86	141.98	231.14	130.24	114.47
2.86	11.19	3.91	-3.86	142.03	231.17	130.28	114.53
1.79	10.1	2.99	-4.85	174.52	263.37	172.1	170.3
2.3	10.6	3.43	-4.46	161.12	252.19	158.98	153.3
2.22	10.52	3.37	-4.54	163.43	254.64	160.44	155.92
2.07	10.4	3.26	-4.62	166.54	256.67	164.22	161.06
1.87	10.18	3.08	-4.77	172.78	262.55	170.43	168.94
2.51	10.83	3.59	-4.22	153.42	242.79	140.85	125.45
2.57	10.81	3.61	-4.13	153.77	245.36	142.32	128.64
2.92	11.22	3.94	-3.87	147.43	234.52	132.29	114.58
2.68	10.93	3.72	-4.05	152.76	243.44	140.5	125.38
3.17	11.4	4.1	-3.66	141.16	224.41	125.42	103.89

2.89	11.06	3.9	-3.87	136.53	216.95	116.45	91.72
3.14	11.4	4.14	-3.6	135.1	215.64	115.16	91.23
3.07	11.42	4.11	-3.61	135.69	217.69	118.08	94.15
3.26	11.47	4.24	-3.55	133.65	213.77	111.58	86.09
3.04	11.23	4.02	-3.68	146.76	241.87	140.91	125.73
2.72	10.94	3.75	-3.94	149.74	247.52	143.71	129.32
3.01	11.28	3.98	-3.75	145.36	239.4	141	124.99
3.15	11.45	4.18	-3.64	144.5	231.64	128.99	110
3.15	11.44	4.17	-3.64	144.33	231.47	128.94	109.94
3.03	11.28	4.06	-3.76	145.69	232.3	130.11	109.96
2.98	11.29	4	-3.79	150.71	240.05	136.82	119.23
3.08	11.38	4.07	-3.67	148.23	236.91	133.51	114.82
3.57	11.85	4.6	-3.16	134.43	219.02	119.54	91.02
3.53	11.77	4.56	-3.28	138.98	220.64	121.58	95.43
2.82	11.07	3.86	-3.86	132.78	212.57	110.87	83.42
2.44	10.68	3.57	-3.98	151.22	253.87	150.33	137.19
2.14	10.41	3.39	-4.1	152.58	254.88	151.95	140.92
2.3	10.49	3.48	-4.1	159.86	261.53	154.94	144.68
2.58	10.78	3.68	-3.95	151.83	252.94	148.52	135.85
2.54	10.75	3.63	-4.02	154.58	253.69	149.72	136.45
1.79	10.03	3.12	-4.37	155.68	260.7	157.4	145.67
1.79	10.03	3.12	-4.37	155.8	260.79	157.45	145.68
1.71	9.95	3.05	-4.39	157.36	263.4	159.3	149.36
1.87	10.04	3.16	-4.32	153.36	258.83	155.57	141.93
1.9	10.13	3.19	-4.27	156.26	260.94	155.49	143.6
2.9	11.12	4.02	-3.59	148.75	247.31	143.8	128.16
1.72	9.95	3.01	-4.37	156.31	261.03	157.42	146.86
2.15	10.36	3.38	-4.1	153.42	255.83	151.94	137.84
1.9	10.14	3.21	-4.26	154.98	260.2	155.36	143.95
1.94	10.16	3.23	-4.23	152.94	257.33	153.71	141.42
1.69	9.92	2.99	-4.4	155.81	261.12	157.01	147.36
1.95	10.18	3.22	-4.23	153.68	257.17	153.93	140.93
2.8	11.03	3.95	-3.74	153.99	249.3	145.91	133.31
3.21	11.57	4.31	-3.46	152.61	242.43	138.43	121.94
3.29	11.61	4.32	-3.35	148.93	242.17	137.41	119.56
3.09	11.42	4.18	-3.46	151.24	245.1	141.28	123.94
3.22	11.5	4.28	-3.35	149.01	243.31	138.22	121.28

2.61	10.77	3.74	-3.87	153.18	253.06	149.93	135.31
3.42	11.67	4.47	-3.41	150.49	240.07	137.27	119.92
3.23	11.52	4.3	-3.56	153.54	241.58	138.79	121.5
3.81	12.09	4.84	-3.03	145.19	235.27	134.84	114.77
3.09	11.41	4.2	-3.74	149.4	235.79	134.48	115.83
2.81	11.16	3.9	-3.97	151.37	238.38	136.41	118.29
3.21	11.54	4.26	-3.47	150.85	242.85	139.11	121.18
3.52	11.81	4.59	-3.27	150.05	239.08	138.28	120.69
3.05	11.34	4.15	-3.47	150.2	244.47	140.88	124.79
3.17	11.47	4.21	-3.41	148.79	243.24	139.42	121.99
3.09	11.47	4.19	-3.52	151.91	243.84	140.59	123.66
3.37	11.69	4.45	-3.36	149.45	240.78	137.29	119.93
3.2	11.53	4.28	-3.39	148.72	243.08	139.02	120.62
3.04	11.39	4.14	-3.62	153.14	244	141.47	123.76
3	11.34	4.1	-3.66	153.71	243.58	141.06	123.48
2.23	10.56	3.39	-4.29	161.94	255.48	151.54	139.95
2.23	10.55	3.39	-4.29	161.76	255.5	151.43	139.75
2.94	11.26	4.01	-3.84	153.12	239.08	137.29	119.85
3.51	11.82	4.59	-3.3	149.05	240.6	138.33	119.74
3.5	11.82	4.58	-3.3	149.08	240.66	138.36	119.81
3.53	11.79	4.6	-3.29	147.64	238.84	136.48	118.17
3.47	11.73	4.53	-3.35	148.92	240.06	137.37	119.9
3.47	11.75	4.58	-3.39	147.14	233.62	132.16	112.57
3.39	11.68	4.53	-3.41	145.04	229.29	129.41	108.11
3.43	11.76	4.56	-3.35	143.55	229.72	130.21	109.48
2.57	10.9	3.7	-4	156.85	251.5	146.48	133.12
1.82	9.77	3.07	-3.99	141.53	221.54	155.04	143.62
1.83	9.79	3.06	-3.98	139.25	218.97	152.77	140.02
1.98	9.93	3.26	-3.86	146.06	233.37	161.95	140.58
1.8	9.94	3.24	-4.11	160.99	265.25	171.79	152.42
1.93	10.09	3.3	-4.02	154.83	260.64	166.19	146.99
2.25	10.35	3.57	-3.78	138.38	236.94	153.42	128.02
1.28	9.37	2.78	-4.46	174.66	275.3	188.61	170.22
1.55	9.62	2.98	-4.29	168.89	269.11	181.22	160.6
1.26	9.35	2.85	-4.36	177.75	274.24	196.58	178.46
1.3	9.36	2.82	-4.33	172.83	271.53	191.7	176.6
1.99	10.13	3.37	-3.92	140.22	236.74	157.52	135.85

0.18	8.49	2.15	-5.24	206.08	299.34	204.83	199.52
0.24	8.58	2.23	-5.23	215.59	304.17	206.75	204.39
2.62	10.83	4.04	-3.66	176.11	270.93	168.84	157.66
0.93	9.27	2.77	-4.86	215.74	304.03	202.13	201.03
1.05	9.33	2.92	-4.68	201.77	293.38	194.83	187.65
2.74	11.03	4.14	-3.63	185.69	276.5	172.43	168.04
1.58	9.92	3.29	-4.46	210.07	296.41	192.98	195.12
1.92	10.21	3.53	-4.24	205.49	293.01	188.04	190.56
3.07	11.34	4.39	-3.38	176.3	269.13	165.24	156.91
3.49	11.77	4.74	-3.06	172.36	263.33	159.48	147.98
1.83	9.76	3.11	-3.97	144.25	224.48	157.71	147.71
3.46	11.59	4.39	-3.41	133.52	219.86	108.21	78.6
3.62	11.76	4.55	-3.33	131.65	216.09	105.78	74.26
3.01	11.25	4.05	-3.82	140.27	223.2	115.22	88.57
3.61	11.91	4.53	-3.3	142.24	221.89	121.66	100.62
2.93	11.21	3.89	-3.98	145.9	230.57	123.41	99.41
2.16	10.52	3.24	-4.63	159.46	244.41	146.94	136.26
2.45	10.82	3.49	-4.37	154.99	238.69	140.2	127.07
2.36	10.68	3.29	-4.49	161.31	251.71	157.96	152.09
3.11	11.45	4.11	-3.7	150.77	232.05	133.78	118.13
2.51	10.84	3.39	-4.39	159.43	247.74	155.82	148.76
2.43	10.77	3.35	-4.44	161.92	249.87	158.66	152.27
3.47	11.74	4.39	-3.35	138.41	217.18	114.15	89.5
3.45	11.78	4.39	-3.48	144.15	223.28	122.63	102.33
3.58	11.88	4.5	-3.3	139.22	220.4	118.24	95.44
3.1	11.42	4.08	-3.8	146.5	228.99	124.77	104.77
3.27	11.58	4.23	-3.65	144.08	226.68	123.34	101.96
3.81	12.02	4.7	-3.07	134.92	212.57	110.08	82.63
2.92	11.21	3.89	-3.98	145.88	230.58	123.39	99.39
2.91	11.17	3.93	-3.97	144.26	227.14	120.96	96.65
2.97	11.21	4.05	-3.94	143.26	225.12	118.56	93.47
3.18	11.4	4.14	-3.78	141.2	222.9	117.9	92.83
3.19	11.41	4.14	-3.76	140.94	222.86	118.06	92.68
2.76	11.01	3.78	-4.14	148	232.71	125.52	100.85
2.55	10.81	3.61	-4.3	151.32	236.45	128.94	104.88
2.9	11.16	3.86	-4.07	147.58	232.58	124.97	100.23
2.74	11	3.76	-4.17	149.32	233.99	126.11	101.89

2.89	11.16	3.86	-4.07	147.51	233.57	125.82	101.08
3.76	11.9	4.68	-3	128.73	207.18	103.57	75.55
3.76	11.9	4.69	-3.08	132.28	210.32	107.39	79.62
3.76	11.9	4.7	-3.08	132.29	210.31	107.39	79.62
3.47	11.59	4.45	-3.26	132.34	217.42	106.69	78.39
3.45	11.6	4.35	-3.4	132.53	214.22	109.61	83.22
3.35	11.51	4.28	-3.45	134.06	212.05	109.04	81.56
3.4	11.55	4.3	-3.43	133.46	211.66	108.62	81.18
4.01	12.15	4.83	-2.85	129.89	208.24	103.22	73.09
3.99	12.14	4.83	-2.92	129.85	209.68	102.49	74.41
3.85	11.99	4.7	-3	132.35	208.81	103.76	74.68
3.29	11.46	4.23	-3.6	134.89	216.55	111.77	85.32
3.43	11.59	4.33	-3.47	135	214.66	109.4	82.26
3.7	11.86	4.57	-3.21	133.19	212.52	105.4	77.36
3.34	11.44	4.33	-3.34	131.06	211.54	104.81	75.78
3.31	11.4	4.29	-3.38	132.15	211.37	105.13	76.18
3.27	11.36	4.25	-3.42	131.81	211.45	104.27	75.84
3.37	11.47	4.37	-3.27	129.65	211.02	104	74.75
3.99	12.09	4.85	-2.85	128.4	206.84	102.84	73.88
3.53	11.68	4.5	-3.17	132	208.21	105.03	77.53
3.62	11.75	4.59	-3.08	129.15	206.06	102.46	74.11
2.27	10.57	3.36	-4.5	159.48	243.84	146.88	135.18
3.12	11.48	4.14	-3.66	150.43	231.72	133	117.42
2.31	10.72	3.4	-4.39	154.7	238.61	139.43	125.23
3.43	11.6	4.39	-3.37	135.49	212.89	109.09	82.93
3.37	11.57	4.32	-3.44	134.07	214.37	110.11	85.02
3.55	11.79	4.54	-3.24	138.01	215.63	111.63	86.66
2.33	10.74	3.22	-4.47	174.83	265.49	181.67	186.26
2.53	10.85	3.4	-4.35	154.25	240.01	147.44	137.8
2.44	10.77	3.35	-4.43	161.74	249.83	158.56	152.15
2.6	10.94	3.46	-4.32	160.73	247.74	155.81	148.17
2.4	10.75	3.33	-4.43	165.12	251.63	164.18	162.86
2.58	10.91	3.47	-4.34	161.42	248.56	159.43	153.67
2.35	10.78	3.24	-4.46	173.35	264.83	179.91	184.73
2.46	10.87	3.35	-4.39	170.06	258.08	174.59	176.67
2.16	10.55	3.15	-4.6	172.86	261.27	178.2	181.22
4.35	12.54	5.27	-2.41	124.27	200.05	98.23	68.42

3.56	11.7	4.51	-3.12	130.74	207.7	103.57	75.96
3.81	12.01	4.79	-3.03	133.88	210.53	108.37	80.46
3.71	11.76	4.69	-3.04	128.33	213.09	103.52	73.27
3.23	11.57	4.01	-3.86	161.55	251.37	146.09	132.16
3.71	12.02	4.45	-3.52	154.87	243.99	141.74	128.01
2.65	11.02	3.5	-4.34	168.02	260.76	151.11	139.07
2.7	11.08	3.55	-4.29	166.44	259.37	149.88	137.39
3.09	11.44	3.87	-3.98	162.51	253.71	146.8	132.71
3.17	11.53	3.98	-3.97	151.7	237.42	131.33	109.53
3.16	11.53	3.98	-3.97	151.75	237.45	131.36	109.58
3.17	11.54	3.99	-3.96	151.13	237.18	130.88	108.93
3.16	11.53	3.98	-3.97	151.81	237.49	131.41	109.64
3.37	11.68	4.14	-3.83	152.66	235.3	129.78	108.95
3.26	11.6	4.04	-3.92	153.27	237.5	131.03	110.66
3.18	11.53	3.99	-3.97	153.7	238.16	131.78	111.49
3.39	11.7	4.15	-3.82	152.38	235.25	129.52	108.97
3.39	11.71	4.15	-3.78	150.38	236.49	129.4	107.81
2.94	11.27	3.93	-4.06	148.55	232.37	128.76	109.08
2.76	11.14	3.75	-4.22	150.85	235.84	135.06	117.59
3.64	12.06	4.46	-3.52	151.04	234.76	139.54	124.46
2.75	11.15	3.72	-4.27	152.4	238.09	138.72	121.15
2.68	11.08	3.67	-4.32	153.72	239.85	140.09	122.68
2.8	11.2	3.8	-4.22	151.54	236.63	137.03	118.37
2.73	11.12	3.75	-4.26	151.54	235.17	134.65	116.78
2.75	11.14	3.75	-4.26	151.15	235.46	135.2	117.3
2.49	10.9	3.55	-4.44	154.36	238.93	137.25	120.63
2.99	11.38	3.89	-4.12	150.52	234.91	134.79	115.29
2.98	11.36	3.9	-4.1	148.98	234.84	136.27	118.66
2.76	11.16	3.66	-4.34	153.56	238.76	138.21	120.01
3.28	11.64	4.04	-3.93	150.26	232.41	131.72	111.36
3.25	11.66	4.01	-3.87	152.24	237.38	137.09	120.23
3.25	11.65	4.01	-3.87	151.98	237.25	136.95	120
3.24	11.64	4.01	-3.88	152.05	237.21	137.02	119.98
3.25	11.65	4.01	-3.87	151.88	237.09	136.87	119.79
3.4	11.82	4.12	-3.78	152.76	238.01	136.87	120.43
2.73	11.14	3.73	-4.27	152.54	236.16	136.52	120.42
2.73	11.14	3.73	-4.26	152.26	236.01	136.42	120.01

2.97	11.32	3.93	-4.07	150.31	232.84	131.6	114.63
2.84	11.22	3.81	-4.19	149.83	233.62	133.65	116.18
3.34	11.76	4.22	-3.66	151.35	237.99	143.51	129.98
2.98	11.36	3.77	-4.05	161.85	246.96	155.63	149.89
3.37	11.84	4.12	-3.83	154.94	241.14	141.14	125.61
3.37	11.85	4.13	-3.82	154.64	240.93	140.96	125.46
3.37	11.84	4.12	-3.83	154.81	241.03	141.05	125.51
3.37	11.85	4.13	-3.82	154.62	240.91	140.95	125.44
3.37	11.85	4.13	-3.82	154.56	240.87	140.92	125.41
3.31	11.91	4.13	-3.92	164.36	250.34	153.85	142.33
2.98	11.33	3.73	-4.07	164.14	251.98	163.2	158.71
2.86	11.23	3.64	-4.17	167.17	254.82	167.98	164.88
3.18	11.5	3.91	-3.96	163.02	249.37	161.94	156.59
3.12	11.44	3.86	-4	164.59	250.22	162.68	156.65
2.84	11.22	3.64	-4.19	164.27	254.2	167.89	165.36
3.34	11.93	4.15	-3.89	160.47	246.88	149.68	137.95
3.34	11.92	4.14	-3.86	160.36	248.68	149.28	136.18
3.24	11.77	4.04	-3.93	162.17	252.03	152.59	141.42
3.47	11.87	4.23	-3.74	151.63	237.69	136.7	119.93
2.94	11.19	4.01	-3.97	143.73	227.59	119.13	94.99
3.48	11.86	4.2	-3.72	150.14	234.02	134.5	115.89
3.34	11.68	4.13	-3.85	151.47	236.76	130.46	109.62
3.15	11.51	3.97	-3.99	153.67	238.31	131.84	111.51
3.36	11.7	4.15	-3.83	151.85	236.43	130.51	109.51
3.23	11.66	4.03	-3.86	159.57	246.05	149.66	140.25
3.23	11.55	3.98	-3.98	151.91	235.71	130.47	110.01
3.2	11.55	3.96	-3.98	153.01	239.03	135.27	115.52
3.46	11.9	4.21	-3.76	151.55	237.67	136.17	119.24
3.64	12.09	4.39	-3.61	150.54	236.23	135.75	118.46
3.6	11.94	4.3	-3.68	149.65	234.29	130.83	110.92
3.12	11.39	3.96	-4.02	143.19	230.58	121.9	96.82
3.76	12.17	4.49	-3.49	152.31	238.06	137.08	120.96
3.4	11.86	4.14	-3.8	153.59	239.79	139.91	123.92
3.36	11.77	4.2	-3.78	156.7	246.69	141.98	126.63
3.23	11.6	4.09	-3.87	158.27	248.67	143.35	128.48
3.24	11.63	4.06	-3.92	154.34	241.22	139.07	121.65
3.13	11.54	3.97	-3.98	154.4	242.51	139.92	122.52

3.53	12.04	4.31	-3.69	152.25	240.42	140.5	124.07
3.53	12.04	4.31	-3.68	152.2	240.34	140.42	123.97
3.48	11.8	4.22	-3.74	143.84	229.81	124.24	100.78
2.22	10.55	3.17	-4.54	179.26	266.58	185.62	192.92
1.97	10.34	2.92	-4.71	181.27	271.18	193.58	203.56
1.97	10.34	2.92	-4.71	181.24	271.15	193.55	203.52
2.63	10.98	3.45	-4.24	167.22	256.82	174.99	175.99
2.12	10.5	3.05	-4.59	177.35	269.04	190.87	199.53
2.59	10.97	3.45	-4.27	168.72	258.57	174.95	177.65
2.29	10.58	3.28	-4.47	175.62	264.3	181.8	187.86
2.17	10.53	3.21	-4.54	177.8	267.12	184	192.24
2.19	10.55	3.23	-4.51	177.22	266.61	182.69	190.74
3.78	11.9	4.94	-3.16	118.94	208.74	99.39	64.9
3.54	12	4.28	-3.67	150.61	238.63	139.19	123.14
2.35	10.75	3.25	-4.46	174	264.55	180.15	184.77
2.17	10.43	3.43	-3.98	151.5	248.23	161.62	156.25
2.01	10.22	3.24	-4.11	139.1	240.1	151	143.39
1.68	9.88	2.99	-4.37	156.08	250.14	168.56	166.52
1.66	9.93	2.98	-4.4	159.95	257.37	171.1	170.03
1.97	10.2	3.23	-4.19	154.39	252.13	166.23	162.23
1.83	10.07	3.12	-4.3	157.78	256.31	170.78	168.69
1.52	9.83	2.88	-4.51	165.39	263.63	178.14	179.68
1.56	9.86	2.91	-4.48	163.83	261.49	175.78	176.4
1.75	9.95	3.02	-4.34	153	247.93	164.9	161.21
1.73	9.93	3.01	-4.36	154.33	249.02	166.76	163.55
1.79	9.98	3.05	-4.31	152.75	247.09	164.86	161.24
2.15	10.33	3.34	-4	148.12	243.57	157.45	151.01
2.06	10.27	3.23	-4.07	152.48	243.33	164.59	160.89
1.75	9.98	3.01	-4.33	156.04	251.43	167.37	164.95
1.9	10.07	3.13	-4.24	150.19	243.57	162.08	156.99
1.83	10.02	3.08	-4.29	151.75	245.69	164.07	159.58
1.74	9.94	3.02	-4.35	153.7	248.4	165.94	162.56
1.98	10.22	3.24	-4.18	152.63	250.07	163.94	159.11
1.8	10.06	3.1	-4.31	156.29	254.06	167.28	165.06
1.84	10.1	3.12	-4.26	150.86	249.65	160.16	156.52
1.69	9.98	3.04	-4.36	154.42	255.15	167.38	165.3
2.02	10.23	3.24	-4.1	138.56	239.3	149.92	141.93

2	10.21	3.23	-4.12	139.5	240.54	151.27	143.75
1.99	10.19	3.26	-4.15	146.94	245.31	159.64	154.81
1.68	9.96	3	-4.39	159.56	256.88	170.66	169.44
1.93	10.17	3.19	-4.22	151.38	249.56	161.42	157.22
1.79	10.06	3.12	-4.29	150.6	251.8	163.53	159.51
1.88	10	3.14	-4.19	142.03	238.07	153.24	148.22
1.87	9.99	3.13	-4.2	141.69	238.91	152.72	147.5
2.01	10.15	3.26	-4.1	137.95	232.8	147.63	139.74
1.85	10.07	3.12	-4.18	139.44	237.7	150.73	143.94
1.55	9.65	2.96	-4.39	138.45	239.45	152.85	144.65
1.86	9.94	3.15	-4.18	127.32	237.35	139.52	127.27
1.85	10.01	3.11	-4.22	140.4	239.38	150.91	144.81
1.83	10.18	3.18	-4.24	169.21	268.03	181.36	182.41
1.82	10.17	3.17	-4.26	170.18	268.78	182.36	183.51
1.82	10.17	3.17	-4.26	170.13	268.87	182.35	183.64
2.08	10.31	3.34	-4.1	170.83	266.71	180.63	181.09
1.99	10.3	3.33	-4.16	170.33	270.67	182.42	181.61
1.87	10.25	3.2	-4.2	170.65	274.9	184.45	182
1.65	10.08	3.04	-4.41	177.8	281.78	191.81	192.48
1.82	10.14	3.19	-4.27	175.21	272.75	188.64	190.41
1.95	10.23	3.23	-4.23	173.02	269.92	182.01	183.15
1.93	10.22	3.22	-4.25	174.14	271.4	183.35	185.21
1.74	10.06	3.09	-4.34	178.24	277.86	191.94	195.56
1.72	10.04	3.08	-4.35	178.85	278.48	192.65	196.64
1.73	10.05	3.1	-4.35	178.86	278.46	192.49	196.77
1.74	10.06	3.11	-4.34	178.37	277.99	191.94	195.96
2	10.37	3.32	-4.06	165.81	269.04	178.4	173.6
1.88	9.95	3.26	-4.13	130.87	231.94	143.98	130.74
2	10.08	3.33	-4.05	130.45	221.83	140.68	126.79
2.04	10.1	3.35	-4	129.27	221.01	140.27	125.11
1.53	9.62	2.88	-4.44	139.35	239.71	151.73	143.61
1.7	9.8	3.1	-4.25	139.62	234.45	150.79	141.03
1.85	10.07	3.12	-4.18	139.45	237.7	150.74	143.95
2.82	11.07	3.62	-4.13	173.82	270.28	162.24	155.21
1.91	10.27	2.94	-4.88	201.01	300.34	188.82	189.48
2	10.34	3.02	-4.82	198.84	297.91	186.44	186.52
1.89	10.26	2.91	-4.89	201	300.33	190.17	191.5

1.87	10.18	2.88	-5.01	207.2	307.88	195.27	199.98
1.91	10.28	2.9	-4.9	204.27	304.4	194.47	201.7
1.38	9.79	2.54	-5.29	218.26	321.88	211.01	220.63
1.85	10.22	2.85	-4.96	208.4	307.07	198.26	206.17
1.87	10.25	2.89	-4.86	200.76	299.83	189.56	190.9
1.97	10.32	2.99	-4.84	199.48	298.66	187.12	187.35
1.33	9.68	2.45	-5.36	220.96	325.36	207.72	213.13
2.19	10.54	3.17	-4.57	196.71	295.55	190.06	194.52
2.18	10.53	3.16	-4.58	197.2	296.1	190.53	195.17
2.44	10.76	3.35	-4.41	187.75	286.29	179.15	179.48
2.45	10.73	3.39	-4.41	193.56	289.96	183.07	185.33
2.59	10.91	3.46	-4.3	183.5	281.72	173.96	171.22
2.76	11.13	3.6	-4.17	179.94	274.62	173.67	174.07
2.39	10.68	3.34	-4.4	191.84	289.92	179.97	179.68
2.36	10.65	3.24	-4.53	180.85	280.69	169.35	164.56
2.52	10.79	3.37	-4.41	175.11	274.22	163.79	156.36
2.52	10.78	3.37	-4.41	175.17	274.31	163.84	156.41
2.52	10.78	3.37	-4.41	175.2	274.31	163.85	156.41
2.22	10.52	3.12	-4.63	183.99	280	169.11	164.4
2.15	10.47	3.09	-4.65	191.67	288.48	177.57	174.49
1.84	10.22	2.89	-4.92	202.5	301.9	190.72	191.94
1.72	10.04	2.77	-5.01	203.08	302.69	187.84	186.87
1.97	10.29	2.96	-4.89	201.95	302.55	190.27	191.69
1.61	10.01	2.7	-5.09	214.91	314.44	206.86	217.54
1.62	10.04	2.73	-5.1	214.16	312.79	206.64	217.45
2.56	10.82	3.41	-4.37	174.45	271.26	161.83	154.14
2.56	10.84	3.41	-4.37	173.41	270.8	160.59	152.04
2.8	11.17	3.61	-4.14	177.48	273.43	170.44	168.27
1.96	10.21	2.83	-5.03	185.88	284.45	166.39	157.82
1.53	9.8	2.47	-5.39	193.68	296.19	173.57	165.26
2.58	10.83	3.42	-4.39	174.74	270.26	159.37	149.84
1.23	9.59	2.44	-5.37	216.64	320.24	201.29	202.63
2.22	10.44	3.08	-4.76	180.02	278.09	160.76	152.28
1.35	9.65	2.33	-5.5	199.93	302.78	181.04	175.53
1.4	9.64	2.39	-5.59	205.43	310.43	183.54	175.07
1.44	9.7	2.41	-5.45	205.53	306.69	184.77	180.95
1.53	9.79	2.51	-5.35	200.93	302.3	180.36	176.39

1.8	10.07	2.73	-5.08	180.52	279.09	158.05	141.85
2.64	10.83	3.4	-4.45	165.53	261.38	147.87	133.62
1.38	9.65	2.35	-5.54	200.07	302.07	177.82	171.63
1.43	9.69	2.38	-5.52	198.8	301.2	176.96	169.78
2.29	10.55	3.19	-4.7	180.62	277.93	164.66	156.32
2.26	10.52	3.16	-4.72	182.09	279.06	165.95	157.86
2.02	10.28	2.92	-4.89	185.13	283.97	165.84	157.53
2.15	10.37	3.02	-4.82	182.43	279.98	164.32	155.78
2.06	10.27	2.98	-4.89	188.47	285.99	169.61	163.47
1.91	10.16	2.83	-4.99	187.67	286.92	167.96	160.6
2.48	10.73	3.34	-4.5	176.82	272.34	160.58	152.61
1.72	10.02	2.62	-5.26	190.11	288.73	168.16	161.72
1.67	9.93	2.71	-5.17	208.45	310.21	189.75	188.91
2.08	10.35	2.97	-4.83	187.43	283.95	171.96	166.47
2.19	10.45	3.08	-4.76	184.55	280.8	168.27	161.63
1.54	9.79	2.46	-5.43	197.51	297.89	176.73	170.74
1.8	10.04	2.72	-5.26	202.25	307.66	185.43	183.3
1.76	9.99	2.72	-5.08	192.33	292.6	171.66	160.84
1.19	9.47	2.26	-5.63	201.98	305.34	178.62	169.23
1.64	9.95	2.55	-5.31	191.19	290.08	169.7	162.05
1.33	9.55	2.34	-5.62	206.06	310.84	182.81	173.5
2.05	10.33	2.98	-4.8	189.76	286.52	173.92	170.65
1.96	10.22	2.83	-4.96	180.19	279.17	159.57	146.41
1.91	10.17	2.8	-4.97	184.06	282.09	163.21	150.94
1.86	10.12	2.77	-5.02	184.07	283.96	163.24	150.64
2.63	10.83	3.45	-4.38	173.51	269.66	156.46	146.38
2.2	10.45	3.08	-4.73	180.12	280.82	165.34	156.38
2.7	10.88	3.44	-4.38	166.25	261.59	148.51	133.95
2.57	10.78	3.34	-4.51	166.36	262.62	148.54	133.79
2.41	10.66	3.28	-4.61	176.27	274.15	160.23	151.2
2.33	10.58	3.14	-4.73	172.11	266.84	149.22	131.63
2.07	10.33	2.9	-4.87	178.55	276.13	157.47	144.56
1.98	10.24	2.84	-4.94	180.21	278.62	159.27	146.52
1.98	10.24	2.84	-4.96	179.11	278.12	156.79	143.01
2	10.26	2.85	-4.95	178.69	277.42	156.4	142.39
1.92	10.18	2.8	-5	181.2	280.98	159.41	146.12
2.28	10.69	3.19	-4.53	180.41	271.14	193.1	201.68

2.65	11.03	3.49	-4.25	174.24	261.43	183.09	186.64
1.93	10.3	2.95	-4.75	191.58	285.7	208.23	227.13
2.27	10.66	3.19	-4.57	188.8	282.08	198.88	214.16
2.29	10.69	3.2	-4.55	187.89	281.35	197.9	212.61
2.57	10.96	3.44	-4.38	181.02	272.72	187.97	197.85
2.35	10.77	3.25	-4.51	184.93	277.9	193.74	204.26
2.41	10.83	3.26	-4.48	184.32	275.75	189.24	196.87
2.38	10.82	3.28	-4.5	184.26	275.2	190.94	201.17
2.31	10.76	3.24	-4.53	183.12	274.45	190.31	199.56
2.7	11.1	3.54	-4.24	175.64	261.85	180.45	186.39
2.71	11.09	3.58	-4.25	174.87	263.16	180.2	183.5
2.17	10.6	3.09	-4.58	182.02	273.3	195.35	206.41
2.67	11.09	3.52	-4.29	177.94	270.59	181.63	186.9
2.67	11.09	3.51	-4.29	178	270.61	181.67	186.95
3.11	11.54	3.86	-4	166.7	258.33	166.84	164.93
3.04	11.46	3.81	-4.03	167.56	259.22	168.06	167.74
3.24	11.71	3.98	-3.91	162.66	254.64	162.36	157.77
3.05	11.44	3.88	-4.04	167.66	258.06	170.42	168.93
1.83	10.21	2.92	-4.77	207.99	307.34	206.99	221.73
2.29	10.74	3.26	-4.48	191.87	287.2	199.36	213.3
2.47	10.82	3.41	-4.29	192.04	290.35	189.11	195.39
2.58	10.91	3.5	-4.22	189.53	287.63	187.22	192.54
2.31	10.7	3.26	-4.41	195.77	293.75	195.3	203.04
2.43	10.78	3.37	-4.32	192.86	291.65	190.5	197.38
2.01	10.4	3.07	-4.64	202.01	301.57	201.69	213.9
1.98	10.37	3.04	-4.66	202.8	302.52	202.5	215.16
1.98	10.37	3.04	-4.66	202.63	302.63	202.66	215
1.74	10.03	2.86	-4.85	213.31	311.63	212.88	229.79
2.47	10.87	3.38	-4.39	186.62	282.17	190.37	197.08
2.44	10.83	3.35	-4.42	189.7	285.72	191.45	199.2
2.23	10.7	3.23	-4.53	194.36	290.05	198.2	210.31
2.61	11	3.48	-4.32	185.42	279.72	186.08	192
2.45	10.83	3.37	-4.39	191.6	287.42	189.86	198.49
2.51	10.89	3.42	-4.35	190.19	285.01	188.14	195.85
1.99	10.4	3.05	-4.68	200.86	297.08	208.19	228.31
2.04	10.47	3.11	-4.62	201.45	299.14	210.32	227.44
1.48	9.93	2.61	-5.04	209.2	306.17	226.58	256.15

1.95	10.28	2.93	-4.75	192.45	286.68	209.47	228.33
2.25	10.64	3.17	-4.58	190.87	283.36	199.73	215.31
1.2	9.58	2.39	-5.21	219.33	314.62	239.01	276.44
1.69	10.11	2.71	-4.94	203.92	298.31	217.5	243.83
1.5	9.92	2.59	-5.03	209.29	307.02	227.07	257.26
1.48	9.92	2.59	-5.04	209.51	306.9	227.16	257.36
1.92	10.33	2.95	-4.79	199.77	295.62	212.05	232.57
2.22	10.62	3.17	-4.59	191.96	285.62	201.18	217.52
2.21	10.62	3.17	-4.59	192.03	285.64	201.28	217.54
1.85	10.26	2.91	-4.82	201.92	297.95	213.99	235.75
1.9	10.32	2.94	-4.79	200.39	296.07	212.09	233.06
1.8	10.21	2.87	-4.85	203.51	299.76	215.7	238.25
1.74	10.15	2.84	-4.88	205.41	302.1	217.75	241.51
1.81	10.21	2.88	-4.84	203.62	300.06	215.85	238.05
1.94	10.32	2.96	-4.79	198.79	294.82	212.31	234.38
1.92	10.31	2.94	-4.8	199.32	295.48	212.88	235.25
1.88	10.26	2.91	-4.83	200.61	297.13	214.35	237.48
1.94	10.33	2.96	-4.79	198.64	294.65	212.19	234.17
1.69	10.09	2.79	-4.96	206.78	304.49	220.66	247.17
1.63	10.02	2.76	-4.94	215.4	309.66	226.62	255.78
1.68	10.1	2.76	-4.91	208.58	303.21	222.82	251.48
2.19	10.54	3.16	-4.58	189.21	282.44	203.61	220.29
1.68	10.09	2.72	-4.92	203.78	300.74	220.16	247.42
1.41	9.87	2.56	-5.08	210.28	307.94	228.24	259.01
2.2	10.56	3.16	-4.59	189.28	282.84	202.16	219.34
2.37	10.79	3.27	-4.5	185.57	278.73	193.9	205.21
2.35	10.77	3.26	-4.51	186.01	279.22	194.5	205.99
2.37	10.79	3.28	-4.47	186.37	279.07	194.31	207.22
1.4	9.73	2.48	-5.37	216.66	322.64	201.42	204.23
1.71	9.94	2.66	-5.38	209.29	315.56	189.83	187.42
1.64	9.87	2.59	-5.4	210.49	317.85	192.46	190.7
1.88	10.14	2.9	-5.01	207.71	312.05	193.63	195.36
1.68	9.99	2.68	-5.15	209.75	311.72	193.46	195.47
1.5	9.83	2.55	-5.29	214.78	320.24	201.74	203.82
1.59	9.9	2.65	-5.24	213.31	319.32	199.51	201.41
1.46	9.75	2.47	-5.47	215.94	323.11	198.92	198.4
1.7	9.93	2.68	-5.31	212.28	319.79	194.92	193.76

1.98	10.22	2.98	-4.9	206.05	308.77	192.66	195.27
1.74	10.04	2.8	-5.05	210.61	314.49	197.95	201.35
1.78	10.14	2.8	-4.87	183.23	276.52	197.86	209.95
1.49	9.87	2.59	-5.03	191.13	286.6	208.94	226.94
1.62	9.97	2.74	-4.94	201.99	300.03	226.15	254.93
1.66	10	2.82	-4.85	201.96	299.15	224.5	253.63
1.72	10.09	2.76	-4.91	184.8	278.21	200.02	213.01
1.9	10.23	2.9	-4.79	180.92	272.48	194.73	204.42
2.1	10.52	3.03	-4.59	181.58	272.92	197.02	207.57
1.72	10.11	2.76	-4.9	184.79	277.39	200.19	212.57
1.58	9.95	2.66	-4.96	189.76	288.79	210.36	227.38
1.57	9.99	2.67	-4.84	185.78	285.77	202.35	215.11
1.45	9.84	2.6	-5.03	192.53	293.14	213.86	233.68
2.16	10.49	3.2	-4.55	177.95	268.29	183.33	190.99
2.36	10.71	3.41	-4.34	168.31	258.38	172.78	173.04
2.09	10.43	3.13	-4.64	179.17	269.1	186.03	193.97
2.01	10.35	3.06	-4.61	176.59	271.42	187.21	194.47
1.59	10.01	2.73	-4.87	188.55	287.62	205.08	218.55
1.62	10.03	2.74	-4.84	188.81	287.56	204.76	219.05
1.59	10	2.69	-4.79	184.21	284.32	201.5	211.32
1.59	10	2.69	-4.79	184.29	284.3	201.58	211.33
1.52	9.94	2.64	-4.83	185.55	286.17	203.95	214.51
1.94	10.36	2.93	-4.75	188.41	282.13	204.47	220.71
1.64	9.96	2.72	-5.11	212.99	318.69	201.02	206.07
1.29	9.73	2.46	-5.38	220.78	322.92	213.63	225.4
0.64	8.98	2.01	-5.53	253.48	358.56	276.37	331.23
1.1	9.43	2.45	-5.14	235.15	341	247.92	286.24
1.58	9.94	2.76	-4.88	220.91	321.17	230.17	260.88
0.9	9.24	2.3	-5.3	239.21	343.17	255.16	297.97
1.82	10.19	2.91	-4.8	209.25	306.88	217.8	244.17
1.16	9.59	2.42	-5.23	224.91	325.14	238.27	273.83
1.22	9.6	2.47	-5.12	228.31	327.72	243.95	284.84
1.08	9.49	2.37	-5.2	232.5	332.35	249.42	292.19
1.34	9.66	2.57	-5.13	224.22	324.9	226.52	248.04
0.7	9.07	2.08	-5.56	240.52	343.07	244.72	271.67
1.08	9.46	2.41	-5.2	232.43	333.86	249.09	290.06
1.03	9.39	2.28	-5.44	237.01	338.44	247.68	280.72

1.12	9.54	2.42	-5.24	226	326.22	239.48	274.04
1.13	9.45	2.38	-5.24	224.68	323.13	246.29	288.55
1.26	9.6	2.46	-5.15	221.48	320.2	242.51	282.27
1.29	9.74	2.49	-5.18	219.32	317.61	233.57	266.72
1.15	9.55	2.42	-5.14	225.6	325.27	248.19	288.53
1.62	9.91	2.7	-5.29	216.73	323.82	200.78	200.42
0.9	9.27	2.16	-5.37	216.56	317.98	240.96	276.69
1.32	9.67	2.58	-4.93	202.48	304.06	218.69	237.92
1.13	9.57	2.37	-5.09	205.34	307.59	223.71	248.31
1.2	9.65	2.45	-5.02	200.26	302.84	219.44	239.66
0.95	9.32	2.23	-5.33	215.43	317.77	234.9	268.89
1.13	9.57	2.38	-5.09	205.08	307.67	223.8	248
1.29	9.72	2.48	-5.03	197.43	299.78	216.82	236.8
1.15	9.59	2.37	-5.12	202.76	306.43	223.51	248.13
1.32	9.76	2.49	-5.02	198.33	300.86	216.56	237.17
0.88	9.31	2.18	-5.35	215.02	317.48	235.94	271.7
0.99	9.43	2.24	-5.3	209.84	312.09	233.79	266.47
0.71	9.11	2.05	-5.47	223.44	326.1	246.76	287.79
1.06	9.23	2.24	-5.62	212.04	319.75	187.07	176.24
3.01	11.44	3.77	-4.01	172.12	263.66	169.68	165.82
3.14	11.54	3.88	-3.92	169.69	262.96	165.05	160.91
2.96	11.23	3.73	-4	167.26	260.86	155.59	144.57
3.1	11.47	3.88	-3.92	171.65	264.57	165.67	162.92
3.45	11.77	4.13	-3.86	147.35	236.41	128.86	106.3
2.54	10.77	3.26	-4.59	164.95	260.03	144.75	128.99
3.41	11.67	4.07	-3.9	148.29	236.8	131.03	110.24
3.41	11.67	4.07	-3.9	148.3	236.68	131.01	109.91
3.23	11.61	3.95	-3.87	165.83	257.67	157.37	150.79
2.61	10.88	3.44	-4.46	166.41	260.5	149.54	135.25
3.18	11.52	3.92	-3.9	160.49	249.89	144.29	129.85
3.18	11.52	3.91	-3.9	160.49	249.91	144.34	129.89
3.4	11.68	4.1	-3.83	148.88	235.47	127.28	104.54
3.16	11.48	3.97	-4.06	147.17	235.76	124.4	99.92
3.15	11.41	4.02	-4	147.15	234.23	124.59	100.56
3.49	11.86	4.19	-3.79	145.89	234.65	127.7	104.86
3.49	11.85	4.19	-3.79	145.93	234.61	127.78	104.86
3.38	11.77	4.13	-3.8	145.91	233.26	126.01	102.22

3.38	11.77	4.13	-3.8	145.9	233.26	126.01	102.23
3.39	11.78	4.13	-3.81	146.55	233.74	126.22	102.52
3.46	11.79	4.21	-3.74	145.04	229.84	123.78	100.77
3.3	11.57	4.06	-3.94	145.64	232.53	125.34	100.28
3.37	11.69	4.14	-3.77	147.88	235.78	128.69	106.69
2.66	10.97	3.53	-4.51	153.57	242.27	128	102.65
2.89	11.16	3.78	-4.24	147.43	240.44	125.19	101.07
2.46	10.76	3.29	-4.54	159.73	250.16	133.02	108.77
3.49	12.01	4.19	-3.83	155.29	247.18	150.39	140.26
3.46	12	4.23	-3.82	156.54	246.75	148.64	138
3.43	11.93	4.17	-3.86	156.53	248.59	151.45	142.84
3.27	11.76	4.02	-3.86	160.87	251.02	158.35	151.05
3.27	11.76	4.02	-3.86	160.85	251.03	158.35	151.03
3.53	11.81	4.29	-3.67	140.62	226.71	119.09	94
3.5	11.81	4.23	-3.73	143.92	230.1	122.68	98.84
3.48	11.82	4.16	-3.83	146.5	235.08	128.88	106.43
3.49	11.83	4.16	-3.83	146.45	235.04	128.82	106.37
3.4	11.74	4.1	-3.94	149.33	236.75	129.89	108.31
3.43	11.75	4.11	-3.88	147.73	235.83	128.93	106.5
3.4	11.73	4.1	-3.93	148.7	236.68	130.13	108.91
3.4	11.73	4.1	-3.93	148.69	236.66	130.11	108.87
3.35	11.68	4.05	-3.93	149.37	237.94	131.44	109.85
3.35	11.68	4.05	-3.93	149.38	237.94	131.46	109.87
3.36	11.68	4.05	-3.93	149.37	237.94	131.43	109.81
3.42	11.74	4.1	-3.91	148.12	236.16	129.91	107.79
2.98	11.24	3.71	-4.33	151.73	240.85	128.05	104.24
2.8	11.07	3.6	-4.4	152.55	241.17	129.35	104.13
3.34	11.68	4.02	-4.04	150.08	238.92	129.38	107.35
3.35	11.68	4.02	-4.04	150.1	238.91	129.36	107.32
2.52	10.77	3.37	-4.5	160.25	255.39	138.6	116.24
2.62	10.88	3.44	-4.44	165.63	258.91	147.46	132.25
3.44	11.76	4.13	-3.86	148.34	235.67	128.44	106.09
3.5	11.84	4.16	-3.82	146.09	234.28	128.05	105.81
3.46	11.87	4.19	-3.75	148.95	236.6	133.32	115.26
3.54	11.74	4.43	-3.45	135.56	221.68	110.71	82.13
3.39	11.59	4.29	-3.57	136.49	224.19	112.68	84.71
3.39	11.59	4.29	-3.58	136.51	224.42	112.87	84.62

3.38	11.6	4.31	-3.57	135.88	224.37	113.27	84.91
3.3	11.5	4.21	-3.7	136.29	225.85	114.26	86.63
3.17	11.37	4.1	-3.8	138.09	227.14	115.56	88.49
3.08	11.3	4.02	-3.9	140.77	229.13	116.36	89.02
3.11	11.31	4.05	-3.85	139.05	228.01	116.25	89.61
3.21	11.42	4.14	-3.79	138.34	227.51	115.68	88.56
3.19	11.41	4.12	-3.8	138.59	227.78	115.91	88.82
3.28	11.5	4.21	-3.66	136.88	225.72	114.02	86.33
3.27	11.5	4.18	-3.71	138.16	225.74	114.01	86.42
3.14	11.42	4.06	-3.91	142.81	231.2	119.67	94.43
2.57	10.88	3.64	-4.3	150.47	238.08	126.3	103.02
2.62	10.92	3.68	-4.29	149.72	240.92	127.43	104.23
3.31	11.53	4.25	-3.59	136.88	222.14	113.29	84.72
2.94	11.24	3.9	-4.03	146.78	232.73	125.97	102.65
2.21	10.44	3.3	-4.75	156.67	248.91	131.72	100.75
2.22	10.5	3.34	-4.75	157.88	248.58	131.93	103.57
2.32	10.59	3.38	-4.67	153	244.49	129	97.29
2.23	10.5	3.26	-4.75	154.7	245.66	129.59	97.36
2.16	10.46	3.23	-4.77	155.99	247.14	130.29	98.57
2.4	10.65	3.45	-4.64	150.04	241.09	126.63	95.11
2.6	10.92	3.58	-4.54	152.01	239.97	126.42	97.67
2.75	11.03	3.71	-4.38	150.15	238.57	125.69	98.29
2.81	11.06	3.75	-4.33	149.84	239	124.99	98.18
2.85	11.1	3.78	-4.29	149.52	238.57	124.34	97.36
3.06	11.37	3.9	-4.12	148.73	237.52	124.49	100.4
2.89	11.21	3.76	-4.25	150.38	240.17	127.19	102.31
2.78	11.09	3.61	-4.38	149.65	239.64	126.77	100.84
2.82	11.13	3.67	-4.34	149.03	238.34	125.09	98.3
2.82	11.17	3.67	-4.38	147.21	235.99	122.05	96.14
2.56	10.87	3.54	-4.59	153.47	242.65	127.34	100.29
2.81	11.16	3.69	-4.41	148.62	235.59	122.59	94.52
2.68	10.97	3.61	-4.5	149.81	238.45	125.11	97.02
2.59	10.9	3.57	-4.56	152.26	240.26	126.8	98.16
2.6	10.93	3.59	-4.5	149.91	239.87	126.37	97.34
2.45	10.76	3.51	-4.65	149.47	241.08	126.58	95.66
2.31	10.6	3.43	-4.7	152.79	243.85	129.29	98.57
1.89	10.15	3.08	-4.98	166.27	256.88	139.99	111.4

2.73	10.96	3.72	-4.26	145.64	233.34	120.12	91.16
2.27	10.61	3.37	-4.63	158.48	246.4	131.32	108.09
3.18	11.41	4.08	-3.88	137.12	227.11	113.55	84.54
3.09	11.3	4.03	-3.97	137.71	227.19	114.35	84.67
2.72	11.01	3.68	-4.45	147.56	236.13	124.34	94.9
2.06	10.3	3.24	-4.86	152.74	244.72	128.14	98.85
2.32	10.61	3.44	-4.58	151.46	240.6	125.9	99.33
2.55	10.85	3.55	-4.46	155.53	243.14	128.95	104.22
2.54	10.83	3.58	-4.47	154	243.49	128.45	101.23
2.78	11.04	3.72	-4.43	146.5	233.15	121.74	93.26
1.95	10.25	3.16	-4.99	157.48	249.22	131.44	102.13
2.44	10.71	3.45	-4.68	149.65	239.71	125.8	96.63
3.04	11.26	4.01	-4.01	139.35	227.15	115.13	84.69
3.14	11.36	4.07	-3.93	137.33	226.26	113.6	83.9
2.94	11.18	3.86	-4.06	142.83	230.67	116.87	88.22
2.73	11.02	3.73	-4.22	144.36	234.08	120.05	92.38
3.03	11.27	3.98	-4	138.39	228.68	115.43	86.36
2.82	11.06	3.79	-4.19	144.62	231.01	118.77	89.67
2.98	11.19	3.96	-4.05	140.53	228.46	115.78	86.61
2.6	10.87	3.61	-4.36	146.93	237.62	123.27	95.61
3.05	11.29	3.9	-3.96	138.66	230.11	116.63	87.51
3.02	11.27	3.87	-3.99	139.09	230.08	116.59	87.95
3.33	11.5	4.16	-3.72	134.11	223.26	112.11	82.12
2.49	10.79	3.49	-4.45	149	239.68	126.93	101.75
2.46	10.76	3.47	-4.48	149.52	240.91	127.83	102.64
2.61	10.89	3.64	-4.3	148.67	237.06	124.48	99.26
2.52	10.82	3.59	-4.34	149.89	238.95	126.34	100.8
2.7	10.85	3.82	-4.31	142.09	230.76	117.92	86.25
2.26	10.43	3.51	-4.63	145.95	236.32	122.5	92.79
2.4	10.58	3.58	-4.54	146.91	236.71	122.6	92.56
2.8	11.01	3.8	-4.21	143.54	231.31	118.68	88.56
2.4	10.61	3.58	-4.53	148.59	239.46	123.91	94.32
2.64	10.87	3.68	-4.39	144.23	231.92	120.11	90.09
2.4	10.57	3.57	-4.5	148.53	240.75	125.31	94.89
2.09	10.27	3.35	-4.8	149.76	243.36	127.31	97.84
2.17	10.34	3.38	-4.74	150.18	243.1	126.58	96.38
2.27	10.43	3.47	-4.67	148.94	240.11	124.32	93.62

2.26	10.45	3.43	-4.7	148	239.07	124.91	93.54
2.07	10.31	3.31	-4.8	152.94	244.77	129.32	99.03
2.08	10.31	3.33	-4.79	152.82	244.63	129.37	98.74
2.18	10.39	3.35	-4.75	150.63	241.61	126.93	95.59
2.15	10.38	3.35	-4.76	151.84	243.35	127.53	96.59
2.16	10.4	3.35	-4.74	151.33	243.5	127.5	96.66
2.42	10.65	3.52	-4.56	149.46	237.79	125.52	94.35
2.46	10.68	3.54	-4.54	149.11	237.25	125.13	93.76
2.48	10.7	3.57	-4.54	148.9	237.6	124.74	93.42
2.33	10.58	3.47	-4.63	151.11	241.46	126.61	96.44
2.23	10.51	3.39	-4.71	151.69	245.12	127.51	99.09
2.24	10.49	3.39	-4.71	150.56	244.66	127.66	99.01
2.25	10.49	3.4	-4.7	150.47	244.55	127.56	98.89
2.4	10.63	3.52	-4.59	150.06	240.81	125.67	95.41
2.24	10.45	3.37	-4.74	150.1	240.94	124.84	94.56
2.27	10.51	3.42	-4.7	148.68	239.63	123.92	92.94
2.14	10.38	3.3	-4.8	149.98	241.17	126.12	95.22
1.93	10.18	3.17	-4.92	155.32	248.02	131.19	100.18
2	10.25	3.2	-4.91	155.12	247.33	129.36	100.63
2.96	11.2	3.86	-4.02	143.31	232.87	121.01	91.36
3.12	11.37	3.95	-3.93	138.89	228.64	115.48	86.43
2.65	10.99	3.65	-4.33	149.09	237.31	122.65	97.15
2.65	10.99	3.65	-4.33	149.1	237.32	122.66	97.18
2.6	10.92	3.6	-4.35	150.43	238.43	123.8	98.46
3.07	11.28	4.01	-4	136.3	227.31	113.47	83.51
3.05	11.27	4	-4.01	136.61	227.66	113.65	83.83
2.61	10.99	3.58	-4.42	156.54	238.14	136.43	118.78
2.68	11.06	3.64	-4.37	155.49	237.19	135.39	117.82
2.68	11.06	3.64	-4.37	155.53	237.21	135.45	117.85
2.89	11.2	3.91	-4.09	146.84	235.98	123.42	99.04
2.73	11.14	3.67	-4.27	152.02	235.56	130.98	110.5
2.34	10.67	3.39	-4.5	158.38	242.29	136.17	117.68
2.41	10.76	3.45	-4.49	156.16	241.16	135.15	115.98
3.02	11.34	3.88	-4.06	146.07	229.84	124.55	100.26
2.75	11.04	3.72	-4.25	147.68	234.25	125.18	101.02
2.43	10.8	3.46	-4.51	158.46	240.27	135.09	116.73
2.82	11.1	3.75	-4.26	152.81	239.04	125.59	100.92

2.72	11.04	3.72	-4.35	153.01	240.98	128.95	105.27
1.6	9.63	3.07	-5.02	139.17	245.81	128.49	92.92
1.92	9.8	3.48	-4.6	134.27	241.03	120.41	86.28
2.53	10.47	3.95	-4.13	124.62	227.06	111.47	75.73
1.99	9.93	3.42	-4.73	135.11	232.05	118.69	84.28
2.09	10.05	3.52	-4.59	133.48	230.33	117.08	82.84
2.35	10.33	3.82	-4.28	126.76	228.44	112.25	77.62
2.02	9.94	3.5	-4.72	134.46	234.15	116.85	82.67
1.7	9.63	3.24	-4.92	138.83	240.33	120.3	87.27
1.74	9.67	3.28	-4.86	136.92	238.65	119.29	85.87
1.69	9.61	3.26	-4.81	132.83	240.8	121.31	87.23
1.84	9.83	3.26	-4.93	138	236.53	122.95	88.25
2.31	10.39	3.51	-4.53	140.32	232.75	117.63	87.55
2.53	10.48	3.78	-4.38	131.73	223.34	112.27	77.83
2.5	10.48	3.72	-4.36	132.98	225.18	113.69	79.43
2.31	10.36	3.56	-4.48	134.66	229.56	117.29	83.15
2.75	10.82	3.86	-4.18	133.19	223.39	112.33	79.59
2.63	10.69	3.77	-4.26	134.48	222.6	113.4	80.54
2.56	10.62	3.76	-4.33	135.27	224.76	113.88	80.06
2.35	10.4	3.6	-4.51	138.87	231.61	118.31	85.34
2.35	10.41	3.61	-4.51	138.82	231.71	118.27	85.33
2.32	10.38	3.59	-4.53	138.92	232.65	119.59	86.3
2.33	10.39	3.59	-4.53	138.82	232.66	119.31	86.2
2.3	10.37	3.58	-4.54	139.37	233.47	120.76	87.25
2.28	10.33	3.53	-4.61	141.3	234.51	121.02	88.85
2.66	10.69	3.82	-4.32	134.6	227.43	115.24	81.01
2.48	10.46	3.74	-4.36	131.02	223.56	113.05	79.55
2.53	10.51	3.78	-4.32	130.51	223.1	112.7	78.88
2.4	10.41	3.63	-4.41	134.15	226.89	115.7	82.25
2.1	10.07	3.46	-4.61	134.97	230.33	117.75	84.01
2.44	10.53	3.63	-4.39	138.21	230.04	117.92	86.16
2.58	10.73	3.72	-4.32	140.05	231.77	117.25	87.4
2.76	10.9	3.86	-4.21	137.04	228.36	113.76	83.32
3.47	11.54	4.48	-3.61	130.06	219.78	107.27	76.18
3.1	11.15	4.13	-3.89	132.25	221.44	110.08	78.38
2.95	10.99	4.03	-4.03	130.47	221.67	110.24	78.16
2.7	10.72	3.87	-4.23	133.78	224.67	113.12	80.29

2.31	10.43	3.53	-4.65	144.62	235.63	121.29	90.02
3.36	11.53	4.23	-3.69	132.95	222.03	109.94	80.31
3.04	11.2	4.09	-3.99	136.2	226.25	114.14	83.16
2.62	10.77	3.78	-4.27	141.07	232.81	118.6	88.39
2.6	10.74	3.74	-4.3	141.57	233.09	118.46	88.06
2.59	10.73	3.74	-4.29	140.73	231.06	115.82	86.11
2.75	10.87	3.88	-4.15	137.76	228.58	113.63	83.38
2.95	11.05	4.03	-4.02	134.95	225.69	111.37	79.69
3.05	11.13	4.12	-3.95	133.32	222.19	109.59	78.03
2.96	11.06	4.04	-4	134.36	224.9	111.16	79.37
2.66	10.79	3.8	-4.22	138.93	230.02	114.96	85.09
2.64	10.77	3.78	-4.24	139.42	230.14	115.28	85.5
2.68	10.8	3.82	-4.2	138.75	229.19	114.86	84.94
2.71	10.82	3.83	-4.19	138.58	228.63	114.73	84.72
2.72	10.83	3.84	-4.18	138.21	228.39	114.4	84.35
2.71	10.82	3.84	-4.18	138.37	228.59	114.57	84.55
2.92	11.05	3.92	-4.14	137.66	228.75	114.44	83.78
2.81	10.95	3.89	-4.18	136.42	227.57	113.19	82.84
2.36	10.48	3.56	-4.61	144.34	234.84	120.79	90.46
2.21	10.38	3.43	-4.64	138.85	232.49	124.66	89.9
2.46	10.6	3.61	-4.55	142.85	234.57	121.65	88.23
2.01	10.13	3.28	-4.81	148.01	240.5	125.33	94.43
2.31	10.47	3.43	-4.67	142	235.31	121.79	88.04
2.22	10.26	3.5	-4.65	143.05	236.62	122.17	90.61
2.67	10.72	3.8	-4.34	133.97	228.66	115.03	81
2.65	10.6	3.79	-4.25	131.99	221.87	113.02	77.98
2.17	10.27	3.37	-4.69	145.17	237.76	123.96	91.34
2.33	10.48	3.56	-4.63	143.36	233.81	121.42	91.16
1.9	10.11	3.19	-4.95	152.3	244.12	129.03	98.49
1.99	10.2	3.28	-4.86	149.85	242.16	127.08	96.54
1.94	10.15	3.22	-4.89	150.13	244.23	127.84	96.92
2.16	10.36	3.38	-4.72	143.59	238.99	123.79	91.76
2.14	10.33	3.36	-4.74	144.1	239.5	124.2	92.33
2.17	10.36	3.39	-4.72	143.51	238.95	123.75	91.69
2.45	10.6	3.57	-4.54	139.8	232.99	118.71	86.57
2.38	10.5	3.52	-4.66	141.07	234.09	122.04	87.69
2.45	10.59	3.57	-4.56	142.41	234.04	121.14	88.08

2.61	10.77	3.72	-4.41	143.59	233.87	121.17	87.75
2.54	10.68	3.62	-4.49	144.16	234.43	121.8	88.94
2.25	10.37	3.45	-4.71	143.7	237.61	124.01	90.91
2.34	10.49	3.49	-4.63	146.87	239.78	125.36	92.77
2.14	10.35	3.39	-4.77	144.48	239.84	128.8	95.17
2.14	10.34	3.38	-4.77	144.57	239.9	128.87	95.25
2.37	10.51	3.55	-4.51	135.82	228.84	120.05	85.52
2.22	10.36	3.45	-4.63	139.78	235.12	124.44	90.44
2.35	10.48	3.51	-4.53	135.66	227.55	119.72	85.39
1.92	10.03	3.25	-4.83	135.91	242.37	127.92	91.54
1.91	10.06	3.27	-4.84	136.23	242.62	128.75	92.58
1.85	9.81	3.32	-4.91	137.28	236.94	122.99	87.54
1.82	9.83	3.21	-4.94	137.02	236.66	124.36	88.96
1.87	9.87	3.23	-4.93	135.98	237.25	123.71	88.47
2.36	10.46	3.55	-4.57	131.23	228.23	119.55	84.19
1.99	10.03	3.27	-4.88	135.81	234.26	120.88	86.81
2.47	10.6	3.65	-4.49	133.9	228.16	119.74	85.65
2.24	10.45	3.47	-4.7	136.94	233.79	124.51	91.56
1.92	9.99	3.23	-4.84	133.64	238	123.49	88.36
2.56	10.65	3.82	-4.35	129.95	229.96	119.76	83.16
2.02	10.16	3.38	-4.65	133.13	241.25	127.11	90.33
1.96	10.08	3.28	-4.81	135.5	241.45	127.63	91.06
2.04	10.2	3.38	-4.74	134.36	239.56	126.99	90.62
2.04	10.19	3.38	-4.74	134.46	239.69	127.13	90.76
1.79	10.06	3.03	-5.09	159.18	254.42	135.73	105.85
2.13	10.42	3.3	-4.82	154.44	246.55	130.27	98.45
2.03	10.26	3.2	-4.89	150.48	243.71	127.69	96.8
2.67	10.95	3.78	-4.37	142.58	235.19	122.67	88.37
2.18	10.47	3.32	-4.81	156.09	247.65	131.95	100.9
1.94	10.2	3.15	-5.01	158.83	250.36	131.7	102.4
2.2	10.41	3.32	-4.77	146.76	239.54	125.23	93.28
2.17	10.39	3.31	-4.77	148.2	240.3	125.08	93.87
2.19	10.41	3.32	-4.76	147.75	239.96	124.99	93.32
2.2	10.4	3.33	-4.75	146.67	240.24	125.16	92.94
2.16	10.36	3.36	-4.79	149.29	242.12	126.66	94.65
2.22	10.43	3.39	-4.77	148.15	239.3	126.13	93.2
2.24	10.48	3.37	-4.77	151.68	242.18	128.37	94.5

2.31	10.55	3.43	-4.74	148.22	237.1	124.99	91.83
2.39	10.6	3.46	-4.7	146.51	237.49	123.6	90.39
2.61	10.87	3.65	-4.48	143.89	233.24	121.02	87.52
2.58	10.82	3.62	-4.49	146.42	233.82	122.14	88.52
2.64	10.86	3.64	-4.44	144.32	232.9	120.81	87.44
2.03	10.33	3.21	-4.95	154.32	247.38	131.58	101.02
2.03	10.33	3.21	-4.95	154.34	247.41	131.6	101.04
2.01	10.31	3.2	-4.96	154.74	247.88	131.93	101.47
2.17	10.36	3.38	-4.77	150.04	242.89	128.94	96
2.55	10.84	3.64	-4.46	143.62	235.45	124.26	90.26
2.29	10.61	3.31	-4.69	154.67	243.88	127.4	95.36
2.16	10.45	3.22	-4.78	156.22	247.59	130.9	99.12
2.16	10.46	3.23	-4.77	155.99	247.1	130.23	98.51
2.27	10.52	3.33	-4.7	154.83	244.72	130.72	98.93
2.33	10.59	3.39	-4.69	151.37	242.93	127.75	96.62
3.47	11.66	4.34	-3.53	133.67	220.09	109.03	79.87
3.3	11.5	4.21	-3.7	136.29	225.86	114.26	86.65
2.26	10.43	3.51	-4.63	145.9	236.32	122.5	92.77
2.4	10.58	3.58	-4.54	146.91	236.71	122.6	92.55
2.26	10.47	3.39	-4.73	149.73	240.68	124.48	94.17
3	11.19	3.98	-4.06	136.4	227.74	113.6	83.82
2.63	10.55	3.95	-4.2	128.9	222.04	109.92	75.7
2.47	10.49	3.67	-4.35	133.38	225.95	115.26	81.21
2.5	10.48	3.71	-4.36	132.96	225.09	113.72	79.4
2.55	10.55	3.75	-4.33	132.85	223.34	113.38	78.93
2.74	10.8	3.85	-4.2	135.33	225.76	113.4	81.57
4.16	12.37	5.27	-2.4	124.89	214.33	105.4	72.37
4.48	12.75	5.6	-2.02	127.37	222.5	113.01	78.97
4.49	12.76	5.6	-2.02	127.25	222.38	112.98	78.86
4.97	13.35	5.97	-1.66	121.38	211.82	105.25	71.25
3.99	12.07	5.22	-2.63	129.94	222.31	111.56	78.04
4.14	12.26	5.24	-2.77	117.85	205.88	98.61	66.05
4.11	12.21	5.22	-2.8	118.57	205.26	98.01	65.42
4.06	12.19	5.15	-2.81	115.56	205.75	97.8	64.01
4.26	12.37	5.28	-2.66	116.64	202.58	95.3	63.77
4.59	12.97	5.71	-2.03	129.95	223.77	119.35	86.08
4.58	12.95	5.69	-1.97	127.38	223.23	116.4	82.81

4.37	12.68	5.49	-2.23	131.95	231.49	125.14	91.85
4.57	12.99	5.68	-2.04	131.8	227.17	125.47	91.3
3.81	11.95	5.01	-3.13	117.96	208.53	100.63	67.93
3.81	11.95	5.01	-3.12	117.89	208.67	100.56	67.91
4.12	12.16	5.15	-2.92	115.56	204.53	97.97	63.98
3.78	11.85	4.92	-3.26	116.36	209.13	100.35	66.09
3.62	11.67	4.71	-3.47	113.65	213.68	102.79	66.08
3.67	11.74	4.76	-3.39	112.7	210.78	100.75	64.92
3.67	11.73	4.72	-3.38	113.48	210.64	100.7	64.76
4.48	12.75	5.6	-2.02	127.37	222.5	113.01	78.97
3.85	11.91	5.04	-2.64	134.62	233.47	121.05	87.97
3.63	11.71	4.88	-2.87	140.62	240.36	125.98	94
3.92	12.03	5.13	-2.62	139.72	240.36	128.21	95.66
4.06	12.19	5.26	-2.48	135.17	233.48	123.76	91.56
4.25	12.36	5.41	-2.34	134.31	231.05	120.08	87.87
3.89	12.06	5.07	-2.67	138.3	238.66	129.39	96.09
3.67	11.74	4.9	-2.76	138.51	236.38	123.63	90.49
3.72	11.78	4.92	-2.76	134.56	233.14	120.9	88.72
4.43	12.6	5.58	-2.16	132.02	229.24	119.53	85.88
4.07	12.12	5.24	-2.48	137.23	235.17	123.08	88.88
4.05	12.18	5.21	-2.51	136.17	234.36	125.09	92.76
3.84	11.9	5.04	-2.67	134.36	231.35	119.89	88.18
4.05	12.17	5.24	-2.5	134.83	229.86	119.57	86.33
4.05	12.16	5.21	-2.52	134.43	229.45	119.07	85.87
4.18	12.26	5.32	-2.41	133.89	230.32	119.14	85.98
3.94	12.01	5.13	-2.64	137.61	234.61	124.57	91.2
4.06	12.16	5.25	-2.53	134.82	232.82	121.14	88.88
3.85	11.97	5.03	-2.69	138	237.42	126.91	94.45
3.71	11.78	4.93	-2.74	137.93	235.03	123.44	90.18
3.73	11.8	4.96	-2.72	138.07	235.02	123.17	89.91
3.54	11.61	4.9	-2.98	136.86	228.95	118.01	86.48
3.46	11.45	4.85	-2.93	143.42	241.52	126.63	94.06
3.51	11.54	4.89	-3.29	131.75	222.86	115.38	83.81
3.33	11.4	4.76	-3.41	133.23	224.15	115.62	84.35
3.54	11.55	4.93	-2.88	142.83	240.93	125.42	92.7
3.55	11.54	4.91	-2.85	143	241.43	126	93.31
3.74	11.81	5.09	-2.85	135.23	227.96	117.2	84.7

3.27	11.27	4.75	-3.04	145.86	243.44	129.65	97.02
3.28	11.3	4.75	-3.05	143.9	242.45	127.93	95.46
3.63	11.67	4.98	-2.97	133.77	226.76	116.07	83.32
4	12.21	5.17	-2.63	143.42	242.31	137.59	105.56
3.64	11.87	4.85	-2.94	142.91	243.71	139.26	107.77
4.08	12.31	5.24	-2.57	142.66	241.04	136.77	104.86
4.17	12.36	5.3	-2.46	137.05	237.4	132.24	97.79
4.08	12.29	5.24	-2.53	136.93	237.52	131.87	97.55
4.06	12.25	5.22	-2.58	143.92	242.53	137.83	105.71
4.04	12.24	5.21	-2.59	143.92	242.86	138.11	106.07
3.56	11.78	4.79	-3	142.97	243.82	139.71	108.29
3.56	11.77	4.78	-3	142.95	243.66	139.46	108.08
4.09	12.31	5.24	-2.57	142.78	241.12	136.86	104.91
3.77	11.94	4.97	-2.84	142.58	242.96	135.55	103.65
4.07	12.24	5.23	-2.53	138.5	237.46	130.86	97.37
3.93	12.1	5.1	-2.66	136.94	238.17	130.08	97.21
3.99	12.18	5.17	-2.59	135.06	235.08	127.09	94.72
3.64	11.73	4.87	-2.85	140.68	241.67	126.36	93.87
3.59	11.67	4.82	-2.75	141.61	242.96	125.12	91.99
3.61	11.77	4.87	-2.85	147.48	249.6	139.28	106
3.58	11.72	4.83	-2.97	144.19	244.53	138.14	106.19
3.57	11.71	4.82	-2.98	144.22	244.46	138.18	106.24
3.02	11.15	4.45	-3.23	164.29	275.29	156.86	127.99
3.23	11.35	4.62	-3.05	163.49	276.66	153.49	125.01
3.26	11.43	4.67	-3.06	158.96	268.74	153.01	124.94
3.65	11.81	4.9	-2.9	143.25	243.93	135.43	103.5
3.93	12.05	5.12	-2.61	139.06	240.67	128.37	95.72
3.31	11.48	4.71	-3.06	159.22	269.83	157.12	129.68
3.31	11.48	4.72	-3.06	158.97	269.78	157.03	129.53
3.75	11.84	4.96	-2.75	139.95	242.51	126.35	93.94
3.6	11.72	4.82	-2.86	143.09	245.4	133.89	101.18
3.55	11.74	4.81	-2.99	144.7	245.67	140.29	109.97
3.85	11.96	5.06	-2.67	138.87	239.85	127.46	94.43
3.82	11.98	5.02	-2.74	138.7	237.89	127.53	95.48
3.71	11.74	4.95	-2.64	140.78	243.87	125.3	91.61
3.76	11.8	4.96	-2.66	137.49	238.35	123.8	90.69
3.75	11.79	4.92	-2.64	137.08	235.72	120.59	88

3.7	11.74	4.86	-2.71	136.27	233.51	120.23	87.86
3.79	11.83	4.96	-2.69	133.67	231.29	117.96	85.19
3.33	11.43	4.72	-2.94	161.27	272.6	150.04	120.07
3.37	11.52	4.69	-2.97	155.34	263.81	148.87	117.69
3.51	11.64	4.89	-2.76	155.71	261.62	141.83	108.65
3.64	11.8	4.88	-2.89	144.48	246.53	137.23	105.48
3.66	11.82	4.9	-2.87	144.74	247.53	137.96	105.82
3.73	11.86	4.96	-2.82	142.17	243.46	134.8	102.65
3.62	11.75	4.85	-2.9	144.92	245.46	134.96	102.48
3.69	11.83	4.91	-2.78	141.6	243.96	132.55	100.02
3.93	12.03	5.12	-2.6	140.91	240.57	129.08	96.86
3.8	11.88	5.01	-2.71	140.54	242.59	127.4	94.2
3.45	11.57	4.87	-2.81	154.43	260.84	140.26	107.63
2.97	10.96	4.52	-3.29	149.51	251.29	134.41	103.22
2.93	10.91	4.49	-3.34	150.11	251.47	134.68	103.31
3.01	11.13	4.45	-3.22	165.5	275.35	154.07	125.64
3.03	11.22	4.44	-3.26	155.96	263.13	150.11	122.57
2.92	10.9	4.57	-3.17	161.91	266.1	147.68	119.79
2.75	10.74	4.39	-3.38	159.07	259.39	144.01	116.5
3.07	11.06	4.61	-3.1	164.29	271.62	149.69	120.23
2.94	10.99	4.52	-3.2	163.08	269.38	148.42	119.51
3.09	11.17	4.58	-3.12	165.78	276.22	150.53	121.2
2.82	10.79	4.43	-3.35	158.76	258.33	142.13	113.42
2.7	10.7	4.38	-3.43	158.01	255	140.15	112.07
2.86	10.84	4.43	-3.31	153.72	256.64	139.42	109.67
2.65	10.66	4.28	-3.59	150.59	249.68	135.53	106.32
3.05	11.05	4.58	-3.25	147.74	246.25	132.11	99.92
2.64	10.66	4.29	-3.81	146.08	235.31	129.51	99.94
2.49	10.53	4.16	-3.94	145.99	234.11	128.71	99.86
2.93	10.92	4.5	-3.22	159.34	262.53	145.1	115.61
2.5	10.54	4.17	-3.94	146	234.13	128.7	99.83
2.63	10.66	4.27	-3.63	148.73	247.22	134	104.02
2.81	10.83	4.37	-3.52	146.47	243.46	131.3	100.14
2.51	10.51	4.21	-3.6	156.26	251.97	140.63	113.67
2.48	10.49	4.18	-3.63	155.94	251.54	140.35	113.33
3.11	11.2	4.58	-3.13	166.92	278.75	152.78	123.18
2.55	10.54	4.21	-3.69	151.72	247.95	136.73	106.88

2.76	10.74	4.42	-3.36	158.77	260.56	144.87	117.22
2.75	10.74	4.39	-3.38	159.23	259.36	144.02	116.54
2.87	10.85	4.45	-3.3	153.76	256.58	139.23	109.47
2.79	10.77	4.4	-3.38	153.98	254.93	139.3	109.32
2.88	10.86	4.46	-3.36	152.78	253.76	137.31	107.31
2.6	10.61	4.25	-3.66	151.28	248.47	135.74	106.34
2.72	10.71	4.32	-3.56	150.98	248.6	134.73	105.3
2.61	10.64	4.24	-3.64	149.69	248.3	134.09	105.21
2.6	10.6	4.21	-3.66	149.18	247.04	134	104.76
2.74	10.74	4.34	-3.54	150.39	248.68	134.24	104.58
2.82	10.83	4.4	-3.49	148.64	247.56	133.91	103.06
2.59	10.58	4.23	-3.67	151.78	247.58	136.32	106.59
2.82	10.82	4.39	-3.57	146.36	243.81	132.45	101.07
2.74	10.74	4.33	-3.5	149.75	249.55	134.1	103.73
2.76	10.79	4.35	-3.52	149.05	244.51	132.99	101.35
2.95	10.96	4.5	-3.38	148.32	245.52	132.17	100.09
3.4	11.4	4.84	-3.04	142.62	241.37	128.21	95.04
2.81	10.84	4.37	-3.52	146.51	243.51	131.52	100.33
3	11.01	4.5	-3.47	143.36	238.36	128.05	96.14
3.2	11.24	4.67	-3.51	140.69	229.64	123.04	92.43
2.82	10.88	4.41	-3.79	141.78	232.3	125.31	95.63
2.56	10.6	4.19	-3.97	143.77	233.34	127.45	97.83
2.56	10.59	4.19	-3.97	143.86	233.44	127.57	97.86
2.55	10.61	4.19	-3.98	142.97	232.99	127.1	97.79
2.52	10.58	4.17	-3.96	143.1	233.85	128.43	98.03
3.41	11.44	4.82	-3.36	134.25	224.33	116.54	85.25
3.41	11.43	4.82	-3.36	134.39	224.26	116.59	85.23
3.5	11.55	4.9	-3.34	133.11	223.27	115.85	84.2
3.25	11.27	4.64	-3.64	134.49	224.39	117.4	86.86
3.47	11.46	4.88	-3.22	135.26	224.9	117.58	86.13
3.27	11.31	4.73	-3.42	135.84	224.77	117.89	87.05
3.25	11.3	4.72	-3.43	135.95	224.88	117.95	87.18
3.21	11.24	4.67	-3.41	135.61	226.3	118.66	87.99
3.21	11.23	4.66	-3.41	135.63	226.25	118.69	87.99
3.44	11.46	4.87	-3.33	136.98	227.14	119.2	88.41
2.99	11.04	4.5	-3.72	138.42	228.33	121.41	91.54
3.24	11.31	4.62	-3.58	132.31	220.82	113.38	82

3.4	11.41	4.76	-2.87	146.55	249.12	131.45	98.84
2.8	10.78	4.36	-3.45	149.67	251.2	134.85	105.42
3.79	11.91	5.09	-2.6	147.66	253.3	135.88	103.77
3.44	11.46	4.84	-2.87	143.93	246.49	128.56	95.75
3.17	11.22	4.69	-3.03	148.44	250.44	131.02	99.73
3.26	11.38	4.75	-2.97	156.69	264.61	144.21	112.55
2.95	10.92	4.49	-3.31	151.3	254.13	136.16	106.16
3.31	11.37	4.82	-2.91	153.71	261.8	140.35	109
4.04	12.09	5.21	-2.56	135.2	232.44	120.25	88.64
4.26	12.41	5.42	-2.29	132.78	229.95	120.07	86.9
3.2	11.2	4.69	-3.11	145.7	243.45	129.51	97.2
3.73	11.86	4.96	-2.82	142.17	243.46	134.8	102.65
3.8	11.88	5.02	-2.71	140.54	242.61	127.43	94.22
3.8	11.88	5.01	-2.71	140.55	242.59	127.4	94.2
3.88	11.84	5.07	-2.57	139.69	239.44	121.81	88.88
4.03	12.17	5.32	-2.4	156.67	265.07	178.42	149.47
3.89	12.04	5.18	-2.51	157.54	265.84	178.04	149.9
3.62	11.82	4.95	-2.79	157.83	264.37	175.54	147.92
3.63	11.82	4.99	-2.73	157.61	262.42	167.33	141.9
3.12	11.28	4.67	-3.14	162	264.33	166.79	146.89
2.98	11.06	4.51	-3.24	166.79	273.17	158.44	132.26
2.92	11	4.45	-3.28	165.59	273.7	155.72	129.01
3.01	11.05	4.53	-3.2	166.29	275.44	153.11	125.95
2.87	11.04	4.36	-3.43	161.41	266.13	158.6	132.52
2.93	11.01	4.44	-3.28	165.64	272.85	157.54	130.88
2.85	11.01	4.35	-3.44	162.22	266.18	158.63	133
3.05	11.09	4.57	-3.16	165.79	275.62	152.6	124.35
3.04	11.07	4.6	-3.14	164.77	273.41	150.78	123.03
2.39	10.36	3.5	-3.62	127.39	204.41	139.3	121.08
2.11	10.08	3.31	-3.83	130.06	209.73	144.08	125.35
2.19	10.16	3.37	-3.77	128.52	208.09	142.55	122.59
2.13	10.1	3.32	-3.81	129.93	209.25	143.63	124.95
2.75	10.94	3.95	-3.57	139.13	245.58	148.58	124.59
2.51	10.72	3.77	-3.68	135.21	241.45	148.31	120.92
2.73	10.92	3.93	-3.59	139.74	246.19	149.23	125.22
2.57	10.72	3.84	-3.51	131.8	236.77	143.72	118.18
3.22	11.48	4.5	-3.27	170.86	265.6	161.55	150.26

2.8	10.97	4.47	-3.34	166.43	265.12	170.03	153.87
3.34	11.55	4.85	-2.92	160.97	264.86	186.84	169.78
2.85	11.02	4.48	-3.14	166.96	257.71	172.41	161.72
2.86	11.01	4.49	-3.13	166.9	257.18	170.65	160.76
2.74	10.93	4.34	-3.41	164.45	260.58	175.36	161.04
2.92	11.11	4.53	-3.15	161.88	258.08	175.78	167.45
2.61	10.83	4.28	-3.41	166.05	260.97	171.77	159.39
3.32	11.37	4.94	-2.66	169.75	274.12	149.9	124.45
3.35	11.38	5	-2.68	167.86	275.16	149.92	123.86
3.22	11.25	4.89	-2.77	166.39	266.94	146.65	122.95
2.5	10.56	4.28	-3.45	156.58	248.96	138.47	112.89
2.58	10.71	4.34	-3.43	154.52	247.85	137.13	110.42
3.32	11.37	4.94	-2.66	169.79	273.87	149.78	124.47
2.96	10.94	4.64	-3.11	158.04	252.21	140.02	112.08
2.44	10.49	4.24	-3.52	156.68	248.88	137.25	113.24
2.47	10.5	4.24	-3.48	157.09	248.74	138.09	113.18
2.55	10.65	4.31	-3.45	154.54	248.46	137.49	111.5
3.33	11.35	4.92	-2.75	166.98	278.97	152.22	125.03
2.42	10.5	4.24	-3.44	161.16	252.99	144	120.1
2.93	11.04	4.63	-2.99	161.63	247.44	148.38	129.7
2.77	10.84	4.5	-3.77	152.87	234.39	133.8	114.5
2.49	10.57	4.36	-3.82	156.56	237.54	134.91	115.53
2.54	10.54	4.31	-3.43	160.75	251.36	141.48	115.88
2.35	10.45	4.15	-3.61	155.07	247.89	137.83	112.94
2.3	10.45	4.14	-3.87	151.62	245.69	136.13	109.77
2.15	10.24	3.93	-4.1	147.12	237.22	132.33	104.08
1.63	9.73	3.52	-4.79	153.65	241.61	135.71	110.4
1.99	10.05	3.68	-4.85	150.24	239.74	132.16	105.05
2.01	10.07	3.7	-4.87	150.35	239.8	131.99	104.83
2.13	10.2	3.92	-4.34	149.89	239.7	133.28	106.39
2.06	10.17	3.85	-4.46	149.07	240.93	131.73	105.08
2.25	10.33	4.02	-4.15	148.71	238.25	131.81	104.85
2.38	10.43	4.18	-3.6	155.99	248.08	137.38	113.23
1.41	9.56	3.31	-5.03	151.62	240.24	136.35	111.31
1.46	9.51	3.24	-5.17	151.68	242.7	136.62	110.81
1.42	9.47	3.21	-5.21	151.99	242.75	136.87	111.21
1.81	9.88	3.53	-4.96	150.57	240.48	133.32	106.72

1.91	9.96	3.63	-4.89	149.9	240.59	132.35	105.65
1.35	9.53	3.16	-5.23	147.4	239.59	133.5	108.61
1.54	9.7	3.33	-5.06	146.48	237.52	132.82	106.25
2.44	10.46	4.28	-3.59	156.58	248.41	139.21	115.91
2.49	10.51	4.29	-3.51	157.82	250.83	139.33	115.84
2.41	10.45	4.33	-4.01	155.58	239.47	135.13	113.76
1.52	9.68	3.25	-5.46	153.99	243.65	133.27	106.04
0.88	9.04	2.7	-5.73	157.16	249.3	140.13	115.92
2.16	10.28	3.82	-4.5	145.12	235.16	128.48	100.48
1.71	9.89	3.37	-5.27	143.75	233.99	129.9	101.66
2.27	10.38	3.85	-4.64	143.27	232.5	126.99	98.04
2.53	10.64	3.99	-4.47	137.43	227.73	122.9	92.62
2.41	10.49	4	-4.56	145.66	234.69	128.07	98.95
2.12	10.19	3.87	-4.29	148.36	237.75	130.96	103.1
1.83	9.95	3.48	-5.14	145.93	236.84	131.26	102.82
2.31	10.43	3.94	-4.65	144.78	235.33	128.31	99.32
2.09	10.22	3.78	-4.77	146.99	237.95	130.01	102.41
2.28	10.38	3.87	-4.71	143.06	232.94	127.63	97.88
2.28	10.38	3.87	-4.71	143.12	232.96	127.64	97.9
1.61	9.75	3.34	-5.05	146.37	237.88	132.71	105.83
1.9	10.04	3.59	-4.87	146.19	237.19	129.9	103.24
1.91	10.04	3.59	-4.86	146.17	237.16	129.86	103.17
1.98	10.11	3.61	-4.77	144.34	234.27	127.99	100.12
2.21	10.34	3.86	-4.55	145.39	235.31	128.49	99.76
2.26	10.38	3.91	-4.47	144.66	235.17	127.92	99.45
2	10.13	3.71	-4.64	146.81	235.74	129.69	102.01
2.22	10.34	3.87	-4.45	144.62	234.44	128.06	99.87
2.54	10.64	4.09	-4.26	142.32	231.28	125.1	96.71
2.61	10.71	4.15	-4.2	142.29	230.76	124.6	95.94
2.07	10.19	3.71	-4.69	143.48	233.14	127.67	99.51
2.57	10.66	4.07	-4.32	140.62	230.49	124.41	95.49
2.7	10.8	4.18	-4.2	140.37	229.71	123.93	94.06
2.78	10.87	4.26	-4.1	139.75	228.57	123.29	93.09
2.85	10.95	4.36	-3.92	140.88	229.62	123.6	94.1
2.98	11.04	4.49	-3.78	142.14	230.68	123.68	93.59
2.38	10.49	4.06	-4.19	146.78	232.93	127.74	98.59
2.34	10.44	4.06	-4.25	147.67	233.59	128.39	99.24

2.68	10.77	4.22	-4.06	140.62	229.33	124.13	93.95
1.45	9.62	3.18	-5.51	152.1	242.28	133.48	106.57
2.02	10.17	3.56	-5.18	139.51	232.81	126.55	97.09
1.56	9.74	3.27	-5.57	146.35	241.23	131.07	102.3
1.82	9.93	3.46	-5.19	149	239.29	130.65	103.13
1.64	9.79	3.31	-5.31	148.72	239.77	131.62	104.78
2.21	10.35	3.77	-4.78	141.65	231.51	125.67	96.64
1.62	9.83	3.31	-5.32	144.12	234.8	130.61	102.69
1.53	9.74	3.21	-5.39	144.97	235.47	131.35	103.66
1.74	9.92	3.39	-5.25	144.15	233.22	129.01	101.06
1.46	9.65	3.15	-5.46	145.74	235.91	132.05	104.51
1.47	9.66	3.16	-5.45	145.46	236.27	131.4	104.65
1.48	9.67	3.17	-5.44	145.35	236.24	131.2	104.47
2.61	10.72	4.14	-4.2	139.44	229.06	123.97	94.02
2.01	10.15	3.6	-5.17	141.55	233.32	128.23	98.15
1.78	9.94	3.42	-5.31	142.96	234.8	129.88	100.35
2.23	10.38	3.74	-4.97	136.99	230.6	123.39	93.44
2	10.19	3.57	-5.21	135.3	232.75	122.98	92.99
2.42	10.58	3.89	-5.03	131.98	227.27	118.97	87.15
2.3	10.44	3.81	-5.04	133.43	228.74	119.8	88.26
2.93	11.01	4.21	-4.55	126.94	225.49	114.12	77.3
2.68	10.77	4.09	-4.71	130.28	228.98	117.29	83.04
2.29	10.46	3.81	-5	132.96	231.72	119.86	88.41
2.17	10.37	3.72	-5.32	139.05	233.93	123.72	92.56
2.64	10.73	4.05	-4.74	130.81	229.3	117.75	83.44
2.47	10.56	3.89	-4.85	130.23	233.06	119.23	83.08
2.37	10.47	3.82	-4.92	130.12	235.31	119.6	83.71
1.97	10.12	3.53	-5.27	132.75	239.01	123.82	90.14
2.58	10.73	4.04	-4.98	127.38	228.66	117.36	83.29
2.61	10.77	4.02	-4.91	128.93	226.62	116.42	84.31
2.2	10.31	3.72	-5.05	130.54	238.79	121.41	85.65
2.43	10.56	3.92	-4.91	132.96	229.2	118.72	86.35
2.32	10.45	3.82	-4.67	134.94	228.02	120.73	91.56
2.47	10.59	3.92	-4.66	133.25	227.04	119.35	88.48
2.58	10.68	3.98	-4.6	133.02	226.46	118.02	87.08
2.47	10.59	3.92	-4.66	133.52	227.09	119.27	88.64
2.3	10.4	3.79	-4.73	134.58	228.56	119.87	90.39

2.99	11.11	4.34	-4.33	128.53	221.91	113.65	80.68
2.76	10.86	4.16	-4.6	129.69	226.36	115.63	81.92
2.42	10.58	3.89	-4.74	133.98	227.58	119.04	88.06
2.28	10.4	3.78	-5.02	129.95	237.45	120.49	84.14
2.32	10.43	3.86	-5.08	133.13	238.53	122.36	84.93
2.35	10.48	3.85	-4.98	129.11	236.77	120.22	82.74
2.32	10.45	3.81	-5.07	131.4	236.61	120.15	84.15
2.32	10.44	3.8	-5.07	131.08	236.41	120.21	84.26
2.24	10.34	3.72	-4.9	130.41	241.65	120.59	83.01
2.3	10.38	3.78	-4.98	130.06	236.87	120.21	84.17
2.36	10.44	3.81	-4.94	129.48	237.75	119.96	83.09
1.98	10	3.57	-5.16	134.89	248.73	125.98	87.87
1.76	9.88	3.43	-5.48	138.36	246.69	129.18	93.75
1.62	9.71	3.32	-5.57	139.89	249.83	130.28	95.22
1.8	9.87	3.43	-5.1	134.67	249.72	125.58	88.37
2.38	10.46	3.89	-5.05	133.09	237.28	121.56	84.33
1.94	9.96	3.53	-5.14	133.64	248.06	125.24	87.44
1.91	9.99	3.5	-5.08	131.7	247.94	124.69	86.83
1.85	9.9	3.47	-5.25	137.2	250.19	127.88	89.75
1.6	9.63	3.21	-4.81	136.17	259.72	129	88.09
1.42	9.5	3.11	-5.22	139.01	260.49	129.49	91.03
1.94	10	3.55	-4.85	133.52	250.03	121.69	83.45
1.55	9.61	3.22	-5.13	138.39	258.09	127.67	89.62
1.72	9.75	3.32	-4.77	134.52	256.54	127.16	84.14
2.31	10.31	3.8	-4.47	129.34	245.61	117.14	76.43
2.97	10.96	4.26	-3.99	121.14	229.64	109.53	70.57
2.09	10.16	3.52	-4.6	130.99	247.35	123.46	80.97
2.26	10.25	3.64	-4.39	127.12	242.92	117.86	75.96
2.27	10.27	3.65	-4.37	127.12	242.57	117.74	75.83
1.8	9.84	3.31	-4.74	133.39	256.69	126.9	85.4
2.22	10.17	3.61	-4.41	129.45	244.61	120.15	78.8
1.32	9.43	3.03	-4.81	151.3	289.59	145.34	107.29
1.79	10.08	3.38	-4.67	153.12	290.88	151.96	108.55
1.66	9.98	3.28	-4.74	156.09	295.94	154.43	113.28
1.26	9.51	2.83	-5.21	145.95	280.02	144.22	104.96
1.69	9.84	3.16	-4.9	144.07	270.37	137.74	94.36
1.42	9.61	2.9	-5.08	144.1	273.77	138.76	97.26

1.7	9.84	3.17	-4.9	144.07	270.19	137.53	94.22
1.66	9.8	3.14	-4.93	144.33	271.17	138.2	95.11
1.5	9.58	3.26	-4.57	145.6	282.93	140.61	101.45
2.51	10.64	4.13	-3.65	138.94	268.56	132.51	89.25
2.36	10.5	3.95	-3.92	144.43	273.79	138.77	96.11
2.83	10.95	3.99	-4.11	126.44	241.22	123.95	79.49
2.88	11.01	4.06	-4.07	126.79	241.11	124.28	78.99
2.89	11.01	4.06	-4.06	126.75	241.02	124.26	78.98
3.25	11.42	4.31	-3.94	139.43	256.51	138.56	90.47
3.26	11.43	4.31	-3.93	138.9	256.44	138.44	90.31
1.67	9.84	2.99	-4.52	151.24	259.22	155.05	145.9
1.5	9.69	2.87	-4.63	156.3	264.38	160.66	152.39
1.47	9.67	2.85	-4.64	157.41	265.05	161.34	153.39
1.25	9.44	2.67	-4.78	155.5	266.4	161.57	154.56
1.72	9.87	3.05	-4.47	147.47	258.62	151.93	141.81
1.34	9.53	2.7	-4.76	145.34	258.44	154.6	146.73
1.93	10.07	3.2	-4.32	143.32	254.14	147.35	136.06
1.42	9.59	2.77	-4.69	145.32	257.47	153.28	145.94
1.87	9.95	3.24	-4.12	130.92	233.69	144.32	131.12
1.68	9.76	3.09	-4.28	134.76	236.18	148.55	138.31
2.34	10.41	3.59	-3.69	126.55	220.26	141.18	118.44
2.42	10.49	3.66	-3.63	125.17	218.85	139.39	116.13
1.77	9.85	3.18	-4.19	137.12	236.85	149.97	138.23
1.74	9.93	2.99	-4.43	129.08	241.85	137.99	126.64
1.49	9.61	2.89	-4.45	143.71	254.87	152.94	139.94
0.93	9.14	2.36	-5.04	154.5	264.01	161.42	153.92
1.07	9.34	2.57	-4.82	162.91	271.3	167.84	162
1.28	9.48	2.65	-4.76	150.19	259.03	156.44	144.75
1.37	9.63	2.77	-4.67	161.05	267.02	164.62	157.59
1.76	9.89	3.16	-4.22	139.37	250.98	147.27	132.16
1.65	9.79	3.07	-4.3	142	253.16	149.78	136.01
1.36	9.53	2.72	-4.69	137.69	251	149.1	140.11
1.23	9.43	2.68	-4.78	157.01	265.65	160.8	153.46
1.09	9.27	2.54	-4.93	158.68	267.48	163.68	156
1.67	9.8	3.09	-4.3	140.9	251.8	148.85	133.92
1.45	9.65	2.85	-4.64	151.05	262.31	157.34	148
1.17	9.38	2.63	-4.82	157.85	265.74	161.65	154.04

1.17	9.38	2.64	-4.82	158.3	266.39	162	155.21
1.27	9.47	2.71	-4.75	156.04	265	159.85	152.07
1.4	9.58	2.82	-4.57	146.07	256.99	153.35	141.48
1.37	9.55	2.86	-4.45	149.72	259.23	158	145.79
1.51	9.73	2.89	-4.55	154.99	262.05	158.62	146.6
1.51	9.7	2.88	-4.56	153.76	260.67	158.3	145.82
1.56	9.76	2.92	-4.52	154.22	261.22	157.86	145.65
1.89	10.09	3.17	-4.3	151.64	258.34	153.81	141.51
1.49	9.69	2.87	-4.58	158.18	263.78	161.31	150.45
1.45	9.66	2.83	-4.61	160.73	266.24	163.22	155.55
1.38	9.58	2.78	-4.63	157.74	262.99	161.06	150.02
2.59	10.67	3.84	-3.52	129.93	236.21	143.04	118.58
2.29	10.38	3.62	-3.72	133.69	240.89	146.95	125.66
1.51	9.7	2.88	-4.56	156.5	262.18	160.37	148.15
1.38	9.56	2.79	-4.59	156.87	262.62	161.62	149.72
1.49	9.68	2.87	-4.55	153.89	259.76	157.77	145.22
1.36	9.53	2.77	-4.63	154.22	260.63	159.82	147.36
1.37	9.61	2.77	-4.68	161.1	267.46	164.89	157.91
1.99	10.08	3.36	-4.05	140.65	248.71	147.84	129.17
2.51	10.6	3.75	-3.61	137	240.6	147.94	123.74
2.18	10.31	3.47	-3.97	142.89	252.5	151.45	131.51
1.97	10.05	3.37	-3.93	140.2	249.41	152.3	134.68
1.46	9.59	2.98	-4.28	147.73	258.66	157.97	145.72
0.73	8.88	2.41	-4.83	167.86	272.36	178.17	173.52
1.27	9.43	2.79	-4.53	147.34	258.68	158.56	147.63
1.01	9.17	2.54	-4.76	153.11	264.62	163.56	155.38
1.04	9.2	2.56	-4.76	152.49	263.86	162.41	153.68
1.01	9.16	2.54	-4.76	152.59	264.22	163.41	155.08
1.29	9.46	2.78	-4.57	146.87	258.08	156.72	145.94
1.37	9.54	2.84	-4.51	144.78	255.93	154.26	142.81
2.09	10.24	3.5	-3.87	134.67	238.57	147.64	128.79
2.04	10.2	3.46	-3.9	135.22	239.39	148.2	129.68
0.9	9.04	2.42	-4.87	155.01	263.6	164.1	156.62
1.28	9.44	2.83	-4.5	150.47	260.57	160.96	149.12
0.72	8.86	2.4	-4.84	168.69	273.01	179.31	174.26
1.1	9.31	2.52	-4.88	147.1	257.6	157.8	151.07
2.18	10.4	3.51	-4.67	132.95	251.41	133.7	95.55

2.19	10.41	3.51	-4.66	132.83	251.23	133.58	95.42
2.13	10.34	3.47	-4.71	134.2	252.79	134.63	96.58
1.97	10.2	3.37	-4.85	137.45	255.94	135.3	98.25
2.48	10.66	3.75	-4.46	129.25	244.54	129.97	89.74
2.38	10.56	3.67	-4.57	130.74	247.17	129.24	91.23
2.55	10.71	3.75	-4.36	128.51	244.7	130.02	88.6
1.75	10	3.17	-5.01	142.03	259.93	140.18	105.05
1.68	9.92	3.14	-5.06	142.53	261.26	140.99	106.06
3.04	11.27	4.18	-4	123.97	224.39	118.99	80.16
2.92	11.17	4.1	-4.1	125.33	229.4	121.45	83.56
2.37	10.55	3.66	-4.58	130.88	247.47	129.41	91.43
2.91	10.88	4.16	-3.81	117.66	222.5	108.81	70.32
2.89	10.87	4.14	-3.84	118.13	222.15	108.86	71.06
2.58	10.56	3.94	-4.08	122.31	229.24	113.37	75.29
2.55	10.53	3.87	-4.08	122.34	230.26	113.22	75.63
2.42	10.39	3.81	-4.29	127.26	238.93	119.59	76.79
2.45	10.43	3.79	-4.33	126.84	239.09	120	76.06
2.55	10.53	3.85	-4.23	126.67	235.89	117.81	75.84
2.47	10.43	3.82	-4.2	125.14	237.06	118.98	77.37
2.38	10.36	3.73	-4.3	127.47	243.61	118.44	75.88
2.28	10.31	3.63	-4.5	128.87	245.6	122.87	79.87
2.58	10.64	3.84	-4.16	124.07	233.63	117	75.05
2.61	10.7	3.88	-4.13	123.89	232.14	116.76	75.02
2.41	10.38	3.75	-4.26	124.48	240.13	116.07	73.87
2.4	10.37	3.76	-4.28	127.22	240.29	118.28	75.92
2.33	10.3	3.71	-4.34	128.57	241.65	119.8	77.59
2.76	10.65	4.06	-4	123.13	233.76	115.8	73.05
2.53	10.47	3.9	-4.16	124.68	235.71	117.18	76.66
2.46	10.51	3.68	-4.23	125.92	231.9	118.33	79.11
2.32	10.34	3.61	-4.35	127.67	234.25	119.39	79.46
2.45	10.48	3.81	-4.34	126.04	235.75	117.7	78.62
2.34	10.36	3.61	-4.36	125.46	234.24	119.12	79.07
2.29	10.38	3.62	-4.54	128.41	247.47	126.87	86.14
2.35	10.36	3.65	-4.4	125.88	234.81	119.04	79.04
2.43	10.5	3.69	-4.3	126.9	235.03	122	82.67
2.54	10.61	3.78	-4.28	125.73	234.27	121.12	82.09
2.65	10.69	3.91	-4.15	122.75	233	117.94	75.28

2.39	10.46	3.7	-4.44	127.72	242.95	124.61	83.37
2.42	10.49	3.72	-4.41	127.17	241.75	124.06	82.77
2.5	10.57	3.76	-4.34	128.61	237.8	123.33	84.49
2.52	10.63	3.75	-4.35	129.02	236.2	123.65	85.04
1.75	9.78	3.16	-4.85	135.17	242.99	126.9	90.18
2.57	10.53	3.99	-4.06	123.72	229.3	112.76	75.03
2.94	10.92	4.24	-3.81	118.81	220.99	108.29	70.2
2.31	10.21	3.8	-4.24	127.97	233.84	117.09	80.03
3.12	11.19	4.37	-3.53	116.11	215.16	103.98	67.69
2.71	10.69	4.11	-3.97	124.81	224.03	109.84	74.03
2.71	10.69	4.11	-3.96	124.89	224.2	109.87	74.18
3.04	11.2	4.06	-3.94	122.06	222.85	117.23	78.05
2.36	10.49	3.64	-4.45	130	238.56	124.26	86.76
2.47	10.61	3.66	-4.42	128.71	238.46	125.53	87.46
2.31	10.46	3.56	-4.53	130.1	240.27	126.38	88.73
2.53	10.64	3.7	-4.37	128.5	237.02	124.05	86.02
2.91	11.11	3.97	-4.06	124.43	225.68	119.1	79.99
3.12	11.34	4.16	-3.88	120.35	221.63	115.46	77.46
2.89	11	4.12	-4.05	125.76	237.73	124.37	81.73
2.62	10.71	3.86	-4.25	127.01	241.96	125.3	82.33
2.76	10.87	4	-4.11	126.73	241.91	123.56	77.99
2.42	10.44	3.67	-4.23	124.71	232.12	118.07	79.18
2.59	10.78	4.11	-3.81	138.98	263.14	135.15	91.39
2.56	10.75	3.66	-4.44	137.38	229.87	120.87	86.55
3	11.27	4	-4.12	130.6	220.58	115.25	80.95
2.96	11.18	3.99	-4.2	132.35	222.58	116.55	81.12
3.01	11.26	4.01	-4.16	130.8	221.36	116.4	81.02
2.93	11.25	3.99	-4.17	134.84	226.97	120.08	83.25
2.27	10.46	3.47	-4.59	138.07	231.47	122.63	88.91
2.76	10.97	3.85	-4.31	130.67	222.96	116.29	82.54
2.88	11.1	3.94	-4.23	129.52	222.52	116.94	81.94
2.47	10.62	3.62	-4.47	132.06	225.86	119.26	84.72
2.6	10.78	3.68	-4.36	129.18	224.3	117.12	82.5
2.71	10.89	3.82	-4.33	130.5	222.27	116.16	81.94
2.71	10.88	3.82	-4.33	129.78	222.04	115.97	81.78
2.65	10.84	3.73	-4.33	128.6	223.35	116.14	81.57
2.7	10.88	3.76	-4.35	128.63	225.39	118.38	83.73

3.07	11.27	4.06	-4.07	124.89	218.28	114.03	79.2
3.54	11.71	4.5	-3.71	119.41	210.34	110.15	74.72
3.14	11.35	4.11	-4.01	123.16	218.09	115.37	79.41
3.49	11.74	4.45	-3.73	118.94	212.54	112.43	75.58
2.91	11.16	3.95	-4.18	127.96	224.94	120.61	85.45
2.91	11.16	3.96	-4.18	127.97	224.92	120.6	85.43
2.91	11.17	3.98	-4.16	128.27	225.15	121.63	86.29
2.6	10.83	3.81	-4.31	128.67	232.08	124.61	88.49
2.36	10.49	3.65	-4.49	132.79	235.04	123.51	87.72
2.91	11.01	4.03	-4.15	126.12	221.74	115.38	79.01
2.73	10.88	3.83	-4.33	127.43	224.4	118.44	82.89
4.07	12.53	4.96	-3.33	120.28	206.05	109.54	76.31
2.7	10.93	3.79	-4.4	129.12	225.8	120.24	85.37
3.23	11.46	4.19	-4	126.08	215.31	112.19	78.84
3.39	11.65	4.29	-3.92	126.25	214.4	112.26	78.39
3.31	11.63	4.35	-3.91	129.19	220.67	118.04	84.78
3.59	11.94	4.55	-3.7	121.06	213.67	114.96	80.45
3.63	11.97	4.57	-3.68	120.52	212.82	114.19	79.88
3.25	11.52	4.22	-3.97	125.84	214.56	112.5	77.79
3.12	11.4	4.12	-4.04	129.81	219.55	115.52	80.82
3.17	11.45	4.17	-4	129.66	219.31	116.02	81.77
2.61	10.8	3.72	-4.39	130.67	225.38	117.66	82.63
2.62	10.79	3.76	-4.39	131.07	223.91	117.5	83.26
2.62	10.78	3.77	-4.39	130.67	223.76	117.72	83.27
3.13	11.34	4.13	-4.09	126.61	217.2	112.63	78.54
2.32	10.45	3.63	-4.51	132.43	234.76	123.73	87.83
2.25	10.38	3.51	-4.62	133.87	233.08	122.46	87.65
2.61	10.77	3.7	-4.4	128.7	226.68	119.94	83.96
3.59	11.78	4.49	-3.83	144.78	258.78	147.49	106.16
3.6	11.79	4.5	-3.83	144.74	258.67	147.41	106.04
3.73	11.92	4.65	-3.72	145.23	255.68	143.93	103.66
3.73	11.92	4.65	-3.72	145.24	255.67	143.93	103.66
1.82	10.01	3.2	-5.39	214.51	347.68	207.23	165.36
2.24	10.49	3.58	-4.9	182.98	306	174.63	124.94
3.4	11.69	4.76	-3.14	150.52	250.83	158.56	129.83
4.04	12.39	5.24	-2.62	143.35	237.88	141.07	112.07
3.41	11.66	4.74	-3.17	145.5	244.19	145.22	114.51

2.41	10.72	3.89	-4.06	147.97	241.93	145.38	117.47
3.13	11.35	4.48	-3.41	146.72	244.49	146.91	116.36
2.96	11.41	4.25	-3.73	156.03	240.57	143.37	123.98
2.88	11.2	4.28	-3.68	145.91	239.81	143.41	114.23
3.07	11.39	4.35	-3.59	144.73	238.4	142.19	113.65
3.08	11.4	4.37	-3.57	144.76	238.61	142.37	113.68
2.71	11.03	3.82	-3.83	145.2	245.88	150.98	140.29
2.43	10.73	3.56	-4.1	156.95	258.92	164.67	159
2.75	11.07	3.85	-3.81	144.79	245.06	150.22	139.02
2.9	11.17	3.99	-3.8	151.06	250.7	154.34	145.93
2.12	10.37	3.29	-4.25	147.99	254.08	151.5	141.04
2.63	10.9	3.77	-3.87	140.55	243.63	145.55	133
2.35	10.65	3.44	-4.21	166.27	263.3	172.8	173.42
2.68	10.95	3.79	-3.8	143.03	245.56	149.49	137.55
2.08	10.42	3.25	-4.38	164.17	267.71	176.7	175.66
2.69	10.95	3.78	-3.75	142.59	244.58	148.04	134.36
2.03	10.46	3.29	-4.14	159.83	261.83	169.45	163.36
2.15	10.49	3.36	-4.19	158.57	263.58	171.31	165.7
2.1	10.46	3.3	-4.26	159.84	265.89	172.84	169.2
2.12	10.46	3.29	-4.31	163.03	265.87	174.45	173.2
1.98	10.38	3.12	-4.47	171.91	272.52	184.65	188.59
1.6	10.03	2.83	-4.68	181.22	283.2	195.79	203.8
1.79	10.18	2.99	-4.56	177.29	278.92	189.64	194.85
2.07	10.44	3.24	-4.4	169.09	269.13	178.47	178.4
2.19	10.52	3.29	-4.31	163.86	265.62	174.28	172.32
2.41	10.77	3.49	-4.19	159.78	257.62	165.26	161.75
2.1	10.43	3.24	-4.43	170.75	266.77	178.47	181.98
2.63	10.89	3.7	-3.97	153.76	251.84	158.21	151.89
2.86	11.13	3.91	-3.86	150.56	248.06	152.24	142.9
2.24	10.52	3.43	-4.03	151.61	256.79	162.25	154.94
2.05	10.34	3.27	-4.14	150.6	254.98	159.74	154.48
1.91	10.21	3.12	-4.23	151.34	255.62	161.61	157.42
2.18	10.47	3.39	-4.02	141.95	245.2	150.6	139.84
1.99	10.36	3.24	-4.19	161.89	267.79	176	171.76
2.08	10.47	3.32	-4.15	160.36	267.43	173.92	169.07
2.26	10.52	3.44	-4.03	146.66	252.73	157.42	147.88
2.24	10.55	3.43	-4	149.55	256.5	160.16	152.86

2.14	10.5	3.35	-4.11	158.71	263.47	171.29	164.43
2.1	10.4	3.29	-4.08	148.79	253.31	158.79	152.54
2.45	10.74	3.6	-3.94	145.04	249.52	153.53	143.14
2.22	10.53	3.43	-4.17	155.12	259.72	167.24	160.68
2.24	10.52	3.43	-4.03	151.62	256.87	162.19	154.9
2.24	10.52	3.43	-4.03	151.61	256.85	162.21	154.9
2.25	10.6	3.43	-3.92	150.49	251.15	159.12	149.57
2	10.33	3.27	-4.19	149.53	256	160.04	152.85
2.03	10.3	3.26	-4.11	146	243.66	154.42	147.13
2.13	10.54	3.35	-4.02	154.57	255.28	162.49	153.75
2.34	10.63	3.52	-3.96	149.33	255.76	159.79	151.91
2.42	10.67	3.57	-3.9	145.98	250.35	155	144.05
2.33	10.6	3.49	-3.97	149.86	256.39	159.09	152.14
2.36	10.64	3.53	-3.95	149.17	255.54	159.55	151.48
2.34	10.63	3.51	-3.96	149.35	255.81	159.84	151.98
2.35	10.63	3.51	-3.97	149.34	255.18	159.67	152.66
2.11	10.47	3.33	-4.13	151.05	256.91	160.74	154
2.21	10.55	3.41	-4.05	152.05	257.7	162.74	155.72
2.26	10.57	3.45	-4.02	151.27	257	162.01	154.78
2.12	10.44	3.33	-4.11	151.21	256.76	161.11	154.16
2.33	10.62	3.49	-4	149.67	255.21	160.37	152.98
2.44	10.71	3.58	-3.93	150.3	253.87	159.82	151.78
2.1	10.47	3.32	-4.13	159.18	264.47	172.2	165.66
2.11	10.48	3.33	-4.13	159.08	264.26	172	165.39
2.1	10.47	3.33	-4.13	159.22	264.25	172.16	165.7
2.02	10.41	3.28	-4.18	160.97	267.3	174.81	170.38
2.09	10.48	3.33	-4.14	159.63	265.61	172.86	167.59
2.52	10.78	3.67	-3.87	148.17	252.51	158.57	149.61
2.52	10.78	3.67	-3.87	148.19	252.51	158.57	149.61
2.47	10.73	3.63	-3.89	148.38	253.26	158.85	150.59
2.09	10.37	3.32	-4.09	148.64	252.19	158.43	153.76
2.06	10.36	3.28	-4.13	150.33	254.75	159.26	154.05
2.07	10.36	3.29	-4.12	150.2	254.35	159.2	153.62
2.14	10.5	3.39	-3.96	158.88	258.77	168.15	160.79
2.02	10.39	3.27	-4.17	162.47	266.31	174.47	169.57
2	10.39	3.26	-4.19	161.83	268.29	175.83	171.66
2.09	10.48	3.32	-4.14	160.28	267.36	173.8	168.89

2.09	10.48	3.33	-4.14	160.29	267.41	173.79	168.88
2.34	10.64	3.41	-4.24	166.47	265.29	172.55	174.3
1.46	9.67	2.83	-4.64	159.62	265.29	163.06	155.95
2.71	10.95	3.85	-3.88	152.66	250.05	148.88	137.17
1.88	10.07	3.16	-4.35	153.1	257.03	154.49	144.94
1.54	9.75	2.9	-4.57	158.1	263.46	161.03	153.49
2.21	10.43	3.43	-4.05	151.59	254.73	150.57	138.92
1.96	10.16	3.21	-4.22	151.35	256.81	152.23	141.6
2	10.21	3.25	-4.19	151.42	255.75	150.82	140.55
2.38	10.59	3.57	-3.93	149.23	251.27	148.16	135.32
2.47	10.68	3.64	-3.88	148.3	249.08	146.89	133.08
2.22	10.47	3.4	-4.12	155.33	257.1	153.14	142.79
2.23	10.47	3.4	-4.12	155.4	257.35	153.3	142.78
2.25	10.48	3.41	-4.14	155.94	258.57	154.08	143.2
2.21	10.44	3.39	-4.18	157.6	260.37	155.19	146.04
2.43	10.67	3.57	-4.02	152.21	255.49	151.4	139.64
2.26	10.49	3.42	-4.12	155.98	257.24	153.43	142.7
2.73	10.95	3.82	-3.83	148.21	248.01	146.06	132.23
2.8	11.02	3.9	-3.82	151.16	248.74	146.59	134.16
2.8	11.03	3.91	-3.82	151.15	248.63	146.53	133.99
2.69	10.91	3.77	-3.88	151.28	249.42	147.55	133.27
3.13	11.32	4.16	-3.59	146.68	240.05	142.17	128.39
2.68	10.94	3.79	-3.91	152.54	247.01	148.56	136.72
2.63	10.88	3.74	-3.95	152.99	248.11	149.44	138.12
2.3	10.58	3.46	-4.11	148.53	252.01	150.75	141.22
3.03	11.28	4.11	-3.65	147.68	242.06	145.67	131.88
2.31	10.53	3.44	-4.13	145.65	248.76	147.03	136.38
1.99	10.21	3.19	-4.36	148.93	255.16	153.41	144.22
2.27	10.53	3.41	-4.05	138.53	247.59	146.41	134.69
2.56	10.87	3.69	-4.03	154.82	254.6	160.7	153.17
2.74	11.04	3.84	-3.84	147.19	247.28	152.94	141.31
2.73	11.04	3.83	-3.83	145.8	245.26	151.12	140.74
2.66	10.97	3.79	-3.78	144.54	245.61	149.77	137.75
2.83	11.1	3.92	-3.92	153.28	247.78	152.43	143.59
2.5	10.8	3.63	-3.97	147.55	247.63	147.92	137.33
2.61	10.88	3.69	-3.92	146.69	247.33	147.53	135.91
2.72	11.02	3.84	-3.92	151.75	252.88	157.17	148.83

2.73	11.03	3.84	-3.9	153.51	253.41	158.2	151.44
2.78	11.08	3.84	-3.99	152.45	245.83	148.62	138.3
2.66	10.99	3.78	-3.85	145.72	246.68	151.64	141.38
2.83	11.05	3.91	-3.81	147.55	243.24	146.71	133.92
2.79	11.02	3.88	-3.85	148.56	243.55	146.83	135.07
2.83	11.05	3.91	-3.82	147.73	243.34	146.51	134.19
2.79	11.02	3.88	-3.85	148.55	243.55	146.83	135.05
2.79	11.03	3.89	-3.85	148.44	243.53	146.8	134.94
2.83	11.06	3.92	-3.81	147.59	243.28	146.52	134.03
2.83	11.06	3.92	-3.81	147.56	243.27	146.5	134.01
3.17	11.36	4.21	-3.58	147.12	239.85	144.12	129.22
3.12	11.39	4.15	-3.6	146.35	236.98	141.83	127.06
3.13	11.39	4.16	-3.6	146.33	236.94	141.78	127
3.13	11.4	4.16	-3.6	146.29	236.87	141.71	126.9
2.39	10.59	3.54	-4.07	151.68	254.6	151.19	140.54
2.9	11.08	3.94	-3.75	146.44	244.18	143.35	128.61
2.32	10.54	3.44	-4.14	151.94	254.81	150.89	140.68
2.32	10.54	3.44	-4.14	152.01	254.83	150.93	140.73
2.01	10.25	3.2	-4.09	135.67	238.78	145.95	136.2
2.01	10.27	3.19	-4.07	133.61	236.81	144.11	133.99
2.07	10.29	3.23	-4.09	133.4	237.9	144.36	132.22
2.23	10.42	3.4	-4.07	127.37	238.5	137.16	124.86
1.87	10.13	3.1	-4.32	132.73	243.65	143.66	134.16
2.17	10.41	3.34	-4.04	135.53	239.27	144.43	132.93
2.17	10.41	3.34	-4.04	135.63	239.25	144.47	132.97
2.21	10.48	3.38	-4.01	139.39	244.26	148.97	138.28
2.14	10.42	3.34	-4.05	142.27	248.28	152.99	143.97
1.9	10.17	3.13	-4.18	145.76	245.7	153.5	146.73
2.02	10.26	3.2	-4.07	133.8	237.5	144.38	134.37
2	10.26	3.2	-4.17	136.53	245.26	147.53	139.54
2.04	10.26	3.17	-4.11	132.4	235.93	142.05	130.48
2.01	10.26	3.19	-4.07	133.71	236.47	144.2	133.92
1.99	10.25	3.22	-4.13	144.03	243.62	153.83	146.6
2	10.27	3.23	-4.13	144.26	243.48	153.86	146.68
2	10.26	3.23	-4.13	144.17	243.53	153.95	146.84
1.9	10.2	3.12	-4.23	151.4	255.7	161.68	157.52
1.91	10.21	3.13	-4.22	150.99	255.16	161.22	156.84

1.95	10.25	3.17	-4.22	150.64	255.25	160.61	155.61
2.05	10.31	3.25	-4.09	141.24	242.88	149.71	140.06
2.02	10.26	3.19	-4.07	133.58	237.51	144.23	134.21
2.04	10.27	3.2	-4.16	127.7	235.64	138.1	126.38
2.06	10.27	3.19	-4.11	131.72	236.05	140.56	128.91
2.18	10.47	3.39	-4.02	142.16	245.38	150.75	140.03
1.49	9.7	2.82	-4.61	140.52	251.47	149.21	140.45
1.65	9.86	2.91	-4.49	133.62	245.16	142.34	132.67
1.8	10.02	3.03	-4.37	131.46	243.31	140.52	130.86
1.67	9.93	2.93	-4.48	132.6	245.26	142.17	133.62
1.77	10.02	2.98	-4.39	129.75	240.51	140.06	130.36
1.64	9.82	2.93	-4.51	133.01	245.73	141.71	131.5
2.09	10.26	3.17	-4.18	120.4	227.93	129.73	116.23
1.6	9.83	2.88	-4.52	135.53	247.71	144.77	135.76
2.03	10.27	3.21	-4.19	128.04	236.86	137.92	126.14
2.03	10.26	3.2	-4.19	128.09	237.03	138.01	126.27
1.83	10.05	3.04	-4.19	132.27	232.94	141.66	132.13
1.64	9.86	2.91	-4.5	134.11	246.03	142.74	133.36
2	10.19	3.1	-4.2	123.45	231.98	133.93	121.5
2.66	10.91	3.7	-4.09	156.46	250.57	155.96	149.74
2.23	10.54	3.3	-4.46	168.17	262.86	174.88	177.09
2.26	10.58	3.29	-4.43	168.61	262.48	175.07	176.57
2.1	10.42	3.16	-4.54	170.97	264.96	180.13	183.59
2.74	11.02	3.81	-4.1	153.23	246.34	149.39	139.93
2.03	10.37	3.11	-4.61	172.14	267.05	180.64	184.97
2.15	10.44	3.17	-4.56	172.12	266.59	179.96	184.53
1.9	10.07	3.17	-4.36	145.01	253.69	148.76	138.57
2.15	10.29	3.35	-4.18	140.01	248.64	144.34	131.33
1.96	10.15	3.18	-4.27	139.4	250.58	146.65	135.03
1.93	10.09	3.2	-4.28	140.76	251.44	145.8	134.67
1.55	9.74	2.84	-4.6	144.06	254.43	151.06	142.14
1.56	9.74	2.84	-4.6	144.01	254.36	151.01	142.05
1.72	9.89	3.01	-4.46	139.07	249.65	144.48	134.45
2	10.18	3.22	-4.27	135.59	247.07	142.14	131.49
1.96	10.17	3.21	-4.34	147.89	254.93	151.57	141.31
2	10.19	3.19	-4.26	138.41	249.27	145.35	134.38
1.93	10.13	3.14	-4.31	138.78	250.61	146.46	135.75

2.28	10.57	3.44	-4.03	146.1	251.64	152.88	143.59
2.38	10.64	3.51	-3.96	143.02	247.85	151.81	141.37
2.53	10.77	3.61	-3.86	140.71	245.34	147.85	136.57
2.55	10.79	3.73	-3.85	140.06	243.45	145.16	132.67
2.16	10.42	3.39	-4.07	139.89	247.84	147.75	138.24
2.53	10.82	3.7	-3.84	139.5	243.15	144.74	132.11
2.16	10.36	3.36	-4.1	130.23	241.03	137.46	124.86
1.95	10.15	3.16	-4.27	138.32	248.8	145.96	136.07
2.24	10.55	3.47	-4.04	139.76	245.79	146.49	135.81
2.21	10.47	3.41	-4.04	137.03	245.01	145.69	136.16
1.95	10.08	3.19	-4.31	144.21	253.59	147.99	136.72
1.84	10.05	3.12	-4.42	151.16	258.24	154.71	146.02
2.12	10.36	3.36	-4.08	134.06	245.61	141.83	131.92
2.17	10.37	3.35	-4.1	130.6	241.25	138.25	125.96
2.07	10.29	3.28	-4.16	135.34	245.96	141.8	131.55
2.13	10.31	3.32	-4.16	139.15	247.9	143.91	131.96
1.83	10.01	3.07	-4.39	137.57	250	144.88	135.29
1.61	9.83	2.92	-4.51	141.77	251.49	148.13	139.98
1.61	9.83	2.92	-4.51	141.73	251.42	148.04	139.79
1.73	9.96	3.03	-4.42	137.92	248.42	145.38	136.71
1.79	10.02	3.07	-4.38	136.52	246.82	144.3	134.71
2	10.13	3.24	-4.24	139.82	250.8	144.4	133.34
2.17	10.44	3.32	-4.05	139.22	246.23	149.87	140.69
2.42	10.69	3.61	-3.93	138.96	242.94	144.12	131.98
2.05	10.24	3.25	-4.17	135.34	245.91	142.04	132.75
1.58	9.8	2.92	-4.58	156.14	263.58	159.61	151.46
1.59	9.81	2.94	-4.57	155.74	263.18	159.09	150.93
1.94	10.13	3.2	-4.36	146.62	254.48	150.73	139.91
2.33	10.61	3.49	-4	144.93	249.71	151.58	141.83
2.21	10.4	3.36	-4.12	137.33	245.07	142.11	129.51
2.3	10.62	3.47	-4.01	145.68	251.13	151.78	141.82
2.46	10.73	3.66	-3.91	140.25	245.15	147.38	134.89
2.23	10.51	3.44	-4.06	139.09	246.65	145.9	136.4
1.97	10.35	3.23	-4.22	164.81	270.13	178.89	175.74
2.23	10.59	3.5	-3.93	158.76	258.62	168.11	161.41
2.23	10.59	3.5	-3.93	158.75	258.59	168.07	161.35
2.17	10.51	3.46	-3.96	160.01	260.61	169.25	162.72

1.99	10.37	3.26	-4.07	161.56	263.67	173.58	168.04
1.91	10.3	3.22	-4.14	165.44	269.17	178.37	175.09
1.93	10.32	3.24	-4.12	165.31	268.88	177.93	174.46
1.72	9.93	3.03	-4.42	155.4	260.34	157.24	148.04
1.76	9.96	3.05	-4.4	155.13	259.61	156.77	146.99
2.63	10.88	3.7	-3.97	153.57	251.76	158.36	151.93
1.71	9.92	3	-4.44	139.14	249.02	144.97	135.41
1.77	9.95	3.03	-4.42	138.29	249.52	145.92	135.38
2.6	10.9	3.66	-4.15	152.23	238.17	137.35	123.86
2.61	10.93	3.67	-4.13	151.9	237.17	137.08	123.5
3.1	11.43	4.09	-3.54	135.64	220.87	120.89	101.31
2.6	10.97	3.65	-4.13	150.68	233.4	134.31	118.07
2.98	11.17	4.1	-3.75	134.44	215.49	113.5	88.65
3.14	11.31	4.23	-3.61	134.26	213.96	112.17	86.37
2.13	10.43	3.19	-4.6	171.15	259.81	168.73	168.27
2.83	11.09	3.88	-4	151.41	245.86	147.85	137.04
2.66	10.92	3.71	-4.06	152.7	243.91	140.61	125.87
3.05	11.4	4.12	-3.62	136.23	218.19	118.7	95.11
2.89	11.2	3.95	-3.76	139.72	221.04	121.13	99.89
3.06	11.27	4.07	-3.68	129.04	206.09	104.06	75.96
3.09	11.31	4.13	-3.6	147.1	239.38	141.64	127.38
3.19	11.4	4.08	-3.64	144.36	235.47	134.91	118.53
2.8	11.08	3.78	-3.87	146.72	242.53	140.81	126.06
2.79	11.03	3.75	-3.92	149.48	242.16	140.37	125.61
2.79	11.04	3.75	-3.92	149.47	242.09	140.34	125.56
2.72	10.95	3.75	-3.93	149.28	247.22	143.47	128.98
3.11	11.32	4.15	-3.58	146.67	239.11	141.41	126.99
3.11	11.33	4.15	-3.58	146.56	239.05	141.35	126.9
2.66	10.91	3.65	-4.05	152.65	245.38	141.3	127.14
2.84	11.12	3.85	-3.9	148.82	236.61	138	123.17
2.89	11.21	3.94	-3.87	146.94	237.11	141.02	126.87
2.92	11.21	3.95	-3.75	151.3	243.52	137.24	121.22
2.91	11.21	3.94	-3.76	151.75	243.77	137.38	121.51
3.1	11.33	4.18	-3.71	141.28	223.79	124.51	100.18
2.55	10.81	3.62	-4.09	153.65	246.06	143.22	129.58
3.25	11.48	4.34	-3.59	142.24	225.58	125.91	104.36
2.75	11.1	3.83	-4.02	151.9	237.39	136.42	116.8

3.4	11.6	4.37	-3.44	141.81	228.47	128.16	106.78
3.37	11.64	4.31	-3.47	142.61	230.35	128.15	107.67
2.96	11.27	3.99	-3.77	152.46	243	138.77	121.75
3.11	11.4	4.14	-3.68	150.88	240.1	137.32	119.17
2.95	11.24	3.97	-3.73	150.82	242.43	136.8	120.63
3.14	11.39	4.12	-3.68	145.5	236.2	132.37	114.27
3.17	11.42	4.14	-3.65	145.24	235.84	132.08	113.74
3.9	12.25	4.92	-2.76	126.64	213	112.06	80.78
2.82	11.08	3.85	-3.85	132.31	212.13	110.62	83.13
3.09	11.29	4.12	-3.66	132.72	215.1	113.17	86.63
3.1	11.28	4.1	-3.69	134.91	215.71	114.48	89.42
2.88	11.14	3.94	-3.88	138.85	220.16	120.68	95.21
2.7	10.89	3.78	-3.85	148.21	249.96	146.28	132.93
2.95	11.12	3.99	-3.67	145.28	244.06	141.61	126.54
2.6	10.85	3.6	-4.09	152.91	248.81	144.05	130.85
2.56	10.76	3.67	-3.94	150.15	252.29	148.82	136.42
2.76	10.93	3.84	-3.85	149.27	249.22	144.73	131.37
2.39	10.58	3.53	-4.06	157.49	259.49	153.35	142.03
2.71	10.89	3.79	-3.84	148.15	249.88	146.2	132.81
2.52	10.73	3.67	-3.89	149.27	250.92	147.22	133.69
2.71	10.87	3.79	-3.88	150.21	250.6	145.57	133.09
2.51	10.72	3.63	-4.01	153.06	254.2	149.36	137.49
2.26	10.48	3.4	-4.23	158.93	257.72	153.27	142.22
2.31	10.52	3.43	-4.19	158.35	257.27	152.47	141.16
2.84	11.02	3.89	-3.78	148.42	246.58	143.98	129.2
2.87	11.05	3.92	-3.75	147.66	245.69	143.53	128.44
2.91	11.08	3.95	-3.72	146.58	244.45	142.91	127.51
2.92	11.09	3.96	-3.72	146.34	244.19	142.76	127.29
2.94	11.11	3.98	-3.71	145.92	243.68	142.51	126.96
2.82	10.99	3.87	-3.74	145.37	247.89	144.62	129.11
2.64	10.87	3.63	-4.03	152.96	248.04	145.04	131.46
1.87	10.07	3.16	-4.31	151.83	258.58	154.05	141.91
1.95	10.17	3.24	-4.24	153.86	257.3	152.81	141.28
2.28	10.49	3.49	-4.01	152.02	254.19	150.25	136.75
2.29	10.52	3.5	-4	152.12	254.48	149.53	136.46
2.3	10.53	3.51	-3.99	151.85	254.28	149.37	136.28
2.55	10.77	3.75	-3.85	153.61	252.66	149.79	137.04

2.66	10.91	3.85	-3.79	153.15	253.12	148	134.82
2.11	10.38	3.36	-4.13	153.25	254.71	152.55	141.14
1.81	10.04	3.13	-4.35	157.19	261.71	156.84	146.02
1.82	10.05	3.14	-4.35	157.01	261.55	156.71	145.75
1.82	10.05	3.14	-4.34	157.06	261.51	156.66	145.81
2.13	10.34	3.36	-4.12	153.45	256.13	152.22	138.25
1.77	10.01	3.12	-4.38	157.55	262.52	157.34	146.41
1.73	9.96	3.01	-4.38	155.52	260.34	156.5	146.59
1.68	9.94	3.05	-4.44	158.48	263.72	158.35	148.19
1.7	9.96	3.06	-4.42	158.28	263.42	158.17	147.95
3.79	12.06	4.81	-3.04	145.17	235.86	134.98	114.98
3.09	11.39	4.17	-3.7	153.06	241.3	138.78	121.5
3.44	11.73	4.53	-3.42	146.59	233.01	131.62	112.21
2.89	11.22	3.99	-3.72	153.79	247.04	142.37	126.73
2.62	10.93	3.75	-3.94	155.05	250.06	144.8	132.24
3.85	12.15	4.88	-2.98	143.27	233.43	133.82	114.08
2.86	11.2	3.95	-3.93	151.09	238.68	136.44	118.17
2.85	11.19	3.93	-3.94	150.92	238.18	136.03	117.54
2.81	11.16	3.88	-3.97	151.32	237.42	135.81	117.02
2.66	10.89	3.84	-3.85	156.35	253.4	149.63	137.3
2.17	10.46	3.32	-4.35	162.41	259.13	153.31	142.8
3.4	11.72	4.46	-3.37	150.37	240.75	137.87	119.66
2.68	11.01	3.79	-3.9	155.04	250.47	145.93	132.11
2.5	10.83	3.63	-4.07	157.93	252.4	148.01	135.18
2.77	10.98	3.85	-3.78	153.82	249.52	144.97	130.5
2.69	11.03	3.82	-3.88	155.18	249.81	145.26	131.87
2.82	11.09	3.91	-3.81	152.47	245.67	140.82	126.54
2.47	10.78	3.63	-4.09	158.7	252.3	147.74	134.35
2.99	11.22	4.03	-3.69	150.25	243.25	137.88	121.87
2.94	11.22	3.98	-3.73	149.88	244.06	137.72	121.18
2.33	10.6	3.46	-4.26	158.88	253.05	148.56	136.92
2.41	10.68	3.5	-4.21	157.14	249.85	146.61	133.83
2.98	11.3	4.1	-3.83	151.09	237.64	136.71	118
2.88	11.22	3.98	-3.9	152.44	238.95	136.89	119.12
2.87	11.21	3.94	-3.92	150.68	237.93	135.8	117.12
2.96	11.28	4.03	-3.83	152.43	238.63	136.58	118.85
2.95	11.28	4.03	-3.83	152.44	238.64	136.59	118.86

3.68	11.96	4.77	-3.11	141.43	225.86	127.78	104.93
2.98	11.31	4.05	-3.67	153.12	244.9	141	124.26
2.82	11.06	3.84	-3.88	149.58	242.45	139.04	124.2
