# Supplementary material for: Eurasian aspen (Populus tremula L.): Central Europe’s keystone species ‘hiding in plain sight’
Source: PLoS One. 2024 Mar 27;19(3):e0301109. doi: 10.1371/journal.pone.0301109 (PMC10971661; doi:10.1371/journal.pone.0301109)
Supplement: S2 Data — (PDF) [file pone.0301109.s006.pdf]

	ecozone	alt	av	slope	t.shape	landf_TPI	Direct Ins	Diurnal An	MRVBF	Negative O	Protection	Texture	TPI	TWI	t_spring	t_summer
os00011	28	568	0.40	4	3	6	6.84	-0.02	1.50	1.54	0.00	97.11	-1.80	7.84	6.41	15.02
os00114	23	586	0.45	14	1	4	6.51	0.01	0.00	1.43	0.00	92.37	3.78	5.42	6.22	14.82
os00248	28	616	0.08	3	3	6	6.94	0.05	1.78	1.55	0.05	81.39	-0.64	8.71	6.01	14.62
os00259	19	537	0.65	8	2	7	6.52	-0.12	0.75	1.54	0.00	96.45	-4.37	6.88	6.38	14.98
os00373	19	640	0.21	2	3	6	6.89	0.02	2.46	1.55	0.00	73.10	-0.29	9.24	5.93	14.56
os00388	28	644	0.40	4	3	6	6.98	0.04	0.63	1.53	0.00	98.91	0.37	7.43	5.93	14.56
os00405	23	506	0.05	13	2	0	6.84	0.17	0.11	1.51	0.11	86.21	-2.95	6.73	6.78	15.41
os00486	23	705	0.94	13	2	0	6.47	-0.16	0.06	1.49	0.20	97.17	-1.47	7.65	4.89	13.63
os00513	33	845	0.05	17	3	5	7.17	0.28	0.00	1.41	0.11	98.19	0.47	5.80	4.76	13.50
os00528	30	636	0.49	11	1	4	6.95	0.04	0.01	1.45	0.06	95.52	4.01	5.78	6.27	15.00
os00531	23	666	0.27	7	3	5	7.04	0.08	0.20	1.48	0.04	96.40	0.79	5.97	6.05	14.79
os00643	23	661	0.25	7	3	9	7.05	0.08	0.28	1.48	0.05	100.00	-0.16	6.66	6.01	14.68
os00682	23	722	0.93	14	1	1	6.51	-0.18	0.00	1.39	0.00	99.53	2.37	6.00	5.64	14.30
os00713	28	609	0.40	1	3	6	6.88	0.00	2.83	1.53	0.02	28.86	-0.57	9.13	6.38	15.09
os00833	19	521	1.00	15	2	7	6.24	-0.23	0.06	1.48	0.15	94.36	-5.55	6.51	6.78	15.45
os00880	23	465	0.77	9	2	0	6.71	-0.05	0.15	1.51	0.10	61.29	-1.69	6.80	7.16	15.86
os00942	28	579	0.78	10	2	3	6.48	-0.11	0.27	1.49	0.09	92.51	-2.77	7.59	6.38	15.07
os00950	27	695	0.37	17	3	5	6.45	0.02	0.00	1.45	0.10	100.00	0.63	6.60	5.55	14.24
os00971	23	777	0.99	3	3	6	6.87	-0.04	1.21	1.53	0.00	96.40	-0.11	7.29	5.11	13.83
os00974	23	785	0.77	2	3	6	6.94	-0.01	0.65	1.54	0.03	96.90	0.24	7.38	5.01	13.77
os00975	23	790	0.66	3	3	6	6.96	-0.01	0.57	1.54	0.03	97.62	0.19	6.70	5.00	13.77
os00978	32	872	0.18	5	3	6	7.10	0.05	0.80	1.50	0.04	98.92	-0.42	7.15	4.48	13.24
os00987	33	877	0.97	3	3	6	6.91	-0.05	0.56	1.51	0.02	98.83	-0.03	7.33	4.47	13.21
os00988	33	897	0.92	0	3	6	7.00	0.01	0.74	1.52	0.01	24.15	0.20	8.72	4.35	13.09
os01022	27	582	0.03	16	2	7	6.78	0.22	0.00	1.49	0.23	99.97	-4.76	7.76	5.66	14.36
os01028	27	791	0.20	13	2	7	7.14	0.15	0.01	1.49	0.14	100.00	-2.72	7.06	4.86	13.60
os01083	27	525	0.00	9	2	7	6.89	0.15	0.62	1.54	0.20	99.64	-6.33	7.99	6.38	15.09
os01087	22	512	0.46	15	1	4	6.35	-0.01	0.00	1.37	0.11	97.26	5.50	5.13	6.99	15.66
os01092	19	511	0.09	12	2	7	6.95	0.13	0.44	1.51	0.17	100.00	-7.28	7.59	6.74	15.44
os01097	33	851	0.65	2	3	6	6.86	-0.05	1.29	1.53	0.02	75.53	-0.35	7.73	4.66	13.48
os01099	33	867	0.74	4	3	6	7.00	0.00	0.52	1.52	0.02	92.31	0.53	7.13	4.68	13.51
os01108	33	906	0.63	4	3	9	6.84	-0.05	0.43	1.51	0.03	93.42	0.08	6.70	4.45	13.27
os01118	33	831	0.77	3	3	6	6.94	-0.03	0.68	1.52	0.04	95.45	0.19	7.57	4.73	13.51
os01128	23	667	0.01	17	2	0	7.01	0.26	0.06	1.44	0.14	99.86	-1.44	6.78	5.65	14.40
os01129	27	657	0.00	11	2	0	7.04	0.19	0.10	1.46	0.14	99.81	-2.21	7.29	5.63	14.39
os01130	23	656	0.53	11	1	2	6.96	0.06	0.00	1.37	0.11	99.76	8.07	5.14	5.69	14.44
os01164	10	376	0.08	5	3	5	6.88	0.06	0.96	1.53	0.06	85.77	0.00	8.14	7.96	16.71
os01226	33	787	0.07	4	3	6	6.95	0.04	0.58	1.53	0.00	98.31	-0.23	6.97	5.08	13.86
os01228	33	802	0.92	1	3	6	6.97	0.02	0.49	1.53	0.00	87.75	0.63	8.70	4.99	13.80
os01229	33	806	0.53	5	3	6	6.87	0.00	0.49	1.52	0.00	90.43	0.60	7.52	4.90	13.70
os01300	23	670	0.35	8	3	5	7.03	0.07	0.39	1.44	0.08	100.00	-0.97	6.49	6.02	14.76
os01386	23	641	0.96	16	3	5	6.13	-0.26	0.00	1.43	0.00	93.29	1.01	5.79	5.98	14.77

os01387	23	619	0.96	16	3	5	6.12	-0.26	0.00	1.46	0.00	86.58	-1.98	5.96	5.97	14.77
os01436	23	686	0.38	20	3	5	6.28	0.01	0.00	1.40	0.19	99.95	-2.31	5.29	6.09	14.96
os01446	14	341	0.05	20	1	4	6.88	0.32	0.00	1.42	0.11	97.59	3.33	5.07	8.19	17.02
os01493	23	658	0.99	3	2	3	6.81	-0.05	2.87	1.53	0.06	92.10	-2.74	8.56	6.23	15.06
os01514	14	460	0.30	9	1	1	7.00	0.12	0.00	1.35	0.06	90.98	8.02	5.23	7.56	16.25
os01521	10	414	0.01	12	3	5	6.99	0.20	0.03	1.49	0.10	99.38	-1.70	6.07	7.61	16.38
os01527	10	378	0.36	6	3	5	6.57	-0.10	0.59	1.51	0.06	99.32	-0.04	6.86	8.12	16.88
os01543	32	741	0.08	3	2	0	7.02	0.05	1.97	1.55	0.07	84.79	-1.30	8.36	5.14	13.91
os01547	9	302	0.98	4	3	6	6.68	-0.04	0.58	1.51	0.03	93.32	0.76	6.81	8.82	17.55
os01584	17	377	0.53	6	2	0	6.63	-0.01	0.46	1.52	0.07	78.01	-1.63	7.50	8.35	17.27
os01636	19	387	0.01	8	2	3	6.93	0.14	0.62	1.48	0.08	99.24	-3.21	7.66	8.35	17.19
os01637	19	387	0.02	8	2	3	6.87	0.11	0.72	1.49	0.08	99.23	-3.37	7.86	8.35	17.19
os01646	17	375	0.26	6	2	3	6.90	0.07	0.90	1.47	0.12	98.95	-4.05	7.01	8.38	17.19
os01685	17	391	0.98	12	3	5	6.45	-0.16	0.09	1.45	0.10	97.15	-1.25	7.21	8.35	17.24
os01690	17	335	0.40	17	2	7	6.80	0.10	0.00	1.47	0.19	82.59	-5.02	6.06	8.49	17.39
os01701	17	387	0.43	6	2	3	6.66	0.00	0.89	1.52	0.08	98.56	-2.72	8.06	8.42	17.35
os01712	19	520	0.53	3	3	6	6.75	-0.02	2.21	1.52	0.04	77.17	-1.54	8.41	7.74	16.72
os01713	19	522	0.23	3	3	6	6.81	0.01	2.64	1.52	0.03	74.07	-0.38	8.19	7.74	16.72
os01718	19	433	0.71	6	3	5	6.78	-0.03	1.14	1.49	0.06	86.89	-1.40	8.25	8.23	17.20
os01739	9	372	0.95	3	3	6	6.67	-0.05	1.91	1.54	0.04	74.57	-1.59	8.66	8.29	17.17
os01751	22	383	0.95	4	3	6	6.67	-0.06	0.57	1.50	0.03	89.97	1.83	7.57	8.31	17.21
os01752	22	381	0.97	7	1	4	6.50	-0.13	0.22	1.48	0.04	90.87	2.07	7.22	8.31	17.21
os01753	22	381	0.97	7	1	4	6.51	-0.12	0.13	1.47	0.04	90.56	2.59	7.07	8.32	17.22
os01807	17	433	0.01	5	3	6	6.92	0.08	1.04	1.51	0.07	97.45	-1.90	7.73	8.10	17.04
os01866	19	396	0.77	3	2	3	6.70	-0.03	2.60	1.56	0.07	86.71	-3.57	8.78	8.29	17.23
os02020	19	284	0.92	0	2	0	6.71	0.00	2.99	1.57	0.12	40.71	-0.79	8.92	8.65	17.42
os02037	17	451	0.80	7	2	3	6.74	-0.05	0.85	1.54	0.09	97.66	-3.14	7.42	8.00	16.90
os02042	17	422	0.38	5	2	3	6.92	0.07	0.89	1.50	0.07	99.24	-2.40	7.26	8.20	17.09
os02043	17	430	0.30	7	2	3	6.92	0.08	0.85	1.50	0.09	99.51	-3.75	7.16	8.18	17.07
os02044	17	463	0.89	10	2	3	6.64	-0.09	0.72	1.52	0.11	97.73	-5.26	8.28	7.99	16.92
os02112	17	440	0.39	13	2	0	6.48	0.02	0.00	1.43	0.11	97.19	1.55	6.77	7.92	16.90
os02160	10	342	0.14	17	2	3	6.59	0.18	0.00	1.44	0.18	99.07	-4.28	5.89	8.44	17.37
os02163	10	346	0.89	25	2	3	5.34	-0.37	0.00	1.40	0.17	94.07	-1.94	5.17	8.51	17.43
os02208	10	456	0.40	2	3	6	6.85	0.01	0.69	1.54	0.05	81.19	0.10	8.81	7.69	16.66
os02303	17	471	0.42	8	3	9	6.92	0.05	0.51	1.48	0.07	99.87	-0.97	6.66	8.00	16.98
os02361	10	381	0.19	11	1	4	6.99	0.15	0.02	1.44	0.10	99.27	3.19	5.71	8.28	17.23
os02364	10	403	0.62	9	3	5	6.44	-0.14	0.19	1.49	0.08	99.14	0.78	6.02	8.22	17.19
os02399	19	500	0.84	6	2	3	6.77	-0.04	1.57	1.54	0.07	77.43	-1.50	8.39	7.47	16.47
os02400	19	499	0.77	3	2	3	6.79	-0.02	2.53	1.56	0.08	77.52	-2.82	8.61	7.46	16.46
os02401	19	502	0.65	7	3	5	6.80	-0.03	0.31	1.52	0.06	77.49	-0.25	8.24	7.47	16.47
os02402	19	502	0.67	6	3	5	6.84	-0.01	0.41	1.53	0.06	77.62	-0.57	8.31	7.47	16.47
os02433	16	528	0.77	5	2	3	6.77	-0.05	1.06	1.54	0.07	94.87	-2.21	6.89	7.23	16.23
os02455	9	377	0.60	8	2	0	6.47	-0.10	0.44	1.52	0.08	99.66	-1.08	7.25	8.26	17.21

os02518	10	419	0.85	7	3	9	6.74	-0.05	0.66	1.47	0.06	99.95	-1.56	7.34	8.30	17.25
os02522	10	384	0.91	3	3	6	6.81	0.01	0.04	1.51	0.02	71.28	1.91	7.81	8.40	17.33
os02524	10	385	0.08	4	3	6	6.87	0.05	0.53	1.49	0.05	95.93	1.08	7.10	8.33	17.29
os02527	17	305	0.05	4	2	7	6.77	0.04	1.76	1.55	0.12	95.27	-5.33	8.41	8.59	17.50
os02530	10	402	0.81	14	1	4	6.17	-0.18	0.00	1.41	0.08	97.83	4.37	5.58	8.29	17.24
os02531	10	377	0.24	9	3	5	6.63	0.02	0.06	1.47	0.06	95.04	1.93	6.67	8.47	17.39
os02546	10	445	0.45	17	2	3	6.86	0.08	0.00	1.42	0.13	99.71	-1.48	5.92	8.10	17.07
os02569	17	353	0.98	8	3	5	6.43	-0.14	0.45	1.50	0.09	97.32	-1.70	7.62	8.53	17.46
os02573	19	409	0.89	5	2	3	6.69	-0.06	1.41	1.53	0.08	97.19	-2.55	8.59	8.17	17.06
os02599	19	447	0.94	3	3	6	6.76	-0.03	2.66	1.53	0.02	72.50	-0.72	8.48	8.04	16.97
os02632	19	386	0.17	5	2	3	6.78	0.04	1.43	1.55	0.07	87.69	-2.71	8.02	8.33	17.23
os02640	10	410	0.77	6	3	5	6.79	-0.02	0.46	1.51	0.04	94.21	0.49	6.43	8.33	17.21
os02661	9	431	0.84	11	3	5	6.33	-0.16	0.03	1.48	0.08	92.89	-0.60	6.62	8.18	17.07
os02677	17	507	0.77	5	3	5	6.72	-0.07	0.89	1.52	0.07	99.84	-2.16	8.29	7.70	16.62
os02721	10	420	0.97	19	3	5	5.85	-0.30	0.00	1.44	0.12	84.49	-0.17	5.39	8.10	17.06
os02748	28	617	0.33	4	3	6	6.82	0.02	1.28	1.52	0.06	99.61	-1.06	7.73	7.22	16.19
os02763	23	615	0.01	8	3	5	6.96	0.12	0.11	1.48	0.05	98.88	0.92	5.89	7.23	16.21
os02764	23	614	0.07	8	3	5	6.92	0.10	0.15	1.49	0.05	98.89	0.60	5.90	7.24	16.21
os02793	28	542	0.40	2	3	6	6.88	0.01	2.75	1.55	0.04	72.73	-0.63	9.14	7.55	16.56
os02800	28	548	0.17	1	3	6	6.84	0.01	0.29	1.53	0.02	87.60	0.47	7.59	7.59	16.61
os02801	28	547	0.13	2	3	6	6.86	0.02	0.28	1.53	0.02	87.21	0.41	7.59	7.59	16.61
os02802	28	546	0.03	1	3	6	6.88	0.03	0.20	1.53	0.02	86.70	0.58	7.55	7.60	16.62
os02809	17	332	0.13	4	2	0	6.79	0.01	1.27	1.53	0.09	87.01	-0.97	8.23	8.57	17.38
os02819	19	427	0.47	9	2	3	6.48	-0.06	0.51	1.53	0.10	95.17	-3.79	7.76	8.09	16.98
os02827	10	398	0.75	7	3	9	6.56	-0.05	0.06	1.46	0.04	99.64	1.88	5.88	8.31	17.11
os02975	17	341	0.23	5	2	3	6.75	0.05	1.38	1.54	0.06	98.68	-2.48	7.86	8.61	17.40
os03031	10	368	0.81	10	2	3	6.66	-0.07	0.50	1.51	0.09	97.84	-3.24	7.01	8.41	17.17
os03047	10	397	0.71	7	3	9	6.58	-0.05	0.06	1.46	0.04	99.62	2.02	5.87	8.32	17.11
os03100	19	308	0.01	9	2	7	6.76	0.14	1.05	1.54	0.18	93.43	-5.91	6.99	8.48	17.22
os03163	10	332	0.18	14	3	5	6.60	0.10	0.00	1.45	0.09	97.68	-0.04	6.60	8.69	17.51
os03164	17	339	0.05	4	2	3	6.68	0.01	1.18	1.51	0.08	97.35	-2.71	8.14	8.66	17.49
os03172	10	354	1.00	15	3	5	6.09	-0.26	0.00	1.45	0.11	98.59	-0.47	6.57	8.50	17.32
os03202	10	422	0.47	6	3	5	6.82	0.00	0.14	1.49	0.03	98.06	2.16	6.10	8.27	17.10
os03270	10	281	0.09	8	2	0	6.71	0.08	0.25	1.50	0.10	71.11	-0.81	6.79	8.67	17.46
os03276	17	390	0.96	6	3	5	6.64	-0.08	0.84	1.49	0.07	99.47	-1.98	7.29	8.20	17.03
os03360	10	383	0.77	5	1	4	6.60	-0.08	0.08	1.49	0.03	95.88	3.03	6.98	8.36	17.17
os03369	19	367	0.70	5	2	3	6.72	-0.03	1.55	1.53	0.09	98.75	-3.80	7.56	8.28	17.08
os03387	10	316	0.92	8	3	5	6.41	-0.13	0.27	1.52	0.09	85.63	-1.14	6.03	8.68	17.44
os03389	9	343	0.79	6	3	5	6.52	-0.08	0.43	1.51	0.05	95.87	-0.32	6.41	8.67	17.43
os03406	10	347	0.16	6	1	4	6.75	0.06	0.01	1.46	0.03	88.41	4.23	7.55	8.60	17.39
os03409	10	312	0.69	3	3	6	6.68	-0.01	1.72	1.54	0.05	89.72	-1.27	8.48	8.69	17.48
os03411	10	351	0.92	4	3	6	6.63	-0.05	1.47	1.53	0.04	86.17	-1.19	7.72	8.56	17.37
os03418	10	317	0.00	7	2	0	6.84	0.11	0.21	1.51	0.08	79.77	0.16	6.71	8.66	17.48

os03436	17	352	0.92	11	3	5	6.27	-0.16	0.01	1.47	0.08	97.08	0.44	7.57	8.52	17.35
os03444	10	448	0.53	9	3	5	6.50	-0.05	0.08	1.48	0.07	98.38	0.87	6.33	8.05	16.94
os03449	17	361	0.81	4	2	3	6.64	-0.06	2.51	1.55	0.07	94.07	-2.71	9.68	8.41	17.27
os03492	17	333	0.27	6	2	0	6.79	0.01	0.42	1.52	0.07	87.48	0.41	8.29	8.57	17.38
os03497	9	411	0.83	9	3	5	6.66	-0.08	0.15	1.50	0.08	97.21	-1.21	6.17	8.21	17.02
os03501	17	354	0.71	7	2	0	6.55	-0.10	0.29	1.51	0.10	98.30	-1.17	8.03	8.33	17.13
os03521	10	438	0.69	10	1	1	6.45	-0.08	0.02	1.45	0.06	95.99	2.90	5.57	8.13	16.97
os03640	10	408	0.83	11	3	5	6.68	-0.06	0.01	1.46	0.06	98.50	1.90	5.78	8.28	17.15
os03652	10	337	0.70	5	2	7	6.63	-0.07	1.23	1.53	0.11	99.06	-2.57	7.77	8.38	17.11
os03671	10	392	0.17	10	1	1	6.98	0.14	0.07	1.46	0.05	93.59	1.97	6.43	8.48	17.34
os03672	10	388	0.19	8	2	3	6.95	0.11	0.30	1.50	0.06	90.84	-1.63	7.50	8.47	17.33
os03674	10	410	0.23	6	3	6	6.76	0.04	0.60	1.52	0.05	92.89	-0.23	6.76	8.32	17.18
os03678	19	390	0.76	5	3	6	6.62	-0.06	1.89	1.54	0.07	86.43	-1.13	8.96	8.26	17.12
os03714	10	321	0.76	2	1	4	6.79	0.03	0.01	1.49	0.02	82.36	3.45	7.97	8.73	17.53
os03723	10	389	0.40	6	3	5	6.87	0.04	0.18	1.48	0.05	99.53	2.50	6.12	8.22	16.97
os03780	17	334	0.75	10	2	3	6.45	-0.07	0.58	1.50	0.11	94.78	-4.55	7.42	8.52	17.27
os03789	19	432	0.81	15	2	3	6.21	-0.17	0.10	1.48	0.14	94.60	-4.68	7.71	7.97	16.77
os03794	17	470	0.97	5	3	6	6.73	-0.06	1.50	1.52	0.07	99.58	-1.74	7.95	7.76	16.61
os03795	17	468	0.96	5	2	3	6.70	-0.07	1.33	1.52	0.07	99.66	-1.88	7.98	7.77	16.61
os03801	17	374	0.43	5	2	3	6.82	0.03	1.65	1.53	0.09	97.68	-4.08	8.06	8.35	17.16
os03802	17	374	0.80	5	2	3	6.75	-0.02	1.40	1.52	0.09	97.76	-3.33	8.05	8.35	17.16
os03803	17	373	0.00	6	2	3	6.83	0.06	1.39	1.53	0.10	97.57	-3.74	8.16	8.35	17.16
os03804	17	372	0.24	6	2	3	6.80	0.04	1.22	1.53	0.10	97.58	-3.59	8.15	8.36	17.16
os03934	19	421	0.15	6	3	5	6.92	0.07	1.06	1.52	0.05	92.22	-2.12	7.96	8.02	16.77
os03937	19	349	0.12	6	2	7	6.73	0.08	1.21	1.54	0.16	93.31	-7.46	6.89	8.18	16.92
os04010	9	311	0.05	6	2	3	6.53	-0.09	1.33	1.52	0.10	95.96	-3.06	7.27	8.49	17.17
os04090	10	318	0.33	9	3	5	6.89	0.08	0.47	1.50	0.09	95.81	-0.31	6.44	8.60	17.35
os04106	10	358	0.78	3	2	3	6.76	-0.01	2.93	1.52	0.06	88.82	-2.84	8.90	8.26	17.06
os04362	9	335	0.41	3	2	3	6.73	-0.01	2.53	1.52	0.10	92.21	-2.55	7.35	8.50	17.35
os04407	10	363	0.43	1	3	6	6.74	-0.01	0.05	1.52	0.01	50.30	1.44	8.68	8.33	17.09
os04446	16	545	1.00	10	3	5	6.50	-0.16	0.23	1.50	0.07	89.58	-1.57	6.39	7.22	16.22
os04681	10	394	0.83	7	3	5	6.50	-0.10	0.32	1.51	0.05	96.26	-0.36	6.33	8.15	16.99
os04682	12	373	0.92	2	3	6	6.68	-0.04	2.06	1.54	0.04	67.77	-0.80	8.15	8.19	17.02
os04730	10	420	0.12	8	2	3	6.95	0.11	0.67	1.50	0.09	99.90	-3.55	6.59	8.12	17.05
os04732	10	415	0.54	7	2	3	6.81	0.00	0.92	1.51	0.09	99.91	-4.52	6.98	8.12	17.05
os04746	10	403	0.15	10	2	3	6.98	0.13	0.06	1.51	0.09	97.75	-2.01	5.88	8.08	17.01
os04787	16	537	0.08	2	3	6	6.89	0.02	2.49	1.55	0.03	53.37	-1.00	8.37	7.31	16.31
os04837	16	430	0.92	0	3	6	6.79	0.00	3.98	1.57	0.02	5.07	0.02	11.51	7.95	16.96
os04876	16	537	0.92	1	3	6	6.84	0.00	1.98	1.54	0.03	76.88	-1.04	8.29	7.36	16.35
os04884	16	499	0.23	3	3	6	6.80	0.01	3.98	1.54	0.02	9.63	0.33	9.59	7.59	16.60
os04964	19	474	0.60	4	3	6	6.68	-0.03	1.34	1.52	0.07	82.45	-1.94	7.74	7.71	16.71
os05023	19	427	0.77	4	2	7	6.74	0.00	1.08	1.56	0.13	68.95	-5.46	6.91	7.87	16.84
os05024	17	430	0.22	5	1	4	6.84	0.07	0.18	1.50	0.05	80.44	2.66	7.41	7.99	16.99

os05047	10	247	0.82	12	2	3	6.52	-0.04	0.52	1.46	0.17	98.30	-5.82	7.34	9.04	17.77
os05081	19	330	1.00	6	2	3	6.56	-0.10	0.95	1.53	0.08	95.16	-2.75	8.11	8.50	17.33
os05082	17	329	0.92	6	2	3	6.52	-0.09	1.04	1.52	0.08	95.23	-2.65	7.90	8.50	17.34
os05103	17	379	0.18	8	3	5	6.92	0.08	0.61	1.51	0.07	98.46	-1.55	7.13	8.26	17.02
os05228	19	395	0.00	4	2	3	6.64	-0.06	1.87	1.53	0.08	97.39	-3.74	7.82	8.24	17.04
os05250	10	395	0.97	11	3	5	6.40	-0.17	0.06	1.49	0.07	94.72	0.35	6.59	8.20	16.99
os05251	10	397	1.00	9	3	5	6.45	-0.15	0.04	1.48	0.06	93.91	1.84	6.52	8.21	17.00
os05261	17	344	0.58	8	3	5	6.57	-0.02	0.73	1.49	0.06	94.16	-1.46	8.38	8.51	17.31
os05270	10	335	0.24	12	3	5	6.93	0.13	0.09	1.51	0.10	77.59	-1.81	8.22	8.45	17.31
os05284	10	303	0.92	4	3	6	6.69	-0.04	0.47	1.51	0.03	94.18	0.88	6.80	8.82	17.55
os05286	9	317	0.20	11	2	3	6.87	0.10	0.05	1.50	0.13	89.10	-3.95	8.58	8.62	17.35
os05425	19	469	0.55	9	3	5	6.56	-0.04	0.15	1.49	0.05	98.17	1.06	6.58	8.22	17.26
os05437	19	435	0.92	2	2	0	6.78	-0.02	2.95	1.56	0.08	91.36	-2.38	8.85	8.26	17.39
os05494	19	461	0.02	9	3	5	6.94	0.12	0.64	1.50	0.09	95.63	-2.22	8.39	8.12	17.11
os05495	19	460	0.00	6	2	7	6.82	0.04	1.80	1.55	0.12	95.77	-5.41	8.33	8.11	17.10
os05523	19	427	0.73	9	2	3	6.42	-0.12	0.43	1.51	0.13	96.96	-5.07	7.82	8.39	17.38
os05593	19	470	0.04	8	2	3	6.57	-0.11	0.88	1.50	0.07	99.11	-2.84	7.22	8.27	17.32
os05614	19	430	0.82	8	2	0	6.50	-0.10	0.72	1.52	0.09	94.67	-2.58	8.67	8.18	17.18
os05619	19	420	0.99	6	2	3	6.59	-0.10	1.05	1.53	0.08	97.87	-1.79	8.04	8.36	17.40
os05663	19	443	0.01	3	3	6	6.86	0.05	1.94	1.56	0.04	46.95	-1.32	8.74	8.14	17.23
os06008	19	406	0.44	8	2	3	6.61	-0.01	0.83	1.47	0.07	91.85	-2.78	6.99	8.36	17.46
os06056	19	483	0.35	2	3	6	6.84	0.02	4.06	1.55	0.02	25.28	-0.22	8.61	8.01	17.12
os06395	19	502	0.65	7	2	3	6.86	0.01	0.88	1.53	0.12	97.33	-5.59	6.68	7.74	16.75
os06463	23	575	0.74	7	3	5	6.62	-0.07	0.36	1.51	0.05	87.41	0.49	7.01	7.38	16.37
os06465	23	604	0.92	13	3	5	6.25	-0.20	0.00	1.46	0.08	97.05	-0.08	5.67	7.32	16.30
os06468	23	642	0.05	2	3	9	6.91	0.02	0.02	1.50	0.01	97.85	1.92	7.42	7.17	16.17
os06471	23	627	0.99	13	3	5	6.39	-0.20	0.03	1.48	0.09	95.81	-1.16	6.75	7.18	16.18
os06478	17	388	0.24	4	2	3	6.65	-0.05	1.94	1.53	0.11	90.83	-5.08	8.28	8.27	17.30
os06479	23	653	0.60	9	1	4	6.61	-0.05	0.02	1.44	0.05	97.68	3.46	5.47	7.07	16.08
os06482	23	624	0.00	13	3	5	7.06	0.21	0.01	1.48	0.08	95.78	-0.86	5.97	7.13	16.12
os06484	23	669	0.22	4	1	1	6.93	0.05	0.09	1.47	0.03	99.91	2.80	6.20	7.02	16.03
os06492	23	573	0.68	11	1	2	6.79	-0.04	0.01	1.46	0.08	95.98	3.06	6.24	7.34	16.32
os06493	23	637	0.01	6	1	1	6.95	0.07	0.03	1.49	0.02	99.40	2.93	6.30	7.22	16.20
os06495	23	633	0.01	5	1	1	6.93	0.07	0.15	1.49	0.02	99.48	2.17	6.37	7.23	16.20
os06597	19	403	0.09	10	2	3	6.96	0.14	0.56	1.50	0.11	88.96	-4.75	8.24	8.33	17.38
os06599	19	406	0.21	4	3	6	6.83	0.06	1.31	1.54	0.04	80.25	-0.73	8.44	8.32	17.37
os07014	10	424	0.53	13	3	9	6.92	0.09	0.00	1.40	0.09	98.33	-0.78	6.20	8.19	17.16
os07085	17	479	0.77	6	2	3	6.82	-0.02	0.58	1.54	0.08	93.29	-2.75	7.45	7.67	16.69
os07112	17	375	0.77	5	2	7	6.74	-0.03	1.06	1.56	0.10	73.07	-3.86	7.86	8.29	17.30
os07165	19	458	1.00	5	3	5	6.65	-0.08	0.77	1.53	0.04	98.39	-1.16	7.08	8.11	17.22
os07167	19	466	0.96	5	3	5	6.69	-0.07	0.58	1.51	0.04	98.18	-0.03	6.83	8.10	17.21
os07243	19	388	0.10	12	2	3	6.46	-0.13	0.95	1.54	0.16	93.42	-7.26	8.82	8.46	17.58
os07480	19	415	0.86	10	3	5	6.48	-0.10	0.31	1.52	0.07	86.63	-0.83	8.65	8.41	17.54

os07490	19	456	0.92	5	3	5	6.62	-0.08	0.84	1.53	0.05	94.45	-1.30	8.13	8.19	17.33
os07601	6	453	0.06	13	1	1	6.87	0.18	0.02	1.38	0.08	96.64	4.94	6.53	8.35	17.46
os07609	11	425	0.85	5	2	3	6.71	0.00	1.48	1.54	0.10	83.83	-4.59	7.03	8.40	17.45
os07628	19	516	0.86	9	2	3	6.68	-0.08	0.36	1.50	0.11	99.85	-2.89	6.80	7.79	16.99
os07673	10	422	0.82	2	3	6	6.75	-0.03	2.97	1.56	0.05	85.80	-1.26	9.00	8.11	17.12
os07684	10	441	0.23	4	3	6	6.77	0.02	0.91	1.51	0.04	73.11	0.02	7.63	8.12	17.13
os07685	10	441	0.58	4	3	6	6.75	0.01	0.70	1.51	0.03	74.02	0.69	7.45	8.13	17.13
os07718	19	381	0.78	5	2	3	6.59	-0.05	0.92	1.53	0.12	94.84	-5.35	7.80	8.64	17.79
os07945	3	430	0.63	3	3	6	6.84	0.01	1.65	1.55	0.03	85.38	-0.53	8.44	8.26	17.36
os07978	19	487	0.98	5	2	3	6.69	-0.07	2.32	1.55	0.06	78.00	-2.54	9.63	7.97	17.05
os08083	11	394	0.60	2	3	6	6.78	-0.01	5.53	1.56	0.06	78.74	-1.17	7.63	8.46	17.53
os08132	19	360	0.02	16	3	5	6.03	-0.24	0.00	1.48	0.14	71.49	-2.37	5.94	8.70	17.82
os08152	8	412	0.40	2	3	6	6.84	0.02	4.56	1.56	0.03	68.08	-0.49	9.70	8.36	17.39
os08317	19	467	0.00	4	2	3	6.71	-0.06	1.98	1.55	0.08	93.41	-3.48	9.40	8.01	17.07
os08319	19	470	0.30	3	2	3	6.74	-0.02	2.14	1.55	0.09	93.43	-3.93	9.46	8.00	17.07
os08321	19	471	0.02	3	2	3	6.73	-0.04	1.98	1.55	0.09	93.47	-3.57	9.44	8.00	17.07
os08323	19	474	0.80	3	2	3	6.76	-0.03	1.92	1.54	0.09	92.90	-4.01	9.45	7.99	17.07
os08442	10	397	0.40	8	3	5	6.88	0.05	0.48	1.49	0.07	98.23	-1.98	6.75	8.56	17.69
os08457	19	381	0.32	8	3	5	6.78	0.09	0.42	1.48	0.08	89.81	-1.03	8.63	8.58	17.69
os08458	19	383	0.53	7	3	5	6.74	0.04	0.74	1.49	0.08	89.70	-0.73	8.45	8.58	17.69
os08461	11	356	0.08	6	2	7	6.89	0.12	0.83	1.55	0.15	77.50	-6.35	7.19	8.60	17.71
os08462	11	356	0.07	6	2	7	6.88	0.12	0.86	1.55	0.15	77.47	-6.56	7.18	8.60	17.71
os08463	11	356	0.08	6	2	7	6.88	0.12	0.86	1.55	0.16	77.43	-6.77	7.16	8.60	17.71
os08539	19	478	0.87	7	3	5	6.71	-0.07	0.35	1.51	0.04	92.49	-0.26	6.73	8.03	17.06
os08587	17	505	0.78	3	3	6	6.73	-0.05	1.46	1.54	0.03	74.79	-0.03	8.32	7.87	16.91
os08683	10	394	0.39	10	2	7	6.54	0.00	0.19	1.50	0.11	85.22	-2.38	7.56	8.49	17.62
os08774	16	523	0.01	2	3	6	6.86	0.02	2.79	1.55	0.02	15.95	-0.09	8.94	7.80	16.86
os08849	10	440	0.79	13	3	9	6.65	-0.07	0.00	1.42	0.08	96.83	0.45	6.49	8.52	17.66
os08850	10	438	0.76	12	3	5	6.63	-0.08	0.01	1.41	0.08	96.30	0.71	6.37	8.52	17.66
os08984	19	389	0.02	10	2	7	6.49	0.06	0.61	1.54	0.26	88.79	-12.95	6.44	8.24	17.25
os08999	11	495	0.68	1	3	6	6.82	0.00	2.93	1.56	0.07	38.34	0.19	10.36	7.04	15.85
os09000	24	494	0.01	2	2	3	6.84	0.03	1.41	1.56	0.09	46.25	-2.48	7.90	7.05	15.85
os09001	24	492	0.92	0	3	6	6.82	0.00	3.38	1.56	0.05	23.98	0.04	11.49	7.07	15.89
os09003	11	494	0.92	0	2	0	6.83	0.00	3.33	1.57	0.07	35.35	-0.39	10.51	7.03	15.84
os09004	24	493	0.08	1	3	6	6.83	0.01	3.85	1.56	0.06	33.35	-0.03	10.92	7.05	15.86
os09032	11	414	0.92	0	3	6	6.80	0.01	3.97	1.56	0.03	24.69	0.11	11.06	7.87	16.83
os09045	24	571	0.64	6	1	4	6.65	-0.06	0.31	1.49	0.06	95.11	2.26	6.75	6.70	15.54
os09051	11	491	0.07	1	3	6	6.84	0.01	2.95	1.57	0.04	24.65	-0.32	8.94	7.08	15.90
os09052	24	491	0.92	0	3	6	6.82	0.00	3.69	1.57	0.04	21.13	0.03	10.11	7.09	15.90
os09086	10	532	0.23	4	1	4	6.79	0.02	0.16	1.51	0.02	88.98	2.02	7.75	7.37	16.35
os09097	8	523	0.21	6	3	6	6.93	0.04	0.18	1.53	0.05	80.15	1.31	8.20	6.95	15.82
os09099	10	510	0.23	10	3	5	6.76	0.08	0.11	1.49	0.07	90.37	-0.26	7.01	7.21	16.09
os09100	10	508	0.23	9	3	5	6.78	0.08	0.22	1.50	0.07	89.93	-1.02	7.10	7.21	16.09

os09103	10	440	0.09	3	2	3	6.87	0.05	2.58	1.56	0.06	61.47	-2.96	7.89	7.78	16.73
os09125	10	484	0.08	1	3	6	6.86	0.02	0.15	1.51	0.01	82.95	1.85	8.42	7.72	16.69
os09168	28	654	0.23	2	3	6	6.90	0.02	1.27	1.55	0.01	54.51	0.07	8.03	6.48	15.38
os09224	19	379	0.92	0	3	6	6.76	0.00	2.68	1.55	0.03	61.32	-1.26	8.87	7.99	16.82
os09225	19	378	0.85	2	3	6	6.71	-0.02	1.94	1.54	0.02	55.40	-0.40	8.60	8.00	16.83
os09226	19	372	0.07	2	3	6	6.70	-0.03	2.20	1.54	0.02	55.70	-0.28	8.20	8.03	16.86
os09227	19	365	0.92	5	3	6	6.60	-0.07	1.77	1.54	0.04	75.63	-1.63	8.81	8.04	16.87
os09229	19	364	0.00	2	3	5	6.72	0.00	2.00	1.54	0.06	88.03	-2.59	9.08	8.00	16.82
os09231	19	371	0.10	4	2	3	6.77	0.03	1.76	1.54	0.05	84.30	-2.06	8.91	7.98	16.80
os09232	19	378	0.97	5	2	3	6.63	-0.08	1.22	1.54	0.05	75.24	-1.25	8.70	7.95	16.77
os09235	11	319	0.08	6	3	5	6.78	0.07	0.42	1.54	0.07	72.41	-2.00	7.75	8.33	17.16
os09236	11	321	0.16	7	3	5	6.75	0.08	0.31	1.52	0.06	73.33	-1.42	7.52	8.33	17.16
os09237	17	305	1.00	5	2	7	6.52	-0.08	1.44	1.56	0.11	88.20	-4.87	7.83	8.36	17.20
os09238	17	306	1.00	7	2	7	6.53	-0.08	1.22	1.55	0.12	88.69	-5.27	7.63	8.35	17.19
os09244	16	369	0.92	1	3	6	6.74	-0.02	3.63	1.56	0.04	72.82	-1.51	9.70	8.03	16.84
os09247	11	349	0.85	6	2	3	6.61	-0.04	1.49	1.55	0.10	94.33	-4.82	8.30	8.10	16.91
os09255	17	374	0.60	6	2	7	6.69	-0.03	2.81	1.57	0.14	86.12	-7.28	8.02	7.80	16.63
os09268	28	493	0.85	4	3	6	6.66	-0.06	0.79	1.53	0.03	91.45	-0.23	7.68	7.36	16.21
os09324	17	340	0.08	4	3	6	6.72	0.02	1.42	1.55	0.06	99.78	-0.95	7.10	8.19	17.03
os09344	19	311	0.83	2	3	6	6.71	-0.03	2.71	1.55	0.02	34.84	-0.09	9.39	8.40	17.20
os09445	19	309	0.66	3	3	6	6.66	-0.04	2.57	1.55	0.02	42.96	-0.86	9.43	8.40	17.20
os09489	8	380	0.23	2	3	6	6.74	0.00	3.13	1.55	0.02	35.00	-0.18	9.79	8.18	17.09
os09490	16	382	0.92	2	3	6	6.72	-0.02	2.94	1.55	0.02	30.74	-0.16	9.41	8.17	17.08
os09577	11	371	0.12	6	2	3	6.79	0.09	1.12	1.54	0.14	87.22	-6.99	7.22	8.23	17.14
os09588	10	268	0.89	9	3	5	6.36	-0.13	0.10	1.50	0.05	94.61	0.67	6.06	8.65	17.42
os09589	14	265	1.00	13	3	5	6.23	-0.21	0.01	1.49	0.07	88.85	-0.29	6.04	8.65	17.42
os09601	14	276	0.15	9	3	5	6.70	0.08	0.12	1.51	0.10	79.57	-1.55	6.89	8.61	17.42
os09614	8	331	0.79	2	3	6	6.68	-0.02	2.78	1.56	0.03	60.26	-1.06	8.14	8.38	17.21
os09731	17	410	0.40	8	2	3	6.87	0.07	0.74	1.51	0.12	99.53	-5.87	7.40	8.11	17.04
os09752	19	454	0.42	3	3	6	6.75	-0.01	1.88	1.54	0.03	84.98	-0.86	8.93	7.98	16.95
os09926	27	462	0.86	2	2	0	6.73	-0.03	2.10	1.56	0.07	62.00	-0.01	8.44	7.00	15.73
os09933	28	592	0.99	6	3	5	6.68	-0.09	0.47	1.50	0.07	99.57	-0.73	6.16	6.51	15.27
os10054	17	544	0.13	6	3	5	6.86	0.06	0.47	1.52	0.05	97.90	-0.51	6.91	6.83	15.52
os10135	28	514	0.99	2	3	6	6.77	-0.03	1.77	1.56	0.00	64.10	-0.11	7.95	6.91	15.61
os10139	27	508	0.23	5	3	5	6.76	0.02	0.19	1.50	0.00	99.19	1.79	5.99	6.79	15.67
os10186	28	516	0.77	9	3	5	6.71	-0.07	0.14	1.50	0.07	99.87	-1.15	6.65	6.76	15.65
os10236	28	429	0.23	2	3	6	6.79	0.02	1.38	1.55	0.00	58.22	0.11	8.62	7.06	15.95
os10301	28	614	0.92	1	2	7	6.79	-0.02	2.98	1.56	0.00	86.12	-2.68	7.47	6.25	14.90
os10302	24	675	0.84	8	3	5	6.59	-0.11	0.25	1.51	0.05	94.65	0.28	6.51	5.88	14.57
os10328	19	569	0.90	7	2	7	6.73	-0.07	0.47	1.54	0.09	85.27	-2.52	7.46	6.47	15.20
os10365	27	528	0.88	7	3	5	6.53	-0.12	0.32	1.51	0.05	73.64	0.01	6.64	6.76	15.53
os10369	28	536	0.67	4	2	0	6.70	-0.05	1.33	1.54	0.06	74.11	-0.88	8.16	6.67	15.41
os10371	28	561	0.92	5	2	0	6.67	-0.08	0.56	1.53	0.07	95.95	-0.38	6.99	6.50	15.25

os10372	28	584	0.79	4	3	6	6.69	-0.07	0.59	1.53	0.05	95.39	-0.39	7.34	6.43	15.19
os10382	17	671	0.34	11	3	5	6.39	-0.19	0.01	1.47	0.08	99.57	0.86	5.88	6.17	14.95
os10387	28	554	0.25	4	3	6	6.93	0.03	0.99	1.54	0.03	93.31	-0.47	7.66	6.71	15.51
os10389	28	593	0.99	8	2	0	6.63	-0.12	0.30	1.51	0.07	99.98	-1.04	6.87	6.41	15.20
os10390	28	616	0.74	7	3	5	6.83	-0.04	0.36	1.50	0.06	99.98	0.43	6.74	6.35	15.15
os10394	24	577	0.77	6	3	5	6.80	-0.05	0.27	1.52	0.04	97.01	0.61	7.48	6.51	15.31
os10403	28	562	0.77	10	3	5	6.72	-0.08	0.09	1.50	0.08	99.05	-0.58	6.01	6.61	15.37
os10419	28	581	0.04	8	3	5	6.93	0.12	0.10	1.50	0.05	97.93	-0.13	5.78	6.66	15.40
os10424	28	600	0.68	3	3	6	6.77	-0.03	1.22	1.53	0.06	99.07	0.25	7.57	6.41	15.17
os10454	23	704	0.02	7	3	9	7.08	0.11	0.22	1.46	0.06	100.00	1.92	6.23	6.06	14.87
os10469	28	662	0.95	5	3	6	6.83	-0.05	0.57	1.52	0.06	94.34	-0.30	7.22	6.05	14.83
os10473	23	620	0.17	9	3	5	6.84	0.08	0.12	1.50	0.06	99.49	0.35	6.40	6.25	15.02
os10493	28	573	0.08	5	3	5	7.00	0.09	0.50	1.51	0.05	93.12	-0.07	7.04	6.52	15.22
os10526	23	558	0.30	8	3	5	6.52	-0.14	0.18	1.50	0.06	95.56	0.36	6.81	6.64	15.37
os10593	28	567	0.60	5	2	0	6.71	-0.03	0.55	1.53	0.07	96.03	-0.31	8.30	6.50	15.23
os10616	28	515	0.40	1	3	6	6.84	0.00	4.80	1.55	0.02	40.76	0.35	8.74	6.87	15.57
os10713	28	595	0.97	6	3	5	6.64	-0.10	0.47	1.52	0.05	98.11	0.37	6.51	6.37	15.13
os10716	17	661	0.92	8	3	5	6.54	-0.13	0.08	1.49	0.07	99.94	0.46	5.89	6.18	14.96
os10717	22	683	0.60	8	1	4	6.65	-0.04	0.03	1.46	0.06	97.61	2.76	6.03	6.14	14.92
os10720	28	537	0.76	5	2	0	6.68	-0.05	0.58	1.53	0.05	75.03	-0.24	8.02	6.67	15.41
os10721	22	583	0.39	9	3	5	6.68	0.01	0.13	1.50	0.06	98.32	-0.48	6.41	6.56	15.34
os10722	22	592	0.36	3	3	9	6.93	0.03	0.29	1.50	0.02	97.37	1.49	6.71	6.55	15.33
os10774	28	616	0.77	7	3	5	6.77	-0.07	0.29	1.51	0.06	99.94	-0.41	6.83	6.37	15.17
os10778	23	574	0.49	5	3	5	6.91	0.01	0.46	1.54	0.00	73.50	-0.09	8.18	6.48	15.14
os10801	28	514	0.77	2	3	6	6.81	-0.02	3.14	1.56	0.04	65.68	-0.24	8.43	6.89	15.59
os10826	28	503	0.92	1	3	6	6.85	0.02	0.70	1.56	0.01	31.96	0.31	8.45	6.93	15.61
os10860	28	601	0.45	6	3	5	6.75	-0.01	0.64	1.53	0.07	99.94	-0.93	7.13	6.23	14.98
os10889	28	661	0.01	5	3	5	6.98	0.08	0.23	1.51	0.04	99.62	0.14	6.53	6.15	14.92
os10919	19	589	0.12	5	3	5	7.00	0.08	0.61	1.54	0.05	93.82	-0.69	8.67	6.31	14.98
os10923	24	559	0.00	6	2	0	6.98	0.10	0.53	1.54	0.08	85.55	-0.97	8.22	6.34	15.01
os10932	27	604	0.95	7	2	0	6.68	-0.10	0.27	1.52	0.07	90.39	0.03	6.67	6.09	14.75
os10975	19	522	0.40	3	2	7	6.87	0.03	2.42	1.56	0.09	87.65	-3.24	8.53	6.47	15.16
os10977	27	593	1.00	5	3	6	6.71	-0.07	1.88	1.53	0.03	51.90	-0.35	8.72	6.42	15.14
os11025	17	546	0.99	9	3	9	6.58	-0.13	0.12	1.44	0.06	95.21	1.18	6.04	6.74	15.46
os11090	24	605	0.04	3	3	6	6.88	0.03	1.92	1.53	0.05	90.56	-1.27	8.13	6.17	14.88
os11121	28	662	0.19	8	3	5	7.05	0.09	0.29	1.50	0.05	99.96	-0.50	6.20	6.05	14.78
os11172	28	598	0.15	5	3	6	7.00	0.08	0.74	1.53	0.06	95.65	-1.30	7.69	6.29	14.96
os11195	28	622	0.58	5	3	5	6.76	-0.02	0.49	1.52	0.04	95.05	-0.07	6.56	6.31	15.09
os11196	28	616	0.99	2	3	6	6.84	-0.03	0.24	1.52	0.03	95.55	0.32	7.81	6.39	15.17
os11229	23	505	0.85	12	3	5	6.64	-0.10	0.02	1.50	0.08	98.92	-0.55	7.22	6.84	15.61
os11230	28	533	0.51	6	3	5	6.90	0.02	0.62	1.52	0.07	99.99	-1.60	8.11	6.75	15.53
os11234	23	598	0.71	9	3	5	6.81	-0.05	0.10	1.49	0.09	95.62	-0.17	6.51	6.41	15.20
os11239	23	574	0.60	6	3	5	6.86	-0.02	0.56	1.52	0.08	98.44	-0.92	8.48	6.46	15.25

os11247	25	605	0.35	6	2	0	6.76	0.01	0.48	1.51	0.09	95.06	0.06	7.21	6.28	15.06
os11319	28	583	0.13	5	2	0	6.89	0.06	0.55	1.53	0.00	98.26	-0.32	6.69	6.52	15.28
os11345	19	506	0.08	2	2	0	6.85	0.01	3.52	1.56	0.00	42.20	-0.20	7.82	6.88	15.62
os11347	23	547	0.62	12	3	5	6.80	-0.02	0.00	1.46	0.09	97.85	-0.17	5.58	6.76	15.49
os11353	24	528	0.23	3	3	6	6.82	0.02	1.47	1.55	0.06	74.98	-0.34	7.87	6.71	15.46
os11356	23	589	0.60	7	2	0	6.65	-0.03	0.14	1.51	0.08	99.72	0.72	8.22	6.33	15.10
os11387	27	614	0.39	7	2	0	6.75	0.01	0.36	1.51	0.00	96.25	-0.16	7.17	6.30	15.07
os11395	27	639	0.92	0	2	0	6.85	-0.01	2.75	1.56	0.00	91.49	-1.63	8.60	6.02	14.80
os11473	28	443	0.37	13	3	5	6.56	0.03	0.01	1.49	0.00	65.93	-1.69	6.87	7.05	15.91
os11543	28	475	0.83	4	2	0	6.64	-0.07	0.56	1.54	0.07	86.10	0.15	7.34	6.94	15.73
os11550	17	485	0.59	8	3	5	6.82	-0.02	0.25	1.52	0.06	99.87	-0.49	6.61	7.04	15.87
os11552	28	463	0.71	2	3	6	6.76	-0.01	3.09	1.55	0.03	53.86	0.50	8.74	6.95	15.83
os11560	28	473	0.04	4	3	6	6.86	0.06	0.58	1.53	0.00	99.41	-0.18	7.58	6.88	15.77
os11610	19	550	0.40	8	2	3	6.95	0.05	0.50	1.52	0.09	99.99	-2.27	6.68	6.67	15.47
os11613	27	504	0.00	4	2	0	6.67	-0.08	0.49	1.53	0.07	93.40	0.80	8.54	6.83	15.59
os11637	17	575	0.60	9	1	1	6.53	-0.08	0.08	1.46	0.05	99.02	2.48	6.07	6.57	15.49
os11692	28	506	0.72	9	3	5	6.74	-0.06	0.15	1.50	0.07	99.21	-0.37	7.79	6.83	15.61
os11719	27	648	0.35	6	1	2	6.78	0.00	0.04	1.47	0.00	89.29	4.26	7.27	6.05	14.83
os11721	27	640	0.05	8	2	0	6.90	0.09	0.30	1.52	0.00	90.55	-1.70	8.21	6.03	14.81
os11727	27	548	0.88	10	2	0	6.65	-0.11	0.06	1.51	0.09	99.98	-0.68	6.78	6.46	15.27
os11728	27	548	0.89	9	2	0	6.65	-0.11	0.07	1.51	0.09	99.98	-0.71	7.05	6.46	15.27
os11731	27	529	0.87	8	2	0	6.70	-0.08	0.39	1.53	0.09	99.85	-1.16	7.53	6.56	15.36
os11732	27	522	0.93	7	2	0	6.68	-0.09	0.76	1.54	0.09	99.76	-1.86	7.87	6.59	15.40
os11741	27	663	0.63	7	3	5	6.90	-0.02	0.32	1.48	0.07	100.00	-0.16	6.29	6.06	14.92
os11751	19	556	0.92	9	3	5	6.49	-0.14	0.24	1.49	0.08	94.86	-0.69	6.59	6.64	15.46
os11757	28	578	0.23	5	2	0	6.80	0.03	0.79	1.53	0.09	99.77	-1.45	8.71	6.36	15.13
os11865	19	514	0.93	8	3	5	6.64	-0.10	0.16	1.52	0.08	78.20	-0.77	8.07	6.83	15.52
os11932	17	578	0.14	15	1	4	6.77	0.15	0.00	1.44	0.07	99.89	1.88	5.43	6.94	15.87
os11980	23	691	0.00	9	2	3	7.07	0.15	0.28	1.49	0.11	100.00	-2.11	7.68	6.74	15.70
os11987	28	609	0.77	5	2	0	6.71	-0.08	0.53	1.53	0.09	99.95	-0.23	7.29	7.07	16.00
os11996	28	692	0.99	5	3	5	6.76	-0.08	0.31	1.49	0.05	99.81	0.15	6.32	6.76	15.75
os12004	24	662	0.38	6	2	3	6.97	0.04	0.87	1.52	0.10	100.00	-2.41	7.99	6.76	15.65
os12031	28	630	0.00	7	2	7	6.99	0.12	0.66	1.52	0.13	99.44	-2.08	7.17	6.80	15.66
os12049	19	497	0.33	9	3	9	6.69	0.03	0.14	1.47	0.06	91.37	0.35	7.18	7.92	16.78
os12102	19	612	0.33	11	2	3	6.73	0.05	0.25	1.51	0.12	99.80	-4.02	7.47	7.04	15.89
os12111	17	628	0.43	16	1	1	6.73	0.00	0.00	1.29	0.06	97.86	13.26	5.61	7.36	16.21
os12127	23	704	0.88	10	3	5	6.48	-0.14	0.04	1.45	0.08	95.29	0.58	5.75	6.67	15.49
os12150	23	785	0.40	10	3	9	7.05	0.06	0.05	1.46	0.07	99.15	-0.78	5.94	6.35	15.20
os12157	23	741	0.01	9	2	3	7.07	0.15	0.19	1.49	0.10	98.77	-2.59	5.82	6.43	15.28
os12163	23	724	0.03	9	3	5	7.09	0.15	0.11	1.49	0.12	99.85	-1.10	5.83	6.43	15.27
os12192	23	704	0.87	11	3	9	6.49	-0.14	0.01	1.45	0.05	99.70	1.83	5.45	6.75	15.56
os12204	23	704	0.23	7	2	0	6.85	0.04	0.38	1.51	0.09	95.42	-0.25	6.87	6.41	15.24
os12299	23	692	0.41	15	2	3	6.91	0.08	0.00	1.47	0.14	97.82	-2.46	5.30	6.56	15.33

os12394	28	589	0.82	2	3	6	6.79	-0.03	2.85	1.55	0.02	62.32	-0.30	8.74	7.05	15.89
os12430	27	612	0.53	14	2	7	6.36	-0.07	0.00	1.48	0.13	99.59	-3.59	8.23	6.99	15.90
os12502	28	604	0.00	7	3	5	7.01	0.12	0.45	1.52	0.07	99.94	-0.87	6.67	7.06	15.89
os12503	23	673	0.17	13	3	5	7.11	0.18	0.00	1.46	0.10	98.81	-1.10	5.60	6.80	15.73
os12504	23	674	0.14	14	3	5	7.12	0.20	0.00	1.45	0.10	99.16	-0.61	5.65	6.80	15.73
os12506	28	614	0.47	5	2	0	6.88	-0.01	0.75	1.54	0.10	99.80	-1.39	7.56	6.94	15.87
os12507	28	615	0.77	6	2	0	6.81	-0.05	0.59	1.53	0.10	99.75	-0.86	7.17	6.95	15.88
os12508	28	612	0.77	5	2	0	6.83	-0.04	0.56	1.53	0.09	99.49	0.20	6.88	6.97	15.90
os12509	28	608	0.77	7	2	0	6.82	-0.04	0.37	1.52	0.09	99.12	-0.14	6.90	6.99	15.92
os12537	19	521	1.00	6	3	5	6.60	-0.11	0.58	1.52	0.05	99.20	-0.81	7.40	7.61	16.52
os12558	17	442	0.89	11	2	7	6.44	-0.12	0.34	1.53	0.20	85.59	-5.83	6.82	7.87	16.74
os12559	17	443	0.87	12	2	7	6.38	-0.14	0.34	1.52	0.19	85.41	-5.97	6.84	7.88	16.74
os12630	22	518	0.73	7	2	3	6.61	-0.06	0.86	1.50	0.07	88.61	-2.42	7.92	7.69	16.54
os12631	22	516	0.78	8	2	3	6.59	-0.05	0.80	1.49	0.08	88.62	-2.70	7.94	7.69	16.54
os12632	22	514	0.69	9	2	3	6.55	-0.06	0.64	1.49	0.09	88.42	-3.00	7.96	7.70	16.55
os12634	19	579	0.87	12	3	9	6.64	-0.11	0.01	1.47	0.07	99.95	0.36	5.65	7.49	16.41
os12635	19	563	0.92	11	3	5	6.62	-0.12	0.03	1.48	0.07	99.87	-1.30	6.27	7.51	16.43
os12664	16	561	0.92	0	3	6	6.85	0.00	2.39	1.55	0.01	5.35	0.07	10.47	7.47	16.33
os12668	19	510	0.26	4	2	3	6.83	0.07	1.21	1.52	0.12	94.92	-5.50	7.73	7.67	16.53
os12669	19	506	0.29	19	2	3	6.84	0.18	0.11	1.48	0.18	95.74	-8.18	6.55	7.68	16.53
os12680	10	496	0.58	23	1	4	6.69	0.02	0.00	1.36	0.11	81.73	3.29	6.15	7.78	16.60
os12682	27	606	0.01	5	2	0	6.95	0.09	0.69	1.53	0.12	99.96	-1.25	8.45	6.91	15.77
os12683	28	605	0.00	5	2	0	6.96	0.09	0.67	1.53	0.12	99.95	-0.79	8.11	6.93	15.78
os12686	28	605	0.01	5	2	0	6.93	0.09	0.77	1.53	0.12	99.94	-0.85	7.67	6.94	15.79
os12711	19	552	0.40	2	3	6	6.81	0.00	2.05	1.54	0.03	80.95	-0.28	8.86	7.47	16.31
os12742	23	622	0.95	14	3	9	6.47	-0.18	0.00	1.45	0.08	99.45	1.51	5.32	7.22	16.15
os12791	19	625	0.79	16	2	0	6.54	-0.14	0.00	1.46	0.13	99.97	-1.45	6.39	6.91	15.77
os12795	19	634	0.43	6	3	5	6.76	0.00	0.52	1.51	0.07	100.00	-0.59	6.96	7.02	15.90
os12798	28	642	0.45	13	3	5	6.54	-0.02	0.00	1.46	0.07	99.77	0.76	5.83	7.02	15.89
os12809	28	653	0.08	5	3	6	7.02	0.09	0.66	1.52	0.09	99.88	-0.80	6.54	6.88	15.76
os12828	28	706	0.82	7	3	5	6.63	-0.12	0.33	1.50	0.06	100.00	-0.30	6.76	6.64	15.57
os12836	28	607	0.81	5	2	0	6.66	-0.10	0.50	1.53	0.10	97.06	-0.28	6.87	6.93	15.82
os12951	27	697	0.35	10	2	7	6.77	0.05	0.40	1.52	0.11	94.55	-2.21	6.88	6.23	15.06
os12962	28	702	0.23	2	3	6	6.92	0.02	2.37	1.55	0.03	52.44	-0.32	8.22	6.37	15.20
os13005	28	666	0.77	2	3	6	6.88	-0.02	1.94	1.54	0.03	68.20	-0.64	8.09	6.62	15.42
os13020	23	620	0.92	4	3	6	6.70	-0.07	0.41	1.54	0.05	82.68	-0.04	8.67	6.78	15.57
os13056	19	497	0.54	11	2	3	6.89	0.04	0.32	1.49	0.10	76.02	-2.63	7.73	7.73	16.60
os13057	19	509	0.80	8	3	5	6.76	-0.05	0.72	1.49	0.08	83.12	-2.18	8.35	7.69	16.56
os13112	28	633	0.89	3	3	6	6.79	-0.03	0.98	1.54	0.03	94.39	0.18	7.13	6.89	15.76
os13278	19	541	0.76	2	3	6	6.80	-0.02	2.92	1.56	0.03	39.20	-1.05	9.03	7.22	16.09
os13296	19	527	0.98	12	1	4	6.43	-0.18	0.00	1.44	0.06	92.89	1.98	6.06	7.52	16.36
os13320	28	614	0.92	1	3	6	6.90	0.02	3.86	1.56	0.04	33.11	-0.69	8.39	6.86	15.63
os13406	19	446	0.48	4	3	6	6.76	0.01	0.59	1.53	0.04	78.99	0.82	8.38	7.87	16.70

os13463	27	641	0.60	6	2	3	6.87	-0.02	0.99	1.54	0.08	90.90	-3.74	8.11	6.64	15.44
os13492	27	617	0.15	6	2	7	6.96	0.08	1.25	1.55	0.12	96.90	-6.19	8.14	6.84	15.68
os13498	28	593	0.60	7	2	0	6.66	-0.05	0.34	1.52	0.08	92.58	-0.96	8.21	6.85	15.71
os13566	28	613	0.92	0	3	6	6.88	0.01	2.51	1.54	0.02	73.13	-0.50	9.17	7.08	15.97
os13577	19	557	1.00	6	2	0	6.62	-0.10	0.62	1.53	0.08	95.26	-1.61	8.26	7.25	16.10
os13604	28	624	0.00	2	3	6	6.91	0.03	1.63	1.54	0.02	38.73	-0.16	8.95	7.00	15.88
os13607	23	681	0.77	6	3	5	6.82	-0.05	0.50	1.52	0.06	99.86	0.17	6.52	6.59	15.47
os13609	27	641	0.98	6	2	0	6.73	-0.08	0.37	1.52	0.08	99.63	-0.59	7.19	6.70	15.54
os13612	28	644	0.49	5	3	5	6.93	0.01	0.95	1.53	0.06	97.61	-1.78	7.01	6.77	15.60
os13613	23	653	0.21	7	3	5	7.03	0.08	0.38	1.51	0.06	97.43	0.87	6.84	6.72	15.54
os13618	28	513	0.81	4	3	6	6.71	-0.06	0.31	1.54	0.06	84.56	0.53	7.95	7.36	16.20
os13622	28	590	0.60	4	3	6	6.72	-0.04	0.70	1.54	0.04	83.28	-0.65	7.81	7.06	15.94
os13630	28	598	0.88	7	3	5	6.60	-0.10	0.36	1.52	0.05	95.66	-0.83	7.33	7.08	15.94
os13662	28	599	0.23	4	3	6	6.86	0.02	1.23	1.52	0.03	76.29	-0.47	7.97	7.13	16.01
os13666	28	584	0.28	6	3	5	6.79	0.03	0.38	1.49	0.04	91.30	1.38	6.74	7.18	16.03
os13681	28	644	0.65	5	3	5	6.90	-0.01	0.50	1.53	0.06	99.58	-0.27	6.89	6.79	15.64
os13684	19	581	0.01	4	2	3	6.95	0.06	1.72	1.55	0.06	91.50	-2.94	8.15	7.10	15.93
os13685	28	582	0.01	7	3	5	6.98	0.10	1.16	1.54	0.06	91.15	-2.15	8.05	7.10	15.93
os13720	27	650	0.08	6	3	5	7.03	0.10	0.13	1.51	0.06	95.01	-0.38	7.91	6.78	15.65
os13721	27	572	0.35	12	2	7	6.60	0.02	0.44	1.53	0.15	96.73	-5.91	6.92	7.01	15.86
os13732	28	483	0.75	5	2	0	6.75	-0.05	0.76	1.52	0.08	93.88	-0.26	7.72	7.64	16.52
os13755	23	727	0.91	7	3	5	6.81	-0.07	0.22	1.50	0.07	99.55	-0.46	5.97	6.33	15.13
os13770	23	780	0.23	6	3	9	6.90	0.04	0.54	1.48	0.04	100.00	1.23	5.99	6.22	15.03
os13785	28	632	0.81	3	3	6	6.75	-0.06	0.97	1.54	0.03	92.69	-0.20	8.07	6.79	15.55
os13794	28	645	0.77	4	3	6	6.86	-0.03	1.08	1.53	0.03	96.75	-0.24	7.15	6.79	15.55
os13799	28	592	1.00	6	3	5	6.64	-0.10	0.97	1.53	0.05	88.11	-1.54	8.93	7.03	15.85
os13801	19	592	0.70	2	3	6	6.80	-0.03	2.62	1.55	0.05	84.22	-1.57	8.95	7.03	15.84
os13819	28	637	0.95	9	3	5	6.63	-0.12	0.35	1.51	0.06	85.64	-0.46	7.70	6.76	15.52
os13831	27	702	0.90	9	3	5	6.69	-0.12	0.17	1.47	0.07	99.98	0.07	6.34	6.46	15.32
os13854	27	607	0.53	7	2	3	6.75	0.00	0.95	1.53	0.08	98.03	-3.30	7.86	6.95	15.80
os13856	19	641	0.49	8	3	5	6.96	0.03	0.62	1.52	0.07	95.90	-2.04	7.06	6.70	15.50
os13857	28	621	0.92	2	2	3	6.78	-0.03	1.85	1.56	0.06	82.95	-2.37	8.49	6.77	15.56
os13860	28	644	0.60	2	3	6	6.82	-0.01	1.96	1.55	0.05	94.42	-1.16	8.34	6.61	15.39
os13862	28	646	0.70	3	3	6	6.79	-0.02	2.00	1.55	0.05	93.59	-0.98	8.40	6.60	15.38
os13865	27	667	1.00	6	3	5	6.67	-0.11	0.42	1.51	0.05	97.71	0.28	6.91	6.63	15.45
os13906	19	584	0.73	3	3	6	6.78	-0.05	1.94	1.55	0.04	93.72	-1.34	8.25	7.16	16.00
os13923	16	505	0.05	9	2	3	6.86	0.12	0.54	1.54	0.10	68.79	-4.27	6.88	7.43	16.32
os13966	27	635	0.27	2	2	7	6.88	0.02	2.23	1.56	0.08	75.24	-2.22	9.26	6.53	15.29
os14009	19	492	0.05	8	3	5	6.92	0.11	0.58	1.52	0.06	94.04	-0.35	7.65	7.66	16.56
os14023	19	458	0.95	10	3	5	6.48	-0.15	0.11	1.50	0.06	90.13	0.42	6.14	7.91	16.77
os14028	17	460	0.14	12	2	3	7.00	0.16	0.42	1.48	0.10	97.34	-3.94	8.07	7.92	16.81
os14040	16	522	0.30	2	3	6	6.89	0.03	1.91	1.54	0.01	60.18	0.30	8.78	7.63	16.50
os14068	17	414	0.42	2	3	6	6.76	0.00	4.08	1.57	0.05	71.60	-2.06	8.39	8.12	16.97

os14069	17	415	0.38	3	3	6	6.72	-0.01	3.98	1.56	0.05	65.43	-1.46	8.51	8.12	16.97
os14070	17	416	0.50	4	3	6	6.72	-0.01	4.29	1.56	0.04	50.90	-1.09	8.35	8.11	16.97
os14074	8	429	0.99	6	3	6	6.63	-0.08	0.56	1.54	0.05	81.47	-0.72	8.93	8.09	16.94
os14089	19	473	0.74	4	2	7	6.70	-0.06	1.70	1.55	0.09	92.36	-3.26	9.05	7.74	16.60
os14094	19	492	0.10	7	3	5	6.80	0.06	0.33	1.50	0.06	79.39	0.16	6.38	7.76	16.63
os14102	28	518	0.90	3	3	6	6.72	-0.03	0.59	1.53	0.02	93.68	0.09	7.44	7.60	16.48
os14118	16	444	0.62	11	3	5	6.46	-0.06	0.11	1.52	0.07	79.49	-0.98	7.57	7.80	16.67
os14130	19	475	0.11	4	2	3	6.88	0.03	1.70	1.54	0.05	93.02	-2.52	8.08	7.72	16.63
os14230	19	500	0.35	4	3	6	6.73	-0.01	0.42	1.53	0.04	79.58	0.82	7.41	7.46	16.36
os14243	24	535	0.40	2	3	6	6.86	0.01	1.73	1.53	0.02	73.32	-0.10	8.48	7.37	16.29
os14276	27	597	0.41	10	3	5	6.65	0.00	0.14	1.50	0.07	96.74	-0.86	6.60	6.89	15.76
os14298	11	441	0.07	2	2	3	6.83	0.02	2.08	1.56	0.08	61.45	-2.23	7.42	7.81	16.68
os14502	17	476	0.40	3	2	0	6.87	0.02	2.70	1.55	0.07	78.90	0.07	8.66	7.87	16.77
os14530	19	518	0.88	8	2	3	6.50	-0.12	0.61	1.52	0.10	99.50	-2.43	7.09	7.43	16.40
os14533	23	598	0.89	12	3	5	6.57	-0.14	0.01	1.46	0.08	99.96	-0.15	5.77	7.03	15.89
os14555	23	596	0.23	10	3	5	6.84	0.10	0.11	1.49	0.09	99.99	-1.56	6.45	6.98	15.89
os14565	23	534	0.98	11	2	0	6.37	-0.19	0.01	1.48	0.09	99.40	0.96	5.75	7.21	16.12
os14590	28	505	0.53	6	3	5	6.89	0.02	0.53	1.52	0.07	100.00	-0.73	6.71	7.61	16.55
os14599	19	549	0.78	2	2	7	6.81	-0.02	2.43	1.55	0.11	93.65	-3.79	8.68	7.20	16.12
os14635	23	569	0.45	12	3	5	6.89	0.02	0.01	1.47	0.09	99.88	-0.07	5.96	7.38	16.37
os14637	23	557	0.83	8	3	5	6.54	-0.13	0.27	1.51	0.08	99.03	0.02	6.18	7.39	16.38
os14638	19	526	0.84	6	3	5	6.61	-0.07	0.35	1.52	0.08	98.62	-0.16	7.43	7.49	16.46
os14658	28	459	0.07	5	2	7	6.64	-0.08	1.37	1.55	0.10	96.32	-3.70	8.16	7.63	16.54
os14836	19	450	1.00	8	3	5	6.54	-0.13	0.43	1.52	0.07	97.59	-1.87	7.00	8.20	17.23
os14908	23	591	0.85	11	2	0	6.33	-0.16	0.02	1.47	0.14	86.84	0.28	6.08	6.92	15.76
os14920	27	792	0.63	9	3	5	6.90	-0.04	0.19	1.50	0.07	100.00	-1.67	8.06	5.84	14.66
os14936	23	752	0.38	10	3	5	7.05	0.07	0.10	1.51	0.09	96.59	-1.63	6.54	6.14	14.96
os14939	27	741	0.85	10	3	5	6.74	-0.10	0.05	1.50	0.08	99.51	-0.58	6.82	6.14	14.96
os14954	23	735	1.00	5	1	1	6.75	-0.08	0.03	1.45	0.02	99.66	4.10	6.11	6.46	15.27
os14960	23	753	0.03	13	3	5	7.17	0.19	0.01	1.46	0.08	99.80	1.15	5.67	6.21	15.04
os14967	27	741	0.77	10	3	5	6.83	-0.06	0.05	1.49	0.08	99.95	-1.02	6.96	6.44	15.27
os14970	23	710	0.07	9	1	1	7.00	0.13	0.05	1.44	0.06	97.22	3.28	5.69	6.68	15.50
os14996	19	631	0.73	5	2	7	6.69	-0.08	1.12	1.55	0.11	99.11	-2.48	7.34	6.78	15.61
os14998	23	665	0.81	13	3	5	6.33	-0.16	0.01	1.48	0.11	100.00	-0.82	5.36	6.67	15.50
os15003	19	696	0.40	10	2	0	6.66	0.00	0.06	1.49	0.11	100.00	-0.11	6.94	6.57	15.43
os15028	23	789	0.20	10	1	1	6.94	0.11	0.01	1.42	0.06	97.76	2.63	5.77	6.16	15.00
os15033	27	732	0.26	7	3	5	6.80	0.02	0.35	1.51	0.11	99.93	-0.76	6.80	6.29	15.14
os15034	23	767	0.31	15	3	5	6.65	0.06	0.00	1.45	0.11	99.84	-0.09	6.19	6.19	15.05
os15035	23	747	0.23	11	3	5	6.78	0.06	0.04	1.48	0.12	99.87	-2.08	6.19	6.23	15.09
os15089	25	827	0.00	8	3	9	7.09	0.13	0.03	1.42	0.04	99.92	1.46	5.62	6.31	15.20
os15118	28	712	0.55	7	2	0	6.95	0.01	0.34	1.52	0.11	99.67	0.08	6.11	6.25	15.09
os15121	23	732	1.00	8	3	5	6.64	-0.13	0.18	1.50	0.08	99.99	-1.14	6.46	6.18	15.05
os15207	19	550	0.60	6	3	5	6.69	-0.02	0.78	1.51	0.08	99.57	-1.81	6.74	7.70	16.65

os15220	19	581	0.02	5	2	0	6.83	0.03	0.96	1.53	0.07	99.28	-1.40	8.67	7.29	16.19
os15267	23	645	0.65	7	2	7	6.85	-0.03	0.68	1.52	0.09	100.00	-2.73	7.78	6.86	15.74
os15277	16	597	0.05	5	2	3	6.94	0.08	1.31	1.54	0.09	72.48	-2.11	8.89	7.18	16.07
os15288	23	747	0.91	8	3	5	6.73	-0.11	0.27	1.49	0.09	100.00	-0.39	6.26	6.39	15.30
os15296	23	679	0.38	10	3	5	7.04	0.08	0.09	1.49	0.09	100.00	-0.36	7.03	6.62	15.47
os15297	27	656	0.92	6	2	0	6.66	-0.10	0.76	1.54	0.10	99.95	-1.67	7.57	6.63	15.47
os15313	23	684	0.01	8	3	5	7.06	0.13	0.19	1.50	0.07	90.77	-0.37	6.24	6.60	15.43
os15321	19	644	0.85	8	2	0	6.55	-0.11	0.20	1.50	0.10	100.00	-0.07	7.38	6.90	15.77
os15326	19	637	0.83	11	2	0	6.44	-0.13	0.03	1.49	0.11	100.00	-1.34	7.55	6.92	15.79
os15346	27	588	0.62	11	2	0	6.87	0.00	0.02	1.50	0.10	88.86	-0.29	6.57	7.15	16.02
os15383	23	811	0.85	18	1	1	6.50	-0.17	0.00	1.38	0.09	100.00	2.18	6.04	6.10	14.96
os15403	23	555	0.98	11	3	5	6.40	-0.18	0.03	1.49	0.08	100.00	-0.99	5.99	7.48	16.39
os15587	28	555	0.31	1	2	0	6.81	0.01	1.99	1.56	0.16	75.50	-1.89	8.22	7.01	15.86
os15608	23	669	0.60	5	3	9	6.73	-0.04	0.51	1.44	0.04	99.96	1.65	6.47	6.91	15.79
os15655	19	570	0.76	8	2	0	6.55	-0.09	0.22	1.51	0.08	99.80	-0.63	6.75	7.41	16.38
os15660	19	584	0.60	7	2	0	6.61	-0.06	0.27	1.51	0.08	99.54	-0.21	6.24	7.26	16.22
os15661	19	585	0.59	7	2	0	6.62	-0.04	0.24	1.50	0.08	99.46	-0.08	6.16	7.28	16.23
os15670	19	623	0.58	5	3	5	6.75	-0.01	0.18	1.49	0.07	100.00	1.19	6.10	7.14	16.06
os15672	28	630	0.71	7	3	5	6.63	-0.07	0.22	1.50	0.07	100.00	0.12	6.14	7.07	16.00
os15691	11	441	0.23	2	2	3	6.73	-0.01	2.76	1.56	0.10	73.86	-3.08	7.56	7.80	16.67
os15732	23	717	0.87	6	3	5	6.69	-0.08	0.48	1.50	0.05	100.00	0.54	6.36	6.38	15.26
os15734	27	668	0.98	9	2	0	6.56	-0.14	0.17	1.51	0.10	99.92	-0.75	7.00	6.49	15.36
os15756	28	726	0.97	6	3	5	6.76	-0.09	0.86	1.48	0.07	99.43	-1.89	7.81	6.43	15.34
os15783	23	729	0.99	9	3	9	6.66	-0.13	0.12	1.46	0.07	99.95	0.53	5.72	6.44	15.31
os15784	23	733	0.93	11	3	9	6.59	-0.16	0.02	1.46	0.08	99.89	-1.15	5.94	6.43	15.29
os15786	23	728	0.99	8	3	9	6.64	-0.13	0.19	1.47	0.07	99.93	-0.85	5.95	6.44	15.30
os15854	28	588	0.40	7	3	5	6.70	0.00	0.23	1.49	0.06	98.02	-0.32	6.74	7.14	16.02
os15862	23	677	0.07	10	1	1	7.09	0.14	0.00	1.41	0.05	98.74	7.45	5.55	6.66	15.53
os15863	23	617	0.77	6	2	0	6.84	-0.03	0.54	1.51	0.10	99.08	-0.84	6.92	6.77	15.64
os15882	27	673	0.90	9	2	0	6.52	-0.13	0.11	1.51	0.07	100.00	-0.68	6.35	6.50	15.36
os15900	23	705	0.44	12	3	9	6.57	-0.02	0.01	1.42	0.08	99.61	1.46	5.87	6.59	15.44
os15933	17	696	0.01	14	3	9	7.09	0.23	0.00	1.43	0.12	100.00	-1.31	5.57	6.55	15.41
os15940	28	694	0.41	4	3	6	6.94	0.00	1.28	1.53	0.09	100.00	-0.72	8.05	6.31	15.14
os15952	17	741	0.99	14	3	9	6.30	-0.24	0.00	1.45	0.09	99.95	0.12	5.76	6.36	15.18
os15953	17	735	1.00	13	3	9	6.36	-0.22	0.00	1.46	0.09	99.97	-0.59	5.82	6.36	15.19
os15972	23	583	0.97	12	1	4	6.44	-0.18	0.00	1.42	0.08	97.59	3.67	5.07	7.11	15.98
os15975	24	618	0.60	6	2	0	6.66	-0.05	0.61	1.53	0.11	97.48	-0.94	7.68	6.61	15.46
os16067	23	768	0.77	5	3	5	6.87	-0.05	0.16	1.49	0.05	98.46	1.99	7.45	6.15	14.96
os16342	23	613	0.09	19	3	5	6.73	0.20	0.00	1.42	0.00	92.15	0.46	6.22	6.83	15.59
os16344	27	604	0.27	22	2	3	6.21	0.04	0.00	1.43	0.00	89.03	-1.50	6.72	6.86	15.62
os16399	23	716	0.71	10	3	5	6.46	-0.17	0.03	1.48	0.11	98.73	-0.33	5.72	6.33	15.13
os16429	28	644	0.08	2	3	6	6.93	0.03	0.88	1.54	0.04	86.39	0.27	8.68	6.74	15.48
os16441	28	631	0.02	8	3	5	6.93	0.11	0.20	1.49	0.07	84.98	0.99	6.77	6.73	15.49

os16447	30	650	0.02	16	3	5	7.00	0.24	0.01	1.44	0.11	94.30	0.01	7.18	6.58	15.36
os16462	28	779	0.74	10	3	5	6.52	-0.13	0.04	1.47	0.09	98.54	-0.35	6.29	6.11	14.92
os16464	27	731	0.83	10	2	0	6.47	-0.15	0.03	1.48	0.10	99.02	0.10	6.96	6.22	15.03
os16479	27	613	0.65	5	2	0	6.88	-0.01	1.86	1.53	0.08	86.34	-0.82	8.02	6.78	15.51
os16505	23	689	0.65	17	3	5	6.79	-0.03	0.00	1.44	0.10	99.88	0.41	5.24	6.60	15.37
os16510	27	529	0.88	21	2	0	6.19	-0.22	0.00	1.41	0.15	81.66	0.48	5.83	7.29	16.02
os16590	23	598	0.02	8	3	5	6.96	0.13	0.37	1.51	0.09	99.31	-1.82	7.39	7.02	15.92
os16600	28	540	0.45	6	2	0	6.68	-0.02	0.61	1.53	0.09	98.27	-1.60	7.07	7.03	15.88
os16601	23	636	0.99	9	3	9	6.52	-0.15	0.11	1.47	0.07	100.00	0.10	5.91	6.67	15.51
os16602	28	542	0.91	3	2	0	6.73	-0.04	3.08	1.56	0.07	35.13	-0.27	9.87	6.89	15.66
os16617	23	668	0.02	4	3	6	6.74	-0.08	0.67	1.53	0.05	91.89	-1.00	6.40	6.44	15.28
os16622	23	637	0.75	3	3	6	6.87	-0.02	1.94	1.51	0.06	98.78	-1.57	7.85	6.47	15.30
os16634	28	563	0.37	3	3	6	6.75	-0.05	0.98	1.55	0.05	57.04	-0.63	8.33	6.72	15.55
os16672	11	499	0.80	5	2	0	6.62	-0.06	0.62	1.55	0.11	48.37	-1.11	7.78	6.86	15.62
os16694	23	650	0.06	16	3	5	6.88	0.21	0.00	1.46	0.12	99.67	-1.84	5.72	6.18	14.97
os16697	11	545	0.22	2	2	0	6.89	0.02	2.85	1.55	0.07	62.71	0.07	7.98	6.72	15.51
os16817	17	605	0.26	14	2	7	7.04	0.17	0.00	1.47	0.13	99.99	-2.35	5.99	6.40	15.21
os16843	24	547	0.92	6	2	0	6.62	-0.09	0.49	1.53	0.11	98.65	-0.15	7.20	6.55	15.38
os16860	24	493	1.00	4	2	7	6.72	-0.05	2.09	1.55	0.10	89.96	-2.66	7.87	7.19	16.09
os16861	24	489	0.92	3	2	7	6.71	-0.05	2.54	1.56	0.11	91.11	-3.38	7.64	7.22	16.12
os16899	17	560	1.00	5	3	5	6.66	-0.09	0.33	1.50	0.05	88.00	0.63	7.34	7.20	16.16
os16927	28	658	0.47	7	3	5	6.95	0.02	0.43	1.51	0.08	99.98	-0.60	6.65	6.22	15.04
os16934	23	661	0.99	11	1	1	6.49	-0.18	0.00	1.44	0.06	99.03	3.33	6.01	6.39	15.24
os16938	23	681	0.02	9	2	0	6.49	-0.17	0.07	1.50	0.11	98.26	-1.58	7.60	5.81	14.57
os16943	19	632	0.81	9	2	3	6.50	-0.12	0.28	1.49	0.12	99.41	-2.96	7.39	6.52	15.36
os16984	28	542	0.35	13	2	0	6.54	0.00	0.00	1.48	0.09	98.55	0.45	5.80	7.05	15.90
os16993	28	533	0.99	6	2	0	6.64	-0.10	0.35	1.53	0.08	98.00	0.13	7.02	7.05	15.91
os17003	28	538	0.92	1	2	0	6.80	-0.02	3.97	1.56	0.07	34.46	0.25	10.29	6.91	15.69
os17037	19	567	0.60	9	2	7	6.54	-0.06	0.56	1.52	0.16	99.45	-6.27	8.13	6.58	15.35
os17043	23	662	1.00	7	2	7	6.61	-0.12	0.63	1.53	0.10	98.19	-2.71	7.22	5.94	14.69
os17093	24	661	0.01	6	3	5	6.65	-0.11	0.42	1.52	0.06	98.29	-0.48	6.80	6.30	15.13
os17113	11	433	0.22	3	2	7	6.80	0.03	1.33	1.56	0.08	50.47	-2.20	7.85	7.63	16.56
os17114	11	434	0.92	1	2	0	6.78	0.00	2.45	1.56	0.07	49.91	-1.01	7.99	7.61	16.54
os17215	28	614	0.98	3	3	6	6.78	-0.04	1.96	1.52	0.06	95.71	-1.08	7.06	6.58	15.40
os17217	28	616	0.92	1	3	6	6.83	-0.02	2.17	1.52	0.06	95.49	-0.85	6.93	6.58	15.40
os17218	24	580	0.99	4	3	6	6.74	-0.07	0.21	1.53	0.05	90.45	0.64	6.98	6.65	15.47
os17219	24	577	0.04	4	3	6	6.72	-0.07	0.29	1.54	0.05	87.65	0.25	7.22	6.66	15.48
os17220	24	579	0.98	5	3	6	6.76	-0.06	0.40	1.53	0.06	89.86	0.20	7.16	6.66	15.48
os17225	27	542	0.91	10	2	0	6.58	-0.13	0.05	1.50	0.10	99.90	-1.12	6.39	6.53	15.44
os17227	28	522	0.99	8	2	0	6.56	-0.13	0.58	1.52	0.11	99.72	-2.13	7.15	6.58	15.48
os17233	28	592	0.08	8	3	5	7.05	0.13	0.34	1.52	0.09	97.83	-1.88	5.98	6.54	15.38
os17243	27	486	0.97	5	2	7	6.63	-0.08	1.27	1.54	0.10	98.91	-2.11	8.02	6.86	15.78
os17267	28	622	0.92	5	3	6	6.67	-0.08	0.90	1.52	0.06	98.65	-1.27	7.23	6.41	15.25

os17348	19	501	0.77	4	2	7	6.81	-0.02	1.95	1.55	0.09	89.63	-3.34	8.71	7.23	16.12
os17433	17	520	0.98	16	2	7	6.25	-0.22	0.01	1.49	0.18	75.04	-4.71	5.54	6.73	15.48
os17437	23	742	0.08	8	3	5	7.12	0.12	0.20	1.47	0.06	99.97	0.91	6.04	5.92	14.72
os17442	19	475	0.92	0	2	0	6.82	0.00	3.99	1.57	0.07	18.69	-0.46	10.15	7.15	15.99
os17496	28	599	0.92	4	3	6	6.71	-0.06	1.63	1.50	0.07	96.11	-0.34	7.48	6.53	15.43
os17526	28	634	0.98	10	3	5	6.56	-0.14	0.17	1.50	0.13	100.00	-1.85	6.55	6.22	15.11
os17528	28	641	1.00	10	3	5	6.46	-0.18	0.05	1.50	0.12	100.00	-1.34	6.69	6.21	15.10
os17572	6	465	0.40	8	2	7	6.43	0.04	0.61	1.56	0.32	84.31	-15.51	6.20	7.64	16.48
os17616	23	776	0.84	10	3	9	6.53	-0.14	0.08	1.48	0.06	99.07	-0.23	5.67	6.22	15.00
os17628	28	733	0.83	7	2	7	6.62	-0.11	0.73	1.52	0.11	99.91	-2.49	8.14	6.22	15.04
os17629	28	733	0.84	9	2	7	6.81	-0.06	0.64	1.53	0.12	99.90	-3.94	8.28	6.21	15.03
os17648	23	802	0.01	9	3	5	7.06	0.15	0.12	1.50	0.07	98.16	-1.40	5.95	5.98	14.80
os17656	28	735	0.79	7	2	7	6.80	-0.07	0.83	1.53	0.11	99.87	-3.26	8.48	6.20	15.02
os17658	23	779	0.23	10	1	4	7.11	0.11	0.00	1.43	0.06	96.45	3.97	5.20	6.19	14.97
os17731	35	775	0.01	4	2	0	7.01	0.07	0.79	1.55	0.08	66.29	-0.56	7.01	5.66	14.38
os17744	27	752	0.08	5	2	3	7.06	0.08	1.33	1.57	0.06	40.95	-2.59	7.86	5.89	14.60
os17752	23	803	0.68	22	2	7	6.61	-0.08	0.00	1.42	0.18	83.24	-3.60	5.62	5.50	14.23
os17760	25	898	0.40	8	2	0	7.10	0.05	0.48	1.50	0.09	100.00	-0.32	6.71	5.01	13.74
os17775	23	873	0.26	9	1	1	7.16	0.11	0.03	1.40	0.08	98.40	2.62	5.57	5.41	14.15
os17814	30	866	0.62	11	2	0	7.01	0.01	0.02	1.48	0.10	99.94	1.50	6.32	5.18	13.96
os17941	33	994	0.01	7	3	5	7.18	0.13	0.22	1.50	0.08	98.25	-0.39	6.13	4.49	13.20
os17942	33	993	0.03	12	2	0	7.24	0.21	0.00	1.47	0.11	94.78	0.91	5.76	4.35	13.07
os17943	33	998	0.01	9	3	5	7.21	0.15	0.12	1.49	0.08	98.58	0.10	6.06	4.51	13.22
os18007	23	791	0.62	9	2	3	6.96	0.01	0.05	1.50	0.10	99.75	-2.57	5.74	5.93	14.74
os18008	27	780	0.55	7	3	5	6.98	0.02	0.34	1.51	0.08	99.71	0.26	6.00	5.95	14.76
os18097	35	811	0.93	11	3	5	6.61	-0.16	0.01	1.48	0.10	100.00	-0.19	6.19	5.64	14.45
os18208	23	813	0.31	5	1	4	6.88	0.01	0.00	1.47	0.03	99.16	3.80	6.40	5.74	14.47
os18227	23	832	0.11	7	3	5	6.96	0.07	0.31	1.52	0.08	97.48	-1.08	6.00	5.59	14.38
os18267	23	723	0.13	11	3	5	6.85	0.10	0.03	1.48	0.11	99.99	-1.92	6.26	6.28	15.10
os18308	35	826	0.70	4	2	0	6.97	-0.01	0.56	1.52	0.05	95.94	-0.05	7.33	5.51	14.30
os18333	23	878	0.87	9	3	9	6.60	-0.12	0.12	1.47	0.06	96.29	0.06	6.17	5.73	14.62
os18340	23	663	1.00	13	3	5	6.38	-0.21	0.00	1.47	0.09	99.61	-0.74	6.15	6.73	15.62
os18346	23	782	0.75	11	3	5	6.51	-0.11	0.02	1.47	0.07	94.98	0.93	6.40	6.02	14.85
os18437	28	850	0.57	7	3	5	7.02	0.01	0.21	1.52	0.07	98.66	-0.12	6.77	5.26	13.99
os18453	28	794	0.12	7	3	5	7.12	0.10	0.36	1.50	0.07	99.52	-0.19	7.71	5.84	14.72
os18456	23	801	0.08	6	3	5	7.10	0.10	0.45	1.49	0.04	98.94	0.65	6.97	5.87	14.74
os18465	28	816	0.60	10	3	5	6.63	-0.07	0.17	1.49	0.09	99.99	-1.35	7.56	5.62	14.41
os18471	27	720	0.77	5	2	3	6.86	-0.04	1.37	1.52	0.09	99.88	-2.34	8.35	6.13	14.94
os18474	28	773	0.55	9	3	5	6.94	-0.01	0.05	1.48	0.07	99.99	0.13	6.97	5.92	14.72
os18479	30	810	0.00	22	3	9	6.97	0.35	0.00	1.40	0.14	99.98	-0.87	5.14	5.83	14.63
os18500	27	836	0.77	8	3	5	6.91	-0.04	0.44	1.49	0.09	99.81	-0.95	7.45	5.56	14.37
os18596	23	698	0.71	8	1	4	6.60	-0.09	0.10	1.44	0.06	99.65	2.81	6.02	6.40	15.17
os18631	27	783	0.88	9	3	5	6.81	-0.08	0.09	1.51	0.00	99.23	-0.43	6.17	5.92	14.73

os18681	27	641	0.17	15	2	7	7.10	0.20	0.00	1.45	0.16	96.27	-2.64	6.20	6.46	15.22
os18682	23	646	0.60	14	2	0	6.87	0.00	0.00	1.46	0.15	98.02	0.20	5.96	6.43	15.19
os18717	23	794	0.08	13	1	1	7.11	0.21	0.01	1.45	0.09	92.33	2.77	5.27	6.12	14.99
os18720	32	726	0.92	1	3	6	6.92	0.01	5.52	1.55	0.01	19.48	0.93	12.06	6.22	15.16
os18721	30	701	0.26	11	2	0	7.09	0.12	0.03	1.48	0.12	97.99	0.16	6.22	6.15	14.90
os18724	28	688	0.90	7	2	0	6.59	-0.11	0.26	1.52	0.11	95.60	0.17	6.94	6.19	14.95
os18732	28	770	0.92	2	3	6	6.89	-0.03	3.66	1.55	0.02	39.51	0.08	8.58	6.16	14.97
os18734	32	769	0.66	1	3	6	6.90	-0.02	3.76	1.55	0.02	38.36	0.01	8.95	6.16	14.97
os18735	28	773	0.77	2	3	6	6.94	-0.01	1.85	1.55	0.02	39.05	-0.59	8.15	6.16	14.97
os18755	23	829	0.35	19	1	4	7.02	0.14	0.00	1.39	0.15	99.45	3.27	4.91	5.49	14.26
os18756	23	814	0.07	7	3	5	7.01	0.10	0.17	1.48	0.05	99.90	1.40	6.25	5.77	14.52
os18822	28	771	0.98	3	3	6	6.90	-0.03	1.55	1.54	0.04	97.40	-0.03	7.26	6.10	14.89
os18846	27	865	0.99	7	3	5	6.79	-0.10	0.39	1.51	0.08	99.98	0.20	7.05	5.35	14.07
os18857	27	865	0.35	6	2	0	7.02	0.01	0.89	1.53	0.12	98.45	-2.28	9.10	5.09	13.87
os18860	27	863	0.00	6	2	0	7.10	0.10	0.44	1.52	0.09	99.90	-0.23	7.29	5.28	14.02
os18878	23	850	0.40	12	3	5	7.10	0.08	0.00	1.47	0.09	99.64	0.01	5.89	5.58	14.33
os18912	23	860	0.09	12	1	4	7.21	0.18	0.01	1.46	0.06	99.62	3.15	5.62	5.58	14.36
os18919	35	755	0.38	5	3	5	7.04	0.05	0.76	1.54	0.06	86.12	-1.10	7.86	6.11	14.90
os18920	23	741	0.40	4	3	6	6.98	0.02	0.39	1.54	0.06	88.76	-0.10	7.78	6.19	14.98
os18931	23	740	0.00	3	3	6	7.02	0.06	0.82	1.54	0.04	87.88	0.04	7.35	6.25	15.10
os18932	23	742	0.99	3	3	6	6.84	-0.05	1.22	1.54	0.04	86.92	-0.35	7.49	6.24	15.04
os18935	23	860	0.91	7	1	1	6.83	-0.08	0.01	1.43	0.05	99.51	5.77	5.87	5.74	14.48
os18939	23	735	0.92	1	3	6	6.91	-0.01	3.66	1.56	0.04	24.29	-0.35	9.82	6.21	14.93
os18940	23	803	0.11	7	1	1	6.98	0.09	0.04	1.45	0.04	98.48	4.21	5.94	6.03	14.83
os19014	24	827	0.05	11	3	5	7.02	0.15	0.02	1.48	0.08	100.00	-0.77	6.13	5.47	14.21
os19066	27	580	0.38	8	2	7	6.54	-0.03	0.49	1.54	0.22	88.75	-2.42	8.60	6.04	14.74
os19068	27	749	0.35	5	2	7	6.87	0.04	1.06	1.54	0.15	97.55	-2.30	7.73	5.45	14.11
os19078	23	703	0.82	19	3	5	5.91	-0.25	0.00	1.41	0.14	97.40	0.86	5.61	5.76	14.48
os19079	27	675	0.36	16	2	0	6.41	0.00	0.00	1.45	0.15	96.09	-0.76	6.56	5.79	14.50
os19082	23	678	1.00	12	3	5	6.41	-0.20	0.00	1.46	0.09	98.97	1.16	5.45	6.21	14.98
os19083	23	708	0.75	7	3	5	6.64	-0.12	0.35	1.51	0.08	97.83	-0.53	6.15	6.00	14.77
os19202	27	644	0.49	8	1	1	6.71	-0.01	0.06	1.45	0.05	99.83	2.72	5.70	6.05	14.89
os19207	24	631	0.12	9	2	0	6.87	0.10	0.10	1.50	0.10	100.00	-0.66	6.34	6.01	14.89
os19224	27	463	0.01	3	3	6	6.87	0.05	1.64	1.54	0.05	72.92	-0.94	8.19	6.96	15.83
os19254	23	673	0.12	13	3	5	6.86	0.15	0.00	1.46	0.11	100.00	-0.33	5.96	5.91	14.81
os19260	23	634	1.00	13	2	0	6.36	-0.21	0.00	1.46	0.10	100.00	1.42	5.96	6.02	14.91
os19289	28	812	0.06	5	3	5	7.01	0.07	1.32	1.50	0.07	97.09	-2.39	8.33	5.36	14.22
os19300	28	688	0.54	12	3	5	6.51	-0.06	0.01	1.47	0.08	100.00	0.89	6.12	5.93	14.80
os19339	24	811	0.07	8	3	5	7.02	0.11	0.21	1.49	0.06	100.00	-0.10	6.32	5.54	14.27
os19365	23	702	0.07	17	3	9	6.88	0.21	0.00	1.44	0.12	100.00	-1.52	5.89	5.98	14.85
os19367	23	647	0.09	11	3	5	6.85	0.12	0.01	1.46	0.09	100.00	-0.30	5.83	6.27	15.15
os19377	23	634	0.28	9	2	0	6.73	0.03	0.09	1.51	0.09	100.00	-1.18	7.18	6.11	14.97
os19379	28	621	0.30	10	2	0	6.72	0.04	0.05	1.50	0.11	99.51	-0.81	6.39	6.14	15.02

os19382	28	644	1.00	8	2	7	6.55	-0.12	0.40	1.53	0.15	96.88	-1.55	8.80	5.71	14.57
os19396	23	702	1.00	12	3	5	6.44	-0.20	0.01	1.47	0.07	89.78	0.36	6.05	5.98	14.87
os19406	23	676	0.13	12	3	5	6.87	0.13	0.01	1.47	0.10	100.00	0.32	6.11	5.99	14.87
os19416	27	724	0.92	1	2	7	6.87	-0.01	2.05	1.55	0.08	89.99	-2.06	8.54	5.65	14.47
os19417	32	740	0.15	7	2	0	6.92	0.06	0.44	1.51	0.08	97.27	-1.17	7.39	5.62	14.45
os19565	23	776	0.60	4	3	6	6.84	-0.02	1.71	1.54	0.04	82.24	-0.64	7.73	6.05	14.85
os19609	27	749	0.92	2	2	7	6.86	-0.01	1.98	1.56	0.13	81.99	-5.33	7.80	5.76	14.53
os19651	10	450	0.84	4	2	3	6.69	-0.06	1.58	1.53	0.07	97.02	-3.58	8.00	8.19	17.07
os19666	14	456	1.00	14	2	3	6.31	-0.20	0.00	1.43	0.10	96.99	1.51	6.58	8.08	16.96
os19689	19	431	0.15	11	2	3	6.70	0.10	0.51	1.52	0.12	76.59	-3.57	7.07	8.30	17.27
os19836	16	465	0.92	0	3	6	6.81	0.00	3.97	1.56	0.03	33.42	-0.07	10.78	7.78	16.71
os19845	10	418	0.77	1	3	6	6.78	-0.01	6.73	1.56	0.01	18.66	0.00	8.69	8.29	17.34
os19851	3	424	0.92	0	3	6	6.79	0.00	6.96	1.56	0.00	0.36	0.12	11.96	8.28	17.35
os19892	10	420	0.92	0	3	6	6.79	0.00	5.96	1.57	0.01	0.46	-0.18	11.76	8.28	17.35
os19894	10	420	0.92	0	3	6	6.78	0.00	5.96	1.57	0.01	0.26	-0.19	9.80	8.28	17.35
os19896	10	421	0.92	0	3	6	6.77	-0.01	6.03	1.57	0.01	4.07	-0.32	9.45	8.27	17.33
os20232	16	512	0.77	2	3	6	6.80	-0.02	0.39	1.54	0.02	57.10	0.73	8.16	7.72	16.66
os20236	8	506	0.49	5	3	6	6.68	-0.04	1.08	1.54	0.04	76.26	-0.90	7.94	7.71	16.65
os20394	11	418	0.92	0	3	6	6.79	0.00	5.97	1.57	0.01	3.18	-0.07	11.80	8.10	17.07
os20401	10	417	0.85	1	3	6	6.75	-0.01	6.96	1.56	0.02	18.30	0.15	9.35	8.19	17.18
os20417	10	427	0.92	2	3	6	6.71	-0.04	1.49	1.55	0.03	73.60	-0.13	8.02	8.27	17.36
os20487	16	418	0.92	0	3	6	6.79	0.00	6.64	1.57	0.01	0.29	-0.34	9.98	8.33	17.44
os20563	19	484	0.08	3	3	6	6.83	0.03	2.41	1.55	0.03	75.25	-0.96	9.24	7.89	16.96
os20567	10	410	0.77	1	3	6	6.79	0.00	6.99	1.55	0.01	5.78	0.61	9.55	8.40	17.44
os20568	10	410	0.92	1	3	6	6.77	-0.01	6.99	1.55	0.01	5.32	0.64	10.10	8.40	17.43
os20569	10	410	0.77	1	3	6	6.77	-0.01	6.99	1.55	0.01	5.56	0.66	9.62	8.40	17.43
os20570	10	409	0.06	1	3	6	6.79	0.00	6.99	1.56	0.01	5.97	0.22	10.20	8.39	17.43
os20571	10	409	0.06	1	3	6	6.77	-0.01	6.99	1.56	0.01	5.51	0.45	9.66	8.38	17.42
os20580	10	417	0.92	0	3	6	6.79	0.00	6.42	1.57	0.01	3.98	0.00	11.71	8.18	17.16
os20581	10	405	0.92	0	3	6	6.78	0.00	6.97	1.57	0.02	0.86	0.00	12.33	8.44	17.53
os20582	10	405	0.92	0	3	6	6.78	0.00	6.97	1.57	0.02	0.76	0.00	12.33	8.44	17.53
os20584	11	406	0.92	0	3	6	6.78	0.00	6.95	1.57	0.02	3.73	0.01	12.24	8.43	17.52
os20586	10	407	0.92	1	3	6	6.78	0.01	2.04	1.57	0.03	38.33	-0.87	8.90	8.41	17.50
os20650	10	400	0.92	0	3	6	6.78	0.00	3.99	1.55	0.03	45.16	0.62	11.76	8.43	17.50
os20653	11	405	0.92	1	3	6	6.76	-0.01	6.92	1.57	0.02	2.63	-0.39	9.74	8.44	17.51
os20654	19	405	0.92	0	3	6	6.78	0.00	6.90	1.57	0.01	7.49	-0.43	10.03	8.43	17.51
os20655	10	409	0.01	1	3	6	6.81	0.02	6.03	1.56	0.02	7.56	-0.33	8.92	8.39	17.48
os20687	3	421	0.92	1	3	6	6.80	0.01	3.71	1.57	0.02	27.76	-0.53	9.80	8.26	17.35
os20718	11	406	0.92	0	3	6	6.78	0.00	6.97	1.57	0.02	3.40	0.00	12.18	8.43	17.52
os20722	24	415	0.92	0	3	6	6.80	0.01	6.47	1.57	0.01	1.38	-0.49	9.56	8.29	17.42
os20725	10	415	0.92	0	3	6	6.78	0.00	6.84	1.57	0.01	0.09	0.13	11.89	8.30	17.43
os20770	2	471	0.92	1	3	6	6.82	0.00	2.96	1.56	0.02	23.13	0.03	9.05	7.73	16.73
os20841	16	521	0.40	1	3	6	6.85	0.01	2.94	1.55	0.03	37.47	-0.95	9.02	7.43	16.43

os20911	8	495	0.16	2	3	6	6.83	0.02	3.43	1.55	0.00	37.60	0.07	8.89	7.16	16.05
os20916	8	463	0.92	0	3	6	6.80	0.00	3.96	1.57	0.02	36.41	-0.69	9.67	7.49	16.43
os21015	8	449	0.92	1	3	6	6.84	0.02	4.35	1.57	0.03	29.49	-0.57	9.05	7.87	16.76
os21017	8	477	0.13	1	3	6	6.78	-0.02	3.34	1.56	0.04	73.15	-1.69	8.40	7.62	16.56
os21021	4	507	0.23	1	3	6	6.82	0.01	0.65	1.55	0.01	10.19	0.11	9.17	7.53	16.48
os21056	24	457	0.92	1	3	6	6.82	0.02	2.57	1.56	0.02	10.06	0.14	8.37	7.72	16.64
os21058	16	478	0.08	3	3	6	6.90	0.05	1.21	1.55	0.02	67.97	0.07	8.42	7.65	16.57
os21085	8	463	0.88	1	3	6	6.79	-0.01	2.82	1.56	0.02	24.77	-0.09	8.29	7.69	16.59
os21086	16	461	0.92	1	3	6	6.80	-0.01	3.86	1.57	0.02	1.44	-0.30	9.62	7.72	16.62
os21165	2	464	0.92	1	3	6	6.79	-0.01	6.19	1.56	0.01	0.67	-0.68	9.35	7.47	16.39
os21167	10	463	0.92	0	3	6	6.81	0.00	5.94	1.56	0.01	7.90	0.16	9.86	7.47	16.38
os21169	2	463	0.99	1	3	6	6.78	-0.01	5.91	1.56	0.01	3.02	-0.05	9.47	7.46	16.37
os21174	10	465	0.92	1	3	6	6.80	-0.01	4.19	1.56	0.01	5.45	0.44	10.49	7.41	16.30
os21181	10	462	0.71	6	3	6	6.63	-0.05	0.66	1.52	0.02	18.49	1.12	8.10	7.40	16.31
os21185	16	473	0.92	0	3	6	6.81	0.00	5.82	1.57	0.01	7.74	0.09	11.37	7.27	16.16
os21191	16	491	0.92	0	3	6	6.82	0.00	2.98	1.55	0.00	22.16	0.42	11.23	7.11	16.04
os21194	10	455	0.92	1	3	6	6.81	0.00	6.02	1.55	0.01	26.16	0.63	9.51	7.60	16.53
os21213	4	468	0.23	2	3	6	6.78	0.00	1.47	1.55	0.02	34.10	0.22	8.88	7.49	16.43
os21357	10	412	0.99	1	3	6	6.77	-0.01	0.61	1.56	0.01	4.56	0.18	8.64	8.57	17.69
os21367	11	398	0.92	0	3	6	6.79	0.01	5.88	1.56	0.01	0.01	-0.12	10.26	8.63	17.74
os21371	11	396	0.08	1	3	6	6.80	0.01	6.70	1.57	0.02	17.80	-0.34	8.96	8.60	17.70
os21372	11	397	0.06	1	3	6	6.81	0.02	6.67	1.56	0.02	10.23	0.04	9.85	8.60	17.70
os21507	16	418	0.92	1	3	6	6.82	0.02	3.73	1.56	0.02	11.72	-0.32	9.85	8.44	17.50
os21564	10	418	0.06	2	3	6	6.69	-0.05	1.65	1.55	0.03	62.08	-0.91	8.09	8.57	17.69
os21592	11	398	0.92	0	3	6	6.78	0.00	5.55	1.57	0.01	0.00	0.00	11.73	8.64	17.77
os21608	16	386	0.01	2	3	6	6.80	0.02	2.79	1.56	0.03	17.80	0.14	9.20	8.68	17.78
os21613	10	403	0.12	7	3	5	6.76	0.06	0.43	1.52	0.07	79.91	-1.56	6.97	8.32	17.33
os21629	10	390	0.23	3	3	6	6.78	0.03	3.96	1.53	0.02	16.50	1.20	9.32	8.62	17.72
os21630	10	388	0.29	4	3	6	6.78	0.03	3.96	1.55	0.03	14.62	-0.12	9.09	8.62	17.72
os21647	10	428	0.92	2	3	6	6.80	0.00	6.13	1.51	0.01	95.05	1.51	6.86	8.53	17.63
os21786	8	420	0.92	0	3	6	6.79	0.00	5.91	1.57	0.01	2.10	0.00	11.73	8.31	17.34
os21838	16	430	0.92	0	3	6	6.79	0.00	6.30	1.57	0.01	0.83	-0.27	10.16	8.26	17.34
os21840	16	430	0.92	0	3	6	6.79	0.00	6.62	1.57	0.01	0.07	-0.38	10.08	8.26	17.35
os22131	8	401	0.92	1	3	6	6.76	-0.01	5.48	1.56	0.02	20.67	-0.22	9.29	8.42	17.48
os22135	19	415	0.31	2	3	6	6.71	-0.03	1.95	1.56	0.04	84.21	-2.02	7.78	8.37	17.41
os22138	2	402	0.01	2	3	6	6.84	0.04	2.33	1.56	0.03	61.32	-0.50	8.13	8.43	17.50
os22143	16	419	0.73	13	3	5	6.30	-0.15	0.04	1.45	0.07	83.44	0.80	5.96	8.38	17.44
os22255	16	421	0.92	1	3	6	6.75	-0.02	5.96	1.56	0.01	0.10	-0.11	9.09	8.30	17.43
os22264	10	419	0.05	2	3	6	6.82	0.02	6.99	1.55	0.01	13.86	0.09	9.03	8.32	17.39
os22265	10	417	0.59	2	3	6	6.81	0.01	6.98	1.56	0.01	4.40	-0.37	8.98	8.32	17.38
os22267	10	416	0.92	0	3	6	6.78	0.00	6.99	1.56	0.01	4.40	-0.23	9.66	8.32	17.38
os22281	2	431	0.60	3	3	6	6.70	-0.03	6.92	1.54	0.02	11.35	0.73	8.00	8.01	16.97
os22282	2	432	0.99	2	3	6	6.73	-0.04	6.96	1.56	0.02	8.82	-0.30	8.24	8.01	16.97

os22283	10	431	0.92	2	3	6	6.77	-0.02	6.96	1.56	0.01	6.94	-0.04	8.62	8.01	16.98
os22311	10	429	0.92	1	3	6	6.82	0.02	6.94	1.56	0.01	7.05	0.27	8.71	8.04	17.00
os22314	4	429	0.08	2	3	6	6.83	0.03	6.90	1.55	0.01	17.03	0.89	8.45	8.04	17.02
os22352	16	434	0.92	0	3	6	6.79	0.00	6.76	1.57	0.00	0.10	0.00	11.68	7.87	16.78
os22362	16	441	0.23	1	3	6	6.80	0.01	3.82	1.57	0.02	15.22	-0.28	8.44	7.78	16.73
os22368	11	436	0.73	1	3	6	6.79	0.00	6.86	1.56	0.01	3.36	-0.03	10.09	7.76	16.68
os22373	2	469	0.92	0	3	6	6.79	-0.01	4.04	1.56	0.01	4.99	-0.36	9.08	7.68	16.64
os22391	4	473	0.92	1	3	6	6.79	-0.01	5.27	1.54	0.00	25.36	0.72	10.17	7.32	16.21
os22395	16	452	0.00	1	3	6	6.83	0.02	4.71	1.56	0.01	3.47	-0.05	9.60	7.75	16.71
os22422	16	442	0.92	0	3	6	6.79	0.00	6.32	1.56	0.01	0.96	0.10	10.48	7.88	16.84
os22501	10	445	0.92	2	3	6	6.77	-0.02	1.58	1.55	0.01	15.12	0.56	9.27	7.62	16.57
os22598	4	458	0.08	1	3	6	6.84	0.02	3.91	1.56	0.01	5.11	-0.15	9.76	7.75	16.70
os22645	2	422	0.23	1	3	6	6.80	0.01	6.91	1.56	0.01	13.23	-0.01	8.61	8.15	17.15
os22649	2	432	0.92	0	3	6	6.79	0.00	3.87	1.57	0.01	16.28	-0.16	10.47	8.02	16.99
os22665	10	431	0.60	2	3	6	6.75	-0.01	6.94	1.55	0.01	26.98	0.08	8.14	8.24	17.29
os22668	10	423	0.92	0	3	6	6.79	0.00	6.98	1.56	0.01	0.39	-0.21	9.69	8.26	17.31
os22669	10	418	0.76	1	3	6	6.75	-0.01	6.98	1.56	0.02	13.22	-0.52	9.19	8.27	17.32
os22675	10	416	0.99	5	3	6	6.66	-0.07	0.96	1.55	0.03	18.74	-0.88	7.94	8.27	17.32
os22681	10	420	0.92	1	3	6	6.75	-0.02	6.94	1.56	0.01	4.18	0.11	9.33	8.28	17.36
os22682	10	420	0.92	1	3	6	6.74	-0.02	6.94	1.56	0.01	3.92	0.04	9.27	8.28	17.36
os22690	10	419	0.92	0	3	6	6.79	0.00	6.99	1.57	0.01	1.05	-0.19	10.43	8.22	17.24
os22691	10	419	0.92	0	3	6	6.79	0.00	6.99	1.57	0.01	0.77	-0.19	9.75	8.22	17.24
os22701	10	422	0.92	0	3	6	6.79	0.00	6.97	1.56	0.01	2.93	0.03	10.95	8.29	17.36
os22703	10	420	0.92	0	3	6	6.79	0.00	6.60	1.57	0.01	0.27	-0.19	11.62	8.27	17.33
os22749	10	458	0.05	2	3	6	6.84	0.02	2.55	1.55	0.02	45.50	-0.65	8.42	7.79	16.77
os22754	10	420	0.08	2	3	6	6.84	0.02	6.96	1.56	0.02	15.91	-0.49	7.86	8.22	17.23
os22762	2	469	0.92	0	3	6	6.79	-0.01	2.25	1.55	0.03	59.08	-0.31	8.65	7.70	16.67
os22763	2	469	0.92	0	3	6	6.80	0.00	2.73	1.56	0.04	57.17	-0.15	8.17	7.70	16.66
os22770	16	441	0.08	1	3	6	6.80	0.01	3.69	1.57	0.01	3.99	-0.16	9.08	7.81	16.75
os22774	16	441	0.92	0	3	6	6.80	0.00	3.84	1.57	0.01	14.17	-0.05	11.38	7.78	16.73
os22779	8	434	0.99	2	3	6	6.77	-0.02	6.85	1.56	0.01	8.10	0.31	8.94	7.95	16.90
os22781	16	436	0.92	0	3	6	6.79	0.00	6.79	1.57	0.01	4.34	-0.28	9.40	7.96	16.90
os22784	16	442	0.92	0	3	6	6.80	0.00	5.95	1.57	0.01	11.00	-0.21	11.39	7.85	16.79
os22785	16	442	0.92	0	3	6	6.79	0.00	5.95	1.57	0.01	8.96	-0.21	10.51	7.85	16.79
os22787	16	445	0.92	0	3	6	6.80	0.00	5.93	1.56	0.01	0.58	-0.06	9.27	7.79	16.72
os22788	16	445	0.92	0	3	6	6.80	0.00	5.93	1.57	0.01	0.60	-0.11	9.57	7.79	16.72
os22789	16	445	0.92	0	3	6	6.81	0.00	5.93	1.57	0.01	0.75	-0.26	9.49	7.79	16.72
os22790	16	445	0.92	0	3	6	6.81	0.01	5.92	1.57	0.01	0.98	-0.32	9.47	7.79	16.72
os22791	16	445	0.92	0	3	6	6.81	0.01	5.92	1.57	0.01	1.07	-0.37	9.44	7.79	16.72
os22792	16	445	0.92	0	3	6	6.81	0.01	5.92	1.57	0.01	1.14	-0.44	9.41	7.79	16.72
os22793	16	445	0.92	0	3	6	6.81	0.00	5.92	1.57	0.01	1.22	-0.47	9.24	7.79	16.72
os22794	16	445	0.77	1	3	6	6.81	0.00	5.91	1.57	0.01	1.34	-0.60	8.96	7.79	16.73
os22802	16	459	0.01	2	3	6	6.72	-0.04	2.07	1.56	0.02	18.18	-0.46	8.55	7.71	16.69

os22828	11	440	0.92	0	3	6	6.80	0.00	6.82	1.57	0.01	5.89	-0.33	9.49	7.66	16.63
os22835	2	465	0.16	1	3	6	6.81	0.01	4.16	1.56	0.02	40.74	-0.17	8.58	7.57	16.56
os22838	3	421	0.92	0	3	6	6.78	0.00	6.98	1.56	0.01	11.85	0.10	9.80	8.20	17.20
os22839	3	421	0.92	0	3	6	6.79	0.00	6.98	1.56	0.01	10.90	0.03	9.83	8.20	17.20
os22870	10	489	0.13	2	3	6	6.84	0.03	1.93	1.55	0.03	45.68	-1.29	8.22	7.45	16.39
os22882	11	415	0.65	3	3	6	6.81	0.00	6.98	1.55	0.02	12.59	0.01	8.42	8.24	17.25
os22887	2	453	0.92	0	3	6	6.80	0.00	6.91	1.56	0.01	13.39	-0.23	10.89	7.58	16.54
os22991	19	442	0.77	2	3	6	6.80	-0.01	4.26	1.56	0.04	57.73	-0.98	9.13	8.01	17.02
os23025	16	437	0.20	2	3	6	6.86	0.03	1.63	1.55	0.02	35.03	-0.35	8.11	8.25	17.13
os23040	10	414	0.92	0	3	6	6.78	0.00	6.92	1.57	0.03	24.24	-0.66	9.09	8.24	17.25
os23041	11	414	0.60	2	3	6	6.75	-0.01	6.98	1.57	0.02	13.21	-0.67	8.58	8.24	17.26
os23044	11	413	0.92	0	3	6	6.79	0.00	6.99	1.57	0.02	10.41	-0.55	8.85	8.26	17.28
os23048	10	410	0.92	0	3	6	6.78	0.00	6.99	1.57	0.01	3.31	0.00	12.04	8.37	17.44
os23075	4	468	0.23	1	3	6	6.80	0.00	4.29	1.56	0.01	0.25	0.04	9.08	7.42	16.36
os23118	11	414	0.70	1	3	6	6.79	0.00	6.27	1.56	0.03	28.29	-1.05	8.16	8.25	17.27
os23131	19	470	0.97	8	3	5	6.62	-0.10	0.40	1.53	0.06	80.33	-1.06	8.08	7.92	16.89
os23132	23	663	0.81	10	1	1	6.78	-0.07	0.00	1.41	0.04	85.24	4.01	5.44	7.20	16.23
os23153	17	484	0.24	6	2	0	6.74	0.02	0.48	1.54	0.07	99.55	-0.43	6.32	7.87	16.87
os23156	23	647	0.60	8	3	5	6.62	-0.05	0.13	1.48	0.06	99.76	-0.18	6.08	7.13	16.16
os23157	23	640	0.55	9	3	5	6.60	-0.05	0.11	1.48	0.06	99.88	-0.02	6.13	7.14	16.17
os23193	19	427	0.92	1	2	3	6.78	-0.01	2.95	1.56	0.05	86.42	-2.20	9.17	8.05	16.98
os23209	19	496	0.11	2	3	6	6.85	0.03	2.59	1.54	0.04	77.46	-0.36	9.73	7.50	16.38
os23212	19	512	0.87	2	3	6	6.74	-0.04	1.89	1.54	0.03	78.66	-0.64	8.57	7.60	16.54
os23247	23	648	0.76	8	3	5	6.55	-0.10	0.08	1.49	0.05	99.79	0.06	6.04	7.12	16.19
os23264	23	625	0.01	6	3	9	6.99	0.10	0.39	1.49	0.05	99.65	0.57	6.13	7.33	16.36
os23272	14	442	0.33	24	2	3	5.97	0.05	0.00	1.44	0.21	88.84	-5.52	5.70	8.11	17.08
os23383	23	611	0.00	8	3	9	7.02	0.13	0.26	1.49	0.05	99.93	0.37	6.41	7.41	16.44
os23392	28	568	0.03	4	3	6	6.88	0.04	1.84	1.54	0.04	84.60	-0.46	9.22	7.49	16.50
os23393	28	569	0.23	4	3	6	6.86	0.03	1.98	1.54	0.04	84.02	-0.61	9.24	7.49	16.50
os23400	28	629	0.11	4	3	6	6.98	0.06	1.30	1.53	0.03	94.17	0.00	7.70	7.15	16.18
os23483	23	554	0.57	9	3	5	6.56	-0.05	0.09	1.50	0.07	100.00	0.04	6.13	7.56	16.59
os23485	23	572	0.37	10	3	5	6.62	0.00	0.02	1.49	0.07	100.00	0.59	6.09	7.50	16.53
os23488	8	515	0.08	1	3	6	6.86	0.01	2.95	1.55	0.03	67.87	-0.96	8.65	7.84	16.91
os23510	23	556	0.91	17	3	5	6.34	-0.20	0.00	1.44	0.13	98.49	1.40	5.42	7.49	16.53
os23512	23	622	0.25	13	3	5	6.70	0.07	0.05	1.48	0.10	98.70	-2.21	7.46	7.09	16.14
os23517	23	645	0.89	8	3	5	6.57	-0.11	0.07	1.47	0.04	99.97	2.01	5.91	7.14	16.17
os23518	23	640	0.51	10	3	5	6.58	-0.05	0.05	1.47	0.06	99.92	-0.16	5.96	7.14	16.18
os23519	23	636	0.58	10	3	5	6.58	-0.06	0.15	1.48	0.06	99.92	-0.42	6.16	7.14	16.18
os23520	23	636	0.60	10	3	5	6.56	-0.06	0.08	1.48	0.06	99.95	-0.57	6.06	7.15	16.18
os23524	23	628	0.97	6	3	5	6.70	-0.09	0.41	1.51	0.07	99.20	-0.25	7.96	7.07	16.09
os23526	23	650	0.09	9	1	4	6.91	0.11	0.01	1.47	0.07	94.25	2.60	6.20	6.97	16.03
os23575	23	601	0.81	8	3	5	6.55	-0.11	0.20	1.50	0.06	97.43	-0.29	7.15	7.32	16.32
os23579	23	675	0.11	6	3	9	6.92	0.06	0.44	1.48	0.03	99.39	0.18	6.30	7.07	16.09

os23591	19	556	0.76	3	2	0	6.88	0.01	1.92	1.55	0.07	97.14	-1.37	8.06	7.39	16.37
os23714	28	500	0.05	6	2	7	6.88	0.08	0.76	1.53	0.13	97.16	-1.82	7.55	7.67	16.65
os23743	23	604	0.82	12	3	9	6.34	-0.17	0.01	1.45	0.06	98.58	1.27	5.50	7.38	16.38
os23745	23	607	0.83	11	3	9	6.38	-0.15	0.01	1.45	0.06	98.46	1.32	5.50	7.37	16.37
os23767	23	581	0.54	10	1	4	6.44	-0.17	0.02	1.48	0.07	99.97	1.74	5.96	7.30	16.31
os23771	25	605	0.65	12	1	1	6.32	-0.20	0.00	1.42	0.09	100.00	5.01	5.90	7.35	16.36
os23779	23	597	0.07	13	3	5	6.89	0.17	0.00	1.44	0.09	99.74	0.78	5.66	7.38	16.39
os23781	28	582	0.85	7	3	5	6.58	-0.09	0.63	1.51	0.09	99.30	-1.98	6.65	7.34	16.34
os23791	23	551	1.00	9	3	5	6.51	-0.15	0.10	1.50	0.06	99.48	-0.16	6.02	7.56	16.56
os23829	24	658	0.77	2	3	6	6.89	-0.01	2.68	1.55	0.03	59.99	-0.75	7.80	6.45	15.44
os23964	28	536	0.97	5	3	5	6.75	-0.06	0.71	1.55	0.05	90.89	-1.39	7.15	7.03	16.02
os23994	28	622	0.83	2	3	6	6.86	-0.02	2.91	1.55	0.04	59.48	-0.51	8.83	6.51	15.51
os24018	28	663	0.23	2	3	6	6.86	0.00	2.68	1.55	0.04	45.73	-1.21	9.33	6.35	15.32
os24048	8	500	0.07	5	2	7	6.92	0.08	0.99	1.55	0.08	77.73	-2.47	8.17	7.14	16.15
os24058	23	570	0.17	10	3	5	7.05	0.14	0.12	1.50	0.07	92.42	-1.37	5.93	7.10	16.12
os24062	23	546	0.00	6	3	5	7.01	0.11	0.42	1.52	0.05	72.91	-0.21	7.16	7.24	16.25
os24080	28	519	0.99	2	2	0	6.80	-0.03	3.97	1.55	0.07	63.64	-0.94	9.05	7.21	16.19
os24108	17	488	0.92	0	3	6	6.83	0.01	4.73	1.56	0.01	22.77	0.48	9.69	7.75	16.79
os24122	17	482	0.92	2	3	6	6.74	-0.03	3.61	1.55	0.02	34.05	0.50	8.66	7.77	16.81
os24123	19	485	0.73	1	3	6	6.82	-0.01	3.12	1.56	0.02	26.40	0.01	9.41	7.78	16.81
os24143	28	530	0.99	5	3	5	6.65	-0.09	0.47	1.52	0.04	96.81	0.22	7.40	7.58	16.64
os24144	19	559	0.92	2	2	3	6.81	0.01	1.99	1.56	0.08	71.02	-4.00	7.48	7.32	16.39
os24170	28	522	0.60	2	3	6	6.79	-0.01	2.98	1.56	0.02	14.73	-0.35	9.10	7.44	16.44
os24192	28	576	0.10	4	3	6	6.89	0.05	0.86	1.54	0.05	87.21	-0.21	7.15	7.24	16.32
os24197	28	565	0.23	2	3	6	6.88	0.03	2.42	1.54	0.03	55.81	-0.65	8.14	7.37	16.42
os24213	28	529	0.23	1	3	6	6.83	0.01	2.45	1.55	0.02	41.35	-0.14	9.09	7.38	16.38
os24224	23	538	0.92	0	3	6	6.84	0.00	2.91	1.55	0.02	62.12	0.22	9.14	7.55	16.58
os24252	28	611	0.01	5	3	6	6.89	0.05	0.59	1.53	0.05	99.89	-0.17	7.49	7.02	16.10
os24263	23	593	0.90	3	3	6	6.83	-0.03	0.20	1.50	0.03	97.27	1.94	6.54	7.33	16.37
os24264	28	574	0.76	6	3	5	6.65	-0.10	0.63	1.53	0.06	98.25	-1.28	6.86	7.27	16.32
os24267	28	592	0.01	3	3	6	6.77	-0.05	1.91	1.54	0.05	93.19	-1.66	8.25	7.08	16.13
os24282	28	559	0.47	2	3	6	6.86	0.00	2.95	1.56	0.03	64.21	-0.96	8.61	7.22	16.26
os24292	19	488	0.39	4	3	6	6.76	0.00	1.63	1.55	0.02	49.99	0.17	8.51	7.81	16.88
os24331	19	507	0.81	5	3	5	6.63	-0.09	0.61	1.54	0.05	89.48	-1.01	7.25	7.68	16.71
os24343	28	510	0.01	2	3	6	6.85	0.02	3.67	1.56	0.03	54.62	-0.70	8.93	7.62	16.66
os24416	23	551	0.91	5	3	6	6.76	-0.06	0.40	1.53	0.05	89.79	-0.22	6.40	7.17	16.17
os24417	23	554	0.98	6	3	5	6.71	-0.08	0.30	1.52	0.06	94.47	-0.70	6.33	7.16	16.16
os24418	28	537	0.43	5	3	6	6.74	-0.01	0.63	1.54	0.04	96.29	-0.31	7.28	7.33	16.32
os24419	28	538	0.51	5	3	6	6.74	-0.01	0.61	1.54	0.04	96.39	-0.28	7.25	7.33	16.32
os24438	23	527	0.92	4	3	6	6.69	-0.07	1.60	1.53	0.03	75.67	-0.40	7.53	7.47	16.50
os24463	28	496	0.08	2	3	6	6.87	0.02	5.02	1.56	0.02	15.91	-0.37	8.40	7.63	16.68
os24478	28	528	0.92	1	3	6	6.86	0.02	1.74	1.56	0.01	18.06	0.11	8.78	7.46	16.48
os24531	23	590	0.60	6	3	5	6.69	-0.04	0.96	1.53	0.04	86.26	-1.10	8.07	7.08	16.11

os24533	28	576	0.36	3	3	6	6.94	0.04	0.93	1.54	0.03	82.75	-0.12	8.07	7.10	16.11
os24538	27	569	0.27	5	3	5	6.97	0.05	0.60	1.53	0.05	72.04	-0.69	7.21	7.10	16.11
os24569	28	550	0.92	0	3	6	6.83	0.00	3.91	1.55	0.02	32.96	-0.02	9.24	7.22	16.24
os24571	23	573	0.09	2	3	6	6.90	0.02	3.00	1.55	0.03	44.63	-0.27	9.11	7.09	16.10
os24572	23	559	0.77	5	3	6	6.81	-0.04	0.20	1.51	0.03	87.78	1.29	7.30	7.26	16.28
os24586	23	578	0.77	1	3	6	6.86	-0.01	2.89	1.55	0.02	40.34	-0.03	8.68	7.10	16.11
os24632	28	643	0.21	4	3	5	6.99	0.05	0.94	1.53	0.05	97.17	-1.11	6.84	6.82	15.83
os24633	23	622	0.00	3	3	6	6.94	0.04	1.81	1.54	0.03	76.25	-0.65	8.03	6.88	15.90
os24636	23	597	0.17	5	3	5	6.86	0.05	0.35	1.52	0.04	87.20	1.01	7.61	6.93	15.93
os24654	23	542	0.77	4	3	6	6.82	-0.03	0.67	1.54	0.03	60.82	-0.14	7.36	7.27	16.28
os24656	23	543	0.75	3	3	6	6.86	0.00	0.50	1.54	0.03	58.88	0.24	7.72	7.28	16.28
os24677	28	543	0.18	1	3	6	6.83	0.00	2.63	1.56	0.03	51.14	-0.50	8.28	7.19	16.17
os24686	23	582	0.72	12	3	5	6.41	-0.09	0.08	1.49	0.08	91.62	-1.79	7.66	6.92	15.93
os24701	27	612	0.17	6	2	3	6.67	-0.09	1.39	1.54	0.11	96.11	-5.29	8.19	6.55	15.54
os24704	27	635	0.61	3	2	3	6.77	-0.04	1.98	1.55	0.07	93.97	-3.59	7.98	6.49	15.48
os24706	24	674	0.01	4	3	6	6.97	0.06	0.79	1.54	0.02	74.74	-0.19	7.66	6.38	15.36
os24712	28	646	0.23	4	3	6	6.89	0.03	0.77	1.54	0.02	82.05	-0.12	7.46	6.58	15.57
os24718	27	632	0.31	8	3	5	6.74	0.01	0.30	1.52	0.00	89.04	-1.78	7.42	6.52	15.56
os24723	23	579	0.57	4	3	6	6.92	0.02	1.26	1.55	0.06	78.29	-1.70	8.32	7.18	16.16
os24726	23	584	0.30	7	3	5	6.75	0.02	0.30	1.52	0.06	68.53	-1.11	8.01	7.14	16.14
os24731	23	605	0.29	9	3	5	7.03	0.09	0.17	1.49	0.06	97.47	0.41	6.67	6.99	15.97
os24765	23	562	0.47	8	3	5	6.67	0.00	0.40	1.49	0.07	97.86	-1.48	7.69	7.58	16.63
os24799	23	595	0.20	3	3	6	6.95	0.04	1.62	1.55	0.03	79.31	-0.65	8.47	7.07	16.06
os24810	23	570	0.05	3	3	6	6.92	0.05	2.30	1.55	0.05	95.92	-1.55	8.66	7.34	16.38
os24815	23	605	0.75	11	2	3	6.41	-0.12	0.01	1.50	0.09	93.17	-2.02	6.35	6.91	15.87
os24816	23	592	0.27	5	1	4	6.82	0.01	0.01	1.48	0.03	95.94	4.83	6.60	7.19	16.19
os24819	28	592	0.35	3	3	6	6.79	-0.03	2.95	1.55	0.06	83.77	-1.27	8.16	7.00	15.98
os24843	23	669	0.23	3	3	6	6.88	0.02	0.15	1.51	0.01	90.68	1.82	7.47	6.86	15.87
os24859	23	542	0.40	4	3	6	6.91	0.03	1.51	1.53	0.05	95.14	-1.20	8.27	7.57	16.62
os24863	27	539	0.70	9	2	3	6.51	-0.14	0.61	1.50	0.10	92.98	-3.74	7.68	7.66	16.67
os24867	23	472	0.63	6	2	0	6.53	-0.11	0.81	1.54	0.11	54.56	-2.10	7.61	7.86	16.90
os24883	28	668	0.40	5	3	6	6.98	0.03	0.80	1.52	0.03	99.87	-0.45	7.33	6.77	15.74
os24910	23	611	0.01	7	3	5	6.97	0.10	0.16	1.51	0.04	89.97	0.77	6.37	6.89	15.84
os24917	23	598	0.56	11	3	5	6.81	-0.04	0.17	1.49	0.08	98.47	-1.42	7.22	7.33	16.33
os24925	28	580	0.36	2	3	6	6.90	0.02	2.98	1.56	0.04	78.81	-0.89	9.42	7.25	16.28
os24937	23	559	0.84	11	2	0	6.36	-0.15	0.01	1.47	0.07	91.51	-0.14	7.75	7.36	16.37
os24948	23	557	0.11	6	3	5	6.89	0.07	0.42	1.50	0.05	90.69	0.77	7.54	7.42	16.48
os24951	23	617	0.92	3	3	6	6.74	-0.05	1.74	1.54	0.05	95.37	-1.09	8.12	7.08	16.14
os24974	28	658	0.08	2	3	6	6.96	0.04	1.76	1.54	0.02	78.29	-0.10	8.38	6.81	15.81
os24977	28	602	0.46	4	3	6	6.93	0.02	3.85	1.56	0.04	57.31	-0.53	8.99	7.02	16.00
os25026	28	607	0.97	4	3	6	6.82	-0.04	1.45	1.54	0.05	97.53	-0.69	7.19	7.03	16.02
os25028	23	637	0.75	7	3	5	6.87	-0.02	0.26	1.51	0.05	94.01	0.54	6.58	6.90	15.88
os25029	27	595	0.44	7	3	5	6.71	-0.02	0.10	1.52	0.05	71.50	-0.38	7.92	7.07	16.06

os25031	23	584	0.01	2	3	6	6.90	0.03	0.35	1.54	0.04	67.56	0.81	9.01	7.13	16.12
os25045	23	555	0.63	4	2	7	6.66	-0.08	1.32	1.56	0.09	74.76	-2.77	7.87	7.38	16.42
os25076	23	573	0.65	9	3	5	6.90	0.01	0.58	1.53	0.08	70.41	-1.91	8.38	7.20	16.17
os25079	28	627	0.92	2	3	6	6.91	0.03	0.66	1.53	0.02	91.23	-0.28	6.68	7.09	16.08
os25082	28	623	0.00	4	3	6	6.98	0.07	0.68	1.52	0.03	93.54	-0.22	6.72	7.08	16.07
os25087	28	671	0.40	5	3	6	6.99	0.04	0.67	1.52	0.03	99.79	-0.16	7.30	6.76	15.73
os25092	23	668	0.28	5	3	5	7.01	0.06	0.53	1.52	0.03	99.99	-0.38	6.95	6.77	15.74
os25095	23	639	0.76	5	3	5	6.88	-0.02	0.51	1.52	0.04	95.08	-0.11	7.05	6.89	15.86
os25098	28	623	0.36	3	3	6	6.94	0.02	2.11	1.55	0.05	86.82	-0.81	7.83	6.90	15.87
os25103	23	661	0.77	6	3	5	6.89	-0.02	0.44	1.51	0.04	98.45	0.39	6.69	6.79	15.77
os25104	23	660	0.76	6	3	5	6.89	-0.02	0.43	1.51	0.04	98.27	0.27	6.71	6.79	15.77
os25105	23	685	0.40	6	3	9	6.99	0.04	0.38	1.51	0.04	100.00	-0.03	6.27	6.73	15.72
os25112	23	594	0.19	10	3	5	7.07	0.13	0.04	1.49	0.08	96.40	-0.30	6.80	6.96	15.95
os25113	23	617	0.39	11	3	5	7.00	0.07	0.03	1.48	0.07	98.18	-0.20	6.31	6.93	15.92
os25116	23	607	1.00	7	1	4	6.64	-0.12	0.08	1.49	0.04	89.10	2.90	6.35	6.93	15.91
os25124	27	640	0.89	5	3	6	6.68	-0.07	0.55	1.52	0.05	92.74	-0.19	7.50	6.68	15.64
os25128	23	594	0.60	2	3	9	6.80	-0.02	0.81	1.51	0.02	69.41	-0.04	7.68	7.17	16.14
os25147	28	648	0.12	6	1	4	6.89	0.05	0.31	1.52	0.02	53.66	1.53	7.45	6.74	15.74
os25152	24	647	0.01	2	3	6	6.91	0.02	2.89	1.55	0.02	48.38	-0.86	8.58	6.68	15.68
os25168	19	490	0.13	5	2	7	6.85	0.08	1.31	1.55	0.13	86.36	-5.04	8.79	7.69	16.72
os25171	28	484	1.00	7	2	3	6.57	-0.11	0.88	1.54	0.06	98.54	-2.49	8.16	7.81	16.83
os25191	23	524	0.15	11	1	4	6.75	0.09	0.01	1.45	0.06	79.89	4.10	5.66	7.76	16.79
os25218	19	510	0.65	3	3	6	6.85	0.00	1.98	1.54	0.04	88.95	-0.63	8.81	7.74	16.79
os25223	25	526	0.71	18	3	5	6.61	-0.08	0.00	1.44	0.12	89.92	-1.65	5.90	7.68	16.73
os25248	28	600	0.11	2	3	6	6.91	0.02	2.65	1.54	0.02	44.37	-0.30	8.15	7.29	16.38
os25257	28	539	0.37	9	3	5	6.72	0.03	0.43	1.50	0.07	86.48	-0.47	8.47	7.54	16.56
os25279	23	584	0.46	8	3	5	6.65	-0.02	0.12	1.49	0.05	90.66	1.20	6.82	7.31	16.33
os25280	23	586	0.57	7	3	5	6.66	-0.02	0.17	1.50	0.05	90.63	1.15	6.55	7.31	16.33
os25289	23	606	0.47	11	2	3	6.61	0.00	0.09	1.50	0.08	95.88	-1.85	7.87	7.23	16.30
os25319	28	542	0.23	3	3	6	6.83	0.02	1.65	1.53	0.02	76.81	0.48	8.54	7.77	16.87
os25348	27	484	0.98	3	3	6	6.73	-0.05	2.24	1.55	0.04	85.21	-1.46	8.71	7.95	17.00
os25354	28	505	0.99	4	3	6	6.71	-0.06	0.60	1.53	0.03	93.71	-0.09	6.95	7.95	16.99
os25355	28	514	0.99	4	3	6	6.70	-0.07	0.52	1.52	0.02	87.86	0.91	7.03	7.94	16.99
os25357	28	496	0.80	3	3	6	6.77	-0.04	0.77	1.54	0.03	93.54	-0.22	7.21	7.95	16.99
os25382	23	602	0.79	4	3	6	6.84	-0.03	1.57	1.54	0.06	96.53	-1.30	8.44	7.20	16.22
os25384	27	608	0.78	3	3	6	6.84	-0.03	1.96	1.52	0.05	97.79	-1.93	7.81	7.29	16.27
os25447	27	526	0.92	5	3	6	6.65	-0.06	0.86	1.54	0.05	96.47	-1.52	8.20	7.49	16.46
os25454	19	515	0.23	2	3	6	6.81	0.01	2.97	1.56	0.04	50.86	-0.35	8.99	7.61	16.63
os25467	23	502	0.33	7	3	5	6.69	-0.01	0.75	1.53	0.06	96.94	-1.13	7.75	7.71	16.74
os25495	23	614	0.77	6	3	5	6.63	-0.08	0.41	1.51	0.04	96.33	0.24	6.89	6.99	15.92
os25506	28	599	0.35	3	3	6	6.82	0.01	1.86	1.54	0.05	94.62	-1.53	7.96	6.96	15.90
os25511	28	654	0.77	3	3	6	6.87	-0.02	2.78	1.55	0.03	61.45	-0.25	8.87	6.60	15.55
os25516	28	573	0.61	2	3	6	6.80	-0.02	3.88	1.56	0.04	63.86	-0.40	9.29	7.21	16.19

os25517	28	586	0.68	3	3	6	6.87	-0.01	0.63	1.53	0.03	77.94	0.31	8.11	7.21	16.20
os25534	28	578	0.77	4	3	6	6.85	-0.02	0.64	1.53	0.05	81.22	-0.31	7.99	7.23	16.21
os25538	27	547	0.69	6	3	5	6.63	-0.06	0.83	1.52	0.06	87.66	-1.19	8.12	7.50	16.52
os25541	23	576	0.23	4	3	6	6.85	0.03	0.89	1.53	0.04	86.30	-0.76	7.21	7.41	16.42
os25546	28	596	0.01	3	3	6	6.95	0.05	1.44	1.52	0.04	89.82	-0.37	6.78	7.32	16.34
os25549	28	588	0.40	2	3	6	6.88	0.00	1.33	1.52	0.02	70.74	1.55	8.46	7.34	16.34
os25567	28	576	0.46	3	3	6	6.82	0.01	1.86	1.55	0.03	64.88	-0.21	8.59	7.29	16.28
os25579	23	537	0.92	2	2	7	6.79	-0.02	2.96	1.56	0.07	93.19	-2.62	8.74	7.43	16.43
os25580	27	540	0.07	3	2	3	6.75	-0.04	2.13	1.55	0.07	93.51	-2.17	8.50	7.42	16.42
os25581	23	541	0.92	3	2	3	6.72	-0.05	1.95	1.55	0.07	93.30	-2.64	8.30	7.42	16.42
os25582	27	541	0.85	3	2	3	6.74	-0.05	2.04	1.55	0.07	93.44	-2.39	8.42	7.42	16.42
os25583	23	552	0.76	6	3	5	6.61	-0.08	0.45	1.52	0.05	92.16	-0.42	7.05	7.41	16.41
os25589	28	563	0.60	4	3	6	6.72	-0.03	0.87	1.54	0.05	94.74	-0.79	7.68	7.30	16.35
os25592	23	581	0.34	10	3	5	6.65	0.03	0.03	1.50	0.09	95.14	-1.72	5.72	7.18	16.17
os25595	23	573	0.23	12	3	5	6.67	0.06	0.03	1.49	0.10	92.96	-1.90	5.73	7.19	16.18
os25602	23	537	0.83	7	2	0	6.55	-0.12	0.31	1.53	0.08	90.41	-0.48	6.64	7.38	16.40
os25612	23	570	0.77	4	3	6	6.83	-0.03	0.72	1.53	0.05	98.08	0.18	7.95	7.23	16.24
os25613	23	578	0.77	5	3	5	6.85	-0.02	0.53	1.53	0.06	98.79	-0.25	7.63	7.19	16.20
os25614	23	579	0.77	6	3	5	6.83	-0.03	0.49	1.52	0.06	98.80	-0.15	7.44	7.18	16.19
os25616	23	583	0.87	8	3	5	6.75	-0.07	0.25	1.51	0.06	98.23	-0.93	7.15	7.17	16.18
os25627	23	594	1.00	5	3	5	6.69	-0.09	0.42	1.50	0.05	98.10	0.55	6.54	7.22	16.21
os25661	23	609	0.82	3	1	4	6.83	-0.03	0.01	1.49	0.02	91.25	3.42	7.07	6.93	15.87
os25675	23	621	0.94	5	3	6	6.75	-0.07	0.69	1.53	0.06	92.97	-0.92	6.81	6.79	15.73
os25678	23	616	0.03	7	3	5	6.96	0.10	0.15	1.50	0.04	91.71	1.60	6.17	6.89	15.83
os25679	23	590	0.12	4	2	0	6.87	0.04	0.56	1.54	0.06	71.16	-0.61	8.86	6.90	15.85
os25680	23	607	0.10	8	3	5	6.88	0.09	0.16	1.51	0.06	85.67	-0.15	6.42	6.89	15.84
os25681	23	610	0.01	7	3	5	6.97	0.10	0.18	1.51	0.04	89.87	0.68	6.44	6.90	15.84
os25689	23	620	0.25	6	3	5	7.01	0.07	0.50	1.52	0.05	98.59	-0.58	7.58	6.82	15.77
os25699	23	615	0.07	6	3	5	6.85	0.05	0.27	1.49	0.03	93.19	1.95	6.61	6.90	15.86
os25700	23	614	0.34	8	3	5	6.77	0.03	0.27	1.51	0.06	94.49	-0.20	6.27	6.88	15.84
os25707	23	588	0.76	1	3	6	6.86	-0.01	3.91	1.55	0.03	30.87	0.04	9.43	6.98	15.93
os25708	23	584	0.19	2	3	6	6.91	0.02	3.95	1.56	0.03	22.39	-0.54	9.34	6.99	15.94
os25724	23	676	0.44	3	3	6	6.95	0.01	0.76	1.54	0.03	98.63	-0.26	7.04	6.56	15.51
os25731	23	674	1.00	6	3	6	6.70	-0.09	0.56	1.50	0.03	87.31	1.00	6.78	6.63	15.58
os25732	23	673	0.99	7	3	5	6.65	-0.11	0.48	1.51	0.05	88.89	-0.17	6.77	6.63	15.58
os25733	23	670	0.79	6	3	5	6.65	-0.11	0.45	1.52	0.05	89.56	-1.03	6.72	6.63	15.59
os25734	23	675	1.00	7	3	5	6.65	-0.11	0.16	1.50	0.03	94.50	1.63	5.92	6.64	15.59
os25737	23	636	0.01	6	3	5	7.00	0.10	0.32	1.53	0.06	84.29	-0.77	7.62	6.73	15.67
os25742	23	674	0.70	3	3	6	6.90	-0.01	0.35	1.52	0.02	98.33	0.86	7.37	6.61	15.56
os25745	23	678	0.00	10	3	5	7.04	0.16	0.03	1.49	0.06	85.29	1.28	6.20	6.61	15.56
os25757	28	648	0.56	3	3	6	6.89	0.03	1.78	1.54	0.03	83.97	-0.45	8.54	6.69	15.63
os25767	23	613	0.81	4	3	6	6.71	-0.06	1.22	1.53	0.04	98.76	-0.85	7.50	7.00	16.01
os25768	23	610	0.15	3	3	6	6.74	-0.06	1.41	1.53	0.04	98.64	-0.32	7.69	7.01	16.03

os25776	23	592	0.53	10	3	5	6.92	0.02	0.06	1.48	0.06	91.89	0.30	5.90	7.16	16.17
os25787	23	571	1.00	6	3	5	6.65	-0.10	0.45	1.51	0.05	94.29	0.41	6.46	7.30	16.27
os25788	23	540	1.00	5	2	0	6.68	-0.08	0.65	1.53	0.07	81.92	-0.51	8.97	7.35	16.32
os25813	23	623	0.84	6	1	1	6.64	-0.08	0.11	1.49	0.03	98.66	2.17	6.03	7.04	16.02
os25831	28	623	0.77	5	2	3	6.86	-0.03	1.52	1.55	0.07	95.25	-2.47	7.81	6.84	15.80
os25843	28	608	0.33	7	3	5	6.73	-0.01	0.31	1.53	0.06	93.58	-0.46	8.72	6.91	15.86
os25854	28	679	0.97	2	3	6	6.85	-0.03	2.54	1.54	0.02	41.57	-0.31	8.42	6.56	15.55
os25872	28	598	0.35	3	3	6	6.94	0.03	1.51	1.55	0.03	80.50	-0.30	8.07	6.98	15.93
os25910	28	628	0.03	2	3	6	6.90	0.03	2.82	1.55	0.03	62.95	-0.96	8.93	6.80	15.78
os25926	23	529	0.03	5	3	6	6.95	0.07	0.75	1.54	0.04	79.01	-0.40	8.11	7.72	16.74
os25933	28	584	0.40	4	3	6	6.93	0.03	1.08	1.54	0.04	93.85	-0.66	8.01	7.38	16.46
os25960	27	570	0.77	3	3	6	6.82	-0.03	2.55	1.55	0.04	85.32	-0.58	8.76	7.40	16.44
os25964	23	600	0.47	4	3	6	6.75	-0.06	1.53	1.54	0.03	89.97	-0.58	8.47	7.33	16.36
os25965	27	600	0.89	3	3	6	6.83	-0.03	2.07	1.55	0.04	88.54	-1.97	8.63	7.33	16.35
os25977	23	591	0.21	4	3	6	6.94	0.03	0.77	1.53	0.02	93.83	-0.10	7.62	7.38	16.43
os25991	23	582	0.33	5	3	5	6.98	0.06	0.21	1.52	0.03	89.31	1.07	7.55	7.35	16.37
os26004	25	543	0.98	3	3	6	6.75	-0.05	0.15	1.52	0.04	93.57	1.74	8.15	7.54	16.59
os26008	23	593	0.41	3	3	6	6.92	0.02	0.30	1.52	0.02	88.74	0.50	7.14	7.44	16.47
os26020	23	616	0.23	4	3	6	6.85	0.03	0.94	1.53	0.04	97.75	-0.57	7.60	7.21	16.26
os26023	23	479	0.47	16	2	0	6.29	-0.05	0.02	1.46	0.10	79.15	-0.53	8.33	7.88	16.92
os26034	23	565	0.60	4	3	6	6.71	-0.04	0.59	1.52	0.03	89.20	0.11	6.94	7.46	16.50
os26065	23	632	0.92	5	3	5	6.68	-0.09	0.45	1.50	0.05	99.91	0.36	7.21	7.06	16.07
os26066	27	622	0.84	9	3	5	6.74	-0.08	0.06	1.47	0.07	99.75	1.74	6.02	7.09	16.09
os26077	23	661	0.23	7	3	5	6.82	0.04	0.15	1.49	0.05	92.49	0.66	6.46	6.94	15.94
os26088	23	673	0.12	5	1	4	7.02	0.07	0.10	1.49	0.02	82.73	2.50	7.01	6.94	15.95
os26089	23	670	0.59	7	1	4	6.96	0.02	0.08	1.49	0.04	86.07	2.42	6.48	6.94	15.95
os26094	23	621	0.40	5	3	6	6.99	0.06	0.61	1.52	0.03	96.33	0.52	7.13	7.15	16.14
os26102	23	666	0.47	2	1	1	6.90	0.01	0.01	1.45	0.00	98.58	5.53	7.45	7.12	16.12
os26105	28	599	0.21	2	3	6	6.91	0.02	2.89	1.55	0.03	59.05	-0.67	8.61	7.25	16.25
os26106	28	598	0.73	2	3	6	6.89	0.01	2.94	1.55	0.03	56.23	-0.69	8.78	7.26	16.25
os26109	23	631	0.23	8	1	1	6.84	0.07	0.03	1.46	0.03	99.42	3.09	5.61	7.27	16.26
os26118	23	629	0.57	5	1	1	6.92	0.00	0.15	1.44	0.02	97.54	2.69	6.22	7.29	16.28
os26119	28	573	0.85	9	3	5	6.67	-0.10	0.13	1.50	0.08	95.95	-1.37	6.05	7.34	16.32
os26120	23	579	0.90	13	3	5	6.55	-0.14	0.00	1.48	0.09	96.84	-0.85	5.81	7.34	16.32
os26121	28	574	0.88	11	2	3	6.63	-0.11	0.08	1.50	0.09	96.42	-1.77	5.92	7.35	16.33
os26124	23	609	0.91	11	3	9	6.62	-0.13	0.02	1.47	0.07	98.80	-0.29	6.46	7.33	16.31
os26126	23	623	0.07	4	1	1	6.96	0.06	0.09	1.46	0.01	98.24	3.24	6.74	7.32	16.31
os26127	27	569	0.37	11	3	5	6.63	0.03	0.02	1.49	0.11	97.32	-1.24	6.90	7.40	16.38
os26128	27	546	0.08	13	2	0	6.78	0.13	0.01	1.48	0.13	94.08	-2.12	7.04	7.45	16.43
os26131	23	597	0.98	12	3	5	6.39	-0.20	0.01	1.45	0.07	99.09	1.30	5.81	7.42	16.40
os26132	23	564	0.77	5	3	6	6.83	-0.03	0.93	1.52	0.07	99.70	-0.99	7.64	7.48	16.46
os26145	23	624	0.54	5	3	5	6.72	-0.04	0.33	1.50	0.03	94.97	1.72	6.69	7.21	16.23
os26152	23	611	0.97	5	3	6	6.77	-0.06	0.28	1.51	0.03	93.98	2.13	6.79	7.30	16.31

os26153	23	609	0.41	4	1	4	6.87	-0.02	0.09	1.51	0.03	95.66	2.33	6.49	7.30	16.31
os26159	23	618	0.32	14	3	5	7.02	0.11	0.00	1.45	0.08	99.64	1.02	5.75	7.27	16.28
os26162	23	608	0.08	8	3	5	6.85	0.08	0.10	1.48	0.05	99.67	1.64	6.02	7.24	16.22
os26164	23	630	0.09	6	1	4	6.87	0.05	0.03	1.46	0.03	99.03	4.20	6.24	7.18	16.17
os26165	23	630	0.01	7	1	4	6.98	0.10	0.03	1.46	0.03	99.08	3.64	6.37	7.18	16.17
os26166	23	625	0.02	9	1	4	6.94	0.11	0.07	1.46	0.04	99.14	2.55	5.77	7.20	16.18
os26172	23	619	0.23	8	3	5	6.80	0.04	0.19	1.49	0.04	99.74	0.97	6.33	7.25	16.22
os26173	23	615	0.03	5	1	1	6.91	0.06	0.02	1.48	0.02	98.51	3.86	6.56	7.32	16.30
os26174	23	616	0.07	4	3	9	6.98	0.07	0.28	1.50	0.02	97.04	1.94	7.03	7.31	16.29
os26192	23	563	1.00	9	2	0	6.53	-0.15	0.06	1.49	0.08	92.41	0.08	6.52	7.37	16.35
os26218	23	632	0.38	6	3	9	6.79	0.01	0.14	1.49	0.04	99.51	2.02	6.32	7.22	16.20
os26222	23	581	0.95	15	3	5	6.35	-0.21	0.00	1.45	0.10	97.16	0.59	5.60	7.47	16.48
os26249	25	647	0.99	10	3	9	6.58	-0.15	0.09	1.46	0.07	99.79	0.21	5.76	7.28	16.31
os26255	23	540	0.57	9	3	5	6.45	-0.16	0.09	1.51	0.08	99.48	-0.93	5.97	7.54	16.55
os26265	28	581	0.47	4	2	7	6.87	0.05	1.39	1.53	0.10	99.38	-3.23	8.36	7.23	16.23
os26268	19	478	0.96	5	2	7	6.70	-0.06	2.29	1.56	0.09	83.16	-3.75	8.23	7.89	16.89
os26292	23	642	0.88	12	3	9	6.36	-0.17	0.01	1.44	0.07	97.80	2.25	5.49	7.28	16.29
os26294	23	682	0.04	5	3	6	6.99	0.08	0.29	1.50	0.05	92.29	-0.68	6.84	6.88	15.90
os26306	27	640	0.06	2	3	9	6.92	0.03	1.90	1.51	0.04	66.38	-0.34	7.66	7.14	16.15
os26307	23	558	0.04	2	2	0	6.92	0.04	2.41	1.55	0.05	75.44	0.03	8.49	7.44	16.46
os26346	19	504	0.17	10	2	3	6.56	-0.04	1.05	1.55	0.15	96.81	-7.03	8.57	7.76	16.76
os26355	19	497	0.92	1	2	3	6.81	-0.01	2.90	1.56	0.08	85.57	-3.16	8.81	7.78	16.78
os26358	27	548	0.36	5	2	3	6.76	0.00	1.50	1.53	0.08	97.91	-3.09	8.29	7.55	16.60
os26365	24	458	0.83	5	3	6	6.83	0.00	3.19	1.55	0.06	64.55	-0.25	8.48	8.04	17.09
os26367	23	631	0.87	13	3	5	6.55	-0.15	0.00	1.45	0.08	99.94	0.51	5.85	7.21	16.20
os26378	19	593	0.23	1	3	6	6.88	0.01	3.99	1.56	0.04	57.95	-1.18	8.80	7.30	16.31
os26379	23	589	0.24	5	3	6	6.81	0.02	0.31	1.52	0.05	65.32	-0.01	7.45	7.35	16.37
os26388	23	670	0.62	2	1	4	6.84	-0.02	0.00	1.50	0.01	88.66	3.30	6.88	6.97	15.98
os26396	28	547	0.27	4	3	6	6.80	0.02	0.66	1.53	0.04	90.07	0.29	8.66	7.62	16.67
os26424	23	604	0.94	14	3	5	6.42	-0.19	0.00	1.46	0.11	100.00	-1.05	5.95	7.31	16.35
os26429	23	636	0.99	11	3	9	6.47	-0.18	0.03	1.47	0.06	97.26	1.11	5.92	7.20	16.25
os26450	25	693	0.92	15	1	1	6.51	-0.16	0.00	1.38	0.09	93.31	5.76	6.12	6.99	16.02
os26456	28	638	0.40	2	3	6	6.93	0.02	0.56	1.53	0.02	55.71	0.24	8.58	7.08	16.07
os26457	28	638	0.32	1	3	6	6.86	0.00	0.50	1.53	0.03	63.18	0.40	8.86	7.08	16.08
os26505	27	639	0.00	2	3	9	6.93	0.03	2.14	1.51	0.04	61.43	-0.32	7.92	7.14	16.15
os26507	23	653	0.89	8	3	9	6.57	-0.12	0.10	1.47	0.06	90.57	0.99	5.95	7.12	16.13
os26508	28	648	0.47	4	3	9	6.81	-0.01	1.75	1.50	0.07	90.27	-1.99	6.47	7.13	16.13
os26513	28	628	0.40	2	3	6	6.90	0.00	1.93	1.54	0.03	69.20	-0.67	7.92	7.13	16.13
os26515	23	618	0.43	4	3	6	6.84	0.02	0.80	1.53	0.05	72.59	-0.69	7.67	7.17	16.17
os26516	28	631	0.53	2	3	6	6.90	0.00	1.53	1.54	0.03	61.74	-0.10	8.02	7.10	16.11
os26517	24	621	0.77	1	3	6	6.88	0.00	2.97	1.54	0.03	61.59	-0.19	8.70	7.10	16.11
os26519	23	653	1.00	6	1	1	6.67	-0.10	0.04	1.47	0.02	96.02	3.45	5.70	7.17	16.20
os26520	23	561	0.08	2	2	0	6.92	0.04	1.50	1.55	0.05	80.99	-0.06	8.37	7.41	16.44

os26538	23	609	0.88	8	3	5	6.68	-0.10	0.35	1.47	0.08	99.25	-0.40	6.65	7.17	16.25
os26569	19	598	0.00	5	2	0	6.99	0.09	0.62	1.53	0.08	99.87	-1.04	7.40	7.16	16.24
os26583	19	527	0.91	9	2	3	6.53	-0.12	1.01	1.53	0.09	95.77	-3.34	8.57	7.71	16.71
os26591	19	503	0.99	8	3	5	6.56	-0.13	0.52	1.52	0.08	90.07	-1.85	7.57	7.75	16.78
os26592	23	504	0.98	11	1	4	6.35	-0.19	0.03	1.47	0.07	90.45	1.61	6.43	7.78	16.80
os26594	23	537	1.00	14	2	7	6.28	-0.23	0.00	1.48	0.10	97.70	-1.14	6.98	7.53	16.58
os26603	28	560	0.13	3	2	3	6.93	0.05	2.23	1.55	0.06	95.32	-2.99	9.18	7.39	16.43
os26606	23	579	0.31	15	3	5	6.54	0.05	0.00	1.46	0.10	94.07	-0.64	5.98	7.40	16.45
os26610	19	485	0.71	7	2	0	6.81	-0.02	0.36	1.54	0.10	66.29	-0.83	8.10	7.74	16.77
os26611	23	487	0.91	9	2	0	6.62	-0.10	0.53	1.53	0.11	74.56	-2.49	8.06	7.76	16.78
os26625	19	509	0.01	5	3	5	6.95	0.09	0.56	1.53	0.05	88.53	-0.63	9.18	7.59	16.66
os26628	28	582	0.60	4	3	6	6.74	-0.03	0.85	1.53	0.03	95.88	-0.14	7.54	7.39	16.47
os26697	23	647	0.02	12	3	9	6.97	0.17	0.00	1.45	0.07	99.83	1.89	5.47	6.27	15.11
os26699	23	566	0.93	12	3	5	6.53	-0.15	0.01	1.48	0.10	97.33	-0.40	6.22	6.60	15.41
os26723	23	642	0.79	9	2	7	6.62	-0.06	0.55	1.50	0.09	99.87	-1.10	8.24	5.96	14.77
os26737	25	524	0.98	7	2	7	6.58	-0.11	0.62	1.54	0.12	76.65	-1.62	7.60	6.47	15.28
os26738	27	543	0.68	11	2	7	6.42	-0.12	0.08	1.51	0.11	91.92	-2.25	7.70	6.45	15.26
os26742	23	502	0.97	12	2	0	6.49	-0.16	0.07	1.52	0.13	63.45	-2.15	6.79	6.74	15.55
os26745	23	516	0.41	15	2	0	6.32	-0.04	0.00	1.44	0.13	65.96	1.82	5.93	6.70	15.54
os26748	23	589	0.14	11	3	9	6.82	0.10	0.03	1.46	0.08	99.03	0.29	5.77	6.54	15.39
os26757	23	550	0.28	10	2	0	7.05	0.13	0.06	1.47	0.11	99.05	0.50	7.37	6.57	15.41
os26758	23	553	0.08	9	2	0	7.05	0.14	0.14	1.50	0.12	99.31	-1.86	7.66	6.54	15.38
os26763	27	588	0.92	5	3	6	6.68	-0.07	1.49	1.51	0.06	98.37	-1.35	7.43	6.51	15.35
os26786	27	634	0.10	5	2	3	6.70	-0.08	1.60	1.54	0.07	98.07	-2.94	9.42	6.05	14.85
os26792	28	587	0.92	4	3	6	6.71	-0.07	0.85	1.54	0.04	92.35	-0.40	8.80	6.32	15.11
os26812	23	599	0.30	10	3	5	7.01	0.09	0.22	1.50	0.08	96.91	-0.99	6.16	6.39	15.21
os26827	25	586	0.60	6	3	5	6.71	-0.03	1.10	1.54	0.06	92.80	-2.04	8.52	6.38	15.17
os26871	28	606	0.04	2	3	6	6.94	0.04	2.86	1.54	0.04	86.87	-0.60	8.57	6.26	15.07
os26873	23	622	0.03	5	3	5	6.99	0.09	0.44	1.50	0.03	93.35	0.39	6.65	6.28	15.10
os26874	28	614	0.60	2	3	6	6.84	0.00	1.61	1.52	0.03	87.02	-0.12	7.83	6.30	15.11
os26884	23	601	0.47	13	3	5	6.95	0.05	0.00	1.45	0.07	95.01	0.98	5.71	6.42	15.26
os26900	27	567	0.16	4	2	0	6.86	0.04	0.98	1.54	0.09	76.05	-1.12	7.40	6.41	15.23
os26908	23	609	0.21	10	3	5	6.79	0.08	0.04	1.45	0.06	95.45	1.20	5.83	6.40	15.24
os26909	25	603	0.13	12	3	5	6.76	0.09	0.01	1.45	0.08	93.97	0.77	5.94	6.40	15.24
os26924	23	582	0.21	10	2	3	6.98	0.07	0.11	1.46	0.08	98.38	0.06	7.29	6.47	15.28
os26927	23	605	0.85	2	3	9	6.81	-0.02	0.05	1.49	0.02	90.03	2.50	8.00	6.46	15.26
os26946	24	586	0.23	1	3	6	6.85	0.00	2.97	1.54	0.04	46.43	-0.15	8.55	6.42	15.27
os26947	28	587	0.88	1	3	6	6.82	-0.02	2.90	1.54	0.03	51.16	-0.33	8.18	6.42	15.26
os26973	23	618	0.56	10	3	5	6.90	0.01	0.12	1.49	0.11	100.00	-1.66	6.26	6.26	15.08
os26986	23	669	0.72	4	3	6	6.71	-0.07	0.30	1.51	0.03	99.88	0.52	6.75	6.12	14.97
os26987	28	656	0.90	4	3	6	6.75	-0.06	1.02	1.52	0.03	99.44	-0.22	7.30	6.14	14.99
os26999	19	531	0.92	5	2	7	6.63	-0.09	1.00	1.55	0.11	85.85	-3.31	7.46	6.60	15.41
os27016	24	666	0.58	3	3	6	6.82	-0.02	1.81	1.54	0.04	83.26	-0.53	7.80	6.06	14.92

os27039	23	518	0.77	4	3	6	6.79	-0.04	1.70	1.54	0.05	90.18	-0.75	9.08	6.83	15.66
os27045	24	555	0.92	3	3	6	6.73	-0.05	2.33	1.54	0.05	90.03	-0.42	8.51	6.53	15.34
os27052	28	567	0.92	2	3	6	6.78	-0.03	0.48	1.52	0.02	69.92	0.37	8.54	6.72	15.54
os27100	28	557	0.92	2	3	6	6.79	-0.02	0.99	1.54	0.01	32.56	0.02	8.76	6.81	15.65
os27143	23	613	0.62	8	1	1	6.57	-0.10	0.08	1.47	0.03	99.74	2.57	6.34	6.74	15.64
os27165	19	439	0.98	3	3	6	6.69	-0.06	0.16	1.54	0.02	80.47	1.08	8.01	7.45	16.30
os27189	23	554	0.23	9	3	5	6.76	0.07	0.08	1.50	0.09	99.86	-0.36	5.58	6.70	15.55
os27190	23	555	0.21	10	3	5	6.76	0.08	0.06	1.49	0.09	99.87	-0.48	5.56	6.70	15.55
os27192	28	617	0.27	2	3	6	6.81	-0.03	1.61	1.53	0.02	81.13	-0.08	7.88	6.38	15.24
os27193	28	616	0.92	2	3	6	6.81	-0.03	1.59	1.53	0.02	76.73	-0.06	8.00	6.38	15.24
os27208	28	602	0.92	0	3	6	6.86	0.00	2.05	1.54	0.00	26.57	0.07	11.09	6.51	15.34
os27217	27	584	0.77	5	3	5	6.67	-0.07	0.87	1.53	0.04	95.82	-1.12	7.75	6.47	15.30
os27225	27	584	0.76	10	2	7	6.41	-0.08	0.44	1.54	0.20	93.72	-10.77	7.94	6.26	15.11
os27227	23	606	0.74	10	3	5	6.44	-0.16	0.02	1.48	0.06	97.24	1.41	6.51	6.32	15.17
os27231	23	569	0.84	3	3	6	6.83	-0.02	1.74	1.54	0.02	65.37	-0.26	7.66	6.61	15.40
os27236	23	536	0.07	2	3	6	6.87	0.02	3.95	1.56	0.02	16.57	-0.02	9.63	6.69	15.53
os27240	23	592	0.00	1	3	6	6.90	0.02	2.95	1.55	0.01	28.97	-0.13	9.13	6.53	15.39
os27241	23	593	0.92	0	3	6	6.86	0.01	2.92	1.54	0.01	26.58	-0.18	9.43	6.53	15.39
os27244	23	541	0.01	5	3	5	6.97	0.09	0.83	1.54	0.06	94.22	-1.66	8.12	6.70	15.57
os27246	27	597	0.00	3	3	6	6.95	0.06	1.85	1.54	0.04	81.58	-0.79	8.53	6.46	15.36
os27249	23	596	0.92	1	3	6	6.85	-0.01	1.81	1.54	0.01	43.48	-0.21	8.29	6.53	15.39
os27263	27	524	0.47	4	2	3	6.80	0.01	1.77	1.55	0.04	89.97	-1.83	8.85	7.08	15.95
os27271	23	541	0.00	5	2	0	6.96	0.09	0.60	1.53	0.05	90.80	-0.75	7.76	6.84	15.68
os27310	19	519	0.00	9	2	3	6.50	-0.14	0.69	1.52	0.11	99.15	-4.67	8.70	6.92	15.71
os27329	23	583	0.40	4	3	6	6.96	0.04	0.40	1.51	0.03	97.50	0.64	7.12	6.63	15.44
os27366	23	530	0.10	13	3	5	7.07	0.18	0.00	1.46	0.09	83.55	1.65	6.06	6.67	15.47
os27370	23	579	0.10	8	3	5	7.04	0.11	0.21	1.48	0.04	98.06	2.06	6.23	6.53	15.35
os27374	23	604	0.62	8	3	5	6.85	-0.02	0.17	1.49	0.06	99.38	0.10	6.04	6.43	15.25
os27395	28	546	0.90	3	3	6	6.73	-0.05	1.70	1.55	0.04	92.61	-0.42	8.69	6.59	15.41
os27401	28	558	0.92	3	3	6	6.73	-0.05	1.30	1.55	0.03	91.78	0.04	7.91	6.55	15.37
os27405	28	606	0.23	2	3	6	6.87	0.02	1.43	1.54	0.02	57.92	-0.15	8.41	6.39	15.20
os27416	23	573	0.99	10	3	5	6.47	-0.16	0.03	1.49	0.07	90.22	0.28	6.90	6.43	15.23
os27417	27	581	0.99	9	2	3	6.53	-0.15	0.28	1.51	0.09	95.97	-2.37	7.50	6.41	15.21
os27422	23	542	0.99	3	3	6	6.78	-0.04	0.67	1.54	0.03	68.87	0.52	8.76	6.63	15.45
os27423	28	537	0.83	1	3	6	6.81	-0.02	2.92	1.56	0.04	59.95	-0.32	8.99	6.65	15.47
os27424	17	536	0.76	6	3	5	6.83	-0.02	0.37	1.53	0.07	67.95	-1.35	7.29	6.63	15.43
os27429	23	552	0.92	0	3	6	6.83	0.00	4.89	1.53	0.02	53.22	1.45	10.55	6.63	15.45
os27431	23	545	0.61	8	3	5	6.59	-0.06	0.43	1.53	0.06	60.43	-0.67	7.91	6.63	15.45
os27453	28	562	0.04	3	3	6	6.86	0.03	0.98	1.55	0.03	51.81	-0.30	7.81	6.47	15.27
os27498	28	610	0.90	4	3	6	6.69	-0.07	0.55	1.52	0.03	98.83	0.43	6.86	6.33	15.16
os27547	28	620	0.03	4	3	6	6.98	0.06	1.78	1.53	0.04	96.99	-1.37	8.62	6.16	14.98
os27549	28	611	0.98	3	3	6	6.81	-0.04	0.58	1.53	0.03	76.00	0.19	8.11	6.24	15.06
os27556	28	601	0.99	6	3	6	6.67	-0.09	0.71	1.54	0.06	81.10	-1.78	8.75	6.25	15.07

os27582	23	524	0.02	3	3	6	6.93	0.06	1.68	1.53	0.03	63.73	-0.50	8.33	7.31	16.19
os27589	28	618	0.82	10	3	5	6.44	-0.13	0.39	1.51	0.09	79.85	-2.22	6.76	6.30	15.16
os27619	27	490	0.01	9	2	3	6.92	0.13	0.56	1.53	0.10	91.01	-3.41	8.82	6.93	15.81
os27645	28	552	0.92	2	3	6	6.80	-0.03	3.87	1.56	0.05	42.07	-0.19	9.03	6.49	15.33
os27655	23	553	0.67	4	3	6	6.76	-0.04	0.32	1.51	0.03	79.11	1.14	7.63	6.80	15.73
os27668	28	560	0.42	5	3	6	6.80	0.02	1.28	1.55	0.04	36.77	-0.76	8.04	6.67	15.54
os27672	27	624	0.00	5	2	3	6.77	-0.01	1.37	1.54	0.12	97.72	-5.50	9.36	6.10	14.93
os27729	23	488	0.63	17	1	4	6.09	-0.13	0.00	1.43	0.09	89.32	3.30	5.96	7.03	15.88
os27732	23	491	0.79	8	1	4	6.54	-0.08	0.03	1.47	0.06	92.29	3.79	6.94	7.01	15.85
os27733	23	494	0.90	7	1	4	6.55	-0.10	0.05	1.47	0.06	94.04	3.63	6.78	7.00	15.85
os27739	23	558	0.08	3	3	6	6.92	0.04	0.70	1.54	0.02	52.22	0.12	7.96	6.75	15.63
os27766	17	501	0.08	2	3	6	6.87	0.03	3.24	1.55	0.04	71.13	-1.33	9.77	7.00	15.86
os27999	23	553	0.25	5	2	3	6.79	0.03	1.27	1.54	0.09	89.30	-3.63	8.12	7.05	15.99
os28040	27	554	0.92	3	3	6	6.78	-0.04	1.68	1.55	0.03	81.63	-0.32	8.56	7.06	15.95
os28049	23	572	0.42	3	3	6	6.91	0.02	0.32	1.52	0.02	99.58	0.66	6.84	7.04	15.94
os28083	28	575	0.60	4	3	6	6.74	-0.03	0.98	1.52	0.04	99.38	-0.42	6.95	6.91	15.81
os28088	23	572	0.60	4	3	6	6.74	-0.03	0.33	1.51	0.03	99.39	0.44	6.95	6.93	15.82
os28093	17	509	0.19	9	3	5	6.75	0.06	0.02	1.48	0.06	94.78	2.64	6.91	7.23	16.11
os28104	23	537	0.72	9	2	3	6.50	-0.10	0.32	1.51	0.08	93.28	-1.74	8.31	7.12	16.01
os28107	19	552	0.24	3	3	6	6.73	-0.06	1.70	1.54	0.04	88.46	-0.53	8.18	7.02	15.91
os28112	23	523	0.70	11	3	5	6.42	-0.10	0.03	1.48	0.07	92.75	0.25	7.09	7.15	16.03
os28113	23	522	0.69	11	3	5	6.41	-0.10	0.03	1.49	0.08	92.38	-0.46	7.13	7.15	16.03
os28115	23	592	0.06	2	3	6	6.91	0.03	1.42	1.52	0.03	85.50	-0.65	7.55	6.88	15.80
os28120	23	545	0.74	7	2	0	6.61	-0.07	0.26	1.52	0.06	94.35	-0.57	7.78	6.97	15.88
os28138	23	617	1.00	5	3	5	6.69	-0.09	0.78	1.53	0.04	92.33	-0.75	7.42	6.68	15.58
os28139	23	615	0.95	5	3	6	6.76	-0.07	0.70	1.53	0.04	94.03	-0.60	7.20	6.68	15.57
os28142	23	550	0.24	10	3	5	7.03	0.11	0.29	1.50	0.06	82.98	0.56	6.38	7.05	15.93
os28144	23	552	0.92	2	3	6	6.79	-0.03	0.21	1.52	0.03	95.75	1.39	7.96	7.05	15.95
os28150	25	487	0.08	3	2	7	6.90	0.04	2.41	1.55	0.07	83.44	-2.34	7.66	7.22	16.10
os28151	23	490	1.00	7	3	5	6.58	-0.11	0.26	1.52	0.06	76.99	0.03	7.75	7.21	16.09
os28152	28	513	0.56	3	3	6	6.86	0.01	2.90	1.54	0.03	80.87	0.79	8.30	7.19	16.07
os28155	28	517	0.96	5	3	6	6.68	-0.07	1.25	1.54	0.04	78.40	-0.34	8.61	7.18	16.06
os28163	23	519	0.77	2	3	6	6.85	0.00	0.32	1.54	0.03	66.72	0.86	9.27	7.10	16.01
os28166	23	593	0.92	6	3	5	6.63	-0.09	0.55	1.51	0.03	90.59	0.01	7.00	6.85	15.77
os28176	28	481	0.40	2	3	6	6.85	0.01	2.92	1.55	0.02	71.04	-0.39	8.86	7.44	16.31
os28187	28	497	0.92	1	3	6	6.78	-0.02	2.81	1.55	0.01	19.02	-0.02	9.05	7.37	16.24
os28200	28	499	0.92	0	3	6	6.82	0.00	3.90	1.55	0.02	6.41	-0.19	10.63	7.23	16.11
os28203	28	542	0.92	2	3	6	6.75	-0.04	0.62	1.54	0.02	61.52	0.37	7.90	7.14	16.05
os28204	28	541	0.92	1	3	6	6.85	0.01	2.78	1.54	0.02	38.70	-0.39	8.76	7.13	16.04
os28214	27	511	0.97	5	3	6	6.70	-0.07	1.21	1.54	0.04	93.06	-1.08	8.63	7.14	16.02
os28267	23	524	0.95	4	3	5	6.78	-0.04	1.53	1.54	0.06	92.32	-1.79	8.69	7.05	15.98
os28294	19	410	0.00	3	3	6	6.82	0.04	1.79	1.54	0.06	80.58	-1.20	8.57	7.75	16.64
os28305	23	512	0.89	3	3	6	6.76	-0.04	2.89	1.55	0.05	83.45	-2.03	9.05	7.13	16.03

os28375	27	477	0.08	6	2	3	6.96	0.09	1.03	1.53	0.08	79.67	-3.38	7.90	7.32	16.19
os28409	23	539	0.73	6	3	5	6.80	-0.04	0.23	1.51	0.03	96.60	0.73	6.69	6.89	15.76
os28411	23	518	0.04	10	3	5	6.84	0.10	0.37	1.50	0.07	91.94	-1.22	7.63	6.88	15.73
os28413	27	469	0.02	3	3	6	6.69	-0.05	2.27	1.55	0.04	87.24	-1.74	8.99	7.51	16.40
os28418	27	499	0.23	4	3	6	6.79	0.02	1.91	1.54	0.04	67.36	-1.75	9.10	7.39	16.29
os28422	23	504	0.81	7	2	3	6.72	-0.06	1.00	1.54	0.09	90.98	-3.34	7.63	7.21	16.08
os28451	28	459	0.92	1	3	6	6.84	0.02	3.94	1.56	0.03	30.94	-0.10	8.32	7.71	16.59
os28452	28	457	0.92	1	3	6	6.83	0.02	4.95	1.56	0.03	24.61	-0.42	8.51	7.72	16.60
os28555	23	552	0.92	1	3	6	6.83	0.00	0.24	1.50	0.01	83.07	1.04	8.64	6.82	15.70
os28579	23	538	0.43	6	3	5	6.74	0.00	0.83	1.52	0.05	74.57	0.81	8.75	6.81	15.69
os28584	28	572	0.92	0	3	6	6.86	0.00	2.00	1.55	0.01	18.07	0.22	8.94	6.64	15.56
os28642	27	628	0.08	3	3	6	6.95	0.04	2.64	1.54	0.03	68.34	-0.07	8.31	6.71	15.64
os28643	23	679	0.11	4	3	6	6.94	0.05	0.19	1.52	0.02	92.87	1.29	6.77	6.56	15.43
os28644	23	599	0.92	5	3	5	6.67	-0.07	0.66	1.53	0.07	96.07	-1.26	7.05	6.79	15.70
os28696	23	549	0.12	7	3	5	6.82	0.06	0.13	1.49	0.03	84.50	1.75	7.53	7.23	16.17
os28770	19	401	0.13	18	2	7	6.94	0.20	0.07	1.49	0.17	96.84	-6.08	7.97	8.13	17.13
os28784	19	490	0.67	5	2	3	6.66	-0.06	2.09	1.54	0.07	91.30	-2.52	8.60	7.94	16.97
os28785	23	495	0.92	5	3	6	6.63	-0.07	0.40	1.53	0.05	91.36	-0.28	8.24	7.93	16.97
os28810	23	640	0.92	1	3	6	6.84	-0.02	1.79	1.52	0.02	73.11	-0.08	8.57	6.81	15.74
os28815	23	525	0.92	6	3	5	6.59	-0.10	0.34	1.51	0.05	98.40	-0.34	7.82	7.48	16.44
os28839	27	499	0.09	4	2	3	6.84	0.04	1.66	1.55	0.08	88.77	-3.77	7.94	7.29	16.19
os28845	17	446	0.08	4	3	6	6.91	0.07	0.54	1.54	0.04	63.91	-0.57	9.09	7.61	16.48
os28847	17	448	0.00	4	3	6	6.90	0.07	0.44	1.54	0.04	66.95	0.32	8.98	7.62	16.49
os28850	27	535	0.97	2	3	6	6.79	-0.03	1.93	1.54	0.02	89.31	-0.43	8.11	7.33	16.23
os28854	19	467	0.18	8	2	3	6.91	0.05	0.77	1.51	0.10	98.70	-3.59	8.13	7.70	16.64
os28928	17	446	0.69	4	2	0	6.83	0.01	1.99	1.55	0.07	81.96	-2.89	7.76	7.71	16.59
os28970	19	436	0.92	2	3	6	6.70	-0.04	1.78	1.55	0.03	89.60	-0.88	8.56	7.81	16.71
os28986	24	461	0.77	1	3	6	6.80	-0.01	2.98	1.55	0.03	38.00	-0.64	8.73	7.64	16.54
os29033	24	588	0.60	5	3	5	6.70	-0.04	0.74	1.53	0.05	95.64	-0.54	7.32	6.99	15.88
os29039	24	594	0.47	5	3	5	6.87	-0.01	0.51	1.53	0.06	98.29	-1.40	7.12	6.95	15.87
os29045	23	623	0.13	3	3	6	6.77	-0.05	1.09	1.55	0.04	88.67	-0.14	8.03	6.72	15.64
os29050	27	601	0.17	2	3	6	6.88	0.02	2.66	1.55	0.04	62.62	-0.56	9.42	6.87	15.77
os29055	27	600	0.01	2	3	6	6.90	0.03	2.73	1.55	0.04	59.21	-0.38	9.46	6.87	15.77
os29068	19	436	0.58	11	3	5	6.48	-0.06	0.36	1.46	0.09	96.24	-0.78	7.05	8.03	17.05
os29128	19	596	0.90	3	3	6	6.74	-0.05	1.73	1.52	0.04	82.00	-0.40	8.02	7.13	16.09
os29132	23	609	0.60	2	3	6	6.82	-0.01	0.22	1.53	0.02	79.62	0.35	8.20	7.09	16.06
os29191	23	519	0.60	6	3	5	6.66	-0.03	0.14	1.51	0.05	98.70	0.42	5.92	7.35	16.31
os29235	23	527	0.71	4	2	3	6.73	-0.02	2.17	1.54	0.05	88.56	-2.02	9.05	7.57	16.56
os29250	23	544	0.01	6	3	5	6.92	0.10	0.36	1.52	0.04	94.12	0.41	7.20	7.46	16.46
os29276	27	575	0.18	4	2	0	6.88	0.04	2.01	1.55	0.06	92.69	-1.69	9.02	6.91	15.82
os29277	27	590	0.09	4	3	6	6.97	0.06	0.59	1.53	0.06	99.04	-0.07	7.23	6.92	15.82
os29278	27	575	0.92	2	2	7	6.87	0.02	2.61	1.56	0.06	94.25	-2.46	9.12	6.91	15.81
os29309	23	565	0.71	8	3	5	6.53	-0.10	0.21	1.48	0.06	89.81	1.02	6.64	7.19	16.13

os29329	23	612	0.75	4	3	6	6.72	-0.05	0.91	1.52	0.05	98.95	-0.27	7.65	6.91	15.83
os29332	23	539	0.23	4	2	0	6.80	0.02	0.52	1.54	0.05	80.84	0.05	7.89	7.24	16.13
os29356	28	487	1.00	5	3	5	6.63	-0.09	0.35	1.53	0.04	71.86	0.49	6.97	7.39	16.45
os29360	23	625	0.01	3	1	1	6.95	0.05	0.04	1.47	0.01	96.34	2.98	6.65	6.84	15.89
os29361	23	624	0.08	4	1	1	6.97	0.06	0.06	1.47	0.01	96.74	2.89	6.42	6.84	15.89
os29364	23	582	0.94	8	3	5	6.64	-0.11	0.47	1.50	0.06	98.00	-0.61	7.35	6.92	15.96
os29376	28	556	0.23	4	2	3	6.84	0.03	1.96	1.54	0.07	94.24	-2.34	8.54	6.89	15.91
os29393	23	590	0.77	6	1	4	6.81	-0.04	0.17	1.49	0.04	96.99	2.27	6.85	6.88	15.95
os29394	23	561	0.89	9	1	4	6.63	-0.12	0.09	1.47	0.05	92.81	2.68	6.27	6.97	16.03
os29400	23	649	0.01	6	1	1	7.01	0.09	0.03	1.48	0.02	98.83	3.56	6.59	6.70	15.73
os29403	23	569	0.23	9	2	7	6.76	0.04	0.51	1.52	0.07	98.11	-1.29	8.25	6.87	15.93
os29412	27	498	0.78	5	2	3	6.62	-0.04	1.22	1.54	0.12	93.07	-5.03	8.72	7.28	16.30
os29427	23	590	0.60	7	1	1	6.65	-0.04	0.01	1.44	0.03	98.58	5.53	5.60	7.05	16.10
os29430	23	618	0.07	7	3	9	7.05	0.12	0.33	1.49	0.04	95.83	0.29	6.34	6.89	15.95
os29436	23	614	0.92	1	3	6	6.83	-0.02	0.04	1.49	0.02	94.64	2.07	7.52	6.84	15.88
os29460	23	657	0.79	2	3	6	6.87	-0.02	0.39	1.54	0.01	55.03	0.63	8.69	6.55	15.60
os29464	23	630	0.27	6	1	1	6.74	0.00	0.13	1.49	0.04	92.23	2.59	6.14	6.86	15.94
os29468	24	574	0.73	7	3	5	6.61	-0.07	0.25	1.51	0.05	92.44	0.12	7.32	6.99	16.01
os29484	23	572	0.99	3	3	6	6.78	-0.04	1.55	1.54	0.03	89.69	-0.35	7.72	6.91	15.86
os29491	27	579	0.92	1	3	6	6.87	0.01	3.30	1.56	0.07	78.32	-1.42	8.07	6.66	15.61
os29492	28	605	0.08	2	3	6	6.93	0.03	2.35	1.55	0.05	82.44	-1.42	8.74	6.61	15.58
os29507	28	627	0.08	3	3	6	6.94	0.04	2.93	1.54	0.04	82.25	-0.99	9.15	6.31	15.21
os29512	27	559	0.92	2	3	6	6.79	-0.02	3.55	1.55	0.02	32.06	0.08	10.36	6.69	15.56
os29522	24	482	1.00	6	3	5	6.60	-0.10	0.76	1.53	0.04	84.88	-1.02	7.97	7.36	16.23
os29530	27	496	0.06	4	2	3	6.85	0.04	1.95	1.54	0.04	91.72	-2.14	8.74	7.17	16.03
os29531	19	459	0.19	3	2	3	6.84	0.01	1.92	1.55	0.05	82.85	-2.44	8.97	7.36	16.23
os29539	24	603	0.85	7	3	5	6.59	-0.10	0.49	1.52	0.06	80.20	-1.60	7.94	6.75	15.64
os29549	23	462	0.04	9	3	5	6.98	0.14	0.11	1.49	0.07	74.86	-0.35	7.02	7.35	16.22
os29554	28	502	0.23	1	3	6	6.82	0.01	1.53	1.55	0.01	35.83	-0.07	8.95	7.30	16.23
os29555	28	551	0.92	1	3	6	6.82	-0.01	4.22	1.54	0.01	18.33	0.17	9.23	7.06	16.04
os29608	23	587	0.92	4	3	6	6.71	-0.07	0.59	1.53	0.04	92.27	-0.41	7.36	6.84	15.84
os29620	23	555	1.00	10	2	7	6.47	-0.16	0.74	1.53	0.12	92.68	-4.76	6.83	6.82	15.79
os29621	23	554	0.99	11	1	4	6.50	-0.16	0.03	1.46	0.06	68.59	3.18	5.89	6.95	15.93
os29622	27	639	0.01	2	3	6	6.93	0.04	2.30	1.55	0.02	77.12	-0.71	8.87	6.57	15.60
os29634	23	588	0.04	4	3	6	6.97	0.07	0.64	1.53	0.04	71.03	-0.06	8.08	6.90	15.91
os29722	23	475	0.27	3	3	6	6.89	0.04	3.33	1.55	0.04	71.52	-1.03	8.60	7.27	16.16
os29742	27	467	0.34	4	2	3	6.71	-0.02	1.86	1.54	0.08	82.85	-3.28	9.29	7.32	16.17
os29749	28	495	0.79	1	3	6	6.81	-0.01	0.18	1.54	0.01	35.56	0.33	8.62	7.26	16.12
os29761	28	533	0.23	2	3	6	6.85	0.02	0.36	1.53	0.02	85.47	0.92	7.85	7.13	16.01
os29767	23	564	0.97	7	3	5	6.68	-0.10	0.37	1.50	0.05	99.11	-0.03	6.43	7.07	15.99
os29776	25	602	0.74	11	3	5	6.37	-0.14	0.01	1.46	0.07	89.69	1.47	6.31	6.84	15.82
os29790	28	495	0.08	2	3	6	6.89	0.04	2.71	1.55	0.03	44.86	-0.23	8.44	7.28	16.22
os29803	23	490	0.39	10	3	5	6.47	-0.15	0.20	1.50	0.06	65.06	-0.18	7.35	7.26	16.16

os29812	28	488	0.92	2	3	6	6.75	-0.04	2.18	1.55	0.02	58.43	-0.38	8.63	7.31	16.22
os29818	23	473	0.99	5	3	6	6.66	-0.08	1.15	1.54	0.05	69.81	-1.37	8.13	7.28	16.17
os29826	28	467	0.00	3	2	7	6.73	-0.03	3.19	1.56	0.08	92.26	-4.30	8.94	7.25	16.14
os29899	23	548	0.00	6	3	5	6.96	0.09	0.93	1.54	0.05	60.79	-0.42	8.72	6.86	15.80
os29902	28	508	0.92	2	3	6	6.84	0.00	0.65	1.53	0.02	66.98	1.15	8.77	7.21	16.16
os29903	28	520	0.92	0	3	6	6.83	0.00	5.70	1.56	0.02	43.01	-0.17	9.99	7.10	16.07
os29911	23	527	0.01	7	3	5	6.94	0.09	1.67	1.54	0.07	86.43	-2.64	8.70	7.09	16.09
os29913	23	541	0.99	10	1	4	6.43	-0.17	0.02	1.43	0.06	76.34	3.73	5.93	7.06	15.96
os29914	28	552	0.77	1	3	6	6.85	0.00	0.23	1.53	0.02	71.82	1.11	8.85	7.06	15.96
os29926	28	579	0.08	1	3	6	6.88	0.01	4.64	1.56	0.04	62.77	-0.55	9.30	6.86	15.75
os29939	23	521	0.12	3	2	3	6.91	0.05	1.87	1.54	0.05	85.76	-1.96	8.61	7.19	16.23
os29966	24	610	0.23	4	2	7	6.85	0.03	1.86	1.54	0.08	91.15	-2.85	7.30	6.62	15.59
os29967	24	612	0.01	4	2	7	6.90	0.04	1.92	1.54	0.08	91.36	-2.53	7.38	6.61	15.59
os29968	28	581	0.77	4	3	6	6.84	-0.03	0.94	1.54	0.04	62.62	-0.63	8.15	6.90	15.83
os29989	23	536	0.05	15	2	3	7.05	0.22	0.00	1.46	0.11	89.89	-0.76	7.32	7.07	16.05
os29992	25	525	0.89	4	2	0	6.75	-0.05	0.51	1.51	0.07	68.95	1.49	8.52	7.15	16.13
os29997	23	589	0.95	5	3	5	6.72	-0.08	0.25	1.53	0.04	94.28	0.53	6.92	6.85	15.85
os30003	27	589	0.99	7	3	5	6.65	-0.11	0.33	1.52	0.05	87.81	-0.37	7.17	6.82	15.82
os30010	23	528	0.23	5	3	5	6.95	0.06	1.44	1.48	0.05	96.53	-1.56	8.69	7.22	16.22
os30019	27	552	0.82	6	2	0	6.77	-0.05	1.34	1.54	0.07	93.63	-2.20	8.51	6.90	15.86
os30069	27	601	0.59	3	3	6	6.78	-0.02	1.59	1.53	0.03	89.00	-1.10	7.12	6.86	15.80
os30072	23	617	0.69	7	1	4	6.63	-0.06	0.04	1.47	0.05	98.00	2.81	5.67	6.81	15.79
os30078	23	525	0.05	9	3	5	7.02	0.14	0.47	1.51	0.07	99.71	-1.45	8.01	7.07	16.00
os30081	23	485	1.00	9	3	5	6.50	-0.15	0.10	1.50	0.07	69.96	-0.89	7.90	7.28	16.22
os30084	23	558	0.04	3	3	6	6.88	0.04	0.69	1.54	0.04	79.48	-0.33	7.23	6.84	15.78
os30093	23	499	0.23	11	3	5	6.67	0.07	0.02	1.48	0.09	99.40	-1.87	7.93	7.20	16.14
os30097	23	558	0.08	4	1	1	6.96	0.06	0.10	1.45	0.03	99.17	2.87	6.08	7.16	16.10
os30098	23	544	0.13	11	3	9	6.79	0.11	0.01	1.47	0.07	99.79	-0.56	6.51	7.16	16.09
os30099	23	545	0.21	9	1	1	6.98	0.07	0.05	1.45	0.05	98.31	2.31	5.93	7.16	16.09
os30106	23	559	0.99	7	3	5	6.66	-0.10	0.37	1.50	0.06	98.42	-0.62	6.46	6.89	15.82
os30111	23	512	0.09	8	3	5	6.83	0.08	0.13	1.48	0.07	97.83	0.72	5.85	7.18	16.12
os30114	23	549	0.25	10	2	3	6.68	0.04	0.11	1.50	0.11	95.17	-2.24	6.75	6.97	15.93
os30115	23	582	0.36	9	3	5	6.69	0.02	0.06	1.50	0.07	88.28	-0.24	7.14	6.77	15.73
os30121	23	615	0.11	8	3	5	6.89	0.09	0.14	1.49	0.04	96.59	1.06	6.12	6.69	15.67
os30126	23	567	0.00	11	3	9	7.02	0.18	0.02	1.46	0.06	99.98	0.87	5.83	7.11	16.07
os30132	23	460	0.12	12	2	7	6.78	0.12	0.32	1.48	0.12	89.91	-2.45	6.41	7.36	16.30
os30136	23	593	0.61	7	3	5	6.68	-0.04	0.61	1.52	0.06	89.21	-0.91	8.53	6.70	15.69
os30145	23	441	0.49	9	2	0	6.83	0.01	0.10	1.52	0.12	89.83	-1.97	6.12	7.50	16.47
os30147	23	560	0.70	8	3	5	6.53	-0.09	0.06	1.45	0.07	83.97	1.85	5.63	6.99	15.98
os30165	28	549	0.92	5	3	5	6.65	-0.09	0.81	1.53	0.06	98.26	-1.91	7.47	6.88	15.81
os30169	28	559	0.08	3	3	6	6.92	0.04	2.61	1.55	0.04	71.22	-0.47	8.42	6.72	15.65
os30172	23	541	0.13	10	1	4	6.77	0.07	0.07	1.48	0.05	82.03	1.12	6.85	6.93	15.84
os30174	23	610	0.76	6	2	3	6.89	0.00	1.36	1.54	0.07	94.21	-2.53	8.48	6.68	15.63

os30192	23	460	0.66	17	3	5	6.05	-0.15	0.00	1.45	0.14	95.94	-2.30	6.01	7.59	16.61
os30193	23	466	0.66	21	3	5	5.85	-0.18	0.00	1.42	0.14	96.20	0.44	5.39	7.59	16.61
os30210	23	560	0.08	14	1	1	6.82	0.16	0.00	1.44	0.06	99.82	3.34	5.15	7.12	16.10
os30214	23	579	0.89	6	2	7	6.70	-0.04	1.87	1.53	0.10	99.61	-4.62	8.36	6.79	15.75
os30221	23	544	0.83	17	1	4	6.18	-0.14	0.01	1.45	0.10	88.44	1.69	5.96	6.94	15.95
os30241	27	595	0.03	2	3	6	6.93	0.04	2.36	1.54	0.04	70.15	-0.73	8.81	6.72	15.70
os30245	23	653	0.54	3	3	9	6.82	-0.01	0.07	1.50	0.01	93.64	1.27	6.92	6.59	15.60
os30256	23	585	0.92	6	1	4	6.66	-0.07	0.02	1.45	0.04	94.67	4.37	6.13	6.96	15.97
os30264	23	579	0.99	5	3	5	6.71	-0.08	0.66	1.52	0.04	97.99	-0.22	6.79	6.91	15.92
os30265	23	585	0.84	4	3	6	6.77	-0.06	0.41	1.52	0.03	97.76	0.41	6.49	6.91	15.92
os30268	23	560	0.80	6	1	4	6.63	-0.07	0.13	1.49	0.04	91.50	3.08	6.59	7.03	16.04
os30269	23	560	1.00	9	1	4	6.53	-0.14	0.11	1.49	0.04	93.77	2.23	6.72	7.04	16.04
os30273	23	595	0.01	7	1	1	6.99	0.12	0.07	1.48	0.04	95.61	2.06	5.94	6.95	15.99
os30295	27	533	0.63	4	2	7	6.73	-0.02	1.91	1.55	0.10	83.22	-3.26	7.85	7.09	16.09
os30296	27	533	0.53	3	2	7	6.74	-0.01	2.18	1.55	0.09	82.72	-2.94	7.96	7.08	16.09
os30347	23	588	0.04	5	3	9	6.88	0.05	0.55	1.50	0.03	96.56	-0.40	6.53	6.85	15.79
os30364	23	532	0.78	3	2	3	6.83	-0.01	1.87	1.55	0.07	93.47	-3.01	8.31	6.99	15.96
os30378	23	520	0.32	13	3	5	6.33	-0.18	0.11	1.50	0.08	83.23	-0.59	6.91	7.03	15.96
os30392	23	547	0.35	10	1	4	6.61	0.00	0.01	1.46	0.06	92.41	2.28	5.63	7.06	16.06
os30395	23	584	0.01	10	3	5	7.05	0.16	0.07	1.49	0.06	97.46	1.22	6.33	6.85	15.87
os30408	27	523	0.95	4	3	6	6.72	-0.06	0.74	1.56	0.06	58.18	-1.00	8.44	6.90	15.80
os30410	23	511	1.00	16	2	3	6.26	-0.22	0.01	1.48	0.10	82.08	-1.44	6.51	7.03	15.92
os30415	23	509	0.99	9	2	3	6.46	-0.15	0.74	1.53	0.13	88.50	-5.46	7.62	7.02	15.93
os30423	23	601	0.34	11	1	1	7.00	0.08	0.00	1.37	0.06	100.00	5.08	5.35	6.93	15.84
os30460	23	638	0.19	8	1	1	7.05	0.10	0.04	1.43	0.04	98.46	3.27	5.58	6.60	15.50
os30461	23	603	0.98	12	1	1	6.50	-0.17	0.01	1.42	0.05	99.76	3.95	5.45	6.74	15.63
os30462	23	606	0.35	6	1	1	6.77	-0.03	0.01	1.41	0.02	99.82	7.36	5.98	6.73	15.62
os30467	25	608	0.97	12	1	1	6.38	-0.19	0.00	1.39	0.05	99.94	5.08	5.34	6.77	15.67
os30475	23	590	0.23	15	3	9	7.05	0.19	0.00	1.39	0.10	100.00	1.67	5.38	6.94	15.84
os30495	23	558	0.20	12	2	3	6.72	0.09	0.05	1.46	0.12	100.00	-3.23	5.77	6.99	15.90
os30500	23	592	0.41	5	1	4	6.76	-0.01	0.11	1.50	0.03	97.44	2.09	6.30	6.85	15.85
os30504	24	518	0.60	3	2	0	6.74	-0.02	1.48	1.55	0.06	77.54	-0.91	8.87	7.08	16.05
os30506	23	590	0.84	7	3	5	6.59	-0.10	0.26	1.50	0.06	99.86	0.58	6.16	6.86	15.85
os30507	28	629	0.24	4	3	5	6.75	-0.07	1.52	1.52	0.05	92.06	-1.99	7.61	6.70	15.70
os30509	28	640	0.99	4	3	9	6.82	-0.05	0.81	1.49	0.03	95.87	0.23	6.71	6.72	15.70
os30514	23	553	0.22	8	2	3	7.02	0.11	0.57	1.52	0.10	98.72	-3.58	7.67	6.97	16.00
os30525	23	532	0.99	4	3	6	6.74	-0.06	0.86	1.54	0.04	63.30	-0.73	8.62	7.05	16.00
os30530	23	511	0.20	9	3	5	6.75	0.05	0.05	1.51	0.06	79.46	-0.22	7.23	7.00	15.93
os30542	23	583	0.03	4	3	6	6.95	0.06	0.43	1.53	0.03	98.11	0.24	6.91	6.64	15.57
os30552	23	575	0.22	11	3	9	7.02	0.11	0.01	1.46	0.06	92.26	2.16	5.62	7.04	16.04
os30571	23	625	0.23	5	3	5	6.85	0.04	0.77	1.53	0.06	99.18	-0.47	6.56	6.05	14.85
os30588	23	612	0.01	6	3	5	6.99	0.09	0.52	1.52	0.04	96.60	-0.69	6.62	6.43	15.34
os30594	23	706	0.01	8	3	5	7.04	0.14	0.09	1.50	0.05	99.12	0.77	6.25	5.78	14.66

os30603	27	619	0.01	4	3	6	6.74	-0.07	0.62	1.52	0.04	98.38	-0.43	6.89	6.30	15.21
os30607	23	632	0.02	5	3	5	6.93	0.07	0.36	1.52	0.04	92.04	0.72	8.36	6.21	15.09
os30608	23	629	0.13	5	3	6	7.00	0.06	1.04	1.53	0.05	90.74	-0.63	9.63	6.24	15.12
os30618	23	586	0.17	13	3	5	6.76	0.11	0.02	1.46	0.06	64.40	1.55	6.42	6.51	15.40
os30621	28	634	0.92	6	3	5	6.67	-0.09	0.35	1.53	0.06	95.10	-0.36	9.00	6.16	15.02
os30630	28	619	0.03	2	3	6	6.79	-0.03	2.74	1.55	0.04	87.82	-1.21	8.86	6.31	15.18
os30661	23	536	0.23	7	3	5	6.82	0.05	0.50	1.52	0.05	89.71	-0.54	8.40	6.82	15.73
os30668	23	528	0.92	6	3	5	6.62	-0.08	0.51	1.53	0.06	74.62	-0.73	7.90	6.88	15.79
os30685	23	504	0.52	9	2	0	6.60	-0.02	0.07	1.51	0.08	84.46	-0.01	8.00	7.03	15.94
os30693	28	640	0.73	4	2	7	6.72	-0.07	1.71	1.55	0.09	98.85	-2.03	8.71	5.95	14.78
os30701	28	682	0.01	6	2	0	7.03	0.09	0.99	1.54	0.07	99.51	-1.81	8.38	5.88	14.78
os30703	23	690	0.00	7	3	5	7.05	0.12	0.28	1.52	0.07	99.76	-0.32	6.25	5.88	14.77
os30711	23	636	0.01	2	2	0	6.92	0.03	2.82	1.56	0.08	84.99	-1.33	7.93	6.14	15.04
os30715	23	640	0.81	7	2	0	6.79	-0.07	0.54	1.52	0.09	99.56	-1.01	7.18	6.00	14.81
os30717	23	675	0.30	10	3	5	7.06	0.10	0.03	1.47	0.08	99.67	0.68	5.88	5.90	14.72
os30719	23	631	0.40	15	3	9	6.49	0.01	0.00	1.40	0.10	99.92	1.29	5.51	6.36	15.21
os30720	23	543	0.79	9	2	7	6.45	-0.13	0.10	1.51	0.13	92.52	-2.51	7.12	6.53	15.35
os30721	23	642	0.09	11	3	5	7.11	0.17	0.02	1.45	0.07	99.58	1.39	5.73	6.30	15.16
os30722	23	566	0.99	9	2	7	6.51	-0.15	0.20	1.53	0.12	93.71	-3.53	7.32	6.28	15.12
os30725	28	630	1.00	6	2	7	6.72	-0.08	1.14	1.54	0.12	97.72	-3.37	8.22	6.04	14.89
os30726	23	639	0.77	4	2	0	6.85	-0.03	1.78	1.54	0.06	84.42	-0.65	8.30	6.14	15.04
os30728	23	660	0.77	9	1	1	6.85	-0.04	0.11	1.47	0.04	99.44	2.14	5.88	6.37	15.28
os30729	28	605	0.92	1	2	0	6.86	-0.01	3.90	1.56	0.05	72.36	-0.95	8.67	6.39	15.30
os30738	23	547	0.43	7	3	5	6.67	0.00	0.21	1.47	0.05	94.86	1.37	6.10	7.01	15.92
os30739	23	513	0.14	12	3	5	6.77	0.10	0.18	1.48	0.07	88.18	0.77	6.71	7.06	15.96
os30741	23	514	0.23	4	1	4	6.77	0.00	0.03	1.44	0.02	89.72	4.44	6.78	7.18	16.12
os30744	23	503	0.47	12	2	3	6.51	-0.05	0.21	1.51	0.09	95.52	-1.42	6.82	7.16	16.07
os30758	28	509	0.34	6	2	3	6.79	0.03	1.50	1.54	0.07	89.09	-1.84	8.32	7.05	15.97
os30763	28	547	0.08	2	3	6	6.89	0.02	2.77	1.56	0.05	80.91	-1.15	8.35	6.72	15.62
os30772	23	623	0.37	7	1	4	6.98	0.05	0.04	1.48	0.04	89.25	2.93	6.65	6.46	15.38
os30773	23	614	0.00	10	3	5	7.01	0.17	0.01	1.50	0.06	89.82	0.67	6.03	6.47	15.39
os30777	23	590	0.69	10	3	5	6.82	-0.04	0.08	1.49	0.07	94.68	1.01	7.46	6.50	15.41
os30778	23	590	0.62	10	3	5	6.85	-0.02	0.06	1.50	0.07	94.09	-0.26	7.84	6.49	15.40
os30785	27	648	0.07	2	3	6	6.93	0.03	2.97	1.55	0.05	78.63	-0.67	8.57	6.13	15.02
os30792	23	595	0.60	4	1	1	6.77	-0.01	0.13	1.48	0.02	93.09	2.59	6.24	6.78	15.71
os30802	23	538	0.07	7	3	5	6.84	0.07	0.32	1.49	0.04	91.79	1.25	6.43	7.10	16.10
os30806	23	555	0.48	13	3	5	6.49	-0.02	0.01	1.45	0.08	99.81	0.38	6.11	6.99	15.98
os30811	23	513	0.80	10	2	3	6.43	-0.12	0.05	1.51	0.10	92.74	-4.11	7.20	7.20	16.19
os30817	23	580	0.60	6	2	3	6.67	-0.05	0.82	1.50	0.11	99.97	-1.80	6.85	6.52	15.37
os30818	23	586	0.95	13	3	5	6.44	-0.19	0.01	1.48	0.09	100.00	-0.91	5.80	6.47	15.32
os30819	23	638	1.00	15	3	9	6.26	-0.24	0.00	1.42	0.08	99.89	0.97	5.63	6.42	15.27
os30835	28	685	1.00	5	3	5	6.75	-0.08	0.81	1.52	0.07	97.53	-1.34	7.47	5.98	14.83
os30838	23	581	0.01	16	2	0	7.01	0.25	0.00	1.47	0.14	99.89	-1.36	6.87	6.33	15.23

os30844	23	655	0.69	13	3	5	6.85	-0.02	0.00	1.45	0.10	99.77	-0.61	5.73	6.16	15.07
os30850	25	505	0.30	13	2	7	6.55	0.04	0.24	1.50	0.16	94.84	-4.04	7.60	6.75	15.63
os30855	23	547	0.47	7	2	7	6.91	0.04	0.86	1.50	0.12	99.38	-5.16	7.92	6.75	15.62
os30862	28	584	0.51	7	2	0	6.96	0.05	0.60	1.48	0.11	99.98	0.04	7.80	6.49	15.37
os30865	28	627	0.96	12	3	5	6.53	-0.16	0.01	1.46	0.11	100.00	-0.88	6.06	6.30	15.20
os30869	23	588	0.29	14	1	1	7.04	0.14	0.00	1.36	0.09	96.15	2.69	5.26	6.83	15.70
os30880	23	675	0.12	4	3	9	6.77	-0.06	1.87	1.41	0.04	97.09	-1.16	7.59	6.53	15.44
os30887	23	424	0.99	20	2	7	5.73	-0.34	0.00	1.43	0.24	95.76	-4.43	5.36	7.03	15.93
os30896	23	536	0.12	9	2	3	6.79	0.07	0.48	1.49	0.10	96.13	-3.85	7.77	6.86	15.81
os30898	23	482	0.19	14	2	7	6.69	0.12	0.01	1.51	0.13	82.69	-3.63	5.84	7.14	16.11
os30899	23	485	0.17	14	2	7	6.65	0.13	0.04	1.49	0.14	85.18	-4.04	6.04	7.12	16.09
os30912	23	590	0.51	9	1	1	6.89	0.00	0.02	1.44	0.04	100.00	3.56	5.47	6.85	15.78
os30919	23	574	0.27	7	2	3	7.00	0.08	0.73	1.51	0.10	96.01	-3.70	8.03	6.64	15.57
os30922	23	580	0.03	9	3	5	6.90	0.10	0.06	1.48	0.07	88.77	1.95	7.43	6.60	15.52
os30923	23	589	0.32	5	2	3	6.96	0.04	1.84	1.53	0.09	92.87	-1.68	8.05	6.58	15.48
os30926	23	590	0.48	12	3	5	6.52	-0.02	0.01	1.46	0.09	96.28	0.39	6.35	6.64	15.53
os30975	23	515	0.62	16	1	4	6.79	0.01	0.00	1.39	0.09	99.74	4.71	5.28	7.14	16.04
os30979	23	459	0.94	25	2	0	5.77	-0.31	0.00	1.41	0.19	95.43	-2.76	5.48	7.20	16.11
os30983	23	521	1.00	18	3	5	6.05	-0.28	0.00	1.45	0.13	99.91	-0.64	5.81	7.01	15.94
os30984	23	519	0.99	17	3	5	6.06	-0.28	0.00	1.45	0.13	99.88	-0.69	5.82	7.01	15.94
os30990	23	618	0.24	8	3	9	7.03	0.09	0.24	1.45	0.08	99.98	-0.88	6.09	6.56	15.43
os31022	23	575	0.85	23	2	8	6.21	-0.21	0.00	1.39	0.18	99.99	-4.98	5.30	6.89	15.76
os31036	23	620	0.00	7	1	1	6.97	0.06	0.00	1.39	0.01	97.80	9.04	6.21	6.66	15.55
os31044	23	570	0.45	12	3	5	6.54	0.01	0.01	1.48	0.10	92.16	-1.79	6.90	6.69	15.56
os31047	23	561	0.10	13	3	5	6.81	0.14	0.01	1.48	0.09	89.88	-2.19	6.81	6.73	15.61
os31060	23	552	0.81	16	3	5	6.12	-0.20	0.00	1.45	0.13	99.85	-1.12	5.85	6.83	15.74
os31067	23	512	1.00	20	3	5	5.88	-0.33	0.00	1.39	0.13	99.33	1.95	5.34	7.09	15.99
os31073	23	653	0.75	5	1	1	6.69	-0.07	0.09	1.46	0.03	99.44	2.07	5.94	6.41	15.35
os31078	23	587	0.07	13	3	9	6.25	-0.21	0.00	1.43	0.07	96.57	1.35	5.51	6.77	15.64
os31080	28	594	0.23	5	3	6	6.79	0.02	0.41	1.51	0.05	99.19	0.67	6.87	6.54	15.44
os31081	23	544	0.76	17	1	2	6.16	-0.16	0.00	1.44	0.10	94.98	2.68	6.78	6.65	15.54
os31082	23	543	0.77	18	2	0	6.05	-0.19	0.00	1.46	0.13	95.04	0.13	6.98	6.65	15.54
os31086	23	618	0.61	9	1	4	6.58	-0.05	0.01	1.44	0.04	99.89	4.55	5.50	6.53	15.43
os31087	23	612	0.28	13	3	5	6.66	0.07	0.00	1.44	0.07	99.85	2.24	5.47	6.53	15.43
os31088	23	620	0.34	12	3	5	6.69	0.05	0.01	1.45	0.07	99.94	0.87	5.81	6.51	15.41
os31097	23	624	0.45	6	3	9	6.90	0.00	0.57	1.48	0.08	99.95	-1.00	6.45	6.52	15.47
os31109	27	646	0.17	7	2	7	6.95	0.05	0.80	1.54	0.11	92.40	-3.63	7.71	6.57	15.59
os31135	23	652	0.08	8	3	5	7.08	0.14	0.08	1.48	0.04	99.79	1.81	6.03	6.67	15.71
os31137	28	523	0.96	3	3	6	6.78	-0.04	0.55	1.53	0.02	87.73	0.53	8.19	7.14	16.12
os31170	23	521	0.08	4	3	6	6.94	0.06	2.37	1.55	0.04	70.81	-1.41	8.88	7.24	16.34
os31171	28	525	0.08	1	3	6	6.84	0.00	2.95	1.56	0.03	76.70	-1.59	8.97	7.20	16.29
os31172	28	524	0.77	2	3	6	6.81	-0.02	2.97	1.55	0.03	67.34	-0.70	9.38	7.23	16.32
os31174	23	531	0.43	14	2	0	6.52	0.02	0.01	1.50	0.11	76.71	-2.35	7.58	7.07	16.02

os31259	28	622	0.39	5	3	5	6.80	0.00	0.92	1.54	0.05	72.20	-1.62	7.51	6.76	15.71
os31261	28	621	0.60	3	3	6	6.81	-0.01	2.79	1.55	0.04	58.97	-0.71	8.02	6.75	15.71
os31273	28	637	0.77	4	3	6	6.81	-0.05	1.70	1.54	0.06	99.67	-1.80	8.29	6.62	15.62
os31308	28	631	0.24	7	3	5	6.81	0.03	0.53	1.52	0.06	93.41	-0.66	6.84	6.65	15.63
os31314	28	581	0.77	4	2	7	6.82	-0.03	2.48	1.55	0.08	92.37	-2.45	7.76	6.84	15.84
os31321	23	606	0.61	10	2	3	6.53	-0.07	0.19	1.52	0.08	93.17	-2.81	7.86	6.65	15.64
os31322	23	603	0.50	6	2	7	6.69	-0.04	1.19	1.55	0.08	92.86	-3.93	8.18	6.66	15.65
os31327	23	639	0.66	7	3	5	6.59	-0.12	0.30	1.51	0.05	88.61	0.01	7.01	6.55	15.54
os31516	23	589	0.81	10	3	5	6.44	-0.13	0.05	1.50	0.08	89.73	-0.94	7.44	6.78	15.79
os31526	23	574	0.40	10	3	5	6.45	-0.15	0.10	1.49	0.08	99.85	-0.92	5.99	7.01	16.05
os31532	23	666	0.99	2	1	1	6.84	-0.03	0.06	1.48	0.01	98.10	2.26	6.74	6.73	15.75
os31534	23	624	0.56	3	3	6	6.92	0.01	0.19	1.52	0.03	94.57	1.05	7.28	6.74	15.75
os31549	24	648	0.83	2	3	6	6.86	-0.02	2.89	1.55	0.03	68.52	-0.94	9.05	6.51	15.53
os31550	23	654	0.88	2	3	6	6.84	-0.03	2.34	1.55	0.02	47.78	-0.43	8.87	6.53	15.54
os31553	23	629	0.23	5	3	5	6.88	0.05	0.39	1.52	0.03	82.36	1.15	8.00	6.66	15.71
os31599	28	594	0.75	7	3	5	6.60	-0.08	0.51	1.50	0.06	92.22	-0.93	7.85	6.93	15.96
os31612	23	613	0.90	1	3	6	6.85	-0.02	2.99	1.56	0.05	71.77	-1.30	8.58	6.74	15.77
os31613	28	619	0.92	1	3	6	6.88	0.00	2.92	1.55	0.04	70.74	-0.89	8.38	6.76	15.79
os31624	28	603	0.09	6	3	5	7.01	0.08	0.17	1.50	0.07	95.57	1.03	6.18	6.76	15.79
os31659	23	536	0.00	10	3	5	6.98	0.16	0.03	1.47	0.06	93.86	0.79	5.93	7.26	16.34
os31667	28	521	0.99	3	3	6	6.79	-0.03	1.58	1.53	0.03	92.65	0.53	8.12	7.27	16.34
os31668	28	520	0.93	4	3	6	6.79	-0.03	2.23	1.53	0.03	92.56	0.41	8.10	7.27	16.34
os31678	28	622	0.79	2	3	6	6.84	-0.02	2.80	1.55	0.04	60.46	-0.50	8.78	6.52	15.52
os31690	28	539	0.77	3	3	6	6.83	-0.02	2.75	1.56	0.04	63.75	-0.55	9.23	7.02	16.06
os31691	24	538	0.53	3	3	6	6.85	-0.01	2.50	1.56	0.04	63.31	-1.06	9.27	7.03	16.06
os31692	28	538	0.77	4	3	6	6.81	-0.03	1.83	1.55	0.04	65.84	-1.34	9.17	7.02	16.06
os31693	28	539	0.92	5	3	5	6.77	-0.05	1.20	1.54	0.04	67.53	-1.23	9.08	7.02	16.06
os31694	28	540	0.91	5	3	5	6.74	-0.06	1.00	1.54	0.04	70.54	-0.63	8.91	7.02	16.06
os31708	23	552	0.00	6	3	5	6.98	0.10	0.59	1.52	0.05	94.61	0.13	7.83	6.96	15.94
os31746	17	527	0.99	12	3	5	6.50	-0.15	0.45	1.51	0.10	85.94	-2.66	6.42	7.14	16.19
os31747	17	532	0.08	13	3	5	7.05	0.20	0.05	1.48	0.09	87.14	-0.68	6.41	7.14	16.18
os31767	23	560	0.05	6	3	5	6.61	-0.11	0.10	1.50	0.05	95.11	1.55	6.13	7.13	16.18
os31768	23	603	0.08	7	3	9	6.93	0.10	0.11	1.48	0.04	97.42	1.24	5.99	7.00	16.04
os31770	23	576	0.25	10	1	4	6.71	0.06	0.01	1.45	0.07	91.42	2.38	5.85	7.05	16.09
os31773	23	542	0.82	13	1	4	6.20	-0.18	0.01	1.42	0.07	83.30	2.95	5.75	7.27	16.34
os31781	23	536	0.97	5	3	6	6.73	-0.06	0.47	1.51	0.03	87.10	0.20	7.33	7.27	16.36
os31809	23	595	0.59	11	2	3	6.52	-0.05	0.13	1.49	0.08	99.78	-1.83	6.82	7.38	16.42
os31820	27	642	0.86	3	3	6	6.84	-0.03	1.92	1.52	0.06	95.76	-2.03	8.29	6.53	15.54
os31829	28	535	0.79	4	3	6	6.78	-0.05	0.80	1.55	0.05	89.82	-1.39	7.22	7.03	16.02
os31830	28	622	0.77	2	3	6	6.85	-0.02	2.89	1.55	0.04	60.02	-0.50	8.82	6.52	15.52
os31840	23	628	0.57	7	3	5	6.93	0.01	0.48	1.52	0.06	94.73	-0.91	6.96	7.01	16.01
os31854	23	594	1.00	5	3	5	6.69	-0.09	0.37	1.50	0.04	98.30	0.73	6.54	7.22	16.21
os31857	23	614	0.34	8	3	5	6.77	0.03	0.27	1.51	0.06	94.49	-0.20	6.27	6.88	15.84

os31858	23	588	0.76	1	3	6	6.86	-0.01	3.91	1.55	0.03	30.87	0.04	9.43	6.98	15.93
os31860	23	678	0.00	10	3	5	7.04	0.16	0.03	1.49	0.06	85.29	1.28	6.20	6.61	15.56
os31861	23	540	1.00	5	2	0	6.68	-0.08	0.61	1.53	0.07	81.83	-0.51	8.98	7.35	16.32
os31886	22	465	0.42	13	2	3	6.92	0.10	0.01	1.49	0.13	88.03	-3.41	6.73	7.15	16.00
os31897	28	513	0.71	3	3	6	6.85	0.00	2.87	1.54	0.03	80.85	0.92	8.29	7.19	16.07
os31901	23	591	0.10	11	3	5	6.89	0.14	0.03	1.49	0.08	99.76	-0.74	6.04	6.47	15.35
os31906	23	546	0.60	7	3	5	6.59	-0.07	0.40	1.52	0.06	94.43	-0.87	7.55	6.97	15.88
os31909	27	575	0.23	3	2	7	6.86	0.02	2.42	1.56	0.06	94.15	-2.31	9.10	6.91	15.81
os31915	27	502	0.37	3	3	5	6.89	0.03	2.15	1.55	0.10	93.46	-2.76	9.12	7.26	16.28
os31919	27	555	0.21	6	2	7	6.98	0.08	0.93	1.50	0.11	99.66	-3.11	7.93	6.73	15.61
os31974	12	179	0.09	1	3	6	6.72	0.01	6.71	1.56	0.01	6.31	-0.51	9.00	9.48	18.18
os31976	12	187	0.92	0	3	6	6.69	0.00	4.13	1.55	0.00	32.52	0.44	10.10	9.47	18.18
os32028	11	184	0.92	0	3	6	6.69	0.00	2.93	1.57	0.05	34.95	-0.46	8.19	9.27	18.02
os32040	10	247	0.01	1	3	6	6.67	-0.03	2.00	1.55	0.01	22.89	-0.12	8.69	9.02	17.78
os32111	3	216	0.99	7	3	5	6.45	-0.11	0.50	1.52	0.05	69.71	-0.78	8.23	9.24	17.99
os32112	3	225	0.09	2	3	6	6.65	-0.03	1.02	1.53	0.01	55.12	0.77	8.73	9.25	17.99
os32122	11	160	0.77	1	3	6	6.67	-0.01	4.96	1.56	0.02	12.29	-0.15	9.72	9.80	18.60
os32123	11	160	0.92	0	3	6	6.67	0.00	4.94	1.57	0.01	7.38	0.02	11.82	9.79	18.61
os32155	9	188	0.31	30	2	7	5.78	0.09	0.00	1.41	0.23	68.13	-7.24	6.28	9.54	18.37
os32158	10	261	0.92	0	3	6	6.72	0.01	1.26	1.54	0.01	40.69	0.02	8.61	8.98	17.73
os32164	10	162	0.92	0	3	6	6.67	0.00	7.94	1.57	0.01	2.70	-0.33	10.29	9.78	18.59
os32167	10	162	0.92	0	3	6	6.68	0.00	7.95	1.57	0.01	2.72	-0.19	11.63	9.78	18.59
os32182	12	252	0.87	3	3	6	6.61	-0.04	0.54	1.54	0.02	73.18	0.47	7.49	8.71	17.41
os32197	12	258	0.02	3	3	6	6.76	0.04	1.68	1.55	0.02	41.90	-0.30	8.13	8.60	17.32
os32201	12	250	0.82	3	3	6	6.68	-0.03	1.31	1.55	0.03	73.77	-0.51	7.65	8.54	17.27
os32214	10	245	1.00	8	3	5	6.48	-0.12	0.13	1.49	0.04	90.52	1.62	5.98	8.99	17.79
os32236	9	228	0.67	6	2	3	6.64	0.07	1.24	1.53	0.15	80.29	-7.38	8.00	8.58	17.30
os32238	9	223	0.01	2	3	6	6.76	0.04	5.91	1.56	0.02	19.38	-0.38	8.80	8.76	17.45
os32244	9	225	0.99	4	3	6	6.61	-0.06	1.60	1.55	0.05	65.94	-2.06	7.93	8.63	17.33
os32245	9	294	0.23	3	2	3	6.77	0.04	1.36	1.50	0.07	98.18	-1.60	6.46	8.47	17.25
os32247	11	259	0.99	2	3	6	6.66	-0.04	1.44	1.55	0.05	79.14	-1.24	7.60	8.48	17.21
os32281	11	229	0.92	0	3	6	6.70	-0.01	6.06	1.57	0.03	38.08	-0.78	9.03	8.72	17.41
os32308	9	268	0.60	8	2	3	6.41	-0.10	0.57	1.52	0.09	99.37	-3.44	7.50	8.44	17.17
os32312	9	265	0.94	6	3	5	6.56	-0.09	0.23	1.51	0.05	98.55	1.06	7.05	8.45	17.18
os32314	11	270	0.81	15	2	3	6.03	-0.19	0.03	1.48	0.13	98.88	-4.76	5.98	8.46	17.19
os32334	11	252	0.85	8	3	5	6.38	-0.12	0.33	1.51	0.07	93.98	-0.96	6.77	8.54	17.25
os32341	11	234	0.38	3	3	6	6.65	-0.01	1.93	1.55	0.06	85.19	-1.58	7.89	8.53	17.24
os32346	10	259	0.81	10	2	3	6.31	-0.16	0.06	1.50	0.09	97.60	-1.69	6.50	8.53	17.28
os32349	9	277	0.97	10	2	3	6.43	-0.14	0.44	1.49	0.11	98.84	-4.49	6.59	8.46	17.19
os32357	10	284	0.62	7	1	4	6.48	-0.06	0.03	1.46	0.06	97.94	3.30	6.16	8.48	17.26
os32360	9	308	0.05	11	3	9	6.77	0.15	0.01	1.47	0.07	97.78	-0.62	5.80	8.45	17.22
os32398	9	251	0.37	5	2	7	6.51	-0.07	1.48	1.54	0.14	95.80	-5.31	7.41	8.74	17.54
os32538	11	227	0.92	0	3	6	6.68	-0.01	5.99	1.56	0.03	24.86	-0.71	8.75	8.70	17.43

os32598	10	224	0.92	1	3	6	6.66	-0.02	5.81	1.55	0.01	28.42	0.84	9.19	8.81	17.51
os32603	10	334	0.36	9	3	5	6.58	0.01	0.23	1.48	0.06	72.27	-0.22	9.02	8.49	17.28
os32608	9	242	0.90	8	2	7	6.41	-0.12	0.67	1.53	0.09	98.35	-2.99	7.76	8.77	17.56
os32612	12	242	0.99	2	3	6	6.65	-0.04	2.20	1.55	0.04	79.10	-0.79	7.75	8.54	17.27
os32616	19	282	0.95	5	3	6	6.61	-0.07	1.23	1.54	0.07	87.53	-1.50	8.54	8.38	17.10
os32638	9	237	0.65	3	2	3	6.60	-0.04	1.95	1.55	0.09	84.27	-4.80	8.06	8.58	17.31
os32640	9	236	0.47	2	3	6	6.68	0.00	1.95	1.55	0.05	82.14	-0.97	7.53	8.59	17.31
os32692	9	265	0.17	7	2	3	6.69	0.05	0.85	1.53	0.09	96.88	-3.04	7.28	8.38	17.11
os32694	9	269	0.37	9	3	5	6.54	0.01	0.38	1.51	0.10	97.61	-2.02	6.91	8.37	17.10
os32705	11	217	0.23	2	3	6	6.68	0.01	2.99	1.55	0.06	22.32	-0.05	8.95	8.56	17.27
os32707	9	223	0.00	2	3	6	6.76	0.04	5.91	1.56	0.02	18.04	-0.33	8.81	8.76	17.45
os32715	11	227	0.92	2	3	6	6.64	-0.03	4.71	1.56	0.03	47.46	-1.06	8.32	8.68	17.42
os32721	9	270	0.19	3	2	3	6.76	0.02	2.63	1.55	0.10	78.03	-5.23	7.95	8.11	16.81
os32722	9	257	0.98	12	2	3	6.32	-0.17	0.28	1.51	0.13	88.30	-5.37	7.69	8.21	16.89
os32870	11	178	0.92	0	3	6	6.68	0.00	6.99	1.56	0.00	2.92	-0.04	11.68	9.46	18.20
os32871	11	179	0.92	0	3	6	6.67	-0.01	6.99	1.55	0.00	11.31	0.56	10.18	9.43	18.15
os32916	8	185	0.92	0	3	6	6.69	0.00	7.89	1.57	0.01	4.49	-0.28	10.71	9.38	18.19
os32948	11	187	0.92	0	3	6	6.69	0.00	7.97	1.57	0.01	17.47	-0.04	11.51	9.30	18.07
os32965	12	184	0.92	0	3	6	6.69	0.00	6.23	1.57	0.00	0.00	0.00	11.63	9.37	18.04
os32966	12	184	0.92	0	3	6	6.69	0.00	6.05	1.57	0.00	0.00	0.00	11.63	9.37	18.04
os32967	12	184	0.92	0	3	6	6.69	0.00	6.15	1.57	0.00	0.00	0.00	11.63	9.37	18.04
os32978	9	275	0.00	12	3	5	6.86	0.19	0.01	1.49	0.08	86.08	-0.95	6.16	8.39	17.10
os32980	12	209	0.92	0	3	6	6.70	0.00	5.61	1.57	0.01	0.58	-0.08	11.65	8.81	17.52
os32990	9	268	0.94	10	3	5	6.46	-0.13	0.74	1.53	0.10	80.92	-2.62	7.75	8.96	17.71
os32991	9	269	0.95	13	2	3	6.38	-0.16	0.13	1.54	0.12	77.87	-3.51	7.70	8.96	17.71
os33003	12	227	0.92	0	3	6	6.70	0.00	6.98	1.57	0.01	0.00	0.01	11.42	8.97	17.68
os33007	10	252	0.26	5	3	6	6.66	0.02	0.54	1.52	0.02	91.36	0.59	6.75	8.90	17.67
os33010	10	239	0.75	1	3	6	6.72	0.00	2.08	1.55	0.02	42.89	0.07	8.56	8.90	17.69
os33015	10	210	0.92	0	3	6	6.70	0.00	4.43	1.56	0.01	0.31	0.29	10.42	9.05	17.78
os33023	9	235	0.23	3	3	6	6.68	0.01	0.50	1.54	0.02	68.66	0.55	7.55	8.88	17.60
os33034	12	242	0.08	3	3	6	6.80	0.05	2.65	1.54	0.02	47.15	0.21	8.84	8.88	17.62
os33041	12	245	0.60	8	3	5	6.51	-0.03	0.36	1.53	0.06	80.02	-1.33	7.58	8.65	17.38
os33046	10	190	0.92	2	3	6	6.62	-0.03	6.36	1.55	0.01	5.18	0.11	8.70	9.42	18.16
os33065	10	214	0.92	0	3	6	6.68	-0.01	5.98	1.54	0.01	37.06	0.43	9.47	9.23	18.02
os33088	12	250	0.08	2	3	6	6.76	0.03	3.33	1.56	0.02	22.98	-0.28	8.67	9.15	17.93
os33089	12	250	0.92	2	3	6	6.66	-0.02	5.06	1.57	0.02	4.68	-0.66	9.20	9.14	17.92
os33172	11	251	0.92	1	3	6	6.74	0.02	3.56	1.56	0.02	20.33	-0.30	9.03	9.13	17.91
os33187	11	230	0.13	4	3	6	6.81	0.06	1.18	1.55	0.06	63.69	-1.72	7.26	9.38	18.27
os33192	9	310	1.00	19	1	4	5.80	-0.31	0.00	1.40	0.13	92.50	1.94	6.15	8.45	17.31
os33198	10	226	0.01	4	3	6	6.81	0.07	0.77	1.54	0.03	80.78	-0.57	7.96	9.08	17.81
os33319	12	220	0.92	0	3	6	6.69	0.00	3.42	1.56	0.01	3.42	0.34	9.51	8.98	17.77
os33321	9	227	0.92	0	3	6	6.70	0.00	2.99	1.55	0.03	58.76	-0.74	11.22	9.08	17.85
os33371	19	249	0.26	9	2	3	6.42	-0.13	0.76	1.51	0.09	95.16	-3.48	8.10	8.72	17.45

os33404	10	210	0.92	1	3	6	6.69	-0.01	1.71	1.53	0.01	44.59	0.50	9.39	9.28	18.10
os33414	10	212	0.99	11	3	5	6.21	-0.20	0.01	1.48	0.08	76.74	-0.42	6.26	9.37	18.19
os33431	10	229	0.87	4	3	6	6.59	-0.06	0.98	1.50	0.02	63.19	0.82	7.64	9.11	17.90
os33446	10	165	0.91	3	3	6	6.58	-0.05	1.63	1.54	0.02	49.31	-0.14	8.94	9.52	18.27
os33457	10	202	0.19	6	3	5	6.70	0.06	0.85	1.52	0.06	64.75	-1.69	8.07	9.31	18.12
os33473	9	248	1.00	8	3	5	6.50	-0.11	0.27	1.53	0.08	63.17	-1.82	6.53	8.24	16.88
os33475	9	275	0.92	1	3	6	6.68	-0.02	1.85	1.56	0.02	42.55	-0.45	8.03	8.26	16.92
os33476	9	275	0.99	3	3	6	6.65	-0.04	1.68	1.55	0.03	53.85	-0.75	7.79	8.26	16.92
os33522	12	275	0.23	2	3	6	6.74	0.02	1.47	1.55	0.03	43.66	-0.50	7.44	8.16	16.90
os33540	12	261	0.92	0	3	6	6.73	0.01	5.27	1.56	0.01	10.20	0.18	10.53	8.33	17.05
os33545	12	280	0.02	2	3	6	6.80	0.04	1.44	1.55	0.01	43.59	-0.11	7.56	8.29	17.06
os33567	12	264	0.16	2	3	6	6.65	-0.04	0.83	1.55	0.02	39.39	0.28	7.89	8.37	17.07
os33571	12	249	0.00	6	3	5	6.85	0.10	0.81	1.54	0.05	62.99	-1.86	8.32	8.45	17.16
os33585	12	236	0.92	0	3	6	6.71	0.00	4.94	1.56	0.01	1.47	0.11	9.41	8.68	17.40
os33616	12	256	0.01	1	3	6	6.75	0.02	2.26	1.56	0.02	16.47	0.02	9.20	8.28	17.09
os33637	12	279	0.92	2	3	6	6.67	-0.03	1.71	1.55	0.02	52.96	-0.41	7.82	8.26	16.93
os33648	12	276	0.13	2	3	6	6.73	0.02	2.79	1.55	0.03	78.09	-0.53	8.56	8.28	16.96
os33732	9	272	0.92	1	3	6	6.71	-0.01	3.90	1.56	0.02	14.82	-0.02	8.28	8.27	16.93
os33742	11	254	0.60	1	3	6	6.70	0.00	4.93	1.56	0.01	1.32	0.21	10.21	8.43	17.10
os33774	12	278	0.92	1	3	6	6.74	0.00	0.36	1.55	0.00	25.99	0.45	9.44	8.25	16.98
os33775	12	278	0.92	0	3	6	6.74	0.00	1.44	1.56	0.01	30.28	-0.07	9.06	8.25	16.97
os33776	12	278	0.77	1	3	6	6.74	0.00	0.71	1.55	0.00	25.20	0.30	9.32	8.25	16.97
os33836	12	205	0.92	0	3	6	6.69	0.00	6.98	1.57	0.00	0.04	0.00	12.54	9.02	17.75
os33845	8	285	0.99	2	3	9	6.69	-0.03	1.78	1.51	0.02	82.08	-0.32	8.12	8.56	17.31
os33849	8	286	0.92	1	3	6	6.75	0.01	0.66	1.54	0.01	68.87	0.14	9.07	8.43	17.18
os33880	12	267	0.00	3	3	6	6.79	0.05	1.57	1.55	0.02	79.44	-0.41	7.67	8.28	16.99
os33881	12	252	0.92	1	3	6	6.71	-0.01	1.99	1.56	0.02	25.16	0.00	8.42	8.42	17.10
os33892	12	276	0.93	3	3	6	6.62	-0.06	0.95	1.54	0.03	73.58	-0.19	7.89	8.29	17.01
os33905	12	258	0.33	10	3	9	6.52	0.02	0.04	1.48	0.06	90.49	0.97	5.91	8.97	17.72
os33942	12	225	0.92	0	3	6	6.70	0.00	2.11	1.56	0.01	0.16	0.17	11.37	8.78	17.56
os33953	12	243	0.88	1	3	6	6.68	-0.02	1.48	1.56	0.01	11.88	-0.12	8.66	8.70	17.42
os33966	8	244	0.92	1	3	6	6.72	0.01	6.81	1.55	0.02	33.48	-0.07	8.69	8.73	17.47
os33978	10	267	0.47	4	1	1	6.76	0.01	0.85	1.50	0.01	88.34	2.59	6.55	8.69	17.45
os33979	10	263	0.88	2	3	6	6.65	-0.02	0.19	1.52	0.02	90.62	0.44	6.80	8.70	17.46
os34011	12	228	0.01	2	3	6	6.76	0.03	2.46	1.56	0.02	28.97	-0.59	8.58	8.78	17.53
os34016	11	229	0.35	2	3	6	6.79	0.05	4.16	1.56	0.04	12.05	-0.57	7.74	8.73	17.51
os34046	11	249	0.92	1	3	6	6.66	-0.02	1.98	1.56	0.01	6.31	-0.06	8.41	8.50	17.30
os34047	12	245	0.92	1	3	6	6.67	-0.02	2.57	1.56	0.01	2.86	-0.31	8.59	8.52	17.31
os34049	12	248	0.35	1	3	6	6.68	-0.02	2.21	1.56	0.01	5.97	0.06	8.95	8.50	17.30
os34059	12	249	0.63	3	3	6	6.60	-0.06	1.69	1.54	0.03	85.08	-0.73	7.36	8.68	17.47
os34070	10	209	0.08	2	3	6	6.77	0.04	6.91	1.56	0.03	7.53	-0.53	8.46	8.87	17.59
os34082	11	247	0.99	2	3	6	6.67	-0.03	4.20	1.56	0.04	69.03	-0.62	8.21	8.54	17.30
os34084	10	245	0.78	3	3	6	6.68	-0.03	1.65	1.55	0.04	66.81	-1.12	8.19	8.54	17.31

os34120	11	238	0.08	1	3	6	6.74	0.01	4.96	1.56	0.01	6.75	0.01	9.77	8.41	17.11
os34121	11	238	0.03	1	3	6	6.73	0.02	4.96	1.56	0.01	6.72	0.00	9.97	8.41	17.11
os34132	12	264	0.77	2	3	6	6.71	-0.02	2.81	1.55	0.02	71.12	-0.03	9.03	8.30	17.02
os34135	12	266	0.76	2	3	6	6.73	-0.01	2.59	1.55	0.02	72.26	-0.08	8.91	8.30	17.02
os34169	12	229	0.92	0	3	6	6.70	0.00	7.66	1.57	0.01	8.07	-0.26	11.57	8.57	17.37
os34186	12	237	0.92	1	3	6	6.66	-0.03	1.57	1.56	0.01	15.16	-0.07	8.26	8.75	17.56
os34189	12	247	0.92	2	3	6	6.68	-0.01	2.73	1.56	0.02	12.74	-0.42	9.16	8.57	17.37
os34197	12	239	0.77	1	3	6	6.70	-0.01	2.89	1.56	0.01	27.19	-0.13	9.49	8.69	17.44
os34202	12	236	0.77	1	3	6	6.69	-0.02	1.88	1.56	0.01	13.07	-0.01	8.64	8.66	17.44
os34230	12	244	0.53	6	3	5	6.58	-0.02	0.66	1.54	0.04	53.43	-0.68	8.02	8.53	17.34
os34231	12	257	0.56	4	1	4	6.64	-0.01	0.18	1.52	0.02	69.83	2.05	7.70	8.55	17.33
os34232	8	235	0.70	2	3	6	6.73	0.02	1.97	1.55	0.04	64.67	-1.42	8.17	8.71	17.55
os34233	11	237	0.40	1	3	6	6.72	0.01	0.03	1.53	0.00	72.05	1.11	8.57	8.72	17.55
os34235	11	236	0.11	2	3	6	6.73	0.02	0.53	1.54	0.01	70.72	0.36	8.36	8.72	17.55
os34245	12	270	0.92	1	3	6	6.71	-0.01	2.64	1.55	0.02	65.09	-0.71	8.58	8.48	17.24
os34258	12	266	0.92	0	3	6	6.72	0.00	6.66	1.56	0.00	0.87	0.00	11.76	8.16	16.89
os34260	12	264	0.23	1	3	6	6.72	0.01	6.55	1.56	0.01	8.83	-0.25	9.33	8.19	16.89
os34271	12	269	0.92	0	3	6	6.72	0.00	6.07	1.56	0.00	0.09	0.04	12.02	8.11	16.86
os34282	10	266	0.92	1	3	6	6.75	0.02	6.69	1.56	0.01	1.75	-0.38	9.78	8.17	16.88
os34333	9	249	0.92	1	3	6	6.68	-0.01	5.85	1.56	0.01	8.53	0.36	9.11	8.22	16.91
os34406	9	258	0.33	1	3	6	6.70	0.00	4.97	1.56	0.02	6.91	-0.17	8.89	8.28	16.91
os34421	12	298	0.92	0	3	6	6.74	0.00	5.73	1.54	0.00	38.99	0.89	9.59	8.11	16.78
os34425	12	266	0.92	0	3	6	6.73	0.01	6.69	1.56	0.01	1.58	-0.36	10.36	8.17	16.88
os34476	9	258	0.39	4	2	3	6.73	0.02	2.28	1.56	0.09	81.30	-4.83	7.84	8.19	16.89
os34487	11	246	0.55	2	3	6	6.65	-0.03	5.88	1.56	0.02	6.92	-0.37	9.03	8.25	16.92
os34516	11	247	0.23	1	3	6	6.73	0.01	5.97	1.56	0.01	7.13	0.23	10.02	8.24	16.92
os34525	12	260	0.92	0	3	6	6.72	0.00	5.97	1.57	0.01	4.00	-0.06	11.65	8.20	16.90
os34581	12	289	0.00	6	3	5	6.87	0.10	0.44	1.53	0.05	39.92	-0.38	7.90	7.99	16.67
os34584	12	288	0.02	8	3	5	6.90	0.13	0.25	1.52	0.07	42.78	-1.18	7.67	7.98	16.67
os34594	12	260	0.92	0	3	6	6.72	0.00	5.95	1.57	0.01	12.67	-0.02	11.26	8.21	16.90
os34596	16	283	0.23	1	3	6	6.72	0.00	2.97	1.56	0.03	49.21	-0.31	8.61	8.09	16.78
os34597	16	283	0.92	1	3	6	6.72	0.00	2.97	1.56	0.03	50.29	-0.42	8.62	8.08	16.78
os34613	9	260	0.92	0	3	6	6.72	0.00	5.97	1.57	0.01	4.03	-0.06	11.63	8.20	16.90
os34671	19	260	0.99	6	2	3	6.52	-0.10	0.68	1.54	0.06	57.15	-2.29	6.99	8.13	16.83
os34711	12	257	0.92	1	3	6	6.69	-0.01	1.71	1.53	0.02	35.56	1.15	9.20	8.35	17.08
os34722	12	276	0.56	3	3	6	6.75	0.00	0.29	1.52	0.01	57.95	1.44	7.61	8.11	16.82
os34760	11	218	0.92	0	3	6	6.72	0.01	6.99	1.57	0.02	8.28	-0.34	9.68	8.79	17.55
os34763	8	218	0.48	1	3	6	6.73	0.01	6.99	1.56	0.01	0.78	-0.05	9.37	8.79	17.55
os34764	11	218	0.08	1	3	6	6.72	0.01	6.99	1.57	0.01	1.66	-0.51	9.65	8.79	17.55
os34771	11	218	0.60	1	3	6	6.68	0.00	6.99	1.56	0.01	1.35	-0.02	9.29	8.79	17.55
os34779	8	219	0.92	0	3	6	6.70	0.00	6.99	1.57	0.01	2.25	0.00	11.66	8.77	17.52
os34822	11	232	0.92	0	3	6	6.71	0.00	6.91	1.56	0.00	12.11	0.19	10.64	8.72	17.54
os34845	8	251	0.92	2	3	6	6.65	-0.03	1.69	1.55	0.04	61.74	-0.07	7.50	8.26	17.03

os34846	12	252	0.66	2	3	6	6.65	-0.02	1.82	1.55	0.04	64.46	-0.38	7.84	8.27	17.05
os34849	12	227	0.92	0	3	6	6.70	0.00	6.91	1.57	0.00	0.34	0.00	11.42	8.49	17.34
os34860	12	236	0.62	1	3	6	6.67	-0.01	6.83	1.56	0.01	0.00	0.04	8.96	8.27	16.93
os34963	16	270	0.04	1	3	6	6.68	-0.03	2.37	1.56	0.03	32.06	0.02	7.53	8.05	16.71
os35030	12	262	0.92	0	3	6	6.73	0.00	3.54	1.56	0.01	15.43	-0.46	9.24	8.33	17.08
os35036	12	264	0.51	2	3	6	6.75	0.01	2.73	1.55	0.04	40.24	-0.79	8.67	8.12	16.82
os35039	10	258	0.10	2	3	6	6.79	0.04	1.26	1.55	0.02	60.07	-0.28	8.14	8.28	16.98
os35056	12	257	0.23	2	3	6	6.69	0.00	2.48	1.56	0.03	62.23	-1.40	8.16	8.31	17.04
os35063	12	306	0.99	2	3	6	6.69	-0.03	2.30	1.55	0.02	32.29	-0.71	8.32	7.96	16.69
os35088	12	221	0.92	0	3	6	6.70	0.00	6.98	1.56	0.00	0.01	0.12	11.84	8.69	17.41
os35089	12	221	0.92	0	3	6	6.70	0.00	6.98	1.57	0.00	0.02	0.06	11.85	8.69	17.41
os35096	12	228	0.08	2	3	6	6.76	0.03	6.98	1.55	0.02	36.56	0.08	8.30	8.76	17.53
os35097	12	228	0.92	1	3	6	6.68	0.00	6.99	1.55	0.01	49.70	-0.20	8.65	8.78	17.54
os35104	12	216	0.60	2	3	6	6.68	-0.01	6.99	1.56	0.01	2.87	-0.05	9.40	8.82	17.60
os35136	10	242	0.08	2	3	6	6.75	0.03	0.98	1.54	0.00	60.71	1.24	8.54	8.52	17.26
os35137	12	237	0.40	3	3	6	6.77	0.02	1.99	1.55	0.03	48.68	-0.99	8.61	8.46	17.23
os35139	10	239	0.64	3	3	6	6.70	-0.01	4.27	1.53	0.01	45.44	1.24	7.66	8.55	17.31
os35167	11	225	0.08	1	3	6	6.72	0.01	5.01	1.57	0.02	7.84	-0.50	9.06	8.65	17.42
os35168	11	224	0.92	1	3	6	6.67	-0.02	6.98	1.56	0.01	3.32	0.38	9.43	8.70	17.49
os35179	11	234	0.99	6	3	5	6.55	-0.08	1.20	1.52	0.02	20.74	1.28	7.39	8.63	17.44
os35180	11	233	1.00	6	3	5	6.56	-0.08	1.19	1.53	0.03	18.74	0.88	7.43	8.63	17.44
os35185	12	227	0.82	1	3	6	6.67	-0.01	6.93	1.57	0.01	9.67	-0.31	9.20	8.70	17.50
os35186	12	228	0.92	0	3	6	6.69	-0.01	6.92	1.56	0.01	12.09	0.03	9.37	8.70	17.50
os35196	12	235	0.92	2	3	6	6.63	-0.04	1.80	1.55	0.03	40.38	-0.60	8.12	8.50	17.35
os35203	8	230	0.92	1	3	6	6.71	0.01	2.76	1.56	0.02	32.25	-0.13	9.29	8.54	17.31
os35211	11	238	0.92	0	3	6	6.71	0.00	6.92	1.57	0.01	0.31	-0.03	11.40	8.25	16.90
os35221	12	247	0.92	0	3	6	6.71	0.00	4.11	1.56	0.01	0.03	0.27	10.82	8.19	16.86
os35250	10	273	0.92	1	3	6	6.74	0.01	3.84	1.57	0.02	17.49	-0.42	9.33	8.27	17.01
os35264	12	222	0.92	0	3	6	6.69	-0.01	0.80	1.55	0.00	19.40	0.46	10.88	8.90	17.65
os35267	12	213	0.92	0	3	6	6.70	0.00	6.98	1.57	0.00	0.00	0.00	11.83	8.86	17.60
os35277	11	217	0.92	0	3	6	6.70	0.00	6.95	1.57	0.01	10.05	0.01	11.76	8.78	17.53
os35280	10	228	0.00	4	3	6	6.80	0.07	0.34	1.53	0.03	70.82	0.92	6.89	8.77	17.52
os35286	12	212	0.92	0	3	6	6.70	0.00	6.94	1.57	0.01	20.46	-0.32	8.89	8.82	17.56
os35291	11	207	0.92	0	3	6	6.68	-0.01	4.70	1.56	0.01	7.00	0.39	10.36	8.98	17.76
os35341	12	239	0.92	1	3	6	6.70	-0.01	3.94	1.57	0.03	37.30	-0.89	8.80	8.65	17.39
os35379	11	207	0.92	0	3	6	6.71	0.01	6.43	1.55	0.01	34.52	0.77	9.76	8.91	17.64
os35395	11	218	0.92	3	3	6	6.61	-0.04	5.18	1.56	0.03	6.68	-0.83	8.05	8.81	17.59
os35400	12	240	0.92	0	3	6	6.71	0.00	2.97	1.56	0.01	1.21	-0.01	11.61	8.68	17.44
os35433	3	216	0.99	7	3	5	6.45	-0.11	0.50	1.52	0.05	69.71	-0.78	8.23	9.24	17.99
os35455	3	329	0.76	5	2	3	6.57	-0.07	1.47	1.52	0.16	92.32	-6.33	6.85	8.10	16.78
os35539	10	310	0.99	11	3	5	6.42	-0.15	0.39	1.51	0.10	86.87	-2.56	7.39	8.18	16.84
os35548	10	323	0.47	7	3	5	6.52	-0.05	0.19	1.48	0.06	97.89	0.23	7.83	8.17	16.85
os35554	19	302	0.83	13	3	5	6.56	-0.09	0.18	1.49	0.12	76.18	-2.69	7.50	8.36	17.04

os35575	3	323	1.00	18	2	3	5.91	-0.29	0.00	1.45	0.15	98.05	-3.76	5.89	8.13	16.79
os35588	3	376	0.73	11	3	5	6.36	-0.14	0.31	1.42	0.11	96.71	-0.45	6.74	7.98	16.69
os35609	22	287	0.02	13	1	4	6.17	-0.21	0.00	1.38	0.08	90.57	5.82	5.96	8.36	17.03
os35617	3	293	0.16	6	2	3	6.59	-0.04	1.08	1.46	0.09	95.58	-0.90	7.17	8.30	16.97
os35625	3	318	0.34	30	2	0	6.69	0.20	0.00	1.36	0.19	98.14	-2.28	5.85	8.08	16.76
os35674	3	373	0.70	13	2	3	6.32	-0.06	0.04	1.46	0.15	96.68	-3.14	6.25	7.95	16.65
os35698	3	282	0.98	12	2	0	6.19	-0.20	0.01	1.44	0.16	95.25	1.35	6.36	8.30	16.97
os35705	3	216	0.00	15	2	7	6.84	0.22	0.43	1.50	0.20	92.96	-5.31	5.83	8.57	17.26
os35720	3	233	0.81	13	2	7	6.03	-0.17	0.10	1.54	0.30	94.79	-12.80	6.06	8.53	17.21
os35733	10	323	0.99	9	1	4	6.45	-0.14	0.05	1.46	0.04	92.23	3.06	6.63	8.33	17.05
os35740	9	257	1.00	3	2	0	6.69	-0.02	2.72	1.55	0.10	85.82	-2.60	7.80	8.50	17.23
os35783	3	360	0.00	8	3	5	6.87	0.11	0.48	1.48	0.06	90.52	1.18	6.88	7.99	16.68
os35784	3	358	0.08	7	3	5	6.93	0.10	0.08	1.49	0.05	86.60	0.70	6.22	7.91	16.60
os35816	19	298	0.46	15	2	7	5.89	-0.25	0.34	1.51	0.21	89.40	-8.73	7.26	8.07	16.74
os35838	3	364	0.88	17	3	5	6.30	-0.18	0.00	1.40	0.11	96.97	0.34	5.92	7.98	16.67
os35844	3	206	0.80	18	2	7	5.76	-0.24	0.00	1.47	0.19	87.48	-5.36	5.60	8.80	17.50
os35845	9	218	0.93	18	2	7	6.03	-0.25	0.00	1.44	0.18	90.43	-2.89	5.77	8.79	17.49
os35857	3	308	0.00	12	2	3	6.86	0.17	0.28	1.41	0.12	88.12	-2.42	7.12	8.32	16.97
os35882	3	282	0.99	15	2	0	6.07	-0.25	0.00	1.41	0.14	92.45	0.32	6.06	8.35	17.05
os35914	3	213	0.67	3	3	6	6.73	0.00	1.12	1.53	0.02	55.18	0.73	8.29	9.10	17.84
os35959	9	235	0.74	6	3	5	6.65	-0.04	1.06	1.52	0.08	75.99	-3.17	8.91	9.02	17.81
os35960	19	296	0.77	9	2	7	6.24	-0.14	0.65	1.54	0.20	89.78	-9.84	7.45	8.07	16.74
os35965	19	300	0.99	6	2	7	6.60	-0.05	2.84	1.55	0.13	94.77	-6.97	7.01	8.22	16.89
os35968	17	336	0.17	12	2	7	6.64	0.10	0.64	1.51	0.20	95.50	-7.18	6.96	8.03	16.71
os35978	10	352	0.80	16	1	4	6.47	-0.11	0.00	1.33	0.07	90.39	7.28	6.22	8.11	16.80
os35979	10	349	0.46	16	1	4	6.74	0.03	0.00	1.33	0.07	90.59	9.91	5.73	8.12	16.80
os35980	10	346	0.11	14	1	4	6.94	0.15	0.00	1.32	0.08	90.43	9.47	5.67	8.12	16.80
os35986	3	266	0.76	12	1	2	6.18	-0.12	0.00	1.40	0.15	87.43	5.16	5.07	8.35	17.01
os35994	3	242	0.09	18	2	7	6.55	0.23	0.00	1.50	0.24	83.34	-6.63	5.94	8.41	17.08
os35997	17	254	0.90	20	2	7	5.77	-0.25	0.00	1.48	0.24	95.24	-6.70	5.51	8.43	17.11
os36001	3	329	0.81	6	3	5	6.53	-0.06	0.58	1.47	0.04	73.15	0.54	7.45	8.41	17.09
os36007	3	237	0.42	17	2	7	5.92	0.01	0.11	1.50	0.27	84.78	-9.52	6.16	8.49	17.14
os36080	19	300	0.96	12	3	5	6.36	-0.16	0.64	1.51	0.13	75.48	-4.46	7.22	8.36	17.04
os36102	10	334	0.86	7	3	5	6.47	-0.10	0.27	1.49	0.04	83.89	0.37	7.55	8.19	16.88
os36144	9	251	0.77	8	2	7	6.63	-0.06	0.72	1.52	0.13	86.86	-2.98	6.61	8.60	17.32
os36167	14	257	0.88	20	2	7	5.79	-0.26	0.00	1.45	0.15	86.73	-4.79	6.10	8.70	17.40
os36168	9	270	0.99	12	2	0	6.24	-0.20	0.02	1.49	0.10	84.33	-1.18	6.87	8.53	17.24
os36193	3	324	0.64	12	1	4	6.40	-0.04	0.00	1.34	0.08	94.55	7.74	5.51	8.33	16.99
os36220	3	352	0.13	16	1	4	6.70	0.15	0.01	1.43	0.08	94.70	0.37	6.03	8.03	16.71
os36228	6	327	0.43	23	3	5	6.72	0.11	0.01	1.35	0.12	96.56	3.68	5.70	8.15	16.83
os36241	3	261	0.50	10	2	7	6.56	0.02	0.78	1.55	0.28	93.78	-12.44	6.40	8.28	16.95
os36251	6	322	0.96	19	2	3	5.80	-0.30	0.00	1.41	0.16	95.87	-4.43	5.61	8.22	16.88
os36275	6	354	0.92	10	2	3	6.44	-0.12	0.66	1.47	0.09	94.94	-2.96	6.96	8.07	16.76

os36291	6	364	0.11	10	1	4	6.75	0.10	0.04	1.38	0.04	95.90	7.22	6.06	8.01	16.69
os36292	3	357	0.45	9	3	5	6.72	-0.03	0.21	1.46	0.10	96.67	-0.80	6.40	8.00	16.68
os36293	6	359	0.61	12	3	5	6.53	-0.12	0.06	1.41	0.08	96.35	2.89	5.98	8.01	16.69
os36298	3	331	0.07	13	1	2	6.90	0.14	0.00	1.42	0.08	91.55	4.99	5.51	8.00	16.68
os36308	3	291	0.64	17	1	4	6.56	-0.05	0.00	1.37	0.12	94.45	4.85	4.77	8.27	16.92
os36315	9	271	0.33	9	2	7	6.58	0.04	0.61	1.54	0.17	93.51	-6.82	6.89	8.22	16.87
os36349	10	289	0.88	12	2	7	6.48	-0.11	0.23	1.53	0.15	87.15	-5.05	7.37	8.26	16.93
os36377	3	258	0.95	8	1	4	6.52	-0.10	0.00	1.40	0.05	73.79	6.96	6.38	8.29	16.94
os36403	10	272	0.92	0	3	6	6.72	0.00	1.12	1.48	0.01	61.88	0.21	11.77	8.45	17.16
os36407	10	254	0.99	13	1	4	6.22	-0.20	0.00	1.38	0.08	67.48	3.73	5.62	8.57	17.27
os36408	17	263	0.98	29	1	4	5.08	-0.44	0.00	1.29	0.10	69.34	7.74	5.80	8.50	17.20
os36458	9	350	0.07	10	1	1	6.80	0.13	0.02	1.39	0.04	99.32	5.12	5.68	8.22	16.94
os36459	10	318	0.15	19	2	3	6.63	0.22	0.00	1.45	0.13	99.30	-3.34	5.58	8.24	16.96
os36510	19	268	0.77	5	2	7	6.48	-0.03	1.36	1.55	0.24	82.35	-10.55	6.73	8.11	16.78
os36590	3	255	0.25	21	2	7	5.82	0.05	0.22	1.51	0.35	86.29	-15.90	7.24	7.93	16.61
os36669	19	361	0.60	5	2	3	6.74	-0.02	1.60	1.53	0.08	94.42	-4.24	8.44	7.77	16.52
os36670	17	362	0.99	10	3	5	6.35	-0.17	0.14	1.48	0.07	96.87	-1.14	7.60	7.78	16.53
os36690	19	348	0.87	9	2	3	6.57	-0.10	0.73	1.52	0.11	95.51	-4.44	7.71	7.79	16.57
os36707	17	325	0.88	12	2	3	6.25	-0.16	0.22	1.46	0.11	96.01	-4.17	6.94	7.99	16.68
os36730	17	322	1.00	11	2	3	6.38	-0.15	0.50	1.50	0.10	97.14	-3.77	7.12	7.97	16.66
os36731	10	307	0.99	16	1	4	6.17	-0.22	0.00	1.30	0.11	94.79	9.44	5.12	8.00	16.68
os36781	3	303	0.00	13	1	4	6.88	0.22	0.00	1.41	0.08	92.19	2.89	6.63	8.05	16.73
os36783	17	295	1.00	19	1	4	6.02	-0.27	0.01	1.39	0.11	89.16	3.14	6.12	8.14	16.81
os36788	9	238	0.97	6	2	7	6.48	-0.10	0.33	1.53	0.17	84.46	-5.13	6.11	8.21	16.88
os36789	9	240	0.84	11	2	7	6.29	-0.17	0.11	1.52	0.18	85.41	-6.28	6.05	8.21	16.87
os36810	17	302	0.47	5	3	6	6.67	-0.01	2.01	1.51	0.04	47.64	-1.18	8.72	8.11	16.81
os36822	8	278	0.51	5	2	3	6.57	-0.03	1.32	1.54	0.15	99.38	-5.94	6.04	8.24	16.94
os36830	10	312	0.77	10	1	4	6.62	-0.07	0.03	1.35	0.06	95.07	7.05	6.18	8.03	16.71
os36840	10	323	0.27	14	2	3	6.89	0.19	0.03	1.47	0.13	98.59	-4.74	5.77	8.05	16.78
os36841	17	310	0.13	16	3	5	6.96	0.22	0.00	1.45	0.12	97.34	-1.56	5.51	8.07	16.79
os36856	10	254	0.99	15	2	7	6.05	-0.24	0.05	1.48	0.14	87.30	-2.98	6.36	8.35	17.03
os36864	10	347	0.04	14	3	5	7.00	0.21	0.02	1.43	0.08	92.81	0.67	6.63	7.71	16.37
os36875	10	366	0.45	13	1	1	6.44	0.00	0.01	1.39	0.07	88.94	1.79	6.14	7.75	16.42
os36883	14	340	1.00	15	3	9	6.16	-0.24	0.00	1.40	0.11	97.82	-0.45	6.37	7.84	16.52
os36901	10	295	0.85	18	3	5	5.85	-0.24	0.00	1.40	0.11	87.90	0.56	6.31	8.24	16.95
os36902	17	316	0.09	4	3	6	6.83	0.07	0.76	1.51	0.04	94.97	0.66	7.93	7.90	16.59
os36912	9	319	0.73	6	2	3	6.72	-0.03	1.32	1.53	0.09	91.49	-4.41	7.73	7.84	16.56
os36913	9	326	0.29	4	3	6	6.87	0.06	0.46	1.51	0.02	95.30	0.82	6.98	7.88	16.59
os36938	10	307	0.88	6	1	4	6.53	-0.07	0.04	1.46	0.05	97.05	2.32	5.92	8.51	17.25
os36945	10	221	0.56	6	2	7	6.65	-0.05	0.80	1.53	0.10	92.87	-1.95	7.27	8.78	17.54
os36970	10	306	0.61	9	3	5	6.59	-0.08	0.24	1.39	0.07	99.56	3.40	5.97	8.51	17.26
os36971	10	305	0.92	17	1	1	5.91	-0.25	0.00	1.36	0.07	99.36	5.70	5.29	8.54	17.28
os36988	10	380	0.71	12	3	9	6.34	-0.10	0.09	1.43	0.09	91.59	-1.79	6.59	8.09	16.86

os37065	10	355	0.07	8	3	5	6.81	0.11	0.09	1.49	0.06	93.00	0.63	6.21	8.07	16.86
os37067	10	300	0.01	2	3	6	6.77	0.03	0.75	1.54	0.01	54.96	0.07	8.10	8.43	17.20
os37111	10	231	0.60	4	2	7	6.58	-0.04	1.48	1.56	0.11	60.82	-3.15	8.07	8.70	17.44
os37112	10	273	0.77	9	1	4	6.38	-0.09	0.03	1.42	0.04	92.02	5.68	5.98	8.61	17.35
os37114	10	268	1.00	6	3	5	6.49	-0.10	0.83	1.50	0.05	85.70	-0.66	7.56	8.64	17.39
os37115	10	290	0.02	6	2	3	6.78	0.09	0.80	1.53	0.10	97.29	-4.64	7.02	8.51	17.25
os37128	10	332	0.03	12	3	5	6.86	0.18	0.10	1.45	0.05	92.78	1.53	6.46	8.38	17.16
os37198	10	259	0.03	23	2	3	6.68	0.32	0.00	1.42	0.17	98.85	-3.02	5.61	8.59	17.33
os37201	10	301	0.03	10	1	1	6.81	0.13	0.00	1.38	0.04	99.49	7.02	5.44	8.56	17.30
os37209	10	292	0.99	17	3	5	6.09	-0.25	0.00	1.39	0.12	99.07	2.01	5.13	8.58	17.31
os37215	3	322	0.98	19	1	1	5.99	-0.28	0.00	1.34	0.11	99.04	2.11	5.38	8.40	17.17
os37216	9	233	0.76	8	2	7	6.35	-0.12	0.53	1.54	0.15	80.64	-6.40	6.96	8.58	17.31
os37224	3	360	0.89	14	1	4	6.09	-0.22	0.01	1.41	0.09	92.49	2.49	6.33	8.00	16.77
os37226	3	358	0.81	14	1	4	6.06	-0.21	0.00	1.42	0.10	93.92	1.36	6.52	7.99	16.76
os37227	10	389	0.92	5	3	9	6.59	-0.07	0.78	1.46	0.02	84.50	1.86	7.11	7.99	16.76
os37228	10	379	0.77	13	1	1	6.19	-0.16	0.00	1.42	0.07	93.73	1.13	5.82	8.10	16.88
os37229	10	389	0.73	7	1	1	6.52	-0.07	0.05	1.42	0.03	92.09	3.83	6.35	8.10	16.87
os37249	6	344	0.27	9	2	3	6.68	0.06	0.53	1.43	0.08	98.03	-1.63	6.81	8.13	16.90
os37251	10	359	0.07	6	3	5	6.81	0.08	0.17	1.45	0.05	97.24	1.48	6.04	8.01	16.78
os37254	17	369	0.06	9	2	3	6.87	0.13	0.61	1.49	0.08	92.35	-2.50	7.56	7.98	16.75
os37265	10	343	0.99	8	1	4	6.58	-0.10	0.08	1.45	0.05	85.82	2.32	6.79	8.14	16.92
os37273	9	310	0.92	7	2	3	6.60	-0.07	1.49	1.53	0.09	94.22	-4.18	8.32	8.26	17.06
os37284	10	296	0.76	9	2	3	6.64	-0.06	0.63	1.51	0.08	96.83	-3.14	7.73	8.40	17.15
os37285	10	281	0.88	6	3	5	6.49	-0.09	0.40	1.49	0.04	92.29	1.08	6.61	8.51	17.23
os37287	10	319	0.59	6	3	5	6.60	-0.02	0.56	1.50	0.04	95.17	0.26	6.72	8.36	17.11
os37313	10	282	0.91	6	2	3	6.55	-0.08	0.78	1.52	0.06	95.76	-1.05	7.58	8.50	17.24
os37342	3	403	0.55	10	1	4	6.64	-0.06	0.01	1.39	0.06	97.76	5.08	5.98	7.74	16.44
os37369	3	311	1.00	5	3	6	6.60	-0.08	0.59	1.53	0.04	72.89	0.58	7.54	8.07	16.76
os37474	6	283	0.35	20	3	5	6.29	0.04	0.00	1.42	0.10	84.26	1.36	5.63	8.18	16.86
os37532	10	352	0.81	9	3	5	6.61	-0.09	0.10	1.50	0.05	99.87	0.09	7.33	7.99	16.73
os37534	10	289	0.00	6	3	6	6.86	0.10	0.63	1.49	0.03	89.69	1.17	6.88	8.33	17.05
os37549	17	312	0.67	16	2	3	6.13	-0.23	0.02	1.46	0.14	93.14	-3.56	6.74	8.11	16.80
os37552	3	333	1.00	15	2	3	6.11	-0.25	0.00	1.42	0.11	96.29	-1.98	6.70	8.13	16.82
os37554	17	366	0.91	10	2	7	6.26	-0.15	0.27	1.50	0.19	90.91	-3.73	7.32	7.70	16.47
os37556	17	365	0.99	14	2	7	6.19	-0.18	0.32	1.50	0.19	91.81	-4.67	7.14	7.71	16.48
os37558	17	337	0.69	14	3	5	6.17	-0.16	0.00	1.46	0.09	99.60	-1.08	5.85	7.99	16.73
os37594	3	349	0.02	12	3	5	6.96	0.19	0.02	1.47	0.10	94.94	1.18	5.88	7.92	16.68
os37623	10	294	0.66	8	1	4	6.42	-0.08	0.03	1.44	0.06	87.17	2.78	7.38	8.34	17.06
os37644	17	313	0.92	6	1	4	6.51	-0.08	1.03	1.43	0.05	86.74	3.76	7.51	8.11	16.82
os37686	3	277	0.93	16	1	4	6.27	-0.19	0.00	1.44	0.11	83.29	1.98	6.21	8.45	17.14
os37706	3	370	0.23	4	3	6	6.77	0.05	0.10	1.48	0.05	87.66	2.02	8.06	7.57	16.32
os37718	19	304	0.16	6	2	3	6.73	0.06	1.11	1.54	0.08	78.82	-3.25	7.20	7.89	16.60
os37732	3	319	1.00	13	2	7	6.21	-0.21	0.20	1.51	0.13	59.01	-3.97	6.46	7.83	16.52

os37761	3	346	0.40	4	3	6	6.63	-0.02	0.29	1.49	0.06	85.86	1.55	7.23	7.64	16.38
os37776	3	348	0.08	8	3	5	6.94	0.12	0.10	1.50	0.06	95.18	0.75	6.72	7.74	16.49
os37807	10	276	0.15	17	2	3	6.84	0.19	0.02	1.49	0.17	73.24	-6.02	6.97	8.26	16.97
os37810	3	261	0.91	11	2	7	6.23	-0.15	0.71	1.53	0.18	74.93	-7.94	7.85	8.24	16.95
os37829	6	369	0.15	11	2	3	6.93	0.12	0.43	1.50	0.14	95.26	-4.45	6.37	7.84	16.61
os37842	17	362	0.05	14	2	3	6.93	0.20	0.00	1.47	0.12	90.39	-1.24	6.59	7.86	16.62
os37855	3	353	0.56	6	3	5	6.56	-0.06	0.90	1.51	0.08	95.26	-1.87	6.60	7.99	16.72
os37870	17	347	0.63	4	2	3	6.86	0.05	1.26	1.52	0.09	97.10	-1.88	6.24	8.04	16.76
os37913	10	308	1.00	9	3	5	6.47	-0.13	0.02	1.44	0.08	98.73	0.97	5.48	8.31	16.98
os37962	10	373	0.61	11	2	3	6.83	0.06	0.47	1.50	0.13	98.15	-4.04	6.13	7.85	16.56
os37977	3	261	0.42	5	3	6	6.54	-0.09	1.22	1.55	0.06	35.77	-1.37	7.98	8.38	17.06
os37985	3	340	0.07	3	3	6	6.83	0.04	1.78	1.55	0.08	86.98	-1.85	7.32	7.62	16.37
os38045	10	298	0.79	3	1	4	6.62	-0.04	0.12	1.51	0.03	85.43	2.22	8.02	8.23	16.94
os38115	19	285	0.87	18	3	5	5.97	-0.24	0.27	1.49	0.14	93.76	-3.47	6.26	8.05	16.75
os38174	9	315	0.40	11	3	5	6.49	-0.02	0.23	1.47	0.08	93.40	-1.33	6.92	8.14	16.84
os38175	10	303	0.77	13	2	3	6.58	-0.08	0.01	1.49	0.12	90.96	-3.39	6.71	8.14	16.86
os38199	3	340	0.01	5	3	9	6.79	0.06	0.39	1.47	0.03	93.44	0.73	6.09	8.17	16.89
os38203	10	305	0.52	5	2	3	6.72	-0.02	1.67	1.55	0.11	95.47	-5.08	7.71	8.20	16.91
os38211	10	318	0.02	17	2	7	6.71	0.20	0.21	1.49	0.14	92.07	-5.06	7.05	8.07	16.76
os38219	10	353	0.81	3	3	6	6.66	-0.05	0.36	1.49	0.03	82.98	1.56	7.04	8.05	16.75
os38233	9	262	0.10	4	3	6	6.62	-0.06	0.47	1.53	0.03	53.38	1.13	8.11	8.34	17.02
os38235	19	324	0.00	15	3	5	6.92	0.25	0.00	1.46	0.08	81.31	0.04	5.51	7.90	16.59
os38259	3	379	0.07	13	2	7	6.69	0.18	0.01	1.47	0.20	91.70	-3.26	5.63	7.60	16.39
os38286	3	320	0.99	16	2	3	6.04	-0.26	0.00	1.45	0.11	97.50	-1.83	5.83	8.20	16.86
os38307	3	285	0.80	17	3	5	6.47	-0.09	0.01	1.39	0.15	91.15	-0.64	5.36	8.33	17.01
os38411	3	315	0.78	12	1	4	6.18	-0.19	0.00	1.33	0.08	93.62	9.14	5.48	8.14	16.82
os38421	10	326	0.98	11	1	4	6.24	-0.20	0.01	1.34	0.06	94.03	6.58	5.55	8.17	16.85
os38431	10	310	0.01	10	1	4	6.91	0.16	0.07	1.41	0.05	90.62	3.88	6.42	8.23	16.91
os38432	3	298	0.95	15	1	4	6.30	-0.18	0.02	1.37	0.09	93.49	3.91	5.69	8.24	16.92
os38440	10	312	0.08	12	3	5	6.85	0.16	0.18	1.51	0.09	81.10	-1.78	7.21	8.16	16.84
os38460	10	301	0.83	20	3	5	5.71	-0.26	0.00	1.37	0.13	89.07	0.53	6.46	8.28	16.96
os38466	3	304	0.03	23	3	5	6.88	0.34	0.00	1.39	0.13	82.18	-0.79	5.90	8.22	16.92
os38468	3	312	0.00	17	3	5	6.90	0.27	0.00	1.43	0.08	81.93	-0.18	6.85	8.19	16.89
os38502	9	269	0.01	8	2	3	6.56	0.03	2.15	1.55	0.16	77.71	-8.48	8.32	8.38	17.09
os38520	3	298	0.76	14	1	4	6.13	-0.16	0.02	1.39	0.06	83.24	5.99	6.28	8.33	17.03
os38571	3	298	0.53	4	3	6	6.69	0.01	1.66	1.55	0.05	87.55	-1.85	8.28	8.14	16.86
os38599	3	278	0.99	9	2	3	6.50	-0.11	1.09	1.54	0.09	82.55	-3.31	7.27	8.25	16.96
os38608	17	313	0.08	6	2	3	6.70	-0.03	1.16	1.50	0.09	95.98	-2.64	6.23	8.16	16.87
os38614	17	382	0.84	13	2	3	6.37	-0.17	0.29	1.45	0.12	94.36	-1.94	7.35	7.88	16.64
os38615	6	396	0.37	18	1	4	5.87	-0.28	0.06	1.41	0.13	92.07	1.33	5.88	7.74	16.51
os38623	10	335	0.03	21	3	5	6.89	0.33	0.00	1.39	0.13	94.86	1.72	5.97	8.03	16.73
os38625	10	330	0.23	9	1	4	6.68	0.08	0.30	1.39	0.05	89.98	4.82	6.33	8.08	16.79
os38653	3	331	0.05	11	3	5	6.82	0.15	0.57	1.51	0.10	93.26	-2.39	7.19	8.10	16.82

os38664	10	325	0.05	4	2	3	6.79	0.02	2.75	1.56	0.06	88.74	-3.10	8.15	8.09	16.80
os38665	10	326	0.27	2	2	3	6.75	0.00	2.98	1.56	0.06	89.48	-3.00	8.36	8.09	16.79
os38672	3	294	0.43	17	3	5	6.79	0.08	0.00	1.42	0.14	86.09	0.20	6.13	8.25	16.94
os38686	6	315	0.01	7	3	6	6.48	-0.10	0.63	1.46	0.08	85.93	0.09	7.57	8.27	16.95
os38696	3	275	0.76	5	3	5	6.51	-0.10	0.39	1.49	0.04	79.41	0.54	7.33	8.50	17.12
os38698	3	283	0.84	18	3	5	6.27	-0.18	0.00	1.40	0.14	94.94	0.31	6.34	8.32	16.99
os38714	3	325	1.00	13	3	5	6.19	-0.23	0.00	1.43	0.09	97.37	0.58	5.75	8.20	16.86
os38722	9	306	0.60	13	3	5	6.22	-0.13	0.01	1.46	0.11	94.26	0.01	6.37	8.19	16.87
os38768	10	304	0.49	4	1	4	6.79	0.02	0.10	1.52	0.04	83.27	2.11	7.52	8.21	16.93
os38774	9	334	0.87	7	2	3	6.51	-0.10	1.52	1.53	0.14	87.30	-7.47	7.82	8.03	16.72
os38797	9	315	0.51	11	3	5	6.41	-0.08	0.32	1.51	0.08	89.02	-1.92	6.73	8.19	16.91
os38798	10	402	0.11	20	1	1	6.96	0.28	0.00	1.35	0.08	96.79	7.05	4.68	8.02	16.76
os38822	3	316	0.15	7	1	4	6.72	0.05	0.16	1.50	0.04	90.74	1.78	6.50	8.07	16.77
os38857	3	364	0.92	13	3	5	6.49	-0.13	0.00	1.44	0.09	88.72	0.60	6.07	8.03	16.80
os38858	17	297	0.00	6	2	7	6.79	0.08	1.65	1.55	0.19	97.03	-8.87	7.24	8.09	16.86
os38877	3	347	0.89	3	3	6	6.77	0.00	2.49	1.52	0.06	81.59	-0.04	8.37	7.60	16.28
os38878	3	329	0.37	20	2	3	6.73	0.17	0.01	1.50	0.16	58.50	-5.80	5.70	7.75	16.48
os38888	3	321	0.00	8	2	3	6.85	0.12	0.71	1.54	0.10	95.73	-4.45	6.78	7.86	16.59
os38899	3	425	0.15	15	2	8	6.96	0.20	0.00	1.45	0.11	100.00	-3.03	5.47	7.60	16.35
os38900	3	428	0.19	14	3	9	7.01	0.17	0.00	1.43	0.08	100.00	1.06	5.30	7.61	16.36
os38937	3	425	0.25	14	3	5	6.92	0.13	0.00	1.46	0.11	93.62	-0.50	5.41	7.13	15.81
os38968	3	361	0.54	22	3	5	5.92	-0.09	0.00	1.41	0.12	79.68	1.58	5.97	7.56	16.20
os38972	3	371	0.09	8	3	5	6.96	0.13	0.10	1.48	0.07	94.47	0.18	6.57	7.49	16.16
os38988	3	327	0.67	12	3	5	6.31	-0.10	0.00	1.47	0.07	78.85	1.33	5.46	7.73	16.43
os39024	3	413	0.77	7	3	9	6.53	-0.07	0.22	1.42	0.06	94.17	0.84	5.68	7.35	15.99
os39169	22	354	0.96	19	1	1	6.05	-0.26	0.00	1.24	0.12	85.11	9.22	4.59	7.92	16.63
os39176	3	417	0.51	7	2	3	6.86	0.03	0.42	1.52	0.09	87.27	-2.33	6.81	7.19	15.86
os39222	3	382	0.21	13	3	5	6.78	0.15	0.01	1.45	0.09	98.41	-1.16	5.98	7.53	16.19
os39296	3	439	0.03	20	2	3	6.78	0.28	0.00	1.43	0.15	99.88	-1.47	6.00	7.07	15.78
os39330	3	369	0.08	5	3	5	6.91	0.09	0.50	1.50	0.03	87.85	1.07	7.11	7.64	16.32
os39397	3	436	0.03	21	3	5	6.78	0.31	0.00	1.41	0.14	99.96	0.75	5.83	7.08	15.79
os39398	6	430	0.08	18	3	5	6.96	0.31	0.00	1.42	0.15	99.89	-0.60	6.03	7.09	15.80
os39435	3	356	0.98	15	1	4	6.09	-0.24	0.00	1.43	0.09	77.25	1.98	5.57	7.63	16.28
os39488	3	466	0.05	11	2	0	6.82	0.14	0.01	1.48	0.09	99.28	0.32	5.62	6.79	15.46
os39489	3	469	0.01	9	2	0	6.94	0.15	0.29	1.51	0.10	98.84	-1.85	5.92	6.76	15.43
os39490	3	419	0.08	15	2	3	6.96	0.22	0.00	1.48	0.14	95.67	-4.94	6.29	7.11	15.74
os39498	3	320	0.14	10	3	5	6.70	0.08	0.05	1.52	0.09	74.69	-1.94	7.10	7.78	16.45
os39509	3	469	0.23	4	2	0	6.78	0.03	1.91	1.54	0.09	93.56	-1.90	8.74	6.79	15.46
os39511	3	466	0.00	2	2	0	6.86	0.04	2.58	1.55	0.07	89.29	-0.25	7.79	6.78	15.45
os39512	3	470	0.40	8	3	5	6.91	0.04	0.04	1.47	0.07	95.34	0.60	5.75	6.95	15.62
os39513	3	469	0.40	5	3	5	6.90	0.03	0.24	1.51	0.04	97.34	0.89	6.65	6.96	15.60
os39531	3	431	0.14	11	2	3	6.96	0.15	0.05	1.50	0.14	95.73	-3.79	5.30	7.10	15.76
os39532	3	425	0.52	18	2	3	6.74	0.07	0.01	1.47	0.15	92.55	-5.31	5.78	7.12	15.80

os39539	3	364	0.34	4	3	6	6.86	0.04	0.84	1.53	0.05	81.90	0.09	8.11	7.48	16.13
os39543	6	388	0.01	5	2	3	6.84	0.08	1.31	1.52	0.13	97.98	-3.52	6.57	7.45	16.11
os39555	3	350	0.01	10	2	3	6.84	0.17	0.33	1.52	0.14	84.27	-4.73	6.86	7.56	16.23
os39556	19	326	0.23	6	2	7	6.74	0.06	1.18	1.55	0.12	87.65	-4.99	7.22	7.70	16.38
os39610	3	388	0.87	9	1	4	6.43	-0.12	0.15	1.49	0.06	78.57	1.28	6.89	7.35	16.00
os39612	3	388	0.98	8	3	5	6.51	-0.12	0.28	1.50	0.06	80.88	0.27	7.25	7.35	15.99
os39625	3	445	0.08	4	1	4	6.91	0.06	0.12	1.50	0.03	81.09	2.04	7.13	7.08	15.74
os39626	3	429	0.01	5	2	3	6.87	0.08	1.60	1.54	0.07	70.67	-2.83	7.43	7.08	15.74
os39632	3	406	0.05	9	3	5	6.99	0.15	0.12	1.50	0.07	94.20	-0.53	6.44	7.20	15.86
os39653	3	451	0.69	12	2	3	6.36	-0.07	0.03	1.47	0.12	97.39	-2.61	5.83	7.01	15.66
os39679	3	396	0.92	0	3	6	6.79	0.01	1.97	1.53	0.02	18.71	-0.24	9.02	7.39	16.04
os39777	3	345	0.99	25	3	5	5.38	-0.41	0.00	1.34	0.17	94.74	1.72	4.97	7.65	16.29
os39784	3	288	0.40	9	2	7	6.61	0.08	0.90	1.54	0.26	98.90	-11.99	8.26	7.80	16.48
os39787	3	277	0.68	14	2	7	6.66	-0.01	0.00	1.49	0.21	87.54	-5.27	7.11	7.86	16.51
os39788	3	343	0.77	5	1	1	6.69	-0.05	0.02	1.31	0.03	94.18	8.66	5.94	7.88	16.54
os39799	3	404	1.00	8	3	5	6.50	-0.14	0.09	1.47	0.04	94.55	1.42	6.77	7.44	16.13
os39800	6	407	0.93	7	1	4	6.62	-0.10	0.12	1.47	0.03	96.35	2.31	6.28	7.44	16.13
os39804	3	362	0.01	14	1	4	6.87	0.21	0.01	1.39	0.08	98.08	4.40	5.51	7.61	16.31
os39818	3	334	0.89	18	1	4	6.21	-0.21	0.00	1.38	0.12	92.08	2.95	5.35	7.82	16.48
os39947	17	315	0.61	6	2	0	6.46	-0.10	0.55	1.51	0.12	84.72	-0.30	7.13	7.60	16.33
os39950	3	386	0.18	9	1	4	6.64	0.06	0.04	1.41	0.05	99.57	5.75	6.44	7.48	16.21
os40017	27	403	0.00	25	2	7	6.78	0.39	0.00	1.39	0.24	99.90	-3.84	5.32	7.13	15.84
os40292	3	333	0.78	10	1	4	6.67	-0.04	0.00	1.39	0.04	95.53	7.89	5.33	7.89	16.57
os40296	10	273	0.88	4	2	0	6.67	-0.03	0.02	1.47	0.10	67.34	1.74	7.02	8.02	16.69
os40299	17	325	0.98	12	3	5	6.26	-0.19	0.04	1.43	0.09	94.26	-1.14	5.98	7.91	16.57
os40305	10	305	0.18	11	2	3	6.81	0.09	0.22	1.47	0.15	95.08	-3.70	5.88	7.99	16.65
os40317	22	272	0.06	21	2	0	6.67	0.27	0.00	1.40	0.17	83.27	-0.74	5.98	8.02	16.71
os40368	10	327	0.08	10	2	3	6.82	0.14	0.43	1.51	0.09	97.33	-3.24	7.37	7.82	16.52
os40388	19	360	0.59	9	3	5	6.51	-0.04	0.08	1.49	0.05	90.00	1.13	8.52	7.58	16.31
os40407	3	296	0.05	11	3	5	6.78	0.17	0.00	1.45	0.14	95.19	0.42	5.52	8.09	16.79
os40410	22	336	0.19	16	3	5	6.96	0.19	0.00	1.40	0.11	99.33	0.14	6.02	7.98	16.68
os40506	10	314	0.76	14	1	4	6.14	-0.15	0.00	1.41	0.07	82.62	4.57	5.94	8.07	16.74
os40570	10	331	0.40	5	2	3	6.80	0.03	1.41	1.52	0.07	79.77	-2.11	8.04	7.95	16.69
os40571	17	321	0.41	13	3	5	6.42	-0.02	0.07	1.49	0.09	81.23	-1.28	7.46	7.95	16.70
os40572	17	321	0.45	11	3	5	6.51	-0.01	0.33	1.51	0.08	78.70	-1.53	7.80	7.94	16.69
os40695	4	316	0.92	1	3	6	6.74	0.00	4.64	1.55	0.00	18.24	0.35	10.03	8.01	16.66
os40696	4	312	0.23	3	3	6	6.74	0.02	1.70	1.55	0.02	37.39	-0.16	8.50	8.01	16.66
os40828	17	405	0.53	19	3	9	6.71	0.02	0.00	1.38	0.12	99.85	0.80	5.27	7.78	16.50
os40925	3	327	0.11	17	1	4	6.75	0.22	0.00	1.33	0.08	98.98	7.54	5.48	8.28	16.94
os40938	3	285	0.01	10	2	3	6.85	0.14	0.59	1.47	0.13	97.95	-6.93	7.65	8.59	17.28
os40947	3	368	0.23	7	3	5	6.63	0.01	0.44	1.44	0.07	94.36	0.12	6.82	7.63	16.29
os40958	3	331	0.05	11	3	5	6.82	0.15	0.57	1.51	0.10	93.26	-2.39	7.19	8.10	16.82
os40959	19	309	0.77	6	2	7	6.73	-0.03	0.87	1.53	0.12	92.86	-2.52	7.73	7.98	16.71

os40966	10	305	0.77	3	3	6	6.74	-0.01	0.10	1.52	0.04	83.59	1.62	7.44	8.21	16.93
os40967	10	304	0.42	4	1	4	6.80	0.02	0.12	1.52	0.04	83.34	2.10	7.51	8.21	16.93
os40969	3	287	0.50	3	3	6	6.73	-0.01	0.82	1.49	0.02	60.71	2.04	8.03	8.20	16.89
os41073	19	213	0.18	9	2	7	6.59	0.05	1.16	1.50	0.19	91.57	-6.81	5.45	8.32	17.02
os41134	23	332	0.00	17	2	0	6.89	0.27	0.00	1.43	0.13	93.62	-0.09	7.31	7.77	16.51
os41153	19	315	0.01	4	2	3	6.77	0.04	2.21	1.53	0.09	84.01	-4.52	9.04	7.98	16.76
os41154	27	315	0.21	7	2	7	6.52	-0.05	1.01	1.53	0.14	95.14	-7.44	8.74	7.96	16.75
os41166	19	295	0.86	7	3	5	6.50	-0.09	0.40	1.49	0.03	86.84	1.05	7.62	8.12	16.86
os41222	23	466	0.80	11	2	3	6.40	-0.13	0.28	1.46	0.12	99.41	-0.97	6.72	7.06	15.84
os41236	23	471	0.66	9	2	7	6.75	-0.04	0.57	1.50	0.14	99.86	-4.15	7.52	6.93	15.72
os41253	28	536	0.77	6	2	0	6.83	-0.01	0.82	1.52	0.00	99.86	-2.10	7.28	6.39	15.19
os41284	27	483	0.00	5	2	0	6.95	0.10	0.60	1.53	0.09	99.80	-0.33	6.86	6.91	15.72
os41303	24	445	0.34	11	2	3	6.60	0.03	0.06	1.49	0.11	99.45	-2.94	6.00	7.20	15.99
os41328	3	348	0.68	28	1	4	5.45	-0.18	0.00	1.34	0.15	87.08	2.87	5.07	7.58	16.24
os41331	3	345	0.23	19	2	3	6.41	0.12	0.00	1.49	0.18	90.01	-5.09	5.80	7.56	16.22
os41341	3	364	0.28	8	3	5	6.76	0.09	0.13	1.49	0.08	95.89	-0.67	6.02	7.50	16.18
os41343	3	389	0.08	7	1	1	6.94	0.10	0.18	1.45	0.03	94.64	2.13	5.92	7.51	16.19
os41412	28	467	0.77	3	3	6	6.75	-0.04	1.75	1.54	0.04	84.05	-0.80	7.76	7.23	15.89
os41450	3	493	0.23	5	3	5	6.79	0.04	0.38	1.52	0.03	71.52	0.98	7.28	6.96	15.59
os41451	28	509	0.14	2	3	6	6.86	0.02	0.34	1.54	0.01	18.48	0.16	8.49	6.96	15.59
os41466	4	494	0.92	0	3	6	6.84	0.01	0.63	1.53	0.01	68.43	0.42	8.69	6.99	15.64
os41504	4	436	0.92	0	3	6	6.79	0.00	5.95	1.57	0.01	1.99	-0.04	11.52	7.43	16.07
os41554	8	455	0.77	2	2	3	6.80	0.00	1.97	1.56	0.09	77.03	-3.76	7.63	7.16	15.86
os41579	3	475	0.97	4	3	6	6.80	-0.02	1.71	1.54	0.05	78.89	-2.11	7.81	7.15	15.81
os41592	19	435	0.23	5	3	5	6.81	0.06	0.92	1.55	0.06	53.03	-2.07	8.31	7.39	16.06
os41622	19	494	0.92	3	3	6	6.73	-0.05	1.78	1.51	0.02	84.03	0.29	7.69	7.24	15.99
os41650	3	459	1.00	14	1	4	6.18	-0.24	0.00	1.43	0.07	92.92	2.55	6.79	7.45	16.21
os41889	3	453	0.08	2	3	6	6.86	0.03	1.24	1.52	0.02	86.38	0.02	7.70	7.56	16.32
os41895	3	457	0.65	9	2	3	6.50	-0.07	0.59	1.50	0.09	92.85	-3.62	7.86	7.36	16.11
os42058	12	487	0.08	2	3	9	6.87	0.03	1.38	1.49	0.01	45.11	0.16	7.78	7.46	16.24
os42213	14	362	0.08	4	3	6	6.89	0.07	0.28	1.53	0.03	62.35	-0.30	7.56	8.19	16.90
os42218	9	321	0.22	3	2	3	6.80	0.05	1.52	1.56	0.06	60.15	-2.17	8.36	8.32	17.09
os42227	10	397	0.03	2	3	6	6.82	0.01	0.96	1.54	0.04	62.22	0.15	8.25	7.83	16.58
os42272	8	255	0.47	4	3	6	6.77	0.01	1.17	1.55	0.05	62.61	-0.73	7.78	8.92	17.73
os42275	8	286	0.01	2	2	3	6.74	0.02	2.04	1.54	0.05	73.76	-2.82	8.40	8.81	17.61
os42387	11	233	0.16	9	1	4	6.70	0.10	0.05	1.49	0.08	83.31	2.14	6.25	8.98	17.90
os42401	11	262	0.92	0	3	6	6.72	0.00	4.94	1.56	0.02	39.85	0.06	10.43	8.81	17.71
os42412	10	220	0.70	4	3	6	6.59	-0.02	0.84	1.55	0.07	42.42	-0.71	8.06	8.96	17.82
os42453	24	437	0.26	5	3	5	6.91	0.05	0.86	1.52	0.00	95.00	-0.85	7.48	7.11	15.87
os42497	28	472	0.23	5	3	6	6.78	0.03	1.74	1.53	0.05	83.41	-1.27	9.22	6.88	15.66
os42511	19	401	0.93	4	2	0	6.74	-0.04	0.83	1.54	0.00	99.94	-0.73	8.06	7.22	15.95
os42596	28	420	0.01	2	3	6	6.83	0.04	1.59	1.54	0.02	57.62	0.51	8.46	7.25	16.02
os42634	28	414	0.99	6	2	7	6.62	-0.09	0.87	1.54	0.09	97.27	-2.72	8.59	7.05	15.85

os42649	23	455	0.25	6	2	3	6.92	0.06	0.74	1.51	0.08	99.95	-2.47	7.65	6.95	15.72
os42662	23	468	0.53	5	3	5	6.61	-0.09	0.50	1.52	0.05	100.00	-0.52	6.61	6.97	15.78
os42715	23	433	0.99	13	2	0	6.20	-0.23	0.00	1.48	0.00	99.77	-0.57	5.75	7.08	15.90
os42717	23	451	0.92	10	3	5	6.35	-0.16	0.06	1.49	0.00	95.45	-1.41	5.70	7.14	15.97
os42759	23	440	0.53	3	3	6	6.74	0.00	0.40	1.53	0.03	85.81	0.39	8.16	7.13	15.89
os42767	23	467	0.40	10	3	9	6.55	-0.02	0.07	1.46	0.07	99.97	-0.11	5.63	7.23	16.03
os42820	28	406	0.77	3	3	6	6.75	-0.03	1.35	1.55	0.03	90.02	-0.20	8.16	7.29	16.09
os42825	28	426	0.92	1	3	6	6.81	0.01	0.20	1.55	0.00	31.03	0.68	9.25	7.24	16.06
os42850	23	457	0.92	13	3	5	6.24	-0.20	0.01	1.47	0.00	95.38	0.00	5.49	7.14	15.97
os42856	23	482	0.60	7	3	5	6.63	-0.03	0.41	1.50	0.00	99.00	0.19	6.66	7.05	15.84
os42857	17	405	0.92	4	3	6	6.65	-0.06	1.50	1.54	0.00	96.75	-0.41	7.56	7.27	15.99
os42858	23	471	0.66	11	3	5	6.76	-0.04	0.03	1.47	0.07	99.76	-0.38	6.81	6.96	15.73
os42859	27	447	0.77	6	3	5	6.76	-0.04	0.73	1.51	0.06	99.98	0.01	7.01	7.03	15.79
os43131	17	352	0.99	6	3	9	6.60	-0.09	0.28	1.49	0.03	90.33	0.80	6.32	7.69	16.36
os43178	19	336	0.60	8	3	5	6.47	-0.07	0.34	1.50	0.06	99.08	-0.42	7.67	7.53	16.19
os43223	3	372	0.98	8	3	5	6.54	-0.12	0.22	1.51	0.00	91.67	0.40	6.87	7.28	15.89
os43239	24	265	0.30	13	3	5	6.52	0.04	0.09	1.50	0.00	86.83	-1.49	7.52	7.89	16.54
os43244	17	277	0.78	11	1	4	6.33	-0.12	0.06	1.48	0.00	97.49	0.88	7.01	7.92	16.60
os43267	17	408	0.99	6	3	9	6.57	-0.10	0.49	1.51	0.03	97.66	0.03	6.50	7.35	16.00
os43314	19	330	0.60	10	3	5	6.39	-0.08	0.11	1.51	0.07	98.87	-1.21	7.59	7.54	16.20
os43437	19	390	0.77	5	2	0	6.76	-0.02	1.14	1.54	0.09	88.64	-1.15	7.90	7.23	15.93
os43479	30	655	0.53	20	3	5	6.11	-0.04	0.00	1.41	0.18	98.60	-0.66	5.09	5.49	14.23
os43487	32	714	0.07	4	2	0	7.02	0.07	0.59	1.52	0.09	78.03	-1.10	7.22	5.32	14.11
os43488	23	718	0.06	4	3	6	7.03	0.07	1.68	1.51	0.08	98.51	-0.85	7.63	5.36	14.16
os43490	25	607	0.96	19	2	0	6.12	-0.27	0.00	1.44	0.16	95.33	-0.45	6.04	5.68	14.41
os43498	23	676	0.01	13	3	5	6.97	0.19	0.03	1.47	0.11	99.05	-1.89	6.84	5.49	14.24
os43577	30	633	0.10	13	2	3	6.75	0.09	0.03	1.49	0.15	94.71	-3.82	7.40	5.83	14.68
os43594	23	517	0.99	6	1	1	6.66	-0.09	0.10	1.41	0.03	95.56	2.98	6.18	6.75	15.49
os43595	23	519	1.00	5	3	9	6.69	-0.07	0.29	1.42	0.03	96.07	2.19	6.64	6.75	15.50
os43600	23	533	0.05	7	2	3	6.83	0.08	1.04	1.54	0.16	96.53	-8.54	6.32	6.46	15.26
os43617	27	447	0.01	10	2	3	6.96	0.15	0.75	1.52	0.12	97.58	-5.68	6.86	7.09	15.90
os43665	24	596	0.23	6	3	5	6.82	0.04	0.35	1.50	0.06	100.00	0.27	6.47	6.21	14.93
os43666	23	586	0.91	14	1	1	6.53	-0.15	0.00	1.39	0.08	100.00	3.17	5.50	6.41	15.16
os43727	30	635	0.88	12	3	5	6.30	-0.19	0.00	1.45	0.08	96.61	1.19	5.75	5.96	14.78
os43730	23	519	0.98	4	3	9	6.71	-0.06	0.56	1.45	0.03	96.47	0.75	6.91	6.75	15.50
os43753	23	590	0.71	11	1	2	6.41	-0.11	0.00	1.43	0.10	98.56	4.54	5.71	5.77	14.51
os43769	23	714	0.01	15	3	5	7.01	0.23	0.00	1.45	0.11	99.94	-0.02	6.23	5.29	14.06
os43771	35	772	0.77	8	3	5	6.63	-0.10	0.29	1.47	0.07	99.71	-0.96	7.02	5.02	13.81
os43772	23	532	0.73	14	2	7	6.26	-0.13	0.01	1.48	0.15	99.97	-3.83	6.82	6.14	14.86
os43805	28	497	0.54	10	3	5	6.48	-0.07	0.20	1.49	0.10	99.97	-2.33	7.18	6.66	15.30
os43851	19	465	0.23	3	2	3	6.77	0.00	1.97	1.56	0.06	94.27	-2.04	8.75	6.74	15.38
os43852	19	472	0.23	4	2	7	6.72	-0.03	1.96	1.56	0.09	96.50	-3.11	8.66	6.61	15.26
os43908	23	604	0.02	16	2	7	6.85	0.24	0.06	1.50	0.17	99.89	-5.52	7.10	5.62	14.40

os43930	23	654	0.76	13	2	0	6.31	-0.15	0.00	1.42	0.15	99.04	2.39	5.55	5.35	14.11
os43933	27	740	0.88	22	3	5	5.77	-0.29	0.00	1.39	0.14	100.00	-1.40	6.28	5.33	14.10
os43935	27	698	0.79	20	2	3	5.89	-0.24	0.00	1.42	0.16	99.94	-2.72	6.71	5.40	14.16
os43979	25	675	0.00	14	2	7	6.52	0.20	0.41	1.51	0.29	98.35	-8.87	7.16	4.90	13.66
os44024	25	571	0.18	14	2	7	7.03	0.24	0.00	1.47	0.19	96.65	-3.18	5.97	5.52	14.26
os44029	27	638	1.00	11	2	7	6.44	-0.18	0.30	1.49	0.18	97.86	-6.56	7.75	5.31	14.06
os44064	23	706	0.30	13	3	5	6.68	0.05	0.00	1.47	0.08	100.00	-0.47	6.05	5.29	14.03
os44136	3	353	0.01	12	2	3	6.96	0.20	0.05	1.44	0.16	99.90	-3.55	5.74	7.56	16.23
os44176	28	445	0.88	2	3	6	6.72	-0.04	2.24	1.54	0.06	85.53	-1.87	8.61	6.90	15.60
os44181	27	425	0.99	12	2	7	6.35	-0.17	0.32	1.51	0.11	92.14	-3.34	7.95	6.98	15.72
os44201	23	771	0.19	10	3	9	7.14	0.13	0.03	1.44	0.06	99.08	0.67	5.88	5.27	14.05
os44232	23	481	0.40	21	2	7	6.78	0.12	0.00	1.42	0.18	99.76	-2.02	5.19	6.57	15.30
os44276	17	450	0.99	12	3	5	6.40	-0.18	0.01	1.42	0.10	99.98	-0.11	6.30	6.92	15.65
os44323	25	552	0.00	8	3	9	6.97	0.12	0.11	1.47	0.03	95.71	1.34	6.11	6.69	15.45
os44331	23	488	0.99	17	1	1	5.98	-0.28	0.00	1.35	0.09	99.88	4.21	4.88	7.12	15.83
os44336	23	620	0.58	10	3	5	6.58	-0.04	0.06	1.47	0.10	99.98	-0.52	6.59	5.82	14.55
os44416	23	747	0.48	13	3	9	6.56	-0.02	0.00	1.42	0.07	99.57	1.80	5.87	5.50	14.27
os44429	23	471	0.53	23	1	2	5.91	-0.06	0.00	1.37	0.15	89.85	4.31	4.92	6.47	15.21
os44437	27	517	0.77	10	2	3	6.74	-0.05	0.38	1.49	0.12	99.65	-3.66	6.90	6.43	15.17
os44444	23	664	0.09	8	2	8	6.86	0.08	0.25	1.46	0.07	100.00	-1.85	5.80	5.91	14.64
os44458	23	459	0.99	13	3	5	6.30	-0.21	0.00	1.42	0.10	98.26	1.93	5.56	6.79	15.49
os44460	27	477	0.34	10	2	7	6.91	0.09	0.61	1.52	0.13	99.98	-5.86	7.95	6.53	15.24
os44470	23	442	0.92	14	1	4	6.52	-0.12	0.00	1.39	0.11	94.49	7.18	5.05	6.78	15.49
os44479	23	453	0.80	5	3	6	6.62	-0.08	0.42	1.40	0.07	98.07	1.18	5.98	6.83	15.56
os44491	23	435	0.51	19	2	7	6.13	-0.05	0.00	1.43	0.17	94.63	-2.40	6.01	6.76	15.49
os44508	23	558	0.40	9	2	0	6.96	0.06	0.32	1.50	0.09	99.98	-0.86	7.52	6.14	14.90
os44509	23	561	0.62	9	2	0	6.91	0.02	0.31	1.49	0.09	99.99	-0.49	7.18	6.11	14.87
os44515	23	649	0.00	9	3	5	7.05	0.16	0.15	1.47	0.08	99.44	-1.29	6.32	5.94	14.71
os44529	23	515	0.12	14	2	7	7.05	0.19	0.00	1.47	0.14	97.09	-2.58	6.42	6.43	15.17
os44533	24	515	0.08	3	3	6	6.92	0.05	1.56	1.53	0.05	71.65	-0.01	8.80	6.47	15.22
os44559	27	423	0.77	7	2	3	6.69	-0.07	0.62	1.49	0.08	99.47	-2.38	7.74	6.97	15.68
os44561	23	319	0.32	6	2	7	6.73	0.03	0.85	1.53	0.21	86.69	-2.52	6.58	7.18	15.89
os44619	27	466	0.01	4	2	7	6.84	0.05	1.68	1.54	0.14	97.30	-7.46	8.20	6.62	15.35
os44636	24	550	0.02	9	2	0	7.04	0.16	0.10	1.49	0.09	100.00	0.84	6.89	6.30	15.07
os44683	27	413	0.80	12	2	7	6.34	-0.06	0.26	1.46	0.17	99.29	-7.26	8.09	7.04	15.75
os44685	27	453	0.54	10	2	3	6.55	-0.03	0.65	1.48	0.13	99.25	-6.24	7.80	6.93	15.67
os44693	27	397	0.30	12	2	0	6.94	0.14	0.11	1.47	0.15	98.70	-1.88	7.50	7.06	15.78
os44731	27	325	0.57	18	2	7	5.72	-0.14	0.05	1.48	0.23	97.52	-8.11	6.05	7.45	16.13
os44744	27	339	0.98	17	2	7	6.08	-0.25	0.00	1.43	0.21	95.26	-5.41	7.27	7.39	16.08
os44836	23	569	0.86	13	1	4	6.26	-0.19	0.00	1.35	0.10	97.85	5.21	5.26	6.29	15.05
os44839	23	605	0.88	19	3	9	5.90	-0.27	0.00	1.40	0.14	99.51	-1.04	5.41	6.19	14.95
os44840	23	582	0.72	18	1	4	6.00	-0.19	0.00	1.40	0.14	98.55	2.11	5.65	6.13	14.90
os44908	23	576	0.30	14	2	7	6.53	0.05	0.01	1.47	0.15	98.89	-4.39	7.01	6.03	14.80

os44932	23	402	0.97	17	2	7	6.12	-0.25	0.00	1.46	0.17	84.20	-2.85	6.11	6.87	15.58
os44953	25	634	0.25	12	2	0	7.05	0.11	0.01	1.48	0.11	99.96	-1.42	6.65	5.61	14.37
os44954	23	635	0.35	12	2	7	7.01	0.08	0.07	1.49	0.11	99.90	-2.53	6.83	5.60	14.37
os44955	23	637	0.45	12	2	7	6.98	0.06	0.05	1.48	0.11	99.84	-2.40	6.88	5.59	14.36
os44976	27	486	0.95	5	3	5	6.69	-0.08	0.52	1.53	0.06	92.56	-0.26	6.92	6.63	15.39
os45006	23	606	0.81	19	3	9	5.94	-0.22	0.00	1.41	0.14	99.48	-0.74	5.59	6.14	14.91
os45007	23	567	0.68	19	1	4	5.89	-0.21	0.00	1.40	0.16	97.86	1.12	5.80	6.15	14.91
os45009	23	604	0.99	21	3	5	5.92	-0.32	0.00	1.37	0.16	99.99	0.84	5.58	6.03	14.81
os45010	23	630	0.99	16	1	1	6.14	-0.26	0.00	1.38	0.11	99.96	2.84	5.27	6.07	14.84
os45016	23	638	0.81	18	3	9	6.01	-0.21	0.00	1.39	0.12	99.90	-0.11	5.38	6.12	14.89
os45017	23	647	0.81	19	3	9	5.95	-0.22	0.00	1.38	0.13	99.95	-0.15	5.32	6.12	14.89
os45019	23	655	0.79	17	1	1	6.14	-0.17	0.00	1.36	0.08	99.98	5.22	5.11	6.15	14.91
os45020	23	650	0.73	14	1	1	6.34	-0.11	0.00	1.37	0.06	99.99	5.98	5.05	6.16	14.93
os45021	23	614	0.27	10	1	1	6.43	-0.15	0.01	1.38	0.06	99.76	4.87	5.50	6.25	15.01
os45023	27	657	0.05	7	2	3	7.06	0.11	0.76	1.49	0.10	99.62	-3.05	7.79	5.76	14.55
os45027	23	589	0.01	18	3	5	6.91	0.28	0.00	1.42	0.12	100.00	-1.30	5.82	6.23	14.98
os45040	23	510	0.25	23	2	0	6.83	0.23	0.00	1.41	0.20	98.37	-2.04	6.22	6.29	15.05
os45042	23	535	0.99	19	3	5	5.87	-0.32	0.00	1.39	0.14	99.81	1.50	5.72	6.47	15.21
os45046	23	511	0.60	20	3	5	5.99	-0.12	0.00	1.40	0.14	92.66	0.87	5.35	6.58	15.31
os45049	23	534	0.84	15	3	5	6.13	-0.20	0.00	1.40	0.12	98.94	0.64	5.64	6.48	15.20
os45052	23	501	0.17	18	1	2	6.52	0.13	0.00	1.39	0.12	97.11	5.66	5.43	6.43	15.16
os45053	17	468	0.78	16	2	0	6.05	-0.19	0.00	1.43	0.15	97.44	-0.59	6.52	6.58	15.31
os45057	23	578	0.87	21	3	9	6.20	-0.23	0.00	1.38	0.15	99.06	0.71	5.59	6.27	15.03
os45082	23	529	0.97	15	3	5	6.13	-0.25	0.00	1.41	0.10	99.09	-0.65	6.54	6.55	15.29
os45122	23	557	0.54	14	3	5	6.35	-0.08	0.00	1.44	0.12	99.87	-0.51	5.53	6.40	15.13
os45124	27	514	0.32	19	2	7	6.34	0.07	0.00	1.43	0.18	97.65	-2.06	6.08	6.26	14.98
os45136	23	543	0.62	17	1	2	6.65	-0.04	0.00	1.40	0.19	95.02	2.06	5.62	6.04	14.77
os45140	23	546	0.53	25	2	0	6.65	0.04	0.00	1.39	0.21	97.81	-1.69	6.38	6.04	14.82
os45147	23	616	0.70	15	1	2	6.73	-0.03	0.00	1.38	0.14	99.11	6.20	5.32	5.82	14.58
os45150	23	613	0.43	21	2	0	6.13	-0.03	0.00	1.42	0.15	99.51	-1.36	6.45	5.86	14.60
os45152	23	566	0.83	21	1	4	5.70	-0.28	0.00	1.38	0.16	97.91	2.44	5.59	6.13	14.90
os45154	23	634	0.89	13	3	5	6.29	-0.18	0.01	1.44	0.12	99.66	0.95	6.87	5.71	14.44
os45163	23	669	0.09	17	3	5	6.82	0.19	0.00	1.41	0.12	99.99	-2.23	6.79	5.74	14.50
os45164	23	701	0.06	6	3	9	6.98	0.09	0.39	1.44	0.04	99.91	0.71	6.61	5.68	14.45
os45182	23	588	0.16	22	1	4	7.03	0.29	0.00	1.35	0.14	98.83	4.37	5.43	5.99	14.71
os45186	23	661	0.39	14	3	5	6.55	0.03	0.00	1.44	0.16	99.98	-2.02	6.44	5.57	14.34
os45213	27	591	0.78	17	2	3	6.08	-0.19	0.00	1.44	0.15	99.90	-4.47	6.27	6.02	14.77
os45218	23	525	0.74	12	1	4	6.27	-0.19	0.00	1.44	0.11	99.47	3.33	5.84	6.27	15.00
os45241	23	595	0.77	2	1	1	6.85	-0.01	0.49	1.40	0.06	99.98	2.04	6.53	6.17	14.89
os45247	23	503	0.40	23	2	7	6.78	0.13	0.00	1.42	0.20	95.17	-1.44	5.33	6.16	14.90
os45314	27	391	0.32	8	2	3	6.87	0.05	0.68	1.48	0.13	100.00	-5.06	7.25	7.22	15.94
os45440	23	568	0.52	3	3	6	6.91	0.02	1.82	1.49	0.04	98.39	-1.56	7.44	6.41	15.14
os45454	27	474	0.70	20	2	0	5.87	-0.19	0.00	1.43	0.17	96.24	-0.99	5.55	6.56	15.28

os45533	23	457	0.92	11	2	7	6.69	-0.06	0.14	1.49	0.16	94.31	-1.51	6.89	6.58	15.30
os45539	23	492	0.01	10	2	3	6.96	0.16	0.17	1.50	0.08	99.13	-2.47	6.48	6.56	15.32
os45554	23	578	0.19	18	2	0	7.03	0.22	0.00	1.42	0.13	98.95	0.15	6.17	6.06	14.83
os45571	23	562	0.40	14	1	4	6.98	0.10	0.00	1.41	0.09	99.60	3.67	5.19	6.22	14.98
os45586	23	619	0.97	16	2	3	6.08	-0.27	0.00	1.46	0.14	99.95	-3.94	6.89	5.90	14.68
os45627	23	621	0.60	8	3	5	6.61	-0.05	0.39	1.48	0.07	99.86	-0.26	6.51	5.93	14.69
os45628	23	617	0.65	9	3	5	6.56	-0.07	0.26	1.48	0.07	99.79	-0.47	6.55	5.93	14.70
os45648	27	631	0.60	9	3	5	6.48	-0.16	0.11	1.47	0.07	99.87	-0.51	6.98	5.90	14.67
os45659	23	610	0.53	9	3	5	6.63	-0.02	0.18	1.42	0.08	97.93	1.16	6.21	6.12	14.86
os45663	23	583	0.07	10	3	5	7.07	0.16	0.21	1.47	0.10	99.93	-2.56	7.06	6.20	14.94
os45679	23	553	0.34	13	3	5	6.59	0.03	0.00	1.45	0.09	95.89	0.64	6.90	6.30	15.05
os45688	23	513	1.00	14	2	0	6.25	-0.23	0.00	1.46	0.12	96.70	-1.34	6.52	6.40	15.15
os45692	23	518	1.00	15	2	0	6.21	-0.24	0.00	1.43	0.11	95.50	1.22	5.95	6.42	15.17
os45696	23	534	0.69	5	1	4	6.90	0.02	0.10	1.49	0.04	99.42	1.99	6.67	6.56	15.30
os45702	23	513	0.30	9	1	4	6.99	0.09	0.03	1.47	0.06	98.85	1.81	6.49	6.63	15.37
os45715	23	443	0.92	15	3	5	6.49	-0.13	0.21	1.46	0.13	98.49	-1.61	7.64	6.96	15.69
os45717	27	458	0.21	5	2	3	6.90	0.06	1.84	1.52	0.08	98.95	-3.66	8.70	6.85	15.58
os45726	23	544	0.21	9	3	5	6.78	0.07	0.09	1.47	0.06	98.41	0.33	6.10	6.51	15.27
os45727	25	505	0.23	13	2	0	6.65	0.09	0.00	1.45	0.10	95.14	-0.12	6.31	6.55	15.30
os45738	17	379	0.44	25	2	7	6.69	0.12	0.00	1.43	0.21	94.29	-4.65	6.12	7.06	15.81
os45756	27	477	0.08	9	2	3	6.96	0.12	0.72	1.50	0.11	99.52	-4.14	7.71	6.81	15.56
os45765	17	388	0.02	9	3	5	6.96	0.14	0.21	1.49	0.10	97.11	-1.07	7.74	7.23	15.96
os45806	27	415	0.39	8	2	3	6.95	0.10	0.64	1.47	0.10	98.01	-2.47	7.97	7.24	15.97
os45871	19	546	0.60	4	3	5	6.67	-0.06	0.57	1.50	0.05	99.63	-0.62	6.81	6.61	15.37
os45944	23	483	0.26	7	2	7	6.96	0.12	0.45	1.51	0.17	95.34	-4.34	6.80	6.43	15.19
os45947	23	498	0.53	11	1	4	6.87	0.02	0.00	1.38	0.07	97.69	5.80	5.24	6.70	15.40
os45952	17	407	0.83	16	1	2	5.87	-0.24	0.00	1.39	0.13	90.02	4.79	6.09	7.01	15.72
os45972	23	510	0.61	15	3	9	6.21	-0.10	0.00	1.43	0.10	100.00	1.02	5.52	6.78	15.52
os45979	27	428	0.80	9	2	7	6.44	-0.13	0.72	1.52	0.11	94.54	-3.94	8.17	6.96	15.69
os45980	27	428	0.98	9	2	7	6.44	-0.14	0.49	1.52	0.10	93.64	-3.16	8.03	6.97	15.70
os45982	27	425	0.88	12	2	7	6.32	-0.18	0.26	1.50	0.11	91.67	-3.01	7.90	6.99	15.72
os45991	23	472	0.01	2	3	6	6.86	0.03	0.48	1.53	0.03	77.98	0.58	8.36	6.84	15.59
os45992	23	510	0.23	3	3	9	6.82	0.02	0.66	1.51	0.02	93.44	0.27	7.17	6.78	15.54
os45993	17	477	0.85	9	2	3	6.46	-0.13	0.57	1.51	0.10	97.96	-2.27	7.23	6.87	15.61
os45997	17	435	0.33	6	2	7	6.75	0.02	1.47	1.55	0.08	90.26	-3.34	7.69	6.91	15.65
os45998	19	476	0.01	3	2	3	6.85	0.02	2.84	1.54	0.06	87.95	-2.58	8.88	6.77	15.50
os46047	23	570	0.08	10	2	8	6.88	0.12	0.35	1.47	0.09	97.36	-3.01	6.60	6.47	15.24
os46049	23	548	0.00	8	1	1	6.98	0.13	0.08	1.48	0.04	94.45	1.78	5.87	6.62	15.37
os46057	23	486	0.25	14	2	3	6.97	0.15	0.18	1.47	0.14	99.99	-4.54	6.81	6.78	15.54
os46065	23	456	0.98	12	3	5	6.41	-0.18	0.00	1.48	0.10	99.65	-0.36	5.85	6.95	15.68
os46094	23	516	0.38	3	3	6	6.88	0.02	1.93	1.52	0.04	97.99	-1.31	8.91	6.49	15.22
os46125	23	505	0.24	5	2	3	6.78	0.04	1.43	1.53	0.11	97.64	-4.57	7.55	6.55	15.27
os46177	27	503	0.81	14	2	3	6.31	-0.17	0.20	1.49	0.10	98.20	-1.51	6.81	6.54	15.26

os46255	17	436	0.28	13	1	4	6.22	-0.19	0.01	1.43	0.08	93.40	3.45	5.98	6.99	15.69
os46286	17	407	0.65	20	3	5	5.93	-0.15	0.00	1.41	0.13	94.16	1.57	5.60	7.11	15.81
os46330	27	403	0.15	5	2	3	6.76	0.03	1.77	1.53	0.07	99.16	-3.10	8.04	7.14	15.85
os46441	17	451	0.32	15	3	5	6.51	0.05	0.00	1.46	0.08	91.19	0.30	6.57	6.86	15.56
os46450	19	440	0.40	5	3	6	6.84	0.01	0.89	1.53	0.06	87.33	-0.72	7.79	6.90	15.59
os46452	19	460	0.68	7	2	3	6.83	0.00	0.72	1.53	0.11	77.61	-3.49	7.39	6.75	15.46
os46488	17	390	0.99	15	2	7	6.17	-0.24	0.00	1.48	0.14	70.59	-3.75	6.05	7.09	15.84
os46598	19	456	0.77	11	2	3	6.35	-0.14	0.62	1.51	0.15	98.24	-5.51	7.11	6.81	15.53
os46647	23	689	0.24	6	1	1	6.86	0.03	0.05	1.43	0.06	98.05	3.76	6.06	5.56	14.30
os46667	23	517	0.47	11	2	3	6.94	0.10	0.34	1.51	0.13	99.06	-5.67	7.23	6.28	14.97
os46723	17	526	0.72	10	2	8	6.82	-0.02	0.16	1.48	0.11	99.24	-3.28	6.79	6.67	15.40
os46739	19	450	0.86	6	2	0	6.74	-0.05	0.89	1.52	0.10	98.75	-1.50	6.98	6.89	15.60
os46751	23	573	0.52	22	2	0	6.02	-0.05	0.00	1.41	0.16	96.37	1.73	5.39	5.89	14.60
os46757	23	527	0.00	8	2	3	6.97	0.12	0.73	1.51	0.07	97.39	-2.86	7.09	6.39	15.08
os46775	17	520	0.15	9	2	7	6.89	0.11	0.22	1.55	0.19	88.60	-7.95	7.09	6.15	14.90
os46788	19	409	0.17	5	2	3	6.87	0.06	1.57	1.53	0.13	98.17	-5.17	8.06	7.06	15.79
os46809	27	458	1.00	9	3	5	6.52	-0.14	0.32	1.50	0.05	94.83	-0.36	7.42	7.00	15.74
os46831	27	456	0.70	6	2	3	6.68	0.01	1.15	1.51	0.11	97.68	-4.22	7.11	6.95	15.72
os46855	27	482	0.82	6	2	3	6.65	-0.04	1.23	1.52	0.07	98.70	-3.09	8.77	6.70	15.43
os46879	19	462	1.00	11	2	3	6.54	-0.13	0.57	1.49	0.10	93.49	-4.45	7.60	6.85	15.56
os46900	19	410	0.03	11	2	3	6.94	0.17	0.27	1.48	0.10	99.28	-3.23	6.99	7.10	15.80
os46909	17	378	0.05	18	1	4	6.76	0.24	0.00	1.41	0.10	74.94	2.02	6.49	7.19	15.92
os46938	23	515	0.62	3	3	6	6.85	0.00	1.94	1.52	0.04	98.13	-1.40	8.93	6.50	15.22
os46963	9	309	0.47	3	2	3	6.80	0.05	1.53	1.53	0.10	79.48	-3.65	8.67	7.74	16.46
os46988	23	475	0.00	11	2	0	6.95	0.19	0.01	1.49	0.12	75.10	-1.08	5.82	6.46	15.16
os47064	10	306	0.10	3	3	9	6.83	0.05	0.60	1.50	0.02	93.87	0.48	6.95	8.07	16.80
os47076	23	484	0.92	1	2	7	6.86	0.04	1.83	1.55	0.10	84.41	-2.69	7.35	6.37	15.07
os47085	23	534	0.77	5	1	4	6.83	-0.02	0.12	1.46	0.02	97.32	2.90	6.36	6.48	15.23
os47086	23	538	0.73	7	1	1	6.58	-0.08	0.03	1.45	0.01	98.03	5.29	5.82	6.54	15.26
os47123	17	479	0.14	13	3	5	7.02	0.16	0.00	1.42	0.10	82.58	3.44	5.97	6.59	15.31
os47124	17	491	0.03	8	1	4	7.00	0.14	0.04	1.42	0.07	92.05	1.51	7.28	6.57	15.30
os47153	23	480	0.94	6	3	9	6.65	-0.09	0.60	1.47	0.04	96.01	1.34	6.48	6.80	15.54
os47163	23	474	0.02	12	3	5	7.02	0.19	0.13	1.48	0.09	99.10	-1.62	7.44	6.55	15.25
os47215	19	395	1.00	9	2	3	6.52	-0.12	0.62	1.51	0.11	98.67	-4.91	7.14	7.24	15.91
os47256	17	387	0.60	18	3	5	6.25	-0.05	0.17	1.47	0.15	99.22	-4.36	6.27	7.20	15.89
os47338	19	280	0.88	7	2	7	6.54	-0.09	0.73	1.55	0.16	72.68	-6.93	7.83	7.72	16.41
os47361	19	332	0.27	3	2	7	6.73	0.02	2.19	1.56	0.19	89.58	-5.87	7.33	7.22	15.95
os47486	19	302	0.23	3	2	7	6.67	0.00	1.90	1.57	0.09	64.58	-3.58	7.63	7.84	16.54
os47487	19	364	0.92	6	3	5	6.55	-0.09	0.76	1.51	0.06	99.95	-0.72	7.13	7.75	16.45
os47521	17	407	0.04	8	2	7	6.91	0.12	1.48	1.53	0.15	94.58	-6.39	7.31	7.09	15.83
os47578	12	311	0.77	2	3	6	6.74	-0.01	1.92	1.55	0.03	65.36	-0.50	8.00	7.89	16.64
os47654	6	407	0.95	11	2	8	6.35	-0.18	0.11	1.50	0.09	87.57	-2.75	6.67	7.50	16.22
os47682	17	514	1.00	10	1	1	6.47	-0.16	0.01	1.37	0.04	98.08	5.27	5.62	6.95	15.67

os47710	23	552	0.94	8	1	1	6.59	-0.13	0.10	1.44	0.04	99.51	3.44	5.93	6.52	15.25
os47744	19	426	0.73	5	2	7	6.65	-0.03	1.54	1.53	0.08	94.21	-2.47	7.77	7.10	15.81
os47793	19	354	0.59	7	2	3	6.77	0.01	1.04	1.55	0.12	93.35	-4.95	8.15	7.52	16.25
os47800	19	503	0.03	11	2	3	6.41	-0.11	0.37	1.48	0.13	99.12	-3.08	7.28	6.75	15.52
os47802	23	577	0.61	10	1	4	6.88	0.00	0.02	1.43	0.06	98.59	4.64	5.61	6.48	15.25
os47813	27	388	0.01	6	2	7	6.55	-0.10	1.03	1.51	0.14	96.69	-3.21	7.83	7.19	15.95
os47843	27	454	0.31	5	3	6	6.76	0.02	0.97	1.51	0.06	99.31	-1.77	7.70	7.02	15.76
os47888	23	566	0.77	8	3	5	6.77	-0.06	0.18	1.48	0.06	98.93	0.98	6.05	6.51	15.28
os47897	23	634	1.00	16	3	9	6.23	-0.25	0.00	1.40	0.08	99.99	0.48	5.90	6.43	15.21
os47898	25	558	0.87	7	3	5	6.75	-0.07	0.32	1.49	0.07	99.93	-0.92	7.21	6.64	15.41
os47899	25	563	0.90	6	2	3	6.82	-0.03	0.78	1.49	0.09	99.98	-2.80	7.41	6.63	15.40
os47946	19	446	0.46	12	2	7	6.33	-0.18	0.14	1.52	0.12	90.29	-4.19	8.30	6.94	15.69
os47951	28	489	0.04	3	3	6	6.90	0.05	0.76	1.52	0.02	73.52	0.07	8.14	6.85	15.60
os47953	27	415	0.87	11	2	3	6.35	-0.13	0.43	1.50	0.14	97.75	-7.02	8.23	7.14	15.87
os47954	27	411	0.80	12	3	5	6.27	-0.16	0.01	1.46	0.10	98.28	-1.10	6.06	7.23	15.96
os47996	9	317	0.10	11	1	4	6.74	0.13	0.02	1.44	0.05	85.70	3.92	6.54	7.85	16.57
os47997	9	315	0.13	14	3	5	6.65	0.13	0.02	1.43	0.07	85.13	2.33	6.61	7.85	16.56
os48018	19	367	0.92	3	3	6	6.73	0.01	1.47	1.53	0.07	91.60	-1.52	8.54	7.46	16.17
os48025	19	365	0.21	5	2	3	6.81	0.06	1.98	1.54	0.08	93.58	-3.27	8.25	7.49	16.21
os48027	11	324	0.92	5	2	7	6.48	-0.10	1.11	1.55	0.16	83.73	-6.05	7.16	7.61	16.33
os48042	27	434	0.07	15	3	5	6.77	0.17	0.00	1.43	0.11	94.30	2.37	6.01	7.06	15.79
os48062	19	386	0.77	4	2	3	6.78	-0.01	1.87	1.52	0.09	93.98	-2.26	8.02	7.43	16.13
os48067	19	440	0.82	9	2	3	6.45	-0.14	0.45	1.48	0.13	97.32	-3.96	7.60	7.00	15.72
os48116	9	283	0.98	12	1	4	6.33	-0.17	0.19	1.45	0.05	62.77	2.12	7.04	7.85	16.55
os48146	19	268	0.27	13	2	7	6.09	-0.21	0.52	1.52	0.19	90.84	-8.65	6.98	7.81	16.50
os48184	9	301	0.92	1	3	6	6.74	0.01	2.00	1.55	0.05	75.33	-1.51	7.52	7.93	16.61
os48218	19	322	0.97	8	2	7	6.60	-0.06	1.64	1.54	0.13	88.32	-6.54	7.21	7.47	16.13
os48276	19	323	0.99	5	3	6	6.62	-0.07	1.67	1.55	0.07	84.15	-2.27	8.76	7.74	16.46
os48294	19	395	0.71	3	2	7	6.82	0.05	2.18	1.56	0.14	88.05	-5.57	7.21	7.18	15.89
os48302	27	418	0.23	8	2	7	6.71	0.06	0.85	1.53	0.14	97.24	-5.97	7.89	7.04	15.77
os48377	19	399	0.47	2	2	7	6.75	-0.01	1.22	1.55	0.14	95.10	-6.42	7.59	7.12	15.85
os48393	19	442	1.00	6	2	3	6.57	-0.10	0.94	1.52	0.11	95.01	-3.06	6.74	6.94	15.64
os48444	28	503	0.00	4	3	6	6.93	0.06	1.77	1.53	0.03	82.85	-0.63	8.30	6.58	15.28
os48445	28	501	0.08	4	3	6	6.93	0.06	2.06	1.54	0.04	79.70	-1.26	8.40	6.58	15.28
os48468	28	465	0.99	4	3	9	6.69	-0.07	0.40	1.49	0.02	86.00	0.96	6.73	6.94	15.65
os48509	17	464	0.98	12	2	3	6.32	-0.18	0.10	1.47	0.16	98.99	-6.96	6.39	6.85	15.60
os48607	27	553	0.83	7	3	5	6.56	-0.10	0.40	1.48	0.08	99.52	-0.84	6.41	6.23	14.91
os48700	17	330	0.94	12	2	7	6.43	-0.15	0.06	1.50	0.10	57.94	-0.63	7.47	7.35	16.06
os48808	17	343	0.80	13	1	4	6.52	-0.11	0.04	1.44	0.06	83.11	2.46	6.90	7.47	16.17
os48814	17	328	0.13	19	2	7	6.55	0.19	0.00	1.45	0.16	96.99	-2.80	6.66	7.38	16.09
os48818	19	342	0.54	11	2	3	6.56	0.00	0.86	1.52	0.10	97.28	-3.81	8.39	7.44	16.18
os48894	17	387	0.99	11	2	3	6.51	-0.10	0.80	1.53	0.00	95.57	-8.57	7.31	7.13	15.85
os48920	16	473	0.47	2	3	9	6.76	-0.03	1.98	1.49	0.04	89.20	-1.85	7.91	6.91	15.64

os48931	19	400	0.48	8	2	3	6.58	-0.01	1.47	1.54	0.15	89.16	-6.62	6.95	7.08	15.82
os49004	23	505	0.25	18	2	3	6.57	0.10	0.00	1.49	0.15	94.47	-4.35	6.60	6.30	14.99
os49005	27	495	0.12	8	2	3	6.73	0.05	0.81	1.53	0.14	93.27	-5.61	7.37	6.31	14.99
os49009	25	541	0.08	6	2	0	6.97	0.10	0.49	1.54	0.00	91.20	-1.82	7.82	6.06	14.74
os49010	23	541	0.01	12	1	4	6.96	0.18	0.00	1.44	0.06	96.03	4.71	5.97	6.19	14.87
os49048	23	590	0.75	15	2	0	6.16	-0.17	0.00	1.45	0.00	99.83	-1.50	5.51	5.74	14.45
os49050	17	450	0.90	13	2	7	6.22	-0.21	0.01	1.49	0.14	83.37	-3.53	7.07	6.45	15.12
os49052	27	486	0.83	13	2	3	6.51	-0.14	0.03	1.47	0.15	98.09	-4.35	6.55	6.42	15.09
os49064	23	595	0.08	3	3	6	6.94	0.06	0.19	1.51	0.00	90.58	0.91	6.69	5.97	14.64
os49065	25	531	0.77	9	2	0	6.51	-0.10	0.35	1.50	0.12	97.21	-1.65	7.56	6.11	14.80
os49066	23	539	0.77	3	3	6	6.84	-0.02	0.47	1.51	0.03	94.40	0.57	7.22	6.18	14.86
os49068	23	516	0.35	18	1	4	6.42	0.06	0.00	1.43	0.11	89.68	1.59	5.24	6.29	14.97
os49069	23	525	0.04	14	3	5	7.05	0.22	0.02	1.48	0.08	91.39	-1.62	6.80	6.29	14.97
os49110	17	503	0.88	6	3	5	6.59	-0.10	0.65	1.50	0.07	94.05	0.69	7.50	6.24	14.93
os49122	17	366	0.79	10	2	7	6.38	-0.11	0.42	1.53	0.11	60.27	-4.72	7.09	7.01	15.73
os49169	23	554	0.66	6	3	9	6.67	-0.04	0.16	1.51	0.00	99.65	0.81	6.30	6.27	14.97
os49173	17	567	0.99	19	2	7	6.00	-0.30	0.00	1.45	0.17	94.55	-2.59	7.33	5.82	14.51
os49176	23	547	0.67	5	1	2	6.64	-0.06	0.09	1.49	0.08	98.17	2.41	6.95	6.00	14.65
os49177	28	543	0.23	10	2	0	6.43	-0.15	0.18	1.50	0.11	97.96	-0.13	7.18	5.96	14.62
os49182	27	557	0.47	12	2	7	6.47	-0.12	1.19	1.51	0.17	97.71	-8.74	6.93	5.88	14.57
os49204	23	479	0.00	22	2	0	6.83	0.37	0.00	1.42	0.18	91.39	-1.04	5.76	6.39	15.06
os49238	23	481	0.09	15	3	5	6.71	0.15	0.00	1.43	0.15	99.69	-1.32	6.15	6.48	15.16
os49258	23	597	0.24	4	1	1	6.82	0.00	0.01	1.45	0.03	99.82	5.50	6.32	6.12	14.77
os49264	23	550	0.04	6	2	7	6.98	0.10	0.97	1.54	0.11	98.11	-4.97	7.39	6.00	14.68
os49265	27	550	0.31	7	2	7	6.76	0.04	0.78	1.52	0.12	98.08	-3.98	7.36	6.01	14.68
os49268	23	598	0.38	10	2	3	6.57	0.01	0.02	1.48	0.11	100.00	-3.36	5.40	6.01	14.68
os49355	19	512	0.39	4	2	7	6.88	0.02	1.46	1.52	0.00	95.35	-1.85	8.33	6.16	14.86
os49362	27	472	0.44	11	2	3	6.46	-0.05	0.71	1.52	0.19	99.37	-8.69	7.69	6.46	15.14
os49379	19	460	0.12	9	2	7	6.72	0.07	0.19	1.51	0.16	73.52	-2.69	7.42	6.38	15.05
os49438	25	546	0.93	11	2	7	6.56	-0.12	0.63	1.50	0.16	97.58	-5.45	7.73	5.91	14.59
os49455	23	588	0.99	14	3	5	6.23	-0.23	0.00	1.45	0.11	98.23	-1.10	6.83	5.79	14.51
os49480	17	415	0.36	7	2	7	6.91	0.07	0.57	1.52	0.13	81.06	-1.81	8.01	6.59	15.29
os49494	17	375	0.32	7	2	3	6.55	-0.05	1.28	1.56	0.00	85.99	-6.03	7.80	7.00	15.68
os49501	19	418	0.02	2	3	6	6.82	0.03	2.92	1.55	0.03	48.94	-0.36	9.17	6.81	15.51
os49507	19	392	0.69	6	2	7	6.63	-0.03	1.52	1.57	0.00	92.31	-4.91	8.45	6.85	15.52
os49513	23	564	0.40	9	3	9	6.97	0.06	0.02	1.46	0.05	99.92	1.75	5.67	6.16	14.86
os49521	28	551	0.23	9	2	0	6.75	0.06	0.11	1.49	0.07	98.71	1.07	6.35	6.04	14.70
os49527	27	502	0.98	2	2	7	6.78	-0.02	1.94	1.54	0.13	85.46	-2.94	7.43	6.08	14.76
os49578	17	537	0.23	5	2	7	6.80	0.03	1.29	1.52	0.11	97.75	-2.35	7.30	6.15	14.88
os49584	17	509	1.00	5	2	0	6.68	-0.08	1.37	1.54	0.00	97.76	-1.01	7.81	6.20	14.91
os49597	17	481	1.00	13	3	5	6.31	-0.21	0.08	1.42	0.07	95.32	2.05	7.07	6.56	15.23
os49611	23	575	0.80	7	2	3	6.75	-0.06	0.69	1.49	0.11	99.41	-4.45	6.52	6.16	14.89
os49617	27	475	0.15	10	2	3	6.43	-0.14	0.52	1.51	0.17	99.42	-6.76	7.68	6.46	15.14

os49672	19	390	0.05	6	2	7	6.89	0.10	1.55	1.55	0.09	88.88	-3.39	7.72	6.94	15.62
os49688	17	492	0.82	22	3	5	6.28	-0.18	0.00	1.37	0.15	96.12	-0.34	5.35	6.43	15.13
os49708	19	368	0.52	7	3	5	6.63	-0.01	0.09	1.50	0.00	71.52	1.44	7.27	7.23	15.94
os49732	23	710	0.26	9	2	0	6.73	0.01	0.04	1.49	0.08	93.90	1.53	7.40	5.29	14.02
os49745	23	660	0.33	3	3	9	6.85	0.01	0.61	1.48	0.03	91.90	1.02	7.09	5.84	14.54
os49761	23	694	0.56	11	3	5	6.92	0.00	0.01	1.48	0.10	99.90	0.31	6.09	5.44	14.16
os49764	27	664	0.23	7	2	0	6.84	0.04	0.18	1.52	0.09	96.41	0.76	7.78	5.45	14.16
os49765	28	699	0.81	7	2	0	6.77	-0.07	0.35	1.51	0.08	97.16	-0.31	7.52	5.31	14.03
os49802	25	714	0.21	11	3	5	6.84	0.10	0.01	1.48	0.09	100.00	-1.04	6.47	5.30	14.03
os49803	23	668	0.63	11	3	5	6.49	-0.09	0.01	1.46	0.10	99.90	0.73	5.98	5.52	14.24
os49867	23	523	0.35	14	1	4	6.61	0.09	0.00	1.40	0.10	94.78	5.96	5.29	6.35	15.10
os49872	23	607	0.88	5	2	3	6.66	-0.06	1.35	1.52	0.11	96.62	-4.11	8.49	5.93	14.74
os49884	23	522	0.71	7	2	7	6.79	-0.04	0.54	1.50	0.12	99.66	-3.00	7.71	6.26	15.01
os49889	25	570	0.64	12	3	5	6.89	0.03	0.18	1.49	0.00	98.85	-2.35	7.24	6.13	14.86
os49900	23	569	0.10	16	2	3	6.82	0.19	0.00	1.47	0.11	89.98	-2.32	7.56	6.22	15.01
os49907	23	565	1.00	16	3	5	6.11	-0.24	0.00	1.41	0.10	95.66	0.68	6.49	6.22	15.02
os49969	23	572	0.91	8	2	0	6.71	-0.08	0.26	1.52	0.08	99.98	-0.92	6.65	5.94	14.73
os49974	23	580	0.77	10	2	0	6.75	-0.07	0.12	1.51	0.08	100.00	-1.07	6.30	5.88	14.67
os49981	9	294	0.92	0	3	6	6.74	0.01	1.19	1.53	0.01	36.33	0.89	10.58	7.97	16.64
os49987	23	646	0.82	15	1	4	6.16	-0.20	0.00	1.42	0.09	92.34	3.45	5.54	5.74	14.47
os50066	9	300	0.00	3	3	6	6.80	0.05	0.97	1.55	0.02	47.26	-0.03	8.81	7.79	16.49
os50102	19	411	0.02	7	2	7	6.50	-0.12	0.90	1.55	0.13	93.32	-6.29	9.29	7.09	15.83
os50116	9	289	0.87	7	3	5	6.49	-0.08	0.78	1.52	0.05	35.42	0.46	8.19	7.91	16.58
os50129	23	659	0.82	13	1	1	6.31	-0.17	0.00	1.43	0.07	98.98	2.49	5.86	5.85	14.56
os50132	10	284	0.94	4	2	3	6.63	-0.05	1.57	1.54	0.06	72.27	-2.03	7.75	7.90	16.59
os50181	17	324	0.60	4	2	3	6.72	-0.02	1.64	1.54	0.08	76.63	-3.95	7.65	7.72	16.45
os50183	19	324	0.87	4	2	3	6.60	-0.05	1.46	1.55	0.09	75.04	-4.12	7.67	7.72	16.45
os50192	17	415	0.12	6	3	5	6.59	-0.09	0.89	1.52	0.05	84.18	-1.37	7.72	7.29	15.98
os50206	27	477	0.16	6	2	7	6.95	0.09	0.91	1.51	0.11	93.56	-2.99	7.28	6.72	15.43
os50209	23	617	0.52	15	3	5	6.38	-0.05	0.00	1.45	0.12	97.10	-0.07	5.90	6.05	14.77
os50210	23	624	0.51	15	1	4	6.35	-0.07	0.00	1.42	0.10	96.70	3.81	5.36	6.04	14.76
os50279	17	392	1.00	12	3	5	6.33	-0.20	0.01	1.48	0.08	84.83	-0.71	7.99	7.32	16.02
os50280	17	390	0.99	13	2	3	6.33	-0.19	0.06	1.50	0.09	84.97	-1.68	8.10	7.32	16.02
os50296	17	452	0.55	4	2	3	6.73	-0.01	1.67	1.53	0.05	96.78	-2.03	8.31	7.00	15.72
os50306	27	457	0.38	6	2	3	6.73	0.03	1.11	1.55	0.15	95.99	-7.28	7.22	6.91	15.64
os50335	25	628	0.23	9	3	5	6.77	0.05	0.04	1.49	0.06	99.43	1.67	5.92	5.99	14.70
os50336	23	593	0.03	11	2	7	6.88	0.13	0.08	1.51	0.11	95.44	-3.74	7.62	6.04	14.75
os50411	27	505	0.08	5	2	3	6.81	0.02	1.79	1.54	0.08	91.20	-4.05	9.04	6.52	15.24
os50475	17	380	0.57	4	3	6	6.71	-0.01	1.98	1.55	0.05	82.81	-1.42	9.23	7.48	16.26
os50499	12	314	0.47	2	3	6	6.70	0.00	1.57	1.56	0.02	39.95	0.01	8.48	7.88	16.58
os50541	28	480	0.23	4	3	6	6.79	0.03	0.88	1.50	0.03	99.86	-0.47	7.76	7.08	15.86
os50549	17	356	0.83	16	2	3	6.48	-0.12	0.08	1.47	0.13	88.02	-3.47	6.36	7.58	16.28
os50570	11	306	0.01	2	3	6	6.77	0.03	2.90	1.56	0.07	77.42	-1.78	8.21	7.87	16.62

os50571	11	305	0.08	1	2	3	6.78	0.03	2.36	1.57	0.07	75.70	-2.23	8.23	7.87	16.63
os50572	11	305	0.08	2	3	6	6.78	0.03	2.33	1.56	0.07	75.32	-2.19	8.21	7.88	16.63
os50573	11	304	0.06	2	2	3	6.78	0.03	2.18	1.56	0.07	75.05	-2.20	8.26	7.88	16.63
os50574	11	304	0.08	2	2	3	6.78	0.03	2.12	1.56	0.08	74.78	-2.34	8.24	7.88	16.64
os50575	11	304	0.09	2	2	3	6.79	0.03	2.12	1.56	0.08	74.55	-2.49	8.17	7.88	16.64
os50576	11	304	0.38	2	2	3	6.78	0.03	1.95	1.57	0.08	74.27	-2.55	8.19	7.88	16.64
os50577	11	304	0.23	2	2	3	6.78	0.02	2.02	1.56	0.08	74.00	-2.40	8.10	7.89	16.64
os50578	11	303	0.13	2	3	6	6.78	0.02	2.14	1.56	0.08	73.49	-2.16	8.12	7.89	16.64
os50579	11	303	0.40	2	3	6	6.78	0.02	2.05	1.56	0.08	72.98	-1.98	8.06	7.89	16.65
os50591	19	409	0.68	7	2	7	6.58	-0.04	0.69	1.54	0.12	83.90	-3.60	7.65	7.15	15.87
os50604	19	408	0.40	3	2	7	6.78	0.00	1.62	1.55	0.16	85.71	-5.04	7.08	7.12	15.85
os50605	19	408	0.58	10	2	7	6.72	-0.01	0.43	1.52	0.16	85.21	-5.27	6.81	7.14	15.87
os50634	23	560	0.60	14	1	4	6.42	-0.04	0.00	1.41	0.07	98.94	5.23	5.44	6.32	15.06
os50635	28	525	0.02	5	3	5	6.92	0.08	0.70	1.52	0.03	99.32	-0.18	7.66	6.60	15.35
os50637	17	450	0.01	5	3	6	6.88	0.08	0.55	1.54	0.05	98.73	0.31	8.18	6.92	15.65
os50638	17	493	0.53	13	2	3	6.36	-0.05	0.05	1.49	0.10	96.18	-3.51	8.76	6.67	15.42
os50639	23	551	0.60	3	3	6	6.89	0.01	1.12	1.43	0.03	91.78	0.93	7.51	6.43	15.15
os50654	19	326	0.70	4	3	6	6.63	-0.03	1.71	1.55	0.06	85.93	-2.04	7.28	7.80	16.55
os50655	17	387	0.02	16	2	3	6.88	0.26	0.00	1.47	0.14	94.38	-2.87	7.56	7.42	16.19
os50666	17	465	0.02	10	2	3	6.88	0.15	0.17	1.48	0.10	99.88	-2.96	7.86	6.83	15.55
os50676	23	501	0.55	11	2	7	6.43	-0.07	0.32	1.53	0.16	95.88	-6.95	6.48	6.46	15.19
os50679	17	497	0.53	8	2	7	6.63	0.00	0.57	1.51	0.13	93.68	-2.58	7.20	6.51	15.23
os50680	23	495	0.11	9	2	7	6.67	0.03	0.71	1.53	0.14	93.68	-4.27	7.23	6.51	15.23
os50684	27	520	0.94	5	3	6	6.71	-0.07	2.17	1.53	0.05	87.91	-1.36	8.92	6.44	15.17
os50685	27	507	0.63	6	2	7	6.73	-0.04	2.20	1.55	0.10	93.77	-4.47	8.51	6.48	15.19
os50689	25	498	0.17	5	2	0	6.83	0.04	1.82	1.55	0.08	91.25	-1.56	8.84	6.51	15.23
os50695	19	428	0.71	4	2	3	6.74	0.00	1.63	1.55	0.07	86.94	-2.66	8.89	7.09	15.82
os50716	17	457	0.20	6	2	3	6.79	0.06	1.09	1.52	0.11	91.75	-5.28	7.72	6.91	15.66
os50718	19	428	0.48	7	2	3	6.63	-0.01	0.85	1.54	0.09	85.83	-4.20	8.70	7.04	15.79
os50720	19	434	0.91	7	2	3	6.60	0.00	0.82	1.54	0.17	85.71	-8.42	8.58	7.03	15.77
os50752	17	398	0.27	5	1	4	6.62	-0.05	0.02	1.44	0.04	49.59	5.24	7.33	7.29	16.06
os50753	17	398	0.98	5	1	4	6.62	-0.08	2.56	1.46	0.04	50.15	4.12	7.92	7.29	16.06
os50773	19	327	1.00	6	3	5	6.54	-0.09	0.65	1.53	0.06	96.25	-1.17	7.32	7.79	16.55
os50781	17	363	0.92	3	2	3	6.83	0.04	2.31	1.54	0.07	96.33	-2.90	9.10	7.60	16.38
os50782	17	362	0.59	3	3	6	6.81	0.02	2.77	1.54	0.06	96.00	-2.05	9.23	7.62	16.39
os50783	9	307	0.13	1	3	6	6.74	0.01	4.43	1.56	0.02	15.15	0.12	9.75	7.96	16.68
os50784	9	307	0.92	0	3	6	6.74	0.00	4.16	1.56	0.02	17.40	0.17	11.79	7.96	16.68
os50785	9	306	0.09	1	3	6	6.76	0.02	4.10	1.56	0.02	14.78	-0.12	9.67	7.96	16.68
os50786	9	307	0.92	1	3	6	6.74	0.00	3.97	1.57	0.03	20.31	-0.26	9.37	7.96	16.67
os50787	9	306	0.06	1	3	6	6.75	0.01	3.99	1.56	0.03	14.96	-0.34	9.62	7.96	16.68
os50789	11	305	0.19	13	3	5	6.63	0.06	0.49	1.48	0.09	88.19	-2.21	6.33	7.97	16.69
os50792	10	333	0.08	4	3	6	6.88	0.08	0.74	1.53	0.04	96.73	-0.52	7.18	7.79	16.53
os50802	19	377	0.92	6	2	7	6.52	-0.09	1.34	1.54	0.10	89.85	-3.62	8.35	7.44	16.23

os50812	23	583	0.28	11	2	3	7.03	0.12	0.05	1.46	0.10	99.79	-2.03	6.37	6.17	15.00
os50835	17	431	0.20	5	3	6	6.85	0.07	0.81	1.54	0.08	96.19	-1.78	7.43	7.11	15.94
os50870	27	649	0.81	10	2	3	6.48	-0.12	0.29	1.50	0.13	99.99	-4.27	7.05	5.68	14.53
os50871	23	655	0.74	13	2	3	6.35	-0.14	0.01	1.47	0.12	99.99	-2.71	6.62	5.67	14.52
os50872	27	661	0.77	12	3	5	6.37	-0.14	0.01	1.46	0.11	100.00	-1.20	6.58	5.66	14.51
os50873	27	667	0.79	11	3	5	6.40	-0.14	0.02	1.47	0.11	100.00	-1.25	6.53	5.66	14.51
os50874	27	678	0.75	12	3	5	6.37	-0.15	0.01	1.46	0.10	100.00	-1.41	6.41	5.65	14.49
os50875	23	651	0.79	13	2	3	6.33	-0.17	0.01	1.48	0.12	99.99	-3.26	6.70	5.69	14.53
os50882	23	574	0.47	9	3	5	6.61	-0.03	0.31	1.48	0.07	95.49	0.74	6.61	6.19	15.02
os50884	27	555	0.41	6	2	7	6.75	0.01	1.03	1.53	0.11	95.95	-3.92	7.91	6.23	15.06
os50889	23	573	0.07	6	3	5	6.85	0.05	0.34	1.48	0.03	95.72	1.54	7.06	6.39	15.20
os50926	19	484	0.70	13	2	3	6.24	-0.18	0.05	1.49	0.11	99.87	-2.23	6.35	6.93	15.71
os50933	19	453	0.30	7	2	3	6.88	0.04	1.06	1.52	0.11	96.49	-5.45	7.59	6.96	15.74
os50934	19	453	0.90	4	2	3	6.66	-0.05	1.73	1.53	0.10	96.58	-3.91	7.41	6.97	15.74
os50935	19	465	1.00	6	2	3	6.62	-0.09	1.20	1.54	0.09	94.71	-4.11	7.17	6.95	15.73
os50936	19	457	1.00	7	2	3	6.59	-0.11	0.73	1.52	0.10	96.29	-4.04	7.41	6.96	15.73
os50941	17	402	0.98	1	3	6	6.74	-0.02	2.87	1.56	0.06	28.76	-0.52	7.66	7.23	16.00
os50951	19	429	0.65	3	2	7	6.76	0.00	1.91	1.55	0.15	95.20	-6.77	7.26	7.00	15.81
os50959	17	467	0.10	4	2	3	6.74	-0.01	1.49	1.53	0.08	94.86	-3.85	7.50	6.94	15.75
os50971	17	417	0.83	15	2	7	6.15	-0.19	0.03	1.49	0.14	95.75	-4.39	6.35	7.14	15.93
os50983	17	395	0.01	2	3	6	6.77	0.01	2.95	1.54	0.05	70.77	-1.46	8.81	7.38	16.14
os51045	27	434	0.14	10	2	7	6.35	-0.16	0.75	1.52	0.17	94.09	-8.53	6.90	6.91	15.68
os51049	17	383	0.07	4	3	6	6.90	0.08	0.55	1.54	0.08	35.36	-1.03	8.22	7.32	16.10
os51078	23	598	0.69	16	1	4	6.10	-0.18	0.00	1.41	0.11	99.99	2.18	6.17	6.09	14.91
os51081	23	665	0.02	9	3	5	7.10	0.16	0.07	1.45	0.08	99.91	0.20	5.86	5.78	14.60
os51084	27	612	0.00	7	2	0	7.00	0.12	0.66	1.50	0.10	98.26	-2.00	8.27	5.84	14.66
os51085	27	657	0.24	3	2	3	6.91	0.04	1.85	1.51	0.10	99.34	-3.69	8.11	5.67	14.51
os51086	23	612	0.51	9	2	0	6.68	0.00	0.44	1.51	0.09	97.79	-1.99	8.25	5.84	14.66
os51104	17	455	0.95	14	2	3	6.50	-0.14	0.19	1.46	0.12	98.54	-3.58	6.32	7.08	15.84
os51183	19	583	0.40	8	3	5	6.97	0.05	0.48	1.51	0.08	100.00	-0.23	6.71	5.80	14.54
os51225	23	637	0.40	8	2	0	7.02	0.07	0.19	1.51	0.10	100.00	-0.44	6.82	5.40	14.14
os51242	23	654	0.63	10	2	0	6.49	-0.15	0.23	1.51	0.12	92.54	-1.97	6.44	4.95	13.71
os51244	23	623	0.04	16	2	7	6.85	0.18	0.00	1.47	0.16	94.21	-1.51	7.27	5.11	13.87
os51247	23	722	0.77	15	2	7	6.65	-0.12	0.00	1.50	0.00	100.00	-2.10	6.30	4.54	13.34
os51248	28	712	0.89	11	2	7	6.67	-0.12	0.02	1.52	0.00	100.00	-2.12	6.57	4.59	13.38
os51343	30	647	0.76	20	1	1	6.63	-0.05	0.00	1.35	0.16	98.66	4.28	4.82	5.47	14.22
os51450	23	748	0.86	17	3	5	6.05	-0.25	0.00	1.41	0.10	99.98	0.45	5.70	4.81	13.61
os51584	30	527	0.92	15	3	5	6.09	-0.24	0.00	1.46	0.12	98.84	-1.18	5.56	6.12	14.94
os51657	19	572	0.46	2	3	6	6.88	0.01	3.96	1.56	0.05	57.81	-0.67	8.88	5.87	14.68
os51665	19	422	0.77	3	2	0	6.84	0.03	1.89	1.55	0.11	80.77	-1.91	7.35	6.57	15.36
os51725	17	563	0.98	14	1	4	6.38	-0.15	0.00	1.33	0.09	98.78	8.37	6.39	6.08	14.84
os51771	19	555	0.65	6	2	3	6.59	-0.08	1.38	1.52	0.14	98.54	-7.38	7.55	6.04	14.78
os51791	19	441	0.55	9	2	7	6.43	-0.02	0.16	1.53	0.19	85.30	-2.76	6.49	6.37	15.10

os51810	19	555	0.02	14	3	5	6.94	0.20	0.00	1.43	0.08	99.62	-0.39	5.84	6.19	14.92
os51834	17	473	0.22	16	1	2	6.65	0.13	0.00	1.39	0.13	66.39	5.33	4.84	6.35	15.05
os51842	17	592	0.18	9	1	4	6.93	0.03	0.03	1.41	0.06	99.31	6.16	5.69	5.84	14.58
os51847	17	491	0.68	19	2	7	6.56	-0.06	0.00	1.46	0.25	94.29	-6.70	6.28	6.10	14.86
os51896	23	596	0.30	15	3	5	6.98	0.09	0.02	1.46	0.10	93.30	-0.39	5.94	5.67	14.44
os51900	17	419	0.92	5	2	7	6.84	0.12	0.83	1.56	0.22	63.98	-7.33	7.39	6.34	15.08
os51918	19	566	0.30	9	2	3	6.70	0.02	0.64	1.49	0.11	97.04	-3.67	7.96	6.00	14.74
os51925	23	626	0.33	13	3	9	6.67	0.07	0.00	1.45	0.07	99.92	-0.02	5.52	5.85	14.60
os51975	23	659	0.77	15	2	0	6.63	-0.11	0.00	1.46	0.13	100.00	-1.09	6.37	5.30	14.04
os51977	25	678	0.23	8	3	5	7.06	0.09	0.18	1.49	0.08	100.00	-0.48	6.26	5.28	14.01
os52079	23	589	0.92	12	3	5	6.31	-0.19	0.01	1.47	0.10	99.53	-1.18	6.32	5.87	14.66
os52084	17	561	1.00	19	1	4	6.09	-0.27	0.00	1.38	0.12	98.56	3.36	5.34	6.07	14.83
os52092	23	656	0.95	12	1	1	6.45	-0.19	0.00	1.38	0.08	99.91	6.15	5.20	5.78	14.52
os52109	27	622	0.93	11	2	7	6.39	-0.14	0.76	1.54	0.22	98.60	-9.40	8.17	5.19	13.97
os52112	25	640	0.11	8	1	1	7.08	0.13	0.15	1.43	0.04	99.64	2.35	5.86	5.83	14.52
os52127	23	760	0.77	6	3	5	6.91	-0.03	0.23	1.50	0.05	95.65	0.51	6.77	4.72	13.51
os52128	27	757	0.76	6	3	5	6.92	-0.02	0.46	1.50	0.05	94.62	0.10	6.81	4.72	13.51
os52163	17	351	0.45	8	1	4	6.53	-0.03	0.04	1.47	0.06	97.84	2.46	6.13	7.50	16.31
os52181	23	675	0.99	15	2	0	6.24	-0.24	0.00	1.46	0.11	91.78	0.04	7.58	5.14	13.95
os52197	28	736	0.93	6	3	5	6.79	-0.08	0.57	1.51	0.05	97.88	-0.47	7.20	4.79	13.63
os52374	19	487	0.89	4	2	7	6.70	-0.06	1.79	1.52	0.11	99.08	-2.52	8.40	6.21	14.91
os52460	19	527	1.00	10	2	7	6.36	-0.15	1.03	1.55	0.24	98.16	-11.98	7.42	5.53	14.23
os52527	19	492	0.92	6	2	7	6.61	-0.09	0.99	1.53	0.12	98.99	-2.60	7.40	6.13	14.89
os52528	23	599	0.11	12	2	7	6.84	0.12	0.06	1.47	0.12	100.00	-3.17	7.22	5.60	14.40
os52529	27	606	0.23	14	2	0	6.65	0.08	0.00	1.44	0.12	100.00	0.04	6.28	5.59	14.40
os52589	22	494	0.06	16	2	0	6.79	0.21	0.00	1.45	0.12	99.67	-1.52	5.75	6.26	14.98
os52664	23	689	0.02	10	1	4	7.10	0.18	0.01	1.43	0.05	95.87	4.13	5.64	5.24	14.04
os52672	27	507	0.08	1	2	0	6.81	-0.01	2.96	1.57	0.11	45.44	-1.30	7.79	5.88	14.63
os52683	23	599	0.71	17	2	0	6.08	-0.15	0.00	1.45	0.15	92.90	1.23	5.81	5.34	14.09
os52744	23	620	0.30	12	3	5	7.07	0.14	0.01	1.43	0.09	100.00	1.36	5.63	5.77	14.54
os52766	19	570	0.99	12	2	7	6.34	-0.20	0.03	1.49	0.13	99.71	-2.93	7.73	5.76	14.56
os52797	23	607	0.51	9	3	5	6.92	0.02	0.40	1.45	0.08	99.98	-0.33	6.03	5.66	14.42
os52813	11	445	0.08	3	2	7	6.86	0.04	1.77	1.57	0.12	50.53	-1.90	7.90	6.55	15.29
os52990	23	598	0.65	11	1	4	6.44	-0.10	0.01	1.44	0.09	97.98	3.65	5.80	5.94	14.73
os53122	23	617	0.70	15	1	1	6.27	-0.12	0.00	1.38	0.08	99.93	2.27	5.61	6.11	14.87
os53159	23	605	0.52	11	2	7	6.86	0.00	0.48	1.50	0.15	99.99	-7.34	7.45	5.69	14.51
os53161	19	490	0.35	13	2	7	6.63	0.10	0.01	1.48	0.18	92.64	-5.63	6.73	6.41	15.24
os53253	17	438	0.64	11	2	3	6.48	-0.04	0.16	1.50	0.09	99.58	-1.96	7.26	6.93	15.74
os53277	23	655	0.23	5	1	1	6.73	-0.05	0.03	1.42	0.04	100.00	5.29	5.87	5.68	14.48
os53309	19	323	0.08	2	3	6	6.78	0.03	1.82	1.54	0.04	90.57	-0.49	8.33	7.73	16.60
os53409	19	339	0.66	4	2	0	6.75	-0.02	1.94	1.55	0.09	96.83	-2.06	8.16	7.36	16.20
os53555	25	646	0.00	11	3	9	7.06	0.19	0.02	1.45	0.07	100.00	-0.52	5.85	5.89	14.71
os53608	23	591	0.79	13	3	5	6.30	-0.21	0.01	1.47	0.10	99.67	-1.26	6.26	5.89	14.67

os53624	11	289	0.62	4	3	6	6.62	-0.05	2.16	1.55	0.06	92.16	-1.61	8.79	7.91	16.78
os53660	9	240	0.12	6	2	3	6.76	0.07	1.33	1.53	0.08	97.10	-3.71	8.53	8.57	17.50
os53667	9	292	0.23	7	3	5	6.68	0.05	0.61	1.50	0.05	95.64	0.31	6.95	8.30	17.26
os53668	9	292	0.23	8	3	5	6.67	0.05	0.55	1.50	0.05	95.85	0.36	6.86	8.30	17.26
os53669	9	291	0.26	9	3	5	6.61	0.04	0.16	1.50	0.06	97.09	-1.98	7.00	8.31	17.26
os53670	9	298	0.30	8	3	5	6.61	0.03	0.17	1.49	0.05	98.03	1.06	6.26	8.32	17.27
os53671	9	296	0.40	8	3	5	6.61	0.03	0.14	1.49	0.05	97.93	0.94	6.30	8.32	17.27
os53672	9	290	0.38	11	3	5	6.54	0.03	0.03	1.48	0.06	97.26	0.91	6.37	8.32	17.28
os53675	9	271	0.11	9	2	3	6.70	0.09	0.36	1.51	0.09	94.51	-3.14	6.90	8.34	17.31
os53676	19	276	0.84	10	2	3	6.37	-0.11	0.53	1.50	0.09	95.78	-2.75	6.68	8.35	17.31
os53815	19	385	0.77	1	2	7	6.76	-0.01	2.60	1.55	0.10	93.43	-3.64	8.08	7.09	15.96
os53824	23	667	0.23	6	3	5	6.86	0.04	0.46	1.51	0.04	97.73	0.32	6.62	5.52	14.39
os53825	23	665	0.23	5	3	5	6.85	0.03	0.58	1.51	0.04	98.31	0.48	7.75	5.50	14.37
os53826	23	665	0.23	5	3	6	6.86	0.04	0.62	1.51	0.04	97.93	0.39	6.50	5.54	14.40
os53840	19	464	0.09	5	2	3	6.94	0.08	1.15	1.53	0.07	99.59	-2.61	8.46	6.74	15.62
os53858	17	486	0.91	7	3	9	6.54	-0.12	0.33	1.49	0.05	99.19	-0.08	6.02	6.82	15.72
os53890	28	562	0.40	2	3	6	6.89	0.02	2.75	1.54	0.05	94.18	-1.58	9.11	6.08	14.94
os53927	23	569	1.00	10	3	5	6.47	-0.17	0.11	1.49	0.09	99.30	-1.33	5.89	6.11	15.00
os53956	28	598	0.12	6	2	3	7.01	0.08	0.97	1.52	0.08	98.94	-2.32	7.74	5.82	14.68
os53963	23	633	0.00	7	3	9	7.03	0.13	0.43	1.50	0.05	97.15	-0.37	6.49	5.83	14.69
os54094	23	554	0.55	5	2	0	6.90	0.01	0.42	1.54	0.08	69.66	-0.86	8.60	6.04	14.88
os54157	23	556	0.96	18	2	7	6.16	-0.24	0.00	1.49	0.18	78.53	-4.45	6.23	6.05	14.88
os54167	23	652	0.19	3	3	6	6.98	0.05	1.19	1.53	0.03	89.27	0.23	7.82	5.53	14.38
os54174	23	647	0.36	5	3	6	6.97	0.03	0.57	1.52	0.03	97.12	-0.02	7.25	5.62	14.49
os54175	28	625	0.01	2	3	6	6.93	0.04	1.68	1.55	0.03	76.36	-0.16	7.92	5.68	14.56
os54182	23	655	0.08	8	3	9	7.07	0.12	0.47	1.47	0.06	99.35	0.27	6.27	5.68	14.55
os54195	19	448	0.26	10	2	7	6.59	0.00	0.50	1.53	0.12	96.60	-3.08	8.72	6.46	15.28
os54202	19	520	0.36	4	2	7	6.64	-0.08	1.54	1.54	0.12	98.92	-3.00	9.02	6.15	15.01
os54206	23	581	0.04	6	3	5	6.93	0.08	0.12	1.47	0.05	94.25	1.45	6.93	6.05	14.88
os54215	17	555	0.79	5	3	5	6.78	-0.05	0.60	1.52	0.04	99.93	-0.24	6.70	6.15	15.01
os54328	19	300	0.01	7	3	6	6.83	0.09	0.20	1.50	0.04	80.77	1.22	7.26	8.08	17.09
os54354	19	447	0.97	5	2	7	6.73	-0.03	2.54	1.55	0.10	75.74	-5.14	7.94	6.89	15.83
os54403	19	459	0.63	8	2	7	6.77	-0.03	0.49	1.53	0.09	93.67	-2.35	8.68	6.72	15.63
os54685	19	321	0.41	4	2	0	6.81	0.02	2.37	1.54	0.08	91.54	-1.55	8.98	7.61	16.51
os55193	19	295	0.21	10	2	7	6.72	0.08	0.72	1.53	0.18	86.25	-8.91	6.54	7.72	16.60
os55273	19	279	0.99	5	2	3	6.57	-0.09	1.14	1.52	0.06	93.16	-1.95	8.58	8.06	16.97
os55373	19	287	0.71	9	2	7	6.72	-0.02	0.70	1.53	0.11	89.09	-2.76	7.80	7.91	16.80
os55488	19	283	0.92	4	3	6	6.64	-0.05	1.70	1.55	0.05	85.54	-0.84	8.82	7.99	16.95
os55489	19	282	0.99	4	3	6	6.65	-0.05	1.78	1.55	0.05	84.80	-0.81	8.84	7.99	16.95
os55517	23	571	0.77	4	3	6	6.82	-0.03	1.32	1.55	0.06	83.14	-0.70	8.10	5.92	14.75
os55519	23	665	0.62	8	3	5	6.93	0.00	0.33	1.50	0.05	98.42	0.34	6.61	5.45	14.30
os55565	19	355	0.08	5	2	7	6.58	0.04	0.91	1.55	0.24	90.45	-4.02	7.54	6.83	15.61
os55596	23	597	0.01	3	3	6	6.92	0.05	1.36	1.53	0.04	91.60	-0.24	9.14	5.84	14.69

os55610	23	658	0.14	8	3	5	7.07	0.11	0.27	1.49	0.04	96.33	1.50	6.90	5.65	14.53
os55626	23	716	0.35	10	3	5	6.73	0.02	0.06	1.47	0.07	99.99	0.49	6.96	5.26	14.11
os55634	23	692	0.40	11	3	5	6.67	0.02	0.02	1.49	0.09	98.86	-1.68	5.63	5.26	14.08
os55639	23	688	0.40	10	3	5	7.01	0.05	0.11	1.50	0.06	96.73	-0.43	6.50	5.34	14.16
os55644	23	671	0.36	13	3	5	6.58	0.03	0.00	1.44	0.08	96.75	1.49	6.11	5.49	14.31
os55664	23	577	0.10	10	1	4	6.89	0.13	0.04	1.48	0.06	80.73	1.76	7.00	5.90	14.73
os55673	23	611	0.47	4	1	4	6.91	0.00	0.07	1.45	0.03	95.67	2.75	6.54	5.78	14.60
os55676	23	597	0.11	9	3	5	6.82	0.08	0.45	1.51	0.08	99.00	-2.14	7.44	5.83	14.69
os55677	23	621	0.37	10	3	5	6.71	0.02	0.06	1.48	0.05	99.82	1.60	6.90	5.84	14.70
os55678	23	626	0.62	7	3	5	6.87	-0.02	0.31	1.50	0.06	97.22	0.10	7.09	5.71	14.59
os55683	23	741	0.31	5	3	5	7.04	0.05	0.65	1.51	0.04	100.00	-0.43	7.25	5.00	13.89
os55727	28	602	0.15	2	3	6	6.92	0.02	0.79	1.54	0.02	75.10	0.21	8.57	5.98	14.88
os55776	28	607	0.40	3	3	6	6.91	0.01	2.42	1.55	0.05	85.48	-1.27	9.05	5.86	14.76
os55822	23	568	0.42	9	2	0	6.96	0.05	0.34	1.51	0.10	89.78	-1.33	6.91	5.92	14.78
os55834	28	608	0.35	4	3	6	6.83	0.02	0.62	1.53	0.05	99.59	-0.07	7.53	5.97	14.84
os55864	23	581	0.08	2	3	6	6.91	0.03	0.96	1.54	0.02	76.50	0.05	7.85	6.10	15.00
os55930	23	621	0.70	4	3	6	6.76	-0.03	1.22	1.47	0.03	95.69	0.26	7.10	6.15	15.07
os55966	19	514	0.52	8	2	0	6.92	0.04	0.37	1.53	0.10	72.08	-1.03	8.12	6.30	15.21
os55969	19	305	0.01	5	2	3	6.73	0.03	1.03	1.54	0.07	87.22	-1.71	8.13	7.95	16.92
os56067	28	639	0.77	4	3	6	6.84	-0.04	1.43	1.51	0.03	86.79	-0.68	7.64	5.85	14.76
os56088	19	452	0.49	7	2	3	6.65	-0.02	0.92	1.48	0.08	88.85	-4.13	8.63	7.23	16.28
os56179	23	544	0.40	6	3	5	6.95	0.05	0.89	1.53	0.05	95.47	-1.32	7.69	6.31	15.18
os56201	17	390	0.73	27	2	7	6.34	-0.07	0.00	1.43	0.23	85.95	-7.13	6.04	7.03	15.87
os56211	19	370	0.92	3	2	7	6.61	-0.04	1.50	1.57	0.27	83.04	-8.65	6.44	6.93	15.82
os56369	23	531	0.67	2	3	6	6.77	-0.04	2.46	1.54	0.04	93.31	-1.56	8.69	6.41	15.28
os56374	23	543	0.40	5	3	5	6.94	0.04	0.92	1.53	0.06	96.67	-1.23	8.00	6.31	15.17
os56376	23	605	0.00	4	3	6	7.00	0.08	0.90	1.49	0.05	95.95	-0.40	7.78	6.04	14.95
os56395	19	270	0.71	3	2	3	6.67	0.00	1.86	1.55	0.10	91.17	-3.88	8.73	8.08	17.10
os56400	19	274	0.92	2	2	3	6.74	0.03	3.17	1.56	0.06	72.11	-2.04	8.50	8.09	17.07
os56424	19	412	0.53	19	2	7	6.02	-0.10	0.15	1.46	0.15	97.76	-4.34	7.50	7.27	16.30
os56441	17	351	0.40	7	2	0	6.89	0.06	0.38	1.53	0.08	99.60	-0.63	6.47	7.61	16.58
os56475	19	387	0.37	5	2	7	6.57	0.00	0.97	1.55	0.19	77.46	-7.24	6.86	6.99	15.86
os56480	23	583	0.08	2	3	6	6.92	0.03	0.37	1.52	0.02	96.05	0.45	7.16	6.13	14.98
os56481	23	584	0.11	2	3	6	6.92	0.03	0.33	1.52	0.02	96.07	0.51	7.08	6.13	14.97
os56485	23	541	0.82	4	3	6	6.79	-0.04	0.78	1.53	0.04	94.78	-0.12	7.41	6.38	15.25
os56486	23	544	0.24	6	3	5	6.98	0.07	0.31	1.52	0.05	96.42	0.21	7.26	6.31	15.17
os56688	19	532	0.19	7	2	0	6.85	0.07	0.37	1.54	0.09	78.83	-1.60	8.62	6.23	15.09
os56734	28	610	0.62	4	3	6	6.90	0.00	0.52	1.52	0.02	98.08	0.39	7.22	6.00	14.87
os56746	23	620	0.86	3	3	6	6.79	-0.05	0.30	1.51	0.02	79.05	0.82	7.86	5.90	14.78
os56747	28	623	0.44	2	3	6	6.86	0.01	1.73	1.51	0.02	72.36	0.20	7.82	5.91	14.79
os56820	17	512	0.92	8	2	7	6.47	-0.13	0.22	1.52	0.13	86.17	-3.30	7.01	6.29	15.12
os56871	23	670	0.77	5	3	6	6.80	-0.06	0.69	1.53	0.04	97.74	-0.20	7.46	5.43	14.32
os56873	23	696	0.65	3	3	6	6.94	0.00	1.42	1.52	0.02	88.92	-0.04	7.45	5.41	14.30

os56959	23	670	0.00	14	1	4	7.04	0.23	0.00	1.45	0.11	99.63	3.34	5.18	5.89	14.59
os57014	19	610	0.19	8	2	7	6.85	0.11	1.09	1.54	0.14	92.49	-6.70	7.90	6.10	14.91
os57044	19	569	0.77	3	2	7	6.81	-0.02	1.99	1.55	0.09	91.53	-3.03	8.51	6.34	15.06
os57077	19	574	0.77	1	2	0	6.86	-0.01	3.20	1.55	0.06	82.76	0.19	9.51	6.34	15.06
os57156	19	588	0.93	4	3	6	6.74	-0.06	2.15	1.54	0.06	90.40	-1.61	8.80	6.36	15.01
os57246	23	745	0.87	9	3	5	6.56	-0.12	0.12	1.45	0.06	99.53	0.00	6.38	5.44	14.19
os57247	23	738	0.78	10	3	5	6.52	-0.13	0.07	1.45	0.07	99.58	0.28	6.50	5.44	14.18
os57251	28	673	0.00	9	2	0	7.05	0.14	0.18	1.52	0.07	97.53	-0.13	7.79	5.71	14.40
os57263	27	593	0.06	6	2	7	6.74	0.06	0.95	1.55	0.19	95.10	-9.16	7.41	6.17	14.87
os57264	23	595	0.23	3	2	7	6.67	0.02	1.61	1.55	0.22	96.05	-11.37	7.39	6.16	14.86
os57312	23	718	0.13	3	1	1	6.96	0.05	0.05	1.46	0.01	96.08	3.85	6.87	5.79	14.53
os57313	23	638	1.00	6	3	6	6.71	-0.09	0.70	1.54	0.07	87.29	-1.23	8.77	6.01	14.74
os57385	23	643	0.95	6	3	5	6.75	-0.08	0.25	1.49	0.05	92.38	1.79	6.40	5.98	14.63
os57403	25	744	0.07	21	2	3	6.96	0.33	0.00	1.41	0.18	97.83	-2.89	5.97	5.57	14.36
os57457	28	556	0.00	6	2	0	6.96	0.10	1.06	1.55	0.11	92.67	-1.96	7.88	6.37	15.13
os57491	23	633	0.98	9	3	5	6.64	-0.12	0.03	1.48	0.08	90.73	1.70	6.23	6.03	14.74
os57497	22	559	0.65	28	1	4	6.47	-0.05	0.00	1.32	0.17	95.66	4.52	5.24	6.49	15.16
os57527	23	648	0.01	9	1	4	6.83	0.07	0.04	1.44	0.06	90.62	3.46	7.06	5.97	14.68
os57541	25	696	0.00	15	1	4	7.08	0.24	0.00	1.43	0.09	99.18	2.97	5.53	5.84	14.54
os57554	23	668	0.07	19	3	9	7.07	0.29	0.00	1.41	0.10	99.83	0.26	5.35	6.08	14.78
os57559	28	664	0.19	2	3	6	6.95	0.03	2.76	1.55	0.03	55.91	-0.34	8.70	5.98	14.77
os57574	25	706	0.43	10	3	5	7.03	0.06	0.04	1.48	0.07	99.80	-0.22	5.95	5.85	14.63
os57582	24	658	0.60	3	3	6	6.81	-0.03	1.54	1.55	0.03	83.67	-0.23	7.45	6.00	14.73
os57583	24	659	0.75	3	3	6	6.79	-0.03	1.11	1.55	0.03	86.45	-0.23	7.30	5.99	14.72
os57586	28	649	0.92	0	3	6	6.87	-0.01	3.95	1.56	0.03	33.87	-1.18	9.07	6.01	14.73
os57616	23	644	0.77	1	3	9	6.87	-0.01	0.47	1.49	0.01	70.64	0.19	8.15	6.21	14.96
os57622	23	715	0.01	11	1	1	7.13	0.18	0.02	1.44	0.04	97.06	4.73	5.85	5.80	14.53
os57640	19	546	1.00	14	2	7	6.26	-0.23	0.01	1.49	0.14	88.82	-2.65	6.30	6.50	15.22
os57663	28	646	0.85	4	3	6	6.79	-0.06	0.81	1.53	0.03	89.47	0.18	7.06	6.13	14.93
os57687	22	592	0.23	13	2	3	6.64	0.05	0.37	1.49	0.14	98.41	-4.18	6.96	6.29	14.97
os57691	28	683	0.72	5	3	6	6.89	-0.02	0.42	1.53	0.03	96.27	-0.01	7.10	5.89	14.61
os57702	23	631	0.00	9	3	5	7.04	0.15	0.16	1.53	0.08	72.12	-1.90	7.74	5.99	14.67
os57713	28	708	0.92	0	3	6	6.91	0.00	2.51	1.55	0.01	14.98	0.14	11.10	5.70	14.39
os57717	28	693	0.92	1	3	6	6.90	-0.01	2.13	1.54	0.02	58.27	0.44	9.21	5.76	14.47
os57718	28	691	0.23	1	3	6	6.92	0.01	3.97	1.55	0.02	17.92	-0.55	9.76	5.73	14.44
os57722	23	637	0.62	8	3	5	6.86	-0.03	0.22	1.52	0.07	64.73	0.08	7.52	6.01	14.67
os57728	23	666	0.05	8	1	4	7.08	0.13	0.05	1.48	0.05	94.23	2.64	7.02	5.90	14.62
os57736	28	680	0.01	3	3	6	6.97	0.05	0.83	1.53	0.03	63.08	-0.25	7.74	5.91	14.65
os57760	23	624	0.00	10	1	4	7.07	0.16	0.05	1.47	0.06	98.99	2.70	6.41	6.14	14.88
os57765	28	663	0.01	4	2	7	6.96	0.06	2.02	1.54	0.11	97.70	-5.60	8.21	5.83	14.62
os57767	23	634	0.09	14	3	5	7.08	0.20	0.00	1.46	0.12	99.76	-1.62	5.83	6.10	14.78
os57780	23	755	0.88	4	3	6	6.85	-0.05	0.45	1.53	0.04	90.32	0.25	8.25	5.24	13.90
os57804	23	674	0.22	6	3	5	7.05	0.08	0.45	1.52	0.06	96.13	-0.79	7.23	5.76	14.42

os57809	23	687	0.23	3	3	6	6.99	0.04	1.94	1.54	0.04	71.96	-1.50	8.73	5.84	14.56
os57810	23	677	0.05	15	2	3	6.98	0.21	0.00	1.47	0.10	94.46	-1.07	6.92	5.86	14.57
os57813	28	661	0.00	3	3	6	6.95	0.05	3.36	1.55	0.04	50.72	-1.16	8.82	5.98	14.77
os57817	23	649	0.29	3	3	6	6.85	0.00	2.28	1.54	0.02	65.41	0.69	8.52	6.04	14.77
os57820	28	622	0.60	2	3	6	6.84	0.00	1.90	1.54	0.03	41.96	-0.38	8.86	6.26	15.03
os57824	28	609	0.92	3	2	3	6.71	-0.05	1.76	1.56	0.09	63.28	-2.85	7.22	6.23	15.02
os57923	28	672	0.92	5	3	5	6.69	-0.07	0.50	1.51	0.04	94.54	-0.04	7.82	5.85	14.57
os57940	23	649	0.00	11	3	5	6.39	-0.18	0.02	1.46	0.09	99.98	-0.98	6.16	5.94	14.64
os57951	23	613	0.38	11	1	4	6.60	0.03	0.02	1.38	0.04	76.63	8.34	5.56	6.32	15.08
os57952	23	621	0.00	4	3	6	6.96	0.07	0.79	1.52	0.03	80.15	-0.24	7.42	6.31	15.07
os57985	23	600	0.99	3	3	6	6.80	-0.04	1.84	1.50	0.03	85.53	-0.58	7.35	6.37	15.16
os58027	23	589	0.83	14	2	0	6.58	-0.13	0.00	1.48	0.11	97.50	-0.14	7.23	6.37	15.17
os58065	17	664	0.47	9	3	9	6.97	0.03	0.12	1.47	0.09	100.00	-1.41	5.73	6.17	14.92
os58066	17	673	0.41	11	3	9	6.99	0.05	0.01	1.46	0.08	100.00	-0.09	5.63	6.13	14.88
os58071	28	641	0.77	3	3	6	6.89	-0.01	1.87	1.54	0.04	93.21	-0.52	7.66	6.20	14.92
os58118	23	642	0.33	11	1	4	6.49	-0.05	0.01	1.43	0.10	99.96	1.93	6.47	6.13	14.98
os58175	19	524	0.76	4	2	7	6.72	-0.05	1.59	1.55	0.11	95.42	-5.41	8.69	6.68	15.40
os58190	23	591	0.89	19	2	0	6.26	-0.22	0.00	1.44	0.14	95.96	-0.80	5.45	6.25	15.00
os58284	23	636	0.76	5	1	4	6.90	-0.01	0.25	1.46	0.03	98.50	2.28	6.20	6.15	14.87
os58313	28	704	0.03	2	3	6	6.95	0.03	0.45	1.53	0.02	59.24	0.50	7.48	5.76	14.52
os58318	25	727	0.76	14	3	9	6.26	-0.17	0.00	1.45	0.09	99.40	-0.84	5.68	5.81	14.55
os58320	25	721	0.97	15	1	1	6.44	-0.20	0.00	1.43	0.07	98.53	3.09	5.36	5.73	14.49
os58457	28	686	0.23	2	3	6	6.90	0.01	1.63	1.54	0.04	71.21	0.06	7.38	5.76	14.51
os58472	28	622	1.00	5	2	7	6.72	-0.08	1.46	1.54	0.08	94.56	-2.50	8.80	6.04	14.77
os58481	17	665	0.63	7	3	9	6.90	-0.02	0.17	1.48	0.05	100.00	0.60	6.01	6.16	14.91
os58483	17	674	0.37	11	3	9	6.71	0.05	0.01	1.46	0.08	100.00	-1.39	5.51	6.13	14.88
os58504	28	642	0.45	3	3	6	6.93	0.01	0.23	1.54	0.02	55.62	0.69	7.90	6.15	14.87
os58544	27	648	0.19	9	2	0	7.03	0.08	0.38	1.50	0.10	98.89	-1.51	7.52	5.93	14.76
os58564	28	652	0.96	4	3	6	6.79	-0.06	1.69	1.52	0.04	95.32	-1.09	8.36	6.14	14.98
os58571	19	552	0.00	5	2	0	6.95	0.08	0.93	1.54	0.11	93.14	-1.08	7.65	6.31	15.06
os58574	23	693	0.92	2	3	6	6.87	-0.01	1.88	1.52	0.04	91.12	0.04	8.40	5.89	14.73
os58606	22	580	0.27	22	2	0	6.47	0.14	0.00	1.43	0.18	94.49	-3.15	6.61	6.23	14.96
os58614	27	577	0.93	6	3	5	6.75	-0.07	0.97	1.52	0.07	98.09	-1.74	7.96	6.46	15.25
os58618	23	680	0.40	6	1	1	7.03	0.06	0.17	1.47	0.03	97.05	2.19	6.25	6.14	14.97
os58619	27	639	0.60	5	2	3	6.96	0.04	1.06	1.52	0.10	99.91	-4.61	7.61	6.17	15.01
os58620	28	591	0.08	4	3	6	6.97	0.06	1.29	1.54	0.06	97.20	-0.32	8.29	6.41	15.23
os58662	19	538	0.27	9	2	3	6.75	0.05	0.87	1.51	0.14	92.29	-6.09	8.60	6.74	15.49
os58687	23	616	0.45	7	3	5	6.74	0.00	0.24	1.47	0.04	89.64	2.06	6.59	6.29	15.03
os58776	28	795	0.66	5	3	6	6.78	-0.08	0.56	1.52	0.03	76.55	-0.10	7.22	5.06	13.76
os58777	32	794	0.45	6	3	5	6.72	-0.09	0.48	1.52	0.03	76.95	-0.07	6.83	5.06	13.75
os58791	23	727	0.67	14	1	1	6.33	-0.13	0.00	1.36	0.06	95.81	9.67	5.42	5.52	14.19
os58792	28	713	0.53	20	3	5	6.12	-0.08	0.00	1.39	0.14	94.88	0.45	5.73	5.51	14.18
os58799	23	653	0.12	21	3	5	6.97	0.31	0.00	1.41	0.14	98.35	-0.78	5.28	5.93	14.60

os58802	27	635	0.03	20	3	5	7.03	0.32	0.00	1.42	0.15	99.44	-1.98	6.58	5.97	14.63
os58817	19	716	0.87	20	3	5	6.32	-0.22	0.00	1.42	0.11	94.33	1.55	6.64	5.51	14.18
os58826	19	713	0.66	6	3	9	6.95	0.00	0.82	1.48	0.05	97.72	-1.53	7.98	5.70	14.37
os59022	24	655	0.92	1	3	6	6.85	-0.02	0.72	1.54	0.03	67.77	0.08	7.68	5.94	14.62
os59029	32	775	0.92	0	3	6	6.94	0.00	3.62	1.53	0.01	28.77	0.36	9.61	5.20	13.90
os59033	28	674	0.92	5	3	6	6.71	-0.08	0.39	1.50	0.03	94.01	0.07	7.37	5.85	14.57
os59058	23	625	0.99	20	1	4	5.99	-0.30	0.00	1.43	0.14	99.95	-0.20	7.36	6.25	14.98
os59060	30	770	0.92	7	1	1	6.84	-0.07	0.22	1.43	0.03	99.20	1.88	5.97	5.60	14.31
os59064	28	800	0.53	5	3	6	6.76	-0.09	0.53	1.51	0.02	71.53	0.65	7.19	5.06	13.76
os59074	19	681	0.00	11	3	5	7.07	0.19	0.02	1.50	0.10	100.00	-0.95	6.17	5.65	14.34
os59075	23	691	0.13	8	2	0	6.53	-0.15	0.11	1.48	0.08	84.10	1.66	6.38	5.52	14.15
os59084	23	662	0.73	8	3	5	6.97	0.03	0.26	1.47	0.08	99.12	-0.72	7.07	5.97	14.67
os59113	28	596	0.92	0	3	6	6.87	0.01	2.31	1.52	0.01	15.41	-0.16	9.81	6.47	15.29
os59131	27	595	0.05	5	3	9	6.89	0.06	0.98	1.48	0.06	91.80	-0.51	7.56	6.63	15.43
os59214	28	425	0.92	7	3	5	6.49	-0.11	0.22	1.51	0.07	99.41	-0.08	6.78	7.35	16.23
os59410	17	369	0.08	2	2	0	6.82	0.04	1.96	1.54	0.10	92.80	-1.69	7.75	8.12	17.13
os59459	27	532	0.83	6	2	3	6.81	-0.03	0.82	1.54	0.07	98.98	-1.93	9.05	6.65	15.59
os59499	17	397	0.37	6	2	7	6.62	-0.01	1.13	1.55	0.13	97.48	-7.06	8.87	7.34	16.27
os59519	10	303	0.64	11	2	3	6.33	-0.10	0.41	1.50	0.12	95.14	-3.33	8.07	8.10	17.02
os59642	28	632	0.77	1	3	6	6.87	-0.01	2.22	1.55	0.01	28.72	0.32	9.34	6.10	15.04
os59644	24	612	0.21	2	3	6	6.92	0.02	2.98	1.55	0.04	82.61	-1.42	9.45	6.13	15.08
os59645	27	632	0.44	2	3	6	6.89	-0.01	1.79	1.55	0.03	81.65	-0.15	8.95	6.08	15.03
os59677	17	476	0.21	9	2	3	6.98	0.10	0.55	1.50	0.08	99.28	-2.69	8.05	6.93	15.86
os59709	17	463	0.98	18	1	1	5.97	-0.30	0.00	1.39	0.09	97.47	2.20	5.54	7.24	16.08
os59744	17	310	0.29	7	3	5	6.92	0.10	0.25	1.49	0.07	91.49	0.62	7.01	7.98	16.84
os59746	17	302	0.09	12	2	0	6.97	0.19	0.01	1.46	0.08	89.70	0.66	6.99	7.98	16.85
os59815	28	600	0.12	2	3	6	6.89	0.03	1.81	1.54	0.02	77.02	0.18	9.18	6.32	15.25
os59817	28	619	0.60	3	3	6	6.81	-0.02	0.23	1.54	0.02	76.70	0.67	8.05	6.25	15.19
os59835	28	610	0.63	3	3	6	6.76	-0.04	0.84	1.53	0.03	98.74	0.15	8.10	6.28	15.18
os59853	17	566	0.61	12	1	4	6.41	-0.09	0.00	1.45	0.07	94.44	3.61	5.52	6.50	15.42
os59887	17	553	0.23	11	3	5	6.72	0.06	0.16	1.48	0.07	95.99	-1.01	6.85	6.70	15.61
os59914	28	542	0.92	2	3	6	6.78	-0.03	2.98	1.54	0.05	62.11	-1.00	8.57	6.60	15.49
os59934	17	580	0.21	8	3	9	6.82	0.07	0.03	1.45	0.04	97.48	2.25	5.74	6.65	15.56
os60014	17	378	0.23	10	2	7	6.82	0.03	0.55	1.52	0.14	94.22	-5.03	8.09	7.48	16.42
os60015	17	382	0.55	12	2	7	6.78	0.01	0.26	1.51	0.14	95.50	-5.02	8.13	7.48	16.41
os60225	17	352	0.72	11	2	3	6.61	-0.06	0.33	1.50	0.14	99.60	-6.39	6.94	8.17	17.12
os60249	17	374	0.81	7	3	5	6.50	-0.10	0.35	1.51	0.09	94.05	-0.42	6.27	7.95	16.91
os60250	17	375	0.65	7	3	5	6.49	-0.10	0.33	1.51	0.09	94.09	-0.60	6.22	7.95	16.91
os60279	17	369	0.09	15	3	9	6.85	0.20	0.03	1.42	0.09	99.28	-1.43	6.33	8.20	17.15
os60280	17	365	0.03	18	2	8	6.79	0.25	0.00	1.41	0.13	99.22	-2.66	6.04	8.20	17.15
os60282	17	301	0.68	14	2	7	6.15	-0.23	0.01	1.52	0.15	70.07	-4.22	7.39	8.31	17.22
os60311	27	480	0.37	5	2	3	6.91	0.05	2.16	1.51	0.10	95.31	-4.54	8.38	7.27	16.26
os60390	23	669	0.73	3	3	6	6.81	-0.03	1.63	1.53	0.04	99.72	-0.22	7.73	6.04	14.99

os60411	28	658	0.74	2	3	6	6.82	-0.02	1.68	1.54	0.03	81.97	-0.39	8.40	6.07	15.02
os60417	10	367	0.66	13	2	0	6.23	-0.12	0.00	1.48	0.11	64.21	-1.49	5.94	7.87	16.74
os60418	10	370	0.66	15	3	5	6.13	-0.12	0.00	1.48	0.11	66.49	-1.76	5.89	7.87	16.73
os60440	23	617	0.47	5	3	5	6.74	-0.02	0.30	1.49	0.04	99.17	1.02	6.25	6.41	15.36
os60442	23	642	0.76	5	3	5	6.71	-0.06	0.74	1.52	0.04	97.43	-0.57	8.16	6.21	15.15
os60467	17	535	0.74	5	2	3	6.65	-0.06	1.27	1.50	0.07	97.60	-2.73	8.09	7.04	16.05
os60618	10	362	0.86	14	3	5	6.48	-0.13	0.01	1.45	0.10	96.93	0.32	7.47	8.18	17.17
os60695	23	593	0.11	3	3	6	6.95	0.04	1.23	1.54	0.03	95.49	-0.52	7.89	6.39	15.32
os60805	17	369	0.08	2	2	0	6.81	0.03	1.96	1.54	0.10	92.80	-1.90	7.74	8.11	17.13
os60807	19	315	0.41	6	2	7	6.68	-0.03	2.00	1.55	0.14	93.91	-6.77	7.48	7.85	16.73
os60827	9	303	0.99	5	3	5	6.58	-0.09	0.58	1.51	0.05	97.43	-0.71	7.56	8.39	17.32
os60843	19	399	0.25	3	2	3	6.84	0.03	1.87	1.54	0.05	90.93	-2.34	7.89	7.41	16.25
os60911	12	287	0.92	1	3	6	6.75	0.02	3.07	1.56	0.02	29.88	-0.58	9.02	8.23	17.02
os60924	12	290	0.92	1	3	6	6.72	0.00	2.89	1.55	0.02	45.71	1.06	9.98	8.21	16.97
os60951	11	278	0.92	1	3	6	6.71	-0.01	3.39	1.56	0.04	17.41	-0.57	9.73	8.31	17.08
os60960	17	344	0.98	2	3	6	6.72	-0.02	0.35	1.54	0.01	92.43	0.33	8.65	7.94	16.74
os60996	17	383	0.70	6	2	3	6.79	0.00	1.00	1.53	0.10	96.85	-4.30	8.29	7.61	16.44
os61066	19	416	0.10	5	2	3	6.76	0.03	1.51	1.55	0.11	95.72	-6.13	7.61	7.22	16.01
os61099	16	397	0.00	2	3	6	6.72	-0.04	1.82	1.55	0.03	72.25	-0.33	8.66	7.35	16.14
os61114	17	402	0.24	5	2	3	6.81	0.06	1.38	1.53	0.08	97.35	-3.50	6.76	7.44	16.26
os61136	17	424	0.05	6	2	0	6.93	0.10	0.43	1.51	0.10	98.82	-0.17	7.84	7.10	15.92
os61182	17	422	0.76	7	2	7	6.77	-0.03	1.04	1.54	0.09	93.70	-3.93	6.96	7.15	15.94
os61183	19	418	0.82	6	2	0	6.64	-0.06	1.45	1.54	0.09	85.86	-1.14	7.53	7.20	15.99
os61387	19	454	0.32	7	2	3	6.86	0.06	1.03	1.53	0.10	97.89	-4.82	7.53	7.03	15.85
os61393	19	431	0.30	5	3	5	6.91	0.07	1.18	1.54	0.05	92.52	-1.52	8.03	7.19	15.99
os61435	17	381	0.22	16	2	3	6.56	0.15	0.37	1.52	0.18	95.49	-8.31	6.27	7.39	16.19
os61505	19	480	0.38	9	2	3	6.61	-0.01	0.58	1.48	0.08	99.28	-2.94	6.84	7.00	15.79
os61561	19	413	0.76	3	2	0	6.78	-0.01	0.61	1.54	0.08	90.46	0.34	7.29	7.22	16.07
os61566	19	425	0.57	10	2	0	6.82	0.01	0.03	1.50	0.09	96.71	-0.43	5.93	7.13	15.96
os61586	19	406	0.95	5	2	3	6.72	-0.04	1.66	1.54	0.08	91.98	-2.87	7.65	7.31	16.08
os61587	19	409	0.12	5	2	3	6.58	-0.09	1.20	1.54	0.10	95.53	-4.35	6.91	7.30	16.08
os61605	17	413	0.76	5	2	7	6.80	0.02	1.48	1.54	0.11	91.40	-3.96	7.12	7.22	16.03
os61682	17	378	0.99	17	3	5	5.99	-0.28	0.00	1.42	0.11	87.64	0.02	5.48	7.55	16.33
os61683	17	381	1.00	15	1	4	6.17	-0.24	0.01	1.42	0.08	89.37	1.25	5.59	7.54	16.32
os61752	17	336	0.23	12	2	7	6.42	0.07	0.77	1.53	0.25	85.74	-5.45	6.79	7.58	16.40
os61768	17	436	0.60	16	3	5	6.22	-0.07	0.00	1.45	0.14	89.99	-2.97	6.99	7.17	16.00
os61784	19	424	0.01	4	2	3	6.87	0.07	1.36	1.54	0.11	90.70	-3.83	7.72	7.21	16.01
os61839	28	457	0.86	2	3	6	6.78	-0.02	3.38	1.55	0.02	41.19	0.48	9.09	7.19	16.07
os61887	17	453	0.70	4	2	7	6.72	-0.02	2.72	1.56	0.13	85.13	-6.64	8.00	7.13	15.96
os62022	17	378	0.91	11	2	0	6.49	-0.11	0.03	1.47	0.15	94.19	-0.88	6.82	7.34	16.22
os62039	17	414	0.01	2	3	6	6.75	-0.03	0.38	1.51	0.03	96.18	1.17	7.18	7.55	16.42
os62071	19	442	0.77	4	2	3	6.79	-0.02	1.65	1.54	0.08	90.20	-3.16	7.79	7.28	16.11
os62103	28	466	0.92	0	3	6	6.81	0.00	3.76	1.54	0.01	54.73	0.26	11.71	7.15	16.02

os62125	19	393	0.89	5	2	7	6.71	-0.04	1.51	1.55	0.12	73.80	-4.00	8.28	7.45	16.29
os62202	19	383	0.77	4	2	7	6.69	-0.05	1.90	1.55	0.11	93.03	-4.81	7.61	7.47	16.32
os62246	17	353	0.92	10	2	3	6.49	-0.06	0.54	1.52	0.18	89.23	-7.97	7.97	7.67	16.51
os62273	17	517	0.15	11	2	3	6.78	0.08	0.40	1.50	0.09	92.17	-2.38	8.75	6.86	15.67
os62291	23	512	0.85	10	3	5	6.63	-0.11	0.05	1.49	0.10	84.01	-1.59	7.39	6.75	15.64
os62310	28	452	0.92	0	3	6	6.80	0.00	3.44	1.56	0.02	24.62	0.02	10.89	7.23	16.12
os62313	28	453	0.08	1	3	6	6.83	0.01	2.94	1.57	0.03	30.60	-0.49	9.00	7.22	16.11
os62329	17	455	0.44	5	2	7	6.63	-0.04	1.74	1.56	0.15	82.79	-8.39	7.29	7.12	15.94
os62389	17	450	0.62	8	2	3	6.46	-0.07	0.60	1.49	0.16	99.46	-7.11	7.55	7.26	16.10
os62435	19	429	0.99	7	2	3	6.45	-0.09	1.22	1.53	0.11	99.07	-3.71	7.04	7.21	16.09
os62442	19	383	0.08	2	3	6	6.82	0.03	3.83	1.56	0.03	74.03	-1.08	9.19	7.50	16.38
os62444	19	383	0.08	1	3	6	6.81	0.02	3.87	1.56	0.03	75.69	-0.44	9.23	7.51	16.38
os62448	19	391	0.73	4	2	3	6.65	-0.06	1.69	1.56	0.08	82.11	-3.06	7.78	7.46	16.35
os62451	19	402	0.72	5	3	5	6.60	-0.09	1.42	1.52	0.05	73.15	0.73	8.96	7.43	16.31
os62459	17	410	0.99	4	2	3	6.71	-0.05	1.67	1.55	0.06	86.89	-2.30	7.77	7.37	16.23
os62464	17	444	0.13	6	2	7	6.57	-0.09	1.08	1.54	0.13	99.93	-4.47	6.51	7.10	16.00
os62491	17	372	0.78	9	3	5	6.44	-0.12	0.56	1.51	0.09	98.92	-2.56	6.96	7.55	16.40
os62507	17	464	0.13	5	2	3	6.78	0.02	1.68	1.52	0.06	99.11	-2.94	8.22	7.14	15.97
os62508	17	453	0.09	6	2	3	6.81	0.06	1.07	1.52	0.06	99.84	-2.57	8.32	7.15	15.98
os62519	19	422	0.00	4	3	6	6.88	0.06	0.63	1.54	0.03	94.69	0.30	7.83	7.40	16.24
os62539	19	372	0.92	12	2	3	6.18	-0.20	0.14	1.51	0.14	98.83	-4.90	5.63	7.56	16.40
os62551	25	437	0.99	8	3	5	6.57	-0.11	0.66	1.49	0.06	98.64	-0.10	7.52	7.32	16.17
os62556	17	377	0.99	8	3	5	6.49	-0.12	0.30	1.53	0.06	75.49	-1.24	7.44	7.57	16.44
os62600	17	357	0.71	14	3	5	6.17	-0.21	0.00	1.47	0.11	96.12	-0.61	6.52	7.69	16.52
os62656	17	309	0.73	15	2	7	6.07	-0.20	0.00	1.46	0.16	75.24	-2.88	5.44	7.79	16.64
os62678	19	391	0.91	7	3	5	6.65	-0.08	0.29	1.52	0.07	100.00	-0.92	7.71	7.52	16.40
os62683	17	418	0.40	4	2	7	6.81	0.03	1.14	1.55	0.13	97.13	-5.62	7.49	7.31	16.17
os62698	19	450	0.99	3	2	3	6.77	-0.02	1.89	1.55	0.07	86.33	-3.72	8.56	7.25	16.09
os62713	17	444	0.93	6	2	3	6.75	-0.04	0.97	1.51	0.08	96.27	-2.42	7.52	7.30	16.13
os62746	17	397	1.00	16	2	0	6.13	-0.25	0.00	1.47	0.14	74.13	-1.57	6.90	7.45	16.31
os62800	19	394	0.20	3	3	6	6.71	-0.04	2.41	1.55	0.05	76.27	-0.64	8.49	7.46	16.34
os62843	23	650	0.01	2	3	6	6.93	0.03	0.83	1.53	0.01	85.72	0.24	7.49	6.20	15.02
os62846	27	596	0.89	5	3	6	6.78	-0.06	0.56	1.54	0.05	80.38	-0.24	7.55	6.35	15.15
os62847	23	647	0.90	7	3	5	6.60	-0.09	0.22	1.48	0.04	99.44	1.07	7.47	6.21	15.02
os62848	23	642	0.87	9	1	4	6.48	-0.13	0.04	1.46	0.05	99.65	2.19	7.08	6.23	15.04
os62850	23	603	0.08	5	3	6	6.66	-0.09	0.50	1.53	0.05	91.09	-0.60	7.18	6.34	15.13
os62862	23	696	0.28	8	1	1	7.04	0.07	0.02	1.44	0.04	99.49	4.38	5.47	6.15	15.03
os62895	28	604	0.08	3	3	6	6.75	-0.06	0.74	1.53	0.05	92.84	0.01	6.69	6.32	15.12
os62898	27	598	0.85	5	3	6	6.77	-0.06	0.63	1.53	0.05	84.84	0.13	7.57	6.34	15.14
os62908	23	562	0.92	1	3	6	6.82	-0.02	2.68	1.55	0.02	53.75	0.00	9.23	6.58	15.43
os62911	25	546	0.92	2	2	3	6.79	-0.02	2.95	1.56	0.06	81.96	-2.17	8.95	6.64	15.47
os62912	25	553	0.92	2	2	3	6.78	-0.01	2.30	1.56	0.08	79.58	-4.06	8.96	6.60	15.44
os62925	23	632	0.99	11	3	5	6.52	-0.16	0.01	1.48	0.13	98.26	-1.43	6.77	6.21	15.08

os62935	17	519	0.64	9	3	5	6.49	-0.09	0.15	1.49	0.06	97.78	-0.45	7.65	6.80	15.58
os62954	19	406	0.77	2	3	6	6.75	-0.02	2.36	1.54	0.04	79.54	-1.30	8.41	7.65	16.48
os62963	19	425	0.01	5	3	5	6.61	-0.08	1.29	1.54	0.07	92.83	-1.54	8.05	7.44	16.23
os62982	17	474	0.77	4	2	3	6.77	-0.03	1.75	1.55	0.06	88.13	-2.25	8.79	7.09	15.87
os62983	19	488	0.23	9	2	3	6.75	0.05	0.50	1.53	0.07	75.48	-1.88	7.90	6.97	15.77
os63032	3	519	0.60	4	3	6	6.72	-0.02	0.21	1.50	0.03	92.62	1.23	6.77	6.85	15.63
os63046	23	584	0.92	4	3	6	6.71	-0.06	0.73	1.54	0.06	84.16	-0.98	7.32	6.41	15.21
os63047	23	609	0.01	8	3	5	7.00	0.12	0.32	1.51	0.05	99.76	-0.56	6.75	6.39	15.18
os63049	23	627	0.31	5	3	9	6.82	0.02	0.55	1.50	0.02	96.44	0.80	6.60	6.40	15.20
os63058	23	596	0.01	10	1	4	6.95	0.15	0.03	1.46	0.06	98.95	2.75	5.64	6.47	15.28
os63082	28	600	0.92	3	3	6	6.76	-0.05	1.41	1.54	0.03	81.50	-0.47	7.26	6.40	15.22
os63089	23	585	0.58	1	3	6	6.87	0.00	3.49	1.55	0.03	56.40	-0.95	9.41	6.44	15.27
os63091	23	581	0.77	2	3	6	6.86	-0.01	3.50	1.55	0.04	67.71	-1.41	8.87	6.45	15.28
os63098	23	549	0.77	1	3	6	6.85	0.00	2.48	1.55	0.05	82.66	-1.25	8.67	6.65	15.46
os63115	25	598	0.04	16	3	5	7.08	0.26	0.00	1.42	0.10	95.60	0.31	5.95	6.39	15.18
os63117	23	589	0.64	19	1	4	5.91	-0.30	0.00	1.41	0.11	90.16	2.82	5.96	6.43	15.22
os63120	23	576	0.07	4	2	0	6.72	-0.07	2.13	1.54	0.07	88.14	-1.80	8.13	6.44	15.22
os63132	23	641	0.24	5	3	6	6.76	-0.07	0.66	1.52	0.03	96.41	0.85	6.98	6.33	15.13
os63143	23	583	0.99	14	2	0	6.34	-0.21	0.11	1.48	0.12	92.42	-0.79	7.54	6.36	15.20
os63146	23	609	0.00	15	3	5	7.01	0.24	0.00	1.41	0.11	95.70	-0.26	5.52	6.40	15.22
os63147	27	574	0.67	12	2	7	6.38	-0.10	0.05	1.48	0.12	93.31	-2.36	6.67	6.47	15.28
os63165	23	657	0.37	11	3	5	7.07	0.11	0.02	1.47	0.07	95.27	1.05	5.85	6.18	15.06
os63166	23	657	0.19	10	3	5	7.08	0.12	0.05	1.48	0.06	95.94	1.44	5.82	6.19	15.06
os63189	27	508	0.10	8	2	3	6.76	0.06	0.86	1.51	0.13	99.56	-6.09	7.64	6.96	15.81
os63201	10	328	0.79	2	3	6	6.67	-0.04	2.45	1.55	0.02	68.08	-0.01	9.10	8.06	16.86
os63304	23	587	0.87	2	3	6	6.79	-0.03	2.94	1.55	0.04	59.93	-0.15	8.02	6.42	15.23
os63358	16	507	0.45	2	3	6	6.74	-0.04	1.77	1.52	0.02	88.83	-0.45	8.02	7.14	15.97
os63408	19	298	0.60	12	2	7	6.16	-0.08	0.41	1.55	0.18	79.37	-6.14	6.76	8.13	16.93
os63439	17	419	0.90	4	3	6	6.64	-0.06	1.44	1.55	0.04	86.46	-1.17	8.98	7.46	16.27
os63448	27	499	0.40	5	3	6	6.75	0.00	1.64	1.52	0.07	99.81	-1.92	7.86	7.05	15.88
os63540	17	408	0.18	11	3	5	7.00	0.16	0.02	1.48	0.08	98.37	0.23	5.81	7.73	16.63
os63923	19	492	0.08	5	3	6	6.94	0.07	1.58	1.53	0.05	95.86	-1.32	8.50	7.07	15.94
os63925	19	455	0.24	9	2	0	6.72	0.05	0.07	1.51	0.08	86.95	-0.38	7.65	7.19	16.06
os63950	17	410	0.11	4	2	7	6.84	0.06	1.88	1.56	0.12	87.86	-5.87	8.59	7.37	16.27
os64043	17	394	0.16	9	3	5	6.97	0.11	0.35	1.51	0.08	94.28	-1.45	7.52	7.70	16.61
os64085	17	405	0.03	8	3	5	6.97	0.11	0.24	1.49	0.06	98.55	0.44	6.66	7.76	16.66
os64184	19	366	0.85	11	3	5	6.30	-0.15	0.02	1.48	0.07	97.18	-0.03	6.85	7.59	16.48
os64200	17	394	0.87	10	2	3	6.63	-0.09	0.60	1.51	0.09	99.46	-3.59	7.48	7.51	16.39
os64247	17	322	0.00	7	2	3	6.90	0.12	0.88	1.52	0.08	92.07	-3.04	6.64	7.86	16.73
os64284	23	657	0.96	15	3	9	6.17	-0.25	0.00	1.41	0.10	100.00	0.18	5.81	6.02	14.82
os64383	19	366	0.68	3	3	6	6.82	0.02	1.52	1.51	0.05	97.73	-1.22	7.50	7.51	16.41
os64399	17	355	0.49	14	2	3	6.39	-0.01	0.25	1.49	0.12	95.84	-4.50	7.79	7.64	16.44
os64411	23	615	0.53	11	1	4	6.49	-0.07	0.01	1.44	0.08	97.81	2.58	5.98	6.03	14.86

os64456	17	364	0.91	7	3	5	6.45	-0.11	0.37	1.48	0.08	94.50	-1.21	7.05	7.62	16.42
os64577	17	431	0.19	9	2	3	6.93	0.11	0.74	1.50	0.14	99.86	-6.49	7.01	7.02	15.87
os64593	19	439	0.35	13	2	7	6.46	0.02	0.01	1.47	0.17	81.90	-3.51	6.27	6.79	15.59
os64596	19	444	0.07	19	2	7	6.78	0.29	0.00	1.48	0.21	92.24	-6.88	7.56	6.69	15.51
os64649	19	484	0.76	11	2	7	6.34	-0.13	0.31	1.51	0.15	94.19	-5.55	7.77	6.60	15.45
os64659	19	483	0.39	6	2	3	6.88	0.03	1.19	1.52	0.11	99.76	-5.19	7.43	6.73	15.59
os64758	28	466	0.92	0	3	6	6.81	0.00	4.09	1.54	0.01	56.68	0.36	11.22	7.15	16.02
os64760	17	498	0.27	11	2	3	6.63	0.05	0.03	1.49	0.10	99.51	-2.60	5.94	7.01	15.87
os64776	19	344	0.19	2	3	6	6.69	-0.03	3.82	1.56	0.04	85.14	-1.43	8.80	7.82	16.67
os64801	17	450	0.00	8	3	5	6.93	0.13	0.48	1.53	0.09	97.73	-2.50	7.21	7.30	16.21
os64820	19	336	0.23	6	2	7	6.77	0.05	0.92	1.54	0.20	99.05	-8.99	6.56	7.45	16.28
os64841	23	615	0.59	11	1	4	6.46	-0.09	0.01	1.43	0.08	97.74	2.82	5.90	6.03	14.86
os65118	19	246	0.39	3	2	3	6.63	-0.04	2.77	1.55	0.09	92.20	-4.87	8.36	8.29	17.17
os65119	19	246	0.86	7	2	3	6.47	-0.10	0.87	1.54	0.08	92.33	-3.97	8.12	8.30	17.17
os65149	19	325	0.63	10	2	3	6.36	-0.09	0.97	1.54	0.12	59.13	-4.59	7.09	7.40	16.18
os65251	17	485	0.96	7	3	5	6.61	-0.11	0.24	1.51	0.00	95.13	1.16	6.55	7.50	16.58
os65252	17	478	1.00	9	3	5	6.50	-0.14	0.13	1.51	0.00	95.37	0.11	6.50	7.50	16.58
os65350	17	317	0.96	26	2	0	5.45	-0.37	0.00	1.41	0.22	82.59	-3.00	5.07	8.56	17.72
os65394	12	394	0.36	2	3	6	6.82	0.01	2.88	1.55	0.02	54.63	-0.79	8.89	8.40	17.59
os65663	19	459	0.56	4	3	6	6.74	0.01	0.28	1.54	0.07	51.23	-1.78	7.93	7.70	16.84
os65664	19	459	0.17	5	2	3	6.76	0.03	0.46	1.54	0.07	51.15	-2.03	7.76	7.71	16.85
os65871	17	518	0.42	7	3	5	6.68	-0.01	0.68	1.51	0.06	90.03	-1.17	7.22	7.19	16.24
os65977	27	494	0.95	8	2	7	6.64	-0.10	0.63	1.53	0.11	97.64	-3.89	7.13	7.22	16.27
os65986	28	536	0.92	0	3	6	6.83	0.00	2.56	1.55	0.01	15.25	0.08	9.37	7.05	16.03
os66077	19	487	0.30	4	2	3	6.76	0.01	1.90	1.55	0.08	79.52	-2.45	7.80	7.38	16.43
os66082	27	518	0.32	7	3	5	6.73	0.01	0.34	1.52	0.06	90.09	-0.04	7.91	7.21	16.27
os66150	27	465	0.92	1	2	7	6.75	0.00	2.55	1.56	0.14	93.95	-6.02	7.19	7.31	16.34
os66246	23	480	0.67	15	2	0	6.34	-0.04	0.04	1.48	0.15	98.65	-1.35	6.27	7.04	15.92
os66275	17	327	0.34	13	2	0	6.41	0.04	0.00	1.47	0.23	94.30	-0.56	5.74	7.87	16.81
os66320	19	309	0.91	2	2	0	6.70	-0.02	2.38	1.56	0.16	46.22	-0.67	8.16	8.10	17.01
os66420	17	430	0.99	7	3	9	6.56	-0.11	0.73	1.45	0.07	96.78	-0.91	7.31	7.97	16.93
os66427	19	423	0.39	4	3	6	6.84	0.01	1.51	1.49	0.05	95.31	-0.49	7.66	7.78	16.78
os66441	10	326	0.71	23	3	5	5.55	-0.24	0.00	1.36	0.21	89.69	1.27	5.04	8.19	17.13
os66445	17	359	0.88	30	1	4	5.80	-0.27	0.00	1.29	0.19	96.00	3.29	4.61	8.16	17.11
os66493	14	450	0.47	29	1	4	6.57	0.11	0.00	1.27	0.14	97.48	11.68	4.50	7.75	16.77
os66563	17	468	0.49	21	3	9	6.01	-0.06	0.00	1.35	0.14	99.18	2.54	5.70	7.61	16.55
os66564	9	310	0.09	8	2	0	6.76	0.10	0.25	1.53	0.13	81.84	-1.93	7.54	8.21	17.15
os66595	10	396	0.24	19	3	9	6.89	0.17	0.00	1.40	0.11	99.74	-0.40	5.43	8.15	17.13
os66631	17	374	0.21	12	2	3	6.83	0.13	0.20	1.49	0.15	99.95	-7.46	7.43	8.08	17.08
os66644	17	398	0.28	18	2	0	6.92	0.19	0.00	1.45	0.14	98.74	-1.74	6.79	7.95	16.97
os66646	22	475	0.03	12	1	1	6.92	0.17	0.01	1.45	0.05	95.91	3.00	5.50	7.82	16.86
os66716	19	452	0.55	15	2	3	6.18	-0.15	0.08	1.47	0.14	95.12	-3.90	7.45	7.62	16.66
os66739	17	455	0.99	11	2	3	6.43	-0.16	0.53	1.49	0.11	86.07	-4.23	7.70	7.50	16.48

os66744	17	439	1.00	10	3	5	6.52	-0.13	0.51	1.48	0.08	98.90	-2.85	7.86	7.60	16.58
os66745	17	441	0.99	9	3	5	6.55	-0.12	0.60	1.49	0.08	98.84	-2.63	7.89	7.60	16.58
os66746	17	441	0.99	8	3	5	6.61	-0.10	0.74	1.49	0.08	98.76	-2.75	8.00	7.59	16.58
os66811	17	492	0.27	17	2	7	6.49	0.09	0.00	1.45	0.15	98.41	-3.62	7.31	6.95	15.84
os66815	23	522	0.61	14	3	9	6.26	-0.10	0.00	1.39	0.09	99.69	1.98	5.28	7.12	16.00
os66824	23	494	0.82	15	3	5	6.08	-0.21	0.00	1.43	0.13	99.43	-0.34	5.54	7.08	16.02
os66982	19	423	0.77	4	2	7	6.76	-0.02	1.34	1.54	0.10	76.39	-2.51	7.93	7.91	17.01
os67137	10	311	0.99	7	3	5	6.49	-0.12	0.28	1.51	0.07	88.09	-1.01	7.19	8.91	18.02
os67175	17	326	0.02	11	2	7	6.94	0.18	0.26	1.51	0.13	95.14	-4.75	6.66	8.61	17.70
os67231	17	415	0.03	16	3	5	6.95	0.16	0.00	1.43	0.14	90.53	-1.13	7.62	7.96	17.06
os67382	10	415	0.23	4	1	4	6.79	0.03	0.16	1.45	0.02	93.03	3.08	6.43	8.14	17.28
os67470	10	322	0.02	5	1	4	6.87	0.09	0.47	1.44	0.06	93.81	4.11	7.33	8.70	17.77
os67478	17	418	0.07	3	3	6	6.87	0.05	2.80	1.54	0.07	93.09	-1.85	8.70	8.18	17.26
os67479	17	418	0.92	1	3	6	6.80	0.01	2.92	1.54	0.06	92.70	-1.62	8.98	8.18	17.26
os67480	17	417	0.02	3	2	3	6.87	0.05	2.79	1.54	0.07	93.19	-2.08	8.66	8.18	17.26
os67481	17	418	0.76	1	3	6	6.83	0.02	2.76	1.54	0.06	92.80	-1.89	8.87	8.18	17.26
os67482	17	418	0.53	2	3	6	6.85	0.03	2.48	1.54	0.06	92.84	-1.98	8.79	8.18	17.26
os67483	17	419	0.30	3	3	6	6.87	0.04	2.19	1.54	0.06	92.93	-1.80	8.69	8.18	17.26
os67504	17	390	0.06	8	2	3	6.92	0.10	0.83	1.53	0.09	97.76	-3.57	8.18	8.32	17.42
os67505	17	389	0.05	7	2	3	6.91	0.09	0.89	1.53	0.09	97.73	-3.45	8.12	8.32	17.42
os67506	17	388	0.03	6	2	3	6.90	0.09	1.01	1.53	0.09	97.71	-3.40	8.10	8.32	17.43
os67507	17	388	0.03	7	2	3	6.90	0.08	1.26	1.53	0.09	97.69	-3.59	8.16	8.32	17.43
os67508	17	378	0.89	7	2	7	6.62	-0.08	1.37	1.54	0.11	94.92	-3.69	7.10	8.34	17.45
os67509	17	378	0.97	8	2	7	6.61	-0.09	1.21	1.53	0.10	95.00	-3.15	7.13	8.34	17.45
os67513	17	384	0.98	17	1	4	6.02	-0.27	0.00	1.43	0.13	94.12	-0.43	6.62	8.25	17.37
os67578	9	265	0.99	6	3	5	6.53	-0.10	0.28	1.53	0.08	91.07	-1.78	7.52	9.16	18.28
os67624	9	308	0.58	7	3	5	6.81	0.02	0.52	1.51	0.06	89.04	-0.39	7.03	8.79	17.84
os67635	9	393	0.00	8	2	3	6.95	0.14	0.64	1.50	0.08	95.40	-3.34	8.38	8.33	17.41
os67643	11	247	0.73	3	2	7	6.59	-0.03	1.81	1.55	0.15	83.76	-4.13	7.45	9.06	18.15
os67646	19	280	1.00	8	2	3	6.45	-0.13	0.62	1.52	0.09	95.29	-3.27	7.03	9.06	18.20
os67666	10	457	0.00	4	3	6	6.89	0.07	0.62	1.53	0.03	75.70	-0.19	7.15	7.84	17.03
os67796	23	577	0.51	5	3	9	6.82	0.02	0.74	1.45	0.04	99.35	-0.16	6.60	6.86	15.74
os67822	27	493	0.89	6	2	3	6.73	-0.04	1.44	1.54	0.11	89.44	-5.65	7.28	7.16	16.07
os67863	23	517	0.37	7	3	5	6.71	0.02	0.53	1.51	0.07	96.61	-0.34	7.64	7.05	15.97
os67865	23	511	0.94	10	1	4	6.50	-0.16	0.01	1.45	0.07	82.80	2.90	5.77	7.09	16.00
os67874	27	585	0.01	6	3	5	6.93	0.09	0.55	1.49	0.06	95.32	-1.13	8.20	6.67	15.62
os67908	17	543	0.92	3	3	9	6.71	-0.06	0.24	1.46	0.03	97.52	1.57	6.66	7.15	16.12
os67929	28	525	0.39	3	3	6	6.90	0.02	3.41	1.55	0.04	73.20	-0.57	8.71	7.09	16.02
os68097	11	460	0.92	0	3	6	6.78	-0.01	4.96	1.57	0.04	51.18	-1.12	8.91	7.57	16.66
os68145	17	502	0.97	7	2	7	6.69	-0.08	0.87	1.54	0.11	89.53	-3.51	8.09	7.35	16.42
os68153	17	486	0.81	9	3	5	6.52	-0.08	0.20	1.48	0.06	94.77	0.48	6.00	7.54	16.63
os68243	17	431	1.00	5	2	3	6.68	-0.07	1.27	1.52	0.05	94.79	-1.90	8.33	7.91	17.01
os68282	10	378	0.08	5	3	5	6.85	0.07	0.19	1.50	0.02	75.14	0.65	7.77	8.78	17.99

os68298	19	358	0.48	6	2	7	6.83	0.08	0.59	1.54	0.16	81.73	-4.26	7.55	8.05	17.11
os68369	17	426	0.98	10	3	5	6.51	-0.14	0.48	1.51	0.08	98.29	-2.02	7.84	8.08	17.23
os68402	17	426	0.39	3	2	3	6.70	-0.04	2.46	1.54	0.07	81.62	-3.75	8.35	7.94	17.02
os68463	9	318	0.92	4	3	6	6.61	-0.05	2.49	1.54	0.05	77.04	-1.31	8.88	9.14	18.34
os68642	9	336	0.30	15	3	5	6.44	0.06	0.00	1.45	0.09	94.23	0.39	6.05	8.97	18.22
os68645	17	372	0.23	4	2	0	6.71	0.01	1.45	1.53	0.09	79.95	-1.70	7.33	8.45	17.67
os68725	9	313	0.92	13	2	7	6.42	-0.15	0.43	1.50	0.15	99.40	-5.34	7.52	8.74	17.84
os68782	17	438	0.35	3	2	3	6.74	-0.01	2.51	1.51	0.07	84.18	-3.39	8.67	7.97	17.05
os68783	17	439	0.41	4	2	3	6.71	-0.03	2.89	1.51	0.06	83.52	-3.31	8.74	7.96	17.04
os68784	17	450	0.08	2	3	9	6.84	0.03	0.12	1.48	0.01	78.26	0.75	8.49	7.97	17.06
os68817	11	192	0.92	0	3	6	6.69	0.00	7.54	1.57	0.00	2.05	0.03	12.24	8.98	17.84
os68831	11	191	0.92	1	3	6	6.68	-0.01	5.96	1.56	0.00	0.60	0.27	10.06	9.07	17.94
os68838	11	190	0.92	0	3	6	6.69	0.00	6.50	1.57	0.00	0.00	0.00	12.06	9.08	17.98
os68898	11	199	0.92	0	3	6	6.69	0.00	6.96	1.57	0.00	0.52	0.00	11.59	8.94	17.82
os68902	11	199	0.92	0	3	6	6.69	0.00	6.97	1.57	0.00	0.15	0.00	11.59	8.93	17.81
os68903	11	199	0.92	0	3	6	6.69	0.00	6.96	1.57	0.00	0.05	-0.01	11.59	8.93	17.80
os68919	11	194	0.92	0	3	6	6.69	0.00	7.31	1.57	0.00	0.00	0.00	12.02	8.98	17.83
os68920	11	210	0.92	1	3	6	6.66	-0.02	4.80	1.57	0.03	32.96	-0.64	8.10	8.83	17.75
os68922	11	209	0.77	6	3	5	6.67	-0.03	3.51	1.53	0.04	30.40	-0.53	7.33	8.82	17.74
os68924	11	211	0.23	1	3	6	6.69	0.00	4.78	1.56	0.02	32.49	-0.43	8.53	8.82	17.74
os68928	11	206	0.92	0	3	6	6.69	0.00	5.76	1.57	0.01	8.34	-0.01	11.59	8.92	17.82
os68930	11	203	0.92	1	3	6	6.66	-0.02	5.51	1.57	0.02	11.08	-0.79	9.01	8.91	17.80
os68932	11	195	0.92	0	3	6	6.69	0.00	7.68	1.57	0.00	0.11	0.00	11.95	8.89	17.74
os68954	9	227	0.77	10	3	5	6.61	-0.06	0.08	1.48	0.05	73.85	1.59	6.74	8.81	17.73
os68955	9	209	0.60	11	2	3	6.71	0.00	0.36	1.53	0.10	58.48	-4.65	7.17	8.82	17.75
os68956	9	227	0.65	11	3	5	6.70	-0.01	0.03	1.48	0.05	70.30	0.71	6.99	8.81	17.73
os68967	11	243	0.28	3	3	6	6.63	-0.05	1.25	1.54	0.02	69.10	0.13	8.03	8.87	17.71
os68969	11	198	0.92	0	3	6	6.69	0.00	6.99	1.57	0.01	0.55	-0.20	9.52	8.97	17.83
os68971	11	196	0.69	1	3	6	6.73	0.02	6.99	1.56	0.03	31.53	-0.78	8.58	8.97	17.83
os68972	11	198	0.68	3	3	6	6.71	0.00	6.99	1.55	0.02	29.98	-0.47	8.54	8.97	17.83
os68973	11	196	0.92	1	3	6	6.65	-0.02	7.00	1.55	0.00	21.25	0.32	8.89	8.93	17.82
os68974	11	195	0.92	1	3	6	6.65	-0.02	7.00	1.56	0.01	20.64	-0.19	8.86	8.93	17.82
os68987	11	194	0.60	1	3	6	6.69	0.00	6.10	1.56	0.00	0.14	0.42	9.55	9.05	17.90
os68988	17	304	0.99	8	2	3	6.50	-0.11	1.03	1.54	0.10	95.82	-3.82	8.16	8.01	16.99
os68989	17	307	0.00	4	2	0	6.83	0.07	1.46	1.54	0.07	88.85	-1.40	8.02	7.96	16.93
os68991	11	269	0.48	1	3	6	6.74	0.01	5.87	1.57	0.01	2.92	-0.27	9.77	8.28	17.23
os68992	11	270	0.40	1	3	6	6.72	0.01	5.86	1.56	0.01	2.44	0.12	9.51	8.28	17.23
os68993	11	271	1.00	3	3	6	6.70	-0.01	5.48	1.53	0.02	11.34	0.98	9.14	8.28	17.23
os69000	11	257	0.92	1	3	6	6.68	-0.02	5.65	1.56	0.02	1.18	0.11	9.78	8.35	17.24
os69027	12	251	0.04	1	3	6	6.69	-0.01	2.63	1.56	0.01	8.78	-0.12	8.95	8.32	17.06
os69102	11	214	0.92	0	3	6	6.70	0.00	5.79	1.57	0.00	0.73	0.08	11.23	8.85	17.92
os69119	11	203	0.92	0	3	6	6.69	0.00	5.98	1.57	0.00	0.51	0.00	11.73	8.78	17.70
os69120	11	203	0.92	0	3	6	6.69	0.00	5.98	1.57	0.00	0.32	0.00	11.73	8.78	17.70

os69127	11	204	0.92	0	3	6	6.69	0.00	5.93	1.57	0.01	0.00	-0.02	11.82	8.79	17.69
os69168	11	287	0.73	2	3	6	6.69	-0.01	4.72	1.56	0.02	1.58	0.11	10.09	7.98	16.85
os69170	11	287	0.92	0	3	6	6.72	0.00	4.67	1.56	0.02	1.98	0.29	11.12	7.98	16.85
os69172	11	287	0.92	0	3	6	6.74	0.00	4.49	1.56	0.02	2.79	0.28	10.91	7.97	16.85
os69192	8	239	0.92	0	3	6	6.71	0.00	5.94	1.57	0.01	2.85	-0.15	11.08	8.46	17.25
os69198	11	254	0.92	0	3	6	6.72	0.00	5.96	1.56	0.01	3.19	0.00	10.51	8.34	17.22
os69199	11	254	0.92	0	3	6	6.73	0.01	5.96	1.56	0.00	0.22	0.29	11.60	8.36	17.23
os69203	11	254	0.92	0	3	6	6.73	0.01	4.99	1.57	0.01	0.00	-0.40	10.50	8.36	17.22
os69213	11	222	0.92	0	3	6	6.70	0.00	6.90	1.57	0.00	0.30	0.10	11.59	8.58	17.40
os69227	11	217	0.01	2	3	6	6.72	0.02	5.99	1.56	0.01	8.23	0.23	9.01	8.73	17.62
os69228	11	217	0.03	1	3	6	6.73	0.02	5.99	1.56	0.01	8.06	0.10	9.01	8.73	17.62
os69240	11	275	0.01	2	3	6	6.75	0.02	4.08	1.56	0.03	0.04	0.00	10.20	8.12	17.04
os69241	8	274	0.92	1	3	6	6.75	0.01	4.99	1.56	0.01	3.97	0.51	10.04	8.14	17.07
os69250	11	255	0.92	0	3	6	6.72	0.00	4.99	1.57	0.01	0.00	0.00	12.62	8.35	17.21
os69252	11	256	0.92	0	3	6	6.72	0.00	3.95	1.57	0.02	2.92	-0.04	12.71	8.30	17.14
os69256	11	255	0.92	0	3	6	6.72	0.00	4.75	1.57	0.01	0.00	-0.08	12.62	8.33	17.17
os69258	11	255	0.92	0	3	6	6.73	0.01	3.96	1.57	0.01	0.32	-0.25	10.62	8.33	17.16
os69262	11	264	0.92	0	3	6	6.72	0.00	4.99	1.57	0.01	0.07	0.09	12.31	8.23	17.13
os69268	11	286	0.23	1	3	6	6.72	0.00	5.52	1.56	0.01	5.21	0.11	9.57	8.00	16.88
os69269	11	285	0.43	1	3	6	6.72	0.00	5.59	1.57	0.02	4.08	-0.38	9.94	8.02	16.89
os69270	11	285	0.92	1	3	6	6.73	0.00	4.99	1.57	0.01	0.00	-0.03	9.99	8.02	16.89
os69274	11	204	0.08	1	3	6	6.73	0.02	5.92	1.56	0.01	2.82	0.08	9.35	8.97	17.89
os69281	11	248	0.92	0	3	6	6.71	0.00	3.48	1.57	0.01	7.59	-0.12	9.80	8.40	17.23
os69324	11	169	0.92	0	3	6	6.68	0.00	6.96	1.57	0.00	7.92	-0.04	10.66	9.57	18.47
os69328	11	169	0.92	0	3	6	6.68	0.00	6.91	1.57	0.00	0.00	0.01	11.92	9.59	18.46
os69338	12	177	0.92	1	3	6	6.70	0.01	3.95	1.57	0.01	3.53	-0.33	9.19	9.62	18.53
os69412	11	174	0.92	1	3	6	6.68	0.01	5.67	1.57	0.01	0.10	-0.34	9.54	9.43	18.38
os69440	12	180	0.23	1	3	6	6.68	0.01	3.93	1.56	0.01	7.36	-0.28	9.05	9.69	18.67
os69454	11	188	0.92	2	2	3	6.59	-0.04	1.32	1.56	0.06	42.76	-2.36	7.73	10.16	19.28
os69456	11	188	0.92	0	3	6	6.69	0.00	6.90	1.57	0.01	4.30	0.05	10.78	10.31	19.49
os69484	11	193	0.92	0	3	6	6.69	0.00	5.86	1.57	0.01	18.79	-0.07	11.58	10.15	19.30
os69510	11	183	0.92	1	3	6	6.65	-0.02	4.81	1.56	0.02	10.40	-0.08	9.28	9.89	19.06
os69511	11	183	0.92	1	3	6	6.66	-0.01	4.76	1.56	0.02	10.20	-0.06	9.41	9.89	19.06
os69513	11	182	0.95	3	3	6	6.62	-0.04	4.79	1.55	0.03	23.01	-0.06	8.14	9.88	19.05
os69515	11	180	0.40	1	3	6	6.71	0.01	5.98	1.57	0.02	3.33	-0.69	9.34	9.96	19.14
os69516	11	182	0.92	0	3	6	6.68	0.00	5.97	1.57	0.00	0.16	0.02	12.13	9.95	19.13
os69525	11	177	0.07	1	3	6	6.72	0.02	6.24	1.56	0.02	15.89	-0.39	9.10	10.11	19.29
os69530	11	175	0.92	0	3	6	6.68	0.00	6.99	1.57	0.01	5.64	0.00	11.39	10.07	19.20
os69531	11	175	0.92	0	3	6	6.69	0.01	6.99	1.57	0.01	7.37	-0.31	9.04	10.07	19.20
os69534	11	176	0.92	1	3	6	6.70	0.01	6.98	1.56	0.01	7.36	0.06	9.23	10.07	19.19
os69539	11	172	0.92	0	3	6	6.68	0.00	6.99	1.57	0.01	6.51	-0.15	11.32	10.06	19.18
os69553	11	169	0.92	0	3	6	6.68	0.00	6.88	1.57	0.01	0.03	0.00	12.20	10.08	19.22
os69565	11	169	0.92	0	3	6	6.68	0.00	6.20	1.57	0.01	5.40	-0.02	12.40	10.05	19.14

os69566	11	169	0.92	0	3	6	6.68	0.00	5.90	1.57	0.01	3.93	-0.01	11.50	10.05	19.13
os69579	11	170	0.92	0	3	6	6.68	0.00	6.81	1.57	0.01	3.89	0.00	12.18	10.30	19.46
os69580	11	170	0.92	0	3	6	6.68	0.00	3.97	1.57	0.02	7.45	0.00	12.18	10.31	19.47
os69597	9	233	0.58	1	2	7	6.69	0.00	2.98	1.56	0.12	85.41	-2.21	8.56	9.62	18.80
os69621	11	164	0.92	0	3	6	6.67	0.00	4.47	1.55	0.01	12.78	0.48	10.55	10.20	19.28
os69625	11	161	0.92	0	3	6	6.68	0.00	3.98	1.57	0.01	1.40	-0.01	11.93	10.18	19.25
os69670	11	157	0.03	1	3	6	6.68	0.01	6.69	1.56	0.01	8.77	-0.23	9.57	10.25	19.35
os69672	11	159	0.92	0	3	6	6.68	0.00	7.69	1.56	0.00	2.45	0.42	11.23	10.23	19.28
os69674	11	158	0.92	0	3	6	6.67	0.00	7.68	1.57	0.00	0.86	0.00	11.75	10.23	19.28
os69682	11	150	0.70	2	3	6	6.69	0.00	7.86	1.56	0.00	14.51	-0.85	8.32	10.23	19.46
os69721	11	207	0.42	5	2	7	6.57	-0.01	1.32	1.55	0.11	83.11	-4.44	7.24	9.59	18.73
os69722	11	207	0.36	5	2	7	6.58	-0.01	1.37	1.55	0.11	83.27	-4.26	7.31	9.59	18.72
os69723	11	207	0.43	5	2	7	6.59	-0.01	1.58	1.55	0.11	83.40	-4.14	7.41	9.59	18.72
os69729	8	211	0.92	0	2	0	6.71	0.01	2.92	1.56	0.10	77.42	-1.66	7.98	9.52	18.66
os69730	8	211	0.08	1	2	0	6.72	0.02	2.90	1.56	0.11	77.07	-1.89	7.98	9.52	18.66
os69731	8	211	0.00	1	2	0	6.72	0.02	2.84	1.56	0.11	76.83	-2.01	7.98	9.52	18.65
os69732	8	212	0.06	2	2	0	6.72	0.02	2.82	1.56	0.11	76.63	-2.06	7.98	9.52	18.65
os69733	8	212	0.23	2	2	0	6.72	0.02	2.63	1.56	0.11	76.37	-2.13	7.98	9.51	18.65
os69734	8	212	0.13	2	2	0	6.71	0.02	2.57	1.56	0.11	76.11	-2.08	8.01	9.51	18.64
os69737	8	215	0.92	0	2	0	6.70	0.00	3.05	1.56	0.11	77.29	-1.46	10.62	9.49	18.62
os69738	8	215	0.92	0	2	0	6.70	0.00	2.85	1.56	0.11	77.86	-1.77	10.23	9.49	18.62
os69739	8	215	0.92	0	2	0	6.70	0.00	2.86	1.56	0.11	78.23	-1.91	11.39	9.48	18.62
os69740	8	215	0.92	0	2	7	6.70	0.00	2.93	1.56	0.11	78.56	-2.15	9.88	9.48	18.61
os69741	8	215	0.92	1	2	7	6.70	0.01	3.04	1.56	0.11	78.91	-2.30	8.45	9.48	18.61
os69742	8	215	0.23	1	2	0	6.70	0.01	3.11	1.56	0.11	79.21	-2.14	8.44	9.48	18.61
os69743	11	216	0.22	2	2	0	6.71	0.02	3.35	1.56	0.11	79.50	-1.98	8.42	9.47	18.61
os69744	11	216	0.23	2	2	0	6.71	0.02	3.50	1.56	0.11	79.77	-1.61	8.43	9.47	18.60
os69745	11	217	0.23	1	2	0	6.71	0.01	3.02	1.56	0.11	80.00	-1.36	8.42	9.47	18.60
os69746	11	217	0.92	1	2	0	6.70	0.00	2.99	1.56	0.10	80.06	-1.20	8.43	9.46	18.60
os69747	8	217	0.92	1	2	0	6.70	0.01	2.98	1.56	0.10	80.18	-1.37	8.43	9.46	18.59
os69748	8	218	0.23	1	2	0	6.71	0.02	2.97	1.56	0.10	80.26	-1.33	8.44	9.46	18.59
os69750	11	219	0.08	2	2	0	6.75	0.04	1.85	1.56	0.11	81.29	-1.97	8.27	9.45	18.58
os69752	11	221	0.08	5	2	7	6.82	0.07	1.49	1.54	0.11	82.75	-2.37	8.13	9.46	18.59
os69753	11	224	0.45	4	2	7	6.77	0.03	1.58	1.54	0.11	85.21	-2.52	7.97	9.46	18.59
os69754	11	225	0.60	4	2	7	6.74	0.01	1.81	1.54	0.12	86.81	-2.41	7.96	9.46	18.60
os69755	11	226	0.00	7	2	7	6.82	0.10	0.88	1.53	0.11	86.65	-2.12	7.73	9.46	18.59
os69756	11	227	0.01	4	2	7	6.79	0.08	1.21	1.54	0.12	87.98	-2.59	7.90	9.46	18.59
os69757	11	228	0.01	5	2	7	6.78	0.08	1.24	1.54	0.12	89.27	-3.04	7.85	9.46	18.59
os69758	8	228	0.19	3	2	0	6.75	0.04	2.16	1.55	0.10	89.61	-1.73	8.16	9.41	18.54
os69759	11	227	0.60	1	2	7	6.67	-0.01	2.18	1.56	0.10	89.43	-2.17	8.21	9.41	18.55
os69779	9	215	0.01	4	2	0	6.77	0.06	1.57	1.55	0.12	86.10	-2.28	7.77	9.56	18.71
os69780	9	215	0.01	4	2	7	6.79	0.07	1.20	1.55	0.12	84.95	-2.63	7.64	9.56	18.71
os69787	9	234	0.65	6	2	7	6.52	-0.04	0.66	1.53	0.11	83.78	-2.08	8.02	9.63	18.81

os69789	11	180	0.92	0	3	6	6.68	0.00	5.98	1.57	0.01	10.64	-0.27	11.65	9.97	19.15
os69791	11	181	0.40	2	3	6	6.72	0.01	5.98	1.56	0.01	3.00	-0.15	9.21	9.96	19.14
os69792	11	181	0.19	2	3	6	6.72	0.01	5.98	1.56	0.01	2.85	-0.23	9.21	9.96	19.14
os69793	11	181	0.10	2	3	6	6.72	0.01	5.97	1.56	0.01	2.72	-0.19	9.18	9.96	19.14
os69795	11	180	0.60	2	3	6	6.66	-0.01	5.98	1.56	0.01	10.38	-0.13	9.75	9.97	19.15
os69796	11	183	0.92	0	3	6	6.68	0.00	5.98	1.57	0.00	0.04	0.08	12.03	9.96	19.13
os69797	11	183	0.92	0	3	6	6.68	0.00	5.98	1.57	0.00	0.00	0.15	12.03	9.97	19.13
os69801	11	184	0.92	0	3	6	6.69	0.00	5.67	1.57	0.01	3.50	-0.08	11.98	9.89	19.00
os69802	11	184	0.92	0	3	6	6.69	0.00	5.68	1.57	0.01	3.81	-0.06	11.99	9.89	19.00
os69803	11	183	0.03	2	3	6	6.73	0.03	5.71	1.56	0.02	23.83	-0.17	9.56	9.89	19.01
os69804	11	183	0.23	2	3	6	6.71	0.02	5.71	1.56	0.02	26.05	-0.46	9.37	9.89	19.01
os69805	11	188	0.05	8	3	5	6.86	0.11	0.15	1.49	0.02	37.04	1.86	7.45	9.90	19.02
os69808	11	171	0.92	0	3	6	6.68	0.00	6.98	1.57	0.01	14.39	-0.12	12.02	10.09	19.22
os69823	11	183	0.92	0	3	6	6.68	0.00	5.51	1.57	0.01	4.07	0.02	11.56	9.90	19.07
os69824	11	183	0.92	0	3	6	6.68	0.00	5.54	1.56	0.01	4.16	0.11	11.75	9.90	19.07
os69829	8	181	0.25	1	3	6	6.72	0.01	3.94	1.56	0.02	16.72	-0.26	9.76	9.90	19.09
os69830	8	181	0.08	1	3	6	6.71	0.01	4.22	1.57	0.02	16.24	-0.56	9.71	9.90	19.09
os69835	8	182	0.92	2	3	6	6.63	-0.02	4.28	1.56	0.02	16.15	-0.14	8.97	9.90	19.09
os69845	11	185	0.92	1	3	6	6.69	0.00	5.98	1.56	0.01	0.05	0.37	10.05	9.69	18.79
os69846	11	185	0.77	1	3	6	6.69	0.00	5.98	1.56	0.01	0.05	0.27	9.90	9.69	18.79
os69849	11	201	0.92	0	3	6	6.69	0.00	4.18	1.57	0.03	44.05	-0.73	9.30	9.44	18.53
os69852	11	187	0.92	0	3	6	6.69	0.00	4.93	1.57	0.01	4.32	0.03	12.02	9.66	18.75
os69857	11	218	0.05	1	3	6	6.72	0.01	3.07	1.56	0.02	34.08	-0.54	8.95	9.45	18.55
os69858	11	218	0.08	1	3	6	6.72	0.01	3.07	1.56	0.02	33.86	-0.50	8.99	9.45	18.55
os69859	11	218	0.08	2	3	6	6.75	0.02	3.07	1.56	0.02	33.14	-0.33	9.06	9.45	18.55
os69861	11	218	0.11	2	3	6	6.75	0.02	3.05	1.56	0.03	32.60	-0.49	9.09	9.45	18.55
os69862	11	218	0.19	2	3	6	6.75	0.02	3.06	1.56	0.03	32.36	-0.69	9.14	9.44	18.55
os69863	11	218	0.08	2	3	6	6.75	0.02	3.05	1.56	0.03	32.13	-0.61	9.11	9.44	18.55
os69864	11	218	0.35	2	3	6	6.74	0.02	3.05	1.56	0.02	31.71	-0.46	9.11	9.44	18.55
os69865	11	163	0.92	1	3	6	6.71	0.02	2.82	1.57	0.00	11.19	-0.90	8.87	9.79	18.76
os69867	11	188	0.92	0	3	6	6.69	0.00	3.17	1.57	0.02	9.69	-0.28	10.12	9.76	18.92
os69961	17	373	0.37	6	2	3	6.59	-0.05	0.92	1.52	0.07	95.53	-2.60	8.16	8.13	17.09
os70178	9	246	0.29	14	2	3	6.06	-0.21	0.01	1.48	0.13	85.24	-3.14	6.37	8.78	17.70
os70183	17	339	0.00	7	2	7	6.90	0.13	0.73	1.51	0.16	100.00	-4.98	7.26	8.27	17.22
os70187	10	357	0.52	11	2	3	6.76	0.00	0.14	1.48	0.14	99.98	-4.15	6.85	8.31	17.26
os70192	10	335	0.65	14	2	7	6.61	-0.01	0.05	1.48	0.20	99.94	-9.90	7.17	8.33	17.28
os70231	10	371	0.69	7	3	9	6.75	-0.03	0.28	1.45	0.05	98.10	1.17	6.27	8.35	17.31
os70232	10	370	0.76	7	3	9	6.75	-0.03	0.30	1.45	0.05	97.97	1.04	6.28	8.35	17.31
os70236	17	278	0.87	15	2	3	6.07	-0.21	0.00	1.48	0.12	95.49	-3.07	6.04	8.43	17.27
os70237	17	280	0.85	5	2	3	6.58	-0.04	1.38	1.54	0.08	94.67	-4.08	8.19	8.41	17.26
os70240	17	347	0.96	9	3	5	6.50	-0.13	0.35	1.48	0.11	98.97	-1.54	6.78	8.29	17.23
os70241	17	371	0.95	17	3	5	6.19	-0.23	0.01	1.45	0.14	99.47	-2.11	6.36	8.21	17.15
os70253	17	245	0.92	3	2	7	6.67	-0.02	2.53	1.56	0.15	88.80	-4.39	7.46	8.36	17.20

os70309	9	224	0.79	11	2	3	6.25	-0.15	0.33	1.51	0.15	98.10	-6.36	7.72	8.84	17.77
os70386	17	315	0.87	7	2	3	6.65	-0.06	1.08	1.52	0.09	98.26	-4.48	7.47	8.39	17.29
os70508	19	256	0.04	2	2	7	6.74	0.00	3.40	1.56	0.09	92.00	-4.45	8.53	8.40	17.29
os70512	11	282	0.22	3	3	6	6.73	0.02	2.13	1.56	0.05	61.84	-1.69	8.43	8.24	17.17
os70535	19	329	0.80	13	2	7	6.20	-0.14	0.22	1.51	0.14	93.48	-4.90	6.97	7.82	16.72
os70542	17	278	0.02	9	3	5	6.38	-0.15	0.26	1.50	0.08	97.13	-0.94	6.77	8.36	17.29
os70555	9	283	0.60	8	3	5	6.51	-0.03	0.18	1.51	0.06	95.70	-0.06	7.08	8.30	17.20
os70596	19	364	0.03	7	3	5	6.89	0.11	0.76	1.51	0.06	96.09	-1.19	7.54	7.72	16.65
os70597	17	293	0.63	6	1	4	6.50	-0.06	0.03	1.43	0.04	86.85	5.57	6.12	8.08	17.01
os70616	19	311	0.92	6	3	5	6.52	-0.08	0.81	1.53	0.08	95.95	-1.56	7.86	7.93	16.91
os70624	17	363	0.85	10	2	3	6.36	-0.12	0.51	1.49	0.12	99.65	-5.78	6.66	7.84	16.84
os70687	17	489	0.01	7	3	5	6.90	0.11	0.23	1.48	0.06	99.20	1.10	6.30	7.23	16.13
os70914	19	420	0.81	5	2	7	6.74	-0.03	1.45	1.55	0.12	91.98	-5.30	7.43	7.42	16.33
os70966	19	504	0.04	7	2	7	6.49	-0.13	0.41	1.51	0.20	99.52	-3.12	7.17	6.69	15.64
os70968	19	569	0.10	16	2	3	6.97	0.22	0.00	1.45	0.14	99.82	-4.73	6.71	6.71	15.65
os71022	17	265	0.21	12	2	0	6.17	-0.20	0.01	1.49	0.09	93.06	-1.40	6.67	8.40	17.25
os71023	19	283	0.34	4	2	7	6.72	0.03	1.70	1.54	0.11	98.05	-3.89	8.29	8.46	17.29
os71024	19	293	0.02	3	2	7	6.62	-0.05	2.14	1.55	0.10	97.94	-2.91	8.26	8.41	17.24
os71174	17	366	0.24	4	3	6	6.62	-0.06	1.38	1.55	0.06	97.98	-0.98	7.68	7.96	16.80
os71217	11	210	0.98	2	2	7	6.64	-0.03	2.78	1.56	0.12	87.23	-3.08	8.08	8.92	17.80
os71277	17	494	1.00	11	2	7	6.42	-0.15	0.45	1.51	0.24	99.30	-11.38	7.47	6.95	15.89
os71416	9	416	0.42	9	3	5	6.60	0.00	0.06	1.51	0.07	99.25	1.22	7.42	7.67	16.52
os71417	9	416	0.40	8	3	5	6.59	-0.02	0.04	1.51	0.07	99.21	1.58	7.44	7.67	16.52
os71545	19	222	0.40	6	2	7	6.82	0.06	1.23	1.55	0.14	92.85	-4.20	7.70	8.74	17.61
os71546	19	221	0.21	4	2	7	6.78	0.04	1.69	1.56	0.13	92.72	-4.04	7.80	8.75	17.61
os71571	9	256	0.45	11	2	0	6.44	0.01	0.02	1.48	0.11	97.39	-0.07	6.82	8.72	17.62
os71572	9	256	0.45	11	2	0	6.43	0.00	0.02	1.48	0.11	97.43	-0.20	6.81	8.72	17.62
os71575	19	225	0.99	2	2	0	6.64	-0.03	2.83	1.55	0.09	86.61	-1.73	8.34	8.89	17.82
os71576	19	233	0.53	5	2	7	6.59	-0.02	1.70	1.54	0.11	95.28	-3.00	7.08	8.85	17.77
os71577	19	232	0.47	5	2	7	6.61	-0.02	1.71	1.54	0.11	95.21	-3.38	7.06	8.86	17.78
os71578	19	231	0.52	5	2	7	6.62	-0.01	1.75	1.55	0.12	95.13	-3.74	7.05	8.86	17.78
os71614	19	404	0.17	11	2	3	6.71	0.09	0.29	1.51	0.10	98.61	-3.83	7.23	7.72	16.56
os71672	19	378	0.69	4	3	6	6.63	-0.05	1.64	1.54	0.08	91.63	-1.33	8.36	7.64	16.53
os71715	17	242	1.00	9	2	3	6.37	-0.15	0.41	1.52	0.10	89.63	-3.20	7.62	8.73	17.61
os71769	17	324	0.79	16	2	0	6.03	-0.19	0.09	1.46	0.15	98.42	-2.23	6.99	7.99	16.86
os71792	9	272	0.99	10	2	0	6.38	-0.16	0.04	1.50	0.08	92.69	0.33	6.86	8.78	17.63
os71857	17	501	0.84	9	2	7	6.45	-0.12	0.63	1.53	0.14	99.35	-4.48	7.89	7.17	15.99
os71878	23	619	0.85	19	3	9	5.93	-0.25	0.00	1.38	0.12	99.98	0.45	5.18	6.60	15.57
os71879	23	631	0.65	19	3	9	6.01	-0.16	0.00	1.37	0.12	99.85	0.07	5.28	6.64	15.61
os71970	17	305	0.80	11	3	5	6.36	-0.08	0.11	1.49	0.08	97.38	-1.62	6.83	8.10	17.12
os71983	19	310	0.49	3	3	6	6.66	-0.04	1.77	1.55	0.03	73.39	-0.71	9.26	8.06	17.01
os72038	19	319	0.23	2	3	6	6.73	0.00	1.97	1.55	0.06	99.35	-1.85	7.66	7.93	16.88
os72156	19	461	0.97	12	2	0	6.31	-0.19	0.07	1.49	0.14	99.80	-0.85	6.35	6.99	15.94

os72200	19	450	0.62	6	3	5	6.59	-0.07	0.66	1.53	0.09	97.46	-1.09	6.85	7.03	15.99
os72217	11	250	0.92	1	3	6	6.70	-0.01	5.94	1.56	0.01	1.92	0.20	10.49	8.30	17.29
os72218	11	250	0.92	0	3	6	6.71	0.00	5.93	1.57	0.01	1.08	0.05	12.00	8.30	17.29
os72221	11	241	0.92	0	3	6	6.71	0.00	3.96	1.57	0.02	6.80	0.00	11.87	8.34	17.30
os72222	11	241	0.92	0	3	6	6.71	0.00	3.96	1.57	0.02	7.65	0.00	11.87	8.34	17.30
os72271	11	251	0.92	0	3	6	6.71	0.00	3.03	1.56	0.01	16.13	0.02	9.81	8.30	17.26
os72274	11	246	0.92	0	3	6	6.71	0.00	5.91	1.57	0.01	3.89	-0.12	11.14	8.30	17.29
os72305	19	268	0.13	4	3	6	6.58	-0.07	0.92	1.53	0.03	85.85	-0.04	7.61	8.34	17.30
os72338	19	311	0.01	4	2	3	6.63	-0.04	1.59	1.55	0.09	90.22	-3.19	7.87	8.04	17.01
os72339	19	314	0.03	5	2	3	6.60	-0.05	1.62	1.54	0.07	90.49	-2.25	7.92	8.03	17.00
os72345	17	297	0.65	14	2	3	6.72	0.00	0.02	1.50	0.11	49.46	-1.62	6.43	8.10	17.08
os72375	11	216	0.08	0	3	6	6.71	0.00	2.51	1.56	0.02	33.14	0.01	9.25	8.60	17.59
os72509	19	304	0.97	7	3	5	6.68	-0.05	0.41	1.51	0.05	87.69	-1.07	7.43	8.26	17.06
os72679	22	239	0.60	3	2	3	6.65	-0.04	1.59	1.55	0.06	82.59	-2.51	7.49	8.61	17.50
os72721	19	260	0.77	1	2	3	6.68	-0.02	3.11	1.56	0.06	74.21	-2.89	8.80	8.28	17.27
os72748	19	349	0.60	8	3	5	6.53	-0.05	0.36	1.51	0.08	97.55	-1.20	7.78	7.83	16.70
os72797	19	287	0.85	6	2	3	6.53	-0.08	0.84	1.54	0.07	88.05	-1.56	7.87	8.29	17.30
os72888	11	207	0.92	0	3	6	6.70	0.00	5.88	1.57	0.00	7.65	-0.13	11.57	8.73	17.50
os72895	11	208	0.92	0	3	6	6.69	0.00	5.89	1.56	0.00	11.04	0.18	9.64	8.73	17.51
os72906	11	218	0.92	1	3	6	6.68	-0.01	5.89	1.56	0.01	15.74	0.16	9.98	8.73	17.57
os72907	11	219	0.92	3	3	6	6.65	-0.03	5.88	1.53	0.02	21.99	1.29	8.29	8.73	17.57
os72915	11	228	0.92	0	3	6	6.70	0.00	4.99	1.56	0.01	15.32	0.15	10.16	8.79	17.72
os72951	19	232	1.00	5	3	5	6.59	-0.07	1.42	1.54	0.05	66.40	-2.00	8.28	8.52	17.48
os73108	19	416	0.81	8	2	7	6.48	-0.12	0.96	1.56	0.13	95.96	-5.86	8.57	7.23	16.04
os73135	19	362	0.21	4	2	3	6.79	0.04	1.74	1.55	0.08	93.79	-2.14	7.45	7.79	16.58
os73190	17	331	0.07	14	3	5	6.13	-0.22	0.00	1.44	0.08	87.63	0.62	5.44	8.06	17.02
os73220	11	284	0.23	6	2	3	6.67	0.04	0.98	1.55	0.10	64.48	-3.89	7.68	8.18	17.06
os73253	19	323	0.92	0	3	6	6.75	0.00	3.95	1.56	0.02	14.16	-0.18	10.93	8.01	16.92
os73258	11	306	0.91	4	3	5	6.65	-0.06	1.12	1.55	0.06	83.25	-1.42	8.43	8.06	16.94
os73280	17	296	0.78	8	1	4	6.45	-0.09	0.10	1.50	0.04	93.38	1.61	7.21	8.26	17.23
os73330	19	371	0.40	11	2	3	6.50	-0.03	0.72	1.53	0.18	98.93	-8.76	7.43	7.72	16.64
os73343	11	326	0.60	1	3	6	6.72	-0.01	2.84	1.56	0.04	25.42	-0.18	8.32	7.98	16.87
os73367	11	386	0.01	5	3	5	6.83	0.07	0.92	1.52	0.04	34.99	0.41	8.42	7.54	16.44
os73416	11	249	0.92	1	3	6	6.70	-0.01	5.97	1.56	0.01	3.88	0.11	9.95	8.30	17.31
os73433	17	285	0.53	2	3	6	6.75	0.01	3.56	1.56	0.06	79.42	-2.22	8.60	8.08	17.06
os73497	17	453	0.99	15	1	1	6.14	-0.25	0.00	1.43	0.08	99.58	4.07	5.91	7.55	16.33
os73560	23	678	0.08	7	3	5	7.07	0.11	0.40	1.51	0.04	99.39	0.78	7.20	5.91	14.65
os73576	22	371	0.36	27	3	5	5.87	0.07	0.00	1.36	0.22	97.06	-0.34	4.59	7.76	16.36
os73581	19	332	0.61	3	3	6	6.78	0.01	2.08	1.56	0.06	77.50	-1.92	7.85	8.12	16.78
os73582	19	311	0.92	1	3	6	6.77	0.02	4.50	1.56	0.07	70.77	-2.09	8.78	8.22	16.89
os73583	11	315	0.92	0	3	6	6.75	0.01	3.94	1.56	0.05	58.96	-0.49	10.68	8.21	16.87
os73584	19	312	0.01	1	3	6	6.78	0.02	4.80	1.56	0.05	68.97	-0.43	8.80	8.21	16.88
os73586	19	332	0.45	6	3	6	6.82	0.02	1.45	1.54	0.06	77.39	-0.78	7.82	8.12	16.77

os73587	11	320	0.92	0	3	6	6.74	0.00	3.57	1.56	0.05	51.02	-0.32	10.67	8.16	16.82
os73589	11	313	0.13	1	3	6	6.77	0.01	4.51	1.56	0.05	65.92	-0.51	8.71	8.20	16.87
os73590	19	313	0.08	1	3	6	6.77	0.02	4.71	1.56	0.05	68.75	-0.34	8.78	8.21	16.88
os73598	19	312	0.01	1	3	6	6.77	0.02	4.81	1.56	0.05	69.10	-0.53	8.87	8.22	16.88
os73605	17	368	0.00	25	2	0	6.78	0.41	0.00	1.39	0.18	96.58	-0.18	5.32	7.65	16.28
os73610	9	341	0.29	6	2	3	6.75	0.07	0.74	1.52	0.15	91.07	-4.93	6.86	8.05	16.67
os73611	19	312	0.92	1	3	6	6.77	0.02	4.85	1.56	0.06	69.50	-0.86	8.87	8.22	16.88
os73648	14	445	0.77	4	1	4	6.83	0.00	0.07	1.49	0.03	95.94	3.01	7.55	7.76	16.60
os73693	23	615	0.87	5	1	4	6.68	-0.07	0.16	1.45	0.04	97.52	3.80	6.00	6.30	14.96
os73754	17	605	0.00	6	2	7	6.94	0.08	1.37	1.53	0.10	98.16	-3.08	8.03	6.16	14.80
os73755	17	596	1.00	15	3	5	6.23	-0.25	0.00	1.43	0.10	99.58	2.27	5.75	6.28	14.93
os73785	23	681	0.35	8	3	5	6.80	0.04	0.20	1.48	0.07	99.99	1.21	6.76	5.82	14.51
os73786	23	653	0.09	10	2	0	6.93	0.14	0.04	1.48	0.10	99.91	-0.42	7.15	5.91	14.60
os73883	25	661	0.19	13	3	5	6.79	0.11	0.00	1.46	0.11	99.92	-0.04	5.94	5.98	14.65
os73891	17	512	0.17	8	2	7	6.91	0.12	0.53	1.51	0.15	98.90	-3.69	7.14	6.70	15.35
os73965	23	669	0.72	5	3	5	6.89	-0.02	0.36	1.51	0.05	99.39	0.29	7.31	6.16	14.96
os73986	28	769	0.77	3	3	6	6.90	-0.03	0.64	1.53	0.04	85.10	-0.19	8.20	5.30	14.03
os73994	17	554	0.01	9	1	4	6.92	0.10	0.09	1.49	0.06	86.49	2.29	8.59	6.78	15.51
os74001	17	486	0.04	4	3	6	6.67	-0.07	0.95	1.51	0.05	96.84	-0.91	7.07	7.37	16.15
os74005	17	484	0.38	3	3	6	6.87	0.02	1.98	1.51	0.06	95.45	-1.57	7.89	7.38	16.15
os74072	22	601	0.01	5	3	6	6.95	0.08	0.95	1.54	0.06	90.16	-1.75	7.31	6.48	15.32
os74073	17	661	0.41	14	3	5	6.68	0.09	0.00	1.45	0.13	93.13	-1.33	5.89	6.17	14.97
os74091	17	486	1.00	17	2	3	6.16	-0.25	0.00	1.48	0.11	89.98	-3.14	7.74	7.24	16.05
os74173	19	439	0.92	1	3	6	6.78	-0.01	2.78	1.54	0.06	78.15	-0.09	9.40	7.15	15.99
os74183	19	417	0.17	6	2	7	6.82	0.06	0.98	1.54	0.07	98.24	-2.40	6.54	7.37	16.21
os74186	19	383	0.76	11	2	7	6.48	-0.09	0.76	1.53	0.13	97.09	-4.18	8.35	7.55	16.35
os74242	19	457	0.02	5	2	3	6.85	0.07	1.47	1.55	0.00	97.86	-2.48	8.00	7.09	15.93
os74272	19	420	0.08	7	2	3	6.86	0.10	0.65	1.53	0.07	98.43	-2.34	6.38	7.36	16.20
os74287	19	405	0.40	4	3	6	6.71	0.01	0.94	1.54	0.06	94.05	-0.76	7.54	7.49	16.33
os74288	19	409	0.54	3	3	5	6.77	0.03	1.42	1.55	0.07	96.48	-1.70	7.00	7.44	16.28
os74304	27	738	0.74	8	2	7	6.44	-0.12	0.71	1.55	0.00	99.28	-11.15	7.35	4.84	13.77
os74395	23	562	0.58	8	2	0	6.63	-0.02	0.18	1.48	0.00	96.41	1.30	6.20	6.31	15.19
os74424	19	344	0.85	4	3	6	6.61	-0.06	2.39	1.54	0.05	72.12	-1.74	8.45	7.95	16.78
os74426	23	658	0.89	8	1	2	6.41	-0.13	0.14	1.44	0.24	99.89	3.02	5.64	5.33	14.30
os74427	28	659	0.90	10	2	0	6.24	-0.17	0.20	1.46	0.25	99.91	-2.33	5.69	5.34	14.31
os74442	23	530	0.64	13	1	4	6.32	-0.11	0.01	1.43	0.10	84.22	3.51	5.92	6.44	15.33
os74444	28	533	0.40	4	3	6	6.92	0.03	0.84	1.51	0.08	95.79	-0.12	6.85	6.43	15.32
os74473	23	719	0.60	12	3	9	6.95	0.03	0.00	1.37	0.12	99.97	0.84	5.69	5.87	14.83
os74488	23	657	0.15	14	2	0	7.04	0.18	0.00	1.45	0.15	99.48	-0.85	6.00	5.39	14.29
os74528	27	526	0.47	6	2	7	6.63	-0.05	0.69	1.54	0.20	89.84	-1.58	6.83	5.85	14.71
os74688	27	662	0.54	14	2	7	6.34	-0.09	0.00	1.46	0.17	99.06	-3.17	6.65	5.52	14.46
os74690	23	652	0.60	13	2	3	6.39	-0.09	0.02	1.44	0.13	98.99	-2.98	5.65	5.85	14.78
os74691	23	710	0.00	8	1	4	7.02	0.12	0.01	1.35	0.06	97.76	9.02	5.45	5.55	14.48

os74693	23	673	0.53	8	2	7	6.66	-0.02	0.40	1.50	0.17	98.68	-2.83	6.73	5.46	14.39
os74739	23	567	0.95	10	3	5	6.64	-0.11	0.27	1.46	0.12	99.68	-0.63	5.90	6.59	15.55
os74746	23	580	0.70	19	3	5	6.00	-0.19	0.00	1.43	0.14	99.55	0.68	5.24	6.50	15.47
os74751	23	612	0.60	21	1	4	6.00	-0.14	0.00	1.40	0.12	99.85	3.14	5.36	6.39	15.36
os74755	23	624	0.40	10	1	1	6.65	0.00	0.01	1.42	0.05	100.00	3.56	5.68	6.56	15.53
os74821	17	357	0.19	25	2	0	6.57	0.28	0.00	1.43	0.21	96.47	-3.42	5.93	7.54	16.46
os74848	17	512	0.45	8	2	3	6.63	-0.01	0.58	1.49	0.15	99.92	-3.55	6.58	6.95	15.92
os74851	19	267	0.69	3	2	3	6.60	-0.06	1.79	1.54	0.07	91.62	-2.78	7.97	8.36	17.26
os74918	19	400	0.98	7	2	7	6.65	-0.06	1.07	1.53	0.17	97.14	-6.76	7.25	7.34	16.25
os74949	9	266	0.15	10	3	5	6.69	0.10	0.04	1.48	0.09	89.81	0.21	6.39	8.43	17.33
os74951	9	269	0.21	10	3	5	6.69	0.10	0.03	1.47	0.08	90.54	0.36	6.37	8.43	17.33
os74959	9	267	0.38	2	2	3	6.78	0.03	2.34	1.56	0.04	86.66	-1.91	8.19	8.49	17.38
os74960	9	267	0.29	3	3	6	6.80	0.04	2.10	1.55	0.04	87.33	-1.21	8.30	8.50	17.38
os74970	9	210	0.40	3	3	6	6.75	0.02	2.72	1.56	0.06	61.21	-0.95	8.37	8.86	17.77
os74975	9	238	0.74	6	2	3	6.50	-0.08	1.21	1.53	0.11	98.38	-4.00	7.78	8.63	17.55
os74986	19	441	0.08	17	2	7	6.64	0.19	0.22	1.48	0.19	96.52	-6.63	5.66	7.06	15.95
os74991	17	335	0.79	11	3	5	6.31	-0.13	0.04	1.48	0.08	92.34	1.61	7.20	7.97	16.86
os74992	17	338	0.67	9	3	5	6.41	-0.10	0.10	1.48	0.07	91.89	1.69	7.24	7.97	16.86
os74995	17	332	0.21	12	2	7	6.68	0.11	0.74	1.53	0.19	94.76	-9.13	6.11	7.79	16.64
os75006	9	272	0.02	7	2	3	6.47	-0.05	0.78	1.51	0.13	98.07	-4.41	7.49	8.37	17.28
os75010	19	284	0.41	6	2	7	6.79	0.03	1.80	1.53	0.13	95.80	-5.94	7.84	8.24	17.20
os75013	19	291	0.41	7	2	7	6.74	0.01	0.86	1.52	0.13	97.19	-6.04	7.76	8.23	17.19
os75014	19	289	0.96	7	2	7	6.60	-0.07	1.24	1.52	0.13	96.55	-5.70	7.76	8.23	17.19
os75019	17	280	0.99	2	2	7	6.65	-0.05	1.85	1.56	0.10	78.17	-3.96	7.74	8.22	17.15
os75022	17	300	1.00	13	3	5	6.20	-0.22	0.00	1.47	0.09	87.04	0.12	6.34	8.25	17.17
os75069	17	362	0.92	7	3	5	6.51	-0.08	0.61	1.48	0.06	94.79	-0.48	7.71	7.97	16.84
os75092	23	693	0.06	9	1	2	7.10	0.14	0.01	1.44	0.10	98.07	2.98	6.39	5.19	14.14
os75145	23	685	0.80	10	1	2	6.47	-0.12	0.01	1.44	0.11	97.59	4.86	5.97	5.23	14.18
os75148	23	706	0.90	15	2	0	6.22	-0.22	0.00	1.46	0.11	99.86	1.05	5.66	5.18	14.13
os75159	19	523	0.49	6	3	5	6.71	-0.01	0.84	1.52	0.10	99.69	-1.34	6.93	6.58	15.49
os75160	23	530	0.60	11	2	7	6.49	-0.09	0.44	1.52	0.10	98.37	-3.53	7.77	6.46	15.38
os75176	27	546	0.88	11	2	0	6.61	-0.12	0.02	1.47	0.16	96.91	-0.44	5.87	6.22	15.14
os75181	23	803	0.40	15	2	8	7.00	0.09	0.00	1.40	0.11	100.00	-3.14	5.60	5.25	14.21
os75199	23	572	0.54	21	2	7	5.93	-0.12	0.00	1.46	0.24	97.12	-5.48	5.56	5.99	14.92
os75200	23	631	0.53	16	1	2	6.12	-0.21	0.00	1.38	0.16	97.56	6.97	5.05	5.69	14.64
os75210	23	744	0.86	19	1	1	6.41	-0.20	0.00	1.34	0.09	99.94	6.15	5.06	5.68	14.64
os75215	23	651	0.48	19	1	4	6.88	0.06	0.00	1.35	0.13	97.43	4.83	5.18	5.92	14.88
os75236	23	667	0.98	20	1	1	5.86	-0.33	0.00	1.32	0.09	99.96	5.35	5.10	6.48	15.38
os75237	23	674	1.00	15	1	1	6.22	-0.25	0.00	1.33	0.07	99.97	6.35	5.19	6.46	15.37
os75250	19	446	0.42	12	2	3	6.92	0.08	0.08	1.46	0.15	88.95	-4.05	6.56	7.14	16.06
os75296	17	522	0.42	3	1	1	6.71	-0.05	0.01	1.42	0.00	100.00	5.50	6.34	7.25	16.20
os75450	23	501	0.20	22	2	0	6.45	0.14	0.00	1.40	0.16	96.57	0.91	6.16	6.66	15.61
os75451	23	529	0.24	6	2	7	6.66	-0.01	1.06	1.53	0.20	99.76	-4.38	7.71	6.35	15.31

os75452	27	459	0.70	7	2	7	6.51	-0.09	0.73	1.53	0.13	98.75	-4.42	7.34	6.92	15.83
os75453	23	545	0.96	17	1	1	6.24	-0.24	0.00	1.35	0.11	99.90	4.93	5.03	6.82	15.74
os75459	25	551	1.00	20	2	3	5.88	-0.32	0.00	1.38	0.17	96.57	-1.16	5.11	6.54	15.45
os75475	23	684	1.00	14	1	1	6.28	-0.24	0.00	1.40	0.08	99.99	2.06	5.51	5.87	14.82
os75482	23	612	0.70	16	2	0	6.22	-0.15	0.12	1.50	0.18	90.72	-2.80	6.39	5.71	14.66
os75484	23	837	0.40	23	3	9	6.17	-0.01	0.00	1.40	0.00	99.58	-0.24	5.63	4.71	13.72
os75494	23	768	0.70	9	1	1	6.62	-0.07	0.02	1.37	0.07	100.00	5.16	5.64	5.40	14.37
os75534	19	456	0.99	9	2	7	6.50	-0.10	1.07	1.54	0.19	95.08	-7.91	7.37	6.75	15.69
os75538	17	514	0.95	16	2	3	6.33	-0.20	0.00	1.43	0.12	99.71	-1.85	6.22	6.85	15.77
os75589	25	580	0.98	21	2	7	5.86	-0.31	0.00	1.41	0.20	99.57	-1.61	5.72	6.15	15.10
os75862	17	410	0.64	14	2	3	6.78	0.02	0.01	1.48	0.12	99.85	-2.63	6.71	7.37	16.29
os75893	9	268	1.00	13	2	3	6.15	-0.22	0.02	1.47	0.14	96.52	-4.48	6.06	8.77	17.63
os75905	9	387	0.91	14	1	4	6.37	-0.18	0.00	1.43	0.08	87.99	2.10	6.08	7.96	16.75
os76031	17	238	0.87	18	2	0	6.31	-0.16	0.00	1.46	0.17	87.96	-1.40	6.13	8.28	16.99
os76042	19	516	0.01	3	3	6	6.90	0.05	1.52	1.53	0.04	77.21	-0.37	9.29	6.98	15.79
os76057	17	296	0.76	20	3	9	5.76	-0.24	0.00	1.36	0.14	99.98	1.20	5.03	8.63	17.43
os76182	17	304	0.62	12	2	3	6.46	-0.10	0.35	1.42	0.14	99.48	-4.54	7.75	8.55	17.38
os76217	9	394	0.46	4	3	6	6.73	0.02	1.72	1.49	0.09	99.81	-1.93	8.08	7.84	16.64
os76219	9	308	0.99	24	2	7	5.42	-0.38	0.00	1.42	0.22	99.33	-5.79	5.92	8.19	17.02
os76296	19	322	0.47	3	2	0	6.62	-0.06	1.92	1.54	0.09	96.40	-2.07	7.50	7.93	16.68
os76341	17	268	0.03	3	1	4	6.80	0.06	0.03	1.49	0.07	97.00	3.45	7.79	8.37	17.17
os76346	17	228	0.35	27	2	7	6.62	0.19	0.00	1.42	0.29	99.59	-8.29	5.73	8.32	17.09
os76360	17	385	0.96	14	2	3	6.08	-0.23	0.07	1.47	0.15	99.63	-6.14	7.41	7.71	16.50
os76374	17	421	0.96	18	3	5	6.12	-0.26	0.00	1.44	0.14	98.72	-1.72	5.14	7.58	16.33
os76384	17	485	0.35	15	2	3	6.89	0.13	0.00	1.46	0.15	99.80	-4.32	5.10	7.20	15.99
os76386	19	517	0.00	2	3	6	6.88	0.03	1.47	1.53	0.04	80.89	-0.13	9.42	6.98	15.79
os76393	19	424	0.03	8	2	3	6.96	0.14	0.55	1.46	0.11	99.94	-3.15	6.93	7.61	16.45
os76409	17	405	1.00	20	2	3	5.88	-0.28	0.01	1.40	0.18	99.76	-6.65	7.32	7.72	16.49
os76455	17	524	0.66	11	3	5	6.94	0.07	0.00	1.43	0.09	99.96	3.56	5.42	7.29	16.12
os76500	9	522	0.01	10	3	5	7.04	0.17	0.04	1.48	0.10	100.00	-0.01	6.04	7.22	16.04
os76554	9	363	0.58	8	2	3	6.57	-0.03	0.89	1.47	0.10	99.54	-4.96	7.82	8.39	17.36
os76632	10	210	0.70	16	2	0	5.89	-0.27	0.00	1.45	0.13	99.14	-0.84	6.23	8.93	17.67
os76633	10	235	0.99	15	2	0	6.08	-0.23	0.00	1.47	0.12	98.62	-0.76	7.60	8.76	17.52
os76796	10	273	0.99	8	2	0	6.32	-0.14	0.06	1.48	0.13	99.29	0.99	6.09	8.48	17.29
os76846	9	190	0.60	8	3	5	6.41	-0.08	0.38	1.51	0.11	98.81	-2.54	7.67	9.12	17.87
os76874	10	347	0.70	11	3	5	6.71	-0.04	0.04	1.49	0.07	97.07	1.33	7.22	7.93	16.68
os76876	27	411	0.98	8	2	0	6.55	-0.12	0.21	1.49	0.09	99.44	0.81	7.01	7.48	16.27
os76887	17	399	0.04	12	2	7	6.98	0.21	0.01	1.49	0.13	98.44	-2.18	5.94	7.52	16.27
os76897	19	392	0.90	6	2	0	6.68	-0.07	0.40	1.51	0.09	99.34	0.24	7.14	7.60	16.35
os76920	19	266	0.09	1	2	3	6.71	0.00	2.61	1.56	0.05	80.10	-2.61	8.08	8.55	17.26
os76930	10	368	1.00	13	2	7	6.24	-0.20	0.11	1.49	0.17	95.01	-4.51	8.09	7.74	16.50
os77011	11	479	0.01	5	3	5	6.90	0.08	0.80	1.51	0.06	99.59	-0.25	7.56	7.48	16.35
os77026	17	555	0.77	6	3	5	6.81	-0.04	0.48	1.49	0.05	100.00	0.31	6.56	7.18	16.03

os77065	9	437	0.01	4	1	1	6.87	0.07	0.14	1.39	0.05	99.55	2.69	6.40	7.92	16.80
os77079	10	415	0.08	9	3	5	6.99	0.14	0.07	1.50	0.07	100.00	0.68	6.32	7.53	16.31
os77094	14	401	0.34	11	3	9	6.92	0.08	0.03	1.46	0.09	98.49	-1.31	5.65	7.95	16.72
os77102	11	357	0.40	2	3	6	6.78	0.00	0.21	1.53	0.04	97.09	0.60	7.59	8.02	16.75
os77107	27	304	0.00	11	2	7	6.87	0.16	0.47	1.52	0.12	100.00	-4.71	7.26	8.27	17.00
os77134	17	411	0.01	9	2	0	6.91	0.14	0.36	1.51	0.13	99.47	-2.66	7.28	7.49	16.27
os77151	19	507	0.19	8	3	5	6.98	0.09	0.37	1.51	0.08	99.34	-1.92	7.87	7.02	15.84
os77187	19	382	0.15	12	2	7	6.67	0.11	0.06	1.49	0.15	99.99	-3.18	7.01	7.81	16.65
os77241	9	539	0.78	3	3	6	6.74	-0.03	0.94	1.52	0.04	92.69	-0.66	6.84	7.34	16.26
os77275	9	228	0.40	23	3	5	6.60	0.08	0.00	1.38	0.15	96.86	0.38	5.98	9.08	17.91
os77294	10	506	0.01	2	3	6	6.85	0.02	0.25	1.52	0.02	83.82	0.21	8.02	7.59	16.48
os77301	9	535	0.23	1	3	6	6.83	0.01	0.99	1.52	0.03	87.86	0.47	8.43	7.39	16.30
os77302	9	536	0.92	2	3	6	6.78	-0.02	0.62	1.52	0.03	90.19	-0.05	7.45	7.38	16.29
os77314	9	372	1.00	12	2	7	6.29	-0.20	0.05	1.47	0.12	98.50	-2.47	7.79	8.04	16.88
os77347	9	311	0.98	8	1	4	6.52	-0.12	0.17	1.49	0.05	82.44	3.05	6.25	8.33	17.06
os77357	11	348	0.94	4	3	6	6.66	-0.06	0.42	1.53	0.05	94.03	0.18	8.10	8.05	16.78
os77378	10	385	0.00	16	1	4	6.97	0.28	0.00	1.43	0.09	99.80	3.60	5.26	7.88	16.64
os77395	27	257	0.58	6	2	7	6.78	0.02	1.67	1.55	0.09	91.36	-4.34	8.47	8.58	17.31
os77416	17	548	0.02	10	3	5	7.05	0.17	0.03	1.45	0.07	100.00	1.90	5.74	7.18	16.03
os77418	17	540	0.21	10	1	4	7.05	0.15	0.01	1.45	0.07	99.99	2.28	5.66	7.26	16.10
os77464	17	318	0.93	15	2	7	6.27	-0.20	0.00	1.45	0.13	98.60	-2.90	7.18	8.17	16.94
os77487	17	525	0.51	22	3	5	6.76	0.07	0.00	1.40	0.15	100.00	-2.24	5.88	7.23	16.08
os77499	17	522	1.00	9	2	3	6.50	-0.14	0.56	1.46	0.12	100.00	-3.92	7.26	7.28	16.14
os77536	17	353	0.39	13	3	9	6.84	0.10	0.00	1.46	0.11	99.42	-1.95	5.39	8.06	16.79
os77638	3	313	0.47	13	3	5	6.88	0.10	0.05	1.47	0.09	97.15	-1.25	5.77	8.03	16.74
os77676	19	308	0.40	5	2	7	6.79	0.05	1.34	1.54	0.12	99.44	-5.01	7.64	7.93	16.66
os77725	9	323	0.43	8	3	5	6.83	0.03	0.11	1.51	0.07	89.54	0.58	6.95	8.02	16.76
os77755	17	278	0.04	14	2	0	6.93	0.23	0.00	1.44	0.19	98.65	0.08	5.45	7.97	16.77
os77764	17	486	0.01	8	2	3	6.98	0.14	0.39	1.52	0.08	97.18	-2.63	6.19	7.27	16.06
os77767	17	272	0.76	19	2	7	5.75	-0.18	0.23	1.45	0.27	100.00	-11.06	6.98	8.42	17.25
os77774	3	369	0.08	3	1	4	6.84	0.03	0.15	1.51	0.02	85.84	2.49	7.85	8.02	16.98
os77946	10	432	0.04	5	3	5	6.63	-0.03	0.23	1.49	0.06	98.78	1.47	6.29	7.51	16.40
os78046	27	568	0.17	9	3	5	7.05	0.14	0.24	1.50	0.11	99.98	-1.83	6.60	6.48	15.42
os78101	3	352	0.00	3	3	6	6.65	-0.05	0.91	1.54	0.02	84.38	-0.15	7.41	8.06	17.01
os78128	10	317	0.75	2	3	6	6.68	-0.02	1.50	1.49	0.03	42.38	0.98	8.50	8.50	17.56
os78152	10	419	0.92	6	3	9	6.54	-0.10	0.42	1.47	0.04	100.00	0.01	6.14	7.85	16.80
os78170	10	449	0.03	4	3	6	6.65	-0.07	0.58	1.52	0.03	88.46	0.07	7.42	7.40	16.29
os78175	10	441	0.01	2	3	6	6.85	0.04	1.44	1.54	0.02	93.62	-0.41	7.61	7.44	16.35
os78181	10	503	0.08	11	3	5	7.05	0.18	0.06	1.48	0.07	96.55	0.41	6.66	7.01	15.94
os78207	9	396	0.92	6	3	6	6.56	-0.10	0.84	1.51	0.05	92.49	-0.40	8.88	7.67	16.65
os78209	10	414	0.37	4	3	6	6.89	0.06	1.27	1.52	0.03	91.89	0.25	7.06	7.71	16.69
os78215	4	419	0.88	5	3	5	6.73	-0.04	0.71	1.51	0.03	92.40	0.90	7.45	7.74	16.76
os78216	4	438	0.39	3	3	6	6.71	-0.04	0.50	1.52	0.02	93.93	0.19	7.30	7.76	16.79

os78228	12	400	0.08	2	3	6	6.82	0.02	2.72	1.55	0.01	21.34	-0.16	8.80	7.82	16.81
os78256	9	380	0.59	13	1	4	6.25	-0.19	0.00	1.45	0.05	87.00	2.14	6.96	7.73	16.72
os78259	10	396	0.94	11	2	3	6.53	-0.12	0.64	1.52	0.12	97.80	-5.20	8.41	7.58	16.54
os78335	3	351	0.92	2	3	6	6.68	-0.04	1.53	1.56	0.03	58.23	-0.39	7.51	7.95	16.88
os78339	12	355	0.03	2	3	6	6.68	-0.04	1.44	1.55	0.03	71.74	-0.04	7.36	7.94	16.88
os78352	12	373	0.12	2	3	6	6.83	0.03	0.55	1.54	0.01	67.12	0.52	7.75	7.93	16.90
os78414	10	465	0.92	3	3	6	6.69	-0.05	1.84	1.54	0.06	96.34	-1.18	7.44	7.18	16.09
os78468	10	425	0.77	7	2	0	6.71	-0.06	0.37	1.54	0.10	54.83	-1.25	7.33	7.25	16.18
os78469	22	456	0.39	4	3	6	6.80	-0.01	0.86	1.52	0.08	85.97	-0.84	7.27	7.28	16.21
os78508	3	363	0.72	5	3	6	6.62	-0.06	0.21	1.53	0.03	55.58	0.83	7.50	7.89	16.89
os78533	10	462	0.92	8	3	9	6.47	-0.13	0.07	1.48	0.05	98.15	0.87	5.76	7.66	16.62
os78539	10	418	0.48	7	3	5	6.63	-0.02	0.52	1.52	0.06	85.38	0.00	7.03	7.35	16.29
os78543	10	445	0.95	7	3	5	6.52	-0.12	0.18	1.49	0.05	77.41	1.58	6.69	7.28	16.22
os78592	10	456	0.54	5	3	6	6.85	0.00	0.24	1.51	0.04	97.83	1.94	6.06	7.27	16.19
os78610	10	460	0.40	8	2	3	6.92	0.06	0.36	1.51	0.11	99.93	-2.42	6.79	7.11	16.05
os78625	27	538	0.92	3	3	6	6.71	-0.05	0.33	1.52	0.04	98.95	0.47	6.53	6.75	15.68
os78627	27	543	0.63	5	3	6	6.66	-0.08	0.56	1.52	0.04	99.57	0.07	6.63	6.71	15.64
os78663	10	441	0.99	7	2	7	6.64	-0.08	1.70	1.54	0.10	88.41	-3.08	8.23	7.16	16.07
os78824	19	397	0.88	2	3	6	6.71	-0.04	1.78	1.55	0.06	95.93	-0.69	8.82	7.58	16.46
os78830	17	592	0.17	11	1	1	7.03	0.13	0.00	1.36	0.06	98.25	8.59	5.26	6.84	15.77
os78973	10	389	0.08	4	3	6	6.88	0.07	1.17	1.53	0.03	90.76	-0.40	7.73	7.96	16.94
os78974	9	365	0.92	1	3	6	6.75	0.00	2.89	1.56	0.04	56.77	-0.47	9.46	7.99	16.94
os79266	10	417	0.06	5	2	3	6.92	0.09	0.90	1.55	0.07	67.42	-2.66	7.89	7.41	16.36
os79276	10	423	0.05	7	3	5	6.95	0.11	0.39	1.52	0.05	89.82	-0.22	6.54	7.43	16.26
os79322	17	405	1.00	6	3	5	6.58	-0.10	0.32	1.52	0.06	95.56	-0.70	6.79	7.48	16.37
os79412	9	411	0.00	2	3	6	6.73	-0.03	2.60	1.54	0.02	56.50	-0.04	8.72	7.55	16.47
os79431	10	393	0.23	4	3	6	6.74	0.02	0.61	1.53	0.03	85.95	0.23	7.25	7.69	16.66
os79433	10	390	0.23	3	3	6	6.75	0.02	1.12	1.54	0.03	83.99	-0.36	7.44	7.69	16.66
os79540	10	453	0.09	3	3	6	6.90	0.05	1.36	1.53	0.02	80.12	-0.12	7.58	7.28	16.18
os79541	10	446	0.01	3	3	6	6.88	0.05	1.78	1.54	0.04	84.74	-1.66	8.01	7.29	16.19
os79542	10	452	0.07	4	3	6	6.91	0.06	1.10	1.53	0.02	80.16	-0.24	7.66	7.29	16.19
os79557	24	441	0.10	2	3	6	6.85	0.03	1.98	1.56	0.04	71.98	-1.67	8.22	7.22	16.08
os79581	10	418	0.09	5	3	5	6.92	0.06	0.34	1.52	0.04	92.91	0.32	7.05	7.46	16.36
os79582	10	401	0.40	4	3	6	6.86	0.03	0.64	1.52	0.03	98.98	0.24	6.74	7.60	16.50
os79776	10	415	0.00	5	3	6	6.88	0.07	0.48	1.52	0.03	77.20	0.95	7.49	7.48	16.41
os79791	10	419	0.60	3	3	6	6.71	-0.02	1.90	1.52	0.04	88.85	-1.59	8.30	7.46	16.36
os79800	10	443	0.23	5	3	6	6.80	0.05	0.46	1.49	0.04	97.98	2.37	6.81	7.37	16.20
os79808	10	421	0.23	3	2	3	6.83	0.05	1.77	1.56	0.09	82.45	-3.89	8.36	7.36	16.24
os79810	10	470	0.99	2	3	6	6.74	-0.04	1.75	1.54	0.02	75.46	-0.04	9.27	7.18	16.10
os79821	10	401	0.08	5	3	6	6.91	0.08	0.73	1.53	0.03	94.20	-0.34	7.05	7.59	16.48
os79911	10	384	0.50	7	3	5	6.86	0.03	0.39	1.52	0.05	96.22	0.05	6.69	7.65	16.55
os79912	10	384	0.51	6	3	5	6.87	0.04	0.22	1.52	0.04	95.73	0.57	6.74	7.65	16.55
os79913	10	390	0.76	4	3	6	6.82	0.01	1.13	1.52	0.04	97.87	-0.18	6.72	7.63	16.53

os79914	10	401	0.21	4	3	6	6.87	0.04	0.58	1.52	0.03	99.09	0.38	6.83	7.60	16.50
os79915	10	408	0.46	3	3	6	6.81	0.00	0.25	1.52	0.01	99.17	1.34	6.72	7.58	16.48
os79916	10	402	0.32	3	3	6	6.85	0.03	0.19	1.52	0.02	99.20	0.92	6.83	7.60	16.50
os80040	19	411	0.08	1	3	6	6.81	0.01	4.98	1.57	0.02	4.93	-0.41	9.91	7.36	16.24
os80133	17	429	0.47	10	3	5	6.91	0.06	0.11	1.50	0.07	93.08	-1.13	7.49	7.50	16.41
os80161	22	461	0.98	8	1	1	6.48	-0.13	0.08	1.46	0.04	99.91	2.37	6.17	7.42	16.32
os80170	28	662	0.56	7	3	5	6.93	0.00	0.29	1.51	0.05	92.97	-0.57	7.04	6.06	14.87
os80181	28	555	0.76	3	3	6	6.85	-0.01	1.69	1.55	0.04	96.61	-0.50	8.79	6.65	15.54
os80182	28	552	0.77	3	3	6	6.84	-0.02	1.71	1.55	0.04	95.10	-0.43	8.78	6.66	15.56
os80205	3	643	0.17	3	3	6	6.96	0.04	0.32	1.54	0.02	76.36	0.75	7.59	6.17	15.01
os80271	17	488	0.43	3	2	3	6.76	0.00	1.77	1.53	0.07	94.42	-2.60	9.05	7.10	16.02
os80325	4	438	0.48	2	3	6	6.84	0.01	1.68	1.54	0.02	84.88	-0.19	8.12	7.46	16.44
os80326	10	426	0.40	3	3	6	6.85	0.02	1.44	1.54	0.03	95.86	-0.16	8.11	7.48	16.46
os80327	10	415	0.38	3	3	6	6.85	0.03	1.64	1.55	0.03	81.78	-0.31	8.44	7.51	16.49
os80328	4	437	0.40	2	3	6	6.84	0.02	1.78	1.54	0.02	88.19	-0.24	8.12	7.47	16.45
os80332	10	439	0.37	3	3	6	6.86	0.02	0.92	1.54	0.03	83.67	0.08	8.62	7.36	16.34
os80382	12	432	0.27	8	3	5	6.69	0.04	0.38	1.51	0.05	100.00	-1.18	6.91	7.51	16.45
os80388	10	394	0.08	2	3	6	6.83	0.03	0.40	1.50	0.02	93.88	0.80	8.11	7.82	16.76
os80468	10	422	0.40	2	3	6	6.84	0.02	0.95	1.54	0.02	76.70	0.14	7.97	7.65	16.61
os80492	3	436	0.01	3	3	6	6.83	0.04	0.33	1.52	0.04	99.99	0.65	6.82	7.49	16.44
os80497	12	450	0.10	3	3	6	6.84	0.05	1.51	1.54	0.04	98.25	-0.76	8.42	7.39	16.35
os80592	3	515	0.01	7	3	5	6.99	0.12	0.36	1.50	0.05	93.67	-0.05	6.58	6.99	15.96
os80627	3	448	0.67	1	3	6	6.76	-0.02	0.13	1.53	0.01	82.28	0.82	8.83	7.38	16.36
os80660	19	416	0.42	8	2	3	6.60	-0.02	0.80	1.53	0.12	96.50	-4.68	8.41	7.54	16.51
os80668	9	371	0.24	9	2	0	6.93	0.08	0.09	1.50	0.07	97.43	0.99	6.60	7.81	16.74
os80669	9	404	0.32	9	2	3	6.70	0.08	0.53	1.53	0.18	99.26	-8.45	7.53	7.70	16.64
os80680	17	421	0.79	11	2	3	6.44	-0.08	0.67	1.50	0.15	95.90	-6.84	8.11	7.53	16.50
os80718	17	469	0.00	10	2	0	6.90	0.12	0.46	1.52	0.13	92.30	-3.56	6.28	7.01	15.89
os80756	3	580	0.77	6	3	5	6.83	-0.03	0.45	1.51	0.03	98.85	1.22	6.87	6.58	15.41
os80810	10	482	0.77	8	3	5	6.79	-0.03	0.36	1.52	0.05	81.66	-0.31	8.43	7.08	16.00
os80825	3	620	1.00	6	3	5	6.66	-0.11	0.12	1.49	0.04	98.29	2.05	6.49	6.31	15.13
os80840	10	517	0.50	6	3	5	6.89	0.01	0.51	1.52	0.06	99.99	-0.52	6.43	6.98	15.86
os80841	17	451	0.44	7	3	5	6.92	0.06	0.72	1.52	0.08	90.96	-0.29	7.54	7.26	16.13
os80871	3	476	0.92	6	3	5	6.58	-0.10	0.46	1.52	0.04	100.00	-0.37	7.61	7.15	16.05
os80872	3	475	0.92	6	3	5	6.59	-0.09	0.60	1.52	0.04	100.00	-0.06	7.39	7.15	16.05
os80873	10	432	0.91	5	3	5	6.64	-0.09	0.86	1.54	0.08	74.16	-0.90	8.57	7.28	16.16
os80883	3	465	0.52	10	1	4	6.53	-0.03	0.05	1.47	0.05	81.96	1.91	6.75	7.34	16.25
os80887	3	525	0.92	5	3	6	6.66	-0.07	0.58	1.53	0.03	97.00	0.14	8.03	6.90	15.84
os80888	3	534	0.23	2	3	6	6.74	-0.04	0.39	1.53	0.02	95.50	0.77	7.68	6.90	15.84
os80890	10	529	0.76	11	3	5	6.38	-0.14	0.02	1.49	0.09	97.74	-1.25	6.56	6.91	15.85
os80891	10	524	0.80	16	3	5	6.11	-0.19	0.00	1.45	0.10	98.77	-1.36	5.87	6.94	15.89
os80907	10	498	0.98	10	3	5	6.53	-0.15	0.06	1.46	0.07	97.02	-0.02	6.74	7.15	16.13
os80908	14	528	0.12	13	1	4	7.07	0.18	0.02	1.39	0.06	97.83	3.94	5.70	7.02	15.99

os80909	14	527	0.10	14	1	4	7.08	0.20	0.00	1.39	0.07	97.86	3.65	5.64	7.02	15.99
os80913	10	531	0.93	5	1	1	6.67	-0.08	0.19	1.48	0.02	88.87	2.20	6.17	7.13	16.12
os80921	10	538	0.01	4	3	6	6.96	0.08	0.56	1.50	0.02	81.05	1.30	7.26	6.96	15.89
os80922	10	536	0.01	6	3	5	6.98	0.10	0.49	1.50	0.03	83.16	1.07	7.00	6.96	15.89
os80928	10	417	0.68	10	2	0	6.77	-0.02	0.04	1.50	0.09	97.94	-0.22	6.76	7.51	16.47
os80983	17	456	0.86	13	2	7	6.24	-0.19	0.10	1.51	0.16	97.61	-5.39	8.54	7.09	16.07
os80984	17	459	0.17	13	2	7	6.25	-0.20	0.09	1.50	0.15	97.65	-4.51	8.33	7.09	16.06
os81006	12	443	0.92	2	3	6	6.73	-0.03	2.66	1.55	0.02	69.76	-0.15	8.59	7.41	16.39
os81007	12	440	0.92	3	3	6	6.70	-0.04	2.41	1.54	0.03	69.90	-0.24	8.94	7.42	16.40
os81051	4	431	0.39	3	3	6	6.86	0.03	1.13	1.54	0.02	93.06	0.26	7.77	7.47	16.44
os81053	4	430	0.23	3	3	6	6.86	0.03	0.95	1.54	0.03	93.69	0.18	7.74	7.47	16.45
os81054	10	429	0.40	4	3	6	6.86	0.03	0.86	1.54	0.03	96.24	-0.01	8.13	7.48	16.46
os81062	10	427	0.40	3	3	6	6.85	0.02	1.40	1.54	0.03	96.28	-0.26	8.15	7.49	16.47
os81067	10	423	0.40	3	3	6	6.85	0.02	1.79	1.55	0.03	95.24	-0.40	8.32	7.49	16.47
os81069	12	423	0.39	2	3	6	6.84	0.02	2.95	1.55	0.04	81.76	-0.34	8.94	7.40	16.38
os81072	3	459	0.99	6	3	5	6.67	-0.08	0.86	1.50	0.05	88.24	-1.50	7.55	7.43	16.42
os81079	10	416	0.19	4	3	6	6.88	0.05	1.29	1.54	0.05	98.77	-1.16	7.55	7.53	16.50
os81090	10	348	0.99	10	3	5	6.37	-0.17	0.05	1.46	0.09	90.22	0.90	6.29	7.92	16.85
os81094	17	436	0.77	7	2	0	6.77	-0.03	0.79	1.51	0.08	99.15	-2.15	8.26	7.41	16.39
os81099	10	367	0.04	16	1	4	7.00	0.26	0.00	1.42	0.08	96.70	3.16	6.09	7.87	16.80
os81101	10	435	0.23	5	3	6	6.79	0.04	1.19	1.53	0.05	98.66	-1.51	8.13	7.45	16.42
os81102	10	457	0.12	6	3	5	6.83	0.07	0.47	1.51	0.04	99.89	-0.12	6.67	7.39	16.37
os81106	3	604	1.00	8	3	5	6.59	-0.13	0.26	1.51	0.06	98.41	-0.26	7.24	6.30	15.14
os81112	10	482	0.22	5	3	5	6.84	0.05	0.15	1.51	0.03	98.42	1.65	7.21	7.22	16.14
os81120	3	391	0.27	5	3	5	6.91	0.07	0.65	1.52	0.03	97.52	0.88	7.19	7.87	16.87
os81156	12	395	0.08	3	3	6	6.78	0.03	1.90	1.54	0.05	98.22	-1.65	8.24	7.81	16.88
os81253	9	356	0.60	6	2	3	6.60	-0.04	0.90	1.55	0.06	79.49	-2.25	7.94	8.22	17.28
os81325	12	361	0.92	0	3	6	6.76	0.00	7.59	1.55	0.00	2.43	0.22	11.29	8.06	17.10
os81416	12	356	0.96	2	3	6	6.73	-0.03	2.57	1.54	0.03	64.42	-0.65	8.87	7.98	16.94
os81466	10	479	0.24	3	3	6	6.89	0.03	0.65	1.53	0.03	90.18	0.02	8.08	7.19	16.08
os81479	10	424	0.92	2	3	6	6.71	-0.04	1.60	1.54	0.02	86.09	-0.07	8.74	7.64	16.65
os81500	3	327	0.24	3	3	6	6.81	0.03	1.93	1.56	0.05	68.38	-1.49	9.28	8.08	17.09
os81521	3	367	0.92	1	3	6	6.76	-0.01	1.67	1.53	0.01	11.30	0.11	8.47	8.01	16.99
os81525	10	420	0.77	6	1	1	6.75	-0.04	0.07	1.47	0.02	98.30	2.41	5.93	7.88	16.91
os81542	10	337	0.01	2	3	6	6.79	0.02	2.90	1.56	0.05	75.27	-1.13	9.01	8.13	17.12
os81574	3	359	0.77	3	3	6	6.75	-0.02	1.34	1.55	0.01	26.21	-0.13	7.82	8.01	16.99
os81600	10	383	0.08	2	3	6	6.82	0.03	2.29	1.55	0.02	74.79	-0.87	9.30	7.81	16.76
os81630	10	429	0.04	3	3	6	6.69	-0.06	1.69	1.54	0.04	99.58	-1.45	7.30	7.65	16.63
os81644	10	429	0.65	4	1	1	6.67	-0.04	0.24	1.45	0.02	98.76	2.87	6.57	7.94	16.91
os81677	3	399	0.95	5	3	5	6.65	-0.08	0.90	1.51	0.07	98.75	-1.38	6.85	7.77	16.82
os81688	3	443	0.00	2	3	9	6.83	0.03	0.14	1.52	0.01	88.60	0.72	7.47	7.72	16.66
os81692	10	406	0.70	4	3	6	6.67	-0.04	1.21	1.52	0.04	98.70	-0.73	7.48	7.87	16.83
os81733	9	418	0.03	8	2	3	6.47	-0.14	0.49	1.54	0.09	57.64	-3.07	8.13	7.44	16.40

os81735	3	422	0.05	3	3	6	6.87	0.06	1.33	1.54	0.04	74.26	0.24	8.25	7.48	16.42
os81848	3	368	1.00	8	2	3	6.50	-0.13	0.81	1.52	0.08	87.92	-3.30	7.47	7.99	16.99
os81849	17	344	0.21	6	2	7	6.89	0.08	1.19	1.54	0.13	94.24	-4.05	8.05	7.90	16.83
os81902	3	408	0.17	3	3	6	6.68	-0.05	1.49	1.54	0.04	76.25	-0.44	8.70	7.59	16.55
os81914	3	411	0.98	5	3	5	6.68	-0.07	0.30	1.49	0.05	95.61	1.89	6.25	7.71	16.73
os81950	10	407	0.22	6	3	5	6.54	-0.10	0.44	1.51	0.04	99.14	0.50	6.74	7.57	16.52
os81954	19	448	0.92	0	3	6	6.79	0.00	2.99	1.54	0.03	34.21	-1.11	9.10	7.35	16.31
os82006	12	358	0.92	1	3	6	6.78	0.00	3.49	1.54	0.01	27.95	-0.03	8.94	8.05	17.07
os82046	3	393	0.02	3	3	6	6.67	-0.05	0.29	1.53	0.01	53.93	1.43	7.94	7.74	16.70
os82048	10	378	0.07	4	3	6	6.88	0.07	0.60	1.53	0.02	67.16	0.54	7.48	7.82	16.81
os82049	12	376	0.06	4	3	6	6.87	0.07	0.75	1.54	0.03	77.55	-0.14	7.48	7.85	16.84
os82053	10	384	0.82	2	3	6	6.75	-0.02	0.78	1.54	0.02	65.19	0.42	8.49	7.79	16.74
os82057	10	396	0.60	2	3	6	6.73	-0.01	0.73	1.54	0.02	84.56	0.28	8.64	7.77	16.79
os82059	12	362	0.92	0	3	6	6.75	-0.01	7.56	1.55	0.00	3.15	0.73	10.13	8.05	17.09
os82060	12	361	0.92	0	3	6	6.76	0.00	7.60	1.55	0.00	2.79	-0.02	10.86	8.05	17.09
os82068	3	368	0.88	4	3	6	6.65	-0.06	1.52	1.54	0.05	91.45	-0.22	7.89	8.12	17.21
os82069	3	367	0.70	4	3	6	6.63	-0.07	1.25	1.53	0.05	90.76	-0.07	8.05	8.12	17.21
os82070	12	370	0.92	4	3	6	6.64	-0.06	1.80	1.54	0.05	91.61	-1.30	7.81	8.11	17.20
os82082	9	296	0.99	12	2	7	6.34	-0.18	0.05	1.51	0.11	69.61	-2.82	7.75	8.30	17.26
os82092	4	442	0.14	2	3	6	6.79	0.00	1.51	1.54	0.03	87.50	-0.06	8.84	7.69	16.70
os82095	4	430	0.01	3	3	6	6.84	0.04	2.74	1.54	0.03	66.42	-0.31	8.70	7.70	16.71
os82106	10	413	0.99	4	3	6	6.64	-0.08	0.45	1.52	0.04	91.75	-0.34	6.36	7.89	16.87
os82107	12	425	0.60	3	3	6	6.72	-0.01	0.94	1.53	0.03	63.21	-0.36	7.33	7.78	16.77
os82119	10	491	0.79	5	3	6	6.64	-0.07	0.56	1.52	0.03	94.25	0.08	6.73	7.44	16.39
os82120	10	447	0.93	9	3	5	6.40	-0.17	0.04	1.49	0.07	93.80	0.05	6.23	7.55	16.51
os82121	10	447	0.99	7	2	3	6.57	-0.11	0.50	1.52	0.07	91.41	-2.56	7.45	7.55	16.50
os82130	10	397	0.85	3	3	6	6.69	-0.05	0.89	1.54	0.04	88.72	0.31	7.95	7.79	16.74
os82143	10	417	0.92	6	3	9	6.55	-0.09	0.51	1.48	0.04	99.99	0.75	6.45	7.83	16.79
os82144	10	420	0.92	6	3	9	6.55	-0.10	0.51	1.47	0.05	100.00	-0.21	6.14	7.84	16.80
os82159	3	373	0.92	0	3	6	6.77	0.00	1.98	1.54	0.00	19.13	-0.03	11.28	7.98	16.95
os82160	3	373	0.92	0	3	6	6.78	0.01	1.95	1.54	0.01	20.09	-0.16	9.62	7.98	16.95
os82163	10	374	0.01	1	3	6	6.78	0.01	0.44	1.53	0.01	39.91	0.45	9.40	8.00	16.96
os82177	3	345	0.12	1	3	6	6.74	-0.01	4.63	1.56	0.01	8.20	-0.12	9.88	7.95	16.93
os82178	12	347	0.99	2	3	6	6.72	-0.03	3.50	1.56	0.02	23.11	-0.22	9.13	7.94	16.92
os82205	3	422	0.11	11	1	4	6.78	0.13	0.10	1.44	0.05	78.25	3.76	6.01	7.55	16.53
os82206	3	419	0.12	14	1	4	6.75	0.16	0.00	1.44	0.07	76.44	3.41	5.93	7.55	16.53
os82208	3	407	0.84	5	3	6	6.61	-0.08	1.08	1.53	0.04	73.03	-0.73	7.62	7.69	16.66
os82232	3	398	0.00	1	2	3	6.79	0.01	2.98	1.56	0.05	79.78	-2.13	8.63	7.64	16.61
os82233	3	389	0.92	1	3	6	6.73	-0.02	3.55	1.56	0.05	81.34	-0.58	9.46	7.68	16.64
os82234	3	398	0.00	1	2	3	6.79	0.01	2.97	1.56	0.05	80.20	-2.19	8.59	7.64	16.61
os82235	3	397	0.92	1	3	6	6.79	0.00	2.98	1.56	0.05	82.31	-1.80	8.60	7.65	16.61
os82236	3	391	0.12	2	3	6	6.70	-0.04	2.34	1.55	0.04	84.80	-0.81	9.07	7.68	16.65
os82237	3	390	0.11	3	3	6	6.69	-0.04	2.38	1.55	0.04	84.71	-0.84	9.07	7.68	16.65

os82238	3	390	0.99	2	3	6	6.70	-0.04	2.76	1.55	0.04	84.30	-0.95	9.13	7.69	16.65
os82248	4	452	0.92	1	3	9	6.76	-0.02	0.42	1.52	0.00	91.04	1.11	7.97	7.61	16.61
os82316	10	326	0.99	14	2	7	6.16	-0.21	0.21	1.52	0.18	90.06	-7.35	8.49	7.93	16.87
os82338	10	400	0.91	4	3	6	6.62	-0.07	0.58	1.52	0.02	91.79	0.18	6.92	7.89	16.85
os82339	10	399	0.37	4	3	6	6.62	-0.07	0.57	1.53	0.02	92.04	0.10	6.93	7.89	16.85
os82340	12	359	0.92	1	3	6	6.74	-0.02	2.98	1.55	0.02	37.59	0.21	9.44	8.06	17.06
os82348	10	369	0.04	7	3	5	6.52	-0.11	0.40	1.52	0.04	97.72	-0.56	7.15	7.95	16.89
os82356	3	382	0.08	3	3	6	6.85	0.04	1.42	1.54	0.03	68.28	-0.70	7.57	8.12	17.19
os82357	12	385	0.27	6	3	6	6.86	0.03	0.93	1.53	0.04	86.48	-0.97	8.21	7.95	16.92
os82372	3	444	0.04	4	3	6	6.83	0.05	0.71	1.52	0.03	95.05	0.03	6.94	7.59	16.58
os82409	10	366	0.67	5	3	6	6.78	-0.01	0.40	1.51	0.04	94.29	-0.40	7.86	8.08	17.06
os82410	10	364	0.98	5	2	3	6.71	-0.04	1.40	1.52	0.05	94.70	-2.01	8.01	8.08	17.06
os82411	10	363	0.91	5	2	3	6.71	-0.04	1.43	1.52	0.05	95.01	-1.78	8.02	8.09	17.07
os82412	10	363	0.89	4	3	6	6.71	-0.04	1.48	1.52	0.05	95.33	-1.55	8.04	8.09	17.07
os82413	9	379	0.99	3	3	9	6.72	-0.04	1.04	1.50	0.01	87.29	0.45	7.56	8.08	17.06
os82432	10	482	0.71	8	3	5	6.80	-0.02	0.32	1.52	0.05	81.48	-0.41	8.56	7.07	16.00
os82433	19	480	0.76	7	3	5	6.78	-0.04	0.52	1.53	0.06	81.57	-1.35	8.59	7.08	16.00
os82454	3	383	0.15	7	2	3	6.70	0.03	0.90	1.54	0.10	89.36	-4.51	7.65	7.68	16.61
os82458	12	465	0.92	1	3	6	6.76	-0.02	2.78	1.55	0.02	57.24	-0.50	9.41	7.26	16.23
os82459	12	465	0.47	2	3	6	6.76	-0.03	2.73	1.55	0.02	59.40	0.09	9.32	7.26	16.24
os82460	12	465	0.92	2	3	6	6.73	-0.04	1.57	1.54	0.02	86.48	0.05	8.36	7.29	16.25
os82462	3	475	0.76	5	3	5	6.85	0.00	0.54	1.53	0.04	98.69	-0.21	7.56	7.19	16.16
os82463	3	484	0.09	4	3	6	6.92	0.05	0.67	1.53	0.03	99.80	-0.07	7.37	7.17	16.14
os82464	3	480	0.47	5	3	5	6.89	0.02	0.62	1.52	0.03	99.40	0.02	7.52	7.18	16.15
os82465	3	477	0.68	5	3	5	6.87	0.01	0.54	1.53	0.04	98.92	-0.02	7.51	7.19	16.16
os82466	3	478	0.73	6	3	5	6.88	0.02	0.45	1.52	0.04	99.03	0.14	7.47	7.18	16.16
os82470	17	491	0.23	4	2	3	6.73	0.04	0.79	1.56	0.12	76.11	-4.79	6.98	6.97	15.76
os82472	19	491	0.23	4	2	3	6.81	0.05	1.45	1.56	0.09	72.20	-3.53	7.17	6.96	15.75
os82491	3	478	0.29	2	3	6	6.88	0.03	2.05	1.55	0.02	42.04	-0.54	8.93	7.14	15.96
os82492	8	473	0.00	6	3	6	6.92	0.09	2.86	1.54	0.04	26.15	-0.48	7.72	7.18	16.00
os82521	17	424	1.00	18	2	7	5.91	-0.30	0.00	1.44	0.20	89.82	-2.97	5.80	7.10	15.91
os82637	8	467	0.92	1	3	6	6.83	0.00	1.14	1.55	0.02	46.36	0.23	8.58	7.17	16.01
os82673	3	517	0.01	4	3	6	6.94	0.08	0.25	1.52	0.03	83.23	1.67	7.70	6.91	15.70
os82680	3	513	0.77	3	3	6	6.80	-0.03	3.10	1.55	0.03	57.85	-0.43	8.98	6.93	15.72
os82747	3	486	0.24	3	3	6	6.90	0.04	1.63	1.54	0.03	76.57	-0.39	7.89	7.07	15.91
os82811	8	493	0.92	1	3	6	6.82	0.00	5.89	1.56	0.01	6.05	0.07	9.31	7.09	15.88
os82843	8	473	0.99	2	3	6	6.77	-0.03	4.97	1.56	0.02	24.05	-0.11	9.13	7.18	16.00
os82857	3	487	0.01	3	3	6	6.91	0.06	0.54	1.54	0.02	45.29	0.02	8.17	7.10	15.90
os82884	3	517	0.01	5	3	5	6.91	0.08	0.17	1.52	0.04	84.29	0.88	7.84	6.90	15.70
os82887	3	494	0.89	8	3	5	6.74	-0.06	0.54	1.53	0.05	73.38	-0.66	8.62	7.03	15.82
os82888	3	494	1.00	9	3	5	6.48	-0.15	0.12	1.50	0.06	84.17	-0.04	6.49	7.04	15.83
os82889	3	489	0.03	8	3	5	7.00	0.13	0.20	1.50	0.04	78.96	1.00	7.34	7.05	15.84
os82890	3	495	0.95	7	3	5	6.63	-0.10	0.18	1.51	0.04	83.60	0.56	6.41	7.03	15.82

os82934	3	503	0.20	7	2	3	6.83	0.07	0.73	1.53	0.07	78.90	-2.22	8.50	6.96	15.76
os82935	6	505	0.08	6	3	5	6.98	0.10	0.28	1.51	0.04	81.22	1.51	7.45	6.99	15.78
os82936	8	494	0.77	4	2	3	6.78	-0.03	1.57	1.56	0.09	69.59	-3.75	8.79	6.99	15.78
os82949	3	484	0.01	5	3	5	6.91	0.09	0.79	1.53	0.06	93.96	-1.05	7.10	7.08	15.86
os82979	2	478	0.92	0	3	6	6.81	0.00	5.97	1.57	0.01	1.65	0.00	11.64	7.13	15.90
os82980	8	474	0.92	0	3	6	6.81	0.00	5.74	1.57	0.03	44.94	-0.79	9.50	7.15	15.97
os82998	3	487	0.89	5	3	5	6.62	-0.08	1.01	1.53	0.06	70.61	-2.28	6.85	7.05	15.91
os83044	23	659	0.24	2	3	6	6.94	0.02	1.82	1.54	0.01	66.28	-0.29	8.64	6.05	14.77
os83155	17	541	0.79	6	2	3	6.76	-0.05	1.15	1.50	0.10	99.12	-4.69	7.99	6.74	15.49
os83188	14	468	0.01	31	2	0	6.58	0.48	0.00	1.33	0.25	91.93	0.91	4.72	6.90	15.72
os83254	28	564	0.92	0	3	6	6.84	0.00	3.18	1.55	0.02	42.64	-0.45	9.40	6.62	15.36
os83385	11	448	0.63	4	2	7	6.65	-0.04	1.09	1.55	0.14	84.10	-6.24	7.58	7.11	15.90
os83391	3	500	1.00	8	3	5	6.59	-0.12	0.35	1.49	0.06	70.80	0.35	7.02	7.05	15.86
os83403	11	474	0.06	8	2	7	6.83	0.10	0.29	1.54	0.11	74.62	-3.64	6.63	7.02	15.78
os83432	4	528	0.40	1	3	6	6.86	0.01	2.95	1.55	0.01	21.74	0.20	9.39	6.86	15.61
os83483	3	559	0.89	3	3	6	6.80	-0.04	1.99	1.54	0.08	94.92	-2.24	8.78	6.53	15.28
os83700	10	496	0.40	8	2	3	6.97	0.08	0.21	1.52	0.08	91.66	-2.20	6.25	7.12	16.04
os84111	17	599	0.12	10	3	5	7.07	0.13	0.08	1.46	0.07	99.56	-0.22	5.96	6.46	15.18
os84135	3	587	0.39	4	3	6	6.82	0.01	0.34	1.51	0.02	52.68	0.75	7.99	6.51	15.27
os84136	3	588	0.92	1	3	6	6.88	0.02	0.43	1.53	0.01	24.20	0.48	9.39	6.52	15.28
os84245	10	375	0.23	11	2	7	6.67	0.08	0.11	1.50	0.11	83.05	-1.91	7.56	7.55	16.42
os84246	9	457	0.07	18	1	4	7.00	0.30	0.01	1.37	0.07	92.04	7.63	5.98	7.22	16.04
os84265	10	483	0.01	4	3	6	6.92	0.07	0.48	1.49	0.05	93.97	0.77	6.77	7.03	15.82
os84371	10	401	0.03	5	3	5	6.60	-0.09	0.48	1.49	0.03	92.44	0.86	7.39	7.64	16.55
os84373	10	486	0.89	3	3	6	6.71	-0.04	1.69	1.54	0.02	83.82	-0.56	7.73	7.22	16.20
os84397	10	426	1.00	9	3	5	6.44	-0.16	0.14	1.51	0.05	82.59	0.09	7.20	7.52	16.48
os84402	10	383	0.66	9	3	5	6.48	-0.08	0.26	1.53	0.06	57.48	-0.97	8.60	7.67	16.64
os84422	10	454	0.55	3	3	6	6.70	-0.05	0.76	1.54	0.03	82.43	-0.03	8.08	7.28	16.17
os84437	10	456	0.40	3	2	3	6.82	0.01	2.67	1.55	0.06	88.78	-2.41	9.14	7.23	16.21
os84438	10	406	0.82	13	1	4	6.18	-0.17	0.00	1.39	0.08	79.47	5.63	5.36	7.49	16.40
os84609	19	506	0.15	13	2	7	6.69	0.10	0.02	1.47	0.12	99.72	-1.17	6.84	6.85	15.65
os84672	10	487	0.92	3	3	6	6.71	-0.05	1.21	1.54	0.02	73.33	-0.08	8.03	7.22	16.19
os84781	10	438	0.92	6	2	3	6.58	-0.08	1.51	1.55	0.10	89.51	-5.05	7.71	7.23	16.08
os84897	10	476	0.08	6	3	6	6.95	0.08	0.71	1.53	0.04	97.89	-0.45	7.78	7.24	16.23
os85036	17	505	0.77	7	3	5	6.68	-0.09	0.30	1.49	0.05	91.75	1.49	6.91	6.93	15.70
os85161	3	488	0.77	3	3	6	6.80	-0.02	1.26	1.54	0.01	55.52	0.00	8.34	7.10	15.94
os85182	10	402	0.92	3	2	3	6.67	-0.05	2.05	1.54	0.08	87.25	-3.87	8.11	7.42	16.31
os85202	10	449	0.56	4	3	6	6.85	0.01	0.55	1.47	0.04	96.89	0.63	6.74	7.29	16.11
os85283	17	466	0.40	3	3	6	6.86	0.02	1.69	1.54	0.02	68.82	-0.01	8.01	7.27	16.24
os85308	10	479	0.92	4	3	6	6.67	-0.06	1.17	1.53	0.03	84.14	-0.63	7.71	7.26	16.22
os85335	10	446	0.72	13	3	5	6.27	-0.14	0.00	1.48	0.08	89.85	-0.48	7.54	7.21	16.07
os85337	10	483	0.92	4	3	6	6.65	-0.07	0.56	1.53	0.02	88.90	0.27	7.64	7.10	15.99
os85419	28	520	0.36	4	2	3	6.89	0.02	1.94	1.54	0.07	96.49	-2.95	8.55	6.73	15.65

os85430	28	553	0.75	7	3	5	6.58	-0.08	0.21	1.51	0.08	100.00	-0.02	6.38	6.61	15.55
os85432	10	481	0.06	7	3	5	6.87	0.09	0.18	1.50	0.04	99.55	1.40	5.94	7.07	16.00
os85480	10	431	0.02	8	3	5	6.98	0.14	0.18	1.51	0.08	55.80	-0.58	7.34	7.23	16.08
os85494	10	449	0.92	1	3	6	6.75	-0.03	2.86	1.56	0.04	81.54	-1.32	8.77	7.09	15.98
os85503	10	491	0.47	8	3	5	6.66	0.00	0.38	1.51	0.08	91.24	-1.11	7.47	6.93	15.82
os85504	10	486	0.43	8	3	5	6.65	0.01	0.16	1.50	0.07	92.42	-0.20	6.92	6.95	15.83
os85529	28	575	0.47	6	2	3	6.88	0.01	1.64	1.55	0.14	95.78	-6.51	8.48	6.49	15.35
os85530	28	577	0.66	7	2	3	6.53	-0.09	0.91	1.53	0.15	96.18	-7.22	8.12	6.48	15.34
os85605	10	494	0.08	2	3	6	6.89	0.04	0.63	1.54	0.02	62.02	0.40	7.73	7.22	16.21
os85788	17	446	0.48	14	3	5	6.83	0.02	0.03	1.47	0.09	98.36	0.14	7.50	7.27	16.14
os85789	4	442	0.48	2	3	6	6.80	0.01	1.47	1.53	0.03	87.60	0.02	8.77	7.69	16.70
os85792	12	349	0.77	1	3	6	6.74	-0.01	1.97	1.56	0.02	57.67	-0.88	8.59	7.93	16.91
os85793	4	343	0.99	2	3	6	6.72	-0.03	3.24	1.54	0.01	71.23	0.70	7.61	8.15	17.11
os85816	19	484	1.00	5	2	7	6.65	-0.08	1.54	1.57	0.07	74.86	-2.31	7.83	7.30	16.21
os85824	28	597	0.21	3	3	6	6.95	0.04	1.59	1.55	0.03	97.61	-0.45	7.77	6.71	15.68
os85825	28	599	0.40	3	3	6	6.95	0.04	1.55	1.55	0.03	98.25	-0.42	7.64	6.69	15.66
os85829	28	554	0.98	3	3	6	6.79	-0.04	1.27	1.55	0.02	72.39	0.03	7.94	7.04	16.00
os85830	28	610	0.44	4	3	6	6.93	0.02	1.24	1.55	0.04	99.47	-0.54	8.03	6.56	15.49
os85831	28	612	0.53	4	3	6	6.93	0.02	0.82	1.54	0.04	99.47	-0.21	7.97	6.55	15.49
os85844	28	566	0.40	2	3	6	6.87	0.00	2.27	1.56	0.02	19.48	-0.19	9.56	6.82	15.71
os86133	17	434	0.68	3	3	6	6.72	-0.02	1.92	1.53	0.05	98.86	-2.02	8.56	7.76	16.70
os86134	17	440	0.78	4	3	6	6.65	-0.05	1.54	1.53	0.06	98.93	-2.02	8.42	7.72	16.67
os86138	19	420	0.71	4	3	6	6.66	-0.06	2.33	1.54	0.05	93.21	-1.01	9.22	7.85	16.80
os86140	11	418	0.81	8	3	5	6.51	-0.09	0.26	1.53	0.06	87.87	-0.68	9.37	7.69	16.61
os86141	19	426	0.92	3	2	0	6.68	-0.04	1.96	1.55	0.05	88.43	-0.40	9.51	7.62	16.53
os86164	28	619	0.73	4	3	6	6.91	0.00	0.58	1.54	0.04	99.38	0.01	7.84	6.51	15.42
os86194	17	451	0.19	7	3	5	6.96	0.09	0.24	1.50	0.08	99.82	-0.71	6.52	7.61	16.44
os86228	24	642	0.23	2	3	6	6.88	0.02	1.66	1.55	0.02	42.22	-0.16	8.02	6.55	15.43
os86233	28	660	0.01	2	3	6	6.93	0.03	0.27	1.54	0.01	79.30	0.28	7.35	6.47	15.38
os86313	23	681	0.85	5	1	1	6.70	-0.07	0.38	1.49	0.03	90.88	2.07	6.66	6.28	15.18
os86368	28	609	0.40	3	3	6	6.92	0.02	1.45	1.55	0.03	94.34	0.13	8.55	6.51	15.41
os86383	28	574	0.77	4	3	6	6.88	0.00	0.37	1.53	0.04	90.85	0.92	9.39	6.84	15.77
os86485	23	661	0.99	4	3	6	6.78	-0.06	0.56	1.51	0.03	99.14	0.87	6.68	6.43	15.33
os86537	19	450	0.92	1	3	6	6.79	-0.01	5.51	1.56	0.02	22.18	-0.30	9.44	7.57	16.47
os86538	19	450	0.92	0	3	6	6.81	0.00	5.36	1.56	0.02	21.12	0.19	10.06	7.57	16.47
os86654	19	443	0.34	3	3	6	6.73	0.00	1.80	1.54	0.05	91.40	-1.01	8.68	7.55	16.52
os86691	17	403	0.88	4	3	6	6.75	-0.03	0.82	1.52	0.04	94.92	-0.36	8.36	7.91	16.85
os86703	19	487	0.92	2	2	3	6.73	-0.05	2.25	1.55	0.09	88.99	-4.66	8.51	7.25	16.24
os86713	17	409	0.92	1	3	6	6.77	-0.01	2.98	1.54	0.04	89.10	-1.76	9.14	7.82	16.78
os86735	17	452	0.08	6	3	5	6.59	-0.10	0.49	1.53	0.06	99.59	-0.87	6.94	7.51	16.48
os86783	17	376	0.68	14	1	4	6.21	-0.09	0.00	1.38	0.07	97.77	5.87	5.75	8.04	16.91
os86937	10	415	0.70	5	3	5	6.83	0.00	0.50	1.51	0.06	99.17	0.03	7.63	7.68	16.65
os86992	19	397	0.49	5	2	7	6.70	0.00	1.82	1.54	0.16	95.76	-7.44	7.94	7.66	16.62

os87001	19	419	0.09	6	2	7	6.92	0.09	1.43	1.53	0.10	95.11	-4.21	7.63	7.61	16.59
os87038	10	425	0.53	4	3	6	6.85	0.02	0.34	1.50	0.03	93.73	0.99	6.83	7.77	16.72
os87049	17	346	0.97	19	2	0	5.82	-0.31	0.00	1.43	0.14	96.69	-0.63	6.64	7.97	16.86
os87054	9	366	0.64	13	3	5	6.69	-0.04	0.00	1.46	0.10	99.57	0.70	7.12	7.98	16.90
os87055	9	428	0.99	11	2	3	6.29	-0.19	0.03	1.48	0.08	95.82	-1.76	7.54	7.75	16.68
os87102	17	390	0.49	6	2	3	6.61	-0.08	1.32	1.52	0.10	94.95	-4.53	8.76	7.92	16.87
os87104	17	388	0.83	3	2	3	6.70	-0.04	2.70	1.53	0.07	96.11	-2.37	8.67	7.93	16.88
os87107	17	387	0.17	3	2	3	6.71	-0.04	2.77	1.53	0.07	96.36	-2.49	8.54	7.93	16.88
os87151	10	390	0.92	0	3	9	6.78	0.00	0.70	1.48	0.01	90.44	0.75	9.90	8.34	17.24
os87163	10	394	0.16	11	1	4	7.00	0.15	0.02	1.45	0.06	74.08	3.31	5.79	8.13	17.05
os87164	9	365	0.25	11	2	7	6.94	0.12	0.10	1.52	0.11	59.34	-2.74	6.99	8.11	17.04
os87183	10	428	0.80	13	3	9	6.19	-0.21	0.05	1.42	0.07	99.65	1.01	6.39	8.16	17.07
os87187	9	349	0.99	3	3	6	6.65	-0.05	1.62	1.53	0.03	85.44	-0.50	8.09	8.29	17.21
os87317	10	403	0.40	5	1	1	6.87	0.03	0.02	1.47	0.01	98.15	3.97	6.28	7.98	16.78
os87355	14	405	0.03	10	3	9	7.00	0.16	0.04	1.44	0.05	94.59	1.19	6.17	7.99	16.81
os87439	12	345	0.75	6	2	0	6.79	0.06	0.60	1.50	0.14	98.70	-3.62	6.94	7.95	16.78
os87459	9	355	0.54	5	2	7	6.63	-0.06	1.37	1.52	0.15	99.14	-5.49	7.83	7.89	16.70
os87468	19	374	0.01	4	3	6	6.77	0.04	1.00	1.52	0.07	98.11	-0.82	7.81	8.00	16.83
os87474	9	408	0.51	6	3	9	6.57	-0.10	0.77	1.47	0.05	99.82	-1.06	6.71	8.06	16.82
os87558	10	453	0.83	8	3	9	6.47	-0.12	0.04	1.45	0.03	99.76	2.18	5.93	7.71	16.55
os87560	9	279	0.83	20	2	7	6.03	-0.21	0.00	1.47	0.22	66.74	-7.81	6.22	8.34	17.10
os87567	19	408	0.64	4	3	6	6.67	-0.03	1.74	1.53	0.05	89.87	-1.62	8.28	7.62	16.44
os87574	10	396	0.99	8	3	5	6.47	-0.14	0.23	1.48	0.05	97.91	-1.09	6.95	7.91	16.73
os87678	10	427	0.08	4	3	6	6.89	0.06	1.20	1.53	0.03	98.16	-0.65	8.05	7.72	16.68
os87679	17	442	0.40	3	3	6	6.85	0.02	1.24	1.52	0.02	82.67	0.14	7.58	7.71	16.65
os87701	17	360	0.33	7	1	4	6.89	0.06	0.26	1.47	0.06	94.11	2.60	6.81	7.98	16.92
os87713	10	432	0.08	3	3	6	6.87	0.04	1.52	1.54	0.02	86.67	0.10	8.21	7.71	16.66
os87720	14	422	0.19	16	2	7	6.63	0.14	0.13	1.49	0.15	94.36	-3.80	7.23	7.58	16.54
os87721	17	431	0.60	9	2	7	6.58	0.00	0.76	1.50	0.12	94.31	-2.88	8.42	7.57	16.53
os87790	10	357	0.70	5	2	7	6.72	-0.03	1.49	1.54	0.10	96.81	-3.32	7.49	7.91	16.85
os87792	17	366	0.18	7	3	5	6.92	0.09	0.52	1.52	0.04	97.34	-0.51	7.95	7.93	16.87
os87855	10	364	0.40	4	3	6	6.83	0.02	1.66	1.49	0.04	96.39	-1.22	8.01	8.11	17.05
os87867	10	416	0.57	4	3	6	6.80	-0.01	0.45	1.52	0.03	90.18	0.89	7.89	7.78	16.74
os87869	10	400	0.99	6	3	5	6.62	-0.09	0.48	1.52	0.05	93.28	-0.95	7.77	7.81	16.77
os87887	10	419	0.95	7	2	3	6.68	-0.07	0.83	1.53	0.07	92.61	-3.04	8.19	7.74	16.71
os87888	10	417	0.88	7	2	3	6.70	-0.06	0.94	1.54	0.07	93.46	-3.40	8.26	7.74	16.71
os87892	17	447	0.08	3	3	6	6.88	0.04	1.65	1.53	0.02	70.87	0.10	7.83	7.67	16.63
os87893	17	446	0.08	3	3	6	6.88	0.05	1.62	1.53	0.02	73.61	0.11	7.85	7.67	16.63
os87894	17	446	0.08	3	3	6	6.89	0.05	1.61	1.53	0.02	76.79	0.12	7.87	7.67	16.63
os87895	17	445	0.08	3	3	6	6.89	0.05	1.49	1.53	0.02	79.47	0.14	7.88	7.67	16.63
os87897	17	444	0.11	3	3	6	6.88	0.04	1.61	1.53	0.02	74.62	0.04	8.05	7.68	16.64
os87911	10	377	0.97	8	3	5	6.54	-0.12	0.20	1.46	0.06	97.14	1.32	6.46	7.87	16.81
os87931	10	433	0.98	9	3	5	6.39	-0.17	0.06	1.45	0.05	91.59	2.22	6.51	7.64	16.60

os87932	17	431	0.45	12	2	3	6.83	0.02	0.43	1.50	0.10	89.92	-3.31	8.36	7.63	16.59
os87934	17	432	0.74	9	2	3	6.73	-0.04	0.78	1.51	0.09	89.79	-3.43	8.43	7.63	16.59
os87935	17	431	0.39	15	3	5	6.91	0.08	0.10	1.45	0.09	89.95	0.40	7.17	7.63	16.59
os87938	17	363	0.04	7	2	3	6.73	0.05	1.17	1.52	0.11	92.26	-5.52	7.78	7.93	16.87
os87943	10	364	0.90	9	3	5	6.52	-0.13	0.09	1.49	0.08	97.23	0.78	6.35	7.93	16.87
os87944	10	359	0.92	10	3	5	6.49	-0.13	0.05	1.48	0.10	97.12	-0.12	6.41	7.93	16.87
os87945	10	366	0.90	9	3	5	6.54	-0.12	0.08	1.49	0.07	97.29	1.02	6.31	7.94	16.87
os87958	17	459	0.40	4	3	6	6.86	0.01	0.23	1.51	0.02	94.68	1.43	7.52	7.56	16.52
os87959	10	458	1.00	6	3	5	6.61	-0.10	0.33	1.49	0.04	94.72	1.69	6.59	7.55	16.49
os87987	17	425	0.01	4	2	3	6.78	-0.01	1.91	1.55	0.08	96.22	-3.97	8.35	7.61	16.57
os87996	17	287	0.87	8	2	0	6.42	-0.13	0.24	1.52	0.11	82.19	-1.98	7.65	8.26	17.17
os87998	17	342	0.33	11	2	3	6.59	0.03	0.45	1.51	0.10	94.28	-2.10	8.11	8.03	16.97
os88011	17	355	0.99	5	3	6	6.60	-0.09	0.78	1.51	0.05	98.03	-0.63	7.11	8.08	17.01
os88160	19	353	0.97	6	2	0	6.63	-0.07	0.38	1.53	0.10	82.12	-1.39	6.78	7.98	16.92
os88162	19	348	0.68	3	2	0	6.77	-0.01	2.19	1.54	0.08	79.08	-0.01	8.22	8.00	16.94
os88168	10	304	0.02	16	2	7	6.69	0.24	0.22	1.50	0.24	90.08	-8.86	5.98	8.10	17.03
os88172	19	403	0.23	1	3	6	6.80	0.02	2.97	1.54	0.05	87.05	-1.83	8.83	7.78	16.73
os88173	10	413	0.06	3	3	6	6.85	0.04	1.70	1.52	0.02	71.56	0.96	8.48	7.78	16.74
os88200	17	367	0.88	10	2	7	6.55	-0.11	0.46	1.49	0.13	97.11	-2.73	8.05	7.98	16.91
os88214	10	423	0.20	20	1	1	6.93	0.24	0.00	1.30	0.08	99.84	8.67	4.74	8.11	16.93
os88218	10	402	0.92	3	3	6	6.77	0.01	2.65	1.52	0.02	43.31	1.42	9.30	7.97	16.89
os88224	14	530	0.77	4	3	6	6.82	-0.02	1.57	1.53	0.03	85.27	-0.64	8.56	7.12	16.12
os88267	19	362	0.02	2	2	3	6.68	-0.03	1.54	1.56	0.09	66.55	-3.00	7.29	8.03	16.90
os88328	11	373	0.27	3	2	3	6.76	0.02	1.83	1.55	0.08	94.79	-3.50	7.23	8.09	16.98
os88335	3	383	0.92	5	3	6	6.61	-0.06	0.80	1.53	0.03	84.70	0.08	7.30	8.12	17.06
os88337	3	386	0.00	4	3	6	6.88	0.07	1.32	1.55	0.05	75.75	-1.49	7.63	7.92	16.83
os88342	3	423	0.88	5	3	5	6.73	-0.05	0.87	1.53	0.03	81.34	-0.43	7.03	7.89	16.79
os88344	17	383	0.01	3	3	6	6.82	0.04	1.68	1.54	0.04	97.11	-0.81	8.35	7.97	16.88
os88376	19	346	0.60	3	2	7	6.64	-0.04	2.01	1.55	0.12	87.26	-3.76	7.01	7.94	16.79
os88390	12	366	0.40	2	3	6	6.80	0.01	1.56	1.55	0.01	54.25	-0.17	9.01	8.12	17.05
os88437	17	423	0.82	3	3	6	6.68	-0.04	1.80	1.54	0.03	96.40	-0.54	8.62	7.71	16.67
os88440	17	470	0.74	15	1	4	6.16	-0.20	0.00	1.40	0.10	99.62	3.51	7.06	7.39	16.34
os88492	12	457	0.30	2	3	6	6.74	-0.01	1.70	1.54	0.03	71.26	-0.38	8.38	7.47	16.32
os88534	10	367	0.21	3	2	3	6.75	0.02	1.77	1.54	0.06	95.84	-2.02	7.07	8.12	17.00
os88537	17	331	0.60	12	2	3	6.36	-0.06	0.08	1.53	0.09	54.65	-3.25	6.89	8.22	17.12
os88538	19	338	0.70	9	2	3	6.45	-0.06	0.33	1.50	0.09	62.42	-2.09	6.03	8.21	17.12
os88539	4	336	0.78	9	2	3	6.47	-0.06	0.73	1.53	0.10	71.84	-3.38	7.62	8.23	17.14
os88550	3	355	0.02	18	1	4	6.89	0.30	0.00	1.39	0.11	73.12	2.23	5.01	8.24	17.17
os88555	10	407	0.17	4	3	6	6.79	0.03	1.14	1.54	0.03	80.37	-0.57	8.29	7.96	16.83
os88557	10	400	0.88	9	3	5	6.43	-0.12	0.23	1.51	0.07	80.05	0.26	7.34	7.95	16.83
os88564	10	363	1.00	8	1	4	6.48	-0.11	0.00	1.42	0.03	48.57	6.45	7.41	8.16	17.06
os88565	10	365	0.06	7	1	4	6.76	0.06	0.00	1.41	0.03	47.34	7.25	7.51	8.16	17.06
os88567	17	388	0.15	7	2	3	6.71	0.03	1.18	1.54	0.08	94.98	-3.77	7.67	8.02	16.90

os88569	17	389	0.00	4	2	3	6.87	0.06	1.69	1.55	0.05	85.30	-2.51	8.75	8.00	16.86
os88571	17	387	0.26	4	2	3	6.84	0.04	1.93	1.55	0.06	83.38	-3.20	9.05	8.01	16.86
os88572	17	386	0.01	5	2	3	6.84	0.06	1.55	1.55	0.06	81.90	-3.26	8.87	8.01	16.86
os88573	17	391	0.17	7	3	5	6.87	0.11	0.72	1.52	0.06	87.25	-1.56	7.82	8.00	16.86
os88583	17	353	0.77	1	3	6	6.76	-0.01	2.57	1.56	0.02	39.75	-0.82	8.48	8.19	17.05
os88592	17	353	0.77	2	3	6	6.75	-0.01	1.96	1.56	0.06	86.48	-1.81	8.97	8.12	17.01
os88598	17	343	0.40	7	3	5	6.69	0.04	0.67	1.52	0.09	74.43	-1.91	7.48	8.18	17.10
os88601	19	365	0.08	4	1	4	6.81	0.05	0.14	1.49	0.03	94.35	2.88	7.36	8.16	17.08
os88608	3	402	0.01	3	3	6	6.87	0.06	1.67	1.54	0.03	96.47	-0.31	7.90	7.91	16.82
os88609	11	389	0.37	4	3	6	6.86	0.04	1.81	1.55	0.05	96.95	-1.75	8.58	7.93	16.84
os88612	3	408	0.00	5	3	6	6.89	0.08	0.44	1.53	0.03	92.07	0.31	7.02	7.92	16.83
os88620	17	387	0.01	4	3	6	6.86	0.07	1.27	1.54	0.05	98.82	-1.75	8.25	7.96	16.87
os88621	17	384	0.23	3	3	6	6.78	0.03	1.64	1.54	0.04	98.09	-0.89	8.33	7.97	16.88
os88637	17	360	0.96	4	3	6	6.60	-0.07	1.38	1.54	0.06	92.89	-1.84	7.97	8.11	17.07
os88640	10	374	0.19	7	1	4	6.78	0.07	0.20	1.49	0.05	88.64	2.21	7.14	8.04	16.97
os88642	10	358	0.32	6	3	5	6.68	0.02	0.54	1.53	0.06	77.10	-1.12	8.62	8.08	17.01
os88644	10	367	0.37	7	3	5	6.68	0.03	0.58	1.52	0.06	87.94	-0.85	7.09	8.08	17.01
os88645	10	390	0.02	5	3	5	6.91	0.09	0.55	1.52	0.05	88.14	-0.44	8.02	7.94	16.90
os88648	10	412	0.60	7	3	5	6.51	-0.08	0.30	1.51	0.06	94.26	0.48	6.96	7.72	16.70
os88651	11	289	0.89	8	2	3	6.45	-0.11	0.31	1.53	0.07	79.92	-1.19	7.91	8.47	17.33
os88655	10	230	0.87	8	3	5	6.39	-0.11	0.23	1.52	0.09	87.01	-1.87	7.22	8.91	17.80
os88657	10	323	0.73	4	3	6	6.60	-0.05	0.56	1.53	0.04	99.08	0.41	8.08	8.28	17.18
os88659	17	314	0.93	7	3	5	6.55	-0.10	0.78	1.52	0.09	96.31	-2.56	7.40	8.30	17.14
os88675	19	357	0.90	10	2	3	6.41	-0.14	0.37	1.52	0.13	92.61	-3.21	6.86	7.94	16.80
os88680	10	339	0.75	7	3	5	6.52	-0.06	0.16	1.49	0.04	90.21	1.76	7.14	8.19	17.04
os88686	19	332	0.84	8	2	3	6.46	-0.10	0.52	1.52	0.08	87.45	-2.76	7.44	8.23	17.08
os88742	10	441	0.92	4	3	6	6.65	-0.06	0.40	1.52	0.02	97.42	0.19	6.53	7.68	16.54
os88799	10	415	0.11	1	3	6	6.75	-0.02	2.46	1.55	0.01	26.69	0.05	8.47	7.76	16.61
os88819	12	402	0.92	0	3	6	6.76	-0.01	1.44	1.54	0.01	50.02	0.66	9.46	7.78	16.62
os88824	10	402	0.06	4	3	6	6.79	0.04	0.62	1.54	0.03	67.84	-0.29	8.32	7.74	16.58
os88851	10	414	0.92	1	3	6	6.80	0.01	2.97	1.55	0.01	5.51	-0.60	9.07	7.72	16.54
os88854	3	423	0.33	6	3	5	6.56	-0.09	0.59	1.52	0.05	90.01	-0.91	6.37	7.71	16.58
os88855	3	423	0.72	5	3	5	6.61	-0.07	0.65	1.53	0.05	89.76	-0.73	6.39	7.71	16.58
os88867	12	431	0.35	9	3	5	6.93	0.07	0.08	1.51	0.05	96.65	-0.23	6.57	7.68	16.53
os88870	12	396	0.00	7	3	5	6.89	0.10	0.38	1.53	0.05	88.11	-0.32	7.64	7.81	16.67
os88874	10	302	0.72	5	3	6	6.64	-0.01	1.41	1.52	0.05	94.43	-1.34	8.27	8.44	17.28
os88968	10	469	0.22	7	3	9	6.83	0.07	0.48	1.47	0.05	95.28	-0.37	6.72	7.39	16.33
os88969	3	404	0.30	9	1	1	6.95	0.10	0.08	1.44	0.06	89.26	3.10	6.73	7.75	16.70
os88970	10	499	0.60	3	3	6	6.74	-0.02	1.60	1.54	0.03	78.32	-0.47	8.57	7.22	16.18
os88975	10	490	0.13	4	1	4	6.83	0.04	0.26	1.46	0.03	95.92	2.50	6.73	7.39	16.35
os88999	3	377	0.57	8	3	5	6.81	0.01	0.41	1.51	0.09	74.51	-0.87	8.77	7.75	16.68
os89000	3	381	0.79	12	3	5	6.58	-0.10	0.01	1.49	0.10	73.13	-0.07	7.44	7.74	16.67
os89001	3	377	0.92	0	2	0	6.77	0.00	3.25	1.56	0.08	62.34	-0.39	8.87	7.66	16.60

os89007	10	547	0.09	2	3	6	6.78	-0.03	1.01	1.53	0.02	80.00	0.30	7.26	6.96	15.96
os89019	17	495	0.96	5	3	5	6.70	-0.07	0.72	1.51	0.05	99.68	-1.02	7.77	7.14	16.04
os89025	17	398	0.48	8	1	2	6.82	0.02	0.02	1.45	0.10	93.34	4.00	6.11	7.52	16.43
os89056	10	488	0.02	16	3	5	7.05	0.24	0.00	1.44	0.07	94.23	1.01	6.70	7.22	16.18
os89057	17	482	0.00	11	2	3	6.97	0.17	0.26	1.50	0.09	93.71	-3.05	7.96	7.21	16.18
os89063	3	421	0.51	6	2	3	6.73	0.01	1.36	1.53	0.08	96.39	-2.82	7.84	7.51	16.43
os89065	10	496	0.53	4	3	6	6.79	0.01	0.70	1.51	0.03	86.31	1.18	7.63	7.19	16.16
os89108	10	468	0.21	7	3	9	6.80	0.06	0.44	1.47	0.06	95.60	-0.58	6.86	7.40	16.34
os89121	17	470	0.08	6	3	5	6.96	0.08	0.49	1.52	0.05	97.04	0.29	7.11	7.29	16.19
os89160	10	406	0.97	15	2	0	6.13	-0.23	0.00	1.47	0.13	82.73	-0.45	6.33	7.37	16.25
os89164	17	433	0.59	10	2	3	6.81	0.01	0.60	1.50	0.16	98.75	-5.23	7.22	7.32	16.21
os89165	17	437	0.40	9	2	3	6.57	-0.08	0.64	1.51	0.18	99.09	-6.42	7.10	7.32	16.21
os89166	17	442	0.01	6	2	3	6.63	-0.06	0.99	1.52	0.15	98.92	-6.59	6.98	7.32	16.21
os89167	17	450	0.64	9	2	3	6.74	-0.05	0.92	1.51	0.14	98.27	-5.09	6.83	7.31	16.21
os89168	10	456	0.56	10	2	3	6.81	0.00	0.63	1.50	0.13	95.43	-4.16	7.39	7.31	16.21
os89199	10	471	0.19	10	3	5	7.00	0.12	0.06	1.48	0.05	97.78	2.04	6.24	7.38	16.31
os89200	3	466	0.17	11	3	5	7.03	0.15	0.01	1.49	0.07	97.77	0.63	6.22	7.37	16.30
os89202	3	455	0.19	10	3	5	7.01	0.14	0.06	1.50	0.07	98.46	0.17	6.25	7.43	16.35
os89205	3	458	0.17	10	3	5	7.01	0.13	0.07	1.49	0.06	98.37	0.61	6.27	7.42	16.35
os89206	3	452	0.15	10	3	5	7.01	0.14	0.06	1.50	0.07	98.48	-0.08	6.28	7.43	16.35
os89207	3	448	0.10	9	3	5	7.00	0.13	0.12	1.50	0.07	98.49	-0.31	6.35	7.44	16.36
os89208	10	443	0.17	9	3	5	6.99	0.12	0.10	1.51	0.07	98.48	-0.77	6.31	7.43	16.35
os89220	10	488	0.36	12	3	5	6.94	0.07	0.03	1.49	0.09	95.82	-1.47	5.77	7.19	16.14
os89222	17	423	0.71	6	3	5	6.80	-0.01	0.99	1.53	0.06	91.51	-0.74	8.32	7.46	16.39
os89223	3	461	0.67	8	3	5	6.49	-0.09	0.18	1.49	0.08	98.47	1.20	6.49	7.27	16.22
os89224	3	467	0.60	6	3	5	6.58	-0.07	0.39	1.49	0.08	98.64	0.88	6.49	7.26	16.21
os89251	10	371	0.57	5	2	0	6.63	-0.03	0.80	1.55	0.08	62.86	-1.59	8.24	7.74	16.67
os89255	3	411	0.01	4	3	6	6.88	0.07	0.45	1.53	0.03	94.21	0.31	7.77	7.67	16.61
os89274	3	382	0.77	4	2	0	6.73	-0.03	1.60	1.55	0.09	79.38	-1.15	8.19	7.62	16.55
os89275	3	384	0.81	10	2	0	6.62	-0.09	0.04	1.50	0.10	71.92	-0.46	7.28	7.65	16.58
os89276	3	383	0.67	9	2	0	6.71	-0.05	0.15	1.51	0.10	69.65	-1.02	7.54	7.66	16.59
os89277	10	391	0.73	10	2	0	6.75	-0.03	0.12	1.52	0.10	86.03	-1.87	6.87	7.61	16.54
os89278	3	380	0.77	10	2	3	6.64	-0.08	0.06	1.52	0.12	69.55	-3.13	7.88	7.70	16.64
os89279	3	382	0.81	12	3	5	6.58	-0.10	0.01	1.48	0.10	73.12	-0.30	7.47	7.72	16.65
os89280	3	377	0.77	10	3	5	6.64	-0.08	0.12	1.51	0.10	71.57	-1.57	8.06	7.74	16.67
os89281	10	492	0.92	5	1	1	6.67	-0.06	0.06	1.45	0.02	96.89	4.70	7.05	7.26	16.22
os89287	10	393	0.03	13	1	2	6.81	0.20	0.01	1.38	0.10	85.97	6.97	5.56	7.54	16.46
os89297	10	505	1.00	4	3	6	6.68	-0.07	0.57	1.53	0.03	81.96	0.41	7.40	7.14	16.11
os89400	17	522	0.94	14	2	0	6.41	-0.18	0.00	1.48	0.11	93.18	-0.98	5.95	6.77	15.61
os89410	19	513	0.17	10	2	7	6.99	0.15	0.59	1.54	0.15	77.08	-6.26	7.31	6.75	15.56
os89497	3	492	0.19	9	3	5	7.01	0.12	0.32	1.50	0.06	94.60	0.02	6.92	7.20	16.10
os89604	10	462	0.91	9	3	5	6.45	-0.13	0.43	1.51	0.07	100.00	-2.17	7.71	7.42	16.31
os89794	17	592	0.03	18	3	9	6.84	0.27	0.00	1.42	0.13	96.07	-1.39	5.38	6.76	15.69

os89819	19	546	0.45	7	3	5	6.67	-0.02	0.35	1.50	0.07	99.81	-1.30	6.43	6.86	15.77
os89873	19	533	0.00	8	2	3	6.89	0.12	1.06	1.52	0.15	96.95	-7.45	7.21	6.79	15.61
os89939	17	535	0.98	5	3	6	6.68	-0.09	0.30	1.50	0.07	95.24	0.80	6.25	6.76	15.60
os90038	28	554	0.21	2	3	6	6.88	0.01	3.87	1.56	0.02	0.04	-0.06	9.87	6.64	15.47
os90039	28	555	0.40	1	3	6	6.86	0.00	3.85	1.56	0.02	0.01	0.07	9.39	6.68	15.52
os90059	8	515	0.86	9	2	7	6.44	-0.15	0.52	1.55	0.17	82.45	-6.62	6.73	6.79	15.64
os90066	28	547	0.48	2	3	6	6.86	0.00	0.40	1.54	0.01	55.59	0.35	8.30	6.81	15.67
os90110	19	517	0.98	5	2	7	6.70	-0.07	1.14	1.55	0.10	89.67	-3.10	8.21	6.77	15.61
os90181	10	404	0.88	7	2	3	6.59	-0.04	1.35	1.50	0.12	98.05	-6.15	7.40	8.01	16.89
os90220	19	405	0.68	8	2	0	6.48	-0.13	0.26	1.52	0.07	92.91	-1.22	6.43	7.58	16.45
os90228	8	400	0.48	3	2	0	6.69	-0.05	1.74	1.55	0.08	87.38	-1.17	8.05	7.64	16.49
os90248	10	397	0.11	6	3	5	6.93	0.08	0.21	1.49	0.04	57.32	0.40	8.02	7.81	16.74
os90347	8	504	0.99	2	3	6	6.77	-0.03	2.98	1.55	0.04	69.12	-0.24	9.33	7.10	16.03
os90393	10	414	0.70	3	3	6	6.80	-0.01	0.22	1.48	0.02	91.87	1.78	6.78	7.95	16.82
os90457	10	296	0.48	2	3	6	6.65	-0.04	1.23	1.54	0.03	98.46	0.05	7.97	8.59	17.43
os90465	17	347	0.23	7	2	3	6.66	0.03	0.71	1.54	0.11	93.69	-3.61	8.32	8.11	16.96
os90501	10	360	0.97	13	2	3	6.32	-0.19	0.00	1.48	0.12	99.95	-2.42	7.11	8.13	17.03
os90521	19	398	0.57	8	2	3	6.86	0.04	0.80	1.51	0.12	100.00	-4.69	6.79	7.87	16.75
os90534	19	376	0.85	10	2	3	6.53	-0.11	0.72	1.52	0.15	96.58	-6.45	7.67	7.89	16.76
os90576	10	395	0.99	7	3	9	6.56	-0.11	0.32	1.49	0.05	99.10	0.02	6.19	8.05	16.89
os90658	10	416	0.54	9	3	5	6.79	0.01	0.10	1.50	0.11	92.19	-1.54	5.74	7.64	16.59
os90660	10	468	0.10	14	2	3	6.75	0.15	0.11	1.49	0.12	91.06	-3.17	6.79	7.40	16.33
os90665	19	428	0.98	7	2	7	6.50	-0.13	0.40	1.53	0.16	94.70	-2.85	7.20	7.37	16.30
os90711	17	482	1.00	22	3	5	5.64	-0.37	0.00	1.42	0.13	94.22	-0.32	6.07	7.46	16.43
os90744	10	497	0.92	1	3	6	6.85	0.01	2.52	1.55	0.02	61.37	-0.11	9.16	7.23	16.17
os90748	10	580	0.74	4	3	6	6.88	-0.01	0.21	1.51	0.02	95.45	1.55	6.51	6.78	15.77
os90750	19	550	0.00	5	3	5	6.97	0.08	1.02	1.53	0.04	88.66	-0.65	9.42	6.87	15.85
os90789	10	277	0.99	8	3	5	6.41	-0.14	0.17	1.50	0.04	94.43	0.72	7.10	8.80	17.64
os90834	11	431	0.99	4	3	6	6.65	-0.07	1.62	1.54	0.06	91.47	-0.77	8.61	7.43	16.33
os90871	10	426	0.01	3	1	1	6.88	0.06	0.37	1.47	0.01	95.37	2.83	6.93	8.08	17.00
os90937	19	429	0.87	5	2	7	6.56	-0.03	1.60	1.55	0.22	94.60	-9.60	7.03	7.36	16.29
os91001	19	456	0.23	1	3	6	6.82	0.02	2.99	1.56	0.05	70.93	-0.35	9.01	7.23	16.17
os91004	19	511	0.88	6	3	5	6.60	-0.09	0.64	1.52	0.06	89.11	-1.08	8.15	7.08	16.01
os91007	2	499	0.60	3	2	3	6.73	-0.03	2.16	1.55	0.06	94.05	-2.67	8.27	7.10	16.03
os91017	4	518	0.99	2	3	6	6.79	-0.02	1.29	1.54	0.02	51.29	0.17	9.16	7.08	16.02
os91025	10	578	0.23	3	3	6	6.85	0.02	0.10	1.51	0.02	84.75	1.46	6.88	6.78	15.76
os91214	19	468	0.81	5	2	7	6.74	-0.04	1.57	1.55	0.10	97.06	-2.82	8.01	7.09	15.96
os91225	22	440	0.08	20	2	7	6.79	0.26	0.06	1.49	0.20	96.55	-7.60	5.91	7.31	16.25
os91277	10	422	0.00	8	1	4	6.93	0.13	0.11	1.46	0.05	98.07	2.60	6.42	7.78	16.69
os91284	19	315	0.23	11	2	3	6.66	0.10	0.24	1.52	0.14	94.32	-5.28	6.98	8.34	17.16
os91309	10	466	0.43	5	3	5	6.65	-0.08	0.78	1.52	0.04	94.93	-0.50	7.67	7.47	16.40
os91314	10	476	0.00	3	3	6	6.69	-0.06	1.76	1.52	0.04	88.82	-0.86	8.62	7.46	16.39
os91315	10	475	0.66	3	3	6	6.82	-0.01	1.98	1.51	0.05	90.84	-1.77	8.27	7.48	16.41

os91317	10	396	0.31	4	3	6	6.71	0.00	0.55	1.52	0.04	97.58	0.53	7.99	7.96	16.86
os91318	10	488	0.10	4	3	6	6.69	-0.06	0.91	1.53	0.02	73.91	0.24	7.56	7.39	16.31
os91320	10	424	0.55	8	3	5	6.83	0.01	0.28	1.51	0.08	99.93	-0.70	6.33	7.66	16.61
os91321	10	411	0.49	5	3	5	6.85	0.02	0.52	1.53	0.07	99.59	-0.77	6.53	7.69	16.64
os91322	10	458	0.10	12	2	3	6.73	0.13	0.33	1.50	0.13	91.92	-4.19	7.57	7.41	16.34
os91332	19	426	0.03	6	2	0	6.57	-0.11	0.43	1.52	0.16	93.65	-0.95	7.12	7.37	16.30
os91333	10	520	0.56	8	1	4	6.57	-0.05	0.04	1.45	0.04	95.65	5.10	5.82	7.19	16.15
os91352	10	411	0.92	3	3	6	6.65	-0.06	1.35	1.54	0.03	88.35	-0.22	7.61	7.83	16.75
os91361	10	408	0.01	3	3	6	6.85	0.05	0.60	1.54	0.02	84.53	0.13	8.48	7.88	16.79
os91367	4	512	0.63	2	3	6	6.78	-0.02	1.09	1.54	0.02	64.71	0.32	9.24	7.11	16.05
os91370	4	502	0.23	3	3	6	6.83	0.03	1.85	1.54	0.03	90.48	-0.48	8.20	7.12	16.06
os91373	19	473	0.01	1	3	6	6.82	0.01	2.09	1.56	0.05	89.50	-1.34	8.00	7.18	16.09
os91374	19	473	0.92	1	2	3	6.83	0.01	1.99	1.56	0.05	90.14	-2.36	7.89	7.18	16.09
os91379	2	502	0.74	4	3	5	6.67	-0.05	1.45	1.54	0.06	94.32	-2.16	7.85	7.10	16.02
os91380	4	506	0.71	3	3	6	6.72	-0.04	1.65	1.53	0.04	96.81	-1.11	6.94	7.11	16.04
os91385	19	511	0.98	8	2	3	6.56	-0.13	0.42	1.52	0.06	77.62	-1.27	8.44	7.07	16.05
os91388	10	570	0.29	5	3	6	6.67	-0.09	0.55	1.53	0.04	86.76	-1.03	7.24	6.89	15.88
os91396	10	546	0.26	2	3	6	6.87	0.01	2.94	1.54	0.04	84.11	-1.61	9.21	6.93	15.94
os91398	16	534	0.16	2	3	6	6.86	0.01	2.49	1.54	0.05	86.96	-1.50	8.92	7.00	15.99
os91399	10	547	0.90	3	3	6	6.80	-0.04	1.67	1.53	0.03	83.60	-0.25	8.25	6.98	15.97
os91400	8	536	0.65	2	3	6	6.85	-0.01	2.46	1.53	0.03	69.27	-0.82	8.58	7.07	16.05
os91405	10	530	0.60	8	3	5	6.60	-0.05	0.39	1.51	0.05	89.64	-0.89	7.05	7.10	16.09
os91412	10	569	0.00	4	3	6	6.94	0.07	0.25	1.52	0.02	74.77	1.37	7.71	6.80	15.78
os91421	19	402	0.92	8	2	3	6.46	-0.12	0.90	1.54	0.12	92.98	-4.41	7.59	7.80	16.69
os91428	10	392	0.07	4	3	6	6.90	0.08	0.56	1.53	0.04	96.48	0.34	8.12	7.94	16.85
os91430	10	403	0.00	5	3	5	6.90	0.09	0.68	1.53	0.04	91.76	-0.35	8.26	7.91	16.82
os91455	22	476	0.41	8	3	5	6.61	0.00	0.05	1.48	0.11	95.55	-0.77	5.48	7.61	16.52
os91462	10	413	0.30	9	3	5	6.64	0.02	0.02	1.50	0.06	83.34	1.14	7.80	7.89	16.77
os91466	10	375	0.31	12	3	5	6.58	0.05	0.08	1.50	0.08	74.70	-1.86	6.51	8.13	16.98
os91499	10	286	0.09	6	3	5	6.74	0.07	0.51	1.53	0.09	65.03	-2.15	6.35	8.57	17.40
os91587	19	281	0.33	5	2	7	6.57	-0.02	1.31	1.56	0.13	91.13	-5.06	8.06	8.41	17.25
os91588	10	313	0.57	10	3	5	6.39	-0.06	0.02	1.47	0.10	91.15	-0.14	6.50	8.50	17.32
os91590	9	261	0.76	16	2	7	6.07	-0.16	0.01	1.47	0.14	78.55	-0.81	6.72	8.62	17.44
os91600	19	500	0.65	2	3	6	6.75	-0.04	2.57	1.55	0.03	54.50	-0.68	8.47	7.17	16.14
os91609	19	436	0.95	7	2	7	6.61	-0.09	0.72	1.53	0.12	99.66	-4.79	8.43	7.47	16.41
os91632	10	409	0.01	7	3	5	6.93	0.11	0.57	1.52	0.06	88.93	-1.42	7.13	7.88	16.80
os04186	7	451	0.02	5	3	5	6.86	0.07	0.52	1.50	0.04	97.92	0.88	7.83	7.89	16.70
os65679	7	432	0.99	10	3	5	6.38	-0.17	0.03	1.48	0.07	81.82	0.69	7.05	8.10	17.28
os37512	7	302	0.99	4	3	6	6.64	-0.06	0.55	1.53	0.04	98.66	0.31	6.60	8.16	16.88
os38508	7	241	0.42	10	2	7	6.77	0.05	0.08	1.53	0.15	81.13	-2.52	7.43	8.38	17.10
os87466	7	433	0.94	6	1	1	6.70	-0.06	0.02	1.40	0.05	99.67	7.46	5.61	7.90	16.73
os37187	7	272	0.52	14	3	5	6.26	-0.03	0.00	1.42	0.10	88.30	1.63	5.19	8.46	17.20
os76142	7	429	0.87	10	1	4	6.39	-0.14	0.01	1.46	0.06	99.08	2.40	6.32	7.68	16.51

os02102	7	384	0.77	6	3	5	6.69	-0.06	0.40	1.47	0.05	95.45	0.48	6.15	8.43	17.35
os76911	7	397	0.84	16	3	9	6.43	-0.15	0.00	1.43	0.10	99.33	-0.93	5.71	7.85	16.61
os87781	7	401	0.00	3	3	6	6.84	0.04	0.36	1.51	0.02	80.72	1.09	7.86	7.86	16.79
os35984	7	327	0.66	12	3	5	6.72	-0.01	0.02	1.44	0.10	92.04	0.02	7.77	8.14	16.81
os91521	7	377	0.99	8	3	5	6.50	-0.13	0.51	1.50	0.07	97.66	-1.58	7.03	8.21	17.08
os04173	7	398	0.98	6	2	3	6.61	-0.09	1.27	1.54	0.07	94.19	-2.60	8.11	8.19	16.99
os76118	7	447	0.04	12	3	5	7.04	0.19	0.01	1.46	0.09	97.30	0.50	7.29	7.37	16.14
os02590	7	375	0.78	8	2	7	6.50	-0.08	0.58	1.53	0.09	95.98	-2.31	8.26	8.30	17.25
os38592	7	309	0.99	5	3	5	6.64	-0.07	0.37	1.52	0.05	99.67	0.09	6.33	8.15	16.86
os38626	7	334	0.23	3	1	4	6.74	0.02	0.19	1.38	0.02	91.06	3.33	6.87	8.14	16.87
os03181	7	413	0.00	7	3	9	6.92	0.12	0.08	1.45	0.04	96.96	1.94	6.11	8.14	16.92
os66472	7	346	0.98	7	3	5	6.56	-0.10	0.04	1.49	0.08	96.52	1.91	6.54	8.17	17.13
os76110	7	370	0.11	10	1	1	6.67	-0.01	0.00	1.27	0.07	98.61	13.48	5.62	8.19	16.88
os91581	7	364	0.32	15	2	3	6.84	0.08	0.03	1.47	0.12	99.66	-3.53	7.07	8.12	16.96
os33437	7	275	0.90	12	3	5	6.18	-0.19	0.01	1.49	0.07	98.90	-0.77	6.42	8.72	17.59
os36659	7	339	0.01	2	3	9	6.80	0.03	1.37	1.51	0.01	62.03	0.57	8.31	8.06	16.75
os70356	7	329	0.60	8	3	5	6.50	-0.06	0.10	1.49	0.05	97.82	1.91	6.25	8.35	17.27
os71574	7	303	0.38	16	3	5	6.33	0.03	0.00	1.42	0.11	96.77	0.48	6.29	8.60	17.50
os02026	7	422	0.09	10	3	5	6.39	-0.16	0.01	1.48	0.07	95.90	0.05	7.23	8.17	17.06
os39922	7	409	0.27	3	2	3	6.68	-0.03	1.37	1.48	0.08	98.79	-3.04	6.87	7.39	16.10
os68246	7	461	0.60	4	3	5	6.67	-0.04	0.65	1.46	0.02	74.21	0.97	7.21	7.89	16.97
os39819	7	290	0.91	23	2	0	6.02	-0.24	0.00	1.42	0.19	86.26	-2.32	5.57	7.84	16.50
os77747	7	282	0.50	18	2	3	6.65	0.05	0.00	1.44	0.16	99.85	-5.86	6.38	8.49	17.26
os84395	7	440	0.08	2	3	6	6.86	0.04	1.56	1.55	0.02	52.33	-0.09	7.75	7.41	16.38
os76914	7	411	0.99	6	3	9	6.61	-0.09	0.20	1.47	0.04	99.48	1.19	6.35	7.92	16.68
os90184	7	371	0.53	3	3	9	6.71	-0.02	0.68	1.52	0.03	84.92	0.28	8.15	8.22	17.17
os37040	7	225	0.89	10	2	3	6.30	-0.14	0.75	1.52	0.12	70.40	-4.79	8.04	8.93	17.71
os65426	7	428	0.01	4	3	6	6.87	0.07	0.30	1.53	0.04	84.60	0.46	7.62	8.14	17.30
os68382	7	441	0.71	5	3	6	6.63	-0.05	1.44	1.53	0.05	93.56	-1.49	8.36	7.95	17.08
os36701	7	357	0.92	1	3	6	6.81	0.03	2.74	1.55	0.04	94.27	-1.27	8.93	7.80	16.59
os67466	7	329	0.00	6	3	5	6.89	0.11	0.49	1.52	0.05	96.50	-0.39	7.13	8.74	17.80
os32672	7	278	0.09	9	3	5	6.76	0.12	0.13	1.50	0.08	90.89	-0.37	6.82	8.28	17.01
os33047	7	189	0.92	0	3	6	6.69	0.00	6.64	1.57	0.01	4.38	-0.19	10.51	9.41	18.16
os09434	7	329	0.08	2	3	6	6.82	0.04	0.51	1.53	0.01	81.19	0.31	7.27	8.30	17.04
os35623	7	343	0.91	19	3	5	5.77	-0.27	0.00	1.37	0.17	99.48	0.34	4.83	8.08	16.76
os76426	7	319	0.00	15	2	7	6.76	0.24	0.21	1.48	0.24	99.81	-8.00	5.75	8.10	16.95
os77227	7	399	0.98	11	3	5	6.31	-0.19	0.02	1.47	0.07	99.65	0.01	5.99	8.09	16.89
os66323	7	330	0.62	27	2	0	5.65	-0.14	0.00	1.40	0.20	96.14	-1.53	6.16	8.14	17.08
os68729	7	306	0.01	5	2	0	6.82	0.07	0.98	1.54	0.11	92.25	-0.80	7.95	8.54	17.58
os77360	7	335	0.21	8	3	5	6.92	0.09	0.11	1.51	0.07	99.91	0.48	5.93	8.08	16.83
os31955	7	176	0.92	1	3	6	6.70	0.01	7.42	1.56	0.01	13.84	0.05	9.08	9.47	18.19
os01769	7	409	0.58	11	3	5	6.46	-0.06	0.13	1.52	0.08	64.43	-1.50	7.66	8.05	16.96
os36046	7	203	1.00	12	2	3	6.13	-0.19	0.14	1.47	0.14	86.47	-3.78	7.07	9.01	17.75

os76562	7	465	0.11	9	3	9	6.80	0.09	0.10	1.44	0.05	98.97	-0.36	6.13	7.88	16.86
os38164	7	322	0.28	8	3	5	6.85	0.05	0.12	1.50	0.11	95.44	-2.01	7.05	8.07	16.76
os23469	7	439	1.00	9	3	5	6.43	-0.16	0.04	1.47	0.07	96.38	0.88	5.58	8.26	17.26
os60945	7	317	0.92	1	3	6	6.73	0.00	3.84	1.55	0.02	22.44	0.11	8.44	8.10	16.89
os87719	7	429	0.00	13	2	0	6.97	0.23	0.00	1.46	0.10	94.67	1.83	5.60	7.59	16.55
os38603	7	365	0.08	8	3	5	6.95	0.13	0.46	1.49	0.06	97.91	-1.25	6.33	7.93	16.68
os76912	7	400	0.99	8	3	9	6.54	-0.12	0.36	1.48	0.08	98.96	-1.85	7.20	7.85	16.61
os68609	7	266	0.95	18	2	3	6.03	-0.26	0.00	1.44	0.13	82.75	-2.18	5.62	9.28	18.39
os02842	7	416	0.71	17	3	5	6.03	-0.19	0.00	1.45	0.10	96.76	-1.30	5.42	8.30	17.18
os02368	7	348	0.76	7	3	5	6.49	-0.11	0.30	1.50	0.07	85.79	0.39	7.50	8.45	17.38
os68435	7	459	0.15	4	3	6	6.92	0.06	0.37	1.47	0.02	73.07	1.45	6.90	7.89	16.97
os71589	7	261	0.12	12	2	7	6.34	-0.12	0.54	1.52	0.12	97.28	-4.98	7.80	8.71	17.62
os42386	7	370	0.18	7	3	5	6.68	0.03	0.09	1.49	0.06	94.78	1.21	6.70	8.11	16.99
os36183	7	268	0.34	7	2	7	6.75	0.05	1.14	1.54	0.16	93.27	-5.89	7.15	8.51	17.23
os42217	7	321	0.92	0	3	6	6.76	0.01	2.42	1.56	0.05	57.35	-0.81	8.66	8.32	17.09
os69443	7	224	0.23	2	3	6	6.70	0.02	2.82	1.55	0.01	18.52	-0.17	8.82	9.37	18.38
os68728	7	306	0.01	4	2	0	6.81	0.07	1.22	1.54	0.11	92.80	-0.78	7.95	8.53	17.57
os37367	7	319	0.94	20	2	3	6.03	-0.26	0.00	1.45	0.16	89.26	-4.20	5.69	8.07	16.78
os35403	7	239	0.23	5	3	6	6.66	0.02	1.17	1.52	0.03	42.33	0.39	8.14	8.72	17.48
os77604	7	368	0.37	9	3	5	6.56	0.00	0.07	1.47	0.07	100.00	0.68	6.24	7.91	16.66
os73661	7	525	0.92	1	3	6	6.80	-0.02	0.69	1.50	0.04	89.62	1.44	7.73	7.14	15.94
os07624	7	462	0.91	7	3	5	6.67	-0.08	0.52	1.53	0.05	79.18	-0.75	8.62	7.85	16.86
os35858	7	317	0.01	15	1	1	6.97	0.26	0.00	1.34	0.07	92.22	8.66	5.59	8.30	16.96
os32223	7	253	0.92	5	3	5	6.51	-0.09	0.31	1.52	0.06	80.19	0.37	8.11	8.76	17.61
os67008	7	403	0.90	4	3	6	6.74	-0.04	1.04	1.51	0.03	92.72	-0.09	7.59	8.33	17.41
os75996	7	321	0.90	16	1	1	6.33	-0.17	0.00	1.39	0.08	100.00	5.56	5.19	8.24	16.99
os91248	7	340	0.96	6	1	4	6.53	-0.09	0.19	1.47	0.02	74.36	2.62	6.32	8.32	17.21
os37155	7	238	0.58	5	2	3	6.58	-0.02	1.11	1.54	0.06	62.93	-1.94	8.00	8.95	17.74
os60058	7	366	0.88	11	3	5	6.49	-0.14	0.01	1.48	0.07	98.48	0.08	6.83	7.85	16.79
os70414	7	290	1.00	5	3	5	6.55	-0.09	0.31	1.51	0.03	98.65	1.15	6.59	8.59	17.51
os71323	7	335	0.21	10	3	9	6.71	0.09	0.05	1.45	0.06	94.37	1.31	6.12	8.37	17.17
os87639	7	421	0.06	3	3	6	6.80	0.03	1.65	1.49	0.03	97.54	-0.40	7.17	7.94	16.79
os33114	7	265	0.40	1	3	6	6.71	0.00	2.51	1.55	0.00	2.73	0.85	9.11	9.18	18.02
os36942	7	249	0.95	21	3	5	5.94	-0.27	0.00	1.38	0.12	93.91	1.07	5.13	8.71	17.45
os32665	7	300	0.12	8	1	4	6.88	0.09	0.07	1.46	0.06	97.42	2.35	6.73	8.24	16.97
os37694	7	299	0.54	9	2	3	6.78	0.01	0.53	1.48	0.11	98.20	-3.90	6.20	8.43	17.15
os38163	7	308	1.00	10	2	3	6.36	-0.17	0.12	1.51	0.08	90.09	-1.83	7.83	8.14	16.84
os21495	7	404	0.92	1	3	6	6.75	-0.02	5.50	1.56	0.02	30.22	-0.45	9.30	8.52	17.61
os85351	7	494	0.60	4	3	6	6.72	-0.02	0.56	1.51	0.06	99.13	-0.42	6.07	7.02	15.94
os37193	7	267	1.00	19	2	3	5.80	-0.31	0.00	1.43	0.17	99.35	-4.25	6.04	8.56	17.30
os36658	7	338	0.01	2	3	9	6.81	0.04	1.24	1.51	0.01	64.69	0.51	7.75	8.07	16.75
os76303	7	336	0.19	12	1	1	6.95	0.13	0.05	1.37	0.08	99.99	4.09	5.99	8.47	17.25
os21522	7	444	0.60	3	3	6	6.74	-0.01	1.40	1.54	0.04	87.84	-0.08	7.62	7.89	16.82

os37058	7	375	0.64	12	1	1	6.28	-0.11	0.00	1.40	0.06	91.98	3.67	5.90	8.06	16.84
os53666	7	282	0.69	13	3	5	6.69	-0.02	0.00	1.44	0.08	91.31	2.51	5.47	8.33	17.30
os87940	7	363	0.03	12	3	5	6.99	0.19	0.01	1.48	0.09	95.49	-1.29	6.36	8.02	16.93
os69606	7	254	0.90	12	2	0	6.19	-0.18	0.01	1.48	0.11	99.90	-0.69	6.50	9.52	18.73
os77095	7	400	0.40	10	3	9	6.92	0.08	0.03	1.46	0.08	98.47	-0.18	5.68	7.95	16.72
os38802	7	328	0.57	8	1	4	6.76	-0.01	0.03	1.41	0.05	96.37	6.10	5.65	8.14	16.82
os32358	7	294	0.06	11	3	5	6.76	0.13	0.09	1.49	0.08	98.97	-1.96	5.86	8.47	17.24
os40676	7	312	0.08	12	1	4	6.97	0.19	0.01	1.43	0.06	81.56	2.00	6.71	8.12	16.80
os40513	7	321	0.73	11	1	1	6.27	-0.14	0.08	1.42	0.06	83.21	2.97	6.59	8.04	16.70
os76312	7	417	0.11	9	2	8	6.78	0.10	0.14	1.46	0.09	99.67	-2.21	5.88	7.93	16.76
os36853	7	269	0.97	16	2	3	6.09	-0.23	0.20	1.50	0.17	92.48	-6.83	5.63	8.28	16.98
os47995	7	323	0.19	4	1	4	6.70	0.01	0.11	1.47	0.02	68.12	4.05	7.91	7.82	16.55
os77384	7	411	0.97	9	3	5	6.53	-0.13	0.11	1.48	0.07	99.98	0.66	6.15	7.73	16.50
os21521	7	449	0.60	4	3	6	6.71	-0.02	0.13	1.52	0.03	80.36	2.01	7.64	7.88	16.81
os33463	7	278	0.89	5	3	5	6.67	-0.05	0.52	1.52	0.04	97.14	0.37	6.52	8.91	17.71
os66656	7	354	0.00	5	2	3	6.87	0.08	1.00	1.51	0.09	99.31	-3.16	6.86	8.41	17.42
os67951	7	374	0.19	3	3	6	6.84	0.05	1.22	1.51	0.05	97.57	0.40	7.08	8.22	17.17
os77363	7	330	0.13	9	3	5	6.96	0.14	0.07	1.50	0.07	100.00	-0.15	7.27	8.11	16.86
os01549	7	235	0.01	4	3	6	6.78	0.06	0.70	1.54	0.04	94.78	0.09	7.04	8.94	17.79
os37604	7	324	0.98	8	2	7	6.55	-0.05	1.79	1.55	0.15	93.67	-8.22	7.58	8.05	16.82
os68530	7	380	0.26	5	1	4	6.68	0.00	0.15	1.48	0.03	98.51	2.72	6.96	8.65	17.78
os76655	7	397	0.52	10	3	9	6.45	-0.05	0.01	1.43	0.10	99.99	0.32	5.28	8.23	17.03
os36181	7	234	0.97	14	3	5	6.05	-0.23	0.00	1.44	0.10	90.35	0.49	6.00	9.03	17.79
os67595	7	256	0.11	9	2	7	6.76	0.12	0.13	1.51	0.10	76.82	-2.31	7.28	9.21	18.33
os35050	7	250	0.39	4	1	4	6.79	0.03	2.88	1.52	0.02	43.11	2.15	8.00	8.40	17.16
os87927	7	433	0.40	15	1	4	6.93	0.09	0.00	1.43	0.08	89.82	1.79	6.97	7.63	16.59
os88662	7	385	0.97	18	3	9	6.08	-0.26	0.00	1.41	0.12	95.11	1.83	6.24	8.07	16.90
os73585	7	335	0.71	6	3	5	6.79	0.00	0.36	1.51	0.05	77.96	0.73	7.64	8.12	16.77
os37307	7	293	0.51	7	3	5	6.77	0.00	0.62	1.51	0.06	92.77	-0.74	7.56	8.43	17.18
os69266	7	280	0.99	7	3	5	6.50	-0.11	0.33	1.54	0.06	73.75	-1.85	7.54	8.07	16.96
os77190	7	262	0.92	14	3	5	6.14	-0.20	0.00	1.48	0.07	96.12	1.10	6.09	8.84	17.65
os01754	7	356	0.80	8	1	4	6.47	-0.08	0.05	1.48	0.05	87.14	2.54	5.71	8.50	17.40
os34609	7	257	0.92	5	3	6	6.66	-0.04	2.23	1.54	0.02	18.31	0.47	8.50	8.20	16.89
os88687	7	307	0.91	7	3	5	6.42	-0.11	0.25	1.48	0.04	79.37	2.03	7.35	8.44	17.29
os69292	7	235	0.06	1	3	6	6.73	0.02	1.87	1.56	0.01	17.97	-0.20	9.44	9.32	18.34
os37541	7	367	0.00	5	3	6	6.86	0.07	0.25	1.49	0.04	96.72	0.98	6.16	7.94	16.70
os77277	7	232	0.92	4	2	3	6.56	-0.06	1.78	1.55	0.09	79.61	-3.32	7.01	8.88	17.66
os04094	7	286	0.01	17	3	5	5.91	-0.25	0.00	1.44	0.12	99.50	-0.50	6.21	8.72	17.45
os87186	7	329	0.68	8	3	5	6.49	-0.05	0.27	1.52	0.06	77.71	-0.22	7.73	8.39	17.30
os71588	7	262	0.58	12	2	7	6.35	-0.14	0.69	1.52	0.12	97.41	-5.10	8.00	8.71	17.62
os35878	7	276	0.68	18	1	4	6.55	-0.04	0.00	1.34	0.11	87.17	5.27	5.95	8.71	17.39
os37225	7	374	0.40	13	3	5	6.20	-0.20	0.00	1.42	0.07	87.91	0.87	6.23	8.00	16.77
os68794	7	234	0.17	8	2	0	6.77	0.12	0.17	1.51	0.08	57.69	-0.76	7.86	8.99	17.98

os36741	7	334	0.17	9	1	1	6.71	0.07	0.19	1.41	0.04	92.00	2.67	6.44	7.99	16.67
os40121	7	276	0.99	6	3	5	6.56	-0.09	0.46	1.49	0.05	85.35	0.71	6.92	8.13	16.86
os76109	7	369	0.01	6	1	1	6.52	-0.04	0.00	1.27	0.07	98.71	12.48	4.85	8.17	16.87
os34768	7	223	0.79	5	3	5	6.68	-0.03	5.25	1.52	0.02	23.11	1.90	7.76	8.77	17.54
os70551	7	286	0.74	6	3	5	6.54	-0.07	0.85	1.53	0.04	88.12	-1.46	8.17	8.32	17.22
os91515	7	370	0.91	6	3	5	6.56	-0.10	0.81	1.51	0.07	97.12	-2.00	7.22	8.22	17.08
os73625	7	454	1.00	7	3	5	6.57	-0.11	0.35	1.51	0.03	96.42	0.60	6.33	7.61	16.41
os14369	7	454	0.99	3	3	6	6.73	-0.05	1.02	1.55	0.02	63.33	-0.04	8.46	8.20	17.24
os63308	7	307	0.98	3	3	6	6.66	-0.05	0.72	1.55	0.02	84.38	0.10	7.79	8.23	17.03
os37026	7	243	0.98	7	2	7	6.42	-0.13	0.23	1.54	0.11	79.48	-3.31	7.10	8.57	17.29
os37951	7	318	0.80	15	1	4	6.03	-0.20	0.00	1.41	0.08	85.01	3.47	5.70	8.11	16.81
os77345	7	383	0.20	9	3	5	6.96	0.11	0.02	1.47	0.05	97.94	2.04	5.96	7.97	16.74
os70554	7	268	0.42	10	3	5	6.62	0.05	0.16	1.49	0.06	73.07	0.50	7.59	8.41	17.31
os32428	7	277	1.00	8	3	5	6.42	-0.14	0.19	1.49	0.06	98.39	0.16	7.11	8.72	17.46
os74957	7	246	0.32	4	3	6	6.80	0.04	2.32	1.55	0.06	89.98	-1.45	8.58	8.55	17.45
os32272	7	256	0.98	10	3	5	6.39	-0.16	0.08	1.49	0.07	92.70	0.31	7.10	8.77	17.51
os66680	7	304	0.99	15	2	3	6.04	-0.26	0.00	1.46	0.12	93.39	-2.30	5.37	8.64	17.64
os05223	7	379	0.17	7	3	5	6.51	-0.11	0.28	1.51	0.05	94.32	-0.48	7.86	8.34	17.10
os21523	7	443	0.47	3	3	6	6.74	-0.01	1.31	1.54	0.04	88.11	-0.03	7.66	7.89	16.82
os36839	7	313	0.02	14	1	4	6.95	0.23	0.00	1.41	0.10	97.14	2.21	5.42	8.07	16.80
os77282	7	427	0.61	9	3	5	6.48	-0.08	0.18	1.50	0.06	99.83	-0.47	7.12	8.03	16.88
os35570	7	388	0.79	7	3	5	6.54	-0.09	0.20	1.48	0.05	94.74	0.50	6.00	7.93	16.64
os77446	7	459	0.01	6	3	5	6.94	0.11	0.57	1.45	0.04	89.27	0.19	6.73	7.92	16.84
os36725	7	311	0.73	22	3	5	6.41	-0.09	0.00	1.36	0.14	92.22	1.35	5.97	8.02	16.71
os77448	7	376	0.74	8	2	3	6.49	-0.06	0.87	1.50	0.11	93.49	-4.27	7.92	8.08	16.90
os21497	7	406	0.92	1	3	6	6.77	0.00	5.14	1.56	0.01	8.57	0.21	10.36	8.51	17.60
os40464	7	315	0.98	18	3	5	5.93	-0.28	0.00	1.43	0.10	98.50	1.84	5.46	8.04	16.77
os60318	7	306	0.98	19	2	3	5.91	-0.28	0.00	1.46	0.15	96.59	-3.60	5.72	8.39	17.39
os21353	7	417	0.08	2	3	6	6.83	0.02	1.12	1.55	0.01	25.18	0.19	8.52	8.51	17.62
os87428	7	358	0.74	9	2	3	6.62	-0.04	0.47	1.49	0.16	99.48	-4.88	7.07	8.09	16.90
os90267	7	401	1.00	7	3	5	6.57	-0.11	0.38	1.52	0.05	91.62	-0.04	6.50	7.75	16.67
os03211	7	343	0.08	11	1	1	6.99	0.18	0.01	1.44	0.05	97.98	3.13	5.69	8.68	17.41
os37543	7	365	0.08	7	3	5	6.94	0.11	0.33	1.49	0.05	97.32	0.37	6.26	7.94	16.69
os37489	7	306	0.22	7	3	5	6.73	0.06	0.29	1.51	0.05	89.45	1.60	7.07	8.05	16.78
os37666	7	299	0.04	16	3	5	6.80	0.23	0.00	1.44	0.09	98.00	0.09	6.15	8.35	17.01
os36322	7	326	1.00	9	1	4	6.41	-0.15	0.11	1.41	0.06	90.55	2.96	6.55	8.14	16.83
os60309	7	353	0.65	12	2	3	6.38	-0.08	0.08	1.49	0.10	99.59	-1.76	6.93	8.07	17.05
os01904	7	362	0.50	16	3	5	6.80	0.06	0.00	1.44	0.11	96.28	1.87	6.13	8.56	17.42
os67619	7	324	1.00	9	3	5	6.42	-0.15	0.09	1.48	0.07	96.97	1.06	6.59	8.72	17.78
os68730	7	306	0.01	5	2	0	6.81	0.07	0.89	1.54	0.11	91.69	-1.06	7.94	8.54	17.58
os89302	7	547	0.15	5	3	6	6.97	0.06	0.51	1.51	0.03	87.74	0.48	6.35	6.98	15.97
os06308	7	366	0.10	8	2	7	6.77	0.07	0.84	1.54	0.17	98.86	-7.77	7.80	8.11	17.14
os33420	7	311	0.92	4	3	6	6.60	-0.07	0.59	1.51	0.03	98.29	0.44	6.99	8.60	17.38

os38662	7	359	0.65	9	3	5	6.73	-0.04	0.10	1.48	0.05	88.18	0.44	5.75	8.06	16.75
os63323	7	349	0.63	4	1	4	6.79	0.00	0.07	1.51	0.02	86.27	2.20	8.51	8.09	16.88
os33049	7	190	0.92	0	3	6	6.69	0.00	5.93	1.57	0.01	2.94	-0.14	10.29	9.42	18.18
os75999	7	278	0.00	12	3	5	6.93	0.20	0.01	1.47	0.10	99.95	-0.29	7.55	8.26	17.02
os77723	7	409	0.36	13	2	3	6.90	0.10	0.02	1.48	0.12	99.44	-2.51	5.66	7.65	16.41
os78916	7	423	0.00	9	3	5	6.40	-0.15	0.09	1.48	0.05	93.23	1.93	6.06	7.59	16.48
os09537	7	279	0.09	5	2	7	6.77	0.05	1.65	1.55	0.14	86.70	-6.50	7.11	8.67	17.50
os65754	7	359	0.33	5	2	3	6.56	-0.09	1.30	1.55	0.08	86.77	-3.11	8.56	8.64	17.81
os77766	7	285	0.89	32	2	3	4.61	-0.44	0.00	1.31	0.26	100.00	-3.47	5.08	8.49	17.31
os02458	7	481	0.00	10	1	4	6.97	0.17	0.02	1.37	0.06	95.82	6.93	5.63	7.94	16.92
os07098	7	469	0.24	8	3	5	6.51	-0.12	0.35	1.52	0.07	67.47	-1.88	7.33	7.86	16.93
os53702	7	297	0.63	18	2	3	6.66	0.01	0.00	1.46	0.13	73.73	-2.85	6.34	8.08	17.05
os87548	7	369	0.60	9	3	5	6.48	-0.05	0.06	1.46	0.09	97.82	-0.70	6.91	7.96	16.76
os90456	7	293	0.34	2	3	6	6.66	-0.04	1.08	1.54	0.03	99.28	0.10	7.88	8.63	17.47
os35093	7	226	0.02	3	3	6	6.60	-0.05	2.03	1.54	0.02	26.55	0.40	8.15	8.76	17.52
os06594	7	405	0.77	7	1	4	6.93	0.08	0.05	1.44	0.03	90.92	2.72	6.58	8.36	17.41
os66305	7	360	0.85	8	3	9	6.67	-0.07	0.22	1.46	0.04	97.33	0.79	6.37	8.53	17.51
os36344	7	311	0.47	7	1	4	6.84	0.04	0.15	1.47	0.06	85.08	2.50	6.25	8.20	16.86
os37922	7	317	0.04	15	2	3	6.81	0.22	0.00	1.47	0.13	82.27	-2.14	6.64	8.10	16.81
os03500	7	383	0.55	10	1	4	6.77	-0.01	0.03	1.38	0.06	83.00	4.26	5.47	8.40	17.18
os77066	7	278	0.87	17	3	9	5.94	-0.24	0.00	1.41	0.09	99.45	0.10	5.13	9.03	17.86
os91242	7	344	0.72	13	2	3	6.33	-0.10	0.08	1.48	0.11	97.15	-2.51	7.70	8.18	17.09
os32673	7	287	0.23	8	3	5	6.71	0.08	0.19	1.49	0.07	92.32	0.31	6.93	8.29	17.02
os34428	7	264	0.22	2	3	6	6.67	-0.03	6.57	1.56	0.01	11.65	-0.41	8.91	8.22	16.93
os33082	7	270	0.65	6	1	4	6.53	-0.04	0.32	1.43	0.03	65.05	5.96	7.01	8.96	17.78
os74947	7	268	0.65	7	3	5	6.49	-0.07	0.16	1.51	0.03	81.51	1.30	7.71	8.49	17.40
os14855	7	450	0.08	1	3	6	6.83	0.01	2.04	1.55	0.02	47.52	0.45	9.55	8.25	17.30
os70606	7	332	0.97	5	2	0	6.65	-0.07	1.10	1.53	0.06	90.51	-1.10	8.99	7.87	16.83
os78051	7	389	0.21	7	1	4	6.73	0.05	0.01	1.41	0.02	83.38	6.97	5.67	7.80	16.69
os88551	7	385	0.17	3	1	1	6.83	0.06	0.09	1.46	0.01	75.43	3.36	6.93	8.20	17.13
os87912	7	377	0.96	8	3	5	6.55	-0.12	0.21	1.46	0.06	97.09	1.39	6.46	7.87	16.81
os88560	7	411	0.99	5	2	3	6.68	-0.06	1.71	1.54	0.07	69.37	-3.00	8.39	7.94	16.85
os70179	7	230	0.77	8	2	7	6.35	-0.11	0.32	1.53	0.15	81.16	-4.79	7.43	8.77	17.69
os36204	7	202	0.18	7	2	7	6.41	-0.12	1.06	1.53	0.10	88.13	-3.97	7.67	9.10	17.88
os87551	7	371	0.46	7	3	5	6.56	-0.02	0.49	1.48	0.09	97.87	-2.16	7.16	7.94	16.75
os88647	7	423	0.96	13	3	5	6.21	-0.22	0.00	1.48	0.09	99.72	-0.93	6.50	7.75	16.72
os67468	7	329	0.00	6	3	5	6.89	0.11	0.54	1.52	0.05	96.31	-0.52	7.21	8.74	17.80
os73636	7	527	0.54	4	3	9	6.70	-0.06	0.41	1.47	0.02	87.05	-0.28	6.80	7.35	16.17
os76928	7	336	0.88	7	3	5	6.62	-0.08	0.34	1.49	0.07	100.00	-0.90	7.59	8.15	16.89
os88584	7	395	0.24	6	3	6	6.74	0.03	0.18	1.51	0.03	65.29	0.97	7.98	8.01	16.92
os76882	7	373	0.83	7	1	4	6.66	-0.07	0.07	1.47	0.07	99.54	2.41	7.17	7.77	16.53
os76918	7	346	0.84	9	1	4	6.67	-0.06	0.03	1.47	0.06	97.79	2.57	6.66	7.96	16.71
os21655	7	416	0.30	2	3	6	6.76	0.00	2.83	1.56	0.02	38.89	0.01	8.79	8.51	17.60

os69187	7	355	0.14	13	3	9	6.73	0.14	0.00	1.46	0.07	99.46	0.58	5.49	7.85	16.65
os38816	7	303	0.95	14	2	7	6.34	-0.18	0.08	1.50	0.12	93.50	-3.97	7.39	8.14	16.83
os34663	7	229	0.92	0	3	6	6.70	0.00	6.91	1.57	0.00	0.00	0.03	11.68	8.67	17.43
os35437	7	248	0.08	1	3	6	6.76	0.02	6.86	1.54	0.01	55.22	0.60	8.15	8.76	17.45
os37103	7	267	1.00	18	3	5	5.86	-0.30	0.00	1.42	0.13	94.69	0.60	5.81	8.54	17.28
os40504	7	305	0.06	17	2	3	6.90	0.26	0.00	1.45	0.13	94.59	-3.00	5.76	8.03	16.71
os69435	7	237	0.60	12	2	3	6.22	-0.12	0.06	1.48	0.11	97.44	-3.82	7.19	9.29	18.34
os74968	7	271	0.17	6	3	5	6.49	-0.10	0.33	1.50	0.04	96.10	0.54	6.45	8.76	17.62
os01749	7	322	1.00	8	1	4	6.47	-0.13	0.04	1.48	0.04	95.39	3.36	6.99	8.62	17.50
os01755	7	329	0.60	2	3	6	6.70	-0.01	0.40	1.54	0.01	63.44	0.42	8.28	8.61	17.48
os07124	7	414	0.15	6	3	5	6.76	0.03	0.41	1.52	0.04	77.98	-0.11	9.01	8.19	17.20
os63550	7	395	0.85	13	3	9	6.19	-0.18	0.00	1.44	0.07	99.68	1.22	5.69	7.99	16.93
os34066	7	212	0.92	1	3	6	6.72	0.01	6.28	1.55	0.01	11.12	0.31	9.26	8.83	17.59
os33879	7	263	0.01	2	3	6	6.79	0.04	0.65	1.55	0.02	67.80	0.30	8.47	8.31	17.02
os34608	7	257	0.77	5	3	6	6.68	-0.03	1.75	1.53	0.02	20.28	0.45	8.55	8.20	16.89
os68356	7	282	0.30	3	3	6	6.81	0.04	1.97	1.55	0.04	76.04	-1.82	7.99	9.40	18.60
os82081	7	298	1.00	6	3	5	6.48	-0.12	0.39	1.52	0.10	70.26	-1.24	7.79	8.30	17.26
os66659	7	317	0.70	10	2	3	6.37	-0.11	0.25	1.52	0.12	97.66	-3.06	6.39	8.50	17.51
os68926	7	258	0.94	6	2	3	6.61	-0.07	1.32	1.52	0.07	97.62	-3.68	7.71	8.78	17.79
os03398	7	337	0.01	6	2	3	6.83	0.09	1.12	1.53	0.08	95.87	-3.00	7.46	8.61	17.38
os91298	7	388	0.84	17	1	4	6.04	-0.17	0.00	1.40	0.09	92.81	3.87	5.73	8.00	16.86
os34154	7	257	0.92	1	3	6	6.72	0.00	1.24	1.55	0.01	43.07	0.16	9.16	8.34	17.16
os34839	7	275	0.92	0	3	6	6.71	0.00	0.78	1.55	0.00	58.64	0.44	8.40	8.42	17.17
os81111	7	502	0.70	12	2	3	6.37	-0.13	0.06	1.51	0.09	63.68	-2.48	7.37	6.91	15.80
os38088	7	295	0.23	13	3	5	6.33	-0.17	0.25	1.49	0.08	83.40	-0.59	7.41	8.12	16.85
os90572	7	456	0.13	19	1	1	7.01	0.26	0.00	1.37	0.09	97.10	4.55	5.28	7.76	16.62
os62936	7	297	0.76	9	3	5	6.70	-0.03	0.09	1.49	0.09	86.24	0.12	6.15	8.26	17.01
os33012	7	222	0.07	8	3	5	6.77	0.11	0.20	1.50	0.05	80.30	1.24	7.18	9.05	17.82
os81252	7	356	0.83	8	2	3	6.48	-0.10	0.68	1.53	0.07	80.77	-2.65	8.02	8.17	17.22
os69279	7	225	0.92	1	2	3	6.68	-0.01	2.96	1.57	0.05	69.86	-2.33	8.12	8.86	17.70
os34587	7	334	0.92	1	3	6	6.77	0.01	2.32	1.54	0.01	12.65	-0.13	9.19	7.89	16.63
os64057	7	373	0.00	9	2	3	6.93	0.14	0.53	1.52	0.11	97.03	-3.61	7.35	7.91	16.85
os76932	7	374	0.35	15	3	5	6.91	0.12	0.00	1.46	0.08	100.00	0.58	5.68	7.97	16.73
os36657	7	339	0.02	2	3	9	6.80	0.04	1.35	1.51	0.01	63.14	0.52	7.91	8.07	16.76
os80591	7	534	0.03	11	3	5	6.92	0.16	0.04	1.49	0.07	87.19	-0.40	7.43	6.89	15.83
os07656	7	438	0.00	13	3	5	6.95	0.21	0.00	1.46	0.08	76.30	3.08	5.80	8.03	16.96
os40677	7	306	0.08	16	3	5	6.98	0.24	0.00	1.42	0.10	82.29	-0.02	6.55	8.12	16.81
os87545	7	365	0.58	9	2	3	6.46	-0.04	0.06	1.46	0.11	97.61	-1.49	7.11	7.97	16.77
os08827	7	439	0.00	6	3	5	6.94	0.11	0.27	1.51	0.04	95.08	0.28	7.33	8.45	17.62
os69870	7	283	0.29	11	2	3	6.85	0.10	0.42	1.50	0.11	95.13	-3.92	7.43	9.07	18.05
os73652	7	512	0.60	9	3	5	6.52	-0.06	0.31	1.48	0.07	92.49	-1.21	6.42	7.31	16.13
os37212	7	263	1.00	11	2	3	6.29	-0.18	0.22	1.47	0.14	93.62	-4.14	5.82	8.56	17.30
os60310	7	356	0.59	11	3	5	6.41	-0.07	0.02	1.48	0.08	99.58	-0.09	6.75	8.07	17.04

os91209	7	372	0.03	9	3	5	6.95	0.13	0.63	1.50	0.08	96.89	-3.17	7.44	8.19	17.03
os80571	7	512	0.61	7	1	4	6.58	-0.05	0.03	1.46	0.04	66.60	4.76	6.13	6.92	15.80
os76839	7	385	0.89	10	3	5	6.36	-0.15	0.30	1.47	0.11	99.87	-1.83	6.83	8.09	16.94
os77279	7	231	0.01	4	2	3	6.59	-0.06	1.90	1.56	0.09	78.66	-3.45	7.06	8.88	17.66
os21517	7	428	0.98	4	3	6	6.73	-0.05	0.36	1.52	0.05	94.16	1.12	7.50	8.11	17.07
os76460	7	405	0.85	13	3	5	6.18	-0.17	0.01	1.45	0.10	95.32	-0.80	5.06	8.07	16.95
os76223	7	303	0.03	22	3	5	6.78	0.37	0.00	1.35	0.18	99.96	0.74	4.80	8.53	17.28
os70548	7	284	0.18	5	2	3	6.54	-0.09	1.25	1.54	0.05	88.56	-2.06	8.28	8.32	17.22
os67606	7	266	0.13	11	2	0	6.93	0.14	0.08	1.47	0.13	83.63	-0.52	6.35	9.21	18.32
os69582	7	239	0.86	11	2	7	6.29	-0.14	0.24	1.52	0.13	99.51	-3.73	7.52	9.57	18.78
os21536	7	454	0.39	3	3	6	6.87	0.03	1.83	1.55	0.03	68.44	-0.11	8.46	8.38	17.53
os67585	7	357	0.04	5	3	9	6.81	0.07	0.63	1.49	0.05	98.33	-0.74	6.92	8.76	17.86
os37582	7	350	0.90	5	3	6	6.56	-0.08	1.37	1.49	0.04	81.02	-0.14	8.44	8.00	16.73
os40473	7	297	1.00	13	2	3	6.23	-0.21	0.01	1.48	0.10	97.59	-2.74	5.66	8.03	16.77
os69444	7	225	0.06	1	3	6	6.71	0.02	2.80	1.55	0.01	19.76	0.26	8.80	9.36	18.38
os38209	7	351	0.00	11	3	5	6.91	0.18	0.02	1.47	0.07	99.90	-0.08	6.41	7.99	16.67
os60908	7	277	0.96	8	2	3	6.40	-0.15	0.23	1.52	0.08	93.92	-1.88	6.15	8.24	16.98
os76104	7	277	0.53	25	2	7	5.58	-0.09	0.00	1.40	0.25	99.74	-5.18	5.33	8.21	16.93
os76906	7	356	0.02	6	3	5	6.92	0.11	0.38	1.52	0.06	99.32	-0.65	6.77	7.90	16.64
os89574	7	392	0.01	12	2	3	6.92	0.19	0.17	1.51	0.10	88.17	-2.63	6.09	7.83	16.68
os62989	7	441	0.08	15	2	3	6.95	0.25	0.00	1.47	0.11	84.69	-2.71	5.73	7.38	16.20
os35304	7	236	0.92	2	3	6	6.64	-0.03	1.48	1.55	0.01	26.10	0.02	8.53	8.75	17.50
os42410	7	284	0.73	12	2	3	6.25	-0.10	0.01	1.49	0.11	81.15	-2.21	6.45	8.74	17.55
os77289	7	335	0.61	6	3	5	6.52	-0.09	0.43	1.51	0.06	99.52	0.17	6.54	8.48	17.31
os80801	7	400	0.98	15	3	5	6.23	-0.23	0.00	1.46	0.09	68.29	0.35	6.44	7.62	16.58
os32054	7	237	0.04	3	3	6	6.60	-0.06	1.19	1.53	0.04	87.58	0.14	8.49	9.02	17.76
os67626	7	300	0.01	5	3	5	6.87	0.10	0.25	1.50	0.07	92.85	0.61	7.26	8.77	17.84
os70490	7	307	0.00	9	1	4	6.92	0.15	0.05	1.45	0.06	97.58	2.35	6.08	8.31	17.20
os76916	7	416	0.78	4	3	9	6.73	-0.05	0.56	1.47	0.04	98.96	0.15	6.51	7.89	16.66
os77285	7	400	0.35	8	2	7	6.58	-0.02	0.28	1.49	0.16	99.26	-2.26	7.05	7.84	16.69
os32674	7	285	0.11	9	3	5	6.74	0.10	0.11	1.49	0.08	92.24	0.23	6.96	8.29	17.01
os35675	7	371	0.04	17	2	3	6.68	0.26	0.29	1.47	0.20	96.44	-7.89	5.55	7.95	16.65
os71647	7	363	0.63	10	3	9	6.43	-0.08	0.08	1.45	0.05	98.94	0.40	6.45	8.33	17.14
os75030	7	254	0.59	15	3	5	6.22	-0.08	0.00	1.47	0.09	63.88	0.13	6.87	8.48	17.38
os71437	7	386	0.51	6	3	5	6.57	-0.09	0.51	1.51	0.05	98.71	0.34	6.56	7.97	16.81
os66798	7	264	0.62	6	2	0	6.53	-0.06	0.43	1.52	0.09	77.02	0.37	6.90	8.72	17.72
os76878	7	370	0.08	5	3	5	6.90	0.07	0.14	1.51	0.06	99.98	1.11	7.00	7.80	16.54
os87930	7	434	0.99	13	1	4	6.31	-0.21	0.00	1.43	0.06	88.27	5.29	5.90	7.63	16.59
os74969	7	270	0.89	6	3	5	6.49	-0.10	0.36	1.51	0.04	95.99	0.40	6.47	8.76	17.62
os21524	7	443	0.47	3	3	6	6.74	-0.01	1.26	1.54	0.04	87.64	-0.05	7.62	7.89	16.82
os66670	7	312	0.54	6	3	9	6.83	0.03	0.42	1.46	0.08	95.35	0.08	6.37	8.73	17.74
os74950	7	282	0.11	9	3	5	6.73	0.10	0.10	1.48	0.07	93.60	0.08	6.32	8.42	17.33
os76226	7	366	0.24	12	3	5	6.95	0.13	0.00	1.46	0.09	99.99	0.40	5.77	8.21	17.04

os02658	7	494	0.94	12	2	3	6.50	-0.15	0.10	1.49	0.08	95.65	-1.76	6.41	7.87	16.78
os07799	7	366	0.01	11	2	7	6.74	0.16	1.12	1.54	0.18	83.96	-6.74	6.80	8.55	17.68
os32989	7	231	0.40	8	2	3	6.80	0.04	1.05	1.54	0.08	63.50	-2.83	8.42	9.20	18.01
os68601	7	245	1.00	7	3	5	6.47	-0.12	0.21	1.49	0.04	82.83	1.84	6.77	9.48	18.61
os69008	7	264	0.23	3	3	6	6.73	0.03	1.53	1.55	0.03	59.02	-0.23	8.45	8.17	16.92
os37565	7	295	0.99	9	1	4	6.45	-0.14	0.02	1.41	0.05	88.86	5.80	5.72	8.28	16.99
os36082	7	305	0.97	12	3	5	6.38	-0.17	0.01	1.47	0.08	73.57	0.76	7.04	8.39	17.06
os84564	7	529	0.15	10	3	5	6.77	0.10	0.08	1.48	0.10	99.30	-1.54	6.05	6.81	15.62
os77045	7	458	0.47	5	3	5	6.71	0.02	0.32	1.50	0.06	100.00	0.97	6.17	7.75	16.62
os87926	7	408	0.11	7	3	5	6.97	0.12	0.38	1.52	0.08	93.78	-0.64	7.64	7.64	16.61
os91511	7	374	0.93	7	2	3	6.51	-0.13	0.32	1.51	0.07	97.47	-2.49	7.00	8.22	17.08
os34592	7	335	0.92	1	3	6	6.78	0.01	1.63	1.54	0.01	9.01	0.20	9.22	7.89	16.62
os37138	7	247	0.01	8	1	4	6.85	0.14	0.12	1.43	0.04	88.85	4.85	6.27	8.79	17.55
os69209	7	245	0.76	2	3	6	6.71	-0.01	3.12	1.57	0.04	26.20	-1.29	8.39	8.35	17.14
os77748	7	285	0.40	22	3	5	6.72	0.16	0.00	1.38	0.15	99.81	-0.52	5.33	8.52	17.29
os07599	7	471	0.08	8	3	5	6.98	0.11	0.03	1.48	0.03	82.89	2.33	6.00	7.96	16.96
os35439	7	231	0.39	8	3	5	6.81	0.05	0.78	1.53	0.07	63.67	-2.19	8.38	9.20	18.01
os71303	7	293	0.45	10	3	5	6.49	-0.01	0.05	1.50	0.08	95.77	-0.65	6.35	8.45	17.28
os91582	7	335	0.95	6	2	7	6.64	-0.07	1.53	1.53	0.13	99.83	-4.85	7.52	8.18	17.01
os39159	7	360	0.94	15	3	9	6.31	-0.19	0.00	1.45	0.10	98.26	0.22	5.84	7.74	16.49
os01758	7	353	1.00	10	2	3	6.48	-0.14	0.17	1.54	0.09	73.04	-1.84	8.05	8.31	17.21
os02482	7	432	0.83	12	2	3	6.61	-0.08	0.02	1.48	0.15	98.75	-4.21	5.54	8.14	17.09
os66626	7	320	0.40	16	3	5	6.31	0.02	0.00	1.43	0.12	99.49	0.41	5.85	8.45	17.43
os77230	7	479	0.05	13	3	5	7.04	0.21	0.01	1.42	0.10	98.34	-0.06	5.36	7.68	16.53
os88871	7	393	0.23	4	3	6	6.75	0.02	1.73	1.54	0.05	86.05	-1.42	7.97	7.82	16.68
os06574	7	391	0.17	10	2	0	6.70	0.09	0.45	1.51	0.14	92.73	-4.04	6.69	8.27	17.31
os37652	7	291	0.00	16	2	7	6.88	0.25	0.00	1.48	0.14	95.23	-3.02	7.17	8.17	16.87
os03231	7	332	0.93	13	2	3	6.39	-0.16	0.56	1.50	0.13	97.54	-5.44	6.78	8.47	17.23
os32865	7	235	0.92	5	3	6	6.53	-0.08	0.55	1.52	0.02	73.35	0.74	7.26	9.38	18.11
os38479	7	299	0.01	7	3	5	6.85	0.12	0.71	1.49	0.05	97.03	0.45	7.72	8.26	16.93
os37609	7	372	0.99	7	3	5	6.58	-0.10	0.85	1.52	0.07	95.81	-2.28	7.27	7.88	16.64
os42313	7	207	0.30	10	3	5	6.84	0.07	0.02	1.48	0.06	64.07	1.67	5.92	9.27	18.22
os01324	7	401	0.03	13	3	5	7.00	0.22	0.00	1.50	0.10	99.86	-1.58	6.29	7.50	16.16
os05272	7	364	0.40	5	3	6	6.79	0.00	1.60	1.54	0.06	87.45	-2.01	8.66	8.37	17.24
os36195	7	284	0.15	23	2	7	6.47	0.30	0.00	1.47	0.25	96.80	-11.09	5.53	8.26	16.93
os33423	7	329	0.86	6	3	9	6.56	-0.09	0.34	1.49	0.03	99.68	1.23	6.32	8.58	17.37
os88660	7	341	1.00	13	3	5	6.20	-0.22	0.06	1.47	0.13	97.89	-1.93	6.67	8.14	16.96
os32987	7	241	0.97	13	2	3	6.16	-0.21	0.01	1.49	0.09	97.37	-2.33	6.67	9.27	17.96
os36043	7	199	0.70	14	3	5	6.14	-0.12	0.30	1.49	0.11	89.48	-2.91	7.31	9.14	17.92
os55983	7	315	0.39	12	2	0	6.91	0.10	0.06	1.50	0.11	95.52	-1.74	6.77	7.65	16.47
os77271	7	454	0.95	13	1	4	6.41	-0.18	0.00	1.44	0.08	99.40	3.36	5.72	7.66	16.52
os77431	7	302	0.92	11	1	1	6.27	-0.17	0.02	1.39	0.06	89.67	2.38	5.59	8.68	17.45
os05281	7	303	0.87	4	3	6	6.61	-0.04	1.20	1.49	0.04	71.95	-0.22	8.35	8.91	17.61

os76925	7	325	0.40	11	3	5	6.88	0.08	0.05	1.50	0.07	99.90	-0.30	6.07	8.11	16.85
os42409	7	280	0.13	3	2	3	6.73	0.04	0.89	1.53	0.08	80.66	-1.80	6.19	8.75	17.56
os36836	7	341	0.17	3	1	1	6.82	0.06	0.08	1.44	0.03	99.14	3.93	6.23	8.18	16.90
os91249	7	330	0.67	6	3	6	6.58	-0.05	0.69	1.51	0.05	93.60	-0.76	7.07	8.34	17.25
os36820	7	285	0.66	9	1	4	6.42	-0.07	0.01	1.45	0.04	90.54	4.83	5.39	8.39	17.10
os37283	7	306	0.00	4	2	3	6.60	-0.06	1.57	1.52	0.11	96.18	-3.89	7.40	8.33	17.08
os77322	7	300	0.73	10	3	9	6.66	-0.05	0.09	1.43	0.09	99.98	-0.77	5.40	8.89	17.68
os67476	7	383	0.12	11	3	5	7.00	0.17	0.01	1.47	0.07	96.79	1.89	6.63	8.41	17.52
os05095	7	298	0.28	6	1	4	6.69	0.03	0.06	1.48	0.04	90.92	2.77	7.80	8.82	17.59
os36205	7	209	0.92	4	2	7	6.55	-0.06	2.48	1.54	0.08	90.77	-3.32	7.93	9.08	17.86
os75913	7	223	1.00	8	3	5	6.41	-0.14	0.18	1.50	0.06	98.02	0.45	6.23	9.06	17.90
os75960	7	246	0.99	11	1	2	6.25	-0.18	0.00	1.46	0.20	99.73	2.45	5.33	8.26	17.02
os77379	7	343	0.26	12	3	5	6.96	0.14	0.01	1.49	0.08	99.97	-0.83	5.89	7.97	16.73
os76934	7	368	0.34	15	3	5	6.92	0.13	0.00	1.47	0.09	100.00	-0.62	5.78	7.98	16.74
os32003	7	193	0.09	3	3	6	6.77	0.04	0.48	1.55	0.02	37.33	-0.22	7.64	9.29	18.02
os67467	7	329	0.01	6	3	5	6.89	0.11	0.57	1.52	0.05	96.45	-0.57	7.18	8.74	17.80
os87928	7	433	0.35	14	1	4	6.92	0.09	0.05	1.44	0.07	89.80	1.54	7.12	7.63	16.59
os39944	7	287	0.02	12	2	3	6.77	0.14	0.12	1.51	0.10	91.31	-4.15	7.11	7.97	16.71
os50586	7	327	0.58	8	3	5	6.56	-0.03	0.54	1.52	0.05	86.72	-1.02	6.93	7.86	16.58
os36909	7	317	0.63	13	1	4	6.71	-0.02	0.09	1.45	0.07	90.05	0.59	7.23	7.88	16.56
os37833	7	386	0.92	9	3	5	6.48	-0.12	0.73	1.48	0.07	94.26	-2.16	6.66	7.86	16.62
os39254	7	311	0.20	25	1	2	6.76	0.26	0.00	1.36	0.19	77.40	4.26	4.55	7.67	16.38
os77309	7	393	0.96	10	3	5	6.47	-0.14	0.08	1.48	0.11	99.97	-2.16	6.51	8.02	16.85
os83469	7	549	0.34	13	2	3	6.65	0.08	0.01	1.50	0.10	90.70	-2.84	5.64	6.65	15.40
os50131	7	280	0.78	15	2	3	6.12	-0.17	0.00	1.49	0.09	34.45	-0.85	6.81	7.91	16.60
os77342	7	381	0.01	8	3	5	6.94	0.14	0.10	1.49	0.06	98.44	1.08	6.46	7.68	16.44
os50588	7	336	0.08	11	3	5	6.97	0.17	0.19	1.50	0.08	75.78	-2.08	7.55	7.90	16.62
os03829	7	386	0.89	8	3	5	6.49	-0.10	0.89	1.50	0.09	96.97	-3.32	7.87	8.25	17.03
os68610	7	263	0.94	17	2	0	6.16	-0.23	0.00	1.43	0.12	75.75	-0.55	5.96	9.25	18.36
os76530	7	331	0.01	7	3	5	6.90	0.12	0.49	1.48	0.06	97.53	-0.23	7.57	8.51	17.42
os36919	7	261	0.99	24	2	7	5.61	-0.34	0.00	1.44	0.20	87.05	-4.28	5.50	8.13	16.82
os36700	7	257	0.99	10	2	0	6.35	-0.16	0.07	1.51	0.07	88.28	-0.90	7.42	8.32	17.06
os77290	7	335	0.92	9	3	5	6.39	-0.14	0.08	1.49	0.06	99.69	0.35	6.37	8.51	17.34
os36812	7	242	0.13	16	2	7	6.57	0.15	0.00	1.51	0.20	72.65	-7.03	6.13	8.20	16.90
os91574	7	384	0.21	18	2	3	6.51	0.14	0.00	1.44	0.14	86.05	-2.82	6.73	8.12	16.94
os33149	7	346	0.92	2	3	9	6.77	0.02	0.10	1.48	0.01	98.54	1.78	7.16	8.85	17.59
os37028	7	315	0.61	9	3	5	6.49	-0.04	0.01	1.47	0.07	96.22	1.94	7.13	8.33	17.08
os70549	7	285	0.91	6	3	5	6.53	-0.08	1.11	1.54	0.05	88.49	-1.91	8.25	8.32	17.22
os76836	7	363	0.03	4	2	7	6.83	0.07	1.40	1.53	0.15	99.33	-2.85	6.30	7.95	16.78
os38604	7	369	0.00	11	3	5	6.97	0.19	0.02	1.48	0.07	98.51	-0.99	6.13	7.92	16.68
os33154	7	349	0.21	10	3	5	6.36	-0.17	0.08	1.48	0.06	76.82	1.14	6.68	8.56	17.48
os69296	7	227	0.92	1	3	6	6.71	0.01	0.65	1.54	0.01	40.67	0.49	8.82	9.36	18.36
os88653	7	338	0.68	5	3	5	6.54	-0.08	0.49	1.50	0.03	91.53	0.93	7.86	8.36	17.23

os02523	7	310	0.89	9	2	7	6.37	-0.10	0.64	1.54	0.14	90.78	-5.22	7.33	8.51	17.44
os77356	7	353	0.83	4	3	6	6.73	-0.03	0.48	1.53	0.04	95.78	0.76	7.70	8.04	16.78
os05154	7	316	0.02	12	3	5	6.97	0.20	0.01	1.46	0.08	94.33	0.94	6.45	8.66	17.42
os71590	7	260	0.99	10	2	7	6.33	-0.13	0.62	1.52	0.13	97.20	-5.80	7.69	8.71	17.62
os07097	7	471	0.96	11	3	5	6.35	-0.18	0.03	1.49	0.08	73.22	-0.99	6.89	7.86	16.93
os38210	7	343	0.03	11	2	3	6.83	0.14	0.56	1.51	0.09	99.75	-3.89	6.95	8.01	16.70
os76452	7	513	0.19	8	3	5	7.02	0.12	0.33	1.50	0.06	99.98	-0.62	7.39	7.33	16.19
os36869	7	269	0.81	4	3	6	6.55	-0.07	0.40	1.53	0.05	93.61	0.70	6.31	8.24	16.94
os37318	7	262	0.01	7	3	5	6.80	0.11	0.80	1.51	0.09	97.26	-2.00	6.98	8.66	17.39
os53648	7	238	0.47	7	2	3	6.55	0.00	1.02	1.54	0.12	90.49	-5.35	7.96	8.52	17.47
os91285	7	332	0.79	11	3	5	6.41	-0.06	0.06	1.49	0.11	96.22	-1.70	6.71	8.30	17.13
os02967	7	331	0.01	14	3	5	6.90	0.20	0.02	1.46	0.07	89.50	1.41	5.68	8.69	17.45
os87929	7	434	0.32	14	1	4	6.92	0.08	0.07	1.44	0.07	89.74	1.32	7.25	7.63	16.59
os03651	7	320	0.19	17	2	7	6.93	0.21	0.00	1.46	0.13	97.17	-2.78	6.31	8.46	17.20
os32983	7	213	0.88	4	3	6	6.61	-0.06	0.68	1.54	0.02	78.53	-0.02	7.71	9.30	18.03
os88360	7	367	0.98	10	1	4	6.46	-0.14	0.02	1.46	0.10	99.37	3.30	5.72	7.97	16.80
os76896	7	407	0.93	6	2	0	6.66	-0.08	0.39	1.51	0.10	99.95	0.30	7.20	7.52	16.29
os04098	7	277	0.93	7	1	4	6.54	-0.10	0.10	1.39	0.09	96.17	1.76	5.70	8.70	17.49
os42271	7	265	0.25	7	3	5	6.84	0.06	0.32	1.53	0.06	78.54	-0.60	6.72	8.95	17.77
os68600	7	244	0.99	7	3	5	6.46	-0.12	0.22	1.49	0.04	83.20	1.45	6.71	9.48	18.61
os76187	7	482	0.07	9	2	3	6.87	0.11	0.61	1.49	0.09	97.35	-2.70	6.66	7.61	16.55
os32359	7	321	0.15	12	3	9	6.67	0.11	0.00	1.44	0.07	98.32	1.63	5.38	8.45	17.23
os60946	7	314	0.23	3	3	6	6.73	0.02	3.45	1.55	0.03	27.07	0.11	8.38	8.10	16.89
os88177	7	403	0.92	4	2	3	6.67	-0.04	1.80	1.53	0.07	96.93	-3.45	8.91	7.73	16.71
os69591	7	299	0.77	2	3	6	6.73	-0.01	0.98	1.51	0.04	97.14	0.06	8.04	9.51	18.71
os84567	7	566	0.40	9	3	5	6.98	0.06	0.13	1.48	0.07	96.95	-0.68	6.01	6.64	15.47
os37146	7	288	0.02	3	2	3	6.69	0.00	2.83	1.55	0.11	91.73	-4.47	7.53	8.43	17.17
os67475	7	381	0.14	11	3	5	7.00	0.18	0.01	1.47	0.08	96.82	1.43	6.66	8.41	17.52
os02974	7	318	0.71	5	2	3	6.64	-0.01	1.32	1.55	0.07	90.21	-3.76	8.19	8.70	17.48
os36316	7	364	0.01	20	1	4	6.87	0.31	0.00	1.37	0.12	98.73	2.40	5.42	7.90	16.58
os88634	7	388	0.50	8	3	5	6.52	-0.06	0.39	1.51	0.06	88.28	-0.30	6.48	8.15	17.11
os09812	7	372	0.23	8	3	9	6.67	0.04	0.04	1.43	0.06	96.35	1.02	5.40	8.50	17.35
os74948	7	268	0.11	9	3	5	6.71	0.10	0.06	1.48	0.08	89.75	0.37	6.48	8.42	17.33
os19663	7	472	1.00	12	1	4	6.33	-0.20	0.00	1.40	0.05	97.91	5.05	5.51	8.09	16.97
os42416	7	285	0.26	6	3	5	6.66	0.02	0.66	1.53	0.04	77.91	-0.46	7.92	8.83	17.70
os37292	7	237	0.73	8	2	0	6.37	-0.11	0.14	1.51	0.11	69.87	-2.07	7.25	8.64	17.38
os02017	7	419	0.75	11	1	1	6.70	-0.05	0.00	1.36	0.05	98.62	5.60	5.20	8.42	17.21
os32959	7	187	0.92	0	3	6	6.68	0.00	6.09	1.57	0.00	9.60	0.10	11.14	9.47	18.24
os68372	7	429	0.52	9	2	3	6.80	-0.01	0.59	1.52	0.12	89.43	-4.43	8.52	8.02	17.16
os75028	7	257	0.80	9	2	3	6.42	-0.10	0.29	1.53	0.09	74.57	-3.16	7.74	8.40	17.28
os38609	7	325	0.05	16	1	4	6.73	0.21	0.00	1.39	0.08	93.97	4.38	6.19	8.12	16.81
os76623	7	399	0.92	11	3	5	6.29	-0.17	0.04	1.47	0.07	98.61	-1.43	5.95	8.01	16.84
os07020	7	372	0.62	6	2	0	6.51	-0.10	0.63	1.52	0.08	99.47	-1.17	7.40	8.26	17.21

os21654	7	416	0.23	2	3	6	6.75	0.00	2.87	1.56	0.02	37.01	-0.22	8.99	8.51	17.60
os77728	7	415	0.81	5	3	9	6.72	-0.05	0.51	1.47	0.04	99.01	0.65	6.55	7.89	16.66
os35963	7	314	0.98	12	2	3	6.36	-0.17	0.54	1.51	0.09	90.77	-3.13	7.10	8.20	16.86
os37815	7	325	0.02	7	2	7	6.70	0.08	0.92	1.54	0.14	91.53	-7.45	6.07	8.03	16.80
os32646	7	268	0.79	6	3	5	6.55	-0.06	0.72	1.52	0.04	74.38	-0.35	7.84	8.46	17.19
os90313	7	433	0.13	7	2	7	6.85	0.09	0.77	1.53	0.12	83.36	-2.49	8.00	7.24	16.13
os07249	7	398	0.79	6	3	6	6.64	-0.03	0.66	1.48	0.05	91.39	0.29	7.97	8.51	17.63
os03205	7	313	0.08	11	3	5	6.96	0.18	0.04	1.50	0.07	97.73	-0.65	6.54	8.73	17.51
os34461	7	265	0.01	4	1	4	6.79	0.05	0.03	1.51	0.02	67.32	1.82	7.68	8.28	16.96
os77318	7	244	0.92	7	3	5	6.44	-0.11	0.16	1.46	0.06	95.20	1.60	6.66	9.02	17.79
os69188	7	318	0.75	9	2	3	6.68	-0.05	0.61	1.53	0.10	99.52	-4.61	7.26	7.91	16.71
os40397	7	266	0.57	13	3	5	6.17	-0.20	0.00	1.47	0.09	89.39	1.06	5.81	8.21	16.94
os37515	7	327	0.08	2	3	9	6.80	0.03	0.47	1.49	0.02	95.65	0.67	7.77	8.14	16.85
os66797	7	266	0.75	5	2	0	6.53	-0.06	0.30	1.51	0.08	77.55	1.21	6.73	8.73	17.72
os76919	7	328	0.63	13	3	5	6.17	-0.20	0.00	1.46	0.07	97.19	0.96	6.69	8.12	16.86
os36120	7	311	0.03	4	3	6	6.79	0.05	1.42	1.50	0.03	61.57	0.73	8.39	8.36	17.05
os38447	7	301	0.08	3	1	4	6.82	0.05	0.32	1.52	0.02	77.71	2.05	8.61	8.24	16.94
os37555	7	375	0.91	9	2	7	6.29	-0.14	0.19	1.48	0.17	92.37	-2.60	6.86	7.73	16.50
os66603	7	379	0.55	5	3	9	6.90	0.08	0.81	1.47	0.03	87.25	1.29	7.16	8.18	17.17
os87942	7	369	0.14	12	3	5	6.98	0.17	0.01	1.48	0.09	95.00	-1.08	6.39	8.00	16.92
os04722	7	390	0.77	4	2	3	6.72	-0.04	1.53	1.52	0.07	96.21	-2.11	7.56	8.22	17.13
os03935	7	337	0.15	9	2	0	6.71	0.07	0.20	1.50	0.08	99.93	-0.74	7.09	8.28	17.00
os69397	7	206	0.10	2	3	6	6.71	0.02	2.23	1.56	0.01	7.62	-0.30	9.06	9.50	18.45
os66579	7	350	0.05	18	2	7	6.90	0.29	0.00	1.46	0.16	98.98	-2.62	5.19	8.04	17.01
os01519	7	370	0.42	5	3	6	6.86	0.04	0.37	1.52	0.05	83.83	-0.38	7.57	8.06	16.82
os37079	7	306	0.77	11	3	5	6.67	-0.05	0.25	1.48	0.07	91.24	-0.56	8.21	8.31	17.09
os67621	7	323	1.00	9	3	5	6.40	-0.16	0.07	1.48	0.07	96.84	0.96	6.52	8.72	17.78
os87514	7	362	0.28	3	2	0	6.83	0.02	2.79	1.54	0.05	52.96	-0.70	7.81	8.14	17.06
os69433	7	243	0.33	13	2	3	6.37	-0.01	0.02	1.48	0.10	96.83	-2.50	7.13	9.28	18.34
os91594	7	309	0.86	5	3	5	6.52	-0.08	0.48	1.50	0.04	78.53	1.30	7.27	8.44	17.29
os37668	7	273	0.99	13	3	5	6.24	-0.21	0.01	1.49	0.12	82.72	-1.69	6.52	8.42	17.06
os68786	7	444	0.65	9	3	5	6.80	-0.01	0.30	1.46	0.07	81.90	0.02	6.71	8.05	17.20
os02309	7	367	0.65	20	2	7	6.55	-0.04	0.00	1.41	0.19	90.73	-1.19	5.24	8.22	17.20
os70516	7	302	0.92	6	3	5	6.48	-0.10	0.19	1.50	0.04	92.51	0.98	6.10	8.26	17.17
os87923	7	393	0.89	13	1	4	6.17	-0.20	0.01	1.41	0.07	69.01	4.44	5.81	7.93	16.88
os03715	7	431	0.41	10	3	5	6.61	0.02	0.06	1.48	0.05	99.19	0.43	5.96	8.28	17.20
os84300	7	541	0.02	8	3	5	6.95	0.12	0.33	1.49	0.07	99.38	0.25	6.12	6.78	15.59
os32124	7	212	0.89	7	2	0	6.41	-0.10	0.39	1.52	0.09	69.35	-1.98	7.60	9.03	17.73
os71587	7	263	0.99	10	2	7	6.40	-0.11	0.70	1.52	0.12	97.54	-5.10	8.14	8.70	17.62
os68210	7	428	0.40	12	1	4	6.53	0.01	0.02	1.40	0.09	83.79	3.21	7.06	8.12	17.26
os66605	7	362	0.88	22	2	7	5.60	-0.31	0.00	1.42	0.18	95.61	-3.71	5.98	7.89	16.90
os71037	7	329	0.92	7	3	5	6.48	-0.10	0.80	1.50	0.05	90.05	-0.58	7.81	8.57	17.49
os87552	7	370	0.60	9	2	3	6.47	-0.06	0.38	1.47	0.09	97.85	-2.00	7.20	7.95	16.75

os02165	7	310	0.85	13	2	0	6.17	-0.18	0.00	1.49	0.16	83.44	-1.42	5.51	8.54	17.46
os87546	7	366	0.51	9	3	5	6.46	-0.04	0.06	1.46	0.10	97.68	-0.95	7.07	7.97	16.77
os38061	7	365	0.11	14	2	3	6.72	0.15	0.04	1.47	0.15	98.33	-4.84	5.95	7.94	16.65
os12893	7	475	0.77	11	3	5	6.77	-0.03	0.18	1.45	0.09	98.69	-0.44	7.20	7.89	16.77
os71844	7	453	0.54	3	3	6	6.76	0.01	0.29	1.50	0.02	98.23	1.49	7.06	7.85	16.68
os77365	7	318	0.06	9	3	5	6.95	0.16	0.10	1.50	0.08	99.97	-1.05	7.62	8.13	16.87
os70547	7	284	0.85	5	2	3	6.54	-0.08	1.24	1.54	0.05	88.63	-1.98	8.28	8.32	17.22
os38612	7	351	0.99	22	3	5	5.79	-0.33	0.00	1.41	0.11	83.09	1.74	6.01	8.05	16.80
os87913	7	376	0.96	8	3	5	6.55	-0.12	0.19	1.46	0.06	97.04	1.57	6.47	7.87	16.81
os38767	7	312	0.99	8	3	5	6.46	-0.13	0.45	1.51	0.06	90.45	-0.44	7.29	8.13	16.83
os70553	7	266	0.37	9	3	5	6.68	0.06	0.39	1.51	0.07	72.99	-1.11	8.07	8.41	17.31
os77112	7	298	0.27	9	1	4	6.91	0.11	0.04	1.46	0.05	99.54	3.40	6.11	8.32	17.06
os03669	7	374	0.47	6	1	4	6.61	-0.02	0.11	1.46	0.04	84.34	4.19	5.96	8.50	17.36
os77078	7	390	0.06	6	3	5	6.95	0.11	0.28	1.51	0.06	99.99	0.25	6.37	7.70	16.45
os90161	7	325	0.99	14	2	3	6.25	-0.20	0.05	1.48	0.11	78.02	-1.15	7.00	8.27	17.16
os36985	7	360	0.66	16	1	4	6.07	-0.14	0.00	1.40	0.10	94.92	2.72	5.84	8.08	16.86
os32572	7	334	0.28	4	3	9	6.87	0.06	0.49	1.48	0.03	99.36	0.76	6.40	8.40	17.16
os91579	7	405	0.50	22	1	1	5.93	-0.07	0.00	1.41	0.13	81.94	0.70	6.82	8.00	16.82
os69436	7	243	0.53	14	3	5	6.25	-0.08	0.03	1.47	0.09	96.88	-1.97	7.22	9.28	18.34
os89214	7	482	0.55	12	2	3	6.75	0.00	0.02	1.48	0.12	97.90	-3.48	5.18	7.30	16.23
os23555	15	479	0.58	8	3	5	6.57	-0.04	0.04	1.49	0.04	99.93	1.39	6.06	8.01	16.93
os64767	15	464	0.07	6	3	5	6.86	0.08	0.65	1.52	0.04	99.10	-0.76	7.33	7.09	15.96
os84143	15	468	0.99	9	2	0	6.54	-0.13	0.12	1.52	0.12	88.48	-1.30	6.99	6.89	15.68
os89799	15	636	0.81	3	3	9	6.77	-0.04	0.21	1.44	0.01	84.89	1.72	7.21	6.66	15.59
os47287	15	407	0.02	5	1	4	6.62	-0.08	0.08	1.49	0.05	95.58	2.70	7.51	7.04	15.77
os72129	15	582	0.40	12	2	7	6.51	-0.05	0.26	1.49	0.15	100.00	-3.10	6.67	6.07	14.98
os15325	15	691	0.10	11	3	5	6.86	0.10	0.05	1.46	0.11	100.00	-0.27	6.11	6.81	15.69
os29527	15	488	0.78	8	1	1	6.49	-0.10	0.05	1.44	0.04	92.57	2.27	5.82	7.26	16.13
os06559	15	447	0.69	9	3	5	6.48	-0.08	0.12	1.51	0.06	72.14	-0.61	7.13	8.03	17.05
os29509	15	556	0.05	2	3	6	6.86	0.02	2.87	1.55	0.03	64.76	-1.05	8.86	6.71	15.58
os54412	15	501	0.27	8	2	0	6.69	0.02	0.39	1.50	0.08	82.99	-0.86	9.35	6.42	15.34
os59650	15	520	0.60	3	2	7	6.67	-0.05	2.80	1.55	0.14	90.16	-2.68	7.93	6.64	15.62
os05417	15	474	0.09	5	3	6	6.82	0.05	0.55	1.51	0.04	97.16	0.05	6.94	8.10	17.10
os40308	15	294	1.00	7	2	7	6.43	-0.13	0.52	1.52	0.12	97.37	-3.71	6.93	7.93	16.60
os67685	15	480	0.03	4	3	6	6.93	0.08	0.41	1.52	0.04	84.65	0.95	7.60	7.41	16.45
os54973	15	529	0.66	7	1	1	6.79	-0.04	0.06	1.45	0.05	98.98	2.32	5.47	6.63	15.55
os77784	15	489	0.00	4	2	7	6.73	0.01	1.75	1.55	0.14	91.95	-7.07	8.67	6.94	15.84
os09755	15	446	0.99	10	2	3	6.56	-0.12	0.50	1.50	0.09	93.91	-4.14	7.43	7.99	16.92
os64810	15	408	0.83	3	3	6	6.71	-0.05	0.54	1.49	0.04	96.83	0.90	7.09	7.68	16.59
os52363	15	433	0.94	9	3	5	6.53	-0.14	0.03	1.48	0.08	97.69	1.38	6.44	6.63	15.32
os61240	15	409	0.00	5	3	5	6.87	0.08	0.53	1.51	0.03	92.56	0.25	7.50	7.33	16.18
os62342	15	450	0.92	1	3	6	6.75	-0.02	1.35	1.55	0.01	36.67	-0.04	8.41	7.32	16.17
os61773	15	422	0.60	6	2	0	6.60	-0.05	0.36	1.53	0.09	76.09	-0.60	7.07	7.17	16.03

os55580	15	501	0.01	6	2	7	6.85	0.06	1.59	1.54	0.13	98.68	-5.15	8.55	6.27	15.08
os72792	15	295	0.23	8	2	3	6.76	0.08	0.79	1.51	0.08	92.46	-2.85	8.21	8.27	17.27
os27794	15	496	0.82	8	1	4	6.52	-0.08	0.07	1.45	0.05	78.90	3.42	7.23	7.08	15.94
os73690	15	531	0.43	14	1	2	6.47	-0.02	0.00	1.43	0.13	97.89	1.84	6.83	6.55	15.22
os53328	15	385	0.09	4	1	1	6.88	0.06	0.06	1.44	0.04	95.84	3.92	5.98	7.53	16.37
os77456	15	299	0.15	8	1	1	6.92	0.10	0.07	1.37	0.05	99.77	2.80	5.96	8.76	17.50
os75549	15	408	0.98	11	2	7	6.31	-0.19	0.06	1.52	0.13	89.15	-3.79	6.78	7.14	16.05
os62753	15	396	0.03	12	2	0	7.00	0.19	0.02	1.50	0.13	81.69	-1.91	6.77	7.36	16.23
os23379	15	429	0.11	10	3	5	6.74	0.10	0.11	1.49	0.10	92.72	-2.13	6.89	8.31	17.28
os61748	15	408	0.70	14	2	3	6.65	-0.06	0.00	1.46	0.10	85.90	0.03	6.73	7.39	16.23
os76399	15	419	0.64	9	1	4	6.47	-0.07	0.02	1.41	0.07	99.59	4.21	5.80	7.67	16.48
os77708	15	360	0.82	7	1	1	6.50	-0.10	0.19	1.46	0.06	99.88	2.97	6.38	8.01	16.74
os87095	15	414	0.90	9	3	5	6.41	-0.14	0.10	1.47	0.05	96.98	-0.09	6.70	7.84	16.70
os37593	15	340	0.68	16	3	5	6.10	-0.23	0.00	1.44	0.11	94.54	-1.42	6.86	7.95	16.71
os53022	15	442	0.01	15	1	4	6.89	0.23	0.00	1.42	0.11	98.15	2.93	5.27	6.88	15.70
os45760	15	376	0.87	28	1	4	5.99	-0.24	0.00	1.34	0.16	85.37	4.50	5.06	7.31	16.03
os46734	15	521	0.08	7	1	1	6.89	0.10	0.25	1.46	0.04	95.76	2.90	6.23	6.80	15.50
os51693	15	547	0.86	15	3	5	6.48	-0.16	0.00	1.44	0.13	99.98	-0.80	5.76	6.13	14.89
os77036	15	503	0.99	8	3	5	6.57	-0.13	0.24	1.50	0.10	99.90	-0.29	5.82	7.29	16.14
os03627	15	435	0.99	8	3	5	6.51	-0.12	0.29	1.51	0.05	90.97	-0.28	6.66	8.00	16.89
os23740	15	528	0.53	8	1	4	6.61	-0.04	0.02	1.38	0.04	98.58	7.06	5.87	7.74	16.73
os37248	15	300	0.28	22	3	9	6.26	0.11	0.00	1.37	0.15	98.65	-2.89	5.33	8.50	17.25
os47255	15	398	0.57	13	1	4	6.36	-0.05	0.02	1.42	0.09	99.16	1.99	5.97	7.21	15.91
os72126	15	472	0.90	13	3	5	6.22	-0.18	0.00	1.48	0.11	90.01	-0.34	7.30	6.85	15.73
os73129	15	423	0.76	13	3	5	6.41	-0.09	0.32	1.45	0.07	94.50	-0.35	7.89	7.49	16.34
os89016	15	482	0.96	7	2	3	6.62	-0.09	0.89	1.50	0.13	98.41	-5.31	8.17	7.16	16.12
os47091	15	489	0.78	4	3	6	6.66	-0.07	0.62	1.53	0.04	68.46	-0.11	8.10	6.65	15.33
os52515	15	421	0.40	4	3	6	6.85	0.02	0.58	1.54	0.00	91.60	0.13	6.99	6.81	15.57
os66076	15	450	0.51	13	1	2	6.39	-0.06	0.00	1.43	0.12	90.66	3.15	5.33	7.53	16.62
os77303	15	555	0.35	9	3	5	6.97	0.06	0.30	1.47	0.08	99.53	-1.12	6.83	7.10	16.00
os17131	15	641	1.00	9	1	1	6.53	-0.15	0.02	1.43	0.07	98.97	3.20	5.62	6.25	15.14
os63611	15	476	0.61	6	3	6	6.71	0.00	1.01	1.48	0.08	98.36	-0.83	7.89	7.30	16.19
os04499	15	523	0.35	10	3	5	6.66	0.03	0.08	1.50	0.08	84.33	-0.81	6.02	7.31	16.29
os10029	15	476	0.65	8	2	3	6.77	-0.04	0.49	1.52	0.08	85.03	-2.20	6.89	7.03	15.76
os47278	15	364	0.53	14	2	0	6.34	-0.06	0.01	1.46	0.09	91.70	1.21	5.75	7.24	15.95
os52959	15	476	0.86	9	3	5	6.44	-0.13	0.12	1.45	0.08	99.39	0.16	6.07	6.82	15.66
os53437	15	305	0.63	4	2	3	6.80	0.02	2.11	1.55	0.08	90.29	-4.33	8.67	7.92	16.75
os52874	15	379	1.00	16	1	2	6.07	-0.26	0.00	1.43	0.15	96.37	2.77	5.18	7.06	15.86
os59798	15	540	0.04	11	2	7	7.06	0.18	0.04	1.49	0.11	91.36	-2.07	7.80	6.50	15.44
os53552	15	501	0.47	18	2	0	6.83	0.07	0.00	1.43	0.16	95.80	-0.17	5.74	6.38	15.19
os73555	15	479	0.33	9	1	1	6.97	0.08	0.03	1.47	0.06	99.43	2.50	6.07	7.32	15.98
os28146	15	551	0.09	3	3	9	6.88	0.04	0.32	1.52	0.02	96.53	0.46	7.02	7.07	15.96
os29224	15	434	0.12	5	2	3	6.86	0.03	1.45	1.54	0.06	97.99	-2.15	8.40	8.11	17.13

os46885	15	416	0.50	14	2	3	6.42	0.00	0.00	1.45	0.10	97.81	0.93	6.46	7.03	15.74
os77571	15	389	0.73	10	3	9	6.73	-0.04	0.05	1.47	0.06	99.46	0.13	5.84	7.81	16.53
os67444	15	431	0.35	4	3	6	6.69	-0.05	2.02	1.54	0.05	74.41	-2.20	8.59	8.06	17.21
os28335	15	499	0.25	5	2	3	6.84	0.00	1.21	1.54	0.08	92.22	-3.96	9.18	7.20	16.08
os65731	15	494	0.92	3	3	6	6.76	-0.04	1.28	1.53	0.03	92.12	-0.17	7.39	7.64	16.84
os82691	15	512	0.92	1	3	6	6.83	0.01	0.26	1.53	0.01	75.65	1.03	8.84	7.00	15.79
os25184	15	502	0.98	5	3	5	6.69	-0.08	0.35	1.47	0.03	98.96	1.18	6.54	7.94	16.98
os91483	15	428	0.05	5	2	0	6.88	0.08	0.31	1.52	0.06	98.00	-0.09	7.55	7.69	16.58
os05705	15	467	0.95	6	3	5	6.70	-0.07	0.69	1.52	0.05	95.99	-1.25	7.30	8.15	17.26
os49079	15	505	0.01	18	2	3	6.81	0.28	0.00	1.47	0.16	98.91	-3.00	4.97	6.32	14.99
os71427	15	394	0.88	4	3	6	6.62	-0.06	0.58	1.52	0.05	99.57	0.16	6.53	7.94	16.78
os65631	15	493	1.00	8	1	4	6.51	-0.14	0.11	1.50	0.05	89.89	1.99	6.90	7.52	16.67
os62293	15	493	0.83	7	1	4	6.54	-0.11	0.18	1.46	0.04	95.67	3.47	7.05	6.93	15.82
os61876	15	489	0.84	16	2	7	6.43	-0.13	0.00	1.47	0.19	100.00	-2.84	5.17	6.62	15.57
os91573	15	427	0.63	6	3	5	6.81	-0.01	0.06	1.48	0.06	98.61	1.71	6.24	7.75	16.57
os08373	15	462	0.92	3	3	6	6.69	-0.05	0.43	1.53	0.04	97.39	0.15	8.72	8.13	17.18
os27309	15	449	0.92	12	1	4	6.24	-0.19	0.00	1.45	0.08	79.96	2.16	6.69	7.20	15.99
os66623	15	422	0.14	5	3	9	6.83	0.06	0.90	1.48	0.03	92.30	-0.27	7.63	8.03	17.03
os89376	15	592	0.92	9	3	5	6.68	-0.10	0.07	1.47	0.08	96.97	-0.09	7.09	6.48	15.26
os61808	15	460	0.54	14	3	5	6.82	0.02	0.00	1.45	0.09	99.95	1.92	5.99	7.16	16.04
os61881	15	398	0.37	3	3	6	6.84	0.02	1.15	1.54	0.03	86.29	0.03	8.29	7.43	16.31
os72720	15	259	0.92	1	2	3	6.73	0.00	3.50	1.56	0.06	80.55	-2.35	8.26	8.29	17.27
os89933	15	474	0.69	11	2	0	6.76	-0.03	0.03	1.50	0.12	68.44	-2.01	6.28	7.08	15.92
os14178	15	531	0.99	7	2	3	6.69	-0.08	1.91	1.54	0.10	88.43	-3.62	8.48	7.44	16.27
os71340	15	330	0.73	10	3	5	6.36	-0.09	0.02	1.48	0.07	93.87	1.27	6.47	8.09	16.92
os48004	15	382	0.13	12	1	1	6.74	0.12	0.01	1.43	0.05	98.93	3.00	5.64	7.66	16.36
os53561	15	589	0.53	6	1	1	6.85	-0.02	0.01	1.41	0.01	99.41	6.25	5.83	6.36	15.18
os65988	15	517	0.01	11	3	5	6.95	0.17	0.01	1.48	0.06	73.23	1.86	6.67	7.09	16.05
os90880	15	357	0.76	8	3	5	6.66	-0.06	0.74	1.52	0.07	97.09	-2.23	7.38	8.19	17.08
os73133	15	373	0.13	10	3	5	6.77	0.12	0.04	1.51	0.07	52.49	-0.81	7.17	7.74	16.59
os47128	15	521	0.71	18	3	9	6.04	-0.16	0.00	1.40	0.11	95.11	1.78	5.03	6.57	15.26
os61618	15	351	0.51	13	3	5	6.78	0.04	0.07	1.51	0.11	49.56	-1.71	6.98	7.60	16.37
os90835	15	445	0.77	4	2	0	6.75	-0.04	0.51	1.53	0.07	93.87	0.79	7.47	7.33	16.23
os01964	15	384	0.87	7	2	3	6.50	-0.10	0.86	1.51	0.06	98.12	-1.87	8.20	8.51	17.44
os61562	15	409	0.89	21	2	3	5.64	-0.30	0.00	1.46	0.17	87.68	-3.99	4.84	7.26	16.13
os64764	15	469	0.96	11	2	0	6.46	-0.12	0.11	1.49	0.11	100.00	-0.22	5.58	6.98	15.88
os07117	15	410	0.99	3	3	6	6.69	-0.05	1.18	1.53	0.02	83.36	0.43	7.46	8.22	17.25
os76704	15	301	0.68	11	1	2	6.60	-0.07	0.00	1.43	0.12	97.22	4.06	5.83	8.16	16.93
os23692	15	532	0.40	12	2	7	6.57	0.01	0.03	1.50	0.12	81.29	-3.10	6.15	7.58	16.58
os67946	15	429	0.97	16	1	4	6.02	-0.27	0.00	1.39	0.09	89.98	3.62	5.95	7.59	16.51
os60029	15	371	0.73	8	3	5	6.74	-0.03	0.14	1.49	0.06	96.84	0.63	6.04	7.68	16.61
os72378	15	237	0.00	6	2	3	6.50	-0.08	1.39	1.54	0.09	87.66	-2.65	8.19	8.36	17.31
os02591	15	398	0.92	8	3	5	6.46	-0.13	0.36	1.51	0.07	96.79	-1.37	6.78	8.28	17.23

os47185	15	386	0.24	22	3	5	6.80	0.23	0.00	1.43	0.17	94.26	-2.68	5.67	7.18	15.87
os76139	15	430	0.43	8	3	5	6.85	0.02	0.12	1.46	0.07	99.79	1.36	6.03	7.61	16.42
os06355	15	527	0.31	13	3	5	7.01	0.13	0.01	1.48	0.10	99.06	-0.43	7.24	7.64	16.66
os29095	15	475	0.30	6	2	3	6.77	0.04	1.17	1.52	0.08	98.15	-2.47	8.14	7.87	16.85
os77490	15	350	0.83	11	2	0	6.25	-0.18	0.02	1.47	0.10	99.13	-0.06	6.43	7.94	16.71
os72267	15	377	0.92	3	3	6	6.68	-0.04	1.27	1.55	0.05	78.53	0.17	7.02	7.61	16.57
os40848	15	371	0.29	10	2	7	6.94	0.11	0.06	1.50	0.16	93.89	-3.67	6.67	7.41	16.05
os49444	15	450	0.38	15	2	7	6.36	-0.01	0.03	1.51	0.00	98.03	-4.90	7.41	6.47	15.11
os09728	15	395	0.87	16	2	0	6.13	-0.19	0.00	1.48	0.13	85.15	-1.08	7.06	8.00	16.91
os36348	15	291	0.99	9	2	0	6.43	-0.14	0.04	1.51	0.11	86.51	-0.90	7.51	8.26	16.93
os42892	15	322	0.51	9	2	3	6.42	-0.11	0.74	1.53	0.10	95.71	-4.32	7.56	7.70	16.34
os64812	15	422	1.00	10	3	5	6.40	-0.17	0.13	1.47	0.08	99.27	0.13	6.06	7.15	15.98
os28075	15	481	0.46	15	1	4	6.39	0.00	0.00	1.43	0.08	80.66	2.24	5.66	7.41	16.31
os66048	15	461	0.78	10	3	5	6.35	-0.14	0.05	1.45	0.08	88.81	2.38	5.71	7.58	16.65
os26156	15	614	0.73	7	1	4	6.59	-0.12	0.09	1.49	0.03	86.02	1.84	6.26	7.28	16.29
os73522	15	512	1.00	27	3	9	5.45	-0.40	0.00	1.37	0.16	99.30	0.07	6.00	7.26	15.95
os29007	15	532	0.55	9	2	3	6.84	-0.01	0.66	1.50	0.08	99.61	-3.17	7.01	7.38	16.32
os87094	15	414	0.86	9	3	5	6.38	-0.14	0.07	1.47	0.06	96.86	-0.14	6.54	7.84	16.70
os10252	15	578	0.99	11	1	1	6.45	-0.18	0.00	1.44	0.08	99.47	2.30	5.25	6.65	15.53
os47087	15	500	0.07	9	3	5	6.87	0.12	0.15	1.49	0.07	94.84	-0.85	7.23	6.53	15.24
os28772	15	408	1.00	11	2	0	6.39	-0.18	0.14	1.49	0.10	91.18	-2.13	8.26	8.17	17.18
os67155	15	389	0.77	3	3	9	6.74	-0.03	0.57	1.49	0.02	94.85	0.58	6.80	8.59	17.69
os07308	15	476	0.93	6	3	9	6.70	-0.07	0.22	1.49	0.03	94.88	1.81	5.98	7.99	17.01
os10183	15	544	0.96	17	1	4	6.27	-0.23	0.00	1.44	0.09	94.33	2.41	5.59	6.54	15.40
os64137	15	579	0.86	17	2	3	6.44	-0.17	0.02	1.45	0.15	99.06	-3.46	5.89	6.67	15.55
os85147	15	477	0.75	2	2	0	6.79	-0.02	1.69	1.55	0.10	91.43	-1.76	7.80	6.90	15.62
os63774	15	429	0.54	12	1	2	6.79	0.02	0.00	1.43	0.11	96.27	1.87	4.97	7.42	16.28
os75719	15	567	0.01	3	1	1	6.90	0.05	0.13	1.42	0.03	99.93	3.08	6.52	6.80	15.74
os59780	15	535	0.35	14	2	7	6.23	-0.21	0.13	1.49	0.13	92.82	-3.54	7.35	6.51	15.46
os77117	15	366	0.23	9	3	5	6.69	0.07	0.20	1.44	0.11	99.28	-0.60	6.85	8.02	16.78
os49886	15	504	0.99	14	2	0	6.17	-0.24	0.00	1.44	0.12	91.54	0.34	5.80	6.50	15.26
os70965	15	528	0.92	18	2	7	5.88	-0.28	0.00	1.42	0.21	99.72	-3.45	5.40	6.69	15.63
os06337	15	570	0.99	10	3	5	6.43	-0.17	0.03	1.47	0.06	99.98	1.22	5.73	7.57	16.57
os83927	15	488	0.01	9	3	5	7.00	0.15	0.21	1.50	0.06	97.20	-0.94	6.88	7.33	16.21
os89371	15	602	0.68	4	3	6	6.86	-0.02	0.64	1.49	0.04	85.62	0.94	7.89	6.42	15.20
os71433	15	391	0.01	4	3	6	6.61	-0.07	0.44	1.51	0.05	99.00	0.79	6.55	7.95	16.79
os50224	15	541	0.01	6	3	5	6.92	0.09	0.76	1.51	0.04	96.44	-1.36	6.86	6.66	15.38
os06662	15	578	0.03	9	1	4	6.94	0.12	0.04	1.47	0.04	97.08	2.90	5.81	7.48	16.51
os70943	15	544	0.11	7	3	5	7.01	0.12	0.46	1.51	0.11	98.42	-2.64	7.02	6.65	15.59
os51527	15	475	0.89	13	3	5	6.24	-0.19	0.00	1.44	0.10	100.00	0.77	5.82	6.76	15.60
os28363	15	482	0.92	6	3	5	6.76	-0.04	0.96	1.52	0.05	98.45	-1.61	7.69	7.38	16.26
os00550	15	553	0.16	14	3	5	7.07	0.20	0.00	1.48	0.09	95.78	-0.37	6.08	6.83	15.71
os07061	15	555	0.02	9	1	1	6.96	0.13	0.02	1.45	0.05	100.00	3.78	5.72	7.66	16.66

os53115	15	435	0.71	19	2	7	6.59	-0.04	0.00	1.44	0.17	99.97	-1.67	6.15	6.55	15.26
os71420	15	626	0.32	21	2	0	6.91	0.16	0.00	1.39	0.00	100.00	2.03	5.32	6.29	15.19
os01665	15	406	0.89	9	1	4	6.60	-0.10	0.03	1.45	0.08	96.01	2.50	5.91	8.24	17.14
os90134	15	427	0.34	5	2	7	6.88	0.06	1.42	1.54	0.10	91.74	-2.37	7.62	7.43	16.32
os03972	15	374	0.92	1	3	6	6.72	-0.02	1.35	1.55	0.02	55.23	-0.08	8.09	8.33	17.09
os55989	15	342	0.01	8	3	5	6.86	0.13	0.31	1.50	0.09	94.02	-0.57	6.12	7.63	16.49
os62506	15	409	0.99	7	2	3	6.53	-0.12	0.47	1.52	0.12	96.22	-3.03	6.27	7.26	16.09
os25373	15	465	0.40	7	2	3	6.53	-0.11	0.80	1.51	0.07	99.22	-2.78	7.83	8.06	17.09
os50612	15	409	0.69	15	2	0	6.14	-0.12	0.03	1.42	0.09	89.71	3.24	5.96	7.23	15.97
os11118	15	522	0.99	7	2	0	6.57	-0.13	0.29	1.52	0.06	85.25	-0.01	6.49	6.60	15.32
os72957	15	232	0.04	7	2	3	6.86	0.11	1.06	1.54	0.09	82.36	-3.34	7.47	8.41	17.35
os77471	15	257	0.98	20	1	4	5.88	-0.30	0.00	1.38	0.13	98.91	4.11	5.12	8.58	17.32
os86480	15	593	0.92	1	3	6	6.88	0.01	1.08	1.54	0.02	60.25	0.05	8.46	6.82	15.72
os11265	15	555	0.30	7	3	5	6.75	0.02	0.35	1.50	0.07	99.86	-0.19	6.59	6.60	15.40
os89997	15	552	0.98	21	3	5	5.95	-0.31	0.00	1.37	0.13	98.99	2.65	5.14	6.79	15.61
os24340	15	522	0.47	2	3	6	6.85	0.00	1.24	1.55	0.02	61.88	0.17	7.96	7.59	16.65
os47186	15	368	0.06	14	2	3	6.76	0.18	0.02	1.49	0.13	76.27	-3.73	5.83	7.21	15.91
os71280	15	309	0.91	7	2	3	6.49	-0.10	0.81	1.50	0.08	99.00	-2.71	7.31	8.46	17.30
os62940	15	360	0.04	13	2	3	6.80	0.20	0.00	1.48	0.18	91.96	-4.36	5.96	7.74	16.54
os19100	15	573	0.13	12	2	8	7.04	0.18	0.01	1.45	0.15	100.00	-3.59	5.60	6.50	15.40
os48319	15	462	0.34	18	3	5	6.30	0.07	0.00	1.43	0.14	96.62	-1.95	4.98	6.98	15.70
os70911	15	489	0.19	4	1	1	6.92	0.05	0.04	1.44	0.02	98.45	3.16	6.08	7.35	16.29
os77458	15	283	0.67	18	1	4	5.85	-0.28	0.00	1.35	0.13	97.24	9.41	4.80	8.35	17.06
os38332	15	385	0.27	12	2	3	6.85	0.07	0.39	1.43	0.12	98.50	-3.56	5.86	7.82	16.50
os46876	15	483	0.11	12	1	4	6.82	0.15	0.00	1.42	0.08	99.02	3.01	5.96	6.80	15.53
os09060	15	558	0.74	8	3	5	6.60	-0.10	0.14	1.48	0.04	93.23	1.55	6.35	7.02	15.91
os48312	15	412	0.08	20	3	5	6.94	0.30	0.00	1.44	0.15	84.49	-1.25	6.03	7.10	15.82
os27804	15	474	0.33	15	3	5	6.49	0.03	0.00	1.45	0.08	89.63	1.19	6.66	7.19	16.03
os77259	15	573	1.00	16	3	9	6.22	-0.25	0.00	1.41	0.09	100.00	-0.61	5.57	7.18	16.15
os74239	15	488	0.46	8	2	3	6.88	0.03	0.71	1.51	0.08	98.59	-1.73	6.94	6.94	15.79
os05468	15	512	0.99	8	3	5	6.60	-0.12	0.26	1.49	0.04	91.69	1.93	7.07	7.82	16.82
os48447	15	430	0.99	7	2	3	6.59	-0.10	0.89	1.54	0.09	95.40	-3.91	7.02	6.84	15.53
os23569	15	511	0.08	11	2	0	6.85	0.15	0.02	1.47	0.12	100.00	-1.69	6.32	7.68	16.66
os53632	15	316	0.29	21	3	5	6.25	0.08	0.00	1.42	0.12	99.65	-0.58	5.81	7.87	16.77
os12713	15	522	0.65	14	1	4	6.15	-0.23	0.00	1.38	0.07	90.40	5.37	5.42	7.62	16.46
os64093	15	403	0.07	11	3	5	7.01	0.18	0.01	1.48	0.09	98.21	0.01	5.81	7.73	16.63
os11642	15	499	0.03	16	3	5	6.89	0.23	0.00	1.45	0.09	96.45	1.05	5.22	6.87	15.80
os64533	15	437	0.79	15	3	5	6.50	-0.12	0.00	1.44	0.12	97.38	-1.79	6.44	7.14	16.02
os17265	15	612	0.97	10	3	5	6.53	-0.16	0.16	1.46	0.08	99.41	-1.04	6.69	6.46	15.32
os78865	15	439	0.60	3	3	6	6.70	-0.02	0.81	1.53	0.04	100.00	-0.24	6.95	7.56	16.48
os61351	15	479	0.57	10	3	5	6.54	-0.03	0.29	1.45	0.14	99.80	0.16	6.68	7.06	15.95
os79458	15	441	0.37	9	2	0	6.66	0.03	0.04	1.50	0.08	84.75	0.24	6.68	7.21	16.00
os76437	15	412	0.92	3	1	4	6.67	-0.04	0.01	1.48	0.03	98.98	4.48	6.52	7.53	16.26

os03680	15	476	0.08	2	3	6	6.86	0.03	0.80	1.54	0.02	43.21	0.13	8.11	7.96	16.88
os05532	15	456	0.60	15	3	5	6.27	-0.06	0.00	1.48	0.10	96.96	-1.30	5.55	8.37	17.46
os49506	15	402	0.19	9	2	7	6.76	0.08	0.86	1.54	0.00	97.17	-4.12	8.03	6.81	15.49
os61353	15	460	0.99	15	3	5	6.17	-0.24	0.00	1.46	0.12	99.79	-0.73	6.44	7.12	16.00
os74310	15	463	0.99	4	2	0	6.68	-0.07	0.51	1.53	0.11	85.55	0.77	6.98	6.73	15.56
os02609	15	426	0.98	12	3	5	6.39	-0.18	0.02	1.49	0.11	89.97	-1.16	5.58	8.14	17.11
os26230	15	580	0.03	13	3	9	7.09	0.20	0.00	1.42	0.06	99.75	2.65	5.67	7.64	16.62
os83724	15	491	0.92	3	3	6	6.70	-0.05	1.19	1.54	0.03	98.71	0.10	7.74	7.33	16.26
os28288	15	444	1.00	5	3	6	6.65	-0.08	0.74	1.53	0.04	92.07	-1.24	7.01	7.70	16.60
os59796	15	541	0.76	11	2	0	6.81	-0.03	0.05	1.48	0.09	97.34	-0.49	7.33	6.56	15.51
os76254	15	317	0.23	9	3	5	6.66	0.06	0.13	1.49	0.07	100.00	0.46	6.59	8.04	16.82
os28308	15	512	0.00	6	3	5	6.98	0.11	0.34	1.51	0.03	96.83	1.19	6.79	7.23	16.12
os62388	15	579	0.12	14	2	3	6.73	0.11	0.02	1.48	0.13	94.11	-5.04	7.37	6.61	15.48
os22116	15	534	0.31	3	3	6	6.71	-0.06	0.44	1.53	0.05	98.93	0.57	8.02	7.67	16.73
os57878	15	459	0.99	12	3	5	6.31	-0.20	0.02	1.43	0.09	94.82	-0.97	5.37	7.41	16.12
os09598	15	375	0.30	6	3	5	6.70	0.02	0.76	1.53	0.06	89.84	-0.99	8.08	8.00	16.81
os12032	15	574	0.52	11	3	5	6.90	0.03	0.01	1.48	0.10	97.71	0.56	7.55	7.22	16.05
os53740	15	313	0.03	5	3	5	6.79	0.07	0.60	1.50	0.03	95.42	1.18	6.99	8.15	17.10
os62759	15	458	0.05	13	3	5	6.89	0.18	0.00	1.45	0.10	97.55	-0.94	5.99	7.24	16.11
os73821	15	471	0.79	20	2	3	5.83	-0.27	0.00	1.46	0.16	96.97	-3.54	5.94	7.23	15.91
os57793	15	584	0.21	9	3	5	6.79	0.07	0.12	1.48	0.08	92.07	0.77	7.29	6.43	15.16
os85035	15	508	0.66	7	1	4	6.82	-0.02	0.10	1.47	0.04	90.28	2.76	6.71	6.93	15.71
os46349	15	367	0.05	20	3	5	6.97	0.30	0.00	1.39	0.14	91.73	1.28	5.71	7.21	15.90
os68212	15	396	0.27	3	3	6	6.76	0.01	1.91	1.56	0.03	71.27	-1.06	7.66	8.29	17.46
os14619	15	583	0.04	8	1	4	6.52	-0.14	0.12	1.47	0.06	96.50	2.05	6.11	7.41	16.41
os48240	15	349	0.44	13	1	4	6.46	0.03	0.00	1.44	0.09	89.83	3.21	5.46	7.67	16.38
os52177	15	461	0.00	11	2	3	6.95	0.16	0.25	1.49	0.12	99.46	-3.56	7.33	6.79	15.62
os68103	15	462	0.07	2	3	6	6.74	-0.04	3.56	1.56	0.04	50.98	-1.47	9.24	7.56	16.66
os39703	15	388	0.56	21	1	4	5.78	-0.17	0.00	1.23	0.08	96.00	16.45	4.66	7.46	16.11
os64243	15	266	0.07	6	2	7	6.61	0.07	0.86	1.54	0.20	79.37	-3.65	7.20	7.88	16.74
os72964	15	244	0.04	6	3	5	6.79	0.09	0.38	1.52	0.05	79.82	-0.58	7.44	8.37	17.34
os62754	15	399	0.08	11	2	0	7.01	0.18	0.01	1.49	0.12	81.33	-1.11	6.43	7.35	16.22
os62786	15	367	0.80	16	3	5	6.01	-0.21	0.00	1.47	0.10	99.08	-1.85	5.43	7.84	16.68
os70967	15	550	0.89	24	3	5	5.49	-0.35	0.00	1.36	0.19	99.83	0.44	5.35	6.80	15.74
os79443	15	468	0.99	4	3	6	6.70	-0.06	1.84	1.53	0.05	96.50	-1.75	8.05	7.19	16.09
os62529	15	527	0.92	1	3	9	6.84	0.00	0.40	1.47	0.01	88.60	1.71	7.64	6.87	15.75
os77404	15	469	0.24	12	2	0	6.71	0.08	0.12	1.47	0.10	97.99	-1.79	6.44	7.23	16.01
os50557	15	377	0.99	11	2	3	6.33	-0.18	0.44	1.50	0.15	98.03	-5.47	7.58	7.45	16.17
os68146	15	471	1.00	10	3	5	6.51	-0.14	0.08	1.52	0.08	79.22	-1.01	8.13	7.54	16.62
os65046	15	269	0.81	11	3	5	6.31	-0.13	0.42	1.50	0.08	92.31	-2.17	6.91	8.05	16.90
os65729	15	508	0.01	7	3	5	6.95	0.11	0.37	1.52	0.03	90.88	0.10	6.49	7.61	16.82
os64934	15	269	0.00	7	3	5	6.88	0.12	0.23	1.50	0.05	85.06	0.22	6.69	8.02	16.84
os03526	15	320	0.92	12	2	0	6.26	-0.18	0.02	1.49	0.12	95.39	-1.53	6.76	8.33	17.16

os62593	15	473	0.98	12	3	5	6.46	-0.16	0.01	1.47	0.11	99.95	1.02	5.50	7.06	15.94
os36306	15	310	0.98	17	3	5	6.02	-0.27	0.00	1.41	0.11	98.47	1.27	5.68	8.24	16.89
os45833	15	490	0.11	2	3	6	6.87	0.02	0.07	1.52	0.02	97.37	1.91	8.15	6.79	15.54
os65179	15	318	0.75	11	3	5	6.63	-0.07	0.29	1.50	0.09	78.22	-1.60	8.04	7.46	16.27
os78818	15	442	0.58	5	3	6	6.65	-0.02	0.64	1.53	0.06	95.98	-1.12	6.69	7.31	16.26
os17525	15	587	0.57	17	3	9	6.78	0.00	0.00	1.44	0.14	100.00	-1.71	6.00	6.56	15.44
os40416	15	337	1.00	11	1	4	6.31	-0.17	0.01	1.42	0.08	95.04	2.88	6.27	7.89	16.59
os63953	15	441	0.98	12	2	3	6.39	-0.16	0.63	1.53	0.15	86.52	-7.16	6.85	7.33	16.22
os71343	15	327	0.65	11	2	0	6.36	-0.09	0.04	1.50	0.09	94.43	-0.29	6.46	8.07	16.90
os64614	15	507	1.00	13	3	5	6.28	-0.22	0.00	1.44	0.10	99.38	1.05	5.55	6.61	15.39
os36318	15	360	0.88	12	3	5	6.28	-0.19	0.10	1.47	0.09	97.41	-1.00	6.13	7.97	16.66
os45286	15	446	0.39	21	3	9	6.12	0.00	0.00	1.40	0.13	98.88	-1.20	5.24	7.19	15.94
os47989	15	409	0.01	14	1	4	6.95	0.24	0.00	1.41	0.09	92.09	5.53	5.63	7.20	15.92
os55023	15	476	0.91	12	2	3	6.48	-0.15	0.03	1.50	0.11	96.56	-4.08	7.51	6.86	15.78
os64106	15	396	0.02	7	3	5	6.94	0.11	0.41	1.50	0.05	96.16	-0.24	7.33	7.77	16.68
os67446	15	462	0.99	4	3	6	6.69	-0.06	0.54	1.53	0.02	76.60	0.20	7.44	7.94	17.12
os89036	15	482	0.01	8	2	0	6.92	0.13	0.22	1.51	0.09	99.78	-1.54	6.06	7.01	15.90
os90644	15	416	0.00	6	2	3	6.92	0.11	1.36	1.52	0.09	94.63	-3.57	7.96	7.75	16.65
os30458	15	573	0.72	10	3	5	6.82	-0.03	0.06	1.47	0.09	99.99	-1.27	7.08	6.83	15.77
os54997	15	349	0.92	7	2	3	6.49	-0.12	0.93	1.52	0.09	99.89	-3.51	8.01	7.59	16.54
os67821	15	501	0.59	4	3	6	6.77	0.00	0.41	1.47	0.03	89.18	2.14	6.92	7.20	16.13
os45288	15	505	0.04	16	1	1	6.90	0.22	0.00	1.41	0.07	99.47	2.96	5.47	7.02	15.76
os76382	15	520	0.41	2	3	6	6.84	0.00	0.26	1.52	0.04	80.05	0.69	9.08	7.00	15.81
os11271	15	552	0.77	9	3	9	6.75	-0.06	0.31	1.50	0.05	99.12	-0.53	6.45	6.78	15.60
os39497	15	334	0.23	1	3	6	6.73	0.00	2.78	1.55	0.04	75.71	-0.24	8.51	7.70	16.39
os50217	15	496	0.58	16	2	3	6.31	-0.06	0.00	1.47	0.12	99.81	-3.32	5.99	6.76	15.49
os73759	15	591	0.70	11	3	5	6.45	-0.11	0.05	1.48	0.08	99.90	-0.42	6.26	6.44	15.07
os24021	15	518	0.40	5	3	6	6.92	0.04	1.89	1.55	0.05	73.77	-1.30	8.24	7.20	16.24
os76212	15	286	0.41	8	3	5	6.55	-0.02	0.14	1.46	0.06	99.97	0.80	6.10	8.49	17.26
os23192	15	449	0.04	7	3	5	6.85	0.09	0.36	1.52	0.04	91.30	0.45	6.91	8.04	16.94
os83311	15	492	0.15	5	3	6	6.86	0.06	0.39	1.53	0.04	58.63	-0.15	7.92	6.98	15.69
os24452	15	470	0.77	1	3	6	6.78	-0.02	4.14	1.55	0.04	70.42	-0.15	8.74	7.74	16.78
os47920	15	423	0.63	14	1	4	6.73	-0.01	0.00	1.41	0.09	78.99	5.28	5.11	7.16	15.87
os40791	15	325	0.07	2	3	6	6.82	0.04	0.43	1.53	0.04	74.46	1.07	7.90	7.77	16.43
os67537	15	420	0.58	6	3	5	6.78	-0.02	0.72	1.51	0.05	88.17	0.21	8.14	8.01	17.05
os50607	15	475	0.01	3	3	6	6.88	0.04	0.87	1.53	0.03	94.75	0.12	7.67	6.92	15.69
os23358	15	513	0.24	12	3	5	7.04	0.14	0.02	1.48	0.08	95.92	-0.61	6.65	7.82	16.88
os54688	15	399	0.46	2	3	6	6.72	-0.03	0.41	1.50	0.01	93.58	0.55	7.64	7.46	16.35
os48876	15	399	1.00	20	2	3	5.73	-0.32	0.00	1.45	0.19	98.80	-5.92	5.51	7.16	15.86
os79631	15	438	0.79	3	3	6	6.73	-0.04	0.19	1.54	0.03	83.71	0.71	8.62	7.28	16.20
os55164	15	395	0.11	15	2	3	6.99	0.23	0.00	1.43	0.13	97.49	-2.45	6.03	7.34	16.28
os47273	15	425	0.92	9	3	9	6.42	-0.13	0.13	1.47	0.06	99.41	-0.42	6.45	7.12	15.82
os48269	15	344	0.01	7	3	5	6.87	0.11	0.28	1.52	0.10	97.28	-0.74	6.19	7.59	16.34

os68665	15	456	0.98	4	2	3	6.72	-0.05	1.44	1.52	0.05	73.68	-2.09	8.91	7.99	17.16
os47205	15	357	0.17	10	3	5	6.97	0.16	0.03	1.49	0.07	70.53	1.45	6.83	7.28	15.97
os20619	15	432	0.87	3	3	6	6.84	0.01	1.90	1.55	0.03	63.38	-0.62	8.76	8.20	17.25
os62706	15	450	0.77	4	3	6	6.75	-0.04	0.34	1.51	0.06	97.25	0.79	6.31	7.25	16.11
os62941	15	396	0.91	11	3	5	6.43	-0.16	0.00	1.47	0.10	99.45	-0.92	5.76	7.59	16.39
os77341	15	549	0.79	3	3	6	6.73	-0.04	0.90	1.51	0.02	96.70	0.08	7.55	7.00	15.81
os46295	15	385	1.00	16	1	4	6.12	-0.26	0.00	1.40	0.09	80.40	4.38	5.60	7.21	15.90
os84032	15	582	0.23	2	3	6	6.87	0.02	2.87	1.55	0.03	81.92	-0.36	9.40	6.50	15.31
os01714	15	480	0.00	6	2	3	6.93	0.10	0.99	1.53	0.06	95.51	-1.47	8.03	7.84	16.82
os05422	15	476	0.03	5	3	5	6.88	0.07	0.54	1.50	0.04	97.10	-0.25	7.04	7.93	16.82
os46297	15	428	0.23	3	3	9	6.68	-0.06	0.28	1.49	0.02	98.22	1.43	6.79	7.22	15.89
os57523	15	591	0.42	13	2	3	6.26	-0.19	0.06	1.45	0.12	96.48	-3.03	7.13	6.38	15.11
os65947	15	519	0.99	8	3	5	6.54	-0.13	0.27	1.50	0.05	90.93	-0.59	6.39	7.41	16.50
os71422	15	395	0.73	5	3	6	6.61	-0.06	0.56	1.52	0.05	99.78	-0.06	6.50	7.94	16.78
os11654	15	542	0.03	9	3	5	6.94	0.12	0.52	1.50	0.08	94.16	-1.19	7.96	6.57	15.50
os60241	15	423	0.47	9	2	3	6.54	-0.03	0.47	1.50	0.12	99.57	-3.74	6.36	7.84	16.81
os56309	15	330	0.85	6	3	5	6.64	-0.06	0.95	1.53	0.07	99.88	-1.90	8.33	7.75	16.72
os71392	15	490	1.00	12	2	0	6.28	-0.21	0.00	1.48	0.13	99.99	-1.03	6.09	7.11	15.99
os40669	15	409	0.09	12	3	5	6.95	0.18	0.01	1.45	0.08	99.88	-0.08	5.71	7.59	16.29
os13631	15	553	1.00	6	3	5	6.62	-0.11	0.54	1.52	0.05	68.32	-1.03	7.98	7.31	16.21
os63226	15	424	0.14	3	3	6	6.70	-0.05	0.20	1.53	0.02	99.78	0.99	7.24	7.57	16.37
os08620	15	399	0.56	11	2	3	6.75	0.04	0.41	1.53	0.15	79.19	-5.76	7.74	8.42	17.49
os11020	15	589	0.03	8	1	1	7.05	0.14	0.20	1.45	0.04	90.18	2.63	5.71	6.58	15.26
os28820	15	486	1.00	10	3	5	6.45	-0.17	0.03	1.45	0.05	93.67	1.82	6.04	7.94	16.98
os77126	15	506	0.91	15	3	9	6.05	-0.23	0.00	1.41	0.14	100.00	-1.46	6.12	7.33	16.15
os85241	15	605	0.22	3	3	9	6.95	0.04	0.44	1.46	0.02	99.50	1.05	7.09	6.48	15.22
os09613	15	398	0.99	4	3	6	6.69	-0.06	1.55	1.54	0.02	94.13	-0.28	8.19	7.99	16.83
os46200	15	352	0.98	13	2	0	6.30	-0.20	0.06	1.49	0.09	77.36	-0.41	7.51	7.26	15.97
os40735	15	327	0.09	10	2	3	6.73	0.10	0.65	1.52	0.12	95.29	-5.25	8.18	7.71	16.35
os36712	15	322	0.15	20	3	5	6.47	0.16	0.00	1.40	0.13	92.12	-0.54	5.78	7.96	16.65
os50397	15	448	0.23	5	3	5	6.79	0.04	0.63	1.49	0.04	99.88	-0.52	7.16	7.27	16.05
os78747	15	499	0.60	13	3	5	6.38	-0.06	0.00	1.48	0.08	99.85	-0.89	5.61	6.97	15.87
os76881	15	505	0.00	17	3	5	6.97	0.29	0.00	1.44	0.14	100.00	-1.78	6.28	7.20	16.00
os71123	15	695	0.01	8	3	9	7.00	0.12	0.18	1.44	0.05	99.75	0.05	6.49	6.38	15.29
os72333	15	301	0.81	3	2	3	6.63	-0.05	1.99	1.55	0.06	85.27	-2.02	8.27	8.08	17.06
os87705	15	367	0.72	10	2	7	6.40	-0.09	0.46	1.50	0.13	97.47	-3.59	7.69	7.86	16.84
os29516	15	437	0.19	6	2	3	6.81	0.06	1.39	1.55	0.07	89.56	-2.82	8.33	7.53	16.40
os17222	15	660	0.47	10	3	5	6.65	-0.02	0.04	1.46	0.06	100.00	1.19	6.09	6.15	15.05
os28343	15	470	1.00	9	2	3	6.56	-0.13	0.51	1.53	0.09	95.98	-3.19	7.34	7.33	16.20
os46438	15	483	0.23	4	3	6	6.81	0.04	0.54	1.52	0.03	96.30	0.16	6.86	6.80	15.49
os49541	15	453	0.11	16	2	0	6.64	0.16	0.00	1.46	0.14	99.57	-1.51	5.71	6.61	15.30
os61747	15	414	0.14	10	3	5	6.71	0.08	0.04	1.48	0.09	94.11	0.04	7.11	7.36	16.20
os62756	15	407	0.13	13	2	0	7.03	0.22	0.00	1.47	0.12	85.67	0.84	5.75	7.35	16.22

os54648	15	297	0.79	12	2	3	6.58	-0.08	0.02	1.49	0.12	94.28	-3.73	7.35	7.85	16.75
os67149	15	405	0.93	9	2	7	6.55	-0.12	0.12	1.51	0.12	92.03	-3.28	7.47	7.92	16.99
os08712	15	457	0.36	4	2	3	6.83	0.00	1.51	1.53	0.07	98.05	-2.85	8.23	8.22	17.39
os70916	15	570	0.65	11	3	5	6.41	-0.18	0.02	1.45	0.09	100.00	1.62	5.50	6.65	15.58
os36720	15	330	0.36	12	1	4	6.89	0.09	0.00	1.41	0.06	94.81	4.38	5.91	7.96	16.66
os56087	15	460	0.92	12	3	9	6.27	-0.18	0.09	1.41	0.06	88.53	2.26	7.04	7.26	16.30
os68126	15	454	1.00	5	3	5	6.65	-0.08	0.50	1.54	0.05	63.45	-0.49	7.87	7.57	16.63
os71622	15	399	0.79	17	1	4	5.99	-0.19	0.00	1.42	0.11	93.22	2.94	5.62	7.81	16.66
os79076	15	497	0.42	4	3	6	6.69	-0.06	0.82	1.52	0.02	71.08	0.40	7.68	7.06	15.99
os83883	15	468	0.16	4	3	6	6.83	0.04	0.68	1.54	0.02	97.25	-0.03	7.67	7.48	16.40
os83870	15	447	0.30	4	3	6	6.90	0.04	0.57	1.52	0.03	100.00	0.14	7.76	7.57	16.52
os70864	15	385	0.00	10	2	0	6.94	0.17	0.04	1.50	0.11	89.02	0.02	6.45	7.60	16.48
os46043	15	503	0.77	10	1	4	6.42	-0.12	0.01	1.46	0.05	98.24	3.40	6.05	6.73	15.49
os22083	15	450	0.85	6	3	5	6.57	-0.08	0.45	1.53	0.06	76.81	-1.40	8.04	8.13	17.16
os48340	15	461	0.52	15	3	5	6.30	-0.06	0.00	1.43	0.09	96.06	1.83	6.00	7.00	15.74
os76099	15	392	0.70	25	3	5	5.26	-0.30	0.00	1.35	0.20	99.80	-2.00	5.19	7.94	16.70
os53391	15	384	0.15	15	1	4	6.99	0.18	0.00	1.40	0.09	93.66	3.61	4.86	7.46	16.30
os06672	15	576	0.83	12	3	5	6.73	-0.07	0.05	1.48	0.07	96.65	0.39	6.16	7.53	16.54
os60084	15	533	0.98	13	2	0	6.47	-0.17	0.00	1.47	0.12	88.35	2.14	5.91	6.67	15.64
os53483	15	377	0.20	8	2	3	6.75	0.07	0.67	1.52	0.09	97.56	-2.91	7.39	7.29	16.16
os48565	15	490	0.01	16	2	3	6.91	0.24	0.00	1.41	0.10	92.13	-1.51	5.88	6.69	15.40
os05966	15	407	0.01	2	3	6	6.79	0.02	0.47	1.52	0.03	90.18	0.74	7.29	8.34	17.47
os74146	15	467	0.40	16	2	7	6.90	0.12	0.05	1.47	0.21	98.61	-6.18	7.59	6.71	15.56
os63629	15	391	0.99	9	3	5	6.51	-0.13	0.05	1.48	0.06	95.91	0.97	6.13	7.80	16.69
os11931	15	578	0.00	11	3	5	7.04	0.18	0.03	1.48	0.07	99.88	-0.02	5.96	6.92	15.85
os72793	15	273	0.77	7	3	5	6.47	-0.08	0.47	1.53	0.07	82.95	-0.87	8.08	8.33	17.33
os14659	15	527	0.91	11	3	5	6.34	-0.16	0.01	1.46	0.07	99.03	1.12	6.19	7.55	16.47
os16856	15	467	0.77	3	2	0	6.78	-0.02	1.37	1.56	0.10	56.87	-1.24	8.05	7.28	16.12
os12633	15	518	0.83	9	1	4	6.48	-0.09	0.00	1.40	0.06	93.33	6.62	5.88	7.70	16.56
os17591	15	526	1.00	15	3	5	6.15	-0.26	0.00	1.45	0.14	93.42	-0.63	6.64	7.39	16.23
os48169	15	351	0.29	7	3	5	6.88	0.06	0.62	1.52	0.04	93.23	-0.42	8.97	7.62	16.31
os48772	15	444	0.88	10	2	3	6.43	-0.13	0.64	1.50	0.14	98.75	-6.35	6.92	6.88	15.57
os59919	15	518	0.27	6	2	3	6.71	-0.01	0.89	1.52	0.07	96.18	-2.47	8.77	6.68	15.57
os73034	15	272	0.93	5	2	3	6.66	-0.04	1.69	1.53	0.07	88.67	-3.42	8.38	8.45	17.44
os53392	15	338	0.07	13	2	3	6.67	0.14	0.11	1.50	0.12	99.36	-2.99	6.67	7.51	16.38
os29760	15	540	0.01	5	3	6	6.97	0.09	0.54	1.52	0.04	96.37	-0.20	6.91	7.09	15.97
os75439	15	435	0.92	2	1	1	6.81	0.02	0.03	1.43	0.03	99.93	3.44	6.88	7.70	16.75
os47317	15	470	0.01	11	3	5	7.00	0.18	0.06	1.47	0.08	98.44	0.58	5.95	6.83	15.57
os40354	15	353	0.68	9	1	4	6.41	-0.10	0.03	1.45	0.04	93.19	3.73	6.21	7.70	16.41
os72070	15	445	0.97	10	1	1	6.44	-0.16	0.03	1.41	0.05	99.98	3.64	5.81	7.64	16.58
os77142	15	494	1.00	11	3	5	6.43	-0.18	0.02	1.47	0.07	100.00	0.31	6.29	7.39	16.18
os54988	15	466	0.92	6	3	5	6.58	-0.10	0.61	1.51	0.05	92.49	-1.25	7.91	6.92	15.85
os76390	15	424	0.99	14	3	9	6.23	-0.23	0.00	1.42	0.12	98.75	-0.79	5.29	7.81	16.66

os14751	15	509	0.08	4	3	6	6.93	0.06	0.84	1.52	0.04	99.22	-0.15	7.17	7.81	16.81
os56463	15	526	0.77	1	3	6	6.82	-0.01	1.44	1.54	0.01	34.90	0.03	9.05	6.57	15.54
os62391	15	536	0.59	6	3	9	6.61	-0.10	0.46	1.47	0.04	93.42	0.42	7.36	6.96	15.82
os71428	15	394	0.92	4	3	6	6.63	-0.06	0.54	1.52	0.05	99.50	0.26	6.53	7.94	16.78
os02780	15	583	0.34	4	3	6	6.73	-0.06	1.01	1.54	0.04	86.67	-0.08	8.22	7.35	16.39
os14815	15	486	0.05	13	3	5	7.06	0.20	0.00	1.46	0.07	99.12	0.90	5.70	8.04	17.04
os17143	15	545	0.63	6	1	1	6.65	-0.04	0.10	1.47	0.03	99.78	2.83	5.93	6.91	15.82
os61606	15	386	0.96	8	3	5	6.55	-0.11	0.26	1.51	0.10	94.31	-2.15	7.33	7.36	16.14
os73338	15	342	0.98	11	2	3	6.39	-0.16	0.48	1.52	0.12	92.17	-4.43	7.58	7.91	16.81
os91471	15	424	0.34	5	3	5	6.90	0.05	0.46	1.52	0.06	97.48	0.21	8.04	7.76	16.65
os76114	15	464	0.35	17	3	5	6.88	0.14	0.00	1.40	0.14	99.79	-1.21	6.11	7.45	16.22
os90192	15	325	0.77	8	2	7	6.65	-0.05	0.77	1.54	0.14	79.73	-4.28	7.70	8.12	17.04
os38557	15	335	0.82	8	2	3	6.67	-0.06	0.69	1.53	0.07	92.87	-2.73	7.94	7.79	16.50
os61204	15	465	0.83	19	3	5	6.35	-0.17	0.00	1.44	0.13	99.73	-1.06	4.91	7.19	16.07
os62503	15	397	0.95	8	2	0	6.54	-0.12	0.09	1.50	0.09	92.73	1.21	6.75	7.27	16.09
os11008	15	488	0.60	4	3	6	6.71	-0.03	0.98	1.54	0.04	61.97	-0.21	8.72	6.99	15.71
os40405	15	346	0.99	19	1	1	5.82	-0.32	0.00	1.31	0.09	96.89	9.46	5.70	7.99	16.69
os76805	15	569	0.87	8	3	5	6.54	-0.11	0.30	1.48	0.08	99.97	-0.62	6.74	6.90	15.77
os50616	15	380	0.63	4	3	6	6.66	-0.03	0.95	1.51	0.04	86.63	0.14	7.81	7.52	16.28
os53695	15	301	0.99	10	2	3	6.31	-0.17	0.25	1.50	0.13	85.40	-4.02	6.93	8.03	17.02
os67971	15	404	0.89	12	2	7	6.09	-0.15	0.81	1.52	0.24	91.75	-10.90	6.79	7.52	16.49
os74182	15	417	0.24	5	3	6	6.73	0.01	0.89	1.54	0.07	97.81	-1.53	6.70	7.46	16.30
os05382	15	475	0.01	4	3	6	6.88	0.07	0.57	1.50	0.03	97.35	-0.19	7.08	7.94	16.83
os07090	15	489	0.92	4	3	6	6.68	-0.06	3.68	1.53	0.04	35.32	0.10	8.65	7.66	16.69
os52979	15	385	0.47	8	2	3	6.78	0.00	0.57	1.51	0.14	98.83	-4.86	6.28	7.26	16.05
os75516	15	520	0.40	14	3	5	6.93	0.08	0.00	1.42	0.11	99.63	1.18	5.42	6.76	15.73
os07149	15	444	0.03	3	3	6	6.73	-0.04	2.29	1.55	0.03	63.20	-0.42	7.63	7.98	17.00
os17223	15	603	0.07	15	3	5	6.84	0.19	0.00	1.45	0.09	100.00	-0.85	5.47	6.44	15.32
os67573	15	323	0.60	4	2	7	6.63	0.01	2.34	1.55	0.16	91.88	-7.62	8.46	8.53	17.58
os91468	15	424	0.09	4	3	6	6.90	0.06	0.46	1.52	0.05	96.78	0.52	7.99	7.74	16.63
os56331	15	389	0.81	3	3	6	6.70	-0.03	0.59	1.53	0.05	86.16	0.43	8.72	7.42	16.42
os77473	15	280	0.89	17	1	4	6.18	-0.22	0.00	1.37	0.12	99.47	3.81	5.18	8.54	17.29
os82189	15	406	0.92	2	3	6	6.83	0.03	2.48	1.54	0.02	62.00	-0.06	8.50	7.59	16.54
os67489	15	422	0.19	3	2	3	6.69	-0.05	1.94	1.54	0.08	92.74	-3.92	8.01	8.15	17.24
os68100	15	464	0.29	3	3	6	6.72	-0.05	2.81	1.56	0.04	46.26	-1.45	8.45	7.56	16.65
os91611	15	373	0.82	10	3	5	6.35	-0.13	0.06	1.49	0.07	79.56	0.33	5.90	8.03	16.92
os71434	15	391	0.11	5	3	6	6.60	-0.08	0.50	1.51	0.05	98.84	0.86	6.54	7.95	16.79
os65589	15	423	0.61	6	3	5	6.65	-0.02	0.57	1.54	0.07	88.00	-1.18	7.49	7.75	16.80
os07962	15	434	0.79	9	3	5	6.47	-0.09	0.17	1.50	0.06	72.08	-0.08	7.81	8.26	17.34
os46052	15	515	0.38	9	3	5	6.98	0.08	0.12	1.49	0.06	93.15	-0.12	7.01	6.71	15.46
os72753	15	277	0.55	9	3	5	6.36	-0.14	0.32	1.49	0.06	95.07	0.08	7.08	8.30	17.28
os78698	15	534	0.92	7	3	5	6.71	-0.08	0.37	1.49	0.07	99.98	-0.07	6.47	6.85	15.77
os70119	15	252	0.92	8	2	3	6.59	-0.08	0.76	1.53	0.09	94.80	-3.42	7.10	8.74	17.67

os52508	15	359	0.21	7	3	5	6.90	0.07	0.63	1.51	0.08	86.36	-0.71	6.44	7.17	15.88
os15994	15	697	0.33	9	1	1	6.74	0.01	0.05	1.45	0.05	99.43	2.41	5.55	6.78	15.70
os49429	15	456	0.65	18	3	5	6.08	-0.15	0.04	1.44	0.13	88.49	-0.79	5.91	6.39	15.09
os58437	15	442	0.47	13	2	0	6.40	-0.03	0.00	1.47	0.16	68.20	-0.01	5.83	7.10	15.79
os65540	15	464	0.01	9	1	4	7.00	0.17	0.05	1.47	0.06	90.43	2.75	6.43	7.79	16.92
os46190	15	473	0.62	5	2	3	6.69	-0.03	1.77	1.53	0.12	98.64	-6.17	7.74	6.66	15.40
os53639	15	305	0.04	8	3	5	6.83	0.11	0.27	1.50	0.04	94.84	1.39	7.27	8.17	17.11
os42745	15	470	0.10	5	3	9	6.93	0.06	0.93	1.51	0.00	94.50	-0.26	6.81	7.06	15.88
os01922	15	384	0.01	15	1	4	6.92	0.25	0.00	1.42	0.09	98.44	3.35	6.07	8.46	17.34
os08981	15	444	0.96	11	1	4	6.54	-0.13	0.01	1.43	0.06	87.58	2.54	6.14	8.22	17.24
os64520	15	302	0.76	3	2	7	6.66	-0.04	1.52	1.55	0.10	97.82	-4.47	7.02	7.84	16.73
os66474	15	374	0.71	16	2	8	6.60	-0.07	0.00	1.43	0.13	97.73	-3.09	6.14	8.26	17.24
os22097	15	480	0.40	3	3	6	6.88	0.02	1.74	1.53	0.05	90.78	-0.41	7.91	8.02	17.03
os91613	15	492	0.61	4	3	6	6.70	-0.04	0.44	1.51	0.03	97.44	0.46	6.70	7.30	16.25
os31640	15	484	0.40	12	2	0	6.94	0.08	0.10	1.51	0.12	84.46	-1.26	7.62	7.29	16.39
os59486	15	398	0.99	1	3	6	6.76	-0.01	0.32	1.48	0.01	67.10	0.87	9.03	7.56	16.46
os43074	15	409	0.09	9	2	3	6.95	0.11	0.28	1.51	0.11	90.10	-4.12	6.65	7.08	15.71
os64658	15	490	0.87	10	3	5	6.70	-0.07	0.12	1.48	0.10	99.65	-0.82	6.82	6.72	15.58
os79241	15	393	0.77	10	2	3	6.42	-0.07	0.41	1.51	0.13	91.08	-5.45	8.13	7.49	16.39
os76026	15	397	0.35	8	2	8	6.92	0.07	0.67	1.46	0.10	99.95	-2.78	6.40	7.83	16.58
os16819	15	569	0.89	14	3	5	6.17	-0.21	0.00	1.46	0.11	99.72	-0.14	5.49	6.70	15.52
os53429	15	336	0.77	9	2	0	6.65	-0.06	0.16	1.50	0.09	98.22	-1.64	6.78	7.55	16.45
os48290	15	406	0.42	10	1	4	6.37	-0.17	0.03	1.46	0.09	88.12	3.72	7.25	7.25	15.95
os88570	15	387	0.09	5	2	3	6.88	0.07	1.59	1.55	0.06	84.28	-3.11	8.87	8.01	16.86
os83947	15	475	0.45	2	3	6	6.77	0.00	1.69	1.55	0.03	76.10	-0.38	7.74	7.42	16.34
os54241	15	546	0.30	14	3	5	6.55	0.05	0.00	1.46	0.09	97.12	0.39	6.48	6.43	15.36
os01724	15	392	0.02	10	2	7	6.98	0.16	0.17	1.49	0.09	96.74	-2.77	8.52	8.24	17.29
os06836	15	396	0.60	10	1	4	6.43	-0.07	0.06	1.44	0.05	92.77	2.82	6.46	8.43	17.50
os48281	15	355	0.02	8	3	5	6.82	0.11	0.24	1.51	0.07	99.75	-0.20	6.58	7.51	16.22
os59857	15	543	0.41	7	3	5	6.93	0.04	0.79	1.53	0.07	99.00	-1.69	8.68	6.62	15.56
os90159	15	341	0.88	9	3	5	6.38	-0.14	0.02	1.49	0.07	97.26	1.32	6.86	8.29	17.15
os40971	15	291	0.57	10	3	5	6.44	-0.06	0.38	1.51	0.00	87.49	-1.66	8.00	7.95	16.65
os72306	15	255	0.30	5	3	6	6.67	0.02	1.26	1.54	0.06	81.11	-1.59	7.93	8.28	17.26
os52183	15	359	0.87	6	2	0	6.67	-0.06	0.44	1.52	0.07	99.39	0.15	7.11	7.41	16.27
os76246	15	488	0.19	7	3	5	6.99	0.10	0.35	1.47	0.05	97.88	0.28	6.77	7.25	16.07
os08980	15	443	0.16	8	1	4	6.96	0.09	0.14	1.45	0.06	88.09	1.60	6.45	8.22	17.24
os17211	15	560	0.61	15	3	5	6.81	0.01	0.00	1.44	0.11	98.42	-1.02	5.85	6.80	15.71
os09612	15	396	0.23	3	3	6	6.74	0.01	1.14	1.52	0.03	73.43	0.05	8.25	8.02	16.86
os47240	15	403	0.73	11	3	5	6.35	-0.11	0.07	1.48	0.08	98.45	0.49	6.69	7.07	15.77
os54916	15	367	0.77	3	3	6	6.76	-0.02	1.19	1.54	0.01	53.16	0.40	8.82	7.45	16.35
os90544	15	382	0.91	5	3	6	6.58	-0.09	0.31	1.51	0.05	96.65	1.41	6.73	7.90	16.77
os08251	15	572	0.19	6	3	5	6.99	0.07	0.44	1.52	0.04	99.29	-0.08	6.79	7.51	16.57
os64714	15	360	0.67	8	1	4	6.74	-0.03	0.04	1.47	0.05	99.00	2.77	6.82	7.50	16.37

os11262	15	564	0.42	8	3	5	6.65	-0.02	0.10	1.49	0.05	99.95	1.20	6.03	6.63	15.43
os54177	15	506	0.45	17	2	0	6.30	-0.03	0.00	1.46	0.12	92.88	-1.89	6.10	6.42	15.30
os77559	15	417	0.17	8	2	0	6.74	0.07	0.18	1.49	0.08	100.00	-0.50	6.71	7.41	16.22
os49445	15	435	0.13	7	1	4	6.80	0.07	0.03	1.48	0.00	85.77	4.20	6.05	6.72	15.33
os73209	15	338	0.64	5	3	6	6.58	-0.06	0.54	1.51	0.03	97.83	0.26	7.10	8.00	16.88
os77734	15	365	0.93	13	3	5	6.41	-0.16	0.01	1.47	0.09	98.18	-1.60	5.65	7.81	16.55
os71945	15	481	0.47	12	3	5	6.90	0.07	0.05	1.48	0.09	99.79	-1.48	6.15	7.02	15.95
os08606	15	438	0.02	8	2	3	6.91	0.12	1.34	1.54	0.10	81.77	-4.08	8.04	8.23	17.28
os89936	15	476	0.90	4	2	7	6.71	-0.06	0.51	1.53	0.13	71.68	-2.57	6.69	7.02	15.87
os25207	15	526	0.23	6	3	5	6.78	0.04	0.60	1.51	0.05	99.98	-0.17	6.73	7.74	16.80
os50743	15	511	0.89	3	1	4	6.74	-0.03	0.24	1.51	0.02	83.87	2.07	8.71	6.56	15.28
os06317	15	573	0.97	14	3	9	6.37	-0.21	0.00	1.44	0.09	99.91	1.00	5.51	7.61	16.61
os48313	15	467	0.80	6	3	9	6.57	-0.08	0.60	1.49	0.04	99.72	0.54	6.76	7.07	15.78
os79562	15	452	0.02	5	3	5	6.91	0.09	0.68	1.53	0.05	87.30	-1.39	7.32	7.19	16.06
os48317	15	482	0.08	4	3	6	6.92	0.06	0.84	1.53	0.04	84.28	0.06	7.54	6.91	15.64
os48143	15	450	0.85	10	2	0	6.66	-0.09	0.09	1.50	0.08	99.94	0.33	6.04	6.87	15.60
os16868	15	586	0.16	11	2	3	7.07	0.16	0.02	1.48	0.10	99.90	-2.26	6.31	6.86	15.75
os62511	15	455	0.04	15	2	3	6.77	0.21	0.00	1.45	0.15	99.52	-3.30	5.61	7.05	15.88
os48451	15	449	1.00	17	2	3	6.06	-0.28	0.02	1.47	0.12	94.17	-3.06	6.82	6.81	15.50
os71389	15	730	0.33	5	3	9	7.05	0.07	0.23	1.42	0.03	99.30	1.28	6.16	6.37	15.28
os47161	15	493	1.00	18	1	1	5.97	-0.29	0.00	1.38	0.10	93.93	2.47	5.52	6.73	15.45
os49421	15	451	0.12	11	1	4	6.78	0.12	0.01	1.42	0.08	85.89	5.10	5.49	6.71	15.39
os66046	15	459	0.05	12	1	4	6.23	-0.20	0.00	1.44	0.08	89.84	3.78	5.85	7.58	16.66
os91330	15	494	0.92	5	3	5	6.62	-0.07	0.52	1.52	0.04	97.81	-0.49	6.67	7.30	16.25
os73927	15	570	0.02	9	2	7	7.02	0.16	0.46	1.51	0.12	99.16	-3.52	7.49	6.55	15.33
os75698	15	502	1.00	16	2	7	6.26	-0.23	0.00	1.43	0.17	98.49	-0.85	5.48	6.79	15.75
os08240	15	437	0.87	8	1	4	6.50	-0.09	0.13	1.48	0.04	71.33	1.99	7.57	8.27	17.34
os50961	15	455	1.00	15	2	7	6.31	-0.20	0.38	1.50	0.17	98.03	-5.28	7.37	6.93	15.72
os64813	15	373	0.92	9	3	5	6.38	-0.15	0.05	1.48	0.06	97.49	1.27	6.18	7.59	16.48
os07584	15	393	0.67	6	3	5	6.56	-0.06	0.31	1.52	0.05	62.77	-0.44	8.18	8.38	17.40
os53302	15	395	0.38	9	2	3	6.79	0.01	0.72	1.50	0.14	99.04	-5.47	6.03	7.25	16.04
os64405	15	339	0.04	13	3	5	6.76	0.15	0.12	1.48	0.10	91.96	-1.07	8.04	7.65	16.46
os76316	15	239	0.93	14	2	3	6.28	-0.18	0.01	1.49	0.15	99.96	-5.75	5.60	8.67	17.48
os23702	15	545	0.02	8	3	5	6.89	0.09	0.41	1.50	0.06	99.64	-1.26	6.36	7.68	16.68
os60988	15	379	0.89	22	1	4	6.03	-0.26	0.00	1.39	0.14	74.05	2.17	6.21	7.66	16.44
os85037	15	499	0.93	11	1	4	6.54	-0.14	0.02	1.48	0.08	90.96	1.27	7.70	6.92	15.70
os56023	15	404	0.60	5	2	3	6.60	-0.05	1.09	1.52	0.10	99.37	-2.44	7.73	7.39	16.36
os48752	15	445	0.99	18	1	4	6.09	-0.27	0.00	1.41	0.08	96.25	3.18	5.84	6.94	15.61
os06500	15	533	0.65	13	3	5	6.47	-0.04	0.02	1.48	0.09	93.30	-0.64	6.98	7.59	16.60
os27853	15	497	1.00	6	3	5	6.62	-0.10	0.35	1.53	0.04	64.67	-0.41	7.74	7.18	16.04
os91555	15	348	0.38	8	3	5	6.59	0.00	0.30	1.48	0.09	97.38	-1.08	6.62	8.29	17.14
os50939	15	396	0.62	5	3	6	6.59	-0.08	0.82	1.54	0.06	64.58	-0.71	8.01	7.27	16.04
os64098	15	395	0.08	10	3	5	7.00	0.16	0.03	1.47	0.09	97.95	0.32	6.06	7.74	16.65

os29267	15	506	0.77	4	3	6	6.77	-0.04	1.10	1.53	0.04	93.74	-0.91	8.40	7.55	16.50
os43327	15	365	0.98	15	1	4	6.21	-0.23	0.00	1.45	0.10	99.99	2.31	5.96	7.29	15.93
os76735	15	359	0.79	7	1	1	6.48	-0.11	0.02	1.36	0.05	99.99	6.34	5.34	8.64	17.58
os89441	15	572	0.70	12	1	4	6.78	-0.04	0.01	1.43	0.08	96.92	3.31	5.98	6.59	15.34
os23328	18	510	0.27	13	1	1	6.24	-0.20	0.00	1.44	0.07	100.00	2.03	5.44	7.97	16.97
os66508	18	438	0.60	11	3	9	6.40	-0.08	0.09	1.45	0.09	98.93	-1.93	6.04	7.82	16.84
os06710	18	469	0.03	9	1	4	6.80	0.07	0.08	1.47	0.04	93.24	2.61	6.37	8.10	17.12
os11639	18	536	0.83	8	3	5	6.51	-0.12	0.20	1.52	0.08	99.95	-1.34	6.74	6.56	15.47
os06861	18	476	0.23	2	3	6	6.80	0.01	0.33	1.54	0.01	41.67	0.84	8.57	8.11	17.25
os26116	18	553	0.23	6	1	4	6.88	0.07	0.11	1.45	0.04	95.74	3.12	5.85	7.61	16.58
os88519	18	354	0.54	5	2	3	6.64	-0.03	1.27	1.55	0.06	80.33	-2.19	7.42	8.05	16.91
os23532	18	620	0.00	10	3	5	6.99	0.15	0.02	1.47	0.07	98.67	0.85	5.81	7.20	16.28
os66119	18	475	0.08	14	3	5	7.05	0.22	0.00	1.46	0.09	97.66	1.09	5.68	7.43	16.48
os82880	18	511	0.92	0	3	6	6.83	0.00	1.98	1.55	0.01	35.86	-0.14	11.31	6.96	15.74
os13935	18	587	0.77	3	3	6	6.86	-0.01	1.18	1.54	0.03	90.54	-0.17	7.52	7.04	15.93
os14631	18	545	0.82	5	3	5	6.63	-0.10	0.55	1.51	0.07	97.57	0.08	6.77	7.44	16.41
os57750	18	567	0.21	17	2	0	7.03	0.19	0.00	1.45	0.13	88.53	0.13	5.89	6.38	15.11
os13122	18	522	0.85	5	2	0	6.65	-0.06	1.28	1.54	0.08	91.43	-1.57	7.75	7.36	16.20
os17033	18	646	0.65	20	3	5	6.72	-0.02	0.00	1.40	0.14	99.96	2.00	5.49	6.39	15.22
os26049	18	532	0.65	7	3	5	6.59	-0.06	0.20	1.50	0.04	87.78	1.36	6.43	7.74	16.79
os39593	18	396	0.28	6	3	5	6.89	0.05	0.44	1.52	0.05	97.11	-0.25	6.86	7.34	16.00
os67767	18	517	0.77	6	1	4	6.64	-0.05	0.01	1.41	0.04	96.93	6.87	6.29	7.32	16.38
os04819	18	542	0.23	7	2	3	6.96	0.06	0.68	1.51	0.06	91.01	-1.66	7.88	7.31	16.30
os36004	18	319	1.00	14	1	4	6.18	-0.23	0.00	1.36	0.08	97.26	6.64	5.98	8.38	17.07
os01259	18	568	0.65	9	1	4	6.82	-0.03	0.03	1.45	0.06	99.72	5.00	5.89	6.62	15.31
os05022	18	455	0.26	11	3	5	7.00	0.15	0.08	1.48	0.08	88.65	-1.07	6.85	7.87	16.85
os53479	18	331	0.30	11	3	5	6.91	0.10	0.03	1.46	0.10	96.01	0.25	5.55	7.72	16.63
os48642	18	400	0.00	11	2	0	6.96	0.20	0.02	1.48	0.13	96.57	-0.80	7.51	6.90	15.58
os59759	18	462	0.82	12	3	5	6.30	-0.15	0.01	1.45	0.08	97.50	0.72	5.76	7.16	16.03
os02356	18	417	0.55	13	2	3	6.77	0.03	0.05	1.46	0.13	99.65	-4.54	6.31	8.15	17.13
os54189	18	501	0.36	13	2	3	6.52	0.04	0.03	1.50	0.11	59.66	-3.19	8.40	6.44	15.35
os88181	18	383	0.28	9	2	3	6.90	0.08	0.87	1.54	0.13	92.41	-6.48	7.83	7.88	16.79
os47148	18	427	0.37	8	2	3	6.88	0.07	0.55	1.53	0.11	98.17	-3.41	7.08	6.90	15.60
os42156	18	470	0.60	3	1	1	6.71	-0.02	0.53	1.41	0.01	82.80	5.05	7.54	7.73	16.58
os46929	18	403	0.93	14	3	5	6.31	-0.20	0.00	1.46	0.12	91.00	-0.25	6.21	7.06	15.78
os61271	18	443	0.10	4	3	6	6.89	0.05	0.18	1.50	0.02	99.15	1.69	6.25	7.16	15.99
os52573	18	418	0.05	11	1	4	6.31	-0.17	0.00	1.41	0.06	87.30	6.40	5.30	6.93	15.62
os63426	18	445	0.92	5	3	5	6.70	-0.06	0.28	1.52	0.03	94.77	1.29	6.95	7.43	16.24
os03696	18	530	0.76	4	3	6	6.70	-0.05	1.21	1.53	0.05	92.91	-1.45	6.99	7.64	16.58
os03924	18	448	0.08	3	3	9	6.88	0.05	0.55	1.51	0.01	91.13	0.59	7.18	8.00	16.73
os21809	18	467	0.23	3	3	6	6.82	0.02	3.87	1.55	0.02	27.39	-0.44	8.80	8.09	17.16
os53478	18	329	0.28	13	3	5	6.91	0.14	0.00	1.45	0.11	96.41	1.30	5.22	7.72	16.63
os41314	18	400	0.64	6	2	3	6.88	0.08	0.92	1.54	0.12	87.83	-3.48	6.60	7.24	15.96

os62370	18	459	0.64	17	3	5	6.70	-0.03	0.00	1.43	0.11	98.93	1.44	5.95	7.27	16.12
os85817	18	484	0.96	5	2	7	6.64	-0.09	1.19	1.56	0.07	76.04	-2.12	7.82	7.31	16.22
os41585	18	454	0.62	16	3	5	6.16	-0.13	0.00	1.47	0.00	58.14	0.05	6.95	7.22	15.85
os29439	18	528	0.08	6	3	5	6.99	0.10	0.44	1.52	0.06	97.23	0.20	6.52	7.13	16.18
os00594	18	490	0.47	5	3	6	6.89	0.02	0.91	1.53	0.05	86.00	-0.30	8.29	7.10	15.85
os66841	18	536	0.00	11	1	1	7.04	0.18	0.00	1.42	0.05	99.96	4.40	5.36	7.18	16.15
os17520	18	631	0.66	9	1	1	6.58	-0.07	0.02	1.47	0.06	100.00	2.47	5.55	6.33	15.22
os36245	18	328	0.19	12	1	4	6.95	0.14	0.01	1.43	0.10	96.56	1.45	5.74	8.17	16.84
os62356	18	470	0.92	3	1	4	6.68	-0.06	0.17	1.46	0.03	97.63	4.36	7.00	7.26	16.10
os78987	18	520	0.21	3	3	6	6.92	0.04	1.30	1.53	0.02	93.65	-0.29	7.26	7.17	16.13
os27785	18	450	0.58	5	2	0	6.66	-0.03	0.12	1.51	0.06	76.41	1.76	7.54	7.28	16.12
os43100	18	417	0.25	10	3	5	6.70	0.07	0.02	1.47	0.07	89.00	1.85	7.01	7.08	15.71
os15278	18	615	0.03	12	3	5	7.08	0.20	0.01	1.46	0.10	84.39	-0.83	6.54	7.20	16.10
os15002	18	700	0.55	11	2	0	6.51	-0.07	0.04	1.49	0.11	100.00	-0.93	5.92	6.55	15.41
os66352	18	488	0.12	15	3	5	6.76	0.16	0.06	1.45	0.14	99.03	-3.10	7.07	7.30	16.27
os30965	18	485	1.00	17	1	4	6.06	-0.28	0.00	1.40	0.09	99.73	4.93	5.33	7.16	16.05
os79687	18	487	0.40	2	3	6	6.85	0.00	2.58	1.54	0.03	81.26	-0.47	8.27	7.02	15.96
os14645	18	589	0.28	12	2	8	7.00	0.12	0.03	1.44	0.11	99.98	-2.90	5.57	7.51	16.49
os85207	18	501	0.79	4	3	6	6.77	-0.04	0.79	1.53	0.03	86.28	-0.20	7.47	7.06	15.89
os27286	18	493	0.71	9	3	5	6.76	-0.05	0.15	1.51	0.06	92.14	-0.32	6.57	7.06	15.84
os27944	18	570	0.23	2	1	1	6.78	-0.02	0.09	1.50	0.01	97.44	2.73	7.16	7.07	15.97
os44574	18	463	0.11	15	1	4	6.72	0.18	0.01	1.36	0.09	95.82	7.67	5.58	6.84	15.57
os37588	18	345	1.00	14	2	0	6.21	-0.22	0.01	1.47	0.12	90.84	-0.12	6.25	7.87	16.63
os84140	18	549	0.79	5	3	9	6.77	-0.05	0.28	1.48	0.03	96.61	1.14	6.13	6.88	15.67
os40293	18	319	0.35	8	2	3	6.80	0.11	0.80	1.50	0.14	95.68	-5.60	5.90	7.88	16.57
os17507	18	539	0.08	13	3	9	7.06	0.21	0.01	1.45	0.10	100.00	-0.55	5.39	6.84	15.71
os38554	18	324	0.49	4	3	6	6.68	-0.01	1.14	1.51	0.03	95.93	-0.38	6.99	8.07	16.77
os10762	18	563	1.00	6	3	5	6.62	-0.11	0.41	1.52	0.06	94.38	-0.19	6.33	6.50	15.23
os80826	18	623	0.92	10	3	5	6.43	-0.17	0.02	1.48	0.08	97.83	-0.11	7.63	6.21	15.03
os23990	18	597	0.09	4	3	6	6.97	0.06	0.33	1.53	0.02	83.15	0.89	7.50	6.79	15.83
os02202	18	488	0.53	4	3	6	6.77	0.01	0.43	1.51	0.04	95.62	0.44	6.95	7.59	16.56
os82824	18	494	0.08	2	3	6	6.87	0.03	3.78	1.55	0.02	26.53	0.29	9.14	7.04	15.82
os67521	18	457	0.08	4	3	6	6.90	0.05	0.72	1.53	0.03	91.66	0.20	8.17	7.88	16.98
os80135	18	431	0.87	15	3	5	6.06	-0.22	0.00	1.45	0.11	97.47	-0.51	5.29	7.55	16.46
os04269	18	528	0.97	6	3	5	6.66	-0.09	0.76	1.53	0.09	98.68	-1.98	7.05	7.30	16.32
os61764	18	484	0.07	4	3	6	6.86	0.05	0.74	1.50	0.03	99.44	0.19	7.23	6.94	15.80
os91440	18	465	0.16	14	3	5	7.03	0.19	0.00	1.44	0.13	88.54	0.17	5.71	7.65	16.52
os06864	18	479	0.92	0	3	6	6.81	0.00	0.70	1.55	0.00	27.41	0.38	11.09	8.11	17.23
os41873	18	401	0.06	5	1	4	6.82	0.07	0.03	1.46	0.03	84.52	4.54	6.87	7.74	16.51
os07007	18	557	0.73	11	3	9	6.40	-0.11	0.01	1.47	0.06	94.90	1.55	5.73	7.63	16.66
os48316	18	483	0.01	10	3	5	6.94	0.15	0.16	1.48	0.07	99.27	-0.85	5.99	6.99	15.72
os61717	18	460	0.92	1	3	6	6.78	-0.02	1.81	1.52	0.03	93.76	-0.73	7.68	7.16	15.96
os57496	18	546	0.65	27	2	0	6.43	-0.06	0.00	1.35	0.20	94.56	-0.44	5.29	6.50	15.17

os11640	18	571	1.00	7	3	5	6.61	-0.12	0.53	1.48	0.05	97.65	0.70	6.45	6.53	15.44
os42832	18	439	0.21	5	3	6	6.91	0.05	1.13	1.53	0.04	79.13	-0.74	7.48	7.16	15.94
os41329	18	341	0.92	10	2	3	6.28	-0.16	0.50	1.51	0.17	89.50	-5.37	6.44	7.57	16.23
os62705	18	460	0.32	5	2	0	6.92	0.05	0.50	1.53	0.07	88.63	0.02	8.07	7.10	15.93
os64108	18	389	0.85	10	3	9	6.53	-0.12	0.04	1.45	0.06	99.08	1.72	6.65	7.93	16.85
os63704	18	444	0.19	9	1	1	6.98	0.11	0.02	1.38	0.05	97.04	4.79	6.03	7.68	16.61
os89845	18	560	0.12	8	2	3	6.87	0.10	0.85	1.51	0.13	94.01	-6.51	7.65	6.68	15.50
os40375	18	353	0.00	2	3	6	6.81	0.04	0.38	1.55	0.03	74.55	0.59	9.61	7.65	16.37
os40665	18	311	0.82	20	3	5	6.50	-0.06	0.19	1.42	0.16	93.41	-2.40	6.08	8.03	16.74
os62354	18	455	0.48	9	2	3	6.50	-0.05	0.65	1.51	0.13	97.08	-5.72	7.94	7.26	16.10
os88465	18	457	0.77	1	3	9	6.80	-0.01	0.72	1.50	0.01	20.87	-0.02	9.06	7.68	16.54
os12621	18	611	0.17	12	1	4	6.40	-0.18	0.01	1.40	0.08	99.91	6.29	5.25	7.25	16.14
os82782	18	541	0.77	3	3	6	6.81	-0.03	1.23	1.51	0.02	98.26	-0.02	7.42	6.87	15.74
os65902	18	496	0.92	2	3	6	6.76	-0.03	0.17	1.50	0.02	92.66	1.51	7.75	7.50	16.55
os05756	18	471	0.60	3	3	6	6.73	-0.02	2.61	1.54	0.03	73.68	-0.31	9.40	8.03	17.13
os07700	18	496	0.08	5	1	1	6.95	0.08	0.15	1.49	0.03	91.20	2.13	6.40	7.81	16.79
os14894	18	473	0.10	11	3	5	6.34	-0.18	0.02	1.48	0.07	97.88	0.10	5.42	7.98	16.99
os42873	18	299	0.09	7	2	3	6.78	0.09	0.65	1.52	0.00	82.47	-2.28	6.80	7.82	16.49
os30929	18	618	0.23	10	3	9	6.79	0.07	0.16	1.45	0.08	99.58	-0.42	6.31	6.59	15.49
os19061	18	610	0.21	15	3	5	7.10	0.21	0.00	1.44	0.13	99.70	1.04	5.93	6.30	15.06
os57015	18	656	0.69	18	3	5	6.10	-0.15	0.00	1.41	0.10	92.81	1.65	5.51	6.11	14.93
os81939	18	464	0.92	6	3	5	6.60	-0.09	0.50	1.52	0.03	96.68	-0.08	7.07	7.34	16.34
os24307	18	505	0.08	3	3	6	6.90	0.05	1.52	1.55	0.03	80.58	-0.67	8.77	7.72	16.78
os47884	18	427	0.33	12	2	7	6.53	0.03	0.16	1.51	0.14	90.32	-5.66	7.77	7.01	15.74
os08100	18	499	0.08	3	3	6	6.89	0.05	1.79	1.54	0.02	74.45	-0.19	8.38	7.94	17.04
os06436	18	576	0.05	13	1	1	6.82	0.13	0.00	1.38	0.04	99.82	9.46	5.12	7.58	16.60
os82560	18	494	0.92	3	3	6	6.70	-0.05	1.90	1.55	0.05	87.61	-1.53	8.13	7.04	15.82
os76865	18	445	0.12	9	2	7	6.73	0.08	0.60	1.50	0.17	99.33	-4.82	6.80	7.37	16.16
os00572	18	587	0.42	6	3	5	6.67	-0.04	0.21	1.48	0.07	96.21	0.82	7.56	6.53	15.30
os23370	18	530	0.03	11	1	4	6.92	0.16	0.00	1.47	0.08	99.25	2.43	5.65	7.75	16.77
os22967	18	541	0.92	2	3	6	6.77	-0.04	1.48	1.54	0.01	86.56	0.18	7.48	7.54	16.46
os90645	18	425	0.37	6	1	4	6.90	0.05	0.06	1.47	0.04	91.70	1.96	7.57	7.76	16.67
os27969	18	477	0.35	3	3	6	6.72	-0.02	0.30	1.51	0.03	80.36	1.57	7.62	7.47	16.38
os40057	18	421	0.33	4	3	6	6.87	0.03	1.11	1.53	0.04	93.67	-0.38	7.79	7.24	15.98
os15669	18	616	0.50	9	3	5	6.60	-0.04	0.10	1.50	0.07	100.00	-0.63	6.28	7.17	16.09
os46128	18	446	0.71	12	2	7	6.32	-0.12	0.59	1.51	0.17	96.09	-6.71	6.08	6.63	15.35
os83375	18	501	0.40	10	1	4	6.93	0.06	0.01	1.46	0.07	99.32	3.04	6.08	6.96	15.73
os89232	18	498	0.23	10	3	5	6.68	0.06	0.08	1.48	0.06	98.11	0.39	7.28	7.17	16.13
os67760	18	488	0.11	8	2	3	6.81	0.09	0.72	1.53	0.13	99.21	-5.56	8.24	7.35	16.42
os43042	18	279	0.72	1	3	6	6.72	0.01	2.98	1.56	0.00	67.73	-1.35	8.97	7.99	16.70
os79930	18	432	0.99	2	3	6	6.73	-0.04	1.33	1.55	0.04	84.68	-0.08	7.83	7.28	16.13
os82836	18	485	0.92	3	3	6	6.72	-0.02	3.63	1.56	0.05	29.92	-0.84	8.33	7.06	15.84
os13616	18	527	0.95	9	2	3	6.54	-0.14	0.50	1.50	0.08	88.90	-1.40	7.26	7.41	16.24

os20233	18	518	0.92	1	3	6	6.82	-0.01	0.36	1.54	0.01	79.49	0.50	7.76	7.71	16.64
os26529	18	536	0.85	3	3	6	6.82	-0.02	2.13	1.54	0.04	72.68	-0.86	8.23	7.74	16.82
os85208	18	483	0.89	3	3	6	6.73	-0.05	0.39	1.53	0.03	99.38	0.72	7.71	7.07	15.91
os14781	18	439	0.01	9	3	5	6.97	0.16	0.09	1.50	0.08	83.92	-0.46	6.15	8.09	17.11
os28097	18	503	0.83	6	3	6	6.64	-0.05	0.16	1.49	0.04	91.72	1.28	6.26	7.31	16.22
os47987	18	449	0.92	9	3	5	6.44	-0.14	0.13	1.46	0.06	97.34	1.10	6.17	7.11	15.82
os50931	18	481	0.23	10	2	3	6.66	0.03	0.55	1.49	0.09	94.64	-1.80	6.31	6.96	15.73
os05016	18	514	0.06	2	1	1	6.87	0.02	0.01	1.51	0.01	89.74	2.59	7.71	7.53	16.53
os21762	18	436	0.74	2	3	6	6.80	-0.01	0.32	1.54	0.01	69.36	0.80	7.91	8.29	17.35
os79273	18	445	0.40	6	2	3	6.90	0.04	0.70	1.54	0.09	85.51	-2.21	7.89	7.22	16.08
os40448	18	387	0.03	10	3	9	6.88	0.16	0.04	1.44	0.07	90.30	1.32	5.54	7.75	16.46
os79784	18	392	1.00	6	2	0	6.59	-0.10	0.71	1.53	0.05	82.35	-0.52	8.20	7.47	16.39
os60496	18	418	0.88	10	1	4	6.56	-0.12	0.01	1.45	0.07	98.00	2.16	5.85	7.78	16.77
os77590	18	377	0.06	2	2	7	6.82	0.03	1.91	1.56	0.09	95.97	-3.56	8.21	7.45	16.18
os62950	18	389	0.65	12	3	5	6.83	0.03	0.01	1.46	0.06	78.49	2.04	7.02	7.85	16.64
os17488	18	515	0.36	9	3	5	6.64	0.00	0.09	1.50	0.07	97.44	0.45	5.89	6.87	15.72
os84820	18	514	0.83	26	2	7	5.35	-0.32	0.00	1.39	0.17	91.94	-1.80	5.82	6.73	15.46
os44655	18	452	0.69	6	3	5	6.59	-0.06	0.80	1.48	0.09	99.77	-1.35	6.92	6.95	15.67
os55866	18	569	0.15	2	3	6	6.90	0.02	0.99	1.53	0.03	89.56	0.20	8.76	6.17	15.07
os65934	18	458	0.11	8	3	5	6.98	0.11	0.63	1.51	0.06	83.02	-0.70	8.53	7.55	16.66
os08733	18	436	0.53	7	3	5	6.88	0.03	0.60	1.51	0.07	87.58	-1.79	7.86	8.28	17.43
os14190	18	452	0.23	12	3	5	6.66	0.08	0.01	1.49	0.10	85.39	-1.67	6.12	7.79	16.64
os46940	18	439	0.00	3	3	6	6.87	0.05	1.22	1.54	0.02	99.64	0.01	8.34	6.97	15.65
os12908	18	512	0.08	1	3	6	6.86	0.02	1.78	1.53	0.02	82.19	0.48	8.08	7.80	16.66
os51037	18	510	0.01	8	3	5	7.02	0.14	0.18	1.47	0.05	97.05	2.02	6.75	6.56	15.37
os84141	18	524	0.96	5	3	5	6.71	-0.07	0.31	1.48	0.04	98.25	0.67	6.80	6.87	15.65
os40043	18	462	0.08	7	3	5	6.98	0.12	0.34	1.49	0.04	97.79	0.54	6.37	7.12	15.86
os82995	18	470	0.00	3	3	6	6.71	-0.05	1.61	1.54	0.03	82.82	-0.51	8.09	7.22	16.00
os02426	18	533	0.13	3	3	6	6.92	0.04	0.55	1.51	0.02	79.62	0.71	7.13	7.40	16.40
os06704	18	442	0.00	3	2	3	6.86	0.04	2.74	1.56	0.05	86.85	-1.90	8.05	8.19	17.24
os48275	18	367	0.19	8	3	5	6.94	0.10	0.16	1.50	0.04	94.46	0.98	6.59	7.56	16.31
os76760	18	486	0.16	12	3	5	7.04	0.16	0.01	1.45	0.09	97.78	1.12	6.55	7.35	16.16
os28909	18	439	0.43	8	3	5	6.48	-0.14	0.18	1.50	0.05	97.36	0.97	6.17	7.96	16.85
os47523	18	358	0.08	7	3	5	6.93	0.10	0.33	1.51	0.03	98.08	0.44	6.49	7.56	16.29
os82158	18	437	0.25	7	3	5	6.92	0.06	0.48	1.51	0.05	99.98	-0.45	6.75	7.72	16.67
os83267	18	552	0.00	11	2	7	6.92	0.17	0.65	1.50	0.14	97.76	-5.51	7.71	6.47	15.20
os40023	18	404	0.92	1	3	6	6.77	-0.01	0.49	1.51	0.02	89.01	1.32	9.24	7.42	16.15
os41573	18	474	0.40	4	3	6	6.90	0.03	0.61	1.54	0.03	80.60	-0.66	7.15	7.15	15.81
os06399	18	536	0.17	5	3	6	6.97	0.07	0.38	1.52	0.04	88.41	-0.05	7.07	7.61	16.67
os47914	18	435	0.92	6	1	4	6.68	-0.07	0.13	1.47	0.04	97.68	3.34	6.40	7.16	15.87
os12704	18	544	0.00	10	3	5	6.97	0.17	0.25	1.45	0.08	87.49	0.85	6.42	7.47	16.29
os37790	18	364	0.40	4	1	4	6.72	0.01	0.09	1.47	0.04	98.55	2.32	6.19	7.87	16.63
os30278	18	552	0.92	0	3	6	6.83	-0.01	0.07	1.53	0.03	81.51	1.10	9.54	7.14	16.19

os83300	18	479	0.96	8	3	5	6.60	-0.11	0.99	1.54	0.07	62.45	-1.39	7.66	7.07	15.81
os39948	18	390	0.00	11	3	9	6.98	0.20	0.02	1.40	0.07	96.69	1.29	6.22	7.53	16.26
os30849	18	503	0.30	11	2	7	6.61	0.04	0.60	1.53	0.16	94.72	-5.72	7.60	6.76	15.63
os36770	18	377	0.01	5	3	9	6.82	0.06	0.58	1.48	0.03	95.98	0.13	6.79	7.87	16.58
os23342	18	461	0.11	7	3	5	6.81	0.08	0.32	1.52	0.09	99.45	-1.61	6.38	8.07	17.08
os25173	18	480	0.57	11	3	5	6.50	-0.04	0.22	1.49	0.07	92.09	-0.64	8.26	7.83	16.86
os61576	18	379	0.34	8	3	5	6.47	-0.13	0.33	1.52	0.05	90.03	-1.89	6.94	7.61	16.48
os07797	18	505	0.06	7	3	5	6.88	0.10	0.24	1.51	0.07	93.74	0.17	7.50	7.75	16.76
os53374	18	508	0.41	9	2	3	6.87	0.04	0.06	1.45	0.10	99.93	-2.03	5.52	6.66	15.49
os79785	18	396	0.99	6	3	5	6.62	-0.09	0.62	1.53	0.05	85.33	0.21	7.83	7.47	16.39
os08314	18	532	0.60	2	3	6	6.80	-0.01	1.16	1.54	0.01	36.23	-0.05	8.75	7.83	16.94
os79352	18	427	0.92	1	3	6	6.78	-0.01	2.71	1.55	0.05	95.28	-1.64	8.62	7.26	16.19
os39392	18	397	0.88	16	3	5	5.99	-0.23	0.00	1.41	0.14	93.21	1.99	5.28	7.28	15.93
os39182	18	430	0.12	4	3	6	6.91	0.07	1.11	1.54	0.06	77.25	-1.43	7.27	7.12	15.77
os43871	18	439	0.60	10	3	5	6.53	-0.04	0.13	1.47	0.07	95.79	0.16	6.32	7.01	15.60
os74208	18	505	0.56	15	2	7	6.78	0.02	0.19	1.52	0.17	97.32	-6.15	7.88	6.47	15.32
os88152	18	453	0.11	9	2	3	6.81	0.10	0.28	1.51	0.09	97.23	-3.02	7.34	7.60	16.49
os44707	18	475	0.02	19	3	5	6.98	0.32	0.00	1.41	0.11	100.00	0.05	6.16	6.85	15.58
os06376	18	482	0.30	4	3	6	6.91	0.04	0.47	1.53	0.02	77.33	0.79	7.42	8.04	17.09
os84120	18	614	0.03	6	3	5	7.01	0.09	0.51	1.52	0.04	95.50	-0.19	7.34	6.25	15.00
os86506	18	416	0.08	8	3	5	6.98	0.14	0.35	1.50	0.08	80.20	-0.02	6.59	7.73	16.62
os11114	18	584	0.47	3	3	6	6.80	0.00	1.78	1.53	0.04	75.88	-0.45	8.29	6.42	15.12
os05105	18	491	0.01	3	3	6	6.88	0.04	1.77	1.51	0.03	68.89	-0.60	7.83	7.69	16.49
os14159	18	563	0.82	6	3	5	6.61	-0.08	0.46	1.51	0.06	95.15	-0.52	6.59	7.27	16.15
os60024	18	437	0.15	17	3	5	6.98	0.19	0.03	1.40	0.10	81.51	2.79	7.42	7.32	16.26
os20787	18	522	0.23	4	3	6	6.79	0.02	0.57	1.53	0.02	93.84	0.04	6.85	7.61	16.63
os51806	18	564	0.73	5	1	1	6.69	-0.05	0.03	1.36	0.02	98.77	6.67	6.07	6.21	14.95
os64270	18	448	0.85	16	1	1	6.01	-0.22	0.00	1.37	0.08	97.80	4.13	5.34	7.24	16.11
os65885	18	582	0.39	2	3	6	6.83	-0.02	1.97	1.53	0.02	70.27	1.15	8.30	6.96	15.99
os79833	18	428	0.17	9	3	5	6.79	0.09	0.10	1.50	0.06	77.92	-0.05	7.60	7.28	16.19
os11841	18	543	0.92	4	3	6	6.76	-0.05	0.60	1.50	0.02	97.74	0.96	6.78	6.85	15.55
os90839	18	460	0.40	7	2	0	6.89	0.05	0.67	1.52	0.09	98.99	-1.21	6.60	7.21	16.11
os17508	18	585	0.41	8	3	9	6.96	0.04	0.07	1.46	0.05	100.00	2.39	5.82	6.84	15.72
os19884	18	466	0.60	6	3	5	6.67	-0.01	0.65	1.54	0.06	70.30	-1.56	7.25	7.71	16.62
os83309	18	527	0.00	2	1	4	6.81	0.00	0.00	1.51	0.01	78.59	2.35	8.43	6.95	15.66
os08312	18	531	0.92	0	3	6	6.83	0.00	1.13	1.54	0.00	24.10	0.30	11.12	7.83	16.94
os10518	18	537	0.01	6	3	5	6.95	0.10	0.12	1.51	0.06	90.42	0.82	7.08	6.76	15.47
os42524	18	367	0.00	6	3	5	6.53	-0.11	0.43	1.51	0.00	97.61	-0.14	6.18	7.54	16.32
os91634	18	428	0.77	4	3	6	6.76	-0.03	0.18	1.52	0.05	97.04	1.55	7.50	7.62	16.57
os44706	18	484	0.04	18	3	5	7.01	0.30	0.00	1.40	0.11	100.00	1.09	6.14	6.84	15.57
os44797	18	447	0.60	10	2	0	6.81	0.01	0.10	1.48	0.10	97.79	0.95	6.57	6.87	15.59
os01542	18	564	0.19	3	1	4	6.92	0.04	0.01	1.48	0.00	79.72	3.21	7.80	6.61	15.25
os13811	18	568	1.00	6	3	5	6.64	-0.10	0.30	1.52	0.04	99.61	0.45	6.60	7.34	16.18

os44792	18	511	0.21	8	3	9	6.99	0.09	0.17	1.46	0.06	100.00	0.93	6.25	6.88	15.62
os42969	18	433	0.68	6	2	7	6.68	0.00	1.13	1.54	0.09	90.83	-2.68	7.49	7.00	15.68
os79790	18	402	1.00	6	3	5	6.60	-0.09	0.48	1.53	0.04	91.93	-0.24	7.24	7.46	16.37
os47915	18	457	0.11	12	2	3	6.81	0.12	0.43	1.49	0.08	94.86	-2.29	7.44	7.04	15.79
os61001	18	427	0.08	2	3	6	6.84	0.03	2.42	1.53	0.06	77.41	0.58	8.71	7.36	16.18
os07791	18	428	0.92	0	3	6	6.78	0.00	3.76	1.55	0.02	53.75	0.31	10.90	8.22	17.32
os90536	18	457	0.01	5	3	6	6.84	0.05	0.17	1.50	0.02	97.25	1.71	6.64	7.74	16.62
os22684	18	430	0.92	0	3	6	6.79	0.00	6.36	1.56	0.01	2.79	-0.06	9.51	8.27	17.31
os40441	18	358	0.83	16	1	4	6.46	-0.11	0.00	1.37	0.10	93.56	5.97	5.55	7.74	16.43
os56917	18	648	0.73	12	3	5	6.45	-0.11	0.02	1.49	0.10	90.87	-1.44	6.51	6.05	14.87
os28249	18	499	0.37	11	1	4	6.37	-0.12	0.01	1.43	0.06	90.21	4.09	6.75	7.19	16.04
os85117	18	583	0.03	6	3	5	6.94	0.09	0.18	1.51	0.04	96.60	0.81	6.81	6.53	15.29
os13592	18	540	0.89	8	2	3	6.55	-0.13	0.44	1.52	0.08	95.01	-2.05	8.53	7.40	16.25
os56833	18	311	0.23	4	3	6	6.74	0.03	0.60	1.54	0.02	80.35	0.83	7.39	7.99	16.97
os61610	18	373	0.39	9	3	5	6.41	-0.14	0.20	1.50	0.10	88.88	-1.14	6.80	7.43	16.21
os84722	18	475	0.23	6	3	5	6.95	0.07	0.38	1.50	0.03	96.07	1.00	6.44	7.22	16.11
os37713	18	353	0.03	11	2	3	6.74	0.17	0.16	1.52	0.14	68.53	-4.00	6.05	7.56	16.28
os41209	18	297	0.19	6	1	4	6.59	0.00	0.02	1.32	0.04	92.52	9.75	5.66	8.21	16.96
os40044	18	464	0.08	6	3	5	6.97	0.10	0.46	1.49	0.04	97.87	0.87	6.34	7.12	15.85
os24475	18	528	0.92	0	3	6	6.83	0.00	1.95	1.55	0.01	39.38	-0.09	10.52	7.46	16.49
os67801	18	591	0.08	2	1	1	6.87	0.01	0.25	1.38	0.02	99.46	3.34	7.11	6.85	15.77
os85747	18	382	0.77	2	3	6	6.76	-0.01	0.31	1.54	0.04	63.52	-0.23	6.98	7.85	16.79
os62396	18	453	0.09	21	2	0	6.68	0.27	0.00	1.40	0.17	99.17	0.60	5.23	7.10	15.92
os14404	18	578	0.99	6	3	9	6.69	-0.09	0.32	1.50	0.05	99.62	-0.34	6.01	7.53	16.54
os63893	18	557	0.78	9	1	1	6.75	-0.06	0.01	1.43	0.04	99.53	3.63	5.26	6.94	15.88
os91075	18	323	0.27	14	2	3	6.53	0.07	0.01	1.51	0.12	88.04	-2.71	7.95	8.28	17.12
os02664	18	559	0.01	6	3	9	6.94	0.09	0.20	1.46	0.03	99.75	1.67	6.17	7.67	16.60
os39461	18	428	0.36	6	2	3	6.88	0.04	0.93	1.52	0.08	96.74	-3.53	7.22	7.14	15.82
os40670	18	403	0.08	11	2	3	6.83	0.14	0.08	1.48	0.09	99.88	-1.31	5.95	7.58	16.28
os66733	18	536	0.84	5	3	5	6.74	-0.06	0.48	1.48	0.04	99.78	1.23	6.05	7.11	16.12
os46851	18	469	0.35	8	3	5	6.93	0.06	0.40	1.52	0.06	85.71	-0.67	6.96	6.78	15.55
os66043	18	504	0.93	11	2	3	6.60	-0.12	0.37	1.48	0.08	99.27	-2.25	7.25	7.38	16.42
os07744	18	463	0.08	2	3	6	6.88	0.04	2.07	1.54	0.05	90.42	-0.63	8.87	8.32	17.55
os40780	18	332	0.99	3	1	4	6.65	-0.06	0.61	1.50	0.02	58.44	2.32	8.56	7.80	16.44
os20025	18	437	0.99	6	3	5	6.63	-0.09	0.44	1.52	0.04	76.46	-0.76	6.61	8.32	17.34
os39785	18	277	0.38	19	2	7	6.73	0.13	0.00	1.45	0.20	84.10	-3.11	5.01	7.86	16.51
os14263	18	564	0.60	9	2	3	6.62	-0.04	0.43	1.52	0.06	85.94	-1.33	8.83	7.13	16.01
os14755	18	587	0.08	11	1	1	7.09	0.18	0.01	1.43	0.05	97.75	4.03	5.39	7.57	16.57
os39637	18	411	0.08	9	2	3	6.97	0.13	0.56	1.51	0.12	93.80	-3.78	7.91	7.15	15.84
os64798	18	382	0.01	9	2	7	6.87	0.15	0.39	1.51	0.12	88.35	-3.01	6.55	7.71	16.61
os66260	18	501	0.77	2	3	6	6.80	-0.02	2.88	1.53	0.04	85.36	-1.72	8.85	7.46	16.50
os06716	18	435	0.02	9	2	3	6.87	0.11	0.96	1.55	0.09	62.80	-4.32	7.92	8.17	17.24
os44315	18	435	0.05	20	1	4	6.72	0.26	0.00	1.38	0.13	99.42	2.86	5.01	7.10	15.85

os83392	18	492	0.94	12	2	3	6.48	-0.16	0.16	1.50	0.10	77.22	-2.91	7.18	7.05	15.86
os86016	18	523	0.38	4	3	6	6.92	0.04	0.77	1.55	0.04	82.18	0.10	8.46	7.17	16.11
os23140	18	450	0.30	18	2	3	6.94	0.17	0.00	1.44	0.14	97.67	-1.49	5.77	8.13	17.13
os61534	18	502	0.98	4	3	9	6.76	-0.05	0.28	1.48	0.01	98.46	1.83	6.92	6.97	15.82
os57134	18	642	0.44	4	3	6	6.96	0.02	0.64	1.52	0.03	96.00	-0.32	7.08	6.15	14.90
os84594	18	554	0.25	2	3	6	6.90	0.02	1.58	1.53	0.01	66.66	0.14	7.45	6.81	15.66
os65531	18	461	0.64	3	2	3	6.71	-0.03	1.85	1.54	0.05	79.64	-1.97	8.38	7.70	16.81
os85146	18	508	0.92	18	2	0	6.30	-0.20	0.00	1.45	0.16	98.11	-2.36	6.53	6.78	15.49
os13055	18	503	0.11	12	1	4	7.04	0.19	0.08	1.41	0.06	73.32	4.80	6.72	7.75	16.62
os39520	18	467	0.01	7	3	5	6.89	0.12	0.16	1.50	0.08	100.00	-0.46	5.73	6.92	15.62
os08833	18	468	0.23	5	3	9	6.81	0.04	0.49	1.50	0.03	94.99	0.06	6.38	8.39	17.58
os47038	18	454	0.08	2	3	6	6.87	0.03	1.71	1.53	0.01	47.31	-0.02	8.40	6.87	15.58
os64742	18	490	0.32	8	3	5	6.68	0.02	0.19	1.46	0.04	86.48	1.71	7.00	7.19	16.03
os27786	18	451	0.60	5	2	0	6.68	-0.03	0.12	1.51	0.05	75.94	2.21	7.56	7.28	16.12
os67729	18	553	0.92	10	2	3	6.65	-0.11	0.31	1.47	0.11	99.03	-3.47	7.00	6.97	15.96
os11004	18	591	0.01	4	3	6	6.92	0.06	0.21	1.52	0.03	91.04	1.17	8.10	6.31	15.01
os06825	18	458	0.47	8	3	5	6.84	0.00	0.48	1.50	0.07	92.65	-1.03	6.99	8.08	17.12
os15641	18	483	0.08	6	3	5	6.96	0.08	0.82	1.53	0.07	93.03	-1.84	8.02	7.97	16.91
os31665	18	544	0.08	3	3	6	6.93	0.05	1.91	1.53	0.04	81.91	-0.23	8.49	7.17	16.26
os48745	18	414	0.98	21	3	5	5.71	-0.34	0.00	1.40	0.14	93.60	1.49	5.44	7.01	15.68
os61697	18	368	0.01	5	3	5	6.88	0.09	0.52	1.53	0.05	90.94	0.20	7.35	7.59	16.39
os77821	18	458	0.45	5	3	6	6.82	-0.01	0.51	1.53	0.03	97.29	0.12	7.31	7.46	16.39
os07102	18	440	0.22	2	3	6	6.81	0.02	2.60	1.55	0.01	11.90	-0.34	8.66	8.05	17.11
os79701	18	424	0.99	5	2	7	6.67	-0.07	1.72	1.56	0.09	79.48	-3.39	8.34	7.28	16.18
os49622	18	483	0.38	20	1	4	6.83	0.09	0.00	1.37	0.12	95.67	4.19	4.89	6.49	15.15
os84100	18	538	1.00	13	1	4	6.32	-0.21	0.01	1.43	0.08	96.94	3.60	5.81	6.73	15.45
os06401	18	546	0.78	2	3	6	6.83	-0.02	0.14	1.52	0.02	85.35	1.12	7.59	7.63	16.69
os41151	18	288	0.47	7	3	5	6.60	-0.01	0.40	1.48	0.05	80.02	0.32	7.87	8.14	16.88
os44711	18	396	0.73	15	1	4	6.12	-0.16	0.00	1.36	0.13	96.96	3.65	5.40	7.19	15.90
os02205	18	491	0.47	4	3	6	6.87	0.01	1.18	1.52	0.03	96.82	-0.23	7.36	7.60	16.57
os05810	18	449	0.48	3	3	6	6.77	0.01	2.35	1.55	0.03	73.18	-0.83	8.99	8.15	17.21
os82981	18	474	0.92	0	3	6	6.82	0.00	5.84	1.57	0.02	40.58	-0.62	9.54	7.16	15.97
os07627	18	506	0.06	7	3	5	6.87	0.09	0.19	1.50	0.07	95.54	0.20	5.94	7.90	17.05
os28968	18	442	0.23	5	1	4	6.80	0.05	0.19	1.48	0.03	72.76	3.49	7.28	7.81	16.71
os46392	18	394	0.93	9	1	4	6.56	-0.12	0.07	1.45	0.06	89.71	5.25	6.06	7.18	15.88
os66714	18	486	0.08	2	3	6	6.87	0.03	2.95	1.54	0.05	86.68	-1.25	9.03	7.50	16.54
os89239	18	568	0.91	8	3	9	6.52	-0.12	0.21	1.47	0.04	93.12	1.45	6.51	6.93	15.91
os14099	18	488	0.81	7	1	4	6.55	-0.10	0.07	1.48	0.06	94.25	2.36	7.51	7.71	16.58
os65599	18	420	0.15	12	2	7	6.81	0.14	0.10	1.50	0.10	76.46	-2.70	7.67	7.87	16.96
os17396	18	523	0.79	8	1	1	6.55	-0.08	0.05	1.46	0.04	97.72	2.81	5.59	6.97	15.83
os84969	18	532	0.89	3	3	6	6.74	-0.05	1.28	1.53	0.03	84.19	-0.12	8.04	6.82	15.59
os59882	18	476	0.83	5	2	3	6.77	-0.04	1.74	1.54	0.07	99.42	-3.10	8.39	6.95	15.86
os40414	18	298	0.12	30	1	2	6.11	0.22	0.00	1.28	0.17	94.21	2.01	6.88	7.96	16.66

os44779	18	489	0.06	19	1	1	6.74	0.23	0.00	1.38	0.09	99.87	3.28	5.92	7.08	15.80
os05727	18	473	0.99	3	3	6	6.74	-0.04	1.15	1.54	0.03	87.12	0.35	8.87	8.05	17.17
os57499	18	587	0.83	25	3	5	6.15	-0.21	0.00	1.37	0.19	99.88	0.03	4.81	6.33	15.01
os16745	18	657	0.19	12	3	9	7.06	0.15	0.01	1.45	0.11	100.00	-2.27	5.63	6.56	15.44
os26460	18	488	0.27	7	2	3	6.71	0.03	1.16	1.54	0.10	75.92	-3.84	7.33	7.81	16.83
os23533	18	620	0.00	10	3	5	7.03	0.17	0.04	1.48	0.07	98.14	0.31	5.91	7.18	16.25
os39690	18	428	0.23	9	1	4	6.40	-0.15	0.01	1.46	0.08	98.25	2.83	5.59	7.21	15.88
os86122	18	491	0.92	4	3	6	6.67	-0.06	1.42	1.53	0.04	99.55	-0.79	7.33	7.32	16.20
os65916	18	468	0.08	7	3	5	6.51	-0.13	0.27	1.51	0.08	97.76	-1.33	7.78	7.51	16.58
os37490	18	319	0.23	5	3	6	6.70	0.02	0.39	1.51	0.04	74.93	0.95	7.43	8.05	16.77
os46892	18	470	0.08	6	3	9	6.96	0.09	0.39	1.47	0.03	98.02	1.59	6.42	6.97	15.69
os90547	18	396	0.01	7	1	4	6.91	0.10	0.11	1.49	0.06	89.85	2.15	7.25	7.87	16.73
os91546	18	430	0.88	10	3	5	6.37	-0.14	0.02	1.47	0.08	97.78	1.64	6.11	7.76	16.62
os23799	18	586	0.91	3	3	9	6.72	-0.05	0.54	1.50	0.02	98.00	1.80	6.72	7.56	16.53
os48738	18	487	0.82	5	3	9	6.72	-0.06	0.38	1.50	0.03	94.04	0.55	6.46	6.83	15.51
os62129	18	522	0.08	3	3	6	6.86	0.03	0.39	1.53	0.02	88.58	0.70	8.88	6.83	15.72
os15808	18	624	0.35	16	1	4	6.45	0.02	0.00	1.44	0.11	99.36	2.34	5.28	6.95	15.86
os22977	18	551	0.00	3	3	6	6.92	0.04	1.84	1.53	0.03	83.33	-0.89	7.40	7.52	16.43
os91195	18	412	0.41	4	3	6	6.67	-0.06	1.67	1.53	0.03	86.72	-0.11	8.22	7.76	16.67
os02432	18	523	0.77	4	3	6	6.82	-0.02	1.84	1.55	0.06	95.26	-1.34	7.49	7.24	16.24
os26818	18	465	0.03	8	3	5	6.83	0.10	0.09	1.49	0.04	72.64	1.50	7.95	7.24	16.03
os06407	18	539	0.40	8	3	5	6.65	-0.03	0.38	1.51	0.05	93.47	-1.01	7.86	7.57	16.64
os65556	18	462	0.91	2	3	6	6.80	-0.01	0.02	1.53	0.01	73.50	1.77	8.59	7.77	16.89
os02691	18	534	0.60	11	3	9	6.83	-0.01	0.03	1.48	0.08	99.44	-1.08	6.03	7.70	16.66
os40438	18	325	0.98	17	2	3	6.37	-0.15	0.29	1.43	0.15	93.26	-3.78	7.33	7.90	16.57
os84960	18	494	0.76	3	3	6	6.83	-0.01	1.31	1.53	0.03	97.49	-0.27	7.98	7.06	15.88
os23613	18	498	1.00	6	1	1	6.60	-0.10	0.46	1.48	0.02	96.91	2.37	6.23	8.08	17.07
os08090	18	468	0.91	7	2	3	6.57	-0.09	0.56	1.52	0.05	79.31	-0.82	7.48	8.05	17.15
os35516	18	346	0.00	3	1	4	6.82	0.05	0.09	1.41	0.04	97.40	3.93	7.09	8.10	16.78
os05821	18	429	0.02	3	3	6	6.84	0.04	2.64	1.55	0.02	52.59	-0.39	9.08	8.22	17.31
os31417	18	508	0.73	6	3	5	6.81	-0.02	0.51	1.54	0.06	63.13	-0.49	8.63	7.32	16.35
os85230	18	516	0.37	14	2	3	6.47	0.00	0.00	1.49	0.11	82.47	-2.41	6.99	6.88	15.66
os84133	18	539	1.00	13	2	0	6.30	-0.21	0.00	1.47	0.11	90.74	-0.57	7.11	6.54	15.27
os06221	18	406	0.07	5	3	6	6.89	0.07	0.14	1.47	0.04	90.93	0.99	7.36	8.38	17.44
os09140	18	450	0.92	3	1	4	6.84	0.04	0.00	1.47	0.02	92.93	5.03	7.12	7.79	16.75
os40010	18	429	0.22	13	2	3	6.67	0.10	0.01	1.46	0.10	96.17	-2.99	6.76	7.16	15.88
os07548	18	459	0.83	3	3	6	6.70	-0.05	0.86	1.54	0.02	64.00	0.00	7.88	7.94	16.90
os59217	18	438	0.49	9	2	3	6.57	-0.02	0.20	1.52	0.07	100.00	-1.56	6.69	7.34	16.22
os67714	18	493	0.67	6	3	5	6.61	-0.10	0.19	1.51	0.03	77.37	1.46	7.25	7.51	16.56
os83277	18	544	0.21	5	2	0	6.88	0.06	0.89	1.54	0.06	79.44	-1.88	7.07	6.62	15.37
os39596	18	394	0.40	3	3	6	6.84	0.02	0.28	1.50	0.03	94.51	1.30	7.26	7.40	16.06
os40442	18	358	0.84	16	1	4	6.39	-0.15	0.00	1.38	0.11	96.88	3.17	6.04	7.72	16.42
os07051	18	545	0.68	7	3	5	6.58	-0.07	0.47	1.51	0.07	97.08	-1.75	7.07	7.60	16.63

os03295	18	429	0.02	12	1	4	6.90	0.18	0.00	1.44	0.06	99.85	2.80	5.43	8.11	16.96
os75318	18	509	0.97	11	3	9	6.48	-0.16	0.02	1.43	0.08	99.99	1.05	5.88	7.05	15.99
os09175	18	588	0.40	9	1	1	6.99	0.06	0.23	1.46	0.04	95.85	1.44	6.52	7.07	16.00
os39184	18	431	0.22	6	3	5	6.94	0.08	0.22	1.50	0.05	90.92	0.94	6.88	7.16	15.82
os20034	18	442	0.99	4	3	6	6.68	-0.07	0.66	1.53	0.03	87.89	-0.51	7.30	8.30	17.32
os82997	18	536	0.87	5	3	5	6.80	-0.04	0.38	1.51	0.02	94.40	1.02	7.04	6.88	15.75
os82645	18	461	0.99	6	3	5	6.61	-0.10	0.47	1.53	0.04	96.55	-0.81	6.61	7.25	16.07
os62737	18	482	0.92	10	3	5	6.35	-0.17	0.16	1.46	0.06	95.04	1.13	7.18	7.14	16.02
os09240	18	361	0.85	6	1	4	6.52	-0.08	0.27	1.47	0.03	91.64	2.84	6.46	8.14	16.96
os38871	18	353	0.01	9	3	5	6.86	0.15	0.06	1.50	0.09	80.51	-0.77	6.57	7.73	16.45
os44710	18	431	0.15	19	1	4	6.55	0.18	0.00	1.32	0.10	98.87	8.11	4.97	7.03	15.76
os89295	18	450	0.99	9	3	5	6.57	-0.12	0.35	1.51	0.08	85.00	-0.49	8.26	7.39	16.37
os12645	18	507	0.42	9	1	4	6.63	0.01	0.03	1.45	0.05	77.46	2.84	7.35	7.74	16.60
os52083	18	582	0.01	19	1	4	7.03	0.32	0.00	1.32	0.11	99.22	8.00	4.80	6.07	14.84
os05775	18	490	0.92	4	3	6	6.69	-0.05	1.12	1.53	0.03	89.16	-0.08	8.53	7.97	17.06
os20642	18	478	0.60	2	3	6	6.77	-0.01	0.19	1.55	0.01	38.73	0.50	8.97	8.01	17.09
os38381	18	385	0.92	2	3	9	6.75	0.00	1.84	1.41	0.02	87.92	0.84	7.69	8.20	16.90
os24881	18	501	0.88	3	3	6	6.73	-0.02	0.35	1.53	0.04	97.68	0.42	7.59	7.77	16.81
os26335	18	625	0.01	10	3	9	7.06	0.16	0.04	1.48	0.05	99.09	1.17	5.90	7.34	16.41
os79218	18	456	0.40	5	3	6	6.89	0.03	0.81	1.52	0.04	97.00	-0.41	7.45	7.12	16.06
os83310	18	506	0.19	7	3	5	6.82	0.06	0.34	1.51	0.04	79.93	1.44	7.10	6.97	15.68
os46073	18	459	0.29	13	1	4	6.52	0.03	0.00	1.34	0.06	93.22	9.34	5.09	6.91	15.64
os74323	18	532	0.55	19	1	2	6.70	-0.02	0.00	1.41	0.12	91.71	3.21	5.27	6.63	15.51
os47231	18	437	0.83	11	3	5	6.59	-0.11	0.06	1.44	0.08	97.86	-0.67	5.53	7.03	15.75
os11064	18	569	0.23	2	2	3	6.85	0.01	2.66	1.54	0.05	88.56	-2.11	8.97	6.61	15.38
os51850	18	452	0.49	18	2	7	6.71	0.04	0.00	1.48	0.20	70.90	-4.37	6.08	6.28	15.02
os52257	18	314	0.66	10	1	4	6.39	-0.09	0.01	1.43	0.05	85.67	5.50	5.94	7.49	16.22
os68189	18	423	0.77	5	2	7	6.75	-0.03	1.02	1.54	0.10	84.47	-2.69	8.63	7.83	16.97
os10511	18	617	0.01	8	3	9	7.00	0.13	0.19	1.49	0.06	99.99	-0.04	5.80	6.48	15.18
os14608	18	615	0.23	8	3	9	6.85	0.07	0.39	1.45	0.09	100.00	-1.80	6.12	7.26	16.19
os61718	18	462	0.01	5	3	5	6.91	0.09	0.38	1.51	0.04	97.10	0.93	6.61	7.17	15.97
os39026	18	428	0.04	18	3	5	6.82	0.24	0.00	1.43	0.10	88.28	2.29	5.47	7.11	15.75
os66688	18	477	0.67	12	3	5	6.78	-0.02	0.01	1.46	0.07	99.13	1.30	5.95	7.59	16.62
os83163	18	509	0.12	11	2	7	6.82	0.11	0.04	1.49	0.10	89.44	-1.39	8.23	6.77	15.56
os35600	18	325	1.00	13	2	7	6.16	-0.21	0.23	1.52	0.18	92.65	-7.78	6.58	7.97	16.65
os06454	18	630	0.38	10	1	1	6.69	0.01	0.01	1.46	0.03	83.88	3.68	5.58	7.34	16.34
os58495	18	614	0.06	19	3	5	6.78	0.25	0.00	1.46	0.14	69.54	-2.31	6.24	6.29	15.01
os28333	18	505	0.83	4	3	6	6.78	-0.04	0.60	1.53	0.04	93.75	-0.49	6.92	7.21	16.06
os64669	18	363	0.24	7	3	5	6.93	0.10	0.25	1.47	0.06	98.63	1.14	5.93	7.45	16.28
os05196	18	391	0.88	3	3	6	6.71	-0.04	0.34	1.54	0.01	62.96	1.11	9.38	8.33	17.16
os83146	18	459	0.18	21	2	0	6.88	0.27	0.00	1.43	0.20	92.80	-2.65	6.04	6.94	15.69
os10901	18	553	0.02	5	3	5	6.90	0.07	1.07	1.51	0.06	93.48	-0.92	7.66	6.60	15.36
os66820	18	465	0.85	14	1	1	6.52	-0.13	0.00	1.41	0.10	99.66	2.26	5.42	7.51	16.39

os82666	18	484	0.77	3	3	6	6.80	-0.02	2.59	1.56	0.04	63.03	-1.27	8.94	7.06	15.85
os04813	18	524	0.05	10	3	9	6.88	0.13	0.03	1.47	0.05	97.31	0.88	5.48	7.53	16.52
os06438	18	561	0.01	16	1	4	6.88	0.24	0.00	1.43	0.10	99.84	2.76	5.04	7.59	16.60
os10397	18	580	0.01	6	3	5	6.95	0.09	0.38	1.51	0.05	93.02	0.74	6.27	6.64	15.43
os90820	18	456	0.04	12	3	5	7.02	0.20	0.02	1.47	0.10	98.23	-1.44	6.11	7.64	16.55
os21092	18	480	0.92	1	3	6	6.84	0.01	2.20	1.55	0.02	49.50	-0.18	8.85	7.58	16.47
os39624	18	451	0.04	7	3	5	6.96	0.11	0.30	1.50	0.04	83.11	0.33	6.37	7.05	15.72
os67790	18	416	0.01	7	1	4	6.95	0.13	0.25	1.48	0.05	79.55	2.99	6.55	7.88	16.92
os66821	18	465	1.00	18	3	9	6.04	-0.28	0.00	1.40	0.14	99.57	0.85	5.37	7.45	16.34
os67334	18	540	0.00	6	3	5	6.91	0.08	0.78	1.51	0.05	89.14	-0.28	8.86	7.06	16.10
os78899	18	477	0.42	3	3	6	6.72	-0.05	0.89	1.52	0.03	97.57	0.06	6.64	7.36	16.24
os66070	18	498	0.91	7	1	4	6.56	-0.08	0.01	1.40	0.04	97.72	7.65	5.69	7.45	16.53
os40641	18	457	0.31	14	1	1	6.53	0.04	0.00	1.38	0.05	98.39	4.35	5.47	7.26	15.91
os43816	18	450	0.23	3	1	4	6.74	0.01	0.03	1.43	0.06	98.64	3.63	6.40	6.87	15.47
os67301	18	506	0.01	1	3	6	6.84	0.01	0.55	1.54	0.01	38.32	1.20	9.19	7.38	16.45
os84310	18	545	0.02	7	3	5	7.01	0.12	0.32	1.50	0.08	99.97	0.42	6.57	6.76	15.58
os02340	18	574	0.82	2	3	6	6.80	-0.02	2.91	1.54	0.05	80.34	-0.83	8.02	7.09	16.09
os06868	18	478	0.92	1	3	6	6.78	-0.02	0.35	1.55	0.01	29.07	0.28	9.05	8.11	17.23
os14522	18	506	0.51	12	2	3	6.44	-0.07	0.04	1.49	0.09	99.25	-2.46	6.79	7.62	16.59
os10131	18	510	0.30	5	3	5	6.74	0.00	0.43	1.52	0.04	96.01	0.99	7.11	6.95	15.72
os12398	18	521	0.17	7	1	4	6.52	-0.13	0.28	1.46	0.04	90.60	2.72	6.67	7.62	16.50
os16015	18	578	0.76	17	3	9	6.07	-0.18	0.00	1.44	0.10	99.96	0.55	5.47	7.38	16.34
os66687	18	477	0.77	11	3	5	6.73	-0.05	0.01	1.47	0.06	99.20	1.22	6.11	7.59	16.62
os60481	18	424	0.05	8	3	5	6.87	0.11	0.21	1.50	0.07	96.00	0.16	6.58	7.59	16.53
os74204	18	605	0.23	7	3	5	6.94	0.08	0.11	1.45	0.13	99.76	1.40	5.65	6.21	15.07
os84976	18	513	0.09	2	3	6	6.88	0.02	1.79	1.55	0.02	70.85	-0.44	8.77	6.91	15.72
os41205	18	348	0.23	3	3	6	6.74	0.02	0.45	1.51	0.03	89.75	-0.09	6.22	7.96	16.73
os14242	18	534	0.87	4	3	6	6.78	-0.04	1.88	1.53	0.03	77.16	-0.68	8.15	7.37	16.29
os28947	18	516	0.92	1	3	6	6.86	0.02	2.57	1.55	0.02	44.23	0.28	9.09	7.24	16.14
os91459	18	457	0.01	7	1	4	6.83	0.07	0.02	1.46	0.05	99.00	5.19	5.78	7.68	16.58
os86123	18	488	0.90	4	3	6	6.66	-0.07	0.49	1.53	0.04	99.64	-0.27	7.30	7.34	16.23
os06684	18	501	0.79	5	3	6	6.65	-0.06	0.31	1.51	0.03	97.24	1.56	6.51	7.94	16.94
os23493	18	510	0.74	4	3	6	6.70	-0.04	1.34	1.53	0.04	92.12	-0.59	7.53	7.90	16.97
os12532	18	524	1.00	8	3	5	6.50	-0.14	0.19	1.51	0.06	99.72	-0.45	6.14	7.61	16.51
os44651	18	480	0.06	9	2	7	7.00	0.14	0.43	1.50	0.09	99.96	-3.05	7.37	6.73	15.49
os59879	18	463	0.22	9	1	4	6.78	0.09	0.11	1.44	0.06	93.29	4.11	6.58	7.17	16.07
os63027	18	499	0.99	5	3	6	6.72	-0.06	1.40	1.53	0.05	81.18	-0.93	7.35	6.97	15.81
os85113	18	607	0.56	3	3	6	6.83	0.00	0.19	1.53	0.02	57.75	0.46	8.12	6.39	15.15
os04060	18	397	1.00	11	1	4	6.34	-0.19	0.01	1.47	0.07	98.36	1.65	5.94	8.08	16.85
os13777	18	604	0.76	5	3	6	6.69	-0.06	0.37	1.51	0.03	99.28	0.40	6.77	7.23	16.09
os13678	18	566	0.93	7	3	5	6.68	-0.10	0.19	1.48	0.04	93.31	1.77	6.51	7.33	16.15
os15806	18	588	0.21	9	1	1	6.81	0.07	0.00	1.40	0.06	98.70	9.09	5.54	7.20	16.09
os17494	18	565	0.17	13	3	9	6.75	0.12	0.00	1.46	0.10	100.00	-1.47	5.63	6.75	15.65

os11798	18	575	0.42	5	3	9	6.77	0.00	0.40	1.48	0.03	88.87	1.97	6.58	6.79	15.49
os14813	18	526	0.20	4	1	1	6.79	0.00	0.03	1.46	0.02	100.00	3.72	6.13	8.00	16.99
os48912	18	454	0.38	7	2	3	6.94	0.09	0.84	1.52	0.08	94.91	-2.83	7.38	6.84	15.56
os83101	18	595	0.99	10	3	5	6.50	-0.16	0.03	1.47	0.06	98.41	1.16	6.27	6.47	15.25
os04404	18	366	0.75	6	3	5	6.53	-0.07	0.57	1.48	0.07	96.35	-0.90	6.30	8.28	17.08
os12640	18	554	0.40	6	3	5	6.95	0.05	0.49	1.51	0.04	82.91	-0.21	6.50	7.51	16.40
os80010	18	437	0.51	8	2	3	6.61	-0.02	0.83	1.53	0.08	98.41	-3.13	7.96	7.16	16.09
os24327	18	500	0.99	2	3	6	6.76	-0.04	1.69	1.55	0.03	76.70	-0.28	7.62	7.68	16.72
os63861	18	480	0.97	11	3	5	6.53	-0.14	0.02	1.45	0.07	97.16	1.43	5.87	7.25	16.16
os01346	18	650	0.53	10	3	5	6.99	0.05	0.18	1.49	0.09	97.89	-0.59	7.56	5.92	14.69
os11689	18	522	0.74	12	3	5	6.73	-0.06	0.01	1.47	0.07	100.00	0.39	5.99	6.87	15.71
os17064	18	621	0.98	17	3	9	6.06	-0.27	0.00	1.40	0.11	99.97	2.27	5.22	6.74	15.54
os62404	18	511	0.50	7	3	9	6.67	-0.01	0.45	1.43	0.05	86.49	0.91	7.07	7.16	16.01
os63796	18	570	0.30	8	1	1	6.72	0.02	0.13	1.42	0.04	98.98	3.03	5.92	6.99	15.90
os50972	18	422	0.81	12	3	5	6.51	-0.13	0.03	1.49	0.09	96.18	-0.49	5.37	7.30	16.07
os61464	18	499	0.40	4	3	6	6.77	0.01	0.59	1.52	0.03	95.17	0.12	7.60	7.03	15.94
os12918	18	520	0.91	5	3	5	6.72	-0.07	0.34	1.52	0.06	95.26	0.65	7.73	7.69	16.69
os61127	18	400	0.89	3	3	6	6.71	-0.04	1.92	1.55	0.06	90.83	-1.18	7.16	7.39	16.20
os06427	18	572	0.99	7	3	5	6.61	-0.12	0.24	1.49	0.04	79.19	0.49	6.03	7.50	16.58
os79783	18	396	0.96	6	3	5	6.61	-0.09	0.54	1.53	0.05	84.78	0.12	7.88	7.47	16.39
os05817	18	439	0.10	3	3	6	6.82	0.04	0.44	1.54	0.02	78.64	0.40	8.48	8.21	17.30
os82594	18	464	0.01	3	3	6	6.84	0.03	4.98	1.55	0.02	34.16	0.07	8.83	7.23	16.05
os02429	18	543	0.88	9	3	5	6.47	-0.14	0.15	1.51	0.07	84.62	-0.41	7.48	7.31	16.30
os10735	18	629	0.75	6	3	5	6.67	-0.06	0.48	1.52	0.06	99.46	-0.79	7.09	6.32	15.09
os82919	18	540	0.31	5	3	5	6.94	0.04	0.58	1.52	0.06	97.97	-0.88	7.56	6.82	15.68
os48723	18	461	0.01	13	3	9	6.91	0.20	0.01	1.42	0.07	99.69	-0.54	5.80	7.00	15.68
os49696	18	384	0.84	2	3	6	6.71	-0.03	1.58	1.54	0.00	74.53	-0.57	7.39	7.22	15.88
os04947	18	515	0.26	7	3	5	6.98	0.08	0.43	1.52	0.06	91.28	-1.52	6.64	7.43	16.40
os80031	18	435	0.76	3	3	6	6.82	0.01	1.89	1.54	0.06	95.43	-2.02	7.66	7.28	16.21
os66732	18	536	0.80	5	3	6	6.74	-0.06	0.50	1.48	0.05	99.78	0.67	6.08	7.10	16.11
os61699	18	474	0.60	5	3	9	6.72	0.00	0.58	1.49	0.03	99.89	-0.27	6.83	7.17	16.00
os79777	18	445	0.23	4	3	6	6.85	0.06	0.93	1.53	0.04	93.82	-0.82	7.14	7.33	16.27
os67664	18	443	0.68	2	3	6	6.82	0.00	2.22	1.52	0.03	60.83	1.71	8.23	7.86	17.00
os14611	18	558	0.75	12	3	5	6.36	-0.13	0.00	1.47	0.08	99.63	1.00	5.78	7.40	16.37
os28595	18	421	0.88	8	3	5	6.65	-0.08	0.35	1.42	0.05	84.58	3.03	6.21	8.27	17.26
os05544	18	544	0.60	3	3	9	6.76	-0.01	0.18	1.51	0.02	94.56	1.07	6.85	7.84	16.86
os14093	18	490	0.13	6	3	5	6.81	0.06	0.40	1.50	0.06	76.89	0.22	6.44	7.76	16.63
os06602	18	464	0.89	8	3	5	6.49	-0.11	0.13	1.49	0.05	76.33	1.55	7.35	8.01	17.07
os39843	18	369	0.31	13	1	4	6.61	0.09	0.00	1.42	0.07	92.32	6.15	5.28	7.49	16.16
os16953	18	533	0.53	15	1	4	6.26	-0.08	0.00	1.42	0.11	84.10	3.00	5.44	7.12	15.96
os66135	18	513	0.00	12	3	5	7.00	0.19	0.01	1.48	0.07	91.32	0.23	5.55	7.35	16.39
os65796	18	491	0.08	4	3	6	6.93	0.06	0.33	1.52	0.02	88.67	0.39	7.25	7.64	16.78
os78756	18	501	0.67	15	2	3	6.19	-0.15	0.00	1.48	0.12	100.00	-3.15	5.49	6.88	15.79

os79213	18	427	0.34	8	3	5	6.96	0.09	0.10	1.49	0.05	96.52	1.50	6.43	7.37	16.28
os07558	18	522	0.43	2	1	1	6.78	-0.03	0.05	1.48	0.01	99.10	2.40	6.71	7.87	16.88
os84142	18	479	0.19	17	1	4	7.00	0.19	0.00	1.44	0.11	85.63	1.34	6.22	6.94	15.73
os65770	18	497	0.83	10	1	4	6.45	-0.12	0.10	1.49	0.05	82.45	1.03	6.71	7.61	16.71
os79899	18	476	0.98	5	3	9	6.64	-0.08	0.60	1.51	0.03	94.15	0.64	6.95	7.15	16.09
os40576	18	380	0.08	7	3	5	6.95	0.11	0.30	1.50	0.05	94.37	0.34	6.98	7.81	16.57
os43077	18	440	0.56	9	3	5	6.84	0.01	0.20	1.47	0.06	94.80	-0.43	6.38	7.04	15.66
os58584	18	583	0.09	7	1	1	7.03	0.11	0.23	1.41	0.05	93.70	2.46	6.14	6.59	15.39
os01091	18	500	0.01	13	2	0	7.03	0.21	0.00	1.46	0.10	100.00	2.23	5.44	6.92	15.61
os43402	18	475	0.35	13	1	4	6.57	0.07	0.00	1.42	0.10	98.98	4.89	5.92	6.88	15.68
os27975	18	462	0.96	4	2	3	6.72	-0.06	1.49	1.55	0.08	96.00	-3.49	7.85	7.52	16.42
os51040	18	510	0.89	8	3	5	6.74	-0.06	0.39	1.51	0.09	98.78	-1.39	7.04	6.52	15.32
os50911	18	433	0.62	11	3	5	6.34	-0.11	0.04	1.48	0.10	91.70	-0.80	6.90	7.12	15.92
os19366	18	655	0.12	11	3	9	6.79	0.09	0.02	1.46	0.09	100.00	-1.14	6.05	6.29	15.17
os20333	18	429	0.67	3	3	6	6.77	-0.02	1.93	1.54	0.04	74.07	-0.82	8.61	8.37	17.38
os36229	18	326	0.52	19	1	4	6.77	0.07	0.00	1.32	0.09	95.99	9.10	5.19	8.15	16.83
os64666	18	423	0.30	12	3	5	6.95	0.12	0.01	1.48	0.08	99.70	0.03	5.33	7.14	16.00
os25108	18	501	0.12	6	3	6	6.84	0.06	0.57	1.55	0.05	59.26	-0.77	8.53	7.56	16.56
os42763	18	391	0.01	9	3	5	6.92	0.16	0.18	1.50	0.09	98.45	-1.91	7.14	7.40	16.20
os06924	18	491	0.97	5	3	5	6.72	-0.06	0.63	1.50	0.04	97.83	0.23	7.25	8.08	17.28
os61763	18	449	0.16	7	1	1	6.97	0.09	0.05	1.39	0.04	98.99	4.52	5.95	7.36	16.20
os83487	18	491	0.01	2	3	6	6.85	0.03	3.83	1.56	0.03	34.92	-0.67	8.89	7.03	15.78
os46895	18	446	0.10	9	3	5	6.85	0.11	0.22	1.49	0.07	98.74	-0.77	6.94	6.95	15.65
os85282	18	450	0.40	4	3	6	6.89	0.04	1.07	1.54	0.03	97.86	-0.47	7.98	7.32	16.29
os42060	18	490	0.92	2	3	9	6.73	-0.04	0.32	1.48	0.01	65.15	1.96	7.29	7.54	16.32
os79589	18	570	0.20	6	1	1	6.97	0.06	0.03	1.48	0.02	96.93	4.22	5.97	6.80	15.68
os07244	18	446	0.99	5	3	6	6.64	-0.08	0.71	1.50	0.05	91.70	0.92	7.41	8.18	17.35
os23349	18	525	0.43	6	3	5	6.73	0.00	0.36	1.53	0.06	95.98	-0.01	7.22	7.67	16.70
os08690	18	460	0.40	3	3	6	6.85	0.01	0.92	1.54	0.02	82.34	0.39	7.58	8.26	17.44
os42661	18	394	0.18	5	2	0	6.61	-0.08	0.72	1.54	0.05	92.31	-0.65	8.18	7.25	16.05
os40638	18	362	0.10	3	3	6	6.67	-0.05	0.61	1.53	0.01	72.54	0.99	7.86	7.73	16.37
os86444	20	548	0.84	5	3	6	6.74	-0.06	0.53	1.53	0.04	100.00	0.26	7.26	7.01	15.93
os08422	20	465	0.99	2	3	6	6.75	-0.03	0.97	1.54	0.02	90.48	0.13	7.65	8.20	17.33
os24221	20	539	0.92	1	3	6	6.81	-0.02	2.97	1.56	0.03	62.60	-0.56	8.75	7.54	16.59
os15631	20	470	0.75	2	3	6	6.82	0.00	1.82	1.55	0.04	82.21	-0.61	8.34	8.03	16.98
os02694	20	554	0.60	6	2	3	6.67	-0.04	0.93	1.51	0.08	99.57	-2.82	7.52	7.55	16.49
os72649	20	250	0.79	9	3	5	6.49	-0.12	0.37	1.53	0.08	66.56	-2.03	7.04	8.53	17.43
os06649	20	543	0.20	3	3	6	6.93	0.05	0.54	1.51	0.02	90.37	1.20	7.91	7.71	16.78
os03594	20	461	0.60	6	3	5	6.64	-0.04	0.56	1.52	0.05	94.69	-0.22	7.30	7.89	16.77
os14182	20	472	0.97	5	2	0	6.77	-0.04	1.56	1.54	0.08	97.50	-1.06	7.99	7.67	16.49
os75928	20	425	0.99	4	3	6	6.66	-0.07	0.59	1.52	0.05	90.90	-0.59	7.76	7.66	16.50
os20877	20	444	0.15	1	3	6	6.80	0.01	3.94	1.56	0.01	2.22	0.26	9.31	7.88	16.86
os79579	20	424	0.93	3	3	6	6.73	-0.04	1.85	1.54	0.03	86.58	-0.88	8.11	7.49	16.40

os82493	20	472	0.85	2	3	6	6.77	-0.03	4.98	1.56	0.02	25.92	-0.39	9.17	7.18	16.00
os20843	20	520	0.08	2	3	6	6.89	0.03	0.79	1.54	0.02	49.91	0.07	8.69	7.43	16.42
os41917	20	462	0.25	10	1	1	6.93	0.08	0.37	1.47	0.06	96.62	1.75	6.37	7.57	16.33
os27387	20	451	0.99	3	3	6	6.69	-0.06	1.18	1.56	0.05	88.69	-0.83	8.13	7.23	16.02
os82667	20	488	0.92	1	3	6	6.84	0.01	1.61	1.56	0.02	66.06	-0.34	8.61	7.07	15.86
os12642	20	558	0.03	7	3	5	6.90	0.09	0.54	1.51	0.06	99.74	-0.72	6.86	7.50	16.39
os59754	20	501	0.92	2	3	6	6.76	-0.03	2.69	1.52	0.02	45.60	-0.36	8.73	6.93	15.86
os74701	20	426	0.23	3	3	6	6.78	0.02	2.00	1.53	0.06	97.59	-0.03	7.29	7.45	16.43
os04943	20	509	0.65	3	3	6	6.74	-0.04	1.88	1.55	0.03	55.63	-0.99	8.37	7.38	16.35
os06650	20	498	0.01	4	3	6	6.90	0.06	1.19	1.54	0.04	94.38	-0.60	8.34	7.85	16.91
os09152	20	516	0.19	2	3	6	6.87	0.01	3.02	1.56	0.05	34.34	0.21	9.12	7.02	15.90
os20639	20	441	0.76	2	3	6	6.83	0.01	2.81	1.55	0.02	47.50	0.25	9.09	8.21	17.26
os66866	20	517	0.92	1	3	6	6.83	0.00	2.98	1.55	0.04	90.59	-0.84	9.20	7.35	16.46
os23296	20	456	0.40	3	2	3	6.85	0.02	2.97	1.55	0.06	85.88	-2.47	9.19	8.10	17.11
os11086	20	606	0.04	4	3	6	6.92	0.06	1.03	1.53	0.03	77.71	-0.22	7.66	6.32	14.99
os22042	20	437	0.23	1	3	6	6.80	0.01	1.91	1.55	0.02	43.55	-0.14	8.37	8.23	17.26
os79672	20	434	0.99	3	3	6	6.73	-0.04	1.06	1.54	0.03	80.97	0.11	7.73	7.29	16.16
os12593	20	544	0.60	3	3	6	6.75	-0.02	0.97	1.53	0.04	99.51	-0.01	7.16	7.58	16.47
os07604	20	409	0.81	3	3	6	6.67	-0.04	1.17	1.55	0.03	79.27	0.19	8.81	8.38	17.42
os42972	20	414	0.36	15	2	3	6.42	0.02	0.17	1.49	0.18	93.41	-7.41	6.83	7.24	16.03
os86700	20	433	0.92	7	2	0	6.53	-0.11	0.71	1.53	0.08	95.37	-1.98	7.11	7.61	16.59
os07951	20	459	0.00	3	3	6	6.86	0.04	2.23	1.55	0.03	59.67	-1.19	8.93	8.10	17.12
os54885	20	369	0.23	3	3	6	6.76	0.02	3.62	1.54	0.02	53.62	-0.07	9.20	7.38	16.29
os62460	20	412	0.19	3	3	6	6.86	0.03	1.82	1.55	0.06	86.45	-1.33	7.92	7.37	16.23
os85521	20	545	0.65	2	3	6	6.85	-0.01	4.97	1.56	0.03	32.82	-0.26	9.24	6.65	15.37
os21732	20	489	0.60	8	2	3	6.55	-0.05	0.06	1.52	0.08	89.80	-2.31	8.41	7.91	16.86
os28417	20	499	0.00	3	3	6	6.89	0.05	2.87	1.54	0.03	60.40	-1.18	9.47	7.39	16.29
os87169	20	385	0.58	3	3	6	6.82	0.01	0.76	1.54	0.04	92.38	0.12	7.27	8.10	17.05
os83181	20	518	0.73	6	2	3	6.59	-0.11	0.93	1.52	0.09	99.71	-3.52	8.56	6.81	15.60
os86049	20	518	0.77	2	3	6	6.83	-0.01	2.81	1.55	0.03	53.40	-0.08	9.28	7.09	15.99
os21636	20	393	0.06	1	3	6	6.81	0.02	3.20	1.57	0.02	40.25	-0.60	8.47	8.57	17.66
os80109	20	413	0.15	5	2	3	6.91	0.07	0.80	1.53	0.06	90.22	-2.24	6.60	7.53	16.43
os06691	20	485	0.00	5	3	6	6.93	0.09	0.97	1.53	0.05	95.61	-1.26	6.97	7.99	17.00
os85632	20	420	0.92	3	3	6	6.68	-0.05	1.43	1.55	0.04	93.64	-0.20	8.38	7.62	16.53
os21530	20	421	0.76	7	2	3	6.51	-0.12	0.32	1.55	0.06	66.68	-2.14	7.55	7.99	16.93
os08309	20	485	0.84	3	3	6	6.74	-0.02	2.31	1.55	0.04	76.57	-1.13	8.65	8.00	17.13
os19641	20	471	0.44	2	3	6	6.82	-0.01	2.76	1.55	0.06	88.66	-1.34	8.48	7.94	16.87
os37993	20	320	0.60	7	3	5	6.45	-0.10	0.56	1.52	0.07	81.09	-1.41	7.07	7.88	16.59
os81713	20	434	0.23	3	3	6	6.79	0.03	0.72	1.53	0.05	64.93	0.00	8.28	7.41	16.40
os86508	20	401	0.15	12	2	0	7.00	0.17	0.01	1.49	0.10	68.40	-1.48	6.70	7.75	16.64
os08671	20	402	0.98	9	2	0	6.57	-0.11	0.33	1.52	0.10	92.21	-1.31	8.08	8.42	17.48
os26803	20	500	0.90	2	3	6	6.78	-0.03	1.26	1.55	0.02	55.41	-0.17	8.77	6.94	15.76
os73092	20	391	0.92	1	3	6	6.73	-0.02	1.41	1.54	0.01	13.94	0.12	8.88	7.65	16.43

os82488	20	493	0.23	1	3	6	6.82	0.01	5.74	1.55	0.01	26.06	0.01	8.18	7.06	15.85
os50719	20	455	0.60	2	3	6	6.77	-0.01	1.54	1.52	0.02	51.88	0.14	8.87	7.03	15.77
os83240	20	531	0.92	0	3	6	6.83	0.00	2.37	1.56	0.01	10.57	-0.10	12.08	6.83	15.58
os85442	20	436	0.01	5	3	5	6.94	0.09	0.75	1.53	0.05	94.18	-1.47	7.44	7.32	16.25
os72775	20	259	0.09	1	3	6	6.68	-0.02	3.97	1.56	0.02	6.04	-0.20	10.21	8.45	17.46
os06117	20	458	0.92	0	3	6	6.79	-0.01	3.81	1.55	0.02	24.06	-0.28	9.11	7.89	16.92
os06651	20	500	0.01	4	3	6	6.90	0.07	1.05	1.54	0.04	94.52	-0.64	8.29	7.84	16.91
os19740	20	498	0.92	2	3	6	6.74	-0.04	1.93	1.54	0.02	57.18	-0.43	9.17	7.74	16.66
os14181	20	435	0.49	5	2	0	6.62	-0.09	0.97	1.55	0.06	54.67	-1.35	7.91	7.81	16.66
os79247	20	383	0.60	2	3	6	6.71	-0.02	2.67	1.56	0.05	78.40	-1.31	8.06	7.55	16.44
os21702	20	434	0.23	2	3	6	6.79	0.01	2.86	1.56	0.03	42.70	-0.65	8.94	8.48	17.64
os20158	20	440	0.05	7	3	5	6.88	0.11	0.31	1.52	0.05	91.11	-0.08	6.89	8.18	17.07
os22052	20	483	0.60	5	2	3	6.75	0.01	1.79	1.55	0.05	94.28	-2.50	8.52	7.98	17.00
os73255	20	348	0.92	1	3	6	6.72	-0.02	0.64	1.56	0.01	15.72	0.25	9.35	7.95	16.85
os03836	20	491	0.92	0	3	6	6.83	0.00	2.45	1.54	0.01	17.31	-0.10	10.84	7.67	16.46
os11260	20	582	0.45	6	3	5	6.73	0.00	0.55	1.50	0.07	99.74	-0.40	7.62	6.52	15.33
os61523	20	447	0.08	6	3	5	6.87	0.09	0.48	1.53	0.05	94.67	-0.49	7.33	7.01	15.84
os19954	20	384	0.92	0	3	6	6.75	-0.01	5.04	1.56	0.02	33.77	-0.50	8.86	8.67	17.67
os61525	20	456	0.00	7	3	5	6.92	0.11	0.67	1.52	0.05	95.76	-0.70	6.67	6.99	15.82
os72497	20	266	0.53	5	2	3	6.63	-0.03	2.44	1.55	0.07	70.23	-2.79	8.15	8.39	17.21
os02306	20	461	0.92	7	2	7	6.53	-0.10	1.02	1.55	0.09	91.55	-4.07	8.42	7.87	16.87
os11500	20	590	0.76	7	3	5	6.82	-0.04	0.68	1.52	0.06	97.79	-1.60	7.47	6.51	15.27
os64910	20	305	0.61	2	3	6	6.76	0.00	0.77	1.53	0.01	82.10	-0.04	7.91	7.91	16.80
os19681	20	419	0.99	4	3	6	6.67	-0.06	1.01	1.55	0.04	79.16	-0.74	7.10	8.39	17.37
os11947	20	466	1.00	6	2	0	6.62	-0.09	0.46	1.54	0.08	79.93	-0.76	7.94	7.33	16.22
os86153	20	457	0.92	4	3	6	6.66	-0.05	0.59	1.53	0.04	99.95	0.23	7.35	7.64	16.49
os43241	20	278	0.35	7	3	5	6.62	0.01	0.62	1.51	0.00	88.61	-1.36	7.51	7.89	16.54
os61818	20	409	0.00	2	3	6	6.84	0.04	3.96	1.54	0.03	53.83	-0.27	8.56	7.39	16.26
os65829	20	534	0.96	6	3	5	6.66	-0.10	0.31	1.51	0.05	84.87	0.17	7.51	7.25	16.34
os21010	20	476	0.98	4	3	6	6.76	-0.04	0.87	1.54	0.03	81.32	-0.48	7.80	7.77	16.66
os22902	20	430	0.60	1	3	6	6.76	-0.01	3.77	1.56	0.01	9.99	0.14	9.27	8.15	17.16
os83908	20	554	0.60	3	3	6	6.76	-0.02	1.48	1.54	0.04	100.00	-0.13	7.26	6.90	15.83
os42888	20	293	0.53	8	2	3	6.58	0.00	0.49	1.53	0.07	95.83	-1.89	7.12	7.83	16.48
os73093	20	391	0.92	1	3	6	6.74	-0.02	1.29	1.54	0.01	13.57	0.09	8.86	7.64	16.43
os11696	20	534	0.61	9	2	0	6.55	-0.07	0.19	1.50	0.07	97.04	0.57	8.14	6.65	15.45
os12923	20	561	0.99	2	3	6	6.80	-0.03	1.48	1.55	0.01	6.04	0.12	8.78	7.45	16.30
os76040	20	548	0.89	3	3	6	6.75	-0.05	1.55	1.53	0.04	95.80	-0.23	6.88	6.91	15.72
os59753	20	501	0.92	2	3	6	6.76	-0.03	2.78	1.52	0.02	44.14	-0.33	8.69	6.93	15.86
os82486	20	493	0.08	1	3	6	6.85	0.02	5.90	1.55	0.00	3.46	0.48	9.09	7.09	15.88
os20121	20	453	0.33	3	3	6	6.84	0.01	1.96	1.55	0.08	94.24	-2.80	8.23	8.10	17.03
os21009	20	493	0.18	7	3	5	6.99	0.10	0.46	1.52	0.05	73.42	-0.75	7.98	7.70	16.61
os21211	20	467	0.60	2	3	6	6.78	0.00	2.93	1.55	0.02	29.50	-0.09	9.09	7.49	16.43
os86601	20	448	0.99	9	1	4	6.42	-0.16	0.06	1.47	0.07	76.96	2.18	7.17	7.46	16.38

os08214	20	453	0.75	5	2	3	6.64	-0.07	1.60	1.55	0.05	81.42	-2.03	8.62	8.15	17.24
os61398	20	417	0.60	2	3	6	6.71	-0.03	3.04	1.56	0.03	56.55	-1.09	8.26	7.33	16.19
os86398	20	646	0.01	2	3	6	6.94	0.04	0.97	1.54	0.01	88.24	0.11	7.64	6.55	15.45
os21631	20	390	0.92	0	3	6	6.77	0.00	6.01	1.57	0.02	20.73	-0.12	9.70	8.62	17.69
os20066	20	435	0.99	2	3	6	6.73	-0.04	1.51	1.55	0.02	78.85	-0.25	7.50	8.32	17.33
os04150	20	399	0.92	3	3	6	6.68	-0.04	1.49	1.54	0.02	62.30	-0.35	8.19	8.15	16.93
os22043	20	436	0.92	1	3	6	6.82	0.02	2.21	1.55	0.02	36.59	-0.34	8.58	8.23	17.26
os22992	20	560	0.01	2	3	6	6.89	0.03	1.82	1.54	0.02	71.75	-0.30	7.64	7.60	16.59
os31811	20	462	0.57	3	3	6	6.71	-0.03	1.84	1.55	0.03	60.07	-0.61	9.14	8.05	17.08
os83718	20	586	0.08	5	3	6	6.98	0.07	0.57	1.53	0.04	100.00	-0.28	6.88	6.83	15.72
os85450	20	558	0.33	7	3	5	6.97	0.06	0.36	1.51	0.07	100.00	-0.92	6.68	6.61	15.54
os64124	20	477	0.77	2	3	6	6.80	-0.02	0.53	1.53	0.03	86.43	0.02	7.06	7.33	16.26
os83911	20	618	0.19	1	3	6	6.89	0.02	1.92	1.54	0.01	21.30	0.04	9.73	6.72	15.62
os83960	20	419	0.76	6	3	5	6.55	-0.09	0.44	1.52	0.04	98.01	0.10	6.31	7.78	16.72
os41424	20	456	0.77	6	3	5	6.77	-0.04	0.77	1.53	0.05	87.60	-0.71	7.00	7.27	15.99
os13970	20	544	0.00	3	3	6	6.74	-0.05	1.60	1.56	0.00	79.57	-0.53	7.25	7.04	15.94
os21855	20	485	0.12	2	3	6	6.85	0.02	3.62	1.55	0.01	8.76	0.11	9.05	8.06	17.14
os83042	20	536	0.59	1	3	6	6.80	-0.02	4.03	1.56	0.01	23.28	0.12	8.93	6.74	15.50
os05590	20	400	0.60	2	2	0	6.72	-0.02	3.93	1.56	0.06	77.18	-1.27	8.71	8.41	17.40
os06683	20	492	0.79	6	3	5	6.59	-0.09	0.48	1.52	0.05	95.61	-0.69	6.88	7.91	16.92
os08213	20	451	0.92	3	2	3	6.70	-0.04	2.05	1.55	0.05	79.40	-2.81	8.73	8.16	17.24
os83018	20	537	0.92	2	3	6	6.87	0.02	4.91	1.54	0.02	52.14	0.71	8.59	6.74	15.47
os83712	20	441	0.07	5	3	6	6.91	0.06	2.16	1.55	0.05	72.52	-1.34	8.63	7.48	16.39
os26585	20	491	0.15	14	2	3	6.69	0.12	0.20	1.52	0.13	90.35	-4.87	7.60	7.88	16.90
os21199	20	479	0.77	2	3	6	6.81	-0.02	1.71	1.55	0.02	54.91	-0.29	8.45	7.44	16.38
os26630	20	479	0.60	2	3	6	6.81	0.01	3.46	1.56	0.03	60.34	-1.46	9.19	7.94	17.00
os85039	20	539	0.08	1	3	6	6.88	0.02	2.94	1.55	0.02	20.17	-0.35	9.30	6.73	15.52
os21962	20	474	0.02	1	3	6	6.84	0.02	3.95	1.56	0.02	12.50	-0.55	9.46	8.05	17.13
os17255	20	578	0.21	3	3	6	6.93	0.03	2.66	1.53	0.06	79.92	-0.58	8.31	6.68	15.59
os22051	20	482	0.53	5	2	3	6.74	0.00	1.97	1.55	0.06	94.54	-2.64	8.56	7.98	17.01
os80044	20	433	0.01	4	3	6	6.91	0.07	0.72	1.52	0.03	81.59	0.15	8.20	7.36	16.30
os21255	20	408	0.92	0	3	6	6.78	0.00	5.47	1.57	0.01	0.06	0.00	11.73	8.50	17.60
os42898	20	290	0.92	1	3	6	6.70	-0.02	2.42	1.54	0.07	86.77	0.22	8.87	7.84	16.47
os64761	20	404	0.27	6	2	3	6.59	-0.09	1.19	1.54	0.05	71.73	-1.78	7.89	7.39	16.28
os05064	20	454	0.92	1	3	6	6.83	0.02	3.10	1.54	0.03	36.60	-0.33	9.36	7.66	16.56
os86163	20	531	0.08	3	3	6	6.92	0.05	1.60	1.55	0.02	57.02	-0.12	8.17	7.17	16.11
os12912	20	461	0.77	3	3	6	6.80	-0.02	2.70	1.56	0.05	83.75	-1.52	9.71	8.01	16.98
os21528	20	419	0.92	1	3	6	6.76	-0.02	2.59	1.56	0.04	60.35	-1.81	8.43	7.99	16.94
os65082	20	255	0.92	3	2	3	6.62	-0.03	1.90	1.54	0.06	92.23	-3.24	8.11	8.21	17.06
os78885	20	464	0.23	2	3	6	6.82	0.03	1.34	1.55	0.04	96.48	0.08	7.80	7.28	16.17
os02209	20	456	0.40	3	3	6	6.87	0.02	1.66	1.54	0.06	83.19	-0.17	9.01	7.68	16.65
os04946	20	514	0.21	2	3	6	6.88	0.03	2.43	1.56	0.04	65.52	-0.95	8.16	7.35	16.31
os21549	20	449	0.01	2	3	6	6.84	0.03	1.48	1.55	0.01	20.69	0.05	8.94	8.43	17.61

os53400	20	380	0.00	2	2	0	6.84	0.04	2.37	1.54	0.06	80.11	-0.05	7.78	7.26	16.12
os27746	20	512	0.08	2	3	6	6.87	0.02	2.95	1.55	0.04	80.53	-1.02	9.09	6.91	15.76
os21875	20	513	0.52	6	3	5	6.57	-0.11	0.49	1.52	0.04	99.00	-0.49	6.47	7.91	16.95
os54924	20	365	0.41	3	3	6	6.78	-0.01	1.69	1.55	0.02	46.38	-0.20	8.45	7.46	16.36
os86469	20	517	0.10	7	3	5	6.54	-0.12	0.31	1.52	0.05	99.03	0.09	7.10	7.20	16.12
os21546	20	452	0.92	1	3	6	6.83	0.02	1.31	1.55	0.01	35.59	0.12	8.76	8.42	17.60
os26802	20	501	0.85	3	3	6	6.77	-0.04	1.05	1.55	0.02	55.53	0.02	8.68	6.94	15.76
os19761	20	431	0.92	0	3	6	6.79	0.00	5.71	1.57	0.01	8.19	-0.23	10.42	8.21	17.24
os86505	20	400	0.02	14	2	7	6.90	0.18	0.04	1.48	0.13	66.18	-2.01	6.43	7.77	16.66
os12707	20	531	1.00	6	3	5	6.63	-0.10	0.22	1.52	0.06	85.39	0.74	7.71	7.45	16.29
os20035	20	433	0.99	3	3	6	6.71	-0.05	1.23	1.54	0.03	67.93	-0.44	7.35	8.32	17.34
os22711	20	433	0.42	2	3	6	6.82	0.00	6.77	1.56	0.01	4.60	-0.04	8.77	7.97	16.94
os83880	20	435	0.01	2	3	6	6.84	0.03	2.55	1.55	0.04	81.84	-0.77	8.83	7.54	16.46
os42057	20	485	0.40	2	3	9	6.87	0.02	1.47	1.47	0.01	72.33	0.15	7.61	7.50	16.26
os22866	20	479	0.92	0	3	6	6.80	-0.01	2.67	1.56	0.01	8.06	-0.10	9.02	7.49	16.42
os87046	20	349	0.97	9	2	7	6.50	-0.11	0.18	1.52	0.13	86.65	-1.57	8.04	7.93	16.85
os19760	20	429	0.92	0	3	6	6.79	0.00	5.79	1.57	0.01	1.87	-0.21	11.41	8.21	17.25
os78995	20	430	0.48	3	3	6	6.73	-0.01	2.42	1.54	0.04	89.74	-1.30	8.21	7.35	16.28
os09586	20	284	0.92	1	3	6	6.73	0.00	3.79	1.56	0.02	25.20	-0.35	9.70	8.48	17.25
os72307	20	284	0.39	3	3	6	6.81	0.03	1.23	1.53	0.02	95.05	0.09	7.44	8.29	17.26
os09392	20	312	0.93	1	3	6	6.71	-0.02	1.74	1.55	0.02	51.08	-0.19	8.51	8.30	17.05
os73346	20	378	0.74	3	3	6	6.68	-0.04	1.45	1.53	0.03	72.47	-0.02	8.06	7.74	16.62
os14183	20	483	0.85	4	2	7	6.72	-0.06	1.46	1.55	0.08	98.42	-2.83	7.92	7.63	16.46
os20949	20	470	0.23	1	3	6	6.80	0.00	5.90	1.56	0.01	19.81	0.32	9.17	7.46	16.34
os78667	20	516	0.90	5	3	6	6.65	-0.09	0.54	1.52	0.05	99.12	-0.54	7.10	6.96	15.88
os08670	20	403	0.92	8	2	0	6.56	-0.11	0.52	1.52	0.10	92.32	-2.07	7.98	8.42	17.48
os20172	20	472	0.08	4	3	6	6.91	0.06	1.58	1.52	0.03	83.99	-0.96	7.21	8.11	17.00
os76385	20	549	0.02	3	3	6	6.73	-0.05	1.17	1.53	0.05	96.62	-0.35	6.63	6.90	15.71
os24297	20	479	0.60	1	3	6	6.79	-0.01	3.57	1.56	0.02	38.01	-0.22	8.57	7.93	16.99
os20170	20	461	0.01	3	3	6	6.88	0.06	0.74	1.52	0.03	94.43	0.27	7.38	8.10	16.99
os20327	20	521	1.00	5	3	5	6.66	-0.09	0.47	1.52	0.04	97.37	-0.18	6.57	7.80	16.77
os07949	20	460	0.04	3	3	6	6.87	0.05	2.05	1.55	0.03	58.29	-1.01	8.83	8.10	17.12
os14124	20	481	0.99	3	3	6	6.72	-0.06	0.44	1.54	0.03	78.58	0.00	8.10	7.64	16.54
os83468	20	498	0.36	2	3	6	6.80	0.00	2.80	1.55	0.02	20.03	-0.30	9.21	7.03	15.80
os21900	20	448	0.01	2	3	6	6.83	0.03	3.95	1.56	0.02	7.10	-0.65	9.36	8.25	17.41
os63895	20	527	0.60	2	3	6	6.79	-0.01	2.69	1.53	0.03	88.34	0.20	8.25	6.95	15.88
os02093	20	477	0.60	5	2	0	6.68	-0.02	0.82	1.54	0.08	99.92	-1.09	7.71	7.78	16.71
os21164	20	465	0.92	0	3	6	6.80	0.00	5.98	1.56	0.00	0.00	0.16	11.14	7.46	16.36
os58064	20	634	0.60	3	3	6	6.82	-0.01	1.83	1.53	0.03	74.97	-0.43	7.80	6.23	15.01
os23012	20	485	0.08	1	3	6	6.84	0.01	2.83	1.55	0.01	1.35	0.25	8.89	7.85	16.80
os73150	20	356	0.92	2	3	6	6.71	-0.02	3.84	1.56	0.02	14.29	-0.19	9.54	7.78	16.65
os43812	20	452	0.00	6	3	5	6.95	0.11	0.56	1.52	0.06	98.48	-0.45	7.33	6.81	15.47
os79950	20	487	0.00	6	3	5	6.96	0.10	0.79	1.52	0.06	99.56	-1.60	7.94	6.96	15.87

os06945	20	454	0.99	2	3	6	6.75	-0.03	1.48	1.55	0.02	53.99	-0.25	8.78	8.24	17.42
os38960	20	358	0.00	5	2	3	6.61	-0.05	1.87	1.53	0.11	83.43	-5.74	7.35	7.57	16.23
os19948	20	384	0.92	1	3	6	6.75	-0.01	5.56	1.54	0.02	30.54	0.40	9.43	8.68	17.67
os81444	20	527	0.40	2	2	3	6.86	0.00	2.51	1.55	0.05	84.62	-2.23	8.67	6.81	15.75
os13428	20	479	0.63	5	2	0	6.62	-0.07	0.76	1.53	0.08	96.74	-0.82	7.38	7.65	16.48
os20495	20	432	0.74	3	3	6	6.69	-0.03	1.56	1.55	0.03	58.56	-0.74	7.57	8.25	17.32
os73534	20	406	0.04	7	2	7	6.77	0.07	0.76	1.51	0.12	98.15	-3.81	8.18	7.50	16.20
os37722	20	363	0.63	6	2	3	6.65	-0.02	1.13	1.52	0.10	90.16	-4.05	7.63	7.61	16.30
os19673	20	452	0.31	4	3	6	6.88	0.03	1.50	1.54	0.04	91.60	-0.67	7.91	8.23	17.20
os61212	20	411	0.98	2	3	6	6.74	-0.03	2.94	1.56	0.03	59.53	-0.83	9.04	7.38	16.26
os78413	20	465	0.92	6	2	3	6.57	-0.10	0.69	1.53	0.07	98.51	-2.14	6.17	7.19	16.09
os14119	20	465	0.92	7	3	5	6.54	-0.10	0.29	1.52	0.04	97.08	0.09	7.14	7.75	16.63
os05735	20	494	0.23	2	3	6	6.84	0.02	1.39	1.54	0.01	75.64	-0.04	7.77	7.99	17.12
os20010	20	397	0.92	0	3	6	6.78	0.00	2.30	1.56	0.01	22.34	-0.37	8.79	8.58	17.61
os65069	20	249	0.62	2	3	6	6.73	0.00	1.76	1.54	0.01	49.57	0.02	8.46	8.36	17.18
os85924	20	612	0.40	2	3	6	6.91	0.02	1.43	1.54	0.01	24.19	-0.17	8.41	6.77	15.77
os23589	20	557	0.76	2	2	0	6.85	-0.01	1.94	1.55	0.07	97.43	-1.49	8.06	7.38	16.36
os20254	20	533	0.99	4	3	6	6.72	-0.06	0.67	1.53	0.02	96.78	0.03	7.22	7.67	16.62
os78033	20	457	0.02	4	2	0	6.87	0.07	0.78	1.53	0.08	97.02	-0.78	7.87	7.10	16.01
os89772	20	501	0.00	7	3	5	6.95	0.11	0.37	1.51	0.08	99.94	-0.31	6.39	7.00	15.92
os09952	20	501	1.00	10	2	3	6.43	-0.16	0.39	1.52	0.10	99.81	-3.81	7.34	6.92	15.68
os19924	20	393	0.77	1	3	6	6.76	-0.01	4.17	1.56	0.02	11.25	0.00	9.65	8.66	17.70
os09025	20	497	0.23	6	3	5	6.81	0.05	0.69	1.53	0.05	70.65	-0.89	7.29	7.36	16.25
os22065	20	451	0.01	3	3	6	6.86	0.04	1.51	1.55	0.02	97.16	-0.05	7.71	8.15	17.19
os56568	20	595	0.79	1	3	6	6.83	-0.01	0.56	1.53	0.01	31.24	0.00	8.61	6.12	14.97
os08453	20	394	0.31	3	3	6	6.74	0.00	2.09	1.51	0.04	84.04	-1.77	8.61	8.58	17.69
os77815	20	520	0.77	2	3	6	6.83	-0.01	2.66	1.54	0.04	74.11	0.02	8.58	7.00	15.92
os78262	20	395	0.35	7	2	3	6.56	-0.02	1.19	1.53	0.13	97.33	-7.11	8.21	7.58	16.54
os78682	20	477	0.52	6	2	3	6.62	-0.03	0.81	1.51	0.12	98.26	-2.22	7.51	7.10	16.01
os06735	20	444	0.99	5	3	6	6.65	-0.08	0.90	1.54	0.04	92.08	-0.58	7.57	8.23	17.30
os06931	20	449	0.53	3	3	6	6.84	0.01	2.05	1.55	0.02	49.82	-0.07	9.06	8.28	17.45
os38255	20	379	0.16	9	2	3	6.75	0.09	0.74	1.50	0.10	90.38	-3.99	7.62	7.84	16.60
os42048	20	437	0.77	1	3	6	6.81	0.00	1.10	1.52	0.03	72.70	0.10	7.77	7.58	16.33
os43274	20	278	0.59	7	2	8	6.57	-0.02	0.91	1.50	0.00	96.27	-2.51	7.62	7.98	16.63
os59065	20	621	0.08	2	3	6	6.95	0.04	1.16	1.55	0.04	83.74	0.04	7.12	6.12	14.79
os79813	20	403	0.24	3	3	6	6.84	0.03	2.07	1.55	0.05	85.22	-1.88	9.44	7.47	16.37
os85441	20	435	0.02	6	3	6	6.94	0.08	0.87	1.53	0.06	93.89	-1.65	7.25	7.33	16.27
os61413	20	407	0.08	3	3	6	6.85	0.04	4.94	1.55	0.03	51.96	-0.15	8.81	7.39	16.26
os48764	20	422	0.06	3	3	6	6.87	0.05	2.39	1.55	0.04	71.47	-1.42	7.76	7.03	15.77
os07759	20	517	0.92	2	3	6	6.77	-0.02	2.96	1.54	0.04	79.67	-0.11	8.68	8.09	17.31
os22066	20	452	0.01	2	3	6	6.86	0.04	1.37	1.55	0.02	97.63	0.07	7.54	8.14	17.19
os08054	20	410	0.90	8	3	5	6.56	-0.12	0.17	1.51	0.07	97.56	-0.91	6.32	8.45	17.61
os72785	20	306	0.29	6	3	5	6.65	0.02	0.40	1.51	0.03	79.82	1.07	7.87	8.15	17.05

os05739	20	492	0.11	2	3	6	6.84	0.02	1.52	1.54	0.01	78.81	0.01	7.86	7.99	17.12
os61217	20	424	0.08	1	3	6	6.84	0.03	0.65	1.54	0.02	56.44	0.65	8.77	7.37	16.23
os20776	20	476	0.99	2	3	6	6.77	-0.03	1.58	1.55	0.01	35.20	-0.05	8.01	7.74	16.74
os21397	20	437	0.99	3	3	6	6.72	-0.04	1.32	1.55	0.02	69.07	0.04	7.69	8.02	16.98
os20721	20	415	0.92	0	3	6	6.80	0.00	6.66	1.57	0.01	0.63	-0.30	9.69	8.30	17.42
os40602	20	333	0.01	13	2	3	6.94	0.21	0.09	1.49	0.10	96.24	-3.35	6.04	7.85	16.56
os77868	20	483	0.99	2	3	6	6.75	-0.04	1.17	1.54	0.04	74.29	0.01	8.29	7.24	16.15
os72266	20	379	0.92	1	3	6	6.71	-0.03	1.79	1.55	0.05	78.58	-0.44	7.54	7.58	16.54
os82842	20	470	0.92	0	3	6	6.81	0.00	4.99	1.56	0.02	25.91	-0.07	9.49	7.21	16.00
os06180	20	404	0.95	3	2	3	6.81	0.01	5.62	1.55	0.05	48.86	-1.90	8.53	8.20	17.20
os20222	20	464	0.91	3	3	6	6.72	-0.03	0.71	1.54	0.02	68.19	0.32	7.62	8.05	16.92
os43229	20	400	0.92	0	3	6	6.78	0.00	0.99	1.55	0.00	25.96	0.50	10.48	7.25	15.87
os21841	20	430	0.92	0	3	6	6.79	0.00	6.02	1.57	0.01	2.62	-0.16	11.38	8.26	17.34
os02419	20	488	0.00	1	3	6	6.85	0.02	4.94	1.53	0.01	61.66	-0.16	8.03	7.69	16.67
os31571	20	547	0.40	1	3	6	6.84	-0.01	3.00	1.56	0.05	83.21	-1.26	9.35	7.10	16.16
os79696	20	401	0.36	5	3	6	6.67	-0.01	0.86	1.53	0.06	98.30	-1.76	7.57	7.49	16.39
os20149	20	456	0.34	6	3	5	6.68	0.00	0.83	1.52	0.06	89.85	-1.56	8.08	8.08	16.94
os26290	20	543	0.09	2	3	6	6.90	0.03	2.80	1.54	0.02	52.69	0.43	8.70	7.53	16.58
os78854	20	434	0.47	3	2	0	6.73	0.00	1.78	1.55	0.05	89.87	-1.05	8.50	7.51	16.45
os22224	20	453	0.92	0	3	6	6.80	0.00	4.72	1.56	0.01	7.93	-0.07	9.53	8.14	17.20
os10542	20	519	0.70	2	3	6	6.84	0.00	4.92	1.55	0.02	45.53	0.21	8.70	6.91	15.61
os24325	20	499	0.99	3	3	6	6.76	-0.04	1.76	1.55	0.03	72.50	-0.40	7.78	7.68	16.72
os40485	20	421	0.00	5	3	9	6.89	0.08	0.55	1.44	0.02	99.46	0.64	6.82	7.51	16.22
os26646	20	485	0.00	3	3	6	6.71	-0.05	1.55	1.53	0.05	94.20	-0.22	7.87	7.43	16.34
os08659	20	417	0.98	7	2	3	6.60	-0.10	0.81	1.53	0.08	95.95	-2.70	6.97	8.40	17.51
os72223	20	247	0.89	7	2	3	6.58	-0.07	1.34	1.54	0.06	74.71	-2.06	8.02	8.32	17.29
os73064	20	388	0.92	1	3	6	6.73	-0.02	1.13	1.54	0.01	24.62	0.49	9.27	7.69	16.47
os79408	20	428	0.99	6	3	5	6.64	-0.08	0.91	1.54	0.04	73.47	-1.24	8.49	7.42	16.37
os85484	20	435	0.69	4	3	6	6.66	-0.05	0.69	1.55	0.03	75.01	-0.36	7.49	7.21	16.06
os86507	20	408	0.04	11	3	5	6.99	0.19	0.07	1.49	0.10	73.67	-1.50	6.56	7.76	16.64
os67374	20	438	0.02	13	2	7	6.97	0.21	0.23	1.51	0.14	93.64	-3.65	7.85	7.68	16.78
os08326	20	492	0.72	2	3	6	6.74	-0.03	2.87	1.55	0.03	80.54	-0.43	9.35	7.91	16.99
os79671	20	422	0.96	2	3	6	6.75	-0.03	1.80	1.55	0.03	85.22	-0.09	8.73	7.35	16.22
os07549	20	455	0.99	2	3	6	6.75	-0.03	3.25	1.55	0.02	48.24	-0.33	8.25	7.93	16.89
os10808	20	517	0.92	1	3	6	6.83	-0.01	4.93	1.55	0.01	42.73	0.11	8.80	6.91	15.61
os22647	20	430	0.21	6	3	5	6.78	0.05	0.26	1.53	0.03	29.27	0.06	7.02	8.05	17.04
os21033	20	489	0.40	3	3	6	6.87	0.02	0.68	1.54	0.02	58.89	0.73	8.75	7.56	16.50
os21706	20	438	0.65	3	3	6	6.71	-0.03	1.53	1.55	0.03	61.37	-0.51	8.71	8.47	17.63
os72014	20	319	0.59	7	3	5	6.75	-0.01	0.52	1.50	0.05	92.63	-0.42	7.12	8.03	17.00
os72778	20	273	0.92	0	3	6	6.71	-0.01	1.36	1.56	0.01	7.16	0.09	9.77	8.34	17.34
os86149	20	453	0.74	5	3	6	6.63	-0.05	0.42	1.52	0.04	99.96	0.35	7.50	7.67	16.52
os73345	20	376	0.66	3	3	6	6.66	-0.05	0.88	1.53	0.03	72.49	0.59	8.05	7.74	16.63
os79170	20	417	0.92	0	3	6	6.79	0.00	4.99	1.57	0.02	1.36	0.04	12.29	7.35	16.20

os43043	20	279	0.92	0	3	6	6.73	0.00	2.96	1.56	0.00	74.81	-0.64	9.18	7.99	16.70
os09985	20	567	0.60	9	3	5	6.86	-0.01	0.14	1.50	0.08	99.75	-1.04	7.36	6.51	15.30
os26775	20	498	0.77	2	3	6	6.80	-0.02	2.57	1.56	0.02	52.09	-0.06	9.03	6.95	15.77
os09514	20	384	0.03	1	3	6	6.82	0.03	2.97	1.55	0.02	21.96	-0.26	9.12	8.18	17.08
os38598	20	280	0.95	3	2	7	6.69	-0.01	2.92	1.56	0.09	80.65	-3.74	7.24	8.21	16.93
os20708	20	431	0.70	2	3	6	6.75	-0.03	6.77	1.55	0.01	24.57	0.32	8.85	8.28	17.42
os19941	20	397	0.92	1	3	6	6.77	0.00	3.94	1.56	0.04	34.72	-0.88	9.12	8.56	17.59
os38523	20	257	1.00	13	2	7	6.18	-0.21	0.24	1.52	0.26	91.14	-11.07	5.45	8.33	17.02
os11684	20	535	0.77	2	3	6	6.82	-0.02	1.99	1.54	0.03	81.79	0.00	7.74	6.82	15.59
os40741	20	302	0.09	2	3	6	6.69	-0.02	2.96	1.56	0.05	47.05	-0.67	9.45	7.91	16.55
os61514	20	410	0.99	6	3	5	6.62	-0.09	0.24	1.53	0.05	71.16	0.27	7.85	7.22	16.04
os72871	20	334	0.73	4	3	6	6.61	-0.05	0.59	1.52	0.02	95.30	0.31	6.90	8.04	16.90
os82110	20	467	0.56	3	3	6	6.81	-0.01	1.50	1.53	0.02	82.01	0.17	8.19	7.48	16.44
os14123	20	482	0.99	4	3	6	6.71	-0.06	0.55	1.54	0.03	79.34	0.08	8.05	7.64	16.54
os14355	20	571	0.90	10	1	4	6.40	-0.16	0.02	1.46	0.06	98.99	2.88	5.75	7.31	16.24
os83030	20	540	0.65	2	3	6	6.80	-0.01	4.97	1.54	0.01	35.95	0.61	8.98	6.74	15.48
os39557	20	332	0.13	9	3	5	6.71	0.08	0.24	1.51	0.11	87.95	-1.98	6.91	7.70	16.38
os19945	20	383	0.92	0	3	6	6.77	0.00	5.06	1.56	0.02	35.51	-0.61	9.03	8.67	17.67
os80851	20	557	0.08	6	3	5	7.00	0.09	0.50	1.53	0.05	98.11	-1.14	7.25	6.63	15.52
os85453	20	531	0.43	5	3	5	6.86	-0.01	0.79	1.53	0.07	99.99	-1.65	7.85	6.70	15.62
os86468	20	550	0.95	4	3	6	6.78	-0.05	0.58	1.53	0.04	100.00	0.32	7.27	7.00	15.92
os82153	20	432	0.92	5	3	6	6.62	-0.07	0.95	1.53	0.04	99.99	-0.88	8.47	7.68	16.64
os11992	20	521	0.92	0	3	6	6.82	0.00	2.66	1.54	0.04	67.78	-0.36	8.05	7.74	16.66
os23067	20	392	0.92	0	3	6	6.77	0.00	5.91	1.56	0.01	0.51	0.16	11.68	8.61	17.67
os19947	20	383	0.93	1	3	6	6.76	-0.01	5.49	1.56	0.02	32.52	-0.34	9.32	8.67	17.67
os43827	20	421	0.87	9	2	3	6.40	-0.14	0.29	1.49	0.10	98.58	-3.17	6.58	7.06	15.66
os61540	20	439	0.01	7	3	5	6.94	0.13	0.45	1.52	0.08	99.16	-2.07	6.95	7.12	15.94
os88240	20	443	0.99	2	3	6	6.74	-0.04	1.73	1.55	0.03	89.50	-0.32	8.00	7.54	16.39
os72546	20	262	0.07	6	2	3	6.70	0.04	2.13	1.55	0.07	58.81	-2.94	8.10	8.49	17.39
os10859	20	601	0.08	7	2	0	7.03	0.12	0.36	1.52	0.07	99.76	-0.70	6.85	6.28	15.02
os27867	20	488	0.01	3	3	6	6.88	0.05	1.90	1.54	0.02	71.78	-0.64	8.33	7.23	16.11
os86520	20	454	0.92	0	3	6	6.80	0.00	4.58	1.56	0.02	29.12	0.04	11.14	7.51	16.39
os55874	20	585	0.99	5	3	5	6.72	-0.08	0.27	1.51	0.03	94.80	0.57	6.87	6.21	15.15
os85433	20	431	0.75	6	3	5	6.58	-0.07	0.47	1.53	0.06	96.40	0.25	7.38	7.16	16.09
os09953	20	492	0.03	8	3	5	6.50	-0.13	0.22	1.52	0.07	99.56	-0.28	6.46	6.95	15.68
os54140	20	541	0.35	4	3	6	6.94	0.05	1.25	1.54	0.04	95.65	-0.82	7.42	6.28	15.14
os73437	20	304	0.12	3	3	6	6.81	0.04	0.84	1.53	0.02	93.17	0.16	7.57	8.15	17.19
os72815	20	301	0.88	9	3	5	6.39	-0.13	0.16	1.49	0.05	88.81	0.36	7.66	8.15	17.00
os27921	20	474	0.08	4	3	6	6.91	0.05	1.06	1.54	0.03	88.88	-0.14	8.15	7.36	16.22
os19882	20	523	0.95	3	3	6	6.79	-0.04	0.58	1.54	0.02	80.43	0.39	7.67	7.66	16.57
os73256	20	347	0.92	0	3	6	6.75	0.00	1.49	1.56	0.01	14.50	0.09	10.25	7.95	16.86
os87108	20	393	0.23	5	3	6	6.74	0.02	1.44	1.49	0.06	94.16	-1.76	7.30	7.98	16.90
os06891	20	470	0.40	2	3	6	6.85	0.01	2.03	1.55	0.02	76.75	-0.10	8.43	8.12	17.23

os86389	20	500	0.90	7	3	5	6.70	-0.08	0.34	1.51	0.05	99.78	0.16	7.30	7.25	16.14
os02081	20	503	0.79	5	3	6	6.78	-0.04	1.13	1.52	0.06	99.82	-1.90	7.82	7.78	16.75
os06605	20	474	0.99	5	3	5	6.66	-0.08	0.59	1.52	0.04	93.38	-0.09	6.93	7.94	17.02
os22666	20	424	0.92	0	3	6	6.77	-0.01	6.98	1.56	0.01	1.17	0.16	10.76	8.26	17.31
os23003	20	510	0.66	2	3	6	6.77	-0.03	0.84	1.54	0.01	49.66	0.31	8.26	7.76	16.69
os23129	20	477	0.92	0	3	6	6.81	0.00	2.95	1.55	0.02	27.51	-0.53	9.07	8.04	17.06
os54056	20	549	0.36	3	3	6	6.91	0.03	1.08	1.53	0.03	66.02	0.31	8.25	6.22	15.12
os56337	20	496	0.83	2	3	6	6.77	-0.03	0.56	1.53	0.02	67.35	0.75	8.62	6.65	15.57
os83716	20	577	0.04	5	3	6	6.98	0.08	0.55	1.53	0.04	100.00	0.28	7.28	6.87	15.76
os00734	20	503	0.87	3	3	6	6.77	-0.04	0.83	1.52	0.02	93.68	0.05	7.58	7.26	16.03
os41052	20	337	0.02	5	3	6	6.87	0.08	0.43	1.53	0.07	97.30	0.34	6.87	7.94	16.65
os43862	20	346	0.77	1	3	6	6.75	-0.01	2.94	1.56	0.04	29.83	-0.10	8.66	7.29	15.89
os59011	20	622	0.08	3	3	6	6.97	0.05	1.13	1.55	0.04	86.50	-0.05	7.05	6.11	14.78
os04944	20	511	0.88	2	3	6	6.76	-0.03	1.90	1.55	0.03	57.90	-0.65	8.54	7.38	16.35
os42878	20	285	0.39	3	2	0	6.78	0.01	2.33	1.57	0.00	80.09	-1.74	8.91	7.86	16.55
os08086	20	454	0.20	5	3	6	6.91	0.05	2.01	1.55	0.04	63.72	-0.85	8.30	8.10	17.19
os15065	20	566	1.00	6	3	5	6.62	-0.10	0.45	1.51	0.06	99.90	0.07	6.28	7.50	16.40
os06652	20	500	0.01	4	3	6	6.90	0.07	0.98	1.54	0.04	94.24	-0.55	8.20	7.84	16.91
os09604	20	349	0.92	1	3	6	6.75	-0.01	2.74	1.57	0.02	38.77	-0.30	8.99	8.26	17.10
os20030	20	435	0.98	4	3	6	6.68	-0.07	0.71	1.54	0.03	74.28	-0.54	7.55	8.32	17.34
os04918	20	514	0.77	1	3	6	6.84	0.00	3.01	1.56	0.03	46.03	-0.10	8.59	7.34	16.30
os06578	20	458	0.08	1	3	6	6.83	0.02	2.90	1.55	0.02	70.86	-0.15	8.99	8.01	17.07
os24367	20	493	0.08	3	3	6	6.86	0.05	1.68	1.55	0.03	53.78	-0.71	8.18	7.71	16.76
os73196	20	321	0.92	1	3	6	6.73	-0.01	0.84	1.55	0.01	29.70	0.12	9.38	8.07	17.02
os76885	20	447	0.92	2	2	7	6.73	-0.03	1.60	1.49	0.15	93.88	-3.30	7.22	7.17	15.97
os17226	20	559	0.96	11	3	5	6.54	-0.15	0.02	1.48	0.10	99.96	0.29	6.45	6.50	15.41
os61103	20	397	0.67	3	3	6	6.83	0.02	1.95	1.54	0.03	81.87	-0.85	9.05	7.37	16.18
os59010	20	621	0.03	2	3	6	6.93	0.03	0.64	1.55	0.03	78.36	0.29	7.41	6.14	14.81
os80240	20	529	0.97	3	3	6	6.78	-0.04	1.73	1.53	0.03	79.42	-0.53	7.92	6.89	15.76
os85443	20	433	0.21	5	2	3	6.61	-0.09	1.38	1.53	0.08	95.36	-3.08	7.69	7.30	16.25
os23341	20	379	0.82	4	2	0	6.72	-0.04	0.75	1.53	0.07	89.76	-0.55	7.13	8.48	17.41
os19946	20	383	0.92	0	3	6	6.76	-0.01	5.51	1.56	0.02	32.43	-0.41	9.60	8.67	17.67
os83108	20	623	0.77	5	3	6	6.86	-0.02	0.39	1.49	0.04	92.88	0.73	7.41	6.40	15.16
os21793	20	415	0.82	3	3	6	6.68	-0.04	4.68	1.55	0.03	19.57	-0.20	8.95	8.26	17.30
os28213	20	492	0.92	2	3	6	6.75	-0.03	2.36	1.56	0.03	56.18	-0.39	8.80	7.23	16.11
os20433	20	425	0.01	7	2	3	6.54	-0.11	0.34	1.54	0.06	47.77	-1.51	7.22	8.22	17.27
os78582	20	461	0.99	4	3	6	6.70	-0.06	1.00	1.54	0.06	96.94	-0.54	7.58	7.13	16.06
os78822	20	409	0.92	2	3	6	6.72	-0.03	3.32	1.56	0.04	34.40	0.13	8.44	7.52	16.36
os16922	20	608	0.17	10	2	0	7.07	0.14	0.06	1.48	0.12	100.00	-1.35	6.38	6.51	15.37
os73254	20	348	0.92	1	3	6	6.72	-0.02	1.11	1.56	0.01	13.90	0.14	9.21	7.95	16.86
os23373	20	486	0.80	4	3	6	6.71	-0.03	0.76	1.54	0.06	73.46	-0.68	7.59	7.92	16.95
os07835	20	426	0.99	4	3	6	6.72	-0.04	2.86	1.55	0.03	59.12	-0.35	9.36	8.27	17.35
os12920	20	516	0.63	4	3	6	6.70	-0.05	1.22	1.54	0.05	89.72	-1.10	6.97	7.74	16.72

os85427	20	483	0.55	6	3	5	6.84	0.00	0.60	1.52	0.08	98.36	-1.08	6.85	7.00	15.92
os38632	20	384	0.01	14	2	3	6.83	0.20	0.31	1.50	0.13	98.59	-4.59	5.79	7.85	16.63
os61313	20	388	0.75	6	3	5	6.76	-0.03	0.71	1.52	0.07	97.49	-1.40	7.20	7.41	16.20
os86477	20	534	0.60	2	3	6	6.77	-0.02	1.26	1.55	0.02	99.69	0.07	8.22	7.15	16.05
os20201	20	465	0.02	7	3	5	6.97	0.10	0.50	1.51	0.04	78.01	-0.19	7.44	8.01	16.87
os84464	20	467	0.61	5	3	6	6.64	-0.08	0.63	1.53	0.05	99.35	-0.89	7.15	7.12	16.01
os43813	20	458	0.00	7	3	5	6.93	0.11	0.39	1.51	0.06	98.05	-0.50	6.92	6.79	15.45
os85263	20	537	0.72	2	3	6	6.85	0.00	2.61	1.54	0.05	89.65	-1.39	8.81	6.74	15.68
os89775	20	517	0.01	8	3	5	6.97	0.13	0.30	1.50	0.10	99.43	-0.39	6.06	6.95	15.87
os19950	20	384	0.77	1	3	6	6.75	-0.01	5.16	1.54	0.02	26.13	1.04	9.17	8.67	17.67
os61814	20	411	0.78	2	3	6	6.78	-0.01	3.71	1.56	0.03	59.25	-0.69	9.18	7.38	16.26
os39130	20	440	0.25	10	2	3	6.68	0.06	0.52	1.52	0.11	94.46	-3.74	7.35	7.13	15.86
os67693	20	491	0.08	3	3	6	6.91	0.04	1.38	1.54	0.02	83.04	-0.06	8.63	7.44	16.47
os72872	20	334	0.89	4	3	6	6.62	-0.05	0.54	1.52	0.02	95.53	0.31	6.90	8.04	16.90
os12591	20	537	0.70	3	3	6	6.76	-0.03	1.29	1.53	0.03	95.28	0.14	7.22	7.61	16.50
os05726	20	482	0.95	5	3	5	6.71	-0.06	1.02	1.53	0.04	92.15	-1.05	8.26	8.00	17.12
os20591	20	437	0.92	4	3	6	6.66	-0.06	0.96	1.54	0.03	50.23	-0.38	7.68	8.22	17.27
os77646	20	303	0.20	14	2	3	6.87	0.16	0.13	1.49	0.13	100.00	-4.37	5.58	8.19	16.93
os79602	20	469	0.13	3	3	6	6.88	0.04	3.03	1.55	0.04	78.83	-0.23	9.51	7.13	16.01
os82986	20	495	0.95	4	3	6	6.72	-0.06	1.73	1.54	0.04	78.06	-1.26	7.79	7.02	15.78
os19875	20	404	0.92	1	3	6	6.76	-0.01	2.97	1.56	0.02	9.23	-0.01	9.20	8.51	17.47
os72520	20	286	0.88	10	3	5	6.35	-0.15	0.26	1.51	0.08	96.40	-2.31	7.31	8.22	17.09
os07654	20	468	0.22	5	2	0	6.60	-0.09	0.48	1.51	0.08	97.99	0.74	6.32	7.73	16.67
os50915	20	399	0.51	5	2	3	6.67	-0.01	1.11	1.55	0.10	86.65	-3.71	6.67	7.32	16.10
os21190	20	493	0.05	3	3	6	6.89	0.04	0.45	1.54	0.00	37.33	0.29	8.65	7.12	16.04
os13655	20	575	0.82	6	3	5	6.61	-0.08	0.36	1.51	0.05	99.57	0.16	7.05	7.23	16.11
os17264	20	647	0.34	8	3	9	6.78	0.04	0.24	1.48	0.07	100.00	-0.50	6.27	6.23	15.14
os78601	20	510	0.99	4	3	5	6.65	-0.08	0.55	1.52	0.04	99.10	-0.40	6.62	7.02	15.98
os36680	20	340	0.92	5	3	5	6.64	-0.07	0.88	1.53	0.08	94.22	-1.93	7.15	7.82	16.58
os61088	20	432	0.66	5	2	3	6.67	-0.01	1.32	1.53	0.10	93.54	-4.08	6.82	7.26	16.05
os22122	20	416	0.23	2	3	6	6.78	0.02	1.68	1.55	0.03	71.96	-0.55	7.74	8.29	17.33
os54243	20	518	0.25	3	3	6	6.78	0.01	1.63	1.53	0.04	88.86	-0.90	7.89	6.58	15.51
os82655	20	462	0.77	5	3	6	6.76	-0.04	0.55	1.54	0.06	47.19	-0.58	8.85	7.13	15.96
os14106	20	462	0.26	2	3	6	6.74	-0.04	1.69	1.55	0.03	73.58	-0.25	8.60	7.81	16.65
os43179	20	336	0.27	4	3	6	6.62	-0.06	1.72	1.54	0.00	83.65	-0.79	7.90	7.60	16.23
os82799	20	484	0.92	0	3	6	6.82	0.00	3.88	1.56	0.03	56.38	-0.36	9.86	7.09	15.93
os80329	20	446	0.08	2	3	6	6.85	0.02	1.22	1.54	0.03	61.77	0.13	8.95	7.37	16.34
os84190	20	591	0.92	1	3	6	6.85	0.01	2.58	1.54	0.02	55.88	-0.35	8.74	6.52	15.43
os05737	20	493	0.07	2	3	6	6.84	0.02	1.54	1.54	0.01	76.41	-0.03	7.80	7.99	17.12
os07422	20	431	0.23	1	3	6	6.79	0.01	0.55	1.54	0.02	56.54	0.26	9.42	8.28	17.34
os72308	20	285	0.10	3	3	6	6.81	0.03	1.28	1.53	0.02	95.32	-0.14	7.37	8.29	17.26
os89321	20	500	0.81	3	3	6	6.73	-0.05	2.21	1.55	0.03	54.13	-0.46	8.35	7.17	16.14
os11683	20	536	0.95	2	3	6	6.81	-0.02	1.54	1.54	0.03	83.92	-0.11	7.54	6.81	15.58

os13259	20	559	0.92	3	3	6	6.75	-0.04	1.06	1.55	0.02	50.49	0.07	8.85	7.07	15.93
os21187	20	490	0.92	1	3	6	6.86	0.02	3.36	1.56	0.00	42.45	-0.27	9.05	7.09	16.02
os01927	20	414	0.30	7	2	7	6.91	0.07	1.08	1.53	0.09	93.96	-2.77	8.44	8.23	17.18
os22145	20	435	0.70	4	3	6	6.68	-0.03	1.86	1.54	0.03	81.16	-1.03	8.84	8.26	17.35
os20104	20	433	0.92	1	3	6	6.78	-0.02	2.58	1.56	0.02	43.37	-0.04	8.26	8.31	17.31
os20892	20	496	0.07	5	3	6	6.65	-0.08	0.52	1.54	0.00	69.69	-0.80	6.79	7.07	15.97
os22578	20	462	0.90	4	3	6	6.67	-0.06	3.91	1.55	0.03	29.12	-0.54	8.15	7.71	16.60
os78665	20	488	0.39	4	3	6	6.79	0.02	1.57	1.54	0.08	99.37	-1.04	7.83	6.99	15.91
os02385	20	475	0.00	3	3	6	6.86	0.04	1.88	1.52	0.05	97.42	-1.38	8.03	7.71	16.72
os08619	20	451	0.03	4	3	6	6.65	-0.07	0.73	1.53	0.03	82.64	-0.41	7.64	8.26	17.33
os14225	20	495	0.96	1	3	6	6.84	0.01	2.75	1.54	0.03	66.57	0.72	9.53	7.53	16.41
os20468	20	471	0.51	2	3	6	6.73	-0.04	1.95	1.55	0.03	35.63	-1.28	8.09	7.74	16.75
os22041	20	438	0.23	1	3	6	6.79	0.00	1.88	1.55	0.02	49.41	-0.21	8.23	8.23	17.26
os02128	20	407	0.77	1	2	0	6.78	-0.01	2.93	1.56	0.05	76.35	-0.81	8.67	8.16	17.14
os20176	20	459	0.06	4	3	6	6.91	0.06	1.32	1.53	0.03	87.82	-0.44	7.63	8.06	16.95
os40206	20	308	1.00	5	2	3	6.54	-0.10	0.99	1.53	0.10	95.92	-3.49	7.23	7.96	16.68
os50711	20	460	0.13	3	3	6	6.86	0.02	1.90	1.54	0.04	61.06	-0.07	8.27	6.96	15.71
os79614	20	480	0.40	2	3	6	6.84	0.00	3.58	1.55	0.03	55.37	-0.67	8.76	7.08	15.98
os83552	20	468	0.23	2	3	6	6.80	0.01	1.79	1.55	0.03	63.42	-0.23	8.34	7.45	16.35
os05992	20	491	0.74	3	3	6	6.85	0.01	3.97	1.55	0.02	23.82	0.13	8.97	7.92	17.02
os19675	20	419	0.96	4	3	6	6.67	-0.07	0.54	1.55	0.04	80.92	-0.58	6.87	8.39	17.37
os46952	20	446	0.77	2	3	6	6.79	-0.02	1.37	1.53	0.01	40.78	0.28	8.93	6.95	15.63
os82812	20	493	0.92	0	3	6	6.82	0.00	5.90	1.56	0.00	4.79	0.37	11.24	7.09	15.88
os82937	20	494	0.83	4	3	5	6.76	-0.04	1.50	1.55	0.07	72.30	-2.14	8.77	7.00	15.79
os08593	20	470	0.01	2	3	6	6.86	0.03	1.55	1.56	0.02	59.39	-0.16	8.35	8.04	17.10
os11740	20	449	0.80	5	3	6	6.65	-0.04	0.54	1.53	0.03	93.23	-0.29	6.67	7.18	16.03
os02439	20	555	0.60	2	3	6	6.87	0.00	1.96	1.53	0.02	72.98	-0.77	8.23	7.20	16.19
os12909	20	531	0.92	1	3	6	6.79	-0.02	2.89	1.55	0.03	58.42	-0.97	9.10	7.65	16.51
os21197	20	496	0.91	2	3	6	6.77	-0.03	2.61	1.55	0.01	1.67	0.05	8.84	7.41	16.35
os80320	20	452	0.61	2	3	6	6.83	0.01	1.50	1.55	0.03	63.56	0.13	8.78	7.36	16.34
os05721	20	482	0.98	4	3	6	6.74	-0.05	1.67	1.54	0.05	91.79	-1.70	8.53	8.00	17.12
os24453	20	488	0.66	3	3	6	6.77	-0.01	2.88	1.55	0.03	72.24	-0.56	8.45	7.71	16.73
os61215	20	417	0.92	1	3	6	6.80	0.00	3.56	1.56	0.03	26.34	-0.11	8.91	7.35	16.18
os85451	20	554	0.40	7	3	5	6.96	0.05	0.64	1.50	0.07	100.00	-0.65	6.70	6.63	15.55
os53856	20	537	0.08	3	3	6	6.93	0.05	1.63	1.53	0.03	85.31	-0.34	8.95	6.35	15.25
os61813	20	418	0.92	1	3	6	6.79	0.00	2.19	1.55	0.02	50.45	0.22	8.88	7.38	16.25
os54926	20	367	0.92	0	3	6	6.73	-0.01	2.69	1.55	0.02	36.34	-0.62	9.56	7.43	16.34
os37309	20	315	0.00	8	2	3	6.88	0.12	0.74	1.52	0.12	86.06	-5.51	7.57	8.31	17.04
os55334	20	348	0.83	2	3	6	6.70	-0.03	3.44	1.54	0.05	96.12	-0.31	7.87	7.57	16.47
os09683	20	421	0.55	2	3	6	6.76	0.00	2.87	1.55	0.03	48.25	-0.95	8.16	8.28	17.29
os21548	20	451	0.01	2	3	6	6.83	0.03	1.25	1.55	0.01	30.12	0.08	8.78	8.42	17.61
os40125	20	322	0.00	9	2	3	6.90	0.13	0.63	1.53	0.08	92.43	-3.35	8.63	7.83	16.54
os12913	20	460	0.59	3	3	6	6.81	-0.01	2.90	1.57	0.05	82.37	-1.78	9.33	8.02	16.99

os01775	20	474	0.95	4	3	6	6.74	-0.05	1.86	1.54	0.06	99.46	-1.15	7.62	7.94	16.89
os21928	20	492	0.91	1	3	6	6.80	0.00	2.56	1.55	0.01	38.77	0.02	8.76	8.01	17.14
os61515	20	418	0.85	2	3	6	6.77	-0.02	0.16	1.53	0.03	79.20	1.38	8.80	7.20	16.01
os83004	20	459	0.02	1	3	6	6.81	0.01	2.95	1.56	0.05	53.94	-0.82	9.02	7.11	15.93
os07763	20	467	0.35	4	3	6	6.90	0.04	0.49	1.54	0.06	82.42	-0.04	8.03	8.24	17.39
os85434	20	433	0.65	5	3	5	6.59	-0.07	0.49	1.53	0.06	96.73	0.28	7.16	7.16	16.09
os02126	20	403	0.08	1	3	6	6.82	0.02	2.90	1.56	0.05	71.21	-0.90	9.65	8.18	17.16
os83882	20	436	0.23	2	3	6	6.81	0.02	2.84	1.55	0.04	77.37	-0.72	8.85	7.55	16.46
os20679	20	440	0.98	4	2	3	6.74	-0.04	1.89	1.54	0.05	76.94	-2.02	8.24	8.18	17.25
os22780	20	437	0.92	1	3	6	6.82	0.02	5.94	1.56	0.01	7.62	-0.11	9.51	7.94	16.91
os04852	20	508	0.31	2	3	6	6.89	0.03	1.90	1.54	0.03	87.65	-0.56	7.46	7.45	16.41
os38212	20	334	0.27	7	2	3	6.64	0.03	0.98	1.52	0.11	99.20	-4.54	7.13	8.03	16.72
os21547	20	449	0.08	2	3	6	6.84	0.03	1.59	1.55	0.01	18.80	0.02	8.99	8.42	17.60
os38405	20	310	0.98	14	3	5	6.05	-0.24	0.05	1.43	0.07	68.89	1.73	7.13	8.13	16.81
os82543	20	472	0.50	4	3	6	6.84	0.01	4.74	1.56	0.05	56.21	-1.44	7.92	7.14	15.96
os27632	20	447	0.09	3	3	6	6.68	-0.06	0.55	1.53	0.02	77.06	0.56	8.03	7.70	16.60
os08654	20	467	0.32	4	3	6	6.66	-0.07	0.44	1.54	0.04	96.44	-0.14	7.58	8.29	17.45
os07593	20	387	0.17	5	3	6	6.84	0.07	1.46	1.53	0.05	86.68	-1.23	7.40	8.53	17.62
os19631	20	413	0.99	2	3	6	6.72	-0.04	1.39	1.56	0.03	62.87	-0.15	7.62	8.38	17.37
os27279	20	461	0.81	4	3	6	6.68	-0.04	0.56	1.55	0.04	97.18	-0.15	7.43	7.21	16.01
os10097	20	482	0.88	11	3	5	6.57	-0.12	0.04	1.51	0.06	86.24	-0.73	6.14	7.04	15.86
os40557	20	377	0.92	9	3	9	6.39	-0.14	0.06	1.48	0.05	99.81	-0.08	6.02	7.74	16.44
os80115	20	392	0.92	1	3	6	6.73	-0.02	2.72	1.56	0.03	43.13	-0.20	9.00	7.65	16.57
os85426	20	475	0.03	6	3	5	6.59	-0.10	0.47	1.52	0.07	99.61	-0.20	6.97	7.01	15.93
os21096	20	483	0.24	1	3	6	6.78	-0.02	3.67	1.57	0.02	25.57	-0.64	8.94	7.51	16.40
os03502	20	353	0.77	6	2	7	6.54	-0.06	0.77	1.53	0.09	98.70	-2.45	7.95	8.32	17.11
os78353	20	448	0.31	4	3	6	6.67	-0.07	1.62	1.54	0.04	90.84	-0.78	8.16	7.22	16.13
os01614	20	408	0.45	3	3	6	6.69	-0.05	0.55	1.54	0.02	88.38	0.45	8.00	8.12	17.04
os21509	20	416	0.92	1	3	6	6.81	0.01	3.71	1.56	0.02	10.31	-0.63	9.57	8.45	17.51
os78431	20	472	0.99	3	3	6	6.73	-0.04	1.97	1.54	0.04	92.05	-1.59	9.16	7.37	16.32
os20134	20	431	0.62	4	3	6	6.67	-0.04	1.04	1.55	0.03	62.14	-0.45	8.85	8.28	17.19
os73301	20	329	0.93	6	3	5	6.59	-0.09	0.30	1.52	0.05	91.08	0.79	7.59	8.06	17.03
os38963	20	331	0.76	9	2	7	6.40	-0.10	0.66	1.54	0.15	87.17	-6.54	7.41	7.60	16.27
os87047	20	360	0.82	5	2	0	6.74	-0.03	0.97	1.53	0.10	92.10	-1.27	7.71	7.92	16.84
os06253	20	497	0.12	3	3	6	6.83	0.03	1.67	1.55	0.05	82.23	-0.78	7.95	7.84	16.94
os84936	20	557	0.08	3	3	6	6.92	0.04	1.50	1.54	0.02	79.22	0.14	8.02	6.70	15.60
os21151	20	467	0.23	1	3	6	6.80	0.01	5.55	1.56	0.01	4.96	0.02	9.06	7.49	16.40
os24387	20	484	0.31	4	3	6	6.78	0.02	0.84	1.54	0.03	63.35	0.05	7.74	7.77	16.85
os02969	20	331	0.30	5	2	3	6.83	0.04	1.31	1.53	0.08	96.34	-3.19	7.41	8.61	17.41
os73961	20	655	0.00	2	3	6	6.94	0.03	2.38	1.55	0.05	81.47	-0.63	8.17	6.03	14.72
os19785	20	393	0.92	0	3	6	6.76	-0.01	4.34	1.56	0.01	8.03	-0.16	10.17	8.66	17.70
os86668	20	641	0.14	2	3	6	6.92	0.03	0.94	1.55	0.01	81.49	0.12	7.77	6.56	15.46
os65164	20	372	0.00	4	3	6	6.64	-0.06	0.37	1.53	0.00	76.18	1.05	7.50	7.15	15.92

os38563	20	349	0.23	4	3	6	6.75	0.03	0.72	1.52	0.03	96.49	0.13	7.83	7.80	16.52
os61824	20	445	0.54	2	3	6	6.77	0.00	2.01	1.55	0.02	36.96	-0.09	8.81	7.32	16.17
os63015	20	390	0.91	4	2	3	6.66	-0.04	2.26	1.56	0.05	56.69	-2.45	9.43	7.61	16.43
os24119	20	480	0.92	2	3	6	6.76	-0.02	3.70	1.56	0.03	36.37	-0.10	8.52	7.76	16.79
os21221	20	446	0.08	2	3	6	6.86	0.04	1.07	1.55	0.04	77.96	-0.15	7.67	8.36	17.50
os41549	20	442	0.01	4	3	6	6.90	0.06	0.61	1.56	0.07	35.17	-2.05	7.63	7.32	16.04
os37485	20	308	0.97	6	3	5	6.61	-0.08	0.51	1.53	0.09	93.83	-1.08	7.57	7.98	16.70
os83249	20	491	0.92	0	3	6	6.83	0.01	2.20	1.55	0.01	25.01	-0.01	8.93	7.07	15.84
os13272	20	533	0.92	1	3	6	6.79	-0.02	1.57	1.55	0.02	43.35	-0.14	7.65	7.37	16.27
os13656	20	572	0.92	5	3	5	6.66	-0.07	0.49	1.52	0.04	99.44	-0.08	7.34	7.24	16.11
os19958	20	404	0.79	3	3	6	6.71	-0.03	3.82	1.56	0.02	2.43	-0.26	8.83	8.53	17.60
os72716	20	274	0.13	4	2	3	6.62	-0.05	1.89	1.55	0.05	37.24	-1.99	8.04	8.26	17.24
os41606	20	443	0.43	11	3	5	6.89	0.05	0.10	1.50	0.08	87.13	-0.32	6.10	7.48	16.21
os55352	20	300	0.40	2	3	6	6.79	0.02	2.38	1.54	0.06	92.64	0.15	8.04	7.87	16.78
os20616	20	449	0.92	1	3	6	6.74	-0.03	3.78	1.56	0.03	52.17	-0.90	8.69	8.09	17.15
os30178	26	645	0.92	0	3	6	6.88	0.00	0.90	1.53	0.01	49.40	0.17	9.71	6.63	15.66
os45581	26	618	0.01	8	3	5	6.99	0.13	0.12	1.49	0.07	98.69	0.52	6.60	5.93	14.70
os58834	26	703	0.02	8	1	4	7.04	0.14	0.06	1.38	0.06	95.11	3.32	6.38	5.66	14.30
os25836	26	592	0.53	3	3	6	6.93	0.02	0.23	1.53	0.05	93.32	0.50	6.99	7.01	15.98
os16051	26	564	0.99	9	3	5	6.49	-0.16	0.06	1.51	0.08	91.89	-0.65	6.51	6.95	15.80
os18389	26	762	0.00	15	2	0	7.09	0.22	0.12	1.48	0.12	93.30	-1.54	7.10	5.83	14.62
os10452	26	663	0.77	3	3	6	6.90	-0.01	0.88	1.52	0.05	97.38	-0.31	7.31	6.10	14.92
os00502	26	750	0.99	22	3	9	5.91	-0.33	0.00	1.36	0.14	99.57	0.15	5.26	5.63	14.37
os18447	26	798	0.92	12	3	5	6.40	-0.19	0.01	1.46	0.08	99.98	-0.03	5.70	6.07	14.96
os13779	26	628	0.01	2	3	9	6.95	0.04	0.40	1.50	0.02	88.89	0.93	6.64	7.15	16.01
os57621	26	709	0.06	16	3	5	7.13	0.24	0.00	1.44	0.08	95.96	1.81	5.73	5.80	14.53
os15801	26	715	0.40	12	1	4	6.68	0.03	0.02	1.45	0.07	93.44	3.11	6.28	6.25	15.08
os43584	26	579	0.46	11	3	5	6.56	-0.01	0.04	1.45	0.08	95.01	0.07	5.98	6.21	15.02
os31515	26	580	0.58	4	2	0	6.91	0.01	2.82	1.55	0.08	86.75	-1.64	9.16	6.79	15.80
os57961	26	617	0.40	2	1	4	6.88	0.02	0.01	1.47	0.02	97.18	4.34	9.06	6.30	15.06
os45923	26	621	0.65	6	1	1	6.65	-0.10	0.04	1.39	0.03	99.95	4.13	5.73	6.19	14.94
os57054	26	701	0.87	5	1	1	6.88	-0.02	0.00	1.46	0.02	96.95	4.50	6.70	5.89	14.63
os01127	26	659	0.23	28	2	7	6.83	0.29	0.00	1.39	0.21	99.83	-1.98	5.72	5.68	14.42
os13946	26	612	0.40	2	3	6	6.91	0.01	0.52	1.54	0.02	54.18	0.02	8.42	6.85	15.72
os31334	26	591	0.46	15	3	5	6.90	0.04	0.00	1.49	0.10	91.03	-0.35	6.16	6.85	15.86
os13788	26	699	1.00	10	3	9	6.54	-0.16	0.06	1.48	0.06	99.58	0.28	5.78	6.62	15.40
os15834	26	679	0.60	6	2	0	6.71	-0.04	0.61	1.51	0.09	98.82	-0.18	6.39	6.60	15.50
os15272	26	649	0.47	5	2	0	6.93	0.01	0.70	1.53	0.07	97.34	-0.30	6.77	6.87	15.77
os13036	26	578	0.63	10	3	5	6.51	-0.07	0.22	1.42	0.08	88.55	1.47	6.38	7.19	16.04
os25040	26	547	0.98	11	3	5	6.43	-0.17	0.08	1.48	0.07	75.40	0.33	8.14	7.43	16.48
os18760	26	822	0.19	10	3	5	6.88	0.08	0.02	1.48	0.06	91.24	0.69	6.03	5.78	14.51
os13722	26	575	0.09	12	2	0	7.08	0.17	0.01	1.48	0.11	94.65	-0.21	6.34	7.02	15.87
os18483	26	796	0.04	11	3	5	7.16	0.19	0.01	1.47	0.09	100.00	-0.96	6.39	5.76	14.57

os26259	26	614	0.00	6	1	4	6.99	0.09	0.01	1.45	0.03	99.78	4.28	5.98	7.28	16.31
os02757	26	571	0.49	8	1	1	6.68	-0.01	0.12	1.47	0.04	97.68	2.36	6.03	7.57	16.56
os15865	26	643	0.28	9	1	1	6.75	0.03	0.12	1.46	0.06	95.40	3.49	6.45	6.90	15.73
os45930	26	604	0.81	12	3	9	6.70	-0.09	0.00	1.41	0.06	100.00	1.27	5.81	6.31	15.05
os30491	26	625	0.80	5	3	9	6.83	-0.04	1.17	1.49	0.04	85.99	-0.58	7.40	6.64	15.58
os23958	26	566	0.47	6	3	5	6.94	0.04	0.63	1.53	0.05	92.18	-0.33	8.62	6.86	15.86
os57267	26	722	0.02	8	3	9	7.02	0.11	0.07	1.46	0.04	99.08	1.31	5.91	5.88	14.58
os25070	26	579	0.92	4	3	6	6.73	-0.06	1.74	1.55	0.05	84.98	-1.79	9.06	7.11	16.13
os25650	26	612	0.01	9	3	5	7.01	0.14	0.11	1.50	0.06	92.01	0.53	7.93	6.84	15.78
os15750	26	767	0.60	8	3	9	6.68	-0.06	0.07	1.46	0.04	95.58	2.50	6.17	6.10	14.87
os18703	26	705	0.21	6	3	6	7.03	0.06	0.39	1.48	0.05	96.16	0.76	6.85	6.53	15.30
os25430	26	532	0.17	18	3	5	6.73	0.16	0.00	1.46	0.11	89.82	-0.66	6.09	7.35	16.28
os52373	26	602	0.97	14	3	5	6.20	-0.23	0.00	1.47	0.09	100.00	0.00	5.87	5.64	14.34
os12444	26	567	1.00	8	3	5	6.56	-0.14	0.20	1.51	0.06	99.40	-0.17	6.36	7.34	16.29
os17677	26	936	0.43	7	3	9	7.09	0.03	0.15	1.47	0.06	100.00	1.35	6.11	5.02	13.73
os02758	26	574	0.00	5	3	9	6.99	0.08	0.27	1.49	0.04	98.23	1.45	6.62	7.53	16.53
os19433	26	734	0.77	15	3	5	6.26	-0.16	0.00	1.45	0.10	100.00	0.39	6.04	5.80	14.65
os45610	26	592	0.73	11	3	5	6.41	-0.12	0.02	1.45	0.10	99.86	-0.52	6.71	6.13	14.90
os19165	26	594	0.31	11	3	5	6.35	-0.19	0.04	1.49	0.11	99.72	-1.45	6.79	6.04	14.90
os12841	26	652	0.81	16	2	0	6.62	-0.11	0.00	1.47	0.13	87.16	-0.55	6.68	6.66	15.43
os25962	26	576	0.40	5	3	6	6.94	0.03	1.22	1.54	0.06	92.19	-1.58	8.91	7.39	16.41
os12735	26	627	0.80	4	3	6	6.76	-0.04	1.47	1.52	0.09	100.00	-0.50	7.17	7.03	15.96
os52124	26	789	0.84	9	1	1	6.81	-0.08	0.02	1.43	0.04	98.76	4.23	5.52	4.74	13.54
os25829	26	636	0.19	9	3	5	7.08	0.12	0.07	1.48	0.06	97.40	2.13	6.89	6.84	15.80
os43493	26	647	0.40	13	2	0	6.99	0.09	0.00	1.48	0.13	99.95	-1.63	6.14	5.56	14.34
os26348	26	607	0.03	11	3	5	6.91	0.13	0.02	1.48	0.06	99.84	1.18	6.22	7.27	16.35
os31194	26	657	0.28	6	1	4	6.79	0.02	0.05	1.49	0.02	93.55	2.93	6.22	6.75	15.70
os23903	26	610	0.63	5	3	6	6.73	-0.08	0.20	1.52	0.03	81.63	1.23	8.29	6.85	15.83
os30060	26	568	0.08	12	3	5	6.86	0.13	0.02	1.49	0.07	87.82	0.10	6.49	7.11	16.08
os13960	26	608	0.47	5	3	6	6.94	0.02	0.48	1.53	0.04	90.53	0.21	7.75	6.81	15.63
os24831	26	584	0.94	6	3	5	6.74	-0.07	0.31	1.51	0.03	99.29	0.99	6.06	7.47	16.48
os29916	26	628	0.26	5	3	5	7.00	0.06	0.65	1.51	0.03	89.65	0.41	7.06	6.73	15.62
os30189	26	607	0.92	12	3	5	6.32	-0.19	0.03	1.49	0.08	97.00	-1.31	6.87	6.74	15.77
os10451	26	695	0.89	10	3	9	6.73	-0.10	0.04	1.47	0.06	99.83	-0.18	6.53	6.03	14.84
os19395	26	727	1.00	12	3	9	6.42	-0.21	0.01	1.46	0.07	98.15	0.47	5.89	5.94	14.83
os59081	26	699	0.18	5	1	4	7.01	0.06	0.01	1.40	0.03	98.42	6.32	6.32	5.78	14.47
os12178	26	759	0.69	9	3	5	6.63	-0.07	0.30	1.49	0.08	99.96	-1.28	6.40	6.38	15.22
os11874	26	641	0.69	4	3	6	6.90	-0.01	0.60	1.53	0.03	97.54	0.31	7.18	6.18	14.89
os16487	26	626	0.00	2	3	6	6.94	0.04	1.18	1.54	0.04	67.50	-0.08	9.37	6.81	15.55
os27436	26	580	0.92	0	3	6	6.86	0.00	4.72	1.53	0.00	53.67	1.46	8.56	6.56	15.37
os31677	26	650	0.99	4	3	6	6.80	-0.05	1.53	1.53	0.05	95.23	-1.95	7.55	6.41	15.43
os29587	26	576	0.51	6	3	5	6.74	0.00	0.62	1.52	0.07	92.59	-1.43	7.87	6.83	15.80
os28124	26	599	0.02	8	3	5	7.04	0.13	0.30	1.50	0.05	92.19	0.14	6.56	6.87	15.79

os13820	26	615	0.71	5	2	3	6.73	-0.02	1.12	1.55	0.09	71.86	-4.72	6.93	6.87	15.63
os29618	26	614	0.81	4	3	6	6.77	-0.06	0.19	1.52	0.02	82.35	0.59	7.67	6.51	15.42
os15914	26	722	0.97	12	3	9	6.42	-0.20	0.01	1.45	0.08	99.64	-0.47	5.93	6.44	15.29
os28351	26	552	0.77	3	3	6	6.81	-0.03	2.89	1.53	0.03	84.71	-0.27	8.85	6.74	15.62
os19567	26	844	0.00	13	3	9	7.10	0.21	0.00	1.47	0.09	99.61	-0.26	5.40	5.77	14.51
os25052	26	591	0.40	3	3	6	6.92	0.02	0.20	1.53	0.03	90.94	0.81	8.76	7.23	16.28
os15781	26	727	0.10	14	3	5	7.15	0.21	0.00	1.46	0.08	99.91	-0.35	5.95	6.33	15.20
os29176	26	572	0.92	1	3	9	6.86	0.01	0.12	1.50	0.01	98.29	1.79	7.59	7.32	16.26
os12148	26	806	0.40	7	1	1	6.83	0.01	0.16	1.45	0.03	98.47	2.83	5.87	6.34	15.19
os51090	26	600	0.58	16	3	5	6.85	0.02	0.00	1.39	0.12	96.85	2.54	5.98	6.02	14.83
os28125	26	609	0.47	7	1	1	7.01	0.07	0.05	1.46	0.03	94.33	3.42	5.67	6.88	15.80
os15542	26	760	0.07	8	1	1	6.99	0.10	0.03	1.43	0.06	99.44	3.60	5.47	6.43	15.31
os26454	26	662	0.59	9	1	1	6.60	-0.06	0.02	1.46	0.04	89.99	2.27	5.60	7.12	16.13
os44442	26	677	0.76	5	3	9	6.93	0.00	0.68	1.45	0.06	99.37	0.26	6.24	5.83	14.57
os00623	26	762	0.98	20	3	5	5.85	-0.34	0.00	1.36	0.16	100.00	0.51	5.56	5.37	14.11
os59063	26	804	0.92	4	3	6	6.82	-0.06	0.65	1.52	0.02	65.35	0.95	7.71	5.05	13.74
os47901	26	539	0.30	7	1	4	6.71	0.00	0.10	1.46	0.06	99.81	2.98	6.47	6.69	15.45
os17959	26	798	0.62	8	2	0	6.97	0.00	0.16	1.50	0.07	98.81	0.83	6.43	5.70	14.47
os24281	26	563	0.92	0	3	6	6.84	-0.01	2.93	1.56	0.03	71.30	-0.80	8.60	7.20	16.24
os30177	26	645	0.92	0	3	6	6.88	0.00	0.84	1.53	0.01	51.33	0.27	10.17	6.63	15.66
os13035	26	601	0.32	4	3	6	6.79	0.00	0.48	1.51	0.03	80.97	0.83	7.51	7.04	15.85
os45909	26	532	0.95	16	1	4	6.29	-0.22	0.00	1.38	0.10	98.82	3.24	5.74	6.45	15.17
os26129	26	540	0.14	15	2	7	6.76	0.16	0.00	1.48	0.13	94.12	-2.41	7.12	7.46	16.45
os28119	26	565	0.74	7	3	5	6.57	-0.09	0.29	1.52	0.06	97.64	-0.38	6.46	6.94	15.85
os31386	26	606	0.08	3	3	6	6.95	0.04	0.74	1.53	0.03	85.72	-0.09	7.48	6.86	15.85
os42830	26	464	0.31	5	3	5	6.94	0.07	0.48	1.52	0.03	88.36	0.12	6.80	7.05	15.84
os58049	26	628	0.23	8	3	5	6.80	0.04	0.46	1.52	0.07	80.93	-1.99	8.14	6.14	14.93
os23848	26	578	0.60	8	2	3	6.92	0.03	0.48	1.54	0.10	79.99	-3.13	8.41	6.85	15.85
os14951	26	783	0.17	9	3	9	7.13	0.12	0.05	1.45	0.05	100.00	1.55	5.70	6.24	15.04
os49718	26	569	0.97	7	3	9	6.69	-0.09	0.36	1.42	0.05	98.15	0.32	6.03	6.32	14.99
os01052	26	655	0.15	14	3	5	7.11	0.20	0.00	1.44	0.10	100.00	-0.22	6.22	5.99	14.68
os13045	26	683	0.63	5	3	9	6.72	-0.06	0.75	1.48	0.04	99.46	-0.24	6.56	6.69	15.51
os16054	26	691	1.00	6	3	9	6.70	-0.10	0.06	1.46	0.03	99.99	2.05	5.72	6.66	15.55
os67828	26	515	0.19	8	3	5	6.79	0.06	0.17	1.49	0.05	83.75	-0.07	6.86	7.14	16.07
os12773	26	589	0.27	6	2	3	6.97	0.05	0.97	1.53	0.10	96.91	-3.14	6.68	7.23	16.09
os29036	26	640	0.73	5	1	1	6.72	-0.04	0.12	1.49	0.02	91.35	2.25	6.68	6.86	15.78
os45239	26	659	0.87	11	3	9	6.70	-0.10	0.04	1.42	0.07	99.76	-0.63	6.22	5.93	14.69
os60406	26	669	0.92	1	3	6	6.86	-0.02	2.89	1.54	0.03	52.00	-0.64	8.84	5.99	14.93
os31709	26	624	0.98	9	1	4	6.52	-0.15	0.13	1.48	0.05	98.02	2.00	6.10	6.70	15.70
os10477	26	647	1.00	7	3	5	6.61	-0.12	0.24	1.50	0.08	97.05	0.04	6.18	6.14	14.93
os43572	26	553	0.40	5	1	1	6.86	0.01	0.01	1.42	0.04	96.53	5.69	5.85	6.51	15.26
os31859	26	644	0.00	4	3	6	6.99	0.07	0.52	1.51	0.02	76.16	1.09	7.13	6.80	15.73
os44489	26	448	0.87	19	3	5	5.78	-0.29	0.00	1.40	0.17	98.21	-0.38	5.38	6.71	15.43

os19456	26	787	0.24	12	1	2	6.81	0.08	0.00	1.45	0.09	88.07	2.30	7.51	5.21	13.88
os25740	26	644	0.00	4	3	6	6.99	0.07	0.52	1.51	0.02	76.16	1.09	7.13	6.80	15.73
os13156	26	661	0.23	6	3	9	6.86	0.04	0.77	1.51	0.04	95.09	-0.12	7.04	6.75	15.55
os15833	26	721	0.58	7	3	9	6.70	-0.04	0.33	1.49	0.08	99.34	-1.31	6.16	6.54	15.44
os29171	26	585	0.93	14	3	9	6.48	-0.17	0.00	1.45	0.07	99.95	0.91	5.87	7.26	16.20
os27511	26	607	0.91	9	3	5	6.63	-0.12	0.20	1.50	0.05	95.98	0.75	7.78	6.23	15.06
os13227	26	702	0.98	10	3	9	6.49	-0.17	0.05	1.48	0.06	98.84	0.48	5.78	6.61	15.39
os24536	26	598	0.08	6	3	5	7.02	0.09	0.48	1.51	0.03	88.34	0.44	6.72	7.06	16.09
os31292	26	603	0.23	6	3	5	6.80	0.02	0.50	1.52	0.05	99.63	-0.22	6.95	6.87	15.85
os30740	26	522	0.66	9	3	5	6.83	-0.02	0.27	1.50	0.05	88.86	-0.48	6.76	7.16	16.09
os02762	26	586	0.08	8	2	3	7.05	0.13	0.26	1.51	0.09	98.29	-2.05	5.88	7.39	16.36
os19147	26	666	0.98	13	3	5	6.44	-0.19	0.00	1.49	0.09	99.75	-0.38	6.21	5.76	14.62
os30180	26	645	0.07	1	3	6	6.89	0.01	0.36	1.53	0.01	45.91	0.21	9.03	6.63	15.66
os30831	26	691	0.08	5	3	9	7.03	0.07	0.52	1.48	0.05	100.00	0.36	6.59	6.14	15.04
os31877	26	518	0.08	6	3	5	6.90	0.08	0.32	1.53	0.05	89.72	-0.67	7.96	7.10	16.01
os13102	26	804	0.25	6	3	5	7.09	0.07	0.61	1.49	0.09	100.00	-1.17	7.27	5.93	14.83
os43532	26	521	0.99	22	1	4	5.81	-0.34	0.00	1.35	0.16	78.11	5.89	5.04	6.32	15.01
os44517	26	622	0.19	8	3	5	7.05	0.10	0.36	1.50	0.07	99.94	-1.14	7.64	6.00	14.77
os15971	26	566	0.85	16	3	5	6.11	-0.21	0.00	1.43	0.10	97.39	1.45	5.44	7.12	16.01
os29483	26	580	0.39	10	3	5	6.66	0.02	0.03	1.47	0.07	95.32	-0.09	6.26	6.99	15.96
os00683	26	696	0.53	22	3	5	6.75	0.04	0.00	1.38	0.00	99.14	1.71	5.34	5.65	14.31
os19405	26	596	0.45	7	3	5	6.68	-0.02	0.48	1.52	0.08	100.00	-0.12	7.07	6.29	15.15
os44862	26	687	0.81	12	3	9	6.38	-0.20	0.01	1.43	0.08	98.91	0.42	5.83	5.70	14.47
os26216	26	608	0.01	11	1	1	6.96	0.16	0.00	1.44	0.05	99.33	1.87	6.09	7.52	16.51
os29981	26	629	0.96	8	3	5	6.67	-0.11	0.19	1.50	0.06	99.25	-0.57	6.36	6.79	15.75
os24976	26	637	0.09	3	2	3	6.96	0.05	1.67	1.55	0.05	89.78	-1.87	9.26	6.87	15.87
os46989	26	516	0.40	6	1	4	6.95	0.06	0.05	1.43	0.05	92.34	3.94	5.68	6.44	15.14
os31289	26	595	0.65	5	3	6	6.72	-0.04	0.66	1.54	0.04	97.90	-0.45	6.93	6.90	15.87
os44554	26	438	0.81	16	3	5	6.49	-0.13	0.00	1.41	0.12	97.85	1.42	5.63	6.91	15.64
os11258	26	570	0.76	7	3	5	6.58	-0.08	0.42	1.51	0.07	99.98	-0.89	6.76	6.47	15.28
os29082	26	476	0.37	5	2	3	6.73	0.00	1.81	1.54	0.06	72.92	-2.24	8.42	7.95	16.98
os45128	26	668	0.00	12	1	4	7.11	0.20	0.00	1.36	0.09	99.93	2.42	5.73	5.69	14.45
os45575	26	597	0.80	9	3	5	6.52	-0.10	0.31	1.47	0.09	99.30	-1.45	7.13	6.05	14.82
os18321	26	735	0.43	14	3	5	6.50	-0.02	0.00	1.45	0.10	99.97	0.31	6.18	6.31	15.19
os31243	26	666	0.40	3	3	6	6.94	0.01	1.86	1.54	0.04	90.57	-0.76	8.47	6.53	15.50
os24912	26	639	0.00	8	3	5	6.56	-0.13	0.13	1.49	0.05	91.16	0.85	8.23	6.69	15.66
os30185	26	626	0.25	6	3	5	7.02	0.07	0.29	1.51	0.04	98.49	0.85	6.20	6.68	15.69
os19432	26	739	0.70	13	3	5	6.34	-0.15	0.00	1.45	0.10	100.00	0.20	6.08	5.79	14.64
os30539	26	608	0.08	15	1	4	6.13	-0.25	0.00	1.40	0.07	99.35	4.70	5.36	6.60	15.48
os26113	26	615	0.92	6	1	1	6.64	-0.10	0.16	1.47	0.03	95.85	2.51	6.24	7.33	16.33
os60165	26	655	0.05	3	3	6	6.97	0.05	0.77	1.52	0.03	93.93	0.95	9.08	6.08	15.02
os25820	26	636	0.08	7	3	5	7.06	0.12	0.22	1.51	0.05	98.88	-0.57	7.34	6.84	15.83
os29119	26	603	0.07	4	3	6	6.98	0.07	0.11	1.51	0.02	77.50	1.92	7.35	7.17	16.14

os29980	26	633	0.99	8	3	5	6.63	-0.12	0.20	1.50	0.06	99.72	-0.77	6.50	6.75	15.72
os18010	26	758	0.01	8	2	0	7.04	0.13	0.35	1.52	0.10	88.75	-2.40	7.21	5.88	14.67
os19439	26	783	0.54	6	2	0	7.03	0.03	0.65	1.51	0.09	97.04	-0.50	7.85	5.33	14.10
os58543	26	654	0.44	10	2	0	6.98	0.04	0.05	1.48	0.08	99.24	1.12	7.15	5.92	14.75
os11871	26	645	0.81	7	3	9	6.77	-0.07	0.23	1.46	0.04	100.00	1.59	6.10	6.39	15.12
os29175	26	568	0.35	3	3	6	6.90	0.02	0.29	1.52	0.03	99.65	0.63	6.91	7.30	16.23
os15743	26	660	0.92	2	1	1	6.79	-0.05	0.15	1.47	0.03	99.15	3.41	7.03	6.78	15.62
os15829	26	689	0.71	16	3	9	6.19	-0.16	0.00	1.44	0.10	100.00	0.47	5.26	6.75	15.65
os19566	26	771	0.30	5	3	6	6.90	0.02	0.59	1.48	0.00	99.83	0.99	6.87	6.25	15.07
os31106	26	702	0.71	9	3	5	6.86	-0.03	0.09	1.50	0.08	96.22	-1.81	6.18	6.33	15.33
os29283	26	575	0.92	0	3	6	6.85	0.00	2.90	1.55	0.03	32.24	-0.21	11.11	7.11	16.05
os44620	26	464	0.13	8	2	7	6.86	0.06	0.94	1.53	0.15	96.44	-7.84	8.14	6.64	15.37
os10314	26	671	0.06	7	3	5	6.98	0.10	0.37	1.51	0.06	98.01	-0.08	6.71	5.96	14.69
os31396	26	551	0.77	6	2	7	6.80	-0.04	0.88	1.54	0.07	62.38	-1.60	7.93	6.98	16.00
os45644	26	614	0.44	11	2	0	6.99	0.07	0.05	1.47	0.10	99.66	-0.04	5.71	5.88	14.63
os43762	26	549	0.01	4	1	4	6.90	0.06	0.07	1.46	0.08	90.80	4.20	6.69	6.22	14.93
os58827	26	689	0.13	7	1	4	7.06	0.09	0.00	1.43	0.03	99.64	5.65	6.27	5.85	14.52
os58616	26	681	0.15	4	3	9	6.99	0.05	0.90	1.49	0.04	99.68	0.10	6.65	6.09	14.92
os25241	26	545	0.50	6	3	6	6.90	0.01	0.73	1.53	0.05	95.74	-1.12	7.87	7.59	16.62
os13462	26	664	0.67	4	3	6	6.89	-0.02	0.69	1.53	0.03	84.05	0.41	7.77	6.64	15.44
os24422	26	561	0.60	5	3	6	6.71	-0.03	0.75	1.53	0.04	99.77	-0.24	6.97	7.26	16.26
os67878	26	624	0.01	4	3	9	6.95	0.06	1.30	1.49	0.02	82.10	0.22	7.46	6.69	15.65
os18484	26	749	0.86	14	3	5	6.25	-0.20	0.00	1.47	0.09	99.05	0.51	7.94	6.04	14.82
os43425	26	695	0.00	11	2	0	7.10	0.18	0.02	1.48	0.14	97.72	-0.42	6.43	5.33	14.11
os31684	26	617	0.77	2	3	6	6.87	-0.01	0.26	1.53	0.02	93.09	0.64	7.25	6.71	15.73
os29274	26	585	0.53	8	3	5	6.96	0.04	0.19	1.50	0.06	90.43	0.00	6.45	7.01	15.97
os30871	26	642	0.88	14	3	9	6.29	-0.21	0.00	1.39	0.08	99.59	1.01	5.24	6.48	15.37
os51342	26	625	0.70	11	2	3	6.43	-0.11	0.04	1.49	0.14	98.89	-2.39	6.80	5.30	14.06
os44475	26	518	0.56	17	1	1	6.16	-0.10	0.00	1.37	0.08	99.79	5.29	5.20	6.66	15.39
os25389	26	594	0.02	4	3	6	6.94	0.06	0.96	1.54	0.05	66.00	-1.48	8.27	7.26	16.25
os23916	26	540	0.32	2	3	6	6.77	-0.02	2.55	1.53	0.02	42.24	1.16	8.67	7.28	16.25
os12793	26	625	0.25	13	3	5	7.07	0.14	0.00	1.46	0.11	99.90	0.89	5.39	7.00	15.81
os12837	26	716	0.55	13	3	5	6.94	0.02	0.00	1.44	0.06	99.99	2.33	5.32	6.57	15.39
os45614	26	511	0.15	14	2	7	6.70	0.13	0.00	1.48	0.16	98.80	-4.00	7.00	6.28	15.04
os16434	26	692	0.60	10	3	5	6.59	-0.06	0.23	1.49	0.07	99.51	-0.82	6.48	6.55	15.32
os16445	26	710	0.03	10	3	9	7.12	0.17	0.04	1.45	0.08	99.66	0.53	5.65	6.52	15.30
os26707	26	680	0.06	9	1	1	7.10	0.14	0.05	1.45	0.05	100.00	2.78	5.61	6.07	14.92
os44521	26	548	0.68	8	3	5	6.84	-0.02	0.07	1.46	0.06	99.85	1.61	5.75	6.34	15.10
os44698	26	546	0.43	14	3	5	6.90	0.05	0.00	1.43	0.09	99.83	0.75	6.17	6.51	15.26
os16448	26	659	0.40	3	1	4	6.94	0.02	0.10	1.45	0.03	98.14	3.29	6.91	6.71	15.47
os11733	26	610	0.38	16	3	5	6.98	0.10	0.00	1.45	0.10	100.00	0.84	5.98	6.28	15.14
os13758	26	643	0.05	8	2	7	6.94	0.13	1.01	1.54	0.13	91.23	-5.37	8.16	6.58	15.34
os27530	26	605	0.14	4	3	6	6.90	0.05	0.88	1.53	0.04	73.99	-0.07	8.66	6.19	14.97

os18659	26	762	0.05	6	3	5	7.08	0.10	0.46	1.52	0.05	94.94	0.03	6.85	6.09	14.95
os25916	26	615	0.06	4	3	9	6.90	0.05	0.17	1.50	0.02	95.45	1.43	6.40	7.38	16.41
os56919	26	686	0.08	4	3	6	7.00	0.06	0.33	1.51	0.02	94.20	1.86	7.91	5.92	14.75
os24987	26	651	0.88	2	1	4	6.81	-0.03	0.02	1.50	0.01	97.02	2.41	6.86	6.96	15.98
os17596	26	599	0.15	12	3	5	6.76	0.09	0.04	1.47	0.09	99.59	-1.45	6.71	7.01	15.95
os25895	26	641	0.19	3	3	6	6.96	0.03	1.80	1.54	0.05	89.55	-0.74	8.74	6.62	15.58
os13236	26	641	0.56	10	3	5	6.57	-0.05	0.04	1.48	0.07	97.24	0.10	6.81	6.78	15.54
os30761	26	562	0.01	7	3	5	6.96	0.10	0.58	1.53	0.04	86.24	-0.77	7.21	6.79	15.72
os44577	26	518	0.13	12	3	5	6.76	0.12	0.01	1.46	0.07	99.55	0.86	5.93	6.63	15.37
os25609	26	538	0.77	7	3	5	6.78	-0.05	0.17	1.51	0.06	84.72	0.94	6.16	7.40	16.39
os80857	26	538	0.15	10	1	4	6.78	0.07	0.01	1.46	0.06	83.69	2.52	8.23	6.72	15.56
os13696	26	599	0.00	11	3	5	7.00	0.18	0.04	1.50	0.09	97.76	-1.76	5.64	7.02	15.88
os16491	26	656	0.05	10	3	5	6.94	0.14	0.06	1.49	0.08	93.15	-1.39	6.90	6.68	15.43
os58155	26	706	0.92	6	3	9	6.66	-0.08	0.28	1.47	0.04	98.77	0.63	6.25	5.94	14.68
os26432	26	633	0.60	8	3	5	6.66	-0.04	0.46	1.51	0.07	99.51	-1.26	6.58	7.15	16.18
os43786	26	633	0.06	7	3	5	6.96	0.10	0.69	1.49	0.08	99.79	-1.27	7.53	5.90	14.67
os31335	26	596	0.44	16	1	4	6.93	0.07	0.00	1.47	0.09	92.59	0.92	6.18	6.85	15.86
os12215	26	647	0.49	10	3	5	6.95	0.03	0.05	1.49	0.10	99.99	-1.16	6.02	6.86	15.67
os06453	26	582	0.98	9	1	4	6.51	-0.14	0.06	1.48	0.07	99.79	2.89	5.74	7.37	16.37
os13707	26	649	0.87	9	1	1	6.52	-0.12	0.05	1.46	0.05	98.35	2.29	5.62	6.94	15.80
os13790	26	581	0.06	7	2	7	6.93	0.11	1.24	1.56	0.15	88.39	-6.38	7.44	6.91	15.69
os45135	26	545	0.77	19	1	2	6.44	-0.13	0.00	1.40	0.19	94.39	2.24	5.19	6.03	14.77
os75158	26	602	0.74	9	1	4	6.60	-0.06	0.03	1.47	0.08	98.77	2.63	5.75	6.12	15.05
os15790	26	743	0.02	10	2	8	7.13	0.18	0.04	1.46	0.10	99.45	-2.78	5.72	6.35	15.23
os15869	26	636	0.27	12	1	4	6.67	0.04	0.01	1.46	0.09	95.33	1.45	5.88	6.90	15.74
os42720	26	485	0.30	4	3	6	6.89	0.03	0.51	1.51	0.00	93.55	0.73	7.56	7.08	15.90
os15585	26	651	0.40	3	3	9	6.95	0.02	0.23	1.49	0.04	87.23	1.70	7.12	6.91	15.76
os19287	26	753	0.99	9	3	5	6.67	-0.13	0.04	1.47	0.06	92.33	2.00	6.00	5.62	14.43
os15850	26	574	0.91	12	3	5	6.56	-0.14	0.01	1.46	0.09	98.80	-1.30	5.95	7.25	16.11
os15791	26	735	0.33	14	1	4	7.09	0.12	0.00	1.43	0.07	99.37	2.90	5.61	6.34	15.23
os15763	26	708	0.10	8	1	1	7.10	0.13	0.02	1.43	0.04	99.99	4.52	5.59	6.71	15.59
os24154	26	529	0.23	2	3	6	6.82	0.01	3.60	1.56	0.05	65.54	-1.39	7.95	7.27	16.31
os25253	26	533	0.04	9	2	7	6.90	0.11	0.61	1.53	0.08	89.36	-2.48	7.88	7.56	16.58
os59040	26	594	0.32	21	2	3	6.87	0.17	0.00	1.43	0.15	100.00	-2.88	5.28	6.37	15.06
os59805	26	561	0.98	4	3	6	6.78	-0.05	1.16	1.53	0.04	98.63	-1.28	7.77	6.65	15.59
os11742	26	634	0.97	7	3	5	6.72	-0.09	0.20	1.47	0.05	100.00	1.87	6.05	6.25	15.11
os23530	26	655	0.03	12	3	5	6.96	0.17	0.00	1.45	0.07	98.41	0.92	5.88	7.08	16.14
os25536	26	592	0.97	7	3	9	6.68	-0.10	0.23	1.49	0.04	98.77	0.54	6.20	7.40	16.45
os24351	26	563	0.02	4	3	6	6.89	0.05	2.14	1.54	0.03	56.84	-0.77	8.33	7.37	16.42
os45484	26	647	0.77	5	1	1	6.85	-0.04	0.09	1.44	0.02	98.26	2.32	6.24	6.14	14.92
os31845	26	654	0.82	7	1	1	6.61	-0.09	0.07	1.48	0.03	97.08	2.95	5.81	6.90	15.83
os15916	26	724	0.96	13	3	9	6.38	-0.21	0.01	1.44	0.08	99.66	-0.10	5.87	6.43	15.28
os59771	26	523	0.95	6	3	5	6.65	-0.10	1.03	1.53	0.06	92.74	-2.25	8.02	6.75	15.69

os26007	26	531	0.85	2	3	6	6.79	-0.03	1.96	1.55	0.05	93.66	-1.54	8.94	7.61	16.65
os10489	26	608	0.34	4	2	0	6.97	0.05	1.33	1.54	0.07	99.99	-1.33	7.73	6.23	14.93
os30341	26	586	0.08	7	3	5	7.03	0.11	0.23	1.51	0.06	100.00	0.47	6.38	6.68	15.65
os30252	26	620	1.00	8	3	5	6.57	-0.14	0.18	1.49	0.06	99.98	-0.26	5.94	6.78	15.80
os31362	26	558	0.10	6	3	5	6.87	0.06	0.88	1.53	0.05	82.74	-1.10	8.79	7.14	16.20
os29271	26	518	0.22	7	3	5	6.84	0.07	0.76	1.53	0.06	90.11	-1.74	8.13	7.49	16.45
os44565	26	583	0.47	3	1	1	6.82	0.00	0.04	1.47	0.01	98.02	2.40	6.73	6.41	15.17
os45486	26	645	0.36	6	1	1	6.99	0.05	0.07	1.42	0.02	99.97	3.47	5.88	6.17	14.94
os58575	26	680	0.16	5	3	5	7.04	0.08	0.35	1.52	0.04	93.82	0.64	7.54	5.98	14.81
os11988	26	624	0.85	7	2	0	6.78	-0.07	0.26	1.51	0.10	99.98	-0.92	6.94	6.97	15.91
os11233	26	625	0.45	6	3	5	6.74	-0.01	0.38	1.48	0.08	97.39	1.88	6.40	6.22	15.03
os24995	26	567	0.49	3	3	6	6.88	0.00	2.42	1.54	0.05	83.08	0.03	9.08	7.23	16.23
os15796	26	733	0.39	9	3	9	6.73	0.01	0.10	1.45	0.05	99.75	0.94	6.16	6.34	15.19
os00115	26	572	0.28	13	2	0	6.66	0.08	0.00	1.46	0.00	88.39	0.27	6.94	6.23	14.83
os30526	26	503	0.77	2	3	6	6.81	-0.02	3.59	1.56	0.04	70.41	-1.07	8.44	7.23	16.14
os57962	26	611	0.06	7	3	5	6.92	0.10	0.17	1.45	0.06	95.84	1.75	6.33	6.33	15.09
os13487	26	761	0.22	8	3	9	6.90	0.06	0.26	1.46	0.06	100.00	1.37	6.18	6.37	15.16
os13701	26	594	0.19	9	3	5	6.48	-0.15	0.09	1.48	0.06	97.04	1.13	6.18	7.11	15.96
os43760	26	575	1.00	13	1	4	6.26	-0.22	0.00	1.39	0.10	98.15	4.37	5.47	6.08	14.81
os29293	26	542	0.76	5	1	4	6.69	-0.05	0.05	1.48	0.02	98.91	3.26	6.10	7.50	16.48
os41244	26	479	0.97	15	3	5	6.09	-0.26	0.00	1.46	0.09	99.93	-0.87	5.85	6.90	15.64
os58091	26	632	0.79	5	1	4	6.67	-0.08	0.17	1.43	0.04	94.65	4.25	6.73	6.20	14.92
os27428	26	552	1.00	5	3	6	6.67	-0.08	0.60	1.52	0.04	90.05	0.27	8.33	6.60	15.43
os30752	26	550	1.00	7	3	5	6.59	-0.11	0.37	1.50	0.06	98.57	0.05	7.18	6.88	15.82
os29949	26	653	0.90	5	3	6	6.72	-0.08	0.70	1.53	0.06	99.58	-0.84	7.65	6.54	15.49
os25728	26	670	0.23	5	3	6	6.86	0.02	0.39	1.51	0.04	80.31	0.60	7.30	6.62	15.57
os18598	26	696	0.91	9	1	4	6.66	-0.12	0.01	1.45	0.04	97.36	4.97	5.40	6.55	15.32
os12122	26	689	0.03	10	3	5	7.02	0.15	0.07	1.49	0.09	97.34	-0.50	6.35	6.67	15.50
os14913	26	736	0.98	11	3	9	6.43	-0.19	0.01	1.46	0.07	100.00	1.35	5.54	6.48	15.38
os31284	26	603	0.23	6	3	5	6.85	0.05	0.56	1.52	0.05	93.43	-0.08	7.28	6.92	15.91
os41319	26	470	0.96	15	3	5	6.05	-0.26	0.00	1.43	0.14	94.24	-0.73	5.04	7.11	15.90
os49233	26	548	0.36	13	1	4	6.62	0.05	0.00	1.42	0.07	99.78	3.56	5.80	6.25	14.93
os28056	26	497	0.92	5	3	6	6.64	-0.06	0.11	1.49	0.04	89.69	2.90	6.96	7.33	16.25
os25639	26	677	0.55	7	3	5	6.93	0.00	0.25	1.50	0.03	98.64	1.45	6.26	6.64	15.59
os12746	26	600	0.05	6	2	0	6.63	-0.11	0.45	1.52	0.09	99.21	-0.63	6.55	7.15	16.09
os24856	26	675	0.23	5	3	6	7.03	0.07	0.53	1.51	0.04	100.00	0.35	7.00	6.88	15.97
os59770	26	528	0.93	6	3	5	6.67	-0.09	0.59	1.52	0.05	94.22	-0.62	7.28	6.74	15.68
os13138	26	598	0.00	10	1	4	7.06	0.18	0.07	1.46	0.06	89.30	2.42	6.89	6.96	15.76
os15748	26	687	0.18	5	2	0	6.93	0.06	0.58	1.52	0.09	78.05	-0.99	7.93	6.19	15.01
os49039	26	574	0.45	4	1	4	6.90	0.01	0.05	1.48	0.00	90.52	3.06	7.29	6.00	14.69
os31522	26	627	0.11	2	3	6	6.93	0.02	2.87	1.54	0.03	30.73	-0.09	9.14	6.65	15.65
os25139	26	566	0.92	3	3	6	6.75	-0.04	1.58	1.55	0.04	90.66	-0.18	7.79	7.15	16.16
os41144	26	467	0.17	15	3	5	6.70	0.14	0.01	1.43	0.11	90.61	0.57	5.93	6.88	15.63

os19069	26	725	0.49	3	1	1	6.85	-0.02	0.02	1.48	0.02	98.34	2.84	6.93	5.99	14.79
os28726	26	529	0.19	5	3	6	6.96	0.06	1.06	1.53	0.04	86.50	-1.16	7.77	7.44	16.40
os57724	26	667	0.20	9	3	5	7.07	0.11	0.24	1.50	0.06	72.76	-0.59	7.16	5.90	14.60
os10370	26	559	0.88	5	3	5	6.75	-0.06	0.51	1.52	0.06	96.09	0.10	6.35	6.65	15.38
os27367	26	523	0.09	13	2	0	7.06	0.20	0.03	1.48	0.10	81.46	-1.47	6.75	6.66	15.47
os12156	26	777	0.00	10	1	1	7.08	0.15	0.00	1.41	0.04	99.28	6.74	5.36	6.46	15.31
os44615	26	579	0.30	5	1	1	6.93	0.03	0.16	1.48	0.02	99.85	2.23	6.47	6.42	15.17
os60391	26	677	0.60	4	3	6	6.79	-0.03	0.60	1.52	0.03	99.59	0.09	7.12	6.01	14.95
os31480	26	638	0.40	8	3	5	6.69	-0.02	0.26	1.52	0.05	95.53	-0.27	6.69	6.69	15.71
os29178	26	560	0.07	6	3	5	7.00	0.10	0.40	1.50	0.04	98.57	1.45	7.35	7.32	16.26
os13269	26	593	0.28	3	3	6	6.93	0.02	2.85	1.54	0.03	62.36	0.01	8.78	6.93	15.81
os12236	26	651	1.00	10	1	4	6.55	-0.15	0.00	1.41	0.05	99.99	6.67	5.33	6.97	15.79
os25998	26	582	0.93	6	3	5	6.76	-0.06	1.19	1.53	0.06	99.47	-2.47	8.24	7.38	16.41
os30018	26	542	0.44	6	2	0	6.70	-0.02	0.34	1.54	0.08	67.85	-1.18	7.31	6.95	15.89
os26607	26	569	0.08	1	3	6	6.89	0.02	2.96	1.55	0.04	59.52	-1.04	9.92	7.43	16.51
os31866	26	606	0.65	9	3	5	6.89	0.00	0.09	1.50	0.05	84.60	0.52	6.14	6.95	15.90
os11700	26	625	0.50	8	1	4	6.71	0.00	0.16	1.47	0.09	97.47	1.91	5.98	6.23	15.04
os58894	26	506	0.76	9	2	0	6.47	-0.10	0.17	1.52	0.09	99.99	-1.85	6.20	6.85	15.58
os12095	26	707	0.14	8	3	9	7.09	0.11	0.09	1.46	0.05	100.00	2.21	5.68	6.75	15.66
os00403	26	575	0.77	3	3	6	6.86	-0.01	0.40	1.51	0.02	92.24	1.11	7.41	6.54	15.17
os15915	26	725	0.99	12	3	9	6.43	-0.20	0.01	1.44	0.08	99.62	0.01	5.90	6.44	15.29
os13119	26	663	0.42	9	3	5	7.00	0.05	0.04	1.48	0.06	97.72	1.72	5.81	6.55	15.40
os29327	26	596	0.60	6	3	5	6.67	-0.05	0.45	1.52	0.05	96.91	-0.27	6.37	6.96	15.88
os47083	26	528	0.04	13	3	5	7.06	0.20	0.03	1.46	0.07	96.91	1.95	6.54	6.46	15.19
os01011	26	593	0.05	12	2	0	6.93	0.18	0.01	1.47	0.13	99.99	-0.66	6.08	6.10	14.81
os44484	26	466	0.45	10	1	4	6.86	0.03	0.00	1.34	0.07	93.66	8.30	5.23	6.75	15.46
os58487	26	607	0.81	10	3	5	6.69	-0.09	0.03	1.45	0.08	98.62	1.51	6.20	6.31	15.02
os24352	26	560	0.92	5	3	5	6.65	-0.09	0.53	1.51	0.05	92.72	-0.43	7.02	7.48	16.53
os25818	26	682	0.87	6	1	1	6.67	-0.09	0.04	1.47	0.02	93.23	3.75	6.00	6.68	15.63
os10666	26	556	0.26	5	3	5	6.76	0.01	0.52	1.52	0.09	92.71	-0.80	6.92	6.64	15.40
os24249	26	543	0.60	5	3	5	6.74	-0.01	0.53	1.52	0.04	84.31	-0.06	6.56	7.45	16.51
os25648	26	653	0.95	8	2	3	6.78	-0.06	0.46	1.51	0.09	97.74	-2.99	6.95	6.68	15.63
os28143	26	529	0.93	7	3	5	6.69	-0.09	0.23	1.52	0.05	89.85	0.61	7.16	6.99	15.90
os28159	26	501	0.57	4	3	6	6.71	-0.03	0.59	1.52	0.03	70.26	0.89	8.69	7.23	16.11
os13663	26	586	0.65	6	3	5	6.87	-0.01	0.21	1.51	0.04	75.66	1.14	7.52	7.14	16.01
os17482	26	650	0.74	8	3	9	6.59	-0.08	0.15	1.48	0.06	100.00	1.31	6.14	6.22	15.12
os28164	26	531	0.95	6	3	5	6.67	-0.09	0.26	1.52	0.05	90.22	0.81	7.03	6.99	15.89
os43916	26	628	0.02	10	1	4	7.08	0.16	0.09	1.45	0.08	99.24	3.15	6.38	5.76	14.52
os57566	26	647	0.25	8	2	0	7.03	0.08	0.04	1.49	0.09	95.93	1.66	7.39	5.93	14.69
os29784	26	638	0.88	5	3	6	6.81	-0.05	0.54	1.53	0.03	88.08	-0.82	6.90	6.63	15.53
os25703	26	629	0.77	5	1	4	6.86	-0.03	0.13	1.49	0.02	97.85	2.40	6.46	6.86	15.81
os19292	26	672	0.06	14	2	0	6.91	0.19	0.00	1.47	0.14	99.67	-0.61	6.04	5.86	14.74
os30672	26	526	0.26	11	3	5	6.34	-0.19	0.01	1.49	0.09	77.72	-1.11	6.84	6.85	15.77

os45672	26	616	0.26	10	2	3	7.04	0.10	0.17	1.47	0.08	98.84	-2.07	7.21	6.09	14.86
os12760	26	607	0.54	7	2	0	6.68	-0.02	0.68	1.52	0.08	99.82	-1.40	7.22	7.12	15.98
os16149	26	621	0.39	8	1	4	6.99	0.06	0.04	1.45	0.06	83.27	3.22	5.80	6.83	15.58
os25596	26	624	0.00	7	3	5	7.03	0.13	0.26	1.50	0.07	91.29	0.39	6.76	6.96	15.95
os30062	26	568	0.11	10	3	5	6.83	0.10	0.04	1.49	0.06	89.55	-0.09	6.55	7.11	16.08
os13196	26	692	0.38	3	3	6	7.01	0.06	1.00	1.52	0.02	88.56	0.58	8.21	6.49	15.34
os01419	26	777	0.00	16	3	5	7.06	0.27	0.00	1.44	0.11	99.98	0.04	5.95	5.55	14.42
os31346	26	642	0.26	8	1	1	7.03	0.09	0.04	1.47	0.03	93.98	3.01	5.81	6.85	15.86
os18347	26	796	1.00	14	3	9	6.37	-0.23	0.00	1.44	0.08	99.00	1.94	5.66	6.19	15.01
os28439	26	488	0.47	2	2	0	6.83	0.00	3.32	1.56	0.05	66.69	-0.39	9.01	7.23	16.13
os10037	26	503	0.00	6	3	5	6.96	0.12	0.35	1.49	0.09	92.34	0.35	6.55	6.87	15.62
os58756	26	670	0.92	4	3	6	6.74	-0.07	0.58	1.54	0.07	96.82	-0.39	6.10	5.71	14.42
os15029	26	758	0.79	6	1	1	6.89	-0.04	0.10	1.42	0.04	99.98	3.14	6.04	6.56	15.47
os16004	26	574	0.86	15	3	9	6.17	-0.21	0.00	1.44	0.08	99.98	0.62	5.56	7.40	16.35
os57822	26	622	0.76	5	3	5	6.86	-0.03	0.42	1.53	0.05	62.38	1.13	7.69	6.25	15.03
os59469	26	602	0.03	12	1	4	6.92	0.16	0.00	1.42	0.05	94.04	6.51	5.50	6.36	15.26
os58887	26	722	0.45	11	3	5	6.63	-0.02	0.01	1.45	0.07	99.94	0.74	5.72	5.84	14.58
os23521	26	626	0.60	10	3	5	6.56	-0.06	0.06	1.47	0.06	99.99	0.23	6.02	7.16	16.20
os45172	26	689	0.47	18	2	7	6.29	-0.04	0.00	1.43	0.15	100.00	-2.14	5.78	5.45	14.23
os25967	26	575	0.49	3	2	3	6.84	-0.02	1.94	1.55	0.06	92.84	-2.96	9.03	7.39	16.41
os45485	26	646	0.53	7	1	1	6.93	0.01	0.09	1.42	0.03	99.95	2.56	5.81	6.15	14.93
os23786	26	565	0.11	8	2	0	6.86	0.09	0.22	1.50	0.10	99.76	-1.56	7.13	7.38	16.40
os10675	26	602	0.33	2	3	6	6.93	0.03	1.18	1.53	0.04	87.66	-0.02	7.50	6.41	15.21
os43528	26	499	0.99	4	2	7	6.71	-0.05	1.44	1.54	0.16	85.49	-2.94	7.27	6.31	15.00
os15262	26	675	0.97	10	2	7	6.64	-0.13	0.10	1.51	0.09	100.00	-2.14	6.46	6.67	15.56
os19434	26	743	0.99	13	1	4	6.33	-0.22	0.00	1.45	0.07	100.00	2.45	5.58	5.86	14.70
os58775	26	792	0.79	4	3	6	6.79	-0.07	0.60	1.52	0.03	84.93	0.21	7.49	5.07	13.78
os15387	26	776	0.27	9	1	1	7.09	0.09	0.02	1.44	0.05	98.23	3.63	5.46	6.27	15.14
os24616	26	561	0.59	23	3	5	6.66	-0.01	0.00	1.39	0.11	75.38	3.13	5.05	7.16	16.17
os24985	26	648	0.15	9	3	5	6.87	0.08	0.14	1.49	0.05	97.23	1.35	5.93	6.96	15.98
os30179	26	645	0.23	1	3	6	6.89	0.01	0.70	1.53	0.01	47.62	0.13	9.27	6.63	15.66
os45637	26	649	0.41	7	3	5	6.77	0.02	0.54	1.49	0.06	96.30	-0.95	7.38	5.85	14.61
os59863	26	564	0.08	3	3	6	6.94	0.05	1.77	1.53	0.03	77.41	-0.56	8.24	6.52	15.45
os43725	26	688	0.22	10	3	5	6.88	0.10	0.04	1.48	0.08	95.13	-0.11	6.11	5.49	14.28
os15851	26	574	0.93	13	3	5	6.50	-0.17	0.01	1.46	0.09	98.77	-1.15	6.08	7.25	16.11
os26428	26	573	0.03	4	3	6	6.72	-0.07	0.58	1.53	0.06	94.05	-0.06	7.22	7.31	16.34
os44461	26	446	0.42	5	1	4	6.88	0.04	0.01	1.44	0.07	96.58	6.26	5.71	6.83	15.54
os29295	26	533	0.08	2	3	6	6.90	0.03	2.11	1.55	0.04	80.52	0.02	8.22	7.39	16.34
os57941	26	664	0.86	12	3	5	6.33	-0.19	0.00	1.44	0.08	99.88	-0.23	6.05	6.02	14.73
os14563	26	578	0.85	15	3	9	6.51	-0.15	0.00	1.43	0.10	100.00	0.39	5.39	7.21	16.12
os31921	26	613	0.19	3	3	6	6.91	0.04	0.39	1.54	0.02	83.94	0.66	8.28	6.42	15.31
os24650	26	541	0.00	3	3	6	6.72	-0.06	1.31	1.55	0.05	75.99	-0.22	7.80	7.16	16.16
os57506	26	630	0.55	18	2	7	6.18	-0.09	0.00	1.45	0.15	99.53	-2.88	5.22	5.98	14.69

os25869	26	605	0.90	4	3	6	6.83	-0.03	0.59	1.54	0.03	90.48	0.28	7.00	6.94	15.88
os44399	26	516	0.37	6	3	5	6.94	0.05	0.49	1.49	0.05	99.68	0.98	6.70	6.62	15.36
os17880	26	906	0.89	9	3	5	6.59	-0.14	0.05	1.47	0.05	97.50	1.71	6.94	5.22	14.05
os11072	26	539	0.10	7	2	0	7.02	0.11	0.48	1.52	0.08	92.60	-0.80	7.66	6.55	15.26
os29136	26	591	0.40	6	3	5	6.95	0.03	0.20	1.51	0.04	93.86	0.43	7.01	7.21	16.19
os12440	26	713	0.95	7	3	9	6.72	-0.11	0.10	1.47	0.06	100.00	1.55	6.34	6.70	15.67
os25375	26	606	0.60	4	3	6	6.89	-0.01	0.36	1.52	0.04	71.39	0.76	9.15	7.19	16.22
os26210	26	618	0.60	8	3	9	6.64	-0.04	0.14	1.45	0.05	98.30	1.16	6.14	7.45	16.44
os57957	26	632	0.99	4	3	6	6.80	-0.05	0.73	1.51	0.03	93.52	0.20	6.91	6.29	15.05
os12443	26	566	0.99	7	3	5	6.60	-0.12	0.28	1.52	0.05	99.51	-0.25	6.52	7.35	16.30
os15890	26	702	0.07	12	2	3	6.89	0.14	0.03	1.48	0.11	98.33	-3.05	6.54	6.46	15.31
os12482	26	700	0.47	12	1	2	6.95	0.03	0.00	1.43	0.09	99.99	3.36	5.77	6.56	15.48
os29126	26	582	0.08	2	3	6	6.91	0.03	3.83	1.54	0.04	65.18	-1.00	9.36	7.17	16.10
os10670	26	678	0.53	3	3	6	6.85	0.00	0.40	1.52	0.05	96.23	0.18	6.49	6.06	14.84
os23924	26	554	0.23	9	3	5	6.74	0.06	0.19	1.52	0.07	99.87	-0.97	6.04	7.04	16.01
os26568	26	625	0.26	6	3	5	7.03	0.08	0.25	1.50	0.06	99.88	0.09	6.04	7.24	16.33
os44487	26	452	0.19	20	1	4	6.99	0.28	0.00	1.30	0.11	92.06	9.86	4.87	6.74	15.45
os44449	26	496	0.92	16	1	1	6.04	-0.24	0.00	1.38	0.11	98.82	2.65	5.62	6.78	15.47
os00673	26	722	1.00	21	3	5	5.89	-0.34	0.00	1.37	0.17	99.11	-1.27	5.41	5.59	14.33
os13937	26	631	0.08	2	3	9	6.93	0.03	0.19	1.52	0.02	68.66	0.62	8.29	6.89	15.79
os25702	26	629	1.00	6	1	4	6.63	-0.10	0.34	1.49	0.04	98.40	1.45	6.37	6.86	15.81
os57420	26	646	0.57	7	1	4	6.68	-0.03	0.11	1.47	0.03	86.29	3.16	6.16	6.15	14.86
os15762	26	664	0.08	10	3	5	7.11	0.17	0.03	1.49	0.09	100.00	-1.37	6.35	6.69	15.58
os31005	26	661	0.99	10	3	9	6.45	-0.18	0.02	1.37	0.05	100.00	2.15	5.61	6.61	15.50
os12425	26	645	0.75	10	3	5	6.48	-0.12	0.08	1.46	0.09	100.00	0.92	6.02	7.00	15.95
os29589	26	677	0.00	6	3	5	7.02	0.10	0.48	1.51	0.04	99.30	0.05	6.62	6.58	15.47
os30749	26	514	0.09	4	3	6	6.87	0.05	3.79	1.53	0.04	70.37	0.99	8.50	7.05	15.96
os25730	26	676	0.23	2	3	6	6.87	0.00	0.20	1.50	0.02	84.07	1.76	7.22	6.62	15.58
os30248	26	620	0.76	5	1	4	6.89	-0.01	0.19	1.48	0.04	99.88	2.76	6.11	6.81	15.82
os24877	26	500	0.98	3	3	6	6.76	-0.04	1.87	1.54	0.04	91.07	-1.02	8.13	7.87	16.89
os24983	26	648	0.95	6	3	5	6.75	-0.08	0.85	1.54	0.05	93.90	-1.07	8.83	6.85	15.87
os29950	26	682	0.12	8	3	9	6.93	0.09	0.21	1.49	0.05	96.67	0.09	6.14	6.59	15.55
os44179	26	615	0.12	6	3	9	6.90	0.07	0.24	1.44	0.03	100.00	1.57	5.98	6.47	15.24
os12431	26	620	0.68	14	2	7	6.33	-0.08	0.00	1.46	0.13	99.68	-1.99	7.65	6.94	15.86
os16343	26	600	0.50	22	3	5	6.02	-0.06	0.00	1.43	0.00	86.57	-1.57	6.43	6.87	15.63
os01462	26	624	0.53	4	3	6	6.93	0.02	0.62	1.53	0.03	82.21	-0.30	7.35	6.46	15.26
os16483	26	626	0.01	2	3	6	6.94	0.04	1.09	1.54	0.04	67.35	-0.06	9.37	6.81	15.55
os11235	26	566	0.60	11	2	0	6.50	-0.06	0.07	1.50	0.11	95.34	-1.68	7.40	6.44	15.23
os15248	26	635	0.84	15	3	5	6.59	-0.13	0.00	1.45	0.10	99.99	0.54	5.17	7.09	15.99
os59158	26	607	0.77	3	1	4	6.85	-0.02	0.15	1.49	0.02	62.62	1.86	7.98	6.48	15.29
os26448	26	708	0.01	9	1	1	7.11	0.13	0.33	1.40	0.05	92.71	3.06	5.99	7.01	16.04
os57826	26	681	0.58	2	3	6	6.85	-0.01	0.38	1.54	0.03	59.13	1.08	8.95	5.92	14.66
os00685	26	723	0.00	17	3	9	7.10	0.28	0.00	1.42	0.10	99.99	-0.14	5.83	5.55	14.23

os25931	26	544	0.73	7	2	7	6.85	0.00	0.90	1.54	0.10	93.32	-3.44	7.50	7.45	16.45
os25553	26	540	0.16	5	3	5	6.65	-0.08	1.13	1.53	0.05	98.77	-1.47	8.20	7.55	16.56
os29262	26	555	0.42	4	3	6	6.78	0.01	1.57	1.53	0.07	85.85	-1.94	8.13	7.28	16.27
os11882	26	662	0.70	10	3	5	6.49	-0.11	0.08	1.49	0.07	99.51	-1.18	6.69	6.25	14.97
os45573	26	579	0.92	18	2	0	5.90	-0.28	0.00	1.40	0.15	98.78	0.18	6.22	6.06	14.83
os29979	26	629	0.99	8	3	5	6.68	-0.11	0.27	1.50	0.05	99.40	-0.33	6.40	6.78	15.75
os43583	26	581	0.30	8	3	5	6.71	0.00	0.70	1.46	0.07	95.01	0.54	6.28	6.21	15.03
os47084	26	535	0.77	2	3	6	6.77	-0.04	1.95	1.53	0.03	82.85	-0.59	7.79	6.41	15.14
os25225	26	560	0.45	6	3	5	6.71	-0.02	1.01	1.54	0.06	92.16	-1.63	8.57	7.44	16.49
os25407	26	564	0.77	5	3	6	6.82	-0.04	1.65	1.54	0.07	95.66	-1.66	8.49	7.39	16.39
os43589	26	551	0.02	12	3	5	6.33	-0.18	0.01	1.44	0.09	94.02	0.31	5.43	6.53	15.33
os43940	26	823	0.82	9	1	1	6.84	-0.07	0.05	1.44	0.06	100.00	2.32	6.19	4.95	13.76
os59803	26	550	0.83	5	3	6	6.78	-0.05	0.55	1.52	0.04	97.10	-0.22	6.91	6.69	15.63
os30770	26	563	1.00	5	3	5	6.67	-0.09	0.68	1.52	0.05	88.38	-0.14	8.75	6.70	15.62
os31287	26	593	0.60	4	3	6	6.73	-0.04	0.71	1.54	0.04	97.12	-0.27	7.29	6.90	15.88
os31288	26	594	0.75	4	3	6	6.73	-0.04	0.83	1.54	0.04	97.62	-0.49	7.14	6.90	15.87
os16481	26	618	0.01	6	3	5	6.98	0.10	0.46	1.50	0.08	96.34	-0.76	5.91	6.89	15.60
os27593	26	601	0.95	10	3	5	6.62	-0.12	0.06	1.49	0.06	91.12	-0.49	5.76	6.94	15.83
os29174	26	568	0.08	2	3	6	6.89	0.01	0.16	1.52	0.03	99.60	1.03	7.14	7.30	16.24
os45480	26	641	0.61	7	3	9	6.62	-0.07	0.26	1.45	0.04	97.86	0.78	6.16	6.11	14.89
os11348	26	558	0.64	16	3	9	6.77	-0.02	0.00	1.44	0.10	98.25	0.54	5.37	6.77	15.50
os15822	26	625	1.00	13	1	1	6.33	-0.21	0.00	1.43	0.06	99.13	2.98	5.29	7.13	16.02
os27473	26	614	0.83	9	3	5	6.77	-0.07	0.09	1.49	0.05	96.11	0.95	6.31	6.29	15.09
os16461	26	786	0.99	16	3	5	6.32	-0.24	0.00	1.45	0.12	98.31	-1.50	5.63	6.04	14.86
os29138	26	598	0.23	7	3	9	6.84	0.07	0.18	1.49	0.05	95.29	-0.24	5.78	7.21	16.16
os25234	26	518	0.99	3	3	6	6.75	-0.04	1.81	1.55	0.04	86.89	-0.68	8.33	7.70	16.74
os31317	26	618	0.40	11	3	5	6.98	0.06	0.03	1.48	0.06	96.89	-0.44	5.97	6.81	15.80
os57555	26	644	0.00	20	3	5	6.98	0.31	0.00	1.41	0.12	97.99	-0.06	5.46	6.10	14.80
os28808	26	730	0.02	9	3	9	7.04	0.14	0.28	1.48	0.07	99.23	-0.66	6.38	6.32	15.30
os58047	26	641	1.00	6	3	5	6.65	-0.11	0.27	1.51	0.03	85.57	0.41	6.47	6.18	14.99
os30651	26	602	0.77	3	3	6	6.86	-0.02	0.33	1.53	0.03	77.95	0.70	8.41	6.50	15.41
os02745	26	644	0.00	5	3	5	7.00	0.09	0.83	1.51	0.05	96.62	-1.02	7.33	7.14	16.12
os31762	26	582	0.76	10	3	5	6.44	-0.10	0.03	1.49	0.08	77.94	-1.37	6.84	6.86	15.91
os43596	26	521	0.23	2	1	1	6.78	-0.03	0.09	1.42	0.01	96.40	2.93	7.37	6.75	15.50
os57360	26	675	0.39	4	1	1	6.84	0.01	0.21	1.42	0.03	99.52	2.50	6.21	6.10	14.76
os25497	26	656	0.67	6	1	1	6.69	-0.05	0.04	1.47	0.02	97.48	3.61	5.96	6.90	15.83
os25825	26	644	0.92	0	3	6	6.89	0.00	3.55	1.54	0.02	66.68	0.75	10.51	6.82	15.79
os57525	26	643	0.91	12	1	4	6.40	-0.16	0.01	1.47	0.08	86.81	1.95	6.59	5.97	14.70
os17581	26	672	0.85	10	2	0	6.45	-0.15	0.04	1.49	0.10	99.92	0.05	5.89	6.70	15.62
os27996	26	598	0.43	5	3	9	6.86	-0.02	0.67	1.50	0.04	95.96	-0.30	6.89	7.04	15.97
os15370	26	719	0.06	13	3	5	7.13	0.21	0.00	1.47	0.10	96.80	-0.87	5.96	6.35	15.16
os19415	26	724	0.86	2	2	0	6.85	-0.04	1.84	1.55	0.08	88.58	-1.39	8.96	5.64	14.47
os23917	26	542	0.22	3	3	6	6.73	-0.06	1.52	1.55	0.05	78.23	-0.28	7.67	7.16	16.15

os43485	26	581	0.02	15	1	2	6.90	0.22	0.00	1.43	0.15	95.60	3.99	6.22	5.86	14.61
os27427	26	567	0.98	7	2	3	6.71	-0.08	0.67	1.53	0.06	96.48	-1.89	7.80	6.54	15.36
os10953	26	599	0.23	15	2	0	6.62	0.08	0.04	1.48	0.11	82.20	-1.43	6.57	6.22	14.91
os12824	26	633	0.34	5	3	6	6.99	0.05	0.64	1.51	0.04	96.58	-0.23	7.04	7.02	15.84
os58393	26	466	0.77	8	1	4	6.49	-0.07	0.00	1.32	0.04	96.75	13.06	5.47	7.32	16.06
os29796	26	502	0.08	2	3	6	6.88	0.03	1.56	1.55	0.02	46.81	0.19	7.98	7.27	16.20
os45987	26	517	0.52	7	1	1	6.89	0.02	0.05	1.46	0.03	99.50	3.94	5.70	6.76	15.52
os59806	26	563	0.77	5	3	6	6.77	-0.06	0.83	1.53	0.04	98.67	-1.27	7.78	6.64	15.58
os13686	26	569	0.49	6	3	5	6.90	0.01	0.33	1.50	0.07	87.71	-0.14	6.80	7.17	16.01
os15956	26	734	0.68	9	3	5	6.59	-0.08	0.15	1.47	0.08	99.90	-0.48	6.50	6.32	15.14
os26334	26	555	0.73	3	3	6	6.84	-0.02	0.73	1.53	0.04	65.30	0.09	10.05	7.55	16.58
os29165	26	561	0.18	3	1	4	6.92	0.03	0.10	1.51	0.02	98.42	2.17	7.78	7.35	16.28
os00408	26	524	0.76	11	2	3	6.50	-0.08	0.75	1.52	0.12	97.88	-4.89	8.09	6.75	15.39
os31707	26	561	0.24	5	2	3	6.85	0.04	1.48	1.53	0.07	91.94	-1.73	8.75	6.86	15.86
os17615	26	755	0.32	14	3	5	7.10	0.13	0.00	1.45	0.11	100.00	0.11	5.70	6.28	15.10
os24273	26	572	0.27	6	3	9	7.00	0.08	0.25	1.49	0.04	99.03	1.58	6.58	7.36	16.41
os51905	26	590	0.76	13	3	9	6.26	-0.16	0.00	1.41	0.08	99.66	0.10	5.53	5.96	14.76
os11300	26	547	0.90	4	2	0	6.79	-0.04	1.55	1.55	0.08	83.64	-0.48	8.41	6.53	15.25
os30547	26	625	0.61	12	3	9	6.44	-0.09	0.01	1.45	0.10	100.00	-0.26	5.95	6.52	15.40
os25781	26	594	0.98	10	3	5	6.59	-0.14	0.08	1.48	0.07	98.83	0.30	6.71	7.11	16.13
os06494	26	629	0.03	6	3	9	7.02	0.10	0.30	1.50	0.03	99.34	0.82	6.03	7.24	16.21
os17955	26	719	0.23	24	2	7	6.89	0.26	0.00	1.43	0.25	92.86	-7.28	5.84	5.86	14.65
os10429	26	576	0.67	14	3	5	6.75	-0.04	0.00	1.45	0.10	95.47	0.51	5.85	6.58	15.35
os31846	26	615	0.08	4	3	6	6.98	0.06	0.87	1.53	0.04	81.27	-0.37	7.64	7.00	15.97
os11703	26	555	0.79	8	2	0	6.54	-0.08	0.16	1.53	0.00	97.75	-0.16	6.54	6.63	15.36
os30065	26	588	0.72	11	2	3	6.44	-0.09	0.09	1.49	0.08	90.23	-2.05	7.20	6.76	15.73
os31001	26	601	0.47	16	3	5	6.94	0.11	0.00	1.44	0.13	99.88	-2.01	5.66	6.64	15.51
os59825	26	640	0.41	4	3	6	6.94	0.02	0.62	1.53	0.03	99.39	-0.01	7.50	6.13	15.04
os02740	26	597	0.62	7	1	1	6.69	-0.03	0.00	1.46	0.02	99.60	5.48	6.28	7.41	16.38
os10443	26	597	0.35	11	3	5	7.01	0.08	0.09	1.49	0.09	99.18	0.13	6.67	6.39	15.16
os18445	26	865	0.69	10	3	9	6.96	-0.02	0.02	1.46	0.06	99.64	0.83	5.76	5.41	14.14
os57762	26	645	0.73	12	1	4	6.79	-0.05	0.00	1.45	0.08	99.98	2.31	5.64	6.12	14.88
os11136	26	662	0.81	10	3	5	6.47	-0.13	0.06	1.49	0.07	99.45	-1.10	6.70	6.26	14.98
os31711	26	595	0.97	4	3	6	6.79	-0.05	1.75	1.53	0.05	95.23	-1.28	7.88	6.77	15.82
os58862	26	644	0.67	19	1	4	6.68	-0.06	0.00	1.40	0.11	99.96	1.74	5.57	6.01	14.67
os11079	26	645	0.69	4	3	6	6.87	-0.02	0.38	1.53	0.03	97.96	-0.47	7.01	6.18	14.88
os27435	26	580	0.92	0	3	6	6.86	0.00	4.96	1.54	0.00	52.47	0.82	9.51	6.56	15.37
os44826	26	573	0.59	10	1	4	6.80	-0.03	0.00	1.35	0.07	98.70	7.80	5.30	6.39	15.14
os59187	26	685	0.24	13	3	5	6.70	0.05	0.04	1.47	0.09	98.98	-0.32	7.03	5.81	14.51
os13871	26	699	0.92	5	1	4	6.74	-0.07	0.34	1.50	0.03	77.16	1.93	7.11	6.39	15.25
os18430	26	765	0.56	12	3	9	6.49	-0.08	0.00	1.46	0.08	99.96	0.57	5.78	5.97	14.73
os50319	26	608	0.81	9	3	9	6.52	-0.08	0.08	1.48	0.07	99.49	-0.49	5.81	6.21	14.93

t_fall	t_winter	tmax_spring	tmax_summe	tmax_fall	tmax_winter	tmin_spring	tmin_summe	tmin_fall	tmin_winter	prec_spring	prec_summe	prec_fall	prec_winter
6.92	-1.31	10.93	20.28	10.57	1.15	1.85	9.76	3.28	-3.81	159.93	242.27	172.20	166.99
6.67	-1.50	10.75	20.06	10.23	0.94	1.68	9.59	3.11	-3.96	156.14	241.31	171.06	168.61
6.47	-1.64	10.40	19.73	9.95	0.81	1.62	9.49	2.96	-4.10	160.53	245.17	175.59	178.38
6.90	-1.32	10.87	20.22	10.49	1.13	1.87	9.76	3.29	-3.80	159.92	242.27	172.48	167.54
6.45	-1.73	10.37	19.70	10.03	0.74	1.49	9.36	2.84	-4.23	169.68	253.76	182.99	191.93
6.44	-1.71	10.29	19.62	9.88	0.74	1.54	9.48	2.99	-4.13	162.60	248.31	179.45	178.43
7.11	-1.30	11.54	20.88	10.92	1.23	1.99	9.95	3.28	-3.84	146.45	232.26	161.64	139.75
5.81	-2.42	8.95	18.27	9.05	-0.06	0.86	9.00	2.55	-4.79	188.79	292.09	199.20	186.26
5.70	-2.44	8.73	18.05	8.92	-0.07	0.74	8.94	2.50	-4.79	192.71	294.33	201.23	191.52
6.78	-1.60	10.78	20.11	10.38	0.87	1.78	9.90	3.22	-4.11	162.36	264.58	173.14	153.21
6.61	-1.74	10.51	19.84	10.18	0.72	1.59	9.73	3.09	-4.22	165.25	268.31	177.68	159.17
6.60	-1.83	10.40	19.70	10.15	0.60	1.60	9.67	3.02	-4.25	168.22	268.94	180.43	159.14
6.33	-1.99	9.99	19.27	9.80	0.37	1.25	9.35	2.84	-4.37	178.29	274.38	197.28	179.06
6.78	-1.59	11.06	20.35	10.49	0.99	1.71	9.84	3.10	-4.15	146.78	242.63	165.02	148.28
7.08	-1.34	11.52	20.81	10.80	1.21	2.02	10.12	3.38	-3.91	140.63	237.74	157.79	135.98
7.39	-1.14	12.11	21.45	11.32	1.52	2.21	10.24	3.46	-3.79	130.85	222.55	147.39	122.36
6.80	-1.56	10.99	20.29	10.44	0.97	1.79	9.86	3.16	-4.10	146.24	243.38	165.36	146.80
6.27	-1.95	9.84	19.14	9.68	0.43	1.28	9.33	2.84	-4.36	173.56	274.98	193.18	178.25
6.07	-2.37	9.28	18.45	9.32	-0.01	0.94	9.19	2.83	-4.71	201.52	297.24	195.97	190.45
5.98	-2.51	9.23	18.45	9.26	-0.15	0.78	9.10	2.72	-4.87	203.54	294.46	197.39	191.39
5.97	-2.51	9.22	18.45	9.25	-0.16	0.77	9.09	2.71	-4.87	203.59	294.54	197.48	191.51
5.53	-2.78	8.69	17.87	8.78	-0.43	0.28	8.59	2.28	-5.13	207.18	300.58	205.06	200.55
5.47	-2.86	8.79	17.94	8.79	-0.45	0.18	8.49	2.15	-5.24	206.08	299.34	204.83	199.52
5.39	-2.91	8.65	17.83	8.67	-0.52	0.07	8.37	2.08	-5.31	206.88	300.97	206.55	201.01
6.39	-2.18	10.14	19.33	9.87	0.26	1.19	9.42	2.91	-4.62	190.01	285.11	186.65	176.07
5.74	-2.55	9.14	18.32	9.05	-0.15	0.57	8.85	2.43	-4.97	199.38	296.40	199.34	192.42
6.97	-1.71	10.85	20.07	10.49	0.72	1.91	10.12	3.46	-4.17	187.67	280.54	180.12	170.27
7.45	-1.30	11.52	20.72	11.00	1.20	2.45	10.64	3.91	-3.78	177.57	272.49	171.67	158.68
7.25	-1.46	11.23	20.43	10.75	1.03	2.23	10.45	3.74	-3.91	181.67	276.50	176.10	164.35
5.67	-2.88	8.96	18.27	9.01	-0.59	0.37	8.70	2.34	-5.20	217.45	301.99	204.54	203.14
5.68	-2.86	8.99	18.30	9.01	-0.54	0.39	8.69	2.32	-5.15	215.27	301.29	203.36	202.10
5.51	-2.92	8.66	17.95	8.79	-0.62	0.24	8.58	2.23	-5.23	215.59	304.17	206.75	204.39
5.69	-2.76	8.95	18.25	9.01	-0.44	0.49	8.80	2.37	-5.11	210.42	300.62	202.13	198.88
6.40	-2.20	10.04	19.28	9.83	0.19	1.28	9.50	2.97	-4.57	198.88	290.48	189.53	185.29
6.39	-2.21	10.03	19.27	9.82	0.18	1.26	9.48	2.96	-4.58	199.05	290.69	189.73	185.51
6.43	-2.17	10.09	19.33	9.87	0.23	1.32	9.53	3.00	-4.55	198.26	290.05	189.09	184.53
8.25	-0.65	12.75	21.98	11.95	1.94	3.19	11.43	4.50	-3.22	167.31	261.43	158.50	144.13
6.04	-2.54	9.21	18.48	9.31	-0.22	0.93	9.27	2.77	-4.86	215.74	304.03	202.13	201.03
5.97	-2.62	9.15	18.44	9.24	-0.30	0.83	9.16	2.70	-4.93	216.47	304.02	202.67	202.11
5.89	-2.64	9.09	18.37	9.18	-0.33	0.73	9.04	2.62	-4.98	214.87	302.44	202.42	201.50
6.67	-1.92	10.45	19.67	10.12	0.50	1.62	9.83	3.23	-4.33	193.23	285.58	185.30	178.71
6.72	-2.13	10.19	19.45	9.95	0.15	1.75	10.10	3.46	-4.42	222.59	307.84	200.00	208.28

6.72	-2.13	10.19	19.45	9.95	0.15	1.75	10.10	3.45	-4.42	222.56	307.87	200.08	208.34
6.66	-2.16	10.14	19.42	9.76	0.10	2.01	10.48	3.55	-4.39	188.26	267.99	171.58	163.96
8.43	-0.35	12.58	21.87	11.84	2.12	3.81	12.18	5.02	-2.82	162.85	249.61	156.98	136.28
6.78	-2.12	10.57	19.80	10.01	0.15	1.89	10.33	3.53	-4.38	164.28	246.97	155.60	137.40
7.90	-0.90	12.20	21.42	11.53	1.64	2.91	11.10	4.28	-3.46	171.19	265.75	164.21	150.53
7.97	-0.86	12.17	21.40	11.58	1.70	3.07	11.34	4.39	-3.38	176.30	269.13	165.24	156.91
8.40	-0.49	12.73	22.01	12.04	2.11	3.49	11.77	4.74	-3.06	172.36	263.33	159.48	147.98
6.09	-2.46	9.41	18.64	9.41	-0.09	0.88	9.19	2.76	-4.81	202.85	293.96	195.86	188.92
8.86	0.07	13.57	22.89	12.63	2.71	4.08	12.19	5.10	-2.55	122.50	207.00	100.02	69.01
8.30	-0.55	13.00	22.70	12.06	2.19	3.69	11.82	4.56	-3.26	131.73	216.32	107.31	78.15
8.39	-0.36	12.94	22.54	12.05	2.35	3.76	11.84	4.69	-3.07	127.77	213.61	104.16	72.40
8.39	-0.36	12.94	22.54	12.05	2.35	3.76	11.84	4.69	-3.07	127.73	213.61	104.16	72.42
8.38	-0.34	12.93	22.48	11.98	2.35	3.82	11.93	4.75	-3.03	130.53	213.30	103.76	73.30
8.32	-0.54	13.01	22.65	12.00	2.15	3.72	11.85	4.65	-3.25	130.23	214.30	105.08	74.66
8.43	-0.46	13.19	22.83	12.15	2.22	3.82	11.95	4.72	-3.15	129.11	213.77	103.65	72.64
8.36	-0.54	13.12	22.84	12.12	2.20	3.70	11.84	4.58	-3.27	131.07	214.81	105.02	73.93
7.79	-0.99	12.33	22.19	11.45	1.74	3.16	11.28	4.12	-3.74	134.02	222.73	110.85	79.91
7.79	-0.99	12.32	22.19	11.45	1.74	3.16	11.28	4.11	-3.74	134.03	222.73	110.85	79.91
8.22	-0.61	12.84	22.69	11.96	2.13	3.59	11.70	4.47	-3.37	129.32	215.57	106.64	74.70
8.42	-0.45	12.98	22.70	12.20	2.31	3.59	11.66	4.64	-3.20	123.36	214.04	102.45	70.16
8.43	-0.45	12.99	22.75	12.28	2.23	3.60	11.68	4.61	-3.16	121.82	211.91	102.21	70.74
8.43	-0.46	12.99	22.75	12.27	2.23	3.60	11.67	4.61	-3.16	121.85	211.93	102.24	70.76
8.43	-0.45	12.99	22.75	12.28	2.24	3.60	11.68	4.62	-3.15	121.83	211.88	102.16	70.66
8.09	-0.72	12.78	22.50	11.85	2.01	3.42	11.58	4.34	-3.46	132.78	219.13	108.77	78.73
8.25	-0.58	12.98	22.69	12.01	2.15	3.60	11.78	4.49	-3.34	133.54	218.65	108.25	79.01
8.66	-0.08	13.15	22.65	12.25	2.53	4.15	12.21	5.05	-2.71	126.36	211.16	101.86	70.24
8.02	-0.81	12.67	22.36	11.79	1.95	3.30	11.48	4.26	-3.57	134.92	217.87	109.86	80.04
8.16	-0.66	12.88	22.56	11.94	2.08	3.49	11.66	4.40	-3.41	131.31	218.17	107.50	77.96
8.15	-0.67	12.86	22.55	11.93	2.07	3.48	11.65	4.39	-3.42	131.43	218.34	107.66	78.15
8.00	-0.79	12.68	22.37	11.77	1.95	3.31	11.47	4.23	-3.56	132.81	219.72	108.98	80.09
7.95	-0.90	12.70	22.36	11.77	1.93	3.14	11.47	4.12	-3.75	145.74	228.13	124.16	103.29
8.41	-0.45	13.26	22.83	12.27	2.41	3.61	11.91	4.53	-3.30	142.24	221.89	121.66	100.62
8.49	-0.36	13.34	22.88	12.34	2.50	3.68	11.99	4.59	-3.20	141.08	221.22	120.67	99.77
7.78	-1.07	12.51	22.12	11.70	1.76	2.88	11.18	3.87	-3.94	146.58	228.17	129.23	111.41
7.99	-0.88	12.82	22.47	11.84	1.99	3.18	11.49	4.13	-3.74	146.26	227.65	125.50	106.11
8.27	-0.53	13.18	22.77	12.24	2.34	3.37	11.68	4.31	-3.41	140.86	222.08	120.23	98.25
8.21	-0.55	13.14	22.76	12.19	2.34	3.29	11.63	4.26	-3.46	142.82	224.45	122.60	101.69
7.57	-1.43	12.49	22.14	11.75	1.59	2.46	10.77	3.37	-4.49	154.98	239.35	144.70	133.80
7.56	-1.44	12.49	22.14	11.75	1.58	2.45	10.76	3.37	-4.50	155.05	239.40	144.75	133.88
7.57	-1.43	12.50	22.15	11.75	1.59	2.46	10.77	3.37	-4.49	154.94	239.30	144.67	133.75
7.57	-1.43	12.50	22.15	11.76	1.59	2.46	10.77	3.37	-4.49	154.90	239.28	144.65	133.72
7.36	-1.55	12.13	21.79	11.48	1.41	2.34	10.66	3.24	-4.51	157.96	247.91	154.75	148.71
8.25	-0.59	13.09	22.69	12.12	2.28	3.44	11.75	4.38	-3.46	143.47	226.45	125.77	105.71

8.32	-0.53	13.13	22.75	12.28	2.32	3.47	11.74	4.39	-3.35	138.41	217.18	114.15	89.50
8.39	-0.46	13.24	22.85	12.31	2.36	3.56	11.83	4.45	-3.32	139.77	218.33	114.36	91.72
8.29	-0.58	13.11	22.73	12.17	2.25	3.53	11.82	4.44	-3.36	141.08	220.84	116.80	94.45
8.53	-0.32	13.43	22.99	12.43	2.47	3.75	12.03	4.64	-3.14	139.10	218.55	113.26	90.19
8.25	-0.61	13.12	22.69	12.11	2.25	3.45	11.78	4.39	-3.48	144.15	223.28	122.63	102.33
8.40	-0.50	13.31	22.91	12.29	2.33	3.58	11.88	4.50	-3.30	139.22	220.40	118.24	95.44
8.09	-0.80	12.89	22.53	11.93	2.03	3.27	11.58	4.23	-3.65	144.08	226.68	123.34	101.96
8.47	-0.47	13.39	23.03	12.47	2.35	3.64	11.85	4.50	-3.28	136.20	215.78	111.75	86.19
8.16	-0.72	12.90	22.48	12.02	2.10	3.40	11.60	4.31	-3.52	138.17	218.80	114.78	87.77
8.05	-0.82	12.80	22.44	11.89	2.00	3.27	11.50	4.20	-3.65	139.48	220.05	116.25	91.35
8.31	-0.60	13.15	22.73	12.19	2.26	3.53	11.70	4.43	-3.46	133.10	215.69	110.07	82.48
8.30	-0.62	13.12	22.73	12.16	2.23	3.52	11.69	4.43	-3.46	135.24	217.00	110.68	83.85
8.16	-0.74	12.97	22.55	11.97	2.13	3.39	11.58	4.35	-3.58	136.60	218.83	112.80	85.91
7.75	-1.07	12.46	22.07	11.52	1.79	2.95	11.20	3.98	-3.95	142.53	226.70	119.57	94.40
8.11	-0.80	12.91	22.58	11.92	2.07	3.31	11.55	4.24	-3.65	140.04	221.85	116.48	91.30
7.33	-1.45	11.82	21.56	11.00	1.38	2.62	10.88	3.66	-4.26	150.29	235.28	128.02	103.70
7.34	-1.46	11.80	21.58	11.00	1.37	2.62	10.88	3.64	-4.28	150.65	235.46	128.55	103.92
7.34	-1.46	11.81	21.59	11.01	1.37	2.62	10.88	3.65	-4.28	150.59	235.39	128.48	103.84
7.54	-1.34	12.25	22.00	11.35	1.51	2.82	11.13	3.76	-4.18	148.54	233.80	126.52	102.40
7.58	-1.31	12.31	22.07	11.42	1.55	2.88	11.17	3.78	-4.15	148.09	232.46	125.63	102.00
7.58	-1.30	12.31	22.07	11.42	1.55	2.88	11.17	3.78	-4.15	148.07	232.43	125.60	101.99
7.59	-1.30	12.32	22.07	11.43	1.56	2.89	11.17	3.79	-4.15	148.05	232.36	125.55	101.95
8.52	-0.27	13.31	22.76	12.32	2.50	3.84	12.01	4.70	-3.04	132.23	209.70	104.93	75.91
8.10	-0.70	12.78	22.36	11.83	2.06	3.40	11.59	4.37	-3.47	135.34	216.92	109.38	80.47
8.41	-0.35	12.88	22.36	12.12	2.39	3.76	11.90	4.69	-3.08	132.28	210.32	107.39	79.62
8.57	-0.25	13.36	22.80	12.39	2.51	3.88	12.03	4.73	-3.01	130.86	210.30	103.68	75.55
8.44	-0.34	13.06	22.47	12.16	2.33	3.80	11.88	4.72	-3.00	128.04	209.92	102.33	72.44
8.41	-0.35	12.88	22.36	12.12	2.39	3.76	11.90	4.70	-3.08	132.29	210.31	107.39	79.62
8.46	-0.26	13.08	22.40	12.18	2.40	3.89	11.98	4.77	-2.93	129.78	208.98	102.03	72.83
8.66	-0.12	13.35	22.83	12.42	2.56	4.04	12.18	4.91	-2.80	128.20	208.17	102.00	71.47
8.64	-0.15	13.32	22.81	12.39	2.53	4.02	12.15	4.88	-2.83	128.54	208.35	102.22	71.72
8.55	-0.23	13.08	22.60	12.18	2.41	3.93	12.03	4.88	-2.86	127.86	211.38	102.99	72.23
8.31	-0.46	12.86	22.41	11.98	2.23	3.68	11.80	4.64	-3.10	130.40	213.45	104.64	75.52
8.73	-0.02	13.20	22.63	12.38	2.70	4.12	12.28	5.04	-2.73	129.99	207.14	104.14	76.27
8.33	-0.36	12.73	22.22	12.00	2.38	3.70	11.85	4.66	-3.10	133.27	210.55	107.58	80.21
8.36	-0.43	13.08	22.52	12.19	2.34	3.66	11.78	4.53	-3.19	131.90	210.52	105.39	77.91
8.28	-0.55	13.01	22.48	12.13	2.23	3.55	11.65	4.43	-3.32	132.40	210.77	106.12	79.00
8.61	-0.12	13.34	22.74	12.39	2.63	4.01	12.15	4.83	-2.85	129.89	208.24	103.22	73.09
8.60	-0.13	13.33	22.74	12.38	2.62	4.00	12.13	4.82	-2.87	129.94	208.54	103.19	73.23
8.54	-0.23	13.33	22.74	12.36	2.53	3.90	12.05	4.76	-2.98	130.04	209.85	103.44	75.03
8.62	-0.16	13.43	22.85	12.45	2.59	3.99	12.14	4.83	-2.92	129.85	209.68	102.49	74.41
8.53	-0.24	13.30	22.72	12.36	2.53	3.85	11.99	4.70	-3.00	132.35	208.81	103.76	74.68
8.62	-0.21	13.39	22.87	12.39	2.51	3.95	12.11	4.83	-2.93	131.18	209.00	102.53	73.43

8.51	-0.35	13.19	22.73	12.25	2.38	3.84	12.00	4.71	-3.06	131.97	210.52	104.21	74.86
8.10	-0.71	12.71	22.33	11.82	2.01	3.41	11.57	4.38	-3.44	134.88	216.95	108.77	79.24
8.40	-0.47	13.09	22.64	12.15	2.26	3.72	11.87	4.65	-3.18	130.59	212.30	105.85	76.10
8.52	-0.27	13.31	22.76	12.32	2.50	3.84	12.01	4.70	-3.04	132.22	209.71	104.92	75.90
8.22	-0.60	12.91	22.45	12.04	2.19	3.50	11.64	4.42	-3.38	133.73	212.66	107.92	80.80
8.33	-0.51	13.07	22.52	12.18	2.26	3.60	11.75	4.51	-3.28	131.97	210.26	106.25	78.10
8.23	-0.57	12.76	22.29	12.00	2.21	3.52	11.68	4.43	-3.34	135.31	214.05	110.50	83.23
8.29	-0.60	13.04	22.62	12.16	2.24	3.53	11.69	4.41	-3.40	133.08	214.24	108.97	81.54
8.37	-0.33	12.94	22.31	12.05	2.33	3.81	11.91	4.69	-3.00	130.88	210.11	103.07	73.49
8.44	-0.41	13.22	22.78	12.31	2.39	3.70	11.86	4.57	-3.21	133.04	212.45	105.33	77.29
8.43	-0.41	13.21	22.77	12.30	2.39	3.70	11.86	4.57	-3.21	133.19	212.52	105.40	77.36
8.30	-0.57	13.12	22.72	12.23	2.29	3.51	11.63	4.37	-3.44	133.18	213.79	109.13	80.58
8.26	-0.53	12.91	22.44	11.95	2.20	3.63	11.81	4.58	-3.26	132.77	215.04	107.45	78.45
8.71	-0.08	13.42	22.85	12.46	2.59	4.09	12.21	4.93	-2.77	128.89	208.37	101.38	71.47
8.24	-0.48	12.80	22.22	11.96	2.18	3.63	11.73	4.54	-3.15	131.22	211.01	103.73	74.51
8.53	-0.25	13.13	22.56	12.22	2.38	3.90	12.02	4.86	-2.87	129.13	210.21	102.47	71.79
8.09	-0.63	12.47	21.99	11.67	2.01	3.45	11.56	4.48	-3.26	131.69	214.15	106.32	76.65
7.93	-0.76	12.25	21.83	11.52	1.90	3.28	11.40	4.34	-3.39	132.74	215.89	107.32	78.70
7.93	-0.75	12.26	21.84	11.53	1.90	3.28	11.40	4.34	-3.38	132.73	215.87	107.27	78.64
8.39	-0.37	12.94	22.47	12.08	2.30	3.74	11.85	4.72	-3.04	128.83	212.57	104.30	73.34
8.40	-0.36	12.94	22.47	12.08	2.30	3.74	11.86	4.73	-3.04	128.77	212.53	104.26	73.29
8.40	-0.36	12.94	22.47	12.08	2.30	3.74	11.86	4.73	-3.04	128.77	212.53	104.27	73.27
8.40	-0.36	12.95	22.48	12.09	2.31	3.75	11.86	4.73	-3.04	128.72	212.50	104.24	73.23
8.09	-0.62	12.67	22.08	11.84	2.07	3.35	11.45	4.36	-3.34	131.67	211.10	104.08	76.13
8.22	-0.49	12.78	22.19	11.95	2.23	3.58	11.68	4.52	-3.18	129.51	210.00	103.90	75.29
8.52	-0.28	13.27	22.57	12.39	2.46	3.70	11.76	4.65	-3.04	129.33	205.55	101.56	71.76
8.59	-0.12	13.23	22.62	12.35	2.62	3.99	12.09	4.85	-2.85	128.40	206.84	102.84	73.88
8.34	-0.37	13.00	22.46	12.19	2.41	3.53	11.68	4.50	-3.17	132.00	208.21	105.03	77.53
8.60	-0.20	13.08	22.56	12.28	2.52	3.93	12.13	4.92	-2.92	132.59	210.28	107.59	79.69
8.37	-0.32	13.03	22.44	12.14	2.46	3.62	11.75	4.59	-3.08	129.15	206.06	102.46	74.11
7.34	-1.55	12.11	21.77	11.46	1.41	2.33	10.66	3.24	-4.52	158.42	248.40	155.12	149.18
8.22	-0.57	12.85	22.37	12.06	2.22	3.43	11.60	4.39	-3.37	134.62	212.72	108.67	82.65
8.25	-0.55	12.89	22.41	12.10	2.24	3.45	11.63	4.42	-3.35	133.86	212.27	108.04	82.14
8.27	-0.48	12.71	22.31	11.99	2.28	3.55	11.79	4.54	-3.24	137.99	215.61	111.63	86.65
8.27	-0.48	12.71	22.31	11.99	2.28	3.55	11.79	4.54	-3.24	138.01	215.63	111.63	86.66
8.17	-0.61	12.78	22.38	12.00	2.22	3.37	11.64	4.34	-3.45	138.83	219.47	114.60	91.72
7.41	-1.33	12.22	21.79	11.52	1.73	2.43	10.84	3.30	-4.40	171.64	261.66	176.63	178.67
7.89	-1.02	12.97	22.60	12.07	2.05	2.91	11.28	3.71	-4.08	160.46	246.23	158.95	153.88
7.42	-1.32	12.24	21.82	11.51	1.73	2.45	10.86	3.33	-4.40	172.06	260.98	174.79	177.41
7.62	-1.21	12.52	22.16	11.74	1.86	2.65	11.02	3.50	-4.25	166.51	254.46	168.34	167.63
7.70	-1.17	12.72	22.34	11.86	1.89	2.71	11.09	3.55	-4.23	162.33	249.05	160.94	156.57
7.86	-1.07	12.83	22.46	12.01	1.92	2.90	11.24	3.70	-4.08	159.61	244.61	155.00	146.51
7.95	-0.97	12.94	22.58	12.07	2.00	3.00	11.35	3.82	-3.96	158.63	241.70	151.29	141.95

9.02	0.32	13.73	22.98	12.78	3.07	4.35	12.54	5.27	-2.41	124.27	200.05	98.23	68.42
8.61	-0.20	13.11	22.59	12.29	2.52	3.92	12.12	4.90	-2.92	132.75	209.67	107.60	79.38
8.61	-0.20	13.11	22.58	12.29	2.52	3.92	12.12	4.90	-2.91	132.68	209.60	107.52	79.32
8.36	-0.37	12.70	22.18	11.91	2.22	3.79	11.86	4.80	-2.97	128.55	210.90	104.27	72.63
8.35	-0.40	12.82	22.31	12.02	2.21	3.67	11.77	4.67	-2.99	126.82	212.63	104.48	72.45
8.31	-0.43	12.67	22.25	11.93	2.18	3.71	11.76	4.69	-3.04	128.33	213.09	103.52	73.27
8.32	-0.43	12.67	22.25	11.93	2.18	3.71	11.76	4.70	-3.03	128.30	213.06	103.47	73.24
8.57	-0.23	13.19	22.74	12.35	2.44	3.81	11.89	4.80	-2.91	124.19	211.32	102.11	70.10
8.55	-0.29	13.10	22.72	12.29	2.35	3.84	11.87	4.79	-2.95	126.18	211.05	103.13	70.34
8.86	0.06	13.56	22.88	12.62	2.71	4.08	12.18	5.10	-2.56	122.53	207.02	100.04	69.03
8.69	-0.07	13.31	22.67	12.41	2.54	3.93	12.02	4.97	-2.69	124.33	208.64	101.19	70.19
8.13	-0.67	12.99	22.71	12.04	2.36	3.44	11.83	4.26	-3.72	158.17	246.51	143.62	129.66
8.14	-0.73	13.15	22.90	12.10	2.37	3.35	11.82	4.18	-3.80	159.74	249.44	148.73	137.02
8.08	-0.82	13.42	23.07	12.51	2.58	2.81	11.17	3.65	-4.20	164.52	257.80	150.12	136.60
8.08	-0.82	13.41	23.07	12.51	2.58	2.81	11.16	3.64	-4.20	164.56	257.90	150.20	136.69
8.26	-0.53	13.14	22.79	12.12	2.48	3.66	11.98	4.41	-3.56	155.80	245.10	142.85	129.51
8.21	-0.65	13.33	23.04	12.43	2.55	3.24	11.60	4.00	-3.85	160.16	250.62	146.79	134.59
8.13	-0.73	13.10	22.74	12.22	2.39	3.27	11.61	4.05	-3.83	161.23	250.46	145.44	131.21
8.24	-0.61	13.16	22.89	12.18	2.42	3.51	11.91	4.31	-3.68	154.87	244.64	141.15	125.95
8.03	-0.89	12.91	22.71	11.89	2.08	3.39	11.73	4.17	-3.80	151.58	236.13	130.05	109.29
8.21	-0.69	13.27	23.04	12.19	2.30	3.47	11.89	4.23	-3.66	156.17	243.21	146.46	136.33
7.90	-1.05	12.86	22.67	11.89	1.88	3.14	11.57	3.93	-3.98	152.33	238.34	138.67	122.28
7.74	-1.23	12.62	22.26	11.62	1.65	2.90	11.27	3.87	-4.14	149.20	232.45	133.42	115.22
7.44	-1.53	12.23	21.84	11.31	1.40	2.51	10.91	3.55	-4.45	154.49	237.75	138.22	119.56
7.38	-1.57	12.16	21.77	11.26	1.37	2.46	10.86	3.50	-4.49	155.16	238.87	138.97	120.57
7.25	-1.64	11.99	21.59	11.10	1.30	2.32	10.75	3.42	-4.57	158.08	240.75	139.84	122.71
7.26	-1.63	11.99	21.60	11.09	1.30	2.32	10.76	3.44	-4.55	158.17	240.51	139.23	121.93
8.34	-0.55	12.95	22.62	12.18	2.35	3.58	11.99	4.52	-3.44	144.35	229.26	131.95	114.42
7.19	-1.70	11.90	21.50	11.01	1.23	2.28	10.66	3.34	-4.60	158.56	242.41	140.75	123.89
7.24	-1.67	11.98	21.56	11.07	1.26	2.32	10.70	3.38	-4.60	158.34	242.00	139.64	122.59
7.14	-1.67	11.75	21.39	10.88	1.20	2.26	10.66	3.39	-4.56	158.66	241.78	138.76	121.75
7.39	-1.55	12.19	21.77	11.25	1.37	2.51	10.88	3.50	-4.47	157.16	239.47	136.77	118.78
7.28	-1.63	12.06	21.64	11.16	1.29	2.39	10.76	3.40	-4.55	158.32	240.71	138.74	121.15
7.29	-1.63	12.06	21.65	11.16	1.29	2.40	10.77	3.41	-4.54	158.26	240.49	138.66	121.08
8.26	-0.67	13.16	22.85	12.16	2.25	3.51	11.92	4.32	-3.61	150.05	234.62	136.03	120.56
8.24	-0.70	13.15	22.85	12.17	2.23	3.50	11.90	4.31	-3.62	150.07	235.03	136.55	121.33
8.24	-0.53	12.97	22.56	12.07	2.33	3.43	11.75	4.42	-3.41	143.37	226.55	126.89	107.12
7.74	-1.23	12.65	22.37	11.90	1.75	2.70	11.01	3.55	-4.22	156.65	243.96	149.21	139.87
8.18	-0.68	13.10	22.74	12.13	2.23	3.49	11.83	4.25	-3.58	154.01	234.21	142.55	129.57
8.05	-0.81	12.90	22.66	11.98	2.13	3.32	11.77	4.15	-3.76	155.61	241.56	146.36	133.68
8.04	-0.82	12.89	22.65	11.97	2.12	3.31	11.76	4.14	-3.77	155.89	241.76	146.63	134.04
8.35	-0.61	13.23	23.03	12.20	2.31	3.72	12.12	4.48	-3.49	149.16	236.18	137.40	122.19
8.23	-0.66	13.26	23.08	12.16	2.31	3.55	12.03	4.34	-3.64	156.76	243.03	146.19	131.50

8.05	-0.81	13.07	22.84	12.01	2.16	3.33	11.79	4.11	-3.79	157.02	244.34	148.98	136.67
8.18	-0.67	13.22	22.96	12.06	2.28	3.51	11.96	4.30	-3.64	159.09	247.74	150.88	141.91
8.22	-0.63	13.19	22.82	12.05	2.34	3.62	12.08	4.37	-3.61	157.94	245.98	151.63	139.93
7.78	-1.05	12.67	22.48	11.71	1.97	2.95	11.46	3.81	-4.03	163.97	251.85	155.33	147.86
8.04	-0.92	13.12	22.80	12.23	2.18	3.11	11.43	3.86	-4.01	164.77	250.53	163.27	157.45
8.05	-0.91	13.13	22.80	12.24	2.18	3.12	11.44	3.86	-4.01	164.62	250.27	162.74	156.72
8.05	-0.91	13.13	22.80	12.24	2.18	3.12	11.44	3.86	-4.00	164.59	250.22	162.68	156.65
8.41	-0.60	13.63	23.45	12.44	2.40	3.63	12.12	4.39	-3.61	153.24	238.40	138.97	122.93
8.10	-0.82	13.04	22.85	12.02	2.09	3.48	11.86	4.20	-3.72	150.14	234.02	134.50	115.89
7.88	-1.02	12.63	22.46	11.69	1.85	3.31	11.65	4.07	-3.89	150.92	236.55	131.78	112.28
8.25	-0.66	13.34	23.13	12.19	2.33	3.58	11.93	4.30	-3.64	143.35	226.82	121.01	97.03
8.46	-0.56	13.70	23.52	12.50	2.44	3.67	12.15	4.43	-3.56	151.96	236.98	137.91	121.40
8.16	-0.80	13.31	23.10	12.24	2.17	3.40	11.72	4.12	-3.76	143.68	229.04	123.59	100.18
7.91	-1.00	12.67	22.49	11.72	1.88	3.34	11.67	4.10	-3.87	149.97	235.73	131.04	111.30
7.90	-1.00	12.66	22.49	11.71	1.87	3.34	11.67	4.10	-3.87	150.15	235.85	131.04	111.38
7.90	-1.00	12.66	22.48	11.71	1.87	3.33	11.67	4.10	-3.87	150.22	235.91	131.10	111.45
7.90	-1.00	12.65	22.48	11.71	1.87	3.33	11.67	4.09	-3.87	150.36	236.02	131.18	111.57
8.35	-0.63	13.47	23.29	12.32	2.37	3.64	12.09	4.39	-3.61	150.54	236.23	135.75	118.46
8.36	-0.60	13.44	23.27	12.32	2.39	3.71	12.12	4.41	-3.57	148.57	233.42	132.83	115.19
8.36	-0.60	13.44	23.27	12.32	2.39	3.71	12.12	4.41	-3.57	148.58	233.42	132.82	115.20
8.37	-0.58	13.46	23.29	12.35	2.40	3.72	12.13	4.42	-3.56	148.36	232.87	132.40	114.81
8.37	-0.58	13.46	23.29	12.35	2.40	3.72	12.13	4.42	-3.56	148.37	232.86	132.39	114.81
8.37	-0.58	13.46	23.29	12.35	2.40	3.72	12.13	4.42	-3.56	148.37	232.85	132.38	114.81
7.92	-1.08	12.93	22.61	11.91	1.84	3.13	11.49	3.96	-3.98	145.76	230.11	123.46	99.40
7.80	-1.15	12.65	22.44	11.71	1.74	3.09	11.39	3.90	-4.04	146.37	231.64	123.43	99.61
8.33	-0.59	13.37	23.15	12.25	2.42	3.64	12.09	4.37	-3.60	154.33	241.35	140.30	125.29
7.79	-0.94	12.47	22.23	11.58	2.07	3.12	11.48	3.99	-3.94	160.69	250.38	145.68	130.70
8.30	-0.69	13.46	23.31	12.30	2.32	3.53	12.04	4.31	-3.69	152.25	240.42	140.50	124.07
8.31	-0.68	13.46	23.32	12.30	2.32	3.53	12.04	4.31	-3.68	152.20	240.34	140.42	123.97
8.08	-0.92	13.18	22.86	12.10	2.01	3.32	11.64	4.04	-3.86	144.58	229.20	124.72	100.72
7.25	-1.47	12.17	21.46	11.58	1.84	1.91	10.26	2.90	-4.76	184.77	279.07	203.47	216.43
7.25	-1.47	12.17	21.46	11.58	1.84	1.91	10.26	2.91	-4.76	184.78	278.96	203.46	216.36
7.28	-1.46	12.20	21.50	11.60	1.85	1.94	10.28	2.93	-4.74	184.76	278.58	202.65	215.49
7.23	-1.48	12.16	21.44	11.57	1.83	1.89	10.25	2.90	-4.77	184.91	279.29	203.79	216.82
7.25	-1.48	12.17	21.46	11.58	1.84	1.91	10.26	2.90	-4.76	184.78	279.32	203.37	216.50
7.86	-1.05	13.15	22.70	12.28	2.16	2.56	10.93	3.40	-4.23	169.15	257.81	178.05	180.52
7.00	-1.62	11.73	21.04	11.24	1.68	1.67	10.04	2.74	-4.92	190.30	286.06	210.01	227.87
7.29	-1.45	12.21	21.51	11.60	1.85	1.95	10.29	2.94	-4.73	184.82	278.33	202.46	215.18
7.29	-1.44	12.21	21.52	11.60	1.85	1.95	10.29	2.95	-4.73	184.85	278.24	202.34	215.04
7.45	-1.38	12.48	22.00	11.68	1.75	2.32	10.72	3.22	-4.50	172.97	263.32	178.80	183.32
7.18	-1.56	12.10	21.45	11.50	1.72	1.82	10.17	2.86	-4.80	184.43	276.10	198.21	209.85
7.34	-1.40	12.46	21.85	11.74	1.88	1.97	10.34	2.92	-4.71	181.27	271.18	193.58	203.56
7.34	-1.40	12.46	21.85	11.75	1.88	1.97	10.34	2.92	-4.71	181.24	271.15	193.55	203.52

7.76	-1.12	12.94	22.47	12.07	2.00	2.63	10.98	3.45	-4.24	167.22	256.82	174.99	175.99
7.72	-1.16	12.84	22.40	12.00	1.95	2.59	10.97	3.45	-4.27	168.72	258.57	174.95	177.65
6.83	-1.83	11.17	20.64	10.70	1.12	1.80	10.09	2.97	-4.78	177.73	269.49	179.73	183.01
8.21	-0.84	12.70	22.31	11.90	1.86	3.27	11.29	4.50	-3.59	123.40	213.09	103.20	70.73
8.22	-0.83	12.71	22.33	11.91	1.87	3.28	11.30	4.51	-3.58	123.17	213.04	103.12	70.63
8.25	-0.81	12.73	22.36	11.93	1.89	3.30	11.32	4.52	-3.57	122.67	212.94	103.00	70.42
8.26	-0.80	12.75	22.38	11.95	1.91	3.32	11.34	4.54	-3.56	122.21	212.81	102.91	70.26
8.22	-0.85	12.68	22.30	11.89	1.85	3.32	11.29	4.51	-3.61	123.22	213.37	103.43	70.66
8.20	-0.87	12.66	22.28	11.87	1.83	3.30	11.28	4.50	-3.62	123.58	213.62	103.70	70.81
8.18	-0.90	12.64	22.25	11.85	1.81	3.28	11.26	4.48	-3.64	123.98	213.91	104.03	71.01
8.47	-0.59	13.13	22.73	12.25	2.16	3.52	11.56	4.70	-3.34	120.43	209.70	102.78	68.07
8.47	-0.59	13.13	22.72	12.25	2.16	3.52	11.56	4.70	-3.34	120.44	209.70	102.78	68.07
8.50	-0.58	13.17	22.77	12.29	2.21	3.53	11.56	4.70	-3.31	120.81	210.65	101.97	68.01
8.50	-0.58	13.17	22.77	12.29	2.21	3.53	11.56	4.70	-3.31	120.84	210.67	102.00	68.03
8.19	-0.83	12.73	22.37	11.90	1.86	3.30	11.32	4.49	-3.56	124.36	214.07	104.62	70.37
8.25	-0.77	12.82	22.45	11.96	1.93	3.37	11.37	4.54	-3.51	123.14	212.58	104.14	69.87
8.03	-1.06	12.47	22.10	11.64	1.60	3.18	11.15	4.43	-3.72	125.98	214.90	105.35	72.06
7.66	-1.41	11.99	21.68	11.24	1.27	2.72	10.72	4.04	-4.07	130.04	221.24	109.84	76.68
8.43	-0.66	12.91	22.52	12.16	2.06	3.50	11.54	4.70	-3.37	123.00	214.76	103.89	69.14
8.57	-0.50	13.19	22.77	12.36	2.24	3.63	11.66	4.78	-3.25	120.21	209.34	101.02	67.46
8.57	-0.50	13.19	22.77	12.36	2.24	3.63	11.66	4.78	-3.25	120.20	209.31	101.01	67.42
8.34	-0.54	12.84	22.57	12.07	2.20	3.54	11.59	4.62	-3.27	123.29	214.33	103.08	70.34
8.34	-0.54	12.83	22.56	12.06	2.19	3.54	11.58	4.62	-3.27	123.45	214.52	103.14	70.39
8.39	-0.49	12.95	22.69	12.19	2.27	3.52	11.62	4.58	-3.26	124.80	214.47	102.90	70.90
8.77	-0.40	13.53	22.95	12.61	2.38	3.78	11.90	4.94	-3.16	118.94	208.74	99.39	64.90
8.77	-0.41	13.53	22.94	12.61	2.38	3.79	11.90	4.94	-3.16	118.80	208.62	99.41	64.86
8.69	-0.36	13.51	23.09	12.59	2.46	3.74	11.76	4.83	-3.15	119.40	207.96	99.40	65.83
8.49	-0.48	13.25	22.84	12.35	2.29	3.52	11.56	4.63	-3.26	121.82	212.02	101.07	67.89
8.13	-0.82	12.68	22.46	11.74	1.88	3.53	11.59	4.49	-3.52	129.42	217.26	105.84	74.46
8.05	-0.85	12.49	22.32	11.60	1.81	3.46	11.59	4.47	-3.54	131.03	220.29	107.74	76.53
7.30	-1.11	11.68	20.94	11.01	1.62	2.34	10.54	3.56	-3.88	151.72	246.14	160.44	154.66
6.90	-1.39	11.10	20.36	10.58	1.38	1.89	10.16	3.21	-4.16	162.65	258.18	175.03	174.51
7.12	-1.29	11.45	20.67	10.78	1.41	2.21	10.40	3.47	-3.96	156.35	248.49	166.98	163.14
7.15	-1.25	11.58	20.79	10.87	1.45	2.25	10.42	3.48	-3.95	155.17	245.44	165.76	161.53
7.11	-1.48	11.93	21.24	11.30	1.67	1.64	10.07	2.93	-4.62	183.36	283.02	196.58	198.82
7.10	-1.41	11.93	21.27	11.38	1.84	1.55	9.99	2.80	-4.65	184.94	283.55	197.82	204.92
7.32	-1.33	12.28	21.63	11.51	1.76	1.85	10.26	3.14	-4.43	176.25	275.56	187.20	184.74
6.63	-1.73	10.76	19.86	10.18	0.92	1.75	9.94	3.07	-4.39	167.57	251.15	179.69	182.38
6.37	-1.82	10.15	19.35	9.77	0.79	1.61	9.78	2.96	-4.43	164.88	253.41	180.02	180.53
6.84	-1.54	11.02	20.29	10.46	1.11	1.93	10.11	3.21	-4.16	147.18	242.48	158.96	154.25
7.05	-1.39	11.49	20.77	10.84	1.35	2.01	10.24	3.27	-4.13	141.67	241.29	152.45	144.43
6.99	-1.43	11.31	20.57	10.68	1.24	2.03	10.25	3.30	-4.13	143.94	245.34	155.95	149.68
6.86	-1.52	11.08	20.34	10.51	1.14	1.91	10.16	3.20	-4.22	148.61	249.92	161.93	157.68

6.82	-1.56	11.02	20.29	10.48	1.13	1.86	10.11	3.15	-4.26	150.19	251.46	163.66	159.73
6.61	-1.71	10.73	20.02	10.28	1.03	1.61	9.92	2.97	-4.42	158.36	258.42	171.98	171.24
7.04	-1.40	11.59	20.92	10.96	1.45	1.85	10.09	3.10	-4.25	149.68	248.28	157.21	152.82
6.81	-1.56	11.08	20.38	10.55	1.19	1.74	10.01	3.05	-4.33	154.68	253.63	165.65	163.18
6.77	-1.60	10.98	20.29	10.48	1.17	1.71	9.98	3.02	-4.36	157.07	255.05	168.01	166.12
6.90	-1.48	11.21	20.52	10.67	1.30	1.84	10.10	3.12	-4.28	153.90	251.71	163.69	160.81
6.96	-1.40	11.21	20.53	10.69	1.38	1.98	10.22	3.24	-4.18	154.89	251.69	165.95	162.01
6.99	-1.39	11.21	20.50	10.66	1.32	2.11	10.30	3.35	-4.10	155.20	249.83	168.02	163.65
6.81	-1.51	10.88	20.19	10.40	1.19	1.92	10.17	3.20	-4.21	161.05	255.58	172.81	170.67
6.54	-1.73	10.56	19.87	10.17	1.03	1.55	9.85	2.90	-4.49	164.67	262.95	177.59	178.87
6.53	-1.73	10.48	19.79	10.10	0.98	1.62	9.90	2.97	-4.43	163.62	262.66	179.44	180.64
6.68	-1.64	10.72	20.00	10.26	1.05	1.81	10.03	3.11	-4.29	157.72	256.99	171.74	169.28
6.85	-1.53	11.21	20.41	10.61	1.22	1.85	10.03	3.09	-4.27	152.01	245.26	164.25	159.83
6.94	-1.44	11.26	20.52	10.64	1.26	2.02	10.20	3.24	-4.12	151.89	248.46	163.95	158.52
6.83	-1.56	11.22	20.47	10.62	1.18	1.78	10.01	3.03	-4.32	153.83	248.64	164.03	160.83
7.09	-1.33	11.64	20.88	10.94	1.40	2.08	10.24	3.25	-4.08	147.12	240.97	158.66	151.70
6.76	-1.59	10.92	20.20	10.40	1.09	1.82	10.08	3.11	-4.29	151.93	253.08	166.02	162.80
6.61	-1.70	10.73	20.02	10.28	1.03	1.62	9.93	2.98	-4.42	157.94	258.17	171.75	170.79
6.58	-1.72	10.68	19.97	10.24	1.01	1.59	9.90	2.95	-4.44	159.32	259.26	173.17	172.64
6.99	-1.43	11.31	20.57	10.68	1.24	2.03	10.24	3.30	-4.13	143.98	245.38	156.00	149.75
6.91	-1.49	11.23	20.51	10.65	1.23	1.90	10.14	3.20	-4.21	146.02	247.34	158.43	152.99
6.90	-1.50	11.23	20.51	10.64	1.23	1.89	10.13	3.19	-4.22	146.42	247.87	159.02	153.46
6.79	-1.58	11.01	20.31	10.51	1.19	1.73	10.00	3.04	-4.34	157.06	254.54	167.75	165.63
6.82	-1.52	10.99	20.17	10.40	1.15	1.97	10.14	3.21	-4.18	160.39	247.45	170.08	169.15
7.11	-1.30	11.64	20.87	10.93	1.41	2.13	10.29	3.31	-4.01	147.33	241.98	158.58	152.18
7.14	-1.25	11.72	20.92	10.99	1.50	2.16	10.33	3.31	-4.01	148.81	242.38	160.00	154.91
6.63	-1.70	10.83	20.09	10.33	1.02	1.63	9.88	2.91	-4.41	159.22	254.31	170.88	170.01
6.58	-1.73	10.68	19.96	10.20	0.96	1.62	9.88	2.93	-4.42	159.45	256.05	172.77	172.14
6.72	-1.56	10.71	19.97	10.26	1.05	1.90	10.01	3.17	-4.16	142.74	237.36	153.96	149.08
6.74	-1.53	10.77	20.00	10.28	1.06	1.92	10.03	3.22	-4.15	144.26	236.44	155.83	151.19
6.55	-1.70	10.41	19.63	9.98	0.88	1.78	9.87	3.10	-4.25	151.83	242.39	165.69	163.06
6.83	-1.50	11.01	20.32	10.56	1.18	1.92	10.03	3.10	-4.19	132.59	231.92	142.28	132.44
6.79	-1.54	11.03	20.34	10.58	1.20	1.80	9.94	3.00	-4.28	132.65	234.22	143.40	135.51
7.04	-1.35	11.50	20.75	10.84	1.37	2.00	10.18	3.22	-4.09	134.29	232.52	145.47	136.70
6.60	-1.76	10.83	20.16	10.33	0.93	1.51	9.60	2.86	-4.46	137.68	240.16	150.77	141.86
6.51	-1.71	10.51	19.80	10.17	0.98	1.60	9.75	2.88	-4.39	144.72	244.87	158.70	154.73
6.70	-1.57	10.69	19.94	10.24	1.04	1.88	9.99	3.16	-4.17	143.15	237.73	154.58	149.69
6.73	-1.54	10.91	20.15	10.43	1.25	1.71	10.00	3.03	-4.32	170.14	264.77	181.86	184.37
6.80	-1.53	11.06	20.28	10.52	1.28	1.75	10.04	3.07	-4.34	167.23	263.73	179.70	181.46
7.17	-1.26	11.56	20.83	10.90	1.52	2.13	10.41	3.40	-4.06	160.53	258.97	170.80	168.28
7.10	-1.31	11.42	20.69	10.79	1.46	2.08	10.37	3.36	-4.10	162.65	260.98	173.42	171.59
6.85	-1.47	10.89	20.16	10.39	1.27	1.91	10.22	3.25	-4.20	170.97	268.56	183.36	183.85
6.87	-1.45	11.01	20.28	10.47	1.31	1.90	10.22	3.24	-4.21	170.57	268.14	181.68	182.29

6.74	-1.54	10.69	19.98	10.26	1.19	1.85	10.16	3.19	-4.26	175.23	272.50	187.25	189.58
6.89	-1.48	11.10	20.35	10.55	1.31	1.96	10.23	3.22	-4.24	175.01	269.69	185.12	188.15
7.17	-1.29	11.64	20.85	10.96	1.52	2.15	10.40	3.41	-4.09	168.14	263.15	175.44	173.66
7.08	-1.32	11.33	20.56	10.72	1.43	2.15	10.40	3.42	-4.05	169.32	264.08	176.02	175.24
7.03	-1.35	11.34	20.57	10.73	1.42	2.09	10.32	3.34	-4.10	170.80	267.33	178.06	177.55
6.75	-1.54	10.88	20.13	10.42	1.25	1.79	10.08	3.08	-4.32	178.79	276.82	189.62	192.45
6.71	-1.58	10.89	20.14	10.42	1.24	1.70	10.01	3.03	-4.40	182.36	279.89	193.61	198.43
6.54	-1.68	10.42	19.69	10.10	1.08	1.63	9.94	3.00	-4.44	187.61	285.75	199.81	206.70
7.34	-1.28	12.16	21.54	11.46	1.77	1.92	10.32	3.22	-4.34	174.10	274.21	184.85	181.82
7.23	-1.21	11.77	21.05	11.06	1.62	2.09	10.39	3.38	-4.04	157.77	257.62	169.42	165.97
7.31	-1.13	11.98	21.26	11.19	1.77	2.10	10.46	3.41	-4.00	161.12	260.15	170.81	165.73
7.28	-1.10	11.94	21.33	11.28	1.85	1.97	10.36	3.30	-4.07	165.99	269.37	178.92	174.26
7.17	-1.44	12.06	21.41	11.38	1.68	1.69	10.13	2.96	-4.55	178.59	279.96	192.02	192.13
7.02	-1.37	11.42	20.73	10.84	1.47	1.91	10.20	3.20	-4.21	159.55	259.16	170.63	168.91
7.12	-1.30	11.60	20.85	10.92	1.52	2.02	10.30	3.30	-4.14	157.38	255.41	168.97	165.57
7.00	-1.39	11.51	20.91	10.94	1.63	1.64	10.08	3.03	-4.42	177.96	281.54	191.58	192.71
7.16	-1.26	11.54	20.81	10.89	1.51	2.12	10.41	3.40	-4.06	160.75	259.28	171.09	168.67
6.56	-1.66	10.44	19.72	10.12	1.10	1.65	9.96	3.02	-4.42	186.89	284.99	198.91	205.29
6.55	-1.67	10.42	19.70	10.10	1.09	1.64	9.95	3.01	-4.43	187.33	285.47	199.44	206.08
6.85	-1.43	11.05	20.35	10.52	1.39	1.83	10.19	3.20	-4.25	175.15	274.97	187.11	188.24
6.85	-1.43	11.05	20.35	10.52	1.39	1.83	10.19	3.20	-4.25	175.16	275.02	187.16	188.31
6.92	-1.39	11.19	20.49	10.61	1.44	1.89	10.24	3.25	-4.21	173.12	273.07	184.69	184.60
6.96	-1.37	11.24	20.55	10.66	1.46	1.91	10.26	3.27	-4.19	172.22	272.26	183.66	183.06
6.58	-1.62	10.51	19.83	10.17	1.20	1.62	9.95	3.01	-4.43	183.30	282.86	197.27	204.03
6.99	-1.35	11.39	20.66	10.75	1.49	1.87	10.24	3.22	-4.22	166.76	267.55	178.93	177.96
6.77	-1.53	10.92	20.18	10.45	1.27	1.81	10.10	3.10	-4.31	178.07	276.04	188.80	191.13
7.12	-1.35	11.67	20.95	10.86	1.34	2.03	10.10	3.36	-4.03	129.51	222.84	139.66	125.18
7.14	-1.61	11.87	21.37	11.20	1.43	2.03	10.38	3.10	-4.63	172.54	264.58	178.45	180.90
7.07	-1.44	11.44	20.94	11.10	1.74	2.02	10.44	3.05	-4.65	200.14	298.32	198.11	205.47
7.35	-1.31	11.92	21.39	11.46	1.92	2.25	10.62	3.21	-4.52	194.02	292.87	191.71	196.29
7.13	-1.49	11.56	21.12	11.24	1.75	1.97	10.38	3.00	-4.72	199.01	301.11	190.77	195.00
7.11	-1.43	11.51	20.91	11.13	1.74	2.02	10.39	3.05	-4.63	202.72	300.77	199.27	208.46
7.17	-1.33	11.45	20.87	11.16	1.87	2.17	10.45	3.20	-4.53	204.02	304.45	193.67	197.01
8.01	-0.85	13.02	22.49	12.39	2.44	2.83	11.08	3.63	-4.12	173.61	269.99	162.00	154.93
7.31	-1.49	12.09	21.43	11.60	1.88	1.99	10.33	3.01	-4.83	199.08	298.19	186.70	186.84
7.51	-1.29	12.54	21.85	11.88	2.11	2.18	10.57	3.14	-4.66	193.98	291.08	184.09	183.83
7.05	-1.70	11.59	20.86	11.24	1.60	1.77	10.12	2.84	-5.01	211.86	312.38	200.39	206.87
6.78	-1.86	11.25	20.55	11.00	1.48	1.43	9.87	2.56	-5.22	217.93	320.24	209.60	217.82
6.88	-1.81	11.33	20.63	11.04	1.53	1.54	9.96	2.68	-5.13	216.82	318.10	206.62	213.72
6.86	-1.82	11.33	20.61	11.04	1.49	1.53	9.94	2.66	-5.16	217.94	319.05	207.30	215.22
7.07	-1.70	11.77	21.01	11.37	1.64	1.69	10.10	2.76	-5.04	210.15	310.63	201.71	209.46
6.83	-1.94	11.41	20.66	11.12	1.39	1.41	9.81	2.56	-5.27	217.44	320.58	209.84	219.67
6.96	-1.77	11.53	20.71	11.23	1.57	1.57	9.98	2.69	-5.12	216.32	315.59	209.16	220.47

7.32	-1.55	12.32	21.69	11.91	2.02	1.79	10.05	2.72	-5.13	192.42	291.27	172.05	166.34
7.28	-1.37	11.75	21.24	11.34	1.80	2.23	10.58	3.20	-4.54	195.67	294.36	189.09	193.19
7.34	-1.25	11.82	21.20	11.42	1.95	2.26	10.56	3.28	-4.48	200.14	299.54	190.08	194.07
7.13	-1.44	11.54	21.01	11.16	1.76	2.06	10.46	3.10	-4.61	199.61	300.08	197.95	205.90
7.13	-1.43	11.54	21.01	11.16	1.76	2.06	10.46	3.10	-4.61	199.50	299.93	197.86	205.75
7.24	-1.36	11.73	21.19	11.30	1.84	2.17	10.56	3.18	-4.54	196.54	296.02	194.51	200.77
7.25	-1.36	11.74	21.20	11.31	1.84	2.18	10.56	3.18	-4.54	196.39	295.85	194.35	200.53
7.27	-1.34	11.77	21.23	11.34	1.85	2.20	10.58	3.20	-4.53	195.90	295.21	193.79	199.70
7.28	-1.33	11.79	21.26	11.35	1.86	2.21	10.59	3.21	-4.52	195.52	294.72	193.35	199.06
7.78	-1.01	12.58	22.09	12.04	2.23	2.66	10.95	3.51	-4.27	180.30	279.60	171.05	167.59
7.97	-0.86	12.91	22.39	12.32	2.40	2.84	11.08	3.63	-4.11	175.43	272.06	164.07	158.23
7.98	-0.86	12.91	22.39	12.32	2.40	2.84	11.08	3.64	-4.11	175.34	271.97	164.02	158.17
7.84	-1.00	12.79	22.27	12.23	2.33	2.57	10.85	3.43	-4.34	179.56	278.10	167.29	162.84
7.85	-1.00	12.79	22.27	12.23	2.33	2.58	10.85	3.44	-4.33	179.46	277.97	167.17	162.68
7.85	-1.00	12.80	22.28	12.24	2.34	2.59	10.86	3.44	-4.33	179.35	277.82	167.05	162.52
7.68	-1.15	12.62	22.13	12.09	2.21	2.34	10.65	3.22	-4.54	181.77	281.85	170.07	166.07
7.69	-1.14	12.65	22.16	12.11	2.22	2.36	10.66	3.24	-4.53	181.27	281.30	169.58	165.40
7.64	-1.26	12.67	22.14	12.15	2.13	2.23	10.53	3.13	-4.63	181.46	280.60	169.36	163.95
7.82	-1.04	12.80	22.27	12.25	2.27	2.52	10.79	3.39	-4.39	180.17	277.55	168.01	163.00
7.83	-1.03	12.81	22.28	12.25	2.28	2.53	10.79	3.40	-4.38	180.09	277.38	167.83	162.80
7.90	-0.95	12.87	22.32	12.29	2.34	2.67	10.91	3.52	-4.25	179.29	275.69	166.65	161.48
7.26	-1.27	11.59	21.01	11.25	1.93	2.24	10.54	3.26	-4.47	201.47	301.00	190.65	193.02
7.27	-1.27	11.61	21.03	11.26	1.94	2.25	10.55	3.27	-4.47	201.17	300.57	190.30	192.55
7.28	-1.26	11.63	21.04	11.27	1.94	2.26	10.55	3.27	-4.46	200.98	300.30	190.08	192.25
7.64	-1.19	12.62	22.04	12.07	2.14	2.33	10.61	3.24	-4.51	186.47	283.21	173.06	167.95
7.46	-1.25	12.13	21.61	11.64	2.00	2.34	10.70	3.27	-4.48	191.72	289.84	187.98	192.25
7.20	-1.55	11.95	21.25	11.47	1.82	1.88	10.26	2.93	-4.90	203.45	303.89	193.65	197.08
7.27	-1.46	12.10	21.47	11.61	1.93	1.91	10.32	2.95	-4.82	199.96	298.14	189.13	189.83
7.28	-1.47	12.10	21.45	11.59	1.92	1.95	10.32	2.95	-4.85	199.42	298.44	188.95	190.10
7.18	-1.41	11.66	21.11	11.26	1.79	2.08	10.45	3.08	-4.63	198.02	298.40	196.50	204.07
7.02	-1.50	11.38	20.84	11.08	1.70	1.91	10.30	2.98	-4.75	203.79	304.69	201.51	209.94
7.24	-1.38	11.72	21.17	11.32	1.83	2.14	10.49	3.14	-4.59	197.61	296.95	195.53	202.44
6.72	-2.14	11.26	20.69	11.19	1.40	1.20	9.44	2.25	-5.68	208.82	314.36	185.79	177.90
6.83	-2.07	11.41	20.85	11.34	1.45	1.31	9.53	2.31	-5.63	206.21	311.24	182.77	173.56
6.95	-1.96	11.88	21.22	11.57	1.68	1.37	9.63	2.33	-5.55	200.71	302.75	178.30	172.31
7.05	-1.78	11.97	21.31	11.57	1.80	1.60	9.86	2.50	-5.36	191.79	292.97	170.67	161.34
7.86	-1.04	12.90	22.38	12.30	2.32	2.55	10.80	3.40	-4.41	175.21	270.85	159.94	150.58
7.83	-1.07	12.86	22.34	12.28	2.30	2.51	10.76	3.36	-4.45	176.09	271.87	161.05	151.99
7.23	-1.57	11.90	21.34	11.55	1.82	1.89	10.16	2.86	-4.95	192.20	291.39	178.96	174.80
7.37	-1.42	12.29	21.81	11.72	1.99	2.11	10.41	2.99	-4.86	173.64	268.58	150.54	132.92
7.63	-1.20	12.64	22.11	12.08	2.19	2.40	10.60	3.19	-4.60	171.01	268.48	152.96	140.92
7.16	-1.88	12.13	21.45	11.78	1.69	1.59	9.83	2.53	-5.41	199.64	301.24	178.52	173.37
7.90	-0.99	12.95	22.44	12.27	2.36	2.77	10.99	3.52	-4.31	164.96	259.86	147.56	132.74

6.96	-1.93	11.86	21.23	11.57	1.69	1.39	9.66	2.36	-5.54	199.68	301.98	177.71	171.01
7.18	-1.57	11.78	21.19	11.46	1.80	1.90	10.13	2.89	-4.96	196.98	293.16	178.24	174.27
7.12	-1.70	12.08	21.47	11.67	1.87	1.65	9.95	2.59	-5.26	192.03	292.04	170.89	163.85
7.35	-1.47	12.17	21.62	11.79	1.96	2.00	10.29	2.94	-4.90	189.27	288.08	174.05	167.58
7.48	-1.39	12.37	21.83	11.93	2.05	2.10	10.39	3.05	-4.83	184.54	281.52	168.94	161.17
7.30	-1.51	12.05	21.50	11.71	1.91	1.96	10.24	2.91	-4.93	190.78	289.46	175.30	169.69
7.00	-1.70	11.47	20.93	11.23	1.67	1.70	10.00	2.76	-5.07	200.69	299.86	184.74	182.33
7.06	-1.64	11.59	21.00	11.32	1.73	1.81	10.05	2.82	-5.02	200.34	297.18	181.60	178.68
7.12	-1.61	11.74	21.12	11.43	1.79	1.82	10.07	2.80	-5.03	199.19	296.77	179.95	176.74
7.08	-1.65	11.66	21.04	11.37	1.76	1.77	10.04	2.77	-5.06	200.99	298.84	181.48	178.59
7.53	-1.30	12.46	21.96	11.97	2.10	2.24	10.46	3.08	-4.69	176.46	274.44	158.06	146.54
7.29	-1.46	12.13	21.62	11.71	1.95	2.00	10.25	2.90	-4.90	183.97	284.58	165.93	156.68
7.32	-1.45	12.18	21.64	11.76	2.00	1.99	10.25	2.90	-4.92	186.02	284.99	166.80	158.58
7.40	-1.46	12.28	21.73	11.86	2.01	2.01	10.26	2.93	-4.92	186.59	285.00	170.55	162.85
7.44	-1.45	12.33	21.78	11.92	2.03	2.03	10.27	2.96	-4.90	186.26	284.54	169.26	161.89
7.15	-1.59	11.72	21.14	11.41	1.77	1.87	10.12	2.88	-4.97	197.33	294.03	179.36	175.79
7.38	-1.47	12.16	21.59	11.77	1.97	2.05	10.26	2.97	-4.88	189.86	286.78	171.49	165.74
7.38	-1.47	12.16	21.60	11.78	1.97	2.05	10.27	2.97	-4.88	189.84	286.76	171.46	165.71
7.08	-1.65	11.76	21.24	11.41	1.80	1.76	10.04	2.72	-5.06	190.91	293.24	171.94	163.76
7.26	-1.55	12.09	21.50	11.66	1.92	1.95	10.19	2.84	-4.98	187.75	286.74	166.45	157.69
7.82	-1.12	12.82	22.29	12.27	2.27	2.49	10.74	3.36	-4.51	176.50	271.39	159.80	151.84
6.78	-1.85	11.15	20.50	11.02	1.54	1.47	9.77	2.58	-5.24	213.68	314.53	195.40	194.17
6.73	-1.92	11.06	20.36	10.94	1.49	1.39	9.69	2.51	-5.31	215.23	319.50	197.91	197.97
7.12	-1.70	11.92	21.20	11.60	1.82	1.65	9.93	2.63	-5.22	200.47	300.86	180.71	177.81
7.10	-1.82	12.04	21.33	11.71	1.74	1.52	9.77	2.48	-5.38	200.75	301.94	180.46	175.97
7.33	-1.52	12.15	21.55	11.79	1.95	1.93	10.16	2.86	-5.00	192.19	291.05	173.97	168.35
7.31	-1.55	12.20	21.57	11.82	1.98	1.86	10.10	2.80	-5.04	192.13	291.82	174.26	168.06
7.06	-1.88	12.04	21.30	11.67	1.69	1.48	9.73	2.45	-5.43	203.03	303.78	182.51	177.68
6.84	-1.73	11.23	20.74	10.97	1.64	1.69	9.93	2.70	-5.12	195.15	295.94	173.67	162.96
7.18	-1.69	12.26	21.61	11.80	1.93	1.66	9.96	2.56	-5.30	190.83	289.53	169.33	161.60
7.01	-1.89	11.95	21.31	11.63	1.74	1.44	9.72	2.39	-5.50	198.37	300.10	176.11	169.65
7.05	-1.84	12.02	21.39	11.67	1.78	1.51	9.78	2.43	-5.45	196.52	297.82	173.82	167.19
6.97	-1.90	11.71	21.07	11.45	1.66	1.52	9.73	2.49	-5.45	198.18	299.45	175.52	166.03
6.96	-1.90	11.70	21.06	11.45	1.65	1.52	9.72	2.49	-5.45	198.29	299.56	175.61	166.10
6.95	-1.93	11.92	21.27	11.62	1.68	1.32	9.62	2.31	-5.54	199.73	303.36	180.48	175.00
7.42	-1.43	12.26	21.67	11.84	1.95	2.05	10.33	2.98	-4.80	189.79	286.57	173.97	170.70
7.53	-1.29	12.46	22.03	11.85	2.11	2.38	10.63	3.18	-4.71	171.25	265.78	148.45	130.81
6.89	-1.90	11.61	20.93	11.32	1.64	1.46	9.65	2.46	-5.47	197.50	298.51	172.30	162.38
7.76	-1.12	12.71	22.27	12.08	2.21	2.62	10.84	3.39	-4.47	167.33	261.50	149.70	134.48
7.98	-0.90	13.05	22.51	12.36	2.43	2.79	11.01	3.58	-4.23	166.48	261.62	149.90	136.84
7.97	-0.87	13.09	22.60	12.39	2.48	2.76	11.00	3.57	-4.22	167.20	260.96	152.22	140.01
7.70	-1.12	12.63	22.20	12.02	2.22	2.58	10.81	3.40	-4.46	168.88	263.65	153.44	138.60
8.15	-0.77	13.31	22.78	12.57	2.56	2.95	11.14	3.72	-4.06	163.71	259.11	150.14	137.22

8.15	-0.77	13.30	22.78	12.57	2.55	2.95	11.14	3.72	-4.06	163.65	259.21	150.12	137.28
8.15	-0.77	13.30	22.77	12.57	2.55	2.95	11.14	3.72	-4.07	163.50	259.43	150.11	137.49
8.13	-0.78	13.26	22.74	12.55	2.53	2.93	11.14	3.71	-4.10	163.46	260.23	150.36	138.12
7.88	-0.99	12.84	22.32	12.27	2.35	2.65	10.86	3.47	-4.35	173.19	269.09	156.03	146.31
7.88	-0.99	12.88	22.38	12.30	2.33	2.63	10.89	3.47	-4.35	173.93	269.34	157.67	148.36
7.76	-1.09	12.67	22.18	12.16	2.27	2.52	10.75	3.37	-4.46	174.82	271.55	158.87	148.30
7.85	-1.03	12.89	22.42	12.23	2.31	2.74	10.93	3.49	-4.37	164.59	258.15	147.08	130.10
7.75	-1.14	12.86	22.44	12.19	2.28	2.57	10.82	3.33	-4.56	164.06	259.70	145.54	129.03
7.55	-1.30	12.62	22.18	12.00	2.15	2.30	10.53	3.08	-4.75	170.46	267.04	149.56	132.77
7.47	-1.30	12.42	22.00	11.81	2.12	2.32	10.58	3.14	-4.74	172.14	267.01	149.39	131.81
7.15	-1.53	11.84	21.34	11.43	1.88	1.98	10.19	2.88	-4.93	185.03	282.98	163.73	151.58
7.85	-1.04	12.88	22.44	12.22	2.30	2.74	10.94	3.49	-4.39	164.73	257.30	146.07	129.75
7.83	-0.94	12.84	22.27	12.00	2.29	2.85	11.24	3.64	-4.16	179.18	270.26	176.41	178.47
7.54	-1.22	12.58	22.10	11.87	2.04	2.33	10.74	3.22	-4.48	179.73	269.55	191.42	199.59
7.23	-1.45	12.00	21.39	11.46	1.82	2.00	10.37	3.00	-4.71	189.25	282.33	205.26	221.70
7.23	-1.46	12.01	21.41	11.48	1.82	1.99	10.35	2.95	-4.74	188.14	283.06	205.08	222.50
7.37	-1.36	12.26	21.69	11.65	1.91	2.13	10.51	3.07	-4.62	184.81	277.25	199.63	212.84
7.66	-1.09	12.69	22.22	11.91	2.15	2.54	10.90	3.38	-4.35	179.06	265.78	186.29	193.44
7.36	-1.34	12.19	21.62	11.56	1.89	2.26	10.63	3.19	-4.58	189.40	282.63	200.01	215.74
7.50	-1.29	12.38	21.95	11.70	1.91	2.37	10.80	3.27	-4.49	181.48	272.91	188.30	197.00
7.50	-1.29	12.39	21.95	11.70	1.91	2.37	10.80	3.27	-4.49	181.71	273.05	187.99	196.85
7.55	-1.21	12.54	22.06	11.80	2.04	2.41	10.84	3.32	-4.46	182.34	272.97	188.52	198.52
7.69	-1.11	12.65	22.16	11.91	2.09	2.61	10.95	3.47	-4.33	179.54	269.99	187.04	196.19
8.07	-0.81	13.17	22.77	12.17	2.35	3.20	11.65	3.96	-3.96	167.64	258.91	165.68	162.25
7.18	-1.44	11.85	21.11	11.36	1.79	1.98	10.38	3.05	-4.69	201.65	298.61	209.27	229.30
6.43	-1.91	10.56	19.79	10.49	1.34	1.14	9.56	2.38	-5.19	225.41	324.80	244.92	284.50
6.68	-1.78	10.73	20.01	10.60	1.38	1.58	9.92	2.76	-4.94	217.42	316.91	219.18	237.78
6.68	-1.79	10.74	20.02	10.61	1.37	1.57	9.91	2.75	-4.95	217.47	317.14	218.86	237.37
6.90	-1.63	11.14	20.43	10.88	1.56	1.78	10.12	2.90	-4.82	211.22	310.19	209.74	224.55
6.70	-1.76	10.90	20.16	10.73	1.48	1.50	9.90	2.69	-5.00	216.75	318.12	214.80	231.35
6.85	-1.63	11.15	20.46	10.90	1.60	1.72	10.09	2.83	-4.85	210.91	311.36	209.65	223.76
7.03	-1.50	11.45	20.72	11.09	1.71	1.92	10.28	2.99	-4.72	206.21	306.05	203.21	215.33
7.11	-1.46	11.53	20.87	11.14	1.72	2.02	10.39	3.08	-4.64	204.13	300.65	201.74	214.86
7.03	-1.52	11.40	20.74	11.05	1.67	1.93	10.30	3.01	-4.70	206.67	303.40	204.61	218.16
6.97	-1.55	11.30	20.64	10.97	1.64	1.84	10.22	2.93	-4.77	207.32	306.92	206.56	221.23
6.69	-1.74	10.72	20.02	10.57	1.39	1.62	9.96	2.85	-4.88	215.33	316.17	218.71	237.31
6.78	-1.64	10.78	20.14	10.58	1.45	1.78	10.14	2.98	-4.73	212.33	313.10	215.73	234.53
6.70	-1.70	10.70	20.04	10.51	1.42	1.66	10.04	2.89	-4.81	214.35	315.55	218.02	236.92
6.73	-1.67	10.73	20.08	10.54	1.43	1.71	10.08	2.93	-4.78	213.61	314.59	217.20	236.04
6.77	-1.68	10.99	20.36	10.76	1.52	1.61	10.03	2.76	-4.92	211.11	313.57	212.78	228.48
6.75	-1.70	10.84	20.12	10.65	1.44	1.66	10.01	2.88	-4.85	213.77	313.83	216.29	234.05
6.71	-1.70	10.70	20.05	10.52	1.42	1.65	10.03	2.88	-4.81	214.26	315.54	217.89	236.83
7.73	-1.04	12.65	22.12	11.83	2.12	2.77	11.19	3.61	-4.22	178.14	272.64	179.00	181.98

7.46	-1.17	12.06	21.48	11.47	1.95	2.52	10.87	3.46	-4.26	191.67	288.58	188.25	193.59
7.16	-1.41	11.61	21.00	11.17	1.77	2.10	10.49	3.13	-4.56	199.33	300.66	199.89	209.26
7.40	-1.24	11.97	21.38	11.41	1.89	2.41	10.75	3.35	-4.34	193.25	292.41	191.32	198.44
6.82	-1.65	11.10	20.48	10.83	1.56	1.71	10.12	2.83	-4.83	208.92	311.56	210.21	223.43
6.99	-1.54	11.35	20.69	11.01	1.65	1.90	10.29	2.99	-4.73	207.03	304.49	205.81	219.33
7.01	-1.54	11.36	20.69	11.02	1.65	1.90	10.27	2.99	-4.72	207.47	304.18	205.60	219.33
6.99	-1.55	11.31	20.63	11.00	1.64	1.88	10.24	3.00	-4.73	208.52	304.63	206.64	220.77
7.19	-1.40	11.67	21.04	11.21	1.78	2.10	10.49	3.13	-4.58	200.05	298.74	199.38	210.44
7.20	-1.39	11.69	21.07	11.23	1.79	2.11	10.50	3.15	-4.56	199.70	298.18	198.90	209.81
7.38	-1.27	11.99	21.33	11.45	1.88	2.33	10.70	3.31	-4.42	195.46	291.87	192.91	202.73
6.65	-1.75	10.61	19.95	10.46	1.38	1.58	9.95	2.83	-4.86	216.24	318.13	219.63	239.25
7.59	-1.14	12.44	21.85	11.76	2.06	2.51	10.94	3.44	-4.34	187.85	283.31	192.45	202.03
7.25	-1.41	12.01	21.32	11.47	1.87	1.99	10.42	3.06	-4.70	199.40	295.45	206.88	224.94
7.19	-1.45	11.87	21.20	11.37	1.83	1.95	10.40	3.02	-4.71	200.50	297.76	209.61	227.57
7.55	-1.10	12.26	21.74	11.60	2.06	2.60	11.04	3.51	-4.25	185.31	279.10	182.31	185.53
7.44	-1.17	12.05	21.53	11.44	1.97	2.49	10.92	3.43	-4.33	189.60	282.95	186.44	190.96
7.45	-1.16	12.07	21.55	11.45	1.98	2.50	10.93	3.44	-4.32	189.28	282.57	186.03	190.41
7.35	-1.23	11.86	21.32	11.34	1.89	2.43	10.81	3.38	-4.34	194.03	289.28	190.30	196.57
7.30	-1.27	11.78	21.25	11.28	1.86	2.37	10.76	3.34	-4.38	195.31	290.77	191.60	198.34
7.84	-1.04	12.87	22.43	12.21	2.30	2.74	10.94	3.49	-4.39	164.90	257.60	146.75	130.15
6.81	-1.77	11.26	20.54	10.94	1.48	1.51	9.94	2.64	-5.04	212.26	309.29	226.48	256.15
6.88	-1.73	11.32	20.66	10.99	1.52	1.66	10.07	2.77	-4.97	207.67	305.60	221.58	248.36
6.82	-1.73	11.37	20.70	11.05	1.61	1.51	9.95	2.57	-5.05	208.55	304.28	223.48	252.50
6.84	-1.69	11.36	20.67	11.05	1.63	1.56	9.96	2.63	-4.99	208.56	305.05	225.84	255.23
6.83	-1.70	11.34	20.65	11.03	1.62	1.55	9.96	2.62	-5.00	208.83	305.48	226.26	255.86
6.83	-1.69	11.34	20.66	11.04	1.62	1.55	9.96	2.62	-5.00	208.71	305.31	226.08	255.58
7.32	-1.42	12.17	21.49	11.56	1.83	2.12	10.53	3.08	-4.66	192.73	288.01	204.50	223.65
6.97	-1.64	11.62	20.91	11.18	1.62	1.70	10.14	2.78	-4.93	204.65	300.52	219.90	245.43
7.07	-1.60	11.73	21.03	11.25	1.66	1.81	10.22	2.86	-4.87	201.87	297.58	216.39	240.19
6.90	-1.74	11.37	20.68	11.01	1.53	1.63	10.03	2.74	-4.99	207.73	306.02	222.18	249.84
6.93	-1.65	11.55	20.79	11.14	1.63	1.65	10.08	2.72	-4.94	208.43	303.82	222.95	252.06
6.93	-1.65	11.49	20.77	11.14	1.66	1.64	10.04	2.72	-4.94	207.86	303.52	224.64	253.10
6.73	-1.74	11.14	20.39	10.89	1.54	1.46	9.89	2.58	-5.02	215.05	311.42	232.04	264.85
6.77	-1.72	11.20	20.47	10.94	1.59	1.48	9.91	2.61	-5.02	212.42	309.40	231.27	262.09
6.78	-1.71	11.22	20.48	10.95	1.59	1.48	9.92	2.61	-5.02	212.21	309.13	231.00	261.71
7.32	-1.39	12.05	21.43	11.48	1.82	2.18	10.52	3.15	-4.59	189.28	282.95	204.53	221.37
6.97	-1.61	11.50	20.83	11.13	1.65	1.73	10.10	2.76	-4.88	202.96	300.23	220.16	246.25
6.67	-2.06	11.12	20.41	11.02	1.44	1.17	9.51	2.31	-5.56	219.66	324.88	201.00	200.89
7.17	-1.90	11.97	21.26	11.70	1.62	1.68	9.91	2.64	-5.40	210.29	316.80	190.82	188.60
7.19	-1.88	12.00	21.29	11.73	1.63	1.70	9.93	2.66	-5.38	209.56	315.87	190.11	187.74
6.81	-1.93	11.25	20.50	11.06	1.44	1.41	9.78	2.53	-5.30	218.38	322.71	206.40	212.64
7.10	-1.71	11.71	20.90	11.40	1.67	1.76	10.05	2.77	-5.09	210.13	312.81	195.95	198.55
7.11	-1.77	11.69	20.95	11.44	1.68	1.77	10.02	2.79	-5.20	212.39	315.95	194.91	194.39

7.02	-1.89	11.54	20.83	11.36	1.55	1.64	9.89	2.67	-5.32	215.49	323.59	198.64	198.01
6.66	-2.00	10.96	20.20	10.88	1.36	1.26	9.64	2.44	-5.37	223.76	330.88	213.00	219.65
6.73	-1.97	11.10	20.36	10.97	1.41	1.33	9.71	2.48	-5.34	220.87	326.46	209.29	216.14
7.16	-1.65	11.69	20.86	11.39	1.65	1.88	10.15	2.91	-4.96	208.60	311.45	195.35	198.73
6.97	-1.81	11.60	20.85	11.34	1.60	1.57	9.91	2.60	-5.23	214.70	316.10	197.90	202.09
7.49	-1.41	12.38	21.61	11.85	1.93	2.18	10.41	3.12	-4.76	196.67	294.41	178.66	178.57
7.22	-1.45	12.05	21.46	11.50	1.84	1.98	10.34	2.94	-4.76	189.59	283.96	204.77	223.20
7.24	-1.43	12.06	21.41	11.46	1.83	1.99	10.36	3.02	-4.70	187.04	281.82	206.41	225.36
6.99	-1.60	11.56	20.87	11.12	1.68	1.77	10.15	2.85	-4.84	195.58	292.85	218.18	241.96
7.18	-1.43	11.72	21.00	11.25	1.72	2.05	10.35	3.11	-4.62	193.01	287.67	210.71	232.00
6.84	-1.65	11.16	20.48	10.84	1.54	1.72	10.05	2.84	-4.84	200.00	296.12	221.68	248.83
6.82	-1.75	11.50	20.73	11.10	1.58	1.45	9.85	2.55	-5.09	197.38	295.86	219.81	242.92
7.00	-1.67	11.78	21.12	11.29	1.60	1.65	10.00	2.73	-4.93	188.71	282.39	204.44	218.90
7.13	-1.53	11.88	21.09	11.42	1.78	1.84	10.14	2.85	-4.86	192.99	289.48	212.20	232.13
6.63	-1.86	11.04	20.26	10.83	1.43	1.30	9.69	2.45	-5.16	203.52	305.27	227.74	256.99
7.03	-1.65	11.75	20.99	11.31	1.68	1.70	10.05	2.74	-4.95	195.07	291.86	213.64	233.79
6.75	-1.80	11.45	20.70	11.09	1.55	1.34	9.73	2.45	-5.16	196.14	295.10	219.13	242.37
6.93	-1.64	11.58	20.89	11.24	1.68	1.52	9.90	2.62	-5.01	193.95	292.11	211.28	231.80
7.32	-1.37	12.42	21.81	11.76	1.99	1.94	10.35	2.90	-4.70	183.15	274.97	199.87	212.92
7.34	-1.35	12.46	21.85	11.78	2.00	1.96	10.36	2.92	-4.69	182.87	274.16	199.26	211.94
7.35	-1.36	12.34	21.83	11.68	1.91	2.07	10.50	3.03	-4.63	182.78	275.56	198.75	211.83
6.64	-1.86	11.17	20.40	10.87	1.47	1.27	9.67	2.43	-5.19	202.84	303.21	226.81	255.11
6.79	-1.79	11.40	20.63	11.02	1.54	1.42	9.82	2.53	-5.11	198.90	298.15	222.01	246.79
6.41	-2.00	10.49	19.66	10.48	1.27	1.16	9.46	2.35	-5.25	213.90	315.29	236.74	273.55
6.88	-1.63	11.36	20.64	10.99	1.65	1.68	10.05	2.79	-4.89	199.64	297.18	223.07	248.46
7.25	-1.43	12.08	21.43	11.47	1.83	2.00	10.37	3.02	-4.70	186.90	281.41	206.06	224.82
7.24	-1.43	12.08	21.44	11.49	1.84	2.00	10.38	3.02	-4.69	186.68	280.24	205.15	223.48
7.19	-1.43	11.75	21.04	11.27	1.73	2.05	10.35	3.11	-4.63	192.08	287.21	210.10	231.24
6.93	-1.59	11.38	20.65	11.02	1.64	1.77	10.10	2.88	-4.86	199.86	296.12	221.25	246.43
6.49	-1.97	10.65	19.82	10.59	1.33	1.23	9.54	2.38	-5.23	211.78	312.32	234.27	268.76
6.76	-1.68	10.95	20.24	10.72	1.51	1.63	9.98	2.81	-4.85	203.07	300.95	226.14	255.97
7.68	-1.13	12.89	22.37	12.10	2.12	2.36	10.74	3.28	-4.39	173.64	265.78	187.13	194.57
7.67	-1.14	12.87	22.34	12.08	2.11	2.36	10.73	3.26	-4.40	174.03	266.29	187.93	195.54
6.90	-1.73	11.62	20.95	11.19	1.55	1.51	9.88	2.62	-5.02	191.76	286.33	208.37	226.21
6.89	-1.73	11.61	20.94	11.19	1.55	1.51	9.88	2.62	-5.02	191.81	286.45	208.51	226.45
6.96	-1.70	11.70	21.04	11.24	1.58	1.59	9.95	2.68	-4.98	190.11	284.35	206.43	222.52
6.96	-1.69	11.71	21.04	11.25	1.58	1.60	9.96	2.68	-4.97	190.03	284.20	206.27	222.23
6.96	-1.69	11.70	21.04	11.25	1.58	1.59	9.96	2.68	-4.98	190.04	284.29	206.37	222.40
6.92	-1.55	11.71	21.05	11.32	1.82	1.34	9.80	2.51	-4.95	189.53	290.12	209.57	222.06
6.95	-1.53	11.77	21.11	11.36	1.85	1.37	9.83	2.53	-4.92	188.72	289.25	208.41	220.30
6.91	-1.70	11.53	20.86	11.17	1.59	1.53	9.91	2.64	-4.98	190.45	287.51	209.50	228.52
7.15	-1.47	12.06	21.47	11.56	1.87	1.68	10.09	2.75	-4.78	181.93	282.44	198.80	209.87
6.81	-1.71	11.38	20.70	11.10	1.64	1.40	9.82	2.53	-5.06	196.30	296.81	216.98	238.45

7.38	-1.40	12.18	21.65	11.47	1.66	2.26	10.61	3.30	-4.46	173.27	262.52	178.45	181.02
7.04	-1.58	11.64	20.88	11.27	1.71	1.78	10.09	2.84	-4.87	197.36	292.05	217.08	237.62
6.44	-1.97	10.77	19.97	10.62	1.32	1.06	9.47	2.27	-5.29	208.01	311.29	234.34	268.16
7.33	-1.39	12.32	21.62	11.70	1.92	1.97	10.35	2.96	-4.71	183.40	279.51	201.72	215.47
6.94	-1.61	11.61	20.99	11.29	1.78	1.46	9.87	2.60	-4.99	194.15	292.90	211.52	229.73
6.70	-1.78	11.20	20.52	10.99	1.59	1.27	9.68	2.43	-5.15	200.20	300.19	219.58	245.18
6.69	-1.79	11.18	20.50	10.98	1.58	1.26	9.67	2.42	-5.16	200.51	300.55	219.97	245.87
7.80	-1.02	12.68	22.13	12.10	2.27	2.58	10.84	3.46	-4.29	183.93	280.24	172.05	167.34
6.71	-2.09	11.08	20.29	10.89	1.17	1.33	9.73	2.50	-5.38	225.21	327.70	221.30	237.84
6.71	-2.03	11.23	20.40	10.99	1.34	1.24	9.66	2.43	-5.38	224.58	325.97	217.74	232.79
6.71	-2.03	11.22	20.39	10.98	1.34	1.23	9.66	2.43	-5.38	224.87	326.30	218.06	233.16
6.54	-2.19	10.88	20.08	10.75	1.14	1.07	9.50	2.33	-5.50	229.38	333.45	226.16	242.39
6.70	-2.04	11.21	20.38	10.98	1.33	1.23	9.65	2.42	-5.39	225.04	326.52	218.23	233.39
6.68	-2.10	11.06	20.26	10.88	1.16	1.30	9.70	2.48	-5.40	225.89	328.57	222.01	238.53
6.32	-2.19	10.21	19.31	10.31	0.96	1.12	9.44	2.36	-5.35	239.61	340.27	255.10	297.79
6.44	-2.17	10.56	19.63	10.49	1.00	1.24	9.60	2.41	-5.33	233.33	334.65	250.60	288.51
6.22	-2.26	10.00	19.10	10.16	0.88	1.02	9.36	2.29	-5.38	243.37	345.28	261.26	308.07
5.86	-2.55	9.40	18.50	9.76	0.55	0.60	8.96	1.96	-5.65	257.88	361.14	277.61	328.74
6.17	-2.29	9.89	18.99	10.09	0.84	0.97	9.30	2.25	-5.41	245.56	347.79	263.18	311.25
6.05	-2.11	9.39	18.64	9.72	1.00	0.96	9.32	2.36	-5.23	241.01	348.25	253.12	292.60
5.56	-2.65	8.78	17.90	9.40	0.48	0.20	8.51	1.73	-5.79	265.82	365.98	282.86	343.45
5.46	-2.74	8.64	17.77	9.30	0.41	0.08	8.40	1.63	-5.88	268.22	369.31	285.29	345.92
5.57	-2.64	8.80	17.92	9.41	0.49	0.22	8.53	1.75	-5.77	265.45	365.51	282.49	343.08
6.52	-1.90	10.47	19.77	10.42	1.26	1.39	9.70	2.61	-5.09	222.48	323.67	224.49	244.81
6.53	-1.89	10.50	19.79	10.44	1.28	1.40	9.71	2.62	-5.08	222.08	323.30	223.98	244.08
6.32	-1.99	10.16	19.39	10.22	1.20	1.13	9.50	2.45	-5.17	231.60	332.50	247.87	288.15
6.34	-2.22	10.37	19.48	10.35	1.01	1.11	9.47	2.32	-5.43	235.93	336.73	249.89	285.69
6.26	-2.25	10.17	19.35	10.23	0.94	1.00	9.39	2.26	-5.41	236.52	337.79	246.58	278.58
6.71	-1.78	10.99	20.24	10.74	1.41	1.53	9.96	2.73	-4.98	217.02	318.58	225.69	250.39
6.20	-2.24	10.06	19.24	10.14	0.94	0.97	9.36	2.24	-5.43	235.84	338.92	246.54	279.67
6.36	-2.05	10.33	19.69	10.34	1.17	1.11	9.54	2.40	-5.28	225.06	325.97	238.65	272.84
7.07	-1.49	11.53	20.88	11.12	1.69	1.91	10.34	3.01	-4.70	204.79	302.98	214.26	232.52
6.56	-1.88	10.70	19.92	10.54	1.30	1.37	9.78	2.58	-5.11	224.17	324.06	232.93	260.93
6.10	-2.15	9.66	18.80	9.93	1.03	0.85	9.14	2.26	-5.33	239.88	340.42	262.35	313.70
6.47	-1.96	10.46	19.79	10.42	1.27	1.23	9.65	2.48	-5.17	221.59	323.35	237.04	269.15
6.47	-1.98	10.54	19.82	10.46	1.26	1.22	9.64	2.48	-5.21	224.21	323.29	237.06	272.09
6.31	-1.98	10.19	19.39	10.27	1.27	1.08	9.46	2.38	-5.18	230.65	331.96	250.39	294.46
6.63	-1.78	10.88	20.12	10.72	1.49	1.39	9.76	2.55	-5.04	215.84	315.06	237.34	272.78
6.49	-1.86	10.58	19.80	10.53	1.39	1.26	9.65	2.48	-5.10	221.44	321.00	243.94	281.81
6.47	-1.91	10.52	19.72	10.50	1.33	1.17	9.57	2.40	-5.16	224.08	323.27	246.26	286.13
6.26	-2.04	10.08	19.29	10.17	1.16	1.04	9.43	2.38	-5.22	233.29	335.08	250.24	291.84
6.89	-1.81	11.14	20.40	11.03	1.51	1.65	9.93	2.76	-5.14	217.70	325.43	205.35	206.93
6.63	-2.12	10.60	19.95	10.76	1.27	1.23	9.53	2.47	-5.48	230.31	345.15	218.02	219.15

6.94	-1.77	11.21	20.46	11.06	1.54	1.73	9.98	2.82	-5.09	215.29	322.92	203.41	205.57
6.91	-1.79	11.16	20.42	11.03	1.53	1.70	9.97	2.81	-5.10	215.90	323.94	204.27	206.55
6.74	-2.14	10.85	20.13	10.81	1.13	1.38	9.85	2.64	-5.42	226.80	333.08	225.14	241.99
6.90	-2.03	10.69	20.05	10.75	1.13	1.76	10.27	3.02	-5.21	225.53	330.99	224.94	240.84
6.74	-1.85	10.69	19.93	10.68	1.37	1.60	9.87	2.79	-5.03	223.96	335.14	213.65	217.61
6.77	-1.83	10.73	19.96	10.70	1.38	1.65	9.91	2.84	-5.00	223.32	334.25	212.80	216.25
6.68	-2.14	11.14	20.35	10.95	1.22	1.18	9.60	2.37	-5.48	223.30	329.00	217.98	229.07
6.68	-2.14	11.14	20.35	10.95	1.22	1.19	9.60	2.37	-5.47	223.30	328.93	217.86	228.95
6.68	-2.15	11.14	20.35	10.95	1.21	1.18	9.59	2.37	-5.48	223.34	329.15	218.16	229.25
6.20	-2.26	10.08	19.21	10.12	0.90	0.95	9.32	2.31	-5.47	240.78	346.81	248.08	277.84
6.38	-2.17	10.43	19.57	10.37	1.02	1.13	9.48	2.41	-5.40	235.16	339.10	242.92	271.64
6.64	-2.08	10.98	20.13	10.81	1.20	1.24	9.64	2.46	-5.37	225.53	330.29	224.23	239.63
6.14	-2.23	9.73	18.91	9.93	0.91	0.93	9.24	2.35	-5.38	242.95	348.41	240.15	265.30
5.99	-2.35	9.43	18.64	9.72	0.79	0.77	9.08	2.23	-5.48	248.62	355.10	246.40	272.54
6.09	-2.29	9.70	18.89	9.92	0.85	0.85	9.17	2.28	-5.44	242.77	347.64	240.43	265.43
6.28	-2.25	10.20	19.30	10.24	0.97	0.95	9.34	2.33	-5.45	239.98	349.74	248.03	274.38
6.33	-2.14	10.09	19.25	10.18	1.03	1.08	9.45	2.48	-5.32	240.58	348.27	244.62	269.91
6.66	-2.10	10.90	20.07	10.80	1.22	1.31	9.74	2.52	-5.42	226.38	332.42	228.73	247.90
6.72	-2.09	11.00	20.17	10.87	1.24	1.37	9.80	2.57	-5.41	224.80	331.17	226.95	246.26
6.78	-2.07	11.06	20.25	10.93	1.24	1.46	9.92	2.66	-5.38	224.67	327.98	224.00	242.23
6.75	-2.05	11.11	20.29	10.94	1.26	1.39	9.80	2.58	-5.35	224.32	327.34	222.36	240.67
6.38	-2.18	10.40	19.49	10.37	1.03	1.07	9.45	2.41	-5.40	237.40	345.00	244.50	270.65
6.69	-2.00	10.97	20.04	10.73	1.21	1.47	9.84	2.68	-5.24	228.24	330.75	232.16	256.57
6.65	-2.05	10.64	19.82	10.58	1.20	1.40	9.81	2.67	-5.30	231.39	336.05	233.46	256.66
6.24	-1.97	9.84	19.01	9.96	1.16	1.11	9.41	2.53	-5.07	229.97	332.19	254.35	301.31
6.57	-1.84	10.67	19.81	10.57	1.40	1.42	9.68	2.56	-5.08	216.82	315.79	238.79	275.44
6.23	-1.99	9.75	18.88	9.92	1.12	1.12	9.35	2.52	-5.08	231.29	332.45	256.87	305.94
6.37	-2.02	10.39	19.54	10.40	1.24	1.16	9.42	2.33	-5.28	220.36	319.56	240.28	277.67
6.38	-2.01	10.43	19.58	10.43	1.25	1.17	9.44	2.34	-5.27	219.65	318.64	239.43	276.23
6.73	-1.67	10.76	20.01	10.60	1.48	1.64	9.94	2.86	-4.81	209.35	308.01	230.12	263.54
6.59	-1.76	10.51	19.76	10.40	1.38	1.48	9.80	2.76	-4.90	214.07	314.24	236.97	273.83
6.64	-1.75	10.81	20.16	10.68	1.47	1.30	9.65	2.58	-4.97	204.30	306.24	219.24	238.21
6.57	-1.78	10.84	20.19	10.68	1.48	1.17	9.59	2.44	-5.05	204.90	308.28	222.59	244.85
7.26	-1.29	12.24	21.57	11.61	1.99	1.67	10.09	2.89	-4.56	181.46	280.37	194.36	199.39
6.50	-1.82	10.73	20.07	10.60	1.45	1.09	9.52	2.37	-5.09	206.42	310.73	225.70	250.01
6.56	-1.77	10.93	20.23	10.74	1.51	1.13	9.56	2.38	-5.08	203.83	307.57	223.99	246.39
6.10	-2.12	9.94	19.21	10.05	1.08	0.77	9.23	2.15	-5.33	221.25	325.22	242.47	280.96
6.48	-1.85	10.78	20.07	10.64	1.46	1.09	9.53	2.34	-5.16	205.84	309.90	226.81	253.69
6.29	-1.94	9.93	19.11	10.03	1.19	1.16	9.45	2.55	-5.06	228.60	330.39	252.52	298.66
6.50	-1.83	10.86	20.15	10.70	1.50	1.10	9.55	2.35	-5.15	204.33	308.21	225.44	251.32
6.71	-1.71	11.25	20.56	10.98	1.65	1.28	9.71	2.46	-5.04	197.90	300.47	217.40	237.85
6.61	-1.77	11.02	20.32	10.82	1.55	1.21	9.66	2.42	-5.09	202.32	305.45	221.67	245.35
6.65	-1.74	11.03	20.35	10.82	1.56	1.27	9.70	2.48	-5.05	202.93	305.19	220.30	243.46

6.37	-1.95	10.39	19.67	10.38	1.31	1.02	9.42	2.36	-5.19	212.60	316.84	231.24	261.93
6.51	-1.90	10.88	20.17	10.76	1.43	1.10	9.55	2.32	-5.24	205.57	306.82	226.39	256.57
6.51	-1.83	10.88	20.17	10.71	1.51	1.11	9.56	2.35	-5.14	204.06	307.91	225.06	250.70
6.30	-2.08	10.39	19.61	10.40	1.20	0.88	9.31	2.18	-5.34	215.12	317.91	236.65	272.75
6.27	-2.10	10.35	19.56	10.36	1.18	0.86	9.29	2.16	-5.35	215.71	318.91	237.98	274.66
6.61	-2.19	10.97	20.17	10.83	1.12	1.13	9.57	2.36	-5.50	228.64	331.73	225.21	240.09
6.37	-2.22	10.45	19.86	10.55	1.20	1.06	9.23	2.24	-5.62	212.09	319.83	187.14	176.29
8.22	-0.68	13.46	22.93	12.67	2.63	2.96	11.23	3.73	-4.00	167.26	260.86	155.59	144.57
8.13	-0.76	13.31	22.78	12.57	2.57	2.86	11.14	3.67	-4.06	169.47	263.44	157.79	148.07
8.22	-0.67	13.34	22.95	12.50	2.52	3.23	11.62	3.98	-3.84	165.83	259.24	159.24	154.26
7.81	-1.09	12.82	22.41	12.11	2.15	2.75	10.99	3.54	-4.35	161.88	255.54	144.09	127.05
8.18	-0.78	13.15	22.90	12.24	2.33	3.45	11.77	4.13	-3.86	147.35	236.41	128.86	106.30
8.19	-0.81	13.16	22.96	12.30	2.33	3.41	11.73	4.09	-3.92	148.69	236.42	130.17	108.24
8.19	-0.77	13.19	23.01	12.33	2.34	3.41	11.67	4.07	-3.90	148.29	236.80	131.03	110.24
8.19	-0.77	13.18	23.01	12.32	2.34	3.41	11.67	4.07	-3.90	148.30	236.68	131.01	109.91
8.18	-0.79	13.14	22.97	12.30	2.33	3.38	11.67	4.05	-3.92	149.24	236.82	131.00	109.45
7.77	-1.13	12.84	22.42	12.11	2.17	2.62	10.90	3.44	-4.45	165.96	260.21	149.48	134.81
7.76	-1.14	12.83	22.40	12.12	2.16	2.61	10.87	3.44	-4.44	166.74	260.23	148.35	134.41
8.00	-0.84	12.90	22.56	11.98	2.18	3.33	11.60	4.02	-3.85	148.46	236.37	126.32	102.41
8.07	-0.81	12.99	22.67	12.08	2.23	3.40	11.68	4.10	-3.83	148.88	235.47	127.28	104.54
8.13	-0.79	13.24	23.00	12.20	2.27	3.34	11.71	4.08	-3.87	147.67	236.33	128.25	105.09
8.21	-0.70	13.23	23.10	12.26	2.27	3.43	11.74	4.15	-3.75	149.64	234.95	130.03	108.75
7.85	-1.06	12.71	22.52	11.73	1.88	3.15	11.41	4.02	-3.99	147.21	234.39	124.57	100.55
8.26	-0.71	13.26	23.02	12.30	2.34	3.50	11.86	4.19	-3.79	145.90	234.64	127.61	104.87
8.26	-0.71	13.26	23.01	12.29	2.33	3.50	11.86	4.19	-3.79	145.89	234.65	127.63	104.86
8.26	-0.71	13.26	23.01	12.29	2.33	3.50	11.86	4.19	-3.79	145.89	234.65	127.65	104.86
8.26	-0.71	13.26	23.00	12.29	2.33	3.49	11.86	4.19	-3.79	145.89	234.65	127.70	104.86
8.26	-0.71	13.25	22.99	12.29	2.33	3.49	11.85	4.19	-3.79	145.93	234.61	127.78	104.86
8.06	-0.79	12.97	22.66	12.04	2.22	3.40	11.68	4.09	-3.82	147.81	235.53	125.74	103.05
8.26	-0.73	13.52	23.29	12.37	2.40	3.38	11.77	4.13	-3.80	145.91	233.26	126.01	102.22
8.26	-0.73	13.52	23.29	12.38	2.40	3.38	11.77	4.13	-3.80	145.90	233.26	126.01	102.23
8.26	-0.73	13.51	23.28	12.36	2.39	3.39	11.77	4.13	-3.80	146.06	233.32	126.02	102.18
8.25	-0.73	13.47	23.25	12.34	2.37	3.39	11.78	4.13	-3.81	146.55	233.74	126.22	102.52
8.24	-0.72	13.38	23.21	12.28	2.31	3.46	11.79	4.21	-3.74	145.04	229.84	123.78	100.77
8.26	-0.73	13.48	23.29	12.37	2.35	3.37	11.76	4.13	-3.80	144.78	232.25	125.29	101.55
8.26	-0.73	13.47	23.29	12.36	2.34	3.37	11.75	4.13	-3.80	144.48	231.76	124.92	101.22
8.23	-0.74	13.41	23.21	12.32	2.30	3.39	11.72	4.14	-3.80	143.96	231.31	124.99	101.19
8.17	-0.80	13.15	23.02	12.20	2.20	3.37	11.69	4.14	-3.77	147.88	235.78	128.69	106.69
8.26	-0.73	13.49	23.27	12.37	2.39	3.39	11.78	4.13	-3.80	146.60	233.70	126.08	102.53
8.19	-0.78	13.27	23.20	12.37	2.28	3.32	11.61	4.04	-3.80	147.62	234.37	128.44	106.03
8.20	-0.77	13.30	23.24	12.40	2.29	3.31	11.59	4.02	-3.81	147.89	234.13	128.48	106.10
7.73	-1.23	12.61	22.33	11.78	1.93	2.84	11.10	3.62	-4.40	151.81	240.31	128.22	102.88
7.47	-1.46	12.23	21.93	11.40	1.61	2.62	10.91	3.54	-4.50	155.54	243.12	128.60	101.56

7.29	-1.35	12.02	21.62	11.48	1.95	2.26	10.46	3.12	-4.64	166.16	259.95	140.05	115.77
7.53	-1.23	12.53	22.13	11.77	2.07	2.45	10.72	3.29	-4.55	159.24	252.68	135.94	111.87
7.86	-1.07	13.01	22.58	12.27	2.32	2.71	10.95	3.45	-4.44	161.55	256.00	142.46	126.13
7.69	-1.16	12.70	22.31	11.99	2.20	2.54	10.81	3.36	-4.54	161.19	257.69	143.58	125.80
7.60	-1.24	12.63	22.22	11.91	2.17	2.44	10.71	3.29	-4.66	161.34	258.12	143.85	125.86
7.76	-1.15	12.87	22.45	12.12	2.29	2.58	10.83	3.38	-4.57	160.32	255.55	140.60	123.72
7.66	-1.19	12.76	22.33	12.00	2.23	2.54	10.78	3.35	-4.61	161.01	257.13	142.21	123.43
7.72	-1.16	12.79	22.35	12.09	2.29	2.61	10.84	3.38	-4.56	162.36	257.69	143.63	124.82
7.75	-1.14	12.83	22.38	12.12	2.31	2.64	10.87	3.41	-4.54	161.57	256.73	142.73	123.98
7.49	-1.29	12.67	22.21	11.87	2.09	2.27	10.56	3.10	-4.65	159.29	250.95	134.66	109.27
7.49	-1.29	12.67	22.20	11.87	2.09	2.26	10.56	3.10	-4.65	159.40	250.18	134.60	108.96
7.48	-1.29	12.66	22.19	11.86	2.08	2.25	10.55	3.09	-4.66	159.65	250.30	134.81	109.23
7.43	-1.31	12.57	22.10	11.79	2.04	2.24	10.51	3.08	-4.66	160.08	251.03	135.49	108.92
7.45	-1.30	12.55	22.09	11.79	2.04	2.28	10.54	3.12	-4.64	159.93	251.00	134.46	108.18
7.37	-1.31	12.25	21.79	11.60	2.01	2.30	10.55	3.13	-4.60	161.45	253.25	135.99	110.27
7.23	-1.44	12.11	21.72	11.50	1.89	2.11	10.36	2.97	-4.76	164.00	258.07	138.94	111.34
7.59	-1.22	12.71	22.30	11.90	2.11	2.46	10.76	3.29	-4.54	159.73	250.16	133.02	108.77
7.53	-1.23	12.54	22.13	11.78	2.08	2.44	10.71	3.29	-4.55	159.15	252.60	135.88	111.69
8.39	-0.63	13.66	23.37	12.58	2.59	3.49	12.01	4.19	-3.83	155.29	247.18	150.39	140.26
8.42	-0.57	13.72	23.46	12.64	2.66	3.53	12.03	4.20	-3.80	155.87	246.66	149.08	138.49
8.40	-0.62	13.69	23.40	12.60	2.61	3.50	12.02	4.20	-3.80	155.85	247.39	151.46	142.00
8.40	-0.61	13.69	23.40	12.60	2.61	3.50	12.02	4.20	-3.80	155.76	247.35	151.34	141.85
8.32	-0.65	13.62	23.27	12.68	2.62	3.28	11.74	3.95	-3.89	161.40	255.72	157.58	151.12
8.37	-0.67	13.64	23.38	12.52	2.53	3.46	11.99	4.23	-3.82	156.57	246.77	148.79	138.00
8.44	-0.55	13.78	23.49	12.67	2.69	3.53	12.03	4.20	-3.80	156.20	247.09	148.37	138.13
8.49	-0.51	13.83	23.50	12.76	2.73	3.55	12.03	4.22	-3.78	156.01	247.39	148.21	137.68
8.19	-0.78	13.32	23.01	12.36	2.31	3.31	11.65	4.04	-3.83	160.93	246.63	158.33	151.29
8.42	-0.58	13.77	23.44	12.66	2.62	3.51	11.98	4.22	-3.76	157.18	246.82	152.59	143.46
8.42	-0.58	13.77	23.44	12.66	2.62	3.51	11.98	4.22	-3.76	157.16	246.80	152.57	143.44
8.36	-0.63	13.64	23.32	12.58	2.57	3.45	11.94	4.16	-3.83	157.79	247.84	152.63	145.01
8.10	-0.77	13.11	22.89	11.99	2.18	3.50	11.78	4.22	-3.72	142.86	226.47	119.26	94.55
8.13	-0.74	13.03	22.85	11.98	2.22	3.52	11.83	4.27	-3.68	151.68	235.27	132.19	112.37
8.13	-0.74	13.03	22.86	11.99	2.22	3.50	11.83	4.27	-3.69	151.91	235.71	132.26	112.53
8.22	-0.67	13.28	23.08	12.13	2.32	3.56	11.90	4.29	-3.66	144.49	228.00	121.59	97.28
8.18	-0.72	13.18	22.98	12.08	2.26	3.57	11.86	4.28	-3.68	144.82	228.83	121.30	97.36
8.20	-0.68	13.29	23.09	12.16	2.32	3.54	11.87	4.27	-3.68	143.16	228.33	122.00	97.80
8.18	-0.73	13.21	23.03	12.11	2.27	3.53	11.85	4.26	-3.71	143.38	229.62	122.08	98.14
8.21	-0.77	13.32	23.29	12.46	2.36	3.30	11.57	4.03	-3.87	149.34	234.81	131.62	109.86
8.23	-0.74	13.19	22.98	12.30	2.32	3.48	11.81	4.15	-3.83	146.69	235.16	129.08	106.69
8.22	-0.74	13.18	22.94	12.30	2.31	3.48	11.82	4.16	-3.83	146.50	235.08	128.88	106.43
8.22	-0.74	13.18	22.94	12.30	2.31	3.49	11.83	4.16	-3.83	146.45	235.04	128.82	106.37
7.92	-0.92	12.77	22.43	11.92	2.20	3.24	11.50	3.92	-4.04	151.38	239.66	129.13	106.93
7.92	-0.92	12.77	22.43	11.92	2.20	3.23	11.50	3.91	-4.05	151.39	239.93	129.35	107.06

7.93	-0.92	12.78	22.44	11.93	2.20	3.24	11.50	3.92	-4.04	151.33	239.72	129.36	107.03
7.97	-0.90	12.76	22.46	11.94	2.19	3.30	11.55	3.99	-4.00	152.09	237.60	129.81	106.83
7.97	-0.91	12.79	22.49	11.95	2.19	3.31	11.56	4.00	-4.01	151.45	238.05	129.96	106.97
7.80	-1.01	12.56	22.19	11.77	2.17	3.15	11.37	3.85	-4.17	150.60	239.16	127.52	104.77
7.71	-1.10	12.49	22.12	11.67	2.09	3.06	11.34	3.77	-4.31	150.88	239.29	127.86	104.21
7.73	-1.03	12.45	22.09	11.68	2.14	3.04	11.27	3.78	-4.21	153.57	243.06	129.63	105.58
7.65	-1.20	12.51	22.19	11.69	1.94	2.84	11.11	3.63	-4.37	151.84	240.65	128.86	104.02
7.39	-1.32	12.36	21.90	11.65	2.01	2.29	10.55	3.12	-4.63	161.91	251.51	134.50	107.99
7.76	-1.06	12.73	22.34	11.91	2.20	2.78	11.04	3.58	-4.33	154.57	247.92	135.73	113.31
7.86	-0.98	12.81	22.42	11.97	2.22	2.97	11.25	3.76	-4.25	152.95	243.80	132.26	110.30
7.65	-1.07	12.35	22.00	11.59	2.12	2.90	11.15	3.71	-4.26	155.04	244.63	131.13	106.55
7.77	-1.07	12.73	22.37	11.96	2.20	2.78	11.06	3.59	-4.33	156.67	250.64	137.74	117.30
8.06	-0.85	12.91	22.63	12.04	2.25	3.39	11.69	4.09	-3.92	148.71	236.94	128.01	106.21
7.95	-0.89	12.74	22.44	11.92	2.16	3.31	11.55	3.99	-3.97	150.25	238.78	129.27	105.50
8.16	-0.83	13.08	22.85	12.22	2.29	3.40	11.74	4.10	-3.94	149.33	236.75	129.89	108.31
8.18	-0.83	13.11	22.87	12.25	2.32	3.40	11.73	4.10	-3.93	148.76	236.13	129.77	107.95
8.18	-0.80	13.12	22.88	12.25	2.33	3.41	11.74	4.10	-3.89	147.93	235.23	129.02	107.01
8.17	-0.79	13.12	22.88	12.22	2.33	3.43	11.75	4.11	-3.88	147.73	235.83	128.93	106.50
8.20	-0.81	13.18	22.98	12.33	2.34	3.40	11.73	4.10	-3.93	148.70	236.68	130.13	108.91
8.20	-0.81	13.18	22.98	12.33	2.34	3.40	11.73	4.10	-3.93	148.69	236.66	130.11	108.87
8.14	-0.83	13.01	22.71	12.14	2.26	3.45	11.76	4.10	-3.90	148.50	237.09	128.09	107.00
8.14	-0.83	13.01	22.72	12.14	2.26	3.45	11.76	4.10	-3.90	148.45	237.09	128.09	107.00
8.20	-0.80	13.17	22.96	12.31	2.33	3.42	11.74	4.10	-3.91	148.12	236.16	129.91	107.79
8.19	-0.80	13.15	22.94	12.30	2.34	3.40	11.71	4.09	-3.93	148.60	237.50	131.09	109.63
7.74	-1.13	12.60	22.28	11.80	2.04	2.98	11.24	3.71	-4.33	151.73	240.85	128.05	104.24
8.14	-0.83	13.02	22.75	12.16	2.27	3.41	11.74	4.10	-3.98	149.37	238.01	129.55	107.00
7.68	-1.17	12.47	22.13	11.69	2.01	2.91	11.22	3.68	-4.36	151.58	240.65	128.88	105.00
7.68	-1.17	12.47	22.13	11.69	2.01	2.91	11.21	3.67	-4.36	151.65	240.72	128.92	105.00
7.75	-1.10	12.52	22.13	11.70	2.10	3.11	11.38	3.81	-4.28	150.81	239.37	127.10	104.47
7.72	-1.10	12.50	22.12	11.67	2.09	3.07	11.34	3.78	-4.30	150.86	239.29	127.78	104.18
7.90	-0.97	12.83	22.44	12.00	2.23	3.04	11.34	3.80	-4.17	151.91	242.59	131.89	109.65
7.91	-0.97	12.84	22.45	12.00	2.24	3.03	11.34	3.80	-4.17	152.70	242.83	132.09	109.89
7.82	-1.01	12.79	22.40	11.96	2.21	2.90	11.18	3.69	-4.27	152.89	246.68	132.47	111.90
7.82	-1.01	12.79	22.40	11.96	2.21	2.90	11.18	3.69	-4.27	152.91	246.71	132.44	111.82
7.78	-1.04	12.74	22.35	11.90	2.20	2.85	11.11	3.65	-4.27	153.03	247.25	133.29	111.86
7.78	-1.04	12.74	22.35	11.90	2.20	2.85	11.11	3.65	-4.27	153.03	247.24	133.35	111.90
7.78	-1.04	12.74	22.35	11.91	2.20	2.85	11.11	3.65	-4.27	153.03	247.22	133.44	111.97
7.78	-1.04	12.74	22.35	11.91	2.20	2.85	11.11	3.65	-4.27	153.03	247.22	133.51	112.03
7.78	-1.04	12.74	22.35	11.91	2.20	2.85	11.11	3.65	-4.27	153.03	247.21	133.54	112.05
7.78	-1.04	12.74	22.35	11.91	2.20	2.85	11.11	3.65	-4.27	153.03	247.21	133.56	112.07
7.78	-1.05	12.74	22.35	11.91	2.20	2.85	11.11	3.65	-4.27	153.03	247.20	133.60	112.09
7.78	-1.05	12.74	22.35	11.91	2.20	2.85	11.11	3.65	-4.27	153.03	247.19	133.66	112.13
7.73	-1.11	12.73	22.35	11.93	2.20	2.73	11.01	3.56	-4.38	156.58	250.30	137.36	115.98

7.68	-1.08	12.40	22.03	11.62	2.12	2.94	11.21	3.75	-4.27	153.48	243.19	130.01	105.09
7.60	-1.20	12.40	22.07	11.60	1.93	2.80	11.05	3.61	-4.38	152.11	242.62	129.45	104.01
8.09	-0.86	13.03	22.75	12.16	2.29	3.34	11.68	4.02	-4.04	150.08	238.92	129.38	107.35
8.09	-0.86	13.02	22.75	12.16	2.29	3.35	11.68	4.02	-4.04	150.10	238.91	129.36	107.32
7.54	-1.22	12.39	22.02	11.71	2.05	2.52	10.77	3.37	-4.50	160.25	255.39	138.60	116.24
8.15	-0.80	13.04	22.77	12.15	2.29	3.45	11.76	4.14	-3.86	148.31	235.66	128.43	106.13
7.59	-1.23	12.73	22.31	11.89	2.10	2.43	10.74	3.29	-4.57	158.76	247.81	132.90	107.79
7.98	-0.96	13.09	22.83	12.26	2.26	2.93	11.23	3.72	-4.14	154.73	246.01	136.42	118.89
8.23	-0.65	13.42	22.92	12.64	2.63	3.06	11.37	3.84	-3.92	164.42	257.94	154.89	145.19
8.15	-0.80	13.04	22.77	12.15	2.29	3.44	11.76	4.13	-3.86	148.34	235.67	128.44	106.09
8.16	-0.79	13.04	22.77	12.15	2.29	3.46	11.77	4.15	-3.86	148.20	235.63	128.34	106.14
8.16	-0.77	13.07	22.82	12.19	2.30	3.44	11.76	4.12	-3.85	148.15	235.77	128.22	105.76
8.27	-0.73	13.24	23.06	12.33	2.35	3.50	11.84	4.16	-3.82	146.09	234.28	128.05	105.81
7.45	-1.31	12.48	22.07	11.68	1.97	2.35	10.63	3.21	-4.60	157.18	248.25	132.19	105.64
8.16	-0.78	13.06	22.81	12.18	2.30	3.44	11.76	4.11	-3.86	148.22	235.74	128.35	106.00
7.92	-0.99	12.72	22.41	11.79	1.83	3.13	11.39	4.08	-3.85	142.02	225.32	116.07	89.72
7.38	-1.38	11.91	21.62	11.07	1.48	2.50	10.82	3.65	-4.25	145.12	231.14	123.43	99.04
7.85	-1.04	12.54	22.28	11.50	1.76	3.19	11.44	4.17	-3.80	142.86	228.67	118.09	90.58
7.23	-1.51	11.69	21.46	10.86	1.29	2.54	10.84	3.62	-4.33	150.88	238.67	127.03	103.71
7.24	-1.50	11.71	21.47	10.87	1.30	2.55	10.85	3.63	-4.32	150.75	238.49	126.85	103.52
8.17	-0.68	12.67	22.44	11.88	2.01	3.42	11.51	4.46	-3.35	129.89	217.32	105.83	75.48
7.69	-1.16	11.89	21.65	11.18	1.45	3.10	11.14	4.18	-3.77	131.53	224.23	108.84	78.44
7.81	-0.97	12.13	21.95	11.45	1.68	3.09	11.15	4.16	-3.64	131.62	220.11	108.92	78.72
7.25	-1.52	11.70	21.51	10.88	1.30	2.55	10.86	3.63	-4.32	151.16	241.28	128.51	104.56
7.39	-1.39	11.89	21.68	11.01	1.38	2.74	11.02	3.77	-4.15	145.83	234.33	121.82	95.81
8.07	-0.82	12.89	22.60	11.88	2.00	3.32	11.58	4.27	-3.61	137.80	223.04	112.85	84.93
7.46	-1.34	11.99	21.77	11.09	1.42	2.81	11.09	3.83	-4.11	145.13	233.25	120.87	94.43
7.53	-1.24	12.05	21.83	11.08	1.52	2.94	11.17	3.93	-4.00	143.05	230.75	119.04	91.39
7.53	-1.25	12.04	21.83	11.08	1.52	2.94	11.17	3.93	-4.00	143.08	230.75	119.09	91.45
7.25	-1.49	11.75	21.46	10.85	1.29	2.58	10.84	3.64	-4.29	148.89	237.41	125.02	98.61
7.57	-1.24	12.22	21.96	11.23	1.57	2.92	11.21	3.96	-4.02	146.13	232.12	121.40	96.48
7.52	-1.28	12.16	21.90	11.17	1.53	2.87	11.16	3.91	-4.06	146.75	233.04	121.99	97.34
7.82	-1.14	12.60	22.44	11.62	1.70	3.10	11.38	4.02	-3.96	143.59	232.42	120.59	95.75
7.52	-1.28	12.11	21.87	11.17	1.52	2.87	11.15	3.90	-4.07	147.66	233.71	122.34	97.94
7.21	-1.53	11.63	21.40	10.81	1.28	2.52	10.84	3.63	-4.33	151.35	240.00	127.99	104.36
7.24	-1.51	11.71	21.48	10.87	1.30	2.54	10.85	3.62	-4.33	150.79	238.50	126.66	103.44
7.25	-1.50	11.71	21.48	10.87	1.30	2.55	10.86	3.63	-4.32	150.73	238.44	126.75	103.46
7.25	-1.50	11.72	21.48	10.87	1.30	2.55	10.86	3.63	-4.31	150.68	238.39	126.74	103.41
7.25	-1.50	11.72	21.48	10.87	1.31	2.55	10.86	3.63	-4.31	150.66	238.35	126.68	103.36
7.18	-1.57	11.66	21.42	10.81	1.26	2.47	10.78	3.56	-4.39	151.60	239.53	127.39	104.19
7.14	-1.59	11.48	21.31	10.69	1.21	2.47	10.78	3.57	-4.40	152.71	243.83	130.07	106.69
7.45	-1.32	11.95	21.65	11.06	1.51	2.71	10.98	3.81	-4.17	146.93	233.19	124.16	98.96
7.24	-1.52	11.64	21.38	10.85	1.33	2.48	10.77	3.60	-4.33	150.82	237.86	127.41	103.16

7.47	-1.37	12.02	21.76	11.19	1.47	2.72	10.99	3.73	-4.19	147.13	233.10	124.51	99.88
7.66	-1.21	12.42	22.05	11.46	1.67	2.92	11.22	3.88	-4.04	148.01	232.17	126.23	104.55
7.46	-1.32	12.04	21.68	11.12	1.48	2.75	11.07	3.78	-4.11	151.35	234.69	129.96	109.83
7.45	-1.33	12.02	21.67	11.10	1.47	2.74	11.05	3.77	-4.12	151.61	234.88	130.21	110.15
7.38	-1.38	11.99	21.63	11.11	1.44	2.64	10.96	3.66	-4.22	152.89	237.11	131.21	111.48
7.39	-1.37	12.04	21.71	11.16	1.47	2.67	10.95	3.66	-4.24	153.27	236.41	131.02	110.49
7.44	-1.41	12.10	21.77	11.20	1.46	2.65	11.00	3.68	-4.26	151.67	237.18	130.04	109.44
7.40	-1.43	12.05	21.73	11.16	1.44	2.62	10.98	3.66	-4.28	152.59	237.45	130.75	109.74
7.59	-1.24	12.28	21.96	11.34	1.59	2.85	11.16	3.83	-4.11	147.61	233.96	126.93	104.53
6.72	-2.08	10.97	20.69	10.37	0.83	1.89	10.15	3.06	-5.00	166.94	256.31	139.70	110.91
7.17	-1.78	11.60	21.34	10.85	1.03	2.44	10.70	3.47	-4.61	151.52	240.95	127.09	93.83
6.78	-2.00	10.89	20.66	10.34	0.80	2.12	10.37	3.21	-4.79	162.88	253.06	135.24	103.30
6.65	-2.13	10.85	20.54	10.26	0.74	1.87	10.12	3.03	-5.00	165.05	257.04	140.20	110.92
7.27	-1.72	11.75	21.54	11.00	1.07	2.53	10.79	3.53	-4.53	149.54	238.88	123.97	90.89
7.21	-1.86	11.81	21.54	10.98	0.99	2.38	10.68	3.45	-4.70	149.21	241.35	127.12	96.64
7.32	-1.66	11.91	21.60	11.04	1.18	2.58	10.91	3.60	-4.52	150.44	240.46	126.34	96.22
7.33	-1.59	11.88	21.59	11.09	1.40	2.53	10.79	3.53	-4.58	154.32	243.71	128.47	100.65
7.70	-1.30	12.70	22.48	11.79	1.73	2.81	11.14	3.67	-4.35	148.41	238.22	124.15	97.34
7.71	-1.30	12.76	22.54	11.83	1.78	2.76	11.09	3.63	-4.39	147.18	236.68	124.05	97.82
7.73	-1.30	12.77	22.55	11.84	1.78	2.77	11.10	3.63	-4.39	147.39	236.59	123.87	97.99
7.57	-1.40	12.41	22.18	11.54	1.65	2.76	11.07	3.64	-4.40	150.83	240.99	126.20	101.21
7.39	-1.51	12.06	21.85	11.20	1.44	2.59	10.89	3.56	-4.47	153.19	244.87	130.41	104.36
7.45	-1.52	12.20	21.92	11.33	1.46	2.68	10.96	3.62	-4.50	150.57	239.36	125.18	97.46
7.33	-1.56	11.96	21.77	11.13	1.39	2.55	10.85	3.53	-4.49	154.37	246.33	130.70	105.40
7.43	-1.47	12.13	21.90	11.32	1.54	2.61	10.92	3.53	-4.47	153.12	245.11	130.20	106.45
7.41	-1.55	12.11	21.84	11.27	1.42	2.64	10.94	3.60	-4.51	151.30	240.15	125.63	98.01
7.54	-1.39	12.31	22.09	11.38	1.53	2.78	11.05	3.73	-4.30	153.09	240.08	126.60	101.32
7.17	-1.67	11.68	21.51	10.91	1.24	2.38	10.69	3.43	-4.57	156.82	249.65	133.36	109.16
7.37	-1.50	12.03	21.83	11.12	1.40	2.62	10.92	3.62	-4.39	153.45	241.67	128.08	103.49
7.34	-1.54	11.96	21.76	11.06	1.36	2.58	10.88	3.60	-4.42	154.25	242.28	128.74	103.98
7.19	-1.65	11.65	21.45	10.84	1.20	2.51	10.79	3.55	-4.52	154.44	244.24	130.58	103.38
7.32	-1.56	11.83	21.64	10.98	1.29	2.63	10.91	3.64	-4.43	153.11	241.16	127.30	100.19
7.79	-1.16	12.63	22.41	11.75	1.83	3.01	11.32	3.85	-4.19	149.41	238.95	126.33	101.16
7.67	-1.35	12.59	22.37	11.70	1.69	2.76	11.08	3.60	-4.39	149.72	239.64	126.86	100.94
7.63	-1.38	12.54	22.32	11.68	1.67	2.71	11.02	3.56	-4.42	151.76	241.62	126.78	102.27
7.28	-1.64	11.87	21.57	11.08	1.37	2.47	10.77	3.49	-4.62	154.84	246.44	129.33	101.56
7.27	-1.64	11.86	21.56	11.07	1.37	2.46	10.76	3.48	-4.63	155.16	246.73	129.54	101.83
7.37	-1.57	12.06	21.77	11.22	1.43	2.57	10.89	3.56	-4.57	153.07	242.24	126.98	99.89
7.37	-1.58	12.06	21.77	11.22	1.42	2.57	10.88	3.56	-4.57	153.10	242.26	127.00	99.91
7.49	-1.49	12.30	22.01	11.39	1.50	2.67	10.97	3.58	-4.52	150.25	239.49	125.56	97.59
7.60	-1.43	12.45	22.30	11.53	1.58	2.79	11.10	3.67	-4.41	147.69	237.21	123.14	95.53
7.45	-1.55	12.23	21.99	11.27	1.37	2.67	10.96	3.63	-4.46	147.86	235.86	123.76	95.19
7.19	-1.80	11.73	21.49	10.91	1.10	2.44	10.74	3.48	-4.67	151.13	241.98	127.41	98.59

7.20	-1.77	11.73	21.49	10.90	1.10	2.46	10.77	3.49	-4.65	151.28	242.56	127.76	98.39
7.20	-1.76	11.71	21.48	10.89	1.11	2.47	10.79	3.50	-4.64	151.14	242.94	127.94	98.49
7.30	-1.68	11.86	21.56	11.02	1.18	2.58	10.90	3.59	-4.54	150.11	241.57	127.13	97.23
7.21	-1.79	11.75	21.46	10.92	1.07	2.43	10.75	3.48	-4.65	151.66	243.19	128.56	98.78
7.33	-1.64	11.92	21.64	11.04	1.21	2.59	10.91	3.62	-4.52	149.69	241.08	125.63	97.17
7.22	-1.81	11.78	21.49	10.94	1.07	2.42	10.74	3.48	-4.66	151.69	242.71	128.02	98.44
6.99	-2.01	11.51	21.26	10.72	0.87	2.11	10.40	3.24	-4.89	153.75	246.74	130.48	101.25
7.03	-1.96	11.60	21.34	10.78	0.91	2.16	10.46	3.29	-4.85	153.04	245.85	129.53	100.71
7.07	-1.95	11.62	21.38	10.83	0.92	2.20	10.52	3.33	-4.81	152.63	245.44	128.78	99.41
7.35	-1.61	11.92	21.63	11.11	1.33	2.62	10.94	3.62	-4.56	151.03	241.24	125.98	98.57
7.35	-1.60	11.92	21.63	11.11	1.33	2.63	10.94	3.63	-4.55	150.93	241.23	125.92	98.55
7.28	-1.66	11.86	21.57	11.01	1.23	2.51	10.81	3.56	-4.56	151.79	241.72	127.10	98.33
7.07	-1.85	11.52	21.25	10.71	0.95	2.32	10.61	3.44	-4.68	153.55	244.05	129.69	99.29
6.81	-1.97	10.96	20.70	10.32	0.83	2.15	10.39	3.31	-4.79	157.98	250.89	133.08	103.36
6.77	-2.02	10.91	20.64	10.25	0.78	2.08	10.32	3.28	-4.82	158.66	251.23	133.69	103.91
6.67	-2.12	10.90	20.59	10.29	0.78	1.88	10.12	3.05	-5.00	166.37	256.23	140.05	111.41
6.82	-1.99	11.22	20.94	10.56	0.98	1.93	10.23	3.08	-4.98	164.12	256.15	138.38	110.69
6.81	-2.04	11.19	20.94	10.59	0.96	1.86	10.12	2.98	-5.04	165.03	255.69	138.30	110.46
7.23	-1.66	11.82	21.52	10.89	1.20	2.53	10.80	3.58	-4.53	152.60	240.44	125.95	98.21
7.21	-1.68	11.78	21.46	10.84	1.18	2.51	10.79	3.56	-4.53	151.72	240.40	125.44	97.77
7.10	-1.88	11.61	21.34	10.81	0.96	2.39	10.60	3.42	-4.71	150.10	241.96	126.07	97.36
7.55	-1.31	12.35	22.14	11.34	1.55	2.85	11.12	3.77	-4.19	145.81	233.28	119.75	92.26
7.16	-1.80	11.73	21.43	10.85	1.07	2.42	10.66	3.46	-4.66	151.55	241.60	126.95	99.09
7.39	-1.57	12.12	21.94	11.17	1.33	2.55	10.78	3.63	-4.42	146.48	233.34	120.23	91.23
7.09	-1.74	11.36	21.09	10.57	0.98	2.46	10.63	3.62	-4.46	147.75	239.90	124.41	94.18
7.29	-1.66	11.88	21.60	11.00	1.19	2.54	10.76	3.61	-4.49	147.26	235.71	123.11	92.39
7.15	-1.76	11.61	21.34	10.78	1.07	2.40	10.62	3.51	-4.60	148.94	240.15	125.69	95.26
7.01	-1.82	11.48	21.22	10.63	1.04	2.26	10.53	3.38	-4.64	153.71	244.06	128.14	101.38
7.55	-1.33	12.34	22.14	11.33	1.58	2.84	11.11	3.76	-4.20	145.62	232.50	119.15	92.78
7.63	-1.18	12.31	22.06	11.33	1.62	3.02	11.28	3.94	-3.97	144.00	231.36	118.95	91.54
7.77	-1.10	12.63	22.45	11.57	1.75	3.12	11.37	3.99	-3.96	141.25	228.78	117.03	87.49
6.93	-1.93	11.35	21.04	10.54	0.92	2.17	10.45	3.32	-4.79	157.46	248.19	131.18	104.32
7.06	-1.87	11.53	21.22	10.69	0.95	2.28	10.45	3.44	-4.69	148.54	239.14	124.68	93.29
7.37	-1.47	12.03	21.77	11.14	1.37	2.58	10.88	3.56	-4.33	146.71	237.40	123.74	98.55
7.30	-1.55	11.93	21.69	11.01	1.32	2.57	10.87	3.59	-4.42	155.84	243.22	127.79	103.05
7.40	-1.45	12.09	21.81	11.17	1.40	2.62	10.92	3.61	-4.30	146.42	236.42	123.34	97.66
7.48	-1.41	12.16	21.96	11.22	1.46	2.75	11.03	3.70	-4.30	151.70	238.33	124.87	100.62
7.20	-1.64	11.67	21.49	10.84	1.20	2.52	10.80	3.54	-4.47	155.75	244.42	128.96	103.94
6.96	-1.82	11.39	21.14	10.56	1.01	2.24	10.52	3.35	-4.66	158.00	246.35	129.83	104.45
7.11	-1.72	11.59	21.33	10.73	1.13	2.43	10.72	3.53	-4.56	155.10	242.18	126.56	100.63
7.12	-1.72	11.62	21.34	10.74	1.11	2.44	10.69	3.52	-4.56	151.41	242.53	125.48	98.30
7.02	-1.79	11.46	21.20	10.61	1.02	2.33	10.58	3.45	-4.64	152.81	245.94	127.40	100.66
7.15	-1.72	11.66	21.35	10.75	1.12	2.47	10.71	3.52	-4.56	152.00	242.57	125.60	99.12

7.20	-1.69	11.77	21.46	10.84	1.16	2.49	10.76	3.54	-4.56	151.59	240.37	125.78	98.32
7.43	-1.46	12.04	21.85	11.14	1.39	2.73	11.01	3.71	-4.31	152.24	240.21	125.83	100.46
7.23	-1.65	11.85	21.54	10.91	1.20	2.54	10.81	3.58	-4.53	152.07	240.15	125.40	97.95
7.16	-1.74	11.76	21.44	10.82	1.13	2.41	10.71	3.48	-4.62	153.14	242.41	126.48	100.38
7.15	-1.75	11.75	21.43	10.81	1.12	2.40	10.70	3.47	-4.63	153.18	242.74	126.53	100.60
6.92	-1.94	11.34	21.03	10.53	0.91	2.16	10.44	3.31	-4.79	157.58	248.32	131.28	104.51
6.93	-1.93	11.34	21.03	10.54	0.91	2.18	10.45	3.32	-4.79	157.42	248.28	131.18	104.21
7.01	-1.87	11.48	21.17	10.64	0.98	2.28	10.54	3.40	-4.73	155.77	246.34	129.82	102.08
7.04	-1.86	11.46	21.17	10.64	0.99	2.33	10.56	3.43	-4.71	154.88	245.86	128.80	101.03
6.96	-1.91	11.34	21.05	10.54	0.93	2.24	10.47	3.36	-4.76	156.29	247.31	130.21	102.99
6.96	-1.91	11.34	21.05	10.54	0.93	2.24	10.47	3.36	-4.76	156.26	247.29	130.19	102.97
6.91	-1.94	11.30	21.00	10.51	0.90	2.17	10.43	3.31	-4.80	157.34	248.33	131.20	104.24
7.08	-1.90	11.59	21.31	10.78	0.95	2.36	10.58	3.40	-4.73	150.54	242.26	126.33	97.98
7.06	-1.92	11.54	21.27	10.75	0.94	2.34	10.55	3.39	-4.74	150.93	242.95	126.52	98.55
7.07	-1.88	11.50	21.22	10.72	0.94	2.38	10.58	3.44	-4.69	151.57	242.98	125.95	97.87
6.88	-2.09	11.30	20.99	10.53	0.74	2.04	10.29	3.21	-4.91	155.60	248.07	130.27	100.45
7.23	-1.82	11.90	21.61	11.01	1.08	2.43	10.69	3.44	-4.71	148.93	241.05	124.45	95.89
6.93	-2.08	11.44	21.16	10.63	0.76	2.06	10.34	3.21	-4.93	154.09	247.75	130.02	100.75
6.90	-2.10	11.37	21.07	10.58	0.73	2.03	10.30	3.21	-4.94	154.59	248.52	130.51	101.61
7.67	-1.24	12.40	22.20	11.37	1.55	3.00	11.21	3.99	-4.04	140.04	228.34	116.07	86.32
7.78	-1.16	12.54	22.36	11.52	1.67	3.11	11.33	4.05	-3.95	137.97	226.76	113.61	83.97
7.71	-1.18	12.44	22.30	11.43	1.61	3.07	11.32	4.00	-3.95	138.98	227.65	115.03	86.21
7.69	-1.20	12.49	22.36	11.48	1.60	3.01	11.26	3.91	-4.01	139.94	230.49	116.71	87.90
7.64	-1.24	12.42	22.26	11.41	1.56	2.95	11.22	3.86	-4.06	141.27	231.08	117.45	89.07
7.37	-1.49	12.01	21.81	11.08	1.34	2.64	10.90	3.64	-4.32	146.45	235.53	121.08	93.81
7.54	-1.38	12.28	22.07	11.27	1.45	2.80	11.05	3.78	-4.20	144.86	231.18	119.07	90.00
7.36	-1.50	11.99	21.77	11.05	1.32	2.65	10.89	3.65	-4.32	147.63	236.41	121.33	94.37
7.36	-1.51	11.99	21.77	11.05	1.32	2.64	10.89	3.65	-4.32	147.64	236.44	121.36	94.39
7.29	-1.56	11.91	21.70	11.04	1.29	2.52	10.85	3.57	-4.43	154.74	241.67	128.31	104.37
7.72	-1.08	12.41	22.37	11.43	1.71	3.13	11.36	4.01	-3.86	140.02	228.32	115.85	86.53
7.89	-0.95	12.56	22.52	11.58	1.82	3.32	11.51	4.17	-3.71	134.91	224.47	113.07	82.62
7.88	-0.96	12.56	22.52	11.57	1.81	3.30	11.49	4.15	-3.73	135.23	224.06	113.04	82.84
7.87	-0.97	12.56	22.51	11.57	1.81	3.29	11.48	4.15	-3.73	135.44	224.14	113.16	82.96
7.88	-0.97	12.57	22.52	11.58	1.81	3.30	11.49	4.16	-3.72	135.01	223.78	112.74	82.67
7.27	-1.55	11.88	21.61	11.02	1.30	2.51	10.81	3.54	-4.39	148.71	239.88	126.21	100.91
7.32	-1.48	11.93	21.66	11.04	1.36	2.60	10.89	3.59	-4.30	148.57	237.23	123.43	97.97
7.54	-1.42	12.15	21.97	11.25	1.40	2.84	11.00	3.85	-4.24	140.11	229.35	116.10	86.14
7.63	-1.33	12.36	22.17	11.40	1.50	2.90	11.09	3.90	-4.18	140.01	227.94	115.53	84.86
7.69	-1.28	12.50	22.32	11.48	1.59	2.93	11.14	3.91	-4.16	140.22	227.31	114.87	84.59
7.18	-1.80	11.64	21.34	10.78	0.98	2.36	10.51	3.57	-4.59	142.76	234.26	121.51	90.61
7.16	-1.81	11.62	21.32	10.77	0.99	2.32	10.48	3.56	-4.59	146.13	235.50	121.44	92.04
6.83	-2.06	11.19	20.89	10.43	0.70	2.00	10.20	3.30	-4.86	149.55	241.40	126.86	96.13
7.33	-1.61	11.85	21.63	10.98	1.20	2.56	10.75	3.69	-4.44	144.86	234.49	120.48	91.02

7.33	-1.61	11.88	21.66	10.99	1.21	2.54	10.74	3.66	-4.45	145.12	234.75	120.73	91.52
7.35	-1.60	11.89	21.68	11.01	1.22	2.56	10.75	3.68	-4.44	144.32	234.21	120.34	90.88
7.51	-1.40	12.23	22.00	11.23	1.44	2.79	10.99	3.80	-4.24	143.16	231.16	118.84	89.10
7.45	-1.46	12.16	21.93	11.16	1.38	2.68	10.90	3.71	-4.31	145.06	233.57	120.31	90.77
7.37	-1.52	12.09	21.86	11.12	1.35	2.57	10.80	3.64	-4.38	146.34	234.07	121.56	92.20
7.37	-1.51	12.10	21.86	11.13	1.36	2.57	10.82	3.66	-4.38	146.18	233.99	121.65	91.67
7.35	-1.58	12.02	21.76	11.09	1.31	2.57	10.79	3.61	-4.43	146.52	235.68	122.25	92.76
7.48	-1.48	12.16	21.94	11.23	1.39	2.68	10.92	3.72	-4.34	144.06	233.42	119.67	89.93
7.47	-1.49	12.15	21.92	11.22	1.39	2.67	10.91	3.71	-4.35	144.09	233.55	119.88	90.11
7.47	-1.49	12.15	21.92	11.22	1.39	2.67	10.91	3.71	-4.35	144.08	233.56	119.90	90.12
7.47	-1.49	12.15	21.92	11.22	1.39	2.67	10.91	3.71	-4.35	144.09	233.56	119.89	90.12
7.47	-1.50	12.15	21.92	11.22	1.38	2.66	10.91	3.70	-4.36	144.07	233.61	119.99	90.21
7.39	-1.57	12.07	21.86	11.12	1.30	2.56	10.78	3.66	-4.41	146.40	234.28	120.09	91.11
7.30	-1.61	11.82	21.58	10.92	1.18	2.54	10.75	3.67	-4.44	146.36	236.41	122.04	91.20
7.31	-1.60	11.83	21.60	10.93	1.18	2.54	10.76	3.67	-4.43	146.23	236.23	122.01	91.07
7.44	-1.52	12.18	21.96	11.21	1.35	2.59	10.84	3.67	-4.40	144.49	233.14	119.38	89.60
7.33	-1.61	11.97	21.71	11.07	1.24	2.52	10.73	3.58	-4.47	145.95	234.94	122.42	92.40
7.30	-1.64	11.92	21.66	11.04	1.22	2.49	10.70	3.56	-4.50	146.53	235.80	122.99	93.03
7.30	-1.65	11.90	21.66	11.03	1.21	2.48	10.69	3.56	-4.50	146.54	236.14	122.97	93.21
7.28	-1.66	11.89	21.63	11.01	1.20	2.47	10.68	3.55	-4.51	146.88	236.27	123.34	93.39
7.33	-1.59	11.92	21.70	11.01	1.21	2.54	10.75	3.64	-4.45	145.80	235.25	121.67	91.30
7.10	-1.86	11.54	21.23	10.71	0.95	2.32	10.50	3.51	-4.63	147.34	238.24	123.75	91.90
6.98	-1.93	11.38	21.10	10.59	0.88	2.19	10.38	3.39	-4.73	148.45	240.66	125.82	94.81
7.06	-1.87	11.52	21.22	10.69	0.95	2.27	10.45	3.44	-4.70	148.55	239.24	124.75	93.36
7.06	-1.87	11.54	21.23	10.70	0.96	2.28	10.46	3.44	-4.69	148.84	239.05	124.70	93.23
7.06	-1.87	11.53	21.22	10.69	0.95	2.28	10.45	3.44	-4.69	148.63	239.17	124.70	93.28
7.06	-1.87	11.53	21.22	10.69	0.95	2.28	10.45	3.44	-4.69	148.53	239.12	124.67	93.29
7.01	-1.91	11.43	21.15	10.62	0.90	2.22	10.39	3.40	-4.73	148.09	240.06	125.56	94.59
7.08	-1.87	11.53	21.25	10.70	0.95	2.27	10.46	3.43	-4.69	147.96	238.74	124.74	93.24
7.06	-1.87	11.51	21.23	10.68	0.94	2.26	10.45	3.43	-4.70	148.00	239.05	124.90	93.52
7.11	-1.82	11.64	21.34	10.78	1.01	2.33	10.51	3.46	-4.66	148.25	237.34	123.64	92.36
7.12	-1.82	11.65	21.35	10.79	1.02	2.33	10.52	3.46	-4.66	148.21	237.11	123.57	92.21
6.80	-2.00	11.09	20.77	10.36	0.78	2.04	10.27	3.28	-4.82	153.57	245.99	130.75	99.98
6.86	-1.97	11.16	20.86	10.41	0.80	2.09	10.31	3.33	-4.79	152.76	244.51	129.31	98.55
6.86	-1.97	11.16	20.86	10.42	0.80	2.09	10.32	3.33	-4.79	152.74	244.46	129.28	98.46
6.86	-1.97	11.16	20.86	10.42	0.80	2.09	10.32	3.33	-4.79	152.72	244.42	129.26	98.40
6.87	-1.97	11.18	20.88	10.43	0.81	2.10	10.32	3.33	-4.79	152.60	244.28	129.12	98.12
6.94	-1.93	11.32	21.00	10.52	0.86	2.14	10.36	3.35	-4.75	152.24	242.61	127.99	96.83
6.84	-1.98	11.15	20.83	10.40	0.81	2.07	10.30	3.30	-4.80	153.34	245.17	129.92	99.09
6.84	-1.99	11.15	20.83	10.40	0.83	2.07	10.30	3.30	-4.81	153.95	245.63	130.37	99.48
6.89	-1.95	11.24	20.94	10.48	0.86	2.12	10.35	3.32	-4.76	152.79	244.98	129.33	98.19
7.15	-1.73	11.66	21.40	10.80	1.08	2.36	10.57	3.48	-4.58	148.50	238.48	124.84	94.79
7.16	-1.72	11.69	21.42	10.83	1.09	2.36	10.58	3.49	-4.57	148.21	238.16	124.62	94.59

7.27	-1.65	11.85	21.58	10.98	1.19	2.49	10.71	3.57	-4.51	148.44	236.35	124.45	93.14
7.38	-1.58	12.03	21.77	11.11	1.27	2.58	10.81	3.64	-4.43	146.21	233.66	121.63	91.12
7.42	-1.55	12.09	21.83	11.16	1.31	2.60	10.84	3.67	-4.41	145.56	232.74	121.01	90.35
7.17	-1.75	11.64	21.37	10.82	1.08	2.43	10.66	3.53	-4.58	149.15	240.47	125.24	94.76
7.00	-1.90	11.37	21.13	10.63	0.94	2.24	10.49	3.39	-4.71	150.70	244.71	127.64	99.04
7.06	-1.84	11.48	21.21	10.68	0.99	2.31	10.55	3.45	-4.67	149.82	243.05	126.46	96.55
6.82	-2.07	11.12	20.83	10.41	0.76	2.00	10.26	3.21	-4.89	156.11	249.37	130.56	102.00
7.11	-1.85	11.67	21.34	10.80	1.01	2.30	10.51	3.42	-4.70	148.22	238.49	124.01	92.58
6.98	-1.95	11.37	21.09	10.59	0.85	2.22	10.48	3.36	-4.77	153.31	245.17	128.00	99.28
7.69	-1.10	12.30	22.09	11.31	1.66	3.15	11.37	4.06	-3.85	141.81	229.23	117.08	88.12
7.43	-1.32	11.99	21.84	11.10	1.47	2.80	11.06	3.75	-4.14	144.21	233.88	121.48	93.13
7.41	-1.43	12.13	21.88	11.15	1.45	2.67	11.00	3.67	-4.32	149.10	236.98	122.45	96.68
7.35	-1.48	12.03	21.80	11.09	1.39	2.61	10.94	3.62	-4.35	149.53	238.22	123.48	97.98
7.35	-1.49	12.03	21.80	11.09	1.39	2.60	10.93	3.62	-4.35	149.61	238.31	123.55	98.04
7.40	-1.43	12.09	21.88	11.13	1.43	2.66	10.98	3.66	-4.33	149.28	237.06	122.69	97.05
7.37	-1.45	11.97	21.80	11.06	1.40	2.68	10.98	3.69	-4.30	151.17	236.94	124.54	98.73
7.52	-1.33	12.29	22.06	11.28	1.52	2.83	11.10	3.77	-4.20	147.24	232.90	119.71	94.30
7.42	-1.42	12.14	21.95	11.19	1.46	2.73	10.99	3.70	-4.27	148.71	234.64	121.30	95.78
7.27	-1.57	11.89	21.70	11.00	1.28	2.53	10.81	3.55	-4.41	149.39	238.35	124.31	97.34
7.77	-1.13	12.65	22.49	11.61	1.72	3.09	11.36	3.94	-3.96	139.55	228.62	115.92	87.03
7.45	-1.40	12.18	21.96	11.19	1.46	2.77	11.03	3.74	-4.25	147.91	234.65	120.88	94.64
7.17	-1.65	11.79	21.43	10.91	1.22	2.30	10.70	3.43	-4.52	158.50	241.29	137.69	120.76
7.19	-1.63	11.82	21.46	10.94	1.24	2.33	10.72	3.46	-4.50	158.21	240.90	137.10	120.18
7.07	-1.68	11.61	21.27	10.79	1.19	2.27	10.62	3.34	-4.53	160.57	243.54	138.43	119.85
7.08	-1.75	11.67	21.33	10.84	1.18	2.24	10.59	3.30	-4.62	161.37	243.17	138.38	120.93
7.08	-1.75	11.67	21.33	10.84	1.18	2.24	10.59	3.30	-4.62	161.31	243.10	138.31	120.89
7.24	-1.64	11.91	21.56	11.03	1.28	2.40	10.74	3.43	-4.53	158.91	240.49	136.33	118.36
7.21	-1.65	11.86	21.50	10.98	1.25	2.39	10.75	3.42	-4.55	157.90	241.13	136.34	117.00
7.29	-1.60	12.03	21.68	11.12	1.32	2.45	10.82	3.47	-4.50	157.94	239.57	134.96	116.39
7.30	-1.59	12.03	21.69	11.13	1.32	2.46	10.83	3.47	-4.49	157.87	239.45	134.91	116.32
7.32	-1.56	12.07	21.70	11.17	1.36	2.48	10.81	3.47	-4.49	157.72	239.14	135.67	116.76
7.33	-1.55	12.12	21.73	11.21	1.35	2.48	10.83	3.50	-4.48	158.22	239.74	136.76	118.60
7.36	-1.53	12.15	21.77	11.24	1.38	2.52	10.87	3.52	-4.45	157.68	238.82	135.89	117.74
7.36	-1.53	12.16	21.77	11.24	1.38	2.52	10.87	3.52	-4.45	157.55	238.73	135.75	117.66
7.37	-1.53	12.16	21.77	11.25	1.38	2.53	10.87	3.52	-4.45	157.53	238.69	135.71	117.61
7.36	-1.54	12.15	21.76	11.24	1.37	2.51	10.86	3.51	-4.46	157.77	239.07	136.09	118.02
7.36	-1.54	12.15	21.76	11.24	1.37	2.51	10.86	3.51	-4.47	157.87	239.41	136.42	118.38
7.43	-1.49	12.23	21.85	11.31	1.42	2.58	10.95	3.54	-4.44	156.80	238.30	135.68	117.57
7.47	-1.47	12.28	21.90	11.34	1.45	2.62	10.99	3.57	-4.42	156.33	237.54	135.25	117.00
7.42	-1.49	12.23	21.85	11.31	1.43	2.59	10.94	3.55	-4.43	156.20	238.20	134.45	116.70
7.46	-1.46	12.31	21.92	11.36	1.48	2.63	10.98	3.58	-4.40	155.20	237.76	133.77	115.69
7.31	-1.63	12.05	21.65	11.16	1.31	2.38	10.76	3.42	-4.54	156.25	241.00	139.16	122.07
7.37	-1.60	12.14	21.74	11.22	1.35	2.45	10.84	3.49	-4.51	156.22	240.58	139.09	121.57

7.38	-1.60	12.15	21.75	11.22	1.35	2.45	10.84	3.50	-4.51	156.24	240.49	138.97	121.47
7.35	-1.61	12.11	21.71	11.20	1.33	2.42	10.81	3.47	-4.51	156.00	240.65	138.55	121.45
7.30	-1.63	12.08	21.67	11.16	1.32	2.41	10.78	3.43	-4.53	158.12	239.89	138.20	120.70
7.26	-1.66	12.03	21.61	11.12	1.28	2.36	10.73	3.39	-4.56	158.75	240.73	138.95	121.53
7.26	-1.66	12.03	21.61	11.12	1.28	2.36	10.73	3.39	-4.56	158.77	240.75	138.96	121.55
7.27	-1.65	12.04	21.63	11.13	1.29	2.37	10.74	3.40	-4.56	158.61	240.54	138.79	121.34
7.30	-1.62	12.09	21.67	11.18	1.31	2.42	10.79	3.43	-4.53	158.05	239.93	138.37	120.75
7.36	-1.58	12.16	21.76	11.24	1.36	2.49	10.86	3.49	-4.48	157.23	238.60	137.47	119.70
7.35	-1.58	12.15	21.74	11.23	1.35	2.48	10.85	3.48	-4.49	157.31	238.84	137.66	119.89
7.41	-1.53	12.21	21.81	11.27	1.39	2.54	10.92	3.52	-4.46	157.18	239.10	136.22	118.40
7.28	-1.63	12.06	21.64	11.16	1.29	2.39	10.76	3.41	-4.54	158.33	240.53	138.70	121.14
7.49	-1.47	12.33	21.94	11.37	1.45	2.62	11.01	3.60	-4.40	155.72	237.43	136.12	118.19
7.36	-1.54	12.09	21.73	11.19	1.38	2.45	10.85	3.52	-4.48	155.27	239.02	136.53	119.39
7.57	-1.35	12.33	21.95	11.35	1.54	2.73	11.12	3.79	-4.20	152.11	234.61	131.49	112.40
7.30	-1.49	11.96	21.60	11.03	1.34	2.49	10.88	3.61	-4.33	155.15	238.28	134.45	115.95
7.82	-1.16	12.62	22.37	11.67	1.69	3.11	11.38	3.97	-4.03	144.18	230.64	122.01	96.96
7.37	-1.55	12.10	21.74	11.20	1.37	2.43	10.85	3.52	-4.49	155.06	239.44	137.79	120.89
7.03	-1.72	11.55	21.22	10.75	1.15	2.22	10.56	3.29	-4.58	161.30	245.02	138.98	120.92
7.24	-1.57	11.84	21.52	10.97	1.29	2.44	10.81	3.51	-4.42	155.70	240.73	133.97	113.20
7.44	-1.45	12.26	21.90	11.29	1.41	2.62	11.03	3.62	-4.34	155.33	236.75	132.57	111.97
7.71	-1.21	12.48	22.23	11.49	1.66	3.03	11.33	3.94	-4.09	147.63	232.34	121.09	97.06
7.75	-1.19	12.50	22.24	11.49	1.68	3.04	11.35	3.97	-4.10	147.14	232.66	121.69	96.83
7.55	-1.36	12.30	22.04	11.32	1.56	2.84	11.15	3.80	-4.23	149.50	235.88	124.43	100.77
7.94	-1.00	12.79	22.64	11.78	1.97	3.27	11.54	4.11	-3.99	146.59	234.68	124.10	99.66
7.28	-1.52	11.92	21.57	11.04	1.38	2.50	10.84	3.53	-4.39	155.38	239.76	133.19	114.11
7.33	-1.56	12.08	21.74	11.14	1.34	2.51	10.87	3.49	-4.47	156.91	238.38	134.55	115.13
7.36	-1.54	12.19	21.84	11.23	1.37	2.53	10.90	3.50	-4.45	156.58	238.04	134.19	114.59
7.11	-1.70	11.72	21.34	10.86	1.19	2.27	10.63	3.31	-4.62	161.87	243.24	138.79	121.17
7.58	-1.38	12.52	22.20	11.51	1.55	2.70	11.09	3.62	-4.35	152.82	236.41	133.22	113.99
7.37	-1.52	12.07	21.76	11.17	1.35	2.54	10.95	3.55	-4.38	154.76	238.03	132.77	111.85
7.28	-1.58	11.95	21.63	11.06	1.28	2.48	10.88	3.50	-4.42	156.36	239.25	133.95	113.60
7.09	-1.64	11.66	21.33	10.85	1.23	2.32	10.65	3.37	-4.53	158.81	242.74	136.63	118.27
7.19	-1.64	11.80	21.44	10.94	1.23	2.37	10.73	3.42	-4.54	157.58	241.47	136.37	117.01
7.18	-1.64	11.79	21.44	10.93	1.23	2.38	10.73	3.42	-4.53	157.27	241.54	136.21	116.87
7.24	-1.57	11.84	21.52	10.97	1.29	2.44	10.80	3.51	-4.43	155.73	240.79	134.04	113.29
7.23	-1.58	11.81	21.50	10.95	1.26	2.43	10.79	3.50	-4.43	156.09	240.95	134.07	113.60
7.23	-1.58	11.82	21.50	10.95	1.26	2.43	10.80	3.51	-4.43	156.03	240.89	134.02	113.50
7.22	-1.61	11.84	21.48	10.97	1.24	2.42	10.79	3.45	-4.49	156.48	240.60	135.52	116.31
7.24	-1.60	11.90	21.54	11.01	1.28	2.44	10.80	3.45	-4.50	156.95	240.28	135.78	116.59
7.21	-1.61	11.79	21.46	10.92	1.23	2.41	10.79	3.48	-4.46	156.45	241.30	134.54	114.33
7.20	-1.61	11.78	21.45	10.92	1.22	2.42	10.81	3.48	-4.46	156.33	241.46	134.48	114.41
7.24	-1.61	11.90	21.58	11.02	1.26	2.44	10.84	3.47	-4.48	158.13	240.03	134.87	115.57
7.42	-1.47	12.23	21.87	11.26	1.40	2.60	11.01	3.61	-4.35	155.88	237.06	132.90	112.34

7.29	-1.51	11.77	21.57	10.91	1.28	2.61	10.92	3.66	-4.31	149.61	240.66	127.69	104.64
7.29	-1.53	11.74	21.55	10.91	1.30	2.58	10.91	3.64	-4.38	151.65	242.53	130.15	106.25
7.69	-1.24	12.42	22.18	11.44	1.63	3.00	11.30	3.92	-4.12	148.32	233.53	121.52	97.55
7.72	-1.22	12.50	22.25	11.49	1.67	3.03	11.32	3.95	-4.09	147.14	232.25	121.83	97.65
7.74	-1.20	12.53	22.28	11.51	1.68	3.05	11.34	3.96	-4.07	146.86	231.83	121.48	97.24
7.54	-1.36	12.29	22.03	11.31	1.56	2.78	11.13	3.77	-4.27	151.00	237.47	124.69	101.54
7.42	-1.44	12.07	21.85	11.17	1.46	2.69	11.00	3.67	-4.34	153.53	241.08	128.23	103.41
7.44	-1.42	12.11	21.90	11.19	1.48	2.69	11.02	3.67	-4.35	153.52	240.69	128.55	104.86
7.70	-1.21	12.45	22.23	11.49	1.71	3.02	11.29	3.91	-4.13	149.46	235.55	123.81	98.57
7.72	-1.20	12.46	22.25	11.51	1.72	3.03	11.30	3.92	-4.12	149.32	235.21	123.63	98.29
7.60	-1.28	12.32	22.12	11.41	1.64	2.90	11.18	3.82	-4.20	148.81	238.55	125.35	101.08
7.46	-1.43	12.13	21.95	11.21	1.52	2.69	11.00	3.69	-4.38	154.08	242.07	129.41	105.96
6.75	-2.22	10.86	20.51	10.33	0.42	1.68	9.69	3.19	-4.87	136.15	242.31	126.32	89.57
6.98	-2.06	11.18	20.84	10.59	0.61	1.99	9.99	3.37	-4.71	134.26	238.28	122.89	86.13
6.52	-2.48	10.42	20.08	9.99	0.18	1.47	9.43	3.02	-5.11	140.75	246.97	127.14	93.49
6.91	-2.11	11.07	20.70	10.46	0.55	1.90	9.89	3.34	-4.76	135.01	239.58	121.60	86.53
6.89	-2.12	11.04	20.67	10.43	0.54	1.88	9.88	3.32	-4.78	135.36	240.09	121.91	87.00
7.08	-1.98	11.35	21.00	10.73	0.69	2.10	10.10	3.46	-4.62	132.93	236.09	121.16	84.00
7.06	-2.00	11.36	20.99	10.73	0.67	2.07	10.06	3.43	-4.64	132.67	236.51	121.13	84.19
6.94	-2.10	11.21	20.83	10.60	0.57	1.92	9.93	3.31	-4.75	134.29	238.74	123.24	86.72
6.96	-2.08	11.21	20.85	10.61	0.57	1.93	9.95	3.30	-4.76	134.47	238.60	123.53	86.75
6.93	-2.10	11.18	20.81	10.58	0.56	1.90	9.92	3.28	-4.78	134.78	239.23	123.88	87.26
6.90	-2.12	11.16	20.79	10.58	0.54	1.89	9.91	3.25	-4.80	133.07	238.47	124.16	87.90
6.68	-2.23	10.46	20.11	10.07	0.34	1.67	9.60	3.29	-4.77	135.50	245.66	124.68	90.11
6.88	-2.04	10.74	20.40	10.29	0.54	1.92	9.80	3.49	-4.61	134.02	241.05	120.08	86.12
6.94	-2.08	10.99	20.68	10.51	0.56	1.82	9.74	3.37	-4.71	130.52	238.15	120.22	85.58
6.94	-2.01	10.91	20.57	10.41	0.54	1.86	9.79	3.45	-4.59	131.75	240.93	121.01	85.71
6.77	-2.23	10.88	20.50	10.37	0.43	1.61	9.62	3.17	-4.88	136.32	243.54	125.64	89.43
6.80	-2.21	10.92	20.54	10.40	0.45	1.64	9.64	3.19	-4.86	135.56	242.83	125.33	88.94
6.80	-2.20	10.94	20.57	10.41	0.45	1.64	9.65	3.20	-4.86	135.59	242.65	125.21	88.72
6.91	-2.13	11.11	20.74	10.53	0.53	1.76	9.77	3.24	-4.80	133.66	241.41	124.00	86.78
6.89	-2.14	11.06	20.71	10.50	0.52	1.74	9.76	3.24	-4.81	134.53	241.68	124.03	87.14
6.90	-2.13	11.08	20.71	10.51	0.52	1.74	9.75	3.24	-4.80	134.11	241.78	124.30	86.94
6.90	-2.13	11.08	20.71	10.51	0.52	1.74	9.75	3.24	-4.80	134.19	241.79	124.31	86.95
6.96	-2.08	11.15	20.77	10.59	0.58	1.79	9.80	3.32	-4.74	133.20	239.70	123.50	86.19
6.92	-2.10	11.15	20.77	10.58	0.56	1.75	9.77	3.27	-4.78	133.39	240.13	123.46	86.42
6.92	-2.12	11.15	20.76	10.57	0.53	1.72	9.75	3.23	-4.81	132.90	240.75	124.84	86.66
6.92	-2.12	11.15	20.76	10.57	0.53	1.72	9.75	3.23	-4.81	132.99	240.68	124.92	86.74
6.74	-2.24	10.81	20.46	10.31	0.42	1.69	9.71	3.20	-4.88	136.34	242.67	125.30	89.41
6.62	-2.38	10.71	20.35	10.22	0.28	1.55	9.62	3.01	-5.02	138.65	246.75	129.83	94.15
6.64	-2.37	10.74	20.38	10.24	0.29	1.57	9.64	3.02	-5.01	138.49	246.46	129.47	93.69
6.98	-2.08	11.28	20.90	10.66	0.60	1.91	9.91	3.33	-4.75	132.25	238.05	122.13	85.64
6.56	-2.44	10.63	20.25	10.16	0.20	1.48	9.59	2.96	-5.05	140.57	248.60	131.50	95.53

7.34	-1.61	11.38	21.04	10.89	1.08	2.30	10.29	3.78	-4.27	131.31	232.29	113.61	79.98
7.02	-2.07	11.15	20.81	10.63	0.66	1.93	9.86	3.42	-4.78	134.55	235.90	117.53	82.72
7.23	-1.71	11.22	20.90	10.74	0.97	2.20	10.18	3.72	-4.39	132.69	231.56	116.78	82.61
7.23	-1.81	11.42	21.15	10.84	0.90	2.19	10.16	3.61	-4.55	132.61	228.40	116.26	81.85
7.02	-1.90	11.33	21.06	10.58	0.83	2.17	10.25	3.45	-4.67	141.46	235.74	121.06	89.33
7.75	-1.40	12.13	21.89	11.42	1.32	2.76	10.70	4.07	-4.14	125.91	218.99	109.11	74.30
7.16	-1.83	11.27	20.97	10.72	0.90	2.13	10.11	3.61	-4.56	134.90	234.37	118.03	83.54
7.16	-1.83	11.26	20.97	10.72	0.90	2.13	10.11	3.60	-4.56	134.92	234.42	118.06	83.58
6.91	-2.05	10.91	20.64	10.45	0.68	1.89	9.85	3.40	-4.78	137.61	238.34	121.19	87.45
6.91	-2.05	10.91	20.64	10.45	0.69	1.89	9.85	3.39	-4.78	137.59	238.39	121.23	87.51
6.99	-2.06	11.14	20.88	10.64	0.74	1.85	9.84	3.34	-4.83	135.86	235.35	118.91	85.23
6.94	-2.09	11.09	20.80	10.54	0.64	1.85	9.78	3.34	-4.84	136.38	233.93	118.13	84.77
6.79	-2.16	10.63	20.40	10.17	0.46	1.91	9.84	3.42	-4.80	139.29	239.22	121.73	87.66
6.85	-2.08	10.69	20.46	10.22	0.53	1.96	9.90	3.44	-4.70	137.14	237.00	120.59	86.74
7.05	-1.98	11.21	20.95	10.66	0.78	1.96	9.90	3.43	-4.71	135.37	233.58	118.06	83.58
7.14	-1.90	11.34	21.06	10.78	0.84	2.09	10.00	3.51	-4.63	132.89	231.40	116.45	81.70
6.99	-2.03	11.16	20.93	10.65	0.72	1.87	9.82	3.32	-4.77	136.69	234.27	119.32	85.33
6.99	-2.03	11.16	20.93	10.65	0.72	1.87	9.82	3.32	-4.77	136.68	234.29	119.34	85.35
7.13	-1.92	11.42	21.18	10.89	0.83	2.02	9.95	3.42	-4.67	134.91	230.99	116.92	83.40
6.95	-2.06	11.12	20.94	10.61	0.64	1.78	9.76	3.27	-4.80	136.62	235.44	119.29	86.30
7.00	-2.03	11.17	20.94	10.66	0.72	1.88	9.83	3.33	-4.76	136.56	234.08	119.20	85.20
7.36	-1.66	11.69	21.43	10.99	1.05	2.46	10.46	3.75	-4.37	132.58	224.46	113.45	81.27
7.19	-1.84	11.46	21.18	10.80	0.88	2.22	10.20	3.57	-4.55	134.48	227.21	116.17	83.11
7.40	-1.53	11.41	21.05	10.90	1.12	2.41	10.35	3.90	-4.18	131.96	227.77	112.93	80.18
7.17	-1.78	11.09	20.79	10.63	0.86	2.17	10.11	3.67	-4.44	134.39	234.17	116.54	83.31
7.16	-1.83	11.10	20.81	10.65	0.83	2.22	10.13	3.70	-4.48	134.71	232.53	116.38	82.04
7.03	-1.99	11.06	20.73	10.60	0.72	2.03	9.92	3.50	-4.69	134.89	234.27	117.07	83.47
6.97	-2.07	10.90	20.63	10.45	0.64	1.94	9.87	3.44	-4.76	137.05	236.33	118.39	84.47
7.10	-1.97	11.24	20.89	10.69	0.67	1.95	9.91	3.48	-4.63	131.15	234.85	117.09	82.66
7.06	-2.00	11.19	20.84	10.65	0.65	1.92	9.88	3.46	-4.66	131.68	235.61	117.53	83.20
6.90	-2.13	11.00	20.67	10.48	0.55	1.79	9.73	3.30	-4.83	136.82	238.62	118.87	85.22
6.96	-2.10	11.02	20.68	10.55	0.63	1.85	9.78	3.37	-4.83	135.85	236.74	118.26	84.23
6.94	-2.11	11.01	20.66	10.52	0.61	1.83	9.77	3.35	-4.84	136.03	237.22	118.52	84.59
7.11	-1.98	11.21	20.92	10.72	0.76	2.04	9.97	3.53	-4.70	133.77	233.44	116.45	82.06
7.13	-1.96	11.25	20.94	10.73	0.77	2.06	9.98	3.53	-4.70	133.07	233.10	116.23	81.69
7.08	-2.02	11.27	20.96	10.73	0.73	1.99	9.92	3.47	-4.75	132.76	234.17	117.52	82.03
7.09	-2.00	11.26	20.98	10.73	0.73	2.00	9.92	3.45	-4.76	133.67	232.89	117.80	82.24
7.09	-2.00	11.27	20.98	10.73	0.73	2.01	9.92	3.45	-4.76	133.63	232.79	117.74	82.33
6.96	-2.11	11.03	20.72	10.51	0.58	1.91	9.81	3.41	-4.80	135.39	235.49	117.64	84.62
6.90	-2.12	10.93	20.61	10.49	0.53	1.76	9.70	3.33	-4.75	131.65	239.17	120.68	86.61
6.72	-2.28	10.64	20.31	10.18	0.38	1.69	9.62	3.23	-4.93	137.72	239.20	120.78	88.69
6.79	-2.21	10.71	20.39	10.21	0.43	1.77	9.69	3.31	-4.86	137.27	238.26	120.13	87.82
6.79	-2.22	10.72	20.40	10.22	0.43	1.77	9.69	3.31	-4.87	137.22	238.08	120.03	87.73

7.52	-1.47	11.89	21.65	11.14	1.21	2.73	10.75	3.94	-4.16	131.91	223.88	110.60	79.93
6.81	-2.29	10.86	20.60	10.34	0.43	1.74	9.72	3.24	-5.02	140.19	239.23	123.81	88.58
7.24	-1.89	11.68	21.48	11.04	0.85	2.19	10.11	3.48	-4.63	133.71	226.04	115.79	81.74
6.98	-1.99	10.92	20.68	10.40	0.64	2.07	9.98	3.54	-4.64	136.02	234.48	118.10	84.45
7.14	-1.98	11.37	21.23	10.81	0.68	2.25	10.26	3.48	-4.67	132.05	232.87	120.11	85.31
7.05	-2.04	11.20	21.03	10.64	0.66	2.11	10.06	3.46	-4.80	135.27	231.04	120.22	85.09
6.65	-2.26	10.42	20.16	9.98	0.33	1.79	9.71	3.33	-4.87	141.27	241.78	124.18	89.63
7.36	-1.75	11.81	21.59	11.16	1.00	2.23	10.17	3.56	-4.50	132.17	225.49	114.41	79.88
7.34	-1.77	11.79	21.57	11.14	0.98	2.20	10.15	3.54	-4.52	132.40	225.84	114.43	80.17
7.34	-1.77	11.78	21.56	11.13	0.97	2.19	10.14	3.54	-4.52	132.47	225.96	114.50	80.26
7.16	-1.93	11.52	21.33	10.93	0.80	1.99	9.91	3.37	-4.66	134.16	230.74	117.68	83.33
7.31	-1.78	11.77	21.58	11.11	0.97	2.23	10.14	3.52	-4.53	131.45	225.29	115.56	80.65
7.28	-1.62	11.59	21.37	10.85	1.13	2.51	10.58	3.68	-4.33	137.93	229.78	116.69	85.52
7.23	-1.70	11.71	21.42	10.87	1.03	2.39	10.48	3.59	-4.45	139.76	230.61	116.86	86.16
7.27	-1.73	11.73	21.48	10.94	1.02	2.36	10.42	3.59	-4.48	137.78	229.13	117.48	85.15
7.16	-1.83	11.59	21.35	10.83	0.95	2.24	10.27	3.50	-4.57	138.73	231.72	118.47	86.14
7.17	-1.82	11.61	21.36	10.84	0.95	2.25	10.28	3.51	-4.57	138.68	231.52	118.34	85.96
7.43	-1.63	11.96	21.71	11.14	1.12	2.50	10.52	3.70	-4.39	136.15	226.29	114.90	81.38
7.33	-1.69	11.79	21.54	10.98	1.06	2.44	10.48	3.65	-4.45	136.98	228.59	115.84	83.42
7.25	-1.74	11.70	21.44	10.90	1.00	2.35	10.41	3.59	-4.50	137.97	229.97	117.64	85.56
7.34	-1.68	11.82	21.57	11.00	1.07	2.46	10.51	3.70	-4.43	137.08	228.18	115.76	82.54
7.34	-1.68	11.82	21.57	11.01	1.07	2.46	10.51	3.70	-4.43	137.06	228.14	115.74	82.52
7.14	-1.80	11.47	21.23	10.72	0.93	2.31	10.36	3.58	-4.54	139.46	231.79	119.28	86.16
7.21	-1.77	11.58	21.33	10.80	0.98	2.37	10.42	3.62	-4.49	138.65	231.25	117.79	84.89
6.96	-1.91	11.12	20.86	10.42	0.80	2.25	10.30	3.52	-4.63	142.48	235.74	121.75	89.78
6.96	-1.91	11.12	20.86	10.42	0.80	2.25	10.29	3.52	-4.63	142.53	235.78	121.76	89.84
7.28	-1.72	11.67	21.43	10.91	1.04	2.42	10.43	3.64	-4.46	138.72	228.37	116.71	83.68
7.30	-1.70	11.67	21.45	10.92	1.05	2.42	10.43	3.65	-4.45	136.91	228.95	116.57	83.11
7.40	-1.58	11.81	21.57	11.04	1.16	2.61	10.61	3.79	-4.35	133.97	224.57	114.87	81.12
7.40	-1.58	11.81	21.58	11.05	1.16	2.60	10.60	3.77	-4.35	133.77	224.47	114.98	81.06
7.38	-1.59	11.76	21.53	10.98	1.16	2.61	10.62	3.79	-4.35	135.22	225.29	115.35	81.31
7.37	-1.60	11.74	21.51	10.96	1.15	2.60	10.62	3.78	-4.35	135.55	225.68	115.54	81.47
7.30	-1.63	11.60	21.36	10.85	1.11	2.61	10.62	3.78	-4.36	136.26	228.90	116.43	82.75
7.12	-1.82	11.41	21.17	10.68	0.92	2.29	10.34	3.57	-4.55	139.65	232.06	119.86	86.60
7.69	-1.32	12.06	21.78	11.31	1.35	2.82	10.84	4.08	-4.00	130.51	221.14	109.65	77.09
7.62	-1.42	12.02	21.76	11.28	1.26	2.69	10.72	3.98	-4.10	131.28	220.99	109.89	77.41
7.53	-1.53	11.90	21.64	11.15	1.17	2.59	10.59	3.89	-4.22	131.87	222.31	111.37	79.26
7.41	-1.59	11.74	21.49	11.04	1.11	2.56	10.58	3.82	-4.29	133.08	224.02	112.70	81.02
7.43	-1.61	11.79	21.55	11.06	1.09	2.52	10.51	3.78	-4.31	132.07	224.03	112.64	80.66
7.42	-1.62	11.79	21.52	11.06	1.08	2.51	10.52	3.80	-4.31	131.86	222.27	112.42	80.07
7.34	-1.66	11.68	21.47	10.99	1.09	2.46	10.48	3.64	-4.37	134.26	227.43	115.21	81.93
7.84	-1.22	12.51	22.27	11.66	1.54	2.99	11.02	4.02	-3.99	128.62	216.30	109.18	75.06
7.38	-1.62	11.76	21.54	11.05	1.12	2.49	10.51	3.68	-4.34	133.14	225.74	115.08	80.96

7.52	-1.53	12.06	21.83	11.30	1.24	2.55	10.54	3.77	-4.31	130.20	222.93	112.63	78.35
7.24	-1.83	11.63	21.41	11.04	0.96	2.14	10.09	3.46	-4.62	132.76	226.60	115.98	81.86
7.25	-1.83	11.62	21.38	11.03	0.93	2.15	10.09	3.47	-4.61	132.58	227.56	116.05	81.64
7.73	-1.36	12.18	21.90	11.36	1.34	2.88	10.91	4.07	-4.04	129.64	220.82	108.66	77.14
7.57	-1.41	11.98	21.73	11.20	1.30	2.81	10.83	3.98	-4.12	132.54	222.57	110.76	79.42
7.46	-1.56	11.80	21.56	11.08	1.15	2.61	10.61	3.83	-4.27	132.72	223.48	112.72	80.13
7.78	-1.22	12.38	22.09	11.47	1.48	3.05	11.11	4.10	-3.94	133.63	221.78	110.15	78.15
7.78	-1.21	12.38	22.10	11.48	1.49	3.05	11.11	4.11	-3.93	133.62	221.71	110.05	78.11
7.15	-1.94	11.47	21.30	10.85	0.77	2.16	10.11	3.45	-4.64	133.77	226.57	117.87	83.81
7.15	-1.95	11.45	21.28	10.84	0.77	2.16	10.12	3.46	-4.64	133.83	226.49	117.98	83.94
7.03	-2.05	11.22	21.04	10.66	0.67	2.10	10.10	3.42	-4.80	133.57	231.84	121.00	85.57
6.97	-1.99	11.28	20.98	10.53	0.79	2.14	10.28	3.38	-4.74	146.59	238.69	123.52	92.99
6.83	-2.08	11.13	20.81	10.38	0.68	1.98	10.11	3.26	-4.83	148.23	240.96	125.11	94.60
7.02	-1.90	11.41	21.11	10.61	0.86	2.18	10.29	3.44	-4.67	143.40	235.85	120.74	90.23
7.44	-1.47	11.79	21.53	11.00	1.24	2.70	10.81	3.89	-4.21	134.67	225.70	113.90	82.51
8.09	-0.92	12.84	22.65	11.86	1.87	3.43	11.60	4.30	-3.70	131.05	218.86	108.01	77.12
7.88	-0.91	12.55	22.49	11.58	1.85	3.30	11.48	4.18	-3.69	136.56	225.26	111.88	82.81
7.87	-0.92	12.55	22.49	11.57	1.85	3.30	11.47	4.18	-3.69	136.61	225.31	111.96	82.84
6.99	-1.92	11.42	21.12	10.61	0.90	2.19	10.39	3.36	-4.75	150.37	241.43	126.72	95.36
7.54	-1.42	12.11	21.90	11.19	1.36	2.84	10.97	3.90	-4.22	139.82	228.84	114.81	84.74
7.50	-1.49	11.86	21.63	11.12	1.23	2.71	10.73	3.87	-4.21	133.55	224.34	112.75	79.71
7.79	-1.25	12.26	21.95	11.44	1.44	3.00	11.02	4.16	-3.93	129.18	220.02	107.73	76.27
7.80	-1.24	12.27	21.96	11.45	1.45	3.01	11.03	4.17	-3.93	129.27	219.98	107.57	76.21
7.52	-1.45	11.91	21.63	11.10	1.27	2.74	10.86	3.94	-4.16	133.44	225.03	111.61	81.27
7.81	-1.15	12.30	22.06	11.43	1.56	3.11	11.21	4.18	-3.89	132.11	222.06	109.16	77.52
7.78	-1.19	12.34	22.06	11.46	1.55	3.09	11.13	4.13	-3.93	131.20	222.21	110.13	76.98
7.87	-1.18	12.50	22.26	11.61	1.56	3.13	11.16	4.14	-3.89	130.89	219.30	108.15	75.62
7.74	-1.28	12.31	22.08	11.44	1.47	2.97	11.01	4.04	-4.02	132.93	221.13	109.95	77.55
7.15	-1.83	11.61	21.30	10.76	0.95	2.35	10.48	3.52	-4.62	144.78	234.66	121.09	90.31
7.14	-1.84	11.57	21.26	10.73	0.96	2.34	10.47	3.53	-4.63	143.82	234.82	120.80	89.92
6.96	-1.98	11.31	21.02	10.54	0.81	2.14	10.28	3.39	-4.74	146.43	238.39	124.21	92.68
7.09	-1.91	11.47	21.19	10.66	0.90	2.27	10.40	3.50	-4.66	144.43	235.69	122.12	90.01
7.09	-1.91	11.47	21.18	10.66	0.90	2.27	10.40	3.50	-4.66	144.55	235.83	122.17	90.16
8.08	-0.81	12.58	22.45	11.70	1.91	3.52	11.67	4.48	-3.53	131.26	220.51	108.06	77.04
7.28	-1.60	11.70	21.50	10.86	1.15	2.54	10.69	3.69	-4.35	141.73	233.11	118.29	88.54
7.24	-1.63	11.69	21.47	10.84	1.13	2.49	10.64	3.67	-4.38	141.76	233.41	118.20	88.77
7.51	-1.42	11.88	21.67	11.07	1.27	2.84	10.91	3.95	-4.09	135.50	227.57	114.14	82.32
7.63	-1.32	12.20	22.04	11.29	1.46	2.92	11.09	3.94	-4.13	137.05	228.29	114.88	84.28
7.53	-1.42	12.09	21.92	11.18	1.35	2.82	10.99	3.87	-4.19	138.45	228.87	116.31	85.12
7.12	-1.82	11.51	21.23	10.70	0.95	2.29	10.42	3.53	-4.59	143.34	233.92	120.33	89.71
7.12	-1.81	11.51	21.24	10.70	0.96	2.30	10.43	3.54	-4.57	143.34	234.05	120.28	89.80
7.11	-1.82	11.50	21.23	10.70	0.95	2.29	10.42	3.52	-4.59	143.38	233.97	120.38	89.75
7.33	-1.62	11.78	21.52	10.90	1.17	2.63	10.72	3.75	-4.40	141.38	231.90	118.08	86.49

7.12	-1.84	11.52	21.23	10.71	0.96	2.31	10.44	3.51	-4.66	145.06	235.44	121.47	91.28
7.35	-1.65	11.87	21.56	10.98	1.14	2.56	10.69	3.73	-4.46	141.32	230.69	118.42	86.94
7.55	-1.51	12.02	21.95	11.23	1.24	2.78	10.97	3.85	-4.25	133.61	225.39	118.12	82.69
7.13	-1.92	11.47	21.40	10.80	0.81	2.21	10.41	3.44	-4.62	139.68	233.10	123.82	90.08
7.13	-1.92	11.47	21.40	10.80	0.80	2.21	10.41	3.44	-4.62	139.69	233.12	123.81	90.11
7.19	-1.87	11.56	21.49	10.87	0.84	2.27	10.45	3.48	-4.58	138.80	231.83	122.83	88.83
7.15	-1.89	11.53	21.41	10.85	0.83	2.25	10.42	3.46	-4.60	138.95	231.58	123.66	88.67
7.16	-1.88	11.48	21.39	10.80	0.82	2.32	10.47	3.52	-4.59	139.17	233.85	123.88	90.09
7.23	-1.81	11.59	21.49	10.89	0.91	2.40	10.56	3.58	-4.52	138.09	231.25	122.30	88.29
7.00	-2.01	11.26	21.17	10.62	0.68	2.12	10.29	3.38	-4.69	140.94	237.03	125.75	92.46
7.15	-1.90	11.44	21.39	10.79	0.79	2.31	10.46	3.52	-4.60	139.34	233.91	124.12	90.10
7.46	-1.67	11.93	21.87	11.18	1.10	2.59	10.74	3.73	-4.44	135.14	227.35	119.32	83.62
7.29	-1.78	11.66	21.60	10.95	0.93	2.45	10.61	3.61	-4.51	138.51	232.11	122.52	88.10
7.14	-1.86	11.47	21.42	10.81	0.85	2.30	10.49	3.49	-4.59	139.48	233.68	124.63	90.87
7.12	-1.90	11.45	21.34	10.77	0.80	2.22	10.41	3.46	-4.64	139.83	232.67	124.65	90.34
6.90	-2.12	11.11	20.98	10.55	0.63	2.03	10.26	3.25	-4.87	148.44	244.96	131.37	97.12
7.14	-1.89	11.41	21.27	10.78	0.86	2.32	10.59	3.49	-4.63	146.68	239.49	127.92	94.07
7.17	-1.87	11.73	21.66	10.91	0.96	2.24	10.37	3.41	-4.71	138.45	232.75	121.52	87.53
7.13	-1.88	11.51	21.27	10.77	0.89	2.35	10.48	3.48	-4.68	141.06	236.34	122.33	88.50
6.94	-1.98	11.09	20.86	10.45	0.75	2.25	10.36	3.44	-4.70	145.35	240.65	125.42	92.19
6.90	-1.98	10.94	20.75	10.33	0.70	2.27	10.40	3.47	-4.66	147.17	241.50	126.34	93.72
6.81	-2.22	10.94	20.79	10.49	0.47	1.66	9.66	3.13	-4.92	139.07	240.41	123.56	89.99
7.10	-1.98	11.44	21.26	10.88	0.76	1.95	9.88	3.33	-4.72	135.50	233.26	118.69	84.56
7.55	-1.50	12.10	21.86	11.33	1.26	2.58	10.57	3.78	-4.28	131.64	222.42	112.62	77.83
7.42	-1.66	11.87	21.63	11.16	1.09	2.46	10.39	3.68	-4.41	131.99	224.51	113.29	79.10
7.55	-1.50	12.10	21.84	11.29	1.24	2.65	10.63	3.82	-4.25	131.14	222.12	112.18	77.95
7.02	-1.89	11.23	20.98	10.51	0.83	2.26	10.30	3.52	-4.62	141.21	234.93	120.93	88.96
7.52	-1.54	12.07	21.87	11.30	1.23	2.62	10.61	3.75	-4.29	131.02	223.09	113.22	78.11
7.44	-1.60	11.94	21.79	11.16	1.18	2.61	10.68	3.73	-4.40	134.39	226.68	115.14	81.01
7.24	-1.82	11.75	21.67	11.00	1.01	2.37	10.44	3.51	-4.62	135.35	230.89	119.00	84.62
7.05	-1.92	11.45	21.28	10.70	0.85	2.24	10.41	3.42	-4.69	141.76	237.06	121.99	90.09
7.08	-1.92	11.33	21.13	10.65	0.82	2.32	10.44	3.49	-4.69	140.44	235.77	123.54	89.71
7.17	-1.86	11.48	21.31	10.78	0.89	2.41	10.56	3.56	-4.61	140.22	234.60	121.74	87.77
6.90	-2.10	11.09	20.91	10.50	0.64	2.06	10.29	3.32	-4.83	146.12	243.51	130.70	97.13
7.10	-1.90	11.56	21.37	10.80	0.90	2.28	10.45	3.42	-4.68	142.03	236.38	122.23	88.85
7.45	-1.58	11.92	21.69	11.14	1.20	2.64	10.63	3.74	-4.33	132.95	224.12	113.80	79.98
7.53	-1.53	12.04	21.76	11.26	1.20	2.60	10.57	3.80	-4.29	130.52	223.45	111.74	77.81
7.47	-1.57	11.96	21.74	11.22	1.18	2.56	10.53	3.71	-4.30	131.98	223.79	113.90	79.41
7.34	-1.65	11.74	21.50	10.98	1.10	2.50	10.52	3.70	-4.41	135.44	227.33	116.06	82.11
7.24	-1.72	11.68	21.41	10.87	1.03	2.50	10.61	3.62	-4.50	138.48	232.68	119.30	85.47
7.05	-1.96	11.51	21.28	10.72	0.86	2.21	10.40	3.36	-4.76	146.38	239.55	125.46	92.66
7.45	-1.56	11.91	21.71	11.10	1.23	2.67	10.73	3.79	-4.35	133.84	228.76	114.76	81.00
7.43	-1.57	11.86	21.65	11.07	1.21	2.67	10.71	3.81	-4.33	133.97	227.67	115.03	81.00

7.47	-1.60	11.98	21.81	11.21	1.18	2.60	10.61	3.71	-4.38	133.28	223.43	114.94	79.59
7.46	-1.57	11.94	21.71	11.17	1.21	2.63	10.63	3.75	-4.32	132.84	223.31	113.35	79.56
7.43	-1.62	11.98	21.78	11.24	1.15	2.52	10.49	3.65	-4.38	132.37	224.75	114.17	80.39
7.13	-1.95	11.53	21.35	10.85	0.81	2.23	10.27	3.43	-4.71	136.19	230.63	120.95	86.01
7.37	-1.71	11.86	21.77	11.12	1.10	2.51	10.54	3.62	-4.50	134.82	226.69	116.14	81.53
7.29	-1.81	11.75	21.67	11.03	0.98	2.41	10.48	3.56	-4.60	134.76	228.19	116.85	83.20
7.29	-1.82	11.80	21.71	11.04	1.01	2.38	10.46	3.51	-4.62	136.15	230.20	117.66	84.19
7.23	-1.69	11.59	21.30	10.79	1.03	2.55	10.63	3.69	-4.43	138.48	231.53	118.42	84.74
7.24	-1.69	11.59	21.31	10.79	1.03	2.54	10.61	3.68	-4.44	138.18	231.59	118.65	84.64
7.06	-1.89	11.45	21.12	10.66	0.87	2.27	10.40	3.46	-4.66	143.53	234.94	121.92	89.81
7.34	-1.70	11.92	21.85	11.11	1.13	2.47	10.57	3.58	-4.53	135.27	231.32	117.28	83.71
6.85	-2.02	11.12	20.88	10.41	0.75	2.09	10.27	3.31	-4.78	149.61	241.95	127.33	95.27
6.85	-2.03	11.12	20.88	10.40	0.75	2.08	10.27	3.31	-4.78	149.63	242.00	127.37	95.32
7.10	-1.87	11.50	21.20	10.71	0.89	2.27	10.43	3.47	-4.64	144.29	236.76	122.47	89.94
7.26	-1.77	11.64	21.44	10.86	0.96	2.50	10.65	3.63	-4.50	142.36	234.21	120.91	86.74
7.32	-1.74	11.74	21.54	10.95	1.02	2.55	10.70	3.69	-4.48	141.67	232.56	119.53	85.84
7.06	-1.92	11.46	21.28	10.71	0.86	2.24	10.42	3.42	-4.69	141.69	237.05	121.98	89.91
7.03	-1.94	11.42	21.24	10.68	0.84	2.22	10.40	3.41	-4.70	142.13	237.22	122.21	90.57
7.37	-1.72	11.96	21.89	11.15	1.09	2.48	10.56	3.61	-4.55	133.88	227.16	116.91	82.68
7.11	-1.92	11.55	21.33	10.81	0.86	2.27	10.41	3.44	-4.71	140.11	234.59	122.26	88.29
7.07	-1.90	11.30	21.07	10.60	0.81	2.40	10.53	3.55	-4.60	144.88	238.54	124.70	91.21
7.03	-1.94	11.25	21.07	10.54	0.77	2.35	10.50	3.50	-4.62	146.81	239.73	125.18	92.45
7.30	-1.83	11.77	21.60	11.03	0.96	2.40	10.43	3.55	-4.57	135.53	227.80	118.81	84.02
7.45	-1.69	12.00	21.88	11.24	1.10	2.56	10.61	3.67	-4.47	134.36	223.99	115.92	81.04
7.12	-1.97	11.49	21.33	10.81	0.79	2.21	10.25	3.41	-4.73	136.23	231.94	121.33	86.54
7.39	-1.76	11.96	21.78	11.19	1.01	2.45	10.53	3.59	-4.53	133.75	226.57	117.46	82.47
7.35	-1.79	11.91	21.74	11.14	0.99	2.42	10.50	3.56	-4.56	133.97	227.02	117.45	82.64
7.35	-1.79	11.90	21.73	11.14	0.98	2.41	10.49	3.56	-4.56	134.09	227.26	117.69	82.80
7.34	-1.79	11.89	21.72	11.13	0.98	2.41	10.49	3.56	-4.56	134.02	227.13	117.52	82.75
7.16	-1.96	11.59	21.40	10.90	0.79	2.19	10.27	3.39	-4.70	137.21	230.69	120.58	86.09
7.35	-1.78	11.89	21.74	11.12	0.98	2.43	10.51	3.58	-4.55	133.99	226.94	117.51	83.07
7.19	-1.93	11.63	21.50	10.92	0.85	2.28	10.37	3.48	-4.67	136.06	230.63	120.40	86.20
7.04	-2.03	11.39	21.24	10.70	0.69	2.14	10.24	3.38	-4.74	139.07	234.87	123.43	90.04
6.98	-2.05	11.29	21.12	10.64	0.65	2.10	10.17	3.36	-4.77	138.49	234.85	123.54	89.62
7.31	-1.82	11.84	21.70	11.09	0.94	2.39	10.46	3.53	-4.59	135.77	229.00	118.68	84.50
7.49	-1.66	12.09	21.91	11.26	1.13	2.63	10.69	3.70	-4.45	131.89	227.08	115.79	81.40
6.96	-2.07	11.31	21.16	10.64	0.73	2.08	10.22	3.30	-4.84	143.09	237.50	125.16	91.33
7.62	-1.57	12.21	22.07	11.38	1.20	2.79	10.88	3.85	-4.33	131.95	223.25	114.68	79.67
7.21	-1.86	11.60	21.42	10.85	0.90	2.42	10.52	3.56	-4.61	139.20	233.39	120.97	87.26
7.15	-1.94	11.61	21.44	10.89	0.81	2.19	10.19	3.43	-4.70	135.33	229.41	119.14	84.73
7.04	-2.05	11.30	21.16	10.65	0.64	2.12	10.13	3.43	-4.71	136.55	231.47	120.97	86.84
7.21	-1.88	11.48	21.31	10.83	0.80	2.37	10.35	3.57	-4.55	134.69	228.16	119.05	84.58
6.98	-2.09	11.30	21.15	10.66	0.63	2.04	10.11	3.29	-4.80	138.26	234.41	123.66	90.19

7.68	-1.44	12.24	22.15	11.39	1.31	2.94	11.06	3.98	-4.21	130.81	222.86	115.40	79.39
7.69	-1.44	12.24	22.15	11.39	1.31	2.95	11.06	3.99	-4.20	130.78	222.83	115.38	79.34
7.30	-1.82	11.82	21.70	11.09	0.97	2.41	10.51	3.54	-4.60	136.00	229.67	119.30	85.14
7.05	-2.04	11.44	21.30	10.77	0.74	2.11	10.21	3.34	-4.79	139.39	234.33	123.51	89.67
7.18	-1.87	11.58	21.47	10.86	0.84	2.32	10.45	3.52	-4.58	138.15	230.95	121.47	87.69
7.01	-2.04	11.35	21.22	10.67	0.67	2.11	10.21	3.34	-4.78	139.70	235.25	124.69	90.89
6.91	-2.11	11.19	21.06	10.55	0.58	2.01	10.13	3.29	-4.80	141.17	237.03	125.87	92.72
7.20	-1.86	11.59	21.48	10.86	0.85	2.34	10.48	3.54	-4.56	138.38	230.42	122.69	87.79
7.16	-1.87	11.55	21.44	10.85	0.85	2.29	10.43	3.49	-4.59	138.75	230.52	122.94	88.31
7.16	-1.88	11.54	21.43	10.84	0.84	2.29	10.43	3.49	-4.59	138.88	230.57	123.16	88.43
7.26	-1.80	11.70	21.58	10.97	0.91	2.37	10.51	3.54	-4.53	137.16	229.36	121.43	86.20
7.27	-1.80	11.71	21.59	10.98	0.92	2.38	10.51	3.54	-4.53	137.03	229.29	121.40	86.18
7.20	-1.86	11.61	21.53	10.90	0.86	2.28	10.46	3.48	-4.58	138.37	231.26	122.84	88.81
7.28	-1.67	11.58	21.42	10.85	1.04	2.58	10.78	3.73	-4.40	138.46	230.94	122.04	87.47
7.28	-1.67	11.58	21.42	10.85	1.04	2.58	10.78	3.72	-4.40	138.49	230.96	122.05	87.49
7.14	-1.92	11.43	21.27	10.85	0.81	2.26	10.31	3.45	-4.61	133.74	231.56	120.62	86.58
7.23	-1.86	11.72	21.55	10.98	0.85	2.27	10.36	3.47	-4.59	133.13	227.81	120.04	85.65
7.27	-1.83	11.64	21.47	10.93	0.85	2.41	10.42	3.63	-4.50	132.65	226.78	117.92	83.31
7.29	-1.80	11.79	21.68	11.08	0.92	2.32	10.44	3.47	-4.53	134.80	226.98	119.25	85.05
7.12	-1.93	11.56	21.44	10.87	0.79	2.14	10.28	3.36	-4.68	136.55	230.63	122.35	88.30
7.21	-1.94	11.56	21.38	10.96	0.79	2.24	10.22	3.48	-4.65	133.17	229.61	118.97	84.17
7.30	-1.82	11.65	21.48	11.03	0.87	2.38	10.39	3.54	-4.55	131.93	227.62	118.72	83.31
7.30	-1.81	11.64	21.46	11.02	0.89	2.41	10.41	3.56	-4.52	131.91	227.78	118.66	83.75
7.24	-1.75	11.41	21.08	10.77	0.91	2.45	10.59	3.71	-4.39	131.68	233.99	123.60	87.74
6.97	-2.00	11.08	20.73	10.48	0.67	2.15	10.31	3.48	-4.64	135.53	239.22	128.31	92.21
7.10	-1.94	11.24	20.87	10.62	0.70	2.26	10.39	3.56	-4.59	133.77	237.15	126.51	90.00
7.09	-1.95	11.23	20.86	10.61	0.69	2.25	10.38	3.56	-4.60	133.90	237.30	126.66	90.18
7.11	-1.87	11.26	20.91	10.63	0.82	2.30	10.46	3.58	-4.51	133.60	237.09	125.52	90.22
7.24	-1.75	11.41	21.08	10.77	0.91	2.46	10.59	3.71	-4.39	131.69	233.92	123.60	87.75
7.27	-1.73	11.48	21.16	10.80	0.93	2.49	10.65	3.71	-4.40	130.46	232.57	123.28	88.27
7.11	-1.98	11.50	21.28	10.85	0.72	2.21	10.41	3.39	-4.67	134.44	232.47	123.89	90.46
7.29	-1.86	11.78	21.54	11.04	0.85	2.39	10.56	3.54	-4.56	132.16	228.89	120.30	86.90
7.12	-1.98	11.51	21.29	10.85	0.72	2.21	10.40	3.40	-4.68	134.42	231.80	123.97	90.53
7.00	-2.06	11.31	21.10	10.70	0.64	2.10	10.31	3.31	-4.76	136.39	235.58	126.05	93.52
7.01	-2.05	11.31	21.10	10.70	0.65	2.11	10.32	3.33	-4.75	136.39	235.67	125.89	93.63
7.24	-1.89	11.52	21.38	10.88	0.80	2.43	10.65	3.64	-4.57	133.83	232.68	123.18	90.07
7.27	-1.85	11.63	21.42	10.93	0.83	2.42	10.55	3.60	-4.53	130.89	226.81	119.23	85.94
7.27	-1.92	11.71	21.49	11.04	0.79	2.31	10.37	3.51	-4.65	131.59	230.24	118.76	83.49
6.98	-2.04	11.14	20.96	10.54	0.62	2.12	10.14	3.40	-4.69	137.18	233.59	122.18	88.79
7.27	-1.87	11.72	21.56	11.02	0.83	2.36	10.52	3.51	-4.59	133.32	228.79	120.18	87.55
6.60	-2.40	10.51	20.16	10.06	0.25	1.56	9.51	3.10	-5.05	139.70	245.07	125.55	91.73
6.83	-2.24	11.09	20.79	10.55	0.48	1.79	9.90	3.14	-4.94	137.54	240.54	126.97	91.76
6.33	-2.67	10.24	19.98	9.80	-0.03	1.32	9.35	2.85	-5.30	144.63	249.12	132.14	98.47

6.78	-2.25	10.84	20.63	10.32	0.49	1.79	9.77	3.26	-5.02	139.36	237.14	123.61	89.31
6.70	-2.36	10.72	20.48	10.21	0.31	1.69	9.66	3.19	-5.01	139.79	241.31	125.30	90.75
6.74	-2.33	10.76	20.52	10.25	0.33	1.72	9.68	3.22	-4.99	139.79	240.54	125.07	90.00
6.95	-2.18	11.12	20.89	10.53	0.52	1.91	9.87	3.35	-4.88	136.09	236.22	121.77	86.66
6.64	-2.40	10.65	20.40	10.13	0.26	1.64	9.61	3.14	-5.04	140.10	242.63	126.17	92.12
6.75	-2.31	10.88	20.66	10.32	0.36	1.74	9.73	3.19	-4.99	138.32	239.74	125.17	90.96
7.17	-2.00	11.49	21.30	10.90	0.70	2.18	10.16	3.44	-4.73	133.09	231.35	119.82	84.54
7.21	-1.96	11.53	21.35	10.94	0.74	2.25	10.21	3.47	-4.67	132.63	229.86	118.97	83.82
7.30	-1.81	11.65	21.48	11.03	0.89	2.41	10.42	3.56	-4.52	131.87	228.20	118.84	83.35
6.51	-2.49	10.43	20.10	9.99	0.16	1.47	9.42	3.04	-5.13	140.92	245.96	127.68	93.31
6.39	-2.63	10.37	20.12	9.91	0.05	1.37	9.42	2.86	-5.28	143.38	246.57	131.18	97.43
6.40	-2.63	10.38	20.12	9.92	0.04	1.38	9.43	2.86	-5.28	143.41	246.64	131.09	97.34
6.60	-2.47	10.73	20.50	10.20	0.21	1.56	9.59	3.00	-5.14	142.15	243.48	128.80	93.84
6.48	-2.49	10.39	20.04	9.91	0.17	1.58	9.59	3.07	-5.10	142.35	246.11	128.81	94.21
6.42	-2.54	10.27	19.91	9.81	0.10	1.50	9.50	3.02	-5.16	143.52	247.21	129.96	95.68
6.80	-2.21	10.89	20.56	10.35	0.45	1.86	9.86	3.25	-4.88	135.97	239.05	124.79	88.97
6.89	-2.15	11.09	20.76	10.52	0.51	1.95	9.95	3.28	-4.82	133.42	237.65	123.54	87.38
6.75	-2.29	10.86	20.53	10.34	0.39	1.77	9.81	3.15	-4.96	135.73	240.98	126.59	90.65
6.72	-2.30	10.77	20.45	10.28	0.36	1.79	9.79	3.19	-4.95	136.87	241.19	125.64	90.90
6.51	-2.45	10.47	20.18	10.02	0.19	1.58	9.62	3.03	-5.11	139.27	246.36	129.04	94.60
6.61	-2.44	10.71	20.45	10.18	0.23	1.57	9.63	3.04	-5.12	141.15	244.40	128.95	94.32
6.76	-2.37	11.04	20.82	10.47	0.35	1.67	9.76	3.09	-5.07	140.27	242.14	126.72	92.18
6.77	-2.35	11.07	20.85	10.47	0.36	1.68	9.77	3.08	-5.05	139.87	241.71	125.98	91.58
7.27	-1.94	11.77	21.52	11.06	0.80	2.25	10.33	3.46	-4.67	131.21	230.93	119.88	84.30
7.31	-1.90	11.84	21.59	11.11	0.83	2.27	10.36	3.48	-4.64	131.37	229.70	119.69	84.09
7.39	-1.83	12.07	21.78	11.29	0.89	2.35	10.47	3.52	-4.59	128.25	226.49	118.10	82.51
7.37	-1.89	11.97	21.70	11.22	0.87	2.32	10.43	3.51	-4.62	130.20	227.14	118.27	82.66
7.30	-1.91	11.82	21.57	11.10	0.82	2.28	10.36	3.48	-4.64	130.92	230.27	119.46	83.70
7.06	-2.15	11.44	21.21	10.84	0.59	2.02	10.04	3.29	-4.88	134.13	232.80	121.54	85.80
6.84	-2.32	11.15	20.95	10.58	0.44	1.77	9.83	3.12	-5.04	137.81	238.49	125.11	89.60
6.86	-2.31	11.16	20.96	10.59	0.45	1.78	9.84	3.13	-5.03	137.49	238.30	124.89	89.45
6.89	-2.24	11.15	20.95	10.59	0.46	1.85	9.86	3.21	-4.98	135.63	237.57	123.89	88.85
6.88	-2.25	11.14	20.94	10.58	0.45	1.85	9.85	3.21	-4.98	135.71	237.70	123.98	88.95
6.59	-2.45	10.65	20.42	10.15	0.24	1.62	9.61	3.05	-5.11	139.11	242.31	127.21	93.30
7.09	-1.98	11.27	21.07	10.67	0.67	2.28	10.37	3.51	-4.63	134.20	231.11	122.06	88.30
7.36	-1.81	11.74	21.59	11.05	0.88	2.47	10.59	3.65	-4.48	132.64	227.89	119.58	84.95
7.27	-1.83	11.54	21.42	10.92	0.86	2.43	10.52	3.60	-4.51	134.57	229.29	120.15	86.18
7.41	-1.77	11.89	21.76	11.16	0.94	2.50	10.63	3.68	-4.48	133.96	227.57	119.41	85.06
6.89	-2.16	11.13	20.80	10.58	0.53	1.90	9.93	3.21	-4.84	134.34	238.16	124.76	88.71
6.87	-2.20	11.07	20.74	10.53	0.51	1.87	9.90	3.20	-4.87	134.69	239.10	125.46	89.13
6.84	-2.23	11.01	20.66	10.47	0.48	1.83	9.86	3.18	-4.91	135.14	240.01	126.07	89.64
6.49	-2.47	10.50	20.12	10.05	0.19	1.45	9.54	2.92	-5.10	142.01	251.15	133.82	98.37
6.77	-2.20	10.87	20.51	10.34	0.43	1.81	9.92	3.19	-4.84	135.37	243.49	128.27	92.87

6.68	-2.25	10.61	20.26	10.18	0.39	1.71	9.87	3.18	-4.89	139.07	246.59	131.97	96.57
7.10	-1.97	11.41	21.01	10.76	0.72	2.12	10.25	3.43	-4.66	133.29	238.06	125.24	88.48
7.11	-1.91	11.26	20.89	10.69	0.77	2.21	10.34	3.53	-4.59	132.60	236.73	125.83	88.62
6.96	-2.02	10.95	20.59	10.45	0.62	2.03	10.15	3.43	-4.65	134.70	241.07	127.32	91.60
6.74	-2.22	10.82	20.47	10.31	0.41	1.79	9.90	3.16	-4.85	135.69	243.94	128.85	93.49
7.16	-1.89	11.37	21.03	10.76	0.76	2.27	10.38	3.59	-4.52	132.07	234.85	121.68	86.27
6.94	-2.05	11.04	20.73	10.51	0.60	2.01	10.16	3.39	-4.70	135.10	240.53	126.03	91.18
7.36	-1.76	11.60	21.28	10.95	0.89	2.49	10.59	3.75	-4.41	130.15	230.83	120.50	84.23
7.16	-2.02	11.57	21.33	10.93	0.68	2.14	10.27	3.37	-4.72	133.02	233.67	123.15	88.07
7.36	-1.87	11.96	21.72	11.21	0.88	2.37	10.48	3.48	-4.61	131.26	228.08	119.58	83.58
7.35	-1.89	11.94	21.70	11.20	0.87	2.35	10.46	3.47	-4.62	131.27	228.57	119.79	83.81
7.18	-1.78	11.31	21.03	10.71	0.87	2.37	10.54	3.64	-4.44	131.50	237.29	126.31	90.94
7.04	-1.95	11.20	20.86	10.60	0.70	2.10	10.27	3.45	-4.59	132.68	241.24	127.91	92.57
6.99	-2.04	11.14	20.81	10.55	0.61	2.06	10.20	3.41	-4.66	134.31	240.99	129.07	92.99
6.96	-2.08	11.14	20.80	10.57	0.56	1.99	10.15	3.35	-4.69	134.64	242.22	128.86	92.79
7.00	-2.05	11.20	20.85	10.61	0.59	2.05	10.18	3.38	-4.67	133.94	240.67	128.50	91.97
7.42	-1.62	11.70	21.36	11.01	1.03	2.59	10.73	3.82	-4.26	128.22	232.48	122.81	86.21
7.47	-1.57	11.78	21.44	11.07	1.08	2.64	10.79	3.86	-4.22	127.54	231.18	122.15	85.40
7.31	-1.69	11.52	21.21	10.87	0.97	2.47	10.65	3.74	-4.35	129.44	234.21	124.45	88.45
7.32	-1.68	11.53	21.22	10.88	0.98	2.48	10.65	3.74	-4.34	129.35	234.09	124.38	88.35
6.93	-2.13	11.21	20.81	10.62	0.57	1.92	10.04	3.27	-4.81	135.56	242.92	127.79	91.90
7.22	-1.84	11.46	21.10	10.84	0.84	2.30	10.44	3.58	-4.52	131.15	234.62	123.67	86.73
7.01	-2.04	11.26	20.91	10.65	0.60	2.03	10.16	3.37	-4.69	133.97	241.06	128.29	91.51
7.04	-2.02	11.28	20.92	10.66	0.62	2.08	10.20	3.40	-4.66	133.34	239.65	128.12	91.16
7.07	-1.99	11.34	20.97	10.69	0.65	2.13	10.25	3.44	-4.64	132.66	238.24	127.69	90.52
7.18	-1.84	11.39	21.02	10.74	0.81	2.29	10.46	3.61	-4.49	130.83	236.78	125.76	89.69
7.38	-1.66	11.65	21.30	10.97	0.99	2.53	10.67	3.79	-4.30	128.60	233.66	123.46	86.69
6.87	-2.10	10.85	20.52	10.34	0.57	1.97	10.14	3.34	-4.74	137.81	245.02	131.60	96.11
7.11	-1.98	11.36	20.99	10.70	0.68	2.19	10.32	3.53	-4.63	133.06	237.90	126.72	89.71
6.93	-2.09	11.07	20.72	10.48	0.55	2.02	10.15	3.38	-4.73	134.88	241.12	129.64	93.30
7.01	-2.04	11.19	20.82	10.55	0.60	2.15	10.26	3.48	-4.66	134.68	238.96	127.43	91.06
7.01	-2.04	11.19	20.82	10.55	0.60	2.14	10.26	3.47	-4.66	134.73	239.03	127.49	91.08
6.91	-2.09	11.05	20.68	10.44	0.55	2.04	10.18	3.40	-4.73	136.04	241.06	129.53	92.82
6.92	-2.09	11.06	20.68	10.44	0.55	2.05	10.18	3.40	-4.72	135.99	240.99	129.46	92.79
6.90	-2.10	11.03	20.66	10.42	0.54	2.03	10.17	3.39	-4.73	136.16	241.29	129.74	93.15
6.94	-2.00	10.97	20.64	10.43	0.64	2.07	10.26	3.43	-4.63	135.82	242.83	130.49	94.92
6.79	-2.18	11.21	20.93	10.47	0.67	1.92	10.23	3.14	-5.02	157.03	250.02	134.13	103.46
6.92	-2.01	11.18	20.97	10.50	0.81	2.16	10.45	3.31	-4.82	154.96	245.93	131.82	100.86
7.35	-1.75	11.83	21.72	11.09	1.03	2.48	10.52	3.61	-4.52	135.79	226.71	117.14	82.53
7.44	-1.64	11.86	21.75	11.13	1.09	2.65	10.92	3.73	-4.40	141.80	232.42	122.88	87.67
7.40	-1.69	11.81	21.70	11.11	1.07	2.60	10.87	3.69	-4.44	142.51	233.44	124.05	88.46
7.42	-1.65	11.82	21.73	11.12	1.09	2.65	10.93	3.72	-4.39	141.16	232.97	123.40	87.87
7.23	-1.83	11.73	21.53	10.95	0.95	2.39	10.53	3.53	-4.63	139.16	232.67	120.06	85.97

6.94	-2.02	11.48	21.18	10.66	0.82	2.05	10.28	3.22	-4.87	150.48	243.42	127.48	96.53
6.93	-2.02	11.47	21.16	10.65	0.81	2.05	10.28	3.22	-4.87	150.76	243.54	127.76	96.80
6.85	-2.05	11.22	20.98	10.50	0.73	2.01	10.24	3.21	-4.87	150.72	244.89	129.61	98.02
6.90	-2.05	11.17	20.95	10.50	0.73	2.11	10.29	3.33	-4.82	151.61	243.76	128.12	95.90
7.05	-1.99	11.44	21.24	10.73	0.78	2.24	10.44	3.38	-4.78	147.43	239.74	125.64	93.08
6.93	-2.05	11.11	20.92	10.45	0.68	2.19	10.35	3.38	-4.78	149.18	241.60	127.65	94.60
6.93	-2.04	11.12	20.92	10.46	0.68	2.20	10.36	3.39	-4.78	149.15	241.48	127.58	94.52
6.86	-2.13	11.02	20.84	10.42	0.62	2.05	10.23	3.29	-4.86	150.24	243.50	129.15	96.19
7.02	-1.96	11.30	21.10	10.58	0.79	2.29	10.48	3.47	-4.69	148.37	238.51	126.77	93.26
7.21	-1.85	11.65	21.46	10.86	0.91	2.39	10.60	3.55	-4.63	147.23	236.99	124.84	91.08
6.98	-2.01	11.28	21.08	10.58	0.77	2.19	10.40	3.40	-4.77	150.09	242.36	129.05	95.68
6.98	-1.99	11.27	21.06	10.57	0.77	2.21	10.42	3.40	-4.76	149.64	242.11	128.60	95.29
6.86	-2.16	11.03	20.88	10.46	0.62	2.00	10.21	3.25	-4.91	149.76	243.67	130.49	97.44
6.87	-2.16	11.04	20.89	10.48	0.62	1.99	10.21	3.25	-4.91	150.02	243.92	130.62	97.39
6.97	-2.10	11.26	21.12	10.67	0.70	2.07	10.31	3.28	-4.87	148.45	243.45	129.39	96.74
7.09	-1.95	11.57	21.35	10.77	0.87	2.30	10.57	3.41	-4.74	152.93	243.96	128.39	97.95
6.96	-2.00	11.27	21.04	10.56	0.80	2.22	10.52	3.36	-4.78	155.21	246.91	131.22	99.96
6.97	-1.99	11.29	21.07	10.58	0.81	2.22	10.52	3.36	-4.78	155.18	247.02	131.05	99.70
7.00	-1.93	11.25	21.02	10.54	0.86	2.31	10.57	3.43	-4.72	154.76	243.40	129.05	97.80
7.40	-1.65	11.87	21.75	11.08	1.07	2.66	10.94	3.73	-4.39	143.95	234.83	122.95	88.60
7.42	-1.64	11.91	21.76	11.14	1.13	2.62	10.91	3.69	-4.42	142.24	233.71	122.75	88.67
7.42	-1.63	11.91	21.77	11.14	1.13	2.62	10.91	3.70	-4.42	142.20	233.66	122.70	88.62
6.78	-2.00	10.89	20.67	10.35	0.80	2.12	10.38	3.21	-4.79	162.84	252.98	135.19	103.20
7.14	-1.83	11.67	21.48	10.98	1.00	2.34	10.60	3.36	-4.67	152.33	242.35	126.81	93.90
7.14	-1.83	11.67	21.49	10.99	1.00	2.34	10.61	3.36	-4.66	152.33	242.29	126.76	93.85
7.14	-1.83	11.67	21.49	10.99	1.00	2.34	10.60	3.35	-4.67	152.40	242.33	126.80	93.90
7.14	-1.83	11.67	21.48	10.99	1.00	2.34	10.60	3.35	-4.67	152.44	242.34	126.82	93.93
7.14	-1.83	11.67	21.48	10.98	0.99	2.34	10.60	3.35	-4.67	152.51	242.40	126.87	94.00
7.11	-1.81	11.50	21.25	10.78	1.02	2.37	10.65	3.42	-4.63	151.74	242.53	127.79	95.46
7.28	-1.75	11.74	21.59	10.98	1.05	2.52	10.78	3.56	-4.56	147.80	238.15	124.26	91.66
7.27	-1.76	11.73	21.57	10.97	1.05	2.52	10.77	3.56	-4.57	147.95	238.23	124.37	91.82
7.28	-1.75	11.72	21.60	10.99	1.01	2.52	10.76	3.57	-4.57	148.77	236.66	124.59	91.72
7.17	-1.86	11.60	21.43	10.87	0.93	2.40	10.66	3.47	-4.64	150.62	238.73	126.67	94.18
7.21	-1.82	11.66	21.49	10.91	0.96	2.44	10.70	3.51	-4.61	149.97	237.81	125.78	93.21
7.44	-1.69	11.91	21.79	11.19	1.08	2.62	10.89	3.69	-4.44	141.89	233.06	122.72	87.70
7.46	-1.67	11.91	21.79	11.18	1.10	2.63	10.92	3.71	-4.42	141.71	233.49	123.24	87.51
7.42	-1.36	12.03	21.77	11.07	1.46	2.75	11.06	3.82	-4.14	147.85	234.97	123.31	99.01
6.79	-2.00	10.96	20.72	10.35	0.82	2.09	10.35	3.25	-4.83	158.95	251.72	134.14	103.21
7.17	-1.77	11.60	21.35	10.86	1.03	2.44	10.70	3.47	-4.61	151.51	240.97	127.02	93.79
6.78	-2.00	10.89	20.67	10.34	0.80	2.12	10.38	3.21	-4.79	162.80	252.97	135.17	103.21
7.13	-1.72	11.62	21.35	10.76	1.10	2.41	10.65	3.49	-4.56	151.44	241.23	125.97	98.77
7.33	-1.59	11.92	21.70	11.01	1.21	2.54	10.75	3.64	-4.45	145.80	235.22	121.64	91.28
7.06	-1.87	11.51	21.23	10.68	0.94	2.26	10.45	3.43	-4.70	148.00	239.05	124.90	93.52

7.11	-1.82	11.64	21.34	10.78	1.01	2.33	10.51	3.46	-4.66	148.25	237.34	123.64	92.36
6.84	-1.99	11.15	20.83	10.40	0.83	2.07	10.30	3.30	-4.81	153.95	245.63	130.37	99.48
7.42	-1.55	12.09	21.83	11.16	1.31	2.60	10.84	3.67	-4.41	145.56	232.74	121.01	90.35
7.42	-1.73	11.91	21.71	11.23	1.02	2.36	10.28	3.62	-4.49	129.46	225.20	112.90	78.84
7.38	-1.59	11.76	21.53	10.98	1.16	2.61	10.62	3.79	-4.35	135.21	225.27	115.34	81.30
6.90	-2.14	10.98	20.82	10.46	0.60	1.94	9.88	3.34	-4.87	137.68	234.76	121.46	86.60
7.21	-1.77	11.57	21.32	10.79	0.98	2.37	10.42	3.62	-4.49	138.65	231.31	117.81	84.92
7.11	-1.82	11.50	21.23	10.70	0.95	2.29	10.42	3.52	-4.59	143.38	233.97	120.38	89.75
7.45	-1.69	11.91	21.84	11.16	1.09	2.57	10.72	3.72	-4.45	135.45	227.84	119.57	84.02
7.10	-1.92	11.25	20.88	10.68	0.76	2.20	10.33	3.53	-4.60	132.75	236.97	126.01	88.79
9.48	0.76	14.11	23.32	13.07	3.32	4.86	13.07	5.87	-1.77	122.55	209.35	102.98	68.64
9.43	0.75	14.06	23.28	13.04	3.31	4.85	13.03	5.87	-1.81	123.07	209.05	102.07	68.80
9.27	0.48	14.03	23.39	13.00	3.05	4.47	12.65	5.51	-2.11	123.87	215.90	105.85	72.35
9.06	0.27	13.87	23.16	12.82	2.95	4.16	12.37	5.27	-2.40	124.89	214.33	105.40	72.37
9.29	0.57	14.01	23.23	13.01	3.15	4.48	12.75	5.60	-2.02	127.37	222.50	113.01	78.97
9.29	0.58	14.02	23.23	13.01	3.15	4.49	12.76	5.60	-2.02	127.25	222.38	112.98	78.86
9.74	1.02	14.62	23.81	13.49	3.69	5.00	13.39	6.02	-1.64	123.35	214.10	107.20	73.08
9.74	1.03	14.61	23.80	13.48	3.68	5.00	13.39	6.03	-1.64	123.12	214.15	107.28	73.09
9.56	0.91	14.08	23.34	13.16	3.50	4.99	13.39	5.97	-1.65	122.09	202.48	99.10	67.00
9.10	0.37	13.65	23.00	12.78	2.90	4.34	12.48	5.44	-2.21	123.40	216.78	108.92	74.03
9.73	1.00	14.61	23.83	13.51	3.68	4.97	13.35	5.97	-1.66	121.38	211.82	105.25	71.25
9.73	1.00	14.61	23.82	13.50	3.68	4.96	13.34	5.97	-1.66	121.37	211.86	105.26	71.26
8.92	-0.03	13.29	22.62	12.53	2.55	4.11	12.17	5.32	-2.59	126.87	218.43	108.81	75.20
8.83	-0.10	13.11	22.45	12.38	2.47	4.07	12.14	5.28	-2.66	126.09	218.26	108.73	75.08
8.80	-0.21	13.12	22.50	12.44	2.39	3.96	12.00	5.17	-2.82	126.33	216.96	108.29	75.00
9.08	0.25	13.72	23.15	12.82	2.89	4.24	12.39	5.34	-2.37	125.50	216.00	105.04	72.75
8.80	-0.05	13.29	22.65	12.49	2.51	3.86	11.97	5.11	-2.63	133.03	227.80	116.98	83.81
8.93	-0.07	13.60	22.98	12.76	2.58	3.90	11.96	5.13	-2.67	134.26	226.84	115.00	82.36
8.83	-0.09	13.43	22.78	12.60	2.52	3.80	11.90	5.04	-2.70	132.64	227.77	116.47	83.65
8.68	-0.19	13.32	22.72	12.46	2.46	3.63	11.76	4.92	-2.78	133.04	226.54	116.54	83.42
8.78	-0.27	13.17	22.57	12.50	2.32	3.78	11.83	5.05	-2.87	128.96	221.07	111.49	78.59
8.91	-0.06	13.62	22.90	12.72	2.56	3.84	11.94	5.09	-2.70	132.71	224.15	112.66	80.79
8.67	-0.23	13.27	22.65	12.44	2.41	3.61	11.72	4.89	-2.82	132.48	227.40	116.38	83.63
8.68	-0.22	13.28	22.66	12.45	2.41	3.62	11.73	4.89	-2.81	132.46	227.44	116.36	83.61
8.68	-0.21	13.28	22.64	12.47	2.43	3.64	11.75	4.91	-2.80	132.38	227.19	117.14	83.79
8.74	-0.16	13.34	22.68	12.53	2.47	3.71	11.83	4.98	-2.75	132.20	227.31	117.33	83.72
8.74	-0.16	13.33	22.68	12.52	2.46	3.70	11.81	4.97	-2.76	132.24	227.29	117.24	83.75
8.75	-0.14	13.35	22.70	12.53	2.48	3.71	11.85	4.98	-2.75	132.23	226.99	117.74	83.58
8.68	-0.21	13.28	22.64	12.47	2.43	3.64	11.75	4.91	-2.80	132.36	227.14	117.31	83.80
8.70	-0.17	13.32	22.70	12.48	2.46	3.65	11.79	4.93	-2.78	132.87	226.63	117.30	83.59
8.66	-0.21	13.31	22.70	12.45	2.45	3.61	11.74	4.90	-2.79	132.98	226.55	116.51	83.49
8.88	0.06	13.68	23.09	12.75	2.74	3.80	11.99	5.03	-2.65	130.06	223.50	111.54	80.14
8.91	-0.06	13.51	22.86	12.67	2.53	3.90	11.98	5.13	-2.67	134.45	229.93	117.81	83.88

8.99	0.00	13.64	22.90	12.77	2.61	3.99	12.07	5.22	-2.63	129.94	222.31	111.56	78.04
8.69	-0.12	13.46	22.86	12.59	2.59	3.52	11.70	4.80	-2.86	129.02	223.02	112.51	80.42
8.91	0.06	13.79	23.19	12.82	2.75	3.75	11.95	5.02	-2.67	128.85	225.72	111.36	79.04
8.84	-0.26	13.25	22.65	12.55	2.33	3.84	11.88	5.09	-2.84	129.04	220.53	112.26	78.97
8.66	-0.29	13.00	22.38	12.30	2.31	3.75	11.83	5.02	-2.88	129.13	220.54	111.15	78.02
8.79	-0.06	13.31	22.68	12.49	2.52	3.83	11.95	5.09	-2.65	133.00	227.54	117.04	83.85
8.79	-0.11	13.39	22.72	12.58	2.51	3.78	11.90	5.03	-2.70	131.99	227.49	117.71	83.45
8.69	-0.36	12.97	22.38	12.31	2.25	3.81	11.84	5.05	-2.97	128.90	218.67	110.27	77.48
8.68	-0.37	12.95	22.37	12.30	2.24	3.80	11.83	5.04	-2.98	128.90	218.75	110.32	77.55
8.84	-0.11	13.20	22.61	12.51	2.43	3.91	11.94	5.16	-2.68	137.61	234.61	118.85	86.61
8.93	-0.07	13.60	22.97	12.76	2.57	3.90	11.96	5.13	-2.67	134.23	226.84	115.00	82.33
8.92	-0.10	13.54	22.88	12.70	2.51	3.85	11.93	5.12	-2.74	132.65	225.39	114.32	81.02
8.52	-0.45	12.73	22.09	12.16	2.06	3.54	11.52	4.88	-2.98	140.80	238.18	124.23	92.08
8.58	-0.41	12.84	22.20	12.26	2.12	3.60	11.59	4.91	-2.95	140.00	237.30	123.74	91.26
9.41	0.59	14.31	23.60	13.20	3.23	4.60	12.80	5.61	-2.07	120.59	206.49	100.20	67.05
9.40	0.55	14.25	23.57	13.22	3.23	4.59	12.75	5.58	-2.10	120.72	207.95	100.53	67.26
9.37	0.36	14.40	23.83	13.32	3.13	4.35	12.56	5.40	-2.42	118.77	206.66	98.98	67.00
9.31	0.24	14.37	23.77	13.31	3.07	4.23	12.41	5.33	-2.53	117.82	206.58	98.93	66.07
9.36	0.53	14.09	23.32	13.07	3.17	4.65	12.79	5.65	-2.06	119.53	205.60	99.59	66.20
9.35	0.53	14.08	23.30	13.06	3.17	4.65	12.79	5.65	-2.05	119.53	205.56	99.56	66.23
9.35	0.53	14.09	23.32	13.07	3.17	4.65	12.79	5.65	-2.06	119.52	205.60	99.59	66.19
8.68	-0.50	13.13	22.47	12.41	2.18	3.67	11.74	4.95	-3.18	127.66	216.39	109.80	77.05
9.02	-0.17	13.59	22.92	12.83	2.54	4.03	12.09	5.20	-2.87	124.48	213.49	106.09	72.24
8.92	-0.02	13.92	23.30	12.87	2.74	4.00	12.11	4.98	-2.76	118.85	206.55	99.09	67.00
8.92	-0.02	13.92	23.30	12.87	2.74	4.00	12.11	4.98	-2.76	118.81	206.50	99.09	67.00
9.01	-0.12	13.84	23.17	12.83	2.63	4.08	12.21	5.21	-2.84	117.41	206.75	98.09	65.02
8.99	-0.07	13.65	23.10	12.72	2.62	4.14	12.26	5.24	-2.77	117.85	205.88	98.61	66.05
8.96	-0.09	13.68	23.14	12.75	2.61	4.11	12.21	5.22	-2.80	118.57	205.26	98.01	65.42
9.07	-0.05	14.00	23.38	12.99	2.72	4.06	12.19	5.15	-2.81	115.56	205.75	97.80	64.01
8.95	-0.23	13.77	23.08	12.82	2.50	3.99	12.12	5.13	-2.95	118.17	207.03	98.71	65.81
8.95	-0.16	13.81	23.16	12.84	2.59	3.95	12.08	5.08	-2.92	118.60	206.81	98.52	66.20
8.87	-0.44	13.50	22.85	12.76	2.29	3.77	11.91	5.01	-3.14	119.40	208.21	102.72	69.24
9.33	0.43	14.49	23.81	13.33	3.20	4.37	12.53	5.31	-2.40	117.12	206.54	98.71	65.00
9.20	0.15	14.19	23.64	13.09	2.96	4.26	12.37	5.28	-2.66	116.64	202.58	95.30	63.77
9.13	0.38	13.91	23.28	12.96	3.08	4.38	12.57	5.33	-2.30	123.09	206.61	101.04	68.01
9.14	0.39	13.91	23.27	12.95	3.06	4.39	12.55	5.32	-2.30	123.06	206.38	100.94	68.00
9.12	0.38	13.90	23.26	12.94	3.07	4.36	12.55	5.32	-2.31	123.01	206.68	101.04	68.03
9.35	0.59	14.20	23.68	13.20	3.31	4.55	12.86	5.51	-2.12	122.98	207.46	99.10	67.45
8.52	-0.25	13.27	22.74	12.37	2.54	3.65	11.92	4.65	-3.03	124.40	202.06	98.00	67.00
9.15	0.36	13.87	23.14	12.86	2.97	4.30	12.48	5.43	-2.25	125.09	216.06	106.00	73.61
9.14	0.19	13.65	23.15	12.86	2.81	4.30	12.39	5.42	-2.42	123.72	216.96	107.23	73.16
9.10	0.30	13.97	23.25	12.90	3.00	4.20	12.41	5.29	-2.37	124.81	213.12	104.62	71.56
8.88	0.05	13.30	22.56	12.51	2.61	4.10	12.34	5.27	-2.55	137.24	235.89	132.03	98.47

9.34	0.58	13.97	23.20	12.96	3.17	4.59	12.97	5.71	-2.03	129.95	223.77	119.35	86.08
9.43	0.67	14.18	23.46	13.19	3.29	4.58	12.95	5.69	-1.97	127.38	223.23	116.40	82.81
9.24	0.40	13.84	23.11	12.98	3.04	4.37	12.68	5.49	-2.23	131.95	231.49	125.14	91.85
9.48	0.66	14.46	23.57	13.27	3.37	4.57	12.99	5.68	-2.04	131.80	227.17	125.47	91.30
9.36	0.61	14.00	23.22	12.98	3.18	4.61	13.00	5.72	-2.01	130.22	223.45	119.41	86.08
8.54	-1.14	13.17	22.38	12.61	1.62	3.30	11.42	4.49	-3.93	117.73	215.21	105.44	69.96
8.53	-1.06	13.10	22.31	12.41	1.69	3.40	11.52	4.67	-3.80	120.24	214.51	107.97	73.35
8.53	-1.06	13.10	22.31	12.41	1.69	3.41	11.52	4.68	-3.79	120.08	214.38	107.78	73.32
8.50	-1.04	12.88	22.28	12.28	1.66	3.46	11.53	4.73	-3.73	124.54	216.14	109.00	76.00
8.62	-0.90	13.18	22.50	12.50	1.83	3.48	11.59	4.76	-3.65	124.03	214.50	107.89	74.88
8.59	-0.89	13.10	22.59	12.42	1.83	3.46	11.55	4.74	-3.63	126.82	215.94	109.95	76.11
8.64	-0.87	13.25	22.54	12.54	1.87	3.48	11.60	4.77	-3.63	124.10	213.50	108.02	74.09
8.73	-0.75	13.32	22.61	12.65	2.02	3.58	11.69	4.79	-3.50	119.92	211.71	104.05	71.91
8.93	-0.47	13.56	22.91	12.84	2.27	3.81	11.90	4.97	-3.19	117.12	208.83	101.06	69.00
8.62	-0.86	12.99	22.56	12.55	1.87	3.55	11.63	4.70	-3.59	116.20	211.90	102.45	67.97
8.55	-1.04	13.12	22.33	12.42	1.70	3.41	11.54	4.68	-3.79	120.03	214.40	108.00	73.21
8.58	-1.02	13.15	22.35	12.45	1.73	3.44	11.55	4.70	-3.76	119.25	213.27	107.65	73.05
8.54	-1.05	13.11	22.32	12.42	1.69	3.42	11.53	4.68	-3.79	120.47	214.70	107.95	73.26
8.70	-0.93	13.38	22.64	12.67	1.83	3.50	11.62	4.74	-3.68	121.07	213.28	107.24	72.99
8.56	-0.96	13.06	22.39	12.40	1.75	3.45	11.54	4.73	-3.70	124.30	215.09	108.88	75.88
8.55	-0.97	13.05	22.38	12.39	1.74	3.45	11.53	4.73	-3.70	124.36	215.15	108.92	75.92
8.55	-0.96	13.06	22.39	12.40	1.75	3.45	11.54	4.73	-3.70	124.32	215.12	108.89	75.90
9.07	-0.07	13.95	23.33	12.97	2.70	4.07	12.18	5.16	-2.80	115.01	205.14	98.00	64.00
8.81	-0.48	13.45	22.80	12.68	2.24	3.70	11.84	4.96	-3.21	121.47	210.26	102.85	70.14
8.72	-0.62	13.25	22.65	12.58	2.10	3.61	11.70	4.86	-3.35	122.22	210.98	104.26	71.94
8.61	-0.96	13.03	22.39	12.49	1.80	3.52	11.61	4.74	-3.70	116.96	212.02	104.18	70.37
8.70	-0.76	13.16	22.42	12.50	1.97	3.69	11.78	4.88	-3.49	117.92	210.32	103.56	68.00
8.60	-0.89	13.04	22.36	12.45	1.86	3.55	11.64	4.76	-3.64	117.51	211.36	103.99	70.37
9.05	-0.08	13.81	23.16	12.85	2.67	4.13	12.28	5.25	-2.81	116.90	207.67	99.79	66.97
8.94	-0.31	13.56	23.05	12.81	2.41	3.98	12.07	5.05	-3.03	116.09	206.03	99.90	64.76
8.92	-0.38	13.58	22.91	12.81	2.31	3.82	11.96	5.03	-3.12	118.42	209.52	102.01	68.89
8.94	-0.38	13.61	22.96	12.84	2.34	3.84	11.97	5.03	-3.10	118.18	209.03	100.86	67.95
8.91	-0.40	13.53	22.93	12.80	2.32	3.81	11.95	5.01	-3.13	117.96	208.53	100.63	67.93
8.91	-0.40	13.54	22.94	12.80	2.32	3.81	11.95	5.01	-3.12	117.89	208.67	100.56	67.91
8.96	-0.31	13.65	23.07	12.87	2.43	3.91	12.03	5.07	-3.05	118.58	207.28	100.98	66.70
8.94	-0.33	13.57	23.01	12.83	2.40	3.90	12.01	5.06	-3.08	118.30	207.64	100.96	67.02
8.77	-0.58	13.26	22.74	12.63	2.14	3.79	11.83	4.93	-3.31	115.34	209.45	100.99	66.26
8.78	-0.57	13.28	22.76	12.65	2.15	3.80	11.85	4.94	-3.30	115.21	209.25	100.93	65.99
8.77	-0.59	13.27	22.75	12.64	2.14	3.78	11.83	4.92	-3.32	115.49	209.61	101.03	66.29
8.88	-0.43	13.46	22.98	12.77	2.32	3.89	11.97	4.97	-3.17	115.57	208.44	99.59	65.18
8.95	-0.18	13.62	23.01	12.75	2.54	4.12	12.16	5.15	-2.92	115.56	204.53	97.97	63.98
8.77	-0.56	13.28	22.77	12.62	2.17	3.78	11.85	4.92	-3.26	116.54	209.45	100.46	66.30
8.78	-0.55	13.30	22.79	12.64	2.19	3.78	11.85	4.92	-3.26	116.36	209.13	100.35	66.09

8.69	-0.98	13.39	22.63	12.77	1.80	3.45	11.60	4.60	-3.76	115.52	213.84	104.00	68.00
8.69	-0.98	13.39	22.63	12.77	1.81	3.45	11.60	4.60	-3.76	115.54	213.84	103.99	68.00
8.62	-0.88	13.04	22.39	12.47	1.86	3.56	11.65	4.78	-3.64	117.49	211.25	103.95	69.65
8.62	-0.88	13.04	22.39	12.47	1.86	3.56	11.65	4.78	-3.64	117.49	211.26	103.95	69.72
8.87	-0.60	13.36	22.93	12.78	2.17	3.81	11.83	4.96	-3.34	115.48	209.00	100.82	65.23
8.96	-0.43	13.59	23.17	12.93	2.30	3.90	11.98	4.97	-3.19	116.98	208.33	100.22	65.01
8.84	-0.55	13.33	22.85	12.72	2.17	3.83	11.89	4.96	-3.29	115.47	210.00	100.73	66.49
8.93	-0.40	13.52	22.91	12.80	2.31	3.85	11.98	5.04	-3.12	117.35	208.01	100.98	67.57
8.91	-0.41	13.46	22.90	12.79	2.31	3.87	11.98	5.02	-3.12	116.79	207.83	101.00	67.16
8.84	-0.63	13.29	22.89	12.76	2.12	3.75	11.78	4.87	-3.37	118.16	210.87	101.07	67.86
8.82	-0.61	13.40	22.90	12.76	2.15	3.71	11.76	4.87	-3.34	118.55	211.19	101.40	67.99
8.95	-0.42	13.54	23.14	12.93	2.34	3.87	11.94	4.98	-3.18	116.48	207.93	101.47	66.02
8.95	-0.41	13.54	23.15	12.93	2.34	3.87	11.94	4.99	-3.18	116.46	207.87	101.40	66.01
8.95	-0.41	13.54	23.15	12.93	2.34	3.87	11.94	4.98	-3.18	116.48	207.87	101.41	66.02
8.78	-0.58	13.30	22.69	12.61	2.13	3.66	11.79	4.92	-3.29	120.28	211.58	103.04	71.08
8.49	-1.17	13.25	22.51	12.57	1.69	3.11	11.28	4.40	-4.01	116.49	219.22	106.00	68.87
8.50	-1.16	13.25	22.51	12.59	1.70	3.12	11.29	4.40	-4.00	116.29	219.36	105.98	68.73
8.46	-1.19	13.14	22.45	12.51	1.67	3.11	11.26	4.40	-4.04	116.22	220.69	106.82	69.49
8.48	-1.15	13.28	22.50	12.58	1.71	3.09	11.26	4.40	-4.04	115.75	219.07	106.02	68.83
8.48	-0.87	13.03	22.40	12.38	1.92	3.42	11.41	4.58	-3.64	117.18	218.26	104.88	66.00
8.50	-1.08	13.50	22.61	12.59	1.85	3.06	11.20	4.40	-4.03	116.39	221.15	106.05	68.25
8.40	-1.24	13.30	22.42	12.44	1.66	2.94	11.12	4.34	-4.13	118.60	222.82	107.76	70.94
8.48	-1.15	13.28	22.50	12.58	1.71	3.09	11.26	4.40	-4.04	115.76	219.07	106.02	68.80
8.47	-1.01	13.13	22.43	12.43	1.85	3.26	11.35	4.51	-3.84	117.19	218.92	105.21	68.43
8.49	-0.83	13.02	22.39	12.40	1.94	3.47	11.46	4.61	-3.60	117.58	218.14	103.66	66.00
8.49	-0.86	13.06	22.42	12.41	1.94	3.43	11.42	4.59	-3.63	117.16	218.30	104.63	66.00
8.42	-0.84	13.03	22.50	12.30	1.96	3.34	11.32	4.50	-3.60	118.45	220.09	105.00	66.00
8.24	-0.95	12.78	22.25	12.05	1.81	3.17	11.10	4.44	-3.72	120.43	227.07	107.52	66.93
8.24	-0.96	12.78	22.25	12.04	1.80	3.17	11.09	4.44	-3.72	120.50	227.14	107.63	66.99
8.42	-0.83	13.04	22.50	12.30	1.96	3.35	11.31	4.50	-3.60	118.53	219.83	105.00	66.00
8.32	-0.86	12.92	22.40	12.17	1.92	3.24	11.17	4.49	-3.64	121.06	221.24	106.19	66.85
8.32	-0.86	12.92	22.40	12.17	1.92	3.23	11.17	4.49	-3.64	121.08	221.29	106.24	66.88
8.42	-0.84	13.03	22.50	12.30	1.96	3.34	11.32	4.50	-3.60	118.45	220.08	105.00	66.00
8.40	-0.89	12.89	22.29	12.27	1.87	3.42	11.38	4.55	-3.66	117.57	220.08	104.94	66.00
8.60	-0.71	13.09	22.54	12.48	2.06	3.60	11.62	4.70	-3.47	116.39	214.21	101.89	65.87
8.36	-0.86	12.84	22.32	12.18	1.87	3.38	11.34	4.55	-3.59	119.38	221.57	105.00	66.00
8.95	-0.34	13.67	23.06	12.88	2.49	3.92	12.04	4.97	-3.16	115.23	206.69	98.73	63.91
8.96	-0.34	13.66	23.05	12.86	2.49	3.93	12.05	4.98	-3.16	115.17	207.08	98.53	63.93
8.96	-0.34	13.66	23.05	12.87	2.49	3.93	12.05	4.98	-3.16	115.18	207.03	98.55	63.93
8.96	-0.34	13.66	23.04	12.86	2.49	3.93	12.06	4.99	-3.16	115.22	207.28	98.45	63.94
8.94	-0.35	13.64	23.02	12.86	2.46	3.92	12.02	4.97	-3.18	114.68	206.62	98.51	63.59
8.86	-0.39	13.74	23.31	12.92	2.45	3.68	11.79	4.77	-3.23	114.22	207.74	98.89	63.00
8.57	-0.78	12.95	22.36	12.45	1.97	3.57	11.68	4.68	-3.53	115.08	214.38	102.66	66.72

8.57	-0.78	12.97	22.38	12.46	1.97	3.57	11.69	4.68	-3.53	115.11	214.35	102.56	66.63
8.77	-0.72	13.34	22.94	12.86	2.07	3.67	11.73	4.67	-3.49	114.22	211.51	100.75	65.00
8.54	-0.80	12.89	22.19	12.41	1.91	3.61	11.67	4.72	-3.52	114.48	213.19	101.95	65.50
8.34	-0.89	12.63	22.05	12.08	1.82	3.46	11.40	4.58	-3.59	118.10	220.04	104.18	66.47
8.55	-0.66	13.20	22.68	12.41	2.07	3.49	11.49	4.68	-3.41	115.04	214.42	103.75	65.30
8.46	-0.85	12.63	22.01	12.22	1.82	3.58	11.59	4.69	-3.52	114.36	215.70	102.81	66.96
8.56	-0.75	12.92	22.31	12.40	1.96	3.62	11.67	4.71	-3.47	113.65	213.68	102.79	66.08
8.54	-0.75	13.10	22.58	12.44	2.02	3.51	11.53	4.64	-3.52	116.95	215.72	103.37	65.87
8.27	-0.96	12.63	22.11	12.03	1.78	3.29	11.26	4.48	-3.66	118.44	220.24	105.53	68.22
8.87	-0.42	13.53	22.88	12.84	2.36	3.87	11.93	4.91	-3.23	113.04	206.36	98.02	64.00
8.87	-0.42	13.53	22.88	12.84	2.36	3.87	11.93	4.91	-3.22	113.02	206.35	98.02	63.99
8.91	-0.37	13.69	23.07	12.90	2.48	3.87	12.00	4.96	-3.18	115.73	207.20	98.93	63.93
8.94	-0.35	13.69	23.08	12.90	2.48	3.88	11.99	4.97	-3.17	115.36	207.24	98.83	63.84
8.97	-0.32	13.76	23.17	12.96	2.53	3.93	12.06	4.97	-3.14	115.56	207.13	98.25	63.12
8.76	-0.60	13.35	22.79	12.76	2.18	3.67	11.74	4.76	-3.39	112.70	210.78	100.75	64.92
8.72	-0.64	13.30	22.71	12.70	2.12	3.67	11.73	4.72	-3.43	113.24	211.64	101.02	64.68
8.77	-0.59	13.44	22.88	12.82	2.23	3.67	11.73	4.72	-3.38	113.48	210.64	100.70	64.76
8.78	-0.51	13.67	23.18	12.94	2.30	3.60	11.64	4.65	-3.36	113.21	208.23	99.06	63.11
8.86	-0.44	13.69	23.20	12.96	2.39	3.67	11.74	4.73	-3.28	113.97	206.63	98.17	63.00
8.77	-0.47	13.71	23.23	12.95	2.40	3.57	11.63	4.66	-3.30	113.94	207.86	99.27	63.00
8.77	-0.47	13.71	23.23	12.95	2.40	3.57	11.63	4.66	-3.30	113.88	207.83	99.29	63.00
8.84	-0.42	13.71	23.27	12.94	2.41	3.66	11.73	4.72	-3.28	113.90	207.00	98.82	63.00
8.84	-0.42	13.71	23.27	12.94	2.41	3.66	11.73	4.72	-3.28	113.91	207.06	98.84	63.00
8.74	-0.68	13.39	22.90	12.78	2.14	3.66	11.81	4.69	-3.47	114.00	211.95	101.00	66.05
8.79	-0.60	13.40	22.83	12.79	2.19	3.69	11.78	4.78	-3.40	112.35	211.05	101.24	64.92
8.54	-0.82	12.93	22.22	12.40	1.90	3.58	11.60	4.67	-3.57	114.18	214.80	101.91	66.00
8.48	-0.86	12.83	22.17	12.31	1.86	3.53	11.56	4.64	-3.58	115.84	215.57	102.06	66.00
8.44	-0.74	13.25	22.79	12.40	2.14	3.27	11.24	4.51	-3.61	118.00	219.02	105.48	66.27
9.00	-0.17	13.76	23.16	12.87	2.58	4.04	12.14	5.12	-2.90	117.11	206.02	98.00	64.04
8.98	-0.23	13.63	23.09	12.82	2.49	4.10	12.14	5.13	-2.93	115.26	205.80	98.01	64.00
8.93	-0.30	13.53	23.00	12.79	2.45	4.00	12.08	5.07	-3.06	113.99	207.32	97.79	63.78
8.92	-0.33	13.51	22.98	12.78	2.44	4.01	12.09	5.07	-3.07	113.19	207.07	97.37	64.00
8.94	-0.25	13.54	22.98	12.76	2.50	4.08	12.14	5.12	-3.00	114.37	206.52	97.05	63.61
9.00	-0.10	13.80	23.24	12.82	2.63	4.14	12.23	5.20	-2.85	115.90	205.29	98.09	63.10
8.90	-0.45	13.54	22.87	12.76	2.26	3.77	11.91	5.02	-3.18	120.37	209.83	102.25	69.60
8.99	-0.19	13.70	23.09	12.80	2.58	4.11	12.19	5.16	-2.95	113.59	204.99	97.00	63.00
8.94	-0.33	13.76	23.19	12.91	2.56	3.86	11.99	4.94	-3.14	114.47	205.79	97.18	63.00
8.82	-0.39	13.55	22.99	12.69	2.44	3.80	11.90	4.92	-3.20	114.57	206.41	98.44	63.88
9.29	0.57	14.01	23.23	13.01	3.15	4.48	12.75	5.60	-2.02	127.37	222.50	113.01	78.97
8.41	-0.25	12.32	21.59	11.73	2.17	3.87	11.98	5.09	-2.67	137.44	235.14	125.24	91.96
8.48	-0.18	12.45	21.72	11.82	2.24	3.92	11.96	5.09	-2.59	136.64	233.76	121.92	88.40
8.46	-0.21	12.50	21.77	11.87	2.22	3.85	11.91	5.04	-2.64	134.62	233.47	121.05	87.97
8.61	-0.13	12.80	22.11	12.16	2.34	3.91	11.98	5.07	-2.62	132.74	230.73	117.04	84.13

8.40	-0.25	12.43	21.70	11.80	2.20	3.79	11.90	5.02	-2.70	139.40	238.92	127.21	94.02
8.31	-0.35	12.27	21.55	11.65	2.09	3.71	11.82	4.94	-2.79	139.81	236.40	125.52	93.78
8.63	-0.12	12.78	22.02	12.10	2.35	3.97	12.06	5.16	-2.57	140.61	240.55	129.22	96.80
8.58	-0.16	12.68	21.93	12.01	2.31	3.92	12.03	5.13	-2.62	139.72	240.36	128.21	95.66
8.37	-0.29	12.41	21.67	11.78	2.18	3.75	11.85	4.99	-2.76	140.44	238.60	127.81	94.57
8.28	-0.37	12.22	21.52	11.60	2.06	3.69	11.82	4.95	-2.80	139.03	236.67	125.07	93.88
8.57	-0.07	12.50	21.76	11.88	2.32	4.06	12.19	5.26	-2.48	135.17	233.48	123.76	91.56
8.80	0.13	12.84	22.08	12.15	2.57	4.30	12.42	5.45	-2.29	133.93	230.17	121.02	88.59
8.78	0.11	12.84	22.03	12.13	2.51	4.25	12.36	5.41	-2.34	134.31	231.05	120.08	87.87
8.57	-0.20	12.76	22.05	12.07	2.27	3.89	12.06	5.07	-2.67	138.30	238.66	129.39	96.09
8.73	-0.07	12.97	22.26	12.27	2.41	4.02	12.20	5.19	-2.56	135.52	235.17	127.23	94.15
8.30	-0.33	12.33	21.61	11.71	2.08	3.67	11.74	4.90	-2.76	138.51	236.38	123.63	90.49
8.24	-0.39	12.18	21.47	11.60	2.05	3.65	11.72	4.88	-2.81	137.83	234.64	123.73	90.46
8.37	-0.25	12.33	21.61	11.72	2.17	3.83	11.88	5.03	-2.67	138.38	234.96	123.44	89.67
8.31	-0.32	12.28	21.56	11.67	2.10	3.72	11.78	4.92	-2.76	134.56	233.14	120.90	88.72
9.01	0.30	13.20	22.42	12.47	2.76	4.43	12.60	5.58	-2.16	132.02	229.24	119.53	85.88
9.00	0.30	13.19	22.41	12.46	2.75	4.43	12.59	5.57	-2.16	132.04	229.24	119.54	85.90
8.59	-0.05	12.59	21.82	11.95	2.35	4.07	12.12	5.24	-2.48	137.23	235.17	123.08	88.88
8.59	-0.09	12.66	21.91	11.97	2.35	4.05	12.18	5.21	-2.51	136.17	234.36	125.09	92.76
9.26	0.53	13.61	22.84	12.77	3.01	4.61	12.85	5.74	-1.96	130.54	226.36	120.05	85.19
9.13	0.38	13.72	22.99	12.77	2.97	4.34	12.60	5.44	-2.18	129.51	225.31	116.65	81.82
8.37	-0.25	12.33	21.61	11.71	2.17	3.83	11.88	5.03	-2.67	138.38	234.96	123.44	89.68
8.51	-0.19	12.56	21.82	11.93	2.26	3.90	11.95	5.08	-2.61	135.66	233.63	121.20	87.90
8.32	-0.31	12.30	21.59	11.71	2.12	3.75	11.81	4.96	-2.75	134.73	232.16	119.86	88.37
8.41	-0.25	12.39	21.66	11.78	2.18	3.84	11.89	5.04	-2.68	134.43	231.39	119.91	88.25
8.41	-0.24	12.39	21.67	11.79	2.19	3.84	11.90	5.04	-2.67	134.36	231.35	119.89	88.18
8.41	-0.24	12.40	21.67	11.79	2.19	3.85	11.90	5.05	-2.67	134.33	231.33	119.88	88.15
8.64	-0.07	12.59	21.83	11.98	2.37	4.13	12.18	5.29	-2.50	135.38	232.76	121.00	88.52
8.68	-0.02	12.69	21.92	12.05	2.41	4.15	12.22	5.30	-2.47	134.46	230.28	119.61	87.11
8.69	0.00	12.73	21.96	12.07	2.43	4.14	12.24	5.30	-2.44	134.55	231.49	119.51	87.19
8.65	-0.04	12.73	21.98	12.09	2.39	4.05	12.17	5.24	-2.50	134.83	229.86	119.57	86.33
8.73	0.01	12.80	22.03	12.15	2.45	4.18	12.26	5.32	-2.41	133.89	230.32	119.14	85.98
8.61	-0.13	12.81	22.12	12.17	2.34	3.91	11.98	5.07	-2.62	132.64	230.67	117.00	84.07
8.46	-0.24	12.59	21.88	11.96	2.23	3.81	11.87	4.99	-2.69	135.03	232.90	120.21	86.45
8.82	0.06	13.12	22.42	12.40	2.59	4.08	12.20	5.24	-2.46	129.92	228.32	114.92	81.50
8.90	0.16	13.24	22.47	12.43	2.67	4.19	12.35	5.38	-2.33	129.24	227.68	114.52	81.05
8.76	0.02	13.01	22.30	12.32	2.52	4.06	12.17	5.22	-2.48	130.06	228.34	115.28	82.48
8.60	-0.04	12.50	21.77	11.88	2.35	4.11	12.23	5.31	-2.43	135.13	234.02	123.24	90.97
8.33	-0.31	12.26	21.55	11.65	2.11	3.76	11.90	5.02	-2.74	138.00	236.07	125.06	93.28
8.44	-0.21	12.37	21.64	11.78	2.22	3.92	12.00	5.12	-2.64	137.81	234.77	124.85	91.32
8.57	-0.11	12.50	21.74	11.89	2.32	4.06	12.16	5.25	-2.53	134.82	232.82	121.14	88.88
8.50	-0.17	12.44	21.69	11.82	2.27	4.01	12.09	5.18	-2.60	136.92	233.47	122.92	90.48
8.36	-0.30	12.37	21.64	11.73	2.16	3.77	11.88	5.00	-2.72	139.42	237.76	126.62	93.99

8.32	-0.32	12.25	21.55	11.65	2.10	3.75	11.87	5.00	-2.76	138.79	236.14	125.09	93.36
8.31	-0.33	12.25	21.54	11.64	2.09	3.74	11.85	4.98	-2.77	139.09	236.18	125.10	93.37
8.32	-0.32	12.25	21.55	11.65	2.10	3.75	11.87	4.99	-2.76	139.03	236.09	125.13	93.27
8.32	-0.32	12.30	21.56	11.68	2.08	3.71	11.78	4.93	-2.74	137.93	235.03	123.44	90.18
8.56	-0.07	12.53	21.76	11.89	2.34	4.01	12.07	5.21	-2.52	136.34	234.64	122.41	88.35
8.50	-0.15	12.48	21.73	11.86	2.27	3.95	12.01	5.15	-2.56	137.73	235.51	123.77	90.02
8.52	-0.17	12.61	21.89	11.99	2.28	3.89	11.95	5.06	-2.59	135.81	233.54	120.85	87.50
8.58	-0.09	12.59	21.94	11.98	2.32	3.96	11.93	5.16	-2.51	137.93	235.71	121.39	87.64
8.70	-0.10	13.03	22.38	12.31	2.41	3.87	11.91	5.11	-2.64	133.84	230.49	118.14	84.75
8.80	-0.05	13.21	22.55	12.46	2.49	3.94	12.00	5.15	-2.60	133.47	229.53	117.49	84.02
8.74	-0.07	13.09	22.44	12.35	2.44	3.89	11.95	5.12	-2.61	133.46	230.03	117.39	84.37
8.53	-0.44	12.91	22.27	12.18	2.12	3.52	11.60	4.89	-3.00	136.83	228.96	118.00	86.62
8.55	-0.43	12.94	22.30	12.21	2.13	3.54	11.61	4.90	-2.98	136.86	228.95	118.01	86.48
8.48	-0.56	12.63	21.97	12.02	1.98	3.58	11.61	4.95	-3.11	134.14	224.90	115.93	83.54
8.36	-0.48	12.41	21.78	11.86	1.98	3.46	11.45	4.85	-2.93	143.42	241.52	126.63	94.06
8.21	-0.96	12.36	21.77	11.78	1.68	3.18	11.28	4.63	-3.56	133.50	224.62	116.26	84.68
8.21	-0.95	12.37	21.78	11.79	1.68	3.18	11.29	4.63	-3.55	133.51	224.52	116.15	84.55
8.22	-0.98	12.39	21.85	11.82	1.65	3.22	11.31	4.61	-3.60	131.25	221.28	113.79	82.00
8.40	-0.67	12.44	21.79	11.85	1.84	3.51	11.54	4.93	-3.19	134.27	223.59	117.07	84.84
8.39	-0.68	12.44	21.78	11.84	1.85	3.50	11.53	4.92	-3.21	132.83	224.52	117.04	84.73
8.41	-0.66	12.47	21.81	11.87	1.84	3.52	11.56	4.94	-3.20	133.66	223.83	117.05	84.53
8.46	-0.40	12.56	21.93	12.00	2.06	3.54	11.55	4.93	-2.88	142.83	240.93	125.42	92.70
8.51	-0.38	12.69	22.04	12.12	2.11	3.55	11.59	4.92	-2.89	140.82	239.92	124.19	91.95
8.57	-0.35	12.80	22.12	12.19	2.16	3.61	11.63	4.97	-2.89	140.53	238.86	123.83	91.38
8.57	-0.35	12.80	22.12	12.19	2.16	3.60	11.62	4.97	-2.89	140.54	238.88	123.84	91.39
8.51	-0.38	12.66	22.04	12.08	2.12	3.56	11.59	4.93	-2.88	141.67	239.60	124.05	92.01
8.58	-0.43	12.96	22.33	12.30	2.15	3.50	11.57	4.87	-3.02	136.01	230.59	119.31	87.51
8.44	-0.40	12.52	21.88	11.96	2.05	3.55	11.54	4.91	-2.85	143.00	241.43	126.00	93.31
8.41	-0.54	12.70	22.04	12.01	2.01	3.43	11.50	4.82	-3.09	135.96	228.04	118.31	87.23
8.42	-0.53	12.71	22.04	12.01	2.02	3.46	11.52	4.84	-3.07	135.81	227.88	118.34	87.00
8.65	-0.33	12.95	22.24	12.22	2.20	3.74	11.81	5.09	-2.85	135.23	227.96	117.20	84.70
8.16	-0.62	12.15	21.47	11.61	1.80	3.27	11.27	4.75	-3.04	145.86	243.44	129.65	97.02
8.21	-0.61	12.22	21.57	11.67	1.84	3.28	11.30	4.75	-3.05	143.90	242.45	127.93	95.46
8.29	-0.57	12.33	21.67	11.77	1.89	3.36	11.36	4.80	-3.02	143.10	242.17	127.43	95.03
8.58	-0.42	12.87	22.24	12.18	2.13	3.63	11.67	4.98	-2.97	133.77	226.76	116.07	83.32
8.35	-0.60	12.47	21.82	11.94	1.92	3.36	11.36	4.76	-3.13	141.45	239.76	125.93	93.42
8.32	-0.67	12.43	21.82	11.93	1.87	3.28	11.28	4.71	-3.21	142.09	238.24	125.95	94.27
8.35	-0.64	12.47	21.85	11.96	1.89	3.31	11.32	4.73	-3.19	141.77	238.41	125.58	93.98
8.72	-0.15	13.08	22.35	12.33	2.41	3.93	12.18	5.12	-2.72	142.75	239.62	137.62	105.02
8.99	0.11	13.33	22.65	12.56	2.64	4.23	12.46	5.38	-2.44	138.98	236.24	134.05	101.84
8.75	-0.11	13.04	22.32	12.32	2.42	4.00	12.21	5.17	-2.63	143.42	242.31	137.59	105.56
8.77	-0.10	13.07	22.34	12.34	2.44	4.02	12.22	5.18	-2.62	143.47	242.23	137.50	105.47
8.40	-0.45	12.55	21.90	11.89	2.05	3.64	11.87	4.85	-2.94	142.91	243.71	139.26	107.77

8.36	-0.44	12.46	21.82	11.83	2.02	3.70	11.87	4.91	-2.87	144.26	244.27	136.47	104.39
8.68	-0.16	12.89	22.23	12.19	2.35	3.98	12.18	5.16	-2.65	139.46	239.07	134.74	102.11
8.90	0.04	13.25	22.51	12.49	2.56	4.17	12.36	5.30	-2.46	137.05	237.40	132.24	97.79
8.83	-0.02	13.15	22.41	12.41	2.49	4.08	12.29	5.24	-2.53	136.93	237.52	131.87	97.55
8.85	0.00	13.19	22.45	12.44	2.52	4.11	12.31	5.26	-2.51	136.80	237.48	131.78	97.54
8.72	-0.11	13.07	22.33	12.32	2.43	3.95	12.18	5.13	-2.63	136.78	236.89	130.48	96.95
8.63	-0.18	12.93	22.21	12.20	2.33	3.84	12.07	5.04	-2.72	136.44	236.93	128.45	96.12
8.82	-0.06	13.15	22.40	12.40	2.49	4.06	12.25	5.22	-2.58	143.92	242.53	137.83	105.71
8.79	-0.08	13.11	22.37	12.36	2.46	4.03	12.23	5.20	-2.60	143.81	242.69	137.93	105.89
8.80	-0.07	13.13	22.38	12.38	2.47	4.04	12.24	5.21	-2.59	143.92	242.86	138.11	106.07
8.65	-0.20	12.91	22.22	12.21	2.32	3.92	12.12	5.10	-2.70	143.17	242.84	138.28	106.33
8.80	-0.07	13.13	22.38	12.39	2.47	4.04	12.23	5.21	-2.59	143.86	243.14	138.42	106.42
8.31	-0.52	12.45	21.80	11.80	1.96	3.56	11.78	4.79	-3.00	142.97	243.82	139.71	108.29
8.30	-0.53	12.43	21.79	11.79	1.95	3.55	11.77	4.78	-3.00	142.98	243.78	139.69	108.32
8.30	-0.53	12.43	21.79	11.78	1.95	3.56	11.77	4.78	-3.00	142.95	243.66	139.46	108.08
8.41	-0.44	12.57	21.92	11.91	2.06	3.66	11.88	4.87	-2.92	142.91	243.68	139.20	107.72
8.41	-0.44	12.56	21.91	11.90	2.06	3.65	11.88	4.86	-2.93	142.89	243.64	139.14	107.66
8.42	-0.42	12.57	21.93	11.91	2.08	3.69	11.90	4.90	-2.90	142.67	243.23	138.29	106.83
8.32	-0.51	12.44	21.78	11.80	1.96	3.61	11.81	4.82	-2.97	143.05	243.30	138.06	107.10
8.29	-0.54	12.40	21.75	11.76	1.92	3.56	11.77	4.79	-3.00	143.02	243.36	138.62	107.69
8.44	-0.39	12.54	21.92	11.91	2.06	3.77	11.94	4.97	-2.84	142.58	242.96	135.55	103.65
8.55	-0.28	12.68	22.06	12.03	2.17	3.87	12.05	5.07	-2.74	141.09	240.88	134.99	102.50
8.64	-0.16	12.88	22.18	12.16	2.32	3.94	12.13	5.12	-2.64	137.21	237.81	129.96	97.84
8.71	-0.07	12.94	22.22	12.21	2.40	4.07	12.24	5.23	-2.53	138.50	237.46	130.86	97.37
8.60	-0.19	12.83	22.12	12.11	2.28	3.93	12.10	5.10	-2.66	136.94	238.17	130.08	97.21
8.71	-0.09	12.98	22.27	12.29	2.41	3.99	12.18	5.17	-2.59	135.06	235.08	127.09	94.72
8.10	-0.52	12.04	21.34	11.47	1.92	3.47	11.54	4.72	-2.98	137.97	236.64	124.79	91.54
8.34	-0.30	12.54	21.88	11.88	2.17	3.59	11.67	4.82	-2.75	141.61	242.96	125.12	91.99
8.49	-0.25	12.83	22.13	12.20	2.32	3.49	11.59	4.81	-2.86	155.65	265.51	146.39	115.92
8.30	-0.44	12.54	21.79	11.89	2.12	3.45	11.66	4.73	-2.99	149.12	253.25	147.10	117.55
8.61	-0.15	12.88	22.11	12.19	2.38	3.82	11.99	5.02	-2.68	147.01	251.60	143.70	112.05
8.41	-0.35	12.60	21.83	11.94	2.18	3.59	11.76	4.86	-2.87	146.90	249.43	137.86	105.61
8.43	-0.33	12.63	21.86	11.98	2.21	3.61	11.77	4.87	-2.85	147.48	249.60	139.28	106.00
8.01	-0.72	12.03	21.40	11.42	1.72	3.37	11.54	4.67	-3.16	145.72	244.27	139.08	108.96
8.02	-0.72	12.03	21.41	11.43	1.73	3.38	11.55	4.67	-3.15	145.74	244.34	139.11	108.98
8.29	-0.47	12.36	21.69	11.74	1.98	3.59	11.79	4.84	-2.94	145.93	245.80	140.90	109.71
8.23	-0.49	12.25	21.58	11.64	1.96	3.62	11.76	4.86	-2.94	144.19	244.75	138.14	106.14
8.58	-0.16	12.65	21.94	11.99	2.28	4.00	12.13	5.19	-2.59	139.65	240.39	131.99	99.81
8.41	-0.35	12.57	21.81	11.94	2.14	3.67	11.83	4.91	-2.84	144.29	246.59	136.79	104.31
8.68	-0.07	12.80	22.05	12.12	2.35	4.10	12.23	5.27	-2.54	139.22	239.15	130.28	97.35
8.02	-0.73	12.11	21.46	11.58	1.74	3.02	11.15	4.45	-3.23	164.29	275.29	156.86	127.99
8.34	-0.49	12.60	21.88	12.02	2.05	3.23	11.34	4.65	-3.04	164.20	276.38	151.18	121.72
8.21	-0.54	12.37	21.70	11.82	1.95	3.23	11.35	4.62	-3.05	163.49	276.66	153.49	125.01

8.07	-0.68	12.19	21.50	11.64	1.82	3.11	11.24	4.52	-3.17	164.06	275.29	156.64	127.88
8.16	-0.57	12.23	21.56	11.66	1.91	3.26	11.43	4.67	-3.06	158.96	268.74	153.01	124.94
8.53	-0.16	12.73	22.10	12.07	2.29	3.82	11.81	5.00	-2.62	137.89	236.99	120.48	87.87
8.52	-0.17	12.72	22.10	12.05	2.28	3.77	11.80	4.98	-2.64	137.67	237.69	121.33	88.12
8.14	-0.57	12.17	21.52	11.57	1.89	3.53	11.67	4.79	-3.01	144.93	244.47	139.03	106.98
8.21	-0.52	12.16	21.50	11.57	1.92	3.57	11.74	4.83	-2.98	143.36	244.68	135.53	104.71
8.32	-0.44	12.33	21.63	11.70	2.03	3.65	11.81	4.90	-2.90	143.25	243.93	135.43	103.50
8.35	-0.41	12.37	21.67	11.74	2.07	3.69	11.84	4.93	-2.87	142.77	243.31	135.06	103.08
8.57	-0.15	12.68	21.92	12.00	2.32	3.93	12.05	5.12	-2.61	139.06	240.67	128.37	95.72
8.20	-0.51	12.32	21.59	11.72	2.00	3.39	11.53	4.69	-3.02	144.97	247.30	134.52	103.47
8.67	-0.05	12.89	22.09	12.21	2.44	3.87	12.01	5.14	-2.55	148.40	252.58	137.12	104.03
8.06	-0.69	12.18	21.49	11.63	1.81	3.09	11.23	4.50	-3.19	164.02	275.16	156.64	127.91
8.54	-0.21	12.67	22.00	12.07	2.27	3.79	11.88	5.00	-2.71	139.73	242.47	126.15	93.84
8.39	-0.34	12.69	22.01	12.10	2.23	3.39	11.49	4.70	-2.93	155.78	265.24	146.23	116.26
8.47	-0.27	12.51	21.84	11.93	2.20	3.76	11.84	4.98	-2.75	140.57	241.88	127.09	94.08
8.48	-0.27	12.53	21.86	11.95	2.21	3.76	11.84	4.98	-2.75	140.68	241.96	127.08	94.06
8.50	-0.26	12.58	21.91	11.98	2.23	3.76	11.84	4.97	-2.75	140.57	242.32	127.09	94.04
8.51	-0.25	12.64	21.98	12.04	2.25	3.75	11.84	4.96	-2.75	139.95	242.51	126.35	93.94
8.37	-0.37	12.57	21.81	11.93	2.15	3.58	11.71	4.84	-2.88	146.01	248.24	135.78	104.60
8.36	-0.36	12.54	21.80	11.90	2.11	3.60	11.72	4.82	-2.86	143.09	245.40	133.89	101.18
8.60	-0.12	13.01	22.28	12.34	2.47	3.65	11.77	4.92	-2.69	152.29	261.07	142.36	111.14
8.32	-0.50	12.53	21.82	11.98	2.02	3.26	11.36	4.66	-3.02	162.29	272.63	150.16	120.76
7.93	-0.80	11.92	21.31	11.33	1.65	3.28	11.46	4.58	-3.23	145.41	243.44	138.97	108.85
8.47	-0.21	12.53	21.80	11.89	2.25	3.85	11.96	5.06	-2.67	138.87	239.85	127.46	94.43
8.59	-0.15	12.76	21.99	12.10	2.32	3.92	12.02	5.11	-2.61	140.99	241.28	129.98	97.12
8.42	-0.24	12.54	21.85	11.91	2.22	3.76	11.80	4.96	-2.66	137.49	238.35	123.80	90.69
8.43	-0.22	12.61	21.91	11.95	2.21	3.72	11.76	4.92	-2.67	138.01	237.01	121.93	88.97
8.49	-0.18	12.72	22.04	12.04	2.25	3.75	11.78	4.92	-2.64	137.19	235.63	120.79	88.00
8.50	-0.17	12.74	22.05	12.05	2.26	3.75	11.79	4.92	-2.64	137.08	235.72	120.59	88.00
8.44	-0.24	12.66	21.96	11.98	2.22	3.67	11.71	4.89	-2.70	137.98	236.51	120.97	88.05
8.52	-0.18	12.81	22.12	12.11	2.28	3.75	11.80	4.94	-2.65	136.07	234.46	119.67	87.52
8.47	-0.23	12.75	22.08	12.08	2.26	3.70	11.75	4.88	-2.71	135.77	233.19	119.66	87.57
8.46	-0.23	12.69	22.01	12.02	2.23	3.70	11.74	4.86	-2.71	136.27	233.51	120.23	87.86
8.63	-0.16	12.98	22.37	12.25	2.36	3.79	11.81	4.96	-2.67	133.36	230.42	117.03	84.38
8.57	-0.18	12.88	22.21	12.19	2.32	3.79	11.83	4.96	-2.69	133.67	231.29	117.96	85.19
8.51	-0.28	12.76	22.07	12.13	2.22	3.51	11.64	4.89	-2.76	155.71	261.62	141.83	108.65
8.53	-0.20	12.78	22.01	12.11	2.34	3.76	11.93	4.94	-2.77	147.98	251.73	144.43	113.63
8.46	-0.28	12.66	21.90	12.02	2.24	3.65	11.80	4.91	-2.79	147.94	250.01	138.65	106.05
8.18	-0.58	12.23	21.58	11.59	1.86	3.53	11.69	4.79	-3.03	145.59	245.57	139.68	109.27
8.05	-0.69	12.07	21.44	11.46	1.75	3.41	11.58	4.70	-3.13	145.69	244.57	139.12	108.94
8.34	-0.42	12.40	21.69	11.78	2.06	3.64	11.80	4.88	-2.89	144.48	246.53	137.23	105.48
8.38	-0.38	12.50	21.76	11.87	2.12	3.66	11.82	4.90	-2.87	144.74	247.53	137.96	105.82
8.40	-0.37	12.47	21.76	11.83	2.13	3.73	11.86	4.96	-2.82	142.17	243.46	134.80	102.65

8.39	-0.37	12.62	21.83	11.93	2.12	3.62	11.76	4.86	-2.89	144.92	245.44	134.89	102.43
8.39	-0.38	12.61	21.83	11.93	2.12	3.62	11.75	4.85	-2.90	144.92	245.46	134.96	102.48
8.53	-0.21	12.68	21.93	12.04	2.26	3.83	11.93	5.04	-2.69	141.14	242.01	130.88	97.92
8.53	-0.23	12.62	21.88	11.96	2.24	3.88	12.01	5.08	-2.69	141.24	240.32	131.02	98.80
8.72	-0.05	12.91	22.12	12.22	2.42	4.06	12.16	5.23	-2.52	140.83	241.14	129.56	96.45
8.60	-0.15	12.73	21.96	12.06	2.32	3.93	12.03	5.12	-2.60	140.91	240.57	129.08	96.86
8.47	-0.21	12.53	21.79	11.89	2.24	3.85	11.95	5.05	-2.67	138.83	239.87	127.44	94.44
8.49	-0.19	12.49	21.75	11.86	2.25	3.93	11.99	5.12	-2.63	138.62	238.20	125.34	92.62
8.54	-0.23	12.61	21.95	12.02	2.26	3.80	11.88	5.01	-2.71	140.54	242.59	127.40	94.20
8.36	-0.35	12.37	21.65	11.78	2.12	3.69	11.77	4.92	-2.82	141.24	241.18	126.85	94.12
8.50	-0.24	12.61	21.89	12.00	2.22	3.78	11.88	5.00	-2.70	141.98	245.25	131.42	99.36
8.38	-0.35	12.41	21.78	11.86	2.12	3.64	11.75	4.90	-2.83	140.71	244.19	129.82	96.59
8.44	-0.33	12.67	21.97	12.01	2.16	3.45	11.57	4.87	-2.81	154.43	260.84	140.26	107.63
8.32	-0.50	12.45	21.81	11.81	1.98	3.58	11.79	4.81	-2.97	143.33	244.34	140.33	109.70
8.39	-0.45	12.55	21.88	11.89	2.04	3.64	11.85	4.86	-2.92	143.60	244.52	141.21	110.77
8.04	-0.70	12.14	21.46	11.64	1.78	3.01	11.13	4.45	-3.22	165.50	275.35	154.07	125.64
8.17	-0.59	12.31	21.62	11.75	1.90	3.20	11.33	4.59	-3.09	164.22	276.30	156.50	127.39
8.23	-0.49	12.35	21.66	11.74	1.99	3.37	11.52	4.73	-3.01	159.56	268.41	154.74	126.85
8.01	-0.72	12.17	21.51	11.56	1.85	3.03	11.22	4.44	-3.26	155.96	263.13	150.11	122.57
8.01	-0.71	12.18	21.52	11.57	1.86	3.04	11.22	4.44	-3.26	155.94	263.05	150.15	122.56
7.76	-0.95	11.53	20.81	11.13	1.45	2.75	10.74	4.39	-3.38	159.07	259.39	144.01	116.50
8.09	-0.65	12.04	21.32	11.56	1.78	3.07	11.06	4.61	-3.10	164.29	271.62	149.69	120.23
8.03	-0.71	12.01	21.30	11.53	1.73	2.94	10.99	4.52	-3.20	163.08	269.38	148.42	119.51
8.21	-0.61	12.39	21.67	11.84	1.93	3.09	11.17	4.58	-3.12	165.78	276.22	150.53	121.20
7.94	-0.83	11.86	21.14	11.41	1.65	2.88	10.85	4.47	-3.31	158.67	259.30	142.48	113.34
8.35	-0.90	12.56	21.93	11.94	1.65	3.31	11.34	4.76	-3.47	139.24	229.25	122.15	90.57
7.79	-1.03	11.66	21.00	11.24	1.45	2.74	10.73	4.34	-3.52	151.10	251.19	135.42	106.27
8.03	-0.78	12.02	21.35	11.47	1.67	3.05	11.05	4.58	-3.25	147.74	246.25	132.11	99.92
7.66	-1.43	11.67	21.03	11.17	1.07	2.49	10.53	4.16	-3.94	145.99	234.11	128.71	99.86
8.12	-0.68	12.27	21.60	11.75	1.85	2.98	11.04	4.48	-3.20	161.61	268.95	147.81	118.58
7.67	-1.42	11.67	21.04	11.17	1.07	2.50	10.54	4.17	-3.94	146.00	234.13	128.70	99.83
7.68	-1.42	11.68	21.05	11.19	1.08	2.51	10.54	4.17	-3.94	146.04	234.21	128.62	99.73
8.14	-0.64	12.22	21.53	11.71	1.85	3.03	11.04	4.55	-3.15	161.46	266.71	145.11	115.98
7.47	-1.21	11.09	20.40	10.76	1.18	2.51	10.51	4.21	-3.60	156.26	251.97	140.63	113.67
7.45	-1.24	11.06	20.37	10.72	1.15	2.48	10.49	4.18	-3.63	155.94	251.54	140.35	113.33
7.74	-0.99	11.48	20.79	11.10	1.44	2.73	10.71	4.38	-3.41	158.94	256.34	141.84	113.49
8.22	-0.59	12.42	21.73	11.90	1.95	3.11	11.20	4.58	-3.13	166.92	278.75	152.78	123.18
7.45	-1.33	11.13	20.47	10.78	1.10	2.43	10.45	4.14	-3.76	152.23	247.21	137.85	108.08
7.44	-1.34	11.12	20.45	10.77	1.10	2.44	10.43	4.13	-3.77	151.78	247.24	137.61	107.35
7.58	-1.21	11.35	20.67	10.94	1.24	2.54	10.55	4.22	-3.68	151.68	248.87	137.16	107.10
7.57	-1.23	11.33	20.67	10.95	1.23	2.55	10.54	4.21	-3.69	151.72	247.95	136.73	106.88
7.72	-0.98	11.46	20.73	11.07	1.42	2.75	10.74	4.40	-3.39	159.57	259.07	144.15	116.76
7.75	-0.96	11.52	20.80	11.12	1.45	2.75	10.74	4.39	-3.38	159.23	259.36	144.02	116.54

8.03	-0.73	12.01	21.29	11.53	1.75	2.95	10.96	4.55	-3.21	158.92	262.10	143.98	114.14
8.01	-0.73	11.92	21.22	11.46	1.70	2.94	10.98	4.54	-3.17	163.27	268.41	147.76	119.63
8.08	-0.70	12.13	21.43	11.64	1.80	2.97	11.03	4.52	-3.19	162.56	269.96	147.74	118.50
8.17	-0.64	12.34	21.67	11.82	1.90	3.03	11.09	4.51	-3.17	160.56	268.73	146.58	117.30
7.93	-0.90	11.82	21.15	11.35	1.59	2.89	10.86	4.47	-3.36	152.80	253.85	137.34	107.33
7.92	-0.90	11.82	21.15	11.35	1.58	2.88	10.86	4.46	-3.36	152.78	253.76	137.31	107.31
7.69	-1.12	11.54	20.87	11.10	1.35	2.63	10.65	4.29	-3.59	150.78	249.75	136.29	106.50
7.69	-1.13	11.55	20.88	11.11	1.36	2.61	10.63	4.25	-3.61	150.36	249.15	135.40	106.18
7.79	-1.07	11.67	21.00	11.25	1.45	2.72	10.71	4.32	-3.56	150.98	248.60	134.73	105.30
7.63	-1.21	11.42	20.74	11.03	1.27	2.59	10.58	4.23	-3.67	151.78	247.58	136.32	106.59
7.93	-1.03	11.96	21.29	11.48	1.51	2.82	10.82	4.39	-3.57	146.36	243.81	132.45	101.07
8.13	-0.68	12.10	21.43	11.55	1.76	3.19	11.19	4.69	-3.12	145.70	243.94	129.99	97.43
8.26	-0.61	12.31	21.61	11.73	1.86	3.32	11.33	4.78	-3.08	143.46	241.61	128.63	95.62
8.31	-0.58	12.39	21.67	11.81	1.91	3.37	11.37	4.82	-3.07	143.20	241.29	128.16	95.35
8.33	-0.55	12.41	21.69	11.83	1.93	3.40	11.40	4.84	-3.04	142.62	241.37	128.21	95.04
7.97	-0.88	11.96	21.29	11.43	1.61	2.94	10.97	4.50	-3.37	147.05	245.00	131.15	99.33
7.97	-0.88	11.97	21.29	11.43	1.61	2.94	10.97	4.50	-3.37	146.98	244.99	131.07	99.26
8.09	-0.75	12.13	21.44	11.58	1.73	3.10	11.13	4.62	-3.25	145.96	243.35	129.35	97.30
8.26	-0.65	12.39	21.69	11.82	1.85	3.23	11.25	4.71	-3.18	145.34	242.44	130.35	97.42
8.09	-0.97	12.21	21.65	11.72	1.56	3.00	10.99	4.51	-3.52	141.92	236.64	127.11	96.00
8.00	-1.04	12.09	21.51	11.59	1.46	2.87	10.90	4.44	-3.58	143.32	237.35	128.20	97.22
7.72	-1.48	11.66	21.04	11.20	1.02	2.58	10.64	4.22	-3.99	143.45	231.40	126.86	97.48
8.30	-0.83	12.38	21.71	11.79	1.73	3.41	11.44	4.82	-3.36	134.25	224.33	116.54	85.25
8.39	-0.79	12.52	21.83	11.92	1.76	3.50	11.55	4.90	-3.34	133.11	223.27	115.85	84.20
8.30	-0.87	12.39	21.72	11.81	1.69	3.39	11.44	4.80	-3.40	133.69	224.24	116.87	85.61
8.35	-0.85	12.50	21.80	11.90	1.72	3.44	11.47	4.84	-3.41	133.48	223.38	115.85	84.59
8.42	-0.71	12.50	21.85	11.91	1.84	3.54	11.57	4.92	-3.27	133.26	222.36	116.01	84.00
8.23	-1.02	12.42	21.79	11.85	1.58	3.25	11.27	4.64	-3.64	134.49	224.39	117.40	86.86
8.05	-1.33	12.18	21.55	11.66	1.27	2.98	11.08	4.46	-3.94	135.49	225.80	120.14	88.14
8.46	-0.61	12.65	22.03	12.02	1.92	3.53	11.55	4.91	-3.14	134.40	225.55	118.01	86.05
8.37	-0.69	12.52	21.89	11.90	1.84	3.47	11.46	4.87	-3.21	134.98	225.63	118.22	86.40
8.44	-0.78	12.67	22.02	12.02	1.77	3.44	11.46	4.87	-3.33	136.98	227.14	119.20	88.41
8.33	-0.94	12.54	21.97	11.97	1.71	3.36	11.41	4.70	-3.54	130.66	221.83	114.43	81.53
8.33	-0.89	12.53	21.99	11.96	1.72	3.34	11.42	4.71	-3.52	130.65	220.97	113.27	82.00
8.32	-0.90	12.52	21.98	11.95	1.71	3.33	11.41	4.70	-3.53	130.67	220.95	113.30	82.00
8.36	-0.22	12.35	21.61	11.75	2.21	3.67	11.71	4.97	-2.64	143.34	247.52	129.42	95.86
8.36	-0.22	12.34	21.60	11.75	2.21	3.68	11.72	4.97	-2.63	143.38	247.59	129.39	96.06
8.23	-0.54	12.35	21.63	11.77	1.96	3.20	11.33	4.70	-3.02	155.26	263.08	144.31	112.26
8.57	-0.13	12.52	21.77	11.93	2.31	4.04	12.09	5.21	-2.56	135.20	232.44	120.25	88.64
8.83	0.13	12.91	22.14	12.21	2.58	4.26	12.41	5.42	-2.29	132.78	229.95	120.07	86.90
8.13	-0.68	12.09	21.43	11.55	1.76	3.20	11.20	4.69	-3.11	145.70	243.45	129.51	97.20
8.40	-0.37	12.47	21.76	11.83	2.13	3.73	11.86	4.96	-2.82	142.17	243.46	134.80	102.65
8.29	-0.45	12.48	21.74	11.84	2.08	3.49	11.69	4.75	-2.99	147.58	251.74	145.82	115.30

8.54	-0.23	12.61	21.95	12.02	2.26	3.80	11.88	5.02	-2.71	140.54	242.61	127.43	94.22
8.54	-0.23	12.61	21.95	12.02	2.26	3.80	11.88	5.01	-2.71	140.55	242.59	127.40	94.20
8.51	-0.14	12.56	21.93	11.93	2.28	3.88	11.84	5.07	-2.57	139.69	239.44	121.81	88.88
8.72	0.03	12.61	21.92	12.15	2.41	4.01	12.13	5.30	-2.39	157.65	259.94	169.77	146.12
8.23	-0.48	12.07	21.40	11.57	1.88	3.47	11.63	4.89	-2.87	159.86	265.49	167.93	146.90
8.42	-0.29	12.23	21.58	11.78	2.05	3.75	11.93	5.07	-2.65	157.53	265.85	177.28	148.99
8.40	-0.31	12.17	21.54	11.74	2.03	3.74	11.93	5.06	-2.66	157.59	265.35	177.88	148.95
8.54	-0.18	12.36	21.71	11.92	2.17	3.88	12.02	5.14	-2.51	156.43	264.84	176.61	149.94
7.56	-1.17	11.36	20.66	10.81	1.17	2.79	11.02	4.34	-3.52	160.28	261.28	159.74	135.94
7.44	-1.30	11.23	20.54	10.68	1.05	2.66	10.91	4.21	-3.65	158.99	259.56	158.43	134.73
7.12	-1.53	10.48	19.92	10.19	0.73	2.34	10.45	4.02	-3.78	162.78	259.03	156.61	134.56
7.45	-1.27	11.17	20.54	10.68	1.12	2.65	10.85	4.19	-3.64	162.46	260.91	158.25	132.85
7.67	-1.08	11.52	20.83	10.95	1.27	2.88	11.13	4.41	-3.45	160.26	262.96	161.58	136.36
8.11	-0.65	12.07	21.37	11.60	1.79	3.07	11.12	4.61	-3.12	165.73	275.65	151.46	123.28
8.10	-0.66	12.03	21.33	11.56	1.78	3.06	11.11	4.62	-3.12	165.43	274.94	150.99	123.21
8.05	-0.71	11.96	21.28	11.51	1.74	3.04	11.09	4.58	-3.16	165.63	275.12	152.33	124.17
8.05	-0.71	11.98	21.29	11.52	1.74	3.05	11.09	4.57	-3.16	165.79	275.62	152.60	124.35
7.33	-0.97	12.08	21.44	11.20	1.69	2.35	10.34	3.48	-3.62	128.00	204.91	140.03	122.26
7.22	-1.20	11.81	21.15	11.11	1.38	2.10	10.03	3.35	-3.76	141.05	226.99	155.24	133.15
7.23	-1.21	11.81	21.15	11.12	1.38	2.10	10.03	3.34	-3.78	141.43	227.57	155.43	133.49
7.26	-1.16	11.85	21.21	11.14	1.41	2.15	10.09	3.38	-3.72	139.04	223.93	153.05	130.52
7.52	-0.96	12.62	21.99	11.66	1.77	2.21	10.17	3.40	-3.68	125.24	207.28	137.76	113.91
7.35	-1.07	12.04	21.41	11.18	1.59	2.30	10.30	3.47	-3.71	124.92	202.59	136.41	117.68
7.30	-1.10	12.18	21.53	11.26	1.65	2.13	10.10	3.32	-3.81	129.93	209.25	143.63	124.95
7.54	-1.03	12.37	21.73	11.50	1.67	2.37	10.41	3.56	-3.76	120.91	211.95	134.01	111.06
7.53	-1.13	12.16	21.48	11.45	1.55	2.34	10.51	3.60	-3.79	141.22	249.21	154.11	129.42
7.71	-1.03	12.25	21.60	11.53	1.59	2.64	10.81	3.88	-3.66	143.18	248.15	150.75	127.99
7.72	-0.96	12.66	22.01	11.75	1.78	2.44	10.65	3.71	-3.73	134.74	241.28	147.60	121.06
7.62	-1.06	12.28	21.60	11.55	1.62	2.44	10.60	3.68	-3.73	139.93	246.83	151.99	126.99
7.61	-0.92	12.40	21.81	11.44	1.69	2.52	10.68	3.80	-3.54	131.86	237.03	144.39	119.09
8.40	-0.41	12.94	22.17	12.13	2.21	3.44	11.62	4.69	-3.07	161.99	258.08	155.24	137.31
8.52	-0.30	13.20	22.48	12.32	2.39	3.47	11.73	4.70	-3.00	160.17	256.51	153.00	135.44
8.14	-0.74	12.43	21.68	11.78	1.83	3.22	11.48	4.50	-3.27	170.86	265.60	161.55	150.26
9.03	0.11	13.76	23.04	12.84	2.85	4.06	12.44	5.19	-2.62	161.19	250.78	150.92	133.43
8.94	0.05	13.61	22.88	12.72	2.75	4.00	12.38	5.15	-2.68	163.32	252.60	152.72	136.02
9.05	0.12	14.20	23.65	13.29	3.05	3.74	12.16	4.83	-2.83	148.95	239.47	138.61	119.67
8.95	-0.06	13.96	23.40	13.12	2.74	3.66	12.01	4.74	-2.84	153.09	245.93	145.06	127.57
9.00	-0.03	13.94	23.35	13.00	2.78	3.97	12.27	5.00	-2.83	141.96	231.97	132.68	112.02
7.74	-0.90	11.21	20.57	10.93	1.43	2.99	11.16	4.54	-3.23	162.61	262.02	178.59	163.58
7.54	-1.15	11.28	20.64	10.86	1.25	2.50	10.68	4.24	-3.57	164.50	263.73	166.74	150.58
7.84	-0.89	11.68	21.00	11.22	1.52	2.75	10.91	4.48	-3.31	165.01	268.71	163.97	148.37
7.88	-0.78	11.66	21.05	11.29	1.65	2.82	11.01	4.47	-3.19	165.43	259.05	172.06	161.62
7.72	-0.92	11.40	20.77	11.10	1.48	2.73	10.93	4.37	-3.33	163.58	258.83	173.07	162.53

7.62	-0.98	11.05	20.44	10.79	1.37	2.83	11.01	4.42	-3.34	164.06	261.36	176.08	161.74
7.64	-0.99	11.20	20.62	10.96	1.40	2.70	10.94	4.35	-3.37	162.46	258.37	174.61	164.35
7.72	-0.97	11.30	20.70	11.03	1.40	2.84	11.10	4.43	-3.36	162.31	261.15	179.89	166.04
7.78	-0.93	11.35	20.74	11.06	1.44	2.93	11.17	4.50	-3.31	162.33	262.62	181.39	165.11
7.76	-0.96	11.53	20.87	11.12	1.47	2.72	10.91	4.37	-3.36	167.56	263.88	169.68	157.15
7.91	-0.71	11.60	21.04	11.30	1.70	2.85	11.03	4.50	-3.12	166.88	258.10	173.10	163.68
7.93	-0.74	11.74	21.17	11.38	1.70	2.83	11.00	4.48	-3.17	167.22	260.64	169.81	158.09
7.86	-0.83	11.80	21.25	11.36	1.66	2.69	10.89	4.37	-3.32	168.20	264.83	166.72	153.12
7.78	-0.93	11.35	20.75	11.06	1.44	2.94	11.18	4.50	-3.30	162.30	262.66	181.41	165.14
7.67	-0.99	11.16	20.56	10.89	1.35	2.91	11.12	4.44	-3.33	161.60	262.30	179.02	163.20
7.88	-0.85	11.72	21.03	11.26	1.56	2.80	10.96	4.52	-3.26	165.63	269.59	163.87	147.90
7.63	-1.04	11.23	20.59	10.82	1.26	2.71	10.85	4.42	-3.34	166.56	266.55	162.71	144.94
7.68	-0.99	11.30	20.66	10.88	1.31	2.77	10.90	4.47	-3.29	166.93	267.62	162.56	144.92
8.28	-0.39	12.10	21.41	11.61	1.96	3.27	11.29	4.93	-2.76	164.45	273.62	148.23	122.19
8.19	-0.66	12.14	21.38	11.65	1.80	2.91	10.96	4.74	-3.13	157.56	245.97	139.37	118.68
8.01	-1.23	11.79	20.96	11.51	1.30	2.77	10.84	4.50	-3.77	152.87	234.39	133.80	114.50
8.47	-0.42	12.58	21.85	12.19	2.20	3.16	11.24	4.73	-3.04	156.74	232.12	139.82	122.12
8.50	-0.20	12.60	21.87	12.19	2.36	3.24	11.32	4.82	-2.76	157.29	238.09	144.45	126.43
8.03	-0.80	11.91	21.17	11.48	1.68	2.77	10.80	4.60	-3.25	157.96	247.18	140.68	119.34
8.20	-0.66	12.15	21.39	11.67	1.81	2.92	10.97	4.75	-3.13	157.55	245.92	139.34	118.67
7.82	-1.17	11.90	21.28	11.33	1.33	2.57	10.63	4.30	-3.67	150.94	243.34	134.24	105.85
6.43	-2.81	9.59	19.04	9.66	-0.41	1.37	9.42	3.20	-5.21	150.84	241.93	137.91	112.59
6.26	-2.86	9.49	18.94	9.47	-0.46	1.14	9.29	3.06	-5.26	153.47	241.97	138.43	114.42
6.31	-2.85	9.54	18.99	9.54	-0.45	1.19	9.31	3.10	-5.26	153.09	242.08	138.38	113.41
6.57	-2.67	9.85	19.24	9.83	-0.27	1.52	9.56	3.32	-5.06	150.54	241.46	137.17	111.09
6.42	-2.85	9.66	19.07	9.67	-0.40	1.33	9.42	3.15	-5.27	152.82	242.39	137.57	111.74
6.69	-2.55	10.14	19.65	9.98	-0.11	1.54	9.66	3.39	-4.96	150.50	240.28	134.87	109.34
7.42	-1.69	11.25	20.65	10.80	0.80	2.25	10.33	4.02	-4.16	148.73	238.28	131.81	104.86
7.42	-1.69	11.25	20.65	10.80	0.80	2.25	10.33	4.02	-4.16	148.72	238.24	131.84	104.86
7.20	-1.99	10.90	20.37	10.57	0.46	2.05	10.16	3.85	-4.45	150.04	240.69	133.16	105.88
7.74	-1.13	11.76	21.23	11.25	1.37	2.43	10.57	4.24	-3.63	152.98	247.17	136.85	110.76
6.98	-1.75	10.39	19.78	10.19	0.63	2.03	10.10	3.81	-4.13	151.10	242.79	136.82	109.55
7.13	-1.71	10.82	20.22	10.49	0.76	2.02	10.12	3.79	-4.15	147.64	240.21	133.62	106.94
6.77	-2.51	10.31	19.80	10.09	-0.08	1.62	9.74	3.43	-4.94	149.36	239.27	133.20	107.79
7.42	-1.69	11.25	20.65	10.80	0.81	2.25	10.33	4.01	-4.15	148.73	238.18	131.89	104.89
6.63	-2.68	10.00	19.41	9.93	-0.23	1.54	9.62	3.32	-5.11	151.59	242.34	135.50	109.49
6.26	-3.10	9.45	18.84	9.53	-0.69	1.12	9.28	2.96	-5.53	154.74	245.67	137.40	112.23
6.02	-3.26	9.12	18.53	9.28	-0.86	0.92	9.07	2.78	-5.68	155.65	247.91	139.51	115.14
6.93	-2.54	10.46	19.81	10.32	-0.08	1.81	9.93	3.55	-5.00	149.83	241.63	132.75	105.37
7.42	-1.34	11.18	20.40	10.79	1.09	2.13	10.19	4.03	-3.79	157.19	247.46	141.06	119.52
7.59	-1.50	11.14	20.33	10.90	0.98	2.33	10.46	4.26	-4.00	156.39	239.34	135.34	116.16
7.49	-1.59	10.99	20.19	10.77	0.86	2.25	10.38	4.20	-4.07	156.25	239.52	135.72	116.31
6.54	-3.08	9.85	19.25	9.91	-0.65	1.40	9.58	3.17	-5.49	152.38	244.24	134.81	108.18

6.30	-3.29	9.51	18.89	9.60	-0.84	1.19	9.36	2.95	-5.72	153.27	245.10	136.53	109.06
6.26	-3.30	9.52	18.87	9.59	-0.84	1.13	9.34	2.91	-5.73	152.97	245.45	135.99	110.21
6.32	-3.26	9.59	18.94	9.65	-0.81	1.19	9.40	2.95	-5.69	152.53	244.74	135.43	109.28
5.97	-3.53	8.93	18.34	9.21	-1.06	0.89	9.01	2.75	-5.98	152.91	259.65	140.27	112.82
6.49	-3.26	9.58	18.99	9.78	-0.79	1.48	9.55	3.21	-5.72	144.86	250.00	133.75	102.37
6.30	-3.35	9.36	18.78	9.57	-0.89	1.27	9.34	3.05	-5.81	147.53	253.13	135.59	105.74
6.28	-3.32	9.35	18.75	9.56	-0.88	1.25	9.32	3.01	-5.79	145.79	254.05	136.87	106.09
8.04	-1.20	12.07	21.39	11.53	1.31	3.02	11.08	4.52	-3.71	141.49	229.92	124.16	93.53
7.52	-1.64	11.45	20.79	11.01	0.86	2.33	10.40	4.04	-4.10	147.55	236.22	129.83	101.02
7.52	-2.33	11.56	20.88	11.18	0.22	2.40	10.55	3.89	-4.89	136.22	229.76	121.72	91.58
6.22	-3.17	9.41	18.84	9.55	-0.75	1.11	9.27	2.89	-5.58	154.00	245.93	137.59	111.82
7.22	-2.61	11.06	20.36	10.79	-0.10	2.08	10.22	3.62	-5.15	142.39	231.65	126.82	97.10
7.52	-2.05	11.45	20.80	11.08	0.43	2.41	10.50	3.93	-4.55	140.30	229.63	124.28	95.19
7.28	-2.37	11.22	20.59	10.86	0.18	2.17	10.31	3.70	-4.92	136.95	231.09	124.33	93.86
7.64	-1.58	11.60	20.94	11.12	0.95	2.59	10.71	4.16	-4.13	141.23	229.52	124.75	95.03
6.66	-2.77	10.10	19.45	10.03	-0.31	1.54	9.70	3.31	-5.21	151.28	242.91	134.48	107.80
6.41	-3.02	9.71	19.11	9.77	-0.60	1.28	9.45	3.04	-5.48	151.56	243.81	135.67	109.65
7.21	-2.50	10.88	20.21	10.70	-0.02	2.06	10.19	3.68	-4.95	143.99	234.78	129.47	100.37
7.18	-2.47	10.85	20.17	10.67	0.02	2.04	10.18	3.69	-4.92	144.30	235.23	129.77	101.30
6.75	-2.77	10.17	19.52	10.14	-0.34	1.60	9.75	3.33	-5.21	149.04	240.74	133.14	106.54
7.44	-2.15	11.26	20.59	10.96	0.34	2.32	10.40	3.91	-4.61	145.60	235.08	128.21	99.67
7.21	-2.29	10.95	20.29	10.70	0.18	2.07	10.20	3.72	-4.78	146.54	236.33	129.35	102.12
7.44	-2.18	11.27	20.60	10.96	0.33	2.31	10.40	3.91	-4.62	144.61	234.94	128.16	99.22
7.49	-2.16	11.32	20.63	11.02	0.31	2.36	10.46	3.94	-4.63	143.05	233.45	127.77	97.70
7.45	-2.21	11.22	20.56	10.96	0.26	2.30	10.42	3.92	-4.67	143.73	234.21	128.32	98.14
6.90	-2.44	10.53	19.93	10.30	0.03	1.75	9.90	3.50	-4.89	145.94	236.15	131.36	104.41
6.88	-2.46	10.50	19.90	10.27	0.01	1.73	9.88	3.48	-4.91	146.00	236.28	131.55	104.64
6.71	-2.62	10.29	19.69	10.10	-0.16	1.56	9.71	3.30	-5.09	146.45	238.39	132.96	106.48
7.14	-2.35	10.87	20.25	10.63	0.13	1.97	10.09	3.63	-4.82	145.96	236.98	129.54	102.42
7.17	-2.31	10.93	20.30	10.68	0.18	2.02	10.12	3.64	-4.77	146.08	236.47	129.03	101.49
7.58	-2.01	11.47	20.80	11.15	0.48	2.45	10.56	4.01	-4.49	142.16	230.22	125.09	95.88
7.74	-1.87	11.71	21.03	11.33	0.63	2.65	10.74	4.15	-4.38	141.39	229.65	123.99	93.57
7.27	-2.03	11.07	20.43	10.73	0.44	2.15	10.28	3.82	-4.51	145.22	235.19	128.55	100.53
7.01	-2.24	10.75	20.13	10.40	0.21	1.83	9.98	3.63	-4.68	148.70	234.73	130.98	103.31
7.60	-1.78	11.56	20.87	11.12	0.74	2.53	10.64	4.08	-4.26	142.30	231.63	125.22	96.96
7.52	-1.86	11.44	20.76	11.03	0.66	2.44	10.55	4.01	-4.34	142.38	231.61	125.60	97.18
7.61	-1.79	11.56	20.88	11.14	0.73	2.56	10.68	4.09	-4.26	141.79	230.53	125.04	95.09
7.91	-1.40	12.00	21.28	11.43	1.13	2.89	10.98	4.38	-3.92	139.97	229.41	123.24	92.96
7.88	-1.54	11.91	21.22	11.41	0.98	2.86	10.95	4.32	-4.06	139.91	228.43	123.14	92.61
7.02	-2.86	10.74	20.05	10.55	-0.31	1.87	10.05	3.49	-5.38	140.90	235.35	128.24	98.89
6.93	-2.91	10.61	19.93	10.44	-0.38	1.78	9.96	3.42	-5.44	142.13	235.99	129.28	99.88
6.90	-2.93	10.55	19.87	10.38	-0.41	1.74	9.92	3.39	-5.47	142.76	236.60	129.62	100.33
6.81	-2.84	10.44	19.82	10.30	-0.37	1.61	9.81	3.30	-5.34	144.24	234.92	130.69	102.90

7.53	-2.12	11.34	20.66	11.05	0.35	2.38	10.49	3.98	-4.60	143.42	233.90	128.01	97.41
6.51	-3.00	9.81	19.21	9.89	-0.57	1.40	9.53	3.13	-5.42	151.63	242.86	134.70	107.99
6.51	-3.00	9.80	19.21	9.88	-0.57	1.39	9.52	3.12	-5.42	151.60	242.84	134.75	108.05
6.50	-3.01	9.80	19.20	9.87	-0.57	1.38	9.52	3.12	-5.42	151.58	242.84	134.79	108.09
7.27	-2.75	11.17	20.48	10.90	-0.19	2.10	10.27	3.66	-5.30	137.89	234.45	124.88	94.46
6.90	-2.93	10.56	19.87	10.39	-0.41	1.74	9.92	3.39	-5.47	142.66	236.46	129.63	100.30
6.91	-2.93	10.56	19.88	10.39	-0.40	1.75	9.93	3.39	-5.46	142.65	236.53	129.53	100.24
6.82	-2.98	10.43	19.76	10.28	-0.47	1.66	9.84	3.33	-5.52	143.73	237.75	130.26	101.18
6.85	-2.96	10.47	19.80	10.31	-0.45	1.68	9.86	3.35	-5.51	143.36	237.25	130.10	100.91
6.89	-2.94	10.53	19.86	10.37	-0.42	1.73	9.91	3.38	-5.48	142.76	236.56	129.78	100.45
6.88	-2.94	10.53	19.85	10.36	-0.42	1.72	9.90	3.37	-5.48	142.77	236.58	129.82	100.48
6.90	-2.93	10.56	19.88	10.39	-0.40	1.74	9.92	3.39	-5.47	142.47	236.28	129.66	100.27
6.91	-2.92	10.58	19.90	10.41	-0.39	1.76	9.94	3.40	-5.46	142.29	236.11	129.54	100.12
6.98	-2.88	10.69	20.00	10.51	-0.34	1.83	10.02	3.46	-5.40	141.39	235.43	128.74	99.27
6.59	-3.15	10.07	19.43	10.03	-0.63	1.42	9.61	3.15	-5.65	145.48	241.84	132.82	104.51
6.96	-2.90	10.65	19.98	10.47	-0.36	1.80	9.99	3.44	-5.42	141.49	235.51	129.10	99.58
7.03	-2.84	10.74	20.06	10.55	-0.31	1.86	10.05	3.49	-5.37	140.47	235.99	127.78	98.88
7.15	-2.74	10.95	20.27	10.71	-0.19	2.01	10.19	3.60	-5.28	138.94	233.40	127.18	96.69
7.24	-2.65	11.08	20.40	10.82	-0.11	2.11	10.26	3.66	-5.21	138.48	233.10	126.26	95.24
7.12	-2.73	10.93	20.25	10.71	-0.17	1.96	10.12	3.53	-5.27	141.83	234.14	126.96	98.16
7.10	-2.76	10.92	20.23	10.68	-0.19	1.95	10.11	3.54	-5.29	142.10	234.80	127.08	98.18
7.23	-2.67	11.09	20.39	10.82	-0.10	2.07	10.23	3.64	-5.20	141.04	233.53	126.16	96.64
6.98	-2.84	10.73	20.05	10.55	-0.29	1.83	10.00	3.44	-5.39	142.75	236.91	128.27	99.03
7.18	-2.62	11.07	20.38	10.78	-0.07	2.05	10.20	3.59	-5.17	139.32	232.75	126.41	96.77
7.06	-2.77	10.84	20.17	10.64	-0.21	1.90	10.06	3.48	-5.31	142.34	234.77	127.53	98.89
6.97	-2.85	10.67	19.99	10.49	-0.31	1.81	9.98	3.43	-5.36	144.29	235.66	128.57	99.73
6.80	-2.98	10.35	19.69	10.26	-0.47	1.69	9.86	3.38	-5.48	145.94	240.20	129.67	101.68
6.81	-2.94	10.44	19.78	10.32	-0.43	1.65	9.83	3.32	-5.49	145.23	238.43	129.68	101.28
6.65	-3.08	10.14	19.48	10.06	-0.60	1.51	9.69	3.22	-5.59	147.11	242.23	131.80	103.19
6.66	-3.07	10.14	19.49	10.09	-0.57	1.53	9.71	3.24	-5.57	146.79	241.90	131.25	103.39
6.90	-2.94	10.55	19.86	10.38	-0.41	1.74	9.92	3.38	-5.47	142.82	236.70	129.62	100.37
6.57	-3.14	9.94	19.27	9.93	-0.67	1.46	9.63	3.19	-5.62	147.61	243.35	132.18	104.09
6.56	-3.10	10.08	19.41	10.01	-0.59	1.41	9.61	3.12	-5.59	148.84	241.12	130.94	105.55
6.51	-3.13	10.02	19.35	9.96	-0.62	1.35	9.56	3.08	-5.63	148.68	241.57	131.57	105.99
6.77	-2.94	10.30	19.59	10.18	-0.51	1.67	9.84	3.36	-5.40	148.17	239.01	130.44	102.92
6.44	-3.16	9.82	19.16	9.81	-0.72	1.33	9.52	3.08	-5.59	151.82	243.47	133.99	107.38
6.81	-2.87	10.39	19.74	10.28	-0.40	1.65	9.81	3.34	-5.32	146.06	237.68	129.31	103.07
7.03	-2.73	10.67	19.99	10.50	-0.26	1.88	10.03	3.52	-5.18	145.82	236.51	128.54	100.96
6.93	-2.77	10.52	19.85	10.41	-0.33	1.83	9.94	3.47	-5.22	147.22	238.01	129.84	102.21
6.93	-2.82	10.51	19.84	10.39	-0.38	1.83	9.96	3.48	-5.25	146.88	236.63	129.66	102.22
7.69	-1.79	11.79	21.14	11.31	0.73	2.63	10.74	4.13	-4.34	137.69	227.84	122.11	92.79
7.08	-2.60	10.90	20.24	10.64	-0.09	1.89	10.06	3.51	-5.11	142.10	231.77	127.64	99.42
7.20	-2.53	11.05	20.36	10.78	-0.01	2.02	10.18	3.64	-5.02	143.03	230.83	127.12	98.39

7.22	-2.51	11.06	20.38	10.80	0.01	2.04	10.19	3.66	-5.00	142.90	230.64	127.04	98.15
7.24	-2.38	11.03	20.39	10.78	0.11	2.11	10.24	3.70	-4.85	143.49	232.05	127.40	98.62
6.84	-2.82	10.48	19.85	10.34	-0.34	1.64	9.83	3.33	-5.31	144.18	234.61	130.51	102.62
6.97	-2.70	10.66	20.01	10.50	-0.21	1.77	9.94	3.44	-5.20	143.58	233.30	129.50	101.17
6.71	-2.94	10.27	19.61	10.18	-0.46	1.53	9.71	3.25	-5.37	146.34	237.35	131.11	104.69
6.69	-2.91	10.33	19.71	10.19	-0.43	1.52	9.72	3.20	-5.41	145.24	235.41	131.41	103.67
6.69	-2.91	10.33	19.71	10.19	-0.43	1.52	9.72	3.20	-5.41	145.22	235.42	131.41	103.68
6.68	-2.96	10.29	19.66	10.17	-0.48	1.51	9.69	3.19	-5.44	144.60	236.22	130.78	103.86
6.84	-2.86	10.53	19.88	10.36	-0.34	1.67	9.84	3.30	-5.38	144.08	234.82	129.31	101.89
6.91	-2.82	10.63	19.96	10.43	-0.32	1.74	9.90	3.37	-5.35	143.69	234.08	129.26	100.93
6.99	-2.70	10.79	20.11	10.56	-0.17	1.82	9.99	3.44	-5.21	143.16	233.60	128.45	100.23
7.06	-2.69	10.90	20.23	10.64	-0.16	1.91	10.08	3.48	-5.23	140.68	233.00	127.01	98.22
7.08	-2.68	10.92	20.25	10.66	-0.15	1.93	10.10	3.50	-5.22	140.31	232.72	126.93	97.91
7.19	-2.58	11.06	20.39	10.79	-0.03	2.05	10.20	3.60	-5.12	139.24	231.38	125.19	97.53
7.24	-2.54	11.13	20.46	10.85	0.01	2.11	10.25	3.64	-5.08	139.10	230.93	124.88	96.79
7.51	-2.31	11.51	20.83	11.15	0.24	2.42	10.54	3.90	-4.84	137.15	228.07	122.55	93.47
7.42	-2.39	11.38	20.71	11.06	0.17	2.32	10.44	3.81	-4.92	137.63	228.87	123.15	94.68
7.15	-2.53	11.04	20.35	10.74	0.00	1.98	10.15	3.55	-5.05	139.60	231.50	126.17	97.53
7.18	-2.50	11.08	20.40	10.78	0.04	2.02	10.18	3.58	-5.02	139.30	231.27	125.71	97.34
7.58	-2.16	11.65	20.97	11.23	0.39	2.48	10.62	3.96	-4.68	136.66	227.43	122.08	92.72
7.38	-2.25	11.38	20.74	11.00	0.30	2.24	10.39	3.77	-4.80	138.05	229.48	123.89	94.54
7.73	-1.95	11.87	21.20	11.37	0.60	2.61	10.74	4.05	-4.51	136.10	227.30	121.61	91.07
7.70	-1.95	11.85	21.20	11.35	0.59	2.61	10.73	4.05	-4.50	133.63	226.82	120.71	89.39
7.23	-1.99	11.09	20.45	10.69	0.58	2.15	10.31	3.80	-4.54	140.27	231.10	125.86	96.92
7.10	-2.67	10.94	20.27	10.68	-0.15	1.95	10.11	3.51	-5.22	140.21	232.60	126.97	97.69
7.29	-2.55	11.23	20.51	10.91	0.00	2.16	10.31	3.69	-5.10	139.19	231.41	125.88	95.42
7.57	-2.34	11.58	20.88	11.22	0.24	2.45	10.57	3.91	-4.88	136.96	229.04	122.83	92.98
7.35	-2.39	11.29	20.65	10.97	0.16	2.27	10.40	3.76	-4.94	136.63	229.68	124.25	94.02
7.50	-2.34	11.53	20.85	11.15	0.21	2.38	10.53	3.87	-4.90	136.39	229.79	121.91	91.80
7.51	-2.34	11.54	20.86	11.17	0.21	2.39	10.54	3.88	-4.90	136.31	229.78	121.82	91.70
7.53	-2.33	11.57	20.88	11.19	0.22	2.40	10.55	3.89	-4.89	136.19	229.76	121.69	91.55
7.41	-2.38	11.38	20.76	11.04	0.19	2.30	10.44	3.78	-4.92	136.12	229.94	123.13	92.52
7.36	-2.43	11.34	20.73	11.00	0.15	2.23	10.36	3.72	-4.98	135.38	229.47	122.90	92.89
7.43	-2.38	11.45	20.81	11.08	0.18	2.29	10.44	3.79	-4.97	135.40	229.14	122.45	91.99
7.47	-2.39	11.49	20.85	11.13	0.18	2.33	10.48	3.84	-4.97	135.80	229.16	122.14	91.63
7.35	-2.53	11.29	20.67	10.99	0.05	2.22	10.36	3.72	-5.06	137.29	231.37	123.39	93.51
7.08	-2.75	11.05	20.39	10.71	-0.14	1.87	10.09	3.46	-5.34	136.47	234.05	123.89	94.80
7.23	-2.58	11.23	20.55	10.85	0.02	2.01	10.20	3.58	-5.20	135.28	232.52	122.95	92.96
7.32	-2.59	11.41	20.72	11.01	0.03	2.14	10.33	3.68	-5.18	134.70	230.87	121.66	90.91
7.47	-2.46	11.58	20.87	11.15	0.16	2.29	10.46	3.80	-5.05	133.60	227.65	119.91	88.86
7.17	-2.63	10.98	20.31	10.79	-0.06	2.01	10.12	3.56	-5.19	131.84	241.02	123.06	88.37
7.20	-2.68	11.03	20.35	10.82	-0.13	2.06	10.18	3.58	-5.23	132.18	239.96	122.97	88.94
7.17	-2.67	11.02	20.31	10.81	-0.13	2.04	10.19	3.58	-5.24	131.71	238.00	122.81	89.27

7.56	-2.62	11.62	20.88	11.24	-0.01	2.38	10.53	3.87	-5.21	134.41	231.69	120.22	87.78
7.64	-2.49	11.76	21.00	11.35	0.12	2.47	10.63	3.92	-5.09	130.38	230.15	118.60	85.92
7.66	-2.37	11.72	20.97	11.31	0.20	2.52	10.69	3.98	-4.97	129.20	229.98	118.08	84.44
7.43	-2.77	11.51	20.75	11.13	-0.19	2.21	10.37	3.73	-5.38	136.06	232.45	122.64	90.07
7.46	-2.75	11.56	20.79	11.17	-0.17	2.23	10.40	3.76	-5.36	135.57	232.03	122.32	89.74
7.36	-2.82	11.36	20.61	11.01	-0.23	2.13	10.33	3.70	-5.40	137.62	233.93	123.99	92.21
7.62	-2.50	11.79	21.08	11.39	0.12	2.44	10.58	3.85	-5.11	131.52	230.01	118.18	86.73
7.39	-2.46	11.31	20.62	11.00	0.09	2.30	10.44	3.78	-5.02	132.20	231.77	119.14	87.24
6.48	-3.31	9.77	19.13	9.81	-0.80	1.36	9.47	3.11	-5.79	147.60	248.96	133.52	104.02
7.03	-2.95	10.61	19.92	10.53	-0.40	1.93	10.05	3.55	-5.48	140.51	241.61	128.35	94.90
7.25	-2.57	11.22	20.54	10.87	0.03	2.12	10.27	3.67	-5.17	133.05	231.32	121.04	90.59
7.43	-2.40	11.44	20.73	11.02	0.21	2.34	10.46	3.84	-4.99	132.82	230.40	119.69	87.93
6.72	-3.15	10.15	19.46	10.14	-0.64	1.61	9.74	3.30	-5.66	145.09	244.42	130.89	100.50
7.13	-2.91	10.77	20.06	10.63	-0.38	1.99	10.09	3.60	-5.47	140.15	240.19	126.42	94.03
6.93	-3.00	10.53	19.84	10.40	-0.44	1.80	9.96	3.48	-5.51	143.58	241.38	128.47	98.51
7.58	-2.55	11.75	21.04	11.34	0.09	2.37	10.55	3.81	-5.14	132.77	229.70	118.29	88.03
7.54	-2.50	11.68	20.98	11.25	0.10	2.34	10.54	3.81	-5.13	132.33	228.40	119.67	89.02
7.50	-2.63	11.65	20.98	11.24	-0.01	2.25	10.46	3.74	-5.24	133.36	230.15	122.11	90.26
7.33	-2.81	11.29	20.58	10.97	-0.21	2.11	10.29	3.67	-5.39	138.93	235.38	124.33	92.90
7.41	-2.62	11.44	20.70	11.09	-0.04	2.24	10.43	3.77	-5.24	131.84	233.40	121.79	88.54
7.63	-2.47	11.75	20.99	11.33	0.13	2.47	10.64	3.93	-5.07	130.10	230.43	118.33	85.66
7.71	-2.41	11.85	21.10	11.44	0.18	2.55	10.72	3.97	-5.03	130.73	228.95	117.51	85.17
7.18	-2.62	10.99	20.32	10.80	-0.06	2.01	10.12	3.56	-5.18	131.80	240.96	123.01	88.28
8.18	-1.63	12.44	21.81	12.11	1.12	3.01	11.10	4.26	-4.37	122.20	223.41	111.78	74.78
7.15	-2.80	10.79	20.11	10.65	-0.28	2.11	10.20	3.65	-5.33	137.99	241.09	126.74	92.91
8.44	-1.30	12.95	22.29	12.51	1.52	3.20	11.27	4.37	-4.14	119.87	219.05	107.46	71.00
7.10	-2.88	10.73	20.00	10.61	-0.32	2.05	10.14	3.60	-5.42	139.82	240.92	127.48	93.49
7.16	-2.66	10.94	20.28	10.77	-0.12	2.03	10.15	3.55	-5.24	132.78	239.57	123.36	89.31
7.19	-2.69	11.04	20.34	10.82	-0.13	2.03	10.16	3.55	-5.25	132.36	240.15	123.35	89.26
7.25	-2.72	10.99	20.30	10.81	-0.18	2.20	10.30	3.72	-5.27	136.22	241.13	124.90	91.22
7.24	-2.73	10.97	20.28	10.80	-0.20	2.18	10.29	3.71	-5.28	136.38	241.27	125.18	91.49
7.41	-2.66	11.36	20.66	11.06	-0.08	2.27	10.42	3.72	-5.25	134.56	238.50	123.05	87.90
7.22	-2.75	11.05	20.32	10.82	-0.18	2.05	10.16	3.62	-5.35	134.95	240.18	125.68	90.73
7.73	-2.31	11.84	21.09	11.43	0.28	2.61	10.75	4.00	-4.90	130.99	226.69	116.10	84.61
7.69	-2.35	11.76	21.04	11.38	0.24	2.57	10.74	4.02	-4.90	129.23	227.69	117.50	84.90
8.20	-1.69	12.51	21.86	12.13	1.07	2.93	10.99	4.24	-4.43	122.63	226.50	111.99	74.03
7.81	-2.06	11.75	21.14	11.54	0.59	2.69	10.76	4.07	-4.69	126.21	231.01	115.61	79.09
8.26	-1.49	12.56	21.87	12.06	1.20	3.14	11.24	4.44	-4.19	122.54	221.76	110.58	76.03
8.19	-1.54	12.46	21.78	11.99	1.15	3.04	11.15	4.37	-4.23	123.12	222.75	111.35	77.05
7.60	-2.13	11.65	21.00	11.21	0.48	2.53	10.68	4.00	-4.76	131.67	228.22	117.72	85.99
8.30	-1.31	12.48	21.88	12.00	1.33	3.31	11.40	4.58	-3.98	126.24	217.71	110.94	77.92
7.97	-1.78	12.17	21.54	11.85	0.98	2.78	10.91	4.08	-4.53	124.78	225.91	113.78	78.36
7.49	-2.30	11.52	20.82	11.10	0.28	2.42	10.53	3.91	-4.92	132.88	229.83	119.01	86.69

7.16	-2.67	11.05	20.34	10.75	-0.09	1.97	10.14	3.58	-5.26	133.25	238.08	123.47	89.73
7.59	-2.15	11.67	20.97	11.21	0.45	2.51	10.64	3.96	-4.74	133.72	226.49	118.47	87.06
7.99	-1.45	12.11	21.46	11.58	1.13	2.96	11.05	4.42	-4.02	134.03	225.21	119.17	87.57
7.32	-2.07	11.23	20.59	10.78	0.48	2.26	10.40	3.85	-4.62	134.23	229.17	121.46	91.52
7.07	-2.29	10.96	20.33	10.54	0.23	2.00	10.16	3.62	-4.83	136.03	231.50	122.75	93.90
7.64	-1.88	11.82	21.19	11.29	0.71	2.58	10.68	4.02	-4.44	135.30	226.85	120.53	90.50
7.53	-2.12	11.61	20.97	11.17	0.43	2.42	10.56	3.89	-4.67	134.67	228.73	122.05	91.54
7.10	-2.28	11.01	20.38	10.58	0.25	2.03	10.18	3.63	-4.82	136.04	231.23	122.51	93.53
7.04	-2.35	10.92	20.30	10.53	0.21	1.98	10.10	3.54	-4.90	134.90	230.97	122.42	93.63
7.21	-2.25	11.16	20.54	10.74	0.29	2.12	10.27	3.67	-4.82	134.03	229.25	121.08	91.92
7.19	-2.26	11.13	20.52	10.72	0.28	2.11	10.25	3.66	-4.83	134.08	229.44	121.21	92.05
7.44	-2.10	11.50	20.86	11.02	0.45	2.38	10.48	3.86	-4.67	134.24	228.73	119.75	89.57
7.40	-2.30	11.43	20.77	11.03	0.27	2.27	10.40	3.78	-4.87	133.29	230.17	122.65	91.63
7.65	-2.06	11.77	21.10	11.30	0.50	2.52	10.65	3.98	-4.62	131.06	227.03	119.31	88.88
7.72	-1.98	11.87	21.19	11.38	0.57	2.62	10.73	4.04	-4.55	130.94	226.52	118.81	88.09
8.26	-1.49	12.66	22.00	12.31	1.27	3.02	11.11	4.25	-4.32	122.36	222.35	111.41	74.20
8.26	-1.49	12.66	22.00	12.31	1.27	3.02	11.11	4.25	-4.32	122.37	222.37	111.40	74.23
7.91	-1.53	12.04	21.37	11.50	1.01	2.89	10.96	4.35	-4.12	134.87	226.15	119.66	87.91
7.96	-1.48	12.07	21.43	11.54	1.10	2.92	11.01	4.39	-4.05	134.71	225.61	119.60	88.08
8.07	-1.35	12.19	21.55	11.66	1.24	3.03	11.13	4.50	-3.94	132.09	224.41	118.34	87.19
7.57	-2.21	11.71	21.04	11.24	0.39	2.40	10.54	3.89	-4.82	131.42	226.86	119.70	89.15
7.90	-1.89	11.98	21.30	11.57	0.77	2.87	10.95	4.24	-4.54	130.28	225.83	114.78	81.13
7.58	-2.21	11.50	20.85	11.16	0.40	2.53	10.61	3.99	-4.81	132.19	228.47	118.06	85.01
8.28	-1.56	12.55	21.84	12.18	1.17	3.16	11.23	4.38	-4.31	121.95	221.25	110.56	74.51
8.26	-1.56	12.52	21.82	12.17	1.17	3.12	11.18	4.34	-4.32	121.30	222.29	110.85	74.72
8.33	-1.44	12.72	21.95	12.25	1.34	3.15	11.25	4.39	-4.21	119.00	217.24	108.20	73.73
8.00	-1.85	11.86	21.17	11.59	0.70	3.07	11.11	4.38	-4.42	125.95	224.99	113.67	78.48
8.13	-1.58	12.34	21.68	11.83	1.07	3.12	11.22	4.41	-4.24	126.95	221.07	112.94	78.97
7.65	-2.02	11.78	21.12	11.30	0.54	2.60	10.70	4.01	-4.60	133.01	226.60	117.53	86.72
7.54	-2.11	11.61	20.93	11.14	0.45	2.49	10.61	3.92	-4.69	133.27	227.11	117.85	86.98
7.62	-2.12	11.69	21.02	11.24	0.49	2.55	10.71	4.02	-4.74	131.57	227.95	117.42	85.84
7.47	-2.33	11.51	20.80	11.08	0.26	2.39	10.51	3.89	-4.94	132.94	229.93	119.42	87.02
7.21	-2.75	11.25	20.48	10.96	-0.15	1.89	10.06	3.47	-5.37	133.20	239.87	123.89	89.50
7.21	-2.76	11.25	20.48	10.96	-0.15	1.90	10.06	3.47	-5.37	133.25	239.94	123.90	89.49
7.50	-2.39	11.60	20.90	11.26	0.27	2.25	10.38	3.75	-5.05	130.28	237.71	120.60	84.49
7.42	-2.60	11.51	20.83	11.14	0.02	2.19	10.38	3.69	-5.19	133.21	239.40	123.25	87.95
6.96	-2.89	10.72	19.95	10.52	-0.28	1.75	9.87	3.43	-5.47	138.23	247.30	129.31	93.77
7.87	-2.03	12.02	21.37	11.65	0.65	2.68	10.75	4.06	-4.75	125.87	233.43	116.76	77.86
8.00	-1.92	12.13	21.53	11.85	0.80	2.81	10.83	4.13	-4.58	124.10	230.28	113.96	76.18
7.88	-1.93	12.02	21.39	11.67	0.77	2.73	10.78	4.11	-4.62	125.64	232.81	116.00	77.65
7.94	-1.84	12.11	21.53	11.72	0.85	2.78	10.82	4.16	-4.54	124.16	232.18	115.16	76.74
7.63	-1.94	11.90	21.27	11.47	0.79	2.36	10.42	3.79	-4.65	127.95	241.10	119.00	78.80
7.50	-2.30	11.49	20.86	11.21	0.34	2.30	10.40	3.78	-4.98	129.43	237.85	120.46	83.57

7.63	-2.16	11.76	21.16	11.40	0.55	2.41	10.49	3.86	-4.84	129.36	238.33	119.11	81.44
7.03	-3.04	10.92	20.15	10.67	-0.44	1.68	9.81	3.36	-5.64	139.10	244.02	127.84	95.13
7.04	-3.04	10.93	20.16	10.67	-0.44	1.69	9.82	3.37	-5.64	139.06	243.92	127.74	95.00
6.82	-3.14	10.67	19.89	10.47	-0.56	1.44	9.59	3.17	-5.73	140.98	248.02	130.06	98.32
6.94	-3.08	10.84	20.03	10.60	-0.49	1.56	9.72	3.27	-5.66	139.92	246.26	129.43	97.24
6.56	-3.25	10.15	19.45	10.04	-0.66	1.33	9.47	3.07	-5.81	145.04	253.29	134.62	102.01
7.16	-2.91	10.99	20.26	10.78	-0.32	1.90	10.00	3.51	-5.53	136.38	241.93	125.57	92.29
7.13	-2.93	10.97	20.23	10.74	-0.33	1.88	9.99	3.49	-5.55	136.40	242.45	125.79	92.84
6.76	-3.19	10.60	19.79	10.41	-0.59	1.34	9.50	3.09	-5.77	142.89	248.20	130.87	99.66
6.87	-3.11	10.73	19.95	10.52	-0.53	1.50	9.65	3.23	-5.70	140.19	247.42	129.57	97.89
6.93	-3.08	10.81	20.02	10.58	-0.50	1.56	9.72	3.27	-5.67	139.90	246.33	129.00	97.15
7.02	-3.04	10.92	20.14	10.67	-0.44	1.67	9.79	3.35	-5.64	138.96	244.07	128.20	95.39
7.02	-3.04	10.92	20.14	10.66	-0.44	1.67	9.80	3.35	-5.64	139.12	244.19	128.13	95.40
6.96	-2.63	10.87	20.19	10.66	0.05	1.60	9.67	3.29	-5.28	136.48	252.00	128.45	91.60
7.58	-1.80	11.91	21.27	11.50	0.98	2.10	10.16	3.66	-4.60	129.43	247.09	122.60	80.45
6.93	-2.45	10.90	20.21	10.61	0.21	1.65	9.74	3.25	-5.10	137.16	256.37	129.29	89.40
6.62	-3.11	10.28	19.57	10.16	-0.52	1.38	9.45	3.09	-5.66	144.70	252.88	133.14	99.10
6.77	-3.05	10.45	19.68	10.26	-0.47	1.55	9.62	3.26	-5.62	141.10	251.57	131.46	96.10
6.74	-3.06	10.41	19.64	10.23	-0.48	1.53	9.60	3.24	-5.63	141.40	252.01	131.81	96.51
6.67	-3.00	10.26	19.56	10.13	-0.42	1.52	9.60	3.22	-5.59	142.20	253.45	133.06	96.67
7.10	-2.87	10.93	20.13	10.70	-0.29	1.86	10.00	3.48	-5.46	138.79	243.37	127.08	92.87
7.18	-2.74	11.01	20.24	10.76	-0.12	1.98	10.10	3.60	-5.33	136.59	243.64	125.68	90.81
6.87	-2.96	10.57	19.79	10.38	-0.38	1.66	9.73	3.35	-5.56	139.68	249.57	129.96	94.43
6.77	-2.98	10.39	19.66	10.23	-0.39	1.59	9.67	3.31	-5.57	142.09	251.19	131.23	95.52
6.77	-2.97	10.40	19.67	10.24	-0.38	1.60	9.68	3.31	-5.56	142.03	251.11	131.20	95.40
6.79	-3.02	10.51	19.72	10.33	-0.44	1.53	9.66	3.26	-5.62	140.68	249.63	131.42	96.69
6.86	-2.50	10.71	20.05	10.48	0.09	1.62	9.69	3.25	-5.11	137.74	257.49	129.77	90.81
7.12	-2.53	10.98	20.29	10.73	0.10	1.93	9.98	3.53	-5.15	134.75	248.15	125.72	87.88
7.08	-2.55	10.93	20.25	10.70	0.07	1.83	9.86	3.46	-5.19	135.99	252.31	125.17	88.89
6.69	-2.90	10.30	19.59	10.17	-0.31	1.54	9.59	3.23	-5.48	141.22	257.08	131.02	96.21
6.61	-2.93	10.16	19.52	10.08	-0.35	1.44	9.51	3.15	-5.50	141.52	259.94	131.77	97.46
7.26	-2.19	11.25	20.55	10.96	0.56	1.96	10.02	3.55	-4.91	134.81	250.98	126.63	87.39
7.58	-1.80	11.89	21.20	11.44	1.01	2.11	10.13	3.66	-4.60	131.52	251.15	123.53	80.08
7.43	-2.08	11.52	20.87	11.18	0.59	2.12	10.17	3.69	-4.79	131.28	247.24	123.95	83.92
7.47	-1.81	11.62	20.96	11.27	0.95	2.08	10.10	3.64	-4.57	131.02	248.86	121.99	81.03
6.92	-2.70	10.83	20.12	10.59	-0.01	1.51	9.60	3.22	-5.36	136.91	254.24	128.88	92.96
6.81	-3.01	10.49	19.71	10.29	-0.44	1.60	9.66	3.29	-5.60	140.75	251.52	130.81	95.57
6.83	-2.87	10.46	19.75	10.30	-0.27	1.71	9.79	3.38	-5.48	140.69	250.74	130.74	94.26
6.86	-2.56	10.69	20.05	10.48	0.05	1.61	9.67	3.26	-5.19	136.10	257.67	130.39	91.66
6.91	-2.45	10.74	20.07	10.49	0.14	1.69	9.74	3.32	-5.05	136.47	256.71	129.14	89.67
7.18	-2.40	11.23	20.53	10.90	0.28	1.88	9.95	3.48	-5.08	133.10	248.72	124.42	86.53
6.89	-2.68	10.65	19.99	10.44	-0.08	1.69	9.76	3.33	-5.28	136.46	252.51	129.62	92.58
7.12	-2.53	10.98	20.29	10.73	0.10	1.93	9.98	3.53	-5.16	134.77	248.18	125.77	87.91

7.48	-2.06	11.69	21.00	11.29	0.63	2.18	10.25	3.66	-4.78	131.03	244.83	121.65	81.43
7.08	-2.38	11.10	20.41	10.78	0.28	1.79	9.85	3.39	-5.01	135.34	253.39	126.72	86.99
7.73	-1.85	12.06	21.36	11.58	0.89	2.46	10.50	3.86	-4.59	128.94	240.63	118.17	77.92
6.13	-2.98	9.71	19.08	9.59	-0.36	0.85	8.94	2.66	-5.59	147.08	273.73	137.52	101.19
6.56	-2.60	10.36	19.74	10.11	0.04	1.30	9.34	3.02	-5.23	143.19	265.75	131.42	92.92
6.25	-2.84	9.88	19.26	9.73	-0.23	1.02	9.06	2.77	-5.47	146.90	270.48	134.66	98.36
6.27	-2.85	9.84	19.24	9.70	-0.23	1.02	9.05	2.81	-5.43	148.03	271.25	135.13	97.77
6.14	-2.95	9.73	19.10	9.60	-0.34	0.87	8.96	2.68	-5.57	147.85	273.35	137.02	100.74
6.14	-2.89	9.66	19.06	9.51	-0.31	0.93	8.99	2.74	-5.46	147.87	275.08	137.38	100.41
6.31	-2.75	9.95	19.33	9.73	-0.15	1.11	9.15	2.89	-5.33	146.06	271.99	134.77	97.10
6.90	-2.16	11.06	20.55	10.55	0.48	1.68	9.63	3.25	-4.81	135.17	257.98	127.96	85.44
6.50	-2.57	10.52	19.98	10.10	0.12	1.32	9.46	2.88	-5.25	140.86	265.75	135.98	96.22
6.80	-2.21	10.90	20.38	10.40	0.40	1.59	9.62	3.19	-4.86	135.86	259.73	129.23	88.24
6.67	-2.36	10.89	20.29	10.35	0.28	1.36	9.42	2.99	-5.02	137.09	262.98	131.18	89.91
6.73	-2.38	10.87	20.37	10.38	0.30	1.56	9.66	3.08	-5.06	139.15	260.94	132.59	91.27
6.73	-2.39	10.89	20.36	10.39	0.30	1.56	9.67	3.06	-5.07	137.92	260.21	131.91	91.47
6.52	-2.42	10.39	19.91	10.01	0.20	1.48	9.56	3.05	-5.07	139.07	265.51	134.78	93.34
6.48	-2.46	10.32	19.84	9.95	0.16	1.43	9.51	3.01	-5.11	139.58	266.49	135.44	94.32
8.31	-1.32	12.96	22.20	12.31	1.53	3.01	11.09	4.33	-4.17	118.42	226.25	110.27	70.37
6.52	-2.70	10.30	19.66	10.09	-0.02	1.18	9.25	2.94	-5.38	141.44	264.58	131.89	95.22
8.21	-1.50	12.53	21.87	12.07	1.28	3.02	11.09	4.36	-4.27	121.32	226.79	111.29	72.71
7.64	-1.80	11.88	21.28	11.42	0.99	2.33	10.38	3.85	-4.60	130.95	244.63	118.34	78.83
8.27	-1.32	12.86	22.16	12.26	1.52	2.97	11.03	4.30	-4.16	121.47	229.98	110.76	71.01
6.59	-2.64	10.42	19.76	10.18	0.08	1.27	9.35	3.01	-5.32	140.76	262.68	131.10	93.36
8.32	-1.44	12.71	22.00	12.24	1.36	3.06	11.19	4.41	-4.25	123.51	224.43	110.01	72.76
8.10	-1.30	12.58	22.02	12.00	1.47	2.88	10.87	4.19	-4.08	121.86	231.45	110.42	71.40
8.11	-1.30	12.59	22.02	12.00	1.47	2.88	10.87	4.19	-4.08	121.87	231.40	110.38	71.35
7.76	-1.66	12.13	21.52	11.60	1.16	2.43	10.45	3.90	-4.46	128.63	242.26	116.37	76.50
7.30	-2.04	11.48	20.86	11.05	0.74	1.96	9.98	3.53	-4.81	133.26	251.08	121.51	82.64
6.74	-2.49	10.70	20.07	10.39	0.24	1.42	9.49	3.11	-5.23	139.09	260.64	129.61	91.23
6.73	-2.49	10.69	20.06	10.38	0.24	1.41	9.49	3.10	-5.24	139.14	260.77	129.69	91.35
7.79	-1.66	12.16	21.54	11.64	1.18	2.47	10.49	3.95	-4.47	127.07	239.89	115.36	76.14
7.79	-1.66	12.16	21.54	11.64	1.18	2.47	10.49	3.95	-4.47	127.09	239.92	115.39	76.15
7.53	-1.87	11.81	21.20	11.32	0.94	2.20	10.24	3.74	-4.66	130.89	245.67	118.63	79.92
7.48	-1.94	11.70	21.10	11.26	0.87	2.14	10.19	3.70	-4.72	131.58	246.28	119.66	80.54
6.69	-2.54	10.62	19.97	10.33	0.19	1.38	9.43	3.07	-5.26	139.54	260.34	129.84	92.46
6.74	-2.51	10.69	20.03	10.38	0.22	1.42	9.46	3.11	-5.23	139.30	259.44	129.26	91.85
7.02	-2.08	11.22	20.76	10.71	0.58	1.81	9.76	3.31	-4.74	135.66	256.94	125.71	83.20
7.84	-1.53	12.47	21.97	11.73	1.30	2.48	10.51	3.95	-4.35	127.29	243.20	114.75	73.89
8.18	-1.27	12.94	22.36	12.20	1.60	2.81	10.83	4.17	-4.15	122.53	234.07	110.91	69.35
7.52	-1.57	11.75	21.38	11.20	1.11	2.39	10.35	3.82	-4.25	129.17	241.01	118.44	78.50
7.95	-1.47	12.58	21.97	11.90	1.38	2.57	10.60	4.01	-4.34	126.47	239.32	114.25	73.07
8.15	-1.22	12.90	22.40	12.13	1.59	2.86	10.84	4.21	-4.08	123.95	233.82	111.58	69.84

8.16	-1.22	12.91	22.41	12.13	1.60	2.87	10.85	4.21	-4.07	123.84	233.65	111.49	69.77
8.16	-1.22	12.91	22.41	12.13	1.60	2.87	10.85	4.22	-4.07	123.80	233.60	111.46	69.74
8.16	-1.22	12.91	22.41	12.14	1.60	2.87	10.85	4.22	-4.07	123.77	233.55	111.44	69.72
8.16	-1.22	12.91	22.42	12.14	1.60	2.87	10.85	4.22	-4.07	123.74	233.51	111.41	69.71
8.17	-1.21	12.91	22.42	12.14	1.61	2.88	10.85	4.22	-4.07	123.71	233.46	111.39	69.69
8.17	-1.21	12.92	22.42	12.14	1.61	2.88	10.86	4.22	-4.07	123.68	233.42	111.37	69.67
8.17	-1.21	12.92	22.42	12.14	1.61	2.88	10.86	4.22	-4.06	123.65	233.37	111.34	69.65
8.17	-1.21	12.92	22.42	12.14	1.61	2.88	10.86	4.22	-4.06	123.61	233.31	111.31	69.63
8.17	-1.21	12.92	22.43	12.15	1.61	2.88	10.86	4.22	-4.06	123.58	233.26	111.28	69.61
7.56	-1.74	12.04	21.51	11.34	1.02	2.26	10.25	3.76	-4.51	130.03	246.62	118.08	77.39
7.55	-1.76	12.01	21.48	11.31	1.01	2.24	10.23	3.74	-4.52	130.20	246.83	118.32	77.61
7.56	-1.75	12.04	21.50	11.34	1.02	2.25	10.25	3.76	-4.51	130.03	246.56	118.08	77.37
6.86	-2.25	11.07	20.53	10.58	0.48	1.59	9.62	3.18	-4.95	138.00	257.84	126.23	86.77
7.10	-2.06	11.40	20.87	10.83	0.67	1.83	9.84	3.40	-4.78	136.27	254.01	123.62	83.39
7.36	-1.84	11.75	21.22	11.12	0.88	2.09	10.08	3.59	-4.57	133.88	250.00	119.97	79.31
7.16	-2.02	11.48	20.96	10.90	0.72	1.90	9.89	3.43	-4.73	135.83	252.91	122.71	82.23
6.95	-2.18	11.17	20.65	10.68	0.54	1.67	9.67	3.21	-4.90	136.22	256.63	124.32	84.83
8.09	-1.26	12.82	22.35	12.01	1.54	2.79	10.79	4.15	-4.05	125.35	234.60	111.47	70.71
7.77	-1.46	12.31	21.89	11.57	1.28	2.54	10.52	3.96	-4.17	126.47	240.30	115.77	74.10
7.24	-1.94	11.63	21.16	11.05	0.76	2.00	9.96	3.42	-4.65	133.35	251.23	120.89	79.94
6.94	-2.18	11.24	20.70	10.72	0.51	1.68	9.66	3.20	-4.88	135.53	256.13	124.34	84.06
6.99	-2.15	11.30	20.78	10.77	0.55	1.72	9.70	3.22	-4.85	135.00	255.23	123.81	83.30
6.99	-2.15	11.30	20.78	10.77	0.55	1.72	9.70	3.22	-4.85	134.98	255.23	123.79	83.29
6.95	-2.15	11.20	20.69	10.67	0.52	1.69	9.67	3.22	-4.84	136.33	256.82	125.36	84.21
6.98	-2.12	11.22	20.73	10.69	0.52	1.72	9.69	3.26	-4.80	135.62	256.87	125.19	83.71
7.01	-2.10	11.25	20.77	10.72	0.54	1.75	9.72	3.28	-4.78	135.42	256.41	125.04	83.30
7.43	-1.76	11.97	21.52	11.31	0.97	2.19	10.12	3.56	-4.51	130.52	247.27	118.92	76.22
7.31	-1.87	11.80	21.33	11.15	0.87	2.03	9.98	3.46	-4.61	132.20	249.98	122.14	79.08
7.42	-1.79	11.97	21.53	11.30	0.96	2.11	10.06	3.51	-4.56	130.17	248.33	121.19	77.85
7.38	-1.81	11.94	21.49	11.27	0.96	2.11	10.07	3.51	-4.54	130.36	249.00	121.42	77.28
7.60	-1.65	12.27	21.83	11.52	1.10	2.33	10.27	3.65	-4.39	128.46	241.38	117.28	74.43
7.60	-1.65	12.27	21.83	11.52	1.11	2.33	10.27	3.65	-4.39	128.45	241.40	117.28	74.44
8.08	-1.17	12.68	22.25	11.90	1.56	2.89	10.84	4.25	-3.91	121.74	231.25	110.93	70.73
7.98	-1.23	12.43	22.01	11.76	1.54	2.83	10.76	4.19	-3.97	122.37	233.42	112.68	71.95
7.99	-1.22	12.44	22.02	11.77	1.54	2.84	10.77	4.20	-3.96	122.35	233.20	112.59	71.78
8.25	-0.96	12.79	22.33	12.08	1.81	3.14	11.04	4.46	-3.76	121.28	226.48	109.00	68.61
8.25	-0.97	12.79	22.33	12.07	1.81	3.14	11.04	4.46	-3.76	121.26	226.58	109.00	68.66
8.25	-0.96	12.79	22.33	12.08	1.81	3.14	11.04	4.46	-3.76	121.34	226.50	109.00	68.65
8.25	-0.97	12.79	22.32	12.07	1.81	3.13	11.04	4.46	-3.76	121.31	226.63	109.00	68.71
8.25	-0.97	12.79	22.33	12.07	1.81	3.13	11.04	4.46	-3.76	121.38	226.54	109.00	68.69
8.25	-1.11	12.89	22.35	12.19	1.72	3.03	11.04	4.30	-3.95	120.97	227.92	109.06	68.84
8.13	-1.20	12.65	22.08	12.00	1.60	2.97	10.96	4.26	-3.99	121.14	229.64	109.53	70.57
7.80	-1.33	12.22	21.82	11.55	1.36	2.69	10.61	4.06	-4.04	125.43	235.83	114.00	74.08

6.68	-2.40	10.81	20.34	10.33	0.30	1.55	9.67	3.06	-5.08	135.64	259.27	132.60	92.27
7.46	-1.74	11.97	21.60	11.28	1.00	2.26	10.29	3.63	-4.51	129.70	245.80	122.64	78.93
6.33	-2.67	10.22	19.73	9.86	-0.06	1.16	9.32	2.84	-5.28	143.38	270.74	137.19	99.13
6.32	-2.67	10.21	19.72	9.85	-0.07	1.15	9.31	2.83	-5.29	143.49	270.94	137.34	99.34
6.32	-2.68	10.20	19.71	9.84	-0.08	1.14	9.31	2.82	-5.30	143.55	271.03	137.42	99.44
6.31	-2.68	10.19	19.71	9.84	-0.08	1.14	9.30	2.82	-5.30	143.60	271.13	137.51	99.55
6.30	-2.69	10.18	19.69	9.82	-0.09	1.13	9.29	2.81	-5.31	143.72	271.34	137.70	99.78
6.33	-2.66	10.23	19.74	9.87	-0.06	1.16	9.32	2.84	-5.28	143.34	270.64	137.15	99.02
6.72	-2.35	10.85	20.41	10.36	0.33	1.54	9.65	3.09	-5.02	139.21	261.18	132.71	91.14
6.75	-2.32	10.90	20.47	10.39	0.35	1.57	9.66	3.11	-5.00	138.82	260.27	132.03	90.57
6.87	-2.24	11.09	20.65	10.55	0.43	1.68	9.76	3.18	-4.96	137.63	257.03	129.99	88.93
7.31	-1.82	11.76	21.38	11.11	0.88	2.10	10.08	3.50	-4.54	130.47	246.51	120.51	79.34
7.33	-1.85	11.83	21.44	11.16	0.87	2.09	10.07	3.48	-4.55	129.00	245.40	120.08	79.03
7.33	-1.85	11.84	21.44	11.17	0.87	2.09	10.07	3.48	-4.55	128.97	245.39	120.01	78.99
7.32	-1.85	11.81	21.42	11.15	0.86	2.09	10.06	3.48	-4.54	128.96	245.58	120.31	79.15
7.32	-1.85	11.83	21.43	11.16	0.86	2.09	10.06	3.48	-4.55	128.97	245.48	120.15	79.07
7.54	-1.69	12.19	21.77	11.45	1.06	2.27	10.23	3.62	-4.42	128.47	241.93	117.85	75.42
7.35	-1.82	11.92	21.52	11.18	0.90	2.09	10.10	3.49	-4.55	128.91	246.13	120.17	78.21
7.30	-1.81	11.76	21.38	11.11	0.89	2.13	10.10	3.52	-4.51	129.29	245.81	120.45	79.70
7.47	-1.72	12.05	21.67	11.32	1.02	2.23	10.21	3.62	-4.43	128.10	244.00	119.01	76.76
7.64	-1.56	12.46	21.99	11.58	1.23	2.31	10.30	3.69	-4.34	126.38	242.75	118.12	74.75
7.28	-1.83	11.69	21.25	11.03	0.88	2.11	10.13	3.57	-4.51	131.47	251.40	122.79	80.39
7.61	-1.55	12.21	21.78	11.45	1.21	2.46	10.42	3.79	-4.30	125.83	241.49	118.33	75.42
6.66	-2.32	10.66	20.20	10.19	0.27	1.55	9.63	3.15	-4.92	138.81	263.80	131.94	91.30
6.42	-2.58	10.31	19.81	9.91	0.02	1.26	9.40	2.93	-5.15	141.82	268.64	135.64	96.63
6.47	-2.55	10.40	19.90	9.98	0.07	1.29	9.44	2.95	-5.14	141.98	267.89	134.96	96.16
6.34	-2.65	10.17	19.66	9.81	-0.06	1.19	9.34	2.87	-5.22	142.57	270.65	136.59	98.53
6.46	-2.56	10.39	19.90	9.98	0.07	1.29	9.44	2.95	-5.14	142.02	267.95	134.98	96.24
7.44	-1.64	11.77	21.38	11.14	1.05	2.35	10.29	3.74	-4.32	128.75	245.79	118.18	77.66
6.42	-2.60	10.42	19.77	10.07	0.08	1.17	9.29	2.78	-5.25	143.35	275.55	139.32	97.16
6.11	-2.82	9.92	19.24	9.67	-0.18	0.87	9.04	2.54	-5.45	147.80	282.57	144.99	103.99
5.79	-2.96	9.01	18.34	9.03	-0.49	0.91	9.07	2.55	-5.41	151.31	290.52	150.65	111.38
5.97	-2.82	9.22	18.68	9.34	-0.26	1.00	9.04	2.63	-5.37	150.82	289.88	145.48	104.74
5.52	-3.13	8.46	17.96	8.72	-0.65	0.64	8.70	2.35	-5.63	154.85	296.30	150.20	113.09
5.56	-3.10	8.51	18.02	8.77	-0.62	0.67	8.73	2.38	-5.61	154.59	295.68	149.66	112.35
6.30	-2.26	9.48	18.89	9.46	0.18	1.46	9.57	3.16	-4.68	149.87	286.98	143.75	104.93
5.81	-2.67	8.79	18.11	8.99	-0.13	0.83	9.11	2.61	-5.24	161.01	308.24	158.56	125.49
6.83	-1.99	10.45	19.80	10.26	0.69	1.81	10.08	3.41	-4.66	154.46	292.49	152.94	111.03
6.53	-2.47	10.49	19.82	10.23	0.22	1.22	9.53	2.82	-5.17	154.06	290.15	152.65	111.48
7.18	-1.77	11.06	20.45	10.76	0.93	2.10	10.31	3.59	-4.46	149.98	282.23	148.66	102.83
6.64	-2.28	10.53	19.88	10.15	0.36	1.62	9.81	3.12	-4.91	142.32	272.53	138.89	96.49
6.63	-2.37	10.49	19.79	10.16	0.24	1.57	9.75	3.08	-5.00	144.78	273.72	140.12	96.97
6.85	-2.15	10.94	20.28	10.52	0.48	1.80	9.92	3.22	-4.82	141.60	267.87	135.29	91.49

6.75	-2.29	10.67	20.00	10.33	0.33	1.68	9.83	3.15	-4.92	144.00	270.79	138.14	94.64
6.86	-2.20	10.89	20.24	10.50	0.43	1.77	9.91	3.22	-4.84	143.05	268.11	136.06	92.31
6.42	-2.57	10.44	19.76	10.12	0.09	1.21	9.40	2.68	-5.22	144.63	275.97	139.93	99.06
6.65	-2.37	10.68	20.01	10.31	0.26	1.53	9.70	3.02	-5.02	143.89	271.82	138.26	95.02
6.33	-2.64	10.21	19.52	9.97	0.01	1.17	9.35	2.68	-5.26	145.74	280.64	142.30	101.33
6.86	-2.16	10.92	20.29	10.49	0.49	1.76	9.91	3.19	-4.82	140.25	267.67	135.26	91.48
6.57	-2.43	10.67	19.98	10.29	0.25	1.35	9.54	2.83	-5.10	143.66	273.14	138.11	96.14
6.43	-2.57	10.49	19.82	10.16	0.11	1.20	9.41	2.68	-5.22	144.69	275.98	139.87	98.82
6.03	-2.83	9.74	19.03	9.54	-0.20	0.87	9.04	2.53	-5.45	149.76	284.41	145.60	105.01
6.01	-2.85	9.72	19.00	9.52	-0.22	0.84	9.02	2.50	-5.47	149.72	284.81	146.06	105.45
6.48	-2.43	10.32	19.68	9.98	0.21	1.41	9.63	2.96	-5.07	144.38	276.24	141.41	99.75
6.63	-2.29	10.52	19.88	10.15	0.35	1.61	9.80	3.11	-4.92	142.41	272.72	139.01	96.70
6.40	-2.53	10.15	19.44	9.91	0.07	1.40	9.58	2.93	-5.14	145.97	278.11	143.28	100.87
6.00	-2.79	9.38	18.79	9.36	-0.21	1.02	9.13	2.64	-5.35	149.78	285.21	144.61	103.57
6.43	-2.53	10.42	19.66	10.07	0.17	1.22	9.38	2.77	-5.23	145.15	275.02	140.09	98.40
5.66	-2.97	8.77	18.12	8.90	-0.42	0.65	8.94	2.42	-5.55	159.88	304.82	160.20	125.07
5.66	-2.97	8.78	18.12	8.90	-0.42	0.65	8.94	2.42	-5.55	159.88	304.78	160.18	125.06
7.82	-1.41	12.09	21.63	11.57	1.34	2.88	11.01	4.08	-4.15	133.44	249.14	128.80	84.31
5.93	-2.86	9.24	18.65	9.24	-0.29	1.02	9.23	2.61	-5.44	155.53	291.61	155.06	115.92
5.66	-3.07	8.81	18.21	8.90	-0.50	0.79	9.04	2.41	-5.59	158.14	296.98	158.14	121.29
7.01	-1.52	10.47	19.80	10.38	1.11	1.98	10.04	3.65	-4.15	139.05	273.11	135.09	93.60
6.45	-2.01	9.59	18.93	9.65	0.55	1.47	9.59	3.24	-4.58	144.64	283.11	141.61	103.89
6.90	-1.66	10.32	19.65	10.23	0.91	1.92	10.10	3.58	-4.24	146.46	282.25	142.40	102.57
6.39	-2.16	9.95	19.24	9.82	0.48	1.22	9.55	2.97	-4.82	151.58	292.04	146.54	110.26
6.39	-2.16	9.95	19.24	9.82	0.48	1.22	9.55	2.97	-4.82	151.67	292.10	146.55	110.36
7.01	-1.50	10.48	19.77	10.29	1.03	2.06	10.19	3.75	-4.06	144.69	276.93	138.94	97.23
6.07	-2.65	9.50	18.80	9.52	-0.03	0.99	9.26	2.65	-5.26	157.71	301.00	156.97	119.35
6.63	-2.06	9.98	19.31	9.91	0.53	1.76	9.92	3.35	-4.60	152.18	290.79	150.00	108.49
6.23	-2.27	9.30	18.60	9.35	0.25	1.41	9.56	3.08	-4.79	154.11	295.64	151.89	114.69
6.44	-2.46	10.09	19.41	9.89	0.18	1.46	9.68	2.99	-5.07	147.97	282.59	144.70	103.48
6.42	-2.46	9.95	19.35	9.75	0.08	1.59	9.77	3.09	-4.98	145.97	280.29	145.40	104.51
6.38	-2.46	9.92	19.25	9.76	0.12	1.42	9.64	2.99	-5.06	149.51	284.67	145.65	104.84
7.06	-1.94	11.01	20.33	10.61	0.70	2.08	10.25	3.50	-4.59	141.71	270.69	136.10	92.85
6.56	-2.33	10.36	19.73	10.04	0.30	1.51	9.72	3.05	-5.00	142.63	273.51	140.57	99.10
6.69	-2.22	10.53	19.89	10.16	0.40	1.70	9.84	3.21	-4.85	141.43	271.10	137.95	95.39
6.32	-2.52	10.03	19.52	9.74	0.09	1.33	9.49	2.91	-5.13	143.48	275.43	140.35	100.46
6.89	-2.18	11.07	20.57	10.55	0.49	1.77	9.90	3.22	-4.87	136.29	260.78	131.90	89.81
7.38	-1.76	11.38	20.92	10.95	0.88	2.43	10.58	3.74	-4.40	136.49	257.12	134.24	89.61
6.34	-2.51	9.86	19.26	9.67	0.02	1.50	9.71	3.02	-5.05	146.68	281.29	146.71	105.84
7.99	-1.26	12.55	22.14	11.88	1.49	2.90	11.00	4.09	-4.03	127.56	244.65	123.12	77.32
7.71	-1.49	11.94	21.45	11.42	1.26	2.80	10.93	3.98	-4.22	135.12	251.24	130.55	86.26
6.53	-2.36	10.08	19.54	9.90	0.22	1.68	9.89	3.19	-4.94	145.14	275.62	144.19	104.44
6.49	-2.42	10.33	19.69	9.99	0.22	1.42	9.64	2.97	-5.06	144.21	275.96	141.22	99.51

8.17	-1.16	12.68	22.29	12.09	1.62	3.15	11.24	4.24	-3.94	128.85	242.17	126.74	79.98
8.66	-0.74	13.52	23.23	12.76	2.15	3.62	11.79	4.58	-3.63	132.80	243.76	132.95	85.00
8.46	-0.92	13.18	22.96	12.52	1.95	3.39	11.56	4.38	-3.84	134.64	248.58	135.45	88.37
8.46	-0.92	13.18	22.96	12.53	1.95	3.40	11.56	4.38	-3.84	134.63	248.56	135.45	88.36
8.47	-0.92	13.19	22.96	12.53	1.95	3.40	11.56	4.39	-3.84	134.74	248.54	135.55	88.42
8.48	-0.91	13.20	22.97	12.53	1.96	3.41	11.57	4.39	-3.84	134.71	248.39	135.55	88.36
8.48	-0.91	13.20	22.97	12.54	1.96	3.41	11.57	4.39	-3.83	134.67	248.33	135.53	88.34
8.48	-0.91	13.21	22.98	12.54	1.97	3.42	11.57	4.40	-3.83	134.54	248.14	135.48	88.24
8.50	-0.90	13.24	23.00	12.56	1.98	3.45	11.60	4.42	-3.81	134.17	247.58	135.30	87.91
8.51	-0.90	13.25	23.01	12.56	1.99	3.45	11.60	4.42	-3.80	134.12	247.46	135.28	87.85
7.63	-1.21	11.70	21.16	11.37	1.49	2.51	10.76	3.91	-3.95	149.00	279.63	148.95	102.30
6.17	-2.83	9.86	19.35	9.61	-0.26	1.18	9.45	2.72	-5.41	157.01	295.47	159.88	120.90
6.15	-2.85	9.83	19.32	9.59	-0.28	1.17	9.44	2.71	-5.42	157.05	295.82	160.09	121.20
6.19	-2.82	9.88	19.37	9.63	-0.25	1.20	9.46	2.73	-5.40	156.91	295.09	159.63	120.57
7.34	-1.52	11.27	20.76	11.02	1.23	2.19	10.46	3.65	-4.30	151.49	287.88	151.57	106.89
7.42	-1.49	11.39	20.92	11.12	1.29	2.28	10.53	3.73	-4.26	151.86	284.88	149.33	103.60
6.74	-2.11	10.56	19.97	10.26	0.60	1.60	9.93	3.22	-4.78	156.25	295.53	156.63	114.81
6.67	-2.36	10.84	20.30	10.42	0.37	1.39	9.70	2.91	-5.09	152.76	289.30	153.09	111.63
6.43	-2.58	10.36	19.78	10.05	0.07	1.29	9.56	2.83	-5.22	152.45	289.08	153.49	113.06
6.42	-2.68	10.47	19.87	10.09	-0.03	1.19	9.50	2.71	-5.33	151.47	287.62	154.72	113.70
6.61	-2.48	10.48	19.96	10.13	0.13	1.60	9.82	3.07	-5.10	154.57	288.20	155.10	112.97
6.61	-2.45	10.61	20.03	10.20	0.12	1.47	9.73	2.99	-5.11	154.00	285.98	153.57	111.20
6.23	-2.78	10.13	19.49	9.87	-0.10	0.94	9.28	2.57	-5.42	155.29	293.26	156.08	116.86
6.24	-2.91	10.12	19.59	9.80	-0.29	1.09	9.40	2.66	-5.50	155.89	290.62	156.28	117.60
6.29	-2.76	10.10	19.61	9.81	-0.17	1.26	9.53	2.79	-5.35	154.59	291.18	157.44	117.08
6.34	-2.72	10.32	19.71	10.01	-0.05	1.04	9.37	2.66	-5.38	154.45	288.77	154.82	114.68
7.05	-1.84	10.90	20.31	10.59	0.82	2.03	10.28	3.53	-4.47	152.40	286.95	151.82	106.64
6.76	-2.13	10.60	20.04	10.29	0.52	1.68	10.00	3.25	-4.76	153.67	290.32	153.72	111.50
6.62	-2.41	10.61	20.04	10.23	0.22	1.49	9.76	3.00	-5.07	153.36	287.32	152.51	110.52
6.79	-2.23	10.56	20.01	10.28	0.40	1.74	10.03	3.27	-4.87	152.16	286.65	152.51	110.35
8.28	-1.05	12.97	22.79	12.33	1.85	3.18	11.39	4.26	-3.93	154.74	272.57	149.48	95.52
7.46	-1.52	11.51	21.15	11.24	1.27	2.28	10.54	3.69	-4.28	160.26	289.78	156.76	108.18
7.28	-1.66	11.26	20.88	10.99	1.07	2.18	10.38	3.62	-4.36	156.07	287.65	153.14	107.74
8.07	-0.96	12.25	21.86	11.84	1.88	2.99	11.17	4.29	-3.81	163.68	285.91	155.09	100.42
8.11	-0.89	12.44	22.02	11.97	1.95	2.98	11.18	4.27	-3.73	156.11	277.03	148.56	95.59
8.41	-0.75	12.88	22.57	12.42	2.21	3.25	11.40	4.44	-3.68	162.99	279.60	151.63	93.81
8.30	-0.77	12.57	22.21	12.16	2.12	3.22	11.38	4.48	-3.68	159.56	278.66	151.80	95.96
8.35	-0.82	12.86	22.58	12.41	2.12	3.14	11.31	4.32	-3.76	164.96	284.21	153.74	95.40
8.35	-0.82	12.86	22.58	12.41	2.12	3.14	11.31	4.32	-3.76	164.94	284.15	153.71	95.35
6.51	-2.62	10.58	19.97	10.23	0.07	1.24	9.51	2.79	-5.30	151.78	282.81	151.90	110.66
6.14	-2.95	9.94	19.34	9.72	-0.27	0.97	9.26	2.56	-5.60	153.08	286.98	154.64	114.49
7.25	-1.92	11.33	20.77	10.85	0.73	2.35	10.47	3.66	-4.58	140.91	261.87	140.57	94.81
6.43	-2.72	10.56	19.97	10.17	-0.07	1.13	9.44	2.67	-5.39	151.95	286.15	152.78	112.72

6.27	-2.76	10.22	19.65	9.91	-0.13	1.08	9.39	2.65	-5.40	152.35	290.52	157.02	116.61
6.00	-2.87	9.40	18.83	9.31	-0.29	1.10	9.38	2.68	-5.45	154.34	288.38	154.15	116.28
6.02	-2.90	9.54	18.94	9.45	-0.25	0.96	9.21	2.56	-5.52	155.62	291.95	155.43	116.12
6.05	-2.91	9.75	19.12	9.60	-0.26	0.92	9.20	2.53	-5.57	154.92	290.16	154.90	116.73
6.17	-2.85	9.99	19.38	9.79	-0.22	0.97	9.27	2.58	-5.51	153.15	288.29	154.98	115.06
6.49	-2.64	10.56	19.94	10.20	0.04	1.22	9.48	2.76	-5.32	151.52	283.40	152.69	111.64
6.40	-2.73	10.31	19.72	10.02	-0.08	1.26	9.53	2.80	-5.39	150.59	281.13	151.39	109.65
6.43	-2.68	10.54	19.93	10.15	-0.05	1.15	9.44	2.69	-5.35	150.12	285.76	154.21	112.76
6.43	-2.66	10.51	19.92	10.13	-0.04	1.18	9.49	2.71	-5.33	150.51	286.70	154.02	113.07
6.32	-2.72	10.29	19.71	9.96	-0.09	1.13	9.43	2.68	-5.37	152.08	290.04	156.42	115.74
5.80	-3.13	9.26	18.67	9.19	-0.51	0.79	9.10	2.42	-5.72	155.96	293.55	158.12	120.98
6.55	-2.56	10.56	20.04	10.24	0.14	1.42	9.71	2.88	-5.32	152.85	285.37	155.05	113.60
6.45	-2.58	10.37	19.86	10.04	0.07	1.35	9.64	2.87	-5.30	162.72	297.97	162.83	120.72
6.49	-2.72	10.50	19.90	10.12	-0.07	1.33	9.64	2.86	-5.40	151.70	282.93	153.03	111.46
6.56	-2.57	10.46	19.90	10.13	0.12	1.49	9.79	2.99	-5.24	151.36	282.54	152.68	112.34
6.65	-2.53	10.72	20.25	10.38	0.21	1.51	9.76	2.90	-5.27	154.02	283.31	154.33	112.32
6.74	-2.24	10.51	20.02	10.24	0.43	1.78	10.13	3.22	-4.92	150.55	280.49	150.70	111.06
6.82	-2.37	10.91	20.44	10.53	0.35	1.72	9.98	3.10	-5.08	150.49	281.52	152.59	109.83
8.20	-1.18	12.69	22.50	12.14	1.68	3.20	11.33	4.23	-4.04	141.34	257.95	140.24	92.06
6.46	-2.66	10.38	19.91	10.10	0.06	1.35	9.65	2.81	-5.36	158.19	291.99	157.44	118.62
7.66	-1.44	11.92	21.72	11.47	1.41	2.55	10.82	3.84	-4.31	169.07	296.05	160.12	108.86
6.82	-2.17	10.94	20.38	10.50	0.56	1.71	9.96	3.16	-4.92	161.10	291.68	159.85	114.95
7.51	-1.55	11.61	21.16	11.22	1.21	2.42	10.59	3.77	-4.28	158.58	283.93	152.30	104.09
7.44	-1.65	11.57	21.16	11.19	1.12	2.29	10.49	3.69	-4.41	160.46	285.68	153.59	105.41
6.91	-2.10	11.11	20.57	10.67	0.66	1.72	9.99	3.17	-4.87	161.52	291.53	158.76	113.30
6.82	-2.17	10.93	20.38	10.50	0.56	1.70	9.95	3.15	-4.92	161.10	291.78	159.98	115.04
6.66	-2.26	10.52	20.02	10.20	0.44	1.55	9.88	3.11	-4.99	160.42	296.51	160.68	118.28
8.38	-0.83	12.97	22.83	12.45	2.11	3.19	11.40	4.29	-3.74	163.01	285.51	152.17	93.50
8.38	-0.83	12.98	22.77	12.45	2.10	3.18	11.34	4.30	-3.78	162.07	280.26	151.56	93.37
7.68	-1.45	11.96	21.78	11.53	1.42	2.54	10.80	3.83	-4.34	167.69	296.29	160.44	108.76
7.93	-1.34	12.39	22.15	11.86	1.54	2.81	11.01	4.00	-4.23	161.42	283.61	154.56	102.80
7.50	-1.55	11.59	21.14	11.21	1.20	2.41	10.56	3.78	-4.27	159.19	284.77	152.64	104.45
6.66	-2.30	10.71	20.11	10.27	0.41	1.54	9.84	3.04	-5.04	161.61	294.24	159.56	117.45
6.66	-2.30	10.70	20.11	10.27	0.41	1.54	9.83	3.04	-5.04	161.63	294.29	159.59	117.49
6.89	-2.13	11.08	20.54	10.64	0.64	1.69	9.97	3.14	-4.89	161.70	291.93	159.02	113.98
6.82	-2.17	10.93	20.38	10.50	0.56	1.70	9.96	3.15	-4.92	161.10	291.75	159.93	115.01
6.74	-2.36	10.83	20.28	10.40	0.40	1.64	9.91	3.07	-5.13	158.95	289.63	157.09	113.68
6.54	-2.49	10.45	19.91	10.11	0.22	1.53	9.81	2.98	-5.24	159.14	292.28	158.38	117.44
6.47	-2.56	10.31	19.78	9.99	0.17	1.49	9.79	2.95	-5.26	159.85	292.88	159.66	119.31
6.48	-2.54	10.30	19.76	9.99	0.17	1.51	9.81	2.97	-5.24	159.54	292.80	159.63	118.99
6.80	-2.25	10.75	20.19	10.33	0.45	1.80	10.06	3.25	-4.94	159.00	289.65	155.95	113.85
6.09	-3.03	9.98	19.42	9.67	-0.42	0.89	9.22	2.50	-5.63	156.22	293.49	159.52	120.52
6.07	-3.06	9.99	19.41	9.66	-0.44	0.83	9.18	2.45	-5.66	157.06	293.86	159.82	120.74

6.38	-2.09	10.65	19.85	10.21	0.70	1.12	9.34	2.52	-4.85	146.55	259.58	156.78	150.78
6.66	-1.91	10.78	20.16	10.45	0.82	1.47	9.66	2.85	-4.65	157.23	265.41	161.56	153.36
6.78	-1.81	11.14	20.41	10.67	0.97	1.53	9.71	2.85	-4.60	142.29	252.83	148.76	139.07
6.79	-1.77	11.02	20.33	10.63	0.96	1.63	9.80	2.96	-4.52	147.21	257.35	153.25	142.86
6.77	-1.57	10.93	20.20	10.38	1.04	1.79	9.85	3.16	-4.20	132.65	236.90	145.27	134.03
6.07	-2.13	9.83	19.14	9.56	0.49	1.07	9.27	2.61	-4.77	156.93	264.93	167.62	164.47
6.07	-2.14	9.82	19.13	9.56	0.49	1.07	9.27	2.61	-4.77	157.05	265.05	167.81	164.62
6.23	-2.16	10.33	19.53	9.96	0.57	1.08	9.26	2.53	-4.86	150.07	258.23	159.50	153.68
6.61	-1.87	10.92	20.14	10.43	0.90	1.43	9.60	2.78	-4.64	135.39	248.76	146.89	137.19
6.60	-1.88	10.91	20.13	10.42	0.90	1.42	9.59	2.77	-4.64	135.67	249.03	147.15	137.52
6.36	-2.19	10.52	19.77	10.19	0.54	1.08	9.26	2.54	-4.94	158.71	267.48	163.77	156.11
6.54	-2.08	10.80	20.05	10.41	0.67	1.25	9.42	2.66	-4.85	152.35	261.79	157.57	148.32
6.46	-2.04	10.83	19.99	10.38	0.69	1.13	9.29	2.57	-4.80	142.52	255.82	152.70	141.80
6.19	-2.21	10.19	19.53	9.94	0.48	0.92	9.20	2.46	-4.92	165.96	273.94	170.95	166.20
6.85	-1.76	11.09	20.38	10.66	0.96	1.66	9.83	3.04	-4.48	148.72	258.46	152.12	141.85
6.57	-2.06	10.76	20.01	10.40	0.69	1.29	9.46	2.70	-4.80	152.86	261.83	157.44	148.49
6.93	-1.58	11.24	20.41	10.68	1.05	1.79	9.91	3.19	-4.22	137.52	248.18	145.66	129.40
6.52	-2.04	10.67	19.92	10.30	0.63	1.25	9.44	2.74	-4.69	163.35	268.66	166.39	155.82
6.34	-2.10	10.59	19.77	10.15	0.69	1.10	9.31	2.51	-4.87	147.23	259.50	158.22	152.27
6.55	-1.93	10.84	20.06	10.37	0.87	1.34	9.53	2.71	-4.72	138.53	252.07	150.02	141.05
6.54	-1.95	10.57	19.92	10.26	0.73	1.37	9.61	2.83	-4.61	158.37	266.75	163.12	154.60
6.43	-2.03	10.48	19.82	10.16	0.66	1.23	9.46	2.70	-4.71	160.92	269.03	165.66	158.33
6.53	-2.04	10.76	20.07	10.38	0.68	1.20	9.43	2.65	-4.79	156.96	265.06	161.33	152.23
6.52	-2.04	10.74	20.06	10.37	0.68	1.19	9.43	2.65	-4.79	157.22	265.28	161.54	152.54
6.53	-2.06	10.80	20.07	10.43	0.67	1.20	9.39	2.65	-4.84	154.18	262.69	159.42	150.04
6.72	-1.86	11.02	20.30	10.63	0.87	1.40	9.60	2.81	-4.60	153.27	260.78	157.80	146.22
6.36	-2.19	10.52	19.77	10.19	0.54	1.09	9.27	2.54	-4.94	158.60	267.37	163.65	155.94
6.94	-1.69	11.26	20.58	10.85	1.06	1.68	9.86	3.02	-4.43	151.59	258.37	155.91	142.26
6.68	-1.89	10.86	20.24	10.52	0.80	1.43	9.66	2.84	-4.60	154.57	263.04	159.84	150.16
6.77	-1.71	10.95	20.14	10.48	0.91	1.64	9.77	3.06	-4.32	141.57	252.53	149.67	135.03
6.38	-2.16	10.74	19.94	10.28	0.63	1.09	9.27	2.51	-4.94	151.89	263.65	159.70	150.45
6.47	-2.10	10.87	20.05	10.41	0.70	1.11	9.28	2.51	-4.88	144.71	257.20	152.55	143.09
6.22	-2.26	10.46	19.68	10.10	0.54	0.92	9.12	2.37	-5.05	152.01	261.08	160.48	153.33
6.27	-2.24	10.55	19.76	10.17	0.58	0.95	9.17	2.39	-5.04	151.17	260.41	159.17	151.68
6.27	-2.22	10.50	19.69	10.12	0.58	0.97	9.20	2.44	-5.02	147.19	258.56	158.74	151.82
6.46	-2.09	10.89	20.08	10.44	0.73	1.11	9.29	2.52	-4.91	145.45	256.76	153.05	143.14
6.43	-2.17	10.66	19.92	10.32	0.60	1.12	9.32	2.55	-4.93	153.88	263.62	160.26	151.65
6.42	-2.14	10.72	19.98	10.33	0.67	1.11	9.31	2.49	-4.95	148.82	260.79	158.36	150.50
6.61	-1.98	10.91	20.19	10.52	0.82	1.39	9.56	2.73	-4.76	145.25	258.09	153.68	145.38
6.43	-2.06	10.40	19.80	10.14	0.65	1.24	9.49	2.71	-4.76	162.27	270.11	166.70	160.41
6.60	-1.80	10.78	19.94	10.33	0.82	1.40	9.59	2.90	-4.44	147.27	257.37	155.84	142.95
5.93	-2.32	9.64	18.84	9.48	0.27	0.83	8.95	2.42	-4.92	158.57	267.60	168.23	164.85
6.30	-2.16	10.54	19.69	10.15	0.57	0.99	9.15	2.45	-4.90	147.16	259.91	157.60	149.27

6.38	-2.20	10.59	19.85	10.27	0.57	1.06	9.27	2.50	-4.97	155.51	264.59	161.94	153.88
6.39	-2.20	10.61	19.87	10.28	0.58	1.07	9.28	2.51	-4.96	154.98	264.16	161.62	153.28
6.54	-1.94	10.56	19.91	10.25	0.73	1.38	9.62	2.84	-4.61	158.41	266.73	162.97	154.64
6.53	-2.03	10.86	20.11	10.42	0.74	1.22	9.41	2.61	-4.83	149.98	260.86	157.20	146.74
6.72	-1.90	11.13	20.47	10.69	0.83	1.37	9.58	2.77	-4.67	151.28	260.22	156.93	144.16
6.74	-1.90	11.08	20.43	10.67	0.82	1.41	9.63	2.80	-4.62	152.62	260.78	157.81	144.92
6.43	-1.90	10.43	19.66	10.06	0.70	1.31	9.44	2.82	-4.47	152.22	261.99	161.11	150.25
6.49	-1.88	10.60	19.78	10.18	0.75	1.30	9.47	2.80	-4.50	151.66	261.07	159.71	148.35
6.79	-1.85	11.22	20.51	10.76	0.91	1.45	9.63	2.82	-4.62	149.93	257.27	154.79	142.53
6.78	-1.87	11.21	20.50	10.76	0.89	1.41	9.60	2.79	-4.64	150.59	258.37	155.82	143.41
6.90	-1.71	11.10	20.46	10.76	1.03	1.68	9.88	3.01	-4.44	154.32	261.23	156.78	145.95
6.88	-1.70	11.08	20.48	10.78	1.02	1.66	9.87	2.99	-4.41	154.87	262.40	157.65	147.38
6.70	-1.87	10.87	20.17	10.54	0.87	1.44	9.66	2.84	-4.61	158.66	264.38	162.87	151.56
6.66	-1.90	10.84	20.14	10.50	0.84	1.40	9.62	2.80	-4.65	159.55	265.10	163.36	152.55
6.71	-1.93	11.01	20.27	10.64	0.79	1.40	9.56	2.79	-4.66	161.77	265.14	164.65	153.07
6.70	-1.83	10.78	20.23	10.53	0.87	1.50	9.71	2.87	-4.56	159.65	265.15	161.94	153.82
7.09	-1.47	11.35	20.63	10.83	1.15	2.02	10.16	3.38	-4.06	143.32	253.66	151.77	132.76
6.74	-1.73	10.90	20.19	10.47	0.88	1.63	9.79	3.04	-4.36	150.53	261.11	158.24	143.38
6.67	-1.90	10.93	20.17	10.54	0.85	1.38	9.58	2.78	-4.62	158.31	264.30	162.49	150.11
6.36	-2.02	10.35	19.63	10.09	0.61	1.21	9.37	2.67	-4.67	162.73	268.63	167.18	156.84
6.41	-1.99	10.31	19.61	10.06	0.62	1.29	9.46	2.74	-4.60	161.16	269.12	165.90	156.41
6.36	-2.03	10.26	19.58	10.03	0.60	1.20	9.40	2.68	-4.66	162.62	270.05	167.05	157.60
6.37	-2.01	10.25	19.56	10.01	0.60	1.24	9.43	2.71	-4.61	162.21	269.05	166.88	157.29
6.57	-1.99	10.80	20.04	10.42	0.74	1.29	9.47	2.73	-4.70	160.51	267.18	164.82	154.06
6.69	-1.87	10.87	20.17	10.53	0.87	1.44	9.66	2.84	-4.61	158.83	264.54	162.84	151.69
6.67	-1.90	10.86	20.16	10.52	0.84	1.40	9.63	2.80	-4.64	158.87	264.41	163.54	152.19
6.66	-1.92	10.90	20.18	10.54	0.80	1.38	9.58	2.78	-4.65	159.37	265.39	163.40	152.42
6.52	-1.96	10.50	19.92	10.28	0.74	1.35	9.59	2.77	-4.65	161.91	268.20	165.83	157.72
6.69	-1.86	10.79	20.23	10.53	0.88	1.47	9.72	2.85	-4.59	158.94	264.76	161.87	154.43
6.81	-1.80	11.00	20.31	10.61	0.94	1.63	9.80	3.00	-4.53	151.71	261.66	155.28	146.27
6.46	-1.99	10.48	19.90	10.23	0.73	1.32	9.54	2.72	-4.71	162.11	269.31	166.29	159.68
6.71	-1.77	10.85	20.17	10.43	0.85	1.60	9.77	3.02	-4.38	151.15	261.64	158.72	144.14
6.97	-1.66	11.19	20.56	10.84	1.07	1.76	9.94	3.07	-4.39	153.27	259.56	155.58	144.00
6.70	-1.81	10.84	20.24	10.56	0.91	1.48	9.73	2.85	-4.56	158.37	265.96	161.22	152.59
6.73	-1.79	10.88	20.29	10.60	0.93	1.51	9.75	2.87	-4.53	158.03	265.41	160.58	151.97
6.91	-1.68	11.15	20.56	10.84	1.07	1.68	9.90	3.00	-4.41	155.01	261.77	157.08	147.26
7.16	-1.40	11.35	20.69	10.87	1.20	2.15	10.29	3.45	-3.99	143.19	252.66	151.77	131.95
6.78	-1.83	11.12	20.41	10.72	0.92	1.47	9.66	2.85	-4.57	152.48	259.61	157.44	145.33
5.85	-2.35	9.40	18.63	9.30	0.13	0.73	8.87	2.41	-4.83	168.27	272.67	178.69	173.89
5.85	-2.35	9.39	18.61	9.30	0.12	0.73	8.86	2.41	-4.83	168.59	272.88	179.17	174.09
6.20	-2.11	9.94	19.17	9.73	0.41	1.08	9.21	2.65	-4.62	155.88	264.33	167.41	157.77
6.19	-2.12	9.93	19.16	9.72	0.40	1.07	9.21	2.65	-4.62	156.15	264.43	167.49	157.95
6.50	-1.90	10.49	19.69	10.15	0.68	1.32	9.48	2.85	-4.49	149.49	259.29	159.12	147.30

6.52	-1.87	10.60	19.77	10.19	0.75	1.31	9.50	2.84	-4.50	149.44	259.00	158.20	146.49
6.19	-2.14	10.00	19.18	9.75	0.43	1.03	9.17	2.59	-4.68	155.74	264.97	166.54	157.81
6.34	-2.03	10.22	19.42	9.94	0.55	1.16	9.35	2.72	-4.58	150.57	261.77	162.05	151.93
6.51	-1.80	10.41	19.69	10.09	0.71	1.44	9.58	2.96	-4.31	147.84	259.24	158.29	146.05
5.96	-2.25	9.55	18.77	9.40	0.20	0.86	9.00	2.52	-4.73	164.83	270.58	176.21	170.28
6.43	-1.90	10.44	19.67	10.06	0.70	1.31	9.44	2.82	-4.47	152.26	262.03	161.16	150.23
6.68	-1.65	10.87	20.13	10.26	0.87	1.62	9.81	3.07	-4.19	144.82	249.14	158.96	144.66
6.28	-2.05	10.06	19.29	9.82	0.49	1.16	9.30	2.75	-4.55	154.00	264.91	166.60	155.91
5.85	-2.35	9.40	18.62	9.30	0.12	0.72	8.87	2.41	-4.83	168.38	272.75	178.83	173.98
6.27	-1.92	9.93	19.22	9.66	0.61	1.36	9.45	2.86	-4.47	150.06	256.49	163.01	156.78
6.15	-2.21	10.07	19.22	9.83	0.42	0.95	9.08	2.47	-4.83	154.51	263.21	163.49	155.81
6.45	-1.99	10.67	19.88	10.23	0.75	1.28	9.46	2.66	-4.74	141.50	253.18	152.02	144.13
6.94	-1.57	11.07	20.47	10.66	1.07	1.88	10.09	3.24	-4.18	150.92	259.64	159.08	141.78
7.04	-1.45	11.23	20.64	10.76	1.16	2.01	10.21	3.35	-4.07	149.68	257.58	157.62	139.60
7.60	-1.54	11.96	21.54	11.23	1.14	2.73	10.89	4.00	-4.19	128.00	238.54	124.93	85.20
8.15	-1.06	12.69	22.38	11.73	1.62	3.55	11.87	4.56	-3.73	121.23	213.67	116.23	81.62
7.07	-2.04	11.21	20.87	10.70	0.70	2.09	10.30	3.44	-4.75	133.32	252.61	134.90	96.29
7.63	-1.50	12.03	21.71	11.39	1.27	2.65	10.83	3.87	-4.28	127.74	242.04	128.91	87.46
8.23	-0.94	13.00	22.62	12.21	1.94	3.20	11.41	4.25	-3.81	122.65	235.24	124.41	81.55
6.62	-2.34	10.35	20.00	10.01	0.29	1.85	10.08	3.20	-4.95	139.24	258.39	140.41	105.40
6.65	-2.33	10.44	20.10	10.08	0.30	1.86	10.08	3.19	-4.94	139.28	257.36	139.90	105.58
6.59	-2.37	10.39	20.04	10.02	0.24	1.81	10.02	3.15	-4.98	140.65	258.94	141.00	106.32
7.29	-1.75	11.48	21.14	10.95	0.96	2.40	10.56	3.64	-4.44	130.90	250.41	131.92	92.14
7.51	-1.54	11.77	21.38	11.17	1.18	2.69	10.81	3.87	-4.23	129.52	244.37	128.58	87.51
8.14	-1.05	12.81	22.39	12.07	1.73	3.16	11.29	4.23	-3.80	124.34	238.06	126.00	81.39
8.14	-1.05	12.81	22.39	12.07	1.74	3.16	11.29	4.23	-3.80	124.30	238.03	125.94	81.39
6.80	-2.24	10.84	20.45	10.37	0.48	1.84	10.05	3.23	-4.97	138.21	257.65	137.08	100.29
6.74	-2.29	10.75	20.37	10.31	0.42	1.79	10.02	3.19	-5.00	138.91	258.63	138.58	101.38
6.75	-2.25	10.69	20.31	10.24	0.45	1.85	10.06	3.25	-4.94	139.43	257.78	137.09	101.38
6.94	-2.09	10.99	20.60	10.48	0.61	2.03	10.23	3.40	-4.82	136.75	253.96	133.45	97.10
7.11	-1.95	11.24	20.84	10.70	0.80	2.16	10.35	3.51	-4.72	133.78	251.53	132.69	94.76
7.03	-2.01	11.08	20.70	10.57	0.73	2.09	10.28	3.47	-4.76	134.07	251.75	133.34	95.72
7.07	-2.00	11.21	20.82	10.68	0.78	2.09	10.29	3.44	-4.76	133.75	252.44	133.33	95.57
7.74	-1.36	12.12	21.82	11.47	1.41	2.87	11.02	4.04	-4.12	126.13	239.02	126.00	85.62
7.74	-1.36	12.11	21.82	11.46	1.41	2.87	11.02	4.03	-4.12	126.15	239.10	126.03	85.66
8.19	-1.09	13.04	22.65	12.02	1.67	3.30	11.58	4.34	-3.88	118.52	216.56	114.77	77.48
8.00	-1.22	12.70	22.33	11.73	1.50	3.20	11.50	4.29	-3.89	122.00	216.93	117.30	79.74
8.00	-1.22	12.70	22.32	11.73	1.50	3.20	11.50	4.29	-3.89	122.00	216.95	117.30	79.75
8.21	-1.08	13.06	22.67	12.03	1.68	3.34	11.62	4.38	-3.86	118.23	215.93	114.72	77.37
8.22	-1.08	13.06	22.67	12.04	1.69	3.35	11.63	4.38	-3.85	118.21	215.87	114.71	77.35
8.27	-0.99	13.12	22.71	12.06	1.74	3.48	11.77	4.50	-3.73	117.73	212.23	112.97	76.25
7.49	-1.64	11.72	21.40	10.99	1.02	2.81	11.10	4.00	-4.31	129.13	232.85	126.44	92.19
6.55	-2.38	10.37	19.99	9.94	0.24	1.71	9.95	3.16	-5.04	142.12	260.64	140.48	105.57

6.57	-2.35	10.32	19.98	9.93	0.24	1.82	10.04	3.20	-4.95	143.08	257.49	140.04	106.67
7.96	-1.27	12.67	22.21	11.72	1.45	3.04	11.27	4.18	-4.00	123.93	224.32	118.95	80.10
7.95	-1.28	12.66	22.20	11.71	1.44	3.04	11.27	4.18	-4.00	123.97	224.39	118.99	80.16
6.84	-2.13	10.75	20.41	10.23	0.51	2.07	10.31	3.45	-4.76	138.07	252.97	135.82	99.91
6.67	-2.28	10.53	20.18	10.05	0.36	1.88	10.14	3.28	-4.92	140.26	256.15	138.28	103.39
7.30	-1.72	11.57	21.31	10.82	0.93	2.52	10.82	3.77	-4.38	131.13	239.49	128.06	92.76
8.23	-0.93	12.60	22.25	11.65	1.76	3.75	12.09	4.76	-3.61	121.67	211.52	116.08	82.35
6.84	-2.20	10.89	20.53	10.43	0.53	1.86	10.10	3.25	-4.93	136.95	256.66	138.02	100.77
8.15	-1.06	12.69	22.38	11.73	1.61	3.55	11.87	4.56	-3.73	121.26	213.71	116.26	81.66
8.02	-1.11	12.61	22.21	11.93	1.66	3.08	11.22	4.16	-3.88	125.05	240.04	126.18	82.82
8.43	-0.82	13.42	23.03	12.50	2.10	3.36	11.60	4.37	-3.73	126.72	235.75	126.74	84.68
7.70	-1.55	12.37	22.03	11.62	1.19	2.46	10.49	3.77	-4.32	127.69	238.22	119.18	76.75
8.41	-0.75	13.27	22.83	12.38	2.13	3.18	11.19	4.46	-3.63	117.91	218.37	106.09	66.26
8.39	-0.77	13.22	22.75	12.35	2.10	3.21	11.18	4.47	-3.64	118.88	220.13	106.07	66.29
8.44	-0.69	13.40	22.98	12.48	2.18	3.19	11.18	4.42	-3.58	114.38	217.00	105.29	66.00
8.16	-0.96	12.89	22.54	12.11	1.83	2.99	10.96	4.22	-3.71	116.94	221.44	107.54	69.14
7.92	-1.17	12.48	22.15	11.80	1.58	2.78	10.75	4.05	-3.92	119.90	224.16	110.23	72.21
7.53	-1.62	12.06	21.66	11.30	1.07	2.38	10.35	3.77	-4.32	127.91	240.07	119.81	77.43
7.63	-1.56	12.22	21.85	11.47	1.16	2.47	10.45	3.80	-4.32	126.66	238.81	119.97	76.03
7.71	-1.53	12.36	22.01	11.61	1.22	2.50	10.51	3.80	-4.27	127.73	235.34	118.37	75.98
7.44	-1.66	11.83	21.47	11.13	1.03	2.37	10.39	3.71	-4.35	127.81	239.51	120.55	79.82
7.49	-1.66	11.95	21.57	11.24	1.02	2.36	10.34	3.73	-4.36	129.44	239.80	120.33	78.15
7.53	-1.63	12.01	21.63	11.29	1.05	2.39	10.38	3.76	-4.33	129.01	238.89	119.83	77.53
7.40	-1.73	11.86	21.45	11.16	0.99	2.22	10.21	3.63	-4.45	128.96	243.17	121.82	79.57
7.49	-1.66	12.04	21.64	11.31	1.06	2.34	10.33	3.70	-4.41	126.62	240.98	120.62	78.20
7.66	-1.50	12.31	21.93	11.49	1.22	2.48	10.44	3.82	-4.22	126.74	238.75	117.54	74.63
7.34	-1.75	11.82	21.41	11.10	0.97	2.17	10.18	3.59	-4.47	129.74	245.04	121.29	80.20
7.55	-1.58	11.94	21.61	11.24	1.12	2.50	10.53	3.86	-4.27	127.11	237.75	119.21	77.97
7.48	-1.63	11.85	21.51	11.17	1.05	2.43	10.46	3.77	-4.30	127.67	238.91	120.13	79.05
7.58	-1.60	12.28	21.88	11.49	1.15	2.34	10.29	3.69	-4.34	127.10	241.13	116.94	75.62
7.58	-1.60	12.27	21.87	11.48	1.14	2.33	10.29	3.69	-4.35	127.13	241.22	117.02	75.69
7.53	-1.64	12.15	21.78	11.38	1.10	2.31	10.26	3.66	-4.37	127.45	241.62	117.66	76.30
7.80	-1.39	12.48	22.12	11.67	1.35	2.61	10.52	3.92	-4.13	124.62	236.50	115.40	72.54
7.79	-1.40	12.47	22.11	11.66	1.35	2.60	10.52	3.91	-4.14	124.63	236.58	115.44	72.61
7.88	-1.24	12.40	22.07	11.66	1.47	2.80	10.72	4.10	-3.95	122.44	231.55	113.80	72.58
7.52	-1.57	11.82	21.49	11.09	1.12	2.55	10.54	3.95	-4.26	127.40	236.74	119.18	78.94
7.56	-1.52	11.85	21.53	11.15	1.11	2.53	10.50	3.94	-4.15	126.55	238.59	118.88	77.36
7.51	-1.59	12.04	21.80	11.38	1.15	2.33	10.35	3.62	-4.35	127.57	234.19	119.29	79.38
7.46	-1.66	11.83	21.51	11.21	1.00	2.42	10.43	3.67	-4.35	126.15	235.41	121.49	82.52
7.65	-1.40	11.90	21.61	11.29	1.24	2.79	10.82	3.99	-4.05	125.46	236.90	121.74	80.88
7.76	-1.36	12.28	21.92	11.59	1.38	2.80	10.89	3.92	-4.09	125.43	233.25	121.46	80.17
7.55	-1.54	11.90	21.53	11.21	1.14	2.65	10.72	3.87	-4.21	126.45	235.05	121.36	81.96
7.46	-1.61	11.96	21.72	11.27	1.12	2.31	10.33	3.66	-4.38	126.21	235.75	119.00	80.01

7.75	-1.30	12.35	22.05	11.58	1.43	2.57	10.55	3.92	-4.02	123.73	229.33	115.41	75.63
7.74	-1.35	12.12	21.80	11.46	1.33	2.80	10.85	4.00	-4.05	125.31	235.72	120.64	79.46
7.89	-1.27	12.47	22.11	11.76	1.48	2.85	10.89	4.03	-4.02	123.27	233.30	119.41	78.00
7.23	-1.79	11.57	21.23	10.92	0.85	2.13	10.11	3.56	-4.42	130.81	237.32	122.15	83.30
7.14	-1.94	11.47	21.20	10.87	0.77	2.01	10.13	3.47	-4.69	130.42	244.38	125.15	85.41
7.52	-1.57	12.09	21.84	11.43	1.17	2.37	10.35	3.61	-4.34	126.56	233.77	118.86	79.18
7.51	-1.58	12.07	21.82	11.41	1.16	2.36	10.35	3.61	-4.34	126.78	233.94	118.98	79.29
7.44	-1.67	11.81	21.49	11.19	0.99	2.41	10.42	3.67	-4.36	126.55	235.79	121.70	82.70
7.51	-1.55	11.91	21.52	11.17	1.14	2.61	10.67	3.81	-4.23	127.04	233.67	121.78	82.26
7.53	-1.51	11.90	21.64	11.28	1.15	2.52	10.55	3.78	-4.20	126.79	238.61	120.55	80.18
7.75	-1.33	12.23	21.96	11.55	1.37	2.76	10.77	3.97	-4.03	122.52	233.57	118.49	76.97
7.75	-1.33	12.25	21.98	11.56	1.38	2.76	10.77	3.97	-4.03	122.60	233.46	118.48	76.96
7.75	-1.33	12.20	21.93	11.54	1.36	2.76	10.76	3.95	-4.03	122.50	234.59	118.07	76.82
7.70	-1.44	12.27	22.00	11.59	1.27	2.58	10.62	3.83	-4.16	124.76	234.44	119.44	77.40
7.65	-1.45	12.12	21.84	11.47	1.23	2.59	10.62	3.84	-4.16	126.14	236.08	119.57	78.50
7.44	-1.61	11.85	21.59	11.24	1.06	2.38	10.43	3.66	-4.33	127.35	239.41	122.59	81.87
7.80	-1.44	12.50	22.14	11.74	1.33	2.59	10.65	3.87	-4.22	122.59	235.32	119.03	77.03
7.45	-1.69	11.86	21.46	11.22	1.05	2.39	10.48	3.73	-4.41	127.97	244.31	124.88	82.95
7.47	-1.68	11.88	21.48	11.24	1.06	2.41	10.49	3.74	-4.40	127.82	244.03	124.76	82.70
7.67	-1.53	12.28	21.88	11.57	1.26	2.52	10.58	3.79	-4.34	126.39	239.24	122.67	80.82
7.79	-1.33	12.27	21.94	11.52	1.39	2.84	10.87	4.04	-4.04	124.36	235.00	119.45	77.87
7.62	-1.47	11.95	21.64	11.28	1.22	2.68	10.73	3.92	-4.15	127.29	238.35	122.19	80.95
7.81	-1.29	12.32	22.04	11.62	1.39	2.81	10.82	4.00	-3.98	122.69	232.31	117.95	76.93
7.91	-1.23	12.38	22.05	11.65	1.50	2.97	11.04	4.15	-3.91	122.03	232.28	118.64	77.32
8.01	-1.13	12.39	22.01	11.62	1.55	3.20	11.27	4.38	-3.83	124.00	230.68	120.72	76.84
7.79	-1.31	12.20	21.86	11.53	1.40	2.83	10.91	4.02	-4.00	122.58	232.59	121.36	79.70
7.59	-1.47	11.89	21.59	11.28	1.18	2.71	10.78	3.93	-4.13	126.16	236.94	122.91	81.80
7.53	-1.55	11.87	21.50	11.18	1.12	2.64	10.70	3.86	-4.22	126.58	235.41	121.48	82.08
7.56	-1.52	11.92	21.55	11.23	1.14	2.68	10.74	3.88	-4.20	126.53	234.30	121.54	81.83
7.65	-1.44	12.10	21.76	11.41	1.23	2.79	10.86	3.92	-4.15	125.91	230.57	121.08	80.95
7.73	-1.48	12.39	22.10	11.66	1.24	2.51	10.58	3.80	-4.21	124.60	233.89	118.12	76.66
6.67	-2.33	10.81	20.39	10.28	0.29	1.59	9.62	3.04	-4.92	135.91	248.07	130.08	93.20
6.78	-2.28	11.03	20.57	10.42	0.34	1.68	9.74	3.11	-4.92	135.90	244.91	127.40	91.27
6.68	-2.36	10.86	20.43	10.30	0.27	1.58	9.65	3.02	-5.00	137.43	247.05	129.18	93.17
6.70	-2.35	10.88	20.45	10.31	0.28	1.59	9.66	3.03	-4.99	137.22	246.80	128.94	92.92
6.76	-2.30	11.01	20.55	10.40	0.33	1.67	9.73	3.11	-4.94	136.45	244.82	127.71	91.65
6.61	-2.37	10.67	20.33	10.18	0.25	1.60	9.70	3.06	-4.98	137.89	249.37	132.74	95.74
6.76	-2.30	10.99	20.54	10.39	0.32	1.66	9.72	3.09	-4.94	136.27	245.34	127.75	91.68
6.78	-2.29	11.02	20.56	10.41	0.33	1.67	9.74	3.11	-4.93	135.99	245.04	127.49	91.38
7.03	-1.97	11.23	20.91	10.68	0.63	1.97	9.96	3.41	-4.55	133.00	240.92	125.34	87.17
7.05	-1.95	11.29	20.94	10.69	0.64	1.97	9.97	3.43	-4.56	132.58	240.53	123.98	86.34
7.02	-1.98	11.25	20.90	10.65	0.61	1.95	9.95	3.41	-4.58	132.77	240.99	124.37	86.78
6.67	-2.29	10.75	20.39	10.25	0.34	1.66	9.77	3.08	-4.92	137.97	249.54	132.36	95.42

7.27	-1.74	11.46	21.12	10.88	0.91	2.12	10.07	3.65	-4.39	129.24	235.36	116.95	80.73
7.98	-1.01	12.45	22.05	11.74	1.75	2.91	10.89	4.23	-3.80	122.13	221.24	108.33	72.14
7.80	-1.20	12.15	21.79	11.52	1.51	2.73	10.69	4.10	-3.92	123.84	225.40	110.85	73.97
7.51	-1.49	11.76	21.42	11.14	1.17	2.40	10.36	3.89	-4.16	126.33	230.76	114.36	77.19
7.40	-1.61	11.61	21.27	11.03	1.05	2.30	10.25	3.80	-4.25	127.41	232.95	115.63	78.57
7.34	-1.62	11.37	21.07	10.86	0.99	2.31	10.21	3.80	-4.24	127.97	233.84	117.09	80.03
6.84	-2.20	11.11	20.72	10.55	0.46	1.68	9.71	3.15	-4.87	135.63	241.28	125.50	88.55
6.83	-2.23	11.06	20.70	10.50	0.45	1.69	9.73	3.15	-4.88	135.32	241.72	124.87	89.32
6.83	-2.21	11.10	20.71	10.53	0.47	1.69	9.72	3.15	-4.88	135.33	241.66	124.56	88.88
6.89	-2.17	11.19	20.78	10.59	0.51	1.76	9.77	3.20	-4.84	134.36	240.37	123.55	87.68
6.84	-2.21	11.10	20.71	10.52	0.45	1.71	9.73	3.14	-4.85	135.10	241.22	125.68	88.89
6.87	-2.18	11.14	20.77	10.56	0.46	1.73	9.78	3.21	-4.81	133.99	241.28	125.30	88.19
6.88	-2.17	11.15	20.78	10.56	0.46	1.74	9.79	3.22	-4.80	133.80	241.38	125.24	87.99
7.05	-2.00	11.47	21.05	10.77	0.66	1.83	9.87	3.33	-4.66	133.74	240.69	123.25	86.67
6.80	-2.22	11.03	20.61	10.46	0.40	1.73	9.74	3.14	-4.86	134.46	243.98	127.74	91.25
6.83	-2.21	11.09	20.67	10.48	0.42	1.76	9.77	3.16	-4.84	134.58	242.87	127.05	90.57
6.83	-2.22	11.12	20.67	10.48	0.41	1.75	9.78	3.16	-4.84	135.04	242.89	126.82	90.10
6.76	-2.30	10.97	20.52	10.38	0.35	1.67	9.72	3.11	-4.93	136.85	244.20	128.34	92.21
6.78	-2.24	10.96	20.56	10.39	0.38	1.74	9.82	3.17	-4.85	136.17	245.17	128.26	92.05
6.81	-2.21	11.01	20.61	10.42	0.40	1.77	9.84	3.19	-4.83	135.04	244.62	127.92	91.31
6.85	-2.18	11.09	20.69	10.48	0.44	1.82	9.87	3.22	-4.80	133.56	242.79	126.93	90.31
6.65	-2.34	10.72	20.36	10.20	0.27	1.64	9.73	3.08	-4.96	137.49	248.74	132.56	95.43
6.65	-2.34	10.72	20.36	10.20	0.27	1.64	9.73	3.08	-4.96	137.44	248.69	132.53	95.39
7.26	-1.78	11.62	21.25	10.95	0.90	2.30	10.35	3.56	-4.45	129.32	236.52	122.93	85.86
8.33	-0.70	12.98	22.52	12.23	2.12	3.12	11.19	4.37	-3.53	116.11	215.16	103.98	67.69
6.85	-2.19	11.13	20.73	10.55	0.46	1.71	9.73	3.15	-4.85	135.29	241.16	125.66	88.36
7.64	-1.31	11.64	21.30	11.10	1.30	2.68	10.64	4.13	-3.93	127.89	225.76	111.03	77.63
8.41	-0.63	12.97	22.53	12.26	2.15	3.31	11.35	4.54	-3.46	117.01	214.27	102.53	66.76
7.86	-1.11	12.06	21.67	11.49	1.58	2.86	10.84	4.23	-3.81	125.00	223.58	107.38	73.31
7.54	-1.42	11.57	21.23	11.06	1.24	2.54	10.50	4.02	-4.04	129.31	226.43	111.37	78.31
7.84	-1.27	12.54	22.16	11.68	1.47	2.92	11.13	3.98	-4.04	123.43	225.25	118.74	79.69
7.36	-1.72	11.70	21.34	11.04	1.01	2.43	10.56	3.67	-4.43	129.55	237.37	124.29	85.70
7.45	-1.65	11.85	21.47	11.14	1.09	2.52	10.64	3.74	-4.38	128.84	235.06	122.33	84.28
7.62	-1.50	12.07	21.72	11.41	1.21	2.72	10.80	3.84	-4.23	126.74	231.71	121.41	82.23
7.82	-1.29	12.52	22.13	11.68	1.44	2.90	11.10	3.97	-4.07	124.68	225.88	119.32	80.22
7.86	-1.25	12.58	22.19	11.71	1.49	2.95	11.15	4.00	-4.04	123.46	224.85	118.49	79.32
7.85	-1.23	12.15	21.87	11.50	1.47	3.03	11.11	4.23	-3.91	124.11	236.96	122.52	79.48
7.78	-1.29	12.07	21.77	11.42	1.41	2.94	11.03	4.15	-3.98	125.07	238.80	123.57	80.94
8.07	-1.07	12.53	22.18	11.81	1.67	3.19	11.28	4.34	-3.77	121.43	232.99	120.37	78.01
6.56	-2.45	10.59	20.03	10.11	0.24	1.47	9.62	2.99	-5.13	138.45	260.83	134.24	95.48
7.77	-1.37	12.20	21.87	11.49	1.36	2.82	10.94	4.04	-4.10	125.33	238.84	123.26	79.40
7.88	-1.33	12.40	22.00	11.66	1.38	2.86	10.88	4.11	-4.08	124.94	235.45	119.08	75.59
6.57	-2.46	10.62	20.10	10.15	0.23	1.45	9.62	2.98	-5.15	137.75	260.61	135.14	95.40

7.88	-1.33	12.37	21.96	11.62	1.38	2.87	10.89	4.12	-4.07	125.00	235.83	119.23	75.69
7.39	-1.70	11.73	21.27	11.13	1.00	2.33	10.47	3.67	-4.41	129.12	250.91	127.10	84.03
7.18	-1.99	11.55	21.07	10.95	0.75	2.04	10.12	3.39	-4.72	130.83	252.64	126.33	83.90
7.09	-2.04	11.43	20.97	10.87	0.69	1.96	10.07	3.33	-4.77	131.36	254.39	127.49	85.35
7.04	-2.08	11.30	20.82	10.74	0.62	1.91	10.05	3.35	-4.76	134.16	255.53	129.56	87.65
7.14	-1.93	11.40	20.96	10.84	0.76	2.06	10.21	3.46	-4.64	131.11	254.81	129.65	87.84
7.46	-1.61	11.95	21.71	11.26	1.12	2.31	10.33	3.66	-4.38	126.17	235.74	119.00	80.07
7.34	-1.69	11.64	21.28	11.04	0.98	2.40	10.48	3.64	-4.37	129.53	241.17	125.87	85.88
7.99	-1.20	12.68	22.31	11.92	1.55	2.97	11.04	4.06	-3.95	122.48	229.95	118.73	76.75
7.53	-1.56	11.98	21.64	11.28	1.17	2.61	10.76	3.77	-4.30	127.46	234.19	123.10	84.35
7.73	-1.38	12.12	21.70	11.43	1.35	2.79	10.85	4.03	-4.12	124.73	239.03	122.14	78.50
6.57	-2.46	10.62	20.10	10.15	0.23	1.44	9.62	2.98	-5.15	137.74	260.62	135.14	95.40
8.60	-0.62	13.14	22.76	12.63	2.35	3.44	11.55	4.57	-3.59	157.95	272.24	150.70	93.62
8.60	-0.62	13.15	22.76	12.63	2.35	3.44	11.56	4.57	-3.59	157.92	272.16	150.69	93.57
7.98	-0.74	12.16	21.59	11.82	2.11	2.66	10.80	4.15	-3.58	133.53	259.07	127.14	83.43
7.57	-1.51	12.23	22.05	11.37	1.34	2.77	11.09	3.78	-4.36	141.95	233.50	120.19	85.19
7.57	-1.50	12.23	22.04	11.37	1.34	2.78	11.09	3.79	-4.35	141.94	233.53	120.26	85.17
8.53	-0.65	13.22	23.14	12.27	2.10	3.88	12.26	4.82	-3.42	125.77	213.97	114.08	78.95
8.39	-0.86	13.17	23.15	12.20	1.95	3.65	12.03	4.59	-3.66	126.00	215.50	115.71	79.54
7.80	-1.32	12.46	22.39	11.61	1.48	2.97	11.27	4.01	-4.12	133.78	224.13	116.78	81.82
7.81	-1.32	12.46	22.39	11.61	1.48	2.97	11.27	4.02	-4.12	133.79	224.09	116.77	81.79
7.39	-1.71	11.95	21.87	11.17	1.02	2.44	10.60	3.57	-4.44	132.18	226.08	118.67	84.18
7.45	-1.72	11.89	21.82	11.23	1.01	2.53	10.68	3.66	-4.43	131.25	224.64	118.72	83.92
7.29	-1.83	11.68	21.59	11.08	0.89	2.36	10.46	3.52	-4.55	133.21	226.10	119.50	84.74
7.54	-1.60	12.12	22.05	11.30	1.17	2.64	10.85	3.75	-4.36	130.09	224.62	116.41	82.35
7.38	-1.70	11.89	21.82	11.10	1.05	2.53	10.73	3.68	-4.44	133.36	227.04	119.51	85.49
7.47	-1.66	12.08	21.99	11.30	1.09	2.52	10.65	3.61	-4.38	129.94	223.61	116.07	82.47
7.32	-1.70	11.56	21.21	10.89	0.97	2.52	10.64	3.77	-4.37	130.76	231.33	121.75	86.07
7.96	-1.19	12.46	22.17	11.59	1.51	3.28	11.47	4.34	-3.87	122.86	215.23	113.24	78.42
8.12	-1.11	12.71	22.41	11.79	1.57	3.48	11.62	4.45	-3.76	120.24	210.94	110.56	73.96
7.99	-1.24	12.67	22.43	11.79	1.48	3.24	11.43	4.20	-3.95	122.16	214.96	112.69	77.33
7.87	-1.35	12.50	22.31	11.67	1.37	3.07	11.27	4.06	-4.07	124.86	218.21	114.02	79.13
8.19	-1.02	12.86	22.57	11.92	1.67	3.54	11.71	4.50	-3.71	119.41	210.34	110.15	74.72
8.16	-1.03	12.83	22.55	11.90	1.66	3.52	11.69	4.47	-3.73	119.78	210.63	110.80	75.23
7.84	-1.38	12.43	22.27	11.63	1.32	3.05	11.24	4.04	-4.09	124.57	220.20	116.11	80.71
7.76	-1.36	12.15	21.87	11.34	1.35	3.04	11.24	4.17	-4.05	125.16	221.09	117.84	81.82
8.23	-0.97	12.84	22.54	11.88	1.74	3.57	11.81	4.59	-3.68	118.06	209.52	111.81	74.79
8.16	-1.03	12.80	22.53	11.86	1.67	3.49	11.74	4.45	-3.73	118.94	212.54	112.43	75.58
8.14	-1.12	12.83	22.51	11.89	1.62	3.35	11.67	4.40	-3.87	122.09	213.74	114.42	79.01
8.02	-1.20	12.51	22.28	11.65	1.50	3.37	11.66	4.37	-3.86	124.40	216.65	116.76	81.18
7.92	-1.26	12.36	22.15	11.56	1.42	3.29	11.58	4.29	-3.94	125.22	219.84	117.75	82.85
7.73	-1.46	12.28	22.09	11.47	1.26	2.97	11.22	4.01	-4.14	127.32	221.67	119.02	84.37
7.69	-1.42	12.14	21.89	11.34	1.30	2.84	11.09	4.01	-4.12	124.88	225.90	119.33	83.29

7.75	-1.38	12.31	22.01	11.46	1.35	2.89	11.16	4.03	-4.09	124.32	223.54	118.56	82.31
7.75	-1.38	12.30	22.01	11.46	1.35	2.88	11.16	4.03	-4.09	124.34	223.57	118.58	82.34
7.75	-1.39	12.30	22.01	11.46	1.35	2.88	11.16	4.03	-4.09	124.37	223.61	118.59	82.36
7.26	-1.81	11.53	21.17	10.87	0.85	2.39	10.52	3.67	-4.46	130.81	233.52	122.81	86.38
7.40	-1.65	11.61	21.29	10.96	1.01	2.60	10.73	3.83	-4.30	129.93	230.03	120.53	84.99
7.36	-1.82	11.74	21.46	11.06	0.86	2.48	10.60	3.66	-4.47	130.08	228.50	120.23	85.03
7.99	-1.21	12.57	22.40	11.69	1.53	3.26	11.61	4.30	-3.97	129.84	220.01	117.13	83.86
8.78	-0.53	13.72	23.50	12.61	2.28	4.07	12.53	4.96	-3.33	120.28	206.05	109.54	76.31
8.52	-0.77	13.40	23.06	12.28	2.05	3.82	12.33	4.74	-3.58	120.86	208.60	114.44	81.25
7.99	-1.32	12.74	22.69	11.84	1.48	3.17	11.44	4.14	-4.09	127.24	217.09	114.40	80.72
8.16	-1.15	12.92	22.89	12.01	1.63	3.39	11.65	4.29	-3.92	126.25	214.40	112.26	78.39
8.64	-0.63	13.44	23.24	12.42	2.16	3.96	12.32	4.83	-3.42	121.74	208.62	109.78	75.59
8.20	-0.96	12.81	22.60	11.86	1.79	3.53	11.93	4.52	-3.75	128.20	216.49	117.33	83.78
8.20	-0.97	12.80	22.60	11.86	1.79	3.53	11.92	4.52	-3.75	128.22	216.51	117.34	83.79
8.20	-0.97	12.81	22.60	11.86	1.79	3.52	11.92	4.52	-3.75	128.22	216.52	117.35	83.79
8.20	-0.97	12.80	22.60	11.85	1.79	3.52	11.92	4.52	-3.75	128.24	216.55	117.37	83.80
8.20	-0.97	12.80	22.60	11.85	1.79	3.52	11.92	4.52	-3.75	128.26	216.58	117.40	83.81
8.19	-0.97	12.80	22.60	11.85	1.79	3.52	11.92	4.52	-3.75	128.28	216.60	117.42	83.82
8.32	-0.90	12.96	22.79	12.01	1.87	3.66	12.05	4.62	-3.65	126.95	213.77	115.36	81.37
8.32	-0.90	12.96	22.79	12.01	1.87	3.66	12.05	4.62	-3.64	126.94	213.73	115.34	81.35
8.32	-0.90	12.96	22.80	12.02	1.87	3.66	12.05	4.63	-3.64	126.92	213.71	115.32	81.33
8.32	-0.90	12.96	22.80	12.02	1.87	3.66	12.05	4.63	-3.64	126.90	213.67	115.30	81.31
8.34	-0.89	12.99	22.82	12.03	1.88	3.68	12.07	4.64	-3.63	126.81	213.25	115.13	80.90
8.34	-0.89	12.99	22.82	12.04	1.88	3.68	12.07	4.64	-3.63	126.79	213.23	115.11	80.88
8.27	-0.94	12.90	22.76	11.96	1.79	3.63	11.97	4.60	-3.68	126.15	214.33	114.96	81.26
8.97	-0.40	14.06	23.82	12.87	2.44	4.27	12.72	5.08	-3.21	117.52	201.93	106.34	73.29
8.65	-0.67	13.60	23.24	12.43	2.13	3.97	12.45	4.87	-3.46	117.92	205.06	111.22	77.69
8.30	-0.89	13.00	22.66	11.95	1.87	3.70	12.13	4.67	-3.65	122.17	211.95	116.12	82.59
8.87	-0.56	14.01	23.71	12.81	2.28	4.11	12.59	4.93	-3.41	114.78	200.15	111.20	76.50
8.86	-0.57	14.07	23.81	12.83	2.30	4.05	12.57	4.89	-3.44	115.81	201.23	111.87	77.83
7.96	-1.20	12.54	22.58	11.74	1.57	3.17	11.45	4.17	-4.00	129.66	219.31	116.02	81.77
7.21	-1.82	11.37	21.00	10.77	0.85	2.33	10.47	3.63	-4.49	132.20	234.24	123.39	87.46
7.38	-1.86	11.96	21.70	11.20	0.88	2.33	10.44	3.52	-4.60	130.34	227.18	118.38	82.65
7.25	-1.94	11.81	21.61	11.14	0.81	2.23	10.35	3.39	-4.70	131.99	229.50	120.56	85.10
7.29	-1.92	11.86	21.63	11.17	0.83	2.28	10.39	3.42	-4.65	131.65	229.60	120.03	84.16
7.00	-2.13	11.31	21.08	10.72	0.57	2.04	10.17	3.27	-4.82	135.44	237.34	125.54	91.43
7.34	-1.87	11.89	21.65	11.17	0.84	2.42	10.57	3.55	-4.58	130.15	227.93	119.74	84.38
7.30	-1.85	11.78	21.48	11.06	0.87	2.41	10.56	3.57	-4.57	130.96	227.95	119.62	85.04
7.65	-1.48	12.32	22.19	11.50	1.36	2.82	11.11	3.83	-4.28	138.72	229.66	118.31	83.30
7.50	-1.58	11.93	21.81	11.18	1.16	2.75	11.02	3.83	-4.35	141.57	233.38	121.44	86.89
7.66	-1.50	12.30	22.18	11.46	1.31	2.78	11.08	3.85	-4.31	140.62	229.50	118.93	84.99
7.94	-1.17	12.61	22.43	11.77	1.74	3.20	11.58	4.11	-4.07	134.64	227.74	118.28	81.73
8.66	-0.62	13.70	23.58	12.56	2.30	3.87	12.42	4.75	-3.59	127.13	215.38	113.81	78.20

8.04	-1.09	12.80	22.53	11.87	1.82	3.30	11.69	4.22	-3.97	133.76	222.89	115.56	78.15
8.12	-1.12	12.84	22.70	11.94	1.78	3.32	11.76	4.29	-4.03	131.57	223.67	118.11	81.47
7.92	-1.28	12.78	22.57	11.82	1.65	3.08	11.46	4.06	-4.16	134.32	225.69	116.31	79.87
8.91	-0.43	14.07	23.92	12.82	2.48	4.16	12.76	5.01	-3.36	124.67	209.61	111.72	76.53
8.81	-0.57	13.93	23.91	12.80	2.36	3.99	12.51	4.83	-3.47	123.14	209.19	111.16	76.12
8.41	-0.84	13.25	23.25	12.27	2.00	3.65	12.09	4.56	-3.70	127.19	217.95	114.76	79.76
8.62	-0.66	13.57	23.28	12.41	2.14	3.91	12.42	4.85	-3.48	118.00	206.30	113.42	79.38
8.00	-1.14	12.66	22.43	11.79	1.78	3.30	11.68	4.19	-4.03	133.76	224.95	116.97	80.76
8.00	-1.14	12.65	22.43	11.79	1.78	3.30	11.68	4.19	-4.03	133.79	224.99	116.99	80.81
8.01	-1.14	12.67	22.44	11.80	1.78	3.30	11.69	4.20	-4.03	133.84	224.98	116.74	80.89
8.94	-0.40	14.04	23.57	13.09	2.67	3.93	12.09	4.81	-3.46	132.48	238.89	131.17	85.00
8.99	-0.40	14.22	23.82	13.20	2.68	3.92	12.08	4.82	-3.44	132.61	238.87	132.58	85.25
9.01	-0.39	14.25	23.88	13.21	2.68	3.92	12.09	4.81	-3.44	132.88	241.16	133.09	86.67
8.93	-0.47	14.02	23.59	13.06	2.57	3.85	12.04	4.80	-3.50	130.49	239.32	131.26	84.25
8.92	-0.47	14.00	23.57	13.06	2.57	3.85	12.04	4.80	-3.50	130.60	239.39	131.36	84.48
8.92	-0.47	13.99	23.55	13.04	2.57	3.85	12.04	4.80	-3.50	130.55	239.59	131.32	84.40
8.97	-0.37	13.89	23.40	12.96	2.62	4.03	12.23	4.99	-3.33	128.56	235.78	129.73	83.46
8.86	-0.57	13.90	23.61	13.02	2.42	3.73	11.89	4.67	-3.54	135.79	245.98	135.13	86.65
8.86	-0.57	13.88	23.60	13.01	2.42	3.74	11.90	4.67	-3.54	135.62	245.96	135.02	86.41
8.86	-0.57	13.89	23.60	13.01	2.41	3.73	11.89	4.67	-3.54	135.97	245.98	135.07	86.52
8.93	-0.53	14.04	23.70	13.13	2.46	3.80	11.97	4.73	-3.53	131.46	240.49	131.99	85.00
8.93	-0.53	14.03	23.70	13.13	2.46	3.81	11.97	4.73	-3.53	131.16	239.36	131.92	84.55
8.91	-0.41	13.83	23.35	12.92	2.58	3.95	12.14	4.90	-3.40	129.68	238.11	130.41	84.92
8.78	-0.58	13.98	23.60	13.00	2.39	3.64	11.87	4.57	-3.56	128.13	236.55	126.61	80.68
8.80	-0.57	14.00	23.62	13.02	2.40	3.66	11.89	4.58	-3.55	127.89	236.41	126.36	80.55
8.79	-0.58	13.99	23.57	13.00	2.39	3.64	11.86	4.56	-3.55	127.89	236.28	126.87	80.57
8.81	-0.60	14.15	23.64	13.05	2.38	3.58	11.83	4.54	-3.60	127.05	237.80	128.11	83.88
8.96	-0.47	13.99	23.60	13.09	2.50	3.89	12.08	4.81	-3.43	126.91	235.73	128.67	82.53
8.97	-0.44	13.93	23.56	13.06	2.54	3.95	12.16	4.87	-3.43	126.67	236.19	129.61	82.03
8.97	-0.44	13.93	23.56	13.06	2.54	3.95	12.15	4.87	-3.43	126.65	236.19	129.58	82.04
8.96	-0.39	13.86	23.43	12.97	2.57	4.02	12.19	4.95	-3.38	128.94	235.45	130.02	82.99
8.96	-0.39	13.86	23.43	12.97	2.57	4.02	12.19	4.95	-3.38	128.94	235.47	130.03	82.99
9.00	-0.36	14.13	23.66	13.11	2.68	3.98	12.17	4.87	-3.38	129.29	235.09	130.02	84.28
8.26	-1.11	12.96	22.69	12.34	1.81	3.07	11.29	4.18	-4.06	157.82	274.82	152.29	97.96
8.21	-1.15	12.89	22.63	12.29	1.77	3.02	11.26	4.14	-4.09	158.74	275.82	152.92	98.96
8.49	-0.95	13.20	22.94	12.56	2.06	3.35	11.52	4.44	-3.98	157.25	270.14	150.47	95.80
8.48	-0.95	13.20	22.94	12.56	2.06	3.35	11.52	4.44	-3.98	157.30	270.18	150.48	95.92
8.48	-0.94	13.20	22.94	12.57	2.05	3.34	11.52	4.43	-3.98	157.13	269.72	150.74	96.47
8.52	-0.93	13.30	22.99	12.62	2.05	3.38	11.53	4.42	-3.87	152.49	265.38	147.10	93.37
8.42	-0.77	13.20	22.59	12.32	2.07	3.44	11.54	4.52	-3.60	121.91	231.78	121.84	76.00
8.85	-0.52	13.95	23.86	13.04	2.43	3.77	12.00	4.69	-3.47	127.08	237.19	127.07	80.18
8.81	-0.60	13.86	23.44	12.98	2.37	3.75	11.90	4.67	-3.53	127.86	237.52	129.22	81.42
8.81	-0.60	13.86	23.44	12.97	2.37	3.75	11.90	4.67	-3.53	127.90	237.48	129.19	81.40

8.81	-0.58	13.82	23.42	12.93	2.38	3.77	11.92	4.68	-3.50	128.78	237.80	128.74	81.51
8.21	-1.13	12.92	22.56	12.18	1.67	3.06	11.15	4.21	-3.95	125.23	238.67	120.09	75.14
8.21	-1.13	12.92	22.56	12.18	1.67	3.06	11.15	4.21	-3.95	125.25	238.69	120.11	75.16
8.20	-1.13	12.91	22.56	12.18	1.67	3.05	11.15	4.21	-3.95	125.32	238.75	120.20	75.20
8.55	-0.76	13.50	22.93	12.61	2.12	3.44	11.54	4.49	-3.63	123.81	231.59	121.91	76.00
8.44	-0.84	13.35	22.97	12.47	1.97	3.33	11.44	4.43	-3.64	120.80	230.40	118.71	73.11
8.46	-0.84	13.41	22.98	12.49	2.00	3.32	11.45	4.43	-3.64	121.85	230.11	118.78	73.52
8.46	-0.85	13.41	22.97	12.49	1.99	3.32	11.46	4.42	-3.65	122.45	230.16	118.96	73.96
8.65	-0.67	13.58	23.07	12.71	2.24	3.60	11.73	4.56	-3.57	125.09	234.44	125.01	78.00
8.80	-0.58	13.74	23.36	12.92	2.38	3.70	11.88	4.66	-3.57	124.72	234.37	126.00	78.02
8.80	-0.58	13.74	23.37	12.92	2.38	3.70	11.88	4.66	-3.57	124.73	234.37	126.00	78.02
8.27	-1.04	13.12	22.84	12.32	1.76	3.15	11.24	4.24	-3.81	123.92	234.01	119.02	73.77
8.29	-1.01	13.14	22.90	12.38	1.77	3.15	11.25	4.24	-3.80	123.88	233.91	118.87	73.18
8.45	-0.86	13.39	22.95	12.47	1.98	3.33	11.46	4.41	-3.66	122.36	230.34	118.96	73.98
8.39	-0.88	13.27	22.85	12.40	1.92	3.32	11.43	4.40	-3.70	122.99	232.03	119.24	74.22
8.43	-0.87	13.32	22.90	12.44	1.95	3.33	11.44	4.40	-3.69	122.56	231.10	119.03	74.05
8.43	-0.87	13.32	22.89	12.43	1.95	3.33	11.44	4.40	-3.69	122.53	231.16	119.05	74.09
8.34	-0.97	13.13	22.85	12.31	1.83	3.29	11.39	4.36	-3.76	121.96	231.35	118.97	73.19
8.21	-1.10	12.97	22.59	12.22	1.69	3.07	11.16	4.20	-3.90	125.01	238.04	120.15	74.10
8.23	-1.10	12.98	22.62	12.23	1.70	3.07	11.17	4.22	-3.90	125.11	237.87	119.93	74.16
8.23	-1.10	12.98	22.62	12.23	1.70	3.07	11.18	4.23	-3.92	125.28	237.75	119.67	74.53
8.93	-0.52	14.11	23.75	13.17	2.50	3.81	11.98	4.73	-3.53	131.87	240.41	132.11	85.00
8.52	-0.87	13.40	22.94	12.64	1.98	3.41	11.52	4.39	-3.78	129.91	240.80	127.54	80.06
9.35	-0.17	14.69	24.30	13.52	2.82	4.43	12.66	5.14	-3.19	138.26	248.64	141.08	96.00
9.36	-0.17	14.70	24.31	13.52	2.83	4.44	12.67	5.16	-3.17	138.72	249.41	141.47	96.00
9.33	-0.07	14.42	23.91	13.19	2.78	4.83	13.14	5.51	-2.94	138.71	249.75	139.92	96.00
9.31	-0.24	14.56	24.22	13.51	2.77	4.35	12.54	5.12	-3.26	136.47	244.74	139.13	93.85
9.40	-0.05	14.56	24.15	13.31	2.82	4.84	13.14	5.50	-2.92	142.61	254.25	141.66	97.06
9.63	0.05	15.45	25.02	13.78	3.05	4.86	13.54	5.50	-2.94	120.76	197.35	108.13	75.04
9.75	0.10	15.51	25.19	13.88	3.03	5.07	13.77	5.67	-2.83	121.90	199.21	111.65	78.55
9.66	0.13	15.46	25.11	13.84	3.16	4.86	13.54	5.49	-2.93	120.33	197.68	107.47	74.00
9.50	-0.14	15.02	24.87	13.63	2.73	4.73	13.25	5.37	-3.03	114.80	198.01	108.29	74.02
9.50	-0.14	15.02	24.87	13.63	2.73	4.73	13.25	5.37	-3.03	114.82	198.01	108.31	74.02
9.49	-0.14	15.02	24.87	13.62	2.73	4.73	13.24	5.36	-3.03	114.37	198.01	108.09	74.00
9.56	-0.10	15.14	24.91	13.65	2.80	4.80	13.35	5.45	-2.97	114.44	197.63	109.74	74.00
9.54	-0.10	15.12	24.91	13.66	2.78	4.77	13.33	5.42	-2.97	114.62	197.35	109.14	74.00
9.65	0.03	15.17	25.00	13.65	2.88	5.03	13.61	5.65	-2.84	116.37	197.26	110.72	76.41
9.63	0.02	14.99	24.69	13.47	2.87	5.15	13.70	5.75	-2.83	116.73	198.30	111.44	77.00
9.63	0.01	14.99	24.69	13.47	2.87	5.15	13.70	5.75	-2.83	116.69	198.30	111.40	77.00
9.63	0.00	15.02	24.74	13.51	2.87	5.09	13.65	5.71	-2.84	117.21	197.46	111.17	76.99
9.63	0.05	14.87	24.58	13.42	2.91	5.25	13.79	5.84	-2.80	117.87	198.73	112.17	77.34
9.69	0.10	14.74	24.45	13.35	2.90	5.43	13.99	6.05	-2.68	120.67	200.27	115.22	80.07
9.63	0.06	14.72	24.40	13.31	2.84	5.34	13.90	5.97	-2.73	118.99	200.54	114.58	79.98

9.63	0.05	14.74	24.41	13.32	2.84	5.31	13.88	5.95	-2.74	118.83	200.62	114.57	79.95
9.81	0.16	15.38	25.06	13.76	3.03	5.24	13.88	5.88	-2.72	120.00	199.30	113.61	78.46
9.82	0.17	15.40	25.07	13.76	3.03	5.21	13.84	5.86	-2.73	119.68	199.39	113.18	78.65
9.30	-0.25	14.38	24.09	12.99	2.54	4.88	13.51	5.60	-3.04	128.32	216.53	126.66	91.73
9.73	0.10	15.27	24.85	13.74	3.00	5.17	13.73	5.67	-2.81	128.98	221.34	126.20	88.31
9.72	0.10	15.24	24.83	13.78	2.99	5.16	13.65	5.64	-2.80	130.97	227.18	127.96	90.73
9.78	0.23	15.25	24.93	13.83	3.10	5.27	13.81	5.76	-2.67	139.53	241.21	134.83	95.36
9.73	0.16	15.29	24.85	13.79	3.07	5.17	13.71	5.67	-2.74	135.28	234.77	131.98	93.00
9.73	0.18	15.29	24.85	13.79	3.08	5.17	13.72	5.67	-2.72	135.39	235.44	132.00	93.00
9.84	0.17	15.24	25.31	14.12	3.09	5.21	13.61	5.60	-2.72	148.13	260.26	144.82	104.49
9.27	-0.31	14.66	24.33	13.23	2.59	4.55	13.13	5.29	-3.22	120.75	210.17	118.70	83.22
9.27	-0.31	14.65	24.32	13.23	2.59	4.55	13.13	5.29	-3.23	120.77	210.20	118.73	83.25
9.27	-0.31	14.65	24.32	13.23	2.58	4.55	13.12	5.29	-3.23	120.79	210.23	118.75	83.28
9.22	-0.36	14.57	24.24	13.17	2.54	4.49	13.07	5.25	-3.27	121.30	211.12	119.40	84.02
9.21	-0.36	14.57	24.24	13.17	2.54	4.49	13.07	5.25	-3.27	121.33	211.17	119.44	84.05
9.21	-0.36	14.57	24.23	13.16	2.54	4.48	13.07	5.25	-3.27	121.35	211.21	119.46	84.08
9.21	-0.37	14.56	24.23	13.16	2.53	4.48	13.07	5.25	-3.28	121.37	211.24	119.49	84.11
9.21	-0.37	14.56	24.23	13.16	2.53	4.48	13.06	5.24	-3.28	121.40	211.30	119.54	84.15
9.20	-0.37	14.55	24.22	13.15	2.53	4.47	13.06	5.24	-3.28	121.43	211.36	119.58	84.19
9.18	-0.39	14.52	24.20	13.13	2.51	4.45	13.04	5.22	-3.30	121.56	211.67	119.83	84.39
9.18	-0.39	14.52	24.19	13.13	2.51	4.45	13.04	5.22	-3.30	121.57	211.73	119.87	84.42
9.18	-0.39	14.52	24.19	13.13	2.51	4.44	13.03	5.22	-3.30	121.58	211.76	119.90	84.43
9.18	-0.39	14.51	24.19	13.12	2.51	4.44	13.03	5.22	-3.30	121.60	211.80	119.94	84.46
9.17	-0.39	14.51	24.18	13.12	2.51	4.44	13.03	5.22	-3.30	121.61	211.84	119.97	84.48
9.17	-0.40	14.51	24.18	13.12	2.50	4.44	13.03	5.21	-3.30	121.62	211.88	120.00	84.50
9.17	-0.40	14.50	24.18	13.11	2.50	4.43	13.03	5.21	-3.31	121.63	211.92	120.04	84.52
9.17	-0.40	14.50	24.17	13.11	2.50	4.43	13.02	5.21	-3.31	121.64	211.98	120.09	84.55
9.16	-0.40	14.49	24.17	13.11	2.50	4.43	13.02	5.21	-3.31	121.65	212.04	120.14	84.58
9.16	-0.41	14.49	24.16	13.10	2.49	4.42	13.02	5.21	-3.31	121.66	212.09	120.19	84.61
9.16	-0.41	14.49	24.16	13.10	2.49	4.42	13.01	5.20	-3.31	121.67	212.14	120.23	84.63
9.16	-0.41	14.48	24.16	13.10	2.49	4.42	13.01	5.20	-3.31	121.68	212.19	120.27	84.66
9.15	-0.42	14.47	24.15	13.09	2.48	4.41	13.00	5.19	-3.32	121.66	212.35	120.42	84.73
9.15	-0.41	14.48	24.16	13.10	2.49	4.41	13.00	5.19	-3.32	121.60	212.14	120.24	84.59
9.16	-0.41	14.49	24.16	13.10	2.49	4.42	13.01	5.19	-3.32	121.56	212.00	120.11	84.50
9.16	-0.41	14.50	24.17	13.10	2.50	4.42	13.01	5.19	-3.32	121.53	211.92	120.05	84.46
9.15	-0.41	14.49	24.16	13.10	2.49	4.41	13.00	5.19	-3.32	121.54	212.05	120.16	84.52
9.16	-0.41	14.49	24.16	13.10	2.49	4.41	13.00	5.19	-3.32	121.52	211.99	120.11	84.48
9.16	-0.41	14.49	24.16	13.10	2.49	4.41	13.00	5.19	-3.32	121.51	211.96	120.09	84.46
9.11	-0.45	14.41	24.10	13.04	2.45	4.38	12.97	5.18	-3.33	121.82	213.32	121.29	85.45
9.11	-0.45	14.41	24.10	13.04	2.45	4.38	12.97	5.18	-3.33	121.86	213.29	121.26	85.45
9.25	-0.34	14.58	24.29	13.19	2.54	4.56	13.13	5.33	-3.23	122.39	214.05	121.99	85.66
9.25	-0.34	14.58	24.29	13.19	2.54	4.56	13.13	5.33	-3.22	122.37	214.01	121.95	85.61
9.31	-0.24	14.38	24.09	12.99	2.54	4.89	13.52	5.61	-3.03	128.14	216.34	126.54	91.60

9.56	-0.10	15.17	24.93	13.66	2.81	4.79	13.35	5.44	-2.97	114.25	197.82	109.91	74.00
9.56	-0.10	15.14	24.91	13.66	2.80	4.80	13.35	5.45	-2.97	114.44	197.61	109.72	74.00
9.56	-0.10	15.14	24.91	13.65	2.80	4.80	13.35	5.45	-2.97	114.46	197.59	109.71	74.00
9.56	-0.10	15.13	24.91	13.65	2.80	4.80	13.35	5.45	-2.97	114.49	197.56	109.70	74.00
9.56	-0.10	15.16	24.93	13.66	2.81	4.80	13.35	5.45	-2.97	114.25	197.82	109.88	74.00
9.55	-0.10	15.16	24.94	13.67	2.80	4.75	13.32	5.40	-2.97	114.48	198.21	109.81	74.00
9.55	-0.10	15.17	24.95	13.67	2.80	4.76	13.33	5.40	-2.97	114.45	198.12	109.89	74.00
9.46	-0.13	15.06	24.82	13.63	2.73	4.71	13.21	5.31	-3.03	112.64	198.17	108.04	73.97
9.46	-0.13	15.06	24.82	13.63	2.73	4.71	13.21	5.31	-3.03	112.62	198.18	108.04	73.97
9.47	-0.13	15.05	24.83	13.63	2.73	4.73	13.23	5.32	-3.03	112.51	198.17	107.73	74.00
9.47	-0.13	15.05	24.83	13.63	2.73	4.73	13.23	5.32	-3.03	112.51	198.17	107.72	74.00
9.48	-0.13	15.05	24.85	13.63	2.73	4.73	13.23	5.32	-3.03	112.65	198.17	107.54	74.00
9.66	0.08	14.87	24.59	13.42	2.92	5.31	13.85	5.90	-2.76	117.97	199.32	112.83	77.77
9.51	-0.12	15.06	24.89	13.64	2.73	4.73	13.26	5.37	-3.01	114.07	198.11	108.06	74.00
9.51	-0.12	15.06	24.89	13.64	2.73	4.73	13.26	5.37	-3.01	114.05	198.12	108.06	74.00
9.47	-0.15	15.03	24.88	13.53	2.75	4.73	13.29	5.39	-3.04	114.95	197.40	111.05	74.33
9.47	-0.15	15.03	24.88	13.53	2.75	4.73	13.29	5.39	-3.04	114.92	197.36	111.04	74.29
9.47	-0.15	15.04	24.88	13.53	2.75	4.74	13.30	5.39	-3.04	114.95	197.38	111.07	74.33
9.33	-0.25	14.85	24.55	13.41	2.66	4.53	13.02	5.23	-3.14	110.23	194.74	108.31	74.00
9.33	-0.25	14.85	24.55	13.41	2.66	4.53	13.02	5.23	-3.14	110.24	194.73	108.32	74.00
9.15	-0.44	14.65	24.33	13.29	2.48	4.23	12.74	5.02	-3.36	111.56	196.97	110.81	75.13
9.32	-0.27	14.80	24.50	13.40	2.65	4.50	13.01	5.23	-3.13	112.39	194.26	107.07	72.02
9.17	-0.45	14.65	24.39	13.33	2.46	4.23	12.74	5.00	-3.37	110.73	194.50	109.60	74.55
9.17	-0.45	14.65	24.39	13.33	2.46	4.23	12.74	5.00	-3.37	110.74	194.50	109.60	74.56
9.16	-0.45	14.65	24.39	13.33	2.46	4.23	12.73	5.00	-3.37	110.75	194.50	109.61	74.57
9.16	-0.45	14.65	24.39	13.33	2.45	4.23	12.73	5.00	-3.37	110.78	194.48	109.62	74.59
9.16	-0.45	14.65	24.39	13.33	2.45	4.23	12.73	5.00	-3.37	110.78	194.47	109.62	74.60
9.16	-0.45	14.65	24.39	13.33	2.45	4.23	12.73	5.00	-3.37	110.79	194.47	109.62	74.61
9.16	-0.45	14.65	24.39	13.33	2.45	4.23	12.73	5.00	-3.37	110.80	194.46	109.62	74.62
9.44	0.00	14.79	24.42	13.49	2.91	4.79	13.10	5.42	-2.90	145.23	257.67	141.87	98.05
9.39	-0.18	14.98	24.74	13.54	2.73	4.58	13.09	5.26	-3.09	113.89	198.01	107.82	73.33
8.18	-1.13	13.10	22.65	12.16	1.73	3.15	11.49	4.21	-4.00	135.38	246.64	137.23	96.80
8.73	-0.67	13.61	23.30	12.73	2.25	3.94	12.07	4.76	-3.60	140.78	250.33	141.88	98.28
8.34	-0.99	12.99	22.68	12.21	1.89	3.55	11.77	4.46	-3.85	145.51	259.80	148.34	107.00
8.38	-0.95	13.03	22.73	12.26	1.93	3.59	11.78	4.49	-3.83	144.78	258.78	147.49	106.16
8.40	-0.94	13.06	22.76	12.28	1.94	3.61	11.79	4.50	-3.83	144.70	258.55	147.30	105.90
8.41	-0.91	12.99	22.71	12.17	1.90	3.73	11.92	4.65	-3.72	145.23	255.68	143.93	103.66
8.41	-0.91	12.99	22.71	12.17	1.90	3.73	11.92	4.65	-3.72	145.24	255.67	143.93	103.66
8.52	-0.76	12.98	22.60	12.22	2.02	3.88	11.93	4.83	-3.57	141.60	252.63	141.69	97.37
8.51	-0.80	13.05	22.66	12.26	2.04	3.79	11.86	4.75	-3.61	140.69	252.96	142.72	98.25
8.36	-0.97	12.84	22.52	12.00	1.78	3.74	11.98	4.69	-3.70	142.83	252.32	141.44	101.19
8.30	-1.02	12.74	22.43	11.93	1.73	3.67	11.91	4.64	-3.75	143.61	253.94	142.66	102.63
8.49	-0.82	12.98	22.61	12.28	2.03	3.77	11.81	4.70	-3.66	142.09	253.68	143.40	100.07

8.86	-0.65	13.88	23.64	13.08	2.36	3.80	11.90	4.63	-3.67	139.68	247.73	140.81	96.26
8.47	-0.84	13.28	22.84	12.39	2.05	3.51	11.74	4.54	-3.73	137.99	250.04	140.41	96.55
8.55	-0.84	13.31	22.98	12.54	2.01	3.48	11.61	4.54	-3.72	141.99	256.04	142.53	94.22
8.46	-0.98	13.15	22.88	12.46	1.93	3.34	11.44	4.45	-3.86	149.45	260.28	145.80	97.08
8.09	-1.20	12.59	22.26	11.99	1.63	3.04	11.23	4.18	-4.06	152.01	272.02	151.18	100.92
8.51	-0.90	13.39	23.06	12.63	2.01	3.34	11.51	4.36	-3.83	143.27	255.65	141.92	92.13
8.46	-0.91	13.20	22.86	12.45	1.95	3.41	11.51	4.49	-3.79	145.33	257.87	143.40	96.35
8.01	-1.24	12.41	22.10	11.85	1.59	3.03	11.21	4.19	-4.07	155.90	274.32	151.45	102.29
8.31	-1.05	12.97	22.66	12.34	1.86	3.20	11.34	4.31	-3.96	152.63	267.89	149.03	96.61
8.20	-1.18	12.88	22.64	12.31	1.78	2.98	11.16	4.11	-4.15	159.13	275.45	155.07	102.32
8.12	-1.21	12.76	22.53	12.18	1.77	2.92	11.16	4.05	-4.22	166.01	283.42	159.81	105.91
7.57	-1.80	11.94	21.59	11.39	1.15	2.52	10.69	3.74	-4.75	170.38	283.66	168.95	126.03
7.65	-1.86	12.41	22.06	11.71	1.19	2.44	10.61	3.60	-4.93	173.18	279.35	171.87	126.86
7.10	-2.34	11.54	21.18	11.08	0.71	1.85	10.11	3.12	-5.40	184.33	299.85	181.93	139.06
7.10	-2.33	11.57	21.21	11.10	0.74	1.84	10.10	3.10	-5.42	184.18	298.89	181.57	139.28
8.56	-0.93	13.24	22.92	12.51	2.02	3.58	11.59	4.57	-3.91	150.09	258.01	147.65	101.18
8.55	-0.93	13.43	23.00	12.61	2.11	3.51	11.58	4.48	-3.95	149.11	253.47	150.92	107.50
8.51	-0.96	13.36	22.94	12.56	2.07	3.47	11.54	4.44	-3.98	150.11	254.52	151.67	108.39
8.15	-1.32	12.85	22.38	12.04	1.64	3.08	11.22	4.28	-4.23	157.69	256.20	159.21	118.91
8.87	-0.66	14.09	23.69	13.09	2.37	3.76	11.90	4.65	-3.69	143.72	247.41	145.41	101.47
7.35	-1.87	11.57	21.22	11.11	1.04	2.36	10.54	3.61	-4.79	170.05	289.32	170.39	130.87
7.91	-1.50	12.47	22.01	11.71	1.40	2.87	11.03	4.11	-4.39	161.00	262.26	162.68	123.87
7.91	-1.50	12.48	22.01	11.71	1.40	2.87	11.04	4.11	-4.39	160.98	262.21	162.64	123.82
8.76	-0.72	13.82	23.45	12.93	2.29	3.66	11.76	4.59	-3.75	144.69	249.44	146.22	103.34
8.76	-0.72	13.82	23.45	12.94	2.29	3.66	11.76	4.60	-3.75	144.67	249.38	146.17	103.29
8.71	-0.81	13.75	23.39	12.82	2.19	3.69	11.83	4.59	-3.80	146.29	252.93	148.85	104.96
8.72	-0.81	13.75	23.39	12.82	2.19	3.69	11.84	4.59	-3.80	146.27	252.88	148.82	104.93
8.84	-0.70	14.08	23.75	13.10	2.36	3.73	11.89	4.62	-3.75	141.55	249.17	144.93	101.00
8.82	-0.71	14.04	23.69	13.06	2.33	3.71	11.87	4.60	-3.77	141.86	249.87	145.20	101.39
8.82	-0.71	14.04	23.70	13.06	2.33	3.71	11.87	4.60	-3.77	141.85	249.86	145.19	101.37
8.82	-0.71	14.04	23.70	13.06	2.33	3.71	11.87	4.60	-3.77	141.84	249.85	145.18	101.36
7.95	-1.46	12.61	22.19	11.94	1.52	2.84	10.94	3.97	-4.46	162.92	266.34	161.96	120.25
7.92	-1.53	12.35	22.00	11.72	1.40	2.95	11.05	4.12	-4.43	164.74	274.76	162.90	116.49
8.77	-0.68	13.67	23.38	12.82	2.29	3.78	11.85	4.69	-3.67	141.72	249.36	143.94	98.46
8.20	-1.17	12.73	22.38	12.05	1.76	3.28	11.34	4.36	-4.09	153.73	265.59	153.31	107.73
8.71	-0.84	14.07	23.55	12.99	2.24	3.50	11.73	4.45	-3.92	147.57	251.33	150.66	108.37
7.43	-1.83	11.71	21.16	11.02	0.94	2.64	10.83	3.82	-4.61	167.28	273.57	172.67	134.75
7.03	-2.33	11.34	21.01	10.89	0.64	1.90	10.14	3.20	-5.29	182.88	301.78	181.66	140.15
7.06	-2.31	11.39	21.05	10.93	0.66	1.93	10.17	3.23	-5.28	182.50	301.32	181.38	139.30
8.28	-1.05	13.03	22.85	12.36	1.86	3.16	11.37	4.25	-3.96	153.09	273.75	149.68	96.16
8.37	-0.95	13.01	22.65	12.45	2.17	3.11	11.37	4.28	-4.06	175.71	294.46	164.19	106.89
8.30	-1.01	12.75	22.42	12.21	1.99	3.10	11.33	4.35	-4.01	177.81	296.61	164.74	105.53
7.54	-1.69	11.80	21.39	11.48	1.44	2.19	10.49	3.60	-4.83	192.35	322.35	184.73	134.25

7.53	-1.65	11.62	21.30	11.22	1.23	2.43	10.71	3.83	-4.59	180.67	308.49	174.70	122.76
8.53	-0.80	13.32	23.07	12.63	2.18	3.33	11.50	4.45	-3.79	158.02	274.36	150.01	91.15
8.52	-0.80	13.31	23.07	12.63	2.17	3.33	11.49	4.45	-3.79	158.05	274.42	150.01	91.24
8.57	-0.71	13.36	23.10	12.69	2.29	3.34	11.52	4.42	-3.71	160.38	277.33	151.85	90.23
8.57	-0.71	13.36	23.10	12.69	2.30	3.34	11.52	4.42	-3.71	160.36	277.42	151.84	90.21
8.53	-0.77	13.33	23.06	12.63	2.20	3.32	11.48	4.42	-3.74	158.38	274.90	150.05	90.66
8.53	-0.76	13.35	23.07	12.64	2.22	3.33	11.49	4.43	-3.72	158.28	274.70	150.02	90.35
8.58	-0.73	13.37	23.08	12.73	2.36	3.31	11.53	4.44	-3.82	167.37	283.76	156.36	94.18
8.33	-0.92	12.94	22.66	12.30	2.11	3.12	11.35	4.34	-3.96	170.91	289.16	161.71	100.72
8.33	-0.92	12.94	22.65	12.29	2.11	3.11	11.34	4.33	-3.96	171.00	289.29	161.84	100.91
8.39	-0.87	13.01	22.75	12.37	2.14	3.19	11.42	4.40	-3.88	168.75	286.94	159.93	98.72
8.81	-0.44	13.59	23.29	12.90	2.59	3.66	11.87	4.73	-3.47	160.03	273.35	148.38	90.13
8.53	-0.99	13.31	22.66	12.78	2.30	3.19	11.47	4.28	-4.26	178.67	293.81	169.61	113.28
8.78	-0.55	13.67	23.21	12.98	2.64	3.55	11.77	4.59	-3.71	163.23	274.98	154.52	98.74
8.57	-0.76	13.24	22.95	12.64	2.33	3.31	11.58	4.46	-3.84	167.54	284.34	155.92	97.32
8.19	-1.39	12.97	22.40	12.54	1.99	2.69	11.00	3.84	-4.74	185.13	305.49	176.73	123.46
8.55	-0.80	13.43	23.14	12.77	2.41	3.18	11.49	4.31	-4.01	173.62	290.06	162.47	104.93
8.90	-0.53	13.83	23.16	13.16	2.67	3.63	11.83	4.61	-3.77	163.24	269.63	153.12	97.20
8.90	-0.54	13.83	23.19	13.17	2.67	3.63	11.83	4.61	-3.77	163.97	270.87	153.59	97.71
8.90	-0.59	13.77	23.23	13.18	2.68	3.67	11.91	4.60	-3.80	165.42	273.94	155.96	100.00
8.90	-0.59	13.77	23.23	13.18	2.68	3.66	11.91	4.60	-3.80	165.30	273.98	155.97	100.00
8.94	-0.57	13.90	23.43	13.26	2.72	3.63	11.96	4.61	-3.86	169.10	281.13	158.24	102.98
8.75	-0.52	13.47	23.16	12.83	2.51	3.57	11.81	4.65	-3.56	161.82	275.56	149.70	90.54
7.76	-1.63	12.04	21.48	11.77	1.62	2.43	10.58	3.74	-4.91	203.17	329.54	195.08	147.62
8.22	-1.30	12.72	22.14	12.33	1.97	2.83	11.04	4.10	-4.54	197.52	318.67	186.31	135.04
8.37	-0.94	13.03	22.68	12.46	2.18	3.09	11.36	4.28	-4.04	176.03	294.08	163.39	105.54
8.46	-1.05	13.27	22.78	12.74	2.22	3.08	11.34	4.17	-4.35	176.75	291.31	166.55	109.12
8.33	-1.13	13.11	22.66	12.57	2.13	2.92	11.20	4.11	-4.39	179.97	297.19	170.17	113.27
8.37	-1.18	13.14	22.66	12.65	2.12	2.97	11.22	4.08	-4.48	180.17	295.35	169.20	113.51
8.54	-0.80	13.32	22.99	12.72	2.31	3.20	11.45	4.36	-3.95	171.33	287.46	158.94	98.83
8.10	-1.31	12.78	22.31	12.28	1.92	2.69	10.97	3.95	-4.53	184.61	304.31	173.64	119.19
8.32	-1.11	13.02	22.59	12.49	2.10	2.91	11.18	4.14	-4.33	180.65	297.80	168.83	113.02
7.97	-1.44	12.45	21.97	12.02	1.73	2.67	10.90	3.93	-4.61	187.75	310.75	179.02	125.72
8.53	-0.78	13.34	23.07	12.65	2.20	3.33	11.50	4.46	-3.76	157.82	274.04	150.01	90.59
8.33	-1.05	12.98	22.76	12.38	1.87	3.18	11.36	4.27	-3.97	159.80	273.59	151.09	95.90
7.84	-0.99	12.48	21.83	11.78	1.67	2.63	10.82	3.86	-3.65	145.25	250.18	152.27	128.27
6.49	-2.11	10.72	19.92	10.36	0.59	1.13	9.33	2.62	-4.82	165.37	270.53	169.07	159.29
8.01	-0.78	12.30	21.45	11.60	1.70	3.22	11.30	4.43	-3.33	153.43	252.75	153.33	132.50
8.33	-0.51	12.84	21.97	12.03	2.08	3.41	11.55	4.66	-3.11	158.01	254.04	151.62	133.56
8.43	-0.44	12.97	22.14	12.17	2.19	3.46	11.62	4.70	-3.07	154.80	251.71	150.15	130.77
8.41	-0.45	12.95	22.11	12.15	2.17	3.46	11.61	4.70	-3.08	155.36	251.72	149.96	130.83
8.42	-0.44	12.96	22.12	12.16	2.18	3.46	11.62	4.70	-3.07	155.14	251.86	150.14	130.91
8.32	-0.52	12.83	21.97	12.03	2.08	3.41	11.55	4.66	-3.12	158.08	254.13	151.70	133.67

8.37	-0.48	12.89	22.03	12.09	2.12	3.44	11.58	4.68	-3.09	156.50	252.13	150.55	131.79
8.41	-0.45	12.95	22.10	12.14	2.17	3.46	11.61	4.70	-3.08	155.51	251.86	150.03	130.97
8.42	-0.44	12.96	22.13	12.16	2.18	3.46	11.62	4.70	-3.07	155.12	251.88	150.18	130.93
8.42	-0.44	12.97	22.13	12.17	2.19	3.46	11.62	4.70	-3.07	155.02	251.85	150.20	130.91
7.94	-0.86	12.24	21.42	11.56	1.66	3.08	11.12	4.28	-3.40	149.82	250.92	153.60	130.42
8.29	-0.59	12.76	21.89	11.99	1.99	3.34	11.50	4.60	-3.18	156.77	252.67	152.47	133.76
8.42	-0.44	12.97	22.13	12.17	2.19	3.46	11.62	4.70	-3.07	154.94	251.80	150.18	130.85
8.00	-0.81	12.70	22.10	12.06	2.08	2.76	11.10	3.96	-3.64	157.64	252.09	149.58	134.26
6.80	-1.88	10.81	19.96	10.31	0.60	1.79	9.96	3.31	-4.34	161.77	267.11	164.76	151.62
6.69	-1.93	10.66	19.79	10.19	0.55	1.65	9.84	3.19	-4.41	165.21	268.81	169.11	156.87
6.78	-1.86	10.78	19.91	10.28	0.62	1.77	9.94	3.31	-4.36	162.50	267.55	166.14	153.82
6.42	-2.16	10.33	19.48	9.94	0.35	1.31	9.53	2.90	-4.64	169.24	275.12	172.99	162.90
6.50	-2.10	10.45	19.60	10.03	0.41	1.39	9.60	2.96	-4.60	167.59	273.58	171.25	160.50
6.56	-1.97	10.52	19.61	10.09	0.53	1.45	9.72	3.01	-4.49	180.20	277.27	179.20	168.86
7.13	-1.55	11.31	20.45	10.77	1.03	2.08	10.24	3.49	-4.11	160.26	262.52	162.91	147.99
6.68	-1.92	11.00	20.31	10.55	0.78	1.33	9.63	2.82	-4.64	171.17	272.10	167.17	159.91
6.01	-2.43	9.98	19.07	9.68	0.22	0.65	8.97	2.33	-5.07	183.13	284.15	180.85	177.63
7.20	-1.46	11.43	20.64	10.92	1.20	2.10	10.37	3.47	-4.10	163.88	265.46	159.82	149.35
7.70	-1.02	12.24	21.48	11.60	1.71	2.54	10.80	3.80	-3.75	159.15	256.76	154.70	140.60
7.71	-1.02	12.24	21.48	11.61	1.71	2.55	10.81	3.81	-3.75	159.23	256.70	154.52	140.39
6.96	-1.74	11.36	20.76	10.92	0.99	1.57	9.87	2.98	-4.48	166.65	267.47	163.05	154.77
6.70	-1.91	10.96	20.28	10.54	0.78	1.37	9.68	2.83	-4.60	172.25	272.09	167.08	160.09
7.60	-1.16	12.06	21.38	11.49	1.61	2.41	10.70	3.69	-3.90	161.57	257.63	154.02	141.42
7.65	-1.81	12.11	21.60	11.82	1.54	2.20	10.39	3.48	-5.15	204.99	331.42	197.46	150.94
7.87	-1.63	12.36	21.79	12.02	1.69	2.39	10.60	3.70	-4.94	204.66	331.82	196.46	148.26
8.02	-1.47	12.48	21.91	12.13	1.80	2.59	10.81	3.89	-4.73	202.28	326.72	191.46	142.57
7.58	-2.05	12.15	21.64	11.91	1.39	2.01	10.24	3.29	-5.47	211.35	336.49	204.96	157.65
7.86	-1.64	12.34	21.78	12.00	1.68	2.37	10.59	3.69	-4.95	204.91	332.25	196.77	148.66
7.96	-1.56	12.49	21.93	12.13	1.77	2.48	10.69	3.78	-4.87	202.70	328.36	194.05	145.18
7.93	-1.59	12.44	21.87	12.08	1.74	2.44	10.66	3.75	-4.90	203.53	329.84	195.06	146.48
5.70	-3.41	9.22	18.85	9.42	-0.28	0.44	8.70	1.99	-6.56	236.29	388.45	231.39	204.85
6.88	-2.79	11.44	20.95	11.19	0.72	1.19	9.47	2.54	-6.29	220.67	353.70	217.82	180.55
8.33	-1.19	12.95	22.37	12.50	2.07	2.95	11.18	4.18	-4.44	191.63	312.34	180.13	127.11
6.18	-2.79	9.64	19.22	9.69	0.12	1.04	9.37	2.66	-5.73	218.13	368.45	212.07	178.99
6.19	-2.79	9.66	19.24	9.70	0.13	1.05	9.38	2.67	-5.73	218.01	368.18	211.93	178.71
7.06	-2.23	10.95	20.55	10.86	0.87	1.94	10.11	3.29	-5.31	204.14	328.81	193.35	146.90
7.05	-2.24	10.94	20.53	10.85	0.86	1.92	10.10	3.27	-5.32	204.00	328.84	193.32	147.13
6.60	-2.47	10.42	19.99	10.30	0.52	1.34	9.68	2.91	-5.48	209.15	352.78	203.90	165.52
6.19	-2.93	9.68	19.29	9.75	0.06	1.09	9.33	2.58	-5.95	213.82	354.73	209.09	173.42
6.61	-2.45	10.25	19.77	10.31	0.60	1.42	9.67	2.93	-5.51	218.38	358.82	210.01	171.11
6.27	-2.90	9.86	19.49	9.88	0.03	1.13	9.39	2.64	-5.87	214.38	349.72	205.68	167.77
6.54	-2.72	10.31	19.95	10.23	0.25	1.35	9.61	2.84	-5.72	210.23	340.51	200.16	159.79
6.28	-2.91	9.90	19.55	9.90	0.04	1.17	9.39	2.64	-5.88	212.95	347.99	205.51	168.15

6.21	-2.97	9.78	19.44	9.82	-0.01	1.13	9.34	2.60	-5.93	213.54	349.97	206.78	170.45
7.00	-2.45	11.50	21.15	11.01	0.62	1.70	9.99	2.96	-5.54	187.40	305.36	184.57	143.13
6.92	-2.51	11.40	21.05	10.94	0.55	1.63	9.93	2.90	-5.60	188.62	308.13	186.10	145.35
6.82	-2.60	11.25	20.90	10.82	0.46	1.53	9.84	2.83	-5.67	190.50	311.62	187.98	148.15
6.97	-2.48	11.49	21.13	11.01	0.60	1.66	9.95	2.91	-5.58	187.97	306.90	185.18	143.96
7.87	-1.42	12.11	21.80	11.55	1.34	3.00	11.13	4.22	-4.18	156.89	274.50	155.03	109.81
7.40	-1.86	11.61	21.33	11.17	0.96	2.28	10.53	3.62	-4.66	170.26	292.91	166.81	120.75
8.56	-0.88	13.08	22.82	12.43	1.99	3.65	11.71	4.68	-3.76	146.53	256.69	147.68	99.58
7.70	-1.65	11.93	21.61	11.47	1.27	2.78	10.88	3.96	-4.53	170.61	283.89	164.16	116.58
8.57	-0.85	13.15	22.90	12.47	2.02	3.70	11.77	4.68	-3.73	145.27	253.81	145.46	98.82
8.57	-0.85	13.15	22.90	12.48	2.02	3.70	11.77	4.68	-3.73	145.27	253.80	145.46	98.81
8.64	-0.79	13.21	22.94	12.51	2.06	3.76	11.80	4.78	-3.65	147.18	255.13	146.37	97.14
8.64	-0.78	13.22	22.95	12.52	2.06	3.77	11.80	4.78	-3.65	147.11	255.00	146.37	97.04
8.89	-0.61	13.84	23.61	12.98	2.34	3.89	11.93	4.80	-3.53	140.96	245.68	140.90	92.80
8.72	-0.72	13.49	23.29	12.75	2.18	3.78	11.83	4.69	-3.64	141.34	250.33	143.33	96.04
7.46	-1.71	11.48	21.08	11.06	1.09	2.68	10.80	3.88	-4.48	166.46	285.89	162.82	119.95
8.21	-1.18	12.66	22.35	12.07	1.69	3.29	11.35	4.34	-4.07	157.38	266.85	153.35	105.31
8.21	-1.18	12.66	22.35	12.07	1.69	3.29	11.35	4.34	-4.07	157.38	266.88	153.35	105.33
8.08	-1.20	12.30	21.92	11.76	1.60	3.27	11.36	4.41	-4.00	156.70	270.44	153.74	107.85
8.53	-0.98	13.23	22.99	12.55	1.98	3.49	11.55	4.50	-3.94	151.28	257.80	147.80	101.03
8.42	-1.10	13.17	22.96	12.49	1.88	3.32	11.40	4.34	-4.09	151.65	260.87	150.65	103.80
8.41	-1.11	13.16	22.95	12.48	1.87	3.31	11.39	4.33	-4.09	151.69	261.04	150.77	103.99
8.42	-1.10	13.17	22.96	12.49	1.88	3.31	11.40	4.33	-4.09	151.66	260.92	150.68	103.87
8.41	-1.11	13.13	22.88	12.46	1.90	3.30	11.39	4.35	-4.11	156.22	263.93	151.68	103.78
8.44	-1.09	13.15	22.91	12.48	1.91	3.33	11.41	4.37	-4.09	156.07	263.55	151.44	103.66
8.19	-1.26	12.71	22.37	12.02	1.67	3.24	11.29	4.38	-4.19	162.76	267.30	157.97	110.47
6.00	-3.14	9.45	19.10	9.53	-0.20	0.95	9.18	2.45	-6.06	217.47	357.20	211.54	178.20
6.03	-3.12	9.49	19.15	9.58	-0.17	0.99	9.21	2.48	-6.05	217.00	356.30	210.81	177.03
5.98	-3.16	9.43	19.08	9.52	-0.21	0.93	9.17	2.44	-6.09	217.94	357.85	211.45	178.55
7.10	-2.28	11.32	20.92	11.04	0.78	1.84	10.06	3.18	-5.35	197.42	322.14	186.93	141.80
6.99	-2.38	11.19	20.80	10.91	0.68	1.71	9.96	3.08	-5.43	197.99	325.32	188.54	144.67
6.79	-2.48	10.84	20.42	10.59	0.53	1.62	9.87	2.99	-5.48	198.85	329.91	189.37	148.90
6.01	-3.10	9.50	19.13	9.49	-0.22	0.99	9.29	2.51	-5.96	211.61	353.66	205.70	173.21
6.60	-2.61	10.53	20.12	10.29	0.37	1.44	9.72	2.89	-5.58	201.13	335.75	192.60	154.20
6.37	-2.77	10.12	19.72	9.97	0.15	1.28	9.58	2.77	-5.70	203.33	342.81	196.49	160.34
6.34	-2.80	10.12	19.73	9.96	0.14	1.23	9.55	2.73	-5.73	203.86	343.12	196.43	160.98
6.53	-2.70	10.46	20.09	10.24	0.31	1.37	9.67	2.80	-5.67	198.87	337.73	192.11	155.76
6.97	-2.32	11.21	20.79	10.86	0.72	1.73	10.00	3.10	-5.38	195.65	324.21	185.38	143.11
6.96	-2.33	11.19	20.77	10.84	0.71	1.72	9.99	3.09	-5.39	195.83	324.57	185.60	143.46
7.58	-1.73	11.83	21.42	11.41	1.31	2.46	10.65	3.74	-4.76	185.12	307.61	175.63	124.56
7.64	-1.72	12.09	21.77	11.62	1.37	2.37	10.63	3.66	-4.82	181.33	302.64	172.68	122.17
7.12	-2.27	11.42	21.02	10.99	0.75	1.93	10.19	3.26	-5.29	188.99	308.81	183.47	139.12
6.86	-2.50	11.06	20.66	10.68	0.51	1.64	9.95	3.03	-5.48	192.52	316.51	187.89	147.00

7.37	-2.03	11.57	21.21	11.17	0.98	2.26	10.44	3.56	-5.02	187.59	303.29	179.64	133.17
7.28	-2.10	11.47	21.11	11.08	0.90	2.17	10.37	3.48	-5.09	188.30	305.58	180.62	135.33
7.06	-2.31	11.13	20.79	10.83	0.68	1.92	10.12	3.28	-5.31	194.63	314.50	186.92	142.81
6.49	-2.81	10.39	20.03	10.21	0.17	1.35	9.59	2.76	-5.79	204.29	333.80	198.57	160.84
6.38	-2.93	10.22	19.90	10.11	0.03	1.17	9.40	2.64	-5.88	209.99	342.26	204.50	167.04
5.53	-3.56	9.01	18.68	9.07	-0.70	0.40	8.75	2.02	-6.43	223.55	368.39	219.93	194.54
6.16	-3.05	9.79	19.42	9.76	-0.13	1.03	9.32	2.52	-5.98	211.75	353.12	205.65	171.25
7.25	-1.96	11.22	20.87	10.82	0.83	2.31	10.52	3.64	-4.77	174.80	296.80	170.54	125.07
7.31	-1.92	11.34	21.00	10.92	0.89	2.37	10.56	3.69	-4.77	174.22	295.07	170.60	123.14
6.69	-2.64	10.80	20.41	10.46	0.36	1.47	9.80	2.91	-5.60	195.14	321.70	190.91	152.13
7.72	-1.57	12.02	21.72	11.48	1.29	2.71	10.85	3.93	-4.44	169.13	287.50	164.54	113.88
8.86	0.01	13.52	22.86	12.61	2.68	3.97	12.45	5.13	-2.65	151.04	244.00	151.47	123.69
8.20	-0.63	12.46	21.66	11.69	1.88	3.46	11.84	4.72	-3.15	155.24	249.01	157.76	131.56
8.55	-0.33	12.79	22.00	12.00	2.11	3.76	11.96	5.08	-2.75	149.09	253.98	156.80	126.47
7.38	-1.56	11.44	20.76	10.78	0.89	2.48	10.79	3.98	-3.98	149.02	242.35	149.44	120.56
8.87	0.06	13.09	22.44	12.36	2.53	4.14	12.42	5.38	-2.41	152.47	254.97	161.93	132.03
8.72	-0.19	13.15	22.49	12.27	2.32	3.96	12.31	5.15	-2.71	149.44	246.08	153.14	123.55
8.20	-0.63	12.22	21.52	11.57	1.78	3.47	11.77	4.82	-3.06	150.71	253.00	160.09	130.80
8.49	-0.29	12.59	21.91	11.88	2.17	3.81	12.12	5.09	-2.74	148.68	248.48	157.34	127.17
8.30	-0.46	12.41	21.73	11.78	1.96	3.44	11.64	4.83	-2.92	156.49	262.93	165.26	139.64
8.69	-0.07	12.77	22.10	12.15	2.36	3.98	12.23	5.25	-2.50	154.67	258.91	168.11	138.03
8.63	-0.18	12.77	22.05	12.09	2.24	3.91	12.14	5.18	-2.62	153.77	255.52	162.71	133.51
8.08	-0.70	12.24	21.54	11.60	1.78	3.20	11.47	4.58	-3.16	152.20	258.00	161.49	134.14
7.93	-0.91	12.03	21.29	11.32	1.47	3.12	11.38	4.51	-3.32	149.27	251.57	155.42	126.51
7.58	-1.27	11.64	20.92	10.93	1.09	2.76	11.05	4.22	-3.64	149.61	246.75	151.21	123.34
7.39	-1.56	11.45	20.77	10.78	0.89	2.48	10.79	3.99	-3.97	149.04	242.45	149.47	120.55
7.98	-0.83	12.00	21.36	11.36	1.60	3.24	11.55	4.61	-3.20	150.60	254.07	160.69	132.79
8.07	-0.78	12.06	21.32	11.39	1.58	3.38	11.67	4.77	-3.17	149.06	249.75	156.11	126.40
7.53	-1.35	11.69	20.96	10.85	1.11	2.89	11.29	4.21	-3.85	151.61	234.64	139.12	116.89
7.46	-1.40	11.55	20.80	10.71	1.01	2.88	11.30	4.21	-3.86	152.28	235.17	139.40	117.16
8.52	-0.29	13.02	22.50	12.11	2.26	3.79	12.23	4.96	-2.84	149.25	242.45	148.12	120.37
9.02	0.18	13.56	22.73	12.61	2.73	4.33	12.65	5.43	-2.38	138.97	233.31	136.99	107.34
8.89	0.03	13.38	22.53	12.44	2.57	4.19	12.51	5.32	-2.53	140.66	234.53	137.86	109.10
8.68	-0.08	12.90	22.23	12.11	2.44	4.04	12.39	5.24	-2.62	143.35	237.88	141.07	112.07
9.20	0.35	13.73	22.95	12.82	2.96	4.50	12.81	5.58	-2.23	138.19	231.81	136.44	105.48
8.22	-0.68	12.46	21.70	11.72	1.83	3.41	11.66	4.74	-3.17	145.50	244.19	145.22	114.51
7.82	-1.09	12.00	21.29	11.27	1.40	2.98	11.25	4.37	-3.58	147.10	243.58	146.21	116.40
7.87	-0.97	11.92	21.21	11.27	1.48	3.08	11.33	4.45	-3.40	147.44	247.66	148.02	118.79
7.95	-0.90	12.01	21.31	11.37	1.57	3.16	11.40	4.51	-3.35	147.02	247.64	147.48	118.00
8.76	-0.13	13.13	22.36	12.38	2.45	3.96	12.18	5.16	-2.68	142.22	244.36	141.08	109.79
8.04	-0.85	12.17	21.41	11.43	1.65	3.29	11.56	4.63	-3.33	145.00	240.70	142.34	112.84
7.68	-1.24	12.02	21.31	11.15	1.31	2.94	11.37	4.22	-3.81	151.24	236.09	139.06	118.48
7.43	-1.45	11.54	20.82	10.71	0.99	2.81	11.23	4.14	-3.92	151.99	234.36	139.29	116.87

8.14	-0.71	12.52	21.85	11.73	1.85	3.33	11.76	4.58	-3.27	154.13	248.41	157.07	129.89
7.90	-0.94	11.98	21.27	11.33	1.51	3.13	11.35	4.48	-3.41	146.72	244.49	146.91	116.36
8.26	-0.58	12.44	21.74	11.81	1.93	3.47	11.68	4.73	-3.09	143.38	244.29	142.95	112.19
8.29	-0.60	12.55	21.78	11.80	1.92	3.48	11.74	4.80	-3.12	145.19	243.41	143.94	113.56
8.51	-0.38	12.81	22.01	12.03	2.18	3.71	11.97	4.98	-2.93	144.17	241.59	141.89	111.16
7.82	-1.07	11.94	21.21	11.22	1.42	3.02	11.32	4.40	-3.57	145.67	240.42	143.32	114.14
7.41	-1.50	11.50	20.85	10.82	0.98	2.51	10.82	3.97	-4.00	147.76	241.58	145.06	117.08
8.04	-0.78	12.29	21.62	11.54	1.78	3.32	11.69	4.54	-3.35	141.87	235.30	138.59	109.35
7.59	-1.38	11.91	21.28	11.06	1.16	2.79	11.26	4.11	-3.90	155.81	238.41	141.95	122.94
9.14	0.22	13.97	23.23	12.99	2.91	4.17	12.59	5.27	-2.46	148.24	236.53	143.44	117.51
7.82	-1.16	12.24	21.59	11.38	1.43	2.96	11.41	4.25	-3.73	156.03	240.57	143.37	123.98
7.64	-1.34	11.97	21.33	11.12	1.20	2.83	11.29	4.14	-3.87	156.04	238.83	142.28	123.16
7.62	-1.35	11.95	21.31	11.10	1.19	2.82	11.28	4.13	-3.88	155.91	238.72	142.11	123.08
8.24	-0.52	12.49	21.82	11.71	2.05	3.59	11.97	4.79	-3.08	142.47	235.72	139.24	111.03
8.58	-0.30	12.88	22.11	12.13	2.23	3.78	12.01	5.02	-2.85	144.02	244.83	143.66	111.90
8.32	-0.57	12.58	21.81	11.83	1.95	3.51	11.76	4.83	-3.09	145.13	243.68	144.06	113.61
8.17	-0.71	12.35	21.60	11.62	1.82	3.38	11.66	4.71	-3.22	144.47	241.13	142.06	111.63
8.76	-0.14	13.15	22.34	12.30	2.39	4.02	12.30	5.22	-2.69	142.01	239.00	140.07	108.12
7.44	-1.45	11.56	20.84	10.73	1.00	2.80	11.22	4.13	-3.94	151.94	233.95	139.02	116.79
7.50	-1.38	11.65	20.92	10.81	1.08	2.86	11.27	4.18	-3.87	151.72	234.47	139.11	116.87
8.41	-0.44	12.49	21.76	11.75	1.99	3.85	12.09	5.07	-2.84	145.37	242.84	146.11	117.46
7.56	-1.26	11.49	20.84	10.80	1.12	2.99	11.33	4.30	-3.66	145.55	237.42	143.27	115.44
7.58	-1.20	11.52	20.90	10.85	1.25	3.00	11.38	4.30	-3.63	144.04	235.19	139.79	112.40
8.36	-0.49	12.55	21.77	11.83	1.95	3.58	11.79	4.91	-2.92	148.61	254.82	154.07	124.15
8.36	-0.40	12.57	21.79	11.91	2.14	3.51	11.71	4.82	-2.95	152.75	260.39	154.88	127.53
8.26	-0.52	12.45	21.67	11.79	1.98	3.41	11.64	4.75	-3.05	151.72	258.19	154.56	126.02
8.33	-0.53	12.49	21.76	11.83	1.96	3.56	11.76	4.84	-3.00	145.89	248.10	146.61	115.87
8.33	-0.45	12.38	21.68	11.77	1.97	3.59	11.85	4.90	-2.88	155.13	257.51	164.77	136.01
7.61	-1.30	11.76	21.03	11.04	1.19	2.77	11.07	4.19	-3.80	146.08	241.61	144.40	115.27
8.64	-0.13	12.79	22.12	11.99	2.31	4.07	12.37	5.27	-2.58	144.88	238.37	143.96	113.80
8.20	-0.73	13.28	22.92	12.57	2.40	2.71	11.03	3.82	-3.83	145.20	245.88	150.98	140.29
7.77	-1.00	12.58	22.04	11.94	2.11	2.43	10.73	3.56	-4.10	156.95	258.92	164.67	159.00
6.95	-1.51	11.41	20.86	11.06	1.62	1.56	9.98	2.85	-4.64	180.30	284.93	194.01	201.95
8.24	-0.71	13.33	22.97	12.61	2.43	2.75	11.07	3.85	-3.81	144.79	245.06	150.22	139.02
8.54	-0.39	13.68	23.47	12.75	2.64	3.29	11.65	4.31	-3.42	144.09	237.99	142.46	128.10
8.08	-0.81	12.80	22.43	12.20	2.15	2.90	11.17	3.99	-3.80	151.06	250.70	154.34	145.93
7.67	-1.02	12.27	21.83	11.71	1.86	2.51	10.78	3.64	-3.90	140.03	245.05	144.31	132.98
7.72	-1.08	12.42	22.02	11.85	1.81	2.47	10.68	3.58	-4.01	141.61	246.35	145.26	133.19
7.37	-1.38	11.92	21.50	11.46	1.48	2.12	10.37	3.29	-4.25	147.99	254.08	151.50	141.04
7.95	-0.95	12.60	22.35	12.10	1.97	2.73	10.96	3.81	-3.89	146.80	243.91	147.82	135.67
7.98	-0.92	12.63	22.36	12.11	2.00	2.80	11.04	3.87	-3.85	146.42	243.74	147.23	134.46
8.00	-0.86	12.71	22.45	12.14	2.09	2.75	11.06	3.86	-3.84	148.12	245.66	148.18	137.36
8.01	-0.85	12.74	22.48	12.15	2.12	2.77	11.08	3.86	-3.82	148.21	246.33	148.20	137.66

8.04	-0.81	13.02	22.64	12.35	2.23	2.63	10.96	3.76	-3.84	145.61	246.74	150.15	139.01
8.00	-0.82	12.78	22.46	12.16	2.13	2.70	10.98	3.85	-3.80	141.10	245.75	146.33	132.68
7.86	-0.94	12.55	22.18	11.92	2.00	2.64	10.90	3.78	-3.86	140.64	243.56	145.60	133.06
8.14	-0.72	13.20	22.82	12.50	2.34	2.68	10.95	3.79	-3.80	143.03	245.56	149.49	137.55
8.13	-0.72	13.19	22.82	12.50	2.34	2.68	10.95	3.78	-3.80	143.03	245.60	149.55	137.58
8.14	-0.76	13.21	22.85	12.53	2.35	2.64	10.94	3.77	-3.87	146.39	247.47	152.35	142.17
7.48	-1.19	12.27	21.77	11.69	1.96	2.08	10.42	3.25	-4.38	164.17	267.71	176.70	175.66
7.55	-1.14	12.38	21.87	11.79	2.01	2.14	10.46	3.30	-4.29	162.74	265.56	173.41	171.78
7.58	-1.13	12.39	21.91	11.80	2.01	2.12	10.48	3.34	-4.28	160.18	266.45	171.45	169.47
8.13	-0.74	13.10	22.81	12.46	2.29	2.69	10.95	3.78	-3.75	142.59	244.58	148.04	134.36
7.91	-0.98	12.68	22.26	12.05	2.03	2.65	10.97	3.76	-4.01	155.39	251.09	157.57	151.20
7.65	-1.08	12.55	22.11	11.93	2.06	2.15	10.49	3.36	-4.19	158.57	263.58	171.31	165.70
7.59	-1.12	12.45	22.01	11.87	2.03	2.10	10.46	3.30	-4.26	159.84	265.89	172.84	169.20
7.57	-1.14	12.39	21.90	11.80	2.02	2.14	10.47	3.30	-4.28	161.31	265.65	172.84	170.30
7.44	-1.21	12.21	21.72	11.67	1.95	2.03	10.37	3.21	-4.36	163.71	269.11	175.68	174.97
7.19	-1.40	11.73	21.21	11.29	1.72	1.75	10.14	3.05	-4.50	174.92	279.29	187.10	190.91
7.16	-1.42	11.69	21.17	11.26	1.70	1.72	10.12	3.03	-4.52	175.49	280.01	187.79	192.16
7.46	-1.20	12.25	21.74	11.68	1.98	2.08	10.40	3.23	-4.36	164.55	268.25	177.04	176.51
7.77	-0.99	12.76	22.21	12.07	2.20	2.40	10.69	3.47	-4.17	165.47	262.20	171.68	171.59
7.19	-1.38	11.84	21.34	11.33	1.78	1.84	10.22	3.08	-4.54	173.20	277.14	185.64	190.70
8.16	-0.79	13.06	22.71	12.39	2.27	2.86	11.13	3.91	-3.86	150.56	248.06	152.24	142.90
8.16	-0.77	13.05	22.67	12.38	2.25	2.89	11.17	3.94	-3.83	151.48	247.43	152.12	142.83
7.70	-0.98	12.46	22.12	11.91	2.01	2.35	10.60	3.51	-3.98	144.52	248.98	153.28	142.65
7.62	-0.92	12.56	21.92	11.80	2.05	2.30	10.60	3.46	-3.94	147.30	249.25	155.43	146.15
7.66	-0.94	12.71	22.12	11.90	2.18	2.20	10.59	3.42	-4.05	153.90	257.00	162.84	155.39
7.80	-0.90	12.65	22.24	12.01	2.10	2.43	10.69	3.58	-3.90	146.05	250.44	155.26	144.76
7.94	-0.86	12.95	22.57	12.27	2.25	2.44	10.74	3.59	-3.94	145.03	249.57	153.49	143.19
7.94	-0.86	12.95	22.57	12.27	2.25	2.45	10.74	3.60	-3.94	145.04	249.52	153.53	143.14
7.57	-1.02	12.36	21.84	11.69	2.03	2.24	10.52	3.43	-4.03	151.62	256.87	162.19	154.90
7.57	-1.01	12.37	21.85	11.69	2.03	2.24	10.53	3.43	-4.03	151.56	256.84	162.22	154.88
7.57	-1.02	12.36	21.84	11.69	2.03	2.24	10.52	3.43	-4.03	151.61	256.85	162.21	154.90
7.48	-1.09	12.30	21.69	11.57	1.84	2.18	10.47	3.35	-4.02	139.86	245.94	149.97	138.93
7.72	-0.91	12.57	22.11	11.92	2.15	2.36	10.63	3.53	-3.97	148.97	255.10	159.88	153.36
7.82	-0.85	12.73	22.26	12.02	2.21	2.47	10.73	3.63	-3.90	148.42	253.34	158.87	150.67
7.77	-0.94	12.57	22.17	11.96	2.06	2.42	10.67	3.57	-3.90	145.98	250.35	155.00	144.05
7.70	-0.91	12.54	22.09	11.90	2.15	2.35	10.63	3.51	-3.97	149.34	255.18	159.67	152.66
7.57	-0.96	12.49	21.84	11.73	2.01	2.26	10.56	3.44	-3.97	147.50	250.03	156.47	147.01
7.60	-0.99	12.44	21.91	11.74	2.05	2.26	10.57	3.45	-4.02	151.27	257.00	162.01	154.78
7.48	-1.05	12.22	21.72	11.58	1.94	2.17	10.49	3.37	-4.08	152.86	258.18	162.93	156.29
7.78	-0.86	12.75	22.25	12.02	2.24	2.44	10.71	3.58	-3.93	150.30	253.87	159.82	151.78
7.86	-0.83	12.77	22.31	12.06	2.23	2.52	10.78	3.67	-3.87	148.17	252.51	158.57	149.61
7.86	-0.83	12.78	22.31	12.06	2.23	2.52	10.78	3.67	-3.87	148.19	252.51	158.57	149.61
7.85	-0.84	12.76	22.29	12.04	2.22	2.50	10.76	3.66	-3.88	148.28	252.80	158.67	149.98

7.82	-0.85	12.73	22.26	12.02	2.21	2.47	10.73	3.63	-3.89	148.38	253.26	158.85	150.59
7.81	-0.86	12.71	22.24	12.01	2.20	2.45	10.72	3.62	-3.91	148.47	253.60	158.99	151.04
7.82	-0.85	12.73	22.26	12.02	2.21	2.47	10.73	3.63	-3.89	148.37	253.28	158.86	150.62
7.56	-1.14	12.78	22.16	12.10	2.20	1.93	10.31	3.01	-4.47	174.49	271.03	185.44	188.12
7.69	-1.06	12.70	22.18	12.02	2.18	2.29	10.59	3.37	-4.28	167.85	266.78	174.03	176.42
7.63	-1.09	12.61	22.11	11.96	2.17	2.20	10.54	3.27	-4.34	167.64	268.12	177.68	179.63
6.60	-1.91	10.69	20.11	10.41	0.81	1.42	9.64	2.80	-4.67	160.05	265.90	163.74	157.07
7.12	-1.53	11.41	20.93	11.07	1.21	1.90	10.10	3.17	-4.30	152.31	257.36	153.69	143.40
7.13	-1.53	11.43	20.95	11.08	1.22	1.91	10.11	3.18	-4.29	152.14	257.22	153.55	143.18
6.70	-1.84	10.79	20.24	10.49	0.91	1.56	9.79	2.91	-4.57	158.09	265.16	161.00	154.57
7.46	-1.28	11.95	21.52	11.48	1.57	2.25	10.50	3.41	-4.14	150.20	254.22	151.59	142.53
7.79	-1.00	12.19	21.89	11.69	1.85	2.78	11.01	3.89	-3.82	151.20	248.96	146.71	134.62
7.81	-0.98	12.21	21.91	11.70	1.87	2.80	11.02	3.90	-3.82	151.21	248.71	146.60	134.16
7.83	-0.97	12.23	21.93	11.73	1.89	2.82	11.04	3.92	-3.81	151.08	248.26	146.27	133.58
7.80	-0.99	12.20	21.90	11.70	1.86	2.79	11.01	3.89	-3.82	151.14	248.90	146.64	134.46
7.70	-1.05	12.03	21.75	11.55	1.79	2.71	10.95	3.85	-3.88	152.66	250.05	148.88	137.17
7.81	-1.05	12.23	21.99	11.82	1.76	2.75	10.94	3.81	-3.85	148.63	246.48	145.63	132.30
8.06	-0.80	12.56	22.23	12.03	2.02	3.08	11.28	4.10	-3.62	146.30	241.58	142.88	127.69
7.93	-0.88	12.38	22.03	11.83	1.94	2.96	11.17	4.02	-3.70	150.71	242.72	145.15	132.14
7.80	-1.06	12.21	21.97	11.80	1.75	2.74	10.93	3.79	-3.86	148.81	246.74	145.85	132.70
7.71	-1.11	12.09	21.87	11.69	1.69	2.67	10.87	3.72	-3.91	150.18	248.38	147.18	134.45
7.40	-1.34	11.69	21.35	11.33	1.49	2.28	10.52	3.44	-4.16	154.76	256.55	153.92	143.61
7.71	-1.05	12.07	21.78	11.58	1.80	2.72	10.96	3.85	-3.88	153.05	249.15	148.32	136.57
7.81	-0.96	12.32	22.02	11.84	1.88	2.75	10.98	3.82	-3.83	152.46	248.42	147.02	133.08
8.05	-0.81	12.61	22.30	12.08	2.06	2.99	11.19	4.00	-3.67	148.53	244.75	143.55	128.52
7.94	-0.88	12.48	22.17	11.97	1.97	2.90	11.09	3.93	-3.74	150.24	246.58	145.15	130.50
7.85	-0.97	12.34	22.05	11.88	1.86	2.75	10.98	3.83	-3.80	149.17	246.93	146.04	131.47
7.42	-1.28	11.79	21.33	11.35	1.45	2.23	10.46	3.44	-4.04	149.70	253.33	149.22	137.47
7.03	-1.59	11.26	20.74	10.88	1.15	1.88	10.07	3.16	-4.35	153.10	257.03	154.49	144.94
7.46	-1.24	11.76	21.39	11.40	1.56	2.39	10.59	3.54	-4.07	151.68	254.60	151.18	140.52
6.81	-1.76	10.97	20.41	10.65	0.97	1.63	9.84	2.97	-4.51	156.13	261.73	159.19	151.31
7.39	-1.31	11.73	21.28	11.32	1.43	2.21	10.43	3.43	-4.05	151.59	254.73	150.57	138.92
7.60	-1.15	12.04	21.59	11.58	1.60	2.44	10.65	3.62	-3.90	148.08	250.15	147.03	133.14
7.52	-1.17	11.96	21.50	11.51	1.57	2.38	10.59	3.57	-3.93	149.23	251.27	148.16	135.32
7.53	-1.17	11.97	21.50	11.51	1.58	2.38	10.60	3.57	-3.93	149.26	251.22	148.10	135.27
7.65	-1.10	12.13	21.65	11.64	1.64	2.47	10.68	3.64	-3.88	148.30	249.08	146.89	133.08
7.68	-1.09	12.16	21.75	11.69	1.71	2.52	10.74	3.66	-3.88	148.97	250.41	147.10	133.25
7.31	-1.34	11.56	21.20	11.21	1.43	2.22	10.47	3.40	-4.12	155.33	257.10	153.14	142.79
7.31	-1.35	11.56	21.20	11.21	1.43	2.23	10.47	3.40	-4.12	155.40	257.35	153.30	142.78
7.33	-1.36	11.58	21.21	11.25	1.41	2.25	10.48	3.41	-4.14	155.94	258.57	154.08	143.20
7.36	-1.34	11.62	21.26	11.29	1.44	2.28	10.51	3.43	-4.13	155.55	257.94	153.50	142.38
7.54	-1.21	11.85	21.57	11.50	1.60	2.45	10.70	3.57	-4.00	152.27	254.77	151.16	138.97
7.43	-1.29	11.70	21.40	11.37	1.51	2.34	10.58	3.50	-4.09	153.92	257.14	153.03	141.90

7.43	-1.29	11.70	21.40	11.37	1.51	2.34	10.58	3.50	-4.09	153.93	257.16	153.04	141.92
7.52	-1.24	11.82	21.56	11.47	1.58	2.43	10.67	3.57	-4.02	152.21	255.49	151.40	139.64
7.36	-1.31	11.64	21.31	11.30	1.46	2.26	10.49	3.42	-4.12	155.98	257.24	153.43	142.70
7.36	-1.31	11.64	21.31	11.30	1.46	2.26	10.49	3.43	-4.12	155.98	257.23	153.39	142.67
7.82	-0.99	12.25	21.99	11.83	1.83	2.73	10.95	3.82	-3.83	148.21	248.01	146.06	132.23
7.49	-1.29	11.76	21.48	11.40	1.53	2.40	10.62	3.53	-4.10	153.30	255.56	152.04	141.51
7.48	-1.29	11.76	21.48	11.40	1.53	2.39	10.62	3.53	-4.10	153.34	255.61	152.07	141.56
7.73	-1.04	12.11	21.82	11.64	1.80	2.72	10.96	3.84	-3.86	151.65	250.21	147.97	136.23
7.74	-1.04	12.13	21.83	11.65	1.81	2.73	10.97	3.85	-3.85	151.47	250.04	147.74	136.01
7.79	-0.99	12.19	21.89	11.69	1.86	2.79	11.01	3.89	-3.82	151.33	248.87	146.74	134.53
7.79	-0.99	12.19	21.89	11.69	1.86	2.79	11.01	3.90	-3.82	151.31	248.84	146.71	134.45
7.81	-0.98	12.21	21.91	11.71	1.87	2.80	11.02	3.90	-3.82	151.16	248.74	146.59	134.16
7.81	-0.98	12.21	21.91	11.71	1.87	2.80	11.02	3.90	-3.82	151.15	248.69	146.56	134.07
7.81	-0.98	12.21	21.91	11.71	1.87	2.80	11.03	3.91	-3.82	151.15	248.63	146.53	133.99
7.74	-1.03	12.10	21.81	11.62	1.80	2.74	10.97	3.86	-3.86	151.99	249.78	147.91	136.23
7.76	-1.05	12.17	21.92	11.76	1.77	2.69	10.91	3.77	-3.88	151.28	249.42	147.55	133.27
7.84	-1.02	12.27	22.04	11.83	1.80	2.79	10.98	3.83	-3.82	147.59	246.82	145.33	131.33
8.13	-0.74	12.76	22.44	12.18	2.14	3.02	11.24	4.05	-3.63	146.53	242.36	141.98	126.57
7.69	-1.07	12.19	21.91	11.70	1.84	2.62	10.89	3.73	-3.95	152.49	248.02	148.81	136.64
8.09	-0.75	12.64	22.27	12.02	2.08	3.13	11.32	4.16	-3.59	146.68	240.05	142.17	128.39
7.74	-1.02	12.25	21.93	11.69	1.84	2.68	10.94	3.79	-3.91	152.54	247.01	148.56	136.72
7.68	-1.06	12.18	21.87	11.65	1.80	2.63	10.88	3.74	-3.95	152.99	248.11	149.44	138.12
6.82	-1.75	10.94	20.40	10.63	0.98	1.68	9.89	3.01	-4.48	156.48	262.96	159.04	151.28
7.55	-1.24	12.13	21.70	11.63	1.63	2.30	10.58	3.46	-4.11	148.53	252.01	150.75	141.22
8.11	-0.78	12.75	22.43	12.13	2.11	3.03	11.28	4.11	-3.65	147.68	242.06	145.67	131.88
8.11	-0.82	12.99	22.78	12.43	2.23	2.68	10.97	3.74	-3.87	146.75	245.55	147.40	136.03
8.31	-0.62	13.50	23.26	12.62	2.49	2.93	11.31	3.99	-3.72	149.01	244.39	149.40	137.28
8.24	-0.66	13.16	22.90	12.49	2.38	2.94	11.26	3.98	-3.70	145.36	245.95	149.03	137.47
8.19	-0.74	13.26	22.91	12.58	2.37	2.69	10.98	3.80	-3.84	145.86	246.80	151.88	141.28
7.47	-1.13	12.11	21.64	11.56	1.79	2.27	10.53	3.41	-4.05	138.53	247.59	146.41	134.69
7.91	-0.97	12.73	22.41	12.16	2.10	2.56	10.87	3.69	-4.03	154.82	254.60	160.70	153.17
8.29	-0.68	13.32	23.01	12.61	2.40	2.87	11.16	3.94	-3.76	145.32	245.38	150.49	139.04
8.23	-0.74	13.30	22.96	12.62	2.39	2.74	11.04	3.84	-3.84	147.19	247.28	152.94	141.31
8.13	-0.83	13.02	22.75	12.39	2.23	2.74	11.05	3.84	-3.88	147.97	250.45	155.00	145.26
8.27	-0.60	13.09	22.76	12.34	2.33	3.16	11.43	4.22	-3.53	145.07	240.17	143.36	129.10
8.21	-0.72	13.29	22.93	12.59	2.40	2.73	11.04	3.83	-3.83	145.80	245.26	151.12	140.74
8.04	-0.77	12.93	22.57	12.29	2.23	2.66	10.97	3.79	-3.78	144.54	245.61	149.77	137.75
7.91	-0.86	12.77	22.39	12.14	2.13	2.56	10.87	3.69	-3.88	146.49	249.33	151.96	141.09
8.11	-0.80	13.01	22.63	12.27	2.22	2.88	11.19	3.96	-3.83	151.35	245.55	150.96	141.02
8.05	-0.86	12.91	22.69	12.35	2.18	2.65	10.94	3.72	-3.89	146.91	246.11	147.49	136.42
7.92	-0.94	12.60	22.21	11.95	2.03	2.83	11.10	3.92	-3.92	153.28	247.78	152.43	143.59
8.05	-0.84	12.86	22.49	12.15	2.15	2.89	11.19	3.97	-3.85	152.09	244.98	151.45	140.63
7.74	-1.11	12.44	22.07	11.92	1.79	2.46	10.74	3.58	-4.02	147.70	248.91	148.69	138.15

7.77	-1.09	12.46	22.08	11.92	1.81	2.50	10.80	3.63	-3.97	147.55	247.63	147.92	137.33
8.21	-0.70	12.96	22.67	12.31	2.24	3.03	11.30	4.07	-3.64	144.78	241.74	144.40	132.19
8.11	-0.76	12.66	22.32	12.05	2.09	3.11	11.33	4.19	-3.61	146.34	240.76	143.56	130.21
7.86	-1.01	12.56	22.22	12.02	1.89	2.61	10.88	3.69	-3.92	146.69	247.33	147.53	135.91
8.00	-0.91	12.77	22.52	12.24	2.10	2.66	10.93	3.76	-3.90	148.07	246.52	148.28	137.51
7.84	-0.95	12.52	22.15	11.90	1.98	2.63	10.89	3.75	-3.87	140.39	243.74	145.48	132.89
7.67	-1.03	12.28	21.90	11.71	1.89	2.45	10.74	3.58	-3.98	141.66	247.30	149.21	137.92
8.27	-0.65	13.19	22.92	12.52	2.38	2.93	11.24	3.98	-3.70	144.82	245.51	148.66	136.46
7.98	-0.83	12.97	22.63	12.33	2.23	2.48	10.77	3.62	-3.90	145.75	248.09	151.61	140.40
8.05	-0.80	13.07	22.75	12.42	2.29	2.56	10.85	3.69	-3.85	144.49	247.30	150.93	139.09
8.08	-0.78	13.13	22.78	12.45	2.31	2.59	10.88	3.70	-3.84	143.27	247.07	150.88	139.05
8.00	-0.81	13.04	22.68	12.36	2.31	2.52	10.82	3.66	-3.90	146.02	249.46	153.08	143.56
8.05	-0.88	12.86	22.56	12.27	2.16	2.72	11.02	3.84	-3.92	151.75	252.88	157.17	148.83
8.24	-0.66	13.15	22.89	12.49	2.38	2.94	11.25	3.97	-3.71	145.18	245.94	149.10	137.48
8.24	-0.66	13.16	22.89	12.49	2.38	2.94	11.25	3.98	-3.71	145.29	245.95	149.08	137.51
8.28	-0.60	13.26	23.06	12.51	2.42	2.98	11.34	4.03	-3.65	145.92	243.33	145.91	135.15
8.28	-0.60	13.26	23.06	12.51	2.42	2.98	11.34	4.03	-3.65	145.91	243.30	145.91	135.15
8.27	-0.61	13.24	23.04	12.50	2.41	2.96	11.33	4.02	-3.66	146.05	243.63	146.24	135.53
8.42	-0.57	13.32	22.96	12.54	2.38	3.29	11.54	4.30	-3.49	143.47	234.92	140.31	124.82
7.89	-0.95	12.71	22.41	12.02	2.08	2.64	11.01	3.77	-3.98	153.92	249.28	154.56	145.54
7.90	-0.94	12.72	22.40	12.02	2.09	2.66	11.02	3.78	-3.96	153.33	248.99	154.08	145.35
8.06	-0.86	13.01	22.66	12.29	2.22	2.78	11.09	3.84	-3.90	151.53	248.51	153.19	143.91
7.99	-0.91	12.88	22.52	12.19	2.15	2.69	11.01	3.77	-3.96	153.13	250.12	155.07	147.02
7.70	-1.16	12.44	22.04	11.88	1.90	2.41	10.74	3.55	-4.21	158.93	254.82	161.43	157.08
7.79	-1.07	12.57	22.17	11.95	1.98	2.53	10.84	3.65	-4.13	156.61	253.53	159.35	153.74
7.78	-1.08	12.56	22.17	11.95	1.97	2.52	10.83	3.64	-4.14	156.71	253.64	159.47	153.93
8.01	-0.90	12.78	22.39	12.17	2.12	2.77	11.05	3.86	-3.92	154.17	249.92	154.96	147.83
8.07	-0.82	12.78	22.41	12.18	2.14	2.89	11.15	3.98	-3.81	151.26	250.94	154.65	146.38
8.08	-0.81	12.80	22.43	12.19	2.15	2.90	11.17	3.99	-3.80	151.08	250.73	154.37	145.98
8.16	-0.75	13.23	22.88	12.53	2.38	2.66	10.99	3.78	-3.85	145.72	246.68	151.64	141.38
8.16	-0.75	13.23	22.88	12.53	2.38	2.66	10.99	3.78	-3.85	145.70	246.75	151.65	141.37
8.18	-0.75	13.25	22.90	12.55	2.39	2.68	11.00	3.80	-3.85	145.50	246.38	151.40	140.97
8.18	-0.69	13.18	22.84	12.51	2.33	2.76	11.00	3.83	-3.72	141.74	244.12	148.17	134.51
8.17	-0.69	13.17	22.83	12.50	2.32	2.74	10.99	3.82	-3.73	141.89	244.24	148.21	134.70
7.85	-1.01	12.39	22.11	11.90	1.87	2.73	10.95	3.83	-3.88	150.29	247.35	146.85	134.75
7.85	-1.00	12.39	22.11	11.90	1.87	2.73	10.95	3.83	-3.88	150.27	247.35	146.82	134.73
7.98	-0.94	12.54	22.29	12.04	1.97	2.83	11.05	3.91	-3.81	147.55	243.24	146.71	133.92
7.93	-0.96	12.49	22.22	12.00	1.93	2.79	11.02	3.88	-3.85	148.56	243.55	146.83	135.07
7.97	-0.94	12.52	22.26	12.03	1.96	2.83	11.05	3.91	-3.82	147.73	243.34	146.51	134.19
7.93	-0.96	12.49	22.22	12.00	1.93	2.79	11.02	3.88	-3.85	148.55	243.55	146.83	135.05
7.93	-0.96	12.49	22.23	12.00	1.93	2.79	11.03	3.89	-3.85	148.44	243.53	146.80	134.94
7.97	-0.93	12.53	22.27	12.03	1.97	2.83	11.06	3.92	-3.81	147.59	243.28	146.52	134.03
7.97	-0.93	12.53	22.27	12.03	1.97	2.83	11.06	3.92	-3.81	147.56	243.27	146.50	134.01

7.97	-0.93	12.53	22.27	12.03	1.97	2.83	11.06	3.92	-3.81	147.55	243.27	146.48	133.99
7.90	-0.97	12.62	22.38	12.14	2.01	2.56	10.85	3.66	-3.96	147.97	247.79	148.60	137.74
8.13	-0.73	12.70	22.37	12.08	2.16	3.17	11.36	4.21	-3.58	147.12	239.85	144.12	129.22
8.11	-0.73	12.71	22.38	12.07	2.16	3.08	11.32	4.15	-3.63	146.83	240.29	144.32	131.04
8.12	-0.73	12.72	22.38	12.07	2.16	3.08	11.32	4.15	-3.63	146.81	240.26	144.29	131.02
8.23	-0.65	12.98	22.67	12.25	2.27	3.16	11.42	4.20	-3.56	146.05	240.29	143.14	130.17
8.17	-0.70	12.75	22.41	12.10	2.17	3.17	11.39	4.21	-3.56	146.32	238.86	143.56	128.97
8.24	-0.61	13.18	22.95	12.37	2.39	3.06	11.40	4.12	-3.60	147.36	242.97	145.42	133.43
8.15	-0.72	12.80	22.51	12.12	2.20	3.08	11.34	4.17	-3.62	147.45	242.40	145.32	131.60
7.88	-0.99	12.61	22.35	12.12	1.97	2.53	10.81	3.65	-3.96	148.13	248.87	148.81	138.02
8.26	-0.68	13.05	22.72	12.35	2.25	3.12	11.39	4.15	-3.60	146.35	236.98	141.83	127.06
8.27	-0.67	13.06	22.72	12.35	2.25	3.13	11.39	4.16	-3.60	146.33	236.94	141.78	127.00
8.27	-0.67	13.06	22.72	12.35	2.25	3.13	11.40	4.16	-3.60	146.31	236.90	141.75	126.95
8.27	-0.67	13.06	22.72	12.35	2.25	3.13	11.40	4.16	-3.60	146.29	236.87	141.71	126.90
8.27	-0.68	13.06	22.72	12.35	2.25	3.12	11.39	4.16	-3.60	146.41	236.92	142.08	127.35
7.46	-1.24	11.76	21.39	11.40	1.56	2.39	10.59	3.54	-4.07	151.68	254.60	151.19	140.54
7.46	-1.24	11.77	21.40	11.40	1.56	2.39	10.59	3.54	-4.07	151.68	254.57	151.16	140.51
7.97	-0.94	12.44	22.18	11.99	1.87	2.90	11.08	3.94	-3.75	146.44	244.18	143.35	128.61
7.60	-1.20	12.16	21.86	11.72	1.72	2.37	10.58	3.47	-4.13	150.17	253.45	150.73	139.52
7.60	-1.20	12.17	21.87	11.72	1.72	2.37	10.58	3.47	-4.13	150.09	253.45	150.68	139.52
7.61	-1.19	12.19	21.89	11.74	1.73	2.38	10.61	3.49	-4.12	149.89	253.32	150.37	139.64
7.54	-1.23	12.06	21.74	11.63	1.67	2.33	10.55	3.44	-4.14	151.86	254.78	150.84	140.61
7.52	-1.24	12.03	21.71	11.60	1.66	2.31	10.53	3.43	-4.14	152.25	255.12	151.17	141.03
7.53	-1.23	12.04	21.72	11.61	1.67	2.32	10.54	3.44	-4.14	152.11	254.91	151.01	140.84
7.53	-1.23	12.06	21.73	11.62	1.67	2.32	10.54	3.44	-4.14	151.94	254.81	150.89	140.68
7.53	-1.23	12.05	21.73	11.62	1.67	2.32	10.54	3.44	-4.14	152.01	254.83	150.93	140.73
7.22	-1.26	11.91	21.25	11.19	1.56	2.02	10.24	3.23	-4.04	133.29	235.23	142.95	131.95
7.22	-1.26	11.90	21.24	11.18	1.56	2.02	10.24	3.23	-4.04	133.32	235.25	142.96	132.04
7.37	-1.17	12.23	21.62	11.49	1.77	2.07	10.29	3.23	-4.09	133.40	237.90	144.36	132.22
7.39	-1.15	12.26	21.66	11.52	1.80	2.10	10.33	3.25	-4.08	133.48	237.47	143.86	131.57
7.34	-1.23	12.06	21.44	11.32	1.66	2.15	10.34	3.34	-4.11	127.34	239.69	138.25	126.17
7.42	-1.13	12.17	21.56	11.48	1.74	2.19	10.46	3.36	-4.02	139.25	244.62	149.39	138.77
7.19	-1.29	11.86	21.22	11.19	1.60	1.96	10.20	3.19	-4.18	145.75	245.25	155.96	148.77
7.18	-1.30	11.93	21.26	11.25	1.62	1.90	10.17	3.13	-4.18	145.76	245.70	153.50	146.73
7.36	-1.19	11.94	21.39	11.33	1.68	2.18	10.43	3.37	-4.06	139.48	247.20	148.48	138.35
7.30	-1.20	12.14	21.48	11.39	1.70	2.02	10.25	3.17	-4.10	132.37	235.29	142.81	131.15
7.39	-1.14	12.26	21.66	11.52	1.80	2.10	10.34	3.25	-4.08	133.67	237.58	143.85	131.65
7.30	-1.20	12.16	21.50	11.41	1.72	2.05	10.27	3.19	-4.11	131.85	235.79	140.50	129.13
7.19	-1.29	11.86	21.21	11.19	1.59	1.96	10.20	3.19	-4.18	145.84	245.30	156.05	148.86
7.27	-1.22	12.03	21.37	11.34	1.68	1.99	10.25	3.22	-4.13	144.03	243.62	153.83	146.60
7.28	-1.21	12.05	21.37	11.36	1.69	2.00	10.27	3.23	-4.13	144.26	243.48	153.86	146.68
7.29	-1.21	12.06	21.39	11.37	1.70	2.00	10.26	3.22	-4.13	144.07	243.44	153.63	146.27
7.27	-1.22	12.03	21.36	11.34	1.68	2.00	10.26	3.23	-4.13	144.17	243.53	153.95	146.84

7.22	-1.26	11.96	21.30	11.28	1.64	1.96	10.22	3.19	-4.16	144.77	244.55	154.78	147.63
7.24	-1.25	11.99	21.33	11.31	1.65	1.97	10.23	3.20	-4.15	144.39	244.16	154.35	147.18
7.24	-1.25	11.98	21.32	11.30	1.65	1.98	10.24	3.21	-4.15	144.28	244.02	154.40	147.27
7.30	-1.21	12.07	21.43	11.34	1.70	2.05	10.31	3.25	-4.09	141.24	242.88	149.71	140.06
7.30	-1.19	12.17	21.53	11.44	1.76	2.06	10.27	3.19	-4.10	131.47	236.20	140.15	128.31
7.35	-1.14	12.22	21.61	11.49	1.77	2.09	10.30	3.22	-4.10	130.87	236.18	140.11	128.54
7.35	-1.20	11.92	21.38	11.33	1.66	2.18	10.43	3.36	-4.06	139.58	247.17	148.24	137.96
6.50	-1.84	10.70	20.00	10.29	0.90	1.40	9.53	2.70	-4.58	140.25	245.64	155.40	147.12
7.05	-1.52	11.58	20.91	10.99	1.32	1.88	10.08	3.09	-4.34	130.12	242.22	138.86	128.49
7.18	-1.39	11.86	21.24	11.21	1.52	1.94	10.18	3.15	-4.27	128.63	238.18	138.61	127.27
6.94	-1.46	11.45	20.74	10.84	1.34	1.78	9.98	3.01	-4.25	136.80	236.64	145.89	138.37
7.31	-1.24	12.12	21.50	11.41	1.69	2.07	10.29	3.22	-4.17	127.21	234.85	136.73	123.20
7.27	-1.25	12.07	21.45	11.36	1.68	2.03	10.27	3.21	-4.19	128.04	236.86	137.92	126.14
7.26	-1.34	11.99	21.34	11.34	1.58	2.03	10.24	3.16	-4.23	122.69	233.74	132.22	119.43
7.10	-1.36	11.88	21.18	11.22	1.55	1.84	10.02	2.99	-4.27	127.06	231.61	138.26	126.88
6.93	-1.65	11.37	20.66	10.85	1.16	1.71	9.92	2.99	-4.45	139.19	249.11	145.06	135.52
7.39	-1.45	12.23	21.76	11.67	1.69	2.02	10.37	3.13	-4.60	173.63	268.45	181.61	186.73
6.85	-1.68	11.27	20.54	10.78	1.14	1.62	9.82	2.90	-4.54	141.95	253.72	150.38	141.72
6.90	-1.63	11.35	20.64	10.83	1.17	1.70	9.90	2.98	-4.45	139.51	250.91	146.87	138.12
6.91	-1.62	11.35	20.65	10.83	1.18	1.71	9.92	2.99	-4.45	139.22	250.93	146.98	138.39
7.79	-1.00	12.48	22.04	11.88	1.88	2.62	10.80	3.72	-3.86	137.11	242.27	141.10	127.49
7.48	-1.16	12.15	21.61	11.56	1.72	2.28	10.48	3.42	-4.05	137.28	244.01	141.38	128.58
7.33	-1.31	11.97	21.35	11.38	1.57	2.12	10.31	3.29	-4.18	136.24	246.24	142.41	130.67
7.87	-0.94	12.59	22.18	11.95	1.98	2.67	10.90	3.78	-3.82	139.27	242.86	143.93	130.50
7.55	-1.08	12.17	21.80	11.66	1.88	2.28	10.57	3.44	-4.03	146.10	251.64	152.88	143.59
7.79	-0.93	12.64	22.34	12.10	2.09	2.38	10.64	3.51	-3.96	143.02	247.85	151.81	141.37
7.93	-0.84	12.82	22.50	12.23	2.17	2.53	10.77	3.61	-3.86	140.71	245.34	147.85	136.57
7.57	-1.07	12.16	21.69	11.61	1.81	2.38	10.66	3.53	-3.96	140.25	246.25	145.19	133.93
7.56	-1.07	12.16	21.79	11.62	1.86	2.31	10.60	3.50	-4.00	144.38	249.13	152.23	141.56
7.76	-0.94	12.44	22.00	11.77	1.97	2.55	10.79	3.73	-3.85	140.06	243.45	145.16	132.67
7.14	-1.37	11.70	21.07	11.04	1.52	1.96	10.22	3.22	-4.22	135.69	246.05	143.97	134.42
7.54	-1.08	12.16	21.80	11.65	1.88	2.27	10.56	3.43	-4.03	146.21	251.78	153.23	144.18
7.50	-1.09	12.12	21.59	11.45	1.76	2.35	10.58	3.54	-3.96	135.76	243.47	143.05	132.33
7.57	-1.06	12.21	21.87	11.70	1.91	2.25	10.58	3.43	-4.05	147.00	252.72	154.43	145.39
7.24	-1.38	11.87	21.21	11.27	1.52	2.01	10.21	3.22	-4.27	136.09	247.53	143.28	131.64
7.36	-1.17	12.02	21.45	11.42	1.73	2.17	10.44	3.32	-4.05	139.22	246.23	149.87	140.69
7.67	-0.98	12.44	21.93	11.73	1.95	2.42	10.69	3.61	-3.93	138.96	242.94	144.12	131.98
7.52	-1.05	12.21	21.62	11.46	1.80	2.37	10.59	3.57	-3.89	132.25	239.54	138.66	126.78
7.61	-1.05	12.25	21.91	11.72	1.90	2.29	10.58	3.46	-4.02	145.38	250.48	152.32	142.79
7.58	-1.06	12.22	21.83	11.68	1.89	2.30	10.62	3.47	-4.01	145.68	251.13	151.78	141.82
7.49	-1.09	12.10	21.58	11.44	1.75	2.34	10.57	3.53	-3.97	135.94	243.56	143.15	132.57
7.42	-1.16	12.02	21.49	11.42	1.72	2.23	10.51	3.44	-4.06	139.09	246.65	145.90	136.40
7.12	-1.34	11.78	21.20	11.16	1.70	1.70	10.14	3.07	-4.41	177.27	279.97	189.19	188.45

7.04	-1.41	11.51	20.98	11.03	1.62	1.73	10.13	3.05	-4.48	177.65	283.60	192.44	195.45
7.39	-1.15	12.17	21.64	11.57	1.91	1.97	10.35	3.23	-4.22	164.81	270.13	178.89	175.74
7.47	-1.00	12.30	21.63	11.49	1.93	2.17	10.51	3.46	-3.96	160.01	260.61	169.25	162.72
7.38	-1.06	12.23	21.60	11.46	1.98	1.99	10.37	3.26	-4.07	161.56	263.67	173.58	168.04
7.23	-1.13	11.95	21.34	11.25	1.82	1.91	10.30	3.22	-4.14	165.44	269.17	178.37	175.09
7.24	-1.12	11.96	21.35	11.26	1.82	1.93	10.32	3.24	-4.12	165.31	268.88	177.93	174.46
7.01	-1.63	11.20	20.69	10.90	1.10	1.79	9.99	3.08	-4.37	154.46	258.94	155.49	145.87
7.00	-1.64	11.20	20.68	10.90	1.09	1.79	9.99	3.08	-4.37	154.54	259.03	155.62	146.02
7.57	-1.21	11.92	21.65	11.51	1.61	2.56	10.76	3.62	-4.02	152.50	250.80	150.44	137.80
7.63	-1.11	12.11	21.63	11.62	1.66	2.44	10.66	3.63	-3.90	149.53	249.99	146.99	133.72
7.89	-0.95	12.71	22.41	12.02	2.09	2.64	11.01	3.77	-3.98	153.91	249.27	154.55	145.52
8.16	-0.70	13.15	22.83	12.50	2.31	2.73	10.98	3.81	-3.74	142.17	244.45	148.22	134.91
8.30	-0.63	13.43	23.05	12.64	2.48	2.86	11.19	3.95	-3.73	144.15	243.70	147.08	134.90
7.50	-1.32	11.98	21.54	11.33	1.50	2.60	10.89	3.66	-4.15	152.29	238.26	137.38	123.91
7.00	-1.79	11.44	21.03	10.92	1.13	2.00	10.30	3.06	-4.71	164.88	252.12	158.21	151.82
6.99	-1.80	11.42	21.01	10.91	1.12	1.99	10.29	3.06	-4.72	165.06	252.32	158.43	152.16
7.24	-1.62	11.82	21.44	11.24	1.33	2.27	10.56	3.24	-4.54	162.36	251.38	158.36	152.18
6.87	-1.85	11.14	20.74	10.71	1.05	1.98	10.23	3.04	-4.73	167.81	256.68	162.62	158.03
6.87	-1.85	11.14	20.73	10.71	1.05	1.98	10.22	3.04	-4.73	167.83	256.76	162.70	158.12
7.06	-1.69	11.45	20.99	10.97	1.22	2.16	10.41	3.18	-4.60	166.96	253.68	163.27	158.30
7.88	-0.99	12.74	22.26	11.92	1.92	2.80	11.13	3.84	-3.90	142.28	231.62	130.70	115.25
7.85	-1.01	12.68	22.22	11.88	1.89	2.77	11.10	3.82	-3.92	142.72	231.96	131.12	115.79
7.94	-0.94	12.86	22.40	12.00	2.00	2.85	11.19	3.87	-3.84	141.81	230.03	129.61	111.98
7.85	-1.00	12.51	22.02	11.71	1.82	2.90	11.19	3.97	-3.83	143.11	229.62	130.38	113.29
7.79	-1.05	12.40	21.90	11.62	1.76	2.87	11.14	3.94	-3.86	143.95	230.16	131.28	114.60
6.82	-1.86	11.08	20.64	10.68	1.03	1.93	10.18	2.99	-4.77	170.68	258.52	166.24	163.01
7.76	-0.92	12.10	21.63	11.30	1.81	3.11	11.28	4.20	-3.64	133.52	214.39	112.08	86.68
6.88	-1.81	11.28	20.74	10.78	1.16	1.83	10.13	3.00	-4.77	176.82	268.41	178.87	182.39
6.82	-1.83	11.17	20.64	10.68	1.12	1.80	10.09	2.96	-4.78	177.74	269.22	179.86	183.20
6.73	-1.97	10.78	20.27	10.46	0.88	1.79	10.10	2.99	-4.85	174.52	263.37	172.10	170.30
6.84	-1.85	11.04	20.59	10.64	1.01	1.98	10.23	3.05	-4.73	169.94	260.04	168.04	165.05
7.08	-1.66	11.46	21.04	10.93	1.26	2.18	10.47	3.22	-4.58	170.07	258.27	167.61	166.72
6.87	-1.88	10.99	20.48	10.63	0.98	1.87	10.18	3.08	-4.77	172.78	262.55	170.43	168.94
7.73	-1.15	12.35	21.85	11.65	1.68	2.78	11.06	3.82	-4.01	148.62	236.94	138.82	125.17
7.73	-1.15	12.35	21.85	11.65	1.68	2.78	11.06	3.82	-4.01	148.66	236.92	138.77	125.16
7.74	-1.19	12.41	22.04	11.72	1.69	2.71	11.00	3.76	-4.09	151.00	242.93	145.09	133.23
8.03	-0.96	12.76	22.36	11.99	1.82	3.09	11.34	4.07	-3.74	144.34	231.76	129.99	110.55
7.52	-1.35	12.00	21.71	11.48	1.45	2.50	10.77	3.56	-4.16	154.66	246.39	143.72	129.89
7.96	-1.03	12.68	22.29	11.92	1.76	3.00	11.27	4.00	-3.80	145.34	232.58	131.00	111.81
7.73	-1.18	12.30	21.96	11.70	1.62	2.72	10.98	3.76	-3.99	151.52	243.55	140.11	124.94
8.15	-0.80	12.78	22.29	12.03	1.93	3.31	11.52	4.25	-3.54	141.07	226.22	125.80	104.71
7.87	-1.15	12.55	22.22	11.94	1.68	2.81	11.06	3.78	-3.98	147.66	239.31	136.42	120.25
7.83	-1.17	12.53	22.22	11.92	1.69	2.77	11.02	3.73	-4.00	146.93	238.91	135.67	120.08

7.81	-1.21	12.50	22.19	11.89	1.66	2.73	10.98	3.70	-4.04	147.55	239.70	135.57	120.82
7.92	-1.12	12.65	22.30	12.00	1.70	2.90	11.16	3.85	-3.94	145.41	231.51	132.50	113.96
8.09	-0.88	12.71	22.27	11.99	1.87	3.21	11.42	4.16	-3.62	141.96	227.29	127.56	106.85
8.08	-0.89	12.76	22.35	12.00	1.86	3.19	11.44	4.16	-3.64	142.53	228.83	128.68	108.11
7.88	-1.09	12.56	22.18	11.82	1.68	2.93	11.21	3.94	-3.83	145.78	232.18	131.44	112.31
8.01	-0.88	12.77	22.38	12.02	1.96	3.08	11.34	4.02	-3.72	144.17	233.22	132.80	116.73
8.02	-0.87	12.78	22.38	12.03	1.96	3.10	11.35	4.03	-3.71	143.98	233.05	132.58	116.38
8.02	-0.87	12.78	22.38	12.03	1.97	3.10	11.36	4.03	-3.71	143.94	233.01	132.55	116.32
8.33	-0.54	13.46	22.93	12.48	2.40	3.22	11.56	4.19	-3.46	134.34	220.95	120.45	98.61
8.18	-0.74	13.24	22.76	12.32	2.20	3.00	11.35	4.02	-3.69	138.96	228.23	127.44	109.23
8.17	-0.75	13.22	22.73	12.30	2.18	3.01	11.36	4.03	-3.70	139.01	228.44	127.67	109.48
8.21	-0.71	13.27	22.73	12.27	2.23	3.05	11.40	4.12	-3.62	136.23	218.19	118.70	95.11
8.34	-0.56	13.42	22.93	12.50	2.35	3.18	11.51	4.16	-3.47	134.87	220.49	120.03	99.38
8.09	-0.81	12.90	22.30	12.08	2.06	3.06	11.27	4.07	-3.68	129.04	206.09	104.06	75.96
8.06	-0.76	12.82	22.21	11.94	2.13	3.16	11.40	4.17	-3.61	131.76	208.77	107.49	80.87
8.03	-0.77	12.70	22.12	11.88	2.03	3.18	11.40	4.20	-3.59	133.97	213.11	111.21	85.37
8.02	-0.77	12.62	22.02	11.87	2.06	3.20	11.37	4.18	-3.59	130.64	209.43	107.71	80.75
8.10	-0.73	12.78	22.22	11.94	2.11	3.25	11.48	4.23	-3.54	132.68	210.78	111.02	83.75
8.09	-0.68	12.79	22.15	11.96	2.15	3.27	11.46	4.27	-3.52	129.56	208.99	106.57	80.12
7.85	-0.97	12.45	21.90	11.68	1.86	2.97	11.20	4.01	-3.79	135.50	214.01	111.95	86.45
8.42	-0.41	13.12	22.44	12.28	2.39	3.61	11.80	4.56	-3.22	128.40	206.79	103.87	76.72
7.81	-1.02	12.18	21.64	11.48	1.68	3.08	11.27	4.08	-3.72	135.62	217.00	116.80	91.55
8.04	-0.84	12.81	22.21	11.99	2.02	3.04	11.24	4.04	-3.72	129.55	206.49	104.41	76.80
7.91	-1.04	12.62	22.29	12.03	1.83	2.81	11.08	3.75	-3.91	149.40	240.27	139.30	124.25
7.88	-1.07	12.60	22.30	12.03	1.81	2.78	11.04	3.73	-3.93	149.47	240.75	139.97	125.17
8.17	-0.74	12.83	22.52	12.24	2.12	3.15	11.35	4.12	-3.60	142.81	237.89	140.08	124.60
7.89	-1.05	12.65	22.34	12.08	1.84	2.76	11.01	3.72	-3.92	149.29	242.62	140.68	126.48
7.77	-1.18	12.45	22.13	11.88	1.66	2.70	10.94	3.67	-4.03	151.91	244.45	140.60	125.78
7.77	-1.18	12.44	22.12	11.87	1.66	2.70	10.93	3.67	-4.03	152.03	244.54	140.70	125.85
8.12	-0.76	12.71	22.37	12.10	2.10	3.09	11.31	4.13	-3.60	147.10	239.38	141.64	127.38
8.13	-0.73	12.75	22.43	12.14	2.11	3.11	11.31	4.13	-3.58	146.51	238.27	141.53	126.97
8.22	-0.78	13.02	22.67	12.38	2.09	3.19	11.40	4.08	-3.64	144.36	235.47	134.91	118.53
7.96	-0.99	12.74	22.39	12.14	1.90	2.80	11.08	3.78	-3.87	146.72	242.53	140.81	126.06
7.99	-0.97	12.77	22.42	12.15	1.92	2.83	11.11	3.81	-3.84	146.31	241.98	140.31	125.42
7.94	-1.03	12.68	22.37	12.09	1.84	2.79	11.03	3.75	-3.92	149.48	242.16	140.37	125.61
7.94	-1.03	12.69	22.38	12.09	1.84	2.79	11.04	3.75	-3.92	149.47	242.09	140.34	125.56
7.86	-1.09	12.56	22.26	12.00	1.79	2.76	11.02	3.72	-3.95	149.48	240.82	140.26	125.28
7.86	-1.09	12.56	22.26	12.00	1.79	2.76	11.02	3.72	-3.95	149.49	240.80	140.25	125.28
7.86	-1.09	12.56	22.26	12.00	1.79	2.76	11.02	3.72	-3.95	149.50	240.78	140.23	125.27
7.86	-1.09	12.56	22.26	12.00	1.79	2.76	11.02	3.72	-3.95	149.51	240.75	140.21	125.27
7.87	-1.08	12.57	22.28	12.01	1.80	2.77	11.03	3.72	-3.94	149.51	240.89	140.18	125.34
8.10	-0.82	12.70	22.41	12.15	2.02	3.04	11.23	4.02	-3.68	146.83	241.95	140.95	125.80
7.87	-1.05	12.57	22.29	12.04	1.82	2.72	10.95	3.75	-3.93	149.28	247.22	143.47	128.98

7.86	-1.06	12.55	22.28	12.02	1.81	2.72	10.94	3.75	-3.94	149.57	247.44	143.66	129.22
7.86	-1.06	12.55	22.28	12.02	1.81	2.72	10.94	3.74	-3.94	149.57	247.45	143.67	129.22
7.86	-1.06	12.55	22.28	12.02	1.81	2.72	10.94	3.75	-3.94	149.56	247.41	143.63	129.20
8.12	-0.77	12.77	22.45	12.17	2.09	3.10	11.30	4.08	-3.64	143.95	238.73	141.31	126.04
8.14	-0.74	12.74	22.40	12.12	2.11	3.11	11.32	4.15	-3.58	146.67	239.11	141.41	126.99
8.14	-0.74	12.74	22.40	12.12	2.11	3.11	11.33	4.15	-3.58	146.64	239.04	141.36	126.91
8.14	-0.74	12.74	22.41	12.12	2.11	3.11	11.33	4.15	-3.58	146.56	239.05	141.35	126.90
7.75	-1.18	12.43	22.12	11.87	1.66	2.68	10.92	3.66	-4.04	151.89	244.99	141.00	126.67
7.73	-1.19	12.39	22.09	11.84	1.65	2.66	10.91	3.65	-4.05	152.65	245.38	141.30	127.14
7.82	-1.16	12.44	22.10	11.84	1.68	2.79	11.04	3.77	-3.99	150.41	240.14	138.75	123.03
8.39	-0.60	13.13	22.83	12.47	2.26	3.34	11.53	4.31	-3.45	141.05	233.50	136.46	120.66
8.21	-0.67	12.87	22.56	12.26	2.17	3.18	11.38	4.20	-3.53	144.95	237.24	140.26	125.12
8.20	-0.80	12.99	22.65	12.34	2.08	3.14	11.37	4.05	-3.66	145.77	235.79	135.26	118.91
8.11	-0.88	12.93	22.55	12.19	2.01	3.04	11.28	4.03	-3.77	144.49	234.88	139.39	123.24
8.12	-0.87	12.94	22.56	12.20	2.02	3.06	11.30	4.05	-3.75	143.90	234.90	138.98	122.85
8.23	-0.74	13.02	22.62	12.29	2.14	3.18	11.42	4.15	-3.62	142.82	233.91	136.39	120.54
7.96	-0.95	12.67	22.33	12.03	1.93	2.88	11.16	3.90	-3.85	146.83	239.87	143.00	128.14
7.96	-0.95	12.67	22.33	12.04	1.93	2.88	11.16	3.90	-3.85	146.81	239.87	142.92	127.95
8.06	-0.83	12.80	22.40	12.06	1.99	3.16	11.41	4.08	-3.66	143.18	232.47	132.04	115.18
8.19	-0.64	12.98	22.31	12.05	2.21	3.29	11.55	4.30	-3.49	131.61	208.18	107.72	81.04
8.08	-0.85	13.02	22.52	12.16	2.09	2.93	11.28	3.99	-3.77	142.20	228.91	130.79	112.60
7.42	-1.42	11.84	21.55	11.34	1.38	2.40	10.67	3.47	-4.22	156.67	249.12	146.12	133.19
8.16	-0.86	12.83	22.31	12.11	1.87	3.23	11.51	4.23	-3.58	144.30	231.30	128.92	108.83
8.22	-0.86	13.02	22.48	12.27	1.95	3.16	11.46	4.16	-3.66	146.63	235.90	131.69	112.80
8.22	-0.76	13.03	22.65	12.30	2.05	3.18	11.47	4.15	-3.62	144.34	233.25	131.26	111.21
8.11	-0.84	12.84	22.41	12.19	2.03	3.01	11.26	4.02	-3.67	148.53	242.20	137.05	120.02
8.06	-0.89	12.85	22.37	12.17	1.99	2.91	11.21	3.94	-3.76	151.75	243.77	137.38	121.51
8.13	-0.81	12.93	22.47	12.24	2.06	3.04	11.28	4.03	-3.66	148.81	240.22	135.80	118.85
8.09	-0.81	12.57	22.06	11.86	1.89	3.30	11.52	4.30	-3.50	135.49	219.92	120.68	92.96
8.25	-0.75	13.00	22.55	12.28	2.04	3.24	11.50	4.21	-3.56	144.29	231.82	130.13	110.14
7.92	-1.05	12.56	22.20	11.91	1.76	2.90	11.14	3.90	-3.89	150.40	240.47	137.95	120.66
7.65	-1.20	12.14	21.80	11.60	1.58	2.64	10.89	3.70	-4.01	151.06	246.25	141.43	128.71
7.66	-1.22	12.00	21.46	11.32	1.43	2.94	11.17	4.01	-3.87	144.75	229.34	128.32	107.93
8.24	-0.75	12.88	22.43	12.16	2.00	3.33	11.59	4.32	-3.47	142.15	229.75	128.97	107.36
8.32	-0.69	13.07	22.63	12.32	2.12	3.38	11.61	4.31	-3.47	142.43	229.23	128.94	107.94
8.32	-0.69	13.06	22.63	12.32	2.12	3.37	11.61	4.31	-3.47	142.47	229.22	128.98	107.99
8.33	-0.68	13.09	22.66	12.34	2.13	3.37	11.62	4.31	-3.47	142.65	229.03	128.89	107.90
8.32	-0.68	13.10	22.69	12.32	2.14	3.37	11.64	4.31	-3.47	142.61	230.35	128.15	107.67
8.08	-0.92	12.95	22.39	12.17	1.93	2.96	11.27	3.99	-3.77	152.46	243.00	138.77	121.75
8.12	-0.93	12.80	22.26	12.08	1.86	3.11	11.40	4.14	-3.68	150.88	240.10	137.32	119.17
8.29	-0.77	13.10	22.56	12.35	2.02	3.24	11.53	4.23	-3.58	145.41	235.52	131.03	111.65
8.29	-0.77	13.10	22.56	12.35	2.02	3.24	11.53	4.23	-3.58	145.35	235.50	130.99	111.60
8.16	-0.94	12.95	22.40	12.19	1.88	3.09	11.39	4.12	-3.73	147.73	235.80	132.17	113.15

8.13	-0.94	12.89	22.35	12.14	1.85	3.11	11.41	4.12	-3.71	146.81	234.92	131.34	111.99
8.13	-0.94	12.89	22.35	12.15	1.85	3.11	11.42	4.13	-3.71	146.76	234.86	131.30	111.91
8.13	-0.93	12.89	22.35	12.15	1.85	3.12	11.42	4.13	-3.71	146.73	234.81	131.28	111.85
8.13	-0.94	12.88	22.34	12.14	1.84	3.11	11.41	4.12	-3.72	146.87	234.87	131.41	111.97
8.32	-0.72	13.07	22.56	12.36	2.05	3.30	11.56	4.28	-3.51	144.55	232.55	130.09	109.82
8.24	-0.75	13.12	22.66	12.41	2.10	3.09	11.38	4.08	-3.62	148.03	236.53	133.69	115.39
8.30	-0.72	13.15	22.72	12.43	2.14	3.21	11.49	4.17	-3.54	146.16	234.60	131.48	112.99
8.28	-0.73	13.14	22.70	12.41	2.13	3.19	11.47	4.16	-3.56	146.52	235.02	131.83	113.35
8.08	-0.87	12.88	22.41	12.20	2.01	2.95	11.24	3.97	-3.73	150.82	242.43	136.80	120.63
8.10	-0.85	12.89	22.43	12.22	2.03	2.98	11.25	4.00	-3.70	149.96	241.77	136.35	120.17
8.09	-0.86	12.89	22.42	12.20	2.02	2.95	11.24	3.97	-3.72	150.61	242.05	136.79	120.32
8.12	-0.82	12.92	22.46	12.23	2.05	3.02	11.27	4.02	-3.67	149.07	240.43	136.01	119.03
8.13	-0.81	12.93	22.47	12.24	2.06	3.04	11.28	4.03	-3.66	148.84	240.19	135.83	118.82
8.21	-0.82	13.04	22.69	12.27	2.00	3.19	11.47	4.15	-3.64	144.58	233.69	129.87	111.24
8.17	-0.87	12.90	22.52	12.23	1.94	3.14	11.39	4.12	-3.68	145.50	236.20	132.37	114.27
8.21	-0.84	12.95	22.57	12.28	1.98	3.17	11.42	4.14	-3.65	145.27	235.96	132.17	113.88
8.21	-0.84	12.95	22.57	12.28	1.97	3.17	11.42	4.14	-3.65	145.24	235.84	132.08	113.74
8.09	-0.94	12.81	22.45	12.15	1.86	3.07	11.33	4.05	-3.75	146.42	236.06	132.73	114.75
7.90	-1.10	12.57	22.24	11.93	1.69	2.89	11.16	3.89	-3.91	148.30	238.09	134.73	117.46
8.56	-0.33	13.33	22.75	12.45	2.42	3.62	11.91	4.66	-3.09	134.03	219.42	121.10	94.07
8.93	0.08	13.92	23.36	12.93	2.90	3.90	12.25	4.92	-2.76	126.64	213.00	112.06	80.78
8.41	-0.48	12.97	22.50	12.24	2.23	3.57	11.83	4.61	-3.18	133.77	218.84	118.57	90.34
8.40	-0.48	12.95	22.40	12.16	2.22	3.64	11.87	4.64	-3.16	135.28	219.77	120.49	93.38
8.11	-0.80	12.60	22.11	11.91	1.93	3.29	11.49	4.31	-3.51	134.88	216.91	117.08	89.50
8.32	-0.60	12.95	22.41	12.16	2.15	3.42	11.67	4.44	-3.34	131.10	214.28	114.95	85.26
8.37	-0.51	12.88	22.30	12.09	2.17	3.56	11.84	4.63	-3.20	137.39	221.68	122.31	96.52
7.79	-1.03	12.55	21.99	11.72	1.81	2.82	11.08	3.85	-3.85	132.31	212.13	110.62	83.13
7.89	-0.92	12.43	21.88	11.63	1.80	3.09	11.29	4.12	-3.66	132.72	215.10	113.17	86.63
7.89	-0.93	12.50	21.95	11.71	1.79	3.08	11.28	4.09	-3.64	133.42	214.68	113.27	86.78
7.88	-0.97	12.40	21.85	11.65	1.75	3.10	11.28	4.10	-3.69	134.91	215.71	114.48	89.42
7.83	-0.94	12.32	21.77	11.53	1.82	3.11	11.33	4.16	-3.65	132.98	214.46	111.89	84.21
7.85	-1.12	12.54	22.04	11.75	1.64	2.88	11.14	3.94	-3.89	138.92	220.17	120.70	95.26
7.85	-1.12	12.54	22.04	11.75	1.64	2.88	11.14	3.94	-3.88	138.85	220.16	120.68	95.21
7.80	-1.05	12.59	22.02	11.75	1.81	2.79	11.05	3.83	-3.88	132.14	211.89	110.01	83.08
7.92	-0.92	12.57	22.01	11.75	1.79	3.07	11.30	4.07	-3.66	134.31	215.05	114.48	87.56
8.51	-0.36	13.19	22.64	12.35	2.41	3.68	11.93	4.65	-3.13	129.59	211.92	111.70	81.02
7.74	-1.06	12.08	21.80	11.70	1.69	2.70	10.89	3.78	-3.85	148.21	249.96	146.28	132.93
8.02	-0.85	12.56	22.25	12.07	1.97	2.95	11.12	3.99	-3.67	145.28	244.06	141.61	126.54
7.52	-1.35	11.93	21.60	11.44	1.46	2.52	10.78	3.59	-4.14	155.57	249.27	145.01	132.27
7.63	-1.26	12.16	21.89	11.69	1.59	2.60	10.85	3.60	-4.09	152.91	248.81	144.05	130.85
8.02	-0.89	12.54	22.23	12.05	1.93	2.95	11.12	3.99	-3.70	145.70	243.45	142.36	126.74
8.01	-0.90	12.52	22.21	12.04	1.92	2.94	11.11	3.98	-3.71	145.93	243.73	142.52	126.95
7.94	-0.94	12.43	22.12	11.97	1.87	2.87	11.05	3.92	-3.75	147.49	245.30	143.48	128.35

7.35	-1.41	11.64	21.38	11.30	1.36	2.30	10.53	3.42	-4.20	157.46	256.26	153.41	142.63
7.50	-1.24	12.00	21.57	11.54	1.57	2.27	10.52	3.45	-4.02	152.54	254.73	151.28	137.85
7.84	-1.00	12.27	21.92	11.82	1.78	2.76	10.95	3.84	-3.78	146.28	247.39	145.29	130.09
7.61	-1.16	11.89	21.61	11.54	1.60	2.56	10.76	3.67	-3.94	150.15	252.29	148.82	136.42
7.60	-1.17	11.89	21.60	11.53	1.59	2.56	10.75	3.66	-3.95	150.40	252.54	148.85	136.72
7.83	-1.05	12.24	21.94	11.84	1.75	2.76	10.93	3.84	-3.85	149.27	249.22	144.73	131.37
7.59	-1.19	11.87	21.58	11.52	1.58	2.54	10.74	3.65	-3.96	150.75	252.94	149.05	137.20
7.75	-1.05	12.09	21.80	11.70	1.70	2.71	10.89	3.79	-3.84	148.15	249.88	146.20	132.81
7.62	-1.16	12.17	21.75	11.69	1.65	2.42	10.65	3.55	-3.95	150.37	252.08	149.11	134.95
7.69	-1.09	12.20	21.78	11.73	1.70	2.52	10.73	3.67	-3.89	149.27	250.92	147.22	133.69
7.66	-1.12	12.14	21.73	11.68	1.68	2.49	10.71	3.64	-3.91	149.42	251.41	147.92	134.50
7.66	-1.12	12.14	21.73	11.68	1.68	2.49	10.71	3.64	-3.91	149.42	251.41	147.92	134.48
7.65	-1.12	12.13	21.72	11.67	1.68	2.49	10.71	3.64	-3.91	149.42	251.42	147.97	134.52
7.65	-1.12	12.13	21.71	11.67	1.68	2.48	10.71	3.63	-3.91	149.42	251.41	148.00	134.53
7.65	-1.12	12.12	21.71	11.66	1.68	2.48	10.71	3.63	-3.91	149.39	251.37	148.01	134.53
7.74	-1.11	12.08	21.78	11.70	1.67	2.68	10.84	3.77	-3.90	150.88	251.18	146.11	133.88
7.73	-1.12	12.07	21.77	11.69	1.67	2.67	10.84	3.76	-3.90	150.98	251.36	146.20	134.06
7.77	-1.09	12.13	21.83	11.75	1.70	2.71	10.87	3.80	-3.88	150.26	250.55	145.64	133.08
7.77	-1.09	12.12	21.82	11.74	1.69	2.71	10.87	3.79	-3.88	150.33	250.61	145.70	133.15
7.77	-1.09	12.13	21.83	11.75	1.70	2.72	10.88	3.80	-3.88	150.21	250.50	145.60	133.00
7.78	-1.09	12.14	21.84	11.76	1.70	2.72	10.88	3.80	-3.88	150.10	250.41	145.52	132.88
7.77	-1.09	12.13	21.83	11.75	1.70	2.71	10.87	3.79	-3.88	150.21	250.60	145.57	133.09
7.57	-1.22	11.86	21.54	11.51	1.56	2.51	10.72	3.63	-4.01	153.06	254.20	149.36	137.49
7.81	-1.07	12.18	21.89	11.79	1.73	2.74	10.90	3.82	-3.86	149.60	250.09	145.11	132.33
7.63	-1.21	11.97	21.65	11.58	1.56	2.59	10.79	3.69	-3.99	153.88	253.12	149.18	135.26
7.62	-1.22	11.96	21.64	11.56	1.55	2.58	10.78	3.67	-4.00	154.01	253.28	149.35	135.61
8.01	-0.90	12.52	22.23	12.04	1.91	2.95	11.12	3.98	-3.71	145.87	243.39	142.56	127.20
7.96	-0.93	12.46	22.15	11.98	1.88	2.88	11.05	3.94	-3.75	147.07	245.96	143.27	128.10
7.90	-0.98	12.37	22.07	11.93	1.84	2.84	11.02	3.89	-3.78	148.42	246.58	143.98	129.20
7.93	-0.95	12.41	22.11	11.96	1.86	2.86	11.04	3.92	-3.76	147.77	245.83	143.60	128.54
7.93	-0.95	12.42	22.11	11.97	1.87	2.87	11.05	3.92	-3.75	147.66	245.69	143.53	128.44
7.90	-0.98	12.36	22.07	11.93	1.83	2.83	11.01	3.89	-3.78	148.43	246.91	144.00	129.33
7.98	-0.92	12.48	22.17	12.01	1.90	2.91	11.08	3.95	-3.72	146.58	244.45	142.91	127.51
7.99	-0.91	12.50	22.18	12.02	1.91	2.92	11.09	3.96	-3.72	146.34	244.19	142.76	127.29
8.01	-0.90	12.52	22.21	12.04	1.92	2.94	11.11	3.98	-3.71	145.92	243.68	142.51	126.96
7.64	-1.16	11.96	21.66	11.58	1.63	2.58	10.77	3.67	-3.95	150.67	252.58	147.80	136.02
7.86	-0.98	12.26	21.95	11.83	1.80	2.82	10.99	3.87	-3.74	145.37	247.89	144.62	129.11
7.48	-1.35	11.78	21.46	11.36	1.41	2.51	10.74	3.60	-4.12	154.45	252.51	148.20	134.95
7.23	-1.51	11.63	21.06	11.23	1.22	1.92	10.15	3.21	-4.25	155.23	260.11	155.26	143.44
7.21	-1.52	11.53	20.92	11.15	1.21	1.95	10.18	3.26	-4.23	152.85	257.63	153.83	141.63
7.55	-1.21	12.08	21.65	11.62	1.60	2.33	10.57	3.49	-3.99	151.43	253.61	150.29	136.57
7.77	-1.02	12.17	21.70	11.66	1.73	2.66	10.91	3.85	-3.79	153.15	253.12	148.00	134.82
7.23	-1.45	11.55	21.09	11.18	1.33	1.98	10.28	3.25	-4.21	155.52	256.96	154.73	145.01

7.31	-1.41	11.66	21.21	11.25	1.37	2.05	10.33	3.31	-4.17	154.03	255.77	152.98	142.26
7.21	-1.50	11.66	21.09	11.25	1.21	1.90	10.13	3.20	-4.26	156.33	260.92	155.35	143.46
7.23	-1.53	11.63	21.05	11.22	1.21	1.91	10.14	3.21	-4.26	154.91	260.15	155.22	143.76
7.11	-1.56	11.39	20.84	11.05	1.18	1.88	10.07	3.16	-4.29	151.69	257.21	153.53	141.67
7.14	-1.53	11.46	20.91	11.12	1.21	1.92	10.10	3.17	-4.26	151.50	257.04	153.04	141.14
7.25	-1.46	11.66	21.11	11.28	1.29	1.93	10.15	3.19	-4.24	152.06	257.28	152.18	141.40
7.27	-1.44	11.69	21.14	11.31	1.31	1.95	10.18	3.21	-4.21	152.03	256.91	151.16	141.16
7.23	-1.52	11.64	21.06	11.24	1.22	1.91	10.14	3.21	-4.26	154.99	260.16	155.16	143.62
8.14	-0.80	12.99	22.40	12.18	2.03	3.03	11.38	4.14	-3.62	153.29	244.06	141.46	123.71
7.90	-0.90	12.33	21.84	11.79	1.81	2.85	11.08	4.01	-3.65	151.05	250.48	145.01	130.96
7.93	-0.88	12.44	21.95	11.87	1.86	2.82	11.07	3.99	-3.66	150.54	250.10	145.48	131.43
8.10	-0.80	12.68	22.26	12.14	2.01	2.93	11.20	4.00	-3.63	150.05	245.06	141.86	126.40
7.46	-1.31	11.73	21.32	11.34	1.45	2.46	10.72	3.59	-4.06	158.48	255.54	150.71	138.20
8.14	-0.78	12.66	22.07	11.92	1.91	3.27	11.55	4.33	-3.49	153.17	242.33	140.64	124.05
8.69	-0.30	13.36	22.79	12.53	2.43	3.79	12.06	4.81	-3.04	145.17	235.86	134.98	114.98
8.23	-0.74	12.94	22.35	12.17	2.03	3.30	11.58	4.33	-3.51	151.63	240.77	138.37	120.05
8.28	-0.64	12.75	22.20	11.96	2.03	3.50	11.84	4.61	-3.32	147.89	239.22	136.81	118.08
8.08	-0.85	12.43	21.87	11.74	1.79	3.29	11.59	4.39	-3.50	151.73	241.69	138.48	121.19
8.11	-0.85	12.47	21.91	11.81	1.79	3.35	11.61	4.41	-3.49	150.69	239.76	136.92	119.55
8.20	-0.77	12.65	22.02	11.86	1.86	3.44	11.73	4.53	-3.42	146.59	233.01	131.62	112.21
7.87	-1.00	12.47	22.08	11.86	1.82	2.83	11.08	3.91	-3.82	152.35	245.83	140.95	126.68
7.68	-1.15	12.14	21.72	11.63	1.65	2.62	10.93	3.75	-3.94	155.05	250.06	144.80	132.24
7.68	-1.17	12.11	21.68	11.61	1.61	2.62	10.92	3.75	-3.98	155.85	250.62	145.38	131.85
7.70	-1.19	12.24	21.90	11.66	1.61	2.70	10.95	3.74	-3.98	151.98	243.97	141.40	126.29
7.51	-1.33	11.90	21.56	11.40	1.44	2.54	10.77	3.60	-4.13	156.69	250.54	145.96	133.01
7.20	-1.60	11.40	21.09	11.06	1.18	2.18	10.47	3.33	-4.35	161.73	259.02	153.23	142.53
7.25	-1.55	11.53	21.22	11.14	1.22	2.22	10.52	3.38	-4.35	161.72	257.72	152.06	140.28
8.86	-0.16	13.74	23.12	12.84	2.62	3.85	12.15	4.88	-2.98	143.27	233.43	133.82	114.08
7.77	-1.01	12.08	21.63	11.61	1.69	2.82	11.03	3.96	-3.73	153.77	249.57	145.86	133.07
8.25	-0.67	13.20	22.64	12.39	2.26	2.99	11.38	4.11	-3.60	152.86	245.03	141.96	125.89
7.66	-1.18	12.10	21.65	11.60	1.61	2.62	10.93	3.74	-3.97	155.97	251.01	145.71	132.15
7.57	-1.25	11.94	21.57	11.50	1.52	2.53	10.75	3.63	-4.00	155.31	254.75	149.26	135.96
7.44	-1.33	11.70	21.32	11.31	1.40	2.46	10.69	3.57	-4.09	159.31	255.19	150.41	138.55
7.46	-1.33	11.73	21.35	11.34	1.41	2.48	10.70	3.58	-4.08	158.91	254.45	149.83	137.97
7.45	-1.31	11.71	21.32	11.32	1.43	2.45	10.70	3.57	-4.07	158.73	255.70	151.00	138.66
7.19	-1.59	11.40	21.07	11.05	1.18	2.17	10.46	3.32	-4.35	162.41	259.13	153.31	142.80
7.49	-1.22	11.58	21.15	11.18	1.46	2.58	10.79	3.78	-3.91	156.94	255.58	150.83	138.86
7.66	-1.18	12.01	21.68	11.61	1.59	2.63	10.82	3.72	-3.96	153.66	252.78	148.78	134.52
8.04	-0.84	12.69	22.20	12.08	2.00	2.89	11.19	3.98	-3.69	153.68	248.60	143.97	127.35
8.42	-0.57	13.24	22.60	12.38	2.23	3.41	11.72	4.47	-3.36	149.82	240.28	136.51	119.03
7.77	-1.07	12.27	21.80	11.74	1.74	2.68	11.02	3.79	-3.88	156.49	250.70	147.12	132.45
7.73	-1.09	12.24	21.77	11.68	1.71	2.69	11.03	3.82	-3.88	155.18	249.81	145.26	131.87
7.74	-1.08	12.26	21.78	11.69	1.72	2.70	11.04	3.83	-3.87	155.17	249.62	145.15	131.64

8.13	-0.85	12.94	22.42	12.20	2.02	2.96	11.28	4.05	-3.72	152.92	243.94	140.45	123.79
7.69	-1.15	12.14	21.65	11.62	1.66	2.66	10.99	3.77	-3.92	155.60	250.80	146.39	132.48
7.89	-1.00	12.51	22.10	11.88	1.83	2.82	11.09	3.91	-3.81	152.47	245.67	140.82	126.54
7.92	-0.98	12.55	22.15	11.91	1.86	2.85	11.12	3.94	-3.79	152.07	245.18	140.36	125.93
7.69	-1.14	12.15	21.73	11.64	1.65	2.64	10.94	3.76	-3.94	155.01	249.87	144.57	131.79
7.68	-1.17	12.11	21.69	11.62	1.62	2.63	10.92	3.75	-3.97	155.79	250.59	145.33	131.80
7.53	-1.31	11.89	21.52	11.45	1.49	2.47	10.78	3.63	-4.09	158.70	252.30	147.74	134.35
8.06	-0.86	12.66	22.31	12.06	1.95	2.99	11.22	4.03	-3.69	150.25	243.25	137.88	121.87
8.06	-0.88	12.81	22.35	12.14	1.99	2.94	11.22	3.98	-3.73	149.88	244.06	137.72	121.18
7.47	-1.30	11.75	21.35	11.36	1.45	2.47	10.72	3.59	-4.05	158.14	255.43	150.68	138.12
7.48	-1.28	11.77	21.38	11.38	1.46	2.47	10.72	3.59	-4.05	157.71	255.39	150.75	137.91
7.51	-1.28	11.83	21.46	11.42	1.47	2.50	10.73	3.62	-4.03	156.45	256.08	149.93	136.51
7.51	-1.28	11.83	21.46	11.42	1.47	2.50	10.73	3.62	-4.03	156.51	256.09	149.96	136.52
7.45	-1.33	11.72	21.35	11.33	1.41	2.47	10.70	3.58	-4.08	159.00	254.44	149.86	138.06
7.46	-1.33	11.74	21.37	11.35	1.41	2.48	10.70	3.59	-4.08	158.72	254.09	149.60	137.73
7.43	-1.42	11.74	21.44	11.32	1.35	2.43	10.67	3.54	-4.19	157.56	253.57	149.02	136.59
7.27	-1.50	11.57	21.23	11.15	1.29	2.23	10.54	3.38	-4.30	161.77	255.62	151.50	139.81
7.30	-1.49	11.61	21.28	11.16	1.30	2.29	10.56	3.43	-4.28	159.22	253.12	148.88	137.44
7.35	-1.45	11.68	21.34	11.21	1.34	2.35	10.61	3.47	-4.25	158.29	252.31	148.26	136.30
7.33	-1.48	11.64	21.31	11.19	1.32	2.33	10.60	3.46	-4.26	158.88	253.05	148.56	136.92
7.37	-1.43	11.77	21.45	11.29	1.36	2.38	10.66	3.50	-4.22	155.26	250.29	145.40	134.00
7.41	-1.43	11.82	21.50	11.33	1.40	2.41	10.68	3.50	-4.21	157.14	249.85	146.61	133.83
7.20	-1.53	11.40	21.07	11.05	1.21	2.21	10.49	3.35	-4.30	162.39	258.74	153.50	143.09
8.01	-0.98	12.54	22.00	11.88	1.75	3.07	11.37	4.14	-3.71	152.29	241.46	139.08	121.82
8.11	-0.92	12.87	22.34	12.17	1.89	3.06	11.34	4.07	-3.72	153.47	240.63	138.69	120.74
8.08	-0.95	12.83	22.30	12.13	1.86	3.03	11.31	4.05	-3.74	153.86	240.85	138.97	121.15
7.81	-1.20	12.39	21.85	11.68	1.56	2.84	11.18	3.94	-3.94	151.16	238.67	136.58	118.32
8.04	-1.04	12.78	22.23	12.03	1.76	2.99	11.31	4.04	-3.81	149.11	235.70	133.93	114.34
8.22	-0.86	13.10	22.53	12.30	1.96	3.14	11.45	4.15	-3.67	146.98	235.57	133.63	114.23
8.65	-0.34	13.46	22.84	12.59	2.44	3.66	11.96	4.71	-3.13	146.70	236.59	136.35	117.52
8.52	-0.45	13.23	22.54	12.33	2.30	3.64	11.94	4.73	-3.16	141.93	227.01	128.32	105.98
8.59	-0.39	13.33	22.65	12.39	2.36	3.68	11.96	4.77	-3.11	141.43	225.86	127.78	104.93
8.68	-0.28	13.47	22.86	12.54	2.46	3.76	12.05	4.84	-3.03	140.12	224.97	127.09	103.21
7.49	-1.38	11.88	21.53	11.42	1.40	2.48	10.73	3.58	-4.17	157.31	252.14	147.83	134.25
7.72	-1.15	12.23	21.88	11.68	1.64	2.71	10.95	3.75	-3.96	150.56	245.42	140.66	127.39
8.07	-0.86	12.81	22.36	12.13	1.98	2.96	11.24	4.02	-3.70	150.25	244.10	138.21	121.56
7.98	-0.74	12.60	22.14	11.83	1.99	3.18	11.28	4.16	-3.45	130.94	211.94	105.65	76.33
8.10	-1.11	12.88	22.91	11.94	1.70	3.32	11.65	4.25	-3.91	129.79	220.26	116.25	80.13
8.50	-0.22	12.78	22.05	12.11	2.32	3.55	11.70	4.85	-2.76	149.22	256.98	140.99	108.42
8.61	-0.13	12.92	22.33	12.23	2.35	3.86	11.87	5.02	-2.59	134.83	231.30	118.45	85.01
7.96	-0.78	12.64	22.09	11.79	2.01	3.14	11.40	4.14	-3.60	135.10	215.64	115.16	91.23
8.67	-0.16	12.97	22.26	12.28	2.35	3.93	12.14	5.10	-2.68	142.71	243.99	139.74	107.98
8.03	-0.79	12.07	21.39	11.41	1.60	3.32	11.64	4.63	-3.21	153.38	253.02	161.42	133.69

8.41	-0.42	13.25	22.80	12.27	2.44	3.61	11.92	4.53	-3.28	141.64	223.08	122.57	101.78
8.17	-0.65	12.33	21.64	11.70	1.85	3.38	11.61	4.68	-3.17	144.24	244.66	144.12	113.61
8.08	-0.78	12.65	22.31	12.04	2.06	3.05	11.27	4.10	-3.62	148.16	239.99	142.21	128.45
8.44	-0.23	12.42	21.74	11.83	2.20	3.83	11.90	5.03	-2.67	134.35	232.34	119.66	86.19
8.35	-0.59	12.88	22.31	12.11	2.11	3.50	11.82	4.58	-3.30	149.08	240.66	138.36	119.81
8.29	-0.48	12.98	22.45	12.13	2.20	3.44	11.55	4.47	-3.16	131.20	210.73	104.01	74.10
7.73	-1.23	11.86	21.07	11.11	1.25	2.89	11.22	4.31	-3.68	147.42	243.30	147.19	118.30
8.27	-0.63	13.15	22.81	12.21	2.20	3.46	11.69	4.33	-3.45	137.40	218.46	114.03	88.75
8.49	-0.23	12.76	22.03	12.09	2.30	3.54	11.69	4.83	-2.77	149.23	256.89	141.00	108.48
8.44	-0.33	12.58	21.87	11.96	2.18	3.70	11.86	4.93	-2.83	145.32	247.97	139.18	106.53
8.22	-0.40	12.77	22.18	11.96	2.38	3.51	11.68	4.48	-3.22	130.51	209.83	105.52	78.03
8.15	-1.09	12.89	22.64	11.96	1.62	3.44	11.64	4.36	-3.81	120.43	212.32	111.46	75.16
8.47	-0.45	12.55	21.73	11.81	1.98	3.81	12.06	5.10	-2.86	148.60	245.31	150.01	119.52
8.27	-0.69	12.83	22.18	11.98	2.02	3.43	11.76	4.56	-3.35	143.55	229.72	130.21	109.48
8.80	-0.20	13.77	23.12	12.77	2.59	3.67	12.05	4.78	-3.00	135.65	222.57	125.59	98.25
8.42	-0.54	12.60	21.94	11.95	2.00	3.53	11.56	4.90	-3.07	135.29	226.65	116.73	84.86
8.41	-0.91	13.33	22.95	12.42	2.02	3.36	11.59	4.38	-3.80	137.59	251.64	140.35	96.34
8.60	-0.89	13.62	23.27	12.73	2.11	3.54	11.71	4.47	-3.90	147.76	255.21	150.73	107.44
8.19	-0.64	12.81	22.44	11.90	2.08	3.51	11.65	4.45	-3.36	132.57	218.09	106.61	77.35
7.92	-1.18	12.00	21.41	11.48	1.33	2.78	10.78	4.37	-3.73	144.13	235.43	127.54	98.08
7.95	-1.16	12.56	22.35	11.73	1.74	3.23	11.59	4.12	-4.08	134.78	226.94	119.03	81.73
8.28	-0.65	12.41	21.70	11.84	1.86	3.24	11.26	4.72	-3.18	145.39	242.45	130.39	97.39
8.76	-0.03	12.88	22.14	12.15	2.42	4.11	12.38	5.38	-2.47	148.30	246.65	155.16	125.02
7.71	-0.98	12.49	22.17	11.94	2.01	2.35	10.60	3.49	-3.99	144.85	249.35	152.91	143.28
8.23	-0.59	12.39	21.70	11.77	1.91	3.45	11.67	4.73	-3.11	143.81	244.67	143.00	112.89
8.39	-0.52	13.35	22.89	12.61	2.42	3.09	11.42	4.18	-3.46	151.24	245.10	141.28	123.94
9.10	0.27	13.59	22.84	12.77	2.83	4.30	12.56	5.42	-2.30	133.39	234.13	125.48	92.72
8.11	-1.06	12.89	22.87	11.94	1.78	3.36	11.71	4.29	-3.91	129.61	220.58	115.02	79.81
7.99	-1.18	12.66	22.54	11.86	1.72	3.25	11.65	4.14	-4.10	135.03	226.38	118.61	82.52
8.25	-0.93	12.38	21.82	11.80	1.67	3.25	11.35	4.66	-3.54	131.84	221.48	113.60	81.98
8.67	-0.62	13.49	23.26	12.45	2.17	4.00	12.35	4.87	-3.41	121.60	207.99	109.33	75.35
8.61	-0.56	12.98	22.36	12.30	2.11	3.59	11.68	4.90	-3.23	127.69	217.52	110.50	77.83
9.33	0.43	14.48	23.80	13.32	3.20	4.37	12.54	5.31	-2.40	117.23	206.73	98.27	65.00
8.52	-0.61	13.06	22.51	12.27	2.14	3.52	11.59	4.77	-3.35	118.90	208.94	101.35	66.85
8.37	-0.31	12.44	21.70	11.81	2.16	3.72	11.82	4.97	-2.78	140.91	239.90	128.15	96.35
8.34	-0.55	12.66	22.00	11.85	1.97	3.55	11.93	4.81	-3.05	151.91	248.79	155.80	126.07
8.25	-0.60	12.70	21.93	11.80	1.94	3.48	11.89	4.70	-3.14	148.54	237.62	143.71	116.81
8.17	-1.07	12.81	22.51	11.86	1.61	3.49	11.65	4.44	-3.77	119.86	211.84	110.75	74.35
8.46	-0.82	13.23	22.92	12.14	1.95	3.83	12.25	4.77	-3.59	118.40	207.32	113.23	78.19
8.35	-0.53	12.58	21.81	11.85	2.02	3.57	11.84	4.86	-3.07	144.25	241.57	142.00	111.01
9.44	0.73	14.08	23.35	13.05	3.27	4.85	13.04	5.85	-1.82	123.50	210.67	102.00	68.45
8.20	-0.65	12.62	22.43	11.88	2.03	3.49	11.52	4.49	-3.35	125.84	213.38	103.69	73.15
9.19	0.47	13.47	22.69	12.68	2.94	4.58	12.81	5.72	-2.01	131.26	227.58	119.38	85.34

8.08	-0.72	12.44	21.91	11.60	1.82	3.33	11.80	4.55	-3.26	148.18	240.56	146.40	120.15
8.39	-0.32	12.41	21.71	11.82	2.15	3.73	11.81	4.96	-2.78	140.93	241.95	127.05	94.38
8.19	-0.75	13.01	22.75	11.97	2.02	3.52	11.76	4.42	-3.51	136.78	221.72	111.04	84.15
8.30	-0.84	13.09	22.73	12.26	2.01	3.09	11.03	4.33	-3.70	118.56	221.18	108.31	68.70
7.78	-1.18	12.46	22.14	11.89	1.67	2.71	10.94	3.68	-4.02	151.71	244.17	140.58	125.45
8.23	-0.57	12.35	21.66	11.71	1.91	3.49	11.69	4.76	-3.04	145.12	245.95	141.51	111.30
8.17	-0.65	12.33	21.64	11.70	1.85	3.38	11.60	4.67	-3.17	144.13	244.57	143.97	113.49
9.00	-0.44	14.33	24.02	12.98	2.44	4.21	12.76	5.02	-3.34	116.53	201.66	110.71	76.54
8.29	-0.62	13.08	22.69	12.17	2.21	3.50	11.67	4.40	-3.44	134.49	215.37	110.90	82.54
8.41	-0.39	13.37	22.96	12.39	2.47	3.52	11.81	4.44	-3.29	139.60	221.70	119.62	97.23
7.94	-1.16	12.56	22.35	11.73	1.74	3.22	11.59	4.12	-4.08	134.84	227.19	119.04	81.88
8.71	-0.80	13.83	23.47	12.89	2.23	3.60	11.75	4.53	-3.83	144.18	251.75	146.38	103.70
8.30	-0.56	13.20	22.63	12.48	2.35	3.05	11.37	4.14	-3.45	152.17	247.52	144.54	127.41
8.76	0.04	12.90	22.14	12.21	2.50	4.10	12.26	5.31	-2.43	134.41	228.93	119.67	86.00
8.52	-0.30	13.20	22.48	12.32	2.39	3.47	11.73	4.70	-3.00	160.08	256.36	152.93	135.32
9.14	-0.32	14.31	24.02	13.14	2.52	4.43	12.76	5.16	-3.17	140.35	255.37	142.77	99.11
8.46	-0.83	13.23	22.92	12.14	1.95	3.83	12.25	4.76	-3.59	118.42	207.38	113.26	78.23
8.39	-0.35	12.48	21.81	11.89	2.16	3.66	11.74	4.89	-2.84	140.77	241.03	126.08	93.31
8.84	-0.36	13.60	23.03	12.73	2.47	3.84	11.94	4.95	-3.18	114.29	205.62	98.04	63.48
8.27	-0.58	12.43	21.68	11.74	1.90	3.41	11.65	4.78	-3.09	151.29	257.00	156.88	126.77
7.51	-1.20	11.91	21.22	11.36	1.55	2.38	10.67	3.64	-3.95	159.90	258.38	154.59	142.63
7.82	-1.05	12.85	22.46	11.96	1.99	2.85	11.23	3.70	-4.06	161.27	245.98	156.01	149.91
8.58	-0.06	12.58	21.81	11.94	2.34	4.05	12.10	5.23	-2.49	137.18	235.08	123.18	88.79
8.82	-0.10	13.80	23.10	12.78	2.71	3.73	12.12	4.84	-2.90	129.12	217.96	118.35	88.60
8.32	-0.86	12.99	22.81	12.09	1.92	3.67	12.01	4.61	-3.65	125.42	213.39	112.85	79.56
8.56	-0.25	12.72	21.96	12.06	2.18	3.75	12.00	5.07	-2.69	152.74	254.90	161.68	132.56
8.49	-0.45	13.40	22.90	12.68	2.45	3.22	11.50	4.28	-3.35	149.18	243.36	138.24	121.24
9.12	0.30	13.59	22.89	12.80	2.88	4.28	12.57	5.42	-2.29	132.25	232.05	123.65	89.95
8.05	-1.12	12.73	22.37	12.02	1.75	2.98	11.19	4.09	-3.97	123.49	239.38	126.55	84.46
8.57	-0.76	13.68	23.27	12.68	2.16	3.49	11.74	4.48	-3.67	133.91	244.77	135.93	91.12
8.48	-1.00	13.39	22.93	12.54	2.02	3.36	11.40	4.43	-4.05	155.07	253.08	153.57	109.04
7.99	-0.77	12.71	22.15	11.85	2.03	3.17	11.42	4.15	-3.59	135.21	217.34	115.64	93.05
9.17	0.42	13.97	23.42	12.98	3.12	4.38	12.62	5.35	-2.29	123.39	207.54	101.41	68.04
8.91	0.04	13.24	22.52	12.46	2.59	4.17	12.38	5.31	-2.50	141.73	239.22	135.22	104.33
8.57	-0.57	12.92	22.29	12.25	2.07	3.56	11.65	4.87	-3.24	127.38	218.50	110.69	78.25
8.66	-0.08	12.86	22.12	12.17	2.36	4.03	12.18	5.20	-2.55	139.72	237.81	128.57	95.57
8.47	-0.27	12.51	21.83	11.92	2.20	3.78	11.85	4.99	-2.75	140.35	242.08	127.18	94.19
8.36	-0.63	13.65	23.37	12.66	2.57	3.37	11.86	4.08	-3.83	157.82	251.18	153.97	146.80
7.35	-1.19	12.04	21.52	11.47	1.82	1.98	10.35	3.26	-4.22	169.23	273.02	181.82	179.14
8.79	-0.08	13.10	22.37	12.36	2.46	4.03	12.24	5.20	-2.60	143.63	242.31	137.58	105.53
8.42	-0.54	12.60	21.94	11.96	2.00	3.53	11.56	4.90	-3.08	135.31	226.65	116.74	84.83
8.74	-0.04	12.86	22.13	12.13	2.41	4.09	12.36	5.36	-2.48	148.41	246.80	155.45	125.31
7.86	-1.01	13.15	22.65	12.27	2.23	2.65	11.02	3.45	-4.24	168.24	259.36	176.62	178.59

8.37	-0.47	12.52	21.87	11.87	2.03	3.61	11.84	4.83	-2.96	142.94	243.83	139.55	108.05
8.48	-0.91	13.22	23.00	12.56	1.97	3.44	11.60	4.40	-3.81	134.19	247.67	135.05	87.84
8.17	-0.73	12.87	22.55	12.25	2.13	3.14	11.36	4.13	-3.59	143.52	237.36	140.27	124.41
9.23	-0.28	14.28	24.00	12.93	2.50	4.78	13.45	5.51	-3.08	130.00	219.68	127.54	93.54
8.26	-0.57	12.44	21.75	11.81	1.94	3.48	11.68	4.73	-3.09	143.36	244.27	142.95	112.15
8.45	-0.26	12.62	21.86	11.99	2.23	3.65	11.79	4.93	-2.76	147.56	250.47	137.10	103.98
8.68	-0.19	13.31	22.71	12.46	2.45	3.63	11.77	4.92	-2.79	133.05	226.57	116.78	83.47
8.41	-0.24	12.56	21.92	11.89	2.21	3.68	11.71	4.93	-2.66	141.13	244.14	125.63	91.85
8.40	-0.81	12.63	21.97	11.99	1.74	3.41	11.44	4.84	-3.36	136.70	227.14	119.20	88.43
8.25	-0.50	12.27	21.62	11.66	1.90	3.57	11.90	4.87	-2.91	153.91	255.71	164.90	134.91
8.59	-0.38	12.89	22.18	12.16	2.16	3.68	11.75	5.03	-2.91	135.29	228.04	117.44	85.41
8.23	-1.51	12.63	21.98	12.27	1.26	2.99	11.09	4.23	-4.34	122.21	223.50	111.84	74.22
8.02	-0.81	12.12	21.40	11.39	1.68	3.32	11.61	4.63	-3.33	144.28	240.36	140.64	110.92
7.85	-1.01	13.14	22.64	12.26	2.22	2.64	11.01	3.45	-4.25	168.34	259.39	176.68	178.74
8.93	0.17	13.72	22.93	12.66	2.85	4.09	12.47	5.23	-2.51	129.39	220.25	117.86	85.18
8.39	-0.94	13.22	22.88	12.17	1.82	3.62	11.96	4.61	-3.70	117.97	209.09	113.09	77.09
8.19	-1.06	12.87	22.51	11.86	1.65	3.51	11.86	4.53	-3.78	120.02	211.93	114.24	79.47
8.38	-0.51	12.63	21.85	11.88	2.04	3.58	11.85	4.87	-3.06	144.40	241.52	142.10	111.21
8.99	-0.11	13.81	23.35	12.82	2.64	4.05	12.20	5.16	-2.87	117.15	206.58	98.21	64.99
8.34	-0.43	12.40	21.78	11.77	2.00	3.70	11.87	4.92	-2.85	143.92	244.30	135.88	104.39
8.56	-0.72	13.47	23.23	12.37	2.11	3.84	12.33	4.73	-3.55	121.47	210.93	114.87	81.46
8.32	-0.55	13.01	22.22	12.04	2.12	3.44	11.84	4.61	-3.23	143.68	230.01	134.96	106.95
9.16	0.40	13.73	22.98	12.81	2.96	4.37	12.64	5.49	-2.16	129.04	222.72	115.51	80.78
8.96	-0.48	14.26	23.95	12.94	2.40	4.16	12.70	4.99	-3.37	117.01	202.31	111.27	77.00
8.64	-0.70	13.15	22.62	12.55	2.10	3.64	11.68	4.72	-3.46	115.37	213.34	101.28	65.57
7.86	-1.06	12.55	22.27	12.02	1.81	2.72	10.94	3.75	-3.94	149.58	247.43	143.65	129.21
8.19	-0.68	12.67	22.10	11.90	1.98	3.48	11.72	4.51	-3.34	139.46	221.18	122.07	96.29
8.32	-0.52	12.83	21.97	12.03	2.08	3.41	11.55	4.66	-3.11	158.04	254.03	151.65	133.58
8.66	-0.15	12.93	22.21	12.23	2.35	3.93	12.13	5.12	-2.65	136.43	237.16	128.23	96.07
8.22	-1.02	12.88	22.59	12.08	1.77	3.22	11.33	4.34	-3.79	121.90	232.41	118.67	74.44
8.91	-0.02	13.65	22.89	12.66	2.62	4.03	12.43	5.15	-2.66	149.45	236.89	143.04	118.90
8.60	-0.34	13.19	22.92	12.43	2.37	3.80	11.85	4.78	-3.02	121.03	210.24	100.32	69.78
8.45	-0.83	12.95	22.38	12.29	1.91	3.44	11.42	4.59	-3.57	117.73	219.83	104.86	66.00
8.53	-0.37	13.26	22.67	12.39	2.38	3.61	11.90	4.66	-3.12	134.49	219.90	121.31	94.45
9.12	-0.33	14.22	23.92	13.08	2.53	4.42	12.75	5.17	-3.17	140.97	256.75	143.51	99.95
8.24	-0.56	12.37	21.68	11.72	1.92	3.50	11.70	4.77	-3.03	145.08	245.93	141.62	111.33
8.93	0.01	13.69	22.90	12.69	2.64	4.07	12.45	5.21	-2.58	148.53	235.50	141.72	117.29
8.73	0.03	13.35	22.69	12.51	2.78	4.07	12.19	4.94	-2.75	127.76	204.95	101.64	73.50
8.42	-0.50	13.53	23.03	12.59	2.43	3.26	11.60	4.24	-3.41	134.30	219.32	119.37	98.51
8.71	-0.80	13.83	23.47	12.89	2.23	3.60	11.75	4.53	-3.83	144.22	251.78	146.39	103.73
8.90	0.15	13.16	22.36	12.42	2.62	4.24	12.40	5.39	-2.30	132.17	228.18	116.97	83.42
8.32	-0.52	12.45	21.81	11.80	1.97	3.56	11.78	4.79	-2.99	142.95	243.80	139.64	108.19
8.82	-0.56	13.73	23.34	12.54	2.19	4.24	12.64	5.09	-3.31	113.62	199.92	108.45	71.79

8.39	-0.70	12.48	21.82	11.86	1.83	3.51	11.56	4.92	-3.25	133.25	223.66	116.63	83.99
8.53	-0.55	12.77	22.24	12.23	2.04	3.49	11.48	4.85	-3.11	139.65	235.61	123.63	91.63
8.45	-0.47	12.53	21.71	11.79	1.96	3.79	12.04	5.09	-2.88	148.62	245.35	150.03	119.57
8.94	-0.35	13.67	23.04	12.87	2.48	3.91	12.02	4.96	-3.17	115.17	206.49	98.76	63.82
8.51	-0.92	13.25	23.00	12.55	1.97	3.38	11.48	4.46	-3.83	147.37	258.78	144.22	94.61
8.36	-0.58	12.88	22.32	12.12	2.12	3.51	11.82	4.59	-3.30	149.05	240.59	138.31	119.71
7.90	-0.90	12.57	21.91	11.93	1.90	2.63	10.92	3.85	-3.73	158.36	253.58	150.55	136.13
8.07	-0.81	13.27	22.87	12.24	2.36	3.15	11.65	3.93	-3.99	166.13	258.40	163.96	160.84
8.49	-0.59	13.12	22.66	12.40	2.22	3.35	11.40	4.57	-3.42	116.25	213.52	101.93	65.94
8.78	-0.04	13.04	22.33	12.32	2.43	4.09	12.28	5.25	-2.52	137.37	237.37	130.71	98.14
8.41	-0.35	12.57	21.81	11.93	2.14	3.67	11.83	4.92	-2.84	144.27	246.33	136.42	104.03
8.28	-0.55	12.45	21.76	11.82	1.96	3.50	11.71	4.76	-3.07	143.47	244.84	142.50	112.13
8.58	-0.87	13.35	23.05	12.60	2.04	3.47	11.55	4.54	-3.75	144.34	256.93	143.38	93.95
8.85	-0.01	13.73	23.06	12.77	2.67	3.69	11.86	4.98	-2.72	128.10	224.29	111.30	79.21
8.69	-0.75	13.28	23.04	12.58	2.10	3.81	11.82	4.80	-3.63	145.69	253.65	146.35	96.08
8.90	0.02	13.79	23.11	12.82	2.71	3.74	11.91	5.02	-2.68	128.13	224.32	111.21	78.94
8.53	-0.81	13.55	23.21	12.44	2.00	3.76	12.11	4.66	-3.61	116.69	202.99	108.88	72.94
8.40	-0.36	12.88	22.36	12.08	2.24	3.78	11.84	4.74	-2.92	127.64	211.44	103.91	72.13
7.86	-1.01	13.15	22.65	12.27	2.23	2.65	11.01	3.45	-4.24	168.25	259.34	176.62	178.58
8.42	-0.53	12.73	22.09	12.04	2.02	3.42	11.49	4.81	-3.09	136.21	228.61	118.08	87.47
8.19	-0.74	12.64	21.94	11.73	1.81	3.40	11.83	4.64	-3.26	154.24	239.04	144.63	122.66
8.27	-0.38	12.21	21.50	11.58	2.04	3.68	11.78	4.92	-2.83	139.53	236.88	125.24	93.88
8.12	-0.70	12.42	21.84	11.59	1.82	3.40	11.82	4.60	-3.22	147.13	239.93	145.98	118.09
8.42	-0.60	12.52	21.87	11.91	1.92	3.53	11.56	4.93	-3.15	133.14	225.49	117.34	84.28
8.26	-0.58	12.67	21.90	11.80	1.94	3.52	11.90	4.73	-3.11	147.29	237.23	142.80	115.71
8.35	-0.63	13.64	23.36	12.66	2.57	3.37	11.85	4.07	-3.83	158.15	251.29	154.37	147.14
8.42	-0.72	12.67	22.12	12.05	1.87	3.38	11.43	4.79	-3.29	136.32	228.24	120.37	88.10
8.35	-0.87	13.09	22.77	11.97	1.83	3.74	12.01	4.75	-3.56	119.33	213.37	112.89	76.87
8.35	-0.65	13.59	23.28	12.52	2.55	3.42	11.93	4.16	-3.88	156.93	248.48	151.06	142.58
8.14	-0.67	12.93	22.30	12.03	2.20	3.22	11.49	4.24	-3.54	131.69	209.02	108.41	81.60
8.02	-0.84	12.59	22.16	12.04	1.97	2.92	11.18	3.99	-3.66	151.30	247.20	142.35	127.21
8.61	-0.12	13.32	22.67	12.34	2.57	4.06	12.17	4.89	-2.81	129.94	207.64	102.58	73.37
8.24	-0.57	12.36	21.67	11.71	1.91	3.50	11.70	4.76	-3.03	145.12	245.97	141.53	111.29
8.42	-0.35	12.73	22.07	12.12	2.24	3.37	11.49	4.71	-2.91	153.43	264.26	144.28	113.91
8.59	-0.17	12.80	22.03	12.12	2.30	3.88	12.00	5.07	-2.65	140.59	242.06	131.14	98.52
8.42	-0.24	12.56	21.86	11.92	2.20	3.73	11.78	4.93	-2.68	137.49	237.36	122.92	89.53
8.11	-1.12	12.73	22.41	11.75	1.59	3.38	11.67	4.44	-3.83	121.22	217.92	117.13	80.71
8.46	-0.41	13.20	22.84	12.21	2.26	3.91	12.01	4.71	-3.08	127.94	213.66	103.23	73.46
8.59	-0.70	13.51	23.15	12.36	2.09	3.93	12.41	4.84	-3.49	118.32	205.79	111.83	78.23
8.47	-0.82	13.24	22.93	12.14	1.96	3.83	12.25	4.77	-3.59	118.37	207.26	113.19	78.14
7.36	-1.40	11.65	21.39	11.31	1.37	2.32	10.55	3.43	-4.19	157.16	255.98	153.22	142.38
8.25	-0.56	12.68	22.31	11.95	2.27	3.56	11.97	4.60	-3.36	146.78	230.64	132.68	116.70
8.76	-0.15	13.31	22.56	12.51	2.52	3.85	12.18	5.02	-2.80	137.80	236.93	134.99	102.91

8.36	-0.42	12.56	21.79	11.90	2.10	3.57	11.71	4.83	-2.92	144.57	245.44	135.50	102.89
8.36	-0.72	12.78	22.38	12.10	1.99	3.36	11.38	4.65	-3.48	120.24	212.21	103.47	68.27
9.33	0.43	14.50	23.81	13.34	3.20	4.37	12.53	5.30	-2.39	117.00	206.00	98.78	65.00
8.57	-0.23	12.74	22.01	12.09	2.20	3.77	12.01	5.08	-2.68	152.86	255.61	161.96	133.21
8.01	-0.85	12.08	21.39	11.45	1.64	3.22	11.45	4.57	-3.30	145.95	247.12	146.46	116.76
7.82	-0.94	12.68	22.18	12.06	2.17	2.48	10.77	3.60	-4.09	157.50	257.02	165.16	159.26
8.73	-0.19	13.54	23.10	12.66	2.60	3.82	11.89	4.82	-3.02	119.40	207.83	99.01	66.73
8.59	-0.72	13.42	23.37	12.42	2.09	3.86	12.26	4.76	-3.52	123.47	213.76	113.30	78.80
8.70	-0.08	12.86	22.19	12.06	2.37	4.14	12.43	5.33	-2.53	144.60	238.28	143.76	113.49
7.95	-0.99	12.79	22.42	11.81	1.92	3.10	11.44	4.06	-3.89	145.45	229.01	127.82	109.35
7.94	-0.92	12.69	22.38	11.89	1.96	3.03	11.46	3.97	-3.85	152.97	239.05	143.71	130.43
8.29	-1.09	12.88	22.71	12.33	1.77	3.30	11.40	4.29	-3.97	139.23	254.20	138.53	90.33
8.04	-0.75	12.67	22.06	11.86	2.05	3.25	11.46	4.24	-3.56	133.67	213.85	111.60	86.12
8.73	-0.27	13.43	22.86	12.58	2.47	3.81	12.09	4.84	-3.03	145.19	235.27	134.84	114.77
8.91	-0.37	13.68	23.05	12.89	2.47	3.86	11.98	4.96	-3.18	115.41	207.05	98.95	63.95
8.29	-0.64	13.19	22.88	12.20	2.28	3.56	11.96	4.36	-3.58	149.75	233.87	135.56	119.87
8.44	-0.88	13.42	23.06	12.35	1.94	3.66	12.01	4.57	-3.67	117.69	204.69	109.94	74.28
8.50	-0.17	12.48	21.74	11.84	2.25	3.92	11.96	5.09	-2.58	136.57	233.50	121.76	88.25
8.41	-0.31	12.56	21.82	11.93	2.15	3.66	11.79	4.93	-2.79	141.52	244.27	132.19	99.58
8.39	-0.45	13.14	22.58	12.25	2.31	3.67	11.81	4.56	-3.23	131.63	209.42	105.34	77.59
9.10	0.20	13.92	23.15	12.95	2.87	4.15	12.55	5.24	-2.48	147.05	235.63	142.38	117.70
8.34	-0.61	13.29	22.73	12.46	2.32	3.09	11.47	4.19	-3.52	151.91	243.84	140.59	123.66
8.62	-0.55	13.00	22.37	12.31	2.12	3.60	11.69	4.91	-3.22	127.82	217.37	110.46	77.77
8.51	-1.13	13.39	22.56	12.62	1.77	3.08	11.25	4.40	-4.03	116.49	219.49	106.00	68.05
8.96	-0.01	13.87	23.39	12.90	2.78	4.03	12.17	5.04	-2.83	118.78	207.53	97.57	64.52
8.65	-0.79	13.24	22.98	12.52	2.06	3.77	11.80	4.78	-3.65	146.98	254.95	145.85	96.62
8.12	-0.78	13.33	22.93	12.30	2.39	3.19	11.69	3.96	-3.96	164.89	257.19	162.59	158.89
8.12	-1.27	12.84	22.57	12.25	1.68	2.91	11.09	4.04	-4.21	157.01	275.29	154.02	102.51
8.01	-0.78	12.83	22.39	12.18	2.22	2.71	10.99	3.80	-3.81	149.72	251.66	155.44	146.66
8.29	-0.70	13.04	22.63	12.27	2.11	3.34	11.61	4.30	-3.49	142.90	230.62	128.55	108.05
8.10	-0.82	12.71	22.42	12.16	2.02	3.04	11.23	4.02	-3.68	146.80	241.91	140.93	125.76
8.09	-0.97	12.92	22.36	12.18	1.86	2.99	11.30	4.00	-3.79	150.65	240.05	136.78	119.15
8.73	-0.67	13.59	23.28	12.71	2.25	3.94	12.07	4.76	-3.60	140.83	250.28	141.96	98.36
9.23	0.53	13.67	22.94	12.81	3.05	4.54	12.82	5.64	-2.03	130.29	225.80	117.76	83.17
8.03	-0.76	12.65	22.05	11.85	2.04	3.24	11.45	4.22	-3.57	133.70	213.96	111.65	86.18
7.92	-1.08	12.60	22.22	11.94	1.71	2.89	11.17	3.91	-3.90	148.61	239.15	135.78	118.74
8.67	-0.62	13.49	23.26	12.45	2.17	4.00	12.35	4.87	-3.41	121.60	207.99	109.32	75.35
7.71	-1.05	12.18	21.53	11.62	1.73	2.52	10.82	3.77	-3.83	159.81	256.22	152.65	140.98
8.42	-0.47	12.69	21.91	11.92	2.07	3.59	11.86	4.89	-3.02	144.71	241.82	142.35	111.84
8.16	-0.86	12.94	22.47	12.21	1.95	3.08	11.38	4.07	-3.67	148.23	236.91	133.51	114.82
8.10	-0.75	12.24	21.50	11.56	1.72	3.34	11.56	4.66	-3.23	146.49	245.30	146.52	115.68
8.27	-0.57	12.45	21.73	11.79	1.93	3.50	11.71	4.78	-3.07	144.71	245.24	144.63	113.88
8.34	-0.65	13.60	23.28	12.53	2.55	3.43	11.93	4.15	-3.86	156.98	248.91	152.07	143.24

8.06	-1.13	12.66	22.13	11.93	1.64	3.03	11.16	4.20	-3.92	128.11	240.44	125.18	81.38
8.43	-0.27	12.54	21.86	11.93	2.21	3.71	11.77	4.95	-2.74	139.30	241.02	124.85	91.33
8.78	-0.50	13.73	23.24	12.97	2.40	3.57	11.63	4.66	-3.30	113.44	207.53	99.86	63.00
8.92	-0.02	13.68	22.96	12.76	2.61	3.82	11.94	5.10	-2.66	133.41	224.97	113.63	81.00
8.76	-0.07	13.07	22.35	12.34	2.43	4.02	12.23	5.18	-2.58	136.98	237.44	131.44	97.39
8.41	-0.81	12.65	21.99	12.00	1.75	3.40	11.42	4.84	-3.36	137.49	227.61	119.69	89.23
9.07	-0.48	14.39	24.19	13.24	2.44	4.20	12.47	4.93	-3.40	139.52	247.97	138.83	94.88
8.77	-0.68	13.81	23.50	12.91	2.30	3.65	11.74	4.60	-3.66	143.01	248.40	141.58	95.46
8.69	-0.24	13.38	23.04	12.54	2.49	3.84	11.92	4.86	-2.98	121.00	208.56	99.58	67.68
8.70	-0.23	13.37	23.00	12.54	2.53	3.85	11.94	4.87	-3.00	122.41	209.09	99.91	67.15
8.09	-0.86	13.26	22.90	12.30	2.17	3.12	11.49	3.87	-3.87	154.69	237.68	145.74	135.06
8.03	-1.18	12.86	22.50	11.92	1.60	3.10	11.34	4.13	-3.96	121.64	222.70	117.79	78.16
8.95	-0.25	13.64	23.05	12.80	2.52	4.04	12.13	5.10	-3.03	113.21	203.25	96.96	63.02
8.63	-0.94	13.07	22.42	12.52	1.82	3.55	11.64	4.76	-3.68	116.87	211.88	104.24	70.07
8.45	-0.83	12.95	22.38	12.29	1.91	3.44	11.42	4.59	-3.57	117.71	219.84	104.86	66.00
9.14	-0.29	14.48	24.33	13.19	2.64	4.31	12.91	5.12	-3.24	122.54	204.84	109.78	75.44
8.42	-0.57	13.33	22.97	12.54	2.38	3.29	11.54	4.31	-3.49	143.40	234.83	140.28	124.73
8.46	-0.88	13.34	23.00	12.26	1.88	3.69	12.03	4.65	-3.66	117.35	206.82	111.69	75.70
8.81	-0.63	13.98	23.75	13.02	2.34	3.60	11.83	4.60	-3.60	137.37	248.98	137.50	89.25
8.55	-0.18	13.30	22.72	12.33	2.55	3.92	12.05	4.77	-2.94	130.01	209.66	102.99	74.38
8.14	-0.83	13.00	22.41	12.19	2.01	3.00	11.34	4.10	-3.66	153.71	243.58	141.06	123.48
8.65	-0.82	13.10	22.70	12.66	1.90	3.58	11.62	4.66	-3.57	114.97	213.22	102.54	67.08
8.58	-0.64	13.51	22.98	12.70	2.22	3.34	11.37	4.49	-3.49	114.30	211.60	101.82	64.84
7.30	-1.35	11.62	21.17	11.21	1.46	2.19	10.43	3.40	-4.15	151.81	256.15	154.23	144.34
8.49	-0.30	12.78	22.13	12.18	2.22	3.47	11.59	4.81	-2.84	154.68	264.11	142.14	111.29
7.93	-1.03	12.43	21.85	11.72	1.63	3.09	11.41	4.20	-3.74	149.40	235.79	134.48	115.83
8.45	-0.74	13.30	22.75	12.45	2.07	3.21	11.25	4.43	-3.57	116.18	215.42	103.69	66.14
9.09	-0.03	14.00	23.45	13.00	2.75	4.10	12.20	5.17	-2.81	116.24	206.52	98.01	64.00
8.26	-0.65	13.45	23.17	12.56	2.45	2.89	11.27	3.95	-3.75	149.41	244.33	149.59	137.77
8.79	-0.62	14.10	23.61	13.04	2.36	3.62	11.82	4.53	-3.61	124.06	235.00	127.35	83.84
8.18	-1.05	12.71	22.24	12.00	1.71	3.06	11.01	4.36	-3.81	121.28	228.22	108.65	68.89
7.98	-1.25	12.81	22.43	11.88	1.55	3.01	11.27	4.06	-4.01	123.17	224.60	118.59	79.48
8.26	-0.63	12.47	21.71	11.74	1.92	3.45	11.73	4.76	-3.17	144.69	241.44	142.29	111.66
8.42	-0.54	12.60	21.95	11.96	2.00	3.54	11.57	4.90	-3.07	135.29	226.64	116.72	84.85
7.30	-1.38	11.53	21.17	11.20	1.40	2.25	10.46	3.41	-4.17	157.31	259.33	154.68	145.14
7.95	-0.98	13.14	22.61	12.18	2.16	2.93	11.30	3.72	-4.10	166.49	252.51	168.98	167.50
8.42	-0.24	12.56	21.92	11.89	2.21	3.69	11.72	4.93	-2.65	141.07	244.10	125.58	91.81
8.05	-0.74	12.68	22.07	11.86	2.06	3.27	11.47	4.25	-3.55	133.64	213.74	111.58	86.09
8.29	-0.68	13.35	23.22	12.25	2.30	3.55	12.04	4.31	-3.68	154.55	240.70	142.80	126.83
8.90	-0.54	13.98	23.50	12.74	2.29	4.22	12.62	5.03	-3.37	128.46	226.78	127.43	90.14
7.68	-1.09	12.13	21.49	11.58	1.69	2.48	10.78	3.73	-3.85	160.03	256.64	152.96	141.17
8.79	-0.08	13.11	22.37	12.37	2.46	4.03	12.23	5.20	-2.60	143.85	243.00	138.25	106.26
8.11	-1.12	12.73	22.40	11.75	1.59	3.37	11.67	4.44	-3.83	121.25	217.97	117.16	80.75

8.32	-0.65	13.08	22.45	12.23	2.15	3.29	11.61	4.37	-3.45	150.84	241.87	139.57	122.64
7.31	-1.34	11.63	21.18	11.22	1.46	2.20	10.43	3.40	-4.15	151.72	256.18	154.13	144.23
8.30	-0.45	12.53	21.88	11.77	2.12	3.63	12.01	4.84	-3.02	142.78	235.63	139.31	111.14
8.94	0.02	13.69	22.91	12.69	2.64	4.07	12.45	5.21	-2.58	148.50	235.47	141.70	117.26
8.00	-0.84	13.27	22.80	12.30	2.38	2.95	11.32	3.74	-4.07	170.50	255.68	174.56	175.59
8.24	-0.75	12.77	22.10	11.85	1.85	3.38	11.81	4.63	-3.33	155.35	241.23	144.06	122.85
8.77	-0.03	12.86	22.11	12.13	2.41	4.19	12.44	5.45	-2.45	147.74	245.92	153.52	123.18
8.51	-0.92	13.25	23.00	12.55	1.97	3.38	11.48	4.46	-3.83	147.34	258.75	144.19	94.57
8.96	-0.48	14.24	23.92	12.92	2.39	4.16	12.70	4.99	-3.37	116.91	202.61	111.12	77.00
9.27	-0.22	14.28	24.01	12.99	2.58	4.88	13.50	5.55	-3.04	129.91	220.29	127.84	93.31
8.25	-0.73	13.43	23.22	12.36	2.46	3.32	11.84	4.12	-3.87	159.92	249.45	151.07	139.29
8.66	-0.61	13.55	23.29	12.46	2.17	3.97	12.40	4.87	-3.41	122.55	208.09	110.98	77.31
8.34	-0.43	12.37	21.67	11.76	2.03	3.64	11.81	4.89	-2.89	145.03	245.74	137.94	106.34
8.41	-0.75	12.65	22.09	12.01	1.81	3.41	11.45	4.83	-3.30	136.76	226.69	120.02	88.23
9.13	-0.32	14.30	24.01	13.14	2.52	4.43	12.76	5.15	-3.17	140.39	255.47	142.81	99.18
8.29	-0.42	12.48	21.72	11.85	2.09	3.51	11.64	4.78	-2.94	146.43	248.09	135.50	104.69
8.42	-0.77	13.16	22.66	12.42	2.01	3.32	11.29	4.46	-3.55	115.13	216.25	104.06	65.93
8.49	-0.40	12.54	21.76	11.82	2.03	3.85	12.11	5.15	-2.82	148.37	245.21	150.58	119.78
8.22	-0.65	12.36	21.63	11.69	1.85	3.44	11.64	4.76	-3.12	145.03	246.62	145.12	115.05
8.10	-0.77	12.78	22.24	12.17	2.05	2.90	11.12	4.02	-3.59	148.75	247.31	143.80	128.16
7.77	-1.34	12.16	21.86	11.57	1.41	2.60	10.57	3.97	-4.07	121.96	228.45	113.12	74.73
8.86	-0.34	13.69	23.06	12.76	2.49	3.83	11.96	4.95	-3.16	115.29	206.93	98.65	63.80
8.79	-0.23	14.00	23.24	13.01	2.71	3.52	11.87	4.61	-3.17	146.73	238.04	136.58	117.17
8.61	-0.39	13.30	22.56	12.38	2.33	3.66	12.07	4.83	-3.07	154.22	240.18	143.64	123.05
7.92	-0.96	12.48	22.21	11.98	1.92	2.79	11.02	3.88	-3.86	149.53	244.49	146.54	134.68
9.06	0.28	13.85	23.13	12.81	2.94	4.18	12.39	5.30	-2.37	125.75	214.98	105.93	72.88
8.62	-0.65	13.58	23.21	12.41	2.13	3.98	12.44	4.89	-3.46	118.23	204.81	111.72	78.23
8.48	-0.93	13.17	22.84	12.40	1.89	3.47	11.56	4.57	-3.80	146.77	257.86	144.24	98.09
8.20	-0.62	12.37	21.68	11.74	1.89	3.42	11.64	4.69	-3.14	143.71	244.39	143.27	112.89
8.01	-0.85	12.38	21.68	11.50	1.65	3.28	11.73	4.53	-3.35	151.90	239.22	143.61	119.81
8.62	-0.55	13.00	22.37	12.31	2.12	3.60	11.69	4.90	-3.22	127.79	217.41	110.47	77.78
8.28	-0.37	12.22	21.52	11.59	2.05	3.69	11.81	4.94	-2.81	139.06	236.73	125.08	93.91
8.45	-1.01	13.28	22.87	12.51	2.01	3.37	11.40	4.38	-4.04	153.93	257.44	152.47	107.73
8.64	-0.84	13.27	23.03	12.61	2.07	3.68	11.73	4.69	-3.74	146.85	255.18	146.53	98.06
8.15	-1.30	12.91	22.42	12.04	1.61	3.05	11.21	4.24	-4.21	156.36	256.05	158.60	118.91
8.61	-0.77	13.65	23.29	12.51	2.05	3.79	12.17	4.69	-3.58	115.90	203.16	110.07	73.77
8.11	-0.78	12.30	21.55	11.58	1.70	3.31	11.54	4.66	-3.27	145.63	244.04	145.75	114.99
7.86	-1.06	12.54	22.27	12.01	1.81	2.72	10.94	3.75	-3.94	149.71	247.50	143.70	129.31
8.77	-0.68	13.81	23.50	12.91	2.30	3.65	11.74	4.60	-3.66	143.00	248.40	141.58	95.45
7.86	-1.01	13.15	22.65	12.27	2.23	2.65	11.02	3.45	-4.24	168.23	259.33	176.60	178.54
8.62	-0.68	13.59	23.31	12.46	2.08	3.88	12.16	4.77	-3.50	115.72	203.18	107.53	71.38
8.57	-0.85	13.15	22.90	12.47	2.02	3.70	11.76	4.68	-3.73	145.30	253.87	145.55	98.87
8.46	-0.40	12.72	22.01	11.93	2.12	3.72	12.08	4.97	-2.90	150.48	247.85	154.79	124.98

7.90	-0.95	12.62	22.26	11.73	1.91	3.10	11.33	4.08	-3.83	139.13	222.90	116.07	91.23
8.36	-0.61	13.46	23.32	12.35	2.37	3.66	12.06	4.38	-3.61	148.33	232.56	132.15	114.21
9.17	0.24	14.19	23.67	13.22	3.04	4.21	12.34	5.14	-2.53	119.02	207.99	98.81	66.37
9.18	-0.29	14.42	24.21	13.11	2.56	4.53	13.00	5.27	-3.11	115.95	199.35	106.07	72.51
8.31	-0.87	13.02	22.40	12.18	1.94	3.31	11.42	4.45	-3.68	123.79	232.95	121.97	76.64
8.57	-0.21	12.73	22.02	12.09	2.30	3.84	11.99	5.04	-2.71	145.59	248.73	139.94	107.56
8.63	-0.12	12.85	22.15	12.21	2.36	3.92	11.99	5.08	-2.62	132.25	230.35	116.84	83.85
7.11	-1.39	11.66	21.04	11.01	1.51	1.93	10.19	3.20	-4.23	136.29	247.17	144.86	135.80
7.90	-1.00	12.30	21.63	11.36	1.48	3.19	11.64	4.48	-3.49	154.70	240.86	143.65	122.91
7.87	-1.06	12.53	22.28	12.02	1.82	2.72	10.93	3.72	-3.93	149.83	246.68	144.13	129.10
8.36	-0.58	12.88	22.31	12.12	2.12	3.51	11.82	4.59	-3.30	149.05	240.60	138.33	119.74
8.18	-1.05	12.71	22.24	11.99	1.71	3.06	11.00	4.35	-3.81	121.26	228.31	108.67	68.91
8.97	0.14	13.41	22.65	12.61	2.68	4.18	12.42	5.31	-2.41	134.43	234.94	126.57	93.41
8.45	-0.75	13.15	22.66	12.28	2.01	3.55	11.63	4.60	-3.47	120.83	229.36	119.00	74.01
8.78	-0.01	12.91	22.17	12.18	2.44	4.13	12.40	5.40	-2.45	148.17	246.55	155.02	124.86
7.91	-0.99	12.96	22.59	12.05	2.05	2.93	11.31	3.75	-4.03	160.22	244.17	153.43	146.50
9.17	0.24	14.19	23.67	13.22	3.04	4.21	12.34	5.14	-2.53	119.02	207.99	98.81	66.36
8.54	-1.00	13.63	23.20	12.71	2.07	3.30	11.40	4.37	-4.10	154.34	249.21	153.34	109.23
8.33	-0.64	12.90	22.23	12.04	2.07	3.48	11.80	4.61	-3.31	142.97	229.25	129.61	108.77
8.22	-1.00	12.41	21.85	11.84	1.58	3.09	11.12	4.57	-3.56	141.42	230.93	124.52	93.05
8.44	-0.43	12.97	22.71	12.19	2.27	3.67	11.71	4.68	-3.16	125.01	212.01	101.40	70.03
8.10	-0.80	12.98	22.60	11.99	2.06	3.30	11.61	4.22	-3.67	143.78	225.22	123.81	103.27
8.41	-0.92	13.24	22.89	12.20	1.84	3.66	11.96	4.64	-3.65	119.50	207.89	110.74	74.42
7.88	-0.95	12.22	21.51	11.36	1.55	3.16	11.61	4.42	-3.47	150.41	237.34	143.43	118.79
7.93	-0.91	12.58	22.02	11.75	1.80	3.08	11.31	4.08	-3.65	134.11	215.00	114.40	87.48
8.20	-0.70	13.08	22.76	12.08	2.21	3.47	11.86	4.28	-3.65	149.53	234.29	136.99	121.90
8.47	-0.30	12.65	21.90	11.99	2.19	3.71	11.85	4.93	-2.77	141.24	243.47	132.13	99.84
8.51	-0.20	13.09	22.49	12.23	2.57	3.86	11.96	4.74	-2.97	129.40	207.14	103.86	75.10
9.31	0.53	14.39	23.59	13.20	3.18	4.38	12.63	5.41	-2.13	120.99	205.75	100.19	68.17
8.51	-0.17	12.78	22.07	12.08	2.27	3.75	11.79	4.94	-2.63	136.47	235.72	120.03	87.94
8.22	-0.51	12.20	21.54	11.59	1.94	3.59	11.75	4.84	-2.96	143.45	244.77	136.78	105.65
9.34	0.40	14.23	23.65	13.29	3.13	4.36	12.77	5.39	-2.35	151.25	237.20	142.68	119.42
7.86	-0.98	12.13	21.30	11.48	1.55	2.86	11.00	4.22	-3.52	164.94	262.06	161.33	143.73
8.44	-0.35	12.98	22.66	12.18	2.28	3.75	11.81	4.74	-3.01	127.37	212.30	104.08	72.54
8.55	-0.10	12.46	21.72	11.82	2.29	4.04	12.15	5.24	-2.50	136.14	234.54	123.82	91.42
8.75	-0.15	13.31	22.56	12.50	2.52	3.82	12.17	5.00	-2.82	137.49	236.47	134.82	102.81
8.25	-0.62	12.74	22.17	11.96	2.04	3.53	11.77	4.56	-3.28	138.98	220.64	121.58	95.43
9.21	0.42	14.31	23.48	13.10	3.10	4.26	12.46	5.28	-2.27	121.17	205.56	100.98	67.74
9.28	0.52	13.74	23.00	12.86	3.07	4.55	12.85	5.69	-2.01	130.45	225.76	120.17	85.80
7.95	-1.29	12.23	21.76	11.67	1.42	3.06	11.18	4.23	-4.01	134.55	248.55	131.94	85.11
7.85	-0.99	12.17	21.44	11.31	1.50	3.15	11.60	4.42	-3.49	150.58	237.78	143.25	119.46
8.81	-0.06	13.30	22.46	12.37	2.48	4.09	12.42	5.23	-2.61	140.96	234.05	137.60	108.83
8.92	0.17	13.65	22.93	12.66	2.82	4.15	12.30	5.20	-2.47	120.46	206.41	100.08	68.52

8.38	-0.51	12.62	21.84	11.87	2.04	3.59	11.86	4.88	-3.05	144.21	241.57	141.98	110.93
8.79	-0.23	13.98	23.23	13.01	2.71	3.53	11.88	4.61	-3.17	146.15	237.58	136.46	117.42
8.50	-0.47	12.87	22.24	12.15	2.09	3.49	11.56	4.86	-3.03	136.82	228.99	118.00	87.01
8.51	-0.45	13.45	23.01	12.71	2.49	3.20	11.53	4.28	-3.39	148.72	243.08	139.02	120.62
8.68	-0.31	13.14	22.49	12.42	2.25	3.65	11.71	4.96	-2.90	136.78	230.25	118.42	86.46
8.57	-0.24	12.84	22.15	12.14	2.27	3.81	12.02	5.02	-2.75	135.99	236.75	129.60	97.68
9.00	0.21	13.50	22.75	12.62	2.81	4.28	12.63	5.40	-2.39	139.45	233.69	137.36	106.84
8.39	-0.84	13.11	22.91	12.11	1.98	3.72	12.12	4.68	-3.61	125.71	211.89	114.56	80.66
8.85	0.04	13.61	23.03	12.69	2.73	4.00	12.14	5.03	-2.65	121.06	207.63	100.78	69.02
9.21	0.51	13.64	22.93	12.78	3.03	4.52	12.79	5.63	-2.05	130.42	225.78	117.85	83.20
9.17	0.26	13.86	23.13	12.93	2.93	4.26	12.66	5.40	-2.41	147.88	236.38	145.93	117.63
8.56	-0.30	12.70	21.96	12.00	2.12	3.82	12.08	5.13	-2.70	150.12	254.12	160.45	131.30
8.26	-0.62	12.47	21.71	11.74	1.92	3.46	11.74	4.77	-3.16	144.55	241.44	142.19	111.50
8.27	-0.62	12.48	21.72	11.75	1.93	3.46	11.73	4.76	-3.16	144.69	241.45	142.29	111.65
9.33	0.63	13.85	23.10	12.87	3.15	4.76	12.95	5.80	-1.88	123.49	212.63	104.16	70.72
8.67	-0.62	13.49	23.26	12.45	2.17	4.00	12.35	4.87	-3.41	121.60	207.99	109.32	75.35
7.86	-1.06	12.55	22.27	12.02	1.81	2.72	10.94	3.75	-3.94	149.59	247.44	143.65	129.22
8.42	-0.69	12.63	22.09	12.09	1.90	3.33	11.32	4.74	-3.26	139.38	234.25	124.94	92.78
8.16	-1.20	12.85	22.34	12.13	1.64	2.87	10.84	4.18	-4.05	122.13	230.95	110.85	69.59
8.33	-0.65	12.44	21.81	11.93	1.90	3.32	11.33	4.73	-3.17	142.07	239.42	125.88	94.10
8.19	-0.54	12.17	21.50	11.57	1.91	3.56	11.71	4.81	-2.99	143.49	244.49	137.10	105.79
8.16	-0.88	12.26	21.67	11.73	1.67	3.08	11.08	4.57	-3.41	144.35	238.82	128.16	96.37
8.23	-0.61	12.58	21.88	11.78	2.00	3.47	11.81	4.68	-3.20	142.00	237.53	140.26	109.47
6.96	-1.58	11.52	20.83	10.96	1.27	1.79	9.97	2.98	-4.42	126.36	239.98	135.53	124.31
8.33	-1.45	12.73	22.02	12.26	1.35	3.06	11.18	4.40	-4.25	123.24	224.34	110.03	72.74
8.02	-0.82	12.12	21.40	11.45	1.64	3.25	11.47	4.58	-3.30	145.80	245.41	145.74	116.02
8.17	-1.19	12.88	22.37	12.15	1.66	2.91	10.87	4.20	-4.02	121.94	230.87	110.43	69.40
8.31	-0.44	12.79	22.30	11.94	2.22	3.66	11.76	4.65	-3.10	131.29	213.19	104.91	74.65
8.98	-0.46	14.29	23.98	12.95	2.42	4.19	12.74	5.00	-3.35	116.73	202.12	110.90	76.76
8.62	-0.29	13.32	22.70	12.38	2.35	3.69	12.14	4.86	-2.96	152.96	241.42	145.63	124.84
8.53	-0.34	12.65	22.00	12.07	2.12	3.64	11.63	4.98	-2.79	142.71	240.85	125.24	92.54
8.67	-0.51	12.99	22.39	12.34	2.13	3.63	11.71	4.98	-3.17	129.92	220.72	112.13	80.00
8.63	-0.36	13.33	22.60	12.41	2.36	3.68	12.09	4.85	-3.05	153.88	240.03	143.31	122.82
8.57	-0.33	12.75	22.13	12.17	2.19	3.66	11.67	4.98	-2.82	141.41	238.90	123.60	91.60
8.27	-0.67	12.76	22.12	11.94	1.98	3.48	11.77	4.61	-3.32	144.12	228.51	128.71	107.20
8.82	-0.06	13.76	23.15	12.78	2.70	3.90	12.03	4.88	-2.82	122.32	207.79	101.50	69.13
8.56	-0.24	12.84	22.14	12.13	2.27	3.81	12.01	5.02	-2.75	135.98	236.77	129.54	97.68
8.51	-0.92	13.25	23.00	12.55	1.97	3.38	11.48	4.46	-3.83	147.35	258.76	144.20	94.58
8.17	-0.66	12.44	21.74	11.67	1.90	3.45	11.81	4.66	-3.24	141.94	235.78	138.82	109.39
8.22	-0.58	12.34	21.65	11.70	1.90	3.48	11.68	4.75	-3.05	145.11	245.92	141.52	111.32
8.56	-0.15	13.42	22.96	12.42	2.65	3.67	12.00	4.70	-2.97	127.57	205.23	98.21	67.33
9.15	-0.28	14.23	23.89	13.08	2.58	4.51	12.82	5.25	-3.13	141.37	256.44	143.35	99.90
8.47	-0.42	13.16	22.64	12.35	2.34	3.57	11.85	4.60	-3.16	134.43	219.02	119.54	91.02

8.42	-0.45	13.35	22.94	12.32	2.35	3.66	11.93	4.57	-3.23	139.55	219.13	115.19	92.45
8.32	-0.57	12.57	21.81	11.83	1.95	3.50	11.76	4.82	-3.09	145.14	243.54	143.95	113.52
8.71	-0.11	13.43	22.84	12.49	2.58	3.90	12.03	4.91	-2.79	122.73	207.99	100.82	69.68
8.71	-0.80	13.83	23.47	12.89	2.23	3.60	11.76	4.53	-3.83	144.16	251.74	146.37	103.68
7.94	-0.92	12.70	22.39	11.90	1.96	3.03	11.45	3.96	-3.85	152.97	239.00	143.78	130.45
8.32	-0.40	12.51	21.75	11.88	2.11	3.53	11.66	4.80	-2.92	146.34	248.07	135.48	104.55
7.55	-1.37	11.83	21.13	10.97	1.18	2.83	11.28	4.11	-3.88	151.24	234.69	138.86	117.88
8.58	-0.43	12.85	22.18	12.16	2.11	3.63	11.67	5.01	-2.99	134.83	226.94	118.11	84.98
8.87	0.15	13.05	22.36	12.38	2.62	4.23	12.41	5.36	-2.34	130.88	230.82	123.63	89.45
8.62	-0.80	13.49	23.22	12.76	2.13	3.54	11.69	4.47	-3.70	134.15	246.09	135.37	86.92
8.39	-0.60	13.20	22.56	12.35	2.20	3.38	11.69	4.44	-3.38	150.23	240.49	136.65	119.47
8.62	-0.11	13.35	22.73	12.40	2.64	4.03	12.16	4.84	-2.83	130.00	207.90	103.22	72.98
7.86	-1.06	12.55	22.27	12.02	1.81	2.72	10.94	3.75	-3.94	149.60	247.45	143.66	129.23
8.46	-0.26	13.04	22.39	12.14	2.39	3.90	11.99	4.78	-2.93	129.91	209.23	102.49	72.55
9.29	0.46	14.16	23.42	13.08	3.13	4.46	12.62	5.46	-2.17	119.63	205.13	100.22	66.65
8.13	-0.76	12.54	21.94	11.77	1.91	3.41	11.64	4.47	-3.42	141.69	222.94	123.55	99.13
7.88	-0.97	11.93	21.23	11.30	1.51	3.09	11.34	4.46	-3.41	146.99	247.13	147.26	117.87
8.74	0.04	13.40	22.78	12.56	2.80	4.01	12.17	4.93	-2.73	127.91	205.31	101.22	73.21
9.06	0.13	13.80	23.08	12.88	2.87	4.09	12.47	5.21	-2.60	161.18	250.76	150.54	133.04
9.18	-0.29	14.42	24.21	13.11	2.56	4.53	13.00	5.27	-3.11	115.95	199.37	106.06	72.50
7.87	-1.00	12.22	21.62	11.45	1.53	3.04	11.51	4.32	-3.52	153.67	246.83	155.52	128.46
8.67	-0.20	13.30	22.69	12.45	2.45	3.61	11.75	4.90	-2.79	133.01	226.59	116.83	83.52
8.30	-0.84	13.09	22.73	12.26	2.01	3.09	11.03	4.33	-3.70	118.61	221.13	108.27	68.67
7.94	-0.98	12.64	22.28	12.01	1.90	2.85	11.13	3.88	-3.87	147.53	240.18	143.44	129.28
9.21	-0.27	14.24	23.97	12.95	2.53	4.80	13.43	5.49	-3.09	131.05	221.32	128.53	94.66
6.98	-1.44	11.44	20.83	10.86	1.42	1.82	10.09	3.11	-4.29	139.76	251.15	148.59	140.78
8.66	-0.15	12.93	22.21	12.22	2.35	3.93	12.13	5.12	-2.65	136.45	237.22	128.29	96.08
8.40	-0.83	13.12	22.91	12.12	1.98	3.73	12.13	4.69	-3.61	125.67	211.80	114.51	80.61
8.64	-0.20	13.44	22.87	12.46	2.57	3.97	12.10	4.80	-2.94	130.18	209.45	102.70	74.57
8.24	-0.41	12.20	21.50	11.62	2.03	3.60	11.68	4.84	-2.85	137.92	235.88	123.98	91.00
8.21	-0.79	13.07	22.73	12.27	2.01	3.21	11.51	4.17	-3.63	144.42	233.66	128.97	110.39
8.60	-0.20	13.09	22.56	12.28	2.52	3.93	12.14	4.92	-2.91	132.56	209.85	107.51	79.48
8.57	-0.85	13.14	22.89	12.47	2.02	3.70	11.76	4.68	-3.73	145.32	253.90	145.54	98.89
8.13	-0.75	13.31	22.78	12.58	2.57	2.87	11.13	3.68	-4.05	168.90	263.43	157.26	147.85
8.90	-0.13	14.09	23.40	13.09	2.74	3.56	12.00	4.71	-2.98	153.03	242.83	144.19	126.27
8.86	0.00	13.18	22.44	12.44	2.51	4.12	12.32	5.27	-2.50	137.01	237.67	132.22	97.76
8.47	-0.24	12.89	22.42	12.05	2.38	3.94	12.03	4.89	-2.86	128.29	213.07	103.37	72.20
9.40	0.49	14.51	23.91	13.43	3.23	4.42	12.59	5.38	-2.29	119.61	206.28	98.49	65.48
8.06	-1.18	12.79	22.65	11.90	1.73	3.24	11.68	4.23	-4.09	132.98	225.03	118.89	82.64
8.57	-0.88	13.18	22.91	12.51	2.01	3.63	11.68	4.62	-3.78	149.33	256.81	148.35	98.76
8.42	-0.34	12.62	21.84	11.96	2.19	3.60	11.76	4.87	-2.86	147.20	249.65	138.79	105.88
8.19	-0.64	12.59	21.84	11.72	1.89	3.46	11.85	4.67	-3.16	146.77	236.56	142.68	115.09
8.25	-0.58	13.10	22.69	12.13	2.29	3.44	11.75	4.38	-3.46	143.38	226.35	125.69	105.61

8.34	-0.65	13.60	23.28	12.52	2.55	3.43	11.93	4.15	-3.87	157.01	248.96	152.10	143.23
8.21	-0.61	12.37	21.68	11.75	1.89	3.42	11.64	4.70	-3.13	143.69	244.40	143.21	112.84
8.50	-0.18	12.50	21.76	11.87	2.24	3.90	11.95	5.07	-2.59	135.51	233.42	121.30	88.05
8.33	-0.44	12.37	21.76	11.74	1.99	3.68	11.86	4.90	-2.86	143.86	244.40	135.76	104.61
8.76	-0.30	13.16	22.56	12.47	2.28	3.74	11.80	5.06	-2.90	130.24	222.72	114.38	80.43
7.63	-1.18	11.89	21.49	11.51	1.53	2.61	10.77	3.74	-3.87	153.18	253.06	149.93	135.31
8.36	-0.60	13.31	23.15	12.27	2.32	3.68	12.12	4.44	-3.53	150.02	235.38	138.32	122.84
8.68	-0.10	13.39	22.84	12.44	2.58	4.07	12.19	4.92	-2.79	128.66	208.63	101.59	71.16
8.56	-0.94	13.20	22.45	12.51	1.85	3.37	11.48	4.57	-3.73	116.95	217.54	103.62	67.63
9.12	0.28	13.65	22.87	12.74	2.90	4.41	12.74	5.50	-2.31	138.36	232.39	136.56	105.93
8.11	-1.10	12.77	22.21	12.01	1.68	3.05	11.19	4.21	-3.91	128.09	239.92	125.27	81.14
8.57	-0.58	12.94	22.37	12.30	2.03	3.47	11.51	4.84	-3.20	135.35	228.28	119.76	87.21
8.47	-0.23	12.73	21.99	12.07	2.28	3.56	11.70	4.85	-2.76	149.46	256.79	139.84	107.72
8.61	-0.77	13.65	23.29	12.51	2.05	3.79	12.18	4.69	-3.58	115.91	203.11	110.06	73.73
8.39	-0.43	12.58	21.87	11.94	2.06	3.64	11.84	4.90	-2.92	144.64	246.56	142.38	112.78
8.59	-0.15	12.89	22.21	12.22	2.34	3.83	11.92	4.97	-2.68	133.22	231.16	116.67	83.91
8.49	-0.20	12.74	22.12	12.07	2.27	3.74	11.75	4.93	-2.68	137.17	235.26	120.47	87.60
8.03	-0.70	12.05	21.43	11.44	1.74	3.40	11.57	4.69	-3.14	145.74	244.47	139.13	108.97
8.22	-1.06	12.92	22.58	11.94	1.63	3.45	11.75	4.49	-3.76	119.37	210.64	113.22	77.13
8.16	-0.74	12.85	22.53	12.24	2.12	3.13	11.34	4.12	-3.60	143.73	237.59	140.54	124.79
8.33	-0.45	12.83	22.42	12.08	2.30	3.60	11.83	4.56	-3.21	136.44	214.64	110.89	85.79
8.33	-0.41	13.04	22.38	12.20	2.36	3.54	11.64	4.50	-3.19	130.32	207.17	102.40	73.90
9.26	-0.16	14.34	23.95	13.15	2.68	4.67	12.97	5.39	-3.01	142.42	256.18	142.42	99.17
8.12	-1.09	12.69	22.40	11.78	1.63	3.42	11.67	4.47	-3.77	119.48	212.04	113.14	77.74
8.32	-0.59	12.86	22.11	12.05	2.02	3.27	11.51	4.55	-3.16	165.82	260.20	157.06	142.27
8.59	-0.24	12.73	22.10	12.09	2.22	3.90	12.09	5.10	-2.70	140.62	240.37	134.85	102.08
8.59	-0.69	13.52	23.15	12.36	2.09	3.93	12.41	4.85	-3.49	118.30	205.76	111.81	78.20
8.15	-0.70	13.19	22.70	12.21	2.20	3.07	11.42	4.11	-3.61	135.69	217.69	118.08	94.15
9.07	-0.48	14.39	24.19	13.23	2.44	4.20	12.47	4.93	-3.40	139.59	247.97	138.86	94.91
8.52	-0.37	13.26	22.67	12.39	2.38	3.61	11.90	4.66	-3.12	134.53	219.93	121.30	94.45
8.64	-0.14	12.86	22.07	12.17	2.35	3.93	12.06	5.12	-2.63	141.06	241.85	131.12	98.30
8.17	-0.96	12.63	22.46	11.81	1.89	3.49	11.95	4.53	-3.80	132.89	224.15	119.22	83.41
8.35	-0.37	12.83	22.45	12.06	2.44	3.60	11.96	4.64	-3.19	144.25	225.20	125.55	106.45
8.47	-0.96	13.16	22.87	12.46	1.92	3.36	11.46	4.47	-3.84	149.68	260.33	145.60	97.30
8.11	-0.84	12.87	22.54	12.25	2.04	3.00	11.23	3.97	-3.72	145.07	240.54	139.46	124.51
8.26	-0.61	13.05	22.69	12.13	2.25	3.50	11.68	4.41	-3.46	135.09	217.23	109.82	81.48
7.13	-1.44	11.64	21.06	11.13	1.45	1.90	10.11	3.12	-4.31	140.64	251.08	147.49	137.77
9.12	0.38	13.70	22.89	12.75	2.93	4.36	12.56	5.48	-2.18	127.76	223.02	113.08	78.10
8.71	-0.80	13.82	23.47	12.89	2.23	3.60	11.75	4.53	-3.83	144.26	251.81	146.41	103.77
8.20	-0.94	12.74	22.56	11.87	1.92	3.54	12.01	4.56	-3.80	131.63	220.76	116.72	79.71
7.99	-1.20	12.57	22.26	11.70	1.50	3.23	11.52	4.29	-3.90	120.25	215.43	115.81	79.53
8.57	-0.92	13.63	23.33	12.75	2.08	3.48	11.67	4.41	-3.93	147.83	255.45	151.63	108.09
8.03	-0.76	12.66	22.06	11.85	2.05	3.24	11.45	4.23	-3.57	133.69	213.93	111.64	86.16

8.51	-0.34	13.37	22.91	12.36	2.52	3.70	12.02	4.62	-3.17	140.78	220.77	120.14	99.28
8.05	-0.74	12.67	22.07	11.86	2.06	3.26	11.47	4.24	-3.55	133.65	213.77	111.58	86.09
8.28	-0.42	12.30	21.62	11.71	2.06	3.58	11.66	4.84	-2.90	140.78	239.97	125.94	93.80
7.98	-0.86	12.98	22.48	12.36	2.43	2.79	11.07	3.60	-4.15	173.63	270.06	162.37	155.36
8.03	-1.39	12.79	22.24	11.94	1.56	2.94	11.17	4.10	-4.32	158.70	264.90	162.04	122.24
8.39	-0.49	12.65	21.87	11.90	2.06	3.59	11.86	4.88	-3.05	144.39	241.51	142.09	111.21
8.51	-0.92	13.25	23.00	12.55	1.97	3.38	11.48	4.46	-3.83	147.33	258.75	144.19	94.56
8.34	-0.48	12.48	21.80	11.82	2.00	3.61	11.81	4.85	-2.95	144.53	245.86	141.36	110.87
8.10	-0.82	12.71	22.42	12.16	2.02	3.04	11.23	4.02	-3.68	146.76	241.87	140.91	125.73
8.46	-0.27	12.50	21.82	11.92	2.20	3.77	11.85	4.99	-2.75	140.38	242.04	127.16	94.17
8.58	-0.86	13.36	23.05	12.60	2.04	3.47	11.56	4.54	-3.74	144.31	256.91	143.37	93.94
8.56	-0.33	12.87	22.06	12.09	2.24	3.77	12.04	5.03	-2.88	143.81	241.48	141.52	110.38
8.45	-0.42	13.25	22.82	12.33	2.40	3.70	11.86	4.58	-3.21	132.83	212.60	105.32	77.22
8.02	-0.87	12.19	21.46	11.48	1.61	3.20	11.46	4.57	-3.35	146.14	243.78	145.69	115.46
8.41	-0.55	13.32	22.75	12.48	2.30	3.24	11.58	4.30	-3.44	151.19	241.66	138.70	121.02
8.39	-0.45	12.55	21.89	11.89	2.04	3.63	11.86	4.85	-2.94	142.95	243.84	139.53	108.04
8.63	-0.25	13.26	22.64	12.41	2.41	3.56	11.68	4.85	-2.83	132.80	226.65	116.36	83.73
8.16	-0.77	12.58	21.98	11.80	1.86	3.39	11.68	4.53	-3.41	145.04	229.29	129.41	108.11
9.07	-0.48	14.39	24.19	13.23	2.44	4.20	12.47	4.93	-3.40	139.58	247.99	138.87	94.92
7.64	-1.15	11.98	21.69	11.61	1.64	2.58	10.78	3.68	-3.95	151.83	252.94	148.52	135.85
8.01	-0.85	12.75	22.38	11.74	2.01	3.25	11.47	4.29	-3.71	139.03	221.01	114.08	87.97
7.44	-1.74	11.87	21.47	11.25	1.03	2.31	10.39	3.62	-4.49	128.53	245.38	125.79	85.14
7.23	-1.41	11.82	21.14	11.24	1.43	2.01	10.19	3.21	-4.29	135.47	246.17	142.05	131.52
7.14	-1.51	11.43	20.98	11.10	1.27	1.88	10.18	3.17	-4.27	157.45	259.32	156.81	147.60
7.60	-2.40	11.66	20.93	11.29	0.20	2.45	10.62	3.94	-4.98	128.12	231.63	118.09	84.51
6.72	-2.38	10.53	20.07	10.31	0.58	1.61	9.89	3.15	-5.31	205.81	342.28	195.27	154.63
7.12	-1.44	11.56	20.95	11.14	1.74	2.03	10.43	3.07	-4.63	201.65	300.96	201.52	212.97
7.52	-1.61	12.05	21.80	11.32	1.16	2.50	10.45	3.72	-4.38	130.29	222.27	112.37	77.72
8.00	-0.93	12.84	22.51	11.90	2.02	3.23	11.61	4.11	-3.86	150.20	234.06	137.17	121.37
7.12	-1.96	11.46	21.28	10.89	0.77	1.97	9.89	3.34	-4.71	135.24	232.96	118.39	84.35
7.05	-1.89	10.89	20.47	10.65	0.83	1.93	10.19	3.45	-4.61	155.44	288.11	154.60	110.02
7.03	-2.10	11.08	20.81	10.60	0.54	2.21	10.43	3.43	-4.74	139.87	250.20	136.71	100.35
8.07	-0.77	13.07	22.74	12.20	2.35	3.12	11.48	3.92	-3.93	162.94	253.64	147.36	133.26
8.31	-0.91	12.49	21.80	11.88	1.68	3.36	11.39	4.75	-3.53	132.32	222.20	116.18	85.14
7.57	-1.63	12.11	21.99	11.39	1.07	2.70	10.89	3.74	-4.36	128.23	225.69	118.16	84.58
7.23	-1.69	11.14	20.80	10.90	1.03	2.10	10.35	3.56	-4.40	158.06	289.94	154.49	109.55
7.30	-1.44	11.81	21.41	11.36	1.40	2.09	10.30	3.29	-4.28	146.12	252.41	150.89	140.42
8.02	-0.93	12.52	22.27	11.54	1.74	3.51	11.55	4.49	-3.59	130.13	218.64	107.15	75.41
7.79	-1.28	12.39	22.04	11.54	1.43	2.95	11.15	4.04	-3.98	122.00	225.39	117.10	80.26
7.33	-1.22	10.81	20.11	10.59	1.33	2.43	10.52	4.06	-3.73	141.63	271.83	135.32	92.99
7.66	-1.58	12.25	21.89	11.50	1.15	2.46	10.46	3.79	-4.33	126.42	238.98	119.91	76.14
7.58	-1.51	12.24	21.93	11.53	1.26	2.40	10.43	3.64	-4.27	125.35	231.16	118.00	79.15
7.51	-1.63	11.90	21.59	11.22	1.06	2.41	10.45	3.79	-4.34	127.08	238.25	119.90	79.15

6.78	-2.29	10.69	20.10	10.30	0.36	1.85	10.07	3.27	-4.97	147.62	273.61	147.58	105.33
8.54	-0.80	13.36	23.01	12.73	2.38	3.20	11.50	4.35	-3.99	172.01	288.17	161.72	103.62
7.38	-1.75	11.85	21.67	11.19	1.01	2.31	10.24	3.61	-4.51	131.42	224.99	114.25	79.63
7.01	-1.73	11.18	20.34	10.62	0.81	1.96	10.12	3.40	-4.23	157.32	262.76	160.22	144.91
7.82	-1.37	12.33	21.88	11.66	1.34	2.75	10.87	3.98	-4.07	128.02	246.34	124.14	79.06
8.93	0.08	13.18	22.39	12.33	2.52	4.35	12.59	5.53	-2.36	146.03	243.75	147.65	117.56
7.54	-1.77	11.71	21.38	11.24	1.09	2.58	10.72	3.84	-4.64	170.88	289.08	167.21	117.85
7.59	-1.49	12.01	21.65	11.32	1.18	2.73	10.78	3.88	-4.19	126.35	231.86	121.74	81.81
8.22	-0.68	13.09	22.82	12.03	2.06	3.53	11.75	4.41	-3.46	134.71	221.67	109.97	82.37
7.72	-1.36	12.15	21.86	11.48	1.36	2.65	10.58	3.96	-4.04	124.58	233.83	116.57	74.96
8.03	-0.79	12.06	21.38	11.41	1.61	3.28	11.60	4.66	-3.17	150.50	253.85	160.32	132.32
8.31	-0.54	12.50	21.72	11.76	1.90	3.54	11.74	4.87	-2.99	149.45	254.95	154.93	124.89
7.95	-0.88	12.51	22.01	11.80	1.87	3.16	11.40	4.10	-3.67	141.19	224.44	125.45	103.93
8.26	-0.47	12.28	21.61	11.67	1.98	3.64	11.78	4.88	-2.92	144.32	244.86	138.25	106.20
7.31	-1.76	11.50	21.00	11.00	0.95	2.22	10.39	3.60	-4.42	135.87	258.54	130.66	86.92
7.76	-1.93	11.93	21.25	11.44	0.63	2.69	10.80	4.10	-4.49	135.34	226.82	121.31	90.59
7.33	-2.47	11.36	20.66	10.94	0.14	2.22	10.37	3.75	-5.06	133.05	230.87	120.06	88.89
6.68	-2.28	10.61	19.97	10.23	0.35	1.62	9.80	3.11	-4.91	141.48	271.66	137.95	95.40
7.50	-1.39	11.65	20.95	10.80	1.04	2.90	11.32	4.21	-3.82	153.99	236.86	140.32	118.82
8.05	-0.78	12.71	22.28	11.87	2.05	3.30	11.48	4.23	-3.57	135.62	216.51	110.58	84.37
7.74	-1.13	12.47	22.08	11.45	1.67	3.01	11.33	4.02	-3.93	147.39	229.90	126.33	105.35
8.74	-0.12	13.02	22.31	12.30	2.41	3.99	12.20	5.16	-2.64	143.31	242.12	137.45	105.42
7.71	-2.33	11.77	21.05	11.39	0.27	2.60	10.75	4.04	-4.88	129.12	227.27	117.22	84.67
7.40	-1.79	11.41	20.98	11.12	1.22	2.26	10.47	3.67	-4.79	196.83	322.25	183.91	134.98
7.94	-1.69	12.59	22.08	12.24	1.71	2.38	10.59	3.63	-5.08	203.60	325.72	195.93	144.92
7.54	-1.21	11.89	21.57	11.53	1.58	2.44	10.68	3.57	-3.98	151.22	253.87	150.33	137.19
7.26	-2.71	11.31	20.54	11.03	-0.08	1.96	10.12	3.53	-5.33	133.01	240.23	123.13	88.26
7.51	-1.29	11.26	20.55	11.11	1.50	2.34	10.60	3.92	-4.07	142.94	272.73	139.79	97.95
7.66	-1.51	12.30	22.25	11.45	1.24	2.78	10.94	3.87	-4.29	129.75	221.38	115.08	80.82
7.40	-1.38	11.41	20.77	10.71	1.08	2.79	11.21	4.11	-3.84	142.86	232.15	137.87	110.11
6.73	-1.68	11.26	20.57	11.00	1.63	1.26	9.69	2.47	-5.01	198.35	300.44	217.27	235.90
7.53	-1.51	11.94	21.60	11.24	1.14	2.65	10.76	3.87	-4.18	126.30	229.92	120.43	82.37
7.47	-1.48	12.21	21.85	11.46	1.42	2.39	10.73	3.47	-4.40	157.35	240.26	141.05	127.69
7.30	-1.11	11.75	21.01	11.08	1.66	2.32	10.52	3.54	-3.89	150.01	245.13	159.00	153.41
7.77	-2.19	11.78	21.08	11.43	0.38	2.69	10.84	4.14	-4.77	127.77	227.18	115.92	81.96
7.26	-1.79	11.44	20.94	10.95	0.92	2.19	10.37	3.57	-4.45	135.92	259.42	131.58	87.93
8.15	-1.07	12.68	22.27	11.99	1.69	3.17	11.27	4.32	-3.87	125.04	238.20	122.19	78.09
7.43	-1.70	11.74	21.24	11.16	1.01	2.36	10.46	3.71	-4.40	130.62	251.93	125.33	82.41
6.93	-2.09	10.99	20.65	10.52	0.61	2.02	10.24	3.35	-4.79	136.72	254.90	136.31	98.70
6.93	-2.07	10.69	20.19	10.39	0.55	2.05	10.22	3.44	-4.66	141.68	266.47	139.34	97.17
7.66	-1.03	11.97	21.17	11.37	1.54	2.70	10.83	3.97	-3.57	154.30	256.83	158.89	137.75
7.31	-1.69	11.69	21.47	10.94	1.08	2.45	10.46	3.66	-4.44	136.67	228.60	116.42	82.73
8.02	-0.94	12.87	22.70	11.83	1.87	3.35	11.56	4.20	-3.75	134.15	220.82	109.25	78.67

7.58	-2.53	11.65	20.90	11.27	0.07	2.40	10.56	3.89	-5.14	130.29	232.15	119.73	86.22
8.15	-0.63	12.29	21.54	11.65	1.87	3.30	11.53	4.65	-3.15	151.97	258.98	155.66	127.54
8.17	-1.01	12.65	22.62	11.89	1.75	3.45	11.77	4.43	-3.77	126.70	218.19	115.35	81.54
7.43	-1.60	11.90	21.65	11.17	1.14	2.50	10.49	3.71	-4.35	132.53	224.71	113.10	79.50
7.79	-1.38	12.25	22.38	11.51	1.39	3.00	11.32	4.03	-4.10	132.80	225.92	120.37	85.39
7.25	-1.27	12.01	21.30	11.26	1.60	2.00	10.25	3.21	-4.13	141.74	242.42	149.64	140.68
7.88	-1.02	12.64	22.52	11.62	1.76	3.25	11.48	4.12	-3.82	138.96	225.09	112.48	83.28
7.88	-1.12	12.44	21.88	11.73	1.62	2.95	11.26	4.02	-3.84	152.89	238.84	137.01	119.46
8.05	-0.90	13.05	22.91	12.02	2.12	3.25	11.60	4.03	-3.90	149.31	235.65	128.61	106.32
7.04	-2.99	10.85	20.11	10.65	-0.41	1.77	9.86	3.41	-5.59	137.69	243.17	126.87	94.25
8.13	-1.32	12.87	22.38	12.00	1.59	3.03	11.19	4.22	-4.23	156.67	256.84	159.11	119.57
7.64	-1.45	12.23	22.20	11.40	1.33	2.84	11.11	3.91	-4.24	132.94	224.86	118.06	83.78
7.31	-1.85	11.65	21.38	11.00	0.89	2.19	10.27	3.61	-4.57	129.89	241.21	122.94	83.23
7.07	-1.94	11.35	21.06	10.80	0.76	1.95	10.07	3.36	-4.65	131.37	247.41	127.23	87.67
7.94	-1.01	12.34	21.72	11.56	1.61	3.17	11.48	4.30	-3.62	148.79	232.94	131.82	112.23
8.00	-0.95	12.83	22.62	11.83	1.95	3.41	11.74	4.17	-3.82	147.95	234.18	129.05	108.99
7.65	-1.32	11.72	21.36	11.17	1.29	2.69	10.65	4.13	-3.95	126.79	223.29	109.86	76.24
8.08	-1.14	12.76	22.45	11.82	1.59	3.32	11.62	4.36	-3.87	123.82	214.96	115.13	79.13
6.99	-1.63	11.16	20.52	10.85	1.04	1.80	10.04	3.12	-4.37	155.83	260.77	157.37	145.52
7.49	-1.56	11.95	21.71	11.21	1.16	2.36	10.37	3.79	-4.29	128.94	238.85	120.12	79.87
7.71	-1.37	12.08	21.78	11.41	1.31	2.80	10.80	4.01	-4.03	122.71	235.04	119.10	78.11
8.57	-0.75	13.25	22.96	12.64	2.33	3.32	11.58	4.47	-3.83	167.36	284.17	155.74	97.17
7.48	-1.33	12.02	21.47	11.58	1.45	2.15	10.36	3.38	-4.10	153.42	255.83	151.94	137.84
7.61	-1.22	12.51	22.00	11.99	2.20	2.33	10.57	3.18	-4.62	176.64	274.08	159.95	149.42
8.26	-1.25	13.07	22.65	12.26	1.74	3.14	11.20	4.24	-4.23	159.18	256.50	157.82	114.50
8.11	-1.26	12.21	21.54	11.65	1.34	3.11	11.20	4.58	-3.86	132.16	224.50	117.20	85.75
6.93	-2.05	10.65	20.13	10.36	0.58	2.07	10.22	3.47	-4.67	143.83	267.88	139.61	97.92
7.34	-1.76	11.74	21.58	11.07	0.94	2.45	10.51	3.62	-4.45	132.20	226.93	118.28	83.66
8.34	-0.59	13.19	22.63	12.41	2.24	3.21	11.54	4.26	-3.47	150.85	242.85	139.11	121.18
8.17	-1.34	12.75	22.20	12.35	1.95	2.75	10.93	4.00	-4.64	197.57	318.94	187.65	137.09
7.26	-2.82	11.01	20.29	10.83	-0.23	2.10	10.24	3.68	-5.40	138.84	238.67	125.81	91.72
7.83	-1.38	12.57	22.17	11.72	1.37	2.62	10.55	3.93	-4.12	124.40	236.22	115.14	72.17
7.68	-1.08	11.94	21.50	11.48	1.62	2.75	10.97	3.90	-3.79	154.98	251.24	147.57	135.08
8.38	-0.52	13.22	22.95	12.18	2.20	3.79	11.94	4.59	-3.23	129.52	216.38	104.85	74.73
7.59	-1.58	12.05	21.70	11.34	1.12	2.47	10.52	3.81	-4.27	126.41	237.48	119.41	77.96
7.34	-1.76	11.81	21.52	11.17	0.95	2.17	10.28	3.54	-4.48	127.80	241.72	123.03	83.21
8.13	-0.78	13.12	22.80	12.21	2.16	3.30	11.66	4.06	-3.71	152.20	236.67	143.64	130.10
8.40	-0.45	12.47	21.75	11.73	1.97	3.84	12.08	5.07	-2.86	145.41	242.92	146.11	117.73
7.60	-1.25	12.27	22.00	11.35	1.57	2.90	11.17	3.86	-4.08	147.94	232.60	125.59	101.16
7.76	-1.30	12.20	21.91	11.42	1.36	2.96	11.11	4.10	-4.01	123.02	225.52	117.69	80.43
7.91	-1.22	12.45	22.11	11.78	1.58	2.93	11.09	4.05	-4.03	123.68	239.27	125.71	84.68
8.61	-0.72	13.35	23.02	12.73	2.36	3.36	11.60	4.50	-3.79	166.54	280.19	153.83	93.94
8.25	-0.65	13.12	22.80	12.19	2.18	3.44	11.66	4.31	-3.47	137.66	218.97	114.54	89.30

7.68	-2.45	11.85	21.10	11.45	0.15	2.49	10.66	3.91	-5.07	128.99	229.93	118.52	83.70
7.94	-0.92	12.04	21.33	11.35	1.50	3.19	11.53	4.53	-3.32	152.87	253.01	162.30	133.27
7.61	-1.36	12.53	22.19	11.53	1.62	2.74	11.15	3.64	-4.32	151.23	235.25	133.83	115.18
7.91	-1.04	12.44	22.28	11.54	1.70	3.32	11.45	4.29	-3.80	133.31	222.58	110.80	79.62
8.21	-0.63	12.25	21.51	11.54	1.80	3.61	11.88	4.86	-3.07	145.37	243.20	145.45	115.86
7.99	-1.30	12.38	22.08	11.82	1.61	2.86	11.08	4.13	-4.19	172.36	291.93	164.20	108.91
7.92	-0.87	11.85	21.21	11.38	1.60	2.92	10.91	4.48	-3.33	150.31	251.86	134.90	103.88
7.13	-2.19	11.23	20.46	10.84	0.53	1.72	9.78	3.41	-4.91	137.31	258.38	128.61	87.22
8.04	-0.97	12.60	22.37	11.67	1.75	3.38	11.46	4.39	-3.68	129.30	219.13	106.24	75.07
8.52	-0.16	12.60	21.88	11.98	2.28	3.90	11.96	5.08	-2.58	136.07	233.65	121.02	87.69
8.32	-0.38	12.24	21.55	11.78	2.08	3.13	11.18	4.84	-2.83	164.46	264.36	145.35	121.29
7.50	-1.61	11.87	21.40	11.24	1.10	2.43	10.57	3.76	-4.33	128.25	248.87	126.05	82.37
7.58	-1.49	12.21	21.95	11.34	1.26	2.64	10.69	3.81	-4.26	132.98	222.88	113.23	78.71
7.69	-1.49	12.36	22.32	11.50	1.26	2.79	10.96	3.86	-4.27	129.62	223.70	115.61	80.13
7.36	-1.61	12.13	21.73	11.21	1.34	2.43	10.83	3.48	-4.53	156.05	240.66	139.49	121.86
7.59	-1.05	11.93	21.18	11.36	1.52	2.58	10.72	3.85	-3.63	153.96	257.26	159.55	138.54
7.50	-1.37	11.96	21.70	11.08	1.35	2.80	10.92	3.91	-4.11	137.39	227.49	113.61	83.29
7.96	-0.88	12.51	22.02	11.80	1.88	3.17	11.40	4.10	-3.66	141.12	224.33	125.37	103.83
7.03	-1.46	11.60	20.99	11.08	1.66	1.69	10.08	2.96	-4.56	184.12	286.04	196.40	201.74
7.18	-2.71	11.04	20.34	10.82	-0.14	2.01	10.15	3.53	-5.27	132.36	240.38	123.50	89.41
8.10	-0.91	12.95	22.76	11.91	1.89	3.40	11.61	4.26	-3.71	132.40	219.05	108.00	77.06
8.53	-0.77	13.35	23.13	12.28	2.02	3.84	12.26	4.75	-3.54	122.36	210.26	113.40	79.23
7.96	-0.95	13.00	22.63	12.08	2.07	3.00	11.37	3.81	-3.95	158.89	241.44	152.32	143.51
6.94	-1.51	11.57	20.88	11.09	1.66	1.50	9.93	2.81	-4.73	189.89	290.25	203.41	212.74
7.01	-2.02	11.32	20.98	10.71	0.67	2.03	10.13	3.35	-4.69	131.66	240.85	126.42	90.22
7.23	-1.41	11.67	21.03	11.17	1.38	2.07	10.22	3.29	-4.21	139.14	249.14	143.24	132.17
7.62	-1.44	12.14	21.77	11.35	1.29	2.70	10.81	3.88	-4.16	125.16	229.65	119.83	81.60
7.21	-2.14	11.39	21.09	10.96	0.79	2.18	10.38	3.47	-5.05	176.74	296.40	175.74	133.27
6.94	-2.08	10.99	20.66	10.53	0.62	2.03	10.24	3.37	-4.78	136.73	254.80	136.07	98.70
8.29	-0.55	12.33	21.60	11.61	1.88	3.71	11.96	4.96	-2.97	145.77	243.13	146.54	117.38
7.00	-2.05	11.15	20.65	10.60	0.58	1.82	9.84	3.38	-4.65	134.32	257.05	127.11	84.79
7.09	-2.34	11.54	21.17	11.08	0.71	1.84	10.10	3.11	-5.41	184.44	299.82	182.01	139.18
7.54	-1.41	12.46	22.09	11.47	1.56	2.68	11.06	3.61	-4.38	153.22	234.86	133.57	114.67
7.56	-1.32	12.37	21.83	11.73	1.75	2.27	10.59	3.35	-4.40	168.36	262.23	173.95	175.54
6.94	-1.67	11.09	20.44	10.79	0.99	1.74	9.97	3.06	-4.41	156.29	261.45	158.18	147.13
8.14	-1.31	12.89	22.39	12.02	1.60	3.04	11.20	4.22	-4.23	156.56	256.55	158.92	119.34
7.21	-2.06	11.43	20.83	10.99	0.70	1.90	9.92	3.45	-4.84	135.60	252.95	122.71	83.86
7.49	-1.47	12.32	21.99	11.40	1.46	2.64	11.03	3.59	-4.44	156.44	239.62	138.26	120.62
7.05	-2.36	11.46	21.11	10.98	0.70	1.81	10.07	3.08	-5.41	182.57	300.08	181.66	141.08
7.36	-1.48	11.24	20.65	10.93	1.24	2.30	10.55	3.80	-4.19	151.81	283.85	148.98	104.87
7.57	-1.48	12.16	21.94	11.34	1.28	2.60	10.61	3.79	-4.28	129.76	221.98	112.49	77.74
7.15	-1.40	11.79	21.29	10.97	1.24	1.86	10.10	3.31	-4.02	145.08	250.00	163.36	141.12
7.66	-1.30	12.51	22.14	11.53	1.61	2.79	11.16	3.80	-4.21	151.54	234.45	133.06	115.07

7.04	-1.93	10.99	20.33	10.55	0.70	2.11	10.16	3.55	-4.53	138.68	264.51	133.20	88.05
6.76	-2.38	10.76	20.25	10.27	0.41	1.85	10.12	3.24	-5.18	177.98	293.30	184.34	150.31
8.23	-0.61	12.83	22.51	11.86	2.08	3.62	11.75	4.59	-3.29	131.10	215.51	106.39	75.75
7.77	-1.01	12.08	21.64	11.62	1.69	2.80	11.03	3.95	-3.74	153.99	249.30	145.91	133.31
8.36	-0.39	13.18	22.62	12.25	2.34	3.50	11.58	4.45	-3.10	128.03	208.01	102.46	72.17
7.94	-1.33	12.32	21.88	11.75	1.43	2.95	11.12	4.10	-4.08	136.13	254.15	134.45	88.94
7.56	-1.59	11.95	21.55	11.24	1.12	2.59	10.63	3.90	-4.30	127.00	242.77	123.33	80.83
7.95	-0.95	12.76	22.63	11.72	1.85	3.35	11.55	4.17	-3.76	134.19	222.34	110.19	80.09
7.62	-1.68	12.13	21.60	11.42	1.08	2.34	10.30	3.80	-4.46	129.53	245.95	117.45	76.44
6.94	-1.46	11.31	20.61	10.80	1.32	1.92	10.02	3.08	-4.22	128.49	230.35	138.53	128.04
8.65	-0.68	13.42	23.08	12.77	2.39	3.41	11.65	4.53	-3.74	165.25	278.17	153.04	92.59
8.77	-0.07	12.96	22.18	12.16	2.35	4.23	12.44	5.40	-2.50	146.11	243.25	146.87	116.92
7.16	-1.70	11.53	21.03	11.08	1.23	2.07	10.39	3.26	-4.62	166.60	256.70	164.27	161.14
6.97	-1.38	11.28	20.55	10.67	1.41	1.91	10.25	3.24	-4.18	165.97	264.89	177.84	177.71
7.24	-1.50	11.63	21.06	11.24	1.25	1.95	10.18	3.22	-4.24	154.50	259.69	155.04	142.43
7.60	-1.40	12.50	22.29	11.65	1.64	2.70	11.00	3.56	-4.43	151.80	241.99	127.52	102.71
7.71	-2.43	11.89	21.13	11.48	0.18	2.53	10.69	3.95	-5.06	129.12	229.80	118.22	83.31
8.54	-0.95	13.59	23.14	12.71	2.10	3.32	11.47	4.37	-4.04	150.46	254.03	152.23	108.25
8.05	-1.03	12.55	22.16	11.88	1.72	2.92	10.91	4.23	-3.80	119.34	220.50	108.07	70.77
6.92	-1.51	11.69	21.00	11.26	1.84	1.33	9.80	2.57	-4.86	190.77	290.38	208.46	219.28
7.50	-2.25	11.57	20.89	11.13	0.35	2.38	10.51	3.88	-4.85	133.89	227.14	119.81	88.68
7.59	-1.91	12.33	22.00	11.65	1.14	2.38	10.54	3.54	-4.97	174.09	280.36	172.31	128.29
8.60	-0.27	12.70	21.90	11.95	2.14	3.99	12.22	5.25	-2.71	147.57	244.71	149.05	118.52
8.16	-0.49	12.10	21.42	11.54	1.95	3.51	11.59	4.76	-2.94	139.49	238.07	125.43	93.35
7.40	-2.70	11.41	20.65	11.06	-0.11	2.21	10.38	3.76	-5.31	134.75	234.44	121.68	89.27
7.21	-1.45	12.18	21.55	11.58	1.86	1.86	10.27	2.82	-4.80	184.51	279.04	204.87	218.37
7.58	-2.12	11.68	20.99	11.21	0.47	2.52	10.65	3.95	-4.68	133.74	226.31	118.13	87.09
7.44	-1.70	11.94	21.73	11.26	1.04	2.42	10.34	3.67	-4.45	129.79	224.49	112.84	78.65
7.49	-1.27	11.46	20.94	10.76	1.13	2.91	11.34	4.22	-3.66	146.22	235.65	142.81	115.32
7.46	-2.14	11.96	21.46	11.75	1.29	1.88	10.11	3.17	-5.55	213.85	341.37	208.16	161.84
7.85	-1.00	13.05	22.67	12.26	2.41	2.60	10.94	3.46	-4.39	167.94	262.44	153.21	140.92
7.44	-2.54	11.54	20.76	11.17	0.07	2.18	10.31	3.68	-5.17	131.97	237.13	121.19	86.13
7.72	-1.06	12.39	22.06	11.41	1.79	2.96	11.23	4.04	-3.94	141.26	225.32	118.45	92.90
8.07	-1.18	12.76	22.41	11.99	1.60	2.99	11.13	4.12	-3.99	124.17	238.90	122.89	78.41
7.77	-1.10	12.77	22.22	12.22	2.22	2.48	10.74	3.35	-4.41	181.98	278.45	168.81	163.68
7.84	-1.27	12.54	22.16	11.68	1.47	2.92	11.13	3.98	-4.04	123.42	225.27	118.75	79.71
7.23	-1.32	12.01	21.42	11.35	1.77	1.76	10.17	3.11	-4.42	174.07	277.64	188.01	185.39
7.47	-1.61	11.76	21.39	11.12	1.11	2.54	10.64	3.81	-4.31	127.70	243.58	126.47	83.87
6.87	-1.66	11.47	20.81	11.16	1.70	1.44	9.84	2.58	-5.06	195.57	294.55	215.21	235.37
7.76	-1.07	12.65	22.18	12.01	2.03	2.44	10.75	3.52	-4.16	162.56	260.25	167.85	165.80
7.41	-1.62	11.83	21.57	11.11	1.10	2.28	10.31	3.74	-4.35	129.98	239.73	121.03	81.17
7.45	-1.08	12.22	21.53	11.44	1.83	2.21	10.49	3.47	-3.97	146.87	246.49	156.13	148.00
7.94	-0.83	11.88	21.17	11.28	1.54	3.15	11.36	4.63	-3.22	159.70	264.79	163.74	137.79

8.00	-0.79	12.65	22.27	11.73	1.98	3.26	11.47	4.25	-3.57	136.25	219.24	110.61	81.09
8.27	-0.69	13.78	23.50	12.72	2.71	2.96	11.35	3.77	-4.08	162.90	254.22	145.87	132.28
7.44	-1.83	11.58	20.92	11.23	0.93	2.06	10.07	3.62	-4.59	131.21	249.35	122.27	81.42
7.46	-1.59	11.90	21.64	11.16	1.14	2.32	10.34	3.77	-4.32	129.45	239.07	120.50	80.50
7.30	-2.05	11.47	20.99	11.31	1.22	1.98	10.16	3.29	-5.31	211.27	339.48	203.56	159.43
8.08	-0.85	12.97	22.60	11.92	1.98	3.29	11.59	4.23	-3.68	143.31	224.66	120.90	98.73
7.59	-1.38	12.49	22.11	11.50	1.55	2.78	11.15	3.72	-4.30	153.77	235.18	133.67	115.74
7.59	-1.30	12.31	21.83	11.72	1.76	2.37	10.67	3.46	-4.36	164.65	255.30	163.73	161.06
7.82	-1.24	12.46	22.22	11.61	1.51	2.95	11.00	4.00	-4.01	129.72	217.41	109.92	75.56
6.99	-2.04	11.06	20.73	10.59	0.66	2.07	10.27	3.40	-4.76	136.30	254.21	135.59	98.02
8.39	-0.40	12.61	21.89	11.94	2.09	3.51	11.75	4.85	-2.87	152.09	259.20	162.22	134.24
7.47	-1.55	11.90	21.70	11.15	1.19	2.58	10.61	3.75	-4.27	132.65	225.15	113.82	80.14
7.00	-1.99	11.15	20.75	10.56	0.69	2.07	10.21	3.43	-4.67	133.65	245.23	128.81	92.24
7.66	-1.26	12.44	22.24	11.42	1.68	2.91	11.25	3.87	-4.20	150.13	237.37	126.00	101.99
7.66	-0.90	12.04	21.40	11.32	1.63	2.80	10.85	4.00	-3.42	135.03	237.88	143.51	118.82
8.18	-0.86	12.70	22.34	11.87	1.84	3.28	11.30	4.47	-3.57	125.37	214.74	104.89	70.51
7.47	-1.17	12.02	21.40	11.57	2.05	2.43	10.70	3.38	-4.38	196.37	293.62	184.58	185.56
8.34	-1.04	12.96	22.74	12.37	1.83	3.35	11.44	4.32	-3.93	140.10	254.19	138.59	90.24
7.50	-1.57	11.86	21.50	11.20	1.11	2.62	10.68	3.80	-4.26	127.79	234.04	122.94	83.27
7.60	-1.07	11.93	21.06	11.28	1.59	2.53	10.76	3.89	-3.73	167.75	264.52	162.39	149.41
6.89	-1.60	11.09	20.38	10.62	1.01	1.78	9.94	3.17	-4.22	147.10	258.58	155.45	139.01
7.25	-1.38	11.88	21.22	11.28	1.52	2.01	10.21	3.23	-4.26	136.00	247.36	143.20	131.58
7.72	-2.26	11.76	21.04	11.40	0.31	2.63	10.78	4.06	-4.83	128.41	227.56	117.09	83.52
8.27	-0.89	13.01	22.90	12.03	1.98	3.57	12.00	4.53	-3.76	130.65	219.40	115.60	78.87
7.53	-1.28	12.46	22.02	11.82	1.96	2.35	10.77	3.21	-4.52	184.06	275.84	188.51	197.34
8.09	-1.44	12.17	21.49	11.65	1.12	3.17	11.28	4.54	-4.05	129.08	220.03	113.47	81.27
7.26	-1.83	11.23	20.74	10.84	0.82	2.35	10.48	3.67	-4.48	138.17	259.61	135.60	91.96
7.64	-1.49	12.33	22.18	11.51	1.36	2.81	11.10	3.81	-4.29	139.04	230.59	118.54	83.62
7.97	-0.90	12.01	21.31	11.49	1.62	2.91	10.92	4.46	-3.42	149.55	246.39	132.72	101.62
8.09	-1.04	12.52	22.16	11.78	1.66	3.24	11.29	4.37	-3.77	121.40	232.96	120.43	77.01
8.62	-0.71	13.39	23.07	12.76	2.39	3.37	11.59	4.50	-3.77	166.90	282.53	155.07	92.32
7.59	-1.49	12.00	21.64	11.32	1.18	2.72	10.77	3.88	-4.20	126.42	231.98	121.81	81.90
7.99	-1.24	12.76	22.32	11.94	1.52	2.91	11.06	4.05	-3.99	122.68	229.22	119.67	78.03
7.18	-2.28	11.68	21.31	11.19	0.79	1.93	10.17	3.18	-5.35	183.54	297.17	180.42	136.85
7.47	-1.08	12.22	21.69	11.60	1.93	2.18	10.48	3.37	-4.09	152.04	256.89	162.43	155.65
7.28	-1.83	11.43	21.08	10.90	0.90	2.31	10.43	3.66	-4.50	129.24	248.86	128.51	87.61
7.60	-1.28	11.60	20.88	10.94	1.14	2.84	11.16	4.28	-3.70	148.83	242.38	148.72	119.92
7.86	-1.44	12.21	21.63	11.66	1.30	2.69	10.70	4.05	-4.19	124.96	234.80	112.41	73.57
7.66	-1.50	12.24	22.11	11.43	1.28	2.85	11.13	3.92	-4.27	138.26	230.11	118.69	83.48
8.43	-0.67	12.88	22.52	12.45	2.19	3.21	11.30	4.41	-3.52	148.08	263.33	145.32	91.78
7.71	-1.44	12.34	22.47	11.56	1.36	2.86	11.16	3.89	-4.23	134.77	227.50	119.02	85.40
8.43	-0.64	12.71	22.31	12.33	2.24	3.33	11.38	4.53	-3.54	153.12	266.31	145.85	93.34
8.37	-0.44	13.00	22.51	12.21	2.36	3.67	11.83	4.54	-3.21	132.57	212.38	110.21	82.62

7.39	-1.63	11.65	21.33	11.06	1.02	2.46	10.53	3.75	-4.29	127.69	241.92	125.15	84.54
8.53	-0.10	12.49	21.73	11.86	2.31	3.98	12.03	5.18	-2.55	136.08	234.51	122.19	88.36
7.37	-2.40	11.33	20.69	11.00	0.15	2.26	10.41	3.76	-4.94	136.49	230.48	123.41	93.15
8.05	-0.72	12.25	21.69	11.91	2.15	2.71	10.82	4.17	-3.57	132.19	256.73	126.15	82.62
7.59	-1.10	12.42	21.96	11.87	2.07	2.22	10.55	3.33	-4.28	163.28	265.04	173.54	171.32
6.96	-1.61	11.64	20.97	11.30	1.75	1.52	9.90	2.64	-4.99	193.41	291.66	210.14	229.97
8.30	-0.77	12.35	21.73	11.77	1.76	3.41	11.43	4.84	-3.27	135.30	224.50	117.80	86.04
7.58	-1.54	11.98	21.65	11.32	1.17	2.69	10.77	3.83	-4.25	126.92	233.66	121.66	83.07
8.25	-1.26	13.04	22.62	12.25	1.73	3.13	11.20	4.23	-4.24	159.30	256.89	157.89	114.60
7.02	-2.11	11.32	20.80	10.74	0.61	1.90	9.99	3.31	-4.81	132.27	254.08	128.18	86.65
8.30	-0.36	12.30	21.58	11.71	2.06	3.64	11.73	4.89	-2.80	138.01	237.02	124.01	90.98
7.69	-1.90	11.81	21.16	11.32	0.66	2.57	10.70	4.04	-4.47	137.35	228.36	121.12	91.58
7.67	-2.02	11.82	21.15	11.33	0.54	2.59	10.70	4.01	-4.59	130.58	224.70	118.43	87.62
7.43	-1.48	11.30	20.92	11.03	1.27	2.41	10.66	3.84	-4.21	168.07	300.70	161.97	112.42
7.86	-1.22	12.52	22.17	11.66	1.47	3.00	11.21	4.10	-3.94	121.56	223.29	116.89	79.06
8.08	-1.08	12.58	22.63	11.85	1.70	3.28	11.62	4.31	-3.86	129.10	219.65	117.38	83.00
7.42	-1.29	11.82	21.35	11.39	1.49	2.20	10.45	3.41	-4.06	152.69	254.04	152.01	139.94
7.96	-0.91	12.61	22.09	11.96	1.90	2.88	11.21	3.99	-3.73	153.96	247.20	142.52	127.02
7.15	-1.85	11.28	20.98	10.62	0.83	2.37	10.55	3.65	-4.54	134.75	234.90	125.58	92.11
8.02	-0.97	12.33	22.03	11.93	1.85	2.85	11.07	4.10	-3.83	159.20	283.42	152.16	100.23
7.40	-1.82	12.07	21.78	11.31	0.89	2.38	10.48	3.54	-4.58	128.72	226.00	118.13	82.02
7.54	-2.05	11.61	20.97	11.17	0.52	2.42	10.55	3.90	-4.60	137.94	228.90	122.43	93.10
7.40	-1.48	11.46	20.79	10.78	0.92	2.54	10.82	4.01	-3.87	149.42	244.68	150.71	122.83
7.10	-1.26	11.61	20.88	10.91	1.59	1.97	10.32	3.30	-4.13	164.29	264.57	176.03	173.29
8.18	-0.62	12.31	21.60	11.79	1.88	3.09	11.16	4.57	-3.14	168.45	278.46	153.05	124.57
7.34	-1.99	11.56	20.93	11.11	0.79	2.00	10.03	3.57	-4.78	133.51	250.66	121.08	81.69
6.91	-1.76	10.96	20.08	10.42	0.71	1.91	10.06	3.40	-4.27	160.35	265.30	163.67	150.20
7.32	-1.72	11.92	21.73	11.15	1.11	2.48	10.77	3.48	-4.57	150.47	239.16	124.03	90.76
8.79	-0.08	12.94	22.22	12.22	2.34	4.05	12.31	5.33	-2.52	149.45	251.30	159.17	128.24
8.17	-0.68	12.68	22.44	11.89	1.99	3.40	11.47	4.41	-3.39	128.98	216.75	105.87	75.63
7.21	-1.31	11.97	21.28	11.33	1.60	1.98	10.12	3.08	-4.23	122.23	229.22	132.36	120.17
7.66	-1.35	12.62	22.41	11.62	1.65	2.85	11.16	3.71	-4.38	147.11	235.04	121.79	93.74
7.63	-2.02	11.75	21.09	11.25	0.53	2.59	10.69	3.99	-4.59	133.14	226.85	117.65	86.84
8.26	-0.46	12.20	21.45	11.67	1.95	3.37	11.38	4.84	-2.88	152.25	258.41	137.76	106.00
8.06	-1.10	12.56	22.34	11.70	1.57	3.45	11.77	4.45	-3.79	124.59	215.62	116.49	81.49
7.39	-1.88	11.81	21.25	11.17	0.89	2.07	10.11	3.63	-4.64	131.70	249.71	120.51	79.92
7.77	-1.01	12.44	22.32	11.44	1.76	3.22	11.44	4.11	-3.79	138.30	227.43	114.15	85.87
7.94	-1.05	12.07	21.66	11.66	1.75	2.86	11.04	4.19	-3.85	163.94	287.78	155.71	103.09
7.67	-1.93	11.89	21.26	11.45	0.77	2.42	10.48	3.87	-4.64	127.46	239.45	118.45	78.90
7.58	-1.02	12.27	21.82	11.67	1.92	2.31	10.58	3.48	-3.94	148.59	253.23	156.56	147.91
7.80	-1.15	12.02	21.62	11.60	1.68	2.65	10.93	3.99	-3.97	162.70	288.63	154.42	102.94
7.66	-2.31	11.68	20.96	11.32	0.27	2.54	10.71	4.00	-4.89	128.24	229.55	117.89	83.99
8.02	-1.57	12.13	21.52	11.65	1.04	3.06	11.17	4.41	-4.19	128.06	221.81	113.24	81.37

8.09	-1.04	12.63	22.50	11.78	1.84	3.35	11.81	4.39	-3.91	135.41	226.20	121.17	85.42
7.77	-2.27	11.86	21.13	11.50	0.31	2.67	10.81	4.05	-4.86	128.34	226.14	116.17	83.21
8.07	-0.91	13.01	22.88	12.00	2.08	3.34	11.63	4.13	-3.90	144.70	231.90	123.78	98.84
7.50	-1.58	11.88	21.47	11.18	1.10	2.63	10.69	3.85	-4.26	127.96	233.70	121.80	82.40
7.93	-1.13	12.36	22.00	11.72	1.63	2.81	10.79	4.15	-3.88	120.90	222.00	109.62	72.42
7.40	-1.50	11.48	20.81	10.80	0.96	2.52	10.81	4.00	-3.97	147.45	242.11	146.45	117.78
7.71	-2.38	11.83	21.08	11.44	0.21	2.57	10.73	3.98	-4.99	130.13	228.57	117.31	84.67
6.94	-1.67	11.23	20.67	10.81	1.10	1.77	9.99	3.08	-4.46	151.75	259.71	155.31	146.71
7.88	-0.91	12.42	22.28	11.54	1.83	3.28	11.40	4.21	-3.65	132.47	221.29	109.81	79.16
7.99	-0.86	13.11	22.62	12.41	2.49	2.77	11.01	3.58	-4.21	166.94	260.55	151.95	139.85
7.70	-2.37	11.81	21.06	11.43	0.21	2.59	10.73	3.99	-4.96	129.78	227.80	117.26	84.22
6.84	-1.68	11.05	20.35	10.59	0.94	1.73	9.89	3.12	-4.30	148.32	259.40	156.12	140.19
7.55	-1.56	12.08	22.02	11.29	1.19	2.75	10.96	3.83	-4.35	132.87	226.09	118.63	83.88
8.12	-1.33	12.87	22.37	12.00	1.59	3.03	11.19	4.21	-4.23	156.71	256.97	159.20	119.66
7.01	-1.41	11.55	20.97	10.99	1.63	1.62	10.08	3.01	-4.45	178.45	282.03	192.61	192.90
7.92	-1.23	12.37	22.01	11.48	1.41	3.29	11.60	4.38	-3.91	123.95	219.59	118.76	83.65
8.13	-1.06	12.56	22.32	12.11	1.85	2.94	11.15	4.14	-3.96	164.89	287.43	155.40	98.43
7.44	-1.91	11.85	21.44	11.25	1.01	2.37	10.54	3.63	-4.83	168.51	278.18	171.70	134.07
7.98	-0.55	11.86	21.21	11.33	1.87	3.32	11.38	4.62	-2.97	141.23	242.75	127.83	96.40
7.52	-1.30	12.44	21.91	11.96	2.13	2.23	10.45	3.08	-4.73	178.68	278.51	161.46	151.95
7.97	-1.02	12.09	21.72	11.52	1.60	3.02	11.00	4.39	-3.65	122.14	220.74	106.75	72.50
8.22	-0.73	13.32	23.12	12.22	2.19	3.52	11.87	4.23	-3.69	143.34	228.21	120.98	97.89
6.90	-1.45	11.18	20.40	10.61	1.25	1.98	10.12	3.23	-4.11	139.27	234.04	148.61	141.76
7.87	-1.03	12.64	22.52	11.62	1.76	3.25	11.47	4.11	-3.83	139.09	225.19	112.55	83.34
7.69	-1.16	11.68	20.96	11.00	1.26	3.00	11.33	4.37	-3.61	147.17	239.77	145.84	116.92
6.92	-1.68	11.20	20.52	10.77	1.08	1.76	9.92	3.05	-4.45	148.44	257.72	152.03	142.50
8.15	-0.78	12.72	22.36	11.87	1.93	3.26	11.29	4.43	-3.49	124.94	214.79	104.60	71.59
7.77	-2.28	11.89	21.13	11.48	0.30	2.64	10.80	4.10	-4.88	126.93	227.95	116.99	82.50
8.14	-0.49	12.11	21.36	11.52	1.89	3.31	11.37	4.75	-2.92	148.02	254.14	133.90	101.20
8.37	-0.68	12.45	21.79	11.82	1.85	3.48	11.51	4.90	-3.18	133.49	224.84	117.15	85.23
7.67	-1.46	12.01	21.63	11.39	1.23	2.51	10.47	3.95	-4.17	126.25	238.75	116.73	76.33
7.30	-1.30	12.10	21.51	11.53	1.88	1.86	10.23	3.05	-4.49	175.99	276.63	187.20	191.68
7.58	-1.27	11.64	20.96	10.99	1.21	2.77	11.04	4.19	-3.74	146.23	242.10	145.44	116.49
6.84	-2.38	10.99	20.54	10.52	0.51	1.79	10.04	3.16	-5.29	177.76	294.15	183.88	149.81
8.38	-0.88	13.00	22.72	12.34	2.15	3.17	11.38	4.36	-3.92	170.18	288.45	160.91	99.78
8.02	-1.00	12.75	22.41	12.10	1.86	2.97	11.23	3.92	-3.84	144.10	233.94	133.43	116.41
7.69	-1.41	12.33	22.10	11.49	1.39	2.73	10.73	3.88	-4.21	129.71	220.46	110.21	75.62
6.65	-1.72	11.19	20.53	10.95	1.65	1.09	9.57	2.31	-5.12	197.35	300.55	220.57	239.28
7.54	-1.52	12.06	21.82	11.30	1.22	2.59	10.58	3.78	-4.30	130.73	222.16	112.06	78.09
7.38	-2.81	11.43	20.67	11.07	-0.22	2.17	10.32	3.70	-5.41	136.81	233.12	123.11	90.67
7.26	-2.59	11.12	20.39	10.84	0.04	2.10	10.20	3.69	-5.23	135.77	243.36	126.10	88.39
7.70	-1.38	12.11	21.83	11.46	1.34	2.63	10.56	3.95	-4.06	124.99	234.03	116.94	75.35
7.58	-1.50	11.99	21.64	11.31	1.18	2.72	10.77	3.88	-4.20	126.48	232.07	121.85	81.96

8.29	-0.80	12.54	22.17	12.14	2.09	3.18	11.33	4.42	-3.70	159.04	279.50	150.96	96.12
8.00	-1.15	12.44	22.27	11.61	1.54	3.40	11.72	4.41	-3.83	124.59	219.10	118.06	82.99
8.13	-0.78	13.02	22.94	12.03	2.23	3.41	11.82	4.19	-3.78	154.48	241.18	139.41	123.00
7.01	-2.33	11.42	21.05	10.92	0.67	1.88	10.12	3.17	-5.34	184.94	299.56	185.12	144.43
8.38	-0.68	12.41	21.77	11.83	1.83	3.49	11.52	4.92	-3.20	134.04	223.70	117.06	84.90
7.68	-1.42	11.95	21.75	11.49	1.43	2.57	10.84	3.86	-4.29	168.80	295.56	159.78	108.30
7.62	-1.51	12.33	22.19	11.45	1.30	2.80	11.08	3.79	-4.32	140.08	230.21	118.37	84.07
8.03	-1.38	12.68	22.27	12.00	1.62	2.95	11.05	4.08	-4.36	162.33	267.66	160.80	118.05
7.37	-1.17	12.09	21.57	11.48	1.86	2.04	10.40	3.27	-4.18	153.75	259.23	163.87	158.98
7.70	-1.16	12.51	22.07	11.89	1.91	2.45	10.74	3.52	-4.25	163.00	254.59	163.21	159.68
7.82	-1.09	12.62	22.23	12.04	1.99	2.53	10.80	3.62	-4.15	158.76	253.41	160.58	156.61
7.82	-1.72	12.59	22.21	11.91	1.34	2.60	10.75	3.73	-4.78	167.68	271.61	164.25	120.63
7.33	-2.55	11.29	20.65	10.96	0.05	2.16	10.33	3.69	-5.12	136.47	230.95	122.27	92.69
8.00	-0.97	12.96	22.72	11.82	1.97	3.30	11.61	4.18	-3.90	143.82	229.33	119.14	95.24
7.50	-2.11	11.56	20.91	11.11	0.45	2.45	10.57	3.89	-4.68	133.47	227.42	118.44	88.05
8.27	-0.60	12.23	21.47	11.54	1.80	3.63	11.93	4.97	-2.99	149.22	246.82	153.02	122.53
7.76	-1.41	12.24	21.78	11.58	1.30	2.70	10.82	3.96	-4.11	128.77	247.64	124.89	79.90
7.52	-1.46	12.36	22.01	11.43	1.48	2.67	11.08	3.63	-4.36	155.09	238.09	137.11	118.69
7.04	-2.08	11.11	20.83	10.61	0.56	2.23	10.44	3.45	-4.73	139.61	249.75	136.43	99.99
7.63	-1.56	11.89	21.51	11.33	1.13	2.67	10.81	3.92	-4.26	130.27	249.31	129.39	85.21
7.32	-2.58	11.24	20.51	10.94	0.04	2.14	10.28	3.69	-5.21	136.40	241.28	123.40	88.87
8.24	-0.68	13.06	22.86	12.06	2.20	3.60	12.03	4.41	-3.57	150.27	237.52	139.13	124.52
7.33	-2.06	11.56	21.05	11.38	1.22	1.85	10.10	3.26	-5.37	215.85	350.04	209.40	165.80
7.95	-1.17	12.57	22.21	11.76	1.55	3.03	11.16	4.15	-3.91	121.10	222.94	116.04	76.98
7.12	-1.62	11.85	21.34	11.18	1.42	2.01	10.37	3.08	-4.64	172.79	264.80	178.85	181.60
8.58	-0.76	13.46	23.15	12.80	2.43	3.23	11.54	4.36	-3.99	173.00	288.36	161.36	103.36
7.62	-1.16	12.56	22.08	11.84	2.06	2.55	10.90	3.41	-4.39	181.46	271.36	188.60	198.98
7.42	-1.31	12.46	21.75	11.77	1.99	2.10	10.49	3.09	-4.60	183.34	277.80	199.41	212.20
7.86	-0.99	12.80	22.28	12.24	2.34	2.60	10.87	3.45	-4.32	179.31	277.78	166.86	162.34
7.58	-1.30	12.53	21.98	12.06	2.15	2.23	10.47	3.11	-4.73	179.54	277.26	162.14	153.02
8.09	-1.66	12.25	21.56	11.89	1.06	2.98	11.07	4.28	-4.38	122.30	223.42	112.52	76.95
7.46	-2.19	11.55	20.88	11.20	0.48	2.19	10.26	3.70	-4.85	130.14	245.62	121.19	82.60
7.08	-1.96	11.19	20.81	10.67	0.79	2.18	10.34	3.53	-4.71	133.35	251.54	133.01	94.20
8.66	-0.66	13.52	23.22	12.88	2.51	3.38	11.64	4.47	-3.82	169.28	284.15	158.35	99.72
7.81	-1.38	12.29	21.87	11.64	1.34	2.75	10.87	3.99	-4.12	129.10	248.38	125.11	79.62
7.32	-1.68	11.70	21.47	10.95	1.09	2.46	10.47	3.67	-4.43	136.49	228.32	116.36	82.52
7.98	-1.34	12.61	22.44	12.00	1.64	2.80	11.06	3.99	-4.33	170.66	289.13	163.27	110.34
7.42	-2.61	11.47	20.76	11.16	-0.04	2.19	10.37	3.67	-5.21	130.21	235.49	121.02	86.85
8.15	-1.27	12.29	21.65	11.75	1.35	3.11	11.20	4.57	-3.86	132.16	224.25	117.76	86.95
8.03	-1.27	12.60	22.18	12.11	1.88	2.71	10.97	3.97	-4.41	182.73	304.83	174.07	118.00
7.71	-1.17	11.77	21.03	11.09	1.30	2.97	11.31	4.35	-3.62	145.68	241.02	144.34	115.62
7.47	-1.51	11.50	21.12	11.21	1.25	2.33	10.57	3.75	-4.26	161.30	290.05	157.33	108.52
8.15	-0.65	12.19	21.58	11.55	1.80	3.41	11.73	4.76	-3.05	151.42	255.32	162.56	133.70

7.82	-1.04	12.74	22.36	11.91	2.10	2.90	11.30	3.72	-4.15	171.24	263.85	176.18	176.40
7.18	-1.90	11.17	20.80	10.89	0.94	2.00	10.25	3.43	-4.73	171.47	302.76	163.48	116.89
7.26	-1.75	11.58	21.19	10.91	0.96	2.34	10.45	3.61	-4.43	130.39	238.56	124.52	86.81
8.13	-1.32	12.87	22.38	12.01	1.59	3.03	11.19	4.22	-4.23	156.65	256.80	159.09	119.54
7.41	-1.44	12.04	21.78	11.15	1.40	2.65	10.99	3.65	-4.29	150.10	236.40	130.04	106.87
7.98	-0.90	13.12	22.70	12.26	2.29	2.94	11.37	3.70	-4.10	170.95	261.95	171.83	172.78
7.22	-1.39	12.19	21.57	11.67	1.97	1.68	10.08	2.77	-4.75	182.63	281.39	197.63	207.42
7.62	-1.56	12.33	21.95	11.54	1.21	2.36	10.33	3.71	-4.30	126.39	241.03	116.32	75.01
8.25	-1.18	12.96	22.52	12.43	2.06	2.86	11.11	4.08	-4.39	182.51	299.48	169.94	114.75
7.93	-1.08	12.53	21.97	11.81	1.67	2.99	11.31	4.05	-3.80	152.13	238.58	136.64	118.73
7.82	-1.08	11.82	21.07	11.15	1.35	3.07	11.40	4.50	-3.49	149.11	243.93	149.66	120.59
8.31	-0.61	13.16	22.67	12.47	2.30	3.12	11.42	4.17	-3.50	151.03	244.00	140.64	122.58
8.22	-0.55	12.37	21.68	11.82	1.94	3.21	11.34	4.60	-3.05	159.88	273.03	149.86	120.95
7.52	-1.53	12.00	21.76	11.25	1.18	2.39	10.40	3.81	-4.25	128.35	238.74	119.86	79.30
7.57	-1.58	11.94	21.55	11.24	1.13	2.61	10.64	3.91	-4.29	126.79	242.57	123.18	80.70
7.21	-1.31	12.00	21.32	11.36	1.62	1.97	10.11	3.07	-4.23	122.47	228.85	132.70	120.02
8.38	-0.69	12.55	21.94	11.93	1.85	3.44	11.46	4.84	-3.21	134.88	226.28	118.74	86.83
7.25	-1.57	11.14	20.47	10.48	0.83	2.66	11.05	3.99	-3.98	144.10	231.84	138.08	110.64
7.83	-1.50	12.50	22.01	11.75	1.30	2.53	10.56	3.97	-4.29	127.39	241.21	113.97	73.43
8.26	-1.12	12.83	22.66	12.28	1.74	3.27	11.38	4.27	-3.99	139.80	255.11	139.01	90.74
7.70	-1.40	12.16	21.92	11.36	1.32	2.85	11.09	4.01	-4.10	124.57	226.27	119.24	82.96
7.94	-1.57	12.46	21.90	12.10	1.76	2.46	10.67	3.77	-4.88	203.18	329.22	194.63	145.93
7.99	-0.86	13.12	22.62	12.41	2.49	2.77	11.02	3.58	-4.20	166.87	260.46	151.88	139.78
7.73	-1.29	12.63	22.31	11.85	1.68	2.68	11.01	3.59	-4.26	152.89	240.87	145.19	131.51
7.63	-1.48	11.89	21.32	11.31	1.21	2.65	10.74	3.95	-4.18	133.65	251.84	126.65	81.87
7.18	-2.22	11.45	21.15	11.00	0.70	2.05	10.29	3.36	-5.15	183.42	300.90	179.96	135.99
7.90	-0.90	12.82	22.46	11.87	2.09	3.15	11.53	3.93	-3.86	159.80	243.92	150.85	142.01
6.84	-1.60	11.52	20.86	11.17	1.74	1.31	9.76	2.51	-4.96	193.27	294.40	212.05	226.84
8.46	-0.75	13.10	22.82	12.05	1.97	3.95	12.34	4.87	-3.46	121.29	210.24	114.40	80.62
7.92	-1.08	12.51	21.95	11.79	1.67	2.99	11.30	4.04	-3.80	152.30	238.70	136.79	118.97
7.82	-1.29	12.16	21.94	11.69	1.55	2.68	10.91	3.95	-4.15	166.79	289.58	156.20	103.66
8.74	-0.11	12.91	22.15	12.12	2.31	4.19	12.41	5.37	-2.53	146.19	243.40	146.94	117.14
7.87	-0.98	12.58	22.21	11.99	1.97	2.61	10.84	3.74	-3.90	141.39	244.49	144.28	131.51
8.18	-0.99	12.78	22.57	11.83	1.77	3.50	11.90	4.50	-3.76	128.54	217.01	117.76	83.97
7.64	-1.49	12.33	22.17	11.50	1.36	2.81	11.10	3.81	-4.29	139.10	230.61	118.40	83.54
8.19	-0.74	12.96	22.43	12.21	2.08	3.08	11.39	4.14	-3.59	151.77	243.82	140.00	123.22
8.14	-1.32	12.88	22.39	12.02	1.60	3.03	11.20	4.22	-4.23	156.57	256.56	158.92	119.36
7.82	-1.32	12.44	22.38	11.56	1.45	3.09	11.24	4.08	-4.07	129.97	221.02	113.80	77.52
8.12	-0.88	13.18	22.97	12.14	2.06	3.38	11.69	4.10	-3.84	144.74	230.51	124.70	102.52
7.29	-2.53	11.33	20.63	10.92	0.09	2.09	10.28	3.65	-5.13	135.06	231.33	121.73	91.66
8.56	-0.78	13.37	23.07	12.74	2.33	3.24	11.49	4.39	-3.90	169.97	285.01	156.80	96.83
7.19	-1.35	11.91	21.30	11.38	1.88	1.81	10.22	3.00	-4.60	175.47	277.63	188.26	195.51
8.78	-0.70	13.67	23.40	12.85	2.25	3.85	11.95	4.70	-3.64	141.79	249.75	142.28	97.30

7.78	-0.84	11.61	20.94	11.29	1.84	2.72	10.83	4.27	-3.52	137.50	263.83	130.36	86.20
7.05	-1.56	11.65	21.09	11.22	1.69	1.88	10.30	2.92	-4.80	197.09	292.51	212.03	233.87
7.13	-2.25	10.88	20.22	10.67	0.42	1.95	9.97	3.59	-4.90	138.16	254.47	129.11	88.80
7.41	-1.11	11.62	20.98	11.03	1.40	2.57	10.63	3.80	-3.63	136.16	243.48	146.73	122.59
7.87	-1.33	12.52	22.55	11.70	1.45	3.03	11.29	4.04	-4.13	128.83	220.03	115.90	80.60
7.27	-2.61	11.22	20.49	10.89	-0.02	2.10	10.27	3.66	-5.19	132.20	236.13	121.06	88.61
8.35	-1.03	12.98	22.76	12.39	1.84	3.36	11.45	4.33	-3.92	139.96	253.92	138.43	90.11
7.80	-0.79	11.09	20.50	10.98	1.50	3.04	11.24	4.63	-3.11	161.74	257.30	183.39	172.17
8.39	-0.50	13.08	22.79	12.12	2.21	3.77	11.90	4.62	-3.20	129.69	215.67	104.92	74.96
8.05	-0.95	13.17	22.85	12.10	1.99	3.28	11.61	4.00	-3.89	144.87	229.64	124.88	101.33
8.02	-1.18	12.64	22.35	11.88	1.59	3.02	11.14	4.17	-3.96	122.63	235.64	120.73	76.96
8.23	-1.01	12.99	22.74	12.03	1.71	3.56	11.75	4.43	-3.73	119.96	209.72	111.11	74.22
7.92	-1.04	12.82	22.58	11.71	1.89	3.21	11.52	4.09	-3.98	144.32	229.65	120.10	96.31
7.62	-1.22	12.03	21.59	11.54	1.58	2.57	10.90	3.70	-4.00	156.85	251.50	146.48	133.12
7.45	-1.64	11.90	21.77	11.11	1.09	2.69	10.97	3.80	-4.36	142.05	234.71	122.44	88.00
7.82	-1.30	12.26	21.95	11.67	1.45	2.87	11.00	4.00	-4.08	126.56	240.75	126.06	85.05
7.75	-1.02	11.76	20.98	11.30	1.49	2.42	10.45	4.19	-3.54	157.83	250.04	139.33	114.10
7.13	-1.94	11.39	20.95	10.84	0.75	2.05	10.21	3.45	-4.65	131.16	254.80	129.68	87.88
7.72	-0.88	12.58	22.13	11.93	2.17	2.38	10.66	3.54	-3.95	149.04	254.97	159.19	151.25
8.16	-0.68	12.31	21.54	11.57	1.73	3.37	11.61	4.73	-3.08	149.74	253.03	158.27	127.74
6.99	-1.65	11.78	21.05	11.31	1.67	1.61	10.00	2.67	-4.98	192.10	289.24	212.74	231.84
7.88	-1.35	12.18	21.84	11.66	1.38	2.95	11.05	4.11	-4.11	130.41	246.98	129.64	83.99
7.70	-2.00	11.85	21.18	11.37	0.57	2.64	10.75	4.05	-4.58	132.49	225.94	117.40	86.39
8.13	-0.94	12.89	22.35	12.14	1.85	3.11	11.41	4.13	-3.71	146.78	234.87	131.32	111.93
7.69	-1.29	12.34	21.89	11.81	1.74	2.48	10.77	3.56	-4.31	157.87	249.15	157.22	151.18
6.93	-2.18	11.02	20.66	10.67	0.57	1.85	10.08	3.21	-4.94	164.57	298.04	162.03	117.49
8.33	-0.44	12.69	22.60	11.90	2.29	3.80	11.97	4.77	-3.17	129.74	216.55	106.01	75.20
8.34	-0.58	13.13	22.85	12.11	2.29	3.73	12.13	4.56	-3.44	148.89	234.25	137.69	121.57
7.97	-1.79	12.09	21.40	11.62	0.84	2.94	11.03	4.31	-4.43	128.16	223.52	113.69	81.06
7.04	-2.06	11.17	20.83	10.67	0.68	2.06	10.27	3.42	-4.77	133.66	253.29	135.41	96.72
8.38	-0.58	13.31	22.69	12.44	2.29	3.26	11.59	4.36	-3.43	151.82	242.24	138.20	121.36
8.50	-0.15	12.61	21.96	12.04	2.32	3.32	11.35	4.95	-2.67	169.11	275.79	150.88	125.27
8.54	-0.77	13.33	23.04	12.70	2.33	3.23	11.48	4.40	-3.89	167.69	286.20	157.32	95.83
7.74	-1.50	12.07	21.67	11.55	1.25	2.76	10.89	3.95	-4.27	132.94	250.80	130.60	85.72
7.67	-1.20	11.61	20.93	10.99	1.18	2.88	11.21	4.33	-3.59	149.20	249.13	155.47	127.49
8.06	-0.94	13.18	22.85	12.10	1.99	3.27	11.61	4.00	-3.89	144.76	229.57	124.88	101.19
7.14	-1.48	11.90	21.31	11.48	1.84	1.72	10.10	2.79	-4.79	183.70	283.18	202.29	215.04
8.17	-0.73	12.69	22.38	11.91	1.97	3.33	11.35	4.46	-3.46	125.27	216.18	103.65	71.23
7.61	-2.35	11.60	20.88	11.29	0.22	2.51	10.65	3.92	-4.91	129.58	229.31	116.93	84.87
7.96	-1.04	12.13	21.81	11.88	1.79	2.75	10.94	4.02	-3.86	149.79	276.12	147.50	97.78
8.12	-0.83	12.44	21.89	11.79	1.81	3.36	11.63	4.45	-3.46	150.21	239.74	136.46	118.52
7.49	-1.44	12.43	22.10	11.43	1.50	2.61	10.98	3.53	-4.41	153.60	238.06	135.06	115.28
7.76	-1.41	12.25	21.89	11.56	1.33	2.73	10.82	3.97	-4.13	126.40	241.47	122.70	78.67

7.00	-1.36	11.33	20.60	10.71	1.43	1.93	10.26	3.25	-4.17	164.99	264.12	176.92	176.63
7.04	-1.82	10.93	20.40	10.63	0.89	1.91	10.19	3.46	-4.53	156.79	291.37	153.37	110.16
7.82	-0.94	11.86	21.21	11.31	1.54	2.98	11.23	4.37	-3.44	148.75	249.71	148.78	119.52
7.32	-1.98	11.54	20.74	11.09	0.79	1.89	9.93	3.54	-4.75	134.68	255.94	126.73	83.88
8.30	-1.12	13.07	22.58	12.53	2.16	2.94	11.19	4.07	-4.38	174.87	294.50	167.59	110.86
8.17	-0.59	12.31	21.63	11.72	1.95	3.28	11.45	4.64	-3.11	156.20	264.98	155.95	128.62
7.51	-1.67	11.69	21.30	11.28	1.29	2.33	10.61	3.73	-4.64	183.55	308.95	176.45	124.45
8.08	-0.95	13.21	22.95	12.16	2.02	3.25	11.62	4.00	-3.90	143.29	228.80	122.27	98.07
7.44	-1.35	11.95	21.43	11.54	1.42	2.11	10.33	3.33	-4.14	153.48	256.70	152.44	138.71
7.68	-1.20	12.49	22.36	11.47	1.60	3.01	11.25	3.90	-4.01	141.03	229.35	115.77	87.45
7.05	-2.06	11.27	20.82	10.75	0.61	1.83	9.78	3.33	-4.72	135.33	256.28	125.22	82.71
7.57	-1.40	12.49	22.13	11.50	1.57	2.72	11.11	3.64	-4.36	153.00	234.31	133.04	114.50
7.57	-2.13	11.63	20.94	11.17	0.45	2.48	10.62	3.94	-4.73	134.00	226.55	118.89	87.58
7.46	-1.11	12.26	21.66	11.54	1.82	2.16	10.45	3.33	-4.03	139.90	246.42	150.48	139.50
7.44	-2.34	11.52	20.85	11.08	0.27	2.28	10.43	3.80	-4.94	132.81	228.41	120.31	89.58
7.50	-2.26	11.28	20.63	11.05	0.26	2.48	10.57	3.94	-4.80	131.09	230.90	119.29	85.17
7.10	-1.51	11.97	21.32	11.43	1.83	1.74	10.18	2.76	-4.84	190.67	285.05	210.26	228.25
7.39	-1.71	11.64	21.25	10.98	0.98	2.44	10.53	3.80	-4.41	129.39	246.48	126.39	84.33
7.41	-2.55	11.50	20.73	11.17	0.09	2.10	10.24	3.63	-5.18	131.33	238.05	121.89	86.06
6.83	-2.39	10.98	20.53	10.51	0.51	1.78	10.03	3.15	-5.30	178.86	294.55	183.96	150.12
7.33	-2.65	11.31	20.62	11.00	-0.07	2.15	10.30	3.68	-5.26	134.13	238.89	124.12	88.84
7.35	-2.68	11.27	20.49	11.00	-0.09	2.19	10.30	3.74	-5.27	135.38	237.98	124.22	88.12
7.69	-1.49	12.36	22.32	11.50	1.26	2.79	10.96	3.87	-4.26	129.71	223.62	115.64	80.13
7.62	-1.22	12.03	21.59	11.55	1.58	2.57	10.90	3.70	-4.00	156.84	251.49	146.48	133.11
7.04	-1.59	11.21	20.49	10.76	1.09	1.91	10.22	3.30	-4.26	167.56	266.85	162.61	153.03
7.21	-2.22	11.53	21.20	11.03	0.73	2.07	10.31	3.38	-5.15	183.15	300.80	180.51	135.70
8.12	-0.88	13.18	22.97	12.15	2.06	3.39	11.69	4.10	-3.84	144.73	230.50	124.67	102.51
7.30	-1.83	11.74	21.39	11.09	0.85	2.09	10.08	3.50	-4.53	129.33	246.10	120.81	79.66
7.86	-1.23	12.16	21.87	11.50	1.47	3.03	11.11	4.23	-3.91	124.13	236.95	122.54	79.49
8.24	-0.74	13.38	23.06	12.40	2.36	3.38	11.70	4.09	-3.80	159.00	245.70	155.76	148.66
7.62	-1.49	11.87	21.31	11.30	1.20	2.63	10.73	3.94	-4.19	133.85	252.07	126.78	82.06
7.89	-1.32	12.43	22.02	11.68	1.38	2.86	10.89	4.11	-4.08	124.92	235.14	118.86	75.46
8.93	0.13	13.20	22.52	12.46	2.63	4.17	12.46	5.41	-2.36	147.71	248.23	157.80	126.77
7.69	-1.14	12.41	22.09	11.47	1.69	2.96	11.26	3.92	-4.00	146.71	232.49	125.82	102.12
7.97	-1.16	12.52	22.11	11.85	1.59	2.80	10.78	4.07	-3.90	119.55	222.93	109.44	72.05
7.24	-1.39	11.87	21.20	11.27	1.51	2.00	10.20	3.22	-4.27	136.18	247.70	143.35	131.71
7.75	-1.45	11.88	21.64	11.41	1.29	2.88	11.10	4.06	-4.18	142.87	262.78	143.61	96.83
7.51	-2.45	11.66	20.88	11.29	0.18	2.24	10.38	3.73	-5.10	131.06	236.62	120.20	84.56
7.58	-1.40	12.43	22.06	11.48	1.54	2.72	11.12	3.73	-4.32	152.75	236.11	136.00	118.28
7.40	-1.65	11.87	21.64	11.15	1.11	2.48	10.44	3.70	-4.38	132.19	224.50	113.95	80.08
8.43	-0.56	12.98	22.33	12.13	2.10	3.64	11.92	4.71	-3.24	146.09	232.56	130.65	111.03
7.58	-1.66	12.24	21.81	11.50	1.09	2.32	10.26	3.64	-4.40	128.58	241.41	117.38	74.62
7.85	-1.26	12.56	22.18	11.69	1.48	2.93	11.14	3.99	-4.04	123.40	225.05	118.62	79.53

7.61	-1.35	12.19	22.00	11.30	1.40	2.90	11.01	3.96	-4.10	136.62	225.86	112.90	82.00
7.97	-0.68	11.62	20.91	11.26	1.68	2.98	10.97	4.67	-3.07	157.89	258.65	142.27	116.78
8.76	-0.04	13.17	22.66	12.24	2.43	4.10	12.47	5.26	-2.51	148.02	242.49	148.03	119.18
7.05	-1.60	11.30	20.61	10.92	1.12	1.87	10.04	3.16	-4.32	153.36	258.83	155.57	141.93
7.94	-0.87	12.52	22.34	11.61	1.86	3.40	11.59	4.30	-3.57	136.43	224.39	112.90	84.38
7.90	-1.31	12.54	22.35	11.68	1.40	3.14	11.35	4.11	-4.02	123.28	218.12	115.47	79.49
8.00	-1.02	12.92	22.64	11.92	1.88	3.27	11.63	4.05	-3.93	149.76	232.25	129.55	110.48
6.99	-1.39	11.47	20.87	10.91	1.63	1.65	10.08	3.04	-4.41	177.80	281.78	191.81	192.48
8.01	-0.97	12.96	22.84	12.01	1.92	3.25	11.66	4.01	-3.87	152.24	237.38	137.09	120.23
7.57	-1.36	12.50	22.11	11.51	1.59	2.74	11.08	3.67	-4.31	152.91	235.34	131.54	112.66
8.17	-0.76	12.70	22.23	12.01	1.95	3.40	11.60	4.37	-3.44	141.81	228.47	128.16	106.78
7.31	-1.48	11.80	21.61	10.94	1.31	2.64	10.94	3.69	-4.28	149.50	240.57	127.18	103.95
7.56	-1.58	12.20	22.12	11.36	1.18	2.65	10.84	3.73	-4.33	128.60	223.35	116.14	81.57
7.21	-1.26	11.92	21.24	11.18	1.54	2.00	10.24	3.23	-4.06	133.46	235.47	143.57	132.36
7.23	-1.49	12.10	21.63	11.64	1.95	1.96	10.22	2.83	-4.96	180.19	279.17	159.57	146.41
7.52	-1.24	12.50	22.01	11.78	2.02	2.38	10.82	3.28	-4.50	184.26	275.20	190.94	201.17
6.85	-1.77	11.08	20.38	10.66	0.95	1.66	9.83	3.03	-4.49	148.70	258.50	151.69	141.63
7.56	-1.31	12.48	21.95	12.02	2.13	2.22	10.44	3.08	-4.76	180.02	278.09	160.76	152.28
6.80	-1.69	11.20	20.48	10.86	1.54	1.62	9.97	2.74	-4.94	201.99	300.03	226.15	254.93
7.74	-1.20	12.45	22.30	11.49	1.61	3.05	11.27	4.00	-4.01	136.61	227.66	113.65	83.83
7.89	-0.85	11.79	21.14	11.38	1.61	2.87	10.85	4.45	-3.30	153.76	256.58	139.23	109.47
7.51	-1.67	12.03	21.94	11.25	1.05	2.64	10.83	3.81	-4.41	131.81	225.21	118.90	84.75
7.43	-1.49	12.18	21.83	11.50	1.49	2.44	10.77	3.35	-4.43	161.74	249.83	158.56	152.15
8.63	-0.08	12.73	21.98	12.10	2.36	4.05	12.16	5.21	-2.52	134.43	229.45	119.07	85.87
7.15	-1.56	11.11	20.31	10.69	0.90	2.11	10.31	3.62	-4.03	184.23	278.12	176.65	165.56
7.86	-1.07	12.82	22.47	12.02	1.91	2.90	11.24	3.70	-4.07	159.59	244.12	154.89	145.77
7.98	-1.32	12.58	22.27	11.91	1.46	2.88	11.01	4.06	-4.07	126.79	241.11	124.28	78.99
7.52	-2.45	11.49	20.74	11.16	0.18	2.32	10.43	3.86	-5.08	133.13	238.53	122.36	84.93
7.46	-1.59	11.70	21.33	11.11	1.13	2.61	10.75	3.81	-4.27	130.08	246.22	129.29	88.34
8.17	-0.64	13.04	22.68	12.13	2.23	3.27	11.57	4.21	-3.51	142.99	225.07	122.16	102.11
7.08	-1.92	10.92	20.46	10.66	0.80	1.96	10.22	3.48	-4.66	155.56	285.90	152.68	108.82
8.02	-0.97	12.82	22.44	12.09	1.94	2.89	11.21	3.94	-3.87	146.94	237.11	141.02	126.87
7.48	-2.63	11.47	20.72	11.16	-0.03	2.33	10.48	3.84	-5.22	133.11	233.98	122.63	88.09
7.83	-0.78	12.82	22.31	11.72	1.87	2.59	10.81	3.91	-3.45	130.69	233.04	142.60	116.11
7.64	-2.20	11.50	20.81	11.21	0.34	2.63	10.73	4.05	-4.74	130.85	229.32	117.79	83.47
7.50	-1.72	12.03	21.67	11.34	1.05	2.32	10.32	3.67	-4.47	128.57	243.64	121.82	78.67
7.55	-1.01	11.31	20.62	11.02	1.63	2.51	10.64	4.13	-3.65	138.94	268.56	132.51	89.25
7.81	-1.23	12.16	21.78	11.49	1.51	2.71	10.69	4.11	-3.97	124.81	224.03	109.84	74.03
7.73	-1.03	12.31	21.94	11.41	1.77	2.97	11.20	4.03	-3.83	140.41	224.70	116.03	88.46
8.07	-0.67	12.71	22.11	11.88	2.05	3.27	11.36	4.25	-3.42	131.81	211.45	104.27	75.84
7.96	-0.94	12.80	22.63	11.82	1.98	3.34	11.68	4.12	-3.86	150.30	236.33	130.45	111.08
7.99	-1.32	12.59	22.28	11.92	1.46	2.89	11.01	4.06	-4.06	126.76	241.05	124.26	78.99
7.79	-0.92	11.54	20.89	11.13	1.43	2.93	11.01	4.44	-3.28	165.64	272.85	157.54	130.88

7.52	-1.53	12.02	21.63	11.26	1.16	2.53	10.62	3.77	-4.23	126.11	233.01	120.33	81.69
7.50	-1.32	11.98	21.55	11.34	1.50	2.60	10.90	3.66	-4.15	152.23	238.17	137.35	123.86
7.32	-0.95	12.10	21.41	11.20	1.69	2.33	10.27	3.45	-3.61	131.45	206.08	141.78	128.52
7.34	-1.73	11.76	21.67	11.01	0.96	2.49	10.67	3.66	-4.47	136.86	229.93	120.86	86.87
7.37	-1.20	11.97	21.33	11.16	1.41	2.25	10.35	3.57	-3.78	138.38	236.94	153.42	128.02
7.43	-1.71	11.80	21.51	11.09	0.93	2.62	10.77	3.78	-4.38	127.29	227.41	118.79	84.48
6.78	-1.67	11.35	20.66	11.05	1.68	1.31	9.75	2.50	-5.03	196.97	297.89	215.90	235.84
8.45	-0.20	12.37	21.62	11.76	2.22	3.96	12.04	5.15	-2.63	136.37	234.03	123.21	90.78
7.50	-1.56	12.04	21.61	11.28	1.15	2.47	10.55	3.74	-4.26	125.61	234.84	120.79	81.95
7.52	-1.33	12.13	21.73	11.63	1.71	2.23	10.54	3.39	-4.35	163.50	260.95	169.02	168.86
7.54	-1.62	12.03	21.78	11.29	1.14	2.53	10.48	3.78	-4.38	131.73	223.34	112.27	77.83
7.74	-1.08	11.74	20.93	11.25	1.36	2.44	10.49	4.24	-3.52	156.68	248.88	137.25	113.24
7.42	-1.22	12.00	21.41	11.43	1.90	2.43	10.78	3.37	-4.32	192.86	291.65	190.50	197.38
6.95	-1.56	11.28	20.61	10.96	1.63	1.83	10.21	2.92	-4.77	207.99	307.34	206.99	221.73
7.48	-1.64	11.87	21.61	11.13	1.04	2.73	10.91	3.84	-4.30	128.24	225.38	119.02	84.73
7.43	-1.64	11.81	21.44	11.12	1.01	2.52	10.62	3.76	-4.29	126.38	230.94	120.85	83.86
7.34	-1.17	12.12	21.58	11.48	1.90	1.94	10.36	3.22	-4.23	162.23	265.30	174.44	169.46
7.55	-1.16	12.59	22.12	11.79	2.09	2.42	10.85	3.32	-4.43	179.34	268.11	187.28	195.83
7.31	-1.18	11.93	21.38	11.24	1.66	2.20	10.41	3.38	-4.04	134.54	242.33	142.81	133.57
7.48	-1.57	11.74	21.36	11.11	1.10	2.35	10.33	3.82	-4.28	126.76	228.44	112.25	77.62
7.30	-1.72	11.81	21.58	11.05	1.06	2.30	10.36	3.56	-4.48	134.72	229.62	117.36	83.24
7.46	-1.94	11.32	20.68	10.97	0.52	2.34	10.45	3.95	-4.45	143.52	234.30	127.18	98.70
8.18	-0.53	12.19	21.53	11.59	1.91	3.57	11.71	4.82	-2.98	144.22	244.46	138.18	106.24
7.21	-1.41	11.79	21.13	11.22	1.42	2.00	10.18	3.22	-4.27	135.59	247.07	142.14	131.49
8.30	-0.82	12.37	21.71	11.78	1.73	3.41	11.43	4.82	-3.36	134.39	224.26	116.59	85.23
7.13	-1.43	12.08	21.43	11.59	1.96	1.59	10.00	2.69	-4.79	184.21	284.32	201.50	211.32
8.38	-0.35	12.75	22.01	12.08	2.25	3.37	11.52	4.69	-2.97	155.34	263.81	148.87	117.69
6.86	-1.52	11.00	20.28	10.45	1.13	1.99	10.19	3.26	-4.15	146.94	245.31	159.64	154.81
6.72	-1.83	10.85	20.28	10.54	0.90	1.54	9.75	2.90	-4.57	158.10	263.46	161.03	153.49
6.98	-1.95	11.40	21.20	10.76	0.88	2.16	10.46	3.23	-4.77	155.99	247.14	130.29	98.57
7.71	-1.18	12.46	22.06	11.62	1.69	2.74	11.07	3.77	-4.04	147.77	230.67	131.49	114.34
7.28	-1.20	12.08	21.40	11.35	1.66	2.01	10.26	3.19	-4.07	133.71	236.47	144.20	133.92
7.98	-1.23	12.40	22.33	11.58	1.47	3.31	11.63	4.35	-3.91	129.19	220.67	118.04	84.78
7.73	-1.02	12.78	22.25	12.08	2.22	2.34	10.65	3.41	-4.24	166.48	265.28	172.54	174.28
7.46	-1.42	12.20	21.85	11.40	1.47	2.44	10.80	3.50	-4.31	152.13	237.02	137.97	122.41
7.33	-1.72	11.58	21.23	10.90	0.95	2.34	10.34	3.76	-4.37	128.94	242.68	121.92	81.22
7.84	-1.12	12.30	21.75	11.62	1.58	2.98	11.30	4.10	-3.83	151.09	237.64	136.71	118.00
8.00	-0.97	12.95	22.83	12.01	1.92	3.25	11.65	4.01	-3.87	152.09	237.31	137.03	120.11
7.89	-0.81	12.81	22.14	11.88	1.91	2.72	10.85	3.89	-3.55	132.13	236.29	142.62	116.50
7.66	-1.28	12.49	22.16	11.57	1.65	2.76	11.14	3.75	-4.22	150.98	235.84	135.19	117.71
7.50	-2.17	11.54	20.85	11.08	0.42	2.42	10.58	3.89	-4.74	133.98	227.58	119.04	88.06
7.52	-1.57	11.92	21.56	11.24	1.16	2.42	10.36	3.81	-4.25	127.55	238.97	118.55	77.65
6.94	-1.58	11.24	20.41	10.69	1.05	1.80	9.92	3.19	-4.22	137.43	248.10	145.56	129.24

6.96	-1.39	11.41	20.80	10.86	1.61	1.64	10.07	3.04	-4.40	178.07	282.52	192.61	193.09
7.80	-0.88	11.62	20.98	11.23	1.55	2.72	10.90	4.37	-3.31	165.59	260.35	171.73	159.92
8.11	-0.66	12.05	21.35	11.58	1.78	3.06	11.11	4.62	-3.12	165.51	275.20	151.03	123.20
7.38	-1.69	11.67	21.26	10.99	1.00	2.50	10.57	3.76	-4.34	128.61	237.80	123.33	84.49
8.02	-1.14	12.75	22.37	11.83	1.59	3.12	11.34	4.16	-3.88	120.35	221.63	115.46	77.46
7.83	-1.25	12.40	22.05	11.59	1.44	2.95	11.13	4.08	-3.97	121.22	225.96	118.00	79.13
7.13	-1.63	11.57	20.97	11.14	1.12	1.81	10.04	3.13	-4.35	157.19	261.71	156.84	146.02
8.11	-1.25	12.27	21.63	11.72	1.35	3.05	11.14	4.51	-3.88	135.84	225.97	119.29	88.19
8.41	-0.21	12.27	21.59	11.74	2.20	3.79	11.91	5.09	-2.60	147.66	253.30	135.88	103.77
7.50	-1.56	12.04	21.61	11.28	1.15	2.46	10.55	3.74	-4.26	125.65	234.82	120.81	81.97
7.88	-0.99	12.21	21.62	11.48	1.70	3.19	11.43	4.30	-3.63	143.51	225.34	126.27	104.11
7.51	-1.13	12.07	21.55	11.65	2.14	2.42	10.71	3.37	-4.37	190.92	288.74	178.81	178.23
7.21	-1.30	11.80	21.22	11.22	1.58	1.97	10.23	3.14	-4.19	142.06	249.24	153.80	145.60
7.61	-1.53	12.16	22.04	11.32	1.22	2.82	11.01	3.88	-4.27	131.85	222.19	116.83	82.77
7.94	-0.95	12.82	22.65	11.87	2.00	3.26	11.60	4.04	-3.92	153.27	237.50	131.03	110.66
7.78	-1.09	12.78	22.38	11.92	2.02	2.84	11.22	3.64	-4.19	164.27	254.20	167.89	165.36
7.95	-0.94	12.93	22.59	12.04	2.13	3.05	11.44	3.88	-4.04	167.66	258.06	170.42	168.93
8.41	-0.28	12.40	21.71	11.92	2.19	3.22	11.25	4.89	-2.77	166.39	266.94	146.65	122.95
6.97	-2.04	11.23	20.87	10.64	0.62	1.97	10.11	3.33	-4.70	133.95	242.79	128.05	91.43
6.75	-1.81	11.14	20.34	10.92	1.48	1.47	9.78	2.55	-5.09	203.26	303.38	223.83	251.36
6.67	-1.90	10.80	20.18	10.47	0.82	1.47	9.67	2.85	-4.64	157.41	265.05	161.34	153.39
7.68	-1.00	12.31	22.00	11.78	1.96	2.37	10.70	3.57	-3.99	145.25	251.88	152.32	142.31
7.69	-1.26	12.52	22.34	11.65	1.77	2.89	11.21	3.76	-4.25	150.38	240.17	127.19	102.31
7.50	-2.07	11.59	20.96	11.12	0.47	2.41	10.52	3.87	-4.63	132.72	227.78	120.25	89.91
7.87	-1.02	12.74	22.60	11.79	1.94	3.15	11.51	3.97	-3.99	153.67	238.31	131.84	111.51
7.59	-1.35	12.43	22.06	11.46	1.55	2.73	11.11	3.74	-4.26	151.68	235.28	134.68	116.86
7.25	-1.24	12.10	21.45	11.32	1.61	1.94	10.19	3.16	-4.11	130.77	233.94	140.24	128.36
7.71	-1.18	11.83	21.11	11.11	1.31	2.89	11.20	4.29	-3.68	145.90	239.83	143.40	114.23
6.99	-1.45	11.16	20.50	10.65	1.11	1.93	10.09	3.30	-4.02	154.83	260.64	166.19	146.99
7.76	-1.07	12.29	22.11	11.35	1.66	3.21	11.42	4.14	-3.79	138.34	227.51	115.68	88.56
7.64	-1.23	12.60	22.19	11.96	2.09	2.48	10.74	3.30	-4.55	168.40	263.92	151.03	136.51
7.98	-0.91	12.63	22.11	11.98	1.91	2.89	11.22	3.99	-3.72	153.79	247.04	142.37	126.73
7.60	-1.40	12.19	21.98	11.36	1.34	2.75	10.79	3.84	-4.16	131.74	221.52	112.81	78.89
7.81	-1.37	11.82	21.23	11.35	1.16	2.67	10.72	4.28	-3.89	142.45	232.97	126.46	96.91
7.37	-1.22	11.89	21.36	11.36	1.90	2.44	10.82	3.40	-4.32	193.72	289.36	189.82	196.10
7.27	-2.68	11.18	20.45	10.92	-0.13	2.09	10.26	3.62	-5.24	130.88	237.83	121.80	88.17
7.22	-1.32	11.86	21.24	11.21	1.55	2.06	10.23	3.24	-4.20	129.24	241.95	139.53	126.40
7.56	-1.27	11.87	21.56	11.49	1.48	2.49	10.71	3.60	-4.04	155.49	254.24	150.27	137.19
7.52	-1.63	12.08	22.01	11.29	1.11	2.62	10.79	3.76	-4.39	131.06	223.90	117.50	83.25
8.54	-0.14	12.65	22.02	12.10	2.38	3.32	11.37	4.94	-2.66	169.79	273.87	149.78	124.47
7.49	-0.94	12.41	21.76	11.59	2.07	2.14	10.50	3.39	-3.96	158.88	258.77	168.15	160.79
7.29	-1.20	12.11	21.42	11.36	1.67	2.02	10.27	3.19	-4.07	133.45	237.24	144.08	134.02
7.58	-1.26	12.53	22.00	12.01	2.13	2.28	10.51	3.13	-4.66	175.82	274.13	157.80	146.87

7.76	-1.13	12.83	22.41	12.10	2.17	2.61	10.88	3.44	-4.46	166.41	260.50	149.54	135.25
7.74	-1.21	12.47	22.31	11.52	1.61	3.02	11.33	3.97	-4.04	144.32	234.07	122.04	98.12
7.33	-1.17	11.95	21.39	11.26	1.67	2.21	10.42	3.40	-4.03	134.34	242.04	142.51	133.13
8.02	-0.87	13.16	22.79	12.25	2.30	3.04	11.46	3.81	-4.03	167.56	259.22	168.06	167.74
7.48	-1.57	12.08	21.81	11.22	1.19	2.56	10.62	3.76	-4.33	135.27	224.76	113.88	80.06
7.59	-2.06	11.70	21.06	11.24	0.52	2.49	10.63	3.94	-4.63	132.12	225.43	119.32	88.42
7.33	-1.84	11.81	21.42	11.15	0.86	2.10	10.07	3.49	-4.54	129.53	245.39	120.22	79.18
7.57	-1.28	12.56	22.16	11.79	1.81	2.48	10.88	3.36	-4.38	167.27	257.32	172.09	174.28
8.10	-0.78	13.08	22.87	11.99	2.11	3.50	11.81	4.23	-3.73	142.48	227.92	119.60	94.79
7.48	-1.07	12.23	21.63	11.55	1.88	2.20	10.49	3.38	-4.02	147.82	251.83	157.02	149.51
8.17	-0.89	12.26	21.67	11.68	1.64	3.21	11.24	4.67	-3.41	135.61	226.30	118.66	87.99
7.71	-0.89	12.58	22.11	11.91	2.16	2.36	10.64	3.53	-3.95	149.10	255.47	159.47	151.31
7.86	-1.25	12.21	21.93	11.33	1.41	3.31	11.60	4.41	-3.91	124.61	220.22	119.68	85.97
7.87	-0.88	11.82	21.12	11.24	1.53	3.05	11.26	4.51	-3.32	161.03	265.39	158.74	133.09
8.12	-0.92	12.64	22.27	11.95	1.89	3.00	10.99	4.29	-3.71	121.11	219.34	106.38	70.36
7.17	-1.43	12.04	21.40	11.57	1.93	1.67	10.05	2.78	-4.80	185.58	284.76	201.81	212.31
7.11	-1.53	11.49	20.82	11.00	1.23	1.95	10.08	3.19	-4.31	144.21	253.59	147.99	136.72
7.54	-1.76	11.45	20.79	11.00	0.75	2.44	10.54	4.08	-4.21	146.85	233.51	127.25	98.40
6.71	-2.49	10.79	20.32	10.44	0.26	1.57	9.83	2.97	-5.19	153.16	282.20	154.46	112.07
7.68	-1.47	12.27	22.17	11.43	1.29	2.88	11.10	3.94	-4.23	129.52	222.52	116.94	81.94
8.16	-0.79	13.18	23.08	12.15	2.23	3.39	11.77	4.16	-3.81	151.27	238.29	136.22	118.77
7.85	-1.00	12.91	22.39	12.25	2.34	2.70	10.88	3.44	-4.38	166.25	261.59	148.51	133.95
7.56	-2.28	11.44	20.75	11.23	0.32	2.47	10.56	3.89	-4.85	130.23	233.06	119.23	83.08
7.87	-1.00	13.00	22.49	12.36	2.36	2.56	10.84	3.41	-4.37	173.41	270.80	160.59	152.04
7.03	-2.05	11.30	20.87	10.72	0.66	1.84	9.88	3.33	-4.71	133.24	256.38	126.65	84.85
7.20	-1.42	11.78	21.10	11.20	1.41	1.99	10.16	3.20	-4.29	135.55	247.22	142.56	131.61
7.70	-1.46	11.68	21.09	11.23	1.06	2.56	10.61	4.20	-3.98	142.99	233.02	127.07	97.75
7.40	-1.16	12.30	21.66	11.56	1.79	2.15	10.34	3.24	-4.09	126.45	230.94	134.82	120.78
7.51	-1.42	12.29	21.96	11.61	1.53	2.51	10.84	3.39	-4.39	159.43	247.74	155.82	148.76
8.05	-0.95	13.01	22.77	12.01	1.97	3.33	11.70	4.08	-3.86	149.70	232.71	130.69	110.67
8.01	-1.69	12.14	21.51	11.64	0.97	2.99	11.11	4.34	-4.33	128.53	221.91	113.65	80.68
7.65	-1.14	11.63	20.95	10.94	1.29	3.06	11.38	4.35	-3.59	144.71	238.17	142.00	113.56
7.98	-0.96	12.42	22.15	11.44	1.69	3.47	11.54	4.48	-3.61	130.06	219.78	107.27	76.18
7.99	-1.74	12.15	21.48	11.66	0.91	2.97	11.08	4.35	-4.37	127.88	222.37	113.81	81.00
7.93	-0.99	12.67	22.27	12.00	2.01	2.78	11.08	3.84	-3.99	152.45	245.83	148.62	138.30
6.86	-1.71	11.32	20.58	10.77	1.08	1.64	9.82	2.93	-4.51	133.01	245.73	141.71	131.50
7.95	-1.25	12.01	21.43	11.52	1.29	2.82	10.88	4.41	-3.79	141.78	232.30	125.31	95.63
7.30	-1.10	12.19	21.53	11.27	1.64	2.12	10.09	3.32	-3.82	129.77	209.41	143.81	124.82
7.63	-1.31	12.49	22.17	11.52	1.58	2.78	11.17	3.76	-4.25	150.31	235.30	137.49	119.41
7.63	-2.04	11.75	21.11	11.27	0.52	2.58	10.68	3.98	-4.60	133.02	226.46	118.02	87.08
7.65	-1.23	12.65	22.03	12.11	2.11	2.29	10.56	3.18	-4.56	185.03	282.16	171.01	165.77
8.23	-0.50	12.50	21.81	11.81	2.04	3.25	11.45	4.61	-3.05	156.60	264.63	152.48	123.09
7.33	-1.75	11.77	21.70	11.01	0.97	2.50	10.69	3.68	-4.44	136.39	228.75	120.72	86.66

7.27	-1.28	12.05	21.37	11.39	1.60	2.09	10.26	3.17	-4.18	120.40	227.93	129.73	116.23
8.05	-0.97	12.13	21.56	11.62	1.56	2.95	10.96	4.45	-3.52	143.15	237.63	128.33	96.47
7.10	-1.97	11.41	21.01	10.76	0.72	2.13	10.25	3.43	-4.66	133.26	238.01	125.20	88.44
8.29	-0.84	12.40	21.78	11.82	1.77	3.33	11.40	4.76	-3.41	133.23	224.15	115.62	84.35
8.04	-0.82	12.76	22.55	11.79	1.92	3.38	11.60	4.31	-3.57	135.88	224.37	113.27	84.91
7.78	-1.14	12.55	22.36	11.52	1.67	3.13	11.35	4.06	-3.94	137.37	226.34	113.68	84.00
7.87	-1.42	12.66	22.34	11.87	1.36	2.58	10.64	3.84	-4.16	124.07	233.63	117.00	75.05
7.73	-1.27	12.61	22.26	11.63	1.64	2.89	11.27	3.84	-4.20	151.34	234.51	135.32	116.17
7.14	-1.89	11.28	20.75	10.81	0.81	2.04	10.21	3.47	-4.56	136.88	261.95	133.10	89.74
7.71	-0.89	12.57	22.11	11.91	2.16	2.36	10.64	3.53	-3.95	149.17	255.54	159.55	151.48
7.79	-1.11	12.49	22.34	11.59	1.80	3.21	11.55	3.96	-3.99	152.92	239.22	135.48	115.64
7.56	-1.04	12.41	21.90	11.75	2.04	2.11	10.48	3.33	-4.13	159.11	264.31	172.06	165.47
7.84	-0.97	11.74	21.08	11.28	1.51	2.81	10.79	4.39	-3.42	149.97	251.12	134.89	104.54
7.72	-1.10	11.58	20.92	11.15	1.38	2.65	10.66	4.28	-3.59	150.59	249.68	135.53	106.32
7.70	-1.11	11.50	20.71	11.07	1.30	2.50	10.50	4.31	-3.51	157.12	250.39	139.07	115.81
7.14	-2.11	11.11	20.59	11.05	1.15	1.82	10.01	3.20	-5.39	214.51	347.68	207.23	165.36
7.75	-1.09	12.40	21.91	11.66	1.76	2.80	11.10	3.84	-3.95	147.55	234.00	137.28	122.26
7.45	-1.95	11.31	20.66	10.96	0.55	2.40	10.49	3.94	-4.45	141.44	231.26	125.42	97.01
7.93	-1.09	12.85	22.60	11.83	1.84	3.21	11.58	3.99	-4.00	149.39	233.04	130.68	110.79
6.70	-1.85	11.01	20.30	10.58	0.94	1.50	9.68	2.81	-4.64	144.81	255.58	151.88	143.75
7.88	-1.06	12.61	22.16	11.89	1.89	2.83	11.09	3.88	-4.00	151.41	245.86	147.85	137.04
6.82	-1.49	10.99	20.28	10.50	1.17	1.85	10.01	3.11	-4.22	140.40	239.38	150.91	144.81
7.88	-0.73	12.05	21.58	11.34	1.83	3.32	11.41	4.44	-3.30	132.67	213.85	107.59	77.50
7.50	-1.33	12.34	21.84	11.90	2.10	2.20	10.45	3.08	-4.73	180.12	280.82	165.34	156.38
7.62	-1.49	12.00	21.68	11.35	1.28	2.67	10.84	3.87	-4.26	128.55	242.44	128.85	88.21
7.62	-1.39	12.58	22.31	11.70	1.75	2.66	10.97	3.53	-4.51	153.57	242.27	128.00	102.65
6.78	-2.29	10.70	20.04	10.36	0.34	1.69	9.84	3.16	-4.90	144.07	270.37	137.74	94.36
7.55	-1.54	11.88	21.53	11.22	1.18	2.62	10.71	3.86	-4.25	127.01	241.96	125.30	82.33
7.20	-1.86	11.62	21.54	10.91	0.86	2.27	10.46	3.47	-4.59	138.07	231.47	122.63	88.91
7.53	-1.02	12.51	21.93	11.72	2.08	2.05	10.48	3.31	-4.13	158.79	260.65	167.98	161.09
7.14	-1.33	11.68	20.98	10.89	1.35	2.04	10.10	3.35	-4.00	129.27	221.01	140.27	125.11
7.57	-1.15	11.78	21.34	11.34	1.54	2.66	10.89	3.84	-3.85	156.35	253.40	149.63	137.30
7.13	-1.43	12.08	21.43	11.59	1.96	1.59	10.00	2.69	-4.79	184.29	284.30	201.58	211.33
7.75	-1.13	12.90	22.50	12.26	2.33	2.54	10.77	3.26	-4.59	164.95	260.03	144.75	128.99
7.19	-1.32	11.93	21.23	11.30	1.58	1.97	10.09	3.08	-4.23	122.37	229.66	132.18	120.17
7.79	-1.11	12.50	22.34	11.59	1.80	3.20	11.55	3.96	-3.98	153.01	239.03	135.27	115.52
7.03	-1.41	11.56	20.77	10.85	1.35	1.97	10.16	3.18	-4.15	147.79	243.64	159.67	154.50
8.16	-0.64	11.92	21.25	11.59	1.81	3.16	11.37	4.71	-3.05	161.58	262.08	185.76	170.89
7.85	-1.09	12.40	22.04	11.81	1.75	2.82	11.06	3.84	-3.88	149.58	242.45	139.04	124.20
7.44	-1.95	11.30	20.65	10.95	0.55	2.39	10.48	3.94	-4.46	141.54	231.29	125.51	97.10
7.48	-1.78	11.39	20.72	10.92	0.70	2.34	10.44	4.06	-4.25	147.67	233.59	128.39	99.24
6.88	-1.37	11.37	20.72	10.67	1.22	1.83	9.76	3.11	-3.97	144.25	224.48	157.71	147.71
7.56	-1.34	12.50	21.95	12.03	2.07	2.19	10.45	3.08	-4.76	184.55	280.80	168.27	161.63

7.50	-1.71	11.38	20.75	10.96	0.79	2.38	10.49	4.06	-4.19	146.78	232.93	127.74	98.59
7.65	-1.15	11.65	20.93	11.17	1.30	2.34	10.42	4.15	-3.62	155.41	247.88	137.62	112.99
7.70	-0.90	12.57	22.10	11.91	2.16	2.34	10.63	3.51	-3.96	149.35	255.81	159.84	151.98
7.55	-2.09	11.64	21.00	11.17	0.47	2.47	10.59	3.92	-4.66	133.52	227.09	119.27	88.64
7.75	-1.35	12.13	21.82	11.53	1.37	2.58	10.56	3.94	-4.08	122.31	229.24	113.37	75.29
8.08	-0.88	13.02	22.85	12.03	2.00	3.40	11.79	4.13	-3.78	149.32	234.31	133.98	115.98
7.94	-1.01	12.39	21.85	11.76	1.69	3.09	11.39	4.17	-3.70	153.06	241.30	138.78	121.50
8.18	-0.80	13.12	22.94	12.27	2.33	3.35	11.68	4.05	-3.93	149.37	237.94	131.44	109.85
8.18	-0.88	12.21	21.59	11.64	1.64	3.27	11.31	4.73	-3.42	135.84	224.77	117.89	87.05
6.61	-1.93	10.70	20.10	10.40	0.80	1.41	9.64	2.81	-4.67	159.58	266.17	162.76	155.66
7.45	-1.63	11.87	21.61	11.15	1.12	2.50	10.48	3.76	-4.34	130.75	223.37	112.94	79.33
6.91	-1.61	11.30	20.63	10.81	1.20	1.72	9.94	3.02	-4.43	138.73	248.85	145.81	137.52
7.60	-1.30	12.53	21.98	12.05	2.12	2.26	10.52	3.16	-4.72	182.09	279.06	165.95	157.86
8.22	-1.11	12.75	22.51	12.13	1.73	3.26	11.43	4.31	-3.93	139.01	256.50	138.47	90.36
7.69	-1.50	12.46	22.04	11.61	1.27	2.41	10.38	3.75	-4.26	124.48	240.13	116.07	73.87
7.52	-1.11	12.21	21.69	11.55	1.82	2.24	10.55	3.47	-4.04	139.76	245.79	146.49	135.81
8.02	-0.73	12.07	21.38	11.53	1.73	3.07	11.19	4.51	-3.18	164.81	277.26	157.39	128.36
8.58	-0.19	12.68	22.00	12.11	2.27	3.74	11.93	5.06	-2.63	155.84	261.77	167.29	141.01
7.70	-1.46	11.67	21.08	11.22	1.05	2.55	10.61	4.19	-3.98	142.96	232.99	127.09	97.78
7.47	-1.53	12.26	22.03	11.30	1.40	2.70	10.99	3.63	-4.45	147.57	235.82	123.71	94.98
7.17	-1.96	11.46	21.15	10.83	0.73	2.25	10.38	3.51	-4.62	133.87	233.08	122.46	87.65
8.06	-0.85	13.06	22.72	12.45	2.25	2.63	10.89	3.72	-3.95	152.40	251.80	158.04	149.36
7.39	-1.66	11.75	21.32	11.03	0.99	2.46	10.53	3.73	-4.35	126.28	236.28	122.44	83.63
7.59	-1.19	12.45	22.06	11.75	1.97	2.60	11.02	3.47	-4.36	178.89	271.39	183.26	188.61
7.25	-1.80	11.60	21.23	10.95	0.95	2.31	10.46	3.56	-4.53	130.10	240.27	126.38	88.73
8.39	-0.59	13.17	22.54	12.32	2.19	3.40	11.72	4.46	-3.37	150.37	240.75	137.87	119.66
7.73	-1.11	12.41	22.07	11.52	1.78	2.91	11.17	3.93	-3.97	144.26	227.14	120.96	96.65
7.73	-1.11	11.65	20.99	11.19	1.40	2.63	10.66	4.27	-3.63	148.73	247.22	134.00	104.02
7.97	-0.56	11.85	21.20	11.32	1.87	3.31	11.37	4.61	-2.98	141.20	242.73	127.74	96.38
7.37	-1.65	11.61	21.40	10.94	1.03	2.60	10.83	3.81	-4.31	128.67	232.08	124.61	88.49
7.39	-2.72	11.42	20.73	11.08	-0.13	2.17	10.37	3.72	-5.32	139.05	233.93	123.72	92.56
7.51	-1.62	12.12	22.05	11.32	1.14	2.60	10.78	3.68	-4.36	129.18	224.30	117.12	82.50
8.15	-0.83	13.31	23.18	12.13	2.24	3.34	11.93	4.15	-3.89	160.47	246.88	149.68	137.95
8.20	-0.48	12.25	21.47	11.60	1.97	3.37	11.37	4.82	-2.90	146.84	250.37	130.79	98.11
8.26	-0.64	13.42	23.04	12.59	2.58	3.23	11.61	3.95	-3.87	165.83	257.67	157.37	150.79
8.31	-0.58	12.39	21.67	11.81	1.91	3.36	11.37	4.81	-3.07	143.52	241.28	128.05	95.48
7.29	-1.48	12.23	21.75	11.72	1.99	2.00	10.26	2.85	-4.95	178.69	277.42	156.40	142.39
7.63	-1.15	12.46	22.07	11.76	2.03	2.67	11.09	3.52	-4.29	177.94	270.59	181.63	186.90
7.74	-1.10	11.66	21.00	11.22	1.41	2.65	10.67	4.27	-3.61	149.06	247.77	134.00	104.30
7.84	-1.29	12.47	22.14	11.70	1.46	2.94	11.09	3.99	-4.04	123.12	224.89	118.29	78.99
7.62	-1.51	12.09	21.89	11.31	1.19	2.83	11.08	3.90	-4.23	127.49	225.55	120.76	86.78
8.04	-0.99	13.12	22.84	12.07	1.96	3.25	11.63	4.03	-3.91	150.67	232.61	131.65	113.06
7.62	-1.90	11.71	21.08	11.25	0.62	2.53	10.64	3.99	-4.47	137.43	227.73	122.90	92.62

7.27	-1.26	12.06	21.44	11.35	1.68	2.03	10.26	3.20	-4.19	128.09	237.03	138.01	126.27
7.37	-1.49	11.92	21.52	11.27	1.36	2.42	10.71	3.48	-4.36	158.40	241.40	143.15	130.87
8.10	-0.73	12.73	22.50	11.77	1.98	3.54	11.74	4.43	-3.45	135.56	221.68	110.71	82.13
7.36	-1.83	11.83	21.46	11.16	0.92	2.16	10.16	3.55	-4.59	128.81	246.58	123.05	80.71
6.64	-1.91	10.89	20.18	10.50	0.87	1.42	9.59	2.77	-4.69	145.32	257.47	153.28	145.94
7.09	-1.35	11.67	21.11	11.02	1.53	1.96	10.22	3.18	-4.20	136.81	246.19	146.64	139.16
7.79	-1.40	12.44	22.43	11.60	1.38	2.96	11.18	3.99	-4.20	132.35	222.58	116.55	81.12
7.13	-1.47	11.52	20.88	11.06	1.30	2.00	10.13	3.24	-4.24	139.82	250.80	144.40	133.34
7.88	-1.02	12.91	22.40	12.32	2.33	2.58	10.83	3.42	-4.39	174.74	270.26	159.37	149.84
7.56	-1.29	11.38	20.71	10.99	1.20	2.49	10.50	4.16	-3.79	150.86	245.48	135.02	106.12
8.22	-0.73	13.31	23.18	12.19	2.27	3.47	12.00	4.26	-3.74	154.85	243.60	144.07	128.45
7.49	-2.31	11.37	20.68	11.16	0.29	2.37	10.47	3.82	-4.92	130.12	235.31	119.60	83.71
7.45	-1.60	11.94	21.57	11.23	1.10	2.43	10.50	3.69	-4.30	126.90	235.04	122.00	82.69
7.54	-1.62	12.03	21.78	11.29	1.14	2.53	10.48	3.78	-4.38	131.73	223.35	112.27	77.83
7.25	-1.90	11.58	21.31	10.91	0.77	2.39	10.56	3.58	-4.57	130.57	231.58	122.38	87.13
6.72	-1.54	10.74	20.01	10.32	1.08	1.87	9.99	3.13	-4.20	141.69	238.91	152.72	147.50
8.01	-0.96	12.94	22.64	11.94	1.99	3.23	11.64	4.09	-3.86	153.17	237.25	137.98	121.51
7.93	-0.86	12.99	22.49	12.15	2.34	2.92	11.33	3.71	-4.08	175.04	266.58	172.36	170.62
7.33	-1.72	11.81	21.67	11.03	1.03	2.53	10.84	3.63	-4.47	144.90	236.73	124.77	90.77
7.58	-2.39	11.73	20.97	11.34	0.22	2.30	10.44	3.77	-5.06	130.55	236.11	119.77	83.75
7.85	-1.28	12.42	22.14	11.64	1.41	2.76	10.65	4.06	-4.00	123.13	233.76	115.80	73.05
7.72	-1.15	12.49	22.06	11.90	1.89	2.44	10.73	3.57	-4.21	160.86	256.37	163.48	160.69
8.07	-0.75	12.77	22.50	11.93	2.13	3.34	11.76	4.22	-3.66	151.35	237.99	143.51	129.98
7.55	-1.04	12.31	21.79	11.66	1.95	2.25	10.55	3.44	-4.02	150.21	254.22	159.63	152.25
7.14	-2.50	11.02	20.32	10.77	0.13	1.94	9.96	3.53	-5.14	133.64	248.06	125.24	87.44
7.09	-1.51	11.46	20.81	11.01	1.24	1.93	10.09	3.20	-4.28	140.76	251.44	145.80	134.67
7.64	-1.30	12.52	22.19	11.54	1.59	2.80	11.19	3.77	-4.23	150.14	235.19	137.42	119.21
8.56	-0.15	12.38	21.72	11.93	2.18	3.89	12.04	5.18	-2.51	157.54	265.84	178.04	149.90
7.72	-1.70	11.69	21.01	11.26	0.81	2.70	10.80	4.18	-4.20	140.37	229.71	123.93	94.06
7.72	-1.16	12.46	22.06	11.62	1.70	2.76	11.09	3.79	-4.02	147.54	230.23	131.07	113.80
8.04	-0.87	12.93	22.72	11.91	2.09	3.39	11.70	4.15	-3.82	152.38	235.25	129.52	108.97
7.35	-1.14	12.22	21.61	11.49	1.77	2.09	10.30	3.22	-4.10	130.82	236.13	140.06	128.51
7.87	-0.99	12.78	22.59	11.80	2.02	3.05	11.53	3.91	-3.97	162.42	251.15	154.02	146.18
7.87	-1.17	12.49	22.27	11.63	1.55	3.13	11.16	4.14	-3.88	130.73	219.35	107.96	75.47
7.73	-2.11	11.60	20.90	11.33	0.43	2.76	10.85	4.15	-4.64	129.32	228.31	116.58	82.38
7.65	-1.48	12.11	21.89	11.33	1.22	2.91	11.17	3.96	-4.18	127.98	224.91	120.60	85.41
7.33	-1.47	11.60	21.31	11.27	1.31	2.26	10.48	3.40	-4.23	158.93	257.72	153.27	142.22
7.85	-1.02	12.80	22.29	12.25	2.33	2.63	10.83	3.45	-4.38	173.51	269.66	156.46	146.38
7.90	-1.29	12.60	22.58	11.69	1.46	3.13	11.35	4.11	-4.05	130.55	219.45	114.10	79.09
7.25	-1.38	12.19	21.54	11.67	1.98	1.73	10.11	2.83	-4.74	183.84	282.42	198.78	208.54
7.11	-1.38	11.62	20.99	11.04	1.45	1.99	10.19	3.21	-4.22	135.59	247.66	143.65	134.23
7.30	-1.80	11.53	21.19	10.97	0.96	2.37	10.55	3.66	-4.58	130.90	247.51	129.43	91.45
8.35	-0.72	12.46	21.83	11.85	1.82	3.47	11.46	4.88	-3.22	135.26	224.90	117.58	86.13

7.62	-1.58	11.58	20.91	11.09	0.96	2.57	10.69	4.15	-4.13	141.12	229.48	125.05	95.09
7.97	-0.95	12.93	22.80	11.94	2.03	3.18	11.55	3.99	-3.95	150.79	237.03	130.58	108.56
6.80	-1.69	11.05	20.22	10.54	0.95	1.64	9.78	3.08	-4.33	140.37	250.69	149.20	134.16
6.91	-1.60	11.51	20.79	11.07	1.71	1.64	10.06	2.75	-4.90	197.88	295.63	220.47	245.80
7.75	-1.18	12.59	22.30	11.63	1.67	3.02	11.34	3.88	-4.06	146.07	229.84	124.55	100.26
7.29	-1.49	11.78	21.58	10.92	1.30	2.62	10.92	3.68	-4.29	149.72	240.92	127.43	104.23
7.79	-1.03	11.68	21.02	11.22	1.45	2.74	10.74	4.33	-3.50	149.75	249.55	134.10	103.73
7.55	-1.31	11.98	21.47	11.34	1.51	2.66	10.95	3.79	-4.11	149.83	235.41	137.26	123.42
7.61	-1.53	12.24	22.17	11.36	1.23	2.80	11.01	3.88	-4.28	129.96	222.07	116.06	81.95
8.40	-0.36	12.69	22.05	12.10	2.21	3.37	11.48	4.70	-2.90	154.23	263.22	143.90	113.00
7.52	-2.60	11.62	20.88	11.23	0.00	2.33	10.50	3.79	-5.19	131.87	231.16	120.45	88.28
8.07	-0.87	12.38	21.84	11.74	1.77	3.33	11.60	4.42	-3.50	150.33	239.26	136.37	118.69
7.93	-1.03	12.37	21.78	11.64	1.64	3.14	11.45	4.25	-3.72	149.44	236.76	135.03	116.30
7.60	-1.18	12.25	21.91	11.32	1.65	2.88	11.13	3.91	-4.03	144.48	231.04	121.15	97.62
7.41	-2.47	11.47	20.75	11.15	0.16	2.17	10.29	3.69	-5.09	130.82	238.79	120.82	85.54
7.24	-1.86	11.43	21.20	10.91	0.80	2.22	10.25	3.57	-4.51	131.33	242.93	125.33	85.42
7.18	-1.46	11.82	21.26	11.31	1.76	2.05	10.45	3.05	-4.70	195.69	289.50	206.12	224.38
7.62	-1.23	12.57	22.16	11.94	2.09	2.48	10.72	3.29	-4.58	168.48	264.46	151.60	137.02
8.02	-0.87	12.59	22.07	11.97	1.96	2.94	11.27	4.03	-3.65	153.30	246.68	143.35	126.83
7.36	-1.54	12.14	21.80	11.49	1.42	2.34	10.66	3.25	-4.50	157.67	247.60	154.52	148.40
7.63	-1.43	11.94	21.57	11.29	1.25	2.53	10.47	3.95	-4.13	124.62	227.06	111.47	75.73
7.60	-1.34	12.43	22.13	11.50	1.56	2.75	11.15	3.72	-4.27	152.40	238.09	138.72	121.15
7.86	-1.37	12.53	22.55	11.69	1.44	3.01	11.26	4.01	-4.16	130.80	221.36	116.40	81.02
7.76	-1.08	12.47	22.08	11.47	1.79	2.97	11.21	4.05	-3.94	143.26	225.12	118.56	93.47
8.30	-0.78	12.35	21.71	11.77	1.74	3.41	11.44	4.83	-3.33	134.58	223.66	117.16	85.45
7.33	-1.17	11.95	21.35	11.26	1.68	2.15	10.40	3.39	-4.04	134.80	244.23	141.76	131.07
8.05	-0.83	12.85	22.57	11.82	1.94	3.29	11.56	4.25	-3.65	139.42	222.81	113.00	85.89
7.96	-0.96	12.83	22.66	11.85	2.00	3.29	11.64	4.08	-3.89	151.55	237.14	131.09	110.14
8.41	-0.24	12.28	21.56	11.70	2.18	3.92	12.01	5.13	-2.65	136.02	234.25	123.56	91.56
8.15	-0.81	13.06	22.91	12.08	2.15	3.40	11.71	4.16	-3.77	150.09	236.48	129.23	107.74
7.42	-1.67	12.02	21.83	11.21	1.13	2.61	10.87	3.65	-4.48	143.89	233.24	121.02	87.52
7.18	-1.37	11.71	21.08	11.12	1.47	2.05	10.24	3.25	-4.17	135.34	245.91	142.04	132.75
6.92	-1.67	11.37	20.65	10.86	1.12	1.70	9.89	2.99	-4.48	139.04	248.15	144.68	134.26
8.27	-0.64	13.10	22.83	12.09	2.25	3.64	12.06	4.46	-3.52	151.04	234.76	139.54	124.46
7.78	-1.09	13.05	22.56	12.17	2.08	2.56	10.92	3.38	-4.26	168.52	256.64	175.78	177.21
7.73	-1.44	11.70	21.10	11.25	1.06	2.59	10.65	4.23	-3.97	142.33	232.36	126.53	97.44
7.90	-1.01	12.90	22.49	12.06	2.06	2.98	11.33	3.73	-4.07	164.14	251.98	163.20	158.71
7.59	-1.55	11.95	21.54	11.22	1.13	2.72	10.88	3.98	-4.20	127.21	238.07	124.63	85.24
7.61	-1.51	12.17	22.08	11.33	1.23	2.82	11.03	3.87	-4.26	131.33	221.94	116.69	82.44
6.95	-1.58	11.49	20.79	10.92	1.25	1.76	9.95	2.98	-4.43	126.10	240.03	135.80	124.81
7.93	-0.81	11.84	21.20	11.43	1.65	2.92	10.90	4.48	-3.26	153.69	257.10	139.20	109.50
8.17	-0.89	12.20	21.58	11.62	1.62	3.25	11.30	4.72	-3.43	135.95	224.88	117.95	87.18
7.64	-1.32	12.46	22.13	11.53	1.61	2.77	11.15	3.75	-4.24	150.56	235.22	135.00	117.86

8.14	-0.66	12.78	22.29	11.95	2.09	3.45	11.60	4.35	-3.40	132.53	214.22	109.61	83.22
7.48	-1.87	11.85	21.47	11.38	1.18	2.24	10.49	3.58	-4.90	182.98	306.00	174.63	124.94
7.29	-1.47	12.00	21.47	11.36	1.60	2.17	10.53	3.21	-4.54	177.80	267.12	184.00	192.24
7.76	-1.06	11.63	20.96	11.19	1.42	2.70	10.70	4.32	-3.54	151.02	250.59	135.75	106.50
8.25	-0.65	13.40	23.02	12.57	2.57	3.21	11.60	3.94	-3.88	166.10	258.05	157.76	151.27
7.22	-1.29	11.81	21.23	11.23	1.58	1.98	10.24	3.15	-4.18	141.93	249.06	153.63	145.30
7.47	-1.10	12.28	21.66	11.54	1.80	2.19	10.48	3.38	-4.00	141.01	245.84	150.90	139.98
7.43	-1.63	11.76	21.42	11.15	1.06	2.52	10.63	3.75	-4.35	129.02	236.20	123.65	85.04
8.30	-0.74	12.87	22.50	12.01	1.96	3.40	11.41	4.57	-3.47	122.31	211.77	103.81	69.41
8.16	-0.59	12.20	21.50	11.65	1.88	3.26	11.40	4.67	-3.06	161.80	274.45	156.23	127.96
7.60	-1.81	11.52	20.85	11.13	0.69	2.57	10.66	4.07	-4.32	140.62	230.49	124.41	95.49
7.67	-1.20	12.16	21.87	11.68	1.61	2.64	10.87	3.63	-4.03	152.96	248.04	145.04	131.46
7.86	-1.04	12.96	22.44	12.36	2.31	2.52	10.79	3.37	-4.41	175.11	274.22	163.79	156.36
6.63	-2.29	10.53	19.88	10.15	0.35	1.60	9.79	3.11	-4.92	142.36	272.94	138.95	96.64
7.88	-1.00	12.75	22.61	11.81	1.96	3.18	11.53	3.99	-3.97	153.70	238.16	131.78	111.50
7.94	-1.01	12.78	22.67	11.85	1.95	3.20	11.47	4.04	-3.98	146.87	232.33	126.34	100.28
8.45	-0.26	12.56	21.85	11.89	2.20	3.82	11.98	5.02	-2.74	138.70	237.89	127.53	95.48
7.75	-1.18	12.49	22.31	11.48	1.64	3.09	11.30	4.03	-3.97	137.71	227.19	114.35	84.67
7.43	-1.43	11.96	21.78	11.10	1.40	2.72	11.05	3.72	-4.28	150.77	240.09	128.64	104.73
7.45	-1.12	12.28	21.78	11.64	1.95	1.99	10.36	3.24	-4.19	161.89	267.79	176.00	171.76
7.20	-1.31	11.96	21.25	11.32	1.59	1.98	10.12	3.09	-4.23	122.39	229.31	132.33	120.08
7.44	-2.43	11.52	20.85	11.08	0.18	2.30	10.44	3.81	-5.04	133.43	228.74	119.80	88.26
7.15	-2.39	11.62	21.14	11.34	0.97	1.67	9.89	2.98	-5.76	214.64	343.59	207.76	167.41
7.56	-2.38	11.58	20.87	11.20	0.21	2.48	10.60	3.92	-4.93	131.19	228.82	117.58	85.66
6.93	-1.45	11.38	20.65	10.74	1.30	1.85	10.07	3.12	-4.18	139.44	237.70	150.73	143.94
6.79	-2.22	10.87	20.20	10.46	0.40	1.71	9.85	3.16	-4.89	142.76	269.39	136.05	92.90
8.03	-0.68	12.06	21.41	11.68	2.12	2.91	11.01	4.37	-3.46	135.50	256.79	128.46	83.50
7.87	-1.33	12.71	22.62	11.78	1.55	2.99	11.33	3.98	-4.20	135.69	226.26	116.00	80.61
6.81	-1.57	11.15	20.39	10.57	1.17	1.79	9.98	3.05	-4.31	152.75	247.09	164.86	161.24
7.41	-1.33	12.28	21.72	11.61	1.93	2.27	10.69	3.20	-4.55	188.12	281.46	197.93	212.48
7.53	-1.56	11.94	21.58	11.25	1.16	2.42	10.36	3.81	-4.25	127.52	238.51	118.46	77.45
7.74	-1.01	11.48	20.81	11.11	1.39	2.70	10.70	4.38	-3.43	158.01	255.00	140.15	112.07
7.72	-1.40	12.13	21.91	11.36	1.26	3.00	11.29	4.06	-4.08	126.60	221.81	119.70	85.53
7.08	-1.48	11.66	21.01	11.07	1.37	1.88	10.09	3.10	-4.33	129.58	241.64	138.43	127.95
8.31	-0.40	12.31	21.61	11.72	2.08	3.63	11.71	4.88	-2.87	140.62	240.36	125.98	94.00
7.41	-1.51	12.16	21.82	11.25	1.43	2.49	10.90	3.55	-4.44	154.36	238.93	137.25	120.63
6.79	-1.89	11.16	20.39	10.74	0.81	1.41	9.61	2.84	-4.62	161.92	265.51	162.78	151.45
7.47	-1.60	11.89	21.64	11.16	1.13	2.54	10.51	3.74	-4.33	131.58	223.84	112.79	79.30
7.73	-1.43	12.19	21.78	11.51	1.27	2.70	10.81	3.96	-4.16	127.09	243.02	124.08	78.74
8.39	-0.45	13.07	22.64	12.20	2.27	3.57	11.65	4.59	-3.15	127.11	211.83	103.62	72.70
7.22	-1.37	11.81	21.16	11.19	1.49	2.06	10.26	3.26	-4.22	129.80	241.78	138.72	126.64
6.94	-1.44	11.19	20.51	10.60	1.22	2.04	10.22	3.32	-4.07	141.42	240.32	153.83	146.11
7.70	-1.49	12.12	21.80	11.36	1.15	2.91	11.01	4.03	-4.15	126.12	221.74	115.38	79.01

7.31	-1.19	12.08	21.40	11.36	1.72	2.03	10.29	3.26	-4.11	145.85	243.64	154.35	147.03
7.61	-1.33	12.54	22.18	11.79	1.64	2.55	10.88	3.40	-4.34	155.76	245.45	151.46	142.69
7.60	-1.35	12.43	22.07	11.46	1.55	2.73	11.12	3.75	-4.26	151.54	235.17	134.65	116.78
6.99	-1.42	11.43	20.77	10.84	1.39	1.85	10.10	3.11	-4.25	151.65	249.43	160.24	156.57
7.84	-1.19	12.45	21.91	11.74	1.58	2.85	11.19	3.93	-3.94	150.92	238.18	136.03	117.54
7.64	-1.18	12.60	22.16	11.95	2.22	2.58	10.80	3.35	-4.54	165.61	260.15	144.47	124.67
7.66	-1.14	11.51	20.84	11.07	1.33	2.60	10.62	4.26	-3.62	150.65	249.33	136.08	106.37
7.93	-1.32	12.62	22.51	11.72	1.45	3.13	11.34	4.13	-4.09	126.61	217.20	112.63	78.54
7.65	-1.53	12.06	21.74	11.31	1.11	2.85	10.95	3.98	-4.19	126.76	222.80	116.04	79.87
7.32	-1.83	11.60	21.47	10.95	0.87	2.54	10.73	3.72	-4.50	132.68	230.83	122.04	88.87
7.61	-1.15	12.43	21.90	11.81	2.00	2.29	10.59	3.38	-4.29	169.85	266.55	175.71	177.31
7.59	-1.55	12.20	22.13	11.38	1.19	2.71	10.89	3.82	-4.33	130.03	222.11	116.02	81.84
7.84	-0.97	11.72	21.06	11.26	1.49	2.80	10.78	4.36	-3.45	149.67	251.20	134.85	105.42
7.59	-1.22	11.27	20.49	10.91	1.16	2.44	10.46	4.28	-3.59	156.58	248.41	139.21	115.91
7.54	-1.67	12.04	21.97	11.33	1.07	2.70	10.92	3.76	-4.37	129.76	225.24	120.09	84.99
7.09	-1.43	11.60	21.06	11.10	1.46	1.89	10.13	3.10	-4.32	142.53	251.50	148.65	139.57
7.27	-1.67	12.00	21.66	11.31	1.26	2.16	10.52	3.24	-4.63	159.46	244.41	146.94	136.26
7.99	-0.98	12.94	22.82	12.00	1.91	3.24	11.64	4.01	-3.88	152.05	237.21	137.02	119.98
7.69	-1.11	12.76	22.30	12.00	2.15	2.50	10.89	3.37	-4.35	177.13	265.67	186.90	192.28
7.26	-1.16	11.76	21.01	11.07	1.65	2.17	10.43	3.43	-3.98	151.50	248.23	161.62	156.25
7.78	-1.12	12.81	22.30	12.26	2.26	2.44	10.71	3.31	-4.49	177.05	273.98	162.76	154.14
7.47	-1.28	12.41	21.88	11.72	1.99	2.35	10.77	3.26	-4.51	186.01	279.22	194.50	205.99
7.72	-1.40	12.13	21.91	11.36	1.26	3.00	11.29	4.06	-4.08	126.60	221.78	119.69	85.52
7.76	-1.42	12.27	21.89	11.39	1.26	2.92	11.17	4.10	-4.10	125.33	229.40	121.45	83.56
6.92	-2.28	10.79	20.28	10.79	0.95	1.62	9.82	3.05	-5.52	217.88	355.28	210.48	171.61
7.23	-1.33	11.75	21.15	11.15	1.47	2.07	10.29	3.28	-4.16	135.34	245.96	141.80	131.55
8.37	-0.34	12.39	21.72	11.85	2.08	3.54	11.72	4.90	-2.78	157.43	263.10	168.78	144.11
7.47	-1.30	12.43	22.00	11.81	2.12	2.33	10.58	3.14	-4.73	172.11	266.84	149.22	131.63
7.47	-1.51	11.79	21.57	11.06	1.21	2.70	10.72	3.87	-4.23	133.78	224.67	113.12	80.29
7.86	-1.17	12.50	21.96	11.78	1.60	2.87	11.21	3.94	-3.92	150.68	237.93	135.80	117.12
7.57	-1.29	12.00	21.49	11.36	1.53	2.68	10.96	3.80	-4.09	149.52	235.08	136.89	122.88
7.85	-1.17	12.81	22.48	11.79	1.77	3.08	11.44	3.92	-4.09	148.85	233.36	130.98	110.90
7.86	-1.10	12.67	22.52	11.67	1.74	3.14	11.42	4.06	-3.91	142.81	231.20	119.67	94.43
7.76	-1.02	12.58	22.11	12.04	2.21	2.65	10.95	3.50	-4.26	181.41	280.04	172.16	168.51
7.37	-1.91	11.22	20.57	10.81	0.57	2.24	10.37	3.94	-4.37	147.39	234.58	128.35	99.82
7.48	-1.66	11.82	21.49	11.22	1.12	2.48	10.66	3.75	-4.46	129.25	244.54	129.97	89.74
7.43	-1.55	11.59	21.32	11.05	1.09	2.39	10.29	3.79	-4.19	128.27	232.14	117.47	79.62
6.82	-1.71	11.17	20.48	10.71	1.09	1.61	9.83	2.92	-4.51	141.73	251.42	148.04	139.79
8.17	-0.58	12.80	22.21	11.94	2.13	3.37	11.47	4.38	-3.26	129.54	211.00	103.97	74.66
7.46	-1.41	12.41	21.82	11.94	1.99	2.08	10.35	2.97	-4.83	187.43	283.95	171.96	166.47
7.53	-1.35	12.50	21.94	12.04	2.11	2.15	10.37	3.02	-4.82	182.43	279.98	164.32	155.78
7.35	-1.36	12.20	21.61	11.56	1.85	2.20	10.60	3.14	-4.60	190.43	284.39	200.95	216.66
7.10	-1.49	11.92	21.30	11.49	1.90	1.59	10.01	2.73	-4.87	188.55	287.62	205.08	218.55

7.08	-1.38	11.58	20.91	10.83	1.31	1.97	10.05	3.29	-4.06	130.44	222.54	142.06	128.34
7.94	-0.93	13.04	22.64	12.20	2.26	2.91	11.34	3.68	-4.13	171.40	262.91	172.92	174.35
7.46	-2.34	11.39	20.75	11.15	0.29	2.29	10.37	3.77	-4.99	130.12	236.94	120.30	84.28
6.83	-1.70	11.30	20.60	10.75	1.09	1.65	9.86	2.91	-4.49	133.62	245.16	142.34	132.67
8.37	-0.35	12.99	22.46	12.20	2.43	3.55	11.70	4.52	-3.14	132.17	207.82	104.95	77.35
7.69	-1.14	12.64	22.17	12.12	2.22	2.35	10.64	3.23	-4.52	181.48	281.10	170.40	165.50
7.46	-1.10	12.30	21.78	11.68	1.97	2.03	10.39	3.27	-4.16	162.51	266.19	174.38	169.48
7.66	-1.35	12.58	22.37	11.69	1.69	2.78	11.09	3.62	-4.37	149.90	240.07	126.87	100.98
7.49	-1.59	11.94	21.59	11.24	1.14	2.55	10.72	3.73	-4.33	128.16	234.93	123.35	85.06
6.64	-2.12	10.25	19.48	9.98	0.23	1.58	9.92	3.29	-4.46	210.07	296.41	192.98	195.12
7.17	-1.22	11.75	21.04	11.06	1.65	2.01	10.34	3.31	-4.10	160.66	260.89	172.26	168.90
7.08	-1.50	11.52	20.81	11.10	1.68	1.95	10.26	3.03	-4.68	196.02	290.43	214.69	238.33
7.42	-1.60	11.77	21.43	11.12	1.08	2.51	10.61	3.75	-4.27	126.79	234.44	122.31	83.58
7.29	-1.73	11.66	21.30	11.00	1.00	2.32	10.50	3.57	-4.49	130.56	238.81	125.83	88.36
7.57	-1.65	12.33	21.89	11.50	1.14	2.25	10.25	3.64	-4.39	127.16	243.08	117.88	76.01
7.44	-1.58	11.86	21.58	11.21	1.15	2.22	10.28	3.68	-4.35	127.52	238.96	120.65	81.39
7.79	-0.96	12.60	22.20	11.99	2.22	2.80	11.17	3.61	-4.14	177.48	273.43	170.44	168.27
7.69	-1.51	12.24	21.87	11.50	1.19	2.55	10.53	3.85	-4.23	126.67	235.89	117.81	75.84
7.55	-1.39	12.35	22.06	11.45	1.53	2.68	11.08	3.67	-4.32	153.72	239.85	140.09	122.68
7.71	-0.89	12.57	22.11	11.91	2.16	2.36	10.64	3.53	-3.95	149.14	255.51	159.52	151.42
8.13	-0.82	13.04	22.89	12.07	2.14	3.39	11.71	4.15	-3.78	150.38	236.49	129.40	107.81
7.41	-1.13	12.28	21.67	11.55	1.78	2.17	10.41	3.34	-4.04	135.63	239.25	144.47	132.97
7.43	-1.49	12.17	21.82	11.49	1.48	2.43	10.77	3.35	-4.44	161.97	249.85	158.67	152.27
6.72	-1.64	10.92	20.21	10.42	1.11	1.69	9.98	3.04	-4.36	154.42	255.15	167.38	165.30
7.16	-1.32	11.70	21.11	11.14	1.59	1.95	10.25	3.17	-4.22	150.64	255.25	160.61	155.61
7.58	-2.39	11.69	20.92	11.33	0.26	2.32	10.45	3.81	-5.07	131.40	236.61	120.15	84.15
7.69	-1.85	12.05	21.35	11.57	0.91	2.41	10.44	3.81	-4.56	128.93	242.25	118.00	77.56
7.51	-1.38	12.30	21.89	11.56	1.59	2.58	10.91	3.47	-4.34	161.42	248.56	159.43	153.67
7.56	-1.08	12.44	21.93	11.77	2.00	2.09	10.48	3.33	-4.14	160.22	267.31	173.68	168.72
7.37	-1.65	11.61	21.39	10.94	1.03	2.59	10.83	3.81	-4.31	128.71	232.15	124.66	88.54
7.52	-1.60	11.94	21.64	11.24	1.11	2.41	10.34	3.78	-4.26	126.26	239.97	119.20	77.71
7.60	-0.97	12.37	21.91	11.75	2.06	2.33	10.60	3.49	-3.97	149.86	256.39	159.09	152.14
7.97	-1.26	12.61	22.59	11.79	1.53	3.12	11.40	4.12	-4.04	129.81	219.55	115.52	80.82
7.50	-1.27	12.45	21.93	11.76	1.99	2.35	10.77	3.25	-4.51	184.93	277.90	193.74	204.26
8.30	-0.66	12.79	22.62	11.92	2.04	3.73	11.88	4.70	-3.34	128.73	216.85	104.76	73.52
7.84	-1.02	13.07	22.75	12.20	2.35	2.59	10.97	3.46	-4.40	168.34	261.25	151.68	139.54
7.89	-0.99	12.88	22.39	12.30	2.34	2.63	10.89	3.47	-4.35	173.89	269.27	157.65	148.31
7.94	-1.02	12.84	22.57	11.89	1.92	3.16	11.54	4.00	-3.95	152.06	235.24	137.12	119.53
8.02	-0.93	12.06	21.41	11.56	1.60	2.91	10.93	4.46	-3.44	147.24	244.92	132.29	100.44
7.31	-1.36	12.19	21.49	11.57	1.92	2.07	10.44	3.07	-4.66	185.63	282.04	204.64	221.01
7.51	-1.62	12.11	22.02	11.29	1.13	2.59	10.75	3.68	-4.37	129.30	224.83	116.84	82.61
7.77	-1.40	12.40	22.36	11.57	1.39	2.91	11.18	3.92	-4.19	132.40	222.13	117.90	83.40
7.24	-1.35	12.00	21.40	11.46	1.84	1.79	10.18	2.99	-4.56	177.29	278.92	189.64	194.85

7.64	-0.97	12.52	22.00	11.83	2.09	2.23	10.58	3.44	-4.04	157.94	261.32	168.12	161.32
7.85	-1.07	12.90	22.50	12.06	2.05	2.86	11.23	3.64	-4.17	167.17	254.82	167.98	164.88
7.27	-1.36	11.88	21.23	11.30	1.48	2.06	10.25	3.24	-4.24	135.35	246.13	141.76	130.90
7.71	-1.44	12.34	22.30	11.49	1.34	2.87	11.13	3.91	-4.23	133.91	222.35	118.61	83.58
7.47	-1.13	12.30	21.80	11.65	1.95	2.00	10.39	3.26	-4.19	161.98	268.45	175.99	171.84
8.22	-0.99	12.38	21.82	11.81	1.63	3.24	11.31	4.62	-3.58	132.31	220.82	113.38	82.00
7.72	-1.04	11.72	20.94	11.28	1.47	2.38	10.42	4.16	-3.57	157.66	249.58	139.50	114.39
7.06	-1.59	11.32	20.70	10.96	1.14	1.87	10.04	3.17	-4.32	151.77	257.91	153.95	141.44
7.39	-1.35	11.43	20.63	10.92	1.15	2.39	10.60	3.87	-3.80	178.93	274.09	173.56	160.77
7.58	-1.35	11.46	20.94	11.04	1.16	2.30	10.45	4.14	-3.87	151.62	245.69	136.13	109.77
7.64	-1.36	12.20	21.96	11.37	1.39	2.84	10.87	3.93	-4.13	131.63	222.16	112.46	77.97
7.00	-2.06	11.26	20.83	10.68	0.62	1.80	9.84	3.31	-4.74	133.39	256.69	126.90	85.40
7.44	-1.75	11.97	21.57	11.27	1.02	2.26	10.27	3.64	-4.50	129.32	244.20	121.40	79.08
6.72	-1.70	11.28	20.60	11.00	1.66	1.29	9.72	2.48	-5.03	197.43	299.78	216.82	236.80
8.25	-0.65	13.52	23.20	12.61	2.58	3.18	11.52	3.91	-3.90	160.49	249.91	144.34	129.89
8.44	-0.21	12.37	21.64	11.77	2.22	3.92	12.00	5.12	-2.64	137.62	234.73	124.74	91.29
7.49	-1.64	11.84	21.41	11.23	1.04	2.44	10.55	3.75	-4.35	128.18	248.51	126.05	82.49
7.54	-1.50	12.34	22.09	11.36	1.45	2.78	11.04	3.72	-4.43	146.50	233.15	121.74	93.26
8.04	-0.61	11.81	21.22	11.47	1.82	2.99	11.16	4.61	-3.01	166.60	257.69	173.81	165.09
8.00	-0.94	12.93	22.84	11.95	2.01	3.25	11.72	4.04	-3.89	156.09	241.94	143.55	128.71
7.65	-1.41	12.21	21.95	11.48	1.31	2.53	10.47	3.90	-4.16	124.68	235.71	117.18	76.66
7.24	-1.29	12.08	21.40	11.42	1.66	1.99	10.16	3.08	-4.22	122.28	230.42	133.25	120.33
7.51	-2.58	11.58	20.85	11.18	0.02	2.32	10.50	3.83	-5.18	131.33	232.14	120.03	87.63
7.65	-1.02	12.32	21.98	11.78	1.93	2.33	10.61	3.49	-4.00	144.93	249.71	151.58	141.83
7.68	-0.88	12.51	21.93	11.52	1.74	2.57	10.72	3.84	-3.51	131.85	236.80	143.76	118.20
7.14	-1.34	11.69	21.10	11.13	1.59	1.93	10.25	3.16	-4.24	150.74	256.19	161.86	157.39
8.09	-0.83	12.96	22.82	11.98	2.08	3.41	11.88	4.21	-3.75	153.61	241.50	142.09	126.53
7.70	-1.08	12.18	22.04	11.26	1.62	3.17	11.37	4.10	-3.80	138.09	227.14	115.56	88.49
8.13	-0.84	13.15	23.03	12.12	2.13	3.40	11.86	4.14	-3.80	153.59	239.79	139.91	123.92
7.92	-0.74	11.58	20.98	11.29	1.65	2.92	11.11	4.53	-3.15	161.88	258.08	175.78	167.45
8.13	-0.45	12.06	21.35	11.49	1.95	3.40	11.41	4.76	-2.87	146.55	249.12	131.45	98.84
7.33	-1.51	11.87	21.37	11.32	1.52	2.17	10.48	3.32	-4.51	168.50	259.25	169.15	168.50
8.06	-0.84	13.00	22.84	11.95	2.10	3.39	11.85	4.15	-3.82	153.03	238.78	137.44	120.98
7.55	-1.35	12.28	22.11	11.40	1.61	2.79	11.07	3.71	-4.30	152.61	240.41	127.06	103.01
7.98	-0.84	13.07	22.58	12.22	2.37	2.96	11.38	3.74	-4.06	173.64	265.16	171.18	168.62
7.62	-1.18	12.27	21.91	11.36	1.69	2.82	11.08	3.89	-4.08	144.93	228.55	120.80	96.80
8.72	-0.71	13.60	23.09	12.98	2.56	3.44	11.78	4.49	-4.00	168.61	283.21	160.71	105.14
7.68	-1.30	12.55	22.29	11.62	1.66	2.86	11.26	3.74	-4.26	152.51	237.56	137.11	118.33
7.95	-0.86	12.59	22.17	11.76	1.93	3.21	11.38	4.16	-3.66	136.83	218.54	113.16	87.00
7.76	-1.07	12.76	22.26	12.13	2.29	2.56	10.77	3.34	-4.46	169.66	266.60	152.15	139.33
7.93	-0.98	12.19	21.47	11.42	1.54	3.11	11.52	4.41	-3.46	154.04	249.87	157.84	131.31
7.82	-1.06	12.69	22.36	11.86	2.10	3.08	11.33	3.77	-4.25	150.58	239.25	127.00	102.74
7.72	-0.97	12.61	22.15	11.91	2.10	2.35	10.64	3.53	-4.08	154.27	258.29	164.03	157.72

7.39	-1.15	12.26	21.66	11.52	1.80	2.10	10.34	3.25	-4.08	133.61	237.55	143.85	131.62
7.47	-1.46	12.21	21.91	11.38	1.60	2.64	10.92	3.55	-4.48	155.55	242.86	128.44	101.34
7.72	-0.85	12.50	21.91	11.53	1.76	2.64	10.76	3.88	-3.47	130.00	235.83	142.74	117.13
7.61	-1.43	11.93	21.55	11.29	1.29	2.54	10.48	3.95	-4.14	125.75	225.91	110.89	75.76
7.31	-1.19	12.08	21.41	11.36	1.73	2.03	10.30	3.26	-4.11	145.99	243.60	154.35	146.94
7.68	-1.13	12.59	22.11	12.08	2.23	2.37	10.69	3.27	-4.48	182.77	282.36	170.74	166.87
7.28	-1.72	11.46	21.15	10.97	0.97	2.38	10.56	3.63	-4.42	131.83	251.14	132.70	93.38
7.82	-1.52	12.31	22.03	11.83	1.55	2.60	10.84	3.81	-4.59	177.24	297.79	169.17	115.82
7.50	-1.40	12.35	21.94	11.65	1.63	2.43	10.76	3.33	-4.46	164.97	252.45	164.18	162.56
7.80	-1.20	12.71	22.45	11.75	1.75	2.98	11.38	3.84	-4.17	151.34	235.98	135.33	116.17
7.24	-1.53	12.14	21.55	11.55	1.74	1.91	10.24	2.92	-4.79	180.60	272.80	193.48	203.25
8.04	-0.91	13.02	22.90	12.03	2.09	3.32	11.61	4.10	-3.91	146.03	232.64	124.71	100.29
7.54	-1.68	11.99	21.93	11.25	1.07	2.70	10.97	3.78	-4.42	133.65	228.17	121.33	88.20
8.03	-0.80	12.76	22.62	11.75	1.97	3.42	11.63	4.31	-3.56	134.33	221.08	109.66	80.66
6.74	-1.60	10.86	20.13	10.34	1.00	1.77	9.84	3.14	-4.21	132.07	238.07	144.62	132.75
8.09	-0.87	13.01	22.78	11.90	2.05	3.48	11.73	4.27	-3.77	141.89	226.42	118.73	94.00
7.51	-0.98	12.47	21.86	11.70	2.10	2.09	10.46	3.35	-4.06	156.91	259.12	167.37	160.34
7.74	-1.08	12.69	22.19	12.15	2.27	2.45	10.77	3.33	-4.43	181.33	280.38	168.57	164.47
8.22	-0.70	13.39	23.04	12.35	2.42	3.36	11.75	4.10	-3.85	161.54	247.23	159.28	152.97
7.85	-1.10	11.94	21.41	11.39	1.43	2.54	10.66	4.33	-3.62	152.01	244.84	134.94	108.01
7.80	-1.22	12.49	22.13	11.80	1.57	2.78	11.08	3.80	-4.01	148.11	237.16	133.98	117.48
8.00	-1.01	12.87	22.67	11.86	1.86	3.33	11.60	4.12	-3.87	142.98	227.28	120.42	94.12
7.89	-1.09	12.06	21.74	11.79	1.71	2.70	10.90	4.00	-3.89	150.53	276.17	147.62	98.13
7.64	-1.46	12.12	21.84	11.47	1.23	2.58	10.62	3.83	-4.17	126.14	236.10	119.65	78.56
6.99	-1.43	11.36	20.68	10.77	1.28	1.95	10.07	3.21	-4.17	121.43	235.04	133.18	119.71
7.89	-0.87	12.89	22.39	12.09	2.30	2.92	11.33	3.72	-4.07	176.03	267.94	173.64	171.91
7.57	-1.41	11.98	21.73	11.20	1.30	2.81	10.84	3.99	-4.12	132.53	222.61	110.69	79.42
8.14	-0.75	13.20	22.74	12.28	2.17	2.98	11.33	3.99	-3.70	139.40	226.77	125.77	107.98
7.12	-1.43	11.72	21.10	11.11	1.44	1.92	10.14	3.13	-4.30	130.38	241.52	139.08	128.88
7.39	-1.43	11.76	21.27	11.20	1.37	2.47	10.74	3.58	-4.26	154.76	241.63	144.40	133.62
8.37	-0.61	13.67	23.36	12.62	2.59	3.47	11.95	4.18	-3.81	157.80	247.17	152.52	144.41
7.73	-1.04	12.78	22.25	12.08	2.19	2.29	10.60	3.37	-4.32	170.04	265.88	175.17	177.25
7.89	-1.13	12.86	22.53	11.84	1.80	3.13	11.48	3.97	-4.06	148.48	233.06	130.30	110.29
7.83	-1.06	12.45	22.02	11.84	1.91	2.77	11.04	3.84	-4.02	152.98	247.38	151.95	143.07
7.95	-0.93	13.20	22.71	12.32	2.28	2.78	11.15	3.58	-4.15	168.93	256.64	177.48	178.31
7.94	-0.96	12.71	22.57	11.77	1.94	3.33	11.69	4.11	-3.88	152.44	238.84	133.76	114.09
8.00	-0.83	12.94	22.50	12.27	2.38	2.92	11.27	3.73	-4.06	171.76	267.33	163.01	157.99
8.26	-0.48	12.42	21.73	11.86	2.00	3.34	11.42	4.68	-3.00	161.66	274.26	151.99	122.52
7.73	-0.98	12.39	22.06	11.82	1.98	2.45	10.77	3.62	-3.95	143.32	249.59	150.53	139.68
7.90	-1.04	12.64	22.18	11.92	1.90	2.85	11.11	3.90	-3.99	151.24	245.54	147.54	136.55
8.26	-0.60	13.22	22.96	12.18	2.43	3.57	12.02	4.36	-3.61	155.10	245.46	142.08	127.44
7.40	-1.68	11.63	21.30	11.05	0.97	2.27	10.21	3.74	-4.32	127.54	231.95	114.79	79.31
8.10	-1.33	12.49	21.91	12.15	1.89	2.79	10.97	4.09	-4.57	196.34	318.52	186.75	136.88

7.30	-1.20	12.14	21.45	11.40	1.69	2.00	10.25	3.17	-4.11	133.87	236.69	143.91	133.02
7.40	-1.80	11.94	21.49	11.28	0.95	2.11	10.05	3.51	-4.55	130.36	248.72	121.57	77.92
7.07	-1.39	11.83	21.13	11.17	1.53	1.83	9.99	2.98	-4.27	127.36	232.14	138.78	127.64
7.59	-1.08	12.50	22.02	11.87	2.05	2.12	10.46	3.31	-4.19	160.95	264.45	173.05	167.72
8.70	-0.67	13.58	23.28	12.91	2.52	3.37	11.64	4.46	-3.85	169.61	283.62	158.08	99.53
7.98	-0.86	12.59	22.26	11.83	2.01	3.16	11.59	4.16	-3.72	150.15	236.87	139.37	125.19
7.79	-1.21	12.70	22.44	11.74	1.74	2.97	11.38	3.83	-4.17	151.38	236.03	135.40	116.24
7.77	-1.14	12.83	22.40	12.12	2.15	2.64	10.88	3.45	-4.43	166.01	259.40	147.71	132.85
7.88	-0.98	12.93	22.41	12.25	2.36	2.72	10.90	3.49	-4.32	165.67	262.44	149.02	135.70
7.78	-0.89	12.52	22.09	11.90	2.08	2.54	10.80	3.67	-3.86	150.67	254.13	158.72	150.05
8.29	-0.72	13.52	23.32	12.39	2.42	3.42	11.94	4.18	-3.85	158.44	247.67	149.38	137.69
8.17	-0.72	13.47	22.95	12.68	2.63	2.91	11.16	3.70	-4.05	167.67	260.09	154.02	143.51
7.90	-1.01	12.69	22.45	11.65	1.86	3.29	11.57	4.13	-3.89	144.26	229.46	122.25	96.92
8.29	-1.21	13.04	22.58	12.54	2.06	2.85	11.13	4.03	-4.51	181.04	299.59	171.51	115.82
7.88	-0.80	12.05	21.56	11.33	1.74	3.26	11.36	4.41	-3.34	131.53	213.23	107.85	77.97
6.91	-1.42	11.18	20.45	10.59	1.37	1.87	10.21	3.21	-4.21	167.90	266.67	179.89	180.36
7.38	-1.83	11.85	21.43	11.17	0.92	2.23	10.24	3.59	-4.55	129.34	247.04	123.19	80.20
8.54	-0.46	13.66	23.31	12.70	2.74	3.69	12.05	4.36	-3.65	155.72	243.92	144.99	132.98
7.36	-1.84	11.83	21.41	11.15	0.91	2.21	10.22	3.58	-4.57	129.43	247.40	123.29	80.47
8.66	-0.87	13.41	22.81	12.88	2.40	3.34	11.63	4.40	-4.13	176.02	289.89	167.15	110.42
7.87	-1.12	12.77	22.39	11.77	1.79	2.99	11.35	3.95	-4.04	147.58	231.53	129.64	111.36
6.86	-1.47	11.18	20.44	10.61	1.35	1.82	10.12	3.15	-4.28	166.90	263.18	178.95	178.64
8.31	-0.74	12.72	22.39	12.32	2.11	3.11	11.23	4.33	-3.63	151.82	267.82	146.93	94.37
8.31	-0.62	13.49	23.07	12.61	2.58	3.27	11.67	4.00	-3.84	164.29	256.90	158.70	153.63
7.45	-1.32	12.30	21.75	11.54	1.71	2.35	10.70	3.40	-4.34	168.65	259.03	173.27	174.10
7.79	-1.00	12.53	21.95	11.73	1.90	2.75	11.04	3.82	-3.90	141.29	223.03	123.02	102.22
8.47	-0.43	12.58	21.85	12.18	2.19	3.16	11.24	4.72	-3.04	156.72	232.25	139.81	122.20
7.70	-1.43	12.21	21.90	11.40	1.30	2.61	10.60	3.98	-4.14	126.02	232.95	117.52	76.00
7.46	-1.74	11.91	21.84	11.21	1.02	2.59	10.82	3.73	-4.48	134.73	226.12	121.77	87.45
7.79	-1.11	12.84	22.42	12.13	2.23	2.67	10.93	3.44	-4.45	163.52	259.07	144.13	127.82
8.10	-0.87	13.00	22.72	12.20	2.31	3.28	11.62	4.02	-4.04	150.28	240.68	133.08	113.49
7.21	-1.54	11.72	21.24	11.20	1.48	2.11	10.40	3.24	-4.55	170.59	263.53	175.03	176.84
8.42	-0.26	12.30	21.56	11.79	2.09	3.37	11.38	5.03	-2.67	164.60	270.06	147.48	120.70
8.10	-1.34	12.49	21.91	12.15	1.89	2.79	10.97	4.09	-4.58	196.40	318.64	186.81	136.98
7.02	-1.35	11.30	20.57	10.68	1.43	2.01	10.32	3.33	-4.13	165.23	263.44	176.07	174.91
7.62	-1.29	12.66	22.12	12.13	2.11	2.22	10.50	3.11	-4.68	181.77	280.40	168.93	163.05
7.32	-1.59	11.35	20.69	10.71	0.82	2.42	10.74	3.93	-4.01	149.56	242.52	149.95	121.11
7.28	-1.72	11.46	21.15	10.97	0.97	2.38	10.55	3.63	-4.42	131.84	251.15	132.70	93.42
7.30	-1.20	12.14	21.48	11.39	1.71	2.02	10.25	3.17	-4.10	132.39	235.31	142.82	131.15
8.13	-0.74	13.20	22.72	12.47	2.51	3.01	11.32	3.77	-4.01	167.38	262.84	159.20	151.78
7.74	-1.14	12.75	22.35	12.05	2.17	2.65	10.88	3.41	-4.44	164.71	260.09	145.94	129.48
7.53	-1.23	12.54	22.13	11.78	2.08	2.44	10.71	3.29	-4.55	159.13	252.65	135.89	111.73
7.68	-1.18	12.24	21.80	11.64	1.73	2.68	10.97	3.71	-4.11	153.11	244.11	148.56	136.45

8.03	-0.94	13.05	22.88	12.03	2.01	3.27	11.59	4.02	-3.90	149.85	231.90	128.16	106.56
7.63	-1.50	12.13	21.80	11.35	1.23	2.55	10.55	3.91	-4.23	124.89	232.83	117.55	76.43
6.89	-1.81	11.30	20.75	10.79	1.17	1.83	10.13	3.00	-4.76	176.48	268.10	178.80	182.41
8.41	-0.60	13.72	23.40	12.64	2.60	3.49	11.96	4.21	-3.79	157.97	247.48	152.50	144.48
8.26	-0.64	13.43	23.04	12.58	2.59	3.22	11.59	3.94	-3.88	165.81	258.12	157.32	150.45
8.33	-0.36	12.68	22.13	11.93	2.18	3.67	11.75	4.76	-2.92	126.31	208.72	103.47	73.34
8.09	-0.87	13.01	22.78	11.90	2.05	3.48	11.74	4.26	-3.76	141.86	226.41	118.75	94.00
7.64	-1.17	12.63	22.27	11.85	2.06	2.58	10.91	3.45	-4.41	167.34	261.91	150.93	137.49
8.00	-0.82	12.71	22.53	11.70	1.93	3.42	11.61	4.31	-3.55	134.01	221.16	109.83	81.12
7.12	-1.64	11.66	21.14	11.12	1.39	1.97	10.29	3.12	-4.64	174.14	264.42	176.65	179.05
7.04	-1.46	11.53	20.99	11.10	1.62	1.66	10.07	2.98	-4.51	177.12	281.24	191.15	196.08
7.56	-1.46	11.95	21.62	11.21	1.18	2.72	10.91	3.89	-4.13	124.59	230.86	120.51	84.43
7.04	-1.70	11.58	21.05	11.02	1.30	1.88	10.20	3.07	-4.72	175.66	265.89	176.64	179.81
7.98	-0.92	12.66	22.26	11.98	2.05	2.89	11.16	3.97	-3.88	151.78	245.39	150.03	139.61
7.43	-1.01	12.18	21.57	11.31	1.67	2.38	10.39	3.54	-3.68	122.49	200.73	134.63	114.25
7.22	-1.47	11.95	21.54	11.43	1.89	2.11	10.34	2.99	-4.83	172.26	267.89	147.40	126.56
7.94	-0.99	12.80	22.63	11.82	1.92	3.29	11.63	4.05	-3.92	151.67	236.66	130.82	110.88
7.05	-1.41	11.62	20.99	11.02	1.42	1.86	10.05	3.05	-4.27	118.72	231.42	130.32	118.43
8.32	-0.58	13.44	23.12	12.51	2.59	3.38	11.71	4.14	-3.73	158.14	247.78	143.75	129.41
7.83	-1.19	12.78	22.45	11.76	1.76	3.05	11.41	3.90	-4.12	149.11	233.51	131.33	111.31
8.04	-0.94	13.06	22.89	12.04	2.02	3.27	11.60	4.02	-3.90	149.76	231.79	128.07	106.43
7.03	-1.45	11.60	20.92	10.97	1.37	1.92	10.04	3.10	-4.27	118.18	232.66	129.32	115.85
7.64	-1.24	12.63	22.09	11.90	1.87	2.36	10.68	3.38	-4.37	166.73	259.93	171.76	171.58
7.81	-1.12	12.65	22.39	11.58	1.78	3.12	11.43	4.04	-4.04	146.32	231.19	121.16	97.22
7.49	-1.25	12.42	22.04	11.70	2.03	2.42	10.70	3.28	-4.55	160.01	254.07	136.88	114.10
7.87	-1.06	12.67	22.53	11.68	1.91	3.20	11.48	4.07	-4.03	148.72	236.03	124.80	100.77
7.10	-1.48	11.60	20.97	11.08	1.39	1.89	10.09	3.13	-4.33	140.14	252.71	147.00	136.37
7.94	-0.98	12.78	22.60	11.80	1.94	3.31	11.65	4.09	-3.90	151.19	237.05	131.07	111.13
7.02	-1.58	11.64	21.05	11.21	1.58	1.71	10.09	2.82	-4.79	185.37	281.32	199.74	213.28
7.91	-1.01	12.69	22.45	11.65	1.86	3.29	11.58	4.14	-3.89	144.18	229.41	122.20	96.85
7.62	-1.04	12.56	22.09	11.92	2.07	2.15	10.49	3.35	-4.16	160.17	262.61	170.67	164.68
8.33	-0.64	13.60	23.31	12.60	2.55	3.39	11.90	4.10	-3.86	158.82	249.35	154.86	147.06
8.42	-0.27	12.28	21.53	11.77	2.08	3.40	11.41	5.05	-2.65	164.18	270.11	147.18	120.24
7.69	-1.35	12.07	21.82	11.44	1.31	2.73	10.74	3.94	-4.04	123.98	236.01	118.26	77.29
7.77	-0.98	12.37	21.90	11.52	1.85	2.95	11.17	4.02	-3.81	137.37	215.01	112.37	88.50
7.36	-1.47	12.05	21.59	11.41	1.52	2.28	10.59	3.28	-4.48	166.53	255.34	167.99	166.70
8.03	-0.82	13.00	22.54	12.23	2.34	3.03	11.39	3.82	-3.96	173.89	266.78	166.70	164.72
7.95	-0.93	13.21	22.72	12.32	2.28	2.78	11.15	3.59	-4.15	168.81	256.50	177.30	178.04
8.54	-0.69	13.01	22.65	12.55	2.28	3.41	11.47	4.53	-3.62	154.94	267.13	149.16	93.20
7.56	-1.22	12.23	21.71	11.67	1.80	2.33	10.64	3.42	-4.27	167.58	263.56	173.77	174.54
7.77	-1.08	12.50	22.11	11.69	1.76	2.87	11.17	3.87	-3.95	146.64	228.28	129.34	111.60
7.44	-1.42	12.21	21.80	11.51	1.54	2.50	10.83	3.38	-4.40	163.40	250.53	162.65	158.26
8.25	-0.73	13.49	23.31	12.37	2.39	3.38	11.92	4.15	-3.86	159.44	249.55	150.05	138.14

7.62	-1.50	11.73	21.37	11.24	1.22	2.78	10.92	4.01	-4.19	132.79	249.63	130.18	87.52
7.24	-1.86	11.69	21.51	11.06	0.90	2.15	10.05	3.46	-4.57	132.07	228.07	115.57	81.71
7.84	-1.11	12.74	22.49	11.66	1.83	3.13	11.43	4.03	-4.04	145.65	231.39	121.78	97.50
7.97	-1.03	12.15	21.82	11.89	1.79	2.76	10.94	4.03	-3.86	149.69	276.30	147.39	97.71
7.49	-1.38	12.15	21.65	11.57	1.67	2.27	10.59	3.40	-4.42	166.31	256.78	165.86	163.40
8.25	-0.74	13.48	23.30	12.36	2.38	3.38	11.92	4.15	-3.87	159.60	249.82	150.08	138.22
7.39	-1.68	11.63	21.30	11.04	0.97	2.27	10.20	3.74	-4.32	127.51	231.96	114.79	79.31
8.14	-0.80	13.09	22.92	12.24	2.33	3.30	11.57	4.00	-3.90	150.52	239.57	133.82	114.04
7.91	-1.03	12.66	22.20	11.94	1.91	2.86	11.13	3.91	-3.98	151.09	245.33	147.31	136.21
7.62	-1.27	12.65	22.05	12.11	2.09	2.23	10.52	3.13	-4.62	183.78	280.03	168.93	164.36
8.26	-0.64	13.42	23.04	12.58	2.59	3.23	11.60	3.95	-3.87	165.87	257.77	157.31	150.68
7.90	-0.95	12.67	22.37	11.86	2.15	3.27	11.51	3.94	-4.03	151.94	238.78	129.40	106.55
7.78	-1.11	12.58	22.12	11.98	2.01	2.50	10.79	3.56	-4.20	159.65	253.15	161.52	156.45
7.64	-0.89	12.42	21.84	11.46	1.71	2.55	10.70	3.82	-3.52	131.25	236.64	143.75	118.49
7.55	-1.20	12.42	22.05	11.69	2.04	2.57	10.80	3.40	-4.46	159.67	252.73	136.24	113.42
8.03	-0.98	12.76	22.39	12.07	1.82	3.08	11.30	4.02	-3.77	143.11	229.68	130.17	110.33
8.13	-0.81	13.08	22.88	12.22	2.33	3.32	11.59	4.03	-3.90	150.49	239.42	133.51	113.51
7.61	-1.05	12.54	22.07	11.91	2.07	2.14	10.48	3.34	-4.17	160.46	263.13	171.39	165.46
8.63	-0.48	13.24	22.70	12.39	2.24	3.71	11.79	4.89	-3.22	119.04	210.28	100.30	66.07
8.56	-0.77	13.30	23.00	12.65	2.32	3.27	11.54	4.46	-3.83	169.63	285.70	157.04	95.35
8.53	-0.59	12.96	22.44	12.20	2.13	3.61	11.68	4.81	-3.30	120.53	209.25	101.11	66.75
8.12	-1.35	12.73	22.29	12.25	1.89	2.74	10.99	3.97	-4.57	185.07	306.00	175.01	120.13
7.73	-1.10	12.72	22.22	12.11	2.28	2.52	10.74	3.32	-4.48	170.56	267.60	152.88	140.37
7.52	-1.20	12.36	21.92	11.68	2.11	2.53	10.74	3.33	-4.50	164.64	258.02	140.34	118.32
7.29	-1.32	11.99	21.49	11.48	1.86	1.89	10.25	3.11	-4.51	171.07	275.25	183.51	186.17
8.26	-0.60	13.22	22.96	12.18	2.43	3.57	12.02	4.36	-3.61	155.14	245.48	142.12	127.48
8.12	-0.76	13.39	22.89	12.61	2.59	2.83	11.11	3.64	-4.11	168.77	261.05	155.01	144.76
7.31	-1.59	11.34	20.68	10.70	0.82	2.41	10.73	3.93	-4.01	149.61	242.55	149.95	121.16
7.89	-1.10	12.81	22.61	11.82	1.90	3.09	11.38	3.93	-4.10	147.19	234.90	124.09	99.60
8.11	-0.76	13.39	22.87	12.60	2.58	2.83	11.11	3.64	-4.11	168.91	261.33	155.05	144.95
7.82	-1.06	12.77	22.44	11.99	2.13	2.80	11.12	3.62	-4.22	162.88	255.01	145.73	130.69
8.00	-1.01	12.87	22.67	11.86	1.86	3.33	11.60	4.12	-3.88	142.96	227.27	120.42	94.12
7.72	-1.14	12.78	22.33	12.15	2.27	2.54	10.76	3.30	-4.54	166.56	262.35	148.25	132.07
7.25	-1.28	12.09	21.43	11.41	1.66	1.95	10.17	3.08	-4.20	126.54	231.97	135.71	123.21
8.14	-0.83	13.17	23.06	12.13	2.20	3.33	11.73	4.12	-3.84	151.76	239.54	136.11	118.04
7.26	-1.79	11.59	21.25	10.96	0.95	2.32	10.46	3.57	-4.52	130.17	239.63	126.26	88.41
7.84	-0.95	12.52	22.15	11.61	1.89	3.05	11.29	4.08	-3.79	139.78	222.73	114.83	87.97
7.49	-1.27	12.63	22.17	11.85	2.09	2.28	10.56	3.11	-4.64	159.49	251.79	135.27	110.66
6.76	-1.87	10.99	20.31	10.66	0.84	1.46	9.70	2.87	-4.59	161.81	266.56	163.02	153.50
7.86	-1.07	12.92	22.52	12.12	2.14	2.77	11.07	3.58	-4.30	160.73	253.18	141.58	124.97
8.19	-1.31	12.75	22.18	12.33	1.91	2.84	11.10	4.05	-4.53	186.03	306.51	177.28	123.67
7.55	-1.20	11.36	20.58	10.95	1.21	2.32	10.34	4.15	-3.65	158.11	249.95	140.60	117.34
7.29	-1.13	12.03	21.46	11.38	1.91	1.92	10.29	3.19	-4.19	165.38	268.37	176.20	172.54

8.11	-0.88	13.14	23.02	12.11	2.09	3.37	11.82	4.10	-3.82	154.52	238.73	138.97	123.33
8.10	-0.69	12.14	21.42	11.62	1.81	2.99	11.01	4.55	-3.17	161.86	267.60	146.23	117.03
8.54	-0.46	13.66	23.31	12.70	2.74	3.69	12.05	4.37	-3.64	155.73	243.88	144.99	132.98
7.21	-1.51	11.64	21.23	11.23	1.30	1.99	10.22	3.19	-4.35	148.83	255.08	153.27	143.95
7.75	-1.09	12.75	22.25	12.13	2.29	2.55	10.76	3.33	-4.47	170.04	266.79	152.25	139.55
8.12	-0.82	13.16	22.99	12.17	2.23	3.34	11.67	4.09	-3.88	145.41	235.05	125.68	101.71
7.80	-0.94	12.24	21.51	11.59	1.64	2.74	10.87	3.98	-3.52	147.86	251.12	154.06	131.45
8.06	-0.70	12.17	21.48	11.66	1.79	3.02	11.14	4.45	-3.21	164.66	274.45	152.74	124.97
8.16	-0.73	13.27	22.84	12.43	2.48	3.16	11.56	3.90	-3.91	168.88	261.95	164.07	159.44
7.69	-1.44	12.22	21.90	11.40	1.29	2.60	10.59	3.97	-4.15	126.21	232.61	117.67	76.00
7.48	-1.19	12.27	21.77	11.69	1.96	2.08	10.42	3.25	-4.38	163.96	267.60	176.52	175.44
7.81	-1.06	12.84	22.38	12.20	2.29	2.69	10.90	3.44	-4.41	165.41	259.32	147.57	131.29
7.92	-0.99	12.83	22.70	11.88	1.98	3.16	11.53	3.98	-3.96	152.89	238.39	132.09	110.68
8.43	-0.51	13.73	23.39	12.75	2.72	3.43	11.85	4.10	-3.74	157.68	250.12	149.99	141.43
8.65	-0.58	13.21	22.77	12.65	2.40	3.51	11.61	4.66	-3.56	156.75	269.59	149.71	94.44
7.05	-1.80	11.58	21.21	11.05	1.14	1.96	10.28	3.01	-4.74	163.04	249.45	154.79	147.17
7.46	-1.38	12.01	21.75	11.18	1.46	2.72	10.98	3.72	-4.20	147.17	233.23	124.61	100.02
7.73	-1.14	12.80	22.41	12.06	2.18	2.56	10.86	3.39	-4.48	167.38	261.43	151.97	138.65
7.36	-1.21	12.29	21.70	11.68	2.06	1.94	10.32	3.08	-4.51	172.51	273.75	185.99	190.56
7.42	-1.27	11.85	21.39	11.42	1.51	2.17	10.46	3.41	-4.05	153.73	255.64	152.04	139.79
7.23	-1.17	11.69	20.94	11.02	1.62	2.16	10.41	3.41	-3.99	152.36	248.55	162.46	157.78
8.49	-0.51	13.66	23.34	12.65	2.68	3.64	12.04	4.31	-3.69	154.23	246.12	147.40	135.24
7.46	-1.33	12.35	21.86	11.66	1.76	2.35	10.68	3.27	-4.44	175.46	262.25	180.86	185.05
8.03	-0.91	12.91	22.66	11.83	1.98	3.42	11.69	4.24	-3.80	142.36	227.37	120.04	94.53
6.66	-2.37	10.52	19.98	10.16	0.37	1.72	9.99	3.15	-5.10	158.59	290.58	158.64	116.32
8.36	-0.61	13.43	23.27	12.32	2.39	3.70	12.11	4.40	-3.57	148.73	233.30	132.65	115.22
7.34	-1.40	11.90	21.43	11.41	1.61	2.09	10.44	3.30	-4.42	167.43	265.70	174.02	176.30
7.86	-0.94	12.55	22.18	11.92	2.00	2.64	10.90	3.78	-3.86	140.69	243.61	145.67	133.15
7.38	-1.22	12.20	21.60	11.60	1.99	2.02	10.40	3.19	-4.45	170.99	272.34	182.33	186.07
8.10	-0.91	13.09	22.88	12.08	2.01	3.35	11.72	4.09	-3.81	149.55	232.34	130.54	112.58
8.14	-0.85	13.16	23.05	12.14	2.12	3.40	11.85	4.13	-3.79	154.04	238.21	138.44	122.29
8.14	-0.62	12.19	21.54	11.55	1.82	3.48	11.66	4.74	-3.07	144.70	245.10	139.50	109.33
7.74	-0.84	12.51	21.87	11.55	1.77	2.67	10.79	3.93	-3.46	130.13	234.88	143.04	116.50
8.55	-0.21	12.65	21.88	12.14	2.34	3.31	11.39	4.93	-2.77	158.32	239.18	140.65	122.90
6.61	-1.68	10.58	19.83	10.12	0.88	1.67	9.74	3.08	-4.26	137.26	243.28	150.34	139.44
7.72	-0.92	12.62	22.14	11.94	2.18	2.32	10.62	3.49	-4.00	149.70	255.30	160.40	153.06
7.60	-1.08	12.52	22.04	11.88	2.05	2.13	10.47	3.32	-4.17	160.78	264.06	172.50	167.08
7.70	-1.44	12.21	21.89	11.39	1.30	2.61	10.60	3.98	-4.14	126.04	232.91	117.52	76.01
7.59	-2.20	11.74	21.14	11.37	0.50	2.33	10.42	3.80	-4.88	129.66	239.60	119.67	81.48
7.94	-0.94	12.96	22.79	11.86	2.14	3.21	11.81	4.06	-3.98	165.73	252.32	155.11	144.07
8.03	-0.92	12.91	22.66	11.82	1.98	3.42	11.68	4.24	-3.80	142.45	227.38	120.07	94.54
8.26	-0.70	13.37	23.23	12.28	2.28	3.56	11.95	4.27	-3.67	146.82	234.82	131.59	112.70
8.44	-0.89	13.13	22.74	12.57	2.29	3.13	11.39	4.29	-4.08	173.88	290.39	163.87	106.08

7.92	-0.98	12.83	22.69	11.88	1.99	3.16	11.53	3.98	-3.96	152.98	238.47	132.13	110.75
7.66	-1.47	12.21	21.89	11.40	1.28	2.55	10.54	3.93	-4.20	126.57	232.50	117.98	76.09
7.73	-1.22	12.64	22.37	11.80	1.93	2.86	11.11	3.66	-4.38	151.27	238.73	127.36	101.72
7.97	-0.88	13.19	22.73	12.29	2.34	2.83	11.24	3.66	-4.10	170.82	257.13	178.26	178.25
8.20	-0.78	13.28	23.22	12.37	2.28	3.32	11.60	4.04	-3.80	147.77	234.17	128.52	106.05
8.24	-1.09	12.44	21.83	11.85	1.54	3.25	11.30	4.60	-3.71	129.01	221.16	112.61	81.34
7.53	-1.19	12.23	21.72	11.68	1.87	2.24	10.57	3.38	-4.29	167.94	266.64	175.07	177.69
7.97	-1.32	12.34	22.05	11.80	1.60	2.84	11.06	4.12	-4.20	172.67	292.53	164.48	109.49
7.40	-1.13	12.28	21.70	11.55	1.82	2.12	10.36	3.27	-4.08	133.08	237.07	143.39	131.57
8.11	-0.80	13.15	22.81	12.21	2.17	3.27	11.63	4.02	-3.74	153.48	236.55	144.00	131.63
8.04	-0.84	13.24	22.76	12.44	2.52	2.83	11.09	3.63	-4.19	163.25	257.17	149.03	135.30
7.97	-1.08	11.85	21.02	11.52	1.51	2.64	10.71	4.40	-3.66	156.06	236.29	136.05	115.97
8.13	-0.74	13.03	22.85	11.98	2.22	3.52	11.83	4.27	-3.68	151.67	235.25	132.18	112.36
7.72	-1.27	12.74	22.37	11.89	1.74	2.63	10.98	3.52	-4.30	153.34	238.76	145.73	134.49
7.30	-1.79	11.76	21.62	11.06	1.00	2.42	10.73	3.57	-4.58	145.23	234.61	124.64	89.97
7.74	-0.90	12.68	22.18	11.96	2.18	2.32	10.63	3.52	-4.00	155.88	258.99	165.36	157.16
8.12	-0.78	13.31	22.76	12.59	2.56	2.85	11.10	3.65	-4.11	169.08	263.58	156.39	147.16
7.55	-1.35	12.27	22.11	11.40	1.60	2.78	11.07	3.71	-4.31	152.73	240.41	127.05	103.05
7.77	-1.11	12.54	22.11	11.96	1.96	2.46	10.78	3.56	-4.20	159.32	258.73	164.32	161.79
8.02	-0.89	12.85	22.68	11.87	2.07	3.40	11.72	4.16	-3.84	151.65	236.35	131.38	110.70
7.14	-1.29	11.65	20.88	10.94	1.42	2.14	10.33	3.34	-4.00	147.83	243.52	157.90	151.27
7.67	-1.34	12.58	22.37	11.70	1.69	2.78	11.10	3.62	-4.37	149.88	240.01	126.81	100.93
8.01	-1.19	12.10	21.44	11.51	1.37	2.96	11.00	4.48	-3.73	137.96	228.82	122.57	92.47
7.60	-1.19	12.17	21.73	11.50	1.66	2.68	10.97	3.71	-4.04	150.51	238.51	141.18	128.06
8.25	-0.63	13.24	23.02	12.15	2.38	3.55	12.02	4.32	-3.64	154.61	244.54	142.07	127.57
8.55	-0.71	13.30	23.07	12.69	2.28	3.31	11.51	4.40	-3.73	160.50	279.28	152.42	90.95
8.14	-1.30	12.54	21.95	12.19	1.92	2.83	11.02	4.13	-4.55	195.53	316.91	185.98	135.31
7.70	-0.97	12.53	22.15	11.96	2.04	2.33	10.59	3.49	-3.96	145.73	250.91	154.91	145.49
7.46	-1.00	12.30	21.64	11.49	1.94	2.13	10.47	3.41	-3.98	160.37	261.21	170.06	163.63
7.90	-1.04	12.64	22.19	11.92	1.90	2.85	11.11	3.90	-3.99	151.19	245.55	147.52	136.53
7.77	-1.49	12.39	22.35	11.56	1.28	2.95	11.23	3.98	-4.26	130.03	221.46	117.34	83.51
7.83	-1.05	12.56	22.39	11.64	1.83	3.26	11.61	4.02	-3.94	151.88	237.94	133.51	114.14
7.56	-0.95	12.57	21.94	11.76	2.14	2.12	10.49	3.38	-4.02	156.46	257.66	165.77	158.36
7.89	-1.02	12.88	22.48	12.05	2.05	2.97	11.32	3.72	-4.08	164.04	252.13	163.18	158.69
7.14	-1.29	11.65	20.88	10.94	1.42	2.14	10.33	3.34	-4.00	147.87	243.57	157.91	151.27
7.98	-0.87	12.78	22.49	11.95	2.18	3.33	11.60	4.02	-3.97	149.76	237.96	129.00	106.23
7.61	-1.23	12.65	22.27	11.91	2.15	2.44	10.73	3.31	-4.63	160.87	256.39	141.62	121.83
8.28	-0.72	13.51	23.31	12.38	2.41	3.41	11.94	4.17	-3.85	158.76	248.12	149.49	137.79
8.29	-1.07	12.92	22.73	12.34	1.85	3.12	11.29	4.25	-4.00	159.27	275.69	153.39	97.00
8.59	-0.74	13.40	23.11	12.78	2.40	3.29	11.57	4.42	-3.89	169.93	284.96	158.10	99.36
7.81	-0.99	12.57	21.98	11.76	1.92	2.77	11.06	3.83	-3.88	141.03	222.75	122.74	101.85
8.13	-1.34	12.74	22.29	12.25	1.89	2.74	10.99	3.97	-4.57	185.05	305.97	175.01	120.11
7.55	-0.94	12.46	21.78	11.59	2.03	2.23	10.59	3.50	-3.88	158.86	257.13	166.63	158.22

8.54	-0.14	12.65	22.01	12.10	2.38	3.32	11.37	4.94	-2.66	169.76	274.10	149.89	124.45
6.90	-1.44	11.22	20.47	10.67	1.38	1.84	10.14	3.15	-4.25	162.93	260.84	175.12	175.29
7.40	-1.67	11.64	21.31	11.05	0.98	2.28	10.21	3.75	-4.32	127.56	231.88	114.70	79.22
8.35	-0.52	12.76	22.54	12.05	2.21	3.59	11.63	4.67	-3.24	123.76	214.99	102.77	70.68
8.49	-0.24	12.74	21.96	12.07	2.30	3.71	11.89	4.91	-2.80	148.13	252.16	144.93	114.34
8.16	-0.79	13.32	23.20	12.32	2.30	3.25	11.60	4.00	-3.88	148.30	235.84	128.81	105.66
8.45	-0.50	13.46	23.10	12.48	2.62	3.69	12.05	4.39	-3.65	156.56	245.67	145.52	132.08
8.56	-0.16	12.89	22.23	12.19	2.33	3.78	11.81	4.96	-2.64	134.14	233.14	118.50	85.72
7.12	-1.29	11.64	20.90	10.98	1.55	1.99	10.28	3.29	-4.15	158.89	256.98	169.96	166.66
8.35	-0.36	12.37	21.60	11.78	2.05	3.48	11.49	4.92	-2.77	152.00	260.00	136.91	104.82
7.54	-1.63	12.09	21.68	11.34	1.11	2.38	10.36	3.74	-4.39	127.22	241.90	119.87	76.96
8.34	-1.10	13.12	22.60	12.60	2.19	2.99	11.24	4.08	-4.38	175.05	293.34	166.49	109.89
7.75	-1.13	12.52	22.09	11.92	1.93	2.45	10.76	3.57	-4.18	157.93	254.02	160.20	155.19
7.72	-1.14	12.78	22.33	12.15	2.27	2.54	10.76	3.30	-4.54	166.57	262.36	148.27	132.09
7.44	-1.31	12.32	21.77	11.65	1.95	2.33	10.73	3.24	-4.52	186.41	280.08	196.59	210.65
7.04	-1.45	11.61	20.94	10.98	1.37	1.90	10.03	3.09	-4.28	118.08	232.78	129.09	115.88
8.17	-0.65	12.34	21.66	11.81	1.90	3.02	11.09	4.51	-3.17	160.58	268.69	146.56	117.29
8.54	-0.46	13.65	23.31	12.70	2.74	3.69	12.05	4.37	-3.64	155.81	243.90	144.98	132.96
7.13	-1.52	11.39	20.92	11.07	1.19	1.90	10.12	3.19	-4.24	153.31	258.63	154.89	144.32
7.11	-1.42	11.64	21.10	11.18	1.67	1.73	10.12	3.04	-4.47	175.97	279.48	189.28	193.03
7.32	-1.51	11.86	21.36	11.32	1.51	2.16	10.47	3.31	-4.52	168.63	259.42	169.33	168.75
7.87	-1.07	12.64	22.28	11.98	1.92	2.69	11.01	3.75	-4.06	152.14	243.73	147.49	136.03
7.86	-0.96	12.74	22.28	12.14	2.31	2.72	11.05	3.56	-4.21	175.88	273.50	167.25	162.50
8.41	-0.60	13.71	23.39	12.66	2.59	3.48	11.96	4.20	-3.77	158.37	247.83	153.55	145.60
8.54	-0.46	13.66	23.31	12.70	2.74	3.69	12.05	4.37	-3.64	155.76	243.90	144.98	132.97
7.74	-1.06	11.61	20.81	11.17	1.36	2.50	10.52	4.31	-3.49	158.15	250.99	139.56	115.73
7.46	-1.72	11.98	21.59	11.27	1.02	2.30	10.30	3.65	-4.49	127.06	242.72	121.96	78.91
7.73	-1.18	12.12	21.57	11.44	1.50	2.98	11.22	4.05	-3.84	143.39	228.59	127.06	106.55
8.70	-0.73	13.57	23.05	12.93	2.55	3.43	11.71	4.48	-4.00	168.72	283.32	160.66	105.15
6.67	-1.68	10.91	20.16	10.38	1.04	1.65	9.89	2.93	-4.40	158.50	253.41	169.73	168.55
7.46	-1.57	11.97	21.71	11.20	1.18	2.51	10.49	3.74	-4.35	132.67	224.85	113.19	78.95
7.70	-1.21	12.23	21.73	11.57	1.62	2.79	11.05	3.83	-4.03	150.20	237.49	140.58	127.01
6.76	-2.36	10.75	20.30	10.41	0.35	1.68	9.97	3.10	-5.09	152.84	281.94	152.49	111.42
7.47	-1.09	12.30	21.78	11.68	1.98	2.05	10.40	3.31	-4.15	163.45	267.84	175.82	172.35
7.25	-1.16	11.67	20.92	11.01	1.63	2.20	10.43	3.44	-3.96	152.25	247.55	161.60	157.31
6.91	-2.01	10.76	20.26	10.49	0.72	1.77	10.05	3.29	-4.74	156.40	292.10	156.12	112.82
8.41	-0.84	13.16	23.00	12.48	2.16	3.14	11.40	4.33	-3.82	166.37	286.28	156.41	96.55
8.42	-0.96	13.18	22.68	12.60	2.26	3.08	11.32	4.19	-4.22	171.84	288.98	163.40	106.67
7.54	-1.51	12.09	21.83	11.27	1.23	2.64	10.62	3.80	-4.26	131.58	222.42	112.52	78.00
7.71	-1.18	12.74	22.33	12.05	2.11	2.56	10.80	3.38	-4.50	167.64	261.86	149.76	135.81
8.29	-1.21	13.05	22.59	12.54	2.07	2.85	11.13	4.03	-4.50	180.96	299.38	171.29	115.73
8.07	-0.82	12.78	22.36	12.04	1.99	3.18	11.42	4.10	-3.65	143.06	232.46	131.96	114.69
8.00	-0.98	12.94	22.80	12.00	1.92	3.26	11.65	4.01	-3.88	151.61	236.76	136.70	119.25

7.53	-1.37	12.17	21.64	11.55	1.65	2.38	10.66	3.50	-4.38	164.35	254.31	163.73	160.76
7.84	-0.93	12.42	22.14	11.52	1.84	3.14	11.34	4.14	-3.71	138.73	223.77	112.30	85.77
7.89	-1.02	12.77	22.47	11.81	1.90	3.10	11.52	3.98	-3.95	152.92	237.43	138.74	122.01
8.17	-0.83	13.11	22.87	12.25	2.32	3.40	11.73	4.10	-3.94	148.89	236.35	129.95	108.16
7.77	-1.13	12.83	22.41	12.04	2.13	2.67	10.97	3.48	-4.38	163.00	256.38	145.65	129.88
8.00	-0.84	12.67	22.54	11.68	1.92	3.38	11.56	4.30	-3.57	134.92	221.19	110.62	81.00
6.90	-2.08	10.65	20.16	10.40	0.59	1.76	10.10	3.37	-4.76	156.12	289.21	153.79	111.64
7.19	-1.90	11.32	20.92	10.95	0.88	1.98	10.21	3.43	-4.70	164.80	291.49	157.30	111.35
7.16	-1.63	11.72	21.20	11.16	1.41	2.00	10.31	3.14	-4.62	173.56	263.50	175.47	177.41
7.45	-1.08	12.23	21.58	11.29	1.58	2.26	10.45	3.61	-3.71	132.72	229.27	148.09	124.24
8.24	-0.63	12.41	21.58	11.68	1.79	3.48	11.73	4.82	-3.07	155.09	255.61	164.36	135.85
8.02	-0.98	11.60	20.80	11.30	1.40	3.00	10.97	4.72	-3.38	154.15	239.99	133.83	112.36
6.60	-1.69	10.56	19.82	10.11	0.87	1.66	9.73	3.07	-4.27	137.52	243.57	150.61	139.78
7.50	-1.40	12.35	21.94	11.65	1.63	2.43	10.76	3.33	-4.46	164.93	252.52	164.21	162.59
8.42	-0.29	12.52	21.88	11.99	2.24	3.16	11.20	4.86	-2.80	164.99	262.80	145.04	122.23
7.99	-0.93	12.87	22.69	11.88	2.02	3.34	11.68	4.13	-3.85	151.32	236.80	130.37	109.61
7.58	-1.13	12.41	21.84	11.70	2.04	2.58	10.98	3.46	-4.32	183.84	278.59	186.67	191.49
7.79	-1.21	12.70	22.44	11.74	1.74	2.97	11.38	3.83	-4.17	151.38	236.04	135.41	116.26
8.38	-0.59	13.10	22.69	12.19	2.19	3.46	11.50	4.59	-3.34	123.68	212.74	101.55	69.33
8.26	-0.64	13.42	23.04	12.59	2.58	3.23	11.61	3.95	-3.87	165.87	257.74	157.32	150.71
7.46	-1.40	12.31	21.88	11.61	1.62	2.36	10.70	3.32	-4.44	166.38	252.17	166.67	164.43
7.97	-0.93	12.80	22.52	11.87	2.00	3.21	11.60	4.06	-3.87	152.22	239.64	138.95	123.90
7.70	-1.30	12.62	22.39	11.70	1.72	2.83	11.14	3.68	-4.34	149.56	238.38	125.35	99.49
8.39	-1.01	13.10	22.75	12.54	2.19	3.02	11.31	4.21	-4.18	176.82	296.52	166.53	109.45
7.57	-1.25	11.56	20.86	10.94	1.18	2.80	11.04	4.21	-3.68	147.72	245.15	146.51	118.01
6.90	-1.56	11.68	21.02	11.29	1.81	1.32	9.78	2.49	-4.96	190.01	290.65	210.24	223.13
7.66	-1.55	12.26	21.90	11.52	1.17	2.49	10.49	3.82	-4.30	126.35	237.95	119.83	76.00
6.62	-1.67	10.60	19.86	10.14	0.89	1.68	9.75	3.08	-4.26	136.85	242.76	149.90	138.89
7.26	-1.43	11.67	21.23	11.26	1.40	2.09	10.32	3.27	-4.25	149.99	256.27	153.91	143.63
7.62	-1.08	12.50	22.05	11.90	2.04	2.13	10.47	3.33	-4.20	159.65	264.70	172.68	167.36
8.39	-0.50	13.12	22.86	12.11	2.21	3.80	11.99	4.65	-3.22	132.36	217.70	107.61	78.45
8.54	-0.46	13.66	23.31	12.70	2.74	3.69	12.05	4.37	-3.64	155.77	243.89	144.98	132.97
6.77	-1.73	11.19	20.54	10.71	1.08	1.57	9.82	2.88	-4.52	136.30	249.11	146.30	137.36
8.11	-0.78	12.98	22.78	11.91	2.15	3.54	11.83	4.29	-3.71	142.33	226.60	119.40	95.68
7.50	-1.55	11.91	21.62	11.16	1.13	2.60	10.59	3.87	-4.23	130.91	220.19	111.11	78.85
8.09	-0.90	13.06	22.91	12.07	2.11	3.35	11.63	4.11	-3.91	144.85	231.63	124.32	99.54
7.46	-1.20	12.20	21.74	11.66	1.94	2.02	10.40	3.23	-4.36	162.85	268.78	175.49	174.21
7.69	-1.05	12.64	22.10	11.97	2.12	2.36	10.65	3.45	-4.24	166.34	265.29	174.01	174.33
6.87	-1.61	11.40	20.67	11.00	1.66	1.62	10.05	2.75	-4.90	199.26	297.13	222.30	248.57
8.29	-1.21	13.04	22.59	12.54	2.06	2.85	11.13	4.03	-4.51	181.00	299.46	171.39	115.76
7.90	-0.94	12.59	22.40	11.63	1.82	3.26	11.49	4.17	-3.70	137.68	226.05	113.85	86.74
8.13	-0.84	13.07	22.86	12.06	2.04	3.47	11.84	4.19	-3.73	147.77	233.85	132.50	114.09
7.82	-0.93	12.63	22.24	12.03	2.25	2.83	11.19	3.63	-4.10	176.56	272.81	168.86	166.30

7.30	-1.24	12.15	21.61	11.47	1.86	1.82	10.25	3.13	-4.34	170.96	275.53	186.14	182.21
8.16	-0.61	12.20	21.56	11.57	1.82	3.47	11.67	4.74	-3.07	143.83	243.89	137.71	107.34
7.72	-1.38	12.19	21.86	11.47	1.33	2.63	10.53	3.95	-4.08	125.85	234.93	115.35	74.40
7.45	-1.52	12.05	21.55	11.49	1.48	2.23	10.54	3.39	-4.52	162.89	253.99	160.07	155.02
8.07	-0.82	13.20	22.68	12.50	2.50	2.82	11.06	3.62	-4.15	167.52	261.97	153.82	142.21
7.40	-1.18	12.10	21.61	11.47	1.79	2.11	10.43	3.35	-4.15	145.39	250.26	153.99	144.85
7.53	-1.22	11.33	20.55	10.93	1.19	2.31	10.32	4.14	-3.67	157.79	249.82	140.48	117.37
7.16	-1.55	11.55	21.15	11.16	1.26	1.95	10.18	3.16	-4.38	149.72	255.79	154.20	145.44
7.37	-1.31	11.79	21.34	11.37	1.48	2.11	10.42	3.36	-4.09	154.49	256.73	153.01	141.18
8.54	-0.46	13.66	23.31	12.70	2.74	3.69	12.05	4.36	-3.65	155.71	243.90	144.99	132.98
7.69	-1.44	12.22	21.90	11.40	1.29	2.60	10.59	3.97	-4.15	126.21	232.62	117.67	76.00
7.71	-1.46	11.68	21.13	11.23	1.09	2.56	10.58	4.18	-3.97	143.90	233.58	127.77	97.90
7.59	-1.61	12.14	22.01	11.39	1.11	2.72	10.94	3.77	-4.34	128.81	226.03	118.79	84.45
8.34	-1.10	13.12	22.60	12.60	2.19	2.99	11.24	4.08	-4.38	175.05	293.35	166.50	109.91
7.77	-1.06	12.71	22.21	12.18	2.28	2.49	10.79	3.36	-4.39	180.80	279.60	168.06	163.70
7.92	-0.98	12.85	22.73	11.89	1.99	3.15	11.52	3.97	-3.98	151.95	237.73	131.51	109.70
8.08	-0.83	13.08	22.90	12.12	2.23	3.31	11.65	4.06	-3.91	146.16	235.62	126.96	102.41
8.49	-0.27	12.79	22.02	12.10	2.29	3.62	11.83	4.88	-2.84	148.58	254.11	147.47	117.52
7.39	-1.14	12.14	21.53	11.49	1.85	2.11	10.44	3.30	-4.08	151.31	254.06	159.69	153.01
7.24	-1.24	12.01	21.35	11.28	1.59	2.00	10.25	3.22	-4.07	132.59	234.98	142.19	130.71
8.42	-0.48	13.68	23.26	12.78	2.79	3.33	11.70	4.05	-3.76	159.86	251.38	148.28	137.46
8.51	-1.01	13.26	22.69	12.80	2.29	3.19	11.45	4.26	-4.30	176.42	291.97	167.77	112.20
7.71	-1.14	12.83	22.31	11.95	2.01	2.62	11.02	3.47	-4.32	171.79	259.29	177.59	178.96
7.60	-1.57	12.17	21.78	11.42	1.16	2.44	10.40	3.78	-4.32	127.21	241.73	118.60	75.86
7.23	-1.43	12.12	21.72	11.50	1.89	2.12	10.38	2.98	-4.74	163.62	257.79	138.74	111.04
7.47	-1.40	12.38	21.83	11.93	2.06	2.11	10.37	3.04	-4.84	183.00	281.39	167.40	159.96
6.71	-1.68	11.32	20.66	11.04	1.69	1.14	9.61	2.35	-5.09	195.15	297.86	218.11	235.08
7.38	-1.24	12.09	21.65	11.62	1.89	1.95	10.32	3.16	-4.37	164.12	272.40	179.21	179.07
8.24	-0.94	12.44	21.86	11.84	1.72	3.20	11.31	4.63	-3.56	133.76	223.18	115.48	83.55
7.56	-1.59	12.11	21.70	11.34	1.09	2.41	10.39	3.80	-4.29	127.35	238.96	119.54	76.69
8.13	-0.76	13.01	22.81	11.94	2.17	3.56	11.84	4.30	-3.69	142.94	226.93	119.89	96.52
7.07	-2.00	11.12	20.71	10.74	0.71	2.07	10.26	3.38	-4.76	162.35	292.64	159.17	113.71
7.38	-1.12	12.07	21.47	11.38	1.73	2.21	10.44	3.38	-4.01	143.01	244.49	151.13	142.18
7.84	-1.03	12.88	22.39	12.21	2.34	2.71	10.93	3.46	-4.36	166.07	260.34	148.21	133.20
8.23	-0.53	12.26	21.49	11.75	1.96	2.93	10.98	4.73	-3.04	162.07	248.38	142.65	120.54
7.32	-1.19	12.11	21.56	11.42	1.76	2.06	10.28	3.22	-4.14	130.53	238.32	141.33	129.87
7.68	-1.08	12.03	21.74	11.58	1.75	2.67	10.91	3.81	-3.91	152.80	251.08	149.41	137.62
6.99	-1.68	11.26	20.83	10.91	1.12	1.78	10.02	3.05	-4.47	152.64	260.04	157.32	150.23
7.92	-0.99	12.83	22.70	11.88	1.99	3.16	11.53	3.98	-3.96	152.92	238.42	132.11	110.71
8.15	-0.77	13.27	23.01	12.28	2.27	3.32	11.67	4.02	-3.82	158.54	244.67	153.27	144.82
8.56	-0.77	13.29	23.00	12.65	2.32	3.27	11.54	4.45	-3.83	169.69	285.76	157.04	95.38
7.49	-1.38	11.88	21.54	11.42	1.40	2.48	10.73	3.58	-4.17	157.31	252.13	147.83	134.25
7.11	-1.30	11.63	20.89	10.97	1.54	1.98	10.28	3.28	-4.16	159.13	257.13	170.24	167.05

7.25	-1.48	12.09	21.60	11.54	1.90	2.03	10.29	2.97	-4.88	173.86	268.46	150.97	131.47
7.21	-1.45	12.08	21.68	11.49	1.88	2.10	10.35	2.96	-4.75	163.83	258.63	139.10	111.73
8.17	-0.68	12.83	22.58	11.87	2.00	3.61	11.75	4.48	-3.38	133.04	219.22	108.44	77.52
8.12	-0.79	13.09	22.93	12.03	2.18	3.45	11.76	4.19	-3.76	144.95	231.10	123.20	99.65
8.27	-0.64	13.45	23.04	12.62	2.62	3.19	11.56	3.94	-3.88	164.73	258.05	155.84	148.24
7.25	-1.41	12.02	21.60	11.47	1.91	2.16	10.39	3.00	-4.72	166.74	259.38	140.60	113.82
7.73	-1.14	12.78	22.34	12.02	2.27	2.62	10.85	3.42	-4.53	161.82	256.09	141.10	121.14
7.32	-1.30	12.04	21.52	11.51	1.88	1.92	10.27	3.13	-4.49	169.23	273.44	181.81	183.99
7.83	-0.96	12.53	22.19	11.70	1.92	2.94	11.26	3.91	-3.84	150.02	231.39	133.21	116.26
8.08	-0.88	13.13	22.94	12.05	2.06	3.35	11.72	4.09	-3.84	144.28	230.19	123.93	100.98
7.60	-1.26	12.68	22.24	12.03	2.18	2.36	10.60	3.13	-4.71	168.98	265.59	148.66	131.19
7.73	-1.21	12.64	22.38	11.80	1.93	2.86	11.11	3.66	-4.37	151.07	238.66	127.28	101.67
8.09	-0.87	13.01	22.78	11.90	2.05	3.48	11.73	4.27	-3.77	141.93	226.42	118.74	94.00
8.15	-0.78	13.01	22.68	12.06	2.12	3.31	11.59	4.22	-3.65	140.09	223.15	119.25	96.43
8.09	-0.78	13.31	22.82	12.56	2.57	2.80	11.07	3.60	-4.15	169.43	261.80	155.50	145.05
8.35	-0.91	12.56	21.92	11.96	1.72	3.37	11.44	4.73	-3.54	130.98	221.41	114.92	81.94
7.33	-1.85	11.88	21.44	11.24	0.92	2.05	10.01	3.45	-4.61	131.34	248.74	121.15	78.62
7.37	-1.17	12.09	21.55	11.46	1.82	2.07	10.39	3.30	-4.15	150.89	257.97	161.58	154.58
7.71	-1.28	12.36	21.91	11.82	1.75	2.49	10.78	3.57	-4.29	157.74	248.78	156.92	150.58
7.82	-1.12	12.75	22.54	11.78	1.80	3.06	11.48	3.88	-4.04	152.46	240.03	139.46	122.40
8.31	-0.62	13.49	23.07	12.61	2.58	3.27	11.68	4.00	-3.83	164.24	256.82	158.54	153.48
7.57	-2.18	11.34	20.63	11.12	0.37	2.59	10.65	4.01	-4.79	130.67	231.27	118.86	83.56
7.30	-1.20	12.14	21.49	11.39	1.71	2.02	10.25	3.17	-4.10	132.37	235.31	142.73	131.06
7.25	-1.24	11.99	21.33	11.31	1.65	1.98	10.24	3.21	-4.15	144.22	243.96	154.30	147.17
7.92	-1.03	12.87	22.64	11.86	1.87	3.21	11.53	3.99	-3.93	144.57	231.14	122.70	98.08
7.43	-1.02	12.25	21.60	11.45	1.93	2.09	10.44	3.38	-4.00	160.76	262.28	171.01	165.08
7.33	-1.56	12.08	21.74	11.43	1.39	2.32	10.65	3.24	-4.53	159.08	249.11	155.72	149.84
7.79	-1.12	12.85	22.37	12.26	2.28	2.41	10.69	3.28	-4.51	178.36	274.66	163.09	156.09
7.49	-1.27	12.36	21.99	11.65	2.02	2.44	10.70	3.30	-4.55	160.88	255.77	138.45	115.55
7.68	-1.08	12.04	21.75	11.59	1.75	2.67	10.90	3.80	-3.91	152.86	251.31	149.30	137.60
7.92	-0.98	12.85	22.72	11.89	1.99	3.15	11.52	3.97	-3.98	152.01	237.77	131.55	109.75
7.63	-1.38	12.56	22.35	11.56	1.62	2.84	11.15	3.69	-4.40	147.00	236.04	121.47	93.84
7.63	-1.49	12.16	21.84	11.37	1.26	2.52	10.52	3.90	-4.23	126.53	232.49	118.07	76.33
7.05	-1.45	11.55	21.01	11.12	1.63	1.67	10.07	2.99	-4.51	176.90	280.93	190.81	195.54
7.01	-1.81	10.81	20.33	10.61	0.88	1.88	10.19	3.41	-4.52	154.73	294.36	156.43	112.40
7.68	-1.45	12.23	21.92	11.41	1.29	2.57	10.56	3.95	-4.17	126.45	232.73	117.81	76.02
7.95	-1.05	12.11	21.79	11.85	1.77	2.73	10.93	4.01	-3.87	149.55	275.98	147.69	97.95
8.56	-0.21	12.76	22.06	12.07	2.26	3.87	12.04	5.05	-2.69	137.64	238.41	129.24	95.98
8.02	-1.00	12.20	21.81	11.79	1.85	2.94	11.11	4.25	-3.85	164.46	287.44	155.89	101.05
8.24	-0.59	12.82	22.71	11.90	2.15	3.70	11.87	4.62	-3.31	129.22	216.15	106.85	76.09
8.25	-0.73	13.48	23.30	12.36	2.39	3.38	11.92	4.15	-3.86	159.55	249.73	150.07	138.19
8.29	-0.72	12.42	21.83	11.93	1.80	3.26	11.26	4.68	-3.26	141.94	238.51	126.39	94.38
8.03	-0.81	13.01	22.55	12.24	2.34	3.04	11.40	3.82	-3.96	173.62	266.56	166.49	164.41

7.98	-0.83	12.60	22.30	11.66	1.94	3.28	11.47	4.26	-3.58	137.33	220.85	111.06	83.25
7.97	-0.94	12.90	22.76	11.97	2.10	3.13	11.50	3.97	-4.00	155.76	242.59	140.28	122.50
7.52	-1.67	12.05	21.64	11.32	1.08	2.35	10.34	3.71	-4.42	127.14	242.38	120.25	77.52
7.36	-1.13	12.04	21.43	11.36	1.71	2.19	10.42	3.37	-4.03	143.33	245.21	151.62	142.99
8.10	-0.80	13.11	22.89	12.01	2.27	3.34	11.93	4.17	-3.87	163.61	251.13	153.64	143.63
7.47	-1.10	12.30	21.77	11.68	1.98	2.05	10.40	3.31	-4.15	163.49	267.90	175.88	172.44
8.17	-0.76	13.03	22.71	12.08	2.13	3.33	11.61	4.23	-3.63	139.80	222.66	118.87	95.92
7.78	-1.11	12.58	22.12	11.98	2.01	2.50	10.79	3.56	-4.20	159.68	253.06	161.57	156.44
8.08	-0.92	13.04	22.88	12.04	2.10	3.34	11.61	4.09	-3.92	145.00	231.77	124.75	99.73
7.91	-0.97	12.87	22.48	12.02	2.23	3.01	11.31	3.79	-4.24	152.45	243.64	132.18	110.66
7.53	-1.35	12.35	21.92	11.63	1.65	2.55	10.91	3.43	-4.35	163.84	250.59	161.66	158.51
8.33	-0.39	12.53	21.77	11.90	2.12	3.55	11.67	4.81	-2.90	146.24	248.11	135.57	104.55
8.25	-0.73	13.49	23.31	12.37	2.39	3.38	11.92	4.15	-3.86	159.46	249.57	150.06	138.17
8.42	-0.24	12.56	21.93	11.90	2.22	3.69	11.72	4.93	-2.65	140.95	243.88	125.46	91.68
7.36	-1.16	12.18	21.60	11.47	1.76	2.10	10.31	3.22	-4.11	129.82	236.50	139.34	127.49
7.80	-1.26	12.43	22.19	11.59	1.51	2.98	11.00	4.03	-4.01	130.20	219.08	110.00	75.18
8.14	-0.79	13.25	23.05	12.18	2.32	3.32	11.84	4.13	-3.86	160.98	250.42	149.85	138.65
8.34	-0.60	13.59	23.38	12.48	2.49	3.46	11.88	4.16	-3.70	157.87	243.81	151.88	143.36
8.31	-0.62	13.49	23.07	12.61	2.58	3.27	11.67	4.00	-3.84	164.35	256.97	158.83	153.75
7.57	-1.45	11.91	21.52	11.26	1.26	2.51	10.45	3.92	-4.16	126.25	226.61	111.25	75.89
7.31	-1.12	11.98	21.28	11.23	1.75	2.11	10.44	3.40	-4.02	157.67	257.85	168.50	163.44
8.16	-1.09	12.29	21.66	11.71	1.53	3.18	11.25	4.59	-3.71	130.10	222.97	115.60	83.43
7.82	-1.01	12.97	22.44	12.20	2.25	2.34	10.67	3.39	-4.27	166.14	263.83	174.01	174.85
7.31	-1.24	12.15	21.61	11.48	1.86	1.82	10.26	3.13	-4.34	171.02	275.44	185.89	182.28
7.59	-1.20	12.51	22.06	11.84	2.17	2.53	10.74	3.33	-4.57	166.97	260.65	144.61	124.89
8.32	-0.52	13.05	22.51	12.16	2.25	3.59	11.73	4.50	-3.29	132.05	210.52	106.38	78.22
7.52	-1.15	12.23	21.77	11.65	1.96	2.19	10.50	3.39	-4.21	159.52	264.85	170.01	166.67
8.28	-0.56	12.73	22.48	11.98	2.13	3.54	11.60	4.57	-3.27	125.90	214.23	103.56	72.09
8.32	-0.64	13.62	23.27	12.69	2.62	3.28	11.74	3.95	-3.88	161.42	255.69	157.54	151.05
7.67	-1.18	12.38	21.97	11.85	1.86	2.37	10.71	3.51	-4.26	162.26	259.75	165.99	163.15
8.25	-0.63	13.41	22.93	12.63	2.63	3.13	11.43	3.89	-3.90	163.70	256.95	154.59	144.06
8.36	-1.02	13.19	22.82	12.60	2.24	2.93	11.26	4.10	-4.30	177.65	296.29	167.91	111.42
8.13	-0.67	12.20	21.50	11.68	1.85	3.00	11.05	4.54	-3.17	162.17	268.39	146.41	117.08
8.02	-0.99	12.75	22.38	12.07	1.81	3.06	11.29	4.01	-3.79	143.20	229.88	130.35	110.56
7.78	-1.13	12.67	22.42	11.69	1.78	3.01	11.42	3.89	-4.06	155.36	239.56	141.10	124.49
7.11	-1.57	11.50	21.06	11.08	1.24	1.91	10.13	3.16	-4.39	149.03	255.57	152.96	144.18
7.51	-1.23	12.53	22.11	11.79	2.10	2.41	10.67	3.25	-4.57	158.41	253.27	135.65	111.81
7.70	-1.33	12.72	22.51	11.74	1.73	2.82	11.17	3.67	-4.38	147.16	235.98	122.05	96.09
8.56	-0.20	13.36	22.81	12.41	2.56	3.88	12.04	4.73	-2.96	132.22	209.11	103.21	73.90
6.56	-2.06	10.84	20.03	10.43	0.65	1.18	9.44	2.70	-4.77	168.88	270.36	168.14	160.14
8.48	-0.51	13.67	23.35	12.66	2.68	3.63	12.04	4.31	-3.70	154.23	246.19	147.49	135.40
6.89	-1.80	11.31	20.77	10.79	1.17	1.84	10.14	3.00	-4.76	176.38	268.00	178.70	182.33
7.80	-0.96	11.62	20.95	11.36	1.79	2.70	10.89	4.21	-3.70	140.10	266.32	134.87	91.50

8.21	-0.53	12.38	21.70	11.84	1.99	3.23	11.34	4.58	-3.04	157.63	269.05	148.44	119.56
7.58	-1.51	12.24	21.93	11.54	1.26	2.40	10.43	3.65	-4.27	125.57	230.95	117.87	79.10
7.92	-1.14	12.51	22.24	11.78	1.58	2.71	10.65	4.06	-3.87	122.56	226.05	111.69	72.80
7.70	-1.31	12.74	22.53	11.82	1.77	2.75	11.08	3.63	-4.39	147.43	237.07	124.34	98.03
8.19	-0.73	13.33	23.08	12.22	2.38	3.39	11.93	4.19	-3.86	160.29	249.36	152.69	142.83
7.49	-0.98	12.24	21.63	11.38	1.70	2.41	10.43	3.60	-3.66	122.42	201.26	134.05	112.99
8.34	-0.39	12.63	21.91	12.01	2.18	3.36	11.47	4.69	-2.96	156.59	266.25	148.68	117.69
7.28	-1.25	12.15	21.48	11.45	1.68	1.98	10.19	3.11	-4.18	127.03	231.21	135.41	122.52
7.48	-1.35	12.49	22.04	11.89	2.08	2.24	10.49	3.05	-4.79	172.81	268.61	150.30	134.36
7.48	-1.39	12.38	21.83	11.93	2.06	2.11	10.37	3.04	-4.83	182.94	281.26	167.36	159.89
8.41	-0.55	13.65	23.31	12.73	2.69	3.43	11.88	4.11	-3.77	159.52	250.65	152.20	144.20
8.54	-0.78	13.22	22.91	12.62	2.31	3.29	11.55	4.43	-3.86	168.16	284.73	156.56	97.68
7.78	-0.99	12.31	21.64	11.66	1.66	2.65	10.77	3.88	-3.65	143.15	248.70	152.02	127.17
8.30	-0.79	12.55	22.19	12.13	2.11	3.20	11.36	4.45	-3.69	159.32	279.51	151.78	96.02
7.99	-0.96	13.00	22.82	12.03	2.10	3.16	11.47	3.94	-4.02	147.37	235.20	126.16	101.18
6.95	-2.05	11.13	20.96	10.52	0.66	2.14	10.35	3.39	-4.77	144.53	239.87	128.84	95.21
6.70	-2.91	10.33	19.71	10.19	-0.43	1.52	9.73	3.20	-5.40	145.09	235.52	131.43	103.75
6.25	-2.12	10.25	19.40	9.96	0.52	1.05	9.20	2.56	-4.76	152.12	263.53	162.00	153.28
7.15	-1.77	11.62	21.35	10.79	1.07	2.40	10.63	3.52	-4.59	150.06	240.81	125.67	95.41
7.21	-1.46	11.94	21.26	11.43	1.80	2.01	10.36	3.01	-4.71	191.55	284.99	207.22	225.68
6.44	-1.90	10.47	19.69	10.44	1.32	1.17	9.57	2.44	-5.16	230.09	328.55	245.39	286.38
6.58	-1.71	10.63	19.94	10.22	1.05	1.56	9.86	2.91	-4.48	163.83	261.49	175.78	176.40
6.39	-1.95	9.88	19.17	9.79	0.48	1.40	9.60	2.98	-4.40	179.79	283.26	186.31	170.75
6.60	-1.92	10.87	20.17	10.69	1.33	1.29	9.74	2.49	-5.18	219.32	317.61	233.57	266.72
7.42	-1.45	12.31	21.72	11.88	1.96	2.02	10.32	2.94	-4.87	188.24	286.13	172.72	168.01
6.37	-2.19	10.52	19.77	10.19	0.54	1.09	9.27	2.54	-4.93	158.68	267.48	163.68	156.00
6.73	-1.78	11.09	20.37	10.89	1.52	1.42	9.79	2.54	-5.07	213.19	308.07	231.29	265.68
6.98	-2.26	10.60	20.13	10.33	0.20	1.80	9.93	3.63	-4.72	149.90	240.01	133.65	107.19
7.05	-1.96	11.29	21.09	10.58	0.77	2.31	10.48	3.49	-4.68	147.56	238.40	125.83	92.35
6.78	-1.81	11.09	20.39	10.70	0.93	1.51	9.70	2.88	-4.56	153.76	260.67	158.30	145.82
6.93	-2.81	10.62	19.94	10.43	-0.33	1.78	9.94	3.42	-5.31	142.96	234.80	129.88	100.35
6.45	-2.06	10.54	19.81	10.24	0.66	1.25	9.44	2.67	-4.78	155.50	266.40	161.57	154.56
6.42	-2.18	10.08	19.32	9.85	0.21	1.30	9.52	2.98	-4.55	198.68	289.95	189.11	185.02
7.12	-1.59	11.83	21.33	11.43	1.84	1.86	10.12	2.77	-5.02	184.07	283.96	163.24	150.64
7.04	-1.96	11.47	21.23	10.72	0.90	2.24	10.48	3.38	-4.77	151.68	242.21	128.36	94.48
6.98	-1.87	11.80	21.05	11.52	1.68	1.45	9.73	2.46	-5.39	206.35	307.35	185.41	182.43
6.96	-1.61	11.40	20.80	11.04	1.60	1.81	10.21	2.88	-4.84	203.62	300.06	215.85	238.05
7.19	-1.39	11.63	21.02	11.20	1.77	2.12	10.49	3.13	-4.55	200.69	300.20	197.94	207.92
7.41	-1.48	12.45	21.87	11.98	2.06	1.96	10.21	2.83	-5.03	185.88	284.45	166.39	157.82
7.48	-1.41	12.16	21.96	11.23	1.46	2.76	11.03	3.71	-4.30	151.63	238.46	124.70	100.42
6.43	-2.00	10.37	19.49	10.39	1.23	1.19	9.52	2.47	-5.26	233.41	338.06	229.03	248.60
7.27	-1.54	12.11	21.52	11.67	1.93	1.96	10.20	2.85	-4.97	187.58	286.37	166.21	157.48
6.40	-1.93	10.37	19.59	10.40	1.31	1.15	9.55	2.42	-5.14	225.60	325.27	248.19	288.53

7.38	-1.47	12.04	21.70	11.12	1.38	2.52	10.92	3.63	-4.35	154.05	237.49	134.13	116.39
7.60	-1.25	12.26	21.98	11.34	1.56	2.90	11.16	3.86	-4.07	147.58	232.58	124.97	100.23
7.17	-1.57	11.83	21.16	11.34	1.72	1.94	10.32	2.96	-4.79	198.79	294.82	212.31	234.38
7.00	-2.76	10.76	20.07	10.53	-0.26	1.85	10.00	3.45	-5.29	143.11	233.34	128.70	99.78
7.00	-2.14	11.27	21.03	10.70	0.56	2.01	10.15	3.24	-4.83	135.43	237.62	125.44	91.71
7.04	-1.86	11.39	21.12	10.69	0.97	2.32	10.59	3.38	-4.67	153.00	244.49	129.00	97.29
6.37	-2.07	10.61	19.81	10.17	0.73	1.15	9.37	2.56	-4.84	144.98	257.56	156.42	149.47
7.21	-1.62	11.69	21.44	10.81	1.21	2.54	10.83	3.58	-4.47	154.00	243.49	128.45	101.23
7.03	-1.90	11.40	21.10	10.57	0.88	2.27	10.43	3.47	-4.67	148.94	240.11	124.32	93.62
6.57	-1.89	10.99	20.15	10.83	1.47	1.20	9.58	2.39	-5.21	219.33	314.62	239.01	276.44
6.98	-1.87	11.42	20.69	11.28	1.54	1.62	9.91	2.70	-5.29	216.73	323.82	200.78	200.42
7.45	-1.51	12.01	21.73	11.11	1.28	2.70	10.85	3.82	-4.31	142.09	230.76	117.92	86.25
6.52	-2.00	9.75	19.10	9.76	0.57	1.50	9.58	3.26	-4.57	145.60	282.93	140.61	101.45
7.56	-1.21	12.23	21.78	11.76	2.00	2.44	10.76	3.35	-4.41	187.75	286.29	179.15	179.48
5.91	-2.45	9.40	18.47	9.77	0.64	0.64	8.98	2.01	-5.53	253.48	358.56	276.37	331.23
7.58	-1.27	12.21	21.94	11.31	1.54	2.86	11.13	3.84	-4.10	147.87	233.12	125.15	101.02
6.39	-2.00	10.61	19.86	10.54	1.30	0.99	9.43	2.24	-5.30	209.84	312.09	233.79	266.47
6.86	-2.79	10.57	19.94	10.38	-0.29	1.69	9.86	3.34	-5.29	144.60	233.86	129.52	101.70
6.63	-1.72	10.80	20.11	10.68	1.50	1.32	9.67	2.58	-4.93	202.48	304.06	218.69	237.92
7.02	-1.74	11.67	20.84	11.31	1.60	1.62	10.04	2.73	-5.10	214.16	312.79	206.64	217.45
7.39	-1.44	12.10	21.86	11.13	1.44	2.65	10.99	3.65	-4.33	149.09	237.31	122.65	97.15
7.33	-1.34	11.88	21.36	11.44	1.90	2.20	10.57	3.17	-4.55	194.87	293.94	192.73	197.53
5.67	-2.96	8.80	18.14	8.92	-0.40	0.68	8.97	2.44	-5.53	159.69	304.34	160.02	124.52
7.01	-1.89	11.38	21.13	10.63	0.94	2.25	10.49	3.40	-4.70	150.47	244.55	127.56	98.89
6.45	-2.80	9.77	19.19	9.74	-0.39	1.34	9.43	3.18	-5.21	151.10	241.76	136.22	111.13
7.37	-1.51	11.94	21.73	11.10	1.37	2.62	10.95	3.64	-4.37	153.36	238.63	127.22	103.38
6.93	-2.03	11.45	21.16	10.65	0.81	2.03	10.26	3.20	-4.89	150.48	243.71	127.69	96.80
7.01	-1.85	11.49	21.14	10.70	1.02	2.22	10.50	3.34	-4.75	157.88	248.58	131.93	103.57
7.24	-1.70	11.69	21.49	10.88	1.05	2.54	10.67	3.62	-4.49	144.15	234.46	121.81	88.97
7.09	-1.68	11.87	21.30	11.44	1.80	1.74	9.98	2.70	-5.13	185.76	286.17	162.13	148.81
7.47	-1.43	12.20	21.98	11.22	1.41	2.73	10.96	3.72	-4.26	145.64	233.34	120.12	91.16
6.96	-1.95	11.27	20.96	10.52	0.79	2.17	10.27	3.37	-4.69	145.17	237.76	123.96	91.34
7.02	-1.98	11.26	21.15	10.63	0.78	2.21	10.38	3.41	-4.69	143.37	238.85	125.87	92.07
6.52	-1.74	10.53	19.83	10.15	1.00	1.52	9.83	2.88	-4.51	165.39	263.63	178.14	179.68
6.49	-1.92	10.82	20.11	10.72	1.41	1.07	9.52	2.30	-5.25	206.49	307.90	227.56	258.36
6.31	-2.13	10.45	19.65	10.05	0.61	1.12	9.31	2.54	-4.85	147.59	256.51	157.15	150.79
6.80	-1.86	11.31	20.58	11.04	1.50	1.43	9.87	2.57	-5.22	217.62	319.06	209.34	217.40
6.61	-1.71	10.81	20.11	10.28	0.95	1.56	9.65	2.97	-4.39	138.38	239.36	152.79	144.55
7.16	-1.65	11.77	20.95	11.43	1.70	1.86	10.13	2.88	-5.00	207.97	310.84	193.71	196.52
7.02	-2.07	11.20	20.91	10.67	0.67	1.91	9.84	3.41	-4.81	135.44	234.51	117.90	83.27
6.72	-2.05	10.79	20.57	10.27	0.78	2.05	10.29	3.15	-4.85	164.86	254.55	136.90	104.99
7.09	-1.92	11.39	21.17	10.70	0.83	2.30	10.42	3.46	-4.68	141.69	236.01	123.15	89.45
7.13	-1.82	11.42	21.18	10.67	0.93	2.32	10.38	3.59	-4.53	138.92	232.65	119.59	86.30

7.16	-1.85	12.13	21.46	11.77	1.70	1.60	9.83	2.54	-5.40	200.10	301.13	178.84	173.71
6.89	-2.09	10.99	20.84	10.47	0.60	2.01	10.04	3.33	-4.79	136.29	234.73	122.31	89.47
6.85	-1.72	11.28	20.54	10.95	1.53	1.63	10.02	2.76	-4.94	215.22	309.44	226.41	255.48
7.13	-1.89	11.38	21.14	10.82	0.87	2.10	10.07	3.46	-4.61	134.97	230.33	117.75	84.01
6.40	-2.17	10.45	19.53	10.40	1.04	1.09	9.48	2.43	-5.39	236.87	343.94	243.75	269.88
7.30	-1.56	11.94	21.71	11.06	1.34	2.55	10.85	3.55	-4.46	155.53	243.14	128.95	104.22
6.77	-1.73	11.20	20.50	10.95	1.57	1.50	9.90	2.57	-5.03	210.88	308.51	229.14	260.56
7.45	-1.48	11.95	21.71	11.08	1.26	2.71	10.82	3.83	-4.19	138.58	228.63	114.73	84.72
6.77	-1.87	11.25	20.53	10.99	1.47	1.41	9.85	2.55	-5.24	218.35	320.39	210.20	218.49
6.59	-2.46	10.61	20.13	10.14	0.18	1.41	9.53	3.03	-5.07	140.74	265.31	133.96	93.97
7.14	-1.82	11.44	21.19	10.68	0.93	2.33	10.39	3.59	-4.53	138.82	232.66	119.31	86.20
6.84	-1.73	11.22	20.58	10.92	1.50	1.63	10.03	2.77	-4.96	208.31	306.14	221.97	248.02
7.21	-1.60	11.82	21.49	10.97	1.25	2.42	10.77	3.46	-4.48	155.95	241.06	134.90	115.72
6.68	-2.81	10.08	19.43	10.07	-0.39	1.54	9.70	3.29	-5.25	149.60	241.06	133.48	107.16
6.19	-2.09	9.57	18.84	9.55	0.33	1.20	9.40	2.82	-4.53	184.76	286.92	190.97	177.19
5.85	-2.36	9.39	18.60	9.29	0.11	0.72	8.86	2.40	-4.84	168.69	273.01	179.31	174.26
7.24	-2.23	11.20	20.57	10.77	0.32	2.15	10.30	3.70	-4.79	134.16	229.67	120.95	92.01
6.35	-1.95	10.28	19.48	10.32	1.27	1.08	9.49	2.37	-5.20	232.50	332.35	249.42	292.19
7.30	-1.57	11.81	21.61	10.96	1.28	2.62	10.90	3.64	-4.44	153.35	241.54	127.78	100.57
6.95	-2.05	11.13	20.96	10.52	0.66	2.14	10.35	3.39	-4.77	144.50	239.86	128.82	95.19
7.27	-1.63	12.37	21.75	11.91	2.01	1.70	9.97	2.60	-5.26	190.71	290.99	170.12	163.38
7.13	-2.66	10.88	20.18	10.65	-0.19	2.01	10.15	3.60	-5.17	141.55	233.32	128.23	98.15
7.48	-1.46	12.30	21.92	11.36	1.46	2.64	11.01	3.58	-4.41	156.10	237.19	135.04	116.74
7.18	-1.79	11.53	21.28	10.75	0.96	2.35	10.41	3.61	-4.51	138.79	231.89	118.29	85.40
7.05	-1.94	11.47	21.23	10.71	0.90	2.27	10.51	3.39	-4.77	152.92	240.90	127.99	95.69
7.71	-0.99	11.50	20.87	11.13	1.47	2.61	10.83	4.28	-3.41	166.05	260.97	171.77	159.39
6.66	-1.84	10.73	20.14	10.43	0.82	1.50	9.70	2.92	-4.53	155.30	263.79	160.89	151.30
7.06	-1.80	11.53	21.23	10.83	1.20	2.17	10.44	3.27	-4.79	159.98	250.82	134.33	106.14
6.74	-1.76	10.86	20.12	10.71	1.42	1.61	9.93	2.77	-4.94	215.80	315.63	216.34	233.15
7.03	-2.65	10.79	20.09	10.57	-0.03	1.85	9.90	3.47	-5.25	137.20	250.19	127.88	89.75
6.63	-1.93	10.43	19.61	10.09	0.52	1.55	9.76	3.16	-4.39	182.73	281.96	181.14	169.88
7.00	-1.82	11.88	21.24	11.50	1.78	1.53	9.80	2.47	-5.39	193.68	296.19	173.57	165.26
7.02	-1.60	11.56	20.94	11.20	1.65	1.79	10.17	2.82	-4.85	197.81	293.46	215.85	239.60
7.35	-1.89	12.01	21.71	11.23	0.87	2.31	10.41	3.49	-4.65	129.56	227.50	118.15	82.94
7.42	-1.37	12.39	21.72	11.78	2.03	2.07	10.45	3.04	-4.73	197.05	295.04	187.03	188.60
7.07	-1.88	11.47	21.17	10.67	0.93	2.27	10.40	3.46	-4.69	145.50	236.11	122.08	91.88
6.76	-2.87	10.25	19.58	10.19	-0.42	1.64	9.79	3.31	-5.31	148.72	239.77	131.62	104.78
6.50	-2.42	10.25	19.88	9.87	0.20	1.71	9.95	3.13	-5.04	143.58	261.32	141.83	107.15
6.92	-1.93	11.18	20.92	10.52	0.90	2.21	10.47	3.32	-4.74	156.15	248.61	131.76	100.53
6.59	-1.69	10.61	19.89	10.17	1.02	1.70	9.96	3.03	-4.37	160.97	260.06	175.39	174.89
7.25	-1.88	10.89	20.30	10.58	0.58	2.13	10.20	3.92	-4.34	149.89	239.70	133.28	106.39
6.98	-1.92	11.42	21.12	10.60	0.89	2.18	10.39	3.35	-4.75	150.63	241.61	126.93	95.59
7.41	-2.23	11.16	20.50	10.92	0.23	2.26	10.39	3.89	-4.70	143.82	234.40	128.55	98.67

6.00	-2.22	9.64	18.70	9.90	0.99	0.82	9.07	2.12	-5.44	235.34	339.55	260.43	309.85
6.98	-1.92	11.42	21.12	10.60	0.89	2.18	10.39	3.35	-4.75	150.63	241.61	126.93	95.59
7.05	-1.87	12.06	21.39	11.72	1.72	1.43	9.71	2.38	-5.47	199.16	301.30	179.77	174.01
6.90	-1.66	11.33	20.73	10.98	1.57	1.74	10.15	2.84	-4.88	205.41	302.10	217.75	241.51
7.39	-1.52	11.88	21.64	11.02	1.23	2.64	10.77	3.78	-4.24	139.42	230.14	115.28	85.50
6.81	-2.19	10.80	20.50	10.37	0.46	1.69	9.61	3.26	-4.81	132.83	240.80	121.31	87.23
6.97	-1.87	11.78	21.04	11.50	1.67	1.44	9.72	2.46	-5.39	206.71	307.56	185.80	182.85
7.18	-1.77	11.68	21.46	10.86	1.11	2.45	10.78	3.49	-4.66	151.36	243.63	128.38	99.08
7.03	-1.97	11.57	21.33	10.76	0.88	2.17	10.37	3.31	-4.77	146.45	240.87	125.44	93.43
7.36	-1.88	12.02	21.73	11.24	0.88	2.33	10.42	3.50	-4.62	129.48	227.33	118.56	83.48
7.45	-1.34	12.02	21.76	11.16	1.46	2.74	10.99	3.76	-4.17	149.32	234.04	126.24	101.97
6.42	-1.84	10.43	19.75	10.39	1.37	1.08	9.51	2.43	-5.04	209.36	310.57	226.05	250.16
6.95	-2.05	11.13	20.96	10.52	0.66	2.14	10.34	3.38	-4.77	144.57	239.90	128.87	95.25
6.60	-2.32	10.65	20.30	10.19	0.30	1.64	9.77	3.05	-4.97	137.79	246.36	131.46	96.21
7.36	-1.64	11.73	21.51	11.03	1.10	2.47	10.49	3.67	-4.35	133.38	225.95	115.26	81.21
6.51	-2.01	10.63	20.06	10.58	1.32	1.23	9.59	2.44	-5.37	216.64	320.24	201.29	202.63
7.10	-2.43	10.63	19.98	10.49	0.03	2.01	10.07	3.70	-4.87	150.35	239.80	131.99	104.83
6.76	-2.59	10.36	19.76	10.15	-0.12	1.61	9.75	3.34	-5.05	146.37	237.88	132.71	105.83
7.32	-1.38	12.06	21.45	11.50	1.83	2.19	10.54	3.16	-4.58	189.21	282.44	203.61	220.29
7.19	-1.82	11.53	21.34	10.77	0.91	2.46	10.60	3.61	-4.55	142.85	234.57	121.65	88.23
6.34	-1.98	10.01	19.29	9.80	0.38	1.26	9.35	2.85	-4.36	177.75	274.24	196.58	178.46
6.74	-1.70	11.26	20.56	10.99	1.64	1.32	9.76	2.49	-5.02	198.33	300.86	216.56	237.17
6.57	-3.09	9.95	19.32	9.95	-0.66	1.45	9.62	3.18	-5.51	152.10	242.28	133.48	106.57
7.51	-1.46	12.36	21.98	11.40	1.48	2.68	11.06	3.64	-4.37	155.53	237.21	135.45	117.85
7.01	-1.92	11.39	21.17	10.65	0.86	2.17	10.36	3.39	-4.72	143.51	238.95	123.75	91.69
7.00	-1.76	11.43	21.17	10.60	1.07	2.32	10.60	3.43	-4.61	157.40	244.70	129.14	103.88
7.14	-2.81	10.76	20.08	10.63	-0.29	2.11	10.20	3.65	-5.34	138.27	241.04	126.97	93.05
7.06	-1.95	11.59	21.35	10.77	0.90	2.20	10.40	3.33	-4.75	146.67	240.24	125.16	92.94
7.52	-2.05	11.42	20.75	11.10	0.43	2.42	10.53	3.98	-4.54	142.28	231.02	125.55	96.53
6.87	-1.45	11.11	20.38	10.54	1.34	1.83	10.18	3.18	-4.24	169.21	268.03	181.36	182.41
7.91	-0.93	12.52	22.44	11.58	1.82	3.36	11.53	4.23	-3.69	132.95	222.03	109.94	80.31
6.55	-3.13	9.94	19.29	9.95	-0.66	1.44	9.62	3.16	-5.60	149.00	243.31	131.46	104.33
6.82	-2.83	10.46	19.84	10.31	-0.35	1.62	9.83	3.31	-5.32	144.12	234.80	130.61	102.69
6.75	-1.78	11.08	20.43	10.83	1.43	1.53	9.95	2.70	-5.03	211.28	310.23	225.23	252.76
6.79	-2.18	11.12	20.83	10.42	0.66	1.94	10.20	3.15	-5.01	158.83	250.36	131.70	102.40
6.90	-2.05	11.35	21.01	10.58	0.78	2.04	10.29	3.21	-4.89	154.35	245.39	128.36	98.83
6.97	-1.94	11.14	20.97	10.52	0.77	2.21	10.42	3.43	-4.69	143.78	239.42	128.38	94.38
6.38	-2.01	10.59	19.84	10.52	1.29	0.98	9.42	2.23	-5.30	210.20	312.52	234.26	267.18
6.95	-2.14	11.27	20.90	10.67	0.56	1.92	10.03	3.25	-4.83	135.91	242.37	127.92	91.54
7.36	-1.54	12.16	21.79	11.22	1.39	2.51	10.87	3.52	-4.46	156.51	238.63	134.35	115.80
6.58	-2.38	10.40	20.02	9.96	0.25	1.75	10.00	3.17	-5.01	142.03	259.93	140.18	105.05
7.04	-1.88	11.43	21.14	10.63	0.96	2.26	10.48	3.41	-4.69	151.29	243.27	127.32	97.58
7.32	-1.56	11.73	21.51	10.86	1.18	2.62	10.77	3.78	-4.27	141.07	232.81	118.60	88.39

6.98	-1.94	11.35	21.12	10.61	0.84	2.14	10.33	3.36	-4.74	144.10	239.50	124.20	92.33
6.47	-1.95	10.40	19.67	10.39	1.21	1.34	9.66	2.57	-5.13	224.22	324.90	226.52	248.04
6.06	-2.25	9.92	19.11	10.06	1.00	0.71	9.11	2.05	-5.47	223.44	326.10	246.76	287.79
6.51	-1.97	10.49	19.91	10.27	0.74	1.35	9.59	2.77	-4.65	161.98	268.34	165.97	157.93
6.78	-1.57	11.07	20.38	10.46	1.06	1.71	9.81	3.11	-4.25	139.19	233.91	150.20	140.23
7.43	-1.50	11.93	21.68	11.06	1.25	2.68	10.80	3.81	-4.20	138.80	229.26	114.90	84.99
7.04	-1.60	11.79	21.06	11.32	1.69	1.77	10.20	2.80	-4.91	202.79	298.67	217.58	242.63
7.06	-1.56	11.59	21.00	11.17	1.66	1.90	10.32	2.94	-4.79	200.39	296.07	212.09	233.06
6.84	-1.93	10.96	20.19	10.85	1.29	1.53	9.97	2.78	-5.12	239.78	348.65	237.44	251.80
6.64	-2.27	10.88	20.59	10.24	0.54	1.79	10.06	3.03	-5.09	159.18	254.42	135.73	105.85
7.28	-1.65	11.65	21.42	10.81	1.12	2.57	10.67	3.75	-4.43	141.98	232.09	118.57	87.43
7.29	-2.02	11.09	20.45	10.75	0.46	2.17	10.29	3.83	-4.50	145.03	235.19	128.47	100.34
6.43	-1.79	10.40	19.65	10.00	0.88	1.53	9.74	2.85	-4.48	163.79	255.10	177.09	178.06
7.15	-1.82	11.54	21.31	10.76	0.97	2.44	10.70	3.54	-4.62	151.01	238.81	126.04	93.13
6.65	-2.97	10.24	19.63	10.13	-0.50	1.48	9.67	3.17	-5.44	145.35	236.24	131.20	104.47
6.99	-2.44	10.54	19.89	10.38	0.01	1.91	9.96	3.63	-4.89	149.90	240.59	132.35	105.65
6.45	-1.95	10.41	19.61	10.09	0.64	1.27	9.43	2.79	-4.53	147.34	258.68	158.56	147.63
6.66	-1.84	10.77	20.17	10.50	0.87	1.43	9.68	2.82	-4.57	158.64	266.49	162.04	153.77
7.59	-1.32	12.31	22.09	11.27	1.48	2.89	11.16	3.89	-4.12	142.51	230.45	117.55	88.02
6.96	-1.94	11.89	21.24	11.58	1.69	1.38	9.65	2.35	-5.54	200.07	302.07	177.82	171.63
7.33	-1.61	11.98	21.69	11.16	1.38	2.53	10.85	3.52	-4.61	154.02	243.18	127.90	100.86
7.00	-2.10	11.29	21.05	10.70	0.60	2.08	10.27	3.33	-4.76	133.66	237.13	124.73	91.37
6.58	-1.85	10.72	19.94	10.60	1.39	1.36	9.73	2.56	-5.07	224.15	323.13	238.65	276.61
6.32	-2.63	9.42	18.86	9.44	-0.22	1.22	9.34	3.21	-5.05	154.51	242.17	139.20	115.46
6.92	-1.99	11.27	21.08	10.65	0.85	2.14	10.41	3.20	-4.80	157.08	248.38	131.66	99.91
7.21	-1.65	11.48	21.28	10.68	1.08	2.53	10.65	3.73	-4.36	142.08	232.14	118.11	88.20
6.89	-2.11	11.01	20.66	10.47	0.53	1.96	10.08	3.31	-4.74	135.25	241.06	126.31	91.48
6.15	-2.40	9.27	18.69	9.27	0.02	1.32	9.43	3.03	-4.81	151.30	289.59	145.34	107.29
7.34	-2.30	11.12	20.45	10.87	0.19	2.23	10.33	3.84	-4.75	143.68	234.31	128.38	98.85
7.30	-1.47	11.91	21.62	10.99	1.37	2.61	10.89	3.64	-4.30	148.67	237.06	124.48	99.26
7.37	-1.59	11.91	21.61	11.12	1.37	2.61	10.91	3.59	-4.53	151.74	242.84	126.64	99.01
7.24	-1.51	12.01	21.31	11.53	1.83	1.98	10.32	2.99	-4.85	202.83	302.05	191.83	193.47
6.98	-1.73	11.57	20.74	11.25	1.61	1.62	10.01	2.70	-5.09	214.88	314.42	206.69	217.39
7.01	-2.69	10.74	20.09	10.55	-0.19	1.83	9.99	3.47	-5.19	143.43	232.51	128.68	100.30
6.96	-1.82	11.54	20.75	11.28	1.61	1.59	9.90	2.65	-5.24	213.31	319.32	199.51	201.41
6.93	-1.95	11.63	20.88	11.36	1.56	1.46	9.75	2.47	-5.47	215.94	323.11	198.92	198.40
6.58	-2.36	10.59	20.22	10.10	0.29	1.60	9.63	3.07	-5.02	139.17	245.81	128.49	92.92
7.06	-2.39	10.77	20.15	10.54	0.08	1.90	10.04	3.59	-4.87	146.19	237.19	129.90	103.24
7.18	-2.19	10.96	20.33	10.67	0.31	2.07	10.19	3.71	-4.69	143.48	233.14	127.67	99.51
7.10	-1.86	11.74	21.01	11.51	1.60	1.70	9.93	2.68	-5.31	212.28	319.79	194.92	193.76
6.75	-1.52	10.84	20.16	10.40	1.31	1.74	10.06	3.09	-4.34	178.24	277.86	191.94	195.56
6.98	-1.74	11.48	20.74	11.26	1.65	1.68	9.91	2.72	-5.12	208.00	311.08	190.78	189.93
6.75	-2.20	10.64	20.29	10.19	0.43	1.77	9.66	3.31	-4.84	137.58	239.20	120.75	87.16

6.76	-2.05	10.57	19.89	10.64	1.09	1.58	10.00	2.84	-5.18	230.19	335.53	224.82	237.78
7.41	-1.42	12.10	21.85	11.15	1.43	2.68	10.98	3.68	-4.29	148.20	234.62	123.25	96.57
6.49	-1.98	10.52	19.93	10.25	0.74	1.34	9.56	2.74	-4.70	161.62	268.84	165.83	159.11
7.10	-1.74	11.59	21.34	10.74	1.09	2.32	10.61	3.44	-4.58	151.46	240.60	125.90	99.33
7.21	-1.45	12.04	21.50	11.50	1.84	1.94	10.36	2.93	-4.75	188.41	282.13	204.47	220.71
6.84	-2.07	11.25	20.92	10.49	0.77	2.00	10.25	3.20	-4.91	155.12	247.33	129.36	100.63
7.09	-1.81	12.02	21.30	11.69	1.74	1.53	9.78	2.50	-5.37	201.09	302.43	180.58	176.48
7.10	-2.05	11.52	21.29	10.86	0.68	2.10	10.17	3.35	-4.78	134.66	233.04	121.68	87.12
7.32	-2.12	11.12	20.46	10.80	0.36	2.14	10.29	3.80	-4.62	144.16	234.67	127.78	100.09
7.45	-1.52	12.16	21.90	11.22	1.35	2.64	10.87	3.68	-4.39	144.23	231.92	120.11	90.09
7.19	-1.52	11.46	20.96	11.12	1.23	1.96	10.16	3.21	-4.22	151.35	256.81	152.23	141.60
7.28	-1.51	12.14	21.59	11.73	1.97	1.91	10.16	2.83	-4.99	187.67	286.92	167.96	160.60
7.08	-1.71	11.62	20.80	11.34	1.63	1.74	10.04	2.80	-5.05	210.61	314.49	197.95	201.35
6.50	-1.82	10.47	19.74	10.11	0.76	1.41	9.60	2.90	-4.42	151.21	264.91	162.25	150.71
7.26	-1.56	11.82	21.54	10.98	1.27	2.49	10.85	3.52	-4.41	153.47	240.96	132.76	112.48
6.69	-2.62	10.26	19.67	10.06	-0.14	1.54	9.70	3.33	-5.06	146.48	237.52	132.82	106.25
7.04	-1.96	11.47	21.23	10.71	0.90	2.24	10.48	3.37	-4.77	151.68	242.18	128.37	94.50
7.14	-1.60	11.89	21.12	11.46	1.75	1.85	10.22	2.85	-4.96	208.40	307.07	198.26	206.17
7.43	-1.47	12.19	21.83	11.27	1.44	2.51	10.92	3.58	-4.43	153.61	238.53	136.01	119.29
7.21	-1.55	12.00	21.47	11.60	1.91	1.89	10.15	2.79	-5.01	188.47	288.69	168.81	161.02
7.18	-1.72	12.17	21.49	11.81	1.85	1.63	9.89	2.54	-5.31	194.76	296.74	176.29	170.57
6.80	-2.98	10.34	19.68	10.26	-0.48	1.68	9.85	3.37	-5.49	146.05	240.31	129.71	101.72
6.72	-2.61	10.72	20.35	10.54	0.42	1.49	9.73	2.91	-5.62	203.76	333.30	194.52	153.45
6.79	-1.74	11.21	20.54	10.94	1.55	1.50	9.92	2.59	-5.03	209.29	307.02	227.07	257.26
7.17	-1.56	11.84	21.17	11.35	1.72	1.94	10.33	2.96	-4.79	198.64	294.65	212.19	234.17
7.71	-0.97	11.23	20.64	10.96	1.39	2.94	11.19	4.48	-3.32	161.54	263.19	179.77	163.83
7.19	-1.44	11.87	21.16	11.37	1.81	1.94	10.37	3.02	-4.72	201.81	297.29	209.45	229.64
6.28	-2.05	10.29	19.54	10.35	1.19	0.95	9.32	2.23	-5.33	215.43	317.77	234.90	268.89
7.42	-1.36	12.32	21.66	11.68	1.88	2.18	10.58	3.11	-4.62	190.58	285.12	201.87	219.58
6.78	-1.74	11.21	20.53	10.94	1.55	1.48	9.92	2.59	-5.04	209.51	306.90	227.16	257.36
7.01	-1.62	11.72	21.04	11.28	1.71	1.70	10.13	2.72	-4.93	203.79	297.50	216.23	241.91
7.35	-1.61	11.95	21.68	11.13	1.30	2.60	10.92	3.58	-4.54	152.01	239.97	126.42	97.67
7.55	-1.37	12.31	22.09	11.29	1.46	2.82	11.06	3.79	-4.19	144.62	231.01	118.77	89.67
6.84	-1.62	11.08	20.26	10.56	1.00	1.70	9.84	3.11	-4.26	141.58	253.67	150.19	135.29
7.07	-1.99	11.19	20.86	10.69	0.72	2.12	10.33	3.46	-4.72	134.41	253.01	134.79	96.76
6.73	-1.53	10.79	20.11	10.36	1.30	1.73	10.05	3.10	-4.35	178.86	278.46	192.49	196.77
7.22	-1.54	11.63	21.45	10.81	1.26	2.54	10.85	3.62	-4.34	151.28	242.52	128.84	105.57
7.45	-1.51	12.21	22.06	11.23	1.39	2.60	10.86	3.68	-4.39	145.03	233.63	119.00	89.38
7.43	-1.47	12.14	21.91	11.32	1.54	2.61	10.92	3.53	-4.47	153.09	244.98	130.10	106.37
6.90	-2.73	10.57	19.93	10.41	-0.24	1.71	9.89	3.39	-5.21	144.44	234.08	129.94	101.87
7.12	-1.86	11.56	21.25	10.72	0.94	2.26	10.43	3.51	-4.63	145.90	236.32	122.50	92.77
6.85	-1.72	11.27	20.53	10.95	1.53	1.63	10.02	2.76	-4.94	215.40	309.66	226.62	255.78
7.15	-1.93	11.30	20.98	10.78	0.79	2.19	10.41	3.51	-4.66	132.83	251.23	133.58	95.42

7.58	-1.30	12.37	22.16	11.36	1.58	2.88	11.14	3.80	-4.17	145.63	232.33	118.74	92.12
6.64	-1.68	10.75	20.01	10.26	1.02	1.72	9.90	3.01	-4.37	156.37	249.35	169.15	167.30
7.02	-1.99	11.21	21.05	10.64	0.71	2.16	10.21	3.39	-4.65	135.96	234.72	122.81	89.31
7.07	-1.96	11.35	21.26	10.69	0.74	2.19	10.34	3.43	-4.64	140.02	235.77	124.92	90.92
7.27	-1.83	11.91	21.78	11.07	1.03	2.39	10.60	3.46	-4.70	146.51	237.49	123.60	90.39
7.57	-1.39	12.15	21.97	11.25	1.38	2.81	10.95	3.89	-4.18	136.42	227.57	113.19	82.84
7.10	-2.28	10.85	20.23	10.60	0.21	1.98	10.11	3.61	-4.77	144.34	234.27	127.99	100.12
6.92	-2.71	10.60	19.96	10.43	-0.22	1.73	9.91	3.41	-5.19	144.25	233.90	129.75	101.59
6.55	-1.95	10.59	20.01	10.33	0.77	1.37	9.61	2.77	-4.68	161.10	267.46	164.89	157.91
7.28	-1.36	11.80	21.26	11.36	1.87	2.18	10.55	3.17	-4.56	195.64	295.24	193.94	199.39
6.69	-1.55	10.62	19.90	10.21	1.16	1.82	10.13	3.18	-4.27	175.46	272.98	188.89	190.75
7.32	-1.59	11.91	21.66	11.03	1.24	2.56	10.81	3.63	-4.45	147.85	236.35	122.52	92.95
6.79	-1.73	11.19	20.50	10.95	1.55	1.49	9.88	2.59	-5.01	209.68	306.50	228.62	259.65
6.68	-1.50	10.77	20.07	10.24	0.94	1.69	9.59	3.12	-3.96	155.96	241.06	170.86	168.32
7.41	-1.78	11.92	21.64	11.16	0.94	2.52	10.65	3.64	-4.49	129.69	225.25	117.91	84.62
6.82	-1.77	11.09	20.41	10.72	0.97	1.56	9.76	2.92	-4.52	154.22	261.22	157.86	145.65
6.83	-1.86	11.33	20.58	11.15	1.59	1.44	9.74	2.53	-5.33	212.09	315.04	193.13	192.32
7.35	-1.48	12.25	21.72	11.83	2.01	1.96	10.22	2.86	-4.95	186.21	285.67	166.54	159.27
6.90	-2.52	10.37	19.74	10.26	-0.08	1.81	9.88	3.53	-4.96	150.57	240.48	133.32	106.72
7.55	-1.39	12.12	21.92	11.17	1.39	2.89	11.02	3.95	-4.17	137.87	229.33	114.66	84.26
7.56	-1.11	11.04	20.41	10.67	1.15	2.74	10.88	4.44	-3.38	163.78	264.55	163.65	144.86
6.70	-1.89	10.98	20.23	10.59	0.86	1.42	9.59	2.81	-4.61	158.57	264.28	161.86	150.25
7.08	-2.00	11.17	20.89	10.68	0.74	2.02	9.94	3.50	-4.72	134.46	234.15	116.85	82.67
7.17	-1.94	11.51	21.26	10.85	0.74	2.29	10.36	3.49	-4.64	131.56	231.10	121.73	86.65
6.81	-2.13	11.13	20.84	10.38	0.67	1.97	10.16	3.24	-4.90	150.57	242.97	127.69	97.13
6.84	-1.97	11.14	20.84	10.40	0.80	2.08	10.31	3.32	-4.79	152.87	244.76	129.45	98.93
6.99	-1.86	11.44	20.71	11.29	1.55	1.63	9.92	2.70	-5.28	216.37	323.17	200.32	200.01
7.04	-1.68	11.59	20.90	11.23	1.63	1.75	10.12	2.83	-5.01	210.83	311.53	199.84	206.02
6.86	-1.69	11.27	20.66	10.95	1.55	1.69	10.09	2.80	-4.91	206.75	304.22	219.46	244.37
7.08	-1.91	11.67	21.39	10.84	0.94	2.19	10.41	3.32	-4.76	147.75	239.96	124.99	93.32
7.64	-1.07	11.39	20.78	10.93	1.33	2.85	11.01	4.35	-3.44	162.22	266.18	158.63	133.00
6.98	-2.88	10.76	19.97	10.55	-0.29	1.76	9.88	3.43	-5.48	138.36	246.69	129.18	93.75
7.50	-1.50	12.05	21.81	11.24	1.22	2.63	10.69	3.77	-4.26	134.48	222.60	113.40	80.54
6.88	-2.03	11.21	20.91	10.46	0.73	2.07	10.26	3.32	-4.81	148.57	241.38	127.16	97.01
7.41	-1.27	12.03	21.50	11.55	1.96	2.31	10.67	3.25	-4.49	192.81	290.92	189.73	193.99
7.04	-1.80	11.48	21.32	10.72	1.09	2.27	10.61	3.37	-4.63	158.48	246.40	131.32	108.09
7.14	-1.94	11.29	20.97	10.78	0.78	2.18	10.40	3.51	-4.67	132.95	251.41	133.70	95.55
7.23	-1.64	12.22	21.56	11.86	1.95	1.70	9.96	2.65	-5.21	193.27	294.78	174.27	168.33
6.68	-1.80	11.05	20.25	10.86	1.50	1.39	9.74	2.50	-5.09	215.58	311.66	235.52	269.74
6.78	-3.10	10.47	19.71	10.32	-0.51	1.53	9.67	3.26	-5.70	143.81	248.46	130.98	97.43
6.91	-2.05	11.11	20.91	10.45	0.74	2.17	10.36	3.38	-4.77	150.04	242.89	128.94	96.00
7.22	-1.80	11.86	21.59	10.99	1.08	2.44	10.71	3.45	-4.68	149.65	239.71	125.80	96.63
7.47	-1.20	11.02	20.36	10.61	1.09	2.76	10.90	4.31	-3.50	160.86	264.63	160.09	137.59

6.51	-1.92	10.80	20.02	10.69	1.35	1.21	9.55	2.37	-5.21	206.84	308.55	231.07	262.10
7.53	-1.42	12.09	21.93	11.20	1.35	2.76	10.90	3.86	-4.21	137.04	228.36	113.76	83.32
6.39	-2.14	10.79	19.99	10.35	0.67	1.01	9.21	2.42	-4.96	149.43	260.80	156.87	147.40
6.97	-1.45	11.24	20.50	10.62	1.21	2.03	10.25	3.29	-4.12	144.87	246.04	156.93	151.22
7.16	-1.83	11.10	20.81	10.64	0.83	2.22	10.13	3.70	-4.48	134.75	232.52	116.36	82.06
6.90	-1.80	11.36	20.66	11.06	1.55	1.56	9.98	2.70	-5.11	215.67	317.06	205.83	212.51
7.11	-2.19	10.86	20.23	10.55	0.29	1.97	10.11	3.70	-4.65	146.96	235.79	129.73	102.25
6.52	-2.40	10.33	19.95	9.92	0.22	1.68	9.92	3.14	-5.06	142.53	261.26	140.99	106.06
6.90	-2.09	11.39	21.10	10.61	0.75	2.03	10.33	3.21	-4.95	154.34	247.41	131.60	101.04
7.45	-1.48	11.96	21.72	11.08	1.27	2.71	10.82	3.84	-4.18	138.37	228.59	114.57	84.55
7.15	-1.62	12.09	21.57	11.59	1.86	1.80	10.07	2.73	-5.08	180.52	279.09	158.05	141.85
7.22	-1.53	11.98	21.28	11.51	1.82	1.95	10.30	2.98	-4.87	203.41	302.79	192.50	194.59
7.40	-1.44	12.05	21.86	11.12	1.43	2.70	10.98	3.67	-4.29	149.84	236.56	123.59	97.52
7.16	-1.89	11.55	21.30	10.80	0.90	2.38	10.50	3.52	-4.66	141.07	234.09	122.04	87.69
7.48	-1.41	12.17	21.98	11.24	1.54	2.72	11.04	3.72	-4.35	153.01	240.98	128.95	105.27
7.09	-1.89	11.64	21.31	10.79	0.96	2.26	10.47	3.39	-4.73	149.73	240.68	124.48	94.17
6.70	-1.55	10.63	19.91	10.22	1.16	1.82	10.14	3.19	-4.27	175.21	272.75	188.64	190.41
7.14	-1.33	11.59	20.91	10.80	1.21	2.09	10.24	3.50	-3.87	134.67	238.57	147.64	128.79
7.10	-1.61	11.78	21.15	11.39	1.78	1.74	10.13	2.81	-4.98	205.51	304.99	193.12	194.93
6.91	-1.43	11.17	20.51	10.61	1.07	1.89	9.84	3.20	-3.92	150.87	235.86	166.66	148.10
6.85	-1.72	11.28	20.54	10.95	1.53	1.63	10.02	2.76	-4.94	215.24	309.47	226.44	255.53
6.92	-1.70	11.36	20.84	11.06	1.67	1.76	9.97	2.75	-5.09	194.00	293.93	171.10	160.19
7.16	-1.81	11.57	21.28	10.74	1.00	2.36	10.48	3.56	-4.61	144.34	234.84	120.79	90.46
7.11	-2.70	10.93	20.23	10.72	-0.14	1.97	10.12	3.53	-5.27	132.75	239.01	123.82	90.14
6.75	-1.93	10.60	19.81	10.26	0.51	1.60	9.83	3.25	-4.35	188.09	283.45	183.29	172.55
7.40	-2.23	11.21	20.54	10.93	0.24	2.28	10.38	3.87	-4.71	143.09	232.99	127.67	97.94
6.80	-1.81	11.13	20.39	10.73	0.95	1.49	9.68	2.87	-4.55	153.89	259.76	157.77	145.22
7.49	-1.44	12.27	22.06	11.44	1.59	2.69	11.01	3.58	-4.43	152.60	242.96	127.69	103.49
6.89	-1.96	11.23	20.94	10.46	0.87	2.10	10.36	3.31	-4.79	153.74	244.83	129.63	99.49
7.01	-1.39	11.29	20.60	10.76	1.39	1.98	10.22	3.24	-4.18	152.63	250.07	163.94	159.11
7.51	-1.46	12.11	21.92	11.27	1.46	2.81	11.06	3.75	-4.33	149.84	239.00	124.99	98.18
6.92	-1.96	11.21	20.91	10.45	0.80	2.17	10.34	3.38	-4.74	150.18	243.10	126.58	96.38
7.25	-1.74	11.63	21.41	10.90	1.01	2.37	10.37	3.61	-4.48	137.38	229.35	117.07	84.04
7.41	-1.57	11.79	21.54	11.02	1.18	2.66	10.69	3.82	-4.32	134.60	227.43	115.24	81.01
7.41	-1.45	12.28	21.72	11.87	2.00	2.03	10.28	2.96	-4.90	186.89	284.92	170.69	163.74
6.70	-1.72	11.20	20.51	10.95	1.63	1.24	9.69	2.44	-5.07	199.22	300.80	218.79	240.55
7.25	-1.74	11.63	21.40	10.89	1.00	2.36	10.37	3.61	-4.49	137.44	229.42	117.13	84.11
6.64	-3.04	9.99	19.39	10.03	-0.63	1.52	9.68	3.25	-5.46	153.99	243.65	133.27	106.04
6.48	-2.06	10.64	19.93	10.29	0.67	1.23	9.43	2.68	-4.78	157.01	265.65	160.80	153.46
6.92	-1.94	11.07	20.81	10.37	0.77	2.22	10.26	3.50	-4.65	143.05	236.62	122.17	90.61
7.04	-1.89	11.47	21.19	10.66	0.92	2.24	10.43	3.41	-4.71	147.79	239.34	125.32	94.00
6.46	-1.87	10.63	19.92	10.53	1.41	1.11	9.52	2.38	-5.14	208.60	313.40	227.59	256.02
7.19	-1.98	11.52	21.33	10.92	0.72	2.22	10.20	3.47	-4.70	132.59	230.49	119.32	84.12

6.82	-2.82	10.52	19.89	10.34	-0.32	1.64	9.82	3.30	-5.32	144.98	234.44	130.29	102.16
7.35	-1.41	12.22	21.58	11.69	1.98	1.99	10.40	3.01	-4.76	197.83	295.67	187.07	187.50
7.18	-1.84	11.94	21.20	11.67	1.63	1.73	9.96	2.69	-5.32	208.64	316.03	191.36	189.12
7.13	-1.73	11.51	21.28	10.69	1.03	2.40	10.61	3.58	-4.53	148.59	239.46	123.91	94.32
7.25	-1.70	11.70	21.49	10.89	1.05	2.54	10.68	3.62	-4.49	144.17	234.39	121.79	88.89
6.92	-2.03	11.58	21.02	11.45	1.52	1.40	9.64	2.39	-5.59	205.43	310.43	183.54	175.07
6.22	-2.56	9.65	18.93	9.38	-0.34	1.42	9.91	3.07	-4.76	201.52	280.25	182.13	179.22
7.06	-1.98	11.44	21.30	10.73	0.85	2.21	10.42	3.36	-4.81	149.80	240.66	126.92	94.21
6.67	-1.83	10.91	20.15	10.68	1.39	1.45	9.85	2.65	-5.04	220.75	320.66	228.75	254.74
7.49	-1.54	11.89	21.63	11.13	1.17	2.62	10.64	3.84	-4.25	132.35	222.13	111.81	79.76
7.19	-1.22	11.43	20.69	10.80	1.50	2.31	10.51	3.56	-3.89	153.42	249.74	164.67	159.41
6.33	-1.91	10.10	19.38	9.84	0.58	1.30	9.43	2.86	-4.39	152.61	262.74	164.23	153.42
6.96	-1.55	11.30	20.66	10.95	1.63	1.86	10.27	2.96	-4.75	205.28	304.95	205.89	221.20
7.49	-1.27	12.43	21.91	11.74	2.00	2.37	10.79	3.27	-4.50	185.57	278.73	193.90	205.21
6.73	-1.90	11.10	20.43	10.67	0.83	1.39	9.61	2.79	-4.65	152.24	260.50	157.49	144.59
6.82	-2.19	10.80	20.41	10.33	0.50	1.92	10.12	3.31	-4.88	138.15	255.91	136.01	100.09
6.38	-1.90	10.32	19.60	9.84	0.57	1.35	9.56	2.91	-4.38	153.10	257.75	167.72	156.63
7.26	-1.49	11.75	21.51	10.89	1.32	2.57	10.88	3.64	-4.30	150.47	238.08	126.30	103.02
6.33	-3.25	9.71	19.03	9.70	-0.77	1.20	9.41	2.96	-5.70	151.32	245.18	133.69	108.94
7.39	-1.44	12.10	21.86	11.13	1.43	2.65	10.99	3.65	-4.33	149.10	237.32	122.66	97.18
6.91	-2.72	10.58	19.94	10.42	-0.23	1.72	9.90	3.40	-5.20	144.33	233.97	129.85	101.72
7.43	-1.39	12.09	21.78	11.18	1.45	2.69	11.03	3.69	-4.21	151.26	237.12	129.22	108.30
6.83	-1.53	11.03	20.34	10.55	1.25	1.80	10.06	3.10	-4.31	156.29	254.06	167.28	165.06
7.08	-2.42	10.62	19.96	10.46	0.04	1.99	10.05	3.68	-4.85	150.24	239.74	132.16	105.05
7.02	-1.50	11.40	20.80	11.03	1.69	1.94	10.35	3.01	-4.67	202.98	305.08	204.04	214.98
6.43	-1.98	10.69	19.94	10.59	1.32	1.03	9.47	2.26	-5.28	208.64	310.53	232.23	264.01
5.86	-2.34	9.42	18.64	9.31	0.14	0.73	8.88	2.41	-4.83	167.86	272.36	178.17	173.52
6.78	-1.64	10.78	20.16	10.57	1.43	1.76	10.15	2.97	-4.74	212.48	313.49	215.92	235.61
7.25	-1.81	11.87	21.61	11.03	1.03	2.45	10.76	3.51	-4.65	149.47	241.08	126.58	95.66
7.10	-1.74	11.60	21.34	10.74	1.09	2.32	10.61	3.44	-4.58	151.50	240.48	125.87	99.25
6.95	-2.05	11.13	20.96	10.52	0.66	2.14	10.35	3.38	-4.77	144.55	239.89	128.86	95.23
6.62	-2.97	10.24	19.60	10.12	-0.49	1.46	9.65	3.15	-5.46	145.77	235.89	132.07	104.52
6.96	-2.08	11.04	20.68	10.55	0.66	2.01	10.23	3.40	-4.83	136.72	255.04	134.66	97.66
6.40	-2.82	9.71	19.15	9.64	-0.40	1.28	9.39	3.14	-5.22	152.70	242.26	136.96	111.90
7.41	-1.36	12.32	21.65	11.68	1.88	2.18	10.58	3.11	-4.62	190.60	285.17	201.91	219.63
7.38	-1.49	12.04	21.72	11.13	1.34	2.61	10.98	3.62	-4.32	154.12	238.29	131.68	111.05
7.48	-2.11	11.30	20.62	11.00	0.36	2.35	10.44	3.95	-4.59	146.07	234.92	128.21	99.53
7.47	-1.48	12.01	21.79	11.07	1.32	2.75	10.91	3.88	-4.24	139.96	230.77	116.85	85.07
6.56	-1.82	10.69	19.88	10.25	0.80	1.37	9.55	2.86	-4.45	149.72	259.23	158.00	145.79
7.38	-1.35	12.26	21.68	11.64	1.91	2.14	10.52	3.08	-4.61	184.65	277.12	199.57	213.05
6.83	-2.25	11.01	20.81	10.43	0.44	1.81	9.79	3.22	-4.95	138.25	237.60	123.89	89.04
7.28	-1.65	11.76	21.46	11.01	1.30	2.53	10.80	3.54	-4.60	153.06	244.53	128.19	100.26
6.51	-1.86	10.54	19.78	10.15	0.75	1.41	9.60	2.85	-4.46	148.02	260.27	157.41	145.42

7.09	-1.87	11.62	21.27	10.76	0.96	2.26	10.47	3.40	-4.71	149.50	240.64	124.79	94.46
7.31	-1.83	11.15	20.49	10.72	0.67	2.12	10.19	3.87	-4.29	148.36	237.75	130.96	103.10
6.05	-2.14	9.54	18.82	9.80	1.00	0.90	9.24	2.30	-5.30	239.21	343.17	255.16	297.97
6.89	-1.48	11.11	20.39	10.54	1.16	1.97	10.17	3.27	-4.12	143.70	240.44	156.31	149.26
7.36	-1.56	11.80	21.62	10.93	1.21	2.61	10.77	3.74	-4.31	140.54	231.70	118.04	87.75
7.09	-1.51	11.44	20.98	11.14	1.68	1.99	10.37	3.02	-4.71	201.93	301.62	193.22	198.85
7.26	-1.58	11.87	21.64	11.05	1.30	2.49	10.79	3.49	-4.45	149.00	239.68	126.93	101.75
7.46	-1.50	12.28	21.90	11.33	1.44	2.61	10.99	3.58	-4.42	156.54	238.14	136.43	118.78
6.78	-1.80	11.04	20.37	10.68	0.94	1.51	9.73	2.89	-4.55	154.99	262.05	158.62	146.60
7.57	-1.21	12.25	21.80	11.77	2.00	2.45	10.77	3.35	-4.41	187.46	286.00	178.82	179.17
6.85	-1.74	11.37	20.65	11.03	1.53	1.56	9.97	2.67	-5.02	212.47	308.82	226.09	255.04
6.96	-1.57	11.24	20.70	10.97	1.65	1.86	10.25	2.95	-4.80	206.36	305.51	202.08	211.56
7.32	-1.57	11.70	21.51	10.87	1.16	2.60	10.74	3.74	-4.30	141.57	233.09	118.46	88.06
6.53	-1.73	10.51	19.82	10.13	0.98	1.60	9.89	2.95	-4.45	163.19	262.91	178.07	179.23
7.19	-1.69	11.68	21.38	10.94	1.30	2.38	10.63	3.41	-4.68	157.87	247.30	131.70	103.36
7.36	-1.47	11.85	21.66	11.01	1.34	2.65	10.98	3.68	-4.32	151.30	241.46	129.36	105.54
7.40	-2.24	11.21	20.54	10.93	0.24	2.28	10.38	3.87	-4.71	143.12	232.96	127.64	97.90
7.45	-2.17	11.23	20.54	10.96	0.28	2.31	10.43	3.94	-4.65	144.78	235.33	128.31	99.32
6.36	-1.97	9.82	19.10	9.75	0.45	1.38	9.57	2.96	-4.41	180.62	283.92	187.05	171.66
7.13	-1.54	11.90	21.42	11.47	1.89	1.91	10.17	2.80	-4.97	184.06	282.09	163.21	150.94
7.04	-1.89	11.47	21.19	10.66	0.92	2.24	10.42	3.41	-4.71	147.88	239.43	125.28	94.02
6.60	-1.96	11.01	20.27	10.55	0.82	1.28	9.48	2.65	-4.76	150.19	259.03	156.44	144.75
7.01	-1.63	11.71	21.01	11.28	1.72	1.69	10.12	2.71	-4.94	203.84	297.89	216.83	242.95
6.98	-2.07	11.25	20.85	10.62	0.61	2.01	10.17	3.36	-4.76	134.79	240.29	127.44	91.11
7.33	-1.36	11.79	21.31	11.39	1.82	2.26	10.60	3.22	-4.53	194.90	293.83	187.55	190.81
6.85	-2.08	11.15	20.83	10.39	0.68	2.00	10.12	3.27	-4.82	148.09	240.65	125.49	94.57
7.29	-1.87	11.72	21.49	11.03	0.84	2.36	10.46	3.55	-4.57	131.23	228.23	119.55	84.19
6.85	-1.97	11.15	20.85	10.41	0.80	2.08	10.31	3.33	-4.79	152.82	244.63	129.37	98.74
7.09	-1.95	11.39	21.29	10.72	0.75	2.22	10.36	3.45	-4.63	139.78	235.12	124.44	90.44
7.80	-1.08	12.55	22.37	11.52	1.68	3.18	11.41	4.08	-3.88	137.12	227.11	113.55	84.54
7.00	-1.82	11.45	21.20	10.62	1.01	2.24	10.54	3.38	-4.65	155.14	245.45	129.57	103.44
6.84	-2.09	11.17	20.87	10.42	0.69	1.99	10.20	3.28	-4.86	149.85	242.16	127.08	96.54
7.16	-1.96	10.95	20.38	10.61	0.55	1.99	10.12	3.74	-4.47	146.83	232.84	129.08	101.33
7.24	-1.39	11.69	21.18	11.30	1.78	2.19	10.54	3.17	-4.57	196.71	295.55	190.06	194.52
7.20	-1.87	12.01	21.30	11.73	1.64	1.71	9.94	2.66	-5.38	209.29	315.56	189.83	187.42
6.98	-1.94	10.88	20.09	10.27	0.35	2.03	10.42	3.69	-4.22	163.41	249.83	157.83	138.15
7.16	-1.65	11.77	20.95	11.43	1.70	1.86	10.13	2.88	-5.00	207.98	310.85	193.72	196.54
6.87	-1.46	10.94	20.21	10.43	1.29	1.92	10.23	3.26	-4.19	170.37	267.98	182.43	182.81
7.32	-1.30	11.88	21.27	11.34	1.86	2.31	10.70	3.26	-4.41	195.77	293.75	195.30	203.04
6.94	-1.55	11.06	20.47	10.65	1.07	1.90	10.11	3.26	-4.15	151.07	259.51	159.14	141.74
7.10	-1.62	11.68	21.35	10.86	1.25	2.34	10.67	3.39	-4.50	158.38	242.29	136.17	117.68
6.46	-2.09	10.66	19.94	10.29	0.65	1.17	9.38	2.63	-4.82	157.85	265.74	161.65	154.04
6.23	-1.93	9.81	19.15	9.65	0.47	1.30	9.36	2.82	-4.33	172.83	271.53	191.70	176.60

7.47	-1.31	12.06	21.82	11.13	1.50	2.85	11.10	3.80	-4.08	145.89	234.21	121.94	93.69
7.56	-1.37	12.34	22.12	11.31	1.47	2.80	11.01	3.80	-4.21	143.56	231.33	118.68	88.59
7.40	-1.52	11.92	21.72	11.04	1.25	2.65	10.81	3.77	-4.28	139.52	230.71	116.48	86.46
6.64	-1.65	10.82	20.08	10.31	1.04	1.67	9.87	2.99	-4.33	148.83	246.58	162.78	159.26
6.83	-2.82	10.48	19.85	10.33	-0.34	1.64	9.83	3.32	-5.32	144.09	234.69	130.53	102.59
7.01	-1.92	11.39	21.16	10.64	0.86	2.16	10.36	3.38	-4.72	143.59	238.99	123.79	91.76
6.98	-2.26	10.61	20.14	10.34	0.21	1.80	9.93	3.64	-4.71	149.87	240.02	133.62	107.13
7.09	-2.70	10.87	20.18	10.68	-0.15	1.96	10.09	3.51	-5.27	133.33	240.18	124.05	90.28
7.46	-1.40	12.14	21.97	11.19	1.39	2.73	11.02	3.73	-4.22	144.36	234.08	120.05	92.38
7.40	-1.35	12.02	21.74	11.08	1.46	2.79	11.04	3.73	-4.16	146.58	234.54	121.62	95.08
7.23	-1.98	10.97	20.48	10.61	0.48	2.06	10.17	3.85	-4.46	149.07	240.93	131.73	105.08
5.98	-3.32	9.00	18.44	9.25	-0.91	0.88	9.04	2.70	-5.73	157.16	249.30	140.13	115.92
7.10	-1.96	11.24	20.92	10.73	0.75	2.15	10.36	3.48	-4.69	133.62	252.33	134.31	96.21
7.04	-2.14	11.43	21.20	10.82	0.58	1.99	10.03	3.27	-4.88	135.81	234.26	120.88	86.81
7.06	-1.94	11.60	21.36	10.78	0.90	2.21	10.41	3.34	-4.75	146.61	240.04	125.01	92.80
7.06	-1.95	11.60	21.36	10.78	0.90	2.21	10.41	3.33	-4.75	146.67	240.18	125.12	92.90
7.23	-1.60	11.78	20.96	11.45	1.70	1.98	10.22	2.98	-4.90	206.05	308.77	192.66	195.27
7.12	-1.79	11.58	21.29	10.76	0.94	2.31	10.39	3.51	-4.53	140.32	232.75	117.63	87.55
7.43	-1.50	11.93	21.69	11.06	1.25	2.68	10.80	3.82	-4.20	138.75	229.19	114.86	84.94
6.88	-2.75	10.53	19.89	10.38	-0.26	1.69	9.87	3.38	-5.22	144.72	234.26	130.18	102.23
7.08	-1.32	11.34	20.57	10.73	1.43	2.15	10.40	3.42	-4.05	169.23	263.93	175.93	175.05
7.31	-1.38	12.04	21.46	11.47	1.82	2.20	10.61	3.16	-4.60	192.41	286.03	201.78	218.17
6.83	-2.18	10.86	20.50	10.38	0.50	1.74	9.67	3.28	-4.86	136.92	238.65	119.29	85.87
6.63	-2.03	10.88	20.12	10.81	1.31	1.22	9.61	2.43	-5.38	225.27	333.01	215.22	221.71
7.35	-1.55	11.82	21.57	10.97	1.20	2.59	10.73	3.74	-4.29	140.73	231.06	115.82	86.11
7.68	-1.20	12.37	22.22	11.38	1.57	3.03	11.27	3.98	-4.00	138.39	228.68	115.43	86.36
7.03	-1.99	11.41	21.19	10.67	0.79	2.22	10.43	3.39	-4.77	148.15	239.30	126.13	93.20
6.55	-1.93	10.83	20.04	10.36	0.85	1.36	9.53	2.72	-4.69	137.69	251.00	149.10	140.11
6.65	-2.24	10.91	20.63	10.22	0.56	1.77	9.99	3.09	-5.03	153.96	246.23	131.20	101.05
6.74	-1.81	10.82	20.23	10.51	0.88	1.54	9.77	2.96	-4.50	154.32	263.40	160.30	150.03
6.90	-2.22	11.11	20.92	10.56	0.49	1.87	9.87	3.23	-4.93	135.98	237.25	123.71	88.47
7.26	-1.50	11.72	21.45	10.92	1.32	2.55	10.81	3.61	-4.30	151.32	236.45	128.94	104.88
7.08	-1.87	11.39	21.20	10.66	0.93	2.35	10.62	3.43	-4.67	152.12	242.35	129.30	97.53
7.42	-1.69	11.26	20.66	10.80	0.81	2.25	10.33	4.02	-4.15	148.71	238.25	131.81	104.85
6.60	-1.83	10.72	19.91	10.32	0.79	1.49	9.61	2.89	-4.45	143.71	254.87	152.94	139.94
7.12	-1.86	11.56	21.25	10.72	0.94	2.26	10.43	3.51	-4.63	145.95	236.32	122.50	92.79
6.99	-1.93	11.35	21.12	10.62	0.90	2.23	10.51	3.39	-4.71	151.69	245.12	127.51	99.09
6.53	-2.02	10.68	19.93	10.31	0.64	1.27	9.45	2.76	-4.67	162.78	268.16	166.14	155.27
7.07	-1.49	11.41	20.86	11.09	1.70	1.98	10.36	3.04	-4.72	203.78	301.74	198.72	206.86
7.26	-1.65	11.61	21.40	10.86	1.11	2.47	10.56	3.66	-4.38	137.52	229.43	117.62	85.49
6.84	-1.67	11.00	20.28	10.81	1.50	1.74	10.03	2.86	-4.85	213.31	311.63	212.88	229.79
6.30	-2.07	10.39	19.60	10.40	1.19	0.88	9.31	2.18	-5.35	215.02	317.48	235.94	271.70
7.28	-1.65	11.75	21.46	11.01	1.30	2.53	10.80	3.54	-4.60	153.08	244.57	128.23	100.31

6.74	-2.38	10.08	19.49	9.96	0.03	1.63	9.73	3.52	-4.79	153.65	241.61	135.71	110.40
7.03	-2.03	11.09	20.81	10.60	0.70	1.98	9.90	3.47	-4.74	135.30	235.16	117.42	83.71
6.62	-1.77	10.87	20.14	10.42	0.99	1.54	9.67	2.83	-4.48	136.64	239.41	148.70	140.59
7.29	-1.52	12.03	21.32	11.55	1.86	1.98	10.31	2.98	-4.87	201.65	301.65	189.69	190.71
7.55	-0.98	12.10	21.44	11.25	1.58	2.59	10.67	3.84	-3.52	129.93	236.21	143.04	118.58
7.43	-1.57	11.88	21.68	11.08	1.23	2.67	10.72	3.80	-4.34	133.97	228.66	115.03	81.00
7.34	-2.43	11.29	20.66	10.97	0.12	2.23	10.38	3.74	-4.97	136.99	230.60	123.39	93.44
7.06	-1.99	11.18	20.85	10.68	0.72	2.11	10.33	3.46	-4.72	134.51	253.11	134.87	96.84
7.44	-1.45	12.32	21.73	11.92	2.02	2.05	10.27	2.97	-4.89	188.13	285.41	169.03	162.72
6.75	-1.73	11.15	20.42	10.91	1.57	1.45	9.89	2.59	-5.03	213.38	310.23	232.51	263.59
7.57	-1.30	12.19	21.95	11.26	1.54	2.93	11.24	3.89	-4.13	148.98	236.00	125.44	100.96
7.47	-1.47	11.99	21.74	11.10	1.28	2.73	10.84	3.85	-4.17	138.03	228.16	114.20	84.11
7.07	-1.32	11.52	20.84	10.89	1.21	1.98	9.93	3.26	-3.86	146.06	233.37	161.95	140.58
7.04	-1.86	11.39	21.11	10.69	0.97	2.31	10.58	3.38	-4.67	153.05	244.54	129.05	97.33
6.75	-2.01	11.31	20.49	11.05	1.36	1.25	9.68	2.43	-5.38	222.96	324.43	216.08	230.62
7.39	-1.57	12.05	21.83	11.14	1.37	2.65	10.95	3.62	-4.50	152.02	239.02	125.54	97.66
6.54	-2.43	10.58	19.91	10.20	0.21	1.39	9.59	2.88	-5.10	144.51	274.19	138.57	97.29
6.89	-1.41	10.96	20.23	10.45	1.28	2.08	10.31	3.34	-4.10	170.83	266.71	180.63	181.09
6.90	-2.14	11.15	20.76	10.56	0.54	1.91	10.06	3.27	-4.84	136.23	242.62	128.75	92.58
7.23	-1.68	11.78	21.53	10.94	1.16	2.46	10.68	3.54	-4.54	149.11	237.25	125.13	93.76
7.30	-1.62	12.07	21.66	11.17	1.30	2.41	10.78	3.42	-4.54	158.07	240.44	138.56	120.92
6.45	-1.89	10.50	19.70	10.46	1.30	1.22	9.60	2.47	-5.12	228.31	327.72	243.95	284.84
6.95	-1.42	11.17	20.49	10.66	1.35	1.97	10.20	3.23	-4.19	154.39	252.13	166.23	162.23
7.20	-1.75	11.60	21.34	10.80	1.03	2.40	10.58	3.58	-4.54	146.91	236.71	122.60	92.55
6.95	-1.44	11.30	20.54	10.71	1.39	1.93	10.22	3.22	-4.25	174.14	271.40	183.35	185.21
7.03	-1.97	11.27	21.06	10.60	0.77	2.25	10.37	3.45	-4.71	143.70	237.61	124.01	90.91
6.98	-2.11	11.32	20.94	10.70	0.58	1.96	10.08	3.28	-4.81	135.50	241.45	127.63	91.06
6.63	-2.35	10.51	20.12	10.12	0.33	1.73	9.96	3.14	-5.04	140.34	260.56	140.07	104.20
7.49	-1.29	12.07	21.76	11.18	1.53	2.79	11.05	3.82	-4.09	147.45	231.99	123.76	99.48
6.80	-1.54	10.91	20.24	10.47	1.21	1.83	10.07	3.12	-4.30	157.78	256.31	170.78	168.69
6.18	-2.07	9.84	18.99	10.03	1.09	0.97	9.30	2.35	-5.26	233.83	337.32	258.60	307.34
6.64	-1.90	10.78	20.07	10.44	0.82	1.45	9.65	2.85	-4.64	151.05	262.31	157.34	148.00
6.65	-1.64	10.83	20.09	10.31	1.05	1.68	9.88	2.99	-4.33	148.67	246.43	162.58	158.99
6.99	-1.89	11.33	21.11	10.64	0.94	2.27	10.52	3.33	-4.70	154.83	244.72	130.72	98.93
6.54	-1.89	10.64	19.79	10.24	0.71	1.37	9.54	2.84	-4.51	144.78	255.93	154.26	142.81
6.61	-1.71	10.80	20.10	10.27	0.95	1.55	9.65	2.96	-4.39	138.45	239.45	152.85	144.65
7.02	-2.07	11.20	20.92	10.67	0.67	1.91	9.85	3.41	-4.81	135.39	234.52	117.91	83.18
7.08	-2.78	10.86	20.17	10.66	-0.23	1.93	10.10	3.52	-5.33	141.78	235.77	127.35	97.96
6.30	-2.13	10.52	19.70	10.10	0.65	1.10	9.31	2.52	-4.88	147.10	257.60	157.80	151.07
6.85	-2.06	11.48	20.92	11.36	1.47	1.33	9.55	2.34	-5.62	206.06	310.84	182.81	173.50
6.54	-1.90	10.70	19.87	10.62	1.38	1.26	9.60	2.46	-5.15	221.48	320.20	242.51	282.27
6.89	-2.39	10.86	20.25	10.56	0.36	1.55	9.61	3.22	-5.13	138.39	258.09	127.67	89.62
