# Supplementary material for: Eurasian aspen (Populus tremula L.): Central Europe’s keystone species ‘hiding in plain sight’
Source: PLoS One. 2024 Mar 27;19(3):e0301109. doi: 10.1371/journal.pone.0301109 (PMC10971661; doi:10.1371/journal.pone.0301109)
Supplement: Attachment — Submitted filename: Response to Reviewers.docx [file pone.0301109.s007.docx]

Dear PLOS ONE Reviewers,

Please accept our revised version of the manuscript *Eurasian aspen (Populus tremula L.): Central Europe’s keystone species ‘hiding in plain sight’* PONE-D-23-20092.

We followed all the suggestions/instruction provided by the **Academic Editor**.

- We edited the text in terms of excess words and the language. Please see the file ‘Revised Manuscript with Track Changes’.
- We extended the legends properly.
- The legends were moved to the end of the manuscript however, it is recommended by the Plos One journal to place them right after they appear for the first time within the manuscript text.
- We remade the figures for the higher resolution, 300 dpi minimum and the font for a larger size.

**The Reviewers’** comments.

There have been ‘no serious comments on the substance of the study, the presentation of the results and their visualisation’ by the Reviewer 1.

We followed all the comments provided by the Reviewer 2.

(1) Many paragraphs in the results section. It is recommended to classify them and write under subtitles. DONE.

(2) What does the value in Figure 2 and Figure 3 mean? please explain them. DONE.

(3) It is recommended to retain 2-3 decimal places for the F and P values in the title of Figure 5. DONE.

**Journal requirements.**

We submit relevant data for both major analytical entities ecoseries and vegtypes as the *Repository_vegtypes* and *Repository_ecoseries* PDF files.

We present written permission from the copyright holders for Fig. 1A, B and Fig. 7 as the ‘Other’ files (Other_Copyright_Permission_Kusbach, Other_Copyright_Permission_Hruban). We also include the demanded text to the figure captions.

We believe this study is a good fit for novel research perspectives favored by *PLOS ONE* readers.

Sincerely

Antonin Kusbach, corresponding author
