# Supplementary material for: Eurasian aspen (Populus tremula L.): Central Europe’s keystone species ‘hiding in plain sight’
Source: PLoS One. 2024 Mar 27;19(3):e0301109. doi: 10.1371/journal.pone.0301109 (PMC10971661; doi:10.1371/journal.pone.0301109)
Supplement: Attachment — Submitted filename: Response.to.reviewer_final.docx [file pone.0301109.s008.docx]

Dear Reviewer,

Please accept our revised version of the manuscript *Eurasian aspen (Populus tremula L.): Central Europe’s keystone species ‘hiding in plain sight’* PONE-D-23-20092R1. We edited the text, please see the file ‘Revised Manuscript with Track Changes.’ We concentrated our revisions on improving the Abstract and clarifying objectives (Introduction) so that they align well with section headings (Results, Discussion), as suggested by you. A detailed accounting of our revisions can be found in notes below, as well as in the Track Changes version of our manuscript included here.

We followed all your suggestions.

***Abstract:*** *The abstract should be concise and clearly state the purpose, significance, methods, results, and conclusions of the study. The abstract is still a bit long in the current version.*

The abstract text body in the review 1 followed the criterion of the 300 words of the PlosOne guidelines. It was not “a bit long” however, we shorten the text to current 236 words along with some rewording. We think, it is concise and states “the purpose, significance, methods, results, and conclusions of the study” enough.

***Introduction:*** *The objectives in the introduction section should be clearly defined. It would be helpful to* *separate the objectives into distinct sections with clear boundaries and clear meanings. The current version is hard to understand.*

We deleted clarified a potential confusion; one sentence before the specific goals. We also made the goals more concise by adjustment of (iii). We believe, goals are clearly defined. We are not sure how do you want to “separate the objectives into distinct sections with clear boundaries and clear meanings”. What distinct section, where? It sounds like corrections in the next chapters as subsection.

***Methods:*** *The methods section is currently lengthy and lacks hierarchy. It would be beneficial to divide this section into more subsections to clearly separate and describe the different content. Each subsection should have a clear objective that aligns* *with the research purpose.*

We created “the hierarchy” for the method section by dividing into subsections: Data sources, Aspen’s ecological characteristics and Analysis. We think the section is structured enough to be aligned with the research purpose.

***Results:*** *The subheadings in the results section should be more aligned with the research objectives stated in the introduction. Currently, it is difficult to see a* *correlation between the subheadings and the objectives. Additionally,* *the results should be presented in a way that clearly explains the significance and contribution of the findings. This will help readers understand the true importance of the numbers, analysis results, and charts.*

We aligned the Result subheadings with the Methods subheadings. There is a correlation between the subheadings and the objectives.

We are not sure, what the reviewer means by “*the results should be presented in a way that clearly explains the significance and contribution of the findings. This will help readers understand the true importance of the numbers, analysis results, and charts.”* The numbers are clearly linked to the analytical methods, charts and properly reported.

***Discussion:*** *The subheadings in the discussion section should also be clearer. There should be less overlap and repetition within the sections. The discussion should focus on outlining the key findings, explaining their biological or ecological mechanisms, speculating on possible reasons, and addressing the study's limitations and future research directions.*

We tried to improve the subheading titles to be more expressive. We think there is no overlap and repetition within the sections; the *Aspen ecological potential section* speaks, besides basic statistics of aspen, about **the broad ecological potential clear from the constructed heatmaps**. This potential was confirmed by the PCA and Random Forest analyses with enumeration of important environmental factors; the *Aspen’s ecological niche section* speaks about delineation of the niche of aspen as the ecological and growth optimum **using the previous heatmap**. There is no overlap with the previous section; the *Aspen current distribution and pathways for improved habitat section* discusses aspen management history, present state and reasons for its current distribution. Then, practical silvicultural practices were suggested for restoration, conservation, and adaptive management.

We think, there is no space for speculations in the Discussion since the analysis is based on the real data!

We believe this study is improved enough for publishing at the *PLOS ONE*.

Sincerely

Antonin Kusbach, corresponding author

et al.
